# Paramutation-like Epigenetic Conversion by piRNA at the Telomere of *Drosophila virilis*

**DOI:** 10.3390/biology11101480

**Published:** 2022-10-09

**Authors:** Ana P. Dorador, Martina Dalikova, Stefan Cerbin, Chris M. Stillman, Molly G. Zych, R. Scott Hawley, Danny E. Miller, David A. Ray, Sergei Y. Funikov, Michael B. Evgen’ev, Justin P. Blumenstiel

**Affiliations:** 1 Department of Ecology and Evolutionary Biology, University of Kansas, Lawrence, KS 66045, USA; 2 Stowers Institute for Medical Research, Kansas City, MO 64110, USA; 3 Divisions of Basic Sciences and Human Biology, Fred Hutchinson Cancer Research Center, Seattle, WA 98109, USA; 4 Department of Molecular and Integrative Physiology, University of Kansas Medical Center, Kansas City, KS 66160, USA; 5 Division of Genetic Medicine, Department of Pediatrics, University of Washington and Seattle Children’s Hospital, Seattle, WA 98195, USA; 6 Department of Laboratory Medicine and Pathology, University of Washington, Seattle, WA 98195, USA; 7 Department of Biological Sciences, Texas Tech University, Lubbock, TX 79409, USA; 8 Engelhardt Institute of Molecular Biology, Russian Academy of Sciences, Moscow 119991, Russia

**Keywords:** paramutation, piRNA, *Drosophila virilis*, transposable element, telomere, retrotransposon, TART

## Abstract

**Simple Summary:**

Paramutation is an epigenetic phenomenon in which one allele triggers an epigenetic conversion of another allele. How paramutation occurs is poorly understood, but it frequently involves small RNAs that can silence an alternate allele *in trans*. In one well-known case in *Drosophila*, small RNAs known as piRNAs mediate paramutation. In this paper, we describe a novel system of epigenetic conversion in *Drosophila virilis* that demonstrates paramutation-like behavior. This occurs at the telomere, where retrotransposon arrays are known to be regulated by piRNAs. This paramutation-like behavior demonstrates that the unique properties of the *Drosophila* telomere may play a role in triggering epigenetic conversion *in trans.* This system promises to reveal new mechanisms that underlie paramutation-like behavior.

**Abstract:**

First discovered in maize, paramutation is a phenomenon in which one allele can trigger an epigenetic conversion of an alternate allele. This conversion causes a genetically heterozygous individual to transmit alleles that are functionally the same, in apparent violation of Mendelian segregation. Studies over the past several decades have revealed a strong connection between mechanisms of genome defense against transposable elements by small RNA and the phenomenon of paramutation. For example, a system of paramutation in *Drosophila melanogaster* has been shown to be mediated by piRNAs, whose primary function is to silence transposable elements in the germline. In this paper, we characterize a second system of piRNA-mediated paramutation-like behavior at the telomere of *Drosophila virilis*. In *Drosophila*, telomeres are maintained by arrays of retrotransposons that are regulated by piRNAs. As a result, the telomere and sub-telomeric regions of the chromosome have unique regulatory and chromatin properties. Previous studies have shown that maternally deposited piRNAs derived from a sub-telomeric piRNA cluster can silence the sub-telomeric *center divider* gene of *Drosophila virilis in trans*. In this paper, we show that this silencing can also be maintained in the absence of the original silencing allele in a subsequent generation. The precise mechanism of this paramutation-like behavior may be explained by either the production of retrotransposon piRNAs that differ across strains or structural differences in the telomere. Altogether, these results show that the capacity for piRNAs to mediate paramutation *in* *trans* may depend on the local chromatin environment and proximity to the uniquely structured telomere regulated by piRNAs. This system promises to provide significant insights into the mechanisms of paramutation.

## 1. Introduction

Paramutation is a phenomenon in which one allele (termed paramutagenic) can impart an altered state of expression or function on another allele (termed paramutable) in the absence of alterations in the DNA sequence. One well characterized system of paramutation is found in maize where the *B’* allele at the *b1* locus has the capacity to silence the expression of purple pigmentation derived from the *B-I* allele [1,2]. After passing through *B’/B-I* heterozygotes, the original *B-I* allele becomes converted to a state designated B’*. This paramutated B’* allele can then in turn paramutate additional *B-I* alleles, converting them to a state that no longer produces purple pigment. This mode of communication and conversion between alleles is mediated by an RNA-dependent mechanism, tandem repeats of noncoding DNA, and the chromatin structure of these repeats [3,4,5]. In addition to maize, paramutation and paramutation-like phenomena are observed in other plants and a number of different animals, such as mice, flies, and nematodes [6]. In mice and nematodes, paramutation-like phenomena also appear mediated by the inheritance of RNA molecules [7,8,9] and, in flies, piwi-interacting RNAs (piRNAs) play a critical role in mediating this mode of epigenetic conversion [10]. Although RNA molecules, DNA methylation, and the state of the chromatin structure seem to be crucial players in the establishment and maintenance of paramutation, the question of how this *trans* effect is established still remains unclear. Understanding the role that each of these epigenetic modifications plays in systems of paramutation may help to elucidate the establishment of this transgenerational signal.

The primary function of piRNAs in *Drosophila* is to silence selfish transposable elements that proliferate within the genome. piRNAs are derived primarily from clusters of transposable elements, known as piRNA clusters, which have been modified at the chromatin level to become a source of transcripts designated for piRNA biogenesis. In the germline, dual-strand piRNA clusters become a source of sense and anti-sense piRNA, and anti-sense piRNAs can target TEs, through reverse complementarity, for either transcriptional, co-transcriptional, or post-transcriptional silencing [11,12,13].

A critical aspect of germline TE regulation in *Drosophila* depends on the maternal provisioning of piRNAs through the female germline. Syndromes of hybrid dysgenesis first revealed that maintenance of TE repression across generations required the maternal deposition of piRNA [14]. Subsequently, it has been shown that this maternal deposition is also required to trigger piRNA biogenesis at piRNA clusters, thus maintaining a continuous line of TE defense across generations [15,16,17]. Interestingly, this mode of epigenetic inheritance also plays a role in epigenetic gene silencing and paramutation through the off-target generation of genic piRNAs. In *Drosophila melanogaster*, a syndrome of paramutation has been identified that is triggered by an array of transgenes [10,18]. This transgene array producing piRNAs in the germline can also paramutate a related copy in *trans* and trigger piRNA biogenesis from a newly paramutated array. The newly paramutated array can in turn trigger piRNA biogenesis in *trans* to new non-paramutated transgene arrays and so on. This silencing mechanism has been observed to persist for up to 50 generations [10]

In the investigation of hybrid dysgenesis in *Drosophila virilis*, a related phenomenon has been described for a genic piRNA cluster residing near the telomere. In particular, one strain of *D. virilis* (the inducer strain, also known as strain 160) was noted to carry several sub-telomeric piRNA clusters that produced piRNAs from genes rather than transposons [19]. One of these sub-telomeric clusters, located on chromosome 2, contained the gene *center divider* (*cdi*), which had been silenced in the germline through conversion into a dual-strand piRNA cluster. Why the *cdi* gene has adopted piRNA cluster status is not known, though it should be noted that the strain carrying the *cdi* dual-strand cluster is also the strain that induces dysgenesis. This strain 160 is the only P-like strain in the *D. virilis* syndrome of dysgenesis and is noted by a large excess of TEs that are seemingly absent or in low abundance in other strains [19,20,21,22,23,24,25,26]. The polymorphic nature of this piRNA cluster (where the active piRNA cluster allele is designated *cdi^A^* and the inactive piRNA cluster allele is designated *cdi^I^*) was used to investigate the manner in which maternal piRNAs could trigger cluster piRNA biogenesis in the next generation [16]. This revealed that maternal deposition was critical for *cdi* cluster maintenance. Paternal transmission of the *cdi^A^* allele cluster led to weak production of *cdi* piRNAs in the germline, whereas maternal transmission maintained the robust expression of *cdi* piRNAs. Strikingly, the maternal transmission of the *cdi^A^* allele triggered piRNA production *in trans* from the paternally inherited non-cluster *cdi^I^* allele, though the production of piRNA *in trans* was weaker compared to that maintained *in cis* from the maternally transmitted allele. Nonetheless, the production of dual-strand piRNA biogenesis could also be maintained in the absence of the original *cdi^A^* allele in subsequent generations [27]. This is as expected from a system of paramutation. 

In this paper, we further investigate this transgenerational epigenetic silencing of the sub-telomeric *cdi* piRNA cluster in *Drosophila virilis*. While previous studies of *cdi* showed the capacity for piRNA biogenesis *in trans* could be maintained independent of the *cdi^A^* piRNA allele, the production of functional polyadenylated *cdi* transcript has not been investigated. In addition, previous studies investigated neither the expression of *cdi* nor piRNA abundance in the first generation of progeny lacking the silencing allele. For this reason, it is not known how penetrant this silencing is in the first generation where the silencing allele has been segregated away. Nor is it even known whether the epigenetic conversion of the *cdi^I^* allele to a piRNA generating cluster is sufficient to lead to genic silencing. In this paper, we show that the silencing of the *cdi^I^* allele *in trans* can persist in females in the absence of the original *cdi^A^* allele piRNA cluster. Though we have yet to examine this persistence in subsequent generations and conversion of the paramutable *cdi^I^* allele into a paramutagenic state, this is consistent with *bona fide* paramutation that depends on the maternal deposition of piRNA. In contrast to the previously defined system of paramutation in *Drosophila melanogaster* [10], this does not depend on the presence of a transgene array. Moreover, an investigation of the telomere structure between the two strains with long reads reveals significant structural differences between the two alleles. This suggests that the sub-telomeric location itself may contribute to epigenetic conversion. Since piRNAs play a critical role in regulating retrotransposons in *Drosophila*, we propose that proximity to the telomere plays an important role in rendering a gene susceptible to epigenetic conversion by piRNA. This is supported by previous studies demonstrating that inducer strain 160, which carries the silent *cdi^A^* genic piRNA cluster, also carries a significantly greater abundance of piRNAs that target the telomeric TART retrotransposon [27]. 

## 2. Materials and Methods

### 2.1. Fly Stocks and Maintenance

Two *Drosophila virilis* strains of the dysgenic syndrome were used: Strain 160 (here indicated as *cdi^A^* strain with the active piRNA cluster) and Strain 9 (here indicated as *cdi^I^* with the inactive cluster) (Lozovskaya et al., 1990). All stocks and crosses were maintained on standard yeast food at 25 °C.

### 2.2. Cross Scheme

The general scheme was to cross *cdi^A^* strain females to *cdi^I^* males, collect F1 hybrid females, backcross them to *cdi^I^* males, and collect *cdi^I^*/*cdi^I^* backcross progeny. Backcross progeny were genotyped to identify *cdi^I^*/*cdi^I^* individuals for RT-qPCR based analysis of *cdi* expression in the ovary. Two experimental replicates were performed with nearly identical conditions, aside from an accidental incubator overheating event whereby Replicate A daughters were exposed to elevated heat for five to ten hours.

### 2.3. Replicate A 

A total of 40 virgin *cdi^A^* strain females (0–3 days old) were selected, aged for one day, and each individually crossed to 2–3 *cdi^I^* strain males. Each of these was designated as a “grandmother replicate”. Vials were flipped two times, every three days, for a total of three broods from each grandmother. From these crosses, the ten most productive vials were selected, and from each of these vials, five to ten F1 virgin females were selected for backcrossing. Backcrosses with 2–3 *cdi^I^* strain males were performed with F1 virgin females collected 0–3 days after eclosion and aged for an additional 5 days. As before, vials were flipped twice, every three days. After laying, ovaries were removed and stored at −80 °C from the 11–14-day-old F1 females. This provided sets of sibling cohorts of five to ten F1 mothers from each grandmother replicate. 

From each of these F1 females (five females per grandmother replicate), eight backcross progeny were selected and mated to *cdi^I^* strain males. The 11–14-day-old backcross progeny were then dissected. Ovaries were removed and stored at −80 °C and carcasses were retained for genotyping to identify *cdi^I^*/*cdi^I^* backcross genotypes. This scheme yielded five grandmother replicates, each of which yielded four F1 mothers. From each F1 mother, one *cdi^I^*/*cdi^I^* daughter was obtained. Due to a power outage and incubator overheating, these daughters experienced a period of elevated heat, estimated to have lasted between five and ten hours. 

### 2.4. Replicate B 

Crosses and collections were performed as in Replicate A with slight modifications. All females were mated at 0–3 days rather than being mated at 5–8 days. Brood vials were flipped twice every three to five days, instead of every three days. Ovaries from F1 mothers were collected 12–15 days post eclosion. Ovaries from backcross daughters were collected 12–16 days post eclosion. Finally, there was no period of elevated heat.

### 2.5. Daughter cdi^I^/cdi^I^ Indel PCR Genotyping Assay and Sanger Sequencing Verification

Since it was essential to measure *cdi* expression in the daughters lacking the *cdi^A^* allele, only *cdi^I^*/*cdi^I^* daughters were selected. A 301 bp deletion was found in the *cdi^I^* strain only, ~73 kb away from the *cdi* locus. This was used to develop an indel PCR genotyping assay using daughter carcasses. The distinct amplicon sizes between alleles were easily distinguished from each other. Eight daughters were genotyped to identify *cdi^I^* homozygotes. Genotype verification was performed by sequencing identified individuals and confirming *cdi^I^* homozygosity with two SNPs inside the *cdi* locus. 

### 2.6. Ovary RNA Extractions 

Ovary RNA extractions were performed using the Zymo Research Direct-zol^TM^ RNA miniprep kit (R2051). A total of 0.4 mL of the Zymo TRI Reagant^TM^ was used to lyse the ovaries. All RNA extractions/purifications were carried out according to the Zymo Research Direct-zol^TM^ RNA miniprep kit user manual. All ovary pairs were extracted individually and labeled according to their corresponding sample number. Upon extraction, all RNA samples were stored at −80 °C. 

### 2.7. DNase Treatment 

All samples were subjected to a DNase treatment (Thermo Fisher, Waltham, MA, USA, TURBO DNA-free^TM^ Kit (AM1907)) to ensure no DNA contamination was present in the RNA. A total of 10 μL of Turbo DNase^TM^ was mixed with 25 μL of 10× Turbo DNase Buffer^TM^ and added to samples, followed by incubation at 37 °C for 20 min, and DNase inactivation.

### 2.8. Reverse Transcription 

All RNA samples were diluted to 100 ng/μL using RNase free water. The samples with an RNA concentration lower than 100 ng/μL were not diluted. RNA was converted to cDNA in half reactions using the Thermo Fisher SuperScript ^®^ IV First-Strand cDNA synthesis reaction kit (11754050) with 1 μL of 50 μM Oligo d(T) (Thermo Fisher: N8080127). Oligo d(T) primer was used to select for functional polyadenylated mRNA transcripts. Reactions were carried out according to the ThermoFisher SuperScript^®^ IV First-Strand cDNA synthesis reaction kit user manual (0.5 μL of Oligo d(T) primer, 0.5 μL of 10 mM dNTP mix, 1 μL of DNase free water, 2 μL of 5× SSIV Buffer, 0.5 μL of 100 nM DTT, 0.5 μL of RNaseOUT^TM^ Recombinant RNase inhibitor, 0.5 μL of Superscript^®^ IV Reverse Transcriptase enzyme (200 U/mL), and 2 μL of RNA template (200 ng)). If sample RNA concentration < 100 ng/μL, 5.5 μL of RNA template was added. 

### 2.9. qPCR Analysis

The expression of *cdi* was measured using quantitative PCR. Primers were designed to amplify the mRNA transcripts of exon 5 in *cdi.* Rp49 was used as a control with primers designed to span an intron between exon 2 and 3. A melt curve analysis for Rp49 was conducted in order to identify any DNA contamination. No DNA contamination was identified. Primers were selected using the primer design guidelines provided in the PowerUP^TM^ SYBR^TM^ Green Master Mix User Guide along with Primer-BLAST. Primer sequences are as follows: cdi_qPCR_1_fwd: GTTTAGAGACTGTTGGTTCA; cdi_qPCR_1_rev: CAGCTTCGATGCGTGCGATA; Rp49_qPCR_1_fwd: TCCAGCATACAGGCCCAAGA; RP49_qPCR_1rev: ACGACGCACTCTGTTGTCAA. 

The RT-qPCR analysis was conducted on an Applied Biosysems^®^ QuantStudio^®^ 3 Real-Time PCR System. The PowerUP^TM^ SYBR^TM^ Green Master Mix was used as the fluorescent dye. Three technical replicates were conducted on each set of experiments. 

### 2.10. Statistical Analysis

Statistical analysis was performed in RStudio. In order to measure the *cdi* expression in the ovary samples, the critical threshold (Ct) value of the *cdi* samples was subtracted from the Ct value of the RP49 samples and taken to the power of 2, assuming 100% primer efficiency (i.e., 2^(Rp49 CT-*cdi* CT)^).

### 2.11. Nanopore Sequencing. TE Library and Telomere Annotation

A new *D. virilis* TE library was generated using RepeatModeler2 [28] on a PacBio assembly of *cdi^A^* strain 160 [29] and a nanopore assembly of *cdi^I^* strain 9 [22]. Additional nanopore reads were further obtained for the *cdi^A^* strain using the same approach as in [30]. In particular, DNA was extracted from a pool of whole adult females using the Qiagen Blood and Cell Culture DNA kit. Sequencing was performed using the Ligation Sequencing Kit (1D) on an Oxford Nanopore Minion and base-calling was performed with Albacore, retaining all reads. These two new assembly specific repeat libraries were further refined using RepeatAfterMe [31], permitting only repeats represented in 5 or more copies within a genome assembly. The consolidation of these two libraries, along with a previous one generated from the reference genome [27], was achieved using CD-hit [32]. The identification of single nanopore reads from the *cdi^A^* and *cdi^I^* strains was achieved by using *cdi* gene sequence in a BLAST query of all nanopore reads. All reads containing *cdi* and further distal sequence were collected and annotated with RepeatMasker using the final TE library. Visualization was performed using custom R scripts.

### 2.12. piRNA Sequences and Mapping

piRNA sequences were obtained from a previous study [27] and mapping was performed with BWA aln, randomly assigning multiple mappers [33]. Mapping was performed to whole genome assemblies from *cdi^A^* strain 160 [29] and *cdi^I^* strain 9 [22]. In the case of the *cdi^A^* strain, the artifictual telomeric TART array (with only a fragment remaining) was removed from the assembly image in Figures 2 and 3 based on being present in only one read among all individual PacBio and nanopore reads and that read most likely being a duplex read. For F1 progeny, mapping was performed on both assemblies combined. To normalize mapping against haploid and diploid assemblies, pure strain courts mapped to a haploid genome were divided by two.

### 2.13. Whole Mount In Situ RNA Hybridization

Ovary dissection, fixation, proteinase K treatment, re-fixation and hybridization steps were performed as described (PMID: 29813067). Matrices for probe preparation were prepared by PCR using cdi_F ATGTCGGAAACACTGCCACT and cdi_R GATAATACGACTCACTATAGGCAACTAACGATCCGATGC primers of genomic DNA of the *cdi^A^* strain. Labeling of RNA probes with DIG-11-UTP (Roche, Switzerland) was made by MAXIscript T7 kit (Ambion, Austin, TX, USA). Anti-DIG-AP antibodies (Roche, Basel, Switzerland) were used in 1:2000 dilution. Images obtained by binocular microscope Nikon Alphaphot-2 YS2 (Tokyo, Japan).

## 3. Results 

### 3.1. Gene Silencing of cdi in the Ovary Is Maintained in the Absence of the Original Silencing cdi^A^ Allele

**Figure 1:**
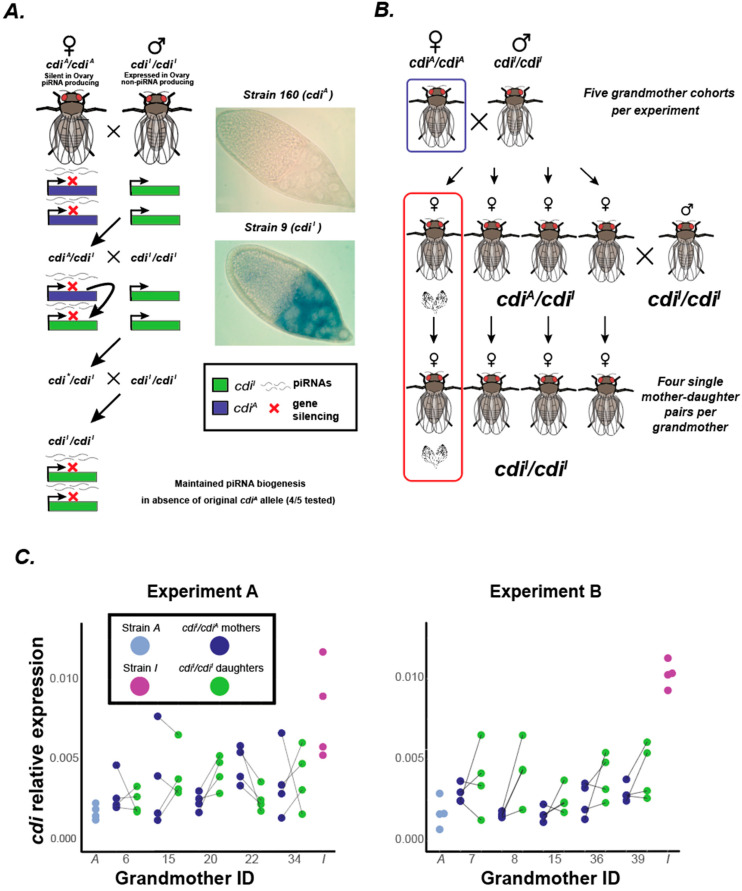
Experimental design and *cdi* expression. (**A**) The system of piRNA-mediated silencing of *cdi* by conversion to a dual-strand piRNA cluster. The *cdi^A^* allele is silenced and produces piRNAs from both strands. Results from previous studies indicate that a maternally inherited *cdi^A^* allele has the capacity to induce piRNA biogenesis from a non-silenced allele *in trans*. In addition, in subsequent generations, genic piRNA biogenesis can be maintained from *cdi* in the absence of the original triggering allele. *In situ* experiments show the expression of *cdi* in the egg chamber of strain 9 (which carries the expressed *cdi^I^* allele) and no expression in strain 160 (which carries the silent *cdi^A^* allele). (**B**) Experimental design for a single cohort derived from one of five grandmothers per experiment. (**C**) Low expression of *cdi* is maintained in the absence of the original triggering allele. Shown are relative expression values for *cdi*, normalized to Rp49. Each experiment presents the results of five cohorts, each daughter within a cohort being a cousin and uniquely derived from a single mother. Values presented are averages of three technical replicates per individual.

In the system of *D. virilis* hybrid dysgenesis, one allele of *cdi* (designated *cdi^A^*) is an active sub-telomeric dual-strand genic piRNA cluster and an alternate allele (designated *cdi^I^*) is not. A previous study [27] showed that matrilineal backcross granddaughters of F1 female progeny carrying the *cdi^A^* allele with males carrying the *cdi^I^* allele could maintain piRNA biogenesis at the *cdi* locus in the absence of the original *cdi^A^* allele (Figure 1A). How this coincides with gene expression is not known. In this paper, we investigated the expression of the gene *cdi* in the absence of the original silent *cdi^A^* allele. This was achieved in two independent experiments (Figure 1B). In each experiment, five grandmothers homozygous (*cdi^A^*/*cdi^A^*) for the silent piRNA cluster *cdi* allele were individually crossed to males homozygous (*cdi^I^*/*cdi^I^*) for the non-silent allele. From each grandmother, four individual F1 female progeny were subsequently backcrossed to *cdi^I^*/*cdi^I^* males. After laying, ovaries from F1 females were collected for real-time qRT-PCR. Multiple backcross progeny were then selected per F1 female and ovaries were also collected. Backcross progeny carcasses were then genotyped using an indel PCR assay to identify *cdi^I^*/*cdi^I^* females and genotypes were confirmed using PCR and Sanger sequencing based on SNPs located within *cdi* that distinguish the two alleles. This genotyping allowed us to identify four mother–daughter pairs per each of the five grandmothers, each daughter having the *cdi^I^*/*cdi^I^* genotype. In total, this yielded 20 mother–daughter pairs per experiment, with *cdi^A^*/*cdi^I^* heterozygous mothers and each *cdi^I^*/*cdi^I^* daughter lacking the original piRNA cluster *cdi^A^* allele. In one experiment (Experiment A), due to equipment failure, these daughters experienced a period of elevated heat of approximately 30 degrees for between five to ten hours one day prior to collection. However, this had no apparent effect on results compared to Experiment B for which there was no period of elevated heat. The expression of *cdi* for all genotypes, as well as homozygous *cdi^A^*/*cdi^A^* and *cdi^I^*/*cdi^I^* controls subjected to the same regime, was evaluated using qRT-PCR on polyadenylated mRNA reverse transcribed with oligo-dT. Expression was measured relative to *rp49* (Figure 1C). 

To identify the determinants of *cdi* expression first we used a linear regression model (linear model: Daughter-relative *cdi* expression ~Plate + Control/Genotype). Control/Genotype included status as being pure strain *cdi^A^*/*cdi^A^*, pure strain *cdi^I^*/*cdi^I^* or *cdi^I^*/*cdi^I^* daughters (Supplemental Document S1). The model was tested on both experimental sets (Experiment A and B) together and separately. When both experimental sets were tested together, there was no significant effect by experiment (Experiment A vs. Experiment B, *p* = 0.2274), but the experiment was retained in the full model (linear model: Daughter-relative *cdi* expression ~ Experiment + Plate + Control/Genotype). In this model, daughters lacking the original *cdi^A^* piRNA cluster allele and homozygous for the *cdi^I^*/*cdi^I^* non-cluster allele showed significantly lower expression than pure strain *cdi^I^*/*cdi^I^* controls (*p* < 0.0001) (Figure 1C). This showed that the epigenetic conversion of *cdi* occurs in a manner similar to paramutation, though the analysis of further generations is required to determine if silencing could persist and if the newly silenced alleles themselves had the capacity to trigger epigenetic conversion and become paramutagenic. Despite this epigenetic conversion, the expression of *cdi* in *cdi^I^*/*cdi^I^* daughters was significantly (*p* < 0.0001) higher than in pure strain *cdi^A^*/*cdi^A^* females (Figure 1C). This indicates that, while epigenetic conversion is occurring, it is not fully penetrant.

Considering the experimental design, we were also able to evaluate several other predictors of *cdi* expression in *cdi^I^*/*cdi^I^* daughters. A model was used to determine if the *cdi* expression of the daughters was influenced by their corresponding grandmothers, or the grandmother ID. From every grandmother, we collected four progeny (designated mothers), and from each mother, we collected one *cdi^I^*/*cdi^I^* daughter. Thus, the mothers from each grandmother were sisters, and the subsequent daughters were cousins. We wanted to test if the variation of *cdi* expression among cousins could be explained by the grandmother from each cohort. In order to test this, a linear model (linear model: Daughter-relative *cdi* expression ~Experiment + Plate + grandmotherID) was used to test for significant differences across cousins that can be explained by the grandmother ID. Our findings initially showed significant differences in *cdi* expression between sets of cousins of different grandmothers (Supplemental Document S1). However, after performing a Tukey’s test to control for multiple comparisons, the adjusted *p*-values showed no significant differences between grandmother cohorts. Thus, the *cdi* expression variation across daughter cohorts is not apparently explained by a transgenerational grandmother effect (Supplemental Document S1).

Since this candidate system of paramutation is driven by a maternal effect, we wanted to test if the *cdi* expression in the ovaries of mothers predicted the *cdi* expression in their corresponding daughter ovaries, lacking the original silencing *cdi^A^* allele. The linear model used (linear model: Daughter-relative *cdi* expression ~Experiment + Plate + grandmotherID + mother relative *cdi* expression) showed no significant effect of mother *cdi* expression (*p*-value = 0.94). This finding suggests that the *cdi* expression of the daughters cannot be predicted by the *cdi* expression of the mothers. One possibility is that genetic variation among recombinant individuals may contribute to variation in *cdi* expression. 

While *cdi^I^*/*cdi^I^* daughters lacked a copy of the *cdi^A^* allele, their mothers were heterozygous *cdi^A^*/*cdi^I^*. We sought to determine if this additional copy of the silent piRNA-cluster allele caused a lower expression of *cdi* in mothers. Using a paired *t*-test of mother–daughter pairs for relative *cdi* expression, we found a significantly higher level of expression in daughters compared to their heterozygous mothers (two-tailed *p* = 0.0367, avg Rp49 norm. expression Daughter: 0.0036, Mother: 0.0029). Thus, epigenetic conversion is not fully penetrant since it appears that daughters lacking the original *cdi^A^* allele have higher expression of *cdi* compared to their heterozygous mothers carrying the *cdi^A^* allele (Figure 1C).

### 3.2. Single-Read Nanopore Sequencing Analysis of the Subtelomeric Regions of cdi^A^ and cdi^A^ Strains

Many species of the *Drosophila* genus use telomeric retrotransposons to maintain chromosome ends [34,35,36] and the piRNA machinery regulates these telomeric retrotransposons [37,38,39,40,41]. In *Drosophila*, the region subterminal to the retrotransposon array is also composed of repetitive telomere-associated sequences (TAS). Recent genetic studies [42] indicate that telomere capping by HipHop plays a role in regulating retrotransposon expression near the telomeres, though apparently through HP1 rather than the piRNA pathway. To determine whether there are structural differences at the telomere that might explain the difference between the *cdi^I^* and *cdi^A^* alleles, we examined available assemblies and single long reads. A previous PacBio assembly of the strain carrying the *cdi^A^* allele [29] contains an array more than 27 kb in length composed of TART elements at the end of this chromosome. However, a manual inspection of individual PacBio reads carrying the *cdi* gene sequence showed that there was no single long read that contained such a TART array connected to *cdi*. Thus, this TART array appeared to be an artifact of the assembly. For this reason, we sought further clarification of the structure of the chromosome ends using nanopore sequencing. The number of reads per each sequencing run was similar (*cdi^I^* strain: 1.16 million reads; *cdi^A^* strain: 1.10 million reads) with similar length distributions (*cdi^I^* strain: 308 K reads > 10 kb; *cdi^A^* strain: 240 K reads > 10 kb). Reads were collected that contained significant BLAST hits to *cdi* as well as some additional distal sequence. These reads were then individually annotated with RepeatMasker with a custom *D. virilis* TE library (Supplemental Document S2). Even though there was a similar number of reads of similar length for each sequencing run, the results from the identified reads were quite different between the two strains (Note: There was a single read from the *cdi^A^* strain that was an apparent nanopore artifact that was removed. This read contained a ~40 kb array that was composed entirely of TART elements. Critically, they were arranged in a tandem inverted orientation with an essentially identical structure in the inverted direction. This is consistent with it being a duplex read and, since no other nanopore reads indicated this telomeric TART array, removal was warranted).

**Figure 2:**
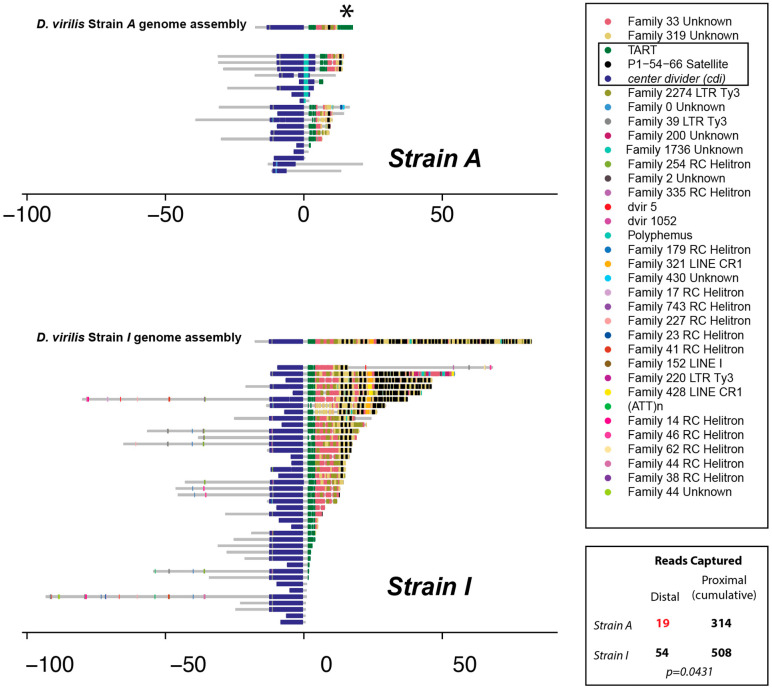
Repeat annotation of individual nanopore reads from Strain A and Strain I. The *cdi* gene sequence was used as a BLAST query for nanopore reads and reads carrying *cdi* along with further distal sequence were annotated with the new TE library and RepeatMasker. One read, a duplex read, was excluded from Strain A. Note the turquoise insertion of Polyphemus in some reads of Strain A. * indicates a fragment of TART that is likely an assembly artifact, but shown for completeness. Bottom right indicates results from nanopore read BLAST capture analysis. There is significant depletion of distal read coverage in the *cdi^A^* strain. This indicates a shorter telomere array.

In particular, many fewer reads carrying *cdi* plus additional distal sequence were found from the *cdi^A^* strain (Figure 2), and no single read attached to *cdi* had a telomeric length greater than 20.7 kb outside of the removed read. In general, half as many reads are expected to cover the very terminal end of a chromosome compared to an internal region because an internal region will have sequence reads initiated from two flanking sides, whereas a terminal end will only be covered by reads initiated from one flanking side. We investigated this by counting the nanopore reads captured by BLAST using the distal most 500 bp non-repetitive fragment from the reference genome and comparing to the number of reads captured by nine additional 500 bp non-repetitive fragments approximately every 100 kb, extending proximal 1 Mb. There is a significant depletion of reads captured by BLAST from the distal end in the *cdi^A^* strain (Figure 2, two-tailed Fisher’s exact test, *p* = 0.0431). Thus, based on read coverage and read length, it appears that the chromosome carrying *cdi* in the *cdi^A^* strain is truncated just distally from *cdi*. 

In addition, the inferred sequence consensus from the gathered reads at the terminus is different between the *cdi^A^* and the *cdi^I^* strains. Even though TART elements are known to be abundant at the telomeres, *D. virilis* differs from this standard arrangement. A previous study found *D. virilis* has satellite arrays at the very terminus rather than retrotransposons [43]. We found the same arrangement in nanopore reads from strain *cdi^I^*. In particular, the end of the *cdi^I^* chromosome is made of an assemblage of “54–66” satellite sequences, the same sequence identified previously [43]. These sequences appear not nearly as extensive in strain *cdi^A^* (Figure 2). Overall, nanopore sequencing indicates that the telomere end itself may be closer to *cdi^A^*, with fewer satellite sequences between *cdi* and the cap. Importantly, the analysis of strain-specific nanopore reads also revealed a TART fragment (green, Figure 2) just flanking *cdi* in *both* strains. We propose that this TART fragment just flanking *cdi* in both strains plays an important role in epigenetic silencing and conversion, due to differences in TART piRNA abundance between strains. While we lack formal evidence, one possibility is that the segregating Polyphemus or proximity of the cap to *cdi* capacitated the production of TART piRNAs and the conversion of *cdi* to a dual-strand cluster in the *cdi^A^* strain. This enhanced TART piRNA production may then mediate paramutation by targeting the TART fragment that is shared in both strains and is just flanking *cdi*.

Further investigation identified a segregating Polyphemus DNA transposon insertion inside *cdi* in the *cdi^A^* strain only (Figure 2, Upper Panel, Turquoise color), located within the sixth exon in the sense orientation. Though not in all reads from the *cdi^A^* strain, we sought to determine if this Polyphemus insertion might be important for the establishment of the genic *cdi* piRNA cluster and silencing of *cdi*. If critical, the insertion would have been present in heterozygous mothers since daughters show a signature of epigenetic conversion. Since the DNA of grandmothers and mothers was not collected, heterozygous first-generation backcross daughters were screened for the segregating Polyphemus insertion using PCR with primers flanking and within the insertion. This allowed the determination of the mother’s Polyphemus insertion status. In experiment A, 14 out of 19 heterozygous daughters screened had a Polyphemus insertion. One cohort (6_4) was excluded from this analysis due to the lack of a heterozygote daughter. In experiment B, 0 out of 17 heterozygote daughters screened had a Polyphemus insertion, and DNA from the remaining three cohorts (36_2, 36_5, and 39_5) was unavailable upon screening. Given that the insertion was missing from all heterozygous daughters tested in experiment B, it is unlikely that the Polyphemus insertion is responsible for the reduced expression of *cdi*, though the segregating insertion may have had a role in cluster establishment. We added inferred Polyphemus insertion status of the mother into the model and found no significant effect with respect to expression level of *cdi* (*p* = 0.891, Supplemental Document S1).

### 3.3. piRNA Mapping to Individual Parental Genome Assemblies of the Telomere

**Figure 3:**
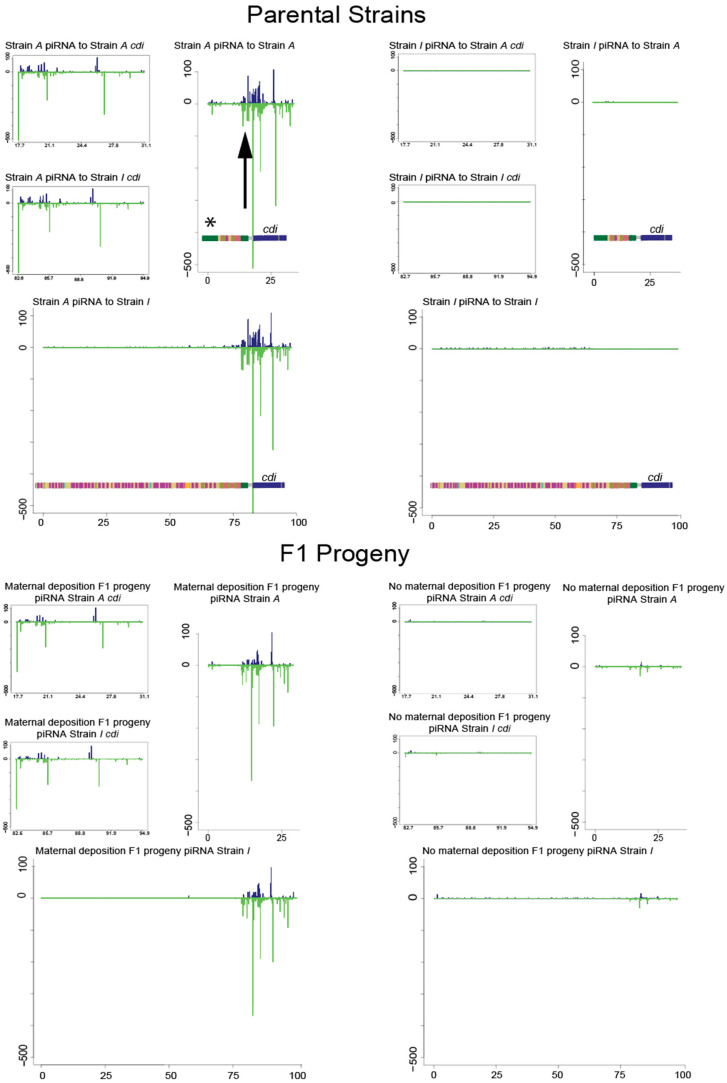
piRNA mappings to unique assemblies. Denoted in the top section are assembly repeat regions of *cdi* and distal sequence. * indicates a fragment of the TART element that is a putative assembly artifact, but shown for completeness. piRNA mappings—from within a strain and also across to the other strain—are shown for pure parental strains in top panel, with a closeup on *cdi* in smaller panels, upper left of each section. The arrow indicates piRNA mapping to the TART fragment just immediately flanking *cdi*. In F1 progeny, the layout is the same as above for parental strains, but piRNAs were mapped to both assemblies at the same time. Despite a different structure of the distal repetitive sequences, there is not abundant piRNA mapping to this satellite array. This long satellite array may function as a buffer from the piRNA-mediated regulation of the telomere end.

The previous investigation of the *cdi* piRNA cluster in *D. virilis* showed that maternal deposition of *cdi* piRNAs triggered *cdi* piRNA cluster activity in *trans* on the opposing allele within the ovary [16,27]. It was also shown that this piRNA cluster activity of *cdi* could be maintained in the absence of the original *cdi^A^* cluster allele in subsequent generations [27]. Given the differences in the telomeric structure, we mapped germline piRNA sequences [27] to the different assemblies obtained from the *cdi^I^* strain [21,22] and the *cdi^A^* strain [29] (Figure 3, excluding the apparent assembly artifact, with a residual distal TART fragment remaining and indicated by an asterisk). This was performed to compare how piRNAs differentially map to the structurally distinct chromosome ends and ensure that piRNA mappings based on a different reference assembly were accurate. We performed this using piRNAs sequenced from pure strains but also the reciprocal female progeny. In the case of reciprocal progeny, we combined two haploid assemblies for mapping. We normalized mapping density between parents (mapped to a haploid genome) and offspring (mapped to a diploid genome) by dividing mapping density by two for haploid mappings. As previously reported, significant differences in piRNA mapping can be seen directly within the *cdi* cluster, but, across the large satellite array, piRNA mapping does not reveal a difference. However, there is a significant difference in piRNA mapping density in the two TART fragments. One of these TART fragments (shown with an asterisk) is attributed to be an assembly artifact in the *cdi^A^* strain. However, another TART fragment that was found in nanopore reads in both strains just distal from *cdi* shows a distinct pattern of piRNA mapping between the two strains. In particular (as indicated with an arrow in Figure 3), there is abundant TART piRNA mapping to the strain with the *cdi^A^* allele, but these piRNAs are not abundant in the strain with the *cdi^I^* allele. This is consistent with the previous observation that TART piRNAs are vastly more abundant in inducer strain 160, which carries the *cdi^A^* allele [27]. By comparing strain specific assemblies, we confirmed that there is a TART piRNA target immediately next to *cdi* in both strains. We propose that excess TART piRNA, perhaps primed by a truncated satellite array, in the *cdi^A^* strain targets this shared TART element and contributes to the paramutation-like behavior at the telomere.

## 4. Discussion

In this paper, we extended prior studies of a genic piRNA cluster residing near a chromosome end in *Drosophila virilis* [16,19,27] and showed that this cluster has paramutagenic behavior with respect to gene expression. In particular, we showed that the females lacking the original silencing allele showed a low expression of *cdi* if their mothers carried the silent allele. Thus, with respect to *cdi*, such homozygous females lacking the silent piRNA cluster allele show reduced expression compared to genetically identical females whose mother lacked the silent allele. However, to prove that this is a complete system of paramutation, it is necessary to show that the converted allele also has the capacity to in turn paramutate another allele. In this paper, we only performed the analysis across two generations. In addition, to show that this is a system of paramutation as seen in plants, it would be important to show that paramutation can occur in both cross directions—where the silencing cluster allele is inherited both maternally *and* paternally. This is unlikely to be the case since the maternal provisioning of piRNA appears critical for the maintenance of the silent state.

### 4.1. Paramutation or a Maternal Effect?

Plants and animals seem to have different mechanisms of paramutation [6]. In plants, the capacity to trigger epigenetic conversion of an alternate allele is independent of parent-of-origin. However, this appears not to be the case in *Drosophila*. In another piRNA-mediated system of paramutation in *Drosophila*, paramutation is mediated by a maternal effect [10] and an allele only has the capacity for paramutation when present in the mother. A male that is heterozygous for the same epi-allele does not transmit a converted allele in the next generation. This reliance on the female germline for paramutation is explained by the fact that only the female germline provisions piRNAs. In this way, the phenomenon of paramutation is entangled in the concept of a maternal effect. Genetically, a maternal effect occurs when the genotype of the mother determines the trait of an offspring. However, while subsequent generations have not been evaluated for this system, the previously described system of paramutation in *Drosophila* does not rely strictly on a maternal effect. This is because, genetically, a paramutated allele can maintain its capacity to paramutate across more than one maternal generation. Since the original triggering allele can mediate its effect in the grandmother, the great grandmother, and so on, this is not strictly a maternal effect but a matrilineal effect. In some sense, one may also argue that this represents a form of transient imprinting since the female always transmits an inactive allele, though this mode of imprinting is lost once an allele is passed through the male germline for one generation.

When considering whether this mode of epigenetic conversion can be considered paramutation, one may also consider the timing of the establishment and maintenance of the epigenetic conversion in *trans*. Maternally provisioned piRNAs play a role in dual-strand cluster establishment [15,16,17] and this is the mechanism by which allelic conversion is maintained across generations. However, it is also clear that epigenetic conversion is occurring in the F1 heterozygous progeny of mothers that are homozygous. This is because studies have found, based on single nucleotide variants that distinguish the alleles [16,27], that piRNAs are derived from both alleles in the germline of F1 progeny. So, in some sense, the establishment of epigenetic conversion is initiated in the female germline and then maintained in the next generation through maternal piRNA deposition. It is unknown whether transient epigenetic conversion is also established in the heterozygous male germline, but it is conceivable that conversion is transiently established in the male germline but not maintained in the next generation due to paternal failure in piRNA transmission. This should be subject to future investigation.

### 4.2. What Drives Epigenetic Conversion of a Sub-Telomeric Genic piRNA Cluster?

While piRNAs are clearly important for the mode of epigenetic conversion of the sub-telomeric gene *cdi*, the properties of the locus and allele that enable this conversion are not clearly understood. In the study that originally identified the *cdi* piRNA cluster, several other genic piRNA clusters were identified near the telomeres of other chromosomes [19]. This suggests that some property of the telomeres in this strain might contribute to this phenomenon. In fact, we investigated a second cluster residing near another telomere, but the germline expression of both alleles was too low to fully evaluate. It is important to note that the sub-telomeric location of these genic piRNA clusters may be purely serendipitous. In this case, the property that confers this behavior may be independent of genomic position. Nonetheless, because telomeres often have unique genetic and epigenetic properties, we believe this association is unlikely to be a coincidence.

If in fact the sub-telomeric position is critical, what specific properties of the telomeres in this system mediate the conversion of *cdi* to a dual-strand cluster in the original *cdi^A^* strain and what property of the alternate telomere allele allows this conversion? Previous studies have shown [27] that there are both greater numbers of TART sequences as well as TART piRNAs in the germline of the *cdi^A^* strain. This may contribute, through some mechanism of spreading, to *cdi* cluster establishment in the *cdi^A^* strain. In this case, the maintenance of the silent state of *cdi* over multiple generations may depend on persisting elevated TART copy numbers and piRNA abundance. This could be tested in subsequent generations of backcrossing. If the capacity to maintain the silent state of *cdi* in recombinant progeny depended on global TART abundance or piRNA levels, then paramutation would not simply be the property of the original allele. Instead, it would rely on an epistatic interaction with variation dispersed through the genome. Though we cannot formally conclude it, we also showed that the telomeric repeat region appears shorter in the original *cdi^A^* strain. Thus, length and proximity to the capping proteins might be contributors. Under this model, only the genetic manipulation of the locus would allow one to distinguish whether simple physical distance or the repetitive sub-telomeric sequences themselves are critical. 

## Data Availability

Nanopore sequence data are available in the SRA under BioProject Number PRJNA884378. Stowers Institute Original data underlying this manuscript can be accessed from the Stowers Original Data Repository at http://www.stowers.org/research/publications/libpb-1741.

## Supplementary Materials

The following supporting information can be downloaded at: https://www.mdpi.com/article/10.3390/biology11101480/s1, Supplemental Document S1: Statistical Tables and Supplemental Document S2: TE Library.
