# Supplementary material for: Paramutation-like Epigenetic Conversion by piRNA at the Telomere of Drosophila virilis
Source: Biology (Basel). 2022 Oct 9;11(10):1480. doi: 10.3390/biology11101480 (PMC9598792; doi:10.3390/biology11101480)
Supplement: Supplementary file 1 [file biology-11-01480-s001.zip › SupplementalDocument S1.pdf]

Table 1.

Determinants of *cdi* expression Experiment A

```
Call:
lm(formula = 2^(DaughterRP49_CT - DaughterCDI_CT) ~ Plate + Control,
   data = studydesigna)

Residuals:
     Min        1Q    Median        3Q       Max
-0.003321 -0.001326 -0.000231  0.001117  0.005916

Coefficients:
                   Estimate Std. Error t value Pr(>|t|)
(Intercept)         0.0039343  0.0003965   9.923 1.78e-15 ***
PlateB              0.0002269  0.0005207   0.436  0.66417
PlateC             -0.0015111  0.0005207  -2.902  0.00482 **
ControlControl_160 -0.0017664  0.0006114  -2.889  0.00500 **
ControlControl_9    0.0044380  0.0006114   7.259 2.52e-10 ***
---
Signif. codes:  0 '***' 0.001 '**' 0.01 '*' 0.05 '.' 0.1 ' ' 1

Residual standard error: 0.00193 on 78 degrees of freedom
  (1 observation deleted due to missingness)
Multiple R-squared:  0.5173,           Adjusted R-squared:  0.4926
```

Determinants of *cdi* expression Experiment B

```
Call:
lm(formula = 2^(DaughterRP49_CT - DaughterCDI_CT) ~ Plate + Control,
   data = studydesignb)

Residuals:
      Min         1Q     Median         3Q        Max
-0.0032843 -0.0011427 -0.0001337  0.0013193  0.0034943

Coefficients:
                   Estimate Std. Error t value Pr(>|t|)
(Intercept)         0.0036045  0.0003286  10.968  < 2e-16 ***
PlateE              0.0010256  0.0004366   2.349 0.021309 *
PlateF             -0.0005231  0.0004366  -1.198 0.234397
ControlControl_160 -0.0020523  0.0005165  -3.973 0.000156 ***
ControlControl_9    0.0065048  0.0005165  12.593  < 2e-16 ***
---
Signif. codes:  0 '***' 0.001 '**' 0.01 '*' 0.05 '.' 0.1 ' ' 1

Residual standard error: 0.001633 on 79 degrees of freedom
Multiple R-squared:  0.7262,           Adjusted R-squared:  0.7123
F-statistic: 52.38 on 4 and 79 DF,  p-value: < 2.2e-16


F-statistic:  20.9 on 4 and 78 DF,  p-value: 9.743e-12

GrandmotherID9_4    1.993e-03  1.290e-03  1.546  0.12679
GrandmotherID9_5           NA         NA     NA       NA
---
Signif. codes:  0 '***' 0.001 '**' 0.01 '*' 0.05 '.' 0.1 ' ' 1
```

```
Residual standard error: 0.001579 on 69 degrees of freedom
Multiple R-squared:  0.7764,	Adjusted R-squared:  0.7311
F-statistic: 17.12 on 14 and 69 DF,  p-value: < 2.2e-16
```

Determinants of *cdi* expression Experiment A and B combined

```
Call:
lm(formula = 2^(DaughterRP49_CT - DaughterCDI_CT) ~ Experiment +
    Plate + Control, data = studydesign_ab)

Residuals:
      Min         1Q     Median         3Q        Max
-0.0042229 -0.0012463 -0.0001406  0.0011827  0.0050139

Coefficients: (1 not defined because of singularities)
                     Estimate Std. Error t value Pr(>|t|)
(Intercept)         0.0038021  0.0003616  10.516  < 2e-16 ***
ExperimentB        -0.0005938  0.0004901  -1.212  0.22744
PlateB              0.0002317  0.0004901   0.473  0.63706
PlateC             -0.0015064  0.0004901  -3.074  0.00249 **
PlateD              0.0005231  0.0004856   1.077  0.28298
PlateE              0.0015487  0.0004856   3.189  0.00172 **
PlateF                     NA         NA      NA       NA
ControlControl_160 -0.0019082  0.0004066  -4.694 5.76e-06 ***
ControlControl_9    0.0054725  0.0004066  13.461  < 2e-16 ***
---
Signif. codes:  0 '***' 0.001 '**' 0.01 '*' 0.05 '.' 0.1 ' ' 1

Residual standard error: 0.001817 on 159 degrees of freedom
  (1 observation deleted due to missingness)
Multiple R-squared:  0.6206,	Adjusted R-squared:  0.6039
F-statistic: 37.16 on 7 and 159 DF,  p-value: < 2.2e-16
```

Determinants of *cdi* expression among cousins explained by Grandmother ID

```
Call:
lm(formula = 2^(DaughterRP49_CT - DaughterCDI_CT) ~ Experiment +
    Plate + GrandmotherID, data = cdi_setAB_test)

Residuals:
      Min         1Q     Median         3Q        Max
-0.0030594 -0.0010731 -0.0000169  0.0009268  0.0034575

Coefficients: (2 not defined because of singularities)
                    Estimate Std. Error t value Pr(>|t|)
(Intercept)        4.885e-03  5.617e-04   8.696 5.07e-14 ***
ExperimentB       -1.077e-03  7.709e-04  -1.397  0.16535
PlateB             2.140e-04  4.957e-04   0.432  0.66677
PlateC            -1.281e-03  4.957e-04  -2.584  0.01114 *
PlateD             2.565e-04  4.888e-04   0.525  0.60079
PlateE             1.148e-03  4.888e-04   2.349  0.02068 *
PlateF                    NA         NA      NA       NA
GrandmotherID15_B -1.749e-03  6.310e-04  -2.772  0.00660 **
GrandmotherID20_  -2.946e-04  6.457e-04  -0.456  0.64915
GrandmotherID22_  -1.993e-03  6.457e-04  -3.086  0.00259 **
```

```
GrandmotherID34_  -6.397e-04  6.457e-04  -0.991  0.32416
GrandmotherID36_  -3.392e-04  6.310e-04  -0.538  0.59199
GrandmotherID39_   1.476e-05  6.310e-04   0.023  0.98138
GrandmotherID6_   -2.109e-03  6.457e-04  -3.267  0.00147 **
GrandmotherID7_   -4.483e-04  6.310e-04  -0.710  0.47905
GrandmotherID8_          NA         NA      NA       NA
---
Signif. codes:  0 '***' 0.001 '**' 0.01 '*' 0.05 '.' 0.1 ' ' 1
```

Table 2.
Adjusted p-value after Tukey Test (Determinants of *cdi* expression among cousins explained by Grandmother ID

```
  Tukey multiple comparisons of means
    95% family-wise confidence level

Fit: aov(formula = grandmother_corr)

$Experiment
          diff          lwr          upr     p adj
B-A 0.0002729953 -0.000288933 0.0008349236 0.3376159

$Plate
            diff           lwr           upr     p adj
B-A  2.670467e-04 -0.0011704893  1.704583e-03 0.9944024
C-A -1.227846e-03 -0.0026653823  2.096897e-04 0.1396734
D-A -5.374255e-04 -0.0019749615  9.001105e-04 0.8862266
E-A  3.543160e-04 -0.0010832200  1.791852e-03 0.9796763
F-A -7.939749e-04 -0.0022315109  6.435611e-04 0.5981907
C-B -1.494893e-03 -0.0029138794 -7.590667e-05 0.0327649
D-B -8.044722e-04 -0.0022234585  6.145142e-04 0.5703896
E-B  8.726929e-05 -0.0013317171  1.506256e-03 0.9999742
F-B -1.061022e-03 -0.0024800080  3.579648e-04 0.2603232
D-C  6.904209e-04 -0.0007285655  2.109407e-03 0.7194194
E-C  1.582162e-03  0.0001631760  3.001149e-03 0.0195966
F-C  4.338714e-04 -0.0009851150  1.852858e-03 0.9487051
E-D  8.917415e-04 -0.0005272449  2.310728e-03 0.4547915
F-D -2.565495e-04 -0.0016755358  1.162437e-03 0.9950692
F-E -1.148291e-03 -0.0025672773  2.706955e-04 0.1841580

$GrandmotherID
                  diff           lwr           upr     p adj
15_B-15_A -2.266551e-03 -4.352390e-03 -1.807116e-04 0.0220415
20_-15_A  -2.914099e-04 -2.377249e-03  1.794429e-03 0.9999864
22_-15_A  -1.989867e-03 -4.075706e-03  9.597273e-05 0.0747335
34_-15_A  -6.364523e-04 -2.722292e-03  1.449387e-03 0.9924638
36_-15_A  -8.566950e-04 -2.942534e-03  1.229144e-03 0.9449060
39_-15_A  -5.026921e-04 -2.588532e-03  1.583147e-03 0.9987413
6_-15_A   -2.106225e-03 -4.192064e-03 -2.038514e-05 0.0457686
7_-15_A   -9.657149e-04 -3.051554e-03  1.120125e-03 0.8905850
8_-15_A   -5.174522e-04 -2.603292e-03  1.568387e-03 0.9984206
20_-15_B   1.975141e-03 -6.485009e-05  4.015132e-03 0.0661735
```

22_-15_B	2.766843e-04	-1.763307e-03	2.316676e-03	0.9999895
34_-15_B	1.630099e-03	-4.098925e-04	3.670090e-03	0.2388298
36_-15_B	1.409856e-03	-6.301352e-04	3.449847e-03	0.4405956
39_-15_B	1.763859e-03	-2.761323e-04	3.803850e-03	0.1517309
6_-15_B	1.603265e-04	-1.879665e-03	2.200318e-03	0.9999999
7_-15_B	1.300836e-03	-7.391551e-04	3.340827e-03	0.5584321
8_-15_B	1.749099e-03	-2.908923e-04	3.789090e-03	0.1599754
22_-20_	-1.698457e-03	-3.738448e-03	3.415344e-04	0.1908050
34_-20_	-3.450424e-04	-2.385034e-03	1.694949e-03	0.9999306
36_-20_	-5.652851e-04	-2.605276e-03	1.474706e-03	0.9963059
39_-20_	-2.112822e-04	-2.251273e-03	1.828709e-03	0.9999990
6_-20_	-1.814815e-03	-3.854806e-03	2.251766e-04	0.1257375
7_-20_	-6.743050e-04	-2.714296e-03	1.365686e-03	0.9866795
8_-20_	-2.260422e-04	-2.266033e-03	1.813949e-03	0.9999982
34_-22_	1.353414e-03	-6.865768e-04	3.393406e-03	0.5009180
36_-22_	1.133172e-03	-9.068195e-04	3.173163e-03	0.7363626
39_-22_	1.487175e-03	-5.528166e-04	3.527166e-03	0.3625166
6_-22_	-1.163579e-04	-2.156349e-03	1.923633e-03	1.0000000
7_-22_	1.024152e-03	-1.015839e-03	3.064143e-03	0.8339458
8_-22_	1.472415e-03	-5.675767e-04	3.512406e-03	0.3769101
36_-34_	-2.202427e-04	-2.260234e-03	1.819748e-03	0.9999986
39_-34_	1.337602e-04	-1.906231e-03	2.173751e-03	1.0000000
6_-34_	-1.469772e-03	-3.509763e-03	5.702190e-04	0.3795146
7_-34_	-3.292626e-04	-2.369254e-03	1.710729e-03	0.9999533
8_-34_	1.190002e-04	-1.920991e-03	2.158991e-03	1.0000000
39_-36_	3.540029e-04	-1.685988e-03	2.393994e-03	0.9999138
6_-36_	-1.249530e-03	-3.289521e-03	7.904617e-04	0.6146522
7_-36_	-1.090199e-04	-2.149011e-03	1.930971e-03	1.0000000
8_-36_	3.392429e-04	-1.700748e-03	2.379234e-03	0.9999398
6_-39_	-1.603532e-03	-3.643524e-03	4.364587e-04	0.2594949
7_-39_	-4.630228e-04	-2.503014e-03	1.576968e-03	0.9992180
8_-39_	-1.476005e-05	-2.054751e-03	2.025231e-03	1.0000000
7_-6_	1.140510e-03	-8.994815e-04	3.180501e-03	0.7291068
8_-6_	1.588772e-03	-4.512188e-04	3.628764e-03	0.2714539
8_-7_	4.482627e-04	-1.591728e-03	2.488254e-03	0.9993971

Table 3.

Effect of mother *cdi* expression to *cdi* daughter expression

```
Call:
lm(formula = DaughterExpression ~ Experiment + Plate + GrandmotherID +
    MotherExpression, data = cdi_expression_testonly)

Residuals:
      Min        1Q    Median        3Q       Max
-0.003064 -0.001082 -0.000030  0.000949  0.003456

Coefficients: (2 not defined because of singularities)
                Estimate Std. Error t value Pr(>|t|)
(Intercept)    4.880e-03  8.017e-04   6.087 1.99e-08 ***
ExperimentB   -1.062e-03  8.915e-04  -1.191  0.23640
```

PlateB 2.119e-04 5.036e-04 0.421 0.67475
PlateC -1.325e-03 5.477e-04 -2.420 0.01727 *
PlateD 2.576e-04 4.934e-04 0.522 0.60272
PlateE 1.155e-03 5.020e-04 2.301 0.02338 *
PlateF NA NA NA NA
GrandmotherID15_B -1.749e-03 6.367e-04 -2.747 0.00711 **
GrandmotherID20_ -2.544e-04 6.987e-04 -0.364 0.71647
GrandmotherID22_ -1.934e-03 6.697e-04 -2.888 0.00472 **
GrandmotherID34_ -5.897e-04 6.725e-04 -0.877 0.38260
GrandmotherID36_ -3.318e-04 6.443e-04 -0.515 0.60770
GrandmotherID39_ 2.591e-05 6.535e-04 0.040 0.96845
GrandmotherID6_ -2.065e-03 6.855e-04 -3.013 0.00326 **
GrandmotherID7_ -4.376e-04 6.521e-04 -0.671 0.50365
GrandmotherID8_ NA NA NA NA
MotherExpression -8.199e-03 1.083e-01 -0.076 0.93979

Signif. codes: 0 '***' 0.001 '**' 0.01 '*' 0.05 '.' 0.1 ' ' 1

Residual standard error: 0.00156 on 103 degrees of freedom
(2 observations deleted due to missingness)
Multiple R-squared: 0.3098, Adjusted R-squared: 0.216
F-statistic: 3.303 on 14 and 103 DF, p-value: 0.0002271

Table 4.

Effect of Polyphemus insertion Experiment A only

Call:
lm(formula = 2^(DaughterRP49_CT - DaughterCDI_CT) ~ poly_mother,
data = Polyphemus_expA)

Residuals:

Min	1Q	Median	3Q	Max
-0.0027548	-0.0013739	-0.0006923	0.0015869	0.0032718

Coefficients:

	Estimate	Std. Error	t value	Pr(>|t|)	
(Intercept)	0.0038408	0.0008383	4.582	0.000307	***
poly_mother1_	0.0001364	0.0009864	0.138	0.891761	

Signif. codes: 0 '***' 0.001 '**' 0.01 '*' 0.05 '.' 0.1 ' ' 1

Residual standard error: 0.001874 on 16 degrees of freedom
(2 observations deleted due to missingness)
Multiple R-squared: 0.001193, Adjusted R-squared: -0.06123
F-statistic: 0.01912 on 1 and 16 DF, p-value: 0.8918

Effect of Polyphemus insertion Experiment A and Experiment B

Call:
lm(formula = 2^(DaughterRP49_CT - DaughterCDI_CT) ~ Experiment + Plate + poly_mother, data = polyphemus)

Residuals:

**Min**	**1Q**	**Median**	**3Q**	**Max**
**-0.0027548**	**-0.0013707**	**-0.0003983**	**0.0015302**	**0.0032718**

```
Coefficients: (1 not defined because of singularities)
              Estimate Std. Error t value Pr(>|t|)
(Intercept)    0.0038408  0.0007543   5.092 1.52e-05 ***
ExperimentB   -0.0002938  0.0008581  -0.342    0.734
PlateD                NA         NA      NA       NA
poly_mother1_  0.0001364  0.0008876   0.154    0.879
---
Signif. codes:  0 ‘***’ 0.001 ‘**’ 0.01 ‘*’ 0.05 ‘.’ 0.1 ‘ ’ 1

Residual standard error: 0.001687 on 32 degrees of freedom
  (5 observations deleted due to missingness)
Multiple R-squared:  0.01528,	Adjusted R-squared:  -0.04627
F-statistic: 0.2483 on 2 and 32 DF,  p-value: 0.7816
```
