# Supplementary material for: Paramutation-like Epigenetic Conversion by piRNA at the Telomere of Drosophila virilis
Source: Biology (Basel). 2022 Oct 9;11(10):1480. doi: 10.3390/biology11101480 (PMC9598792; doi:10.3390/biology11101480)
Supplement: Supplementary file 1 [file biology-11-01480-s001.zip › SupplementalDocument S2.refTElib_01_25_2022.fasta.pdf]

>Ref_Ulysses
TGTTGCATTTGACGTTATCGGCAGCACCGAGGGTTACTCAGTTGGTTGCGTGCGATATTATGGGCCACTGC
CGATATCTTTTTGGCATATGGTCGGAGTATGCCGGGTGAAGCTGGGCCCACGGCGGACGCAAAATTTTGA
AGGGTGGCAACGCCAGACATGCGATCCACGGCGCCAAAAGCGACGATTGCTATTGGCCGCCCGTTTCGCT
CTTAAAGGGAATACGAGGAGAGTGGATCGCATGCCCTTGGATATGCCCTTGGCCTTCATTTGGGCCGTTG
ACCCAAAAAAAATGCAGCTGTGCAGATGAGCAGCAACGGTGCCCGCCTCGCAAATGTATGCGTGTGCCGAA
ATTCGGCACCGATGGTGTGGCTGAAAAAAAATGTCGAGGAGTTCGATTTGTTCAGCGGTTAACGGCGGGC
GTGCCAGCTCGAAGTGAGCAAAAAAGTTGGACATTGCATTTCTGCTTGACAGAGTAAAATTTTTTCCCTTC
TGAAAAATTAAGCCGGCACATAATACCAATGGCTTAACAGACGAATAAAAGTTTTGTTTTTGTGTTCTTTG
CCATGTAATAAATTTATATAAATCAAACCGCTCGAATCACAAAGAAAATATAATAAAAAAATTGAAAGAG
ACTTAAAAATTTAAGTTGCGACGAGAGACGAACCAAATAAGCCAAAAATTAACGGCTCCGGCACCAAGG
AGACCGGCTCGCGCACCAGAGAGGCCCACGAAATATTCAAGGCCAGGATTCAGTGCAGCGGCGCAAACC
AGCCATATCCGCGACGTCCGCCAGCAAGCGTCGGCGCAAACGTGAAGGCGACGAGACGAGCCACCAACG
ACCGGGCTCCAACAACGAAGAAGTCGGTCCACAGGCGTAGACCCCGGTCCCACATCCACCGTAGTGAGTC
CGTTCCATTGTTCTGGCCCATCCCTCGGTTCGAGCCCCTGGCATGAGTTCAGAAGAATTGACTCCTAATTG
CACTCCCTGAAAAGGAGTAGATTTTTTTTTATCGTTAAATTTGGTTAATTTGTATTTATAAAATATATTCAT
ACATACATAGGCACTTACGCCGAGCTTCACTTTAGAGTGCGAGCGGTGAGGAAAACTCCAACGGTGATGC
ATCTGCGCCTGTAAAATAAAAAAAAAAAAAAAAATCTAAAATTAACTCTTGCGCGAACTATGCTCACATAC
TTACCTAATTTGAATTTACGCATTTATATCGCCCCTTACCACCCCCCAGAGAGCTCTTTAGGTAGGGTAAG
GTAGAATTAAGCGATTTAATACATACAATACACAATACGTGACATACCCAGTTTTAAGTAATTGTGCGGG
CCATTGTTCCTAAATCGGGGCAACGATTGCCTCTAAAGTGCCTCATTGCGCGTTTCTTTTGCGGCGACTAG
CCAATATCCTTGTAGTGTTTCGTCGCCTGTTCCGTTCAGTGTTGTAAAAAAAAAAAAAAAAAAATTAATTCT
ATAAGATAAGAGAAAAATTTAGGAATTAATGCGCTGTGTCAATTAGTATAAGTATATGCAGCAGTAATAC
GCACCTGTGAAGTTAATGAATTATCCGCTTGCATGAGATCCTGAACAGCTGCAAGATAAATGCCATAAGC
AATGATTATGATTGTTATACATTTTAAGAAAACGCAACCAATTATACGCACCTATTGAAAGAAATCATGTC
CGGCTAAAATAATATTGCTGGGGAGAGTGGGCATTATGGCCGACACGGGCTAATATAGGCCATTTGGTGG
CCAAAATGAGGGAGACGAGTGGAGGTGATCGTCTCATGGTAGGGCGGGTACGCTGGGTCCGGAACCGGG
GCCCGATCTGGAAGAGTAAGGTGGATAAATAGTGTTTTTTTTTGTTAATTTATATTGAGTGTCTTACCTTTG
TTGTTTACATTTTCATAGTTGTTACAGCTGTGGATTTTGTTCATGAGTTAGTAGTTCCGAATATTTGTTATC
GATTATGCGGTTCATTTTGAGTAGTGTTGTGTGAGTCTATTTTGCTACAGACGTGTAGCTTTGTTGTTATCG
ATTGTGCGGACTGCGGGCCAGGGATGGGACCACACCCAGAGAATCCGATGAGCCAGCCGGCGCGGCCCA
CTGAGAAACAAGCAGTTCCCTAACAATATGGCGCCCAAATTGAAGAAGCACTCGGCTTCTTTGAAGTCGG
TGTGTAATCAAAGAAAAATAAATACGATAAAAATGAAGGTACAGAGGAAAGGACTCCCTTGGCTTCAGG
ACCCCACACCGCTGAAGTCTTATGTCGGCAGAGTAGGTAGCTCATAATGCGCACGGTGTAGTGGAATCCG
AGAAGCACGACGGAAGTAGAGGTTATCTGAATATCCATCCATAAGCATGAGGGGTTTTTTTTGTAGTGGT
TGATGGCCGTTCGCATGTTGGCGAAACATTTGCGACTATCCATCGTTCGCCTGGTGGTAGCCGCAACGACA
AAGGCATCTCATGGTTAAAAAAATGTGATATTAGGGTAGTTTAGTCAGATGTTATTTATTCAATTAGTTGT
TTTTGTCTCATTTTGTGTTAATGTTGATGTATGCTGGCCGTAGTTATACGAGCTTCGGAGACCGGTTGACCC
AGACGATATAGAGCCGAGCCTGCGCCGCATCTGTCACACAGCATATCCCAAGAATGGCCAGAAGAGCGA
CGGAGAACGGATCTTGCCATGGTGTCCATTGACATGGGGTGTTTTCTTGTCGCGGTTGATGACTGGTAAGT
CACGTTTGCTAGTTGGTGAACGTTCGCGACCGTCAATCGTAGCCTGGTGGTAGCCGCCATGACAAAATAA
CCTGTGAACGAAATGTGATATTAGGGTAGGTCTGACTAGAGTTTGTTTAATGTTTTCAATTTGTTTTGTCCC
ACTCTGTGTTAACGTTTGGTGTGTGCTGGCCGTAGTTAAATGAGCTTCGGAGGCAGGTGACCCAGGACGA
TATAGAGCCGATCCTGGCGCCGCACCTGTCACGCAGCATACTCTAAGGGTGCACAGAAGTGCGACAGAAA
CCAATAGCCTTCAAGGTAGCGTTCCTTGCATGCCGTCCGTCAGTCAAACTGACGAACTGAGCATGGGACG
AATGCCTTAAGTGTTGTCCACTGTCCAGATTATGCGTAGATTATTTAGCCTGTGTTAGGATTTGTCGTCTCT
CCCCAAGTATTTGAAATTAATTAGAAAGAAAACAAAATTTTTGTGAAGTGGTTTTTGTGGATCAATGTGTA
TTAATTTATTATTCTTTATTAGTAAGATTTGTTAGTGGGATGTTTATTTATTTATTAATTTATTAGAAATATT
AGTAAAGTTTTTATAAGGGTTGTACGCCTTGTAGAAGATAGGGGGTACTGTTCTCAGAAGGAATAAGTAT
GGCTGAGAAACAGGTGTCTGATTGGGCTTCTGACGACTGATGGGGCGTCGCTCTGCCACCGCCGAGCCCA
GCTGGAGGGCTGGTGCGAACGCCAATGAATTTCCGGGGAGTTTAATGAGAGAGACAGATCCCTGTAAAG
GAGGGTCACCTGGTAGACTTGGAGGAAAGAATTGGCGCAACAGCCTAGACCTCCACGGAACCTGGAGTTC
CCAAAAGCCCCGAAAGACTCAGTGCTCAAATCCGGCGGCAAACGCGGCGATCGCTCAGGCCCACCAAAG

GATATCTGCATGCTCTGCCATCTGGCATCAGCGAGTCCATCCGCGAGGAGATGCGAGCTGGCTTCATGGA
AATGATGAAGGGCATCCATTGATGTGCTTAGGCCGCCAGGGAGCCAAACCCGCCGAAACTAACACTGCGC
CGAAGCCGCCAAGGACATGTCGAGCCGGGACCGCGAACAGGCGCCATCCCGAAAGAACGGAAGTGTTCC
AGCGCGAGCATTCCACCACCGTGCCACCGAAGCCACGTAGGCAAGCGCACAGGGCGGAGTATTCGTCCA
GCAGCCCAGTTTACGTGGCAGATGCTCAGTCGAGGAACTGGCGTAACCCGAATAACCGCCATTTCCGAGT
AGAGGACCGGAGAGCGGATGATTTTCAACCGGAGGAGGCGTACGCCAACGTGCGAGAAACTGCGTACAT
GAAGCTCGAGCGGTGGAACGTCAAGTTTGACGGCGAGGACGCGATGAATTCGGTCGAGGACTTCGTGTTC
CGCCTAGAGTTCCTGCAGAGGCAGTATCAGTGCCCGTGGAAGGAGGTCCTGCGCGGTTTCCACCTGCTCCT
GACCGGCCGCGCCCGCGAGTGGTACTGGATGCACGTGCGGCATTCTAGGGTCGACAGCTGGATGCAACTG
CGGCATGCCCTCTTGGACAGGTTCCGGGGCTACCAGACGGAACACGAGGTAATGCAGGAGCTTCTACAAC
GAGAGCAACAGGCCAGCGAAGGGGTGGATGATTATATCCACCACATGCGTCAACTCGCCGCGCGATTCCA
GAAGCCGCTGAGAGACCGAGAGCTGGTGAGGATTATAAAGCGTGGTTTAAAAGAAAGTCTGGCGAAATA
TATTTATGCCATGGACGTACTCACCGTGGATGAGCTGCGCCAAGAGTGCCTAGAAGTGGAGAGGCACATG
GGTCGCAGAAGCCGGACTGGGTACCTTCAGCCGTCGCGTTGTCCACAAGGAACGAGGCCCGTAGTCCACG
AGGTTGAAGTTCCACCGCATCTTACAGAAACGCCACCGGGAGAACTGGAGGAGGCATTCGTGCGAACGA
GGAACTCATCCGAATTGTATGCTGGAACTCGAGACAGTTCGACCACGTCTTTAGAGACTGCCTGTCCAAG
GAGCGGAAAATATTCTGCTATAGGTGTGGAAAGCCGGACACGTTCTGCTCGCAGTGTGAAAACTGTCCGG
GAAACCCCAGAGGGAGCGCGGTGATGGCGGGACAGACGCGTTCCGGGACGGCGACCGCGGGAAAGCAG
GAGAGGGGCACCTACCAGAGCCAATAATCATTGGAAAGGAGACGGAGATTTGGAATAAGGATCAGGATA
AGGCTAATTATAATAATAGTATAAGCAAAGGAGGAACACTTAGGGGCTTACCATACGAAGAACGTGTTCG
GGCATACATGTCGGCCCGAAATAGAATTTTTGGTGAGCGACAGCTGGAGGGGATGACGCTAGCCACCCGG
AGAATGGTGAAGGCACGTGCGCGCTTCCGCAGACGCAGGATTACCAGGCGCCAGGTCGTCGAAGCGGTG
AGACGGGAGGAGAGTATAGACCCACGAGTATTCGCCGAAGTGGAGGTCGCTGGAGCCAAAATGAAAGGG
TTGCTGGACACGGGGGCCTCAGTCAGTCTGTTGGGACAAGGATGCCGGGAGTTAGTGGAGAAACTGGGAT
GGGAAGCGCGGCCATACGAATCGATGGTGAGGACAGCATGCATGGGTGCCAATCGCCCAATTTTAGGTCG
CGTTGTTCTGCCTGTGAAATACGGAATAGAACGGTTAGATATCGTATTTTACATGTGCCCGGACCTGCGAC
AGGAACTATACCTGGGAATCGACTTCTGGCGAGCCTTTGAGATCGCACCAGAGCTGCTCGGTCCGGCCAG
AAAGTCCGAAACACCACCAGAGGCATCTGAGGTAACGGTAGCGAACCCAGAGGTGGCTTATTACCGGGA
CGACGACGACTGCGTAACGGATCCGGAAATGTGGGACCTGGACAACGATCAGAGGAGTCAGTTGGAAAG
CGTGAAGCGCAGATTTCTCCAATTTGAGAAGGATGGCCTAGGGAAAACTCACCTGCTGCAGCACCGAATT
CAGCTGATCGAGGGAGCAGAACCCGTGAAAGACAGACTTAATCCGCTGTCCCCGGCCAAACAGGAGATC
GTGTGGGCCGAGGTGGATAAGATGCTAAAGTTGGGCATCATCGAGGAGAGTGACAGCCCCTGGAGCAAC
CGGACGACGGTGGTGATGAGGCCTGGGAAGAACAGGTTTTGCTTAGACGCCAGGAAATTAAACAGTGTA
ACGGTAAAAGACGCGTACCCGCTTCCATGCATCGAGGGCATCCTATCGCGATCGACGAGACTCATTTTAT
CTCTAGCGTCGACCTTAAGTTCGCGTTCTGGCAATAGAGATGGAGGAGAAGAGCAGGGCGTATACGGCGT
TTACTGTACCAGGAGGCCGCTGTACCAGTTCCGCCACATGCCATTCGGGCTCTGCAACGCCGCTCAACACT
TCGAGGCTCATGACAAGGTAATCCCGGCGAATCTGAGGTCCAACGTATTCGTATACCTGGACGACTTGCT
GATAATATCGGCAGATTTTCCAACGCACTTAAAATACTTGGAATTGGTGGCCGAGTGTCTGAGGAACGCG
AACCTCACCATAGGCATGGCGAAGTCGAAATTCCTGTTCCGCAACCTAAACTACCTGGGTTTCATTCAATT
AAGGCGGCGGACGTGGCGCATGGATCCGGGAAGGGTAGAAGCGATCCGGAACATCCCGAATCCGAGGAC
GGTCAAGGAACTACGAAGCTTCTTAGGTACGGCGGGGTGGTACCGCCGATTCATAAAGAATTTCGCTGAG
ATATCGGTACCGTTGACTGATGCCCTTAAGAAGAGAACGGGTAGATTTGTGTTGAGCGACGAGGCCATAG
AAGCCATAGAGAGCTTAAAGTTAGCCCTCACCACAGCCCCGGTGTTAGTTCACGCAGATTTTCGAAGACC
ATTCTTCATCCAATGCGACGCATCTCACTACGGAGTAGGAGCTGTGTTGTTCCAGCTAGACGACGAACAA
CAGGAAAGGCCGATCGCATTCTTCTCGGCCAAACTCAACAAGCACCAGATCAACTATTCGGTGACCGAGA
AGGAATGCCTAGCCGCCAAACTGGCCATACATCGATTCCGGCCGTACGTGGAGATGATGCCGTTCACGGT
AATCACCGATCATGCGAGTCTGCAGTGGCTTATGAGTCTCAAAGACTTGAGCGGGAGATTAGCCAGGTGG
TCCCTCGAACTGCAAGCGTTCCCTTTCTCCATGCAGTACCGCAAGGGAGCCGACAACGTGTGCAGACACA
TTGTCCGAAGCGTGGAAGAGGTCGAACTGACCCCGGAAGATCTGTTGGGATTTCAGACCCCGGAGTTCGA
GAGTCCGAATATCGAGGAGCTGATAAGAGAGGTGATGAGCCAACAAGGGAAGTTTCCGGACCTTAGCAG
TGGCAGGACGGACTTCTCTTCAAGAGGGACCGTGCACGAGAGCTTGGAGGATGAGGTCGAAGGCACAAG
TTGGAAGCTCTGGGTGCCGGAGTCGCTGACGGCAGGACTCATCCAACAAGCGACACGGCGGACGAGACG
CTCAGCTCATGGAGGCATGAGGAAAACGCTGCACGCGCTGGCCAGGCAATATTATTGGCCCAACATGGCC
ATACAAGTCAGGGACTATGTTCGGAAATGCGATACATGCAAGGAGACCAAGGCACAGAACTACCGAATG

CAGGTCGGAATTGGGGAAGAAGTGCGCACCGACCGCCCCTTCCAGAAGCTATATATCGACTTCTTGGGGA
AGTATCCACGGTCGAAACGAGGGCATGCGTGGATATTCGTCGTCGTAGACCACTTCTCGAAGTTTACCTTC
CTGAAGGCCATGAGGGAAGCCACCGCGGCGGATGTCGTGAACTTCCTAGTGCATGAGGTGTTCTTCAAAT
TTGGCGTGCCGGAAGTGATCCATTCGGATAATGGACGACAATTCGTGTCAAAGTCGTTCGATGCGATGGT
CCAGGCGTTTGGCATTACTCACCTGCGCACACCAGTGTATTCGCCTCAGAGCAACGCCGCCGAACGCGTG
AACCGCACAGTGCTGTCGGCAATCCGAACCTACCTGGGTCAGGATCATCGGGAATGGGACGCGTACCTAC
CAGAGGTGGAAGTGGCGATCCGGAATGCGGTCCACAGCGGCTACGGGAGTCACTCCGTTCTTCGCGGTCT
TTGGACAGCAGATGTACCTGAATGGTTCCAGTTACAAACTGGCCAGGAAGCTGTAGATCATTGGCCGACC
ACAGTATTTCTGACCTTGACGCAAAGGACAGACTGGCTGTAATCCGAAGCCAAGTCAAGGACCACCTGCA
CACCGCATACGAGCGAAGTCGCCAACGGTACGACCACCGCGCGCCGACAGCTCCATCTGGAACCGGGAC
AGGAAGTGTGGAGGCGCAACTTCGCACTAAGCAGCTTCGGCAAAGCCTTCAATGCCAAGTTCGCCCGGAA
GTTCTTGAAGAGCCGCGTCGTCCGAGCCGTGGGAACCAACGCCTACGAGTTGGAAGATCTCCAGGGACGC
GTGCTAGGCGTCTTCCACGCAAAAGATATACGAACATAATCGTAATTTGCTCACCTCCTTGCCAGGCAACG
GCGGCGGCAGCGCCAGTCCCCCCCTTTTTTTTTGGGTCAGCAAAAAAAAAAAAAATATAGATTACTTGTCGT
GGGTGTGGCCTACGTTGGGCATCGTTATTAGCCGGCTGGGTCAATCCATGAAATGCGCACGCAGATTCCTT
GTAACTGTCCTTCATGTCGTTACAATAATCTGCGTGGTGATGTTGCATTTGACGTTATCGGCAGCACCGAG
GGTTACTCAGTTGGTTGCGTGCGATATTATGGGCCACTGCCGATATCTTTTTGGCATATGGTCGGAGTATG
CCGGGTGAAGCTGGGCCCACGGCGGACGCAAAATTTTGAAGGGTGGCAACGCCAGACATGCGATCCACG
GCGCCAAAAGCGACGATTGCTATTGGCCGCCCGTTTCGCTCTTAAAGGGAATACGAGGAGAGTGGATCGC
ATGCCCTTGGATATGCCCTTGGCCTTCATTTGGGCCGTTGACCCAAAAAAATGCAGCTGTGCAGATGAGCA
GCAACGGTGCCCGCCTCGCAAATGTATGCGTGTGCCGAAATTCGGCACCGATGGTGTGGCTGAAAAAAAA
TGTCGAGGAGTTCGATTTGTTCAGCGGTTAACGGCGGGCGTGCCAGCTCGAAGTGAGCAAAAAAGTTGGA
CATTGCATTTCTGCTTGACAGAGTAAAATTTTTTCCCTTCTGAAAAATTAAGCCGGCACATAATACCAATG
GCTTAACAGACGAATAAAAGTTTTGTTTTTGTGTTCTTTGCCATGTAATAAATTTATATAAATCAAACCGC
TCGAATCACAAAGAAAATATAATAAAAAAATTGAAAGAGACTTAAAAATTTAAGTTGCGACGAGAGACG
AACCAAATAAGCCAAAAATTAACGGCTCCGGCACCAAGGAGACCGGCTCGCGCACCAGAGAGGCCCACG
AAATATTCAAGGCCAGGATTCAGTGCAGCGGCGCAAACCAGCCATATCCGCGACGTCCGCCAGCAAGCGT
CGGCGCAAACGTGAAGGCGACGAGACGAGCCACCAACGACCGGGCTCCAACAACGAAGAAGTCGGTCCA
CAGGCGTAGACCCCGGTCCCACATCCACCGTAGTGAGTCCGTTCCATTGTTCTGGCCCATCCCTCGGTTCG
AGCCCCTGGCATGAGTTCAGAAGAATTGACTCCTAATTGCACTCCCTGAAAAGGAGTAGATTTTTTTTAT
CGTTAAATTTGGTTAATTTGTATTTATAAAATATATTCATACATACATAGGCACTTACGCCGAGCTTCACTT
TAGAGTGCGAGCGGTGAGGAAAACTCCAACGGTGATGCATCTGCGCCTGTAAAATATAAAAAAAAAAAA
AAATCTAAAATTAACTCTTGCGCGAACTATGCTCACATACTTACCTAATTTGAATTTACGCATTTATATCG
CCCCTTACCACCCCCCAGAGAGCTCTTTAGGTAGGGTAAGGTAGAATTAAGCGATTTAATACATACAATA
CACAATACGTGACATACCCAGTTTTAAGTAATTGTGCGGGCCATTGTTCCTAAATCGGGGCAACGATTGCC
TCTAAAGTGCCTCATTGCGCGTTTCTTTTGCGGCGACTAGCCAATATCCTTGTAGTGTTTCGTCGCCTGTTC
CGTTCAGTGTTGTAAAAAAAAAAAAAAAAAAATTAATTCTATAAGATAAGAGAAAAATTTAGGAATTAATG
CGCTGTGTCAATTAGTATAAGTATATGCAGCAGTAATACGCACCTGTGAAGTTAATGAATTATCCGCTTGC
ATGAGATCCTAAACAGCTGCAAGATAAATGCCATAAGCAATGATTATGATTGTTATACATTTTAAGAAAA
CGCAACCAATTATACGCACCTATTGAAAGAAATCATGTCCGGCTAAAATAATATTGCTGGGGAGAGTGGG
CATTATGGCCGACACGGGCTAATATAGGCCATTTGGTGGCCAAAATGAGGGAGACGAGTGGAGGTGATC
GTCTCATGGTAGGGCGGGTACGCTGGGTCCGGAACCGGGGCCCGATCTGGAAGAGTAAGGTGGATAAAT
AGTGTTTTTTTTTGTTAATTTATATTGAGTGTCTTACCTTTGTTGTTTACATTTTCATAGTTGTTACAGCTGT
GGATTTTGTTCATGAGTTAGTAGTTCCGAATATTTGTTATCGATTATGCGGTTCATTTTGAGTAGTGTTGTG
TGAGTCTATTTTGCTACAGACGTGTAGCTTTGTTGTTATCGATTGTGCGGACTGCGGGCCAGGGATGGGAC
CACACCCAGAGAATCCGATGAGCCAGCCGGCGCGGCCACTGAGAAACAAGCAGTTCCCTAACA
>Ref_dvir_266
CACGACAGGCGACAAGAATAAAAAAAAAAAAAAAGGTAGACAGAGTTAAGGTATGACGGAATACCACGA
TATATAAGGAGCGACTACGATCTGCAAGCAGAATATATCATATAGAGCCGAGGAGCTAAGATGTTGGCTA
CCCTCGGTCTTCACCCACCCTGCCTGTAGCTGCATCTGCGATTGTTGCAACTACCCTTTTGTATGCGATATT
ATTTACCTAAGGTGCCCAAAAAGGAATTAAGATTTCATTTTATCATATAATTGTATTCAAAGAAGATTTAA
ACTCATGATCGTACTTCATTATACATTACGTGAGAAGAACAACGTTTTTCTTTTTTTTTTTTTTATAAAACT
AAACTCAATCTGGGCAAAAACACACATGAATGAGATTACACTTCGAAACTAATATGAAGTTAAATCACTG
CTGATCTGAATGATCCACAAAAGTTATCTGTAATTACGAAGGCTGATTAGGAGTACACCCGCTGACTATA

ACCGCGATTCAAACAATACGTACAATTCGATATGTTGGCTTATTACCTTTTGTTCTTTCACACAGTACTCAC
TACTTTAGTATGATCCAACGATGTCAGCAGGCCTGTTGACGACGATGATGTGCTGTGGACTCTTAATTTGA
ACTGTGTAACGTAGAAATATAGTAGTCAGCGACATATACATGCTAAATCAGGATCGAAAGACAATGTCAG
CGATATAAAACGGAAAGAATTGCAATTTAAATTTCATGTGTACTTATGCCCTTCGCTTATACCTACGGCCA
GTATGCCGTTTAACAATTATATCAGATGTTGTAAATCCGAGTGAGGGTGCGCACTGGTTAGCTATCCACCA
GATCTGCGCTCAAGAATGTCCGGGAATCAATGGGTCGAACACGTGGTCACGTTGGTCTGCCTGTGCGTGC
AGCAAGGAACTATTGGTTAGCGAGGTTCAAAGCTTATCTGCAGAAAATGAGACGATGCTGAAATCTTGGT
TCGGCCTTAGGAAATTCGCTCCCTGCTACCAGTGTAGATTGCCGCTTTTTGGTGCTTTGGGCTTTGGCTTTG
GCTCCGATAAGGACTACGCGTTATGCGTAATTTTTCTTTTTGGCACGCAATAACGCAGCCCCGCGCTTTAG
CCTGCACCTTCCTACGACTACCACACACAGGAAAGGCACCCCACGACTCCAGTAACGATCCCTTAGTGAA
GATTTATCAGTGTCCGAATTCTGAAAATTTTCCCTTTTCTTTCCGGGGACCGTGGACCTTTTTTCCTTCCCTT
TTATTTATTTATTAATTTTTTTGTTTTTCCGAGCTTGAGTGCCGATACACGTGGGTTTATTACGCCCACGTG
AAAAATTGAATTTTATAATTTTTAAAGCAATCTAAACCGACTGTACTCGTTGGCGATTAAAATGGAAAAG
AATGACGCCACGCCAAAACTAAAAGAGGCAATTAAGCATTCACCCAAGACACGATATTTTCATTCGCAAC
GGCGTCATTAACATAGGTCATGGTCACTCTGGTTATGTTATTAACCTCCGCTGATCATATGTAATTGCATA
AATGTTATGGTGCAGTGCTGCCTATTCAAGCTGATTGCGATCAGCTGATCATGCCAATGGCAATGTATGTT
TATGTTTACAGAAATAAAACTCATCACCCCATCACAACGCAGGTTTAATCGAAAGGATTAACTAATACTG
AGATAAAAGCTGAAAAAGGGGATTACTTACATTCCCAAATGTCTTTAGCATGGTAATGACCAAGTTCTTTT
CCCTTGAGGTCTTCTAACAAATATATACAATTACCAATTTTTCGTTTGATACGTGCCTTAGTTCCAACTGGT
GCAAGTTTTGCGTTGAAATGCTTAGCCAAATTGCTTAAAACGAAGTTGCGTTTTATTACTATTTGACCGAC
TTCAAAAGAACGAGGGCGTGTGCGTAAGTTATACGTTCGCAAATTCTTTTCATATGCCTTATCCATGTGTT
TTTGAATATTAGAACGTATTAAGGGAAATTCGTCTGCTCGATCGAGTTTTAATTGCCCTTCATTCAGCATG
TTCAGGCTTTTGAGCAACTTGTAATCATTGCCGTGCTTAATCATATTTTGCCCAAACACAACGAAGTATGG
AGAAATCCCAATAGATTGGTGTATGGTGTTTCGTAACGAACTATTTATACTGCTAATGTATACATCCCATA
GGCTTTGGTCGGACCGAATATATGTGCGCAAAGCAGCATTAATCGAACGATTAACTCTTTCCACCGCGTTT
GCCTGTGGCGAGTATACACCGGTACACATATGCTGTATGCCGTACTTCGACAAGAGTGAGGAGAATTCCA
GGCTTTTAAATTGAGTGCCATTGTCACTCACTATTATCTCGGGTACTCCAAAACAGTCAAAAATATCTTCT
TGCAGTATATCTACCACTACTTTGGCATTGAGCCTTTTTATGGGCTTGAGAAATGTGAACTTTGAGTAGTG
ATCCAGAATGACGAAAATACCAATGTTTCGTTTTTTTGATGGTGGGTACGGACCAACAAAGCGAAATACC
TTTGGAAAAGCCTTTTGCTCATCACCCCTTGGTTTCCCATGGGTGGTTTGAGGCATCGATTAGGAACTTTC
GACGTTTGACAGAACTCGCAGTGGCGTATGTACTCGCGTACGTCAACCACCATGTTCGGCCAGTACAAAT
GTCTGCGAATACATTCCAAACATTTGGCCATCCCACAATGCGCGGATGTTGGTTGATCATGTGCTGCTTTC
ATAACATTTAAGCGTAATTCAGATGGCACAAATAATTTCCAACCCTCCATGTTCTCATCTACACTACTAGC
TGAGGGTTCATTTCGGAAATATATATAAATCATCTACCACTCGAAAATCTGGCAAGTTGGAACGAATAAT
ATTATTTTTTAGCCCGGCAGTATTCGTTGGATCGAAATGCCCTCCGAAGAACAAGTCGATTTCTCAGGGTA
TACCTCTAGGTCCACCGCAGCTACTTCGTCTGCTGGCTCAAAGGCACGGGAAAGCGTGTCTGCGACCACA
TTCTCACTACCCTTGCGATGTTCGATAGAAAAATTATATCCTTGCAATTTGAATGACCATCTAGCTAGTCT
ACCACTTAGATCTTTTTGGTTCATTAGCCAACGTAAACTTGCATGATCAGTCACGACTTTAAAATCGTGTC
CTTCGATGTACATGCGAAATTTTCTTACAGCCAATACAACGGCAAGACATTCGAGTTCCGTAACGGAATA
ATTTCGCTGCGCTTTATTTAGTTTTTTCGACATGTATGCGATGGGTCGCTCGATTCCTGACTCATCACATTG
GGCTAACAGACCGCCCACGCCATGCATGGAAGCGTCGCATTGGACTATAAACGGCTTCTCATAGTTTGCG
TTTATCAAGAACGGTGCTGAGGATTAATTTTCTTTTGATTTCTTCAAAACGAAATTTGAGCATTATCATTCC
AAAGCAAATGACGTGCCCTTTAGCAATTCTGTTAGGTGAAAGATAATCGACGAATACTGAGGGATAAACC
GTTGATACCAACCGGTCATTCCTAGGAATCGTCTCAGCTGTCTTGTTGAGGCACTGAATCTGCAACTGCTT
GAACTTTAGCCGGATTAGGCTGCAAGGTGCCTTCACCTACGATGTAACCAAGATATTCGACTTTTGTTAGA
CAAAAATTACTTTTACGGATATTAATGGTCAAACCTGCCCTACGCAATCGTGTTGCGATCTCAGCCAAATG
AATTAAATGTTCATCAAAACTTTGCGATAGTACAAGTAAATCATCCAAATAAACCATAACATGACCTTTCA
TGTCGTAAGGAATTACTTGATCCATCAATCGGCTCATGGTCTGCGGTGCATTGCACAATCCGAATGGCATC
CGCTTAAATTGATATAGCGGCCTGTTCGGGATAGTGAATGCGGTTTTTGCCCGCGATGATTCATCCAAGCA
GATTTGCCAAAAGGCATCTTTTAAATCGATTTTCGACACACAATGTACAGGAGGAAGTCTACTTATCAAGC
CATCTAAATTTGGTATAGGATAAGCATCCTTACTGGTCACTTCATTTACCTTGCGGCAGTCTAAACAAAAT
CTGACTTTTCCAGGCTTAACAATCAATATCGCGGGAGATGACCATGCGGAATATGGAGCTTCTTCAATGAC
TCCAAGCTTGATCATCCTATCTACTTCTTCGCACAACAATTTTTCCCTTGCTGGTGATAACGGATAAAACCT
CTGGTTTTATGGGCTTGGCGTCCCCAGTATCGATGATATGTTCTACAAGAGTAGTCTTTCCTAGACCATCA

ATTTCAAACGAAGGAAAGGAAGTGATTACTGATTGTAAGCGTTTCTTTTGGGCTAGTGTTAACGACAACT
GATTCGACTCCACTTCTTCGAAATCCACTGAGTTCAATGTATTCGAATGATGTACTGAAATGCCAAACGCT
CTCCAGAAATCTATTCCACAGAGAACGTTTTCCTTGATAGACGGGACAATTAAGAATTCGAAATTATTTGC
AATGGAGTTATAAACGATAGGAATTGAAATGGAACCTACAACATGTTGTTTTTGTCCGTCGGCGGTTCTCA
CATCGCCTTTTATTTTGTTAAAGTCAATTTTCGAATCGAGAATTTTATTCGCTAAGCTCCCTCCAATCACGC
TTTTCGTAGCCCCACTATCTAACAAGGCTAAGTAATGCTGATTGAATATTTCTAAATTCGTGAACGGTCGC
ACATCGTTTGATCGATGATCAATCGCTGAAATACGATGCTTATTTAGCGAGCGTCGCTGAGTCCATAGGTT
TCTTAGTCTCTCTGACGAACCGAGGCTTAAATTCAACTTTGGGTTCTGAAGCGAAAATAGTTGCTCTACGG
TCTAAATATTCGTGTAAACGGACGTGGTAAGGTTTAAGTTTTGTCTGACACGGTTTTGATAAGTCTGGTGT
GAAGGTCGAATCAGGTGTTAAGGTATCTTTATTAAAGTCTGGATCCGAGGCTATTGACGACGGACATTCG
CGTTTAAGAGACGACGAAAGTCCTCCGGAGAGTTTTCCGAAACAGGGTTACACTTTCTACAAGTGGGTTT
GTAAGTGTCAACGGCACCACAGCCGTAACAAAATACACGACGCGCTTTTAAACATTCTTGATATCTGTGTC
CCTTTTCCTCGCAGTTCCAGCAAATCAATTTATCCGGAGAATGTATAGCATTTAAGTCCCCAGTTTCTACC
AGATTCTGCGTAAGATCCGTCATTGTGGTATCTTCATGGACTACCTCGTTGACTACTCGTTGTGGTTTAATT
GGTCGAGAAATTGGCTTGGAAAGGTAATTCGAATAGAACGCTTCATGCTTGTGACATTCTTTGCGTAATGT
CGCAAGACTCGGAGTATTTACATGAAGTAGTTCGTGTCTCAATTCTGGTTTTAGGTTGCGCTTGACATTGA
TCACTAACTCTGAGTCTGACATTGGTTCCCGTAACGCATCTGACAAGGCAAGAATCGTATCTAAAAATTCA
TCAAAGCTTTCGGACCCTCGTTGTTTGCGTCTTCTAACCATGTCTTTTATGTCTTCGTCTGATCTGTGGTCTT
TATATCTTTCTCGCAAGCATGTGCACAAATTGAACCAGCCTGTTTGGTCAGTCGTACGGTGATATCTCCAA
AAAAATGTCAAAGCTGGTCCGGTAAACAGCAAATTGGCAAACTGATATAACAACCTAAAATTGCCATTTA
AACATTGTGAAGTCAAGCAATTTATGCGATATATAAAGTCGTCGACTGGGATATCACTAGCCACGCCTGA
AAATCTGACATCCCAATTGGATATGACACTCGATATTTTGTCGGGCCTATCGACTGCGTATTCACGTCTCG
CATCAGACTGAACACTAGCATTCGGTAATCTCGAAGCGTTGTTCGATCGCGGTGCATCACGTGGAAAATC
ATTTTCCCATTCTAATTGGGGTTCCTCACGTGGCTCCTGATACAAGTTTAAATTTCTAACTGCGGCATTTAA
TTCTTCGGACAAATAGGACGCCATTTCAGCCTTATGGGCTTCCAATGTCTTACTGATAAGCAACTGCACAC
GCTGTTCTGACAAACCAAGCGATTCCTTAGAAGGTTGGGTGTTTCGCGATCCCGCTTGCTCCCGAGAGTTG
GTCGGCCCACTAGGACGACCTCTGGCGGCTTTAGGAGTATTTCGAGCATTACTTCGGGTAATAGTTCGGGA
CCGAGGAACGGCCCCCCGTGTTTGTAGGGTCTTTGTGAAGCTACTGCATTCAAGTCACTGCTATCTTCGTA
TAATTCTCGACTACAAACTCGTTTGCAAGTGGGACATGTCTCATTGCTTCTTAACCATTGATTTAGGCAATT
TCGATGAAATTTATGAGCACATGGAGTTTCTACAGTGTCTGCATCACCAATCGATGATTCATTGCAGATAG
TACACACTGCAGGTGCTTCGCTGAGAGTTAAATTTTCCTGACTACGAACGACAGCCATCATCAATAAATCG
ATTATAATTGGTCAAAGAGTTAAATGGGAATTGATCCCGAAAGGATTTTAATTTTAAGATATATACGAGA
AGCGCTAATGAGCGATAATTATGAAAATCGGAAACGGTTTTAAGGCAGAAAGAAAAGGTTTAAGGCACT
TCGATGTTAATACAACAATATTATAACATTTTATAACATGGGACTTATCCTTAGGTAAATACATCGCATAA
TCTAAATCTCTATGTTTGATATATCTTCTTACGAAAAGCAAGCAAACTATTTTACTAAATCAGTGTTGAGTT
GTCTGAACTAAGTTGAAGAGGGACGAGTCGAAATAACCCAACCAGAACAATTCTCAATTCAACTAGAACA
AACGGAATGTTATTATGCCCTACCAACCTTCTATCACTTGGTAGAGAACCGACTCGAAATCAGATCTAAA
AGTCCTAAATGAATCAAAAGTGGCGAAAACATAATAAGATTTCTAGTGGAACAGTATAACTGATCCACTA
AATAAATCGCTCGACTATTTACTTCCTGGTCTGGCTCCAACAAAAGACATCAGAATACCAGCAGTTCCAA
GAGAGGATAAATGTCCGTCACGGAAACTAAAATTATTTGGATGGTATATTGTGATAAGGATAGGAAGAAG
GGTGAAAGCTAGCATGGATTTAACGTAGGATCGTCCACATTGGCTCGTGACAAATCATGTAACGAACAAT
GCTATCGGCTCTCTCCTAAGTTCTTGTGGGCCAATTCCGGATCTCGGGGGTGAGAAGCGGAAGAGCAGAA
GCTCGGGCCCCACGTTGGGCGCCATTTTTCTTATTATTTTATCATGTAACGAACACGACGGCTACGCTGGG
CGCGTCTTGCCTAACACTCGTGTTGTGCATTGGGAGCGTACTCCGTCTACGGGTTCCGACCGTGCTCAGGG
AAGTGGCGTGGGTTCTTTTGCTCCCTAACACACTCGACGCGTCAAGTATAATAAAGAAAAAAAAAGGTAG
ACAGAGTTAAGGTATGACGGAATACCACGATATATAAGGAGCGACTACGATCTGCAAGCAGAATATATC
ATATAAGAGCCGAGGAGCTAAGATGGTGGCTACCCTCGGTCTTCACCCACCCTGCCTGTAGCTGCATCTGC
GATTGTCGTTGCAACTACCCTTTTGTATGCGATATTATTTACCTAAGGTGCCCAAAAAGGGAATTAAGATT
TCATTTTATCATATAAGTGTATTCAAAGAAGATTTAAACTCATGATCGTACTTCATTATACATTACGTGAG
AAGAACAACGTTTTTCTATTTTTTTTTTTTTTTTTATAAAACTAAACTCAATCTGGGCAAAAACACACATGAA
TGAGATTACACTTCGAAACTAATATGAAGTTAAATCACTGCTGATCTGAATGATCCACAAAAGTTATTTGT
AATTACGAAGGCTGATTAGGAGTACACCCGCTGACTATAACCGCGATTCAAACAATACGTAACAATTCGA
TATGTTGGCTTATTATCTTTTGTTCTTTCACACAGTACTCACTACTTTAGTATGATCCAACGATGTCAGCAG
GCCTGTTGACGACGATGATGTGCTGTGGGCTCTTAATTTGAACTATGTAACGTAGAAATATAGTAGTCAGC

GACATATACATGCTAAATCAGGATCGAAAGACAATGTCAGCGATATAAAACGGAAAGAATTGCAATTTA
AATTTCATGTGTACTTATGCCCTTCGCTTATACCTACGGCCAGTATGCCGTTTAACAATTATATCAGATGTT
GTAAATCCGAGTGAGGGTGCGCACTGGTTAGCTATCCACCAGATCTGCGCTCAAGAATGTCCGGGAATCA
ATGGGTCGAACACGTGGTCACGTTGGTCTGCCTGTGCGTGCAGCACGAACTATTGGTTAGCGAGGTTCAA
AGCTTATCTGCAGAAAATGAGACGATGCTGAAATCTTGGTCCGCCCTTAGGAAATCGCTCCCTGCTACCA
GTGTAGATTGTCGCTTTTTGGTGCTTTGGGGCCTTGGCTTTGGCTCCGATAAGGACTACGCGTTATGCGTA
ATTTTTCTTTTTGGCACGCAATAACGCAGCCCCGCGCTTTAGCCTGCACCTTCCTACGACTACCACACACA
GGAAAGGCACCCCACGACTCCAGTAACGATCCTTTAGTGAATATTTGTCAGTGTCCGAATTCTGAAAATTT
TCTTTATTCCGGTGAAATGTTATAGCCAAGCTCTTGGGAGCGCATGATGTCTATATTACCCCTTTTCTTTCT
GGGGACCTTGGACCTTTTTTCCTTCCCTTTTATTTAATTTTTTTTTTTTTTTTTGAGCTTGAGTGCCGATACA
CGTGGGTTTATTACGCCCACGTGAAAAATTGAATTTTATAATTTTTAAAGCAATCTAAACCGACTGTACTC
GTTGGCGATTAAAATGGAAAAGAATGACGCCTCGCCAAAACTAAAAGAAGCAATTTAGCATTCACCCAA
GACACGATATTTTCATTCGCAACGGCGTCATTAACATAGGTCATGGTCACTCTGGTTATGTTATTAACCTC
CGCTGATCATATGTAATTGCATAAATGTTATGGTGCAGTGCTGCCTATTCAAGCTGATTGCGATCAGCTGA
TCATGCCAATGGCAATGTATGTTTATGTTTACAGAAATAAAACTCATCACCCCATCACAAAGGTTAAAAG
GGTTACTTACATTAAATGTCTTTAGCATGGTATGACCAAGTTCTTTTCCCTTGAGGTCTTCTAACAAATATA
TACAATTACCAATTTTTCGTTTGATACGTGCCTTAGTTCCAACTGGTGCAAGTTTTGCGTTGAAATGCTTAG
CCAAATTGCTTAAAACGAAGTTGCGTTTTATTACTATTTGACCGACTTCAAAAGAACGAGGGCGTGTGCGT
AAGTTATACGTTCGCAAATTCTTTTCATATGCCTTATCCATGTGTTTTTGAATATTAGAACGTATTAAGGGA
AATTCGTCTGCTCGATCGAGTTTTAATTGCCCTTCATTCAGCATGTTCAGGCTTTTGAGCAACTTGTAATCA
TTGCCGTGCTTAATCATATTTTGCCCAAACACAACGAAGTATGGAGAAATCCCAATAGATTGGTGTATGGT
GTTTCGTAACGAACTATTTATACTGCTAATGTATACATCCCATAGGCTTTGGTCGGACCGAATATATGTGC
GCAAAGCAGCATTAATCGAACGATTAACTCTTTCCACCGCGTTTGCCTGTGGCGAGTATACACCGGTACAC
ATATGCTGTATGCCGTACTTCGACAAGAGTGAGGAGAATTCCAGGCTTTTAAATTGAGTGCCATTGTCACT
CACTATTATCTCGGGTACTCCAAAACAGTCAAAAATATCTTCTTGCAGTATATCTACCACTACTTTGGCAT
TGAGCCTTTTTATGGGCTTGAGAAATGTGAACTTTGAGTAGTGATCCAGAATGACGAATATACCAATGTTT
CGTTTTTTTGATGGTGGGTACGGACCAACAAAGTCGACATATAACCTTTGGAAAGGCCTTTCGCTGACCAC
TTGGTTTCCCATGGTTGGTTTGAGGCATCGATTAGGAACTTTCGACGTTTGACAGAACTCGCAGTGGCGCT
CGCGTACGT
>9_rnd-1_family-127__LTR___Gypsy
ATATAAGTTAACAACTAAGCATAAATATATTGCCCTAGCAATTAAGTAGCTTCGTATTAACAATGCTGACG
CGCCAGAACTGAGTTCAGCGCTCTGCGCTAAGAACGGCCAGCAGCGGTAAGCTGACACTGCATGGAATGC
TGAGTCGGCTTGCCGACCATGCTTTGATGGCGTGATGCATTGAGGCTTAGGGTATTTGTATAATTTGCTTG
TCCCCTAGTTTTTAGTTTTATATGAAGCGCGCAACACATAAGTCGCCGAAATAAATATACTGCTCCGGCAT
TGCCGATAAAACATATCTTTCGTAATATTACTGGATCAAGGAGAACATATTTTGATCAAGGGCAGCGCGG
GATCAACGCAACTTACCCAAGATAACATTGTGTTACATCGCAGAACATACATTAATGAATAACTGGCGCC
CAACCAATAAATAGGAACCCATACCATGTAAGTTCATAAGTGATTTCAGTTTGAAGAAATTATTCACGAA
CGCCACGTACCGATACGTGTGATTTTTAAAAAAAAAAAAAAAAAATTGTAAAGTAAAGTAAATATTGCTGCT
CTCTTCTGCCAGCATTTCATTCCTTTAAATCATTGCATACTTTCACTAAAAACTGTTGCATACTTTTACCAG
CGTTCCTTGTCAATAAAGCATTGCATACCTTAAACGAATAATTATTGCATACTTTCGGCAGCATTACATTTT
TGCTGCATACCCCTAACAATTTTTATAATAAATATTGCATACCCCAAGATTCACCAGTTTCCACTATTGCC
AACAGCGTTACCTGCCGATCTTTCTCGATCGGCCTAAGTTTGAAAAGTGCCAACCATAAGCCAATGAGTTG
GAATTATAGGGAGATAAAAGTCGAATATAATAGTGACGATAGCTGGGAGGGGAAATCAATTGCTCAGTA
GATCCTACTCCTGCAGATACCGTTGTAACTGAAGAAATGGAGCCGGCTCAAGTTCAGAATCTCATTGAGG
GTGCCATCAGGCAAGCTTTAGCGCAACAACAAAATCAGTTTCAAGCACAAATAACCGCTTTAGCTTCACA
AGTACAGAGTTTGCAGGTGGAGGCGCCGCAAATCGTCACATACGAGAAAATATCCGTCGATCCNAACGCC
AAATGCGATATTCCCTTGGACATTATTAAATCTGTGCCGGATTTCTCCGGCGTACAGGATGAGTATGTAGC
GTGGAGGCAAGCAGCCATCTACGCGTACGAACTATTCAAGAACTATCCAAAGAGCACTGCACACTACCAA
GCGGTAGCGATCATTAGGAACAAAATCAAAGGCCCAGCTGGAGCCTTACTAGTGTCTCACAACACTGTCC
TAAATTTCGATGCAATACTAGCTAGGCTAGATTGCACATACTCGGACAAAACATCCTTGCGCTTGCTGAGA
CAATGTTTGGAGTTAGTTACGCAGGGCGAAATGTCCCTGATGCAATACTACGATGACGTCGAAAAGAAAC
TCACGCTCGTAACTAACAAAATTGTAATGTCTCACGACCAAGAAGGCGCGGATTTGCTCAACCGGGAGGT
CAGAGACGACGCCTTGCACGCCTTTATCTCCGGTCTTAGGAAGCCGTTGAGAGCGGTTGTGTTCCCTGCAC
AACCAAAAGATCTACCATCCGCTCTGGCCCTTGCCAGAGAGGCAGAAGCTAGCATTGAGAGAAGCATGTT

CGCGAACTCTTACGCTAAAGCCTTGCAAGACCGAGCGCAAATTGCTGAAAGTAACAAAACCGTGCCCAAG
GCAAGCAAACCAGATTTAACAAAGATGAGCAAAATCAGGAGAGAAATCCCCATTTTGTGAAACGCCAAA
AGGGCGGTCCCCAGCCCCAGGCCCCTAAAGATAATCAAACTGAGGCTCCACAGCCCATGGAAGTTGACTC
TTCCTCCAGATTTAGACAAAAACCGACTACAGCAAGCGTCAGGCTAATGAGTCCAACGCACCAAAACGCA
AGAATTCGTCAGACCGTTCAACAGGTCAAAGACGACAACGCGTTAACAATGTCGTACAATCAGATTCCAA
AGAAGCCGTAACAGAATTCGAGAAGGCGGCCAAAGAGGCAGTTGAAGAACTAGAGAGTGAGAACGAGT
ACGCACCTGGTGACGACTCGATTAATTTTTAGGGAACACTCCCGGCTTCCGTACATTGAACGACGGTTGGC
TGGGAGAACTCTGAAGATGTTGATTGACACTGGCGCGGCAAAAATTATGTGAAGCCCTTAACAGAGCTAA
AGACCATAACGCCGGTCGCCAAGTCATTCTCGGTAAACTCGATCCACGGGTCTACCGAAATCAAGCGCAA
ATGCTTGATGAACATTTTCCAACATACCTCCCCGTTTTTCTCCTCGATACCCTAAGTTCTTTCGACGCCATC
ATAGGCTTCGACTTGCTAACACAAGCCGGAGTCAAATTGAACTTGGCAAAAACACTCTGGAATACCAGAG
TACATCCGAAAAACTCCAGTACTATCAATGTGCTGATGTGAACTTCACCGATGTCAACGACATCGTAGTCC
CCCTTCCGTAAAGAAAGAGTTCAGGGACATGATACTCAGAAACAAAAGAGCCTTCTCAAATGCAGACGA
AGCTCTCCCTTTTAATACTGCTGTTATCGCTACAATCCGAACAGTAGACAACGAGCCTGTGTATTCAAGAT
ACCCGAGTCTCATGGGTGTTTCTGACTTCATAAACAAAGAAGTCAAACAATTACTGGACGATGGCATAAT
CAGGCCCTCCAGATCGCCTTACAACAGCCCAACATGGGTGGTTGATAAAAAGGCACTGACTCATACGGTA
ACCCAAAAAAGGTTGGTAATCGACTTCAGGAAACTGAACGAAAAAACCATACCGGACCGCTACCCCATG
CCGAGCATCCCCATGATCTTAGCAAATCTAGGAAAGGCGAAATACTTCACTACCCTCGACCTAAAGTCGG
GGTACCATCAAATTTACCTTGCGGAGCATGACCGCGAAAAGACTTCATTTTCAGTAAGCAGCGGCAAGTA
CGAGTTCTGTCGCCTACCATTCGGCCTGCGCAATGCAAGCAGCATATTCCAAAGGGCAATAGACGATATC
CTGAGGGAACATATAGGAAAGATTTGCTTTGTCTATGTAGACGACGTCATAATTTTTCTAAAAACGAAAC
GGAGCACCTTCAACACATTAACATTGTGTTAAAATGTCTAATCGACGCAAACATGCGTGTAGGCCCCGAA
AAGACTCGTTTCTTCAAGGAAAGTATCGAATTCCTGGGTTTCATTGTAACCAAAGACGGAGCAACATCCG
ACCCCAAAAAGTTAAAGCCATTCAGGAATTCCCTGAACCAAAGAATGTCTACAGCGTTAGGTCCTTCTTG
GGGTTAGCCAACTACTATAGGGTCTTTATTAAGGACTTTGCCGCTATAGCGCGTCCGATATCGGACATACT
TAAAGGGGAAAATGGTTCAGTTAGTAGGCACGTATCAAAGAAAATCCCTGTTGAATTTAATGAAGTTCAA
CGCGATGCATTTCACCGTCTGCGGAGTATTTTGGCATCAGAAGACGTCATGTTAATGTATCCAGACTTCCA
GAAACCGTTCGATTTAACCACTGATGCTTCGGCCAGTGGAATTGGCGCGGTTCTTTCCCAAGGCAATAGGC
CAATAACAATGATTTCTCGCGCCTTAAAACAGGCCGAACAAAACTATGCCACTAACGAAAGAGAATTGCT
GGCCATTGTGTGGGCTTTAGGCAGATTGCAGAACTTTCTGTACGGTTCTAGGGAAATTAACATTTTCACGG
ACCACCAGCCGCTCACGTTCGCTGTGTCGGACAAGAACACGAATTCCAAGATTAAAAGATGGAAATCTTA
CATTGACCAGCACAATGCCAAAATGTTTTATAAACCGGGCAAAGAGAATCTGGTGGCGGATGCCCTTTCA
AGGCAAAACATTAATGCTCTGCAAGATGGGCCCCGTTCGGACGCTGCAACCGTTCACAGCGAACTTTCCC
TGACCTACACGGTCGAGACAACAGACAAACCTTTGAATTGCTTTAGGAACCAGATTGTCCTAGAGGAATC
GCGTTTTCCGCTAAAGCGAAACTTGTTGCTGTTTCGCAGCAAAACACGCCACTTGATCAGTTACACTGACA
AGAGCNCAATTCTCAAAACCCTCAAAGAAGTTGTGAATTCTGGAGTCGTTAATGCAATACATTGCGATCTT
CCCACGTTGGCAAGTTTCCAACACGACCTGATTTTGTATTTCCCTGCTACCCAATTCTGGCATTGCAAAAA
TCTTGTGCAAGATATAACGGACAAAAACGAGCAGTTAGAAATCATCACTGCCGAGCACAATCGAGCGCAC
AGAGCAGCTCAGGAAAACATTAAACAAGTACTTCGGGACTATTATTTTCCCAATATGGCCAGTCTGGCCA
AAGAAGTGGTTGCAAACTGCAGGGTCTGCACTAAAGCCAAATACGATAGGCACCCGAAGAAACAAGAAC
TCGGGGAAACGCCTATACCAAGTTATACTGGCGAAATGTTGCATATTGACATTTTCTCGACAGACAAGAA
ACAGTTCTTGACCTGCATAGACAAATTCTCAAAATTTGCAGTCGTCCAACCGATATTATCCAGAACGATAA
TCGATGTCACAAGCCCTCTACTGCAAATTGTTAACCTTTTTCCCGCAACCAAGACAATTTATTGCGATAAC
GAGGCGGCCTTCAATTCCGAGACCATCACTTCGATGTTCGAAACGCCTACGGCATTTGCATTGTGAATGCG
GCTCCGCTTCACAGCACCTCAAACGGCCAGGTGGAGCGCTTTCATAGCACCCTGACCGAAATTGCGAGAT
GCCTCAAGTTAGACAAGAAAATCAGCGACACTACAGAATTAATCTTAAAGGCTACGATAGAGTACAACA
AAAGTTTGCACTCAGTAACCCTCGAGAAACCAATTGAGGTCGTTCATGCAGCAGCTTCTGATCGTCGCGG
GAACGTCAAAAACAGGCTAATTAAGGCACAGCAAGACAATATCAAAAGGTGCAATGCATCCAGGCAAAA
CCGCGTTTTCGAGGTAGGAGAAAAGGTGTTCCAGAAGAACAACAAGAGGCTAGGGAACAAGTTAACTCC
CTTATGCTCAGAACAAAAGGTCGAAGCAGACCTGGGAACGTCTGTCCTCATTAAGGGGAGGGTGGTCCAT
AAGGACAACCTGAAATAAGTTTTCCTCCACAATTAGCTAGTAAGCCAACTCAGAGTTTAAACACCGTCTT
ATATTGTTCGTCCATTTCAGGTTTGCACTCACTTTGCTTGTGGCACTAGCAGCGGTAAATGCTCGGATCAC
CGACTTCTCGCATGCCAACTACATTCCGGTCTTAGACGGGGAAGTACTGGTGTTCGATCAACGTAGCTACT
TGAGGCACTCAAGTAACATTTCCGAATTTGTTAGTATGATAGACGAGACGGAAAGACTGTCCGATTCCTTT

CCACAGTCGCATATGCGCAAACTACTAGACGTAGATACAGATCACCTTAGAACATTGTTGTCCGTTCTTCA
AGTGCATCACAGATTTGCACGAAGCCTCGATTTCTTAGGCACTGCCTTGAAGGTTGTTGCTGGCACCCCTG
ATGCTTCAGATTTCTTAAGGATCCGCGTTACAGAGACGCAGTTGGTGGAGGCTAACTCCAAACAGATACT
AATAAACTCCGAAACTCAAAAACAAATAAACAGACTAACGGACACTATCAATAAAATTATCAGTTCCCGC
AAAGGCGATTTGGTTGACACTCCTCATTTATTCGAGACCCTGTTGGCAAGAAATAGAATACTGAACACAG
AAATTCAAAATTTAGTACTCACCATAACGTTGGCAAAAGCAAGCATAGTAAATCCTACAATCCTTGATCA
TACCGATCTAAAATCACTTATCGAACAAGATACCCCAATAGTTAGCTTAATAGAAGCTTCTAAGATCAAG
GTCCTTCAGTCCGAGAATATTATCCACATATTAATAGCCTACCCTAAAGTTGAGTTTAGGTGCCAAAAGGT
CTCCGTCTACCCCGTTTCACACCAACAAACCATTCTGCGACTCGACGAAGACACGTTGGCGGAATGCGAA
AAGGATACCTTTGCAGTTACTGGATGCACCGTAACTACGACCTACGCCTAAAAACCATTAACAGCTATAA
AATAACCGGGAACGGTCAAAAGCTTGAGAGAGGAGTAGTCAACACATAAGAATTGCACTTCATATTTATG
TCATATAGAACACACTTTTGTTTATAAGAACGCAAGAATGTTGCAGTGCACTTTGCTTTACTGCGACATTC
TGCTGAGCTACGAGTTCAGTGCCCGGCCACTGCGGCCAGCAGCGGTAACCTGACACTTGCCTATGCCGCA
AGCGACAGTGTTGGAGTATGCCCAATCGGCTTGCCGGATATAGTAGCTAAGACTTTATGCGCCTAGCCTTA
GGGTTCAGATCTAATTCAATGTTTTAATTCTAGTTTGGTTTGTAACCAACTTTGTAGGAACAACTTCTTCAA
ATATACTGCTCCGGCACTTGCCGTTATCAGTCCGTTATACTAGTTGTTTCATTGCTGAGCCACAAGGCCAG
TGATTTTTGGAAGGCTAGTTGCTTTCCGAACATAATCTACAAGTAAACTTTACCAATCTCAGCACTTGTGA
CGAGGGTCTGAATATACAACATCAACATCTACAATTGAAAAATCAAATAACTTACAAGGTGTGAGCTATA
TTATTCAGGTAAAAAGCTTATATGCTACTGGATACTAATTTATAAAAGTTGCTGCGCACGCACCACCGAAT
TTCGCAGCTGCTATTAGGTACCAACGACGGAAGACAGACAACTGTTCATCAGTCAATTCTGGCCATATTG
GGTGTATGAATACGTGCGTGATCTTGCGCGGTACATGAAGTGATTTGTCAAACGAACACCTCCATCGTCCT
GTAAGCAAACGTGATCATTATCAAGCAAACCATCCTGGAAAAAGGCAAGGAGACAATACGAACAGTCGT
CGGGATAGCTATCGAGATCCTTCAATGACCTGTGACCAAAGTCGGTGTGCTAGGCGCTGAACATATTAGA
GGTAAACAAGCTCCAGAAGAGAATGCGGAAACTGCAGGTGCTTGTTACCAAGGCCTCAACATTAAATGA
AAAATATTGTGATCTTTTCTAAATAGTACCTAGATGATATTGGTCAAATCTTTATAGTAGTTCTGGATAAC
AGACCCACTATCGAGACAATACGTTAACTAAATCTACACATGTTTTCTGGACTTGCCTAATCAGAGGCAAC
TCACGCCGCTCCTGGTTGGTTACGAGCCACTGCCATAAAGTCGAACAACTCTTGTTCATTAGCGAGCACTA
GGTNCAATAGACTGTATTGACTCCCTTTTATTAATATAATAAATGTAGGTATAGATTTATTTACTTATTATT
TTTAAATTTCGAAAGCAGGTTCGTTGCTTTGTATGCGGTGGTAAAGTCCATGACCGACATAATGCAAACAA
TTCAAACAGAAAACATTTCATTCCGCGTTCTTTATTCTAAATTCAATTGCAACCTATCCGCAATTATATTAA
TCTAGCAACATATTCCTGCGTAATATATAGTATATACAGCAATATATTTGTATATTGTTTGTAAGACCATTT
AAGAGTCAACACTGTTATTTTACGGGGTTAACCTTTGTGTTACTTAAACATATTTTTTATTTAACGATTTTC
ATGTTCCAAGATTATTGGAGATTGGAGCCGCTTGCCTCCGTCAGGCTACGCATATATTGAGCTTGCATATA
TCTTCCATATCTACAAATGGTGTGTGCATTTCTGCAGCAATAAACCGTTTACTCGCTGCTATGTCTAAGCGT
GGTGCTACTTTCGAACTGTTCGTTTACTTTTCGCGTGCTAACATCTCTCGAATTGCTCGACCCAAATCGTGT
AAAATATAGTGCTAGGGAAAATTATCAGTCATTAAATAATTTTTTTAATAAACAATTTAATTTTAATTTTTT
TTGTATTTTGATTTGCTAGCTCGAGGGCCAGGGTTTAGTTTATTGAAAACTGAAAAGTTAAAAAAAATTGG
CTTTATTCGATTGCTTATCGGCAACCGTCTTCAGGGCACTAACACCAAGAAACAATAAACAAATAACATT
AAAATAAATTAATTTAAGCAATAAATTAAACATTTAACATACGTCTGCGCTTCTTGACTTGGAGATTGAAA
TTCTTTGGCTATTTAATAAGTCTCAACACAAGCGAGCGCAATTTTATGTGTATTTGTTTAAGTTTACTCTTT
AGTTTGTTGTGTAGCTATTTTATTGTATGCTGTATGTTGAAGTGTTGCAGGATTATTGTTGTTTCCTTGTCAT
ACAGTCGGTCATTGGTGCTAATTTTATACCCTGAACCCATTAAAATGCGTAAAACAAGTATAATGTATTTA
TGTAAATATATGAAACAGGCAGAAGGTAGAAGCACCGATCCCATAAAGTACATACATATNNTCTTGATCA
GCATCAACAGCCGAATCAATCCAGCCATGTCCGTCCGTCTGTCTGTCTAACCGCCCACCCGTCTATCCGAA
CGTATGAACGCGAGGATATCAGAAACTATAGCAGTTAGAGACAGATTTTCCGATATTCGATAACTTGGTC
CGTTTCCAAGTAATCGATAAGAATCGACATCAAAATCCTTTTTTTTTACCAATTTTGGTAAATAATAAGAG
CTAGAGTAAAATAATTGATATATTGCTTTTAGACTAGTATATATATATATATATATATATACATATATATA
TATATATATCAAGGTCCTCTCAGTTAATACCTATTGCCACCTTTCCGCTACCACCGCAGAGCTATAAATCA
AGTTAATAACCACACTTATATTGCCCATGAATTTGAGCCACATTTTAAATGCAATTTTCTTTTTGAGTTCAA
TAAGATGTTCTGATGGTTCAATAAGATGTACTGTAGAACATTTTATTTAGATTGGTTAAGAAAATCCCATA
AGTTATGTGTCTTACTGCGCAGTTGGATGGGGTCAAATTCCAAGAATTTCTGTATACATGTACACACACAC
ACATTCATGCGCATCTGCTGCGAAATCTGTCAACAGCATTGCGATTGCGATTGTTTCTTGTGCTGATATTTT
AATTCAATAAGACACAATTATTGGTGTGACTGTTGAGTAGAGGTGGTGCGGTCTGTCGCGAATTCGAGAA
TTCTTAATCTGAAGTCCGATTTGTGTCGCATTGTCATTGTTTTTAGTTTACACTCTATTGAAATTATTGTTGG

TGACCCCGCAACAATGTATTTTGTAAATTACATTACTTCTATGTATTGTAGTTGTTGCGCTATTGAGCTTTT
TCAATAGCTTTCACAAATAGAAAGATTGAACTATTTATTTTTTGTGTTCAAAATTGTTTTATATTTCTTAAA
GTTACGAGAAGCAGGCAGACCCGAATTGGTCTGTGCTTATTATTTAAGAACAATAGTTTGTATATAGGCA
AGCGAACCCGAATAAGAACGTGCCAATTTTAGCGAAGAGACAATTATTAGTTATAATTTTGAGTAATGCA
AGTGGCCGATGCGTACCAAATGCATGAAGCGAACGAACTTAATCTAACNCGATGCGCAGCATCGAGGCCT
TCGCATAAGTAAAGCTAGGGCAATAAAACGATCGTTACAGCGAAGAGCGGCAGCTCTATGTAAATGAATT
CTTAAGAGCGAAATTTAGGCCCTTGCTTTGCAGAGCGAGTATTATAAGATAAAATTAAGATGCTGCTCTTA
TCAGTTGCGAAGT
>Ref_NewTE8
TGTAATGGTGGGGAGGTTCGGGTTCAGTTTTTTTGCCTTTCGTTTGGCCATCTACCAAAAATAAAAACAAA
CGGCCGTGGCTGTTGCCTAACCGGGTGCATAGGGATTGGAGGTTCGCACTTCTTGCTAATACTCGGACAAT
AGCTCGTCGGACTGGTCGGGGTTTTTCCTTTCCCTTTTGCACTTACCATAAACACATAGTTTAAGTGCGAT
CAAAAAAAATAAAAAAAAACTTATTGATTTAAGTAAATCCATTGAAACAAAAAAAAACCGGATTCTAGT
ATGGGTACCATTAATGTGTTCCGACCGAACACCCAAATTCAAACTATTCAATTTCGCGTAAGTGAGAGAA
CTAAGGTGTTGATCGCCTCTCTTTCTCTTTACCCTCCCTACCTTGACAAGCAAAAAAAAAATGGAATGTGG
ATGCTTGTCCCTATTTCAGGTTCTTAAACCATCACTATAAATTGCAAGCGGCTAACATGGGTACTTGACAG
ATAAAACAATTAATTATGGAATTAGGATATCATATCATATAAGCTAATATAATTCGTTTATTCACGCATTG
TTCAATTTTGTTGCCAATTAATAGCAAGGTGAGTATAACTCTATTTACAAGAAAAAATATACAGTTTCCAA
ACAAAATTTGAATCCAAAGTTAATATGATGAATGTGCAATAACTAGTTTAAATCCCATAAAGAAATTAAG
GGGAAGGAGAAATTCACCAACATTATCTTGAGACTTGACGGATAGTAATTAATTTACCGTCTTGCTAAATT
AACTGCATTTAAATGTTCATGTGTGTATATAACTACATATATCTATAGGCATTGGTTTAAACTCTTACTCAC
GCGATATCCTTCGAAGCAGACGTTGGGCCCAATGTATACGACGATTATGATCGCTGTTGCTAAATATTACA
AAAGAAATTTATAAGTATATAAACACATGTATATATATATATATATATATATATATATATATGAGCAACGG
TCAAGGTCGGTGGTGTAGGAATTTCCTCTCGCTTTAACTTATTTAGGCATATTCATTGCACATTTTGTTTTA
AAGAAATTAGTAAAAGAAAAAAAAAAATTAAAGGAAATAAAAATTAAAACTAAGGATCTATTAGCCGTCC
ATCTTGGCAACGTTAAACAATTAATGGAATTATATGGCAATGCTGCTAAGCAAGCGAAAGGGGTACACCA
AAAGTAATACAGACTCGGCTCCTTTGTTACTCTCTGCAGGTTACACTATATGCTTAGTGCATGCGGTCCCG
ATGGTGCTGCATAAATAGTAAGCGTTTCACCCGCTTGACGCCGTTTTCCGAGGCTATGCCAGATGTTGTTC
TTCGCAAGACAGCGGCTGATAAGTAAAATAGCAACAAAATTGGTTCTGCTGGGTTGCTAAGTGCGACACG
TGCTCTTGGTGTTGCCCTTGTCTGGTCCGATCTCCCCCTCTGGTAGTGCCGACTAGTACGTCTAAATCTAGT
CAACTGAGCAGGCACGGAAACTGTGGAAGCCTAATTAGTTCCAAGTCTCAACACTAGCACATATAAATAC
CTTTTCTTTTAAGGGTCACCCCGAATCACAATTAGGTTAGTGGGTCGAGTTGTTATGAAGGTTTTTGGTTTC
TATTAGCGTACCAAAAGAACACTTCCAAACTGTACCACAATTGCAGCCTTGGTCGTTGATGTCTTTGCAAA
AATTATTTGCAAAGAAAGAAAAAATGTTATGAACTTAACTCGCACGCGCTTCAAATATATCTGACATGCT
GAGCTGTACTTTTAACCTTTACTACTTCCCAACGACAAAAAAAAATAAGGAAGCTTTCAAAAGCTCTACATC
ACGCGATTATATTTTAAAACGGTACTATGGCCTGATCTTATCACCTTTTTCCTCTCTATTCGCCCTATTCGG
TTGCGCATGCCCTCAGCTGTGCAATAAACGAAGAATTGGCTTCCAGCGCGGTCGGTAAAGCAGTAACTAA
ACTAGAGCAGCTGATCAACAAGCAGATTCGTTTGGGGAGTGCAAAGCAGCCACTACACCACAGTTTATGC
AGCGCTACTTGCTAGCGGGACATAAACTGAAATAACTATAATAACTGTTGGGTTAGAACTTGTTACTAGTT
TGTATATCTTTGAGATGAAAAGTACCGATGCATTTACCTGTTTCATTAGCTAGTTGATAGTAGGCTGGGCT
GACTTTTTGTACAACAATCGCCGGAATGAAAACTGGTGCAAGCTTAGAACTAAACTTCTTTTGCGCGTTAC
TCTGACAAAAATTGCGGGCGAAGACTTTCGTACCGGCAGTAAGACTTATGTTTCGGCTGCGCAAATTATAT
ATCTTAGCATTTCTATTATGTGACTGAGCTACATTGTCTTTAGCTATCTGACGGATTACTTGTAAGGCTGTG
GGCCTCTCAATAGCAGTGTCTTCGGACAAGAGGTTAAGTCGCTTTAGCAAGTCATAGTCTTTACCGTTTAA
CATCATATTCTGTCCAAACGCTAAAAAATATGGAGAGTACCCTGTACTTTGGTGAACGCTGGCTCTCAAGG
CAGCACAAATGGCGTACAGCTTTTTATCCCAATTTGAATGATCTTTTCCGATATAGGCTCTAATAGCCGCT
AATACTGATCTATTTACTCGCTCACTCGCATTACTTTGTGGCGAGTATATTGCCGTTCGAATGTGGATGATT
CCATACCGGGTAAGAAAAGCTTTAAAGACACTAGAAATGAACTGAGAGCCATTGTCGGTCAGAATAGTTT
CTGGCACGCCGAAAGTGCAAAACAGTGCGTTTTCTAGATAGTCGATAATTTTGTTGGTAGTAAACGCGCG
AAGAGGCCACATAAAATGAAATTTAGTATTGTGGTCTACAACCATCAGGAGACCTATATTGCCGTTCTTG
GACCGAGGATATGGCCCTAGAAGATCCATGTAGAGCTTCTGAAATGGCCGTTCTGACGTCATAGGCTTGC
CCATTGGAGGTCTCAACACTACATTGGGGCTTTTCGTTTGCCGACAAACTTTACAATTGACTATATATTCA
TGAATTTGGGCCGACATTTTTGGCCAAAACAAGTATCTCTTGAGTTTCTCTATTGTTTTTCCGATGCCACTG
TGTGCAGCGTTAGGGGGATTGTGGGCTCTCTCTATCACTTGTATGGTGAGAGACGAAGGTATCCAGAGTTT

CCAGGATTGTTTCTCCTGAATGCTATATCCCGTCGCTGGCTCAGTACGTTTATATATAAAACCGTCGATTAT
CTGTAAATCCGGTAATCTGGATTGGTTAGAAGAAATACTGTCTATCAGATCCAGATAGTTTGGCGATTTGA
ATTCGGGTGAAGACAGGTCAACTATGGGACCTTGCTCCTCGAATTCCGCAAGCTCTGTCTCATTTTGACGC
GATAGCGCATCAGCGACAACATTTTGTGTACCGCTACGATGCGAGACTTTAAACTTGAAGCCCTGTAATTT
GATCACCCATCTACCTTGACGTCCTCTGAGATCCTTCTGTCTCATGAGCCATTGTAAGGAAGCATGATCAG
TTATTACTTCGAAGTCCTGTCCTTCTATATACGCTCTAAATTTAAAAATACCTCTTATTACGGCTAAGCATT
CCAGATCGGTCACACTATAATTCCGTTGGGCCTTTGACAGTTTCTCTGACATGTATGCTATTGGAGCCTCCT
CTTCCTGTTCATTTAGTTGAGCTAGAACGCATCCTAAACCAGTTGTGCTAGCATCGCAAACTAATCTAAAA
GGTTTTGAGAAGTCGGGTGTAGCCAATACAGGTGCAGAAGTTAAAAGTGTTTTTAAGGTGTTGAAAGCGG
AATCAGCATCAGTGTTCCATTTCAATACTTTCGATTTCTTTAGCAACTCTGTAAGCGGAAAGCTAACTGTT
GCATAATGATTAACGAAACGTCTATACCATCCAGCGAGTCCAAGAAAACGTCTCAGTTGTTTTACACTTTT
TGGTACAGGGAATTCGTTAATAGCTTGTATTTTGCTAGGGTCCGTCTTTAGTTGACCATACCCAATTATAA
AGCCCAAATATTTAATTTCCCGCATACAAAATTTTGATTTTGCGATGTTGATTGTCAAGTTCGCTTTACGCA
AGTGCAGCGCTACTTCCTGCAAAAGCAACATATGCGAATCGAAGTCCTCTGATAGCAGAAGTAAATCATC
TAAGTAGACAAATACGCGGGTCCGCAAATGGGCTGGTATCACGCGTTCCATTAGTCTACAAAGCGTTTGA
GGAGCGTTACACAATCCAAACGGCATTACTTTGTATTGAAATAATGGACGATTAGGGACTGTAAAAGCGG
TATACTGACGTGATTTTTCTTCTAGGGGTATCTGCCAGAATGCGTGCTTCATATCGAGGCCCGTTATATATT
TAGCCGGCGGCAGACGACTAAGAATTCCGTCGATATGGGGTAGAGGGTACGCATCTTTACGAGTGTACTT
ATTTATGACTCGAGAATCTAGACACAGCCTGTTTTTATCCTTTCTTTGCACAAGGACGCAGTTGCTGCTCC
AGGGGCTATGCGATACCTCTATTATATCTAAAGCTAGCATTTTCTCGATCTCGTCAAACATTAGCTTTTCA
ACGGCTGGCGAAATAGGCCAATGTCTTTGTTTGACGGGGGTCACATTATCTATTTCAATGCGATGTTCGAT
TAACTCAGTGCGTCCTAATCCTTCCTTTTCATAAGAATAGAAATTAGAAATGATCGAATTTAATCGTGACT
TTTCTTCAGCGGTTAGCTGATGCATTTTCGCTGTTTCCTGGATATCCTGTTCGTTACTGTTGTCTAATTCATT
TACGGACTCATCAGGACGAATTAAATTCGTTAACAGTCCAAAGTCCTGCCAAAAATTTATGCCTAAGTAA
ACATCTTGTTTCAAATCGGGTATAACATAAAAATCAACATTATGCACTTGTTTTCGAAATTCAACTTTAAG
TGTCAGTTTGCCTACCACTGGGCAAATAGCGTTGTTAGCTGTTCGGATTGATCCAGAGCATTTGCGCGCTT
TGGGATGTGACAACAGTGACTTAGCTGCAACATTACCGAGGCAACTAATCGTCGCTCCGGAATCTAATAA
TGCTACATATTTTTCTCCCTCTAGGCTTAACTCAGTGTACAAGCGGTTATCTGAGCTCATAAACACTGATTG
AACGAGCTTCTTACGAAGACTTTTAATATTTTTCCAGAATTTCCTAAGTCGAACAGTAGAGCGTTTTGGCC
GACTGAGAAATTCGATGGGTTCTATGCTGCTAAAAATTCGTCTTCGTGTGTCAAGATAGTGTTTTACTCTA
ATATGGTACGGTACGTACTTATTTTCAATATCAGTTTGAGTTGCAGTCTCGGCGACTGAACATGTATTTGC
CATATAAGTTGTAAATATAGTATTTGGAGTTGATTGCTCCAAACTACCATATGACTGTATTTCTGGCAAGG
ACTTTATTCTTTTGTGGGATGCGTCAAACTGGTGTTGGTCTGCGCATCCCGCTTCCGGTTTCCCAAAAGATT
GCATTTGCTACAATTGGGTTTAAAAGTATTTTTAGCACCGCAACCGTAGCAGAATATACGCCTTTCTCCCA
TGCAGTCATCGAATCGGTGTCCCACTTTATCGCAATTCCAGCACACTATTTTCTTGTTCTGTATTTCTGAAA
TATTATCTTCTATATCATTATTTTCCAGTAACTCAGAGACATTACCTCTACCGTATTTGGTTCGATTGGGAA
ACGAGCCAGTTTCTCTACAGAAACTTTCATGTTTCTTAACTTCCTCTCTTAATTCTGCTCGAGTTCTAATCT
TCAAATGCAATAGTTCGTACCTGAGCGCTGTTCTCGCATTGCGTTTTAATAGGTCAACAAGCTTGTCTTCG
CTTATTTCGTTAGATAATTGGCTAACCAGGCCTTCAATCGCTGATTGAAAATCATCAAATGTCTCCTGATC
TTTCTGGCGTCTTTTCCTAATTAGTTCCCACAAGTCAAAGTCGTCTTTAACGTCCTCGAATCTAAGTCGGAA
ATCCGTACAGAAAGCCAACCAGGTATACTGTGGGTTTTTCTTGTGGAAGCTCCAAAACCAATCATTGGCTT
TCCCGAAGAAAAGTAAATGAAGATTGTCACAAAGCAGCCTATAGTCGCCGTTCAAATTTTGAGCCGTTAA
TGAACGAATTCGATACAAGAATTCATCTATCTGCATAGAGTCTTTCGAACCGTCAAATTTAAGTCGCCAGG
TTTGCATAATGCTAGCTGCTTTTTCTGAACTCAAACTTGAGTTTGATCGATTGCTACCTCTGGGGCTGTATG
GTCCGCAGTCAAGGGGTATGTCAGAGTTCTGGGCACTATTAGGGAGAGTATCAGCACCTAATCCCCTTGG
AGACTGAACTAATCTAGAGCGTGTTGATTCAGCTTGCTCACTTCGTACATTTAATGTTGCTAACTTCTCTTG
CAGTGATTGTTCAACAGAAGCTTTAATAACGTTTTTGAGTTCTGCTATTAAAGTCCTTTGCTGAGATTGTA
GAGCTTCGTTGATATAGGTAATTGTCTCTACATTTGGACCATTGTCCACTGACTTATTTCCGTTGCTGCCTA
AAGACATGCAACTACCTAAATATTCTCTATGATTTCGATTCATAGACCTAGTTTGCATACTTCGCTTGGGC
GTGGCTCCTCGCCCTTTTCCGAATCGCTGACCTCTCCTGATACCTGTAGTAGTCGAACCATCTTGGCAAGG
TAGAGTATTTCGCGATAGAGTTTCGTCTGTTTCAGCATTACCAGCTGAGTGTAACTCCGCTGCCAGTGTAT
TGTTCATAAATATTAGTTGCCCTGGGTTGCAATCTGCCTCACATACTGGACATTTTGGGGTTGTCTTTAGTG
TATTGCTTATGCAAACGTCGTGAAAACGGTGTTGACAAGGAGTAGTCGCAGATAGCTGTTCTGCTTGCAAT
TGCTCATGGCAAATGCCACAAAATGGATCTTGCGCGTTAATCGCTGCAACTATGCTCTCTGTGCTTCGTCT

CACTGCCATAATGAATGCAAACTTTTGCTTGTTTCCAAACAACAAAAGGAAAACTAAATAGGACTTGTAT
TATTAAGTCTTGGTTATTTATTCTATCTGTCTTTTTCCCACACGTTATTGCTTACAATCCTTAAGCAAACCTG
AGTAGTTGAATTTGTGCTATTTATTTTACTGGAGTCTATTTCTGATTTGGATTTTTACTGCTCATGGACTAC
TAGACAACATCCGAAATATTTCCAGTTTGTTGTAATTCCAAATAGTCCTTGAAACAATTAACCCAAATTTG
ATTATTATTCCCTCTTTTCCGATCTTACCCGCTGCTTAAAGTACTAGTAATTAATTGTGGTCGACTAATCCA
GCAATGCCTGAATTACAATCGCAAATTCCTACAACTAACTACTGTCTGAACACAACGTATTTTCCCGAGAA
AGGGGCCTAACAGGGTAATTAAAAAATAATAGGTAGTAATTGATATGGTATAACTTCGAGTTTCGATTGG
GGATTGTTGTGCACTAGGTGTTAGTCGTGCTTACGCAAGAAGTAAAAGTTTTACGTTCTTGCTAGGGCCCC
ACGTTGGGCGCCATATATGTAATGGTGGGGAGGTTCGGGTTCAGTTTTTGCCTTTCGTTTGGCCATCTACC
AAAAATAAAAACAAACGGCCGTGGCTGTTGCCTAACCGGGTGCATAGGGATTGGAGGTTCGCACTTCTTG
CTAATACTCGGACAATAGCTCGTCGGACTGGTCGGGGTTTTTTCCTTTCCCTTTTGCACTTACCATAAACAC
ATAGTTTAAGTGCGATCAAAAAAAATAAAAAAAAACTTATTGATTTAAGTAAATCCATTGAAACAAAAAA
AAACCGGATTCTAGTATGGGCACCATTAATGTGTTCCGACCGAACACCCAAATTCAAACTATTCAATTTCG
CGTAAGTGAGAGAACTAAGGTGTTGATCGTCTCTCTTTCTCTTTACCCTCCCTACCTTGACAAGCAAAAAA
AATGGAATGTGGATGCTTGTCCCTATTTCAGGTTCTTAAACCATCACTATAGATTGCAAGCGGCTAACATG
GGTACTTGACAGATAAAACAATTAATTATGGAATTAGGATATCATATCATATAAGCTAATATAATTCGTTT
ATTCACGCATTGTTCAATTTTGTTGCCAATTAATAGCAAGGTGAGTATAACTCTATTTACAAGAAAAAATA
TACAGTTTCCAAACAAAATTTGAATCCAAAGTTAATATGATGAATGTGCAATAACTAGTTTAAATCCCATA
AAGAAATTAAGGGGAAGGAGAAATTCACCAACATTATCTTGAGACTTGACGGATAGTAATTAATTTACCG
TCTTGCTAAATTAACTGCATTTAAATGTTCATGTGTGTATATAATTACATATATCTATAGGCATTGGTTTAA
ACTCTTACTCACGCGATATCCTTCGAAGCAGACGTTGGGCCCAATGTATACGACGATTATGATCGCTGTTG
CTAAATATTACAAAAGAAATTTATAAGTATATAAACACATGTATATATATATATATATATATATATATATA
TATGAGCAACGGTCAAGGTCGGTGGTGTAGGAATTTCCTCTCGCTTTAACTTATTTAGGCATATTCATTGC
ACATTTTTGTTTTAAAGAAATTAGTAAAAGAAAAAAAAATTAAAGGAAATAAAAATTAAAACTAAGGATC
TATTAGCCGTCCATCTTGGCAACGTTAAACAATTAATGGAATTATATGGCAATGCTGCTAAGCAAGCGAA
AGGGGTACACCAAAAGTAATACAGACTCGGCTCCTTTGTTACTCTCTGCAGGTTACACTATATGCTTAGTG
CATGCGGTCCCGATGGTGCTGCATAAATAGTAAGCGTTTCACCCGCTTGACGCCGTTTTCCGAGGCTATGC
CAGATGTTGTTCTTCGCAAGACAGCGGCTGATAAGTAAAATAGCAACAAAATTGGTTCTGCTGGGTTGCT
AAGTGCGACACGTGCTCTTGGTGTTGCCCTTGTCTGGTCCGATCTCCCCCTCTGGTAGTGCCGACTAGTAC
GTCTAAATCTAGTCAACTGAGCAGGCACGGAAACTTTTCTTTAAGGGTCACCCCGAATCACAATTAGGTTA
GTGGGTCGAGTTGTTATGAAGGTTTTTGGTTTCTATTAGCGTACCAAAAGAACACTTCCAAACTGTACCAC
AATTGCAGCCTTGGTCGTTGATGTCTTTGCAAAAATTATTTGCAAAGAAAGAAAAAATGTTATGAACTTAA
CTCGCACGCGCTTCAAATATATCTGACATGCTGAGCTGTACTTTTAACCTTTACTACTTCCCAACGACAAA
AAAAATAAGGAAGCTTTCAAAAGCTCTACATCACGCGATTATATTTTAAAACGGTACTATGGCCTGATCTT
ATCACCTTTTTCCTCTCTATTCGCCCTATTCGGTTGCGCATGCCCTCAGCTGTGCAATAAACGAAGAATTGG
CTTCCAGCGCGGTCGGTAAAGCAGTAACTAAACTAGAGCAGCTGATCAACAAGCAGATTCGTTTGGGGAG
TGCAAAGCAGCCACTACAA
>160_rnd-1_family-89__LTR___Pao
TGTCTGGTTACGCAGCCAAAACAACTGGAGCGACCTCGCAACTGATAAGAGCAGCATCTTAATTTTATCTT
ATAATACTCGCTCTGCAAAGCAAGTGCCTAAATTTCGCTCTTAAGAATTCATTTACATAGAGCTGCCGCTC
TTCGCTGTAACGATCGTTTTATTGCCCTAGCTTTACTTATGCGAAGGCCTCGATGCTGCGCATCGAGTTAGT
TAAGTTCGTTCGCTTCATGCATTTGGTACGCATCGGCCACTTGCATTACTCAAAATTATAACTAATAATTGT
CTCTTCGCTAAAATTGGCACGTACTAATTCGGGTTCGCTTGCCTATATACAAACTATTGTTCTTAAATAATA
AGCACAGACCAATTCGGGTCTGCCTGCTTCTCGTAACTTTAAGAAATATAAAACAATTTTGAACACAAAA
AATAAATAGTTAATTATTTCTATTTGTGAAAGCTATTGAAAAAGCTCAATAGCGCAACATTTGGTGACCCC
GACGTGATTTTTATTTTTGTTGTTGTAAGATTATTTGCTTTACATTAAAGCAATAATCTTAAAAGGCACCAC
CTGGCATTTCGTTTTGTGCACTTTATATACATACATACTTATCGCCGTCGCTTTGCCTACTCTCTCGGTCGC
TTAAAGTCTCTGCTCATTCGTGCGGTACTTATGTAAAAGCTATTGCCGTTGCTTCATTACATAGCTTGACAC
TTGCGTTCGCCGAAGTTCTTATTGTACAACTATTGTTTTTTGAACGGTTTGCATGTACTAGCGATCACCGTT
GTTAGTTACTCAGCTAGTTTCGCACGCGCTTTCTATCGCGCTTCTAATGGCAACCGAAAATAATGCCTCTA
GTGCAGTGCCCTCTGCAAACAGGGTGACCTTTCTGAAGCGCAAAGCCATTTCAATTTATGACAGTTTGCAA
ACACTTCGAAATGCTCTAACTAATTCGCCACTTAGTGCAATTGATGAGTGCGGTCTTAGTGTTAGGCTGAA
GCAAATCGAGCGGCTTCAGACCTCATTTGATATTACTCACACTAGCCTCGAAGAACTAGACTTTGATGAA
ATCGGTAACCAATTGCGTGAGGACTTCGACGAGTTAGCAGTTTCTATGGAAATTTCGGTTCGGAGTGAAA

TTGCGAAACGTGTCTCAATATTGCCCTGCTCAACATTTCGCGACCAATCTGTGCTGCCCTCGCAGACAGTT
AGAGCTAAGGCACCGCCAACACTACCCTCAATAAAGTTGCCTACGTTCAGTGGTGGTTACACAGAATGGG
CCGACTTTTATTCTATGTTCACAACCGTCATAGAAAATCATCCTGATCTAGCAGATATCGAAAAGCTGCAG
CATCTTCGATCGTGCCTGAAGGATACAGCGCTGGACGCGATTCGATCATTGGAAATAACAAACGATAATT
ATGCCATAGCACTTAATTTATTGGAGAAAAGGTTTAATAATAGACGTTTGGTTTTTCAGGCACACATACTT
GAGATATTGGGGCTCAAGAGGGTGGCAGGTGGATCGGCCGTGTCGCTAAGGGAGTTATCCGACCAATTCA
ACGCACATTTTCGCGCGTTAAAGAGCATGGGCAACTCCGAACAAATCGCTGGATGCATCATTGTGCAGAT
TATTTTACAAAAGCTGGACCCAATATCACAAGCCAAATGGGAAGAAAGGCTAGAGGATTCGTCGTTCGTT
AATTTAATACCATCGTGGGAGCATATGGCTACGTTTCTGGAGCAGAGATGCCGAACAATGGAAACCGTAA
ATTTTGCTATGGCAAGCTATGCCCCTAGCCATCAGGTGGGAAAAACTAGAGCAATTAACACCAGTAGATC
GTCATTTATCGCCACTAACCACGACGCCCCTGTCTGCGTCTTTTGTGACAGCCCTGGTCATAGTATATATA
ATTGTTCTCGATTTGGAAATTTGACCCCTGCATTACGGCTTAGGGAGGCAAAACGAGTTGCATTGTGCTTG
AATTGCCTAAAGAAAGGTCATCAATTGCGATCATGCAGCTCTGGATGCTGTCGGACCTGCGGGGCGAAAC
ATCATTCTCTGCTGCATTTAAACAATTCCGTATCTCCGTCTGGTCAGCTGACTGAGCCTGCAAATTCATCGC
CGATAGCCCATGCCTCTTTCGCTAAGTCATCGCCATCTACCTCCACATCTCTTGTTGCCCAGTATTTCAGTA
GTGATGTTGTCGTCTTGGCTACGGCTATGATCTTTGTAAGAAATCGCTCCGGCACTTTGATACCTTGTCGTG
CCCTTTTGGATTCTGGTTCTCAGCTTCACCTTATAACGTCGCGCCTTGCTCAACAGCTTCAGCTAAAGAAG
CACAAGTCCTCAGCCATTGTCTCTGGTCTAGGAGATTCTCGTTTTGAAACAGAAGGAAACACGGTCACTAT
CACAATACAGTCTCAAGTCTCTGAATACTCAACATGCCTTACAGCTCTGGTAGCGCCTAACATAACTGATA
GTCAACCGAGTTTTACTATCAACACGACGAATTGGAACATTCCCGATAACATAAAACTTGCAGATCCGTCT
TTCTTTAAACCTCAACGCGTGGACTTGCTACTTGGAGCTAGCCTGTTTTACGACCTTCTCAGCGTCGGCCA
GATAAGGCTTCTCCCAGGACTACCGTGTCTTCAAAAAACACGGCTTGGTTGGGTAGTTTCTGGAGGCGGA
CCCCGCTTGAGTGGTTCTATGTTAATGGCAAAGCATACTGCGGGCAAACTTGAGTCAAGTTCTGAAGAAC
GTGCTGATGAATTGGTTCGAAGATTTTGGGAGATAGAATCCTGCTTGGAAAGTACACCTACCACTTCAAA
GGAAGAAAACATCTGTGAAGAACATTTCGTAAAGACTCATATCAGATTGCCTAGTGGCCAATATTCAGTT
CGTTTACCCTTCAAGCGAAGTTTGGAATGTCTGGGGACGTCATACCAACAAGCCTTTCGTCGGTTCCTGAC
CCTGGAACGCAAACTACAAAATCGTCCAGATCTGAAGATTCAATATTCAAACTTTCTAAAGGAATATTTG
GATCTTGGTCATATGTCGCCTGTTGCTCCAGAGTCTATAGGACTTTGTCAATACTTTCTTCCTCATCACTGT
GTCTTTAAAGAAGACAGTTCAACGACAAAATTGCGAGTCGTTTTCGACGGCTCAGCAGCTACCTCGTCAG
GTTATTCATTAAATGAACTATTGATGGCTGGACCTGTTATTCAAAGCTCGCTGTTTCATATCCTTCTTCGCT
TTCGTAATTTCCCGATCGCCCTAACAGGAGATATTGGAAAAATGTATCGCTGCGTACGCGTTTCAGCACCG
GATGATTTTCTCCAATGCATTCTGTGGCGAGACTCGCCAGAACAGGATATAAAGATTTTTAAGCTGGATAC
AGTTACATACGGAACAAAGCCAGCTTCATTCCTCTCTATCCGTGCTATGCATCAGCTGTCTATGGATGAGG
AAACTAATTTTCCAATTGGATCTCGGATTTTGCGGCGTGACTTCTACGTTGATGACCTCATTTCAGGGGGT
GATTCAATTCAAGAAGCAAAGGAACTTCTTAATCAGACGGCTGGAATCTTGGCAAGTGGGGGCTTCAAGT
TGCGAAAGTGGTGCTCAAACAATTCTGCTGTACTGAAAGGGATACCCGTAGAGGACAGAGAATCTTCCCT
AAAGTTTGATGATGGTAGCGACATCACCAAAACTCTTGGACTCGCTTGGGACTCAGCGTCGGATCGATTG
TTGTTTTCGTTTTCGTCCATTCAGCAGAGATCTAAACCATCNAAAAGGTCAATACTATCGACCATAGCTCG
CTTGTATGACCCGCTTGGACTCATAGGGCCGGTAATTGCCAAGGCTAAAATCTTTATGCAGCAGATCTGGA
AGGAGAAGCTTGAGTGGGACGAGAGCTTGCCATCATCACTGGCAACAGCTTGGCATGATCTTTGCTGCAG
TTTTGAGAAGGCACAGTTTCTTAAATTTCCTCGTCTCGTGTCAATACCTGCAGCAAGAGTTAGAAGTTCAC
GGTTTCTGCGACGCCAGCATCGAAGCATATGGGGCCTGTGTCTACGTCCTTTCTCGACGAACATCAGCCAG
TTGTCAGCTGTTGTGTTCCAAATCGCGTGTAGCTCCTCTGAAAACAATCACAGTTCCAAAGCTAGAGCTCT
GTGGTGCCGTCTTATTATCTAATCTCATGCGTGAGATATCTAAAATGAATCTGTTCGATGGAAAATTCTAC
TGTTGGTCGGATTCTTCTGTATGCCTTTCCTGGATAAGGACCGAGCCCTGCAAGCTCAATGTGTTTGTGGC
TAATCGCGTCACAGCAGTTCAAGAGCTAACTAATGGCATGGAATGGCGTTACGTGCCTACTTCACTCAAC
CCGGCAGATATACTCTCGAGAGGATCTACGCCCAGTGAATTAGATCAATCGTCACTGTGGTTCAACGGCC
CAGAATTTCTAGTTAAAAACGAATTGGACTGGCCTGCTTCTTGCTTGTCAGAAAGGACAGAACTCGAACTT
AAAAAAAGGGTCCTTATTGCGTCATCTCCCTTCGTTGATATAACTGTCGCTTGTAAATACGTCAACTCATT
TGATACAATGCAGCGTATATTCGGATATATTTATAAATTCGTACACAGAAGCAGGCGTCCTGGACTAACA
GTGTCTGATATACAACATGGGACCCAACTCTTACTAAGGCTCGTCCAGAGAGCACACCTGTGGGAAGAGA
TCAAATCATTGCAATCGAGAAGAGTTGTTAAACCGTCCAGTTGTATCGCATCACTTAACCCTTTTTTGGAT
GATTTCGGAATTCTTAGAGTTGATGGGCGCTTGAAAAACTCTTCGCTGGATTTCGACGCTCGCCATCCAAT
AATTCTCCCGTGTCACCACCCCGTCACAAACTCGGTCATCGCACGGTTTCACAATCGGGACTTACACGCTG

GTCCACGGGCACTTCTTGCTCATATTCGGTTGCAGTATTGGCCGATTGGTGGAAGGAAGACCGTATCCAAG
GTGGTTGGAAAATGCGTGAGATGCTTTCGTGCCAAGCCTCGATTAATAGAGCATATAATGGGTGATCTGC
CCGAAGAACGTTTGACTGCTTGTCGAGCCTTTGAAGTCTCTGGTGTTGACTATTGTGGACCATTTTTCTATA
AGCCAGACGTACGAAATAAAGCTCCAGTCAAATGTTACATGGCTGTCTTCGTNTGCTTCGCGACTAAAGC
GGTTCACCTGGAACTGGTCAAGGATCTGTCGACAGCTTCTTTCTTGAATGCGCTTAAGAGGTTTATATCGA
CTCGAGGCAGGCCATCTCAGATATGGTCGGATAACGCTACCAACTTCGTCGGCGCCAAAAACGAGCTTCC
GGATCTCAAGCGTCTATTCACGAGTCGTAATCATATCGAGTCAGTGCATAATTTTTGCATGGCTGATTCCA
TTGATTGGAAATTTATACCTCCTCGTTCACCGCATTTTGGCGGCCTTTGGGAAGCAGCAGTGAAGACAGCG
AAACATCATTTTTATCGTGCTGTTGGATCTTGCGTTCTCGACTTCGAAGAGTTTCGCACCCTAGTCTGCCAC
ATAGCGTCGATAATTAATTCCAGGCCATTGTTTCCTCTTTCAGAAGATCCAGCAGATCTAGACGTGCTCAC
TCCAGCTCATTTCTTGATTGGTGGACCCAGCTCATCGTTCTTTGAACCTGATGTGACGCACCTTCAATTCAA
TCGTCTGGATGGTTGGCAACGCGTAACGTACCTCCAACAACTGTTTTGGTCTCGCTGGCGCGAAGAATACT
TAACGCTGCTTCAGCAGCGCTCCAAATGGCGTACTCCGAAACCAGGCCTATGCGTCAACGACGTAGTCCT
CGTAAAGGATGAAAATCTGCCGCCTCTAAGGTGGCCACTGGCGAGAATCACAGAGCTAATCGCGGGAAA
GGATGGAGTCGCTCGAGTAGCCGTTTTGCGCACCGCGACAGGAGAAACGAGNAGAGCTGCAAACAAGCT
ATGCCTATTGCCCATCAGGGAATCTGTTGAAAGCCCATGCCTTCCAACGGGGGGAGAATGTCTGGTTACG
CAGCCAAAACAACTGGAGCGACCTCGCAACTGATAAGAGCAGCATCTTAATTTTATCTTATAATACTCGCT
CTGCAAAGCAAGTGCCTAAATTTCGCTCTTAAGAATTCATTTACATAGAGCTGCCGCTCTTCGCTGTAACG
ATCGTTTTATTGCCCTAGCTTTACTTATGCGAAGGCCTCGATGCTGCGCATCGAGTTTAGTTAAGTCAGTTC
GCTTCATGCATTTGGTACGCATCGGCCACTTGCATTACTCAAAATTATAACTAATAATTGTCTCTTCGCTAA
AATTGGCACGTACTAATTCGGGTTCGCTTGCCTATATATAAACTATTGTTCTTAAATAATAAGCACAGACC
AATTCGGGTCTGCCTGCTTCTCGTAACTTTAAGAAATATAAAACAATTTTGAACACAAAAAATAAATAGTT
AATTATTTCTATTTGTGAAAGCTATTGAAAAAGCTCAATAGCGCAACAGTCATAAACATACACTTTGAACA
ATTTAGATGCAATTGATTGGTCGAACACCAAAGTTGCATAGCGTTCAAATCGTCTTGTAGACGAGAATGA
TGTAGGGAATTAGTGTATGATAGAAAAAGTTTGACATTGTCCGCATACATGAGTACACGGGCATGTGATA
TAACAGAGGGGAGATCATTTATAAAAAGAGTAAAGAGTAAAGGTCCAAGAAGATTAGAGTTAGAGTTAG
AAGCTCACGGTTTCTGCGACGCCAGCATCGAAGCATATGGGGCCTCTGTCNACGTCCTTTCTCGACGAACA
TCAGCCAGTTGTCAGCTGTTGTGTTCCAAATCGCGTGTAGCTCCTCTGAAAACAATCACAGTTCCAAAGCT
AGAGCTCTGTGGTGCCGTCTTATTATCTAATCTCATGCGTCAGATATCTAAAATGAATCTGTTTGATGGAA
AATTCTACTGTTGGTCGGATTCTTCTGTATGCCTTTCCTGGATAAGGACCGACCCTGCAAGCTCAATGTGTT
TGTGGCTAATCGCGTCACAGCAGTTCAAGAGCTAACTCATGGCATGGAATGGCGTTACGTGCCTACTTCAC
TCAACCCGGCAGATATACTCTCGAGAGGATCTACGCCCAGTGAATTAGATCAATCGGCACTGTGGTTCAA
CGGNCCAGAATTTCTAGNTAAAAACAAATTGGANTGGCCTGCTTCTTGCTTGTCAGAAAGGACAGAACTC
GAACTTAAGAAAAGGGTCCTTATTGCGTCATCTCCCTTCGTTGATATAACTGTCACTTGTAAATACGTCAA
CTCATTTGATACAATGCAGCGTATATTCGGATATATTTATAAATTCATACACAGAAGCAGGCATCCTGGAC
TAACAGTGTCTGATATACAACATGGGACCCAACTCTTACTAAGGCTCGTCCAGAGAGCACACCTGTGGGA
AGAGATCAAATCATTGCAATCGAGAAGAGTTGTTAAACCGTCCAGTTGTATCGCATCACTTNACCCTTTTT
TGGATGATTTCGGAATTCTTAGAGTTGATGGGCGCTTGAAAAACTCTTCGCTGGATTTCGACGCTCGCCAN
CCAATAATTCTCCCGTGTCACCACCCCATCACAAACTCGGTCATCACACGGTTTCACAATCGGGACTTACA
CGCTGGTCCACGGGCACTTCTTGCTCATATTCGGTTGCAGTATTGGCCGATTGGTGGAAGGAAGACCGTAT
CCAAGGTGGTTGGAAAATGTGTGAGATGCTTTCGTGCCAAGCCTCGATTAATAGAGCATATAATGGGTGA
TCTGCCCAAAGAACGTTTGACTGCTTGTCGAGCCTTTGAAGTCTCTGGTGTTGACTATTGTGGACCATTCTT
CTATAAGCCAGACGTACGAAATAAAGCTCCAGTCAAATGTTACATGGCTGTCTTCGTCTGCTTCGCGACTA
AAGCGGTTCACCTGGAACTGGTCNAGGATCTGTCGACAGCTTCTTTCTTGAATGCGCTTAAGAGGTTTATA
TCGACTCGAGGCAGGCCATCTCAGATATGGTCGGATAACGCTACCAACTTTGTCGGCGCCAAAAACGAGC
TTCTGGATCTCAAGCGTCTATTTACAAGTCGTAATCATATTGAGTCAGTGCATAATTTTTGCATGGCTGATT
CCATTGATTGGAAATTTATACCTCCTCGTTCACCGCATTTTGGCGGCCTTTGGGAAGCAGCAGTGAAGACA
GCGAAACATCATTTTTATCGTGCTGTTGGATCTTGTGTTCTCAACTTCGAAGAGTTTCGCACCCTAGTCTGC
CACATAGCATCGATAATTAATTCCAGGCCATTGTTTCCTCTTTCAGAAGATCCAGCAGATCTAGATGTGCT
CACTCCAGCTCATTTCTTGATTGGTGGACCCAGCTCATCGTTCTTTGAACCTGATGTGACGCACCTTCAATT
CAATCGTCTGGATGGTTGGCAACGCGTAACGTACCTCCAACAACTGTTTTGGTCTCGCTGGCGTGAAGAAT
ACTTAACGCTGCTTCAGCAGCGCTCCAAATGGCGTACTCCGAAACCAGGCCTATGCGTCAACGACATAGT
CCTCGTAAAGGATGAAAATCTGCCGCCTCTAAGGTGGCCACTGGCGAGAATCACAGAGTTAATCGTGGGA
AAGGATGGAGTCGCTCGAGTAGCCATTTNGCGCACCGCAACAGGAGAAGAGCTGCAAACAAGCTATGCC

TATTGCCCATCAGGGAATCTGTTGAAAGCCCATGCCTTCCAACGGGGGGAGAATGTCTGGTTACGCAGCC
AAAACAACTGGAGCGACCTCGCAACTGATAAGAGCAGCATCTTAATTTTATCTTATAATACTCGCTCTGCA
AAGCAAGTGCCTAAATCTCGCTCTTAAGAATTCATTTACATAGAGCTGCCGCTCTTCGCTGTAACGATCGT
TTTATTGCCCTAGCTTTACTTATGCGAAGGCCTCGATGCTGCGCATCGAGTTTAGTTAAGTCNGTTCGCTTC
ATGCATTTGGTACGCATCGGCCACTTGCATTACTCAAAATTATAACTAATAATTGTCGCTTCGCTAAAATT
GGCACGTACTAATTCGGGTTCGCTTGCCTATATACAAACTATTGTTCTTAAATAATAAGCACAGACCAATT
CGGGTCTGCCTGCTTCTCGTAACTTTAAGAAATATAAAACAATTTTGAACACAAAAAATAAATAGTTAATT
ATTTCTATTTGTGAAAGCTATTGAAAAAGCTCAATAGCGCAACAGTCATAAACATACACTTTGAACAATTT
AGATGCAATTGATTGGTCGAACACCAAAGTTGCATAGCGTTCAAATCGTCTTGTAGACGAGAATGATGTA
GGGAATTAGTGTATGATAGAAAAAGTTTGACATTGTCCGCATACATGAGTACACGGGCATGTGATATAAC
AGAGGGGANATCATTTATAAAAAGAGTAAAGAGTAAAGGTCCAAGAAGATTA
>160_rnd-1_family-116__LTR___Gypsy
ATATAAGTTAACAACTAAGCATAAATATATTGCCCTAGCAATTAAGTAGCTTCGTATTAACAATGCTGACG
CGCCAGAACTGAGTTCAGCGCTCTGCGCTAAGAACGGCCAGCAGCGGTAAGCTGACACTGCATGGAATGC
TGAGTCGGCTTGCCGACCATGCTTTGATGGCGTGATGCATTGAGGCTTAGGGTATTTGTATAATTTGCTTG
TCCCCTAGTTTTTAGTTTTATATGAGGCGCGCAACACATAAGTCGCCGAAATAAATATACTGCTCCGGCAT
TGCCGATAAAACATATCTTTCGTAATATTGTCTGAGATCAAGGAGAACATATTTTGATCAAGGGCAGCGC
GGGATCAACGCAACTTACCCAAGATAACATTGTGTTACATCGCAGAACATACATTAATGAATAACTGGCG
CCCAACCAATAAATAGGAACCCATACCATGTAAGTTCATAAGTGATTTCAGTTTGAAGAAATTATTCACG
AACGCCACGTACCGATACGTGTGATTTTAAAAAAAAAAAAAAAAAAAAAAAAAAAAAAAAAAAAATTGTAA
AGTAAAGTAAATATTGCTGCTCTCTTCTGCCAGCATTTCATTCCTTTAAATCATTGCATACTTTCACTAAAA
ACTGTTGCATACTTTTACCAGCGTTCCTTGTCAATAAAGCATTGCATACCTTAAACGAATAATTATTGCAT
ACTTTCGGCAGCATTACATTTTTTGCTGCATACCCCTAACAATTTTTTATAATAAATATTGCATACCCCAAG
ATTCACCAGTTTCCACTATTGCCAACAGCGTTACCTGCCGATCTTTCTCGATCGGCCTAAGTTTGAAAAGT
GCCAACCATAAGCCAATGAGTTGGAATTATAGGGAGATAAAAGTCGAATATAATAGTGACGATAGCTGG
GAGGGGGAAATCAATTGCTCAGTAGATCCTACTCCTGCAGATACCGTTGTAACTGAAGAAATGGAGCCGG
CTCAAGTTCAGAATCTCATTGAGGGTGCCATCAGGCAAGCTTTAGCGCAACAACAAAATCAGTTTCAAGC
ACAAATAACCGCTTTAGCTTCACAAGTACAGAGTTTGCAGGTGGAGGCGCCGCAAATCGTCACATACGAG
AAAATATCCGTCGATCCNAACGCCAAATGCGATATTCCCTTGGACATTATTAAATCTGTGCCGGATTTCTC
CGGCGTACAGGATGAGTATGTAGCGTGGAGGCAAGCAGCCATCTACGCGTACGAACTATTCAAGAACTAT
CCAAAGAGCACTGCACACTACCAAGCGGTAGCGATCATTAGGAACAAAATCAAAGGCCCAGCTGGAGCC
TTACTAGTGTCTCACAACACTGTCCTAAATTTCGATGCAATACTAGCTAGGCTAGATTGCACATACTCGGA
CAAAACATCCTTGCGCTTGCTGAGACAATGTTTGGAGTTAGTTACGCAGGGCGAAATGTCCCTGATGCAA
TACTACGATGACGTCGAAAAGAAACTCACGCTCGTAACTAACAAAATTGTAATGTCTCACGACCAAGAAG
GCGCGGATTTGCTCAACCGGGAGGTCAGAGACGACGCCTTGCACGCCTTTATCTCCGGTCTTAGGAAGCC
GTTGAGAGCGGTTGTGTTCCCTGCACAACCAAAAGATCTACCATCCGCTCTGGCCCTTGCCAGAGAGGCA
GAAGCTAGCATTGAGAGAAGCATGTTCGCGAACTCTTACGCTAAAGCCTTGCAAGACCGAGCGCAAATTG
CTGAAAGTAACAAAAACCGTGCCCAAGGCAAGCAAACCAGATTTAACAAAGATGAGCAAAATCAGGAGA
GAAATCCCCATTTTGTGAAACGCCAAAAGGGCGGTCCCCAGCCCCAGGCCCCTAAAGATAATCAAACTGA
GGCTCCACAGCCCATGGAAGTTGACTCTTCCTCCAGATTTAGACAAAAAACCGACTACAGCAAGCGTCAG
GCTAATGAGTCCAACGCACCAAAACGCAAGAATTCGTCAGACCGTTCAACAGGTCAAAGACGACAACGC
GTTAACAATGTCGTACAATCAGATTCCAAAGAAGCCGTAACAGAATTCGAGAAGGCGGCCAAAGAGGCA
GTTGAAGAACTAGAGAGTGAGAACGAGTACGCACCTGGTGACGACTCGATTAATTTTTTAGGGAACACTC
CCGGCTTCCGTACATTGAACGACGGTTGGCTGGGAGAACTCTGAAGATGTTGATTGACACTGGCGCGGCA
AAAAATTATGTGAAGCCCTTAACAGAGCTAAAGACCATAACGCCGGTCGCCAAGTCATTCTCGGTAAACT
CGATCCACGGGTCTACCGAAATCAAGCGCAAATGCTTGATGAACATTTTCCAACATACCTCCCCGTTTTTT
CTCCTCGATACCCTAAGTTCTTTCGACGCCATCATAGGCTTCGACTTGCTAACACAAGCCGGAGTCAAATT
GAACTTGGCAAAAAACACTCTGGAATACCAGAGTACATCCGAAAAACTCCAGTACTATCAATGTGCTGAT
GTGAACTTCACCGATGTCAACGACATCGTAGTCCCCCCTTCCGTAAAGAAAGAGTTCAGGGACATGATAC
TCAGAAACAAAAGAGCCTTCTCAAATGCAGACGAAGCTCTCCCTTTTAATACTGCTGTTATCGCTACAATC
CGAACAGTAGACAACGAGCCTGTGTATTCAAGATACCCGAGTCTCATGGGTGTTTCTGACTTCATAAACA
AAGAAGTCAAACAATTACTGGACGATGGCATAATCAGGCCCTCCAGATCGCCTTACAACAGCCCAACATG
GGTGGTTGATAAAAAAAGGCACTGACTCATACGGTAACCCAAAAAAAAAGGTTGGTAATCGACTTCAGGAA
ACTGAACGAAAAAACCATACCGGACCGCTACCCCATGCCGAGCATCCCCATGATCTTAGCAAATCTAGGA

AAGGCGAAATACTTCACTACCCTCGACCTAAAGTCGGGGTACCATCAAATTTACCTTGCGGAGCATGACC
GCGAAAAGACTTCATTTTCAGTAAGCAGCGGCAAGTACGAGTTCTGTCGCCTACCATTCGGCCTGCGCAA
TGCAAGCAGCATATTCCAAAGGGCAATAGACGATATCCTGAGGGAACATATAGGAAAGATTTGCTTTGTC
TATGTAGACGACGTCATAATTTTTTCTAAAAACGAAACGGAGCACCTTCAACACATTAACATTGTGTTAAA
ATGTCTAATCGACGCAAACATGCGTGTAGGCCCCGAAAAGACTCGTTTCTTCAAGGAAAGTATCGAATTC
CTGGGTTTCATTGTAACCAAAGACGGAGCAACATCCGACCCCAAAAAAGTTAAAGCCATTCAGGAATTCC
CTGAACCAAAGAATGTCTACAGCGTTAGGTCCTTCTTGGGGTTAGCCAACTACTATAGGGTCTTTATTAAG
GACTTTGCCGCTATAGCGCGTCCGATATCGGACATACTTAAAGGGGAAAATGGTTCAGTTAGTAGGCACG
TATCAAAGAAAATCCCTGTTGAATTTAATGAAGTTCAACGCGATGCATTTCACCGTCTGCGGAGTATTTTG
GCATCAGAAGACGTCATGTTAATGTATCCAGACTTCCAGAAACCGTTCGATTTAACCACTGATGCTTCGGC
CAGTGGAATTGGCGCGGTTCTTTCCCAAGGCAATAGGCCAATAACAATGATTTCTCGCGCCTTAAAACAG
GCCGAACAAAACTATGCCACTAACGAAAGAGAATTGCTGGCCATTGTGTGGGCTTTAGGCAGATTGCAGA
ACTTTCTGTACGGTTCTAGGGAAATTAACATTTTCACGGACCACCAGCCGCTCACGTTCGCTGTGTCGGAC
AAGAACACGAATTCCAAGATTAAAAGATGGAAATCTTACATTGACCAGCACAATGCCAAAATGTTTTATA
AACCGGGCAAAGAGAATCTGGTGGCGGATGCCCTTTCAAGGCAAAACATTAATGCTCTGCAAGATGGGCC
CCGTTCGGACGCTGCAACCGTTCACAGCGAACTTTCCCTGACCTACACGGTCGAGACAACAGACAAACCT
TTGAATTGCTTTAGGAACCAGATTGTCCTAGAGGAATCGCGTTTTCCGCTAAAGCGAAACTTGTTGCTGTT
TCGCAGCAAAACACGCCACTTGATCAGTTACACTGACAAGAGCNCAATTCTCAAAACCCTCAAAGAAGTT
GTGAATTCTGGAGTCGTTAATGCAATACATTGCGATCTTCCCACGTTGGCAAGTTTCCAACACGACCTGAT
TTTGTATTTCCCTGCNACCCAATTCTGGCATTGCAAAAATCTTGTGCAAGATATAACGGACAAAAACGAGC
AGTTAGAAATCATCACTGCCGAGCACAATCGAGCGCACAGAGCAGCTCAGGAAAACATTAAACAAGTAC
TTCGGGACTATTATTTTCCCAATATGGCCAGTCTGGCCAAAGAAGTGGTTGCAAACTGCAGGGTCTGCACT
AAAGCCAAATACGATAGGCACCCGAAGAAACAAGAACTCGGGGAAACGCCTATACCAAGTTATACTGGC
GAAATGTTGCATATTGACATTTTCTCGACAGACAAGAAACAGTTCTTGACCTGCATAGACAAATTCTCAAA
ATTTGCAGTCGTCCAACCGATATTATCCAGAACGATAATCGATGTCACAAGCCCTCTACTGCAAATTGTTA
ACCTTTTTCCCGCAACCAAGACAATTTATTGCGATAACGAGGCGGCCTTCAATTCCGAGACCATCACTTCG
ATGCTCCGAAACGCCTACGGCATTTGCATTGTGAATGCGGCTCCGCTTCACAGCACCTCAAACGGCCAGG
TGGAGCGCTTTCATAGCACCCTGACCGAAATTGCGAGATGCCTCAAGTTAGACAAGAAAATCAGCGACAC
TACAGAATTAATCTTAAAGGCTACGATAGAGTACAACAAAAGTTTGCACTCAGTAACCCTCGAGAAACCA
ATTGAGGTCGTTCATGCAGCAGCTTCTGATCGTCGCGGGAACGTCAAAGACAGGCTAATTAAGGCACAGC
AAGACAATATCAAAAGGTGCAATGCATCCAGGCAAAACCGCGTTTTCGAGGTAGGAGAAAAGGTGTTCC
AGAAGAACAACAAGAGGCTAGGGAACAAGTTAACTCCCTTATGCTCAGAACAAAAGGTCGAAGCAGACC
TGGGAACGTCTGTCCTCATTAAGGGGAGGGTGGTCCATAAGGACAACCTGAAATAAGTTTTCCTCCACAA
TTAGCTAGTAAGCCAACTCAGAGTTTAAACACCGTCTTATATTGTTCGTCCATTTCAGGTTTGCACTCACTT
TGCTTGTGGCACTAGCAGCGGTAAATGCTCGGATCACCGACTTCTCGCATGCCAACTACATTCCGGTCTTA
GACGGGGAAGTACTGGTGTTCGATCAACGTAGCTACTTGAGGCACTCAAGTAACATTTCCGAATTTGTTA
GTATGATAGACGAGACGGAAAGACTGTCCGATTCCTTTCCACAGTCGCATATGCGCAAACTACTAGACGT
AGATACAGATCACCTTAGAACATTGTTGTCCGTTCTTCAAGTGCATCACAGATTTGCACGAAGCCTCGATT
TCTTAGGCACTGCCTTGAAGGTTGTTGCTGGCACCCCTGATGCTTCAGATTTCTTAAGGATCCGCGTTACA
GAGACGCAGTTGGTGGAGGCTAACTCCAAACAGATACTAATAAACTCCGAAACTCAAAAACAAATAAAC
AGACTGACGGACACTATCAACAAAATTATCAGTTCCCGCAAAGGCGATTTGGTTGACACTCCTCATTTATT
CGAGACCCTGTTGGCAAGAAATAGAATACTGAACACAGAAATTCAAAATTTAGTACTCACCATAACGTTG
GCAAAAGCAAGCATAGTAAATCCTACAATCCTTGATCATACCGATCTGAAATCACTTATCGAACAAGATA
CCCCAATAGTTAGCTTAATAGAAGCTTCTAAGATCAAGGTCCTTCAGTCCGAGAATATTATCCACATATTA
ATAGCCTACCCTAAAGTTGAGTTTAGGTGCCGAAAGGTCTCCGTCTACCCCGTTTCACACCAACGAACCGT
TCTGCGACTCGACGAAGACACGTTGGCGGAATGCGAAAAGGATACCTTTGCAGTTACCGGATGCACCGTA
ACTACGCATAACACCTTCTGCGAACGAGCACGGCGCGAAACCTGTGCTAGCTCCCTTCACGCGGGGGTA
CAGCCCATTGCCAAACGCAGCCGAGTCATCTTGCCCCTGTCACCATAGTGGACGACGGCGTCGTCGTCATT
AACGATCGTAGCGCAATACTCGCCTACGACGATTGCGCTGCGATGAAGATAGATGGGACAAACCTCGTCA
CCTTCTCGTCCTATGCCACCGTAAATGGCACGCGATACTTCAATCGCAAGAGCGTGCAAAATCGGTTCCCG
GTGGTAGCGGCATCACCGTTGCTTAACATCAGCGGCCATGACTCGATACTCAGCCTTCCCTACATTCACCG
CCTGAGTGAAAACAACCTTAACTACATCAAAGAGGTCAGAGACAGTATTGCAGTCGGAAATACATTGGCG
ATTTCCTTCGGCATAATGCTGTTGGCCTGTGCTGTGGGGGCTGGAGTCTACTGGCTCCATAGGCTGACAAT
CAAGCGTCGAAACTCCCGGAAGATCTCGAAGATATTGGAAAACTTAAATGCGACCGAGGACGGTCGATCC

TCGAGAGGGGGAGTAGTTAACCACTGACTATTGGTATGTGCTGACCCAGCGTTCTGGGGAAGCCAATGAC
CAGAGTCATGTGGCGACGGATATGTCGCAACTATGTCACCGGCCGCGACGGTCATCCGACACCGCACACG
AGGCGCTAACGCTGCACTTTTGCGAGAGCGGCGGCAGCGGTTCTACAGCAGCAACGGCGGCTCTGGGGCA
GCGACGGCGGCTCTGCAGGCATTGCTTAGATTGATAAAGATTGTATTTTTTAGTCTAAGTTAGGAAACAAT
AAAAGCGCGACGTATCGTCCCTTAAAGTCTCCCGTTAGTTAACTCATTTTTTATATGCATTTTTANATGCAA
CCGGCAAACCCAAAGTTGCAGACATNAATGCACTAATTTGCACACAACCTAAAAAACCCAATACAAAAA
AAAAACGCGATAAAACGGTTTCAAAACTCCAGTTGTGACGGCAGCGGTGGAATTCATCAAAATACACAA
ATACACAACAATAGAATCATGTAAGTGATTTGTGAAGTGAGAATAGAAAATTTCGAGAACAATTTTTTTT
TCGAATACGCACAAATTAATTTTGTTAAAGTAAGAAAAGGAGTGTCTATTTCTGATTGAGGAAAAAATTT
ATGAGAATTATTTAAAAAAAAAAAAAAAAAAGAAAAATAAAAATCCCATTAGCTTAACCTTTTATGTGTTG
TTCGGTTTCATACAGAGGTTTAAATTCGAATGAGCGACTGTGACTCCGACGTAGACGCGCATGAACACTA
CGATAGCGACGACAGTATTTTAGCGAGGTTAATAGGGGGATTTGGAACATTGCAGGTATCAGGTTGTGTC
ACCCCAACTGAACATAATACAGAAACAGACTCAGTTATGGACCCAGAGCAGTTGCGTGCGATAATTGAGG
CCGCAGTGCGGAGCGCACTCACAGCGCAGGCGCAAGAATTCGCGAATAGGGAAGCTACGCTAAGGCAAG
AGCTTCAACAACAAATTGCGCAGGTATCGAATAAGCTAAGAAACACACAACCTTCAGTCCCGGTAGTAAA
GGTTTACAGGGAAGTTGAAATCATAGACGGGGTGGAATGTAATGAGCCGCTCGACGTGGTAAAATGTCTT
ACCGAGTTCCATGGGACTCAAGACACTTACGTGTCCTGGAGGCAGGCCGCGCATGCCGCCTATAAAATTT
TCAAAAAATACGACGGTAGTTCACGTCACTACCAAGCCACCGCAATCATTAGGAGTAAAGTGAGAGGCCC
GGCAGACGCGGTTCTCGCCTCGTTTAACACGCCGTTAAACTTTGAGGCCATCATAGCGAGGATGGACTTC
ACTTATAGTGATAAGCGGCCTATCCATGTAGTTGAACAGGAAATGGGTACGCTTAGGCAGGGCGGCATGT
CCATTCTACAGTACTACGATGAGGTTGAAAAGAAACTCACGTTACTCACAAATAAAGCCCATATGTCATA
TGACAGCAATCTGGCAGCGGGAATGTGTGAAAAATTCAGGGGTGACGCACTTCGCGTATTTATCTCTGGA
TTAAAACGCAGTCTAACCGATGTGTTGTTTTCGGCTCACCCCTCGGATCTACCGACGGCACTAGCGCTAGC
CCAGGAGGTCGAGGCTAATCACGAGAGATATGCCTTCGCGACAAGTTTTGCTAGGGGGCAGGAAGAGCG
ATCTCGTCCAGAGCAGCGCCCACAAGGGCGCCAGCAACAACAACAGCAAGAAAAAAAGCAATTCTCTCA
GGGTGGCCAGGGCAAGAATCCTCATTTCAAGAAGCAGAACGGCCAATCCACAGACAAAGCCCAGAATAG
AGCTGGGGCCGATGCCCCAGTTCCCATGGAAGTCGACCCTTCTTCTTCCAGGTTTAGGCAACCCACAAACT
TTTCGAATTCCCAGGGCTCGGCTAAGTTTAACGCCAGCCAAGGTCAGGGAAATAAACGCTCCGCCGGATC
GGAACGCGCGACAGGCCAAAGGAGGCAAAGGGTAGACCACGTCCAGGCGAGCGAGGCCGAGAGCGCAG
GGTACACCGACGCGGCACAAGAGGCGTCAGACGAACAACAAGACGAACATAGCCCTGATTATGATAGCG
ATAGCGCTAATTTTTTAGGGGAAGGTCCCTGCTACCGTTCATTCGACGGAGAATAGCGGGGGTAGATATG
AGATTTTTAATCGACACGGGTGCGTCAAAAAATTACATCAGACCGCACAAACGCCTTAAAGGCGTCGTCC
CGGTCGAGTCCCCTTTCGTTGTCCACTCCATTCATGGGTCCAACAACATCGCCGAAAAATGCAAGTTATCC
ATGCTTGGGTGCGACACATATTTTTTCCTACTAGATTCTTTATCCACTTTTGATGGAATCATAGGGCTGGAC
CTGCTAAAGCAGGTGGGAGCGGTTTTATGCCTAAAAAAAGGTATGTTAATATTTGGTGCGACTTCGGAAG
CGTTGCAGTACCACAAATGTGTGAGCGTTAATCACACGAAAAATAAGGAAGTAGAGGCACCACCTGCGGT
GAAGGCAGATTTCCTGCGAGTACTTCATCGAAACAACAAGGCTTTTGCGGACTCCAACGAGGCGCTGCCT
TATAACACGGCTGTTGTGGCCACGATCCGTACGACCGACGAAAATGTGGTCTACTCAAAGCTATACCCCT
ACCCTAGGGGTGTAGAGGATTTTGTCGTTTCAGAAGTCAACGAACTACTAAAAAACGGTATAATTAGAAA
GTCGAAATCGCCCT
>160_rnd-1_family-246__LTR___Gypsy
CGTCGTGAAGTGAGCTTCGCTGATCGCACAGCTTTTCTAGATAAACTCCTTGACTTGATTAATCCAAATGC
GGTTAATGACCTCTATTGCAACCTGCATACATTATCACAGCTGAGCGTGGCTTAGTTCGCATGTTTCCAGG
CACAAAGTTCTGGCACTGCAAAAACCGAGTTGCAGATGTCTTCAACGTTGACGAGAGGAGAGAAATTCTC
GTTGCCGAACATACTAGAGCGCATAGAGCAGGTCAAGAAAATGTTAAGCAAGTGTTATCAGAATACTATT
TCCCTAAAATGGCTAAGTTGGCTAAAGAAATTGCGGAAAGTTGTAGAATCTGCTAATCAGTCAAATACGA
TAGGCACCAGAAAAGACAGGAGCTGGTAGAAACTTCGATCCATGAGACTCCTGGGGAATTTCTACACATT
GATATATTTCCCACGGACAAACAATTTTTTTTGTCCTGTGTTGATGACGATGTTTGGTTAAGTAACCATCA
GAGACAACACCGACTGCGTGCAGTAACAGCAGAAGACAACAATCATACTTGCAAACTAGAGCAAATATT
GTTTGGGAGCTGTTTGTAAGCTGCTGCCCATTCTATTTCGTAGGCCCTTTGCCTTCTGCACGAATGCCCGCT
GCCCACCGTTTTGAGCGCAGCTCCATTTCGTTACAAGTCATTAGTTTTTCATTTGATCGCAACCGAGGCAC
AACTATTTCGAGTTGGCAAACAATATTAACAATAATTGCTAATAAGTGTAAGTCGAAACACGAGGGAAGC
GCATGAGCCGCAAGCGGCCGTTGTGAAAAGCACACAATAGCTTAGGTAATGTTGATCAATCTTTTGTAAA
TTTAGNTTTTAATCCGGCTTTTACTGAGNCACATCTCAGCGACTTTGCGCATAAATTTAGCGTATCGGCCT

AGTGTTTTACAATAATAAAATAATCAAAATAAAGTCTTATAACGAAAATCCGCGAGGACTTCTTATTTA
AATTTTAATTTGCGCCAAACGTTTTTCGCTTTTTGTGCTATAATTATATTGTGTGCGGTGCAGTGTAAAGAA
CTAATCTACCAGNACAACACACTTCGCGTTGTGTAATAGCAATACCCAACCTACCAGTGCAAGCAAAAAA
GCTGTGCCTGACCTATTCTATTTACAGCACAAGCCCAACAAAAAGGCATTTGATAGCGACTTGTCACCAA
GCAAGCGGGTTTGGGCAGCGAAGTAAGCCAACACCCATAGACTTCAGCGAGGGATCATAAGAAAGCATT
CACGTCTGCAGCGTGGGAAGCAGTAGTTTTTAGACGTAGAGCATACTGCCCTTGTAGGATCAAAAAAATG
TAAGTGGCCCATCATTTGGCACAAACTTTTTTCAATATGATTGATTTATTTCAAATAAAATCCAAAGTCTG
AAATATAAAAAATTGCAATAATTGTGCTACCAACTAATTTTGAGTAACACAATTTTGCATCCATCAGCAAT
TTTCAATAAACTCTCCTAATTCAGCATTCATTTAATTTATTGTTGTTGATTTGCCCTTGAAATTGTGTTGTTA
TTATTGTTGACAGTATAGTGGATGAACAGCTCGTTTCTCAGGTACAGAGACAGCTCTGCGAGGGGATTTG
AGTAAAACGACTTCAACACCTTAAATAGGGAAGCCGTCCCGCAAGAAATCGGTGACAACAGGTGTAAAA
TGGAAATGGAGCCTTAGCAGCTTAGGGCTTTGGTGCAGGCCGCAGTGGCCTCCGCCTTTGAAGCCGAAAG
CGCAGTATTGAAGCAAAGAGATGTAGAACTGAGGGCTTCCTTAAACGCTTGGATACAAGAATTGGAAGGC
AGAATATCTGCCAGTAGTATAGAAACACAGTAGTATAGAAACCACCGTCCAGGCATTTGAGCCGGTGGAA
ATCAGAAAGGACGTCGAATGTAATGAACCGTTGGACGCCGTTAAATGCTTACCAGAATTTACGGGGGTGC
CAGAGACGTACGCCTTTTGGAGACAAGCTGCGATAGCGGCATATAAAATGTACAAGCCATACGACGGCA
GCTCACGCCACTACCAGGAGGTCATTATTATTAAGAGCAAAATTAGAGGGGCATTAGAGCGACTTTTTGG
ATCAGTTTGGCATAGTAATGAATTTTGATGCCATAATAAGCCGTCTCGATTTAACATACAGGGATAAGAG
AGGCATTAACGTCGTAGAGCAAGAGATGAGCACCCTAAGACAATTTTATGACAAGGTTGACAAAAAGCTT
ACTCTTCTCACCAATAAGGTCAACATGTGCTAGAAACCAAGCATGGCACAAGGGCTCTGTACGAAGATCA
GAGATGGTGCGTTGCGTGATTTTGTCTCAGACCTAAGGCGTAGCCTGACAGATGTCCTGTTCGCGGCAAA
GCCTAAGGGCTTACCGTCGGCCCTAGCTCTAGCGCAAGAGGTAGAGGAAAACCATGAGAGGTACGTATTT
GCCGCTAGCTTCGCTCAAGGGCAAGAAGATAGGGAACGAAAAGTGACCCCGAGGCCGCAGGTCCGTCAG
CAAGATAAGGGTCAACAGCTGGGGGATCCGCAACCCAGCGGTTCCAAAAATCCTCGCTTCAAAAAACACC
AGAAACAGCAATAAAGTCAATTTGAGCAACAGAGTGGCAGGTGTGTTCTACAACAACCGTAGGCACCAG
CCTACTTTAACTCTTGCGGTCCTCAAGCCTACAAAAGTACGGCCACGTCTGGGCGCTTAAGCGGACAGAG
GAGGCGGATAGTCAATCACACAGCTCACGAGCTTGACCAAGATTCAGGTTCGTATGCTAGCTCTGCAGCA
GCAGCAGAACAGCAAATTGCCGACGACAATGGTAGCGGGTATGATTCAGACACTCTCAATTTTTTAGGGG
AAGCTCCCTGCTGCCGTACATCAGTGGAGTAGCAATGATCCAGATGAGATTTCTCATCGACACGGGCGCT
TCCAAGATATTTATCAGGTCATATAAAATCTAAAATGTGTGTGCCCAGTAAATTCTTCCTTCCCTACCCATT
CTGTACACGGCACCACGAAGATCACTCATAAGTGCCTCCTTATATCTCTGTTCGACAAAACTGCCAAATTT
TTCTTACTCCCAGACCTATCCACATTTGATGGAATTGTGGGGCTCGCCTTGCTCACGCAAGCAGGAGTGTC
GCTTTGTCTTTCTTCTGGCCAACTCAAATGTGGCGCAGCAACTGAACAAATCGAATTCCACAGGTGTGCGG
ATTTTTACTTTACCAATTTTGACTGCGCCGACGCGCCCGATGCTGCAAAATATGCATTCCTAAAGATGCTA
AAAAGCAAACATAAGGCCTTCGCGGATCCGGAANAAGCACTTCCATATAACACATCAGTGGTGGCCACTA
TCAGGACCACTGATGAACAACCAGTGTCGGACATAACAGTACCCCTATCCCATGGGAGCAGCGGAATTCA
TTAATAAAGAGATTCAAAGCCTTCTGAAGAACGGTATCTTAAAAAAATCTGTTTGCTCCTACAAAAACCC
CATTTGGGTGATGAACAAGAAGGGGACGGACGAGTTTGGTGACAAGAATATGCGTCTCGTAATGGATTTT
CGCAAGCTTAACGAGAAAACCATCGCAGACAGATACCCTATGCCTAACATTCCAATGATTTTGGGGAATC
TTGGCAAAGCTAAATACTTTTCGACATTGGCTATCACCAGATCACGCTAGCAGAGCGTGACCGTGAAAAA
ACAGCCTTTGCAGTTAACTTGGGGAAGTACGAATTTCGTCGACTTTCCTTTGGGTTGAAGAATGCGGCCAG
TATCTTCCAAAGAACTGTTGACGTCGTACTGCGTGAGCAATTTGGCAAATCTTGCTATGTCTATGTTGACG
ATGTCATCGTCTTCTCAAAAGATGAAAATTCTCATGTCCAACATTTGGAGTGGGTCCTAAAGAGCTTGTAC
GAGGCAAATATGAGAATCTCCAAGGAAAAATCGTGTTTTTCTAAGAAAAGCGTAGGTTTTCTTGGTTTCGT
GGTAACTAGTAATGGAGCAACAACGCACCCTGAAAAAGTCAACGCGATTCAGGAGTTCCCAGTACCAAA
GAATATATTCGAGATCAGATCATTTTTAGGACTGGCCAGTTACTATAGGTGTTTCATCAAAGGCTTTGCCT
CAAGAGCAAAACCCATTTCTGATCTTCTCAAGGGCGAGGATGGGTCAGTAAGTAAACATAGAAGGCGAA
ACATCCCGGCTGAATTCTCTGAGCTACAGCGCCACGCATTCCAAAAGCTTCGCAGCATTTTGTCATTAGAA
AACGTCATGGTCAGATACCCTGACCTTAAGACACCGTTTGATCTAACGCTTCAGCTTACGGCATAGGGGC
AGTGCTTTCTCAAGAGGGCCATCTCAATGATTTCCAGAACATTGAAACCCCCTATATGGGGTACCTATATG
GGGTAAACGACATTAACATTTTCACGTCGTGAAGTGAGCTTCGCTGATCGCACAGCTTTTCTAGATAAACT
CCTTGACTTGATTAATCCAAATGCGGTTAATGACCTCTATTGCAACCTGCATACATTATCACAGCTGAGCG
TGGCTTAGTTCGCATGTTTCCAGGCACAAAGTTCTGGCACTGCAAAAACCGAGTTGCAGATGTCTTCAACG
TTGACGAGAGGAGAGAAATTCTCGTTGCTGAACATACTAGAGCGCATAGAGCAGCTCAAGAAAATGTTAA

GCAAGTGTTATCAGAATACTATTTCCCTAAAATGGCTAAGTTGGCTAAAGAAATTGCGGAAAGTTGTAGA
ATCTGCTAATCAGTCAAATACGATAGGCACCAGAAAAGACAGGAGCTGGTAGAAGCTTCGATCCATGAG
ACTCCTGGGGAATTTCTACACATTGATATATTTCCCACGGACAAACAATTTTTTTGTCCTGTGTTGATAAG
TTCTCTAAATTTGCAGTCGCACAACCAATTCAGTCACGCACTATCGGGGACTTAAAAGCTACACTTCTGCA
GCACATGAATTTCTTTTCACGAGCCGGAAAAAGAGATATGGACCACCGCAAACGTTGAGAAATGTCTACG
CAAGTGAGGAAGAAGTTTTTGTCTGTGAAAAGATATCAATTTGTAACATTTCTCCCGTTAGTTGGGAGACT
GACGTCTGGCCGAGCTCATTTTTCGGGTGTCTGTTGTACTTTGATTTTGTGCAAACTTTACAATTTAAGACA
ACTTCGTTAGAAAATTTAATGTTATTTTGGGAAAATAGTATTCCGACACGACCTGTTTCACAAGTCCCTTA
GTAACTAGTATTGGAAACTAGCCAAAGCTGGAAGACAACATTTAAATGCATTAACCACGTTTGGGTTAAT
GACGATGTTTGGTCAAGAGACAACACCGACTGCGTGCAGTAACAGCAGAAGACAACAATCATACTTGCA
AACTAGAGCAAATATTGTTTGGGAGCTGTTTGTAAGCTGCTGCCCATTCTATTTCGTAGGCCCTTTGCCTTC
TGCACGAATGCCCGCTGCCCACCGTTTTGAGCGCAGCTCCATTTCGTTACAAGTCATTAGTTTTTCATTTGA
TCGCAACCGAGGCACAACTATTTCGAGTTGGCAAACAATATTAACAATAATTGCTAATAAGTGTAAGTCG
AAACAAAGAATATTGTTCATTTGATTGCAAAAAGGCTTGCAACTACAAGTTAACAGAGCCCAAGTACACC
AGCTCCAGATGCTGATATAGCACTGNGCCGGATGTTCTNACGAGTTAACAGGGGTCAGCAAAATGCAATN
TAACACCAGCCACAGATGCTGACATAGCTCTGCGCCGGATGTTCTTGCACGAGGGAAGCGCACGAGCCGC
AAGCGGCCGTTGTGAAAAGCACACAATAGCTTAGGTAATGTTGATCAATCTTTTGTAAATTTAGTTTTTAA
TCCGGCTTTCACTGAGGCACATCCCAGCGACTTCGCGCATAAATTTAGCGTATCGGCCTAGTGTTTTACAA
AAATAATAAAATAATCAAAATAAAGTCCTATAACGAAAATCCGCGAGGACTTCTTATTTAAATCTTAATT
GGCGCCCAACGTTTTTCGGACCATTTTGTTCTATAATTATTTTGTGTGCTGTGCAGTGTAAAGAACTAATCT
ACCAGTACAAGACACTTCGCGCTGTGTAANAGCAATACCCAACCTACCAGTGCAAACAATAAAAGCTGTG
CCTGACCTACATCAATTTACGTCAGCGGCGCAAGCCCAATAAAAAGCCATTTGATACACCGAAGCCGCTG
GAGCTCTTCGGGCAGCGACTTGTCACCAAACCAGCGCGCAAGCGGGTTTGGGCAGCGAAGTAAACCAAC
ACCCATAGACTTCAGCGAGGGATCATAAAAAAGCATTCGCGACTGCAGCGTGGGAAGCAGTAGTTTTAGA
CGTAGAGCATACAACCCTTNTAGGATCAAATAAAATGTAAGTGGGCCATCATTTGGCACAAACTTTTTTCA
ATTTGCGTGATTTATTTAAAATAAAATCCAAAAATACATAAGTGAGAAATATTAAAATTGCAGTAATTGT
GTTCCCAACTTACTTTGAGTAACACAATTTTGCATCCATCAGCAATTTTCAATAAACTCTCCTCTGTCAGCA
TTCATTTAATTTACTGTTGTTGATTTGCCCTTGAAATTGTGTTGTTATTATTGTTGACAGTATAGTCCATGA
ACAGCTCGTTTCTCAGGTACAGAGACCGCTCTGCGAGGGGATCTGAGTCAGACGACTCCACCACCTTAGA
TAGGGAAGCCGTCCCGCAAAAAATCGCTGACAACAGGTGTAAAATAGGAATGGAGCCTGAGCAGCTTAG
GGCTTTGGTGCAGGCCGCAGTGGCCTCCGCCTTAGAAGCACAGAGCGCAGTATTGAAGCAAAGAGATGCA
GAGCTGAGGGCTTCCTTAAACGCTCGGATACAAGAATTGGCAGGCAGAATATCTGCCAGTAGTATAGAAA
CACCGCCGGTCCAGGCATTTGAGCCGGTGGAAATCAGAAACGACGTCCAATGTAATGAACCATTGGACGC
CGTTAAATGCTTACCAGAATTTACGGGGGTGCCAGAGACGTACGCCTCTTGGAGACAAGCTGCGATAGCG
GCATATAAAATTTACAAGCCATACGACGGCAGCTCACGCCACTACCAGGCGGTCATTATTATNAAGAGCA
AAATTAGAGGGGCAGCTGACGGCGTTTTGGGTCAGTTTGGCACAGTAATGAATTTTGATGCCATAATAAA
CCGTCTCGATTTCACATACAGGGATAAGAGAGGCATTCACGTCATAGAGCAAGAGATGAGCACCCTAAGA
CAGGGCTCGTTAACAATTTTACAATACTATGACGAGGTTGAGAAAAAGCTTACTCTTCTCACCAATAAGGT
CAACATGTCCTACGAACCAAGCATGGCACAAGGGCTCTGTACGAAGTTCAGAGATGATGCGTTGCGTGTT
TTTGTCTCAGGCCTAAAGCGTAGCCTGACGGATGTCCTGTTCGCGGCAAAGCCTAAGGACTTACCGTCGGC
CCTAGCTCTAGCGCAAGAGGTAGAGGCAAACCATGAGAGGTACGCCTTTGCCGCTAGCTTCGCTAAAGGG
CAAGAAGATAGGGAACGAAAAGTGACCCCGAGGCCGCAGGTCCGTCAGCAAGATAGGGGTCAACAGCAG
GGGGATCCGCAACCCAGCGGCTCCAAAAATCCTCACTTCACAAAACAACAGAAACAGCAACAAAGTCAA
TTTGAGCAACAGAGTGGCAGGTGGAACAAAGGTGGCGGGTCACAGGAACCAATGGACGTCGATCCATCT
GTGTCAAAAATTCTACAACCATCGCAAGCACCAGCCTACGTTAACTCTAGCGCTCCTCAAGCCTACAAAA
GAACGGCCACGTCTGGGCGCTTAAGCGGACAGAGGAGGCAGAGAGTCAATCACACAGCTCACGAGCCTG
ATCAAGATTCAGGTTCGTATGCTAGCTCTGCAGCAGCAGCAGAACAGCAAATTGCCGACGACAATGGTAG
CGGGTATGACTCAGACACTCTCAATTTTTTAGGGGAAGCTCCCTGCTGCCCTACATCAGTAGGAGAGTAGC
AGGGATCCAGATGAGATTTCTCATCGACACGGGCGCTTCCAAGAATTATATCAGGCCACATAAAAATCTA
AAAGGTGTGTGCCCAGTAAATTCTTCCTTCTCTACCCATTCTATACACGGCACCACGAAGATCACTCATAA
GTGCCTTATATCTCTGTTCGGCAAAACTGCCACATTTTTCTTACTCCCAGACCTGTCCACATTTGATGGAAT
TGTGGGCCTCGACTTGCTCACGCAAGCAGGAGTGTCGCTTTGTCTTTCTTCTGGCCAACTCAAATGTGGCG
CAGAAACTGAACAAATCGAATTCCACAGGTGCGCGGATGTTAACTTTACCAATGTTGACTGCGCCGACGC
GCCCGATGCTGTAAAAGATGCATTCCTAAAGATGCTAAAAAGCAAACATAAGGCCTTCGCGGATCCGGAA

GAAGCACTTCCCTATAACACATCAGTGGTGGCAACCATCAGGACCACTGATGAACAACCAGTTTATGTCC
GACTGTACCCCTACCCCATGGGAGCAGCGGAATTCGTTAATAAAGAAATTCAAAGCCTTCTGAAGAACGG
TATCATCAAAAAATCTGTTTCCCCCTACAACAACCCCATTTGGGTGGTGGACAAGAAGGGGACGGACGAG
TTTGGTAACAAGAATATGCGCCTCGTAATGGATTTTCGCAAACTTAACGAGAAAACCATCGCAGACAGAT
ACCCTATGCCTAACATTCCAATGATTTTGGGGAATCTTGGCAAAGCTAAATACTTTTCGACATTGGACCTA
AAATCCGGCTATCACCAGATCACGCTAGCAGAGCGTGACCGTGAAAAAACAGCCTTTGCAGTTAACGGGG
GGAAGTACGAATTTTGTCGACTTCCCTTTGGGTTGAAGAATGCGGCCAGCATCTTCCAAAGAACTGTTGAC
GACGTACTGCGTGAGCAAATTGGCAAATCTTGCTATGTCTACGTTGACGATGTCATCGTCTTCTCAAAAGA
CGAAAATTCTCATGTCCAACATGTAGAGCGGGTCTTAAAGAGCCTGTACGAGGCAAATATGAGAATCTCC
AAGGAAAAATCGCGTTTTTTAAGAAAAGCGTAGGTTTTCTTGGTTTCGTGGTAACCAGTAATGGAGCTAC
AACGGACCCCGAAAAAGTCAAAGCGATTCAGGAGTTCCCAGAACCAAAGAATATATTCGAGATCAGATC
ATTTTTAGGACTGGCCAGTTACTATAGGTGTTTCATCAAAGACTTTGCCTCAATAGCAAAACCCATTTCTG
ATCTTCTAAAGGGCGAGAATGGGTCAGTAAGTAAACATAGATCGCGAAACA
>9_rnd-6_family-112__LTR___Gypsy
TGTGATGGGGTGATGAGTTTTATTTCTGTAAACATAAACATACATTGCCATTGGCATGATCAGCTGATCGC
AATCAGCTTGAATAGGCAGCACTGCACCATAACATTTATGCAATTACATATGATCAGCGGAGGTTAATAA
CATAACCAGAGTGACCATGACCTATGTTAATGACGCCGTTGCGAATGAAAATATCGTGTCTTGGGTGAAT
GCTAAATTGCTTCTTTTAGTTTTGGCGAGGCGTCATTCTTTTTCCATTTTAATCGCCAACGAGTACAGTCGG
TTTAGATTGCTTTAAAAATTATAAAATTCAATTTTTCACGTGGGCGTAATAAACCCACGTGTATCGGCACT
CAAGCTCAAAAAAAAAAAAAAATAATTAAATAAAAGGGAAGGAAAAAGGTCCAAGGTGCCCAGAAAGAA
AAGGGGTAATATAGACATCATGCGCTCCCAAGAGCTTGGCTATAACATTTCACCGGAATAAAGAAAATTT
TCAGAATTCGGACACTGACAAATATTCACTAAAGGATCGTTACTGGAGTCGTGGGGTGCCTTTCCTGTGTG
TGGTAGTCGTAGGAAGGTGCAGGCTAAAGCGCGGGGCTGCGTTATTGCGTGCCAAAAAGAAAAATTACG
CATAACGCGTAGTCCTTATCGGAGCCAAAGCCAAGGCCCCAAAGCACCAAAAAGCGACAATCTACACTG
GTAGCAGGGAGCGAATTTCCTAAGGGCGGACCAAGATTTCAGCATCGTCTCATTTTCTGCAGATAAGCTTT
GAACCTCGCTAACCAATAGTTCGTGCTGCACGCACAGGCAGACCAACGTGACCACGTGTTCGACCCATTG
ATTCCCGGACATTCTTGAGCGCAGATCTGGTGGATAGCGAACCAGCGCGCACCCTCACTCGGATTTACAA
CATCTGATATAATTGTTAAACGGCATACTGGCCGTAGGTATAAGCGAAGGGCATAAGTACACATGAAATT
TAAATTGCAATTCTTTCCGTTTTATATCGCTGACATTGTCTTTCGATCCTGATTTAGCATGTATATGTCGCT
GACTACTATATTTCTACGTTACATAGTTCAAATTAAGAGCCCACAGCACATCATCGTCGTCAACAGGCCTG
CTGACATCGTTGGATCATACTAAAGTAGTGAGTACTGTGTGAAAGAACAAAAGATAATAAGCCAACATAT
CGAATTGTACGTATTGTTTGAATCGCGGTTATAGTCAGCGGGTGTACTCCTAATCAGCCTTCGTAATTACA
AATAACTTTTGTGGATCATTCAGATCAGCAGTGATTTAACTTCATATTAGTTTCGAAGTGTAATCTCATTCA
TGTGTGTTTTTGCCCAGATTGAGTTTAGTTTTATAAAAAAAAAAATAGAAAAACGTTGTTCTTCTCACGTAA
TGTATAATGAAGTACGATCATGAGTTTAAATCTTCTTTGAATACACTTATATGATAAAATGAAATCTTAAT
TCCTTTTTGGGCACCTTAGGTAAATAGTATCGCATACANAAGGGTAGTTGCAACGACAATCGCAGATGCA
GCTACAGGCAGGGTGGGTGAAGACCGAGGGTAGCCAACATCTTAGCTCCTCGGCTCTATATGATATATTC
TGCTTGCAGATCGTAGTCGCTCCTTATATATCGTGGTATTCCGTCNTACCTTAACTCTGTCTACCTTTTTTTT
CTTTATTATACTTGACGCGTCGAGTGTGTTAGGGAGCAAAAGAACCCACGCCACTTCCCTGAGCACGGTC
GGAACCCGTAGACGGAGTACGCTCCCAATGCACAACACGAGTGTTAGGCAAGACGCGCCCAGCGTAGCC
GTCGTGTTCGTTACATGATAAAATAATAAGAAAAATGGCGCCCAACGTGGGGCCCGAACTTCTGCTCTTC
CGCTTCTCACCCCCGAGATCCGGAATTGGCCCACAAGAACTTAGGAGAGAGCCGATAGCATTGTTCGTTA
CATGATTTGTCACGAGCCAATGTGGACGATCCTANGTTAAATCCATGCTAGCTTTCACCCTTCTTCCTATCC
TTATCACAATATACCATCCAAATAATTTTAGTTTCCGTGACGACATTTATCCTCTCTTGGAACTGCTGGTAT
TCTGATGTCTTTTGTTGGAGCCAGACCAGGAAGTAAATAGTCGAGCGATTTATTTAGTGGATCAGTTATAC
TGTTCCACTAGAAATCTTATTATGTTTTCGCCACTTTTGATTCATTTAGGACTTTTAGATCTGATTTCGAGT
CGGTTCTCTACCAAGTGATAGAAGGTTGGTAGGGCATAATAACATTCCGTTTGTTCTAGTTGAATTGAGAA
TTGTTCTGGTTGGGTTATTTCGACTCGTCCCTCTTCAACTTAGTTCAGACAACTCAACACTGATTTAGTAAA
ATAGTTTGCTTGCTTTTCGTAAGAAGATATATCAAACATAGAGATTTAGATTATGCGATGTATTTACCTAA
GGATAAGTCCCATGTTATAAAATGTTATAATATTGTTGTATTAGCATCGAAGTGCCTTAAACCTTTTCTTTC
TGCCTTAAAACCGTTTCCGATTTTCATAATTATCGCTCATTAGCGCTTCTCGTATATATCTTAAAATTAAAA
TCCTTTCGGGATCAATTCCCATTTAACTCTTTGACCAATTATAATCGATTTATTGATGATGGCTGTCGTTCG
TAGTCAGGAAAATTTAACTCTCAGCGAAGCACCTGCAGTGTGTACTATCTGCAATGAATCATCGATTGGTG
ATGCAGACACTGTAGAAACTCCATGTGCTCATAAATTTCATCGAAATTGCCTAAATCAATGGTTAAGAAG

CAATGAGACATGTCCCACTTGCAAACGAGTTTGTAGTCGAGAATTATACGAAGATAGCAGTGACTTGAAT
GCAGTAGCTTCACAAAGACCTACAAACACGGGGGCCGTTCCTCGGTCCCGAACTATTACCCGAAGTAATG
CTCGAAATACTCCTAAAGCCGCCAGAGGTCGTCCTAGTGGGCCGACCAACTCTCGGGAGCAAGCGGGATC
GCGAAACACCCAACCTTCTAAGGAATCGCTTGGTTTGTCAGAACAGCGTGTGCAGTTGCTTATCAGTAAG
ACATTGGAAGCCCATAAGGCTGAAATGGCGTCCTATTTGTCCGAAGAATTAAATGCCGCAGTTAGAAATT
TAAACTTGTATCAGGAGCCACGTGAGGAACCCCAATTAGAATGGGAAAATGATTTTCCACGTGATGCACC
GCGATCGAACAACGCTTCGAGATTACCGAATGCTAGTGTTCAGTCTGATGCGAGACGTGAATACGCAGTC
GATAGGCCCGACAAAATATCGAGTGTCATATCCAATTGGGATGTCAGATTTTCAGGCGTGGCTAGTGATA
TCCCAGTCGACGACTTTATATATCGCATAAATTGCTTGACTTCACAATGTTTAAATGGCAATTTTAGGTTGT
TATATCAGTTTGCCAATTTGCTGTTTACCGGACCAGCTTTGACATTTTTGGAGATATCACCGTACGACTGA
CCAAACAGGCTGGTTCAATTTGTGCACATGCTTGCGAGAAAGATATAAAGACCACAGATCAGACGAAGAC
ATAAAAGACATGGTTAGAAGACGCAAACAACGAGGGTCCGAAAGCTTTGATGAATTTTTAGATACGATTC
TTGCCTTGTCAGATGCGTTACGGGAACCAATGTCAGACTCAGAGTTAGTGATCAATGTCAAGCGCAACCT
AAAACCAGAATTGAGACACGAACTACTTCATGTAAATACTCCGAGTCTTGCGACATTACGCAAAGAATGT
CACAAGCATGAAGCGTTCTATTCGAATTACCTTTCCAAGCCAATTTCTCGACCAATTAAACCACAACGAGT
AGTCAACGAGGTAGTCCATGAAGATACCACAATGACGGATCTTACGCAGAATCTGGTAGAAACTGGGGA
CTTAAATGCTATACATTCTCCGGATAAATTGATTTGCTGGAACTGCGAGGAAAAGGGACACAGATATCAA
GAATGTTTAAAAGCGCGTCGTGTATTTTGTTACGGCTGTGGTGCCGTTGACACTTACAAACCCACTTGTAG
AAAGTGTAACCCTGTTTCGGAAAACTCTCCGGAGGACTTTCGTCGTCTCTTAAACGCGAATGTCCGTCGTC
AATAGCCTCGGATCCAGACTTTAATAAAGATACCTTAACACCTGATTCGACCTTCACACCAGACTTATCAA
AACCGTGTCAGACAAAACTTAAACCTTACCACGTCCGTTTACACGAATATTTAGACCGTAGAGCAACTATT
TTCGCTTCAGAACCCAAAGTTGAATTTAAGCCTCGTTCGTCAGAGAGACTAAGAAACCTATGGACTCAGC
GACGCTCGCTAAATAAGCATCGTATTTCAGCGATTGATCATCGATCAAACGATGTGCGACCGTTCACGAA
TTTAGAAATATTCAATCAGCATTACTTAGCCTTGTTAGATAGTGGGGCTACGAAAAGCGTGATTGGAGGG
AGCTTAGCGAATAAAATTCTCGATTCGAAAATTGACTTTAACAAAATAAAAGGCGATGTGAGAACCGCCG
ACGGACAAAAACAACATGTTGTAGGTTCCATTTCAATTCCTATCGTTTATAACTCCATTGCAAATAATTTC
GAATTCTTAATTGTCCCGTCTATCAAGGAAAACGTTCTCTGTGGAATAGATTTCTGGAGAGCGTTTGGCAT
TTCAGTACATCATTCGAATACATTGAACTCAGTGGATTTCGAAGAAGTGGAGTCGAATCAGTTGTCGTTAA
CACTAGCCCAAAAGAAACGCTTACAATCAGTAATCACTTCCTTTCCTTCGTTTGAAATTGATGGTCTAGGA
AAGACTACTCTTGTAGAACATATCATCGATACTGGGGACGCCAAGCCCATAAAACAGAGGTTTTATCCGT
TATCACCAGCAAGGGAAAAATTGTTGTGCGAAGAAGTAGATAGGATGATCAAGCTTGGAGTCATTGAAG
AAGCTCCATATTCCGCATGGTCATCTCCCGCGATATTGATTGTTAAGCCTGGAAAAGTCAGATTTTGTTTA
GACTGCCGCAAGGTAAATGAAGTGACCAGTAAGGATGCTTATCCTATACCAAATTTAGATGGCTTGATAA
GTAGACTTCCTCCTGTACATTGTGTGTCGAAAATCGATTTAAAAGATGCCTTTTGGCAAATCTGCTTGGAT
GAATCATCGCGGGCAAAAACCGCATTCACTATCCCGAACAGGCCGCTATATCAATTTAAGCGGATGCCAT
TCGGATTGTGCAATGCACCGCAGACCATGAGCCGATTGATGGATCAAGTAATTCCTTACGACATGAAAGG
TCATGTTATGGTTTATTTGGATGATTTACTTGTATTATCGCAAAGTTTTGATGAACATTTAATTCATTTGGC
TGAGATCGCAACACGATTGCGTAGGGCAGGTTTGACCATTAATATCCGTAAAAGTAATTTTTGTCTAACAA
AAGTCGAATATCTTGGTTACATCGTAGGTGAAGGCACCTTGCAGCCTAATCCGGCTAAAGTTCAAGCAGT
TGCAGATTTTCCAGTGCCGTCAACAAAAAGACAGCTGAGACGATTCCTAGGAATGACCGGTTGGTATCAA
CGGTTTATCCCTCAGTATTCGTCGATTATCTTTCACCTAACAGAATTGCTAAAGGGCACGTCATTTGCTTGG
AATGATAATGCTCAAATTTCGTTTGAAGAAATCAAAAGAAAATTATCCTCAGCACCGTTCTTGATAAACG
CAAACTATGAGAAGCCGTTTATAGTCCAATGCGACGCTTCCATGCATGGCGTGGGCGGTCTGTTAGCCCA
ATGTGATGAGTCAGGAATCGAGCGACCCATCGCATACATGTCGAAAAAACTAAATAAAGCGCAGCGAAA
TTATTCCGTTACGGAACTCGAATGTCTTGCCGTTGTATTGGCTGTAAGAAAATTTCGCATGTACATCGAAG
GACACGATTTTAAAGTCGTGACTGATCATGCAAGTTTACGTTGGCTAATGAACCAAAAAGATCTAAGTGG
TAGACTAGCTAGATGGTCATTCAAATTGCAAGGATATAATTTTTCTATCGAACATCGCAAGGGTAGTGAG
AATGTGGTCGCAGACACGCTTTCCCGTGCCTTTGAGCCAGCAGACGAAGTAGCTGCGGTGGACCTAGAGG
TATACCCTGAAATCGACTTGTCTTCGGAGGCATTTCGATCCAACGAATACTGCCGGCTAAAAAATAATATT
ATTCGTTCCAACTTGCCAGATTTTCGAGTNGTAGATGATTATATATATTTCCGAAATGAACCCTCAGCTAG
TAGTGTAGATGAGAACATGGAGGGTTGGAAATTATTTGTGCCATCTGAATTACGCTTAAATGTTATGAAA
GCAGCACATGATCAACCAACATCCGCGCATTGTGGGATGGCCAAATGTTTGGAATGTATTCGCAGACATT
TGTACTGGCCGAACATGGTGGTTGACGTACGCGAGTACATACGCCACTGCGAGTTCTGTCAAACGTCGAA
AGTTCCTAATCGATGCCTCAAACCAACCATGGGAAACCAAGTGGTCAGCGAAAGGCCTTTCCAAAGGTTA

TATGTCGACTTTGTTGGTCCGTACCCACCATCAAAAAAACGAAACATTGGTATATTCGTCATTCTGGATCA
CTACTCAAAGTTCACATTTCTCAAGCCCATAAAAAGGCTCAATGCCAAAGTAGTGGTAGATATACTGCAA
GAAGATATTTTTGACTGTTTTGGAGTACCCGAGATAATAGTGAGTGACAATGGCACTCAATTTAAAAAGC
CTGGAATTCTCCTCACTCTTGTCGAAGTACGGCATACAGCATATGTGTACCGGTGTATACTCGCCACAGGC
AAACGCGGTGGAAAGAGTTAATCGTTCGATTAATGCTGCTTTGCGCACATATATTCGGTCCGACCAAAGC
CTATGGGATGTATACATTAGCAGTATAAATAGTTCGTTACGAAACACCATACACCAATCTATTGGGATTTC
TCCATACTTCGTTGTGTTTGGGCAAAATATGATTAAGCACGGCAATGATTACAAGTTGCTCAAAAGCCTGA
ACATGCTGAATGAAGGGCAATTAAAACTCGATCGAGCAGACGAATTTCCCTTAATACGTTCTAATATTCA
AAACACATGGATAAGGCATATGAAAAGAATTTGCGAACGTATAACTTACGCACACGCCCTCGTTCTTTTG
AAGTCGGTCAAATAGTAATAAAACGCAACTTCGTTTTAAGCAATTTGGCTAAGCATTTCAACGCAAAACT
TGCACCAGTTGGAACTAAGGCACGTATCAAACGAAAATTGGTAATTGTATATATTTGTTAGAAGACCTCA
AGGGAAAAGAACTTGGTCATTACCATGCTAAAGACATTTGGGAATGTAAGTAATCACCCCTTTTTCAGCTT
TTATCTCAGTATTAGTTAATCCTTTCGATTAAACCTGCGTTGTGATGGGGTGATGAGTTTTATTTCTGTAAA
CATAAACATACATTGCCATTGGCATGATCAGCTGATCGCAATCAACTTCAATGGGCAGCACTGCACCATA
ACATTTATGCAATTACATATGATCAGCGGAGGTTAATAACATAACCAGAGTGACCATGACCTATGTTAAT
GACGCCGTTGCGAATGAAAATATCGTGTCTTGGGTGAATGCTTAATTGCCTCTTTTAGTTTTGGCGNGGCG
TCATTCTTTTCCATTTTAATCGCCAACGAGTACAGTCGGTTTAGATTGCTTTAAAAATTATAAAATTCAATT
TTTCACGTGGGCGTAATAAACCCACGTGTATCGGCACTCAAGCTCGGAAAAAAAAAAAAAATAANTAAATA
AAAGGGAAGGAAAAAGGTCCAAGGTCCCCGGAAAGAAAAGGGTAATATAGACATCATGCGCTCCCGAGA
GCTTGGCTATAACATTTCACCGGAATAAAGAAAATTTTCAGAATTCGGACACTGACAAATCTTCACTAAG
GGATCGTTACTGGAGTCGTGGGGTGCCTTTCCTGTGTGTGGTAGTCGTAGGAAGGTGCAGGCTAAAGCGC
GGGGCTGCGTTATTGCGTGCCAAAAGAAAAATTACGCATAACGCGTAGTCCTTATCGGAGCCAAAGCCAA
GGCCCCAAAGCACCAAAAAGCGACAATCTACACTGGTAGCAGGGAGCGAATTTCCTTAAGGGCGGACCA
AGATTTCAGCATCGTCTCATTTTCTGCAGATAAGCTTTGAACCTCGCTAACCAATAGTTCGTGCTGCACGC
ACAGGCAGACCAACGTGACCACGTGTTCGACCCATTGATTCCCGGACATTCTTGAGCGCAGATCTGGTGG
ATAGCTAACCAGTGCGCACCCTCACTCGGATTTACAACATCTGATATAATTGTTAAACGGCATACTGGCCG
TAGGTATAAGCGAAGGGCATAAGTACACATGAAATTTAAATTGCAATTCTTTCCGTTTTATATCGCTGACA
TTGTCTTTCGATCCTGATTTAGCATGTATATGTCGCTGACTACTATATTTCTACGTTACACAGTTCAAATTA
AGAGCCCACAGCACATCATCGTCGTCAACAGGCCTGCTGACATCGTTGGATCATACTAAAGTAGTGAGTA
CTGTGTGAAAGAACAAAAGATAATAAGCCAACATATCGAATTGTACGTATTGTTTGAATCGCGGTTATAG
TCAGCGGGTGTACTCCTAATCAGCCTTCGTAATTACAAATAACTTTTGTGGATCATTCAGATCAGCAGTGA
TTTAACTTCATATTAGTTTCGAAGTGTAATCTCATTCATGTGTGTTTTTGCCCAGATTGAGTTTAGTTTTAT
AAAAAAAAAAAAGAAAAACGTTGTTCTTCTCACGTAATGTATAATGAAGTACGATCATGAGTTTAAATCT
TCTTTGAATACACTTATATGATAAAATGAAATCTTAATTCCTTTTTGGGCACCTTAGGTAAATAGTATCGC
ATACATAAGGGTAGTTGCAACGACAATCGCAGATGCAGCTACAGGCAGGGTGGGTGAAGACCGAGGGTA
GCCAACATCTTAGCTCCTCGGCTCTATATGATATATTCTGCTTGCAGATCGTAGTCGCTCCTTATATATCGT
GGTATTCCGTCCTACCTTAACTCTGTCTACCTTTTTTTTCTTTATTATACTTGACGCGTCGAGTGTGTTAGGG
AGCAAAAGAACCCACGCCACTTCCCTGAGCACGGTCGGAACCCGTAGACGGAGTACGCTCCCAATGCACA
ACACGAGTGTTAGGCAAGACGCGCCCAGCGTAGCCGTCGTGTTCGTTACA
>9_rnd-1_family-230__LTR___Gypsy
GCTGCAGAGGCAGACTTCTAATGGCTACGGAAGCGGCACGGCGGGAACGTGCAAGAGAAAGGACGGAGT
AGGAGCGATGACCAGAATCCACACGGACATAGAAGGCCAGGAGCGATCGCCGGAATCGAGCGGCCATGG
AGTTCTGTCTGGAGTCGAAAGTGGTNCCAGGATGGGGCGANAACGGCATCGAAGAGGCGGANTGGCCAG
CGGGTCTGGACCCAGAACTGAGGGAGTTCCTGGAGTCAGAGCTGGCTCTCTTCGAGGGGCTGCAGGGAAT
CTCGCACATCGCGGAGCACCGAATCCGGCTGAGGGATGACAAGCCGTTGAAACAAAGGTACCACCCCGA
GAATCCGGCCATGCAGAGGGTGATCGACGAGCAAGTGGATGAGTTGATCCGGGCAAATGCCATCGAGCC
GTCGCGTAGTCCGCACAGCGCCCCGATCGTCTTGGTCAAGAAGAAGACTGGCGAGTGGCGTATGTGCATT
GACTACCGACAACTCAACGCGCATTCCATTCCGGACGCTTATCCGGTGCCTCGTATTAACCACATCCTGGA
GCGGTTGCGCCACGCCCGGTTCATCTCCACTCTGGATTTGAAGCACGGGTACTGGCAGATACCGATGGCA
GCCGATAGCCGGGAGTGTACGGCGTTCACAGTCCCTGGAAGAGGTCTTTCCAATGGCGGGTGATGCCATT
CGGCTTACACTCGGCGTGCGCGACATTCCAGCGCGCTTTGGATACGGTAATAGGGCCGCAGATGGAGCCT
CATGCGTTCGCTTACCTAGACGACATCGTGGTAATAGGAGCGACGAAAACACAGCATGTAGAGAATTTGA
GGGAGGTATTCCGCCGGCTAAGGGCGGCCAATCTCCGGTTAAATAGGAAAAATGCAGCTTCTTCCGGGAG
AGGTTAGTCTACCTAGGACATGTAATTTGCGGAGAAGGGATACGGACCGATCCGGAGAAAGTGGAGGCG

ATACGAAACCTGAGGGCCCCAACAAATTGCAAAGAACTGCGACAGTGTCTCGGGATGGCGTCATGGTACA
GGCGTTTCGTACCCAATTTCGCGACCCAGGTACAGCCACTCACAGCGCTCTTAAAGAAGGGGCAGAAATG
GGCATGGGAGCAAGAACAGGAGGAGGCATTGCGTCAATTGAAGGAGAGCTTGACGACTGCCCCTGTGTT
GGCTTGCCCCGACTTTTCCGCCAAGTTCGTCTTGCAGACTGACGCGAGTGAGTATGGCCTCGGTGCAGTGC
TGACGCAGACGATCGATGGACAAGAGCGGGTGATAGCATACGCGAGCCGAAAGTTGCTGAAGGCCGAGG
TAAACTATTCCGCCACTGAGAAAGAGTGCCTGGCAATCGTGTGGTCGATCCGGAAGTTGCGTTGCTACCT
AGAAGGGTACCGATTCGACGTAATAACGGACCATCTAGCGCTCAAGTGGCTGAATTCGATCGAGAGCCCG
TCAGGAAGGATTGCGCGATGGGCATTGGAACTGCAGCAATTCCAATTTGACGTTCATTATCGTCGAGGGA
AGCTCAACGTCGTGGCCGATGCCCTGTCGAGGCATCCTCTAGAGACCTGTCAACAAGCAGTGGTGGCCGA
GGCCCCTGCAAATGGGTCGCGAGGATGCGTGTTAGAATCGCCGAAGAGCCAGCCAAGTTCCCGGATTACA
CCGAGGAAAACGGACAGCTATATAGGCATTTGGGTCACCGGATAGACGATGAGGACTACATCCCTTGGAA
ACTATGCGTAGGCAGTGGTCAACGCCAGAGGGTACTGCGGGAATGTCGCGATGCTCCCACGGCCGGACAT
CAAGGTGTCAGAAAAACGGTGGCGAGGTTAGCCCAGCGGTACTACTGGCCGGGTATGTTCCGCGATGCCG
CCAGATACGTGAAATGTTGCGAAGACTGCCAAAAATTCAAGAGTGAGCAACTCAAACCGGCTGGTCGCAT
GCTGACGAGGCAGGTGGCAGAGCCGATGGCGGTACTTTGCGCGGACTTCGTCGGACCCCTTCCACGGTCG
AAACATGGAAACACGATGCTATTGGTGTTCCATGACGCTTTCTCGAAATGGGTGGAGTTGGTCCCTCTCAG
AAGAGCCACGGCAGCACACCTGCAAACTGCGTTTCGAGAGAGGATTCTGAGTAGGTTCGGGGTACCTAGA
AAGTTTGTGTGCGACAACGGGGTACAATTTACGAGTCGGAGCTTCAAGTCATTTTTGGGGTCCCTGGGTGT
AGAGATCCAGTATACCGCCCCGTACTGTCCGCAAGAGAATCCCACTGAGAGGACTAACCGGACTGTAAAG
ACCATGATAGCGCAATTTATTGAGGGCCACCAGAGTTCATGGGATGAACTCTTGCCCGAGATCTCGCTGG
CGGTCAACACGAGCGTGGCAGATTCAACGGGCTTTAGTCCCGCTTTCCTGATGCAGGGCAGGGAACCTCG
GTTACCCGCAGCTCTGTACGATCAGGTAACTCCGGGGTCCGCAACTAGTGCGCTCGACCCTAGGACAAAG
GCGGATAGGCTAAAAAGAAATTTTTGATATCGTCCACAGCAACCTCCAGCGCGCATCAAAGGACCAGGGC
AGGCATTACAACTTGCGGAGACGAGAGTGGCGGCCCTCGTTGGACTCGATGGTCCTGTTGAGGCAGCACC
AGTTGTCCAACGCTGCCGAAGGTTTCGCCGCAAAGTTGGCACCCAAATTTGACGGCCCCTACAAAGTCGT
CAAATTCATATCCCCCAATATTGTGCGGCTGGTGAAATCAGACGACCGCAAGAAGAGGGTGGCCAACATC
TCACAATTGAAACCATTCTACAGAAAGACGACGAGCGACGAAGAAGACGAAGATCCAGGGCACGCAAAT
GCCCACGAGTAAGCGAAGGATGAGAGCAACACCCGTCGCCCAACGAAACTATTATGATTAAGCTTAGATA
AAACTAACATTAACAACATAATTCAGAATAGGCATAGTAAAGTGTGAGCCGCACTGAGAGAATAGGGCA
TTAACAAATAAGTAAAGAGTAAGTTAACCAATAAGCTGAACTTTCAGATGGAACCCGAAGTTATCGACAT
ATCGTCAGACGAAGACAACAGCAGCGGGGACACCGAGCCTTTCCCCGGGCCGGGAGCCGCAGGCCGTCC
GCGCGACCCACGCCAGGGGCGTGAGTACTATCGAAGAGAACGGCAAGAAGGACCGCCTCGCCAGTTCCG
CCATACACTACTCCCCCGGCGGACCGGCATAANGGAGAGGCTAGACGGGATGGTCTTGCTGATGGCAGTC
GAGGTCACGACAATGTTCGTGGAGGCACCGTTCGCCCGGAACTCGCCACCGTACGCGGAAGAGCCCGCAT
CTCCGCCCCTCGCATCCCACTCGGCACAATCATCAGAGCCGGCAACCCCAACCGCGGCCGGCGAGATGGA
CGAGGAAGAGTCCGCACCGTTCGGAACGGAGGAGCACACACCACCAGCGCAGGACGTGGAGGCGGTAGG
AGAAGAGATATCCCCGTCGGCCGGAGACGCGGAGTCGCCACCGACGGAGGTGCCGAACGGCATCCGCGC
AAACGACGGGGAAGCAGACCCGCTGAGCCTCGACCCAGAACCCTTAGTGGGAGGGGACCCGTCAACGGA
GGACGAGCCGGAATTCCGTCGCCGCTTTGCCACCCACCGTCTGGAGGTAGACCCGGGCAGCCAGTTTCCC
AGGATAACGCTGCGGTACCTACCAAAAGTCCCCGCGGTAGCCAACAAACCCCGTCTGAACCCCGCAAAGC
GCGAATGGGGGTGCCGAAGAAGGCCCCGGCGGAAAGCGCCGACGCAGCCGCCATCGTCGACGGAGAGCC
GCCAGGGCGAGCGTCGCCGGAGTGGAGGCCATCGCCGACCCAGTCCCGAGGGAGCATCCTCGTCGGCCA
CGAAGAGGCTTCGCGCGGAGACCGAGAAGCCCGAAGGATCGCAGCGGTGCGTGCCGCGTTCGCCCCGCG
AGCGCCAGGAGGGTCCTCCCCAGCTCGGGAAGGATAACCGCCGCTGCNAGATCGCTTCTCGAGGAGGGC
ATCCCGAACCCTGGCCGATACCGATTGCGGTCTGCCAGATGGTCGTGGAGGAGCACAATCTCAGCACCAA
GGACGCGGCGAACTCGGCCGTTTCCCCAACCCGCGCCGAGGCGCCAGACGAAACCTTTCCTAAGGAATCG
GAGGAGGAGTGGCTCGACGACGAGGACGAGATGCCCGCGCTCGTGCCTATTGCCCGCGCCGAGACACCC
GACACCATCGCCCGGCGGGAGTTAGAAGACGCCTGGTTCGGCCAAGAAGGCAAGGCGCCATCCCGAAAA
GGTTCCTTCACGGTTCGCCAGGAAGGCCGGGATGAAGTCTGGTCCGAGGAAGAGGACGCGGTGGTGACG
GCTTCGTCACCCAAGCTTTCGAGTCCAACCCAGGATTCCTCCCCATGGCTCTAAAGACTCCCACGGAGAGC
GAGTTGTGGGACGACATAGTGGACCTTTTCGACGGCATCGACGGGTGGGACACTAGCCTGGACTCAGTGC
TGGAGGAGGAACCCGACACCGAGGTCGACACGGTCGCCCGCAGTGCCGACGTGAGGGTCACACCGGAGA
GCCACCCCAAGAAACCCGACACCGAGGTCGAGGCGATCGCCCACAGTGCTGACGTTGGGGTAAAACCGG
AGGGCCCCGTCGAGGAACCCGTGCCCGATTCCACCCCCTCAGGCTGGAAGGTGTTTCCTCTTGGAACACC

GTTGTCGTCGGGCGTGGTGGACCTAATCGCCAGAGAGGCGCGAATCCGCCGCTTCCGGTGCCGACGTACT
CGGTTCCACGTGTCCGACGGGGAGAGCGAGTACAGCGTTACGCTGAACGCGAAAGGCGACGTGACCGTC
ACCTGTCCGAAGTGGGGGGGGTGTGAGGCGGCTAATGAGACCAGCCACCCCACAGTTTTCACGGCAAGG
AGCCGTCATCCCTTTGGCCAAATTACGGCAAGGAGCCGCTGGCGGGACAACGCATTAATTTTCTTTAGTTA
TAAGTATTAATTATATTAGGTTAAGATCTTAGTGAAATAAGAGGCAAGGAGCCATCAGTAATATAAGCAG
TTCGATTAGTAGCAAAGAGGCAAGGAGCCGCGAATATTATAATTGATTTAAATAGTGAGTTTGAATTTAA
CCGCCGGCCGCCGCTGCAACCGTGCGTTGTCGATCGGTTGCTCAACACCGTCTTTGAGAGCGCAACTTCAA
TTCGGCGGCGAAGAGCGAAAGCTCCGAGGCGAAGTTGCGAAAGCTCGCGGCGAAGTTGAGCAGCCGAAG
CGAGCGCATGGCTTGCCGGCGAGCGTGCCGGCACTTGTGATCGAACGTTAAGGGTGAGTAGAAGTGCTGC
GCGAGATAAACGAAATCATAGATGGTATAGTGGAGCGCTAGGGCTCCAGAAAACAGTGCGGCAGGGTTT
AGCTTAGGATTTACGATAAGGTCGGAATGAATAATAGTGAATAAAGTGCCAACGTTGTAATTATCTAGAA
AGTATTGGATTATTCCCTGTTTCCTGGTAACCCGGCTTACCTTAACATCGGAACTAACCGGTGTTCGACCT
AACCTCGAACGAGGGGCATGCGGTACGAGATTGTGGCTCCGGGGTGGGCATTGCCGGTTGGTGTTTAGGT
AGTGGAGTTGGTGCGAGGCCACGGCCCGCTAGTGTAGTTGGTGCGAGGCCACGGCCCGCTAGCATAGTGG
TACGAGGCCACGGCCCGGCAGAAAGATAGAGCGNTTAGGTAGTGGAGTTGGTGCGAGGCCACGGCCCGC
TAGTGTAGTTGGTGCGAGGCCACGGCCCGCTAGCATAGTGGTACGAGGCCACGGCCCGGCAGAGAGATA
GGGTGTTTAGGTAGTGGAGTTGGTGCGAGGCCACGGCCCGCTAGTGTGGTTGGAGCGAGGCCACGGCCCG
CTAGCATAGATGGTACGAGGCCACGGCCCGATGGTGTAGGTGTGTAGAGGTAGATCTGGGTTTGGTTTGG
TCAGGATACTCACCCCCTTAAGCCCTCTCTCCCAGCGAAATTATAAATAACTTGTAACTCGCTAGACCTGG
TAAGACTGAGAATTATACCCCGGCCAGAAGCTTCCTTACAAGTGGCGCCCGAGCAGGGACCAGAGGCGTT
TAGGGGTGTGGCATGTCGGCGGATTAAAGCCCGCAAGCATGCCCGAATAGGTATTTTCCAAAAATATATA
TAGGGTATTTCTAGTCGGGTATACCCTAGAGGGAAGGTAACCTAGCAGAGAGCAAGAAAATGAGAAGGT
CATGTATGTGCGAAATATGAAAATAGGGTATTAAGTAGTCCGGTATACCCCAGAGAACATTCCTATACCT
TGTGTTAGGCATAGCCAGAAAATGAACACGTGCAGCTGGTGAGTGACAAAAATGCTAGCAGCAAGCCGG
CAAGTAAGATAAAGAGACGAAAACATAACGGTCGGTCGTGAGAGTACAGCGCGCGCGCGCCAGCCGGGC
GACGTCGAGACCACGCTCGCACACGGGTGCCCACACGCGCGGCGCAGTCGACGTCGGCGTCGAAGGACC
GTTAAGGCGTCGCGGCAGCGGTTGTACAGTGGGGCGAATCCAACTCACGCGCGGTGAAAAGTTGAATTCG
CGAGTACAGCTGTCGGGGCAGTGAAGTGGTCACCCTTCAAGTGAATGTGAACAAAGGGAACAATAAGAG
CGGAACAAGTGCCAAATAAAAAAATAAGTGGATAATAATAAAATTTTTTTTTTTATAAATAAAATTAATT
AAACAAAAAAAAATAAAAAAATAAGTGGATAATAATAAAAATTTTTCTTTTATAAATAAAATTAATTAAA
CAAAAAAATAAATAAAAAAATAAGTGAATAATAATAAAAATTTTCTTTTAANAAANAAAATTAATTAAAC
AAAAAAATAAATAAAAAAATGAGTGGATAATAATAAAAATTAAAAATAAACCGAGGTTCACAATAAAGA
TAAAGGCAGCGACCACATAAGGAACAGCCTAAAAAGCGGAAAAAGTGAGAAAAGAAAATTTTTGAAATA
CCTTTTGTTGAGTTTTTTTAAAAAAGGGGGTAAATACTTTTGTGGAATATTGTTTTGTATTTTTGTTTGGTG
AAAGATATAAAGACAAAAAAAAAAGAAAAAANTAAAGAAGAAAAACTAAAAAGGAACCAAAGTGGCTGA
GCAAGATCGGCGTAAAAGTGAAAAAGACACCAACACCAAGAAAAGGAGAGAGGAATAGTAAAGGGACC
GACGGAACCCCTAGAACGGGAAGAGAGGGAAACAGCGACGTCGACTAGCAAGGAAGTATACAATAAAG
GATGGAGGCAGGTGAGTGCCAAAACCCGGGGTAGGCGTCCGCTGGATTCACCAGCGACGCAAAGAGGAG
TTAGTGGCGATCGCCCAGGAGTTTGGGCTGGAAAAGGAGGGGCGCGTGGAGGAGCTACGTAAGCGGCTG
TCCACCTACGTGCAGGAGGGCGGTCATGGTCGGCTCGCCGAGCTGGAATCCCAGTATGCTAAAACTCTTG
AGCCGCCATCTTGGGGCGTCCCACCCGACTTTGGTGGACGGCCACGGCAAAAGTCGGAACCTGACCTCAG
AAGCTCCCAGCTGTACGGGCTTTTGCAGGCGCCCACCCCGAGTTTGACCGTCTCGGGCGTGGAGGCAGTT
CCTGCTCTCGGTGGAAATGAAGACCGCCGAGCAACTGCGGTCAACGCAGAGGTGGGGAGGTCCCCTGTTG
AACCGGGTGGTGGTCCCTGCTACACTAGGCCGCACCATCCATCGGAGGCGAGGCCACTTCCCAATGTCCC
GGAGCAAGCTCGGAAGTGGGGTATGAGGTACGATGGACGCTCCGACCCCCTTGGGTTCTTAGAGGACCTC
GAGGAGCGCGCTATCACCTATGGTATAGCCCTCGACCGTATGCCCAGGGCAATGAGCGAGGTATTCTGCG
ATAGGGCTGCTCGTTGGTTTCTGAGCAGTGGGCTAAGGGATGTGCCGTGGGCCGTGTTTAGGAGGGATTT
CTTGGAGTTTTTCTTGCCCCCTCGTTACTTCGAGAGGCTTGAGGACGAGATCCGATCCCGGAGGCAGCGCG
TAGGTGAAGCGTTCAAGGAATATTTGATAGAGCTGAGGGCTCTTATGCACCACGCTAGATACGAGCCGGC
ACAAGAGCTGCATCGGATCTACGAGAACGCCGCTCCGGAATACAGGCTCTATGTGCGACGGCAAGACTTT
TCTACCCTATCCCAGCTAACTCAACTGGCCGTAGAATACGAGACCGTCGTGAGGCAGAGGGCGGAGTACT
CAGGCAGCCAACCCTTGGGTCAGGGTGTCTCTAGTTCAAATAGTGCAGAGTGGGCGAGGACGGCGCAGTC
CTCGGGACATCTCAATCCGTTCCGGTCTGCTCCCAGCGGATCCGGGACGACCGTTGTGGACAACAGGGTC
TCCACCCCACCCGCTGCAACTCACGCAGAGGTGGCCCATCAGATGATGAGCCAAGAGGCCGGGAACGGA

TATGATCCGCGACGGGCGTGTCGTAGGTGCGCACAGGAGGGGCATTTTGCCAGGGAATGTCCCAACGCCC
CTGTCTTATTTTGTTGGGTATGTGGAAGCCGGGGCCGAAGTAGTCGGGAGTGTTGCCGCCGCGCGGAGAC
GGGAAACGGGCAACGGCGTCGTCCGGACCAGGATCGGGTCGGCGCCAATACACACGGAACGCAGCAATA
GGACACCCACTGAAGGTGGACGGTGGCCGGATCGTCGCTACGGTAGAGATTGGCGGACGAACGATGCCC
GCCACGATCGATACCGGAGCCACTCGTAGCTTCATGAGTGAGGACTGCGTACGCAAATGGACGATCCGGG
GAAGAGCGGAGGAAATACAGGCACGTATCCGCCTGGCAGATGGGTCCACCCTAGAGGTAGAAAAGGCTT
CGAGAGTAGACGCCAGCTTAGGGGGAAAAACGATCAGCATGACGATGCTCATCATGCCCCTCAATGCTGG
ACCATGTAATCCTGGGTATGGATTTTTTGGGGGCGATGAATACCACAATACGCTGCGGCGAGGCGGAACT
GGTCCTAGAAGCAGCGACGACACCGGGCGTGAGGCCAGGAACGAGCGAGGAGTCGGAGGAAGGCCGCC
CAAGGCATGAAAAGGGAGCACCTGCTCGGCTACAGAGGGAGCCGGAGGAAGGCCTCCCAAGGCATGGAA
AGGGAGCACCTGCTCGGCTACAGAGGGAGCCGGAGGAAGGCCTCCCAAGTCATGATGGGGGGCACCTGC
TCGGCTACAGAGGGAGCCGGAGGNAGNCCTCCCAAGTCATGAGAAGGGAGCACCTGCTCGGC
>Ref_dvir_611
AATCCGGGCAGGGAGCTTGATTTGCCTCGTAGGTGGCTCTTTGCAGGCCTTAGCAAAACTAATGTTTGCAT
TCACTTTGCTGCAGTTTAGTGGTAGTCCATCTCTTGGAGCTGTCTGTCTGTTTGCCGTTAAGAGGCTCTTTG
CCTTCTTATAAGCTGGGCATCCTTTGTATGAGGCCACATGGGGACCCTGGCAGTGCAGGCAGAAGGCCGG
GTCACTGCTCTTCTTTGAGCATTCCGACGTGGAGTGTCCGCCTCCACATTTCACACATCTATGTTCCAGGTG
ACAGTATCTTTGAGTGTGTCCGTATCCTTGGCAGCGATAGCATTGGGGGACATCGTTAAACTTCAGTGGAG
GCTCGACTGTAACCACAGAGCAGCAAAGCCTTTTGATTTTGTATGCGTCTTTGTTATTTTTCGCCGGCTCGA
GATTTACGAAGAATATGTTTAGGGGTTTTTTTGTGCTTTTTTGTGTGAGGTTATAAAGGTCTCTTACTTCGT
GCCCGTAGTTGCTTAAACCTTCCTTGATCTCGACCAGCTCAGTAGAGTGGTGCAGGCCTTTGACCACTATC
CGGTAGGCTCTTTCGTCTCTGGGCTGAAAGGTCTGATACCGTTTGTTGTTTTCATTGAGAAATGCCTTAAGT
GCAGTGAAGCTAGCTTTATCTGGCGTCATTACTCTAATGTTGTTGTTTTTAGTGGCTTTATATGTAAATTCT
ACTGCTTTGCCAAGGGCGCCAGTTATGGCATTGGTAAGCGCTTTGATATTGGTCACGTCTGGAATAAAAAT
TGGTGGTGGCCTGATCGTCTTAGGTTCTTTAAGCAGGCTCCTTATTTGGTTGGCCCTTAGCTAGTCCTATGA
CTTCAGTGGAGTCATCGCTGGCCGAAGATTCTTCGTCGTCTTCATCTGTAGAGAGTGAAGCAAATCTGTTA
CTTGTTAGATTTTCTCTAGCAGCTTTGCTGGTGCTTGGTCCGTCAGGTAGGTGTACGCGGGAAGGCTGTTT
GCGCTGTTTGCGAACGGGCTTCCTCCTTATTTCTCCAGGTTCACTGGTGCTTGGTCCGTCAGGTAGGTGTAC
GCGGGAAGGCTGTTTGCGCTGTTTGCGGACGGGCTTCCTCCTTATTTCTCCAGGTTCACTAGTGCAACTCG
AATTCTCAGAGTCAGGGTTGCTAGTTCTGATGTTGGTCTTCTTCCTCTTGGTGCTGCTAGTTTTGATGAGCT
GTGTAGCACGTGGCTCGGCTTGCCAGTCCATCTTGTCCATCTGGTTGGTGAGGGGTACGGGGGAGCGGGA
GCAAACAAGCTGCCCGTCTGGCGAGCGTAAGCAGCGAGTGCTAGTCTGTGTTATTGTTTGTTTTTGTATTC
TAATTGTCGTGTGTGTGTTGAAATAACGCGCACAAGCATGTAAGCGTGTGCGTGTGTAAGTATTAGTGCGC
CGTTATTTGCGCACGGCACGCAAGCTGAAAGAGCGCAGTAACGGTTGCAGCGACCAATCAGAGCGCAGCT
GGCCTGAAAATTTCGCCGCAACTAACTGTTGTTTATGTTGCTTGTGTATTTGGAATGCTTCGCAGCGGTCC
GCAGGCACGTCTTTACTCAACGAGTGTTCGATCGCGACTAAAAAACCTATTCATGGTGAGCAGTGTGTGCT
CATCACATTATACGCTGAAAGCGTATAATTTTAAAAGTTTGTCATATACTCGTTATTTGAGTTAGATATCG
TTATTTGTTATGTTGTATAACATTTTGGCTTACGATATTAAGAAAAAAAGATAATTGTGAAACTTTCAAGC
CAAGATTGTGTTGTTAGTAATAATTTTATTGTTACTACATCGTGTAAACAAATGGGTTTCCTTTTATCGTAA
AATTTAACAATTGCATCCAGTGGAAATTCGATTCATACAGATGTATGGACGATTAGTACGCTGGAAAAAA
AGTTTCTAAAATTTTTGTAATTATTTTAAATATTTGTCATAAATGAAACTTTAACATTTTACATACTATTTA
ATATAGTATGTGACTTGTTTACTTAATATATTTTTATCGCATAATTATGTTTGCAAGTTATGCATACATAA
TTTATACATATAAATAAATTATCAAGTTTGTCATCAAAATTAAAGTGTTTATTCCGTTATGGTATTAAGCA
ATGTGTGATAAAATATATATTAAGTAAGTGTACATATAAAGATATTTTTGAACGAATTGTATATATTTTCA
TATAGATATAATATGTGGTGAATCTTAAAAAATGTGAAACGTAAATTAACGAACATGAAATGCTATGTAA
CAATGGCCATATTCGTTTATATATAACATTAATATCTATAGTATATTTTTTATTCAAAAATATATTAAGTTG
TAATGTAAATACATTTTGTAAATATGTATGTATATTTTGTACATACAAATTATAAAAACATTATTCTGGTTG
ATCCTGCCAGTAGTTATATGCTTGTCTCAAAGATTAAGCCATGCATGTCTAAGTACACACGAATTAACAGT
GAAACCGCAAAAGGCTCATTATATCAGTTATGGTTCCTTTAGATCGTTAACAGTTACTTGGATAACTGTG
GTAATTCTAGAGCTAATACATGCAATTTAAAACACGGACCTTTTGGAACGTGTGCTTTTATTAGGCTAAAA
CCAAGCGATCGCAAGATCGTTTTATTGGTTGAACTCTAGATAACATGCAGATCGTATGGTCTTGTACCGAC
GACAGATCTTTCAAATGTCTGCCCTATCAACTTTTGATGGTAGTATCTAGGACTACCATGGTTGCAACGGG
TAACGGGGAATCAGGGTTCGATTCCGGAGAGGGAGCCTGAGAAACGGCTACCACATCTAAGGAAGGCAG
CAGGCGCGTAAATTACCCACTCCCAGCTCGGGGAGGTAGTGACGAAAAATAACAATACAGGACTCATATC

CGAGGCCCTGTAATTGGAATGAGTACACTTTAAATCCTTTAACAAAGGACCTATTGGGAGGGCAAGTCTG
GTGCCAGCAGCCGCGGTAATTCCAGCTCCAATAGCGTATATTAAAGTTGTTGCGGGTTAAAAACGTTCGT
AGTTGAACTTGTGCTTCATACGGGGTAGTACAACTTACAATTGTAGTTAGTACTATACCTTTATGTATGTA
AGCGTATTACCGGTGGAGTTTCTTATATTTAATTAAATACTTGTATTTTTTTATATATTCCTCCTATTTAAA
AACCTGCATTAGTGCTCTTCATCGAGTGTTATTGTGGGCCGGTACAATTACTTTGAACAAATTAGAGTGCT
TAAAGCAGGCTTCAAATGCCTGAATATTCTGTGCATGGGATTAATGAAAATAAGACCTCTGTTTTCTGCTT
TCATTGGTTTTCAGATCAAGAGGTAATGATTAATAGAAGCAGTTTGGGGGCATTAGTATTACGACGCGAG
AGGTGAAATTCTTGGACCGTCGTAAGACTAACTTAAGCGAAAGCATTTGCCAAAGATGTTTTCATTAATCA
AGAACGAAAGTTAGAGGTTCGAAGGCGATCAGATACCGCCCTAGTTCTAACCATAAACGATGCCAGCTAG
CAATTGGGTGTAGCTACTTTTATGGCTCTCTCAGTCGCTTTCCCGGGAAACCAAAGCTTTTGGGCTCCGGG
GGAAGTATGGTTGCAAAGCTGAAACTTAAAGGAATTGACCGGAAGGGCACCACCAGGAGTGGAGCCTGC
GGCTTAATTTGACTCAACACGGGAAAACTTACCAGGTCCGAACATAAGTGTGTAAGACAGATTGATAGCT
CTTTCTCGAATCTATGGGTGGTGGTGCATGGCCGTTCTTAGTTCGTGGAGTGATTTTGTCTGGTTAATTCCG
ATAACGAACGAGACTCAAATATATTAAATAGATATCTTCAGGATTATGGTGTTGAAGCTTATATAGCCTTC
ATTCATAGTGGCAGTAAAATGTTTATTGTGTTTGAATGTGTTTATATAAGTGGAGCCGTACCTGTTGGTTT
GTCCCATTATAAGGACACTAGCTTCTTAAAATGGACAAATTGCGTCTAGCAATAATGAGATTGAGCAATA
ACAGGTCTGTGATGCCCTTAGATGTCCTGGGCTGCACGCGCGCTACAATGAAAGTATCAACGTGTATTTCC
TAGGACCGAGAGGTCCGGGTAAACCGCTGAACCACTTTCATGCTTGGGATTGTGAACTGAAACTGTTCAC
ATGAACTTGGAATTCCCAGTAAGTGTGAGTCATTAACTCGCATTGATTACGTCCCTGCCCTTTGTACACAC
CGCCCGTCGCTACTACCGATTGAAATTATTTAAGTGAGGTTCTCCGGACGTGATCACTGTGACGCCTTGTG
TGTTGCGGTTGTTTCGCAAAAGTTGACCGAACTTGATTATTTAGAGGAAGTAAAAGTCGTAACAAGGTTTC
CGTAGGTGAACCTGCGGAAGGATCATTATTGTATAATGTTCATTTCCGTTAATAAAAACAATTTTATATAT
AGAAATATATAAAATTTTTCTGAATATAAATGAATAATCATTTAAAAACATATACACATACTTATTCCCAA
TTAATATATATTTATATAATATCTGTCATTTTCAATTTGGGATTTGTCTGTGTTTATGCGTTTTAATGATGA
AAAACTTGCGTGTATATGTACCATAATATACATGCGTTGCACAGATGTATTGTTCATTATTATATGCGCAT
ACATTATATAAACGCAACAACCTAAATTTTATGTGTTGTACCTGTTTTCAGGTTAATGCTTTGCATATTTCT
ATAAAAATATTTAATTCTTCTATCTATATAAAAATTTTATTCATACCATATTTTTAAAGTATATGTAAAACT
AAGACATTTCGCAACACAATTTAGGTATAAACAATTTTATTGAAGGAATTGATATATGCCAGTAAAATGG
TGTATTTTTAATTTCTTTCAATAAAAAAACATTTGTGACGTAATGAAATAAACCAATATAAATTATCACTCT
AAGCGGTGGATCACTCGGCTCATGGGTCGATGAAGAACGCAGCAAACTGTGCGTCATCGTGTGAACTGCA
GGACACATGAACATCGACATTTTGAACGCATATCGCAGTCCATGCTGTTTTGTACTTTAATTAATTTTATA
GTGCTGCTTGGACTACATATGGTTGAAGGGTTGTAAGACTTTGCTAACTAAGTTGTTTAAAATTTTTCGAA
GTTTTAAGCATATGGTATATTATTGGATAGATTATGTGTGTCTCTGATATGCATAAATTATTCCATAATATT
AATATATATATGGATGTACTACTCATTGTATATTTTGTATGAGAAAACTGAAGAATTATATTTTCTTTTTTC
AAATCAAATAATACTGAGGAATGTCTAGCATAAAAGAAAAATATATTTTTCCTTAATCTAGAATTGCCTCT
TATTAATGATGTGTGATAAAAGAATTGTTGTAAAATTTATGGAACTTCAGAACAAAAGTATTATCCATTAT
ATTGAACAATAAATAATGTTCAATTAACATTGTTTTATACAACCTCAACTCATATGGGACTACCCCCTGAA
TTTAAGCATATTAATTAGGGGAGGAAAAGAAACTAACAAGGATTTTCTTTAGTAGCGGCGAGCGAAAAGA
AATCAGTTCAGCACTAAGTCACTTTGTCTATATGGCAAATGTGAGATGCAGTGTACGGAACATCAACATTC
TAGTATGAGAAGTTAACGATTTAAGTCCTTCTTAAATGAGGCCATTTACCCATAGAGGGTGCCAGGCCCGT
ATAACGTTAATGATTACTAGATGGTGTTTCCAAAGAGTCGTGTTGCTTGATAGTGCAGCACTAAGTGGGGT
GGTAAACTCCATCTAAAACTAAATATAACCATGAGACCGATAGTAAACAAGTACCGTGAGGGAAAGTTG
AAAAGAACTCTGAATAGAGAGTTAAATAGTACGTGAAACTGCTTAGAGGTTAAGCCCGATGAACCTGAAT
ATCCGTTATGGAAAATTCATCATTAGAATTGTAATATTTTAAACAATATTATAATAATAGTGTGCATTTTTT
CCATATAAGGACATTGTAATCTATTAGCATAAATATTTATCATAAAATTATGTCTGATTGCTTATTTGAATT
ATATGCTTGGCATTTTAACATAAAATAAAAGATATAAATTTGATAAAGTGCTGATAGATTTATATGATTAC
AGTGCGTTAATTTTTCTGGGAATTATATAATGGCATAATTATCATTGATATTTGTGTTTATTATATGCATTT
GTATGATTAACAATGCGAAAGATTCAGGATACCTTCGGGACCCGTCTTGAAACACGGACCAAGGAGTCTA
ACATATGTGCAAGTTATTGGGATATAAACCTAATAGCGTAATTAACTTGACTATTATAATGGGATTAGTTT
TTTAACTATTTATAGTTAATTAACGCAATCCCGGGGGCGCTCTATATAGTTATGTATAATAATATTTATATT
ATTTATGCCTCTAACTGGAACGTACCTTGAGCATATATGCTGTGACCCGAAAGATGGTGAACTATACTTGA
TCAGGTTGAAGTCAGGGGAAACCCTGATGGAAGACCGAAACAGTTCTGACGTGCAAATCGATTGTCAGAA
TTGAGTATAGGGGCGAAAGACCAATCGAACCATCTAGTAGCTGGTTCCTTCCGAAGTTTCCCTCAGGATA
GCTGGTGCATTTAACTGTTGTATAAAATAATCTTATCTGGTAAAGCGAATGATTAGAGGCCTTAGGGTCGA

AACGATCTTAACCTATTCTCAAACTTTAAATGGGTAAGAACCTTAACTTTCTTGATATGAAGTTTAAGGTT
ATGATATAATGTGCCCAGTGGGCCACTTTTGGTAAGCAGAACTGGCGCTGTGGGATGAACCAAACGTAAT
GTTACGGTGCCCAAATTAACAACTCATACAGAAACCATGAAAGGCGTTGGTTGCTTAAAACAGCAGGACG
GTGATCATGGAAGTCGAAATCCGCTAAGGAGTGTGTAACAACTCACCTGCCGAAGCAACTAGCCCTTAAA
ATGGATGGCGCTTAAGTTGTATACCTATACATTACCGCTAAAGTAGATGGTTTATATTACTTGTAATATAA
ATTTTGAAACTTTAGTGAGTAGGAAGGTACAATGGTGTGCATAGAAGTGTTTGGCGTAAGCCTGCATGGA
GCCGCTATTGGTACAGATCTTGGTGGTAGTAGCAAATAATCGAATGAGACCTTGGAGGACTGAAGTGGAG
AAGGGTTTCGTGTGAACAGTGGTTGATCACGAGTTAGTCGGTCCTAAGTTCAAGGCGAAAGCCGAAAATT
TTCAAGTAAAGAAAAATCTAAATATATTTGTCATCTAACCGATTTTTATACACTTGAATAATTTTGAACGA
AAGGGAATACGGGTTCCAATTCCGTAACCTGTTGAGTATCCGTTTGTTATTAAATATGGGCCTCGTGCTCA
TCCTGGCAACAGGAACGACCATAAAGAAGCCGTCGAGAGGTATCGGAAGAGTTTTCTTTTCTGTTTTATA
GCCGTACTACCATGGAAGTCTTTCGCAGAGAGATATGGTAGATGGGCTAGAAGAGCATGACATATACTGT
TGTGTCGATATTTTCTCCTCGGACCTTGAAAATTTATGGTGGGGACACGCAAACTTCTCAACAGGCCGTAC
CAATATCCGCAGCTGGTCTCCAAGGTGAAGAGTCTCTAGTCGATAGAATAATGTAGGTAAGGGAAGTCGG
GCAAATTAGATCCGTAACTTCGGGATAAGGATTGGCTCTGAAGATTGAGATAGTCGGGCTTGATTGGGAA
ACAATAACATGGTTTATGTGCTCGTTCTGGGTAAATAAAGTTTCTATCATTTATGGTAGTTTCTTGTTCCCC
GGATAAGTTTAGTTACGTAGCCAATTGGTGGAACTTTCTTGCTAAAAATTTTTAAGAATACTAGTTTGGGC
TACCAAATTAGTTCTTTTAAATTATAACGATTATCAATTAACAATCAATTCAGAACTGGCACGGACTTGGG
GAATCCGACTGTCTAATTAAAACAAAGCATTGTGATGGCCCTAGCGGGTGTTGACACAATGTGATTTCTGC
CCAGTGCTCTGAATGTCAAAGTGAAGAAATTCAAGTAAGCGCGGGTCAACGGCGGGGAGTAACTATGACT
CTCTTAAGGTAGCCAAATGCCTCGTCATCTAATTAGTGACGCGCATGAATGGATTAATAACGAGATTCCTA
CTTACTGTCCCTATCTACTCAGTTCGTTTCAGACAGTCGTTGGGAACAGACGTGTTTATTTCATCGCTCGCT
TCGCAAACAATTTTAAGATAACGGTGCTTTCAAGCAACGGTATCTTGTTTACATTTTGTGCGCTTATCAGC
AATAAAGTTTTATTGCTTTGTTTACGTCGTTGCTGCGTGAGCAACAGGCGTTCGTGTGTGTGTGTGAAACC
GCCAGCTTCTACGTGTGTGTGTGTGTGTGCGTGCTATTTATATATTAAATAGATAAGCGCACACCTGCGGA
CGATGCCGCCACGCAAAAAGAAGCCCGCTGAGAAGGGGGCTTTTGAAAATTGTAGGATGACGTCGGAAG
ATGAGGTGACCTTGTCGGAGTCGTCATCGAACAGCGAGGTAAGGCGGAGGAGCAAGCAGCGCCTGAGGA
AGGCAACTTCCGAAAGCGCTGTCAGCTCAGTTGGTGCGCGTGAGGGGAGCGCTGTTCCCGCGGGAGCCGC
AGATGGCTCGCCCGGAGCAGCGGGTGCAAGTGTGGAGGAGCGGGTTTTTGCCGTTCCTCATGCCGTGAGC
GTTTTGGATGTGTCTGAACCTGCCGTGGAGGGGCCGTCGACAAGTGCGGCGGCTTTGAGGAAGCGGAAAG
GAACAGCTGTCGAGCAGATGAAGGGGATAACGGGAGCTTTATTGCAGCTTGCACTCAAGGAGAGAGCCA
CGGGGTCGCTCATTTGCAAGTTAGTCGATTATACAGGGCAGTATGAGCAGCTCCTGTTCGCGCTTGTGGCG
CAGAATGAGCGGCTGCAGGGGCGCTTGGAGGCCGTTTGCGGGGGTGCAGGCGTTTCGCATGAATTGCATG
TTCCAGCCGGCCAAGCGCGGGCTCCGTCGGTGAACGCCGCCAGAGGGGGTCCCAGGTCGGCAGTGCCGTC
TACCCCGGAGATGCCTCGGCCAGTTGAGACATGGTCCTTGGTTGTGCGCAGCAAGGCTGCTGGCAAAACC
GCTAAGGACGTGGTGGAGCAGGTGGTGAAGGAGGTAGGTCCCTCTCTTGGTGTGCGGATACATGAGGTGA
AACCTCTAAGGGATGGAGGTGCGTTAATTCGGACTCC
>9_rnd-1_family-88__LTR___Gypsy
CGAATCCGGCTGAGGGATGACAAGCCGTTNAAGCAAAGGTACCACCCCGAGAATCCGGCCATGCAGAGG
GTGATCGACGAGCAAGTGGATGAGTTGATCCGGGCAAATGCCATCGAGCCGTCGCGTAGTCCGCACAGCC
CCCCGATCGTCTTGGTCAAGAAGAAGACTGGCGAGTGGCGTATGTGCATTGACTACCGACAACTCAACGC
GCATTCCATTCCGGACGCTTATCCGGTGCCTCGTATTAACCACATCCTGGAGCGGTTGCGCCACGCCCGGT
TCATCTCCACTCTGGATTTGAAGCACGGGTACTGGCAGATACCGATGGCAGCCGATAGCCGGGAGTGTAC
GGCGTTCACAGTCCCTGGAAGAGGTCTTTTCCAATGGCGGGTGATGCCATTCGGCTTACACTCGGCGTGCG
CGACATTCCAGCGCGCTTTGGATACGGTAATAGGGCCGCAGATGGAGCCTCATGCGTTCGCTTACCTAGA
CGACATCGTGGNAATAGGAGCGACGAAAACACAGCATGTAGAGAATTTGAGGGAGGTATTCCGCCGGCT
AAGGGCGGCCAATCTCCGGTTAAATAAAAAAAATGCAGCTTCTTCCGGGAGAGGTTAGTCTACCTAGGAC
ATGTAATTTGCGGAGAAGGGATACGGACCGATCCGGAGAAAGTGGAGGCGATACGAAACCTGAGGGCCC
CAACAAATTGCAAAGAACTGCGACAGTGTCTCGGGATGGCGTCATGGTACAGGCGTTTCGTACCCAATTT
CGCGACCCAGGTACAGCCACTCACAGCGCTCTTAAAGAAGGGGCAGAAATGGGCATGGGAGCAAGAACA
GGAGGAGGCATTGCGTCAATTGAAGGAGAGCTTGACGACTGCCCCTGTGTTGGCTTGCCCCGACTTTTCCG
CCAAGTTCGTCTTGCAGACTGACGCGAGTGAGTATGGCCTCGGTGCAGTGCTGACGCAGACGATCGATGG
ACAAGAGCGGGTGATAGCATACGCGAGCCGAAAGTTGCTGAAGGCCGAGGTAAACTATTCCGCCACTGA
GAAAGAGTGCCTGGCAATCGTGTGGTCGATCCGGAAGTTGCGTTGCTACCTAGAAGGGTACCGATTCGAC

GTAATAACGGACCATCTAGCGCTCAAGTGGCTGAATTCGATCGAGAGCCCGTCAGGAAGGATTGCGCGAT
GGGCATTGGAACTGCAGCAATTCCAATTTGACGTTCATTATCGTCGAGGGAAGCTCAACGTCGTGGCCGA
TGCCCTGTCGAGGCATCCTCTAGAGACCTGTCAACAAGCAGTGGTGGCCGAGGCCCCCTGCAAATGGGTC
GCGAGGATGCGTGTTAGAATCGCCGAAGAGCCAGCCAAGTTCCCGGATTACACCGAGGAAAACGGACAG
CTATATAGGCATTTGGGTCACCGGATAGACGATGAGGACTACATCCCTTGGAAACTATGCGTAGGCAGTG
GTCAACGCCAGAGGGTACTGCGGGAATGTCATGATGCTCCCACGGCCGGACATCAAGGTGTCAGAAAAA
CGGTGGCGAGGTTAGCCCAGCGGTACTACTGGCCGGGTATGTTCCGCGATGCCGCCAGATACGTGAAATG
TTGCGAAGACTGCCAAAAATTCAAGAGTGAGCAACTCAAACCGGCTGGTCGCATGCTGACGAGGCAGGT
GGCAGAGCCGATGGCGGTACTTTGCGCGGACTTCGTCGGACCCCTTCCACGGTCGAAACATGGAAACACG
ATGCTATTGGTGTTCCATGACGCTTTCTCGAAATGGGTGGAGTTGGTCCCTCTCAGAAGAGCCACGGCAGC
ACACCTGCAAACTGCGTTTCGAGAGAGGATTCTAAGTAGGTTCGGGGTACCTAGAAAGTTTGTGTGCGAC
AACGGGGTACAATTTACGAGTCGGAGCTTCAAGTCATTTTTGGGGTCCCTGGGTGTAGAGATCCAGTATA
CCGCCCCGTACTGTCCGCAAGAGAATCCCACTGAGAGGACTAACCGGACTGTAAAGACCATGATAGCGCA
ATTTATTGAGGGCCACCAGAGTTCATGGGATGAACTCTTGCCCGAGATCTCGCTGGCGGTCAACACGAGC
GTGGCAGATTCAACGGGCTTTAGTCCCGCTTTCCTGATGCAGGGCAGGGAACCTCGGTTACCCGCAGCTCT
GTACGATCAGGTAACTCCGGGGTCCGCAACTAGTGCGCTCGACCCTAGGACAAAGGCGGATAGGCTAAA
AGAAATTTTTGATATCGTCCACAGCAACCTCCAGCGCGCATCAAAGGACCAGGGCAGGCATTACAACTTG
CGGAGACGAGAGTGGCGGCCCTCGTTGGACTCGATGGTCCTGTTGAGGCAGCACCAGTTGTCCAACGCTG
CCGAAGGTTTCGCCGCAAAGTTGGCACCCAAATTTGACGGCCCCTACAAAGTCGTCAAATTCATATCCCCC
AATATTGTGCGGCTGGTGAAATCAGACGACCGCAAGAAGAGGGTGGCCAACATCTCACAATTGAAACCAT
TCTACAGAAAGACGACGAGCGACGAAGAAGACGAAGATCCAGGGCACGCAAATGCCCACGAGTAAGCG
AAGGATGAGAGCAACACCCGTCGCCCAACGAAACTATTATGATTAAGCTTAGATAAAACTAACATTAACA
ACATAATTCAGAATAGGCATAGTAAAGTGTGAGCCGCACTGAGAGAATAGGGCATTAACAAATAAGTAA
AGAGTAAGTTAACCAATAAGCTGAACTTTCAGATGGAACCCGAAGTTATCGACATATCGTCAGACGAAGA
CAACAGCAGCGGGGACACCGAGCCTTTCCCCGGGCCGGGAGCCGCAGGCCGTCCGCGCGACCCACGCCA
GGGGCGTGAGTACTATCGAAGAGAACGGCAAGAAGGACCGCCTCGCCAGTTCCGCCATACACTATTCTCC
CGGCGGACCGGCATAATGGAGAGGCTAGACGGGATGGTCTTGCTGATGGCAGTCGAGGTCACGACAATG
TTCGTGGAGGCACCGTTCGCCCGGAACTCGCCACCGTACGCGGAAGAGCCCGCATCTCCGCCCCTCGCAT
CCCACTCGGCACAATCATCAGAGCCGGCAACCCCAACCGCGGCCGGCGAGATGGACGAGGAAGAGTCCG
CACCGTTCGGAACGGAGGAGCACACACCACCAGCGCAGGACGTGGAGGCGGTAGGAGAAGAGATATCCC
CGTCGGCCGGAGACGCGGAGTCGCCACCGACGGAGGTGCCGAACGGCATCCGCGCAAACGACGGGGAAG
CAGACCCGCTGAGCCTCGACCCAGAACCCTTAGTGGGAGGGGACCCGTCAACGGAGGACGAGCCGGAAT
TCCGTCGCCGCTTTGCCACCCACCGTCTGGAGGTAGACCCGGGCAGCCAGTTTCCCAGGATAACGCTGCG
GTACCTACCGAAAGTCCCCGCGGTAGCCAACAAACCCCGTCTGAACCCCGCAAAGCGCGAATGGGGGTGC
CGAAGAAGGCCCCGGCGGAAAGCGCCGACGCAGCCGCCATCGTCGACGGAGAGCCGCCAGGGGCGAGCG
TCGCCGGAGTGGAGGCCATCGCCGACCCAGTCCCGAGGGAGCATCCTCGTCGGCCGCGAAGAGGCTTCGC
ACGGAGACCGAGAAGCCCGAAGGATCGCAGCGGTGCGTGCCGCGTTCGCCCCGCGAGCGCCAGGAGGGT
CCTCCCCAGCTCGGGAAGGATAACCGCCGCTGCAAGATCGCTTCTCGAGGAGGGCATCCCGAACCCTGGC
CGATACCGATTGCGGTCTGCCAGATGGTCGTGGAGGAGCACCGTCTCAGCACCAAGGACGCGGCGAACTC
GGCCGTTTCCACAGCCCGCGCCGAGGCGCCAGACGAAACCTTTCCTAAGGAATCGGAGGAGGAGTGGCTC
GACGACGAGGACGAGATGCCCGCGCTCGTGCCTGTTGCCCGCGCCGAGACACCCGACACCATCGCCCGGC
GGGAGTTAGAAGACGCCTGGTTCGGCCAAGAAGGCAAGGCGCCATCCCGAAAAGGTTCCTTCACGGTTCG
CCAGGAAGGCCGGGATGAAGTCTGGTCCGAGGAAGAGGACGCGGTGGTGACGGCTTCGTCGCCCAAGCT
TTCGAGTCCAACCCAGGATTCCTCCCCATGGCTCTAAAGACTCCCACGGAGAGCGAGTTGTGGGACGACA
TAGTGGACCTTTTCGACGGCATCGACGGGTGGGACACTAGCCTGGACTCAGTGCTGGAGGAGGAACCCGA
CACCGAGGTCGACACGGTCGCCCGCAGTGCCGACGTGAGGGTCACACCGGAGAGCCACCCCAAGAAACC
CGACACCGAGGTCGAGGCGATCGCCCACAGTGCTGACGTTGGGGTAAAACCGGAGGGCCCCGTCGAGGA
ACCCGTGCCCGATTCCACCCCCTCAGGCTGGAAGGTGTTTCCTCTTGGAACACCGTTGTCGTCGGGCGTGG
TGGACCTAATCGCCAGAGAGGCGCGAATCCGCCGCTTCCGGTGCCGACGTACTCGGTTCCACGTGTCCGA
CGGGGAGAGCGAGTACAGCGTTACGCTGAACGCGAAAGGCGACGTGACCGTCACCTGTCCGAAGTGAGG
AGGGGGTGTGAGGCGGCTAATGAGACCAGCCACCCCACAGTTTTTCACGGCAAGGAGCCGTCATCCCTTT
GGCCAAATTACGGCAAGGAGCCGCTGGCGGGACAACGTATTAATTTTTCTTTAGTTATAAGTATTAATTAT
ATTAGGTTAAGATCTTAGTGAAATAAGAGGCAAGGAGCCATCAGTAATATAAGCAGTTCGATTAGTAGCA
AAGAGGCAAGGAGCCGCGAATATTATAATTGATTTAAATAGTGAGTTTGAATTTAACCGCCGGCCGCCGC

TGCAACCGTGCGTTGTCGATCGGTTGCTCAACACCGTCTTTGAGAGCGCAACTTCAATTCGGCGGCGAAG
AGCGAAAGCTCCGAGGCGAAGTTGCGAAAGCTCGCGGCGAAGTTGAGCAGCCGAAGCGAGCGCATGGCT
TGCCGGCGAGCGTGCCGGCACTTGTGATCGAACGTTAAGGGTGAGTAGAAGTGCTGCGCGAGATAAACG
AAATCATAGATGGTATAGTGGAGCGCTAGGGCTCCAGAAAACAGTGCGGCAGGGTTTAGCTTAGGATTTA
CGATAAGGTCGGAAATGAATAATAGTGAATAAAGTGCCAACGTTGTAATTATCTAGAAAGTATTGGATTA
TTCCCTGTTTCCTGGTAACCCGGCTTACCTTAACATCGGAACTAACCGGTGTTCGACCTAACCTCGAACGA
GGGGCATGCGGTACGAGATTGTGGCTCCGGGGTGGGCATTGCCGGTTGGTGTTTAGGTAGTGGAGTTGGT
GCGAGGCCACGGCCCGCTAGTGTAGTTGGTGCGAGGCCACGGCCCGCTAGCATAGTGGTACGAGGCCACG
GCCCGGCAGAAAGATAGGGTGTTTAGGTAGTGGAGTTGGTGCGAGGCCACGGCCCGCTAGTGTAGTTGGT
GCGAGGCCACGGCCCGCTAGCATAGATGGTACGAGGCCACGGCCCGATGGTGTAGGTGTGTAGAGGTAG
ATCTGGGTTTGGTTTGGTCAGGATACTCACCCCCCTTAAGCCCTCTCTCCCAGCGAAATTATAAATAACTT
GTAACTCGCTAGACCTGGTAAGACTGAGAATTATACCCCGGCCAGAAGCTTCCTTACAAGTGGCGCCCGA
GCAGGGACCAGAGGCGTTTAGGGGTGTGGCATGTCGGCGGATTAAAGCCCGCAAGCATGCCCGAATAGG
TATTTTCCAAAAAATATATATAGGGTATTTCTAGTCGGGTATACCCTAGAGGGAAGGTAACCTAGCAGAG
AGCAAGAAAATGAGAAGGTCATGTATGTGCGAAATATGAAAATAGGGTATTAAGTAGTCCGGTATACCCC
AGAGAACATTCCTATACCTTGTGTTAGGCATAGCCAGAAAATGAACACGTGCAGCTGGTGAGTGACAAAA
ATGCTAGCAGCAAGCCGGCAAGTAAGATAAAGAGACGAAAACATAACGGTCGGTCGTGAGAGTACAGCG
CGCGCGCCAGCCGGGCGACGTCGAGACCACGCTCGCACACGGGTGCCCACACGCGCGGCGCAGTCGACG
TCGGCGTCGAAGGACCGTTAAGGCGTCGCGGCAGCGGTTGTACAGTGGGGCGAATCCAACTCACGCGCGG
TGAAAAGTTGAATTCGCGAGTACAGCTGTCGGGGGCAGTGAAGTGGTCACCCTTCAAGTGAATGTGAACA
AAGGGAACAATAAGAGCGGAACAAGTGCCAAATAAAAAAATAAGTGGATAATAATAAAATTTTCTTTTA
TAAATAAAATTAATTAAACAAAAAAAAAAAAAAAAAAATAAGTGGATAATAATAAAAATTTTTCTTTTAT
AAATAAAATTAATTAAACAAAAAAATAAATAAAAAAATAAGTGAATAATAATAAAAATTTTCTTTTANAA
AAAAAAATTAATTAAACAAAAAAATAAATAAAAAAATGAGTGGATAATAATAAAAATTAAAAANAAACC
GAGGTTCACAATAAAGATAAAGGCAGCGACCACATAAGGAACAGCCTAAAAAGCGGAAAAAGTGAGAA
AAGAAAATTTTTGAAATACCTTTTGTTGAGTTTTTTTAAAAAAGGGGGTAAATACTTTTGTGGAATATTGT
TTTGTATTTTTGTTTGGTGAAAGATATAAAGACAAAAAGAAAAAAAAAAAANAAAAGAAAAAAACTAAAA
AGGAACCAAAGTGGCTGAGCAAGATCGGCGTAAAAGTGAAAAAGACACCAACACCAAGAAAAGGAGAG
AGGAATAGTAAAGGGACCGACGGAACCCCTAGAACGGGAAGAGAGGGAAACAGCGACGTCGACTAGCA
AGGAAGTATACAATAAAGGATGGAGGCAGGTGAGTGCCAAAACCCGGGGGTAGGCGTCCGCTGGATTCA
CCAGCGACGCAAAGAGGAGTTAGTGGCGATCGCCCAGGAGTTTGGGCTGGAAAAGGAGGGGCGCGTGGA
GGAGCTACGTAAGCGGCTGTCCACCTACGTGCAGGAGGGCGGTCATTCGACTGGGATTTGGGCTCGGCTC
GCCGAGCTGGAATCCCAGTATGCTAAAACTCTTGAGCCGCCATCTTGGGGCGTCCCACCCGACTTTGGTGG
ACGGCCACGGCAAAAGTCGGAACCTGACCTCAGAAGCTCCCAGCTGTACGGGCTTTTGCAGGCGCCCACC
CCGAGTTTGACCGTCTCGGGCGTGGAGGCAGTTCCTGCTCTCGGTGGAAATGAAGACCGCCGAGCAACTG
CGGTCAACGCAGAGGTGGGGAGGTCCCCTGTTGAACCGGGTGGTGGTCCCTGCTACACTAGGCCGCACCA
TCCATCGGAGGCGAGGCCACTTCCCAATGTCCCGGAGCAAGTTCGGAAGTGGGGTATGAGGTACGATGGA
CGCTCCGACCCCCTTGGGTTCTTAGAGGACCTCGAGGAGCGCGCTATCACCTATGGTATAGCCCTCGACCG
TATGCCCAGGGCAATGAGCGAGGTATTCTGCGATAGGGCTGCTCGTTGGTTTCTGAGCAGTGGGCTAAGG
GATGTGCCGTGGGCCGTGTTTAGGAGGGATTTCTTGGAGTTTTTCTTGCCCCCTCGTTACTTCGAGAGGCTT
GAGGACGAGATCCGATCCCGGAGGCAGCGCGTAGGTGAAGCGTTCAAGGAATATTTGATAGAGCTGAGG
GCTCTTATGCACCACGCTAGATACGAGCCGGCACAAGAGCTGCATCGGATCTACGAGAACGCCGCTCCGG
AATACAGGCTCTATGTGCGACGGCAAGACTTTTCTACCCTATCCCAGCTAACTCAACTGGCCGTAGAATAC
GAGACCGTCGTGAGGCAGAGGGCGGAGTACTCAGGCAGCCAACCCTTGGGTCAGGGTGTCTCTAGTTCAA
ATAGTGCAGAGTGGGCGAGGACGGCGCAGTCCTCGGGACATCTCAATCCGTTCCGGTCTGCTCCCAGCGG
ATCCGGGACGACCGTTGTGGACAACAGGGTCTCCACCCCACCCGCTGCAACTCACGCAGAGGTGGCCCAT
CAGATGATGAGCCAAGAGGCCGGGAACGGATATGATCCGCGACGGGCGTGTCGTAGGTGCGCACAGGAG
GGGCATTTTGCCAGGGAATGTCCCAACGCCCCTGTCTTATTTTGTTGGGTATGTGGAAGCCGGGGCCGAAG
TAGTCGGGAGTGTTGCCGCCGCGCGGAGACGGGAAACGGGCAACGGCGTCGTCCGGACCAGGATCGGGT
CGGCGCCAATACACGCGGAACGCAGCAATAGGACACCCACCGAAGGTGGACGGTGGCCGGATCGTCGCT
ACGGTAGAGATTGGCGGACGAACGATGCCCGCCACGATCGATACCGGAGCCACTCGTAGCTTCGCGAGTG
AGGACTGCGTACGCAAATGGACGATCCGGGGAAGAGCGGAGGAAATACAGGCACGTATCCGCCTGGCAG
ATGGGTCCACCCTAGAGGTAGAAAAGGCTTTGAGAGTAGACGCCAGCTTAGGGGGAAAAACGATCAGCA
TGACGATGCTCATCATGCCCCTCAATGCTGGACCATGTAATCCTGGGTATGGATTTTTGGGGGCGATGAA

TACCACAATACGCTGCGGCGAGGCGGAACTGGTCCTAGAAGCAGCGACGACACCGGGCGTGAGGCCAGG
AACGAGCGAGGAGTCGGAGGAAGGCCNCCCAAGGCATGGAAAGGGAGCACCTGCTCGGCTACAGAGGG
AGCCGGAGGAAGGCCTCCCAAGGCATGGAAAGGGAGCACCTGCTCGGCTACAGAGGGAGCCGGAGGAA
GGCCTCCCAAGGCATGGAAAGGGAGCACCTGCTCGGCTACAGAGGGAGCCGGAGGAAGGCCTCCCAAGT
CATGGGAAGGGAGCACCTGCTCGGCTACAGAGGGAGCCGGAGGAAGGCCTCCCAAGNCATGGAAAGGGA
GCACCTGCTCGGCTACAGAGGGAGCCGGAGGAAGGCCTCCCAAGTCATGGATGGGGAGCACCTGCTCGG
CTACAGAGGGAGCCGGGAGGAAGGCCTCCCAAGTCATGATGGGGGAGCACCTGCTCGGCTACAGAGGGA
GCCGGAGGAAGGCCTCCCAAGGCATGGAAAAGGGAGCACCTGCTCGGCTACAGAGGGAGCCGGAGGAA
GGCCTCCCNAGGCATGGAAAGGGAGCACCTGCTCGGC
>9_rnd-1_family-368__LTR___Gypsy
TGTAATGGTCGCAATTTTCCTATTTGAATTAAACCGCGTAAGCGATCCTAACAGCTTGCGTTTGTGTAACG
ACGCTATAGCGATCAGCTTTTGTCTGTCACGGTCAGCTGCGAATGATTCTGCCGCTGCTATAAACCGCCTG
CGTGCACGTTGGCAGCGCAGCTATCTTCAATCTAATCCGCGAAAGAAAAAGCTCTGAGACCCTATATAAG
CCTGTAGGTCCCGGATATAACCCTTCTTTTTTCAAAAATTTCTTTACCATCGAAGGTCTGGCTACGGCGGTT
CGGACATAGCCCCACCAAAAAAATTAAACTAAAAGCATCAGTCGTTTAATTCGTTGAATTTAAATTCCAA
CTAAATTGAACCAAAAAAAAAAAAAAAAAAAAAAAAAAAAGGAAAACCCTAAAAGTGGCCGATGGGCGA
AATATCAATTTTTTTGTTCGTTTGATGATAAACCTTTCGCTACGCCAGCCGGCCACCAGCACCTCACCGGC
ATATATTTCTTTGTGCCAAGATCTGGTCACTGTTTTAACCAGTTCTTGTCTGCCTGCCGAAAGTAATTTTTG
GAGGAATCAATCTTGCCGCCGCCGTCTCTGACATAAAACCCACTTATTTTGAGGCAACCGCCACGTGTGCC
CATTGGACGGCACTGTAATTTCTTGGGACGGCAATGTCATTTCTGCCCCCATTAAATAGACGTCAGCCTCA
ACTGCGGATCAGCTGCCATTCCCTTGCTCATGCCAGCAAGAACGGGCAGGCGCCGTTTCCACAGTCTGGA
ATCCGGAAGAGGGAGTAGCCTTTTGGGCCCCATTGACGCGCCGCTGATCAGAAGCCCAGATGACATCGCC
CATTAAGTTGGCCGGAGCGTGCAGATGCATGAAAATTGCTGTTAAATGTTTTTAGTAAAAAAATTGTTAAT
TAAATCTTGTGAGAGACTTCCAAAAGTTTGTGCAAAGTGCATTNATTAGGGCCCTCAAAAACCGCGTGCA
AGTGCAGGGAAAAAAAAAAATTTACATCTATATACATATCTTTATATATATAGAAAGGAATAATAGTATA
AATCTATCCCCCTTCTTGTTGGGGTAGAGTCCAAAGTCATTTCTGAATTTTTTCCACTTAGTTTCGGAGAGT
ATCTTCTAAGTACTCACAATTTAAAAACCCTCCTGTGCCATAATAATATAATCAAAACAATTTAAAGCCCC
ACAGAAGACAAAAGAAAACAAATGAAAGAAAAAAAAATAAAATTTGCTGCAAACTCTCCGCTGGAAGGC
CTTTCATCGAGGAATACATGTAAGTCTGTACCTTTTGTTTTGAGTATATTTTCTTATTTTGGCACAGATAAG
CACAGCCAAGCTGACATGCGCACCTGCTGAGTTTTTTTCGACTTTCTGTTTCACATTACGCTTTAGCTCCGA
TCAATATGTTTATGACCGTGTCAGTCTGGTGAATGCTTATCATCTTTGATTCTACCTACACCCCGCTCAATT
ATTAGCTGAAACAGAAGCAACCTAAAAGAAAAGATTTTTAACAGAATCAAATATATGTACACAAAGATGT
ATCATCGTATTCACTGATCAGCTGGTTGCGCTGCGCTCCGATTTGCCCATGCGCTTCGAGCTTAACCTTCTG
TTGCCCATATTTTTTTCGACCATACAATTAACCATCAACTACAATTATATTGACTCTATTATAATAAATTTT
TTTGCTTTTGCCGAATACACTTTAGTTCAATTTACTAATGTCATTTTTTTCTTGCCTAGCCTTATTCTATAAT
TATTAAAATGCACTTTAGCGAAATTTTGAGATTTTTTTTTTATTTTGCTCAATAAGAGAAAAAAAATATTA
ACTGAATAGTTTATAAAATGAATTATTTTTGAAGAATTTTTTGAGCCATTGTAAAAATATTAACTGACTAG
TTTTAGTTACTGAATTATTGTTGAATCATTAATGCCACTTGTAATAAGATGCAGATTATCCAACGGAAGGA
TTCAATTTCATTGAATAATGCTTTTGTTTATAATAAATATTAATTACGGATTTTGATTATGAATTGCTAAGT
TTTCTTTCCTGGTGACATGGTGATATAATGTCGTAGAAGANTGTTTTTTTGGGATCTTCAAAACCTTAAAC
GCTGAAGATCATGGTTGGGAGGGTATCGAACGCGAGAGTCAGCTTACATCCAAGACTTCGCCTCTTCGGA
ATATATATGCCCCTAATGAAAGCTATCACCATTGTATTGGCACTTTAAGTGCTCGTTAAGTTATGCCTGTTT
GAGATTTTGTTTCTTCGGAGTATCGGTCTCCAATTTAGATTCTGTAGGGATCCTAAAATAAAATTGTGTAT
GCAGGTCGAGACATGGCAAAAAGAACCCCCACCCGAAAGAGCTAGGGGAGCAAGAGGATCCCGCCCTGC
CATGCTAACTCAACCACGAGGGGCTCGGTGAATACTTCCCGAATTATTTTGTGAAGATTGGCTGCCAACC
GCCGTCACCGTTGCTACCCGATATGTATTGCTAGAGTAGCTTCACCACAGTACATGCTACATAAATTTTTG
GCGCCCAATCATGGGGCCCGAGGTTGTCTATATAAAAAAAAAAGAGTCTTTATATTATTGTCCACGCACG
CTGACGTCGATGTGACTGGTTTAGTCTATCTTCCGCATGCGCAGATACTGGTAGTATACAAGATAGTAATT
AGGACTACGATGCTTACAATTCAGAGCTTGAATTATGCCGAGAATCTGACCTTACTATATTGTTGAGTGCC
TATATATAAACACGACTATAGTTATTAACACCAGCTATACAATCGGTCCCATACATTTGCTCGTTTTCTTTC
TTATAATTTTGGAGAAACAACAGATTAGGTTTGCTTAGGTAGAAATACTTTCATATGCAATTTTAGAATAC
TGCATATTTTGATAAGATATCAGTACTGATTGCTACTTGATAGCTTGTGGATGGTTAAGTAAATCTCAGTC
TTCGTGGAGCCAAAAGTTATAAAGTTAGATTGAATGGTTTTGTATTAACGATACTATCAAAGATAGTTAGA
ATGTTGCCCGGAACTTGGCCATATGCTGCTTTGCATATCCTTGCCGAACATTTCTGACTGGATTAAAATGT

TGAATGTTACAAACCGCTTTCTAAAGTAGTTTCAATAAACCAGATAAATTACCTACTTTCATTGAGGCGAT
TATATTTCGTAATTAAAAAAAAAAAAAAAAAAAAAAAAAAAAAAACTTCTACTCTATTATGTTACCAAACCAA
TGTCACGAATCAGGAAAAACTTAATTAATCATGTTGCCTTTACGCAGCCCACAAAATGTACAGCCGTCGTC
AGAGAGAGCTGATAACCACTGTGTTCTATGCCAGTTAGTAATGGACAATACACAGTCTTGCTATTGCACCC
CTTGTAGGCATGTTTTTCACAAACTTTGCTTGGAAGGTTGGTTGGTAAACCATGCTAAATGTCCCAGTTGT
AGTGCTGTGATTGACCCAGCCTCAATTAAATTAGTTGTTGGAGATTGCTCGCCGAGAGGAAAACAATCTG
CTCCACCTCCACCTACGAACATAGGGAGAACGGTAACGCGCAGTCTAGCAAAATCTCTTAATCCTGGTCC
GACCCAGAGTGGCGAGAATCCAACAGCAAGGTTATTAGATGTTAGTGATGTACCACAATCAGGACTCGAC
GCTAGCAATAGCACGAATCCACCTGAAATCGCTCCTGCACGACCAGCTCCTGGTGTAAGAAGAAGAGGAA
GACCCACTCAGTCCAATAATACCCGCCAAAATCTGGGAGTGGACTATAGGATAATTCAGGAGATGATCGA
ACAATCAATCACTCGCGTTATGTCATCACTGCAAATGCAAGCAACACCCTTTCCAAGAGTGTTGCCAACCC
CGATCCCGGTGTCGCCTAGTAGTTCGTCATTCCGAAATGGAAAAATTGCTGACACCATGCAAAAATGGAA
TCTTAATTTCGATGGGTCTACCGAAGGATTAGGGGTAGAAGAATTTCTATATAGAGTCAAAACATTGACT
GATGAAACTTTAGACAGCGACTTTATGGGTCTCTGTAAGCACTTACATGTGCTATTTTNGGCAAAGCCCGT
GACTGGTACTGGCGGTACCACAAGCAGGTAGACCACATAGTCTGGACTGAATTTTGTACTGCACTTCGCC
ATCAGTACAAAGATTATCGCTCCGATTTTATGAGCAAGGAACTAGTAAGAGCTAGAAAGCAAAACCCTGG
GGAATCATTTGTGTCGTTCCATGACGCTGTAGCGTCACTGATCGATAAATTTGCAATTAAAATTGACGAGG
AGGAGTTAATCGAAATTTTAAAAAATAATTTATTACCCGAAACCCGACATAAACTGCTATACCAGCCAAT
AAGTTCAGTTGGCCATCTACGTAGATTGGTCCAAATGAACGAGAATTTAGTTCATGAGCTCAGTTGTCGCA
CTGATGGTATGAATAAATCCAGGACTATTGGCCGTCGCCAAGTATATGGACTAGAAGAGTCTTTTATTGA
ACCGGAGGTAGAAAATTCAGAAGATACAAACTTTGACATCGCAGCAATTCGTAGATGTCATGCCAACTTT
AAATGTTGGAACTGCGAAGAGCAAGGGCATCTGTGGGAAAATTGCCTTGCTGACCGCCGTATATTCTGTT
ACGGATGCGGGGCACCCAACATTTATAAGCCCCAGTGCCAAAACTGTATCCGTAAAGCTTCGGAAAACCA
ACAGAAGGGTGCATCCAGCAGCAGTCGGATGCCCCCAAAAATCTAATTAATTCCCATACGAACTGTGTAC
CTGTGGACCGAAGCAAAAGCTTGGTCCCTACACCGACTTTAAAGTCACATTCCAACAATTTAGAAATAGC
TAAAACTATTAAATTACGATTTTTACCGTATCATGAACGGTTAGCTAATTATTTGCATGTCCGAAATAAGA
TTTTTTATGAAGAAAAACCCCGTATTTCTCAGCGAACGCAGCGCTTGAAAAATTATTATTCAAAAACAAGC
AACATAGCAAGCTTTTAGTTTCGACAGTTTTTGCAATCCCACCGATATGCGCAGCTATGCCGAAGTGTCAT
TTTTAACCTTTACCGAGCTTGGTCTATTGGATACAGGCGCGAATATTAGCTGCATAGGTTCTACGCTTGCG
CAACAAGATTTTACCGCAATGCCGGAATTTAAGCCTATTAAATCGCATGTCAGAACAGCAGATGGCCATA
GTCAGAAAGTCATCGGAGTGCTTGAAGTTAAAATGCAGTATAAATCGGCTACCAAGTCCTTGAAACTGTA
CGTGATTCCTTCTATAAACCAAAAATCTAATTTTGGGTCATGATTTTGAGAGCCTTTGAGCTTGCGCCAAG
CATTATAAATTCTGTAGAACTAAAATCAATGGAAAAAGAGCAGGAAACAGAGTCGATTGCATACCCCTTA
GATAGCCAGCAAACGCAACAGCTGCACGTAGTAAAGCAGTTGTTCCCCAACGCTGCTGATAGATTAGGCC
GCACAACATTGATCCAACACCGTATAGATATAGGAGACGCACGTCCAGTGAAACAAAGATTCTATCCTGT
AAGTCCGGCCGTAGAAAAGTTGTTATATGCAGAAATTGATCGCATGTTGCAGCTTGGAGTTATTGAGCCG
TCTGTAAGCGCTTGGAGCTCGCCTATGCGACTCGTCCTTAAGCCCAACAAGGTGCGCCTTTGTCTGGATGC
TCGAAGACTGAATGACGCCACAAAAAAGATGCTTATCCTTTGCCGAGCATCGATGGCATTTTTTCGCGGCT
ACCTAAGGCGAAAATAATTTCCAAACTAGATTTGAAAGACGCCTATTGGCAAATAGAACTTTCCGAGGAT
TCAAAGCCTCTCACTGCTTTTACGGTCCCCGGAAGACCGCTATATCAGTTTAAGGTCATGCCCTTTGGCCT
TTGTAACGCGCCTTCTACCATGTGTCGGTTAATGGACGAGATAATTCCTTCTGACCTGCGCTATTGCGTGTT
TGGTTATTTAGATGATCTGTGTATTGTGTCAGAAGATTTCAGTTCACATCTGGCGGTATTGGTGCGCATCG
CTGAGCAGTTTAGAAAAGCCAACTTGACTTTAAATATTTCGAAAAGTCAATTCTGTGTAACAAGTATCAAT
TATCTTGGTTACGTGATTGGTAGCGGAGGTATTTGCACAGATCCGGAAAAAGTAGCTTGTATTGTAAATTG
GCCGCCGCCAAAAACTTGAAGCAAGTTCGAGGATTTCTAGGAGTTTGCGGTTGGTATCGTCGGTTTATATG
CAACTTTTCCGATCTCACTTGCCCCATAACGGACATATTATCGACAAAGAAAAAGTTTGAGTGGACCCCTG
AAGCACAGACCGCAATGGACAACTTAAAAACAGTGCTAACATCCTCGCCGGTTTTAAAAAATGCTGACTT
TAAGAAGAAATTTTTCTTACATTGTGACGCTAGTGATTTTGGCATTGGCGCCGTTTTAGTACAGATGTCTG
AAGNTAATGAAGAACAACCGATAGCTTTCATGTCAAAAAAATTAAGCCGTGCGCAGCGAAACTATAGTGT
TACCGAGCGTGAATGCCTAGCAGTCATATTAGCCATCGAGAAGTTCCGATGTTATTTGGAACTCCAGGAA
TTTGAAGTGGTTACCGACCATTCTAGTTTGCTATGGCTTATGCGCCAGCAAAATATATCGGGTAGACTGGC
ACGATGGGTTTTCAGACTGCAACAATTTAAATTTTCCGTCTCCCATCGGAAAGGCAAAGAACACATTGTTC
CCGACGCTTTGTCTCGGTTGCCTGAGAATGAAATCGCTCAATTAGACATGGGTCCTGTAATAGATCTTGAG
TCCGATACATTTATGGACGCTAGCTATGTACAAATTAAAGACAAATTTTCCCAAAATNTTACTCAGTTTCC

GGACATCAAAATCGTTGATAAGTTTTTGTATGTACGCACCGAACATTCGAGGGGAGACTCGACACCAGAA
AGTCCTTCATGGAAACTATGGGTGCCTGATCGACTTAAAGTCGATGTGCTCAAACAAGCCCATGACAGCA
TAACGTCGTCTCACGCCGGCATGCAAAAACCATTGAGAAAATCCGCAGACATTTGTTCTGGCCAGGATTA
GTTAAAGATGTACGTGAATACATACGAAATTGCGAGATATGTAAAGAAACTAAGGCTCCAAATTATACCC
TTCGGCCACCTATGGGTGCTCACATCCCAACTTGCAGACCATTTCAAAGGCTTTATATTGACCTGCTTGGG
CCATACCCTAGAAGCAAAAGCGGTCATGTGGGATTGTTAATAGTCTTAGATCACTTTAGCAAGTATCATTG
GTTGCAGCCCCTGAAGAAATTCACCTCAGCTGTGATACAAAAATTTCTTCTTGAACAAATTTTCCATTGTT
TTGGTGTGCCACAAACTATAATAAGTGACAACGGCACCCAGTTCAAAGCTCGTGAGTTCAATGCCTTCTTA
GCCGAACTTGGCATTGAACATCTGTATACAGCTCTATATTCACCGCAGAGCAATGCTGCAGAACGAGTTA
ACCGCTCAATACTCGCCGCCATTCGCTCGTATTTAAAACAGGATCAGACCCATTGGGATGAACATATTACA
GGGATTTCATGCTCCTTAAGGTCTGCTCTGCACCAATCGATTGGGTGTTCCCCATACCGTGCCCTATTCGG
GGTGGATATGATCACTCATGGTACCGATTACAAATTATTGAAGGAGTTGTCCTTATTGGAAGAACCGCTG
GTCCCCCTGTTGCGGTCTGACCATTTGGCATTGCTAAGGACAGATATTCAGAAAAACATCAGGAAAGCCT
ATGAGCGCAATGCGGCTCAATATAATTTACGCAGCAAGCCTGTTTCTTTTCGTGAAGGACAGGAGGTGTTC
TGTCGAAATTTTGCGCTTAGTAAATTCTCACACAATTTTAATGCTAAACTGGCTCCCCAGTTTATTAAATGC
CGTGTCAAAAAGAAACTCGGCAATTGTTTTTATTCCCTTGAAAATCAGCAAGGAAAAGAAATTGGCACTT
TTCATGCCAAGGACATTAGAGTCTAATTCCCGCTAATATACCCGTCTAACGGTAGGATATTATCGGGTGGT
GTAATGGTCGCAATTTTCCTATTTGAATTAAACCGCGTAAGCGATCCTAACAGCTTGCGTTTGTGTAACGA
CGCTATAGCGATCAGCTTTTGTCTGTCACGGTCAGCTGCGAATGATTCTGCCGCTGCTATAAACCGCCTGC
GTGCACGTTGGCAGCGCAGCTATCTTCAATCTAATCCGCGAAAGAAAAAGCTCTGAGACCCTATATAAGC
CTGTAGGTCCCGGATATAACCCTTCTTTTTTTAAAAATTTCTTTACCATCGAAGGTCTGGCTACGGCGGTTC
GGACATAGCCCCACCAAAAAAATTAAACTAAAAGCATCAGTCGTTTAATTCGTTGAATTTAAATTCCAAC
TAAATTGAACCAAAAACAAAAAAATAAAAACAAAAAGNAAAAGAAAGGCGAAATATCAATTTTTTTGTT
CGTTTGATGATAAACCTTTCGCTACGCCAGCCGGCCACCAGCACCTCACCGGCATATATTCTTTGTGCCA
AGATCTGGTCACTGTTTTAACCAGTTCTTGTCTGCCTGCCGAAAGTAATTTTTGGAGGAATCAATCTTGCC
GCCGCCGTCTCTGACATAAAAACCCACTTATTTTTGAGGCAACCGCCACGTGTGCCCATTGGACGGCACTG
TAATTTCTTGGGACGGCAATGTCATTTCTGCCCCCATTAAATAGACGTCAGCCTCAACTGCGGATCAGCTG
CCATTCCCTTGCTCATGCCAGCAAGAACGGGCAGGCGCCGTTTCCACAGTCTGGAATCCGGAAGAGGGAG
TAGCCTTTTGGGCCCCATTGACGCGCCGCTGATCAGAAGCCCAGATGACATCGCCCATTAAGTTGGCCGG
AGCGTGCAGATGCATGAAAATTGCTGTTAAATGTTTTTTTAGTAAAAAAATTGTTAATTAAATCTTGTGAG
AGACTTCCAAAAGTTTGTGCAAAGTGCATTNATTAGGGCCCTCAAAAAAACCGCGTGCAAGTGCAGGGGA
AAAGAAAAAAATTTACATCTACATACATATCTTTTATATATATAGAAAGGAATANTAGTATAAATCTATCC
CCCTTCT
>9_rnd-6_family-342__LTR___Copia
ACTGCTCGACTCTGCCGGGACTGCCGATCTGCCGACTGCCCGACTGCCGGACTGCCGACTTGCCGGGACT
GCCGACCTGCCGACCTGCCGGACTGGCCGATCTGCCGACTCGCCGGGACTGCCGACCTGCCGATCTGCCG
GGACTGCCGGACCTGCCGACTGCCGACTCTGCCGGGACTGCCGGACTGCCGATCTGCCGGACCCGCCGGC
TGCCGCCGGACTGCCGGACCGCCGACCGCCGACTGCCGATCTGCCGGGACTGCCGGATCTGCCGGACTGC
CGGACTGCCGATCTGCCGAGACTGCCGCCGGATCTGCCGGACTGCCGATCTGCCGACTGCCGGGACTGCC
GACTGCCGGATCTGCCATCTGCCGGACTGCCGACTGCCGGACCGCCGACTGCCGGACTGCCGGCCGACTG
CCGGACCGCCGATCTGCCGGACTGCCGACTCCGCCGGACTGCCGACTGCCGATCTGCCGGACCGCCGATC
TGCCGGANCCGCCGGACTGCCGGACTGCCGATCTCCGATCTGCCGACTGCCGATCTGCCGGATCTGCCGG
ACTGCCGATCTGCCGGATCCGGCCGACCGCCGGACTGCCGGNCCGCCGACTTGCCGACTGCCGGACTGCC
GGATCTGCCGATCTGCCGGACTGCCGGTCTGCCGACCGCCGATCTGCCGGACTGCCGATCTGCCGACTGCC
GGATCTGCCGATCTGCCGGACTGCCGACTGCCGATCTGCTCGAGACTGCCGGACTGCCGATCTGCCGATCT
CCGGACTGCCGCGATCTGCCGGACTGCCGACCTGCCGACCTGCCGACTGCCGATCTGCCGATCTGCCGGN
CTGCCGACCTGCCGATCTGCCGGACTGCCGACTGCCGACCGCCGACTCGCGCCGACCGCCGACTGCCGAT
CCTGCCGGACTGCCGGCCGCCGGGACTGCCGATCTGCCGATCTGCCGACTGCCGACCGCCGATCTGCCGG
GACTGCCGACTGCCGACCGCCGATCTGCCGGACTGCCGATCTGCCGGACTGCCGATCTGCCGGACTGCCG
GACTGCCGATCTGCCGGACTGCCGGGCCGACTCGCCGGACTGCCGATCTGCCGGACTGCCGACCGCCGAC
CGCCGGACTGCCGATCTGCCGANCTGCCGACTCGCGACTGCCGATCTGCCGATCTGCCGGACTGCCGGAC
TGCCGGGACTGCCGATCTGCCGACCTGCCGGGACTGCCGATCTGCCGACTGCCGATCTGCCGGACTGCCG
GACTGCCGATCTGCCGGGACTGCCGGGACTGCCGGNCTTGCCGACCGCCGATCTGCCGGACTGCCGGACC
GCCGGACTGCCGATCTGCCGACCGTCGACCGCCGATCTGCCGGGACTGCCGATCTGCCGGGATCTGCCGA

CCTGCCGATCTGCCGGACTGCCGATCTGCCGATCTGCCGACTGCCGGACTGCCGANCTGCCGACTGCCGA
CCTGCCGACGACTGCCGGACTGCCGATCCGCCGGANCTGCCGGATCTGCCGGACTGCCGGACTGCCGATC
TGCCGGACTGCCGATCTGCCGATCTGCCGGACTGCCGANCCGCCGACCTGCCGATCTGCGGACTGCCGAC
TGCCGACTTGCCGATCTGCCGACTGCCGGGCCGACTGCCGACGACTGCCGANCTGCCGGACCTGCCGGAC
TGCCGACGCCGGGATCTGCCGATCGCGACGCCGCCGACCGCCGACTGCCGACTGCCGGGACTGCCGGCCG
ACTTCGCCGACTGCCGATCTGCCGGACGCCGATCTGCCGGGACTGCCGGACCGCCGATCTTGCCGACTGC
CGGACTGCCGGACTGCCGGACTGCCGATCTGCCGGACTGCCGACCGCCGATCTGCCGGGACTGCCGACTG
CCGATCTGCCGGACTGCCGGACTGCCGACCGCCGACTGCCGATCTGCCGGGACTGCCGGGACTGCCGGNC
TGCCGATCCCGACGCCGACCTGCCGGACACCCGGCCGACCCGCCGACTGCCGACCGCCGATCGCTGACGC
CGACCGCCGACTGCCGGACTGCCGATCTGCCGACTGCCGGACTGCCGACTGCCGGACCGCCGGACTGCCG
ATCCGTGCCGGGACTTCGGACTGCCGATCTGGCCGGGACTGCCGATCTGCCGACCTGCCGGGACTGCCGT
CACTGCCGGGACCGCTCGACCTGCCGGACTGCCGGACTGCCGACTGCCGACTGCCGGGACTGCCGGGACT
GCCGGGACTGCCGACCTGCCGATCTGCCGGACTGCCGATCGCCGACCCTGCCGACCTGCCGACTGCCGGA
TCTTGCCGGACTGCCGGACCTGCTCGACGGTCTGCCCTGCCGGACCTGCCGACTGCCGGGACTGCCGGGA
CCGCCCGACTGCCGATCTGCCGACGGGACTGCCGGACTGCCGACTCTGCCGGACTGCCGACCTGCCGATC
TGCCGGACTGCCGATCTGCCGGACTGCCGATCCGCCGACCGACTGCCGGGNCGCCGACTGCCGACTGCCG
GACTGCCGGNCTGCCGATCTGCCGGANCTGCCGACTTCGCCGGACTGCCGACCGCCGACTGCCGACCTGC
CGACTGCCGGAATCTGCCTGCCGACTTCTGCCGGACTGCCGATCTGCCGGACCGCCGACCGCCGGACTGC
CGGACCTGCCGATCTGCCGATCTGCCGGGACTGCCGACCGCCGATCTGTGCGGGCTGCCGGAACTGCCGA
CTGCCGANCTGCCGANCTGCCGAGCCGACTGCCGATCCCGACTTGCCGGATCTGCCGGGACCGGACTGCC
GGACTGCCGATCTGCCGAGNCTGCCGGACTGCCGACCTGCCGACTGCCGGACTGCCGATCGCCGGGTCTG
CCGGACTGCCGATCTGCCGGGACTGCCGANCTGCCGGGACTGCCGATCCGCCGACTGCCGGANCTGCCGA
CCTGCCGATCTGCTGCCGATCTGCCGGACCTGCCGGACTGCCGATCTGCCGGACTGCCGGACCGCCGATCT
GATCGGCTCCTGCCGGGACCGCCGACCTTGCCGACCTGCCGGGACCGCCGATCTGCCGATCTGCCGACCT
GCCGGACTGCCGACCGACTGCCGGACCTGCCGGACCGGCCGACTGCCGGAATCTGCCGGACTGCCGACTG
CCGACCGCCGACCGCCGACCCGGCCGATCTGCCGGACTGCCGACCTGCCGACCTGCCGGACTGCCGATCT
GCCGGACTGCCGACTGCCGATCTGCCGGACTGCCGGACTGCCGACTGCCGATCTGCCGGACTGCCGACCT
GCCGANCTGCCGGACTGCCGATCTGCCGACTGCCGGACTGCCGACCTGCCGACTGCCGACTGCCGACTGC
CGGGACTGCCGGACTGCCGATCTGCCGACCTGCCGGACTGCCGATCTGCCGGATCTGCCGGACTGCCGAT
CTGCCGGACTGCCGACTGCCGATCTGCCGACTGCCGGGATCTGCCGGACCGCCGGACTGCCGACCTGCCG
GACTGCCGACTGCCGACCTGCCGGACTGCCGGACTGCCGGACTGCCGACTGCCGGGACTGCCGACTGCCG
ACTGCCGGACTGCCGATCTGCCGACTGCCGGNCTGCCGGACTGCCGACTGCCGACTGCCGATCTGCCGGA
CTGCCGACTGCCGGCTGCCGACTGCCGACCGCCGACTGCCGGACTGTCGATCTGCCGGGACTGCCGACTG
CCGATCTGCCGATCTGCCGGACTGCCGACCTGCCGGACCTGCCGACTGCCGGACTGCCGACTGCCGGNCT
GCCGGACTGCCGATCTGCCGACTGCCGGACTGCCGGACTGCCGACTGCCGGACTGCCGGATCTGCCGGAC
TGCCGATCTGCCGGACTGCCGACTGCCGATCTGCCGATCTGCCGGATCTGCCGACTGCCGACCGCCGGGA
CTGCCGATCTGCCGATCTGCCGACTGCCGATCTGCCGGGACTGCCGATCTGCCGACTGCCGACTGCCGATC
TGCCGACTGCCGACTGCCGGACTGCCGACTGCCGGACTGCCGATCTGCCGACTGCCGACTGCCGATCTGC
CGGACTGCCGATCTGCCGACTGCCGACTGCCGACTGCCGACCTGCCGACTGCCGACTGCCGACTGCCGAT
CTGCCGGACTGCCGGNCTGCCGGGACTGCCGATCTGCCGGACTGCCGATCTGCCGGACTGCCGACTGCCG
GGACTGCCGACTGCCGATCTGCCGGACTGCCGGCTCGCCGACTGCCGGACTGCCGGACTGCCGATCTGCC
GACTGCCGACTGCCGGACTGCCGATCTGCCGGATCTGCCGGACTGCCGGGACTGCCGATCTGCCGGNCTG
CCGACTGCCGATCTGCCGACTGCCGATCTGCCGGACTGCCGATCTGCCGATCTGCCGACTGCCGATCTGCC
GGACTGCCGGACTGCCGACTGCCGATCTGCCGGGACTGCCGATCTGCCGGACTGCCGGATCTGCCGANCT
GCCGACTGCCGGACTGCCGATCTGCCGATCTGCCGACTGCCGATCTGCCGGACTGCCGGACTGCCGACTG
CCGACTGCCGATCTGCCGACCTGCCGGACTGCCGATCTGCCGGACTGCCGATCTGCCGATCTGCCGACTGC
CGGACTGCCGACTGCCGACTGCCGACTGCCGGACTGCCGGACTGCCGATCTGCCGATCTGCCGGACTGCC
GATCTGCCGATCTGCCGACTGCCGATCTGCCGATCTGCCGACTGCCGGGACTGCCGATCTGCCGATCTGCC
GATCTGCCGACTGCCGACTGCCGACCGCCGACTGCCGATCTGCCGACTGCCGATCTGCCGGACTGCCGGA
CTGCCGATCTGCCGACTGCCGGACTGCCGACTGCCGATCTGCCGACTGCCGGACTGCCGATCTGCCGANCT
GCCGGACTGCCGACTGCCGGACTGCCGGACTGCCGATCTGCCGGACTGCCGATCTGCCGACTGCCGGACT
GCCGGACTGCCGATCTGCCGACTGCCGGATCTGCCGACTGCCGGACTGCCGGATCTGCCGACTGCCGATC
TGCCGGACTGCCGATCTGCCGGACTGCCGATCTGCCGGNCTGCCGGGACTGCCGATCTGCCGACTGCCGG
ACTGCCGATCTGCCGATCTGCCGACCTGCCGACTGCCGATCTGCCGGACTGCCGGACTGCCGATCTGCCGG

GNCTGCCGGACTGCCGACTGCCGACTGCCGGACTGCCGGACTGCCGATCTGCCGACTGCCGACTGCCGAC
TGCCGATCTGCCGACTGCCGGACTGCCGGNCTGCCGACTGCCGACTGCCGGGACTGCCGATCTGCCGGGA
CTGCCGACCTGCCGATCTGCCGGGACTGCCGGACTGCCGACTGCCGGACTGCCGGACTGCCGATCTGCCG
GACTGCCGGGACTGCCGATCTGCCGACTGCCGGACTGCCGACCTGCCGGACTGCCGACTGCCGGNCTGCC
GACTGCCGACCGCCGGNCTGCCGGCTGCCGACCGCCGATCTGCCGACTGCCGATCTGCCGATCTGCCGAC
TGCCGGACTGCCGATCTGCCGGACTGCCGACTGCCGGACTGCCGACTGCCGATCTGCCGACTGCCGGACT
GCCGATCTGCCGACTGCCGGACTGCCGACTGCCGATCTGCCGGACTGCCGACTGCCGGGACTGCCGATCT
GCCGACTGCCGACTGCCGGACCTGCCGACTGCCGGACTGCCGGACTGCCGGACTGCCGATCTGCCGGACT
GCCGGACTGCCGATCTGCCGACTGCCGGACCGCCGACTGCCGACTGCCGACTGCCGACTGCCGGNCTGCC
GACTGCCGGGACTGCCGGACTGCCGATCTGCCGACCTGCCGACCTGCCGGACTGCCGACTGCCGATCTGC
CGATCTGCCGACTGCCGATCTGCCGGACTGCCGGGACTGCCGGACTGCCGACCGCCGACTGCCGGACTGC
CGATCTGCCGATCTGCCGACTGCCGACTGCCGACTGCCGATCTGCCGGACTGCCGGGCTGCCGACTGCCG
ATCTGCCGACTGCCGATCTGCCGACTGCCGACTGCCGGACTGCCGACTGCCGGACCGGCCGATCTGCCGA
CTGCCGGACTGCCGGGNCTGCCGATCTGCCGACCTGCCGGACTGCCGGNCTGCCGACTGCCGATCTGCCG
ATCTGCCGGACTGCCGACTGCCGACTGCCGACCGCCGACTGCCGGNCTGCCGATCTGCCGGGACTGCCGG
GACTGCCGATCTGCCGACTGCCGACTGCCGATCTGCCGGNCTGCCGGCCGCCGATCTGCCGACTGCCGAC
TGCCGGACTGCCGGGACTGCCGACTGCCGATCTGCCGGACTGCCGACTGCCGGGACTGCCGANCTGCCGG
ACTGCCGACTGCCGACTGCCGATCTGCCGGACTGCCGATCTGCCGGNCTGCCGGGACTGCCGGACTGCCG
ACTGCCGACTGCCGACTGCCGGGACTGCCGGGACTGCCGACCTGCCGACTGCCGACTGCCGACTGCCGGA
CTGCCGACCTGCCGACTGCCGGACTGCCGACTGCCGATCTGCCGGACTGCCGGACTGCCGACCTGCCGAT
CTGCCGATCTGCCGACCTGCCGGNCTGCCGACCTGCCGATCTGCCGGGACTGCCGATCTGCCGACCTGCCG
GACTGCCGACTGCCGANCTGCCGACTGCCGGGACTGCCGANCTGGCCGGACTGCCGACTGCCGATCTGCC
GGACTGCCGATCTGCCGGACTGCCGACTGCCGANCTGCCGATCTGCCGGACTGCCGACTGCCGACTGCCG
ATCTGCCGGGACTGCCGATCTGCCGGACTGCCGACTGCCGGACCTGCCGATCTGCCGGNCTGCCGATCTG
CCGGACTGCCGATCTGCCGACTGCCGGGACTGCCGATCTGCCGACTGCCGGACTGCCGATCTGCCGGACT
GCCGATCTGCCGGACTGCCGATCCGCCGACTGCCGATCTGCCGACTGCGACTGCCGACTGCCGATCTGCC
GGGACTGCCGGACTGCCGATCTGCCGGACTGCCGGGACTGCCGATCTGCCGGGACTGCCGANCTGCCGGA
CTGCCGATCTGCCGACCGCCGGACTGCCGACTGCCGGGACTGCCGATCTGCCGGACTGCCGACTGCCGAC
TGCCGGNCTGCCGACTGCCGGACTGCCGGGACTGCCGGACTGCCGGNCTGCCGGNCTGCCGGACTGCCGA
CTGCCGATCTGCCGGACTGCCGATCTGCCGACTGCCGGGACCGCCGATCTGCCGACTGCCGGACTGCCGG
GTCTGCCGACTGCCGACTGCCGGACTGCCGATCTGCCGGACTGCCGGACTGCCGACTGCCGATCTGCCGA
NCTGCCGACTGCCGGGACTGCCGACCTGCCGACTGCCGGACTGCCGACCTGCCGGCTGCCGATCTGCCGG
ACTGCCGGGACTGCCGATCTGCCGGACTGCCGGNCTGCCGGNCTGCCGACTGCCGATCTGCCGATCTGCC
GATCTGCCGACTGCCGGACTGCCGATCTGCCGATCTGCCGACTGCCGACTGCCGGNCTGCCGACTGCCGA
TCTGCCGACTGCCGGACTGCCGGACTGCCGGACTGCCGGACTGCCGATCTGCCGACTGCCGGACTGCCGG
ACTGCCGACTGCCGACTGCCGATCTGCCGACCTGCCGGACTGCCGACCGCCGGACTGCCGACTGCCGACT
GCCGATCTGCCGATCTGCCGGGACTGCCGANCTGCCGATCTGCCGGATCTGCCGATCTGCCGGACTGCCG
GATCTGCCGACTGCCGACTGCCGGGACTGCCGACTGCCGATCTGCCGGNCTGCCGATCTGCCGATCTGCC
GGACTGCCGATCTGCCGGACTGCCGACTGCCGATCTGCCGGACTGCCGACCTGCCGGACTGCCGATCTGC
CGGACTGCCGATCTGCCGGACTGCCGACTGCCGACCTGCCGACCTGCCGACTGCCGATCTGCCGACTGCC
GGACTGCCGATCTGCCGACTGCCGGNCTGCCGGGATCTGCCGGACTGCCGGACTGCCGATCTGCCGACCT
GCCGGACTGCCGACTGCCGATCTGCCGGGACTGCCGACTGCCGATCTGCCGACTGCCGGGACTGCCGATC
TGCCGATCTGCCGGACTGCCGATCTGCCGATCTGCCGACTGCCGATCTGCCGATCTGCCGGACTGCCGATC
TGCCGACCTGCCGGGACTGCCGACTGCCGACTGCCGATCTGCCGGACTGCCGACTGCCGACCTGCCGACC
TGCCGACTGCCGGATCTGCCGATCTGCCGGACTGCCGACTGCCGATCTGCCGGACTGCCGGGACTGCCGG
CCGTCGATCTGCCGACTGCCGGGACTGCCGACTGCCGGACTGCCGACTGCCGACTGCCGACTGCCGATCT
GCCGGACTGCCGATCTGCCGGGCTGCCGATCTGCCGGACTGCCGACTGCCGACTGCCGGGACTGCCGACT
GCCGGACTGCCGATCTGCCGGACTGCCGGACTGCCGACCGCCGGTCTGCCGACTGCCGGACCGCCGATCT
GCCGATCTGCCGACTGCCGANCTGCCGACTGCCGATCTGCCGGACTGCCGGACTGCCGATCTGCCGGGNC
TGCCGGACTGCCGACTGCCGACTGCCGATCTGCCGACTGCCGGTGACTGCCGANCTGCCGGACTGCCGAC
CTGCCGANCTGCCGGACTGCCGATCTGCCGATCTGCCGGGACTGCCGATCTGCCGGACTGCCGGACTGCC
GACCGATCTGCCGACTGCCGGGATCTGCCGGACTGCCGATCTGCTCGATCTGCCGGACTGCCGACCGCCG
ACTGCCGGACTGCCGATCTGCCGGACTGCCGATCTGCCGACTGCCGGGACTGCCGATCTGCCGACTGCCG
ATCTGCCGACTGCCGGACTGCCGATCTGCCGGNCTGCCGGGACTGCCGACTGCCGACTGCCGATCTGCCG

GGATCTGCCGGACTGCCGATCTGCCGATCTGCCGACTGCCGACTGCCGACTGCCGACTGCCGGNCTGCCG
GACTGCCGACTGCCGATCTGCCGACTGCCGGACTGCCGATCTGCCGGGACTGCCGATCTGCCGACTGCCG
ACTGCCGGACTGCCGATCTGCCGGACTGCCGACTGCCGGATCTGCCG
>160_rnd-1_family-82__LTR___Gypsy
ATATGTCGTACACGGACCGCGAGCAGGCGCCATTCCGAAAGAGCAAGAAGGGGTACAGCGCGAGGAATT
TCCTCCACCAGTGCCATCGAAGCCACACCGACAGGGCGCGCAAGGAGCGGAGTACTCATACAGCAGCCC
AGTATATGTTGCAGCTGCTCAGGCAAGGAGCTGGCGGGGGCCGAATAATTGCCATTTCCGAGTAGAGGAC
CGCAGAGCGGATGACTTTCAGCCGGAGGAAGCGTATGCCAACGTACGAGAAGCGACGTACCTAAGGCTC
GAGCGGTGGAACGTCAAGTTCGATGGCGAGGACGCAATGAATTCTGTCGAGGACTTCGTGTTTCGCCTTG
AGTTCCTGCAGAGGCAGTATCAATGCCCGTGGAAGGAGGTCCTGCGTGCCTTTTTCCTGCTCCTGACCGGC
CGTGCCCGCGAGTGGTACTGGATGCACCTAAAAAACTGGAGGAGGCATTCATGCGAGACAGGACATCATC
CCGAGTAGTGTGCTGGAACTGAAGGCAGTTCGACCATGTCTTTAGATACCGCCTATCTAAGAAGAGTTAG
ATATTCTGCTACAGACGCGGAAAGCCGGGCACATTCTGTGCTGAATGCGAAAATTGCCCGGGAAACCACA
GAGGAAGCGCAATGGTAGCGGGACAGACGCGTTCAGGGACGGCGATCGCCGAAAAACAAGAGAGGAAC
ACCTACCATAAACAATAACACTGGCAGAGGAGAAGAATATAGACCATAAGGACCAGGATAAGGTTATAT
ATAAGGAGATAAAAATGAAGGAAAACCATTAAGAGGCTTACCATATGAAGAACGTGTCCGGGCATACAT
GTCGGCCCGGAATAGGATTTTTGGTGAGCGACAGCTGGAGGGGATGACATTGGCCACCAGAAGAGTGCTC
AAGGCTCGGGCGCGCTTCCGCAGACGCAAGATGACGAGGCGCCAGGTCATCGAAGCTGTGAAACGGGAG
GATAGCCTAGATCCGCGCGTATTCGCCGAAGTGGAGGTCGCTGGAATCAAAATGAAAGGGTTGCTAGACA
CCGGAGCCTCGGTCAGCCTGCTGGGACAGTGGATATTAGTCGTTTGTTGGTTGAAGCTTTTATATGTGCAT
CATTTAAAATGCAGCCGATTGTCGCAGTTTTGTTCTTGTTGTAAAATTGTTGATTCATCAATGTTTTGTGTG
TCGTTGCTTGTTACGAAACTGATTGTTTCTCGGTGGGCCGCGCGGCTGGCTCATCGGTTTCTCTTTGCTTCT
GTGGCACACAGCGCCTGAATCCAGCGACGGAGGACGCTGGCCAGATCAAACCAGATTCGGCGTAACCAT
CACGACGGCGAGACGAGCCATCACGGACGGGACTCCAACGACGAAGAGGCGGCCGTAGGCGAGACCACG
CCCACTTCATCACTGAGTGAGTCTGTTCTACGTCGGCCTTCCTCGCCTGAGTNAAAGGCAGAGGCTGAAGC
AGACCCATAGTTGAGGCTCCCTGAAGGAGACCAGATATTTTTTTTTGGAATTGTTAACTCTAATTTTTTTTT
TGTAAATTGAATACATAATATACTCACGACTAGTCGCAATTAAGGAAATTTCCGAAAGTGCGAACAAAGT
GCGAAAGTGCGAAATGGCCCAAAGATTATTGCGTCTGCCAAGATTTTGGTCAACTTTATTGGATTGATTGT
CTCCCCTTTCTTTTATTTCTTTGTTATTTTATATTAACATCTCACCTTAGATGGTTACATTTACTTATCGTCA
CAGCTGTTTATGTTGTTCATCTGTTAGAAGTTCCGATTGTTTCTTGTTATCAATTTGTGACCAAATATTTTTT
TTTTGGAATTGTTAACTCTAATTTTTTTTTTGTAAATTGAATACATAATATACTCACGACTAGTCGCAATTA
AGGAAATTTCCGAAAGTGCGAACAAAGCGCGAAAGTGCGAAATGGCCCAAAGATTATTACGTCTGCCAC
GATTTTGGTCAACTTTATTGGATTGATTGTCTCCCCTTTCTTTTATTTCTTTGTTATTTTATATTAACATCTC
ACCTTAGATGGTTACATTTACTTATCGTCACAGCTGTTTATGTTGTTCAGCTGTTAGAAGTTCCGATTGTTT
ATTGTTATCAATTTGTGGTTCGTTTTGCAGAGTGTTGTGTAAGTCTGTTTTGCCACAGACGTGTAGCTTTGT
TGTAATCGATTGTGCCGACTGCGGGCCAGGGATGGGACTACACCCAGAAAAGCCGATAAGCCAGCCTGCG
TGGCCCACCGAGAAACAAGAAGTTTCGTAACATTATGGCGCCCAATNAGGCGCGCGAGGGGCGGAGTAT
TCGTCCAGCAGCCCAGTTTACGTNGCAGATGCTCAGTCGAGGAACTGGCGTAACCCGAATAACCGCCATT
TCCGAGTAGAGGACCGGAGAGCGGATGATTTTCAACCGGAGGAGGCGTACGCCAACGTGCGAGAAACGG
CGTACATGAAGCTCGAGCGGTGGAACGTCAAGTTCGACGGCGAGGACGCGATGAATTCGGTCGAGGACTT
CGTGTTCCGCCTGGAGTTCCTGCAGAGGCAGTATCAGTGCCCGTGGAAGGAGGTCCTGCGCGGTTTCCAC
CTGCTCCTGACCGGCCGCGCCCGCGAGTGGTACTGGATGCACGTGCGGCATTCTAGGGTCGACAGCTGGA
TGCAACTGCGGCACGCCCTCTTGGACAGGTTCCGGGGCTACCAGACGGAACACGAGGTGATGCAGGAGCT
NCTACAACGAGAGCAACAGGCCAGCGAAGGGGTGGATGATTATATCCACCACATGCGTCAACTCGCCGC
GCGATTCCAGAAGCCGCTGAGAGACCGNGAGCTGGTGAGGATTATAAAGCGTGGTTTGAAAGAAAGTCT
GGCGAAATATATTTATGCCATGGACGTACTCACCGTGGATGAGCTGCGCCAAGAGTGCCTAGAAGTGGAG
AGGCACATGGGTCGCAGAAGCCGGACTGGGTACCTNCAGCCGTCGCGTTGTCCACAAGGAACGAGGCCC
GTAGTCCACGAGGTTGAAGTNCCACCGCATCTTACAGAAACGCCGCCGGGAGAACTGGAGGAGGCATTC
GTGCGAAGCAGGAACTCATCCCGAATTGTATGCTGGAACTGCAGACAGTTCGACCACGTCTTTAGAGACT
GCCTGTCCAAGGAGCGGAAAATATTCTGCTATAGGTGTGGAAAGCCGGACACTTTCTGCTCGCAGTGTGA
AAACTGTCCGGGAAACCCCAGAGGGAGCGCGGTGATGGCGGGACAGACGCGTTCCGGGACGGCGACCGC
GGGAAAGCAGGAGAGGGGCACCTACCAGAGCCAATAATCATTGGAAAGGAGACGGAGATTTGGAATAAG
GATCAGGATAAGGCTAATTATAATAATAGTATAAGCAAAGGAGGAACACTTAGGGGCTTACCATACGAA
GAACGTGTTCGGGCATACATGTCGGCCCGAAATAGAATTTTTGGTGAGCGACAGCTGGAGGGGATGACGC

TAGCCACCCGGAGAGTGGTGAAGGCACGTGCGCGCTTCCGCAGACGCAGGATTACCAGGCGCCAGGTCGT
CGAAGCGGTGAGACGGGAGGAGAGTATAGACCCACGAGTATTCGCCGAAGTGGAGGTCGCTGGAGCCAA
AATGAAAGGGTTGCTGGACACGGGGGCCTCAGTCAGTCTGTTGGGACAAGGATGCCGGGAGTTAGTGGA
GAAACTGGGATGGGAAGCGCGGCCATACGAATCGATGGTGAGGACAGCGGCGGGTGCCAATCGCCCAAT
TTTAGGTCGCGTTGTTCTGCCTGTGAAATACGGGAATAGAACGGAAGATATCGTATTTTACATGTGCCCGG
ACCTGCGACAGGAACTATACCTGGGAATCGACTTCTGGCGAGCCTTTGAGATCGCACCAGAGCTGCTCGG
TCCGGCCAGAAAGTCCGAAACACCACCAGAGGCATCTGAGGTAACGGTAGCGAACCCAGAGGTGGCTTA
TTACCGGGACGACGACGACTGCGTAACGGATCCGGAAATGTGGGACCTGGACAACGATCAGAGGAGTCA
GTTGGAAAGCGTGAAGCGCAGATTTCTCCAATTTGAGAAGGATGGCCTAGGGAAAACTCACCTGCTGCAG
CACCGAATTCAGCTGATCGAGGGAGCAGAACCCGTGAAAGACAGACATTATCCGCTGTCCCCGGCCAAAC
AGGAGATCGTGTGGGCCGAGGTGGATAAGATGCTAAAGTTGGGCATCATCGAGGAGAGCGACAGCCCCT
GGAGCAACCGGACGACGGTGGTGATGAGGCCTGGGAAGAACAGGTTTTGCTTAGACGCCAGGAAATTAA
ACAGTGTAACGGTAAAAGACGCGTACCCGCTTCCATGCATCGAGGGCATCCTATCGCGAATCGACGAGAC
TCATTTTATCTCTAGCGTCGACCTTAAGTTCGCGTTCTGGCAAATAGAGATGGAGGAGAAGAGCAGGGCG
TATACGGCGTTTACCGTACCAGGGAGGCCGCTGTACCAGTTCCGCCACATGCCATTCGGGCTCTGCAACGC
CGCTCAACAGCTTTGCAGGCTCATGGACAAGGTAATCCCGGCGAATCTGAGGTCCAACGTATTCGTATAC
CTGGACGACTTGCTGATAATATCGGCAGATTTTCCAACGCACTTAAAATACTTGGAATTGGTGGCCGAGTG
TCTGAGGAACGCGAACCTCACCATAGGCATGGCGAAGTCGAAATTCTGCTTCCGCAACCTAAACTACCTG
GGTTTCATCATAGGCGGCGGGACGCTGCGCATGGATCCGGGAAGGGTAGAAGCGATCCGGAACATCCCG
AATCCGAGGACGGTCAAGGAACTACGAAGCTTCTTAGGTACGGCGGGGTGGTACCGCCGATTCATAAAGA
ATTTCGCCGAGATATCGGTACCGTTGACTGATGCCCTTAAGAAGAGAACGGGTAGATTTGTGTTGAGCGA
CGAGGCCATAGAAGCCATAGAGAGCTTAAAGTTAGCCCTCACCACAGCCCCGGTGTTAGTTCACGCAGAT
TTTCGAAGACCATTCTTCATCCAATGCGACGCATCTCACTACGGAGTAGGAGCCGTGTTGTTCCAGCTAGA
CGACGAACAACAGGAAAGGCCGATCGCATTCTTCTCGGCCAAACTCAACAAGCACCAGATCAACTATTCG
GTGACCGAGAAGGAATGCCTAGCCGCCAAACTGGCCATACATCGATTCCGGCCGTACGTGGAGATGATGC
CGTTCACGGTAATCACCGATCATGCGAGTCTGCAGTGGCTTATGAGTCTCAAAGACTTGAGCGGGAGATT
AGCCAGGTGGTCCCTCGAACTGCAAGCGTTCCCTTTCTCCATGCAGTACCGCAAGGGAGCCGACAACGTC
GTCGCAGACACATTGTCCCGAAGCGTGGAAGAGGTCGAACTGACCCCGGAAGATCTGTTGGGATTTCAGA
CCCCGGAGTTCGAGAGCACCGAATATCGAGAGCTGATAAGAGAGGTGATGAGCCAACAAGGGAAGTTGC
CGGACCTTAGAGTGCAGGACGGACTTCTGTTCAAGAGGACCGTGCACGAGAGCTTGGAGGATGAGGTCG
AAGGCACAAGTTGGAAGCTCTGGGTGCCGGAGTCGCTGACGGCAGGACTCATCCAACAAGCACACGCGG
ACGAGACGTCAGCTCATGGAGGCATGAGGAAAACGCTGCACGCGCTGCGCAGGCGATATTATTGGCCCA
ACATGGCCATACAAGTCAGGGACTATGTTCGGAAATGCGATACATGCAAGGAGACCAAGGCACAGAACT
ACCGAATGCAGGTCGGAATTGGGGAAGAAGTGCGCACCGACCGCCCCTTCCAGAAGCTATATATCGACTT
CTTGGGGAAGTATCCACGGTCGAAACGAGGGCATGCGTGGATATTCGTCGTCGTAGACCACTTCTCGAAG
TTTACCTTCCTGAAGGCCATGAGGGAAGCCACCGCGGCGGATGTCGTGAACTTCCTAGTGCATGAGGTGT
TCTTCAAATTTGGCGTGCCGGAAGTGATCCATTCGGATAATGGACGACAATTCGTGTCAAAGTCGTTCGAT
GCGATGGTCCAGGCGTTTGGCATTACTCACCTGCGCACACCAGTGTATTCGCCTCAGAGCAACGCCGCCG
AACGCGTGAACCGCACAGTGCTGTCGGCAATCCGAACCTACCTGGGTCAGGATCATCGGGAATGGGACGC
GTACCTACCAGAGGTGGAAGTGGCGATCCGGAATGCGGTCCACAGCGCTACGGGAGTCACTCCGTTCTTC
GCGGTCTTTGGACAGCAGATGTACCTGAATGGTTCCAGTTACAAACTGGCCAGGAAGCTGAGATCATTGG
CCGACCACAGTATTTCTGACCTTGACGCAAAGGACAGACTGGCTGTAATCCGAAGCCAAGTCAAGGACCA
CCTGCACACCGCATACGAGCGAAGTCGCCAACGGTACGACCACCGCGCGCGACAGCTCCATCTGGAACCG
GGACAGGAAGTGTGGAGGCGCAACTTCGCACTAAGCAGCTTCGGCAAAGCCTTCAATGCCAAGTTCGCCC
GGAAGTTCTTGAAGAGCCGCGTCGTCCGAGCCGTGGGAACCAACGCCTACGAGTTGGAAGATCTCCAGGG
ACGCGTGCTAGGCGTCTTCCACGCAAAAGATATACGAACATAATCGTAATTTGCTCACCTCCTTGCCAGGC
AACGGCGGCGGCAGCGCCAGTCCCCCCTTTTTTTTTGGGTCAGCAAAAAAAAAATATAGATTACTTGTCGT
GGGTGTGGCCTACGTTGGGCATCGTTATTAGCCGGCTGGGTCAATCCATGAAATGCGCACGCAGATTCCTT
GTAACTGTCCTTCATGTCGTTACAATAATCTGCGTGGTGATGTTGCATTTGACGTTATCGGCAGCACCGAG
GGTTACTCAGTTGGTTGCGTGCGATATTATCGGCCACGCCGATATCTTTTTGGCATATGGTCGGAGTATGC
GGGGTGAAGCTGGGCCCACGGCGGAGCCCAAAATTTTGAAGGGTGGCAACGCCAGACATGCGATCCACG
GCGCCAAAAGCGACGATTGCTATTGGCCGCCCGTTTCGCTCTTAAAGGGAATACGAGGAGAGTGGATCGC
ATGCCCTTGGATATGCCCTTGGCCTTCATTTGGGCCGTTGACCCAAAAAAATGCAGCTGTGCAGATGAGCA
GCAACGGTGCCCGCCTCGCAAATGTATGCGTGTGCCGAAATTCGGCACCGATGGTGTGGCTGCCAAAAAT

GTCGAGGAGTTCGATTTGTTCAGCGGTTAACGGCGGGCGTGCCAGCTCGAAGTGAGCAAAAAAGTTGGAC
ATTGCATTTCTGCTTGACAGAGTAAAATTTTTTCCCTTCTGAAAAATTAAGCCGGCACATAATACCAATGG
CTTAACAGACGAATAAAAGTTTTGTTTTTGTGTTCTTTGCCATGTAATAAATTTATATAAATCAAACCGCTC
GAATCACAAAGAAAATATAATAAAAAAATTAAAAGAGACTTAAAAATTTAAGTGCGACGAGAGACGAAC
CAAATAAGCCAAAAATTAACGGCTCCGGCACCAAGGAGACCAGCTCGCGCACCAGAGAGGCCCACGAAA
TATTCAAGGCCAGGATTCAGTGCAGCGGCGCAAACCAGCCATATCCGCGACGCCGCCAGCAAGCGTCGGC
GCAAACGTGAAGGCGACGAGACGAGCCACCAACGACCGGGCTCCAACAACGAAGAAGCGGCCACAGGC
GAGACCCCGCCCACATCCACCGAGTGAGTCCGTTCCATTGTTTTGCCCATCCTCGTTCGAGCCCCTGGCAG
AGTCAGAAGAAGACTCCTAATTGCACTCCCTGAAAAGGAGTAGATTTTTTTTTATCGTTAAATTTGGTTAA
TTTGTATTTATAAAATATATTCATACATACATAGGCACTTACGCCGAGCTTCACTTTAGAGTGCGAACGGT
GAGGAAAACTCCAACGGTGATGCATCTGCGCCTGTAAAATATAAAAAAAAAAANCTAAAATTAACTCTTG
CGCGAACTATGCTCACATACTTACCTAATTTGAATTTACGCATTTATATCGCCCCTTACCACCCCCAGAGA
GCTCTTTAGGTAGGGTAAGGTAGAATTAAGCGATTTAATACATACAATACACAATACGTGACATACCCAG
TTTTAAGTAATTGTGCGGGCCATTGTTCCTAAATCGGGGCAACGATTGCCTCTAAAGTGCCTCATTGCGCG
TTTCTTTTGCGGCGACTAGCAATATCCTTGTAGTGTTTCGTCGCCTGTTCCATTCAGTGTTGTAAAAAAATA
AAAAAAATTAATTCTATAAGATAAGAGAAAAATTTAGGAATTAATGCGCTGTGTCAATTAGTATAAATAT
ATGCAGCAGTAATACGCACCTGTGAAGTTAATGAATTATCCGCTTGCATGAGATCCTAAACAGCTGCAAG
ATAAATGCCATAAGCAATGATTATGATTGTTATACATTTTAAGAAAACGCAACCAATTATACGCACCTATT
GAAAGAAATCATGTCCGGCTAAAATAATATTGCTGGGGAGAGTGGGCATTATGGCCGACACGGGCTAATA
TAGGCCATTTGGTGGCCAAAATGAGGGAGACGAGTGGAGGTGATCGTCTCATGGTAGGGCGGGTACGCTG
GGTCCGGAACCGGGGCCCGATCTGGAAGAGTAAGGTGGATAAATAGTGTTTTTTTGTTAATTTATATTGAG
TGTCTTACCTTTGTTGTTTACATTTTCATAGTTGTTACAGCTGTGTATTTTGTTCATGAGTTAGTAGTTCCGA
ATATTTGTTATCGATTATGCGGTTCATTTTGAGTAGTGTTGTGTGAGTCTGTTTTGCTACAGACGTGTAGCT
TTGTTGTTATCGATTGTGCGGACTGCGGGCCAGGGATGGACCACACCCAGAGAATCCGATGAGCCAGCC
GGCGCGGCCCACTGAGAAACAAGCAGTTCCGTAACA
>160_rnd-1_family-273__LTR___Pao
TTGTTGAAAGGTCCAGATACACTAGCATCTTTGATGGGGGTGCTAATACGCTTCCGAGAGCGTCCTATCGC
AGTATCCGGCGACATAAGGGAAATGTTTCATCAAGTCAAGGTGCGCCTAGAAGACCAACCGGCACAGAA
GTTTCTCTGGCGTGATGGAGATTCGTCACGCTTCCCCGAAACATACGTTATGCAAGTCATGACGTTTGGAG
CATCCTGCTCGCCAGCTCTAGCAAATTACGTCGAGAATCGCAATGCCGAGCGGTTTGTAGCGGAGCATCC
GGATGCCGTAAAGGCGATTTGCGAAAACACATTTGTCGACGACTGGCTGCAGTCGGTGGATACTGGAGCT
GAAACGATACAGTTAGCCGAGACTGTAAAAAGGATTCATGCTAGTGGCGGCTTCGAGATGCGCCGCTGGA
CATCAAATTCGAAGCAAGTTATACAGGCGCTGGAAGACGACTGTGAGGAGTTGGACAAGCGTATCAGCG
CTCAGGATGAAGCGCAAGAGAAGGTCTTAGGCCTGTGGTGGCTACCTGGACAAGATCTGTTGACGTTTGT
GGTGACGCCGAACTTGCTCGCGAAGGCATCTAAGGAGCGCCCAACTAAAAGACGTGTTCTGAGTGTGATC
ATGTCTATTTTCGACCCGCTCGGGCTGCTAGGATTCTTTAATATACGCGCTAAGATCATCCTTCAGAACAT
ATGGCGATCCAATATCGGATGGGATGACGACCTCACCAACGAAGACGAAGCCGATTGGCAACACTGGCTT
GATCTAGTTTCAAATTTGAATGCCGTCCGCATTCCACGGTGCATGAAGTGGGTGAGCCGAACCCAGGCTG
TGCAGATGCATACATTCGTTGACGCCAGTATGAATGCTTACGCCGCAGTTGTATTTTTGCGGGCTGAACTT
GATGGCCAAGTACATTGCAGTTTGGTCGCCTCGAAAACGAGAGTAGCACCCCTAAAGCCCGTGTCCATAC
CTCGAATGGAACTGATGGCCGCGGTCTTAGGTCTGAGACTGGCCAAATGTATCCAAAAGGAAATGTCGGT
ACGNATACATAGACGAACATTCTGGACAGATTCAAAGGATGTGCTCTACTGGATCCGTTCCGATGCTCGA
AAGTTCCAGCAATTCGTAGCGCTTCGGATAGGAGAGATTTTAGAAGAATCAGATGTGGACAGCTGGCGAT
GGGTACCATCGGCTCAAAACGTGGCAGACGATGGTACGAAATGGACTAAAACGCCTGAAATTCATGGCTC
AACCAGGTGGTTCAATGGACCACCATTTTTGTATCTAGAAGAATCGCAGTGGCCACAATCGGAAATAGGC
CCTGCATTAAACATGTTGTACCATGCTGAAGAACAACCCAATCACACGTCGTCCTGGAGATGTATTCTGCC
GGATCTGCAGCGTTTCAGCAAGCTAGAAAAATTGAGAGCCGTACAGCTATGCGTACTGGATTTCCTACGG
AATATTACTAAGAAGGTCAGATTGGACGGAGGACTGGATCTGCAGAAGACGCTGCTCCTTAAAAGAAGC
AATGAAATGGATGTGATTTTTATCCGGATTTGTCAAGAAGAGGAGTTTTATAGTGAGATCACCTGCTTAAG
GTCGGGGCGCCGCTTAACCGATCGCAAAAGCTTGCTGTTTAAGTGCTCCCCTTATGTGGATGACTCGGGCA
TTCTACGTATTAAAGGACGGATAGACAATATAGAAGGAGTCGAAGTCTGCGTAAAACGCCCGATAATTCT
GCCAAGACGACATCAATTGACGTATCTGCTGGTTGAGTTTTATCACCGCAGATATCATCACCTACACAACG
AAATCGTCGTAAATGAACTGCGGCAGCGGTACTGGGTTTGTGGCTTACGAGCTTTGGTGAGAGAAGTTTC
CAATACCTGCCCAGCGTGCCGCATACGACGCGCCCGCCCAAAGCCGCCAGGGATGGGCGACCTGCCAATC

GAACGATTATCGCCCTATACTCCACCGTTCACATATACTGGAGTGGATTACTTCGGCCCCTACGACATTGT
CGTTGGACGGCGTCGCGAGAAGCGCTGGGGCGTGCTATTTACATGCCTCACGGTGCGCGCCGTTCACCTA
GATATAGCTACGTCGCTCTCAACTGACTCATACTTATGCGTTCTGAAGTCGTTCGTGGCCAGACGCGGCTG
CCCCCGCCGCATGCTGTCGGACAATGGCACAAATTTCAGAGGCGCTAGCAGAGTATTAAAAGATGAAATT
GAACGTATATCGCCGGCTCTGATCGAGCGACAGTACCCAGAACTCGAGTTTACCTTTATCCCGCCAGGATC
ACCCCACATGGGAGGATCGTGGGAACGAATGGTGCGCTCTACAAAGTCAATACTGTCGGAAATTCTGCCG
CCCTCTGGGTTACGAGAGGAGGTGCTACGAGCAGCATTGGCTGACGTAGAAAGCGTGCTCAACTCAAGGC
CACTCACTTATGTTCCCTTGGAGACATCTGAGTCCGAAGCCCTGACTCCAAATCATTTCTTATAGGTCATT
CAAGCGGATTGCGTGAGAGAGGATTACGGGAGCAAGGCGGAGCTAGCTTGGCACGAGGCTTCCGAGTCG
CCAGTCAATTGGCTGACCAATTTTGGAAGAGATGGCTGCGCGAGTATCTGCCTACACTCACCAGACGTAC
TAAGTGGTTTCAACCGCCACTCGAACCAATATCAGTCGACGACATCGTCATAATAGTGGACGACGGTGCC
AAGCGAAACTGTTGGACAAGAGGAGTGGTCGTAGACGTGCACCGTTCAAAGGATGGCCAGGTCCGAAGT
GCCGTGGTGCGAACCGCCGATGGAATTATTACTCGCCCCGCCGTCAAGCTAGCTAAGCTTGACATTAAAG
TTGGTAACACACTGCACGGACGTTCGTGAATTACGGGGTGGGGAATGTTACGGGGCGAAGTGAAAATAGC
TGTTGACTAAGTCCTACTGGGTCTCGCATTTTTTCTGTAAGCAGCCCTCGCTGCTTACACTGGAAGCGCAC
CGTTAGAATTAGTTTTTACATGATAAGTCGATCGCACGCAGCTGCACAAGCTGCATTTTCGTCGAGGTTCA
TTTTCGTTTTGTGCTTGCAGCTGAGCAGAAGAGTGAAAGAGAACAGAAGCGCGTGCGGAGCACGAATGTA
AGTCTCATAANCNTGTAAAATATAGGTCGTTAAATAATGAAAGAATAATAAAGATTTCAGCCGTTCGAAT
CGTATCGTATCGTACCGCCTACGAATCGAATCGAATCGAGTTGTTTCTTTCGACGCTAGAAGTCCCTGCCG
AATACGCGCCCCGGCGCGAGTCTCGGCGCCAACATTCTGAAATATTCTCTGCNGCAGAGAGTGTCCGAGG
TATTCTCTGCTACGGCGGAGATTTCTGCGAAGCAAGCGACTTGCAAAGTCACAACCTGAGCTGCCTATAG
AGAGACCTGGTCAAGAGCTAGACTCACACGTAGCTGCCATAACCTCAAAGCGGACTTCACGGTATCGGTG
TGGCGAGATGGCTGACGGTGATGAACAAGATGCAAGTGCGAGAAGGACGACAAGTAGTAATCAACCCGG
TGGAACTGCAACGCTTGTAACGGCATGGTCAACGGGACAGGCTGACGGATCGCCTGTCCCTGCTGGAGTT
CAAGATAAAGTCGCCGGCGTAGAAAAGGGTAGCGGAGCAGCAAACATAGAAATTTATAGCGGCTGTCCG
TCTACGTCGTCCGCAGCTACTGGTCCAAGGGAAAGTTCAACACCGACGCAGCAAAGAACTGAGAAAGAG
GGAGCATTCTTCGACCCTTTGGGATACCAGAACGTGATCTGCAAGCAGAAACAGGTTGCGGCTAATTGGA
GTGTACACCCCAGTTTGCCATATCAAGCAAACACGGCGGGTGTTTAACCAGGCTACGGAGCCCAGTACTG
CCGCGTACATGGGCCACACAGGTCTGGGAGCGCTCAATAGATCGATAGAGCACGCGCCGCGGCAGCAATT
CGATAATGTACATTGTAATGNTGCGACGCCGTGGAATGGACCGCTGACATCTGCGCAAATCAGCGCGCGA
CACGTNATCAGCAAAGATTTACCAATNTTTACNGGCAAACCAGAGGAATGGTTGATCTTTATCAGCAATT
ATGAGCAATCCACAGAGAGATGTGGATTCAGCAACGAGGAAAATCTCATCCGGCTTCAGAAGTGCCTCAG
AGGGCAAGCCCTGGATGCGGTGCGCGGAAAGCTTATGATNCCAGCTACAGTGCCGTATGCTATAGGCACA
TTGCGAATGCTGTATGGCCGCTCTGAAATAGTACATGCTGCGCTGCAGCAAAAGCTCCGAGAGGAACCAG
CAATAAAGTCGGACAACTTGAACACCGTTATCCGACTGGCCCTAGCTGTCCAAAACTACTGCGCAACGAT
AGCGGCCATTGGTATGCACGAATATCTTTACGATCCCGCGCTGCTGAACGAGCTTGTTGCTAAGATGCCCA
GCAATATGAAATTGGACTGGGGCCGNCATCGAATGTCGAATGANGGTAGCAGTTTGGCCACCTTTGATAA
CTGGCTGTTCAATGTGGCNATGTGCGCCACTATGGTCACGCCCTACGAGACACCACGGGCTGATAACGAA
AAGAAGAGCTCCACGAAAACCACTAGAGAGCGAATTTTTGTGCACAACACGGTAGATAACCCTGAGGGTT
CGCAGCAACAGCGTTATCAACAGGAACAGGAGAGAGTTTCATGTGCGAAGTGCGCCGGTAACCACCGTCT
ATCAGATTGCGACGACTTCAGAGCCATGGGTACAAAACAGCGGTGGGAACTTGTTAAGGAAAAGCGGCT
CTGCTTTTGCTGCTTTATGCGACATCGTGCACAACAGTGTAAAACAAAGAAGAAGTGTCACGTGGATGGT
TGCGAGTCAACGCACCATGTGCTGCTGCATACGCCTACCACGAGCGGTAGTAAAGTCGAGCACACGGGCC
AAGGTAGCAACCACCGGATTAAGGCAAGTGAGTCTGCCGTTATGTTTCACGGCAGCTCCGCAAGTAAGAC
ACTGTTTCGATATGTTCCAATCACGCTCTATAGTAAATCAAAAAGCGTGCGCGCATATGCATTAGTGGATG
AAGGAGCTGAGTGTACGCTAATAGAAAGAGCGCTTGCAGACGAGCTGGAACTAGATGGTCCGGCGACCC
AGCTGTGTCTTAAATGGACAGGGGACATAACCAAGAACGAACCTGAGTCGATGTCCGTGGCTGTGAGCAT
TTCAGCTCCAGAACATAACGCGATACAGTATGCNTTAAAAAACGTGCGAACNGTAGCGAACTTGGACTTA
CCAGAGCAAAGTATCGGGCCACAGTTGCTCAAGTCTCGGAAGTACTTATCTACGCTTCCCATTCTGCCGTA
TAACAACGTAAAGGCTCAGCTTATTATCGGCTTGGACAACATCAAAATAACTGCACCCCTGGAGATACGA
GAAGGTGAAAACGACGACGTCATAGCAGCGCGAAGCAGACTAGGATGGGCCGTATATGGCCGCCCCATG
GTGGGTGAAAGCACCACGCCTCGTCTGCTACATATTTGCCAGTGCAGTGCGGCGCGTGGGATGGATGAGG
CTCTGAAGAACTATTTTGCCCTGGAGTCGCTAGGTGTGAGCGTCTCCTCAAATCCGCTGAGATCCAAAGAG
GACGAACGCTCGATGCAGATAATGGAGGCAACAACAACCTATCTGGAAGACGAAAAGCGCTGGCAGACC

GGCCTGTTATGGNGGTTCGATCGCATTCACATGCCAGACTCTTACCAGATGGCGGTTAAGCGCCTGAAAT
GTCTAGAGGCAAAGATGTCNAAAGACCCACCACTGAAGGAGTTCATGATGAACACGATGCACGACTACA
AACAGAAGGGATATATCCGCCGGCTGAAGGATAGTGAGTTAGTAGCTAACACGACGTCGTGGTTTCTTCC
TATCTTTACAGTCACCAATCCGAACAAGCAAAAGACGCGCTTGGTTTGGGATGCGGCCGCCAAAGTTAGT
GGCACGTCGCTGAACGATTGTTTGCTGAAAGGCCCAGATACACTAGCATCTTTGATGGGCGTGCTAATAC
GCTTCCGAGAGCGCCCTATCGCCGTATCCGGCGACATACGGGAAATGTTTCATCAGGTCAAGGTGCGCCG
AGAAGACCAACCGGCNCAGAAGTTTCTCTGGCGTGATGGAGATTCGTCACGCTCCCCCGAAACGTACGTT
ATGCAAGTCATGACGTTCGGAGCATCCTGTTCGCCAGCCCTAGCAAATTACGTCAAGAATCGCAATGCCG
AGCGGTTCGTAGCGGAGCATCCGGATGCCGTAAAGGCGATTTGCGAAAACACATTTGTAGACGACTGGCT
GCAGTCGGTAGATGCAGAATCCGAAATGATGCAGTTAGCTGAGACTGTAAAAAGGATTCATGCTAGTGGC
GGCTTCGAGATGCGCCACTGGACATCAAATTCGAAGCAAGTTATGCAGGCGCTGGAAGACGACTGTGAGG
GCTTGGACAAGCGTATCAGCGCTCAGGATGAAGCGCAAGAAAAGGTCCTGGGCCTGTGGTGGCTACCTGG
ACAAGATCTGTTGACGTTTGTGGTGAAGCCGAACTTGCTCGCGAAAGCATCTAAGGAGCGCCCAACTAAA
AGACGCGTTCTGAGCGTGGTCATGTCTATTTTCGACCCGCTCGGGCTGCTAGGATTCTTTAATATACGCGC
TAAGATCATCCTTCAGAACATATGGCGATCCAACATCGGATGGGATGACGACCTTACCAAAGAAGACGAA
GCGGATTGGCGACACTGGCTAGATCTAGTTTCAAAGTTGAATACCGTCCGCATTCCACGGTGCATGAAGT
GGGTGAGCCGAACCCAGGCTGTGCAGATGCATACATTCGTTGACGCCAGTATGAATGCTTACGCCGCAGT
TGTATTTTTGCGGGCTGAACTTGATGGCCAAGTACATTGCAGTTTGGTCGCCTCGAAAACGAGAGTAGCAC
CCCTAAAGCCCGTGTCCATACCTCGNATGGAACTGATGGCCGCGGTCTTAGGTCTGAGACTGGCCAAATG
TATCCAAAAGGAAATGTCGGTACGCATACATAGACGAACATTCTGGACAGATTCAAAGGACGTGCTCTAC
TGGATCCGNTCCGATGCTCGAAAGTTCCAGCAATTCGTAGCGCTTCGCATCGGAAAGATTTTAGAAGAAT
CAGATGTGGACAGCTGGCGCTGGGTCCCATCAGCTCAAAACGTGGCAGACGATGGCACGAAATGGACCA
AAACGCCCGAAATTCACGGCTCAACCAGGTGGTTCAATGGACCACCATTTTTGTATCTAGAAGAATCGCA
GTGGCCACAACAGGAAATGGCCCTGCAATAAACATGTTGTACCATGCTGAAGAACAANCCAATCACACGT
CNTCCTGGAGATGTATCCTGCCGGATCTGCAGCGCTTCAGCAAGCTGGAGAAATTGAGAGCCGTACAGCT
ATGCGTACTGGATTTCCTACGGAATATTACTAAGAAGGTCAGATTGGACGGAGGACCGGATNCGCAGAAG
ACGCTGCTCCTTAAAAGAAGCAATGAAATGGATGTGATTTTTATCCGGATTTGTCAAGAAGAGGAGTTTT
ATAGTGAGATCACCTNCTTAAGGTCGGGGCGCCGCCTAACCGATCGCAAAAGCTTGCTNTTCAAGCGCTC
CCCTTATGTGGATGACTCGGGCATTCTACGTATTAAGGGACGGATAGACAGTATAGAAGGAGTCGAAGTC
TGCGTAAAACGCCCGATAATTCTGCCAAGACGACATCGATTGACGTATCTGCTNGTTGAGTTTTATCACCG
CAGATATCATCACCTGCACAACGAAATCGTCGTAAATGAACTGCGNCAGCGGTACTGGGTTTGTGGCTTG
CGAGCTTTGGTGAGAGAAGTCTCCAGTACCTGCCCAGCGTGCCGCATACGACGCGCCCGCCCAAAGCCGC
CAGGGATGGGCGACCTGCCAATCGAACGATTATCGCCCTATACTCCACCGTTCACATATACTGGAGTGGA
TTACTTCGGNCCCTACGACATTGTCGTTGGACGGCGTCGTGAGAAGCGCTGGGGCGTGCTGTTCACATGCC
TCACGGTACGCGCCGTTCACCTAGATATAGCCACGTCGCTCTCAACTGACTCATACTTATGCGTTCTGAAG
TCGTTCGTGGCCAGACGCGGCTGCCCCCGCCGCATGCTGTCGGACAATGGCACAAATTTCAGAGGCGCTA
GCAGAGTATTAAAAGATGAAATTGAACGTATATCGCCGGCTCTGATCGAGCGACAGTACCCGGAACTNGA
GCTTACCTTTATCCCGCCAGGATCACCCCACATGGGAGGATCGTGGGAACGAATGGTGCGCTCTACAAAG
TCAATACTGTCGGAGATTCTGCCGCACTCTGGGCTACGAGAGGAGGTGCTACGAGCAGCATTGGCAGACG
TAGAAAGCGTGCTCAACTCAAGGCCGCTCACTTATGTTCCCTTGGAGACATCTGAGTCCGAAGCCCTGACT
CCGAACCATTTTCTTATAGGTCATTCAAGCGGATTGCGTGAGAGAGGNTCACGGGAGCGGGGCGCAGCTA
GCTTGGCACGAGGCTTCCGAGTCGCCAGCCAATTGGCTGACCAATTTTGGAAGAGATGGCTGAGCGAGTA
TCTGCCTACACTCACCAGACGTACTAAGTGGTTTCAACCGCCANTCGAACCAATATCAGTCGACGACATC
GTCATAATAGTGGACGACGGTGCCAAGCGAAACTGTTGGACAAGAGGAGTGGTCGTNGACGTGCACCGTT
CGAAGGATGGCCAGGTCCGAAGCGCCGTGGTGCGAACCGCCGATGGAATTATTACTCGCCCCGCCGTCAA
GCTAGCTAAGCTTGACATTAAAGTTGGTAACACACTGCACGGACGTTCGTGAATTACGGGGTGGGGAATG
TTACG
>9_rnd-1_family-274__LTR___Pao
CTTCACTCTTGACCCTGCATCATGGAACATTCCGTCAAACATACAACTAGCTGATCCTGAATTCTTCAAAT
CTCAGCAAATCGACATGTTGATCGGAGCCAGTCTGTTTTTCGATCTGCTCTGCGTTGGCCAGATAAAACCA
GCTGCTGGACTGCCGATANTGCGAAAGACTCGCTTTGGTTGGGTCGCGACTGGAGGCGCTGCACATGCAG
GAAAATCATCGCTCGTGGCTAGGCGATTAGNGATGAATCCAGATCTGCTGGTAGACTCGCATCTGCAGAC
GAATGCACAGATTGACGAATTAATCCGTCGGTTTTGGGAGCTGGAATCTTGCACCGAGCCGGAGTCATCA
CCGAACAGGGGGGAGCGTGACTGCGAGGCACACTTTCAGGCCAATTTTAAGCGTCTGCCGACTGGCGAGT

ATTCAGTTCGCTTACCGCTTCGNCTAAGCCTGGATCAGCTGGGTGACTCCTATCAACAGGCGCTTCGCCGA
TTTCTTAATTTAGAAAGGAAACTAGACCGCAACCCAACTTTGAAAACCCAGTATGCAGCATTTATCAAGG
AATATCTTGACTTAAATCATATGTCACTTGTTAGCTTAGCTGCACTGGGCCAATGCAAATACTATCTGCCA
CATCACTGCGTGCTGAAGGAGGATAGTACTACAACCAAGCTAAGGGTCGTGTTTGATGGATCAGCAGCTA
CTACCTCTGGACACTCGCTGAATGATGCACTGATGGCAGGTCCTACCATCCAACCGAAACTGTTTTCGATA
CTGATGCGTTTTCGTACATTCGCAGTCGCCCTCACAGGCGACATATGCAAAATGTATCGATGCGTACGAGT
CGAACCAGCAGACAGCTATCTTCAATGTATCCTGTGGCGTGATTCTCAGCATCAGAAGATACAGACTTAC
AAATTAGACACCGTCACCTACGGCACAAAACCAGCATCGTTTCTCTCAGTGCGGGCTATGCACCAACTGG
CCATGGATGAGCAGCAGGCATTTCCTATTGGCGCTGACATTATTAAAAGGGATTTCTACGTGGATGATCTC
ATCTCTGGTGGTAGCTGTGTTCAGGAAGCAGTCGAGATATTGAAGCAAACGTCTGGACTACTCGCCAAGG
GCAACTTTAAGCTGCGAAAATGGTGTTCTAGCGACGCTACTGTACTCCAAAGCATACCGGAAGAGGATAG
AGAATCGCTGCTTAAGTTCAACGATGGCAGCGATATCACGAAAACGTTAGGCCTCGTTTGGGATCCCGCC
TCAGACTGTTTTCTTTTTTTTTTTTTCTCCACTGAGGCTACCCTCGAGACTGACAAAACGATCAATCCTCTCC
TCAATTGCTCGTTTTTACGACCCCCTTGGTCTTGTTGGNCCTGTAATAACAAAATCTAAAATTTTTATGCAG
GATCTCTGGAGAGAAAAACTGGACTGGGATGAGAGCTTGCCTGTACACTTAAGCACAGCCTGGGTCAACT
TCTGCGCTGATTTTGAGCAGACTCAGCAATTCCAGTATCCNCGTCGTGCACTCTCATCAGACAGCACAGTT
GAGATTCACGGATTTTGTGATGCCAGCCTGAGCGCTTATGGAGCATGCGTCTATACAGTTTCAAGGTGCCA
TGGCAACACAAGCGTACGTCTCTTGTGTTCCAAGTCGCGTGTCGCGCCTGTGAAAACCATCACTGTACCAA
AGCTGGAACTCTGCGGAGCTGCATTATTGGCTGAACTTCTCAGCGAAATCTGCCAACTGAAAGTGTTTAAC
TGTCGTTATTACTGTTGGTCAGACTCTGCTGTAGTTTTGGCTTGGATTCGCAATGATGCTTCCAAATTCAAT
ATTTTCGTTGCCAATCGGGTCGCAGCCATCCAAGAGCTCACCACTGGCATGGAATGGCATCATATTCCCAC
TGAATTAAGCCCGGCGGATATAATTTCACGAGGAGCGCTGCCCAGTGAGCTTTCCAGGTCCCCGTTATGG
GCCCATGGCCCGAGCTTTCTCAGAAAGGAAAAGGAAGAGTGGCCTGCCTCCTGTGTACCTGTCGAATCCT
TGCCAGAACTCCGACACAAGGTATTACTCGGAACTGCAGCGCAACTTGATCTGTCAATTGGCTGCAAGTT
CATCAACTCGTTCTCCAAGCTGCAACGCGTCTTTGCTTACATTTACAAGTTTGTAAATCGAATCCGAGGAT
CTGAGGTAACTGTAAGCCACGTGCATCATGGCACACATCTGTTGCTGCGGTCAGTGCAAATGGCTACTCTT
GGCGATGACTACAAGGCACTGATGGAAGGAAGGCCCGTCAAACGGTCTAGCTCAATGGCTTCTCTCGCGC
CATTCCTTGACGACTTCGGCTTACTCCGCATCGGTGGACGGCTGAAAAATTCGTCGTTGGACTTCTCAGCG
CGACATCCGATTATATTGCCTCGCCAGCATCCTGTGACCCGAGCAATAATAATCCACTTTCATAAACGGAA
TTTACATGCTGGACCTCGCGCCGTATTGGCTTCGATTCGGCTCCAATACTGGCCCATTGGCGGTCGTAAAA
CAGTCTCTAGCATTGTTGCCAAATGCACAATCTGTTTTCGTGCCAAGCCACGATTGGCAGAGCATATAATG
GCAGATCTACCAGCTGATCGTCTCGACACATCGTATCCTTTTATGGTCACTGGCGTTGATTACTGTGGACC
ATTCTTCTACAAAAGCGAAGTCCGCAACAGGCCTCCCGTGAAATGCTACATTAGCCTGTTCATATGCTTCG
CTACAAAGGCAGTACACTTAGAACTTGTAAAGGATTTGTCTACAATATCGTTTCTAAACGCCCTAAAACG
GTTCATCCTGACTCGCACTCGACCTTCAAGGATCTGGTCTGACAATGCGACAAACTTCGTAGGCGCTAAGA
ATGAGCTGGCAGATCTGAGCCGCCTGTTCCTGAGTGACGAGCATGTCAAAGCTGTTCACGAGTTTTGCCTA
TCCGATTCAATTGAATGGTCGTTCATCCCTCCTCGCTCTCCACACTTTGGCGGGCTATGGAAGCCGCTGT
GAAGACTGCGAAGCACCATTTTTATCGCTCTGTCGGCCCTTCCATCTTGGACTTCGATTCGCTGCGCACGC
TCGTCTGCCACATCACGGCAATTATTAATTCTCGTCCATTACTTCCACTTTCAGAGCATCCCGATGATCTTG
ACGTCTTGACACCCGCACACTTCCTGGGTACAGCGCCATCTTCGTCATACATTGAGCCTGATCTAAGGAAG
CTTAATTTCAATCGGCTTAGCTATTTTCAGCGCGTCACATACCTCCAACAAGTATTTTGGGCCCGTTGGCG
CGAAGAGTATTTAACGCTTCTTCAACAGCGCTCCAAATGGCGCACTCCTCAGCCAGGACTTTCCATCAACG
ATGTCGTCCTTGTAAAGGATGAGAACCTACCGCCGCTGAAGTGGCCATTAGCTAGAGTTCTAGAGCTGAT
CTCGGGATCTGATGGGGTATCTAGAGTTGCAGTGCTTCAGACTGCGACTGGAGTCATACGCAGAGCCGTA
CGAAAGCTGTGTTTGCTGCCAAAACAGGATGATGTTGAAAGTCCCTTGCCTTCCAACGGGGGAGGATTGT
TTGGTCAAGTGACCGGTAGAGATAACAGCGACGGCTGCGTGCAGTAACAGCAGAAGACAATAANCATAA
TTACAGCTTAAGAGCAAATGATGTTTGCTCTTTGTAAGTTGCTGCCTACTCTCTTCATCGGCCCTTTGCCTC
CTGCATTGACGCCCGCTGCCCATCGTTCTGAGCGGAGCTCAATTTCGTTCGTTTTGCATATAGTTTAGTTTT
TCGTTTGATCGCAAGCAAGGCACAACTATTTCGAGTTGACTGCCGAGCAATATTATAACATTTCGTAAATT
ATAATAAGTGCCCTGTGCGGTAAATAAATACATTCGAAACAAAATATAAAACTTCGAATTTCGTTGCACA
CAAAGCAGTGCTAATTAATTAGTTGCAAAAAGGCTTGTAACTACACATTTTGGGTGACCCCGACGTGATTT
TTTTTTTTTTTCGTTTCCCTTCTGTTCGCATTTTGTTTTATAATTTCATTTTATAATTTCATTGCGCATATTTA
CAAAAGGTTGTGCGTTAAGTGCTGCGAGCCTCAACATAAAGTGATCAATTTAACCAAGTCTCTCTAATAA
CTGTACTTAACCGCNTGCTTGTGCATATGCATACAGTTTGTCGTTGTACATAGACGCCGTAGNAAAATCGC

CGTTAATCGTTGCGCGCTTTAACCGGCTATACGCTATCTCGCTCGAACAAACACTTGTGCGCGCAAATTTT
GTATTGTAGTTCGGCTGTGTGAACTTTGTTGTGCGCTACATTAACAATGTTTGATGAAGGTGCAAGTGCAA
ATGAAAATGAGGCGACATCTGCTAATCAAGTGGCGGTTGGAACACAGGCTGCAGTTCTTAAAGTAAAGAT
TAAGAATTTAAGTGATCGAGTCAATAGATTGTCCTCTGAACTTGATCCCGCGCGACTTCGCGATGTTGATG
ACTACGAGCTGCAAGATTACATATGCATGGCATCTGACTTGCAGGCGAAATTTGAAACAATCTATGATAG
TTTGTTGGATGTGAGTCAGGCCAGCGTTGAAGAGGATCTTCAGTCAAGCTTTGAGTCAACCATTAGGCGG
CTACGAGTGTCCCTTCAACGCGAGCGCGGAAATCGAAGCAAGATTCAGCAGACTCCACATTGCTCCACTT
TCAATTCCGCCGCAGCCGATGATCCGCGCTCTACTTTTGTTGTTCCAAACCACTCTCGATTGCCTCAACTTA
AATTGCCGGAGTTTAGTGGAGGCTACACAGAATGGTCTGACTTCTCGAATATGTTCACCACGGTCATTGAT
AAGGATCCGTATTTGACCAACATTGAAAAACTTCAGCATCTACGGTCATGCCTCAAAGGAACAGCGCTGG
ATACAATTCGCTCGTTGGAAATGTCAAACGCTAATTATGCTGCCGCCTTAGAACTGCTTGATAAGCGTTTT
AATAACAAGCGTCTTGTTTTTCAGGCACATATTGCTGAAATTTTGGGTTTAAGAAAGGTGGATAAGGGTGC
GACGACACAACTGCGCGAATTCTCAGATAAGGTCAACTCTCATTTACGTGCGTTAAAATCGATGGGCAGT
GTGGAACAGATCGCCGGCTGCATCATAGTACACACACTGCTGCAAAAACTAGATAGCGCCACGCAAGCTA
GCTGGGAGGATGATGCGCCGTTGGACGTCATACCATCATGTGAGCGGCTTACAGCTTTCATTGAGAAGCG
TTGTCAAAGGCTGGAAAATGCGGATCACGCGACGGCAATGTACACGCCTGGCTCCCAGGTGGGCCAGAAC
AACAGTAGNAGAAGAACGTTTGTGGTGACCAAGAATGGAACATATGCATGTGTATTTTGTGAAGCCGCAG
GCCACTCCATTTATAAATGTCTGCAATTCGCAAAATTATCGCCCTCGCTGCGCCTTCATGAAGCCAAACGG
CTTGCGCTGTGCCTTAATTGCCTGCAAAGGGGACATCAGCTGAGAGTCTGCGGTTCCAGCACTTGCAGAGT
CTGTGGAAGCAAACATCATAGCCTGTTGCATCTTGGCAACACAAGCAGTCACAGCGTTGCTTCTAGCTCAC
ACAATGTTCAAGAAGCCGAAACTTATACGTCATCTCAAAACATCCTGGCGGCAACTTCATCGTCGACACT
CTCCATCGACCAGCATCTTAAGCACGATGTCGTCCTACTTGCCACTGCCGTCATCAATGTAAAAAATCGCG
CTGGCTCCTTGGTGCCCTGCCGTGCGTTGCTCGACTCTGGGTCGCAATTGCACATCATCACCTCTCGTCTTG
CTCATCAGCTCCAGCTGCGCAAATTCAAGTCCACAGCAGTCGTCTCTGGCATTGGTGATGCAGCGTTTTCG
TCAGATGGTTTTTCGGTAAACATCAATGTCCAATCTCGAGTGTCTGAATACTCCACATGCATCCCGGCATT
GATTGCACCATCCATCACCGATAATCAGCCTGGCTTCACTCTTGACCCTGCATCATGGAACATTCCGTCAA
ACATACAACTAGCTGATCCTGAATTCTTCAAATCTCAGCAAATCGACATGTTGATCGGAGCCAGTCTGTTT
TTCGATCTGCTCTGCGTTGGCCAGATCAAACTAGCTGCTGGACTGCCGATACTGCAAAAGACTCGCTTTGG
TTGGGTCGCGACTGGAGGTGCTCCACATGCAGGAAAATCATCGCTCGTGGCTAGGCGATTAGTGATGAAT
CCAGATCTGCTGGTAGACTCGCATCTGCAGACGAATGCACAGATTGACGAATTAATCCGTCGGTTTTGGG
AGCTGGAATCTTGCACCGAGCCTGAGTCATCACCAAACAGGGAGGAGCGTGACTGCGAGGCACACTTTCA
GGCCAATTTTAAGCGTCTGCCGACTGGCGAGTATTCAGTTCGCTTACCGCTTCGACTAAGCCTGGATCAGC
TGGGTGACTCCTATCAACAGGCGCTTCGCCGATTCCTTAATTTAGAAAGGAAACTAGACCGCAATCCAAC
TTTGAAAACCCAGTATGCAGCATTTATCAAGGAATATCTTGACTTAAATCATATGTCACTTGTTAGCTTAG
CTGCACTGGGCCAATGCAAATACTATCTGCCACATCACTGCGTGCTGAAGGAGGATAGTACTACAACCAA
GCTAAGGGTCGTGTTTGATGGATCAGCAGCTACTACCTCTGGACACTCGCTGAATGATGCACTGATGGCA
GGTCCTACCATCCAACCGAAACTGTTTTCGATACTGATGCGTTTTCGTACATTCGCAGTCGCCCTCACAGG
CGACATATGCAAAATGTATCGATGCGTACGAGTCGAACCAGCAGACAGCTATCTTCAATGTATCCTGTGG
CGTGATTCTCAGCATCAGAAGATACAGACTTACAAATTAGACACCGTCACCTACGGCACAAAACCAGCAT
CGTTTCTCTCAGTGCGGGCTATGCACCAACTGGCCATGGATGAGCAGAAGGCATTTCCTATTGGCGCTGAC
ATTATTAAAAGGGATTTCTACGTGGATGATCTCATCTCTGGTGGTAGCTGTGTTCAGGAAGCAGTCGAGAT
ATTGAAGCAAACGTCTGGACTACTCGCCAAGGGCAACTTTAAGCTGCGAAAATGGTGTTCTAGCGACGCT
ACTGTACTCCAAAGCATACCGGAAGAGGATAGAGAATCGCTGCTTAAGTTCAACGATGGCAGCGATATCA
CGAAAACGTTAGGCCTCGTTTGGGATCCCGCCTCAGACTGTTTTCTTTTTTCTTTTTCTCCACTGAGGCTAC
CCTCGAGACTGACAAAACGATCAATCCTCTCCTCAATTGCTCGTTTTTACGACCCCCTTGGTCTTGTTGGTC
CTGTAATAACAAAATCTAAAATTTCTATGCAGGATCTCTGGAGAGAAAAACTGGACTGGGATGAGAGCTT
GCCTGTACACTTAAGCACAGCCTGGGTCAACTTCTGCGCTGATTTTGAGCAGACTCAGCAATTCCAGTATC
CCCGTCGTGCACTCTCATCAGACAGCACAGTTGAGATTCACGGATTTTGTGATGCCAGCCTGAGCGCTTAT
GGGGCATGCGTCTATACAGTTTCAAAGTGCCATGGCAACACAAGCGTACGTCTCTTGTGTTCCAAGTCGCG
TGTCGCGCCTGTGAAAACCATCACTGTACCAAAGCTGGAACTCTGCGGAGCTGCATTATTGGCTGAACTTC
TCAGCGAAATCTGCCAACTGAAAGTGTTTAACTGTCGTTATTACTGTTGGTCAGACTCTGCTGTAGTTTTG
GCTTGGATTCGCAATGATGCTTCCAAATTCAATATTTTCGTTGCCAATCGGGTCGCAGCCATCCAAGAGCT
CACCACTGGCATGGAATGGCATCATATTCCCACTGAATTAAGCCCGGCGGATATAATTTCACGAGGAGCG
CTGCCCAGTGAGCTTTCCAGGTCTCCGTTATGGGCCCATGGCCCGAGCTTTCTCAGAAAGGAAAAGAAAG

AGTGGCCTGCCTCCTGTGTACCTGTCGAATCCTTGCCAGAACTCCGACACAAGGTATTACTCGGAACTGCA
GCGCAACTTGATCTCTCGATTGGCTGCAAGTTCATCAACTCGTTCTCCAAGCTGCAACGCGTCTTTGCTTA
CATTTACAAGTTTGTAAATCGAATCCGAGGATCTGAGGTAACTGTAAGCCACGTGCGTCATGGCACACAT
CTGTTGCTGCGGTCAGTGNAAATGGCTACTCTTAGGGATGACTNCAAGGCACTGATGGAAGGAAGGCCCG
TCAAACGGTCTAGCTCAATGGCCTCTCTCGCGCCATTCTTTGACGACTTCGGCTTACTCCGCATCGGTGGA
CGGCTGAAAAAATTCGTCGTTGGACTTCTCAGCGCGACATCCGATTATATTGCCTCGCCAGCATCCTGTGA
CCCGAGCAATAATAATCCACTTTCATAAACGGAATTTACATGCTGGACCTCGCGCCGTATTGGCTTCGATT
CGGCTCCAATACTGGCCCATTGGCGGTCGTAAAACAGTCTCTAGTATTGTTGCCAAATGCACAATCTGTTT
TCGTGCCAAGCCACGATTGGCAGAGCACATAATGGCAGATCTACCAGCTGATCGTCTCGACACATCGTAT
CCTTTTATGGTCACTGGCGTTGATTACTGTGGACCATTCTTCTACAAGAGCGAAGTCCGCAACAGGCCTCC
CGTGAAATGCTACATTAGCCTGTTCATATGCTTCGCTACAAAAGGCAGTACACTTAGAACTTGTAAAGGAT
TTGTCTCCANTATCGTTTCTAAACGCCCTAAAACGGTTCATCCTGACTCGCACTCGACCTTCAAGGATCTG
GTCTGACAATGCGACAAACTTCGTAGGCGCTAAAGAATGAGCTGGCAGATCTGGAGCCGCCTGTTCCTG
>9_rnd-6_family-1051__LTR___Gypsy
TTCACAGTAGAATACCTAAAGGGGAAAGATAATTTCGTAGCAGACGCGCTTTCGCGTATTAATATNAAAG
GAACTCAAANATATGCAACATAAAGTCCTGAAAGTCACTACCAGGCGACAAAGTAGACAAAANAAAAAC
TGTACAGTAGAAAAATTATGAACTTCTGCCAAGGCAAAGTCATAAAAATGTATCCAAGCCCAACGTACAT
GAAGTCATTACAAATGATGAAGTACGAAAAGTAGTGACCTTGCGCATAACAGAATCTAAATGTTTATTTA
AACATGGAAATAAAATTATCGCAAGAATTGNAGTTAGCGATTTATATACCAATGGAATTCTCGACCTAGG
TCAGTTCTTCCAAAGGCTCAAAGAAGCGGCCGGTATATTAAAAATCAGCCAACTCAAGGTGGCACCGAGC
GAAAANATCTTTGAATCAGTTTCAATAGATTCCTTTTAAAAATATGGGCAATAAAATATTAAAATCTTTAA
GAGTAGCGCTACTCCANCCGGTGACCCAAATAAAAGATGGAAAAGAGATACAACAAATATTGTCTACAT
ACCATGACGACCCAATTCAAGGAGGTCANAGCGGCATAACAAGAACGTTAGCGAAAATAAAAAGACACT
ACTACTGGAAAAATATGACACGTCATATAAAACGNANGTACGTGTCAAAAAATGCTAAATGTCTAAAGCA
AAGACGCACACAAAAACTCTTCTGANTCGCACAGAAACACCTGTGAATGCTTTCGATATAGTGATAGTGG
ATACAGTTGGTCCGCTACCCAAATCGGAAAAATGGTAATGAATACATAGTCACATTAATATGCGACCTTA
CAAAATATTTAGTAGCCATTCCAAAAAAAGCAAAAGCGCAAATAATGTCGCAAAAGCTATATTCGAAAAT
TTCATACTCAAGTTTGGTCCAATGAAGACGTTCATCTCGGACATGGGAACAGAGTATAAAAACCAGATTT
TAACTGACTTATGCAAGTATTTGAAGATCAAGAATCTCACCTCTACTGCATATCATCATCAAACATTAGGG
ACAATAGAACGAAGTCATCGAACTTTTAATGAATACATTCGTTCATACATCTCTGCAGACAAATCTGATTG
GGATGTCTGGGTTCAATATTTCACTTATTGCTTCAACACGACACCATCAGCAACGCATGAGTATTGTCCAT
ACGAACTAGTATTTGGCAGACTACCGGAAACAGTTCATAGATTTAAACAAAACTAATTACATAGATCCTA
TTTATAATTTAGATGACTATTCTAAGGAAATAAAGTTTAGATTAGAAATAGCATACAAAGGAGCTAGAAT
TATGTTAGAAAAGCTAAGGCTTATAGGAAATTAGCATATGACGAGAAAAACAAAAAACTTGAATTAAAA
GTAGGANATAAAGTACTTATAAGANATGAAACAGGGCACAAATTAGACCCAGTATATTTAGGACCATATA
TAGTAGAGAAAATAGAAAATAATGACAACATTTTTTATTAAAGATAGTAAAGATAAAAAAACAAAAAG
TTCATAGGAATAGATTAACAATTTTTAATTCTTAATAAAATAAAATAAAAAAATATATGACGACCTGATC
AACCGAATGAAAAAATTTTTTACAAATAAACGAATTATAAATTTTTATACCCTAAATAATTTTTGTATTTTT
ATACTCCAACATTTAAGATTTCTTATTATATAAAAAAAATTTTCAATTTTTTATAAAAAACAAACCCACAA
TAATTAAGTTCATTTAATAACTACACATTCATAATGTTACGTTATTTCTCTAAAAGGGAGGTGTAGTGCGT
ATATGCCCTTACAGAATACCCCGTACTTAAACATAAAGTATGCAGCACTTTGTTGCATACAATAACAATAA
TATAGTAAACGAGCCCAGAGCCACATTATGTATTGGCTCGGTCTCATATTACGCAGCGCGGTTCACTAGCC
TCACTAGCTGACCGTTTATGCTTATGATGCATATCCCACGCTCCGTCTGAGCGCATTCCGTGAGCAATATA
TATGCTCACTAATACAGGCATATGCGAGCTACTCACTCTCCCTCTCTTTGTAGCTGTTAACGAATAACAGC
ATAAGTATATATGTATATCCATAGGCTAAGCGAACATTATAACAAGTGAATAAATTCATTCTGAACAAAG
ATACAAAACAGCACTGACGTGCTGACTTNTTTTCATTGACAATAAGTGCTCATTTTACATGGCGACCGTG
ACTTGGTCACGCATCCCTGCCTGGAGGAAGTAGACCAAGTGAAAAGGAACTTTTCATGGCCAACAAAGAG
GCCATGTATAATATACAATTTGAAAAAGGAACTTCATGGCCAACAAAGAGGCCATGTATAATATCCAAGT
GAAAAAGGAACTTCATGATCAACGAGAGGCATCCCGTATTCTGAGTGAGTAGAGAGTGAGTGTTATGGTT
CATAAACGCGGTGGCTTAAAAAATAAATTAATAAAAGAATAGCGCACCTAAGGTGGCTGCTATACACAA
AACAGCCAACACCCGCATGGTCGACAGCTCAGAAGTTACAAAATGAGCTCGACTATCTGCAAAATGAAAT
TAAGCAGAGAAAACCAAACACCACTACTCACTCAAATATAAAAGTTCTAATAAACCAAACAACTAATAA
AGTTACACCCATTGACGCAACCAGCCTACCAAAAGAAAAACGAATGTGCCCACAGGATTGCGAAGGCCC
ACTCGACAGCCCCTTCTGCGAATATACGTGCAGACACTGCACAGCCAGCACCTCCAAACATTACATTACA

ACCAACAAATAAACACCTGTGCGAAGACCAGCACCGGTACCGCCAGCGCAACAAGCTGTGCCATCGGAA
TAAGTGCGAGAGGCACACCGCCACTTGCGCATACGAGGGAGCATCAGCGTGCCACATCCGGCGACGCCC
AGCTGATGCAACGATGAACAACAGTTGCAGCTATATCCTTAACTACTTTTAATTAAATATAAAAAAATTTT
TGTAAAATTGAGCGGAAACACTTCCGCCCGATTAGCAGCATAGCCCGTAAAGCTATGCGAAATGCTAGGG
TAGTATTTTGTGTTATAAGAAATATTGAAACCTTGATTCTAAAAACCCAAGTCAACCAGCACTAACGTGCT
TTTGTGAAATCATTATTAAATCCAAACATTACCAACATATTAAATTAAATAGTAATAAATAAAACTTAAAA
ATTGTAATGCCCGACGACAATTGAGTCGACCGGCAAGACGTTTGGCCAGTACAGCCATTCGAAACAATAG
GTAGGAAAAATACAGATTGTAAAAAAAATTTTTTAGATGCGTTTTAAGCAGAAAACAAACAAGCAAAAC
AAAACCCGACTTGTAAAACAATAAAAGCATCAAATTCAATAAATATAGTAATAAAACCTCAAATGAACG
ATACAAACAACAACTTGTCTTGTAAAAGTAAACAAAAACAAATTTAACATTACGCATTTGTAAATTTTTTA
AGATAATTCCTAAGACCTCAAATGAACGATACAAACAACAAATATATGAATATTAATATCAAAAAGAGG
AAATTAAAAGTTATAATTCGTTCAGTTTGTCACATTAGGTAATTCAAAATTATGATCAAGAATTAGCATGA
TTAAGGACAAGATTAACAAGGTATATAACGACTATACAGACTTTCGTATGCGAGCCATACAAATTTTGCA
AAGGCATAGAGAATTCATACCAGTATATGAGTTCGAATACGAATATATAGAAACATTCGGCACAGTGACA
AGTCCACTCTATTCATGTTCTAAAATAGAGAGCGGAATAATCTTGTCCACACTACCCTACGTCAAAATAAA
AACAATTATTGACGAGCATCCGATTCTAGGGCAGACAACAATTGTTTTAGTACGTCTGCATAGGTAGTAGT
CAAATCAAAAAGCAAGAATATTTATATAGGAATATGGGGGAAAAGAATCAAAGGCAGATGATAACACT
GCCAATGTAGTTAACGTAGTAGAAGTCGTAGACACCACTCCCCATGTGCAGTTAACAAACATACTGCTAA
TAATACTCATTGTAATAGCTTCAGTAGACATCCTTCTGAAGATTTATCTTCTTCATAAAAANCACTGAGGA
AGCGTTACATGAGTCGAGCCAACGACTTAGACAAGGTCTAAGCCACTGCTTAAATAAAATATTTAAGTAG
CGGAAGCTTACATGGACTGGTCCCAAATAGTAGAAAAGATAAAGCGGCATAGGGAAAATTTCGATAAAT
CATACAAGTGCCTTAATAAAAGCGCACCAGTTTTAAGACCCACCATAATTAAACATACTGAGATCCTTGT
ATATGAGTACAATGAAATCAGGAGTCTAGTTACCGAGTGTCAAAATAAACTGACAAGTTTACACAGAGTC
CAAACTTCAGACGTTTTACAGCGTCTAAGGGATAATCTGGTAAACTTAAATAAAAAGTATGATCTCCAGC
TTTATCTTCCATCGGTGTTAAATACACCGGTAACATTTGATAAGTCACTACTGACAAACGACCAAATAGAA
GATACAAAATTAGTAAACATGGCACAAACAGTTGTAGAATTTATCAAGCTAGCCACATCTCTGATACCAG
AGTTTGACGGTAAGCCGGAAAATCTACAAAGTTTTCTAGATTCTTTGAATCTATTAGAAACCTTAAAAGGC
ACACATGAGCTAACGGCCATAAGCCTTATAAAAACAAAGCTAAAAGGCCATGCGAGAAATTTGATAAGT
AATGAGCAGACAATTGCTGCAATCATTACGCAACTATCAAATGCAGTAAAAGGAGAATCGGTAGAAGTTG
TATCTGCTAAGCTTCTTAATTTGCAACAGAAAAATAAAACGGCTAACCAATACACCCAAGAGGTGGAAAA
ACTGACAAAGGCCCTTGAAGGCGCCTATATCAGTGAAGGTCTAAGCCAATCACTTGCAAACAAATACAGC
ACTACAACAGCTGTAAAAAGCTATGACCCAAAATTGTTCGATTGATAAGGTAAAACTTATCATGCAGGCA
GGCACATTCACAAATATGAATGATGCCGTATCCAAATTTGTCAATAGCTGCACGGAGATAACAGGTCAAA
GTAACACTGTCCTGTATTATCAACGAGGTGCAAACAATAATAATAGAGGAGGCCGAGGTTATAATCGTGG
CAGAAATTACAACCACAACAATTATAACCGAGGCGGTAACAACAATAACAATAATTACAATAACCGTGG
AGGAAGACGAGGCCAAAATAGAGGAAGAGGCCGAGGTAACTCCAACCAAGGTTATAATAGTAACAATGT
GAGAATGGCGCAAAATACGCCGGAAAACTCTCAGAACCCTTTAGGAAACAACCAGTAAATGTAAAGGTT
CATTCCATCAATTACAGTCTTAATATATTTGTCACTTTTATAATCATTCAACTGAAAATAAACTCACATTCC
TAATAGATACTGGTGCAGATATCTCACTTTTAAAAATAAATTCAGACAATTTCAAAATTCAAGAAGAAAA
AATTATAAACATCCAAGGCATAGGCCAAGGTGTAATAAAATCTCAAGGAACAACGTCAATAGAACTTCAA
TACACAAAATATATTATTCCACACGAATTTCATTTAGTAGATTCTGATTTTTCAATACCGTGTGATGGAAT
AATAGGCATTGATTTTATTAAAAAATTCAATTGTCAATTAGATTTCAAACCAAGTGAAGACTGGTTTATAA
TTAGACCTCAAAATTTAAATTATCCAATTCATGTTCCGATAACATATAGTGCTGGCAACAACACAGTTCTC
TTGCCAGCCAGATCCCAAGTAATTCGAAAAATAGACATTAATACAGTAAATGATTATATATTTGTTCCTAA
TCAAGAAATACATAATGGAATTTATGTTGCAAATACAATAGCAGCATCCAAACATGTATACATTCGCCTTC
TAAACACAACTAATTTCGACCAATTGGTCAAAGTAAACAAAATCCAATATGAAAATTTAAAAGATTACGA
CATTCATAGCACTACTCCAGAAAATAGAAGCGAACAAGTACTTTCAAAATTAAAGAAAAATTTTCCAGAA
CAATTCAAAAGTCAACTAACAGAATTATGCACCAAGTATAGTGATGTGTTCGGACTGGAAACCGAACCAA
TAGCGACAAATAATTTCTATAAACAAACATTAAGACTTAAAGATGATGAACCCATTTATATTAAAAACTA
CAGAAGCCCGCATAGCCACGTTAAAGAAATTCAAAACAAGTAGGGAAATTAATAAACGACAAAATCGTA
GAACCGTCTGTATCCGAGTATAACAGCCCACTCTTGTTAGTTCCGAAAAATCATTACCAAATTCGGAAGA
AAAGAAATGGCGATTAGTAATTGACTATCGCCAAATCAACAAAAACTACTTTCTGATAAATTCCCGCTCC
CCAGAATTGATGATATTCTAGACCAACTGGGCAGAGCTAAATACTTTTCATGCCTTGACTTAATGTCAGGT
TTCCACCAAATTGAACTTGAAGAAAATTCAAGAAATATAACATCTTTTTCAACGAGCAATGGCTCATATCG

CTTCACGCGATTACCATTTGGTCTTAAAATAGCACCAAATTCGTTTCAGAGAATGATGACTATAGCATTCT
CGGGTTTAGAACCCTCCCAGGCATTCCTTTATATGGATGACTTAATGGTGATAGGATGTTCCGAAAACACA
TGATTAAAAACTTAACTGATGTTTTTAATGTATGTAGGAAATATAACCTGAAGTTGCATCCAGAAAAATGT
TCATTTTTCATGCATGAAGTGACATTTTTAGGTCACAAATGCACTGACAAAGGAGTATTGCCAGATGACAA
GAAGTTTGACGTCATCAAAAATTATCCTGTCCCTCATGATGCGGACAGCGCGAGACGATTTGTAGCATTCT
GCAATTATTATCGTCGTTTTATAAAAAACTTCGCCGATTATTCACGGCACATAACTAGATTATGTAAAAAG
AATGTTCCTTTTGAATGGTCAAGTGAATGCCAAAACGCATTCCAGTACCTTAAAGAAAAGCTTATGCACCC
CACATTATTACAATATCCTGATTTTCGCAAAGAATTTTGCATTATAACAGATGCCAGTAAACAAGCTTGCG
GAGCAGTTCTAACTCAGAACCGTGACGGAATTCAGCTCCCAATAGCTTATGCATCTCGTTCATTTACAAAG
GGGAAAGCAATAAGAGCACAACAGAACAAGAGTTAGCGGCAATTCATTGGGCAATTACCCATTTTAGAC
CATATATTTATGGCAAACATTTTACTATTAAAACGGATCACAGACCATTGACATATCTTTTTTCTATGACTA
ATCCCAGTTCTAAATTAACTCGCATGCGGCTAGAGCTTGAAGAATACGACTTCACAGTAGAATACCTAAA
GGGGAAAGATAATTTCGTAGCAGACGCACTTTCGCGTATTAATATAAAGGAACTCAAAGATATGCAACAT
AAAGTCCTGAAAGTCACTACCAGGCGACAAAGTAGACAAAATAAAAACTGTACAGTAGAAAATTATGAA
CTTCTGCCAAGGCAAAGTCATAAAAATGTATCCAAGCCCAACGTACATGAAGTCATTACAAATGATGAAG
TACGAAAAGTAGTGACCTTGCGCATAACAGAATCTAAATGTTTATTTAAACATGGAAATAAAATTATCGC
AAGAATTGAAGTTAGCGATTTATATACCAATGGAATTCTCGACCTAGGTCAGTTCTTCCAAAGGCTCGAA
AAAGAGGCCGGTATATTAAAAATCAGCCAACTCAAGGTGGCACCGAGCGAAAAGATCTTTGAATCAGTTT
CAATAGATTCTTTTAAAAATATGGGCAATAAAATATTAAAATCTTTAAGAGTAGCGCTACTCCAGCCGGT
GACCCAAATAAAAGATGAAAAAGAGATACAACAAATATTGTCTACATACCATGACGACCCAATTCAAGG
AGGTCATAGCGGCATAACAAGAACGTTAGCGAAAATAAAAAGACACTACTACTGGAAAAATATGACACG
TCATATAAAAAGAGTACGTACGTAGATGTCAAAAATGCCAAATGTCTAAAAAAAAAGACGCACACAAAAA
CTCCTCTGACTCGCACAGAAACACCCGTGAATGCTTTCGATATAGTGATAGTGGATACAGTTGGTCCGCTA
CCCAAATCGGAAAAAATGGTAATGAATACATAGTCACATTAATATGCGACCTTACAAAATATTTAGTAGCC
ATTCCAATAAAAAGCAAAAGCGCAAATAATGTCGCAAAAGCTATATTCGAAAANTTCATACTCAAGTTTG
GTCCAATGAAGACGTTCATCTCGGACATGGGAACAGAGTATAAAAACCAGATTTTTACTGACTTATGCAA
GTATTTGAAGATCAAGAATCTCACCTCTACTGCATATCATCATCAAACATTAGGGACAATAGAACGAAGT
CATCGAACTTTTAATGAATACATTCGTTCATACATCTCTGCAGACAAATCTGATTGGGATGTCTGGGTTCA
ATATTTCACTTATTGCTTCAACACGACACCATCAGCAACGCATGAGTATTGTCCATACGAACTAGTATTTG
GCAGACTACCGAAACAGTTCATAGATTTAAACAAAACTAATTACATAGATCCTATTTATAATTTAGATGAC
TATTCTAAGGAAATAAAGCTTTAGATTAGAAATAGCATACAAAAAGAGCTAGAATTATGTTAGAAAAAGC
TAAGGGCTTATAGGAAATTAGCATATGACGAAAAAANAAAAAACTTTGAATTAAAAGTAGGAGATAAAG
TACTTATAAGAAACGAAACAGGGCACAAATTAGACCCAGTATATTTAGGACCATATATAGTAGAGAAAAT
AGAAAATAATGACAACATTTTTTATTAAAGATAGTAAAGATAAAAAAACAAAA
>9_rnd-5_family-302__Unknown
CTCGCTCTCCGTGGGAGTCTTTAGAGCCATGGGGGAGGAATCCTGGGTTGGACTCGAAAGCTTGGGTGAC
GAAGCCGTCACCACCGCGTCCTCTTCCTCGGACCAGACTTCATCCCGGCCTTCCTGGCGAACCGTGAAGGA
ACCTTTTCGGGATGGCGCCTTGCCTTCTTGGCCGAACCAGGCGTCTTCTAACTCCCGCCGGGCGATGGTGT
CGGGTGTCTCGGCGCGGGCAATAGGCACGAGCGCGGGCATCTCGTCCTCGTCGTCGAGCCACTCCTCCTC
CGATTCCTTAGGAAAGGTTTCGTCTGGCGCCTCGGCGCGGGCTGTGGAAACGGCCGAGTTCGCCGCGTCC
TTGGTGCTGAGATGGTGCTCCTCCACGACCATCTGGCAGACCGCAATCGGTATCGGCCAGGGTTCGGGAT
GCCCTCCTCGAGAAGCGATCTTGCAGCGGCGGTTATCCTTCCCGAGCTGGGGAGGACCCTCCTGGCGCTC
GCGGGGCGAACGCGGCACGCACCGCTGCGATCCTTCGGGCTTCTCGGTCTCCGTGCGAAGCCTCTTCGTG
GCCGACGAGGATGCTCCCTCGGGACTGGGTCGGCGATGGCCTCCACTCCGGCGACGCTTGCCCCTGGCGG
CTCTCCGTCGACGATGGCGGCTGCGTCGGCGCTTTCCGCCGGGGCCTTCTTTGGCACCCCCATTCGCGCTT
TGCGGGGTTCAGACGGGGTTTGTTGGCTACCGCGGGGACCTTTTGGTAGGTACCGCAGCGTTATCCTGGG
AAACTGGCTGCCCGGGTCTACCTCCAGACGGTGGGTGGCAAAGCGGCGACGGAATTCCGGCTCGTCCTCC
GTTGACGGGTCCCCTCCCACTAAGGGTTCTGGGTCGAGGCTCAGCGGGTCTGCTTCCCCGTCGTTTGCGCG
GGATGCCGTTCGGCACCTCCGTCGGTGGCGACTCCGCGTCTCCGGCCGACGGGGATATCTCTTCTCCTACC
GCCTCCACGTCCTGCGCTGGTGGTGTGTGCTCCTCCGTTCCGAACGGTGCGGACTCTTCCTCGTCCATCTCG
CCGGCCGCGGTTGGGGTTGCCGGCTCTGATGATTGTGCCGAGTGGGATGCGAGGGGCGGAGATGCAGGCT
CTTCCGCGTACGGTGGCGAGTTCCGGGCGAATGGTGCCTCCACGAACATTGTCGTGACCTCGACTGCCATC
AGCAAGACCATCCCGTCTAGCCTCTCCATTATGCTGGTCCGCCGGGAGAATAGTGTATGGCGGAACTGGC
GAGGCGGTCCTTCTTGCCGTTCTCTTCGATAGTACTCACGCCCCTGGCGTGGGTCGCGCGGACGGCCTGCG

GCTCCCGGCCCGGGAAAGGCTCGGTGTCCCCGCTGCTGTTGTCTTCGTCTGACGATATGTCGATAACTTC
GGGTTCCATCTGAAAGTTCAGCTTATTGGTTAACTTACTCTTTACTTATTTGTTAATGCCCTATTCTCTCAG
TGCGGCTCACACTTTACTATGCCTATTCTGAATTATGTTGTTAATGTTAGTTTTATCTAAGCTTAATCATAA
TAGTTTCGTTGGGCGACGGGTGTTGCTCTCATCCTTCGCTTACTCGTGGGCATTTGCGTGCCCTGGATCTTC
GTCTTCTTCGTCGCTCGTCGTCTTTCTGTAGAATGGTTTCAATTGTGAGATGTTGGCCACCCTCTTCTTGCG
GTCGTCTGATTTCACCAGCCGCACAATATTGGGGATATGAATTTGACGACTTTGTAGGGGCCGTCAAATTT
GGGTGCCAACTTTGCGGCGAAAACCTTCGGCAGCGTTGGACAACTGGTGCTGCCTCAACAGGACCATCGA
GTCCAACGAGGGCCGCCACTCTCGTCTCCGCAAGTTGTAATGCCTGCCCTGGTCCTTTGATGCGTGCTGGA
GGTTGCTGTGGACGATATCAAAAATTTTTTTAGCCTATCCGCCTTTGTCCTGGGGTCGAGCGCACTAGTT
GCGGACCCCGGAGTTACCTGATCGTACAGAGCTGCGGGTAACCGAGGTTCCCTGCCCTGCATCAGGAAAG
CGGGACTAAAGCCCGTTGAATCTGCCACGCTCGTGTTGACCGCCAGCGAGATCTCGGGCAAGAGTTCATC
CCATGAACTCTGGTGGCCCTCAATAAATTGCGCTATCATGGTCTTTACAGTCCGGTTAGTCCTCTCAGTGG
GATTCTCTTGCGGACAGTACGGGGCGGTATACTGGATCTCTACACCCAGGGACCCCAAAAATGACTTGAA
GCTCCGACTCGTAAATTGTACCCCGTTGTCGCACACAAACTTTCTAGGTACCCCGAACCTACTTAGAATCC
TCTCTCGAAACGCAGTTTGCAGGTGTGCTGCCGTGGCTCTTCTGAGAGGGACCAACTCCACCCATTTCGAG
AAAGCGTCATGGAACACCAATAGCATCGTGTTTCCATGTTTCGACCGTGGAAGGGGTCCGACGAAGTCCG
CGCAAAGTACCGCCATCGGCTCTGCCACCTGCCTCGTCAGCATGCGACCAGCCGGTTTGAGTTGCTCACTC
TTGAATTTTTGGCAGTCTTCGCAACATTTCACGTATCTGGCGGCATCGCGGAACATACCCGGCCAGTAGTA
CCGCTGGGCTAACCTCGCCACCGTTTTTCTGACACCTTGATGTCCGGCCGTGGGAGCATCATGACATTCCC
GCAGTACCCTCTGGCGTTGACCACTGCCTACGCATAGTTTCCAAGGGATGTAGTCCTCATCGTCTATCCGG
TGACCCAAATGCCTATATAGCTGTCCGTTTTCCTCGGTGTAATCCGGGAACTTGGCTGGCTCTTCGGCGAT
TCTAACACGCATCCTCGCGACCCATTTGCAGGGGGCCTCGGCCACCACTGCTTGTTGACAGGTCTCTAGAG
GATGCCTCGACAGGGCATCGGCCACGACGTTGAGCTTCCCTCGACGATAATGAACGTCAAATTGGAATTG
CTGCAGTTCCAATGCCCATCGCGCAATCCTTCCTGACGGGCTCTCGATCGAATTCAGCCACTTGAGCGCTA
GATGGTCCGTTATTACGTCGAATCGGTACCCTTCTAGGTAGCAACGCAACTTCCGGATCGACCACACGATT
GCCAGGCACTCTTTCTCAGTGGCGGAATAGTTTACCTCGGCCTTCAGCAACTTTCGGCTCGCGTATGCTAT
CACCCGCTCTTGTCCATCGATCGTCTGCGTCAGCACTGCACCGAGGCCATACTCACTCGCGTCAGTCTGCA
AGACGAACTTGGCGGAAAAGTCGGGGCAAGCCAACACAGGGGCAGTCGTCAAGCTCTCCTTCAATTGAC
GCAATGCCTCCTCCTGTTCTTGCTCCCATGCCCATTTCTGCCCCTTCTTTAAGAGCGCTGTGAGTGGCTGTA
CCTGGGTCGCGAAATTGGGTACGAAACGCCTGTACCATGACGCCATCCCGAGACACTGTCGCAGTTCTTT
GCAATTTGTTGGGGCCCTCAGGTTTCGTATCGCCTCCACTTTCTCCGGATCGGTCCGTATCCCTTCTCCGCA
AATTACATGTCCTAGGTAGACTAACCTCTCCCGGAAGAAGCTGCATTTTTTTCTATTTAACCGGAGATTGG
CCGCCCTTAGCCGGCGGAATACCTCCCTCAAATTCTCTACATGCTGTGTTTTCGTCGCTCCTATTACCACGA
TGTCGTCTAGGTAAGCGAACGCATGAGGCTCCATCTGCGGCCCTATTACCGTATCCAAAGCGCGCTGGAA
TGTCGCACACGCCGAGTGTAAGCCGAATGGCATCACCCGCCATTGGAAAAGACCTCTTCCAGGGACTGTG
AACGCCGTACACTCCCGGCTATCGGCTGCCATCGGTATCTGCCAGTACCCGTGCTTCAAATCCAGAGTGGA
GATGAACCGGGCGTGGCGCAACCGCTCCAGGATGTGGTTAATACGAGGCACCGGATAAGCGTCCGGAAT
GGAATGCGCGTTGAGTTGTCGGTAGTCAATGCACATACGCCACTCGCCAGTCTTCTTCTTGACCAAGACGA
TCGGGGCGCTGTGCGGACTACGCGACGGCTCGATGGCATTTGCCCGGATCAACTCATCCACTTGCTCGTCG
ATCACCCTCTGCATGGCCGGATTCTTGGGGTAGTACCTTTGTTTCAACGGCTTGTCATCCTTCAGCCGGATT
CGGTGCTCCGCGATGTGCGAGATTCCCTGCAGCCCCTCGAAGAGAGCCAGCTCTGACTCCAGGAACTCCC
TCAGTTCTGGGTCCAGACCCGCTGGCCAATCCGCCTCTTCGATGCCGTTATCGCCCCATCCTGGGACCATT
TCGATTCCAGATAGAACTCCGTGGCCGCTCGATTCTGGCGATCGCTCCTGGCCTTCTATGTCCGTGTGGAT
TCTGGTCATCGCTCCTACTCCGTCCTTTCTCTTGCACGTTCCCGCCGTGCCGCTTCCGTAGCCATTAGAAGT
CTGCCTCTGCAGCTCTATGCGCCACCTCTTGGGTTCTCCAGGCTGTCGATTCGGGCCGGACTTTACTCCCCT
TTTCCTGACCTTTTCCGTCGAAGGCCGAGCAGGTACTCCCTTCCCATGACTTGGGAGGCCTGCCTCTGGCT
CCCTCTGTAGCCGAGCAGGTGCCCCCATCATGACTTGGGAGGCCTTCCTCCGGCTCCCTCTGTAGCCGAGC
AGGTGCTCCCTTTCCATGCCTTGGGAGGCCTTCCTCCGGCTCCCTCTGTAGCCGAGCAGGTGCTCCCTTTTC
ATGCCTTGGGCGGCCTTCCTCCGACTCCTCGCTCGTTCCTGGCCTCACGCCCGGTGTCGTCGCTGCTTCTAG
GACCAGTTCCGCCTCGCCGCAGCGTATTGTGGTATTCATCGCCCCCAAAAAATCCATACCCAGGATTACAT
GGTCCAGCATTGAGGGGCATGATGAGCATCGTCATGCTGATCGTTTTTCCCCCTAAGCTGGCGTCTACTCT
CAAAGCCTTTTCTACCTCTAGGGTGGACCCATCTGCCAGGCGGATACGTGCCTGTATTTCCTCCGCTCTTCC
CCGGATCGTCCATTTGCGTACGCAGTCCTCACTCATGAAGCTACGAGTGGCTCCGGTATCGATCGTGGCGG
GCATCGTTCGTCCGCCAATCTCTACCGTAGCGACGATCCGGCCACCGTCCACCTTCAGTGGGTGTCCTATT

GCTGCGTTCCGTGTGTATTGGCGCCGACCCGATCCTGGTCCGGACGACGCCGTTGCCCGTTTCCCGTCTCC
GCGCGGCGGCAACACTCCCGACTACTTCGGCCCCGGCTTCCACATACCCAACAAAATAAGACAGGGGCGT
TGGGACATTCCCTGGCAAAATGCCCCTCCTGTGCGCACCTACGACACGCCCGTCGCGGATCATATCCGTTC
CCGGCCTCTTGGCTCATCATCTGATGGGCCACCTCTGCGTGAGTTGCAGCGGGTGGGGTGGAGACCCTGTT
GTCCACAACGGTCGTCCCGGATCCGCTGGGAGCAGACCGGAACGGATTGAGATGTCCCGAGGACTGCGCC
GTCCTCGCCCACTCTGCACTATTTGAACTAGAGACACCCTGACCCAAGGGTTGGCTGCCTGAGTACTCCGC
CCTCTGCCTCACGACGGTCTCGTATTCTACGGCCAGTTGAGTTAGCTGGGATAGGGTAGAAAAGTCTTGCC
GTCGCACATAGAGCCTGTATTCCGGAGCGGCGTTCTCGTAGATCCGATGCAGCTCTTGTGCCGGCTCGTAT
CTAGCGTGGTGCATAAGAGCCCTCAGCTCTATCAAATATTCCTTGAACGCTTCACCTACGCGCTGCCTCCG
GGATCGGATCTCGTCCTCAAGCCTCTCGAAGTAACGAGGGGGCAAGAAAAACTCCAAGAAATCCCTCCTA
AACACGGCCCACGGCACATCCCTTAGCCCACTGCTCAGAAACCAACGAGCAGCCCTATCGCAGAATACCT
CGCTCATTGCCCTGGGCATACGGTCGAGGGCTATACCATAGGTGATAGCGCGCTCCTCGAGGTCCTCTAA
GAACCCAAGGGGGTCGGAGCGTCCATCGTACCTCATACCCCACTTCCGAACTTGCTCCGGGACATTGGGA
AGTGGCCTCGCCTCCGATGGATGGTGCGGCCTAGTGTAGCAGGGACCACCACCCGGTTCAACAGGGGACC
TCCCCACCTCTGCGTTGACCGCAGTTGCTCGGCGGTCTTCATTTCCACCGAGAGCAGGAACTGCCTCCACG
CCCGAGACGGTCAAACTCGGGGTGGGCGCCTGCAAAAGCCCGTACAGCTGGGAGCTTCTGAGGTCAGGTT
CCGACTTTTGCCGTGGCCGTCCACCAAAGTCGGGTGGGACGCCCCAAGATGGCGGCTCAAGAGTTTTAGC
ATACTGGGATTCCAGCTCGGCGAGCCGAGCCCAAATCCCAGTCGAATGACCGCCCTCCTGCACGTAGGTG
GACAGCCGCTTACGTAGCTCCTCCACGCGCCCCTCCTTTTCCAGCCCAAACTCCTGGGCGATCGCCACTAA
CTCCTCTTTGCGTCGCTGGTGAATCCAGCGGACGCCTACCCCCGGGTTTTGGCACTCACCTGCCTCCATCCT
TTATTGTATACTTCCTTGCTAGTCGACGTCGCTGTTTCCCTCTCTTCCCGTTCTAGGGGTTCCGTCGGTCCCT
TTACTATTCCCCTCTCCTTTTCTTGGTGTTGGTGTCTTTTCACTTTTACGCCGATCTTGCTCAGCCACTTTGG
TTCCTTTTTAGTTTTTTTTCTTATCTTTTTGTCTTTATATCTTTCACCAAACAAAAAAATACAAAACAATATT
CCACAAAAGTATTTACCCCCTTTTTTAAAAACTCAAAAAGGTATTTCAAAAATTTTCTTTTCTCACTTTTCC
GCTTTTTAGGCTGTTCCTTATGTGGTCGCTGCCTTTATCTTTATTGTGAACCCTCGGTTTATTTTAATTTTAT
TTATTATCCACTCATTTTTTATTTATTTTTTTGTTTAATTAATTTTATTTATAAAAGAAAATTTTATTATTATT
CACTTATTTTTATTTATTTTTGTTTTAATTAATTTTATTTATAAAAGAAAAATTTTATTATTATCCACTTATT
TTTTTATTTATTTTTTTGTTTAATTAATTTTATTTATAAAAAGAAAAATTTTATTATTATCCACTTATTTTTAT
TTGGCACTTGTTCCGCTCTTATTGTTCCCTTTGTTCACATTTCACTTGAAGGGTGACCACTTCACTGCCCCG
ACAGCTGTACTCACGAATTCAACTTTTCACCGCGCGTGAGTTGGATTCGCCCCACTGTACAACCGCTGCCG
CGACGCCTTAACGGTCCTTCGACGCCGACGTCGACTGCGCCGCGCGTGTGGGCACCCGTGTGCGAGCGTG
GTCTCGACGTCGCCCGGCTGGCGCGCGCGCGCTGTACTCTCACGACCGACCGTTATGTTTTCGTCTCTTTAT
CTTACTTGCCGGCTTGCTGCTAGCATTTTTGTCACTCACCAGCTGCACGTGTTCATTTTCTGGCTATGCCTA
ACACAAGGTATAGGAATGTTCTCTGGGGTATACCGGACTACTTAATACCCTATTTTCATATTTCGCACATA
CATGACCTTCTCATTTTCTTGCTCTCTGCTAGGTTACCTTCCCTCTAGGGTATACCCGACTAGAAATACCCT
ATATATTTTTTTTTGGAAAATACCTATTCGGGCATGCTTGCGGGCTTTAATCCGCCGACATGCCACACCCCT
AAACGCCTCTTGGTCCCTGCTCGGGCGCCACTTGTAAGGAAGCTTCCGGCCGGGGTATAATTCTCAGTCTT
ACCAGGTCTAGCGAGTTACAAGTTATTTATAATTTCGCTGGGAGAGAGGGCTTAAAGGGGGTGAGTATCC
TGACCAAACCAAACCCAGATCTACCTCTACACACCTACACCATCGGGCCGTGGCCTCGTACCATCTATGCT
AGCGGGCCGTGGCCTCGCNCCAACCACACTAGCGGGCCGTGGCCTCGCACCAACTCCACTACCTAAACAC
CCTATCTCTCCGCCGGGCCGTGGCCTCGTACCGCTATGCTAGCGGGCCGTGGCCTCGCACCAACTACACTA
GCGGGCCGTGGCCTCGCACCAACTCCACTACCTAAACACCAACCGGCAATGCCCACCCCGGAGCCACAAT
CTCGTATCGCATGCCCCTCGTTCGAGGTTAGGTCGAACACCGGTTAGTTCCGATGTTAAGGTAAGCCGGGT
TACCAGGAAACAGGGAATAATCCAATACTTTCTAGATAATTACAACGTTGGCACTTTATTCACTATTATTC
ATTCCGACCTTATCGTAAATCCTAAGCTAAACCCTGCCGCACTGTTTTCTGGAGCCCTAGCGCTCCACTAT
ACCATCTATGATTTCGTTTATCTCGCGCAGCACTTCTACTCACCCTTAACGTTCAATCACAAGTGCCGGCA
CGCTCGCCGGCAAGCCATGCGCTCGCTTCGGCTGCTCAACTTCGCCGCGAGCTTTCGCAACTTCGCCTCGG
AGCTTTCGCTCTTCGCCGCCGAATTGAAGTTGCGCTCTCAAAGACGGTGTTGAGCAACCGATCGACAACG
CACGGTTGCAGCGGCGGCCGGCGGTTAAATTCAAACTCACTATTTAAATCAATTATAATATTCGCGGCTCC
TTGCCTCTTTGCTACTAATCGAACTGCTTATATTACTGATGGCTCCTTGCCTCTTATTTCACTAAGATCTTA
ACCTAATATAATTAATACTTATAACTAAAGAAAATTAATACGTTGTCCCGCCAGCGGCTCCTTTGCCGTAA
TTTGGCCAAAGGGATGACGGCTCCTTGCCGTGAAAACTGTGGGGTGGCTGGTCTCATTAGCCGCCTCACA
>160_rnd-5_family-2385__LTR___Gypsy
CCGCGAGCTTTTAAACTTTAAGGAAAGAAGCTTTATCATAACCATAGCTGACTTGCAGCATAAGTTCACA

ATTGTACTTTAGTTCTAATCTTGGATTCAAATAAATCGGAAGCCTTCAGCCTCCCACTTAAAAATATATTTT
TTTCCAATTAGTAGCCACTAATCGGTTAATTGGCGCCCAACGTGGGGCTCGCGATTATAAACACAAAAAA
AGGATAATTAAAAGAAACAAAATCCTTAGTGATTCGCGAGTGAGCAAAAGTGCCGATTTACCAGTCCAAG
ATTGTCTCAAAAGAGCATCAAAAATTTTCTAAGAACGAAAATATCGAGTGCCTCTAAAAGAAACATTAAA
AATTTCCGACGAACGGAAATATCCTGTTTCTGAAAACTTGAAGAGTAGTGAAAATACCCTGAGCTGGAAA
TATTAATTGTTCACAAGACTTAAAAGTGAAATCACATTTCCCGTTCAAGTTAAATGAAAACATTCCAAAAA
ATTAATCGATCAAAGAAGCGAAACGAATATTTTCGCCAACAACATTTTTTCACCAATCAAAAACTTTTAAC
CAAATAAAAAATATCACGTCAACCTGAGACCCCCATAAAAGAAGACCCTAAAACCCAGAAGGAAGGAAG
GAAGGAAGACTTGAACGACATATGTATAATCACCCATAAAGAAAGACCTTTTACGTGAGCCATCATCATT
TACTTTAAAAAAAATTTTTTTTACTTAAGTAATGAGAGTTTAAATTTGTGTAAGCATTTGTTAGAATAATA
AGTGTTATAAGCTGGATAACCATATAGAAAGTTTAATTGAAGATTTTCAAAATTTAAAAATGGCTAACAA
TAATAACTTGACAGCTGATTTGGTAGCTCAGTTGATTGCGACTCAGACCAGAGCGCTTCAAGAGCAAATT
GCAGAATTGAAGTTACAAATCACTCTCTCATCTAATAATGAACAAGAATATAAGATTACTGAAATAAATG
ACCTTATTCGTTGCGATGAATCTTTTGATGTTATCAAGTCCATACCAGAATTCTCAGGCAGCTATAATGCA
TATGTTAGTTGGCGCGAAGCCGCTTGCTGTGCCATGCGAATGTATAAAGAAGGAAGTAGGAAGTACGTAG
GTGCTTTAACAATATTAAGAAATAAAATAACAGGTAAAGCTAATGACACCTTGACTAATCACGGTACCGT
TCTTAATTTTGAAGCTATACTTGCAAGGTTGGATTTCGCCTACAGTGATAGACGACCAATCTACATAATAG
AACAGGAGTTAAGTATTTTACGACAGGGTAACTTATCCGTTCTCGAATACTACAATCTAGTTAATAAAAA
ATTAACCCTTTTGATAAACAAAACTATTATGACGTATGGTGACAAAGCCNACATCATTACGGAAATGAAC
AAGAAAAACAGAGACAACGCTTTAAGAATTTTCATCACAGGTCTTAACGGTTCTTTATCAGAAGTTTTGTT
CTCCCTTAAACCTGAAAATTTGCCTAATGCTTTGGCAAAAGCTCAGGAACTGGAAGCGAATTACCAAAGG
GCTAACTTCGCTTACCACCATTTCGCAAATACCCATCAACGTAACAATCTAAACCCAGGGCATAACAATC
AGCAAAAGAAAGCTGAATCCCCAAAACAACCTAATAAAAGATATGACAATCACTTAAGGTTCAATCAAG
TTCCGCAAAATCAAATTTTTGCGCAAAGCAACAAGACTCCTCAACCTCCACCGGAGCCCATGGATGTAGA
CAAATCCATACAACTACAGAATACACCTTATCCGTATCCCCAAAATCGAAATTCGGCTCAACCAAAGAAA
CGGGAATACGCTCCCCCTTCGGTTCAGCAACCTAACAAACTCATGCGTATCAATAACCTCGAGGAAGACC
ATTTTTTAGAAGAAGAGGGGGAGAACTCCCCTACATTTTCAGAATAGACAAAAAAATAAATAAAATTTTA
AAAATATTAATTGACACCGGTGCCACCGCTTGTTATATAAAAGAGGGTATATACGATAACGCAGAAGAAT
TAGTTATAAAAAAAGGAATTAAAACTGTAAATGGCTACAGTATCATAACTCATTTTCATAACATAAACAT
ATTTGGCAAACGCCACAGATTTTATGAAATTAATAATCTAGACGCAGATCTATTAGTCGGTTATAATCTAC
TGAAGGAGATTAAGGCAGTAATCGACTTGTCAAAGGGAACTTTGTTTTATAATAACAAAAAGGAAAATAT
TAATTTTACAAAACATGTCCAAGAAACGGTTGGCCATTTAAAAAGTTCAGACGAAGCCTATAACGTTGCT
TCGTATTTGAACTACCAAAGTGGGAAATGTAAATCATTTTCTTTATTTAATGTATCCGATACAGAATCATC
GTCTTTAAGTGACGCTGATAACCCACGTATCGAAAAAACTAAAGTTGACGATATTCTTAATGAAGGAAAG
CTAAAGAAATTAAAACTCAAAAAGAGCAGACGTCGAAAGATGTCGGTATCCTCTACAACAAGTTCTTCTT
TATGCAAAATAAATCATTCCATTAATGAAATGGATAATTTGAATATCCTTGAGCAACAAGTCGTTCAACA
AATAGAAACAAAGCATTCTCAGATTCAAACATGTCTCCCTTTTAGAACAGATATTAGAGGAGAGATAAGA
ACATTAAACGAAAATGCAATTTACAGTAAACAATATCCTTATCCTCATTCAGCTAACAATTTTGTTAGTCA
GGAAATAAACAGGATGTTAAATGAAAATATAATAAGGCCCAGCAGAAGTCCTTATAATGCCCCGGTATGG
GTAGTAGGGAAAAAAGGTTTTAATGAAGATGGAACGCCTAAGAATCGATTGGTAATAGATTTTAAGAAAC
TGAATAACGATACAATCCCCGATAGGTATCCAATGCCTGATCCTTCAGTCATTCTATCCAACCTAGGCAAA
GCCAAAATGTTTTCGACAATTGACTTAGAATCGGGATTTCATCAGATTCTAATGAAAGAAGAAGACATCG
AAAAAACAGCTTTTTCCATTAACAATGGAAAATATGAATTTCTTCGTTTGCCATTTGGGCTTGCGAACGCG
CCAAGCATCTTTCAGAGAGCAATGGACAATATATTACGTGAAAAAATCGGAAAAACTTGTCACATTTATA
TGGACGACATAATAGTTTTTTCAAATACCCCGGAACAACATTTTGATGATTTACAAGAAATCATTGAAATT
CTATTCAAAGCAAACATGAAAATATCCTTGGAAAAATCAAAATTCTTTAGAAAAGAAATAGAATTTTTAG
GATACATAGTATCATACAATATCATTAAAACTGATCCGAAAAAAATAGAATCAATCATGAAATACCCTCC
TCCTAATAATTTAAGGGAATTACGAGGTTTTCTTGGCTTAACAGGCTACTACAGGAAGTTTGTAAAAAACT
ACGCGACTATAGCAAAACCACTCACTAAATTGTTACAGGGACATAACGGTATGGTTTCAAAACATATGTC
CAAAAAAACAGAAATATCTCTAGACGAAGAAGCAATCTCTGCGTTTGAAAACTTAAAATCACACTTGATT
GCAAACATCGAACTAACTCAGCCAGATTATACAAAAAAGTTCGTCCTCACCACAGACGCTTCCGACATCG
CCATAGGCGCCGTCCTTTCGCAGGAAAACAAACCAATCACCTTTGTTTCAAAAACCTTAAATAAAACAGA
AAGAAATTACGCCACAAATGAAAGGGAATTATACGCCATTGTATGGGCATTAAAAAACCTTAGGAACTAC
TTGTATGGTGTTACAAATTTAGAAATCCAAACAGACCATCAACCACTTTCTTTTGCGGTTTCTGCACGAAA

CCCTAACTCAAAAATGAAAAGATGGCAATGCTTTCGTAGAAGAATTTTCCCCTAAAATATTTTACAAACC
AGGATCTACCAATGTCGTCGCTGACGCGTTATCAAGGGTAATGATCAATCATATTTCCAATGAAAGTAATC
TCGTGTCCGAAGAAGAACCGTCGTCCGATAATGAAACTATCCATTCAGCCGACAGTAGTTATAAAAGCGT
AATTGAAGAAACACACAAACCATTGAACCAATTTAAACAACAATTACTTGTTTCTAAAAACAACATAACA
ATTCATGAAAAATATGAAGTTTTCAACAAAACAAGACATATTATAGAATACGACACAGTACACAATTTAA
TAATCATACTTAAAGAATTTCTTCAACCAAACCTGACTACAGGAGTTCATTGCAGTCTCGAAGATTTATAC
GAAATCCAAAACTCATTAAAAAACACTTTTAACAACAAATTTTTATTTACTAAAAAGTTTGTCCTAGACAT
CGAAAACAAAAATGACCAAGAAATTATAATAGAAGAAACACATGCTCGTGCTCATAGAGGTTTGGACGA
AAATCTTAAGCAAATTCTCAAATACTATTATTGGCCAACACTATATAAAACCCTTAAGCAACACATACGCT
ACTGCGAAATTTGTAATAAAAATAAATATAACAGAAACCCTAAGCAAATTCCTATTTCTAAAGCCCCTATT
CCGGAGAGAGAAGGTGAACAATTACATATTGACATTTTTTATGCTCAAAAATTAACATTCTTGACATGCCT
AGACGCATATTCTAAATTTTTAGTGATTAAGGAAATCAATGACAAATCAGATATGGCAAACAAAGTGTTG
GAACTTATCCAAACTTTTCCTTTCGCTAAATCCATAATGATGGACAACGAACCTACGTTTTCCTCGACACA
ATTCAAATCGTCAATGTCTCGTTCAGGTATAGCGATTTATCATGCAGACCCCAGACACAGTTTAAGCAACG
GTCAGGTAGAAAGAGTTCATTCAACAATTATCGAAATTTCTCGTTGTATTCAAGAAGAATACAATATAAT
GGATCATTCCGAAGTCGTATACCGAGCAGTTAAAGAGTATAACAACACAATTCAATCGACAACTCAACAA
AAACCATGTGACATATTATTTAACAAAATAGAACACCCAAAATTGTCAGAAAAATTAAAATCAGCCCAAG
CCAAAATGTTAGAGTACCATAACAAAAAGAGGTCACCAAAAAATTACCAAGTAGGTGAAATAATTTATG
AAAAAAAAAAACATGGCGAACGAAACAAACTACAACCTAGATTTAAAAAACAGGTAGTTAAAGAAGACT
TAGGAAATAAAGTCAAAATAAACAATAGGGATCGTATTATTCACAAAGACAATATCAAATTCTAACCTAT
TTTATGTTTTTTTTTAATGTATTTTTAGAATGCACATTTTATGTATCTTATCTGTTNTACTGCCGTTTTGCG
GTGCAGACATTATCAATTATTCAAATGATGACTATGTCCTACTTGAGGATACAGATAATCCTTTTTTATAC
GAAGCATACGGAGAAGTTTTTCACGTTACTAATCTAAGTTTTTATAACGATATTTTTGAAAAAGAATTAAA
TTTTTTTAAAAACAAAAACAATACAATTGATCGGGAAGTAACCTTGGAATTGAATTTAATCAAAACACTC
CTTTCTGAACTTACACCTCACAAAATTGCCAAACGTAGTATCAATGAACTAGGCACAATTTTTAAATGGAT
CACAGGAACACCAGACCATGATGACATGATAACTATTCAAACTAAAATCAATGAACTTGTTCAAAACAAT
AATATGCAAAATCAAATCAATTCTGCAATTTTCAAAGAAGTCACTAAAATCACAAATCTTTTAAGAGATA
TTAAATTTAATCAGGAATTATTTATGAAAAAATATCGACTTAAGTTGTTATCACTTGACCTACAAAATCTG
CTGGATACTATCACATTTTCCAAAGCCGCAATATTGAATCCAAAAATCTTAAATAAACAAGACATGATAA
ATATATTTAATCACGAAACACTCAATATCACTCTAATCGATCTTTTAGACATTTCCTCTTTTTCGATCGTTT
CATACCAAGGTTTAATCATAGTTTTTATAAAATACCCTACAATTAATTACAATTGTAAGTTATACCACGCT
AAAGCCATATCCCAAACAGATGGAAAATTTTTCGTTAACGACAAAATTGCTAAATGTAATAACACGTTTT
ACCTCATTGATAACTTTAAAAGAGAAATTTATAATAGTTTTGCCCACATTAATTTTGAACCAAACTGTTTT
GTAAATCTCCTTAATAAAAAATTTACAAGTTGTATAAAATTATCCGAAAAAAATAAACCAATAGAATTTA
TAACAGACGGCGCTATTTTAGTATCAGGCACAAACTGTATTAACGAAACCTGGCTTACAGGCATTTACCTT
GTTACATTTAAAACCATAGTAACCATAAACAATGTTACCTACACCAATGTAAAAGAAAAAATTCTTAATT
ATGTAAAACACAATAAATTCACAAACTACTTAGTTAGTGAATATCAAGTTACTAATAATACTGAACTTCA
ATTAGAAAATACAAATATACTATCAAAAATTGTTATAAAATTAGAAAGTAATCATTCAAAAATAACCTTT
TTATTATTTATACTAATTTTTATTTTTACTATGGTAAAGCTTCTTAAACGCTGCCCTTTAATAAATCTAAAT
CCCCTCAAAATACTGAAATTGAAATCGTCAAGTAATCAAAAGTACCATAACACTCAAACTACAGAAACAG
AGCTTGACATATTAAACCAATTAAACGAACAAATTGAATCTTTAAAAAGAAATCATTAAGAATCGGGGAC
GATTCTTTTTAAGAAAGGGAGGAGTTATCACCCAGCCCGCAAGGCAATTCTTATTGCAATCATAAACAAC
ATTTCAGTATAAGTCGCTATCCCAATTACTTAGCCCTTTCAGACTTATTAGACCCGTTTCAATCACTACGTC
CACTTAACGCACAGCTGCAGCTCTGCTAACATAAATATCCAGATTCCCGGCTCTAACATAAACATAACAG
CGCAACCACTTAACATAAAGATTCATATTCTCATGCGCAATTCATAACGTAAACTAAAGACCCAGTCTCTA
GAGCGCAATTGCTAACGTAAACATGACTCAAAGCGTCTAGCTTACGCAAGCCACCCCCGCGAGCTTTTAA
ACTTTAAGGAAAGAAGCTTTAGCATAAACACAGCTGATTCGCAGCATAAGATCACCGCGAGCTTTTGAGC
TTTAAGAGAATAAGCTTTATCATAACCATAGCTGACTTGCAGCATAAGTTCACAATTGTACTTTAGTTCTA
ATCTTGGATTCAAATAAATCGGAAGTCTTCAGCCTCCCACTTAAAAAAATATTTTTTTTCCAATTAGTAGC
CACTAATCGGTTAATTTGCGCCCAACGTGGGGCTCGCGATTATAAACACAAAAAAAGGATAATTAAAAGA
AACAAAATCCTTAGTGATTCGCGAGTGAGCAAAAGTGCCGATTTACCAGTCCAAGATTGTCTCAAAAGAG
CATCAAAAATTTTCTAAGAACGAAAATATCGAGTGCCTCTAAAAGAAACATTAAAAATTTCCGACGAACG
GAAATACCCTGTTTCTGAAAACTTGAAGAGTAGTGAAAATACCCTGAGCTGGAAATATTGATTGTTCACA
AGACTTAAAAGTGAAATCAAATTTCCCCGTTCAAGTTAAATGAAAACATTCCAAAAAATTAATCGATCAA

AGAAGCGAAACGAATATTTCGCCAACAAAANTTTTTCACCAATCAAAAACTTTTAACCAAATAAAAAAT
ATCACGTCAACCTGAGACCCCCATAAAAGAAGACCCTAAAACCCAGAAGGAAGGAAGGAAGGAAGACTT
GAACGACATATGTATAATCACCCATAAAGAAAGACCTTTTACGTGAGCCATCATCATTTACTTTAAAAATA
AATTTTTTTTACTTAAGTAATGAGAGTTTAAATTTGTGTAAGCATTTGTTAGAATAATAAGTGTTATAAACT
GGATAACCATATAGAAAGTTTAATTGAAGATTTTCAAAATTTAAAAATGGCTAACAATAATAACTTGACA
GCTGATTTGGTAGCTCAGTTGATTGCGACTCAGACCAGAGCGCTTCAAGAGCAAATTGCAGAATTGAAGT
TACAAATCACTCTCTCATCTAATAATGAACAAGAATATAAGATTACTGAAATAAATGACCTTATTCGTCGC
GATGAATCTTTTGATGTTATCAAGTCCATACCAGAATTCTCAGGCAGCTATAATGCATATGTTAGTTGGCG
CGAAGCCGCTTGCTGTGCCATGCGAACGTATAAAGAAGGAAGTAGAAAGTACGTAGGTGCTTTAACAATA
TTAAGAAATAAAATAACAGGTAAAGCTAATGACACCTTGACTAATCACGGTACCGTTCTTAATTTTGAAG
CTATACTTGCAAGGTTGGACTTCGCCTACAGTGATAGACGACCAATCTACATAATAGAACAGGAGTTAAG
TATTTTACGACAGGGTAACTTATCCGTTCTCGAATACTACAATCTAGTTAATAAAAAGTTAACCCTTTTGA
TAAACAAAACTATTATGACGTATGGTNACAAAGCCCACATCATTACGGAAATGAACAAGAAAAACAGAG
ACAACGCTTTAAGAATTTTCATCACAGGTCTTAACGGTTCTTTATCAGAAGTATTGTTCTCCCTTAAACCTG
AAAATTTGCCTAATGCTTTGGCAAAAGCTCAGGAACTGGAAGCGAATTACCAAAGGGCTAACTTCGCTTA
CCACCATTTNGCAAATACCCATCAACGTAACAATCTAAGCCCAGGGCATAACAATCAGCAAAAGAAAGCT
GAATCCCCAAAACAACCTAATAAAAGATATGACAATCACTTAAGGTTCAATCAAGTTCCGCAAAATCAA
>160_rnd-1_family-124__LINE___CR1
AGTGGGATTTGAGCGACCACAACATGATCACTGTTGTGATGTCGCCATCCCCAAATGCATATGCTCCTGTG
CTGTCCTGGAAGCTCCCTAAGGCTGATTGGCAAATGTTCGAACCGGAGCTGGTAGAGGAAATCGATGAGC
ACCTTTTCCAGGAGGACATTGGGATGGTGCCGTTAGATTCCGCTTAGTCTGCACTACGCCTTGTCGTACAG
TCTGTGTGCGACAGAGTGTTATGCCGCAGTCCGTCGAGAGCCCAGGGAAAAGTTAAATGGTGCACTGCCG
AACTGAGTACCAAACGCCGGGAGGTCAGAAGACCGAGGCGGAGTCTCCAGGATGCACGGCGCAGAGGCT
ACGAATCTGCGCAGTTTCTTGCAGCTCATCTGCCGACAGCCGTCCGCGAGTTCAGGACGCTTATCCTGAAG
ACGAAGGAGGAAAACTGGCGACGATTCGTTGGAGACCACAGAGATGATGATGGTGTCATGTCTACCGGA
TATTCCGGGGACAAAAACGGTCCACCGATTTAAGGTGCCTTCGGGCGGATGGCAGGCTGGTTATAACATG
GTACGACTGCGCGAGTTTTCTCCTCCTTAACTTTTTTCTAGTTGCGGAGTCGAGTTAAGACATCGTCATTCC
GGACGAAGCTGCTCCAGCGCTCGATGCAGCTTCCTGTCGGGGCATCTGCCTATTGCCAGTGTTTAGCAAAG
TGCTTGAGGGGATCTTGGTGGATCGGTTGAAGGAGGTGCTTCTTGATGGAAATAGATGGCAATTTGGCTTT
TTACCTGAACTCTGTGTCGACGATGCCAGGAAGCACGTATTGACCAGCTTTGCCGCCAGCAACGCGCAGC
CTAATCGGTGCGGGATGCAAGGAAGCTACCTCTTGGAGAGGTTTCTCATGTGGCTGCGGTACGGTTTGCA
GGAGCAAGCCTAACATATGTCAACAAAGGAGCGAGTTCTCAGTTGAGAGTGATTGACGTGTTTTACTTGT
GCTCCAAGATTTTTATATATTTTTGCGCTTTAATCCTGCTTTTTATTTAAAAATTTTGCATTTTTCGGTGCTT
TATGTAAAAAGCTTTTATAAACTGTGCAAAGTGTTATCTGTTGTGCTGCGTAAGTCTGTTTATTATTGCATT
TATAAAACGTGGCGTCAGTTCCGTGCTGTTGTTATTGCCTGTTTTGATTCTTTTGCCTTGGTGCTCCCCTGT
GCATACACTTGTTTGTGCGTGTGTTCGCTGCATACTTTCTGCTTGTGCATCGCGCTCTCGCAAATTTTTTCT
ATTGCTTTTCGCTCCAAACAATTTGTTTGTGTACGAATAATGGTTTTTTGAGTGTTTACTTGCTCTATCGGT
AGCGAAGAAGATTATGGAGGAGGTTGTACCATCACTTGGTGTGCGTGTACACGACGTGCGAGAGCTTAAA
CACCCCGTCGCCATCATTCGCACGCCATCGGTAAGCGAATTTCAAAAGGTGGTGGCGAATAAAAAGTTTG
CAGAGGTTGGACTACATATCCAAGCAAACAGGAGTCTGAGACCAAAGGTCATAGTATACGACGTGGACA
CGACGATCTTGCCGGAGATCTTCATGGAGGAGCTTTATAAAAATAATATTAAGGAGGAAATGACCTCAGA
GGCTTTCAAGAAAGTCGATTCCGGTCACTCGTCCTCACCTATGCTTCCCATAGATGTGTCGGCTTTGACAA
TATCTCCGAGTGGCGCTTCAAGCAGTACGTATGTAGATAATGTGGGCAGATGGGGCACATCGAAGTCAAG
TGGGTAAATCCGGTGGACTGCCGGAATTGCCGCTTCAAGAGGATGCCCTCAGGGCATTATATGCTTTCATG
GTCACGCCCGATCTATGGCGCTGTGTTGGCGAGGGTGCAAGCTAAACATTAATGTTTAGCTTCATCCAAGC
CAACTGTGACCGGGGCCGCGCTGCGTGTATCGATATCGGTGTCTGTATGAGTGACCACGGCCACTTCTTTG
CGCTGCTGGAGGAGCCATATGTAGACGTCGGTGAAAAGATTACGAGCTTCGCACCCCGTTTTTGGAAGCC
ACACTGTCGGAGACCGGAAAGGACCGCTGCTATTATACATTACCCGGATGCCATCAGCCTACCAGTGAAG
TAGCTGACCACCGAGGCTGGAGTATGCGTGGGGGTGACTGTGGCACGGTTTAATTGACCCCATATACTGC
CATGTTCATGCTGATTTGATGAGGATATTGTGCTATATGAGCTATATTGAGCTATATATGCCTTGCTGCTAC
TGGCGAACAATACGCCGGTAATTTTTAGCCAGGGCGCAAATGCTGTGTCTCCCATGTGGTTCAGCAAATTC
TTTTGTGGCCAGCTGAGCTACAGACGAGGCGAGCAATTGGGTAGTCGTGCCTGCGTGCCAAACTAAGTCA
GTGAGGCTTACACGTTTGATAATCGAAGAAGACAGAGCGATATCGACGTAATCTTCAACGAAGCAGCGTC
TACGTGGAACACGTATGACGGGAGTGTGGACGAGTGGGATTTGAGCGACCACAACATGATCACTGTTGTG

ATGTCGCCATCCCCAAATGCATATGCTCCTGTGCTGTCCTGGAAGCTCCCTAAGGCTGATTGGCAAATGTT
CGAACCGGAGCTGGTAGAGGAAATCGATGAGCACCTTTTCCAGGAGGACATTGGGATGGTGCCGTTAGAT
TCCGCTTAGTCTGCACTACGCCTTGTCGTACAGTCTGTGTGCGACAGAGTGTTATGCCGCAGTCCGTCGAG
AGCCCAGGGAAAAGTTAAATGGTGCACTGCCGAACTGAGTACCAAACGCCGGGAGGTCAGAAGACTGAG
GCGGAGTCTCCAGGATGCACGGCGCAGAGGCTACGAATCTGCGCAGTTTCTTGCAGCTCATCTGCCGACA
GCCGTCCGCTAGTTCAGGACGCTTATCCTGAAGACGAAGGAGGAAAACTGGCGACGATTCGTTGGAGACC
ACAGAGATGATGATGGTGTCATGTCTACCGGATATGCCGGGGACAAAAACGGTCCACCGATTTAAGGTGC
CTTCGGGCGGATGGCAGGCTGGTTATAACATGGTACGACTGCGCGAGTTTTCTCCTCCTTAACTTTTTTCTA
GTTGCGGAGTCGAGTTAAGACATCGTCATTCCGGACGAAGCTGCTCCAGCGCTCGATGCAGCTTCCTGTCG
GGGCATCTGCCTATTGCCAGTGTTTAGCAAAGTGCTTGAGGGGATCTTGGTGGATCGGTTGAAGGAGGTG
CTTCTTGATGGAAATAGATGGCAATTTGGCTTTTTACCTGAACTCTGTGTCGACGATGCCAGGAAGCACGT
ATTGACCAGCTTTGCCGCCAGCAACGCGCAGCCTAATCGGTGCGGGATGCAAGGAAGCTACCTCTTGGAG
AGGTTTCTCATGTGGCTGCGGTACGGTTTGCAGGAGCAAGCCTAACATATGTCAACAAAGGAGCGAGTTC
TCAGTTGAGAGTGATTGACGTGTTTTACTTGTGCTCCAAGATTTTTATATATTTTTGCGCTTTATTCCTGCTT
TTTATTTAAAAATTTTGCATTTTTCGGTGCTTTATGTAAAAAGCTTTTATAAACTGTGCAAAGTGTTATCTG
TTGTGCTGCGTAAGTCTGTTTATTATTGCATTTATAAAACGTGGCGTCAGTTCCGTGCTGTTGTTATTGCCT
GTTTTGATTCTTTTGCCTTGGTGCTCCCCTGTGCATACACTTGTTTGTGCGTGTGTTCGCTGCATACTTTCTG
CTTGTGCATCGCGCTCTCGCAAATTTTTTCTATTGCTTTTCGCTCCAAACAATTTGTTTGTGTACGAATAAT
GGTTTTTTGAGTGTTTACTTGCTCTATCGGTAGCGAAGAAGATTATGGAGGAGGTTGTACCATCACTTGGT
GTGCGTGTACACGACGTGCGAGAGCTTAAACACCCCGCTGCCATCATTCGCACGCCATCGGTAAGCGAAT
TTCAAAAGGTGGTGGCGAATAAAAAGTTTGCAGAGGTTGGACTACATATCCAAGCAAACAGGAGTCTGA
GACCAAAGGTCATAGTATACGACGTGGACACGACGATCTTGCCGGAGATCTTCATGGAGGAGCTTTATAA
AAATAATATTAAGGAGGAAATGACCTCAGAGGCTTTCAAGAAAGTCGATTCCGGTCACTCGTCCTCACCT
ATGCTTCCCATAGATGTGTCGGCTTTGACAATATCTCCGAGTGGCGCTTCAAGCAGTACGTATGTAGATAA
TGTGGGCAGATGGGGCACATCGAAGTCAAGTGGGTAAATCCGGTGGACTGCCGGAATTGCCGCTTCAAGA
GGATGCCCTCAGGGCATTATATGCTTTCATGGTCACGCCCGATCTATGGCGCTGTGTTGGCGAGGGTGCAA
GCTAAACATTAATGTTTAGCTTCATCCAAGCCAACTGTGACCGGGGCCGCGCTGCGTGTATCGATATCGGT
GTCTGTATGAGTGACCACGGCCACTTCTTTGCGCTGCTGGAGGAGCCATATGTAGACGTCGGTGAAAAGA
TTACGAGCTTCGCACCCCGTTTTTGGAAGCCACACTGTCGGAGACCGGAAAGGACCGCTGCTATTATACAT
TACCCGGATGCCATCAGCCTACCAGTGAAGTAGCTGACCACCGAGGCTGGAGTATGCGTGGGGGTGACTG
TGGCACGGTTTAATTGACCCCATATACTGCCATGTTCATGCTGATTTGATGAGGATATTGTGCTATATGAG
CTATATTGAGCTATATATGCCTTGCTGCTACTGGCGAACAATACGCCGGTAATTTTTAGCCAGGGCGCAAA
TGCTGTGTCTCCCATGTGGTTCAGCAAATTCTTTTGTGGCCAGCTGAGCTACAGACGAGGCGAGCAATTGG
GTAGTCGTGCCTGCGTGCCAAACTAAGTCAGTGAGGCTTACACGTTTGATAATCGAAGAAGACAGAGCGA
TATCGACGTAATCTTCAACGAAGCAGCGTCTACGTGGAACACGTATGACGGGAGTGTGGACGAGTGGGAT
TTGAGCGACCACAACATGATCACTGTTGTGATGTCGCCATCCCCAAATGCATATGCTCCTGTGCTGTCCTG
GAAGCTCCCTAAGGCTGATTGGCAAATGTTCGAACCGGAGCTGGTAGAGGAAATCGATGAGCACCTTTTC
CAGGAGGACATTGGGATGGTGCCGTTAGATTCCGCTTAGTCTGCACTACGCCTTGTCGTACAGTCTGTGTG
CGACAGAGTGTTATGCCGCAGTCCGTCGAGAGCCCAGGGAAAAGTTAAATGGTGCACTGCCGAACTGAGT
ACCAAACGCCGGGAGGTCAGAAGACCGAGGCGGAGTCTCCAGGATGCACGGCGCAGAGGCTACGAATCT
GCGCAGTTTCTTGCAGCTCATCTGCCGACAGCCGTCCGCTAGTTCAGGACGCTTATCCTGAAGACGAAGG
AGGAAAACTGGCGACGATTCGTTGGAGACCACAGAGATGATGATGGTGTCATGTCTACCGGATATNCCGG
GGACAAAAACGGTCCACCGATTTAAGGTGCCTTCGGGCGGATGGCAGGCTGGTTATAACATGGTACGACT
GCGCGAGTTTTCTCCTCCTTAACTTTTTTCTAGTTGCGGAGTCGAGTTAAGACATCGTCATTCCGGACGAA
GCTGCTCCAGCGCTCGATGCAGCTTCCTGTCGGGGCATCTGCCTATTGCCAGTGTTTAGCAAAGTGCTTGA
GGGGATCTTGGTGGATCGGTTGAAGGAGGTGCTTCTTGATGGAAATAGATGGCAATTTGGCTTTTTACCTG
AACTCTGTGTCGACGATGCCAGGAAGCACGTATTGACCAGCTTTGCCGCCAGCAACGCGCAGCCTAATCG
GTGCGGGATGCAAGGAAGCTACCTCTTGGAGAGGTTTCTCATGTGGCTGCGGTACGGTTTGCAGGAGCAA
GCCTAACATATGTCAACAAAGGAGCGAGTTCTCAGTTGAGAGTGATTGACGTGTTTTACTTGTGCTCCAAG
ATTTTTATATATTTTTGCGCTTTATTCCTGCTTTTTATTTAAAAATTTTGCATTTTTCGGTGCTTTATGTAAA
AAGCTTTTATAAACTGTGCAAAGTGTTATCTGTTGTGCTGCGTAAGTCTGTTTATTATTGCATTTATAAAAC
GTGGCGTCAGTTCCGTGCTGTTGTTATTGCCTGTTTTGATTCTTTTGCCTTGGTGCTCCCCTGTGCATACACT
TGTTTGTGCGTGTGTTCGCTGCATACTTTCTGCTTGTGCATCGCGCTCTCGCAAATTTTTTCTATTGCTTTTC
GCTCCAAACAATTTGTTTGTGTACGAATAATGGTTTTTTGAGTGTTTACTTGCTCTATCGGTAGCGAAGAA

GATTATGGAGGAGGTTGTACCATCACTTGGTGTGCGTGTACACGACGTGCGAGAGCTTAAACACCCCGCG
CCATCATTCGCACGCCATCGGTAAGCGAATTTCAAAAGGTGGTGGCGAATAAAAAGTTTGCAGAGGTTGG
ACTACATATCCAAGCAAACAGGAGTCTGAGACCAAAGGTCATAGTATACGACGTGGACACGACGATCTTG
CCGGAGATCTTCATGGAGGAGCTTTATAAAAATAATATTAAGGAGGAAATGACCTCAGAGGCTTTCAAGA
AAGTCGATTCCGGTCACTCGTCCTCACCTATGCTTCCCATAGATGTGTCGGCTTTGACAATATCTCCGAGT
GGCGCTTCAAGCAGTACGTATGTAGATAATGTGGGCAGATGGGGCACATCGAAGTCAAGTGGGTAAATCC
GGTGGACTGCCGGAATTGCCGCTTCAAGAGGATGCCCTCAGGGCATTATATGCTTTCATGGTCACGCCCG
ATCTATGGCGCTGTGTTGGCGAGGGTGCAAGCTAAACATTAATGTTTAGCTTCATCCAAGCCAACTGTGAC
CGGGGCCGCGCTGCGTGTATCGATATCGGTGTCTGTATGAGTGACCACGGCCACTTCTTTGCGCTGCTGGA
GGAGCCATATGTAGACGTCGGTGAAAAGATTACGAGCTTCGCACCCCGTTTTTGGAAGCCACACTGTCGG
AGACCGGAAAGGACCGCTGCTATTATACATTACCCGGATGCCATCAGCCTACCAGTGAAGTAGCTGACCA
CCGAGGCTGGAGTATGCGTGGGGGTGACTGTGGCACGGTTTAATTGATCCCATATACTGCCATGTTCATGC
TGATTTGATGAGGATATTGTGCTATATGAGCTATATTGAGCTATATATGCCTTGCTGCTACTGGCGAACAA
TACGCCGGTAATTTTTAGCCAGGGCGCAAATGCTGTGTCTCCCATGTGGTTCAGCAAATTCTTTTGTGGCC
AGCTGAGCTACAGACGAGGCGAGCAATTGGGTAGTCGTGCCTGCGTGCCAAACTAAGTCAGTGAGGCTTA
CACGTTTGATAATCGAAGAAGACAGAGCGATATCGACGTAATCTTCAACGAAGCAGCGTCTACGTGGAAC
ACGTATGACGGGAGTGTGGACGAGTGGGATTTGAGCGACCACAACATGATCACTGTTGTGATGTCGCCAT
CCCCAAATGCATATGCTCCTGTGCTGTCCTGGAAGCTCCCTAAGGCTGATTGGCAAATGTTCGAACCGGAG
CTGGTAGAGGAAATCGATGAGCACCTTTTCCAGGAGGACATTGGGATGGTGCCGTTAGATTCCGCTTAGT
CTGCACTACGCCTTGTCGTACAGTCTGTGTGCGACAGAGTGTTATGCCGCAGTCCGTCGAGAGCCCAGGG
AAAAGTTAAATGGTGCACTGCCGAACTGAGTACCAAACGCCGGGAGGTCAGAAGACCGAGGCGGAGTCT
CCAGGATGCACGGCGCAGAGGCTACGAATCTGCGCAGTTTCTTGCAGCTCATCTGCCGACAGCCGTCCGC
TAGTTCAGGACGCTTATCCTGAAGACGAAGGAGGAAAACTGGCGACGATTCGTTGGAGACCACAGAGAT
GATGATGGTGTCATGTCTACCGGATATNCCGGGGACAAAAACGGTCCACCGATTTAAGGTGCCTTCGGGC
GGATGGCAGGCTGGTTATAACATGGTACGACTGCGCGAGTTTTCTCCTCCTTAACTTTTTTCTAGTTGCGG
AGTCGAGTTAAGACATCGTCATTCCGGACGAAGCTGCTCCAGCGCTCGATGCAGCTTCCTGTCGGGGCAT
CTGCCTATTGCCAGTGTTTAGCAAAGTGCTTGAGGGGATCTTGGTGGATCGGTTGAAGGAGGTGCTTCTTG
ATGGAAATAGATGGCAATTTGGCTTTTTACCTGAACTCTGTGTCGACGATGCCAGGAAGCACGTATTGACC
AGCTTTGCCGCCAGCAACGCGCAGCCTAATCGGTGCGGGATGCAAGGAAGCTACCTCTTGGAGAGGTTTC
TCATGTGGCTGCGGTACGGTTTGCAGGAGCAAGCCTAACATATGTCAACAAAGGAGAGAGTTCTCAGTTG
AGAGTGATTGACGTGTTTTACTTGTGCTCCAAGATTTTTATATATTTTTGCGCTTTTATCCTGCTTTTTATTT
CAAAATTTTGCATTTTTCGGTGCTTTATTTAAAAAGCTTTTATAAACTGTGCAAAGTGTTATCTGTTGTGCA
GCGTGTCTGTTAGCGTGTCAATAACCAAATTTAATTTCTCCACCCCTCGCGAAATATCATCATTCAAAATA
AATGTTTCACATGATATGTTCCCAATCATTTGTGCGTACATTCTCCGAGATGGCCAATCCTGACGCTTACA
CGACCTCGTGTATGCATTCAGCAATTCGCGTTGACAGGCACTGCTCAGGTGTTACATAAACAATGCTCAAG
GTTAAATTTTGCAAAAA
>160_rnd-5_family-3741__LTR___Pao
TTTATAATTGATTTTTTGAGAATATACTGATTGCACAAAAAGATCTACTGTTAAAAATAAAGATCTGTTAA
GAAACACAAAATTTATTATAGGAAACGAGTTTTGACTGGCTTCGTGTGTACGGCAACTAGCGCAAACTAT
AAGAATTTCTGTACGCATGTACAAACACATTCATAGGTTTCTTCAATGAATTCTATCAACAGCAATGCAAT
TGCGATCGTATTGTTTTCTCTGCTGCTATTTTAATCCGTTTTTCTAATTAAGGAGAATCTTATATCATTCCTC
TTCAAGAAGAAGGTCTGAAGGTCGGATATACAAAATTACAGGGGCATTGCAAAACTGTCCACTATTTGTA
CATTCCTAAAAAAATAATCACATCGAATTTGCAGCATTTCTGTAAATCAGTGAATACTGTTCTTGCATCTG
GTCTCGGCAGTATAGCAATAGCCCGGATCGTATTGAATCTGTTCAGAAGCAATTTCTCGAAGCAATCAATC
TCCGGTGGCCATCCCACTCCAGTAGGTTTATTTTTCTTGATCTTCCATCGTTAACCAACCGTAGGACACTCC
TCGATATTGTCTCCCTACAGAAGTGGATTCTACTTTTCTGCTAGGGCGTCTTCAGTTTTCTATTCCGTCTAG
ACCAACCAGGAATTATCGCCGCTTGTTCCTACCAACGTGCACAACAGATTTCACTACGCACAACTCTTTTT
ACGAGCATTGTCTACCCGTAATAAAATCGTTGCTTTCAGGATACCTACGGAAACTCACTGACCATTCCCAG
CAATAGGTAGGGGCATAGCTTTCCGGACAAACCTAAAATTTTCCCCCTCGGTTCAGTGATTTCCCATCACT
ATCTCTTTATCCTACTTATATAACACTCTTTTTATTAGTATACATTTCTTTACTTTATTAACAACTTCTTTCTT
TTGTATTCTTTTTAAGATTATTTTTAATTTTTGCTTTTAGACTCTATTGTTCCTTCTGTCACCCTTTTTCTTAG
CTGTAGGCTCTAATGGCGTCACTGACAAAATTAGCTGTGAAAAGGGGCATTGGTGGCCGGACGTTCAAAT
AAAACACCTCCATCGGTCGGTGATACTATTAGTAAATTGTGTATTCTAGCTAAGCTCTTAGCATTTAATTTT
CATAATAAATGTATAATGTATTCAAAAATGATGAAGAAACAGAATGAAAGAATGAGTATCTAAAAGAGC

GAAATAAATAGCGAACCCTACAAGGGACGTCCAAGTCGCTGGCATGGTTGTTGTCATGGAAGATAGCCTA
CCAGTAAATAAATGGGGCCTTGGCTGAATACGTCTCGGCACAAAAGAATAACGCATCTCATAAATTGCAT
TATCCAGTTTACTCCTCTGCATTGAAACAATATGGCACCTTCATCCAAGCGATACAACCAAATCCAGGGTA
CGTATTCCTTCAGATGCCGGGTGTGCAGAGAAATTCATGGATGACGCGTCTAATGAAGCTTACGTCCTAG
AAGCGGCTCCAAGCAGTCCTTAAAAACAAATATTTCTCCTACTGACCGACTCACCTAAACACAGGGCAGT
CATGTCCCTGCGGAGCAAAATCAAACATCTTCAAGAAAGATCCCCACAGCCCGCTCCACATGCGAAATCT
CCAAAGCTCTCGTCCCTCTCAGTCAACCATGAAGCCTTACTTAGAGAGCTTCTGGCTCCTCGCCCTCCTCT
GGCTCCGCGGCTCACCATAATAACGGCGAAGCACTTTCACCATGCGGATGTCGCTTACGTCCCTTCTCCAG
CACAATAAGATCAGTATCCTACCCACCGCGACTCTGCTCTCCGGACAGACCTATGTCAAACGCATTGCCTG
TGCTACGCTCCAGGACCACTCAAATGACCTGAGGCAACGTTTCAAAGGATGAACCCATCAAGGCAGATAT
GAAGGAACAGTTCAGCAATGTCCTCCCCTCCATATCGCTCACTCTCTTTCTTTAATTTTAATTGTCAATGTC
GTCACACGAAAATAACGAAAAAACGCGCTTTTCGCTTCTACTTATTGTTTATACTCTCCGCCAAGCAACTT
CCGTATACTCGACCACATATGTAACTGTTAAACAGAACAGTTTGCATTGCTCAACCAGAAACATAAAACA
GACTACACAAGCGTAACGAAATAAAATGTTTTACGAAGGACGCACCGGAATCTTCTTCATTTATTTTTGGT
TACTTGTGTGCAAATCGCATTGAAAATAGTGACTGTTATTTGCGAGCTCTGGTTTTTGATAAGCTTGGATTT
CAGCCAATGCGGTGGGCGTGCTTTCGAGATGTCCTCTTAAATGCTTTATTTGGCGGCTAAATTCTTCATTA
ATGTTTCTATCATTCAGCACAATTTATCGCCCATATTGAGCCAATCTACCGCAACATCTTTTATTTGACACA
AATCATATAGATATTGAAACCTTAATAACGTCTTTAAGAATTTTTATTCAATCTGAACAGGTCATTCTGGA
GCTTTAATTGGAATTTAATTTGTTACCGCCCATACTTTTCTTATAAAAGTTTTTAAAGTTATGTCAATTTAA
GAAGGAAGCATCTCCATCACTCCATCAGAATCACAAGCCGAGTGGCTCGATCGACTCGTATGTATAAACG
CAAGGATCTCAAATCCTATAAGAGCTGAAGACTCGAAATTGTATGTGTAGGTCCACATAGTGCCCGCGCA
GAACGAGTTTTTCCAATATTCGATATTTTGCCCCGTTTTTATGTAATTAATAAATAACGATATCGTATTTAT
AATTGATTTTTTGAGAATATACTGATTGCACAAAAAGATCTACTGTTAAAAATAAAGATCTGTTAAGAAA
CACAAAATTTATTATAGGAAACGAGTTTTGACTGGCTTCGTGTGTACGGCAACTAGCGCAAACTATAAGA
ATTTCTGTACGCATGTACAAACACATTCATAGGTTTCTTCAATGAATTCTATCAACAGCAATGCAATTGCG
ATCGTATTGTTTTCTCTGCTGCTATTTTAATCCGTTTTTCTAATTAAGGAGAATCTTATATCATTCCTCTTCA
AGAAGAAGGTCTGAAGGTCGGATATACAAAATTACAGGGGCATTGCAAAACTGTCCACTATTTGTACATT
CCTAAAAAAATAATCACATCGAATTTCCAGCATTTCTGTAAATCAGTGAATACTGTTCTTGCATCTGGTCT
CGGCAGTATAGCAATAGCCCGGATCGTATTGAATCTGTTCAGAAGCAATTTCTCGAAGCAATCAATCTCC
GGTGGCCATCCCACTCCAGTAGGTTTATTTTTCTTGATCTTCCATCGTTAACCAACCGTAGGACACTCCTCG
ATATTGTCTCCCTACAGAAGTGGATTCTACTTTTCTGCTAGGGCGTCTTCAGTTTTCTATTCCGTCTAGACC
AACCAGGAATTATCGCCGCTTGTTCCTACCAACGTGCACAACAGATTTCACTATGCACAACTCTTTTTACG
AGCATTGTCTACCCGTAATAAAATCGTTGCTTTCAGGATACCTACGGAAACTCACTGACCATTCCCAGCAA
TAGGTAGGGGCATAGCTTTCCGGACAAACCTAAAATTTTCCCCCTCGGTTCAGTGATTTCCCATCACTATC
TCTTTATCCTACTTATATAACACTCTTTTTATTAGTATACATTTCTTTACTTTATTAACAACTTCTTTCTTTTG
TATTCTTTTTAAGATTATTTTTAATTTTTGCTTTTAGACTCTATTGTTCCTNCTGNCACCCTTTTTCTTAGCTG
TAGGCTCTAATGGCGTCACTGACAAAATTAGCTGTGAAAAGGGGCATTGGTGGCCGGACGTTCAAATAAA
ACACCTCCATCGGTCGGTGATACTATTAGTAAATTGTGTATTCTAGCTAGGCTCTTAGCATTTAATTTTCAT
AATAAATGTATAATGTATTCAAAAATGATGAAGAAACAAGAATGAAAGAATGAGTATCTAAAAGAGCGA
AATAAATAGCGAACCCTACAAGGGACGTCCAAGTCGCTGGCATGGTTGTTGTCATGGAAGATAGCCTACC
AGTAAATAAATGGGGCCTTGGCTGAATACGTCTCGGCACAGAAGAATAACGCATCTCATAAATTGCATTN
TCCAGTTTACTCCTCTGCATTGAAACAATATGGCACCTTCATCCAAGCGATACAACCAAATCCAGGGTACG
TATTCCTTCAGATGCCGGGTGTGCAGAGAAATTCATGGACGACGCGTCTAATGAAGCTTACGTCCTAGAA
GCGGCTCCAAGCAGTCCTTAAAAACAAATATTTCTCCTACTGACCGACTCACCTAAACACAGGGCAGTCA
TGTCCCTGCGGAGCAAAATCAAACATCTTCAAGAAAGATCCCCACAGCCCGCNCCACATGCGAAATCTCC
AAAGCTCTCGTCCCTCTCAGTCAACCATGAAGCCTTACTTAGAGAGCTTCTGGCTCCTCGCCCTCCTCTGG
CTCCGCGGCTCACCATAATAACGGCGAAGCACTTCCACCATGCGGATGTCGCTTACGTCCCTTCTCCAGCA
CAATAAGATCAGTATCCTACCCACCGCGACTCTGCTCTCCGGACAGACCTATGTCAAACGCATTGCCTGTG
CTACGCTCCAGGACCACTCGAATGACCTGAGGCAACGTTTCAAAGGATGAACCCATCAAGGCAGATATGA
AGGAACAGTTCAGCAATGTCCTCCCCTCCATATCGCTCACTCTCTTTCTTTAATTTTAATTGTCAATGTCGT
CACACGAAAATAACGAAAAAACGCGCTTTTCGCTTCTACTTATTGTTTATACTCTCCGCCAAGCAACTTCC
GTATACTCGACCACATATGTAACTGTTAAACAGAACAGTTTGCATTGCTCAACCAGAAACATAAAACAGA
CTACACAAGCGCAACGAAATAAAATGTTTTACGAAGGACGAACCGGAATCTTCTTCATTTATTTTTGGTTA
CTTGTGTGCAAATCGCATTGAAAATAGTGACTGTTATTTGCGAGCTCTGGTTTTTGATAAACTTGGATTTC

AGCCAATGCGGTGGGCGTGCTTTCGAGATGTCCTCTCAAATGCTTTATTTGGCGGCTAAATTCTTCATTAA
TGTTTCTATCTATATTTCATTCNGCACAATTTATCGCCCATATTGAGCCAATCTACCGCAACATCTTTTATT
TGACACAAATCATATAGATATTGAAACCTTAATAACGTCTTTAAGAATTTTTATTCAATCTGAACAGGTCA
TTCTGGAGCTTTAATTGGAATTTAATTTGTTACCGCCCATACTTTTCTTATAAAAGTTTTTAAAGTTATGTC
AATTTTAAGAAGGAAGCATCTCCATCACTCCATCAGAATCACAAGCCGAGTGGCTCGATCGACTCGTATG
TATAAACGCAAGGATCTCAAATCCTATAAGAGCTGAAGACTCGAAATTGTATGTGTAGGTCCACATAGTG
CCCGCGCAGAACGAGTTTTTCCAATATTCGATATTTTGCCCCGTTTTTATGTAATTAATAAATAACGATATC
GTATTTATAATTGATTTTTTGAGAATATACTGATTGCACAAAAAGATCTACTGTTGAAAATAAAGATCTGT
TAAGAAACACAAAAGTTATTATAGGAAACGAGTTTTGACTGGCTTCGTGTTTACGGCAACTAGCGCGAAC
TATAAGAATTTCTGTACGCATGTACAAACACATTCATAGGTTTCTTCAATGAATTCTATCAACACAATGCA
ATTGCGATCGTATTGTNTTCTCTGCTGCTATTTTAATCCGTTTTTCTAATTAAGGAGAATCTTATATCATTC
CTCTTCAAGAAAAAGGTGGAAGGTCTGATATANAAAANTACAGGGGCATTGCAAAACTGTCNACTATTTG
TACATTTTTAAAAAAATAATCACATCGAATTTGCAGCATTTCTGTAAATCAGTGAATACTGTTCTTGCATC
TGGTCTCGGCAGTATAACAATAGCCCGGATCGTATTGAATCTGTTCAGAAGCAATTTCTCGAAGCAATCA
ATCTCCGGTGGCCATCCTACTCCAGTAGGTTTATTTTTCTTGATCTTCCATCGTTAACCAACCGTAGGACAC
TCCCCGATATTGTCTCCCTACAGAAGTNGATTCTNCTTTTCTGCTAGGGCGTCTTCAGTTTTNTATTCCGTC
TAGACCAACNAGGAATTATCGCCCCTTGTTCCTACCAANGTGCACAACAGATTTCACTATGCACAACTCTT
TTTGCGAGCATTCTCTACCCGTAATAAAATCGTTGCTTTCAGGATACCTACGGAAACTCACTGACCATTCC
CAGCAATAAGTAGGGGCATAGCTTTCCGGACAANCCATAAATTTTCCCCCTCGGTTCAGTGATTTCCCATC
ACTATCTCTTTATCCTATCTGTATAACACTCTTTTTATTAGTATACATTTCTTTACTTTATTAACAACTTCTT
TCTTTTTTTTTCTTTTGAAGATTATTTTAAATTTTTTCTTTAGACTCTATTTTTCCTACTGACACCCTTTTTCT
TAGCTGTAGGCTCTAATGGCGTCACTGACAAAATTAGCTGTGAAAAGGGGCATTGGTGGCCGGACGTTCA
AATAAAACACCTCCATCGGTCGGTGATACTATTAGTAAATTGTGTATTCTAGCTAGGCTCTTAGCATTTAA
TTTTCATAATAAATGTATAATGTATTCAAAAATGATGAAGAAACATGTTTTGTCGATCAATAATAAATAAA
TAAATGTTAATACATGTAAAAGCAATCCTCATCGCGCTCCTCACATGACTTGAAGACCCTCTTCCACCACA
TCTTCAAAAGCATACATTTTAGAAGTACTCGTTTCTCCTGTCCAAGCTTGGAGCTCCGACCGATTTATTGG
CTCTAAGTTCAGGACACTTCCTAGTTGGTGGCTTACCATTTTCAGCAAACGAGCCAGTAATCAAAGTAGAC
ATCCCCATATAAACCGATGGCAGTGTATCAAAGCTCGCCATCAGCAATTCTGCTTTCGGTGAAAGAATGA
GTATTTAAAAGAACGAAATAAATGGCGGCCCCTACAAGGGACGTCCAAGTCGCTGACATGGTTGTTATCA
TGGAAGATAGCCTACCAGTAAATAAATGGGGTCTTGGCTGAATACGTCTCGGCATAAAAGAATAACGCAT
CTCATAAATTGCATTCTCCAGTTTACTCCTCTGCATTGAAACAATATGGCACCTTCATCCAAGCGATACAA
CCAAATCCAGGCTACGTATTCCTTCAGATGCCGGGTGTGCAGAGAAATTCATGGACGGCGCGTCTAATGA
AGCTTACGTCCTAAAAGCGGCTCCAAGCAGTCCTTAAAAACAAATATTTCTCCTACTGACCGACTCACCTA
AACACAGGGCAGTCATGTCCCTGCGGAGCAAAATCAAACATCTTCAAGATAGATCCCCACAGCCCGCTCC
ACATGCGAAATCTCCAAAGCTCTCGTCCCTCTCAGTCAACCAGGAAGCCTTACTTAGAGAGCTTCTGGCTC
CTCGCCCTCATCTGGCTCCGCGGCTCACCATAATAACGGCGAAGCACTTCCACCATGCGGATGTCGCTTAC
GTCCCTTCTCCAGCACAATAAGATCAGTATCCTGCCCACCGCGACTCTGCTCTCTGGACAGACCTATGTCA
AACGCATTGCCTGTGCTACGCTCCAGGACCACTCGAATGACCTGAGGCAACATTTCAAAGGATAAACCCA
TCAAGGCAGATATGAAGGAACAGTTCAGCAATGTCCTCCCCTCCATATCGCTCACTCTCTTTCTTTAATTTT
AATTGTCAATGTCGTCACACGAAAATAACGAAAAAACGCGCTTTTCGCTTCTACTTATTGTTTATACTCTC
CGCCAAGCAACTTCCGTATACTCGACCACATATGTAACTGTTAAACAGAACAGTTTGCACTGCTCAACCA
GAAACATAAAACAGACTACACAAGCGTAACGAAATAAAATGTTTTACGAAGGACGCACCGGAATCTTCTT
CATTTATTTTTGGTTACTTGTGTGCAAATCGCATTGAAAATAGTGACTGTTATTTGCGAGCTCTGGTTTTTG
ATAAACTTGGATTTCAGCCAATGCGGTGGGCGTGCTTTCGAGATGTCCTCTTAAATGCTTTATTTGGCGGC
TAAATTCTTCATTAATGTTTCTATAATTCAGCACAATTTATCGCCCATATTGAGCCAATCTACCGCAACATC
TTTTATTTGACACAAATCATATAGATATTGAAACCTTAATAACGTCTTTAAGAATTTTTATTCAATCTGAAC
AGGTCATTCTGGAGCTTTAATTGGAATTTAATTTGTTACCGCCCATACTTTTCTTATAAAAGTTTTTAAAGT
TATGTCAATTTAAGAAGGAAGCATCTCCATCACTCCATCAGAATCACAAGCCGAGTGGCTCGATCGACTC
GTATGTATAAACGCAAGGATCTCAAATCCTATAAGAGCTGAAGACTCGAAATTGTATGTGTAGGTCCACA
TAGTGCCCGCGCAGAACGAGTTTTTCCAATATTCGATATTTTGCCCCGTTTTTATGTAATTAATAAATAAC
GATATCGTATTTATAATTGATTTTTTGAGAATATACTGATTGCACAAAAAGATCTACTGTTGAAAATAAAG
ATCTGTTAAGAAACACAAAAGTTATTATAGGAAACGAGTTTTGACTGGCTTCGTGTTTACGGCAACTAGC
GCGAACTATAAGAATTTCTGTACGCATGTACAAACACATTCATAGGTTTCTTCAAT
>160_rnd-1_family-226__LTR___Gypsy

AAAAAAAAAACTGAAAGTCTGAGTGAGTAGAGTGCGAGTAATGGGCAAGCGCGGAGGCCAGAAAAACA
AAATAAACAAAAGAATAGCACACATTAAGTGGCTATTATACACGAATACGCCGCCCACATGGGCAACGG
CACAAAAATTAACTGCCGAACTTGATTTTCTGAAAGAAAGCCTCCAGCAAAGAAAGCCAAATACCGCAAC
TCACTCAGCAAATATCAAAGTTATAAATGAAAAACTAATGAAAAAACCACCATCTCCAAAGCCGTTGCCG
AAAAAACCTGACGTCTGCCCGAACGACTGCCCTGGACCACTATACGCCCCAGCATGCAAATCCACATGCT
TGGGACCAAAGACCAGTACGAAGACCGGCACCGGCCCCGCCTGAGCCAATAGGCCACGGATATCAGCAG
CACTGAAGGCAACAGCGCGAGGGGCACTCCGCCACTTTCGCCTACGAAGGAGCGTCAGCGTAGCCAAAC
CGGCGACGACCAGCTGACGCGGAGGACAACTGGACATTTGGAGCAACAGTTAACATTATTATAATAAAAT
TTGTAAAATTGTGCGGAACAATCTCCGCTCAATTAGAAGTATAGCCAGTAAAGCTATACAAACCAATAGG
TAGAAAAACTTGTTAACGTAAATGCAAAGCAATAATTTTAAAATATATATATATATATATATTATCGAAAC
AATTTATGTAAAATTGAGCAGAAAATCTTTTGCCCAATCAGAGGTGAGGCCCATAAAGCCAAACTAAAAA
ATTAGGCAGGTTGCAATGTAAAAAAAAAAAAAAAAAATTTTACACGTCTCAGGCACTGGGTAAATTACCCA
GTAGCAAACAAATTAATCCAAATCTTAAATTCCAAATAAATTTGAAATTTTAAAATGAAAAAAAAAATAAT
AATAAGTTTTCAGTTCAGTCAGTTAAATCAGAAANAAAAAAAAAAAAAAACAAATTGAATCAAATTTATGA
ATAGTAATACAATAAGTAAATTTTCCGAAGATTTTCTAATTGAATCAAATCATAATCCTGAACAGAACTCC
AAAATGGGATTATTAGACGTTAAGTCAGTGGATTCCACTGCTAACGTTATAAATAAAGTTGAATCAAACA
ACACAGATTTAGAATTAACAAATATATTACTGGTAATCATAGTTGTTATAATGTGTGCAAATTGTTTTTAT
AAAATATATAAACTGCATAACAAATGCCTAAAAAAAAGATTCATGAGCCGAGCAAATGACTTGGACAAA
ATTTAAATTTGCACAGCACAAACAACAATGATTAATCATAATTGACAATATCATAATAACTATAAGATAC
CAATTAAACTAAATAAATAAAAAAAAAAAAAATAAATAAATAAATAATAATGAACACACAGTATGATGAA
TAGTAAGAATATCCCAGAATAATCTTGAAAAACAAAGTAGCTTGTCAATATTATGGAATGGCCAGAACTT
TCTGAGCGGATCCAAAATATTAAAACGACATTTGAAAAAGCGTATAAATGTCTAACACAAAATAGACAAA
CGCATATAGATACAGTTAATAGGCACTCCATAACATTGATCGACAGCTTTAACGAAGCTCGAATATTGAT
ATATCAACATAAAGATGTATTAACCAATGAACATTGGTCAGAAACGTCAAGGTTGCTAATAAGACTGAGA
AATAATATACAAACAGTTAAAGACAAGCATAATTTAAATTTAATACTACCTTCTATTTTGAATACACCTGC
AAAGTTGGAGCTCGGCGAAAACCTTGAAGTTCGGACTGAGGATATTCAATTAGAAGAAGAAGATTTAACA
AATCTAACAATCCCTGCTGTTATCACACTGGATTCCAAAGATATTTTATCCGAAAGTGAAGAAAGTGCTGA
TAATAGCCGTATAATGGTTGACACAGCCGCTGCCCAGAGGGCATATATTAGGGAAATTGCACAAGCAGTC
CCAGAATTTGACGGAAAGAAAACCCGTCTTGAAAAATTCTTAACGGCATTAAGACTTGTCAACTTAACAA
AGGGAACTTATGAGGAAATTGCCATAGAGTTGATTAAATCAAAAATAACGGGCACAACCCTCAACAAAA
TAAAAAATGAAACCACAATAGACGGAATTATTGCAAAATTAAAAGCCGTAGTTGTCGGTGAAACACCCGC
CAACATAAAGGCAAAAATGGACACTTTAACGCAGAAGGGCAAAACAGCCACTCAGTTCACAACTGAACT
AGAAAATCTGCGACAGCTTCTTGAAGCTGCTTATATTGACCAAGGCATACCATCTGAAAATGCTGACACA
CTCAGTACTACAGAAGCCGTTAACACAATGATTAGTAAAACTAGTCACGAAAGTGTTAAAACCGTTATGG
AGTCTGGTATTTTCACTACCATGGATGCTGCCGTTAGTGCATACATCCGTACTAGTACAAGACTTACTGGT
AGTGTGAATACAGTGATGTACTCTAAAAGAGGGAATAACAACTACCGCGGTAGTAACAATCGTGGTAACT
ATCGTGGTAAAGGCAATGGTAACTACCAAAACAGAGGAAATAATAACTACGGTAATCAAAACCGTAGAG
GCCGAGGAAATGGAAACAACAATTCCAACCGAGGCCGAAACAACCAAAATAATAATAATACAAACAATG
TTAGAGTAACCCAGGACAATCAGGGAAACTCTCGACAACCCTCAGATATTCAGCAGTAGGAAATTACGCA
GTTCATACCATCAATCTTAGTTTGAATATATTTGTAAACTTTTTCAATGTGAAGACAAATAAAAAACTTGT
ATTTTTAATAGATACTGGTGCAGACATATCCCTGTTAAAGGAAAATTCTGATATCTTTCATGATATCCAAA
AAAATAAGAAGATCAATATTCAAGGCATAGGCCAAGATATAATACAGTCCAAAGGACTGATTTCAATAG
AGATACAGACAAGTAAATACATAATTCCACACGATTTTCACTTAGTAGATTTGCATTTCGCAATACCTTGT
GATGGAATAATAGGCATTGACTTCATTAAGAAATTCAATTGCCAGTTAGATTTCAAACCAAATGAAGATT
GGCTCATAATGAGACCAAATAATCTAAATTTCCCAATTTATGTTCCAATAACATACAGTTCTGGTAATAAT
TCCATTGTTCTACCAGCAAGATCCCAAGTAGTCAGAAAAATTCACCTATCATCAGATGAAGATAGTGTACT
AATTCCAAACCAGGAAATTAAAGAAGGCATTTATGTCGCAAACACAGTTGCAACAACCAAAAATGCATTT
GTCCGATTACTGAATACGACCAGTACCGATCAAGTAGTAGATATTAAAAATTTGTCATATGAACCACTTTC
GAACTACGACATAGTTCAAAACAATCCAGAAAATAGAGAAAAAACTGTCTTATCTCAATTAGCTAAAAAT
TTTCCGCCACAATTCAAAAACGAATTGACTAACTTATGCACCCGGTTTAGTGACGTATTCGGACTCGAAAC
CGAATCGATCACNACCAATAACTTCTACAAGCAAAAACTAAGATTGAAGGATGATGAACCNGTCTACATA
AAAAATTATAGAAACCCTCANAGTCAAATAGACGAGATNCAAGTNCAAGTACAAAAATTAATCGACAAT
AAAATAGTCGAACCGTCNGTNTCCCCNTATAACAGCCCACTTTTATTAGTTCCGAAGAAATCTCTTCCGGG
CTCTGACAAGAAAAAATGGCGATTAGTAATTGACTATCGTCAAATTAATAAAAAGCTATTGTCCGACAAA

TTTCCGCTGCCTAGAATTGATGATATTCTAGATCAACTAGGTAGAGCAAAATATTTCTCATGCCTNGACTT
AATGTCNGGATTCCACCAAATTGAACTCGAAAAAAGTTCTAGAGATATAACGTCNTTNTCAACAAGCAAT
GGCTCGTATCGCTTCACGCGATTGCCATTCGGNTTAAAAATAGCGCCGAATTCTTTCCAGAGAATGATGAC
TATAGCATTTTCCGGNCTAGAACCNTCNCAAGCATTCCTTTATATGGATGATCTAATAGTAATCGCTTGTT
CCGAGAAACATATGCTCAAAAACTTAACTGATGTTTTCGACAAATGTAGGAAACNCAACCTGAAACTACA
TCCNGAAAAATGTTCATTTTTCATGCATGAAGTCACATTTTTGGGACACAAATGCACNGATAAGGGAATCT
TGCCAGACGACAAAAAAATACGACGTCATTAAAAATTACCCAGTCCCACACGATGCGGACAGCGCCAGAC
GATTCGTTGCATTCTGCAACTACTATAGGCGCTTTATCAAAAACTTTGCCGACTATTCNCGTCACATAACA
AGATTATGTAAAAAAGACGTAGAATTCGAATGGACGNCTGAATGCCAGATGCTTTCGAACATCTCAAAAC
GGCACTAATAAACCCGACNTTGTTACAATATCCAGACTTCACCAAAGAATTCTGCATAATAACAGATGCA
AGTGAGCAAGCGTGTGGAGCAGTTTTAACTCAAAACTNNAATGGACTCCAACTTCCAGTTGCNTACGCAT
CAAGATCCTTTACTAAAGGAGAAAGTAACAAGAGTACAACNGAACAAGAATTAACAGCTATTCACTGGG
CAATAACACACTTTNGACCATATATTTATGGTAACCATTTCACGGTAAAAACGGATCACAGACCACTAAC
ATCTATTTTCAATGGTCAATCCAAGTTCACAATTAACGCGTATGAGGCTTGATTTGCAGGAATATACCTTT
ACGGTAGAATATCTGAAGGGTAAAGACAGTTACGTAGCGGATGCGTTATCGAGAATAACCATCAAGGAG
CTACAAAATATAACAGGAAACATAATGAAAGTCACTACAAGACANGCACGTAGACAAGAATCCTGCGCA
GGAGAAAAAGAAATAGAATTGCCNAGGCAATCTATAGAAAAAGCTTCAAAGCCCAAAGTGTACCAAGTC
ATTAACAATGACGAAGTACGNAAAGTAGTGACCTTGCATGTAAACAATATGACATGTTTTTTTAAACATG
GNAGACAAATTACAGCAAGATACGATGTTACCGATTTATATACTAATTGAACCATTGATTTAGATCAANT
CTTTCAAAGGCTTGAAACGCAAGCCGGTATATATAAAATCAGCCAACTCAAAGTGGCACCGTGGGAAAAT
ATCTTTGAACACGTTTCAATGGATAATTTCAAAAANATGGGCAATCAAATATTAAANTCNTTGAGAGTAG
CGCTACTCAACCCGGTGACCCAAATAAANNCTGTAAAAGAAAAAGAAGCTATATTGTCTANACTACATGA
TGATCCAATACNAGGAGGTCACACAGGCATTACAAAAACCTTGGCCAAGGTCAAACGACATTATTNTTGG
AAAGGTATGNCTCGAGANATAAAAGAGTACATCCGCAAATGTCCAAAATGCCAAAAAGCAAAAACTACA
AAAAACACAAAAACTCCTCTAACAATAACAGAAACACCAATATCCGCTTTCGACAGAGTAATCGTNGACA
CAATTATTACCACTACCTAGANCAGAGAATGGGAACGAATACGCAGTCACACTCATTTGTGATTTNACGA
AATACCTNGTCGCGATTCCGATCCCAAATAAAAGCGCAAACACTGTCGCTAAAGCTATATTCGAATCTTTT
ATTCTGAAGTACGGTCCAATGAAGACGTTCATTACGGACATGGGNACGGAATATAAGAATTCAATTATAA
ACGATTTGTGCAAGTATNTGAAAATAAANAACATAACATCAACTGCACATCATCACCAGACAGTTGGAAC
AGTAGAAAGAAGTCACAGGACTTTTAACGAATACATACGATCATATATATCAGTTGATAAAACTGATTGG
GGCGTATGGATCNAATACNTCGTNTACTGTTTCAACACGACCCCCTCTATGGCACACAATTATTGTCCATA
TGAGCNAGTTTTTGGTAAACACCCAATTTACCAATATAAATAGNATNGACCCACTATATAATGTAGAAGA
TTATGCTAAAGAAGNTAAGTTTAGATTAGAANCAGCATATAAAAGGTCTAGGATAATGCTTGAAAAACAT
AAAGCAAGAAGTAAGGAACTTTNTGATAAAAAAGTTATAGATTTTCAAGTAAAAATAGGAGATAAAGTC
TTATTAAGAAACGAAACTGGCCATAAATTGGATCCAGAATATATAGGCCCATATACAATAGTTAACATAG
AAAATAGGGATAACATTCTAATTAAAGATAACAAAAATAGGACCCAAAAAGTTATAAGAATAGACTAAA
AATTTTTAACGAATAAAACATATAAATGTTCTATATGAAAGTCTTTATGTTCCTTTATGAATTCACAACAA
ATAACAAGTAAGAGGTTCTAGTCGGGAGCTCCCGACTAGGGGATACCCTGAACCCTCTTCTTCCAACATC
AAATGCATATATAATCTATTTTAGAAGCTATATGTCAAGTTTGGTTCTAGTCGGGAGCTCCCGACTATGGG
ATACCCTGAACCCTCTTCTTCCAACATCAAATGCATATATATTCTATTTTAGAAGCTATATGTCAAGTTTGG
TGACTCTAGCTCTTATTATTTACCAAAATTGCCCAAAAAAAGGATATCGATATTGATTTTTATCGATTGCTT
GGAAACGGAGTAAGCTATCGATTATCGGAAATAAACTCGATCTGCGCGGGAACTAGGAGCACCTACATCT
AAAATTTCAAGTCTCTAGCTCTTATATGTTCTGAGATCCTTGCGTTCATACATACGGATGGACGGACGGAC
GGACGGACGGACAGACGGACATGGCTAGATCGACTCGGCTATTGATGCTGATCAAGAATATATATACTTT
ATGGGGTCGGAGATGCTTCCTTCTGCCTGTTACATACATTTGGATTTTGCACAAATACAATATACCCTTAT
ACCCATTTTTAATGGGTTCAGGGTATAAAAATACATATACATTGGCCTGATCAACCAACTAAAAAAAAAT
CAATCCTTGAAAAATACAAGTTACTATAATGAATTTTACGTTTTTATATTTGATATCTTTATACTTTGAAAT
GTTTACATTAGTATATTCCCATAAATCTTAAATAATTTTTTTAAATTCATATTATTTTTATCATTTTTTTACA
TTTTATTATTTTTATAGCAAAAAAAAAAATTTATANACAAAAGATTTTGTTCAAAATAATTAAGTTCATTAAA
TAACTACACATCCATAATGTTACGTTATTTCTTTGAAAAGGGAGCTGTAGTGTGTATATGCCTTAAAAGAA
CACNCTATATACAAATACAAAGTATAAACAAGCCCGGAGCCACTTTGTATATTGGCTCTGGCTCATATTAC
GCAGCGCAGTTCACTAGCCTCTCAGCTGATGCAACGATGAACAACAGTTGCAGTTATATCCTTAATTACTT
TTAATTATAAAAAAAAAAATTTTTTGTAAAATTGAGCAGCATAGCCCGTAAAGCTATGCGAAATGCTAGGT
AGTATTTTGTGTTATTAGCAATATTGAAAACTTGAAACTAAAAACATTGGCATTTAAAGTTAAAAACACTA

TATGTTACATAAAAACATTATATGAATTTAAAAACATTACATCAACTTATGTAAGAAATTTTAATTAAAAT
GGGTTCGAACGAATCCAACGTTGAAAAATCAGCCGCAAATGTTATAAATAACATAAAAGTAATTGACCAT
AAAGAATAATTACTACAATATCTCTTGTATCACTAGTATTGAAAGTGTATGCAATGCATACAAAGAAAAT
TAAGAAACGCTATAGCAGTAGAGCAGACGGACTAGATAAGGTATGAATATATTTGAATATGTCAGACATA
GAGCCAATCATTAGGTTTACGTATAGAGATGAGCCAGACTTTCGTACTCGCGTATTAAACGTACTGCGATT
ACATAGAGATTTTATAAAAGTGTGTTATTTCGAATACGAATACACGGACATGTTCAATACTCATATTAATG
GTGCGACTGCATCGATACATATCTATTATAATAATATGGAATGGAGTATATTAGTGAAAAATATCAATGA
CTTTAAACACCAATTTGACAAGTCATATAAATGCATCAATAAAGGCATACCTCTAAAGGTAGAAACACTA
ATATACCATGTACAAATATTGATAAAACAATATAACAATATAGTACATGCAGTAGATTCCATTAAAGAAA
AACTAACACAAGAACATGCCAAACAATGCCNTAAGGTCATACAATCTTTAAATACAAGATNTNAAAACAT
TGGCATTAGACAGAATATCATAATAAATGTTCCAGAAGATCTATTTTTACCAGCAAAGTTTGAACCAAAC
CAGCTGCAAGATCTGAATGAGTCGAAGCACTAGCCAAAGAACATATAGACTGGACGATGGAAAGTAGGC
ACAAAAAGTATCGGTGGCATCTGGTCCAAAATCGCGAGTAAGGGAGTAAGATATACAAATGATGTATTTT
TGAACCGCTTACTCTACAGGAAAGTGCTTGGCGCACTCTCTTCCATATGATTTGTGCGATAGAACGACAAC
GCTGTTAACTTTTATCTCGCTCGCACTTACTCTCAGCTCTCAATGCTCTCTCTTTATGTTTAGGGATCGTTTA
AATGTGTGTGTGTGTGTATTTGTCCGACAAGCAAAACTGGGCAAAAATTCAATTTTTCCCAAATGCTGAGG
CATTTCTACCCTTTTGAAGGCACGAGTAATATGGAATAGAGAAAGGGTTTGCCACGTCGATGCGAA
>9_rnd-5_family-1307__LTR___Pao
GCACACAAGCGCATAAAAGCTTCTATTCATATACAAAATATGCAAGTATGCGTGAACAGCGAGTGGAAAA
TATTTTGAATGCACACATGGCAACATTTAGGTACCCTTACCAACGATAACGAGCTAACATGCAAGAATTTC
ACATCGACGTGGCAAACCCTTTCTCTATTATATATTACTCGTGCCTTCAAAAGGGTAGAAATGCCTCAGCA
TTTAAAAAATATTGAATTTGTGCGGAGTTTTGCTTGTCGGACATACACACACACACACATTTAAACGATCC
CTAAACAGAATGAGAGAGAATTGAGAGCTGACAGTATGTGCGAGCGAGATAAANATTAACAGCGTTGCG
GTTCTATCACACAAATTATATGGAAGAGAGTGCGCCAAGCACGTTCCTGTAAGGTAAGCGGTTCAAAAAT
ATATCATATGTATATCTTACTCCATTACTAGAGTGCTTGGACCAGATGCCTCCGAAACATTTTGTGCCAAC
GTTTCTGTTTCCGTTTTCGACGTTGGGTAAGCTTGGTTTGGAAAGAGGCTATATCTGCACCCCTGCGAAGA
AGTTCTACCGCAGGAGGGTGATATTCTGATGAGTCCAGCGTGTCGATTTGTAGAGGCAGATGATTCGATA
GTTCGTATAGTGACTTATTGCGAATCGTTCCATGGCATATTCCTTTATATGGCGAAAAGTCTATTGCTGAT
AGAGCCCAGCCCCGATGATTTGGGACGTGGGTAGCGCTACCTGTTGCCTTGCTGCAGCCTCTGATGTTGTC
CCACTAGTGATGTTTTGCATTCCAATCTCCAGCGAATTTGTATTTGTTACCCTCGAGTCAAAAAGGTCATG
AAATTTATTAGTTCCGATGGAATTGTACCTGGAGGACAATATATAGACGGTAGCACTAGATCGCCTTGT
GATGAATCTATTCCGATCACTGCTACTTGCATGAAACTAAGTGTGATGGGGCTAAGAGAATATTGTTTAAT
ATAATTTAGTAATTAATTATAAGTTTAGCAGCGCCTCCCTGTGGGGCGTCATTAAATTGGTTAGCTTTATG
GAGAGGGTATACTGGAACTCGTATGAATTTTCTGGAACAGATGTGGGTTTCATTAATTAGTAAGGAATCT
GGGCTGTGTTGTTCTATGAATGTTTTAATTTCAAGCGCTCTACTCTATACCGAACAGGCGTTCCAAAAACA
CTATTTTCCACTATTTTTGGTGGCAAGGTCAATGAGGCTCCTAATATCATTGACCATACCTCTAATCTTCT
CTAGGAGACGCTGGTTTGATCTTTCCATCATTTGAGACATTTTTGTGAAGGATTGGGTCAGCATTGAGATC
ATGCTACGAGTCCGTAGAGTGGTGGTGGCCGAATTTTTTAAAGAGGCTACGGGGGATGCTTAGATGCTTTT
GAGAATGTTGTTTGTGGCTTAAATTGCCATTTGGAGGACACGACCTCTGACTGCATCTGCAAAGGTGGTGC
CGGTAGATATTTGTGAACCCTGTTGAGATCTTGATCCAGCTTGTGGGTTGTAGGTCGAGTAATCGATTAGA
AAAATTACTGGGAGTATTTCTTTATGCTGCCGTCTGATTGGCTGTTTTTTGTAGACTTTGCATCCCAGATGG
TTGGCTGGGTGTTCTTCGCCGCAGTTAGCGCATGGTCAAATATTATTACCTGTTAAAGTGCACTCAGCTGA
TCGGTGCTGGTTGCCACATTTAACACATACTGGTCTTCTGTTGCAGTATGCTTTGGTATGCCTAAAGGCTTG
GTAGCGATGGTACTGCGCAAGCAGATTGCTTTTCTAGGCATGTTCCGGATAAAACCTCATTCTGCAAAGTT
GGGTAATATTAAATTCCTCTTTATTATTTGCGGCTTTGGCAGCATCAACAAGGCACAGGCTAATTTTCTTCT
TTTTTCGTTTTTATCATATGGCTGGTACATGTTAACTACGTGGTGGATTTTCTAAATGACAAATCTAAATGG
TCTGTCTTCGCGCAGCTTGTAGTGACGGAGCAGCATATTAGTGCTCTCAGAATTCTTACATTGGATGAACG
TGTCGCCCCTCATATTTGTGTGAGCAGAGAAATTGTCTAAGCCGATGGCGAGATCCATGTTCAGAAGGCG
TCTTAATTGCTTACTCATGGGATTATGATCCTCGGTGGACGATGATTCGTTCTTGCCGGTGATGGATTNTTT
TATTTTTCTTCTGCAGTTGCTTCCGTCAGGAACCTTCTTCAATGTTTATTGGGGATGCGTTGGCGACGCGTT
TAATGAATAGAACAGGGGAGCCTGCTTGGTGGTTCCGTTTAACTGCTCCTTTCATTATTTTGATGGGTTGA
ACCCAAGGTGAAACAGTTTACCAGCGATAGCCGACTTTGGTATACGCAGAATTGTTTCTAATATTTTTGGT
AGGCATTGGGTTAACTTTGGGTGGACTTTCGCCTGGGGTGCAGCTTAAGGGATATGGTTGTCTAGAAGAG
TGAGTACAAATCCCAAAGTAAAATTCTCCATGCGGGGGCTGGTAATGGTGCTATTGTAAGTTGTGAATGT

AGTGGTATTTGCACTATGGATATCCGTATGGATACCTGTGGAAGATAGGTTGGCATTAGCAATTGTGGTGA
TAATTGTGCAGCTAAGAGTATGGCAAGCTCCGCAATTGGAATTATTGTTGTTAAAGGCGTTTGGGTTGATT
GGGCCGACAGGTATCAATTCGGCGCTAATGTCTTCTTCTATTTAGTCGACGGCATTTGATTTTATTGGCCAT
TAATTTACATGTTTTTTCGCTTTTCTTGTCTATATAATATAGGGTTGAGCTGAGTTGTTAACTCTAACTATA
TTTTTTTTTATTATTTCTTACAACTATTGATTGTTGTGTCACTTTTAAAAATTAAGTTTAAGCACAATAAGT
GTTTTGCGGGATAACTTTGCCTTTACCGAAAAGGCAAATTAGTTGGATGTGTGCGCAACAGCACTGGGTTT
CAGAACTACCGCTGTAACCGCTACCGGGTAAGGCATTCCCGACATCCTAGTTTTTCAATTGATTCGCAGCA
AAGCGGCCAAAGCGTATTAAGAAAAGCTAGCTGGTAGCTACATTGGCAAGACGTGTTACATTTGTGTTAG
CGCACATGGGTTAAGAGCACTTGCAAAATTATCACCTTTGCAACGCTATCAGTAGGTTAAAAGGCGTGCG
ATACCCTAAAATGTTTTAGTAAGCAGCATATAATGAGGAGTTGCGATAGCTAACACTGTACCATATGCAA
CAAGCCTCAAATTGCTTTATGGTAATGTCTCGGGTAGCGTAGGTGACGGTGTGCATAATGGCGAATTGCA
ATAGGGGAACAGTTCTTTATATAGCGGACTATTAACAAATAGCGGGCTATTGGAAGTCTTCTAGCGACCG
TCGTGGTTTTGATGAAAGCTAATGATAATGATTAATCATAACTGTAGATGTATGCTATAATCTGGTAGTCA
AAATAATTTTCTTACTTCTACATTAGTATCGAGCTAATATCTGGAAATGTTTGAATAAAGTATGCGAATCA
GTGGGATCGACAATACGACATAACACATTATTGGTGATGTGAATGCCATTTTTTTAATCCAGAGGGACAA
GGTTTGCAGCCACGGACAATTTGTATGTGTTGGCCGAAATTAGGCAATATAATCCGACAAGCCCCTCTCTA
GGCTTAAATTGACAACCACTCAACGGAGTAACACTTGCGGATTTGACATTTGACTCTTCTCAAAGCATTTA
TATGCCATTGAGAGTTTATTTATACTTCAAGCTGATTGTTGCATGTCAAATCAAACTAGGATAAACATTGC
CAACCACCCTTCGCTGTTGGGCTGGGTAGTCGTTGGCGAACTTACGCCGTACGACACCCTAACGTGTCGCT
GCTAGCCAAGGAGAGGAGGCAAAATGGGCTAAGCTCAAATAAGCTGCAAGAACTAGAGCAACGGTATTG
TAAATTTGAGGAAATATAGGTTGCGCTGTTGATAAATTTAATGAAAAGAAAAAACTATGCAAAAACAGCT
TCGTTTTCAATGTTGGACAATCTGAAGATGGCCAATTAGCTACAGCACTTCCATTCAAAAGTCCCAAGAAA
ATACTAGAGTATTGCCCTTAAATTTAGTAATGAAGTTGAATCGCAATCCTCCCCTTAAGGAGCAGTTTTCA
GAATTTATTGAAGAATACAAAAATGTTGTGTCATTTGGAAATGTTATACGCCGGTAGGTCGCATCGCAACT
TCCAGTGCACATTGTGGTGGTTTGACATGAACACGATAAGGCGCTTATCTAGCAGAAAAATATGGAAAC
AGTATCCAATGGCAGTTGAAGCGATTTGATGTAGTGTCTAACTGGAACATATGTTGTGTGCTGCAGACACC
GTAAGCAACCTTGTGGAATTGAACCAGGAAGTGAGCAAGTGAGCTGTTAGAACTTGGCAGATTTTGGCTA
CACACGTGGTGTAGTAATAATCATGAAGTCCTCAGCAAAGCGGACGCTAAGAGCCTCGAAGGCTTAAAAC
TGAAGATGCAGCTAAAGCTCTTGGAATATATTGGGTGAGTCAAAGCGGCCTAGTTTATTTATCGTTTAATG
TCAAATGCTTTACTATTACGAAACGAACTGTGCTATCAAAATTAGATCAAGTGTTCCATGCCTTGGGATGC
TTGAGCCCTATACTGCAGGATATTTGTGCAAGAGTTCTGGTGGCGTCAAGTCACGCGGCTCTATGGCAAA
GATACCGAGAGGAACTGAAGCTCACAAATCAGTGTTCGCTGCCATGATGGACTGATTAGAGCATTTCGGT
TTCTCTGACGCGTCCAATTGAGCTTACGGTGGAGCGATTCATGCTCGCGTGTATAATAAAGACGGAACGG
TTTCAGTGCACCTAGTGACTGCGAAATCTAAGGTAGCTCCAGTCAGAGTGACTTCCTTGCCCCGCTTTGAG
CTGCAAAGAGCACTTTTAATGGCAAGACTTAATGCAAGGATTCTCAATTGTATTCATCTAAAGGTTTCGCA
GCACGACATTTTACAGACTACTCAATTGTGCTCAACTGGTTGTCATCGCATGCTAGCCGATGGACGACTTT
CGTAACTAACCCGATGGCTGAATTACAATAGTTAAGCAATGTTCAGAATTGGCATACACTAGATTTAAAA
TTAAACCCAGCTGATATGTATCCATGGAATTGTATCCAGCGGACTTAAACAATGCAACCTTGTGGCGGGA
GGCACTTGTTGGGGCATCCAGTAGATGACTGGGCTAACCCGCGATGGCCCTGCAGCTTCGAAGCAATATC
TGAACAGAGGAAGGGTATGTTTTCACTTGCCTTGNGACGAGGATGACTTATGATGTTATCGGGAAATGTA
AATTTACTAACGACTTACGACGGACCAAAGACAATATAAACACTAAGATGAGTTAGGCCCATACATTTGA
ATGCGACGCAAAATTTGTAGGCTGGCCGCTCATCTGGCCTTTGAGGCTTCGCACAACATGCTTGCCGACTG
GCTGTTCGTTCTCCGAAAACGGAGAAGTATATTGAGCGAAAAGTTTTAAACTAACGATTGACACAAAGTA
TCTTTGCTTTATCCATGAAATTCATGCTTTATTTAAATGGTATATTACCCTAGATACTAACACTAACCAAAC
AACATATAGACTGGACATCACATTGAAGAAACGTTGGCACAAAAAGGATCGGAGGCATCTGGTCCAAAC
TCGCGAGTAAGGGATTAAGATATACATATGATGTATTTTTGAACCGCTTACTCTACAGGAGAGTGCTTGGC
GCACTCTCTTCCATATGATTTGTGTGATAGAACGGCAACGCTGTTAATTTTTATCTCACTCGCACTTACTCT
AAGCTCTCAATTCTCTCCCTTTATGTTTAGGGATTAAGTGTGTGTGTATGTGTCCGACAAACTAAACTGCG
CACAAATTCAACTTTTCTTAATTGCTGAGGCATTTCTACTGTTTTGAAGGCACGAGTAATATGAAATAGAG
AAAGGGTTTGCCACGTCGATGCGAAATTCTTGCATGTTAGCTCGTTATCGTAGGTAAGGGTACCTAAGTGT
TGCCATGTGTATTTTTGAAAATATTTTGGACTCGCTATTCACGCATACTTACATATTTTGTATATGAATAGA
AGCATTTATGCGCTTGTGTGCCTGTTGCTTGCCTGTCCCCGATGCAAATTTAACAAGATTTTGTGATTTCTT
ATTTTCGATCTCTACACATAGACACTCACATACAAATGTCNTTGAATGGGCATGGGCGCAAAAGAAAATA
CATTATTGGCACGCGTCTGACGCGCGCCCTTCTTTTTAAGATTTTATTGAATGAATGTTGACCCCTTATTAC

CTGTAGAAGCCCGTTGGGCCGAAAGTGTCATATTTTGGCAAGCACCTGCACCCGCATATGTAGGTCAAAT
GACTGATCACCAAAATTATTACAACTGTAAATACATATAAAATCGCTCTTTTTTTTTTAATTTGTTATATTT
GAATATATATATATATGTGNTTTTATTTTTACACCAATTTCATTCTTAATCTTANAATAGCAGGGCGCGCG
TCAGACGCCCGGGCGCTGCTATACTGCTGATCTGATCTGCCCATTCGTAGACATTTGTATGTGTGTGTCTA
TGTGTAGAGGTCAAAAATAAGAAATCACAAAATCTTGTAAAATTTGCATCAGGGACAGGCAAGCAACAG
GCACACAAGCGCATAAAAGCTTCTATTCATATACAAAATATGCAAGTATGCGTGAANAGCGAGTGGAAA
ATNTTTTGAATGCACACATGGCAACATTTAGGTACCCTTACCAACGATAACGAGCTAACATGCAAGAATT
TCACATCGACGTGGCGAACCCTTTCTCTATTTCATATTACTCGTGCCTTCAAAAGGGTAGAAATGCCTCAG
CATTTAAAAAATATTGAATTTGTGCGGAGTTTTGCTTGTCGGACATATACACACACACACATTTAAACGAT
CCCTAAACAGAATGAGAGAGAATTGAGAGCTGACAGTATGTGCGAGCGAGATAAAAATTAACAGCGTTG
CGGTTCTATCACACAAATTATATGGAAGAGAGTGCGCCAAGCACGTTCCTGTAAGGTAAGCGGTTCAAAA
ATACATCATATGTATATCTTACTCCNTTACTAGNGTGCTTGGACCAGATGCCTCCGAAACATTTTGTGCCA
ACGTTTCTGTTTCCGTTTTCGATGTTGGGTAAGCTTGGTTTGGAAAGAGGCTATATCTGCACCCATGCGAA
GAAGTTCTACCGCAGGAGGGTGATATTCTGATGAGTCCAGCGTGTCGATTTGTAGAGGCAGATGATTCGA
TAGTTCGTATAGTGACTTATTGCGAATCGTTCCATGGCATATTCCTTTATATGGCGAAAANTCTATTGCTG
ATAGAGCCCAGCCCCGATGATTTGGGACGTGGGTAGCGCTACCTGTTGCCTTGCTGCAGCCTCTGATGTTG
TCCCACTAGTGATGTTTTGCATTCCAATCTCCAGCGAATTTGTATTTGTTACCCTCGAGTCAAAAAGGTCAT
GAAATTTATTAGTTCCGATGGAATTGTACCTGGGAGGACAATATATAGACGGTAGCACTAGATCGCCTTG
TGATGAATCTATTCCGATCACTGCTACTTGCATGAAACTAAGTGTGATGGGGCTAAGAGAATATTGTTTAA
TATAATTTAGTAATTAATTATAAGTATAGCAGCGCCTCCCTGTGGGGCGTCATTAAATTGGTTAGCTTTAT
GGAGAGGGTATACTGGAGCTCGTATAAATTTTCTGGAACAGATGTGGGTTTCATTAATTAGTAAGGAATC
TGGGCTGTGTTGTTCTATGAATGTTTTAATTTCAAGCGCTCTACTCTATACCGAACAGGCGTTCCAAAACA
CTATTTTCCACTATTTTTTGGTGGCAAGGTCAACGAGGCTCCTAATATCATTGACCATACCTCTAATCTTCT
CTAGGAGACGCTGGTTTGATCTTTCCATCATTTGAGACATTTTTGTGAAGGATTGGGTCAGCATTGAGATC
ATGCTACGAGTCCNTAGAGTGGTGGTGGCCGAATTTTTTAAAGAGGCTACGGGGGGATGCTTAGATGCTT
TTGAGAATGTTGTTTGTGGCTTAAATTGCCATTTGGAGGACACGACCTCTGACTGCATCTGCAAAGGTGGT
GCCGGTAGATATTTGTGAACCCTGTTGAGATCTTGATCCAGCTTGTGGGTTGTAGGTCGAGTAATCGATTA
GAAAAATTACTGGGAGTATTTCTTTATGCTGCCGTCTGATTGGCTGTTTTTTGTAGACTTTGCATCCCAGAT
GGTTGGCTGGGTGTTCTTCGCCGCAGTTAGCGCATGGTCAAATATTATTACCTGTTAAAGTGCACTCAGCT
GATCGGTGCTGGTTGCCACATTTAACACATACTGGTCTTCTGTTGCAGTATGCTTTGGTATGCCTAAAGGC
TTGGTAGCGATGGTACTGCGCAAGCAGATTGCTTTTCTAGGCATGTTCCGGATAAAACCTCATTCTGCAAA
GTTGGGTAATATTAAATTCCTCTTTATTATTTGCGGCTTTGGCAGCATCA
>160_rnd-1_family-280__LTR___Gypsy
TGTTGCATTTGACGTTATCGGCAGCACCGAGGGTTACTCAGTTGGTTGCGTGCGATATTATCGGCCACGCC
GATATCTTTTTGGCATATGGTCGGAGTATGCGGGGTGAAGCTGGGCCCACGGCGGAGCCCAAAATTTTGA
AGGGTGGCAACGCCAGACATGCGATCCACGGCGCCAAAAGCGACGATTGCTATTGGCCGCCCGTTTCGCT
CTTAAAGGGAATACGAGGAGAGTGGATCGCATGCCCTTGGATATGCCCTTGGCCTTCATTTGGGCCGTTG
ACCCAAAAAAATGCAGCTGTGCAGATGAGCAGCAACGGTGCCCGCCTCGCAAATGTATGCGTGTGCCGAA
ATTCGGCACCGATGGTGTGGCTGCCAAAAATGTCGAGGAGTTCGATTTGTTCAGCGGTTAACGGCGGGCG
TGCCAGCTCGAAGTGAGCAAAAAAGTTGGACATTGCATTTCTGCTTGACAGAGTAAAATTTTTTCCCTTCT
GAAAAATTAAGCCGGCACATAATACCAATGGCTTAACAGACGAATAAAAGTTTTGTTTTTGTGTTCTTTGC
CATGTAATAAATTTATATAAATCAAACCGCTCGAATCACAAAGAAAATATAATAAAAAAATTAAAAGAG
ACTTAAAAATTTAAGTGCGACGAGAGACGAACCAAATAAGCCAAAAATTAACGGCTCCGGCACCAAGGA
GACCAGCTCGCGCACCAGAGAGGCCCACGAAATATTCAAGGCCAGGATTCAGTGCAGCGGCGCAAACCA
GCCATATCCGCGACGCCGCCAGCAAGCGTCGGCGCAAACGTGAAGGCGACGAGACGAGCCACCAACGAC
CGGGCTCCAACAACGAAGAAGCGGCCACAGGCGAGACCCCGCCCACATCCACCGAGTGAGTCCGTTCCAT
TGTTTTGCCCATCCTCGTTCGAGCCCCTGGCAGAGTCAGAAGAAGACTCCTAATTGCACTCCCTGAAAAGG
AGTAGATTTTTTTTTATCGTTAAATTTGGTTAATTTGTATTTATAAAATATATTCATACATACATAGGCACT
TACGCCGAGCTTCACTTTAGAGTGCGAACGGTGAGGAAAACTCCAACGGTGATGCATCTGCGCCTGTAAA
ATATNAAAAAAAAAAAATCTAAAATTAACTCTTGCGCGAACTATGCTCACATACTTACCTAATTTGAATTTA
CGCATTTATATCGCCCCTTACCACCCCCAGAGAGCTCTTTAGGTAGGGTAAGGTAGAATTAAGCGATTTAA
TACATACAATACACAATACGTGACATACCCAGTTTTAAGTAATTGTGCGGGCCATTGTTCCTAAATCGGGG
CAACGATTGCCTCTAAAGTGCCTCATTGCGCGTTTCTTTTGCGGCGACTAGCAATATCCTTGTAGTGTTTCG
TCGCCTGTTCCATTCAGTGTTGTAAAAAAAAAAAAAAAAAATTAATTCTATAAGATAAGAGAAAAATTTA

GGAATTAATGCGCTGTGTCAATTAGTATAAATATATGCAGCAGTAATACGCACCTGTGAAGTTAATGAAT
TATCCGCTTGCATGAGATCCTAAACAGCTGCAAGATAAATGCCATAAGCAATGATTATGATTGTTATACAT
TTTAAGAAAACGCAACCAATTATACGCACCTATTGAAAGAAATCATGTCCGGCTAAAATAATATTGCTGG
GGAGAGTGGGCATTATGGCCGACACGGGCTAATATAGGCCATTTGGTGGCCAAAATGAGGGAGACGAGT
GGAGGTGATCGTCTCATGGTAGGGCGGGTACGCTGGGTCCGGAACCGGGGCCCGATCTGGAAGAGTAAG
GTGGATAAATAGTGTTTTTTTGTTAATTTATATTGAGTGTCTTACCTTTGTTGTTTACATTTTCATAGTTGTT
ACAGCTGTGTATTTTGTTCATGAGTTAGTAGTTCCGAATATTTGTTATCGATTATGCGGTTCATTTTGAGTA
GTGTTGTGTGAGTCTGTTTTGCTACAGACGTGTAGCTTTGTTGTTATCGATTGTGCGGACTGCGGGCCAGG
GATGGGACCACACCCAGAGAATCCGATGAGCCAGCCGGCGCGGCCCACTGAGAAACAAGCAGTTCCGTA
ACAATATGGCGCCCAATTTGAAGAAGCACTCGGCTTCTTTGAAGTCGGTGTGTAATCAAAGAAAAATAAA
TACGATAAAAATGAAGGTACAGAGGAAAGGACTCCCTTGGCTTCAGGACCCACACCGCTGAAGTCTTATG
TGGCACGAGTAGGTAGCTCATAATCGCGCAGCGTGTAGTGGAATCCGAGAAGCACGACGGAAGTAGAGG
TTATCTGAATATCCATCCATAAGCATGAGGGGTTTTTTTGTAGTGGTTGATGGCCGTTCGCATGTTGGCG
AAACATTTGCGACTATCCATCGTTCGCCTGGTGGTAGCCGCAACGACAAAGGCATCTCATGGTTAAAAAA
ATGTGATATTAGGGTAGTTTAGTCAGATGTTATTTATTCAATTAGTTGTTTTTGTCTCATTTTGTGTCAATG
TTGGATGTATGCTGGCCGTAGTTATACGAGCTTCGGAGACCGGTGACCCAGGACGATATAGAGCCGAGCC
TGCGCCGCATCTGTCACACAGCATATCCCAAGAATGGCCAGAAGAGCGACGGAGACGGATCTTGCCATGG
TGTCCATTGACATGGGGTGTTTTCTTGTCGCGGTTGATGACGGTAAGTCACGTTCGCTAGTTGGCGAACGT
TCGCGACCGTCAATCGTAGCCTGGTGGTAGCCGCCATGACAAAATAACCTGTGACGAAATGTGATATTAG
GGTAGGTCTGACTAGAGTTTGTTTAATGTTTTCTATTTGTTTTGTCCCACTCTGTGTTAACGTTTGGTGTGT
GCTGGCCGTAGTTAAATGAGCTTCGGAGGCAGGTGACCCAGGACGATATAGAGCCGATCCTGCGCCGCAC
CTGTCACACAGCATACTCTAAGGGTGCACAGAAGTGCGACAGAAACCAAATAGCCTTCAAGGTAGCGTTC
CTTGCATGCCGTCCGTCAGTCAAACTGACGAACTGAGCATGGGACGAATGCTTAAGTGTTGTCCACTGTCC
AGATTATGCGTAGATTATTTAGCCTGTGTTAGGATTTGTCTTCTCTCCCCCAAGTATTTGAAATTAATTAGA
AAGAAAAAAAATTTAATTTTGTGAGTGTTTTTGTGGTCGATGTGTATTAATTTATTATTCTTTATTAGTAAG
ATTTGTTAGTGGGTGTTTATTTATTTATTAATTTATTAGAAATATTAGTAAAGTTTTTATAAGGGTTGTAAG
CCTTGTAGAAGAGAGGGGTACTGTTCTCAGAAGGGATAAGTATGGCTGAGGAACAGGTGTCTGATTGGGC
TTCTGACGCTGATGGGGCGTCGGCTCTGCCACCGCCGCGCCCAGCTGGAGGGCTGGTGCGAACGCCAATG
AATTTCCGGGGATTTCCAGAGAGACAGATCCCGAAAGAGGGTCACCTGGTAGACTTGGAGAAAGAATTG
GCGCAACAGGATAGACCTCCACGGAACCTGGAGTTCCCAAAAGCCCCGAAAGACTCAGTGCTCAAATCG
GCGGCAAACGCGGCGATCGCTCAGGCCCACCAAACATATCTGCATGCTCTGCCATCTGGCATCAGCGAGT
CCATCCGCGAGGAGATGCGAGCTGGCTTCATGGAAATGATGAAGGGCATCATTGATGTGCTTAGGCCGCC
AGGGAGCCAAACCGCCGAGACTAACACTGCGCCGAAGCCGCCAAGACATGTCGAGCCGGGACCGCGAAC
AGGCGCCATCCCGAAAGAACGGGAAGTGTTCCAGCGCGAGGCATTTCCACCACCGGTGCCACCGAAGCC
ACGTAGGCAAGGCGCACGAGGGGCGGAGTATTCGTCCAGCAGCCCAGTTTACGTGGCAGATGCTCAGTCG
AGGAACTGGCGTAACCCGAATAACCGCCATTTCCGAGTAGAGGACCGGAGAGCGGATGATTTTCAACCGG
AGGAGGCGTACGCCAACGTGCGAGAAACTGCGTACATGAAGCTCGAGCGGTGGAACGTCAAGTTCGACG
GCGAGGACGCGATGAATTCGGTCGAGGACTTCGTGTTCCGCCTAGAGTTCCTGCAGAGGCAGTATCAGTG
CCCGTGGAAGGAGGTCCTGCGCGGTTTCCACCTGCTCCTGACCGGCCGCGCCCGCGAGTGGTACTGGATG
CACGTGCGGCATTCTAGGGTCGACAGCTGGATGCAACTGCGGCATGCCCTCTTGGACAGGTTCCGGGGCT
ACCAGACGGAACACGAGGTAATGCAGGAGCTTCTACAACGAGAGCAACAGGCCAGCGAAGGGGTGGATG
ATTATATCCACCACATGCGTCAACTCGCCGCGCGATTCCAGAAGCCGCTGAGAGACCGAGAGCTGGTGAG
GATTATAAAGCGTGGTTTAAAAGAAAGTCTGGCGAAATATATTTATGCCATGGACGTACTCACCGTGGAT
GAGCTGCGCCAAGAGTGCCTAGAAGTGGAGAGGCACATGGGTCGCAGAAGCCGGACCGGGTACCTTCAG
CCGTCGCGTTGTCCACAAGGAACGAGGCCCGTAGTCCACGAGGTTGAAGTTCCACCGCATCTTACGGAAA
CGCCGCCGGGAGAACTGGAGGAGGCATTCGTGCGAGGCAGGAACTCATCCCGAATNGTGTGCTGGAACT
GCAGACAGTTCGACCACGTCTTTAGAGACTGCCTGTCCAAGGAGCGGAAAATATTCTGCTACAGGTGCGG
AAAGCCGGACACNTTCTGCTCGCAGTGCGAAAACTGTCCGGGAAACCCCAGAGGGAGCGCGGTGATGGC
GGGACAGACGCGTTCCGGGACGGCGACCGCGGGAAAGCAGGAGAGGGGCACCTACCAGAGCCAATAATC
ATTGGAAAGGAGACGGAGATTTGGACCATAAGGACCAGGATAAGGCTAATTATTATATATAANGGTANA
AGCGAAGGAGGANCACTNAGGGGCTTACCATACGAAGAACGTGTCCGGGCATACATGTCGGCCCGGAAT
AGAATTTTTGGTGAGCGACAGCTGGAGGGGATGACGCTGGCCACCNGGAGAGTGGTGAAGGCTCGGGCG
CGCTTCCGCAGACGCAGGATGACGAGGCGCCAGGTCGTCGAAGCTGTGAGACGGGAGGATAGCCTAGAC
CCACGCGTATTCGCCGAAGTGGAGGTCGCTGGAGCCAAAATGAAAGGGTTGCTAGACACCGGGGCCTCG

GTCAGCCTGTTGGGACAAGGTGGCCGGGAGTTAGTGGAGGAACTGGGANNNGAGGCGCGGCCGTACGNN
TCGATGGTNAGGACNGCTGGAGGNGAGGATAAATCAATCATAGGTAGGGTAGAGCTCCTAATCTCGTACA
GGGAGCAGCATAAAAACATAACGTTTTTATCTGCCCCTACCTGAAACCAGCGAGCCTATTTGGAATTGA
TTTCTGACGAGCTTTTAACCTAGCCCCTGATATTTTGGGCCCCGAGGAGATAACCTCCACAGAACACTATG
CGGAAGACGAAGAGGGTGAAAATGAGCCAGACTCATGGGAGTTGTCTCGGGAGGAAAGGGTAGAGTTGG
AGGCGGTAAAGCAGGGGTTTTTGCCTTTTGAGCATAGCGGATTAGGAACGCCAGACTTAGAGAAGCACAC
TATCGACTTAGTGGAAGGTGCCCAACCATTCAAAGATAGACCCTACCCATTATCACCGGCGATGCAGAAG
GTAGTGGATGACGAAGTAGACAAAATGTTGAGTTTGAAGGTTATAGAAGAGTGCAACAGCCCATGGAGT
AACCGCACAACCGTAGTGCGAAGACCGGGGAAACCCGTTTTTGCCTCGACGCACGCAAGCTCAACAATTT
CACGGTGAAAGACGCGTATCCACTACCCTGTATAGAAGGCATCCTATCTAGAATNTACCAGACCCACTAC
ATCTCCAGTGTGGATCTGAAGTTCGCGTATTGGCAGGTCGAACTAGACGACAAAAGCAAGCCCTATACCG
CATTTACCGTACCAGGTAGGCCGCTATACCAGTTTCGCGTCATGCCGTTTGGTTTATGCAATGCAGCCCAA
CGTCTAGTTCGTCTGATGGACAAGGTAATTCCCAACGAGTTGCGGTCGAACGTCTTTGTGTACTTGGTCGA
CTTATTAGTCATTTCCCTTGATTTCCNAACGAATTTGAAATATTTGCGACAAGTGGGAAAATGCTTAAGAG
AAGCCAAGCTCTCNATCGGACTGCACAAATCCAAATTTTGCTTCCGGGCATTAAAATACCTAGGATTCATT
ATTGGTGGTGGCACGCTTCGCATGGACCCCGAACGGGTTANAGCTTTTAAGCGAATCCCCGTGCCTCGGTC
GCGCAAAGAGGTTCGCTCTTTTTTGGGTACCGCAGGTTGGTACCGGCGCTTTATGCGAGATTTCGCGGCAA
TGGCCGCCCCACTGACTGAAATGCTAAAAAAGGATAGCGGGAAGCGCGTATTGAGATCTNACGTAGGCG
GTTTTACGGGCCCGGCATGGTTTATCAGGTAAGGGATTGGGTGAGACGATGTGTGACATGCAAGGAAGTC
AAGGCACCAAACTTTCGCATGCAAGTGCGTCTAGGAGAAGAGGTAGTGACCGACCATTCCAAAAACTATA
TATCGACTTTCTGGGAAAGTACCCACGTTCGAAGCGAGGGCACGCGTGGATCTTCGNGGTAGTCGACCAT
TTTTCAAAATCTACCTTCTTGAAAGCGATGCGGGAGGCTACGGCTCTTAACGTGGTGGAGTTTTTGGTCAG
CGAGGTTTTTTATAAGTTTGGAGTACCCGAGGTCATCCACTCTGATAACTCTGGCCATCCGGGCGTATCTA
GAGCAAGATCATCGCGAGTGGGACCTCTACCTTCCGGAAATAGAGGTGTCCATACGAAATGCCATACACA
CCGCGACCGGGGTGACCCCCTTCTTTGCTGTTTTCGGACATCATATGTTCCTTAACGGGGCATGCTACAGA
TTGGCTCGTAGGTTAGCGTCTCCGAGTGATCATGAGATCTCCCATTTGCACCCCGCAGACAAAATACACCT
GATTAGACGGCGAGTCCAAGGCCAACTACATCAGGCTTACGAAAGAAGTCAGCGGCAGTACAATGCTCG
AGCCCGGGTGTTCCAGGCGGAACCCGGCCAAGAAGTTTACCGACGCAATTTTGTGCTGAGTGACTTTGGA
AAAGCTTTNAACGCCAAGTTTGCAAGAAAGTTCCTTAAGGCCAGAGTAGAGCGTCGAGTAGGCAACAACT
CGTACCTGCTGGAAGATTTGAAAGGGAAGCCCTTAGGAGTGTTCCATGCGAAGGACATACGAGTGTAGAT
CAGGGTCGTGGGTGTGGCCTACGTTGGGCGTTCTATTTGGCCGGCTGGGCGAACCGGATAGGANATGCGC
ACGCAGATTTATAGTAACGGTCGGGTTCGCGCTTACAATAATCTGCGTGGCGATGCAACATTACATTTGAC
ATATCGACGACATCGATATTGCCCGTAATTGGTTGCGTTCTTTATTATCGGCCGCGCCGATATCTTTGGTCA
TAGGGCCAGAGTATCGGGGTGAAGCCGGACACAGAACGGCGTTTAAAATATCGAGGAGTGGCAACACCG
AACTTGCGATCCACGACGCCAAAGGAGGCGATTGCTATTGGCTGCCCGATTCGCNCCCAAAGAGATAAAA
GAGAGTGGATCGCATGAAGATGCCCTTGGCCGTGCGGCCGTTGACCCGGAGAACAGCNGTGCCGATGAG
CAGCAACGGTAACCATATTTAAATGTACGCGGGTACCGAATTTCGGCACCTTTGATGTGCTGCCCAATGTA
CAGTGNGGGAGTTCGACTTTGTCAGCGGTCCACAGCGGACGTACCGGCCACGGAGCATNTGCTTAAACAA
ATCAAAGCGCATACATTAGTTAATTGACAAGATAATTCCTTATGTGCTAACGTATTGCAAAAGAANTAGT
GCACAAGAGCGATAATACACGAAGCAATAATACACTAGAAACACACTGTCGTCATTTAAAACCACTATAA
AAGCGATACTCTATTAGAGCAGCCGCAGCCATTCTGTGGCACACANCGCTTGAANCCCGCGCCGGAGGAC
GCTGGCCAGATCAACCCAGATATTCGCGGGCCGGATTTTCAAGGCACGGCCAAGTATCGGCGTAACCATG
CGACGGAGAGACGAGCCATCACGGACGGGACTCACACGACGAAGAGGCGGCCACGGGCGAGACCACGC
CCATATCATCCGAGTGAGTCTGTTCCATTTCGTGCCTCCTCGCCTGAGTAAACGGCAGAGTCCGAAGCAGA
CCCCTNATTGTGCTCCCTGAAAGAGAACCGATTTTTTTTCGGAATTTTTAACTCTAGTTTTTTTTTGTTATTA
AATAGATAAAAATACTCACGACTAGTCTAAATTAAGTAAATTTCCGAAAGTGCGAACCGGGAAGGAAAG
GGCCCAAAGATTATTGCGTCTGCCAAGTAAAGAATGAAATTAAAAATTTAGATTTTGGTCAACTTATGCA
CGAGTGATACCCACTTATACGTATATATATATAT
>9_rnd-1_family-235__LTR___Gypsy
AAAAGTGAAAAGACACCAACACCAAGAAAAGGGGAGAGGAATAGTAAAGGGACCGACGGAACCCCTAG
AACGGGAAGAGAGGGAAACAGCGACGTCGACTAGCAAGGAAGTATACAATAAAGGATGGAGGCAGGTG
AGTGCCAAAACCCGGGGGTAGGCGTCCGCTGGATTCACCAGCGACGCAAAGAGGAGTTAGTGGCGATCG
CCCAGGAGTTTGGGCTGGAAAAGGAGGGGCGCGTGGAGGAGCTACGTAAGCGGCTGTCCACCTACGTGC
AGGAGGGCGGTCATTCGACTGGGATTTGGGCTCGGCTCGCCGAGCTGGAATCCCAGTATGCTAAAACTCT

TGAGCCGCCATCTTGGGGCGTCCCACCCGACTTTGGTGGACGGCCACGGCAAAAGTCGGAACCTGACCTC
AGAAGCTCCCAGCTGTACGGGCCTTTTGCAGGCGCCCACCCCGAGTTTGACCGTCTCGGGCGTGGAGGCA
GTTCCTGCTCTCGGTGGAAATGAAGACCGCCGAGCAACTGCGGTCAACGCAGAGGTGGGGAGGTCCCCTG
TTGAACCGGGTGGTGGTCCCTGCTACACTAGGCCGCACCATCCATCGGAGGCGAGGCCACTTCCCAATGT
CCCGGAGCAAGTTCGGAAGTGGGGTATGAGGTACGATGGACGCTCCGACCCCCTTGGGTTCTTAGAGGAC
CTCGAGGAGCGCGCTATCACCTATGGTATAGCCCTCGACCGTATGCCCAGGGCAATGAGCGAGGTATTCT
GCGATAGGGCTGCTCGTTGGTTTCTGAGCAGTGGGCTAAGGGATGTGCCGTGGGCCGTGTTTAGGAGGGA
TTTCTTGGAGTTTTTCTTGCCCCCTCGTTACTTCGAGAGGCTTGAGGACGAGATCCGATCCCGGAGGCAGC
GCGTAGGTGAAGCGTTCAAGGAATATTTGATAGAGCTGAGGGCTCTTATGCACCACGCTAGATACGAGCC
GGCACAAGAGCTGCATCGGATCTACGAGAACGCCGCTCCGGAATACAGGCTCTATGTGCGACGGCAAGA
CTTTTCTACCCTATCCCAGCTAACTCAACTGGCCGTAGAATACGAGACCGTCGTGAGGCAGAGGGCGGAG
TACTCAGGCAGCCAACCCTTGGGTCAGGGTGTCTCTAGTTCAAATAGTGCAGAGTGGGCGAGGACGGCGC
AGTCCTCGGGACATCTCAATCCGTTCCGGTCTGCTCCCAGCGGATCCGGGACGACCGTTGTGGACAACAG
GGTCTCCACCCCACCCGCTGCAACTCACGCAGAGGTGGCCCATCAGATGATGAGCCAAGAGGCCGGGAAC
GGATATGATCCGCGACGGGCGTGTCGTAGGTGCGCACAGGAGGGGCATTTTGCCAGGGAATGTCCCAACG
CCCCTGTCTTATTTTGTTGGGTATGTGGAAGCCGGGGCCGAAGTAGTCGGGAGTGTTGCCGCCGCGCGGA
GACGGGAAACGGGCAACGGCGTCGTCCGGACCAGGATCGGGTCGGCGCCAATACACGCGGAACGCAGCA
ATAGGACACCCACTGAAGGTGGACGGTGGCCGGATCGTCGCTACGGTAGAGATTGGCGGACGAACGATG
CCCGCCACGATCGATACCGGAGCCACTCGTAGCTTCATGAGTGAGGACTGCGTACGCAAATGGACGATCC
GGGGAAGAGCGGAGGAAATACAGGCACGTATCCGCCTGGCAGATGGGTCCACCCTAGAGGTAGAAAAGG
CTTTGAGAGTAGACGCCAGCTTAGGGGGAAAAACGATCAGCATGACGATGCTCATCATGCCCTCAATGCT
GGACCATGTAATCCTGGGTATGGATTTTTGGGGGCGATGAATACCACAATACGCTGCGGCGAGGCGGAAC
TGGTCCCTAGAAGCAGCGACGACACCGGGCGTGAGGCCAGGAACGAGCGAGGAGTCGGAGGAAGGCCGC
CCAAGGCATGGAAAGGGAGCACCTGCTCGGCTACAGAGGGAGCCGGAGGAAGGCCTCCCAAGGCATGGA
AAGGGAGCACCTGCTCGGCTACAGAGGGAGCCGGAGGAAGGCCTCCCAAGGCATGGAAAGGGAGCACCT
GCTCGGCTACAGAGGGAGCCGGAGGAAGGCCTCCCAAGGCATGGAAAGGGAGCACCTGCTCGGCTACAG
AGGGAGCCGGAGGAAGGCCTCCCAAGGCATGGAAGGGGAGCACCTGCTCGGCTACAGAGGGAGCCGGA
GGAAGGCCTCCCAAGGCATGGAAAGGGAGCACCTGCTCGGCTACAGAGGGAGCCGGAGGAAGGCCTCCC
AAGGCATGGAAAGGGAGCACCTGCTCGGCTACAGAGGGAGCCGGAGGAAGGCCTCCCAAGGCATGGAAA
GGGAGCACCTGCTCGGCTACAGAGGGAGCCGGAGGAAGGCCTCCCAAGTCATGAAGGGGGCACCTGCTC
GGCTACAGAGGGAGCCGAAGGCAGGCCTCCCAAGTCATGAGAAGGGAGTACCTGCTCGGCCTTCGACGG
AAAAGGTCAGGAAAAGGGGAGTAAAGTCCGGCCCGAATCGACAGCCTGGAGAACCCAAGAGGTGGCGC
ATAGAGCTGCAGAGGCAGACTTCTAATGGCTACGGAAGCGGCACGGCGGGAACGTGCAAGAGAAAGGAC
GGAGTAGGAGCGATGACCAGAATCCACACGGACATAGAAGGCCAGGAGCGATCGCCAGAATCGAGCGGC
CACGGAGTTCTGTCTGGAGTCGAAGTGGTCCCAGGATGGGGCGATAACGGCATCGAAGAGGCGGANTGG
CCAGCGGGTCTGGACCCAGAACTGAGGGAGTTCCTGGAGTCAGAGCTGGCTCTCTTCGAGGGGCTGCAGG
GAATCTCGCACATCGCGGAGCACCGAATCCGGCTGAAGGATGACAAGCCGTTGAAACAAAGGTACTACC
CCAAGAATCCGGCCATGCAGAGGGTGATCGACGAGCAAGTGGATGAGTTGATCCGGGCAAATGCCATCG
AGCCGTCGCGTAGTCCGCACAGCGCCCCGATCGTCTTGGTCAAGAAGAAGACTGGCGAGTGGCGTATGTG
CATTGACTACCGACAACTCAACGCGCATTCCATTCCGGACGCTTATCCGGTGCCTCGTATTAACCACATCC
TGGAGCGGTTGCGCCACGCCCGGTTCATCTCCACTCTGGATTTGAAGCACGGGTACTGGCAGATACCGAT
GGCAGCCGATAGCCGGGAGTGTACGGCGTTCACAGTCCCTGGAAGAGGTCTTTTCCAATGGCGGGTGATG
CCATTCGGCTTACACTCGGCGTGTGCGACATTCCAGCGCGCTTTGGATACGGTAATAGGGCCGCAGATGG
AGCCTCATGCGTTCGCTTACCTAGACGACATCGTGGTAATAGGAGCGACGAAAACACAGCATGTAGAGAA
TTTGAGGGAGGTATTCCGCCGGCTAAGGGCGGCCAATCTCCGGTTAAATAGGAAAAAATGCAGCTTCTTC
CGGGAGAGGTTAGTCTACCTAGGACATGTAATTTGCGGAGAAGGGATACGGACCGATCCGGAGAAAGTG
GAGGCGATACGAAACCTGAGGGCCCCAACAAATTGCAAAGAACTGCGACAGTGTCTCGGGATGGCGTCA
TGGTACAGGCGTTTCGTACCCAATTTCGCGACCCAGGTACAGCCACTCACAGCGCTCTTAAAGAAGGGGC
AGAAATGGGCATGGGAGCAAGAACAGGAGGAGGCATTGCGTCAATTGAAGGAGAGCTTGACGACTGCCC
CTGTGTTGGCTTGCCCCGACTTTTCCGCCAAGTTCGTCTTGCAGACTGACGCGAGTGAGTATGGCCTCGGT
GCAGTGCTGACGCAGACGATCGATGGACAAGAGCGGGTGATAGCATACGCGAGCCGAAAGTTGCTGAAG
GCCGAGGTAAACTATTCCGCCACTGAGAAAGAGTGCCTGGCAATCGTGTGGTCGATCCGGAAGTTGCGTT
GCTACCTAGAAGGGTACCGATTCGACGTAATAACGGACCATCTAGCGCTCAAGTGGCTGAATTCGATCGA
GAGCCCGTCAGGAAGGATTGCGCGATGGGCATTGGAACTGCAGCAATTCCAATTTGACGTTCATTATCGT

CGAGGGAAGCTCAACGTCGTGGCCGATGCCCTGTCGAGGCATCCTCTAGAGACCTGTCAACAAGCAGTGG
TGGCCGAGGCCCCTGCAAATGGGTCGCGAGGATGCGTGTTAGAATCGCCGAAGAGCCAGCCAAGTTCCCG
GATTACACCGAGGAAAACGGACAGCTATATAGGCATTTGGGTCACCGGATAGACGATGAGGACTACATCC
CTTGGAAACTATGCGTAGGCAGTGGTCAACGCCAGAGGGTACTGCGGGAATGTCATGATGCTCCCACGGC
CGGACATCAAGGTGTCAGAAAAACGGTGGCGAGGTTAGCCCAGCGGTACTACTGGCCGGGTATGTTCCGC
GATGCCGCCAGATACGTGAAATGTTGCGAAGACTGCCAAAAATTCAAGAGTGAGCAACTCAAACCGGCT
GGTCGCATGCTGACGAGGCAGGTGGCAGAGCCGATGGCGGTACTTTGCGCGGACTTCGTCGGACCCCTTC
CACGGTCGAAACATGGAAACACGATGCTATTGGTGTTCCATGACGCTTTCTCGAAATGGGTGGAGTTGGT
CCCTCTCAGAAGAGCCACGGCAGCACACCTGCAAACTGCGTTTCGAGAGAGGATTCTAAGTAGGTTCGGG
GTACCTAGAAAGTTTGTGTGCGACAACGGGGTACAATTTACGAGTCGGAGCTTCAAGTCATTTTTGGGGTC
CCTGGGTGTAGAGATCCAGTATACCGCCCCGTACTGTCCGCAAGAGAATCCCACTGAGAGGACTAACCGG
ACTGTAAAGACCATGATAGCGCAATTTATTGAGGGCCACCAGAGTTCATGGGATGAACTCTTGCCCGAGA
TCTCGCTGGCGGTCAACACGAGCGTGGCAGATTCAACGGGCTTTAGTCCCGCTTTCCTGATGCAGGGCAG
GGAACCTCGGTTACCCGCAGCTCTGTACGATCAGGTAACTCCGGGGTCCGCAACTAGTGCGCTCGACCCT
AGGACAAAGGCGGATAGGCTAAAAGAAATTTTTGAATATCGTCCACAGCAACCTCCAGCGCGCATCAAA
GGACCAGGGCAGGCATTACAACTTGCGGAGACGAGAGTGGCGGCCCTCGTTGGACTCGATGGTCCTGTTG
AGGCAGCACCAGTTGTCCAACGCTGCCGAAGGTTTCGCCGCAAAGTTGGCACCCAAATTTGACGGCCCCT
ACAAAGTCGTCAAATTCATATCCCCCAATATTGTGCGGCTGGTGAAATCAGACGACCGCAAGAAGAGGGT
GGCCAACATCTCACAATTGAAACCATTCTACAGAAAGACGACGAGCGACGAAGAAGACGAAGATCCAGG
GCACGCAAATGCCCACGAGTAAGCGAAGGATGAGAGCAACACCCGTCGCCCAACGAAACTATTATGATT
AAGCTTAGATAAAACTAACATTAACAACATAATTCAGAATAGGCATAGTAAAGTGTGAGCCGCACTGAGA
GAATAGGGCATTAACAAATAAGTAAAGAGTAAGTTAACCAATAAGCTGAACTTTCAGATGGAACCCGAA
GTTATCGNCATATCGTCAGACGAAGACAACAGCAGCGGGGACACCGAGCCTTTCCCCGGGCCGGGAGCC
GCAGGCCGTCCGCGCGACCCACGCCAGGGGCGTGAGTACTATCGAAGAGAACGGCAAGAAGGACCGCCT
CGCCAGTTCCGCCATACACTATTCTCCCGGCGGACCGGCATAATGGAGAGGCTAGACGGGATGGTCTTGC
TGATGGCAGTCGAGGTCACGACAATGTTCGTGGAGGCACCGTTCGCCCGGAACTCGCCACCGTACGCGGA
AGAGCCTGCATCTCCGCCCCTCGCATCCCACTCGGCACAATCATCAGAGCCGGCAACCCCAACCGCGGCC
GGCGAGATGGACGAGGAAGAGTCCGCACCGTTCGGAACGGAGGAGCACACACCACCAGCGCAGGACGTG
GAGGCGGTAGGAGAAGAGATATCCCCGTCGGCCGGAGACGCGGAGTCGCCACCGACGGAGGTGCCGAAC
GGCACCCGCGCAAACGACGGGGAAGCAGACCCGCTGAGCCTCGACCCAGAACCCTTAGTGGGAGGGGAC
CCGTCAACGGAGGACGAGCCGGAATTCCGTCGCCGCTTTGCCACCCACCGTCTGGAGGTAGACCCGGGCA
GCCAGTTTCCCAGGATAACGCTGCGGTACCTACCAAAAGTCCCCGCGGTAGCCAACAAACCCCGTCTGAA
CCCCGCAAAGCGCGAATGGGGGTGCCGAAGAAGGCCCCGGCGGAAAGCGCCGACGCAGCCGCCATCGTC
GACGGAGAGCCGCCAGGGGCGAGCGTCGCCGGAGTGGAGGCCATCGCCGACCCAGTCCCGAGGGAGCAT
CCTCGTCGGCCACGAAGAGGCTTCGCACGGAGACCGAGAAGCCCGAAGGATCGCAGCGGTGCGTGCCGC
GTTCGCCCCGCGAGCGCCAGGAGGGTCCTCCCCAGCTCGGGAAGGATAACCGCCGCTGCCAGATCGCTTC
TCGAGGAGGGCATCCCGAACCCTGGCCGATACCGATTGCGGTCTGCCAGATGGTCGTGGAGGAGCACCAT
CTCAGCACCAAGGACGCGGCGAACTCGGCCGTTTCCCCAGCCCGCGCCGAGGCGCCAGACGAAACCTTTC
CTAAGGAATCGGAGGAGGAGTGGCTCGACGACGAGGACGAGATGCCCGCGCTCGTGCCTATTGCCCGCG
CCGAGACACCCGACACCATCGCCCGGCGGGAGTTAGAAGACGCCTGGTTCGGCCAAGAAGGCAAGGCGC
CATCCCGAAAAGGTTCCTTCACGGTTCGCCAGGAAGGCCGGGATGAAGTCTGGTCCGAGGAAGAGGACG
CGGTGGTGACGGCTTCGTCGCCCAAGCTTTCGAGTCCAACCCAGGATTCCTCCCCCATGGCTCTAAAGACT
CCCACGGAGAGCGAGTTGTGGGACGACATAGTGGACCTTTTCGACGGCATCGACGGGTGGGACACTAGCC
TGGACTCAGTGCTGGAGGAGGAACCCGACACCGAGGTCGACACGGTCGCCCGCAGTGCCGACGTGAGGG
TCACACCGGAGAGCCACCCCAAGAAACCCGACACCGAGGTCGAGGCGATCGCCCACAGTGCTGACGTTG
GGGTAAAACCGGAGGGCCCCGTCGAGGAACCCGTGCCCGATTCCACCCCCTCAGGCTGGAAGGTGTTTCC
TCTTGGAACACCGTTGTCGTCGGGCGTGGTGGACCTAATCGCCAGAGAGGCGCGAATCCGCCGCTTCCGG
TGCCGACGTACTCGGTTCCACGTGTCCGACGGGGAGAGCGAGTACAGCGTTACGCTGAACGCGAAAGGCG
ACGTGACCGTCACCTGTCCGAAGTGAGGGGGGGGGCGGCTAAGGAGACCAGCCACCCCACAGTTTTCACG
GCAAGGAGCCGTCATCCCTTTGGCCAAATTACGGCAAGGAGCCGCTGGCGGGACAACGCATTAATTTTCT
TTAGTTATAAGTATTAATTATATTAGGTTAAGATCTTAGTGAAATAAGAGGCAAGGAGCCATCAGTAATA
TAAGCAGTTCGATTAGTAGCAAAGAGGCAAGGAGCCGCGAATATTATAATTGATTTAAATAGTGAGTTTG
AATTTAACCGCCGGCCGCCGCTGCAACCGTGCGTTGTCGATCGGTTGCTCAACACCGTCTTTGAGAGCGCA
ACTTCAATTCGGCGGCGAAGAGCGAAAGCTCCGAGGCGAAGTTGCGAAAGCTCGCGGCGAAGTTGAGCA

GCCGAAGCGAGCGCATGGCTTGCCGGCGAGCGTGCCGGCACTTGTGATCGAACGTTAAGGGTGAGTAGA
AGTGCTGCGCGAGATAAACGAAATCATAGATGGTATAGTGGAGCGCTAGGGCTCCAGAAAACAGTGCGG
CAGGGTTTAGCTTAGGATTTACGATAAGGTCGGAATGAATAATAGTGAATAAAGTGCCAACGTTGTAATT
ATCTAGAAAGTATTGGATTATTCCCTGTTTCCTGGTAACCCGGCTTACCTTAACATCGGAACTAACCGGTG
TTCGACCTAACCTCGAACGAGGGGCATGCGGTACGAGATCGTGGCTCCGGGGTGGGCATTGCCGGTTGGT
GTTTAGGTAGTGGAGTTGGTGCGAGGCCACGGCCCGCTAGTGTAGTTGGTGCGAGGCCACGGCCCGCTAG
CGTAGTGGTACGAGGCCACGGCCCGGCAGAAAGATAGAGCGTTTAGGTAGTGGAGTTGGTGCGAGGCCA
CGGCCCGCTAGTGTGGTTGGNGCGAGGCCACGGCCCGCTAGCATAGATGGTACGAGGCCACGGCCCGAC
GGNGTAGGTGTGTAGAGGTAGATCTGGGTTTGGTTTGGTCAGGATACTCACCCCCCTTTAAGCCCTCTCTC
CCAGCGAAATTATAAATAACTTGTAACTCGCTAGACCTGGTAAGACTGAGAATTATACCCCGGCCGGAAG
CTTCCTTACA
>9_rnd-1_family-231__LTR___Gypsy
AAACTAAAAAGGAACCAAAGTGGCTGAGCAAGATCGGCGTAAAAAGTGANAAGACACCAACACCAAGA
AAAGGAGAGAGGAATAGTAAAGGGACCGACGGAACCCCTAGAACGGGAAGAGAGGGAAACAGCGACGT
CGACTAGCAAGGAAGTATACAATAAAGGATGGAGGCAGGTGAGTGCCAAAAACCCGGGGGTAGGCGTCC
GCTGGATTCACCAGCGACGCAAAGAGGAGTTAGTGGCGATCGCCCAGGAGTTTGGGCTGGAAAAGGAGG
GGCGCGTGGAGGAGCTACGTAAGCGGCTGTCCACCTACGTGCAGGAGGGCGGTCATTCGNCTGGGATTTG
GGCTCGGCTCGCCGAGCTGGAATCCCAGTATGCTAAAACTCTTGAGCCGCCATCTTGGGGCGTCCCACCC
GACTTTGGTGGACGGCCACGGCAAAAGTCGGAACCTGACCTCAGAAGCTCCCAGCTGTACGGNCTTTTGC
AGGCGCCCACCCCGAGTTTGACCGTCTCGGGCGTGGAGGCAGTTCCTGCTCTCGGTGGAAATGAAGACCG
CCGAGCAACTGCGGTCAACGCAGAGGTGGGGAGGTCCCCTGTTGAACCGGGTGGTGGTCCCTGCTACACT
AGGCCGCACCATCCATCGGAGGCGAGGCCACTTCCCAATGTCCCGGAGCAAGTTCGGAAGTGGGGTATGA
GGTACGATGGACGCTCCGACCCCCTTGGGTTCTTAGAGGACCTCGAGGAGCGCGCTATCACCTATGGTAT
AGCCCTCGACCGTATGCCCAGGGCAATGAGCGAGGTGTTCTGCGATAGGGCTGCTCGTTGGTTTCTGAGC
AGTGGGCTAAGGGATGTGCCGTGGGCCGTGTTTAGGAGGGATTTCTTGGAGTTTTTCTTGCCCCCTCGTTA
CTTCGAGAGGCTTGAGGACGAGATCCGATCCCGGAGGCAGCGCGTAGGTGAAGCGTTCAAGGAATATTTG
ATAGAGCTGAGGGCTCTTATGCACCACGCTAGATACGAGCCGGCACAAGAGCTGCATCGGATCTACGAGA
ACGCCGCTCCGGAATACAGGCTCTATGTGCGACGGCAAGACTTTTCTACCCTATCCCAGCTAACTCAACTG
GCCGTAGAATACGAGACCGTCGTGAGGCAGAGGGCGGAGTACTCAGGCAGCCAACCCTTGGGTCAGGGT
GTCTCTAGTTCAAATAGTGCAGAGTGGGCGAGGACGGCGCAGTCCTCGGGACATCTCAATCCGTTCCGGT
CTGCTCCCAGCGGATCCGGGACGACCGTTGTGGACAACAGGGTCTCCACCCCACCCGCTGCAACTCACGC
AGAGGTGGCCCATCAGATGATGAGCCAAGAGGCCGGGAACGGATATGATCCGCGACGGGCGTGTCGTAG
GTGCGCACAGGAGGGGCATTTTGCCAGGGAATGTCCCAACGCCCCTGTCTTATTTTGTTGGGTATGTGGAA
GCCGGGGCCGAAGTAGTCGGGAGTGTTGCCGCCGCGCGGAGACGGGAAACGGGCAACGGCGTCGTCCGG
ACCAGGATCGGGTCGGCGCCAATACACGCGGAACGCAGCAATAGGACACCCACTGAAGGTGGACGGTGG
CCGGATCGTCGCTACGGTAGAGATTGGCGGACGAACGATGCCCGCCACGATCGATACCGGAGCCACTCGT
AGCTTCATGAGTGAGGACTGCGTACGCAAATGGACGATCCGGGGAAGAGCGGAGGAAATACAGGCACGT
ATCCGCCTGGCAGATGGGTCCACCCTAGAGGNGGAAAAGGCTTTGAGAGTAGACGCCAGCTTAGGGGAA
AAACGATCAGCATGACGATGCTCATCATGCCCTCAATGCTGGACCATGTAATCCTGGGTATGGATTTTTGG
GGGCGATGAATACCACAATACGCTGCGGCGAGGCGGAACTGGTCCTAGAAGCAGCGACGACACCGGGCG
TGAGGCCAGGAACGAGCGAGGAGTCGGAGGAAGGCCGCCCAAGGCATGAAAAGGGAGCACCTGCTCGG
CTACAGAGGGAGCCGGAGGAAGGCCTCCCAAGGCATGGAAAGGGAGCACCTGCTCGGCTACAGAGGGAG
CCGGAGGAAGGCCTCCCAAGGCATGGAAGGGGAGCACCTGCTCGGCTACAGAGGGAGCCGGAGGAAGGC
CTCCCAAGGCATGAAAAGGGAGCACCTGCTCGGCTACAGAGGGAGCCGGAGGAAGGCCTCCCAAGGCAT
GGAAAGGGAGCACCTGCTCGGCTACAGAGGGAGCCGGAGGAAGGCCTCCCAAGTCATGAAGGGGGCACC
TGCTCGGCTACAGAGGGAGCCGGAGGCAGGCCTCCCAAGTCATGAGAAGGGAGCACCTGCTCGGCCTTCG
ACGGAAAAGGTCAGGGAAAGGGGAGTAAAGTCCGGCCCGAATCGACAGCCTGGAGAACCCAAGAGGTG
GCGCATAGAGCTGCAGAGGCAGACTTCTAATGGCTACGGAAGCGGCACGGCGGGAACGTGCAAGAGAAA
GGACGGAGTAGGAGCGATGACCAGAATCCACACGGACATAGAAGGCCAGGAGCGATCGCCAGAATCGAG
CGGCCACGGAGTTCTGTCTGGAGTCGAAGTGGTCCCAGGAGGGGGCGATAACGGCATCGAAGAGGCGGA
TTGGCCAGCGGGTCTGGACCCAGAACTGAGGGAGTTCCTGGAGTCAGAGCTGGCTCTCTTCGAGGGGCTG
CAGGGAATCTCGCACATCGCGGAGCACCGAATCCGGCTGAAGGATGACAAGCCGTTGAAACAAAGGTAC
TACCCCAAGAATCCGGCCATGCAGAGGGTGATCGACGAGCAAGTGGATGAGTTGATCCGGGCAAATGCC
ATCGAGCCGTCGCGTAGTCCGCACAGCGCCCCGATCGTCTTGGTCAAGAAGAAGACTGGCGAGTGGCGTA

TGTGCATTGACTACCGACAACTCAACGCGCATTCCATTCCGGACGCTTATCCGGTGCCTCGTATTAACCAC
ATCCTGGAGCGGTTGCGCCACGCCCGGTTCATCTCCACTCTGGATTTGAAGCACGGGTACTGGCAGATACC
GATGGCAGCCGATAGCCGGGAGTGTACGGCGTTCACAGTCCCTGGAAGAGGTCTTTTCCAATGGCGGGTG
ATGCCATTCGGCTTACACTCGGCGTGTGCGACATTCCAGCGCGCTTTGGATACGGTAATAGGGCCGCAGA
TGGAGCCTCATGCGTTCGCTTACCTAGACGACATCGTGGTAATAGGAGCGACGAAAACACAGCATGTAGA
GAATTTGAGGGAGGTATTCCGCCGGCTAAGGGCGGCCAATCTCCGGTTAAATAGGAAAAAATGCAGCTTC
TTCCGGGAGAGGTTAGTCTACCTAGGACATGTAATTTGCGGAGAAGGGATACGGACCGATCCGGAGAAA
GTGGAGGCGATACGAAACCTGAGGGCCCCAACAAATTGCAAAGAACTGCGACAGTGTCTCGGGATGGCG
TCATGGTACAGGCGTTTCGTACCCAATTTCGCGACCCAGGTACAGCCACTCACAGCGCTCTTAAAGAAGG
GGCAGAAATGGGCATGGGAGCAAGAACAGGAGGAGGCATTGCGTCAATTGAAGGAGAGCTTGACGACTG
CCCCTGTGTTGGCTTGCCCCGACTTTTCCGCCAAGTTCGTCTTGCAGACTGACGCGAGTGAGTATGGCCTC
GGTGCAGTGCTGACGCAGACGATCGATGGACAAGAGCGGGTGATAGCATACGCGAGCCGAAAGTTGCTG
AAGGCCGAGGTAAACTATTCCGCCACTGAGAAAGAGTGCCTGGCAATCGTGTGGTCGATCCGGAAGTTGC
GTTGCTACCTAGAAGGGTACCGATTCGACGTAATAACGGACCATCTAGCGCTCAAGTGGCTGAATTCGAT
CGAGAGCCCGTCAGGAAGGATTGCGCGATGGGCATTGGAACTGCAGCAATTCCAATTTGACGTTCATTAT
CGTCGAGGGAAGCTCAACGTCGTGGCCGATGCCCTGTCGAGGCATCCTCTAGAGACCTGTCAACAAGCAG
TGGTGGCCGAGGCCCCCTGCAAATGGGTCGCGAGGATGCGTGTTAGAATCGCCGAAGAGCCAGCCAAGTT
CCCGGATTACACCGAGGAAAACGGACAGCTATATAGGCATTTGGGTCACCGGATAGACGATGAGGACTA
CATCCCTTGGAAACTATGCGTAGGCAGTGGTCAACGCCAGAGGGTACTGCGGGAATGTCATGATGCTCCC
ACGGCCGGACATCAAGGTGTCAGAAAAACGGTGGCGAGGTTAGCCCAGCGGTACTACTGGCCGGGTATG
TTCCGCGATGCCGCCAGATACGTGAAATGTTGCGAAGACTGCCAAAAATTCAAGAGTGAGCAACTCAAAC
CGGCTGGTCGCATGCTGACGAGGCAGGTGGCAGAGCCGATGGCGGTACTTTGCGCGGACTTCGTCGGACC
CCTTCCACGGTCGAAACATGGAAACACGATGCTATTGGTGTTCCATGACGCTTTCTCGAAATGGGTGGAGT
TGGTCCCTCTCAGAAGAGCCACGGCAGCACACCTGCAAACTGCGTTTCGAGAGAGGATTCTAAGTAGGTT
CGGGGTACCTAGAAAGTTTGTGTGCGACAACGGGGTACAATTTACGAGTCGGAGCTTCAAGTCATTTTTG
GGGTCCCTGGGTGTAGAGATCCAGTATACCGCCCCGTACTGTCCGCAAGAGAATCCCACTGAGAGGACTA
ACCGGACTGTAAAGACCATGATAGCGCAATTTATTGAGGGCCACCAGAGTTCATGGGATGAACTCTTGCC
CGAGATCTCGCTGGCGGTCAACACGAGCGTGGCAGATTCAACGGGCTTTAGTCCCGCTTTCCTGATGCAG
GGCAGGGAACCTCGGTTACCCGCAGCTCTGTACGATCAGGTAACTCCGGGGTCCGCAACTAGTGCGCTCG
ACCCTAGGACAAAGGCGGATAGGCTAAAAGAAATTTTTGATATCGTCCACAGCAACCTCCAGCGCGCATC
AAAGGACCAGGGCAGGCATTACAACTTGCGGAGACGAGAGTGGCGGCCCTCGTTGGACTCGATGGTCCTG
TTGAGGCAGCACCAGTTGTCCAACGCTGCCGAAGGTTTCGCCGCAAAGTTGGCACCCAAATTTGACGGCC
CCTACAAAGTCGTCAAATTCATATCCCCCAATATTGTGCGGCTGGTGAAATCAGACGACCGCAAGAAGAG
GGTGGCCAACATCTCACAATTGAAACCATTCTACAGAAAGACGACGAGCGACGAAGAAGACGAAGATCC
AGGGCACGCAACTGCCCACGAGTAAGCGAAGGATGAGAGCAACACCCGTCGCCCAACGAAACTATTATG
ATTAAGCNTAGATAAAACTAACATTAACAACATAATTCAGAATAGGCATAGTAAAGTGTGAGCCGCACTG
AGAGAATAGGGCATTAACAAATAAGTAAAGAGTAAGTTAACCAATAAGCTGAACTTTCAGATGGAACCC
GAAGTTATCGNCATATCGTCAGACGAAGACAACAGCAGCGGGGACACCGAGCCTTTCCCCGGGCCGGGA
GCCGCAGGCCGTCCGCGCGACCCACGCCAGGGGCGTGAGTACTATCGAAGAGAACGGCAAGAAGGACCG
CCTCGCCAGTTCCGCCATACACTATTCTCCCGGCGGACCGGCATAATGGAGAGGCTAGACGGGATGGTCT
TGCTGATGGCAGTCGAGGTCACGACAATGTTCGTGGAGGCACCGTTCGCCCGGAACTCGCCACCGTACGC
GGAAGAGCCTGCATCTCCGCCCCTCGCATCCCACTCGGCACAATCATCAGAGCCGGCAACCCCAACCGCG
GCCGGCGAGATGGACGAGGAAGAGTCCGCACCGTTCGGAACGGAGGAGCACACACCACCAGCGCAGGAC
GTGGAGGCGGTAGGAGAAGAGATATCCCCGTCGGCCGGAGACGCGGAGTCGCCACCGACGGAGGTGCCG
AACGGCACCCGCGCAAACGACGGGGAAGCAGACCCGCTGAGCCTCGACCCAGAACCCTTAGTGGGAGGG
GACCCGTCAACGGAGGACGAGCCGGAATTCCGTCGCCGCTTTGCCACCCACCGTCTGGAGGTAGACCCGG
GCAGCCAGTTTCCCAGGATAACGCTGCGGTACCTACCAAAAGTCCCCGCGGTAGCCAACAAACCCCGTCT
GAACCCCGCAAAGCGCGAATGGGGGTGCCGAAGAAGGCCCCGGCGGAAAGCGCCGACGCAGCCGCCATC
GTCGACGGAGAGCCGCCAGGGGCGAGCGTCGCCGGAGTGGAGGCCATCGCCGACCCAGTCCCGAGGGAG
CATCCTCGTCGGCCACGAAGAGGCTTCGCACGGAGACCGAGAAGCCCGAAGGATCGCAGCGGTGCGTGC
CGCGTTCGCCCCGCGAGCGCCAGGAGGGTCCTCCCCAGCTCGGGAAGGATAACCGCCGCTGCNAGATCGC
TTCTCGAGGAGGGCATCCCGAACCCTGGCCGATACCGATTGCGGTCTGCCAGATGGTCGTGGAGGAGCAC
NATCTCAGCACCAAGGACGCGGCGAACTCGGCCGTTTCCACAGCCCGCGCCGAGGCGCCAGACGAAACCT
TTCCTAAGGAATCGGAGGAGGAGTGGCTCGACGACGAGGACGAGATGCCCGCGCTCGTGCCTGTTGCCCG

CGCCGAGACACCCGACACCATCGCCCGGCGGGAGTTAGAAGACGCCTGGTTCGGCCAAGAAGGCAAGGC
GCCATCCCGAAAAGGTTCCTTCACGGTTCGCCAGGAAGGCCGGGATGAAGTCTGGTCCGAGGAAGAGGA
CGCGGTGGTGACGGCTTCGTCGCCCAAGCTTTCGAGTCCAACCCAGGATTCCTCCCCCATGGCTCTAAAGA
CTCCCACGGAGAGCGAGTTGTGGGACGACATAGTGGACCTTTTCGACGGCATCGACGGGTGGGACACTAG
CCTGGACTCAGTGCTGGAGGAGGAACCCGACACCGAGGTCGACACGGTCGCCCGCAGTGCCGACGTGAG
GGTCACACCGGAGAGCCACCCCAAGAAACCCGACACCGAGGTCGAGGCGATCGCCCACAGTGCTGACGT
TGGGGTAAAACCGGAGGGCCCCGTCGAGGAACCCGTGCCCGATTCCACCCCTCAGGCTGGAAGGTGTTTC
CTCTTGGAACACCGTTGTCGTCGGGCGTGGTGGACCTAATCGCCAGAGAGGCGCGAATCCGCCGCTTCCG
GTGCCGACGTACTCGGTTCCACGTGTCCGACGGGGAGAGCGAGTACAGCGTTACGCTGAACGCGAAAGGC
GACGTGACCGTCACCTGTCCGAAGTGAGGGGGGGGTGTGAGGCGGCTAANGAGACCAGCCACCCCACAG
TTTTCACGGCAAGGAGCCGTCATCCCTTTGGCCAAATTACGGCAAGGAGCCGCTGGCGGACAACGCATT
AATTTTCTTTAGTTATAAGTATTAATTATATTAGGTTAAGATCTTAGTGAAATAAGAGGCAAGGAGCCATC
AGTAATATAAGCAGTTCGATTAGTAGCAAAGAGGCAAGGAGCCGCGAATATTATAATTGATTTAAATAGT
GAGTTTGAATTTAACCGCCGGCCGCCGCTGCAACCGTGCGTTGTCGATCGGTTGCTCAACACCGTCTTTGA
GAGCGCAACTTCAATTCGGCGGCGAAGAGCGAAAGCTCCGAGGCGAAGTTGCGAAAGCTCGCGGCGAAG
TTGAGCAGCCGAAGCGAGCGCATGGCTTGCCGGCGAGCGTGCCGGCACTTGTGATCGAACGTTAAGGGTG
AGTAGAAGTGCTGCGCGAGATAAACGAAATCATAGATGGTATAGTGGAGCGCTAGGGCTCCAGAAAACA
GTGCGGCAGGGTTTAGCTTAGGATTTACGATAAGGTCGGAATGAATAATAGTGAATAAAGTGCCAACGTT
GTAATTATCTAGAAAGTATTGGATTATTCCCTGTTTCCTGGTAACCCGGCTTACCTTAACATCGGAACTAA
CCGGTGTTCGACCTAACCTCGAACGAGGGGCATGCGGTACGAGATTGTGGCTCCGGGGTGGGCATTGCCG
GTTGGTGTTTAGGTAGTGGAGTTGGTGCGAGGCCACGGCCCGCTAGTGTAGTTGGTGCGAGGCCACGGCC
CGCTAGCGTAGTGGTACGAGGCCACGGCCCGGCAGAAAGATAGAGCGNTTAGGTAGTGGAGCTGGTGCG
AGGCCACGGCCCGCTAGTGTAGTTGGTGCGAGGCCACGGCCCGCTAGCATAGTGGTACGAGGCCACGGCC
CGGCGGAGAGATAGGGTGTTTAGGTAGTGGAGTTGGTGCGAGGCCACGGCCCGCTAGTGTGGTTGGAGCG
AGGCCACGGCCCGCTAGCATAGATGGTACGAGGCCACGGCCCGATGGTGTAGGTGTGTAGAGGTAGATCT
GGGTTTGGTTTGGTCAGGATACTCACCCCCTTTAAGCCCNCTCTCCCAGCGAAATTATAAATAACTTGTAA
CTCGCTAGACCTGGTAAGACTGAGAATTATACCCCGGCCGGAAGCTACCTTACA
>160_rnd-1_family-0__Unknown
TTTGGAACGTCATATCTCCGCGCGGAGATATGNCGTTTCGAAACGNCATAACTCCCCGCGATATGNCGTTC
CGGANCGTCATAAATATCTCCGCGNAGATAATGTCTGATTTGTTNAAAGCTTATACCTTTTTGCCAATGCG
TACGGATCCGTACCATCCAAAAGGTATAGCATTCGAACGAATTAATCATCACGAGATATTAAAAAAAAAT
CGTCGAAAAAGTTTCGATTTTCGCGCCGAACCTCGAGTTNTGTCGTTTTGGAACGTCATATCTCCGCGCGG
AGTTATGTCGTTTTGGAACGTCATATCTCCGCGCGGAGTTATGTCGTTTTGGAACGTCATATCTCCGCGCG
GAGTTATGACGATTTGGAAGGTCATATCGCGNGGAGTTATGTCGTTTCGNAACGTCATATCTCCGCGCGG
AGCTATGTAACCCCTTGCCATTTTGTCGATTTGGGTCAAAATTTTGGATTTCCTTTTTTTGATGCCAATCGA
TAGAACTGATCCTTACGAGTCAAAAATGGTATAAAATTCCAAAATCGGACATTATTTGACGGAAATATTC
CAAAAAATCATCAAAAAAGGTTGAATTTACGAACGAATCGGTTTTTTGTCAACAACATTTTTCAACCCATC
GATGATTAATTTGTTTGAATGCCAATTGATGGTAGGGATCCGTACGCATTCAAAAAGGTATAACATTTTAA
AATCAGACATTATTTACCCGAGATATTANAAAAAAATCATCGAAAAAGTTTCGATTTTCGCACCGACCTC
GATTTTGAAAAAAAACGTGATGATTTGGAAGGTCATATCTCCGCGCGGAGTTATGTCGTTTTGGAACGTCA
TATCTCCGCGCGGAGNTATGTCGTTTTGGAACGTCATATCTCCGCGCGGAGCTATTACCCGAGATATTAAA
AAAAATCATCGAAAAAGTTTCGATTTTCGCACCGACCTCTATTTTGAAAAAAACGTGATGTAACCCCTTGC
CATTTTGTCGATTTGGGTCAAAATTTTGGATTTCCTTTTTTTGATGCCAATCGATAGAACTGATCCTTACGA
GGTCAAAAATGGTATAAAATTCCAAAATCGGACATTATTTGACGGAAATATTCCAAAAAATCATCAAAAA
GGTTGAATTTACGAACGAATCGGTTTTTTGTCAACAACATTTTTCAACCCATCGATGATTAATTTGTTTGAA
TGCCAATTGATGGTAGGGATCCGTACGCATTCAAAAAGGTATAACATTTTAAAATCAGACATTATTTACCC
GAGATATTAAAAAAAAATCATCGAAAAAGTTTCGATTTTCGCACCGACCTCGATTTTGAAAAAAATCGTG
ATGATTTGGAAGGTCATATCTCCGCGCGGAGTTATGTCGTTTTGGAACGTCATATCTCCGCGCGGAGTTAT
GTCGTTTTGGAACGTCATATCTCCGCGCGGAGTTATGTCGTTTTGGAACGTCATATCTCCGCGCGGAGTTA
TGTCGTTTTGGAACGTCATATCTCCGCGCGGAGTTATGTCGTTTTGGAACGTCATATCTCCGCGCGGAGTT
ATGTCGTTTTGGAACGTCATATCTCCGCGCGGAGCTATGTCGTTTTGGAACGTCATATCTCCGCGCGGAGT
TATGTCGTTTTGGAACGTCATATCTCCGCGCGGAGTTATGTCGTTTTGGAACGTCATATCTCCGCGCGGAG
CTATTACCCGAGATTTTGGAACGTCATATCTCCGCGAAAGTTTCGATTTTCGCACCGACCTCGATTTTGAA
AAAAATCGTGATGATTTGGAAGGTCATATCTCCGCGCGGAGTTATGTCGTTTTGGAACGTCATATCTCCGC

GCGGAGTTATGTCGTTTTGGAACGTCATATCTCCGCGCGGAGCTATGTCGTTTTGGAACGTCATATCTCCG
CGCGGAGTTATGTCGTTTTGGAACGTCATATCTCCGCGCGGAGTTATGTCGTTTTGGAACGTCATATCTCC
GCGCGGAGTTATGTCGTTTTGGAACGTCATATCTCCGCGCGGAGTTATGTCGTTTTGGAACGTCATATCTC
CGCGCGGAGTTATGTCGTTTTGGAACGTCATATCTCCGCGCGGAGTTATGTCGTTTTGGAACGTCATATCT
CCGCGCGGAGCTATGTCGTTTTGGAACGTCATATCTCCGCGCGGAGCTATGTCGTTTTGGAACGTCATATC
TCCGCGCGGAGTTATGTCGTTTTGGAACGTCATATCTCCGCGCGGAGTTATGTCGTTTTGGAACGTCATAT
CTCCGCGCGGAGCTATGTCGTTTTGGAACGTCATATCTCCGCGCGGAGCTATTACCCGAGATATTAAAAAA
AAATCATCGAAAAAGTTTCGATTTTCGCACCGACCTCGATTTTGAAAAAAAACGTGATGTAACCCCTTGCC
ATTTTGTCGATTTGGGTCAAAATTTTGGATTTCCTTTTTTTGATGCCAATCGATAGAACTGATCCTTACGAG
TCAAAAATGGTATAAAATTCCAAAATCGGACATTATTTGACGGAAATATTCCAAAAAAATCATCAAAAAGG
TTGAATTTACGAACGAATCGGTTTTTTGTCAACAACATTTTTCAACCCATCGATGATTAATTTGTTTGAATG
CCAATTGATGGTAGGGATCCGTACGCATTCAAAAAGGTATAACATTTTAAAATCAGACATTATTTACCCG
AGATATTAAAAAAAAATCATCGAAAAAGTTTCGATTTTCGCACCGACCTCGATTTTGAAAAAAATCGTGA
TGATTTGGAAGGTCATATCTCCGCGCGGAGCTATGTCGTTTTGGAACGTCATATCTCCGCGCGGAGTTATG
TCGTTTTGGAACGTCATATCTCCGCGCGGAGTTATGTCGTTTTGGAACGTCATATCTCCGCGCGGAGTTAT
GTCGTTTTGGAACGTCATATCTCCGCGCGGAGTTATGTCGTTTTGGAACGTCATATCTCCGCGCGGAGCTA
TGTCGTTTTGGAACGTCATATCTCCGCGCGGAGCTATGTCGTTTTGGAACGTCATATCTCCGCGCGGAGTT
ATGTCGTTTTGGAACGTCATATCTCCGCGCGGAGNTATGTCGTTTTGGAACGTCATATCTCCGCGCGGAGN
TATGTCGTTTTGGAACGTCATATCTCCGCGCGGAGNTATGTCGTTTTGGAACGTCATATCTCCGCGCGGAG
NTATGTCGTTTTGGAACGTCATATCTCCGCGCGGAGNTATGTCGTTTTGGAACGTCATATCTCCGCGCGGA
GCTATTACCCGAGATATTAAAAAAAAACATCGAAAAAGTTTCGATTTTCGCACCGACCTCTATTTTGAAAA
AAAGCGTGATGTAACCCCTTGCCATTTTGTCGATTTGGGTCAAAATTTTGGATTTCCTTTTTTTGATGCCAA
TCGATAGAACTGATCCTTACGAGTCAAAAATGGTATAAAATTCCAAAATCGGACATTATTTGACGGAAAT
ATTCCAAAAAANCATCAAAAAGGTTGAATTTACGAACGAATCGGTTTTTTGTCAACAACATTTTTCAACCC
ATCGATGATTAATTTGTTTGAATGCCAATTGATGGTAGGGATCCGTACGCATTCAAAAAGGTATAACATTT
TAAAATCGGACATTATTTACCCGAGATATTAAAAAAAATCATCGAAAAAGTTTCGATTTTCGCACCGACCT
CGATTTTGAAAAAAATCGTGATGAAGTCGATCTCCGCGGGGAGTTCTGTCGTTTTGGAACGTCATATCTCC
GCGCGGAGTTATGTCGTTTTGGAACGTCATATCTCCGCGCGGAGNTATGTCGTTTTGGAACGTCATATCTC
CGCGCGGAGNTATGTCGTTTTGGAACGTCATATCTCCGCGCGGAGTTATGTCGTTTTGGAACGTCATATCT
CCGCGCGGAGTTATGTCGTTTTGGAACGTCATATCTCCGCGCGGAGTTATGTCGTTTTGGAACGTCATATC
TCCGCGCGGAGCTATTANCCGAGATATTAAAAAAAAATCATCGGAAAAGNTTCGATTTTCGCACCGACCT
CTATTTTGAAAANAAGCGTGATGTAACCCCTTGCCATTTTGTCGATTTGGGTCNAAATTTTGGATTTCCTTT
TTTTGATGCCAATCGATAGAACTGATCCTTACGAGTCAAAAATGGTATGAAATTCCAAAATCGGACATTAT
TTGACGGAAATATTCCAAAAAAACATCAAAAAGGTTGAATTTACGAACGAATCGGTTTTTTGTCAACAAC
ATTTTTGAACCCATCGATGATTAATTTGTTTGAACGCCAATTGATGGTAGGGATCCGTACGCATTCAAAAA
GGTATAACATTTTAAAATCAGACATTATTTACCCGAGATATTAAAAAAAAATCATCGAAAAAGTTTCGAT
TTTCGCACCGACCTCGATTTTGAAAAAAATCGTGATGGTTTGGAAGGTCATATCTCCGCGCGGAGTTCTGT
CGTTTTGGAACGTCATATCTCCGCGCGGAGNTATGTCGTTTTGGAACGTCATATCTCCGCGCGGAGTTATG
TCGTTTTGGAACGTCATATCTCCGCGCGGAGNTATGTCGTTTTGGAACGTCATATCTCCGCGCGGAGNTAT
GTCGTTTTGGAACGTCATATCTCCGCGCGGAGNTATGTCGTTTTGGAACGTCATATCTCCGCGCGGAGTTA
TGTCGTTTTGGAACGTCATATCTCCGCGCGGAGCTATGTCGTTTTGGAACGTCATATCTCCGCGCGGAGCT
ATTACCCGAGATATTAAAAAAAAATCATCGAAAAAGTTTCGATTTTCGCACCGACCTCGATTTTGAAAAA
AANCGTGACGATTTGGAACGTCATATCTCCGCGCGGAGTTATGTCGTTTTGGAACGTCATATCTCCGCGCG
GAGCTATTACCCGAGATATTAAAAAAAATCATCGAAAAAGTTTCGATTTTCGCACCGACCTCTATTTTGAA
AAAAACGTGATGTAACCCCTTGCCATTTTGTCGATTTGGGTCAAAATTTTGGATTTCCTTTTTTTGATGCCA
ATCGATAGAACTGATCCTTACGAGTCGAAAATGGTATAAAATTCCAAAATCGGACATTATTTGACGGAAA
TATTCCAAAAAATCATCAAAAAGGTTGAATTTACGAACGAATCGGTTTTTTGTCAACAACATTTTTCAACC
CATCGATGANTAATTTGTTTGAACGCCAATTGATGGTAGGGATCCGTACGCATTCAAAAAGGTATAACAT
TTTAAAATCAGACATTATTTACCCGAGATATTAAAAAAAAATCATCGAAAAAGTTTCGATTTTCGCACCGA
CCTCGATTTTGAAAAAAATCGTGATGATTTGGAAGGTCATATCTCCGCGGGGAGTTATGTCGTTTTGGAAC
GTCATATCTCCGCGCGGAGCTATGTCGTTTTGGAACGTCATATCTCCGCGCGGAGTTATGTCGTTTTGGAA
CGTCATATCTCCGCGCGGAGCTATGTCGTTTTGGAACGTCATATCTCCGCGCGGAGCTATGTCGTTTTGGA
ACGTCATATCTCCGCGCGGAGNTATGTCGTTTTGGAACGTCATATCTCCGCGCGGAGCTATGTCGTTTTGG
AACGTCATATCTCCGCGCGGAGTTATGTCGTTTTGGAACGTCATATCTCCGCGCGGAGCTATGTCGTTTTG

GAACGTCATATCTCCGCGCGGAGTTATGTCGTTTTGGAACGTCATATCTCCGCGCGGAGTTATGTCGTTTT
GGAACGTCATATCTCCGCGCGGAGCTATGTCGTTTTGGAACGTCATATCTCCGGCGGATATTACCCGAGAT
ATTAAAAAAAAACATCGAAAAAGTTTCGATTTTCGCACCGACCTCTATTTTGAAAAAAAGCGTGATGTAA
CCCCTTGCCATTTTGTCGATTTTGGTCAAAATTTTGGATTTCCTTTTTTTGATGCCAATCGNTAGAACTGAT
CCTTACGAGTCAAAAATGGCATAAAATTCCAANATCGGACATTATTTGACGGAAATATTGCAAAAAATCA
CGAAAAAGGTTGAATTTACGAACGAATCGGTTTTTTGNCAACAACGTTNTTCAACCCATCGATGATTAATT
TGTTTGAATGCCAATCGATGGTAGGGATCCGTACGCATTCAAAAAGGTATAACATTTTAAAATCGGACAT
TATTTACCCGAGATATTAAAAAAAAATCATCGAAAAAGTTTCGATTTTCGCACCGACCTCGATTTTGAAAA
AAATCGTGATGTTTGGAAGGTCATATCTCCGCGGGGTTCTGTCGTTTTGGAACGTCATATCTCCGCGCGGA
GNTATGTCGTTTTGGAACGTCATATCTCCGCGCGGAGTTATGTCGTTTTGGAACGTCATATCTCCGCGCGG
AGCTATGTCGTTTTGGAACGTCATATCTCCGCGCGGAGCTATGTCGTTTTGGAACGTCATATCTCCGCGCG
GAGTTATGTCGTTTTGGAACGTCATATCTCCGCGCGGAGTTATGTCGTTTTGGAACGTCATATCTCCGCGC
GGAGTTATGTCGTTTTGGAACGTCATATCTCCGCGCGGAGTTATGTCGTTTTGGAACGTCATATCTCCGCG
AAAAAGTTTCGATTTTCGCACCGACCTCTATTTTGAAAAAAAGCGTGATGTAACCCCTTGCCATTTTGTCG
ATTTNGGTCAAAATTTTGGATTTCCTTTTTTTGATGCCAATCGTTAGAACTGATCCTTACGAGTCAAAAATG
GCATAAAATTCCAANATCGGACATTATTTGACGGAAATATTGCAAAAAATCATGAAAAAGGTTGAATTTA
CGAACGAATCGGTTTTTTGTCAACAACATTTTTCAACCCATCGATGATTAATTTGTTTGAATGCCAATTGAT
GGTAGGGATCCGTACGCATTCAAAAAGGTATAACATTTTAAAATCAGACATTATTTACCCGAGATATTAA
AAAAAAATCATCGAAAANGTTTCGATTTTCGCACCGACCTCGATTTTGAAAAAAATCGTGATGAAGGTCA
TATCTCCGCGGGGAGTTNTGTCGTTTTGGAACGTCATATCTCCGCGCGGAGTTATGTCGTTTTGGAACGTC
ATATCTCCGCGCGGAGTTATGTCGTTTTGGAACGTCATATCTCCGCGCGGAGTTATGTCGTTTTGGAACGT
CATATCTCCGCGCGGAGCTATGTCGTTTTGGAACGTCATATCTCCGCGCGGAGTTATGTCGTTTTGGAACG
TCATATCTCCGCGCGGAGTTATGTCGTTTTGGAACGTCATATCTCCGCGCGGAGTTATGTCGTTTTGGAAC
GTCATATCTCCGCGCGGAGTTATGTCGTTTTGGAACGTCATATCTCCGCGCGGAGCTATGTCGTTTTGGAA
CGTCATATCTCCGCGCGGAGCTATGTCGTTTTGGAACGTCATATCTCCGCGGAAAAGTTTCGATTTTCGCA
CCGACCTCTATTTTGAAAAAAAGCGTGATGTAACCCCTTGCCATTTTGTCGATTTNGGTCAAAATTTTGGA
TTTCCTTTTTTTGATGCCAATCGATAGAACTGATNCTTACGAGTCAAAAATGGTATGAAATTCCAAAATCG
GACATTANTTGACGGAAATATTCCAAGAAAAAACATCAAAAAGGTTGAATTTACGAACGAATCGGTTTTT
TGTCAACAACATTTTTCAACCCATCGATGATTAATTTGTTTGAACGCCAATTGATGGTAGGGATCCGTACG
CATTCAAAAAGGTATAACATTTTAAAATCAGACATTATTTACCCGAGATANTAAAAAAATCATCGAAAAA
GTTTCGATTTTCGCACCGACCTCGNTTTTGAAAAAAATCGTGATGAAGGTCGTATCTCCGCGGGGTTCTGT
CGTTTTGGAACGTCATATCTCCGCGCGGAGTTATGTCGTTTTGGAACGTCATATCTCCGCGCGGAGTTATG
TCGTTTTGGAACGTCATATCTCCGCGCGGAGNTATGTCGTTTTGGAACGTCATATCTCCGCGCGGAGNTAT
GTCGTTTTGGAACGTCATATCTCCGCGCGGAGNTATGTCGTTTTGGAAAAGATTCGATTTTCGCACCGACC
TCTATTTTGAAAACAAGCGTGATGTAACCCCTTGCCATTTTGTCGATTTGGGTCNAAATTTTGGATTTCCTT
TTTTTTATGCCAATCGATAGAACTGATCCTTACGAGTCAAAAATGGTATGAAATTCCAAAATCGGACATTA
TTTGACGGAAATATTCCAAAAAANCATCAAAAAGGTTGACTTTACGAACGAATCCGTTTTTTGTCTACAAC
ATTTTTNAACCCATCGATGATTAATTTGTTTGAACGCCAATTGATGGTAGGGATCCGTACGCATTCAAAAA
GGTATAACATTTTAAAATCAGACATTATTTACCCGAGATATTANAAAAAAATCATCGAAAAAGTTTCGAT
TTTCGCACCGACCTCGATTTTGAAAAAAATCGTGATGAAGGCCATGTCTCCGCGGGGAGTTCTGTCGTTTT
GGAACGTCATATCTCC
>Ref_TART
CAATTTAAAAAAAACACAACTTAAAAATCAGAATTTTTCAATTCTAATAATTAACCAAGCTTGCAAATAT
ATTTGTGAAGTTATCTGAGTAATTGCTAAACAGGCATAGTGGCAAGCAAATACAATAAATATTTAAAAGG
TGCAATAGTGGTAAAGACAAACATAACTGCACCAGAAATAATATATAAAGTGCATAAATGCAAAAACCA
ACTGTGTCTAAAAGCCCGCCTAAAAGTCCGCATAACGCTTCTGAAATTACCGCAAACTCGGCTGTCCCCCC
CCACCGCGCAACCGAAGAATGCAGCATAAGTGAAAGAGCGAAAAGTGAACTTTCACAGTTGCAGCAACA
GCTCACAAAAAATCCGCAAAAGCAGCTCAGCCGAGAACACCGGCGACAGCAGTTGCTAAGGCTATGCGA
AGAGGAGGAAAGGTTAATGCCCGATGTCCCAGCGCAGCAGCCGACGCTTACGCTCAAGCATAAGCCGAA
GCTGAAAACAGTTCTCGCCAACTCTTCACCGCCCAGCTCGGCACCGCTGAGAATGTCTCTGACGAAGTCGT
CGAAAGCTAAGGCAAGGAAAGAGAGCCAATCATCTGCCTGCAGAGAGAAAAGTCCAGAAGAGAACACCT
ATGTCAATGTGTTGGAGTCTGACGATGAAAGCGACATAGCAGCCAATAAAAATTATGACCAGCCGCAACA
GCAGACAACTATTGCACAGGTACATAATTTCAATACAAATACTAGCATAACTAGCACTCCCAAAGCAGCC
TCTCACTGTGATCCATCGCCAAACGTATCGGCACACGAAGTTGAACTTCATAATGTTGATAACACAAGAG

AAGCTAGCTATGTTAATAACATTGATGAACTAACAGACGTCTTTGACGACGAAGATGATGATGATGACTG
TTACCGGCCCGCTGCTGTTATTAACAGCTCAGTTGCTGACATGCTTAACAGAACAACTACTGTTGAGCATA
ACAGCACAGCCACTGCTAACAGAGGGGAAATTCTTGCAAATGTTAAAACATCTGATGCATTTGCTGCCAA
TAAAATTAATAGTATTACTTTTGAAAAAATCGAACCAATTCCACAAACAATAACGCCAACAGTAGCAAAA
GGACCTAAATTTAACTGCTCTATGCTACCTACAACTTCCCTGTCTGCCAAGAAAAGTTTAAAACGTAAAGG
TACACTGCCGCAACAAAGTAAACAAAAGCGAGCACAGACACTGGCAGTACAAGGAAACTCACGACCAGG
CATGAAGATACAGGACTTCTTGAAGTCATCAACAAGAGTAACTAAGGCAACAAATAAAGCAGCTATAATC
AACAAATATAAAAAAAGCAATCGTAATACTAAAAAAGTAAACCCCTCTAGCAGCACTAAACTCAACAAC
AGCAGTATGGATATTGACCTGGATAGCAGCGACATATCAGCAAATAACATAGGCTCTGATGATGACATTG
TTCAGATCCAGAGCAATGCACAAGAGGTTACAAACCACGCCACCAAAGTCCAGCAACAACAACAGCATC
GACAGCAGCAACAACATCACCAGCAGCAGCAACAACAGCAACAGCAGCAGCCAACTAAAAGCACAAAA
ATTCCAACAATTTTTCTACCCAGCATAAGTGACATACAACAAATAATTGATATGCTCAATAATACAGTGGG
TGCAAATAAATATACAACAAAGTGCACACAAAACGATGGAGTCAGAGTGCAATGCAGCGACTTACTATC
GTATAATGCAGCTATAGCACTGCTCGGCGCCAATACGGAAATACAGATGCACACCCACCAGATGCGAAGC
GAGCGAGGATACCGTGTTTTACTTAAAAATGTCCACCACTCCACGCCATGCGAGCAAATCCGAGCAGAAT
TGGCGAAACATGGACACACGGTTCGCTTTGCCAGCGTCATTAAACATCGATTTGAGCGACGGCCACTTAA
CATGTTTGAAGTCGAACTGGCACCAAACGGCGACACCAACGACAAAGTACTGGAACTGAAAACTCTGGG
AAACCAACACATTGAAGTAGAAAGACAGCTGAAGCGAGATGAGCCTGTACAGTGTCATCGGTGCCAATC
GTTTGGGCATAGCAAGAACTACTGTCGTAGGCCTTTTGCATGTCTCAAATGCGGTGAACAGCACCCGACT
ACAACGTGTACTAAACCCAGAAACACACCTGCTAAGTGCGTCAACTGCAAGGCTGACCACATAGCCAGCT
TTAAGGGATGCAGTGTGTACAAAATGGAGCGGGAAAAACTAGCAGCAAACCGTGTCCGGGCGGCAATTG
ATAGGCAACAGCAGCACCAACAACAGCAGCAACAGCAACAACAACAGCAGCAGCAGCAGCAGCAACAG
CAGCATCAGCAACAGCAACAGCAACAACAACAGCAACAACAGCAGCAGCAACAACAACAACAAAATCG
GCAGCTACAACAACATCAGCAGCAGCAGCAACAACAACATCGGCAGCTACAACAACATCATCAGCAGCA
GCAGCAACTACAACACCAACACCAGCAGCCACAATACCTAGCAATTAATACTTCAGCTCCTAGTGGAATT
AAGTCGACTAAGGAACGGAAGCAACAGCAACAGCAACTTCGGCAACAACAGCAAAAGCAGCAGCAGCA
ACAACAGCAGCAACATCAACAATTGAAGCCAAACAATCAACTGACTTATAGCCAAGTAGCCAGTGGACA
AGCAAATACGAGCCTCAGTCACAACCCAGCCATGCAACACCTCAAGAAGTACCAGCAACAGCTACAACT
GGAGCAGCAACAGCAGCAACGACAACAGCAGCAACAACAACAGCAGCAGCTACAGCCCGAGCAGCAGC
AGCAACTTCAGAGGCAACAGCGACTGAGGCAACAACAACTACCTGAGCAGCAAAAACGGCAGCAATATC
AACGACAGCAAGAGCAGCAATCACATGAGCAACAGCAACGACACCAGCAGCAATCGCAACAACAACAAC
ATAGACAACAGGCGGACTCACCACTCTTGGAGGCGCTGCAGCAAAATGTGATAAGTATAAATGAGATGTC
CAAGAAAATAGACATGCTAATTAGCCTGTTGCTCACAATGGCAGCAAGCAACACTAATATTAATAAACAA
GGAGAACAACAAACAATCCCACTAACAACATCAACCTCTGATACCTTGAACAATGTCAATAAAAAAAAC
AATTTTCCTAATTTAATGAGTACATTGATTAGCACAGCACAGCCAATAAACAACAGCCAATCGGCAAACG
CCAATTGTGAACACATAGCCACTGACAGTAGCCAGTATGTATAGAGCGAACAACACCAACAATGCCAGCG
CGACTGCCACATTGAGTCAAGTCAATGATGGCAGACACACCACTCACACTACTAGTGACACCACAACTAC
TACAACAACACATTTGTCCAACCTTAACAGTCCATTGCTTAACAGGAGAAGGCCAAACATTATTCCAAAT
GACGAACCACAGCAGCGACCAATATGGCAACTCTTTTAAAATTGCATATTGGAACGCTGCTGGCGTTAGA
AACAAACTAAATGAATTAGAGGTTTTCATGAATCGACATAAAATTGATATTATGATGCTCGTCGAGCTAC
GATTGGGCCCCAGCGCTCTCAATCCAGGTCCCCCAATTACTATTAACGGCTACCATACATATGCGGCAACT
CGCCCACCACCACACCATAGATGCGGGGGTGTTGCCACTTTAGTAAAACACGGAATCAAACACATTGCCC
TGGAAGCAATTGTGTTTGAATCCATGCAATCTGCACCAGTAGCTGTGACCCTGGCAAAAAATGAGACCAT
TGTCCTAGCTCCAATTTATTGCCCACCGCAATATAATTGGAGCGCTGACATGTTTGCCAAACTTTTCCAAC
ATTTTGAAAAATTGAGCGGGTCCGGGTTCATTTTATGTGGCGACTGGAACGCCAAACATTCCTGGTGGGG
CAACCAACGTGCTTGTCGACGAGGGGTAGAGCTGCTAAGAAGCATTCACAGCAACAAGAAGCTGAACAT
TCTTGCTACAGGTGGGGCTACACACTTCCCATACACAAGTAGGAACAGGCCATCCGCTATCGATATTGCG
GTCTACAGGGCATTAACAATGACCGGACTACAAACACACTCTACAATCGATCTAGAATCGGATCATCTAC
CTATACATATAGGCCTCAGAGTAGGCCGATTCCCGTATAAACAAACAACAATGCGATTGCTACCGATAAA
TGCAAACGTAAAAAAATTTCAAAACCATTTAGATAAAAACGTTCGTCTTAATACAGAAATAATATCGGGA
CCCGATATTGAAGACGCCATCGACATATTAAATAAAAACATATATAATGCTTCCTTAGCAGCAACACACC
CACCTCGTAGACATCAACAGCAACAAAGACAAGGCACAAATACAAATCGCATGAACCATGGCCGTTTTAA
ATTAACAAACGAAACAATTAGACTTTTGGCAATAAAACGGCAGCGCAAGAGAGAACATATGGTTATGCG
AACACCTCTCACGCGAAGCCGACTTAGCCAGGCCCAAAACAAACTAAAAAAAGCGCTTCGTATGGACAA

file:///E/10月/10.9/PDF/4. biology-1907542/biology-1907542-supplementary/SupplementalDocument2.refTElib_01_25_2022.fasta.txt[2022/10/9 15:38:02]

GAAGAAACAAACGAACAAAATGTTTGAACAAATAGATGCCACTGATCGCTACAAGATACAAAAGCTTTG
GCGCATCACAAACAACATCAAAAGGCAACCAGAGCCAAATTGGCCACTTAAAATACAAGCGACTGACAA
CAACAACAGTCGCAGCAATAGAACACAATGGACTAAAACTAGTAAAGAAAAGGCTGAGGTCTTCGCTGC
ATACCTGGAGCAACGTTTCAGCCCCATATTCTCAAATACGGCTGAATACAGACTCCAGGTGAATAATGAG
ATAAACACTAGACAGCAAACTAACGATTGCGACAGCGGCGCTGGCGTCGCTGGTGTTATCCCTTTTCGCC
CAATTACATATACTGAGGTGACAAAAGAGATTGGCTGCTTGACAATAAAAAAGGCTGCAGGCATCGATAA
CATAGACAATAGAGTCATTAAAGCACTACCAAAGAAAGCAATACTGTACCTTGTCATGATATACAACTGC
ATACTCAGACATGGCCATTTCCCGCGACAGTGGAAATGCGCTGCCATAAAAACAATTTTAAAGCCTGGAA
AACCCACAGAAGATGTCGCCTCATATCGACCAATAAGCCTGCTTGCAGGTTTCTCCAAAATCTTTGAAAG
GCTTCTCATGTACAGGCTCTATGAATGCTCTGAATTTGCAAAGGCCGTGCCACTACATCAATTTGGCTTTC
GCAAAGATCATGGCACTGAGCAGCAACTAGCACGGGTAACCCAATTCATCCTGAGTGCATTTGAAAAGAA
ACAATACTGCTCAGCCGTCTATATCGATATACGCGAAGCATTTGATCGAGTATGGCACGAAGGATTGCTCT
TGAACTTGGCAAGATACTGCCTAATAGGCTGTATAATGGTTCTCAAGAGCTATCTACTTGATCGCACATTC
ATCGTACATGGAAACGAGGGCATTAAATCTAGAATAGGCAAAGTTAGCGCTGGAGTGCCCCAGGGGAGC
GTACTTGGGCCAATTCTATATATCATCTACACATCAGATATGCCATTACCACACATTGCAACACCTCCCAA
TCCAACAACAACAGCAGCAACAATAACAAATACAGAAACGTCCATAACTAACCCATACAACTGCAGCAT
GCTACTGTCAACGTTTGCCGATGACACAGTCATAATGAGCTCTGCTGACATACTGCAAACATCATACAGTG
CAAATCAAACGTATCTGCGCCAATTCACCATTTGGTGCAATCGCTGGTGCATCCAAATTAATGACAGCAA
ATCAGCTCATGTAGTACATACTCTACGCAATTTAAACAGCCGAATGAATTACCTGACTCCACGACTCAATG
GGCAAGAAATCCCCTCTAAACCAAGGCAAAAGTACCTAGGTGTCCATCTGGACCGAAAGCTCCACATGCA
ACACCATGTTACGCAGCTACGATGCCGTCTTAAAGCACTCTATAATAAACTAGAGTGGCTAATAGGAAAC
AAAAGCGTACTCTCTGTAGAATGCAAAATCACTATCTACAAGCAGATGATTGCGCCGATATGGAGATATG
CTTTGCCGATCTGGGGTGCCATGATCTCTGACACACAGCTACGCCGAATAGAATCCACACAGAACTGGAT
TCTAAGGAAGATAGTGAAGGCCTCCTGGTGGACTAGAAACAAAGACATACGTGATACTTACGACATAGGC
ACAGTGGACGAAATATTCCATACTACAAGCAAAAGATTCGCCGACTCACTTGCTATTCATCCTAATGCCA
ACGCTCGTAAGCTAATCGCAAGTCCGTATGTTCCAATTCGTCTTGATCGGCAAAGGTATAGTCTGCAACTA
CAACAACATGTTCGCCCTCTACAGCAACTAGTGCAGCAATCGCAAGAACAACAACAGCTGCCAACTCTGC
TTCGCATGGAGCTTGAAGAAGAAGAAGCAAACTCGCTCAGAATGCAACAGCAGCAGCAACAGCAGACCC
CACAACAACAGCCAGCCAGATTCAGCGAAATGAGGATAAATTCGCTAAGAAGGCACTACAGAGAGGGAC
GAATTACGCTCGAAGAGCTAAAACTAGCAATTAGGGAACAGCCATTGGTGATTCAGCAATTAGTACTGCC
TCGCGAGCTGGCTATTCAAATATACCAGCAGCAACAACAGCAGCAGCAACAGCAACAACAGCAGCAGCA
ACAGCAACAACAGCAGCAGCAACAGCAACAACAGCAGCAGCAACAGCAGCAGCAACAGCAACAAGAGC
AGCAGCAACAACAGCAGCAGCAGCAACACCAGTACCAACAGCAGCATCAGCAACAGCAACAACAGCAA
ATACAGCAGCAGCAACAACAGCTCGAGGGACTCCAGCATCAGCCAAATCAGTCGCCTGACGTCGAAATA
GTGCTAGATACACCACAGCAGCAACAAGAGCAACAACTAGAACGGCCACTTCCACAACCTCAGCAAGCC
AATTTACAACAGCAACGGCTAACTGAGGACGAGCAGCAGCTTGAGCACCGACTTGAAAGACTCAGGCAG
CAACTTGAGCGCGAGATGGACGAGGCTGCAAATAACGAGCCAACTCAACAACAGCGAACACCAGAGCAG
CAGCAATCAAACCAACAGCAACACACAATAGCCTTAACTGAAAATAAAAATACAAATATTAATCCAAGC
AACTCAGTCTGTGCCAATATAACAAATTTTTCTATTTCACAGAGCATAATAATAGCCCCATACAGCCTCAT
AAACATTGCCACAGATGCTCCTTGGCAACAAAATCAGCCCACAACCACACAACAGCTCCGCCAGCAGCAC
TCGAACCTTCACCAGCAGCACCTACAGCAACAACGGGAGGTAGTGGAGGCGCCCAGCAGCAACAACGCA
CAGTCAACAGTCAGGGGGGCTAAAAGAAGAAGAAGCAGTTCAACAAGCTGTATTGAACCCAAAAGACGC
TCACCTGCAGCTCCTGACAACCAGCAACAGCGGCAACTCCACACTCTACTCGGCAAAAGGCACTCGCAAC
AACGTCAAGCATGCCTTCGCAGAACACGGCTGGGTACATGGCAGCTGCAAATGCCCCACAGGCTGGGAAT
ATGGCTCCTACTCTCCTGCTTTGGCATGCAAACACACCATAAGGTGCCCCAGAAGCTTGTGTGGGCACCTC
CAGCAGACTACGGTCAATCCTCGACGAACGGCAGACACCTCCAAATTGCCAGAAGGCGCCCGTGGCACTC
CTGGCAGCGGCTCCAACGTCGATGGTCCAATTGCGGGCGGCAAACACACAGGAAGGCCAGGCCGTATAA
CATATTAATCCATGAGACCTGAAATAGATAAATAATAAGTAAATTTGCACCAACTAGAATTATAAATTCC
AACAAATCCTTAAATTCGCCCCTCTGGCAAACTATGTTTACAATTTAACTCAATCAATATGTTAACTCAGC
TGTTCATAACGCAATCGAAAGCAGCCCTGATAACATTAACAGAGGTATGTAATAACAGAACCATTGCAGT
AATTGCTCAACTGTTCTGAACGCAATCGCCAACAACCCTGATAACGTTAGACAGAGGCACTGTTAATAAC
AGAAATAGTACCGCCAACGTGCTAAAAATTCAAATAAGCCTGCTCAAGCATAGTATTAAGTAATGTTAAA
TGTAAAAAATAACTGTTAAATGTTTATTTATCGTAATTCGTCACAATTTTCTTTTATGTTGTGATATGCATG
TAAAATTTTGTTAAAATATGTCATACATGCCAGTAATTTGTCAGCTAGTCCGTTTCACAATTAGACCTATTT

CGTTCCAATTCTGTCTCATATATTGCATTGGAACTAGAATTAAGCAATGAAAAAGACAATAAAATGCGAC
TACTAGCTTAATCGCTAGAAGTTCGCCGTAGCGCACGCATGCGCAGCAATTTAAAAATTA
>160_rnd-6_family-3320__DNA___TcMar-Tc1
CCACTTAAGTTTCTTGGAGCACGACTGATTTTGCCACAAAATTTATGGTGCGTGAGATAGAAGGAGGTGA
TCTTTCATCCAAAGTGTCATGTAAGACATGTTTGATAAGCAAAACACACGTGATGTACGTGCATTTTCTAA
TTCATCGGGAAATAGAGCCAGTAAACTTTTCGCGCTAGTTCATTCGGATGTGCGTAGACTTTTTGAGACCA
GATCAATAGCGGTAATTGATATTACCTTCATATATTTCATTAAAATGAAAGATGATGTGTTATTTTTTGCAT
TTAAGCATTTGATGGAGAGTGGTAGACGAGTAACGTGTATTCGTAGCGACAATGAGCGTGAATACGTGAA
CAACACGTTTGAGGAGTATCTCAATAATAAGGATATAGCACGACAACTGACAATACCGTATTCGCCGCAG
CAGAAAGAAGTGGTTGAAAGGGCGAACCGAACATTGGTTGTAATGTCACGATGCCTGCTTGTACATTCTG
CTTTGAAGGAGTCTTTTATAGGCGAAGGCACTATTCAGAGCGGTTAGAAATATGACGCCAATAGAAGTAT
GGAATGTTAAAGAACCCTCCATTTGTCACTTTAATGTTGGGATAATAACAAAACTATCGGATATGCGAGC
AATTAAAAAGTATATCGTTTGTATAATTCAAACTCTGGACAAGTGATCGAGCAGAGGGATGCTCTGTTCG
ATAAGCAATACAGCAGTGAACTGTACGATACTGGAGCCATTACATTTGATCTTACAACGACGAATATCAA
CGTCGGAATAAAAAATGACAGCAGCAGCGACAATTTAATGAGTGCACTCAAGGTACCAGGTTTGGGACC
AGGTAGACAAAAATTAGTTCGCTATGGAAAGCGAGGACGAACCAAGAAGCAGTACAGTATTCTTGGCGC
TTTGGAAAGTACCACGAATACCTACATGCTACGAGTCTATTAGCTGTTCCAAAGGTTCACTTGACAATGAA
CATAGAATACGACCCTCTGATGTTGAATACAACTATGCGCATTATCAATAAAGATCAGCACTTTGTTAAAG
TATCAGGTGTTGATCAAAATTGATCCTTGATTGCACCAGCATAAGAAAGATAGTCGCAATGAATAATAAA
TGAATACGTGATTTGTTTTGTCGACAAAGCAATGAAAACTGCACGCCTTTGGATGCTGGATACCAAAATGT
TTTCATAAATAAGGAGTGTAGCAAAGTGAATGCGTCGCAATACCAATAATTTATGGAGAAACTAATGTGG
CTGACGACGATTAGGCCGGACATTTTGCACTCAGTAACAAAATTAACGCAGTGCAATAGGGAACCACATA
GCTAACACCTTGCAGGCGTTAAACATGCTCTAAGGTATCTGGCATCAATAACACTTGAAGCTGCACTACA
AAGAAAAAATAAATCTTGTATATTGCATGGCTTTCGAGTAGTAGCGTAGCGCGACATACATTTGTATAATT
AGTCGCCCATACATTTGAAAGTGACGCAAAATCTCTTGGCTGGCTTCTCATCTGGCTTTCCCCACTTAACC
AGACATGCCAGCCGACTGACTTGTCCGCTCTCCAAAAGCCAGACGAACGACTTATACAAAATGCATTCAT
ATGTAAATACAATTTGGGGAAGGTCGTGGTCAGCGCTAAAAAACTAATAAAATGTATTATTTTGTCGGTA
ACAGTCCAAGCAGTAGTGCATAACAATACACTCAATTATGCTGTTATGTATAAACACGCATACAAACATA
ATTGCCTGCAAGGGTCTTACATTCTATTTTTGGCTTTTTCGGTCTTTCGGTCGGGAAAGCGCAATGATACTG
AATTCGTATTCGTTCCCAATTTAAAAAAAATCTAGCTGCAAAGTTGTATTTTGTTGTATGGCGATACCTAT
CTTAGTGTTTATATTGTCTGTGGTTTATGGAATTCCCAAGTAGAGTTCTTCCTACAAGAGCCTCTTCTCTGT
GTTTTTATACCCTTGCAGAGGGTATTATAATTTTGTCGCGAAATGTGTAACGCATAGAAGGAGACATCTCC
GACCCCATAAAGTATATATATATTCTCTATCAGCATCAACAGCTGAGTCGATAAAGCCATGTTCCTCTGTC
CGTCTGTCTGTTTCTATGGGAACTAGTCTCTCAGTTTTTAAGCTATTTAAAAATAAAACTTTGCAGAAGTCC
CTCTTTCTGTTGCACTCAGCACATATGTCAAACCCAAAATTACACATGTATAAGAACTTTAAAGTATGTAT
GTATGCGTTGTTATTCACAGCTCTGGCTTTAGCTGACAAGAAATTAATTTTGTTAGTTTTGTAGTGCAGATT
ATGAGCTATCCCAAATTTGTGTTGATAGATGAGTGCATTTTGTGTACTGAGGTTGGCTCCGTAGAAAATAT
GTATTTCTAAGTCGATGCAAATATTTACATAATTTCTTTAAATTAAATTAATATTATTTTTATTCTCCAATT
AAGAAATAAAAGAGTGCTAAAAAATACAATTACATATTTATTTGTATAAATAAATATCACTTTTGATTCAA
TATTTAGTTAGATATTTCAATTTAGATTAGTGCGCTTGGGATTGGGGGTAGGTCGTGTTCTGCGCTAAATA
AGTAAGAAAATTGTTTCTCCGCCAGCCAGAGACTTTGCCAGAGCTATCCACTGCAACACATTCAAATATG
CTGTGTTGTATGTATATTCAAACACATACATACATAATTGCTTGCACTTCCCTCATTATTTCTTTAATTTATT
TTATATAACTTAAAAATAATAATAATTTAATTAATAATATCAAATATAAAAAATTACATAAATATTTGCAT
CGACTTAAAAATACATATTTTGTATAGAGCCAACCTCAGTACACAAAATGCATGTATATATCAACATAAA
TTTTGGGTAGGTCGTGATTTGCGCTAAAAAAAACTAAGAAAGTTGTTTTTTTGCCAGCCAGAGACTTTGCC
AGAGCAGTGGATAACAACACATTCAAATATGCTGTTATGTATGTATATTCACACGCATACAAACATAATG
GCTTGCACAGTCGTCACAGAGCCGAAGCTGAGAGCAAGTTCTCAAGCAAATTTCAAAAAGTCAAGCTGCA
TAGTAGCATTTTGTTGTATGGCGTTACTCATCTTAGTGTTTGTATTGTCTTTGGTTTCACTATGCTCCTCATA
TTTATGCAAAATCCTATTAAGATCGGACCACTATATCATATAGCTACCATAGGAACGATCGGTTGAAAACT
AAGTTGTTGTATGAACAAACAATTTGTTTTTCAAGATATCTTGACCAAATTCGGCATTTATTAGTTTTTTTA
TGCTCCTCATATATAGGCAAAATACTATTAAGATCGGCCACCAATATCATATAGCTGCCATAGGAACGAT
CGGTCGAAAATTAAGTTGTATAAAAAAACATTTTGGTTCTCAAGATATCTTTTCTAAACTCGGCATTTATT
AGTTTTACTATACTCCTCATATATAGGCAAATTAGTGGCGCCAAGTCACGTTCATACAGACAGACGGACGT
TTTCAACCTTTTGACCATTTTCCCTCGTGCCGTTGTTCAGCGGACTGGCTGCACAGAAGGCATAGTAGAAA

AAGGCCAATATTAGATAGTTTTATTAAAGGGGGTTGGTCCATTGGGACAGTCTTCTTTTCTAAACTCGGC
ATATATTAGTTTTACTATACTCCTCATATATAGGCAAATTAGAGGCGCCAAGTCACATTCATACATACGGA
CAGACAGACGGACGTTTTCAATCTTTTAACCATTTACCCTCGTGCCGTTGTTCAGCGGACTGGCTGCACAG
AAGGCATAGTATAACAAGGCCAATATTATATAGTTTTTATTAAAGGGGGTTGGTCCATTGGGACAGTCTTA
AGTAAGATTAATATATTTATAAATTTTGTATATAGATAATGAAACTCGCAAGCTTTCTGTATCAGCAGTCT
GGTGAGTAAGTTCTCTGTACATCCATTTTGTGTCCGATTATCATTTCTCAAGCTGCTTTCTTCTTATTCTTTT
CAAATTTCTTCGTCACTAATGTGAATGTTTCTTTTTCATGCTGGCCCTAACTGCAAGGCTCAAATTCAAGA
ACCGGGCGGTCGTGCGATAGCGAAATGGCGGGGCGTTGCCGTGTTCCTCCAAAATGTTGGGGCTGATTCT
GCGATACCCTAAATACAGTGAAAATGGGGAAAAGTTGGTTTTGAATCATAGTTTTGTGTAAATATATGTA
ACAGTAAGAAGGAAGCACCTCCGATACCATAAAGTATATACATTCTTGGCCAACATTAATAATCAAGTCG
ATCTCGTTTCAGTATGTTGAAGGTTGTTCAAGCCCTCTCAGTTTGTTTCCGATAGTCGGTAACATCCACAAT
TCCAAGCAATTGATATAAATCTATATTAAATTCCTGTTCTTTATGAATACCTCTTAAGCTTCAAANGCTATG
CCACAATACAAAGATATACCAAACAGCCTTTGTATCGGATAACGATAAATCGCAACGAGGGAACCCCAA
GGGTGCCTGCGACTATTTTAGTGTGCCCAGACAGAATAAGACTGCTGAGTGTGGCAGCCGCTGAGCGAGT
TGCGGTTTTATACTGAACGTTCGGCTCTCGAGGCGGGCGTTCGGCTGCCACTCTCAGCAGTCCTACTCTGT
ATGGGCACACTAAAATAGTCACAGGTACCATTTAGGTTCCCTCGTTGCGATTTATGGTTATCCGATAACAA
GGCTGTATGGTTAGTGGCTATGCTCAACAACCCCGTAAAATCAAACAAATAGAAGTTCCTATTGGAATCA
CACAATGTAAAACTAAATTTTAAGTCTATTGCCGTTTTAAAAATAGACGATTAGTCAATGCACAATAGGAT
CTAGTTCAGGAAGATCGAGTAAGAAACACAGCAGCTATAAACAAAACGTGTGGTTATATTGGTTAGCCGC
TTGGAGGCCAACGCAAAAGTAGTTTCTGCATGCATGGTGTATTGTATTATAATATCCTTGCAGAGGGTATA
ATAATTTTGTCGTGAAATCTGTAACGCATAGAATGAGACATCTCCGACCCCATAAAGTATATATATTCTTT
ATCAGCATCAACAGCCGAGTCAGGTCCCTCTGTCCGTCTGTCTGTTTCTATGGGACCTCGTCTCTCAGATTT
TAAGCTATCTTAATGAAACGTTGCAGAAGTCCTTTTTTTGTTGCACGCAGCACATATGTCAAAACCAGCTG
GATCGGACCACTATATCATATAGCTGCCATAGGAACGATCGGTCGAAAATTAAGGTTTTTTATGAAAAAG
CATTTTGTTCATCAAGATATCTTGACCAAACTCGGCATGTACAGCTGTGAAAACAAATAATAGCACCACTA
CAACACATTTTGTTCTTGATGAAAAAAAAAATGCGATTACTGTTCCGATTTTTGTGAAANTTGATTTTATTTT
ATACTGTTTGGNATTCCCCATAAACAAAAACAGTGTTAGCGTTCTTCAAATTTCTGTCTATATTTTCTTTTT
GGGGGATTTTAAGAAAAAACCCCCCAAAATGCAGAAAAAAAAAGTAGCACCATTAGCCNCCTCCTTAGCG
AATCCTCGTTATATCGCGGAATTGCCTTTGCAGAATGAACGTGCTTATTAGTTTGACTAAGATATCATTTT
TTCTATCATAAAAGTTAATAATAATCATTACGATGGAACGTGGAATGCACTGCACTGCANAGAAGCGCGA
TCTTATCAAAAAATTGATATTGGACGGAAAAACTTACAGAGATGTTGGTCAATTTGTAGGATNCTCCAAT
AAAATAATTAAAAACGTCTTAAACTATCAAAAAAATATTGAAACACGCGGACGATAACGATCTATGACCC
CTCTTCATGTCAAANGACTGATTCGTCAGAACACGAAGGATCCCTTCAAGCCTGCTACCGAGCTTAAAAA
AAGAGCTTAACGTAAAAGTGACGGAACAAACAGTTCGCTAATATCTTAGGCAACATAACCTAAAAGACTG
CAAACCAAGAAAGGTACCACTTTTAAACGCGACACCTATTACTAAGCGCATGAAGTTTGCCCAGGGATCG
TGCAAATTGGACATCCCAACAATGGAGGAATATTCTATGGTCAGATGAGTCCAAGGTGGCTATGATTGGT
GGAAAGGGATCTCGATCATACGTTAGACGTCCAAGGAACACAGAATACAAGCCCCAGTACACCACNAAA
CCCATTAAGCATGGAGGGTGTAGTGTAATGGTATGGGCCTGCTTTTCCTACTATGGAGTAGGTCCAATACA
TTGGAGAAAACCATCATGGATCAGCACGTGTATGTGAATATCATGGAGACAGTTATGCTACCCTTTGTAG
AATACGAAGTGCCCCTCGTACGGGTNTTTCAACAGGACAATGACCCCAAACACACCAGCAAGAAAGCAA
AGCAATGGTTCAGGGCAAACTTGGAACGTTTGCTGCTGAGTCGCCTGACCTGAATCCCATCGAAAATCTG
TGGTCCGACGTCAAGACGTCAAGGCAGTTGCTGCTTCCAAACCAACCACTATCGATCACTTTGGAAGAAA
TTAAAGAGTCCTGGTCCAAAATTCCCCTAAAACGATGCCAGGACTCGGCTGATTCCATGCCAAGACGCTG
TGCAGCTATTATTGCTAATAAAGGACACTGTACTAAATATTAAATAATAAATATAGATTAAGTATTAATAG
ATTAAAAAATGAATTCCTCACTTATTAGCCATTTCTATATTTTAAGTCGATTTTTGAAAACTGGTGCTATT
ATTTTTTTCGGNTAATTTAATCAATATCAATTTCAGCACATTTTCTTAAATAACTTAAGAACGTTAAGCAAT
AGGATGATTTTCTTTTCATTCCCATNTTTGTAAGAGTCTTCCCTTTCTAAAAATGTGTTTCAAGTTCTAAAA
ATGTGTCAAGTTTCCTTACTGTTAAAAATGTGGGTGTGGCAACTATTCAAAAATNNNTTGTGGTAGTGGTG
CTATTATTTTTTTCTNAACTGTTATGCCAATTTTAAATCCTTACAGCCCAAAAACATATATGTGTATGCATG
TGCCTCGCTCTCACTCATTGCTTCGCGCACTTCCATCCATCTCTTTCTCCCTGCATAGCTTCCCACAAAACC
CCGAGATCTACAAACATTCATATGTAAGTTAACAACTAAGCATAAATATATTGCCCTAGCAACTAAGTAG
CTTCGTATTAACAATGCTGACGCGCCAGAACTGAGTTCAGCGCTCTGCGCTAAGAACGGCCAGCAGCGGT
AAGCTGACACTGCATGGAATGCTGAGTCGGCTTGCCGACCATGCCTTGATGGCGTGATGCATTGAGGCTT
AGGGTATTTGTATAATTTGCTTGTCCCCTAGTTTTAGTTTTATATGAGGCGCACAACACATAAGTCGCTGA

AATAAATATACTGCTCCGGCATTGCCGATAAAACATATCTTTCGTTATTGTCTGAGCCCAAGGCCAGCAAT
AGAAGCCAACTTTAAAACATAATCTTCAGTAATATTACTGGATCAAGGATAACTATATTTGGATCAAGGG
CAGCGTACAGCTGTGAAAACAAATATAGCACCACTACTACACATTTTGTTCTTGATGAAAAAATGCGATT
ACTGTTCCGATTTTTGTGAAATTTGATTTTTTTTTATACTGTTTGGGATTTCCCATAAACAAAAACAGTGTT
AGCGTTCTTCACATTTCTGTCTATATTTTTTTTGGGGGGATTTTAAGAAAAAACCCCCAAAAATGCTGAAA
AAAAAGGTAGCACCATTTGCCCTTTTCTTAGCGAATCCTCTTTATATCGTGGAATTGCCTTTGCAGAATGA
ACGCGCTTATTAGTTTTGACTAAAATATCATTTTTTCTATTATAAAAGTTAATAATAATCTTAACGATGGA
ACGTGGAATGCACTGCACTACAAAGAAGCGCGATCTTATCAAAAAATTTATATTGGACGGAAAAACTTAC
AGATTTGTTAGTCAATTTGTAGGATCCTCCAATAAAATAATTAAAAACATCTTAAACTATCAAAAAAATAT
TGAAACACGCTGACGATAACGATTTATGACCCCTCTTCATGTCAAATGACTGATTCGTCAGAACACGAAG
GATTCCTTCAAGCCTGTTACCGAGCTTAAAAAAGAGCTTAACGTAAAAGTGACGGAACAAACAGTTCTCT
AATATCTTAGGGAACATAACCTAAAAGACAGCAAACCAAGAAAGGTACCACTTTTAAAAGCGACACCTAT
TACTAAGCGCATGAAGTATGCCAAGGATCGTGCAAATTGGACATCTCAACAATGGAGGAATATTGTATGG
TCAGATGAGTGCAAGGTGGCTATAATTGGTGGAAAGATATCTCTATCATACGTTAGACAACCAAGGAACA
CAGAATACAAGCCCCAGTACACCACTTAATCCATTAAGCATGGAGGGTGTATTGTAATGGTATGGGCCTG
>9_rnd-1_family-388__Unknown
TTTATTAGAAGTTTCTGCTGCATATAACGAACTCTGCTGGTTAAGTGGTTTTCACATATTTTTGGTTGGAGC
GATGGAGGTGGAAAAGGAGACCACAAGGAGAGTGCTGAGCGCATCTGGGATCGACTTAGTGAATGAGTC
TCTCTCAGTGAGTGAGTCTGTCTCACTGAGTGACCTCTCACGCTGAGTGAGGNTCAGTCAATGCTGTGTTC
ACACTAGCACCGAACNGGCATGAACATTCTCCCCCTGGCAATATGCAGATTGCAAGAATTCCTATTGCGT
GCATGTGCCAGAGAGTATCCATCCAAACTTAGAATTTTGAGCCATCGGCAGACCGCCTTGGCTCGGGAGG
ATACCTGGTAGGATGATGTCTGGGTAGACGTCGGCGCCGAGTACCAAAGGGACTGGAGATGGACGGAAC
CATTCGTTGTCCGCCNAAGCGACGTTGCTGAACGGGTCCTTCATGTCCGCTCTGATGGCTCGGGCTGGAGT
TATGCAGCTGAGTTGCGGGTCAGCCACGAAGACCACTTCTCGAGACATCTGAGTTGTCTTGGGCGTAGCA
CGGCTGTGCACGCTCCTTCTGCGCCAATGCGTGTGACGGAAAGGCCATAGGCCCTTGCCACGGTTGCATTA
ATGCGGCTTACTGAACAACATGGATCGATCAGGGCCCGAGTCTCAAGGGTCTTTTTCCGGTGTCGAGCAG
CACTATGGCGGTGGAGCAGTATACCGACCCCCTTGTGCTGGAGAAGGGACGTAAGCGACGTCCGTATAGT
AGCACGTNTCGGGCTTGGGGGTGATGTGCCGCTGGAGGGCGAGGGGCCAGAAGCTCTTCTTGTGGGCCTG
TTGTCTGACCGAGAGGGTCGAGCGCTACGGGGATCACGCATGTGGAGTAAGGTGTGGTGGTCCTTTTTGC
AAATTTTGCACTTGGCTCCACTGCGGCATGATTCCCCTGAATGCTGATGAGCCAGGCAGTTGGAGCAATAT
TTGTTAATTAACACGGCTCGGAGACGCTTCTCGGGCGTGAGCTTCAAGAAGCGCTGACACTTGCGTAGCG
CATGGATGCCTCTACACACCCGGCATCTAAAGGAGTCCGTTCCTTGACGTTGATTGGATGCCTTCGTTGAC
GGTGCCATGCTGGTTCTCTACAGAGGAGTGAACTGGAGAATAAGATGTAGGANAGGTGTTATTATGTGAT
GGTTTTGTTTTGAAAGAGGGATTAGTTGCTTTTAAGGATTAGTCGGTGGTGCTATTTGCCTCAGTAGGAAG
AAGGACCAATTTGGCTATTGGCCTTCGGATGACACCACGAGCGGTAAGCACGTCCACTACGCGGACCTTG
TCATCTGTGCCTGGGCAAGCCAGCTGGACTCGGCCAAGGCGCCATTCATTTATTGGTATGTTATCTTCCTT
GATAATCACCATATCACCGACTTGGAGGTCCCTTGTAGGGGACTGCCATTTGTTTCGCTTATGGAGCTCTT
TCAAATACTCATTCTTCCACCGTAAGCAAAATTGCTGGTGGAGCGCTTTGAGTCGCTGCCAGCGGTTAATT
ATAGAAATAGCGTCTTCTTTGATTACTGGCTCGTTAGTTGACAGAAGAGGGCCACCAACTAGGAAGTGGC
CTGGACTNAGAGCCAATAAATCAGTCGGATCTTCCGACATTGGAGAGATTGGCCTGGAGTTAAGGCAGGC
TTCAATCTTGGAGAGGAGAGTCGCGAACTCCTCAAATGTATATTTTGAGGATGCGGTGGACTTGTAGAAG
TGGGTCTTAAAGCTTTTGACACCTGCTTCCCACAATCCGCCCATGTGAGGAGCGCCAGGAGGATTGAAAT
GCCAACACAAGTTTTGATGGCTATGCTTGGATAAGATGAGCTCTTTGGTCGCTTGCAAGAAATCTCTCGAA
AGAACGGATGACGCTCCCACAAATGTTTTCCCATTATCTGAGTACATCTGTTGTGGAAGGCCACGTCTTGC
TATAAATCGCGAAAATGCGGCCAGGAATCTCTCCGTGGTTAGATCTGTGGTGGCTTCTAAATGGATTGCTT
TCGTGCTGAAACAAACGAAGACACAGACATAACCCTTGGTAATTTTGCATGCTCGACCAATGTAGCTTTTA
ATATCGAAAGGTCCGGCAAAGTCTACACCCGTATGTGTGAAGGGTCGGGAAAAGGTTGATCTCGCCCTTG
GGAGTTCGCCCATAAGTTGGGTCTGGAGTCGTCTACGGTGTATTACACAGACCTTGCAGGAGTTTATGGTA
GATTTTACCAGGACCTTGAGCTTTGGGATCCAGTATTTGGCTCTTATGAGGCGCATTACCAACTGATTGCC
TCCGTGAAAACAAATGAGGTGGGTAAATCGGACCAGGAGGCGTGAAAAATCACAATTGGCCGGAATGAT
TATGGGGTGTCGCTCATCATAGCCTAACGAGGTTGACGCTCTCACCCGTCCACAGGATCGCAGAATACCC
CTTTGATCGATGAAAGGGGTTAAATTGCGAATGCTGCTTGATCCTTGAATTGGCTTCTTATCGAGCAAACA
GCTATACTCCTTAGGATAAGCTTGGCGTTGAGTATACTCGATAAGTCGGAACTGAACTCTCGTAAGTTCTT
CATTACGGAGCTCGGCGGGAAAGTGGATCGCTGGATTTCTACAGCGCTGGATAAAGCGACAGACATATGC

GATAACTCGCAATGCCTTGTCGAATCTGGAGAATCGTTCCAGAAGGTTATAGGAGGGTAGCTGTGAGACA
TTGCACTTCACTGCGCGTTGTTCAATCTCTGTCTCAAGTACCGTAACAGGAACTGGCCATTGGTTTTGTGGT
AGTTGTAACCATTCCGGTCCATGCCACCACAAAGTGCTGCCCAATAGGTCCCGAGGAGAAACCCCCGACT
AGCTAGGTCAGCTGGGTTATGTTCGGATCGAACATGGGACCATTGCCATTGGTTGATGATATCTTGGCTAC
CCGGTTAGCCACAAAGGTAGACCATTTGCATGGAGGTTTATCCAACCAGGCAAGAACAATAGTGGAGTCT
GTCCAATTGAAATTTTCNTTACTCTGGATGGGGAGCTGAGGAATAAGAGCCGTGGTGAGCTCGGAAAGAA
GGACGGCGCCACACAGCTCTAAGCGTGGTAGTGATACCGTCTTAACTGGCGCGACTCTCGTTTTTGCTGCG
AGAAGGTGAGTGGAGATGCATCCGTTGATCTCGACGCGCAAATAAAGTGCAGCACCATATGCGCGCTGGG
ACGCGTCGCAGAAGCCGTGAAACTGCACCTTTGCCTTTTGCTGAAAATCGACCCATCTTGGAATACGGATC
TCTTCGAGAGAGGGATAGTCGGTTAGGAAGTCATGCCAACGTTGAACGAGATCTTCCGGCAGGGAATCAT
CCCACCCCAGCTCTTGGAGCCAGATCTCTTGCATGAAAATCTTTGCTCGAACTATCACAGGTGCTAGCCAT
CCAGCAGGATCGAACAATTTCGCAATCTGTGATAGTACTTCACGTTTAGTGAGCGAAGGCTGAGATACCA
TTTTGGACGGAGCAAAGAAAAACTGGTCTGTTGTGGCTTGCCATCGAATGCCTAGCGTTTTAGCGACGCTG
GCCTCTTCGATTTCTAAGAAATCCGCACACAGTAAGTGGTCCCTTGGTATTCCTTTTAACACTTCCTTTGTG
TTGGATGTCCACTTTCTCAGAGGGAACCCAGCTCTGTCGAGTGCCATGCGGAGTTCTGCAATGGCTGAAAT
TGCTTGTGGCTTCGTGTGTGCCCCTGCTAGAACATCATCGACATACATATAATTTTCAATTATATCGGATG
CGATGGGACAATGTTCTCGTATATCACGAGCAAGTTGACGGAGGACCCGGATTGCGAGGTATGGGGCACA
GTTTACCCCAAAGGTAACTGTATTGAGTTCGAAGTCGCAAAGTTCTTCGTTCTCATTGCGAAATAGTATTC
GTTGGTAGGGCGTATGTCTTGGATCGACCTGGATTTGCCGATACATCTTGGTTATATCGGCATTGAAAACG
TAGCGGTAGAATCGCCACTTTAGTATCTGAATCGTCAGATCGGATTGGAGTACTGGTCCCGTGTGTAGGAT
ATCATTTAGACTTTTGCCATTGGAAGACGGGCTGGAGGCGTTAAACACAACGCGAACCTTTGTTGTGGTGC
TGTCGGGCTTAAAAACAGCATGATGCGGCAGGTAAAAATTATTGGGGTTGCTGTTTGGAGAGACTTGTGT
CATATGACCTAAGTCGAAGTACTCCCGAATCACGGAGTCGTACTGCTCTTTCAAAGGAGGGTTTCTTTTTA
AACGATTCTCGTTCTTTAGAAACTGAGCTAGTGCGATAGATCTTGAATGTCCTAAGTCTATGTGATTAGGT
TCTTGGAAAGGTAGGGTGACTACATATCTGCCATTCTCGTTCCTTGTTGTTGTTCGGATGAAATTACTCTCA
CAAAAGGAGTCGGAGCTCTTTACCAGTTTGACTGGAATGTCCTCCACCTCCCAAAATTTTGTGAGAATGAC
GTCTAATTGCTCTTCTTGAGAGACAGATATTCGAGTCGAGAATGACGAGATTGCGGTGGATGCGGTCTTTG
AAACTGGGCCTGTTAAAATCCACCCGAAAATAGTTTCCTGGCCCAGCAACGTGCCACATATATTAGGATG
GGAGCCGCCCGTTAGTATGGACGGTAGGATATCAGCGCCAATCAGCACATCTATCTGCGAACTCTTGTGG
AAATTAGGGTCCGCTAACTGGAGTTTTGGCAGGGTTGTCAATAAACTTTGGGGGATCGAACATGATGGTA
GATTCCCTGCCAAATGTGGTAGGACGTAGGCTGATGCCTCGATTTGGATACGAGGCTTGGAAGGAGAGCC
AATATTAAAATGGCAGAGCTTGCGAGGCTGGGCTGCAACAGCTTGATTTAATCCCGATACTTGTGCCTGTA
CGGATTTGTACGGCAGCTTTATCAGGTGGAAGAGTCGCTCGGAAATAAATGTTGCCTCTGATCCCGAGTC
GATCAGTGCACGAGCTGTATAATTTACACCCAAATGGCGAACATATATAACAGCCGTACTTAGGAGAACA
CCGTGGGTGTTAATTGCAACATAATTGTGAACATTCGTTTGTTGGGATGGAGTGGACTGTATGGATTGATT
GGAGGGAGCTGTTGCTTGTGGCATGGAGACTACTGTAGGGCTCAATTGTGGAGTGTCACTACGATGCAAG
AGCGTATTGTGACGACCCTTGCACGTAAAACAATTGTGAGCACTTGTGCAATCCCTTAACTGATGACCCCT
AGCGAAACAGTTCAAACATAGTTTTTGCTGTTTGATGTAAGTTGCCCGTTCGCTGACGGGCATTTGGAGAA
ATCGTGGGCACAGACGGATAGGGTGATTCTCCCTGGAGCACAATTTGCAGGCTTGCGGCTTGTTAGCGAC
CTTATTCTCAAATGAGTTAAGCCTTCTAGTGGTAGGAGGTTCTGTTCGAACAATTCTGGAGTGCGCTGGAG
TATTTGCGCTTGGCTTAAGCTCTTCTATCGCTTCTAGAGTGCGATAGCGTTCTAAGAGAAAGGCGTTCATC
TCAAGCCAAGATGAGATGTCGTTTTTTCTGGAGATGGACTGTTCCCATAAGGAGAGTGTTAATTTGGGTAA
TTTGGAGGAACATAGGAACACGAGGATACAGTCCCAGTTTTCTATGTTGATTTCGGATAACTCCAGAGCT
GTTAAGCAACCTTGAATGGTGCTCTGAAGTTCCTTAATTGCCGTTCCAGACTCTTGGGAGATTGGCTGCAG
GTTGAAAAGAATCTTGAGCTGGCTGTTGACTAACAATTTTCGGTTCTCAAAACGCTCAGTCAGATTGGCCC
AGGCTGATCGGAAGCCATCGTTAGTCAAAGGTGACTTCGATACAATCGCCTGAGCCTCACCGCTGGTTTTC
GCATTGAGATGGAATAATTTCTCGACCTCTGATAACCTAGGATTATTTATGTAAATTGCGGTAAAGAGGTC
TCGAAATGTGGGCCAACGTATGTAATCTCCGCTGAAGATTTCTGTGTCGCAAGGAGGTAAGCGACACCCT
GTGGGAACATATGTCGGAATGGTGGACTGAGCGGATGGTCTATTTTGGGCGTTAGCGGTATCGATTTGCTC
GCTAAGTTTAGCGGCGCATCTCTCATATACCATGTAACAATAGTCGTACTTGGACTGCATAACTGAAATGG
TTTCCAGTGAACCGGCTTCCGAGATTATTGTTGAGCCGACCTCGTACTCTTTTTCAACCTTGTCCCACAAGG
CTCGCACTTGGTCCCGACGGACCTGGCACGTATAGAGAGACGGGTCAGAGCCTCCAGGAGTGTTTATTTTT
GCCTCAAACTGACTTAGCCGATCAGTGACAGCAATAAACTTGATTAAGGCCGCGTCTGTCATATTTTGGGC
CGTTTTGGTATTAGCTCGCGTGGAAATCGGACTAGATCTTGTGCTAATGGTTGCCTTAGTGCGTCCGACTG

CAGTGGTCAGGGATGCTTCGCTTTTTAGCTTTGGACGGCGGGCGACTGATTTTTATCAGTGGACTTGGCCT
CGAACTAAGAGGTGCCTTAACAGTTCCCCTTGGCGTGGACGCGGATGATTCGCTTTTAGTCCGGGACGAT
GTTTCCCTTTCGACTCTAAGGGCTGCTGTTGGAGACGGAGAAGTTCTGGAGGTGCTTGCGGACGGAACTG
CTAATTGATGCTTTGTTTCTTCCCCTTTGCCAGTTGGCATACTAGGCGGAAGCGCTTGGGTAGGAAAAACA
AAGGTGCTCGTTTCTAGTAGTAGAGGTTGCGATGCTTAAAACAAAGAAACACTGGAATGCCGGTACAGGT
AAATAATCGAGTTTATAATTCGATTGAGATTGCGGAAGATTTAAATAATTTTGCTTAAAATCAAAATTTAT
TTGATTTAATTAAATTTAATTAATAAATTTATTTGACTTGGTAATTTAAAAAAATACGATTATTTATTAAACA
CGGGAAATAATAATAAAGAATTCGATGCTCGTTTTCGATAATTTTATTATTATATTAGTCTTTTGAATACG
GTAACGGTTGGTTTATTTTACTTTTTGTGGTACTCATAAAAATACCCACTATTGTGGCTGAGTACACACCG
CTGACAAAATGTAGAACTTCGAAGTTAATGTGTAGTGTTTTTGTTTATTTTACAATAAATTAGTAGTATTAT
TAATCTATTAATTATGATTGTTGTGTTTTTATTTGGTGCGTCTCGGAACAAAGAGTAATTGTATGGGAATGT
GTGCAATTGCATAAAAACGTGTAATTGGAGTACGGATCGGCAGCTGTGGTTTTTACATATGTGTGTGGTGT
GTACATACGCACATACGCCGGTTGAAATTGCAATTTATTACTATAATATTGCTGTGTTATAATATTGCTGT
GGATTGTGCACTAAAAATATTATATGTGGACACACATAGTACATATGATTTGTGGATTTATTTATCGGATG
TATGTACTATGTGGTTGCACAAATATATTCGTTTGCACTATGCACAATTATAAGTTAAGAAACTGAATTGG
AGTTTGTGTTTATTTTTATTAAGTAATACAGGGTGCAATAAATTATAAGTTAAGAGAATGAATTGGAGTTT
GTGTTTATGTTTATTAAATAATAAGGTTAAATATAGTGTCTATTCAAAATGCATATGGGCAATTATATTCG
GATTGAATATATGTATGTATGTATGTATGTACAATACGTGCTGGTGAACCTGGTGTATGTTTGTGTGTCGG
ACCAAGAACATGAATTAACAATATTAATTAGGAAACAATATTGTGAAGCAACTTGTCGGATTAAGTGCGG
ACACGTAATTAAAAAAATNTTTTTTATAGTTAGGAAAGTGCACGAGTGCGTACGTATGTACATACAAAAA
ATGTTGAAGTGGATTTCTTTTGTAGTTGGAAGTAACTAACACAAAATTTAACTAAACTGTTTTTAGTTTTGT
GGTCGGATAATTTATATATATTCAGAAATATATATTGCCAATCAGTTTTCTTAAAGAAAAAAAATATGGCG
GAAAAATATGGCGGATATTGCCGAATATATATAGAAAGGAAATTTTGTATTTACTTATCTTTGCCAAAAA
ACTTGTGTAGGGGCGATCGGTTTATATATAATCCGGCTCGAAGGACCAATGTTTGTGGGGGTGGCGTCAG
ATAAATAATCCTTGATTATTGTTGTTGGTGAAAAAACCACTTTATTAGAAGTTTCTGCTGCATGTAACAAA
CTCTGCTGGTTAAGTGATTTTACATATTTTTGGTTGGAGTGATGGAGGTGGAAAGGAGACCACAAGGAGA
GTGCTGAGCGCATCTGGGATCGACTTAGTGAGTGAGTCTCTCTCAGTGAGTGAGTCTGTCTCACTGAGTGA
CTCTCTCACACTGAGTGAGGCTCAGTCAATGCTGTGTTCACACTAGCACTGAACTGGCATGAACA
>9_rnd-1_family-306__LTR___Pao
TGTTCATGCCAGTTCAGTGCTAGTGTGAACACAGCATTGACTGAGCCTCACTCAGTGTGAGAGAGTCACTC
AGTGAGACAGACTCACTCACTGAGAGAGACTCACTCACTAAGTCGATCCCAGATGCGCTCAGCACTCTCC
TTGTGGTCTCCTTTCCACCTCCATCACTCCAACCAAAAATATGTAAAATCACTTAACCAGCAGAGTTTGTT
ACATGCAGCAGAAACTTCTAATAAAGTGCTTTTTTCACCAACAACAATAATCAAGGATTATTTATCTGACG
CCACCCCCACAAACATTGGTCCTTCGAGCCGGATTATATATAAACCGATCGCCCCTACACAAGTTTTTTGG
CAAAGATAAGTAAATACAAAATTTCCTTTCTATATATATTCGGCAATATCCGCCATATTTTTCCGCCATAT
ATTTTTTCTTTAAGAAAACTGATTGGNAATTTTCTGAATATATATAAATTATCCGACCACAAAACTAAAAA
CAGTTTAGTTAAATTTTGTGTTAGTTACTTCCAACTACAAAAGAAATCCACTTCAACATTTTTTGTATGTAC
ATACGTACGCACTCGTGCACTTTCCTAACTATAAAAAAAATTTTTTTAATTACGTGTCCGCACTTAATCCG
ACAAGTTGCTTCACAATATTGTTTCCTAATTAATATTGTTAATTCATGTTCTTGGTCCGACACACAAACATA
CACCAGGTTCACCAGCACGTACGTACATACATACATACATACATATATTCAATCCGAATATAATTGCCCAT
ATGCATTTTTGAATAGACACTATATTTAACCTTATTATTTAATAAANATAAACACAAACTCCAATTCANTC
TCTTAACTTATAATTGTGCATAGTGCAAACGAATATATTTGTGCAACCACATAGTACATACATCCGATAAA
TAAATCCACAAATCATATGTACTATGTGTGTCCACATATAATATTTTTAGTGCACAATCCACAGCAATAT
TATAACACAGCAATATTATAGTAATAAATTGCAATTTCAACCGGCGTATGTGCGTATGTACACACCACAC
ACATATGTAAAAACCACAGCTGCCGATCCGCACTCCAATTACACGTTTTTATGCAATTGCACACATTCCCA
TACAATTACTCTTGTTCCGAGACGCACCAAATAAAAACACAACAATCATAATTAATAGATTAATAATACT
ACTAATTTATTGTAAATAAACAAAAACACTACACATTAACTTCGAAGTTCTACATTTGTCAGCGGTGTGTA
CTCAGCCACAATAGTGGGTATTTTTATGAGTACCACAAAAAGTAAAAATAAACCAACCGTTACCGTATTCA
AAGACTAATATAATAATAAAATTATCGAAAACGAGCATCGAATTCTTTATTATTATTTCCCGTGTTTAATA
AATAATCGTTTTTTTAAATTACCAAGTCAAATAAATTTATTAATTAAATTTATTAAATCAAATAAATTTTGA
TTTTAAGCAAAATTATTTAAATCTTCCGCAATCTCAATCGAATTATAAACTCGATTATTTACCTGTACCGGC
ATTCCAGTGTTTCTTTGTTTTAAGCATCGCAACCTCTACTACTAGAAGCGAGCACCTTTGTTTTCCTACCCA
AGCGCTTCCGCCTAGTATGCCAACTGGCAAAGGGGAAGAAACAAAGCATCAATTAGCAGTTCCGTCCGCA
AGCACCTCCAGAACTTCTCCGTCTCCAACAGCAGCCCTTAGAGTCGAAAGGGAAACATCGTCCCGGACTA

AAAGCGAATCATCCGCGTCCACGCCAAGGGGAACTGTTAAGGCACCTCTTAGTTCGAGGCCAAGTCCACT
GATAAAAATCAGTCGCCCGCCGTCCAAAGCTAAAAGCGAAGCATCCCTGACCACTGCAGTCGGACGCACT
AAGGCAACCATTAGCACAAGATCTAGTCCGATTTCCACGCGAGCTAATACCAAAACGGCCCAAAATATGA
CAGACGCGGCCTTAATCAAGTTTATTGCTGTCACTGATCGGCTAAGTCAGTTTGAGGCAAAAATAAACAC
TCCTGGAGGCTCTGACCCGTCTCTCTATACGTGCCAGGTCCGTCGGGACCAAGTGCGAGCCTTGTGGGACA
AGGTTGAAAAAGAGTACGAGGTCGGCTCAACAATAATCTCGGAAGCCGGTTCACTGGAAACCATTTCAGT
TATGCAGTCCAAGTACGACTATTGTTACNTGGTATATGAGAGATGCGCCGCTAAACTTAGCGAGCAAATC
GATACCGCTAACGCCCAAAATAGACCATCCGCTCAGTCCACCATTCCGACATATGTTCCCACAGGGTGTC
GCTTACCTCCTTGCGACACAGAAATCTTCAGCGGAGATTACATACGTTGGCCCACATTTCGAGACCTCTTT
ACCGCAATTTACATAAATAATCCTAGGTTATCAGAGGTCGAGAAATTATTCCATCTCAATGCGAAAACCA
GCGGTGAGGCTCAGGCGATTGTATCGAAGTCACCTTTGACTAACGATGGCTTCCGATCAGCCTGGGCCAA
TCTGACTGAGCGTTTTGAGAACCGAAAATTGTTAGTCAACAGCCAGCTCAAGATTCTTTTCAACCTGCAGC
CAATCTCCCAAGAGTCTGGAACGGCAATTAAGGAACTTCAGAGCACCATTCAAGGTTGCTTAACAGCTCT
GGAGTTATCCGAAATCAACATAGAAAACTGGGACTGTATCCTCGTGTTCCTATGTTCCTCCAAATTACCCA
AATTAACACTCTCCTTATGGGAACAGTCCATCTCGAAAAAAACGACATCTCATCTTGGCTTGAGATGAAC
GCCTTTCTCTTAGAACGCTATCGCACTCTAGAAGCGATAGAAGAGCTTAAGCCAAGCGCAAATACTCCAG
CGCACTCCAGAATTGTTCGAACAGAACCTCCTACCCACTAGAAGGCTTAACTCATTTGAGAATAAGGTCG
CTAACAAGCCGCAAGCCTGCAAATTGTGCTCCAGGGAGAATCACCCTATCCGTCTGTGCCCACGATTTCTC
CAAATGCCCGTCAGCGAACGGGCAACTTACATCAAACAGCAAAAACTATGTTTGAACTGTTTCGCTAGGG
GTCATCAGTTAAGGGATTGCACAAGTGCTCACAATTGTTTTACGTGCAAGGGTCGTCACAATACGCTCTTG
CATCGTAGTGACACTCCACAATTGAGCCCTACGGTAGTCTCCATGCCACAAGCAACAGCTCCTCCAATCA
ATCCATACAGTCCACTCCATCCCAACAAACGAATGTTCACAATTATGTTGCAATTAACACCCACGGTGTTC
TCCTAAGCACGGCTGTTATATATGTTCGCCATTTGGGTGTAAATTATACAGCTCGTGCACTGATCGACTCG
GGATCAGAGGCAACATTTATTTCCGAGCGACTCTTCCACCTGATAAAGCTGCCGTACAAATCCGTACAGG
CACAAGTATCGGGATTAAATCAAGCTGTTGCAGCCCAGCCTCGCAAGCTCTGCCATTTTAATATTGGCTCT
CCTTCCAAGCCTCGTATCCAAATCGAGGCATCAGCCTACGTCCTACCACATTTGGCAGGGAATCTACCATC
ATGTTCGATCCCCCAAAGTTTATTGACAACCCTGCCAAAACTCCAGTTAGCGGACCCTAATTTCCACAAGA
GTTCGCAGATAGATGTGCTGATTGGCGCTGATATCCTACCGTCCATACTAACGGGCGGCTCCCATCCTAAT
ATATGTGGCACGTTGCTGGGCCAGGAAACTATTTTCGGGTGGATTTTAACAGGCCCAGTTTCAAAGACCG
CATCCACCGCAATCTCGTCATTCTCGACTCGAATATCTGTCTCTCAAGAAGAGCAATTAGACGTCATTCTC
ACAAAATTTTGGGAGGTGGAGGACATTCCAGTCAAACTGGTAAAGAGCTCCGACTCCTTTTGTGAGAGTA
ATTTCATCCGAACAACAACAAGGAACGAGAATGGCAGATATGTAGTCACCCTACCTTTCCAAGAACCTAA
TCACATAGACTTAGGACATTCAAGATCTATCGCACTAGCTCAGTTTCTAAAGAACGAGAATCGTTTAAAA
AGAAACCCTCCTTTGAAAGAGCAGTACGACTCCGTGATTCGGGAGTACTTCGACTTAGGTCATATGACAC
AAGTCTCTCCAAACAGCAACCCCAATAATTTTTACCTGCCGCATCATGCTGTTTTTAAGCCCGACAGCACC
ACAACAAAGGTTCGCGTTGTGTTTAACGCCTCCAGCCCGTCTTCCAATGGCAAAAGTCTAAATGATATCCT
ACACACGGGACCAGTACTCCAATCCGATCTGACGATTCAGATACTAAAGTGGCGATTCTACCGCTACGTTT
TCAATGCCGATATAACCAAGATGTATCGGCAAATCCAGGTCGATCCAAGACATACGCCCTACCAACGAAT
ACTATTTCGCAATGAGAACGAAGAACTTTGCGACTTCGAACTCAATACAGTTACCTTTGGGGTAAACTGTG
CCCCATACCTCGCAATCCGGGTCCTCCGTCAACTTGCTCGTGATATACGAGAACATTGTCCCATCGCATCC
GATATAATTGAAAATTATATGTATGTCGATGATGTTCTAGCAGGGGCACACACGAAGCCACAAGCAATTT
CAGCCATTGCAGAACTCCGCATGGCACTCGACAGAGCTGGGTTCCCTCTGAGAAAGTGGACATCCAACAC
AAAGGAAGTGTTAAAAGGAATACCAAGGGACCACTTACTGTGTGCGGATTTCTTAGAAATCGAAGAGGCC
AGCGTCGCTAAAACGCTAGGCATTCGATGGCAAGCCACAACAGACCAGTTTTTCTTTGCTCCGTCCAAAAT
GGTATCTCAGCCTTCGCTCACTAAACGTGAAGTACTATCACAGATTGCGAAATTGTTCGATCCTGCTGGAT
GGCTAGCACCTGTGATAGTTCGAGCAAAGATTTTCATGCAAGAGATCTGGCTCCAAGAGCTGGGGTGGGA
TGATTCCCTGCCGGAAGATCTCGTTCAACGTTGGCATGACTTCCTAACCGACTATCCCTCTCTCGAAGAGA
TCCGTATTCCAAGATGGGTCGATTTTCAGCAAAAGGCAAAGGTGCAGTTTCACGGCTTCTGCGACGCGTCC
CAGCGCGCATATGGTGCTGCACTTTATTTGCGCGTCGAGATCAACGGATGCATCTCCACTCACCTTCTCGC
AGCAAAAACGAGAGTCGCGCCAGTTAAGACGGTATCACTACCACGCTTAGAGCTGTGTGGCGCCGTCCTT
CTTTCCGAGCTCACCACGGCTCTTATTCCTCAGCTCCCCATCCAGAGTAAAGAAAATTTCAATTGGACAGA
CTCCACTATTGTTCTTGCCTGGTTGGATAAACCTCCATGCAAATGGTCTACCTTTGTGGCTAACCGGGTAG
CCAAGATATCATCAACCAATGGCAAATGGTCCCATGTTCGATCCGAACATAACCCAGCTGACCTAGCTAG
TCGGGGGTTTCTCCTCGGGACCTATTGGGCAGCACTTTGTGGTGGCATGGACCGGAATGGTTACAACTACC

ACAAAACCAATGGCCAGTTCCTGTTACGGTACTTGAGACAGAGATTGAACAACGCGCAGTGAAGTGCAAT
GTCTCACAGCTACCCTCCTATAACCTTCTGGAACGATTCTCCAGATTCGACAAGGCATTGCGAGTTATCGC
ATATGTCTGTCGCTTTATCCAGCGCTGTAGAAATCCAGCGATCCACTTTCCCGCCGAGCTCCGTAATGAAG
AACTTACGAGAGTTCAGTTCCGACTTATCGAGTATACTCAACGCCAAGCTTATCCTAAGGAGTATAGCTGT
TTGCTCGATAAGAAGCCAATTCAAGGATCAAGCAGCATTCGCAATTTAACCCCTTTCATCGATCAAAGGG
GTATTCTGCGATCCTGTGGACGGGTGAGAGCGTCAACCTCGTTAGGCTATGATGAGCGACACCCCATAAT
CATTCCGGCCAATTGTGATTTTTCACGCCTCCTGGTCCGATTTACCCACCTCATTTGTTTTCACGGAGGCAA
TCAGTTGGTAATGCGCCTCATAAGAGCCAAATACTGGATCCCAAAGCTCAAGGTCCTGGTAAAATCTACC
ATAAACTCCTGCAAGGTCTGTGTAATACACCGTAGACGACTCCAGACCCAACTTATGGGCGAACTCCCAA
GGGCGAGATCAACCTTTTCCCGACCCTTCACACATACGGGTGTAGACTTTGCCGGACCTTTCGATATTAAA
AGCTACATTGGTCGAGCATGCAAAATTACCAAGGGTTATGTCTGTGTCTTCGTGTGTTTCAGCACGAAAGC
AATCCATTTAGAAGCCACCACAGATCTAACCACGGAGAGATTCCTGGCCGCATTTTCGCGATTTATCGCAA
GACGTGGCCTTCCACAACAGATGTACTCAGATAATGGGAAAACATTTGTGGGAGCGTCATCCGTTCTTTCG
AGAGATTTCTTGCAAGCGACCAAAGAGCTCATCTTATCCAAGCATAGCCATCAAAACTTGTGTTGGCATTT
CAATCCTCCTGGCGCTCCTCACATGGGCGGATTGTGGGAAGCAGGTGTCAAAAGCTTTAAGACCCACTTCT
ACAAATCCACCGCATCCTCAAAATATACATTTGAGGAGTTCGCGACTCTCCTCTCCAAGATTGAAGCCTGC
CTTAACTCCAGGCCAATCTCTCCAATGTCGGAAGATCCGACTGATTTATTGGCTCTAAGTCCAGGCCACTT
CCTAGTTGGTGGCCCTCTTCTGTCAACTAACGAGCCAGTAATCAAAGAAGACGCTATTTCTATAATTAACC
GCTGGCAGCGACTCAAAGCGCTCCACCAGCAATTTTGCTTACGGTGGAAGAATGAGTATTTGAAAGAGCT
CCATAAGCGAAACAAATGGCAGTCCCCTACAAGGGACCTCCAAGTCGGTGATATGGTGATTATCAAGGAA
GATAACATACCAATAAATGAATGGCGCCTTGGCCGAGTCCAGCTGGCTTGCCCAGGCACAGATGACAAGG
TCCGCGTAGTGGACGTGCTTACCGCTCGTGGTGTCATCCGAAGGCCAATAGCCAAATTGGTCCTTCTTCCT
ACTGAGGCAAATAGCACCACCGACTAATCCTTAAAAGCAACTAATCCCTCTTTCAAAACAAAACCATCAC
ATAATAACACCTGTCCTACATCTTATTCTCCAGTTCACTCCTCTGTAGAGAACCAGCATGGCACCGTCAAC
GAAGGCATCCAATCAACGTCAAGGAACGGACTCTTTTNAGATGCCGGGTGTGTAGAGGCATCCATGCGCT
ACGCAAGTGTCAGCGCTTCTTGAAGCTCACGCCCGAGAAGCGTCTCCGAGCCGTGTTAATTAACAAATAT
TGCTCCAACTGCCTGGCTCATCAGCATTCAGGGGAATCATGCCGCAGTGGAGCCAAGTGCAAAATTTGCA
AAAAGGACCACCACACCTTACTCCACATGCGTGATCCCCGTAGCGCTCGACCCTCTCGGTCAGACAACAG
GCCCACAAGAAGAGCTTCTGGCTCCTCGCCCTCCAGCGGCACATCACCCCCAAGCCCGAGACGTGCTACT
ATACGGACGTCGCTTACGTCCCTTCTCCAGCACAAGGGGGTCGGTATATTGCCCACCGCCATAGTGCTGCT
CGACACCGGAAAAAGACCCTTGAGACTCGGGCTCTGATCGATCCATGTTGTTCAGTAAGCCGCATTAATG
CAACCGTGGCAAGGGCCTATGGCCTTTCCGTCACACGCATTGGCGCAGAAGGAGCGTGCACAGCCGTGCT
ACGCTCCAAGACAACTCAGATGTCTCGAGAAGTGGTCTTCATGGCTGACCCGCAACTCAGCTGCATAACT
CCAGCCCGAGCCATCAGAGCGGACATGAAGGACCCGTTCAGCAACGTCGCTTTGGCGGACAACGAATGGT
TCCGTCCATCTCCAGTCCCTTTGGTACTCGGCGCCGACGTCTACCCAGACATCATCCTACCAGGTATCCTC
CCGAGCCAAGGCGGTCTGCCGATGGCTCAAAATACCAAGTTTGGATGGATACTCTCTGGCACATGCACGC
AATAGGAATTCTTGCAATCTGCATATTGCCAGGGGAGAATGTTCATGCCAGTTCAGTGCTAGTGTGAACA
CAGCATTGACTGAGCCTCACTCAGTGTGAGAGAGTCACTCAGTGAGACAGACTCACTCACTGAGAGAGAC
TCACTCACTAANTCGATCCCAGATGCGCTCAGCACTCTCCTTGTGGTCTCCTTTCCACCTCCATCACTCCAA
CCAAAAATATGTAAAATCACTTAACCAGCAGAGTTCGTTACATGCAGCAGAAACTTCTAATAAAGTGCTT
TTTCACCAACAACAATCAAGGATTATTTATCTGACGCCACCCCACAAACA
>9_rnd-1_family-324__LTR___Pao
TGTTCATGCCAGTTCAGTGCTAGTGTGAACACAGCATTGACTGAGCCTCACTCAGTGTGAGAGAGTCACTC
AGTGAGACAGACTCACTCACTGAGAGAGACTCACTCACTAAGTCGATCCCAGATGCGCTCAGCACTCTCC
TTGTGGTCTCCTTTCCACCTCCATCACTCCAACCAAAAATATGTAAAATCACTTAACCAGCAGAGTTTGTT
ACATGCAGCAGAAACTTCTAATAAAGTGTTTTTTTCACCAACAACAATAATCAAGGATTATTTATCTGACG
CCACCCCCACAAACATTGGTCCTTCGAGCCGGATTATATATAAACCGATCGCCCCTACACAAGTTTTTTGG
CAAAGATAAGTAAATACAAAATTTCCTTTCTATATATATTCGGCAATATCCGCCATATTTTTTTTTCTTTA
AGAAAACTGATTGGCATTATTTTCTGAATATATATAAATTATCCGACCACAAAACTAAAAACAGTTTAGTT
AAATTTTGTGTTAGTTACTTCCAACTACAAAAGAAATCCACTTCAACANTTTTTGTATGTACATACGTACG
CACTCGTGCACTTTCCTAACTATAAAAAAATTTTTTTAATTACGTGTCCGCACTTAATCCGACAAGTTGCTT
CACAATATTGTTTCCTAATTAATATTGTTAATTCATGTTCTTGGTCCGACACACAAACATACACCAGGTTC
ACCAGCACGTATTGTACATACATACATACATACATACATATATTCAATCCGAATATAATTGCCCATATGCA
TTTTGAATAGACACTATATTTAACCTTATTATTTAATAAAAATAAACACAAACTCCAATTCANTCTCTTAA

CTTATAATTGTGCATAGTGCAAACGAATATATTTGTGCAACCACATAGTACATACATCCGATAAATAAATC
CACAAATCATATGTACTATGTGTGTCCACATATAATATTTTTAGTGCACAATCCACAGCAATATTATAAC
ACAGCAATATTATAGTAATAAATTGCAATTTCAACCGGCGTATGTGCGTATGTACACACCACACACATAT
GTAAAAACCACAGCTGCCGATCCGTACTCCAATTACACGTTTTTATGCAATTGCACACATTCCCATACAAT
TACTCTTGTTCCGAGACGCACCAAATAAAAACACAACAATCATAATTAATAGATTAATAATACTACTAATT
TATTGTAAATAAACAAAAACACTACACATTAACTTCGAAGTTCTACATTTGTCAGCGGTGTGTACTCAGCC
ACAATAGTGGGTATTTTATGAGTACCACAAAAAGTAAAATAAACCAACCGTTACCGTATTCAAAGACTAA
TATAATAATAAAATTATCGAAAACGAGCATCGAATTCTTTATTATTATTTCCCGTGTTTAATAAATAATCG
TATTTTTTTAAATTACCAAGTCAAATAAATTTATTAATTAAATTNATTAAATCAAATAAATTTTGATTTTAA
GCAAAATTATTTAAATCTTCCGCAATCTCAATCGAATTATAAACTCGATTATTTACCTGTACCGGCATTCC
AGTGTTTCTTTGTTTTAAGCATCGCAACCTCTACTACTAGAAACGAGCACCTTTGTTTTCCTACCCAAGCGC
TTCCGCCTAGTATGCCAACTGGCAAAGGGGAAGAAACAAAGCATCAATTAGCAGTTCCGTCCGCAAGCAC
CTCCAGAACTTCTCCGTCTCCAACAGCAGCCCTTAGAGTCGAAAGGGAAACATCGTCCCGGACTAAAAGC
GAATCATCCGCGTCCACGCCAAGGGGAACTGTTAAGGCACCTCTTAGTTCGAGGCCAAGTCCACTGATAA
AAATCAGTCGCCCGCCGTCCAAAGCTAAAAGCGAAGCATCCCTGACCACTGCAGTCGGACGCACTAAGGC
AACCATTAGCACAAGATCTAGTCCGATTTCCACGCGAGCTAATACCAAAACGGCCCAAAATATGACAGAC
GCGGCCTTAATCAAGTTTATTGCTGTCACTGATCGGCTAAGTCAGTTTGAGGCAAAAATAAACACTCCTGG
AGGCTCTGACCCGTCTCTCTATACGTGCCAGGTCCGTCGGGACCAAGTGCGAGCCTTGTGGGACAAGGTT
GAAAAAGAGTACGAGGTCGGCTCAACAATAATCTCGGAAGCCGGTTCACTGGAAACCATTTCAGTTATGC
AGTCCAAGTACGACTATTGTTACATGGTATATGAGAGATGCGCCGCTAAACTTAGCGAGCAAATCGATAC
CGCTAACGCCCAAAATAGACCATCCGCTCAGTCCACCATTCCGACATATGTTCCCACAGGGTGTCGCTTAC
CTCCTTGCGACACAGAAATCTTCAGCGGAGATTACATACGTTGGCCCACATTTCGAGACCTCTTTACCGCA
ATTTACATAAATAATCCTAGGTTATCAGAGGTCGAGAAATTATTCCATCTCAATGCGAAAACCAGCGGTG
AGGCTCAGGCGATTGTATCGAAGTCACCTTTGACTAACGATGGCTTCCGATCAGCCTGGGCCAATCTGACT
GAGCGTTTTGAGAACCGAAAATTGTTAGTCAACAGCCAGCTCAAGATTCTTTTCAACCTGCAGCCAATCTC
CCAAGAGTCTGGAACGGCAATTAAGGAACTTCAGAGCACCATTCAAGGTTGCTTAACAGCTCTGGAGTTA
TCCGAAATCAACATAGAAAACTGGGACTGTATCCTCGTGTTCCTATGTTCCTCCAAATTACCCAAATTAAC
ACTCTCCTTATGGGAACAGTCCATCTCCAAAAAAACGACATCTCATCTTGGCTTGAGATGAACGCCTTTCT
CTTAGAACGCTATCGCACTCTAGAAGCGATAGAAGAGCTTAAGCCAAGCGCAAATACTCCAGCGCACTCC
AGAATTGTTCGAACAGAACCTCCTACCACTAGAAGGCTTAACTCATTTGAGAATAAGGTCGCTAACAAGC
CGCAAGCCTGCAAATTGTGCTCCAGGGAGAATCACCCTATCCGTCTGTGCCCACGATTTCTCCAAATGCCC
GTCAGCGAACGGGCAACTTACATCAAACAGCAAAAACTATGTTTGAACTGTTTCGCTAGGGGTCATCAGT
TAAGGGATTGCACAAGTGCTCACAATTGTTTTACGTGCAAGGGTCGTCACAATACGCTCTTGCATCGTAGT
GACACTCCACAATTGAGCCCTACGGTAGTCTCCATGCCACAAGCAACAGCTCCCTCCAATCAATCCATAC
AGTCCACTCCATCCCAACAAACGAATGTTCACAATTATGTTGCAATTAACACCCACGGTGTTCTCCTAAGT
ACGGCTGTTATATATGTTCGCCATTTGGGTGTAAATTATACAGCTCGTGCACTGATCGACTCGGGATCAGA
GGCAACATTTATTTCCGAGCGACTCTTCCACCTGATAAAGCTGCCGTACAAATCCGTACAGGCACAAGTAT
CGGGATTAAATCAAGCTGTTGCAGCCCAGCCTCGCAAGCTCTGCCATTTTAATATTGGCTCTCCTTCCAAG
CCTCGTATCCAAATCGAGGCATCAGCCTACGTCCTACCACATTTGGCAGGGAATCTACCATCATGTTCGAT
CCCCCAAAGTTTATTGACAACCCTGCCAAAACTCCAGTTAGCGGACCCTAATTTCCACAAGAGTTCGCAG
ATAGATGTGCTGATTGGCGCTGATATCCTACCGTCCATACTAACGGGCGGCTCCCATCCTAATATATGTGG
CACGTTGCTGGGCCAGGAAACTATTTTCGGGTGGATTTTAACAGGCCCAGTTTCAAAGACCGCATCCACC
GCAATCTCGTCATTCTCGACTCGAATATCTGTCTCTCAAGAAGAGCAATTAGACGTCATTCTCACAAAATT
TTGGGAGGTGGAGGACATTCCAGTCAAACTGGTAAAGAGCTCCGACTCCTTTTGTGAGAGTAATTTCATCC
GAACAACAACAAGGAACGAGAATGGCAGATATGTAGTCACCCTACCTTTCCGAGAACCTAATCACATAGA
CTTAGGACATTCAAGATCTATCGCACTAGCTCAGTTTCTAAAGAACGAGAATCGTTTAAAAAGAAACCCT
CCTTTGAAAGAGCAGTACGACTCCGTGATTCGGGAGTACTTCGACTTAGGTCATATGACACAAGTCTCTCC
AAACAGCAACCCCAATAATTTTTACCTGCCGCATCATGCTGTTTTTAAGCCCGACAGCACCCACAACAAA
GGTTCGCGTTGTGTTTAACGCCTCCAGCCCGTCTTCCAATGGCAAAAGTCTAAATGATATCCTACACACGG
GACCAGTACTCCAATCCGATCTGACGATTCAGATACTAAAGTGGCGATTCTACCGCTACGTTTTCAATGCC
GATATAACCAAGATGTATCGGCAAATCCAGGTCGATCCAAGACATACGCCCTACCAACGAATACTATTTC
GCAATGAGAACGAAGAACTTTGCGACTTCGAACTCAATACAGTTACCTTTGGGGTAAACTGTGCCCCATA
CCTCGCAATCCGGGTCCTCCGTCAACTTGCTCGTGATATACGAGAACATTGTCCCATCGCATCCGATATAA
TTGAAAATTATATGTATGTCGATGATGTTCTAGCAGGGGCACACACGAAGCCACAAGCAATTTCAGCCAT

TGCAGAACTCCGCATGGCACTCGACAGAGCTGGGTTCCCTCTGAGAAAGTGGACATCCAACACAAAGGAA
GTGTTAAAAGGAATACCAAGGGACCACTTACTGTGTGCGGATTTCTTAGAAATCGAAGAGGCCAGCGTCG
CTAAAACGCTAGGCATTCGATGGCAAGCCACAACAGACCAGTTTTCTTTGCTCCGTCCAAAATGGTATCT
CAGCCTTCGCTCACTAAACGTGAAGTACTATCACAGATTGCGAAATTGTTCGATCCTGCTGGATGGCTAGC
ACCTGTGATAGTTCGAGCAAAGATTTTCATGCAAGAGATCTGGCTCCAAGAGCTGGGGTGGATGATTCC
CTGCCGGAAGATCTCGTTCAACGTTGGCATGACTTCCTAACCGACTATCCCTCTCTCGAAGAGATCCGTAT
TCCAAGATGGGTCGATTTTCAGCAAAAGGCAAAGGTGCAGTTTCACGGCTTCTGCGACGCGTCCCAGCGC
GCATATGGTGCTGCACTTTATTTGCGCGTCGAGATCAACGGATGCATCTCCACTCACCTTCTCGCAGCAAA
AACGAGAGTCGCGCCAGTTAAGACGGTATCACTACCACGCTTAGAGCTGTGTGGCGCCGTCCTTCTTTCCG
AGCTCACCACGGCTCTTATTCCTCAGCTCCCCATCCAGAGTAAAGAAAATTTCAATTGGACAGACTCCACT
ATTGTTCTTGCCTGGTTGGATAAACCTCCATGCAAATGGTCTACCTTTGTGGCTAACCGGGTAGCCAAGAT
ATCATCAACCAATGGCAAATGGTCCCATGTTCGATCCGAACATAACCCAGCTGACCTAGCTAGTCGGGGG
TTTCTCCTCGGGACCTATTGGGCAGCACTTTGTGGTGGCATGGACCGGAATGGTTACAACTACCACAAAAC
CAATGGCCAGTTCCTGTTACGGTACTTGAGACAGAGATTGAACAACGCGCAGTGAAGTGCAATGTCTCAC
AGCTACCCTCCTATAACCTTCTGGAACGATTCTCCAGATTCGACAAGGCATTGCGAGTTATCGCATATGTC
TGTCGCTTTATCCAGCGCTGTAGAAATCCAGCGATCCACTTTCCCGCCGAGCTCCGTAATGAAGAACTTAC
GAGAGTTCAGTTCCGACTTATCGAGTATACTCAACGCCAAGCTTATCCTAAGGAGTATAGCTGTTTGCTCG
ATAAGAAGCCAATTCAAGGATCAAGCAGCATTCGCAATTTAACCCCTTTCATCGATCAAAGGGGTATTCT
GCGATCCTGTGGACGGGTGAGAGCGTCAACCTCGTTAGGCTATGATGAGCGACACCCCATAATCATTCCG
GCCAATTGTGATTTTTCACGCCTCCTGGTCCGATTTACCCACCTCATTTGTTTTCACGGAGGCAATCAGTTG
GTAATGCGCCTCATAAGAGCCAAATACTGGATCCCAAAGCTCAAGGTCCTGGTAAAATCTACCATAAACT
CCTGCAAGGTCTGTGTAATACACCGTAGACGACTCCAGACCCAACTTATGGGCGAACTCCCAAGGGCGAG
ATCAACCTTTTCCCGACCCTTCACACACACGGGTGTAGACTTTGCCGGACCTTTCGATATTAAAAGCTACA
TTGGTCGAGCATGCAAAATTACCAAGGGTTATGTCTGTGTCTTCGTNTGTTTCAGCACGAAAGCAATCCAT
TTAGAAGCCACCACAGATCTAACCACGGAGAGATTCCTGGCCGCATTTTCGCGATTTATNGCAAGACGTG
GCCTTCCACAACAGATGTACTCAGATAATGGGAAAACATTTGTGGGAGCGTCATCCGTTCTTTCGAGAGA
TTTCTTGCAAGCGACCAAAGAGCTCATCTTATCCAAGCATAGCCATCAAAACTTGTGTTGGCATTTCAATC
CTCCTGGCGCTCCTCACATGGGCGGATTGTGGGAAGCAGGTGTCAAAAGCTTTAAGACCCACTTCTACAA
GTCCACCGCATCCTCAAAATATACATTTGAGGAGTTCGCGACTCTCCTCTCCAAGATTGAAGCCTGCCTTA
ACTCCAGGCCAATCTCTCCAATGTCGGAAGATCCGACTGATTTATTGGCTCTAAGTCCAGGCCACTTCCTA
GTTGGTGGCCCTCTTCTGTCAACTAACGAGCCAGTAATCAAAGAAGACGCTATTTCTATAATTAACCGCTG
GCAGCGACTCAAAGCGCTCCACCAGCAATTTTGCTTACGGTGGAAGAATGAGTATTTGAAAGAGCTCCAT
AAGCGAAACAAATGGCAGTCCCCTACAAGGGACCTCCAAGTCGGTGATATGGTGATTATCAAGGAAGAT
AACATACCAATAAATGAATGGCGCCTTGGCCGAGTCCAGCTGGCTTGCCCAGGCACAGATGACAAGGTCC
GCGTAGTGGACGTGCTTACCGCTCGTGGTGTCATCCGAAGGCCAATAGCCAAATTGGTCCTTCTTCCTACT
GAGGCAAATAGCACCACCGACTAATCCTTAAAAGCAACTAATCCCTCTTTCAAAACAAAATCATCACATA
ATAACACCTGTCCTACATCTTATTCTCCAGTTCACTCCTCTGTAGAGAACCAGCATGGCACCGTCAACGAA
GGCATCCAATCAACGTCAAGGAACGGACTCTTTTAGATGCCGGGTGTGTAGAGGCATCCATGCGCTACGC
AAGTGTCAGCGCTTCTTGAAGCTCACGCCCGAGAAGCGTCTCCGAGCCGTGTTAATTAACAAATATTGCTC
CAACTGCCTGGCTCATCAGCATTCAGGGGAATCATGCCGCAGTGGAGCCAAGTGCAAAATTTGCAAAAAG
GACCACCACACCTTACTCCACATGCGTGATCCCCGTAGCGCTCGACCCTCTCGGTCAGACAACAGGCCCA
CAAGAAGAGCTTCTGGCTCCTCGCCCTCCAGCGGCACATCACCCCCAAGCCCGAGACGTGCTACTATACG
GACGTCGCTTACGTCCCTTCTCCAGCACAAGGGGGTCAGTATACTGCCCACCGCCATAGTGCTGCTCGACA
CCGGAAAAAGACCCTTGAGACTCGGGCTCTGATCGATCCATGTTGTTCAGTAAGCCGCATTAATGCAACC
GTGGCAAGGGCCTATGGCCTTTCCGTCACACGCATTGGCGCAGAAGGAGCGTGCACAGCCGTGCTACGCT
CAAGACAACTCAGATGTCTCGAGAAGTGGTCTTCATGGCTGACCCGCAACTCAGCTGCATAACTCCAGCC
CGAGCCATCAGAGCGGACATGAAGGACCCGTTCAGCAACGTCGCTTTGGCGGACAACGAATGGTTCCGTC
CATCTCCAGTCCCTTTGGTACTCGGCGCCGACGTCTACCCAGACATCATCCTACCAGGTATCCTCCCGAGC
CAAGGCGGTCTGCCGATGGCTCAAAATACCAAGTTTGGATGGATACTCTCTGGCACATGCACGCAATAGG
AATTCTTGCAATCTGCATATTGCCAGGGGGGAGAATGTTCATGCCAGTTCAGTGCTAGTGTGAACACAGC
ATTGACTGAGCCTCACTCAGTGTGAGAGAGTCACTCAGTGAGACAGACTCACTCACTGAGAGAGACTCAC
TCACTAATTCGATCCCAGATGCGCTCAGCACTCTCCTTGTGGTCTCCTTTCCACCTCCATCACTCCAACCAA
AAATATGTAAAATCACTTAACCAGCAGAGTTTGTTACATGCAGCAGAAACTTCTAATAAAGTGCTTTTTCA
CCAACAACAATCAAGGATTATTTATCTGACGCCACCCCCACAAACA

>160_rnd-1_family-300__LTR___Gypsy
AAATAAAAATAAGTGAATAATAATAAAATTTTCTTTTATAAATAAAATTAATTAAACAAAAATAAATAAA
AATGAGTGGATAATAATAAAATTAAAATAAACCGAGGTTCACAATAAAGATAAAGGCAGCGACCACATA
AGGAACAGCCTAAAAGCGGAAAAGTGAGAAAAGAAAATTTTGAAATACCTTTTGTTGAGTTTTTTTTTTTT
AAAAAAGGGGGTAAATACTTTTGTGGAATATTGTTTTGTATTTTTGTTTGGTGAAAGATATAAAGACGAA
AAAGAAAAAAAAAAAAAAAGAAAAAAAAAAAAAAAACTAAAGAAGAAAACTAAAAGGAACCAAAGTGGC
TGAGCAAGATCGGCGTAAAAGTGAAAAGACACCAACACCAAGAAAAGGAGAGAGGAATAGTAAAGGGA
CCGACGGAACCCCTAGAACGGGAAGAGAGGGAAACAGCGACGTCGACTAGCAAGGAAGTATACAATAA
AGGATGGAGGCAGGTGAGTGCCAAAACCCGGGGGTAGGCGTCCGCTGGATTCACCAGCGACGCAAAGAG
GAGTTAGTGGCGATCGCCCAGGAGTTTGGGCTGGAAAAGGAGGGGCGCGTGGAGGAGCTACGTAAGCGG
CTGTCCACCTACGTGCAGGAGGGCGGTCATTCGACTGGGATTTGGGCTCGGCTCGCCGAGCTGGAATCCC
AGTATGCTAAAACTCTTGAGCCGCCATCTTGGGGCGTCCCACCCGACTTTGGTGGACGGCCACGGCAAAA
GTCGGAACCTGACCTCAGAAGCTCCCAGCTGTACGGGCTTTTGCAGGCGCCCACCCCGAGTTTGACCGTCT
CGGGCGTGGAGGCAGTTCCTGCTCTCGGTGGAAATGAAGACCGCCGAGCAACTGCGGTCAACGCAGAGG
TGGGGAGGTCCCCTGTTGAACCGGGTGGTGGTCCCTGCTACACTAGGCCGCACCATCCATCGGAGGCGAG
GCCACTTCCTAATGTCCCGGAGCAAGTTCGGAAGTGGGGTATGAGGTACGATGGACGCTCCGACCCCCTT
GGGTTCTTAGAGGACCTCGAGGAGCGCGCTATCACCTATGGTATAGCCCTCGACCGTATGCCCAGGGCAA
TGAGCGAGGTATTCTGCGATAGGGCTGCTCGTTGGTTTCTGAGCAGTGGGCTAAGGGATGTGCCGTGGGC
CGTGTTTAGGAGGGATTTCTTGGAGTTTTTCTTGCCCCCTCGTTACTTCGAGAGGCTTGAGGACGAGATCC
GATCCCGGAGGCAGCGCGTAGGTGAAGCGTTCAAGGAATATTTGATAGAGCTGAGGGCTCTTATGCACCA
CGCTAGATACGAGCCGGCACAAGAGCTGCATCGGATCTACGAGAACGCCGCTCCGGAATACAGGCTCTAT
GTGCGACGGCAAGACTTTTCTACCCTATCCCAGCTAACTCAACTGGCCGTAGAATACGAGACCGTCGTGA
GGCAGAGGGCGGAGTACTCAGGCAGCCAACCCGTGGNCCCGGGTGTCTCGAGTTCGAATAGTGCAGAGT
GGGCGAGGACGGCGCAGTCCCCGAGGGGACATCTCAATCCGCTCCGGTCTGCTCCCAGCGGATCCGGAAC
GACCGTTGTGGACAACAGGGTCTCCGCCCCACCCGCTGCAACNCACGCAGAGGTGGCCCATCGGATGATG
AGCCAAGAGGCCGGGAACGGATATGATCCGCGACGCGCGTGTCGTAGGTGCGCACAGGAGGGGCACTTC
GCCAGGGAATGTCCCAACGCCCCCGTCTTATTTTGTTGGGTATGTGGAAGCCGGGGCCGAAGTAGTCAGG
ACCAGGATCGGGTCGGCGCCGATACACACGGAACGCAGTAGTAGGACACCCGCTGAAGGTGGACGGTGG
CCGGATCGTCGCTACGGTAGAGATCGGCGGACGAACGATGCCCGCCACGATCGATACTGGAGCCACTCGT
AGCTTCATGAGTGAGGACTGCGTACGCAAGTGGACGATCCGGGGAAGAGCGGAGGAAATACAGGCACGC
ATCCGCCTGGCGGATGGGTCCACCCTAGAGGTGGAGAAGGCCTTGAGAGTAGACGTCAGCTTAGGGGGA
AAAACGATCAGCATGACGATGCTCATCATGCCCTCAATGCTGGACCACGTAATCCTGGGTATGGATTTCTT
GGGTGCGATGGGCACCACAATACGCTGCGGCGAGGCGGAACTGGTCCTAGAAGCAGCGACGACACCGGG
CGCGGGGCCAGGAACGAGCGAGGAGTCGGAGGAAGGCCTCCCAAGGCATGAAAAGGGAGCACCTGCTCG
GCTACAGAGGGAGCCGGAGGAAGGCCTCCCAAGGCATGGAAAGGGAGCACCTGCTCGGCTACAGAGGGA
GCCGGAGGAAGGCCTCCCAAGTCATGAAAAGGGAGCACCTGCTCGGCTACAGAGGGAGCCGGAGGAAGG
CCTCCCAGGTCATGGAAAGGGAGCACCTGCTCGGCNNCCGACGGAAAAGGTCAGGAAAAGGGGAGTAAA
GTCCGGCCCGAATCGACAGCCTGGAGAACCCAAGAGGCGGCGCGTAGAGCTGCAGAGGCAGACTTCTGN
TGGCTACGGAAGCGGCACGGCGGGAACGTGCCAGGGAAAGGAGGGAGTAGGAGCGATGGCCAGAATCC
ACACGGACACAGGGGGCCAGGAGCGACTGCCAGAATCGAGCGGTCATGGAGTTCTGTCTGGAGTCGAAG
TGGTGCCAGGAGGGGGCGANAACGGCATCGAAGAGGCGGAGTGGCCAGCGGATCTGGATCCAGAACTGA
GGGAGTTCCTGGAGTCAGAGCTGGCTCTCTTCGAGGGGCTGCGGGGAGTCTCGCACATCGCGGAGCACCG
AATCCGGCTGAGGGATGACAAGCCGTTGAAACAGAGGTACTACCCCAAGAATCCGGCCATGCAGAGGGT
GATCGACGAGCAAGTGGATGAGCTGATCCGGGCAAATGCCATCGAGCCGTCGCGTAGTCCGCACAGCGCC
CCGATCGTCTTGGTCAAGAAGAAGACTGGCGAGTGGCGTATGTGCATTGACTACCGACAACTCAACGCGC
ATTCCATTCCGGACGCTTATCCGGTGCCTCGTATTAACCACATCCTGGAGCGGTTGCGCCACGCCCGGTTC
ATCTCCACTCTGGATTTGAAGCACGGGTACTGGCAGATACCGATGGCAGCCGATAGCCGGGAGTGTACGG
CGTTCACAGTCCCTGGAAGAGGTCTTTTCCAATGGCGGGTGATGCCATTCGGCCTACACTCGGCGTGTGCG
ACCTTCCAGCGAGCNTTGGATACGGTAATAGGGCCGGAAATGGAGCCCCATGCGTTCGCTTACCTAGACG
ACATCGTGGTAATAGGAGCGACGAAAGAACAGCATGTAGAGAATTTGAGGGAGGTATTCCGCCGGCTAA
GGGCGGCCAATCTCAGGTTAAATAGGAAAAAATGCAGCTTCTTCCGGGAGAGGTTAGTCTACCTAGGACA
TGTAATTTGCGGGGAAGGGATACGGACCGATCCGGAGAAAGTGGAGGCGATACGNAACCTGAGGGCCCC
GACAAATTGCAAAGAACTGCGGCAGTGTCTCGGGATGGCGTCATGGTACAGGCGCTTCGTNCCCAACTTC
GCGACCCAGGTACAGCCACTCACAGCGCTCTTAAAGAAGGGGCAGAAATGGGCATGGGAGCAAGAACAG

GAGGAGGCATTGCGTCAATTGAAGGANAGCTTGACGACTGCCCCTGTGTTGGCTTGCCCCGACTTTTCCGC
CAAGTTCGTCTTGCAGACTGACGCGAGTGAGTATGGCCTCGGCGCAGTGCTGACGCAGACGATCGATGGA
CAAGAGCGGGTGATAGCGTACGCGAGCCGAAAGTTGCTGAAGGCCGAGGTGAACTATTCCGCCACTGAG
AAAGAGTGCCTGGCAATCGTGTGGTCGATCCGGAAGTTGCGTTGCTACCTAGAAGGGTACCGGTTCGACG
TGATAACGGACCATCTAGCGCTCAAGTGGCTCAACTCGATCGAGAGCCCGTCAGGAAGGATCGCGCGATG
GGCGTTGGAACTGCAACAGTTCCAATTTGACGTCCATTATCGNCGAGGGAAGCTCAACGTCGTGGCCGAT
GCGCTATCGAGGCATCCTCTAGAGACCTGTCAGCAAGCAGTGGTGGCCGAGGCCCCCTGCAAATGGGTCG
CGAGTATGCGTGCTAGAATCGCCGAAGAGCCAGCCAAGTTCCCGGATTACACCGAGGAAAACGGACAGC
TATATAGGCATTTGGGTCACCGGATAGACGATGAGGACTACATCCCCTGGAAACTATGCGTAGGCAGTGG
TCAACGCCAGAGGGTACTGNGGGAATGTCATGATGCTCCCACGGCCGGACATCAAGGTGTCAGGAAAAC
GGTGTCGAGGTTAGCCCAGCGGTACTACTGGCCGGGTATGTTCCGCGATGCCGCCAGATACGTGAAATGT
TGCGAAGACTGCCAAAAATTCAAGAGCGAGCAACTCAAACCGGCTGGTCGCATGCTGACGAGGCAGGTG
GCAGAGCCGATGGCGGTACTTTGCGCGGACTTCGTCGGACCCCTTCCACGGTCGAAACATGGAAACACGA
TGCTACTGGTGTTCCATGACGCTTTCTCGAAGTGGGTGGAGTTGGTCCCTCTCAGGAGAGCCACGGCAGCA
CACCTGCAAACTGCGTTTCGAGAGAGGATTCTAAGTAGGTTCGGGGTACCTAGAAAGTTTGTGTGCGACA
ACGGGGTACAATTTACGAGCCGGAGCTTCAAGTCGTTTTTGGGGTCCCTGGGTGTAGAGATCCAGTATAC
CGCCCCGTACTGTCCGCAAGAGAATCCCACTGAGAGGACCAACCGGACTGTAAAGACCATGATAGCGCA
ATTTATTGAGGGCCACCAGAGTTCATGGGACGAACTCTTGCCCGAGATCTCGCTGGCGGTCAACACGAGC
GTGGCAGATTCAACGGGCTTTAGTCCCGCTTTCCTGATGCAGGGCAGGGAACCTCGGTTACCCGCAGCTCT
GTACGATCAGGTGACTCCGGGGTCCGCAACTAGTGCGCTCGACCCTAGGACAAAGGCGGATAGGCTAAA
AGAAAATTTTTGATATCGTCCACAGCAACCTCCAGCGCGCATCAAAGGACCAGGGCAGGCATTACAACTT
GCGGAGACGAGAGTGGCGGCCTTCGTTGGACTCGATGGTCCTGTTGAGGCAGCACCAGTTGTCCAACGCC
GCCGAAGGTTTCGCCGCAAAGTTGGCACCCAAATTTGACCGGCCCCTACAGGGTCGTCAAATTCATATCC
CCCAATATTGTGCGGCTGGTGAAATCAGGCGANCGCAAGAAGAGGGTGGCCAACATCTCACAGTTGAAA
CCATTCTACCGAAAGACGACGAGCGACGAAGAAGACGAAGATCCAGGGCACGCAAATGCCCACGAGTAG
GCGAAGGATGAGAGCGACACCCGTCGCCCAACGAAACCATTATGATTAAGCTTAGATAAGACTAACACTA
ACAACATAATTCAGAATAGGCNTAGTAAAGTGTGAGCCGCACTGAGAGGATAGGGCATTAACAAATAAG
TAANGAGTAAGTTAACCAATAAGCTGAACTTTCAGATGGAACCCGAAGTTATCGACATATCGTCAGACGA
AGACAACAGCAGCGGGGACACCGAGCCTTTCCCCGGGCCGGGAGCCGCAGGCCGTCCGCGCGACCCACG
CCAGGGGCGTGAGTACTATCGAAGAGAACGGCAAGAAGGACCGCCTCGCCAGTTCCGCCATACACTATTC
TCCCGGCGGACCAGCATAATGGAGAGGCTAGACGGGATGGTCTTGCTGATGGCAGTCGAGGTCACGACA
ATGTTCGTGGAGGCACCATTCGCCCGGAACTCGCCACCGTACGCGGAAGAGCCTGCATCTCCGCCCCTCG
CATCCCACTCGGCACAATCATCAGAGCCGGCAACCCCAACCGCGGCCGGCGAGATGGACGAGGAAGAGT
CCGCACCGTTCGGAACGGAGGAGCACACACCACCAGCGCAGGACGTGGAGGCGGTAGGAGAAGAGATAT
CCCCGTCGGCCGGAGACGCGGAGTCGCCACCGACGGAGGTGCCGAACGGCATCCGCGCAAACGACGGGG
AAGCAGACCCGCTGAGCCTCGACCCAGAACCCTTAGTGGGAGGGGACCCGTCAACGGAGGACGAGCCGG
AATTCCGTCGCCGCTTTGCCACCCACCGTCTGGAGGTAGACCCGGGCAGCCAGTTTCCCAGGATAACGCT
GCGGTACCTACCAAAAGTCCCCGCGGTAGCCAACAAACCCCGTCTGAACCCCGCAAAGCGCGAATGGGG
GGGTGCCGAAGAAGGCCCCGGCGGAAAGCGCCGACGCAGCCGCCATCGTCGACGGAGAGCCGCCAGGGG
CGAGCGTCGCCGGAGTGGAGGCCATCGCCGACCCAGTCCCGAGGGAGCATCCTCGTCGGCCACGAAGAG
GCTTCGCACGGAGACCGAGAAGCCCGAAGGATCGCAGCGGTGCGTGCCGCGTTCGCCCCGCGAGCGCCA
GGAGGGTCCTCCCCAGCTCGGGAAGGATAACCGCCGCTGCNAGATCGCTTCTCGAGGAGGGCATCCCGAA
CCCTGGCCGATACCGATTGCGGTCTGCCAGATGGTCGTGGAGGAGCACNATCTCAGCACCAAGGACGCGG
CGAACTCGGCCGTTTCCACAGCCCGCGCCGAGGCGCCAGACGAAACCTTTCCTAAGGAATCGGAGGAGGA
GTGGCTCGACGACGAGGACGAGATGCCCGCGCTCGTGCCTATTGCCCGCGCCGAGACACCCGACACCATC
GCCCGGCGGGAGTTAGAAGACGCCTGGTTCGGCCAAGAAGGCAAGGCGCCATCCCGAAAAGGTTCCTTC
ACGGTTCGCCAGGAAGGCCGGGATGAAGTCTGGTCCGAGGAAGAGGACGCGGTGGTGACGGCTTCGTCG
CCCAAGCTTTCGAGTCCAACCCAGGATTCCTCCCCCATGGCTCTAAAGACTCCCACGGAGAGCGAGTTGT
GGGACGACATAGTGGACCTTTTCGACGGCATCGACGGGTGGGACACTAGCCTGGACTCAGTGCTGGAGGA
GGAACCCGACACCGAGGTCGACACGGTCGCCCGCAGTGCCGACGTGAGGGTCACACCGGAGAGCCACCC
CAAGAAACCCGACACCGAGGTCGAGGCGATCGCCCACAGTGCTGACGTTGGGGTAAAACCGGAGGGCCC
CGTCGAGGAACCCGTGCCCGATTCCACCCCCTCAGGCTGGAAGGTGTTTCCTCTTGGAACACCGTTGTCGT
CGGGCGTGGTGGACCTAATCGCCAGAGAGGCGCGAATCCGCCGCTTCCGGTGCCGACGTACTCGGTTCCA
CGTGTCCGACGGGGAGAGCGAGTACAGCGTTACGCTGAACGCGAAAGGCGACGTGACCGTCACCTGTCC

GAAGTGAGGGGGGGGTGTGAGGCGGCTAATGAGACCAGCCACCCCACAGTTTTCACGGCAAGGAGCCGT
CATCCCTTTGGCCAAATTACGGCAAGGAGCCGCTGGCGGGACAACGTATTAATTTTCTTTAGTTATAAGTA
TTAATTATATTAGGTTAAGATCTTAGTGAAATAAGAGGCAAGGAGCCATCAGTAATATAAGCAGTTCGAT
TAGTAGCAAAGAGGCAAGGAGCCGCGAATATTATAATTGATTTAAATAGTGAGTTTGAATTTAACCGCCG
GCCGCCGCTGCAACCGTGCGTTGTCGATCGGTTGCTCAACACCGTCTTTGAGAGCGCAACTTCAATTCGGC
GGCGAAGAGCGAAAGCTCCGAGGCGAAGTTGCGAAAGCTCGCGGCGAAGTTGAGCAGCCGAAGCGAGCG
CATGGCTTGCCGGCGAGCGTGCCGGCACTTGTGATCGAACGTTAAGGGTGAGTAGAAGTGCTGCGCGAGA
TAAACGAAATCATAGATGGTATAGTGGAGCGCTAGGGCTCCAGAAAACAGTGCGGCAGGGTTTAGCTTAG
GATTTACGATAAGGTCGGAATGAATAATAGTGAATAAAGTGCCAACGTTGTAATTATCTAGAAAGTATTG
GATTATTCCCTGTTTCCTGGTAACCCGGCTTACCTTAACATCGGAACTAACCGGTGTTCGACCTAACCTCG
AACGAGGGGCATGCGATACGAGATTGTGGCTCCGGGGTGGGCATTGCCGGTTGGTGTTTAGGTAGTGGAG
TTGGTGCGAGGCCACGGCCCGCTAGTGTAGTTGGTGCGAGGCCACGGCCCGCTAGCGTAGTTGGTGCGAG
GCCACGGCCCGCTAGCGTAGTGGTGCGAGGCCACGGCCCGCTAGTGTAGTTGGTGCGAGGCCACGGCCCG
CTAGCGTAGTGGTACGAGGCCACGGCCCG
>9_rnd-1_family-236__LTR___Pao
TGTTGCGGGACGAATTAATTTATCGATTGTTTAGTATTAAGTATATTCTGAAAGTATGTATATTTGCCCTCC
GAATGTACGGGCACACTGTATTTGGCTTGTTGCCGCCATTACTTAAATGTTAAGCTCATTATTGTCGTGAG
TTCGAAGGCCGTAGGAGGAAAGACTGCGGTAAGATTCCGAGGGAATATATATCTAAGTTCTAACCTATGT
AATCCGTTTAGAATAAAACACTTGCACTAGCACGTTTTGCATTACGCTGTCTCTAGTAACATCGGAGTAAC
ATGGCCGGGGGCCAGGCGTAAACTTGCAGTGTTCAAATTGCAACTCCACCTTCATTTGGCACGCGACATG
ATCTTTTATTCGCAGTTCTTATTGTAAGAAACTGCAAGGGCGACTTGCGCTGAAGACAAGACGTGGTGTAA
CATTGGTGTTACAGTGTGTGTACATAGACACAGCACAGGCAGGGCCTCCACCCAGTTATAGAGTCACCAT
TAGTACGCAGCCAGCGTAAAATATAGCAGAAACCATCTCCGAGCTGAGTAGGATGACACAACGGTCACCC
AGGAAAAGCCCACGCTTAAACGATGGCCAGGAGATAGCTACCACGGCGAGTGGGACGCAGACAAAGCGG
ACAAGTAGAATGGTGAGCACTGTATCACAAATGCAAGTTCCCGCTGTTAGCGCACCACAAAGCCCCAGCA
CCGTGGATGCGGGCACAAGTGTACAACCTGCCGGCACTGTGGCCGCGGGAGTGAGTGCACGAGCCAGCA
CATTGACAGGAGCGCCAAGCCCAAGCACGCAACTCTCAAGTACGCAAGTCATGGATTTGATAAAGAGAGT
CGCTGTGCTAGAGCACGAGTTGCAGAGGTCAAAAACTGGTCAAGCACATGTGAGTACAGTTACTGATCCC
GTTAAGTTGAGTAGTGCTGGCATGAGTGGTACAGCTAACCTGCCGCCATCTTTGATCGGAGCGGAGAGCG
GAGAGCCATCGTCGAGTGGAAGTTTGAGTGAAACTGCAACTATGAGTGTAGTCCCAAATTCAAAAGTAAG
TTTGAGTGAAACTGCAATTTTGAGTGGAACTACCATTTTGAGCAGATCGCCATCTTTGAGTGAGGCACTGC
CTACGTCATTGAGTGGAAATTTGGGAACACAGTTGCAAACGGTAGAACGGGCTACAGTTATGGATAACTG
CTCTACAACGTCAAGCCGCTCGACGCCATATTGCTTTGCAGGCATTGCACAGCCAACGCTCAGCTACACTC
CGGCACAGGAGAACCAACAAACGATAATTTTGGAGCCAACGCAGGCAACTGGGAACTTCGCATGGACGA
CTCCCAGGAACGATGTATGCCTGCCACGCAAATTACCAGATCTACCCGAGTTTGGAGGTCAACCTGAGGA
TTGGCCTATATTCTTCTGCGCGTTTACGGAAACAACTTTGGCGTACAACTGTACAAATCTGGAGAATAACC
AGCGATTGTTGAAGGCACTCAAAGGCGAAGCACGTGACACTGTGAAGTCGTTGCTCATACATCCCAGGAA
TGTGAACAACGTAATTGAACAATTACGTTTTAGATACGGCCGCCCAGAACAGCTAATACGCAGCCAGCTA
ATTAGTATTCGAGAGGTGCAGCCAATTCAGGAGCACAGCATAGCTAAAATCGTGCCATATGCTACACGCG
TAAGTAACCTTGCCGCCTTTCTACAATCGGCTAACGGGGAACAGCATTTAGCGAATCCTACACTGATGGA
GGATTTGGTCGCTAAACTACCACCAAGCAAGAGGGTGGAATGGGCCAGACATGCAGCTACGATTAGGCCT
TTCCCGACGGTCGTACATTTTAGCGCGTGGTTAACGGACTACGCCAATGTGGTATGTACGATTGTGGACGT
CGATAGTAAGGAGCAAAGGCGTAGAGTGCTGCATGCCAGCATTGACCAGAATAATGAAGGGCGTCACCG
AGGTCGCACCAAACAGTGTCCAATCTGTAATGGACAACACGCAGTCAGGGACTGCAACGAGTTTAACTTA
GCCTCTCCGCTAAAGAGGTGGACAGTCGTAAAAAGGCACCGGCTTTGTTTTTCGTGTTTACGAGTTGGCCA
CATGGCAAGAGTCTGCAATAGGGCCAACGAATGCCAAGTTAATGGATGCCGGAAGAGACATCATCGACT
CTTACATTATCATGATGCTATGAGTCACAATGCACCGCAACCAGCAGGTGATAGAGTGATGAATACTGGA
CGGATGCCGCAGCCAGCGGATCCTCACACAAGGGCACCGCAGCCAGCGGATGCTCAATCGACGCAACAA
AGGAACTTAAGTTGTGTCGATTCGGATGGAGAACGCCTACTATTCCGCATATTGCCAGTGACATTGCACG
GGGCCAATAAGCGGATCGATACGTACGCGCTCCTCGACGAGGGCTCGTCTGTAACGATGATCGACGACGA
ACTACTGCGCGATCTAGATATCAAAGGGGAACGCAGGCAACTAAATATACAGTGGTTTGGTGGAAGAGC
AACCAGAGAGAGCCGACCACAGTGGTAAGCATGGAAGTTAGTGGAGCGGACAAGCGCAAACGGCATGTGTT
GCGAAATGTGTACGCCGTGTCCAACCTCAGCTTACCAATGCAAAGCCTGCATCAACGTGACGTCCAAATG
TGGGGCAACGGTGCACGTCTTCCAATGAAACCGTATCGCGGGGCGGTACCAAAACTTCTGATTGGACTTG

ACCATGCGCATCTTGGACTGCCAATGCAAACAAAAAGGTTTGCACCACAGGGACCATATGCCGCAGCCAC
CGAACTTGGATGGGTGGTTTTTGGGCCGGCAAGAGATCAACCGACGGCAACGTCATCAAAGTCATGCCTC
CTAGCAGTGTCGCAGGAGGATGCAATCCAAAAGATGGTAAACGACTATTTTGATATCGAAAACTTCGGNG
TGAAGCTTACGCCACCAGTCGCAGCCAGCGACGATGCACGAGCACAGAGGATACTCGAAGACACCACGG
TGAAAATAGGAGAACGTTACCAGACGGGTTTATTATGGAACCGTGATAATGTTGAGTTGCCACGAAGCTA
TGATATGGCATACAAAAGATTGGTCAACATTGAGAGAAAGATGAAGCGTAACGATCAGTTTAAACAAGC
CTATATGAAAATTATGGACGATTATGTTCACAAAGGATATGCTCGACGATTGAAGCAGCATGAGGTCGCT
TTAGCCAACAGCGACAAACTTTGGTATCTTCCGCACTTTGGAGTTGAAAATCCAAATAAGCCCGGTAAGA
TAAGACTGGTGTTCGACGCGGCAGCAAAGGTTGGTGATATTTCATTGAATTCGGCATTATCCAAGGGGCC
GCAGCACTATAAGTCGCTGCCGGCTGTTCTCTTCCATTTTCGGGAAGGTGCCGTTGGCGTTTGTGGAGACA
TCCGAGAGATGTTCCATCAAGTGTTGATTCGACCGCAGGACAGATGCGCCCAGCGATTCCTGTGGCGAAA
CGGCGATGATCGCCGGGAGCCTGATGTTTATGAGATGAGGGTAATGACGTTTGGAGCAGCATGTTCGCCA
AGCGCCGCACACTATGTGAAGACCTTGAATGCCCTGCAGTTTCAGGATTCAGACCCTCGCGCGGTTAAAG
CTATTCTTGAATGCCACTATGTGGACGACTATGTGGATAGTTTTGATAGTGAAGGCGAAGCCATCACCATA
TCGACAAGAATAAGAGAAATACATGCAAATGCTGGATTCGAATTGTGCCAGTTTACTTCCAGTTCGCCAA
CTGTAGCTGACGCGCTGGGCCAACATGGGGCTGCGAGGAGCATCGGATGGGGCGAAGCTGAAGAAAAGA
TTCTAGGCATGTGTTGGCAACCAGCCACGGACGATTTCAAATTCAACATGAAGTACCACAAAGTACCCCG
ATGTGTGCTAAATTTGGAGCGAATTCCTACGAAGAGGGAATTCTTGAGCTTGATCATGTCAACATTCGATC
CTCTGGGATTTCTTAGCTGCCTCATGATAACTGGAAAGTTACTAATGCGTGAGATTTGGCGACGAAATGTG
CAATGGGATGAACCCTTACCAGATGAGATCGCCCGGGCCTTTAGCAAATGGCTTCAGGATATGAACAACG
TGGAAGGATTTCGGAGCTCGCGCCATTACTTCGGCCCAACACCTGTGCGGGCAATACAGCTGCACGTTTTT
GTCGATGCTAGCCAGTCAGCATTCGCAGCCGTGACTTACTTGAGGGTCACCTACGACAACAACGACGTGC
GAGTATGCTTCGTATGTGCCAGGACGAAGTGTGCCCCAATGAAGACGATGTCAATTCCACGACTGGAATT
GCAAGCAGCCGTGTTAGGAACGAGGCTGATGGACACTGTCAAAAAGGAGCACAGTATAGCGATCAGCGA
CGCTATACTATGGACTGACTCCAAAACNGTGCTGCGTTGGATAGGCAGCACCCACCGTCGATATAAGCAG
TTCGTCGGAAATCGAGTGGGAGAAATTTTGGAGTCAACAAAGGTTTCCCAATGGAGATGGGTACCTACTG
CTGAAAATGCAGCAGACGACGCAACGAGGCCGCATTGCCACGTAGACCTCAGCCAACGGTCACGATGGTT
AGACGGGCCAACATTTTTGCGGCAGCCGGAAAGCAGCTGGCCGCAGTCCGAACCGGGCACGGAACACTC
ATCCTACGAAGATGAAGAAGAGATGCCGAGTGAATTTGCCTTAGTTGCAGCAAATAACTTTTTCATTTCAT
TTCAGAGATTCTCTAGCTACAGTCGGCTGGTGAGGACGACGGCTTGGGTTTTAAGGTTCACGCGCTGGAG
CCGTGGACAGAGGACTGAGCTTGAGAGATTCGGCCTCACCGCGACGGAGTGTGAGAACGCTGAGAACTT
GCTGATACGGCGAGCGCAATGCGAAGCGTTCCCTGACGAGGTTCGAGCCTCATACAATGAAGAATCAGTC
GGTCATCGAAGCGACATAAGAGGGCTGGCGCCNTACTTGGATGACAATGGAGTCTTGCGTGCGTATGGCA
GGATCGATGCCGCACTGTGTATACCGCATAGTGCGAGAAGGCCAATAATCTTGTCACACCGGCACGCACT
GACGGAACTGATTGTGTATCACTACCATGTGAAAATGAAGCATCAAAACGTGGATGCAACGATAGGCGAC
ATCAGGACCAGATTTTGGATAACAAAGCTGCGTCGACTGTTGCGTACAGTAATATCTGGATGTAGTGTGT
GCAAACTACATAGAGCGCAGCCAGCGCCACCTATAATGGGTCCTTTGCCAGAGGACAGACTGGAGGCCA
ATGGATGGCCATTTAAGTTTACGGGTCTGGACTATTTTGGACCACTCCTTGTGACGATCGGACGTCGAAAG
GAAAAGAGATGGGTCGCCTTGCTTACGTGTCTGACAACAAGGGCCATACATTTGGAGCTGGCACATGACC
TGTCAACTGATTCCTGCATAATCGTGATCGGGAACTTTCTTTGCCGTCGCGGACCTGTACTGAGGTTGCGC
AGTGACAACGGAAAGAACTTTGTTGGGGCCGACAGAGAAGCCAAAAGGCTNATAGATGTATTCGAGCCG
GAGAAGATTCAGGGTGAGCTATTTCTAGAGGCATTGAATGGATCTTCAACTGCCCAGCGAACCCAGCTGA
AGGGGGAGCCTGGGAAAGGATGGTGCAGTGTGTCAAGAGAGTGCTACGCCACACAGTAAAGGAGGTGGC
ACCAAAGCAACACGTACTGGAGAGTTTCCTGATTGAGGCCGAGAACGTTGTGAATTCTCGCCCGCTTACC
CACTTGCCGGTGTCTGTGGACCAGGAAGCCCCCTAACACCGAACGATCTACTCAAAGGAGTGGCCAATCT
GCCTGACACGCCCGCTATCCACGAGCAGCCGAACGAGAGACGCGCCACGAGGAAGCAGTGGCGCATGGC
GCGGCTGTTACGAGACCGTTTTTGGAAGCGGTGGGTCTTAGAGTACCTGCCTGCGCGCGTTGAGCCGATA
CGCCATGGTGATATGGTGTTTATATGCGACCCGGCCGTGCCACGCAGAGAGTGGCGAAGGGGCATAGTGG
AGGAGCTCTACCCTGGACCAGATGGAGTGCCTCGACGCGCCAGCGTCCGCGTGGCGGACAACAATCGAGT
GCGACTGATGACTCGTCCTCGTGGCTAAACTAGCCATACTGGATGTGAGTGAAGCAGGGCCTTCACGGGG
GCGGGGATGTTGCGGGACGAATTAAGTTATCGATTGTTTAGTATTAAGTATATTCTGAAAGTATGTATATT
TGCCCTCCGAATGTACGGGCACACTGTATTTGGCTTGTTGCCGCCATTACTTAAATGTTAAGCTCATTATTG
TCGTGAGTTCGAAGGCCGTAGGAGGAAAGACTGCGGTAAGATTCCGAGGGAATATATATCTAAGTTCTAA
CCTATGTAATCCGTTTAGAATAAAACACTTGCACTAGCACGTTTTGCATTACGCTGTCTCTATTAACATCG

GAGTAACATGGCCGGGGCCAGGCGTAAACTTGCAGTGTTCAAATTGCAACTCCACCTTCGTTTGGCACGC
GACACTTAGTAAAACTTTATCTCCCCTGAATAAAATTATGTCCTTAGTTTTAAGTCATATAGCTCTTTATTT
TTTTGTTTGCGTAGTTCAAGCATATTTTTTGCTCTTTGGTATGCTACTTCTAATCAATACTTACTTTCTTTAG
CGAAATCTTCTATGTTCTATTTAGGTTCTATATTACCTATGGTATCTAGTATTTCGGCAGATTACTAGTCTT
ACCAAAACTAACTCATATGGACAATAATTATGTGCCACATNTATGATTTATGAGGATGCCATGAGTCACA
ATGCACCGCATCAAGCAGGTGATACAGTGATGAGTACTGGACGGATGCCGCAGCCAGCGGATCNTCACAC
AANGGCACCGCAGTCAGNNGATGCTCAATTGACTCAATTGACAAAGGAACTTAAGTTGTGTCGATTCGGA
TAGGGAACNCCGACTTTTTCCGCATATTGCCAGTGACGTTGCACGGGGCCAATAAGCTGACCGATACGTA
CGCGCTCCTTGACGGTGACAAACGCAAGCGTCATGTATTGCGAAATGTGTACGCTGTGTCCAACCTGAGC
TTACCAATGNAAAGCCTGCATCGACGTCAAGTCCAAATGTCGGGCAACGGCGCACGACTTCCAATGAAAC
CGTATCGCGGTGCGGAACCAAAACTTCTGATTGGACTTGACCACGCACANCTTGAACTGCCAATGCAAAC
AAAAATATTNGCACCACAGGGACCATATGCCGCAGCCTCCGAACTTGGATGGGTGGTTTTGGACTGGTAA
GAAGTCAACCGACGGCAACGTCATCAAAAACATGCCTCCTAGCAGTGTCGCAGGAGGATGCAATCCAAA
AGATGGTAAACGACTATTTTGATATCGAAAACTTCGCCACCAGTCGAAGCCAGCGACGATGCACGAGCAC
AGAAGGTACTCGAAGACACCATGGTGAAAATAGGGAAACGTTACCAGACAGGTTTATTATGGAACCGTG
ATAATGTTAAGTTGCCACGAAGCTATGATATGGAATACAAAAGGTTCGTCAACATTGAGAGAAAGAGAA
AGCGTAACGATCGGTTTAAACAAGCCTAAATGAAAATTATGGACGTTTATGTTCATAAAGGATATGCTCG
ACGATTGAAGCAGCATAAGGTCGCTTTAGCCAACACGACAAACTTTGGTATCTTCCGCACTTTTGAGTTAA
AAGTCCAAATAAGCCCGGTAAGATAAGACTGGTGTTCGACTCAGCATTGATCATGTCAACATTCGATCCT
CTGGGATTTCTTAGCCGCCTCATGATAACTGGAAAGTTATTAATGCCTGAGATTTGAAGACGAAATGTGCA
ACGGGATGAACCTACCATATGAGATCGCCCGGGCGCAAATAACTCTNTCATTTCATTTCAGAGATAATAA
TAATAATAATTTTGAGTGTTAACGTTGAGCTCAAAATTTAATTCTCCTAAAATTATTAAAATAATCGTTTA
ACAACCGTCCCTATTGGCTAGAAGCACGCGTTCTCACCAACTCGTTCGCCCGCGCGTACGTGCGCAATTAA
GAATAACGTAGACAAACAAATAANTTACAAGAAAATAACCGAGTCTTGATTGATACTGGGTAGCTGGGTA
ACGTTCACGCCCCAGGACCCCCCTTTTTTTCCATCACGATGGACTGGCAAACTTCGCCTGATAATATTGGC
GA
>9_rnd-1_family-417__Unknown
CTGAGTGAGGCTCAGTCAATGCTATGTTCACACTAGCACCGAACTGGCATGAACATTCTCCCCCTGGCAAT
ATGCAGATTGCAAGAATTCCTATTGCGTGCATGTGCCAGAGAGTATCCATCCAAACTTGGTATTTTGAGCC
ATCGGCAGACCGCCTTGGCTCGGGAGGTTACCTGGTAGGATGNTGTCTGGGTAGACGTCGGCGCCGAGTA
CCAAAGGGACTGGAGATGGACGGAACCATTCGTTGTCCGCCAAAGCGACGTTGCTGAACGGGTCCTTCAT
GTCCGCTCTGATGGCTCGGGCTGGAGTTATGCAGCTGAGTTGCGGGTCAGCCACGAACCACTTCTCGAGA
CATCTGAGTTGTCTTGGGCGTAGCACGGCTGTGCACGCTCCTTCTGCGCCAATGCGTGTGACGGAAAGGCC
ATAGGCCCTTGCCACGGTTGCATTAATGCGGCTTACTGAACAACATGGATCGATCAGAGCCCGAGTCTCA
AGGGTCTTTTTCCGGTGTCGAGCAGCACTATGGCGGTGGGCAATATACCGACCCCCTTGTGCTGGAGAAG
GGACGTAAGCGACGTCCGTATAGTAGCACGTNTCGGGCTTGGGGGTGATGTGCCGCTGGAGGGCGAGGA
GCCAGAAGCTCTTCTTGTGGGCCTGTTGTCTGACCGAGAGGGTCGAGCGCTACGGGGATCACGCATGTGG
AGTAAGGTGTGGTGGTCCTTTTTGCAAATTTTGCACTTGGCTCCACTGCGGCATGATTCCCCTGAATGCTG
ATGAGCCAGGCAGTTGGAGCAATATTTGTTAATTAACACGGCTCGGGGACGCTTCTCGGGCGTGAGCTTC
AAGAAGCGCTGACACTTGCGTAGCGCATGGATGCCTCTACACACCCGGCATCTAAAAAGAGTCCGTTCCT
TGACGTTGATTGGATGCCTTCGTTGACGGTGCCATGCTGGTTCTCTACAGAGGAGTGAACTGGAGAATAA
GATGTAGGACAGGTGTTATTATGTGATGATTTTGTTTTGAAAGAGGGATTAGTTGCTTTTAAGGATTAGTC
GGTGGTGCTATTTGCCTCAGTAGGAAGAAGGACCAATTTGGCTATTGGCCTTCGGATGACACCACGAGCG
GTAAGCACGTCCACTACGCGGACCTTGTCATCTGTGCCTGGGCAAGCCAGCTGGACTCGGCCAAGGCGCC
ATTCATTTATTGGTATGTTATCTTCCTTGATAATCACCATATCACCGACTTGGAGGTCCCTTGTAGGGGACT
GCCATTTGTTTCGCTTATGGAGCTCTTTCAAATACTCATTCTTCCACCGTAAGCAAAATTGCTGGTGGAGC
GCTTTGAGTCGCTGCCAGCGGTTAATTATAGAAATAGCGTCTTCTTTGATTACTGGCTCGTTAGTTGACAG
AAGAGGGCCACCAACTAGGAAGTGGCCTGGACTAAGAGCCAATAAATCAGTCGGATCTTCCGACATTGG
AGAGATTGGCCTGGAGTTAAGGCAGGCTTCAATCTTGGAGAGGAGAGTCGCGAACTCCTCAGATGTATAT
TTTGAGGATGCGGTGGACTTGTAGAAGTGGGTCTTAAAGCTTTTGACACCTGCTTCCCACAATCCGCCCAT
GTGAGGAGCGCCAGGAGGATTGAAATGCCAACACAAGTTTTGATGGCTATGCTTGGATAAGATGAGCTCT
TTGGTCGCTTGCAAGAAATCTCTCGAAAGAACGGATGACGCTCCCACAAATGTTTTCCCATTATCTGAGTA
CATCTGTTGTGGAAGGCCACGTCTTGCGATAAATCGCGAAAATGCGGCCAGGAATCTCTCCGTGGTTAGA
TCTGTGGTGGCTTCTAAATGGATTGCTTTCGTGCTGAAACACACGAAGACACAGACATAACCCTTGGTAAT

TTTGCATGCTCTACCAATGTAGCTTTTAATATCGAAAGGTCCGGCAAAGTCTACACCCGTATGTGTGAAGG
GTCGGGAAAAGGTTGATCTCGCCCTTGGGAGTTCGCCCATAAGTTGGGTCTGGAGTCGTCTACGGTGTATT
ACACAGACCTTGCAGGAGTTTATGGTAGATTTACCAGGACCTTGAGCTTTGGGATCCAGTATTTGGCTCT
TATGAGGCGCATTACCAACTGATTGCCTCCGTGAAAACAAATGAGGTGGGTAAATCGGACCAGGAGGCGT
GAAAAATCACAATTGGCCGGAATGATTATGGGGTGTCGCTCATCATAGCCTAACGAGGTTGACGCTCTCA
CCCGTCCACAGGATCGCAGAATACCCCTTTGATCGATGAAAGGGGTTAAATTGCGAATGCTGCTTGATCCT
TGAATTGGCTTCTTATCGAGCAAACAGCTATACTCCTTAGGATAAGCTTGGCGTTGAGTATACTCGATAAG
TCGGAACTGAACTCTCGTAAGTTCTTCATTACGGAGCTCGGCGGGAAAGTGGATCGCTGGATTTCTACAGC
GCTGGATAAAGCGACAGACATATGCAATAACTCGCAATGCCTTGTCGAATCTGGAGAATCGTTCCAGAAG
GTTATAGGAGGGTAGCTGTGAGACATTGCACTTCACTGCGCGTTGTTCAATCTCTGTCTCAAGTACCGTAA
CAGGAACTGGCCATTGGTTTTGTGGTAGTTGTAACCATTCCGGTCCATGCCACCACAAAGTGCTGCCCAAT
AGGTCCCGAGGAGAAACCCCCGACTAGCTAGGTCAGCTGGGTTATGTTCGGATCGAACATGGGACCATTT
GCCATTGGTTGATGATATCTTGGCTACCCGGTTAGCCACAAAGGTAGACCATTTGCATGGAGGTTTATCCA
ACCAGGCAAGAACAATAGTGGAGTCTGTCCAATTGAAATTTTCTTTACTCTGGATGGGGAGCTGAGGAAT
AAGAGCCGTGGTGAGCTCGGAAAGAAGGACGGCGCCACACAGCTCTAAGCGTGGTAGTGATACCGTCTT
AACTGGCGCGACTCTCGTTTTTGCTGCGAGAAGGTGAGTGGAGATGCATCCGTTGATCTCGACGCGCAAA
TAAAGTGCAGCACCATATGCGCGCTGGGACGCGTCGCAGAAGCCGTGAAACTGCACCTTTGCCTTTTGCT
GAAAATCGACCCATCTTGGAATACGGATCTCTTCGAGAGAGGGATAGTCGGTTAGGAAGTCATGCCAACG
TTGAACGAGATCTTCCGGCAGGGAATCATCCCACCCCAGCTCTTGGAGCCAGATCTCTTGCATGAAAATCT
TTGCTCGAACTATCACAGGTGCTAGCCATCCAGCAGGATCGAACAATTTCGCAATCTGTGATAGTACTTCA
CGTTTAGTGAGCGAAGGCTGAGATACCATTTTGGACGGAGCAAAGAAAAACTGGTCTGTTGTGGCTTGCC
ATCGAATGCCTAGCGTTTTAGCGACGCTGGCCTCTTCGATTTCTAAGAAATCCGCACACAGTAAGTGGTCC
CTTGGTATTCCTTTTAACACTTCCTTTGTGTTGGATGTCCACTTTCTCAGAGGGAACCCAGCTCTGTCGAGT
GCCATGCGGAGTTCTGCAATGGCTGAAATTGCTTGTGGCTTCGTGTGTGCCCCTGCTAGAACATCATCGAC
ATACATATAATTTTCAATTATATCGGATGCGATGGGACAATGTTCTCGTATATCACGAGCAAGTTGACGGA
GGACCCGGATTGCGAGGTATGGGGCACAGTTTACCCCAAAGGTAACTGTATTGAGTTCGAAGTCGCAAAG
TTCTTCGTTCTCATTGCGAAATAGTATTCGTTGGTAGGGCGTATGTCTTGGATCGACCTGGATTTGCCGATA
CATCTTGGTTATATCGGCATTGAAAACGTAGCGGTAGAATCGCCACTTTAGTATCTGAATCGTCAGATCGG
ATTGGAGTACTGGTCCCGTGTGTAGGATATCATTTAGACTTTTGCCATTGGAAGACGGGCTGGAGGCGTTA
AACACAACGCGAACCTTTGTTGTGGTGCTGTCGGGCTTAAAAACAGCATGATGCGGCAGGTAAAAATTAT
TGGGGTTGCTGTTTGGAGAGACTTGTGTCATATGACCTAAGTCGAAGTACTCCCGAATCACGGAGTCGTAC
TGCTCTTTCAAAGGAGGGTTTCTTTTTAAACGATTCTCGTTCTTTAGAAACTGAGCTAGTGCGATAGATCTT
GAATGTCCTAAGTCTATGTGATTAGGTTCTTGGAAAGGTAGGGTGACTACATATCTGCCATTCTCGTTCCT
TGTTGTTGTTCGGATGAAATTACTCTCACAAAAGGAGTCGGAGCTCTTTACCAGTTTGACTGGAATGTCCT
CCACCTCCCAAAATTTTGTGAGAATGACGTCTAATTGCTCTTCTTGAGAGACAGATATTCGAGTCGAGAAT
GACGAGATTGCGGTGGATGCGGTCTTTGAAACTGGGCCTGTTAAAATCCACCCGAAAATAGTTTCCTGGC
CCAGCAACGTGCCACATATATTAGGATGGGAGCCGCCCGTTAGTATGGACGGTAGGATATCAGCGCCAAT
CAGCACATCTATCTGCGAACTCTTGTGGAAATTAGGGTCCGCTAACTGGAGTTTTGGCAGGGTTGTCAATA
AACTTTGGGGGATCGAACATGATGGTAGATTCCCTGCCAAATGTGGTAGGACGTAGGCTGATGCCTCGAT
TTGGATACGAGGCTTGGAAGGAGAGCCAATATTAAAATGGCAGAGCTTGCGAGGCTGGGCTGCAACAGC
TTGATTTAATCCCGATACTTGTGCCTGTACGGATTTGTACGGCAGCTTTATCAGGTGGAAGAGTCGCTCGG
AAATAAATGTTGCCTCTGATCCCGAGTCGATCAGTGCACGAGCTGTATAATTTACACCCAAATGGCGAAC
ATATATAACAGCCGTACTTAGGAGAACACCGTGGGTGTTAATTGCAACATAATTGTGAACATTCGTTTGTT
GGGATGGAGTGGACTGTATGGATTGATTGGAGGGAGCTGTTGCTTGTGGCATGGAGACTACTGTAGGGCT
CAATTGTGGAGTGTCACTACGATGCAAGAGCGTATTGTGACGACCCTTGCACGTAAAACAATTGTGAGCA
CTTGTGCAATCCCTTAACTGATGACCCCTAGCGAAACAGTTCAAACATAGTTTTTGCTGTTTGATGTGAGT
TGCCCGTTCGCTGACGGGCATTTGGAGAAATCGTGGGCACAGACGGATAGGGTGATTCTCCCTGGAGCAC
AATTTGCAGGCTTGCGGCTTGTTAGCGACCTTATTCTCAAATGAGTTAAGCCTTCTAGTGGTAGGAGGTTC
TGTTCGAACAATTCTGGAGTGCGCTGGAGTATTTGCGCTTGGCTTAAGCTCTTCTATCGCTTCTAGAGTGC
GATAGCGTTCTAAGAGAAAGGCGTTCATCTCAAGCCAAGATGAGATGTCGTTTTTTTCTGGAGATGGACT
GTTCCCATAAGGAGAGTGTTAATTTGGGTAATTTGGAGGAACATAGGAACACGAGGATACAGTCCCAGTT
TTCTATGTTGATTTCGGATAACTCCAGAGCTGTTAAGCAACCTTGAATGGTGCTCTGAAGTTCCTTAATTG
CCGTTCCAGACTCTTGGGAGATTGGCTGCAGGTTGAAAAGAATCTTGAGCTGGCTGTTGACTAACAATTTT
CGGTTCTCAAAACGCTCAGTCAGATTGGCCCAGGCTGATCGGAAGCCATCGTTAGTCAAAGGTGACTTCG

ATACAATCGCCTGAGCCTCACCGCTGGTTTTCGCATTGAGATGGAATAATTTCTCGACCTCTGATAACCTA
GGATTATTTATGTAAATTGCGGTAAAGAGGTCTCGAAATGTGGGCCAACGTATGTAATCTCCGCTGAAGA
TTTCTGTGTCGCAAGGAGGTAAGCGACACCCTGTGGGAACATATGTCGGAATGGTGGACTGAGCGGATGG
TCTATTTTGGGCGTTAGCGGTATCGATTTGCTCGCTAAGTTTAGCGGCGCATCTCTCATATACCATGTAAC
AATAGTCGTACTTGGACTGCATAACTGAAATGGTTTCCAGTGAACCGGCTTCCGAGATTATTGTTGAGCCG
ACCTCGTACTCTTTTTCAACCTTGTCCCACAAGGCTCGCACTTGGTCCCGACGGACCTGGCACGTATAGAG
AGACGGGTCAGAGCCTCCAGGAGTGTTTATTTTTGCCTCAAACTGACTTAGCCGATCAGTGACAGCAATA
AACTTGATTAAGGCCGCGTCTGTCATATTTTGGGCCGTTTTGGTATTAGCTCGCGTGGAAATCGGACTAGA
TCTTGTGCTAATGGTTGCCTTAGTGCGTCCGACTGCAGTGGTCAGGGATGCTTCGCTTTTAGCTTTGGACG
GCGGGCGACTGATTTTTATCAGTGGACTTGGCCTCGAACTAAGAGGTGCCTTAACAGTTCCCCTTGGCGTG
GACGCGGATGATTCGCTTTTAGTCCGGGACGATGTTTCCCTTTCGACTCTAAGGGCTGCTGTTGGAGACGG
AGAAGTTCTGGAGGTGCTTGCGGACGGAACTGCTAATTGATGCTTTGTTTCTTCCCCTTTGCCAGTTGGCA
TACTAGGCGGAAGCGCTTGGGTAGGAAAACAAAGGTGCTCGTTTCTAGTAGTAGAGGTTGCGATGCTTAA
AACAAAGAAACACTGGAATGCCGGTACAGGTAAATAATCGAGTTTATAATTCGATTGAGATTGCGGAAGA
TTTAAATAATTTTGCTTAAAATCAAAATTTATTTGATTTAATNAATTTAATTAATAAATTTATTTGACTTGG
TAATTTAAAAAATACGATTATTTATTAAACACGGGAAATAATAATAAAGAATTCGATGCTCGTTTTCGATA
ATTTTATTATTATATTAGTCTTTGAATACGGTAACGGTTGGTTTATTTTTACTTTTTGTGGTACTCATAAAAT
ACCCCACTATTGTGGCTGAGTACACACCGCTGACAAATGTAGAACTTCGAAGTTAATGTGTAGTGTTTTTG
TTTATTTACAATAAATTAGTAGTATTATTAATCTATTAATTATGATTGTTGTGTTTTTATTTGGTGCGTCTCG
GAACAAGAGTAATTGTATGGGAATGTGTGCAATTGCATAAAAACGTGTAATTGGAGTACGGATCGGCAGC
TGTGGTTTTTACATATGTGTGTGGTGTGTACATACGCACATACGCCGGTTGAAATTGCAATTTATTACTAT
AATATTGCTGTGTTATAATATTGCTGTGGATTGTGCACTAAAAATATTATATGTGGACACACATAGTACAT
ATGATTTGTGGATTTATTTATCGGATGTATGTACTATGTGGTTGCACAAATATATTCGTTTGCACTATGCAC
AATTATAAGTTAAGAGANTGAATTGGAGTTTGTGTTTATNTTTATTAAATAATAAGGTTAAATATAGTGTC
TATTCAAAATGCATATGGGCAATTATATTCGGATTGAATATATGTATGTATGTATGTATGTACAATACGTG
CTGGTGAACCTGGTGTATGTTTGTGTGTCGGACCAAGAACATGAATTAACAATATTAATTAGGAAACAAT
ATTGTGAAGCAACTTGTCGGATTAAGTGCGGACACGTAATTAAAAAAATTTTTTTATAGTTAGGAAAGTG
CACGAGTGCGTACGTATGTACATACAAAAAATGTTGAAGTGGATTTCTTTTGTAGTTGGAAGTAACTAAC
ACAAAATTTAACTAAACTGTTTTTAGTTTTGTGGTCGGATAATTTATATATATTCAGAAAATATGCNNATC
AGTTTTCTTAAAAAAAAATATGGCGGAAAAATATGGCGGATATTGCCGAATATATATAGAAAGGAAATTT
TGTATTTACTTATCTTTGCCAAAAACTTGTGTAGGGGCGATCGGTTTATATATAATCCGGCTCGAAGGACC
AATGTTTGTGGGGGTGGCGTCAGATAAATAATCCTTGATTATTGTTGGTGGAAAAAACCACTTTATTAGAA
GTTTCTGCTGCATGTAACAAACTCTGCTGGTTAAGTGATTTTACATATTTTTGGTTGGAGTGATGGAGGTG
GAAAGGAGACCACAAGGAGAGTGCTGAGCGCATCTGGGATCGAATTAGTGAGTGAGTCTCTCTCAGTGA
GTGAGTCTGTCTCACTGAGTGACTCTCTCACACTGAGTGAGGCTCAGTCAATGCTGTGTTCACACTAGCAC
TGAACTGGCATGAACA
>Ref_dvir_750
CCTCTTGTCGTTAAATTATATGAGATTGTCCCATTTAGAGCCATTTAGTAAAATTTCAAACTTCAACCCCGC
ATGCCAATGAGATTTACATCAAAGTGAATATCATTATATTACTGATACATTTAATGAAATATTAATGATCT
TGTTCTTGATCATTATATCAACGCTTATACCGACTATTATATTTAACAATATATGTTTTCTTTCTCAAACGA
ACTGATTTTTAAAGTTTTGAAACTTTATATGCTAGAAGAGCATGCTATCTTGATCATGTCTTTAATTAACTT
GTTTTTGCTCATTGAGGATACGATACAACTTACGTGCGAATATAGCGTTTTACATATGATGAAGTATGAAA
TTTAAATGATGTCGGTGAAAGATGATTTAGTAAAGTGATGTTTGAGCTCAAATCAGCACCATCTGTAAATG
AATAAGACTTTAGTTTACGTATATATGAATTAGCTTATATTCCAAAAGCAAGTATACCGTTCCTTGCAAAA
ATCATTGTAAAAAAACATGATCAGTTAATATTTTAACTTGGCTTACAAAAAAATTATATATACTAGGAATA
ATTCAAGGGCAGGGCGGCCACGCTGCACCATTAAGAATTTGGATTTACGAAGATCTTATTTTGAAATTGCT
GTTAGTTTTCTTTAGCATATTTCATAACGAAGGAGGGCTGCTGCTTATATATATGTAGATGGTTTTTCAGA
GTCCATTTTCACATTGGCGTTCCGAAGATATAGGTTATGTGAAGCACGCGCTAAATCTGAGCATTGAGGCT
ATATGCGAGGCCATACGTGCTTGTTGCTCCTTTCACTGTGAAATTTAAGCTGTTACTGTGGCCGTCGAAGC
TACTTATGCCAACAGCATTCCGTCGAAATTTTCGCCTTTCCGTGGATAGAGAAAGTTCTTCTAGAATTCCA
CACTCGTGGTGGCAGACTCTCACGGTGGACTTCACACCCGTGGCTATGGCAACACGGCCTAAACTTCGCC
GGCTGTTATATTGGCCTACTGATCAGTATTCGGTTGGTGGTAAATTTCTTCCACGTGTCTTTAGACAACCG
GTCTTCTCGGCTTGCAAAAGATTACTTGATGAGCTCTAAAATGCACGTCTGCTTCGTAGTGCCGCCGAAAT
TAAAAAGAAATCACGCCACGCGATTTGGTTTGGTCTCAGCATCGGTATACAAAAAAAAGCTCTCCAATCA

ACGGGATCCTTCCAATGCTTTTGACATCTAGCGTTGCCAGATCACCCGATTTACAATTTTCTCTTACCAGAT
AAGGTTACCACTTAAATGTAAATTATTAAGAATCAGCTGACACTTTCTGACTACGTTAAAATAAACAAAC
ATCTTTTTCCCTTGCTGCTGTTCCTATTTTTTTAATTCGACATTTGATATATGCTGGTAAAAGTTTTGCATT
AATGCCTTTACTAGCATTGCTTAGAGTAAAATTCTTTTTAAAAATTTCCTGTCCTTTGTAATTCACCCATCT
CGATCTTCGATTATAACATTTTTCTGATTTTAAATGTGCTTCCAATAGATTGTCTTTTATTTTGTCATGTAGC
AGTTGCATTTTATTACAATGTGGCATTACATTCACCTGATTATCTCGCAGCGCTTCTAATTGTTGTAACAAT
TTATAACTTTTTCCATGCCTTAACATCGGCTGACCAAACATGCTAAAATATGGAGTACATTTTAGAGAGCT
GTGATAATCGCTACGCAAGACACTACAAATTTCTGACAAATGCTTATCCCACTGATCGTTTTTTTTCTTAA
GCAAAATCCTTAGTTTAATTAAAATTTCTCTATTTACTCTCTCACTGACGTTTGCTTGAGGAGAATATAAGC
CTGTACGTATTTGTGCAATCCCATATTTATGTAAAAACTCTACCATTTGAGAAGATACAAACTGTTTACCA
TTGTCGCTATGAATATATTGTGGAACACCAAAAGTATGAAATATGTGTTCTTCTAAATACTGCATAATATT
TATGCTATTGGCTTTTTTTAGTGCTTTTAAGATATGTACTTCGTAAAATGGTCAATAGCAATAAATACCATC
GAATTGGTCTCTCTGTAATTATTTGTTTTCCCATGGGCGGTCTTTGAATAATGTTACTTGGCTTTGCCATCT
TACAATTTTCACATTGCTCTACAAATACCTCAACTTCCTTACACATATTGGGCCAATAATACTTTTCTCTAA
TTCGCGCGAGAGTTTTACCAAAACCTGCATGACACGATGAGTCCTCAAGATTACTTGCAATAACTTGAGCT
CGTAACTCACTAGGTAAAAACAATCGCCATAAGCTAACTTCATCTTCAAATTCGTTTGGTATACGAAATTG
CACGAAACACAAGTATCTGCAACGAATTGCCATTTACTAACTAGCGTTCTCAATTTGTCATACTCGGGTGA
TTGAAAGCATTGATTTTCCAAATCAATTTCTGATAGCTTCTGGGGTGTTTCTAAACAATCTAAATATCCCCT
AGACAGAGTATCGGGTACTACATTAAGTGAGCCTTTACGGTACTCAATTTTGAAATCATATTGTTGCAATT
TTAAGCTCCATCTGCCTAGTCTCCCTATTACATCTTTTTTTGTCATAAGCCATTTAAGACTGACATGATCAG
TTATCACTTTAAATGGTAAGCCTTCTACGTAGGGTCTAAATCTTTTTAAACACACTATAGCTGCTAAGCAT
TCTAGTTCCGTGACTGTATAATTTTTTCACGTTTCTTTAATTTCTGAGAGACGTAAGCTATAGGGTGTTCCT
TGCCGTTTATATCGCATTGTGTTAAAACGCCACCTACTCCTGCCCGCGAAGCGTCACATTGAATAGTAAAC
TCTTTATTAAAGTCTGGCTGTACCAACACTGGGGCGGTTATCAGTGCTTGTTTAAGATTTGCAACTGCCTG
TACTGCTTCAGGCAATAATGCGAACTTCAATGATTTCTGCAGAGAATCCGTTAATGGTGCTGCTAATTCAG
CATAATTTGGGATGAACTGTCTATAATATCCAGTCATGCCTAGAAATCGTCTGACTTGCCGTTGTGTTGAT
GGTACTGGAAAACGAACGATATCCTCTACTTTCCCCGGGTCAGTTTTCAAGGTTCCTTGACCCACAATATA
ACCTAAGTATTTAATTTCTTGTTGACAAAATTTACTCTTCTCGACATTAATAGTTAATTGAGCCTTTTTCAA
ACAATCCGCTACTTGTCTTAGTGTTTTAAGGTGACTATCGAAATCCGAATTGCATACTAGCAAATCATCAA
GATAAATAAAAACTTTTTCTCTCAACGACCCTGGTATTACCTTATGCATTAATCTACTCATTCTTTATGCTG
AATTACACAAGCCGAAGGGCATAACAGTGAATTGGTACAAAGGTCTTCCTGGTATAGCGAAAGCCGTTTT
TTCTCTCGATGCTTCACCTAACGTTATTTGCCAATAAGCATCTTTCAGATCCATACCTGAGATGAAGTACG
TATCTTGCAATCGACTGAGTAATCCATTGATGTGTGGCAGTGGATATGCATCTTTCTTAGTAATAAGAATT
TAATCCGACGAGAATCAATACACAGCCTGACCTTTTCCAGGTTTCCTAACTAATACTATGGGGACTGCACC
AAGGAGAATTTGCTTTTCTTCTATTACTCCCAAAACTAACATACGATCTAATTCTGAATAAACTTCTTTTTG
TCTGGGTAGTGACAAAGGATAGTGCTTGAGCTTAAATGGCTCTGATGTCGCCGTATCAATATCATGGATAT
CTAGATTGGTACGACCTAGTCCTAATTTAGTAAACGATGGAAATTCTTCAATAACTTCCTTAAGTTGTCTG
GTTTGAGTATTCGACAACGATTTGCTTTTAATCATTGGAACATTATCTGGTTGCACTTCTAATGCAGAAAT
CAATTTTGGAGCTATATTAAACTCTTTCCAAAAATTAACTCCTAAATAAACTTCTTGTTGTAGACTGGGTA
CTAAATAAAATAAAATGTTTTTCTCTAATCCGTTATAAGAAACCCCTGTTTCTACCGTTCCCAAAATGTCT
GTAACTTTCCATCAGCTGTTTTAATTGAGCATTTTAATTCTTTGATTGATAAACGCATTTTATCCATTCTCTC
CAAACAATTTTTTCCTAATACACTAACTGATGCTCCTGAGTCTAATAAACCATATACTTCTTCATCGCCTAT
CCGAATTTTTGCATATGGCCTATTATCATTGCTCTTGCTGCAAATAATAGTTTCTAATTCCTTTCGTAAATG
CATTCTATTATCTTTCTCTACATTTTAATATTTTAGATGATATTTTTCTTAGACTTAACTTGCACTTAAGCTG
CTCTAAACGTAATGTATGTAATTCATTCACTTTAAATTCACTTAATTCTTCTGTTTTAGATTTATCTAATTGT
TTGATTGGGGTTTCTTTCTCGAACGAGGTAACCCCTTTAATCCCACGTTCGGCTAATTGTTTCCCTTATGCT
TAGGACAAGTAAGGTTACATTTTTCTCTCCACATCGGTAACAGAAAAGAATCCTTGTTGCAGACGGACAG
TCATAAAACGAGTGACCTATTTGATCACAGTTCCAACACCTATACTTGGACTTATCTACTGCCTTTTCATAC
TTTTCTCGAATTTCTTAAAAGCGGATAATTCAATTTCTTTCTCGCTGTCAGATTCTTCTGCCTTTAGATCCG
ATACTTCTATTTCATTTGCAAATTTACCAGTTGTCTGCTGCAAGCGTCTAATTTGTTTACGAGCCATAAACT
TTTCGGCCTTACGAGCTTCCGACAAAAGGTGATCTAAACTAAAAATGCTTTGGGCGAACAATAGGTTGCC
TAAAGATTCTCTAACATTTCCTTTTATAATATCTGCTAGTTTTGCATCACTTAACGGTTCTTTCAGACGGGA
ATTCTTTGTGACTACATCCATATAAAATTCATCAAACGTTTCCTTAATTGCTGTTGTCTCCAATAACACGCA
TCAACCTTTCAGATTCGTTGTCTGTCTCTCCGAACTTGTGAGTAATAGCTAATTTTAAATCCTTGAAAGTAG

CTTCTGGTTCTTGCTTCAGATAGAACCAATACCATTTTTCGGCTTTCCCTCGTATCAGTACATGAAACTCTG
CTAACAATCGAGATTCCGAGCCCTTTTTACTAATGTAGCTACTTTGAATAAAAATTCTTCTACATCATCACT
TCCATCATAAGAAATATTCCACTTATCCACTTGCACTTTGTCATTGCTCGCTATAGGAAAATTATTGGAATT
ATTCAAATTACGTGACTGGTTACGACTAGAAGTGCCATTTTCATTAGGCAATTCAAGATAATTGTTAAAAT
TTTCTAATGAAGGTCTAGCTCTTCCTTCGCGTACTGCTTCTACTAAGTTACGTTGCATATTAGCAATCTCTC
TCATCATCGTATTGTGGTTAATTGTAAGAACTCTTTCTAACACTGCACTTAGAGTTTCACCTAAGCCACTA
GCTTCTGGACCTTCCGCCATATTAATGTTTGGAAAACTTTCTACTTCGACTGTAGGCTATTCTGCTGAGCTC
GGACTATATGTGCGCCGTCTTCCTCTACCTTGCGCGTTTCCTTCCTCGTCAAACGTGTATTCTACCTGTGAT
TGATTGGTCTGAGTACTTTTTGTTTTGCGCTTTCTTGTTCTCATACTCCAGTTTTTTTTTTTTTTTGCTATGG
ATTTTTAATGCTTATTTTCTTAATATAATTATATTATTAAATTCTGTTATTAATATTACTAACAATTTTCTTA
CAATTCATAATTTTAGAGGCATACAAATACTTCCGTACAGACTGTTTTCGCAGTTTAAAAAAAAATCTTCT
ATCGTGACAATAATTATTTCTGTGAATTACTATACTTGACGATTACAAATTGGTTGAGCTGTCACTATGGC
AACATTGTTTCTGATTCTTTCAAGTGACAAGGGACTCATATGACAACACCAAGGAATTTAAATTGAATCGC
TTCATGTAGCAATATATTGTAACGCTGGTGGCTGTAGGCGGCTAGAATAGTGCACTGATATATGCATGATA
CCTAGGGCTGTTTAACTTATTGATGCTCTATTCTTATGAGTGTTCGTTTCCTGCTTGACACGTCTGGTTGCG
TTGCTTAGCTCGCCGGATGGGGTGTGAGATCTTGAAACTAACAAAACTCATAGCCTAAGAATGCTACTGC
TACCGAAACAATAAATAACAAATACACGTGTCAAGACTGGTGCTTATAAAGCGGTATTAACTGAAAAGCG
GACAGACGTTATCGTTCACGATAGTTTTTGGCTCAGTTGGATGGCATAACTGAGGCTACACACTTAATTTT
CCCTCCCTATAAATTGCTCTGATCAAAGCAACGCCATGACAATCCTCGTGTGTAAAATTATATGAGATTGT
CCCTTTTAGTCTATTTAGTTACATTTTAAACTTCTACCCCGTCTTTATACTTCCACATCTGCATGCCTCAAAT
GAGATTTACATCAAAGTGAATATCATTATATTACTGATACATTTAATGAAATATTAATGATCTTGTTCTTG
ATCATTATATCAACGCTTATACCGACTATTATATTTAACAATATATGTTTTCTTTCTCAAACGAACTGATTT
TTAAAGTTTTGAAACTTTATATGCTAGAAGAGCATGCTATCTTGATCATGTCTTTAATTAACTTGTTTTTGC
TCATTGAGGATACGATACAACTTACGTGCGAATATAGCGTTTTACATATGATGAAGTATGAATTTTAAATG
ATGTCGGTGAAAGATGATTTAGTAAAGTGATGTTTGAGCTCAAATCAGCACCATCTGTAAATGAATAAGA
CTTTAGTTTACGTACATATGAATTAGCTTATATTCCAAAAGCAAGTATACCGTTCCTTGCAAAAATCATTG
TAAGAAAAAATGATCAGTTAATATTTTAACTTGGCTTACAAAAAAATTATATATACTAGGAATAATTCAA
GGGCAGGGCGGCCACGCTGCACCATTAAGAATTTGGATTTACGAAGATCTTATTTTGAAATTGCTGTTAGT
TTTCTTTAGCATATTTCATAACGAAGGAGGGCTGCTGCTTATATATATGTAGATGGTTTTTCAGAGTCCATT
TTCACATTGGCGTTCCGAAGATATAGGTTATGTGAAGCACGCGAGCATTGAGGCTATATGCGAGGCCATA
CGTGCTTGTTGCTCCTTTCACTGTGAAATTTAAGCTGTTACTGTGGCCGTCGAAGCTACTTATGCCAACAG
CATTCCGTCGAAATTTTGCCTTTCCGTGGATAGAGAAAGTTCTTCTAGAATTCCACACTCGTGGTGGCAGA
CTCTCACGGTGGACTTCACACCCGTGGCTATGGCCACACGGCCTAAACTTCGCCGGCTGTTATATTGGCCT
ACTGATCAGTATTCGGTTGGTGGTAAATTTCTTCCACGTGTCTTTAGACAACCGGTCTTCTCGGCTTGCAA
AAGATTACTTGATGAGCTCTAAAATGCACGTCTGCTTCGTAGTGCCGCCGAAATTAAAAAGAAATCACGC
CACGCGATTTGGTTTGGTCTCAGCATCGGTATACAAAAAAAAAGCTCTCCAATCAACGGGATCCTTCCAATG
CTTTTGACATCTAGCGTTGCCAGATCACCCGATTTACAATTTTCTCTTACCAGATAAGGTTACCACTTAAAT
GTAAATTATTAAGAATCAGCTGACACTTTCTGACTACGTTAAAATAAACAAACATCTTTTCATATTTTACA
AGGCAGCCACCTTCTTCGCAGGCTTCTAAGCCGAAGAAAAGGAAGAAACGGTTGACTTATCCTCCTTTAC
AATTTTTCTCTCGTGGGGCTCGACCACAACCTGAGATAAAGCCGGGCACATTACATAGCTTTTAGATCTTT
AGCATGAAATTTTCCTACAAATTTCCCTTGCATATTTTCTATTTCATATAGTGCTGTTCCTATTTTTTTTTTT
GATTCGACATTTTATATATGCTGGTAAAAGTTTTACATTAATGCCTTTACTAGCATTGCTTTGAGTAAAATT
CTTTCTAAAAATTTCCTGTCCTTCTTTGTAATTCACCCATCTCGATCTTCGATAATAACATTTTTCTGATTTT
AAATGTGCTTCCAATAGATTGTCTTTTATTTTTGTCATGTA
>Ref_dvir_303
TGGTGACATGGTGAATATAATGTCGTAGAAGAATGTTTTTTTTTTGGGATCTTCAAAACCTTAAACGCTGA
AGATCATGGCTGGGAGGGTATCGAACGCGATATTGGCACTTTTGCTCGTTAAGTTATGCCTGTTTAGATTT
TGTTTCTTCGGAGTATCGGTCTCCAATTTAGATTCTGTAGGGATCCTAAAATAAAATTGTGTATGCAGGTC
GAGACATGGCAAAAAGAACCCCCCCACCCGAAAGAGCTAGGGAGCAAGAGGATCCCGCCCTGCCATGCT
AACTCAACCACGAGGAGCCTCGGTGAATACTTCCCGAATTATTTTGTGAAGATTGGCTGCCAACCGCCGTC
ACCGTTGCTACCCGATATGTATTGCTAGAGTAGCTTCACCACAGTACATGCTACAATTTTTGCGAGGTGTC
TAACAAGATAGTAATTAGGACTACGATGCTTACAATTCAAGCTTGAATTATGCCGAGAATCTGACCTTACT
ATATTGTTGAGTGCCTATATATAAACACGACTATAGTTATTAACACCAGCTATACAATCGGTCCCATACAT
TTGCTCGTTTTCTTTCTTATAATTTTGGAGAAACAACAGATTAGGTTTGCTTAGGTAGAAATACTTTCATAT

GCAATTTTAGAATACTGCATATTTTGATAAGATATCAGTACTGATTGCTACTTGATAGCTTGTGGATGGTT
AAGTAAATTTCAGTCTTCGTGGAGCCAAAAGTTATAAAATTAGATTGAATGGTTTTGTATTAACGATACTA
TCAAAGATAGTTAGAATGTTGCCCGGAACTTGGCCATATGCTGCTTTGCATATCCTTGCCGAACATTTCTG
ACTGGATTAAAATGTTGAATGTTACAAACCGCTTTCTAAAGTAGTTTCAATAAACCAGATAAATTACCTAC
TTTCATTGAGGCGATTATATTTCGTAATTAAAAAAAAAAAAAAAAAAAAAAATAACTTCTAGTCTATTATGTTA
CCAAACCAATGTCACGCATCAGAATTTAATTAATCATGTTGCCTTTACGCAGCCCACAAAATGTACAGCCG
TCGTCAGAGAGAGCTGATAACCACTGTGTTCTATGCCAGTTAGTAATGGACATACACAGTCTTGCTATTGC
ACCCCTTGTAGGCATGTTTTTCACAAACTTTGCTTGGAAGGTTGGTTGGTAAACCATGCTAAATGTCCCAG
TTGTAGTGCTGTGATTGACCCAGCCTCAATTAAATTAGTTGGAGATTGCTCGCCGAGAGGAAAACAATCT
GCTCCACCTCCACCTACGAACATAGGGAGAACGGTAACGCGCAGTCTAGCAAAATCTCTTAATCCTGGTC
CGACCCAGAGTGGCGAGAATCCAACAGCAAGGTTATTAGATGTTAGTGATGTACCACAATCAGGACTCGA
CGCTAGCAATAGCACGAATCCACCTGAAATCGCTCCTGCACGACCAGCTCCTGGTGTAAGAAGAAGAGGA
AGACCCACTCAGTCCAATAATACCCGCCAAAATCTGGGAGTGGACTATAGGATAATTCAGGAGATGATCG
AACAATCAATCACTCGCGTTATGTCATCACTGCAAATGCAAGCAACACCCTTTCCAAGAGTGTTGCCAACC
CCGATCCCGGTGTCGCCTAGTAGTTCGTCATTCCGAAATGGAAAAATTGCTGACACCATGCAAAAATGGA
ATCTTAATTTCGATGGGTCTACCGAAGGATTAGGGGTAGAAGAATTTCTATATAGAGTCCAAACATTGACT
GATGAAACTTTAGACAGCGACTTTAAGCACTTACATGTGCTATTTTTTAGGCAAAGCCCGTGACTGGTACT
GGCGGTACCACAAGCAGGTAGACCACATAGTCTGGACTGAATTTTGTACTGCACTTCGCCATCAGTACAA
AGATTATCGCTCCGATTTTATGAGCAAGGAACTAGTAAGAGCTAGAAAGCAAAACCCTGGGGAATCATTT
GTGTCGTTCCATGACGCTGTAGCGTCACTGATCGATAAATTTGCAATTAAAATTGACGAGGAGGAGTTAA
TCGAAATTTTAAAAAATAATTTATTACCCGAAACCCGACATAAACTGCTATACCAGCCAATAAGTTCAGTT
GGCCATCTACGTAGATTGGTCCAAATGAACGAGAATTTAGTTCATGAGCTCAGTTGTCGCACTGATGGTAT
GAATAAATCCAGGACTATTGGCCGTCGCCAATTGAACCGGAGGTAGAAAATTCAGAAGATACAAACTTTG
ACATCGCAGCAATTCGTAGATGTCATGCCAACTTTAAATGTTGGAACTGCGAAGAGCAAGGGCATCTGTG
GGAAAATTGCCTTGCTGACCGCCGTATATTCTGTTACGGATGCGGGGCACCCAACATTTATAAGCCCCAGT
GCCAAAACTGTATCCGTAAAGCTTCGGAAAACCAACAGAAGGGTGCATCCAGCAGCAGTCGGATGCCCCC
AAAAATCTAATTAATTCCCATACGAACTGTGTACCTGTGGACCGAAGCAAAAGCTTGTCCCTACACCGAC
GTTATTATTCAAAAATCAAGCAACATAGCAAGCTTTTAGTTTCGACAGTTTTTTGCAATCCCACCGATATG
CGCAGCTATGCCGAAGTGTCATTTTTAACCTTTACCGAGCTTGGTCTATTGGATACAGGCGCGAATATGAG
CTGCATAGGTTCTACGCTTGCGCAACAAGATTTTACCGCAATGCCAATCGCAGTCAGACAGCAGATGGCC
ATAGTCAGAAAGTCATCGGAGTGCTTGAAGTTAAAATGCAGTATAAATCGGCTACCAAGTCCTTGAAACT
GTATGTGATTCCTTCTATAAACCAAAATCTAATTTTGGGTCATGATTTTTGGAGAGCCTTTGAGCTTGCGCC
AAGCATTATAAATTCTGTAGAACTAAAATCAATGGAAAAAGAGCAGGAAACAGAGTCGATTGCATACCC
CTTAGATAGCCAGCAAACGCAACAGCTGCACGTAGTAAAGCAGTTGTTCCCCAACGCTGCTGATAGATTA
GGCCGCACAACATTGATCCAACACCGTATAGATATAGGAGACGCACGTCCAGTGAAACAAAGATTCTATC
CTGTAAGTCCGGCCGTAGAAAAGTTGTTATATGCAGAAATTGATCGCATGTTGCAGCTTGGAGTTATTGAG
CCGTCTGTAAGCGCTTGGAGCTCGCCTATGCGACTCGTCCTTAAGCCCAACAAGGTGCGCCTTTGTCTGGA
TGCTCGAAGACTGAATGACGCCACAAAAAAAGATGCTTATCCTTTTGCCGAGCATCGGATGGCATTTTTTC
GCGGCTACCTAAGGCGGAAAATAATTTCCCAAACTAGATTTTTTGAAAGACGCCTATTGGCAAATAGAAC
TTTCCGAGGATTCAAAGCCCTCTCACTGCTTTTACCGGTCCCCGGAAGACCGCTATATCAGTTTAAGGTCA
TGCCCTTTGGCCTTTGTAACGCGCCTTCTACCATGTGTCGGTTAATTGGACGAGATAATTCCTTCTGACCTG
CGCTATTGCGTGTTTGGTTATTTAGATGATCTGTGTATTGTGTCAGAAGATTTCAGTTCACATCTGGCGGTA
TTGGTTCGCATTGCTGAGCAGTTTAGAAAAGCCAACTTGACTTTAAATATTTCGAAAAGTCAATTCTGTGT
AACAAGTATCAATTATCTTGGTTACGTGATTGGTAGCGGAGGTATTTGCACAGATCCGGAAAAAGTAGCT
TGTATTGTAAATTGGCCGCCGCCAAAAAACTTGAAGCAAGTTCGAGGATTTCTAGGAGTTTGCGGTTGGT
ATCGTCGGTTTATATGCAACTTTTCCGATCTCACTTGCCCCATAACGGACATATTATCGACAAAGAAAAAG
TTTGAGTGGACCCCTGAAGCACAGACCGCAATGGACAACTTAAAAACAGTGCTAACATCCTCGCCGGTTT
TAAAAAATGCTGACTTTAAGAAGAAATTTTTCTTACATTGTGACGCTAGTGATTTTGGCTTTGGCGCCGTT
TTATTACAGATGTCTGGAAGCTAATGAAGAACAACCTGATGGTTTTCTTGTCAAAAAAATTAAGCCGTGC
GCAGCGAAACTATAGTGTTACCGAGCGTGAATGCCTAGCAGTCATATTAGCCATCGAGAAGTTCCGATGT
TATTTGGAACTCCAGGAATTTGAAGTGGTTACCGACCATTCTAGTTTGCTATGGCTTATGCGCCAGCAAAA
TATATCGGGTAGACTGGCACGATGGGTTTTTAGACTGCAACAATTTAAATTTTCCGTCTCCCATCGGAAAG
GCAAAGAACACATTGTTCCCGACGCTTTGGTCTCGGTTGCCTGAGAATTGAAATCGCTCAATTTAGACATG
GGTCCTTGTAATAGATCTTGAGTCCGATACATTTATGGACGCTAGCTATGTACAAATTAAAGACAAATTTT

CCCAAAATTTTACTCAGTTTCCGGACATAAAAATCGTTGATAAGTTTTTGTATGTACGCACCGAACATTCG
AGGGGAGACTCGACACCAGAAAGTCCTTCATGGAAACTATGGGTGCCTGATCGACTTAAAGTCGATGTGC
TCAAACAAGCCCATGACAGCATAACGTCGTCTCACGCCGGCATGCAAAAAACCATTGAGAAAATCCGCAG
ACATTTGTTCTGGCCAGGATTAGTTAAAGATGTACGTGAATACATACGAAATTGCGAGATATGTAAAGAA
ACTAAGGCTCCAAATTATACCCTTCGGCCACCTATGGGTGCTCACATCCCAACTTGCAGACCATTTCAAAG
GCTTTATATTGACCTGCTTGGGCCATACCCTAGAAGCAAAAGCGGTCATGTGGGATTGTTAATAGTCTTAG
ATCACTTTAGCAAGTATCATTGGTTGCAGCCCCTGAAGAAATTCACCTCAGCTGTGATACAAAAATTTCTT
CTTGAACAAATTTTCCATTGTTTTGGTGTGCCACAAACTATAATAAGTGACAACGGCACCCAGTTCAAAGC
TCGTGAGTTCAATGCCTTCTTAGCCGAACTTGGCATTGAACATCTGTATACAGCTCTATATTCACCGCAGA
GCAATGCTGCAGAACGAGTTAACCGCTCAATACTCGCCGCCATTCGCTCGTATTTAAAACAGGATCAGAC
CCATTGGGATGAACATATTACAGGGATTTCATGCTCCTTAAGGTCTGCTCTGCACCAATCGATTGGGTGTT
CTCCATACCGTGCCCTATTCGGGGTGGATATGATCACTCATGGTACCGATTACAAATTATTGAAGGAGTTG
TCCTTATTGGAAGAACCGCTGGTCCCCCTGTTGCGGTCTACCATTTGGCATTGCTAAGGACAGATATTCAG
AAAAACATCAGGAAAGCCTATGAGCGCAATGCGGCTCAATATAATTTACGCAGCAAGCCTGTTTCTTTTC
GTGAAGGACAGGAGGTGTTCTGTCGAAATTTTGCGCTTAGTAAGTTTATTAAATGCCGGGTTAAAAAAAA
ACTCGGAAATTGTTTTTATTCCCTTGAAAATCAGCAAAGAAAATTGGCACTTTTCATGCCAGGACATTAGA
GTCTATTCCCGCTATATACCCGTCTAACGGTAGGATATTATCGGGTTGTAATGGTCGCAATCCTATTGAAT
TAAACCGCGTAAGCGATCCTAACAGCTTGCGTTTGTGTAACGACGCTATAGCGATCAGCTTTTGTCTGTCA
CGGTCAGCTGCGAATGATTCTGCCGCTGCTATAAACCGCCTGCGTGCACGTTGGCAGCGCAGCTATCTTCA
ATCTAATCCGCGAAAGAAAAAAAGCTCTGAGACCCTATATAAGCCTGTAGGTCCCGGATATAACCCTTCT
TTTTTTCAAAAATTTCTTTACCATCGAAGGTCTGGCTACGGCGGTTCGGACATAGCCCCACCAAAAAAATT
AAACTAAAAGCATCAGTCGTTTAATTCGTTGAATTTAAATTCCAACTAAATTGAACCAAAAACAAAAAAA
AAAAAAAAAAAAAAAATAATAAAAAGGAAAACCCTAAAAGTGGCCGATGGGCGAAATATCAATTTTTTT
GTTCGTTTGATGATAAACCTTTCGCTACGCCAGCCGGCCACCAGCACCTCACCGGCATATATTTCTTTGTG
CCAAGATCTGGTCACTGTTTTAACCAGTTCTTGTCTGCCTGCCGAAAGTAATTTTTGGAGGAATCAATCTT
GCCGCCGCCGTCTCTGACATAAAAACCCACTTATTTTGAGGCAACCGCCACGTGTGCCCATTGGACGGCA
CTGTAATTTCTTGGGACGGCAATGTCATTTCTGCCCCCATTAAATAGACGTCAGCCTCAACTGCGGATCAG
CTGCCATTCCCTTGCTCATGCCAGCAAGAACGGGCAGGCGCCGTTTCCACAGTCTGGAATCCGGAAGAGG
GAGTAGCCTTTTGGGCCCCATTGACGCGCCGCTGATCAGAAGCCCAGATGACATCGCCCATTAAGTTGGC
CGGAGCGTGCAGATGCATGAAAATTGCTGTTAAATGTTTTTAGTAAAAAAATTGTTAATTAAATCTTATGA
GAGACTTCCAAAAGTTTGTGCAAAGTGCATTTATTAGGGCCCTCAAAAAACCGCGTGCAAGTGCAGGGAA
AAAGAAAAATTTACATCTATATACATATCTTTATATATATAGAAAGGAATAATAGTATAAATCTATCCCCC
TTCTTGTTGGGGTAGAGTCCAAAGTCATTTCTGAATTTTTTTTATATTTTTTTTTTAATTTTTTCCCACAATT
AAAAACCCTCCGGGCCCTAAATATATAACCAAAACATTTAAAGCCCCCCGAAAAAAAAAAAAAAAACAAT
TGGAAGAAAAAAACAATAAAATTTGCTGCAAACTCTCCGCTGGAAGGCCTTTCATCGAGGAATACATGTA
AGTCTGTACCTTTTGTTTTGAGTATATTTTCTTATTTTGGCACAGATAAGCACAGCCAAGCTGACATGCGC
ACCTGCTGAGTTTTTTTTTTCGACTTTCTGTTTCACATTACGCTTTAGCTCCGATCAATATGTTTATGACCGT
GTCAGTCTGGTGAATGCTTATCATCTTTGATTCTACCTACACCCCGCTCAATTATTAGCTGAAACAGAAGC
AACCTAAAAGAAAAGATTTTTAACAGAATCAAATATATGTACACAAAGATGTATCATCGTATTCACTGAT
CAGCTGGTTGCGCTGCGCTCCGATTTGCCCATGCGCTTCGAGCTTAACCTTCTGTTGCCCATATTTTTTTTC
AACCATACAATTAACCATCAACTACAATTATATTGACTCTATTATAATAAATTTTTTTTGCTTTTGCCGAAT
ACACTTTAGTTCAATTTACTAATGTCATTTTTTTTTCTTGCCTAGCCTTATTCTATAATTATTAAAATGCACT
TTAGCGAAATTTTGAGATTTTTTTTTTATTTTGCTCAATAAGAAAAAAAATATTAACTGAATAGTTTATAA
AATGAATTATTTTTGAAGAATTTTTGAGCCATTGTAAAAATATTAACTGACTAGTTTTAGTTACTGAATTAT
TGTTGAATCATTAATGCCACTTGTAATAAGATGCAGATTATCCAACGGAAGGATTCAATTTCATTGAATAA
TGCTTTTGTTTATAATAAATATTAATTACGGATTTTGATTATGAATTGCTAAGTTTTCTTTCCTGGTGACAT
GGTGATATAATGTCGTAGAAGAATGTTTTTTTTTTGGGATCTTCAAAACCTTAAACGCTGAAGATCATGGC
TGGGAGGGTATCGAACGCGAGAGTCAGCTTACATCCAAGACTTCGCCTCTACGGAATATATATGCCCTAA
TGAAAGCTATCACCATTGTATTGGCACTTTAAGTGCTCGTTAAGTTATGCCTGTTTGAGATTTTGTTTCTTC
GGAGTATCGGTCTTCAATTTAGATTCTGTAGGGATCCTAAAATAAAATTGTGTATGCAGGTCGAGACATG
GCAAAAAGAACCCCCCCACCCGAAAGAGCTAGGGAGCAAGAGGATCCCGCCCTGCCATGCTAACTCCAC
CACGAGGAGCCTCGGTGAATACTTCCCGAATTATTTTGTGAAGATTGGCTGCCAACCGCCGTCACCGTTGC
TACCCGATATGTATTGCTAGAGTAGCACAGTACATGCTACA
>160_rnd-4_family-280__LTR___Gypsy

AGTTAACACTTAAGAATTGCACCAAAATAACGCTGTATAAGAAACTTGCACAATACTTATATTGACCCATT
TACAACCAAGCACATTCCGCTGACACATGAGTTCAGCGGCCTGACCGCAACGGTCAGCAGCGGTAACCTG
ACACTTGACTATGCTGCAAGTATCCGCGTTGTCGCTTATGCCGAGTCGGCTTGCCGACTGTAGCTTTGTAG
TTTATAGATCCAAGTTTTAGGTATAATTCCAATTCAATTTTATACCACAATTCAGTTTTGTATTTGACGTTT
AAGAATAAACTTGCACTACTCCGGCACGCTGCCGTAAATATCATTTGTTATATATTACAAAAACATTGCTG
AGCCACAAGGCCAGTAATTAGCAAAGAGAGTGAAGTCAAACTCTCCACCGTAATCGTCCTAAATATCGCT
CGACTTGGCATCGCTGGACCAGCAGATTGATATAATTGGCGCCCAACTCGTGGTAATAACGAAAGAAAAT
TACCCACGCGAGCGTTCGTTAATAACTGAAACACGCTAACAAATACATTACGTGATTGCTGACCTGCGTC
AATACAGGCAGCAACAGTGCAACAACTTCGCCCTCGTAAAGACACATTGGCAGAAGTGCACCGAGGCCA
CTGAAGCTATCTGGACAGCGAAGACTGCGGCGTTGGAGCTTAATTCCCCTTTAGTAGTTGTCTTAGAATCA
TGTAAGTGGGCTTTTCCTTAACTCTTTTCAAGTCAATTCAAAAGAAATTTAAAAAAAAAGCACATTATATT
TTGCATTACCTATTACTCGATTATAAATCTATAAAGAAAAATAATAATAATAAGTTATACGGCATTGAGAG
CTTTGATTGATAGATAAGCTAGATATTTGTCACTTGTATTTAAAGCAAATTGCAAGAACGCATATTTTGTC
GGCTTGCTTCCCACTCTATTCGTTAGTTGTCTTTACGTAACTTTGTAGTTAAACGCTCTGGCGTAACATGAC
ACGCCAGCGTAGAGGAACTCACGCTTAATCATCAAAATATTGAATTGTTTATCTGTAGCGAAGGCATATG
AGCTCTTCATTCTCACAATACAGAACGCCCGGCCACTGTTCAAGTGACTCAGAATCTGACTGTGACACTCA
GGCCCTTATTCGCAGACCAAAACTGGAACCCGTTGTTCCCCCAGCGCCTAGCAACATGAACGCTGAACAA
CTCAGGGCAATAATGCAGACGGCCATCTCCAGCGCCTTAGCTGAGCAAGCTGCTGCAAACCAACAGAGAG
AACAAGAGTTAATTCAAAGAATTAACCAAATGTCTGAACAGATAGCGGCTGTGCGCATTACTACGCCAGA
AGTGCAAACGTATGTGGCCATTGAAATAAACAATGAGATACGCTGCGACGAGCCACTTGACGCCGTCAAA
TGCTTGCCCGAGTTTACCGGAGCACAAGAATCTTATGTGTCGTGGCGGCAAGCAGCGCTGGCAGCCTATC
AAATTTTCAAACCATATAATGGCAGTTCACGCCACTATCAGGCGGTTATCATTATCAGAAGCAAGGTCAG
GGGCCCTGCTGATGCAGTACTAGCTTCGTTTGGCACAGTATTGAACTTTGATGCGATTATAAATCGCCTCG
ATTTCACGTATAGTGACAAACGACCCATCCATGTAATAGAACAGGAGTTAGGCACCTTGAGGCAGGGGGG
CCTATCGCTACTTCAGTATTACGACGAAGTAGAAAAAAAAACTGACTTTACTGACCAACAAGGTTAATATG
TCATATGAGCCAATTATGGCTAAAGGCTTATGCGAAAAATTCCGTGATGACGCATTGCGCGTTTTTATATC
AGGTCTCAAACGCAACCTCACAGACGTGCTTTTCGCGGCTAAGCCAAAGGACTTACCCTCGGCACTCGCC
CTTGCACAGGAGGTTGAATCTAACCATGAGAGATATGCCTTTGCGGCTAACTACGCTAGAAGCCAGGAGG
AAAAAGAGCGTAGACCAAGCACTAAAGTGCAGAATAGCCAGCAGGGGGAGCCACAGTCAAAATGGCCA
GAAAAACCCACACTACAATAAGCAGGCCAGGACGCAAAGCCAAGCCGATCAAAAGAGCGGCAGACAAC
AGAATAACAATACGGCACCAGTGCCCATGGAAGTTGACCCTTCCATATCGAGGATGCTACAGCCCTCACA
AGCCCTAGCTTATGTAAACAAGAGATCAGCCCAATCTGATCGCACAAGTGCTCAGAGAAAGCAGCAAAG
GGTAAACCACGTCGCGCAGGGCGCACCAGGGGGGTCAGAGTCGTATGCCGGCCGCCGATAGCGCGGCGT
CGCAAATTGACGACGACGGCGACAGCGATGCTGGTTCNGATGTGCTCAATTTTTTAGGAGCAAGTCCCTG
CTACCTCTCATCCGACGAAGAGTAGCGGGGACTGATATGAAATTCCTCATCGATACGGGCGCGTCGAAAA
ATTTCATTCGACCATATGAAGGATTAAAAGGCGTTTGCCCGGTTGACAAATTTCACAATTCATTCNATCCA
CGGCGAGACACAAATTACACACAAATGCTTTGTTTCGATGTTCGGGTCAAAGGCAACCTTCTTTATTCTGC
CGGACTTGTCAACTTTTGATGGGATCGTAGGCCTTGACCTGCTAACGCAGGCAGCNGCTTCGCTTTGCCTA
GCTTCAGGACAACTTAAGTGGGGTACGGAGGTCGAACAAATCACCTTCCACACGTGCGCNAACGTAAATT
TCACAAATGTAGACTGTGCAGAGGCATCCCCATTAGCTAGAAGTTCTTTTTTGAAAATGCTAAAGACCAG
ACAAAAGGCCTTTGCAGACCCAGACGAGGCGTTGCCGTATAACACATCAGTCGTGGCTACCATTAGGACC
ACNGATGAGCAACCGATCTATGCCAAGCTTTACCCATATCCAATGGGAGCAGCTGATTTCGTTAATAAAG
AAATCAAGGATCTGCTAAAAAATGATATAATTCAAAAGTCCGTCTCTCCCTACAATAACCCAATATGGGT
AGTAGACAAGAAGGGCACTGATGAAGCAGGCAATAGGAAAATGAGGTTGGTAATGGACTTTCGTAAACT
TAACGAAAGAACCGTGCCTGACAAATACCCTATGCCAAATATATCAATGATATTGGGGAATTTGGGGAGA
GCCAAATTCTTTTCAACATTAGACCTCAAGTCTGGTTATCACCAGATCATACTCGCTGAACGCGACCGTGA
AAAAACCTCTTTCTCTGTGAACGGGGGGAAATACGAGTTCAAAAGACTCCCCTTTGGATTAAAGAATGCA
GCAAGCATCTTCCAAAGAACCATCGACGACATACTGCGAGAGCAGATTGGCAAATCTTGCTATGTTTATG
TCGATGACGTAATCATCTTTTCAGAAGATGAGGAATCCCACATTAAACACGTGGATTGGGTCCTAAAAAG
CTTACACGAAGCAAACATGAGGGTCTCCGTCGAAAAGTCACGTTTTTTTAAGAAAAGCGTGAACTTTCTTG
GTTTCATAGTCACCAGCACTGGCGCCACGACGGATCCTGAAAAGGTTAGGGCAATACAGGAGTATCCAGA
ACCTAAAACACTGTTCGAGGTCAGATCATTTCTAGGCCTCGCAAGTTATTACAGATGTTTCATTAGGGACT
TCGCCGCAATAGCAAGACCCATCTCGAATATCTTGAAGGGTGAAAATGGAACAGTCAGTAAGCATAGGTC
CAGAAATATAAAGGTTCAATTCTCTGAGGTGCAGCAAAGGGCGTTTCAGAAGTTGCGCGATATTTTGTCA

TCCGAGAATGTAATGCTCAGGTACCCCGATTTTAAGAAGCCATTCGATCTAACGACCGACGCTTCGGCTCA
CGGCATAGGTGCAGTTTTGTCTCAAGAGGGTCGTCCTATAACAATGATTTCAAGAACATTGAAGGACAGC
GGAGGTTAACTATGCAACAAATGAGAGAGAGTTATTGGCCATCGTATGGTCNCTTGCCAAGTTAAGGCAC
TATCTGTACGGGGCGAAAGACATTAACATCTTCGCNGTTTCGGAATCCAACCCGAATGCGAAGATTAAAA
GGTGGAAAGCGCGCATTGACGAGTCTAATGCACGNATNTTCTATAAACCNGGCAAGGAGAATCTGGTTGC
GGATGCCCTGTCAAGACAGCACGCTCAACGCTCTGAATGAACAAGATGGGCCGAGTCCTGGACGCAGCA
ACGGTCCACAGCGAGCTNTCCCTNACCNACACCGTCGAGACAACAGACAAACCCTTGAATTGCTTTCGGA
ACCAGATNGTCCTAGAGGAGGCGCGTTTCCCCCTAAAGCGAAGCTTNATTCTTTTTGGNAACAAGANACG
CCACTTGATCAGCTACACTGACATCGAACCGCTNCTAGAAGAGCTCGAAGANGTNGTTTCACCCGAATGC
GTNAATGCNCTCCACTGCGATCTNCACACGCTNGCAANGNTNCAAGACGACCTTAGTTCGNANNTACCCG
GCCACAAAATTCTGGCACTGCAAAAATCTTGTGCGAGATATAGCGGACGNANNCGAGCGGTTGGAAAGN
ANAACACTCCGAGCACAATAGAGCGCACAGGGCAGCNCAGGAAAACATTAAGCAGGTACTCTCGGANTA
CTATTTTCCNAANATGGCNAGNNTGGCCAAAGAAGTGGTGCAAAACTGCAGGGCCTGCGCTAGGGCCAA
ATACGATAGGCACCCGAAGAAACAAGAGCTCGGGGAGACGCCGATCCCAAGTCATATGCNGGNGAGATG
TTGCACATNGACATTTTCTCCACNGACAGGAAACANTTCCTGACCTGCATCGACAAGTTCTCNAAGTTTGC
AGTCGTCCAACCGGTNCCNTCNAGAACNATNGTGGATNTCANAAGCCGCTCTACTGCAAACTGNTGAACT
TTTTCCCGNAGCCAGGACAATTTACTGCGACAACGAGCCGTCNTTGAATTCNGAGACCATCGGACTTCGA
TGCTCCGAAACGACTACGGCGNTCAGCATTGCGAATGCGCCNCCGCTGCACAGCACCTCGAACGGCCAGG
TNGAGCGCTTTCATAGCACCCTGNCCGAANTNGCGAGATGCCTCAAGNTNGACAAGAANATCAGCGACA
CCGCAGAANTNATCTTACNGGCTACGATNGAGTACAACAAGAGTTCGATCACTCGGTNACCNNCGAGAG
ACCAGTTGANGTCGTTCATGCCGCACCCNGATGAGCCGCAAGGGAACGTACAAAAACAGGCTAATTAGG
GCACAGCAAGACGACGTCAAACTAGGTGGAACGCATCNACGGCAGAACNGCGTCTTCGAGGTAGGNGAA
AAGGTGTTCCAGAAGAACAACAAGAAGGCTAGGGAACAAGCTAACTCCCTTATGCGCAGAACAGGNGGT
CGAAGCAGACCTGGGAACGTCTGTCCTCATTAAGGGGAGGGTGGTCCACAAGGACAACTTGAGGTAATCA
CCACGAGAATAAGGCCCTTGAAGTTTTTTCTTATAATACCAATCTGTTTAGCCGCTTGGCTCGCTTCTTCCA
ATTAATTATAAGACTTAGATATTAGCGTGTGCCCTCAGTTTAAGTTCTGGTGGTGGTGGGTTAAACAATAT
TAAAACACAAAAATAGCTAAAATTCCCATTCCACAGGTTTGAACTAATTCTCATCTTGTCCCTGTCCCTGG
CTTCGGCGCGCGTCACCGATTATTCAAACGCAAGGTACATCCCGATAGTGGATGGACGAATCCTGGTGTG
GGAGGATTTCGCCTACATTAAACACTCGGCGAATCTTTCAGAATATGCACGTGTGGTGGAGGAAACGGTC
AGTTTGCTTGACATGTTCCCACAATCTCACATGAAGAAGCTCCTAAAGGTGGACACAGCTCACCTGCAGG
ACCTGCTGGAAGCGTTGGGCGTACACCATAGGGTGGCTAGAAGTTTGGATCTCCTAGGGTCCATGCTGAA
AGTAGTAGCAGGCACTACGGATGCCAACGATTTAGAAAGAATAAGATTTACAGAACAACAACTAATACA
TGAAAACAATAGACAAATAGAGATTAATTCAAAAGTACAAGACCAAGTTAATAAGCTTACAATTACTGTC
AATCAAATTCTCAAGGCCGCGAAAAACACACAAGTAAACACTGAACACTTGTATGAAACGCTATTGGCTA
GAAATAGGATGCTGATGATGAAATTGCAAAATTTAATGCTTGCAATATCATTAGCAAAAGCAAACATTGT
AAGCCCGAACATTTTAGACCATATCGACTTGAAATCAGTTTGGCTTGAGGAGCCCACCGACACCCCCATA
GCAGAACTTTTGTCCGTTTCGTCCGTTAAGATACTACAATCGAACAACGCCTTATACTTCATTATTAGGTTC
CCAAAAGTTAAGCTAGCTTGTAAGAAAATCACAATTTATCCCGTCATTCAGAAAGGCGTAATACTGCGAA
TAACAGACAACACTGTGGCAGAGTGTGATGAACACGTTCACGCTCTCGAAAACTGCACCGCATCCCTTGG
AGCGACGTTCTGCAGACTCTCAAAGGAAAGCTCATGCGCCAGAGAGCTACACGCCGGGGGGTGGCTAACT
GCGAAACCCAGCCTAGCAACCTGGAGCCAATTACTCAGGTGGACGAAGGGATGCTAATTATCAACGACA
AGCTAGCAGAAGTGCAAGTTGATAACGGCCCTAAGACCTGGCTCCACGGCACACACCACATTACGTTCGC
TGATCGGGCTAACGTAAATGGAACTCTTTTCATAAACCATAATAACATCTTGGATAGAATCCCAGGAACG
GCGAACTCTCCACTGCTGAACATTACGGCTAGCCGAGATATACTAAGTCTTCCATATCTCCATCTATTGAA
CGAACGTAATGTGGAGGCAATTCAACAGTTCAGGGAGCAAATCAATGAGGACCGGGCCTACCGAGTTTCG
TGCATAGCAGGAGCTATCTGTTGCGCAACCGTTTTTATCGGGTTGACACTTTGGCGAATCACCAAGAAGA
GGAAGACTGCAAGGCGGCTGAGAGAGGTGATTGCCGGCATAGGGTCGACCGAGGACGGTCATAAACTCG
AGGGGGGAATAGTTAACACTTAAGAATTGCACCAAAATAACGTTGTATAAGAAACTTGCACAATACTTAT
ATTGACCCATTTACAACCAAGCACATTCCGCTGACACATGAGTTCAGCGGCCTGACCGCAACGGTCAGCA
GCGGTAACCTGACACTTGACTATGCTGCAAGTATCCGCGTTGTCGCTTATGCTGAGTCGGCTTGCCGACTG
TAGCTTTGTAGTTTATAGATCCAAGTTTTAGGTATAATTCCAATTCAATTTTATACCACAATTTAGTTTTGT
ATTCGACGTTTAAGAATAAACTTGCACTACTCCGGCACGCTGCCGTAAATATCATTTGTTATATATTACAA
AAACATTGCTGAGCCACAAGGCCAGTAATTAGCAAAGAGAGTGAAGTCAAACTCTCCACCATAATCGTCC
TAAATATCACTGGAGTAGGCATCGCTGGACCAGCAGATTGATATAATTGGCGCCCAGCTCGTGGTAATAA

CGAAAGAAAATTACCCACGCGAGCGTTCGTTAATAACTGAAACACGCTAACAAATACATTACGTGATTGC
TGACCTGCGTCAATAGAGGCAGCAACAGTGCAACAACTTTGCCCTCGTAAAGACACATTGGTAGAAGTGC
ACCGAGGCCACTGAAGCTATCTGGACAACGAAGACTACGGCGTTGGAGCTTAATTCCCCTTTAGTAGTTG
TTTTAGAATCATGTAAGTGGGCTTTTCGTTAACTCTTTTCAAGTCAATTCAAAAGAAATTTAAAAAAAAAG
CACATTATATTTTGCATTACCTATTACTCGATTATAAATCTATAAAGAAAAATAATAATAATAAGTTATAC
GGCATTGAGAGCTTTGATTGATAGATAAGCTGCATATTTGTCACTTGTATTTAAAGCAAATTGCAAGAACG
CATATTTTGTCGGCTTGCTTCCCACTCTATTTGTTAGTTGTCTTTATGTAACTTTGTAGTTAAACGCTCTGGC
GTAACATGACACGCCAGCGTACAGGAACTCACGCTTAATCATCAAAATATTGAATTGTTTATCTGTAGC
>9_rnd-1_family-372__LTR___Gypsy
TGTAGTGCGTATATGCCCTTACAGAATACCCCGTACTTAAACATAAAGTATGCAGCACTTTGTTGCATACA
ATAACAATAATATAGTAAACGAGCCCAGAGCCACGTTATGTATTGGCTCGGTCTCATATTACGCAGCGCG
GTTCACTAGCCTCACTAGCTGACCGTTTATGCTTATGATGCATATCCCACGCTCCGTCTGAGCGCATTCCGT
GAGCAATATATATGCTCACTAATACAGGCATATGCGAGCTACTCACTCTCCCTCTCTTTGTAGCTGTTAAC
GAATAACAGCATAAGTATATATGTATATCCATAGGCTAAGCGAACATTATAACAAGTGAATAAATTCATT
CTGAACAAAGATACAAAACAGCACTGACGTGCTGACTTATTTTTCATTGACAATAAGTGCTCATTTTACAT
GGCGACCGTGACTTGGTCACGCATCCCTGCCTGGAGGAAGTAGACCAAGTGAAAAGGAACTTCATGGCCA
ACAAAGAGGCCATGTATAATATACAATTTGAAAAAGGAACTTCATGATCAACGAGAGGCATCCCGTATTC
TGAGTGAGTAGAGAGTGAGTGTTATGGTTCATAAACGCGGTGGCTTAAAAAATAAAATTAATAAAAGAAT
AGCGCACCTAAGGTGGCTGCTATACACAAAACAGCCAACACCCGCATGGTCGACAGCTCAGAAGTTACAA
AATGAGCTTGACTATCTGCAAAATGAAATTAAGCAGAGAAAACCAAACACCACTACTCACTCAAATATAA
AAGTTCTAATAAACCAAACAACTAATAAAGTTACACCCATTGACGCAACCAGCCTACCAAAAGAAAAAC
GAATGTGCCCACAGGATTGCGAAGGCCCACTCGACAGCCCCTTCTGCGAATATACGTGCAGACACTGCAC
AGCCAGCACCTCCAAACATTACATTACAACCAACAAATAAACACCTGTGCGAAGACCAGCACCGGTACCG
CCAGCGCAACAAGCTGTGCCATCGGAATAAGTGCGAGAGGCACACCGCCACTTGCGCATACGAGGGAGC
ATCAGCGTGCCACATCCGGCGACGCCCAGCTGATGCAACGATGAACAACAGTTGCAGCTATATCCTTAAC
TACTTTTAATTAAATATAAAAAAATTTTTGTAAAATTGAGCGGAAACACTTCCGCCCGATTAGCAGCATAG
CCCGTAAAGCTATGCGAAATGCTAGGTAGTATTTTGTGTTATAAGAAATATTGAAACCTTGATTCTAAAAA
CCCAAGTCAACCAGCACTAACGTGCTTTTGTGAAATCATTATTAAATCCAAACATTACCAACATATTAAAT
TAAATAGTAATAAATAAAACTTAAAAATTGTAATGCCCGACGACAATTGAGTCGACCGGCAAGACGTTTG
GCCAGTACAGCCATTCGAAACAATAGGTAGGAAAAATACAGATTGTAAAAAAAATTTTAGATGCGTTTT
AAGCAGAAAACAAACAAGCAAAAACAAAACCCGACTTGTAAAACAATAAAAAGCATCAAATTCAATAAA
TATAGTAATAAAACCTCAAATGAACGATACAAACAACAACTTGTCTTGTAAAGTAAACGAAAACAAATTT
AACATTACGCATTTGTAAATTTTTTAAGATAATTCCTAAAACCTCAAATGAACGATACAAACAACAAATAT
ATGAATATTAATATCAAAAAGAGGAAATTAAAAGTTATAATTCGTTCAGTTTGTCACATTAGGTAATTCAA
AATTATGATCAAGAATTAGCATGATTAAGGACAAGATTAACAAGGTATATAACGACTATACAGACTTTCG
TATGCGAGCCATACAAATTTTGCAAAGGCATAGAGAATTCATACCAGTATATGAGTTCGAATACGAATAT
ATAGAAACATTCGGCACAGTGACAAGTCCACTCTATTCATGTTCTAAAATAGAGNGCGGAATAATCTTGT
CCACACTACCCTACGTCAAAATAAAAACAATTATTGACGAGCATCCGATTCTAGGGCAGACAACAATTGT
TTTAGTACGTCTGCATAGGTAGTAGTCAAATCAAAAAGCAAGAATATTTATATAGGAATATGGGGAAAA
GAATCAAAGGCAGATGATAACACTGCCAATGTAGTTAACGTAGTAGAAGTCGTAGACACCACTCCCCATG
TGCAGTTAACAAACATACTGCTAATAATACTCATTGTAATAGCTTCAGTAGACATCCTTCTGAAGATTTAT
CTTCTTCATAAAAATCACTGAGGAAGCGTTACATGAGTCGAGCCAACGACTTAGACAAGGTCTAAGCCAC
TGCTTAAATAAAATATTTAAGTAGCGGAAGCTTACATGGACTGGTCCCAAATAGTAGAAAAGATAAAGCG
GCATAGGGAAAATTTCGATAAATCATACAAGTGCCTTAATAAAAGCGCACCAGTTTTAAGACCCACCATA
ATTAAACATACTGAGATCCTTGTATATGAGTACAATGAAATCAGGAGTCTAGTTACCGAGTGTCAAAATA
AACTGACAAGTTTACACAGAGTCCAAACTTCAGACGTTTTACAGCGTCTAAGGGATAATCTGGTAAACTT
AAATAAAAAGTATGATCTCCAGCTTTATCTTCCATCGGTGTTAAATACACCGGTAACATTTGATAAGTCAC
TACTGACAAACGACCAAATAGAAGATACAAAATTAGTAAACATGGCACAAACAGTTGTAGAATTTATCAA
GCTAGCCACATCTCTGATACCAGAGTTTGACGGTAAGCCGGAAAATCTACAAAGTTTTCTAGATTCTTTGA
ATCTATTAGAAACCTTAAAAGGCACACATGAGCTAACGGCCATAAGCCTTATAAAAACAAAGCTAAAAG
GCCATGCGAGAAATTTGATAAGTAATGAGCAGACAATTGCTGCAATCATTACGCAACTATCAAATGCAGT
AAAAGGAGAATCGGTAGAAGTTGTATCTGCTAAGCTTCTTAATTTGCAACAGAAAAATAAAACGGCTAAC
CAATACACCCAAGAGGTGGAAAAACTGGCAAAGGCCCTTGAAGGCGCCTATATCAGTGAAGGTCTAAGC
CAATCACTTGCAAACAAATACAGCACTACAACAGCTGTAAAAGCTATGACCCAAAATTGTTCGATTGATA

AGGTAAAACTTATCATGCAGGCAGGCACATTCACAAATATGAATGATGCCGTNTCCAAATTTGTCAATAG
CTGCACGGAGATAACAGGTCAAAGTAACACTGTCCTGTATTATCGACGAGGTGCAAACAATAATAATAGA
GGAGGCCGAGGTTATAATCGTGGCAGAAATTACAACCACAACAATTATAACCGAGGCGGTAACAACAAT
AACAATAATTACAATAACCGTGGAGGAAGACGAGGCCAAAATAGAGGAAGAGGCCGAGGTAACTCCAAC
CAAGGTTATAATAGTAACAATGTGAGAATGGCGCAAAATACGCCGGAAAACTCTCAGAACCCTTTAGGAA
ACAACCAGTAAATGTAAAGGTTCATTCCATCAATTACAGTCTTAATATATTTGTCACTTTTTATAATCATTC
AACTGAAAATAAACTCACATTCCTAATAGATACTGGTGCAGATATCTCACTTTTAAAAATAAATTCAGAC
AATTTCAAAATTCAAGAAGAAAAAATTATAAACATCCAAGGCATAGGCCAAGGTGTAATAAAATCTCAA
GGAACAACTTCAATAGAACTTCAATCCACAAAATATATTATTCCACACGAATTTCATTTAGTAGATTCCGA
TTTTTCAATACCGTGTGATGGAATAATAGGCATTGATTTTATTAAAAAATTCAATTGTCAATTAGATTTCA
AACCAAGTGAAGACTGGTTTATAATTAGACCTCAAAATTTAAATTATCCAATTCATGTTCCGATAACATAT
AGTGCTGGCAACAATACAGTTCTCTTGCCAGCCAGATCCCAAGTAATTCGAAAAATAGACATTAATACAG
TAAATGATTATATATTTGTTCCTAATCAAGAAATACATAATGGAATTTATGTTGCAAATACAATAGCAGCA
TCCAAACATGTATACATTCGCCTTCTAAACACAACTAATTTCGACCAATTGGTCAAAGTAAACAAAATCCA
ATATGAAAATTTAACAGATTACGACATTCATAGCACTAATCCAGAAAATAGAAGCGAACAAGTACTTTCA
AAATTAAAGAAAAATTTTCCAGAACAATTCAAAAGTCAACTAACAGAATTATGCACCAAGTATAGTGATG
TGTTCGGACTGGAAACCGAACCAATAGCGACAAATAATTTCTATAAACAAACATTAAGACTTAAAGATGA
TGAACCCATTTATATTAAAAACTACAGAAGCCCGCATAGCCACGTTAAAGAAATTCAAAAACAAGTAGGG
AAATTAATAAACGACAAAATCGTAGAACCGTCTGTATCCGAGTATAACAGCCCACTCTTGTTAGTTCCGA
AAAAATCATTACCAAATTCGGAAGAAAAGAAATGGCGATTAGTAATTGACTATCGCCAAATCAACAAAA
AACTACTTTCTGATAAATTCCCGCTCCCCAGAATTGATGATATTCTAGACCAACTGGGCAGAGCTAAATAC
TTTTCATGCCTTGACTTAATGTCAGGTTTCCACCAAATTGAACTTGAAGAAAATTCAAGAAATATAACATC
TTTTTCAACGAGCAATGGCTCATATCGCTTCACGCGATTACCATTTGGTCTTAAAATAGCACCAAATTCAT
TTCAGAGAATGATGACTATAGCATTCTCGGGTTTAGAACCCTCCCAGGCATTCCTTTATATGGATGACTTA
ATGGTGATAGGATGTTCCGAAAAACACATGATTAAAAACTTAACTGATGTTTTTAATGTATGTAGGAAAT
ATAACCTAAAGTTGCATCCAGAAAAATGTTCATTTTTCATGCATGAAGTGACATTTTTAGGTCACAAATGC
ACTGACAAAGGAGTATTGCCAGATGACAAGAAGTTTGACGTCATCAAAAATTATCCTGTCCCTCATGATG
CGGACAGCGCGAGACGATTTGTAGCATTCTGCAATTATTATCGTCGATTTATAAAAAACTTCGCCGATTAT
TCACGGCACATAACTAGATTATGTAAAAAGAATGTTCCTTTTGAATGGTCAAGTGAATGCCAAAACGCAT
TCCAATACCTTAAAGAAAAGCTTATGCACCCCACATTATTACAATATCCTGATTTTCGCAAAGAATTTTGC
ATTATAACAGATGCCAGTAAACAAGCTTGCGGAGCGGTTCTAACTCAGAACCGTGACGGAATTCAGCTCC
CAATAGCTTATGCATCTCGTTCATTTACAAAAGGGGAAAGCAATAAGAGCACAACAGAACAAGAGTTAGC
GGCAATTCATTGGGCAATTACCCATTTTAGACCATATATTTATGGCAAACACTTTACTATTAAAACGGATC
ACAGACCATTGACATATCTTTTTTCTATGACTAATCCCAGTTCGAAATTAACTCGCATGCGGCTAGAGCTT
GAAGAATACGACTTCACAGTAGAATACCTAAAGGGGAAAGATAATTTCGTAGCAGACGCACTTTCGCGTA
TTAATATAAAAGAACTCAAAGATATGCAACATAAAGTCCTGAAAGTCACTACCAGGCGACAAAGTAGAC
AAAATAAAAACTGTACAGTAGAAAATTATGAACTTCTGCCAAGGCAAAGTCATAAAAATGTATCCAAGCC
CAACGTACATGAAGTCATTACAAATGATGAAGTACGAAAAGTAGTGACCTTGCGCATAACAGAATCTAAA
TGTTTATTTAAACATGGAAATAAAATTATCGCAAGAATTGAAGTTAGCGATTTATATACCAATGGAATTCT
CGACCTAGGTCAGTTCTTCCAAAGGCTCGAAAAAGAGGCCGGTATATTAAAAATCAGCCAACTCAAAGTG
GCACCGAGCGAAAAGATCTTTGAATCAGTTTCAATAGATTCTTTTAAAAATATGGGCAATGAAATATTAA
AATCTTTAAGAGTAGCGCTACTCCAGCCGGTGACCCAAATAAAAGATGAAAAAGAGATACAACAAATATT
GTCTACATACCATGACGACCCAATTCAAGGAGGTCATAGCGGCATAACAAGAACGCTAGCGAAAATAAA
AAGACACTACTACTGGAAAAATATGACACGTCATATAAAAGAGTACGTACGTAGATGTCAAAAATGCCA
AATGTCTAAAACAAAGACGCACACAAAAACTCCTCTGACTCTCACAGAAACACCTGTGAATGCTTTCGAT
ATAGTGATAGTGGATACAGTTGGTCCGCTACCCAAATCGGAAAATGGTAATGAATACATAGTCACATTAA
TATGCGACCTTACAAAATATTTAGTAGCCATTCCAATAAAAAGCAAAAGCGCAAATAATGTCGCAAAAGC
TATATTCGAAAATTTCATACTCAAGTTTGGTCCAATGAAGACGTTCATCTCGGACATGGGAACAGAGTATA
AAAACCAGATTTTAACTGATTTATGCAAGTATTTGAAGATCAAGAATCTCACCTCTACTGCATATCATCAT
CAAACATTAGGGACAATAGAACGAAGTCATCGAACTTTTAATGAATACATTCGTTCATACATCTCTGCAG
ACAAATCTGATTGGGATGTCTGGGTTCAATATTTCACTTATTGCTTCAACACGACACCATCAGCAACGCAT
GAGTATTGTCCATACGAACTAGTATTTGGCAGACTACCGAAACAGTTCATAGATTTAAACAAAACTAATT
ACATAGATCCTATTTATAATTTAGATGACTATTCTAAGGAAATAAAGTTTAGATTAGAAATAGCATACAA
AAGAGCTAGAATTATGTTAGAAAAAGCCAAGGCTTATAGGAAATTAGCATATGACGAAAAAAAAAAAAA

CTTTGAATTAAAAGTAGGAGATAAAGTACTTATAAGAAACGAAACAGGGCACAAATTAGACCCAGTATAT
TTAGGACCATATATAGTAGAGAAAATAGAAGATAATGACAACATTTTTNTTAAAGATAGTAAAGATAAAA
AAAAAAAAGTTCATAAGAATAGATTAAAAATTTTTAATTCTTAATAAAATAAAATAAAAAAATATGACGG
CCTGATCAACCGAATGAAAAAAACAAATAAACGAATTATAAATTTTTATACCTTAAATAATTTTTGTATTT
TTATACTCCAACATTTAAAATTTCTTATTATATAAAAAAATTTTCAATTTTTACAAAAAAAAAACCCACAA
TAATTAAGTTCATTTAATAACTACACATTCATAATGTTACGTTATTTCTCTAAAAAGGAGGTGTAGTGCGT
ATATGCCCTTACAGAATACCCCGTACTTAAACATAAAGTATGCAGCACTTTGTTGCATACAATAACAATAA
TATAGTAAACGAGCCCAGAGCCACATTATGTATTGGCTCGGTCTCATATTACGCAGCGCGGTTCACTAGCC
TCACTAGCTGACCGTTTATGCTTATGATGCATATCCCACGCTCCGTCTGAGCGCATTCCGTGAGCAATATA
TATGCTCACTAATACAGGCATATGCGAGCTACTCACTCTCCCTCTCTTTGTAGCTGTTAACGAATAACAGC
ATAAGTATATATGTATATCCATAGGCTAAGCGAACATTATAACAAGTGAATAAATTCATTCTGAACAAAG
ATACAAAACAGCACTGACGTGCTGACTTATTTTCATTGACAATAAGTGCTCATTTTACANNGCGACCGTG
ACTTGGTCACGCATCCCTGCCTGGAGGAAGTAGACCAAGTGAAAAGGAACTTCATGACCAACAAAGAGG
TCATGTATAATATACAATTTGAAAAGGAACTTCATGGCCAACAAAGGGGCCATCTATAATATCCAAGTGA
AAAAGGAACTTCATGATCAACGAGAGGCATCCCGTATTCTGAGTGAGTAGAGAGTGAGTGTTATGGTTCA
TAAACGCGGTGGCTTAAAAAATAAAATTAAAAAAAGAATAGCGCACCTAAGGTGGCTGCTATACACAAA
ACAGCCAACACCCGCATGG
>160_rnd-6_family-4002__LTR___Gypsy
TAATGGTCGCAATTTTCCTNTTTGAATTAAACCGCGTAAGCGATCCTAACAGCTTGCGTTTGTGTAACGAC
GCTATAGCGATCAGCTTTTGTCTGTCACGGTCAGCTGCGAATGATTCTGCCGCTGCTATAAACCGCCTGCG
TGCACGTTGGCAGCGCAGCTATCTTCAATCTAATCCGCGAAAGAAAAAAAGCTCTGAGACCCTATATAAG
CCTGTAGGTCCCGGATATAACCCTTCTTTTTTCAAAAATTTCTTTACCATCGAAGGTCTGGCTACGGCGGTT
CGGACATAGCCCCACCAAAAAAAATTAAACTAAAAGCATCAGTCGTTTAATTCGTTGAATTTAAATTCCAA
CTAAATTGAACCAAAAACAAAAAAATAAAAAAAAAATAAAATAAAGAAAAGGAAAACCCTAAAAGTGG
CCGATGGGCGAAATATCAATTTTTTTGTTCGTTTGATGATAAACCTTTCGCTACGCCAGCCGGCCACCAGC
ACCTCACCGGCATATATTTCTTTGTGCCAAGATCTGGTCACTGTTTTAACCAGTTCTTGTCTGCCTGCCGAA
AGTAATTTTTGGAGGAATCAATCTTGCCGCCGCCGTCTCTGACATAAAAACCCACTTATTTGAGGCAACC
GCCACGTGTGCCCATTGGACGGCACTGTAATTTCTTGGGACGGCAATGTCATTTCTGCCCCCATTAAATAG
ACGTCAGCCTCAACTGCGGATCAGCTGCCATTCCCTTGCTCATGCCAGCAAGAACGGGCAGGCGCCGTTT
CCACAGTCTGGAATCCGGAAGAGGGAGTAGCCTTTTGGGCCCCATTGACGCGCCGCTGATCAGAAGCCCA
GATGACATCGCCCATTAAGTTGGCCGGAGCGTGCAGATGCATGAAAATTGCTGTTAAATGTTTTTAGTAA
AAAAATTGTTAATTAAATCTTGTGAGAGACTTCCAAAAGTTTGTGCAAAGTGCATTTATTAGGGCCCTCAA
AAAACCGCGTGCAAGTGCAGGGAAAAAAGAAAAATTTACATCTATATACATATCTTTATATATATAGAAA
GGAATAATAGTATAAATCTATCCCCCTTCTTGTTGGGGTAGAGTCCAAAGTCATTTCTGAATTTTTTTATA
TTTTTTTAATTTTTCCCACTTAGTTTCGGAGAGTATCTTCTAAGTACTCACAATTTAAAAACCCTCCTGTGC
CATAATAATATAATCAAAACAATTTAAAGCCCCACAGAAGACAAAAGAAAACAAATGAAAGANAAAAAA
AATAAAATTTGCTGCAAACTCTCCGCTGGAAGGCCTTTCATCGAGGAATACATGTAAGTCTGTACCTTTTG
TTTTGAGTATATTTTCTTATTTTTGGCACAGATAAGCACAGCCAAGCTGACATGCGCACCTGCTGAGTTTTT
TTTTTCGACTTTCTGTTTCACATTACGCTTTAGCTCCGATCAATATGTTTATGACCGTGTCAGTCTGGTGAA
TGCTTATCATCTTTGATTCTACCTACACCCCGCTCAANTATTAGCTGAAACAGAAGCAACCTAAAAGAAAA
GATTTTTAACAGAATCAAATATATGTACACAAAGATGTATCATCGTATTCACTGATCAGCTGGTTGCGCTG
CGCTCCGATTTGCCCATGCGCTTCGAGCTTAACCTTCTGTTGCCCATATTTTTTTTCGACCATACAATTAAC
CATCAACTACAATTATATTGACTCTATTATAANAAATTTTTTTTGCTTTTGCCGAATACACTTTAGTTCAAT
TTACTAATGTCATTTTTTTTTCTTGCCTAGCCTTATTCTATAATTATTAAAATGCACTTTAGCGAAATTTTGA
GATTTTTCTTTTATTTTGCTCAATAAGAAAAAAAATATTAACTGAATAGTTTATAAAATGAATTATTTTTGA
AGAATTTTTGAGCCATTGTAAAAATATTAACTGACTAGTTTTAGTTACTGAATTATTGTTGAATCATTAAT
GCCACTTGTAATAAGATGCAGATTATCCAACGGAAGGATTCAATTTCATTGAATAATGCTTTTGTTTATAA
TAAATATTAATTACGGATTTTGATTATGAATTGCTAAGTTTTCTTTCCTGGTGACATGGTGATATAATGTCG
TAGAAGAATGTTTTNTTTGGGATCTTCAAAACCTTAAACGCTGAAGATCATGGTTGGGAGGGTATCGAAC
GCGAGAGTCAGCTTACATCCAAGACTTCGCCTCTTCGGAATATATATGCCCTAATGAAAGCTATCACCATT
GTATTGGCACTTTAAGTGCTCGTTAAGTTATGCCTGTTTGAGATTTTGTTTCTTCGGAGTATCGGTCTCCAA
TTTAGATTCTGTAGGGATCCTAAAATAAAATTGTGTATGCAGGTCGAGACATGGCAAAAAGAACCCCCCC
ACCCGAAAGAGCTAGGGAGCAAGAGGATCCCGCCCTGCCATGCTAACTCAACCACGAGGGGCCTCGGTG
AATACTTCCCGAATTATTTTGTGAAGATTGGCTGCCAACCGCCGTCACCGTTGCTACCCGATATGTATTGC

TAGAGTAGCTTCACCACAGTACATGCTACATAAATTTTTGGCGCCCAATCGTGGGGCCCGAGGTTGTCTAT
ATCAAAAAAAAAAAAAAAAAAAATGAGTCTTTATATTATTGTCCACGCACGCTGACGTCGATGTGACTGG
TTTAGTCTATCTTCCGCATGCGCAGATACTGGTAGTATACAAGATAGTAATTAGGACTACGATGCTTACAA
TTCAGAGCTTGAATTATGCCGAGAATCTGACCTTACTATATTGTTGAGTGCCTATATATAAACACGACTAT
AGTTATTAACACCAGCTATACAATCGGTCCCATACATTTGCTCGTTTTCTTTCTTATAATTTTGGAGAAACA
ACAGATTAGGTTTGCTTATGGTAGAAATACTTTCATATGCAATTTTAGAATACTGCATATTTTGATAAGAT
ATCAGTACTGATTGCTACTTGATAGCTTGTGGATGGTTAAGTAAATTTCAGTCTTCGTGGAGCCAAAAGTT
ATAAAATTAGATTGAATGGTTTTGTATTAACGATACTATCAAAGATAGTTAGAATGTTGCCCGGAACTTGG
CCATATGCTGCTTTGCATATCCTTGCCGAACATTTCTGACTGGATTAAAATGTTGAATGTTACAAACCGCT
TTCTAAAGTAGTTTCAATAAACCAGATAAATTACCTACTTTCATTGAGGCGATTATATTTCGTAATTAAAA
AAAAAAAAAAAAAAAAAAAAAAAAAAAACTTCTACTCTATTATGTTACCAAACCAATGTCACGAATCAG
GAAAAACTTAATTAATCATGTTGCCTTTACGCAGCCCACAAAATGTACAGCCGTCGTCAGAGAGAGCTGA
TAACCACTGTGTTCTATGCCAGTTAGTAATGGACAATACACAGTCTTGCTATTGCACCCCTTGTAGGCATG
TTTTTCACAAACTTTGCTTGGAAGGTTGGTTGGTAAATCATGCTAAATGTCCCAGTTGTAGTGCTGTGATT
GACCCAGCCTCAATTAAATTAGTTGTTGGAGATTGCTCGCCGAGAGGAAAACAATCTGCTCCACCTCCAC
CTACGAACATAGGGAGAACGGTAACGCGCAGTCTAGCAAAATCTCTTNATCCTGGCCCGACCCAGAGTGG
CGAGAATCCAACAGCAAGGTTATTAGATGTTAGTGATGTACCACAATCAGGACTCGACGCTAGCAATAGC
ACGAATCCACCTGAAATCGCTCCTGCACGACCAGCTCCTGGTGTAAGAAGAAGAGGAAGACCCACTCAGT
CCAATAATACCCGCCAAAATCTGGGAGTGGACTATAGGATAATTCAGGAGATGATCGAACAATCAATCAC
TCGCGTTATGTCATCACTGCAAATGCAAGCAACACCCTTTCCAAGAGTGTTGCCAACCCCGATCCCGGTGT
CGCCTAGTAGTTCGTCATTCCGAAATGGAAAAATTGCTGACACCATGCAAAAATGGAATCTTAATTTCGAT
GGGTCTACCGAAGGATTAGGGGTAGAAGAATTTCTATATAGAGTCAAAACATTGACTGATGAAACTTTAG
ACAGCGACTTTATGGGTCTCTGTAAGCACTTACATGTGCTATTTTTAGGCAAAGCCCGTGACTGGTACTGG
CGGTACCACAAGCAGGTAGACCACATAGTCTGGACTGAATTTTGTACTGCACTTCGCCATCAGTACAAAG
ATTATCGCTCCGATTTTATGAGCAAGGAACTAGTAAGAGCTAGAAAGCAAAACCCTGGGGAATCATTTGT
GTCGTTCCATGACGCTGTAGCGTCACTGATCGATAAATTTGCAATTAAAATTGACGAGGAGGAGTTAATC
GAAATTTTAAAAAATAATTTATTACCCGAAACCCGACATAAACTGCTATACCAGCCAATAAGTTCAGTTG
GCCATCTACGTAGATTGGTCCAAATGAACGAGAATTTAGTTCATGAGCTCAGTTGTCGCACTGATGGTATG
AATAAATCCAGGACTATTGGCCGTCGCCAAGTATATGGACTAGAAGAGTCTTTTATTGAACCGGAGGTAG
AAAATTCAGAAGATACAAACTTTGACATCGCAGCAATTCGTAGATGTCATGCCAACTTTAAATGTTGGAA
CTGCGAAGAGCAAGGGCATCTGTGGGAAAATTGCCTTGCTGACCGCCGTATATTCTGTTACGGATGCGGG
GCACCCAACATTTATAAGCCCCAGTGCCAAAACTGTATCCGTAAAGCTTCGGAAAACCAACAGAAGGGTG
CATCCAGCAGCAGTCGGATGCCCCCAAAAATCTAATTAATTCCCATACGAACTGTGTATCTGTGGACCGA
AGCAAAAGCTTGGTCCCTACACCGACTTTAAAGTCACATTCCAACAATTTAGAAATAGCTAAAACTATTA
AATTACGATTTTTACCGTATCATGAACGGTTAGCTAATTATTTGCATGTCCGAAATAAGATTTTTTATGAA
GAAAAACCCCGTATTTCTCAGCGAACGCAGCGCTTGAAAAATTATTATTCAAAAATCAAGCAACATAGCA
AGCTTTTAGTTTCGACAGTTTTTTGCAATCCCACCGATATGCGCAGCTATGCCGAAGTGTCATTTTTAACCT
TTACCGAGCTTGGTCTATTGGATACAGGCGCGAATATGAGCTGCATAGGTTCTACGCTTGCGCAACAAGA
TTTTACCGCAATGCCGGAATTTAAGCCTATTAAATCGCATGTCAGAACAGCAGATGGCCATAGTCAGAAA
GTCATCGGAGTGCTTGAAGTTAAAATGCAGTATAAATCGGCTACCAAGTCCTTGAAACTGTACGTGATTCC
TTCTATAAACCAAAATCTAATTTTGGGTCATGATTTTTGGAGAGCCTTTGAGCTTGCGCCAAGCATTATAA
ATTCTGTAGAACTAAAATCAATGGAAAAAGAGCAGGAAACAGAGTCGATTGCATACCCTTTAGATAGCCA
GCAAACGCAACAGCTGCACGTAGTAAAGCAGTTGTTCCCCAACGCTGCTGATAGATTAGGCCGCACAACA
TTGATCCAACACCGTATAGATATAGGAGACGCACGTCCAGTGAAACAAAGATTCTATCCTGTAAGTCCGG
CCGTAGAAAAGTTGTTATATGCAGAAATTGATCGCATGTTGCAGCTTGGAGTTATTGAGCCGTCTGTAAGC
GCTTGGAGCTCGCCTATGCGACTCGTCCTTAAGCCCAACAAGGTGCGCCTTTGTCTGGATGCTCGAAGACT
GAATGACGCCACAAAAAAAGATGCTTATCCTTTGCCGAGCATCGATGGCATTTTTTCGCGGCTACCTAAG
GCGAAAATAATTTCCAAACTAGATTTGAAAGACGCCTATTGGCAAATAGAACTTTCCGAGGATTCAAAGC
CTCTCACTGCTTTTACGGTCCCCGGAAGACCGCTATATCAGTTTAAGGTCATGCCCTTTGGCCTTTGTAACG
CGCCTTCTACCATGTGTCGGTTAATGGACGAGATAATTCCTTCTGACCTGCGCTATTGCGTGTTTGGTTATT
TAGATGATCTGTGTATTGTGTCAGAAGATTTCAGTTCACATCTGGCGGTATTGGTGCGCATCGCTGAGCAG
TTTAGAAAAGCCAACTTGACTTTAAATATTTCGAAAAGTCAATTCTGTGTAACAAGTATCAATTATCTTGG
TTACGTGATTGGTAGCGGAGGTATTTGCACAGATCCGGAAAAAGTAGCTTGTATTGTAAATTGGCCGCCG
CCAAAAAACTTGAAGCAAGTTCGAGGATTTCTAGGAGTTTGCGGTTGGTATCGTCGGTTTATATGCAACTT

TTCCGATCTCACTTGCCCCATAACGGACATATTATCGACAAAGAAAAAGTTTGAGTGGACCCCTGAAGCA
CAGACCGCAATGGACAACTTAAAAACAGTGCTAACATCCTCGCCGGTTTTAAAAAATGCTGACTTTAAGA
AGAAATTTTTCTTACATTGTGACGCTAGTGATTTTGGCATTGGCGCCGTTTTAGTACAGATGTCTGAAGCT
AATGAAGAACAACCGATAGCTTTCATGTCAAAAAAAATTAAGCCGTGCGCAGCGAAACTATAGTGTTACCG
AGCGTGAATGCCTAGCAGTCATATTAGCCATCGAGAAGTTCCGATGTTATTTGGAACTCCAGGAATTTGA
AGTGGTTACCGACCATTCTAGTTTGCTATGGCTTATGCGCCAGCAAAATATATCGGGTAGACTGGCACGAT
GGGTTTTCAGATTGCAACAATTTAAATTTTCCGTCTCCCATCGGAAAGGCAAAGAACACATTGTTCCCGAC
GCTTTGTCTCGGTTGCCTGAGAATGAAATCGCTCAATTAGACATGGGTCCTGTAATAGATCTTGAGTCCGA
TACATTTATGGACGCTAGCTATGTACAAATTAAAGACAAATTTTCCCAAAATTTTACTCAGTTTCCGGACA
TCAAAATCGTTGATAAGTTTTTGTATGTACGCACCGAACATTCGAGGGGAGACTCGACACCAGAAAGTCC
TTCATGGAAACTATGGGTGCCTGATCGACTTAAAGTCGATGTGCTCAAACAAGCCCATGACAGCATAACG
TCGTCTCACGCCGGCATGCAAAAAACCATTGAGAAAATCCGCAGACATTTGTTCTGGCCAGGATTAGTTA
AAGATGTACGTGAATACATACGAAATTGCGAGATATGTAAAGAAACTAAGGCTCCAAATTATACCCTTCG
GCCACCTATGGGTGCTCACATCCCAACTTGCAGACCATTTCAAAGGCTTTATATTGACCTGCTTGGGCCAT
ACCCTAGAAGCAAAAGCGGTCATGTGGGATTGTTAATAGTCTTAGATCACTTTAGCAAGTATCATTGGTTG
CAGCCCCTGAAGAAATTCACCTCAGCTGTGATACAAAAATTTCTTCTTGAACAAATTTTTCATTGTTTTGGT
GTGCCACAAACTATAATAAGTGACAACGGCACCCAGTTCAAAGCTCGTGAGTTCAATGCCTTCTTAGCCG
AACTTGGCATTGAACATCTGTATACAGCTCTATATTCACCGCAGAGCAATGCTGCAGAACGAGTTAACCG
CTCAATACTCGCCGCCATTCGCTCGTATTTAAAACAGGATCAGACCCATTGGGATGAACATATTACAGGG
ATTTCATGCTCCTTAAGGTCTGCTCTGCACCAATCGATTGGGTGTTCCCCATACCGTGCCCTATTCGGGGTG
GATATGATCACTCATGGTACCGATTACAAATTATTGAAGGAGTTGTCCTTATTGGAAGAACCGCTGGTCCC
CCTGTTGCGGTCTGACCATTTGGCATTGCTAAGGACAGATATTCAGAAAAACATCAGGAAAGCCTATGAG
CGCAATGCGGCTCAATATAATTTACGCAGCAAGCCTGTTTCTTTTCGTGAAGGACAGGAGGTGTTCTGTCG
AAATTTTGCGCTTAGTAAATTCTCACACAATTTTAATGCTAAACTGGCTCCCCAGTTTATTAAATGCCGTGT
CAAAAAGAAACT
>9_rnd-1_family-116__LTR___Gypsy
AAAGGAACTTCATGGCCAACAAAGAGGCCATNTATAATATCCAAGTGAAAAAGGAACTTCATGATCAAC
GAGAGGCATCCCGTATTCTGAGTGAGTAGAGAGTGAGTGTTATGGTTCATAAACGCGGTGGCTTAAAAAA
TAAAATTAATAAAAGAATAGCGCACCTAAGGTGGCTGCTATACACAAAACAGCCAACACCCGCATGGTCG
ACAGCTCAGAAGTTACAAAATGAGCTTGACTATCTGCAAAATGAAATTAAGCAGAGAAAACCAAACACC
ACTACTCACTCAAATATAAAAGTTCTAATAAACCAAACAACTAATAAAGTTACACCCATTGACGCAACCA
GCCTACCAAAAGAAAAACGAATGTGCCCACAGGATTGCGAAGGCCCACTCGACAGCCCCTTCTGCGAATA
TACGTGCAGACACTGCACAGCCAGCACCTCCAAACATTACATTACAACCAACAAATAAACACCTGTGCGA
AGACCAGCACCGGTACCGCCAGCGCAACAAGCTGTGCCATCGGAATAAGTGCGAGAGGCACACCGCCAC
TTGCGCATACGAGGGAGCATCAGCGTGCCACATCCGGCGACGCCCAGCTGATGCAACGATGAACAACAGT
TGCAGCTATATCCTTAACTACTTTTAATTAAATANAAAAATTTTAGTAAAATTGAGCGGAAACACTTCCGC
CCGATTAGCAGCATAGCCCGTAAAGCTATGCGAAATGCTAGGGTAGTATTTTGTGTTATAAGAAATATTG
AAACCTTGATTCTAAAACCCAAGTCAACCAGCACTAACGTGCTTTTGTGAAATCATTATTAAATCCAAACA
TTACCAACATATTAAATTAAATAGTAATAAATAAAACTTAAAAATTGTAATGCCCGACGACAATTGAGTC
GACCGGCAAGACGTTTGGCCAGTACAGCCATTCGAAACAATAGGTAGGAGAAAAATACAGATTGTAAAA
AAAAAATTTTTTTAGATGCGTTTTAAGCAGAAAACAAACAAGCAAAACAAAACCCGACTTGTAAAACAAT
AAAAGCATCAAATTCAATAAATATAGTAATAAAACCTCAAATGAACGATACAAACAACAACTTGTCTTGT
AAAAGTAAACGAAAACAAATTTAACATTACGCATTTGTAAATTTTTTAAGATAATTCCTAAAACCTCAAAT
GAACGATACAAACAACAAATATATGAATATTAATATCAAAAAGAGGAAATTAAAAAGTTATAATTCGTTC
AGTTTGTCACATTAGGTAATTCAAAATTATGATCAAGAATTAGCATGATCGAGGACAAGATTAACAAGGT
ATATAACGACTATACAGACTTTCGTATGCGAGCCATACAAATTTTGCAAAGGCATAGAGAATTCATACCA
GTATATGAGTTCGAATACGAATATATAGAAACATTCGGCACAGTGACAAGTCCACTCTATTCATGTTCTAA
AATAGAGAGCGGAATAATCTTGTCCACACTACCCTACGTCAAAATAAAAACAATTATTGACGAGCATCCG
ATTCTAGGGCAGACAACAATTGTTTTAGTACGTCTGCATAGGTAGTAGTCAAATCAAAAAGCAAGAATAT
TTATATAGGAATATGGGGGAAAAGAATCAAAGGCAGATGATAACACTGCCAATGTAGTTAACGTAGTA
GAAGTCGTAGACACCACTCCCCATGTGCAGTTAACAAACATACTGCTAATAATACTCATTGTAATAGCTTC
AGTAGACATCCTTCTGAAGATTTATCTTCTTCATAAAAAAATCACTGAGGAAGCGTTACATGAGTCGAGCCA
ACGACTTAGACAAGGTCTAAGCCACTGCTTAAATAAAATATTTAAGTAGCGGAAGCTTACATGGACTGGT
CCCAAATAGTAGAAAAGATAAAGCGGCATAGGGAAAATTTCGATAAATCATACAAGTGCCTTAATAAAA

GCGCACCAGTTTTAAGACCCACCATAATTAAACATACTGAGATCCTTGTATATGAGTACAATGAAATCAG
GAGTCTAGTTACCGAGTGTCAAAATAAACTGACAAGTTTACACAGAGTCCAAACTTCAGACGTTTTACAG
CGTCTAAGGGATAATCTGGTAAACTTAAATAAAAAGTATGATCTCCAGCTTTATCTNCCATCGGTGTTAAA
TACACCGGTAACATTTGATAAGTCACTACTGACAAACGACCAAATAGAAGATACAAAATTAGTAAACATG
GCACAAACAGTTGTAGAATTTATCAAGCTAGCCACATCTCTGATACCAGAGTTTGACGGTAAGCCGGAAA
ATCTACAAAGTTTTCTAGATTCTTTGAATCTATTAGAAACCTTAAAAGGCGCACATGAGCTAACGGCCATA
AGCCTTATAAAAACAAAGCTAAAAGGCCATGCGAGAAATTTGATAAGTAATGAGCAGACAATTGCTGCA
ATCATTACGCAACTATCAAATGCAGTAAAAGGAGAATCGGTAGAAGTTGTATCTGCTAAGCTTCTTAATTT
GCAACAGAAAAATAAAACGGCTAACCAATACACCCAAGAGGTGGAAAAACTGACAAAGGCCCTTGAAGG
CGCCTATATCAGTGAAGGTCTAAGCCAATCACTTGCAAACAAATACAGCACTACAACAGCTGTAAAAGCT
ATGACCCAAAATTGTTCGATTGATAAGGTAAAACTTATCATGCAGGCAGGCACATTCACAAATATGAATG
ATGCCGTATCCAAATTTGTCAATAGCTGCACGGAGATAACAGGTCAAAGTAACACTGTCCTGTATTATCG
ACGAGGTGCAAACAATAATAATAGAGGAGGCCGAGGTTATAACCGCGGCAGAAATNACCGCCGCAACNA
TCGTGGCCGAGGCAATGGTAACTACCAAAACAGAGGAAACAATAACTACGATAACCAGGCGGAGGAAGN
GGAGGCAANAATTCCGACCAAGAGGCCGAAACAACCAAGGTAATAATAATACAAACAATGTNAGAGTGG
CCCAGAACANGCCGGGAAACTCTCAGCAACCCTCGGATATTCAGCAGCAGGAAATTATGCGGTTCATNCC
ATCAATCNCAGTCTGAATATATTTGTAAACTTTTNCAATGTGAAGACGAAAAAAAACTCGCATTTCTAATA
GATACTGGTGCAGACATNTCCCTTTTAAAGAAAAATTCNGATANTTTCATGANATCCAAGAAGATAAAAA
NATNAACATCCAAGGCATAGGCCAAGGCGTAATAAAATCCAAAGGACCGACTTCAATAGAGATACAGAC
AAGTAAATACATNATTCCACACGAATTTTCACTTAGTAGATTCGCATTTCGCAATACCNTGTGATGGAATA
ATAGGCATTGACTTCATTAAGAAATTCAATTGCCAGTTAGATTTCAAACCAAGTGAAGACTGGCTCATAAT
NAGACCNAANAATCTAAATTNCCCAATTTATGTTCCGATAACATACAGTNCTGGCAACAATNCCGTTCTTC
TGCCAGCNAGATCCCAAGTAGTCNGAAAAATNCACNCTANCNTCAGATGAATGATNGTGTACTNGTTCCA
AACCAGGAAATNNANGANGGNATTTATGTTGCAAACACAGTNGCAGCANCCAAAAAATGCATNCGTCCG
NCTNCTGAACACGACCAGTNCCGACCAAGTGGTCGANGTAAAAAATCCGTNATATGAACCACTTTCGAAC
TACGACATAGTTCATAAAACTAATCCAGAAAAATAGAAGCGAANAACTGTCTTATCTCAATTAGCNAAAA
ATTTTCCACNACAATTCAAAAACGAACTGACAGANTTATGCACCAAGTNTAGTGACGTGTTCGGACTCGA
AACCGAACCAATAGCNACNAATAACTTCTACAAACAAANACTAAGATTGAAGGATGATGAACCAGTCTA
CATNAAAAATTACAGAAGCCCNCAAAGTCAAGTAGANGAGATNCAAGTACAAGTAGGAAAATTAATCGA
CGACAAAATNGTCGAACCGTCTGTATCCCNNTATAACAGCCCACTTTTGTTAGTTCCGAAGAAATCNCTNC
CAGGTTCNGACAAGAAAAAATGGCGATTAGTAATTGACTATCGCCAAATCAACAAAAAACTGCTGTCGGAT
AAATTCCCGCTGCCTAGAATTGATGACATTCTGGACCAACTGGGAAGAGCNAAATACTTTTCATGCCTTGA
CTTAATGTCAGGNTTTCATCAAATTGAACTCGAAGAAAATTCAAGAGATATAACGTCTTTCTCAACGAGC
AATGGCTCGTATCGCTTCACGCGATTGCCATTCGGTCTNAAAATAGCGCCAAATTCNTTCCAGAGAATGAT
GACTATAGCATTCTCGGGNTTAGAACCTTCTCAGGCNTTCCTTTATATGGATGATTTAATGGTCATNGGNT
GTTCCGAAAAACACATGCTCAAAAACTTAACTGATGTTTTTGAGGNATGTAGGAAATACAATCTNAAGTT
GCATCCNGAAAAATGTTCATTTTTCATGCATGAAGTCACATTTTTGGGNCACAAATGCACGGACAAAGGA
ATNTTGCCNGACGACAAGAAGTNCGACGTCATTAAAAATTACCCAGTTCCACATGATGCGGACAGCGCCA
GACGATTCGTNGCATTCTGCAACTACTATAGNCGTTTTATCAAAAATTTCGCCGACTATGCNCGNCACATA
ACNAGATTATGTAAAAAGGATGTNNANTTCGAATGGNCAGCTGANTGCCAGCACGCNTTCGAACACCTCA
AATCGGCACTNATGAATCCGAATTTATTACAATACCCNGATTTCAGCAAAGAATTTTGCATAATAACAGA
TGCAAGTAAACAAGCATGCGGAGCAGTTCTAACTCAGAACCGNGACGGACTCCAGCTCCCAGTNGCTTAT
GCATCNNGNTCGTTTACTAAGGGTGAAAGCAATAAGAGCACAACAGAACAAGAGTTGGCGGCAATTCAT
TGGGCAATNACCCATTTCAGACCATATATTTATGGCAGACACTTCANTGTCAAAACGGATCACAGACCAT
TGACATACTTGTTCTCNATGGTNAATCCNAGCTCNAAACTAACNCGCATGNGGCTNGAGCTNGAGGAGTA
CGACTTCACAGTNGAATACCTGAAGGGNAAAGACAATTTCGTAGCGGACGCGTTATCGAGAATAACCATT
CAACAGCTACAACAAATAAATGGATCAATAAAGAAAGTCACTACNAGACGTCAAAGTAGACAAAATTCC
TGCGCAGGAAAAGAAAANTATGAACTTTTGCCANGGCAATCTATAGAAAAAGCTTCNAAGCCCAACGTA
CATGAAGTCATTAACAATGACGAAGTACGNAAAGTAGTGACCTTGCGCGTAAAAGNATGTNATGTTTTTT
TAAACATGGTAAGAAAATTACCGCAAGATATGANGTTAGCGATTTATATACCAATGGAACTCTCGACTTA
GGTCAATTTTTCCAAAGGCTCGAAACGCAAGCCGGTATAAATAAAATCAGCCAACTCAAAGTGGCACCGT
GGGAAAATATCTTTGAACACGTTTCAATNGATAATTTCAAAAATATGGGCAATAAAATATTAANNTCNTT
GAGAGTAGCGCTACTCAACCCGGTGACCCAAATAGAAACAATAAAAGAAAAAGAGNTCTANTGTCTACG
TTCCATGACGATCCAATACAAGGAGGTCACACAGGCATCTCAAAAACCTTGGCCAAGGTCAAAAGACATT

ATTATTGGAAAGGTATGACNCGAGATATTACTGAGTACGTACGAAAATGTCAAAAATGCCAAAAGTCAAA
AGTGACAAGAAAAATAAAAACTCCTTTGACNATAACNGANACGCCAATACACGCTTTCGACAGGGTNAT
AGTAGACACCGTTGGTCCGCTACCCAAGTCNGAAAATGGNAATGAATACGCAGTCACATTAATNTGCGAC
CTGACNAAATATCTAGTCGCGATTCCAATTCCAAACAAAAGCGCTAACACAGTAGCAAAAGCGATNTTCG
AGAATTTTATTCTAAAGTACGGTCCAATGAAGACGTTCATTACGGACATGGGAACAGAATACAAAAATTC
NATAATAAATGATTTGTGCAAGTATTTACAAATAAAGAACATAACATCAACTGCACACCACCACCAGACA
GTTGGAACAGTCGAAAGAAGTCACCGAACTTTTAATGAATACATACGTTCATATATATCGGTTGATAAAA
CTGATTGGGACGTATGGATCCAATATTTCGTTTACTGTTTCAACACGACCCCATCTACGGCGCATGACTAT
TGTCCATACGAACTTGTTTTCGGTAAAACAAACAATTTATTAAAACATTTTAATAGCACAGACACCATAGA
ACCTTTATATAGTATAGATGATTATGCTAAGGAAAGTAAATATAGATTAGAAATAGCATATAAAAGAGCT
AGAATTATGTTAGAAGAAAATAAGAGAAATAATAAGGAATTATATGATAAAAAANAANAAACATAGATA
TATCGGTAGGAGATAAAGTCCTACTTAAGAATGAAACAGGACATAAGTTAGATCCAACTTATTTAGGACC
CTATACAGTAGAAAAGTTAGAAGATAACGATAATATAATAATTAGGGATACTAAAAAGAAAAAACAAAA
AGTACATAAGGATAGATTAAAANTTTATAATAATTGAATTGAAAACTCTAATATAAACAAAGAAAANTGT
TCGAACAAAATAATACAAAACAATAAAAAGGAAAATGGAATTTAAGAAAAGGAAACTCAAAAAGAAAG
AAAAATTCTTTTCTTTTATAAAAAAAAAGACAACATTTAAAANAAAATACTAACACATACATATATTTTTTT
AAATACACATAGTTAAACATATACATTTACACATACATATTAACAAACATTTTTATATTAAAATAATATAA
ACAAAAAAAAAAAAAAAAAAACACAAACAAAAATAATTAACATTAATACTTAAAATAACTACATAAATTA
CGTTATTTTTTAAAAGGAGGGAGATGTAGCATGCGTACATATGTATATACATACATATGAATAAGGCTGC
AACACTTTGTTGTTATGCCAACAGAATGCAAACATAAGCAGCGACTTTGTCGCAGCGCGCCTAACGCTAA
GTGCCCTCTGCGCATATTCAAGTGCAGACGAAATATGATCCGCACTTGATAGCAACGACCGCCCAAATAG
CTAAGTCAGCGTTCTCACGCTTGACCGTCGCTGTGTATACATATGTGCGCGCATGACTCTCTCTGACTCTCT
TGCTGTATATAATAAGCCTAAAAACCATGTATGTAAAACGGCTGCTCACTGCTTCACGGTGGACAGCTCTA
ACTCCAAAAAACATTGAATAAAGAACCATCAAGGAAGACTGACGTCTTTTCATCAGGGACTATTATATGG
CGACCGTGACAGGACTGGATCAGCTCCTGTTTTAGCTGATCGAACCGCTGTATGTACTGTAACTATTGAAG
AGGACGAAGGAGGCAACAACAACAAGCAAAGGCGGAACAAAAACAAAAAGCTGAGTGAGTAGAGTGTA
GTGAGTGTTATGGGTCGCGGTGGCCGGGTTAAAAATTGAATAAAGAAATTGCTCACATTANGTGGCAATT
ATATTCACCCAAAGCCAACACACCGTGGAAAGCAACAGCAAGGCTACAGGCCATGCTTGACTTTTATCAG
CAACAGCTGAAACAGATAAAAGAAAAACCCTACACCACATANTCACTCAGTGAATATTAAAGTTGTTAAT
GACAAAACTATAACCACACCACCTCCCAAAAAGCAAAGTGCCTCAGTACGTCAAGCAATGCCCGGCAGAT
TGCCCTGGACCTCAGGACAGCCCACACTGCAGATCGACTTGCCTGGTAAGGCGGTNACCTGTGCGAAGAC
CGGCACCGGTTCCGCCTGAGCCCATAGGCCCGGGACAACAGCAGCTGAGAAATCAACAAGCGCGAGAGG
CACCCCAGCCCCTTGAGCCNTCGAAGGGAGCGTCAGCGCAACCATACTGGTGATGACCANCTGACGCGGA
AGAAC
>160_rnd-6_family-2274__LTR___Gypsy
ACTGCCAACATTTATACACAACAGCAAAAGCAGCAGAGCAACAGGCAAAAGCTCTGGCAGGTGCTTGTAT
TAATGATTGCTTAAATTCAGATTTAGCCGCCAAATATAGCACAGCCCAAGCAGTAAAAGCTATGACTAAA
AACTGCGCTATAGATAAGATAAAACTTATCATGCAGGCAGGCACATTCCATAACATGAATGATGCCATAT
CAAAATTTGATGGTAGTTGCACAGAAGCAACCGGACAAACTAATACTGTCTTTTATTACCAAAATTACTCT
GATCGTAGTGGAAACCGAGGCAGAGGTAATTATAGAGGCCAACCATACAACAGAGGCGTCTACAATAAT
AACTCTAGAGTCCGTGGCAGCTCGAACCGAGGCCACAACAATAACAATAGAGGCAATGTCAGGGTAGCC
NAAGGAATTTCGGGAAACCAACGACCCCCCTCAGGTGCACAGAGCCAATAAATGGCTCAATAAAATTCA
ATAAACCTCAACCTCAGCATTTTTGTCTCTTTTTAAAACGTAGAAACAGTGAAAGAAAAGGTACTTTCATG
GCACACTTGATAGATACAGGTGCAGATATTTCAATTTTAAAGGAAGGTTCTGATATTCTCAACGACATCCA
GTTGAATAGCACTATAGACATTCAAGCTATAGGTCAAGGAATTATAAAATCCAAAGGCTTGGCCCAAATT
GAGCTACAAGCTGATAACTATATAATCCATCACAATTTGCATATAGTCGATGCGAATTTCGCGATACTGTG
TGACGGAATAATAGGCATAAATTTCATAAAAAGTCTTAATTGCAAAATTGACCTAGAACCGTACAAAGAT
TGGTTTATAAAAAGATTAAACAATATAAATGTCCAAATTTATGTCCCAATAACATACAATTCTGGTAACAA
TTCAATACTTTTAGCAAGATCAGAAGTTGTCCGAAAAATTGAAATAAACTCAAGTGAAGATAGTATATTC
ATTCGAAATCAGGAAATTCGTTGCAAACACAGTTTCAACACAAAACAATTAAGAGGTTCTAGTCGGGAGC
TCCCGACTAGGGGATACCCTGAACCCTATTCTTCCAACTACAAATGCATATATATATTCTATTTTAAAAGC
TATATTTCAAGTTCGGTGACTCTAGCTCTTATTATTTACCAAAATTGTCCAAAAAACAGGATATCGATGTC
GATTTTTATCGATTGCTTGGAAACGGAGTAAGTTATCGATTATCGGAAACAAAGGTGTAACATTTAAAAA
TCAGACATTATTTACCCGAGATATTAAAAAAAAATCATCGAAAAAGTTTCGATTTTCGCACCGACCTCGAT

TTTGAAAAAAAAAAACGTGATGATTTGGAAGNTCATATCTCCGCGCGGAGATTTTGAAAGAAAAAAAAACCA
TTTTGTCGATTTGGGTCAAAATTTGGATNTTCATATTTTTGATGCCAATCGTTAGAACTGATCCTTACGAGT
CAAAAATGGTATAAAATTCCAAAATCGGACATTATTTGACGGAAATATTCCAAAAAATCATCAAAACAGG
TTGACTTTACGAACGAATCGGTTTTTTGTCAACAACATTTTTNAACCCATCGATGTTTAAANTGTTTGAATG
CCAATTGATGGTAGGGATCCGTACGCATTCAAAAAGGTATAACATTTTAAAATCAGACATTATTTACCCG
AGATATTAAAAAAANATCATCGAAAAAGTTTCGATTTTCGCGCCGACCTCGATTTGGAAAAAAAAAGCGT
GATGATTTGGAAGNTNATATCCCCGCGCGGAGATTTTGAAAAAAAAAAAACGTGCCATTTTGTCGATTCG
GGTCAAAATTTTGGATATTCCTATTTTTTTTGATGCCAATCGTTAGAACTGATCCTTACGAGTCATAAAATG
GTATAAAATTCCAAAATCGGACATTATTTGGCGGATTTGATNCCAAAATCATCAAAACAGGTTGACATTT
ACGAACGAATNGGTTTTTNGTCAACAACNTTTTTTAACCCATCGATGTTTAANNTGTTTGAATGCCAATTG
ATGGTAGGGATCCGTACGCATTCAAAAAGGTGTAATATTTTGAAACCAGACATTATTTACCCGAGATATT
AAAAAATATCATCGAAAAAGTTTTGATTTTCGAGCCGACCCCGGTTTTGGCAAAAAACGTGATGTAACCC
CTTGCCATTTGGTCAATTTTGGTCAAAATTTAGATATTCATATTTTTGATGCCAATCGTTAGAACTGATCCT
CACGAGCATAAAATGGTATACAATTCCAAAATCGGCAATTTTTCGGCTGAGATATTACAAAAAATCATCA
AAACAGGTTGACCTTACAATCGAATGGGTTTTTGGTCAACAACTTTTTTTAGCCCATCGATATTTAAAATG
TTTGAATGACAATTTGTAGTAGGGATCCGTACGAATTNAAAAAGGAACTNTAATATTTTGACAGAAANNA
GTATANTANNNACCCGTANANATTCAAAAAAAACGTAATATTTCGAAACAAGTTTCGATTTTCGCACCGAC
CTCGATATTAAAAAAANATCGTCGAAAAAGTTTCGATTTTCGCANCGACCTCGATTTGGAAAAANGTCGT
NATGGAANGTCATATCTCCGCGCGGAGTTATGTCGTTTTGGAACGTCATATCTCCACGCGGAGTTATGTCG
TTTTGGAACGTCATATCTCCGCGCGGAGTTATGTCGTTTTGGAACGTCATATCTCCGCGCGGAGTTATGTC
GTTTTGGAACGTCATATCTCCGCGCGGAGTTATGTCGTTTTGGAACGTCATATCTCCGCGCGGAGTTATGT
CGTTTTGGAACGTCATATCTCCGCGCGGAGTTATGTCGTTTTGGAACGTCATATCTCCGCGCGGAGTTATG
TCGTTTTGGAACGTCATATCTCCGCGCGGAGTTATGTCGTTTTGGAACGTCATATCTCCGCGCGGAGTTAT
GTCGTTTTGGAACGTCATATCTCCGCGCGGAGTTATGTCGTTTTGGAACGTCATATCTCCGCGCGGAGCTA
TTACCCGAGATATTAAAAAATATCATCGAAANAGTTTTAATTTTCGAGCCGACCCCGGTTTTGGCAAAAA
ACGTGATGTAACCCCTTGCCATTTGGTCAATTTTGGTCAAAATTTAGATATTCATATTTTTGATGCCAATCG
TTAGAACTGATCCTTACGAGCATAAAATGGTATACAATTCCAAAATCGGCAATTTTTCGGCTGAGATATTA
CAAAAAATCATCAAAACACGTTGACCTTACAATCGAATGGGTTTTTGGTCAACAACTTTTTTTAGCCCATC
GATATTTAAAATGTTTGAATGACAATTTATAGTAGGGATCCGTACAAATTCAAAAAGGTGTAATATTTTGA
AACCAGACATTATTTACCCGAGATATTAAAAAAACATCATCGAAAAAGTTTCGATTTTCGCACCGACCTC
GATTTAAAAAAAAATCGTGATGGAAGGTCATATCTCCGCGCGGAGTTATGTCGTTTTGGAACGTCATATCT
CCACGCGGAGTTATGTCGTTTTGGAACGTCATATCTCCGCGCGGAGTTATGTCGTTTTGGAACGTCATATC
TCCGCGCGGAGTTATGTCGTTTTGGAACGTCATATCTCCGCGCGGAGTTATGTCGTTTTGGAACGTCATAT
CTCCGCGCGGAGTTATGTCGTTTTGGAACGTCATATCTCCGCGCGGAGTTATGTCGTTTTGGAACGTCATA
TCTCCGCGCGGAGTTATGTCGTTTTGGAACGTCATATCTCCGCGCGGAGTTATGTCGTTTTGGAACGTCAT
ATCTCCGCGCGGAGCTATTACCCGAGATATTAAAAAATATCATCGAAANAGTTTTAATTTTCGAGCCGACC
CCGGTTTTGGCAAAAAACGTGATGTAACCCCTTGCCATTTGGTCAATTTTGGTCAAAATTTAGATATTCAT
ATTTTTGATGCCAATCGTTAGAACTGATCCTTACGAGCATAAAATGGTATACAATTCCAAAATCGGCAATT
TTTCGGCTGAGATATTACAAAAAATCATCAAAACANGTTGACCTTACAATCGAATGGGTTTTTGGTCAACA
ACTTTTTTTAGCCCATCGATATTTAAAATGTTTGAATGACAATTTATAGTAGGGATCCGTACAAATTCAAA
AAGGTGTAATATTTTGAAACAAGACATTATTTACCCGAGATATTAAAAAAACATCATCGAAAAAGTTTCG
ATTTTCGCACCGACCTCGATTTAAAAAAAAATCGTGATGGAAGGTCATATCTCCGCGCGGAGTTATGTCGT
TTTGGAACGTCATATCTCCACGCGGAGTTATGTCGTTTTGGAACGTCATATCTCCGCGCGGAGTTATGTCG
TTTTGGAACGTCATATCTCCGCGCGGAGTTATGTCGTTTTGGAACGTCATATCTCCGCGCGGAGTTATGTC
GTTTTGGAACGTCATATCTCCGCGCGGAGTTATGTCGTTTTGGAACGTCATATCTCCGCGCGGAGTTATGT
CGTTTTGGAACGTCATATCTCCGCGCGGAGTTATGTCGTTTTGGAACGTCATATCTCCGCGCGGAGTTATG
TCGTTTTGGAACGTCATATCTCCGCGCGGAGTTATGTCGTTTTGGAACGTCATATCTCCGCGCGGAGTTAT
GTCGTTTTGGAACGTCATATCTCCGCGCGGAGCTNTTACCCGAGATATTAAAAAATATCATCGAAANAGTT
TTAATTTTCGAGCCGACCCCGGTTTTGGCAAAAAACGTGATGTAACCCCTTGCCATTTGGTCAATTTTGGT
CAAAATTTAGATATTCATATTTTTGATGCCAATCGTTAGAACTGATCCTTACGAGCATAAAATGGTATACA
ATTCCAAAATCGGCAATTTTTCGGCTGAGATATTACAAAAAATCATCAAAACAGGTTGACCTTACAATCG
AATGGGTTTTTGGTCAACAACTTTTTTTAGCCCATCGATATTTAAAATGTTTGAATGACAATTTATAGTAG
GGATCCGTACAAATTCAAAAAGGTGTAATATTTTGAAACCAGACATTATTTACCCGAGATATTAAAAAAA
CATCATCGAAAAAGTTTCGATTTTCGCACCGACCTCGATTTNAAAAAAAAATCGTGATGGAAGGTCATATCT

CCGCGCGGAGTTATGTCGTTTTGGAACGTCATATCTCCACGCGGAGTTATGTCGTTTTGGAACGTCATATC
TCCGCGCGGAGTTATGTCGTTTTGGAACGTCATATCTCCACGCGGAGTTATGTCGTTTTGGAACGTCATAT
CTCCGCGCGGAGTTATGTCGTTTTGGAACGTCATATCTCCGCGCGGAGTTATGTCGTTTTGGAACGTCATA
TCTCCGCGCGGAGTTATGTCGTTTTGGAACGTCATATCTCCGNGCGGAGTTATGTCGTTTTGGAACGTCAT
ATCTCCGCGCGGAGCTATGTCGTTTTGGAACGTCATATCTCCGCGCGGAGCTATTACCCGAGATATTAAAA
AATATCATCGAAAAAGTTTTAATTTTCGAGCCGACCCCGGTTTTGGCAAAAAACGTGATGTAACCCCTTGC
CATTTGGTCAATTTTGGTCAAAATTTAGATATTCATATTTTTGATGCCAATCGTTAGAACTGATCCTTACGA
GCATAAAATGGTATACAATTCCAAAATCGGCAATTTTTCGGCTGAGATATTACAAAAAATCATCAAAACA
CGTTGACCTTACAATCGAATGGGTTTTTGGTCAACAACTTTTTTTAGCCCATCGATATTTAAAATGTTTGAA
TGACAATTTATAGTAGGGATCCGTACAAATTCAAAAAGGTGTAATATTTTGAAACAAGACATTATTTACCC
GAGATATTAAAAAAACATCATCGAAAAAGTTTCGATTTTCGCACCGACCTCGATTTAAAAAAAAATCGTG
ATGGAAGGTCATATCTCCGCGCGGAGTTATGTCGTTTTGGAACGTCATATCTCCACGCGGAGTTATGTCGT
TTTGGAACGTCATATCTCCACGCGGAGTTATGTCGTTTTGGAACGTCATATCTCCACGCGGAGTTATGTCG
TTTTGGAACGTCATATCTCCGCGCGGAGTTATGTCGTTTTGGAACGTCATATCTCCGCGCGGAGTTATGTC
GTTTTGGAACGTCATATCTCCGCGCGGAGTTATGTCGTTTTGGAACGTCATATCTCCGCGCGGAGTTATGT
CGTTTTGGAACGTCATATCTCCGCGCGGAGTTATGTCGTTTTGGAACGTCATATCTCCAAGCGGAGTTATG
TCGTTTTGGAACGTCATATCTCCGCACGGAGTTATGTCGTTTTGGAACGTCATATCTCCGCGCGGAGTTAT
GTCGTTTTGGAACGTCATATCTCCGCGCGGAGCTTTTACCCGAGATATTAAAAAATATCATCGAAATAGTT
TTAATTTTCGAGCCGACCCCGGTTTTGGCAAAAAACGTGATGTAACCCCTTGCCATTTGGTCAATTTTGGT
CAAAATTTAGATATTCATATTTTTGATGCCAATCGTTAGAACTGATCCTTACGAGCATAAAATGGTATACA
ATTCCAAAATCGGCAATTTTTCGGCTGAGATATTACAAAAAATCATCAAAACAGGTTGACTTTACAATCG
AATGGGTTTTTGGTCAACAACTTTTTTTAGCCCATCGATATTTAAAATGTTTGAATGACAATTTATAGTAG
GGATCCGTACAAATTCAAAAAGGTGTAATATTTTGAAATCAGACATTATTTACCCGAGATATTAAAAAAA
NATCATCGAAAAAGTTTCGATTTTCGCACCGACCTCGATTTANGAAAAAATCGTGATGGAAGGTCATATC
TCCGCGCGGAGTTATGTCGTTTTGGAACGTCATATCTCCACGCGGAGTTATGTCGTTTCGGAACGTCATAT
CTCCGCGCGGAGTTATGTCGTTTTGGAACGTCATATCTCCAAGCGGAGTTATGTCGTTTTGGAACGTCATA
TCTCCGCGCGGAGTTATGTCGTTTTGGAACGTCATATCTCCGCGCGGAGTTATGTCGTTTTGGAACGTCAT
ATCTCCGCGCGGAGTTATGTCGTTTTGGAACGTCATATCTCCGCGCGGAGTTATGTCGTTTTGGAACGTCA
TATCTCCGCGCGGAGCTATGTCGTTTTGGAACGTCATATCTCCGCGCGGAGCTTCCCGAGATATTAAAAAA
TATCATCGAAAAAGTTTTAATTTTCGAGCCGACCCCGGTTTTGGCAAAAAACGTGATGTAACCCCTTGCCA
TTTGGTCAATTTTGGTCAAAATTTAGATATTCATATTTTTGATGCCAATCGTTAGAACTGATCCTTACGAGC
ATAAAATGGTATACAATTCCAAAATCGGCAATTTTTCGGCTGAGATATTACAAAAAATCATCAAAACACG
TTGACCTTACAATCGAATGGGTTTTTGGTCAACAACTTTTTTTAGCCCATCGATATTTAAAATGTTTGAATG
ACAATTTATAGTAGGGATCCGTACAAATTCAAAAAGGTGTAATATTTTGAAACAAGACATTATTTACCCG
AGATATTAAAAAAACATCATCGAAAAAGTTTCGATTTTCGCACCGACCTCGATTTAAAAAAAAATCGTGA
TGGAAGGTCATATCTCCGCGCGGAGTTATGTCGTTTTGGAACGTCATATCTCCACGCGGAGTTATGTCGTT
TTGGAACGTCATATCTCCACGCGGAGCTATGTCGTTTTGGAACGTCATATCTCCACGCGGAGTTATGTNGT
TTTGNAACGTCATATCTCCGCGCGGAGTTATGTCGTTTTGGAACGTCATATCTCCGCGCGGAGTTATGTCG
TTTTGGAACGTCATATCTCCGCGCGGAGTTATGTCGTTTTGGAACGTCATATCTCCGCGCGGAGTTATGTC
GTTTTGGAACGTCATATCTCCGCGCGGAGTTATGTCGTTTTGGAACGTCATATCTCCGNGCGGAGTTATGT
CGTTTTGGAACGTCATATCTCCGCGCGGAGTT
>Ref_dvir_765
TTTAGTTTTTTTTTAGTTTTTTTTAAGTTGAGTGCATCCGAGCACAATATATGTCAGCAGTGGAATGAAATT
CCTCCGTGACTTGTTTTATTCTTTTCCATTACTTTCACTTTAGCAACATATAAGAACATTATTTGGCCATAG
AATGTGTCTGTATTGAACTTACGTACTAAATACAATAAATACTTATACTAAAATACTAAAAGACTAAAAG
TACTAATTCCCTAATAATACTAAATTGCTTTAACAACTTAAAACTGTCAGTGTGGCAACACTGCATAGGTG
GCAACGCTACGCAATATACAGTTAGATTTGACTACATGTGCTCTGCATATACATTTTTTTTTTTTTCCCGTC
GCAACGTGGAAATCGAAAACTTTTTAACAGAGGTAAGTCGCATTTTTGCAAAAATTAATTTCTTAACATAT
AACTAATTATAAATATTTCTTTTTACAGATTTATGTCGAAATTGCTATCAGATTTGATAATTGCGCTCAGAG
GTCGCGCTTTTCTATGCGCCAGGCGCCGCCAGCCATGTACAGGCCGCCACACAGGCGCCGACGCATTCAT
TTGCTGATGCGCAATAAGCCTCGCATGGGCATTTGACCGCATCCGACGACGCCTCCGCTGGCTTATTGGAT
TGTGCTGCCGCTTCTTATGGACACCACTATGCAGACAGGAGTCGCTGCATATAAAAGTGTCGTGCACATG
GTGTGCATTTTGGATTCCATATGGGCCACGCTCAGTCGGCCAGTTTGGACTTCTTCGAGGCCGGAGCATGG
CCATTGACCATTGCAGCTCCGTCTGCAGGGGCGCCCGCACGAGTTCCGCGTGTGCTGGAGGCAGGTACG

TGCTTTTTTCCTACATTGCGGAGTCCGTCTAAATTGCCTTTTTCGTTTTTCAGGTATGGCTGCTGTGAAAAA
CATCGGATATGGCCCAGCCACAGCCAGTGCCTCTGGCCGATACGCTGCTGCTGCCACTGCCGCTTGTCAGC
AGCTCATCTTGGAGCGGCCCTTGCAGTCGCCTCCGAATTGACGCCATTTGGTAAGTGCATCTACGTTTTGC
AGGCAGCATTAAATTCTCAACCTTTCGCTTTACAGGCGTCGATTGTCGTTGCTGCAAATCATGCTCGGTCA
TCGATGGCGGCCGTGATGGCCCAGGCTGCCCCCGCACAGGCACCAGCTGCATTTCCAGCTTCTCAGGGGG
GCCAGGCTCCATCAGCTGCTGCGTCGTTGGACATCTGTCAACCAGGCGCTCCACAATGAGCCCAGGCAAA
TCCAGTGGCAACTGCACGGAATTTGTATTGTTTTGCCGCCGCTCAGGAAGCGAATCAGGCTGCAGTTGCTG
CTGCATCGTTGGATGGACATAGTTATGCCACCGCGGAGAAAGCGGGCCGGGCTCAGTCTCAGGCTGCAAC
GAGGGATGTAGATCGTTTTGCCGCCGCAGGGAGAGCGGGCCAGGCTGTACTATATAGACCAGCGAGGGA
TATAAGTTATTTTGCCGCCGCATGGAGAGCGGGCCAGGCTGTACTGAGTGTACCAACGAGGGATTCAATT
CAGCCTTCTTCTGCAGGTAAGTTGACAAGAACAATTCAAATTCATCATTATCAAAATCACTATCATTATCA
TTGTTTACATTGTTAGTATTAGTAGCATTTGTATTGTTAGTGCTGCTGCCGCATAGCCGTTGGCCATGCTGC
ATTTGCAGCTCATTTGTATTCAGCTGCACTGACTCATTGTCTGCGAGCCGCTGCTGCATTTCAATTTGAATA
TTATTTCCTGCAATAATGAGCGCTTCATCCATCTCCTGTCCCAGTGCCTGCTCCAACATATCAAGGCTGCG
CTCTAGTTGCAATTCATCGGTATCTATAGCCGATTGTATTATGGCCCCTGGGGCCGGCTCTGCCGTCCGTA
TTAGTTCCTGATGCTGCCGCTGCAGGTGTTCGTGCTGCAGCTCAGGTAGAATTAGCCATTGTATCTGTGGT
GGCTGGTTGTCGATTAGCAGCATTAAGTTTTCGCGAGTTATTGTGCCCGCTTTAAGTTTGCGACGAAGCAT
GTTTATTGGCATTCATCAAAGCGTTTTGTTCCCACTCGGCAAGCATAGCGGCCCTCCTTTCAGCCTCTTGCT
CATCTTCTTCTAGCTGCAGCAGCGTTGGCAACCTCTCCTCAGCTACCTCTCTCTGCTGTGTTAACTGCTGCT
GCCGTAGTGGCGCCATGCATCTATCCAATTGCTCCGTGTACCTGGGCCTTAGTAGCCGCACTGGTATAAAT
GGCTCTAGTATCAGTTTCCGAGCTTCAATGTTCGGATGACAGCTTAGTGACTTCAAATCTATCGCTAGCCA
ACTGATATACTTATCTACGCTTTTTATCCCATACGTATCCCTCTTGTATACCAACTGGCATTCATGCATGCC
CGCAAGATTTTATTCTGCATTGTTTCTATCCTTTTTATTTGCGAATTTGATACCAGTGCTCCCCATACAGGA
AGCGCATATTGCCATATAGGTGCGATCAGCTGCTTATATACCACAGCCTTGCAATTTCTAGCTAGCCTACT
ATTTTTTCCCAGCAACCAGTCCAGCTTTTTGTGTACTCCACGCAGCCTTATGCAAAGCTGAGTTATGTGTAT
ATTCAGTGTCAGTTTTCTATCCAATACTAAGCCCAAGTATCGGTGCCGCCCGTTATTTTCGATCGTTTGTCC
ATTTATCATTGGACACAGCCTTACTAGTGTTGCATCCAGCCTCCGCGTGCTAAACATTACATGCGCTGTTTT
GCTACTATTTATCTTTATGCACCATTTCTCTGCCCAACGCATCAAATGTATTAAGTATTTTTCATTTAGATT
CACTGCCTTTCATTACAAGTCTAGGTAGTGGCATGTCTGAAGAAAATATTGTATATAGTATAGGCCCGAGC
ACACTGCCCTGGGGTACGCCTGCACACACTTTTCCAGGCCTTGACTTTATGCCGTCGTTGCATTTTACAAT
AAATATTCGCTCAGCCAAGTAGCTTTCCAGCACGTTGTACAGTGCAAGTGGTAGCAATTTTATTAATTTTA
CTAATAGCCCAGCGTGCCATACACGGTCAAACGCTTCGCTGATATCAATAAATACAGCCGAGCAATATTG
TCGCTTCTCGTAAGCATTCAATATATATTGAGTCACACGGGCCAGCTGCTGCTCGGTACCATGGTCTTTTCT
GAACCCAAATTGGTGGCTTGGTATAGCATTTGCAAACTCTTTGCATTCGAATAGCCTATTCAGCAGCAGTT
TCTCAAACAATTTTGAGAAACCTGCTAGTAGACTAATTGGTCTATATGACGATATCTCTCCTGTACTTTTAC
CAGGCTTTAATATCATACTGATCTCTGCACATTTCCATTGCCTAGGAAAAAACCCATACCTCAATATACAA
TTGAATATAAGTACTAAGTATATAACCGCCTTCTCAGGCAACGATTTAATAACCCTATTGTCAATATTGTC
CATGCCAGGAGACTTATTTAACTCTAGCATATCTATCTCTAGCATAGTCTCGCTAACACTTATCGGACGAA
ATGGCAGAACGGACATACTATCTGTATTCTCTTCGAATTGCTCCAGCTTTTTTCGCTTTAGCTCTAATAGCT
CATCTGCAATCAGGTTACGCACAACCTGTGTATTTGTCAGATTTGGTGTGAAGCGTTTCTCCAAATGACAT
GCAAACGCTTCAGCCTTCTCCTCATTACTTCGTGTCCACGTTATATTGCCGTTTCTCCTACAACTACTGCCA
CTAGCTATCTCTAACTTTAGCGGCCAATTTGGTTGCGGCTGCCTTTTCAAATTATTTGTTAGCCTCCATAAT
TTTTGCATTTTATAGCGATCCTGAACATCAATCTGCTTCAATAACTCATTCATAGTATTCACTTTTGACAGA
CATAGCTCTTTCTTTAGCCGATTTTGTACTTGTCTATACATTTTTCTTGCATTCGGACAACGCAAAAGCATT
AATTGCCTTTTATATACCTGCTTCATCTTTATCAGCCCTCGTAAGTGCCCATTAAGCCTATATTGTCCGCTA
TTTCGTTCGTCGGTACAGTTTATCCTGCCCATGCCGATGTTCCTCGTATTGAGTCTGCTGCTGCCTCATATA
AATTCTTATTTAAGAAGTCAAAAGCGTCCTCTATATCGGCGCCTGATCTTATCTCAATGTTTAGCAATATG
CGTCGATTTAAGTACTCTTGGAACTTTTTTATGTTCGCATTCACTGGCAGCAATATTTTACGATTTCCTTTC
ATCATATTAGCAACATTTAGCCCTTCTATGCCCATTTCAATATGAACAGGCAAGTGGTCCGAATCTAAGTC
TGTCGTCGTATACGTTCTTAGTCGCGCATTTTGTAGTCCGCCATATACGGCAAAGTCAATTGCCGACGGCA
TTTTCCGCCTGTCATATGGATAGTGAGTGGCACCGCCGGTAGCTAGGATATTATAGCGCCTATTCTTGTGT
ACGCTATCCATTAGCGCCTTCCCGCGTTTGCATCCTCGTATATTGCCCCACCAGGTATGCTTAGCGTTCCAA
TCCCCACATAGCATAAAGCCAGATGAGCCATTGCATCCATCGTCTATGTGTTTGAATAGCCTGCTAAATTG
CTCTGAAGACCATTTAGGCTGTGGTGGGCAGTATATAGCTGCAATTATTATTCTATTTTTATTCCTCTCGCT

ACTCATCAAGGCAATTGGAGCACACTGCATAAAATTTTCCGATATTGGCTGCAAAGCGATATGCTTAATAT
CATTTTTAACTAAGATTGCTACACCTCCACTTTTGTAGTTATCTGGATGGTAGCAAAGTATGTGAGGTAC
CCATTAATCTTCAAAACATTCGAGCTAATAATTGTATTTGCGCTAGTATTAATACGAGTCTCTATTATCATC
ATTATATCAATTTTGTGGCGTTGGATAAACAGTTCTAATTCTAGTAACTTATTCCCCACCCCTGCAGCATTC
CAGTAGGCAATCCTAAAGGGGCTGCCTACCTGATTGCCGCTATATGCATCTAAGTTGCTATTAGTGGTAGA
AGTTGTATTCAAATTATTAGGGCTACTATTCAGATTATTCATTATTATTCGTGTCATTGGCTTCATACATAG
TGTGGCTGCCTGCCATTTGACAATCGCTATCAATAGGTCTATTTTCTACAATGGTACTATTCTGTATAGGCA
TGGGCATATGTAATGGTTGGGCATTTTTAAACGTATTGGCGACAAATTTAAATAATACATTTAGTAAATTA
TCAATCTTTTTACCCATCTCCTCGACGATATTGGCGTTCCGCTCCAACGCTCTCATAAGCGGACAATCAAT
CTGCGGAGTTTGGTGTTGCTGCTGTTGTTGTGATTGCTCGTTGTGTGTTGGCACCTGTACAATTCGCGTGGG
CTGTTGTTGTTGTTGTTGGGTTTGTTGACTTGGGAAGCGCTGCTGTTGGTTTTGGATACGCTGCTGCTGGTT
TTGGTTGCGCTGCTGCTGCTGAGGATGGCGCTGCTCTTGATGGCGCTGCTGGATTTGGCGATAGTGTTCCT
GTTGCTGCCACGGATGTTGTTGTTGCTGCCGTTTTTGCTGCTGCTCCATACGAAGCTGCTCCTGTAGCTTTT
CAAGATGCCTTGATGCAGGATTACGGATTTTAACACCACCAACACCACGGGCTACTTGGCTGTATGTCATA
CTTTTTGTGTTTGGATTTGTATTCGGGTTTGATCTCTGCTGAGACGCCTGCTGCCGTATTGGAGACTCCGGC
TGACGTTGCTGCTGCTTTGGAGCTTTTGTTGCACCGTACCGTTGACTTGCATATTGTTCGGCAGCCGTTGCT
GTTGTACGTTTCGTTGTCGCTGTGTGTTATCATCGTTGCATTGCTGTTGTTGCTGATTTACTACTTGTTGCCG
TTGTTGTTGTTGCTGTTGCTGCTGCTGCTGTAATATTGACTGTATTTCGTTGCTGCTGCTGTTGCTGCTTTTT
TTGTTGCCGGCCACGAATACGATTTATGTTTATTAAACTTTGCGCATGTTTGAATACAGCGCATCCTTTGTA
ACTGGCAATGTGCCCCTCTCCACAGTTGGCGCATTTAGCAATTGTGTCCCTATCCTTGGCACACATTGTTGT
TGGGTGCTCACCGGCACATTTAAGGCAGACAAATGGCCGACGGCAATAGTTTTTGCTGTGACCAAATGCC
TGACACCTATGACACTGTACTGGGTCAGCACTTTTATGTTGTCTTTCCACAGCGACATGCTGATTACCTAA
TTCGTTTAATTTACATATTTTTGTATGACCACCATCCACTCTTGGCTCAATCTCTATTTCAAATATGTTCAAT
GGCTTGCCACTAAATCGATGCTTCATGACGTTAACGTATCTGACAACATAACCCAATTCTCCCAATTGGGT
ACGCAACCAGTCGCATGGAGTCGAACTGTGCAAATGTTTTAATATAACCCTGTAGCCACGCTCACTTCTCG
GCTGGTGTGTGTGCATAGGTATATTTTCTGTATTTAATAAGCCCAATATGGCGTTATATGTGCTGAAATCTT
TACATTGTACTCTAATCCCATCACCCTGGGTACATTTTGTTGTAAATTTATTGACGCCCTGTGCGTTATTTA
ATTTCTCCATTAGTGGCTGAATTTCTTTTACATTGGGCACAAAAAGTGTTGGAATTTTAATACTTTGACCTA
ACTGCGGTTGCGCTTCCTGAACTTCACTAGCTACCTGCTTGCCGCCTATTTGCTGCTCTGTTGCTTCATCAT
CCGAAATAATTACCTCCGAATATCCATCACTACTGGTGTTGGCATCAATGTCCATGTTCATGTTGCCTTCCT
CCCCACCGGGCATGGCATTTTTGGCTAAATTATTTTTTGGTCTTGCAATATTGTATAATCTAACAATAGGCT
GCAAGTTAGATTGCATTGCATTTTGCAAATTATTAGGGCCAAGAGAGCTAGCAGCGCGCATTACTTTACGC
TGCTGCTTCATTGGCATAGTCACTCTACGCTTAGCGCCATTTATTTTTGCCGCCATTGTCACCTTAGCAGCA
TCACGCAAGCGACCACTTGTTGTTCCTGTTGCACATTCACTTAACCGTTGGCGCCAATTTTTGCGCGTCACC
TGTTGCAAAGATGGCGGAACATTGCCTTCTAGGTAGCTATGACATTTGCGCTAGTGGTTGTTCTTGTACTT
GTTGTAGTTGCTGCCCCTGTTGACTCAGGCAGAAATGGCGGATCATTTAGCGGCACTTCTGCCGCGAGAAT
TGGCGTCTGTTGCTGTTGTTGCACTTGCTGGCAAGTAGATCCTGTAGTTGTTGTTGCTGCTCCCACCGCTCT
TCTCTCAATTCCAACTATAGCATTTGTCCAGTTAGTGTCCTCATCGCTATCACAGAACATATGAGAAAGCG
CCGTCTCAGAGAATGCTGAGGTAGCACTTTCGCAAGTTAAGATAGTCTCATCAGAAATATCACCCTGTATT
TCACTTCTCTCTGTTGTGCGCTTCACATTTTCACCTGCACTTGTATTAGTGACGCCAGCGGCTGCCATATTT
GCCGGCACATAACCAAGACGTTTGGCCTCATTATGCACACGATTTATATCCTCAGATTGCACCTGCACAAT
CCCAGGATCTCTATTTAGCCACTCCTCAGAGGGCTTTCCACTTTTATTTGCATTGTGTTCCATATTTACTAG
TTGATTCGATAGCGCATTTCCATTTTATATGCGCACAATATATATTTTATATGCCGAGTATGTGTGTTTGTG
TGCCACATAAATTGCACTTGCACTGTACGAGCGCAATGTCTTTATTTGATTTTAATTTCTTTTTGGTTTGGT
TTTGGTTTAATTGACTTTTTATTTATTTATTTTATTTTATTTTATTTATTTTTTTTTTTTTGTTTTAAATAGATG
AGTGCGTCCGAGCGCAATCAAGCCTTTCGGTTTTTTTAAGTTGAGTTTTTTTTTTTTTTAATCTAAGTTTAAG
TTTAATTTTTTT
>160_rnd-1_family-152__LTR___Gypsy
CCCCAGCCAAACAGCCCTAGGTTATGGGCTAATATTCTGAGCGCCATTTCATAAAGCACGTCTGCAGTGCC
AATTGGAGCATCCGACGGGTACAGAAGATATTGACGCTCATGGCGGTCTCATCAACCGAATCCGGGTCCC
CATCGCAAACCCAGTGCAAAACAAAAAAAACTACCGACTTAAATGATTAGCTAAAATTTCTAAATTTATT
GGGTGCAGTGCCATATGAGTTGAGTGCCCTAGCATTAAATAAGAAATATCGACATTTTTATTCCATTTTTT
TTGTATATATACGAAAGCAAGTCAGTACAAAGCAAGTCAGCAGTGCTCTCAAGCTAAAAAAAAAAACGTTG
CAACAAAAGAAAAATGAAAATATAACTTAAAAAAATTTGCTGTATAAATAAATAGTACAAAGAAAAATC

ATAAAAATAATATGCATAATTAACAACTTTTTAATGCTCACGTGATGAAAGAAAGTTTTCTCATTGCGTCT
TCGTATCTGCATACAGACATACATTCATACAGCTTTTAGTTAGGTAATAGTTAGCACTAACCAAACAACAT
CTAGACTGGACATCAGTGCAGGAAAGGTGGCACAAAAAGTATCGGTGGCATCTGGTCCAAGCTCGCGAGT
ATGGGAGTAGAAGATATACAGTTGATGTATTTTTGAACCGCTTACTCTACAGGAAAGTGCTTAGCGTACA
CTCTTCCATATGATTTGTGCGATGATGTTTAGGGATCGCTTAAATGTGTGTGTGTGCAAATTATTAATTAAT
GTATGTCCTGCGATGTAACACAATGTTATCATGGGTAAGTTGCGTTGATCCCGAGCTGCTGTAGTGCGTAT
ATGCCCTTACAGAATACCCCGTACTTAAATATAAAGTATGCAGCACTTTGTTGCATACAATAACAATAATA
TAGTAAACGAGCCCAGAGCCACATTATGTATTGGCTCGGTCTCATATTACGCAGCGCGGTTCACTAGCCTC
ACTAGCTGACCGTTTATGCTTATGCTGCATATCCCACGCTCCGTCTGAGCGCGTTCCGTGAGCATACATAT
ATGCTCACTAATACAGGCATATGCGAGCTACTCACTCTCCCTCTCTTTGTAGCTGTTAACGAATAACAGCA
TAAGTATATATGTATATCCATAGGCTAAGCGAACGTGATAACAAGTGAATAAATGCATTCTGAACAAAGA
TACAAAACAGCACTGACGTGCTGGCTTATTTTTCATTGACAATAAGTGCTCTTTTTACATGGCGACCGTGA
CAAGTGTCACGAACATGTTAAAAAGTAATAGTAAAATGCTCACCATAAAGTAGACACACACACAGAAAA
TAATAACTACAACCTGCTTTGAGTTGGACTGGAGCTGAAAAAAAAAAAAACAAAAATATACTGAGTGAGT
ACAGGGCATGAGCTCTAAGGCAAAAAATAAGCCAAGACGCAGTGGCATTAAGGAGCGAATCAGACGGAG
AAAGTCTGAAATCGTAAGACTCCTTTATAAATGCAAAAATCCCCTCCCATGGGACACTGCAGTGAAACTA
AATGACGAACTCGAACATCTCGATTCGTTGTTAAAATCAAAGAGCGAGCCCTGTCCCTCAGATATAAATA
TAAAACAACTAACAAAGAATCATCGTCCACCGCCAAGACAGATACCAGCACGTCCACGCAGATGCCCACC
AGATTGTGAGGGCCCCCTTCACAGCCCCCATTGCGAGGGCACCTGCCGCACTAAGGAAGCAGAGACAACC
AACGCATGAGAGGCAAACCGCCGCTTATGCCTACGAGGGAATTCTAAGCAACTAAGACGAGTTGCATGA
GAGGCAAACCGCCGCTTATGCCTACGAGGGAATACTAAGCAACTAAGACGAGTTGCATGAGAGGCAAAC
CGCCGCTTATGCCTACGAGGGAATACTAAGCAACTAAGACGAGTTGCATGAGAGGCAAACCGCCGCTTAT
GCCTACGAGGGAATACTAAGCAACTAAGACGAGTTGCATGAGAGGCACACCGCCCCTTATGCCTACGTGG
GAAGAAGAAGAAGAAGACCCTATAACAACAGTACCGGCAGCGCCCAACATATTAAATTAAATAGTAATA
AATAAAACTTAAAAATTGTAATGCCCGACGACAATTGAGTCGACCGGCAAGACGTTTGGCCCGTACAGCC
ATTCGAANCAATAGGTAGGAAAAATACAGATTGTAAAAAAAAAAAATTTTTTTAAGATGCGTTTTAAGCAG
AAAACAAACAAACAACAACTTGTCTTGTAAAAGTAAACGAAAACAAATTTAACANTACGCATTTGTAAAT
TTTTTTTAAGATAATTCCTAAAACCTCAAATGAACGATACAAACAACAACTTGTCTTGTAAAGTAAACGAA
AACAAATTTAACATTACGCATTTGTAAATTTTTTTTAAGATAATTCCTTTTTACTTTTATGTATGATCCCAT
ACAATATATGAATATTAATATCAAAAAGAGGAAATTAAAAAGTTATAATTCGTTCAGCTTGTCTCATTAG
GTAATTCAAAATTATGATCAAGAATTAGCATGATCGAGGACAAGATTAACAAAGTATATAACGACTATAC
AGACTTTCGTATGCGAGCCATACAAATTTTGCAAAGGCATAGAGAATTCATATCAGTATATGAGTTCGAA
TCCGAATATATAGAAACATTCGGCACAGTGACAAGTCCACTCTATTCATGTTCTAAAATGGAGTGCGGAA
TAATCTTGTCTACACTACCCTACGTCAAAATAAAAACAATTATTGACGAGCATCCGATTCTAGGGCAGAC
AACAATTGTCTTAGTACGTCTGCATAGGTAGTAGTCAAATCAAAAAGCAAGAGTATTTATATAGGAACAT
GGGGGGAAAACAATCAAAGGCAGATGATAACACTGCCAATGTNGTTAACGTAGTAGAAGTCGTAGACAC
CACTCCCCATGTGCAGTTAACAAACATACTGCTAATAATACTCATTGTAATAGCTTCAGTAAACNTCCTTC
TGGAGATTTATCTTCNTCATAAAAAATCACTGAGGAAGCGTTACATGAGTCGAGCCAACGACTTAGACAA
GGTCTAAGCCACTGCTCTAATAAAATATTTAAGTAGCGGAAGCTTACATGGAGCCGGTCCCAAATAGTAG
AAAAGATAAAGCGGTATAGAGAAAATTTCGATAAATCATACAAGTGCCTTAATAAAAGCGCACCAGTTTT
AAGACCCACCATAATTAAACATACTGAGATCCTTGTATATGAGTACAATGAAATCAGGAGTCTAGTTACC
GAGTGTCAAAATAAACTGACAAGTTTACACAGAGTCCAAACTTCAGACGTTTTACAGCGCTAAGGGATAA
TCTGGTAAACTTAAATAAAAAGTATGATCTCCAGCTTTATCTACCATCGGCGCTAAATACACCGGTAACAT
TTGATAAGTCACTACTGACAAACGACCAAATAGAAGANACAGAATTAGTAGACATGGCACAAACAGTTG
TAGAATTTATCAAGCTAGCCACATCTCTGATACCAGAGTTTGACGGTAAGCCGGAAAATCTACAAAGTTTT
CTAGATTCTTTGAATCTATTAGAAACCTTAAAAGGCACACATGAGCTAACGGCCGTAAGCCTTATAAAAA
CAAAGCTAAAAGGCCATGCGAGAAATTTGATAAGTAATGAGCAGACAATTGCTGCAATCATTACGCAACT
ATCGAACGCAGTAAAAGGAGAATCGGTAGAAGTTGTATCTGCTAAGCTTCTAAATTTGCAACAGAAAAAT
AAAACGGCTAACCAATACACCCAAGAGGTGGAAAAACTGGCAAAGGCCCTTGAAGGCGCCTATATCAGT
GAAGGTCTAAGCCAATCACTTGCAAACAAATACAGCACTACAACAGCTGTAAAAGCTATGACCCAAAATT
GTTCGATTGATAAGGTAAAACTTATCATGCAGGCAGGCACATTCACAAATATGAATGATGCCGTCTCCAA
ATTTGTCAATAGTTGCACGGAGATTAACAGGTCAAAGTAACACTGTCCTGTATTATCGACGAGGTGCAAA
CAACAATAATAGAGGAGGCCGAGGTTATAATCGTGGCAGAAATTACAACCACAACAATTACAACCGAGG
CGGTAATAACAACAACAACAATAATTACAATAACCGTGGAGGAAGACGAGGCCAAAATCGAGGAAGAGG

CCGAGGTAATCCAACCAAGGTNATAATAATAACAATGTGAGAGTGGCGCAAAATGCGTCGGAAAACTCT
CAGAACCCTTTAGGAAACAACCAGTAAATGCAAAGGTTCATTCCATCAATTACAGTCTTAATATATTTGTC
ACTTTTTATAATCATTCAACTGAAAATAAACTCACATTCCTAATAGATACTGGTGCAGATATCTCACTTTT
AAAAATAAATTCAGATAATTTCAAAATTCAAGAAGANAAAATTATAAACATCCAAGGCATNGGCCAGGG
TGTAATAAAATCNCAAGGAACAACTTCAATAGAACTTCAATCAACAAAATATATTATTCCACACGAATTT
CATTTAGTAGACTCNGATTTTTCAATACCGTGTGATGGAATAATAGGCATTGATTTTATTAAAAAATTCAA
TTGTCAATTAGATTTCAAACCAAGTGAAGACTGGTTTATAATTAGACCTCAAAACTTAAATTATCCNATTT
ATGTTCCGATAACATATAGTGCTGGCAACAATACANTTCTTCTGCCAGCCAGATCCCAAGTAATTCGAAA
AATAGACATTAATNCAGTAAACGATTATATATTTGTTCCNAATCAAGAAATACATAATGGAATTTATGTTG
CAAATACAATAGCAGCATCCAAACATGTATACATTCGCCTTCTAAACACAACTAATTTCGACCAATTGGTC
AAAGTAAACAAAATCNAATATGAAAATTTAACAGATTACGACATTCATAACACTAATCCAGAAAATAGA
AGCGAACAAGTACTTTCAAAGTTAAAGAAAAATTTTCCAGAACAATTCAGAAGTCAACTAACAGAATTAT
GCACCAAGTATAGTGATGTGTTCGGACTGGAAACCGAACCAATAGCGACAAATAATTTTTATAAACAAAC
ATTAAGACTTAAAGATGATGAACCCATTTATATNAAAAACTACAGAAGCCCGCATAGCCAAGTTGANGAA
ATCCAAAAACAAGTAGGGAAATTAATAAACGACAAAATCGTAGAACCGATCTGTATCCGAGTATAACAG
CCCACTCTTGCTAGTTCCGAAAANATCATTACCAAATTCGGAAGAAAAGAAATGGCGATTAGTAATTGAC
TATCGCCAAATCAACAAAAAATTACTTTCTGACAAATTCCCGCTCCCCAGAATTGATGATATTCTAGACCA
ACTGGGCAGAGCTAAATACTTTTCATGCCTTGACTTAATGTCAGGTTTCCACCAAATTGAACTTGAAGAAA
ATTCAAGAAATATAACATCTTTTTCAACGAGCAATGGCTCATATCGCTTCACGCGATTACCATTTGGTCTT
AAAATAGCACCAAATTCATTTCAGAGAATGATGACTATAGCATTCTCGGGTTTAGAACCCTCTCAGGCATT
CCTTTATATGGATGACTTAATGGTGATAGGATGTTCCGAAAAACACATGATTAAAAACTTAACTGATGTTT
TTAATGTATGTAGGAAATATAACCTAAAGTTGCATCCAGAAAAATGTTCATTTTTCACGCATGAAGTGACA
TTTTTAGGTCACAAATGCACTGACAAAGGAGTATTGCCAGATGACAAGAAATTCGACGTCATCAAAAATT
ATCCTGTCCCTCATGATGCGGACAGCGCGAGACGATTTGTAGCATTCTGCAATTATTATCGTCGATTTATA
AAAAACTTCGCCGATTATTCACGGCACATAACTAGATTATGTAAAAAGAATGTTCCTTTTGAATGGTCAAG
TGAATGCCAAAACGCATTCCAATACCTTAAAGAAAAGCTTATGCACCCCACATTATTACAATATCCTGATT
TTCGCAAAGAATTTTGCATTATAACAGATGCTAGTAAACAAGCTTGCGGAGCGGTTCTAACTCAGAACCG
TGACGGAATTCAGCTCCCAATAGCTTATGCATCTCGTTCATTTACAAAAGGGGAAAGCAATAAGAGCACA
ACAGAACAAGAGTTAGCGGCAATTCATTGGGCAATTACCCATTTTAGACCATATATTTATGGCAAACACTT
TACTATTAAAACGGATCACAGACCATTGACATATCTTTTTTCTATGACTAATCCCAGCTCGAAATTAACTC
GCATGCGGCTAGAGCTTGAAGAATACGACTTCACAGTAGAATACCTAAAGGGGAAAGATAATTTCGTAGC
AGACGCACTTTCGCGTATTAATATAAAAGAACTCAAAGATATGCAACATAAAGTCCTGAAAGTCACTACC
AGGCGACAAAGTAGACAAAATAAAAACTGTACAGTAGAAAATTATGAACTTCTGCCAAGGCAAAGTCAT
AAAAATGTATCTAAGCCCAACGTACATGAAGTCATTACAAATGATGAAGTACGAAAAGTAGTGACCTTGC
GCATAACAGAATCTAAATGTTTATTTAAACATGGAAATAAAATTATCGCAAGAATTGAAGTTAGCGATTT
ATATACTAATGGAATCCTCGACCTAGGTCAGTTCTTCCAAAGGCTCGAAAAAGAGGCCGGTATATTAAAA
ATCAGCCAACTCAAAGTGGCACCGAGCGAAAAGATCTTTGAATCAGTTTCAATAGATTNTTTTAAAAATA
TGGGCAATGAAATATTAAAATCTTTAAGAGTAGCGCTACTCCAGCCGGTGACCCAAATAAANGATGAAAA
AGAGATACAACAAATATTGTCTACATACCATGACGACCCAATTCAAGGAGGTCATAGCGGCATAACAAGA
ACGCTAGCGAAAATAAAAAGACACTACTACTGGAAAAATATGACGCGTCATATAAAAGAGTACGTACGT
AGATGTCAAAAATGCCAAATGTCTAAAACAAAGACGCACACAAAAACTCCTTTGACTCTCACAGAAACAC
CTGTGAATGCTTTCGATATAGTGATAGTGGATACAGTTGGTCCGCTACCCAAATCGGAAAATGGCAATGA
ATACATAGTCACATTAATATGCGACCTTACAAAATATTTAGTAGCCATTCCAATAAAAAGCAAAAGCGCA
AATAATGTCGCAAAAGCTATATTCGAAAATTTCATACTCAAGTTTGGTCCAATGAAGACGTTCATCTCGGA
CATGGGAACAGAGTATAAAAACCAGATTTTAACTGATTTATGCAAGTATNTGAAGATCAAGAATCTCACC
TCTACTGCATATCATCATCAAACATTAGGGACAATAGAACGAAGTCATCGAACNTTTAATGAATACATTC
GTTCATACATCTCTGCAGACAAAACTGATTGGGATGTCTGGGTTCAATATTTCACTTATTGCTTCAACACG
ACACCATCAGCAACGCATGAGTATTGTCCATACGAACTAGTATTTGGCAGACTACCGAAACAGTTCATAG
ATTTAAACAAAACTAATTACATAGATCCTATTTATAATTTAGATGACTACTCTAAGGAAATAAAGTTTAGA
TTAGAAATAGCATACAAAAGAGCTAGAATTATGTTAGAAAAAGCCAAGGCTTATAGGAAATTAGCATATG
ACGAAAAAACAAAAAACTTTGAATTAAAAGTAGGAGATAAAGTACTTATAAGAAACGAAACAGGGCACA
AATTAGACCCAGTATATTTAGGACCATATATAGTAGAGAAAATAGAAGATAATGACAACATTTTTATTAA
AGATAGTAAGGATAAGAAACAAAAAGTNCATAAGGATAGGTTAAAAATNTNTAATCCATAAAA
>160_rnd-1_family-272__Unknown

GATCGGACCACTATATCATATAGCTGCCATAGGAACGATCGGTCGAAAATTAAGTTTTTGTATGAAAAAA
CATTTTGTTTATCAAGATATCTTGACCAAACTCGGCATTTATTAGTTTTACTTTACTCCTCATATATATGCA
AAATCCTATNAAGATCGGACCACTATATCATATAGCTGCCATAGGAACGATCGGTCGAAAATTAAGTTTT
TGTATGAAAAAACATTTTGTTTTTCAAGATATCTTGACCAAACTCGGCATTTATTAGTTTTACTTTACTCCT
CATATATATGCGAAATCCTATNAAGATCGGACCACTATATCATATAGCTGCCATAGGAACGATCGGTCGA
AAATTAAGTTTTTGTATGAAAAAACATTTTGTTTNTCAAGATATCTTGACCAAACTCGGCATTTATTAGTTT
TACTTTACTCCTCATATATATGCAAAATCCTATTAAGATCGGACCACTATATCATATAGCTGCCGTAGGAA
CGATCGGTCGAAAATTAAGTTTTTGTATGAAAAAACATTTTGTTTTTCAAGATATCTTGACCAAACTCGGC
ATTTATTAGTTTTACTTTACTCCTCATATATATGCAAAATCCTATTAAGATCGGACCACTATATCATATAGC
TGCCGTAGGANCGATCGGTCGAAAATTAAGTTTTTGTATGAAAAAACATTTTGTTTATCAAGATATCTTGA
CCAAACTAGGCATTTATTAGTTTTACTTTACTCCTCATATATGTGCAAAATCCTATAAAGATCGGACCACT
ATATCATATTGCTGCCATAGGAACGATCGGTCGAAAATTAAGTTTTTGTATGAAAAAACATTTTGTTTNTC
AAGATATCTTGACCAAACTCGGCATTTATTAGTTTTACTTTGCTCCTCATATATATGCAAAATCCTATTAAG
ATCGGACCACTATATCATATAGCTGCCATAGGATCGATCGGTCGAAAATTAAGTTTTTGTATGAAAAAAC
ATTNTGTTTTTCAAGATATCTTGACCAAACTCGGCATTTATTAGTTTTACTTTACTCCTCATATATGTGCAA
AATCCTATAAAGATCGGACCACTATATCATATAGCTGTCATAGGAACGATCGGTCGAAAATTAAGTTGTA
TGAAAAAACATTTTGTTTATCAAGATATCTTGACCAAACTCGGCATTTACTATTTTCCCGGTACTTCTTAGA
TAGAGGCAAAGCACTATGAGCATTATGAAAAGGTTGGGTCTGCAAGGGTATTAGATCTTTGGCGTGCCGA
AGATAGCCCTTCTTTCTCGTTAGTTTTTAGTTTTAAAAACTCAGTACAATTTCAAAAAATTTTAACAATGTA
CTAACGACTAAGTTATGTCAAAATTTAAAATTCCTTTATTTACCAGTTCGTTTGAAAAAAATGTTCATTCAT
TTTCAATAGCTTTTAGGTAAAAAACAAGTAAGAATGCTNAGTTGGGAGTGCACGGCTAGGGGATACCAT
TCCTACCAACAACAAACGCCGCTATAGCTATAGAATAAAAGATACCTGATTTGCATGGACAGGCAGAGGC
ACATGGCTAGATCGACTAGGCTATTGATGCTGATCAAGAATACATACTTTAAGTGGTTGAAGATCCTTTCT
TCTGCCTGTTACATACGGCTGCACAAATACATTATACCCCTTTTACCCATTTTTAATGGGTTCAGGCTTTAA
AAAGGCGAACACAATCAGTCAATAAATAAAATCTTGCCGTGTTGCGTTTCCTGGAGCCATTTCAAACTTGT
TAGATTAAAATATAAACAAATAAATAACAAATGTTATAAATATATATGTTCATATGTATAAATATGCATAT
AAATTTCATTTGAATTAAATAGCATGGAATCCAAAAGTGACGCTATGTTAGGGGCTGCCAACTTATGTAG
AATGCTAACCTATGGGAACATAAAGATGCCACCTTGTGGAAGTACATTGACATTGTTAGTATATTTAGCCA
AAAAATTGTTAAATTTAAAATAATTTTCTACTTACATGTCTTTAGCGTGCCCATTTGATTTTTATGTATTCT
TGCCATTCACACGACAGCATTGCCAGCTGATCGTGCAGAAATTTCCCCCAGCTAACTGACCTAAGACAAT
ATGTTAAATGTACAGAGTCTGTTATGTCATGTTATGTTTATATGTAGGTCAGTCGAGATTAAATGTTAAAT
GTATCTCTTGTCATGTCCCTCTCCACCTTGCAAACTGGTGAACGTTGCATAGTTTTAAGTGCTCCCAAAACT
GTTCTAAAATTTCTGTTTGCAATTGAAATCATATGTAGAAATGAGCAACAATAAAAATCTCTCTGTGGCGG
TCGAGGCGAGCGGGCGTGTTTGCGGACAGCGCCCGGTCGGCCAATCAGACGAACAACTAAAAATTGTGA
AAGTACATAAGTAAAAGTACACATGACGATGAAACATGTTATTTACATATATGTTTATAAGTTAAACATG
TTATTTATATAAAATAAATACTTTAAATAGTTGTTAAAACATTCTACAATTTGTATTATTATCTGTGTATAT
ATAAATCTATATACTTCGTTTTGAACACTCCCGTTCTCATTGCCTGCGTTGGCTGTACTGACGAATTCAACT
TTTCACCGCACGCGAGTTGAATTCGTCCCACTGTACAGCCTCTGTCGCGACGCCTTAACGGCCCTTCGACG
CCGACGTCGGCTGCGGCGCGCATCTGTGCACACGTATGCAAGCGTGGTCTCACTGTCGCCCGTCTGGCGC
GCGCGCGCTGTACTTTTTTGATCGCCGCTATATTCCCGATGTTCACTCTGTATGTGCGTCAACTTAGCGGCG
GTGACTTAGCGTCGATTTTTGGTACTTGGTATCCGAAAAGGAATCGGTCACTTTTAAATGCATTTATTTAT
GTTTTTAAGCACAGAACCTAATAAATTTAAATGCAGAATTGTAAAAAATTAAATTATTTACTGCCTACATT
CATTTGTTTTTGAATTTTAATTTATAATATGGGTAAACCAAGGGGTTTAAATATTAGGTTTCCACCATTCCA
TAAATATCCAAATTTAGGGATAATGGCCCCGAAAACCGCATATACACGATTAAAGCCTATAGTTTCACCT
AGACAACTGTGAAAATTTGATTAAAATCTTCGGTACCATTTTTAAGATATTTGGAAAAGAGTGCAACAAC
CCCCATGTCAGCCCCATATAAAATGACCGCAAAAAATCAACCTTCTTATTTCTTAACTCAGAGACAATGTC
CCTCAATGCACGCTATACCCTCTTGTAGATACATATTTTAAGAAGCTTTAGGCACTAAATTTAAGTAAATG
GACGGTACAGATTTTCAGAAAATTATTTACTTGTAAACCAACCTCATATAGTTCATTCATAAAGTCTATCG
AACTGCTATTTCCTACGTGAACTGAATCACTGCCTGGCATCAGTGTTGAACAATTTTGTACAGAATTGGGC
GGGAACCAATCCAATTTGAATAATATGTTATCATGAAAATTACTTATTGTTAACAATAAGTTTATAGAGCG
AAATTAGACAGATTTTCAACTGGCAGATAATGTTAAGAATCGAAAAATTATTACGAAAAGTTTTAAGTTC
GTAAGAAATATTAGAGCTTTAATATTTTGTATAACCCTTATTGGTGAGTTGTTTTACCCGTTTCAGGCTGCC
TACTCAAAAGAACGCCAGGACGTATCTAGTGGTAGTAATCATGTTAGCAGTAAACTAGAAATTAGCAATA
ATATTAGTGATATGTCTAGCATAGAAAATAATAATTCTAATTATAGTCTTAATGAGCCTACAAATTGTCCG

TCTGAGTTTAATAATGACGCATGCTTTGTCGAAGCGACATTAAAATGCTTTCGGAAAAAATACCACAATTT
TCTTGATAGCTTAAACAGAGAAGTTCAGTTAGTTAGTAATTTGAAAGGATGCCGCGCAGCACTTTCAGGTT
ACAACAATTTGGATACTGAAATGAGTATCCAAGAATTTATGGCCACGTTAAATCAATTGTGTGCGATTAA
AAAAAAAAAAACATTGAACACATTATTCGAGAAGCATACACTCTTCATAGCAATGCATGTGTTTTGCACT
TTAAGTCAATTAGACTTAACACAAACTCTTTTGCTGCATATGGTTACTGCAAAGATCCATCATGCAACTAC
TTCTTGTTCCATGGCAGCCAACATATCTCCCATACAATTGGCCTTAAAAAGTCAACGCAATGCAGAACAAT
AAATAGATTTATAGAAAAGGCTTCCATTAGAAACTCTTCTGCGTTCGAGTATAGATCAAAAAAAAAAAAN
AACAAAATCAATATGTCTCGAGCCAGAGTTGGTAATGATCAGATTACTAATGAGAAACTACCCTTTTATA
GAAATTTAATTTCGGAGGAAAATTGACTTTGACAACTCACGGATAAATCGTCCTTGCTTTAAGCCGTTTTT
ATTATTTGTCTTTATAAAACTATATTTAAGCGTTTTAAAGAATGATTCTATAGGCCCATTACTGGGTCTTTT
ATTAGATTTATCAATAAAAGTAATGATAATATTGGTTCAAAAAGGTGCGTAAACAATATATTTATTTAAAA
TTAGATCCAGCATTTCTGCACTTAAATAAATATTTGACGTACAGAAGTTGCTTTTATATTGTGATACAAGT
TCTGTAACTTCTGTACGTATTTCTTGAAGATTAAGTTTAAATGGACTATTTTCGGCAATGGACAGCAACGT
TGGATCATTGACGACTTCCTTACTTACTCCCAATTTTATACTGTCTATCAAATAAAATGTGGATGGCTCTTC
ACAATATTTAGTAAAATAATTAGCGGCATCATCCACAATATTATTAACATATGGAGAAAGAAGTATTATT
GCAATATTTCGGAACCATTTTTCTAATTTTCCAATATTATTTATGGAATATGCACAACCCAAGGCATTTCTT
AAAAATGATTTAAGATCAATATTGTCGAAAGCAACTACTTCATCTATGTTGCGACACTTAATTTTGCTGTA
GTGGCTCATACAAAACTGAACCCAGACTTTTGGAAATGTGCGATTTAAATTATACAAAAAACTAAAACAA
ATTTCATTGTACGTTGCAAGATCAATGTTGTTATATTCTTTTAAAATGGCTCCAATTATTGCAAAACTGACA
TCAGTTATAATTCTTTTGCAAATAGGCCATTTGAATTTATTTTTTATGCAAAACTCGCGCAGCTCACATAGA
AATCTGCCTATATCTGACGATTTATGATTGGAGAGAATCGCATGTGCGACAGGAAAAACGATTCGAAGCT
CTGGAATATAAGCAACCAATGAATATAAAAATGTACATTTTTTGATCAAGTTCATTGGCTGTACTACATTC
CCAGTCGCATCAAATATGCAGTGTCTGTCCCATAAAAATATTTCTAGTTTCATAGAAAGTAACTGAATAAA
GTTGGCATACACATTAGTAATCATATCGAAGGTATCTTTAAATGGATCTTTGTCAAAATCAAATTTTCCAT
TTTCCTTCGAAATTAAATTTCTATAAAAGGGTAGTTTCTCATTAGTAATCTGATCATTACCAACTCTGGCTC
GAGACATATTGATTTTTTTATTTTTCTTTTTTGATCTATATTCAAACGCAGAAGAGTTTCTAATGGAAGCCT
TTTCTATAAATCTATTTTTCGTTCTGCACCGCGTTGACTTTTTAAGGCCAATTGTATGGGAGATATGTTGGC
TGCTTGTGTATACACTTACAACACCGTCAAATGTCCCATGGAACAAAACGTAATTGCATGACGGATCTTTG
CAGTAACCATATGCAGCAAAAGAATTTTTGTTAAGTCTAATTGACTTAAAATACAAAACACATGCGTTGTT
ATGAGAAGTGTATGCTTCTCGAATAATGTGNTCAATGCTTTTTTTTTTAAAATCGCACACAATTGATTTAA
CGTGGCCATAAATTCTTGGATACTCATTTCAGTATCCAAATTGTTGTAACCTGAAAGTGCTGCGCGGCATC
CTTTCAAATTACTAACTAACTGAACTTCTCTGTTTAAGCTATCATGAAAATTGTGGTATTTTTTCCGAAAGC
ATTTTAATGTCGCTTCGACAAAGCATGCGTCATTATTAAACTCAGACGGACAATTTGTAGGCTCANTAAGA
CTATAATTAGAATTATTATTTTCTATGCTAGACATATCACTAATATTATTGCTAATTTCTAGTTTACTGCTT
ACATGATTACTACCGCTAGATACGTCCTGGCGTTCTTTTGAGTAGGCAGCCTGAAAGGGGTAAAACAACT
CACCAATTAGGGTTATAAAAAATATTAAAGCTTTATGATTACTTACGCGTTTAAAACTTTTCGTAATAATT
TTTCGATTCTTAACATTATCTGCCAGTTGAAAATCTGTCTAATTTCGCTCTATAAACTTATTGTTAACAATA
AGTAATATAGATTGAAAATCAGTTGGACTTAAAGTTTCCAACTGGAGATATTATGCTTCTAGCAATGGCTC
TTTATGGCATCACGAAATCCAAGAGAGATTGTCCTTAAAAAATTGTTTATATATTAATGGTAAAATTAAAT
CGAATAAGTTAAGCAACAATTTTATTATCTACCCTTGCAGAGGGTATTATAATTTTGTCGTGAAATGTGTA
ACGCATAGAAGGAGACATCTCCGACCCCATAAAGTATATATATTCTTAATCAGCATCAACAGCCGAGTCG
ATATAGCCATGTCCGTCTGTCCGTTTGTCTATTTCTATGCGAACTAGTCCCTCAGTTTTAAAGCTCTCTTAA
TGAAACTTTGCAGAACTCCCTCTTTCTGTTGCACGCAGCATATATGTGAAAACCAGCTGGATCGGACACTA
TATCATATAGCTGCCATAGGAACGATCGGTCGAAAATTAAGTTTTTGTATGAAAAAACATTTTGTTTATCA
AGATATCTTGACCAAACTCGGCATTTATTAGTGTTACTTTACTCCTCATATATGTGCAAAATCCTATTAAG
ATCGGACCACTATATCATATAGCTGCCATGGGAACGATCGGTCGAAAATTAAGTTTTTGTATGAAAAAAC
ATTTTGTTTTTCAAGATATCTTGACCAAACTCGGCATTTATTAGTTTTACTTTACTCCTCATATATGTGCAA
AATCCTATAAAGATCGGACCACTATATCATATAGCTGCCATAGGAACGATCGGTCGAAAATTAAGTTTTT
GTATGAAAAAACATTTTGTTTATCAAGATATCTTGACCAAACTCGGCATTTATTAGTTTTACTTTACTCCTC
ATATATATGCAAAATCCTATTAAGATCGGACCACTATATCATATAGCTGCCATAGGANCGATCGGTCGAA
AATTAAGTTTTTGTATGAAAAAACATTTTGTTTNTCAAGATATCTTGACCAAACTCGGCATTTATTAGTTTT
ACTTTACTCCTCATATATGTGCAAAATCCTATAAAGATCGGACCACTATATCATATAGCTGCCATAGGAAC
GATCGGTCGAAAATTAAGTTTTTGTATGAAAAAACATTTTGTTTTTCAAGATATCTTGACCAAACTCGGCA
TTTATTAGTTTTACTTTACTCCTCATATATATGCAAAATCCTATNAAGATCGGACCACTATATCATATAGCT

GCCATAGGAACGATCGGTCGAAAATTAAGTTTTTGTATGAAAAAACATTTTGTTTATCAAGATATCTTGAC
CAAACTCGGCATTTATTAGTTTTACTTTACTCCTCATATATGTGCAAAATCCTATAAAGATCGGACCACTA
TATCATATAGCTGTCATAGGAACGATCGGTCGAAAATTAAGTTTTTGTATGAAAAAACATTTTGTTTATCA
AGATATCTTGACCAAACTCGGCATTTACTA
>160_rnd-1_family-189__LTR___Gypsy
TGTGCCTGTTCATCAAAATCATTAAAANGAAGGGCGCGCGTCAGACGCGNGCCAATAATGATTTGTATGT
ATTTTCTTTTGCGCCCATGATCTGCCCATTCACAGATATTTGTATGTGTGTGTCTGTGTGTAAAGGTCCAAA
ATCAGAAATCCTAAAATCTTGTTAAATTTGCATCGGGGACAGGCAGGCAGGCAAGCAACAGGCACACAA
GCGCATAAATGCTTCTATTCATACACAAAATATGCAAGTATGCGTGTATAGCGAGTCCAAAATATTCTAA
ATGCACACACGGCAACGCTTGGGTACCCTTACCAACGATAGCGAACTAAAATGCCAGAATTTCGCATCGA
CGTGGCAAACCCTTTCTCTATTTCATATTAGCCGTGCCTTCAAAAGGGTAGAAATGCCTCAGCATTTGAGA
AAAATTGAATTTGTGCGCAGTTTTGCTCGTCGGACAAATACACACACACAGACATTGAAACGATCCCTAA
ACATAGAGAGAGCATTGAGAGCTGAGAGTAAGTGCGAGCGAGATAAAAGTTACCAGCGTTGCCGTTCTAT
GGCACAAATCATATGGAAGAGAGTGTGCCAAGCACTTTCCTGTAGAGTAAGCGGTTTAAAAATACATCAT
ATGTATATCTTACTCCCTTACTCGCGAGTTTGGACCAGATGCCTCCGATACATTTTGTGACTGCTTCCCGGT
ACTCATGTCCAGTCTATATGTTGTTTGGTTAGCGGTGGTAGCCACAACGACAAAGACATCTTGTGATGAAA
AAAAGGTGACATATGGGTAGTTTTTGTTAGAGTTTGTCTATTGTGTTTGCCTCTATTTGTTTTGTTTCACTTT
GTGTTTACGTTTGGTGTGTGTTGGCCCCAGCAATCTGAGTTTCGGAGACAGGTGACCCAGGACGATATTTT
TTCGATCTAGCGCCACATCTATCACACAGCATACCCCAAAAGTGCGACAGATACCAATTGGCCTTCAAGG
CCTTCAAGTATTTTATATTTATTAGAAAAACAATATAAAAAATCATTGTTGTGAGTTTTTTCTGTTGTCGAT
ATTTATTTGATCATTTATTTATTATTCATTATTAGTAAGATTTGTTCGTTGGTGTTTATTTATTTATATATTT
GATAGGAATTATTTGTGTAGTTTTTGTATTAGGGATTAGTATGGCCCAGGAACAGGTGTCTGATTGGGGCT
CTGTTGCTGAGAGAATATGTCGTACACGGACCGCGAGCAGGCGCCATTCCGAAAGAGCAAGAAGGGGTA
CAGCGCGAGGAATTTCCTCCACCAGTGCCATCGAAGCCACACCGACAGGGCGCGCAAGGAGCGGAGTAC
TCATACAGCAGCCCAGTATATGTTGCAGCTGCTCAGGCAAGGAGCTGGCGGGGGCCGAATAATTGCCATT
TCCGAGTAGAGGACCGCAGAGCGGATGACTTTCAGCCGGAGGAAGCGTATGCCAACGTACGAGAAGCGA
CGTACCTAAGGCTCGAGCGGTGGAACGTCAAGTTCGATGGCGAGGACGCAATGAATTCTGTCGAGGACTT
CGTGTTTCGCCTTGAGTTCCTGCAGAGGCAGTATCAATGCCCGTGGAAGGAGGTCCTGCGTGCCTTTTTCC
TGCTCCTGACCGGCCGTGCCCGCGAGTGGTACTGGATGCACCTAAAAAACTGGAGGAGGCATTCATGCGA
GACAGGACATCATCCCGAGTAGTGTGCTGGAACTGAAGGCAGTTCGACCATGTCTTTAGATACCGCCTAT
CTAAGAAGAGTTAGATATTCTGCTACAGACGCGGAAAGCCGGGCACATTCTGTGCTGAATGCGAAAATTG
CCCGGGAAACCACAGAGGAAGCGCAATGGTAGCAGGACAGACACGTTTAGGGACGGCGATCGCCGAAAA
ACAAGAGAGGAACACCTACCATAAACAATAACACTGGCAGAGGAGAAGAATATAGACCATAAGGACCAG
GATAAGGTTATATATAAGGATAAAAATGAAGGAAAACCATTAAAAGGCTTACCATATGAAGAACGTGTC
CGGGCATACATGTCGGCCCGGCATAGGATTTTTGGTGAGCGACAGCTGGAGGTGATGACATTGGCCACCA
GAAGAGTGCTCAAGGCTCGGGCGCGCTCCCGCAGACGCAAGATGACGAGGCACCAGGTCATCGAAGCTG
TGAAATGGGAGGATAGCCTAGATCCGCGCGTATTCACCGAAGTGGAGGTCGCTGGAATTAAAATGAAAG
GGTTGCTAGACACCAGAGCCTCGGTCAGCCTGCTGGGACAGTGGATATTAGTCGTTTGTTGGTTGAAGCTT
TTATATGTGCATCATTTAAAATGCAGCCGATTGTCGCAGTTTTGTTCTTGTTGTAAAATTGTTGATTCATCA
ATGTTTTGTGTGTCGTTGCTTGTTACGAAACTGATTGTTTCTCGGTGGGCCGCGCGGCTGGCTCATCGGTTT
CTCTTTGCTTCTGTGGCACACAGCGCCTGAATCCAGCGACGGAGGACGCTGGCCAGATCAAACCAGATCG
GCGTAACCATCACGACGGTGAGACGAGCCATCACGGACGGACGCCAACGACGAAGAGGCGGCCGTAGGC
GAGACCACGCCCACTTCATCACTGAGTGAGTCTGTTCTACGTCGGCCTTCCCTCGCCTGAGTNAAAGGCAG
AGGCTGAAGCAGACCCATAGTTGAGGCTCCCTGAAGGAGACCAGATATTTTTTTTTTTGGAATTGTTAACT
CTAATTTTTTTTTTGTAAATTGAATACATAATATACTCACGACTAGTCGCAATTAAGGAAATTTCCGAAAG
TGCGAACAAAGTGCGAAAGTGCGAAATGGCCCAAAGATTATTGCGTCTGCCAAGATTTTGGTCAACTTTA
TTGGATTGATTGTCTCCCCTTTCTTTTATTTCTTTGTTATTTTATATTAACATCTCACCTTAGATGGTTACAT
TTACTTATCGTCACAGCTGTTTATGTTGTTCATCTGTTAGAAGTTCCGATTGTTTCTTGTTATCAATTTGTGA
GACCAGATATTTTTTTTTTTGGAATTGTTAACTCTAATTTTTTTTTTGTAAATTGAATACATAATATACTCAC
GACTAGTCGCAATTAAGGAAATTTCCGAAAGTGCGAACAAAGCGCGAAAGTGCGAAATGGCCCAAAGAT
TATTGCGTCTGCCANGATTTTGGTCAACTTTATTGGATTGATTGTCTCCCCTTTCTTTTATTTCTTTGTTATT
TTATATTAACATCTCACCTTAGATGGTTACATTTACTTATCGTCACAGCTGTTTATGTTGTTCATCTGTTAG
AAGTTCCGATTGTTTCTTGTTATCAATTTGTGGACCAAATATTTTTTTTTTTGGAATTGTTAACTCTAATTTTT
TTTTGTAAATTGAATACATAATATACTCACGACTAGTCGCAATTAAGGAAATTTCCGAAAGTGCGAACAA

AGCGCGAAAGTGCGAAATGGCCCAAAGATTATTACGTCTGCCACGATTTTGGTCAACTTTATTGGATTGAT
TGTCTCCCCTTTCTTTTATTTCTTTGTTATTTTATATTAACATCTCACCTTAGATGGTTACATTTACTTATCGT
CACAGCTGTTTATGTTGTTCAGCTGTTAGAAGTTCCGATTGTTTATTGTTATCAATTTGTGGTTCGTTTTGC
AGAGTGTTGTGTAAGTCTGTTTTGCCACAGACGTGTAGCTTTGTTGTAATCGATTGTGCCGACTGCGGGCC
AGGGATGGGACTACACCCAGAAAAGCCGATAAGCCAGCCTGCGTGGCCCACCGAGAAACAAGAAGTTTC
GTAACATTATGGCGCCCAATTAGGTCGCGCGGGGAGCGAAGTACTCATCCAGCAGCCCAGTATATGTCGC
CGATGCTTAGCGAGGAACTACCGGAATCCGAATAATCGCCATTTCCGAGTGGAGGACCAGAGAGCGGAT
GACTTTCAGCCGGAGGAAGCGTATGCCAACGTACGAGAAGCGACGTACCTAAAGCTTGAGCGGTGGAAT
GTCAAGTTCGATGGCGAGGACGCAATGAATTCTGTCGAGGACTTCGTGTTTCGCCTGGAGTTCATGCAGA
GGCAGTTTCAATGCCCGTGGAATGAGGTCCTGAGCGGCGCTCACGTGCTCCTAACCGGCCGTGCCCGCGA
GTGGTACTGGATGCACGTTCGGCATTCCAGGGTCGTTAGCTGGATGCAACTGTAGCACACACTCTTAGAC
AGGTTCCTGAGATATCAGACGGAACAGCGAAGGGGTGGACGACTATATTCACCACATGCGTCAACTCATC
GCGTGGTTCCGGAAGGTTCCAGAAGGTTCCGCTGAGAGACCGCGATCTGGAGAAAATTTAAAGCGTGGCT
TGCAGGAGATCTGCGCAATGGATGTACTCAGCGTGGATCAGTTGCGCCAAGAATGCATAGAGAGTGTAGA
GGCACGTTTGTCGCTGTAGCCGGACTGGGTACGTACAGCCGTCGCGTTAGTCACCAGGAACAAGGCCCAC
AGTTCACATAAACGCCTACTAGTGAACTAGAGGAGGCATTCGTGCTGTAGGCAGTTCGATCATGCCTTTA
GATACTGCCTATCAAGCGGAAGATATTCTGCTACAGATGCGGAAAGCCGAACACATTCTGCTTGAAATGC
GAAACTATCCGGGAAACCCCAGAGGGAGCGCGATAGTAGCGGGACAGGCGCGTTCAGGGACGGAGAACA
CGGAGAAGCAGGAGAGGAGCCCCTACTAAAGCCAATAGTCGTTGGAAAGGAGTCGGATATTTGAGATAA
GGACCAGGATAAGGGTATTTATAAGATATAAACAAAAGAGGTACATTAAGGGGCTTACCATACGAAGAA
TGTGTCCGGGAATACATGTCGGTCCGAAATAGATTTTTTGGTATGCGACAGTTTGATGGGATGACGCTGGC
CACCAAAAGAGTGGTGAAAGCTAGGGCGCGCTTTCGTAGACGCAGGATGACGAGGCGCCAGGTCATTGA
AACTGTAGCCGGGAGGATCCAAAAGAAAAGGGTTATTAGACACCGGAGCTTCGGCCAGTCTTTTGGGAAA
GGGTGGTCGAGCATTAGTTATTTAATTATTCATTAGGAGTTAAGGTGCAACCGTACGTTTCGGTAGTCACG
AACGCTGGAGGGGAGGATAAATCAATAATTGATAGGGTAGAACTCCTAATCTCGTACGGGGCAGCATAA
AAAGATAACGTTATAATCTGCCCCTACCTGAAACAGTGGGCCTATTTTGGAATTGATTCCTGCGAGCTTTT
AATCTAGCCCCTGCTGTGTTGGACCCGAGAAGGTGATCTCCACGGAACACCAAGGCGAAGAGCTCTTGGT
TGTGGCGATGGCACACTATGCGGAAGAAGACGACGGACACAAATACGAGCCAGATTCATGGGAGTTGTC
TCGGGAAGAAGGGATAGAATTGGAGGCAGTAAAGCAGGAGTTTTATCTTTGAGCATAGCGGACTAGGAT
CGACAGACTTAGAGAAGCACACTATCGACTTATTGGAAGGTGCACAACCATTCAAAGATAGACCCTACTC
ATTATCGCCGCGATGAAGAAGGTAGTGGATGATGAAGTGGACAAAATGTTGATTTTGAAGGTTATAGAAG
TGCAACAACCCATTGAGTAATCGCACAGCCGTATTGCGAAGACCGGGGAAACCCCGTTTTTGCCTCGCTG
CACGCAACCTCAACAATTTGATGGTGCAAGACGCGTATCCACTACCCTGTATAGAATAGACCGAATCCAC
TACATCTCCAGTGCGGATCTGAAGTTCGCATATTGGCAGGTCGAACTAGTCGACAAAAGCAACCCCTATA
CCGTATTTACCGTACCAGGTAGGCCGCTATACCAGTTTCGCGTCATGCCGTTTGGCTTATACAATGAAGCC
CAATGTCTAGTTCGTCTGATGGTCAAGGTAATTCCCAACGAGTTACGGTCCAACGTTTTGTTGTACTTGAT
TTCCAAACGCAATTGAAATATTTGCGACAAGTGGGCAAATGCTTAAAAGAAGCCAAACTCTATATCGGAT
GAAAAAATCCAAATTTGGCCTATGGGCATTAAAATACCTAGGATTCATTATAGGTGGTGGCACGATTCGC
ATAGACCCCGAACGGGTTATAGCTATTAAACGAATCACCGTGGCTCGGTCACGGAAAGAGTTTCGCTCTT
TTTGGGTTGGTACCGGCGCTTTGTGCGAGATTTCGCGGCAATAGCCGCCCCACTCACTGAAATGCTAAAAA
AGGATAGCGGAAAGTTTGTATTGAGTGACGAGGTAACACGAGCCGATTATCAGTTGAAGCCCACGCTCCC
AAACAAAAACAAAATGGTATGTTGGAAAGTTACGTATATATATATATATATATATATATATATATATATAT
ATATATATATATAAGAAAGTAAATAAGTGAGAATTAAATCGCTTAATTTATACATTGCCTACCTAAAGAG
CTTTGTGGGATGCTTAGGGCGATGGAAATAAATATAAAGATTATTTCATTATTTATAAATATATATATGGA
TATGTATATATATATGTATATATATATATATATATATATATATATATACATGTATGCATGTATGGCCGGT
GTGGAAGCTTAAAACACCATTTTTTTTACTCCTTTTTCGCAGTTGACAGGTGCTATAACTTTTATTTATTTA
TTTATTTATTTATTTATTTATTAATGGTCGACAAAACGAGTGTCTTCCTCATTATTCAAAAGATTGTACAAT
AGAATTATATGCTAAAAGCCTAGTTAAAGAATACAATTTTGTAAATAGCATCACCGACCGTCCGGCCAGC
TGTGCCCCTTTTCCCAGCTAATTTTGTCAGTGACGCTATTAGAGCCTACATCTAAGCAGAAGGTTGTAAGT
AGGAAAAAGGGTGATCTCAAGTGAAATTAAAAATAATCTTAATTTGCTATATTAATAAAGAAAATACAAT
AAATAGGAAAAAAGAAAGTAAGAAAATTGTTAATAAAGTAAAGCAATGTAAGTTAGATAAAGAGTGTTA
AGTGGATAGGACAAAGAGAAAAGTAATGGGAAATCACCGACACGGTGGGGGAAAATTTTTCAGCCTGTC
CGGCTAGCTATGCCCCTGCTCATAGCTGGGAATGGTCAGCGACTTTCCGAAGGTATCCTGAAAGCAACGA
TTTTATTAGGGGTAGAGACAGTTCGGTAGAGAAAACGTGATGCAAACTATTATAATTTTTACATAGCACG

CGAAAAGAATTGTGCATAGCGTAATCTGTTGTGCACGTAGATAATAATAACGGGCGATAATTTCTGGTTG
GTCTAGCCGGAACAGAGAATTGAAGACGCCCTAGCAGAAAAGGAGAATCTATGTCGCCAATAATCAACTT
ATGTGGAAGGACAACACCGAGAATTGTCCTACGGTTGATTAACGACGGAAGGTTGAGAAGAAGCAGTCT
GCTGGAGTAGGACGGCAACCGAAGATTAGGGTCCCAATTTAAACCTCGTAAGGCAAAACGCAGAAATTG
CTTCTGAACAGATTCAATACGATCCAGGCTGTTGATATACTGTGGAGACCAGATGCAAGAGCAGTATTCA
AGGATAGGGCGAACAAGAGAGATGTACAGAAGTTTTGTTATAAAAGGATCGTTAAACTCTTTAGATCATC
GTTTAATGAATCCAAGTACACCTTTTGCCTCATTAACAATGGTATCTATTTGAAGATTGAAACAGAGTTTA
GAATCAAAAATAACGCCTAGATCCTTAACTGTTAGTATACGTTGCAAAGGGATATTGTCGATTGTATAACT
GACAAGATAAGGTGTAGTACGATTAAATGTCATAAACATACACTTTGAACAATTTGGATGCAGTTGATTG
GCCGAACACGAAAGTTGCATAGCGTTCA
>9_rnd-1_family-269__LTR___Pao
TAGTAGCGGGCAGGTCGCGATGGGTATATGAATGATACTGGACATANATATACTATTCTATTGTAAACAT
GTCGATTTTGGTGGATCTAGCTCTTATTATTTACAAAATTTCCTCAAAAAAGAAGATTTCGTTATCGATTTG
TATCAATTGATTGGAAACAGTGGAGGTTATCGATTATCGGAAAGAAACTCGATCTGAGCATGAACTAAGA
GGACCTACAGCTTAAATTTCACGTCTCTAGCCTTTATAGTTACTAAGATCTTGCGTTCATACAAACGGACG
GACGAAAAAGACGGACAGACAGACGAACATGGCAAGATTGACTCGGCTGTTTATGCATATCAAGAATAT
ATATACTTTATGGGGTCGGAAAAGCTGCCTTCTACCTTTTACATGCATTTCACATTTTGCACAAATACAAT
ATACCTATTTAATCCATTTTTCAATGGGCTCAGAGTATAAAAAAAGTAAGTTCCCTTGGCGGGGTTCGAGC
TCGCAACTAACTGAACCACTTGCCACCGACTCCACTCAACAACCAAACTAATAATCGAGTATTAGTGAGA
AACAAAACAATTAACATAGTAATACGAACAACAATACAATCGCAATTTTAATTCTGTTAATAAATACGAA
GCAGACGCGCATAAATTGTGTGAGTACATGCATACAGATATTATTCGAATTTGCGCTGGTGCCGCAAACA
CGTGTTTCTTAATCGATCTTCATTATATTTTCTACAGTATTCCTCAATATACCATAGAATACCTGTGTATTC
TTTAAAAGTAAATTGCATTAAAATTGTAGTTTTAATTGGTGAGCAAAGAAGGTTGGATTATTAATTTAATT
TAAAGCCATAAAGTAGTAGCGGGAAGGTCCCGATAGGTACGTGAATGATACTGGATATAAATATAGTATT
CTAGTAAACATGTCAATTGTGGTGGATCTAGCTCTTATTGTTTATTCAATTTGCTGAAAATTCGGATTTCGA
TATCGATTTGTATTGATTGCTTGGNAACGGAGGAAGTTATCGATTATCGGAAACAAACTCGATCTGCGCAT
GTACTAGCGGATCTACACCTATAATTTCACATCTCTAGCTCTTATAGTTACTTAGATCTTGCGTTCATACAC
TGGCCATCGCCTCCTCTCAACAACCACACTAATAATCGAGTATTAGTGCGAAAAAACCAATAACATAATA
ACTCAGAACAATACAATCGCAATTATAATGCTGTTGATAAACACGAAGCAGGCGCGTATAAATGTTTGTG
TNCGTGCACGTGCATACAGATATTCTTCGAATTTGGGCTGGTGCCGCAAACACGTGTTTCTTAATGTCTTC
CTGAAAATTTCTACGGTATATCNTAATATACCATAGCATTTGTGTATTCTCAAAAGTAAATTGCATTAAAA
TTGTGGCTTTAATTGGTGATCAATGGGAGTTGGGTTTGTTACGGGGCGAAGTGAAAATAGCTGTTGACTAA
GTCCTACTGGGTCTCGCATTTTTCTGTAAGCAGCCCTCGCTGCTTACACTGGAAGCGCACCGTTAGAATTA
GTTTTTACATGATAAGTCGATCGCACGCAGCTGCACAAGCTGCATTTTTGTCGAGGTTCATTTTCGTTTTGT
GCTTGCAGCTGAGCAGAAGTGAAAGAGAACAGAAGCGCGTGCGGAGCACGAATGTAAGTCTCATAATCA
TGTAAAATATAGGTTGTTAAATAATGAAAGAATAATAAAGATTTCAGCGTTCGAATTATATTATATCGTAC
CGCCTCGTACGAATCGAATTGAATCGAGTTGTTTCTTTCGACGCTAGAAGTCCCTGCCGAATACGCGCCCC
GGCACGAGTCTCGGTGCCAACATTCTGAAATATTCTCTGCAGCAGAGAGTGTCCGAGGGATTCTCTGCTAC
GGCGGAGATTTCTGCGAAGCAAGCGACTTGCAAAGTCACAACCTTAGCTGCCTATAGAGAGACACGTGGT
ACAAGAGCTAGACTCACACCTAGCAGCCATAACCTCAAAGCGGATTTCATAGTATTGGTGTGGCGAGATG
GCTGACGGTGATGAACAAGATGCAAGTGCGAGAAGGACGACAAGTAGTATTCAACCCGGTGGAACTGCA
ACGCTTGTAACGGCATGGTCAACGGGACAGGCTAACGGATCGCCTGTTCCTGCTGGAGTACAAGATAAAG
TCGCCGGCGTAGAAAAAGGTAGCGGGGCAGCAATCATAGAAACTTATAGCGGCTGTCCGTCTACGTCGTC
CGCTGCTACTGGTCCAAGGGAAAGTTCAACACCAACGCAGCAAAGAACTGAGAAAGTGGGAGCTCTTTTC
GACCCTTTGGGATACCAGAACGTGATCTACAAGCAGAAACAGGTTGCGGCTAATTGGAGTGTACACCCCA
GTTTGCCATATCAAGCAAATACGGCGGTGTTTAACCAGGCTACGGAGCCCAGTACTGCCGCGTACATGGG
CCACACAGGTCTGGGAGCGCTCAATAGATCGATTGAGCACGTGCCACGGCAGCAATTTGACAATGTGCAT
TGTAATAATGCGACGCCGTGGAATGGATCGCTGACACCTGCGCAAATCAGCGCGCGACATGTCATCAGCA
AAGATTTACCAATTTTTACAGGCAAACCAGAGGAATGGTTGATCTTTATCAGCAATTATGAGCAATCCAC
AGAGAGATGTGGATTCAGCAACGAGGAAAATCTCATCAGGCTTCAGAAGTGCCTCAGAGGGCAAGCCCT
GGATGCGGTGCGCGGAAAACTTATGATCCCAGCTACAGTGCCGTATGCTATAGGCACATTGCGAATGTTG
TATGGCCGCTCTGAAATAGTACATGCTGCTCTGCAGCAAAAGCTCCGAGAGGAACCAGCAATAAAGTCGG
ACAACTTGAACACCGTTATCCGACTGGCCCTAGCTGTCCAAAACTACTGCGCAACGATAGCGGCCATTGG
TATGCACGAATATCTTTACGATCCCGCGCTGCTGAACGAGCTTGTTGCTAAGATGCCCAGCAATATGAAAT

TGGATTGGGGCCGGCATCGAATGTCGAATGAAGGTAGCAGTTTGGCCACCTTTGATAACTGGCTGTTCAA
TGTGGCAATGTGCGCCACTATGGTCACGCCCTACGAGACACCACTGGCTGGTAACGAAAAGAAGAGCTCC
ACGAAAACCACTAGAGAGCGAATTTTTGTGCACAACACGGTAGATAGCCCTGAGGGTTCTCAGCAAACAA
CGTTCACAACAGGAACAGGAGAGGGTTTCATGTGCGAAGTGCGCCGGTAACCACCGTCTATCAGATTGCG
ACGACTTCAGATCAATGGGTACAAATCAGCGCTGGGAACTTGTTAAGGAAAAGAGGCTCTGTTTTTGCTG
CTTTCTACGTCATCGTGCACAACAGTGTAAATCAAAGAAGAAGTGTCATGTAGATGGTTGCGAGTCAACG
CACCATGTGCTGCTGCATACGCCTACCATGAGTGGTAGTAAAGTCGAGCACACGGGCCAAGGTAGCAACC
ACCGGATTAAGGCAAGTGAGTCTGCCGTTATGTTTCACGGCAGCTCCGCAAGTAAGACACTGTTTCGATAT
GTTCCAATCACGCTCTATAGTAAATCAAAAAGCGTGCGCGCATATGCATTAGTGGATGAAGGAGCTGAGT
GTACGTTAATAGAAAGAGCGCTTGCAGACGAGCTGGAATTAGATGGTCCTGCGACCCAGCTGTGTCTTAA
ATGGACAGGGGACATAACCAAGAATGAACCTGAGTCGATGTCCGTGGCTGTGAGCATTTCAGCTCCAGAA
CATAACGCAATACAGTATGCCTTAAAAAACGTGCGAACAATAGCGAACTTGGACTTACCAGAGCAAAGTA
TCGGGCCACAGTTGCTCAAGTCTCGGAAGCACTTATCTACGCTTCCTATTCTGCCGTATAACAACGTCAAG
GCTCAGCTTATCATTGGTTTAGACAATATCAAAATAACTGCACCCTTGGAGATACGAGAAGGTGAAAACG
ACGACGTCATAGCAGCGCGAAGCAGACTAGGATGGGCCGTATATGGCCGCCCCATTGTGGGAGACAGCA
CCACGCCTCGGCTGCTACATATTTGCCAGTGCAGTGCGGCGCGTGGGATGGATGAAGCTCTGAAGAACTA
TTTTTCAATTGAGTCACTAGGTGTGAGCGTTTCCTCACATCCGCTGAGATCCAAAGAGGACGAACGCTCGA
TGCAGATTATGGAGGCAACAACAACCTATCTGGAAGACGAAAAGCGCTGGCAGACCGGCCTGTTATGGA
GGTTCGATCGCATGCACCTGCCAGACTCTTATCAGATGGCTGTTAAGCGCCTGAAATGTTTAGAGGCAAA
GATGTCAAAAGACCCACCACTGAAAGAGTTCATGATGAACACGATGCACGATTACAAACAGAAGGGATA
TATCCGCCGGTTGAAGGATAGTGAGTTAGTAGCTAACACTACATCGTGGTTTCTTCCTATCTTTACAGTCA
CCAATCCGAACAAGAAAAAGACGCGCCTGGTTTGGGATGCGGCTGCCAAAGTTAGTGGCATGTCGCTGAA
CGATTGTTTGTTGAAAGGTCCAGATACACTAGCATCTTTGATGGGGGTGCTAATACGCTTCCGAGAGCGTC
CTATCGCAGTATCCGGCGACATAAGGGAAATGTTTCATCAAGTCAAGGTGCGCCNAGAAGACCAACCGGC
ACAGAAGTTTCTCTGGCGTGATGGAGATTCGTCACGCTTCCCCGAAACATACGTTATGCAAGTCATGACGT
TTGGAGCATCCTGCTCGCCAGCTCTAGCAAATTACGTCAAGAATCGCAATGCCGAGCGGTTTGTAGCGGA
GCATCCGGATGCCGTAAAGGCGATTTGCGAAAACACATTTGTCGACGACTGGCTGCAGTCGGTGGATACT
GGAGCTGAAATGATACAGTTAGCTGAGACTGTAAAAAGGATTCATGCTAGTGGCGGCTTCGAGATGCGCC
GCTGGACATCAAATTCGAAGCAAGTTATACAGGCGCTGGAAGACGACTGTGAGGAGTTGGACAAGCGTA
TCAGCGCTCAGGATGAAGCGCAAGAGAAGGTCTTAGGCCTGTGGTGGCTACCTGGACAAGATCTGTTGAC
GTTTGTGGTGACGCCGAACTTGCTCGCGAAGGCATCTAAGGAGCGCCCAACTAAAAGACGTGTTCTGAGT
GTGATCATGTCTATTTTCGACCCGCTCGGGCTGCTAGGATTCTTTAATATACGCGCTAAGATCATCCTTCA
GAACATATGGCGATCCAATATCGGATGGGATGACGACCTCACCAACGAAGACGAAGCCGATTGGCAACA
CTGGCTTGATCTANTTTCAAATTTGAATGCCGTCCGCATTCCACGGTGCATGAAGTGGGTGAGCCGAGCCC
AGGCTGTGCAGATGCATACATTCGTTGACGCCAGTATGAATGCTTACGCCGCAGTTGTATTTTTGCGGGCT
GAACTTGATGGCCAAGTACATTGCAGTTTGGTCGCCTCGAAAACGAGAGTAGCACCCCTAAAGCCCGTGT
CCATACCTCGAATGGAACTGATGGCCGCGGTCTTAGGTCTGAGACTGGCCAAATGTATCCAAAAGGAAAT
GTCGGTACGCATACATAGACGAACATTCTGGACAGATTCAAAGGATGTGCTCTACTGGATCCGTTCCGAT
GCTCGAAAGTTCCAGCAATTCGTAGCGCTTCGGATAGGAGAGATTTTAGAAGAATCAGATGTGGACAGCT
GGCGATGGGTACCATCGGCTCAAAACGTGGCGGACGATGGTACGAAATGGACTAAAACGCCTGAAATTC
ATGGCTCAACCAGGTGGTTCAATGGACCACCATTTTTGTATCTAGAAGAATCGCAGTGGCCACAATCGGA
AATAGGCCCTGCATTAAACATGTTGTACCATGCTGAAGAACAACCCAATCACACGTCGTCCTGGAGATGT
ATTCTGCCGGATCTGCAGCGTTTCAGCAAGCTAGAAAAATTGAGAGCCGTACAGCTATGCGTACTGGATT
TCCTACGGAATATTACTAAGAAGGTCAGATTGGACGGAGGACTGGATCTGCAGAAGACGCTGCTCCTTAA
AAGAAGCAATGAAATGGATGTGATTTTTATCCGGATTTGTCAAGAAGAGGAGTTTTATAGTGAGATCACC
TGCTTAAGGTCGGGGCGCCGCTTAACCGATCGCAAAAGCTTGCTGTTTAAGTGCTCCCCTTATGTGGATGA
CTCGGGCATTCTACGTATTAAAGGACGGATAGACAATATAGAAGGAGTCGAAGTCTGCGTAAAACGCCCG
ATAATTCTGCCAAGACGACATCAATTGACGTATCTGCTGGTTGAGTTTTATCACCGCAGATATCATCACCT
ACACAACGAAATCGTCGTAAATGAACTGCGGCAGCGGTACTGGGTTTGTGGCTTACGAGCTTTGGTGAGA
GAAGTTTCCAATACCTGCCCAGCGTGCCGCATACGACGCGCCCGCCCAAAGCCGCCAGGGATGGGCGACC
TGCCAATCGAACGATTATCGCCCTATACTCCACCGTTCACATATACTGGAGTGGATTACTTCGGCCCCTAC
GACATTGTCGTTGGACGGCGTCGTGAGAAGCGCTGGGGCGTGCTATTTACATGCCTCACGGTGCGCGCCG
TTCACCTGGATATAGCTACGTCGCTCTCAACTGACTCATACTTATGCGTTCTGAAGTCGTTCGTGGCCAGA
CGCGGCTGCCCCCGCCGCATGCTGTCGGACAATGGCACAAATTTCAGAGGCGCTAGCAGAGTATTAAAAG

ATGAAATTGAACGTATATCGCCGGCTCTGATCGAGCGACAGTACCCAGAACTCGAGTTTACCTTTATCCCG
CCAGGATCACCCCACATGGAGGATCGTGGAACGAATGGTGCGCTCTACAAAGTCAATACTGTCGGAAA
TTCTGCCGCCCTCTGGGTTACGAGAGGAGGTGCTACGAGCAGCATTGGCTGACGTAGAAAGCGTGCTCAA
CTCAAGGCCACTCACTTATGTTCCCTTGGAGACATCTGAGTCCGAAGCCCTGACTCCAAATCATTTTCTTA
TAGGTCATTCAAGCGGATTGCGTGAGAGAGGGGAGCAAGGCGGAGCTAGCTTGGCACGAGGCTTCCGAG
TCGCCAGTCAATTGGCTGACCAATTTTGGAAGAGATGGCTGCGCGAGTATCTGCCTACACTCACCAGACG
TACTAAGTGGTTTCAACCGCCACTCGAACCAATATCAGTCGACGACATCGTCATAATAGTGGACGACGGT
GCCAAGCGAAACTGTTGGACAAGAGGAGTGGTCGTAGACGTGCACCGTTCAAAGGATGGCCAGGTCCGA
AGTGCCGTGGTGCGAACCGCCGATGGAATTATTACTCGCCCCGCCGTCAAGCTAGCTAAGCTTGACATTA
AAGTTGGTAACACACTGCACGGACGTTCGTGAATTACGGGGTGGGGAATGTTACGGGGCGAAGTGAAAA
TAGCTGTTGACTAAGTCCTACTGGGTCTCGCATTTTTTTCTGTAAGCAGCCCTCGCCGCTTACACTGGAAG
CGCACCGTTAGAATTAGTTTTACATGATAAGTCGATCGCACGCAGCTGCACAAGCTGCATTTTTGTCGAG
GTTCATTTTTCGTTTTGTGCTTGCAGCTGAGCAGAAGTGAAAGAGAACAGAAGCGCGTGCGGAGCACGAA
TGTAAGTCTCATAATCATGTAAAATATAGGTTGTTAAATAATGAAAGAATAATAAAGATTTCAGCGTTCG
AATTATATTATATCGTACCGCCTCGTACGAATCGAATTGAATCGAGTTGTTTCTTTCGACGCTAGAAGTCC
CTGCCGAATACGCGCCCCGGCACGAGTCTCGGTGCCAACA
>9_rnd-1_family-299__LTR___Pao
ACAAGGCATTGCGAGTTATTGCATATGTCTGTCGCTTTATCCAGCGCTGTAGAAATCCAGCGATCCACTTT
TCCCGCCGAGCTCCGTAATGAAGAACTTACGAGAGTTCAGTTCCGACTTATCGAGTATACTCAACGCCAA
GCTTATCCTAAGGAGTATAGCTGTTTGCTCGATAAGCAGCCAATTCAAGGATCAAGCAGCATTCGCAATTT
TAACCCCTTTCATCGATCAAAGGGGTATTCTGCGATCCTGTGGACGGGTGAGAGCGTCAACCTCGTTAGGC
TATGATGAGCGACACCCCATAATCATTCCGGCCAATTGTGATTTTTCACGCCTCCTGGTCCGATTTACCCA
CCTTATTTGTTTTCACGGAGGCAATCAGTTGGTAATGCGCCTCATAAGAGCCAAATACTGGATCCCAAAGC
TCAAGGTCCTGGTAAAATCTACCATAAACTCCTGCAAGGTCTGTGTAATACACCGTAGACGACTCCAGAC
CCAACTTATGGGCGAACTCCCAAGGGCGAGATCAACCTTTTCCCGACCCTTCACACATACGGGTGTAGAC
TTTGCCGGACCTTTCGATATTAAAAGCTACATTGGTAGAGCATGCAAAATTACCAAGGGTTATGTCTGTGT
CTTCGTGTGTTTCAGCACGAAAGCAATCCATTTAGAAGCCACCACAGATCTAACCACGGAGAGATTCCTG
GCCGCATTTTCGCGATTTATCGCAAGACGTGGCCTTCCACAACAGATGTACTCAGATAATGGGAAAACAT
TTGTGGGAGCGTCATCCGTTCTTTCGAGAGATTTCTTGCAAGCGACCAAAGAGCTCATCTTATCCAAGCAT
AGCCATCAAAACTTGTGTTGGCATTTCAATCCTCCTGGCGCTCCTCACATGGGCGGATTGTGGGAAGCAGG
TGTCAAAAGCTTTAAGACCCACTTCTACAAGTCCACCGCATCCTCAAAATATACATTTGAGGAGTTCGCGA
CTCTCCTCTCCAAGATTGAAGCCTGCCTTAACTCCAGGCCAATCTCTCCAATGTCGGAAGATCCGACTGAT
TTATTGGCTCTAAGTCCAGGCCACTTCCTAGTTGGTGGCCCTCTTCTGTCAACTAACGAGTAATCAAAGAA
GACGCTATTTCTATAATTAACCGCTGGCAGCGACTCAAAGCGCTCCACCAGCAATTTTGCTTACGGTGGAA
GAATGAGTATTTGAAAGAGCTCCATAAGCGAAACAAATGGCAGTCCCCTACAAGGGACCTCCAAGTCGGT
GATATGGTGATTATCAAGGAAGATAACATACCAATAAATGAATGGCGCCTTGGCCGAGTCCAGCTGGCTT
GCCCAGGCACAGATGACAAGGTCCGCGTAGTGGACGTGCTTACCGCTCGTGGTGTCATCCGAAGGCCAAT
AGCCAAATTGGTCCTTCTTCCTACTGAGGCAAATAGCACCACCGACTAATCCTTAAAAGCAACTAATCCCT
CTTTCAAAACAAAATCATCACATAATAACACCTGTCCTACATCTTATTCTCCAGTTCACTCCTCTGTAGAG
AACCAGCATGGCACCGTCAACGAAGGCATCCAATCAACGTCAAGGAACGGACTCTTTTAGATGCCGGGTG
TGTAGAGGCATCCATGCGCTACGCAAGTGTCAGCGCTTCTTGAAGCTCACGCCCGAGAAGCGTCTCCGAG
CCGTGTTAATTAACAAATATTGCTCCAACTGCCTGGCTCATCAGCATTCAGGGGAATCATGCCGCAGTGGA
GCCAAGTGCAAAATTTGCAAAAAGGACCACCACACCTTACTCCACATGCGTGATCCCCGTAGCGCTCGAC
CCTCTCGGTCAGACAACAGGCCCACAAGAAGAGCTTCTGGCTCCTCGCCCTCCAGCGGCACATCACCCCC
AAGCCCGAGACGTGCTACTATACGGACGTCGCTTACGTCCCTTCTCCAGCACAAGGGGGTCAGTATATTG
CCCACCGCCATAGTGCTGCTCGACACCGGAAAAAGACCCTTGAGACTCGGGCTCTGATCGATCCATGTTG
TTCAGTAAGCCGCATTAATGCAACCGTGGCAAGGGCCTATGGCCTTTCCGTCACACGCATTGGCGCAGAA
GGAGCGTGCACAGCCGTGCTACGCTCCAAGACAACTCAGATGTCTCGAGAAGTGGTCTTCATGGCTGACC
CGCAACTCAGCTGCATAACTCCAGCCCGAGCCATCAGAGCGGACATGAAGGACCCGTTCAGCAACGTCGC
TTTGGCGGACAACGAATGGTTCCGTCCATCTCCAGTCCCTTTGGTACTCGGCGCCGACGTCTACCCAGACA
TCATCCTACCAGGTATCCTCCCGAGCCAAGGCGGTCTGCCGATGGCTCAAAATACCAAGTTTGGATGGAT
ACTCTCTGGCACATGCACGCAATAGGAATTCTTGCAATCTGCATATTGCCAGGGGGGAGAATGTTCATGC
CAGTTCAGTGCTAGTGTGAACACAGCATTGACTGAGCCTCACTCAGTGTGAGAGAGTCTCACTCAGTGAG
ACAGACTCACTCACTGAGAGAGACTCACTCACTAANTCGATCCCAGATGCGCTCAGCACTCTCCTTGTGGT

CTCCTTTCCACCTCCATCACTCCAACCAAAAATATGTAAAATCACTTAACCAGCAGAGTTTGTTACATGCA
GCAGAAACTTCTAATAAAGTGCTTTTTTTCACCAACAACAATAATCAAGGATTATTTATCTGACGCCACCC
CCACAAACATTGGTCCTTCGAGCCGGATTATATATAAACCGATCGCCCCCTACACAAGTTTTGGCAAAGA
TAAGTAAATACAAAATTTCCTTTCTATATATATTCGGCAATATCCGCCATATTTTTCCGCCATANTTTTTTT
TTTTAAGAAAACTGATTGGCAATTTTCTGAATATATATANATATATATATATACGAATATATATAAACTAA
AAACAGTTTAGTTAAATTTTGTGTTAGTTACTTCCAACTACAAAAGAAATCCACTTCAACATTTTTTTGTAT
GTACATACGTACGCACTCGTGCACTTTCCTAACTATAAAAAAAATTTTTTAATTACGTGTCCGCACTTAAT
CCGACAAGTTGCTTCACAATATTGTTTCCTAATTAATATTGTTAATTCATGTTCTTGGTCCGACACACAAAC
ATACACCAGGTTCACCAGCACGTATTGTACATACATACATACATACATATATTCAATCCGAATATAATTGC
CCATATGCATTTTGAATAGACACTATATTTAACCTTATTATTTAATAAACATAAACACAAACTCCAATTCA
TTCTCTTAACTTATAATTTGTTGCATNGTGCANACGAATATATTTAGTAAAAACACACAGCTCCCGATCCG
GTANNTAAATCCACAATTTTGCATAGTGCAANCGNATATATTTGTGTACAACCACATAGTACATACATCCG
ATAAATAAATNCACAAATCATATGTACTATGTTGTCCACATGCAATATTTTTAGTGCACAATCCACAGCAA
TATTATAACACAGCAATATTATAGTAATAAATTGCAATTTCAACCGGCGTATGTGCGTATGTACACACCAC
ACACATATGTAAAAACNACAGCTGCCGATCCGTACTCCAATTACACGTTTTTATGCAATTGCACACATTCC
CATACAATTACTCTTGTTCCGAGACGCACCAAATAAAAACACAACAATCATAATTAATAGATTAATAATA
CTACTAATTTATTGTAAATAAACAAAAACACTACACATTAACTTCGAAGTTCTACATTTGTCAGCGGTGTG
TACTCAGCCACAATAGTGGGTATTTTATGAGTACCACAAAAAGTAAAAATAAACCAACCGTTACCGTATT
CAAAGACTAATATAATAATAAAATTATCGAAAACGAGCATCGAATTCTTTATTATTATTTCCCGTGTTTAA
TAAATAATCGTATTTTTTAAATTACCAAGTCAAATAAATTTATTAATTAAATTNATTAAATCAAATAAATT
TTGATTTTAAGCAAAATTATTTAAATCTTCCGCAATCTCAATCGAATTATAAACTCGATTATTTACCTGTAC
CGGCATTCCAGTGTTTCTTTGTTTTAAGCATCGCAACCTCTACTACTAGAAACGAGCACCTTTGTTTTCCTA
CCCAAGCGCTTCCGCCTAGTATGCCAACTGGCAAAGGGGAAGAAACAAAGCATCAATTAGCAGTTCCGTC
CGCAAGCACCTCCAGAACTTCTCCGTCTCCAACAGCAGCCCTTAGAGTCGAAAGGGAAACATCGTCCCGG
ACTAAAAGCGAATCATCCGCGTCCACGCCAAGGGGAACTGTTAAGGCACCTCTTAGTTCGAGGCCAAGTC
CACTGATAAAAATCAGTCGCCCGCCGTCCAAAGCTAAAAGCGAAGCATCCCTGACCACTGCAGTCGGACG
CACTAAGGCAACCATTAGCACAAGATCTAGTCCGATTTCCACGCGAGCTAATACCAAAACGGCCCAAAAT
ATGACAGACGCGGCCTTAATCAAGTTTATTGCTGTCACTGATCGGCTAAGTCAGTTTGAGGCAAAAATAA
ACACTCCTGGAGGCTCTGACCCGTCTCTNTATACGTGCCAGGTCCGTCGGGACCAAGCGCGAGCCTTGTG
GGACAAGGTTGAAAAAGAGTACGAGGTCGGCTCAACAATAATCTCGGAAGCCGGTTCACTGGAAACCAT
TTCAGTTATGCAGTCCAAGTACGACTATTGTTACATGGTATATGAGAGATGCGCCGCTAAACTTAGCGAGC
AAATCGATACCGCTAACGCCCAAAATAGACCATCCGCTCAGTCCACCATTCCGACATATGTTCCCACAGG
GTGTCGCTTACCTCCTTGCGACACAGAAATCTTCAGCGGAGATTACATACGTTGGCCCACATTTCGAGACC
TCTTTACCGCAATTTACATAAATAATCCTAGGTTATCAGAGGTCGAGAAATTATTCCATCTCAATGCGAAA
ACCAGCGGTGAGGCTCAGGCGATTGTATCGAAGTCACCTTTGACTAACGATGGCTTCCGATCAGCCTGGG
CCAATCTGACTGAGCGTTTTGAGAACCGAAAATTGTTAGTCAACAGCCAGCTCAAGATTCTTTTCAACCTG
CAGCCAATCTCCCAAGAGTCTGGAACGGCAATTAAGGAACTTCAGAGCACCATTCAAGGTTGCTTAACAG
CTCCGGAGTTATCCGAAATCAACATAGAAAACTGGGACTGTATCCTCGTGTTCCTATGTTCCTCCAAATTA
CCCAAATTAACACTCTCCTTATGGGAACAGTCCATCTCCAGAAAAAAGACATCTCATCTTGGCTTGAGATG
AACGCCTTTCTCTTAGAACGCTATCGCACTCTAGAAGCGATAGAAGAGCTTAAGCCAAGCGCAAATACTC
CAGCGCACTCCAGAATTGTTCGAACAGAACCTCCTACCACTAGAAGGCTTAACTCATTTGAGAATAAGGT
CGCTAACAAGCCGCAAGCCCGCAAATTGTGCTCCAGGGAGAATCACCCTATCCGTCTGTGCCCACGATTT
CTCCAAACGCCCGTCAGCGAACGGGCAACTTACATCAAACAGCAAAAACTATGTTTGAACTGTTTCGCTA
GGGGTCATCAGTTAAGGGATTGCGCAAGTGCTCACAATTGTTTTACGTGCAAGGGTCGTCACAATACGCT
CTTGCATCGTAGTGACGCTCCACAATTGAGCCCTACAGTAGTCTCCATGCCACAAGCAACAGCTCCCTCCA
ATCAATCCATACAGTCCACTCCATCCCAACAAACGAATGTTCACAATTATGTTGCAATTAACACCCACGGT
GTTCTCCTAAGCACGGCTGTTATATATGTTCGCCATTTGGGTGTAAATTATACAGCTCGTGCACTGATCGA
CTCGGGATCAGAGGCAACATTTATTTCCGAGCGACTCTTCCACCTGATAAAGCTGCCGTACAAATCCGTAC
AGGCACAAGTATCGGGATTAAATCAAGCTGTTGCAGCCCAGCCTCGCAAGCTCTGCCATTTTAATATTGGC
TCTCCTTCCAAGCCTCGTATCCAAATCGAGGCATCAGCCTACGTCCTACCACATTTGGCAGGGAATCTACC
ATCATGTTCGATCCCCCAAAGTTTATTGACAACCCTGCCAAAACTCCAGTTAGCGGACCCTAATTTCCACA
AGAGTTCGCAGATAGATGTGCTGATTGGCGCTGATATCCTACCGTCCATACTAACGGGCGGCTCCCATCCT
AATATATGTGGCACGTTGCTGGGCCAGGAAACTATTTTCGGGTGGATTTTAACAGGCCCAGTTTCAAAGA
CCGCATCCACCGCAATCTCGTCGTTCTCGACTCGAATATCTGTCTCTCAAGAAGAGCAATTAGACGTCATT

CTCACAAAATTTTGGGAGGTGGAGGACATTCCAGTCAAACTGGTAAAGAGCTCCGACTCCTTTTGTGAGA
GTAATTTCATCCGAACAACAACAAGGAACGAGAATGGCAGATATGTAGTCACCCTACCTTTCCAAGAACC
TAATCACATAGACTTAGGACATTCAAGATCTATCGCACTAGCTCAGTTTCTAAAGAACGAGAATCGTTTAA
AAAGAAACCCTCCTTTGAAAGAGCAGTACGACTCCGTGATTCGGGAGTACTTCGACTTAGGTCATATGAC
ACAAGTCTCTCCAAACAGCAACCCCAATAATTTTTACCTGCCGCATCATGCTGTTTTTAAGCCCGACAGCA
CCACAACAAAGGTTCGCGTTGTGTTTAACGCCTCCAGCCCGTCTTCCAATGGCAAAAGTCTAAATGATATC
CTACACACGGGACCAGTACTCCAATCCGATCTGACGATTCAGATACTAAAGTGGCGATTCTACCGCTACG
TTTTCAATGCCGATATAACCAAGATGTATCGGCAAATCCAGGTCGATCCAAGACATACGCCCTACCAACG
AATACTATTTCGCAATGAGAACGAAGAACTTTGCGACTTCGAACTCAATACAGTTACCTTTGGGGTAAACT
GCGCCCCATACCTCGCAATCCGGGTCCTCCGTCAACTTGCTCGTGATATACGAGAACATTGTCCCATCGCA
TCCGATATAATTGAAAATTATATGTATGTCGATGATGTTCTAGCAGGGGCACACACGAAGCCACAAGCAA
TTTCAGCCATTGCAGAACTCCGCATGGCACTCGACAGAGCTGGGTTCCCTCTGAGAAAGTGGACATCCAA
CACAAAGGAAGTGTTAAAAGGAATACCAAGGGACCACTTACTGTGTGCGGATTTCTTAGAAATCGAAGAG
GCCAGCGTCGCTAAAACGCTAGGCATTCGATGGCAAGCCACAACAGACCAGTTTTTCTTTGCTCCGTCCAA
AATGGTATCTCAGCCTTCGCTCACTAAACGTGAAGTACTATCACAGATTGCGAAATTGTTCGATCCCGCTG
GATGGCTAGCACCTGTGATAGTTCGAGCAAAGATTTTCATGCAAGAGATCTGGCTCCAAGAGCTGGGGTG
GGATGATTCCCTGCCGGAAGATCTCGTTCAACGTTGGCATGAATTCCTAACCGACTATCCCTCTCTCGAAG
AGATCCGTATTCCAAGATGGGTCGATTTTCAGCAAAAGGCAAAGGTGCAGTTTCACGGCTTCTGCGACGC
GTCCCAGCGCGCATATGGTGCTGCACTTTATTTGCGCGTCGAGATCAACGGATGCATCTCCACTCACCTTC
TCGCAGCAAAACGAGAGTCGCGCCAGTTAAGACGGTATCACTACCACGCTTAGAGCTGTGTGGCGCCGTC
CTTCTTTCCGAGCTCACCACGGCTCTTATTCCTCAGCTCC
>9_rnd-1_family-333__LTR___Gypsy
TGTAGTGCGTATATGCCCTTACAGAATACCCCGTACTTAAACATAAAGTATGCAGCACTTTGTTGCATACA
ATAGCAATAATATAGTAAACGAGCCCAGAGCCACATTATGTATTGGCTCGGTCTCATATTACGCAGCGCG
GTTCACTAGCCTCACTAGCTGACCGTTTATGCTTATGATGCATATCCCACGCTCCGTCTGAGCGCATTCCGT
GAGCAATATATATGCTCACTAATACAGGCATATGCGAGCTACTCACTCTCCCTCTCTTTGTAGCTGTTAAC
GAATAACAGCATAAGTATATATGTATATCCATAGGCTAAGCGAACATTATAACAAGTGAATAAATTCATT
CTGAACAAAGATACAAAACAGCACTGACGTGCTGACTTATTTTTCATTGACAATAAGTGCTCATTTTACAT
GGCGACCGTGACTTGGTCACGCATCCCTGCCTGGAGGAGGNAGTAGACCAAGTGAAAAGGAACTTCATG
GCCAACAAAGAGGCCATGTATAATATACAATTGAAAAAGGAACTTCATGATCAACGAGAGGCATCCCGT
ATTCCGAGTGAGTAGAGAGTGAGTGTTATGGTTCATAAACGCGGTGGCTTAAAAAATAAAATTAATAAAA
GAATAGCGCACCTAAGGTGGCTGCTATACACAAAACAGCCAACACCCGCATGGTCGACAGCTCAGAAGTT
ACAAAATGAGCTTGACTATCTGCAAAATGAAATTAAGCAGAGAAAACCAAACACCACTACTCACTCAAAT
ATAAAAGTTCTAATAAACCAAACAACTAATAAAGTTACACCCATTGACGCAACCAGCCTACCAAAAGAAA
AACGAATGTGCCCACAGGATTGCGAAGGCCCACTCGACAGCCCCTTCTGCGAATATACGTGCAGACACTG
CACAGCCAGCACCTCCAAACATTACATTACAACCAACAAATAAACACCTGTGCGAAGACCAGCACCGGTA
CCGCCAGCGCAACAAGCTGTGCCATCGGAATAAGTGCGAGAGGCACACCGCCGCTTGCGCATACGAGGG
AGCATCAGCGTGCCACATCCGGCGACGCCCAGCTGATGCAACGATGAACAACAGTTGCAGCTATATCCTT
AACTACTTTTAATTAAATATAAAAAAATTTTTGTAAAATTGAGCGGAAACACTTCCGCCCGATTAGCAGCA
TAGCCCGTAAAGCTATGCGAAATGCTAAGGTAGTATTTTGTGTTATAAGAAATATTGAAACCTTGATTCTA
AAAACCCAAGTCAACCAGCACTAACGTGCTTTTGTGAAATCATTATTAAATCCAAACATTACCAACATATT
AAATTAAATAGTAATAAATAAAACTTAAAAATTGTAATGCCCGACGACAATTGAGTCGACCGGCAAGACG
TTTGGCCAGTACAGCCATTCGAAACAATAGGTAGGAAAAATACAGATTGTAAAAAAAAAAATTTTTTAGA
TGCGTTTTAAGCAGAAAACAAACAAGCAAAACAAAACCCGACTTGTAAAAAACGAAAAGCATCAAATTC
AATAAATATAGTAATAAAACCTCAAATGAACGATACAAACAACAACTTGTCTTGTAAAGTAAACGAAAAC
AAATTTAACATTACGCATTTGTAAATTTTTTAAGATAATTCCTCTAAACTTTTATGNACGATCCCANACAA
CAAATATATGAATATTAATATCAAAAAGAGGAAATTAAAAAGTTATAATTCGTTCAGTTGGATATAAAGA
AATGTGCTGTCNCATTAGGTAATTCAAAATTATGATCAAGAATTAGCATGATCGAGGACAAGATTAACAA
GGTATATAACGACTATACAGACTTTCGTATGCGAGCCATACAAATTTTGCAAAGGCATAGAGAATTCATA
CCAGTATATGAGTTCGAATACGAATATATAGAAACATTCGGCACAGTGACAAGTCCACTCTATTCATGTTC
TAAAATAGAGNGCGGAATAATCTTGTCCACACTACCCTACGTCAAAATAAAAACAATTATTGACGAGCAT
CCGATTCTAGGGCAGACAACAATTGTCTTAGTACGTCTGCATAGGTAGTAGTCAAATCAAAAAGCAAGAG
TATTTATATAGGAATATGGGGGAAAANAATCAAAGGCAGATGATAACACTGCCAATGTAGTTAACGTAGT
AGAAGTCGTAGACACCACTCCCCATGTGCAGTTAACAAACATACTGCTAATAATACTCATTGTAATAGCTT

CAGTAAACATCCTTCTGAAGATTTATCTTCTTCATAAAAATCACTGAGGAAGCGTTACATGAGTCGAGCCA
ACGACTTAGACAAGGTCTAAGCCACTGCTTAAATAAAATATTTAAGTAGCGGAAGCTTACATGGACTGGT
CCCAAATAGTAGAAAAGATAAAGCGGCATAGGGAAAATTTCGATAAATCATACAAGTGCCTTAATAAAA
GCGCACCAGTTTTAAGACCCACCATAATTAAACATACTGAGATCCTTGTATATGAGTACAATGAAATCAG
GAGTCTAGTTACCGAGTGTCAAAATAAACTGACAAGTTTACACAGAGTCCAAACTTCAGACGTTTTACAG
CGTCTAAGGGATAATCTGGTAAACTTAAATAAAAAGTATGATCTCCAGCTTTATCTACCATCGGTGTTAAA
TACACCGGTAACATTTGATAAGTCACTACTGACAAACGACCAAATAGAAGATACAAAATTAGTAAACATG
GCACAAACAGTTGTAGAATTTATCAAGCTAGCCACATCTCTGATACCAGAGTTTGACGGTAAGCCGGAAA
ATCTACAAAGTTTTCTAGATTCTTTGAATCTATTAGAAACCTTAAAAGGCACACATGAGCTAACGGCCATA
AGCCTTATAAAAACAAAGCTAAAAGGCCATGCGAGAAATTTGATAAGTAATGAGCAGACAATTGCTGCA
ATCATTACGCAACTATCAAATGCAGTAAAAGGAGAATCGGTAGAAGTTGTATCTGCTAAGCTTCTTAATTT
GCAACAGAAAAATAAAACGGCTAACCAATACACCCAAGAGGTGGAAAAACTGGCAAAGGCCCTTGAAGG
CGCCTATATCAGTGAAGGTCTAAGCCAATCACTTGCAAACAAATACAGCACTACAACAGCTGTAAAAGCT
ATGACCCAAAATTGTTCGATTGATAAGGTAAAACTTATCATGCAGGCAGGCACATTCACAAATATGAATG
ATGCCGTNTCCAAATTTGTCAATAGCTGCACGGAGATAACAGGTCAAAGTAACACTGTCCTGTATTATCG
ACGAGGTGCAAACAACAATAATAGAGGAGGCCGAGGTTATAATCGTGGCAGAAATTACAACCACAACAA
TTATAACCGAGGCGGTAACAACAATAACAATAATTACAATAACCGTGGAGGAAGACGAGGCCAAAATAG
AGGAAGAGGCCGAGGTAACTCCAACCAAGGTTATAATAGTAACAATGTGAGAATGGCGCAAAATACGCC
GGAAAACTCTCAGAACCCTTTAGGAAACAACCAGTAAATGTAAAGGTTCATTCCATCAATTACAGTCTTA
ATATATTTGTCACTTTTTATAATCATTCAACTGAAAATAAACTCACATTCCTAATAGATACTGGTGCAGAT
ATCTCACTTTTAAAAATAAATTCAGACAATTTCAAAATTCAAGAAGAAAAAATTATAAACATCCAAGGCA
TAGGCCAAGGTGTAATAAAATCTCAAGGAACAACTTCAATAGAACTTCAATCNACAAAATATATTATTCC
ACACGAATTTCATTTAGTAGATTCCGATTTTTCAATACCGTGTGATGGAATAATAGGCATTGATTTTATTA
AAAAATTCAATTGTCAATTAGATTTCAAACCAAGTGAAGACTGGTTTATAATTAGACCTCAAAATTTAAAT
TATCCAATTCATGTTCCGATAACATATAGTGCTGGCAACAATACAGTTCTCCTGCCAGCCAGATCCCAAGT
AATTCGAAAAATAGACATTAATACAGTAAATGATTATATATTTGTTCCTAATCAAGAAATACATAATGGA
ATTTATGTTGCAAATACAATAGCAGCATCCAAACATGTATACATTCGCCCCTTCTAAACACAACTAATTTC
GACCAATTGGTCAAAGTAAACAAAATCCAATATGAAAATTTAACAGATTACGACATTCATAGCACTAATC
CAGAAAATAGAAGCGAACAAGTACTTTCAAAATTAAAGAAAAATTTTCCAGAACAATTCAAAAGTCAACT
AACAGAATTATGCACCAAGTATAGTGATGTTGTTCGGACTGGAAACCGAACCAATAGCGACAAATAATTT
CTATAAACAAACATTAAGACTTAAAGATGATGAACCCATTTATATTAAAAACTACAGAAGCCCGCATAGC
CACGTTAAAGAAATTCAAAAACAAGTAGGGAAATTAATAAACGACAAAATCGTAGAACCGTCTGTATCC
GAGTATAACAGCCCACTCTTGTTAGTTCCGAAAAAATCATTACCAAATTCGGAAGAAAAGAAATGGCGAT
TAGTAATTGACTATCGCCAAATCAACAAAAAACTACTTTCTGATAAATTCCCGCTCCCCAGAATTGATGAT
ATTCTAGACCAACTGGGCAGAGCTAAATACTTTTCATGCCTTGACTTAATGTCAGGTTTCCACCAAATTGA
ACTTGAAGAAAATTCAAGAAATATAACATCTTTTTCAACGAGCAATGGCTCATATCGCTTCACGCGATTAC
CATTTGGTCTTAAAATAGCACCAAATTCATTTCAGAGAATGATGACTATAGCATTCTCGGGTTTAGAACCC
TCCCAGGCATTCCTTTATATGGATGACTTAATGGTGATAGGATGTTCCGAAAAACACATGATTAAAAACTT
AACTGATGTTTTTAATGTATGTAGGAAATATAACCTAAAGTTGCATCCAGAAAAATGTTCATTTTTCATGC
ATGAAGTGACATTTTTAGGTCACAAATGCACTGACAAAGGAGTATTGCCAGATGACAAGAAGTTTGACGT
CATCAAAAATTATCCTGTCCCTCATGATGCGGACAGCGCGAGACGATTTGTAGCATTCTGCAATTATTATC
GTCGATTTATAAAAAACTTCGCCGATTATTCACGGCACATAACTAGATTATGTAAAAAGAATGTTCCTTTT
GAATGGTCAAGTGAATGCCAAAACGCATTCCAATACCTTAAAGAAAAGCTTATGCACCCCACATTATTAC
AATATCCTGATTTTCGCAAAGAATTTTGCATTATAACAGATGCCAGTAAACAAGCTTGCGGAGCGGTTCTA
ACTCAGAACCGTGACGGAATTCAGCTCCCAATAGCTTATGCATCTCGTTCATTTACAAAAGGGGAAAGCA
ATAAGAGCACAACAGAACAAGAGTTAGCGGCAATTCATTGGGCAATTACCCATTTTAGACCATATATTTA
TGGCAAACACTTTACTATTAAAACGGATCACAGACCATTGACATATCTTTTTTCTATGACTAATCCCAGTT
CNAAATTAACTCGCATGCGGCTAGAGCTTGAAGAATACGACTTCACAGTAGAATACCTAAAGGGGAAAG
ATAATTTCGTAGCAGACGCACTTTCGCGTATTAATATAAAAGAACTCAAAGATATGCAACATAAAGTCCT
GAAAGTCACTACCAGGCGACAAAGTAGACAAAATAAAAACTGTACAGTAGAAAATTATGAACTTCTGCC
AAGGCAAAGTCATAAAAATGTATCCAAGCCCAACGTACATGAAGTCATTACAAATGATGAAGTACGAAA
AGTAGTGACCTTGCGCATAACAGAATCTAAATGTTTATTTAAACATGGAAATAAAATTATCGCAAGAATT
GAAGTTAGCGATTTATATACCAATGGAATTCTCGACCTAGGTCAGTTCTTCCAAAGGCTCGAAAAAGAGG
CCGGTATATTAAAAATCAGCCAACTCAAAGTGGCACCGAGCGAAAAGATCTTTGAATCAGTTTCAATAGA

TTCTTTTAAAAATATGGGCAATGAAATATTAAAATCTTTAAGAGTAGCGCTACTCCAGCCGGTGACCCAA
ATAAAAGATGAAAAAGAGATACAACAAATATTGTCTACATACCATGACGACCCAATTCAAGGAGGTCAT
AGCGGCATAACAAGAACGCTAGCGAAAATAAAAAGACACTACTACTGGAAAAATATGACACGTCATATA
AAAGAGTACGTACGTAGATGTCAAAAATGCCAAATGTCTAAAACAAAGACGCACACAAAAACTCCTCTG
ACTCTCACAGAAACACCTGTGAATGCTTTCGATATAGTGATAGTGGATACAGTTGGTCCGCTACCCAAATC
GGAAAATGGTAATGAATACATAGTCACATTAATATGCGACCTTACAAAATATTTAGTAGCCATTCCAATA
AAAAGCAAAAGCGCAAATAATGTCGCAAAAGCTATATTCGAAAATTTCATACTCAAGTTTGGTCCAATGA
AGACGTTCATCTCGGACATGGGAACAGAGTATAAAAACCAGATTTTAACTGATTTATGCAAGTATTTGAA
GATCAAGAATCTCACCTCTACTGCATATCATCATCAAACATTAGGGACAATAGAACGAAGTCATCGAACT
TTTAATGAATACATTCGTTCATACATCTCTGCAGACAAATCTGATTGGGATGTCTGGGTTCAATATTTCACT
TATTGCTTCAACACGACACCATCAGCAACGCATGAGTATTGTCCATACGAACTAGTATTTGGCAGACTACC
GAAACAGTTCATAGATTTAAACAAAACTAATTACATAGATCCTATTTATAATTTAGATGACTATTCTAAGG
AAATAAAGTTTAGATTAGAAATAGCATACAAAAGAGCTAGAATTATGTTAGAAAAAGCCAAGGCTTATA
GGAAATTAGCATATGACGAAAAAAAAAAAAACTTTGAATTAAAAGTAGGAGATAAAGTACTTATAAGAA
ACGAAACAGGGCACAAATTAGACCCAGTATATTTAGGACCATATATAGTAGAGAAAATAGAAGATAATG
ACAACATTTTTATTAAAGATAGTAAAGATAAAAAACAAAAAGTTCATAAGAATAGATTAAAAATTTTTAA
TTCTTAATAATNCAAAATTCTTTATGAATTCGAACTCAAAAAAAAATATATAAAATAAAATATGACGGCC
TGATCAACCGAATGAAAAAAAAATTTTTTTACAAATAAACGAATTATAAATTTTTATACCTTAAATAATTT
TTGTATTTTTTATACTCCAACATTTAAAATTTCTTATTATATAAAAAAAAAATTTCAATTTTTACAAAAAAA
AAAAAAANCCTCAATAATTAAGTTCATTTAATAACTACACATTCATAATGTTACGTTATTTCTCTAAAAAG
GAGGTGTAGTGCGTATATGCCCTTACAGAATACCCCGTACTTAAATATAAAGTATGCAGCACTTTGTTGCA
TACAATAACAATAATATAGTAAACGAGCCCAGAGCCACATTATGTATTGGCTCGGTCTCATATTACGCAG
CGCGGTTCACTAGCCTCACTAGCTGACCGTTTATGCTTATGCTGCATATCCCACGCTCCGTCTGAGCGCAT
TCCGTGAGCAATATATATGCTCACTAATACAGGCATATGCGAGCTACTCACTCTCCCTCTCTTTGTAGCTG
TTAACGAATAACAGCATAAGTATATATGTATATCCATAGGCTAAGCGAACATTATAACAANTGAATAAAT
TCATTCTGAACAAAGATACAAAACAGCACTGACGTGCTGGCTTATTTTTCATTGACAATAAGTGCTCTTTT
TACA
>160_rnd-4_family-338__DNA___P
TTGTATGTTTTGTATATGAATAGAAGCATTTATGCGCTTGTGTGCCTGTTGCTTGCCTGCCTGCCTGTCCCC
GATGCAAATTTAACAAGATTTTAGGATTTCTGATTTTCGACCTCTACACACAGACACACACATACAAATGT
CTGTGGCTGATCCGCCATACTCTACTAGGCCCATCATTGTAGTAAAGCATCATTTTTGTTTCATCATAAAAT
ATGATTGTCCCAATAATCAATTGGATAAGAAATGTGCATATAGGCGAAGCACAATCGTTTTTCAATGTTTA
TTTTAGAAAGGAGCGGTTTCTTGCGCGCAGCTCTTTACTTGTAATCATTGCCAATTATTGCTTGCCTTACTG
TTTCATAAGAAACCGCAAATTCAAATTTATCATTCAGCTCCCTGGGAAGAGCCCTGGTTAAGATCTGAGG
ATTTAGAGCCACCTTCCTCATCATGAAACGCTCTGTAAGCTCCGCTATTTTAGGCTTAGGTCCGCCTCCTCT
TCATTTTCAATTCGTTTTAAGACGTTGTATATAGTCTTTCGTGTAACAGAAAACATTGCTTATAGCTCAAGC
ACACTTTTTCCCATCTGGTGGTTATAGAATATAAGCTGGACAACTTCAAATTATAATCTTTTGCCTGGCATC
TTTTAAAAATCAAATAGCTTCTCAAAGCACGTGATATCATCGAAAAGCATCGCCGAATTAAATGAAATGA
AATGCATTGAAAACAATGCAAAGCAAAGAATAGATATCGGTAAATGTACCGTTGTTCTTGGAATATTGAA
AAAAGGGAGAAGGAAAAGAATGTGTAAAGAGTTTCTTGACAGTAGTCAAACCAAAGACAATATAAACAC
TAAGATGAGTAACGCCCATACATAGCCTGGCCTTTCATCTGGCCTTTGAGGCTTCGTACGTCTTGCCTGCC
GACTGGTTGTTCGTTCTCCAAAGACCAGACGAACGAATGCCGAAGTCGCGGCTTGCTGATGCTGACACGA
GCTACGAAGTTAGTCGCCAGCCTGCCTTCAGCAAAGCTGGTCGATTCTGCTTTGCGTCGCTTCGTTTTCGA
AGTACATTGAGCGAAAAAGTTTAGAACTTACGATCGACGCAAAGTGCTTGTGTTTTATCCATGAAATGCA
TGCTTTATTTAAATGATATATTACCCTAGATATTAGCGATTAATTTTACAGGAAAATTTAAGAATTTATAA
AATTTTTACACAAGGCGCTTCTTGCGCAATAACTATGTTAAGAAAATAGCACACATAGATTGATTTCACTT
TTCTTTTTGGACAGTGTATGCTCGAGCAAAGTTCTTGTCTCAGCCAATAGCGTGCGTGCACCTTTCGTTGTA
TGCGTGAATTATACATGCATAAGAACTTTAAGGTATGTGTTGTTATTCACTGCTCTGGCTTCAGCTGGCAA
GAAATTAATTTTGTTTGTTTTGTAGCGCAGATCATGACCTATCCCAAATTTGTGTTGATATATGAATGCATT
TTGTGTACTGAGGTTGGCTCCGCAGAAAATATGTATTTCTAAGTCGATGCAAATATTTACATAATTTTTTTA
AATTGAATTAATATTATTTTTATTCTCCAATTAAGAAATAAAAGAGTGCTAAAAAAACAATTACATTTCAA
GTCGACTCGAAGGTCATATTTAGTTATCACCCTATTATAATTATAATGGCCTCCTCGCGGTTTTCCCTGCGT
ACTTCTTTATAATTCGAACAAAAAATAATAAAATAAAAANAAATAAAAATTAATTAAATGTATCTTTGAA
AACAACAAATTTGCACTTTTATTTCCTTAATTTATTTTGTATTACTTAAAAATAATAATAATTTAATTAATA

ATTTCAAATGTAAAAAATTACATAAATATTTGCATAGACTTAAATATACAAATTTTTTTGTACGGAGCCAA
CCTCAGTACACAAAATGCACCCATAAGTATATCCACAGAANTTTTGGGTAGGTCGTGATCTGCGCTAAAA
AACNAAGAAAATTGTTTCTTTGCCAGCCAGANACTTTGCCAGAGCAGTGGATAACAACACATTCAAATAT
GCTGTTATGTATGTATATTCACACGCATACAAACATAATTGCTTGCACGGCCCTCATAGAGCCGAAGCTGA
GAGCAAATTATATTTTTAGCTTTTTCGTTCTCTCGGCTGTGAGAGCGCAATGCTACAGATTTCGTTTTCGTT
CTCAATTTTCAAAAAATCAAGCTGCATAGTAGCATTTTGTTGTATGGCGTTAGTCATCTTAGAGTTTATATT
GTCTTTGGTCAAACTGAGTCATTTTTGTATTTGTGTTTTTTTTTTTTTTTGGTTTCTTATTGTTAACATTTCATT
TTGATGCTACTGTTTCATTAAAAATATCATCACTTCCTATAAATTCCCAAAAGCAAGTTTAAAATGTCAGG
CGGGTGTTCATATTTTTAGTCCAATAGATGAAAAAATATATTTTTATTCAATGTGTAAACTCATTCTTGACA
TACTGTATATACTATACAATGCTTATACCCTATAATTACAATTATGAACTGTGGGGTAGCTATCCTAGTGG
CGTCCTCACAAATTTTCTTGTAAAAGTTATCGTTAGTATCTAGGGTATTATATCATTTTAATAAAGCATGCG
TTTCATGGATAAATCAAAAGTGCTTTGCGTCGATTGTAAGTTCAAAACTTTTTCGCTCAATGTACTTCGAA
AACGAGGCCATGCAAATCACCATCGACCAGCTTTGCTGAAGGCAGGTAGGCAACTAATTTCTCTGCTCGT
GTCAGAATCAGAGAGCCGCGACTTTGGCATCCATGTCGTGCGAAATCGTAAAAGCCAGTTTAGAAAGTCA
GGCTATAAATTTTGCGTTGCATGCAATTGGCGTTACTCATCTTAGTGTTTAGTTTTTCTTTGGTATAGTATT
ATCGTGAATGTATGTATACACACATATGGTATGTACATGTCTCACAATTATGTACGCATCCTTTGTCGAAA
AATGTGTAACGCATAGAAGGAGACTTCTCCGACCCCATAAAGTATACATATTCTTTATCAGCATCAACAG
CCGAGTCGATATAGCCATGTCTGTCTGTCCATCTGTCTGTTTCTATGCGAACTAGTCTCTCAATTTTAAAGC
TATCTAAATGAAACTTTGCAGAAGTCCCTCTTTCTGTTGCACGCAGAACATATGTCAAAAAAAGCTGGATC
GTACCACTATATCCCACCACTATATATAAAATTAAATTGTTGTATGAAAAAACATTTTGTTTTAAGATAC
CTTGACCAATCTCGGCATTTATTAGTTTTACTATACTCCTCATATATATGCAAAATCCTATTAAGATCGCAC
CACTATATCATATACCTGCCTTACGAACGATCGGTCGAAAATTAAGTTGTTGTATGAAAAAACATTTTGTT
TATCAAGATATCTTAACCAATCTCGACATTTATTAGTTTTATTGTACTCCTCATATAAATGCAAAATCCGAT
TTAGATCGGACTACTATATCATATATCTGGCATAGGAACGATGGGTTGAAAACAAGTTTTTTGATTAAAAA
CCTTTCTTGTTTATCAAGATATCTTGACCAAACTCGGCATTATCTATTTTCCTCTGCTTCTAAGATACGGGC
GAAGCACTATAAGCANTATGACAAGGTTGGGTCGGCAAGGCTATCAGATCTTCGGCGTGNCGCTAAGGCC
ACTGATTTTATCCGACACGGCATAAGTAAGTCGTGTCACCTAGCTTTAATACCCTACTTCTAATATAAATT
TGTTGCTCATTGAGAGTGAACAACTACAGCCTGCACGACTTCCCTATTNTTAATTCAAATTCAATATTTGA
GTAATTCACATTTTTTGTTTCGTTAACCCCCGCACAGTTTGAGCAAAGGAAACCAAATGAGATCAAGCAAT
AACGAAGATCAGTGCAAGGCAGCACACTTTTGGCACGTGCCAATGAAAATTTGATTACTACTGACAAATA
AAGTATCGATGTACATATTTCATTATGATGTTTTTTTTTTTTTTGGGTACAACACAGAATAACTTCTTTATTA
TATAATAATGAATACAACTCTCTTAATTGTCACATAAACATTTAATACATTTTTCATTGGCTGTGTGTAATC
GGTTAAGAATTTCTCATGGTGCATTTAAAATTTAGGAAACAACCGAGAATAAAGAGATGCCGAAAACGAA
AAATAACGGACAAGTCAATATACAAATACAGTGGTTGTTACATCAAATTAATTGAGTTGGCACTTTGTATT
CAATATAGAGTTTGTATTCGGGAGCGGCGTTTTCGTAGATCCGGTGCAACTCCTGTGCCGGTTCGTATCTG
GCGTGATGCATCAGAGCTCTCAGCTCTAACAGGTACTCCATGAATTCCTCCCCCTCGCGTTGCCTGCGGGA
CCGGATTTCGTCCTCAAGCCCTTCGAAGTAGCGTGGGGCAGGAAGAACAGGAAGAAGCAAAAACCCCTC
CTGAACACCGACCATAGCACATCTCTAAGCCCACCGACCGGCCCTGTCGCAGAACACCTCACTCATCGGC
CGGGGCAATCTATCGAGGGCGATGGCGCGTTTCTCTAGGTCTCCAAGAACTCGAGGGGGTCGGAGCGATC
ATCGTATCTCATGCCCCACCTCCGAACTTGTTCCGGAGCGTTTGTGAAAGGCCTAACCTCCGACCGAGAAT
ATGGTGGTTGGGGCCCCACGCCTACGTTGGTCGTGGTTGCACGGCGGTCTCAATTTTCCTTGGGATCGGGA
GCCTCTTCCACACCCGTGACGGCCACACCAGGGATAGGCGGCCGCGGACGCCTGTACAATTGTGAGCTCC
TATGATCAGGCTCTGACTTTTGGCACGGCCGTCCAGCCACGTCAGATGGGACGCCCCAGGATAGCAGCTC
TAACGTTTTCGCGAATTGAGACTCCAGCTCGGCAAGCCGAGCCCAGATCTCAGACGAATGGCTGCCCTCC
TGCACGGAGGTGGATAGCCGCCTACGCAGTTCCTCCACGCACCCTTCCTTATCTAGCCCGAACTCCTGGGC
GATGGCCACCAACTCTTCCTTGCGCCGCTGATAGATACAACCACTAACCAAACAACATATAGACTAGACA
TGAGTACCAGAAAGTAGGCACAACATGTATCGGAGGCATCTGGTCCAAACTCGCGAGTAAGGGAGTAAG
ATATACATATGATGTATTTTTGAACCGCTTACTCTACAGGAAAGTGCTTGGCGCGCTCTCTTCCATATGATT
TGTGCGATAGAAAGGCAACGCTGTTAACTTTTATCTCGCTCGCTCTTACTCTCAGCTCTCAATGCTCTCTCT
TTATGTTTAGGGATCGTTTAAATGCGTGTGTGTGTATGTGTCCGACAAGCATAACTGCGCAAAAATTCAAT
TTTTCCCAAATGCTGAGGCATTTCTACCCTTTTGAAGGCACGAGTAATATGAAATAGAGAAAGGGTTTGCC
ACGTTGTATGCGAAATTCTTGCATATTTGGACTCGCTATTCACGCATACTTGTATGTTTTGTATATGAATAG
AAGCGCTTGTGCGCCTGTGTGCCTGTTGCTTGCCTGCCTGCCTGTCCCCGATGCAAATTTAACAAGATTTT
AGGATTTCTGATTTTCGACCTCTACACACAGACACACACATACAAATGTCTGTGGCTGATCATGTCTGTGG

CTGATCCGCCATACTCTACTAGGCCCATCATTGTAGTAAAGCATCATTTTTGTTTCATCATAAAATATGATT
GTCCCAATAATCAATTGGATAAGAAATGTGCATATAGGCGAAGCACAATCGTTTTTCAATGTTTATTTTAG
AAAGGAGCGGTTTCTTGCGCGCAGCTCTTTACTTGTAATCATTGCCAATTATTGCTTGCCTTACTGTTTCAT
AAGAAACCGCAAATTCAAATTTATCATTCAGCTCCCTGGGAAGAGCCCTGGTTAAGATCTGAGGATTTAG
AGCCACCTTCCTCATCATGAAACGCTCTGTAAGCTCCGCTATTTTAGGCTTAGGTCCGACGCACCTTCATTT
TCGATTCGTTTTAAGACGTCGTATATAGTCTTTCGTGTAACAGAAAACATTGCTTATAGCTCAAGCACACT
TTTTCCCATCTGGTGGTTATAGAATATAAGCTGGACAACTTCAAATTATAATCTTTTGCCTGGCATCTTTTA
AAAATCAAATAGCTTCTCAAAGCACGTGATATCATCGAAAAGCATCGCCGAATTAAATGAAATGAAATGC
ATTGAAAACAATGCAAAGCAAAGAATAGATATCGGTAAATGTACCGTTGTTCTTGGAATATTGAAAAAAG
GGAGAAGGAAAAGAATGTGTAAAGAGTTTTCTTGACAGTAGTCAAACCAAAGACAATATAAACACTAAG
ATGAGTAACGCCCATACATTAGCCTGGCCTTTCATCTGGCCTTTGAGGCTTCGTACGGCATGCCTGCCGAC
TGGTTGTTCGTTCTCCAAAGACCAGACGAACGAATGCCGAAGTCGCGGCTTGCTGATGCTGACACGAGCT
ACGAAGTTAGTCGCCAGCCTGCCTTCAGCAAAGCTGGTCGATGCTGCTTTGCGTCGCTTCGTTTTCGAAGT
ACATTGAGCGAAAAAGTTTAGAACTTACGATCGACGCAAAGTGCTTGTGCTTTATCCATGAAATGCATGC
TTTATTTAAATGATATATTACCCTAGATATTAACGATTAATTTTACAGGAAAATTTAAGAACTTATATAAT
TTTTACACAAGGCGCTTCTTGCGCAATAACTATGTTAAGAAAATAGCACACATAGATTGATTTCACTTTTC
TTTTTGGACAGTGTATGCTCGAGAAAAGTTCTTGTCTCAGCCAATAGCGTGCGTGCACCTTTCGTTGTATG
CGTGAATTATACATGCATAAGAACTTTNAAGTATGTATGTATGTGTTGTTATTCACTGCTCTGGCTTTAGCT
GGCAAGAAATTAATTTTGTTAGTTTTCTAGCGCAGATCATGACCTATCCCAAATTTGTGTTGATATATGAA
TGCATTTTGTGTACTGAGGTTGGCTCCGCAGAAAATATGTATTTCTAAGTCGATGCAAATATTTACATAAT
TTCTTTAAATTAAATTAATATTATTTTTATTCTCCAATTAAGAAATAAAAGAGTGCTAAAAAATACAATTA
CATTTCAAGTCGACTCGAAGGTCATATTTAGTTATGACCCCATTATAATTATAATGGCCTCCTCGCGGTGT
TCCCTGCGTACTTTGTTCTTTATAATTCGAACAAAAAATAATAAAATAAAAAAAAAATAAAAATTAATTAA
ATGTATCTTTGAAAACAACAAATTTGCACTTTTATTTCCTTAATTTATTTTGTATTACTTAAAAATAATAAT
AATTTAATTAATAATTTCAAATATAAAAAATTACATAAATATTTGCATCGACTTAAAAATACAAATTTTTT
TGTACGGAGCCAACCTCAGTACACAAAATGCATTCATATATATCAACAGAAATTTTGGGTAGGTCGTGAT
CTGCGCTAAAAAACTAAGAAAATTGTTTCTTTGCCAGCCAGAGACTTTGCCAGAGCAGTGGATAACAACA
CATTCAAATATGCTGTTATGTATGTATATTCACACGCATACAAACATAATTGCTTGCACGGCCCTCATAGA
GCCGAAGCTGAGAGCAAATTCTATTTTTAGCTTTTTCGTTCTCTCGGCTGTGAGAGCGCAATGCTACAGAT
TTCGTTTTCGTTCTCAATTTTCAAAAAATCAAGCTGCATAGTAGCATTTTGTTGTATGGCGTTACTCATCTT
AGTGTTTATATTGTCTTTGGTC
>9_rnd-1_family-115__LINE___R1
ACACATACACACACACACACACACACACACACACACACACACACACACACACACACACACAGTGGACGCG
AGATTGTTTTCATAGAGTGCGGACGCGATTTGTAACTGCTCCGTGAAAATAAACAAATAAACCTGTGAAA
TTAAAGTGAAGTTAGTGCACGTGTGCAGGGAACCGAGACAGGCCTAAGACACCAAAAGGTGCGTGGTGG
TACTCACTGCGAAGCCCGTGCAGACCATTCCTGGGGTGGCGTCTACAGTTCCACGATCCAGGCGAGGGGA
GTGGAGGCGTCTACTTGGCGTGTTTGTGCGCTTTTTGTGAGTTTTACCACTCAATTGTATTAGCCGTCTCGC
AAAGCTATTTGTGCAGTTGGTTGCATTCACTCTAACAGCTGTTAGTAGTGTTGGCTAGCGCAGTTCACAAC
CAGCTGATCTGCACTATAGAGCCAGCTGCCAAACTGCGTAGCGGAGATACCAACCAGGCGATAAGCGGTA
ACTGGCAGAAAGTACAGCATACTTTCGGGCTTACGCTGTTCTTGCTAAGCATTTAAGCTTTGGAGAAAGA
ACAGGCAAAGAAGCCGATATCGGTGCTCACTACAGGCTCCAGGGCAAAAAAGGAGCAAAAGGTCGCGGA
ACAGACCAATTTCCAACCCCCGCCGCGGCGGTGCCCATAAAAACGGGGCTCTGGTGACCGAGTCGGAGCG
GTATAACTCCCAGCGGTTAATAGCCAGAGGGAAACCGTTGGCCACCGAGGGACTTTCCTGTAGAGCCCTG
ATAGTTAGAGCAGGTGCCCAGAGAAAAATGGAGGATGAGCTGCATGCCTTTGCGCGCCGCTCTGCACTAC
AGCGATCACCGTCACACCAGGCGGTCACGCCAGTGCAGCAAAGAGTGGCTGCGTCGTCGCCGGCAAAAG
CGAGTGAGGAGCTGCCAAGAACGCTAAGAGGCACGATCGAGAAGATGGGCTTAGTCCTAAACGGCTTAA
CGGCGCTGTTCAACGGACAACGCCACATTAACGGGCTGATGCGAGAGGAACTCGCGAGAGTAGAAAGCC
TGCAGAAGGTGGCGCTCAAGCTCTGTGCGCCGGAAACGAAGTGCGACGAGGCGCAAACGACGCCGTCAT
TAGCCAGACGTGCGGAGCCGAACGTGCTAAAGACGCGCCCAACGAAGCTCGGGGGCCAAAAAGAGGCAA
GCACGCCCAAACGGCCGAGGGATGGCGTGCCAGAGCAGCGCAGAACCCCCTCCAAGAAGGCTAAACGCC
AAGGTCAAAACCCAGCGAAGCAGGCGCTGCTCACGCATCAAAAGACAGCCCCTGCAAACTCCAACTTAG
CTGGCCATAATGGCAACCCAGAGAGCTGGACAGCAGTGAACGGACGTAAGCGGACGCAGAAGCGTCCCA
GAGCCGACGCCATTACAGTCCACGGAGTGGGTGACTGCACATATGCCGATATGCTCAGGCTCGTGAAGAG
CGACCCGGCCCTCAAGCACCTAAGTGGCGATGTGCATGGCATTCGCAAGACCGCGAAGGGCGATCTTCTG

CTGCGCCTCAATAGGAAGCCGGAGCACTCCGCAGATGAGCTTCAAGAAGCTGTGGGAAAGGCATTGGGT
GACCGGGCGGTAGTAAGAACGCTAACCGAAACGTCCCAGGTGGAGATCAAGGACCTGGATGAGCTGGCG
ACTAAGGAGGAAGTGCTCGAAGCGATTAAAAGTGCCTTACAAGACGATTCCTTGCCGATTGAAACGATCA
AATCGCTGCGACCAGCATTTGGCGGCCAGCAAAGGGCCACAGTTACTCTCCCAGCGCCAAAAGCCAGGAC
GCTTGTCGAGAAGGCCAAAGTGAGGATCGGATGGGTTGTCTGCCGTATTCGTGCAATAGACCAACCAAAG
CGATGCTTTAAATGCCAGGGTTTTGGGCACATTGCGGCGAACTGCACCAGCGGAGACGATATGAAAGGCG
CATGCTTCAGATGCGGGGACACAGGTCACGAAGCAAAGAGCTGCTCCAAGCCACCAAAATGTAGCCTTTG
CATACGGAAAGGAGAAAAATCAACGGACCACGCTACAGGAAGCTTTAAATGTCCGGCGTATCGCGAGGC
AGTACGCAAAATGAAATGTTAAGGTTACTCCAAATAAACCTTAACCACTGCATAGCTGCTCAAGCCATCT
TGGAGCAGACTGTTAGGGAGATGCGCATTGATATTGCCATTATCAGCGAACCCCACCACCGAGAAACCAA
CTGCCCGAGAATATGGACCGAGGATCAGGATGGAAAAGCTGCGCTCTGGGTATGTGGCGACCCATCGACA
TCGCTGACAAAAACAAAGTCCTTCACTGGGTTTGTACGCGCACAATACGGCAGCCTGTGGATATACAGCT
GCTACCTGGCCCCAAGCATGCAGCTCTGCGAATTTACAGCTGCATTAGATGCATTAGTAGATGACGCAAG
GGGGCGGAAGGTCATTATTGCTGGTGACTTCAACGCCTGGGCAGTGGAATGGGGCAGCCAAGCAACTAAC
GCAAGAGGTCGTGCCCTGTTGGAAAGCTTCGCAGCCCTAGATATAGCCCTGCTAAACTTCGGCTCGGAAA
ATACCTTCACTCGAGGTGGTATCGGATCCATAATCGATCTTACCTTCGCAAGCTCGTCACTTTTTAGCGCA
ACAAAATGGCAACTAAGCGACTTATACACCGCAAGCGACCACTCCGCAATAATATGCACGATTGGTGAAG
GAACTGCAGCCAGCACTAGAAGCCTAAGTCGGAAAGCTTACCGCCTGGATACTTTCGACCCCGTGGCCTT
TTCGAATGCAGTCCACTCGCTTGTCACTGCCGGCAACGCAGAGAGAAGAGCCTCCCAAATGGCATGCCAG
CTATCGCGTGCCTGTGACGAGAGCATGCAAACCCGGAAAACGTTTCGGCGCCACCATGTACCCACGTACT
GGTGGAACGAAAATATTGCCGAAGCACGGCGGAACTGCATCCAAGCCAGGAGGAGATACCAGCGAGCCC
GAGGTAGACCCAACTATGCCGAACAGCAGCTAAGGTATCAGAGTGCCAGAAAAATGCTAAAAAGTCCAT
AAGAGATAGCAAACGAGAATGTTTCCTGGAGCTATGCGACTCAGCCGAATATGATCCTTGGGGCAAGGCG
TACAAAACTGTCGTTAAAAGACTCTACGCCGGCAAACAAGCGCAGCCTTCTGACCCAAGTGAGCTGAAGT
TGATTGTGGAAACGCTATTCCCAATCAGACCCGACGTATTACACACGCAAGCCATACCATCGAGCCACGT
AGCAGCCATGCCGTCTCTCATCGACGAAGTCTCAGTCGAAGAGGTGCTGACGATCGCCAGAAGCCTAAAG
CCCAGTAAGGCACCGGGTCCGGACGGAATCCCCAACCGAGCACTGAAACTAGCCATGTCCTTGCAGCCCG
CGCAGTTCGTGCAGCTTTTAACATGTGCCTAATGGAGAGAACTTTCCCCACAATATGGAAACTCCAAACG
CTAGTTTTATTACCGAAGCCTGGTAAGCCGCCCGACGACCCATCGTCATACAGGCCGATTTGCCTCCTGGA
CACGTTAGGCAAAGTGCTGGAGAAGCTAATCTGCAACAGGCTTCAGGTAGCTGTCGACAACAATGGTGGC
CTGTCCCAAAGCCAATTTGGTTTCACCAAGGCAAAGTCTACAATAGACGCTCTGGAGAAAGTGGTCGGCA
TAGCAACAAACGCCATCCAAGGATCGAGATGGAAAAGCGGCACAAAAAAGTACTGCCTCATCGTCACGC
TGGATGTTAAAAACGCCTTTAACACAGCCAGGTGGGACAAAATATTGGATGCATTGTCAACGTTCCGCGT
CCCATATTATCTTCAGGAGACGATACGCAGCTACTTCACTGGAAGACTGCTGCAATACGACACCGCAGAC
GGCCCCACTACACATCAAGTGTCCGCTGGAGTGCCCCAAGGCTCAGTTCTAGGACCCCTCCTCTGGAACAT
CATGTACGATGGCATCCTCCGACTGGAACTCCCGATCGATACAAGTATCATCGGCTTCGCGGACGACGTG
GCCCTTGTAGTCGTCGGCAAGGAATTGAAGGACGTAGAGGAATCGTGCAACCACAGTATTGACCGTGTGC
ACCAATGGCTGGCAGCAGCTGGACTCGAGCTGGCTGCTCACAAAACAGAAGCGGTGTTAGTCAGCAGTCG
AAAAAGAGTGGAAACAGCGCACATAAGAGTCGGCAGTGCGACAATAGAATCAGCAAGGGCGCTAAAGTA
CCTGGGTGTCATGCTAGACACAAGAATGTCGTTCAAAGAGCACTTGGAGTACGTGCACAAGAAAGCAGCA
GAGTCCTGTCGAGCGCTATCCAGGATTATGCTGAACACACGGGGACCGAAACAGCAGAGACGCCGTCTCC
TTATGACCGTAAACCGAGCGGTAATAATGTACGCGTCCCCCATCTGGAGCAGAGCTATGGCTGTTCCCAG
CTACAGCCGGGGCGTACACTCTATATATCGCCTAAGCGCTCTACGGGTCAGCTGCGCATTCCGGACGGTAT
CGGACGAAGCGGCGCTGGTTATCGCAGGAATGGTCCCGCTAAGCGAACTGGCAACAGAAGCTGTAGTTTG
CTATAGAGCCCACGATGGAAGCAACACAGCAAACTCGATTCGCACNGCGGCAAGGATGCAGAGCGTGGC
ACGATGGCAAGCACGCTGGGATCGCGCATCTAAAAGGACGCTGGACGCATAGGCTTATCCCAAACTTGGA
AGCCTGGACAAACCGCAAGTATGGCAATACTAATTTTTACTTAACGCAGCTCCTATCNGGCCACGGATGC
TTCAGGAGCTACTTGAAGCGATTCGGCCACGACAACACAGACGAGTGTTCTTGGTGCGGCAGNGGAATCG
CAGAGGACGCCAACCACGTCATNTTCGAGTGTGGANGNTTCNCAGCGAACAGACAGGAACTGGAGGACA
NCNTGGGCAGACCTATCACGGTGGACAACCTGGTCACGAGCATGNTGGAGACCGCACAAAAGTGGGACG
CTGCCNGCACCTTCGCAGCAGAAGTGATGCGGGAACTCAGGAGAGTTGAGCGCAGCAGACGGAGAGCGG
CGGANGTTTAGGTAGCAAGAACATGCCTGTGAAGCAATGCTTCGCGGCCGTTCCGCAGGCAATCCACGCT
ACCTAANCATCACGTACCCCTCCTCTTCTATGTAATCTTGCTTCCCAAACAAAAATTGAAACATGCCGCTT
ACCTTCGTTGATATATCAGCTGTTTATGTTTACGGTACTTTACCATTTTTCAAACATCGATAGAAATCTCGA

TAAATCATCGTAAAAAACTCAATATGTTCCGCGCGAATTTACACATTTCTCAAAAATGTGCCTTTTTGAAT
GATGTGTGAAATGTGTTTTCTTATCTGGAAACGCACAGGGCGCATTTGTAGGGACCAACTGGCACAAAAA
TTTATCATTTCATCCCATTACACCAGCACCCGTTCACAGTCAGAAAAGTCACCGAAATATTATGCATGCAT
CCCATCGTAGTCGCTATTTGCTTTCGCACGACGCTGCATGATTTCGCTATCTCAGCGTCGGTGCGTTTCTTC
ACTCGCACTTTCGTGAGAATGAAGGAATGCTCGATAGATCAGGCCACATCACCGTTATAATTTAGATGTG
GTGAATAATAATTGTGTTTGTATTTGTGTTTTGCTGAAATTGAAGAAAAGTAGAAGGTCCGATTGCCACGC
TTCGTTCTTTTTAATTCGACAGCGGAACAAGCAAGTCGCATATTTTGAGCGCGCATAAATCATAGTAATTA
ACGGTGCAGTGGCTGTACACCGCTTCGGATTTTTAAGGGCATATCGAGCCCAACTCTTTTTAAAAGTCCCC
AGTGCTCATACAGTTCGCGTTCAGCAGATGTTATTCGCTTTTAATTAATCAAGCGCGTAATTCTGAATTAA
CCCCTTTTTTAGTGGATAAAATCTTACTTAGCTACCGGCCAGCCATTGCGCAACGACGCGTTCGTGTATTC
ATATATATATACACATATAAATTCAGCGTGGCTGGTCGGTCGTAGCCAATTTGTGAGTTGCAACGGCTCTA
AGGGAGTGGAAAATATCAAAAGGGCCACGGATTTCTTAAGGATCACTACAGATTAACACTGCGGCGCCAC
CGGCACATAGCGGCTGAGTCTATACCCCGCGACCGCGTGTTTAGCCATTGATAACCAAGCTTTGCTGATCT
ACTTCTACTTTGCCGCCCCTTTTTGATCAACGGAGTGTCTCCAACAAGGTATAGGACCGCTTAAGTGTCCG
CAGATCGAGATCGAATATACGTGCTGGGCGATTGGTTGGAATTTTTGCGTACACGCTTCGATCTAAATGNT
CATGCCATTTAGCCATTGATAACCAAGCTTTGCTGATCTACTTCTACTTTGCCGTCCCTTTTTGATCAACGG
AGTGTCTTCAACAAGGAATCGGACCGCTTAAGTGTGCGCAGATCGAGATCGAATATATGTGCTGGGCGAT
TGGTTGGAATTTTTGCGTACACGCTTCGCAGCAGAAGTGATGCTGGAACTCAGGAGAGTTGAGTGCAATA
GATGGAGAGCGGAGGAAGTTTAGGTGGCAACAACATGCCTATGAAGCAATGCTTTGCGGCCGTTCCGCAG
GCAATCCACGCCACCTAATCATCACGTACTATCTACATCCTAAGCATAAATTATTTATTATTTATATATTTA
TGTGCAAATAAACAGAATACAAAAAAACACACACACACACAATGGAAACATAACACCCCTTCCACCCCTC
CCCCACCGCCCCTTAATCCTTAACACCCCCGCCACCCCTTTACCTTGCTTAACAACACTTGACCCTGATAAT
AAATGTAAACAAACATACTGTGCCAGAACATACAGTCCCCACCACGGTGACATCACTTCCTGTGCCAGAA
CATACAGTTCCTATCTACTATCCTATGTAGACATAAGAATATAATAAAAACGTGTTTCATCGACTTGGGAA
ATTCCAATTACTGCCCGATCAGTTTCGAAATCATTTTCAAGAAAAGGCCAGGTTGTGTTTTTTTTTGTTTGT
ACATACAAACATAGCCATGATAATTGTATAAGGCCATTAGAATCGAATAAATTCACAGCATTAATTTGTA
GGATTACTCAACTGAATAAGTTTCTNTGCTTCGAAATTTTTTTAACAAAAAAGTGACATTTTTGACCCAAT
AAAATGTATATATTCCTTATCCGCATGGACATAGGAGTCCGTATTGTATTAATATTTTAAGCGTACGCAGT
ACTATGTTCAAGTTTATATTATATCTATTCTAAAAACTCTACAAGATTAAAATATATTCATAGATATCGAT
ATTGATAAAAAATTTTTTTTATACCCTGAACCCATTAAAAATGGGTATAAGGGTATATTGTATTTGTGCAA
AATCCAAATGTATGTAACAGGCAGAAGGAAGCATCTCTGACCCCATAAAGTATATATATTCTTGATCAGC
ACCAATAGCCGAGTCGATCTAGCCATGTCCGTCTGTCTGTCCGTCCGTCCGTCCGNATGTATGAACGCAAG
GATCTCAGAACCTATAAGATCTAGAGACGAAATTAGATGTAGGTGCTCCTAGTTCCCGCGCAGATCGAGT
TTGTTTCCGATAATCGATAACTTACTCCGTTTCCAAGCAATCGATAAAAATCGATATCGGCATCCTGTTTTT
TTTTAGCAAATTGGGTAAATAATAAGAGCTAGAGCCACCAAA
>160_rnd-1_family-44__Unknown
GTAAAAGGAGAGTCAGTAGAAGTTGTTTCTGCTAAAATTCTAAACTTACAACAAAAGAGCAAAACGGCCA
ATCAATATACCGAAAAGGTAGAAAAATTGAAGGAGCCTATATCAGTGCAGGTCTAAGCCAATCACTTGCA
AAACAAATGCAGAACNAAAACAGCTGTAAAAGCTATGACCCAAAATTGTTCGATTGATAAGGTAAAACTT
ATCATACATGTCAGGCACATTCACAAATATGAATGATGCCGTCTCCAAATTTGTCAATGGTTGCACGGAG
GTAACAGGTCAAAATAACATCGTCCTGTATTATCGATGAGGTGCAAATCACAATAATACAGGAGGCCGAG
GTTATAATCGTGGCAGAAATTACAACCACAACAATTACAACCGAGGCGGTAATAAAAACAACAACAATA
ATTACAATAACCGTGGAGAAAGACGAGGCCAAAATCGAGGAAGAGGCCAACCAAGGTAACAATAATAAC
AATGTGAGAGTGGCGCAAAGTGCGTCGGAAAACTCTCAGAACCCTTTAGGAAACAATCAGTAAATGCAA
AGGTTCATTCCATCAATTACAGTCTTAATACATTTGTCACTTTTTATAATCATTCAACTGAAAATAAACTCA
CATTCCTAAAAGATAATGATGCAGATATCTCACTTTTAAAAATAAATTCAGATAATTTCAAAATTCAAGGA
GACAAAATTATAGACACCCAAGGCATCGGCCAGGGTGTAATAAAATCGCAAGGAACAACTTCAACAGAA
CTTCAATCAACAAAATATAATATTCCACACGAATTTCATTTCGTAGACTCAGATTTTTCAATACCGTGTGA
TGGAATAATAGGCATTGATTTTATTAAAAAATTCAATTGTCAATTAGATTTCAAACCAAGTGAAGACTGGT
TTATAATTAGACCTCAAAACTTAAGTTATCCTATTTATGTTCCAATAACATATAGTGCTGACAACAATGCA
TTTCTTCTGCCAGCCAGATCCCAAGTAATTCGAAAAATAGACATAAATTCAGTAAACGATTATATATTTGT
TCCAAATCAAGAAATACCTANTTGAATTTATGTTGCAAATACAATAGCAGCATCCAAACATGTATACATTC
GCCTTCTAAACACAACTAATTTCGACCAATTGGTCAAAGTAAACAAAATCGAATATGAAAATTTAACAGA
TTACGATATTCATAACACTAATCCAGAAAATAGAAGCGTACAAGTACTTTCAAAGTTAAAGAAAAATTGT

CCATAACAATTCAGAAATTAATTAACAGAATTATGCACCAAGTATAGTGATGTGTTCGGACTGGAAACCG
AACCAATAGCGACAAATAATTTTTATAAACAAACATTAAGACTTATAGATGANGAACCCATTTATATAAA
AAACTACAGAAGCCCGCATAGCCAANTTGATCCAATTGTGAAATCCAAAAACAAGTAGGGAAATTAATA
AACGACAAAATCGTNGAACCGTCTGTATCCGAGTATAACAGCCCACTCTTGCTAGTTCCGAAAATATCATT
ACCAAATTCGGAAGAAAAGAAATGGCGATTAGTAATTGACTATCGCCAAATCAACAAAAAATTACTTTCT
GACAAATTAACTCTCCCCAGAATTGATGATATTCTAGACCAACTGGGCAGAGCTAAATACTTTTCATGCCT
TGACTTAATGTCAGGCTTCCACCAAATTGAACTTGAAGAAAATTCAAGAAATATAACATATTTTTCAACGA
GCAATGGCTCATATCGCTTCACGCGATTACCATTTGGTCTTAAAATAGCACCGAATGCATTTCAGAGAATG
ATGACTATAGCATTCTCGGGTTTAGAACCCTCTCAGGCATTCCTTTATATGGATGACTTAATGGTGATAGG
ATGTTCCGAAAAACACATGATTAAAAACTTAACTGATGTTTTAAATGTATGTAGGAAATATAACCTAAAG
TTGCATCCAGAAAAAATGTTAATTTTTCACGCATGAAGTGACATTCTTAGGTCACAAATGCACTGACAAAG
GAGTATTGCCAGATGACAAGAAATTCGACGTCATCAAAAATTATCCTATCCCCCATGATGCGGACAGCGC
GAGACGATTTGTAGCATTCTGCAATAATTATCGTCGATTTATAAAAAACTTCGCCGATTATTCACGGCACA
TAACTAGATTATGTAAAAAGAATGTTCCTTTTGAATGGACGGNCGAGTGCCAAAACGCATTCCAATACCT
CAAAGAAAAGCTTATGCACCCCACATTATTACAATATNCTGATCTTCGCAAAGAATTTTGCATTATAACAG
ATGCTAGTAAACAAGCTTGCGGAGCGGTTCTAACTCAGAACCGTGACGGAATTCAATTCCCATGCAGCTT
ATGCATCTCGTTCATTTACAAAAGTGGGAAAGCAATAAGAGCACAACAGAACAAGAGTTGGCGGCAATTC
ATTGGGAAATTACCCATTTTAGATCATATATTTATGACAAACACTTTACTATTAAAACGGATCACAGACCA
TTGACATATCTTTTTTCTATGACTAATCCCAGCTCGAAATTAACTCGCATGCGGCTAGAGCTTGAAGAAAA
CCACTTCACAGTAGAATACCTAAAGGGAAAAGATAATTTCGTAGCAGACGCACTTTCGCGTATTAATATA
AAAGAGCTCAAGGATATGCAACATAAAGTCCTGAAAGTCACTACCAGGCGACAAATTAGACAAAAAAAA
CTGTACAGTAGAAAATTATGAACTTCTGCCAAGGCAAAGTCATAAAAATGTATCTAAGCCCAACGTACAT
GAAGTCATTACAAACGATGAAGTACGAAAAAAAGTGACCTTGCGCATAACAGAATCTAAATGTTTATTTA
AACATGGAAATAAAATTATCGCAAGAATTGAAGTTAGCGATTTAAATACTAATGGAATCCTCGACTTAGT
TCAGTTCTTCCAAAGGCTTGAAACAGAAGCCGGTATATTAAAAATCAGCCAACTCAAAGTGGCACCGAGT
GAAAAGGTCTTTGAATCAGTTTCAATAGATTATTTTAAAAATTTGAGCAATATAATATTAAAATCTTTAAG
AGTAGCGCCACTCCAGCCGGTGACCCAAATAAACGATGAAAAAGAGATACAACAAATACTGTCTACATA
CCATGACGACCCAATTCAAGGAGGCCACAGTGGCATAACAAGAACGCTAGCAAAAATAAAAAGACACTA
CTACTGGAAAAATATGACGGGTCATATAAAAGACGTACGTACGTGCAGATGCCAAATGTCTAAAATAAAG
ACACACACAACAAGTAAGAGGTTCTAGTCGGGAGCTCCCGACTAGGGGATACCCTGAACCCTCTTCTTCC
AACATCAAATGCATATATATATTCTATTTTAGAAGCTATATGTCAAGTTTGGTGACTCTAGCTCTTATTATT
TACCAAAATTGCCCAAAAAACAGGATATCGATATCGATTTTTATCGATTGCTTGGAAACGGAGTAAGTTA
TCGATTATCGGAAACAAACTCGATCTGCGCGGGAACTAGGAGCANCTACATCTAAAATTTCAAGTCTCTA
GCTCTTATAGGTTCTGAGATCCTTGCGTTCATACATACGGACGGACGGACGGACGGACGGGGACAGACAG
ACGGACATGGCTAGATCGACTCGGCTATTGATGCTGATCAAGAATATATATACTTTATGGGGTCGGAGAT
GCTTCCTTCTGCCTGTTACATACATTTGGATTTCGCACAAATACAATATACCCTTATACCCATTTTTAATGG
GTTCAGGGTATAAAAAGACATAATTTAATAAAGAGTTAATAGAATATATATTTAATGGAATGGAATGAAA
TTGTTTCTATATTAAGCAAACGTTCTGAGCTTGATAGATCATTTAAGTGCCTCAATAAAAGTGCACTAATA
TCAAACCAAACAAAAATAAAACATGTTCAAGTTCTGGTAGAATCATACAACAGAGTNCGTGACATTGTAA
ACAACAATGGACGTAGGTTAAGCGATTTATATTTAACACAATCAATTGACGTGTTAAAAAGGCTACGTGA
AAATATTAGACATATTTCGGAAAGACATAATTTTCAAATATTGTTCCGCATGCGCTAAATACACCAGCAAC
ATTCGAAATCACTGATTTAATGATATCAGTAGAAGAAGAACTTCGAACAAAAGCAAGAACAAATCGAAA
TAGTCGACATGGCTCAAACACCAGTAGATTTTCTTAAGTTAGCCTCATCGCTCTTACCAGAGNTTGATGGC
AAGGCAGAAAATTTAAAAAGCTTCATAGATGCTCTGAATCTGGTAGATTCTCTAAAAGAAACACACGAAG
CACTTGCTGTAAGTCTTTATTAAGACAAAACTAAAGAGCCATGCCAGGAACCTGATAAGTGATGAGAACA
CTATTGCAGCTCTCATATCGAAACTATCGGACAAAGTAAAAGGAGAGTCAGTAGAAGTTATTTCTGCTAA
ACTTCTAAACTNACAACAAAAGAGCAAAACGGCCAATCAATATACCCAAAAGGTAGAAAAATTGAAGGA
GCCTATATCAGTGAAGGTCTAAGCCAATCACTTGCAAAACAAATACAGCACTACAACAGCTGTAAAGGCT
ATGACCCAAAATTGTTCGATCGATAAGGTAAAACTTATCATACAGGCAGGCACATTCACAAATATGAATG
ATGCCGTCTCCAAATTTGTCAATAGTTGCACGGAGGTAACAGGTCAAAGTCAGCACCGCTCTATACCNCC
GACGAGGTAAGACGTGTTACAGCAAAATAATAGANGAGGCCGAGGTTATAATCGTGGCAGAAATTACGA
CCACAACAATTACAACCGAGGCGGTAATAACAACAACAACAACAATAATTACAATAACCGTGGAGGAAG
ACGAGGCCAAAATCGGGGAAGAGGCCGCGGGAACCNNAACCAAGGTAACAATTATAACAATGTGAGAGT
GGCGCAAAACGCGTCGGAAAACTCTCAGAACCCTTTAGGAAACAATCAGTAAATGCAAAGGTTCATTGCA

TCAATTACAGTCTTAATATATTTGTCACTCTTTATAATCATTCAACTGAAAATAAACACACATTCCTAATA
GATACTGGTGCAGATATCTCACTTTTAAAAATAAATTCAGATAATTTCAAAATTCAAGAAGACAAAATTA
TAAACATCCAAGGCATCGGCCAGGGTGTAATAAAATCGCAAGGAACAACTTCAACAGAACTTCAACCAA
CAAAATATATTANTCCACACGAATTTCATTTAGTAGACTCCGATTTTTCAATACCGTGTGATGGAATAATA
GGCATTGATTTTATTAAAAAATTCAATTGCCAATTAGATTTCAAACCAAGTGAAGACTGGTTTATAATTAG
ACCTCAAAACTTAAATTATCCTATTTATGTTCCAATACGTAATTGGCAACAATGCATTTCTTCTGCCAGCC
AGATCCCAANTAATTCGAAAAATAGACATTAATTCAGTAAACGATTATATATTTGTTCCAAATCAAGAAA
TACATAATGGAATTTATGTTGCAAATACAATAGCAGCATCCAAACATGTATACATTCGCCTTCTAAGCACA
ACTAATTTCGACCAATTGGTCAAAGTTAACAAAATCGAATACGAAAATTTAACGGATTACGATATTCATA
ACACTAATCCAGAAAATAGAAGCTAACAAGTACTTTCGAAGTTAAAGAAAAATTTTCCAGAACAATTCAG
AAATCAATTAACAGAATTATGCACCAAGTATAGTGATGTGTTCGGACTGGAACCGAACCAATAGCGACA
GATAATTTTTATAAGCAAACATTAAAACTTAAAGATGATGAACCCGTTTATATNAAAAACTACAGAAGCC
CGCATAGTCAAGTTGAGGAAATCCAAAAACAAGTAGGGAAATTAATAAACGACAAAATCGTAGAACCAT
CTGTATCCGAGTATAACAGCCCACTCTTGCTAGTTCCGAAAAAATCATTACCGCATTCGGAGGAAAAGAA
ATGGCGATTAGTAATCGACTATCGCCAAATCAACAAAAAATTACTTTCTGACAAATTCCCNCTCCCCAGA
ATTGATGATATTCTAGACCAACGGGGCAGAGCTAAATACTTTTCATGCCTTGACTTAATGTCAGGTTTCCA
CCAAATTGAACTTGAAGAAAATTCAAGAAATATAACATCTTTTTCAACGAGCAATGCCTCATATCGCTTCA
CGCGATTACCATTTGGTCTTAAAATAGCACCAAATTCATTTCAGAGAATGATGACNATAGCGTTCTCGGGT
TTAGAACCCTCTCAGGCATTCCTTTATGTGGACGATTTAATGGTGATCGTGATTAGAAACNCAACTGATGT
TTTTAATGTATGTAGANAATATAACCTAANGTTGCATCCAGAAAAATGTTCATTTTTCATGCACGAAGTGA
CATTTTTAGGTCACAAATGCACTGACAAAGGAGTATTGCCAGATGACAAGAAATTTGATGTCATCAAAAA
TTATCCTGTCCCTCATGATGCGGACAGCGCGAGACGNTTTGTAGCATTCTGCAATTATTATCGTCGATTTA
TAAGAAACTTCGCCGATTATTCACGGCACATAACTAGATTATGTAAAAAGAATGTTCCTTTTGAATGGTCA
AGTGAATGCCAAAACGCATTCCAATACCTTAAAGAAAAGCTTATGCATCCCACATTATTACAATATCCTG
ATATTCGCAAAGAATTTTGCATTATAACAGGTGCTAGTAAACAAGCCTGCGGAGCGGTTCTAACTCAGAA
CCGTGACGGAATTCAGCTCCCAATAGCTTATGCATCTCGTTCGTTTACAAAAGGGGAAAGCAATAAGAGC
ACAACAGAACAAGAGTTAGCGGCAATTCATTGGGCAATTACCCATTTTAGACCATATATTTATGGCAAGC
ACTTTACTATTAAAACAGAACACAGACCATTGACGTATCTTTTTTCTATGACTAATCCCAGCTCGAAATTA
ACTCGCATGCGGCTAGAGCTTGAAGAATACGACTTCACAGTAGAATACCTAAAGGGGAAAGATTATTTCG
TAGCAGATGCACTTTCGCGTATTAATATAAAAGAACTCAAAGATATGCAACATAAAGTCCTGAATGTCAC
TACCAGGCGACAAAGTAAACAAAATAAAAACTGTATAGTAGAAAATTATGAACTTCTGCCAAGGCAAAG
TCATAAAAATGTATCCAAGCCCAACGTACATGAAGTCATCACAAATGATGAAGTACGAAAAGTAGTGACC
TTGCGCATAACAGAATCTAAATGTTTATTTAAACATGGAAATAAAATTATCGCAAGAATTAAAGTTAGCG
ATTTATATACCAATGGAATTCTCGACCTAGGCTAGTTCTTCCAAAGGCTCGAAAAAGAGACCGGTATATTA
AAAATCAGCCAACTCAAAGTGCCGCCGAGCGAAAAGATCTTTGAATCAGTTTCAATAGATTGTTTTAAAA
ATATGGGCAATGAAATATTCAAATCTTTAAGAGTAGCGCTACTCCAGCTGGTGACCCAAATAACAGATGA
TAAAGAGATACAACAAATATTGTCTACATACCATGACGACCCCCTATTACGACCTCAAGGAGGTCATAGC
GGAATAAGAAGAACGCTAGCGAAAATAAAAAGACACTACTACTGGTAAAATATGACACGTCATATAAAA
GAGTACGTACGTAGATGTCAAAAATGACAAA
>9_rnd-5_family-13__DNA___Crypton-V
AATGCATGTCATATGAAAAATTTTAAATACGGTAAATTCATATGACTGTGGAGTGTTGCCTTGCCGGCATA
TGTTTATAAAAAGAATAATGAAGTTATAAAAGTGTGTTATTAAATTAGTACATGAAGACATTAAGGTGAA
TGGTAGCATTTGGAAAATATATCGCCATTATAGATGATGTGTCAAAAAAACCTATATAGGGAGTGGTGTC
GCGCAAGGCATGTCATATGAAAAATTTTTAAATACGGTAAATTCATTATGACTGTGGAGTGTTGCCTTGCC
GGCATAAGTCAATTATGTTTATACAAAAAGAATAATGAAGTTATAAAAGTGTATTATTAAATTGGTACAT
GAAGACATTAAGGTGAATGGTAGGTGAAAATAATGGTGAAATGGTGAAAATAAGAATCTAATAAAAAAT
GNGGCTTAATTTTTTTTTCCAATTAAAACAGTTATTTCCTCATAACAGTCATTCTTTTTGACCGCTTTCGTG
AGCACTATCCAAACTATTTTATTAAGTTAGAGTCGTTAGTTTCAAGTTGCTTACAATTAATCGGGCATATG
TCGGGAATTTAGCTAAACAATTATTTTATATTTATAAGCATTTTTATTAACTTAGTTTATGCATCAAGTATA
AAATTTATTTTCTTCTCGTAATGTGATGTAGTGCAACACTTTGATTTACATATAAGTGAGATATCGATAAT
AGCATGAAAATAAGAATCTAATAAAAAAATGCGGCTTNATTTTTTTTCCAATAAAAACAGTTATTTCCTCA
TAACAGTTATTCTATTTGACCGCTTTCATTTTTACAATCCATCATCAATTTAATATCTCTCTCACATCTTCAT
TTTAATTGCATAAATATCGACTAGTTTTTACCATAACGCGAGCAAAATGAACGCGTTTCGAGTATTCAATT
TGATAGTGCAGAGCTGCCTTAAAAGTACATTTGAAGAAATGACTTGACTTGCTCGTCGCGTAACTCGAAA

ACTTGGCGCCATTAAATTTCTGGAAACTTTGCGAGAGGATGTATTTCTTAATTAAGTGCGAGCAATATTAT
GTATTGCATAAAAAAAAATTAAAATAGTGGAAGGAAGTAAAATGTGCATTGCAAAATTTAGCAAAGGCA
GTTTCGAAGCGGAGATTATTGATTCAAGCGAGGTAAGTTGAGCTTGACATTTCAATAATTATATTTGTCTG
TATATGTACTTTTATTTCATTAGAAGATCGTGAAATACTGGACCAGCAATGTCAAGAACTTAATAAGGGTA
TGTACATAAATCAATAACAGTTTTGTCATTTTCGCATATGCATATTTATACAATAGTCTATATACATATGTA
CATAGGCAATGCAATAATCGTTTTTTAATTTAAATTAAAATTAATAATACATTAAATATTTATAGACTTAC
TAGAAGCAGNCACTAAGTTAAATGAAAGTGAAGCAACAACGAACGATGATAAATCCGGTAAGTACAATA
CTAATGTGCCTTTGAAATAAATTAATGAGCATTGTCTTTTAAAAGAGGAGCCATTAGCAGCTTATAAGTCA
AATACTGACACAACAAATAAAAGCAACCTGGGACAATTTGAAGCTTTCGGTATGTGTGCAATTATTAAAA
CTAAGCCAGGACTAAATAAAAAATCATTTGCATTACACTTCTGACAGAGTTATGTTTTGTAATGTATTTAT
TATTATATGTTAAGTCTTAAATATGCATAATTGTGAGTACCACAGTTTTAAGGCCAGAGTCGGAAAATATC
AAAAGATTTATAGCCTTATTAGTTGTTGAAAACAATTTAATTATCATTTGTATTATGAGTTAATCACATAT
GCAAAATCAGATCAATTTATTATAAAAAGCTACACAAAAGACACAATCGGTCATTACTTTTCTAGGAATA
GTGTATTCTCTTGTACGCTATATGCCTCCGGACGTACAAACCTTTTTTAGTATTCAAACAGTTTTTGCTAAT
TTGTATTTTAACTAAAGCTAACATTGTATACTGATAATACTCGCCTTTTGGATTGTTACCTAATTTGTGCTA
GTACCAGCTATTTTGTGTTCGTTTCAGGTGTCTTGTGCCCGTCTTATATTAACATGTAAAATAAATAAATGT
ATTAATAAAATACTTATACCTTAACATAATAGACAAAGACTGGCCTCTAAATGGCTAGATTCCCCAGCAT
AAGCATTTTATACATTTAAATCGCTTGGTAATTTTCCACATTCTATTCAAGGAGTCGATTCCAACTATACTC
CGGATATAACTAATCAGTCCAGCACTTATATACTTCCAAGTTTAACTTACACAATGTAACTCCACTAAGTG
ATATAAGGGATTTGATTATTCCTAATTGTGAAAACTTTTTTTGCTTGTTTTGTAATAAATTGCAATCAAAAT
TCGTTCGTCACTTGCATAATAATAAGCCACAAGTAAAAAGTGTTTATGCCTGTCGAAAAATGACAAAACT
CGAAGAGTAGTTATTAATGAGATTTGGAGTAATGGCGATTAAATCGACAATATCGATGCATCGTTAAATA
ACGAAATTTTAAAGCGAATTAACTCTAAAAACGAATATGTTGTCATATGTTGCAAGGGATGTTCTGGATTT
TTCCCCAAAAGTACGATACGCAGTCATATAAATAATTGCATAGGCGGCGATCATAAGAAAGGAACTCCTG
ACTTGCTTAAGCAAGGTCGCTCTAAAACCGGCTCTATGCATAAAAGTGCAAGTGACGTTCTCTTGTAAGCT
ATATGCCTCCTGACGTATTTAAACCGTTTTTGTTAATTTTTTTATTTATTTACTAAGGCTATTTTTATATACT
TGATATTACTCAAATTTTATTTTGTTAACTGATTCGTGTTCATTCCAGCTAGTTTGTGTTCGTTTCAACTGTC
TTGTGCTCGTCTTATATTTATATGTTTCTAACAAAGGTAATGAAAAGTATAAATACATTTTTTTTATATATA
TAAATAAAATCCTTATCATGATCAGTACAGATATACATTTTTTTAACAACATTCATGCTGCTTAACGTTAT
ATAAAAATGATTGTATTAAAATATAAGATGCAATAGAGACACATCCGAATGATCATAGCAAATGCCAATT
AACTGCAACTGAAGTGGAGGTATACACTATGCCATATCCTTAAAGGTCTGAAAGTTAAGCAAAATAAATG
TTCTTAAATNTGTATATGGCACTGCTCCTAGTGCTACTATATTGAATACAAAATATTTACTTAAAATATTG
AATTACGTCCATTAATAATTAAAATAGATTCCTTTTTATCTATTAATTATTAAATATATTCAAANAGATGC
ATCCGAACAATTTATAAACACAACTCAAAATGCATCGGCTAATAGTGGATCTTTAGGACTTAACGACTTTG
GCTCTAATTGTTTAGATTCATCGGCAAAAATCAAAATCATTCCACGATAAACGATTCATTTCAATCGCTTA
ATACTAATTGTCCAGATTCCACTCAAGGATTCGATTCCAGCTATGCTCCGGATATAACTAACCGGTCCAGC
ATTTTANATAATTCCAACATTAAACAAGTTTTTCTTACACAACTGNAACTCCACTAAGTGATGTAAGGGAT
TTGATTATTCCAACTGCGAAAACCCAAACAAATTGTAAGAAACTTTTTGCTTGTTTTGCAATAAATTGCAA
NCAAAATTCGCTCGTCACTTGCAGATTGTTCATAANAATAAGCCGCAAGTACAAAAGTGTTTGTGCCTGTC
GAAAAATGACAAAACTCGAAGAGTAGTTATTGATGAGATTTGGAGTAATGGCGATTAAATCGACAATATC
GATGCATCGTTAAATAACGGAACTCTNAAGCGAGCCCGTCAAACTCTAAAAACGAATATGTTGATTTCAT
ATGTTGCAAGGGATGTTCCGGATTTTTCCCCAAAAGCACAATACGCAGTCATATAAATAATTGCATAGGC
GGCGATCATAAGAAAGGAACTCCTGACTTGCTTAAGCAAGGTCGCTCTAAAACCGGCTCTATGCATAAAA
GTGCAAGTGACGTTCTCTTGTAAGCTATATGCCTCCTGACGTATTTAAACCGTTTTTGTTAATTTTTTTATTT
ATTTACTAAGGCTATTTTTATATACTTGATATTACTCAAATTTTATTTTGTTAACTGATTCGTGTTCATTCCA
GCTAGTTTGTGTTCGTTTCAACTGTCTTGTGCTCGTCTTATATTTATATGTTTCTAACAAAGGTAATGAAAA
GTATAAATACATTTTTTTTATATATATAAATAAAATCCTTATCATGATCAGTACAGATATACATTTTTTAAC
AACATTCATGCTGCTTAACGTTATATAAAAATGATTGTATTAAAATATAAGATGCAATAGAGACACATCC
GAATGATCATAGCAAATGCCAATTAACTGCAACTGAAGTGGAGGTATACACTATGCCATATCCTTAAAGG
TCTGAAAGTTAAGCAAAATAAATGTTCTTAAATATGTATATGGCACTGCTCCTAGTGCTACTATATTGAAT
ACAAAATATTTACTTAAAATATTGAATTACGTCCATTAATAATTAAAATAGATTCCTTTTTATCTATTATTA
AATATATTCAAATAGATGCATCCGAACAATTTATAAACACAACTCAAAATGCATCGGCTAATAGTGGATC
TTTAGGACTTAACGACTTTGGCTCTAATTGTTTAGATTCATCGGCAAAAATCAAAATCATTCCACGATAAA
CGATTCATTTCAATCGCTTAATACTAATTGTCCAGATTCCACGCAAGGATTCGATTCCAGTTATGCTCCGG

ATATAACTAACCGGTCCAGCATTTTAAATAATTCCAACATTAAACAAGTTTTTCTTACACAACTGAAACT
CCACTAAGTGATGTAAGGGATTTAATTATTCCAACTGCGAAAACCCAAACAAATTGTAAGAAACATTTTG
CTTATTTTGCAATAAATTGCAAACAAAATTTGCTCGTCACTTGCAGATTGTTCATAAAAATGAGCCGCAAG
TACAAAAATTTTTGTTCCTGTCGAAAAATGACAAAACTCGCAGATTACTTATTGATGAGATTAGGAAAAA
TGGTGATTATATCCATNATATCGATGCATCGTTAAATAACGGAACTCTAATCGTGCCTCGTCAAAAGCGTG
TAAACTCTAAAAACGAAGCGGTTGATTTCATATGTTGCAAGGGATGTTCCGGATTTTTCCCCAAAAGCACA
ATACGGAGTCATATTAATAATTGCAAAGGGAGCGATCATAAGAAAGGAACTCGTGACTTGCTTAAACAAG
GTCGCTCCAAAACCGGCTTCATCCATAAAAGTGCAAATGACGTTGTGCGCACAAAAATAATGCCTGTTTT
AAATGATGATCCCATTTCTAGAAATCTTATATATGATGAATTGATTGTTTTATTCGCAAACGCAGAATCTA
TGAAGCACGGCGAACTTCAGTACGATATGATCAGAGCAAACATGAGATTGCTCAGCAGACTATTACATGA
AATAAAAAAATCAGAATCTAATGAGATACAGGAACTTAAAGACGCCATTAATCCCAAACATTTGGATTTG
ATGATATCTTGTATCAGAAAAGTGGCTCAGTGGGACTCTTCTTCGTTGTTTTTAAAACACCTTCAGTTGCA
ACGAACCTTACAACTCTAATTAAAAGNGCGCAAAAAATATTGTGGACTCATTGCATTAAAACACAAAATCA
TGCAAAAAAATTAATTAAAGATTTTCTTGTTCTATGGAAACCGAAATTCCTCTTTTTATAAACAAAAAAA
GCTATAGAGGACCAGTCAAAGAAAAAAGAGCCCTACCAACTATTTTACCATGTAAAGAAGACATCAAAA
ACTGTTTGATTATGTGATAAAAAACGGAAAGCGCTCTGAGTGTGCTAAAAATTAAGTTTGATTTTTTATGG
ACACCGTTGATACAAAGCACACTAATTGGCCTTCAAATTTTTAACCGCCGTAGAGCCGGTGAATTTGAAA
GACTAACAATTGAGAACTATCTAACAGGGCACGCCATNGATGAAAATGTAGATAANNAAAACTTCGAAC
GTCTCACGAAAGAAACTAGAGAAATCGCTCAAAAATACACAAGATTGACTATTCGTGGAAAACTCAATCG
AACTGTGCCAGTGCTGCTCACACCGTTTGACAAGTATTGTATAGAAACTATTCTCAAACATCGTAAAAGA
GCTGGTGTTAAAAAAATAATGATTACATATTTAGCGTGCCTAACGCCAAGCCCTTAGCAAATCAATATGT
AAGGGCTTGTCCGATTCTGAAAAAATTTGCCCAGGAATGTGGGGCTCGCTGCCCAGGGGCTTTACGGGGA
ACGATGCTTCGAAAGCATTTAGCTACCTATACATCGATGCTTAACGTCAAAGATGTCCAAATTGATAGGCT
TGCCAATTTTATGGGTCATCATAAAGATATACATAAAGAAATCTACAGAATCCCTGTAACAGTAGCGGAG
ATAGCCGAAGTATCCCAGATGCTTATGGCAGCTCTTGGAAATAATGTTGAGGATGAAAACACAGTAGCCC
TTGATCAGTTCGAAGAAAGTAAGTACGTAGATTCATCAAGTTTGTGGGTGATACTAAGAGTAGTTATGGT
GAAAANTGTAATATCTTAACTTAACTAATAATTACTTGTCGGGCTTATTGTGAATGTTTTTACATTGTACCT
GAAATCCATTTTGCAGATGATCCTTTAGAGGATTCGGAATCAGATTATTCTGAACAGCGATCAGAAAGCG
ACTTCAATATTTCCGATGATGANATTGTAGAAAATCGAATAAAAAGGGAAGCGCCCAAGTAGTAAGTATT
AAATTGCAACCTACATATTTATGTCACGTGCTTACATGCATCGCGTTTAGGCTATCCTATTTATCCTCATTG
ACAAAGTAAAAAGAGATATCGCGCTTGTGAAATGTCCGATCGTCTGTTCAAACGAACGCGATATTTTGCT
GGCCCATTAAAGTTATATCAACGAGAAGTAAAGTGCTAAAATTATATAATGACGACCACTGCGGCGTGGT
AAATCGCAAAAATCGTCCTAAATAACAAACCTTATACAATCATAGAGATTGACTAACTTTTAGTTGAAGA
CGAAGATGTTTAACTGGGTGCGTGGCGAATGTTTAAAATTAATATCACCTAATAAATTGTTTTGGTAGGCT
GTATTAAGCTGTTATTGGTCTGTGATTGAGTATTGCGACGGTTGCAACCGACTGCGACGCTTCTAATTGAT
TTTTATTTATTACTAAAACGTTAATCTTTTGCACACTAAATTATATTAAACTTTATTATTTAACAGGATCAT
CATTTGGAAAAACCATAAGAAACCGTTGGTCGAATGAAGAGCGTGAAGAAATATACCAGGCTTTTGGAG
ACAACTGTTGTCTCTTAAAGGATTGCCGTCGCTTAAGCAATGCAACGCAGTTATAAATAATTCTACATATC
TAAAATGTAGGACACCGGCGCAAGTAAAAGCATGGATAGATAATCAGCGCAAAGCTAAATCAAGAAAAT
CAACTCCGCAAATACATTAAATAATGAATATGTTGACCATATTTAGAACTAAATTGACTATACTTTTTCGT
ATTTTTTATGTTAATATTAATTTATGCTAAGAATATTTNGAATTTGTTAAAAATGCATACTATAAATGTATT
TAATAACGTCATTTATGTAAATT
>9_rnd-5_family-632__LTR___Gypsy
TGCAAGTTATCACCCAGCCCGCAAGGCAATTCTTATTGCAATCATAAACAACATTTCAGTATAAGTCGCTA
TCCCAATTACTTAGCCCTTTCAGACTTATTAGACCCGTTTCAATCACTACGTCCACTTAACGCACAGCTGC
AGCTCTGCTAACATAAATATACAGATTCCCGGCTCTAACATAAACATAACAGCGCAACCACTTAACATAA
AGATTCATATTCTCATGCGCAGTTCATAACGTAAACTAAAACCCAGTCTCTAGAGCGCAACCGCTTAACGT
AAACATGACTCAAAGCGTCTAGCTTGCGCAANCCACCCCCGCGAGCTTTTAAAGACCCANTCTCTGGAAC
GCAATTGCTAGCGTAAACACGGCTGATTCGCAGCATAAGATCACCGCGAGCTTTTGAGCTTTAAGAGAAN
AAGCTTTATCATAACCATAGCTGACTTGCAGCATAAGTTCACAATTGTACTTTAGTTCTAATCTTGGATTC
AAATAAATCGGAAGCCTTCAGCCTCCCACTTAAAAATATATTTTTTTCCAATTAGTAGCCACTAATCGGTT
AATTGGCGCCCAACGTGGGGCTCGCGATTATAAACACAAAAAAGGATAATTAAAAGAAACAAAATCCTT
AGTGATTCGCGAGTGAGCAAAAGTGCCGATTTACCAGTCCAAGATTGTCTCAAAAGAGCATCAAAATTTT
CTAAGAACGAAAATATCGAGTGCCTCTAAAAGAAACATTAAAAATTTCCGACGAACGGAAATACCCTGTT

TCTGAAAACTTGAAGAGTAGTGAAAATACCCTGAGCTGGAAATATTGATTGTTCACAAGACTTAAAAAGT
GAAATCAAATTTCCCGTTCAAGTTAAATGAAAAACATTCCAAAAAATTAATCGATCAAAGAAGCGAAACG
AATATTTTCGCCAACAAAATTTTTTCACCAATCAAAAACTTTAACCAAATAAAAAATATCACGTCAACCTG
AGACCCCCATAAAAGAAGACCCTAAAACCCAGAAGGAAGGAAGGAAGGAAGACTTGAACGACATATGTA
TAATCACCCATAAAGAAAGACCTTTTACGTGAGCCATCATCATTTACTTTAAAAAAAANTTTTTTTACTT
AAGTAATGAGAGTTTAAATTTGTGTAAGCATTTGTTAGAATAATAAGTGTTGTTATAAGCTGGATAACCAT
ATAGAAAGTTTAATTGAAGATTTTCAAAATTTAAAAATGGCTAACAATAATAACTTGACAGCTGATTTGGT
AGCTCAGTTGATTGCGACTCAGACCAGAGCGCTTCAAGAGCAAATTGCAGAATTGAAGTTACAAATCACT
CTCTCATCTAATAATGAACAAGAATATAAGATTACTGAAATAAATGACCTTATTCGTTGCGATGAATCTTT
TTGATGTTATCAAGTCCATACCAGAATTCTCAGGCAGCTATAATGCATATGTTAGTTGGCGCGAAGCCGCT
TGCTGTGCCATGCGAATGTATAAAGAAGGGAAGTAGAAAGTACGTAGGTGCTTTAACAATATTAAGAAAT
AAAATAACAGGTAAAGCTAATGACACCTTGACTAATCACGGTACCGTTCTTAATTTTGAAGCTATACTTGC
AAGGTTGGATTTCGCCTACAGTGATAGACGACCAATCTATATAATAGAACAGGAGTTAAGTATTTTACAG
GGTAACTTATCCGTTCTCGGAATACTACAATTTAGTTAATAAAAACTAACCCTTTTGATAAACAAAACTAT
TATGACGTATGGTGACAAAGCCCACATCATTACGGAAATGAATAAGAAAAAAACAGAGACAACGCTTTT
AAGAATTTTTCATCACAGGTCTTAACGGTTCTTTATCAGAAGTTTTGTTCTCCCTTAAACCTGAAAAATTTG
CCTAATGCTTTGGCAAAAGCTCAGGAACTGGAAGCGAATTACCAAAGGGCTAACTTCGCTTACCACCATT
TCGCAAATACCCATCAACGTAACAATCTAAACCCAGGGCATAACAATCAGCAAAAGAAAGCTGAATCCCC
AAAAACAACCTAATAAAAGATATGACAATCACTTAAGGTTCAATCAAGTTCCGCAAAAATCAAATTTTTG
CGCAAAGCAACAAGACTCCTCAACCTCCACCGGAGCCCATGGATGTAGACAAATCCATACAACTACAGAA
TACACCTTATCCGTATCCCCAAAATCGAAATTCGGCTCAACCAAAGAAACGGGAATACGCTCCCCCTTCG
GTTCAGCAACCTAACAAACTCATGCGTATCAATAACCTCGAGGAAGACCATTTTTTTAGAAGAGGGGAGA
ACTCCCCTACATTTTTCAGAATAGACAAAAATAAATAAAATTTTAAAAAATATTAATTGACACCGGTGCC
ACCGCTTGTTATATAAAAAGAGGGTATATACGATAACGCAGAAGAATTAGTTATAAAAAGAATTAAAACT
GTAAATGGCTACAGTATCATAACTCATTTTTCATAACATAAACATATTTGGCAAACGCCACAGATTTTATG
AAATTAATAATCTAGACGCAGATCTATTAGTCGGTTATAATCTACTGAAGGAGATTAAGGCAGTAATCGA
CTTGTCAAAGGGAACTTTGTTTTTATAATAACAAAAAGGAAAAATATTAATTTTTACAAAAACATGTCCAA
GAAACGGTTGGCCATTTAAAAGTTCAGACGAAGCCTATAACGTTGCTTCGTATTTGAACTACCAAAGTGG
GAAATGTAAATCATTTTCTTTATTTAATGTATCCGATACAGAATCATCGTCTTTAAGTGACGCTGATAACC
CACGTATCGAAAAAACTAAAGTTGACGATATTCTTAATGAAGGAAAGCTAAAGAAATTAAAACTCAAAA
AGAGCAGACGTCGAAAGATGTCGGTATCCTCTACAACAAGTTCTTCTTTATGCAAAATAAATCATTCCATT
AATGAAATGGATAATTTGAATATCCTTGAGCAACAAGTCGTTCAACAAATAGAAACAAAGCATTCTCAGA
TTCAAACATGTCTCCCTTTTAGAACAGATATTAGAGGAGAGATAAGAACATTAAACGAAAATGCAATTTA
CAGTAAACAATATCCTTATCCTCATTCAGCTAACAATTTTGTTAGTCAGGAAATAAACAGGATGTTAAATG
AAAATATAATAAGGCCCAGCAGAAGTCCTTATAATGCCCCGGTATGGGTAGTAGGGAAAAAGGTTTTAAT
GAAGATGGAACGCCTAAGAATCGATTGGTAATAGATTTTAAGAAACTGAATAACGATACAATCCCCGATA
GGTATCCAATGCCTGATCCTTCAGTCATTCTATCCAACCTAGGCAAAGCCAAAATGTTTTCGACAATTGAC
TTAGAATCGGGATTTCATCAGATTCTAATGAAAGAAGAAGACATCGAAAAAAACAGCTTTTTCCATTAAC
AATGGAAAATATGAATTTCTTCGTTTGCCATTTGGGCTTGCGAACGCGCCAAGCATCTTTCAGAGAGCAAT
GGACAATATATTACGTGAAAAAATCGGAAAAACTTGTCACATTTATATGGACGACATAATAGTTTTTCAA
ATACCCCGGAACAACATTTTGATGATTTACAAGAAATCATTGAAATTCTATTCAAAGCAAACATGAAAAT
ATCCTTGGAAAAATCAAAATTCTTTAGAAAAGAAATAGAATTTTTAGGATACATAGTATCATACAATATC
ATTAAAACTGATCCGAAAAAAATAGAATCAATCATGAAATACCCTCCTCCTAATAATTTAAGGGAATTAC
GAGGTTTTCTTGGCTTAACAGGCTACTACAGGAAGTTTGTAAAAACTACGCGACTATAGCAAAACCACTC
ACTAAATTGTTACAGGGACATAACGGTATGGTTTCAAAACATATGTCCAAAAAAACAGAAATATCTCTAG
ACGAAGAAGCAATCTCTGCGTTTGAAAACTTAAAATCACACTTGATTGCAAACATCGAACTAACTCAGCC
AGATTATACAAAAAAAGTTCGTCCTCACCACAGACGCTTCCGACATCGCCATAGGCGCCGTCCTTTCACA
GGAAAACAAACCAATCACCTTTGTTTCAAAAACCTTAAATAAAACAGAAAGAAATTACGCCACAAATGA
AAGGGAATTATACGCCATTGTATGGGCATTAAAAAACCTTAGGAACTACTTGTATGGTGTTACAAATTTA
GAAATCCAAACAGACCATCAACCACTTTCTTTTGCAGTTTCTGCACGAAACCCTAACTCAAAAATGAAAA
GATGGCATGCTTTCGTAGAAGAATTTTCCCCTAAAATATTTTACAAACCAGGATCTACCAATGTCGTCGCT
GACGCGTTATCAAGGGTAATGATCAATCATATTTCCAATGAAAGTAATCTCGTGTCCGAAGAAGAACCGT
CGTCCGATAATGAAACTATCCATTCAGCCGACAGTAGTTATAAAAGCGTAATTGAAGAAACACACAAACC
ATTGAACCAATTTAAACAACAATTACTTGTTTCTAAAAACAACATAACAATTCATGAAAAATATGAAGTTT

TCAACAAAACAAGACATATTATAGAATACGACACAGTACACAATTTAATAATCATACTTAAAGAATTTCT
TCAACCAAACCTGACTACAGGAGTTCATTGCAGTCTCGAAGATTTATACGAAATCCAAAACTCATTAAAA
ACACTTTTAACAACAAATTTTTATTTACTAAAAAGTTTGTCCTAGACATCGAAAACAAAAANGACCAAGA
AATTATAATAGAAGAAACACATGCTCGTGCTCATAGAGGTTTGGACGAAAATCTTAAGCAAATTCTCAAA
TACTATTATTGGCCAACACTATATAAAACCCTTAAGCAACACATACGCTACTGCGAAATTTGTAATAAAA
ATAAATATAACAGAAACCCTAAGCAAATTCCTATTTCTAAAGCCCCTATTCCGGAGAGAGAAGGTGAACA
ATTACATATTGACATTTTTATGCTCAAAAATTAACATTCTTGACATGCCTAGACGCATATTCTAAATTTTTA
GTGATTAAGGAAATCAATGACAAATCAGATATGGCAAACAAAGTGTTGGAACTTATCCAAACTTTTCCTT
TCGCTAAATCCATAATGATGGACAACGAACCTACGTTTTCCTCGACACAAATCGTCAATGTCTCGTTCAGG
TATAGCGATTTATCATGCAGACCCCAGACACAGTTTAAGCAACGGTCAGGTAGAAAGAGTTCATTCAACA
ATTATCGAAATTTCTCGTTGTATTCAAGAAGAATACAATATAATGGATCATTCCGAAGTCGTATACCGAGC
AGTNAAAGAGTATAACAACACAATTCAATCGACAACTCAACAAAAACCATGTGACATATTATTTAACAAA
ATAGAACACCCAAAATTGTCAGAAAAATTAAAATCAGCCCAAGCCAAAATGTTAGAGTACCATAACAAA
AAAGAGGTCACCAAAAAATTACCAAGTAGGTGAAATAATTTATGAAAAAAAACATGGCGAACGGAACAA
ACTACAACCTAGATTTAAAAAACAGGTAGTTAAAGAAGACTTAGGAAATAAAGTCAAAATAAACAATAG
GGATCGTATTATTCACAAAGACAATATCAAATTCTAACCTATTTTATGTTTTTTTTAATGTATTTTTAGAAT
GCACATTTTATGTATCTTATCTGTTNTACTGCCGTTTTGCGGTGCAGACATTATTAATTATTCAAATGATGA
CTATGTCCTACTTGAGGATACAGATAATCTTTTTTATACGAAGCATACGGAGAAGTTTTTTCACGTTACTA
ATCTAAGTTTTTATAACGATATTTTTNTAAAAAGAATTAAATTTTTTTAAAAACAAAAAAACAATACAATT
GATCGGGAAGTAACCTTGGAATTGAATTTAATCAAAACACTCCTTTCTGAACTTACACCTCACAAAATTGC
CAAACGTAGTATCAATGAACTAGGCACAATTTTTAAATGGATCACAGGAACACCAGACCACGATGACATG
ATAACTATTCAAACTAAAATCAATGAACTTGTTCAAAACAATAATANGCAAAATCAAATCAATTCTGCAA
TTTTCAAAGAAGTCACTAAAATCACAAATCTTTTAAGAGATATTAAATTTAATCAGGAATTATTTATGAAA
AAAATATCGACTTAAGTTGTTATCACTTGACCTACAAAAATCTGCTGGATACTATCACATTTTCCAAAGCC
AGAATATTGAATCCAAAAATCTTAAATAAACAAGACATGATAAATATATTTAATCACGAAACACTCAATA
TCACTCTAATCGATCTTTTAGACATTTCCTCTTTTCGATCGTTTCATACCAAGGTTTAATCATAGTTTTTATA
AAATACCCTACAATTAATTACAATTGTAAGTTATACCACGCTAAAGCCATATCCCAAACAGATGGAAAAT
TTTTTTCGTTAACGACAAAATTGCTAAATGTAATAACACGTTTTACCTCATTGATAACTTTAAAAGAGAAA
TTTATAATAGTTTTGCCCACATTAATTTTTGAACCAAACTGTTTTGTAAATCTCCTTAATAAAAAATTTACA
AGTTGTATAAAATTATCCGAAAAAATTAAACCAATAGAATTTATAACAGACGGCGCTATTTTAGTATCAG
GCACAAACTGTATTAACGAAACCTGGCTTACAGGCATTTACCTTGTTACATTTAAAACCATAGTAACCATA
AACAATGTTACCTACACCAATGTAAAAGAAAAAGTTCTTAATTATGTAAAAACACAATAAATTCNCAAAC
TACTTAGTTAGTGAATATCAAGTTACTAATAATACTGAACTTCAATTAGAAAATACAAATATACTATCANA
AATTGTTATAAAATTAGAAAGTAATCATTCAAAAATAACCTTTTTATTATTTATACTAATTTTTATTTTTAC
TATGGTNAAGCTTCTTAAACGCTGCCCTTTAATAAATCTAAATCCCCTCAAAATACTGAAATTGAAATCGT
CAAGTAATCAAAAGTACCATAACACTCAAACTACAGAAACAGAGNTTGACATATTAAACCAATTAAACG
AACAAATTGAATCTTTAAAAGAAATCATTAAGAATCGGGGACGATTCTTTTAAGAAAGGAGGAGTTATCA
CCCAGCCCGCAAGGCAATTCTTATTGCAATCATAAACAACATTTCAGTATAAGTCGCTATCCCAATTACTT
AGCCCTTTCAGACTTATTAGACCCGTTTCAATCACTACGTCCACTTAACGCACAGCTGCAGCTCTGCTAAC
ATAAATATCCAGATTCCCGGCTCTAACATAAACATAACAGCGCAACCACTTAACATAAAGATTCATATTCT
CATGCGCAATTCATAACGTAAACTAAAGACCCAGTCTCTAGAACGCAATTGCTAACGTAAACATGACTCA
AAGCGTCTAGCTTACGCAAGCCACCCCCGCGAGCTTTTAAACTTTAAGGAAAGAAGCTTTAGCATAAACA
CAGCTGATTCGCAGCATAAGATCACCGCGAGCTTTTGAGCTTTAAGAGAATAAGCTTTATCATAACCATA
GCTGACTTGCAGCATAAGTTCACAATTGTACTTTAGTTCTAATCTTGGATTCAAATAAATCGGAAGTCTTC
AGCCTCCCACTTAAAAANATATTTTTTCCAATTAGTAGCCACTAATCGGTTAATTTGCATAC
>9_rnd-1_family-70__LTR___Gypsy
AAATAAAAAAATAAGTGAATAATAATAAAANTTTTCTTTTTATAAATAAAAATTAATTAAACAAAAAAAT
AAATAAAATGAGTGGATAATAAAAAAATTAAAATAAACCGAGGTTCACAATAAAGATAAAGGCAGCGAC
CACATAAGGAACAGCCTAAAAGCGGAAAAGTGAGAAAAAGAAAANTTTTGAAATACCTTTTGTTGAGTTT
TTTTAAAAAAAGGGGGTAAATATTTTTGTGGAATATTTTTTTGTATTTTTGTTTGGTGGAAGATATAAAGA
CAAAAAGAAAAAAAAAAACTAAAGAAAAAAAAACTAAAAGGAACCAAAGTGGCTGAGCAAGATCGGCGT
AAAAGTGAAAAGACACCAACACCAAGAAAAGGAGAGAGGAATAGTAAAGGGACCGACGGAACCCCTAG
AACGGGAAGAGAGGGAAACAGCGACGTCGACTAGCAAGGAAGTATACAATAAAGGATGGAGGCAGGTG
AGTGCCAAAACCCGGGGGTAGGCGTCCGCTGGATTCACCAGCGACGCAAAGAGGAGTTAGTGGCGATCG

CCCAGGAGTTTGGGCTGGAAAAGGAGGGGCGCGTGGAGGAGCTACGTAAGCGGCTGTCCACCTACGTGC
AGGAGGGCGGTCATTCGACTGGGATTTGGGCTCGGCTCGCCGAGCTGGAATCCCAGTATGCTAAAACTCT
TGAGCCGCCATCTTGGGGCGTCCCACCCGACTTTGGTGGACGGCCACGGCAAAAGTCGGAACCTGACCTC
AGAAGCTCCCAGCTGTACGGGCTTTTGCAGGCGCCCACCCCGAGTTTGACCGTCTCGGGCGTGGAGGCAG
TTCCTGCTCTCGGTGGAAATGAAGACCGCCGAGCAACTGCGGTCAACGCAGAGGTGGGGAGGTCCCCTGT
TGAACCGGGTGGTGGTCCCTGCTACACTAGGCCGCACCATCCATCGGAGGCGAGGCCACTTCCCAATGTC
CCGGAGCAAGTTCGGAAGTGGGGTATGAGGTACGATGGACGCTCCGACCCCCTTGGGTTCTTAGAGGACC
TCGAGGAGCGCGCTATCACCTATGGTATAGCCCTCGACCGTATGCCCAGGGCAATGAGCGAGGTGTTCTG
CGATAGGGCTGCTCGTTGGTTTCTGAGCAGTGGGCTAAGGGATGTGCCGTGGGCCGTGTTTAGGAGGGAT
TTCTTGGAGTTTTTCTTGCCCCCTCGTTACTTCGAGAGGCTTGAGGACGAGATCCGATCCCGGAGGCAGCG
CGTAGGTGAAGCGTTCAAGGAATATTTGATAGAGCTGAGGGCTCTTATGCACCACGCTAGATACGAGCCG
GCACAAGAGCTGCATCGGATCTACGAGAACGCCGCTCCGGAATACAGGCTCTATGTGCGACGGCAAGACT
TTTCTACCCTATCCCAGCTAACTCAACTGGCCGTAGAATACGAGACCGTCGTGAGGCAGAGGGCGGAGTA
CTCAGGCAGCCAACCCTTGGGTCAGGGTGTCTCTAGTTCAAATAGTGCAGAGTGGGCGAGGACGGCGCAG
TCCTCGGGACATCTCAATCCGTTCCGGTCTGCTCCCAGCGGATCCGGGACGACCGTTGTGGACAACAGGG
TCTCCACCCCACCCGCTGCAACTCACGCAGAGGTGGCCCATCAGATGATGAGCCAAGAGGCCGGGAACGG
ATATGATCCGCGACGGGCGTGTCGTAGGTGCGCACAGGAGGGGCATTTTGCCAGGGAATGTCCCAACGCC
CCTGTCTTATTTTGTTGGGTATGTGGAAGCCGGGGCCGAAGTAGTCGGGAGTGTTGCCGCCGCGCGGAGA
CGGGGGAAACGGGCAACGGCGTCGTCCGGACCAGGATCGGGTCGGCGCCAATACACGCGGAACGCAGCA
ATAGGACACCCACTGAAGGTGGACGGTGGCCGGATCGTCGCTACGGTAGAGATTGGCGGACGAACGATG
CCCGCCACGATCGATACCGGAGCCACTCGTAGCTTCATGAGTGAGGACTGCGTACGCAAATGGACGATCC
GGGGAAGAGCGGAGGAAATACAGGCACGTATCCGCCTGGCAGATGGGTCCACCCTAGAGGTAGAGAAAG
GCTTTGAGAGTAGACGCCAGCTTAGGGGGAAAAACGATCAGCATGACGATGCTCATCATGCCCTCAATGC
TGGACCATGTAATCCTGGGTATGGATTTTTTGGGGGCGATGAATACCACAATACGCTGCGGCGAGGCGGA
ACTGGTCCTAGAAGCAGCGACGACACCGGGCGTGAGGCCGGAGGAAGGCCTCCCAAGGCATGAAAAGGG
AGCACCTGCTCGGCTACAGAGGGAGCCGGAGGAAGGCCTCCCAAGGCATGGAAAGGGGAGCACCTGCTC
GGCTACAGAGGGAGCCGGAGGAAGGCCTCCCAAGGCATGGAAAGGGGAGCACCTGCTCGGCTACAGAGG
GAGCCGGAGGAAGGCCTCCCAAGGCATGGAAAGGGAGCACCTGCTCGGCTACAGAGGGAGCCGGAGGA
AGGCCTCCCAAGGCATGGAAAGGGGAGCACCTGCTCGGCCTACAGAGGGAGCCGGAGGAAGGCCTCCCA
AGGCATGGAAGGGGAGCACCTGCTCGGCTACAGAGGGAGCCGGAGGAAGGCCTCCCAAGNCATGANAAG
GGGGCACCTGCTCGGCTACAGAGGGAGCCGGAGGAAGGCCTCCCAAGNCATGAGAAGGGAGCACCTGCT
CGGCCTTCGACGGAGAAGGCCGGGAAAAGGGGAGTAAAGTCCGGCCCGAATCGACAGCCTGGAGAACCC
AAGAGGTGGCGCATAGAGCTGCAGAGGCAGACTTCTAATGGCTACGGAAGCGGCACGGCGGGAACGTGC
AAGAGAAAGGACGGAGTAGGAGCGATGACCAGAATCCACACGGACATAGAAGGCCAGGAGCGATCGCC
AGAATCGAGCGGCCATGGAGTTCTGTCTGGAGTCGAAGTGGTGCCAGGAGGGGGCGAGAACGGCATTGA
AGAGGCGGAGTGGCCAGCGGATCTGGATCCAGAACTGAGGGAGTTCCTGGAGTCAGAGCTGGCTCTCTTC
GAGGGGCTGCAGGGAATCTCGCACATCGCGGAGCACCGAATCCGGCTGAAGGATGACAAGCCGTTGAAA
CAAAGGTACTACCCCAAGAATCCGGCCATGCAGAGGGTGATCGACGAGCAAGTGGATGAGTTGATCCGG
GCAAATGCCATCGAGCCGTCGCGTAGTCCGCACAGCGCCCCGATCGTCTTGGTCAAGAAGAAGACTGGCG
AGTGGCGTATGTGCATTGACTACCGACAACTCAACGCGCATTCCATTCCGGACGCTTATCCGGTGCCTCGT
ATTAACCACATCCTGGAGCGGTTGCGCCACGCCCGGTTCATCTCCACTCTGGATTTGAAGCACGGGTACTG
GCAGATACCGATGGCAGCCGATAGCCGGGAGTGTACGGCGTTCACAGTCCCTGGAAGAGGTCTTTTCCAA
TGGCGGGTGATGCCATTCGGCTTACACTCGGCGTGTGCGACATTCCAGCGCGCTTTGGATACGGTAATAG
GGCCGCAGATGGAGCCTCATGCGTTCGCTTACCTAGACGACATCGTGGTAATAGGAGCGACGAAAACACA
GCATGTAGAGAATTTGAGGGAGGTATTCCGCCGGCTAAGGGCGGCCAATCTCCGGTTAAATAGGAAAAA
ATGCAGCTTCTTCCGGGAGAGGTTAGTCTACCTAGGACATGTAATTTGCGGAGAAGGGATACGGACCGAT
CCGGAGAAAGTGGAGGCGATACGAAACCTGAGGGCCCCAACAAATTGCAAAGAACTGCGACAGTGTCTC
GGGATGGCGTCATGGTACAGGCGTTTCGTACCCAATTTCGCGACCCAGGTACAGCCACTCACAGCGCTCTT
AAAGAAGGGGCAGAAATGGGCATGGGAGCAAGAACAGGAGGAGGCATTGCGTCAATTGAAGGAGAGCT
TGACGACTGCCCCTGTGTTGGCTTGCCCCGACTTTTCCGCCAAGTTCGTCTTGCAGACTGACGCGAGTGAG
TATGGCCTCGGTGCAGTGCTGACGCAGACGATCGATGGACAAGAGCGGGTGATAGCATACGCGAGCCGA
AAGTTGCTGAAGGCCGAGGTAAACTATTCCGCCACTGAGAAAGAGTGCCTGGCAATCGTGTGGTCGATCC
GGAAGTTGCGTTGCTACCTAGAAGGGTACCGATTCGACGTAATAACGGACCATCTAGCGCTCAAGTGGCT
GAATTCGATCGAGAGCCCGTCAGGAAGGATTGCGCGATGGGCATTGGAACTGCAGCAATTCCAATTTGAC

GTTCATTATCGTCGAGGGAAGCTCAACGTCGTGGCCGATGCCCTGTCGAGGCATCCTCTAGAGACCTGTCA
ACAAGCAGTGGTGGCCGAGGCCCCCTGCAAATGGGTCGCGAGGATGCGTGTTAGAATCGCCGAAGAGCC
AGCCAAGTTCCCGGATTACACCGAGGAAAACGGACAGCTATATAGGCATTTGGGTCACCGGATAGACGAT
GAGGACTACATCCCTTGGAAACTATGCGTAGGCAGTGGTCAACGCCAGAGGGTACTGCGGGAATGTCATG
ATGCTCCCACGGCCGGACATCAAGGTGTCAGAAAAACGGTGGCGAGGTTAGCCCAGCGGTACTACTGGCC
GGGTATGTTCCGCGATGCCGCCAGATACGTGAAATGTTGCGAAGACTGCCAAAAATTCAAGAGTGAGCAA
CTCAAACCGGCTGGTCGCATGCTGACGAGGCAGGTGGCAGAGCCGATGGCGGTACTTTGCGCGGACTTCG
TCGGACCCCTTCCACGGTCGAAACATGGAAACACGATGCTATTGGTGTTCCATGACGCTTTCTCGAAATGG
GTGGAGTTGGTCCCTCTCAGAAGAGCCACGGCAGCACACCTGCAAACTGCGTTTCGAGAGAGGATTCTAA
GTAGGTTCGGGGTACCTAGAAAGTTTGTGTGCGACAACGGGGTACAATTTACGAGTCGGAGCTTCAAGTC
ATTTTTGGGGTCCCTGGGTGTAGAGATCCAGTATACCGCCCCGTACTGTCCGCAAGAGAATCCCACTGAG
AGGACTAACCGGACTGTAAAGACCATGATAGCGCAATTTATTGAGGGCCACCAGAGTTCGTGGGATGAAC
TCTTGCCCGAGATCTCGCTGGCGGTCAACACGAGCGTGGCAGATTCAACGGGCTTTAGTCCCGCTTTCCTG
ATGCAGGGCAGGGAACCTCGGTTACCCGCAGCTCTGTACGATCAGGTAACTCCGGGGTCCGCAACTAGTG
CGCTCGACCCTAGGACAAAGGCGGATAGGCTAAAAGAAATTTTTGATATCGTCCACAGCAACCTCCAGCG
CGCATCAAAGGACCAGGGCAGGCATTACAACTTGCGGAGACGAGAGTGGCGGCCCTCGTTGGACTCGAT
GGTCCTGTTGAGGCAGCACCAGTTGTCCAACGCTGCCGAAGGTTTCGCCGCAAAGTTGGCACCCAAATTT
GACGGCCCCTACAAAGTCGTCAAATTCATATCCCCCAATATTGTGCGGCTGGTGAAATCAGACGACCGCA
AGAAGAGGGTGGCCAACATCTCACAATTGAAACCATTCTACAGGAAGACGACGAGCGACGAAGAAGACG
AAGATCCAGGGCACGCAACTGCCCACGAGTAAGCGAAGGATGAGAGCAACACCCGTCGCCCAACGAAAC
TATTATGATTAAGCGTAGATAAAACTAACATTAACAACATAATTCAGAATAGGCATAGTAAAGTGTGAGC
CGCACTGAGAGAATAGGGCATTAACAAATAAGTAAAGAGTAAGTTAACCAGTAAGCTGAACTTTCAGAT
GGAACCCGAAGTTATCGTCATATCGTCAGACGAAGACAACAGCAGCGGGGACACCGAGCCTTTCCCCGGG
CCGGGAGCCGCAGGCCGTCCGCGTGACTCCACGCCAGGGGCGTGAGTACCATAGAAGAGAACGGCAAGA
AGGACCGCCTCGCCAGTTCCGCCATACACTATTCTCCCGGCGGACCGGCATAATGGAGAGGCTAGACGGG
ATGGTCTTGCTGATGGCAGTCGAGGTCACGACAATGTTCGTGGAGGCACCGTTCGCCCGGAACTCGCCAC
CGTACGCGGAAGAGCCTGCATCTCCGCCCCTCGCATCCCACTCGGCACAATCATCAGAGCCGGCAACCCC
AACCGCGGCCGGCGAGATGGACGAGGAAGAGTCCGCACCGTTCGGAACGGAGGAGCACATACCACCAGC
GCAGGACGTGGAGGCGGTAGGAGAAGAGATATCCCCGTCGGCCGGAGACGCGGAGTCGCCACCGACGGA
GGTGCCGAACGGCACCCGCGCAAACGACGGGGAAGCAGACCCGCTGAGCCTCGACCCAGAACCCTTAGT
GGGAGGGGACCCGTCAACGGAGGACGAGCCGGAATTCCGTCGCCGCTTTGCCACCCACCGTCTGGAGGTA
GACCCGGGCAGCCAGTTTCCCAGGATAACGCTGCGGTACCTACCAAAAGTCCCCGCGGTAGCCAACAAAC
CCCGTCTGAACCCCGCAAAGCGCGAATGGGGGTGCCGAAGAAGGCCCCGGCGGAAAGCGCCGACGCAGC
CGCCATCGTCGACGGAGAGCCGCCAGGGGCGAGCGTCGCCGGAGTGGGAGGCCATCGCCGACCCAGTCC
CGAGGGAGCATCCTCGTCGGCCACGAAGAGGCTTCGCACGGAGACCGAGAAGCCCGAAGGATCGCAGCG
GTGCGTGCCGCGTTCGCCCCGCGAGCGCCAGGAGGGTCCTCCCCAGCTCGGGAAGGATAACCGCCGCTAC
CAGATCGCTTCTCGAGGAGGGCATCCCGAACCCTGGCCGATACCGATTGCGGTCTGCCAGATGGTCGTGG
AGGAGCACAATCTCAGCACCAAGGACGCGGCGAACTCGGCCGTTTCCCCAACCCGCGCCGAGGCGCCAG
ACGGAACCTTTCCTAAGGAATCGGAGGAGGAGTGGCTCGACGACGAGGACGAGATGCCCGCGCTCGTGC
CCGTTGCCCGCGCCGAGACACCCGACACCATCGCCCGGCGGGGAGTTAGAAGACGCCTGGTTCGGCCAAG
AAGGCAAGGCGCCATCCCGAAAAGGTTCCTTCACGGTTCGCCAGGAAGGCCGGGATGAAGTCTGGNCCG
AGGAAGAGGACGCGGTGGTGACGGCTCCGTCGCCCAAGCTTTCGAGTCCAACCCAGGATTCCTCCCCCTC
GGCTCTAAAGACTCCCACGGAGAGCGAGTTGTGGGACGACATAGTGGACCTTTTTCGACGGCATCGACGG
GTGGGACACTAGCCTGGACTCAGTGCTGGAGGAGGAACCCGACGCCGAGGTCGACACGGTCGCCCGCAG
TGCCGACGTGAGGGTCACACCGGAGAGCCACCCCGAGAAACCCGACACCGAGGTCGAGGCGATCGCCCA
CAGTGCTGACGTTGGGGTAAAACCGGAGGGCCCCGTCGAGGAACCCGTGCCCGATTCCACCCCCTCAGGC
TGGAAGGTGTTTCCCCTCGGAACACCGTTGTCGTCGGGCGTGGTGGACCTAATCGCCAGAGAGGCGCGAA
TCCGCCGCTTCCGGTGCCGACGTACTCGGGTTCCACGTGCCGACGGGGAGAGCGAGTACAGCGTTACGCT
GAACGCGAAAGGCGACGTGACCGTCACCCGTCCGAAGCGAG
>9_rnd-1_family-439__LTR___Gypsy
TGTAGCATGCGTACATATGTATATACATACATATGAATAAGGCTGCAACACTTTGTTGTTATGCCAACAGA
ATGCAAACATAAGCAGCGACTTTGTCGCAGCGCGCCTAACGCTAAGTGCCCTCTGCGCATATTCAAGTGC
AGACGAAATATGATCCGCACTTGATAGCAACGACCGCCCAAATAGCTAAGTCAGCGTTCTCACGCTTGAC
CGTCGCTGTGTATACATATGTGCGCGCATGACTCTCTCTGACTCTCTTGTTGTATATAATAAGCCTNAAAA

CCATGTATGTAAAACGGCTGCTCACTGCTTCACGGTGGACAGCTCTAACTCCAAAAACATTGAATAAAGA
ACCATCAAGGAAGACTGACGTCTTTTCATCAGGGACTATTATATTATATGGCGACCGTGACTAAGGTCAC
AACAGACCTGGACATGGAAGGACAGCAGCAACGGCAGTAACAAAAACACACAACAACAACTATACAAA
ACAACAACAACTATGAATCGTGTTACAAGTGATGACCAGCAGTCAACAAGTGTGAGAGGCAATCCGTCCC
TTACGCCAGCGCGGGAGACTTAGAGATTGAAAGGAAAAAAAAAAAACTGAAAGTCTGAGTGAGTAGAGTG
CGAGTAATGGGCAAGCGCGGAGGCCAGAAAAACAAAATAAACAAAAGAATAGCACACATTAAGTGGCTA
TTATACACGAATACGCCGCCCGCNTGGGCAACGGCACAAAAGTTAACTGCCGAACTTGATTTTCTGAAAG
AAAGCCTCCAGCAAAGAAAGCCAAATACCGCAACTCACTCAGCAAATATCAAAGTTATANGTGAGAAAC
AAATAANAANACCACCACCTCCGAAGCCGTTGCCGAAAAACCTGACGTCTGCCCGAACGACTGCCCTGGA
CCGCTATNCGCCGCAGCATGCAANTCCACGTGCTTGGGACCAAAGAACGACCNGTGCGANGACCGGCAC
CGGCCCCGCCTGAGCCAATAGGCCCNGGACATCAGCAGCAGCGAAGCAACAAGCGCGAGGGGCACNCCG
CCACTTNCGCCNACGAGGGAGCGTCAGCGTAGCCAAATCCGGCGACGACCAGCTGACGCGGCAGGACAA
CTGGACATTTGGAGCATAACTACATTATTATAATAAAATTTGTAAAATTGTGCGGAACAATCTCCGCTCAA
TTAGAAGTATAGCCNGTAAAGCTATACAAACCGATAGGTAGAAAAACTTGTTAACGTAAATGCAAAGCA
ATAATTTTTAAAATATATATATATATATATATTATCGAAACAATTTATGTAAAATTGAGCAGAAAATCTTT
TGCCCAATCAGAGGTGAGGCCCATAAAGCCAAACTAAAAAATTAGGCAGGTTGCAATGTAAAAAAAAA
ATTACACGTCTCAGGCACTGGGTAAATTACCCAGTAGCAAACAAATTAATCCAAATCTTAAATTCCAAAT
AAATTTGAAATTTTAAAATGAAAAAAANAATAATAAGTTTTCAGTTCAGTCAGTTAAATCAGAAATAAAA
AAAAAAAAAAAAAATTGAATCAAATTTATGAATAGTAATACAATAAGTAAATTTTCCGAAGATTTTCTAAT
TGAATCAAATCATAATCCTGAACAGAACTCCAAAATGGGATTATTAGACGTTAAGTCAGTGGATTCCACT
GCTAACGTTATAAATAAAGTTGAATCAAACAACACAGATTTAGAATTAACAAATATATTACTGGTAATCA
TAGTTGTTATAATGTGTGCAAATTGTTTTATAAAATATATAAACTGCATAACAAATGCCTAAAAAAAGATT
CATGAGCCGAGCAAATGACTTGGACAAAATTTAAATTTGCACAGCACAAACAACAATGATTAATCATAAT
TGACAATATCATAATAACTATAAGATACCAATTAAACTAAATAAATAAAAAAAAAAAATAAATAAATAAA
TAATAATGAACACACAGTATGATGAATAGTAAGAATATCCCAGAATAATCTTGAAAAACAAAGTAGCTTG
TCAATATTATGGAATGGCCAGAACTTTCTGAGCGGATCCAAAATATTAAAACGACATTTGAAAAGCGTAT
AAATGTCTAACACAAAATAGACAAACGCATATAGATACAGTTAATAGGCACTCCATAACATTGATCGACA
GCTTTAACGAAGCTCGAATATTGATATATCAACATAAAGATGTATTAACCAATGAACATTGGTCAGAAAC
GTCAAGGTTGCTAATAAGACTGAGAAATAATATACAAACAGTTAAAGACAAGCATAATTTAAATTTAATA
CTACCTTCTATTTTGAATACACCTGCAAAGTTGGAGCTCGGCGAAAACCTTGAAGTTCGGACTGAGGATAT
TCAATTAGAAGAAGAAGATTTAACAAATCTAACAATCCCTGCTGTTATCACACTGGATTCCAAAGATATTT
TATCCGAAAGTGAAGAAAGTGCTGATAATAGCCGTATAATGGTTGACACAGCCGCTGCCCAGAGGGCATA
TATTAGGAAATTGCACAAGCAGTCCCAGAATTTGACGGAAAGAAAACCCGTCTTGAAAAATTCTTAACGG
CATTAAGACTTGTCAACTTAACAAAGGGAACTTATGAGGAAATTGCCATAGAGTTGATTAAATCAAAANA
ACGGGCACAACCCTCAACAAAATAAAAAATGAAACCACAATAGACGGAATTATTGCAAAATTAAAGCCG
TAGTTGTCGGTGAAACACCCGCCAACATAAAGGCAAAATGGACACTTTAACGCAGAAGGGCAAAACAGC
CACTCAGTTCACAACTGAACTAGAAAATCTGCGACAGCTTCTTGAAGCTGCTTATATTGACCAAGGCATAC
CATCTGAAAATGCTGACACACTCAGTACTACAGAAGCCGTTAACACAATGATTAGTAAAACTAGTCACGA
AAGTGTTAAAACCGTTATGGAGTCTGGTATTTTCACTACCATGGATGCTGCCGTTAGTGCATACATCCGTA
CTAGTACAAGACTTACTGGTAGTGTGAATACAGTGATGTACTCTAAAAGAGGGAATAACAACTACCGCGG
TAGTAACAATCGTGGTAACTATCGTGGTAAAGGCAATGGTAACTACCAAAACAGAGGAAATAATAACTAC
GGTAATCAAAACCGTAGAGGCCGAGGAAATGGAAACAACAATTCCAACCGAGGCCGAAACAACCAAAAT
AATAATAATACAAACAATGTTAGAGTAACCCAGGACAATCAGGGAAACTCTCAACAACCCTCAGATATTC
AGCAGTAGGAAATTATGCAGTTCATACCATCAATCTTAGTTTGAATATATTTGTAAACTTTTTCAATGTGA
AGACAAATAAAAACTTGTATTTTTAATAGATACTGGTGCAGACATATCCCTGTTAAAGGAAAATTCTGAT
ATCTTTCATGATATCCAAAAATAAGAAGATCAATATTCAAGGCATAGGCCAAGATATAATACAGTCCAAA
GGACTGATTTCAATAGAGATACAGACAAGTAAATACATAATTCCACACGATTTTCACTTAGTAGATTTGCA
TTTCGCAATACCTTGTGATGGAATAATAGGCATTGACTTCATTAAGAAATTCAATTGCCAGTTAGATTTCA
AACCAAATGAAGATTGGCTCATAATGAGACCAAATAATCTAAATTTCCCAATTTATGTTCCAATAACATAC
AGTTCTGGTAATAATTCCATTGTTCTACCAGCAAGATCCCAAGTAGTCAGAAAAATTCACCTATCATCAGA
TGAAGATAGTGTACTAATTCCAAACCAGGAAATTAAAGAAGGCATTTATGTTGCAAACACAGTTGCAACA
ACCAAAAATGCATTTGTCCGATTACTGAATACGACCAGTACTGATCAAGTAGTAGATATTAAAAATTTGTC
ATATGAACCACTTTCGAACTACGACATAGTTCAAAACAATCCAGAAAATAGAGAAAAAACTGTCTTATCT
CAATTAGCTAAAAATTTTCCACCACAATTCAAAAACGAATTGACTAACTTATGCACCCAGTTTAGTGACGT

ATTCGGACTCGAAACCGAATCGATCACTACCAATAACTTCTACAAACAAAAACTAAGATTGAAGGATGAT
GAACCAGTCTACATAAAAATTATAGAAACCCTCAAAGTCAAATAGATGAGATACAAGTACAAGTACAAA
AATTAATCGACAATAAAATAGTCGAACCGTCTGTATCCCCTTATAACAGCCCACTTTTGTTAGTTCCGAAG
AAATCTCTTCCAGGTTCTGACAAGAAAAAATGGCGATTAGTAATTGACTATCGTCAAATTAATAAAAACTG
TTGTCGGATAAATTCCCGCTGCCTAGAATTGATGACATTTTGGATCAACTAGGAAGAGCAAAATACTTCTC
ATGCCTTGATTTAATGTCAGGATTTCATCAAATTGAACTCGAAAAAGTTCAAGAGATATAACGTCTTTCTC
AACAAGCAATGGCTCGTATCGCTTCACGCGATTGCCATTCGGTTTAAAAATAGCGCCGAATTCTTTCCAGA
GAATGATGACTATAGCATTTTCCGGATTAGAACCTTCTCAAGCTTTCCTTTATATGGATGATTTAATAGTC
ATTGGTTGTTCCGAAACACATATGCTCAAAAACTTAACTGATGTTTTTGAGAAATGTAGGAAATACAATCT
TAAATTACATCCTGAAAAATGTTCATTTTTCATGCATGAAGTCACATTTTGGGACACAAATGCACGGATAA
AGGAATCTTGCCTGACGACAAAAAAAACGACGTCATTAAAAATTACCCAGTTCCACATGATGCGGACAGC
GCCAGACGATTCGTTGCATTCTGCAACTACTATAGACGTTTTATCAAAAATTTTGCCGACTATGCTCGTCA
CATAACAAGATTATGTAAAAGAGATGTAGAATTCGAATGGACAGCTGATTGTCAGCATGCTTTCGAACAT
CTCAAATCGGCACTAATAAATCCGAATTTATTACAATACCCAGATTTCAGCAAAGAATTTTGCATAATAAC
AGATGCAAGTAAACAAGCATGTGGAGCAGTTTTAACTCAAAACAAAAATGGACTCCAACTTCCAGTTGCT
TATGCATCAAGATCGTTTACTAAGGGTGAAAGTAATAAAAGTACAACAGAACAAGAATTGGCAGCAATTC
ATTGGGCAATAACTTATTTCAGACCATATATTTATGGTAGACACTTCAATGTCAAAACAGATCACAGACCA
TTAACATACTTGTTCTCAATGGTAAATCCAAGCTCAAAGCTAACGCGTATGAGACTTGAACTAGAGGAGT
ATAATTTTACAGTCGAATATCTGAAGGGTAAAGACAATTATGTAGCGGATGCGTTATCAAGAATAACCAT
TCAACAGCTACAACAAATAAATGGATCAATAAAGAAAGTCACTACAAGATATCAAAGTAGACAAAATTC
CTGCGCAGGAAAAGAAAAATAGAATTGCCATGGCAATCTATAGAAAAAGCTTCAAAGCCCAACGTATAT
AAAGTCATTAACAATGACGAAGTACGTAAAGTAGTGACCTTGCATGTAAAAATATGCTATGTTTTTTAAA
CATGGTAAGAAAATTACTGCAAGATATGATGTTAGCGATTTATATACCAATGGAACTCTTGACTTAGGTCA
ATTTTTCCAAAGGCTTGAAACGCAAGCCGGTATAAATAAAATCAGCCAACTCAAAGTGGCACCGTGGGAA
AATATCTTTGAACACGTTTCAATCGATAATTTCAAAAATATGGGCAATAAAATATTAATTTCATTGAGAGT
AGCGCTACTCAACCCGGTGACCCAAATAGAAACAATAAAAGAAAAAGAAGCTCTATTGTCTACGTTCCAT
GATGATCCAATACAAGGAGGTCACACAGGCATCTCAAAAACCTTGGCCAAGGTCAAAAGACATTATTATT
GGAAAGGTATGACTCGAGATATTACTGAGTACATACGAAAATGTCCAAAATGCCAAAAGTCAAAAGTGA
CACGAAAAATAAAAACTCCTTTAACAATAACTGATACGCCAATACACGCTTTCGACAGGGTTATAGTAGA
CACCATTGGTCCACTACCCAAGTCTGAAAATGGAAATGAATACGCAGTCACATTAATTTGTGATCTGACTA
AATATCTAGTCGCGATTCCAATTCCAAACAAAAGCGCTAACACAGTAGCAAAAGCGATTTTTGAGAATTT
TATTCTAAAGTACGGTCCAATGAAGACGTTCATTACGGACATGGGAACAGAATACAAAAATTCTATAATA
AATGATTTGTGCAAGTATTTACAAATAAAGAACATAACATCAACTGCACACCACCACCAGACAGTTGGAA
CAGTCGAAAGAAGTCACAGAACTTTTAATGAATACATACGTTCATATATATCGGTTGATAAAACTGATTG
GGACGTATGGATCCAATATTTCGTTTACTGTTTCAACACAACCCCATCTATGGCACATGACTATTGTCCAT
ATGAACTTGTTTTCGGTAAAACAAACAATTTATTAAAACATTTTAATAGCACAGACACCATAGAACCTTTA
TATAGTATAGATGATTATGCTAAGGAAAGTAAATATAGATTAGAAATAGCATATAAAAGAGCTCGAATTA
TGTTAGAAGAAAATAAGAGAAATAATAAGGAATTATATGATAAAAAANAATAAACATAGATATATCGGT
AGGAGATAAAGTCCTACTTAAGAATGAAACAGGACATAAGTTAGATCCAACTTATTTAGGACCCTATACA
GTAGAAAAGTTAGAAGATAACGATAATATAATAATTAGGGATACTAAAAAGAAAAAAAAAGAGTACATA
AGGATAGATTAAAATTTTATAATAATTGAATTGAAAACTCTAATATAAACAAAGAAAATTGTTCGAACAA
AATAATACAAAACAATAAAAAGGAAAATGGAATTTAAGAAAAGGAAACTTAAAAAGAAAGAAAAATTCT
TTTCTTTTATAAAAAAGAGACAACATTTAAAATAAAATACTAACACATACATATATTTTTTTAAATACACA
TAGTTAAACATATACATTTACACATACATATTAACAAACATTTTTATATTAAAATAATATAAACACAAAAA
AAAAAAAAAAAACAAACAAAAATAATTAACATTAATACTTAAAATAACTACATAAATTACGTTATTTTTT
AAAAGGAGGGAGATGTAGCATGCGTACATATGTATATACATACATATGAATAAGGCTGCAACACTTTGTT
GTTATGCCAACAGAATGCAAACATAAGCAGCGACTTTGTCGCAGCGCGCCTAACGCTAAGTGCCCTCTGC
GCATATTCAAGTGCAGACGAAATATGATCCGCACTTGATAGCAACGACCGCCCAAATAGCTAAGTCAGCG
TTCTCACGCTTGACCGTCGCTGTGTATACATATGTGCGCGCATGACTCTCTCTGACTCTCTTGTTGTATATA
ATAAGCCTAAAAACCATGTATGTAAAACGGCTGCTCACTGCTTCACGGTGGACAGCTCTAACTCCAAAAA
ACATTGAATAAAGAACCATCAAGGAAGACTGACGTCTTTTCATCAGGGACTATTATATTATA
>9_rnd-1_family-145__LTR___Gypsy
TGTGTAAGTTAACAACAAATAAATTCATACAGTCCCAATAAGAAGCATTGCAACACCTCATAAGAAGTGC
AGCAAAATGCTGACTTGGTGTCTGTGCCATTTCCTCCACGCGCCGACTGGCAACCGCCGAAAATTCCAAG

AAGAATTGCCCAACGGGCCAACAACATCAGCAGCGACGCCGCTGCCAACGCCGCTGCCGCTAAGCCACA
GGTTACTAGGGCTAGAATTGATTTAAGTTTATTCAGTTGCAAATTAACATCAAATAAAGGAAGTCTTGCTT
CCCTAAAAATTATATTTTTTCATCGTTTAATAAACTTTGGAATTAACTGGCGCCCGAGCAGGGACCGGCTC
GTGTTACAACAGAACAAAACGCTTCTACGTACGCGTTAGTCGGTTATACGCGAAAATAAAACCAGTGCAA
CTCTCGTCGATTAAATTCAACGATCGGTTAAATTCGCGAAAGGCAATTGGAACTTAATTGTTCAATTTTGC
CCATCGGTGGACAACGCATCTTGTAAGGCCCGCCCGCAGGATACTCTCAACAAAGACTGGTCGCAGGATC
CCCAGTAGATACCCAAAGGCTTCAAAAGGATGAAATCCGGACCGGCGGGGAGGTTGCCACATCAGGAAT
AGTACTGTAAGTTAGAAATTAAGTGAAAAAAAAAAAAAAACAAACGAACAACTGANACAAAGTTATAAGA
ATAATAAATACTAAAAATTGTAGAACGATAAGCAAGATAAAAAAGTGTAAAACGATCAACAAATATAAA
GACTACGAACGTAGTATAAATTATTTCCAACTTTGCGACTCCCAAGGGACCCTCCAAAGTAGAAGGAAGA
AACCAGCCACTGGTTTTAAAAAGAAAACAAAACGATCAAAAATTCAAGTAAAATATAGAGTAAAAACTA
ACACTAGATTTAAATATACTTCGAAAATATCTTAAAATATACAATATAAGTAATATATCTCGTTTATGGCT
AATGAAAGCAATTTAGTTAGGCCCCCAATAGTATCAGATCAACCACAGGTAGGCGGACATAACTCGAACA
GACAGCAACAACGACATATCGACAGACAAAACGAACATTTACAAAATAGAGTCATGGCGAATAACGCAG
AACAGATAATAGCAGCCACGGTTAGCCAGATTTTGGCACAACAACTGCCGACATTGGTAAAGCAGTTAGT
CAACCCCTCTAGGGAGTTTAGTGACGTAGACGACCAAACAATAACTGCCAACCACAATTTGAATGACTTA
GACAGAGTTCCGGACATAGTTAAAAGCCTCAGAGAATTCTCCGGTAATCCAGCAGAATTTAGCTCATGGA
AGAAGAGTGTAGATCGTATACTCTCAGCCTATGCAGCTGAGGCAGGTTCGCCAAAATACTTTGGCATATT
GCATACAGTGAGAAACAAAATCACTGGCAATGCCGACACGGCCCTCGAGTCGTATAATGTTCCCCTAAAT
TGGAGCGCTATCTCCAAATGTCTCACGCTGCACTACGCAGACAAAAGAGATCTGGGAACATTAGAATATC
AGATGACAATGTTAGTGCAAGGTAACAACCAAACAGTAGAAGAATTTTACCAGGTCGTTTACAACCACTT
GTCGCTGATATTAAATAAAGTAGGCTGCATGGAATTAAATAGGGAATCCGAAACCCTTATGACAAAAATA
TACCGAGATAAAGCATTAGATACCTTCATTAGAGGTTTGAAGGGAGATTTACCACGGATTCTTGGAATAA
AGGAGCCAGCAGATCTACCTTCAGCCCTTCACTATTGTTTAAAACTAGAGAATCAAAGCTATCGCACTAA
CATGGCTTTGAATAGAAATCAACAGACACATGCCAGAAACCCAAATAGAAATATACCACCACCTACACTG
CCACCTCGAACAGCTCAGCCTCATTTCGCTCAAACTTTTAGGCCACCACCCATTAGCTTCAGGCCCCAACT
CCCGATCCATACACCTTGGCCAACCAATACAAACCATGGACCACCGACTTACAACAATAATGGACCGATT
CCACAATATCCCAACCAACGAACCTACCAGACACAACCACAACAGGTATACATACCCAATAGACCTTCTG
CACCAAAACCCCAACCACGTCCTGAGCCAATGGACGTGGACCAAAGCATCCGCACGAGGATGATAAACT
ACATGAACAGACCCAATTTTGAAGTAGGGAAAAGACCTCCACCACCAACAAACGCCACGGACAAAAACA
AAGAAATTTCAACATACAAACAGGAGAAGAACAGACGGAGACAGAAAACACCGACAACTATGTGACTAT
GGAGGAATACGTACAATCAATGCTAAATTACGAACAGCAGACTGAATACGAACAGCCATTAAACGAATA
CGCCCAACAAATTGAAGAAACAGATGACGGACAAAATACCCAATACGAACACTCAGACATTAATTTTTTA
GGATAAAGAGCTCCTCTCTTCCTTACATAGAATGTAAGATGGGGAGCGGAAAATTACTCAAAATACTAAT
AGATACAGGATCAAACCGAAATTACGTCCAACCAAAATTCGTGTCCGAGCCTATTCCAAACGAAAAACCC
TTTGAAGCCGTCACAGTCGGCGGAAGCATTAAAATTACACACCACAAGTTCGCTAATCTTTTTAATAGAA
ACGATATAAAAATAAAATTCTTTCTCTTACCCACCCTAAAATCATTTGACGCCATATTAGGTAACGATAGT
CTAAAGGAACTAGATGCTGTGATACAAAGCAGCAAGAATCTACTAATAATCAAGAACAACATTTGCATAC
CCATTAAGCAGAAAAAAATAGAGGCGGTCAACACGGTAATACCACGGACAGAACATATGACAAACGAACA
AAAAATAGCCTTAATCAACTAGTTAAAATGCACCCACACCTATTTGCCAATCCGGACGAAAAACTGACAT
ACACAACCAACGTCAGAGCAGAGATTCGAACAAAAACGACTCCCCATATATTCAAAATTTTACCAATTTC
CAATGTCTTTAAAAGACGAAGTACACAGCCAAATCAAAGAACTTTTAGAGGACGGAATCATCCGACCCTC
CCGATCCCCGTACAATTCACCTGTGTGGATTGTTCCGAAAAAATTAGATGCCTCCGGGCAAAAGAAATAT
CGCATGGTTATAGACTACAGGAAACTAAATAAAGAAACTACCGCAGACAAATACCCTATACCCGAAATCA
ACGAGGTACTAGCACAGCTAGGCAATAACAAAGTTTTCTCTGTCTTAGATTTGAAAAGTGGCTTTCATCAA
ATACCCCTTAAGGAGTCAGACATAGAAAAACTGCCTTCTCCGTTAACAACGGAAAATACGAGTTTACACG
ACTCCCATTCGGTCTCAAGAATGCGCCCTCAATTTTCCAGAGAGCTCTTGACGACATCCTTCGTGAGCATA
TTGGAAAAATATGCTTCATTTATATTGACGATATCATTATCTTTAGCAAAGATGATGAATCGCATGCGCAG
CACCTCGCCGCCATATTCAGAACTCTACAAGAAGCCAACATGAAATGTCAGTTGGACAAATGCGAGTTCT
TTAAAAGCAGAGTAGAATTCCTGGGATTTGTCATCTCAGACAAGGGGATAGAAACTAATCCAAACAAAAT
TGAGACAATAAGAAAGTACCCACTTCCAAAAACACTCAAAGATTTAAGATCGTTTATTGGCCTTGCCAGC
TATTATAGGAGGTTTATAAAAGACTTCGCTAAGCTGGCAAAGCCCCTAACCTCTCTTTTAAGAGGGGAAG
ATGGACGCGTGTCCAAAAATCAATCAGCTAAAAAGCTCATTAATCTCAACGAAGAAGCGATCGAAGCATT
CAATAAAATAAAAATTCCCTAGTTTCGAATGAGGTAATACTTCATTACCCAGACTTCAGCAAAGAATTTCA

TCTGACAACAGATGCATCCAACTTTGCTATTGGGGCTGTCCTATCCCAGAACAAGCGACCCATTTGTTTCC
TCTCTAGAACTCTCTCTAAAACAGAGGAGAACTACGCCACCAATGAAAGGGAAATGTTGGCAATAATTTG
GGCCTTAAAAGCCCTCAAGAATTACCTCTACGGTAAAGCGAGTGTAAAATATACACCGACCATCAACCCT
TAACCCACTCTCTTAGCAGTTGGAACGGCAACGCTAGGATAAAGAGGTGGAAAGCTTACCTAGAAGAGTT
TGATTACGAACTCCTCTATAAGCCAGGTAGGGATAACATTGTAGCCGATGCGCTATCACGTATCCCTGTGA
TCAATAATATTGGATCAGTAGCCTCCACCCAGCACAGTGCAGACAGTTCAAGCCACAACTTAATTCCGAG
CATAGAAGCTCCAATCAACGTATTCAAAACCAGATATTTCTCAGTAGAGCCAACGAAACAAATTACGAAT
TCCATATCCCATTCCCAACATTCCACAGGCATACAATTACTCGACCAAACTTCTCAGAACACGACCTTACG
CAAATACTGAAAGCTCATCTAAACCCATCCGTAATCAACGGAATAATAACTTCAGAAGAAATAATGAACC
AAATTCAGCAGATATATCCCCAACATTTTAGGAACTTCAAGATTAGATTCACACAGTCTCAAGTAAAAGA
TTTAACAAATGAAACAGAACAAGATGAGAAGATATTGGTTACGCACAACAGAGCCCACCGCAACGCACT
AGAAAACAAAATGCAATTGTCCGAAAGATTCTATTTCCCAAAAATGAAGCAACGAATAACAGAATTAGTC
AAACAGTGTCAGGTTTGTAAAGAAGAAAAATACGATAGGCATCCCACTAACCCAAAACTCAAAGAAACA
CCAATCCCCGAATACCCAGGACATACAATCCACATCGACATATACACGACAGAAAAAAACTTAGTACTAA
CAGCAGTAGACAAATTCTCGAAATTAGCTCAGGGACGAATTATCAAATCTAGGGCAATAGAAGACATTAA
AAACCACTTCATGAAATCCTTTTTTATTTTGGCGTACCTAAAGTAGTTGTAATAGATAACGAAAAATCCCT
TAATTCAGCCTCAATCACTTTCATGATGCAGGACCAATTAAATATAGAAGTTTATAAAACACCACCCTACA
AAAGCGCAGTGAACGGTCAGGTAGAACGATTTCATTCAACACTATCAGACATAATGAGATGCAACAAAA
GAAAACAGGAATACAGAACATTCGAAGAACTACTCAACAAATCAATATACGACTACAACTACACTATAC
ACTCGACAACTAACAAGAGACCATTAGACATATTCTTCGGCAGAAACGTGACTACGGACCCAGACCAATA
CGAAAAAGCTAGGAAGGAGAACATTGACAGACTCAAACAGAAACAATCGACCGACATGCAGAATCATAA
CAAAAACGTAACGACATACGAACTTACATACCTGGTCAAGAAATATTCGTCAAAGTCAATACCAGATTAG
GTACCAAACTGTCACCCAGATACAAAAAGGAAATAGTTAAAGAAAATAGAAACACAACCGTACTAACAA
CAACTGACAAAATAATACATAAAAGCAATATAAAAATACATAAAAATTACAGATTCCTATTAATGATGAC
AATAAAAGCAACAGAAGGACACTTGGAAATTTTGAACTACACAGACGCACAAACAATTACAATTCTAAA
AGGAATAGGGAAACTACAGACATCGACAACACGAATCGTACAAGTCATAGAGCTAGAACAGATTAAAAA
CGCATTAGATAATCTCCTTTTTACACCGAAAAGACTTAACAACCAATACTTTATACCACACTTTACGTCAC
GAAATAACGCAAGCTATTGTCACTTTCGACACAATATCACTTTTCCATAAACGAAAGACTAGAGCGATCA
ACATTATAGGCACAGCTTGAAGTATGTAGCCGGCAGCCCGGACAATGACGACTTAATAATGCTTACTGAC
AACATAAACAAACTTATAGAAAACGACAATAAACAGCTTATCATAAACAGGCAATTAGAAGATCGAATT
AATAAACTCACGAAAATCTCAAACGATTTAACCAATAGCATAAGAAAGGAATCTCTCATAAGTGATGAGA
TAGCTATTAGCTTACAAAATCAAATTAGGCTCATAAAAGAAGAAATAATTAATATTAAGTACGCCATACA
ATGGGCGAAATTGAATTTAATAAATACATTCCTACTTGGCGAAAAAGAAATAGAGATAGTAAACGATAAC
TTTAATAAAAGCAACATGCCACCTTTAAATATTGAAGAAATGCTAGAATTCGCAGATGTATCCATTTTGCA
TAATGGAACCACTCTGTTTTATATTGTAAAAATTCCAAATTTAGAAGAAATAGAATGCAAAAATACTATA
ATAAAGCCAGTAGTGAAAAACAACAATATAATAAATTTAGAATTTAACGAACTGTTTCATTGTAATAAAA
CAATCTATGGCATAAACAACAATTGTAAAAGATACCATAATATTAAAATTTGTAACAAATCAAAAATTGT
AAACTTGAACAATAATAATTGTATACCTAAGTTAGTTAGCGGAGAGCAAGCGCTCTGTGGCTTTAGCAAT
GCAGATCATATCCCGGAGATCGAGGAAATCGACGAGGGACTTATACTAGTGAACGACTTCAACGGCACTA
TTAGATGGCAAGAAGAGAAGAAACGCGTGAGAGGCACCTACATGATTAACTTCTGAACGAAACCTTATTC
ATCAACAAACAAGAGTTCAGCAACCTAGAGGTCACCGTCATGACCCCAGGAAATCCCACAATACAAATA
ACCCCAATAGAAAACGAAAGAATAAAGATTCTTTCTCTGGAAGCATTAGAAGAATTGCACATCAACAACA
TTAAAAACATGCAAAACTTCAGACGCACTCTATAGCAAATAGAGTCGGTTTCATAACTCTGTTCACAGTTG
CCATCCTTCTTACCTGTGTATTACACGTAATGAAGAAAAATAAGAAAAATATAATACTGCAACTGAACAA
AGAACAAGAGAGACCCAAAAAGAACCAGCACATATCCAAATACAAAATACAGATGCCAACAACCTCAGG
TTCAACAACATACCTTTCTTCTGATGATCGCTTGAGGACAAGCTTAAATCAAAGGGGAAGAGTTAACAAC
AAATAAATTCATACAGTCCCAATAAGAAGCATTGCAACACCTCATAAGAAGTGCAGCAAAATGCTGACTT
GGTGTCTGTGCCATTTCCTCCACGCGCCGACTGGCAACCGCCGAAAATTCCAAGAAGAATTGCCCAACGG
GCCAACAACATCAGCAGCGACGCCGCTGCCAACGCCGCTGCCGCTAAGCCACAGGTTACTAGGGCTAGAA
TTGATTTAAGTTTATTCAGTTGCAAATTAACATCAAATAAAGGAAGTCTTGCTTCCCTAAAAATTATATTTT
TTTCATCGTTTAATAAACTTTGGAATTAACTTATACACCAAGTGCGAAAAACAATTTCACTAACCGCGGAT
GCCAGTAATATTGNCATACGAGNAGTGTTATCACAAGAGCATAAACCGATCCATGCAAGCAGAACACTGA
ACGAACACGAAATAAATAGAAATAGAAT
>9_rnd-1_family-257__LTR___Gypsy

TGTGAGGCGGCTAATGAGACCAGCCACCCCACAGTTTTCACGGCAAGGAGCCGTCATCCCTTTGGCCAAA
TTACGGCAAGGAGCCGCTGGCGGGACAACGTATTAATTTTCTTTAGTTATAAGTATTAATTATATTAGGTT
AAGATCTTAGTGAAATAAGAGGCAAGGAGCCATCAGTAATATAAGCAGTTCGATTAGTAGCAAAGAGGC
AAGGAGCCGCGAATATTATAATTGATTTAAATAGTGAGTTTGAATTTAACCGCCGGCCGCCGCTGCAACC
GTGCGTTGTCGATCGGTTGCTCAACACCGTCTTTGAGAGCGCAACTTCAATTCGGCGGCGAAGAGCGAAA
GCTCCGAGGCGAAGTTGCGAAAGCTCGCGGCGAAGTTGAGCAGCCGAAGCGAGCGCATGGCTTGCCGGC
GAGCGTGCCGGCACTTGTGATCGAACGTTAAGGGTGAGTAGAAGTGCTGCGCGAGATAAACGAAATCAT
AGATGGTATAGTGGAGCGCTAGGGCTCCAGAAAACAGTGCGGCAGGGTTTAGCTTAGGATTTACGATAAG
GTCGGAATGAATAATAGTGAATAAAGTGCCAACGTTGTAATTATCTAGAAAGTATTGGATTATTCCCTGTT
TCCTGGTAACCCGGCTTACCTTAACATCGGAACTAACCGGTGTTCGACCTAACCTCGAACGAGGGGCATG
CGGTACGAGATTGTGGCTCCGGGGTGGGCATTGCCGGTTGGTGTTTAGGTAGTGGAGTTGGTGCGAGGCC
ACGGCCCGCTAGTGTAGTTGGTGCGAGGCCACGGCCCGCTAGCGTAGTGGTACGAGGCCACGGCCCGGCA
GAGAGATAGGGCGTTTAGGTAGTGGAGTTGGTGCGAGGCCACGGCCCGCTAGTGTGGTTGGNGCGAGGC
CACGGCCCGCTAGCATAGATGGTACGAGGCCACGGCCCGATGGTGTAGGTGTGTAGAGGTAGATCTGGGT
TTGGTTTGGTCAGGATACTCNCCCCCTTTAAGCCCTCTCTCCCAGCGAGAAATTATAAATAACTTGTAACT
CGCTAGACCTGGTAAGACTGAGAATTATACCCCGGCCAGAAGCTTCCTTACAAGTGGCGCCCGAGCAGGG
ACCAAGAGGCGTTTAGGGGTGTGGCATGTCGGCGGATTAAAGCCCGCAAGCATGCCCGAAATAGGTATTT
TCCAAAAAAAAATATATATAGGGTATTTCTAGTCGGGTATACCCTAGAGGGAAGGTAACCTAGCAGAGAG
CAAGAAAATGAGAAGGTCATGTATGTGCGAAATATGAAAATAGGGTATTAAGTAGTCCGGTATACCCCAG
AGAACATTTCTATACCTTGTGTTAGGCATAGCCAGAAAATGAACACGTGCAGCTGGTGAGTGACAAAAAT
GCTAGCAGCAAGCCGGCAAGTAAGATAAAGAGACGAAAACATAACGGTCGGTCGTGAGAGTACAGCGCG
CGCGCGCCAGCCGGGCGACGTCGAGACCACGCTCGCACACGGGTGCCCACACGCGCGGCGCAGTCGACG
TCGGCGTCGAAGGACCGTTAAGGCGTCGCGGCAGCGGTTGTACAGTGGGGCGAATCCAACTCACGCGCGG
TGAAAAGTTGAATTCGTGAGTACAGCTGTCGGGGGCAGTGAAGTGGTCACCCTTCAAGTGAATGTGAACA
AAGGGAACAATAAGAGCGGAACAAGTGCCAAATAAAAAAAAAGTGGATAATAATAAAATTTTCTTTTTA
TAAATAAAATTAATTAAACAAAAAAAAATAAAAAAANAAGTGGATAATAATAAAATTTTTTTTTATAAAT
AAAATTAATTAAACAAAAAAATAAATAAAAAAATAAGTGAATAATAATAAAAATTTTCTTTTAANAAAA
AAAAATTAATTAAACAAAAAAATAAATAAAAAAATGAGTGGATAATAATAAAAATTAAAAATAAACCGA
GGTTCACAATAAAGATAAAGGCAGCGACCACATAAGGAACAGCCTAAAAAGCGGAAAAAGTGAGAAAA
GAAAATTTTTGAAATACCTTTTGTTGAGTTTTTTTATAAAAAAGGGGGTAAATACTTTTGTGGAATATTGTT
TTGTATTTTTGTTTGGTGAAAGATATAAAGACAAAAAAAAAAAAAAAAAAAAAAAAAAAAAAAAAAAGGAA
CCAAAGTGGCTGAGCAAGATCGGCGTAAAAGTGAAAAAGACACCAACACCAAGAAAAGGAGAGGAATA
GTAAAGGGACCGACGGAACCCCTAGAACGGGAAGAGAGGGAAACAGCGACGTCGACTAGCAAGGAAGT
ATACAATAAAGGATGGAGGCAGGTGAGTGCCAAAACCCGGGGGTAGGCGTCCGCTGGATTCACCAGCGA
CGCAAAGAGGAGTTAGTGGCGATCGCCCAGGAGTTTGGGCTGGAAAAGGAGGGGCGCGTGGAGGAGCTA
CGTAAGCGGCTGTCCACCTACGTGCAGGAGGGCGGTCATTCGACTGGGATTTGGGCTCGGCTCGCCGAGC
TGGAATCCCAGTATGCTAAAACTCTTGAGCCGCCATCTTGGGGCGTCCCACCCGACTTTGGTGGACGGCCA
CGGCAAAAGTCGGAACCTGACCTCAGAAGCTCCCAGCTGTACGGGCTTTTGCAGGCGCCCACCCCGAGTT
TGACCGTCTCGGGCGTGGAGGCAGTTCCTGCTCTCGGTGGAAATGAAGACCGCCGAGCAACTGCGGTCAA
CGCAGAGGTGGGGAGGTCCCCTGTTGAACCGGGTGGTGGTCCCTGCTACACTAGGCCGCACCATCCATCG
GAGGCGAGGCCACTTCCCAATGTCCCGGAGCAAGTTCGGAAGTGGGGTATGAGGTACGATGGACGCTCCG
ACCCCCTTGGGTTCTTAGAGGACCTCGAGGAGCGCGCTATCACCTATGGTATAGCCCTCGACCGTATGCCC
AGGGCAATGAGCGAGGTATTCTGCGATAGGGCTGCTCGTTGGTTTCTGAGCAGTGGGCTAAGGGATGTGC
CGTGGGCCGTGTTTAGGAGGGATTTCTTGGAGTTTTTCTTGCCCCCTCGTTACTTCGAGAGGCTTGAGGAC
GAGATCCGATCCCGGAGGCAGCGCGTAGGTGAAGCGTTCAAGGAATATTTGATAGAGCTGAGGGCTCTTA
TGCACCACGCTAGATACGAGCCGGCACAAGAGCTGCATCGGATCTACGAGAACGCCGCTCCGGAATACA
GGCTCTATGTGCGACGGCAAGACTTTTCTACCCTATCCCAGCTAACTCAACTGGCCGTAGAATACGAGACC
GTCGTGAGGCAGAGGGCGGAGTACTCAGGCAGCCAACCCTTGGGTCAGGGTGTCTCTAGTTCAAATAGTG
CAGAGTGGGCGAGGACGGCGCAGTCCTCGGGACATCTCAATCCGTTCCGGTCTGCTCCCAGCGGATCCGG
GACGACCGTTGTGGACAACAGGGTCTCCACCCCACCCGCTGCAACTCACGCAGAGGTGGCCCATCAGATG
ATGAGCCAAGAGGCCGGGAACGGATATGATCCGCGACGGGCGTGTCGTAGGTGCGCACAGGAGGGGCAT
TTTGCCAGGGAATGTCCCAACGCCCCTGTCTTATTTTGTTGGGTATGTGGAAGCCGGGGCCGAAGTAGTCG
GGAGTGTTGCCGCCGCGCGGAGACGGGAAACGGGCAACGGCGTCGTCCGGACCAGGATCGGGTCGGCGC
CAATACACGCGGAACGCAGCAATAGGACACCCACTGAAGGTGGACGGTGGCCGGATCGTCGCTACGGTA

GAGATTGGCGGACGAACGATGCCCGCCACGATCGATACCGGAGCCACTCGTAGCTTCATGAGTGAGGACT
GCGTACGCAAATGGACGATCCGGGGAAGAGCGGAGGAAATACAGGCACGTATCCGCCTGGCAGATGGGT
CCACCCTAGAGGTAGAAAAGGCTTTGAGAGTAGACGCCAGCTTAGGGGGAAAAACGATCAGCATGACGA
TGCTCATCATGCCCTCAATGCTGGACCATGTAATCCTGGGTATGGATTTTTGGGGGCGATGAATACCACAA
TACGCTGCGGCGAGGCGGAACTGGTCCTAGAAGCAGCGACGACACCGGGCGTGAGGCCAGGAACGAGCG
AGGAGTCGGAGGAAGGCCNCCCAAGGCATGAAAAGGGAGCACCTGCTCGGCTACAGAGGGAGCCGGAG
GAAGGCCTCCCAAGGCATGGAAAGGGAGCACCTGCTCGGCTACAGAGGGAGCCGGAGGAAGGCCTCCCA
AGGCATGGAAGGGGGCACCTGCTCGGCTACAGAGGGAGCCGGAGGAAGGCCTCCCAAGGCATGGAAAGG
GAGCACCTGCTCGGCTACAGAGGGAGCCGGAGGAAGGCCTCCCAAGGCATGGAAAGGGAGCACCTGCTC
GGCTACAGAGGGAGCCGGAGGAAGGCCTCCCAAGTCATGANGGGGGAGCACCTGCTCGGCTACAGAGGG
AGCCGGAGGAAGGCCTCCCAAGTCATGAGGGGGGGNACCTGCTCGGCCTTCGACGGAGGGAGCCGGGAA
AAGGGGAGTAAAGTCCGGCCCGAATCGACAGCCTGGAGAACCCAAGAGGTGGCGCATAGAGCTGCAGAG
GCAGACTTCTAATGGCTACGGAAGCGGCACGGCGGGAACGTGCAAGAGAAAGGACGGAGTAGGAGCGAT
GACCAGAATCCACACGGACATAGAAGGCCAGGAGCGATCGCCAGAATCGAGCGGCCACGGAGTTCTATC
TGGAATCGAAATGGTCCCAGGATGGGGCGATAACGGCATCGAAGAGGCGGATTGGCCAGCGGGTCTGGA
CCCAGAACTGAGGGAGTTCCTGGAGTCAGAGCTGGCTCTCTTCGAGGGGCTGCAGGGAATCTCGCACATC
GCGGAGCACCGAATCCGGCTGAAGGATGACAAGCCGTTGAAACAAAGGTACTACCCCAAGAATCCGGCC
ATGCAGAGGGTGATCGACGAGCAAGTGGATGAGTTGATCCGGGCAAATGCCATCGAGCCGTCGCGTAGTC
CGCACAGCGCCCCGATCGTCTTGGTCAAGAAGAAGACTGGCGAGTGGCGTATGTGCATTGACTACCGACA
ACTCAACGCGCATTCCATTCCGGACGCTTATCCGGTGCCTCGTATTAACCACATCCTGGAGCGGTTGCGCC
ACGCCCGGTTCATCTCCACTCTGGATTTGAAGCACGGGTACTGGCAGATACCGATGGCAGCCGATAGCCG
GGAGTGTACGGCGTTCACAGTCCCTGGAAGAGGTCTTTTCCAATGGCGGGTGATGCCATTCGGCTTACACT
CGGCGTGTGCGACATTCCAGCGCGCTTTGGATACGGTAATAGGGCCGCAGATGGAGCCTCATGCGTTCGC
TTACCTAGACGACATCGTGGTAATAGGAGCGACGAAAACACAGCATGTAGAGAATTTGAGGGAGGTATTC
CGCCGGCTAAGGGCGGCCAATCTCCGGTTAAATAGAAAAAAATGCAGCTTCTTCCGGGAGAGGTTAGTCT
ACCTAGGACATGTAATTTGCGGAGAAGGGATACGGACCGATCCGGAGAAAGTGGAGGCGATACGAAACC
CGAGGGCCCCAACAAATTGCAAAGAACTGCGACAGTGTCTCGGGATGGCGTCATGGTACAGGCGTTTCGT
ACCCAATTTCGCGACCCAGGTACAGCCACTCACAGCGCTCTTAAAGAAGGGGCAGAAATGGGCATGGGA
GCAAGAACAGGAGGAGGCATTGCGTCAATTGAAGGAGAGCTTGACGACTGCCCCTGTGTTGGCTTGCCCC
GACTTTTCCGCCAAGTTCGTCTTGCAGACTGACGCGAGTGAGTATGGCCTCGGTGCAGTGCTGACGCAGA
CGATCGATGGACAAGAGCGGGTGATAGCATACGCGAGCCGAAAGTTGCTGAAGGCCGAGGTAAACTATT
CCGCCACTGAGAAAGAGTGCCTGGCAATCGTGTGGTCGATCCGGAAGTTGCGTTGCTACCTAGAAGGGTA
CCGATTCGACGTAATAACGGACCATCTAGCGCTCAAGTGGCTGAATTCGATCGAGAGCCCGTCAGGAAGG
ATTGCGCGATGGGCATTGGAACTGCAGCAATTCCAATTTGACGTTCATTATCGTCGAGGGAAGCTCAACG
TCGTGGCCGATGCCCTGTCGAGGCATCCTCTAGAGACCTGTCAACAAGCAGTGGTGGCCGAGGCCCCCTG
CAAATGGGTCGCGAGGATGCGTGTTAGAATCGCCGAAGAGCCAGCCAAGTTCCCGGATTACACCGAGGA
AAACGGACAGCTATATAGGCATTTGGGTCACCGGATAGACGATGAGGACTACATCCCTTGGAAACTATGC
GTAGGCAGTGGTCAACGCCAGAGGGTACTGCGGGAATGTCATGATGCTCCCACGGCCGGACATCAAGGTG
TCAGAAAAACGGTGGCGAGGTTAGCCCAGCGGTACTACTGGCCGGGTATGTTCCGCGATGCCGCCAGATA
CGTGAAATGTTGCGAAGACTGCCAAAAATTCAAGAGTGAGCAACTCAAACCGGCTGGTCGCATGCTGACG
AGGCAGGTGGCAGAGCCGATGGCGGTACTTTGCGCGGACTTCGTCGGACCCCTTCCACGGTCGAAACATG
GAAACACGATGCTATTGGTGTTCCATGACGCTTTCTCGAAATGGGTGGAGTTGGTCCCTCTCAGAAGAGCC
ACGGCAGCACACCTGCAAACTGCGTTTCGAGAGAGGATTCTAAGTAGGTTCGGGGTACCTAGAAAGTTTG
TGTGCGACAACGGGGTACAATTTACGAGTCGGAGCTTCAAGTCATTTTTGGGGTCCCTGGGTGTAGAGAT
CCAGTATACCGCCCCGTACTGTCCGCAAGAGAATCCCACTGAGAGGACTAACCGGACTGTAAAGACCATG
ATAGCGCAATTTATTGAGGGCCACCAGAGTTCATGGGATGAACTCTTGCCCGAGATCTCGCTGGCGGTCA
ACACGAGCGTGGCAGATTCAACGGGCTTTAGTCCCGCTTTCCTGATGCAGGGCAGGGAACCTCGGTTACC
CGCAGCTCTGTACGATCAGGTAACTCCGGGGTCCGCAACTAGTGCGCTCGACCCTAGGACAAAGGCGGAT
AGGCTAAAGAAATTTTTGATATCGTCCACAGCAACCTCCAGCGCGCATCAAAGGACCAGGGCAGGCATTA
CAACTTGCGGAGACGAGAGTGGCGGCCCTCGTTGGACTCGATGGTCCTGTTGAGGCAGCACCAGTTGTCC
AACGCTGCCGAAGGTTTCGCCGCAAAGTTGGCACCCGAATTTGACGGCCCCTACAAAGTCGTCAAATTCA
TATCCCCAATATTGTGCGGCTGGTGAAATCAGACGACCGCAAGAAGAGGGTGGCCAACATCTCACAATTG
AAACCATTCTACAGAAAGACGACGAGCGACGAAGAAGACGAAGATCCAGGGCACGCAAATGCCCACGAG
TAAGCGAAGGATGAGAGCAACACCCGTCGCCCAACGAAACTATTATGATTAAGCTTAGATAAAACTAACA

TTAACAACATAATTCAGAATAGGCATAGTAAAGTGTGAGCCGCACTGAGAGAATA
>160_rnd-3_family-200__Unknown
TTTGGAANGTCATATCTCCGCGGGGAGTTATGTCGTTTTGGAACGTCATATCTCCGCGCGGAGTTATGTCG
TTTTGGAACGTCATATCTCCGCGNGGAGTTATGTCGTTTTGGAACGTCATATCTCCGCGNGGAGTTATGTC
GTTTTGGAACGTCATATCTCCGCGNGGAGTTATGTCGTTTTGGAACGTCATATCTCCGCGGGGAGTTATGT
CGTTTTGGAACGTCATATCTCCGCGCGGAGTTATGTCGTTTTGGAACGTCATATCTCCGCGCGGAGCTATT
ACCCGAGATATTACAAAAAAANAATCATCGAAAAAGTTTCGATTTTCGCACCGATCTCCATTTTGTCGATT
TGGGTCAAAATTTTGGATTTCCTTTTTTTGATGCCAATCGATAGAACTGATCCTTACGAGTCAAAAATGGT
ATAAAATTCCTAAATCGGACATTATTTGACGGAAATATTCCAAAATATCATCAAAAAGGTTGACTTTACG
AACTAATGGTTTTTTTGTCTTNAACATTTTTGAACCCATCGATGATTGATTTCTTTGAATGCCAATTGATGG
TAGGGATCCGTACGCATTCAAAAAGGTATAACATTTTAAAATCAGACATTATTTACCCGAGATATTAAAA
AAAAATCATCGAAAAAGTTTCGATTTTCGCAAAAAAACGTGGTGATTTGGAAGGTCATATCTCCGCGCGG
AGTTATGTCGTTTTGGAACGTCATATCTCCGCGCGGAGTTATGTCGTTTTGGAACGTCATATCTCCGCGCG
GAGTTATGTCGTTTTGGAACGTCATATCTCCGCGCGGAGTTATGTCGTTTTGGAACGTCATATCTCCGCGC
GGAGTTATGTCGTTTTGGAACGTCATATCTCCGCGCGGAGTTATGTCGTTTTGGAACGTCATATCTCCGCG
CGGAACTATTACCCGAGATATTACAAAAAAAAATCATCGAAAAAGTTTCGATTTTCGCACCGATCTCCATTT
TGTCGATTTGGGTCAAAATTTTGGATTTCCTTTTTTTGATGCCAATCGATAGAACTGATCCTTACGAGTCAA
AAATGGTATAAAATTCCTAAATCGGACATTATTTGACGGAAATATTCCAAAATATCATCAAAAAGGTTGA
CTTTACGAACTAATGGTTTTTTTGTCTTNAACATTTTTGAACCCATCGATGATTGATTTCTTTGAATGCCAA
TTGATGGTAGGGATCCGTACGCATTCAAAAAGGTATAACATTTTAAAATCAGACATTATTTACCCGAGAT
ATTAAAAAAAAATCATCGAAAAAGTTTCGATTTCGATTTCGNAAAAAAACGTGGTGTTTGGAAGGTCATA
TCTCCGCGGGGAGTTATGTCGTTTTGGAACGTCATATCTCCGCGCGGAGTTATGTCGTTTTGGAACGTCAT
ATCTCCGCGNGGAGTTATGTCGTTTTGGAACGTCATATCTCCGCGNGGAGTTATGTCGTTTTGGAACGTCA
TATCTCCGCGNGGAGTTATGTCGTTTTGGAACGTCATATCTCCGCGCGGAGTTATGTCGTTTCGGAACGTC
ATATCTCCGCGCGGAGCTATTACCCGAGATATTACAAAAAAAAATCATCGAAAAAGTTTCGACTTTCGCA
CCGATCGCCATTTTGTCGATTTGGGTCAAAATTTTGGATTTCCTTTTTTTGATGCCAATCGATAGAACTGAT
CCTTACGAGTCAAAAATGGTATAAAATTCCNAAATCGGACATTATTTGACGGAAATATTCCAAAAAATCA
TCAAAAAGGTTGAATTTACGAACNAATCGNTTTTTTGTCNNCAACATTTTTCAACCCATCGATGATTGATT
TNTTTGAATGCCAATTGATGGTAGGGATCCGTACGCATTCAAAAAGGTATAACATTTTAAAATCAGACATT
ATTTACCCGAGATATTAAAAAAAAATCATCGAAAAAGTTTCGATTTTCGCAAAAAAACGTGGTGATTTGG
AAGGTCATATCTCCGCGGGGAGTTATGTCGTTTTGGAACGTCATATCTCCGCGCGGAGTTATGTCGTTTTG
GAACGTCATATCTCCGCGAGGAGTTATGTCGTTTCGGAACGTCATATCTCCGCGCGGAGTTATGTCGTTTT
GGAACGTCATATCTCCGCGGGGAGTTATGTCGTTTTGGAACGTCATATCTCCGCGCGGAGTTATGTCGTTT
TGGAACGTCATATCTCCGCGCGGAGTTATGTCGTTTTGGAACGTCATATCTCCGCGCGGAGTTATGTCGTT
TCGGAACGTCATATCTCCGCGCGGAGTTATGTCGTTTCGGAACGTCATATCTCCGCGCGGAGATATGGCGA
TTCGGAAGGTCATATCTCCGCGGGGAGTTATGTCGTTTTGGAACGTCATATCTCCGCGCGGAGTTATGTCG
TTTTGGAACGTCATATCTCCGCGCGGAACTATTACCCGAGATATTACAAAAAAATAATCATCGAAAAAGA
CGCGATGGAACCNATCGCCATTTTGTCGATTTGGGTCAAAATTTTGGATTTCCTTTTTTTGATGCCAATCGN
TAGAACTGATCCTTACGAGTCAAAAATGGTATAAAATTCCAAAATCGGACATTATTTGACGGAGATATTC
CAAAAAATCATCGAAAAGGTTGACTTTACGAACGAATCGNTTTTTTGTCAACAACATTTTTGAACCCATCG
ATGATTGATTTNTTTGAATGCCAATCGATGGTAGGGATCCGTACGCATTCAAAAAGGTATAACATTTTAAA
ATCGGACATTATTTACCCGAGATATTAAAAAAAAATCATCGAAAAAGTTTCGATTTTCGCAAAAAAACGC
GGTGATTCGGAAGGTCATATCTCCGCGGGGAGTTATGTCGTTTTGGAACGTCATATCTCCGCGCGGAGTTA
TGTCGTTTTGGAACGTCATATCTCCGCGCGGAGTTATGTCGTTTTGGAACGTCATATCTCCGCGCGGAGTT
ATGTCGTTTTGGAACGTCATATCTCCGCGCGGAGTTATGTCGTTTTGGAACGTCATATCTCCGCGCGGAGT
TATGTCGTTTTGGAACGTCATATCTCCGCGCGGAGTTATGTCGTTTCGGAACGTCATATCTCCGCGCGGAG
CTATTACCCGAGATATTACAAAAAAAAATCATCGAAAAAGTTTCGATTTTCGCACCGACCTCTATTTTGAAA
AAAAACGTGATGTAACCCCTCGCCATTTTGTCGATTTGGGTCAAAATTTTGGATTTCCTTTTTTTGATGCCA
ATCGATAGAACTGATCCTTACGAGTCAAAAATGGTATAAAATTCCGAAATCGGACATTATTTGACGGAAA
TATTCCAAAAAATCATCAAAAAGGTTGAATTTACGAACGAATCGGTTTTTTGTCAACAACATTTTTCAACC
CATCGATGATTGATTTGTTTGAATGCCAATTGATGGTAGGGATCCGTACGCATTCAAAAAGGTATAACATT
TTAAAATCAGACATTATTTACCCGAGATATTAAAAAAAAATCATCGAAAAAGTTCGATTTTCGAAACCTCG
ATTTTCGCGAAAAAACGTCGTGATTTGGAAGGTCATATCTCCGCGNGGAGTTATGTCGTTTTGGAACGTCA
TATCTCCGCGCGGAGTTATGTCGTTTCGGAACGTCATATCTCCGCGCGGAGTTATGTCGTTTTGGAACGTC

ATATCTCCGCGCGGAGTTATGTCGTTTCGGAACGTCATATCTCCGCGCGGAGTTATGTCGTTTCGGAACGT
CATATCTCCGCGCGGAGNTATGNCGTTTTGGAACGTCATATCTCCGCGCGGAGTTATGTCGTTTTGGAACG
TCATATCTCCGCGCGGAGTTATGTCGTTTTGGAACGTCATATCTCCGCGCGGAGTTATGTCGTTTTGGAAC
GTCATATCTCCGCGCGGAGTTATGTCGTTTTGGAACGTCATATCTCCGCGCGGAGTTATGTCGTTTTGGAA
CGTCATATCTCCGCGCGGAGCTATTACTCGAGATATNAAAAAAAAAATCATCGAAAAAGTTTCGATTTTCG
CACCGACCCATTTTGAAAAAAAGCGTGATGTAACCCCTTGCCATTTTGTCGATTTGGGTCAAAATTTTGGA
TTTCCTTTTTTTGATGCCAATCGNTAGAACTGATCCTTACGAGTCAAAAATGGCATAAAATTCCAAAATCG
GACATTATTTGACGGAAATATTGCAAAAAATCATCAAAAAGGTTGANTTTACGAACGAATCGGTTTTTTGT
CAACAACATTTTTCAACCCATCGANGATTAATTTGTTTGAATGCCAATTGATGGTAGGGATCCGTACGCAT
TCAAAAAGGTATAACATTTTAAAATCAGACATTATTTACCCGAGATATTAAAAAAAAAATCATCGAAAAAG
TTTCGATTTTCGCACCGACCTCGATTTTGAGAAAAACGTGGTGATTTGGAAGGTCATATCTCCGCGNGGAG
TTATGTCGTTTTGGAACGTCATATCTCCGCGCGGAGTTATGTCGTTTTGGAACGTCATATCTCCGCGCGGA
GTTATGTCGTTTTGGAACGTCATATCTCCGCGCGGAGCTATGCCGTTTCGGAACGTCATATCTCCGCGCGG
AGTTATGTCGTTTCGGAACGTCATATCTCCGCGCGGAGTTATGTCGTTTCGGAACGTCATATCTCCGCGCG
GAGTTATGTCGTTTCGGAACGTCATATCTCCGCGCGGAGTTATGTCGTTTTGGAACGTCATATCTCCGCGC
GGAGTTATGTCGTTTTGGAACGTCATATCTCCGCGCGGAGTTATGTCGTTTTGGAACGTCATATCTCCGCG
CGGAGCTATTACCCGAGATATNAAAAAAAAAATCATCGAAAAAGTTTCGATTTTCGCACCGACCTCGATTT
TGAAAAAAAGCGTGATGTAACCCCTTGCCATTTTGTCGATTTGGGTCAAAATTTTGGATTTCCTTTTTTTGA
TGCCAATCGATAGAACTGATCCTTACGAGTCAAAAATGGTATAAAATTCCNAAATCGGACATTATTTGAC
GGAAATATTCCAAAANATCATCAAAAAGGTTGANTTTACGAACGAATCGGTTTTTTGTCAACAACATTTTT
CAACCCATCGATGATTAATTTGTTTGAATGCCAATTGATGGTAGGGATCCGTACGCATTCAAAAAGGTATA
ACATTTTAAAATCAGACATTATTTACCCGAGATATTAAAAAAAAAATCATCGAAAAAGTTTCGATTTTCGCA
CCGACCTCGATTTCGAAAAAACGTGGCGATTTGGAAGGTCATATCTCCGCGNGGAGTTATGTCGTTTTGGA
ACGTCATATCTCCGCGCGGAGTTATGTCGTTTTGGAACGTCATATCTCCGCGCGGAGTTATGTCGTTTTGG
AACGTCATATCTCCGCGGGAGNNATGTCGTTTTGGAACGTCATATCTCCGCGCGGAGTTATGTCGTTTTGG
AACGTCATATCTCCGCGNGGAGTTATGTCGTTTTGGAACGTCATATCTCCGCGCGGAGTTATGTCGTTTTG
GAACGTCATATCTCCGCGCGGAGTTATGTCGTTTTGGAACGTCATATCTCCGCGCGGAGTTATGTCGTTTT
GGAACGTCATATCTCCGCGCGGAGTTATGTCGTTTTGGAACGTCATATCTCCGCGCGGAGTTATGTCGTTT
TGGAACGTCATATCTCCGCGCGGAGCTATTACCCGAGATATNAAAAAGAAATCATCGAAAAAGTTTCGAT
TTTCGCACCGACCTCNATTTTGAAAAAAAGCGTGATGTAACCCCTTGCCATTTTGTCGATTTGGGTCAAAA
TTTTGGATTTCCTTTTTTTGATGCCAATCGATAGAACTGATCCTTACGAGTCAAAAATGGTATAAAATTCC
AAAATCGGACATTATTTGACGGAAATATTCCAAAAAATCATCAAAAAGGTTGAATTTACGAACGAATCGG
TTTTTTGTCAACAACATTTTTCAACCCATCGATGATTAATTTGTTTGAATGCCAATTGATGGTAGGGATCCG
TACGCATTCAAAAAGGTATAACATTTTAAAATCAGACATTATTTACCCGAGATATTAAAAAAAAAATCATC
GAAAAAGTTTCGATTTTCGCGAAAAAACGTGGCGATTTGGAAGGTCATATCTCCGCGCGGAGTTATGTCG
TTTTGGAACGTCATATCTCCGCGCGGAGTTATGTCGTTTTGGAACGTCATATCTCCGCGCGGAGTTATGTC
GTTTTGGAACGTCATATCTCCGCGCGGAGTTATGTCGTTTTGGAACGTCATATCTCCGCGCGGAGTTATGT
CGTTTTGGAACGTCATATCTCCGCGCGGAGTTATGTCGTTTTGGAACGTCATATCTCCGCGCGGAGTTATG
TCGTTTTGGAACGTCATATCTCCGCGCGGAGTTATGTCGTTTTGGAACGTCATATCTCCGCGCGGAGTTAT
GTCGTTTTGGAACGTCATATCTCCGCGCGGAGTTATGTCGTTTTGGAACGTCATATCTCCGCGCGGAGTTA
TGTCGTTTTGGAACGTCATATCTCCGCGCGGAGTTATGTCGTTTTGGAACGTCATATCTCCGCGCGGAGTT
ATGTCGTTTTGGAACGTCATATCTCCGCGCGGAGTTATGTCGTTTTGGAACGTCATATCTCCGCGCGGAGC
TATTACCCGAGATATTAAAAAAAAAATCATCGAAAAAGTTTCGATTTTCGCACCGACCTCATTTTGAAAAA
AACGTGATGTACCCCCATTTTGTCGATTTGGGTCAAAATTTTGGATTTCCTTTTTTTGATGCCAATCGATA
GAACTGATCCTTACGAGTCAAAAATGGTATAAAATTCCNAAATCGGACATTATTTGACGGAAATATTCCA
AAANATCATCAAAAAGGTTGACTTTACGAACNAATCGGTTTTTTGTCNACAACATTTTTNAACCCATCGAT
GATTNATTTNTTTGAATGCCAATTGATGGTAGGGATCCGTACGCATTCAAAAAGGTATAACATTTTAAAAT
CAGACATTATTTACCCGAGATTATTAAAAAAAAATCATCGAAAAAGTNTCGATTTTCGAAAAAAAACGTGG
TGATTTGGAAGGTCATATCTCCGCGCGGAGTTATGTCGTTTTGGAACGTCATATCTCCGCGCGGAGTTATG
TCGTTTTGGAACGTCATATCTCCGCGCGGAGCTATGTCGTTTTGGAACGTCATATCTCCGCGCGGAGNTAT
TACAAAAAAACATCGAAAAAGTTTCGATTTTCGCACCGATCTCATTTAAAAAACGTGATTACCTCCATTTT
GTCGATTTGGGTCAAAATTTTGGATTTCCTTTTTTTGATGCCAATCGATAGAACTGATCCTTACGAGTCAA
AAATGGTATAAAATTCCTAAATCGGACATTATTTGACGGAAATATTCCAAAATATCATCAAAAAGGTTGA
CTTTACGAACTAATGGTTTTTTTGTCGANCATTTTTNAACCCATCGATGATTGATTTCTTTGAATGCCAATT

GATGGTANNNNNNNNNNNNTATTACTATTTTAGGGNCAACATATCATGTTTCGTGACTCTA
>160_rnd-5_family-470__LTR___Gypsy
ATATAAGTTAACAACAAATAAATTCATACAGTCCCAATAAGAAGCATTGCAACACCTCATAAGAAGTGCA
GCAAAATGCTGACTTGGTGTCTGTGCCATTTCCTCCACGCGCCGACTGGCAACCGCCGAAAATTCCAAGA
AGAATTGCCCAACGGGCCAACAACATCAGCAGCGACGCCGCTGCCAACGCCGCTGCCGCTAAGCCACAG
GTTACTAGGGCTAGAATTGATTTAAGTTTATTCAGTTGCAAATTAACATCAAATAAAGGAAGTCTTGCTTC
CCTAAAAATTATATTTTTTTCATCGTTTAATAAACTTTGGAATTAACTGGCGCCCGAGCAGGGACCGGCTC
GTGTTACAACAGAACAAAACGCTTCTACGTACGCGTTAGTCGGTTATACGCGAAAATAAAACCAGTGCAA
CTCTCGTCGATTAAATTCAACGATCGGTTAAATTCGCGAAAGGCAATTGGAACTTAATTGTTCAATTTTGC
CCATCGGTGGACAACGCATCTTGTAAGGCCCGCCCGCAGGATACTCTCAACAAAGACTGGTCGCAGGATC
CCCAGTAGATACCCAAAGGCTTCAAAAAGGATGAAATCCGGACCGGCAGGGGAGGTTGCCACATCAGGA
ATAGTACTGTAAGTTAGAAATTAAGTGAAACAANAAAAAAAAACAAACGAACAACTGAAATAGAAGTTA
TAAGAATAATAAATACTAAAAATTGTAGAACGATAAGCAAGATAAAAAAGTGTAAAACGATCAACAAAT
ATAAAGACTACGAACGTAGTATAAATTATTTCCAACTTTGCGACTCCCAAGGGACCCTCCAAAGTAGAAG
GAAGAAACCAGCCACTGGTTTTAAAAAGAAAACAAAACGATCAAAAATTCAAGTAAAATATAGAGTAAA
AAACTAACACTAGATTTAAATATACTTCGAAAATATCTTAAAATATACAATATAAGTAATATATCTCGTTT
ATGGCTAATGAAAGCAATTTAGTTAGGCCCCCAATAGTATCAGATCAACCACAGGTAGGCGGACATAACT
CGAACAGACAGCAACAACGACATATCGACAGACAAAACGAACATTTACAAAATAGAGTCATGGCGAATA
ACGCAGAACAGATAATAGCAGCCACGGTTAGCCAGATTTTGGCACAACAACTGCCGACATTGGTAAAGCA
GTTAGTCAACCCCTCTAGGGAGTTTAGTGACGTAGACGACCAAACAATAACTGCCAACCACAATTTGAAT
GACTTAGACAGAGTTCCGGACATAGTTAAAAGCCTCAGAGAATTCTCCGGTAATCCAGCAGAATTTAGCT
CATGGAAGAAGAGTGTAGATCGTATACTCTCAGCCTATGCAGCTGAGGCAGGTTCGCCAAAATACTTTGG
CATATTGCATACAGTGAGAAACAAAATCACTGGCAATGCCGACACGGCCCTCGAGTCGTATAATGTTCCC
CTAAATTGGAGCGCTATCTCCAAATGTCTCACGCTGCACTACGCAGACAAAAGAGATCTGGGAACATTAG
AATATCAGATGACAATGTTAGTGCAAGGTAACAACCAAACAGTAGAAGAATTTTACCAGGTCGTTTACAA
CCACTTGTCGCTGATATTAAATAAAGTAGGCTGCATGGAATTAAATAGGGAATCCGAAACCCTTATGACA
AAAATATACCGAGATAAAGCATTAGATACCTTCATTAGAGGTTTGAAGGGAGATTTACCACGGATTCTTG
GAATAAAGGAGCCAGCAGATCTACCTTCAGCCCTTCACTATTGTTTAAAACTAGAGAATCAAAGCTATCG
CACTAACATGGCTTTGAATAGAAATCAACAGACACATGCCAGAAACCCAAATAGAAATATACCACCACCT
ACACTGCCACCTCGAACAGCTCAGCCTTATTTCGCTCAAACTTTTAGGCCACCACCCATTAGCTTCAGGCC
CCAACTCCCGATCCATACACCTTGGCCAACCAATACAAACCATGGACCACCGACTTACAACAATAATGGA
CCGATTCCACAATATCCCAACCAACGAACCTACCAGACACAACCACAACAGGTATACATACCCAATAGAC
CTTCTGCACCAAAACCCCAACCACGTCCTGAGCCAATGGACGTGGACCAAAGCATCCGCACGAGGATGAT
AAACTACATGAACAGACCCAATTTTGAAGTAGGGAAAAGACCTCCACCACCAACAAACGCCACGGACAA
AAAACAAAGAAATTTCAACATACAAACAGGAGAAGAACAGACGGAGACAGAAAACACCGACAACTATG
TGACTATGGAGGAATACGTACAATCAATGCTAAATTACGAACAGCAGACTGAATACGAACAGCCATTAAA
CGAATACGCCCAACAAATTGAAGAAACAGATGACGGACAAAATACCCAATACGAACACTCAGACATTAA
TTTTTTAGGATAAAGAGCTCCTCTCTTCCTTACATAGAATGTAAGATGGGGAGCGGAAAATTACTCAAAAT
ACTAATAGATACAGGATCAAACCGAAATTACGTCCAACCAAAATTCGTGTCCGAGCCTATTCCAAACGAA
AAACCCTTTGAAGCCGTCACAGTCGGCGGAAGCATTAAAATTACACACCACAAGTTCGCTAATCTTTTTAA
TAGAAACGATATAAAAATAAAATTCTTTCTCTTACCCACCCTAAAATCATTTGACGCCATATTAGGTAACG
ATAGTCTAAAGGAACTAGATGCTGTGATACAAAGCAGCAAGAATCTACTAATAATCAAGAACAACATTTG
CATACCCATTAAGCAGAAAAAAAATAGAGGCGGTCAACACGGTAATACCACGGACAGAACATATGACAAA
CGAACAAAAAAATAGCCTTAATCAACTAGTTAAAATGCACCCACACCTATTTGCCAATCCGGACGAAAAA
CTGACATACACAACCAACGTCAGAGCAGAGATTCGAACAAAAAACGACTCCCCCATATATTCAAAATTTT
ACCAATTTCCAATGTCTTTAAAAGACGAAGTACACAGCCAAATCAAAGAACTTTTAGAGGACGGAATCAT
CCGACCCTCCCGATCCCCGTACAATTCACCTGTGTGGATTGTTCCGAAAAAATTAGATGCCTCCGGGCAAA
AGAAATATCGCATGGTTATAGACTACAGGAAACTAAATAAAGAAACTACCGCAGACAAATACCCTATACC
CGAAATCAACGAGGTACTAGCACAGCTAGGCAATAACAAAGTTTTCTCTGTCTTAGATTTGAAAAGTGGC
TTTCATCAAATACCCCTTAAGGAGTCAGACATAGAAAAAACTGCCTTCTCCGTTAACAACGGAAAATACG
AGTTTACACGACTCCCATTCGGTCTCAAGAATGCGCCCTCAATTTTCCAGAGAGCTCTTGACGACATCCTT
CGTGAGCATATTGGAAAAATATGCTTCATTTATATTGACGATATCATTATCTTTAGCAAAGATGATGAATC
GCATGCGCAGCACCTCGCCGCCATATTCAGAACTCTACAAGAAGCCAACATGAAATGTCAGTTGGACAAA
TGCGAGTTCTTTAAAAGCAGAGTAGAATTCCTGGGATTTGTCATCTCAGACAAGGGGATAGAAACTAATC

CAAACAAAATTGAGACAATAAGAAAGTACCCACTTCCAAAAACACTCAAAGATTTAAGATCGTTTATTGG
CCTTGCCAGCTATTATAGGAGGTTTATAAAAGACTTCGCTAAGCTGGCAAAGCCCCTAACCTCTCTTTTAA
GAGGGGAAGATGGACGCGTGTCCAAAAATCAATCAGCTAAAAAGCTCATTAATCTCAACGAAGAAGCGA
TCGAAGCATTCAATAAAATAAAAAATTCCCTAGTTTCGAATGAGGTAATACTTCATTACCCAGACTTCAGC
AAAGAATTTCATCTGACAACAGATGCATCCAACTTTGCTATTGGGGCTGTCCTATCCCAGAACAAGCGAC
CCATTTGTTTCCTCTCTAGAACTCTCTCTAAAACAGAGGAGAACTACGCCACCAATGAAAGGGAAATGTT
GGCAATAATTTGGGCCTTAAAAGCCCTCAAGAATTACCTCTACGGTAAAGCGAGTGTAAAAATATACACC
GACCATCAACCCTTAACCCACTCTCTTAGCAGTTGGAACGGCAACGCTAGGATAAAGAGGTGGAAAGCTT
ACCTAGAAGAGTTTGATTACGAACTCCTCTATAAGCCAGGTAGGGATAACATTGTAGCCGATGCGCTATC
ACGTATCCCTGTGATCAATAATATTGGATCAGTAGCCTCCACCCAGCACAGTGCAGACAGTTCAAGCCAC
AACTTAATTCCGAGCATAGAAGCTCCAATTAACGTATTCAAAAACCAGATATTTCTCAGTAGAGCCAACG
AAACAAATTACGAATTCCATATCCCATTCCCAACATTCCACAGGCATACAATTACTCGACCAAATTTCTCA
GAACACGACCTTACGCAAATACTGAAAGCTCATCTAAACCCATCCGTAATCAACGGAATAATAACTTCAG
AAGAAATAATGAACCAAATTCAGCAGATATATCCCCAACATTTTAGGAACTTCAAGATTAGATTCACACA
GTCTCAAGTAAAAGATTTAACAAATGAAACAGAACAAGATGAGAAGATATTGGTTACGCACAACAGAGC
CCACCGCAACGCACTAGAAAACAAAATGCAATTGTCCGAAAGATTCTATTTCCCAAAAATGAAGCAACGA
ATAACAGAATTAGTCAAACAGTGTCAGGTTTGTAAAGAAGAAAAATACGATAGGCATCCCACTAACCCAA
AACTCAAAGAAACACCAATCCCCGAATACCCAGGACATACAATCCACATCGACATATACACGACAGAAA
AAAACTTAGTACTAACAGCAGTAGACAAATTCTCGAAATTAGCTCAGGGACGAATTATCAAATCTAGGGC
AATAGAAGACATTAAAAAACCACTTCATGAAATCCTTTTTTATTTTGGCGTACCTAAAGTAGTTGTAATAG
ATAACGAAAAATCCCTTAATTCAGCCTCAATCACTTTCATGATGCAGGACCAATTAAATATAGAAGTTTAT
AAAACACCACCCTACAAAAGCGCAGTGAACGGTCAGGTAGAACGATTTCATTCAACACTATCAGACATAA
TGAGATGCAACAAAAGAAAACAGGAATACAGAACATTCGAAGAACTACTCAACAAATCAATATACGACT
ACAACTACACTATACACTCGACAACTAACAAGAGACCATTAGACATATTCTTCGGCAGAAACGTGACTAC
GGACCCAGACCAATACGAAAAAGCTAGGAAGGAGAACATTGACAGACTCAAACAGAAACAATCGACCGA
CATGCAGAATCATAACAAAAAACGTAACGACATACGAACTTACATACCTGGTCAAGAAATATTCGTCAAA
GTCAATACCAGATTAGGTACCAAACTGTCACCCAGATACAAAAAGGAAATAGTTAAAGAAAATAGAAAC
ACAACCGTACTAACAACAACTGACAAAATAATACATAAAAGCAATATAAAAAATACATAAAAATTACAG
ATTCCTATTAATGGATGACAATAAAAGCAACAGAAGGACACTTGGAAATTTTGAACTACACAGACGCACA
AACAATTACAATTCTAAAAGGAATAGGGAAACTACAGACATCGACAACACGAATCGTACAAGTCATAGA
GCTAGAACAGATTAAAAACGCATTAGATAATCTCCTTTTTTACACCGAAAAAGACTTAACAACCAATACT
TTATACCACACTTTACGTCACGAAATAACGCAAGCTATTGTCACTTTCGACACAATATCACTTTTCCATAA
ACGAAAGACTAGAGCGATCAACATTATAGGCACAGCTTGGAAGTATGTAGCCGGCAGCCCGGACAATGA
CGACTTAATAATGCTTACTGACAACATAAACAAACTTATAGAAAACGACAATAAACAGCTTATCATAAAC
AGGCAATTAGAAGATCGAATTAATAAACTCACGAAAATCTCAAACGATTTAACCAATAGCATAAGAAAG
GAATCTCTCATAAGTGATGAGATAGCTATTAGCTTACAAAATCAAATTAGGCTCATAAAAGAAGAAATAA
TTAATATTAAGTACGCCATACAATGGGCGAAATTGAATTTAATAAATACATTCCTACTTGGCGAAAAAGA
AATAGAGATAGTAAACGATAACTTTAATAAAAGCAACATGCCACCTTTAAATATTGAAGAAATGCTAGAA
TTCGCAGATGTATCCATTTTGCATAATGGAACCACTCTGTTTTATATTGTAAAAATTCCAAATTTAGAAGA
AATAGAATGCAAAAATACTATAATAAAGCCAGTAGTGAAAAACAACAATATAATAAATTTAGAATTTAAC
GAACTGTTTCATTGTAATAAAACAATCTATGGCATAAACAACAATTGTAAAAGATACCATAATATTAAAA
TTTGTAACAAATCAAAAATTGTAAACTTGAACAATAATAATTGTATACCTAAGTTAGTTAGCGGAGAGCA
AGCGCTCTGTGGCTTTAGCAATGCAGATCATATCCCGGAGATCGAGGAAATCGACGAGGGACTTATACTA
GTGAACGACTTCAACGGCACTATTAGATGGCAAGAAGAGAAGAAACGCGTGAGAGGCACCTACATGATT
AACTTCTGGAACGAAACCTTATTCATCAACAAACAAGAGTTCAGCAACCTAGAGGTCACCGTCATCAACA
AACAAGAGTTCAGCAACCTAGAGGTCACCGTCATCAACAAACAAGAGTTCAGCAACCTAGAGGTCACCGT
CATGACCCCAGGAAATCCCACAATACAAATAACCCCAATAGAAAACGAAAGAATAAAGATTCTTTCTCTG
GAAGCATTAGAAGAATTGCACATCAACAACATNAAAAACATGCAAAAACTTCAGACGCACTCTATAGCA
AATAGAGTCGGTTTCATAACTCTGTTCACAGTTGCCATCCTTCTTACCTGTGTATTACACGTAATGAAGAA
AAATAAGAAAAATATAATACTGCAACTGAACAAAGAACAAGAGAGACCCAAAAAAGAACCAGCACATAT
CCAAATACAAAATACAGATGCCAACAACCTCAGGTTCAACAACATACCTTTCTTCTGATGATCGCTTGAG
GACAAGCTTAAATCAAAGGGGGAAGAGTTAACAACAAATAAATTCATACAGTCCCAATAAGAAGCATTG
CAACACCTCATAAGAAGTGCAGCAAAATGCTGACTTGGTGTCTGTGCCATTTCCTCCACGCGCCGACTGGC
AACCGCCGAAAATTCCAAGAAGAATTGCCCAACGGGCCAACAACATCAGCAGCGACGCCGCTGCCAACG

CCGCTGCCGCTAAGCCACAGGTTACTAGGGCTAGAATTGATTTAAGTTTATTCAGTTGCAAATTAACATCA
AATAAAGGAAGTCTTGCTTCCCTAAAAATTATATTTTTTTCATCGTTTAATAAACTTTGGAATTAACTTATA
CA
>160_rnd-6_family-2343__LTR___Gypsy
ATATATAAGTTAACACTTAAGAATTGCACCAAAATAACGCTGTATAAGAAACTTGCACAATACTTATATT
GACCCATTTACGACCAAGCACATTCCGCTGACACATGAGTTCAGCGGCCTGACCGCAACGGTCAGCGGCG
GTAACCTGACACTTGACTATGCTGCAAGTTTCCGCGTTGTCGCTTATGCTGAGTCGGCTTGCCGACCGTAG
CTTTGTAGTTTATAGATCCAAGTTTTAGGTATAATTCCAATTCAATTTTATACCACAATTCAGTTTTGTATT
TGACGTTTAAGAATAAACTTGCACTACTCCGGCACGCTGCCGTAAATATCATTTGTTATATATTACAAAAA
CATTGCTGAGCCACAAGGCCAGTAATTAGCAAAGAGAGTGAAGTCAAACTCTCCACCATAATCGTCCTAA
ATATCACTGGACTAGGCATCGCTGGACGAGAGACGGATCAACTATCGCATCGCTGGACCAGCAGATCGAT
ATAATTGGCGCCCAACTCGTGGTAATAACGAAAGAAAATTACCCACGCGAGCGTTCGTTAATAACTGAAA
CACGCTAACAAATACATTACGTGATTGCTGACCTGCGTCAATACAGGCAGCAACAGTGCAACAACTTCGC
CCTCGTAAAGACACATTGGCAGAAGTGCACCGAGGCCACTGAAGCTATCTGGACAGCGAAGACTGCGGC
GTTGGAGCTTAATTCCCCTTTAGTAGTTGTCTTAGAATCATGTAAGTGGGCTTTTCCTTAACTCTTTTCAAG
TCAATTCAAAAGAAATTTAAAAAAAAAGCACATTATATTTTGCATTACCTATTACTCGATTATAAATCTAT
AAAGAAAAATAATAATAATAAGTTATACGGCATTGAGAGCTTTGATTGATAGATAAGCTAGATATTTGTC
ACTTGTATTTAAAGCAAATTGCAAGAACGCATATTTTGTCGGCTTGCTTCCCACTCTATTCGTTAGTTGTCT
TTACGTAACTTTGTAGTTAAACGCTCTGGCGTAACATGACACGCCAGCGTAGAGGAACTCACGCTTAATC
ATCAAAATATTGAATTGTTTATCTGTAGCGAAGGCATATGAGCTCTTCATTCTCACAATACAGAACGCCCC
GCCACCGTTCNAGCGACTCAGANTCCGACTGTGACACTCAGGCACCNTATTCGCGGACACAAGACTGGNN
CCCGCNGTTCCCCCAGCGCCTAGCAACATGGACGCNGANCAGCTCAGGGCAATAGTGCGGACGGCCATCN
CCAGCGCCTTAGCCGAGCAGGCCGCTGCAAACCAGCAGAGGGAGCAAGNGTTGACTCAAAGAATTAACG
AGNTGNCTGAACAGATAGCGGCNGCGCGCGTTGCCGCCCCACAAGTGCNAGCGTATGCGGCCATNGGAA
TNAGNAACGAGATACGCTGCGACGAGCCGCTAGACGCCGTCAAATGCTTGCCCGAGTTTGCNGGAGCACA
GGAATCTTATGTGTCGTGGCGGCAGGCAGCGCTGGCAGCCTACCAGATCTTCAGACCACATAATGGCAGT
TCACGCCACTATCAGGCGGTTATCATCATCAGAAGCAAGGTCAGGGGCCCCGCNGACGCGGTGCTGGCCT
CGTTTGGCACAGTATTGAACTTTGATGCGATNATAAATCGCCTCGATTTCACGTACAGTGACAAGCGACCC
ATCCATGTNATNGAACAGGAGTTAGGCACCTTGAGGCAGGGGGGCCTGTCGCTGCTTCAGTATTACGACG
AAGTAGAGAAGAAACTGACTCTNCTCACCAACAAGGTTAACATGTCATATGAGCCGGCTTTGGCAAAGGG
CCTATGCGAAAAATTCCGCGACGACGCGTTGCGCGTGTTCATATCGGGTCTCAAACGCAGCCTCACAGAC
GTGCTCTTCGCGGCTAAGCCAAAGGACTTGCCCTCGGCGCTAGCCCTCGCGCAGGAGGTCGAGTCNAACC
ATGAGAGGTACGCCTTCGCGGCTAACTNCGCNAGAAGCCNGGAGGAAAAAGAGCGCAGGCCGAGCGCNA
AAGCGCAGAGTAGCCAGCAGGGGGAGCATTCCCGCCGCACAGTCAAGCGGACAGAAGAACCCGCACTNC
AGCAAGCAGGCCAGGACGCAANGCCAGACCGACCAACGGAGCGGCAGGCAGCAGAATAACAATACGGC
ACCGGTGCCCATGGAGGTCGACCCTTCCNTNTCGAGGGTNCTGCAGCCCNCNCAGGCCCCNGCTTACGCG
AACAGGGAGCANCCCGCATCTGACCGCACAGGCGGTCAGAGAAATCAGCAGAGGTTGAACCACGTCGCG
CAGGGCGCGGGACAGGGGGCAGAGGCGTATGCCGATGCCGCCTCCAGCGCGGCGGCGCAAATCGACGAC
GATGCCGGTTATGACTCTGATGCCCTCAATTTTTTAGGAGCAAGTCCCTGCTACCCGTCATCAGACGAAGA
GTAGCGGGGACTCACATGCGATTCCTCATAGACACGGGCGCGGCCAAGAATTTCATTCGGCCACATGAAG
GGTTAAAGGNCGTCCGCCCGGTTGACAAGAGTCCCCCTTCAAAATTCATTCCATTCACGGCGAGACACAA
ATTACACACAAATGCTTTGTTTCGATGTTCGGGNCAAAGGCNACCTTCTTTATTCTGCCGGACTTGTCAAC
TTTTGATGGGATCGTAGGCCTTGACCTGCTAACGCAGGCAGCNGCCTCGCTTTGCCTAGCTTCAGGACAAC
TTAAGTGGGGTACGGAGGTCGAACAAATCACCTTCCACACGTGCGAAAACGTAAATTTCACAAANGTAGA
CTGTGCAGAGGCATCCCCATTAGCTAGAAGTTCTTTCTTGAAAATGCTAAAGACCAGACAAAAGGCCTTT
GCAGACCCAGACGAGGCGTTGCCGTATAACACATCAGTCGTGGCTACCATTAGGACCACNGATGAGCAAC
CGATCTATGCCAAGCTTTACCCATATCCAATGGGAGCAGCTGATTTCGTTAATAAAGAAATCAAGGATCT
GCTAAAAAATGATATAATTCAAAAGTCCGTCTCTCCCTACAATAACCCAATATGGGTAGTAGACAAGAAG
GGCACTGATGAAGCAGGCAATAGGAAAATGAGGTTGGTAATGGACTTTCGTAAACTTAACGAAAGAACC
GTGCCTGACAAATACCCTATGCCAAATATATCAATGATATTGGGGAATTTGGGGAGAGCCAAATTCTTTTC
AACATTAGACCTCAAGTCTGGTTATCACCAGATCATACTCGCTGAACGCGACCGTGAAAAAACCTCTTTCT
CTGTGAACGGGGGGAAATACGAGTTCAAAAGACTCCCCTTTGGATTAAAGAATGCAGCAAGCATCTTCCA
AAGAACCATCGACGACATACTGCGAGAGCAGATTGGCAAATCTTGCTATGTTTATGTCGATGACGTAATC
ATCTTTTCAGAAGATGAGGAATCCCACATTAAACACGTGGATTGGGTCCTAAAAAGCTTACACGAAGCAA

ACATGAGGGTCTCCGTCGAAAAGTCACGTTTTTTTAAGAAAAGCGTGAACTTTCTTGGTTTCATAGTCACC
AGCACTGGCGCCACGACGGATCCTGAAAAGGTTAGGGCAATACAGGAGTATCCAGAACCTAAAACACTG
TTCGAGGTCAGATCATTTCTAGGCCTCGCAAGTTATTACAGATGTTTCATTAGGGACTTCGCCGCAATAGC
AAGACCCATCTCGAATATCTTGAGAAGGGTGAAAATGGAACAGTCAGTAAGCATAGGTCCAGAAATATA
AAGGTTCAATTCTCTGAAGTGCAGCAAAGGGCGTTTCAGAAGTTGCGCGATATTTTGTCATCCGAGAATGT
AATGCTCAGGTACCCCGATTTTAAGAAGCCATTCGATCTAACGACCGATGCTTCGGCTCACGGCATAGGT
GCAGTTTTGTCTCAAGAGGGTCGTCCTATAACAATGATTTCAAGAACATTGAAGGACAGCGAGGTTAACT
ATGCAACAAATGAGAGAGAGTTATTGGCCATCGTATGGTCACTTGCCAAGTTAAGGCACTATTTGTATGG
GGTGAAAGACATTAATATCTTCACTGACCATCAACCCTTGACCTTCGCAGTTTCGGAATCCAATCCAAATG
CGAAGATTAAAAGGTGGAAAGCGCGCATTGACGAGTCTAATGCACGTATCTTCTATAAACCTGGCAAGGA
CAATCTGGTTGCGGATGCCTTGTCAAGACAGCAGCTCAACGCTGTAAATGAACAAGAAGTTGAGTCCTGC
GCAGCAACGATCCACAGTGAGGTATCTCTCACCAACACCATTGAGACAACAGACAAACCCTTGAATTGTT
TTCAAAACCAAATAGTGCTAGAAGAGGCGCGTTTCCCCTTAAAACGAAGTTTTATTCTTTTTGGAAATAAG
AAACGCTACTTGATCAGCTATACTTGCATCGAATCGCTGCTAGACGAGCTCGAAGATGTAGTTTCACCCAA
ATGCGTAAATGCTCTCCACTGTAATTTACACACGCTTGCAATGATACAAGATGCATTGGTTCGAAGATACC
CGGCCACAAAATTCTGGCACTGCAAAAATCGCGTAGTGGACGTATTTGCGGTTGGAGAAAGAAGGGAAA
TCCTCACAGCCGAGCATAATAGGGCCCATAGGTCTGCACAGGAAAATATTAAGCAGGTACTCTCGGAGTA
CTACTTTCCAAAGATGGCAAAAATAGCCAATGAAATAGTACAAAATTGCAAAACATGTGCTAGGGCTAAA
TATGATAGACACCCCAAAAAACAAGAGCTTGGGGAGACACCGATCCCCTCACATGTAGGAGAGATGTTAC
ACATAGACATTTTCTCCACTGACAGGAAATACTTTCTCACTTGCATCGACAAGTTCTCTAAGTTTGCTATTG
TCCAACCGGTCCCTTCAAGAACCATTGTGGATCTGAAGCCAGCTTTAATGCAACTAATGAATTTTTTTCCA
AAAGCCAGATCCATTTACTGTGACAATGAACCGTCATTGAATTCAGAGACCATCGTGACTNTGCTCGCAA
ACAACTATGGGGTCAGCATTTCAAATGCACCACCATTGCATAGCATCTCGAATGGACAGGTTGAGCGCTT
TCACAGCACTCTGTTAGAACTAGCCAGATGCCTAAAGATCGATAAAGGCATAAGTGACACCGTAGAAGTC
ATTTTACTGGCTACAACTGAATACAATAAGTCGATCCATTCGGTCATCGACNAGAGACCAGTCGACGTGG
TTCAGGCGCACCCAGACGAGCCACAAGAGAANATACAAGANAGGATCAGAGACGCCCAGACGGCGCTCA
GAGCTAGGGAGAACGCNAANCGGCAGAATAGGGTCTTCGACGTTGGTGACAAAGTTTTGGTCAAGTCCA
ACCGAAGGCTCGGGAACAAACTCACACCTTTATGTGATGAGAAGGCCGTNGAAGCAGACCTGGGGACCG
AGGTCCTCATTAGAGGGAGGGTGGTCCACAAGGACAACCTGAGGTGATCACCACGAGAATAAGGCCCTT
GAATTTTTTCTTACANCTTACCAATCTGTTTAGCCGCTTGGCATAGCTTCTTCCAATTAATTATAAGACTTA
GATATTAGCGTGTGCCCTCAGTTTAAGTTCTGGTGGTGGTGGGTTAAACAATATTAAAACACAAAAATAG
CTAAAATTCCCATTCCACAGGTTTGGACTNATTCTCATCTTGTCCCTGTCCCTGGCTTCGGCGCGCGTCACC
GATTACTCAAACGCCAGGTACATCCCNATNGTGGACGGNCGAATCCTGGTGTGGGAGGANTTCGCCTACG
TTAGGCACTCGGCGAATCTTTCAGAGTACGCGCGNGTGGTGGAGGAAACGGTCGGNTTGCTTGACATGTT
CCCGCAATCTCACATGAAGAAGCTCCTAAGNGTGGACGCGGCTCACCTGCAGGACCTGCTGGAAGCGTTG
GGCGTACACCATAGGGTGGCTAGAAGTTTGGATTTCCTAGGGTCCATGCTGAAAGTAGTAGCGGGACACC
CGATGCCAGCGATTTAGAAAGANTAAGATTCACTGAACGACAACTAATAAATGAAAACAATAGACAAAT
AGAGATNAACNCNAAAGTNCAAGACCAAGTTAATAAGCTNACAATCACTGTCAATCAAATTCTCAAGGCC
GCGAAAAACACACAAGTAGACACTGGNCATTTATATGAAACGCTATTGGCTAGAAATAGAATGCTGATGA
TGGAATTGCAAAATTTAATGCTTGCNATTACNCTTGCGAAAGCNAACATCGTAAGCCCGAACATTTTAGA
CCACNCAGATTTGAAATCAGTTTGGCTNGAGGAACCCACCGACACTCCCATAGCAGANCTTATGTCCGTT
GCGTCCGTNAAAGTACTACAATCNATTAACGCTNTACACTTTATNATTAAGTTCCCAAAAATTAAAATAGC
TTGCAGAAAAATCACGATTTATCCCGTCGTCCACAAAGACGTAATATTGCGATGACAATGGCNGAGTGCG
ATGGANACGTCCACGCNCTCGAGAATTGCGCCGCNTCGCCTGGAGCGACGTTTTGCCGACTNTCANCGGA
GAGCTCATGCGCCAGAGAGCTNCACGCCGGTGGCGCGGCNAACTGCGAAATCCAGCCGAGCAACCTGGA
GCCAATCACCCGGGTGGACGACGGAATNCTNATCATCAACGACGGGCCCGCAAAAGTTCGNGTTGACAA
CGGCACTGAGACCCGGGTCCAAGGCACGCATCTCATCACGTTCGNTGATCGGGCCGACGTNAACGGAACT
CTGTTCTTNAATCGCAACAACGTCCGGAATAGAGTCCCGGGNATNGCGAANTCTCCCCTGCTGAACATCA
CCGGCCAGCCGAGATGTNCTNAGTCTCCCNTACCTTCATCGNTTGAACGAACGTAATNTGGAGGCGATTC
ANCAGTTCAGGAAGTCAANGACGAAACCGGAAGCGCCGACCGAGCGTCGTGATAGCTTCTCGTTGGGAG
CTATNTGTTGCGCNATAATTTGCGTCGGNCTACACTTCGGCGGATCACCAAGAAGAGGAAGNCTGCAAGG
CGGCTGAGAGAGGTGATCGCCGGGATAGGGTCGGCCGAGGACGGCCATTAACTCGAAGGGGGAGCAGTT
AACAGAGGCCAACGACACCAGCTCCAGATGCTGATATAGCACTGAGCCGGATGTTCTTACGAGCTAACAG
GGGTCAGCAAAATGCAACATAACACCAGCCGCAGATGCTGACATAGCTCTGGGCCGGATGTTCTTGCACG

ACGGCGGCGCACGAGGCGCAAGCTAAGCATTCTAGCGGCCGTTGTGAAAGCACACAATAGCTTAGGTATT
TTTGATCAATCTTTTGTAAATTTAGTTTTTAATTCGACTTTCACTGCGGCACATCGGCCCAGCGACTTCGTG
TATAATTTAACTCACTCACTACGGCGTGTCGGCCTAGTGTTTTATAAATCATTATCAAAATAAAGTTCTAT
CAAAATAATCCGCGAGGACTTATTATTTAAATCCTAATTTATA
>9_rnd-5_family-541__LINE___R1
CGGAAGAGGAGCACCTTGCTACTGGTGGAATGAGCAAATCAGCGACCTAAGGAAGGTCTGCCTGCGTGCT
AGAAGGCGCTCTCAGCGTCAGGAGAAGCATTAAACGAAGCAAATCCAGCTGTTCCAGAAAAAATGCGAT
GAGGCCGATGTTACCGCGTGGGGCACGGCATACAAGGTCGTCACGAAGAAGATCCGCAGCCAGGTGACC
TGCCCTACCGCACTTGCCACTATAGTTGAGACACTGTTCCCCCAACACCCACGACTGGAAGTAGCCAACC
GCTCACTGAGCACGGAGCTAGTGCAGCAGGTGTCGGCAGACGACGTGTTAGCGGCAAGCAAAAAACTAG
GCGACAAAAAGGCTCCAGGACCAGATGGGGTGCCAAACAAGGCGCTAAAGTTAGCAATGCTAACAAAAC
CCGACGCCTTCGCCGAGACATATACTAGATGTCTACAGGACGGTGTATTCTCTAAGAGATGGAAGCGACA
GAAACTAATCCTTCTACCGAAAGGGGACAAGCCAATAGAGGAACCCTCGGGATACAGGCCAATCTGCCTA
CTGGACACGGCGGGAAAGGTACTGGAAAGGATTGTGTATGACCGACTACTTAGTGTCGTCGAAGAGAAA
GAGGCCCTGTCCAAACAGCAATACGGATTTCGCAAGGCAAGGTCTACGGTCGATGCAATTAAGGCGGTGG
TAGATATTGCCGCTAAAGCAATAGAGGGAACCAGATGGAGGTGGGGAGACAAGGAGTATTGCGCGGTGA
TTACGTTGGACATCCGCAATGCCTTTAACTCAGCAAGCTGGTCNCGTATACTGGAAGCCTTAGACCGAATA
GGGATACCGTCCTCCNTTTTCCGTATAATATCCAACTACCTGTCCGATCGGGTATTGAGTTATGATACGGA
TGACGGTGCTAGGAGTTACACGGTCTCAGCAGGGGTCCCACAGGGGTCGGTCCTGGGACCACTCCTGTGG
AACATAATGTACGACGAGGTGCTCAGAGTGCAATTGCCCCAGGGATGCAGAATCATAGGATTCGCGGATG
ACCTAGCTCTAGTAGTGGTGGGCAAAGAAATAGAAGACGTGGAAGCGGCTGCGAATGCAGCCATAAGGA
TCGTGGCCAGCTGGATGCGTGGCGCTAGCCTGGAGCTGGCAGGCCATAAAACAGAGGCGGTCCTCATCAC
AAGCCGCAAAATAGTCGAGTTTGCTAGATTTCAGGTAGACAGCGTTAGTATCGAGTCAAAGGCTTCCATT
AAGTATCTGGGGGTCATGTTAGACAACAGGCTTAGCTTTCGACCGCACGTAGATTACGTTAGCCAGAAAG
CAGCCAAAATGCAGGCGGCCCTTTCGAGGATGCTACCCAACATAGGAGGACCTAAAGCAGGTAGACGCC
TGCTCCTCGCGAGAGTTGTAGCCTCCGTGCTGCTCTATGCTGCACCGATATGGGCTTCAGCGGTTGCAAGC
CAGAAAGGCCTCCGAAGGAAGATGTCAATCCCCTATAGGCTCAGTGCTCTGCGCACCATTTCTGGATTCC
GGACGGTGTCGGACGAGGCAGCCACAGTATTGGCAGGAATGCTGCCAGTAGATATATACTGGCCAAGGA
AATGGAGAAAATTTACATGGCCCGAATAGCCACGGGAGACGCCGTCTCAATGGACGTCATCCGGTCGGAG
AGAGCAACATCAATGGCAACTTGGCAGGAGAGATGGAACACGGCGAACAAGGGGAGATGGACACATAA
ACTGATCCCCAATCTGGAGCAATGGATTAGGAGACGGAATGGCGAAATGAACTTCCACCTAACACAGTTT
TTCACTGGACACGGCGGGTATAGGAAGTATCTCCATAGGTTCAGGCATGAGGACTCTCCAGAGTGCCCGA
ACTGCCCAAATCTGGACGAAGACCCGGAACACGTGCTGTACCAATGCTCGAGGTACAGAGAGGTAGTGG
GCTCTGTACCTCCACCGGAGGGACTGGTAGCGTTCATGCTGGAAGGAGAGGACAAGTGGAGAAATATAA
GCCAGCTCATAATAGGCATCCAGAGGGATCTGAGACGATGCGAAAAGGAGCGACGACTAATGGATACTG
CCTAAAATAGGCAACCCATCCCCGCGAAGTAATACTTTGCGGTGATCCCGCGGGGAATGTGGTGTGTGAG
TGGGGGGTGAGTTTAGTACGTAGGTGCTGACGGAGTCAGCAAATCGTGCATACGGTTGGCCCGGGCCATC
AGTATCTTACGAAGATTTTCACCTCCGTGCCGTAAGCAAAAAAAAACACACACACACACGACTACGATCG
TTTTCATAGAGTGCGGACGCGATTTGTAACTGCTCCGTGAAAATAAACAAATAAACCTGTGAAATTAAAG
TGAAGTTAGTGCACGTGTGCAGGGAACCGAGACAGGCCTAAGACACCAAAAGGTGCGTGGTGGTACTCA
CTGCGAAGCCCGTGCAGACCATTCCTGGGGTGGCGTCTACAGTTCCACGATCCAGGCGAGGGGAGTGGAG
GCGTCTACTTGGCGTGTTTGTGCGCTTTTGTGAGTTTTACCACTCAATTGTATTAGCCGTCTCGCAAAGCTA
TTTGTGCAGTTGGTTGCATTCACTCTAACAGCTGTTAGTAGTGTTGGCTAGCGCAGTTCACAACCAGCTGA
TCTGCACTATAGAGCCAGCTGCCAAACTGCGTAGCGGAGATACCAACCAGGTGATAAGCTGTAACTGGCA
GAAAGTACAGCATACTTTCGGGCTTACGCTGTTCTTGCTAAGCATTTAAGCTTTGGAGAAAGAACAGGCA
AAGAAGCCGATATCGGTGCTCACTACAGGCTCCAGGGCAAAAAGGAGCAAAAGGTCGCGGAACAGACCA
ATTTCCAACCCCCCGCCGCGGCGGTGCCCATAAAAACGGGGCTCTGGTGACCGAGTCGGAGCGGTATAAC
TCCCAGCGGTTAATAGCCAGAGGGAAACCGTTGGCCACCGAGGGACTTTCCTGTAGAGCCCTGATAGTTA
GAGCAGGTGCCCAGAGTGAAAAATGGAGGATGAGCTGCATGCCTTTGCGCGCCGCTCTGCACTACAGCGA
TCACCGTCACACCAGGCGGTCACGCCAGTGCAGCAAAGAGTGGCTGCGTCGTCGCCGGCAAAAGCGAGT
GAGGAGCTGCCAAGAACGCTAAGAGGCACGATTGAGAAGATGGGCTTAGTCCTAAACGGCTTAACGGCG
CTGTTCAACGGACAACGCCACATTAACGGGCTGATGCGAGAGGAACTCGCGAGAGTAGAAAGCCTGCAG
AAGGTGGCGCTCAAGCTCTGTGCGCCGGAAACGAAGTGCGACGAGGCGCAAACGACGCCGTCATTAGCC
AGACGTGCGGAGCCGAACGTGCTAAAGACGCGCCCAACGAAGCTCGGGGGCCAAAAAGAGGCAAGCAC

GCCCAAACGGCCGAGGGATGGCGTGCCAGAGCAGCGCAGAACCCCCTCCAAGAAGGCTAAACGCCAGGG
TCAAAACCCAGCGAAGCAGGCGCTGCTCACGCATCAAAAGACAGCCCCTGCAAACTCCAACTTAGCTGGC
CATAATGGCAACCCAGAGAGCTGGACAGCAGTGAACGGACGTAAGCGAACGCAGAAGCGTCCCAGAGCC
GACGCCATTACAGTCCACGGAGTGGGTGACTGCACATATGCCGATATGCTCGGGCTCGTGAAGAGCGACC
CGGCCCTCAAGCACCTAAGTGGCGATGTGCATGGCATTCGCAAGACCGCGAAGGGCGATCTTCTGCTGCG
CCTCAATAGGAAGCCGGAGCACTCCGCAGATGAGCTTCAAGAAGCTGTGGGAAAGGCATTGGGTGACCG
GGCGGTAGTAAGAACGCTGACCGAAACGTCCCAGGTGGAGATCAAGGACCTGGATGAGCTGGCGACTAA
GGAGGAAGTGCTCGAAGCGATTAAAAGTGCCTTACAAGACGACTCCTTGCCGATTGAAACGATCAAATCG
CTGCGACCAGCATTTGGCGGCCAGCAAAGGGCCACAGTTACTCTCCCAGCGCCAAAAGCCAGGACGCTTG
TCGAGAAGGCCAAGGTGAGGATCGGATGGGTTGTCTGCCGTATTCGTGCAATAGACCAACCAAAGCGATG
CTTTAAATGCCAGGGTTTTGGGCACATTGCGGCGAACTGCACCAGCGGAGACGATATGAAAGGCGCATGC
TTCAGATGCGGGGACACAGGTCACGAAGCAAAGAGCTGCTCCAAGCCACCAAAATGTAGCCTTTGCATAC
GGAAAGGAGAAAAATCAACGGACCACGCTACAGGAAGCTTTAAATGTCCGGCGTATCGCGAGGCAGTAC
GCAAAATGAAATGTTAAGGTTACTCCAAATAAACCTTAACCACTGCATAGCTGCTCAAGCCATCTTGGAG
CAGACTGTTAGGGAGATGCGCATTGATATTGCCATTATCAGCGAACCCCACCACCGAGAAACCAACTGCC
CGAGAATATGGACCGAGGATCAGGATGGAAAAGCTGCGCTCTGGGTATGTGGCGACCCATCGACATCGCT
GACAAAAACAAAGTCCTTCACTGGGTTTGTACGCGCACAATACGGCAGCCTGTGGATATACAGCTGCTAC
CTGGCCCCAAGCATGCAGCTCTGCGAATTTACAGCTGCATTAGATGCATTAGTAGATGACGCAAGGGGGC
GGAAGGTCATTATTGCTGGTGACTTCAACGCCTGGGCAGTGGAATGGGGCAGCCAAGCAACTAACGCAAG
AGGTCGTGCCCTGTTGGAAAGCTTCGCAGCCCTAGATATAGCCCTGCTAAACTTCGGCTCGGAAAATACCT
TCACTCGAGGTGGTATCGGATCCATAATCGATCTTACCTTCGCAAGCTCGTCACTTTTTAGCGCAACAAAA
TGGCAACTAAGCGACTTATACACCGCAAGCGACCACTCCGCAATAATATGCACGATTGGTGAAGGAACTG
CAGCCAGCACTAGAAGCCTAAGTCGGAAAGCTTACCGCCTGGATACTTTCGACCCCGTGGCCTTTTCGAAT
GCAGTCCACTCGCTTGTCACTGCCGGCAACGCAGAGAGAAGAGCCTCCCAAATGGCATGCCAGCTATCGC
GTGCCTGTGACGAGAGCATGCAAACCCGGAAAACGTTTCGGCGCCACCATGTACCCACGTACTGGTGGAA
CGAAAATATTGCCGAAGCACGGCGGAACTGCATCCAAGCCAGGAGGAGATACCAGCGAGCCCGAGGTAG
ACCCAACTATGCCGAACAGCAGCTAAGGTATCAGAGTGCCAGAAAAATGCTAAAAAGTCCATAAGAGAT
AGCAAACGAGAATGTTTCCTGGAGCTATGCGACTCAGCCGAATATGATCCTTGGGGCAAGGCGTACAAAA
CTGTCGTTAAAAGACTCTACGCCGGCAAACAAGCGCAGCCTTCTGACCCAAGTGAGCTGAAGTTGATTGT
GGAAACGCTATTCCCAATCAGACCCGACGTATTACANACGCAAGCCATACCATCGAGCCACGTAGCAGCC
ATGCCGTCTCTCATCGACGAAGTCTCAGTCGAAGAGGTGCTGACGATCGCCAGAAGCCTAAAGCCCAGTA
AGGCACCGGGTCCGGACGGAATCCCCAACCGAGCACTGAAACTAGCCATGTCCTTGCGGCCCGCGCAGTT
CGTGCAGCTTTTAACATGTGCCTAATGGAGAGAACTTTCCCCACAATATGGAAACTCCAAACGCTAGTTTT
ATTACCGAAGCCTGGTAAGCCGCCCGACGACCCATCGTCATACAGGCCGATTTGCCTCCTGGACACGTTA
GGCAAAGTGCTGGAGAAGCTAATCTGCAACAGGCTTCAGGTAGCTGTCGACAACAATGGTGGCCTGTCCC
AAAGCCAATTTGGTTTCACCAAGGCAAAGTCTACAATAGACGCTCTGGAGAAAGTGGTCGGCATAGCAAC
AAACGCCATCCAAGGATCGAGATGGAAAAGCGGCACAAAAAGTACTGCCTCATCGTCACGCTGGATGTTA
AAAACGCCTTTAACACAGCCAGGTGGGACAAAATATTGGATGCATTGTCAACGTTCCGCGTCCCATATTA
TCTTCAGGAGACGATACGCAGCTACTTCACTGGAAGACTGCTGCAATACGACACCGCAGACGGCCCCACT
ACACATCAAGTGTCCGCTGGAGTGCCCCAAGGCTCAGTTCTAGGACCCCTCCTCTGGAACATCATGTACG
ATGGCATCCTCCGACTGGAACTCCCGATCGATACAAGTATCATCGGCTTCGCGGACGACGTGGCCCTTGTA
GTCGTCGGCAAGGAATTGAAGGACGTAGAGGAATCGTGCAACCACAGTATTGACCGTGTGCACCAATGGC
TGGCAGCAGCTGGACTCGAGCTGGCTGCTCACAAAACAGAAGCGGTGTTAGTCAGCAGTCGAAAAAGAG
TGGAAACAGCGCACATAAGAGTCGGCAGTGCGACAATAGAATCGGCAAGGGCGCTAAAGTACCTGGGTG
TCATGCTAGACACAAGAATGTCGTTCAAAGAGCACTTGGAGTACGTGCACAAGAAAGCAGCAGAGTCCTG
TCGAGCGCTATCCAGGATTATGCTGAACACACGGGGACCGAAACAGCAGAGACGCCGTCTCCTTATGACC
GTAAACCGAGCGGTAATAATGTACGCGTCCCCATCTGGAGCAGAGCTATGGCTGTTCCCAGCTACAGCCG
GGGCGTACACTCTATATATCGCCTAAGCGCTCTACGGGTCAGCTGCGCATTCCGGACGGTATCGGACGAA
GCGGCGCTGGTTATCGCAGGAATGGTCCCGCTAAGCGAACTGGCAACAGAAGCTGTAGTTTGCTATAGAG
CCCACGATGGAAGCAACACAGCAAACTCGACTCGCACTGAGGCAAGGATGCAGAGCGTAGCACGATGGC
AAGCACGCTGGGATCGCGCATCTAAAGGACGCTGGACGCATAGGCTTATCCCAAACTTGGAAGCCTGGAC
AAATCGCAAGTATGGCAATACTAATTTTTATTTAACGCAGCTCCTATCAGGCCACGGATGCTTCAGGAGCT
ACTTAAAGCGATTCGGCCACGACAACACAGACGAGTGTTCTTGGTGCGGCAGAGGAATCATAGAGGACG
CCAATCACGTCATATTCGAGTGTGGACGTTTCGCAGCGAACAGACAGGAACTGGAGGATATCATGGCAG

ACCTATCACGGTGGANAACCTGGTCACGAGCATGATAGAGACCGCACAAAAGTGGGACGCTGCCAGCAC
CTTCGCAGCAGAAGTAATGCGAGAACTCAGGAGAGTTGAGCGCAGCAGACGGAGAACCGCGGATATTTA
GGTAGCAAGAACATGCCTGTGAAGCAATGCTTCGCGGCCGTTCCGCAGGCAATCCACGCTACCTAAACAA
TANCCCANCTATAACCTAAGCTAAATAACTAAGNATAAATAAACTGAATACAAAAAAAAAAACACACAC
ACACACACACACATACACACACACACACACACACACACACACACACACACACACACACACACACACA
CACA
>160_rnd-1_family-91__LTR___Gypsy
TATAAGAGTTAACAACAAATAAATTCATACAGTCCCAATAAGAAGCATTGCAACACCTCATAAGAAGTGC
AGCAAAATGCTGACTTGGTGTCTGTGCCATTTCCTCCACGCGCCGACTGGCAACCGCCGAAAATTCCAAG
AAGAATTGCCCAACGGGCCAACAACATCAGCAGCGACGCCGCTGCCAACGCCGCTGCCGCTAAGCCACA
GGTTACTAGGGCTAGAATTGATTTAAGTTTATTCAGTTGCAAATTAACATCAAATAAAGGAAGTCTTGCTT
CCCTAAAAATTATATTTTTTTCATCGTTTAATAAACTTTGGAATTAACTGGCGCCCGAGCAGGGACCGGCT
CGTGTTACAACAGAACAAAACGCTTCTACGTACGCGTTAGTCGGTTATACGCGAAAATAAAACCAGTGCA
ACTCTCGTCGATTAAATTCAACGATCGGTTAAATTCGCGAAAGGCAATTGGAACTTAATTGTTCAATTTTG
CCCATCGGTGGACAACGCATCTTGTAAGGCCCGCCCGCAGGATACTCTCAACAAAGACTGGTCGCAGGAT
CCCCAGTAGATACCCAAAGGCTTCAAAAAGGATGAAATCCGGACCGGCAGGGGAGGTTGCCACATCAGG
AATAGTACTGTAAGTTAGAAATTAAGTGAAACAAAAAAAAAAAAAAACAAACGAACAACTAACACAAAAG
TGAAATAGAATTATAAGAATAATAAATACTAAANATTGTAGAACGATAAGCAAGATAAAAAAGTGTAAA
ACGATCAACAAATATAAAGACTACGAACGTAGTATAAATTATTTCCAACTTTGCGACTCCCAAGGACCC
TCCAAAGTAGAAGGAAGAAACCAGCCACTGGTTTTAAAAAGAAAACAAAACGATCAAAAATTCAAGTAA
AATATAGAGTAAAAAACTAACACTAGATTTAAATATACTTCGAAAATATCTTAAAATATACAATATAAGT
AATATATCTCGTTTATGGCTAATGAAAGCAATTTAGTTAGGCCCCCAATAGTATCAGATCAACCACAGGTA
GGCGGACATAACTCGAACAGACAGCAACAACGACATATCGACAGACAAAACGAACATTTACAAAATAGA
GTCATGGCGAATAACGCAGAACAGATAATAGCAGCCACGGTTAGCCAGATTTTGGCACAACAACTGCCGA
CATTGGTAAAGCAGTTAGTCAACCCCTCTAGGGAGTTTAGTGACGTAGACGACCAAACAATAACTGCCAA
CCACAATTTGAATGACTTAGACAGAGTTCCGGACATAGTTAAAAGCCTCAGAGAATTCTCCGGTAATCCA
GCGGAATTTAGCTCATGGAAGAAGAGTGTAGATCGTATACTCTCAGCCTATGCAGCTGAGGCAGGTTCGC
CAAAATACTTTGGCATATTGCATACAGTGAGAAACAAAATCACTGGCAATGCCGACACGGCCCTCGAGTC
GTATAATGTTCCCCTAAATTGGAGCGCTATCTCCAAATGTCTCACGCTGCACTACGCAGACAAAAGAGAT
CTGGGAACATTAGAATATCAGATGACAATGTTAGTGCAAGGTAACAACCAAACAGTAGAAGAATTTTACC
AGGTCGTTTACAACCACTTGTCGCTGATATTAAATAAAGTAGGCTGCATGGAATTAAATAGGGAATCCGA
AACCCTTATGACAAAAATATACCGAGATAAAGCATTAGATACCTTCATTAGAGGTTTGAAGGGAGATTTA
CCACGGATTCTTGGAATAAAGGAGCCAGCAGATCTACCTTCAGCCCTTCACTATTGTTTAAAACTAGAGA
ATCAAAGCTATCGCACTAACATGGCTTTGAATAGAAATCAACAGACACATGCCAGAAACCCAAATAGAA
ATATACCACCACCTACACTGCCACCTCGAACAGCTCAGCCTCNTTTCGCTCAAACTTTTAGGCCACCACCC
ATTAGCTTCAGGCCCCAACTCCCGATCCATACACCTTGGCCAACCAATACAAACCATGGACCACCGACTT
ACAACAATAATGGACCGATTCCACAATATCCCAACCAACGAACCTACCAGACACAACCACAACAGGTATA
CATACCCAATAGACCTTCTGCACCAAAACCCCAACCACGTCCTGAGCCAATGGACGTGGACCAAAGCATC
CGCACGAGGATGATAAACTACATGAACAGACCCAATTTTGAAGTAGGGAAAAGACCTCCACCACCAACA
AACGCCACGGACAAAAAACAAAGAAATTTCAACATACAAACAGGAGAAGAACAGACGGAGACAGAAAA
CACCGACAACTATGTGACTATGGAGGAATACGTACAATCAATGCTAAATTACGAACAGCAGACTGAATAC
GAACAGCCATTAAACGAATACGCCCAACAAATTGAAGAAACAGATGACGGACAAAATACCCAATACGAA
CACTCAGACATTAATTTTTTAGGATAAAGAGCTCCTCTCTTCCTTACATAGAATGTAAGATGGGGAGCGGA
AAATTACTCAAAATACTAATAGATACAGGATCAAACCGAAATTACGTCCAACCAAAATTCGTGTCCGAGC
CTATTCCAAACGAAAAACCCTTCGAAGCCGTCACAGTCGGCGGAAGCATTAAAATTACACACCACAAGTT
CGCTAATCTTTTTAATAGAAACGATATAAAAATAAAATTCTTTCTCTTACCCACCCTAAAATCATTTGACG
CCATATTAGGTAACGATAGTCTAAAGGAACTAGATGCTGTGATACAAAGCAGCAAGAATCTACTAATAAT
CAAGAACAACATTTGCATACCCATTAAGCAGAAAAAAATAGAGGCGGTCAACACGGTAATACCACGGAC
AGAACATATGACAAACGAACAAAAAAATAGCCTTAATCAACTAGTTAAAATGCACCCACACCTATTTGCC
AATCCGGACGAAAAACTGACATACACAACCAACGTCAGAGCAGAGATTCGAACAAAAAACGACTCCCCC
ATATATTCAAAATTTTACCAATTTCCAATGTCTTTAAAAGACGAAGTACACAGCCAAATCAAAGAACTTTT
AGAGGACGGAATCATCCGACCCTCCCGATCCCCGTACAATTCACCTGTGTGGATTGTTCCGAAAAAATTA
GATGCCTCCGGGCAAAAGAAATATCGCATGGTTATAGACTACAGGAAACTAAATAAAGAAACTACCGCA
GACAAATACCCTATACCCGAAATCAACGAGGTACTAGCACAGCTAGGCAATAACAAAGTTTTCTCTGTCT

TAGATTTGAAAAGTGGCTTTCATCAAATACCCCTTAAGGAGTCAGACATAGAAAAAACTGCCTTCTCCGTT
AACAACGGAAAATACGAGTTTACACGACTCCCATTCGGTCTCAAGAATGCGCCCTCAATTTTCCAGAGAG
CTCTTGACGACATCCTTCGTGAGCATATTGGAAAAATATGCTTCATTTATATTGACGATATCATTATCTTTA
GCAAAGATGATGAATCGCATGCGCAGCACCTCGCCGCCATATTCAGAACTCTACAAGAAGCCAACATGAA
ATGTCAGTTGGACAAATGCGAGTTCTTTAAAAGCAGAGTAGAATTCCTGGGATTTGTCATCTCAGACAAG
GGGATAGAAACTAATCCAAACAAAATTGAGACAATAAGAAAGTACCCACTTCCAAAAACACTCAAAGAT
TTAAGATCGTTTATTGGCCTTGCCAGCTATTATAGGAGGTTTATAAAAGACTTCGCTAAGCTGGCAAAGCC
CCTAACCTCTCTTTTAAGAGGGGAAGATGGACGCGTGTCCAAAAATCAATCAGCTAAAAAGCTCATTAAT
CTCAACGAAGAAGCGATCGAAGCATTCAATAAAATAAAAAATTCCCTAGTTTCGAATGAGGTAATACTTC
ATTACCCAGACTTCAGCAAAGAATTTCATCTGACAACAGATGCATCCAACTTTGCTATTGGGGCTGTCCTA
TCCCAGAACAAGCGACCCATTTGTTTCCTCTCTAGAACTCTCTCTAAAACAGAGGAGAACTACGCCACCA
ATGAAAGGGAAATGTTGGCAATAATTTGGGCCTTAAAAGCCCTCAAGAATTACCTCTACGGGGTAAAGCG
AGTGTAAAAATATACACCGACCATCAACCCTTAACCCACTCTCTTAGCAGTTGGAACGGCAACGCTAGGA
TAAAGAGGTGGAAAGCTTACCTAGAAGAGTTCGATTACGAACTCCTCTATAAGCCAGGTAGGGATAACAT
TGTAGCCGATGCGCTATCACGTATCCCTGTGATCAATAATATTGGATCAGTAGCCTCCACCCAGCACAGTG
CAGACAGTTCAAGCCACAACTTAATTCCGAGCATAGAAGCTCCAATTAACGTATTCAAAAACCAGATATT
TCTCAGTAGAGCCAACGAAACAAATTACGAATTCCATATCCCATTCCCAACATTCCACAGGCATACAATT
ACTCGACCAAATTTCTCAGAACACGACCTTACGCAAATACTGAAAGCTCATCTAAACCCATCCGTAATCA
ACGGAATAATAACTTCAGAAGAAATAATGAACCAAATTCAGCAGATATATCCCCAACATTTTAGGAACTT
CAAGATTAGATTCACACAGTCTCAAGTAAAAGATTTAACAAATGAAACAGAACAAGATGAGAAGATATT
GGTTACGCACAACAGAGCCCACCGCAACGCACTAGAAAACAAAATGCAATTGTCCGAAAGATTCTATTTC
CCAAAAATGAAGCAACGAATAACAGAATTAGTCAAACAGTGTCAGGTTTGTAAAGAAGAAAAATACGAT
AGGCATCCCACTAACCCAAAACTCAAAGAAACACCAATCCCCGAATACCCAGGACATACAATCCACATCG
ACATATACACGACAGAAAAAAACTTAGTACTAACAGCAGTAGACAAATTCTCGAAATTAGCTCAGGGAC
GAATTATCAAATCTAGGGCAATAGAAGACATTAAAAAACCACTTCATGAAATCCTTTTTTATTTTGGCGTA
CCTAAAGTAGTTGTAATAGATAACGAAAAATCCCTTAATTCAGCCTCAATCACTTTCATGATGCAGGACCA
ATTAAATATAGAAGTTTATAAAACACCACCCTACAAAAGCGCAGTGAACGGTCAGGTAGAACGATTTCAT
TCAACACTATCAGACATAATGAGATGCAACAAAAGAAAACAGGAATACAGAACATTCGAAGAACTACTT
AACAAATCAATATACGACTACAACTACACTATACACTCGACAACTAACAAGAGACCATTAGACATATTCT
TCGGCAGAAACGTGACTACGGACCCAGACCAATACGAAAAAGCTAGGAAGGAGAACATTGACAGACTCA
AACAGAAACAATCGACCGACATGCAGAATCATAACAAAAAACGTAACGACATACGAACTTACATACCTG
GTCAAGAAATATTCGTCAAAGTCAATACCAGATTAGGTACCAAACTGTCACCCAGATACAAAAAGGAAAT
AGTTAAAGAAAATAGAAACACAACCGTACTAACAACAACTGACAAAATAATACATAAAAGCAATATAAA
AAATACATAAAAATTACAGATTCCTATTAAACGATGACAATAAAAGCAACATGACACCTGGAAATTTTGA
ACTACACAGACGCACAAACAATTACAATTCTAAAAGGAATAGGGAAACTACAGACATCGACAACACGAA
TCGTACAAGTCATAGAGCTAGAACAGATTAAAAACGCATTAGATAATCTCCTTTTTTACACCGAAAAAGA
CTTAACAACCAATACTTTATACCACACTTTACGTCACGAAATAACGCAAGCTATTGTCACTTTCGACACAA
TATCACTTTTCCATAAACGAAAGACTAGAGCGATCAACATTATAGGCACAGCTTGGAAGTATGTAGCCGG
CAGCCCGGACAATGACGACTTAATAATGCTTACTGACAACATAAACAAACTTATAGAAAACGACAATAAA
CAGCTTATCATAAACAGGCAATTAGAAGATCGAATTAATAAACTCACGAAAATCTCAAACGATTTAACCA
ATAGCATAAGAAAGGAATCTCTCATAAGTGATGAGATAGCTATTAGCTTACAAAATCAAATTAGGCTCAT
AAAAGAAGAAATAATTAATATTAAGTACGCCATACAATGGGCGAAATTGAATTTAATAAATACATTCCTA
CTTGGCGAAAAAGAAATAGAGATAGTAAACGATAACTTTAATAAAAGCAACATGCCACCTTTAAATATTG
AAGAAATGCTAGAATTCGCAGATGTATCCATTTTGCATAATGGAACCACTCTGTTTTATATTGTAAAAATT
CCAAATTTAGAAGAAATAGAATGCAAAAATACTATAATAAAGCCAGTAGTGAAAAACAACAATATAATA
AATTTAGAATTTAACGAACTGTTTCATTGTAATAAAACAATCTATGGCATAAACAACAATTGTAAAAGAT
ACCATAATATTAAAATTTGTAACAAATCAAAAATTGTAAACTTGAACAATAATAATTGTATACCTAAGTTA
GTTAGCGGAGAGCAAGCGCTCTGTGGCTTTAGCAATGCAGATCATATCCCGGAGATCGAGGAAATCGACG
AGGGACTTATACTAGTGAACGACTTCAACGGCACTATTAGATGGCAAGAAGAGAAGAAACGCGTGAGAG
GCACCTACATGATTAACTTCTGGAACGAAACCTTATTCATCAACAAACAAGAGTTCAGCAACCTAGAGGT
CACCGTCATGACCCCAGGAAATCCCACAATACAAATAACCCCAATAGAAAACGAAAGAATAAAGATTCTT
TCTCTGGAAGCATTAGAAGAATTGCACATCAACAACATNAAAAACATGCAAAAACTTCAGACGCACTCTA
TAGCAAATAGAGTCGGTTTCATAACTCTGTTCACAGTTGCCATCCTTCTTACCTGTGTATTACACGTAATG
AAGAAAAATAAGAAAAATATAATACTGCAACTGAACAAAGAACAAGAGAGACCCAAAAAAGAACCAGC

ACATATCCAAATACAAAATACAGATGCCAACAACCTCAGGTTCAACAACATACCTTTCTTCTGATGATCGC
TTGAGGACAAGCTTAAATCAAAGGGGGAAGAGTTAACAACAAATAAATTCATACAGTCCCAATAAGAAG
CATTGCAACACCTCATAAGAAGTGCAGCAAAATGCTGACTTGGTGTCTGTGCCATTTCCTCCACGCGCCGA
CTGGCAACCGCCGAAAATTCCAAGAAGAATTGCCCAACGGGCCAACAACATCAGCAGCGACGCCGCTGC
CAACGCCGCTGCCGCTAAGCCACAGGTTACTAGGGCTAGAATTGATTTAAGTTTATTCAGTTGCAAATTAA
CATCAAATAAAGGAAGTCTTGCTTCCCTAAAAATTATATTTTTTTCATCGTTTAATAAACTTTGGAATTAAC
TTATA
>160_rnd-1_family-165__LTR___Gypsy
ACAATGATAAGCGACAAAATAAAATAAATAAATAAAATGAAATTTCAAGAAATATATATATATTCTTATT
ACCAGAAATGCCTATTAATAAGTTTGCGATAGAAGTATATTTAGAAGCAATATATAATAGCGACGCAGAC
TTTAGAATTCGTGCATTGAATATTCTACAACAATAAGATAAAAGGCATAATTTTAAATACACTGCCATATG
TTGGCATTAAAGAAATAGTGGTCCAGTGGACGGCGAGTGCCCAATTATAATGGTAAAACTTCATAAATAG
AGAACTATGGAATGGAGTGAATTAGCGAAAAAAATAAATAATTTCAAAGATAAATTCGAAAAATCTTATA
AGTGTGTTAATAAGAACGCACCTATAAGGGTTGAAACGCTAATTCACCACATAAACATATTAGTGCTAAA
CTATAATAACATAGTTCAACTTTATTTAAGTAAAGAAAATAGGCTTACAGAGCAGCATGTAAATCAATGT
AAGAAAATAATCAAATCACTTGGGACTAGAATATCGAATATCAGCACTAGGCACAACATAGTAATAGATA
TCCCAACAGATTTAAAATTAATGGCAACTTTTGAACCAGATCAACTAGAAGAACTGAATGAGTCTAAGCC
AACACTAAATAGTGACCCTGATATTGAAGCTGATAGCGACATTGACCCACTTGAATTAACAGAACCAAAC
ATGGCAGTAAATCAAGTGGCCTTAGACCGCGAATATGTAAGACAGGTATCGTCTACAGTCCCGGAATTTG
ACGGCGAAAAGCCGTCACTGGCCAGATTTCTAACGGCTTTAAGACTNGTCGACCGNACNAAGGGTACACG
AGAAGACTTAGCCGTAGAGGTCATAAAGTCNAAAATAATTGGCCCAATTCTGTACAAAGTACAAAATGAA
ACNNCNATTANCGGNATTATNAATAAATTAAANACCGATGTCAAAGGCGAGTCAACCGACGTTATCAAG
GCNAAGTTGCTTAACATAAAGCAGAAAGGCAAGNCAGCCNCGCAATNTACAGCTGAAATTGACAGTTTG
CGTAAGCAACTNGAAGCAGCCTATATTGACGATGGACTAGATTCTGATAACGCNGAGAAATTTNCTACTA
AAGAAACAATATCTGCCGTGACAAAGAATTGCGAATACGAAAAGCTTCGCGTAATATTAGAAGCGGGCG
ATTCCGGCANTTTCAATGATGCCGTGGGTGAATATATCCAGTGCAGCACTGAAATGACTGGAAGCGCGAG
TACAGTATTGTACTACAAAGGCGGAAACGACCGTGGAAATTACAGCCGTAACAACCAAAGNGGTAGAGG
CAATGGCCGCGGCAATGGTAACTNCCACTACAANGGTAATAACAACGACAGGGGTAATTTCAGAGGAAA
TTACCGNGGTGGCAACCGAGGAAGAAACAATTCCAACCGAGGCGGCAACCAAAACCAAAACAATAACAA
TAGAGGTAATGTCCGAGTAACTCAAGGTAATTCGGAAAACCAACAAAACCCCTCAGATGTTCAGAGCCAA
TAAATGGCGCAATAAAAACCATCAACCTTAATCTNAGCATATTTGTAAAAATTAACAATGAAAGAACAGG
TAAATCACTTGCACTGTTAGTAGATACTGGTGCAGATATATCCATATTAAAAGAAAATNCAGATATATTCG
AAAATGTAAACGAAAAAATTACTACCCAAATATCGGGCATNGGCGAAGGCATAATTCNTTCAAAAGGCTT
AGCCTTTATAGAACTCACTACAGGTAAATACATAATTCCACATAACTTTCATTTAGTCGANCAAAANTTCC
CAATACCTAGTGATGGCATACTAGGCATAGATTTCATAAAACAATNTAATTGCCAACTAGATTTCACNCA
AAGTGGCGACTCGCTCATTCTTCGCCCAAACAACTTAAATTATCCAATTCAATTACAAATACTTCATACTT
GTGACAATAATGCCACAGTACTCCCAGCNNGTTCCGAAGTTGTCCGTGAAATACAAATACCCTCAATANA
AAATCGAATTCTNATACCAAATCAAGAAATTGANGAAGGAATTTACGTCGCAAACACGATATCCAACNAC
AAAAATGCATNTGTCCGATTACTAAATACAACAAATACCGATCAAATAGTAAACTTAAATAAATTAAAAT
ATGAACCAACTTCGAACTACGATATAGTTCAAAAGAAACCAACGAAGAAAGAGANAACTNCGTTATGNA
AAAATTAACTAAAAACTTTCCACCNCTATTCAAAAATCAATTAAAAGATTTATGCACCAAGTATACNGAC
ATATTCGGACTNGAAACCGAATCGATAACAACCAATAATTTTTACAAGCAAAAACTNAGATTGAAGGATG
ATGAGCCCGTATACATAAAAAAATTATAGAAGTCCTCACAGCCAAGTAGANGAAATTCAAGCCCAAGTNCA
AAAATTAATCGACGATAAAATAGTCGAACCGTCNGTCTCAGAATATAACAGCCCACTTTTATTAGTTCCG
AAGAAGTCTCTTCCGGGCTCTGANAAGAAGAAATGGCGATTAGTAATTGACTATCGTCAAATTAATAAAA
AGCTATTGTCCGATAAATTTCCGCTNCCTAGAATTGATGATATTCTAGATCAACTAGGTAGAGCAAAATAT
TTTTCATGCCTNGACTTAATGTCNGGCTTTCACCAAATTGAACTCGAAAAAAGTTCTAGAGATATAACGTC
NTTTTCAACGAGCAATGGCTCGTATCGCTTCACGCGATTACCATTCGGTTTAAAAATAGCGCCAAACTCNT
TTCAAAGAATGATGACNATAGCATTTTCCGGNCTAGAACCATCNCAAGCNTTCCTTTATATGGATGATCTA
ATAGTAATCGGTTGTTCCGAAAAACATATGGTCAAAAACTTAACTGATGTTTTTGACAAATGTAGGAAAT
NCAACCTGAAACTACATCCGGAAAAATGTTCATTTTTCATGCATGAAGTCACATTTTTGGGACACAAATGC
ACTGATAAAGGAATCTTGCCAGACGACAAAAAATACGACGTCATNAAAAATTACCCAGTCCCACACGATG
CGGACAGCGCTAGACGATTCGTTGCATTTTGCAATTACTATAGACGCTTTATNAAAAATTTTGCCGACTAT
TCACGTCACATAACAAGATTATGTAAAAAAGACGTNAAATTCGAATGGACANCTGAATGCCAGAATGCTT

TCGAACATCTCAAAACGGCACTAATAAACCCNACCTTGTTACAATATCCAGATTTCACCAAAGAATTCTGC
ATAATAACAGATGCAAGTAAGCAAGCGTGTGGAGCGGTTTTAACTCAAAACCATAATGGACTCCAACTTC
CAGTTGCATACGCATCAAGATCNTTTACTAAAGGAGAAAGTAACAAGAGTACNACNGAGCAAGAATTAG
CGGCNATTCATTGGGCAATAACGCATTTCCGACCATATATTTATGGTAANCATTTCACGGTNAAAACGGA
CCACAGACCACTAACATACCTATTTTCAATGGTAAATCCAAGTTCAAAATTAACGCGTATGAGGCTTGAAT
TAGAGGAGTATAATTTTACGGTAGAATATCTGAAGGGTAAAGACAATTTCGTAGCNGATGCGTTATCAAG
AATAACCATCAAAGAGCTACAAAAAATAACAGGAAAAATAAAGAAAGTCACTACAAGATATCAAAGTAG
ACAAAAATCCTGCGCAGGAAAAAAAGAAATAGAATTGCCAAGGCAATCTATAGAAAAAGCTTCAAAGCC
CAACGTATACGAAGTCATTAACAATGACGAGGTACGNAAAGTAGTGACCTTGCATGTAAAAGATATGCTA
TGTTTCTTTAAACATGGTAAGAAAATTACTGCAAGATATGACGTTAGCGATTTATATACTAATGGAACTAT
TGACTTAGGTCAATTNTTCCAAAGGCTCGAAACGCAAGCCGGTATATATAAAATCAGCCAACTCAAAGTG
GCACCGTGGGAAAATATCTTTGAACACGTTTCAATAGATAATTTCAAAAATATGGGCAATAAAATATTGA
ANNCATTAAGAGTAGCGCTACTCAACCCGGTGACCCAAATAAATAAAAAAGAGAAAAAGAAGCTATATT
GTCTACACTNCATGACGATCCAATACAAGGAGGTCACACAGGCATTACAAAAACCTTGGCCAAGGTCAAA
AGACATTATTTTTGGAAAGGTATGACTCGAGATATAACAGAGTACATACGNAAATGTCCAAAATGCCAAA
AAGCAAAAACNACANAAACACAAAAACCCCTCTAACAATAACNGAAACACCAATANNCGCTTTCGACAG
AGTAATAGTNGACACAATTGGTCCACTACCNAGATCAGAGAATGGNAACGAGTACGCAGTCACACTNATT
TGTGACTTNACNAAATACCTAGTCGCNATTCCGATCCCAAATAAAAGCGCAAACACTGTCGCTAAAGCTA
TATTCGAATCTTTTATTCTGAAGTACGGTCCAATGAAGACGTTCATTACGGACATGGGAACGGAATATAA
GAATTCAATTATAAACGATTTGTGCAAGTATATGAAAATAAACAACATAACATCNACTGCACATCATCAC
CAGACAGTTGGAACAGTAGAAAGAAGTCACAGAACTTTTAACGAATACATACGATCATATATATCAGTTG
ATAAAACTGATTGGGACGTATGGATCCAATACTTCGTNTACTGTTTTAACACGACCCCCTCTATGGCACAC
AATTATTGTCCATATGAGCTAGTTTTTGGTAAAACACNCAATTTACCAAAATANTTTAATAGNATAGACAC
ACATAGAACCTATATAATGTAGANGATTATGCTAAAGAAGGTAAGTNTAGATTAGAAGTAGCATATAAAA
GGGCTAGGATAATGCTNGAAAAACATAAGANAAAAAGTAAGGAACTNTNTGATAAAAAAGTNATAGATT
TTCAAGTANAAATAGGAGATAAAGTCTTATTAAGAAACGAAACNGGCCATAAATTGGATCCAGAATATAT
AGGCCCATATACAGTAGNNAACATAGAAGATAGGGATAACATNCTAATTAAAGATAACAAAAATAGGAC
NCAAAAAGTTCATAAGGATAGACTAAAAATTTTTAACGAATAAAACATATAAATGTTCTATATGAAGTCT
TTATGTTCCTTTATGAATTCACAACAAATAACAAGTAAGAGGTTCTAGTCGGGAGCTCCCGACTAGGGGA
TACCCTGAACCCTCTTCTTCCAACATCAAATGCATATATAATCTATTTTAGAAGCTATATGTCAAGTTTGGT
TCTAGTCGGGAGCTCCCGACTATGGGATACCCTGAACCCTCTTCTTCCAACATCAAATGCATATATATTCT
ATTTTAGAAGCTATATGTCAAGTTTGGTGACTCTAGCTCTTATTATTTACCAAAATTGCCCAAAAAAAGGA
TATCGATATCGATTTTTATCGATTGCTTGGAAACGGAGTAAGCTATCGATTATCGGAAATAAACTCGATCT
GCGCGGGAACTAGGAGCACCTACATCTAAAATTTCAAGTCTCTAGCTCTTATATGTTCTGAGATCCTTGCG
TTCATACATACGGATGGACGGACGGACGGACGGACGGACAGACGGACATGGCTAGATCGACTCGGCTAT
TGATGCTGATCAAGAATATATATACTTTATGGGGTCGGAGATGCTTCCTTCTGCCTGTTACATACATTTGG
ATTTTGCACAAATACAATATACCCTTATACCCATTTTTAATGGGTTCAGGGTATAAAAATACATATACATT
GGCCTGATCAACCAACTATAAAAAAAAATCAATCCTTGAAAAATACAAGTTACTATAATGAATTTTACGT
TTTTATATTTGATATCTTTATACTTTGAAATGTTTACATTAGTATATTCCCATAAATCTTAAATAATTTTTTT
AAATTCATATTATTTTTATCATTTTTTTACATTTTATTATTTTATAGCAAAAAAAAAATTATATACAAAAGA
TTTTGTTCAAAATAATTAAGTTCATTAAATAACTACACATCCATAATGTTACGTTATTTCTTTGAAAAGGG
AGCTGTAGTGTGTATATGCCTTAAAAGAACACCCTATATACAAATATAAAGTATAAACAAGCCCAGAGCC
ACTTTGTATATTGGCTCTGGCTCATATTACGCAGCGCAGTTCACTAGCCTCTCAGCTGATGCAACGATGAA
CAACAGTTGCAGTTATATCCTTAATTACTTTTAATTATAAAAAAAAAATTTTTTGTAAAATTGAGCAGCAT
AGCCCGTAAAGCTATGCGAAATGCTAGGTAGTATTTTGTGTTATTAGCAATATTGAAAACTTGAAACTAA
AAACATTGGCATTTAAAGTTAAAAATACTATATGTTACATAAAAACATTATATGAATTTAAAAACATTAC
ATCAACTTATGTAAGAAATTTTAATTAAAATGGGTTCGAACGAATCCAACATTGAAAAATCAGCCGCAAA
TGTTATAAATAACATAAAAGTAATTGACCATAAAGAATAATTACTACAATATCTCTTGTATCACTAGTATT
GAAAGTGTATGCAATGCATACAAAGAAAATTAAGAAACGCTATAGCAGTAGAGCAGACGGACTAGATAA
GGTATGAATATATTTGAATATGTCAGACATAGAGCCAATCATTAGGTTTACGTATAGAGATGAGCCAGAC
TTTCGTACTCGCGTATTAAACGTACTGCGATTACATAGAGATTTTATAAAAGTGTGTTATTTCGAATACGA
ATACACGGACATGTTCAATACTCATATTAATGGTGCGACTGCATCGATACATATCTATTATAATAATATGG
AATGGAGTATATTAGTGAAAAATATCAATGACTTTAAACACCAATTTGACAAGTCATATAAATGCATCAA
TAAAGGCATACCTCTAAAGGTAGAAACACTAATATACCATGTACAAATATTGATAAAACAATATAACAAT

ATAGTACATGCAGTAGATTCCATTAAAGAAAAACTAACACAAGAACATGCCAAACAATGCCATAAGGTC
ATACAATCTTTAAATACAAGATATTAAACCATTGGCATTAGACAGAATATCATAATAAATGTTCCAGAAG
ATCTATTTTTACCAGCAAAGTTTGAACCAAACCAGCTGCAAGATCTGAATGAGTCGAAGCACTAGCCAAA
GAACATATAGACTGGAAAGTAGGCACAAAAAGTATCGGTGGCATCTGGTCCAAAATCGCGAGTAAGGGA
GTAAGATATACAAATGATGTATTTTTGAACCGCTTACTCTACAGGAAAGTGCTTGGCGCACTCTCTTCCAT
ATGATTTGTGCGATAGAACGACAACGCTGTTAACTTTTATCTCGCTCGCACTTACTCTCAGCTCTCAATGCT
CTCTCTTTATGTTTAGGGATCGTTTAAATGTGTGTGTGTGTGTAT
>160_rnd-5_family-3953__LTR___Gypsy
AGTTAACAAGAATACGTTAAAATTATTCAAAAATTACCTTAGCTCGAGCATAATTTTTATTCAAGGCCTGA
TCTGCATTAAACAACTTAAAAATTAAAATTTCTTAACAATTTTAAAATTGTATAATATTTATCATTTCACTA
TTTTTACGTCATTCAAAATAAAATAACTCCAAACGATACAAATGATGCGATACAATAAAAATATACATAT
ATCCGCGCATTTAGAAATAAAACAAGTTCTAGTCGGGTGCTGACGAGCATCAATGTGTTTGGGTGCATGT
ATACAGAAATTCTTGTAATTTGCGCTAACTGAAGCAAACACGATTTTAAAGAAATATTCTACAGTATAAC
GTTTTGTACCATAGAATATTTGCGCATCTCAAAAAGTAAATTGTATTAAAATTATTTCTTTAATTGGTGGG
CAATATAAGTTGGGTTGAGGTAGTAGCGTGGCGATAGTAGTAGGTGGTGAAAGGTATAAACTGAAAGAT
ACTTGATATATATATACTATTCTAGAGTAAACATGTCACTTTTGGTTGCTTTAGTTCTTATTTTTAACCAAT
TTTCTCAAAAGACAGGATTTTGATATCGATTTTTATCGATTGCTTGAAAACGGAGTAAGTTATCGATCATC
GGAAACAAATCGATCTGCGCATGAACTAGGAGGACCTACAGCTAAAATTAAAAGCCTCTAGCTTGTTTAC
CCTGTTTACCCATTTCAATGGGTTCAGAAAACATATAAAAAAAAATTTATTTTAATAAATTTTACCTCAACT
TTTATATCATTATTATTATTATTATTATAATTCATTTAATTACCACACATATATAATATTAGGTTATTTCTCT
AAAAAGGCAGGTGTAGTGTACTTATACACATATGAATATAAATACATAACTCAACTTGTAACATTTTGCTG
CAATTCAAAACAAATATCAATGTATAAAGCCAAGCGCATAGAATATATATGATCATGGATCCATGATGCC
ACTTGAGCAGGCCACGGCAGAGTCAGAACGTTGACCCTGATCTTGCACCTTACCCGCTCCGAGAATGAGA
ATTTATATATGAGTACACCCTCTCTCTCTCTCTCTCTCTCTCTCTTTGTAGCTGTTAGCCGATAACAGCATA
AGCATATACATGTATCCTTAGGATAAGTACATTCTACTGACTTTCTATAAATTCATTTCGAAAGACGTATG
AAAACCAGCACTTGACGTGCTTTACCACAACACAGTTGAAGTGGACGATTTTTCTTACACGCCGCTACAA
ACCGCTCGGCATTGCGATGCTCCGAACGTCATGACCTGCATAACGTAAGTTTCGGGGAAGGGTGACGAAT
CTCCATCACGCCAGAGAAATTTCTGTGCCGCTTGGTCTTCTCGGCGCACCTTGACCTGATGAAACATTTCC
CGTATGTCGCCGGATACGGCGATAGGGCACTCTCTGAAGCGAATTTGCACACCCATCAAAGATACTAGTG
TATCTGGACCCTTCAGCAAACAATCGTTCATTGAAACGAGTCTTTTTATTGTTCGGATTGATGACTGAAAA
TATAAGGAGAAACCACGATGTCGTGGTCAGCCATAAATCGAAATGAGTCTAAAAAACGTGTCTCCAAATT
TGTATGCTTTGTAATATATGTGTATTTTGAATGGGAAATGTAAATCTCTTTATTGAGAGGTAGAACATTAA
TTTCACCTTCAGCTTTTGACAACTCTCTTACAAACAAAGAGAGTAAAATATTATGAAAAGGTACGGGGAT
AAATGATTAAGTAATTAACATTACAGGCATCATGAGCGGCCCCTCTGAATTTACCAGTTAAGTGGCAACA
TATTAATGCAAATGTCGCAATTTACAGCATTTTGAAATTTATGGTCATCTTCTTAACTTATTTCTGTTGGCT
TTACAGTAGATAAATATTTGCTATAAATCTCTCTTGCATCCGCAATAACCGATGTTACAAAATGTAATCCT
ACATTTTCTCTACTATGTTGTACGAATTTATCTAAACTAGAATCATTTGCAGATTATATAAAATATGCGTAT
ACAACAAAAATGTGTTCTTGAATGTTTATTACATTCTCGCTCACACTCCTGTTGATAGGTTTTAAAACGCC
CTCGAGATCTGCATAAATTATAAAAGGTATATCTAAACTATTTACATAATTCTTAAATTTTAAACTGCTTT
ATCGTTTGTTGAAAGCTTCGCCACCACTTTTCCACACTGCTCGCCACGCATCCTGTCGTTATGCGAATAGTT
TAAGCATACATTACAGAAAAGTAATTTCCTCGTATGCTACGTCAGTTGATTGTGTGCAAGCCTGGAGGAG
AAAATATGCCTGAAAATAAAAAAGAAATGTATTTTTTTTTGTTTTCTGAACATAACTTGATAGATTTTTAG
TCAGTAGTAATTTTAGTAATGTGAAATATCATGCATTTCCAAAAGAAATAGGAAAACTCCTTGGTGCTCTT
TAGTTATTTAGAGTGATCGAATAATTTCAATAATATCTTTATCATAGCCAAAAACATTTATACTAAAGTTG
GTATTTTAAAGCTCAAGCAATTAAATGTTTTTAAGCTTTAAAGGAAATTCCATATAATGATATTTGAGTGG
AAATATCTTATCAATTTCAATAATTGGACTCCTTTTATCATCGATATGGAGCACGCCTATAAGCATGGGAA
TACCCTGAACCCTCTTATTCGAACATCTTAACATTTAAAACAAGTAAGAGGTTGTAGTCGGGAGCTCCCGA
CTAGGGGATACCCTGAACCCTCTTCTTCCAACATCAAATGCATATATATATATTCTATTTTAGAAGCTATA
TGTCAAGTTTGGTGACTCTAGCTCTTATTATTTACCAAAATTGCCCAAAAAACAGGATATCGGTATCGATT
TTTATCGATTGCTTGGAAACGGAGTAAGTTATCGATTATCGGAAACAAACTCGATCTGCGCAGGCACTAG
GAGCACCTACATCTAAAATTTCAAGTTGTGAGGCGGCTAATGAGACCAGCCGCCCCACAGTTTTCACGGC
GAGGAGCCGTCATCCCTTTGGCCAAATTACGGCAAGGAGCCGCTGGCGGGACAACGTATTAATTTTCTTT
AGTTATAAGTATTAATTATATTAGGTTAAGATCTTAGTGAAATAAGAGGCAAGGAGCCATCAGTAATATA
ATCAGTTCGATTAGTGAGCAAAGAGGCAAGGAGCCGCGAANATTATAATTGATTTAAATAGCGAATTTGA

ATTTAACCGCCGGCCGCCGCTGCAACCGTGCGTTGTCGATCGGTTGCTCAACACCGTCTTCGAGAGCGCAA
CTTCAATTCGGCGGCGAAGAGCGAAAGCTCCGAGGCGAAGTTGCGAAAGCTCGCGGCGAAGTTGAGCGG
CCGAGGCGAGCGCATGGCTTGCCGGCGAGCGCGCCGGCACTTGGGANCGAACGCTAAGGGTGAGTAGAA
GTGCNGCGCGAGACGAGCGAGGTCATAGATGGTACAGCGGAGCGCTAGGGCTCCAGAAAACAGTGCGGC
AGGGTTTAGCTCGGGATTTACGATAAGGTCGGAATGAATGAGTAGTGAATAAAGTGCCNACGTTGTAATT
ATCTAGAAAGTATCGGATNATTCCCTGTTTCCTGGTAACCCGGCTTACCTTAACATCGGAACTAACCGGTG
TTCGACCTAACCTCGAACGAGGGGCATGCGGTACGAGATTGTGGCTCCGGGGTGGGCATTGCCGGTTGGT
GTCTAGGTAGTGGAGTTGGTGCGAGGCCACGGCCCGCTAGTGTAGTTGGTGCGAGGCCACGGCCCGCTAG
CGTAGTGGTACGAGGCCGCGGCCCGGCGGAAAGATAGGGTGTTTAGGTAGTGGAGTTGGTGCGAGGCCA
CGGCCCGCTAGTGTAGTTGGCGCGAGGCCACGGCCCGCTAGCGTAGTGGTACGAGGCCACGGCCCGGTGG
TGTAGGCGTGCAGAGGTAGATCTGGGCTTGGTCTGGTCAGGATACTCACCCCCCCCCCCTTAAGCCCTCTC
TCCCAGCGAAATTATAAATAACTTGTAACTCGCTAGACCTGGTAAGACTGAGAATTATACCCCGGCCAGN
AGCNTCCTTACAAGGTGGCGCCCGAGCAGGGACCAGAGGCGTTTAGGGGTGTGGCATGTCGGCGGATTAA
AGCCCGCAAGCATGCCCAAATAGGTATTTTCAAAAAAAAAAAGTTTTATTTTATAAAGGGGTATTTTTAGT
CGGGTATACCCTAGAGGGAAGGTACCCTAGCGGAGAGCAAGAGAATGAGGATATGGTATATGTGCGAAA
TATGAAANTAGGGNATTAAGTAGTCCGGTATACCCCAGCGAACATTTCTATACCCTGTGTTAGGCANAGC
CAGAAAATGAACACGTGCAGCNNNTGAGTGACGAAAATGCTNGCAGNAANCCGGCANGCAAGGCAAGG
AGGACGAAAACATAACGGTCGGTCGTGAGAGTACAGCGCGCGCGCCAGCCGGGCGACGTCGAGACCA
CGCTCGCACACGGGTGCCCACACGCGCGGCGCAGTCGACGTCGGCGTCGAAGGACCGTTAAGGCGTCGCG
GCAGCGGTTGTACAGTGGGGCGAATCCAACTCACGCGCGGTGAAAAGTTGAATTCGTGAGTACAGCTGTC
GGGGGCAGTGAGGTGGTCGCCCTTTAAGATTGAATGCGAACGAAGTGAACAATAAGAGCGGAACAAGCG
CCAAATGAAAACGAACGCGGGTTAAAGTGTCAAGAACGTCGAGAGAATTACTTTTTTTTTTTTTTCGTGAA
ATGGCTAAANTTAAAAAAAAAAATANNTGGATAAAAATAAAATTTTTTATAAATAAAATTAATTAAACAAA
AATAAATAAAAGAAATAAAACAAACAGTTAAGTACACAAGAATAAACCAAGGTAACTGGGTAAAGGCGA
AGGCAGCGACCAAATGAGGAACGGCCTAAAAGCGGAAAAGTGAGAAGGAAAATTTAATTACCTTTTGTT
GAGTTTTTTTTTTATAAACAAAAGAAAAAAAAATACTTTTGTGGAATATTNTTTTGTATTTTTGTTTGGTGAA
AGATATAAAGGCAAAANAAAAAAAAAAAAAAAAAGGAACAAAAGTGGCTGAGTAAGATCGGCGTAAAAG
TGAAAAGACACCAACACCAAGAAAAGGAGAGAGGAATAGTAAAGGGACCGACGGAACCCCTAGAACGG
GAAGAGAGGGAAACAGCGACGTCGACTAGCAAGGAGGTATACAATAAAGGATGGAGGCAGGTGAGTGC
CAAAACCCGGGGGTAGGCGTCCGCTGGATTCACCAGCGACGCAAAGAGGAGTTAGTGGCGATCGCCCAG
GAGTTTGGGCTGGAAAAGGAGGGGCGCGTGGAGGAGCTACGTAAGCGGCTGTCCACCTACGTGCAGGAG
GGCGGTCATTCGACTGGGATTTGGGCTCGGCTCGCCGAGCTGGAATCCCAGTACGCTAAAACTCTTGAGC
CGCCATCTTGGGGCGTCCCACCCGACTTTGGCGGACGGCCACGGCAAAAGTCGGAACCTGACCTCAGAAG
CTCCCAGCTGTACGGGCTTTTGCAGGCGCCCACCCCGAGTTTGACCGTCTCGGGCGTGGAGGCAGTTCCTG
CTCTCGGTGGAAATGAAGACCGCCGAGCAACTGCGGTCAACGCAGAGGTGGGGAGGTCCCCTGTTGAACC
GGGTGGTGGTCCCTGCTACACTAGGCCGCACCATCCATCGGAGGCGAGGCCACTTCCCAATGTCCCGGAG
CAAGTTCGGAAGTGGGGTATGAGGTACGATGGACGCTCCGACCCCCTTGGGTTCTTAGAGGACCTCGAGG
AGCGCGCTATCACCTATGGTATAGCCCTCGACCGTATGCCCAGGGCAATGAGCGAGGTGTTCTGCGATAG
GGCTGCTCGTTGGTTTCTGAGCAGTGGGCTAAGGGATGTGCCGTGGGCCGTGTTTAGGAGGGATTTCTTGG
AGTTTTTCTTGCCCCCTCGTTACTTCGAGAGGCTTGAGGACGAGATCCGATCCCGGAGGCAGCGCGTAGGT
GAAGCGTTCAAGGAATATTTGATAGAGCTGAGGGCTCTTATGCACCACGCTAGATACGAGCCGGCACAAG
AGCTGCATCGGATCTACGAGAACGCCGCTCCGGAATACAGGCTCTATGTGCGACGGCAAGACTTTTCTAC
CCTATCCCAGCTAACTCAACTGGCCGTAGAATACGAGACCGTCGTGAGGCAGAGGGCGGAGTACTCAGGC
AGCCAACCCTTGGGTCAGGGTGTCTCTAGTTCAAATAGTGCAGAGTGGGCGAGGACGGCGCAGTCCTCGG
GACATCTCAATCCGTTCCGGTCTGCTCCCAGCGGATCCGGGACGACCGTTGTGGACAACAGGGTCTCCAC
CCCACCCGCTGCAACTCACGCAGAGGTGGCCCATCGGATGATGAGCCAAGAGGCCGGGAACGGATATGA
TCCGCGACGGGCGTGTCGTAGGTGCGCACAGGAGGGGCATTTCGCCAGGGAATGTCCCAACGCCCCTGTC
TTATTTTGTTGGGTATGTGGAAGCCGGGGCCGAAGTAGTCGGGAGTGTTGCCGCCGCGCGGAGACGGGAA
ACGGGCAACGGCGTCGTCCGGACCAGGATCGGGTCGGCGCCAATACACGCGGAACGCAGCAATAGGACA
CCCGCTGAAGGTGGACGGTGGCCGGATCGTCGCTACGGTAGAGATCGGCGGACGAACGATGCCCGCCAC
GATCGATACCGGAGCCACTCGTAGCTTCATGAGTGAGGACTGCGTACGCAAATGGACGATCCGGGGAAG
AGCGGAGGAAATACAGGCACGTATCCGCCTGGCAGATGGGTCCACCCTAGAGGTAGAAAAGGCTTTGAG
AGTAGACGCCAGCTTAGGGGGAAAAACGATCAGCATGACGATGCTCATCATGCCCTCAATGCTGGACCAC
GTAATCCTGGGTATGGATTTTTTGGGGGCGATGAATACCACAATACGCTGCGGCGAGGCGGAACTGGTCC

TAGAAGCAGCGACGACACCGGGCGTGAGGCCAGGAACGAGCGAGGAGTCGGAGGAAGGCCGCCCAAGG
CATGAAAAGGGAGCACCTGCTCGGCTACAGAGGGAGCCGGAGGAAGGCCTCCCAAGGCATGGAAAGGGA
GCACCTGCTCGGCTACAGAGGGAGCCGGAGGAAGGCCTCCCAAGTCATGGAAGGGGAGCACCTGCTCGG
CTACAGAGGGAGCCGGAGGAAGGCCTCCCAAGTCATGGGAAGGGAGCACCTGCTCGGCTACAGAGGGAG
CCGGAGGAAGGCCTCCCAAGTCATGAAAAGGGAGCACCTGCTCGGCTACAGAGGGAGCCGGAGGAAGGC
CTCCCAAGTCATGANAAGGGAGCACCTGCTCGGCTACAGAGGGAGCCGGAGGAAGGCCTCCCAAGTCAT
GATGGGGGAGCACCTGCTCGGCTACAGAGGGAGCCGGAGGAAGG
>160_rnd-1_family-2__Unknown
TTATGTCGTTTTGGAACGTCATATCTCCGCGGGGAGTTNTGTCGTTTTGGAACGTCATATCTCCGCGCGGA
GTTATGTCGTTTTGGAACGTCATATCTCCGCGCGGAGTTATGTCGTTTTGGAACGTCATATCTCCAGCGAG
GAGTTATGTCGTTTCGGAACGTCATATCTCCGCGCGGAGTTATGTCGTTTTGGAACGTCATATCTCCGCGC
GGAGTTATGTCGTTTTGGAACGTCATATCTCCGCGCGGAGTTATGTCGTTTTGGAACGTCATATCTCCGCG
CGGAGTTATGTCGTTTTGGAACGTCATATCTCCGCGGGGAGTTCCGAGGTTTTGGAAAATCTCGAAAAAGT
TTCGATTTTCGCACCGACCTCTATTTTGAAAAAAAGCGTGATGTAACCCCTTGCCATTTTGTCGATTTGGGT
CAAAATTTTGGATTTCCTTTTTTTGATGCCAATCGTTAGAACTGATCCTTACGAGTCAAAAATGGCATAAA
ATTCCAAAATCGGACATTATTTGACGGAAATATTGCAAAAAATCATCAAAAAGGTTGAATTTACGAACGA
ATCGGTTTTTTGTCAACAACATTTTTCAACCCGTCGATGATTAATTTGTTTGAATGCCAATCGATGGTAGG
GATCCGTACGCATTCAAAAAGGTATAACATTTTAAAATCAGACATTATTTACCCGAGATATTAAAANAAA
TCATCGAAAAAGTTTCGATTTTCGCNCCTACCTCGATTTTGAANAATGTCGTTTTGAAGGTCATATCTCCG
CGGGGAGTTATGTCGTTTTGGAACGTCATATCTCCGCGCGGNGTTATGTCGTTTTGGAACGTCATATCTCC
GCGCGGAGTTATGTCGTTTTGGAACGTCATATCTCCGCGCGGAGTTATGTCGTTTTGGAACGTCATATCTC
CGCGCGGAGTTATGTCGTTTTGGAACGTCATATCTCCGCGCGGAGTTATGTCGTTTTGGAACGTCATATCT
CCGCGCGGAGTTNTGTCGTTTTGGAACGTCATATCTCCGCGCGGAGTTATGTCGTTTCGGAACGTCATATC
TCCGCGCGGAGCTATTACTCGAGATATCGCAAAAAATCATCGAAAAAGTTTCGATTTTCGNACCGACCTCT
NTTTTGNCAANAAGCGTGNTNCAACCCCTTGCCATTTTGTCGATTTGGGTCAAAATTTTGGATTTCCTTTTT
TTGATGCCAATCGATGGNACNGATCCTTACGCAGTCAAAAATGGTATAAAATTCCAAAATCGGACATTAT
TTGNCCGAGATATTACAAAAAATCATCGAAAAGGTTTCGANTTTACGAACGAATCGGTTTTTTGTCAACA
ACATTTTTCAACCCATCGATGATTAATTTGTTTGAATGCCAATTGATGGTAGGGATCCGTACGCATTCAAA
AAGGTATAACATTTTAAAATCAGACATTATTTACCCGAGATATTAAAAAAAAATCATCGACAGACCTCAT
TTGAAAAAAGTCGTGATTTGGAAGGTCATATCTCCGCGAGGAGTTGTGTCGTTTTGGAAGGTCATATCTCC
GCGGGGAGTTATGTCGTTTTGGAACGTCATATCTCCGCGCGGAGTTATGTCGTTTTGGAACGTCATATCTC
CGCGCGGAGTTATGTCGTTTTGGAACGTCATATCTCCGCGCGGAGTTATGTCGTTTTGGAACGTCATATCT
CCGCGCGGAGTTATGTCGTTTTGGAACGTCATATCTCCGCGCGGAGTTATGTCGTTTTGGAACGTCATATC
TCCGCGCGGAGTTATGTCGTTTTGGAACGTCATATCTCCGCGCGGAGCTATTACCCGAGATATCAAAAAA
AATCATCGAAAAAGTTTCGATTTTCGCACCGACCTCNATTTTGAAAAAAAGCGTGATGTAACCCCTTGCCA
TTTTGTCGATTTGGGTCAAAATTTTGGATTTCCTTTTTTTGATGCCAATCGNTAGAACTGATCCTTACGAGT
CAAAAATGGTATAAAATTCCAAAATCGGACATTATTTGACGGAAATATTNCAAAAAATCATCAAAAAGGT
TGAATTTACGAACGAATCGGTTTTTTGTCAACAACATTTTTCAACCCATCGATGATTAATTTGTTTGAATGC
CAATCGATGGTAGGGATCCGTACGCATTCAAAAAGGTATAACATTTTAAAATCGGACATTATTTACCCGA
GATATTAAAAAAAATCATCGAAAAAGTTTCGATTTTCGCACCGACCTCGATTTCGAAAAAAAACGTGACG
ATTTGGAANGTCATATCTCCGCGGGGAGTTATGTCGTTTTGGAANGTCATATCTCCGCGNGGAGTTATGTC
GTTTTGGAACGTCATATCTCCGCGCGGAGTTATGTCGTTTTGGAACGTCATATCTCCGCGCGGAGTTATGT
CGTTTTGGAACGTCATATCTCCGCGCGGAGTTATGTCGTTTTGGAACGTCATATCTCCGCGCGGAGTTATG
TCGTTTTGGAACGTCATATCTCCGCGCGGAGTTATGTCGTTTTGGAACGTCATATCTCCGCGCGGAGCTAT
TACCCGAGATATTAAAAAAATATCATCGAAAAAGTTTCGATTTTCGCACCGACCTCGATTTTGAAAAAAA
GCGTGATGTAACCCCTTGCCATTTTGTCGATTTGGGTCAAAATTTTGGATTTCCTTTTTTTGATGCCAATCG
NTAGAACTGATCCTTACGAGTCAAAAATGGTATAAAATTCCAAAATCGGACATTATTTGACGGAAATATT
NCAAAAAATCATCAAAAAGGTTGANTTTACGAACGAATGGGTTTTTTGTCAACAACATTTTTCAACCCATC
GATGATTAATTTGTTTGAATGCCAATTGATGGTAGGGATCCGTACGCATTCAAAAAGGTATAACATTTTAA
AATCAGACATTATTTACCCGAGATATTAAAAAAAAATCATCGAAAAAGTTTCGATTTTCGCACCGACCTCG
ATTTTGAAAAAAACGTCGTGATTTGGAAGGTCATATCTCCGCGCGGAGTTATGTCGTTTTGGAACGTCATA
TCTCCGCGCGGAGTTATGTCGTTTTGGAACGTCATATCTCCGCGCGGAGTTATGTCGTTTTGGAACGTCAT
ATCTCCGCGCGGAGTTATGTCGTTTTGGAACGTCATATCTCCGCGCGGAGTTATGTCGTTTTGGAACGTCA
TATCTCCGCGCGGAGTTATGTCGTTTTGGAACGTCATATCTCCGCGCGGAGTTATGTCGTTTTGGAACGTC

ATATCTCCGCGCGGAGTTATGTCGTTTTGGAACGTCATATCTCCGCGCGGAGTTATGTCGTTTTGGAACGT
CATATCTCCGCGCGGAGTTATGTCGTTTTGGAACGTCATATCTCCGCGCGGAGTTATGTCGTTTTGGAACG
TCATATCTCCGCGCGGAGTTATGTCGTTTTGGAACGTCATATCTCCGCGCGGAGTTATGTCGTTTTGGAAC
GTCATATCTCCGCGCGGAGCTATTACCCGACGTATTAAAACAAATCATCGAAAAAGTTTCGATTTTCGCAC
CGACCTCTATTTTGAAAAAAAGCGTGATGTAACCCCTTGCCATTTTGTCGATTTGGGTCAAAATTTTGGAT
TTCCTTTTTTTGATGCCAATCGTNAGAACTGATCCTTACGAGTCAAAAATGGTATGAAATTNCAAAATCGG
ACATTATTTGACGGAAATATTCCAAAAAATCATCAAAAAGGTTGAATTTACGAACGAATCGGTTTTTTGTC
AACAACATTTTTCAACCCATCGATGATTAATTTGTTTGAATGCCAATTGATGGTAGGGATCCGTACGCATT
CAAAAAGGTATAACATTTTAAAATCAGACATTATTTACCCGAGATATTAAAAAAAAATCATCGAAAAAGT
TTCGATTTTCGCACCGACCTCGATTTTGAAAAAAATCGTTNTGGAACGTCATATCTCCGCGCGGAGTTATG
TCGTTTTGGAACGTCATATCTCCGCGCGGAGTTATGTCGTTTTGGAACGTCATATCTCCGCGCGGAGTTAT
GTCGTTTTGGAACGTCATATCTCCGCGCGGAGTTATGTCGTTTTGGAACGTCATATCTCCGCGCGGAGTTA
TGTCGTTTTGGAACGTCATATCTCCGCGCGGAGTTATGTCGTTTTGGAACGTCATATCTCCGCGCGGAGTT
ATGTCGTTTTGGAACGTCATATCTCCGCGCGGAGTTATGTCGTTTTGGAACGTCATATCTCCGCGCGGAGT
TATGTCGTTTTGGAACGTCATATCTCCGCGCGGAGTTATGTCGTTTTGGAACGTCATATCTCCGCGCGGAG
TTATGTCGTTTTGGAACGTCATATCTCCGCGCGGAGTTATGTCGTTTTGGAACGTCATATCTCCGCGCGGA
GTTATGTCGTTTTGGAACGTCATATCTCCGCGCGGAGTTATGTCGTTTTGGAACGTCATATCTCCGCGCGG
AGTTATGTCGTTTTGGAACGTCATATCTCCGCGCGGAGTTATGTCGTTTTGGAACGTCATATCTCCGCGCG
GAGTTATGTCGTTTTGGAACGTCATATCTCCGCGCGGAGTTATGTCGTTTTGGAACGTCATATCTCCGCGC
GGAGTTATGTCGTTTTGGAACGTCATATCTCCGCGCGGAGTTATGTCGTTTTGGAACGTCATATCTCCGCG
CGGAGCTATTACCCGAGATATTAAAAAAAAATCATCGAAAAAGTTTCGATTTTCGCACCGACCTCGATTTTG
AAAAAAANCGTGATGTAACCCCTTGCCATTTTGTCGATTTGGGTCAAAATTTTGGATTTCCTTTTTTTGATG
CCAATCGTTAGAACTGATCCTTACGAGTCAAAAATGGTATAAAATTCCAAAATCGGACATTATTTGACGG
AAATATTCCAAAAAATCATCAAAAAGGTTGAATTTACGAACGAATCGGTTTTTTGTCAACAACATTTTTCA
ACCCATCGATGATTAATTTGTTTGAATGCCAATTGATGGTAGGGATCCGTACGCATTCAAAAAGGTATAAC
ATTTTAAAATCAGACATTATTTACCCGAGATATTAAAAAAAAATCATCGAAAAAGTTTCGATTTTCGCACCG
ACCTCGATTTTGAAANANGTCGTTTTGGAAGGTCATATCTCCGCGCGGAGTTATGTCGTTTTGGAACGTCA
TATCTCCGCGCGGAGTTATGTCGTTTTGGAACGTCATATCTCCGCGCGGAGTTATGTCGTTTTGGAACGTC
ATATCTCCGCGCGGAGTTATGTCGTTTTGGAACGTCATATCTCCGCGCGGAGTTATGTCGTTTTGGAACGT
CATATCTCCGCGCGGAGTTATGTCGTTTTGGAACGTCATATCTCCGCGCGGAGTTATGTCGTTTTGGAACG
TCATATCTCCGCGCGGAGTTATGTCGTTTTGGAACGTCATATCTCCGCGCGGAGTTATGTCGTTTTGGAAC
GTCATATCTCCGCGCGGAGTTATGTCGTTTTGGAACGTCATATCTCCGCGCGGAGTTATGTCGTTTTGGAA
CGTCATATCTCCGCGCGGAGTATTACCGACGTTTTGGAAAAATATCATCGAAAAAGTTTCGATTTTCGCAC
CGACCTCTATTTTGAAAAAAAGCGTGATGTAACCCCTTGCCATTTTGTCGATTTGGGTCAAAATTTTGGAT
TTCCTTTTTTTGATGCCAATCGTTAGAACTGATCCTTACGAGTCAAAAATGGCATAAAATTCCAAAATCGG
ACATTATTTGACGGAAATATTGCAAAAAATCATCAAAAAGGTTGAATTTACGAACGAATCGGTTTTTTGTC
AACAACATTNTTCAACCCATCGATGATTAATTTGTTTGAATGCCAATTGATGGTAGGGATCCGTACGCATT
CAAAAAGGTATAACATTTTAAAATCAGACATTATTTACCCGAGATATTAAAAAAAAATCATCGAAAAAGTT
TCGATTTTCGCACCGACCTCGATTTTGAAAAAAATCGTGATGAAGGTCATATCTCCGCGGGGAGTTATGTC
GTTTTGGAACGTCATATCTCCGCGCGGAGTTATGTCGTTTTGGAACGTCATATCTCCGCGCGGAGTTATGT
CGTTTTGGAACGTCATATCTCCGCGCGGAGTTATGTCGTTTTGGAACGTCATATCTCCGCGCGGAGTTATG
TCGTTTTGGAACGTCATATCTCCGCGCGGAGTTATGTCGTTTTGGAACGTCATATCTCCGCGNGGAGTTAT
GTCGTTTTGGAACGTCATATCTCCGCGCGGAGTTATGTCGTTTTGGAACGTCATATCTCCGCGCGGAGTTA
TGTCGTTTTGGAACGTCATATCTCCGCGCGGAGTTATGTCGTTTTGGAACGTCATATCTCCGCGCGGAGTT
ATGTCGTTTTGGAACGTCATATCTCCGCGCGGAGTTATGTCGTTTTGGAACGTCATATCTCCGCGCGGAGT
TATGTCGTTTTGGAACGTCATATCTCCGCGCGGAGCTATTACCCGAGATATTAAAAAAAAATCATCGAAA
AAGTTTCGATTTTCGCACCGACCTCGATTTTGAAAAAAAACGTGATGTAACCCCTTGCCATTTTGTCGATT
TGGGTCAAAATTTTGGATTTCCTTTTTTTGATGCCAATCGATAGAACTGATCCTTACGAGTCAAAAATGGT
ATAAAATTCCAAAATCGGACATTATTTGACGGAAATATTCCAAAAAATCATCAAAAAGGTTGACTTTACG
AACGAATCGGTTTTTTGTCAACAACATTTTTCAACCCATCGATGNTTAATTTGTTTGAATGCCAATTGATG
GTAGGGATCCGTACGCATTCAAAAAGGTATAACATTTTAAAATCAGACATTATTTACCCGAGATATTAAA
AAAAATCATCGAAAAAGTTTCGATTTTCGCACCGACCTCGATTTTGAAAAAAATCGTGATGATTTGGAAG
GTCATATCTCCGCGCGGAGTTATGTCGTTTTGGAACGTCATATCTCCGCGCGGAGTTATGTCGTTTTGGAA
CGTCATATCTCCGCGCGGAGTTATGTCGTTTTGGAACGTCATATCTCCGCGCGGAGTTATGTCGTTTTGGA

ACGTCATATCTCCGCGCGGAGTTATGTCGTTTTGGAACGTCATATCTCCGCGCGGAGTTATGTCGTTTTGG
AACGTCATATCTCCGCGCGGAGTTATGTCGTTTTGGAACGTCATATCTCCGCGCGGAGCTATTACCCGAGA
TATTAAAAAAATCGTCGAAAAAGTTTCGATTTTCGCACCGACCTTCATTTTGAAAAAAAGTCGTGATGATT
TGGAAGGTCATATCTCCGCGCGGAGTTATGTCGTTTTGGAACGTCATATCTCCCGCGCGGAGTTATGTCGT
TTTGGAACGTCATATCTCCGCGCGGAGCTATGTCGTTTTGGAACGTCATATCTCCGCGCGGAGTTATGTCG
TTTTGGAACGTCATATCTCCGCGCGGAGTTAT
>160_rnd-5_family-6782__LTR___Gypsy
TATAAGTTAACAGAGGCCAACGACACCAGCTCCAGATGCTGATATAGCACTGAGCCGGATGTTCTTACGA
GCTAACAGGGGTCAGCGAAATGCAACATAACACCAGCCGCAGATGCTGACATAGCTCTGGGCCGGATGTT
CTTGCACGACGGCAGCGCACGAGGCGCAAGCTAAGCATTCTAGCGGCCGTTGTGAAAGCACACAATAGCT
TAGGTATTTTTGATCAATCTTTTGTAAATTTAGTTTTTAATTCGACTTTCACTGCGGCACATCGGCCCAGCG
ACTTCGTGTATAATTTAACTCACTCACTACGGCGTGTCGGCCTAGTGTTTTATAAATCATTATCAAAATAA
AGTTCTATCAAAATAATCCGCGAGGACTTATTATTTAAAACCTAATTGGCGCCCAACGTTTTTCGGACCAT
TTTGTTCCATAATTATTTTGTGTGCTGTGCAGTGTAAAGAACTAACCTACCAGTGCAAACAATAAAAGCTG
TGCCTGACCTACATCAACTTACGTCAGCGGCACAAGCACAATAAAAACCATTTGATACACCGAAGCCGCT
GGAGCTCTTCGGCCAGCGACTTGTCACCAAACCAGCGCGCAAGCGGGTTTGGGCAGCGACGTAAACCAAC
ACCACAGACTTCAGCGAGGGATAAAAAAAAAGCATTCGCGACTGCAGCGTGGGAATCAGTAGTTTTATAC
GTAGAACATACAACCTTTCTAGGATCAAATAAAATGTAAGTGGGCCATCATTTGGCACAAACTTTTTTCAA
TTTGCGTGATTTATTTAAAATAAAATCGTAAAAAAATATAAGTAGAAATATTAAAATTGCAGTAATTGTGT
TCCCAACTAACTTTGAGTAACACAATTTTGCGTCCATCAGCAATTTTCAATAAACTCTCCTCTGTCAGCATT
CATTTAATTTACCGTTGTTGATTTGCCCTTGAAATTGTGTTGTTATTATTGTTGACAAGTATAGTCCATGAA
CAGCTCGTTTCTCAGGTACAGAGACCGCTCTGCGAGGGGATCTGAGTCAGACGACTCCACCACCTTAGAT
AGGGAAGCCGTCCCGCAAAAAATCGCTGACAACAGGTGTAAAATAGGAATGGAGCCTGAGCAGCTTAGG
GCCTTGGTGCAGGCCGCAGTGGCCTCCGCCTTAGAAGCACAGAGCGCAGTATTGAAGCAAAGAGATGCA
GAGCTGAGGGCTTCCTTAGACGCTCGGATACAAGAATTGGCAGGCAGAATATCTGCCAGTAGTATAGAAA
CACCGCCGGTCCAGGCATTTGAGCCGGTGGAAATCAGAAACGACGTCCAATGTAATGAACCATTGGACGC
CGTTAAATGCTTACCAGAATTTACGGGGGTGCCAGAGACGTACGCCTCTTGGAGACAAGCTGCGATAGCG
GCATATAAAATTTACAAGCCATACGACGGCAGCTCACGCCACTACCAGGCGGTCATTATTATTAAGAGCA
AAATTAGAGGGGCAGCTGACGGCGTTTTGGGTCAGTTTGGCACAGTAATGAATTTTGATGCCATAATAAA
CCGTCTCGATTTCACATACAGGGATAAGAGAGGCATTCACGTCATAGAGCAAGAGATGAGCACCCTAAGA
CAGGGCTCGTTAACAATTTTACAATACTATGACGAGGTTGAGAAAAAGCTTACTCTTCTCACCAATAAGGT
CAACATGTCCTATGAACCAAGCATGGCACAAGGGCTCTGTACGAAGTTCAGAGATGATGCGTTGCGTGTT
TTTGTCTCAGGCCTAAAGCGTAGCCTGACGGATGTCCTGTTCGCGGCAAAGCCTAAGGACTTACCGTCGGC
CCTAGCNCTAGCGCAAGAGGTAGAGGCAAACCATGAGAGGTACGCCTTTGCCGCTAGCTTCGCTAAAGGG
CAAGAAGATAGGGAACGAAAAGTGACCCCGAGGCCGCAGGTCCGTCAGCAAGATAGGGGTCAACAGCAG
GGGGATCCGCAACCCAGCGGTTCCAAAAATCCTCACTTCACTAAACAACAGAAACAGCAACAAAGTCAAT
TTGAGCAACAGAGTGGCAGGTGGAACAAAGGTGGCGGGTCACAGGAACCAATGGACGTCGATCCATCTG
TGTCAAAAATTCTACAACCATCGCAAGCACCAGCCTACGTTAACTCTAGCGCTCCTCAAGCCTACAAAAG
AACGGCCACGTCTGGGCGCTTAAGCGGACAGAGGAGGCAGAGAGTCAATCACACAGCTCACGAGCCNGA
TCAAGATTCAGGTTCGTATGCTAGCTCTGCAGCAGCAGCAGAACAGCAAATTGCCGACGACAATGGTAGC
GGNTATGACTCAGACACTCTCAATTTTTTAGGGGAAGCTCCCTGCTGCCCTACATCAGTAGGAGAGTAGC
AGGGATCCAGATGAGATTTCTCATCGACACGGGCGCTTCCAAGAATTATATCAGGCCACATAAAAATCTA
AAAGGTGTGTGCCCAGTAAATTCTTCCTTCTCTACCCATTCTATACACGGCACCACGAAGATCACTCATAA
GTGCCTTATATCTCTGTTCGGCAAAACTGCCACATTTTTCTTACTCCCAGACCTGTCCACATTTGATGGAAT
TGTGGGGCTCGACTTGCTCACGCAAGCAGGAGTGTCGCTTTGTCTTTCTTCTGGCCAACTCAAATGTGGCG
CAGAAACTGAACAAATCGAATTCCACAGGTGCGCGGATGTTAACTTTACCAATGTTGACTGCGCCGACGC
GCCCGATGCTGTAAAAGATGCATTCCTAAAGATGCTAAAAAGCAAACATAAGGCCTTCGCGGATCCGGAA
GAAGCACTTCCCTATAACACATCAGTGGTGGCAACCATCAGGACCACTGATGAACAACCAGTTTATGTCC
GACTGTACCCCTATCCCATGGGAGCAGCGGAATTCGTTAATAAAGAGATTCAAAGCCTTCTGAAGAACGG
TATCATCAAAAAATCTGTCTCCCCCTACAACAACCCCATTTGGGTGGTGGACAAGAAGGGGACGGACGAG
TTTGGTAACAAGAATATGCGCCTCGTAATGGATTTTCGCAAACTTAACGAGAAAACCATCGCAGACAGAT
ACCCTATGCCTAACATTCCAATGATTTTGGGGAATCTTGGCAAAGCTAAATACTTTTCGACATTGGACCTA
AAATCCGGCTATCACCAGATCACGCTAGCAGAGCGTGACCGTGAAAAAACAGCCTTTGCAGTTAACGGGG
GGAAGTACGAATTTTGTCGACTTCCCTTTGGGTTGAAGAATGCGGCCAGCATCTTCCAAAGAACTGTTGAC

GACGTACTGCGTGAGCAAATTGGCAAATCTTGCTATGTCTACGTTGACGATGTCATCGTCTTCTCAAAAGA
CGAAAGTTCTCATGTCCAACATGTAGAGTGGGTCTTAAAGAGCCTGTACGAGGCAAATATGAGAATCTCC
AAGGAAAAATCGCGTTTTTTAAGAAAAGCGTAGGTTTTCTTGGTTTCGTGGTAACCAGTAATGGAGCTAC
AACGGACCCCGAAAAAGTCAAAGCGATTCAGGAGTTCCCAGAACCAAAGAATATATTCGAGATCAGATC
ATTTTTAGGACTGGCCAGTTACTATAGGTGTTTCATCAAAGACTTTGCCTCAATAGCAAAACCCATTTCTG
ATCTTCTAAAGGGCGAGAATGGGTCAGTAAGTAAACATAGATCGCGAAACATCCCGGTAGAATTCTCTGA
GCTTCAGCGTCACGCATTCCAAAAGCTACGCAACATTTTGTCATCAGAAAACGTCATGCTCAGATACCCTG
ACTTTAAGAAGCCGTTTGATCTAACGACAGACGCTTCAGCTTACGGCATAGGGGCAGTGCTTTCTCAAGA
GGGTCGCCCCATCTCAATGATTTCCAGAACATTGAAACCCCCGGAGGTGAACTACGCCACCAACGAGCGG
GAACTTTTGGCTATAGTTTGGGCCCTTAGCAAGCTACGTCACTACCTATATGGGGTAAAAGACATTAACAT
TTTCACGGACCACCAACCGTTAACTTTTGCCGTTTCTGAGTCAAACCCGAACGCCAAAATTAAAAGATGG
AAGGCGCGCATTGAGGAAACGAATGCGCGAATATTTTATAAGCCAGGCAAGGAGAATTTAGTTGCTGATG
CGCTATCGCGACAACAGCTCAACGCTCTTGACGAGCTGAATACTGATTCCTGCGCAGCCACAGTCCACAG
CGAGCTGTCCTTGACCTATACAATTGAGTCGACCGAAAAGCCACTTAATTGTTTCCAAAATCAAATTGTCA
TTGAAGAGGCACGCGTCCCCACCAAACGCAACTTCATTCTTTTCGGGGACAAGAGGCGTCATGAAGTGAG
CTTCACTGATCGCACAGCCCTTCTAGATGAACTCCTTGACTTGATTAATCCAAATGCGGTCAATGCCCTCT
ATTGCAACCTGCACACACTAGCACAGGTGCAGGATGGCTTAGTTCGCATGTTTCCAGGCACAAAGTTCTG
GCACTGCAAAAACCGAGTTGCAGATGTCTTCAACGTTGATGAGAGGAGAGAAATTCTCGTTGCCGAACAT
AATAGAGCGCACAGAGCAGCTCAAGAAAATGTTAAGCAAGTGTTATCAGAATACTATTTCCCTAAAATGG
CGAAGTTGGCTAAGGAAATTGCGGAAAGTTGTAGAATCTGCTCAGCAGCCAAATACGATAGGCACCCGA
AAAGACAGGAGCTGGGAGAAACTCCGATCCCTGAGACTCCTGGGGAATTTCTACACATTGATATTTTTTCC
ACGGACAAAAAATTTTTTTTGACCTGTGTTGATAAGTTCTCTAAATTTGCAGTCGTACAACCAATTCAGTC
GCGCACTATCGGGGACTTAAAAGCTCCACTACTGCAGCTCACGAATTTCTTTCCACGAGCCAGAACAATTT
ATTGCGACAACGAGCCTTCGATGAATTCTCAAACGATAAAGACCCTGTTGTTAAACAACTACGGAGTCCA
TATTGTTAATGCGCCGCCGCTGCATAGCACCTCCAATGGCCAAGTCGAGCGCTTCCATAGCACTCTAGCCG
AGCTTGCACGTTGCCTCAAGCTGGAGAGAAACATCAGTGACTCAGTCGAAGTCATAATGCTAGCCACGAT
TGAGTATAACAAGACAATACATTCGGTCATCAACAAGAGACCTGTTGATGCCCTCCATGTGCCCTCAGGC
GAACCCGAAGGGGAAATATCAGCAAAGTTAAGAAGTGCACAAGATGCGCTCCGGGCTAGGCTGAACGAC
CAGCGACAGAATAGAGTCTTTGAAGTAGGCGAAAAAGTNCTAGTAAAAAGAAACCGCAGGCTTGGCAAT
AAACTCACGCCCTTATGTGAAGAACGGATCGTAGAGGCAGACCTTGGCACGACCGTTCTCATTGGAGGGA
GGGTGGTCCACAAGGACAACCTTAAGTAGGGCGCGAGAGCTGAAATAAAACATCGATTCTATGCATCTTG
AATTTAACTATAAGTTTCAACATTTGAGCCACTAGGCTTCACCTTTAAGTTTTAATATTTACGCCACTTGGC
ATTNCCTTTAGGAAGACACGTNGTAATAGTTTGGTCCGTTTTGTCTGTCTTCCGCAGGTTCGGGTTTCTGCT
ATTTCTATTTTTTGCAAGTGCGGCAGCGCGTGTGACTAATTACTCACACGCCAAGTATATTCCCATTGTTG
AGGGCCAAGTTCTGGTCTGGGAGGAATTCAATTTCGTCAGGCACACAGCAAACATCTCTGAGTACGAGAA
AATGGTAAACGAAACAATGGAGCTATGTGAAATGTTTCCGCAGTCACACATGAGAAAGCTTCTAAGCGTA
GATGCGTCCCACCTCAGAGATCTGTTAGAAGCTCTTGGCGTGCACCACAGGGTGGCAAGAAGTTTAGATT
TCTTGGGCACAGCCCTCAAAGTAGTTGCGGGAACACCTGACGCCAGTGACTTCGAGAAGATCAAATTCAC
TGAGTTACAGTTGGTGAACTCTAACAACNGACAGGTAGCTATCAACACAAAAGTTCAGGAACAAATCAAT
CAACTCACTACAAGTGTCAACACATTACTAAAAGCTGCAAAAAAGAATCAGATAGATTCCGGGCATTTAT
ACGAAACTCTTTTAGCTCGGAATAGAATGTTGCTGATGGAAATGCAGAATTTGATGCTCGCTGTTACCCTT
GCAAAAATTAATATCATAAGCCCAAATATATTAGATCACGCAGATTTGAAATCTGTTTGGGTCGATGAAC
CCACAGGTACTTCAATAGCTGACCTTATGTCCGTTGCGTCCGTGAAAGTATTACAATCTGTTAATGCCCTT
CACTTTATCATTAAGTTCCCGAAAATCAAAATGGCTTGCAGAAAGGTGATGCTGTATCCAGTTGCACATGA
GAATGCTGTGCTACAAATTGACGACAACATTATAGCTGAGTGCGGAGATCAAGTGCTTGCCCTTAAGAAT
TGCACTTCCACGCCGGGAGGAACCTTTTGTCAACTTTCACCGACCAGCTCTTGTGCCAGAGAGCTGCATGC
TGGTGGCTTGGCAAACTGCCAAATGCAGCCGAGTCACTTAGACCCGATCACATGGGTCGATGACGGAATT
CTGATCATCAATAACCGGCCTGCTAAAGTTTGCGCTGACAACGGTACTGAGACTTGGGTTCACGGAACGA
ATCTAATCACGTTCAATAATTGGGCTCTGATCAACGAAACTAAGTACTTGAATCGCAACAACGTCCAGAG
TAAGTTCCCCGGCATCGCGGCATCTCCCCTTCTGAACGTCACCGGCCACCAAGACGTGCTGAGTCTCCCCT
ACCTTCATCGCCTGAATGAACGTAACCTGGAGATCATCAAGGAAGTCAAGGACGAAATTGGAAGCGTCGA
CAGCGTGGTAATAGCCTTCTTGTTGGGTGCTGTGTGTTGCACCATAATTTGCGTCGGAGTCACCTACCGTC
GGTTCACCAAAAACATGGCATCGGCCAAGGAGATTAAGAAGGTGATCGCCGAGATAGGAGCGGCCGAGG
GCGGCCTTAACTTAAAAGGGGAGCAGTTAACAGAGGCCAACGACACCAGCTCCAGATGCTGATATAGCA

CTGGGCCGGATGTTCTTACGAGCTAACAGGGGTCAGCAAAATGCAACATAACACCAGCCGCAGATGCTGA
CATAGCTCTGGGCCGGATGTTCTTGCACGACGGCAGCGCACGAGGCGCAAGCTAAGCATTCTAGCGGCCG
TTGTGAAAGCACACAATAGCTTAGGTATTTTTGATCAATCTTTTGTAAATTTAGTTTTTAATTCGGCTTTCA
CTGAGGCACATCGGCCCAGCGACTTCGCGTATAATTTAACTCACTCACTACGGCGTGTCGGCCTAGTGTTT
TATAAACCATNATTAAAATAAAGTTCTATCAAAATAATCCGCGAGGACTTATTATTTAAATCCTAATTTAT
ATAT
>9_rnd-5_family-6356__LTR___Gypsy
GTGTAGCATGCGTACATATGTATATACATACATATGAATAAGGCTGCAACACTTTGTTGTTATGCCAACAG
AATGCAAACATAAGCAGCGACTTTGTCGCAGCGCGCCTAACGCTAAGTGCCCTCTGCGCATATTCAAGTG
CAGACGAAATATGATCCGCACTTGATAACAACGACCGCCCAAATAGCTAAGTCAGCGTTCTCACGCTTGA
CCGTCGCTGTGTATACATATGTGCGCGCATGACTCTCTCTGACTCTCTTGCTGTATATAATAAGCCTAAAA
CCATGTATGTAAAACGGCTGCTCACTGCTTCACGGTGGACAGCTCTAACTCCAAAAACATTGAATAAAGA
ACCATCAAGGAAGACTGACGTCTTTTCATCAGGGAACTATTATATGGCGACCGTGACAGGACTGGATCAG
CTCCTGTTTTAGCTGATCGAACCGCTGTATGTACTGTAACTATTGAAGAGGACGAAGGAGGCAACAACAA
CAAGCAAAGGCGGAACAAAAAAAAAAAAGCTGAGTGAGTAGAGTGTAGTGAGTGTTATGGGTCGCGGTG
GCCGGGTTAAAAAATTGAATAAAGAAATTGCTCACATTAAGTGGCAATTATATTCACCCAAAGCCAACAC
ACCGTGGAAAGCAACAGCAAGGCTACAGGCCATGCTTGACTTTTATCAGCAACAGCTGAAACAGATAAA
AGAAAACCCTACACCGCATACTCACTCAGTGAATATTAAAGTTGTTAATGACAAAACTATAACCACACCA
CCTCCCAAAAGCAAAGTGCCTCAGTACGTCAAGCAATGCCCGGCAGATTGCCCTGGACCTCTGGACAGCC
CACACTGCAGATCGACTTGCCTGGTAAGGCAGTNACCTGTGCGAAGACCGGCACCGGTTCCGCCTGAGCC
CATAGGCCCAGGACAACAGCAGCTGAGAAATCAACAAGCGCGAGAGGCACCCCAGCCCCTTGAGCCTTC
GAAGGGAGCGTCAGCGCAACCATACTGGTGATGACCAGCTGACGCGGAAGAACAGGAACAGCAGACACC
AGCAATAATTTTTAAAATAAATATATATATATTATATTATCGAAACAATTTATGTAAAATTGAGCAGAAAA
ATCTTTTGCCCAATCAGAAGTGAGGCCCATAAAGCCACACTAAAAAAATTAGGCAGGTTGCTATGTAAAA
AAAAAAAAATTTTTACACGTCTCAGGCACTGGGTAAACTACCCAGTAGCAAACAAATTAATCCAAATCTT
AAATTCCAAATAAATTTTTGAAATTTTAAAATGAAAAAAAAATAATAAGTTTTTCAGTTCAGTCAGTTTAA
TCAGAAATAAAAAAAAAAAAACAAATTGAATCAAATTAAAGCCATAGGCAGGAAAAAATGTTCTATTTA
TGAATAGTAATGCAATAAGTAAGTTTTTCCGAAGATTTTCTAATTGAATCAAATCATAATTCTGAACAGAA
CTCCAAAATGGGATTATTAGACGTTAAGTCAGTGGATTCCACTGCTAACGTTATAAATAAAGTTGAATCA
AACAACACAGATTTAGAATTAACAAATATATTACTGGTAATCATAGTTGTTATAATGTGTGCAAATTGTTT
TTATAAAATATATAAACTGCATAACAAATGCCTAAAGAAAAGATTCATGAGCCGAGCAAATGACTTGGAC
AAAATTTAAATTTGCACAGCACAAACAACAATGATTAATCATAATTGACAATATCATAATAACTATAAGA
TACCAATTAAACAAAATAAATAATAATGAACACACAGTATGATGAATAGTAAGAATATCCCAGAATAATC
TTGAAAACCAAAGTAGCTTGTCAATATTATGGAATGGCCAGAACTTTCTGAGCGGATCCAAAATATTAAA
ACGACATTTGAAAAAGCGTATAAATGTCTAACACAAAATAGACAAACGCATATAGATACAGTTAATAGGC
ACTCCATAACATTGATCGACAGCTTTAACGAAGCTCGAATATTGATATATCAACATAAAGATGTATTAACC
AATGAACATTGGTCAGAAACGTCAAGGTTGCTAATAAGACTGAGAAATAATATACAAACAGTTAAAGAC
AAGCATAATTTAAATTTAATACTACCTTCTATTTTGAATACACCCGCAAAGTTGGAACTCGGCGAAAACCT
TGAAGTTCGGACTGAGGATATTCAATTAGAAGAAGAAGATTTAACAAATCTAACAATCCCTGCTGTTATC
ACACTGGATTCCAAAGATATTTTATCCGAAAGTGAAGAAAGTGCTGATAATAGCCGTATAATGGTTGACA
CAGCCGCTGCCCAGAGGGCATATATTAGGGAAATTGCACAAGCAGTCCCAGAATTTGACGGAAAGAAAA
CCCGTCTTGAAAAATTCTTAACGGCATTAAGACTTGTCAACTTAACAAAGGGAACTTATGAGGAAATTGC
CATAGAGTTGATTAAATCAAAAATAACGGGCACAACCCTCAACAAAATAAAAAATGAAACCACAATAGA
CGGAATTATTGCAAAATTAAAAGCCGTAGTTGTCGGTGAAACACCCGCCAACATAAAGGCAAAAATGGA
CACTTTAACGCAGAAAGGCAAAACAGCCACTCAGTTCACAACTGAACTAGAAAATCTGCGACAGCTTCTT
GAAGCTGCTTATATTGACCAAGGCATACCATCTGAAAATGCTGACACGCTCAGCACTACAGAAGCCGTTA
ACACAATGATTAGTAAAACTAGTCACGAAAGTGTTAAAACCGTTATGGAGTCTGGTATTTTCACTACCATG
GATGCTGCCGTTAGTGCATACATCCGTACTAGTACAAGACTTACTGGTAGTGTGAATACAGTGATGTACTC
TAAAAGAGGGAATAACAACTACCGCGGTAGTAACAATCGTGGTAACTATCGTGGTAGAGGCAATGGTAA
CTACCAAAACAGAGGAAATAATAACTACGGTAATCAAAACCGTAGAGGCCGAGGAAATGGAAACAACAA
TTCCAACCGAGGCCGAAACAACCAAAATAATAATAATACAAACAATGTTAGAGTAACCCAGAACAATCA
GGGAAACTCTCAACAACCCTCAGATATTCAGCAGTAGGAAATTATGCGGTTCATACCATCAATCTTAGTTT
GAATATATTTGTAAACTTTTTCAATGTGAAGACAAATAAAAACTTGTATTTTTAATAGATACTGGTGCAGA
CATATCCCTGTTAAAGGAAAATTCTGATATCTTTCATGATATCCAAATAAATAAGAAGATCAATATTCAAG

GCATAGGCCAAGACATAATACAGTCCAAAGGACTGATTTCAATAGAGATACAGACAAGTAAATACATAA
TTCCACACGATTTTCACTTAGTAGATTTGCATTTCGCAATACCTTGTGATGGAATAATAGGCATTGACTTC
ATTAAGAAATTCAATTGCCAGTTAGATTTCAAACCAAATGAAGATTGGCTCATAATGAGACCAAATAATC
TAAATTTCCCAATTTATGTTCCAATAACATACAGTTCTGGTAATAATTCCATTGTTCTACCAGCAAGATCCC
AAGTAGTCAGAAAAATTCACCTATCATCAGATGAAGATAGTGTACTAATTCCAAACCAGGAAATTAAAGA
AGGCATTTATGTTGCAAACACAGTTGCAACAACCAAAAATGCATTTGTCCGATTACTGAATACGACCAGT
ACCGATCAAGTAGTAGATATTAAAAATTTGTCATATGAACCACTTTCGAACTACGACATAGTTCAAAACA
ATCCAGAAAATAGAGAAAAACTGTCTTATCTCAATTAGCTAAAAATTTTCCACCACAATTCAAAAACGAA
TTGACTAACTTATGCACCCAGTTTAGTGACGTATTCGGACTCGAAACCGAATCGATCACTACCAATAACTT
CTACAAACAAAAGCTAAGATTGAAGGATGATGAACCAGTCTACATAAAAAATTATAGAAACCCTCAAAG
TCAAATAGATGAGATACAAGTACAAGTACAAAAATTAATCGACAATAAAATAGTCGAACCGTCTGTATCC
CCTTATAACAGCCCACTTTTGTTAGTTCCGAAGAAATCTCTTCCAGGTTCTGACAAGAAAAATGGCGATTA
GTAATTGACTATCGTCAAATTAATAAAAACTGTTGTCGGATAAATTCCCGCTGCCTAGAATTGATGACATT
TTGGATCAACTAGGAAGAGCAAAATACTTTTCATGCCTTGATTTAATGTCAGGATTTCATCAAATTGAACT
CGAAAAAGTTCAAGAGATATAACGTCTTTCTCAACAAGCAATGGCTCGTATCGCTTCACGCGATTGCCATT
CGGTTTAAAAATAGCGCCGAATTCTTTCCAGAGAATGATGACTATAGCATTTTCCGGATTAGAACCTTCTC
AAGCTTTCCTTTATATGGATGATTTAATAGTCATTGGTTGTTCCGAAACACATATGCTCAAAAACTTAACT
GATGTTTTTGAGAAATGTAGGAAATACAATCTTAAATTACATCCTGAAAAATGTTCATTTTTCATGCATGA
AGTCACATTTTGGGACACAAATGCACGGATAAAGGAATCTTGCCTGACGACAAAAAAACGACGTCATTAA
AAATTACCCAGTTCCACATGATGCGGACAGCGCCAGACGATTCGTTGCATTCTGCAACTACTATAGACGTT
TTATCAAAAATTTTGCCGACTATGCTCGTCACATAACAAGATTATGTAAAAAAGGTGTAGAATTCGAATG
GACAGCTGATTGTCAGCATGCTTTCGAACATCTCAAATCGGCACTAATAAATCCGAATTTATTACAATACC
CAGATTTCAGCAAAGAATTTTGCATAATAACAGATGCAAGTAAACAAGCATGTGGAGCAGTTTTAACTCA
AAACAAAAATGGACTCCAACTTCCAGTCGCTTATGCATCAAGATCGTTTACTAAGGGTGAAAGTAATAAA
AGTACAACAGAACAAGAATTGGCAGCAATTCATTGGGCAATAACTTATTTCAGACCATATATTTATGGTA
GACACTTCAATGTCAAAACAGATCACAGACCATTAACATACTTGTTCTCAATGGTAAATCCAAGCTCAAA
GCTAACGCGTATGAGACTTGAACTAGAGGAGTATAATTTTACAGTCGAATATCTGAAGGGTAAAGACAAT
TTTGTAGCGGATGCGTTATCAAGAATAACCATTCAACAGCTACAACAAATAAATGGATCAATAAAGAAAG
TCACTACAAGATATCAAAGTAGACAAAATTCCTGCGCAGGAAAAGAAAAATAGAATTGCCATGGCAATCT
ATAGAAAAAGCTTCAAAGCCCAACGTATATAAAGTCATTAACAATGACGAAGTACGTAAAGTAGTGACCT
TGCATGTAAAAAATATGCTATGTTTTTTAAACATGGTAAGAAAATTACTGCAAGATATGATGTTAGCGATT
TATATACCAATGGAACTCTTGACTTAGGTCAATTTTTCCAAAGGCTTGAAACGCAAGCCGGTATAAATAA
AATCAGCCAACTCAAAGTGGCGCCGTGGGAAAATATCTTTGAACACGTTTCAATCGATAATTTCAAAAAT
ATGGGCAATAAAATATTAATTTCATTGAGAGTAGCGCTACTCAACCCGGTGACCCAAATAGAAACAATAA
AAGAAAAAGAAGCTCTATTGTCTACGTTCCATGATGATCCAATACAAGGAGGTCACACAGGCATCTCAAA
AACCTTGGCCAAGGTCAAAAGACATTATTATTGGAAAGGTATGACTCGAGATATTACTGAGTACATACGA
AAATGTCCAAAATGCCAAAAGTCAAAAGTGACAAGAAAAATAAAAACTCCTTTAACAATAACTGATACG
CCAATACACGCTTTCGACAGGGTTATAGTAGACACCATTGGTCCACTACCCAAGTCTGAAAATGGAAATG
AATACGCAGTCACATTAATTTGTGATCTGACTAAATATCTAGTCGCGATTCCAATTCCAAACAAAAGCGCT
AACACAGTAGCAAAAGCGATTTTTGAGAATTTTATTCTAAAGTACGGTCCAATGAAGACGTTCATTACGG
ACATGGGAACAGAATACAAAAATTCTATAATAAATGATTTGTGCAAGTATTTACAAATAAATAACATAAC
ATCAACTGCACACCACCACCAGACAGTTGGAACAGTCGAAAGAAGTCACAGAACTTTTAATGAATACATA
CGTTCATATATATCGGTTGATAAAACTGATTGGGACGTATGGATCCAATATTTCGTTTACTGTTTCAACAC
AACCCCATCTATGGCACATGACTATTGTCCATATGAACTTGTTTTCGGTAAAACAAACAATTTATTAAAAC
ATTTTAATAGCACAGACACCATAGAACCTTTATATAGTATAGATGATTATGCTAAGGAAAGTAAATATAG
ATTAGAAATAGCATATAAAAGAGCTAGAATTATGTTAGAAGAAAATAAGAGAAATAATAAGGAATTATA
TGATAAAAAAGTAATAAACATAGATATATCGGTAGGAGATAAAGTCCTACTTAAGAATGAAACAGGACA
TAAGTTAGATCCAACTTATTTAGGACCCTATACAGTAGAAAAGTTAGAAGATAACGATAATATAATAATT
AGGGATACTAAAAAGAAAAAACAAAAAGTACATAAGGATAGATTAAAATTTTATAATAATTGAATTGAA
AACTCTAATATAAACAAAGAAAATTGTTCGAACAAAATAATACAAAACAATAAAAAGGAAAATGGAATT
TAAGAAAAGGAAACTCAAAAAGAAAGAAAAATTCTTTTCTTTTATAAAAAAAGAGACAACATTTAAAAN
AAAATACTAACACATACATATATTTTTTAAATACACATAGTTAAACATATACATTTACACATACATATTAA
CAAACATTTTTATATTAAAATAATATAAACAAAAAAAAAAAAAAAAAAACAAAAAAAAAATAATTAACATT
AATACTTAAAATAACTACATAAATTACGTTATTTTTNAAAGGAGGGAGATGTAGCATGCGTACATATGTA

TATACATACATATGAATAAGGCTGCAACACTTTGTTGTTATGCCAACAGAATGCAAACATAAGCAGCGAC
TTTGTCGCAGCGCGCCTAACGCTAAGTGCCCTCTGCGCATATTCAAGTGCAGACGAAATATGATCCGCACT
TGATAACAACGACCGCCCAAATAGCTAAGTCAGCGTTCTCACGCTTGACCGTCGCTGTGTATACATATGTG
CGCGCATGACTCTCTCTGACTCTCTTGCTGTATATAATAAGCCTAAAACCATGTATGTAAAACGGCTGCTC
ACTGCTTCACGGTGGACAGCTCTAACTCCAAAAAACATTGAATAAAGAACCATCAAGGAAGACTGACGTC
TTTTCATCAGGGACTATTATA
>160_rnd-5_family-8838__LINE___R1
ACACACACACACACACACACACACACACACACATCACACCGTGTGGCAAGCGACCCACAGAATACTACC
ACAGCTTGTCATTGCTTGTCGTACGAGGCGACTGAAATGATACGGTGATGTAATTGCTCGGAGAGGTCCCT
CTAGTAAGCGAAAAGATTCTGACTGCCATTAACCTGGCAATGGCCAGATCGATTCCGTTCCTGGGCAAGT
AAAGCAACTTGTGACCTCCGGCAGTGGCCAGGTATCCCCTGGGTAGGCGCTGCACACCCGTGATGCGCCT
AGCTAATACGAGAAGGCGACTAACATTGAGACCAGTAACGTCTTAAGTTGCACACTCTGGACGACTCTAC
ACGACAAGACGACCCCTGTGCTGACGGCTGACAGTATTCTAGCATCTCAGTAAGCGGAGTGAAATCTGGC
CGACAGGCCGAGGCGCTGAGGGGTCAAATCCAAACACTGGACATNNCCCCAGAAAATATCAGTCTCCGA
AAGGCGTACGGCCAAACACAGACGGCGACGATAAANCTGCCAGCGGGAAGCGCGAAAAAGGCCCTTTCA
GTAGGTTTCCTGAAAGTAGGCTGGGTCAACTCAAGAATCCGCGAACGGATAAACATCACCAGATGTTTCA
GATGCCTCGAGTTTGGACATCTAGCGAGACACTGCAAAAGTGATCTGGACAGATCGAATGTGCCGACGAT
GCGGAGGAAAGGACCATCTGGCTAAGGACTGCAAACAAGAGCCACAGTGCATGCTGTAAGGAAAGGAAT
GCCGACAGAAAATGAAGTGGACACAGCTGAACCTCAACCACTGCGAGGCTGCTCAGGCTCTACTGACACA
ATCAATACGGGAGAGAAATACGGACGTCGCAATAATCTGCGAGCCATACAAGCCTATCGCTGGAAGTGG
CTGGGTGGCAAGTAAATGTGGAAAAGCGGTCATTTGGTCTCTCAAATCACCGGTCCAGGAGGTCAGAGCA
GCAAACGACGGATTCGTGAGAGCAAAAATTAATGGAATCACCGTTTACAGTTGCTACGCACCCCAAGCTG
GGAACTGCGGCAATTTCAGACCATGCTGGAAGAAATTTCAGAAGACGCTCGCTATAGAGATCCTCTCGTC
ATAGCCGGAGACTTTAACGCCTGGGCCGTGGAATGGGGAAGCAAGGAAACTAACGCGAGAGGGACCACC
CTGCTAGAAGAGTTCGCGGCGCTCGACTTGGGCCTTGCAAACGACGGGCAGGAGCTCACGTACTGCAAGG
CCGGTAGAGGCTCTATTATAGACCTTACCCTGCTCAGCAGCTCACTNTATAGGCTAACTACATGGAAAGTC
AGTAATGAATTCACATTCAGCGACCACCGAGCAGTGCATTTCGAGATCCACGGGAGAGGACGGAGAAAA
CAGCAGCCCAAAACAACAGGCCCCAAATGGAAGGACAACGGCCTAGACGAGGAAACCTACACGGACTCC
TTACGTCATGCTCAATGGACTTGGGCCACGGAGTTAGCAAACGACATGGCGTGGCAGCTCATGGAAAACA
TCTCGGATGCGTGCGACTTATCCGTGCCCAGGAGGATGCCAAACAAAAGAGGAGTACCTTGCTACTGGTA
GAATGAGCAAATCAGCGATCTAAGGAAGGCCTGCCTGCGTGCTAGAAGGCGCTCTCAGCGTGTCCGAGGC
CACCCAGACTTTGCATCTAGGCTGGAACACCTGCGTGCAGCCAAGCGAGACCTCAAGAGTAGTAGAGATA
CAGCCCAATCTGCCTACTGGACACGGTGGGAAAGGTACTGGAGACGATTGTATATGAGCGATTTCCTAGT
GTCGTCGAAGACAAAGAGGCCCTGTCCAAACAGCAATACGGATTTCGCAATGCAAGGTCTACGGTCGATG
CAATTAAGGCGGTGGTAGATATTGCCGCTAAAGAAATAGAGGGAAGCAGATGGAGGTGGGGAGATAAGG
AGACTTGCGCGATGATTACGTTGGACATCCGCAATGCCTTTAACTCAGCATGCTGGTCTCGTATACGGGAA
GCCCTAGACCGAATAGGGATACCGTCCTCCGCTTTCCGTATAATATCCAACTACCTGTCCGATCGGGTATT
GAGTTATGATACGGATGACGGTGCTAGGAGTTACACGGTCTCAGCAGGGGTCCCACAGGGTCGGTCCTGG
GACCACTCCGGTGGAACATAATGTACGACGAGGTGCTCAGAGTGCAATTGACCCAGGGATGCAGAATCAT
AGGGTTCGCAGATGACCTAGCTCTAGTAGTGGTGGGCAAAGAAATAGAAGACGTGGAAGCGGCTGCAAA
TGAACCCATAAGAATCGTGGCCAGCTGGATGCGTGGCGCTAGCCTGGAGCTGGCAGGCTATAAAACAGA
GGCGGTCCTCATCACAATCCACAAAATAGTCGAGTTTGCTAGATTTCAGGTAGACAGCGTTAGTAGGGTG
ATGTTAGACAGTAGGCTTAGCTTTCGACCGCACGTAGATTACGTTCGCCAGAAAGCAGCCAAAATGCAGG
CGGCCCTTTCGAGGATGCTACCCAACATAGGAGGAGAGAGTTGTAGCCTCCGTGCTGCTCTATGCTGCAC
CGATATGGGCTTCAGCGGTTGCAAGCCAGAAAGGCCTCCGGAGGAAGATGTCAATCCCCTATAGGCTCAG
TGCTTTGCGCACCATTTCTGGATTCCGGACGGTGTCGGACGCCACAGTATTGGCAGGAAAGCTGCCAGTA
GATATACTGGCCAAGGAAATGGAGAACATTTACATGTTCCGAATATCCACGGGAGACGCCGTCTCAATCA
ATGGCAACTTGGCAGGAGAGATGGAACACGGCAAACAAGGGGATATGGACACATAAGCTGATCCCCAAT
CTGGAGCAATGGATTAGGAGCAGAATGGCGAAATGAACTTCCACCTAACACAGTTTTTCACTGGACACGG
CGGGTATAGGAAGTATCTCCATAGGTTCAGGCATGAGGACTCTCCAGAGTGTCCGAACTGCCCAAATCCG
GACGAAGACCCGGAACACGTGCTGTACCAATGCTCAAGGTACAGAGAGGTAGTAGGCTCTGTACCTCCAC
CGGTAGGGACTGGGAGCGTTCATGCTGGAAGGAGAGGACAAGTGGAGAAATATAAGTCAGCTCATAATA
GGCATCTAGAGGGATCTGAGACGATGCGAAAAGGAGCGACGACTAATGGATACTGCCTAAAATAGGCAA
CCCATCCCCGCGAAGTAATACTTTGCGGTGATCCCGCGGCAAATGTGGTGTGTAAGTGGGGGGTGAGTTT

AGTACGTAGGTGCTGACGGAATCAGCAAATCGTGCATACGGAGATTTTCACCTCCGTGCCGTAAGCAAAA
AAAAAAAAAAAAAAAAAAAAAAACACACACACACACATACGACACGACTACGTCGTTTGATAGAGTGCGG
ACGCGTTATTAACTGCCACTCGAAAAATAAACAAATCGGTAGTGAGAGGCACGCTCGAAAAGATGGGCTT
AGTTCTAGACATCATATTCGAATGTGGAAGGTTCTCAGCGAACAGGCAGGAACTGGAGGACAGCTTGGGG
AGACCTATCACGGTGGACAACTTGGTCAGGAACATGTTGGAACATGTTGGAGAACGCACAAAGTTGGGA
AGCAACCTGCACTTTGCAGCAGAAGTGATGCTGGAACTCAGGAGAGTTGAGCGCAATAGACGGAGAGCG
GAGGAAGTTTAGGTAGCAAAAACATGCCTGTGAAGCTTCGCGGCCGATCCGCAGGCAATCTACGCTACCT
AATTCACCACGTAACCCCCCTTACTATGTACATNCTTAGCATAANTTATNTATTATNNACATNCNTATATN
CGTACAAATACACATACACATACACATACACACACACACACATACACACACACACACACACACAC
ACACACACACACACACACACACACACACACACACAGCCCCGTGTGGCAAGCGGCCCACAGAATACTACC
ACAGCTTGTCATTGCTTGTCGTACGAGGCGACTGAAATGATACGGGGATGTAATTGCTCGGAGAGGTCCC
TCTAGTAGGCGAAAAGATTCTGACTGCCATTAACCTGGCAATGGCCAGATCGATTCCGTTCCTGGGCAATT
AAAGCAACTCGTGACCTCCGGCAGTGGCCAGGTATCCCCTGGGTAGGCGCTGCACACCCGTGACGCGCCT
AGCTAATACGAGAAGGCGACTAAGACTGAGACCAGTAACGTCTTAGGTTGCAAACTCTGGACAACCCTAC
ACGACAAGACGACCCCTGGGCTGACGGCTGACAGTATTCTAGCATCTCAGTAAGCGGAGTGAAATCTGGC
CGAAATGACTGCTAGGTTAATGCTGTTACCGCCCCCGTCCCGTTAAAACATGGCAAATCTCCTAGCACGCT
CCTCGAATCGTCGCCATTTAGTTGGCAGTATAGGTTCAGATGTGATATTCCTGATTAACGCGGATCTGGCT
CTGAACACCCGACCCCATGAAGGCGGGTGTCAACCCTCGCATGGCCTCACTGCTTGCAAAGACAACCATG
AGATCTAAACGCGACTGTACAACGACCAAGGACAACGGCCTAGGAGTTGGGACCCAATAAAATGAGTAA
CAATGAAACCCCACAAACGAAGGAGAGCTGTGATGCTTTTGCAAAGAGCAGCAGAATGGCGAGATCACC
ACTAGGTGTTCCAACAGCCGCCCCGACAGAGACGAACTCAGAACGGGAGCCAANGACGGCAAACCAGAG
CAGCTCAGCACTACGAGAACTCGGGCAGGAGATCTCAAAGCTGGTAGTCATGCTCGAAGATGGCAAGCG
ACGCTCCATCCACCAGCCAATGCGAGACTCGATAGAGAGCATCAGAGTGCTCTACGAGCTGGCGGCAATC
CAGCTGCACGACGAAGGAGCCAAGGAGATCATCAGGACGCACCAGTCGTCCACCATCGCCNCTGTTCAGG
ACGACGATAGACGTCAAGCGGAAGCAGGCCACAGCACGGACACCACAAACAAAGCGAATCCGAGGACC
AGCCGGTAGCCAGGCTACCGTATCAACGGCGCAGGAGACACCGAGCCCCAAAGCGGATGGCGACAAGTG
GAAGGAGGTCACATACAAGAAAAGGAAGGAGGTCACCAGGAAAAACAGCAAGGGATTGCTCAAGGAAA
AGGCGCGACCGGATGCTATAGTCATCTCGGCAAAAGGCGAAGCGAGCTACTCAGAGATACTGAGGAAAG
TAAAGCGGACGAAAAACTGGGCGAGCTCGGAAAAGCGGTGACAAGAATAAGGAGGACACAAAAAGGAG
ACCTACTACTCCAGCTNAACCACTCGGGAGAGAATACCATAGCATTTGGAAACCTGGTAGGCGAAACGTT
AGGCGAAAATGCAGAGGTCCGGACGCTGTCCCACCGAGTGACCGTTGAGTGCAAGGACCTGGACGAGAT
CACGACCAGGGAGGATATCTGTGAGGCGCTGAGGGNTCAAATCCAAATACTGGACATAGCCCCTGAGAA
TATCAGCCTCCGAAAGGCGTACGGCCGAACACAGACGGCGACGATAAGACTGCCAGCGGAAGCGCGAA
AAAGGCCCTTTCAGTAGGTTTCCTGAAAGTAGGCTGGGTCAACTCGAGAATCCGCGAACGGATNAACATC
ACCAGATGTTTCAGATGCCTCGAGTTTGGACATCTAGCAAGACACTGTAAAAGTGATCTAGACAGATCGA
ATCTGTGCCGACGATGCGGAGGAAAGGACCATCTGGCTAAGGACTGCAAACAAGAGCCACAGTGCATGC
TCNGTAAGGANAGGAATGCCGACTCCAAACACNTTGCCGGCAGCGGCAGATGCCCCGTGTTTAGGAGCNC
CCTGGCCAACAGAAAATNAAGTGGACGCAGCTGAACCTCANCCACTGCGAGGCTGCTCAGGCCCTGCTGA
CGCAATCAATACGGGAGAGCAATACGGACGTCGCGATAATCTGCGAGCCATACAAGCCTATCGCTGGAA
GTGGCTGGGCTCGACTTGGGCCTTGCAAACGATGGGCAGGAGCTCACGCACTGCAAGGCCGATGGAAGCT
CTATTATAGACCTCATCTACATGAAAAGTCGGCAATAAATTCACATTCAGCGCCCACCGATCAGTACATTC
CGAGATCCACGGGAGAGGACGGAGAAAACAGCAGCCCAAAACAACAGGCGCTAAATGGAAGGACAACG
GCCTGGACGAGGAAACCTACACGGACTTCTTACGTCATGCTCAATGGACTTGGGCCACGGAGTTAGCAGA
AGACATGGCGCGGCAGCTCATGGAAAACATCTCTGACGCGTGCGACTTATCCATGCCAGGAGGATGCCAA
ACAGAAGAGCAGCACCTTGCTACTGGTGGAATTATCAAATCAGCGACCTAAGGAAGGCCTGCCAGCGAG
CTAGAAGGCGCTCCCAGCGTGCCCGAGGCCACCCAGACTTTGCATCTTTACGTCAGCCAAGCGAGACCTG
AAGAGAAGCATCAAACAAGCAAATCCAGCTACTTCCAGAAAATATGCGATGAGGCCGATGTTAACCCGT
GAGGCACGACATACACGGTCGTCACGTAGAAGATCCGCAGCCAGGGAACAACACGGGTTGTTTCTGCCCT
ACCCCACTTGCCACTAACTAGTGCAGCAGATATCGGTAAACGAGTGGAAAGTGGTAGCGGCAAGCAAAA
ACTAGGCGACAAAAACGCCAGATAGAGGACCTGATGGAGTGCAAAACGAGGCGCTAAGGTTAGCCATGC
TAACGAAACCCGACGCCTACGCCGAGACATATACTAGATGTCTACATGACGGTGTATTCCCTAAGAGATG
GAAGCGGCAGAAACTAATCCTTCTACCGAAAGGGGACAAGCCAATAGAGGAACCCTCGGGATACAGGCC
AATCTGCCTACTGGACACGGCGGGAAAGGTACTGGAAAGGATTGTGTATGACCGACTACTTAGTGTCGTC
GAAGAGAAAGAGGCCCTGTCCAAACAGCAATACGGATTTCGCAAGGCAAGGCTTACGGTCGATGCAATT

AAGGCGGCGGTAGATATTGCCGCTAAAGCAATAGAGGGAACCAGATGGAGGTGGGGAGACGAGGAGTAT
TGCGCGGTGATTACGTTGGACATCCGCAATGCCTTTAACTCAGCAAGCTGGTCTCGTAANCTGGAAGCCTT
AGACCGAATAGGGATACCGTCCCCCATTCTCCGTATAGTATCCAACTACCTGTCCGATNGGGTATTGAGTT
ATGATACGGATGACGGTGCTAGGAGTTACACGGTCTCAGCAGGGGTCCCACAGGGGTCGGTCCTGGGACC
ACTCCTGTGGAACATAATGTACGACGAGGTGCTCAGAGTGCAATTGCCCCAGGGATGCAGAATCATAGGA
TTCGCGGATGACCTAGCTCTAGTAGTGGTG
>9_rnd-1_family-297__LTR___Gypsy
TGTAGCATGCGTACATATGTATATACATACATATGAATAAGGCCGCAACACTTTGTTGTTATGCCAACAGA
ATGCAAACATAAGCAGCGACTTTGTCGCAGCGCGCCTAACGATAAGTGCCCTCTGCGCATATTCAAGTGC
AGACGAAATATGATCCGCACTTGATAACAACGACCGCCCAAATAGCTAAGTCAGCGTTCTCACGCTTGAC
CGTCGTTGTGTATACATATGTGCGCGCATGACTCTCTGACTCTCTTGCTGTATATAATAAGCCNAAAACCA
TGTATGTAAAACGGCTGCTCACTGCTTCGCGGTGGACAGCTCGAACTCGAAAAGAAAAACAGATAATAAA
GAACCATCAAGAAAGACGGACGTCTTTTTAATCTTGGAACCATTACATGGCGACCGTGACAGGACGTCAC
AAAACATGAAAACGTTAAGATACATAACTTCAACTCTAATTTTAAACCAGTCATAAAAGTAATTAAAGGA
AGTAAATTAAAAGGAAATAACACTCAAAACAACTGAAACAACGAAGCATCTGGAACGGCACACACCCTG
GAGAATCAAAAATTAACTGAGTGAGTACAGGGGGCATGAGCTCTAAAAATACTTTAAAGCTAAAAAGGC
GCAGTGGCATTAAAGAGCGGATCAGGCAAAGAAAGTCTGAGATCGTAAGACTCTTATATAAGTCCCAAA
ACCCAATACCTTGGGAAACTGCAGTCAAGCTACATGACGAACTCAAACATCTTGAGTCGTTAAACAAAGT
AAAGAGCCCCCCTGTCCCTCAGAGGAAAAACTTATTAATCCAGNTNCACATCGACCACAGCCACAACCGA
TCCCGAGAAGGCAACGTGAATGCCCAGCAGACTGCGAGGGACCGCTTTACAGCCAACATTGCCTGGGCAC
ATGTCAGCGGCCACCCACGAGTAAAAACTAGTACGAGAGGCAAACCGCCCCTTGTACCAACGCTGGAAC
AAGAAGAAGTACGAGAGGCAGACCGCCCCTTGTACCAACGCTGGAAAACACCAAGGGTGAACTGACCGA
CAGCGCCACAGAGGGCGCACAGCAAAAAAAAATTTATAATTATAATTATTATTAAAATATTTGTAAAACT
ATTGTAATGCCCGACGACAATTGAGTCGACCGGCAAGACGTTTGGCCCGTAAAGCCATTCGAAATACTAG
GCAGGACAAAATGTACATTCAGTAAAAACCGAATTAATATTAAATTTCTCCCCGCCCACGGGCGTAAATA
CAAAAAATTTTAAATATAAACTCAAACTACAAACCTTATAAAGTTAATGAATAGCGTTAACAAAACTATA
AGTTCATATATAAATCAAGGACCCAAGTATATATTAAGAATAACAACCCCAAATATGGGTTTGGTAGACG
TTAAGACTGTTGACTCGACGGCTAACGTAATAAATAAAGTTGAGGTCAGCAACACAGATGTAAAATTGAT
AACAATTTTTGTCGTTATGATATTTGTCATATTAGTAGGACATCTATTGTACAAATTATATACGTATCATAA
TAAATGCATNAAAAGAGATACCAGAGTCACGCAAATGACCTGGATAAAATCTAAATTTTTTCTTGAACAA
TGATAAGCGACAAAATAAAATAAATAAATAAAATGAAATTTCGAGAAATATATATATATTCTTATTACCA
GAAATGCCTATTAATAAGTTTGCGATAGAAGTATACTTAGAAGCAATATATAATAGCGACGCAGACTTTA
GAATTCGTGCATTGAATATTCTACAACAATAAGATAAAAGGCATAATTTTAAATACACTGCCATATGTTGG
CATTAAAGAAATAGTGGTCCAGTGGACGGCGAGTGCCCAATTATAATGGTAAAACTTCATAAATAGAGAA
CTATGGAATGGAGTGAATTAGCGAAAAAAATAAATAATTTCAAAGATAAATTCGAAAAATCTTATAAGTG
TGTTAATAAGAACGCACCTATAAGGGTTGAAACGCTAATTCACCACATAAACATATTAGTGCTAAACTAT
AATAACATAGTTCAACTTTATTTAAGTAAAGAAAATAGGCTTACAGAGCAGCATGTAAATCAATGTAAGA
AAATAATCAAATCACTTGGGACTAGAATATCGAATATCAGCACTAGGCACAACATAGTAATAGATATCCC
AACAGATTTAAAATTAATGGCAACTTTTGAACCAGATCAACTAGAAGAACTGAATGAGTCTAAGCCAACA
CTAAATAGTGACCCTGATATTGAAGCTGATAGCGACATTGAGCCACTTGAATTAACAGAACCAAACATGG
CAGTAAATCAAGTGGCCTTAGACCGCGAATATGTAAGACAGGTATCGTCTACAATTCCGGAATTTGATGG
CAAAAGCTATCACTGGCCAGATTTCTAACGGCTTTAAGACTAGTCGATCGTACTAAAGGTACACAAGAAG
ACTTAGCCGTAGAAGTCATAAAGTCNAAAATAATTGGCCCAATTTTGTACAAAGTACAAAACGAAACATC
CATTATTGGTATTATAAATAAATTAAATACCAATATCAAAGGCGAGTCAACTGACGTTATCAAAGCTAAG
TTGCTTAACGTAAAGCAGAAAGGCAAGTCAGCCTCGCAATATACAGCTGAAATCGACAGTTTGCGTAAGC
AACTGGAAGCAGCCTATATTGACGATGGACTAGATTTTGATAACGCAGAGAAATTTTCTACTAAAGAAAC
AATATCTGCCATGACAAAGAATTGCGAATACGAAAAGCTTCGCATAATATTAGAAGCGGGCAATTTCAGC
AATTTCAATGATGCTATGGGTAAATATATCCAATGCAGCACTGAAATGACTGGAAGTGCCCAAGCACAAT
ATTGTACTACAAAGGCAGAAACGATCGTGGAAATTACAGCCGTAACAACCAAAGAGGTAGAGGCAATAG
CCGCGGTAATTTCCACTATAACGGTAATANAACAACAATAGGGCAATGGCAGAGGACATTATAGAGTAAT
GCCCGTGGTGGCAACCGAGGAAGGAACAATTCNAACCGAGGTAGCNACCAAAAATAGTAACAATAGAGG
TAATGTCCGAGTAACTGAAGGTAACTCGGAAAACCAACGAGACCCCTCAGATGTTCAGAGCCAATAAATG
GCNCAATAAAAACCATCAACCTCAACCTCAGCATGTTTGTAAAAATTAAGAATGAAAGAACAGGTAAATC
AATTGCACTGTTGGTNGATACTGGTGCAGATATATCCATATTAAAAGAAAATGCAGATATATTCGAAAAC

GTAAACGAAAGAGTTACTACCCAAATATCGGGNATTGGCGAAGGCACAATTCCTTCAAAAGGCTTAGCCT
TTATAGAACTCACTACAGGCAAATATATAATTCCGCATAACTTTCATTTAGTCGANCAAAANTTCCCAATA
CCGAGTGATGGCATACTAGGCATAGATTTCATAAAACAATATAATTGCCAACTAGATTTCACTCAAAGTG
GCGACTCACTTATTCTTCGCCCAAACAACTTAAATTATCCGATTCGATTAAAAATACTTCATACTTGTGAC
AATAATGCCACAGTACTCCCAGCTCGTTCCGAAGTTGTCCGTGAAATACAAATNCCCTCAATACAAAATC
AAATTCTTATATTAAATCAAGAAATTGATGAAGGAATTTACATCGCAAACACGATATCCAACCACAAAAA
TACATATGTCCGTATTATAAATACAACAAATGCCAATAAGATAGTAAACTTAAATAAAATAAAATATGAA
ACTTTGAACAATTATGATATAGTAAAAGAAACCAACGAAGGAAGAGACAACTTCGTTATGGAAAAATTA
ATCAAAAACTTTCCGCCTTTATTTAAAAATCAATTAAAAGATTTATGCACCAGATATACCGACATATTCGG
ATTAGAAACCGAATCGATAACAACTAATAATTTTACAAGCAAAAGCTTAGATTGAAGGATGATGAGCCCG
TATACATAAAAATTATAGAAGTCCTCACAGCCAAGTACAAGAAATTCAAGCCCAAGTGCAAAAATTAATA
GACGACAAAATAGTCGAACCATCAGCCTCAGAGTATAATAGCCCACTTTTATTAGTTCCGAAGAAGTCCC
TTCCGGACTCTGACAAGAAGAAATGGCGATTAGTAATTGACTATCGTCAAATTAATAAAAAGCTATTGTC
CGATAAATTTCCGCTACCGAGAATTGATGATATTCTAGATCAGCTAGGTAGAGCAAAATATTTTTCATGCC
TAGACTTAATGTCTGGCTTTCACCAAATCGAACTCGAAAAAGTTCTAGAGATATAACGTCATTTTCAACGA
GCAATGGCTCGTATCGCTTTACGCGATTACCATTTGGCTTGAAAATAGCCCCAAACTCCTTTCAAAGAATG
ATGACAATAGCATTTTCTGGTCTCGAACCATCTCAGGCCTTCCTTTATATGGATGATCTGATAGTAATCGG
TTGTTCCGAAAAACATATGGTCAAAAAACTTAACTGAAGTTTTTGACAAATGTAGGAAATTCAACCTGAA
ACTACATCCAGAAAAATGTTCATTTTTCATGCATGAAGTCACATTTTTGGGACATAAATGCACTGATAAAG
GAATCTTGCCAGACGACAAAAAATACGACGTCATAAAGAACTACCCAGTCCCACATGATGCGGACAGCG
CTAGGCGATTCGTTGCATTTTGCAATTACTATAGGCGCTTTATTAAAAATTTTGCCGACTATTCACGTCACA
TAACAAGATTATGTAAAAAGACGTTAAGTTCGAATGGACATCTGAATGCCAGAATGCCTTCGATCACCTC
AAAACGGCTCTTATAAACCCAACCTTGTTACAATATCCAGATTTCGCTAAAGAATTCTGCATAATAACTGA
TGCAAGTAAGCAAGCGTGTGGAGCGGTTTTAACTCAAAACCATAATGGACTCCAACTTCCAATTGCATAC
GCATCAAGATCCTTTACTAAAGGAGAAAGTAACAAGAGTACTACTGAGCAAGAATTAGCGGCTATTCATT
GGGCAATAACGCATTTCCGACCATATATTTATGGTAACCATTTCACGGTTAAAACTGACCATAGACCGCTA
ACCTACTTATTTTCAATGGTAAATCCAAGTTCAAAATTAACACGTATGAGGCTTGAATTAGAGGAATATAA
TTTTACGGTAGAATATCTGAAGGGTAAAGATAATTTCGTAGCCGATGCGTTATCAAGAATAACCATAAAA
CAGCTACAAGAAATAACAGGGACAATCCACAAAGTCACTACAAGATATCAAAGTAGACAAAATTCCTGC
GCAGGAAAATATCAGGTAGAATTGCCAAGGCAATCTACAGAAAAAGCTTCACAGCCCAACGTATACGAA
GTCATTAACAATGACGAGGTACGCAAAGTAGTGACCTTGCATATAAAAGATACGCTTTGTTTCTTTAAACA
TGGCAAGAAAATTATTGCAAGAATTGACATTGGTGATCTATATACAAATGGAAATATTGACTTAGGTCAA
CTATTCCCAAGGCTCGAAAATGAAGCCGGTATATTGAAAATAAGCCAATTGAAAATGGCACCGTGGAAA
ATATCTTTGATACAATTTCAATAGATAATTTCAAAAATATGGGCAATAAAATATTGACTAAATTAAGGGTA
GCGCTACTCAACCCGGTGACCACAATAACAAATAATAAAGAAAAAGAAGCTATATTGTCTACACTACATG
ACGACCCAATACAAGGAGGTCATACTGGCATTACAAAAACCTTGGCCAAGGTCAAACGACATTACTTTTG
GAAAGGTATGACTCGAGATATTACTGAGTACATACGTAAATGTCACAAATGCCAGAAAGCTAAAATAACT
AAGCATAGTAAGACCCCATTAATTATAACTGATACACCTGTACATGCGTTCGATAGGGTTATAGTGGACA
CAATTGGTCCGCTACCCAAATCTGATAATGGTAATGAGTACGCAATCACATTAATTTGTGACTTGACTAAA
TACCTAGTCGCGATACCAATACCAAACAAAAACGCTAGTACAGTAGCCAAAGGTATATTCCAATCTTATA
TTCTAAAGTACGGTCCAATGAAGACGTTCATTACGGACATGGGAACAGAATACAAGAACTCAATTATAAA
TGATTTGTGCAAGTTTATGAAAATAGACAACATAACATCAACTGCACATCATCACCAGACAGTTGGAACA
GTCGAAAGAAGTCACAGAACTTTTAACGAATACATACGATCTTATATATCAGTTGATAAAACTGATTGGG
ACGTATGGCTCCAATACTTTGTCTATTGTTTTAATACGACCCCTTCTACGGCACATAATTATTGTCCGTATG
AATTAGTATTTGGTAAGACATGTAATCTACCAAAATACTTCAATAGCATAGCAAACATAGAACCAATATA
TAACATAGATGATTATGCTAAGGAGAGTAAGTATAGATTAGAAGTAGCATACAAAAGAGCAAGAAATAT
GCTAGAATTAAATAAGATGAAACAAAAGAAGCGATATGACTTAAATAGTAAGGAATTAAAATTATCCAT
AGGAGATAAGGTTTTATTAAAAAATGAAGTAGGACATAAACTAGATTTTNAAATACACAGGGCCCTATAG
AGTAGAAAAAAATAGAAGATAGGGATAATATAGTTATTTCCGGAAATAAGAATAAAACGCAAACAGTCC
ATAAGGACAGATTAAAAATTTTTAATTCATAATCAAGCATGCAAAAATACATGAAAAAAATATATATAAA
AAAAAAAAAAAAATATATATATAAAAAAAAAAAATAATAATAATAAAANAAATAATTATTAAGGAAAATA
ACTCCATTGTATTACGTTATTTTTTTAAAAGGAGGGAGATGTAGCATGCGTACATATGTATATACATACAT
ATGAATAAGGCTGCAACACTTTGTTGTTATGCCAACAGAATGCAAACATAAGCAGCGACTTTGTCGCAGC
GCGCCTAACGNTAAGTGCCCTCTGCGCATATTCAAGTGCAGACGAAATATGATCCGCACTTGATAACAAC

GACCGCCCAAATAGCTAAGTCAGCGTTCTCACGCTTGACCGTCGCTGTGTATACATATGTGCGCGCATGAC
TCTCTCTGACTCTCTTGCTGTATATAATAAGCCAAAAACATGTATGTAAAACGGCTGCTCACTGCTTCGCG
GTGGACAGCTCGAACTCGAAAAGAAAATGAATAAAGAACCATCAAGGAAGACGGACGTCTTTTAATCTT
GGAACCATTACAAT
>160_rnd-1_family-291__LTR___Gypsy
ATATATAAGTTAACACATAACACTTAATATAGGATATTTCATAAGTTATAATCAACCCTCGCTATCGCGTG
CTTCATAAGTTAAGGAACAGCCCCCGCAATTGCGACACTGCATACTTTGTATCAGCGCGCAATAGCTGCA
ATGGTCAGCGGCGGTAACCGGACACTTTTGTTTTGCTGCAAGAGTTTCCGTTATTGCCTAAGATTTTGCTG
AGTCGGCTTGCCGACCATGACATTGTGGCGTGATGCGAGTTTTGGCTTAGCTTAAGTAAAACTTTCTGTGC
ATTAAGTTTCTTTAGTTTTGAATTCGGCGCTCTTCCAATAAGTCGCTTAAATAAATATTCACTCCGGCACTT
AGCCGTTAAATAATTTGTTTTATTGTCTGAGCCCGAGGCCAGCAATAAGAGGCGCAGTCGCCTCTCAACCT
AATCTTCAGCCACTACAGGACTGAAGGATGTTAATTGGCGCCCAACTAGTGGTCGGACTCCACAACCAAA
ACAAGTAAAACGGCAGCGCAGGAGCTGCAACAACCAACAACAATTAAGACAGCAGCGCGGGAGCTGCAA
CAACCAACAACAATTAAGACAGCAGCGCGGGAGCTGCGAGTGACCAATTTTATAAAGAAAATTTTTTTTG
TGTTAATTGAATCATGTAAGTGGATACTCTAGCCAAAATTTTTCTTCAAACACAAAATTTTTTTTTATTTTA
TAAAAATTAGTTAGTACTAGTGAGTATTTTATATTGCAACAGATCATACAAAAAAATTTTTTTTTTAAATA
ATAATAATCAGAACCAAGTTTCTGAAACACGTGAGAGTGACTGCTTTGAGAATTTTAGTTGAAACAATTT
ATCATCCAGATCGTTAATAAAATTACTTTGTGTTACTTTCTATGTGTCTAAAATTGTGCTATAACAATACAT
ACAGTTCTCGTGGTTCAGAGCTGCGACGTAATTCGACCCATCTTTTGACGGCCCGTTTTATTTTTCGCGGCC
ACTGGCCTGCGCATGTCACGGTTCAGCCAATATAGAAACCCTCGCAAGCAGTCTAGCGACTCAGATTCCG
ACTGTGACATTCGCATCAGAGTCCCGGCTACAAGACTTCTCCCTGCAGTTCCCCCACCATCAGGTAAAATG
GATCCAGATCAGCTTAGGGAAGTAGTAAGGACGGCTATCAGCAATGCTTTAGCCGAACAGGCCGCTGCAA
ACCAGCAGAGGGAGCAAGCGTTGACTCAAAGAATTAATGAGTTAGCTGAACAAATAGCAGCAGCAAACG
TTGCCGCCCCACAAGTAGTAGCATATGCTGCTATAGGAATTAGAAACGACATTCGCTGTGACGAGCCGCT
AGATGCCGTCAAGTGCTTACCTGAGTTTGCAGGAGTACAGGAATCTTATGTATCTTGGAGGCAGGCAGCA
TTAGCCGCTTACGAGATCTTCAGACCACATAATGGCAGTTCTCGTCATTATCAGGCTGTTATCATCATAAG
AAGCAAGATTAGGGGCCCCGCAGACGCGGTGCTGGCCTCATTTGGTACTGTATTGAATTTTGATGCGATA
ATAAATCGCCTAGATTTCACATACAGTGACAAGAGACCAATTCATGTCATTGAACAGGAATTAGGTACCT
TGAGGCAAGGTGGCCTGTCCCTGCTTCAATATTACGATGAGGTAGAGAAGAAACTCACTCTTCTCACTAA
CAAAGTTAACATGTCATACGAGCCGGCTTTAGCAAAGGGCCTATGTGAAAAATTCCGCGACGATGCGTTG
CGTGTGTTCATATCGGGTCTCAAGCGCAGTCTTACAGATGTGCTCTTTGCAGCAAAGCCCAGAGATTTGCC
TTCAGCGCTAGCTCTCGCGCAAGAAGTCGAGTCAAATCATGAAAGGTACGCATTCGCGGCTAATTTCGCA
AGAAGTCTAGAAGACAAAGAGCGCAAGCCGCATGCAAAAGCGCAGGGTCACCAACAGGAGAGGCATTCG
CAACAAGCCGAAGCACAAGCTAGCGGTACAAAGAATCCGCACTTTAGCAAGCAAAGCAAGGCCCCAGTG
CAGACTGACCAACGAAGCAGCAGGCAGCACAATAATAATGGCGCACCGGTGCCCATGGAGGTCGATCCT
TCCTTCTCAAGGGTCTTGCAGCCCACTCAGGCCCCTGCTTACGCGAATAGGAAGCACCCCGCATCTGACCG
CACAGGCGGTCAGAGAAATCAGCAGAGGTTGAATCACGTCACGCAAGACGCGGGACAGGGGGCAGAGGC
GTATGCTGATGCCGCCTCCAGCGCAGCGGCACAAATCAATGACGATGCTAGTTATGACTCTGATGCCCTC
AATTTTTTAGGAGCAAATCCCTGCTACCCGTCATCAGACGAAGAGTAGTAGGGACTCACATGCGATTCCTC
ATAGACACGGGCGCGGCCAAGAATTTCATTCGGCCACATGAAGGGTTGACCGTCCGCTCGGTAGAGTCCC
CCTTCAAAATTCATTCCATTCACGGCGAGACACAAATTACACACAAATGCTTTGTTTCAATGTTTGGGTTA
AAGGCAACCTTTTTTATTCTGCCGGACTTGTCAACTTTTGATGGGATTGTAGGCCTTGACCTGCTAAAGCA
GGCAGCTGCTTCGCTTTGTCTAGCTTCAGGACAACTTAAGTGGGGTACGGAGGTCGAACGAATCACCTTCC
ACACGTGCAAAAATGTAAATTTCACAAATGTAGACTGTGCAGAGGCATCCCCATTAGCTAGAAGTTCTTTT
TTGAAAATGCTAAAGACCAGACAAAAGGCCTTTGCAGACCCAGACGAGGCGTTGCCGTATAACACATCAG
TCGTGGCTACCATTAGGACCACAGATGAGCAACCGATCTATGCCAAGCTTTACCCATATCCAATGGGAGC
AGCTGATTTCGTTAATAAAGAAATCAAGGATCTGCTAAAAAATGATATAATTCAAAAGTCCGTCTCTCCCT
ACAATAACCCAATATGGGTAGTAGACAAGAAGGGCACTGATGAAGCAGGCAATAGGAAAATGAGGTTGG
TAATGGACTTTCGTAAACTTAACGAAAGAACCGTGCCTGACAAATACCCTATGCCAAATATATCAATGAT
ATTGGGGAATTTGGGGAGAGCCAAATTCTTTTCAACATTAGACCTCAAGTCTGGTTATCACCAGATCATAC
TCGCTGAACGCGACCGTGAAAAAACCTCTTTCTCTGTGAACGGGGGGAAATACGAGTTCAAAAGACTCCC
CTTTGGATTAAAGAATGCAGCAAGCATCTTCCAAAGAACCATCGACGACATACTGCGAGAGCAGATTGGC
AAATCTTGCTATGTTTATGTCGATGACGTAATCATCTTTTCAGAAGATGAGGAATCCCACATTAAACACGT
GGATTGGGTCCTAAAAAGCTTACACGAAGCAAACATGAGGGTCTCCGTCGAAAAGTCACGTTTTTTTAAG

AAAAGCGTGAACTTTCTTGGTTTCATAGTCACCAGCACTGGCGCCACGACGGATCCTGAAAAGGTTAGGG
CAATACAGGAGTATCCAGAACCTAAAACACTGTTCGAGGTCAGATCATTTCTAGGCCTCGCAAGTTATTA
CAGATGTTTCATTAGGGACTTCGCCGCAATAGCAAGACCCATCTCGAATATCTTGAAGGGTGAAAATGGA
ACAGTCAGTAAGCATAGGTCCAGAAATATAAAGGTTCAATTCTCTGAAGTGCAGCAAAGGGCGTTTCAGA
AGTTGCGCGATATTTTGTCATCCGAGAATGTAATGCTCAGGTACCCCGATTTTAAGAAGCCATTCGATCTA
ACGACCGATGCTTCGGCTCACGGCATAGGTGCAGTTTTGTCTCAAGAGGGTCGTCCTATAACAATGATTTC
AAGAACATTGAAGGACAGCGAGGTTAACTATGCAACAAATGAGAGAGAGTTATTGGCCATCGTATGGTC
ACTTGCCAAGTTAAGGCACTATTTGTATGGGGTGAAAGACATTAATATCTTCACTGACCATCAACCCTTGA
CCTTCGCAGTTTCGGAATCCAATCCAAATGCGAAGATTAAAAGGTGGAAAGCGCGCATTGACGAGTCTAA
TGCACGTATCTTCTATAAACCTGGCAAGGACAATCTGGTTGCGGATGCCTTGTCAAGACAGCAGCTCAAC
GCTGTAAATGAACAAGAAGTTGAGTCCTGCGCAGCAACGATCCACAGTGAGGTATCTCTCACCAACACCA
TTGAGACAACAGACAAACCCTTGAATTGTTTTCAAAACCAAATAGTGCTAGAAGAGGCGCGTTTCCCCTT
AAAACGAAGTTTTATTCTTTTTGGAAATAAGAAACGCTACTTGATCAGCTATACTTGCATCGAATCGCTGC
TAGACGAGCTCGAAGATGTAGTTTCACCCAAATGCGTAAATGCTCTCCACTGTAATTTACACACGCTTGCA
ATGATACAAGATGCATTGGTTCGAAGATACCCGGCCACAAAATTCTGGCACTGCAAAAATCGCGTAGTGG
ACGTATTTGCGGTTGGAGAAAGAAGGGAAATCCTCACAGCCGAGCATAATAGGGCCCATAGGTCTGCACA
GGAAAATATTAAGCAGGTACTCTCGGAGTACTACTTTCCAAAGATGGCAAAAATAGCCAATGAAATAGTA
CAAAATTGCAAAACATGTGCTAGGGCTAAATATGATAGACACCCCAAAAAACAAGAGCTTGGGGAGACA
CCGATCCCCTCACATGTAGGAGAGATGTTACACATAGACATTTTCTCCACTGACAGGAAATACTTTCTCAC
TTGCATCGACAAGTTCTCTAAGTTTGCTATTGTCCAACCGGTCCCTTCAAGAACCATTGTGGATCTGAAGC
CAGCTTTAATGCAACTAATGAATTTTTTTCCAAAAGCCAGATCCATTTACTGTGACAATGAACCGTCATTG
AATTCAGAGACCATCGTGACTTTGCTCGCAAACAACTATGGGGTCAGCATTTCAAATGCACCACCATTGC
ATAGCATCTCGAATGGACAGGTTGAGCGCTTTCACAGCACTCTGTTAGAACTAGCCAGATGCCTAAAGAT
CGATAAAGGCATAAGTGACACCGTAGAAGTCATTTTACTGGCTACAACCGAGTACAATAAGTCGATCCAT
TCGGTCATCGACCAGAGACCAGTCGATGTGGTTCAGGCGCACCCAGATGAGCCACAAGAAAAGATACGA
GAAAAGATCAGAAACGCTCAGACGACGCTCAGAGCTAGGGAGAACGCAAATCGGCAGAATAGGGTCTTC
GACGTTGGTGACAAAGTTTTGGTCAAGTCCAACCGACGGCTCGGAAACAAACTCACACCTTTATGTGATG
AGAAGGCCGTGGAAGCAGACCTGGGGACCGAGGTCCTCATTAGAGGGAGGGTGGTCCACAAGGACAACC
TAAGGTGACTCCCTTGTTTTTTCACACCTTTTTTTTTAGCCACTTGGCATAGTTTTTATAAAACATTTTTGGT
GGTGGGCAAACCTCTTTAAAAAAAGAAAAACAAAAAAGTTGCTAAAAAGAAAGAAAACAACTTCACTTC
ACAGGTTTGGATCCATATTACTCCTTTCCTTATCAGTGGCTTCGGCACATGTCACCGACTACTCGCGTGCC
AAGTACATTCCCATCGTCGACGGTGAAATCTTGGTATGGGAAGAATACGCCTATGTTAGGCACTCGGCAA
ACCTCTCAGAGTACGTGCGAATGGTGGAAGAAACGGTCAAATTAATTGACATGTTCCCGCTATCCCACAT
GAAAAAGCCTCTAAGTGTGGACACGGCACATCTACAAGACCTATTGGAGGCGTTAGGCACACACCATAGA
GTCGCCAGGAGTTTAGATTTCCTAGGATCCATACTGAAGGTTGTGGCGGGTACACCCGATGCCGGTGATTT
AGAGCAGACTAGGTTCACTGAAAGGCAACTAATAAATGCCAACAATAGACAAATAGAAATAAACACTAG
AGTTCAAGACCAGCTTAACCAGTTAACAATCACTGTCAATAAAATTCTCAAGATAACCAAAAACTCACAA
GTTGACACTAGCCATTTATTTGAAACACTACTGACTAGAAACAGAATGCTGATGATGGAGTTGCAAAATT
TAATGCTTGCTATTACTCTTGCGAAGGCTAACATTGTTAGCCCAAACATTTTGGACCACTCAGATTTGAAA
TCAATTTGGCTAGAGGAACCCACCAACACTCCCATAGGAGATCTTATGTCCGTTGCGTCCGTAAAAGTTCT
TCAATCCATTAACACTATACATTTTATAATTAAATTCCCAAAGATTAAAAAAGCTTGCAGAAAAATTACGA
TTTATCCTGTCGTCCACAAGAACTTCATATTGCGATTGTTTGACAACGTCATAGCCGAATGCGATGGAGAA
ACCCACACACTGCAGAGTTGCGCCGCTTCACCTGGAGCCACATTTTGTCGTCTGGCATTAGAGAGTTCATG
TGCCAAAGAGCTCCATGCAGGTGGCACGGCACACTGCGAGATCCAGCCGAGCAACCTGGAAATAATCAC
CCATGTAGATGACGGAATCATCATCCTCAACGACGGGTCCGCAAGGATTCAGGTGGACAACGGCACTGAG
ATCCAGGTACAAGGCACGCATCTCATCACGTTCGGAGACCGAGTCGCAATTAACGGACTCTGTTTTACA
ATCACAACAACGTACGCAATAGAGTCCCGGGTATTGCCAATTCTCCCTTGCTGAACATCACTGCCAGCCA
AGATGTCCTCAGCCTCCCTTACCTTCATCGTTTGAGTGAACGTAATTTGGAGGTGATACAGCAATTCAAGA
AAGACGCCGGAACTGATCAAGCTTATCGTGTGGCATTCATCATTGGAGCCATATGCTGTGCTTTAATCTGC
ATTGGCCTCACTCTTTGGCGGATCATCGACGCAAAGAAGTCTGCGAAGCAGTTACGAGAAGTCGTCGCCG
GGATTGGGTCGGCCGAGGACGGCCTTATAATTGAAGGGGGAGTAGTTAACACATAACACTTAATATAGGA
TATTTCATAAGTTATAATCAACCCTCGCTATCGCGTGCTTCATAAGTTAAGGAACAGCCCCCGCAATTGCG
ACACTGCATACTTTGTATCAGCGCGCAATAGCTGCAATGGTCAGCGGCGGTAACCGGACACTTTTGTTTTG
CTGCAAGAGTTTCCGTTATTGCCTAAGATTTTGCTGAGTCGGCTTGCCGACCATGACATTGTGGCGTGATG

CGAGTTTTGGCTTAGCTTAAGTAAAATTTTCTGTGCATTAAGTTTTTTAGTTTTGNATT
>9_rnd-1_family-250__LTR___Gypsy
TGTAGCATGCGTACATATGTATATACATACATATGAATAAGGCTGCAACACTTTGTTGTTATGCCAACAGA
ATGCAAACATAAGCAGCGACTTTGTCGCAGCGCGCCTAACGATAAGTGCCCTCTGCGCATATTCAAGCGC
AGACGAAATATGATCCGCACTTGATAACAACGACCGCCCAAATAGCTAAGTCAGCGTTCTCACGCTTGAC
CGTCGTTGTGTATACATATGTGCGCGCATGACTCTCTCTGACTCTCTTGCTGTATATAATAAGCCAAAACC
ATGTATGTAAAACGGCTGCTCACTGCTTCGCGGTGGACAGCTCGAACTCGAAAAGAAAACANATAATAAA
GAACCATCAAGGAAGACGGACGTCTTTTTAATCTTGGAACCATTACATGGCGACCGTGACAGGACGTCAC
AAAAACATGAAAACGTTAAGATACATAACTTCAACTCTAATTTTAAACCAGTCATAAAAGTAATTAAAGA
AAGTAAATTAAGGAAATAACACTCAAAAACAACTGAAACAACGAAGCATCTGGAACGGCACACACCCTG
GAGAATCAAAAATTAACTGAGTGAGTACAGGGGGCATGAGCTCTAAAAATACTTTAAAGCTAAAAAGGC
GCAGTGGCATTAAAGAGCGGATCAGGCAAAGAAAGTCTGAGATCGTAAGACTCTTATATAAGTCCCAAA
ACCCAATACCTTGGGAAACTGCAGTCAAGCTACATGACGAACTCAAACATCTTGAGTCGTTAAACAAAGT
AAAGAGCCCCCCTGTCCCTCAGAGGAAAAACTTATTAATCCAGNTNCACATCGACCACAGCCACAACCGA
TCCCGAGAAGGCAACGTGAATGCCCAGCAGACTGCGAGGGACCGCTTTACAGCCAACATTGCCTGGGCAC
ATGTCAGCGGCCACCCACGAGNAAAAAACTAGTACGAGAGGCAAACCGCCCCTTGTACCAACGCTGGAA
CAAGAAGAAGTACGAGAGGCAGACCGCCCCTTGTACCAACGCTGGAAAACACCAAGGGTGAACTGACCG
ACAGCGCCACAGAGGGCGCACAGCAAAAAAAATTTATAATTATAATTATTATTAAAATATTTGTAAAACT
ATTGTAATGCCCGACGACAATTGAGTCGACCGGCAAGACGTTTGGCCCGTAAAGCCATTCGAAATACTAG
GCAGGACAAAATGTACATTCAGTAAAAACCGAATTAATATTAAATTTCTCCCCGCCCACGGGCGTAAATA
CAAAAAATTTTAAATATAAACTCAAACTACAAACCTTATAAAGTTAATGAATAGCGTTAACAAAACTATA
AGTTCATATATAAATCAAGGACCCAAGTATATATTAAGAATAACAACCCCAAATATGGGTTTGGTAGACG
TTAAGACTGTTGACTCGACGGCTAACGTAATAAATAAAGTTGAGGTCAGCAACACAGATGTAAAATTGAT
AACAATTTTTGTCGTTATGATATTTGTCATATTAGTAGGACATCTATTGTACAAATTATATACGTATCATAA
TAAATGCATAAAAAGAGATACCAGAGTCACGCAAATGACCTGGATAAAATCTAAATTTTTCTTGAACAAT
GATAAGCGACAAAATAAAATAAATAAATAAAATGAAATTTCGAGAAATATATATATATTCTTATTACCAG
AAATGCCTATTAATAAGTTTGCGATAGAAGTATACTTAGAAGCAATATATAATAGCGACGCAGACTTTAG
AATTCGTGCATTGAATATTCTACAACAATAAGATAAAAGGCATAATTTTAAATACACTGCCATANGTTGG
CATTAAAGAAATAGTGGTCCAGTGGACGGCGAGTGCCCAATTATAATGGTAAAACTTCATAAATAGAGAA
CTATGGAATGGAGTGAATTAGCGAAAAAAATAAATAATTTCAAAGATAAATTCGAAAAATCTTATAAGTG
TGTTAATAAGAACGCACCTATAAGGGTTGAAACGCTAATTCACCACATAAACATATTAGTGCTAAACTAT
AATAACATAGTTCAACTTTTATTTAAGTAAAGAAAATAGGCTTACAGAGCAGCATGTAAATCAATGTAAG
AAAATAATCAAATCACTTGGGACTAGAATATCGAATATCAGCACTAGGCACAACATAGTAATAGATATCC
CAACAGATTTAAAATTAATGGCAACTTTTGAACCAGATCAACTAGAAGAACTGAATGAGTCTAAGCCAAC
ACTAAATAGTGACCCTGATATTGAAGCTGATAGCGACATTGAGCCACTTGAATTAACAGAACCAAACATG
GCNGTAAATCAAGTGGCCTTAGACCGCGAATATGTAAGACAGGTATCGTCTACAATTCCGGAATTTGATG
GCAAAAGCTATCACTGGCCAGATTTCTAACGGCTTTAAGACTAGTCGATCGTACTAAAGGTACACAAGAA
GACTTAGCCGTAGAAGTCATAAAGTCTAAAATATTGGCCCAATTTTGTACAAAGTACAAAATGAAACATC
CATTATTGGTATTATAAATAAATTAAATACCAATATCAAAGGCGAGTCAACTGACGTTATCAAAGCTAAG
TTGCTTAACATAAAGCAGAAAGGCAAGTCAGCCTCGCAATATACAGCTGAAATTGACAGTTTGCGTAAGC
AACTGGAAGCAGCCTATATTGACGATGGACTAGATTTTGATAACGCAGAGAAATTTTCTACTAAAGAAAC
AATATCTGCCATGACAAAGAATTGCGAATACGAAAAGCTTCGCATAATATTAGAAGCGGGCAATTTCAGC
AATTTCAATGATGCTATGGGTAAATATATCCAATGCAGCACTGAAATGACTGGATGCCCAAGTACAATAT
TGTACTACAAAGGCAGAAACGATCGTGGAAATTACAGCCGTAACAACCAAAGAGGTAGAGGCAATAGCC
GCGGCAATAGTAATTTCCACTATAATGGTAATAACAACAATAGGGGTAATTTCAGAGGAAATTACCGAGG
TGGCAACCGAGGTAGAAACAATTCAAACCGAGGTGGCTACCAAAACCAGAACAGTAACAATAGAGGTAA
TGTCCGAGTAACTCAAGGTAACTCGGAAAACCAACAGACCCCCTCAGATGTTCAAAGCCAATAAATGGCT
CAATAAAAACCATCAACCTTAATCTTAGCATGTTTGTAAAAATTAAGAATGAAAGAACAGGTAAATCAAT
TACACTGTTAGTAGATACTGGTGCAGATATATCCATATTAAAAGAAAATACAGATATATTCGAAAATGTA
AACGAAACAATTACTACCCAAATATCGGGTATTGGCGAAGGCATAATTCCTTCAAAAGGCTTAGCCTTTA
TAGAACTCACTACAGGCAAATATATAATTCCACATAATTTTCATTTAGTCGAACAAAACTTCCCAATACCG
AGTGATGGCATACTAGGCATAGATTTTATAAAACAATATAATTGCCAACTAGATTTCACTCAAAGTGGCG
ACTCACTTATTCTTCGCCCAAACAACTTAAATTATCCAATTCGATTAAAAATACTTCATACTTGTGACAAT
AATGCCACAGTACTCCCAGCTCGTTCCGAAGTTGTCCGTGAAATACAAATTCCCTCAATACAAAATCAAAT

TCTTATATTAAATCAAGAAATTGATGAAGGAATTTACATCGCAAACACGATATCCAATCACAAAAATACA
TATGTCCGTATTATAAATACAACAAATGCCAATAAGATAGTAAACTTAAATAAAATAAAATATGAAACTT
TGAACAATTATGATATAGTAAAAGAAACCAACGAAGGAAGAGACAACTTCGTTATGGAAAAATTAATCA
AAAACTTTCCACCTTTATTTAAAAATCAATTAAAAGATTTATGCACCAGATATACCGACATATTCGGATTA
GAAACCGAATCGATAACAACTAATAATTTTACAAGCAAAAGCTTAGATTGAAGGATGATGAGCCCGTATA
CATAAAAATTATAGAAGTCCTCACAGCCAAGTACAAGAAATTCAAGCCCAAGTGCAAAATTAATAGACG
ACAAAATAGTCGAACCATCAGCCTCAGAGTATAATAGCCCACTTTTATTAGTTCCGAAGAAGTCCCTTCCG
GACTCTGACAAGAAGAAATGGCGATTAGTAATTGACTATCGTCAAATTAATAAAAAGCTATTGTCCGATA
AATTTCCGCTACCGAGAATTGATGATATTCTAGATCAGCTAGGTAGAGCAAAATATTTTTCATGCCTAGAC
TTAATGTCTGGCTTTCACCAAATCGAACTCGAAAAAAGTTCTAGAGATATAACGTCATTTTCAACGAGCAA
TGGCTCGTATCGCTTTACGCGATTACCATTTGGCTTGAAAATAGCCCCAAACTCCTTTCAAAGAATGATGA
CAATAGCATTTTCTGGTCTCGAACCATCTCAGGCCTTCCTTTATATGGATGATCTGATAGTAATCGGTTGTT
CCGAAAAACATATGGTCAAAAATTTAACTGAAGTTTTTGACAAATGTAGGAAATTCAACCTGAAACTACA
TCCAGAAAAATGTTCATTTTTCATGCATGAAGTCACATTTTGGGACATAAATGCACTGATAAAGGAATCTT
GCCAGACGATAAAAAATACGACGTCATAAAGAACTACCCAGTCCCACATGATGCGGACAGCGCTAGGCG
ATTCGTTGCATTTTGCAATTACTATAGGCGCTTTATTAAAAATTTTGCCGACTATTCACGTCACATAACAA
GATTATGTAAAAAGACGTTAAGTTCGAATGGACATCTGAATGCCAGAATGCCTTCGATCACCTCAAAACG
GCTCTTATAAACCCAACCTTGTTACAATATCCAGATTTCGCTAAAGAATTCTGCATAATAACTGATGCAAG
TAAGCAAGCGTGTGGAGCGGTTTTAACTCAAAACCATAATGGACTCCAACTTCCAATTGCATACGCATCA
AGATCCTTTACTAAAGGAGAAAGTAACAAGAGTACTACTGAGCAAGAATTAGCGGCTATTCATTGGGCAA
TAACGCATTTCCGACCATATATTTATGGTAACCATTTCACGGTTAAAACTGACCATAGACCGCTAACCTAC
TTATTTTCAATGGTAAATCCAAGTTCAAAGTTAACACGTATGAGGCTTGAATTAGAGGAATATAATTTTAC
GGTAGAATATCTGAAGGGTAAAGATAATTTCGTAGCCGATGCGTTATCAAGAATAACCATAAAACAGCTA
CAGGAAATAACAGGGACAATCCACAAAGTCACTACAAGATATCAAAGTAGACAAAATTCCTGCGCAGGA
AAATATCAGGTAGAATTGCCAAGGCAATCTACAGAAAAAGCTTCACAGCCCAACGTATACGAAGTCATTA
ACAATGACGAGGTACGCAAAGTAGTGACCTTGCATATAAAAGATACGCTTTGTTTCTTTAAACATGGCAA
GAAAATTATTGCAAGAATTAACATTGGTGATCTATATACAAATGGAAATATTGACTTAGGTCAACTATTCC
CAAGGCTCGAAAATGAAGCCGGTATATTGAAAATAAGCCAATTGAAAATGGCACCGTGGGAAAATATCTT
TGATACAATTTCAATAGATAATTTCAAAAATATGGGCAATAAAATATTGACTAAATTAAGGGTAGCGCTA
CTCAACCCGGTGACCACAATAACAAATAATAAAGAAAAAGAAGCTATATTGTCTACACTACATGACGACC
CAATACAAGGAGGTCATACTGGCATTACAAAAACCTTGGCCAAGGTCAAACGACATTACTTTTGGAAAGG
TATGACTCGAGATATTACTGAGTACATACGTAAATGTCACAAATGCCAGAAAGCTAAAATAACTAAGCAT
AGTAAGACCCCATTAATTATAACTGATACACCTGTACATGCGTTCGATAGGGTTATAGTGGACACAATTG
GTCCGCTACCCAAATCTGATAATGGTAATGAGTACGCAATCACATTAATTTGTGACTTGACTAAATACCTA
GTCGCGATACCAATACCAAACAAAAACGCTAGTACAGTAGCCAAAGGTATATTCCAATCTTNTATTCTAA
AGTACGGTCCAATGAAGACGTTCATTACGGACATGGGAACAGAATACAAGAACTCAATTATAAATGATTT
GTGCAAGTTTATGAAAATAGACAACATAACATCAACTGCACATCATCACCAGACAGTTGGAACAGTCGAA
AGAAGTCACAGAACTTTTAACGAATACATACGATCTTATATATCAGTTGATAAAACTGATTGGGACGTAT
GGCTCCAATACTTTGTCTATTGTTTTAATACGACCCCTTCTACGGCACATAATTATTGTCCGTATGAATTAG
TATTTGGTAAGACATGTAATCTACCAAAATACTTCAATAGCATAGCAAACATAGAACCAATATATAACAT
AGATGATTATGCTAAGGAGAGTAAGTATAGATTAGAAGTAGCATACAAAAGAGCAAGAAATATGCTAGA
ATTAAATAAGATGAAACAAAAGAAGCGATATGACTTAAATAGTAAGGAATTAAAATTATCCATAGGAGA
TAAGGTTTTATTAAAAAATGAAGTAGGACATAAACTAGATTTTAAATACACAGGGCCCTATAGAGTAGAA
AAAATAGAAGATAGGGATAATATAGTTATTTCCGGAAATAAGAATAAAACGCAAACAGTCCATAAGGAC
AGATTAAAAATTTTTAATTCATAATCAAGCATGCAAAAATACATGAAAAAAAAAAAAAAAAAAAATATAT
ATATATAAAAAAAAATAATATATAATAATAATAAAAATAAATAATTATTAAGGAAAATAACTCCATTGTA
TTACGTTATTTTTTAAAAGGAGGGAGATGTAGCATGCGTACATATGTATATACATACATATGAATAAGGCT
GCAACACTTTGTTGTTATGCCAACAGAATGCAAACATAAGCAGCGACTTTGTCGCAGCGCGCCTAACGAT
AAGTGCCCTCTGCGCATATTCAAGTGCAGACGAAATATGATCCGCACTTGATAACAACGACCGCCCAAAT
AGCTAAGTCAGCGTTCTCACGCTTGACCGTCGTTGTGTATACATATGTGCGCGCATGACTCTCTCTGACTC
TCTTGCTGTATATAATAAGCCAAAAACCATGTATGTAAAACGGCTGCTCACTGCTTCGCGGTGGACAGCTC
GAACTCGAAAAAAAAACAGATAATAAAGAACCATCAAGAAAGACGGACGTCTTTTAATCTTGGAACCAT
TACA
>9_rnd-1_family-93__LTR___Gypsy

TGTAGCATGCGTACATATGTATATACATACATATGAATAAGGCTGCAACACTTTGTTGTTATGCCAACAGA
ATGCAAACATAAGCAGCGACTTTGTCGCAGCGCGCCTAACGATAAGTGCCCTCTGCGCATATTCAAGTGC
AGACGAAATATGATCCGCACTTGATAACAACGACCGCCCAAATAGCTAAGTCAGCGTTCTCACGCTTGAC
CGTCGTTGTGTATACATATGTGCGCGCATGACTCTCTCTGACTCTCTTGCTGTATATAATAAGCCAAAACC
ATGTATGTAAAACGGCTGCTCACTGCTTCGCGGTGGACAGCTCGAACTCGAAAAGAAAAACAGATAATAA
AGAACCATCAAGAAAGACGGACGTCTTTTAATCTTGGAACCATTACATGGCGACCGTGACAGGACGTCAA
AAAACATGAAAACGTTAAGATACATAACTTCAACTCTAATTTTAAACCAGTCATAAAAGTAATTAAAGGA
AGTAAATTAAAAGGAAATAACACTCAAAAACAACTGAAACAACGAAGCATCTGGAACGGCACACACCCT
GGAGAATCAAAAATTAACTGAGTGAGTACAGGGGGCATGAGCTCTAAAAATACTTTAAAGCTNAAAAGG
CGCAGTGGCATTAAAGAGCGGATCAGGCAAAGAAAGTCTGAGATCGTAAGACTCTTATATAAGTCCCAAA
ACCCAATACCTTGGGAAACTGCAGTCAAGCTACATGACGAACTCAAACATCTTGAGTCGTTAAACAAAGT
AAAGAGCCCCCCTGTCCCTCAGAGGAAAAACTTATTAATCCAGTTTCACATCGACCACAGCCACAACCGA
TCCCGAGAAGGCAACGTGAATGCCCAGCAGACTGCGAGGGACCGCTTTACAGCCAACATTGCCTGGGCAC
ATGTCAGCGGCCACCCACGAGTAAAAAACTAGTACGAGAGGCAAACCGCCCCTTGTACCAACGCTGGAA
CAAGAAGAAGTACGAGAGGCAGACCGCCCCTTGTACCAACGCTGGAAAACACCAAGGGTGAACTGACCG
ACAGCGCCACAGAGGGCGCACAGNAAAAAATTTATAATTATAATTATTATTAAAATATTTGTAAAACTAT
TGTAATGCCCGACGACAATTGAGTCGACCGGCAAGACGTTTGGCCCGTAAAGCCATTCGAAATACTAGGC
AGGATAAAATGTACATTCAGTAAAAACCGAATTAATATTAAATTTCTCCCCGCCCACGGGCGTAAATACA
AAAAATTTTAAATATAAACTCAAACTACAAACCTTATAAAGTTAATGAATAGCGTTAACAAAACTATAAG
TTCATATATAAATCAAGGACCCAAGTATATATTAAGAATAACAACCCCAAATATGGGTTTGGTAGACGTT
AAGACTGTTGACTCGACGGCTAACGTAATAAATAAAGTTGAGGTCAGCAACACAGATGTAAAATTGATAA
CAATTTTTGTCGTTATGATATTTGTCATATTAGTAGGACATCTATTGTACAAATTATATACGTATCATAATA
AATGCATAAAAAGAGATACCAGAGTCGCGCAAATGACCTGGATAAAATCTAAATTTTTCTTGAACAATGA
TAAGCGACAAAATAAATAAATAAAATGAAATTTCGAGAAATATATATATATTCTTATTACCAGAAATGCC
TATTAATAAGTTTGCGATAGAAGTATACTTAGAAGCAATATATAATAGCGACGCAGACTTTAGAATTCGT
GCATTGAATATTCTACAACAATAAGATAAAAGGCATAATTTTAAATACACTGCCATANGTTGGCATTAAA
GAAATAGTGGTCCAGTGGACGGCGAGTGCCCAATTATAATGGTAAAACTTCATAAATAGAGAACTATGGA
ATGGAGTGAATTAGCGAAAAAAATAAATAATTTCAAAGATAAATTCGAAAAATCTTATAAGTGTGTTAAT
AAGAACGCACCTATAAGGGTTGAAACGCTAATTCACCACATAAACATATTAGTGCTAAACTATAATAACA
TAGTTCAACTTTATTTAAGTAAAGAAAATAGGCTTACAGAGCAGCATGTAAATCAATGTAAGAAAATAAT
CAAATCACTTGGGACTAGAATATCGAATATCAGCACTAGGCACAACATAGTAATAGATATCCCAACAGAT
TTAAAATTAATGGCAACTTTTGAACCAGATCAACTAGAAGAACTGAATGAGTCTAAGCCAACACTAAATA
GTGACCCTGATACTGAAGCTGATAGCGACATTGAGCCACTTGAATTAACAGAACCAAACATGGCNGTAAA
TCAAGTGGCCTTAGACCGCGAATATGTAAGACAGGTATCGTCTACAATTCCGGAATTTGACGGNAAGAAA
AGCTATCACTGGCCAGATTTCTAACGGCTTTAAGACTAGTCGATCGTACTAAAGGTACACGAGAAGACTT
AGCCGTAGAAGTCATAAAGTCTAAAATAACTGGCCCAATTTTGTACAAAGTACAAAATGAAACATCCATT
ATTGGTATTATAAATAAATTAAATACCGATATCAAAGGCGAGTCAACCGACGTTATCAAAGCNAAGTTGC
TTAACATAAAGCAGAAAGGCAAGTCAGCCTCGCAATATACAGCTGAAATTGACAGTTTGCGTAAGCAACT
GGAAGCAGCCTATATTGACGATGGACTAGATTTTGATAACGCAGAGAAATTTTCTACTAAAGAAACAATA
TCCGCCATGACAAAGAATTGCGAATACGAAAAGCTTCGCATAATATTAGAAGCGGGCAATTTCAGCAATT
TCAATGATGCTATGGGTAAATATATCCGATGCAGCACTGAAATGACTGGANGCCCGAGTACAATATTGTA
CTACAAAGGCAGAAACGATCGCGGAAATTACAGCCGTAACAACCAAAGAGGTAGAGGCAATAGCCGCGG
CAATAGTAATTTCCACTATAATGGTAATAACAACAACGGGGGTAATTTCAGAGGAAATTACCGAGGTGGA
GGCCGAGGAATGGAAACAATTCAAACCGAGGCGGCTACCAAAACCAGAACAGTAACAATAGAGGTAATG
TCCGAGTAACCCAAGGCAACTCGGGAAACCNNCAGAACCCCTCAGATGTTCANAGCCAGTAAATGGCTCA
GTTAAAACCATCAACCTTAGTCTTAGCATGTTTGTAAAANTTNANAATGTGAAGAACAGGTAAAAACTTG
CACTNTTAGTAGATACTGGTGCAGACATATCCATGTTAAAGGAAAATNCAGATATATTCGANAATGTCCA
AACGAAAAAATCACTACCCAAATATCGGGCATNGGCGAAGGCATAATNCCGTCCAAAGGGCTNAGCTCT
NTATGGGACNCACTACAGGCAAATACATAATTCCACACGATTTTCACTTAGTCGANCAGAATTTCCCAAT
ACCNNGTGATGGCATANTAGGCATNGACTTTATNAAACAATNCAATTGCCAACTAGATTTCANNCAAAGT
GGNGACTCGCTTATNNTTNGCCCAAACAACTTAAATTATCCAATTCGTGTTCCAATAACACACAGTTCTGG
CAATAATNCCACAGTNCTNCCAGCNNGNTCCGAAGTNGTCCGNGAAATACAAATTCCNTCAGATGAAGAT
CGTGTNCTNATTCCAAACCAAGAAATTGANGAAGGNATTTACGTCGCAAACACGATTGCAACAACCAAAA
ATGCATATGTCCGTATTACTAAATACGACAAATGCCGATCAAGTAGTGAAATTAAAAAATTANAATATGA

ACCACTTCGAACAATTACGACATAGTCAAAAGAAACCAACGAAGGAAGAGACAACTTCGTTATCNAAATT
AACCAAAAACTTTCCACCNCNATTCAAAAATCAATTAAAAGACTTATGCACCAGATATANCGACGTATTC
GGATTNGAAACCGAATCGATAACNACCAATAACTTTTACAAGCAAAAGCTNAGATTGAAGGATGATGAG
CCCGTATACANAAAAATTATAGAAGCCCTCACAGCCAAGTANANGAAATNCAAGCNCAAGTGCAAAAAT
TAATCGACGACAAAATAGTCGAACCGTCNGCCTCAGAGTATAATAGCCCACTTTTATTAGTTCCGAAGAA
GTCCCTTCCGGGCTCTGACAAGAAGAAATGGCGATTAGTAATTGACTATCGTCAAATTAATAAAAAGCTA
TTGTCCGATAAATTTCCGCTGCCNAGAATTGATGATATTCTGGATCAACTAGGNAGAGCAAAATACTTTTC
ATGCCTTGACTTAATGTCAGGCTTTCACCAAATCGAACTCGAAAAAGTTCNAGAGATATAACGTCTTTTTC
AACGAGCAATGGCTCGTATCGCTTCACGCGATTACCATTCGGCTTAAAAATAGCGCCAAACTCCTTTCAAA
GAATGATGACNATAGCATTTTCCGGNCTAGAACCNTCTCAGGCTTTCCTTTATATGGATGATCTGATAGTN
ATCGGTTGTTCCGAAAAACATATGNTCAAAAACTTAACTGATGTTTTTGAGAAATGTAGGAAATNCAACC
TGAAACTACATCCAGAAAAAATGTTCATTTTTCATGCATGAAGTCACATTTTTGGGACACAAATGCACNGAT
AAAGGAATCTTGCCAGACGANAAAAAATACGACGTCATNAAGAACTACCCAGTCCCACATGATGCGGAC
AGCGCTAGACGATTCGTTGCATTCTGCAATTACTATAGGCGTTTTATCAAAAATTTTGCCGACTATTCACG
TCACATAACAAGATTATGTAAAAAAGACGTTAAGTTCGAATGGACAGCTGAATGCCAGAATGCCTTCGAN
CACCTCAAAACGGCNCTNATAAACCCGACCTTGTTACAATATCCAGATTTCGGCAAAGAATTTTGCATAAT
AACNGATGCAAGTAAGCAAGCGTGTGGAGCGGTTTTAACTCAAAACCATAATGGACTCCAACTTCCAATT
GCATACGCATCAAGATCCTTTACTAAGGGAGAAAGTAACAAGAGTACTACNGAGCAAGAATTAGCGGCN
ATTCATTGGGCAATAACGCATTTCCGACCATATATTTATGGTAANCACTTCACNGTCAAAACNGACCACA
GACCGCTAACATACTTATTTTCAATGGTAAATCCAAGCTCAAAGCTAACGCGTATGAGGCTTGAACTAGA
GGAGTATAATTTTACGGTNGAATATCTGAAGGGTAAAGACAATTTCGTAGCCGATGCGTTATCAAGAATA
ACCATNAAACAGCTACAAGAAATAACAGGGACAATCNAGAAAGTCACTACAAGATATCAAAGTAGACAA
AATTCCTGCGCAGGAAAAAANAAGTAGAATTGCCAAGGCAATCTATAGAAAAAGCTTCAAAGCCCAACG
TATACGAAGTCATTAACAATGACGAGGTACGTAAAGTAGTGACCTTGCATGTAAAAGATACGCTATGTTT
CTTTAAACATGGCAAGAAAATTACTGCAAGANNTGACGTTGGCGATTTATATACCAATGGAANTNTTGAC
TTAGGTCAATTNTTCCAAAGGCTCGAAAAGGAAGCCGGTATANNNAAAATCAGCCAACTCAAAGTGGCA
CCGTGGGAAAAATATCTTTGANACAGTTTCAATAGATAATTTCAAAAATATGGGCAATAAAATATTGACT
ACATTAAGGGTAGCGCTACTCAACCCGGTGACCACAATAANAAACAATAAAGAAAAAAGAAGCTATATT
GTCTACGCTCCATGACGACCCAATACAAGGAGGTCATACNGGCATTACAAAACCTTGGCCAAGGTCAAAN
GACATTACTTTTGGAAAGGTATGACTCGAGATATTACTGAGTACATACGNAAATGTCAAAAATGCCAGAA
AGCTAAAATAACTAAGCATAGTAAGACCCCNTTAACNATAACTGATACGCCNGTACACGCTTTCGACAGG
GTTATAGTGGACACNATTGGTCCGCTACCCAAATCTGANAATGGNAATGAGTACGCAGTCACATTAATTT
GTGACTTGACTAAATACCTAGTCGCGATNCCAATNCCAAACAAAAGCGCTAGCACAGTAGCCAAAGCNAT
NTTCGAATCTTTTATTCTAAAGTACGGTCCAATGAAGACGTTCATTACGGACATGGGAACAGAATACAAG
AATTCAATTATAAATGATTTGTGCAAGTNTNTGAAAATAGACAACATAACATCAACTGCACACCACCACC
AGACAGTTGGAACAGTCGAAAGAAGTCACAGAACTTTTAACGAATACATACGNTCATATATATCGGTTGA
TAAAACTGATTGGGACGTATGGCTCCAATACTTCGTCTACTGTTTCAACACGACCCCNTCTACGGCACATA
ATTATTGTCCGTATGAACTAGTATTCGGTAAGACANGCAATCTACCAAAACACTTCAATAGCATAGNNAA
CATAGAACCNNTATATAATATAGATGATTATGCTAAGGAAAGTAAGTATAGATTAGAAGTAGCATACAAA
AGAGCTAGAANTATGCTAGAATAAATAAGATGAAACAATAAAGAAGCTATATGACTTAAANAGTAAGGA
ATTAAAAATTATCGTAGGAGATAAAGTCTTATTAAGAAATGAAGCAGGACATAAAGCTAGATCCTAAATA
CNCAGGGCCCTATANAGTAGAAAAATAGAAGATAGGGATAATATAGTTATTNCGGATATAAGAATAAAA
NACAAACAGTCCATAAGGACAGATTAAAAATTTTTAATTCATAATCGAGCATGCAAAACTCTAATATAAA
AAAAAAAAAATATATATAAAAAAAAAAAAATACAAAATAATAAAAAAAAATAAATAATTATTAAGGAAAA
NNAACTCAATTGNATTACGTTATTTTTTTAAAAGGAGGGAGATGTAGCATGCGTACATATGTATATACATA
CATATGAATAAGGCTGCAACACTTTGTTGTTATGCCAACAGAATGCAAACATAAGCAGCGACTTTGTCGC
AGCGCGCCTAACGCTAAGTGCCCTCTGCGCATATTCAAGTGCAGACGAAATATGATCCGCACTTGATAAC
AACGACCGCCCAAATAGCTAAGTCAGCGTTCTCACGCTTGACCGTCGCTGTGTATACATATGTGCGCGCAT
GACTCTCTCTGACTCTCTTGCTGTATATAATAAGCCAAAAACCATGTATGTAAAACGGCTGCTCACTGCTT
CGCGGTGGACAGCTCTAACTCNAAAAAAAAAATAAAGAACCATCAAGGAAGACTGACGTCTTTTCATCAGG
GAACCATTACA
>9_rnd-1_family-286__LTR___Gypsy
TGTAGCATGCGTACATATGTATATACATACATATGAATAAGGCTGCAACACTTTGTTGTTATGCCAACAGA
ATGCAAACATAAGCAGCGACTTTGTCGCAGCGCGCCTAACGATAAGTGCCCTCTGCGCATATTCAAGTGC

AGACGAAATATGATCCGCACTTGATAACAACGACCGCCCAAATAGCTAAGTCAGCGTTCTCACGCTTGAC
CGTCGTTGTGTATACATATGTGCGCGCATGACTCTCTCTGACTCTCTTGCTGTATATAATAAGCCAAAACC
ATGTATGTAAAACGGCTGCTCACTGCTTCGCGGTGGACAGCTCGAACTCGAAAAGAAAAACAGATAATAA
AGAACCATCAAGAAAGACGGACGTCTTTTAATCTTGGAACCATTACATGGCGACCGTGACAGGACGTCAC
AAAAACATGAAAACGTTAAGATACATAACTTCAACTCTAATTTTAAACCAGTCATAAAAGTAATTAAAGG
AAGTAAATTAAAAGGAAATAACACTCAAAAACAACTGAAACAACGAAGCATCTGGAACGGCACACACCC
TGGAGAATCAAAAATTAACTGAGTGAGTACAGGGGGCATGAGCTCTAAAAATACTTTAAAGCTAAAAAG
GCGCAGTGGCATTAAAGAGCGGATCAGGCAAAGAAAGTCTGAGATCGTAAGACTCTTATATAAGTCCCAA
AACCCAATACCTTGGGAAACTGCAGTCAAGCTACATGACGAACTCAAACATCTTGAGTCGTTAAACAAAG
TAAAGAGCCCCCCTGTCCCTCAGAGGAAAAACTTATTAATCCAGTTTCACATCGACCACAGCCACAACCG
ATCCCGAGAAGGCAACGTGAATGCCCAGCAGACTGCGAGGGACCGCTTTACAGCCAACATTGCCTGGGCA
CATGTCAGCGGCCACCCACGAGTAAAAACTAGTACGAGAGGCAAACCGCCCCTTGTACCAACGCTGGAAC
AAGAAGAAGTACGAGAGGCAGACCGCCCCTTGTACCAACGCTGGAAAACACCAAGGGTGAACTGACCGA
CAGCGCCACAGAGGGCGCACAGCAAAAAAAAATTTATAATTATAATTATTATTAAAATATTTGTAAAACT
ATTGTAATGCCCGACGACAATTGAGTCGACCGGCAAGACGTTTGGCCCGTAAAGCCATTCGAAATACTAG
GCAGGACAAAATGTACATTCAGTAAAAACCGAATTAATATTAAATTTCTCCCCGCCCACGGGCGTAAATA
CAAAAAATTTTAAATATAAACTCAAACTACAAACCTTATAAAGTTAATGAATAGCGTTAACAAAACTATA
AGTTCATATATAAATCAAGGACCCAAGTATATATTAAGAATAACAACCCCAAATATGGGTTTGGTAGACG
TTAAGACTGTTGACTCGACGGCTAACGTAATAAATAAAGTTGAGGTCAGCAACACAGATGTAAAATTGAT
AACAATTTTTGTCGTTATGATATTTGTCATATTAGTAGGACATCTATTGTACAAATTATATACGTATCATAA
TAAATGCATAAAAAAGAGATACCAGAGTCACGCAAATGACCTGGATAAAATCTAAATTTTTCTTGAACAA
TGATAAGCGACAAAATAAAATAAATAAATAAAATGAAATTTCGAGAAATATATATATATTCTTATTACCA
GAAATGCCTATTAATAAGTTTGCGATAGAAGTATACTTAGAAGCAATATATAATAGCGACGCAGACTTTA
GAATTCGTGCATTGAATATTCTACAACAATAAGATAAAAGGCATAATTTTAAATACACTGCCATANGTTG
GCATTAAAGAAATAGTGGTCCAGTGGACGGCGAGTGCCCAATTATAATGGTAAAACTTCATAAATAGAGA
ACTATGGAATGGAGTGAATTAGCGAAAAAAATAAATAATTTCAAAGATAAATTCGAAAAATCTTATAAGT
GTGTTAATAAGAACGCACCTATAAGGGTTGAAACGCTAATTCACCACATAAACATATTAGTGCTAAACTA
TAATAACATAGTTCAACTTTATTTAAGTAAAGAAAATAGGCTTACAGAGCAGCATGTAAATCAATGTAAG
AAAATAATCAAATCACTTGGGACTAGAATATCGAATATCAGCACTAGGCACAACATAGTAATAGATATCC
CAACAGATTTAAAATTAATGGCAACTTTTGAACCAGATCAACTAGAAGAACTGAATGAGTCTAAGCCAAC
ACTAAATAGTGACCCTGATACTGAAGCTGATAGCGACATTGAGCCACTTGAATTAACAGAACCAAACATG
GCNGTAAATCAAGTGGCCTTAGACCGCGAATATGTAAGACAGGTATCGTCTACAATTCCGGAATTTGATG
GCAAAAGCTATCACTGGCCAGATTTCTAACGGCTTTAAGACTAGTCGATCGTACTAAAGGTACACAAGAA
GACTTAGCCGTAGAAGTCATAAAGTCTAAAATAATTGGCCCAATTTTGTACAAAGTACAAAATGAAACAT
CCATTATTGGTATTATAAATAAATTAAATACCAATATCAAAGGCGAGTCAACTGACGTTATCAAAGCTAA
GTTGCTTAACATAAAGCAGAAAGGCAAGTCAGCCTCGCAATATACAGCTGAAATTGACAGTTTGCGTAAG
CAACTGGAAGCAGCCTATATTGACGATGGACTAGATTTTGATAACGCAGAGAAATTTTCTACTAAAGAAA
CAATATCTGCCATGACAAAGAATTGCGAATACGAAAAGCTTCGCATAATATTAGAAGCGGGCAATTTCAG
CAATTTCAATGATGCTATGGGTAAATATATCCAATGCAGCACTGAAATGACTGGATGCCCAAGTACAATA
TTGTACTACAAAGGCAGAAACGATCGTGGAAATTACAGCCGTAACAACCAAAGAGGTAGAGGCAATAGC
CGCGGCAATAGTAATTTCCACTATAATGGTAATAACAACAATAGGGGTAATTTCAGAGGAAATTACCGAG
GTGGCAACCGAGGTAGAAACAATTCAAACCGAGGTGGCTACCAAAACCAGAACAGTAACAATAGAGGTA
ATGTCCGAGTAACTCAAGGTAACTCGGAAAACCAACAGACCCCCCAGATGTTCAAAGCCAATAAATGGCT
CAATAAAACCATCAACCTTAATCTTAGCATGTTTGTAAAAATTAAGAATGAAAGAACAGGTAAATCAATT
ACACTGTTAGTAGATACTGGTGCAGATATATCCATATTAAAAGAAAATACAGATATATTCGAAAATGTAA
ACGAAACAATTACTACCCAAATATCGGGTATTGGCGAAGGCATAATTCCTTCAAAAGGCTTAGCCTTTAT
AGAACTCACTACAGGCAAATATATAATTCCACATAACTTTCATTTAGTCGAACAAAACTTCCCAATACCGA
GTGATGGCATACTAGGCATAGATTTTATAAAACAATATAATTGCCAACTAGATTTCACTCAAAGTGGCGA
CTCACTTATTCTTCGCCCAAACAACTTAAATTATCCAATTCGATTAAAAATACTTCATACTTGTGACAATA
ATGCCACAGTACTCCCAGCTCGTTCCGAAGTTGTCCGTGAAATACAAATTCCCTCAATACAAAATCAAATT
CTTATATTAAATCAAGAAATTGATGAAGGAATTTACATCGCAAACACGATATCCAATCACAAAANACATA
TGTCCGTATTATAAATACAACAAATGCCAATAAGATAGTAAACTTAAATAAAATAAAATATGAAACTTTG
AACAATTATGATATAGTAAAAGAAACCAACGAAGGAAGAGACAACTTCGTTATGGAAAAATTAATCAAA
AACTTTCCACCTTTATTTAAAAATCAATTAAAAGATTTATGCACCAGATATACCGACATATTCGGATTAGA

AACCGAATCGATAACAACTAATAATTTTACAAGCAAAAGCTTAGATTGAAGGATGATGAGCCCGTATACA
TAAAAATTATAGAAGTCCTCACAGCCAAGTACAAGAAATTCAAGCCCAAGTGCAAAANTAATAGACGAC
AAAATAGTCGAACCATCAGCCTCAGAGTATAATAGCCCACTTTTATTAGTTCCGAAGAAGTCCCTTCCGGA
CTCTGACAAGAAGAAATGGCGATTAGTAATTGACTATCGTCAAATTAATAAAAAGCTATTGTCCGATAAA
TTTCCGCTACCGAGAATTGATGATATTCTAGATCAGCTAGGTAGAGCAAAATATTTTTCATGCCTAGACTT
AATGTCTGGCTTTCACCAAATCGAACTCGAAAAAGTTCTAGAGATATAACGTCATTTTCAACGAGCAATG
GCTCGTATCGCTTTACGCGATTACCATTTGGCTTGAAAATAGCCCCAAACTCCTTTCAAAGAATGATGACA
ATAGCATTTTCTGGTCTCGAACCATCTCAGGCCTTCCTTTATATGGATGATCTGATAGTAATCGGTTGTTCC
GAAAAACATATGGTCAAAAATTTAACTGAAGTTTTTGACAAATGTAGGAAATTCAACCTGAAACTACATC
CAGAAAAATGTTCATTTTTCATGCATGAAGTCACATTTTTGGGACATAAATGCACTGATAAAGGAATCTTG
CCAGACGATAAAAAATACGACGTCATAAAGAACTACCCAGTCCCACATGATGCGGACAGCGCTAGGCGA
TTCGTTGCATTTTGCAATTACTATAGGCGCTTTATTAAAAATTTTGCCGACTATTCACGTCACATAACAAG
ATTATGTAAAAAGACGTTAAGTTCGAATGGACATCTGAATGCCAGAATGCCTTCGATCACCTCAAAATGG
CTCTTATAAACCCAACCTTGTTACAATATCCAGATTTCGCTAAAGAATTCTGCATAATAACTGATGCAAGT
AAGCAAGCGTGTGGAGCGGTTTTAACTCAAAACCATAATGGACTCCAACTTCCAATTGCATACGCATCAA
GATCCTTTACTAAAGGAGAAAGTAACAAGAGTACTACTGAGCAAGAATTAGCGGCTATTCATTGGGCAAT
AACGCATTTCCGACCATATATTTATGGTAACCATTTCACGGTTAAAACTGACCATAGACCGCTAACCTACT
TATTTTCAATGGTAAATCCAAGTTCAAAGTTAACACGTATGAGGCTTGAATTAGAGGAATATAATTTTACG
GTAGAATATCTGAAGGGTAAAGATAATTTCGTAGCCGATGCGTTATCAAGAATAACCATAAAACAGCTAC
AGGAAATAACAGGGACAATCCACAAAGTCACTACAAGATATCAAAGTAGACAAAATTCCTGCGCAGGAA
AATATCAGGTAGAATTGCCAAGGCAATCTACAGAAAAAGCTTCACAGCCCAACGTATACGAAGTCATTAA
CAATGACGAGGTACGCAAAGTAGTGACCTTGCATATAAAAGATACGCTTTGTTTCTTTAAACATGGCAAG
AAAATTATTGCAAGAATTAACATTGGTGATCTATATACAAATGGAAATATTGACTTAGGTCAACTATTCCC
AAGGCTCGAAAATGAAGCCGGTATATTGAAAATAAGCCAATTGAAAATGGCACCGTGGAAAATATCTTT
GATACAATTTCAATAGATAATTTCAAAAATATGGGCAATAAAATATTGACTAAATTAAGGGTAGCGCTAC
TCAACCCGGTGACCACAATAACAAACAATAAAGAAAAAGAAGCTATATTGTCTACACTACATGACGACCC
AATACAAGGAGGTCATACTGGCATTACAAAAACCTTGGCCAAGGTCAAACGACATTACTTTTGGAAAGGT
ATGACTCGAGATATTACTGAGTACATACGTAAATGTCACAAATGCCAGAAAGCTAAAATAACTAAGCATA
GTAAGACCCCATTAATTATAACTGATACACCTGTACATGCGTTCGATAGGGTTATAGTGGACACAATTGGT
CCGCTACCCAAATCTGATAATGGTAATGAGTACGCAATCACATTAATTTGTGACTTGACTAAATACCTAGT
CGCGATACCAATACCAAACAAAAACGCTAGTACAGTAGCCAAAGGTATATTCCAATCTTTTATTCTAAAG
TACGGTCCAATGAAGACGTTCATTACGGACATGGGAACAGAATACAAGAACTCAATTATAAATGATTTGT
GCAAGTTTATGAAAATAGACAACATAACATCAACTGCACATCATCACCAGACAGTTGGAACAGTCGAAAG
AAGTCACAGAACTTTTAACGAATACATACGATCTTATATATCAGTTGATAAAACTGATTGGGACGTATGG
CTCCAATACTTTGTCTATTGTTTTAATACGACCCCTTCTACGGCACATAATTATTGTCCGTATGAATTAGTA
TTTGGTAAGACATGTAATCTACCAAAATACTTCAATAGCATAGCAAACATAGAACCAATATATAACATAG
ATGATTATGCTAAGGAGAGTAAGTATAGATTAGAAGTAGCATACAAAAGAGCAAGAAATATGCTAGAAT
TAAATAAGATGAAACAAAAGAAGCGATATGACTTAAATAGTAAGGAATTAAAATTATCCATAGGAGATA
AGGTTTTATTAAAAAATGAAGTAGGACATAAACTAGATTTTAAATACACAGGGCCCTATAGAGTAGAAAA
AAATAGAAGATAGGGATAATATAGTTATTTCCGGAAATAAGAATAAAACGCAAACAGTCCATAAGGACA
GATTAAAAATTTTTAATTCATAATCAAGCATGCAAAAATACAAAAAAAAAATATATATATAAAAAAAAAA
AAANAATATATAATAATAATAGAAATAAATAATTATTAAGGAAAATAACTCCATTGTATTACGTTATTTTT
TAAAAGGAGGGAGATGTAGCATGCGTACATATGTATATACATACATATGAATAAGGCTGCAACACTTTGT
TGTTATGCCAACAGAATGCAAACATAAGCAGCGACTTTGTCGCAGCGCGCCTAACGATAAGTGCCCTCTG
CGCATATTCAAGTGCAGACGAAATATGATCCGCACTTGATAACAACGACCGCCCAAATAGCTAAGTCAGC
GTTCTCACGCTTGACCGTCGTTGTGTATACATATGTGCGCGCATGACTCTCTCTGACTCTCTTGCTGTATAT
AATAAGCCAAAAACCATGTATGTAAAACGGCTGCTCACTGCTTCGCGGTGGACAGCTCGAACTCGAAAAA
GAAAACAGATAATAAAGAACCATCAAGAAAGACGGACGTCTTTTAATCTTGGAACCATTACA
>160_rnd-1_family-188__LTR___Gypsy
ATATAAGTTAACAACTAAGCATAAATATATTGCCCTAGCAACTAAGTAGCTTCGTATTAACAATGCTGAC
GCGCCAGAACTGAGTTCAGCGCTCTGCGCTAAGAACGGCCAGCAGCGGTAAGCTGACACTGCATGGAATG
CTGAGTCGGCTTGCCGACCATGCTTTGATGGCGTGATGCATTGAGGCTTAGGGTATTTGTATAATTTGCTT
GTCCCCTAGTTTTTAGTTTTATATGAGGCGCGCAACACATAAGTCGCCGAAATAAATATTCGTACTGTCTG
AGCCCAAGGCCGGCANTAGAAGCCGACTTCAAAACATAATCTTCCGTAATATTACTGGATCAAGGAGAAC

ATATTTTGATCAAGGGCAGCGCGGATCAACGCAACTTACCCAAGATAACATTGTGTTACATCGCAGAAC
ATACATCAATNGATAACTGGCGCCCAACCAATAAATAGGAACCCATACCATGTAAGTTCATAAGTGATTT
CAGTTTGAAGAAATTATTCACGAACGCCACGTACCGATACGTGTGATTTTTATAAAAAAAAAAAAAAATT
GTAAAGTAAAGTAAATATTGCTGCTCTCTTCTGCCAGCATTTCATTCCTTTAAATCATTGCATACTTTCACT
AAAAACTGTTGCATACTTTTACCAGCGTTCCTTGTCAATAAAGCATTGCATACCTTAAACGAATAATTATT
GCATACTTTCGGCAGCATTATATTTTTTGCTGCATACCCCTAACAATTTTTTATAATAAATATTGCATACCC
CAAGATTCACCAGTTTCCACTATTGCCAACAGCGTTACCTGCCGATCTTTCTCGATCGGCCTAAGTTTGAA
AAGTGCCAACCATAAGCCAATGAGTTGGAATTATAGGGAGATAAAAGTCGAATATAATAGTGACGATAG
CTGGGAGGGGGAAATCAATTGCTCAGTAGATCCTACTCCTGCAGATACCGTTGTAACTGAAGAAATGGAG
CCGGCTCAAGTTCAGAATCTCATTGAGGGTGCCATCAGGCAAGCTTTAGCGCAACAACAAAATCAGTTTC
AAGCACAAATAACCGCTTTAGCTTCACAAGTACAGAGTTTGCAGGTGGAGGCGCCGCAAATCGTCACATA
CGAGAAAATATCCGTCGATCCTAACGCCAAATGCGATATTCCCTTGGACATTATTAAATCTGTGCCGGATT
TCTCCGGCGTACAGGATGAGTATGTAGCGTGGAGGCAAGCAGCCATCTACGCGTACGAACTATTCAAGAA
CTATCCAAAGAGCACTGCACACTACCAAGCGGTAGCGATCATTAGGAACAAAATCAAAGGCCCAGCTGG
AGCCTTACTAGTGTCTCACAACACTGTCCTAAATTTCGATGCAATACTAGCTAGGCTAGATTGCACATACT
CGGACAAAACATCCTTGCGCTTGCTGAGACAATGTTTGGAGTTAGTTACGCAGGGCGAAATGTCCCTGAT
GCAATACTACGATGACGTCGAAAAGAAACTCACGCTCGTAACTAACAAAATTGTAATGTCTCACGACCAA
GAAGGCGCGGATTTGCTCAACCGGGAGGTCAGAGACGACGCCTTGCACGCCTTTATCTCCGGTCTTAGGA
AGCCGTTGAGAGCGGTTGTGTTCCCTGCACAACCAAAAGATCTACCATCCGCTCTGGCCCTTGCCAGAGA
GGCAGAAGCTAGCATTGAGAGAAGCATGTTCGCGAACTCTTACGCTAAAGCCTTGCAAGACCGAGCGCAA
ATTGCTGAAAGTAACAAAAACCGTGCCCAAGGCAAACAAACCAGATTTAACAAAGATGAGCAAAATCAG
GAGAGAAATCCCCATTTTGTGAAACGCCAAAAGGGCGGTCCCCAGCCCCAGGCCCCTAAAGATAATCAAA
CTGAGGCTCCACAGCCCATGGAAGTTGACTCTTCCTCCAGATTTAGACAAAAAACCGACTACAGCAAGCG
TCAGGCTAATGAGTCCAACGCACCAAAACGCAAGAATTCGTCAGACCGTTCAACAGGTCAAAGACGACA
ACGCGTTAACAATGTCGTACAATCAGATTCCAAAGAAGCCGTAACAGAATTCGAGAAGGCGGCCAAAGA
GGCAGTTGAAGAACTAGAGAGTGAGAACGAGTACGCACCTGGTGACGACTCGATTAATTTTTTAGGGAAC
ACTCCCGGCTTCCGTACATTGAACGACGGTTGGCTGGGAGAACTCTGAAGATGTTGATTGACACTGGCGC
GGCAAAAAATTATGTGAAGCCCTTAACAGAGCTAAAGACCATAACGCCGGTCGCCAAGTCATTCTCGGTA
AACTCGATCCACGGGTCTACCGAAATCAAGCGCAAATGCTTGATGAATATTTTCCAACATACCTCCCCGTT
TTTTCTCCTCGATACCCTAAGTTCTTTCGACGCCATCATAGGCTTCGACTTGCTAACACAAGCCGGAGTCA
AATTGAACTTGGCAAAAAACACTCTGGAATACCAGAGTACATCCGAAAAACTCCAGTACTATCAATGTGC
TGATGTGAACTTCACCGATGTCAACGACATCGTAGTCCCCCCTTCCGTAAAGAAAGAGTTCAGGGACATG
ATACTCAGAAACAAAAGAGCCTTCTCAAATGCAGACGAAGCTCTCCCTTTTAATACTGCTGTTATCGCTAC
AATCCGAACAGTAGACAACGAGCCTGTGTATTCAAGATACCCGAGTCTCATGGGTGTTTCTGACTTCATAA
ACAAAGAAGTCAAACAATTACTGGACGATGGCATAATCAGGCCCTCCAGATCGCCTTACAACAGCCCAAC
ATGGGTGGTTGATAAAAAAGGCACTGACTCATACGGTAACCCAAAAAAAAGGTTGGTAATCGACTTCAGG
AAACTGAACGAAAAAACCATACCGGACCGCTACCCCATGCCGAGCATCCCCATGATCTTAGCAAATCTAG
GAAAGGCGAAATACTTCACTACCCTCGACCTAAAGTCGGGGTACCATCAAATTTACCTTGCGGAGCATGA
CCGCGAAAAGACTTCATTTTCAGTAAGCAGCGGCAAGTACGAGTTCTGTCGCCTACCATTCGGCCTGCGC
AATGCAAGCAGCATATTCCAAAGGGCAATAGACGATATCCTGAGGGAACATATAGGAAAGATTTGCTTTG
TCTATGTAGACGACGTCATAATTTTTTCTAAAAACGAAACGGAGCACCTTCAACACATTAACATTGTGTTA
AAATGTCTAATCGACGCAAACATGCGTGTAGGCCCCGAAAAGACTCGTTTCTTCAAGGAAAGTATCGAAT
TCCTGGGTTTCATTGTAACCAAAGACGGAGCAACATCCGACCCCAAAAAAGTTAAAGCCATTCAGGAATT
CCCTGAACCAAAGAATGTCTACAGCGTTAGGTCCTTCTTGGGGTTAGCCAACTACTATAGGGTCTTTATTA
AGGACTTTGCCGCTATAGCGCGTCCGATATCGGACATACTTAAAGGGGAAAATGGTTCAGTTAGTAGGCA
CGTATCAAAGAAAATCCCTGTTGAATTTAATGAAGTTCAACGCGATGCATTTCACCGTCTGCGGAGTATTT
TGGCATCAGAAGACGTCATGTTAATGTATCCAGACTTCCAGAAACCGTTCGATTTAACCACTGATGCTTCG
GCCAGTGGAATTGGCGCGGTTCTTTCCCAAGGCAATAGGCCAATAACAATGATTTCTCGCGCCTTAAAAC
AGGCCGAACAAAACTATGCCACTAACGAAAGAGAATTGCTGGCCATTGTGTGGGCTTTAGGCAGATTGCA
GAACTTTCTGTACGGTTCTAGGGAAATTAACATTTTCACGGACCACCAGCCGCTCACGTTCGCTGTGTCGG
ACAAGAACACGAATTCCAAGATTAAAAGATGGAAATCTTACATTGACCAGCACAATGCCAAAATGTTTTA
TAAACCGGGCAAAGAGAATCTGGTGGCGGATGCCCTTTCAAGGCAAAACATTAATGCTCTGCAAGATGGG
CCCCGTTCGGACGCTGCAACCGTTCACAGCGAACTTTCCCTGACCTACACGGTCGAGACAACAGACAAAC
CTTTGAATTGCTTTAGGAACCAGATTGTCCTAGAGGAATCGCGTTTTCCGCTAAAGCGAAACTTGTTGCTG

TTTCGCAGCAAAACACGCCACTTGATCAGTTACACTGACAAGAGCACAATTCTCAAAACCCTCAAAGAAG
TTGTGAATTCTGGAGTCGTTAATGCAATACATTGCGATCTTCCCACGTTGGCAAGTTTCCAACACGACCTG
ATTTTGTATTTCCCTGCNACCCAATTCTGGCATTGCAAAAATCTTGTGCAAGATATAACGGACAAAAACGA
GCAGTTAGAAATCATCACTGCCGAGCACAATCGAGCGCACAGAGCAGCTCAGGAAAACATTAAACAAGT
ACTTCGGGACTATTATTTTCCCAATATGGCCAGTCTGGCCAAAGAAGTGGTTGCAAACTGCAGGGTCTGCA
CTAAAGCCAAATACGATAGGCACCCGAAGAAACAAGAACTCGGGGAAACGCCTATACCAAGTTATACTG
GCGAAATGTTGCATATTGACATTTTCTCGACAGACAAGAAACAGTTCTTGACCTGCATAGACAAATTCTCA
AAATTTGCAGTCGTCCAACCGATATTATCCAGAACGATAATCGATGTCACAAGCCCTCTACTGCAAATTGT
TAACCTTTTTCCCGCAACCAAGACAATTTATTGCGATAACGAGGCGGCCTTCAATTCCGAGACCATCACTT
CGATGCTCCGAAACGCCTACGGCATTTGCATTGTGAATGCGGCTCCGCTTCACAGCACCTCAAACGGCCA
GGTGGAGCGCTTTCATAGCACCCTGACCGAAATTGCGAGATGCCTCAAGTTAGACAAGAAAATCAGCGAC
ACTACAGAATTAATCTTAAAGGCTACGATAGAGTACAACAAAAGTTTGCACTCAGTAACCCTCGAGAAAC
CAATTGAGGTCGTTCATGCAGCAGCTTCTGATCGTCGCGGGAACGTCAAAAACAGGCTAATTAAGGCACA
GCAAGACAATATCAAAAGGTGCAATGCATCCAGGCAAAACCGCGTTTTCGAGGTAGGAGAAAAGGTGTT
CCAGAAGAACAACAAGAGGCTAGGGAACAAGTTAACTCCCTTATGCTCAGAACAAAAGGTCGAAGCAGA
CCTGGGAACGTCTGTCCTCATTAAGGGGAGGGTGGTCCATAAGGACAACCTGAAATAAGTTTTCCTCCAC
AATTAGCTAGTAAGCCAACTCAGAGTTTAAACACCGTCTTATATTGTTCGTCCATTTCAGGTTTGCACTCA
CTTTGCTTGTGGCACTAGCAGCGGTAAATGCTCGGATCACCGACTTCTCGCATGCCAACTACATTCCGGTC
TTAGACGGGGAAGTACTGGTGTTCGATCAACGTAGCTACTTGAGGCACTCAAGTAACATTTCCGAATTTGT
TAGTATGGTAGACGAGACGGAAAGACTGTCCGATTCCTTTCCACAGTCGCATATGCGCAAACTACTAGAC
GTAGATACAGATCACCTTAGAACATTGTTGTCCGTTCTTCAAGTGCATCACAGATTTGCACGAAGCCTCGA
TTTCTTAGGCACTGCCTTGAAGGTTGTTGCTGGCACCCCTGATGCTTCAGATTTCTTAAGGATCCGCGTTAC
AGAGACGCAGTTGGTGGAGGCTAACTCCAAACAGATACTAATAAACTCCGAAACTCAAAAACAAATAAA
CAGACTAACGGACACTATCAATAAAATTATCAGTTCCCGCAAAGGCGATTTGGTTGACACTCCTCATTTAT
TCGAGACCCTGTTGGCAAGAAATAGAATACTGAACACAGAAATTCAAAATTTAGTACTCACCATAACGTT
GGCAAAAGCAAGCATAGTAAATCCTACAATCCTTGATCATACCGATCTGAAATCACTTATCGAACAAGAT
ACCCCAATAGTTAGCTTAATAGAAGCTTCTAAGATCAAGGTCCTTCAGTCCGAGAATATTATCCACATATT
AATAGCCTACCCTAAAGTTGAGTTTAGGTGCCAAAAGGTCTCCGTCTACCCCGTTTCACACCAACAAACCA
TTCTGCGACTCGACGAAGACACGTTGGCGGAATGCGAAAAGGATACCTTTGCAGTTACTGGATGCACCGT
AACTACGCATAACACCTTCTGCGAACGAGCACGGCGCGAAACCTGTGCTAGCTCCCTTCACGCGGAAAC
ATCGCCAACTGCCACACTCAACCTAGCCACCTGAAAGCGATAATGCCTGTAGATGACGGCGTCGTGGTTA
TCAACGAAGCGACAGCGCGCGTCCGAACAGATGATGACGCAGAGGTAACCGTCTCAGGAACCTTCCTGAT
AACTTTCGAACGGTCTGCAACTATCAATGGCACAGAATTTGTAAACCTGCGCAAATCACTAAGCAAGCAG
CCTGGCATCGTGAGGTCACCGCTCCTAAACATCATCGGACATGACCCGGCACTAAGCATACCACTCCTGC
ATCGTATGAACATCAACAACTTGCTATCAATTCGAGATTTCAAGGAGGAGGTAGTTGCGGCTGGCTCGCC
TAAATTCTGGTTCGCGATTGGCGCGGTCCTGAATGTCGGACTAATTGGCTCATTCTTCCTTTTTCTGGTGCT
AAGGAAAAGACGAGCCTCCATGCAGATGCAGAAGGCGATTGACAAATACGACATGCCCGAGGACGGCCA
TCATCCTGAGGGGGGGGTAGTTAACAACTAAGCATAAATATATTGCCCTAGCAACTAAGTAGCTTCGTAT
TAACAATGCTGACGCGCCAGAACTGAGTTCAGCGCTCTGCGCTAAGAACGGCCAGCAGCGGTAAGCTGAC
ACTGCATGGAATGCTGAGTCGGCTTGCCGACCATGCTTTGATGGCGTGATGCATTGAGGCTTAGGGTATTT
GTATAATTTGCTTGTCCCCTAGTTTTTAGTTTTATATGAGGCGCGCAACACATAAGTCGCCGAAATAAATA
TACTGCTCCGGCATTGCCGATAAAACATATCTTTCGTTATTGTCTGAGCCCAAGGCCAGCAATAGAAGCCA
ACTTCAAAACATAATCTTCAGTAATATTACTGGATCAAGGANAGCGCGTTTGGATCAAGGGCAGCGCGGG
ATCAACGCAACTTACCCAAGATAACATTGTGTTACATCGCAGAACATACATCAATTGATAACTTATA
>160_rnd-1_family-360__LTR___Gypsy
ATAACTTCTCCCGTTTACCATCCGGCGAGTATTCAGTGCGGTTGCCTCTGAAGCGGAGTGCAGAACTTCTA
GGAGAGTCTTATGGCCAGGCTCATCTTCGATTTCTCAGTCTTGAGCGAAAATTGGGTCGGAATTCGCATCT
GAAGGAGCAGTATTCGGCAGCACCACAACCAAGTTGCGTGTTGTGTTTGATGGCTCAGCAACTACTGATT
CTGGACATTCTCTTAATGATGTACTGATGGCGGGACCAATCATTCAACCTAAGTTGTTTCACATTCTGATT
AGAGGAACCCTCGGGATACAGGCCAATCTACCTACTGGACACGGCGGGAAAGGTCCTGGAAAGGATTGT
GTATGACCGTTTACTTAGTGTCGTCGAAGAGAAAGAAGCTCTGTCCAAACAGCAATACGGATTTCGCAAG
GCAAGGCTTACGGTCGATGCAATTAGGGCGGCGGTAGATATTGCCGCTAAAGCAATAGAGGGAACCAGA
TGGAGGTGGGGAGACGAGGAGTATTGCGCGGTGATTACGTTGGACAACCGCAATGTCTTAAACTTAGTAA
GCTGGTCTCGAAATCTGGAAGCCTTAGACCGAATAGGGATACCGTCCCCCATTCTCCGTATTGTAGTATGT

GCATATATTGAGGGTACAAGGTACCAATAAAGTAACATCGTTTAAGCATAAATAAATCAGCAACACATTG
TTGCAGTACACAAACAAAACCTTTTCGAGTGAGCGTGTAATATGATCGCCCACTTATGAGCTACGCTAAGT
CAGCAGTCCCACGCTGACCATTCGTAGTACATATATACATACATGCAAATATGTATATAAGACTCTCTCTC
TCCCGATTATGCTGATAAGACAAAATATGTATGAAGCCGCTCCGCTGCTGGCGTAACCGGCAGCGCAGCT
ATGCGATCTTAGTCATAAGAATATATTGAATAAAACATTCAAAAAACTAAAGTCAAACGGCACAGACGTG
CCCGTGTGTTGAACTATTTGAATCGATGACATTTACCATTACATGGCGACCGTGACTAAGGTCAAAACAG
ACCTGGATATGGAAGGATAGCAGCAACGGCAGTAACAACAACAACAGCAACAACAGCAGGAACAACCA
AACAAAAAAAAAAAAAAAAAAAACCAGTGAGTAGAGTGTGATGAGTGCGAGGCGAAGCGGTGTGGAGTA
AACAGCAAAAATGTCATAAGTTGGGACAACTCGGTCAAGTTACATGCCGAAGTTGAATACATAAAAAAAT
TACTTTCAACTCGAAAAAGTCAAACACCAGCCACGCACACATCCACCTACCCTGTTATCAAAAATATTGC
AAAGCAAAAATTAATCCAACCTTCACCAAAATTAAAGGAGATTCCGAATCAAAAAAAGACCTGCACGAA
CGACTGCGAGAGACCGCTATTTATTCTACATTACCGATACACCTGTGCAGCCAGCACTGGCGCCCCATCAA
AGAATTAACAACCTGTGCAGCCAGCACCGATCCCGTCTGACGCACCAAGTCAGGCCAAAAATAAATGAAC
CGACAGCGCTTCCAAGAGCGCATTTTTTCCCATTGCATACTTATTGTTACNATTTTTATAACAAGTATTATA
ATACCAGTACATCTATTATATAAATTGTATAAGTACCATAATAAATGCATTAAAAAGAGATATCAAAGCC
AGGCAAACGATCTGCATAAAATTTAAAATAAAATTACTAATAATCAGCAACAAAGATTGCTTAAAGTTAT
TGAAAATAAATTAATTAAAAATATATAACTATTTGATTGAAAATTTGTATATGTCTTTGCACATTTTAATA
ATTTATTTTAAAATGAAATGGAATGGCAAAACATTAATGCCGCATTAATTAAAATAAAAAAAAATGTTGA
TAAATCATACAAGTGTCTAGCACAAAATAGGAATATTTCAAACGAGACTATAAGTAGACACGCAGCTATA
CGAGTTGCATGTTTTAACCAAGCTCGTTCCCAGATACACGACAATAAAGAAAAGTCAAATACTCAGCATT
GGTCGTATGTATCAAGATTTTTGCATAGATTAAACACAAACCTAAATAGCATTAAATTAAAATATTCGATA
GACATAACTATACCTTCGATTTTGAATACTCCAGCATTAGAGGAGGAAGAAGCAAACACAGACTTAGAGA
CAGAAAGCGAATCTGAAATCGCAAGCGGTTCAGATAAACATATAAAAGAAGAAGATCTAAGCGATCTTA
CGATACCAGCGGTAATCACACTAGCCGAACTATCTGAGTCTGAACCAGAGTCTATTACTGAAGTAAAAAC
TGTAAATATGGCCATGGCACAAGTAGAATTTCTTAAGCTAGCCTCTTCACTCATACCTGAGTTTAGTGGAA
AGTCTGAGAATCTACAAAGTTTCATTGATGCTCTTAGTTTAGTGGATTCACTGAAAGGAACACATGAAACT
ACAGCAGTCAGCCTAATAAAAACTAGACTCAAAGGCCATGTTCGAAACTTCATCAATACTGAACCAACAA
TAGTGACGATAATAGACAAACTAAAAAGCGGCATAAAAGGCGAATCAGCTAAAGAGCATTATCCGCTAA
ATTGTTCAATCTGCAGCAAAAGCAAAATACAGCAAGTCAATATATTCAAGAAGTTGAACAGTTGACAAGG
GCTTTGCAATCAGCACATATGATTAACGGTGTATCGCAAGAATATGTCGAAGAACTGAGCCTACGAAGTT
GGGTCTGTAATGAAATCAATCATAGCAAACAGCACAATTGACGAAGTTAGGTCTGTAATGAAATCACGAA
ATTTCAATACANTGAGTGAGATTACAACTGTGTTCGTCGATAGTTGCACAGACGCAGTAGGACACCTAAA
TAGAGTCCAATATGCTGCGCAGCAACAAAGATATAGACCGAACCGAGGATATAACCGCGGAAACTACCG
TGGGATACTGGTGCAGACATATCTCTGTTAAAAGACAACTCAGACACTTTTAATGATATAAACATGGACA
ACAAAATAAATATTCGGGGAATCAATCAAGGATTAATTCAATATAAAAGACTGACATCCTTAGAAATACA
AATAACTAAATATATAATTCCACACAACTTCCATATTGTCGAATCAACCTTTTCCATACCGTGTGATGGAA
TTTTAGGAATAGATTTTATTAAAAAATATAATTGTCAACTAGATTTCACACTTTCAAAAGATCTGCTCATA
ATAGGACCAGACAATCTATACTATCCTATATATGTTCCCATAAATTACAGCTCTGGTAACAATACAATAGT
GTTACCAGCAAGATCCGAAGTTGTTCGAAAAATTAAGTTAATTTCAAATGAAGATAGCATTTTAATTCCGA
ACCAGGAAATTCAACCTGGTATATATGCAGCATACACAGTCGTAACAACTGCAAATGCATTCGTCCGTTT
ACTAAATACAACTAGTACCAATCAAATGATTAATATAAATAAGTTGAATTATGAACCATTAGCAAATTAT
GATATATCCGAAATAAACAATGAAACTAGAACGAACGATGTTATGTCAAAGTTAACTAAAAATTTTCCAA
AACTATTGTTGGATTCTGCAGCAGCCTCATCAAATTAGCATAAGCAAACATTCATGTAAACATAAGAAGA
GGTTGTAATTTTGGTCTAGTGGACCATATAAACAAGGTTGGCAAGCCTTTCTTGCACTTGTCGCAGCTGCT
ATAAATTGCATATTAGTTTTTTGTCTGCTCTCTTAGTTGTGTGCTCTGTTTGGTGTTCTGATTTTGTCCTTGT
GAAAAAATGATGCAAGGCGAGAACACAGATGATATGCAGTCAAACAGGGTGTCTTTTCTTAAGCGCAAA
GCGGTTGCATTGTTTGAGCGAGCCAAGTGTGTTGAAAATGAATTATCTGAGGATAAAATTAACAACTCTT
GTGCCGTCGACTTAGAAGTGCGCATTTTGCTAATTGAACAAACTAAGTCTGCCTTTAATACCGCGCATAGC
TCGCTAGAGGAGCTTAATTTCGATGAAATTGGCAGCAAACTACGCGATGATTTTGACGAGTTGATCGCCA
TGGTTAAAGCTCGCGTAAATATTCAGCGTAGTCAATCTAACGGCCCGCCCCACTACCGGGATGCATAATA
GTCCACGCGCTGCTTCAAAGGTTGGACACCCAGACTCAGACCAAATGGGAGGAGGCCGCAGGCATTGAC
AAAATCCCGACAGCCGAAGGGCTCTTCACATTTCTGGAGAGGCGTAGTCAAAGGCTGGAGAGCGTGCAAC
ATGCCATGTCATCCCATATAACCAATTATCAGGTGGGCAAATCCAACCATAACACTAACCACAGCAAAAG
AGTGCTTGTAGCTGAGACTAACCAAAATGGGTGCAGTTTTTGTGATGCGTCTGGTCACGGATCTACACAT

GTGGTAAATTCGGAAAGTTGTCTCCATCGCTTTGGCATAAGGAAGTCAAGAAACTGGCTCTTTGTTATAAC
TGCCTAAGGAAGGGACACCAAACTCGATCTTGCAGTAGTAGTCATTTTCGTACTTGTGGAGAAAAGCATC
ATACACTGTTCCATTTTGTCACTTCAAGAGAAGCTGACGCGCCGACACAATTACATACATCGGCTGATTTG
GGCGCCGCCACTTCGACGCACTCTCACTCCACTACGTCTCTTGCTGTCCAAGGTCTTCAGTCTGGTGTTGTG
CTCCTGGCCACTGCAGTGGTACTGGTAAAGAATAGTTCTGGTTCCTTGTTTCCCTGTAGGGCCTTATTAGA
CTCAGGCTCGCAGTTGCACATGGTCACATCCCGTTTCGCACAGAGACTTCAGTTAAGGAAAACTAGGTCG
TCATGGGTCTCGGAGACACAAGCGTCTCTACTGAAGGATCAGCCGTTAGGATTAACATGAAGTAACAAGA
CTCAGATTATTCAGCCATCTTTGATGCTCTTGTGACTCCAACAATTACTGATAGTCAGCCCAGCTTCGTCAT
CGATACATCAGACTGGAAAATTCCCTCGAACATAAGGTTGGTTTGATCATGCGTTCAACAAACCACAGCG
AGTGGATTTCTTAATCGGAGCGAGCCTTTTCAATGAGCTTTTGTCTGTTGGGCAAATAGTGCTTTTGCCAG
GACTACCTCTTCTGCAAAAAACTCGTCTTGGCTGGGTCGTGACCGGTGGATTTGAGAGACCGCGCAATGC
CTCGTTGTTGGCTGCCGGCCGCACTCCTAATAGCGAAGCTAGTCAGTTGGACGTTCGTCTGAATGAGTTGG
TTCGGCGGTTTTAAGAAATTGAAATCTTGGAAGAGCCTACAGTAAAGGCCACCAAAGAAGAGGCGGAAT
GCGAGTAGCATTTTGCCAATGACTTCTCCCGCTTACCATCCGGCGAGTATTCAGTGCGGTTGCCTCTGAAG
CGGAGTGCAGAGCTTCTAGGAGAGTCTTATGGCCAGGCTCATCTTCGATTTCTCAGTCTTGAGCGAAAATT
GGGTCGGAATTCGCATCTGAAGGACCAGTATTCGGCAGCACCACAACCAAGTTGCGTGTTGTGTTTGATG
GCTCAGCAACTACTGATTCTGGACATTCTCTTAATGATGTACTGATGGCGGGACCAATCATTCAACCTAAG
TTGTTTCACATTCTGATTAGAGGAACCCTCGGGATACAGGCCAATCTACCTACTGGACACGGCGGGAAAG
GTCCTGGAAAGGATTGTGTATGACCGTTTACTTAGTGTCGTCGAAGAGAAAGAAGCTCTGTCCAAACAGC
AATACGGATTTCGCAAGGCAAGGCTTACGGTCGATGCAATTAGGGCGGCGGTAGATATTGCCGCTAAAGC
AATAGAGGGAACCAGATGGAGGTGGGGAGACGAGGAGTATTGCGCGGTGATTACGTTGGACAACCGCAA
TGTCTTAAACTTAGTAAGCTGGTCTCGAATTCTGGAAGCCTTAGACCGAATAGGGATACCGTCCCCCATTC
TCCGTATTGTAGTATGTGCATATATTGAGGGTACAAGGTACCAATAAAGTAACATCGTTTAAGCATAAAT
AAATCAGCAACACATTGTTGCAGTACACAAACAAAACCTTTTCGAGTGAGCGTGTAATATGATCGCCCAC
TTATGAGCTACGCTAAGTCAGCAGTCCCACGCTGACCATTCGTAGTACATATATACATACATGCAAATATG
TATATAAGACTCTCTCTCTCCCGATTATGCTGATAAGACAAAATATGTATGAAGCCGCTCCGCTGCTGGCG
TAACCGGCAGCGCAGCTATGCGATCTTAGTCATAAGAATATATTGAATAAAACGTTCAAAAAACTAAAGT
CAAACGGCACAGACGTGCCCGTGTGTTGAACTATTTGAATCGATGACATTTACCATTACATGGCGACCGT
GACTAAGGTCAAAACAGACCTGGATATGGAAGGATAGCAGCAACGGCAGTAACAACAACAACAGCAACA
ACAGCAGGAACAACCAAACAAAAAAAAAAAAAAAAAAAACCAGTGAGTAGAGTGTGATGAGTGCGAGG
CGAAGCGGTGTGGAGTAAACAGCAAAAATGTCATAAGTTGGGACAACTCGGTCAAGTTACACGCCGAAG
TTGAATACATAAAAAAATTACTTTCAACTCGAAAAAGTCAAACACCAGCCACGCACACATCCACCTACCC
TGTTATCAAAAATATTGCAAAGCAAAAATTAATCCAACCTTCACCAAAATTAAAGGAGATTCCGAATCAA
AAAAAGACCTGCACGAACGACTGCGAGAGACCGCTATTTATTCTACATTACCGATACACCTGTGCAGCCA
GCACTGGCGCCCCATCAAAGAATTAACAACATGTGCAGCCAGCACCGATCCCGTCTGACGCACCAAGTCA
GGCCAAAAATAAATGAACCGACAGCGCTTCCAAGAGCGCATTTATTTCCCATTGCATACTTATTGTTACAA
TTTTTATATAACAAGTATTATAATACCAGTACATCTATTATATAAATTGTATAAGTACCATAATAAATGCA
TTAAAAAGAGATATCAAAGCCAGGCAAACGATCTGCATAAAATTTAAAATAAAATTACTAATAATCAACA
ACAAAGATTGCTTAAAGTTATTGAAAATAAATTAATTAAAAATATATAACTATTTGATTGAAAATTTGTAT
ATGTCTTTGCACATTTTAATAATTTATTTTAAAATGAAATGGAATGGCAAAACATTAATGCCGCAT
>9_rnd-1_family-424__LTR___Gypsy
ATATAAGTTAACAACTAAGCATAAATATATTGCCCTAGCAACTAAGTAGCTTCGTATTAACAATGCTGAC
GCGCCAGAACTGAGTTCAGCGCTCTGCGCTAAGAACGGCCAGCAGCGGTAAGCTGACACTGCATGGAATG
CTGAGTCGGCTTGCCGACCATGCTTTGATGGCGTGATGCATTGAGGCTTAGGGTATTTGTATAATTTGCTT
GTCCCCTAGTTTTTAGTTTTATATGAGGCGCGCAACACATAAGTCGCCGAAATAAATATACTGCTCCGGCA
TTGCCGATAAAACATATCTTTCGTTATTGTCTGAGCCCAAGGCCAGCAATAGAAGCCAACTTCAAAACAT
AATCTTCAGTAATATTACTGGATCAAGGAGAACGTATTTGGATCAAGGGCAGCGCGGGATCAACGCAACT
TACCCAAGATAACATTGTGTTACATCGCAGAACATACATTAATTGATAACTGGCGCCCAACCAATAAATA
GGAACCCATACCATGTAAGTTCATAAGTGATTTCAGTTTGAAGAAATTATTCACGAACGCCACGTACCGA
TACGTGTGATTTTTAAAAAAAAAAAAAAAATTGTAAAGTATAAAGTAAATATTGCTGCTCTCTTCTGCCAGC
ATTTCATTCCTTTAAATCATTGCATACTTTAAAAATGTTGCATACTTTCACTAAAAACTGTTGCATACTTTT
ACCAGCGTTCCTTGTCAATAAAGCATTGCATACCTTAAACGAATAATTATTGCATACTTTCGGCAGCATTA
TATTTTTGCTGCATACCCCTAACAATTTTTATAATAAATATTGCATACCCCAAGATTCACCAGTTTCCACTA
TTGCCAACAGCGTTACCTGCCGATCTTTCTCGATCGGCCTAAGTTTTGAAAAGTGCCAACCATAAGCCAAT

GAGTTGGAATTATAGGGAGATAAAAGTCGAATATAATAGTGACGATAGCTGGGAGGGGAAATCAATTGC
TCAGTAGATCCTACTCCTGCAGATACCGTTGTAACTGAAGAAATGGAGCCGGCTCAAGTTCAGAATCTCA
TTGAGGGTGCCATCAGGCAAGCTTTAGCGCAACAACAAAAATCAGTTTCAAGCACAAATAACCGCTTTAG
CTTCACAAGTACAGAGTTTGCAGGTGGAGGCGCCGCAAATCGTCACATACGAGAAAATATCCGTCGATCC
TAACGCCAAATGCGATATTCCCTTGGACATTATTAAATCTGTGCCGGATTTCTCCGGCGTACAGGATGAGT
ATGTAGCGTGGAGGCAAGCAGCCATCTACGCGTACGAACTATTCAAGAACTATCCAAAGAGCACTGCACA
CTACCAAGCGGTAGCGATCATTAGGAACAAAATCAAAGGCCCAGCTGGAGCCTTACTAGTGTCTCACAAC
ACTGTCCTAAATTTCGATGCAATACTAGCTAGGCTAGATTGCACATACTCGGACAAAACATCCTTGCGCTT
GCTGAGACAATGTTTGGAGTTAGTTACGCAGGGCGAAATGTCCCTGATGCAATACTACGATGACGTCGAA
AAGAAACTCACGCTCGTAACTAACAAAATTGTAATGTCTCACGACCAAGAAGGCGCGGATTTGCTCAACC
GGGAGGTCAGAGACGACGCCTTGCACGCCTTTATCTCCGGTCTTAGGAAGCCGTTGAGAGCGGTTGTGTT
CCCTGCACAACCAAAAGATCTACCATCCGCTCTGGCCCTTGCCAGAGAGGCAGAAGCTAGCATTGAGAGA
AGCATGTTCGCGAACTCTTACGCTAAAGCCTTGCAAGACCGAGCGCAAATTGCTGAAAGTAACAAAACCG
TGCCCAAGGCAAACAAACCAGATTTAACAAAGATGAGCAAAATCAGGAGAGAAATCCCCATTTTGTGAA
ACGCCAAAAGGGCGGTCCCCAGCCCCAGGCCCCTAAAGATAATCAAACTGAGGCTCCACAGCCCATGGA
AGTTGACTCTTCCTCCAGATTTAGACAAAAACCGACTACAGCAAGCGTCAGGCTAATGAGTCCAACGCAC
CAAAACGCAAGAATTCGTCAGACCGTTCAACAGGTCAAAGACGACAACGCGTTAACAATGTCGTACAATC
AGATTCCAAAGAAGCCGTAACAGAATTCGAGAAGGCGGCCAAAGAGGCAGTTGAAGAACTAGAGAGTGA
GAACGAGTACGCACCTGGTGACGACTCGATTAATTTTTAGGGAACACTCCCGGCTTCCGTACATTGAACG
ACGGTTGGCTGGGAGAACTCTGAAGATGTTGATTGACACTGGCGCGGCAAAAATTATGTGAAGCCCTTAA
CAGAGCTAAAGACCATAACGCCGGTCGCCAAGTCATTCTCGGTAAACTCGATCCACGGGTCTACCGAAAT
CAAGCGCAAATGCTTGATGAATATTTTCCAACATACCTCCCCGTTTTTCTCCTCGATACCCTAAGTTCTTTC
GACGCCATCATAGGCTTCGACTTGCTAACACAAGCCGGAGTCAAATTGAACTTGGCAAAAACACTCTGGA
ATACCAGAGTACATCCGAAAAACTCCAGTACTATCAATGTGCTGATGTGAACTTCACCGATGTCAACGAC
ATCGTAGTCCCCCTTCCGTAAAGAAAGAGTTCAGGGACATGATACTCAGAAACAAAAGAGCCTTCTCAAA
TGCAGACGAAGCTCTCCCTTTTAATACTGCTGTTATCGCTACAATCCGAACAGTAGACAACGAGCCTGTGT
ATTCAAGATACCCGAGTCTCATGGGTGTTTCTGACTTCATAAACAAAGAAGTCAAACAATTACTGGACGA
TGGCATAATCAGGCCCTCCAGATCGCCTTACAACAGCCCAACATGGGTGGTTGATAAAAAGGCACTGACT
CATACGGTAACCCAAAAAAGGTTGGTAATCGACTTCAGGAAACTGAACGAAAAACCATACCGGACCGCT
ACCCCATGCCGAGCATCCCCATGATCTTAGCAAATCTAGGAAAGGCGAAATACTTCACTACCCTCGACCT
AAAGTCGGGGTACCATCAAATTTACCTTGCGGAGCATGACCGCGAAAAGACTTCATTTTCAGTAAGCAGC
GGCAAGTACGAGTTCTGTCGCCTACCATTCGGCCTGCGCAATGCAAGCAGCATATTCCAAAGGGCAATAG
ACGATATCCTGAGGGAACATATAGGAAAGATTTGCTTTGTCTATGTAGACGACGTCATAATTTTTCTAAAA
ACGAAACGGAGCACCTTCAACACATTAACATTGTGTTAAAATGTCTAATCGACGCAAACATGCGTGTAGG
CCCCGAAAAGACTCGTTTCTTCAAGGAAAGTATCGAATTCCTGGGTTTCATTGTAACCAAAGACGGAGCA
ACATCCGACCCCAAAAAGTTAAAGCCATTCAGGAATTCCCTGAACCAAAGAATGTCTACAGCGTTAGGTC
CTTCTTGGGGTTAGCCAACTACTATAGGGTCTTTATTAAGGACTTTGCCGCTATAGCGCGTCCGATATCGG
ACATACTTAAAGGGGAAAATGGTTCAGTTAGTAGGCACGTATCAAAGAAAATCCCTGTTGAATTTAATGA
AGTTCAACGCGATGCATTTCACCGTCTGCGGAGTATTTTGGCATCAGAAGACGTCATGTTAATGTATCCAG
ACTTCCAGAAACCGTTCGATTTAACCACTGATGCTTCGGCCAGTGGAATTGGCGCGGTTCTTTCCCAAGGC
AATAGGCCAATAACAATGATTTCTCGCGCCTTAAAACAGGCCGAACAAAACTATGCCACTAACGAAAGAG
AATTGCTGGCCATTGTGTGGGCTTTAGGCAGATTGCAGAACTTTCTGTACGGTTCTAGGGAAATTAACATT
TTCACGGACCACCAGCCGCTCACGTTCGCTGTGTCGGACAAGAACACGAATTCCAAGATTAAAAGATGGA
AATCTTACATTGACCAGCACAATGCCAAAATGTTTTATAAACCGGGCAAAGAGAATCTGGTGGCGGATGC
CCTTTCAAGGCAAAACATTAATGCTCTGCAAGATGGGCCCCGTTCGGACGCTGCAACCGTTCACAGCGAA
CTTTCCCTGACCTACACGGTCGAGACAACAGACAAACCTTTGAATTGCTTTAGGAACCAGATTGTCCTAGA
GGAATCGCGTTTTCCGCTAAAGCGAAACTTGTTGCTGTTTCGCAGCAAAACACGCCACTTGATCAGTTACA
CTGACAAGAGCACAATTCTCAAAACCCTCAAAGAAGTTGTGAATTCTGGAGTCGTTAATGCAATACATTG
CGATCTTCCCACGTTGGCAAGTTTCCAACACGACCTGATTTTGTATTTCCCTGCNACCCAATTCTGGCATTG
CAAAAATCTTGTGCAAGATATAACGGACAAAAACGAGCAGTTAGAAATCATCACTGCCGAGCACAATCG
AGCGCACAGAGCAGCTCAGGAAAACATTAAACAAGTACTTCGGGACTATTATTTCCCAATATGGCCAGT
CTGGCCAAAGAAGTGGTTGCAAACTGCAGGGTCTGCACTAAAGCCAAATACGATAGGCACCCGAAGAAA
CAAGAACTCGGGGAAACGCCTATACCAAGTTATACTGGCGAAATGTTGCATATTGACATTTTCTCGACAG
ACAAGAAACAGTTCTTGACCTGCATAGACAAATTCTCAAAATTTGCAGTCGTCCAACCGATATTATCCAG

AACGATAATCGATGTCACAAGCCCTCTACTGCAAATTGTTAACCTTTTTCCCGCAACCAAGACAATTTATT
GCGATAACGAGGCGGCCTTCAATTCCGAGACCATCACTTCGATGTTCGAAACGCCTACGGCATTTGCATTG
TGAATGCGGCTCCGCTTCACAGCACCTCAAACGGCCAGGTGGAGCGCTTTCATAGCACCCTGACCGAAAT
TGCGAGATGCCTCAAGTTAGACAAGAAAATCAGCGACACTACAGAATTAATCTTAAAGGCTACGATAGAG
TACAACAAAAGTTTGCACTCAGTAACCCTCGAGAAACCAATTGAGGTCGTTCATGCAGCAGCTTCTGATC
GTCGCGGGAACGTCAAAAACAGGCTAATTAAGGCACAGCAAGACAATATCAAAAGGTGCAATGCATCCA
GGCAAAACCGCGTTTTCGAGGTAGGAGAAAAGGTGTTCCAGAAGAACAACAAGAGGCTAGGGAACAAGT
TAACTCCCTTATGCTCAGAACAAAAGGTCGAAGCAGACCTGGGAACGTCTGTCCTCATTAAGGGGAGGGT
GGTCCATAAGGACAACCTGAAATAAGTTTTCCTCCACAATTAGCTAGTAAGCCAACTCAGAGTTTAAACA
CCGTCTTATATTGTTCGTCCATTTCAGGTTTGCACTCACTTTGCTTGTGGCACTAGCAGCGGTAAATGCTCG
GATCACCGACTTCTCGCATGCCAACTACATTCCGGTCTTAGACGGGGAAGTACTGGTGTTCGATCAACGTA
GCTACTTGAGGCACTCAAGTAACATTTCCGAATTTGTTAGTATGATAGACGAGACGGAAAGACTGTCCGA
TTCCTTTCCACAGTCGCATATGCGCAAACTACTAGACGTAGATACAGATCACCTTAGAACATTGTTGTCCG
TTCTTCAAGTGCATCACAGATTTGCACGAAGCCTCGATTTCTTAGGCACTGCCTTGAAGGTTGTTGCTGGC
ACCCCTGATGCTTCAGATTTCTTAAGGATCCGCGTTACAGAGACGCAGTTGGTGGAGGCTAACTCCAAAC
AGATACTAATAAACTCCGAAACTCAAAAACAAATAAACAGACTAACGGACACTATCAATAAAATTATCA
GTTCCCGCAAAGGCGATTTGGTTGACACTCCTCATTTATTCGAGACCCTGTTGGCAAGAAATAGAATACTG
AACACAGAAATTCAAAATTTAGTACTCACCATAACGTTGGCAAAAGCAAGCATAGTAAATCCTACAATCC
TTGATCATACCGATCTAAAATCACTTATCGAACAAGATACCCCAATAGTTAGCTTGATAGAAGCTTCTAAG
ATCAAGGTCCTTCAGTCCGAGAATATTATCCACATATTAATAGCCTACCCTAAAGTTGAGTTTAGGTGCCA
AAAGGTCTCCGTCTACCCCGTTTCACACCAACAAACCATTCTGCGACTCGACGAAGACACGTTGGCGGAA
TGCGAAAAGGATACCTTTGCAGTTACTGGATGCACCGTAACTACGCATAACACCTTCTGCGAACGAGCAC
GGCGCGAAACCTGTGCTAGCTCCCTTCACGCGGGAAACATCGCCAACTGCCACACTCAACCTAGCCACCT
GAAAGCGATAATGCCTGTAGATGACGGCGTCGTGGTTATCAACGAAGCGACAGCGCGCGTCCGAACAGA
TGATGACGCAGAGGTAACCGTCTCAGGAACCTTCCTGATAACTTTCGAACGGTCTGCAACTATCAATGGC
ACAGAATTTGTAAACCTGCGCAAATCACTAAGCAAGCAGCCTGGCATCGTGAGGTCACCGCTCCTAAACA
TCATCGGACATGACCCGGCACTAAGCATACCACTCCTGCATCGTATGAACATCAACAACTTGCTATCAATT
CGAGATTTCAAGGAGGAGGTAGTTGCGGCTGGCTCGCCTAAATTCTGGTTCGCGATTGGCGCGGTCCTGA
ATGTCGGACTAATTGGCTCATTCTTCCTTTTCTGGTGCTAAGGAAAGACGAGCCTCCATGCAGATGCAGAA
GGCGATTGACAAATACGACATGCCCGAGGACGGCCATCATCCTGAGGGGGNAGTTAACAACTAAGCATA
AATATATTGCCCTAGCAACTAAGTAGCTTCGTATTAACAATGCTGACGCGCCAGAACTGAGTTCAGCGCTC
TGCGCTAAGAACGGCCAGCAGCGGTAAGCTGACACTGCATGGAATGCTGAGTCGGCTTGCCGACCATGCT
TTGATGGCGTGATGCATTGAGGCTTAGGGTATTTGTATAATTTGCTTGTCCCCTAGTTTTTAGTTTTATATG
AAGCGCGCAACACATAAGTCGCCGAAATAAATATACTGCTCCGGCATTGCCGATAAAACATATCTTTCGT
AATNTTACTGGACCAAGGAGAACATATTTTGATCAAGGGCAGCGCGGGATCAACGCAACTTACCCAAGAT
AACATTGTGTTACATCGCAGAACATACATTAATGAATAACTTATAT
>160_rnd-1_family-163__LINE___CR1
TTTGATGAATGAAATTTGNGTAAAAATAAAAATCTTTAAAATATTAAAATATACCAAATTAAAAAAAAGG
AGCGATTATATATTTATGGTTTTAATAATTTTGGTGATCAGTCACTTGACCTCCATATGCAGGTGCTTGCCA
AAAGTTGACCCATTCGACCCAACGAGCTGCTGCAGGTAATAAGGGGTCAACATTCATTCATCAAAATCNT
AAAAAAGAAGGGCGCGCGTCAGACGCGTGCCAATAATGATATTTTCTTTTGCGCCCATGATCAGCCCATT
CACAGACATTTGTATGTGTGTGTCTGTGTGTAGAGGTCCAAAATCAGAAATCCTAAAATCTTGTTAAATTT
GCATCGGGGACAGGCAGGCAGGCAAGCAACAGGCACACAAGCGCATAAATGCTTCTATTCATATACAAA
ATATGCAAGTATGCGTGAATAGCGAGTCCAAAATATTTTAAATGCACACATGGCAACGCTTGGGTACCCT
TACCAACGATAGCGAGCTAAAATGCAAGAATTTCGCATCGACGTGGCAAACCCTTTCTCTATTTCATATTA
GCCGTGCCTTCAAAAGGGTAGAAATGCCTCAGCATTTGAGAAAAATTGAATTTGTGCGCAGTTTTGCTCGT
CGGACAAATACACACACACACACACACATTGAAACGATCCCTAAACATAGAGAGAGCATTGAGAGCTGAGA
GTAAGTGCGAGCGAGATAAAAGTTAACAGCGTTGCCGTTCTATCGCACAAATCATATGGAAGAGAGTGCG
CCAAGCACTTTCCTGTAGAGTAAGCGGTTCAAAAATACATCATATGTATATCTTACTCCCTTACTCGCGAT
TTTGGACCAGATGCCTCCGATACTTTTTGTGACAACGCTGTTTGTATGTGATGTCCAGTCTATATGTTGTTT
GGTTAGTGNTTGTATNTAGACTCTCTAGCCTTTAATTTTCAAATTATCGCCTTCACTGAGACCTGGCTGAA
GCCGGACATCTCAGACTCTTCAATTTTTACCTCCAAATACACCACTTACAGGTGTGATCGAGCTTCCCGNA
TCGGNGGAGGAGTACTCATAGCNGTCGAGTCTGCTCTTTCGTCGGAACGCATTGATCTTTCCATCTCAGAG
ATTGAGCTGGTGGCTGTAAGAGTTGTATTTGGAAGTTTCTCTGTCTTTATTTCATGTTCTTATATGCCTTNT

ATCTAACTTGTCCACCTATCAAACCCATCTTAATGCTATTAAATCTATCTCCTCCCTACTTTCTGATAGAGA
CCTTTTAGTAGTTCTTGGGGACTTTAATCTTCCAGATGTCGAGTGGTCATCGCCATCAGACTTAAACTTTCT
GTTGCCGTCTTCGTCTAACGAATTTATTGATGGTCTACATGAAATGTCCTTACGGCAGGTTAATCGTGTGC
GTAATTCAAGAAACAGATCTCTTGATCTTATTTTCGTGTTGAATTCGTTCAACAGTGTTGTGCACAGGATC
AATAAGCTTACACTTCCTGAAGACCCTTTTCATCCAACTCTGGAAATGGTCATTGGTTTACCAGCCCGAGT
CCCTATCAAGGATCCGCAATTAGTTGAACGTTTGCGTTGTTTCCGAAAGGCTTATTTTCATAAGCCAAATG
ATCTTATTCTGCAAGTCCACTGGTCCCAGTTGTACAATTGTGCAGACATTGATGTTGCCATTAAACTTTTTT
ATGATACCTTGNACTCTATTTTTGATGTCTGCGTNCCTCTTGATCACATAGCGAGGCCTGATAGGCCTCCCT
GGTTCTCAAAGGAACTTATAAGGTTAAAAAATGTGACGTCGAAGTGTTATAAAAAGTTTAAGAAATCGGG
AAATCACTCTGATTCATCACGTTATGCAGTTGCCCGATAAAATTTTTCCACTCAAAATTCCATCTGTTTTAA
GGATTACCTTCGGCGTTGTCGGCTTCAATTTTCACACAATCCTTGTCAATTCTAAACGAAAGACATATATT
TTGCCTTCTTCGGTTAAGTTTGCAAATGAGAGCGCGAACACTGATCCGGGCATAGCCGATCTGTTTGCAAA
CTTTTTATTCCCTGAACACATTAAAAATGGGTATAAGGGTATATTGTATTTGTGCAAAAGCCAAATGTATG
CAACAGGCAAGAAAATATCCCCGACCCCATAAAGTATATATATTCATGATCAGCACCAATAGCCGAGTCG
ATCCGTCTGTATAAACGCAATGATCTCAGAGTCTATAAGAGCTAGAGACTTGAAATTTTAGATGTAGGTG
CTCCTAGTGCCTGCACAGATCAAGGTTGTCTCCGATAATCGATAACTTACTCAGTTTCCAAGCAATCGATT
TAGCGACCTTAACACTATCAAGCCTGGTGGTGTTCCTGCTTGCGTGCTTAGGTACTGCGCTGCAGCATTAT
GTAAGCCTGTTTTACAACTTTTTGAATTGTCCGTAAACACNTCANCATCCGACCATTTGGAAGGAATCATA
CACTATCTCTCTCCAAAAGAAAGTGACTAAGTCCTATGTTGAAAACTAAAGAGGTTTTTTCAAATTATCCG
CTATACCTAAAGCTATTTAGCCATTTATGTAGTTCTATTATTTCACCTTCTCAGCATGGGTTCGTGAAGCGC
AGATCTACAACCAATAACCTTCTTAAGTTTACTTCGTTTGTAATTGATGGGTCTAAGAAAAGAATGCAGAC
TGACGCCATCTACACCGATATCAGTAAGGCTTTCGACTTTGTCAACCAATCCTTGCTGCTTCATAAATTAT
GCCTTCTCGGCTTCCCAGTCAATCCTTTGAACTGGATTTCAGAGTATTTGCGTGATAGAACCCAAAAGGTT
CTTTTCAGAAATTCGTTTTCTAAACGTATTCTGGTGACTTCCGGTGTTCCTCAAGGTAGTGATCTTGGCCCG
CTACTCTTTTCATTAATGATCTTCCCTCGATACTGTCTCACTCTCGAGTATTAATGTATGCTGATGATGTAA
AATTATGCTTTGCGCATAAAGACATGGCATCATGTCATCTTACAAGCGGACATTGATGCGTTTCAGGCTTG
GTGTCTAGCCAACCTTTTAAACTCATACTGCTCTAAATGTAGGGTAATGACCTTCTATAAAAGCCCTCAAT
TGGTTTCCTACATCCTTGATAGCTTACCGGGCGAGCGTGTTAACGGTACTTCTAATTAAGTTAATGATCTA
GGTGTCATCCTTGCTCATAAACTTAAATTCGACATGTGGCCGTGGTCAATAAGGCCAGAATAGTTATGGGT
TTCATCAAGCGATGGTCGAAGGAGTTTGACGATCCGTACACGACAAAAACACTTTATATATCTTTGGTACG
AGCTATTCTTGAGTATGCCTAATGTAACTGGTCACCACAATATGCCATCCACCAGGATAGCATTGAATCCG
TTCAGAAACAATTCTTGTTGTTTGCCCTTCGTGGTTTAAATTGGGATCCTCGCGTGCGGTTACCGTCATACT
CAGCATACAGCAGACTTCTGATGATTAACCTTCCTTCCTTGATTAATCGTAGAACCATGTTGGGTGTAATA
TTTATTCATAAACAAATTACTGCTGAAATCAAATCTTCTGTGTTGACTAGCCAATTAAATTTCTCTATACCT
AATAGACTAACACGTAATTTTGTACCCCTATCGCTTTAATTTGGCTCAACTAATTATGCTTTGCATGAACC
GTTCAGGGTTCTTTGTGATGACTATAACAAGCATTACAGAGTAATTTGGTCGGTATCTTCACTTCCTCGTAT
AAAGTCTTCTATTCTAAACTATTTAGCAAGTAGATAGTCTTATTTTTATTTGTAATTGTATTTGATGTTTTTT
TTCTTATTTTTTTTTTTATTTAATTTTCATTTCGTGTCGTACGTTATACGACTGCGCCCCCCGGTCGGTTGGG
CTGGAGGTGGGATGCTGTACGTTGCATCAGGATTCGCGTAATAAAAAGCAATATAAATGGATTATTAAAC
TCTTTAGATCATCGTATAATGAATCCAAGTACACCTTTTGCCGATTGAAGCTGTCAAGTTTAAAAAGTAAA
ATGCCATGGTGGACAGAGTCAAACGCCTTACTGAAGTCGGTGTAAATGAAATCAAATTACATTTTCGAAA
GGAAAGCATCATTTGCCAAGGAAGTAAGCTCTAGTAGATTGGTCGTAGTTGACCGATTCTTTAAGAATCC
ATGTTGATTTGCAGAATAGCTGCAAATTCGTAGTGATTTTTTTTTTTAGGTATAGCGGACAGTTTTGCAATG
CCTCTGTAATTTTGTATATCAGTCAATTTCACCTTTCTTGGGGAGAGGTATGATATAAGATTCTTTCCAGAG
ATGGAAAGCCTGAAGTTTCAAGGAACAGATTAGCAATGGATGAAAAATGGAAGTACTATACCCCTTGAG
AAGACAATTAGAAACATTGTTAGATTAAGAAGAAGAAGCCTACTGGAGTAAGATGGCAACCGAAGATTA
GGGTCCCAATTTAAACCCCATCTGGCAAAAAGCAGAGACCAGATGCAAGAGNAGTTTTCAAGAATAAGG
CGAACAAAAAACTCTGGTTGCAATCGAAGCGAACAGACGAGTTTTTTTTTTGTGCTCTCTTGTTATGATTT
TAATCAAACACCGGCCTTTTAATATTTATATTTAAAATATTTCCATTATTCTGTTTGCCTTCTCGGTTAATTT
TTTTTATTCTATCGCGCGTTCATGCTTGTGGCCTGTGCATATTATCATTTAAATATTGTTACTTGTAAATAT
GTCTTCTTGTGCACTTTTGTTTGTGCGCTTGCGCTATTAGCTTCTATAGGTAAGCTTCTTGTTTAACTGCAC
ATCTCTTATCTTGGTGTGCAAGAATTTGTGAGCAGGCAAGCAAATTAAATATAGACTATTCTCGCTCTCGC
GCTCTCTCACTGTTTTTTTCAACTTCCGCTTTGATATTGTTCTACTTTCTGCCGCTTGGTGCTCTATACGCTA
TTAATCTAAACTGCTCTCTTTTTTAATTTGGCTGCGTTATTAGAGTAGAGACCCACGATAATGGACCATAA

AATTGACTTCTTCAAGAAAACTTGCCAAATGCAAATTACGGCAAGTCAATTCAGCATACCTTGTTGGCTGT
ATCTAAATGTCGTGCATTCAAAATGTACGGGTTACACTGGCCTAGTGGGGATGCTTTTGCCAAACACAAT
GGTTTGCACTATAGTTGTGACTCGTGTAGGGAGATTAATGATAAAATGCGGGCGATTATACGTCAGACCA
GAAGTGGCTTTAAGGGTCTGATTGTTGGTGTTCGTAAACTAAACGATAAGCTCACAAGCTTGGATACCCA
ATTTAATATCCCAAAACTCTTGGATGAGTCTCCTGGGACTAAAAAGCCCATTCCTTGTGATTTGACATTGC
CTAATACCCCTCAGCCTCCGAATGCAAATCTGCAGCCTACATCTCGTTTATTACTCCGGTAGCTGGGAGGT
CTGCTGTGGACACTGATTCTGACCTAAGCTGTGTGAAATCGGCACGAGAAAACCAGTCAATTTATCCGTGT
CGTCCGCTGTCATAGAAGTTACGAAAGGGCGATGGCAACAATTAAATTTACAACAGCCCCTAGAAGGCTC
GTCGTCCGGACTACCGGCTGCTTTAGGTACCACAGCTAATAGTGTTCCCACTGCCCTACCTGTGGCGCCTA
CGCCTGTAGTGGGTGTTTTGTTTCTAGGCTTTCCCCTTACTTCTCATGAGATGGTGTAAAAGCCTTTATACA
GGTAAAAATGCAAGCCCCAGGTATAAAGGTGGAGAAATTTAAACGCGCCTATGAAAAGGATATTGCATC
CTTTAATTTTCAAACTAACGCCTCTACTGAGACCTGGCTGAAGCNGGACATCTCAGACTCTTCAATTTTTA
CCTTCAAATACACAACTTACAGATGTGATCGAGCTTCCCGTATCGGTGGAGGAGTACTCATAGCTGTCGAT
TTTGCTCTTTCGTGGGAACGCACTGATCTTTCCATCTCAGAGATTGAGCTGGTGGCTGTAAGAGTTGTATTT
GGAAGTTTCTCTGTCTTTATTTCATGTTCTTATATGCCTTATATCTAACTTGTCCACCTATCAAACCTATTTT
CTGATAGAGACCTTTTAGTACTTCTTGGGGACTTTCATCTTCCATATGTGGAGTGGTCATCGCCATCAGAC
TTAAACTTTCTGTTGCCGTCTTCGTCTAACAAATTTATTGATGGTTTACATGAAATGTCCTTACGGCAGGTT
AATCGTGTGCGTAATTCAAGAAACAGATCTCTTGATCTTATTTTCGTGTTGAATTCGTTCAACAGTGTTGTG
CACAGGATCAATAAGCTTACACTTCCTGAAGACCCTTTTCATCCAACTCTGGAAATGGTCATTGGTTTACC
AGGCCGAGTCCCTATCAAGGATCCGCAATTAGTTGAACGTTTGCGTTGTTTCCAAAAGGCTTATTTTCATA
AGCCAAATGAACTTATTCTACAAGTCCACTGGTCCCAGTTGTACAATTGTGCAGACATTGATGTTACCATT
AAACTTTTTTATGATACCTTGAACTCTATTTTTGATGTCTGCGTACCTCTTGATCACATAGCGAGGCCTGAT
AGGCCTTCGTGGTTTTCAAAGGAACTTATAAGGTTAAAAAATGTGACGTCGAAGTGTTATAAAAAGTTTA
AGAAATCGGGAAATCACTCTGATTCATCACGTTATGCAGTTGCCCGATAAAATTTTTCCACTCAAAATTCC
ATCTGTTTTAAGGATTACCTTCGGCGTNGTCGGCTTCAATTTTCACACAATCCTTGTCAATTCTAAACGAA
AGACATATATTTTGCCTTCTTCGGTTAAGTTTGCAAATGAGAGCGCGAACACTGATCCGGGCATAGCCGAT
CTGTTTGCAAACTTTTTATTCCCTGAACACATTAAAAATGGGTATAAGGGTATATTGTATTTGTGCAAAAG
CCAAATGTATGCAACAGGCAAGAAAATATCTCCGACCCCATAAAGTATATATATTCATGATCAGCACCAA
TAGCCGAGTCGATCCGTCTGTATAAACGCAATGATCTCAGAGTCTATAAGAGCTAGAGACTTGAAATTTT
AGATGTAGGTGCTCCTAGTGCCTGCACAGATCAAGGTTGTCTCCGATAATCGATAACTTACTCAGTTTCCA
AGCAATCGAT
>160_rnd-1_family-96__LTR___Gypsy
ACAATGATAAGCGACAAAATAAAATAAATAAATAAAATGAAATTTCAAGAAATATATATATATTCTTATT
ACCAGAAATGCCTATTAATAAGTTTGCGATAGAAGTATATTTAGAAGCAATATATAATAGCGACGCAGAC
TTTAGAATTCGTGCATTGAATATTCTACAACAATAAGATAAAAGGCATAATTTTAAATACACTGCCATATG
TTGGCATTAAAGAAATAGTGGTCCAGTGGACGGCGAGTGCCCAATTATAATGGTAAAACTTCATAAATAG
AGAACTATGGAATGGAGTGAATTAGCGAAAAAAATAAATAATTTCAAAGATAAATTCGAAAAATCTTATA
AGTGTGTTAATAAGAACGCACCTATAAGGGTTGAAACGCTAATTCACCACATAAACATATTAGTGCTAAA
CTATAATAACATAGTTCAACTTTATTTAAGTAAAGAAAATAGGCTTACAGAGCAGCATGTAAATCAATGT
AAGAAAATAATCAAATCACTTGGGACTAGAATATCGAATATCAGCACTAGGCACAACATAGTAATAGATA
TCCCAACAGATTTAAAATTAATGGCAACTTTTGAACCAGATCAACTAGAAGAACTGAATGAGTCTAAGCC
AACACTAAATAGTGACCCTGATATTGAAGCTGATAGCGACATTGACCCACTTGAATTAACAGAACCAAAC
ATGGCAGTAAATCAAGTGGCCTTAGACCGCGAATATGTAAGACAGGTATCGTCTACAGTCCCGGAATTTG
ACGGCGAAAAGCCGTCACTGGCCAGATTTCTAACGGCTTTAAGACTNGTCGATCGTACTAAAGGTACACG
AGAAGACTTAGCCGTAGAGGTCATAAAGTCNAAAATAATTGGCCCAATTCTGTACAAAGTACAAAATGAA
ACNNCNATTANCGGNATTATNAATAAATTAAANACCGNNGTCGNAGGCGAGTCAACCGACGTTATCAAG
GCNAAGTTGCTTAACATAAAGCAGAAAGGCAAGNCAGCCNCGCAATNTACAGCTGAAATTGACAGTTTG
CGTAAGCAACTNGAAGCAGCCTATATTGACGATGGACTAGATTCTGATAACGCNGAGAAATTTNCTACTA
AAGAAACAATATCCGCCATGACAAAGAATTGCGAATACGAAAAGCTTCGCATAATATTAGAAGCGGGCA
ATTTCAGCAATTTCGATGATGCCGTGGGTGAATACATCCGATGCAGCACNGAAATGACTGGAAGCCCGAG
TACAGTATTGTACTACAAAGGCAGAAACNATCGCGGNAATNACAGCCGTAACAACCAAAGAGGTAGAGG
CAATGGCCGCGGCAATAGTAATTTCCACTACAANGGTAATAACAACNACAGGGGTAATTTCAGAGGAAAT
TACCGNGGTGGCAACCGAGGNAGAAACAATTCNAACCGAGGCGGCNACCAAAACCAAAACAATAACAAT
AGAGGTAATGTCCGAGTAACTCAAGGTAATTCGGAAAACCAACAAAACCCCTCAGATGTTCAGAGCCAAT

AAATGGCGCAATAAAAACCATCAACCTTAATCTNAGCATATTTGTAAAANTTAACAATGAAAGAACAGGT
AAATCANTTGCACTNTTAGTAGATACTGGTGCAGATATATCCATATTAAAGGAAAATNCAGATATATTCG
AAAATGTAAACGAAAAAATTACTACCCAAATATCGGCATTGGCGAAGGCATAATTCNTTCAAAAGGCTT
GGCCTTTATAGAACNCACTACAGGTAAATACATAATTCCACATAACTTTCATTTAGTCGATCAGAANTTCC
CAATACCTAGTGATGGCATACTAGGCATAGATTTCATAAAACAATATAATTGCCAATTAGATTTCACTCAA
AGTGGCGACTCGCTCATTCTTCGCCCAAACAACTTAAATTATCCAATTCGATTACAAATACTTCATACTTG
TGGCAATAATGCCACAGTACTCCCAGCTNGTTCCGAAGTTGTCCGTGAAATACAAATACCCTCAATANAA
AATCGAATTCTGATACCAAATCAAGAAATTAANGAAGGAATTTACGTCGCAAACACATATCCAACCACAA
AAATGCATNTGTCCGATTACTAAATACAACAAGTACCGATCAAATAGTAGACATTAAATAANTTGAAATA
TGAACCANTTTCGAACTACGATATAGTTCAAAAGAAACCAACGAAGGAAGAGACAACTNCGTTATGNCA
AAATTAACTAAAAACTTTCCACCNCTATTCAAAAATCAATTAAAAGACTTATGCACCAGATATACCGACA
TATTCGGACTNGAAACCGAATCGATAACAACCAATAATTTTTACAAGCAAAAGCTNAGATTGAAGGATGA
TGAGCCCGTATACATAAAAAATTATAGAAGTCCTCACAGTCAAGTAGACGAAATTCAAGCCCAAGTNCAA
AAATTAATNGACGACAAAATAGTCGAACCGTCAGCCTCAGAATATAATAGCCCACTTTTACTAGTCCCGA
AGAAGTCTCTTCCGGACTCTGANAAGAAAAAATGGCGATTAGTAATTGACTATCGTCAAATTAATAAAAA
GCTATTGTCCGATAAATTTCCGCTNCCTAGAATTGATGATATTCTAGATCAACTAGGTAGAGCAAAATATT
TTTCATGCCTNGACTTAATGTCNGGNTTTCACCAAATTGAACTCGAAAAAAGTTCTAGAGATATAACGTCN
TTTTCAACGAGCAATGGCTCGTATCGCTTCACGCGATTACCATTTGGCTTGAAAATAGCGCCAAATTCNTT
TCAAAGAATGATGACNATAGCATTTTCTGGNCTNGAACCATCNCAAGCNTTCCTTTATATGGATGATCTAA
TAGTAATCGGTTGTTCCGAAAAACATATGGTCAAAAACTTAACTGATGTTTTTGACAAATGTAGGAAATN
CAACCTGAAACTACATCCNGAAAAATGTTCATTTTTCATGCATGAAGTCACATTTTTGGGACACAAATGCA
CNGATAAAGGAATCTTGCCAGACGACAAAAAATACGACGTCATNAAAAATTACCCAGTCCCACACGATG
CGGACAGCGCTAGACGATTCGTTGCATTTTGCAATTACTATAGGCGCTTTATNAAAAATTTTGCCGACTAT
TCACGTCACATAACAAGATTATGTAAAAAAGACGTTAAATTCGAATGGACAGCTGAATGCCAGAATGCCT
TCGAACATCTCAAAACGGCACTAATAAACCCAACCTTGTTACAATATCCAGATTTCACCAAAGAATTCTGC
ATAATAACAGATGCAAGTAAGCAAGCGTGTGGAGCGGTTTTAACTCAAAACCATAATGGACTCCAACTTC
CAATTGCATACGCATCAAGATCCTTTACTAAAGGAGAAAGTAACAAGAGTACNACTGAACAAGAATTAGC
GGCTATTCATTGGGCAATAACNCATTTCCGACCATATATTTATGGTAACCATTTCACGGTTAAAACGGATC
ACAGACCACTAACATACTTATTTTCAATGGTAAATCCAAGTTCAAAATTAACGCGTATGAGGCTTGAATTA
GAGGAGTATAATTTTACGGTAGAATATCTGAAGGGTAAAGACAATTNCGTAGCCGATGCGTTATCAAGAA
TAACCATNAAAGAGCTACAAGANATAACAGGAAAAATAANGAAAGTCACTACAAGATATCAAAGTAGAC
AAAANTCCTGCGCAGGAAAAAANGAAGTAGAATTGCCAAGGCAATCTATAGAAAAAGCTTCAAAGCCCA
ACGTATACGAAGTCATTAACAATGACGAAGTACGTAAAGTAGTGACCTTGCATGTAAAAGATATGCTATG
TTTCTTTAAACATGGTAAGAAAATTACNGCAAGATATGATGTTAGCGATTTATATACNAATGGAANTATTG
ACTTAGGTCAACTNTTCCAAAGGCTCGAAAAGGAAGCCGGTATATATAAAATCAGCCAACTCAAAATGGC
ACCGTGGGAAAATATCTTTGAACANGTTTCAATAGATAATTTCAAAAATATGGGCAATAAAATATTGAAT
ACATTGAGAGTAGCGCTACTCAACCCGGTGACCACAATAACAAACGATAAAGAAAAAGAAGCTATATTG
TCTACACTACATGACGATCCAATACAAGGAGGTCACACAGGCATTGCAAAAACCTTGGCCAAGGTCAAAA
GACATTATTNTTGGAAAGGTATGACTCGAGATATNACTGAGTACATACGNAAATGTCNAAAATGCCAGAA
AGCAAAAATAACAAAGAATANTAAGACCCCTTTAACAATAACTGATACGCCGATACACGCTTTCGACAGA
GTNATAGTGGACACAATTGGTCCGCTACCNAAATCAGAAAATGGNAATGAGTACGCAGTCACGCTAATTT
GTGACTTGACTAAATACCTAGTCGCGATACCGATCCCAAATAAAAGCGCNAGCACNGTNGCNAAAGCNAT
ATTCGAATCATTTATTTATTCTGAAGTACGGTCCAATGAAGACGTTCATTACGGACATGGGAACGGAATAT
AAGAATTCAATTATAAACGATTTGTGCAAGTATNTGAAAATAAAACTAACATCAACTGCACATCATCACC
AGACAGTTGGAACAGTAGAAAGAAGTCACAGAACTTTTAACGAATACATACGATCATATATATCAGTTGA
TAAAACTGATTGGGACGTATGGCTCCAATATTTCGTTTACTGTTTCAACACGACCCCCTCTATGGCACATA
ATTATTGTCCATATGAGCTAGTNTTTGGTAAGACANACAATCTACCAAAATATTTTAATAGCATAGATAAC
ATAGAACCNNTATATAACATAGANGATTACGCTAAAGAAAGTAAGTATAGATTAGAAGTAGCATACAAA
AGAGCTAGAANTATGCTNGAANNAAATAAATAGAAAAATAAGGAATTATATGATTTAAAAACTAGGAGA
TAAAGTCTTACTAAGAAATGAAGCAGGCCATAAGCTAGATCCTAAATACACNGGGCCCTATAGAGTAGAA
AAAATAGAAGATAGNGATAATATAATAAAATNAGAAATAAGAATAAAACGCAAACAGTTCATAAGGATA
GATTAAAAATTTTTAATTCATAAAAGACATACAAGAAAAAAATGAATTATACAAGGTACGCAAAGAAAA
AATAAAAAAAACTAAATACATTATAAATACATTACAAATATAATAAATCTTCAAATATCAAAATTATTTT
AATATAAAACAAATTTAAGTAAAATAACTCCATTTAGTACGTTAGGAGGGAGATGTAGTATGTGCATATA

TTGAGGGTACACTGTACCAATAAAGTAACATCGTTTAAGCATAAATANATCAGCAACACATTGTTGCAGT
ACACAAACAAAACCNTTTCAAGTGAGCGTGTAATATGATCGCCCGCTTATGAGCTACGCTAAGTCAGCAG
TCCCACGCTGACCATTCGTAGCACATATATATATATATATATATATATATATACATGCAAATATGTACATA
AGACTCTCTCTCTCCCGATTATGCTGATAAGACAAAATATGTATGAAGCCGCTCCGCTGCTGGCGTAACCG
GCAGCGCAGCTATGCGGTCTTAGCCATAAGAATATATTGAATAAAACATTCTAAAAACTAAAGTCAAACG
GCACAGACGTGCTAGATTCATTAGCTCGTACCTATTACGTACTACCTCGAGTTTACAAGAGAGCACTTGGA
GTACGTGCACAAAAAAGCAGCAGAATCCTGTCGAGCGCTATCCAGGATTATGCTCAACACTCGAGGTCCA
AAACAGCAGAGACGCAGTCTTCTCATGACAGTAAACAAAGCGGTAATAATATACGCGTCCCCCATCTGGA
GCAGAGTAATGGCTGTTCCTAGCTACAGCCGGAGTATAAATTCTATATATCGCCTATGCGCTCTACCGGTC
AGCTGCGCATTCCGGACGGTATCAGGATAAGCAACGCTGGTTTTCGCAGGGATGGTCCCGTTCAATGAAC
TGGCAACAGAAGCGGTAGTTTTCTACAGAGCTCTGAATGGAAACAGCACAGCAAACTCGGTTCGCACAGC
AGCAAGGATGCAAAGCATGGAACGATGGCATCCACGTTGGGATCGCTCATCTAAAGGACGCTGGACACA
TAGGCTTATCCCTAACTTGGAAGCCTGGATAAACCGCAAGTATGGAAACACTAATTTCCACTTAACGCAG
CTCCTATCTGGCCACGGATGCTTCTAGAGCTACTTGAACCGATTCGGCCTCGATAACACAGACGAGTGTTC
TTGGTGCGGCAGTGAAATCGCAGAAAGGCTCCTCACTGTAGGAGGGAAAAACCAGCAGTGCGGCATATC
AACCTAGATGTGAAAGATGATATTGCCAGGGTGAAGGTGATACAGATGGCAGCAACTGCTAAACTACCA
AGTGAGCCAACTCCACTAGGAAGCCGACGCCAGCAGATCCGAGAAAACCACACCGGCTAAATGTAGATA
AACTCAGGATCACAGAAAAAAATCAGCTCACAAAAAACAACAACAGAGGGCCTGGAAATCACAAATGTT
TAAAAGGCGGTCCCAAGAGGGGTTGAAGCCTTACCCGAGCTAAGGAAAGCATATCGCAGAACACAGACG
GCTACCATGCTTCTACCCAAGATCGCAGCAAACGAACTTCTGAAGAAAGCGAAGAAACGCATTGGCTGGG
TTGTGTACCGTATATGCCAAAAATTGGAACCAAGAAGATGTTTCCAGTGCCTGGACCTTGGGCACACGGC
TGCGCGATGCATTAAAGGTTTGCATATGCAAGCCAATGCATAATCTGCACAAGGCACGAGACTGCGAACA
CGGCAAACAACACCCTCAGCCATGCCTGCTCATGGTTAATCAAGGCGTCTCAGGCAACAATCAAAAATTA
AGTTAATACAAATTAACCTGAACTATTGCAGAGCGGTCCAGGACCTACTCGCTCAAACTACACGGAAGA
GAACGCTTACTTCGCCATCGTCAGTAAACCACACCAGTCAAACATGATGGCATCTGGATTCAGAGCAACG
GCGGCCAAGTATTGCGAAATATGTATATATATATATATATATATAGTTGCTATATTGCACCCAGCATAGGA
ATTACCGCGTTCGCCGATATACTG
>160_rnd-5_family-10007__RC___Helitron
AGTTGTCAGTTTTGAATAAAGCTCGTAGACAACAAGCTAGAAATGAGCTAAATAAGTCTAATTCTGAATA
TGCTGAGCATGAACGTATCAACAATATAGAGTCCCAACGAGAGCGTAGGGAAAATGAAGGTTTTAGCCA
AACTGAGAGAAATAGGAATAGGCAAAGAAACCAAGTTCGCAGACAATACAGAGTTTACAGGGCAAACGA
ACAAAGACAAAATACTGTAGACCACTCGAGTCGACGAGCTAATTCTCCAGTTAGAGAACAGGAGCAATTG
GCTAATACTTCGCGTCGATCGACACGCAGACGAGACCCAGCCTACCGGGCAAACGAACAAAGACAAAAT
ACTGTAGACCACTCGACTCGACGAGCTAATTCTCCAGTTAGAGAACAGGAGCGATTGGCTAATACTTCGC
GTCGATCGACACGCAGACAAGACCCAGTCTACCGGGTAAACGAACAAAGACGAAATACTGTAGACCACT
CGACTCGACGAGCTAACTCTCCAGTTAGACAACAGGCTAATACTTCGCGTCGATCGAGACGCAGACAAAA
CCCAGTTTATTGGGCAGACGAACAAGAACAAGACAATATTCGTCGAGGTCGCCATCGACAAGACATAGA
ACGACGACTTTTAGAAAGAACTAGGGATCGGAATCACAGGTTAAAGAGAATCCGATTCGTAGGATAATTG
AGCAGGAACGTAACACAAGTGTAAGGCGAAGTGCTCGCATTCGTGCACGACAGGAGGCTTTTGACGCCA
ATAGAAGTTTGTCCCAGCGAGTTAGCCAGTGTAGGAGTAATACAGGTAATAGGATAGCAGAGAATAGGC
GCAATGCGACGAGGAACAGGGGTGAACGTGCGATTAGTTTTTCAGAGGATAGAATCTTTCCAGCTATTAG
GATTAACTTTGATGGTCTGATAGCTGGATTCAATAGAACAGTTAAGTAGGGTCCCGATTAGGTTTGCTTTT
GTTGCGAGGGCTTATGGTTTCCATATCAAGTGGACAAATTTTCTCGTGAGGTTATTGAAAGGAAGTGTAGT
GCGGAAGTAAGCTCCGACACTTTATATCTTTTCACTATATTTCCCTCTGTTAATAATAAATATAGTTTTTGC
AAAACGTGTAGTCGTAGCATTAAATTAGGCAAAAAACGTAAAACTTCTATTGCAAATGGCCTAGATATTC
CCGATCTGCCAGATTGTTTGAACGGACATGGCTATATCGACTCGGCTGTTGATGCTGATCAAGAATATATA
TACTTTATGGGGTCGGAGATGTCTCCTTCTATGCGTTACACATTTCACGACCAAATTATAATATCTCTGCA
AGGGCATAAAATGTCATATAGTAAAATTTGTAATTTAAATTAATTAATTCCAATAAATTATTTCCCATATA
ATTTATTAAAATTATACTATATGTATGTATTGATATATATATTTATTATTTTGTTATATGTCTGGAATAAAT
AAAAAGTTGGTTATATTTGTATAGTTTTAAAAAAAAAAGACTAAAACTAAAGTTATGTTTAAGAACTTGA
GATTTTTGCTATTTCGCATAAATGCATAAATNCTTGCACATACACGCCAAATTGTAACGGCCGAAAATTAT
GTTTGTCGATAATTCACTTCTTTTTTTTTAAATGGTCACAAAACACTTCAATTTATTTATTTTCAACCGCTTG
AAACCGATAGAAAAGTTTATTTGAATAAAAATCGTTGTTAGATCAGTAAGAAACGCAAAAATCACAATTC
GGCGATCGAAAATCGACAACACGCAANATTCTGTAGTGTCACTGGCCAAACAGCATAAAACAGAAAGTC

TAATCTGTAGTANTTTAGTAGTTTTTCAAACATTGTTTTTGATTTCCCAGATTTTATGGCAAATTTATGAA
TTTGCAGCTGGTCAGATGCAGATGCAGGTGCTTTCCATAAGTTGACCCTTTCGGCCCAAAATGCTGATACA
GATACAGGTCATGCACCCATGATCTGCCCATTCACACATTTATGGGTGTGTGTGTCGTAAATCAGAAATCC
CAAAATATAGTCAAATGCGCATTGGACAGGTAGGCAGGCACCAAAAGGCACAACTTTTTTGTACCCTTTG
GGTTAGTGGTAGTGTGATGGCAATCGCAGGCTACAGACTAAATCGATTACTGCTTCTTCGGAGCACATTG
AAAGAGAGTGTTCGGAAATATTCACTCTCCTACCGCCACGATTTAGTATACATATCGAACTGCGCGCGCTA
CTTAGCTTATTATTATAATCACATATTAAACATCACATTATTATTATAAGCACCTACATCTAAAATTTCAAG
TCTCTAGCTCTTATAGGTTCTGAGATCCTTGCGTTCATACATACGGACGTAAGGACCCAATGGGAATAAAT
GTATATTGCATTTGTGCAAAATCCAAATGTATGTAACAGGCAGAAGGAAGTATCTCCGACCGCATAAAGT
ATATATATTCTTGATCAGCATCAATAGCCGAGTCGATCTAGGCATGTCCGCCCGTCTGTCTGTCTGTCCGT
CCGTCCGTATGTATGAACGCAAGGATTTCAGAACCTAAAAGAGATGTAGGTGCTCCTAGTGGCGAGTTTA
TTTCCGAAATTCGATAACTTACTCCGTTTCCAATCAATCGATAAAAATCGATGTCGATATCCTTTTTTTGGG
CAATTTTGGTAAATAATAAGAGCTAGAGTCACCAAACATGATATGTTGCTTTTAGAATATTATATATATAT
CAAGTTTCTTCAATTTATACCTAACGCCACCTCCCCGTTAACACCGCAGCTCTGTAAAGCAAGTTAATAAC
CCAGCCTTTATTGTCAACCAATTTAAGCCACAATTTAAATTCAATTGACTTTTTTAAAATACACAAATATTC
TATACTACCATAAGATATACTGTAGAAAATTTCATAAAGATCGGTTAAGAAAAAAACAAAGTTATATGTA
ACTGCGCAATTGGATGGGCAGCTAGCGAAAACATCAATGCAACAACTGCATTTGGTGTTGGAATAAGAGA
GTTCAGCGTATTCCCTAGTCGGGAGCTCCCGACTAGAACCTCCTACTTGTTTTCATATTGCCGTAAAGAAT
TGCAACATTTAAGTTGTATGTTAAGACATTTTGATTTTCAAGATATCTTAACCAAACTCGGTATTTATTAGT
ATGACTATACTCCTCATATATAGGCAAAATCTTATTAAGATCGGACCACTATATCATATAGCTGCCACTGG
AAAGATCGGTCGAAAATTCAGTTCTTGTATGAAAAAATTGTTTGGCTCTCACGAAATTTTGAACAAATAA
ATTTTGTTCCTGTGCTTCTTAGATATGAGCATTATGAGTCATCAGTGGAATGATAATTAACGTCGGATTAA
TAAAGGCTCTTTTTATACCCTTGCAGAGGGTATTATAATTTGGTCGCGAAATGTGTAACGCATAGAAGGAG
ATATCTCCGACCCCATAAAGTATATATATTCTTGATCAGGATCAACTGCCGAGCCGATATAGCCGTGTCCG
TCTGTCCATCTGTATATTTCTATGCGAACTAGTCTCTCAGTTGTAAAGCNATCTTAATGAAACTTTGCAGA
AGTCCCTCTTCATGTTGCACGCAGCACATATGTTAACACCAGCTGGATCGGACCACTAAGTTGAAAATTAA
GTTTCTGTATGAAAAAACATTTTGTTTATGAAGATATCTTGACCAAACTCGGTATTTATTAGTTTTACTATG
CTCCTCATATATATGCAAGATCCTATTAAGATCGGACCACTATATCATATAGCTGCCATAGGAACGATCAG
TAGAAAATTAAGTTTTTGTATGACAATACATTTTGTTTATCAAACACGGCATTCAGTAGTTTTACTATGCTC
CTCATATATATGCAAGATCCTATTAAGATCGGACCACTANATCATATAGCTGCCATAGGAACGATCGGTA
GAAAATTAAGTTGTTGTATGAAAAAACATTTTGTTTATCAAGATATCTTGACCAAACTCGGTATTTATTAG
TTTTACTATGCTCCTCATATATATGCAAAAACCTTTTAAGATCGGACCATTATTTCATATAGCTGCCATAGA
AACGATCGGTCGAAAATTAAATTTTTGTATGAAAAAACATGTTGTTTATCAAGATATCTTGACCAAACTCG
GCATTTATTAGTTTTACTATGCTCCTCATATAAATGCAAGATCCTATTAAGATCGGACTACTATATCATATA
CCTGCCACAGGAACGGTCGGTCGAAAATTAAATTTTTGTATGAAAAACATTTTCTTTATCAAGATATCTTG
ACCAAACTCGGTATTTATTAGTTTTACTATGCTCCCCANATATATGCAAAATCCTTTTAAGATCGGACCAC
TATATCATATAGCTGCCATAGAAACGATCGGTAGAAAATTAAGTTTTTGTATGAAAAACATTTTGTTTATC
AAGATATCTTGACCAAACTCGGCATTTATTAGTTTTACTATGCTCCTCATATATATGCAAGATCCTATTAA
GATCGGACCACTATATCATATAGCTGCCATAGGAACGATCGGTAGAAAATTAAGTTTTTGTATGACAAAC
ATTTTGTTTATCAAGATATCTTGACCAAACNCGGCATTTATTAGTTTTACTATGCTCCTCATATATATGCAA
GATCCTATTAAGATCGGACCACTATATCATATAGCTGCCATAGGAACGATCGGTNGAAAATTAAGTTTTTG
TATGAAAAACATTTTGTTTATCAAGATATCTTGACCAAACTCGGTATTTATTAGTTTTACTATGCTCCTCAT
ATATATGCAAAATCCTATTAAGATCGGACCACTATATCATATAGCTGCCATAGGAACGATCGGTAGAAAA
TTAAGTTTTTGTATGACAANACATTTTGTTTATCAAGATATCTTGACCAAACNCGGCATTCANTAGTTTTAC
TATGCTCCTCATATATATGCAAGATCCTATTAAGATCGGACCACTATATCATATAGCTGCCATAGGAACGA
TCGGTAGAAAATTAAGTTTTTGTATGAAAAAACATTTTGTTTATCAAGATATCTTGACCAAACTCGGTATT
TATTAGTTTTACTATGCTCCTCATATATATGCAAAATCCTNTTAAGATCGGACCACTATATCATATAGCTGC
CATAGGAACGATCGGTAGAAAATTAAGTTTTTGTATGAAAAACATTTTGTTTATCAAGATATCTTGACCAA
ACTCGNCGTTTATTAGTTTTACTATGCTCCTCATATATATGCAAGATCCTATTAAGATCGGACCACTANAT
CATATAGCTGCCGTAGGAACGATCGGTAGAAAATTAAGTTTTTGTATGANAAAACATTTTGTTTATCAAGA
TATCTTGACCAAACTCGGTATTTATTAGTTTTACTATGCTCCTCATATATATGCAAGATCCTATTAAGATCG
GACCACTATATCATATAGCTGCCGTAGGAACAATCGGTGGAAAATTAAGGTNTTGTATGGAAAACNTTTT
GTTCATTCAAGATATCTTGACCAAACACGGCATTTANNGGTTTCACTATGCTCCCCNCATNTNTGCAAAAT
CCTATTNAGATCGGACTACTATATCATATANCTGCCATAGGAACGATCGGTAGAAAATTAANTTTTTGTAT

GAAAAACATTTTGTTTATCAAGATATCTTGACAAAACTCGGTATTTATTAGTTTTACTATGCTCCTCATATA
TATGCAAGATCCTATTAAGATCGGACCACTATATCATATAGCTGCCATAGGAACGATCGGTAGAAAATTA
AGTTTTTGTATGAAAAACATTTTGTTTATCAAGATATCTTGACCAAACTCGGCATTTATTAGTTTTACTATG
CTCCTCATATATATGCAAGATCCTATTAAGATCGGACCACTATATCATATAGCTGCCATAGGAACGATCGG
TAGAAAATTAAGTTTTTGTATGAAAAACATTTTGTTTATCAAGATATCTTGACCAAACTCGGTATTTNTTA
GTTTTACTATGCTCCTCATATATATGCAAGATCCTATTAAGATCGGACCACTATATCANATAGCTGCCATA
GGAACGATCGGTAGAAAATTAAGTTTTTGTATGACAAAACATTTTGTTTATCAANATATCTTGACCAAACT
CGNCGTTTANTAGTTTTACTNTGCTCCTCATATACATGCAAAATCCTATTAAGATCGGACCACTATATCAT
ATAGCTGCCATAGGAACGATCGGTAGAAAATTAAGTTTTTGTATGAAAAACATTTTGTTTATCAAGATATC
TTGACCAAACTCGGTATTTATTAGTTTTACTATACTCCTCATATATATGCAAGATCCTATTAAGATCGGACC
ACTATATCATATAGCTGCCATAGGAACGATCGGTAGAAAATTAAGTTTTTGTATGACAAACCATTTTGTTT
ATCAAGATATCTTGACCAAACNCGGCGTTTAATAGTTTCACTATGCTCCTCATATATATGCAAAATCCTAT
TAAGATCGGACCACTATATCATATAGCTGCCATAGGAACGATCGGTAGAAAATTAAGTTCTTGTATGAAA
AACATTTTGTTTATCAAGATATCTTGACCAAACTCGGTATTTATTAGTTTTACTATGCTCCTCATATATATG
CAAGATCCTATTAAGATCGGACTACTATATCATATAGCTGCCATAGGAACGATCGGTAGAAAATTAAGTT
TTTGTATGACAAAACATTTTGTTTATCAAGATATCTTGACCAAACTCGGCGTTTAATAGTTTCGCTATGCTC
CTCATATATATGCAAAATCCTATTGAGATCGGACTACTATATCATATAGCTGCCATAAGAACGANCGGTA
GAAAATTAAGTTCTTGTATGACAAAACATTTTGTTTANCAAGATATNTTGACCAAACTCGNCGTTTANTAG
TTTTACTATACTCCTCATATATATGCAAGATTCTATTANGATNGGACCACTATATCATATAGCTGCCATAG
GAACGATCGGTCGAAAATTAAGTTTTTGGAAGACAAAACNTTTTGTCNNTCAAGATATCTTGACCAAACN
CGGCATTTAANGGTTTCACTATGCTCCCCNCATNTNTGCAAAATCCTATTNANATCGGACTACTATATCAT
ATANCTGCCATAA
>Ref_DROGYPSYZ
AGTTAACAACTAAGCATAAATATATTGCCCTAGCAATTAAGTAGCTTCGTATTAACAATGCTGACGCGCC
AGAACTGAGTTCAGCGTCTGCGCTAAGAACGGCCAGCAGCGGTAAGCTGACACTGCATGGAATGCTGAGT
CGGCTTGCCGACCATGCTTTGATGGCGTGATGCATTGAGGCTTAGGGTATTTGTATAATTTGCTTGTCCCCT
AGTTTTTAGTTTTATATGAAGCGCGCAACACATAAGTCGCCGAAATAAATATACTGCTCCGGCATTGCCGA
TAAAACATATCTTTCGTAATATTACTGGATCAAGGAGAACATATTTTGATCAAGGGCAGCGCGGGATCAA
CGCAACTTACCCAAGATAACATTGTGTTACATCGCAGAACATACATTAATGAATAACTGGCGCCCAACCA
ATAAATAGGAACCCATACCATGTAAGTTCAATAAGTGATTTCAGTTTGAAGAAATTATTCACGAACGCCA
CGTACCGATACGTGTGATTTTTAAAAAAAAAAAAAAAAAAAAAAAAATTGTAAAGTAAAGTAAATATTGCTG
CTCTCTTCTGCCAGCATTTCATTCCTTTAAATCATTGCATACTTTCACTAAAAACTGTTGCATACTTTTACC
AGCGTTCCTTGTCAATAAAGCATTGCATACCTTAAACGAATAATTATTGCATACTTTCGGCAGCATTACAT
TTTTTGCTGCATACCCCTAACAATTTTTTATAATAAATATTGCATACCCCAAGATTCACCAGTTTCCACTAT
TGCCAACAGCGTTACCTGCCGATCTTTCTCGATCGGCCTAAGTTTGAAAAGTGCCAACCATAAGCCAATGA
GTTGGAATTATAGGGAGATAAAAGTCGAATATAATAGTGACGATAGCTGGGAGGGGGAAATCAATTGCT
CAGTAGATCCTACTCCTGCAGATACCGTTGTAACTGAAGAAATGGAGCCGGCTCAAGTTCAGAATCTCAT
TGAGGGTGCCATCAGGCAAGCTTTAGCGCAACAACAAAATCAGTTTCAAGCACAAATAACCGCTTTAGCT
TCACAAGTACAGAGTTTGCAGGTGGAGGCGCCGCAAATCGTCACATACGAGAAAATATCCGTCGATCCAA
ACGCCAAATGCGATATTCCCTTGGACATTATTAAATCTGTGCCGGATTTCTCCGGCGTACAGGATGAGTAT
GTAGCGTGGAGGCAAGCAGCCATCTACGCGTACGAACTATTCAAGAACTATCCAAAGAGCACTGCACACT
ACCAAGCGGTACAGTTCATTAGGAACAAAATCAAAGGCCCAGCTGGAGCCTTACTAGTGTCTCACAACAC
TGTCCTAAATTTCGATGCAATACTAGCTAGGCTAGATTGCACATACTCGGACAAAACATCCTTGCGCTTGC
TGAGACAATGTTTGGAGTTAGTTACGCAGGGCGAAATGTGCCTGATGCAATACTACGATGACGTCGAAAA
GAAACTCACGCTCGTAACTAACAAAATTGTAATGTCTCACGACCAAGAAGGCGCGGATTTGCTCAACCGG
GAGGTCAGAGACGACGCCTTGCACGCCTTTATCTCCGGTCTTAGGAAGCCGTTGAGAGCGGTTGTGTTCCC
TGCACAACCAAAAGATCTACCATCCGCTCTGGCCCTTGCCAGAGAGGCAGAAGCTAGCATTGAGAGAAGC
ATGTTCGCGAACTCTTACGCTAAAGCCTTGCAAGACCGAGCGCAAATTGCTGAAAGTAACAAAAACCGTG
CCCAAGGCAAGCAAACCAGATTTAACAAAGATGAGCAAAATCAGGAGAGAAATCCCCATTTTGTGAAAC
GCCAAAAGGGCGGTCCCCAGCCCCAGGCCCCTAAAGATAATCAAACTGAGGCTCCACAGCCCATGGAAG
TTGACTCTTCCTCCAGATTTAGACAAAAACCGACTACAGTAAGTCGTCAGGCTAATGAGTCCAACGCACC
AAAACGCAAGAATTCGTCAGACCGTTCAACAGGTCAAAGACGACAACGCGTTAACAATGTCGTACAATCA
GATTCCAAAGAAGCCGTAACAGAATTCGAGAAGGCGGCCAAAGAGGCAGTTGAAGAACTAGAGAGTGAG
AACGAGTACGCACCTGGTGACGACTCGATTAATTTTTTAGGGAACACTCCCGGCTTCCGTACATTGAACGA

CGGTTGGCTGGGAGAACTCTGAAGATGTTGATTGACACTGGCGCGGCAAAAAATTATGTGAAGCCCTTAA
CAGAGCTAAAGACCATAACGCCGGTCGCCAAGTCATTCTCGGTAAACTCGATCCACGGGTCTACCGAAAT
CAAGCGCAAATGCTTGATGAACATTTTCCAACATACCTCCCCGTTTTTTCTCCTCGATACTCTAAGTTCTTT
CGACGCCATCATAGGCTTCGACTTGCTAACACAAGCCGGAGTCAAATTGAACTTGGCAAAAAACACTCTG
GAATACCAGAGTACATCCGAAAAACTCCAGTACTATCAATGTGCTGATGTGAACTTCACCGATGTCAACG
ACATCGTAGTCCCCCCTTCCGTAAAGAAAGAGTTCAGGGACATGATACTCAGAAACAAAAGAGCCTTCTC
AAATCGAGACGAAGCTCTCCCTTTTAATACTGCTGTTATCGCTACAATCCGAACAGTAGACAACGAGCCT
GTGTATTCAAGATACCCGAGTCTCATGGGTGACTCTGACTTCATAAACAAAGACGTCAAACAGTTACTGG
ACGATGGCATAATTAGGCCCTCCAGATCGCCTTACAACAGCCCAACATGGGTGGTTGATAAAAAAGGCAC
TGACTCATACGGTAACCCAAAAAAAAAGGTTGGTAATCGACTTCAGGAAACTGAACGAAAAAACCATACC
GGACCGCTACCCCATGCCGAGGATCCCCATGATCTTAGCAAATCTAGGAAAGGCAAAATACTTCACTACC
CTCGACCTAAAGTCGGGGTACCATCAAATTTACCTTGCGGAGCATGACCGCGAAAAGACTTCATTTTCAGT
AAGCAGCGGCAAGTACGAGTTCTGTCGCCTACCATTCGGCCTGCGCAATGCAAGCAGCATATTCCAAAGG
GCAATAGACGATATCCTGAGGGAACATATAGGAAAGATTTGCTTTGTCTATGTAGACGACGTCATAATTTT
TTCTAAAAACGAAACGGAGCACCTTCAACACATTAACATTGTGTTAAAATGTCTAATCGACGCAAACATG
CGTGTAGGCCCCGAAAAGACTCGTTTCTTCAAGGAAAGTATCGAATTCCTGGGTTTCATTGTAACCAAAG
ACGGAGCAACATCCGACCCCAAAAAAGTTAAAGCCATTCAGGAATTCCCTGAACCAAAGAATGTCTACAG
CGTTAGGTCCTTCTTGGGGTTAGCCAACTACTATAGGGTCTTTATTAAGGACTTTGCCGCTATAGCGCGTC
CGATATCGGACATACTTAAAGGGGAAAATGGTTCAGTTAGTAGGCACGTATCAAAGAAAATCCCTGTTGA
ATTTAATGAAGTTCAACGCGATGCATTTCACCGTCTGCGGAGTATTTTGGCATCAGAAGACGTCATGTTAA
TGTATCCAAACTTCCAGAAACCGTTCGATTTAACCACTGATGCTTCGGCCAGTGGAATTGGCGCGGTTCTT
TCCCAAGGCAATAGGCCAATAACAATGATTTCTCGCGCCTTAAAACAGGCCGAACAAAACTATGCCACTA
ACGAAAGAGAATTGCTGGCCATTGTGTGGGCTTTAGGCAGATTGCAGAACTTTCTGTACGGTTCTAGGGA
AATTAACATTTTCACGGACCACCAGCCGCTCACGTTCGCTGTGTCGGACAAGAACACGAATTCCAAGATT
AAAAGATGGAAATCTTACATTGACCAGCACAATGCCAAAATGTTTTATAAACCGGGCAAAGAGAATCTGG
TGGCGGATGCCCTTTCAAGGCAAAACATTAATGCTCTCGAAGATGGGCCCCGTTCGGACGCTGCAACCGT
TCACAGCGAACTCTCCCTGACCTACACGGTCGAGACAACAGACAAACCTTTGAATTGCTTTAGGAACCAG
ATTGTCCTAGAGGAATCGCGTTTTCCGCTAAAGCGAAACTTGTTGCTGTTTCGCAGCAAAACACGCCACTT
GATCAGTTACACTGACAAGAGCTCAATTCTCAAAACCCTCAAGGAAGTTGTGAATTCTGGAGTCGTTAAT
GCAATACATTGCGATCTTCCCACGTTGGCAAGTTTCCAACACGACCTGATTTTGTATTTCCCTGCTACCCA
ATTCTGGCATTGCAAAAATCTTGTGCAAGATATAACGGACAAAAACGAGCAGTTAGAAATCATCACTGCC
GAGCACAATCGAGCGCACAGAGCAGCTCAGGAAAACATTAAACAAGTACTTCGGGACTATTATTTTCCCA
ATATGGCCAGTCTGGCCAAAGAAGTGGTTGCAAACTCGAGGGTCTGCACTAAAGCCAAATACGATAGGCA
CCCGAAGAAACAAGAACTCGGGGAAACGCCTATACCAAGTTATACTGGCGAAATGTTGCATATTGACATT
TTCTCGACAGACAAGAAACAGTTCTTGACCTCGATAGACAAATTCTCAAAATTTGCAGTCGTCCAACCGAT
ATTATCCAGAACGATAATCGATGTCACAAGCCCTCTACTGCAAATTGTTAACCTTTTTCCCGCAACCAAGA
CAATTTATTGCGATAACGAGGCGGCCTTCAATTCCGAGACCATCACTTCGATGCTCCGAAACGTCTACGGC
ATTTGCATTGTGAATGCGGCTCCGCTTCACAGCACCTCAAACGGCCAGGTGGAGCGCTTTCATAGCACCCT
GACCGAAATTGCGAGATGCCTCAAGTTAGACAAGAAAATCAGCGACACTACAGAATTAATCTTAAAGGCT
ACGATAGAGTACAACAAAGTTTTGCACTCAGTAACCCTCGAGAAACCAATTGAGGTCGTTCATGCAGCAG
CTTCTGATCGTCGCGGGAACGTCAAAAACAGGCTAATTAAGGCACAGCAAGACAATATCAAAAGGTGCA
ATGCATCCAGACAAAACCGCGTTTTCGAGGTAGGAGAAAAGGTGTTCCAGAAGAACAACAAGAGGCTAG
GGAACAAGTTAACTCCCTTATGCTCAGAACAAAAGGTCGAAGCAGACCTGGGAACGTCTGTCCTCATTAA
GGGGAGGGTGGTCCATAAGGACAACCTGAAATAAGTTTTCCTCCACAATTAGCTAGTAAGCCAACTCAGA
GTTTAAACACCGTTTTATATGGTTCGTCCATTTCAAATTTGCACTCACTTTGCTTGTGGCACTAGCAGCGGT
AAATGCCTCGGATCACCGACTTCTCGCATGCCAACTACATTCCGGTCTTTGACGGGGAAGTACTGGTGTTC
GATCAACGTAGCTACTTGAGGCACTCAAGTAACATTTCCGAATTTGTTAGTATGATAGACGAGACGGAAA
GACTGTCCGATTCCTTTCCACAGTCGCATATGCGCAAACTACTAGACGTAGATACAGATCACCTTAGAACA
TTGTTGTCCGTTCTTCAAGTGCATCACAGATTTGCACGAAGCCTCGATTTCTTAGGCACTGCCTTGAAGGTT
GTTGCTGGCACCCCTGATCCTTCAGATTTCTTAAGGATCCGCGTTACAGAGACGCAGTTGGTGGAGGCTAA
CTCCAAACAGATACTAATAAACTCCGAAACTCAAAAACAAATAAACAGACTAACGGACACTATCAATAA
AATTATCAGTTCCCGCAAAGGCGATTTGGTTGACACTCCTCATTTATTCGAGACCCTGTTGGCAAGAAATA
GAATACTGAACTCAGAAATTCAAAATTTAGTACTCACCATAACGTTGGCAAAAGCAAGCATAGTAAATCC
TACAATCCTTGATCATACCGATCTAAAATCACTTATCGAACAAGATACCCCAATAGTTAGCTTGATAGAAG

CTTCTAAGATCAAGGTCCTTCAGTCCGAGAATATTATCCACATATTAATAGCCTACCCTAAAGTTGAGTTT
AGGTGCCAAAAGGTCTCCGTCTATTTTGTTTCACACCAACAAACCATTCTGCGACTCGACGAAGACACGTT
GGCGGAATGCGAAAAGGATACCTTTGCAGTTACTGGATGCACCGTAACTACGCATAACACCTTCTGCGAA
CGAGCACGGCGCGAAACCTGTGCTGACTCCCTTCACGCGGGAAACATCGCCAACTGCCACACTCAACCTA
GCCACCTGAAAGCGATAATGCCTGTAGATGACGGCGTCGTGGTTATCAACGAAGCGACAGCGCACGTCCG
AACAGATGATGACGCAGAGGTAACCGTCTCAGGAACCTTCCTGATAACTTTCGAACGGTCTGCAACTATC
AATGGCACAGAATTTGTAAACCTGCGCAAATCACTAAGCAAGCAGCCTGGCATCGTGAGGTCACCGCTCC
TAAACATCATCGGACATGACCCGGCACTAAGCATACCACTCCTGCATCGTATGAACATCAACAACTTGCT
ATCAATTCGAGATTTCAGGGAGGAGGTAGTTGCGGCTGGCTCGCCTAAATTCTGGTTCGCGATTGGCGCG
GTCCTGAATGTCGGACTAATTGGCTCATTCTTCCTTTTTCTGGTGCTAAGGAAAAGACGAGCCTCCATGCA
GATGCAGAAGGCGATTGACAAATACGACATGCCCGAGGACGGCCATCATCTGAGGGGGGGAGTAGTTAA
CAACTAAGCATAAATATATTGCCCTAGCAATTAAGTAGCTTCGTATTAACAATGCTGACGCGCCAGAACT
GAGTTCAGCGTCTGCGCTAAGAACGGCCAGCAGCGGTAAGCTGACACTGCATGGAATGCTGAGTCGGCTT
GCCGACCATGCTTTGATGGCGTGATGCATTGAGGCTTAGGGTATTTGTATAATTTGCTTGTCCCCTAGTTTT
TAGTTTTATATGAAGCGCGCAACACATAAGTCGCCGAAATAAATATACTGCTCCGGCATTGCCGATAAAA
CATATCTTTCGTAATATTACTGGATCAAGGAGAACATATTTTGATCAAGGGCAGCGCGGGATCAACGCAA
CTTACCCAAGATAACATTGTGTTACATCGCAGAACATACATTAATGAATAACT
>9_rnd-1_family-348__LTR___Gypsy
TGTAGTGCGTATATGCCCTTACAGAATACCCCCGTACTTAAACATAAAGTATGCAGCACTTTGTTGCATAC
AATAACAATAATATAGTAAACGAGCCCAGAGCCACGTTATGTATTGGCTCGGTCTCATATTACGCAGCGC
GGTTCACTAGCCTCACTAGCTGACCGTTTATGCTTATGATGCATATCCCACGCTCCGTCTGAGCGCATTCC
GTGAGCAATATATATGCTCACTAATACAGGCATATGCGAGCTACTCACTCTCCCTCTCTTTGTAGCTGTTA
ACGAATAACAGCATAAGTATATATGTATATCCATAGGCTAAGCGAACATTATAACAAGTGAATAAATTCA
TTCTGAACAAAGATACAAAACAGCACTGACGTGCTGACTTATTTTTCATTGACAATAAGTGCTCATTTTAC
ATGGCGACCGTGACTTGGTCACGCATCCCTGCCTGGAGGAAGTAGACCAAGTGAAAAGGAACTTCATGGC
CAACAAAGAGGCCATGTATAATATACAATTTGAAAAAGGAACTTCATGATCAACGAGAGGCATCCCGTAT
TCCGAGTGAGTAGAGAGTGAGTGTTATGGTTCATAAACGCGGTGGCTTAAAAAATAAAATTAATAAAAGA
ATAGCGCACCTAAGGTGGCTGCTATACACAAAACAGCCAACACCCGCATGGTCGACAGCTCAGGAAGTTA
CAAAATGAGCTCGACTATCTGCAAAATGAAATTAAGCAGAGAAAAACCAAACACCACTACTCACTCAAAT
ATAAAAGTTCTAATAAACCAAACAACTAATAAAGTTACACCCATTGACGCAAACCAGCCTACCAAAAGAA
AAAACGAATGTGCCCACAGGATTGCGAAGGCCCACTCGACAGCCCCTTCTGCGAATATACGTGCAGACAC
TGCACAGCCAGCACCTCCGGACAGCCCACACTGCAGACCGACGAATAAACACCTGTGCGAAGACCGGCA
CCGGTNCCGCCTGAGCCCATAGGCCCAGGACAACAGCAGCTGTGAAATCGGAACAAGCGCGAGAGGCAC
NCCGCCACTTGCGCCTACGAGGGAGCGTCAGCGTAGCCACATCCGGCGACGNCCAGCTGACGCAACGAC
GAACAACAGNCGCAGCTATATCCTTAACTACTTTTTAATTAAATATAAAAAAATTTTTGTAAAATTGAGCG
GAAACACTTCCGCCCGATTAGCAGCATAGCCCGTAAAGCTATGCGAAATGCTAGGTAGTATTTTGTGTTAT
AAGAAATATTGAAACCTTGATTCTAAAAACCCAAGTCAACCAGCACTAACGTGCTTTTGTGAAATCATTAT
TAAATCCAAACATTACCAACATATTAAATTAAATAGTAATAAATAAAACTTAAAAATTGTAATGCCCGAC
GACAATTGAGTCGACCGGCAAGACGTTTGGCCAGTACAGCCATTCGAAACAATAGGTAGGAAAAATACA
GATTGTAAAAAAAATTTTTTAGATGCGTTTTAAGCAGAAAACAAACAAGCAAAACAAAACCCGACTTGTA
AAACAATAAAAGCATCAAATTCAATAAATATAGTAATAAAACCTCAAATGAACGATACAAACAACAACT
TGTCTTGTAAAGTAAACAAAAACAAATTTAACATTACGCATTTGTAAATTTTTTTTAAGATAATTCCTAA
AACCTCAAATGAACGATACAAACAACAAATATATGAATATTAATATCAAAAAGAGGAAATTAAAAAGTT
ATAATTCGTTCAGTTTGTCACATTAGGTAATTCAAAATTATGATCAAGAATTAGCATGATTAAGGACAAGA
TTAACAAGGTATATAACGACTATACAGACTTTCGTATGCGAGCCATACAAATTTTGCAAAGGCATAGAGA
ATTCATACCAGTATATGAGTTCGAATACGAATATATAGAAACATTCGGCACGGTGACAAGTCCACTCTATT
CATGTTCTAAAATAGAGAGCGGAATAATCTTGTCCACACTACCCTACGTCAAAATAAAAACAATTATTGA
CGAGCATCCGATTCTAGGGCAGACAACAATTGTTTTAGTACGTCTGCATAGGTAGTAGTCAAATCAAAAG
CAAGAATATTTATATAGGAATATGGGGGAAAAGAATCAAAGGCAGATGATAACACTGCCAATGTAGTT
AACGTAGTAGAAGTCGTAGACACCACTCCCCATGTGCAGTTAACAAACATACTGCTAATAATACTCATTG
TAATAGCTTCAGTAGACATCCTTCTGAAGATTTATCTTCTTCATAAAAANTCACTGAGGAAGCGTTACATG
AGTCGAGCCAACGACTTAGACAAGGTCTAAGCCACTGCTTAAATAAAATATTTAAGTAGCGGAAGCTTAC
ATGGACTGGTCCCAAATAGTAGAAAAGATAAAGCGGCATAGGGAAAATTTCGATAAATCATACAAGTGC
CTTAATAAAAGCGCACCAGTTTTAAGACCCACCATAATTAAACATACTGAGATCCTTGTATATGAGTACA

ATGAAATCAGGAGTCTAGTTACCGAGTGTCAAAATAAACTGACAAGTTTACACAGAGTCCAAACTTCAGA
CGTTTTACAGCGTCTAAGGGATAATCTGGTAAACTTAAATAAAAAGTATGATCTCCAGCTTTATCTTCCAT
CGGTGTTAAATACACCGGTAACATTTGATAAGTCACTACTGACAAACGACCAAATAGAAGATACAAAATT
AGTAAACATGGCACAAACAGTTGTAGAATTTATCAAGCTAGCCACATCTCTGATACCAGAGTTTGACGGT
AAGCCGGAAAATCTACAAAGTTTTCTAGATTCTTTGAATCTATTAGAAACCTTAAAAGGCACACATGAGC
TAACGGCCATAAGCCTTATAAAAACAAAGCTAAAAGGCCATGCGAGAAATTTGATAAGTAATGAGCAGA
CAATTGCTGCAATCATTACGCAACTATCAAATGCAGTAAAAGGAGAATCGGTAGAAGTTGTATCTGCTAA
GCTTCTTAATTTGCAACAGAAAAATAAAACGGCTAACCAATACACCCAAGAGGTGGAAAAACTGACAAA
GGCCCTTGAAGGCGCCTATATCAGTGAAGGTCTAAGCCAATCACTTGCAAACAAATACAGCACTACAACA
GCTGTAAAAGCTATGACCCAAAATTGTTCGATTGATAAGGTAAAACTTATCATGCAGGCAGGCACATTCA
CAAATATGAATGATGCCGTATCCAAATTTGTCAATAGCTGCACGGAGATAACAGGTCAAAGTAACACTGT
CCTGTATTATCAACGAGGTGCAAACAATAATAATAGAGGAGGCCGAGGTTATAATCGTGGCAGAAATTAC
AACCACAACAATTATAACCGAGGCGGTAACAACAATAACAATAATTACAATAACCGTGGAGGAAGACGA
GGCCAAAATAGAGGAAGAGGCCGAGGTAACTCCAACCAAGGTTATAATAGTAACAATGTGAGAATGGCG
CAAAATACGCCGGAAAACTCTCAGAACCCTTTAGGAAACAACCAGTAAATGTAAAGGTTCATTCCATCAA
TTACAGTCTTAATATATTTGTCACTTTTTATAATCATTCAACTGAAAATAAACTCACATTCCTAATAGATAC
TGGTGCAGATATCTCACTTTTAAAAATAAATTCAGACAATTTCAAAATTCAAGAAGAAAAAATTATAAAC
ATCCAAGGCATAGGCCAAGGTGTAATAAAATCTCAAGGAACAACGTCAATAGAACTTCAATACACAAAA
TATATTATTCCACACGAATTTCATTTAGTAGATTCTGATTTTTCAATACCGTGTGATGGAATAATAGGCATT
GATTTTATTAAAAAATTCAATTGTCAATTAGATTTCAAACCAAGTGAAGACTGGTTTATAATTAGACCTCA
AAATTTAAATTATCCAATTCATGTTCCGATAACATATAGTGCTGGCAACAACACAGTTCTCTTGCCAGCCA
GATCCCAAGTAATTCGAAAAATAGACATTAATACAGTAAATGATTATATATTTGTTCCTAATCAAGAAAT
ACATAATGGAATTTATGTTGCAAATACAATAGCAGCATCCAAACATGTATACATTCGCCTTCTAAACACA
ACTAATTTCGACCAATTGGTCAAAGTAAACAAAATCCAATATGAAAATTTAAAAGATTACGACATTCATA
GCACTACTCCAGAAAATAGAAGCGAACAAGTACTTTCAAAATTAAAGAAAAATTTTCCAGAACAATTCAA
AAGTCAACTAACAGAATTATGCACCAAGTATAGTGATGTGTTCGGACTGGAAACCGAACCAATAGCGACA
AATAATTTCTATAAACAAACATTAAGACTTAAAGATGATGAACCCATTTATATTAAAAACTACAGAAGCC
CGCATAGCCACGTTAAAGAAATTCAAAACCAAGTAGGGAAATTAATAAACGACAAAATCGTAGAACCGT
CTGTATCCGAGTATAACAGCCCACTCTTGTTAGTTCCGAAAAATCATTACCAAATTCGGAAGAAAAGAAA
TGGCGATTAGTAATTGACTATCGCCAAATCAACAAAAACTACTTTCTGATAAATTCCCGCTCCCCAGAATT
GATGATATTCTAGACCAACTGGGCAGAGCTAAATACTTTTCATGCCTTGACTTAATGTCAGGTTTCCACCA
AATTGAACTTGAAGAAAATTCAAGAAATATAACATCTTTTTCAACGAGCAATGGCTCATATCGCTTCACGC
GATTACCATTTGGTCTTAAAATAGCACCAAATTCATTTCAGAGAATGATGACTATAGCATTCTCGGGTTTA
GAACCCTCCCAGGCATTCCTTTATATGGATGACTTAATGGTGATAGGATGTTCCGAAAAACACATGATTAA
AAACTTAACTGATGTTTTTAATGTATGTAGGAAATATAACCTGAAGTTGCATCCAGAAAAATGTTCATTTT
TCATGCATGAAGTGACATTTTTAGGTCACAAATGCACTGACAAAGGAGTATTGCCAGATGACAAGAAGTT
TGACGTCATCAAAAATTATCCTGTCCCTCATGATGCGGACAGCGCGAGACGATTTGTAGCATTCTGCAATT
ATTATCGTCGTTTTATAAAAAACTTCGCCGATTATTCACGGCACATAACTAGATTATGTAAAAAGAATGTT
CCTTTTGAATGGTCAAGTGAATGCCAAAACGCATTCCAGTACCTTAAAGAAAAGCTTATGCACCCCACATT
ATTACAATATCCTGATTTTCGCAAAGAATTTTGCATTATAACAGATGCCAGTAAACAAGCTTGCGGAGCA
GTTCTAACTCAGAACCGTGACGGAATTCAGCTCCCAATAGCTTATGCATCTCGTTCATTTACAAAGGGGAA
AGCAATAAGAGCACAACAGAACAAGAGTTAGCGGCAATTCATTGGGCAATTACCCATTTTAGACCATATA
TTTATGGCAAACATTTTACTATTAAAACGGATCACAGACCATTGACATATCTTTTTTCTATGACTAATCCCA
GTTCTAAATTAACTCGCATGCGGCTAGAGCTTGAAGAATACGACTTCACAGTAGAATACCTAAAGGGGAA
AGATAATTTCGTAGCAGACGCACTTTCGCGTATTAATATAAAGGAACTCAAAGATATGCAACATAAAGTC
CTGAAAGTCACTACCAGGCGACAAAGTAGACAAAATAAAAACTGTACAGTAGAAAATTATGAACTTCTGC
CAAGGCAAAGTCATAAAAATGTATCCAAGCCCAACGTACATGAAGTCATTACAAATGATGAAGTACGAA
AAGTAGTGACCTTGCGCATAACAGAATCTAAATGTTTATTTAAACATGGAAATAAAATTATCGCAAGAAT
TGAAGTTAGCGATTTATATACCAATGGAATTCTCGACCTAGGTCAGTTCTTCCAAAGGCTCGAAAAAGAG
GCCGGTATATTAAAAATCAGCCAACTCAAGGTGGCACCGAGCGAAAAGATCTTTGAATCAGTTTCAATAG
ATTCTTTTAAAAATATGGGCAATAAAATATTAAAATCTTTAAGAGTAGCGCTACTCCAGCCGGTGACCCA
AATAAAAGATGAAAAGGAGATACAACAAATATTGTCTACATACCATGACGACCCAATTCAAGGAGGTCA
TAGCGGCATAACAAGAACGTTAGCGAAAATAAAAAGACACTACTACTGGAAAAATATGACACGTCATAT
AAAAAGAGTACGTACGTAGATGTCAAAAATGCCAAATGTCTAAAAAAAAGACGCACACAAAAACTCCTC

TGACTCGCACAGAAACACCCGTGAATGCTTTCGATATAGTGATAGTGGATACAGTTGGTCCGCTACCCAA
ATCGGAAAAATGGTAATGAATACATAGTCACATTAATATGCGACCTTACAAAAATATTTAGTAGCCATTC
CAATAAAAAGCAAAAAGCGCAAATAATGTCGCAAAAGCTATATTCGAAAAATTTCATACTCAAGTTTGGT
CCAATGAAGACGTTCATCTCGGACATGGGAACAGAGTATAAAAACCAGATTTTTAACTGACTTATGCAAG
TATTTGAAGATCAAGAATCTCACCTCTACTGCATATCATCATCAAACATTAGGGACAATAGAACGAAGTC
ATCGAACTTTTAATGAATACATTCGTTCATACATCTCTGCAGACAAATCTGATTGGGATGTCTGGGTTCAA
TATTTCACTTATTGCTTCAACACGACACCATCAGCAACGCATGAGTATTGTCCATACGAACTAGTATTTGG
CAGACTACCGAAACAGTTCATAGATTTAAACAAAACTAATTACATAGATCCTATTTATAATTTAGATGACT
ATTCTAAGGAAATAAAGCTTTAGATTAGAAATAGCATACAAAAAGAGCTAGAATTATGTTAGAAAAAGCT
AAGGCTTATAGGAAATTAGCATATGACGAAAAAANAAAAAACTTTGAATTAAAAGTAGGAGATAAAGTA
CTTATAAGAAACGAAACAGGGCACAAATTAGACCCAGTATATTTAGGACCATATATAGTAGAGAAAATA
GAAAATAATGACAACATTTTTTTAAAGATAGTAAAGATAAAAA
>160_rnd-1_family-108__LTR___Gypsy
ATATAAGTTAACAACTAAGCATAAATATATTGCCCTAGCAACTAAGTAGCTTCGTATTAACAATGCTGAC
GCGCCAGAACTGAGTTCAGCGCTCTGCGCTAAGAACGGCCAGCAGCGGTAAGCTGACACTGCATGGAATG
CTGAGTCGGCTTGCCGACCATGCTTTGATGGCGTGATGCATTGAGGCTTAGGGTATTTGTATAATTTGCTT
GTCCCCTAGTTTTTAGTTTTATATGAGGCGCGCAACACATAAGTCGCCGAAATAAATATACTGCTCCGGCA
TTGCCGATAAAACATATCTTTCGTAATATTGTCTGAGNCCAAGGCCAGCAATGGCCCCAGAACATATCTTC
GCGGATCAAGGGCAGCGCGGGATCAACGCAACTTACCCAAGATAACATTGTGTTACATCGCAGAACATAC
ATTAATTGATAACTGGCGCCCAACCAATAAATAGGAACCCATACCATGTAAGTTCATAAGTGATTTCAGTT
TGAAGAAATTATTCACGAACGCCACGTACCGATACGTGTGATTTTAAAAAAAAAAAAAAAAAAAAAAAA
AAAAAAAAAAATTGTAAAGTAAAGTAAATATTGCTGCTCTCTTCTGCCAGCATTTCATTCCTTTAAATCAT
TGCATACTTTCACTAAAAACTGTTGCATACTTTTACCAGCGTTCCTTGTCAATAAAGCATTGCATACCTTAA
ACGAATAATTATTGCATACTTTCGGCAGCATTATATTTTTTGCTGCATACCCCTAACAATTTTTTATAATAA
ATATTGCATACCCCAAGATTCACCAGTTTCCACTATTGCCAACAGCGTTACCTGCCGATCTTTCTCGATCG
GCCTAAGTTTGAAAAGTGCCAACCATAAGCCAATGAGTTGGAATTATAGGGAGATAAAAGTCGAATATAA
TAGTGACGATAGCTGGGAGGGGGAAATCAATTGCTCAGTAGATCCTACTCCTGCAGATACCGTTGTAACT
GAAGAAATGGAGCCGGCTCAAGTTCAGAATCTCATTGAGGGTGCCATCAGGCAAGCTTTAGCGCAACAAC
AAAATCAGTTTCAAGCACAAATAACCGCTTTAGCTTCACAAGTACAGAGTTTGCAGGTGGAGGCGCCGCA
AATCGTCACATACGAGAAAATATCCGTCGATCCTAACGCCAAATGCGATATTCCCTTGGACATTATTAAAT
CTGTGCCGGATTTCTCCGGCGTACAGGATGAGTATGTAGCGTGGAGGCAAGCAGCCATCTACGCGTACGA
ACTATTCAAGAACTATCCAAAGAGCACTGCACACTACCAAGCGGTAGCGATCATTAGGAACAAAATCAAA
GGCCCAGCTGGAGCCTTACTAGTGTCTCACAACACTGTCCTAAATTTCGATGCAATACTAGCTAGGCTAGA
TTGCACATACTCGGACAAAACATCCTTGCGCTTGCTGAGACAATGTTTGGAGTTAGTTACGCAGGGCGAA
ATGTCCCTGATGCAATACTACGATGACGTCGAAAAGAAACTCACGCTCGTAACTAACAAAATTGTAATGT
CTCACGACCAAGAAGGCGCGGATTTGCTCAACCGGGAGGTCAGAGACGACGCCTTGCACGCCTTTATCTC
CGGTCTTAGGAAGCCGTTGAGAGCGGTTGTGTTCCCTGCACAACCAAAAGATCTACCATCCGCTCTGGCCC
TTGCCAGAGAGGCAGAAGCTAGCATTGAGAGAAGCATGTTCGCGAACTCTTACGCTAAAGCCTTGCAAGA
CCGAGCGCAAATTGCTGAAAGTAACAAAAACCGTGCCCAAGGCAAACAAACCAGATTTAACAAAGATGA
GCAAAATCAGGAGAGAAATCCCCATTTTGTGAAACGCCAAAAGGGCGGTCCCCAGCCCCAGGCCCCTAA
AGATAATCAAACTGAGGCTCCACAGCCCATGGAAGTTGACTCTTCCTCCAGATTTAGACAAAAAACCGAC
TACAGCAAGCGTCAGGCTAATGAGTCCAACGCACCAAAACGCAAGAATTCGTCAGACCGTTCAACAGGTC
AAAGACGACAACGCGTTAACAATGTCGTACAATCAGATTCCAAAGAAGCCGTAACAGAATTCGAGAAGG
CGGCCAAAGAGGCAGTTGAAGAACTAGAGAGTGAGAACGAGTACGCACCTGGTGACGACTCGATTAATT
TTTTAGGGAACACTCCCGGCTTCCGTACATTGAACGACGGTTGGCTGGGAGAACTCTGAAGATGTTGATTG
ACACTGGCGCGGCAAAAAATTATGTGAAGCCCTTAACAGAGCTAAAGACCATAACGCCGGTCGCCAAGTC
ATTCTCGGTAAACTCGATCCACGGGTCTACCGAAATCAAGCGCAAATGCTTGATGAATATTTTCCAACATA
CCTCCCCGTTTTTTCTCCTCGATACCCTAAGTTCTTTCGACGCCATCATAGGCTTCGACTTGCTAACACAAG
CCGGAGTCAAATTGAACTTGGCAAAAAACACTCTGGAATACCAGAGTACATCCGAAAAACTCCAGTACTA
TCAATGTGCTGATGTGAACTTCACCGATGTCAACGACATCGTAGTCCCCCCTTCCGTAAAGAAAGAGTTCA
GGGACATGATACTCAGAAACAAAAGAGCCTTCTCAAATGCAGACGAAGCTCTCCCTTTTAATACTGCTGT
TATCGCTACAATCCGAACAGTAGACAACGAGCCTGTGTATTCAAGATACCCGAGTCTCATGGGTGTTTCTG
ACTTCATAAACAAAGAAGTCAAACAATTACTGGACGATGGCATAATCAGGCCCTCCAGATCGCCTTACAA
CAGCCCAACATGGGTGGTTGATAAAAAAGGCACTGACTCATACGGTAACCCAAAAAAAAAGGTTGGTAAT

CGACTTCAGGAAACTGAACGAAAAAACCATACCGGACCGCTACCCCATGCCGAGCATCCCCATGATCTTA
GCAAATCTAGGAAAGGCGAAATACTTCACTACCCTCGACCTAAAGTCGGGGTACCATCAAATTTACCTTG
CGGAGCATGACCGCGAAAAGACTTCATTTTCAGTAAGCAGCGGCAAGTACGAGTTCTGTCGCCTACCATT
CGGCCTGCGCAATGCAAGCAGCATATTCCAAAGGGCAATAGACGATATCCTGAGGGAACATATAGGAAA
GATTTGCTTTGTCTATGTAGACGACGTCATAATTTTTTCTAAAAACGAAACGGAGCACCTTCAACACATTA
ACATTGTGTTAAAATGTCTAATCGACGCAAACATGCGTGTAGGCCCCGAAAAGACTCGTTTCTTCAAGGA
AAGTATCGAATTCCTGGGTTTCATTGTAACCAAAGACGGAGCAACATCCGACCCCAAAAAAGTTAAAGCC
ATTCAGGAATTCCCTGAACCAAAGAATGTCTACAGCGTTAGGTCCTTCTTGGGGTTAGCCAACTACTATAG
GGTCTTTATTAAGGACTTTGCCGCTATAGCGCGTCCGATATCGGACATACTTAAAGGGGAAAATGGTTCA
GTTAGTAGGCACGTATCAAAGAAAATCCCTGTTGAATTTAATGAAGTTCAACGCGATGCATTTCACCGTCT
GCGGAGTATTTTGGCATCAGAAGACGTCATGTTAATGTATCCAGACTTCCAGAAACCGTTCGATTTAACCA
CTGATGCTTCGGCCAGTGGAATTGGCGCGGTTCTTTCCCAAGGCAATAGGCCAATAACAATGATTTCTCGC
GCCTTAAAACAGGCCGAACAAAACTATGCCACTAACGAAAGAGAATTGCTGGCCATTGTGTGGGCTTTAG
GCAGATTGCAGAACTTTCTGTACGGTTCTAGGGAAATTAACATTTTCACGGACCACCAGCCGCTCACGTTC
GCTGTGTCGGACAAGAACACGAATTCCAAGATTAAAAGATGGAAATCTTACATTGACCAGCACAATGCCA
AAATGTTTTATAAACCGGGCAAAGAGAATCTGGTGGCGGATGCCCTTTCAAGGCAAAACATTAATGCTCT
GCAAGATGGGCCCCGTTCGGACGCTGCAACCGTTCACAGCGAACTTTCCCTGACCTACACGGTCGAGACA
ACAGACAAACCTTTGAATTGCTTTAGGAACCAGATTGTCCTAGAGGAATCGCGTTTTCCGCTAAAGCGAA
ACTTGTTGCTGTTTCGCAGCAAAACACGCCACTTGATCAGTTACACTGACAAGAGCACAATTCTCAAAACC
CTCAAAGAAGTTGTGAATTCTGGAGTCGTTAATGCAATACATTGCGATCTTCCCACGTTGGCAAGTTTCCA
ACACGACCTGATTTTGTATTTCCCTGCNACCCAATTCTGGCATTGCAAAAATCTTGTGCAAGATATAACGG
ACAAAAACGAGCAGTTAGAAATCATCACTGCCGAGCACAATCGAGCGCACAGAGCAGCTCAGGAAAACA
TTAAACAAGTACTTCGGGACTATTATTTCCCAATATGGCCAGTCTGGCCAAAGAAGTGGTTGCAAACTGC
AGGGTCTGCACTAAAGCCAAATACGATAGGCACCCGAAGAAACAAGAACTCGGGGAAACGCCTATACCA
AGTTATACTGGCGAAATGTTGCATATTGACATTTTCTCGACAGACAAGAAACAGTTCTTGACCTGCATAGA
CAAATTCTCAAAATTTGCAGTCGTCCAACCGATATTATCCAGAACGATAATCGATGCTCCGAAACGCCTAC
GGCATTTGCATTGTGAATGCGGCTCCGCTTCACAGCACCTCAAACGGCCAGGTGGAGCGCTTTCATAGCA
CCCTGACCGAAATTGCGAGATGCCTCAAGTTAGACAAGAAAATCAGCGACACTACAGAATTAATCTTAAA
GGCTACGATAGAGTACAACAAAAGTTTGCACTCAGTAACCCTCGAGAAACCAATTGAGGTCGTTCATGCA
GCAGCTTCTGATCGTCGCGGGAACGTCAAAAACAGGCTAATTAAGGCACAGCAAGACAATATCAAAAGG
TGCAATGCATCCAGGCAAAACCGCGTTTTCGAGGTAGGAGAAAAGGTGTTCCAGAAGAACAACAAGAGG
CTAGGGAACAAGTTAACTCCCTTATGCTCAGAACAAAAGGTCGAAGCAGACCTGGGAACGTCTGTCCTCA
TTAAGGGGAGGGTGGTCCATAAGGACAACCTGAAATAAGTTTTCCTCCACAATTAGCTAGTAAGCCAACT
CAGAGTTTAAACACCGTCTTATATTGTTCGTCCATTTCAGGTTTGCACTCACTTTGCTTGTGGCACTAGCAG
CGGTAAATGCTCGGATCACCGACTTCTCGCATGCCAACTACATTCCGGTCTTAGACGGGGAAGTACTGGT
GTTCGATCAACGTAGCTACTTGAGGCACTCAAGTAACATTTCCGAATTTGTTAGTATGATAGACGAGACG
GAAAGACTGTCCGATTCCTTTCCACAGTCGCATATGCGCAAACTACTAGACGTAGATACAGATCACCTTA
GAACATTGTTGTCCGTTCTTCAAGTGCATCACAGATTTGCACGAAGCCTCGATTTCTTAGGCACTGCCTTG
AAGGTTGTTGCTGGCACCCCTGATGCTTCAGATTTCTTAAGGATCCGCGTTACAGAGACGCAGTTGGTGGA
GGCTAACTCCAAACAGATACTAATAAACTCCGAAACTCAAAAACAAATAAACAGACTAACGGACACTAT
CAATAAAATTATCAGTTCCCGCAAAGGCGATTTGGTTGACACTCCTCATTTATTCGAGACCCTGTTGGCAA
GAAATAGAATACTGAACACAGAAATTCAAAATTTAGTACTCACCATAACGTTGGCAAAAGCAAGCATAGT
AAATCCTACAATCCTTGATCATACCGATCTAAAATCACTTATCGAACAAGATACCCCAATAGTTAGCTTGA
TAGAAGCTTCTAAGATCAAGGTCCTTCAGTCCGAGAATATTATCCACATATTAATAGCCTACCCTAAAGTT
GAGTTTAGGTGCCAAAAGGTCTCCGTCTACCCCGTTTCACACCAACAAACCATTCTGCGACTCGACGAAG
ACACGTTGGCGGAATGCGAAAAGGATACCTTTGCAGTTACTGGATGCACCGTAACTACGCATAACACCTT
CTGCGAACGAGCACGGCGCGAAACCTGTGCTAGCTCCCTTCACGCGGGAAACATCGCCAACTGCCACACT
CAACCTAGCCACCTGAAAGCGATAATGCCTGTAGATGACGGCGTCGTGGTTATCAACGAAGCGACAGCGC
GCGTCCGAACAGATGATGACGCAGAGGTAACCGTCTCAGGAACCTTCCTGATAACTTTCGAACGGTCTGC
AACTATCAATGGCACAGAATTTGTAAACCTGCGCAAATCACTAAGCAAGCAGCCTGGCATCGTGAGGTCA
CCGCTCCTAAACATCATCGGACATGACCCGGCACTAAGCATACCACTCCTGCATCGTATGAACATCAACA
ACTTGCTATCAATTCGAGATTTCAAGGAGGAGGTAGTTGCGGCTGGCTCGCCTAAATTCTGGTTCGCGATT
GGCGCGGTCCTGAATGTCGGACTAATTGGCTCATTCTTCCTTTTTCTGGTGCTAAGGAAAAGACGAGCCTC
CATGCAGATGCAGAAGGCGATTGACAAATACGACATGCCCGAGGACGGCCATCATCCTGAGGGGGGGT

AGTTAACAACTAAGCATAAATATATTGCCCTAGCAATTAAGTAGCTTCGTATTAACAATGCTGACGCGCC
AGAACTGAGTTCAGCGCTCTGCGCTAAGAACGGCCAGCAGCGGTAAGCTGACACTGCATGGAATGCTGAG
TCGGCTTGCCGACCATGCTTTGATGGCGTGATGCATTGAGGCTTAGGGTATTTGTATAATTTGCTTGTCCCC
TAGTTTTCAGTTTTATATGAAGCGCGCAACACATAAGTCGCCGAAATAAATATACTGCTCCGGCATTGCCG
ATAAAACATATCTTTCGTAATATTACTGGATCAAGGAGAACATATTTTGATCAAGGGCAGCGCGGGATCA
ACGCAACTTACCCAAGATAACATTGTGTTACATCGCAGAACATACATTAATGAATAACTTATA
>160_rnd-1_family-23__RC___Helitron
GATCGGACCACTATATCATATAGCTGCCATGGGAACGATCGGTCGAAAATTAAGTTTTTGTATGAAAAAA
CATTTTGTTTTTCAAGATATCTTGACCAAACTCGGCATTTATTAGTTTTACTTTACTCCTCATATATGTGCA
AAATCCTATAAAGATCGGACCACTATATCATATAGCTGCCGTAGGAACGATCGGTCGAAAATTAAGTTTT
TGTATGAAAAAACATTTTGTTTTTCAAGATATCTTGACCAAACTCGGCATTTATTAGTTTTACTTTACTATT
CATATATATGCAAAATCCTATTAAGATCGGACCACTATATCATATAGCTGCCGTGGGATCGATCGGTCGA
AAATTAAGTTTTTGTATGAAAAAACATTTTGTTTATCAAGATATCTTGACCAAACTAGGCATTTATTAGTTT
TACTTTACTCCTCATATATGTGCAAAATCCTATAAAGATCGGACCACTATATCATATTGCTGCCATAGGAA
CGATCGGTCGAAAATTAAGTTTTTGTATGAAAAAACATTTTGTTTTTCAAGATATCTTGACCAAACTCGGC
ATTTATTAGTTTTACTNNGCTCCTCATATATATGCAAAATCCTATTAAGATCGGACCACTATATCATATAG
CTGCCATAGGATCGATCGGTCGAAAATTAAGTTTTTGTATGAAAAAACATTNTGTTTTTCAAGATATCTTG
ACCAAACTCGGCATTTATTAGTTTTACTTTACTCCTCATATATGTGCAAAATCCTATAAAGATCGGACCAC
TATATCATATAGCTGTCATAGGAACGATCGGTCGAAAATTAAGTTGTATGAAAAAACATTTTGTTTATCAA
GATATCTTGACCAAACTCGGCATTTACTATTTTCCCGGTACTTCTTAGATAGAGGCAAAGCACTATGAGCA
TTATGAAAAGGTTGGGTCTGCAAGGGTATTAGATCTTTGGCGTGCCGAAGATAGCCCTTCTTTCTCGTTAG
TTTTTAGTTTTAAAAACTCAGTACAATTTCAAAAAATTTTAACAATGTACTAACGACTAAGTTATGTCAAA
ATTTAAAATTCCTTTATTTACCAGTTCGTTTGAAAAAAATGTTCATTCATTTTCAATAGCTTTTAGGTAAAA
AACAAGTAAGAATGCTATAGTTGGGAGTGCACGGCTAGGGGATACCATTCCTACCAACAACAAACGCCGC
TATAGCTATAGAATAAAAGATACCTGATTTGCATGGACAGGCAGAGGCACATGGCTAGATCGACTAGGCT
ATTGATGCTGATCAAGAATACATACTTTAAGTGGTTGAAGATCCTTTCTTCTGCCTGTTACATACGGCTGC
ACAAATACATTATACCCCTTTTACCCATTTTTAATGGGTTCAGGCTTTAAAAAGGCGAACACAATCAGTCA
ATAAATAAAATCTTGCCGTGTTGCGTTTCCTGGAGCCATTTCAAACTTGTTAGATTAAAATATAAACAAAT
AAATAACAAATGTTATAAATATATATGTTCATATGTATAAATATGCATATAAATTTCATTTGAATTAAATA
GCATGGAATCCAAAAGTGACGCTATGTTAGGGGCTGCCAACTTATGTAGAANGCTAACCTATGGGAACAT
AAAGATGCCACCTTGTGGAAGTACATTGACATTGTTAGTATATTTAGCCAAAAAATTGTTAAATTTAAAAT
AATTTTCTACTTACATGTCTTTAGCGTGCCCATTTGATTTTTATGTATTCTTGCCATTCACACGACAGCATT
GCCAGCTGATCGTGCAGAAATTTCCCCCAGCTAACTGACCTAAGACAATATGTTAAATGTACAGAGTCTG
TTATGTCATGTTATGTTTATATGTAGGTCAGTCGAGATTAAATGTTAAATGTATCTCTTGTCATGTCCCTCT
CCACCTTGCAAACTGGTGAACGTTGCATAGTTTTAAGTGCTCCCAAAACTGTTCTAAAATTTCTGTTTGCA
ATTGAAATCATATGTAGAAATGAGCAACAATAAAAATCTCTCTGTGGCGGTCGAGGCGAGCGGGCGTGTT
TGCGGACAGCGCCCGGTCGGCCAATCAGACGAACAACTAAAAATTGTGAAAGTACATAAGTAAAAGTAC
ACATGACGATGAAACATGTTATTTACATATATGTTTATAAGTTAAACATGTTATTTATATAAAATAAATAC
TTTAAATAGTTGTTAAAACATTCTACAATTTGTATTATTATCTGTGTATATATAAATCTATATACTTCGTTT
TGAACACTCCCGTTCTCATTGCCTGCGTTGGCTGTACTGACGAATTCAACTTTTCACCGCACGCGAGTTGA
ATTCGTCCCACTGTACAGCCTCTGTCGCGACGCCTTAACGGCCCTTCGACGCCGACGTCGGCTGCGGCGCG
CATCTGTGCACACGTATGCAAGCGTGGTCTCACTGTCGCCCGTCTGGCGCGCGCGCGCTGTACTTTTTTGA
TCGCCGCTATATTCCCGATGTTCACTCTGTATGTGCGTCAACTTAGCGGCGGTGACTTAGCGTCGATTTTTG
GTACTTGGTATCCGAAAAGGAATCGGTCACTTTTAAATGCATTTATTTATGTTTTTAAGCACAGAACCTAA
TAAATTTAAATGCAGAATTGTAAAAAATTAAATTATTTACTGCCTACATTCATTTGTTTTTGAATTTTAATT
TATAATATGGGTAAACCAAGGGGTTTAAATATTAGGTTTCCACCATTCCATAAATATCCAAATTTAGGGAT
AATGGCCCCGAAAACCGCATATACACGATTAAAGCCTATAGTTTCACCTAGACAACTGTGAAAATTTGAT
TAAAATCTTCGGTACCATTTTTAAGATATTTGGAAAAGAGTGCAACAACCCCCATGTCAGCCCCATATAAA
ATGACCGCAAAAAATCAACCTTCTTATTTCTTAACTCAGAGACAATGTCCCTCAATGCACGCTATACCCTC
TTGTAGATACATATTTTAAGAAGCTTTAGGCACTAAATTTAAGTAAATGGACGGTACAGATTTTCAGAAA
ATTATTTACTTGTAAACCAACCTCATATAGTTCATTCATAAAGTCTATCGAACTGCTATTTCCTACGTGAAC
TGAATCACTGCCTGGCATCAGTGTTGAACAATTTTGTACAGAATTGGGCGGGAACCAATCCAATTTGAAT
AATATGTTATCATGAAAATTACTTATTGTTAACAATAAGTTTATAGAGCGAAATTAGACAGATTTTCAACT
GGCAGATAATGTTAAGAATCGAAAAATTATTACGAAAAGTTTTAAGTTCGTAAGAAATATTAGAGCTTTA

ATATTTTGTATAACCCTTATTGGTGAGTTGTTTTACCCGTTTCAGGCTGCCTACTCAAAAGAACGCCAGGA
CGTATCTAGTGGTAGTAATCATGTTAGCAGTAAACTAGAAATTAGCAATAATATTAGTGATATGTCTAGCA
TAGAAAATAATAATTCTAATTATAGTCTTAATGAGCCTACAAATTGTCCGTCTGAGTTTAATAATACGCAT
GCTTTGTCGAAGCGACATTAAAATGCTTTCGGAAAAAAATACCACAATTTTCTTGATAGCTTAAACAGAGA
AGTTCAGTTAGTTAGTAATTTGAAAGGATGCCGCGCAGCACTTTCAGGTTACAACAATTTGGATACTGAA
ATGAGTATCCAAGAATTTATGGCCACGTTAAATCAATTGTGTGCGATTAAAAAAAAAAAAACATTGAACA
CATTATTCGAGAAGCATACACTCTTCATAGCAATGCATGTGTTTTGCACTTTAAGTCAATTAGACTTAACA
CAAACTCTTTTGCTGCATATGGTTACTGCAAAGATCCATCATGCAACTACTTCTTGTTCCATGGCAGCCAA
CATATCTCCCATACAATTGGCCTTAAAAAGTCAACGCAATGCAGAACAATAAATAGATTTATAGAAAAGG
CTTCCATTAGAAACTCTTCTGCGTTCGAGTATAGATCAAAAAAAAAAAAGGACAAAATCAATATGTCTCG
AGCCAGAGTTGGTAATGATCAGATTACTAATGAGAAACTACCCTTTTATAGAAATTTAATTTCGGAGGAA
AATTGACTTTGACAACTCACGGATAAATCGTCCTTGCTTTAAGCCGTTTTTATTATTTGTCTTTATAAAACT
ATATTTAAGCGTTTTAAAGAATGATTCTATAGGCCCATTACTGGGTCTTTTATTAGATTTATCAATAAAAG
TAATGATAATATTGGTTCAAAAAGGTGCGTAAACAATATATTTATTTAAAATTAGATCCAGCATTTCTGCA
CTTAAATAAATATTTGACGTACAGAAGTTGCTTTTATATTGTGATACAAGTTCTGTAACTTCTGTACGTATT
TCTTGAAGATTAAGTTTAAATGGACTATTTTCGGCAATGGACAGCAACGTTGGATCATTGACGACTTCCTT
ACTTACTCCCAATTTTATACTGTCTATCAAATAAAATGTGGATGGCTCTTCACAATATTTAGTAAAATAAT
TAGCGGCATCATCCACAATATTATTAACATATGGAGAAAGAAGTATTATTGCAATATTTCGGAACCATTTT
TCTAATTTTCCAATATTATTTATGGAATATGCACAACCCAAGGCATTTCTTAAAAATGATTTAAGATCAAT
ATTGTCGAAAGCAACTACTTCATCTATGTTGCGACACTTAATTTTGCTGTAGTGGCTCATACAAAATTGAA
CCCAGACTTTTGGAAATGTGCGATTTAAATTATACAAAAAACTAAAACAAATTTCATTGTACGTTGCAAG
ATCAATGTTGTTATATTCTTTTAAAATGGCTCCAATTATTGCAAAACTGACATCAGTTATAATTCTTTTGCA
AATAGGCCATTTGAATTTATTTTTTATGCAAAACTCGCGCAGCTCACATAGAAATCTGCCTATATCTGACG
ATTTATGATTGGAGAGAATCGCATGTGCGACAGGAAAAACGATTCGAAGCTCTGGAATATAAGCAACCAA
TGAATATAAAAATGTACATTTTTTGATCAAGTTCATTGGCTGTACTACATTCCCAGTCGCATCAATATGCA
GTGTCTGTCCCATAAAAATATTTCTAGTTTCATAGAAAGTAACTGAATAAAGTTGGCATACACATTAGTAA
TCATATCGAAGGTATCTTTAAATGGATCTTTGTCAAAATCAAATTTTCCATTTTCCTTCGAAATTAAATTTC
TATAAAAGGGTAGTTTCTCATTAGTAATCTGATCATTACCAACTCTGGCTCGAGACATATTGATTTTTTTAT
TTTTCTTTTTTGATCTATATTCAAACGCAGAAGAGTTTCTAATGGAAGCCTTTTCTATAAATCTATTTTTCG
TTCTGCACCACGTTGACTTTTTAAGGCCAATTGTATGGGAGATATGTTGGCTGCTTGTGTATACACTTACA
ACACCGTCAAATGTCCCATGGAACAAAACGTAATTGCATGACGGATCTTTGCAGTAACCATATGCAGCAA
AAGAATTTTTGTTAAATCTAATTGACTTAAAATACAAAATACATGCGTTGTTATGAGAAGTGTATGCTTCT
CGAATAATGTGATCAATGCTTTTTTTTTTTAAATCACACACAATTGATTTAACGTGGCCATAAATTCTTGGA
TACTCATTTCAGTATCCAAATTGTTGTAACCTGAAAGTGCTGCGCGGCATCCTTTCAAATTACTAACTAAC
TGAACTTCTCTGTTTAAGCTATCATGAAAATTGTGGTATTTTTTCCGAAAGCATTTTAATGTCGCTTCGACA
AAGCATGCGTCATTATTAAACTCAGACGGACAATTTGTAGGCTCAATAAGACTATAATTAGAATTATTATT
TTCTATGCTAGACATATGACTAATATTATTGCTAATTTCTAGTTTACTGCTTACATGATTACTACCACTAGA
TACGTCCTGGCGTTCTTTTGAGTAGGCAGCCTGAAAGGGGTAAAACAACTCACCAATTAGGGTTATAAAA
AATATTAAAGCTTTATGATTACTTACGCATTTAAAACTTTTCGTAATAATTGTTCGATTCTTAACATTATCT
GCCAGTTGAAAATCTGTCTAATTTCGCTCTATAAACTTATTGTTAACAATAAGTAATATAGATTGAAAATC
AGTTGGACTTAAGTTTCCAACTGGAGATATTATGCTTCTAGCAATGGCATCACGAAATCCAAGAGAGATT
GTCCTTAAAAAATTGTTTATATATTAATGGTAAAATTAAATCGAATAAGTTAAGCAACAATTTTTTATACC
CTTGCAGAGGGTATTATAATTTTGTCGTGAAATGTGTAACGCATAGAAGGAGACATCTCCGACCCCATAA
AGTATATATATTCTTAATCAGCATCAACAGCCGAGTCGATATAGCCATGTCCGTCTGTCCGTCTGTCTGTTT
CTATGCGAACTAGTCCCTCAGTTTTAAAGCTATCTTAATGAAACTTTGCAGAACTCCCTCTTTCTGTTGCAC
GCAGCACATATGTGAAAACCAGCTGGATCGGACCACTATATCATATAGCTGCCATAGGAACGATCGGTCG
AAAATTAAGTTTTTGTATGAAAAAACATTTTGTTTNTCAAGATATCTTGACCAAACTCGGCATTTATTAGT
TTTACTTTACTCCTCATATATATGCAAAATCCTATTAAGATCGGACCACTATATCATATAGCTGCCATAGG
AACGATCGGTCGAAAATTAAGTTTTTGTATGAAAAAACATTTTGTTTTTCAAGATATCTTGACCAAACTCG
GCATTTATTAGTTTTACTTTACTCCTCATATATATGCAAAATCCTATTAAGATCGGACCACTATATCATATA
GCTGCCATAGGAACGATCGGTCGAAAATTAAGTTTTTGTATGAAAAAACATTTTGTTTATCAAGATATCTT
GACCAAACTCGGCATTTACTANTTTCNCNGTACTCCTCANATANGGGCAAAGCACTATGAGCATTATGAA
AAGGTTGGGTCTGCAAGGGTATTAGATCTTTGGCGTGCCGAAGATAGCCCTTCTTTCTCGTTTTTAT
>Ref_dvir_685

TTCTAAATTCATACACAGAAGCAGGCATCCTGGACTAACAGTGTCTGATATACAACATGGACCCAACTC
TTACTAAGGCTCGTCCAGAGAGCACACCTGTGGAAGAGATCAAATCATTGCAATCGAGAAGAGTTGTTA
AACCGTCCAGTTGTATCGCATCACTTAACCCTTTTTTGGATGATTTCGGAATTCTTAGAGTTGATGGGCGCT
TGAAAAACTCTTCGCTGGATTTCGACGCTCGCCATCCAATAATTCTCCCGTGTCACCACCCCATCACAAAC
TCGGTCATCACACGGTTTCACAATCGGGACTTACACGCTGGTCCACGGGCACTTCTTGCTCATATTCGGTT
GCAGTATTGGCCGATTGGTGGAAGGAAGACCGTATCCAAGGTGGTTGGAAAATGTGTGAGATGCTTTCGT
GCCAAGCCTCGATTAATAGAGCATATAATGGGTGATCTGCCCAAAGAACGTTTGACTGCTTGTCGAGCCTT
TGAAGTCTCTGGTGTTGACTATTGTGGACCATTTTTCTATAAGCCAGACGTACGAAATAAAGCTCCAGTCA
AATGTTACATGGCTGTCTTCGTCTGCTTCGCGACTAAAGCGGTTCACCTGGAACTGGTCAAGGATCTGTCG
ACAGCTTCTTTCTTGAATGCGCTTAAGAGGTTTATATCGACTCGAGGCAGGCCATCTCAGATATGGTCGGA
TAACGCTACCAACTTTGTCGGCGCCAAAAACGAGCTTCTGGATCTCAAGCGTCTATTTACAAGTCGTAATC
ATATTGAGTCAGTGCATAATTTTTGCATGGCTGATTCCATTGATTGGAAATTTATACCTCCTCGTTCACCGC
ATTTTGGCGGCCTTTGGGAAGCAGCAGTGAAGACAGCGAAACATCATTTTTATCGTGCTGTTGGATCTTGT
GTTCTCAACTTCGAAGAGTTTCGCACCCTAGTCTGCCACATAGCATCGATAATTAATTCCAGGCCATTGTT
TCCTCTTTCAGAAGATCCAGCAGATCTAGATGTGCTCACTCCAGCTCATTTCTTGATTGGTGGACCCAGCT
CATCGTTCTTTGAACCTGATGTGACGCACCTTCAATTCAATCGTCTGGATGGCTGGCAACGCGTAACGTAC
CTCCAACAACTGTTTTGGTCTCGCTGGCGTGAAGAATACTTAACACTGCTTCAGCAGCGCTCCAAATGGCG
TACTCCGAAACCAGGCCTATGCGTCAACGACATAGTCCTCGTAAAGGATGAAAATCTGCCGCCTCTAAGG
TGGCCACTGGCGAGAATCACAGAGTTAATCGTGGGAAAGGATGGAGTCGCTCGAGTAGCCATTTTGCGCA
CCGCAACAGGAGAAACGAGAAGAGCTGCAAACAAGCTATGCCTATTGCCCATCAGGGAATCTGTTGAAA
GCCCATGCCTTCCAACGGGGGGAGAATGTCTGGTTACGCAGCCAAAACAACTGGAGCGACCTCGCAACTG
ATAAGAGCAGCATCTTAATTTTATCTTATAATACTCGCTCTGCAAAGCAAGTGCCTAAATTTCGCTCTTAA
GAATTCATTTACATAGAGCTGCCGCTCTTCGCTGTAACGATCGTTTTATTGCCCTAGCTTTACTTATGCGAA
GGCCTCGATGCTGCGCATCGAGTTAGTTAAGTTCGTTCGCTTCATGCATTTGGTACGCATCGGCCACTTGC
ATTACTCAAAATTATAACTAATAATTGTCTCTTCGCTAAAATTGGCACGTACTAATTCGGGTTCGCTTGCCT
ATATACAAACTATTGTTCTTAAATAATAAGCACAGACCAATTCGGGTCTGCCTGCTTCTCGTAACTTTAAG
AAATATAAAACAATTTTGAACACAAAAAATAAATAGTTAATTATTTCTATTTGTGAAAGCTATTGAAAAA
GCTCAATAGCGCAACATTTGGTGACCCCGACGTGATTTTTATTTTTGTTGTTGTAAGATTATTTGCTTTACA
TTAAAGCAATAATCTTAAAAGGCACCACCTGGCATTTCGTTTTGTGCACTTTATATACATACATACTTATC
GCCGTCGCTTTGCCTACTCTCTCGGTCGCTTAAAGTCTTTGCTCATTCGTGCGGTACTTATGTAAAAGCTAT
TGCCGTTGCTTCATTACATAGCTTGACACTTGCGTTCGCCGAAGTTCTTATTGTACAACTATTGTTTTTTGA
ACGGTTTGCATGTACTAGCGATCACCGTTGTTAGTTACTCAGCTAGTTTCGCACGCGCTTTCTATCACGCTT
CTAATGGCAACCGAAAATAATGCCTCTAGTGCAGTGCCCTCTGCAAACAGGGTGACCTTTCTGAAGCGCA
AAGCCATTTCAATTTATGACAGTTTGCAAACACTTCGAAATGCTCTAACTAATTCGCCACTTAGTGCAATT
GATGAGTGCGGTCTTAGTGTTAGGCTGAAGCAAATCGAGCGGCTTCAGACCTCATTTGATATTACTCACAC
TAGCCTCGAAGAACTAGACTTTGATGAAATCGGTAACCAATTGCGTGAGGACTTCGACGAGTTAGCAGTT
TCTATGGAAATTTCGGTTCGGAGTGAAATTGCGAAACGTGTCTCAATATTGCCCTGCTCAACATTTCGCGA
CCAATCTGTGCTGCCCTCGGTGACAGTTAGGGCAAAGGCACGCCAACACTACCCTCAATAAAGTTGCCTA
CGTTCAGTGGTGGTTACACAGAATGGGCCGACTTTTATTCTATGTTCACAACCGTCATAGAAAATCATCCT
GATCTAGCAGATATCGAAAAGCTGCAGCATCTTCGATCGTGCCTGAAGGATACAGCGCTGGACGCGATTC
GATCATTGGAAATAACAAACGATAATTATGCCATAGCACTTAATTTATTGGAGAAAAGGTTTAATAATAG
ACGTTTGGTTTTTCAGGCACACATACTTGAGATATTGGGGCTCAAGAGGGTGGCAGGTGGATCGGCCGTG
TCGCTAAGGGAGTTATCCGACCAATTCAACGCACATTTTCGCGCGTTAAAGAGCATGGGCAACTCCGAAC
AAATCGCTGGATGCATCATTGTGCAGATTATTTTACAAAAGCTGGACCCAATATCACAAGCCAAATGGGA
AAGAAAGGCTAGAGGATTCGTCGTTCGTTAATTTAATACCATCGTGGGAGCATATGGCTACGTTTCTGGA
GCAGAGATGCCGAACAATGGAAACCGTAAATTTTGCTATGGCAAGCTATGCCCCTAGCCATCAGGTGGGA
AAAACTAGAGCAATTAACACCAGTAGATCGTCATTTATCGCCACTAACCACGACGCCCCTGTCTGCGTCTT
TTGTGACAGCCCTGGTCATAGTATATATAATTGTTCTCGATTTGGAAATTTGACCCCTGCATTACGGCTTA
GGGAGGCAAAACGAGTTGCATTGTGCTTGAATTGCCTAAAGAAAGGTCATCAATTGCGATCATGCAGCTC
TGGATGCTGTCGGACCTGCGGGGCGAAACATCATTCTCTGCTGCATTTAAACAATTCCGTATTTCCGTCTG
GTCAGCTGACTGAGCCTGCAAATTCATCGCCGATAGCCCATGCCTCTTTCGCTAAGTCATCGCCATCTACC
TCCACATCTCTTGTTGCCCAGTATTTCAGTAGTGATGTTGTCGTCTTGGCTACGGCTATGATCTTTGTAAGA
AATCGCTCCGGCACTTTGATACCTTGTCGTGCCCTTTTGGATTCTGGTTCTCAGCTTTCCACCTTATAACGT
CGCGCCTTGCTCAACAGCTTCAGCTAAAGAAGCACAAGTCCTCAGCCATTGTCTCTGGTCTAGGAGATTCT

CGTTTTGAAACAGAAGGAAACACGGTCACTATCACAATACAGTCTCAAGTCTCTGAATACTCAACATGCC
TTACAGCTCTGGTAGCGCCTAACATAACTGATAGTCAACCGAGTTTACTATCAACACGACGAATTGGAA
CATTCCCGATAACATAAAACTTGCAGATCCGTCTTTCTTTAAACCTCAACGCGTGGACTTGCTACTTGGAG
CTAGCCTGTTTTACGACCTTCTCAGCGTCGGCCAGATAAGGCTTCTCCCAGGACTACCGTGTCTTCAAAAA
ACACGGCTTGGTTGGGTAGTTTCTGGAGGCGGACCCCGCTTGAGTGGTTCTATGTTAATGGCAAAGCATAC
TGCGGGCAAACTTGAGTCAAGTTCTGAAGAACGTGCTGATGAATTGGTTCGAAGATTTTGGGAGATAGAA
TCCTGCTTGGAAAGTACACCTACCACTTCAAAGGAAGAAAACATCTGTGAAGAACATTTCGTAAAGACTC
ATATCAGATTGCCTAGTGGCCAATATTCAGTTCGTTTACCCTTCAAGCGAAGTTTGGAATGTCTGGGGACG
TCATACCAACAAGCCTTTCGTCGGTTCCTGACCCTGGAACGCAAACTACAAAATCGTCCAGAATCTGAAG
ATTCAATATTCAAACTTTCTAAAGGAATATTTGGATCTTGGTCATATGTCGCCTGTTGCTCCAGAGTCTATA
GGACTTTGTCAATACTTTCTTCCTCATCACTGTGTCTTTAAAGAAGACAGTTCAACGACAAAATTGCGAGT
CGTTTTCGACGGCTCAGCAGCTACCTCGTCAGGTTATTCATTAAATGAACTATTGATGGCTGGACCTGTTA
TTCAAAGCTCGCTGTTTCATATCCTTCTTCGCTTTCGTAATTTCCCGATCGCCCTAACAGGAGATATTGGAA
AAATGTATCGCTGCGTACGCGTTTCAGCACCGGATGATTTTCTCCAATGCATTCTGTGGCGAGACTCGCCA
GAACAGGATATAAAGATTTTTAAGCTGGATACAGTTACATACGGAACAAAGCCAGCTTCATTCCTCTCTAT
CCGTGCTATGCATCAGCTGTCTATGGATGAGGAAACTAATTTTCCAATTGGATCTCGGATTTTGCGGCGTG
ACTTCTACGTTGATGACCTCATTTCAGGGGGTGATTCAATTCAAGAAGCAAAGGAACTTCTTAATCAGACG
GCTGGAATCTTGGCAAGTGGGGGCTTCAAGTTGCGAAAGTGGTGCTCAAACAATTCTGCTGTACTGAAAG
GGATACCCGTAGAGGACAGAGAATCTTCCCTAAAGTTTGATGATGGTAGCGACATCACCAAAACTCTTGG
ACTCGCTTGGGACTCAGCGTCGGATCGATTGTTGTTTTCGTTTTCGTCCATTCAGCCAGAGATCTAAACCA
TCCAAAAGGTCAATACTAATCGACCATAGCTCGCTTGTATGACCCGCCTTGGACTCATAGGGCCGGTAATT
GCCAAGGCTAAAATCTTTATGCAGCAGATCTGGAAGGAGAAGCTTGAGTGGACGAGAGCTTGCCATCAT
CACTGGCAACAGCTTGGCATGATCTTTGCTGCAGTTTTGAGAAGGCACAGTTTCTTAAATTTCCTCGTCTC
GTGTCAATACCTGCAGCAGAGTTAGAAGTTCACGGTTTCTGCGACGCCAGCATCGAAGCATATGGGGCCT
GTGTCTACGTCCTTTCTCGACGAACATCAGCCAGTTGTCAGCTGTTGTGTTCCAAATCGCGTGTAGCTCCTC
TGAAAACAATCACAGTTCCAAAGCTAGAGCTCTGTGGTGCCGTCTTATTATCTAATCTCATGCGTGAGATA
TCTAAAATGAATCTGTTTGATGGAAAATTCTACTGTTGGTCGGATTCTTCTGTATGCCTTTCCTGGATAAGG
ACCGAGCCCTGCAAGCTCAATGTGTTTGTGGCTAATCGCGTCAACAGCAGTTCAAGAGCTAACTAATGGC
ATGGAAAATGGCGTTACGTGCCTACTTCACTCAACCCGGCAGATATACTCTCGAGAGGATCTACGCCCAG
TGAATTAGATCAATCGTCACTGTGGTTCAACGGCCCAGAATTTCTAGTTAAAAACAAATTGGACTGGCCTG
CTTCTTGCTTGTCAGAAAGGACAGAACTCGAACTTAAAAAAAGGGTCCTTATTGCGTCATCTCCCTTCGTT
GATATAACTGTCACTTGTAAATACGTCAACTCATTTGATACAATGCAGCGTATATTTCGGATATATTTATA
AATTCATACACAGAAGCAGGCATCCTGGACCTAACAGTGTCTGATATACAACATGGACCCAACTCTTAC
TAAGGCTCGTCCAGAGAGCACACCTGTGGAAGAGATCAAATCATTGCAATCGAGAAGAGTTGTTAAACC
GTCCAGTTGTATCGCATCACTTAACCCTTTTTTGGATGATTTCGGAATTCTTAGAGTTGATGGGCGCTTGAA
AAACTCTTCGCTGGATTTCGACGCTCGCCATCCAATAATTCTCCCGTGTCACCACCCCATCACAAACTCAG
TCATCACACGGTTTCACAATCGGGACTTACACGCTGGTCCACGGGCACTTCTTGCTCATATTCGGTTGCAG
TATTGGCCGATTGGTGGAAGGAAGACCGTATCCAAGGTGGTTGGAAAATGTGTGAGATGCTTTCGTGCCA
AGCCTCGATTAATAGAGCATATAATGGGTGATCTGCCCAAAGAACGTTTGACTGCTTGTCGAGCCTTTGAA
GTCTCTGGTGTTGACTATTGTGGACCATTTTTCTATAAGCCAGACGTACGAAATAAAGCTCCAGTCAAATG
TTACATGGCTGTCTTCGTCTGCTTCGCGACTAAAGCGGTTCACCTGGAACTGGTCAAGGATCTGTCGACAG
CTTCTTTCTTGAATGCGCTTAAGAGGTTTATATCGACTCGAGGCAGGCCATCTCAGATATGGTCGGATAAC
GCTACCAACTTTGTCGGCGCCAAAAACGAGCTTCTGGATCTCAAGCGTCTATTTATAAGTCGTAATCATAT
TGAGTCAGTGCATAATTTTTGCATGGCTGATTCCATTGATTGGAAATTTATACCTCCTCGTTCACCGCATTT
TGGCGGCCTTTGGGAAGCAGCAGTGAAGACAGCGAAACATCATTTTTATCGTGCTGTTGGATCTTGTGTTC
TCAACTTCGAAGAGTTTCGCACCCTAGTCTGCCACATAGCATCGATAATTAATTCCAGGCCCCTCTTTCAG
AAGATCC
>9_rnd-1_family-415__LTR___Gypsy
TATATATATAAGTTAACCACTGACTATTGGTATGTGCTGACCCAGCGTTCTGGGGAAGCCAATGACCAGA
GTCATGTGGCGACGGATATGTCGCAACTATGTCACCGGCCGCGACGGTCATCCGACACCGCACACGAGGC
GCTAACGCTGCACTTTTGCGAGAGCGGCGGCAGCGGTTCTACAGCAGCAACGGCGGCTCTGGGGCAGCGA
CGGCGGCTCTGCAGGCATTGCTTAGATTGATAAAGATTGTATTTTTTTAGTCTAAGTTAGGAAACAATAAA
AAGCGCGACGTATCGTCCCTTAAAGTCTCCCGTTAGTTAACTCATTTTTTTATATGCATTTTTACATGCAAC
CGGCAAACCCAAAGTTGCAGACATTAATGCACTAATTGGCGCCCAACCTAAAAACCCAATACAAAAAAA

AAACGCGATAAAACGGTTTCAAAACTCCAGTTGTGACAGCAGCGGTGGAATTCATCAAAATACACAAATA
CACAACAATAGAATCATGTAAGTGATTTGTGAAGTGAGAATAGAAAATTTCGAGAACAATTTTTTTTCGA
ATACGCACAAATTAATTTTGTTAAAGTAAGAAAAGGAGTGTCTATTTCTGATTGAGGAAAAAATTTATGA
GAATTATTTAAAAAAAAAAAAAAAAAAAAAAAAAAATCCCATTAGCTTAACCTTTTATGTGTTGTTCGGTTTCA
TACAGAGGTTTAAATTCGAATGAGCGACTGTGACTCCGACGTAGACGCGCATGAACACTACGATAGCGAC
GACAGTATTTTAGCGAGGTTAATAAGGGGATTTGGAACATTGCAGGTATCAGGTTGTGTCACCCCAACTG
AACATAATACAGAAACAGACTCAGTTATGGACCCAGAGCAGTTGCGTGCGATAATTGAGGCCGCAGTGCG
GAGCGCACTCACAGCGCAGGCGCAAGAATTCGCGAATAGGGAAGCTACGCTAAGGCAAGAGCTTCAACA
ACAAATTGCGCAGGTATCGAATAAGCTAAGAAACACACAACCTTCAGTCCCGGTAGTAAAGGTTTACAGG
GAAGTTGAAATCATAGACGGGGTGGAATGTAATGAGCCGCTCGACGTGGTAAAATGTCTTACCGAGTTCC
ATGGGACTCAAGACACTTACGTGTCCTGGAGGCAGGCCGCGCATGCCGCCTATAAAATTTTCAAAAATAC
GACGGTAGTTCACGTCACTACCAAGCCACCGCAATCATTAGGAGTAAAGTGAGAGGCCCGGCAGACGCG
GTTCTCGCCTCGTTTAACACGCCGTTAAACTTTGAGGCCATCATAGCGAGGATGGACTTCACTTATAGTGA
TAAGCGGCCTATCCATGTAATTGAACAGGAAATGGGTACGCTTAGGCAGGGCGGCATGTCCATTCTACAG
TACTACGATGAGGTTGAAAAGAAACTCACGTTACTCACAAATAAAGCCCATATGTCATATGACAGCAATC
TGGCAGCGGGAATGTGTGAAAAATTCAGGGGTGACGCACTTCGCGTATTTATCTCTGGATTAAAACGCAG
TCTAACCGATGTGTTGTTTTCGGCTCACCCCTCGGATCTACCGACGGCACTAGCGCTAGCCCAGGAGGTCG
AGGCTAATCACGAGAGATATGCCTTCGCGACAAGTTTTGCTAGGGGGCAGGAAGAGCGATCTCGTCCAGA
GCAGCGCCCACAAGGGCGCCAGCAACAACAACAGCAAGAAAAAGCAATTCTCTCAGGGTGGCCAGGGCA
AGAATCCTCATTTCAAGAAGCAGAACGGCCAATCCACAGACAAAGCCCAGAATAGAGCTGGGGCCGATG
CCCCAGTTCCCATGGAAGTCGACCCTTCTTCTTCCAGGTTTAGGCAACCCACAAACTTTTCGAATTCCCAG
GGCTCGGCTAAGTTTAACGCCAGCCAAGGTCAGGGAAATAAACGCTCCGCCGGATCGGAACGCGCGACA
GGCCAAAGGAGGCAAAGGGTAGACCACGTCCAGGCGAGCGAGGCCGAGAGCGCAGGGTACACCGACGC
GGCACAAGAGGCGTCAGACGAACAACAAGACGAACATAGCCCTGATTATGATAGCGATAGCGCTAATTTT
TTAGGGGAAGGTCCCTGCTACCGTTCATTCGACGGAGAATAGCGGGGGTAGATATGAGATTTTTAATCGA
CACGGGTGCGTCAAAAAATTACATCAGACCGCACAAACGCCTTAAAGGCGTCGTCCCGGTCGAGTCCCCT
TTCGTTGTCCACTCCATTCATGGGTCCAACAACATCGCCGAAAAATGCAAGTTATCCATGCTTGGGTGCGA
CACATATTTTTTCCTACTAGATTCTTTATCCACTTTTGATGGAATCATAGGGCTGGACCTGCTAAAGCAGGT
GGGAGCGGTTTTATGCCTAAAAAAAGGTATGTTAATATTTGGTGCGACTTCGGAAGCGTTGCAGTACCAC
AAATGTGTGAGCGTTAATCACACGAAAAATAAGGAAGTAGAGGCACCACCTGCGGTGAAGGCAGATTTC
CTGCGAGTACTTCATCGAAACAACAAGGCTTTTGCGGACTCCAACGAGGCGCTGCCTTATAACACGGCTG
TTGTGGCCACGATCCGTACGACCGACGAAAATGTGGTCTACTCAAAGCTATACCCCTACCCTAGGGGTGT
AGAGGATTTTGTCGTTTCAGAAGTCAACGAACTACTAAAAAACGGTATAATTAGAAAGTCGAAATCGCCC
TACAACAATCCACTGTGGGTGGTCGATAAGAAAGGCACAGACGAGGTAGGCAATAGAAATAAACGTCTC
GTAATAGACTTTAGGAAGCTCAACCTCAAGACGGTTGCCGACAAATATCCAATGCCAAGTATCACAATGA
TACTGGCTAATCTGGGTAAAGCCAAGTACTTCACGACGCTCGACCTAAAGTCAGGTTATCACCAGATTAC
GCTGGCGGAGCAGGATCGTGAGAAGACGGCCTTTTCGATCAATGGCGGGAAGTACGAATTCTGTCGACTT
CCCTTCGGTTTGAAGAATGCAGCAAGCATTTTTCAGAGAACCATAGATGACGTCCTGCGGGAGCAGATTG
GAAAATCTTGCTACGTTTATGTAGATGACGTCATCGTCTTTTCGGAAAACGAGTCGGACCATGTCAGACAT
GTCGACTGGGTATTAGGGAGCCTTTTAAACGCGAACATGCGCGTGGCGCACGAGAAGTCACAGTTTTTTA
AAGAAAGCGTCGAGTACCTCGGCTTCATAGTGACTAAAGACGGTGCAAAGTCAGATCCAGAAAAGGTAA
AGGCCATTCGGGAATATGCTGAGCCGACCACGGTGTTTGAAGTAAGATCCTTTTTGGGGCTGGCAAGCTA
TTACCGCTGCTTTATCAAAGATTTTGCAGCAATAGCGAAACCAATCTCCGACATCCTGAAAGGGGAAAAC
GGATCCATTAGCAAGTACAGGTCTAGAAAGGTCCCTATACAATTCAACGATGCTCAACGCAACGCGTTCC
AGAAGCTTAGGAACATATTAGCGTCGGAAGACGTCATACTGATGTACCCGGACTATAAAAAACCATTTGA
CTTGACCACAGACGCTTCAGCCTATGGCATAGGTGCTGTCTTGTCGCAAAATGGTCGCCCCATTACTATGA
TATCCCGGTCCTTAAAAGATAGGGAAGCAAACTTCGCTACCAACGAGCGGGAGTTGCTTGCCATCGTCTG
GGCATTGGGTAAGTTACGGCACTTCCTTTACGGGGTTAGGGACATAAATATCTTCACTGATCACCAGCCTC
TCACGTTCTCGGTATCCGAGAGCAATCCCAATGCAAAAATAAAGCGCTGGAAAGCGCGCATTGATGAAAC
AGGGGCTAAGCTTTTCTATAAGCCTGGTAAAGAGAATCATGTAGCGGACGCGCTGTCGCGCCAGAACGTG
AACGCCCTGCAGGGTAGCCGCGATGAACCAGAATCTGACGCGGCCACGATTCACAGTGAATTGTCCCTGT
CTTACACAATCGATTCGACGGAGAAGCCCTTAAACTGCTTCCGAAACCAGGTTGTATTAGAGGAATCTCG
CTTTCCTTTAAAAAGAAATTTCATACTTTTCGGCGAGAAAACCCGCCATTTGATAAACTTTACCGATACGG
TCGCCCTCATCGAAACGATAAAGGCCATCATTAACCCAAACGTGGTTAATGCGTTTCACTGTACTCTCCCA

ATCTTGGCCAGTTTCCAGCACGAGTTGGTAAGAGCCTTTCCGACTACGAAGTTTTGGTATTGCAAAAACTT
CGTGGCCGACGTGTTCGACGTCAGTGATAGAAGAGAAATCATCACTAGGGAACACAACCGAGCCCATAG
GGCGGCTCAGGAGAATGTAAAACAGGTCATGACGGAGTACTATTTCCCTAAAATGACCAAATTAGCTAAT
GAAGTGGTGCTTAACTGTAGAATTTGCACGAAAGCAAAATACGACAGACACCCAAAGAAGCAGGAACTC
GGCCAGACGCCACTTCCCTCACAGACTGGAGAAATGTTGCACATTGACATTTTCTCCACGGACAAAAAGT
TCTTCCTTACTTGTGTTGACAAGTTCTCGAAGTTTGCGGTAGTCCAGCCTATCGCGTCTCGAACGATAGAA
GACACGAGACCACCTCTGCTGGTCTTAATGAATATTTTCCCTTCGGCTAAGCAAGTGTATTGCGACAATGA
GCTCGCATTCAAGTCAGAGACTATCAGGTCTCTGCTATTGAATAACTACGGCGTAAGCGTGATAAATGCG
CCTCCCCTTCACAGCACGTCCAACGGTCAGGTAGAGCGCTTTCACAGCACTCTTCTGGAGATAGCTAGGTG
CTTAAAGCTCCAAAGGGGTATAGTTGACACAGTCGACCTTATCCTGCAGGCCACTATCGAGTATAACAAT
TCCATTCACTCAGTGGTTGAGAAGAAGCCTAAGGACATCCTGCTTGACTCGCTTCCGGAATCCACCCACGA
GGTTAGAGGCAGGATAGAAAGGGCCCAAATCCAGCAGAACGCTAGGGTTAATAAAATGCGTCGGAACAG
AGTCTTCGAGGTAGGAGAAAAGGTCCTGGTAAAGACGAACCGCAGACTAGGCAACAAGCTATCGCCTTTG
TGCATTGAAAAAAAGGTTGAAGCGGACTTGGGGACAACGGTCCTCATTGGGGGGAGGGTGGTCCACAAG
GACAACCTCCGTTGAATTGAAAGCACAGTTTTTTTCATTACCATTCCGATAACAGTAGTTTCAACATTTTA
GGATTACGTTCACCATACTTTCAATCATGTCCATGACAACGGCTCGGGTGACGGACTACTCGCACGCGGA
CTACATACCGATTATGGACGGGGACATTCTACTTTGGGACAGTTACGGATACTTACGTCACACTACCAATC
TCACGGACTATAGGACAATGACTGAGGAGACCATGAAGCTGACGGGAATGTTTCCACAATCCCATATGAG
GAAATTGCTGGAAATTGACACTGGACATGTACTGGACATGTTGTCCGCTCTCGATGTGCACCATCGCAAG
GTAAGAAGCCTTGACTTCTTGGGAACAGCCCTTAAGGTTGTAGCGGGCACCCCGGACGCAAACGACTTCA
GGAAAACAGAACTGACTCAGATGCAGCTGGTCACTGCAAGCAACAGACAAATTTCTATAAATTCCAGGAT
GCAGGAAAAGATCAATCAACTGACCGACACCGTTAACAGAATTATTAAGTCCTCAAAAGAAAAGCAGGC
CGACACTGGCCACCTCTTTGAAACCTTAAGCACACGCAATAGAATGATAATAACAGAAATAGGGAACCTT
ATGCTTACTTTAACCCTAGAAAAAATTAACGTAATAAACCCAACAATAATAGATCATTCTGATCTTATGAA
TATTCTCACTGACCAAATCACAGGGACCCCTATCACTGACATATTGAAAGTATCTAATATTAAGGTAGAGC
GTAAGGATAATGTAATAAGTTTGATAATCGAATTCCCTATAATAAGTAAGCAATGCAGGAAGATAACAAT
TTTCCCAGTCGCCCACAACGGTTCTGTGCTCCAACTCCACGACAATCTTGTGGCCGATTGCGACGGACAGA
TGTTGGCCGTAGCCAATTGCTCACAAACTGCGACTGCCACATTCTGCAAGCGTTCCAAGAGCATCAGTTGC
GCACAAGGCCTCCATGCAGGGGGTACAGCCCATTGCCAAACGCAGCCGAGTCATCTTGCCCCTGTCACCA
TAGTGGACGACGGCATCATCGTCATTAACGATCGTAGCGCAATACTCACCTACGACGATTGCACTGCGAT
GAAGATAGATGGGACAAACCTCGTCACCTTCTCGTCCTATGCCACCGTAAATGGCACGCGATACTTCAAT
CGCAAGAGCGTGCAAAATCGGTTCCCGGTGGTAGCGGCATCACCGTTGCTTAACATCAGCGGCCATGACT
CGATACTCAGCCTTCCCTACATTCACCGCCTGAGTGAAAACAACCTTAACTACATCAAAGAGGTCAGAGA
CAGTATTGCAGTCGGAAATACATTGGCGATTTCCTTCGGCATAATGCTGTTGGCCTGTGCTGTGGGGGCTG
GAGTCTACTGGCTCCATAGGCTGACAATCAAGCGTCGAAACTCCCGGAAGATCTCGAAGATATTGGAAAA
CTTAAATGCGACCGAGGACGGTCGATCCTCGAGAGGGGGAGTAGTTAACCACTGACTATTGGTATGTGCT
GACCCAGCGTTCTGGGGAAGCCAATGACCAGAGTCATGTGGCGACGGATATGTCGCAACTATGTCACCGG
CCGCGACGGTCATCCGACACCGCACACGAGGCGCTAACGCTGCACTTTTGCGAGAGCGGCGGCAGCGGTT
CTACAGCAGCAACGGCGGCTCTGGGGCAGCGACGGCGGCTCTGCAGGCATTGCTTAGATTGATAAAGATT
GTATTTTTNAGTCTAAGTTAGGAAACAATAAAAGCGCGACGTATCGTCCCTTAAAGTCTCCCGTTAGTTAA
CTCATTTTTTATATGCATTTTTACATGCAACCGGCAAACCCAAAGTTGCAGACATTAATGCACTAATTTAT
ATA
>9_rnd-1_family-468__Unknown
TAAACAACATTAAACTGTATATATAATAATAGTAATAATATAACAATTGACATTTCGAAAATACATTGGA
AAATAATTGTGGCTATGAAATTTTGATATATTGTATATAATTATTGATATATGATTATTAATATACAAATA
AAATTATAAATGACATTAATCTTTATTTATTTATTAACTTATATAAAATTATATTGAATTGTTATAAAATTA
GTACATGAAAACGTTACAGTGCATGGCAGCATTTGGAAAAAATATCGCCATTATAGATGATGTGTCAAAA
ACCTATATAGGGATGGTGTCGCGCAATGCATGTCATATGAAAAATTTTAAATACGGTAAATTCATATGACT
GTGGAGTGTTGCCTTGCCGGCATATGTTTATAAAAAGAATAATGAAGTTATAAAAAGTGTGTTATTAAATT
AGTACATGAAGACATTAAGGTGAATGGTAGCATTTGGAAAATATATCGCCATTATAGATGATGTGTCANA
AAAACCTATATAGGGAGTGGTGTCGCGCAAGGCATGTCATATGAAAAAAATTTTTAAATACGGTAAATTC
ATATGACTGTGGAGTGTTGCCTTGCCGGCATAAGTCAATTATGTTTATACAAAAAGAATAATGAAGTTATA
AAAGTGTATTATTAAATTGGTACATGAAGACATTAAGGTGAATGGTAGGTGAAAATAATGGTGAAATGGT
GAAAATAAGAATCTAATAAAAAATGNGGCTTAANTTTTTTTCCAANTAAAACAGTTATTTCCTCATAACA

GTCATTCTTTTTGACCGCTTTCGTGAGCACTATCCAAACTATTTTATTAAGTTAGAGTCGTTAGTTTCAAGT
TGCTTACAATTAATCGGGCATATGTCGGGAATTTAGCTAAACAATTATTTTATATTTATAAGCATTTTTATT
AACTTAGTTTATGCATCAAGTATAAAATTTATTTTCTTCTCGTAATGTGATGTAGTGCAACACTTTGATTTA
CATATAAGTGAGATATCGATAATAGCATGAAAATAAGAATCTAATAAAAAAAATGCGGCTTAATTTTTTTTT
CCAATAAAACAGTTATTTCCTCATAACAGTTATTCTTTTTGACCGCTTTCATTTTTACAATCCATCATCAAT
TTAATATCTCTCTCACATCTTCATTTTAATTGCATAAATATCGACTAGTTTTTACCATAACGCGAGCAAAAT
GAACGCGTTTCGAGTATTCAATTTGATAGTGCAGAGCTGCCTTAAAAGTACATTTGAAGAAATGACTTGA
CTTGCTCGTCGCGTAACTCGAAAACTTGGCGCCATTAAATTTCTGGAAACTTTGCGAGAGGATGTATTTCT
TAATTAAGTGCGAGCAATATTATGTATTGCANAAAAAAATTAAAATAGTGGAAGGAAGTAAAATGTGCAT
TGCAAAATTTAGCAAAGGCAGTTTCGAAGCGGAGATTATTGATTCGCGAGGTAAGTTGAGCTTGACATTT
CAATAATTATATTTGTCTGTATATGTACTTTTATTTCATTAGAAGATCGTGAAATACTGGACCAGCAATGT
CAAGAACTTAATAAGGGTATGTACATAAATCAATAACAGTTTTGTCATTTTCGCATATGCATATTTATACA
ATAGTCTATATACATATGTACATAGGCAATGCAATAATCGTTTTTTAATTTAAATTAAAATTAATAATACA
TTAAATATTTATAGACTTACTAGAAGCAGATACTAAGTTAAATGAAAGTGAAGCAACAACGAACGATGAT
AAATCCGGTAAGTACAATACTAATGTGCCTTTGAAATAAATTAATGAGCATTGTCTTTTAAAAGAGGNGC
CATTAGCAGCTTATAAGTCAAATACTGACACAACAAATAAAAGCAACCTGGGACAATTTGAAGCTTTCGG
TATGTGTGCAATTATTAAAACTAAGCCAGGACTAAATAAAAAATCATTTGCATTACACTTCTGACAGAGTT
ATGTTTTGTAATGTATTTATTATTATATGTTAAGTCTTAAATATGCATAATTGTGAGTACCACAGTTTTAAG
GCCAGAGTCGGAAAATATCAAAAGATTTATAGCCTTATTAGTTGTTGAAAACAATTTAATTATCATTTGTA
TTATGAGTTAATCACATATGCAAAATCAGATCAATTTATTATAAAAAGCTACACAAAAGACACAATCGGT
CATTACTTTTCTAGGAATAGTGTATTCTCTTGTACGCTATATGCCTCCGGACGTACAAACCTTTTTTAGTAT
TCAAACAGTTTTTGCTAATTTGTATTTTAACTAAAGCTAACATTGTATACTGATAATACTCGCCTTTTGGAT
TGTTACCTAATTTGTGCTAGTACCAGCTATTTTGTGTTCGTTTCAGGTGTCTTGTGCCCGTCTTATATTAAC
ATGTAAAATAAATAAATGTATTAATAAAATACTTATACCTTAACATAATAGACAAAGACTGGCCTCTAAA
TGGCTAGATTCCCCAGCATAAGCATTTTATACATTTAAATCGCTTGGTAATTTTCCACATTCTATTCAAGGA
GTCGATTCCAACTATACTCCGGATATAACTAATCAGTCCAGCACTTATATACTTCCAAGTTTAACTTACAC
AATGTAACTCCACTAAGTGATATAAGGGATTTGATTATTCCTAATTGTGAAAACTTTTTTGCTTGTTTTGTA
ATAAATTGCAATCAAAATTCGTTCGTCACTTGCATAATAATAAGCCACAAGTAAAAAAGTGTTTATGCCTG
TCGAAAAATGACAAAACTCGAAGAGTAGTTATTAATGAGATTTGGAGTAATGGCGATTAAATCGACAATA
TCGATGCATCGTTAAATAACGAAATTTTAAAGCGAATTAACTCTAAAAACGAATATGTTGTCATATGTTGC
AAGGGATGTTCTGGATTTTTCCCCAAAAGTCACAATACGCAGTCATATAAATAATTGCATAGGCGGCGAT
CATAAGAAAGGAACTCCTGACTTGCTTAAGCAAGGTCGCTCTAAAACCGGCTCTATGCATAAAAGTGCAA
GTGACGTTCTCTTGTAAGCTATATGCCTCCTGACGTATTTAAACCGTTTTTGTTAATTTTTTTATTTATTTAC
TAAGGCTATTTTTTATATACTTTGATATTACTCAAATTTTATTTTGTTAACTGATTCGTGTTCATTCCAGCTAG
TTTGTGTTCGTTTCAACTGTCTTGTGCTCGTCTTATATTTATATGTTTCTAACAAAGGTAATGAAAAGTATA
AATACATTTTTTTTATATATAAATAAANTCCTTATCATGATCAGTACAGATATACATTTTTTTAACAACATT
CATGCTGCTTAACGTTATATAAAAATGATTGTATTAAAAAATATAAGATGCAATAGAGACACATCCGAAT
GATCATAGCAAATGCCAATTAACTGCAACTGAAGTGGAGGTATACACTATGCCATATCCTTAAAGGTCTG
AAAGTTAAGCAAAATAAATGTTCTTAAATNTGTATATGGCACTGCTCCTAGTGCTACTATATTGAATACAA
AATATTTACTTAAAAATATTGAATTACGTCCATTAATAATTAAAATAGATTCCTTTTTATCTATTAATTATT
AAATATATTCAAANAGATGCATCCGAACAATTTATAAACACAACTCAAAATGCATCGGCTAATAGTGGAT
CTTTAGGACTTAACGACTTTGGCTCTAATTGTTTAGATTCATCGGCAAAAATCAAAATCATTCCACGATAA
ACGATTCATTTCAATCGCTTAATACTAATTGTCCAGATTCCACGCAAGGATTCGATTCCAGTTATGCTCCG
GATATAACTAACCGGTCCAGCATTTTAAATAATTCCAACATTAAACAAGTTTTTTCTTACACAACTGAAAC
TCCACTAAGTGATGTAAGGGATTTAATTATTCCAACTGCGAAAACCCAAACAAATTGTAAGAAACATTTTT
GCTTATTTTGCAATAAATTGCAAACAAAATTTGCTCGTCACTTGCAGATTGTTCATAAAAATGAGCCGCAA
GTACAAAAATTTTTGTTCCTGTCGAAAAATGACAAAACTCGCAGATTACTTATTGATGAGATTAGGAAAA
ATGGTGATTATATCCATAATATCGATGCATCGTTAAATAACGGAACTCTAATCGTGCCCCGTCAAAAGCGT
GTAAACTCTAAAAACGAAGCGGTTGATTTCATATGTTGCAAGGGATGTTCCGGATTTTTCCCCAAAAGCAC
AATACGGAGTCATATTAATAATTGCAAAGGGAGCGATCATAAGAAAGGAACTCGTGACTTGCTTAAACAA
GGTCGCTCCAAAACCGGCTTCATCCATAAAAGTGCAAATGACGTTGTGCGCACAAAAATAATGCCTGTTT
TAAATGATGATCCCATTTCTAGAAATCTTATATATGATGAATTGATTGTTTTATTCGCAAACGCAGAATCT
ATGAAGCACGGCGAACTTCAGTACGATATGATCAGAGCAAACATGAGATTGCTCAGCAGACTATTACATG
AAATAAAAAAAATCAGAATCTAATGAGATACAGGAACTTAAAGACGCCATTAATCCCAAACATTTGGATTT

GATGATATCTTGTATCAGAAAAGTGGCTCAGTGGGACTCTTCTTCGTTGTTTTTAAAACACCTTCAGTTGC
AACGAACCTTACAACTCTAATTAAAAAGTGCGCAAAAATATTGTGGACTCATTGCATTAAAACACAAAAT
CATGCAAAAAAAAATTAAAGATTTTCTTGTTCTATGGGAAACCGAAATTCCTCTTTTTATAAACAAAAAAA
GCTATAGAGGACCAGTCAAAGAAAAAAGAGCCCTACCAACTATTTTACCATGTAAAGAAGACATCAAAA
ACTGTTTGATTATGTGATAAAAAATCGGAAAGCGCTCTGAGTGTGCTAAAAATTAAGTTTGATTTTTTATC
ATGGACATCGTTGATACAAAGCACACTAATTGGCCTTCAAATTTTTAACCGCCGTAGAGCCGGTGAATTTG
AAAGACTAACAATTGAGAACTATCTAACAGGGCACGCCATCGATGAAAATGTAGATAAAGAAAACTTCG
AACGTCTCACGAAAGAAACTAGAGAAATCGCTCAAAAATACACAAGATTGACTATTCGTGGAAAACTCA
ATCGAACTGTGCCAGTGCTGCTCACACCGTTTGACAAGTATTGTATAGAAACTATTCTCAAACATCGTAAA
AGAGCTGGTGTTAAAAAAATAATGATTACATATTTAGCGTGCCTAACGCCAAGCCCTTAGCAAANCAATA
TGTAAGGGCTTGTCCGATTCTAAAAAAATTTGCCCAGGAATGTGGGGCTCGCTGCCCAGGGGCTTTACGG
GGAACGATGCTTCGAAAGCATTTAGCTACCTATACATCGATGCTTAACGTCAAAGATGTCCAAATTGATA
GGCTTGCCAATTTTATGGGTCATCATAAAGATATACATAAAGAAATCTACAGAATCCCTGTAACAGTAGC
GGAGATAGCCGAAGTATCCCAGATGCTTATGGCAGCTCTTGGAAATAATGTTGAGGATGAAAACACAGTA
GCCCTTGATCAGTTCGAAGAAAGTAAGTACGTAGATTCATCAAGTTTGTGGGTGATACTAAGAGTAGTTA
TGGTGAAAAATGTAATATCTTAACTTAACTAATAATTACTTGTCGGGCTTATTGTGAATGTTTTTACATTGT
ACCTGAAATCCATTTTGCAGATGATCCTTTAGAGGATTCGGAATCAGATTATTCTGAACAGCGATCAGAA
AGCGACTTCAATATTTCCGATGATGANATTGTAGAAAATCGAATAAAAAGGAAAGCGCCCAAGTAGTAA
GTATTAAATTGCAACCTACATATTTATGTCACGTGCTTACATGCATCGCGTTTAGGCTATCCTATTTATCCT
CATTGACAAAGTAAAAAGAGATATCGCGCTTGTGAAATGTCCGATCGTCTGTTCAAACGAACGCGATATT
TTGCTGGCCCATTAAAGTTATATCAACGAGAAGTAAAGTGCTAAAATTATATAATGACGACCACTGCGGC
GTGGTAAATCGCAAAAATCGTCCTAAATAACAAACCTTATACAATCATAGAGATTGACTAACTTTTAGTTG
AAGACGAAGATGTTTAACTGGGTGCGTGGCGAATGTTTAAAATTAATATCACCTAATAAATTGTTCTGGTA
GGCTGTATTAAGCTGTTATTGGTCTGTGATTGAGTATTGCGACGGTTGCAACCGACTGCGACGCTTCTAAT
TGATTTTTATTTATTACTAAAACGTTAATCTTTTGCACACTAAATTATATTAAACTTTATTATTTAACAGGA
TCATCATTTGGAAAAACCATAAGAAACCGTTGGTCGAATGAAGAGCGTGAAGAAATATACCAGGCTTTTG
GAGACAATTCTGTTGTCTNTTAAAGGATCGCCGTCGCTTAAGCAATGCAACGCGGTTATAAATAATTCTAC
ATATCTAAAATGTAGGACACCGGCGCAAGTAAAAACATGGATAGATAATCAGCGCAAGGCTAAATCAAG
AAAATCAACTCCGCAAATAGATTAAATAATGAATATGTTGACCATATTTAGAACTAAATTGACTATACTTT
TTCGTATTTTTTTATGTTAATATTAATTTATGCTAAGAATATTTGGAATTTGTTAAAAATGCATACTATAAA
TGTATTTAATAACGTCATTTATGTAAATTCATATTGTTAATTTTACTTTTAACCTAAGATGATTTGAAACTG
CATTCAAAATGTATTTGAGTTTAATTTCAACTAAGCCAGCCTGGCACTGAATTTAAAATGTATAGATTTAA
TAAAATATTGCTGAGTTTTATAGGCGTTATACTTACATTCGAGTTTTGTTGTATTTCACCGTTCCTGTGTCC
TCATACCCTCTATATAATCGCTATATATATAAAATATTTGCATCTTCCTCAGTACGTTATTTGGGGACAGCC
ATTTGATTGTTATTTACTAAATTGGGGTGTGAACTGG
>160_rnd-6_family-772__RC___Helitron
CCACCGCAGAGCTATAAATGAAATTAATAACCCAACTTTAATTGCCAACCAATTTAAACCACAATTTAAA
TGTAATTGTCTTTTTCAGAATATACAAATATTCTATGGTACAATAAGATATACTGTAGAAAATTTTATAAA
GATCGGTTAAGAAAAACCAAAGTTATATGCAACTGCGCAATTGAATGGGGCAGCAGCTTTAAGAATTTCT
CTATACATGTACACACACACATTCATGCGCGTCTGCTTCGCATTCTAGCAGCATGGTAATTGAATTTTTTTT
GTAATGTTATTTTAGTTCCTTTTTTATCATAAGTACTTAATTGTTGGTGTGGCTGCTGAGTAGAGGTATAGC
AGGTGGTGCGGTCTGCCGCGAGTTCGAGCCCTACCGGGGAAAGTATTATTATTTATATTTTTTTTGCTGGT
GTGGCTGTTGAGTAGAGTCGACAGCAAGTAATGCGGTCAGTTGCGAGTTCGAGCCCTGCCAAGGGAACTT
TTTTTTATTAATTTATTTGATGCTGGAATAAGAGGGTTCAGGGTATCCCCTAGTCGGGAGCTCCCTAGCTC
CCCTAGAACCTCTTACTTGTTTTTTTATCAANTCGGTTTCCTATATAGGCTACATGGTTTAGATATATTTAA
TTTTTCGAATACCTCGGTACAAATTTTCAACAGTTGTTATAAAAGTAAGGATATGCATTACATAAGGTGAA
GTGGGCAGACACTTCCACAATTTTGTCATAAAATATATTCATGAAAAACTCTTAAGGCTTTTAATATAAAA
TATTCTTTACTTTACTTCTTTGCAAATATTATTTACACAACATCTGAATAACCATTTTTAATTACTTATTTTT
ATCGCACAGTAGACTTTACTTTTTTAAATACTAAAATCTTATAATTTATGGAGTCAACTGTTTCAGACATC
ACATACTATCCAGATAAATCATCGTCATTTATAAATTGTACATATTTTTCTCGCAAACCAATGGCACCGTT
AGATGATGTTAGAAAAATTACTGTGCCAATTACTAAAAGTCAGAAACAATGTAGACGGCACTTTTACATA
TTTTGTAAAACATTGCAAACAAAATTTGCACGCCACATAGAGTTTAAATATAGAGTGGAACCAGTTAGAG
AGATCAGCAGTTTATCAAAAGACAAACAGCAAAGAATGAAATTGCTTGAGGGATGCTACTTACAATGAA
GGAACTTTTATAGTTTCACGTCAATTAAGGGAAAACTCAAAAAGAGGGCTTTGGATTTTGTGTGCTCCAAT

GGATGTAAAGCCTACTTACCGAAGACAACAACTCGGAATCAAATTAAAAATTAATGTAGTAATATCCATA
AAATATGCAACCGAGATATATTAACCTCTGCACGAAGTGCTGCAAATAATATTGCGAAATAAAATAATGC
CTGTCCTTAATGACGTCGCCATTACTAGGACAATATTGTATGATCACCTAATTATATTGTTTAAAAATTTA
GAAGCAATGAAGCACACGGAAGTACAGCATGATATGATAACAGCAATTATGCGTTATCTAAGTCGCCGTT
TGATAGTAATAAAATACGTTATTTCCCCTAAATATTATGATTCGGTTAAGTCTGGAATTCGTTATGTTGCTG
AGTTTGATTATGATAATATTCGTTTTATGAAGCCTACTGCAGCGACAACTCTAACATGCTTGGTAAAAATA
GTACATGTGCGAGAATATTAAAAGCACAGATCATAAAATATTGCGACGATAGCATAAAATATTTAAAAAA
ATAAAAGATTTTGTTCAGGATCTACCTGCTAAAGCTTTGAATGAGCTAAACTAGAAATTCAACATATCATC
ATTAACCTCGTTATATCAGAGCAGAGGTAGCGAACTTGAGAGTCTTACTCTTAAGAGCTACAGAACTGCT
CATGCAATTGGGGCTATCGACGATAGAGCTAATTCTGACCTGTATGAAGGCACCTCAGCCAAAATGAAAA
ACATCACCAGGGTGATTATTCGTGCCAAAAAAACCGAACATTTCCAGTTCTTTTTACACCATGCGATCGGT
ATTGCATAGCCAATATACTCGAATATGGCAAACGCACAATAAATAAACCAGCTACTACACTAAAGATATA
TTTTAGTGTGCCAAGCCCGTGGCCACTAAGAAAAGAAAATATTCGTGAGTGTCCTATCCTAGAGAAATTC
GCAAATGAATGTGGTACACGTTGCCCTGAGTCCCTTTCAGGCACAGACCTGCGAAAGCAATTGACCACAC
ATACCGCTTTGTTCAACGTTAAGGACATTCAAATTGATTATTTGGCGAGCTTAATGGGTGATCATAAAGAT
ATTCATAAAAATTACTATCGTGTTGCTGCCTCTGTAGCGGGGGTGACCCAAGTTTCTCAATTACTTATGTC
AACAATGAACATGGAAGAGGAAAATGCTGAAATTGCAGATGCTAATCTCGAGAGTGAGTTAATTTATTTA
GCCCGTCTGTTGCGAAGAAAATTTTAATAATCGCTGCAAGTTTTGGACCTGGAACTGTCATATTCGGAATA
CTTCAATAGAAGTTTTTCAAGTATCGGAACTTCGATACAGTAGAAGATACATTCAGTATTTAAGAAATTAG
TAATGGTTTTGGCGACCTATCCATATTTAAAATATTTTGCTGGCGGGACCTCGGCCCGAGTTTTTTTTTAA
ATTAATAGATTAAAACAATCTAATTTAATACTTATATTGCTTATTTTATTTAAAGCAAAATATTAAATAAA
ATATTTTTCAACTGTTTATTTAAACGAGCCCGAACCTCGACCACGCCAACATAAATATTTAAAAACATGCG
CATTCCGGACGGTATCAGACGAAGCAGCGCTGGTTATCGCAGGGATGATCCGCACAGCAGCAAGGATGC
AAAGCATGGAACGATGGCAAGCACGTTGGGATCGCTCATCTAAAGGACGCTGGACACATAGGCTTATCCC
AAACTTGGAAGCCTGGATAAGCCGCAAGTATGGTAATACTAATTTTTACTTAACGCAGCCTATTTGGCCAC
GGATGCTTCAGAAGCTACTTGAAGCGATTCGGCCACGATAACACAGACGAGTGTTCTTGGTGCGGCAGTG
AAATCGCAGAGGACGCCAACCACACCATCTTTGAATGTGGAAGGTTCTCAGTGAACAGGCAGGGCCTGGA
GGACAGCTTGAGGAGACCTATCACGGTGGACAACTTGGTCAGGAACAAGTTGAAGACCGCACAAAAATG
GGAAGCTACCTACACTTTTGCAGCAGAAGTGATGCTGGAACTCAGGAGAGTTGAGCGCAATAGATGGAG
AGCGGAGGAAGTTTAGGTGGCAACAACATGCCTGTGAAGCAATGCTTTGCGGCCGTTCCGCAGGCAATCC
ACGCCACCTAATCATCACGTACCCCCCTCCTCCTCTATGTAATCTTGCTTCCCAAACAAAAATTGAAACAT
GCCGCTTACCTTCGTTGATATATCAGCTGTTTATGTTTACGGTACTTTACCATTTTTCAAACATCGATAGAA
ATCTCGATAAATCATCGTAAAAAACTCAATATGTTCCGCGCGAATTTACACATTTCTCAAAAATGTGGCCT
TTTTGAATGATGTGTGAAATGTGTTTTCTTATCTGGAAACGCACAGGGCGCATTTGTAGGGACCAACTGGC
ACAAAAATTTATCATTTCATCCCATTACACCAGCACCCGTTCACAGTCAAAAAAGTCACCGAAATATTATG
CATGCATCCCATCGTAGTCGCTATTTGCTTTCGCACGACGCTGCATGATTTCGCTATCTCAGCGTCGGTGC
GTTTCTTCACTCGCACTTTCGTGAGAATGAAGGAATGCTCGATAGATCAGGCCACATCACCGTTATAATTT
AGATGTGGTGAATAATAATTGTGTTTGTATTTGTGTTTTTGCTGAAATTGAAGAAAAGTAGAAGGTCCGAT
TGCCACGCTTCGTTCTTTTTAATTCGACAGTGGAACAAGCAAGTCACATATTTTGAGCGCGCATAAATCAT
AGTAATTAACGGTGCAGTGGCTGTACACCGCTTCGGATTTTTAAGGGCATATCGAGCCCAACTCTTTTTAA
AAGTCCCCAGTGCTCATACAGTTCGCGTTCAGCAGATGTTATTCGCTTTTAATTAATCAAGCGCGTAATTC
TGAATAAACCCCTTTTTTTAGTGGATAAAATCTTACTTAGCTACCGGCCAGCCATTGCGCAACGACGCGTT
CGTGTATTCATATATATATACACATATAAATTCACCGTGGCTGGTCGGTCGTAGCCAATTTGTGAGTTGCA
ACGGCTCTAAGGGAGTGGAAAATATCAAAAGGGCCACGGATTTCTTAAGGATCACTACAGATTAACACTG
CGGCGCCACCGGCACATAGCGGCTGAGTCTATACCCCGCGACCGCGTGTTTAGCCATTGATAACCAAGCT
TTGCTGATCTACTTCTACTTTGCCGCCCCTTTTTGATCAACGGAGTGTCTTCAACAAGGAATCGGACCGCTT
AAGTGTGCGCAGATCGAGATCGAATATATGTGCTGGGCGATTGGTTGGAATTTTTGCGTACACGCTTTGCA
GCAGAAGTGATGCTGGAACTCAGGAGAGTTGAGTGCAATAGATGGAGAGCGGAGGAAGTTTAGGTGGCA
ACAACATGCCTATGAAGCAATGCTTTGCGGCCGTTCCGCAAGCAANCCACGCCACCTAATCATCACGTAC
TATCTACATCCTAAGCATAAATTATTTATTATTTATATATTTATGTGCAAATAAACAGAATACAAAAAAAA
ACACACACACACACAATGGAAACATAACACCCCTTCCACCCCTCCCCCACCACCCCTTAATCCTTAACACC
CCCCGCCACCCCTTTACCTTGCTTAACAACACTTGACCCTGATAATAAATGTAAACAAACATACTGTGCCA
GAACATACAGTCCCCACCACGGTGACATCACTTCCTGTGCCAGAACATACAGTTCCTATCTACTATCCTAT
GTAGACATAAGAATATAATAAAAACGTGTTTCATCGACTTGGGAAATTCCAATTACTGCCCGATCAGTTTC

GAAATCATTTTCAAGAAAAGGCCAGGTTGTGTTTTTTTGTTTGTTTGTACATACAAACATAGCCATGATAA
TTGTATAAGGCCATTAGAATCGAATAAATTCACAGCATTAATTTGTAGGATTACTCAACTGAATAAGTTTC
TGTGCTTCGAAATTTTTTAAAAAAAAGTGACATTTTTGACCCAATAAAATGTATATATTCCTTATCCGCAT
GGACATAGGAGTCCGTATTGTATTAATATTTTAAGCGTACGCAGTACTATGTTCAAGTTTATATTATATCT
ATTCTAAAACTCTACAAGATTAAAATATATTCATAGATATCGATATTGATAAAAATATTTTTTTTATACCCT
GAACCCATTAAAAATGGGTATAAGGGTATATTGTATTTGTGCAAAATCCAAATGTATGTAACAGGCAGAA
GGAAGCATCTCCGACCCCATAAAGTATATATATTCTTGATCAGCACCAATAGCCGAGTCGATCTAGCCAT
GTCCGTCTGTCTGTCCGTCCGTCCGTCCGTATGTATGAGCNCAAGGATCTCAGAACCTATAAGAGCTAGAG
ACTTGAAATTTTAGATGTAGGTGCTCCTAGTTCCCGCGCAGATCGAGTTTGTTTCCGATAATCGATAACTT
ACTCCGTTTCCAAGCAATCGATAAAAATCGATATCGACATCCTGTTTTTTTAGCAAATTGGGTAAATAATA
AGAGCTAGAGCCACCAAACATGATATGTTGCTTCTAGAATATTATAGATATTTCAAGTATCTTTCATTTCA
TACCTATCGCCACCTCCCCGCTACCACCCCAGAGCTATAAATCAAGTTAATAACCCAACTTTTATTGCCAA
CCAATTTAAACCACAATTTAAATGCAATTGACTTTTTCAGAATACACAAATATTCTATGGTACAATAAGAT
ATACTGTAGAAAATTTCATAAAGATCGGTTAAGAAAAACCAAAGTTATATGCAACTGCGCAATTGAATGG
GGCAGCAGCTTTAAGAATTTCTGTATACATGTACACACACACATTCATGCGCGTCTGCTTCGCATTCTAAC
AGCATGGTAATTGCCATCTTTTTTTTGTAATGTTATTTTAGTTCCTTTTTTTATCATAAGTACTTAATTGTTG
GTGTGGCTGCTGAGTAGAGGCATAGCAGGTGGTGCGGTCTGTCGCGAGTTCGAGCCCTACCGGGGAAAGT
ATTATTNTTCGAGCCCTATCAAGGGAGCTTTTACTTTTATTAATTTATTTGGTGTTGGAATAAGAGGGTTCA
GGGTATCCCCTAGTCGGGAGCTCCCGACTAGAACCTCTTACTTGTTTTTTTTTTTCGCTGGTGTGGCTGTTG
AGTAGAGTCGACAGTAAGTAATGCGGTCAGNTGGAAATTCGAGCCCTGCCAAGGGAACTTTTTTTTATTA
ATTTATTTGGTGTTGGAATAAGAGGGTTCAGGGTATTCCCTAGTCGGGAGCTTTCGACTAGAACCTCTTAC
TTGTTTTAAATTTAAATCTTGTCCAGATCATTTGCTGGCTCATATACTTCTTTTTTAAGCATTTTTTATGCAA
CTTGTATAGTTTATAAAAACAATCGGCGCACATTATAACAACAATTATTATCAACAGTGTGTTTACTAGTT
GTAAGTCCATGTTACTTGACTCTACTTTATTAATGACATAGGCAGTGGAGTCCACTGTCTTTAAGTCTAAA
ATGGGACCATTTTGGGTTCGTATGTAATTAAATTATAATTATAACTATTAGGCAAATTTTTACCAAATTTG
CTTATTGAATTGCTTTGCATTTACGCTTACAGTTTTCCTGCCAATATGTAGGTTTGGTTTCGTATTGCTTTAC
>Ref_Slicemaster
ACGGAAAGTCAAAAAACGAGCATACGGAAAGTCAAAAACGGAAAGTCAAAAAAAAGTCAAAAAAAAAA
TCAAAAATTGAAAATTACTAAAGTTGGTGGCAATGTGTCCGCGAGAGTGCGCGGCGCACGTGAAAAATCA
AAAATTGTGAAACCACATACATTTTGTCTGAAAACTATCAAATAGAAAATTGCACATTTTTCGGTGCAGCA
AAGGGCAAAGGGCACTAGGCCCAGCCGTATTTGTGCTAGTGCTGTAAATAAAAACTTATCGATAGGACGA
TATAAAACACATGTAATAACAGAAAGAATCGATATCACATGATCTATTAAATAATAGTGAGACCAGACTT
CTTACTTTCTCGTATGCCTCTCAAACTGTTTACATGGCACAGCGTCAACATAACAATAAACAAATCACGAC
ACCGATTGATGAGGAAGACTCGGACATTGAAATTCAGCTAATCAAAGCTGGCCGCGGTCCATTCTCAGCT
AATAATGCGTTGAAAATCCCTGCAATGCAAATTGATGCCAATCAAAATAACAATGAGCAGCTAAATCATG
CGCCCAGCACCTCGCGTGCCGCAGGCTTAGCTGCTGCTAATAAAGTTGCCCCCGACGCAGCCGCAACTAC
TAAGAAGTCAACTCTTGCTGACTTGCTGACCAAAATTGCTAGTGATAAAAAACTCAAGGGTAAGGTTTCT
AAACGCCATGCAATTCCAGATAACAGCGCCAAGCGAATCAAGATGCTAGAAGCCGATATAAGCAAGGCT
GCTGCTAATGATGCCGCCCTGAGTGAAGAATTAGAAAATCTCCTTGGGAACGGCCAACAGCATGAACTCT
GGGTTACAGACCATCCTGATGAAGACATGCAGATTGACGCTGTTGATCCAGTAACCGATGCGCCTGCCCC
AAACATTACTAATGTAAATGTAAACAACAACACTACGGTAAATCATGCAAATTCTGCTGGTGTTGACGTA
AGGGCCCCAGTACCCAAGGTACAAAAAATCAAACCAGCGCCACTTTTCATACCTTTTGTTCCGGAAATAA
TGCCATTAATTGAAGGCATTAATGATGTCATTGGTGAGGGCACCTATGCAACTAAGCCTTCTGCTGGTCAG
GGCCTGCAAGTGCTTTGCAACGATGTAGATTCTTACCGAAAGCTAGTACAGTACTTCCAAGATAACTCCAT
TGCTTCACATACATACCAACTGAAGCACGAACGTGGCTTCAAAATTATCATGCGCCATTTACATCGCTCTA
CTCCGGCGGATTGGATTCGCACTAAATTGTCTGACCTGGGGCACAAAGCCCGATTCGTTAGTGTTATTAAA
AAACGATTCACTAGGGAGCCAATGGACATGTTTGAGATAGAATTGGAGCCCAAGGCAAATAACAGCGAA
ATTTTAAGGCTCAACATGCTCGGCGGCCAAAAGGTCAAAGTTGAGCTACCACTACGAACTACCGATATAG
TGCAATGTCATCGCTGCCAACAATTTGGGCATACGAAGAATTATTGCAGGCGTGCATTCGTATGCGTCAA
ATGTAGCGGGAAACATCCTTCTACTGAGTGCAGCATAACGGCTAACGATCGGCCAAAATGCGCTAATTGT
GAAGGCCCACATGCGGCTAGCTACAAAGGGTGTCAGTCCTTCAAGGATGCCTGCAAGCAAAAAGAGTCTG
AGAGCGTGGTCAAAGCCAATGTCCTACTTGCTCAGCTAGGACAGCCAACATTGCCAGTTATGCGTAGTCG
CCAAAGAAAACGGTCACAACAGCGACAGCAGCAGCCGCCGAAATCGGTACAGCAGCAAGATCAACGTCA
ACGCTCAAAGACGCGCCAACGGGTACATATTGATCAGCAGCAACAACAACAACAACAACAAACTGTGAA

CTTTGCTGCAAATTCTTATGCGTCGGCCTTGCGTTCTGGCTTGCCGGATATGCATTCGCAAAAAGCAGCAA
CAACAGAAGCTAAAGCGGCAGACAGCCCAACAGCAGCATCGCCAATCACGCCCTAAGCAACAACAACTC
CCGACTCGCAGGCAAATGGATGGTCAACAAACGCTGGCACGCCAGCAAAATAGAAGCCGCAGCTGCAGT
ACGCGACGGAGCGCTACTAACTTAAACACAAACTGCGACATTGGTCAAAAACTGGACACTCTTATCACTT
TAATGACTGAAGATCGTTTGAGAGCCAACTCCAACGTTGAAAACAAGCTAGATAAACTTCTGAACTTACT
TACCAACTTGACATCTTGCCTAATGGCAAAGGAGCAAGTTACTAGCAAACAAAATGTTTAACAACCATAT
ACCTAGCACAGTCCGTCAAGATAGCAGTAATCGCAGATCAAACGGGCTCAAAATCGCCTCATGGAATGCA
TGTGGGTTGCGAAACAAAATGTTAGAAATGGAGGCATTCATCTATCAGAACAATATTGACATCATGATCA
TAGTTGAAATTCGCCTGCGCGGACAGACGACATCAATATCGACGGCTTCTATGCATATCACGCCCTTCAC
CCATTCATACACAACGAGGAGGCGCAGCTATACTGGTCAAGCACAGTCTACAGCATATCGCTCTTCCTCTC
ACTAGCGCTATATCAATGCAGTGCGCGGCAACAAAAATCACTCTGCTTAATGGGTCTGCTATTAACATCGC
AGCAGTATATTGCCCGCCCCTGATTGAACTCGAAGATGAGCTGTTTGCTGCTCTTCGTGAGCTCGGCAACA
AGTTCATCATTGCAGGAGATTGGAATGCGAAAAGTCATTGGTGGGGCAATGCTAGATCCTGCAGACGAGG
TGGTAATCTTCTCAGATATGTACAGCAAAATAATTGTGAGATTCTTGCAACTGGGTCCTCAACACACTTCC
CGTACAATGCTAGACAATCGCCGTCAGCAATAGACCTTTGCTGTTCTATAAGGGACTGCAGAGAGAACTC
CTACAGATAAGCGCTACACACGACCTTGGCTCTGATCATCTCCCTCTATTAATCGAACTTCTTGAAACCCC
GGCATTTAACAAGTCAAGGTGCTATCTACTACCACTTAACGCGAGTGTTGCTCGCTTTCAAGCCTGGCTAA
ACCAGCATGTACTGCTGGATACAGAAATCCACTCAGCTGCAGATATTGAGGATGCCACAGATATTCTTGA
GCGTAATATTCGAGCAGCAGCACAATATGCAACAAACTCTCACGCAATTCAATACCACCAACGAGCGCAA
AACCAGTGCCCACTCAGCAGGTCTCTGGAATTAATGATCAAGGCCAAAAGACTGCTCAAACGTCATTTTC
TTGTGAACCGACACCCATTAGCCAAAAGAATTCATGACAGTCTCGCAAACCAGGTCAGAAGAACTTTAAA
AGAAGCCAAGGAGAGACGTCTGAATAGCTTGCTTGAATCTATAGATCCTAGTGACCGCTACAACTTTCAA
AAGCTGTGGCGACTAACAAACGGCATTAAACGGCAGCCTGCGCCAAATGTGCCAGTTAAAAAGTTCATTC
AACCTGGCAGCAGCGCAGAAGACAGCAACGCGTGGTGTAAAACTACGCAAGAAAAAGCGGAAGCGTTTG
CTCAAAATTTAGAAGAGAGATTCAAGCCTATTATTACTGCCTCAACAGCTGACAGAGAGCACATCTCGAG
TCAACTACAGCTACCTCCAACGCACGCAGAGATGCATTTTCGGCCAATAACAGATGCTGAGGTAATAGCA
GAAATCAAGGCGCTTAAACGTAAAAAGTCTCCTGGGCTTGATACAATTGACAACCGGACTATAAAGGCCT
TGCCGTTAGTTGCTCTCCAGTACCTTAGCCTCATTTATAATAGCATACTGCGCTTGGGCTACTTTCCTAGCA
AATGGAAGTGTGCTGTGGTCATTATGATTCCTAAGCCTGGCAAGCCGCAAGAGCAACTAAATTCATACAG
ACCAATAAGTCTGCTATCAGGTCTTTCAAAAATATTCGAGCACTTGCTGCTTAGACGAATGTACGAATCAG
ATGGTTTTATACATGCTATTCCAAAGCATCAATTTGGCTTTCGCAGCGAACACGGAGCAGACCAGCAGATT
GGCCGAGTCACGCAATTTATACTCGAAGCATATGAAAGGAACCAGTACTGTGCTGCTACATATATGGATA
TCCAAGAGGCCTTTGATCGAGTATGGCATGATGGGCTACGATATAAGCTACGGATGATTCTGCCGTATGCT
ACATACAATGTAGTTAGCCACTACCTCGCCGAGAGAACATTCATGGTAGAATGCAGCGGGGGAATCCGAT
CTAGCACTAAGCGAATTGCGGCTGGAGTGCCGCAAGGAAGTGTACTTGGTCCTGTGCTTTATACGCTATAT
ACAGCTGACATGCCCATCCCGGGACCAATAAGTCCAACAACTGGATATGTGAGGCATATGTACCGCAAAG
AAACATTGCTTGCAACATATGCAGACGATACCGTCATACTAACTGTAGGCAGAACGCAGACAGATGCAGT
TAATCGAATGAACGAGTATCTCGAAGTCTTGTCTGCTTGGGCCACTAAATGGTGTATAACAATAAACGCA
ACCAAGACGGTGCACGTCATGCACACTTTGCGCAGGCTTCAAATGCAGACAGATGCCCAAACTGCACACA
TAGGCGGGCAAGCGCTACTCAACCAAACGGTGCACACTTACCTCGGTGTAAAATTGGATACACAGCTGCA
ATTTAATAGACACATATCTGCTGTAATTAACAAATTGAGAACGAGGTTTAAAAAAATGCAATGGCTGATT
GATAGCACAAGCAAACTACCCCTCAAAACAAAAGTTATAATTTATAAACAACTCATCGCGCCAATTTGGC
AATATACATTGCCCGTCTGGGGACCGCTTGCAAGTCAAACACAAATCGATCGCATACAGACTATGCAAAA
TAAAATTCTCAGATCGATCGCAGGTGCACCATGGTACGTGCGCAATAGCACCATAAGCAATGATCTGGAA
GTTGCAAGTGTAGATGACGTCTACAATGAGGCATGTAGCAAATATGTGCAGCGCCTTTTGAATCACAGCA
ACTGCCTGGCTAAAGACCTAGCAATCAACCCATATGTACCGCAGCGGCTAACTAGGGAGAGGTACAGCCG
AATGCTACAAAGACATATGCCCCATGTGCAGCGACAGCAAGAGCAGCAGCTGCTAGAAGCTCAACAACG
AATGGCAGAAATTGAGGCACACCGACCTATACATGGAAGATCTGTTGAACGGCCACTAACTGCTCTGCAG
CAAAGCATTTTGGAGCGCCTTAGTAGGCAAAGAAGCTATCAACAACAGCGCGCCGAACAACGAGAAGCA
GAACGCGCACAGCGGAGAATAGAGAGGCAACAAAGAGAAGAGCAACATAGACAGGAAATGCTTCGAGA
ACATCAAGAGTCGATACAAATATATGCTCAAATATATGCGCAAAGAGAGCAGGCACTACAGCGGCTAGA
AGAGCGACTACAAGCAAGACGTCTGGCAAGGCAAGCGTACTTCGCTATGCCACCGGCCCAACAAGAAGC
CGTGCCGATAACGAGTATTTTTATATTTCCAGCGAACTCTGCAGCAGCCAACCGCAGCGCAATGGGCATTT
CTCCAACGACGAGCACCCACACAAACACAACAACAGCACAGGCAACCAATACGGCGTCGGCTATCAGCT

TGTCTGTGAGGCACACAATTGACGGCATACCAGATGTGCAAAATGGCGAAGGATTTACTGATCTCGTTAT
CATGGTTACGTTTAGGTGCATTCAGCGCTCAAATCAGACAACTCGACCAGAGCAGCTTCCACGTCAATGG
CGTCTGATCGAACGGCAACTGCAACAAAGATTGAGAATCAGAAGATATGCTGCAAGAAGCTATTATCTGA
TGCATACTACCAGACGACAGCGGCTAGCACGTTTGCAGCAATTCAGACTCCAATATCCAGCACAGTGGCG
ATCAATCTGGCGACGGCTGCTCCTGCGAACTTTGGACTCGAATTTGTGGTACCTAGATCAGCGCTGGTCAA
CATGGCGACTCCAGTTAATCAGGCTGCAAAATATATTCCGCTGGTTGACCAACAGACGACATGGTGGTAG
CAGCCGCTACTTATCTGACCGGTAACAACGGCTGAGATGCAAGGATTGCATGTTTTATAATCCACTAATTC
AAACAAAGATCAACTACTCACACTTCGAAGCAGTCGTTATGATCAAATTGGCTATATCGTTTAGCGCCTTC
TGAAGAGTGCAAATATGCACATCGTCCACCTTCTCCAACAAATGCAGCACGAAGGAAACCTGAAAAGAA
GACCATGCAGCATAAATATAAGATTATTAAAATTAAATTCAAATTTAACTTACCTTTGAAAGACACAATA
GCTAAAAGCTGTGCCTGTTAACAGCGGAAAACTTGTTTACCTTTTTTAATGTTAAATTGTCCTAGTGATGG
GACTTGCTAATATGTGCCTGTAAATAACATAATGTTAAATTGTAAGTTAACAGAAATGTTAAAATGTAGG
CGCTAAAAAATTCAATTAAAATATGTCCAAATATTAATGTTGAATAAAGAATACCAACACTAGCTGCACC
CCCCCTCCTGCTATGCAGGTGCGGATTGGTGCAATTTGAATGTTGGTCTAGTGCGTGTGTCAACGAGCATA
CGGAAAGTCAAAATCAAAAATTGTGAAACCACATACATTTTGTCTGAAAACTATCAAATAGAAAATTGCA
CATTTTTCGGTGCAGCAAAGGGCAAAGGGCACTAGGCCCAGCCGTATTTGTGCTAGTGCTGTAAATAAAA
ACTTATCGATAGGACGATATAAAACACATGTAATAACAGAAAGAATCGATATCACATGATCTATTAAATA
ATAGTGAGA
>160_rnd-1_family-217__LTR___Gypsy
TATATATATATATATATATATATATATATATATATATATATATATATAGTGACATATCCATAGTCGCACCCACCCT
TTAACATAACTTAGCACATAAGCATAAGCACAANTATAAACATTTCTAATATTGTAAACAAAGAGCCTGG
TGATAACATCGACATTGGTCAAGATCAAACTGTCCCCCTTTGGTAGACATAAACAATTCACCCTAAATATC
ACGCCACTGTCAATGTTCCCATCAGAGCACTATCCCACGCATAATTGCTGACGCAGCTCAAACTTCACTCA
ATTTTGACTCAAACTCTCTCAAAGCGAAGTTTGCTAAGCGACCGCAACAGTTAGATAAATCAGTTCATATT
CGGGAAGCAAATCTGAATGTACTCAACAAAAGTAGCCGTTAAGTGAAAATAATAAATTGAAATATATTTT
TTTATAGTAACCTAACGTGTAAAACTTAATTGGCGCAGCCGGTAGGATACGTCGGGAAAAAGTAAAGATC
GCTAAAGGATAATTAATTCCCAGATCTAACGCACAATCGCGACCGAAAGTGTTGTCGGAGTGTAATAAAC
ATTTTCTCGAATACGTATCCGCACAAGAACCTTCGGGTCACTAGTAGCCTAATAATATTGACCCTTTGGGC
AATAAAGCTACTACAGTTAATAACGACTGGCTTTGCATATCGTTTGATCCCTGCAATACGTTATTACTGGG
TCTTTTTAAGACACACACACAAAAAAAAAAAAAAAAACATTTAAACAATAATCCATCACAAACTTTAACCT
AAAATAAAAGTGCAAAATCAAAGAACAATAAAGTTCAAACCTACAAACTCAAAACTATTCAAAAAAAAG
TGTATTACACCAAGATGACNATGGAATTATCAGAACAACACCTCAACCAGGCCCTTTCACAACTTAGACA
GGTGCCAAGCTTTGACGGATCAACGGATCAACTAAACGCCTTCATTAAAAGGATAGATTACATCCTACAC
CTGTATCCAACCCGAGATGTTAGACAACACAGCATCTTATACGGAGCCATCGAATTGCAAGTCACAGGAG
ATGCTCAGAGAATATCACAAAGGACAGCAGCCAACACTTGGCAGGAGCTGAGAAACGCATTGATTGAAG
AATACAAAGTGCAGACACCATTTGAAGAACTCCTTCGACGCTTATACAACACGAATTACCAAGGAAACGT
CCGTAAGTTCATCGAGGAGCTCGAGAATAAATCTTTTGTTATATTAAATAAGTTAGCACTAGAAAATATAC
CTAGCAATACGACCCTTTATACAAATGCTATGAATAACACAATCAAAGATGTTATTACAAAAAAATTACC
AGATAGGCTTTTCATGATGTTAGCCAGACACGACATTACGTCGACACAAAAACTAAAGCAAGTAGCTCAA
AGAGAGGGATTATATGAAAATAGCGTTACTGAAAAACCTAAAAATAATAATGTTCAGGGAAATCNAAAT
AACAATCGTAGGAACATGGGAAATTATCAGCAAAATGCTAACCCAACCACCATTTCAAGTGGTTATTCCA
ACACGAATCAGAGTTATCACGCACAAAATAAACAGCACAATAAGTCCGAAGACAATCAGAAAGCTCACC
ACGAGTTCCAACAGAAATTAAGTCAAGGTAGATACCAAAATCCCTTAAACTACCAAAATCAGTCCCATTC
ACGCTTGAATGCGCCAAACCCTCCGACTAAACGTCAAAGAGATAGTAACAGTGGGCAGATGAAAATGGA
TACATCCGAAAATTTTCATCAGCAAGCCTCGGAACAAACAGAAGAAATCCATTCATAAAATTGATTATGA
ATGACGAAGTTCTAAAATGTGTCATCGACACAGGTTCAACCATAAATCTAATGAAAACAAACCGCTTAAA
TTTTCCAGTTTACAACGAAACATTAAAGGTTCACACCATAAATGGTGTTATTGAATTAAAACAAAGTATAC
GATTAGGAGCAAGCAAAATTTGTCCGAGCAAACAAAAATTCTATATTCACGACTTCTCAGAGCATTATGA
TGTTCTGATTGGGAGAGAATACCTTGAGGCATGTCAAGCAAAAATAGACTATGCACAAGGATCCGTAACC
TTAGGAGAATTTAACTTTTGTTTTAGATATAACGACGAAGAGGTCGAAGAGGACATGACCGCACAAGAGT
GCCTTGATCCACCCTCAACAGAGGACAGACCATTTAACTTCGCTATTAATAACGAATTAATAGAAAACAA
TGAGTTTAGGCTAGAGCACCTAAACTCAGAAGAAAAAGAAAAAATNAAAAAAGTTTTACATGAATTTCGT
GATATCCAGTACCATGAAGGTGACAATTTGACTTTCACTAGTACAATTAAACACAAAATTCTAACAAAAC
ATGAGGACCCAATTTACAAGCGTCCATACAAATATCCTCAAATATACGATGAGGAAGTAAATAAACAAAT

AAGCGATATGATAAAACAAAATATCATACGTAAATCCAAATCCCCTTACTGCTCACCAGTAATAATCGTT
CCAAAGAAAAACGATGCATCTGGAAAGCAAAAGTTCCGGTTAGTCATAGATTACCGCAATCTCAATGAAC
TAACTATTAATGATAAATACCCTATCCCGATCATGGATGAAATATTAGACAAACTTGGAAAATGTCAATA
TTTCACAACCATTGATCTAGCCAAAGGCTTCCACCAAATCCAAATGGATCCTGGTTCAATACCCAAGACCG
CATTTTCGACTAAACATGGCCATTACGAGTACACTCGCATGCCATTTGGTCTTAAGAATGCACCTGCAACG
TTCCAACGTTGTATGAACAATCTCTTAGAAGATTTAATTTTTAAGGATTGTCTGGTCTACTTAGACGACATT
ATTATTTTTTCCACTTCATTGGAGGAACACATTTTGTCGCTACAAAAGGTATTTAAGAAGCTTAGAGAAGC
TAACTTAAAACTACAGTTGGATAAATGCGAGTTTATGAGAAAAGAAACTGAATTTCTCGGTCACATCATC
ACAACTGAGGGTATAAAACCAAATCCCAACAAGATACAAGCAATTGTCAAATTCCCAATTCCAAAAACCC
CAAAAGAAATTAAATCATTTCTAGGATTATGTGGTTTCTATAGGAAATTTATACCAAATTTCGCTAATATA
GTAAAACCATTAACACTTAAATTAAAAAAGGGAGCAAAAATCAATATAAAAGACAGAGACTACGAACTA
GCGTTTGAAAAGCTAAAAGTACTCATAACATCCGACCCAATTTTAATATACCCTAACTTCGAAAAACCATT
TTCATTAACTACAGACGCGAGTAATATGGCAATAGGGGCCGTCCTTTCGCAAGAGCATAAGCCTATATGC
TATGCAAGTAGAACTCTCAACGAGCATGAGTTAAATTATTCAACGATTGAAAAAGAATTATTAGCAATCG
TTTGGGCCACTAAATACTTTAGGTCCTATCTCTTTGGTCGACAATTTCAAATCCTTAGTGATCACAGACCA
CTCGTCTGGTTAAATAATATGAAAGAACCTAACATGAAATTACAAAGGTGGAAGATCAAGCTAAATGAGT
TTGATTTCCAAATTAAATACGTCCCAGGAAAAGAAAATTATGTGGCAGATGCTCTGTCCAGAATTCAATTA
AACGAAAACTTCTTAGGCGAAGATACAATTAGCACAAGAGCAACAATACATAGTGCTCAAGAAGACAAC
AGTAACCATTTACAAATCACAGAAAGACCACTTAATTATTATAATCGACAAATAGAATTTGAAAAAGGAA
CCGAGAACGAAACAAAAGTTACGAATTACTTTCACAAAACCAACATAAAAATTACGTACAAAGACATGA
CTAACGCGCATGCCAAAGAATTAATAAAAGAATACTTATGCACAAAGAAAAGTGTGCTATACTTCCACAA
CGAAGCAGATTTTCCAATTTTTCAAGAAGCCTACTTAGAAATAATAAGTCCGAATAATTCAACAAAGGCA
ATGAAAACTAGTACAAAATTAATAGACCTTCAAACATACGCAGAATTCAAAGAATTAATATTAAAAAAGC
ACAAAGAATTATTACATCCAGGAATTGAAAAAACTATCAATTGGTTCAAAGAAACCCACTATTTCCCAGA
TTATCAAAATTTAATTCAAAATCTAATAAATGAATGTGAGATTTGCAACATCGCAAAAACAGAACACAGA
GATACAAAATTAACTTTCGAAATAACTCCGGAAATCGCTAATATCCGAGAAAAATATGTAATGGATTTTT
ACATAGTAGGAGATAAACAATTTTTATCTTGCATTGATATCTATTCAAAATTTGCATCATTAATAGAAATA
AAAAGTAGAGACTGGCTAGAAACCAAACGAGCTATATTACAAGTGTTCAACCAAATGGGTAAACCCATC
GAAATAAAGGCAGACAAAGACTCAGCTTTTATGTGCACAGCTTTACAATTATGGCTAAAATCAGAAGCAG
TAAATATTAATATAACCACTAGTAAAAATGGTATATCCGACGTAGAACGATTTCATAAGACAGTTAACGA
AAAACTAAGAATCATTAACAGCGACTCAGACGTCGAAAATAAACTTACAAAATTTGAAACTATACTCTAC
ACGTACAATCATAAAACAAAGCACAAAACGACTAACAGAACACCAGCGGACATATTCATATACGCAGGT
ACACCAGAGTATGACACTCAAGCTAATAAAGAAAAACTAATAAATAACCTCAACAAAAAACGAACAAAT
TATGAAATTGACACTAGATACAAACACTCACCGTTAGTTAAATCAAAGACGACAACCCCGTTCAAGAAAA
CAGGAGAACTAAGACAAATCGACGACAAACATTTTGAGGAAACTAACAGAGGCAGGAAAATAACACATT
ACAAAACCAAATTCAAAAAGAAAAAGAAGACTAATCAAAGCAAATATAACAATTACAGGTCAACAACAG
ACAGCGATCAAAACATACAAGCACCAGCTTAAATTAATTATTATAATATTACTATCAATAACTGTAAACC
ATATAAATGCACAGAGTATTGAAATTAATCCTATCAAAGCCCATAACGGATATCTCATATTTAAAACAGG
AACCATAGACATTCCGACAGATTACGAATTCCACTGTTTAACGGTTAATATAACAAAAACCGAAGAAACT
TTTAACAATTTAATTGAACAAAGTAAAGAATTCAATAACCTTATACAAATCGAATACCTAGTAGAAAAAT
TAAACAGAGAAATAAACGGCTTAAAAATAGCCAAACGCAGCAAAAGAGGTTTAGTTAACATAGTAGGAA
CAGCATATAAATACTTATTTGGAACATTAGATCAAGAAGACAAAGCAGAAATTGAACAAAAAATAAGCA
ACTTAGCAGAAAACAGCGTTCAGGTTAACGAATTAAATTACATAATAGAAGCTATAAACAAAGGAATAG
AAATCATGAACGAACTAGATCAAGAGAAACAAAAAGGAAAGAAATTAGATATCCTTATATTTAACTTACA
ACACTTTACAGAATATATAGAAGACATTGAGATGGGAATGCAGCTCACAAGATTAGGAATTTTTAACCCA
AAATTACTAAAACATGACTATTTGTTGCATGTTAATTCTGAGAAATTGTTGAATACAAAAACTTCCACTTG
GTTTAAATCAGATACAAACGAAATACTGATAATATCCCATATTCCTCGAGAAATAATAAAAAGCCCGGTA
TTCGAAATAATTCCATACCCGGATGAAAATAATAATATTTTGACAGAAATAGCTCATGAAAAATATTTTAC
CCAAGATAAAAAAGTTTATAGCAGAGAAACAAAAAAATTAATTAATAATGAATGTTTAACAGGAATTTTA
AACCAAATAACATCAGAATGTAGTTATACTAAAATTTTGCAAAATTTTCAAATCAACTACGTAGAACCAA
ATATAATTTTAACTTGGAATTTACCAAAAACTATATTAAACCATAATTGTATAAATAACGAAATAACAATA
GAAGGAAACAATATAATAAAAATCTTTAATTGTTCACTGCAAATTAATGAAATTTCAATTTCAAACAATAT
GTTAGATTATACTCAAAGCATTTACGTAAGCAATGATGTTATAAAATTAGAACCACTTTCGTTTGTACAAA
CGAAACAATTATAAATGCACATACAAAATTTAACAATGTATTCCAGATAATTACAATAACCATATTTGT

AATCATATTAATAAGTTTAACACTGTATTTGACATATAAGTTCAAAAGCATACCTAAGAAATTAATTATAA
AATATAAAAAACCCAAAGTAGAAGAAACACCTACAATAATAGGAGATATACCAATTGTAAAAGAAGAAA
ATGTTACACTATATCCAAACTTAAACACCTGAGGACAGGCACTTTTCTAATGGTTGGGGGAGTGACATATC
CATAGTCGCACCCACCCTTTAACATAACTTAGCACATAAGCATAAGCACAATTATAAACATTTCTAATATT
GTAAACAAAGAGCCTGGTGATAACATCGACATTGGTCAAGATCAAACTGTCCCCCTTTGGTAGACATAAA
CAATTCACCCTAAATATCACGCCACTGTCAATGTTCCCATCAGAGTACTATCCCACGCATAATTGCTGACG
CAGCTCAAACTTCACTCAATTTTGACTCAAACTCTCTCAAAGCGAAGTTTGCTAAGCGACCGCAACAGTTA
GATAAATCAGTTCATATTCGGGAAGCAAATCTGAATGTACTCAACAAAAGTAGCCGTTAAGTGAAAATAA
TAAATTGAAATATATTTTTTTATAGTAACCTAACGTGTAAAACTTAATTATATATATATATATATA
>Ref_Tv1
AGTGACATATCCATAGTCGCACCCACCCTTTAACATAACTTAGCACATAAGCATAAGCACAATTATAAAC
ATTTCTAATATTGTAAACAAAGAGCCTGGTGATAACATCGACATTGGTCAAGATCAAACTGTCCCCCTTTG
GTAGACATAAACAATTCACCCTAAATATCACGCCACTGTGGTAGACATAAACAATTCACCCTAAATATCA
CGCCACTGTCAATGTTCCCATCAGAGTACTATCCCACGCATAATTGCTGACGCAGCTCAAACTTCACTCAA
TTTTGACTCAAACTCTCTCAAAGCGAAGTTTGCTAAGCGACCGCAACAGTTAGATAAATCAGTTCATATTC
GGGAAGCAAATCTGAATGTACTCAACAAAAGTAGCCGTTAAGTGAAAATAATAAATTGAAATATATTTTT
TTATAGTAACCTAACGTGTAAAACTTAATTGGCGCAGCCGGTAGGATACGTCGGGAAAAATTAAAGATCG
CTAAAGGATAATTAATTCCCAGATCTAACGCACAATCGCGACCGAAAGTGTTGTCGGAGTTGTAATAAAA
ATTTTCCTCGAATACGTATCCGCACAAGAACCTTCCGGTCCCTAGTTAGCTTAATAATATTGACCCTTTGG
GCAATAAAGCTACTACAGTTAATAACGACTGGCTTTGCATATCGTTTGATCCCTGCAATACGTTATTACTG
AGTCTTTTAAGACACACACACAAAAAAAAAAAAACATTTAACCAATAATCCATCACAAACTTTAACCTAA
AATAAAAGTGCAAAATCAAAGAACAATAAAGTTCAAACCTACAAACTCAAAACTATTCAAAAAAAAGTG
TATTACACCAAGATGACAATGGAATTATCAGAACAACACCTCAACCAGGCCCTTTCACAACTTAGACAGG
TGCCAAGCTTTGACGGATCAACGGATCAACTAAACGCCTTCATTAAAAGGATAGATTACATCCTACACCT
GTATCCAACCCGAGATGTTAGACAACATAGCATCTTATACGGAGCCATCGAATTGCAAGTCACAGGAGAT
GCTCAGAGAATATCACAAAGGACAGCAGCCAACACTTGGCAGGAGCTGAGAAACGCATTGATTGAAGAA
TACAAAGTGCAGACACCATTTGAAGAACTCCTTCGACGCTTATACAACACGAATTACCAAGGAAACGTCC
GTAAGTTCATCGAGGAGCTCGAGAATAAATCTTTTGTTATATTAAATAAGTTAGCACTAGAAAATATACCT
AGCAATACAACCCTTTATACAAATGCTATGAATAACACAATCAAAGATGTTATTACAAAAAAATTACCAG
ATAGGCTTTTCATGATGTTAGCCAGACACGACATTACGTCGACACAAAAACTAAAGCAAGTAGCTCAAAG
AGAGGGATTATATGAAAATAGTGTTACTGAAAAACCTAAAAATAATAATGTTCAGGGAAATCCAAATAAC
AATCGTAGGAACATGGGAAATTATCAGCAAAATGCTAACCCAACCACCATTTCAAGTGGTTATTCCAACA
CGAATCAGAGTTATCACGCACAAAATAAACAGCATAATAAGTCCGAAGACAATCAGAAAGCTCACCACG
AGTTTCCAACAGAAATTAAGTCAAGGTAGATACCAAAATCCCTTAAACTACCAAATCAGTCCCATTCACG
CTGAATGCACCAAACCCTCCCGACTAACGTCAAGAGATAGTAACAGTGGGCAAATTAGAATCTGGGATAA
CATCGGGAAATTTTTATTCAGCAAGCCTCGGGAACCAAACAGGAACAAATCCATTCATAAAAATTGGATT
ATGGAATTGACGAAGTTCTTAAAATGTGTCATCGACACAGGTTCAACCATAAATCTAATGAAAACAAACC
GCTTAAATTTTCCAGTTTACAACGAAACATTAAAGGTTCACACCATAAATGGTGTTATTGAATTAAAACAA
AGTATACGCTTAGGAGCAAGCAAAATTTGTCCGAGCAAACAAAAATTCTATATTCACGACTTCTCAGAGC
ATTATGATGTTCTGATTGGGAGAGAATACCTTGAGGCATGTCAAGCAAAAATAGACTATGCACAAGGATC
CGTAACCTTAGGAGAATTTAACTTTTGTTTTAGATATAACGACGAAGAGGTCGAAGAGGACATGACCGCA
CAAGAGTGCCTTGATCCACCCTCAACAGAGGACAGACCATTTAACTTCGCTATTAATAATGAATTAATAG
AAAACAATGAGTTTAGGCTAGAGCACCTAAACTCAGAAGAAAAAGAAAAAATAAAAAAAGTTTTACATG
AATTTGTGATATCCAGTTACCATGAAGGTGACAATTTGACTTTCACTAGTTACAATTTAAACACCAAATTC
CTAACCAAACATGAGGACCCCATTTACAAGCGTTCCCATACCAATATCCTCCAATTATCGAATGAAGAAG
TTATTCCATTTAGCGATTTGATTAAACCAATTCCTAATGGTTTACCAGTAATAATTGTTCCAAAGAGAAAC
GATGCATTTGGGAAGCCAAAGTTCCGGTTAGTCATAGATTACCGCCATTTCAATGAACTAACTATTAATGA
TAAATACCCTATCCCGATCATGGATGAAATATTAGACAAACTTGGGAAATGTCAATATTTCACAACCATTG
ATCTAGCCAAAGGCTTCCACCAAATCCAAATGGATCCTGGTTCAATACCCAAGACCGCATTTTCGACTAA
ACATGGCCATTACGAGTACACTCGCATGCCATTTGGTCTTAAGAATGCACCTGCAACGTTCCAACGTTGTA
TGAACAATCTCTTAGAAGATTTAATTTTTAAGGATTGTCTGGTCCACTTAGACGACATTATTATTTTTTCCA
CTTCATTGGAGGAACACATTTTGTCGCTACAAAAGGTATTTAAGAAGCTTAGAGAAGCTAACTTAAAACT
ACAGTTGGATAAATGCGAGTTTATGAGAAAAGAAACTGAATTTCTCGGTCACATAATCACAACTGAGGGT
ATAAAACCAAATCCCAACAAGATACAAGCAATTGTCAAATTCCCAATTCCAAAAACCCCAAAAGAAATTA

AATCATTTCTAGGATTATGTGGTTTCTATAGGAAATTTATACCAAATTTCGCTAATATAGTAAAACCATTA
ACACTTAAATTAAAAGGGAGCAAAATCAATATAAAAGACAGAGACTACGAACTAGCGTTTGAAAAGCTA
AAAGTACTCATAACATCCGACCCAATTTTAATATACCCTAACTTCGAAAAACCATTTTCATTAACTACAGA
CGCGAGTAATATGGCAATAGGGGCCGTCCTTTCGCAAGAGCATAAGCCTATATGCTATGCAAGTAGAACT
CTCAACGAGCATGAGTTAAATTATTCAACGATTGAAAAAGAATTATTAGCAATCGTTTGGGCCACTAAAT
ACTTTAGGTCCTATCTCTTTGGTCGACAATTTCAAATCCTTAGTGATCACAGACCACTCGTCTGGTTAAATA
ATATGAAAGAACCTAACATGAAATTACAAAGGTGGAAGATCAAGCTAAATGAGTTTGATTTCCAAATTAA
ATACGTCCCAGGAAAAGAAAATTATGTGGCAGATGCTCTGTCCAGAATTCAATTAAACGAAAACTTCTTA
GGCGAAGATACAATTAGCACAAGAGCAACAATACATAGTGCTCAAGAAGACAACAGTAACCATTTACAA
ATCACAGAAAGACCACTTAATTATTATAATCGACAAATAGAATTTGAAAAAGGAACCGAGAACGAAACA
AAAGTTACGAATTACTTTCACAAAACCAACATAAAAATTACGTACAAAGACATGACTAATACGCATGCCA
AAGAATTAATAAAAGAATACTTATGCACAAAGAAAAGTGTGCTATACTTCCACAACGAAGCAGATTTTCC
AATTTTTCAAGAAGCCTACTTAGAAATAATAAGTCCGAATAATTCAACAAAGGCAATGAAAACTAGTACA
AAATTAATAGACCTTCAAACATATGCAGAATTCAAAGAATTAATATTAAAAAAGCACAAAGAATTATTAC
ATCCAGGAATTGAAAAAACTATCAATTGGTTCAAAGAAACCCACTATTTCCCAGATTATCAAAATCTAAT
AAATGAATGTGAGACTTGCAACATCGCAAAAACAGAACACAGAGATACAAAATTAACTTTCGAAATAAC
TCCGGAAATCGCTAATATCCGAGAAAAATATGTAATGGATTTTTACATAGTAGGAGATAAACAATTTTTAT
CTTGCATTGATATCTATTCAAAATTTGCATCATTAATAGAAATAAAAAGTAGAGACTGGCTAGAAACCAA
ACGAGCTATATTACAAGTGTTCAACCAAATGGGTAAACCCATCGAAATAAAGGCAGACAAAGACTCAGCT
TTTATGTGCACAGCTTTGCAATTATGGCTAAAATCAGAAGCAGTAAATATTAATATAACCACTAGTAAAA
ATGGTATATCCGACGTAGAACGATTTCATAAGACAGTTAACGAAAAACTAAGAATCATTAACAGCGACTC
AGACGTCGAAAATAAACTTACAAAATTTGAAACTATACTCTACACGTACAATCATAAAACAAAGCACAAA
ACGACTAACAGAACACCAGCGGACATATTCATATATGCAGGTACACCAGAGTATGACACACAAGCTAATA
AAGAAAAACTAATAAATAATCTCAACAAAAAACGAACAAATTATGAAATTGACACTAGATACAAACACT
CACCGTTAGTTAAATCAAAGACGACAACCCCGTTCAAGAAAACAGGAGAACTAAGACAAATCGACGACA
AACATTTTGAGGAAACTAACAGAGGCAGGAAAATAACACATTACAAAACCAAATTCAAAAAGAAAAAGA
AGACTAATCAAAGCAAATATAACAATTACAGGTCAACAACAGACAGCGATCAAAACATACAAGCACCAG
CTTAAATTAATTATTATAATATTACTATCAATAACTGTAAACTATATAAATGCACAGAGTATTGAAATTAA
TCCTATCAAAGCCCATAACGGATATCTCATATTTAAAACAGGAACCATAGACATTCCGACAGATTACGAA
TTCCCACTGTTTAACGGTTAATATAACAAAAACCGAAGAAACTTTTAACAATTTAATTGAACAAAGTAAA
GAATTCAATAACCTTATACAAATCGAATACCTAGTAGAAAAATTAAACAGAGAAATAAACGGCTTAAAA
ATAGCCAAACGCAACAAAAGAGGTTTAGTTAACATAGTAGGAACAGCATATAAATACTTATTTGGAACAT
TAGATCAAGAAGACAAAGCAGAAATTGAACAAAAAATAAGCAACTTAGCAGAAAACAGCGTTCAGGTTA
ACGAATTAAATTACATAATAGAAGCTATAAAACAAGGGGATAGGAATCATGAACGAACTAGATCAAGAG
AAACAAAAAGGGAAGAAATTAGATATCCTTATATTTAACTTACAACACTTTACAGAATATATAGAAGACA
TCGAGATGGGAATGCAGCTCACAAGATTAGGAATTTTTAACCCAAAATTACTAAAACATGACTATTTGTT
GCATGTTAATTCTGAGAAATTGTTGAATACAAAAACTTCCACTTGGTTTAAATCAGATACAAACGAAATA
CTGATAATATCCCATATTCCTCGAGAAATAATAAAAAGCCCGGTATTCGAAATAATTCCATACCCGGATG
AAAATAATAATATTTTGACAGAAATAGCTCATGAAAAATATTTTACCCAAGATAAAAAAGTTTATAGCAG
AGAAACAAAAAAATTAATTAATAATAAATGTTTAACAGGAATTTTAAACCAAATAACATCAGAATGTAGT
TATACTAAAATTTTGCAAAATTTTCAAATCAACTACATAGAACCAAATATAATTTTAACTTGGAATTTACC
AAAAACTATATTAAACCATAATTGTATAAATAACGAAATAACAATAGAAGGAAACAATATAATAAAAAT
CTTTAATTGTTCATTGCAAATTAATGAAATTTCAATTTCAAACAATATGTTAGATTATACCTCAAAGCATTT
ACGTAGGCAATGATGTTATAAAATTAGAACCACTTTCGTTTGTACAAACGAAACAAATTATTATATGCAC
ATACAAAATTTACCAATGTATTCCAGATAATTACAATAACCATATTTGTAATCATATTAATAAGTTTAACA
CTGTATTGACATATAAGTTCAAAAGCATACCTAAGAAATTAATTATAAAATATAAAAAACCCAAAGTAGA
AGAAACACCTACAATAATAGGAGATATACCAATTCTAAAAGAAGAAAATGTTACACTATATCCAAACTTA
AACACCTGAGGACAGGCACTTTTCTAATGGTTGGGGGAGTGACATATCCATAGTCGCACCCACCCTTTAA
CATAACTTAGCACATAAGCATAAGCACAATTATAAACATTTCTAATATTGTAAACAAAGAGCCTGGTGAT
AACATCGACATTGGTCAAGATCAAACTGTCCCCCTTTGGTAGACATAAACAATTCACCCTAAATATCACGC
CACTGTGGTAGACATAAACAATTCACCCTAAATATCACGCCACTGTCAATGTTCCCATCAGAGTACTATCC
CACGCATAATTGCTGACGCAGCTCAAACTTCACTCAATTTTGACTCAAACTCTCTCAAAGCGAAGTTTGCT
AAGCGACCGCAACAGTTAGATAAATCAGTTCATATTCGGGAAGCAAATCTGAATGTACTCAACAAAAGTA
GCCGTTAAGTGAAAATAATAAATTGAAATATATTTTTTTATAGTAACCTAACGTGTAAAACTTAATT

>160_rnd-1_family-335__LTR___Gypsy
ATTTTACATGGCGACCGTGACTTGGTCACGCATCCCTGCCTGGAGGAAGTAGACCAAGTGAAAAGGAACT
TCATGGCCAACAAAGAGGCCATGTATAATATACAATTTGAAAAGGAACTTCATGGCCAACAAAGAGGCC
ATCTATAATATCCAAGTGAAAAAGGAACTTCATGATCAACGAGAGGCATCCCGTATTCTGAGTGAGTAGA
GAGTGAGTGTTATGGTTCATAAACGCGGTGGCTTAAAAAATAAAATTAATAAAAGAATAGCGCACCTAAG
GTGGCTGCTATACACAAAACAGCCAACACCCGCATGGTCGACAGCTCAGAAGAAAATTAAATTAAGCAG
AGAAAACCAAACACCACTACTCACTCAAATATAAAAGTTCTAATAAACCAAACAACTAATAAAGTTACAC
CCATTGACGCAACCAGCCTACCAAAAGAAAAACGAATGTGCCCACAGGATTGCGAAGGCCCACTCGACA
GCCCCTTCTGCGAATATACGTGCAGACACTGCACAGCCAGCACCTCCAAACATTACATTACAACCAACAA
ATAAACACCTGTGCGAAGACCAGCACCGGTACCGCCAGCGCAACAAGCTGTGCCATCGGAAATAAGCGC
GAGAGGCACACCGCCACTTGCGCATACGAGGGAGCATCAGCGTGCCACATCCGGCGACGCCCAGCTGAT
GCAGACGATGAGCAGCAGATTGAGCTAAACTTTTATAACTTACATACACTAAACATATAAANAAATTTTG
TAAAATTGAGCGGAATCANTTCCGCCCGATTAGAAGCGTAGCCCGTAAAGCTATGCGAAANNATAGGTAG
GCAAATGTTAAATAGAAACATCCAGATTGTGAAAAAAAAATTGCTTTAACAAAAATTAATGCAAGACACA
ATTAAAGAGAATATATATTTGTAACCAGTTCTAAGAATATAATAAATTTGAAAATGGGTTCGTGTGGCTCA
GATGATAGTCAAACAAAAGATAATACAGCCTTTGTGGTCAATAAAGTAAAAATAGTTGATCACACAGAGG
ACATTCAAGCTCTGTGGATTTTAACATTTATATTACAAGCTCAATTTTTCCTCACTATGTGTGTAAAATAAA
ATAAAATTATTAAAAGAAAAACAGGTAAGAGGTTCTAGTCGGGAGCTCCCGACTAGGGGATACCCTGAA
CCCTCTTCTTCCAACATCAAATTCATATATATATTCTATTTTAGAAGCTATATGTCAAGTTTGGTGACTCTA
GCTCTTATTTTTTACCAAAATTGCCCAACAAACAGGATATCGATATCGATTGCTTGGAAACGGAGAAACTT
ATCGATTATCGGAAACAAACTCGATCTGCGCAGGCACTAGGAGCACCTACATTTCAAGTCTCTAATTCTTA
TAGGTTCTGAGTTACTTGCGTTCATACATACGGACGGACGGACAGACAGACGGACGGATAGACAGGCGTT
CATGGCTAGATCGACTCGCCTCTTGATGCTGATCAAGAATATATGTATTTTATGGGGTCGGAGATGCTTCC
TTCTGCCTGTTACATACATTTGGATTTTGCACATATACAATATAGCCCTATATCCATTTTTAATGGGTTCAG
GGTATAAAAAGTATCAATGTTTAATTGATAACATTCATTGGAGCCATGAATGAAATGATAGTGTCCAAAT
CAAATAATAACACACAAAATTGTCCGCAAATCAAAGAAATAAGATCAAAATTTGACAAACCATATAAAT
GTCTAACGCAATACAAAGAAACACAGCAAAGTACAANAAACAAACACGCAAATATATTAGTAAAGTTTT
TAAATGAAGCACGAACATTATATTCGACCATAGAGAAAGATNACCAATCAACATTTGTCACAAGCCTCAA
AGGTCTTAACCAAATTTCGAATAAATTAAGTTGCGATAAGAGTAAAGAATAATTTGGATAAATCGATTCC
AACTATACTGAACACAGCTTTAACAATCGAAACAGCTAAACAGCCTGAAGATAAAGAAAAGAAAGAAAG
TGAATCTGATACAGTCACTGACATGGAACTAGAAGTTAAAGACACACATAATCTTACAACTTCAGCGATA
ATTGCATTACCTGAATTGTCAGAGTGTTCTGATTCTGAAGCAAACACCAGTGTAATAGAATTAAAACCTAT
TACGATGGAAGACACTTCTGCCGCCTCAAGGGCTTCCATAAAAGAAATTGTACAGGCCACACCGGAAATC
GATGGAATAAAATCCATCTACAAATTTATTGCTGCAATACAATTAATAAACCTAACANAAGGTCCATACG
AAGAAATTGTAATTGAAGTAATAAAATCCAAAATAGTTGGTTCAACTCTTTATAAAATACAAGATGAAAT
CACAATAAACGCGATGCTATCTAAATTGCAGACTATGATTTTCGGCGATTTTCCGATGTAGTAGAGGCAA
AAACGGCTTATTTCTACCAAAAATGTAAATTAGCCACACAACTCGCAAACTAAATCGATAATTTGGAATC
TTCTTATATCGATGAAGGACGAAAGGCAGAACATGCTGATACGTTCTGTACCAAAGAAGCCATTTCAGTC
ATGATCAAAATTTTGAGCATGACAAACTAAAAGCAATACTTTACGCAGGCACATTCAAAACGATGCACAA
AATATATCCAATACAGTATAGTTATGACAGGCAATGCATGCTCGGTATTGTATTCTTGTATTGTAATAACT
GTAGCAGGCTTCTAGCAACACAACAGTGAAGCTGCACGTCCTATCCATAGGTAGCTGTTAAGTATTGTTCG
ATAACTAAGAATAAGAAATAAGAAGAAGAAGAGTGTTAGACAATTGTAAAAGATCTTGGAGTCAGACCT
TAGCAGAGAAGCGAGTAAAGTAGACGTGTTTTGGACTTGCTGAAGCTAAAAACTTTTGTATTGTAACGGC
TCCTACATAACTATCGTGGTAATTACCGAGGCAGAGAAAACTCTCGTGGTTATAATAACAATTATAACAA
TAATTGTGGAAACTATAGAGGTAACAACCGCCTAAACGGAGATTACAATAATGGAAACAACGCCTCAAAT
AATAATAATAATAATAGCAACAACTACAGACACAACGGTAATGTCCGTCATGTCCAAGACTTAGCAGAAA
AATGAACGAAAAGTAAATTTTTAGAAAATAAATTTTTAGAATATAAATTGTTAGAAGATAAATTGTTAGA
GAGTAAATTGTTAGAAGATAATTTATTAAGAAGCAAATTGTTAGAAGATAAATTGCTAGCAGTCAACGAC
AAATTGTTAGGTTACGAATTAATAACAGAGAAGATCGTTAGATAAAAAAAAAGTTAGATAAATCGTTAGA
AAAGCAGAAAAGGGAGAAAGGAACAGAGGAGGACACAATTAATAGAGAGGATTTTAAAATTTTAGAATA
GGATTTTTAATTAAAATGATATAGATACAGAAGTTAGTGAAAAGGTTTATAACATTGATAAGCAGGTAGA
GTATGCATTAAGAAACAAATTTATACAGATGCTAGATGCTACATACACAAAGGCAAAGAGACTAGATAGC
CCTAAAATTAGATACGAAATATAGATTACAGTCGAGAAAGCGAAGCCACTTAGTTGCTTGCCAAGGCGAT
TATCGTACACAGAAAAGAGAAATTACAAATAATGTTAGACGAATACCTTAGAGATGGAACATCCAACCCA

GTAGCTAATAATATGCTTCTGCAATCGTTTTAGTTAAGAAGTACACCGATGATTTGAGAATGTTTATTGCC
TTCTGAAGACTAAAATAGATCACGATTAAAGACAATTATCCGTTGCGTTTAATAGACGACTTGTTAGATAG
GCTTGCAGGTAAAACCATATTTACTAAACTTGATATGAAAAACGGCTATTTTCACGTTTTTGCTGAGAAGG
AATCTATTAAACACATCTTTCGTGACCCTTTGAGGACAATTTGAATTTTGAAGAATGCCCATGGGATTGAA
AACGGCCTCTCAAAAATTTCAAAGATTTATTAATAATATCTTTTCGGACATGATAAGGGATAATATAATCG
TATACATGGATGNTATTATGATTGCCGGCAGGAATACTCACGAGCACTTAGAAACCCTAAGGGTAGTGTT
TAAGCGTTTAGTAGACAATAAGCTAGAGTTGAGGCTGGATTTTATGCAAGACCATATAAAATATATAGGG
TTCGAAGTCTACGAAGACGGCAGGAAAGCCGATGCAAAAGGTTCGGAGGCGATAAACAATTTTCCGATA
CCGGATAAAATTTAAGCAGTTCAACGTTTTTTGGGACTTTGCGTGTACTTTAGGAGATTTATTAAGGATTT
CTCAACAATCGCAAAACCTTAACATGATTTGTTGAGAAAAGACAAAATATTTCAGTTTGGGGAAAACGAG
CTAAGATGTTTTGTGTATCTTAAGCAGAGATTGTTAAAAGCACCGGTACTATCGATTTATGATCACAGAGA
TGAAATTGAGTTGCATTGTGATGCTGGTGCCTTAGGATTTGGAGCAGTATTGATGCAAACAAGATGTTAG
ATTGCATCCGATATTTTATTTCACGAAAGCGAAACACCACAGCGTTGAGCTGGAGACCTTAGCCATTATAT
ATGCATTACGAAGATTCTGGATATACGCGCAAGGGAAACGGTTCACATTCATATCTGATTGCAATTCACTG
ACACTGACCTTGAACAAGATTGNCCTTAATCCCAGAATTGCAAGATGGGCGTTAGAGCTACAAGAATTCG
ATTATGAGATAGTGCATAGAGCAGGTAAGCAAATGCCACACGTTCATGCATTGTCGAGATGTCATAACAC
GATTATGATAACGGAAGCAAATAACTTGGAAACCAATTTAGTAATATGTCAAACANGAAACGAGAAAAC
AAGGGATATTAGGAAATTGTTGGAGGTAGGTGAGCTTAAATTCTTTGAAATGAGAAATGGAGTAATATAT
AAAAAGACGAATGACAAAAGATTAAATTTCTATGTGCCAGAAAATATTATATAAAAACCATGACGAGCTT
GGGCATATTGTTAGGGACAAGACGTTAAACGCGATAGTTAAAACTTACTGGTTCCCGAACGTGAAAAGAA
AAATATTGAGTCACATCGAAAACTGTTTATGGTGCATATCATTTTCACCTAAGACAGGAAAAGAAGATGG
GCTATTGCACAGCATACCAAAGGGCAACAAATCTTTTGACTTAGTTCATGTAGATCATTACGGACCAGNA
GACAACAGCAGAGCAAAGAAATATATATTTGTAGATGTAGATGGGTTCACAAAATTTGTTAGATTATATA
CAACAAAGACATGAAACAGCAATGAAGTTATCGGCGCATTGAACGATNACTTTTGAGCCTACTGTAGGCC
AAAATGCATAATATCTGATCGAGGAAGCTGTTTCACATCGGCCGAATTTGAACAATTTGTAGAAAAATTA
AACGTGAAACATTAATATAGGCACAGGCAAGCCAATGGACAAGAAGAGCGAATTAAAAGAAGTTTAGGA
CCAATTATTGCGAAACTAACCGAACCTGAAAAGGGACAGCCATGGGATATTATCATCTAAAAAGTAGAAT
ACACTTTGAATACTCATACACTTTGAACACTCAGTATAGTAGCACCAGGTTTTTTCTTAGCACAATGTTATT
TGGGTTACCACAAAAAGGAATAATTATTGACGAACTAAGTAAAAAGTTAGAGGAAACCAAAATATTAGA
CGCAGCAGACGAGCGAAACTTAGATGATATTAGACAAAATCGCGTGGGGGGCCGCTCCTAGCGCTCCGCT
GCACTCTTTGCACTTCGTCGCTCACACTGCTCTTCGCTGCAGGTCTACTCATCCTGATAGTCTTTCTAAAAG
TGCCAGCACGCTCGCTAGCGAGCAGGGCGCTTGCCTCGGCCGCTCGAGTTCCCCGCAAGCTTTCGCAACT
ACACCTTGCCCTTCGCCACTGAATTGTAGTTGTGCTCTCGAACGCGGTGTTGAGCAACAAATCAGCAACGC
AAGGTTTCTGCGACGGCCGGCGGTTAAGTTCAAACTCAATTCAAACTAGGCTCCATGACCTTAAGTAGCTT
CAATACATTAGCACCTTGCCTTAACTGTTCTTATTACTAATACAATGCTTATATACTATACAATGCTTATAC
CCTTTTATTATAATTATGAACTGTGGGGTGGCTACCTTAGTGGCCGTCTCACAACCGCCAAAACGGAGATT
ACAAAAATGGAAACAACGTCTCAAATAATAATAATAATAATATGAAAAATAATAATAATAGTAATAATA
ATAATAATAATATTAANAATAATATTGATAATAATAATAATAATAATAATAATAATAATAATAATAATAA
TAATAATAATAATAATGGAGCATCAGCGTGCCACATCCGGCGACGCAGAGCTAATGTAGATGNTGAGCAG
CAGATTAAGCTAAACTTTTATAACTTACATACACCANACATATAAATAAATTTTTTAAAATTGAGCGAAAT
CAATTCTGCCCGATTAGAAGTGTAGCCCGTNAAGCTATACCAAAACATAGGTAGGCAAATGTTAAATAGA
AACATCCAGATTGTGAAAAAAAAATTGCTTAACAAAAATTAATGCAAGACACAATTAAAGAGAATATAT
ATTTGTAACCAGTTCTAAGAATATAATAAATTTGAAAATGGGTTCGTGTGGCTCAGATGATAGTCAAACA
AAAGATAATACGGCCNTTGTGGTCAATAACGTAANAATAGTTGATCACACAGGGGACATTCAAGCTCTGT
GGAGTTTAACACNTATAAGTATTACAAGATCAATTTTTCCTCACTATGTTTGTAAAA
>9_rnd-6_family-393__LTR___Gypsy
TATATATATAGTGACATATTCACAACAACAGCATTTTGAACCACTGTTATTCATAAGCTAATTGCATGGCA
TAACCCATTGTGTGTTTACAAACTGAATAATCATAACAATAACATGCCTTCTGCATCCGAATTCAAATAAC
GCTAACAGCAGCAAAAATCCTAAGAGCAGAATGCCTTCTGCAGCCAAGCTCACATATTGTCAACAATCAT
TTATAAACAAAAACTGATCAACAGAAAGGCGCTAGCATAAACAAGCTGCAGCCGCTGTCCCTTTGCAAAA
AGTCACGTACTCCAATTATTATTTAGCTTTAAATATAGCGTAAGCCAATTTTGGTAGCTCAGTCTTACTCCA
GATATTCAACATAATAAACCGATACAGATTCACGGTCGGGAAATATAGTTTTTATTTCCAAACTTATAACT
ACGTTATTGGCGCAGCCGGTAGGATAGCATTTTGTGAAAAGAAATAAATTAAAGTTCGTGAGTGATACGA
TCAATTAAAGAGAGGCTTATCCATACAACTGGTGTCTTCTCTGATCAACAAGTGTAATTGTGCACAGTATT

ACAACTAAATCGAATATATTTCAACTCCAAAATTCGCTATCCGCAAAAGAACTTAACGTGTGAAAACAAT
TCAAGCAACGTATAAATCCAATTCGGTCGTTGCAACAAAGTGAATTTCGTGACACAAAAAAAAAAAAAATG
GAGCAACCAGAAGGCTCTCATGTGGCTGCACTTGCAGCAATGAATAAATTAAACGACCAAAATTTAAACC
AATTGCTTCAACAAATACGGAACGTGCCAGCCTTTGAGGGAGACCCTATAACCCTGGGTCCATTTATCAG
AAGGATGGATTACCTGCTTCAACTGTACTCTACTCACAATGAACGACAACAATGCATCATATTCGGGGCG
ATTGAGCTCCAGATATCCGGAGATGCCCAGAAGGTTCCTAGAATGCTGCAAATCAACAACTGGTCCCAAC
TGAGGGAAGCCTTAATCTCCGAATACAAGACGCAAACACCCGTGGAAGAACTACTGCGAAGACTATATA
ATACCAATTATCAAGGTAATTTACGTAAATTTTGTAGTGAGGTAGAGGCTAAGATAATAATTATAACTGA
CAAGTTAGAATTAGAAGGGAATTTAGATAATTCCAGATTGTACACAAACGCTATGCATAACACTGTAAAA
AACTTGATAAATAAAAAAATTACCAGATAGACTCTTTATGTCACTCGCAAGACATGATATATCCACTATTT
CCAAATTAAAACAAGTAGCTCAAATGGAGGGCTACTATGAGGAAAAATTATTATAACAATAATAAAAAA
ACAAAATACTAACAATGCTATAAATTATACAGGCAATACTAAACCTCAATTTACCCCAAGTTACATAACT
AACAATAATCAAAGCCACAATTTTTCACATCACCCTCCGAACAACCAAACTTATTTTAACCAAAATAAGC
AACTAGCTCAAGAATTCAAACAGAATTTACAACAAGCACAAATAAATAACCCCATAAATCAAAAATATTT
CTCAAGGTAGATATATACCAAATCAACCGTACAAGCGTATACGAGAAAATACTAGTGGTTCCCAAAAAAT
GCAGGTTGATGAAAATTATCAACAATCCGAAAACTATCAGGGTAACTATGTACAAGAAGAGTATGGTTGT
CAGAACAACCAATATGAGGTGTTTCAGAATAATGAAATAAAAGACGAAAATTTTCCAATTGGAGCCTCGG
GACAAGCCAGTACATAGAAATTAAAATTAATAACAGACGCTACAGATGTCTCATTGACACTGGGTCGTCA
ATAAATATGATGAGGGAAAACTTCCTTAATATACCAGTTTATACAAGACCTACTTCGATTAGAACCATGA
CAGGGATATCAAACTAAAACCATTATGTATCCACTGCACCTAATAAACTCTTTAGGCAAGATCAGATATTT
TACATACATGATTTTTCACCTCATTATGATATACTTTTAGGCAGAAATTTATTAAAGGCAAACAACGCCAT
AATTGACTACGCTCATGACAGAATAATAATTAGCGAGAGATCGTTCAATCAGAAGAAATTAGTTTGGGAT
CCAAATTCAGAAAAAAACTTCGAAGCATATTTAAAGCCAGAAAGTTATACACTAGAAATTAATTACGCAA
TAAGTAATGAAATAACATCGGGAGAAGATTACCGAGTTGATCATCTAAACGTTGAAGAAAAATCAAAACT
ATTAAAATTACTGAAAAATTTCAGTGACATTCAATATCGAGAAGGTGACAATTTGACCTTCACGAGCATT
GTTAAGCACGAAATTAATACACATCATGAAAATCCAATTTACAAGAAACCATACGGTTACCCTTTCGCTTT
CGAACAAGAAGTCGAAAAGCAAATTAAAGATATGCTAACCCAAGGTATTATTCGGCATAGCCAGTCGCCT
TATTGTAGTCCCTTATGGATCGTACCAAAAAGGCAGACGCCTCAGGAAAGGCAAAATTTAGATTAGTGGT
CGATTACAGAGGATTAAACGACATTACAATCAATGATAAATTCCCCATTCCAAACATGGACGAGATTTTG
GGAAAATTAGGAAGATGTTCATACTTTACAACAATAGATTTAGCAAAAGGTTTCCATCAGATTGAGATGG
ACAAGAACTCAATTAATAAAACAGCATTTTCTACCAAATACGGACATTACGAGTATACTCGAATGCCATT
TGGACTTAAAAATGCACCAGCAACATTCCAAAGGTGCATGAATCACGTTCTTCGTGATTTAATTAACAAG
CATTGTCTTGTTTATTTGGACGACATTATTGTCTTCTCCACATCACTTGAAGAACATTTAAATTCAATAAAT
AAAGTTTTTGAAAAATTAAGACAGGCAAATTTGAAACTTCAATTAGATAAGTGCGAGTTTTTAAAAAAGG
AAACAGAATTCTTAGGCCATATTATAAGTCCAGAAGGCATACGGCCAAACAAATTGAAAGTAAAAAGCA
ATACAAGAATTTCCTTTGCCAAAAACATCAAAGGAAATTAAATCATTTCTAGGTTTATGTGGTTATTATCG
AAAGTTCATTCCAGATTTTGCAAAAATTGCAAAACCCATGACTCTTTACCTAAAGAAAGCGGCAAAAATT
GATATCAAAAATGTTGAATACAATAAAGCCTTTGAAAAATTAAAAATATTAATTATAAACGACCCAATAT
TAGTATGTCCAGACTTTAATAAAAAGTTTCAGGTAACAACCGACGCAAGTAATTTTGCAATAGGAGCTGT
GTTGTCACAAGATAACAAGCCAATTAGTTTCTCAAGTAGAACGTTAAACGATCATGAACAAAATTACAGT
ACAATAGAAAAAGAGCTTTTAGCAATAGTGTGGGCAGTAAAATATTTTCGCCCATATTTATATGGCAGAC
CATTTGACATTTTAAGCGATCATAAGCCACTCGTTTGGCTAAACAACATTAAAGAACCCAACATGAAATT
ACAACGTTGGCGTATTAAACTTAGTGAATATAATTTCTCAATCAAATACTTAGAAGGAAAATTAAACCAC
GTAGCAGATGCTTTATCACGCATCAAAATTGAAGAAAATATGCTAGGCGAACAAGATATTAACACACAAG
CTACAGCACATAGCGCTGAAGAAGATAATGAAAATTTAATACATTTAACAGAAAAACCATTAAACTATTT
TAGAAGACAAATAGAAATGATTAGAAGTGATACCGAGCAAATAGAAGTTGCACCATATTTTAAAAAATTA
AGAATAAAAATCGAATATTTAAACTTAAACATTGAAAAAGCTAAACAGATATTGATAAAACATTTTTGCA
ACAATTCAAGCGTATTATACATTGAATACGATTCCGATTTTGAGATAATACAACAAGCCTTTAAAGAAAT
AATGAACCCCAAAACAACTCGAATATTACGAACAAATATTAAACTGAAAGACATTAGGAGCTATGCAGA
ATTTAAAGAAATAATAGTTAGCAACCACAAGAAACTTTTACATCCAGGCATCGAAAAGATGGTCAGACTT
TTTAAAGAAACATATTATTACCCAGACTATCAAAATTTAATCCAAAACATAATTAACGAATGTGAAGTTTG
TAATCTAGCTAAAATTGATCACAGGAACACATCTTGTAAATTTAAAATTACACCAGAAGTAGAAAACTGT
AGAGAAACTTACGTAGTAGACATTTATTCAATAAAAACCACAGCTATTTAACATGTATTGATTTATATTCA
AAATTTGCAAATGTAATTGAACTTAACGGCAAGGATTGGATGGAAGTTAAAAGATCCAAATATTTAACAA

CATGGGAAAACCTATAACTCTGAAGGCAGATAGGGACAGTGCTTTTATGAGTCTTGCCCTGAAAGAATGG
TTGAATAAGGAAAAAGTTGAAATTCAAATAACAACAAGTAAAAATGGTGTAGCGGATATAGAAAGACTA
CATAAGACAATTAATGAAAAAATACGCATTATAAATACTGACGATGACGAAGAAAATAGACTCATGAAA
ATTTGAGGTAATTTTATACACTTATAATCATAAGCTAAAACATAACTCTACTGGAAGAACTCCAGCTGACA
TTTTCATTTACGCAGGTACACCTAATTATAATACCCAAATTATAAAAGAGACCAGAATAAATAATCTTAAT
AAAACCGAAATGAATTTCGCAGTAAATACAAAGTTTAGGAAAGCACCCATAATTCACGGTAAATTACATA
ACCCTTACAAACCCACTGGGAAAATAGAACAAATTGACGAAGATCATTTTGAGGAGACTAACAGAGGTC
GAAAAATTAAGCATTATAAAACAAATTATCAAAAACAAAAGAAATTAAATAAGAGCAAATATTTACAGG
AGGACGAAGTTCCAAAACAGGACATATAACNTTCATTTTTCTGGCACTAATGATGTCAACACTATTACCA
GGAAGTAATAACCAGTTTATAGATATTAACCCCTATTAAATCAAATAATGGATACTTACTCTTTAAAATAG
GCATGATAGACATACCCGATAATTACGAGCACCACTGCCTCACAATAAATTTAACAAAGACAGAAGACAC
ATATGACAAATTAATGTTACAGACAAACAAATNTAAGGACAGAATACAAATTCAATATTTGACAGAAAA
GTTGCAAAGAGAAATAAACGGTATCCGAATAGTAAAAAGAAGTAAGAGAGGATTACTTAATTTCGTAGG
AACAACATACAAATACTTGTTTGGAACTTTAGATAACAACGATAAGGAGGAACTTGAAGAAAAAAATGA
ATTCAATAGCTCAAAATAGTATTCAAATAAACGAACTCAACCATGTTATTGAAGCGGTCAACCAAGGAAT
AGAAATAGTAAACAAAGAAAGAGAAGAAAGAGAAGAATTTAAAGTACTGGAACTACTTGCATTCAACTT
ACAACAATTCACAGAATATATTGAAGATTTAGAAATGGGAATGCAACTAACTAGATTGGGAATTTTAATC
CAAAATTATTAAAACACGACTATCTCACAAACGTTAATTCCGAAAAAGCTACTTAACATAAAAAACATCT
ACTTGGCTTAGATCAGATACGAATGAAATTTTGATTATTTCACACATTCCTAGAGAAATAAATAAAACCCC
ATTATACGAAATNCTACCCTACCCGGACGATGATAAAAAAATTTACAAGACGAAACACAATACAAATACT
ACTCACATAATAATAATTTATACAACAAGGACAAGAAAAATTAACTATTAACCAATGCATATTAGGTATA
ATAAAACAAACTAACACTAGTTGCAGATTTTCAAAAACACATCTAATAACACAAACTAACTATATCGAAC
CGAATATACTTATAACATGGAACTTACCCAAAATCACATTGAATCAAAACTGTATTAAAAATGATATCTTA
ATTGAGGGAAATAACATAATAAAAATTTCATAATTGCTCAGCAATACTTAATGAGATATCTATAAGCAAT
TTGAACTCAGACTACACACAGAGTATATACGTAAGAAATAATATTACTAAATTAAAGCCACTTTCGTACA
TTCAAACAAAAGAAATAATTTTCGAAAATATTAAAGAAATGAAAAATATTCCAAATTATAATAAATTACA
GTATTTGTAATCATCATATTAATCATAATAATGTATCTAGCATATAAGTTTAAAAATATACCCAAACAAAT
ATTTATTAAGAAAGTAAATGTTATTGATCAACCACCTGCACAACTCCCAGTTGAACTTGAAATCGAAAAT
GAACAAGAAAATTTAAATATGTTACCAAAATTGTATCCTAGCATTTAAAGAATATTTTTTATTATACTTAC
CTTTTTCATATACCCTACACGCGTGAGGACACGCTTTATTTTTGTGGGGAGGAGTGACATATTCACAACAA
CAGCATTTTGAACCACTGTTATTCATAAGCTAATTGCATGGCATAACCCATTGTGTGTTTACAAACTGAAT
AATCATAACAATAACATGCCTTCTGCATCCGAATTCAAATAACGCTAACAGCAGCAAAATCCTAAGAGCA
GAATGCCTTCTGCAGCCAAGCTCACATATTGTCAACAATCATTTATAAACAAAACTGATCAACAGAAAGG
CGCTAGCATAAACAAGCTGCAGCCGCTGTCCCTTTGCAAAAAGTCACGTACTCCAATTATTATTTAGCTTT
AAATATAGCGTAAGCCAATTTTGGTAGCTCAGTCTTACTCCAGATATTCAACATAATAAACCGATACAGAT
TCACGGTCGGGAAATATAGTTTTTATTTCCAAACTTATAACTACGTTATTATATATAT
>160_rnd-1_family-52__RC___Helitron
TACACCAGAAATGTTTGCGGAGAAAATATATGAGCAAAGCTAACGATCTAGATAAAATCTAAAAACTAA
AAATAAAATGTTAACAAAAGCCGCTACATCGATTATTGTTATCATTGTGCCTCAATAAAAGTGCACTATTA
TCAAACAAACAAAAATAAAACATGTTCGAGTTCTAGTGGNATTATATAACAAAGTCCTTGACATTGTAAA
CAACAATGAACGTAGGTTAAGCGATTTACATTTAACACAATCAATTGACGTGTTAAATAGCCTGCGTGAA
AATATTAGACATATTTCGGAAAGACATAATTTTCAAATATTTGTTCCAAATGCGCTAAATACACCAGCAAC
ATTCGAAATCACTGATTTCATGATATCAGTAGAAGAATAACTTAAACAAAAGCAAGAACAAATCGAAATA
GTCGACATGGCTCAATCACCAGTAGATTTTCTTAAGATAGCCTTATCGCTCTTACCAGAGTGTGATGGCAA
GGCAGAAAATTTAAAAAGCTTCATAGATGCTCTGAATCTGGTAGATTCTCTAAAAGAAACACACGAAGCA
CTTGCTGTAAGTCTTCTTAAGACAAAACTAGAAGGCCATGCCAGGAACCTGATAAGTGATGAGAACACTA
TTACAGCTCTCATATCGAAACTATCGGACAAATTAAAAGGAGAGTCAGTAGAAGTTATTTCTGCTAAACTT
CTAAACTTACAACAAAAGAGCAAAACGGCCAATCAATATACCCAATAGGCTTAAAATTGAAAAAAAAAG
TCCCTTGAAGGAGTCTATATCAGTGAAGGTCTAAGCCAATCACTTGCAAACACTACAAGAGCCGTAAAAC
CTATGACCCAAAATTGTTCGATTGATAAGGTAAAACTTATCATGCAGGCAGGCACATTCACAAATATGAA
TGATGCCGTCTCCAAATTTGTCAATAGTTGCACGGAGGTAACAGGTCAAAGTAACACTGTCCTGTATTATC
GACGAGGTGTAAATCAAAATAATAGACGAGGCCGAAGTTATAATCGTGGCAAAAATTGCAACCACAACA
ATTGCAACTGCGGCGGTAATAACAACAACAACAACAATAATTACAATAACCGTGCAGGAAGACGAGGCC
AAAATCGGGGAAGAGGCCGCGGGAACTCCAACCAAGGTAACAATTATAACAATGTGAGAGTGGCGCAAA

ATGCGTCGGAAAACTCTCAGCACCCTTTAGGAAACAATCAGTAAATGCAAAGGTTCATTGCATCAATTAC
AGTCTTAATATATTTGTCACTCTTTATAATCATTCAACTGAAAATAAACACACATTCCTAATAGATACTGG
TACAGATATCTCACTTTTAAAAATAAATTCAGATAATTTCAAAATTCAAGAAGACAAAATTATAAACATC
CAAGGCATCGACCAGGGTGTAATAAAATCGCAAGGAACAACTTCAATAGAACTTCAACCAACAAAATAT
ATTAATCCACACGAATTTCATTTAGTAGACTCCGATTTTTGAATACCGTGTGATGGAATAATAGGCATTAA
TTTTATTAAAAAATTCAATAGCCAATTAGACTTCAAACCAAGTAAAGACTGGTTTATAATTAAACCTCAAA
ACTTAAATTATCCTATTTATGTTCCAATGCAACAATGCATTTCTTCTGCCAGCCAGATCCCAACTAATTCGA
AAAATAGACATTAATTCAGTAAACGATTATATATTTGTTCCAAATCAAGAAATACATAATGGAATTTATGT
TGCAAATACAATAGCAGCATCCAAACATGTATACATTCGCCTTCTAAGCACAACTAATTTCGACCAATTGG
TCAAAGTTAACAAAATCGAATATGAAAATTTAACAGATTACGATATTCATAACACTAATCCAGAAAATAG
AAGCTAACAAGTACTTTCAAAGTTAAAGAAAAATGTTCCAGAACAATTCAGAAATCAATTAACAGAATTA
TGCACCAAGTATAATGATGTGTTCGGACTGGGAACCGAACCAATAGCGACAGATCATTTTTATAAACAAA
CATTAAAACTTAAAGATGATGAACCCATTTATATCAAAAACTACAGAAGCCCGCATAGTCAAGTTGAGGA
AATCCAAAAACAAGTAGGGAAATTAATAAACGACAAAATCGTAGAACCATCTGTATCCGAGTATAACAG
ACCACTCTTGCTAGTTCCGAAAAAATTATTACCACATTCGGAGGAAAAGAAATGGCGATTAGTAATTGAC
TATCGCCAAATCAACAAAAAATTACTTTCTGACAAATTCCCACTCCCCAGAATTGATGATATTCTAGACCA
ACGGGGCAGAGCTAAATACTTTTCATGCCTTGACTTAATGTCAGGTTTTCACCAAATTGAACTTGAAGAAA
ATTCAAGAAATATAACATCTTTTTCAACGAGCAATGCCTCATATCGCTTCACGCGATTACCATTTGGTCTT
AAAATAGCACCAAATTCATTTCAGAGAATGATGACAATAGCATTCTCGGGTTTAGAACCCTTTCAGGCATT
CCTTTATGTGGATGATTTAATGGTGATCATGATTAAAAACTTAACTGATGTTTTTAATGTATGTAGATAAT
ATAACCTAATGTTGCATCCAGAAAAATGTTCATTTTTCATGCATGAAGTACATTTTTAGGTCACAAATGCA
CTGACAAAGGAGTATTGCCAGATGACAAGAAATTTGATGTCATCAAAAATTATCCTGTCCCTCATGATGC
GGATAGCGCGAGACGATTTGTAGCATTCTGCAATTATTATCGTCGATTTATAAGAAACTTCGCCGATTATT
CACGGCACATAACTAGATTATGTAAAAAGAATGTTCCTTTTGAATGGTCAAGTGAATGCCAAAACGCATT
CCAATACCTTAAAGAAAAGCTTATGCATCCCACATTATTACAATATCCTGATATTCGCAAAGAATTTTGCA
TTATAACAGGTGCTAGTAAACAAGCCTGCGGAGCGGTTCTAACTCAGAACCGTGACGGAATTCAGCTCCC
AATAGCTTATGCATCTCGTTCATTTACAAAAGGGGAAAGCAATAAGAGCACAACAGAACAAGAGTTAGC
GGCAATTCATTGGACAATTACCCATTTTAGACCATATATTTATGGCAAGCAGTTTACTATTAAAACAGAAC
ACAGACCATTGACATATCTTTTTTCTATGACTAATCCCAGCTCGAAATTAATTCGCATGCGGCTAGAGCTT
GAAGAATACGACTTCACAGTAGAATACCTAAAGGGGAAAGATTATTTTGTAGCAGATGCACTTTCGCGTA
TTAATATAAAAGAACTCAAAGATATGCAACATAAAGTCCTGAATGTCACTACCAGGCGACAAAGTAAACA
AAATAAAAACTGTATAGTAGAAAATTATGAACTTCTGCCAAGGCAAAGTCATAAAAATGTATCCAAGCCC
AACGTACATGAAGTCATCACAAATGATGAAGTACGAAAAGTAGTGACCTTGCGCATAACAGAATCTAAAT
GTTTATTTAAACATGGAAATAAAATTATCGCAAGAATTAAAGTTAGCGATTTATATACCAATGGAATTCTC
GACCTAGGCTAGTTCTTCCAAAGGCTCGAAAAAGAGACCGGTATATTAAAAATCAGCCAACTCAAAGTGC
CGCCGAGCGAAAAGATCTTTGAATCAGTTTCAATAGATTGTTTTAAAAATATGGGCAATGAAATATTCAA
ATCTTTAAGAGTAGCGCTACTCCAGCTGGTGACCCAAATAACAGATGATAAAGAGATACAACAAATATTG
TCTACATACCATGACGACCCCCTATTACGACCTCAAGGAGGTCATAGCGGAATAAGAAGAACGCTAGCGA
AAATAAAAAGACACTACTACTGGTAAAATATGACACGTCATATAAAAGAGTACGTACGTAGATGTCAAA
AATGACAAATGTCTTAAACAAAGACGCACACATAAACTCCTTTGACTCTCACAGAAACACCTGTGACTGC
TTTCGATATAGTGATAGTGGATACAGTTGGTCCGCTACCTAAATCGGAAAATGGCAATGAATACATAGTT
ACATTAATATGCCACCTTACAAAATATTTAGTAGCCATTCCAATAAAAAGCAAACGCGCAAATATTGTCG
CAAAAACTATATTCGAAAATGTAATACTCAAGTTTGGTCCAATTAAGACGTTCATCTCGGACATGGGAAC
AGAAAAAACCAGATTTTAACTGATTTATGCAAGTAAATGAAGACCAAGAATCTCACCTCTACTGCATATT
ATCATCAAACATTAGGGAGAATCGAACGAAGTCATCGAACATTTAATGAATACATTCGTTCATACATCTTT
GCAGACAAAACTGACTGGGATGTCTGGATTCAATATTTCACATATTGCTTCAACACTTCAACACCATCAGC
AACGCATGAGTATTGTACATACGAACTAGTATTTGGCCGACTACTAAATAGTTCATAGATTTAAATAAAA
CTAATTACATAGATCCTATTTATAATCTAGATGACTACTCTAAGGAAATAAAGTTTCGATTAGAAATAGCA
TATAAAAGAGCTGGAATTATGTTAGAAAAAGCCAAGGCTTATAGGAAATTAGCATACGATAAAAAAACA
AAAAATTTTGAATTAAAAATAGGAGATAAAGTACTTAAAAGAAATGAAACAGGCCACAAATTAGACCCA
GTATATTTAGGACCATATATAGTAGAAAAAATAGAAGATAATGATAACATTTACATTAAAGATAGTAAGA
ATAAGAAACAAAAAGAACATAAGGATAGGTTACAAATATATAATCAATGAAACGTTTCATGAAAAAAAG
GGTCTGATCAACCTAAAACAAAAAATGTTTAGCATAATGAAATATAAGCATAATGAAAAACAAATGGGA
ATAAAATAAAGAAAATTTAAATTCTTCTAGGAAAGTCGAATGAAAAATATAAAGTTGAATGGAGTTAAGG

TATCTCAAATATATTCAAATAAAAAAAATATTGTATCAATGAACACAAATATTAAAACAAAAAAGAAAAG
AGAGATAACACATTTTAATACACCTAATATATTTTACACATTCTTAATTACACTTTTTTATACATTTTTAAA
ACACATAAAATAAGATACACAAAAAAATAACACAAAAAAAAATTAAAAATTAATATTACTATTAATTTCA
ACATTTTTTACCGACTTTAACACTCTCTAAATATTGCGTAATTTCTTTAAAACGGTAGGTGTAGTATACCTA
CCCTGCCAGTAATACAAATCAAGGGGTATAACAGTAAATACATACATTGTGACACTCAGTAATAATAATC
ATAAATATACAAATACCCAAAAGACCACACTTAAACATATAAACACACCAGCGCATATGTCATACGATCC
ACCACTTACTCCCGACTAGAACCTCTTACTTGTTTTTTTTTTTGCTGGTGTGGCTGTTGAGTAGAGTCGACA
GCAAGTAGTGCGGTCAGTCGCGAGTTCGAGCCCTGCCAAGGGAACTTTTTTTTATTAATTTATTTGGTGCT
GGAATATGAGGGTTGACAGTAGGTATCCCGGCTAGTCGGGAGCTCGAGCCCCGACAAGGGAACCTCTTAC
TTGTTTTATTAATTTATTTGGTGTTGGAATAAGAGGGTTCAGGGTATCCCCTAGTCGGGAGCTCCCGACTA
GAACCTCTTACTTGTTTTATATAAGTACTTATTGTTGGTGTGGCTGCTGAGTAGAGGCGACAGCAAGTGGT
GCGGTCNGTCGCGAGTTCGAGCCCTACCGGGGAAAGTNTTNTTATTTTTATACCCTGAACCCATTAAAAAT
GGGTATAAGGGTATATTGTATTTGTGCAAAATCCAAATGTATGTAACAGGCAGAAGGAAGCATCTCCGAC
CCCATAAAGTATATATATTCTTGATCAGCACCAATAGCCGAGTCGATCTAGCCATGTCCGTCTGTCCGTCC
GTCCGTCCGTCCGTCCGTCCGTATGTATGAACGCAAGGATCTCAGAACCTATAAGAGCTAGAGACTTGAA
ATTTTAGATGTAGGTGCTCCTAGTTCCCGCGCAGATCGAGTTTGTTTCCGATAATCGATAACTTACTCCGTT
TCCAAGCAATCGATAAAAATCGATATCGACATCCTTTTTTTTTTAGCAAATTGGGTAAATAATAAGAGCTA
GAGCCACCAAACATGATATGTTGCTTCTAGAATATTATAGATATTTCAAGTATCTTTCATTTCATACCTATC
GCCACCTCCCCGCTACCACCCCAGAGCTATAAATCAAGTTAATAACCCAACTTTTATTGCCAACCAATTTA
AACCACAATTTAAATGCAATTGACTTTTTCAGAATACACAAATATTCTATGGTACAATAAGATATACTGTA
GAAAATTTCATAAAGATCGGTTAAGAAAAACCAAAGTTATATGCAACTGCGCAATTGAATGGGGCAGCA
GCTTTAAGAATTTCTGTATACATGTACACACACACATTCATGCGCGTCTGCTTCGCATTCTAACAGCATGG
TAATTGCATCTTTTTTTTGTAATGTTATTTTAGTTCCTTTTTTTATCATAAGTACTTAATTGTTGGTGTGGCT
GCTGAGTAGAGGCATAGCAAGTGGTGCGGTCTGTCGCGAGTTCGAGCCCTACCGGGGAAAGTATTTTTTT
TTTTTTTTTGCTGGTGTGGCTGTTGAGTAGAGTCGACAGCAAGTAATGCGGTCAGTTGCGAGTTCGAGCC
CTGCCAAGGGAACTTTTTTTTATTAATTTATTTGGTGTTGGAATAAGAGGGTTCAGGGTATCCCCTAGTCG
GGAGCTCCCGACTAGAACCTCTTACTTGTTTTTTT
>160_rnd-1_family-274__LTR___Pao
TTACATACGTTGGCCCACATTTCGAGACCTCTTTACCGCAATTTACGTAAATAATCCTAGGTTATCAGAGG
TCGAGAAATTATTCCATCTCAATGCGAAAACCANCGGTGAGGCTCAGGCGATTGTATCGAAGTCACCTTT
GACTAACGATGGCTTCCGATCAGCCTGGGCCAATCTGACTGAGCGTTTTGAGAACCGAAAATTGTTAGTC
AACAGCCAGCTCAAGATTCTTTTCAACCTGCAGCCAATCTCCCAAGAGTCTGGAACGGCAATTAAGGAAC
TTCAGAGCACCATTCAAGGTTGCTTAACAGCTCTGGAGATATCCGAAATCAACATAGAAAACTGGGACTG
TATCCTCGTGTTCCTATGTTCCTCCAAATTACCCAAATTAACACTCTCCTTATGGGAACAGTCCATCTCCAG
AAAAAACGACATCTCATCTTGGCTTGAGATGAACGCCTTTCTCTTAGAACGCTATCGCACTCTAGAAGCGA
TAGAAGAGCTTAAGCCAAGCGCAAATACTCCGGCGCACTCCAGAATTGTTCGNACAGAACCTCCTACCAC
TAGAAGGCTTAACTCATTTGAGAATAAGGTCGCTAACAAGCCGCAAGCCTGCCAATTGTGCTCCAGGGAG
AATCACCCTATCCGTCTGTGCCCACGATTTCTCCAAATGCCCGTCAGCGAACGGGCAACTTACATCAAACA
GCAAAAACTATGTTTGAACTGTTTCGCTAGGGGTCATCAGTTAAGGGATTGCACAAGTGCTCACAATTGTT
TTACGTGCAAGGGTCGTCACAATACGCTCTTGCATCGTAGTGACATTTCACAATTGGGCCCTACGGTAGTC
TCGATGCCACAAGCAACAGCTCCCTCCAATCAATCCATACAGTCCACTCCATCCCAACAAACGAATGTTC
ACAATTATGTTGCAATTAACACCCACGGTGTTCTCCTAAGCACGGCTGTTATATATGTTCGCCATTTGGGT
GTAAATTATACAGCTCGTGCACTGATCGACTCGGGATCAGAAGCAACATTTATTTCCGAGCGACTCTTCCA
CCTGATAAAGCTGCCGTACAAATCCGTACAGGCACAAGTATCGGGATTAAATCAAGCTGTTGCAGCCCAG
CCTCGCAAGCTCTGCCATTTCAATATTGGCTCTCCTTCAAAGCCTCGTATCCAAATCGAGGCATCAGCCTA
CGTCCTACCACATTTGGCAGGGAATCTACCATCATGTTCGATCCCCCAAAGTTTATTGACAACCCTGCCAA
AACTCCAGTTAGCGGACCCTAATTTCCACAAGAGTTCGCAGATAGATGTGCTGATTGGCGCTGATATCCTA
CCGTCCATACTAACGGGCGGCTCCCGTCCTAATATATGTGGCACGTTGCTGGGCCAGGAAACTATTTTCGG
GTGGATTTTAACAGGCCCGGTTTCAAAGACCGCATCCACCGCAATCTCGTCATTCTCGACTCGAATATCTG
TCTCTCAAGAAGAGCAATTAGACGTCATTCTCACAAAATTTTGGGAGGTGGAGGACATTCCAGTCAAACT
GGTAAAGAGCTCCGACTCCTTCTGTGAGAGTAATTTCATCCGAACAACAACAAGGAACGAGAATGGCAGA
TATGTAGTCACCCTACCTTTCCGGGAACCTAATCACATAGACTTAGGACATTCAAGATCTATCGCACTAGC
TCAGTTTCTAAAGAACGAGAATCGTTTAAAAAGAAACCCTCCTTTGAAAGAGCAGTACGACTCCGTGATT
CGGGAGTATTTCGACTTAGGTCATATGACACAAGTCTCTCCAAACAGCAACCCCAATAATTTTTACCTGCC

GCATCATGCTGTTTTTAAGCCCGACAGCACCACAACAAAGGTTCGCGTTGTGTTTAACGCCTCCAGCCCGT
CCTCCAATGGCAAAAGTCTAAATGATATCCTACACACGGGACCAGTACTCCAATCCGATCTGACGATTCA
GATACTAAAGTGGCGATTCTACCGCTACGTTTTCAATGCCGATATAACCAAGATGTATCGGCAAATCCAG
GTCGATCCAAGACATACGCCCTACCAACGAATACTATTTCGCAATGAGAACGAAGAACTTTGCGACTTCG
AACTCAATACAGTTACCTTTGGGGTAAACTGTGCCCCATACCTCGCAATCCGGGTCCTCCGTCAACTTGCT
CGTGATATACGAGAACAATGTCCCATCGCATCCGATATAATTGAAAATTATATGTATGTCGATGATGTTCT
AGCAGGGGCACACACGAAGCAACAAGCGATTTCAGCCATTGCAGAACTCCGCATGGCACTCGACAGAGC
TGGGTTCCCTCTGAGAAAGTGGACATCCAACACAAAGGAGGTGTTAAAAGGAATACCAAGGGACCACTT
ACTGTGTGCGGATTTCTTAGAAATCGAAGAGGCCAGCGTCGCTAAAACGCTAGGCATTCGATGGCAAGCC
ACAACAGACCAGTTTTTCTTTGCTCCGTCCAAAATGGTATCTCAGCCTTCGCTCACTAAACGTGAAGTACT
ATCACAGATTGCGAAATTGTTCGATCCTGCTGGATGGCTAGCACCTGTGATAGTTCGAGCAAAGATTTTCA
TGCAAGAGATCTGGCTCCAAGAACTGGGGTGGATGATTCCCTGCCGGAAGATCTCGTTCAACGTTGGCA
CGACTTCCTAACCGACTATCCGTCTCTCGAAGAGATCCGTATCCCAAGATGGGTCGATTTTCAGCAAAAGG
CAAAGGTGCAGTTTCACGGCTTCTGCGACGCGTCCCAGCGCGCATATGGTGCTGCACTTTATTTGCGCGTC
GAGATAAACGGATGCATCTCCACTCATCTTCTCGCGGCAAAAACGAGAGTCGCGCCAGTTAAGACGGTAT
CACTACCACGCTTAGAGCTGTGTGGCGCCGTTCTTCTTTCCGAGCTCACCACGGCTCTTATTCCTCAGCTCC
CCATCCAGAGTAGAGAAAATTTCAATTGGACAGATTCCACTATTGTTCTTGCCTGGTTGGATAAGCCTCCA
TGCAAATGGTCTACCTTTGTGGCTAACCGGGTAGCCAAGATAACATCAACCAATGGCAAATGGTCCCATG
TTCGATCCGAACATAACCCAGCTGACTTAGCTAGTCGGGGGGTTTCTCCTCGGGACCTATTGGGCAGCACT
TTGTGGTGGCATGGACCGGAATGGTTACAACTACCACAAAACCAATGGCCAGTTCCTGTTACGGTGCTTG
AGACAGATATTGAACAACGCGCAGTGAAGTGCAATGTCTCACGGCTACCCTCCTATAACTTTCTGGAACG
ATTCTCCGGATTCGACAAGGCATTGCGAGTTATCGCATATGTCTGTCGCTTTATCCAGCGCTGTAGAAATC
CAGCGATCCACCTCCCCGCCGAGCCCCGTAATGAAGAACTTACGAGAGTCCAGTTCCTACTTATCGAGTAT
ACTCAACGCCAAGCTTATCCTAAGGAGTATAGCTGTTTGCTCGATAAGAAGCAAATTCAAGGATCAAGCA
GCATTCGCAACTTAAACCCTTTCATCGATCAAAGGGGTATTCTGCGATCCTGTGGACGGGTGAGAGCGTC
AACCTCGTTAGGCTATGATGAGCGACACCCCATAATCATTCCGGCCAATTGTGATTTTTCACGCCTCCTGG
TCCGATTTACCCACCTCATTTGTTTTCACGGAGGCAATCAGTTGGTAATGCGCCTCATAAGAGCCAAATAC
TGGATCCCAAAGCTCAAGGTCCTGGTAAAATCTACCGTAAGCTCCTGCAAGGTCTGTGTAATACACCGTC
GGCGACTCCAGACCCAACTTATGGGCGAGCTCCCAAGGGCGAGATCAACCTTTTCCCGACCCTTCGCACA
CACGGGTGTAGACTTTGCCGGACCTTTCGATATTAAAAGCTACGTTGGATCGAGCATGCAAAATTACCAA
GGGTTATGTCTGTGTCTTCGTTTGTTTCAGCACGAAAGCAATCCATTTAGAAGCCACCGCAGGTCTTACGA
CGGAGAGTTNTTTCTGGCCGCATTTTCGCGATTTATAGCGAGACGTGGGCCTTCCCCAACAGATGTACTCA
GATAATGGGAAAACATTTGTGGGAGCCTCATCCGTTCTTTCGAGAGATTTCTTGCAAGCGACCAAGGAGC
TCATCTTATCCAAGCACGGCCGTCAAAACTTGTGTTGGNAATTTANNCCTCCTCGCGCNCCGCACNTNGGC
GGNCTNTGGGAAGCAGCNGTGAAGAGCNGCGAAGCACCACTTCTATCGNTCCGCCGGATCGTCCNCAATA
TACATTCGAGGAGTTTCGCGACCCTNGTCTGCCACATAGCGTCGATAATTAATTCCAGGCCATTGTTTCCT
CTTTCGGAAGATCCGGCAGATCTAGATGTGCTCACTCCAGCTCATTTCTTGATTGGTGGACCCAGCTCATC
GTTCTTTGAACCTGATGTGACGCACCTTCAATTCAATCGTCTGGATGGTTGGCAACGCGTAACGTACCTCC
AACAACTGTTTTGGTCTCGCTGGCGTGAAGAATACTTAACACTGCTTCAGCAGCGCTCCAAATGGCGTACT
CCGAAACCAGGCCTACGCGTCAACGACATAGTCCTCGTAAAGGATGAAAATCTGCCAATAAATGAATGGC
GCCTTGGCCGAGTCCAGCTGGCTTGCNCAGGCACAGATGACAAGGTCCGCGTAGTGGACGTGCTTACCGC
TCGCGGTGTCATCCGAAGGCCAATAGCCAAATTGGTCCTTCTTCCTACTAAGGCAAATAGCACCACCGACT
AATCCTTAAAAGCAACTAATCCCTCTTTCAAAAACAAAACCATCACATAATAACACCTGTCCTACATCTTA
TTCTCCAGTTCACTCCTCTGTAGAGAACCAGCATGGCACCGTCAACGAAGGCATCCAATCAACGTCAAGG
AACGGACTCTTTTAGATGCCGGGTGTGTAGAGGCATCCATGCGCTACGCAAGTGTCAGCGCTTCTTGAAG
CTCACGCCCGAGAAGCGTTCCCGAGCCGTGTTAATTAACAAATATTGCTCCAACTGCCTGGCTCATCAGCA
TTCAGGGGAATCATGCCGCAGTGGAGCCAAGTGCAAAATTTGCAAAAAGGACCACCACATCTTACTCCAC
ATGCGTGATCCCCGTAGCTCTCGACACTCTCGGTCAGACAACAGGCCCACAAGAAGAGCTTCTGGCCCCT
CGCCCTCCAGCGGCACATCACCCTCAAGCCCGATACGTGCTACTATACGGACGTCGCTTACGTCCCTTCTC
CAGCACAAGGGGGTCAGTATATTGCCCACCGCCATAGTACTGCTCGACACCGGAAAAAAGACCCTTGGGC
TCTGATCGATCCATGTTGTTCAGTAAGCCGCATTAATGCAACCGTGGCAAGGGCCTATGGCCTTTCCGTCA
CACGCATTGGCGCAGAAGGAGCGTGCACAGCCGTGCTACGCTCCAAGACAACTCAGATGTCTCGAGAAGT
GGTCTTCGTGGCTGACCCGCAACTCAGCTGCATAACTCCAGCCCGAGCCATCAGAGCGGACATGAAGGAC
CCGTTCAGCAACGTCGCTTTGGCGGACAACGAATGGTTCCGTCCATCTCCAGTCCCTTTGGTACTCGGCGC

CGACGTCTACCCAGACAAAATCCTACCAGGTAACCTCCCGAGCCAAGGCGGTCTGCCGATGGCTCAAAAT
ACCAAGTTTGGATGGATACTCTCTGGCACATGCACGCAATAGGAATTCTTGCAATCTGCATATTGCCAGGG
GGGGGAGAATGTTCATGCCAGTTCAGTGCTAGTGTGAACACAGCATTGACTTAGCCTCACTCAGCGTGAG
CGAGTCACTCAGTGAGACAGACTCACTCACTGAGAGAGACTCATTCACTAAGTCGATCCCAGATGCGCTC
AGCACTCTCCTTGTGGTCTCCTTTCCACCTCCATCACTCCAACCAAATATATGTTAAATCACTTAACCAGCA
GAGTCTGTTACATGCAGCAGAAACTTCTAATAAACAACAATAATCAAGGATTATTTATCTGACGCCACCC
CCACAAACAGGAACTATCCGTTAAAAATTAAGTTGTATGAAAACACATTAAGTTTTTGAATGAAAAAACA
TTATGTTTATCAAGATATTTTGACAAAACTCGGCATTTATTAGTTTTACCATGCTCCCCATATATATTCAAA
ATCCTATAAAGATCGGACCACTATATCATATAGCTGCCATAGAAACGATCGGTCGAAAATTAAGTTGTTG
TATGAAAAAACATATTGTTTATCAAGATATCTTGACCAAACTCTGCATTTATTAGTTTTATTTTTCTCCTTA
TATATTTGCAAAATCGTATTAAGATCGGACCACTATGTCATATGGCTGCCATAGGAACGATCGGTAGAAA
ATTAAGTTGTTGTATGAAAAACATTTTGTTTTTCAAGATATCTTGACCAAACTCGGCATTTATTAGGTTTAC
TATTCTCCTAATATATATGCAAAATCATTTTAAGATCGGACCACTATATCATATAGCTGCCACAGGAACGA
TCGGTCGAAAATTAAGTTGTTATATGAAAAAATATATTGTTTATCAAGATAGTTTGACCAAACTCTGCATT
TATTAGCTTAACTTTACTCCTCATATACATTGAAAATCCTATTGACATCGGACCACTATATCATATAGGTG
CCATAGAAACGATCGGTCGAAAATTAAGTTGTTGAATGAAAAACATATTGTTTATCAAGATATCTTGACC
AAACTCTGTATTTATTAGTTTAACTATTCTCCTAATATATTTGCAAAATCCTATTAAGATCGGACCACTATA
TCATATAGCTGCC
>Ref_dvir_734
GGGAAAAAGGAAATGTACTACTTTTGAATTATTTTCAGATGGCAACTCTAAGTTATCCATCTGAGTAACAT
GCTTGGAATTTTTGGGCACGCTGTGTTTGTTTTTACTACGCGCTATTGATGCGCGTAGCAGCGTGTGCCCTC
TGTAATATTTAGCATTTGAGTTGCCTTTTTGGCTACGGCTGAGTGGAGGAGAAACATATTCAGTAGAATAC
TCTAGTAAGTAATTTGTAGTTTCGATAGCATCCTTGGTGCTAAAATTAAAATAGCTTTCCAAGGCTGCTTG
AATGTCATGACTGACGCATTTGCTTAGTAAACAGATAGCTTCTGGGTCCGATAATAGGCATTTTATTATAG
TCGCCTTATCTATAGTACGGCTGAAAGCCATTTTTGAGTATATGAATTTTTCGCTGATTTTATCAATATTGA
ATGCAGTTATCCGATTTGGAGTAAATCTGCTATATTCTGAATTTTTAATGAATTGTATGTTTCGAGAGTTTG
TTGTAAGCTCCTTCACTCTATGGCCGTATCTCAGCATATTACTTTTTTTACTCATTTCGAGTATCATTGGCG
TCTGAGGAGCTACTAGCCGTTTAAACTAGCTAGCGTCCTAGTGGCTGTTCCGTGCAATTTGGTTGCAGGAT
ATGCATTAATTTTATTTTTGGCCACTATTTTCAAAAGTAGTTCAACTACTTGGTCCAATTTCGTGGTCATTT
GCATAATTGCAGCCATGTTGCTATTTAAAGCATCCAATGGCTGCATTATTGACCGGCTCTTGGCTTTCTTTT
TGTTGCCGTTGCTTATTTTTCTGTTGCTTTTGCTGCTGCTGCTGCTGATGATGACTAGCGCCTGTTGTGTTTG
AAGCATTCGCTGCTCCTGATTGATTTTTCTGGCGCTCTTCCTCTTGTTCCATCTGAAGTTTCCTTCTGGAACT
TTAGGAGGTGTTGCATGGCCGGATTATGCATCCGACCAGTATTGAACTCGCCCGCTGGTCACCTGACCTAT
AAGTCATCGTCGAGTTGGGCAATGTTTGCTTTGCATCTTCTGGTTCTTCTGCTGCTGATGTTGTTCACGGTC
CTGGTTGGACACCACGTTTTGCGTTCCATGTTGACGCTTATTGACTGATGGCTGCTTGTGCTCGTGCCTGGA
TGGGCATGACTCGTGCCCGGTTTGCTGATAGCCTAGCTCGCTCATCTTTAAAAGCTTTGCCAGCCTTTATA
ATTGGCTACATGCTGGCCCCTGACAATTGGCCGCACTTTGAAGGGCTCGCCTCTTGTCTTGGAGCATGTCG
TGGTCGGGTGTCCTTGTCCGCATTTAAGGCATACAAATGGTCGTCGGCAATAGTTTTTACTATGCCCGTAT
GCCTGGCAACGGTGGCACTGAACAGGATCAGTGCGCTTCCGCTGCCTCTCCACTTTGATTTCCTGGCTGCC
AAGTTGTTTCAGTTCCAGTATCTTAGTGTTGTTCTCGTTTTTCCACTAGCTCAATTTCGAACGTATTGATCG
GTTTGGCAGTGAAGCGTTGTTTTATCACGCCCACATACCTAACCTCATGCCCCAATCTTCGCAATTCCATG
CGTACCCAGTCGCATGGAGTTGTGTGGTGTAAATGGCGAATAATGATTCTAAATCCGCGCTCATTGCGCAT
TTGATGGGTGTGCCGGTGAACATTAGAGGCATCAAGCAACGCAATTGTTGCATTGTATGCCTCAATGTCCG
CACATTGCACTCTCACACCACCTGTCTGCGTACTTTTAGTAGCAAACTTATTAGCACCCACATTATTTGAG
ATATCTTTGATAATTCTTTGAATATCATCAATATTGGGCAGGTATATGGTGGGCACCTTGACCCGTTGAGC
TAGATTCAGCTGCATTGTCTGACCAGAGATTACATTTGGATTGCAATCTGTTGCCTCGTCTTCAACTTCCAT
GATGTCAATGTCATCACTGTCCTCGTTGTTGTTGTCGTTAGCAACGAATGTAGCATTGTTAGTTTTGCCAAC
ATTCGTGCCATTATTTGTCTGCGTGCTCTTTCTAGCTTTAGCTGCTGGTATATTATTGTCGCTTGACAGATT
GTCGGATACTTTATTGGCTGTATTTGATTTAAAGAAATCAGAATACGAAGGAGTATCCCTGACACTGTCTG
CGTTCGTAATATACTCAGCGTATGCTAATAGTAGTGCACGCTTGTCACTCTTATTCGCTGGCATAGTTGGT
GAGAATTTGCGCTTAATAGGTCGTTTAATGACCGTATTGGCTTTGGGTGATCCACAGATTCTAGTACCCAA
CATATCCTCATTTATTGGCAGCGATTGTTGCCCACTCGCCTCCAGTTCATTTTCTGAAACTTGCATAATTTC
AGCTGGCATCATTCCCGCATAATTATTAGGAATTAATTGACTAGCCGATGGAGCAGCATCGGCATTATTTT
TATTAATTTCCTCCATTTCCTGGCTATGTGTGACACAGATAATTTTCTGAGTCAGAATTGGTTGGGCTATCA

GCAAGCATGTTGCTAATGAACTTGCGCTCTAACAACATGTTGCTGTAGCCCCATACTGTCGCTCTCAACAT
TTTGTTCGCAGCTCGAGACGAACATGTTGCCATGATCGCTCTCACTACGTTCATAGCTGCTGCTGCTGCTG
AATTTGCTCGTGTTCGATGCCGCTCTGCTGCCGACACTCAAACGCTCGATTGGCGCTTCTGCCGACGCAAG
CGCCGACAGCGACTGAGCCGTAGAGAGCATTGGCGCTCTCTTGTGCTTCGGCTTCGGCTTTCTCCGTTTCA
GCTGGTGTTGATGTTGTTGCTGCTGCTTGGGTAGCTGGTGCCGTTGCTGGTGTTGAGCTGTAAGCTGCTGC
TTGCTGATGCTGCAAGCTGCAGCTGGTATTGGTGCGCCAAATGGCGACATTTTCAGCAACAGGTGCCGGG
GGGTATGGGGGGGGGCTATAGCTTTTGTAGCTTTGGATGCAGCAATGGCCGCCGGCTTGGACGCCGCCTT
TTCGCGCATTTTTTTCCTTATTTACTTGTCTATGTACTTGCCCTTTTTGGCCTTATATACATATCATTATGTA
TTATATTTATGAGTTTTTTTTGGCGTAGTTTTCCCCTCGCTGTCGTCAGGTATCTCGTCTGTGATGGCTTCTT
TTGTTAATAGAATTAACAATATGTTGCTGCTCTGCTGTATATAAGTATATATACAGCACACAGGTTAGGTT
TTATATATTTAATTTTTATTTATTTATTTTATTTTATTGTTTAGCATTTAAAAATGCTGATTGGCCCGTATTT
AATTTGTTAGAATACTTTATTTTATTAGTTTTTCACTTTTTCTCTCCATTGTGGCTTTTATTTAAATTTTTTTT
TTTTTTTTTTTGAATTTATTTTTAGCATTTAAAAATGCTGATTGGCAGTTTATTTGTTTAAACTTTATTACTT
TTAATTGTTTGGCACAAGCATTTTATTTTATTTATTTTATTTAATTTAATTTTAATTTACTGGCAGAAATAA
AGACACTTGTAAAAATTTTTTTAGTTGTTCGTCTGGTTGGCCGACCGGGCGCTGTCCGCAAACACGTCCGC
TCGCCACGACCGCCACAGAGAGATTTTTATTGTTGCTCATTTCTACATATAGTTTTGCAAACAGAAATTTT
AGAACATTTATGGGAGCACTTAAAACCTATGCAACGATCTCCAGTTTGCAAGGTGGAGAGGGACATGACG
GGGGAGATACATTTAACATTTAATCTCGACTGACCTACATTTAAACATAACATAACATAACAGACTCTGTT
CGATTAACATTATTGTCGTAGGTCAGTTCGCTGGGGGAAATTTATGCACGATCAGCTGGCAATGCTGTCGT
GTGAATGGCAAGAATAACATAAGTATCAATTGGGCACGCTAAGTACATGTGAGTGGACAAAAATTTTAAA
TTTAACAATTTTATGGCTAAATAATCTAATTAATTAATTTATTTTATATCGCACCGCCTTACGATGCGCTGG
CCGCCTGCCTCTGGCCTTGCGGTGGACAATTGGCCTCCACCTGGTACCTGGCCGGCTTATTGTCGCATAAA
TTTGCAATTTGCATTGCGTGATGGCTGCCGCTACATCTGTTGGAAGTTGCATCGCTTTGGAAGGGCATAAC
TTCAGCTGCTGGAGCGGATGACTGGCAATGAGCAACACCATCCACACACCCAGGATGCATGGATAGCGGA
TCGCCGTGTTATCGCGCCTGTTTTTGCCGGAGGGCGGAGTGATATGCAGCCTGCGCTGTATCATTTTTGCA
CGTACGGGTGGCGCTGTGTCTGGCCACTTCGGCGTCGAGTGGATGACGTTTCGCCGGCTTCCGTGTGTGCT
GAGCAGGCCGCTGACGCATTCGCTGGTCCATTTGTGCAGGTAAGTGGATACATGGCATTTTTTGCAATTTG
CATTTTTTAACATTTCTGTATTGCTTGCAGGTACGGCGTCTGCTGGGCTGGATAGATGGCCTCTGGGTAGT
CATCCTTCGCCTGCATCTTCGTCGCCGCTCCAATTTGGCAGTTTGTGCCATTTTCAGCTGGATTTGGTGGCC
ATTTATTTGCCGTAGTGGACCACCGTTCGTGCGGTGCCGTGTTGTGCAAGATGCATGCCCGGCTGGGCATT
CTGCTCAATTTGGAGTCGCTGCGTCTGGCCAAGGGAGGCACCGTTTTGACCACAATGTCCCCATTGCTGGA
GAAGCTGCTGCCCCTTTAAGGACTGGATGATCAATAAGGGGCTGTGCTTTTTAGTGCCAAAGGGAGTCCA
GTGAGGACGCCGTCGACTGATGCTGGTTGTGTCTGCCTGTACCCAGGTACAGATTGTGCATTTTGCGGCTC
GTGCTTGTGCCCGTAGGTGGGCGTGGCGGCTGCCGTCGGCTGCTGCTGCACGTTCTGCTGCTCCCATAGGA
GGGGCTGCAGATTTGGCGGGCGTTGCTGGTGACCATAGGAGGCATTGGAGGCTCCTATTGACCATCGCTG
CTGTGCTTGCTGCCCCCATAGGAGAGGCTGCGGATTTAGCGGCTGCTGCTGGTGCCCAAATAAGGGCGTG
GTGGATGCCGTCGGCTGTTGCTGCGTGCTCAGCTGTCCCCATAGCACGGACCGAGGATTTTGCGGCTGCTG
CTGGTGTCCATAGGCGGCACTGGAGGCTCCTATTGACCATCGCTGCTGTGCCTGCTGCCCCCATAGACGAG
GCTGCAGATTTTGCGGCTGCTGCTGGTGCCCAGAGGAGGGCTGGTGGATGCCGTCGGCTGTTGCTGCATG
CTCGGCTGCCCTAGCACGGACTGAGGATTTTGCGGCTGCTGCTGGTGACCATAGGCGGCACTGGAGGCTC
CAATTGACCATCGCTGCTGTTCTTGCTGCTCCCATAGGAGAGGCTGCGGATTTTGCGGCTGCTGCTGGTGA
CCATAGGCGGCACTGGAGGCTCCAATTGACCATCGCTGCTGTTCTTGCTGCTCCCATAGGGGAGGCTGCG
GATTTTGCGGCTGCTGCTGGTGCCCAAATGAGGGCGTGGTGGATGCCGTCGGCTGTTGCTGCATGCTCGGC
TGTCCCCATAGCACGGACTGAGGATTTTGCGGCTGCTGCTGGTGACCATAGGAGGCATTGGAGGCTCCAA
TTGACCATCGCTGCTGTTCTTGCTGCTCCCATAGGCGAGGCTGCGGATTTTGCGGCTGCTGCTGGTGACCA
TAGGCGGCACTGGAGGCTCCTATTGACCATCGCTGCTGGTTCTGCTGCTCCCATAGGAGAGGCTGCGGATT
TTGCGGCTGCTGCTGGTGCCCAAATAAGGGCGTGGTGGATGCCGTCGGCTGCTGCTTCGCCGAATGGAGT
TGCTGGTGCTGTGGATGCCCTTCAGGCCGCTGGATAGGCCAATATTGGGTAAGTGAAGTCTGGAATGTATT
CAGATTTGAATTAGAGTTTATGTCCGTGGTCGAGTCCGAATTTGTCAGTTTGTATTGTTCTGTCCGACAGTG
CTCGCTGTCAGTTAGTGGTTGCTTGGCTTCGTAATTGTTACCTAGTTGCATCATACCCTGCAACAGGTTCTC
AAGCATAAGTTTTTCGTGCGGGTCCGTATTATCTACGTCCCGTACAAGTTTTTTCTCCAAGACGTTGAAGA
TATGTTCGACTAGTGGGTCAGCTGGCTGCAGATTGTCAATTTCTAGGTCATCAGTCAGTTGATTTTGTTGC
ACATTGTGCATGAGTGTCTGTTGAGTAGTTGGTAGTTGAACTTTGTTGTCTTTTGGTGTCCATTCTAAGTTG
GACACCTGAGTAGACTGCCAATTAGTTTTGGGCACGCTATATTTATTTTTACTACGCACTATAGGTGCGCG

TAGCAGCGTGTGCCCTCTATATTGTTTGACATATGATTTGCCTTTTTGGCTACGGCTGCGTGGGGGAGTAC
CATGTTGATTATGAGTGAATAATTCATTTAAAAATTCGATTGCATCCTCAGAGTTGAAGTTAAATTCAAAC
TCCAAGGCTGCTTGCATATCATTATATATATGATTGTTTATTTCGTCGATAGATTCTGTATATTCTAGATAG
CGTTTTTTAATATTCGCCTTATTAGTGGGCTGGCCGATAGCCAGTTGTTGATCATTGGCATAATTGCCGATG
TTTGCAAAGTTAAACGCAGGTAGCCGATTACCTTGTTTTAAGGGATAGGTTGCGGCTCTGCCTGTTTCTAA
ATTAATGACTGTTTGTTTGTTCCGAGAGTTTTTTGTGTACTCCCTCACTCGCTGGCCGCATATAAGACTGGC
CCTTGCAATACTCACCTCAAGTAGCATTGCCCCCTGAGCAGCTACTACCAGTTAAACTAAGCTAGCGTCCT
AGTG
>9_rnd-1_family-267__LTR___Gypsy
ACTAAGCAACTAAGACGAGTTGCATGAGAGGCAAACCGCCGCTTATGCCTACGAGGGAATNCTAAGCAA
CTAAGACGAGTTGCATGAGAGGCAAACCGCCGCTTATGCCTACGAGGGAATACTAAGCAACTAAGACGA
GTCGCATGAGAGGCAAACCGCCGCTTATGCCTACGAGGGAATACTAAGCAACTAAGACGAGTTGCATGA
GAGGCAAACCGCCGCTTATGCCTACGAGGGAATACTAAGCAACTAAGACGAGTTGCATGAGAGGCAAAC
CGCCCCTTATGCCTACGTGGGAAGAAGAAGAAGACCCTATAACAACAGTACCGGCAGCGCCCAACA
TATTAAATTAAATAGTAATAAATAAAACTTAAAAATTGTAATGCCCGACGACAATTGAGTCGACCGGCAA
GACGTTTGGCCNGTACAGCCATTCGAAACAATAGGTAGGAAAAATACAGATTGTAAAAAAAAATTTTTAA
GATGCGTTTTAAGCAGAAAACAAACAAGCAAAACAAAACCCGACTTGTAAACAATAAAAGCATCAAATT
CAATAAATATATGTAATAAAACCTCAAATGAACGATACAAACAACAACTTGTCTTGTAAAGTAAACGAAA
ACAAATTTAACATTACGCATTTGTAAATTTTTTAAGATAATTCCTTTTTAATTTTATGTATGATCCCATACT
ATATATGAATATTAATGATACAAAAGAGGAAANTAAAAGTNNTAATTCGTTCAGTTGGAATANAAAGAA
ATAATGCGCTCNGCTTGTCTCATTAGGTAATTCAAAATTATGATCAAGAATTAGCATGATCGAGGACAAG
ATTAACAAAGTATATAACGACTATACAGACTTTCGTATGCGAGCCATACAAATTTTGCAAAGGCATAGAG
AATTCATACCAGTATATGAGTTCGAATCCGAATATATAGAAACATTCGGCACAGTGACAAGTCCACTCTA
TTCATGTTCTAAAATAGAGNGCGGAATAATCTTGTCTACACTACCCTACGTCAAAATAAAAACAATTATTG
ACGAGCATCCGATTCTAGGGCAGACAACAATTGTCTTAGTACGTCTGCATAGGTAGTAGTCAAATCAAAA
AGCAAGAGTATTTATATAGGAATATGGGGGAAAACAATCAAAGGCAGATGATAACACTGCCAATGTAGT
TAACGTAGTAGAAGTCGTAGACACCACTCCCCATGTGCAGTTAACAAACATACTGCTAATAATACTCATT
GTAATAGCTTCAGTAAACATCCTTCTGAAGATTTATCTTCTTCATAAAAAATCACTGAGGAAGCGTTACAT
GAGTCGAGCCAACGACTTAGACAAGGTCTAAGCCACTGCTTAAATAAAATATTTAAGTAGCGGAAGTTTA
CATGGACTGGTCCCAAATAGTAGAAAAGATAAAGCGGCATAGAGAAAATTTTTCGATAAATCATACAAGT
GCCTTAATAAAAGCGCACCAGTTTTAAGACCCACCATAATTAAACATACTGAGATCCTTGTATATGAGTAC
AATGAAATCAGGAGTCTAGTTACCGAGTGTCAAAATAAACTGACAAGTTTACACAGAGTCCAAACTTCAG
ACGTTTTACAGCGTCTAAAGGGATAATCTGGTAAACTTAAATAAAAAGTATGATCTCCAGCTTTATCTACC
ATCGGTGTTAAATACACCGGTAACATTTGATAAGTCACTACTGACAAACGACCAAATAGAAGATACAAAA
TTAGTAAACATGGCACAAACAGTTGTAGAATTTATCAAGCTAGCCACATCTCTGATACCAGAGTTTGACG
GTAAGCCGGAAAATCTACAAAGTTTTCTAGATTCTTTGAATCTATTAGAAACCTTAAAAGGCACACATGA
GCTAACGGCCATAAGCCTTATAAAAACAAAGCTAAAAGGCCATGCGAGAAATTTGATAAGTAATGAGCA
GACAATTGCTGCAATCATTACGCAACTATCAAATGCAGTAAAAGGAGAATCGGTAGAAGTTGTATCTGCT
AAGCTTCTTAATTTGCAACAGAAAAATAAAACGGCTAACCAATACACCCAAGAGGTGGAAAAACTGGCA
AAGGCCCTTGAAGGCGCCTATATCAGTGAAGGTCTAAGCCAATCACTNGCAAACAAATACAGCACTACAA
CAGCTGTAAAAGCTATGACCCAAAATTGTTCGATTGATAAGGTAAAACTTATCATGCAGGCAGGCACATT
CACAAATATGAATGATGCCGTATCCAAATTTGTCAATAGCTGCACGGAGATAACAGGTCAAAGTAACACT
GTCCTGTATTATCGACGAGGTGCAAACAATAATAATAGAGGAGGCCGAGGTTATAATCGTGGCAGAAATT
ACAACCACAACAATTATAACCGAGGCGGTAACAACAATAACAATAATTACAATAACCGTGGAGGAAGAC
GAGGCCAAAATAGAGGAAGAGGCCGAGGTAACTCCAACCAAGGTTATAATAGTAACAATGTGAGAATGG
CGCAAAATACGCCGGAAAACTCTCAGAACCCTTTAGGAAACAACCAGTAAATGTAAAGGTTCATTCCATC
AATTACAGTCTTAATATATTTGTCACTTTTTATAATCATTCAACTGAAAATAAACTCACATTCCTAATAGAT
ACTGGTGCAGATATCTCACTTTTAAAAATAAATTCAGACAATTTCAAAATTCAAGAAGAAAAAATTATAA
ACATCCAAGGCATAGGCCAAGGTGTAATAAAATCTCAAGGAACAACTTCAATAGAACTTCAATCCACAAA
ATATATTATTCCACACGAATTTCATTTAGTAGATTCCGATTTTTCAATACCGTGTGATGGAATAATAGGCA
TTGATTTTATTAAAAAATTCAATTGTCAATTAGATTTCAAACCAAGTGAAGACTGGTTTATAATTAGACCT
CAAAATTTAAATTATCCAATTCATGTTCCGATAACATATAGTGCTGGCAACAACACAGTTCTCTTGCCAGC
CAGATCCCAAGTAATTCGAAAAATAGACATTAATACAGTAAATGATTATATATTTGTTCCTAATCAAGAA
ATACATAATGGAATTTATGTTGCAAATACAATAGCAGCATCCAAACATGTATACATTCGCCTTCTAAACAC

AACTAATTTCGACCAATTGGTCAAAGTAAACAAAATCCAATATGAAAATTTAACAGATTACGACATTCAT
AGCACTACTCCAGAAAATAGAAGCGAACAAGTACTTTCAAAATTAAAGAAAAATTTTCCAGAACAATTCA
AAAGTCAACTAACAGAATTATGCACCAAGTATAGTGATGTGTTCGGACTGGAAACCGAACCAATAGCGAC
AAATAATTTCTATAAACAAACATTAAGACTTAAAGATGATGAACCCATTTATATTAAAAACTACAGAAGC
CCGCATAGCCACGTTAAAGAAATTCAAAAACAAGTAGGGAAATTAATAAACGACAAAATCGTAGAACCG
TCTGTATCCGAGTATAACAGCCCACTCTTGTTAGTTCCGAAAAAATCATTACCAAATTCGGAAGAAAAGA
AATGGCGATTAGTAATTGACTATCGCCAAATCAACAAAAACTACTTTCTGATAAATTCCCGCTCCCCAGAA
TTGATGATATTCTAGACCAACTGGGCAGAGCTAAATACTTTTCATGCCTTGACTTAATGTCAGGTTTCCAC
CAAATTGAACTTGAAGAAAATTCAAGAAATATAACATCTTTTTCAACGAGCAATGGCTCATATCGCTTCAC
GCGATTACCATTTGGTCTTAAAATAGCACCAAATTCATTTCAGAGAATGATGACTATAGCATTCTCGGGTT
TAGAACCCTCCCAGGCATTCCTTTATATGGATGACTTAATGGTGATAGGATGTTCCGAAAAACACATGATT
AAAAACTTAACTGATGTTTTAATGTATGTAGGAAATATAACCTAAAGTTGCATCCAGAAAAATGTTCATT
TTTCATGCATGAAGTGACATTTTTAGGTCACAAATGCACTGACAAAGGAGTATTGCCAGATGACAAGAAG
TTTGACGTCATCAAAAATTATCCTGTCCCTCATGATGCGGACAGCGCGAGACGATTTGTAGCATTCTGCAA
TTATTATCGTCGATTTATAAAAAACTTCGCCGATTATTCACGGCACATAACTAGATTATGTAAAAAGAATG
TTCCTTTTGAATGGTCAAGTGAATGCCAAAACGCATTCCAATACCTTAAAGAAAAGCTTATGCACCCCACA
TTATTACAATATCCTGATTTTCGCAAAGAATTTTGCATTATAACAGATGCCAGTAAACAAGCTTGCGGAGC
AGTTCTAACTCAGAACCGTGACGGAATTCAGCTCCCAATAGCTTATGCATCTCGTTCATTTACAAAAGGGG
AAAGCAATAAGAGCACAACAGAACAAGAGTTAGCGGCAATTCATTGGGCAATTACCCATTTTAGACCATA
TATTTATGGCAAACATTTTACTATTAAAACGGATCACAGACCATTGACATATCTTTTTTCTATGACTAATCC
CAGTTCTAAATTAACTCGCATGCGGCTAGAGCTTGAAGAATACGACTTCACAGTAGAATACCTAAAGGGG
AAAGATAATTTCGTAGCAGACGCACTTTCGCGTATTAATATAAAAGAACTCAAAGATATGCAACATAAAG
TCCTGAAAGTCACTACCAGGCGACAAAGTAGACAAAATAAAAACTGTACAGTAGAAAATTATGAACTTCT
GCCAAGGCAAAGTCATAAAAATGTATCCAAGCCCAACGTACATGAAGTCATTACAAATGATGAAGTACG
AAAAGTAGTGACCTTGCGCATAACAGAATCTAAATGTTTATTTAAACATGGAAATAAAATTATCGCAAGA
ATTGAAGTTAGCGATTTATATACCAATGGAATTCTCGACCTAGGTCAGTTCTTCCAAAGGCTCGAAAAAG
AGGCCGGTATATTAAAAATCAGCCAACTCAAGGTGGCACCGAGCGAAAAGATCTTTGAATCAGTTTCAAT
AGATTCTTTTAAAAATATGGGCAATGAAATATTAAAATCTTTAAGAGTAGCGCTACTCCAGCCGGTGACC
CAAATAAAAGATGAAAAAGAGATACAACAAATATTGTCTACATACCATGACGACCCAATTCAAGGAGGT
CATAGCGGCATAACAAGAACGTTAGCGAAAATAAAAAGACACTACTACTGGAAAAATATGACACGTCAT
ATAAAAGAGTACGTACGTAGATGTCAAAAATGCCAAATGTCTAAAACAAAGACGCACACAAAAACTCCT
CTGACTCTCACAGAAACACCTGTGAATGCTTTCGATATAGTGATAGTGGATACAGTTGGTCCGCTACCCAA
ATCGGAAAATGGTAATGAATACATAGTCACATTAATATGCGACCTTACAAAATATTTAGTAGCCATTCCA
ATAAAAAGCAAAAGCGCAAATAATGTCGCAAAAGCTATATTCGAAAATTTCATACTCAAGTTTGGTCCAA
TGAAGACGTTCATCTCGGACATGGGAACAGAGTATAAAAACCAGATTTTAACTGACTTATGCAAGTATTT
GAAGATCAAGAATCTCACCTCTACTGCATATCATCATCAAACATTAGGGACAATAGAACGAAGTCATCGA
ACTTTTAATGAATACATTCGTTCATACATCTCTGCAGACAAATCTGATTGGGATGTCTGGGTTCAATATTTC
ACTTATTGCTTCAACACGACACCATCAGCAACGCATGAGTATTGTCCATACGAACTAGTATTTGGCAGACT
ACCGAAACAGTTCATAGATTTAAACAAAACTAATTACATAGATCCTATTTATAATTTAGATGACTATTCTA
AGGAAATAAAGTTTAGATTAGAAATAGCATACAAAAGAGCTAGAATTATGTTAGAAAAAGCCAAGGCTT
ATAGGAAATTAGCATATGACGAAAAAAAAAAAAAACTTTGAATTAAAAGTAGGAGATAAAGTACTTATAA
GAAACGAAACAGGGCACAAATTAGACCCAGTATATTTAGGACCATATATAGTAGAGAAAATAGAAGATA
ATGACAACATTTTTATTAAAGATAGTAAAGATAAAAAAACAAAAAGTTCATAAGAATAGATTAAAAATTT
TTAATTCTTAATAAAAAAAAATAAAAAAATATATGACGGCCTGATCAACCGAATGAAAAAAATTTTTACA
AATAAACGAATTATAAATTTTTTACCTTAAATAATTTTTGTATTTTTATACTCCAACATTTAAAATTTCTTA
TTATATAAAAAAATTTTCAATTTTTACAAAAAANCANAATAATTAAGTTCATTTAATAACTACACATTCAT
AATGTTACGTTATTTCTCTAAAAAGGGGGTGTAGTGCGTATATGCCCTTACAGAATACCCCGTACTTAAAC
ATAAAGTATGCAGCACTTTGTTGCATACAATAACAATAATATAGTAAACGAGCCCAGAGCCACATTATGT
ATTGGCTCGGTCTCATATTACGCAGCGCGGTTCACTAGCCTCACTAGCTGACCGTTTATGCTTATGATGCA
TATCCCACGCTCCGTCTGAGCGCATTCCGTGAGCAATATATATGCTCACTAATACAGGCATATGCGAGCTA
CTCACTCTCCCTCTCTTTGTAGCTGTTAACGAATAACAGCATAAGTATATATGTATATCCATAGGCTAAGC
GAACATTATAACAAGTGAATAAATTCATTCTGAACAAAGATACAAAACAGCACTGACGTGCTGACTTATT
TTTCATTGACAATAAGTGCTCTTTTTACA
>9_rnd-1_family-249__LTR___Gypsy

ACTAAGCAACTAAGACGAGTTGCATGAGAGGCAAACCGCCGCTTATGCCTACGAGGGAATACTAAGCAA
CTAAGACGAGTTGCATGAGAGGCAAACCGCCGCTTATGCCTACGAGGGAATACTAAGCAACTAAGACGA
GTTGCATGAGAGGCAAACCGCCGCTTATGCCTACGAGGGAATACTAAGCAACTAAGACGAGTTGCATGAG
AGGCAAACCGCCGCTTATGCCTACGAGGGAATACTAAGCAACTAAGACGAGTTGCATGAGAGGCAAACC
GCCCCTTATGCCTACGTGGGAAGAAGAAGACCCTATAACAACAGTACCGGCAGCGCCAACATATTAAATT
AAATAGTAATAAATAAAACTTAAAAATTGTAATGCCCGACGACAATTGAGTCGACCGGCAAGACGTTTGG
CCNGTACAGCCATTCGAAACAATAGGTAGGAAAAATACAGATTGTAAAAAAAAAAATTTTTAAGATGCGT
TTTAAGCAGAAAACAAACAAGCAAAACAAAACCCGACTTGTAAAACAATAAAAGCATCAAATTCAATAA
ATATATGTAATAAAACCTCAAATGAACGATACAAACAACAACTTGTCTTGTAAAGTAAACGAAAACAAAT
TTAACATTACGCATTTGTAAATTTTTTAAGATAATTCCTTTTTACTTTTATGTACGANCCCATACAATATAT
GAATATTAATATCAAAAAGAGGAAATTAAAAAGTTATAATTCGTTCAGCTTGTCNCATTAGGTAATTCAA
AATTATGATCAAGAATTAGCATGATCGAGGACAAGATTAACAAAGTATATAACGACTATACAGACTTTCG
TATGCGAGCCATACAAATTTTGCAAAGGCATAGAGAATTCATACCAGTATATGAGTTCGAATCCGAATAT
ATAGAAACATTCGGCACAGTGACAAGTCCACTCTATTCATGTTCTAAAATAGAGNGCGGAATAATCTTGT
CCACACTACCCTACGTCAAAATAAAAACAATTATTGACGAGCATCCGATTCTAGGGCAGACAACAATTGT
CTTAGTACGTCTGCATAGGTAGTAGTCAAATCAAAAAGCAAGAGTATTTATATAGGAATATGGGGGAAAA
NAATCAAAGGCAGATGATAACACTGCCAATGTAGTTAACGTAGTAGAAGTCGTAGACACCACTCCCCATG
TGCAGTTAACAAACATACTGCTAATAATACTCATTGTAATAGCTTCAGTAAACATCCTTCTGAAGATTTAT
CTTCTTCATAAAAAATCACTGAGGAAGCGTTACATGAGTCGAGCCAACGACTTAGACAAGGTCTAAGCCA
CTGCTTAAATAAAATATTTAAGTAGCGGAAGCTTACATGGACTGGTCCCAAATAGTAGAAAAGATAAAGC
GGCATAGGGAAAATTTCGATAAATCATACAAGTGCCTTAATAAAAGCGCACCAGTTTTAAGACCCACCAT
AATTAAACATACTGAGATCCTTGTATATGAGTACAATGAAATCAGGAGTCTAGTTACCGAGTGTCAAAAT
AAACTGACAAGTTTACACAGAGTCCAAACTTCAGACGTTTTACAGCGTCTAAGGGATAATCTGGTAAACT
TAAATAAAAAGTATGATCTCCAGCTTTATCTACCATCGGTGTTAAATACACCGGTAACATTTGATAAGTCA
CTACTGACAAACGACCAAAAATAGAAGATACAAAATTAGTAAACATGGCACAAACAGTTGTAGAATTTAT
CAAGCTAGCCACATCTCTGATACCAGAGTTTGACGGTAAGCCGGAAAATCTACAAAGTTTTCTAGATTCTT
TGAATCTATTAGAAACCTTAAAAGGCACACATGAGCTAACGGCCATAAGCCTTATAAAACAAAGCTAAAA
AGGCCATGCGAGAAATTTGATAAGTAATGAGCAGACAATTGCTGCAATCATTACGCAACTATCAAATGCA
GTAAAAGGAGAATCGGTAGAAGTTGTATCTGCTAAGCTTCTTAATTTGCAACAGAAAAATAAAACGGCTA
ACCAATACACCCCAAGAGGTGGAAAAACTGACAAAGGCCCTTGAAGGCGCCTATATCAGTGAAGGTCTA
AGCCAATCACTTGCAAACAAATACAGCACTACAACAGCTGTAAAAGCTATGACCCAAAATTGTTCGATTG
ATAAGGTAAAACTTATCATGCAGGCAGGCACATTCACAAATATGAATGATGCCGTATCCAAATTTGTCAA
TAGCTGCACGGAGATAACAGGTCAAAGTAACACTGTCCTGTATTATCGACGAGGTGCAAACAATAATAAT
AGAGGAGGCCGAGGTTATAATCGTGGCAGAAATTACAACCACAACAATTATAACCGAGGCGGTAACAAC
AATAACAATAATTACAATAACCGTGGAGGAAGACGAGGCCAAAATAGAGGAAGAGGCCGAGGTAACTCC
AACCAAGGTTATAATAGTAACAATGTGAGAATGGCGCAAAATACGCCGGAAAACTCTCAGAACCCTTTAG
GAAACAACCAGTAAATGTAAAGGTTCATTCCATCAATTACAGTCTTAATATATTTGTCACTTTTTATAATC
ATTCAACTGAAAATAAACTCACATTCCTAATAGATACTGGTGCAGATATCTCACTTTTAAAAATAAATTCA
GACAATTTCAAAATTCAAGAAGAAAAAATTATAAACATCCAAGGCATAGGCCAAGGTGTAATAAAATCTC
AAGGAACAACTTCAATAGAACTTCAATCCACAAAATATATTATTCCACACGAATTTCATTTAGTAGATTCC
GATTTTTCAATACCGTGTGATGGAATAATAGGCATTGATTTTATTAAAAAATTCAATTGTCAATTAGATTT
CAAACCAAGTGAAGACTGGTTTATAATTAGACCTCAAAATTTAAATTATCCAATTCATGTTCCGATAACAT
ATAGTGCTGGCAACAACACAGTTCTCTTGCCAGCCAGATCCCAAGTAATTCGAAAAATAGACATTAATAC
AGTAAATGATTATATATTTGTTCCTAATCAAGAAATACATAATGGAATTTATGTTGCAAATACAATAGCAG
CATCCAAACATGTATACATTCGCCTTCTAAACACAACTAATTTCGACCAATTGGTCAAAGTAAACAAAATC
CAATATGAAAATTTAACAGATTACGACATTCATAGCACTANTCCAGAAAATAGAAGCGAACAAGTACTTT
CAAAATTAAAGAAAAATTTTCCAGAACAATTCAAAAGTCAACTAACAGAATTATGCACCAAGTATAGTGA
TGTGTTCGGACTGGAAACCGAACCAATAGCGACAAATAATTTCTATAAACAAACATTAAGACTTAAAGAT
GATGAACCCATTTATATTAAAAACTACAGAAGCCCGCATAGCCACGTTAAAGAAATTCAAAAACAAGTAG
GGAAATTAATAAACGACAAAATCGTAGAACCGTCTGTATCCGAGTATAACAGCCCACTCTTGTTAGTTCC
GAAAAATCATTACCAAATTCGGAAGAAAAGAAATGGCGATTAGTAATTGACTATCGCCAAATCAACAAA
AAACTACTTTCTGATAAATTCCCGCTCCCCAGAATTGATGATATTCTAGACCAACTGGGCAGAGCTAAATA
CTTTTCATGCCTTGACTTAATGTCAGGTTTCCACCAAATTGAACTTGAAGAAAATTCAAGAAATATAACAT
CTTTTTCAACGAGCAATGGCTCATATCGCTTCACGCGATTACCATTTGGTCTTAAAATAGCACCAAATTCA

TTTCAGAGAATGATGACTATAGCATTCTCGGGTTTAGAACCCTCCCAGGCATTCCTTTATATGGATGACTT
AATGGTGATAGGATGTTCCGAAAAACACATGATTAAAAACTTAACTGATGTTTTTAATGTATGTAGGAAA
TATAACCTGAAGTTGCATCCAGAAAAATGTTCATTTTTCATGCATGAAGTGACATTTTTAGGTCACAAATG
CACTGACAAAGGAGTATTGCCAGATGACAAGAAGTTTGACGTCATCAAAAATTATCCTGTCCCTCATGAT
GCGGACAGCGCGAGACGATTTGTAGCATTCTGCAATTATTATCGTCGATTTATAAAAAACTTCGCCGATTA
TTCACGGCACATAACTAGATTATGTAAAAAGAATGTTCCTTTTGAATGGTCAAGTGAATGCCAAAACGCA
TTCCAATACCTTAAAGAAAAGCTTATGCACCCCACATTATTACAATATCCTGATTTTCGCAAAGAATTTTG
CATTATAACAGATGCCAGTAAACAAGCTTGCGGAGCAGTTCTAACTCAGAACCGTGACGGAATTCAGCTC
CCAATAGCTTATGCATCTCGTTCATTTACAAAAGGGGAAAGCAATAAGAGCACAACAGAACAAGAGTTAG
CGGCAATTCATTGGGCAATTACCCATTTTAGACCATATATTTATGGCAAACATTTTACTATTAAAACGGAT
CACAGACCATTGACATATCTTTTTTCTATGACTAATCCCAGTTCTAAATTAACTCGCATGCGGCTAGAGCT
TGAAGAATACGACTTCACAGTAGAATACCTAAAGGGGAAAGATAATTTCGTAGCAGACGCACTTTCGCGT
ATTAATATAAAAGAACTCAAAGATATGCAACATAAAGTCCTGAAAGTCACTACCAGGCGACAAAGTAGA
CAAAATAAAAACTGTACAGTAGAAAATTATGAACTTCTGCCAAGGCAAAGTCATAAAAATGTATCCAAGC
CCAACGTACATGAAGTCATTACAAATGATGAAGTACGAAAAGTAGTGACCTTGCGCATAACAGNATCTAA
ATGTTTATTTAAACATGGAAATAAAATTATCGCAAGAATTGAAGTTAGCGATTTATATACCAATGGAATTC
TCGACCTAGGTCAGTTCTTCCAAAGGCTCGAAAAAGAGGCCGGTATATTAAAAATCAGCCAACTCAAGGT
GGCACCGAGCGAAAAGATCTTTGAATCAGTTTCAATAGATTCTTTTAAAAATATGGGCAATGAAATATTA
AAATCTTTAAGAGTAGCGCTACTCCAGCCGGTGACCCAAATAAAAGATGAAAAAGAGATACAACAAATA
TTGTCTACATACCATGACGACCCAATTCAAGGAGGTCATAGCGGCATAACAAGAACGTTAGCGAAAATAA
AAAGACACTACTACTGGAAAAATATGACACGTCATATAAAAGAGTACGTACGTAGATGTCAAAAATGCC
AAATGTCTAAAACAAAGACGCACACAAAAACTCCTCTGACTCTCACAGAAACACCTGTGAATGCTTTCGA
TATAGTGATAGTGGATACAGTTGGTCCGCTACCCAAATCGGAAAATGGTAATGAATACATAGTCACATTA
ATATGCGACCTTACAAAATATTTAGTAGCCATTCCAATAAAAAGCAAAAGCGCAAATAATGTCGCAAAAG
CTATATTCGAAAATTTCATACTCAAGTTTGGTCCAATGAAGACGTTCATCTCGGACATGGGAACAGAGTAT
AAAAACCAGATTTTAACTGACTTATGCAAGTATTTGAAGATCAAGAATCTCACCTCTACTGCATATCATCA
TCAAACATTAGGGACAATAGAACGAAGTCATCGAACTTTTAATGAATACATTCGTTCATACATCTCTGCAG
ACAAATCTGATTGGGATGTCTGGGTTCAATATTTCACTTATTGCTTCAACACGACACCATCAGCAACGCAT
GAGTATTGTCCATACGAACTAGTATTTGGCAGACTACCGAAACAGTTCATAGATTTAAACAAAACTAATT
ACATAGATCCTATTTATAATTTAGATGACTATTCTAAGGAAATAAAGTTTAGATTAGAAATAGCATACAA
AAAGAGCTAGAATTATGTTAGAAAAAGCCAAGGCTTATAGGAAATTAGCATATGACGAAAAAAAAAAAA
CTTTGAATTAAAAGTAGGAGATAAAGTACTTATAAGAAACGAAACAGGGCACAAATTAGACCCAGTATAT
TTAGGACCATATATAGTAGAGAAAATAGAAAATAATGACAACATTTTTATTAAAGATAGTAAAGATAAAA
AAACAAAAAGTTCATAAGAATAGATTAAAAATTTTTAATTCTTAATAAAAANAAATTAAAAATATATGAC
GGCCTGATCAACCGAATGAAAAAAAATTTTTTTTTACAAATAAACGAATTATAAATTTTTTATACCTTAAA
TAATTTTTGTATTTTTATACTCCAACATTTAAAATTTCTTATTATATAAAAAAAATTTCAATTTTTACANAA
AAAGAAGAAAACCCCAATAATTAAGTTCATTTAATAACTACACATTCATAATGTTACGTTATTTCTCTAAA
AAGGAGGTGTAGTGCGTATATGCCCTTACAGAATACCCCGTACTTAAACATAAAGTATGCAGCACTTTGTT
GCATACAATAACAATAATATAGTAAACGAGCCCAGAGCCACATTATGTATTGGCTCGGTCTCATATTACG
CAGCGCGGTTCACTAGCCTCACTAGCTGACCGTTTATGCTTATGATGCATATCCCACGCTCCGTCTGAGCG
CATTCCGTGAGCAATATATATGCTCACTAATACAGGCATATGCGAGCTACTCACTCTCCCTCTCTTTGTAG
CTGTTAACGAATAACAGCATAAGTATATATGTATATCCATAGGCTAAGCGAACATTATAACAAGTGAATA
AATTCATTCTGAACAAAGATACAAAACAGCACTGACGTGCTGACTTATTTTCATTGACAATAAGTGCTCNT
TT
>9_rnd-1_family-434__LTR___Gypsy
AAAAACTATGCCAAGTGGCTAAAAAAAGGTGTGAAAAAACAAAGGAGTCACCTTAGGTTGTCCTTGTGG
ACCACCCTCCCTCTAATGAGGACCTCGGTCCCCAGGTCCGCTTCCACGGCCTTCTCATCACATAAAGGTGT
GAGTTTGTTTCCGAGCCGTCGGTTGGACTTGACCAAAACTTTGTCACCAACGTCGAAGACCCTATTCTGCC
GATTTGCGTTCTCCCTAGCTCTGAGCGTCGTCTGAGCGTTTCTGATCTTTTCTTGTATCTTTTCTTGTGGCTC
ATCTGGGTGCGCCTGAACCACATCGACTGGTCTCTGGTCGATGACCGAATGGATCGACTTATTGTACTCAG
TTGTAGCCAGTAAAATTACTTCTACGGTGTCAGTTATGCCTTTATCGATCTTTAGGCATCTGGCTAGTTCTA
ACAGAGTGCTGTGAAAGCGCTCAACCTGTCCATTCGAGATGCTATGCAATGGTGGTGCATTTGAAATGCT
GACCCCATAGTTGTTTGCGAGCAAAGTCACGATGGTCTCTGAATTCAATGACGGTTCATTGTCACAGTAAA
TGGATCTGGCTTTTGGAAAAAATTCATTAGTTGCATTAAAGCTGGCTTCAGATCCACAATGGTTCTTGAAG

GGACCGGTTGGACAATAGCAAACTTAGAGAACTTGTCGATGCAAGTGAGAAAGTATTTCCTGTCAGTGGA
GAAAATGTCTATGTGTAACATCTCTCCTACATGTGAGGGGATCGGTGTCTCCCCAAGCTCTTGTTTTTGGG
GTGTCTATCATATTTAGCCCTAGCACATGTTTTGCAATTTTGTACTATTTCATTGGCTATTTTTGCCATCTTT
GGAAAGTAGTACTCTGAGAGTACCTGCTTAATATTTTCCTGTGCAGACCTATGGGCCCTATTATGCTCGGC
TGTGAGGATTTCCCTTCTTTCTCCAACCGCAAATACGTCCACTACGCGATTTTTGCAGTGCCAGAATTTTGT
GGCCGGGTATCTTCGAACCAATGCATCTTGTATCATTGCAAGCGTGTGTAAATTACAGTGGAGAGCATTTA
CGCATTTGGGTGAAACTACATCTTCGAGCTCTTCTAGCAGCGATTCGATGCAAGTATAGCTGATCAAGTAG
CGTTTCTTATTTCCAAAAAGAATAAAACTTCGTTTTAAGGGGAAACGCGCCTCTTCTAGCACTATTTGGTT
TTGAAAACAATTCAAGGGTTTGTCTGTTGTCTCAATGGTGTTGGTGAGAGATACCTCACTGTGGATCGTTG
CTGCGCAGGACTCAACTTCTTGTTCATTTACAGCGTTGAGCTGCTGTCTTGACAAGGCATCCGCAACCAGA
TTGTCCTTGCCAGGCTTATAGAAGATACGTGCATTAGACTCGTCAATGCGCGCTTTCCACCTTTTAATCTTC
GCATTTGGATTGGATTCCGAAACTGCGAAGGTCAAGGGTTGATGGTCAGTGAAGATATTAATGTCTTTCAC
CCCATACAAATAGTGCCTTAACTTGGCAAGTGACCATACGATGGCCAATAACTCTCTCTCATTTGTTGCAT
AGTTAACCTCGCTGTCCTTCAATGTTCTTGAAATCATTGTTATAGGACGACCCTCTTGAGACAAAACTGCA
CCTATGCCGTGAGCCGAAGCGTCGGTCGTTAGATCGAATGGCTTCTTAAAATCGGGGTACCTGAGCATTA
CATTCTCGGATGACAAAATATCGCGCAACTTCTGAAACGCCCTTTGCTGCACTTCAGAGAATTGAACCTTT
ATATTTCTGGACCTATGCTTACTGACTGTTCCATTTTCACCCTTCAAGATATTCGAGATGGGTCTTGCTATT
GCGGCGAAGTCCCTAATGAAACATCTGTAATAACTTGCGAGGCCTAGAAATGATCTGACCTCGAACAGTG
TTTTAGGTTCTGGATACTCCTGTATTGCCCTAACCTTTTCAGGATCCGTCGTGGCGCCAGTGCTGGTGACTA
TGAAACCAAGAAAGTTCACGCTTTTCCTANAAAAACGTGACTTTTCGACGGAGACCCTCATGTTTGCTTCG
TGTAAGCTTTTTAGGACCCAATCCACGTGTTTAATGTGGGATTCCTCATCTTCTGAAAAGATGATTACGTC
ATCGACATAAACATAGCAAGATTTGCCAATCTGCTCTCGCAGTATGTCGTCGATGGTTCTTTGGAAGATGC
TTGCTGCATTCTTTAATCCAAAGGGGAGTCTTTTGAACTCGTATTTCCCCCGTTCACAGAGAAAGAGGTTT
TTCACGGTCGCGTTCAGCGAGTATGATCTGGTGATAACCAGACTTGAGGTCTAATGTTGAAAAGAATTTG
GCTCTCCCCAAATTCCCCAATATCGTTGATATATTTGGCATAGGGTATTTGTCAGGCACGGTTCTTTCGTTA
AGTTTACGAAAGTCCATTACCAACCTCATTTTCCTATTGCCTGCTTCATCAGTGCCCTTCTTGTCTACTACC
CATATTGGGTTATTGTAGGGAGAGACGGACTTTTGAATTATATCATTTTTAGCAGATCCTTGATTTCTTTGT
TAACGAAATCAGCTGCCCCCATTGGATATGGGTAAAGCTTGGCATAGATCGGTTGCTCATCTGTGGTCCTA
ATGGTAGCCACGACTGATGTGTTATACGGCAACGCCTCGTCTGGGTCTGCAAAGGCCTTTTGTCTGGTCTT
TAGCATTTTCAAAAAGAACTTCTAGCTAATGGGGATGCCTCTGCACAGTCTACATTTGTGAAATTTACATT
TTTGCACGTGTGGAAGGTGATTTGTTCGACCTCCGTACCCCACTTAAGTTGTCCTGAAGCTAGACAAAGCG
AAGCAGCTGCCTGCTTTAGCAGGTCAAGGCCTACAATCCCATCAAAAGTTGACAAGTCCGGCAGAATAAA
AAAGGTTGCCTTTAACCCAAACATTGAAACAAAGCATTTGTGTGTAATTTGTGTCTCGCCGTGAATGGAAT
GAATTTTGAAGGGGACTCTACCGAGCGGACGGTCAACCCTTCATGTGGCCGAATGAAATTCTTGGCCGCG
CCCGTGTCTATGAGGAATCGCATGTGAGTCCCTACTACTCTTCGTCTGATGACGGGTAGCAGGGATTTGCT
CCTAAAAATTGAGGGCATCAGAGTCATAACTAGCATCGTCATTGATTTGTGCCGCTGCGCTGGAGGCGGC
ATCAGCATACGCCTCTGCCCCCTGTCCCGCGTCTTGCGTGACGTGATTCAACCTCTGCTGATTTCTCTGACC
GCCTGTGCGGTCAGATGCGGGGTGCTTCCTATTCGCGTAAGCAGGGGCCTGAGTGGGCTGCAAGACCCTT
GAGAAGGAAGGATCGACCTCCATGGGCACCGGTGCGCCATTATTATTGTGCTGCCTGCTGCTTCGTTGGTC
AGTCTGCACTGGGGCCTTGCTTTGCTTGCTAAAGTGCGGATTCTTTGTACCGCTAGCTTGTGCTTCGGCTTG
TTGCGAATGCCTCTCCTGTTGGTGACCCTGCGCTTTTGCATGCGGCTCGCGCTCTTTGTCTTCTAGACTTCT
TGCGAAATTAGCCGCGAATGCGTACCTTTCATGATTTGACTCGACTTCTTGCGCGAGAGCTAGCGCTGAAG
GCAAATCTCTGGGCTTTGCTGCAAAGAGCACATCTGTAAGACTGCGCTTGAGACCCGATATGAACACACG
CAACGCATCGTCGCGGAATTTTTCACATAGGCCCTTTGCTAAAGCCGGCTCGTATGACATGTTAACTTTGT
TAGTGAGAAGAGTGAGTTTCTTCTCTACCTCATCGTAATATTGAAGCAGGGACAGGCCACCTTGCCTCAAG
GTACCTAATTCCTGTTCAATGACATGAATTGGTCTCTTGTCACTGTATGTGAAATCTAGGCGATTTATTATC
GCATCAAAATTCAATACAGTACCAAATGAGGCCAGCACCGCGTCTGCGGGGCCCCTAATCTTGCTTCTTAT
GATGATAACAGCCTGATAATGACGAGAACTGCCATTATGTGGTCTGAAGATCTCGTAAGCGGCTAATGCT
GCCTGCCTCCAAGATACATAAGATTCCTGTACTCCTGCAAACTCAGGTAAGCGCTTGACGGCATCTAGCG
GCTCGTCACAGCGAATGTCGTTTCTAATTCCTATAGCAGCATATGCTACTACTTGTGGGGCGGCAACGTTT
GCTGCTGCTATTTGTTCAGCTAACTCATTAATTCTTTGAGTCAACGCTTGCTCCCTCTGCTGGTTTGCAGCG
GCCTGTTCGGCTAAAGCATTGCTGATAGCCGTCCTTACTACTTCCCTAAGCTGATCTGGATCCATTTTACCT
GATGGTGGGGGAACTGCAGGGAGAAGTCTTGTAGCCGGGACTCTGATGCGAATGTCACAGTCGGAATCTG
AGTCGCTAGACTGCTTACGAGGGTTTCTATATTGGCTGAACCGTGACATGCGCAGGCCAGTGGCCGCGAA

AAATAAAACGGGCCGTCAAAAGATGGGTCGAATTACGTCGCAGCTCTGAACCACGAGAACTGTATGTATT
GTTATAGCACAATTTTAGACACATAGAAAGTAACACAAAGTAATTTTATTAACGATCTGGATGATAAATT
GTTTCAACTAAAATTCTCAAAGCAGTCACTCTCACGTGTTTCAGAAACTTGGTTCTGATTATTATTATTTAA
AAAATTTTTTATATGATCTGTTGCAATATAAAATACTCACTAGTACTAACTAAAATAAAAAATTTTTGTGT
TTGAAGAAAAATTTTGGCTAGAGTATCCACTTACATGATTCAATTAACACAAAAAAAAATTTCCTTTATAA
AATTGGTCACTCGCAGCTCCCGCGCTGCTGTCTTAATTGTTGTTGGTTGTTGCAGCTCCCGCGCTGCTGTCT
TAATTGTTGTTAGTTGTTGCAGCTCCTGCGCTGCTGTTTTACTTGTTTTGGTTGTGGAGTCCGACCACTAGT
TGGGCGCCAAATTAACATCCTTCAGTCCTGTAGTGGCTGAAGATTAGGTTGAGAGGCGACTGTGCCTCTTA
TTGCTGGCCTCGGGCTCAGACAATAAAACAAATTATTTAACGGCCTAAGTGCCGGAGTGAATATTTATTTA
AGCGACTTATTGGAAGAGCGCTGAATTCAAAACTAAAGAAACTTAATGCACAGAAAGTTTTACTTAAGCT
AAGCCAAAACTCGCATCACGCCACAATGTCATGGTCGGCAAGCCGACTCAGCAAAATCTTAGGCAATAAC
GGAAACTCTTGCAGCAAAACAAAAGTGTCCGGTTACCGCCGCTGACCATTGCAGCTATTGCGCGCTGATA
CAAAGTATGCAGTGTCGCAATTGCGGGGGCTGTTCCTTAACTTATGAAGCACGCGATAGCGAGGGTTGAT
TATAACTTATGAAATATCCTATATTAAGTGTTATGTGTTAACTACTCCCCCTCAANTATAAGGCCGTCCTC
GGCCGACCCAATCCCGGCGACGACTTCTCGTAACTGCTTCGCAGACTTCTTTGCGTCGATGATCCGCCAAA
GAGTGAGGCCAATGCAGATTAAAGCACAGCATATGGCTCCAATGATGAATGCCACACGATAAGCTTGATC
AGTTCCGGCGTCTTTCTTGAATTGCTGTATCACCTCCAAATTACGTTCACTCAAACGATGAAGGTAAGGGA
GGCTGAGGACATCTTGGCTGGCAGTGATGTTCAGCAAGGGAGAATTGGCAATACCCGGGACTCTATTGCG
TACGTTGTTGTGATTGTAAAACAGAGTCCCGTTAATTGCGACTCGGTCTCCGAACGTGATGAGATGCGTGC
CTTGTACCTGGATCTCAGTGCCGTTGTCCACCTGAATCCTTGCGGACCCGTCGTTGAGGATGATGATTCCG
TCATCTACATGGGTGATTATTTCCAGGTTGCTCGGCTGGATCTCGCAGTGTGCCGTGCCACCTGCATGGAG
CTCTTTGGCACATGAACTCTCTAATGCCAGACGACAAAATGTGGCTCCAGGTGAAGCGGCGCAACTCTGC
AGTGTGTGGGTTTCTCCATCGCATTCGGCTACGACGTTGTCAAACAATCGCAATATGAAGTTCTTGTGGAC
GACAGGATAAATCGTAATTTTTCTGCAAGCTTTTTTAATCTTTGGGAATTTAATTATAAAATGTATAGTGTT
AATGGATTGAAGAACTTTTACGGACGCAACGGACATAAGATCTCCTATGGGGGTGTTGGTGGGTTCCTCT
AGCCAAATTGATTTCAAATCTGAGTGGTCCAAAATGTTTGGGCTAACAATGTTAGCCTTCGCAAGAGTAAT
AGCAAGCATTAAATTTTGCAACTCCATCATCAGCATTCTGTTTCTAGTCAGTAGTGTTTCAAATAAATGGC
TAGTGTCAACTTGTGAGTTTTTGGTTATCTTGAGAATTTTATTGACAGTGATTGTTAACTGGTTAAGCTGGT
CTTGAACTCTAGTGTTTATTTCTATTTGTCTATTGTTGGCATTTATTAGTTGCCTTTCAGTGAACCTAGTCTG
CTCTAAATCACCGGCATCGGGTGTACCCGCCACAACCTTCAGTATGGATCCTAGGAAATCTAAGCTCCTGG
CGACTCTATGGTGTGTGCCTAACGCCTCCAATAGGTCTTGTAGATGTGCCGTGTCCACACTTAGAAGTTTT
TCATGTGGGATAGCGGGAACATGTCAATTAATTTGACCGTTTCTTCCACCATTCGCACGTTACTCTGAGAG
GTTTGCC
>9_rnd-1_family-335__LINE___R1
CAGAGCTATAAATGAAATTAATAACCCAACTTTTTATTGCCAACCAATTTAAACCACAATTTAAATGTAAT
TGTCTTTTTCAGAATATACAAATATCTCTAANGGTACAATAAGATATACTGTAGAAAATTTTTATAAGGAT
CGGTTAAGAAAACCAAAGTTATATGCAACTGCGCAATTGAATGGGGCAGCAGCTTTAAGAATTTCTGTAT
ACATGTACACANACACATTCATGCGCGTCTGCTTCGCATTCTAGCAGCATGGTAATTGCATCTTTTTTTGTA
ATGTTATTTTAGTTCCTTTTTTATCATGGGGGNACTTAATTGTTGGTGTGGCTGCTGAGTAGAGGTATNGCA
GGTGGTGCGGTCTGCCNCNNTGNTCGAGCCCTACCGGGGAAAGTATTATTATTTTTTTTTTTTTTGCTGGTG
TGGCTGTTGAGCAGAGTCGACAGCAAGTATTNCTGGTCGGTAAGCGAGTCCGAGCCCTGCCAAGGGAACT
TCTTTTATTGATTTATTTGATGTTGGAATAAGAGGGTTCAGGGTATCCCTAGNTCAGGGGGAGCTCCCTAG
CTCCCCTAGAACCTCTTTTACTTTTTTTNTTATCAAGTCAGTTTCCTATATAGGCTACATGGTTTAGATATA
TTTAATTTTTCGAATACCTCGGTACAAATTTTCAACAGTTGTTATAAAAGTAAGGATATGCATTACATAAG
GTGAAGTGGGCAGACACTTCCACAATTTTGTCATAAAATATATTCATGAAAATAAGGCTTTTAATATAAA
ATATTCTTTACTTTACTTCTTTGCAAATATTATTTACGCAACATCTGAATAACCATTTTTAATTACTTATTTT
TATCGCACAGTAGACTTTACTTTTTTAAATACTAAAAATCTTATAATTTATGGAATCAACTGTTTCAGACAT
CACATACTATCCAGATAAATCATCGTCATTTATAAATTGTACATATTTTTCTCGCAAACCAATGGCACCGT
TAGATGATGTTAGAAAAAATTACTGTGCCAATTACTAAAAGTCAGAAACAATGTAGACGGCACTTTTACA
TATTTTGTAAAACATTGCAAACAAAATTTGCACGCCACATAGAGTTTAAATATAGAATGGAACCAGTTAG
AGAGATCAGCGGTTTATCAAAAGACAAACAGCAAAGAATGAAATTGCTTGAGGGATGCTACTTACAATG
AAGGAACTTTTATAGTTTCACGTCAATTAAGGGAAAACTCAAAAAGAGGGCTTTGGATTTTGTGTGCTGC
AATGGATGTAAAGCCTACTTACCGAAGACAACAACTCGGAATCAAATTAAAATNTTAATGTAGTAATATC
CATAAAATATGCAACCGAGATATATTAACCTCTGCACGAAGTGCTGCAAATAATATTGCGAAATAAAATA

ATGCCTGTCCTTAATGACGTCGCCATTACTAGGACAATATTGTATGATCACCTAATTATATTGTTTACAAA
TTTAGAAGCAATGAAGCACACGGAAGTACAGCATGATATGATAACAGCAATTATGCGTTATCTAAGTCGC
CCTTTGATAGTAATAAAATATGTTATTTCCCCTAAATATTATGATTCTGTTAAGTCTGGAATTCGTTATGTT
GCTGAGTTTGATTATGATAATATTCGTTTTATGAAGCCTACTGCAGCGACAACTCTAACATGCTTGGTAAA
AATAGTACATGTGCGAGAATATTAAAAGCACAGATCATAAAAATATTGCGACAATAGCATAAAATATTTA
GAAAAATAAAAGATTTTGTTCAGGATCTACCTGCTAAAGCTTTGAATGAGCTAAACTAGAAATTCAACAT
ATCACCATTAACCTCGTTATATCAGAGCNGAGTTAGCGAACTTGAGAGTCTTACTCTTAAGAGCTACAGA
ACTGCTCATGCAATTGGGGCTATCGACGATAGAGCTAATTCTGACCTGTATGAAGGCACCTCAGCCAAAT
GGAAAACATCACCAGGGTGATTATTCGTGCCAAAAACCGAACATTTCCAGTTCTTTTTACACCATGCGATC
GGTATTGCATAGCCAATATACTCGAATATGGCAAACGCACAATAAATAAACCAGCTACTACACTANAGAT
ATATTTTAGTGTGCCAAGCCCGTGGCCACTAAGAAAAGAAAATATTCGTGAGTGTCCTATCCTAGAGAAA
TTCGCAAATGAATGTGGTACACGTTGCCCTGAGTCCCTTTCAGGCACAGACCTGCGAAAGCAATTGACCA
CACATACCGCTTTGTTCAACGTTAAGGACATTCAAATTGATTATTTGGCGAGCTTAATGGGTGATCATAAA
GACATTCATAAAAATTACTATCGTGTTGCTGCCTCTGTAGCGGGGTGACCCAAGTTTCTCAATTACTTATG
TCAACAATGAACATGGAAGAGGAAAATGCTGAAATTGCAGATGCTAATCTCGAGAGTGAGTTAATTTATT
TAGCCCGTCTGTTGCGAAGAAAATTTTAATAATCGCTGCAAGTTTTGGACCTGGAACTGTCATGTTCGGAA
TACTTCAATAGAAGTTTTTCAAGTATCGGAACTTCGATACAGTAGAAGATACATTCAGTATTTAAGAAATT
AGTAATGGTTTTGGCGACCTATCCATATTTAAAATATTTTGATGGCGGGACCTCGGCCCGAGTTTTTTTTA
ATTAATAGATTAAAACAATCTAATTTAATACTTATATTGCTTATTTTATTTAAAGCAAAATATTAAATAAA
ATATTTTTCAACTGTTTATTTAAACGAGCCCGAACCTCGCCGCCCGACNNCGCCAACATAAGCGCTCTACG
GGTCAGCTGCGCATTCCGGACGGTATCAGACGAAGCAGCGCTGGTTATCGCAGGGATGGTCCGCACAGCA
GCAAGGATGCAAAGCATGGAACGATGGCAAGCACGTTGGGATCGCTCATCTAAAGGACGCTGGACACAT
AGGCTTATCCCAAACTTGGAAGCCTGGATAAGCCGCAAGTATGGTAATACTAATTTTTACTTAACGCAGCC
TATCTGGCCACGGATGCTTCAGAAGCTACTTGAAGCGATTCGGCCACGATAACACAGACGAGTGTTCTTG
GTGCGGCAGTGAAATCGCAGAGGACGCCAACCACATCATCTTTGAATGTGGAAGGTTCTCAGCGAACAGG
CAGGGNCTGGAGGACAGCTTGGGGAGACCTATCACGGTGGACAACTTGGTCAGGAACATGTTGGAGACC
GCACAAAAATGGGAAGCNACCTGCACTTTTGCAGCAGAAGTGATGCTGGAACTCAGGAGAGTTGAGCGC
AATAGACGGAGAGCGGAGGAAGTTTAGGTGGCAACAACATGCCTGTGAAGCAATGCTTTGCGGCCGTTCC
GCAGGCAATCCACGCCACCTAATCATCACGTACCCTACTATCTACATCCTNAGCATAAATTATTTATTATT
TATATACTTATATTTGTGCAAATAAACAGAATACAAAAAAACACACACACACACAATGGAAACATAACAC
CCCTTCCACCCCTCCCCACCCGCCCCTTAATCCTTAACACCCCCGCCACCCCTTTACCTTGCTTAACAACAC
TTGACCCTGATAATAAATGTAAACAAACATACTGTGCCAGAACATACAGTCCCCACCACGGTGACATCAC
TTCCTGTGCCAGAACATACAGTTCCTATCTACTATCCTATGTAGACATAAGAATATAATAAAAACGTGTTT
CATCGACTTGGGAAATTCCAATTACTGCCCGATCAGTTTCGAAATCATTTTCAAGAAAAGGCCAGGTTGTG
TTTTTTTGTTTGTTTGTACATACAAACATAGCCATGATAATTGTATAAGGCCATTAGAATCGAATAAATTC
ACAGCATTAATTTGTAGGATTACTCAACTGAATAAGTTTCTGTGCTTCGAAATTTTTTAACAAAAANGACA
TTTTTGACCCAATAAAATGTATATATTCCTTATCCGCATGGACATAGGAGTCCGTATTGTATTAATATTTTA
AGCGTACGCAGTACTATGTTCAAGTTTATATTATATCTATTCTAAAACTCTACAAGATTAAAATATATTCA
TAGATATCGATATTGATAAAAATTTTTTTTATACCCTGAACCCATTAAAAATGGGTATAAGGGTATATTGT
ATTTGTGCAAAATCCAAATGTATGTAACAGGCAGAAGGAAGCATCTCTGACCCCATAAAGTATATATATT
CTTGATCAGCACCAATAGCCGAGTCGATCTAGCCATGTCCGTCTGTCTGTCCGTCCGTCCGTCCGTATGTA
TGAACGCAAGGATCTCAGAACCTATAAGATCTAGAGACGAAATTAGATGTAGGTGCTCCTAGTTCCCGCG
CAGATCGAGTTTGTTTCCGATAATCGATAACTTACTCCGTTTCCAAGCAATCGATAAAAATCGATATCGGC
ATCCTGTTTTTTTTAGCAAATTGGGTAAATAATAAGAGCTAGAGCCACCAAACATGATATGTTGCTTCTAG
AATATTGTATATATGTCAAGTATCTTTCATTTTGTACCTATCGCTACCTCCCCGCTACCACCGCAGAGCTAT
AAATGAAATTAATAACCCAACTTTTATTGCCAACCAATTTAAACCACAATTTAAATGTAATTGTCTTTTCA
GAATATACAAATATTCTATGGTACAATAAGATATACTGTAGAAAATTTTATAAAGATCGGTTAAGAAAAA
CCAAAGTTATATGCAACTGCGCAATTGAATGGGGCAGCAGCTTTAAGAATTTCTGTATACATGTACACAC
ACACATTCATGCGCGTCTGCTTCGCATTCTAACAGCATGGTAATTGCATCTTTTTTTGTAATGTTATTTTAG
TTCCTTTTTTATCATAAGTACTTAATTGTTGGTGTGGCTGCTGAGTAGAGGTATAGCAGGTGGTGCAGTCT
GCCACGAGTTCGAGCCCTACCGGGGAAAGTATTATTATTTTTTTTTTTTGCTGGTGTGGCTGTTGAGTAGA
GTCGACAGCAAGTATTGCGGTCAGTTGCGAGTTCGAGCCCTGCCAAGGGAACTTTTTTTTATTAATTTATT
TGATGTTGGAATAAGAGGGTTCAGGGTATCCCCTAGTCGGGAGCTCCCTAGCTCCCCTAGAACCTCTTACT
TGTTTTTTTATCAAGTTAGTTTCCTATATAGGCTACATGGTTTAGATATATTTAATTTTTCGAATACCTCGG

TACAAATTTTCAACAGTTGTTATAAAAGTAAGGATATGCATTACATAAGGTGAAGTGGGCAGACACTTCC
ACAATTTTGTCATAAAATATATTCATGAAAAACTCTTAAGGCTTTAATATAAAATATTCTTTACTTTACTT
CTTTGCAAATATTATTTACACAACATCTGAATAACCATTTTTAATTACTTATTTTTATCGCACAGTAGACTT
TACTTTTTAAATACTAAAATCTTATAATTTATGGAGTCAACTGTTTCAGACATCACATACTATCCAGATAA
ATCATCGTCATTTATAAATTGTACATATTTTTCTCGCAAACCAATGGCACCGTTAGATGATGTTAGAAAAA
TTACTGTGCCAATTACTAAAAGTCAGAAACAATGTAGACGGCACTTTTACATATTTTGTAAAACATTGCAA
ACAAAATTTGCACGCCACATAGAGTTTAAATATAGAATGGAACCAGTTAGAGAGATCAGCAGTTTATCAA
AAGACAAACAGCAAAGAATGAAATTGCTTGAGGGATGCTACTTACAATGAAGGAACTTTTATAGTTTCAC
GTCAATTAAGGGAAAACTCAAAAAGAGGGCTTTGGATTTTGTGTGCTGCAATGGATGTAAAGCCTACTTA
CCGAAGACAACAACTCGGAATCAAATTAAAAATTAATGTAGTAATATCCATAAAATATGCAACCGAGATA
TATTAACCTCTGCACGAAGTGCTGCAAATAATATTGCGAAATAAAATAATGCCTGTCCTTAATGACGTCGC
CATTACTAGGACAATATTGTATGATCACCTAATTATATTGTTTAAAAATTTAGAAGCAATGAAGCACACGG
AAGTACAGCATGATATGATAACAGCAATTATGCGTTATCTAAGTCGCCTTTTGATAGTAATAAAATACGTT
ATTTCCCCTAAATATTATGATTCGGTTAAGTCTGGAATTCGTTATGTTGCTGAGTTTGATTATGATAATATT
CGTTTTATGAAGCCTACTGCAGCGACAACTCTAACATGCTTGGTAAAAATAGTACATGTGCGAGAATATT
AAAAGCACAGATCATAAAATATTGCGACAATAGCATAAAAATATTTAGAAAAAATAAAAGATTTTGTTCA
GGATCTACCTGCTAAAGCTTTGAATGAGCTAAACTAGAAATTCAACATATCATCATTAACCTCGTTATATC
AGAGCAGAGTTAGCGAACTTGAGAGTCTTACTCTTAAGAGCTACAGAACTGCTCATNCAATTGGGGCTAT
CGACGATAGAGCTAATTCTGACCTGTATGAAGGCACCTCAGCCAAAATGAAAAACATCACCAGGGTGATT
ATTCGTGCCAAAAAAACCGAACATTTCCAGTTCTTTTACACCATGCGATCGGTATTGCATAGCCAATATA
CTCGAATA
>160_rnd-1_family-183__LTR___Gypsy
GGCAAACCGCCGCTTATGCCTACGGGGGAATACTAAGCAACTAAGACGAGTTGCATGAGAGGCAAACCG
CCCCTTATGCCTGCGTTGGAAGAAGAAGAAGACCCTATAACAACAGTACCGGCAGCGCCCAACATAT
TAATTTAAATAGTAAGAAATAAAACTTAAAAATTGTAATGCCCGACGACAATTGAGTCGGCCGGCAAGAC
GTTTGGCCCGTACAGCCATTCGAAACAATAGGTAGGAAAAATACAGATTGTAAAAAAAAAAAAAAAAAA
ATTTTAAGATGCGTTTTAAGCAGAAAACAAACAAGCAAAACAAAACCCGACTTGTAAACAATAAAAGCA
TCAAATTCAATAAATATATGTAATAAAACCTCAAATGAACGATACAAACAACAACTTGTCTTGTAAAAGT
AAACGAAAACAAATTTAACATTACGCATTTGTAAATTTTTTAAGATAATTCCTTTTTAATTTTATGTATGAT
CCCATACTATATATGAATATTAATATTAAAAAGAGGAAATTAAAAAGTTATAATTCGTTCAGTTGGAATAT
AAAGAAATAAGTGCTCCGCTTGTCTCATTAGGTAATTCAAAATTATGATCAAGAATTAGCATGATCGAGG
ACAAGATTAACAAAGTATATAACGACTATACAGACTTTCGTATGCGAGCCATACAAATTTTGCAAAGGCA
TAGAGAATTCATATCAGTATATGAGTTCGAATCCGAATATATAGAAACATTCGGCACATTGACAAGTCCA
CTCTATTCATGTTCTAAAATAGAGTGCGGAATAATCTTGTCTACACTACCCTACGTCAAAATAAAAACAAT
TATTGACGAGCATCCGATTCTAGGGCAGACAATATAGGAATATGGGGGGAAAACAATCAAAGGCAGATG
ATAACACTGCCAATGTAGTTAACGTAGTAGAAGTCGTAGACACCACTCCCCATGTGCAGTTAACAAACAT
ACTGCTAATAATACTCATTGTAATATCTTCAGTAAACATCCTTCTGAAGATTTATCTTCTTCATAAAAAATC
ACTGAGGAAGCGTTACATGAGTCGAGCCAACGACTTAGACAAGGTCTAAGCCACTGCTTAAATAAAATAT
TTAAGTAGCGGAAGCTTACATGGACTGGTCCCAAATAGTAGAAAAGATAAAGCGGTATAGAGAAAATTTC
GATAAATCATACAAGTGCCTTAATAAAAGCGCACCAGTTTTAAGACCCACCATAATTAAACNTACTGAGA
TCCTTGTATATGAGTACAATGAAATCAGGAGTCTAGTTGCCGAGTGTCAAAATAAACTGACAAGTTTACA
CAGAGTCCAAACTTCAGACGTTTTACAGCGTCTAAGGGATAATCTGGTGAACATAAATAAAAAGTATGAT
CTCCAGCTTTATCTACCATCGGTGTTAAATACACCGGTAACATTTGATAAGTCACTTCTGACAAAAGACCA
AATAGAAGATACAAAATTAGTAAACATGGCACAAACAGTTGTAGAATTTATCAAGCTAGCCACATCTCTG
ATACCAGAGTTTGACGGTAAGCCGGAAAATCTACAAAGTTTTCTAGATTCTTTGAATCTANTAGAAACCCT
AAAAGGCACACACGAGCTAACGGCCGTAAGCCTTATAAAAACAAAGCTAAAAGGCCATGCGAGGAACCT
GATAAGTGATGAGCAGACNATTGCNGCAATCATTACGCAACTATCGGACGCAGTAAAAGGAGAGTCGGT
AGAAGTTGTNTCTGCTAAGCTTCTNAACTTGCAACAGAAGAGCAAAACGGCCAACCAATACACCCAAGAG
GTGGAAAAACTGGCAAAGGCCCTTGAAGGNGCCTATATCAGTGAAGGTCTAAGCCAATCACTTGCAAACA
AATACAGCACTACAACAGCTGTAAAAGCTATGACCCAAAATTGTTCGATTGATAAGGTAAAACTTATCAT
GCAGGCAGGCACATTCACAAATATGAATGATGCCGTCTCCAAATTTGTCAATAGTTGCACGGAGGTAACA
GGTCAAAGTAACACTGTCCTGTATTATCGACGAGGTGCAAATCACAATAATAGAGGAGGCCGAGGTTATA
ATCGTGGCAGAAATTACAACCACAACAATTACAACCGAGGCGGTAATAACAACAACAACAATAATTACA
ATAACCGTGGAGGAAGACGAGGCCAAAATCGAGGAAGAGGCCGAGGTAACTCCAACCAAGGTNACAATA

ATAACAATGTGAGAGTGGCGCAAAATGCGTCGGAAAACTCTCAGAACCCTTTAGGAAACAACCAGTAAA
TGCAAAGGTTCATTCCATCAATTACAGTCTTAATATATTTGTCACTTTTTATAATCATTCAACTGAAAATAA
ACTCACATTCCTAATAGATACTGGTGCAGATATCTCACTTTTAAAAATAAATTCAGATAATTTCAAAATTC
AAGAAGACAAAATTATAAACATCCAAGGCATAGGCCAGGGTGTAATAAAATCGCAAGGAACAACTTCAA
TAGAACTTCAATCAACAAAATATATTATTCCACACGAATTTCATTTAGTAGACTCNGATTTTTCAATACCG
TGTGATGGAATAATAGGCATTGATTTTATTAAAAAATTCAATTGTCAATTAGATTTCAAACCAAGTGAAGA
CTGGTTTATAATTAGACCTCAAAACTTAAATTATCCNATTTATGTTCCGATAACATATAGTGCTGGCAACA
ATACAGTTCTTCTGCCAGCCAGATCCCAAGTAATTCGAAAAATAGACATTAATACAGTAAACGATTATAT
ATTTGTTCCNAATCAAGAAATACATAATGGAATTTATGTTGCAAATACAATAGCAGCATCCAAACATGTA
TACATTCGCCTTCTAAACACAACTAATTTCGACCAATTGGTCAAAGTAAACAAAATCCAATATGAAAATTT
AACAGATTACGACATTCATAACACTAATCCAGAAAATAGAAGCGAACAAGTACTTTCAAAGTTAAAGAA
AAATTTTCCAGAACAATTCAGAAGTCAACTAACAGAATTATGCACCAAGTATAGTGATGTGTTCGGACTG
GAAACCGAACCAATAGCGACAAATAATTTTTATAAACAAACATTAAGACTTAAAGATGATGAACCCATTT
ATATTAAAAACTACAGAAGCCCGCATAGCCANGTTGAAGAAATCCAAAAACAAGTAGGGAAATTAATAA
ACGACAAAATCGTAGAACCGTCTGTATCCGAGTATAACAGCCCACTCTTGCTAGTTCCGAAAAAATCATT
ACCAAATTCGGAAGAAAAGAAATGGCGATTAGTAATTGACTATCGCCAAATCAACAAAAAACTACTTTCT
GACAAATTCCCGCTCCCCAGAATTGATGATATTCTAAGACCAACTGGGCAGAGCTAAATACTTTTCATGCC
TTGACTTAATGTCAGGTTTCCACCAAATTGAACTTGAAGAAAATTCAAGAAATATAACATCTTTTTCAACG
AGCAATGGCTCATATCGCTTCACGCGATTACCATTTGGTCTTAAAATAGCACCAAATTCATTTCAGAGAAT
GATGACTATAGCATTCTCGGGTTTAGAACCCTCTCAGGCATTCCTTTATATGGATGACTTAATGGTGATAG
GATGTTCCGAAAAACACATGATTAAAAACTTAACTGATGTTTTTAATGTATGTAGGAAATATAACCTAAA
GTTGCATCCAGAAAAAATGTTCAATTTTTCACGCATGAAGTGACATTTTTAGGTCACAAATGCACTGACAAA
GGAGTATTGCCAGATGACAAGAAATTCGACGTCATCAAAAATTATCCTGTCCCTCATGATGCGGACAGCG
CGAGACGATTTGTAGCATTCTGCAATTATTATCGTCGATTTATAAAAAACTTCGCCGATTATTCACGGCAC
ATAACTAGATTATGTAAAAAGAATGTTCCTTTTGAATGGTCAAGTGAATGCCAAAACGCATTCCAATACCT
TAAAGAAAAGCTTATGCACCCCACATTATTACAATATCCTGATTTTCGCAAAGAATTTTGCATTATAACAG
ATGCTAGTAAACAAGCTTGCGGAGCGGTTCTAACTCAGAACCGTGACGGAATTCAGCTCCCAATAGCTTA
TGCATCTCGTTCATTTACAAAAGGGGAAAGCAATAAGAGCACAACAGAACAAGAGTTAGCGGCAATTCAT
TGGGCAATTACCCATTTTAGACCATATATTTATGGCAAACACTTTACTATTAAAACGGATCACAGACCATT
GACATATCTTTTTTCTATGACTAATCCCAGTTCGAAATTAACTCGCATGCGGCTAGAGCTTGAAGAATACG
ACTTCACAGTAGAATACCTAAAGGGGAAAGATAATTTCGTAGCAGACGCACTTTCGCGTATTAATATAAA
AGAACTCAAAGATATGCAACATAAAGTCCTGAAAGTCACTACCAGGCGACAAAGTAGACAAAATAAAAA
CTGTACAGTAGAAAATTATGAACTTCTGCCAAGGCAAAGTCATAAAAATGTATCTAAGCCCAACGTACAT
GAAGTCATTACAAATGATGAAGTACGAAAAGTAGTGACCTTGCGCATAACAGAATCTAAATGTTTATTTA
AACATGGAAATAAAATTATCGCAAGAATTGAAGTTAGCGATTTATATACTAATGGAATCCTCGACCTAGG
TCAGTTCTTCCAAAGGCTCGAAAAAGAGGCCGGTATATTAAAAATCAGCCAACTCAAAGTGGCACCGAGC
GAAAAGATCTTTGAATCAGTTTCAATAGATTNTTTTAAAAATATGGGCAATGAAATATTAAAATCTTTAAG
AGTAGCGCTACTCCAGCCGGTGACCCAAATAAAAGATGAAAAAGAGATACAACAAATATTGTCTACATAC
CATGACGACCCAATTCAAGGAGGTCATAGCGGCATAACAAGAACGCTAGCGAAAATAAAAAGACACTAC
TACTGGAAAAATATGACACGTCATATAAAAGAGTACGTACGTAGATGTCAAAAATGCCAAATGTCTAAAA
CAAAGACGCACACAAAAACTCCTCTGACTCTCACAGAAACACCTGTGAATGCTTTCGATATAGTGATAGT
GGATACAGTTGGTCCGCTACCTAAATCGGAAAATGGCAATGAATACATAGTCACATTAATATGCGACCTT
ACAAAATATTTAGTAGCCATTCCAATAAAAAGCAAAAGCGCAAATAATGTCGCAAAAGCTATATTCGAAA
ATTTCATACTCAAGTTTGGTCCAATGAAGACGTTCATCTCGGACATGGGAACAGAGTATAAAAACCAGAT
TTTAACTGATTTATGCAAGTATNTGAAGATCAAGAATCTCACCTCTACTGCATATCATCATCAAACATTAG
GGACAATAGAACGAAGTCATCGAACATTTAATGAATACATTCGTTCATACATCTCTGCAGACAAAACTGA
CTGGGATGTCTGGATTCAATATTTCACTTATTGCTTCAACACGACACCATCAGCAACGCATGAGTATTGTC
CATACGAACTAGTATTTGGCAGACTACCGAAACAGTTCATAGATTTAAACAAAACTAATTACATAGATCC
TATTTATAATTTAGATGACTACTCTAAGGAAATAAAGTTTAGATTAGAAATAGCATACAAAAGAGCTAGA
ATTATGTTAGAAAAAGCCAAGGCTTATAGGAAATTAGCATATGACGAAAAAACAAAAAACTTTGAATTAA
AAGTAGGAGATAAAGTACTTATAAGAAACGAAACAGGGCACAAATTAGACCCAGTATATTTAGGACCAT
ATATAGTAGAGAAAATAGAAGATAATGACAACATTTTTATTAAAGATAGTAAAGATAAGAAACAAAAAG
TNCATAAGGATAGGTTAAAAATTTTTAATTCATAAAAATCCAATAAAAAATTCTCTTTATGAATTCGAACT
NCAAAAAAAAAAAAATAAAATAAAAATATATGACGGCCTGATCAACCGAATGAAAAAAAAAAAAATTTT

TTTTACAAATAAACGAATTATAAATTTTTATACCTTAAATAATTTTGTATATTTATACTCCAACATTTAAAA
TTTTTTATTATATAAAAAAAAAAAAAANATTTTACAAAAAAAAAAAAAAAACCTCAATAATTAAGTTCATTT
AATAACTACACATTCATAATGTTACGTTATTTCTCTAAAAAGGGAGGTGTAGTGCGTATATGCCCTTACAG
AATACCCCGTACTTAAATATAAAGTATGCAGCACTTTGTTGCATACAATAACAATAATATGGTAAACGAG
CCCAGAGCCACATTATGTATTGGCTCGGTCTCATATTACGCAGCGCGGTTCACTAGCCTCACTAGCTGACC
GTTTATGCTTATGCTGCATATCCCACGCTCCGTCTGAGCGCATTCCGTGAGCAATATATATGCTCACTAAT
ACAGGCATATGCGAGCTACTCACTCTCCCTCTCTTTGTAGCTGTTAACGAATAACAGCATAAGTATATATG
TATATCCATAGGCTAAGCGAACGTTATAAAAACTGAATAAATTCATTCTGAACAAAGATACTAAACAGCA
CTGACGTGCTGGCCTTTTTTNATTGACAATAAGTGCTCTTTTTACA
>160_rnd-5_family-2276__LTR___Pao
TGTTCGGTCATATACCTCATATATACATTAGTCAGGGTATCAATAGCAATGTCAAAGCAGATTATGTTAAG
ATTATGCTAATTCTCTTCTATTTGAATGTCATAAGCACTCATACACATGTACGAGGCCTCGCTCTCTTTCGA
ACTCTGTAATTGCTCGGTCCCTTGAATCTTAATTTATGTTAAGAAATCAGTTCTGTAAGCATCATCATAGA
AGTCAGTCGTGTTGCTCATCAATTTGTAAAACCTTTATTAAAACTTCATACCATCCACACACGCCCACTTG
AGATAAGATTCTTAAAAGGTCAAAAAAACGCTTTTGATCCAACATTTTGGCGCCCAACATTGGGGATAGT
GTGTGGAAATTGCCGGCATATCGTTGCCGCACAACATTTCGTTGGAAAAAGGTTTCATCGAACTCCTCGCA
AAATACAGCATCGACAGCAACATTTGGCCAGCTCTTCTTTCTTGTTGCCGTTGCTTCGTCGCTGGAAACTC
GCTACCTTCGCTTCGCTACTGCATTCTTGTAGCCGTTACTTCGCGCTGGATACGCTCTGAGTTTGATTGCTC
ATTGCCTTTGCCTCACATCTCCTTTCTCGTTGCCGTTGCTTCGTCGCTGGAAACTCGCTGCCTTCGCTTCGCT
GCTGCATTCTCGTTGCCGTTGCTTCGTCGCTGGAAGCTCGCTGCCTCTGCTTGTTCTTTGCATTTTGCTTCAT
CACTGCAAGCGACCCTCACATCGGCGAGCGCGACATTTACAAGTCTTAGCTGTTCTGTTAACAGTGGGAG
CATTTGAACTCGCTCGCTCACATTGCTGAGAGCGACATTATCAGGTTAAAGTTAGGCTGTGAAAATTCTGC
AGTATCAAAACTGCCGCTCGGTCACATCGGTGATCACAACATTCTCGATACATAATGTCAACTCTTGAAGC
AAACTTACGTAAGCGAGCGAACATAAAACGCAATGTCGCACACTTAGAGGATGTTGTAAGAGTGTCTACT
GAGGTCGATGGCAGTGAAAAAAGATTATCACACGGTGAGCTACAATTACGCTTGGACATGTTGGAGATAT
ACATTAAGGAGGTTTTGGAAGTACAATCTGAAATTGAATTGGAAGATCCTCAGGACAATGGACGTGCAGA
GGTGGAAGACACATATGTGAGTATCATCAATTGTCCATCCAATCCCAACTGGATGATGTACTGCACAGCA
CCTCATTTTCTAACCCTGCTCCAATTCGCTCTTCGCCGCCATCGAGTCTTCCTCGATTGACTCCGCCTGTAT
TTGATGGGAAACGAGCAGAGTTTAAAAACTTCATTACATCATTCAATCAGATTATCGGTCGTGAGTACTCT
CTTTCGAACATTGAAAAGTTTAATCATCTGCTGAATTGCTTGAAGGGCCCTGCCCTACAGGCAGTAAAGGC
ATTCCAAGTNACTCAGGAAAATTATCCCAAGGCTCTTGCTCGTTTAAGTGAACGTTATGACAATCCTACAC
TTACATTTTTAGACACAATAGAATCACTATTCAATCTACAGCCAGTCGACAAACCGGACGGAAATCAGCT
GCGCAGTCTGCTGGACAATGCGTCAGCCCTGTATTCATCATTATCGTCATTGGGTACCGGATCAGATATTG
CCCAAGCCATGTTGATATTCATCGTCATCGGAAAATGTGACCAGCAAACTCGTAACAAGTGGAATGAGTC
TCTTGACTTACGTACATTGCCAAATTGGGCTCACTGCACAACTATTTTGGAACGTCATTGTCAGTTTCTGCA
ATCAAGCGGCATTGCATCGTATCGAATGGCTGATTCTTCGAAACAAAAACACGTGTACAAGCCTAGCAAG
GGATTATCATTTGCTATTACTAATCCNANATGCATTGTTTGTTCCAGCTCCGACCATAAAATTGTACAATG
TTCTCAGTTTCAAGACATGAGTCCTAAACTCAAGTACGGAGTCGTAAAAAATGCTGGACTTTGCATTAATT
GCCTTCAATTAGGACATCGGATGGCTCAGTGTCCGTCTAACNATCNTTGNCGAAGCCGCAAGCATCGCCA
TCGCACGCTTCTGCATCTTGGCCAACCGCCNGCGTCANCGTCGTCNACANCGACTCCAGCTACAACGCACT
CGCATATGAATCAATCGCCAGGCGGACAAATAATTCTTGCTACTGCAAAGGTACTAGTGCGAGATTCGTC
AGGGGCGTATAAACAAGGCAGAGCATTGCTTGACTCATGTTCCCAAGTCAATTTCATAACTGATCAATTC
GCCCAACGGCTGCATCTACGCCGTGATAAGCATCATTTCGAAATCCGCAGCATAGGGGAGACCTCGACTA
AAATTAACAACTGTACAACTACTGGAATTAANTCTCGTTTGTCACCATTTGAAGTAACACTACAGTTCTGC
ATAACGTCTCACATTGCCTACCAGCCGGATTCGGAAATCGACACCTCAGATTGGAGGTTGCCCGCGAATA
CACCACTTGCGGACGAAACTTTCCACAGGTCAGGTCGGATTGATCTCCTTCTCGGTACCGAGGCATTTTTC
GAATGCCTGGCTGTTGGACAGATTAAGCTCGGAGACAATTTGCCAGTCCTTCAGAAAACGTTGCTTGGAT
GGGTTGTGTCTGGCAAGTATCGTCATCAGTCAGCTAAGCAGTCATCAACGTGCTTGCTTTCTGTGGAGGAG
TCAATTGACGAAAATCTTCAATTACTGTGGAGCATTGATGCTCTAGAAAATTCTCCAAAGTCAATCAATCC
AGAACATCGGCTCTGTGAAGATATGTNTTCAAGGACAACACAGATGAATAACGGCAGGATTGTTGTGCGT
CTACCTTTCAAGGGAGATCCAATGTGCCTTGGCAATTCAAGCGATGTTGCACGTCGTCGTTTTTTATCGCT
CGAGAGGCGCCTCAGTCAGCAGCCTACTGTTCAATCGCAATATGTAACCTTCATGAAAGAATATGAGGAT
CTTGGCCATATGACTTTAGTGACGAATCCGAGATTAAGTGAGCCACACTTTTATATACCTCACCATTGTGT
TTTTAAACCCAGCAGTGCATCAACGAAGTTACGAGTCGTGTTTGACGCCTCGTGCACTTCATCGTCCCAGC

ATTCATTGAATGATTTGCTTCTCGTTGGCCCAACACTCCAAGAAGATCTGTATATACTTCTCCTTCGTTTTC
GATTGGTTCGTTACGCCATCACAGCGGACGTTACGAAAATGTATAGACAAGTTTTAGTCAGCCCAGTGGA
TCGGAAATTTCAATATATATTTTGGAGAGCATCCCCTGATGCTGATTTGCAGACCTTTGAGCTAAATACAG
TGACGTATGGAACGGCCTCGGCCCCATACTTAGCAGTTCGCAGTTTGCAATATCTGGCAGATCTGCATGTG
AACGAGTTTCCCATTGGCGCATCCATTGTAAAATCATCGTTTTATGTTGATGATCTGCTATGCGGAGCAAA
CGAGCTCATCGAGTTGCAGAACATCAAACACGAGGTCACCGAGATACTTCGCCGAGGACACTTCCCATTG
GTTAAGTGGCACTCCAATCATCCCGCCGTCAGGAACGACGAATCGGTAAAGGATTTAAACTTGGATGAAA
ACGCTGTCACTAGCGCACTTGGCGTAGCTTGGGATCAACGTCGTGATGTGCTACTATTTGAATTTAAACCC
AAAAAATTGTATTCAACAGTCACAAAGAGGACTATTCTCTCGACTGCGTCTTCTCTGTTCGATCCATTAGG
ATTGCTGGCACCAATCATTATCGTCTCGAAAATCATCATACAGGAGCTATGGCTGCTAAAACTCGCCTGGG
ATGAATCTGTTCCGCAATCACTTCTACAAGCATGGAGAAACTGCGTAACGTCTCTCAAGGAATTGTCGTCG
ATTTCAGTACCCCGTTTTTGCTTGCAAGCTGAGCTCACAGACATACAACTGCACGCTTTCTGTGACGCATC
AATACGTGCTTACGGCTGTTGCGTATATGTGCGTTCCACGGACTCGTATGGCAATGTCCAGGTTCAGCTTC
TGACTGCTAAATCTCGGGTGGCTCCTGTCAAGAAGCTGACGCTTCCTCGATTAGAGTTAAGCGGAGCTCA
ACTTCTGAGTAAGTTATATACAACTGTCAAAAACATATTCATCGGCAAGCCGCACAGCGCGCATTTCTGGT
CTGATTCGCAGATTGTCTTGCACTGGCTGCAACAACACTCTATCACCTTGTCGACATTTGTTGGAAATAGA
GTATCCATTATACAAGATCAAACAGCAGATGGAAAGTGGAGACATGTACCGTCAAAATTGAACCCTGCTG
ACATCGTGTCACGTGGATGCCCAGCTAATGAGCTCCAGCAATCAATTTGGTTTACAGGACCTGCGTTTCTG
CGTGATTCCGAGACTCAATGGCCCTGCCAACAATTCGACAATGATTTGGACATGGACATCATCGATGCTG
AAAAACGGAAGTCTACATTTGCTGCAATTATCGAAAGTAATTCTATTCTGGACTGGCTGAACGGAACTAG
TTCATACATTCGGGCGCTTCGAGTTATGGCATATATGTTCCGGTTTTACCATATTGCCAAAAAGGAACACC
CTGTCGAAAGTTCGCCGCCATGCCCAGAAGAACTTCGCCACGCGTTGCACTGCATCATCAGGATCATCCA
ACAGCACTATTTCAAGGAAGATTTGCAATTGCTAAGGAAACAAAGAGATTTGAAAAGCAATCTGAAATTT
CTCAGCCCCTTCATAGATGACTCTTCTGGATACGAGCTCATTAAGGTTGGCGGCAGACTCGAGCATGCTGA
TATCCCCTCTCGTCAAAGGCATCCTTTACTTGTTCCCAAGGAATCGCAATTTGTCCACAATTATGTTCGGCA
TCTTCACTTGCGCAATTACCATGCAGGACCAAAGGCATTAGTTGGACTGCTGCGGCTTCAATTCTGGGTCA
TAAACGCAAGAGCTTTAGCACGCAGCATTGTTCGTTCTTGTGTACACTGTGTCAGATATCGTCCAGTTCTA
CTTCGTCAAGTCATGGGTTCCCTGCCACCTGAGCGCTTATCCACAGACAAGCCATTTCGCCACGTTGGAGT
CGACTTCTGCGGTCCAGTGAACACCTATCTTCGAATACGTGGCAAGGGTCCCACAAAATCCTATATCGCCA
TATATGTCTGCATGGCCGTAAAGGCCGTTCACATTGAAATCGTGTCAGATCTTTCAACGAATAAGTTTTTG
GAATCGTTGAAACGCATGGTTGCCCGTCGTGGACCAGTATCGCACATCTATTGTGACAACGCCACAAATTT
TGTCGGAGCATCCAACAAGCTACTCGAGTTAAAGAAGTTCATGTTCAACACTAGCACTCAAGATGCTATC
CTATCATATTGTTCCTCTGAAGAGATAACCTTTCATTTCTTGGGAGGCCGCTGTCAAGAGCGCAAAGAGTC
TGCTGGTGCGTACCCTCGCTAACACCCGGTTCACCTTTGAGGAACTGAACACCGCAGTCGTTGAGATAGA
AGCAGTACTCAACTCTCGCCCGCTGTCACCGCTATCATCTGATCCCAACGACTTCAGCGCTCTCACTGCAG
GACACTTTCTAAATGGAGATGCACCGCGCGCTTTGCCCGAGCGTGAGATTATCACCAACAACATCTCCAA
CTTGGATCGTTACGAGCAGATCACAGCTGTTAAACAGCAATTCTGGCGTCGTTGGTCTGCGGAGTATGTGA
ACGAGCTTCGAGCCAGGACAAAATGGACTTCGGCTTCCCCAAATCTCACAGTTGACTCTATGGTCATCATA
CATGAGGACAACACACCGCCATTACACTGGAAATTAGGGCGTGTTGAGTCTGTTGTGAGTGGCAAAGATG
ACCGGGTTCGTGTCGCATATATTCGCACTGCCTCTGGACTCTGTTGCCGGCCAGTACATAAGCTGGCTGTT
CTACCTGTCTCTTGAAAGATAATTCTTTCAAGGCGGCCGGGATGTTCGGTCATATACCTCATATATACATT
AGTCAGGGTATCAATAGCAATGTCAAAGCAGATTATGTTAAGATTATGCTAATTCTCTTCTATTTGAATGT
CATAAGCACTCATACACATGTACGAAGCCTCGCTCTCTTTCGAACTCTGTAATTGCTCGGTCCCTTTAATCT
TAATTTATGTTAAGAAATCAGTTCTGTAAGCATCATCATAGAAGTCAGTCGTGTTGCTCATCAATTTGTAA
AACCTTTATTAAAACTTCATACCATCCACACACGCCCACTTGAGATAAGATTCTTAAAAGGTCAAAAAAA
CGCTTTTGATCCAACATTTTGGCGCCCAACATTGGGGCTAGTGTGTGGAAATTGACGGCATATCGTTGCCG
CACAACATTTCGTTGGAAAAAGGTTTTATCGAACTCCTCGCAAAATACAGCATCGACAGCAACATTTGGC
CAGCTCTTCTTTCTTGTTGCCGTTGCTTCGTCGCTGGAAACTCGCTACCTTCGCTTCGCTACTGCATTCTTG
NAGCCGTTACTTCGCGCTGGANACGCTCTGAGTTTGATTGCTCATTGCCTTTGCCTCACATCTCCTTTCTCG
TTGCCGTTGCTTCGTCGCTGGAAACTCGCTACCTTCGCTTCGCTGCTGCATTCT
>9_rnd-1_family-463__LTR___Pao
TGTTGCGGGACGAATTAANTTATCGATTGTTTAGTATTAAGTATATTCTGAAAGTATGTATNTTTGCCCTCC
GAATGTACGGGCACACTGTATTTGGCTTGTTGCCGCCATTACTTAAATGTTAAGCTCATTATTGTCGTGAG
TTCGAAGGCCGTAGGAGGAAAGACTGCGGTAAGATTCCGAGGGAATATATATCTAAGTTCTAACCTATGT

AATCCGTTTAGAATAAAACACTTGCACTAGCACGTTTTGCATTACGCTGTCTCTANTAACATCGGAGTAAC
ATGGCCGGGGGCCAGGCGTAAACTTGCAGTGTTCAAATTGCAACTCCACCTTCGTTTGGCACGCGACATG
ATCTTTTATTCGCAGTTCTTATTGTAAGAAACTGCAAGGGCGACTTGCGCTGAAGACAAGACGTGGTGTAA
CNTTGGTGTTACAGTGTGTGTACATAGACACAGCACAGGCAGGGCCTCCACCCAGTTATAGAGTCACCAT
TGGTACGCAGCCAGCGTAAAATATAGCAGAAACCATCTCCGAGCTGAGTAGGATGACACAACGGTCACCC
AGGAAAAGCCCACGCTTAAACGATGGCCAGGAGATAGCTACCACGGCGAGTGGGACGCAGACAAAGCGG
ACAAGTAGAATGGTGAGCACTGTATCACAAATGCAAGTTCCCGCTGTTAGCGCACCACAAAGCCCCAGCA
CCGTGGATGTGGGCACAAGTGTACAACCTGCCGGCACTGTGGCCGCGGGAGTGAGTGCACGAGCCAGCA
CATTGACAGGAGCGCCAAGCCCAAGCACGCAACTCTCAAGTACGCAAGTCATGGATTTGATAAAGAGAGT
CGCTGCGCTAGAGCACGAGTTGCAGAGGTCAAAAACTGGTCAAGCACATGTGAGTACAGTTACTGATCCC
GTTAAGTTGAGTAGTGCTGGCGTGAGTGGTACAGCTAACCTGCCGCCATCTTTGATCGGAGCTTTGAGCGG
AGAGCCATCGTCGAGTGGAAGTTTGAGTGAAACTGCAACTATGAGTGTAGTCCCAAATTCAAGAGTAAGT
TTGAGTGAAACTGCAATTTTGAGTGGAACTGCCATTTTGAGCAGATCGCCATCTTTGAGTGAGGCACTGCC
TACGTCATTGAGTGGAAATTTGGGAACACAGTTGCAAACGGTAGAACGGGCTACAGTTATGGATAACTGC
TCTACAACGTCAAGCCGCTCGACGCCATATTGCTCTGCAGGCATTGCACAGCCAACGCTCAGCTACACTCC
GGCACAGGAGAACCAACAAGCGATAATTTTGGAGCCAACGCAGGCAACTGGGAACTTCGCATGGACGAC
TCCCAGGAACGATGTATGCCTGCCACGCAAATTACCAGATCTACCCGAGTTTGGAGGCCAACCTGAGGAT
TGGCCTATATTCTTCTGCGCGTTTACGGAAACAACTTTGGCGTACAACTGTACGAATCTGGAGAATAACCA
GCGATTGTTGAAGGCACTCAAAGGCGAAGCACGTGACACTGTGAAGTCGTTGCTCATACATCCCAGGAAT
GTGAACAACGTAATGGAACAATTACGTTTCAGATACGGCCGCCCAGAACAGCTAATACGCAGCCAGCTAA
TTAGTATTCGAGAGGTGCAGCCAATTCAGGAGCACAGCATAGCTAAAATCGTGCCATATGCTACACGCGT
GAGTAACCTTGCCGCCTTTCTACAATCGGCTAACGGGGAACAGCATTTAGCGAATCCCCTACACTGATGG
AGGATTTGGTCGCTAAACTACCACCAAGCAAGAGGGTGGAATGGGCCAGACATGCAGCTACGATTAGGC
CTTTTCCCGACGGTCGTACATTTTAGCGCGTGGTTAACGGACTACGCCAATGTGGTATGTACGATTGTGGA
CGTCGATAGTAAGGAGCAAAGGCGTAGAGTGCTGCATGCCAGCATTGACCAGAATAATGAAGGGCGTCA
CCGAGGTCGCACCAAACAGTGTCCAATCTGTAATGGACAACACGCAGTCAGGGACTGCAACGAGTTTAAC
TTAGCCTCTCCGCTAAAAAGGTGGACAGTCGTAAAAAGGCACCGGCTTTGTTTTTCGTGTTTACGAGTTGG
CCACATGGCAAGAGTCTGCAATAGGGCCAACGAATGCCAAGTTAATGGATGCCGGAAGAGACATCATCG
ACTCTTACATGATCAATGATGCCATGAGTCACAATGCACCGCAGCCAGCAGGTGATAGAGTGATGAATAC
TGGACGGATGCCGCAGCCAGCGGATCCTCACACAAGGGCACCGCAGCCAGCGGATGCTCAATCGACGCA
ACAAAGGAACTTAAGTTGTGTCGATTCGGATGGAGAACGCCTACTATTCCGCATATTGCCAGTGACATTG
CACGGGGCCAATAAGCGGATCGATACGTACGCGCTCCTCGACGAGGGCTCGTCTGTAACGATGATCGACG
ACGAGCTACTGCGCGATCTAGATATCAAAGGGGAACGCAGGCAACTAAATATACAGTGGTTTGGTGGAA
GAGCAACCAGAGAGCCGACCACAGTGGTAAGCATGGAAGTTAGTGGAGCGGACAAGCGCAAACGTCATG
TGTTGCGAAATGTGTACGCCGTGTCCAACCTGAGCTTACCAATGCAAAGCCTGCATCAACGTGACGTCCA
AATGTGGGGCAACGGTGCACGTCTTCCAATGAAACCGTATCGCGGGGCGGTACCAAAACTTCTGATTGGA
CTTGACCATGCGCATCTTGGACTGCCAATGCAAACAAAAAGATTTGCACCACAGGGACCATATGCCGCAG
CCACCGAACTTGGATGGGTGGTTTTTGGGCCGGTAAGAGATCAACCGACGGCAACGTCATCAAAGTCATG
CCTCCTAGCAGTGTCGCAGGAGGATGCAATCCAAAAGATGGTAAACGACTATTTTGATATCGAAAACTTC
GGTGTGAAGCTTACGCCACCAGTCGCAGCCAGCGACGATGCACGAGCACAGAGGATACTCGAAGACACC
ACGGTGAAAATAGGAGAACGTTACCAGACGGGTTTATTATGGAACCGTGATAATGTTGAGTTGCCACGAA
GCTATGATATGGCATACAAAAGATTGGTCAACATTGAGAGAAAGATGAAGCGTAACGATCAGTTTAAACA
AGCCTATATGAAAATTATGGACGATTATGTTCACAAAGGATATGCTCGACGATTGAAGCAGCATGAGGTC
GCTTTAGCCAACAGCGACAAACTTTGGTATCTTCCGCACTTTGGAGTTGAAAATCCAAATAAGCCCGGTA
AGATAAGACTGGTGTTCGACGCGGCAGCAAAGGTTGGTGATATTTCATTGAATTCGGCATTATCCAAGGG
GCCGCAGCACTATAAGTCGCTGCCGGCTGTTCTCTTCCATTTTCGGGAAGGTGCCGTTGGCGTTTGTGGAG
ACATCCGAGAGATGTTCCATCAAGTGTTGATTCGACCGCAGGATAGATGCGCCCAGCGATTCCTGTGGCG
AAACGGCGATGATCGCCGGGAGCCTGATGTTTATGAGATGAGGGTAATGACGTTTGGAGCAGCATGTTCG
CCAAGCGCCGCACACTATGTGAAGACCTTGAATGCCCTGCAGTTTCAGGATTCAGACCCTCGCGCGGTTA
AAGCTATTCTTGAATGCCACTATGTGGACGACTATGTGGATAGTTTTGATAGTGAAGGCGAAGCCATCAC
CATATCGACAAGAATAAGAGAAATACATGCAAATGCTGGATTCGAATTGTGCCAGTTTACTTCCAGTTCG
CCAACTGTAGCTGACGCGCTGGGCCAACATGGGGCTGCNAGGAGCATCGGATGGGGCGAAGCTGAAGAA
AAGATTCTAGGCATGTGTTGGCAACCAGCCACGGACGATTTCAAATTCAACATGAAGTACCACAAAGTAC
CCCGATGTGTGCTAAATTTGGAGCGAATTCCTACGAAGAGGGAATTCTTGAGCTTGATCATGTCAACATTC

GATCCTCTGGGATTTCTTAGCTGCCTCATGATAACTGGAAAGTTACTAATGCGTGAGATTTGGCGACGAAA
TGTGCAATGGGATGAACCCTTACCAGATGAGATCGCCCGGGCCTTTAGCAAATGGCTTCAGGATATGAAC
AACGTGGAAGGATTTCGGAGCTCGCGCCATTACTTCGGCCCAACACCTGTGCGGGCAATACAGCTGCACG
TTTTTGTCGATGCTAGCCAGTCAGCATTCGCAGCCGTGACTTACTTGAGGGTCACCTACGACAACAACGAC
GTGCGAGTATGCTTCGTATGTGCCAGGACGAAGTGTGCCCCAATGAAGACGATGTCAATTCCACGACTGG
AATTGCAAGCAGCCGTGTTAGGAACGAGGCTGATGGACACTGTCAAAAGGGAGCACAGTATAGCGATCA
GCGACGCTATACTATGGACTGACTCCAAAACNGTGCTGCGTTGGATAGGCAGCACCCACCGTCGATATAA
GCAGTTCGTCGGAAATCGAGTGGGAGAAATTTTGGAGTCAACAAAGGTTTCCCAATGGAGATGGGTACCT
ACTGCTGAAAATGCAGCAGACGACGCAACGAGGCCGCATTGCCACGTAGACCTCAGCCAACGGTCACGA
TGGTTAGACGGGCCAACATTTTTGCGGCAGCCGGAAAGCAGCTGGCCGCAGTCCGAACCGGGCACGGAA
CACTCATCCTACGAAGATGAAGAAGAGATGCCGAGTGAATTTGCCTTAGTTGCAGCAAATAACTTTTTCAT
TTCATTTCAGAGATTCTCTAGCTACAGTCGGCTGGTGAGGACGACGGCTTGGGTTTTAAGGTTCACGCGCT
GGAGCCGTGGACAGAGGACTGAGCTTGAGAGATTCGGCCTCACCGCGACGGAGTGTGAGAACGCTGAGA
ACTTGCTGATACGGCGAGCGCAATGCGAAGCGTTCCCTGACGAGGTTCGAGCCTCATACAATGAAGAATC
AGTCGGTCATCGAAGCGACATAAGAGGGCTGGCGCCATACTTGGATGACAATGGAGTCTTGCGTGCGTAT
GGCAGGATCGATGCCGCACTGTGTATACCGCATAGTGCGAGAAGGCCAATAATATTGTCACACCGGCACG
CACTGACGGAACTGATTGTGTATCACTACCATGTGAAAATGAAGCATCAAAACGTGGATGCAACGATAGG
CGACATCAGGACCAGATTTTTGGATAACAAAGCTGCGTCGACTGTTGCGTACAGTAATATCTGGATGTAG
TGTGTGCAAACTACATAGAGCGCAGCCAGCGCCACCTATAATGGGTCCTTTGCCAGAGGACAGACTGGAG
GCCAATGGATGGCCATTTAAGTTTACGGGTCTGGACTATTTTGGACCACTCCTTGTGACGATCGGACGTCG
AAAGGAAAAGAGATGGGTCGCCTTGTTTACGTGTCTGACGACAAGGGCCATACATTTGGAGCTGGCACAT
GACCTGTCAACTGATTCCTGCATAATCGTAATCAGGAACTTTCTTTGCCGTCGCGGACCTGTACTGAGGTT
GCGCAGTGACAACGGAAAGAACTTTGTTGGGGCCAACAGAGAAGCCAAAAGGCTAATAGATGTATTCGA
GCCGGAGAAGATTCAGGGTGAGTTATCTTCTAGAGGCATTGAATGGATCTTCAACTGCCCAGCGAACCCA
GCTGAAGGGGGAGCCTGGGAAAGGATGGTGCAGTGTGTCAAGAGAGTGCTACGCCACACAGTAAAGGAA
GTGGCACCAAAGCAACACGTACTGGAGAGTTTCCTGATTGAGGCCGAGAACGTTGTGAATTCTCGCCCGC
TTACCCACTTGCCGGTGTCTGTGGACCAAGAAGCCCCCCTAACACCGAACGATCTACTCAAAGGAGTGGC
CAATCTGCCTGACACGCCCGCTATCCACGAGCAGCCGAACGAGAGATGCGTCACGAGGAAGCAGTGGCG
CATGGCGCGGCTGTTACGAGACCGTTTTTGGAAGCGGTGGGTCTTAGAGTACCTGCCTACACTTGTGCGAC
GCGTGAAATGGTGTGCGCGCGTTGAGCCGATACGCCATGGTGATATGGTGTTTATATGCGACCCGGCCGT
GCCACGCAGAGAGTGGCGAAGGGGCATAGTGGAGGAGCTCTACCCTGGACCAGATGGAGTGCCTCGACG
CGCCAGCGTCCGCGTGGCGGACAACAACCGAGTGCGACTGATGACTCGTCCCGTGGCTAAACTAGCCATA
CTGGATGTGAGTGAAGCAGGGCCTTCACGGGGGCGGGGATGTTGCGGGACGAATTAATTTATCGATTGTT
TAGTATTAAGTATATTCTGAAAGTATGTATATTTGCCCTCCGAATGTACGGGCACACTGTATTTGGCTTGTT
GCCGCCATTACTTAAATGTTAAGCTCATTATTGTCGTGAGTTCGAAGGCCGTAGGAGGAAAGACTGCGGT
AAGATTCCGAGGGAATATATATCTAAGTTCTAACCTATGTAATCCGTTTAGAATAAAACACTTGCACTAGC
ACGTTTTGCATTACGCTGTCTCTAGTAACATCGGAGTAACATGGCCGGGGCCAGGCGTAAACTTGCAGTGT
TCAAATTGCAACTCCACCTTCGTTTGGCACGCGACA
>9_rnd-1_family-480__LTR___Pao
TGTTGCGGGACGAATTAATTTATCGATTGTTTAGTATTAAGTATATTCTGAAAGTATGTATATTTGCCCTCC
GAATGTACGGGCACACTGTATTTGGCTTGTTGCCGCCATTACTTAAATGTTAAGCTCATTATTGTCGTGAG
TTCGAAGGCCGTAGGAGGAAAGACTGCGGTAAGATTCCGAGGGAATATATATCTAAGTTCTAACCTATGT
AATCCGTTTAGAATAAAACACTTGCACTAGCACGTTTTGCATTACGCTGTCTCTAGTAACATCGGAGTAAC
ATGGCCGGGGGCCAGGCGTAAACTTGCAGTGTTCAAATTGCAACTCCACCTTCATTTGGCACGCGACATG
ATCTTTTATTCGCAGTTCTTATTGTAAGAAACTGCAAGGGCGACTTGCGCTGAAGACAAGACGTGGTGTAA
CATTGGTGTTACAGTGTGTGTACATAGACACAGCACAGGCAGGGCCTCCACCCAGTTATAGAGTCACCAT
TAGTACGCAGCCAGCGTAAAATATAGCAGAAACCATCTCCGAGCTGAGTAGGATGACACAACGGTCACCC
AGGAAAAGCCCACGCTTAAACGATGGCCAGGAGATAGCTACCACGGCGAGTGGGACGCAGACAAAGCGG
ACAAGTAGAATGGTGAGCACTGTATCACAAATGCAAGTTCCCGCTGTTAGCGCACCACAAAGCCCCAGCA
CCGTGGATGCGGGCACAAGTGTACAACCTGCCGGCACTGTGGCCGCGGGAGTGAGTGCACGAGCCAGCA
CATTGACAGGAGCGCCAAGCCCAAGCACGCAACTCTCAAGTACGCAAGTCATGGATTTGATAAAGAGAGT
CGCTGTGCTAGAGCACGAGTTGCAGAGGTCAAAAACTGGTCAAGCACATGTGAGTACAGTTACTGATCCC
GTTAAGTTGAGTAGTGCTGGCGTGAGTGGTACAGCTAACCTGCCGCCATCTTTGATCGGAGCGGAGAGCG
GAGAGCCATCGTCGAGTGGAAGTTTGAGTGAAACTGCAACTATGAGTGTAGTCCCAAATTCAAGAGTAAG

TTTGAGTGAAACTGCAATTTTGAGTGGAACTACCATTTTGAGCAGATCGCCATCTTTGAGTGAGGCACTGC
CTACGTCATTGAGTGGAAATTTGGGAACACAGTTGCAAACGGTAGAACGGGCTACAGTTATGGATAACTG
CTCTACAACGTCAAGCCGCTCGACGCCATATTGCTTTGCAGGCATTGCACAGCCAACGCTCAGCTACACTC
CGGCACAGGAGAACCAACAAGCGATAATTTTGGAGCCAACGCAGGCAACTGGGAACTTCGCATGGACGA
CTCCCAGGAACGATGTATGCCTGCCACGCAAATTACCAGATCTACCCGAGTTTGGAGGCCAACCTGAGGA
TTGGCCTATATTCTTCTGCGCGTTTACGGAAACAACTTTGGCGTACAACTGTACGAATCTGGAGAATAACC
AGCGATTGTTGAAGGCACTCAAAGGCGAAGCACGTGACACTGTGAAGTCGTTGCTCATACATCCCAGGAA
TGTGAACAACGTAATTGAACAATTACGTTTTAGATACGGCCGCCCAGAACAGCTAATACGCAGCCAGCTA
ATTAGTATTCGAGAGGTGCAGCCAATTCAGGAGCACAGCATAGCTAAAATCGTGCCATATGCTACACGCG
TGAGTAACCTTGCCGCCTTTCTACAATCGGCTAACGGGGAACAGCATTTAGCGAATCCTACACTGATGGA
GGATTTGGTCGCTAAACTACCACCAAGCAAGAGGGTGGAATGGGCCAGACATGCAGCTACGATTAGGCCT
TTCCCGACGGTCGTACATTTTAGCGCGTGGTTAACGGACTACGCCAATGTGGTATGTACGATTGTGGACGT
CGATAGTAAGGAGCAAAGGCGTAGAGTGCTGCATGCCAGCATTGACCAGAATAATGAAGGGCGTCACCG
AGGTCGCACCAAACAGTGTCCAATCTGTAATGGACAACACGCAGTCAGGGACTGCAACGAGTTTAACTTA
GCCTCTCCGCTAAAGAGGTGGACAGTCGTAAAAAGGCACCGGCTTTGTTTTTCGTGTTTACGAGTTGGCCA
CATGGCAAGAGTCTGCAATAGGGCCAACGAATGCCAAGTTAATGGATGCCGGAAGAGACATCATCGACT
CTTACATNATCATGATGCCATGAGTCACAATGCACCGCAGCCAGCAGGTGATAGAGTGATGAATACTGGA
CGGATGCCGCAGCCAGCGGATCCTCACACAAGGGCACCGCAGCCAGCGGATGCTCAATCGACGCAACAA
AGGAACTTAAGTTGTGTCGATTCGGATGGAGAACGCCTACTATTCCGCATATTGCCAGTGACATTGCACG
GGGCCAATAAGCGGATCGATACGTACGCGCTCCTCGACGAGGGCTCGTCTGTAACGATGATCGACGACGA
GCTACTGCGCGATCTAGATATCAAAGGGGAACGCAGGCAACTAAATATACAGTGGTTTGGTGGAAGAGC
AACCAGAGAGCCGACCACAGTGGTAAGCATGGAAGTTAGTGGAGCGGACAAGCGCAAACGGCATGTGTT
GCGAAATGTGTACGCCGTGTCCAACCTGAGCTTACCAATGCAAAGCCTGCATCAACGTGACGTCCAAATG
TGGGGCAACGGTGCACGTCTTCCAATGAAACCGTATCGCGGGGCGGTACCAAAACTTCTGATTGGACTTG
ACCATGCGCATCTTGGACTGCCAATGCAAACAAAAAGGTTTGCACCACAGGGACCATATGCCGCAGCCAC
CGAACTTGGATGGGTGGTTTTTGGGCCGGTAAGAGGTCAACCGACGGCAACGTCATCAAAGTCATGCCTC
CTAGCAGTGTCGCAGGAGGATGCAATCCAAAAGATGGTAAACGACTATTTTGATATCGAAAACTTCGGTG
TGAAGCTTACGCCACCAGTCGCAGCCAGCGACGATGCACGAGCACAGAGGATACTCGAAGACACCACGG
TGAAAATAGGAGAACGTTACCAGACGGGTTTATTATGGAACCGTGATAATGTTGAGTTGCCACGAAGCTA
TGATATGGCATACAAAAGATTGGTCAACATTGAGAGAAAGATGAAGCGTAACGATCAGTTTAAACAAGC
CTATATGAAAATTATGGACGATTATGTTCACAAAGGATATGCTCGACGATTGAAGCAGCATGAGGTCGCT
TTAGCCAACAGCGACAAACTTTGGTATCTTCCGCACTTTGGAGTTGAAAATCCAAATAAGCCCGGTAAGA
TAAGACTGGTGTTCGACGCGGCAGCAAAGGTTGGTGATATTTCATTGAATTCGGCATTATCCAAGGGGCC
GCAGCACTATAAGTCGCTGCCGGCTGTTCTCTTCCATTTTCGGGAAGGTGCCGTTGGCGTTTGTGGAGACA
TCCGAGAGATGTTCCATCAAGTGTTGATTCGACCGCAGGATAGATGCGCCCAGCGATTCCTGTGGCGAAA
CGGCGATGATCGCCGGGAGCCTGATGTTTATGAGATGAGGGTAATGACGTTTGGAGCAGCATGTTCGCCA
AGCGCCGCACACTATGTGAAGACCTTGAATGCCCTGCAGTTTCAGGATTCAGACCCTCGCGCGGTTAAAG
CTATTCTTGAATGCCACTATGTGGACGACTATGTGGATAGTTTTGATAGTGAAGGCGAAGCCATCACCATA
TCGACAAGAATAAGAGAAATACATGCAAATGCTGGATTCGAATTGTGCCAGTTTACTTCCAGTTCGCCAA
CTGTAGCTGACGCGCTGGGCCAACATGGGGCTGCGAGGAGCATCGGATGGGGCGAAGCTGAAGAAAAGA
TTCTAGGCATGTGTTGGCAACCAGCCACGGACGATTTCAAATTCAACATGAAGTACCACAAAGTACCCCG
ATGTGTGCTAAATTTGGAGCGAATTCCTACGAAGAGGGAATTCTTGAGCTTGATCATGTCAACATTCGATC
CTCTGGGATTTCTTAGCTGCCTCATGATAACTGGAAAGTTACTAATGCGTGAGATTTGGCGACGAAATGTG
CAATGGGATGAACCCTTACCAGATGAGATCGCCCGGGCCTTTAGCAAATGGCTTCAGGATATGAACAACG
TGGAAGGATTTCGGAGCTCGCGCCATTACTTCGGCCCAACACCTGTGCGGGCAATACAGCTGCACGTTTTT
GTCGATGCTAGCCAGTCAGCATTCGCAGCCGTGACTTACTTGAGGGTCACCTACGACAACAACGACGTGC
GAGTATGCTTCGTATGTGCCAAGACGAAGTGTGCCCCAATGAAGACGATGTCAATTCCACGACTGGAATT
GCAAGCAGCCGTGTTAGGAACGAGGCTGATGGACACTGTCAAAAAAAGGAGCACAGTATAGCGATCAGC
GACGCTATACTATGGACTGACTCCAAAACAGTGCTGCGTTGGATAGGCAGCACCCACCGTCGATATAAGC
AGTTCGTCGGAAATCGAGTGGGAGAAATTTTGGAGTCAACAAAGGTTTCCCAATGGAGATGGGTACCTAC
TGCTGAAAATGCAGCAGACGACGCAACGAGGCCGCATTGCCACGTAGACCTCAGCCAACGGTCACGATG
GTTAGACGGGCCAACATTTTTTGCGGCAGCCGGAAAGCAGCTGGCCGCAGTCCGAACCGGGCACGGAAC
ACTCATCCTACGAAGATGAAGAAGAGATGCCGAGTGAATTTGCCTTAGTTGCAGCAAATAACTTTTTCATT
TCATTTCAGAGATTCTCTAGCTACAGTCGGCTGGTGAGGACGACGGCTTGGGTTTTAAGGTTCACGCGCTG

GAGCCGTGGACAGAGGACTGAGCTTGAGAGATTCGGCCTCACCGCGACGGAGTGTGAGAACGCTGAGAA
CTTGCTGATACGGCGAGCGCAATGCGAAGCGTTCCCTGACGAGGTTCGAGCCTCATACAATGAAGAATCA
GTCGGTCATCGAAGCGACATAAGAGGGCTGGCGCCNTACTTGGATGACAATGGAGTCTTGCGTGCGTATG
GCAGGATCGATGCCGCACTGTGTATACCGCATAGTGCGAGAAGGCCAATAATNTTGTCACACCGGCACGC
ACTGACGGAACTGATTGTGTATCACTACCATGTGAAAATGAAGCATCAAAACGTGGATGCAACGATAGGC
GACATCAGGACCAGATTTTGGATAACAAAGCTGCGTCGACTGTTGCGTACAGTAATATCTGGATGTAGTG
TGTGCAAGCTACATAGAGCGCAGCCAGCGCCACCTATAATGGGTCCTTTGCCAGAGGACAGACTGGAGGC
CAATGGATGGCCATTTAAGTTTACGGGTCTGGACTATTTTGGACCACTCCTTGTGACGATCGGACGTCGAA
AGGAAAAGAGATGGGTCGCCTTGTTTACGTGTCTGACGACAAGGGCCATACATTTGGAGCTGGCACATGA
CCTGTCAACTGATTCCTGCATAATCGTAATCAGGAACTTTCTTTGCCGTCGCGGACCCGTACTGAGGTTGC
GCAGTGACAACGGAAAGAACTTTGTTGGGGCCAACAGAGAAGCCAAAAGGCTAATAGATGTATTCGAGC
CGGAGAAGATTCAGGGTGAGCTATCTTCTAGAGGCATTGAATGGATCTTCAACTGCCCAGCGAACCCAGC
TGAAGGGGGAGCCTGGGAAAGGATGGTGCAGTGTGTCAAGAGAGTGCTACGCCACACAGTAAAGGAAGT
GGCACCAAAGCAACACGTACTGGAGAGTTTCCTGATTGAGGCCGAGAACGTTGTGAATTCTCGCCCGCTT
ACCCACTTGCCGGTGTCTGTGGACCAGGAAGCCCCCTAACACCGAACGATCTACTCAAAGGAGTGGCCA
ATCTGCCTGACACGCCCGCTATCCACGAGCAGCCGAACGAGAGATGCGTCACGAGGAAGCAGTGGCGCA
TGGCGCGGCTGTTACGAGACCGTTTTGGAAGCGGTGGGTCTTAGAGTACCTGCCTACACTTGTGCGACGC
GTGAAATGGTGTGCGCGCGTTGAGCCGATACGCCATGGTGATATGGTGTTTATATGCGACCCGGCCGTGC
CACGCAGAGAGTGGCGAAGGGGCATAGTGGAGGAGCTCTACCCTGGACCAGATGGAGTGCCTCGACGCG
CCAGCGTCCGCGTGGCGGACAACAACCGAGTGCGACTGATGACTCGTCCCGTGGCTAAACTAGCCATACT
GGATGTGAGTGAAGCAGGGCCTTCACGGGGGCGGGGATGTTGCGGGACGAATTAAGTTATCGATTGTTTA
GTATTAAGTATATTCTGAAAGTATGTATTTTTGCCCTCCGAATGTACGGGCACACTGTATTTGGCTTGTTGC
CGCCATTACTTAAATGTTAAGCTCATTATTGTCGTGAGTTCGAAGGCCGTAGGAGGAAAGACTGCGGTAA
GATTCCGAGGGAATATATATCTAAGTTCTAACCTATGTAATCCGTTTAGAATAAAACACTTGCACTAGCAC
GTTTTGCATTACGCTGTCTCTAGTAACATCGGAGTAACATGGCCGGGGGCCAGGCGTAAACTTGCAGTGTT
CAAATTGCAACTCCACCTTCGTTTGGCACGCGACA
>160_rnd-1_family-193__LTR___Gypsy
TCAAGAATATATATACTTTATGGGGTCGGAGATGCTTCCTTCTGCCTGTTACAAACGTTTGGATTTTGCAC
AAATACAATATACCCTTATACCCATTTTTAATGGGTTCAGGGTATAAAAAGGGACTTAAATAGCATTACA
GAAAAAAGTCGCAATAACCATGCTGCTAAAATGCCAAGCAGACGCGCATGAACGTGTGTTTGTACATGTA
TACAGAAATTCTTAAAGATGCTGCTCTATTCAATTGCGCAGTTGCATATAACTTTGGTTTTTTTTTNACCGA
TCTTTATGAAATCTACAGTATATCTTATTGTATCATAGAATATTTGTGTATACTGAAAAAGTCATTTGCAA
ATTAATTTTGGCTTAAATTGGTTGGCAATAAAAGTTGGGTTATTAACTTGATTTATAGCTCTAGGGTGGTA
GCGGAGAGGTGGCGATAGGTATAAAATGAAAGATACTATATAATATTTTAGAAGCNACATGTCANGTTTC
GTGACTCTAGCTCTTATTATTTACCAAAATTGCCCAAAAAAAAGGATATCGATATTGATGTTTATCGATTG
CTTGGAAACGGAGTAAGCTATCGATTATCGGAAACAAACTCGATCTGCGCGGGCACTAGGAGCACCTNCA
TAAAATTTCAAGTCTATAGCACTTATATGTTCTGAGATCCTTGCGTTCATACATACGGACGGACGGACGGA
CAGACAGACGGACATGGCTAGATCGACTCGGCTATTGATGCTGATCAAGAATATATATACTTTATGGAGT
CGGAGATGCTTCCTTCTGCCTGTTACATACATTTGGATTTTGCACAAATACAATATACCCTTATACCCATTT
TTAATGGGTTCAGGGTATAAAAATACATATACATTGNCCTGATCAACCAACTGAAAAAAAAAATCAATCC
TTGAAAAAAACAAATTACTATAATGAATTTTACATTTTTATATTTTATATCTTTATATTTTGAAATGTTCAC
ATTAGTATATTCTCATAAATCTTAAATAATTTTTTTAAATTCATATTATTTTTATCATTTTTTTACATTTTAT
TATTTTATAAAAAAAAAATTATATACAAAAAATTTTGTTCAAAATAATTAAGTTCATTAAATAACTACACA
TCCATAATGGTACGTTATTTCTTTGAAAAGGGAGCTGTAGTGTGTATATGCCTTAAAAGAACACCCTATAT
ACAAATATAAAGTATAAACAAGCCCAGAGCCACTTTGTATATTGGCTCTGGCTCATATTACGCAGCGCAG
TTCACTAGCCTCTCAGCTGATGCAACGATGAACAACAGTAGCAGTTATATCCTTAATTACTTTTAATTATA
AAAAAAAAATTTTTGTAAAATTGAGCAGCATAGCCCGTAAACCTATGCGAAATGCTAGGTAGTATTTTGT
GTTATTAGCAATATTGAAACCTTGAAACTAAAAACATTGGCATTTAAAGCTAAAAACACTATATGTTACAT
AAAAACATTATATGAATTTAAAAACATTACATCAACTTATGTAAGAAATTTTAATTAAAATGGGTTCGAA
CGAATCCAAGGTTGAAAAATCAGCCGCAAATGTTATAAATAACGTAAAAGTAATTGACCATAAAGAATA
ATTACTACAATATCTCTTGTATCACTAGTATTGAAAGTGTATGCAATGCATACAAAGAAAATTAAGAAAC
GCTATAGCAGTAGAGCAGACGGACTAGATAAGGTATGAATATATTTGAATATGTCAGACATAGAGGCAAT
CATTAGGTTTACGTATAGAGATGAGCCAGACTTTCGTACTCGCGTATTAAACGTACTGCGATTACATAGAG
ATTTTATAAAAGTGTGTGATTTCGAATACGAATACACGGACATGTTCAATACTCATATTAATGGTGCGACT

GCATCGATAGATATCTATTATAATAATATGGAATGGAGTATATTAGTGAAAAATATCAATGACTTTAAAC
ACCAATTTGACAAGTCATATAAATGCATCAATAAAGGCATACCTATAAAGGTAGAAACACTAATATACCA
TGTACAAATATTGATAAAACAATATAACAATATAGTACATGCAGTAGATTCCATTAAAGAAAAACTAACA
CAAGAACATGCCAAACAATGCCTTAAAGTCATACAATCTTTAAATACAAGATTGAAAAACATTGGCATTA
GACAGAATATCATAATAAATGTTCCAGAAGATCTATTTTTACCAGCAAAGTTTGAACCAAACCAGCTGCA
AGATCTGAATGAGTCGAAGCACTAGCCAAAGAACATATAGACTGGACATGAGTACAGGAAAGTAGGCAC
GAAAAGTATCGGTGGCATCTGGTCCAAAATCGCGAGTAAGGGAGTAAGATATACAAATGATGTATTTTTG
AACCGCTTACTCTACAGGAAAGTGCTTGGCGCACTCTCTTCCATATGATTTGTGCGATAGAACGACAACGC
TGTTAACTTTTATCTCGCTCGCACTTACTCTCAGCTCTCAATGCTCTCTCTTTATGTTTAGGGATCGTTTAAA
TGTGTGTGTGTGTGTATTTGTCCGACAAGCAAAACTGGGCAAAAATTCAATTTTTCCCAAATGCTGAGGCA
TTTATACCCTTTTGAAGGCACGAGTAATATGCAATAGAGAAAGGGTTTGCCACGTCGATGCGAAATTCCT
GCATTTTAGCTCGCTATCGTTGGTAAGGGTACCCAAGTGATGCCATGTGTGCATTTAAAATATTTTGGACT
CGTTATTCACGCATACTTGCATATTTTGTATATGAATAGAAGCATTTATGCGCTTGTGTGCCTGTTGCTNGC
CTGTCCCCGATGCAAATTTAACAATATTTTGGNATTTCTTATTTTCGACCTCTACACATAGGCATAGACAT
ACAAGTGTCTGTGAATGGGCAGATCATGGGCGCAAAAGAAAATATCATTATTGGCACGCGTCTGACGCGC
GCCCTTGAAATGAACAACAATAGCAATGGCAGAGGACATTATAGACGTAATGCCCGTGGTAATAACCGA
GGAAGGAATAATTCCAACCGAGGTAACAACCAAAATAATAACAATAGAGGTAATGTCCGAGTAACTGAA
GGTAATTCGGAAAACCATTGAGACCCCTCAGATGTTTAGAGCCAATAAATGGCACAATAAAAACCATCAN
CCTCAACCTCAGCATATTTGTAAAANTTAACAATGAAAGAACAGGTAAATCCTTTGCACTGTTGCTTGATA
CTGGTGCAGATATATCTATATTAAAAGAAAATGTAGATATATTTGAAAACGTAAACGAAAGAGTTACTAC
CCAAAATCGGGAATTGGCGAAGGAACAATTTTTTCAAAANGCTTGGCCTTTATAGAACTCACTACAGGTA
AATACATAATTCCGCATAACTTCCATTTAGTCGATCAAAAATTCCCAATACCTAGTGATGGCATACTAGGC
ATAGATTTCATAAAACAATATAATTGCCAATTAGATTTCACTCAGAGTGGGCGACTCACTAATTCTTCGCC
CAAACAACTTAAATTATCCGATTAAATTACAAATACTTCATACTTGTGACAATAATGCCACAGTACTCCCA
GCTCGTTCCGAAGTTGTCCGTGAAATACAAATACCCTCAATAAAAAATCAAATTTTGATATTAAATCAAG
AAATTGATGAAGGAATTTATATCGCAAATACCAATATCCAACCACAAAAATACTTGTGTCCGAATTTTAA
ATACAACAAATACCAATAAGATCGTCAATTTAAATAAAATAAAATATGAAACCTTAAATGATAAAAGAA
ACTAACGAAGAAAGAGACAACTTCGTTATGAATAAATTAATTAAAAACTTTCCGCCTTTATTCAAAAGTC
AATTAAAAGATTTATGTACCAGGTATACAGACATATTCGGACTAGAAACCGAATCAATAACAACAAATAA
TTTTTACAAACAAAAGCTTAGACTGAAGGATGATCAGCCCGTATAAATAAAAAATTATAGGAGTCCTCAT
AGCCAAGTACAAGAAATTCAAGCCCGAGTACAAAAATTAATAGACAATAAAATAGTCGAACCGTCAGTC
TCATAATATAATAGCCCACTCTTACTAGTCCCGAAGAAGTCTCTTCCGGACTCTGAAAAGAAAAAATGGC
GATTAGAAATTGACTATCGTCAAATTAATAAAANGCTATTGTCCGACAAATTTCCACTACCTAGAATTGAT
GATATTCTAGATCAACTAGGTAGAGCAAAATATTTTTCATGCCTAGACTTAATGTCTGGCTTCCACCAAAT
TGAACTTGAAAAAAGTTCTAGAAATATAACGTAATTGTCAACAAGCAATGGCTCATATCGCTTCACGCGA
TTACCATTTGGATTAAAAATAGCATTTTCTGGTCTACAACCATCACAAGCATTTCTTAANATGGATGATCT
AATAGTAATCGCTTGTTCCAAGAAACATATGGTCAAAAACTTAACTGATGTTTTCGACCAATGTAAGAAA
CTCAACCTGAAACTACATCCGGAAAAATGTTCATTTTTTATGCATGAAGTCACATTTTTGGGACACAAATG
CACTGATAAGGGAATCTTGCCAGACGACAAAAAATACGACGTGATAAAAAATTACCCAGTCACACACTAT
TCGCGTCGCGTAACAAGATTAGTTGTAAAAAATACGTAAGATTCGAATGGACGCCTGAATGCCAGAACAT
CTCAAAACAGCACTAATAAACCTAAACTTGCTACAATATCCAGACTTCACCAAAGAATTCTGCATAATAA
CAGATGCAAGTGAGCAAGCGTGTGGAGCAGTTTTAACTTAAAACTTGACTGGACTCCAACNTCCAATTGC
ATACGCATCAAGATCCTTTACTGAAGGAGAAAGTAACAAGAGTACTACTGAACAAAAATTAACAGCTTTT
CTCTGGGTAATAACACACTTTCGACCATATATTTATGGTAACCATTTCACGGTAAAAACGGATCACAGACC
ACTAACATCTATTTTCAATGGTCAATCCAAGTTCACAATTAACACGTATGAGGCTTGATTTGCAGNAATAT
ACCTTTACGGTAGAATATCCGAAGGGTAAAGACAGTTACGTAGCAGATGCGTTATCGAGAATAACCATCA
AGGAGCTACAAAATATAACAGGAAACATAATGAAAGTCACTACAAGACAAGCACGTAGACAAGAATCCT
GCGCAGGAGAAAAAGAAATAGAATTGCCTAGGCAATCTATAGAAAAAGCTTCAAAGGCCAAAGTGTACC
AAGTCATTAACAATGACGAGGTACGAAAAGTAGTGACCTTGCATGTAAACAATATGACATGTTTTTTAAA
CATGGNAGACAAATTACAGCAAGATACGATGTTACCGATTTATATACTAATTAAACCATTGATTTGGATC
AAATCTTTCAAAGGCTTGAAACGCAAGCCGGTATATATAAAATCAGCCAACTCAAAGTGGCACCGTGGGA
AAATATCTTTGAACACGTTTCAATGGATAATTTATAGAAAATGGGCANTCAAATATTAAAATCTTTAAGA
GTTGCGCTACTCCACCCGGTGACCCAAATAAATTCTGAAAAATAAAAANAAGCTATCTTGTCTAAACTAC
TTGATGATCCCATACTAGGAGGACAGGCATTATAAAAACCTTGGCCAAAGTCAAACGACATTATTTTGG

AAAGGTATGTCTAAACAAATAAAAGACTACATCCGCAAATGTCCAAAATGCCAGAAAGCAAAAACTACA
AAGAACACAAAAACTCCTCTAACAATAACAGAAACACCAATATCCGCTTTCGACAGAGTAATCGTTGACA
CAATTATTACCATTACCTAGAACAGAGAATGGGAACGAGTATGCAGTTACACTCATTTGTGATTTTACGAA
ATACCTCGTCGCAATTCCGATCCCAAATACAAGTGCAAACACTGTCGCTAAATCTATATTCGAATCTTTTA
TTCTGAAGTACGGTCCAATGAAGACGTTCATTACGGACATGGGTACGGAATATAAGAATTCAATTATAAA
CAATTTGTGCAAGTATATGAAAATAAACAACTAACATCAACTGCACATCATCACCGGACAGTTGGAACAG
TAGAAAGAAGTCACAGGACTTTTAACGAATACATACGATCATATATATCAGTTGATAAAACTGATTGGGG
CGTATGGATCAAATACATCGTATACTGTTTTAACACGACCCCCTCTATCGCACACAATTATTATGAGCAAA
TTTTTGGTAAACACCCAATTTACCAATATAAATAGAATCGACCCACTATATAATGTAGAAGATTATGCTAA
AGAAGNTAAGTTTAGATTAGAAATAGCATATAAAAGGTCTAGGATAATGCTTGGAAAACATAAAGCAAG
AAGTAAGGAACTTTTTGATAAAAAAGTTATAGATTTTCAAGTAAAAATAGGAGATAAAGTCTTATTAAGA
AACGAAACTGGCCATAAATTGGATCCAGAATATATAGGCCCATATACAATAGTTAACATAGAAAATAGGG
ATAACATTCTAATTNAAGATAACAAAAATAGGACCCAAAAAGTTTATAAGAATAGACTAAAAATTTTTAA
CGAAT
>9_rnd-1_family-413__RC___Helitron
CGCAGACAAGACCCAGTCTACCGGGCAAACGAACAAAGACAAAATACTGTAGACCACTCGATCGACGAG
CTAATTCTCCAGTTAGAGAACAGGAGCAATTGGCTAATACTTCGCGTCGATCGACACGCAGACAAGACCC
AGTCTACCGGGCAAACGAACAAAGACAAAATACTGTAGACCACTCGACTCGACGAGCTAATTCTCCAGTT
AGACAACAGGAGCAATTCGCTAATACTTCGCGTCGATCGACACGCAGACAATACATAGTTTACAGGGCAA
ACGAACAAAGACAAAATACTGTAGACCACTCGACTCGACGAGCTAATTCTCCAGTTAGAGAACAGGAGC
AATTGGCTAATACTTCGCGTCGATCGACACGCAGACAAGACCCAGTCTACCGGGCAAACGAACAAAGAC
AAAATACTGTAGACCACTCGACTCGACGAGCTAATTCTCCAGTTAGACAACAGGAGCAATTGGCTAATAC
TTCGCGTCGATCGACACGCAGACAAGACCCAGTCTACCGGGCAAACGAACAAAGACAAAATACTGTAGA
CCACTCGACTCGACGAGCTAATTCTCCAGTTAGACAACAGGAGCAATTGGCTAATACTTCGCGTCGATCG
ACACGCAGACAAGACCCAGTCTACCGGGCAAACGAACAAAGACAAAATACTGTAGACCACTCGACTCGA
CGAGCTAATTCTCCAGTTAGAGAACAGGAGCAATTGGCTAATACTTCGCGTCGATCGACACGCAGACAAG
ACCCAGTCTACCGGGCAAACGAACAAAGACAAAATACTGTAGACCACTCGACTCGACGAGCTAATTCTCC
AGTTAGACAACAGGAGCAATTGGCTAATACTTCGCGTCGATCGAGACGCAGAGAAAACCCAGTTTATAGG
GCAGACGAACAAGAACAAGACAATATTCGTCGAGCTCGCCATCGACAAGACATACAACGACGACTTTTA
GAAAGAGCTAGGGATAGGAATCAAAGATCACAGGTTAGAGAGAATCCGATTCGTAGGATAATTGAGCAG
GCACGTAACACAAGTGTGAGGCGAAGTGCTCGCATTCATGCACGACAGGAGGCTTTTGACGCCAATAGAA
GTTTGTCCCAGCGAGTTAGCCAGTATAGGGGTAATACAGGTAATAGGATAGCAGAGAATAGGCGCAATGC
TACGAGGAACAGGGGTGAACGTGCGATTAATTTTTCAGAGGATAGAATCTCTCCAGCTATTAGGAGTAGC
TTAGATGGTCTGATAGCTGGATTCAATAGAACAGTTAAGCAGGGTCCCGATCAGGTTTGCCTTTGTTGCGA
AGGCTTATGGTTTCCACATCAAGTGGAAAAAATTTCTCGTGAGGTTATTTTGAAAGCAAGTGTAGTGCGG
AAGTAAGCTCCGATATTTTATATCTTTCCCTCTATATTTCCCTCTGATAATAATAAATATGATTTTTGCAAA
ACGTGTAGTCGTAGCATTAAATTAGGCAAAAAACCTAAAACTTCTATTACAAATGGCCTAGATTTTCCCGA
TCTGCCAGATTGTTTGAAAGGCTTAACGCCTTTAGAGGAAAGGCTGATTTCCCCTCGCCTTCCTTTCATGA
TTATTAAGTCATTAGGGATATGGTCGACAAAATGCAATTAAAGGAGCAGTGGTCAATGTACCCATTCCTG
TGGCCAATGTTGTAACGTCCCTTCCGAGGGCGTTTAATGAGAGCGAGGTAGTGCAAATTCATCTCAAACG
TAGGATGGAATATATCCATGATTTCATGGCTGAAACCATTAGGCCTTCTAAAGTTGCTGAAGCAATAAAA
TTTTTAGTCAACACCGAGCTTTATAGAAAGCATCAAATTACTATTAATGAACAGTGGTTGTCGTCTTTTTC
GTCTGACGAGGTTCCGTTTGTTGCATCTGCTGATGACAGGGACTTAGTATCGCAGTTAGTGGCAAGATCTG
CTCAAGCCTCCATAGATGAGCAGAATAATATAGAAAGAAGATATTAATCCAGGAGGCCAGGAAACATTA
TTAGAAAACGTCCCTGTCGAAAAATATTGCCATGCAAAGAATAGTTATAGCCCCTGGAGAGGGTCAGCGG
CCTTTAGACATGATCGAAGATTGTGATTCAGAGGAGCTGGCCTTCCCAACTATTTTTGCTGGCATTAAGAG
AAAGTGTGCAGAAAGTTTTACAACCATAGTTAGGTCAGAACTTAGAAACTTTGATAGGAGAGGTTGTAGA
ACAGATAAATTATTTCTCAATTTTAAAAAATTGGAAATGATTAATATAAGGAACAGTACTTCCATTTGCTT
ACGTAAGCACAGCGCCACTCGAGGCATTACGGCAGCACAAGTCCTTAATGAGGAGTATTTAGAAAATTTA
ATACGTCACGATGATTGCTATAGGGTTTTGAAAAATATTCGCACTTCACCTGCTCATTGGGAAGGAGAAA
AGAAGAAGGTCATGGCGATGATAAGGCAGATTGGTCTTCCGACATTTTTTATAACCTTTTCGGCAGCAGA
GACTAGATGGGCAGAGCTGTTAGTTTTACTTTCTAAGAATGTAGATAAGCTAGAAATTTCAGAAGAATTG
GCAGCTAATTTAAGTTTTGCCAATAAAGCCCGTCTAATTAGAGCCGACCCTGTTACTTGCGCTAGGTATTT
TGATTTTCGTTACAGGCAGGTTTTAAAACTGATGAAAAAGGAAGGCGGTGTTTTTGGTAGTCACAGAGTT

ATTAAGTATTATTGGCGAGTTGAGTTTCAGCAAAGAGGCTCCCCCACGTTCATGGCATGTTTTGGCTGAA
AGATGCTCCGATAGTTGACTTGGCTGATGAGTCAAGCATTCCGTCTGTAAAGTCCTTTATTGATCAGTTTA
TCACAACTGAAACAAATAACCCGGATGTGGCTCCATTTTTGGAGTATCAAACTCACAATCACGGGCACTC
ATGTAGGCGAGAAGTTAGGGCACAAAATATATGTAGATTTGGTATTCCATACCCTCCCATGCCCCAAACA
GAAATTCTATTCCCATTAGAAGAAGATGCTGAAAATAAAGAAGTGCATCGCAGAAATTTCGAAAAGATTC
AGGGAGTTTTAGAATATCCGTTTGATCGAGAAAGTATAGATTACCTTAGCGATTTCAATAACTTTTTGACC
CACGATCAACTTAACTTAAGTTATGAGTGCTATATAAATGCACTCAGGTCGAGTATAAAGAAGCCCAAAA
TATATTTAAAGCGATCATTCGCAGCAATTAAGCTTAACGCTTATAATACTCACCTCCTTCTACTGCAGCGC
GCGAATATAGATATCCAGTTTATATTAGATCCCTATGCTTGCTGCAGTTACGTCGTTAATTATATTAACAA
GTCCCAGAGAGGCATTTCAAAGCTAATGCGTGAAGCTGTAAGTGAGATAAGCAGGGGCAATGTAACTTTA
AAGCAAAGGTTACAGCATATAGGGCACAAGTTTATTTCGGGTACGGAAATATCCGCCCAGGAAGCCGTTT
ACTGCTGTTTAGGTATGTCTTTGTCCGAAAGCAGTAACGCAGTGGTTTTTATTAATACCTCATTACCAGAG
AATCGGGTTGGGTTAAGGAAGTCTAAGCAACAGTTGCAAAACTTACCCGATGACTCAGTAGCAATATTGG
AAATTGGACTTATAGACCATTATGTTCAGCGCCCCGACGCTTTAGATAGTTTATGCCTAGCTGATTTTGCT
GCTTTTTACAGCTTTAGCAAGCGGCATAGTGGTACTCGCCAAAATATAGATGATGCTGATGACATCGAAA
CATCCAGGGCTTCCCAGCAATTAGTTTACGCACTTCGCGATGGTAGTGGGTTCGTAAAGAAGAGGAACAC
TCCTATTATAATTAGGTATAGGAGATTTAATATTAATACTGATAGGAGCAATTATTTTCGAGAATTAGTTA
TGTTGTATGTCCCATGGAGGGATGAAAATGTAGAACTAGTAGCAAGAGACTGCGAATCGTTTTGCGGCCA
GAATCATGAGTTAATTGAGAGCAATCGCAGGAAATACAATAGCTTAAATGACAGTGAGCTTCAGCATGCT
CTTGAAAATGCAGTGCAGGCAGAAAATGAAGAAAATGTTGAAAATCAAGAAGTAGTAGACGATAGATTT
CGCGTATTAGGATTACCAGAAATAAATCCTCATTTAAATGTCTTAAACATTAATGGAAATGACGAGAATC
TAGATTCAGAAGATAACAATGTCCGATTAATAAAACTTCCAGCTATAGTTTCAGCTGAAAGCTTAGCAGA
TATAGTCAGGTCACTTAATTTAAAGCAGAAAACTTATTTTACACATGTTTTACACAATGCCAAAAACAAAT
CCGTCTTCTACGAATTTGTTGGAGGTGGTGCGGGTGTCGGAAAGAGCCGACTTATTTTAGCTCTTTTTCAA
TCTTTGTCCGTTATGTACAATAGTAGGCCAGGAAGTGATCCAAATTCTCCTAAAATAATATTGTGTGCTCC
AACAGGTAAAGCTGCTTTCGGAATCAGTGGCTCAACACTGCATTCCATGTTTTCCTTACCAGTAAATCAAT
CTGGTTCCGATTTGAGGGCTTTAAGTAATGATTTAGTAAATACTTTGCATTCAAGGTTAATCGACCTTAAA
CTAATTATTATAGATGAAATATCTATGGTAGGCTCCAAAATGTTTTCCCAGTTGGACTCCCGTTTAAAAAC
AAATTTTTAAAAATACTTCTCCATTCGGTGGTATTTCTGTCATAGTTTTCGGGGATCTACGACAATTATCTC
CAGTAGGTGACCGTTGGATTTTCGAATCCAACTCTAATGATCCCTACAGTGCAATTTATGCTTCATTGCTG
TGGGACCTTTTCAAGTATTTTGAACTCACAGAAATAATGCGTCAACGGGAGGACCAACCGTTTGCTTCTGC
TTTAAATAATATGGCATCGGGAAACATGACAGCCGATGATATTAGTCTTGTCCGATCTCGCATTTGTCAGC
AATCGGATGTGCCCGATGATGCCATTCATCTATTTAGTACAAATGCGGCGACAGATAGTTATAACACGAT
AAAGCTAAACTCAATTCCGACTGATCAATTTATTTCTACCGCTGATGATTTTATAAAAGCAACACATTTAA
CTGCGGCTTGTAGACAGAGAATTTTAGAAGCCGTTAGTAGCTTTAGGTCGTCCGAAACACAGGGATTGTG
CATGTCCCTTCTTCTAAAAACCACTGCGAAATATATGATTACGGTCAATGTAGATACTGGGGATGGATTAG
TTAATGGGGCAACAGGCATGTTAATGCAAATAGATTTCGATGCATCCAGCACTCCCATTATTTTATGGATG
AAATTTTCAGATGATACCGTAGGAGTACTTGCTCGCTCAAAGTGTACACATCGTACAGAACCTTTCTGGAC
CCCAATTCAGAAAATAGTTAAAAGTTTTCAATATAGAAGAAATGAGCAAGTTTCTATTGACCGCAGACGG
TTTCCAGTTGTACCTGCAGAAGGTATAACTATTCACAAAAGTCAGGGAGCTACGTATAGCAAAGTGGTGG
TTCACACTTGCTCTCGTATGCAGCGTGCTGCAATATACGTAGCTTGTAGTAGAGCAACAACTGCTTCCGGG
TTGTTTATAATAGGAGATTTTGTTCCTCCTAAGCCTCCGAAAAATGACGATAAGGTCCAAAATGAGATAG
AGAAATTGCGGACTGGAAAACTACTTCGAACACATTATGATCACTTAGTTAACAGTTCCGCCCTAGACATT
TTCTTTCATAATACTGAAAGCATTCACTGTCATATTGAAGATATTCGATCAGATAAGCTTATGCTTCAGTC
GTCTGTTCTATGTTTCGTTGAGGCCTCAACATATTCAAATGAAAATATTGAAATTCCAGGGTTTACTGTTGT
TGTCCGTCTTGATTGCGAGGTAATACCGAATGCACCGCGACCTAAGAGGGGAATCGTCATTTATATTAAA
AATGCAATTTTAAACAATATATCTAATTGCTTTAGCGGTAGGCAAATTTCTGGGTCATTCGTTTTTGAGCA
TGCAATATTTCGTTATAAGTCTGTATGTTTTTAGTTATTTACAAAAGTCCGTCATATCCGCTTGGTTTGTTT
AAAACAGAATTTGAGGTTTTATTTCAGCAATATTTATTTCTAAGGGAGCGTTGCATTGTTTTAGGAGACTT
TAATTTATGCTTGAGCAAATTTGATAGATGTAGGGCTCTTTACGATGAATTGCTCAGAAAGGGATTAAAG
GTGCCTATTAGATTTAAAACGCCCACTACCAATTGTGACTCTCACATTGATTGGGCTTTTTCAAATCTCTCC
AGTGAGGAAGTTAGCGCTAGGACACTCGAGACAGTGTATAGTTACCACAGTGGTATCTCTCTAACGGTTC
AGGATAGGGTAAATTAAGTTTCTATATATATGTCTATATAATTACAATATGTTTAATTTAAACTTAAGAAT
TTTTTATGAATATAAATAAATATATTACCTATATTTATATTTAAAAGTTGTTTTGTGGTCTTGTACTTGGGT

TCAGGGTATTTTCTAGTCGGGCACCCCGACTAGAGCACTCTACTT
>Ref_dvir_315
GACCAAAAAGACGTACCCCACCGGATAGGAGAGATTTTAGAAGAATCAGATGTGGACAGCTGGCGCTGG
GGTACCATCGGTCTCAAAACGTGGCAGACGATGGTACGAAATGGACCAAAACACCTGAAATTCATGGCTC
AACCAGGTGGTTCAATGGACCACCATTTTTGTATCTAGAAGAATCGCAGTGGCCACAATCGGAAATAGGC
CCTGCATTAAACATGTTGTACCATGCTGAAGAACAACCCAATCACACGTCGTCCTGGAGATGTATTCTGCC
GGATCTGCAGCGTTTCAGCAAGCTAGAAAAATTGAGAGCCGTACAGCTATGCGTACTGGATTTCCTACGG
AATATTACTAAGAAGGTCAGATTGGACGGAGGACTGGATCTGCAGAAGACGCTGCTCCTTAAAAGAAGC
AATGAAATGGATGTGATTTTTATCCGGATTTGTCAAGAAGAGGAGTTTTATAGTGAGATCACCTGCTTAAG
GTCGGGGCGCCGCTTAACCGATCGCAAAAGCTTGCTGTTTAAGTGCTCCCCTTATGTGGATGACTCGGGCA
TTCTACGTATTAAAGGACGGATAGACAATATAGAAGGAGTCGAAGTCTGCGTAAAACGCCCGATAATTCT
GCCAAGACGACATCAATTGACGTATCTGCTGGTTGAGTTTTATCACCGCAGATATCATCACCTACACAACG
AAATCGTCGTAAATGAACTGCGGCAGCGGTACTGGGTTTGTGGCTTACGAGCTTTGGTGAGAGAAGTTTC
CAATACCTGCCCAGCGTGCCGCATACGACGCGCCCGCCCAAAGCCGCCAGGGATGGGCGACCTGCCAATC
GAACGATTATCGCCCTATACTCCACCGTTCACATATACTGGAGTGGATTACTTCGGACCCTACGACATTGT
CGTTGGACGGCGTCGTGAGAAGCGCTGGGGCGTGCTATTTACATGCCTCACGGTGCGCGCCGTTCACCTA
GATATAGCTACGTCGCTCTCAACTGACTCATACTTATGCGTTCTGAAGTCGTTCGTGGCCAGACGCGGCTG
CCCCCGCCGCATGCTGTCGGACAATGGCACAAATTTCAGAGGCGCTAGCAGAGTATTAAAAGATGAAATT
GAACGTATATCGCCGGCTCTGATCGAGCGACAGTACCCAGAACTCGAGTTTACCTTTATCCCGCCAGGATC
ACCCCACATGGGAGGATCGTGGGAACGAATGGTGCGCTCTACAAAGTCAATACTGTCGGAAATTCTGCCG
CCCTCTGGGTTACGAGAGGAGGTGCTACGAGCAGCATTGGCTGACGTAGAAAGCGTGCTCAACTCAAGGC
CACTCACTTATGTTCCCTTGGAGACATCTGAGTCCGAAGCCCTGACTCCAAATCATTTTCTTATAGGTCATT
CAAGCGGATTGCGTGAGAGAGGATTACGGGAGCAAGGCGGAGCTAGCTTGGCACGAGGCTTCCGAGTCG
CCAGTCAATTGGCTGACCAATTTTGGAAGAGATGGCTGCGCGAGTATCTGCCTACACTCACCAGACGTAC
TAAGTGGTTTCAACCGCCACTCGAACCAATATCAGTCGACGACATCGTCATAATAGTGGACGACGGTGCC
AAGCGAAACTGTTGGACAAGAGGAGTGGTCGTAGACGTGCACCGTTCAAAGGATGGCCAGGTCCGAAGT
GCCGTGGTGCGAACCGCCGATGGAATTATTACTCGCCCCGCCGTCAAGCTAGCTAAGCTTGACATTAAAG
TTGGTAACACACTGCACGGACGTTCGTGAATTACGGGGTGGGGAATGTTACGGGGCGAAGTGAAAATAGC
TAGTTGAACTAAGTCCTACTGGGTCTCGCATTTTTTCCTGTAAGCAGCCCTTCGCTGCTTACACTGGAAGC
GCAACCGTTAGAATTAGTTTTTACATGCATAAGTCGATCGCACGCAGCTGCACAAGCTGCATTTTTGTCGA
GGTTCATTTTCGTTTTGTGCTTGCAGCTGAGCAGAAGTGAAAGAGAACAGAAGCGCGTGCGGAGCACGAA
TGTAAGTCTCATAATCATGTAAAATATATGTAGGTTGTTAAATAATGAAAGAATAATAAAGATTTCAGCG
TTCGAATGTAGTATATTATATCGTACCGCCTCGTACGAATCGAATTGAATCGAGTTGTTTCTTTTCGACGCT
AGAAGTCCCTGCCGAATACGCGCCCCGGCACGAGTCTCGGTGCCAACATTCTGAAATATTCTCTGCTGCA
GAGAGTGTCTGAGGGATTCTCTGCTACGGGGAGATTGAGATGGCTGACGGTGATGAACAAGATGCAAGTG
CGAGAAGGACGACAAGTAGTATTCAACCCGGTGGAACTGCAACGCTTGTAACGGCATGGTCAACGGGAC
AGGCTAACGGATCGCCTGTCCTGCTGGAGTACAAGATAAAGTCGCCGGTGTAGAAAAAGGTAGCGGGGC
AGCAATCATAGAAACTTATAGCGGCTGTCCGTCTACGTCGTCCGCTGCTACTGGTCCAAGGGAAAGTTCA
ACACCAATGCAGCAAAGAACTGAGAAAGTGGGAGCATTCTTCGACCCTTTGGGATACCAGAACGTGATCT
ACAAGCAGAAACAGGTTGCGGCTAATTGGAGTGTACACCCCAGTTTGCCATATCAAGCAAATACGGCGGT
GTTTAACCAGGCTACGGAGCCCAGTACTGCCGCGTACATGGGCCACACAGGTCTGGGAGCGCTTAATAGA
TCGATTGAGCACGTGCCACGGCAGCAATTCGACAATGTACATTGTAATAATGCGACGCCGTGGAATGGAC
CGCTGACATCTGCGCAAATCAGTGCGCGACACGTAATCAGCAAAGATTTACCAATATTTACTGGCAAACC
AGAGGAATGGTTGATCTTTATCAGCAATTATGAGCAATCCACAGAGAGATGTGGATTCAGCAATGAGGAA
AATCTCATCCGGCTTCAGAAGTGCCTCAAAGGGCAAGCCCTGGATGCGGTGCGTGGAAAGCTTATGATAC
CAGCTACAGTGCCGTATGCTATAGGCACATTGCGAATGCTGTATGGCCGCTCTGAAAATAGTACATGCTG
CGCTGCAGCAAAAGCTCCGAGAGGAACCAGCAATAAAGTCGGACAACTTGAACACCGTTATCCGACTGG
CCCTAGCTGTCCAAAACTACTGCGCAACGATAGCGGCCATTGGTATGCACGAATATCTTTACGATCCCGCG
CTGCTGAACGAGCTTGTTGCTAAGATGCCCAGCAATATGAAATTGGATTGGGGCCGGCATCGAATGTCGA
ATGAAGGTAGCAGTTTGGCCACCTTTGATAACTGGCTGTTCAATGTGGCAATGTGCGCCACTATGGTCACG
CCCTACGAGACACCACTGGCTGATAACGAAAAGAAGAGCTCCACGAAAACCACTAGAGAGCGAATTTTT
GTGCACAACACGGTAGATAGCCCTGAGGGTTCTCAGCAACAACGTTCACAACAGGAACAGGAGAGGGTT
TCATGTGCGAAGTGCGCCGGTAACCACCGTCTATCAGATTGCGACGACTTCAGATCAATGGGTACAAATC
AGCGCTGGGAACTTGTTAAGGAAAAGAGGCTCTGTTTTTGCTGCTTTCTACGTCATCGTGCACAACAGTGT

AAATCAAAGAAGAAGTGTCATGTAGATGGTTGCGAGTCAACGCACCATGTGCTGCTGCATACGCCTACCA
TGAGTGGTAGTAAAGTCGAGCACACGGGCCAAGGTAGCAACCACCGGATTAAGGCAAGTGAGTCTGCCG
TTATGTTTCACGGCAGCTCCGCAAGTAAGACACTGTTTCGATATGTTCCAATCACGCTCTATAGTAAATCA
AAAAGCGTGCGCGCATATGCATTAGTGGATGAAGGAGCTGAGTGTACGTTAATAGAAAGAGCGCTTGCA
GACGAGCTGGAATTAGATGGTCCTGCGACCCAGCTGTGTCTTAAATGGACAGGGGACATAACCAAGAATG
AACCTGAGTCGATGTCCGTGGCTGTGAGCATTTCAGCTCCAGAACATAACGCAATACAGTATGCCTTAAA
AAACGTGCGAACAATAGCGAACTTGGACTTACCAGAGCAAAGTATCGGGCCACAGTTGCTCAAGTCTCGG
AAGCACTTATCTACGCTTCCTATTCATAACAACGTCAAGGCTCAGCTTATCATTGGCTTAGACAATATCAA
AATAACTGCACCCCTGGAGGATACGAGAAGGTGGAAAACGACGACGTCATAGCAGCGCGAAGCAGACTA
GGATGGGCTGTATATGGCCGCCCCATGGTGGGAGAAAGCACCACGCCTCGGCTGCTACATATTTGCCAGT
GCAGTGCGGCGCGTGGGATGGATGAAGCTCTGAAGAACTATTTTTCAATTGAGTCACTAGGTGTGAGCGT
TTCCTCACATCCGCTGAGATCCAAAGAGGACGAACGCTCGATGCAGATTATGGAGGCAACAACAACCTAT
CTGGAAGACGAAAAGCGCTGGCAGACCGGCCTGTTATGGAGGTTCGATCGCATGCACCTGCCAGACTCTT
ATCAGATGGCGGTTAAGCGCCTGAAATGTCTAGAGGCAAAGATGTTCAAAGACCCACCACTGAAAGAGTT
CATGATGAACACGATGCACGACTACAAACAGAAAGATATCCGCCGGTTGAAGGATAGTGAGTTAGAGCT
AACACGACATCGTGGTTTCTTCCTATCTTTACAGTCACCAATCCGAACAAGATAAAGACGCGCTTGGTTTG
GGATGCAGCTGCCAAAGTTAGTGGCATGTCGCTGAACGATTGTTTGCTGAAAGGTCCAGATACACTAGCA
TCTTTGATGGGCGTGCTAATACGCTTCCGAGAGCGTCCTATCGCCGTATCCGGCGACATACGGAAATGTT
TCATCAGGTCAAGGTGCGCCGAGAAGACCAACCGGCACAGAAGTTTCTCTGGCGTGATGGAGATTCGTCA
CGCTTCCCCGAAACATACGTTATGCAAGTCATGACGTTTGGAGCATCCTGCTCGCCAGCTCTAGCAAATTA
CGTCAAGAATCGCAATGCCGAGCGGTTTGTAGCGGAGCATCCGGATGCCGTAAAGGCGATTTGCGAAAAC
ACATTTGTCGACGACTGGCTGCAGTCGGTGGATACTGGAGCTGAAATGATACAGTTAGCTGAGACTGTAA
AAAGGATTCATGCTAGTGGCGGCTTCGAGATGCGCCGCTGGACATCAAATTCGAAGCAAGTTATACAGGC
GCTGGAAGACGACTGTGAGGAGTTGGACAAGCGTATCAGCGCTCAGGATGAAGCGCAAGAGAAGGTCTT
AGGCCTGTGGTGGCTACCTGGACAAGATCTGTTGACGTTTGTGGTGACGCCGAACTTGCTTGCGAAGGCA
TCTAAGGAGCGCCCAACTAAAAGACGTGTTCTGAGTGTGATCATGTCTATTTTCGACCCGCTCGGGCTGCT
AGGATTCTTTAATATACGCGCTAAGATCATCCTTCAGAACATATGGCGATCCAATATCGGATGGGATGAC
GACCTTACCAACGAAGACGAAGCCGATTGGCAACACTGGCTTGATCTAGTTTCAAATTTGAATGCCGTCC
GCATTCCACGGTGCATGAAGTGGGTGAGCCGAACCCAGGCTGTGCAGATGCATACATTCGTTGACGCCAG
TATGAATGCTTACGCCGCAGTTGTATTTTTGCGGGCTGAACTTGATGGCCAAGTACATTGCAGTTTGGTCG
CCTCGAAAACGAGAGTAGCACCCCTAAAGCCCTGTCCATACCTCGAATGGAACTGATGGCCGCGGTCTTA
GGTCTGAGACTGGCCAAATGTATCCAAAAGGAAAATGTCGGTACGCATACATAGACGAACATTCTGGACA
GATTCAAAGGGATGTGCTCTACTGGATCCGTTCCGATGCTCGAAAGTTCCAGCAATTCGTAGCGCTTCGGA
TAGGAGAGATTTTAGAAGAATCAGATGTGGACAGCTGGCGATTGGGTACCATCGGGCTCAAAACGTGGCA
GACGATGGTACGAAATGGACTAAAACGCCTGAAATTCATGGCTCAACCAGGTGGTTCAATGGACCACCAT
TTTTGTATCTAGAAGAATCGCAGTGGCCACAATCGGAAATAGGCCCTGCATTAAACATGTTGTACCATGCT
GAAGAACAACCCAATCACACGTCGTCCTGGAGATGTATTCTGCCGGATCTGCAGCGTTTCAGCAAGCTAG
AAAAATTGAGAGCCGTACAGCTATGCGTACTGGATTTCCTACGGAATATTACTAAGAAGGTCAGATTGGA
CGGAGGACTGGATCTGCAGAAGACGCTGCTCCTTAAAAGAAGCAATGAAATGGATGTGATTTTTATCCGG
ATTTGTCAAGAAGAGGAGTTTTATAGTGAGATCACCTGCTTAAGGTCGGGGCGCCGCTTAACCGAATCGC
AAAAGCTTGCTGTTTAAGTGGCTCCTCCCCTTATGTGGATGACTCGGGCTATTCTACGTATTAAAGGACGG
ATAGACAATATAGAAGGAGTCGAAGTCTGCGTAAAACCCCGATAATTTTGCCAAGACGACATCAATT
>9_rnd-1_family-123__LTR___Pao
TGTCTGGTTACGCAGCCAAAACAACTGGAGCGACCTCGCAACTGATAAGAGCAGCATCTTAATTTTATCTT
ATAATACTCGCTCTGCAAAGCAAGTGCCTAAATTTCGCTCTTAAGAATTCATTTACATAGAGCTGCCGCTC
TTCGCTGTAACGATCGTTTTATTGCCCTAGCTTTACTTATGCGAAGGCCTCGATGCTGCGCATCGAGTTAGT
TAAGTTCGTTCGCTTCATGCATTTGGTACGCATCGGCCACTTGCATTACTCAAAATTATAACTAATAATTGT
CTCTTCGCTAAAATTGGCACGTACTAATTCGGGTTCGCTTGCCTATATACAAACTATTGTTCTTAAATAATA
AGCACAGACCAATTCGGGTCTGCCTGCTTCTCGTAACTTTAAGAAATATAAAACAATTTTGAACACAAAA
AATAAATAGTTAATTATTTCTATTTGTGAAAGCTATTGAAAAAGCTCAATAGCGCAACATTTGGTGACCCC
GACGTGAATTTTATTTTTGTTGTTGTAAGATTATTTGCTTTACATTAAAGCAATAATCTTAAAAGGCACCAC
CTGGCATTTCGTTTTGTGCACTTTATATACATACATACTTATCGCCGTCGCTTTGCCTACTCTCTCGGTCGC
TTAAAGTCTCTGCTCATTCGTGCGGTACTTATGTAAAAGCTATTGCCGTTGCTTCATTACATAGCTTGACAC
TTGCGTTCGCCGAAGTTCTTATTGTACAACTATTGTTTTTGAACGGTTTGCATGTACTAGCGATCACCGTTG

TTAGTTACTCAGCTAGTTTCGCACGCGCTTTCTATCGCGCTTCTAATGGCAACCGAAAATAATGCCTCTAG
TGCAGTGCCCTCTGCAAACAGGGTGACCTTTCTGAAGCGCAAAGCCATTTCAATTTATGACAGTTTGCAAA
CACTTCGAAATGCTCTAACTAATTCGCCACTTAGTGCAATTGATGAGTGCGGTCTTAGTGTTAGGCTGAAG
CAAATCGAGCGGCTTCAGACCTCATTTGATATTACTCACACTAGCCTCGAAGAACTAGACTTTGATGAAAT
CGGTAACCAATTGCGTGAGGACTTCGACGAGTTAGCAGTTTCTATGGAAATTTCGGTTCGGAGTGAAATT
GCGAAACGTGTCTCAATATTGCCCTGCTCAACATTTCGCGACCAATCTGTGCTGCCCTCGCAGACAGTTAG
AGCTAAGGCACCGCCAACACTACCCTCAATAAAGTTGCCTACGTTCAGTGGGGTGGTTACACAGAATGGG
CCGACTTTTATTCTATGTTCACAACCGTCATAGAAAATCATCCTGATCTAGCAGATATCGAAAAGCTGCAG
CATCTTCGATCGTGCCTGAAGGATACAGCGCTGGACGCGATTCGATCATTGGAAATAACAAACGATAATT
ATGCCATAGCACTTAATTTATTGGAGAAAAGGTTTAATAATAGACGTTTGGTTTTTCAGGCACACATACTT
GAGATATTGGGGCTCAAGAGGGTGGCAGGTGGATCGGCCGTGTCGCTAAGGGAGTTATCCGACCAATTCA
ACGCACATTTTCGCGCGTTAAAGAGCATGGGCAACTCCGAACAAATCGCTGGATGCATCATTGTGCAGAT
TATTTTACAAAAGCTGGACCCAATATCACAAGCCAAATGGGAAGAAAGGCTAGAGGATTCGTCGTTCGTT
AATTTAATACCATCGTGGGAGCATATGGCTACGTTTCTGGAGCAGAGATGCCGAACAATGGAAACCGTAA
ATTTTGCTATGGCAAGCTATGCCCCCTAGCCATCAGGGTGGGAAAAACTAGAGCAATTAACACCAGTAGA
TCGTCATTTATCGCCACTAACCACGACGCCCCCTGTCTGCGTCTTTTGTGACAGCCCTGGTCATAGTATATA
TAATTGTTCTCGATTTGGAAATTTGACCCCTGCATTACGGCTTAGGGAGGCAAAACGAGTTGCATTGTGCT
TGAATTGCCTAAAGAAAGGTCATCAATTGCGATCATGCAGCTCTGGATGCTGTCGGACCTGCGGGGCGAA
ACATCATTCTCTGCTGCATTTAAACAATTCCGTATCTCCGTCTGGTCAGCTGACTGAGCCTGCAAATTCATC
GCCGATAGCCCATGCCTCTTTCGCTAAGTCATCGCCATCTACCTCCACATCTCTTGTTGCCCAGTATTTCAG
TAGTGATGTTGTCGTCTTGGCTACGGCTATGATCTTTGTAAGAAATCGCTCCGGCACTTTGATACCTTGTCG
TGCCCTTTTGGATTCTGGTTCTCAGCTTCACCTTATAACGTCGCGCCTTGCTCAACAGCTTCAGCTAAAGAA
GCACAAGTCCTCAGCCATTGTCTCTGGTCTAGGAGATTCTCGTTTTGAAACAGAAGGAAACACGGTCACT
ATCACAATACAGTCTCAAGTCTCTGAATACTCAACATGCCTTACAGCTCTGGTAGCGCCTAACATAACTGA
TAGTCAACCGAGTTTTACTATCAACACGACGAATTGGAACATTCCCGCTAACATAAAACTTGCAGATCCGT
CTTTCTTTAAACCTCAACGCGTGGACTTGCTACTTGGAGCTAGCCTGTTTTACGACCTTCTCAGCGTCGGCC
AGATAAGGCTTCTCCCAGGACTACCGTGTCTTCAAAAACACGGCTTGGTTGGGTAGTTTCTGGAGGCGGA
CCCCGCTTGAGTGGTTCTATGTTAATGGCAAAGCATACTGCGGGCAAACTTGAGTCAAGTTCTGAAGAAC
GTGCTGATGAATTGGTTCGAAGATTTTGGGAGATAGAATCCTGCTTGGAAAGTACACCTACCACTTCAAA
GGAAGAAAACATCTGTGAAGAACATTTCGTAAAGACTCATATCAGATTGCCTAGTGGCCAATATTCAGTT
CGTTTACCCTTCAAGCGAAGTTTGGAATGTCTGGGGACGTCATACCAACAAGCCTTTCGTCGGTTCCTGAC
CCTGGAACGCAAACTACAAAATCGTCCAGATCTGAAGATTCAATATTCAAACTTTCTAAAGGAATATTTG
GATCTTGGTCATATGTCGCCTGTTGCTCCAGAGTCTATAGGACTTTGTCAATACTTTCTTCCTCATCACTGT
GTCTTTAAAGAAGACAGTTCAACGACAAAATTGCGAGTCGTTTTCGACGGCTCAGCAGCTACCTCGTCAG
GTTATTCATTAAATGAACTATTGATGGCTGGACCTGTTATTCAAAGCTCGCTGTTTCATATCCTTCTTCGCT
TTCGTAATTTCCCGATCGCCCTAACAGGAGATATTGGAAAAATGTATCGCTGCGTACGCGTTTCAGCACCG
GATGATTTTCTCCAATGCATTCTATGGCGAGACTCGCCAGAACAGGATATAAAGATTTTTAAGCTGGATAC
AGTTACATACGGAACAAAGCCAGCTTCATTCCTCTCTATCCGTGCTATGCATCAGCTGTCTATGGATGAGG
AAACTAATTTTCCAATTGGATCTCGGATTTTGCGGCGTGACTTCTACGTTGATGACCTCATTTCAGGGGGT
GATTCAATTCAAGAAGCAAAGGAACTTCTTAATCAGACGGCTGGAATCTTGGCAAGTGGGGGCTTCAAGT
TGCGAAAGTGGTGCTCAAACAATTCTGCTGTACTGAAAGGGATACCCGTAGAGGACAGAGAATCTTCCCT
AAAGTTTGATGATGGTAGCGACATCACCAAAACTCTTGGACTCGCTTGGGACTCAGCGTCGGATCGATTG
TTGTTTTCGTTTTCGTCCATTCAGCAGAGATCTAAACCATCAAAAAGGTCAATACTATCGACCATAGCTCG
CTTGTATGACCCGCTTGGACTCATAGGGCCGATAATTGCCAAGGCTAAAATCTTTATGCAGCAGATCTGGA
AGGAGAAGCTTGAGTGGGACGAGAGCTTGCCATCATCACTGGCAACAGCTTGGCATGATCTTTGCTGCAG
TTTTGAGAAGGCACAGTTTCTTAAATTTCCTCGTCTCGTGTCAATACCTGCAGCAGAGTTAGAAGTTCACG
GTTTCTGCGACGCCAGCATCGAAGCATATGGGGCCTGTGTCTACGTCCTTTCTCGACGAACATCAGCCAGT
TGTCAGCTGTTGTGTTCCAAATCGCGTGTAGCTCCTCTGAAAACAATCACAGTTCCAAAGCTAGAGCTCTG
TGGTGCCGTCTTATTATCTAATCTCATGCGTGAGATATCTAAAATGAATCTGTTCGATGGAAAATTCTACT
GTTGGTCGGATTCTTCTGTATGCCTTTCCTGGATAAGGACCGAGCCCTGCAAGCTCAATGTGTTTGTGGCT
AATCGCGTCACAGCAGTTCAAGAGCTAACTAATGGCATGGAATGGCGTTACGTGCCTACTTCACTCAACC
CGGCAGATATACTCTCGAGAGGATCTACGCCCAGTGAATTAGATCAATCGTCACTGTGGTTCAACGGCCC
AGAATTTCTAGTTAAAAACAAATTGGACTGGCCTGCTTCTTGCTTGTCAGAAAGGACAGAACTCGAACTT
AAAAAGGGTCCTTATTGCGTCATCTCCCTTCGTTGATATAACTGTCACTTGTAAATACGTCAACTCATTTG

ATACAATGCAGCGTATATTCGGATATATTTATAAATTCATACACAGAAGCAGGCATCCTGGACTAACAGT
GTCTGATATACAACATGGACCCAACTCTTACTAAGGCTCGTCCAGAGAGCACACCTGTGGAAGAGATC
AAATCATTGCAATCGAGAAGAGTTGTTAAACCGTCCAGTTGTATCGCATCACTTAACCCTTTTTGGATGAT
TTCGGAATTCTTAGAGTTGATGGGCGCTTGAAAAACTCTTCGCTGGATTTCGACGCTCGCCATCCAATAAT
TCTCCCGTGTCACCACCCCATCACAAACTCGGTCATCACACGGTTTCACAATCGGGACTTACACGCTGGTC
CACGGGCACTTCTTGCTCATATTCGGTTGCAGTATTGGCCGATTGGTGGAAGGAAGACCGTATCCAAGGT
GGTTGGAAAATGTGTGAGATGCTTTCGTGCCAAGCCTCGATTAATAGAGCATATAATGGGTGATCTGCCC
AAAGAACGTTTGACTGCTTGTCGAGCCTTTGAAGTCTCTGGTGTTGACTATTGTGGACCATTTTTCTATAA
GCCAGACGTACGAAATAAAGCTCCAGTCAAATGTTACATGGCTGTCTTCGTATGCTTCGCGACTAAAGCG
GTTCACCTGGAACTGGTCAAGGATCTGTCGACAGCTTCTTTCTTGAATGCGCTTAAGAGGTTTATATCGAC
TCGAGGCAGGCCATCTCAGATATGGTCGGATAACGCTACCAACTTTGTCGGCGCCAAAAACGAGCTTCTG
GATCTCAAGCGTCTATTTACAAGTCGTAATCATATTGAGTCAGTGCATAATTTTTGCATGGCTGATTCCATT
GATTGGAAATTTATACCTCCTCGTTCACCGCATTTTGGCGGCCTTTGGGAAGCAGCAGTGAAGACAGCGA
AACATCATTTTTATCGTGCTGTTGGATCTTGTGTTCTCAACTTCGAAGAGTTTCGCACCCTAGTCTGCCACA
TAGCATCGATAATTAATTCCAGGCCATTGTTTCCTCTTTCAGAAGATCCAGCAGATCTAGATGTGCTCACT
CCAGCTCATTTCTTGATTGGTGGACCCAGCTCATCGTTCTTTGAACCTGATGTGACGCACCTTCAATTCAAT
CGTCTGGATGGTTGGCAACGCGTAACGTACCTCCAACAACTGTTTTGGTCTCGCTGGCGTGAAGAATACTT
AACACTGCTTCAGCAGCGCTCCAAGTGGCGTACTCCGAAACCAAGCCTATGCGTCAACGACATAGTCCTC
GTAAAGGATGAAAATCTGCCGCCTCTAAGGTGGCCACTGGCGAGAATCACAGAGCTAATCGTGGGAAAG
GATGGAGTCGCTCGAGTAGCCATTTTGCGCACCGCAACAGGAGAAACGAGAAGAGCTGCAAACAAGCTA
TGCCTATTGCCCATCAGGGAATCTGTTGAAAGCCCATGCCTTCCAACGGGGGGAATGTCTGGTTACGCAG
CCAAAACAACTGGAGCGACCTCGCAACTGATAAGAGCAGCATCTTAATTTTATCTTATAATACTCGCTCTG
CAAAGCAAGTGCCTAAATTTCGCTCTTAAGAATTCATTTACATAGAGCTGCCGCTCTTCGCTGTAACGATC
GTTTTATTGCCCTAGCTTTACTTATGCGAAGGCCTCGATGCTGCGCATCGAGTTAGTTAAGTTCGTTCGCTT
CATGCATTTGGTACGCATCGGCCACTTGCATTACTCAAAATTATAACTAATAATTGTCTCTTCGCTAAAAT
TGGCACGTACTAATTCGGGTTCGCTTGCCTATATACAAACTATTGTTCTTAAATAATAAGCACAGACCAAT
TCGGGTCTGCCTGCTTCTCGTAACTTTAAGAAATATAAAACAATTTTGAACACAAAAAAATAAATAGTTA
ATTATTTCTATTTGTGAAAGCTATTGAAAAAGCTCAATAGCGCAACA
>9_rnd-1_family-214__LTR___Pao
TGTCTGGTTACGCAGCCAAAACAACTGGAGCGACCTCGCAACTGATAAGAGCAGCATCTTAATTTTATCTT
ATAATACTCGCTCTGCAAAGCAAGTGCCTAAATTTCGCTCTTAAGAATTCATTTACATAGAGCTGCCGCTC
TTCGCTGTAACGATCGTTTTATTGCCCTAGCTTTACTTATGCGAAGGCCTCGATGCTGCGCATCGAGTTAGT
TAAGTTCGTTCGCTTCATGCATTTGGTACGCATCGGCCACTTGCATTACTCAAAATTATAACTAATAATTGT
CTCTTCGCTAAAATTGGCACGTNCTAATTCGGGTTCGCTTGCCTATATACAAACTATTGTTCTTAAATAATA
AGCACAGACCAATTCGGGTCTGCCTGCTTCTCGTAACTTTAAGAAATATAAAACAATTTTGAACACAAAA
AATAAATAGTTAATTATTTCTATTTGTGAAAGCTATTGAAAAAGCTCAATAGCGCAACATTTGGTGACCCC
GACGTGATTTTTATTTTTGTTGTTGTAAGATTATTTGCTTTACATTAAAGCAATAATCTTAAAAGGCACCAC
CTGGCATTTCGTTTTGTGCACTTTATATACATACATACTTATCGCCGTCGCTTTGCCTACTCTCTCGGTCGC
TTAAAGTCTCTGCTCATTCGTGCGGTACTTATGTAAAAGCTATTGCCGTTGCTTCATTACATAGCTTGACAC
TTGCGTTCGCCGAAGTTCTTATTGTACAACTATTGTTTTTGAACGGTTTGCATGTACTAGCGATCACCGTTG
TTAGTTACTCAGCTAGTTTCGCACGCGCTTTCTATCGCGCTTCTAATGGCAACCGAAAATAATGCCTCTAG
TGCAGTGCCCTCTGCAAACAGGGTGACCTTTCTGAAGCGCAAAGCCATTTCAATTTATGACAGTTTGCAAA
CACTTCGAAATGCTCTAACTAATTCGCCACTTAGTGCAATTGATGAGTGCGGTCTTAGTGTTAGGCTGAAG
CAAATCGAGCGGCTTCAGACCTCATTTGATATTACTCACACTAGCCTCGAAGAACTAGACTTTGATGAAAT
CGGTAACCAATTGCGTGAGGACTTCGACGAGTTAGCAGTTTCTATGGAAATTTCGGTTCGGAGTGAAATT
GCGAAACGTGTCTCAATATTGCCCTGCTCAACATTTCGCGACCAATCTGTGCTGCCCTCGCAGACAGTTAG
AGCTAAGGCACCGCCAACACTACCCTCAATAAAGAGTTGCCTACGTTCAGTGGTGGTTACACAGAATGGG
CCGACTTTTATTCTATGTTCACAACCGTCATAGAAAATCATCCTGATCTAGCAGATATCGAAAAGCTGCAG
CATCTTCGATCGTGCCTGAAGGATACAGCGCTGGACGCGATTCGATCATTGGAAATAACAAACGATAATT
ATGCCATAGCACTTAATTTATTGGAGAAAAAGGTTTAATAATAGACGTTTGGTTTTTCAGGCACACATACT
TGAGATATTGGGGCTCAAGAGGGTGGCAGGTGGATCGGCCGTGTCGCTAAGGGAGTTATCCGACCAATTC
AACGCACATTTTCGCGCGTTAAAGAGCATGGGCAACTCCGAACAAATCGCTGGATGCATCATTGTGCAGA
TTATTTTACAAAAGCTGGACCCAATATCACAAGCCAAATGGGAAGAAAGGCTAGAGGATTCGTCGTTCGT
TAATTTAATACCATCGTGGGAGCATATGGCTACGTTTCTGGAGCAGAGATGCCGAACAATGGAAACCGTA

AATTTTGCTATGGCAAGCTATGCCCCTAGCCATCAGGTGGAAAAACTAGAGCAATTAACACCAGTAGAT
CGTCATTTATCGCCACTAACCACGACGCCCCTGTCTGCGTCTTTTGTGACAGCCCTGGTCATAGTATATAT
AATTGTTCTCGATTTGGAAATTTGACCCCTGCATTACGGCTTAGGGAGGCAAAACGAGTTGCATTGTGCTT
GAATTGCCTAAAGAAAGGTCATCAATTGCGATCATGCAGCTCTGGATGCTGTCGGACCTGCGGGGCGAAA
CATCATTCTCTGCTGCATTTAAACAATTCCGTATCTCCGTCTGGTCAGCTGACTGAGCCTGCAAATTCATCG
CCGATAGCCCATGCCTCTTTCGCTAAGTCATCGCCATCTACCTCCACATCTCTTGTTGCCCAGTATTTCAGT
AGTGATGTTGTCGTCTTGGCTACGGCTATGATCTTTGTAAGAAATCGCTCCGGCACTTTGATACCTTGTCGT
GCCCTTTTGGATTCTGGTTCTCAGCTTCACCTTATAACGTCGCGCCTTGCTCAACAGCTTCAGCTAAAGAA
GCACAAGTCCTCAGCCATTGTCTCTGGTCTAGGAGATTCTCGTTTTGAAACAGAAGGAAACACGGTCACT
ATCACAATACAGTCTCAAGTCTCTGAATACTCAACATGCCTTACAGCTCTGGTAGCGCCTAACATAACTGA
TAGTCAACCGAGTTTTACTATCAACACGACGAATTGGAACATTCCCGNTAACATAAAACTTGCAGATCCG
TCTTTCTTTAAACCTCAACGCGTGGACTTGCTACTTGGAGCTAGCCTGTTTTACGACCTTCTCAGCGTCGGC
CAGATAAGGCTTCTCCCAGGACTACCGTGTCTTCAAAAAAACGGCTTGGTTGGGTAGTTTCTGGAGGCGG
ACCCCGCTTGAGTGGTTCTATGTTAATGGCAAAGCATACTGCGGGCAAACTTGAGTCAAGTTCTGAAGAA
CGTGCTGATGAATTGGTTCGAAGATTTTGGGAGATAGAATCCTGCTTGGAAAGTACACCTACCACTTCAA
AGGAAGAAAACATCTGTGAAGAACATTTCGTAAAGACTCATATCAGATTGCCTAGTGGCCAATATTCAGT
TCGTTTACCCTTCAAGCGAAGTTTGGAATGTCTGGGGACGTCATACCAACAAGCCTTTCGTCGGTTCCTGA
CCCTGGAACGCAAACTACAAAATCGTCCAGATCTGAAGATTCAATATTCAAACTTTCTAAAGGAATATTT
GGATCTTGGTCATATGTCGCCTGTTGCTCCAGAGTCTATAGGACTTTGTCAATACTTTCTTCCTCATCACTG
TGTCTTTAAAGAAGACAGTTCAACGACAAAATTGCGAGTCGTTTTCGACGGCTCAGCAGCTACCTCGTCA
GGTTATTCATTAAATGAACTATTGATGGCTGGACCTGTTATTCAAAGCTCGCTGTTTCATATCCTTCTTCGC
TTTCGTAATTTCCCGATCGCCCTAACAGGAGATATTGGAAAAATGTATCGCTGCGTACGCGTTTCAGCACC
GGATGATTTTCTCCAATGCATTCTATGGCGAGACTCGCCAGAACAGGATATAAAGATTTTTAAGCTGGATA
CAGTTACATACGGAACAAAGCCAGCTTCATTCCTCTCTATCCGTGCTATGCATCAGCTGTCTATGGATGAG
GAAACTAATTTTCCAATTGGATCTCGGATTTTGCGGCGTGACTTCTACGTTGATGACCTCATTTCAGGGGG
TGATTCAATTCAAGAAGCAAAGGAACTTCTTAATCAGACGGCTGGAATCTTGGCAAGTGGGGGCTTCAAG
TTGCGAAAGTGGTGCTCAAACAATTCTGCTGTACTGAAAGGGATACCCGTAGAGGACAGAGAATCTTCCC
TAAAGTTTGATGATGGTAGCGACATCACCAAAACTCTTGGACTCGCTTGGGACTCAGCGTCGGATCGATT
GTTGTTTTCGTTTTCGTCCATTCAGCAGAGATCTAAACCATCNAAAAGGTCAATACTATCGACCATAGCTC
GCTTGTATGACCCGCTTGGACTCATAGGGCCGGTAATTGCCAAGGCTAAAATCTTTATGCAGCAGATCTGG
AAGGAGAAGCTTGAGTGGGACGAGAGCTTGCCATCATCACTGGCAACAGCTTGGCATGATCTTTGCTGCA
GTTTTGAGAAGGCACAGTTTCTTAAATTTCCTCGTCTCGTGTCAATACCTGCAGCAGAGTTAGAAGTTCAC
GGTTTCTGCGACGCCAGCATCGAAGCATATGGGGCCTGTGTCTACGTCCTTTCTCGACGAACATCAGCCAG
TTGTCAGCTGTTGTGTTCCAAATCGCGTGTAGCTCCTCTGAAAACAATCACAGTTCCAAAGCTAGAGCTCT
GTGGTGCCGTCTTATTATCTAATCTCATGCGTGAGATATCTAAAATGAATCTGTTCGATGGAAAATTCTAC
TGTTGGTCGGATTCTTCTGTATGCCTTTCCTGGATAAGGACCGAGCCCTGCAAGCTCAATGTGTTTGTGGC
TAATCGCGTCACAGCAGTTCAAGAGCTAACTAATGGCATGGAATGGCGTTACGTGCCTACTTCACTCAAC
CCGGCAGATATACTCTCGAGAGGATCTACGCCCAGTGAATTAGATCAATCGTCACTGTGGTTCAACGGCC
CAGAATTTCTAGTTAAAAACAAATTGGACTGGCCTGCTTCTTGCTTGTCAGAAAGGACAGAACTCGAACTT
AAAAAGGGTCCTTATTGCGTCATCTCCCTTCGTTGATATAACTGTCACTTGTAAATACGTCAACTCATTTG
ATACAATGCAGCGTATATTCGGATATATTTATAAATTCATACACAGAAGCAGGCATCCTGGACTAACAGT
GTCTGATATACAACATGGACCCAACTCTTACTAAGGCTCGTCCAGAGAGCACACCTGTGGGAAGAGATC
AAATCATTGCAATCGAGAAGAGTTGTTAAACCGTCCAGTTGTATCGCATCACTTAACCCTTTTTGGATGAT
TTCGGAATTCTTAGAGTTGATGGGCGCTTGAAAAACTCTTCGCTGGATTTCGACGCTCGCCATCCAATAAT
TCTCCCGTGTCACCACCCCATCACAAACTCGGTCATCACACGGTTTCACAATCGGGACTTACACGCTGGTC
CACGGGCACTTCTTGCTCATATTCGGTTGCAGTATTGGCCGATTGGTGGAAGGAAGACCGTATCCAAGGT
GGTTGGAAAATGTGTGAGATGCTTTCGTGCCAAGCCTCGATTAATAGAGCATATAATGGGTGATCTGCCC
AAAGAACGTTTGACTGCTTGTCGAGCCTTTGAAGTCTCTGGTGTTGACTATTGTGGACCATTTTTCTATAA
GCCAGACGTACGAAATAAAGCTCCAGTCAAATGTTACATGGCTGTCTTCGTATGCTTCGCGACTAAAGCG
GTTCACCTGGAACTGGTCAAGGATCTGTCGACAGCTTCTTTCTTGAATGCGCTTAAGAGGTTTATATCGAC
TCGAGGCAGGCCATCTCAGATATGGTCGGATAACGCTACCAACTTTGTCGGCGCCAAAAACGAGCTTCTG
GATCTCAAGCGTCTATTTACAAGTCGTAATCATATTGAGTCAGTGCATAATTTTTGCATGGCTGATTCCATT
GATTGGAAATTTATACCTCCTCGTTCACCGCATTTTGGCGGCCTTTGGGAAGCAGCAGTGAAGACAGCGA
AACATCATTTTTATCGTGCTGTTGGATCTTGTGTTCTCAACTTCGAAGAGTTTCGCACCCTAGTCTGCCACA

TAGCATCGATAATTAATTCCAGGCCATTGTTTCCTCTTTCAGAAGATCCAGCAGATCTAGATGTGCTCACT
CCAGCTCATTTCTTGATTGGTGGACCCAGCTCATCGTTCTTTGAACCTGATGTGACGCACCTTCAATTCAAT
CGTCTGGATGGTTGGCAACGCGTAACGTACCTCCAACAACTGTTTTGGTCTCGCTGGCGTGAAGAATACTT
AACACTGCTTCAGCAGCGCTCCAAGTGGCGTACTCCGAAACCAAGCCTATGCGTCAACGACATAGTCCTC
GTAAAGGATGAAAATCTGCCGCCTCTAAGGTGGCCACTGGCGAGAATCACAGAGCTAATCGTGGGAAAG
GATGGAGTCGCTCGAGTAGCCATTTTGCGCACCGCAACAGGAGAAACGAGAAGAGCTGCAAACAAGCTA
TGCCTATTGCCCATCAGGGAATCTGTTGAAAGCCCATGCCTTCCAACGGGGGGAGAATGTCTGGTTACGC
AGCCAAAACAACTGGAGCGACCTCGCAACTGATAAGAGCAGCATCTTAATTTTATCTTATAATACTCGCTC
TGCAAAGCAAGTGCCTAAATTTCGCTCTTAAGAATTCATTTACATAGAGCTGCCGCTCTTCGCTGTAACGA
TCGTTTTATTGCCCTAGCTTTACTTATGCGAAGGCCTCGATGCTGCGCATCGAGTTAGTTAAGTTCGTTCGC
TTCATGCATTTGGTACGCATCGGCCACTTGCATTACTCAAAATTATAACTAATAATTGTCTCTTCGCTAAA
ATTGGCACGTNCTAATTCGGGTTCGCTTGCCTATATACAAACTATTGTTCTTAAATAATAAGCACAGACCA
ATTCGGGTCTGCCTGCTTCTCGTAACTTTAAGAAATATAAAACAATTTTGAACACAAAAAATAAATAGTTA
ATTATTTCTATTTGTGAAAGCTATTGAAAAAGCTCAATAGCGCAACA
>9_rnd-1_family-183__LTR___Pao
TGTCTGGTTACGCAGCCAAAACAACTGGAGCGACCTCGCAACTGATAAGAGCAGCATCTTAATTTTATCTT
ATAATACTCGCTCTGCAAAGCAAGTGCCTAAATTTCGCTCTTAAGAATTCATTTACATAGAGCTGCCGCTC
TTCGCTGTAACGATCGTTTTATTGCCCTAGCTTTACTTATGCGAAGGCCTCGATGCTGCGCATCGAGTTAGT
TAAGTTCGTTCGCTTCATGCATTTGGTACGCATCGGCCACTTGCATTACTCAAAATTATAACTAATAATTGT
CTCTTCGCTAAAATTGGCACGTACTAATTCGGGTTCGCTTGCCTATATACAAACTATTGTTCTTAAATAATA
AGCACAGACCAATTCGGGTCTGCCTGCTTCTCGTAACTTTAAGAAATATAAAACAATTTTGAACACAAAA
ATAAATAGTTAATTATTTCTATTTGTGAAAGCTATTGAAAAAGCTCAATAGCGCAACATTTGGTGACCCCG
ACGTGATTTTTATTTTTGTTGTTGTAAGATTATTTGCTTTACATTAAAGCAATAATCTTAAAAGGCACCACC
TGGCATTTCGTTTTGTGCACTTTATATACATACATACTTATCGCCGTCGCTTTGCCTACTCTCTCGGTCGCTT
AAAGTCTCTGCTCATTCGTGCGGTACTTATGTAAAAGCTATTGCCGTTGCTTCATTACATAGCTTGACACTT
GCGTTCGCCGAAGTTCTTATTGTACAACTATTGTTTTTGAACGGTTTGCATGTACTAGCGATCACCGTTGTT
AGTTACTCAGCTAGTTTCGCACGCGCTTTCTATCGCGCTTCTAATGGCAACCGAAAATAATGCCTCTAGTG
CAGTGCCCTCTGCAAACAGGGTGACCTTTCTGAAGCGCAAAGCCATTTCAATTTATGACAGTTTGCAAACA
CTTCGAAATGCTCTAACTAATTCGCCACTTAGTGCAATTGATGAGTGCGGTCTTAGTGTTAGGCTGAAGCA
AATCGAGCGGCTTCAGACCTCATTTGATATTACTCACACTAGCCTCGAAGAACTAGACTTTGATGAAATCG
GTAACCAATTGCGTGAGGACTTCGACGAGTTAGCAGTTTCTATGGAAATTTCGGTTCGGAGTGAAATTGC
GAAACGTGTCTCAATATTGCCCTGCTCAACATTTCGCGACCAATCTGTGCTGCCCTCGCAGACAGTTAGAG
CTAAGGCACCGCCAACACTACCCTCAATAAAGTTGCCTACGTTCAGTGGTGGTTACACAGAATGGGCCGA
CTTTTATTCTATGTTCACAACCGTCATAGAAAATCATCCTGATCTAGCAGATATCGAAAAGCTGCAGCATC
TTCGATCGTGCCTGAAGGATACAGCGCTGGACGCGATTCGATCATTGGAAATAACAAACGATAATTATGC
CATAGCACTTAATTTATTGGAGAAAAGGTTTAATAATAGACGTTTGGTTTTTCAGGCACACATACTTGAGA
TATTGGGGCTCAAGAGGGTGGCAGGTGGATCGGCCGTGTCGCTAAGGGAGTTATCCGACCAATTCAACGC
ACATTTTCGCGCGTTAAAGAGCATGGGCAACTCCGAACAAATCGCTGGATGCATCATTGTGCAGATTATTT
TACAAAAGCTGGACCCAATATCACAAGCCAAATGGGAAGAAAGGCTAGAGGATTCGTCGTTCGTTAATTT
AATACCATCGTGGGAGCATATGGCTACGTTTCTGGAGCAGAGATGCCGAACAATGGAAACCGTAAATTTT
GCTATGGCAAGCTATGCCCCTAGCCATCAGGTGGGAAAAACTAGAGCAATTAACACCAGTAGATCGTCAT
TTATCGCCACTAACCACGACGCCCCTGTCTGCGTCTTTTGTGACAGCCCTGGTCATAGTATATATAATTGTT
CTCGATTTGGAAATTTGACCCCTGCATTACGGCTTAGGGAGGCAAAACGAGTTGCATTGTGCTTGAATTGC
CTAAAGAAAGGTCATCAATTGCGATCATGCAGCTCTGGATGCTGTCGGACCTGCGGGGCGAAACATCATT
CTCTGCTGCATTTAAACAATTCCGTATCTCCGTCTGGTCAGCTGACTGAGCCTGCAAATTCATCGCCGATA
GCCCATGCCTCTTTCGCTAAGTCATCGCCATCTACCTCCACATCTCTTGTTGCCCAGTATTTCAGTAGTGAT
GTTGTCGTCTTGGCTACGGCTATGATCTTTGTAAGAAATCGCTCCGGCACTTTGATACCTTGTCGTGCCCTT
TTGGATTCTGGTTCTCAGCTTCACCTTATAACGTCGCGCCTTGCTCAACAGCTTCAGCTAAAGAAGCACAA
GTCCTCAGCCATTGTCTCTGGTCTAGGAGATTCTCGTTTTGAAACAGAAGGAAACACGGTCACTATCACAA
TACAGTCTCAAGTCTCTGAATACTCAACATGCCTTACAGCTCTGGTAGCGCCTAACATAACTGATAGTCAA
CCGAGTTTTACTATCAACACGACGAATTGGAACATTCCCGCTAACATAAAACTTGCAGATCCGTCTTTCTT
TAAACCTCAACGCGTGGACTTGCTACTTGGAGCTAGCCTGTTTTACGACCTTCTCAGCGTCGGCCAGATAA
GGCTTCTCCCAGGACTACCGTGTCTTCAAAAAACACGGCTTGGTTGGGTAGTTTCTGGAGGCGGACCCCGC
TTGAGTGGTTCTATGTTAATGGCAAAGCATACTGCGGGCAAACTTGAGTCAAGTTCTGAAGAACGTGCTG

ATGAATTGGTTCGAAGATTTTGGGAGATAGAATCCTGCTTGGAAAGTACACCTACCACTTCAAAGGAAGA
AAACATCTGTGAAGAACATTTCGTAAAGACTCATATCAGATTGCCTAGTGGCCAATATTCAGTTCGTTTAC
CCTTCAAGCGAAGTTTGGAATGTCTGGGGACGTCATACCAACAAGCCTTTCGTCGGTTCCTGACCCTGGAA
CGCAAACTACAAAATCGTCCAGATCTGAAGATTCAATATTCAAACTTTCTAAAGGAATATTTGGATCTTGG
TCATATGTCGCCTGTTGCTCCAGAGTCTATAGGACTTTGTCAATACTTTCTTCCTCATCACTGTGTCTTTAA
AGAAGACAGTTCAACGACAAAATTGCGAGTCGTTTTCGACGGCTCAGCAGCTACCTCGTCAGGTTATTCA
TTAAATGAACTATTGATGGCTGGACCTGTTATTCAAAGCTCGCTGTTTCATATCCTTCTTCGCTTTCGTAAT
TTCCCGATCGCCCTAACAGGAGATATTGGAAAAATGTATCGCTGCGTACGCGTTTCAGCACCGGATGATTT
TCTCCAATGCATTCTGTGGCGAGACTCGCCAGAACAGGATATAAAGATTTTTAAGCTGGATACAGTTACAT
ACGGAACAAAGCCAGCTTCATTCCTCTCTATCCGTGCTATGCATCAGCTGTCTATGGATGAGGAAACTAAT
TTTCCAATTGGATCTCGGATTTTGCGGCGTGACTTCTACGTTGATGACCTCATTTCAGGGGGTGATTCAATT
CAAGAAGCAAAGGAACTTCTTAATCAGACGGCTGGAATCTTGGCAAGTGGGGGCTTCAAGTTGCGAAAGT
GGTGCTCAAACAATTCTGCTGTACTGAAAGGGATACCCGTAGAGGACAGAGAATCTTCCCTAAAGTTTGA
TGATGGTAGCGACATCACCAAAACTCTTGGACTCGCTTGGGACTCAGCGTCGGATCGATTGTTGTTTTCGT
TTTCGTCCATTCAGCAGAGATCTAAACCATCCAAAAGGTCAATACTATCGACCATAGCTCGCTTGTATGAC
CCGCTTGGACTCATAGGGCCGGTAATTGCCAAGGCTAAAATCTTTATGCAGCAGATCTGGAAGGAGAAGC
TTGAGTGGGACGAGAGCTTGCCATCATCACTGGCAACAGCTTGGCATGATCTTTGCTGCAGTTTTGAGAAG
GCACAGTTTCTTAAATTTCCTCGTCTCGTGTCAATACCTGCAGCAGAGTTAGAAGTTCACGGTTTCTGCGA
CGCCAGCATCGAAGCATATGGGGCCTGTGTCTACGTCCTTTCTCGACGAACATCAGCCAGTTGTCAGCTGT
TGTGTTCCAAATCGCGTGTAGCTCCTCTGAAAACAATCACAGTTCCAAAGCTAGAGCTCTGTGGTGCCGTC
TTATTATCTCTAATCTCATGCGTGAGATATCTAAAATGAATCTGTTCGATGGAAAATTCTACTGTTGGTCG
GATTCTTCTGTATGCCTTTCCTGGATAAGGACCGAGCCCTGCAAGCTCAATGTGTTTGTGGCTAATCGCGT
CACAGCAGTTCAAGAGCTAACTAATGGCATGGAATGGCGTTACGTGCCTACTTCACTCAACCCGGCAGAT
ATACTCTCGAGAGGATCTACGCCCAGTGAATTAGATCAATCGTCACTGTGGTTCAACGGCCCAGAATTTCT
AGTTAAAAACAAATTGGACTGGCCTGCTTCTTGCTTGTCAGAAAGGACAGAACTCGAACTTAAAAAGGGT
CCTTATTGCGTCATCTCCCTTCGTTGATATAACTGTCACTTGTAAATACGTCAACTCATTTGATACAATGCA
GCGTATATTCGGATATATTTATAAATTCATACACAGAAGCAGGCATCCTGGACTAACAGTGTCTGATATAC
AACATGGGACCCAACTCTTACTAAGGCTCGTCCAGAGAGCACACCTGTGGGAAGAGATCAAATCATTGCA
ATCGAGAAGAGTTGTTAAACCGTCCAGTTGTATCGCATCACTTAACCCTTTTTTGGATGATTTCGGAATTC
TTAGAGTTGATGGGCGCTTGAAAAACTCTTCGCTGGATTTCGACGCTCGCCATCCAATAATTCTCCCGTGT
CACCACCCCATCACAAACTCGGTCATCACACGGTTTCACAATCGGGACTTACACGCTGGTCCACGGGCAC
TTCTTGCTCATATTCGGTTGCAGTATTGGCCGATTGGTGGAAGGAAGACCGTATCCAAGGTGGTTGGAAA
ATGTGTGAGATGCTTTCGTGCCAAGCCTCGATTAATAGAGCATATAATGGGTGATCTGCCCAAAGAACGT
TTGACTGCTTGTCGAGCCTTTGAAGTCTCTGGTGTTGACTATTGTGGACCATTTTTCTATAAGCCAGACGTA
CGAAATAAAGCTCCAGTCAAATGTTACATGGCTGTCTTCGTNTGCTTCGCGACTAAAGCGGTTCACCTGGA
ACTGGTCAAGGATCTGTCGACAGCTTCTTTCTTGAATGCGCTTAAGAGGTTTATATCGACTCGAGGCAGGC
CATCTCAGATATGGTCGGATAACGCTACCAACTTTGTCGGCGCCAAAAACGAGCTTCTGGATCTCAAGCG
TCTATTTACAAGTCGTAATCATATTGAGTCAGTGCATAATTTTTGCATGGCTGATTCCATTGATTGGAAATT
TATACCTCCTCGTTCACCGCATTTTGGCGGCCTTTGGGAAGCAGCAGTGAAGACAGCGAAACATCATTTTT
ATCGTGCTGTTGGATCTTGTGTTCTCAACTTCGAAGAGTTTCGCACCCTAGTCTGCCACATAGCATCGATA
ATTAATTCCAGGCCATTGTTTCCTCTTTCAGAAGATCCAGCAGATCTAGATGTGCTCACTCCAGCTCATTTC
TTGATTGGTGGACCCAGCTCATCGTTCTTTGAACCTGATGTGACGCACCTTCAATTCAATCGTCTGGATGG
TTGGCAACGCGTAACGTACCTCCAACAACTGTTTTGGTCTCGCTGGCGTGAAGAATACTTAACACTGCTTC
AGCAGCGCTCCAAATGGCGTACTCCGAAACCAGGCCTATGCGTCAACGACATAGTCCTCGTAAAGGATGA
AAATCTGCCGCCTCTAAGGTGGCCACTGGCGAGAATCACAGAGTTAATCGTGGGAAAGGATGGAGTCGCT
CGAGTAGCCATTTTGCGCACCGCAACAGGAGAAACGAGAAGAGCTGCAAACAAGCTATGCCTATTGCCCA
TCAGGGAATCTGTTGAAAGCCCATGCCTTCCAACGGGGGGGAATGTCTGGTTACGCAGCCAAAACAACTG
GAGCGACCTCGCAACTGATAAGAGCAGCATCTTAATTTTATCTTATAATACTCGCTCTGCAAAGCAAGTGC
CTAAATTTCGCTCTTAAGAATTCATTTACATAGAGCTGCCGCTCTTCGCTGTAACGATCGTTTTATTGCCCT
AGCTTTACTTATGCGAAGGCCTCGATGCTGCGCATCGAGTTAGTTAAGTTCGTTCGCTTCATGCATTTGGT
ACGCATCGGCCACTTGCATTACTCAAAATTATAACTAATAATTGTCTCTTCGCTAAAATTGGCACGTACTA
ATTCGGGTTCGCTTGCCTATATACAAACTATTGTTCTTAAATAATAAGCACAGACCAATTCGGGTCTGCCT
GCTTCTCGTAACTTTAAGAAATATAAAACAATTTTGAACACAAAAAAAAAATAGTTAATTATTTCTATTTGT
GAAAGCTATTGAAAAAGCTCAATAGCGCAACA

>9_rnd-6_family-38__LINE___R1
CACACACACACACACACACACACACACACACACAATAACACACACACACACACACACACACACACACACAC
ACACACACACACACACACACACACACACACACACACACACACACACACACACACACACACACACACACACA
GAGTACATGAGTGAAAGTGAAAGTGAGTTGACGCGTCTCGACTGTCGCTCCGGCTGTTCTCTGTCGTGGG
CCGCAGCTCCGGCAGGGAAAGCAAAGCGATGATGTGCAATCCATTGGCGAAGGGGCTGAGCACGTGGAG
ACCCACGCTCACATACGCGATTCGCAAGCGGCGGATGCCAGGGTGCGTCAAACCAAAGATGACTACATAC
GCACACGGAGAGACGCAGGTGGCACGACTGGCACGACTGGTGGTACACCTGCAAGACTGTGGAGGTGAA
CACGAGAATGGAGCAAACCCGCGCACTCACCTGGATGACGGGATGATGGCGAGGACTACCTGATGGTAT
ATATCCAACCCTAAACCTGATCCAAGTAAGCAAGTTGAGCACTTTCTCAGCGAAACTATCATGGCGACAT
TGTTTATGCTCAAGCACATGAGCAAGACAAAAGCTCTGGACAGCTGGTTGGTTGTACAGTGCTGCCAACC
GAAGTACATGCGTCAACGAAAAAGCAATTACGCGCGAAAGTATGCCACGCTGTGTGGGTATACATGAGG
ACAAAGGAAGATATTGCATGCTTTTAGGCTTTTACGTTTTACTCGCATTGCAGCTGAAGACTCGAGGCAAG
AAATTGTACAGAAGGCCACGGATCCGACCAAGTCAACTCCTGCCGCATAGCGGTGTACTAAAAGGGGCGC
TGGCGACCGAGCTGGAGCGGTTAAACTCCCATCTGTCAAACGCCAGTGAGAAACCATTGGCCACCGTTGG
AGCCACAAATCAGCAGCGATGAGCGCCACAACTGGCAGCACGCCCGTCAATGCAAGCAAGCAAACTCCA
CCCCCACACTGAGCCCAGTAAAGTGTGGAAGCGACCCACTGGACCCCCCTTACGTAACTGTAGAAATGGG
CTGACGAGTCGGGCATTCTAGGACCTTAATAGGCAAGCTGACACCTTGTCGAAACGGCTCCTAGGCTAAT
GCTAGCCTCGCCCCCGTCCCGCTAAAACCTTGGCAGGCCTCGTAGAACGCTTATCGTATGTCGCCATTCAG
TTGGCCGTGCAGGCTCAGTTGATATTCCCTGACTAGCACTGATCTGGCTCTGAACTCCGTCTCCCATAAGG
ACCGGAGTCAACCCTCGCGTGGACCTCACTCCATGGAAAGGCAACCACGGGATCGCGAAGGTGACTGCAC
AATACACACGACAACAACGGCTTCGAGTTGGGACCCAATTAATCACAATGATGAACCGAAACAACAAAA
GCACCACCCCACTAGGTGGACAAGAGGAAGAAGGAGTATGCAAGGCCGACCCGTTCGCTAGGCGATCAT
GCCTGGCGAGCACGCCCCCGGCTACACCAGTAAGGGCCCCTCAGCCCACTGCACCATGCAGGGAAATTGA
AGAGAAAGAGACCCCGAAAAGGCTTAGGGACCCGGCCAGCCCTACGAACAAGCCACGGAGCACCCCGCC
CAAGAAGAGCAAGGCAAATACCCAGGAGATCGAAGAGATTGGCTCGATACTGGACGAGCTACTGTTCAT
GTATCATGGCAAGCAGCCAGCGATGAGGCACATAAACTTGGCCACTAAGAGCATGCTAGCGAGACTAAA
AGAGCTGCAAGAAGTAGTCAAAGCGCAGCTCACGGAAAACCTACGCATGCGATGCAGCGAAGAGTCCCA
TAAGCAGATGAGCCATGCTGAAGAAAGCGCCGGAAGCAATTCTAAATGCGACACGGCAACGCAGACGGA
AGAAAGCGGTGCATGCAGTAAACCACCGACAGCGGCGCAAAACCGACGAACGAGGTTACAGGAAAAATC
AGATCTTGGCCCCAAGCAACTCCAAGCCGGCACCAACTCGCAAGCAGCAACGTTCACAGCCGGCAGAGG
GCAAGGTACCGACCCAAGCGCGAGAGGCAAAAACACCGGTCAAAAATACCCGAGAGGTAAAAAATAAAT
GGACTGAGGTGAAGGGCAAGCTCACCAGGCGGAAACAGCCCAACCGACCGGATGCGATCCTGATCACGT
GTACAGAGAGCATGCCATACGCAGATGTGCTCAAAATGGTGAAAACAGCTCCTGCACTGCAGGCGCTCAA
AAATAATGTACAGGGCATCCGCAAATCAGCAGCAGGAGAACTGATCCTCAGGCTCCAGAAGCCATCGGA
CCCAGCTACCGACCAGTTGCGTGTGGCAATCAGCAGCGCACTGGCTGGAAAAGCTGCCGTTAGAACCTTG
CAGGAAACGGTGCAAATGGAGGTTCTAGATATCGATGAGTCCACGACAGCAGATGAGGTCCTCACAGCTC
TCTCTGCCTCGGTGGAAGGAGACATACCAGTAGAAGCGACGCCCACTATGAGGGCGGCCTACGGAGGAA
CACAGATCGCATCGCTGGCCCTGCAACCGAGCCTAGCCACGAAACTGCTCGAAACCGGCAAGGTCCGCAT
AGGGTGGGTTATATGCCGAATACGCCAAAAGATCGAGCCAAAGAGATGCTTCAGATGCATGGGATTCGGC
CATACATCCACCAGGTGCAAGAGCCAGCATCCGGTTGGAAAGGAGACATGCTTTAAATGCGCAGGTACTG
GGCACAAGTCTGGACAGTGCACGCATGAGGCAAAGTGCATCCTCTGCACAAGAATGGGCACGGGCACTA
ACCAAGCCGCGCACTCAACTCTGAGCAGGAACTGCCCGGAGTATAAGAAGGCCTACAAAGCGCTTAGAA
ATGGTTAAGTTCCTGCAACTTAACCTAAACCACTGCAGAGCAGCCCAGGATCTGCTCGCACAAACGACCC
GGGAGACGAGAATTGATGTAGCCATCATTAGCGAACCGTATACACCCAAACATATCGGAGTGTGGCACGA
AAGCCTAGACGCTGGAGCAGCACTATGGAGCTGCGGACAGGCACCATACCAGCTCACTCAACGGATGGCT
CGCAAAGGGTTTGCTAGGGCAAAATGTGGTCGCATATACGTTTATAGCTGCTACATACCACCAAGCATAC
CCCTGGCAGAGTACACAGCCATTATTGAAGACATCGCACAGGACGCGCACGGAAAATCTCCAGTGATAAT
CGCCGGGGACTTCAACGCGTGGGCCACAGAATGGGGCAGCAGCAGAACGACACCAAGGGGAACCATCCT
GCTGGACATATTTGCTTCGCTAAATGTCTGTCTGCTGAACATCGGCACAAGAAGCACTTACAGCAAAGCG
GGTCGCGAGTCGATCATAGACCTGACGTTTGCCAGCCCAGACGTTGCCCGAGACGCCAAATGGCATGTAT
CGGATGTATACACCCACAGCGACCACTTTGCGGTGATAACGGACACCTNTCAACTTGGAAGCAGAGGCGC
CAGTAGAAGGCTCCAACACACGGGATACAAGATGGACACTATGGACCTGGAAAGACTCCTCCCTATGGTA
GAAGGACTTGGCATAAGAGGCGACGCCAACAGCTCTGCTGACGCGATAGCAGAGACTATCACCAACGCA
TGTGACGCTATACTGACAAAAGTTCGCAAGGGTGGAAACGCAACATAGACCGGTAGCCTGGTGGAACGA

GGACATAGCCAAAGCGCGCAAAGAGTGCCTCGCAGCTAGGCGGAAGAGCCAACGGTCAAGACCGTCCCC
CAATTTTGAGCACCATCTGCAGAACTTCAGAGCCAAGCGGAAATTACTCAAGGAAGCTATCAGACGCAGC
AAATCCCGATACTTCCAGGAGCTCTGCGACGCTGCGGACGCGGAGCCCTTTGGACTGGCTTACAAGCTAG
TCATGGGAAGGCTATGCAGGCAACCAATGCCAACAAACCCGGAACAGCTCAAACAAATAGTCCTAACGC
TCTTTCCTCGACAGACACCTGGAATTGCACCGCAACTACCAGCAAACGGAATAAACGACTGTGCACCCAC
CGACGACAATGAGGTACTGAGCATTGCTGCGAAGCTAAAGGCCGACAAATCCCCTGGACCAGACGGAAT
ACCGAACGGGGTAATAAAAGCTGTCATTACTGCACAGCCCAGAAGCTTCGTGGAGTTGTACAATCAATGT
ATAGCGGAATGCACAGTACCACGTAGATGGAAACTCCAGCGACTTGTGCTCATCCCGAAGCCCGCGGCAA
GGAACTGGATGATCCCTCCTCATACCGCCCCCTGTGTATGCTGGACACGATGGGAAAAATATTTGAGCGG
ATAATATGCAATCGCCTAGAATGCAAGCTTGCTGAAGTGCGCGGTCTGTCTGAACGNCAATTCGGCTTCA
GGAAACACAGGAGCACCACGGATGCCATCAGGGCCGTGGCTGAACTAGCAGAAGACGCTATCCAAGGCA
CAAGATGGACAGGTGGCACCAAACAGTACTGCCTAGTCTGTACGTTGGATGTCAGGAACGCGTTCAACTC
AGCCAATTGGACAAAAATACTAGAAGCGCTAACGGCCAGACACATCCCGAGCCAACTAATAAAGTTAGT
GACCAGCTACTTCCAGGACAGAGTGCTAAATTACGACACGGAAGACGGCCAACGACAGTACAGGGTAAC
TGGAGGCGTTCCTCAGGGCTCTGTTCTCGGGCCGCTACTGTGGAACGTCATGTACGACGGAGTACTGCGCC
TTTGCCTACCAGAGGGCAACACAATCATTGGTTTCGCCGATGACATCGCTGTTGTTACTGTGGCGAAGAAA
CTCGATGAGGTCACTCTTAGGAGCAGTGCGGCCATCGACAGGATAATTGACTGGCTATCCCAGAACGGAC
TCACGCTAGCGGAGCAGAAGTCGGAGGCAGTCTTAATAAGCAGCAGAAAGATAGTTGAGACGACATCGT
TGCGCGTTGGCAGTGCTATCATTAAGTCCAAGCCCGCCATCAAATACCTGGGCGTCATGATAGACCACAG
ACTGTCGTATAAACAGCACCTGGAGTATATTAGCCACAAAGCGGCCAGTGTTACAACAGCCATTAGCCGA
ATGTTGGCGAACACCAAAGGACCAAGGCAGAGGAGCAGAAGGCTAATCGCTGGAGTGGTGACGTCCACA
ATCCTCTACGGATCCAACATATGGGCCCCCGCAATGGAGGTTGCATCTTACAGCCGAGGATGCAATGCTG
CATACCGACGATGTGCATTGCGTGTAGCCACCTGCTTCCGCACTGTATCGGAAGACGCGGCCTTAGTACTG
GCAGGCATGATCCCACTCGGCCTGATGGCNGCCGAAAGGCAAAGTGGGGCAACCACCAACAGGCAACTG
AGGGATAACACCATCGANCAATGGCAAAGCAAATGGGATGCGAGCTCAAGTGGCAGATGGACCCACAGA
CTAATCCCAAACCTGAAGCTTTGGCTACAGCGGCGACACGGCGATGTAGACCACTACCTGGCACAAATAT
TTACTGGGCATGGCTGCTTTAAAGCGTACCTGCACAGGTTTAATCATGAGGACGACCCCTTTTGCGAGCAC
TGTGGGTACGGTGTGATTGAGGACGCGGAGCACGCATTGTTCTACTGCCCACTCTATAGTCGCGAGCGAG
ACAAAGCGACGGCGGGCAGAAATGATTTGTCTCCGGAGAATCTCGTGGCGCGAATGGTGGCCACTGAAA
ATGGATGGACAGCCGTTGCTAACATGGCATCGGCTATAATGAAGGAGCTGCGACGACAGGAGAGGATCC
GACGCGGCATCGACTGATGAGCGGCTGATATACAGCCGTACCCCGCCCTGCGAAGTAGTACTTTGCGGTT
GTCCCGCAGGAGCGGGCTACAACTTTCTACTCCCCACAAAATACCCTTTCCTTCTTTGTTTGTCTTAGTTCC
CCTTTGTAAAATGTTAAGCCGTTATTTTCTTTTCTTCCAAAATAAAAAAAAAAAACACACACACACACAC
ACACACACACACACACACACACACACACACACACACACACACACACACACACACACACACACACACACACA
CACACACACACACACACACACACACACACACACACACACACACACACACACACACACACACACACACACAC
ACACACACACACACACACACACACACACACACACACACACACACACACACACACACACACACACACACACA
CACACACACACACACACACACACACACACACACACACACACACACACACACACACACACACACACACACAC
ACACACACACACACACACACACACACACACACACACACACACACACACACACACACACACACACACACACA
CACACACACACACACACACACACACACACACACACACACACACACACACACACACACACACACACACACAC
ACACACACACACACACACACACACACACACACACACACACACACACACACACACACACACACACACACACA
CACACACACACACACACACACACACACACACACACACACACACACACACACACACACACACACA
>9_rnd-1_family-381__LTR___Gypsy
TATAAGTTAACAGAGGCCAACGACACCAGCTCCAGATGCTGATATAGCACTGAGCCGGATGTTCTTACGA
GCTAACAGGGGTCAGCAAAATGCAACATAACACCAGCCGCAGATGCTGACATAGCTCTGGGCCGGATGTT
CTTGCACGACGGCAGCGCACGAGGCGCAAGCTAAGCATTCTAGCGGCCGTTGTGAAAAGCACACAATAG
CTTAGGTAATTTTGATCAATCTTTTGTAAATTTAGTTTTTAATCCGGCTTTCACTGAGGCACATCGGCCCAG
CGACTTCGCGCATAATTTAACTCACTCACTACGGCGTATCGGCCTAGTGTTTTATAAATAATAAAATAATC
AAAATAAAGTCCTATAACGAAAATCCGCGAGGACTTCTTATTTAAATCTTAATTGGCGCCCAACGTTTTTC
GGACCATTTTGTTCTATAATTATTTTGTGTGCTGTGCAGTGTAAAGAACTAATCTACCAGTACAACACACT
TCGCGCTGTGTAATAGCAATACCCGACCTACCAGTGCAAGCAAAAAAGCTGTGCCTGACCTACATCNATT
TACGTCAGCGGCACAAGCCCAATAAAACGCATTTGATACACCGAAGCCGCTGGAGCTCTTCGGCCAGCGA
CTTGTCACCAAACCAGCGCGCAAGCGGGTTTGGGCAGCGANGTAAACCAACACCCACAGACTTCAGCGA
GGGATCATAAAAAAGCATTCGCGACTGCAGCGTGGGAANCAGTAGTTTTAGACGTAGAGCATACAACCCT
TGTAGGATCAAATAAAATGTAAGTGGGCCATCATTTGGCACAAACTTTTTTCAATTTGCGTGATTTATTTA

AAATAAAATCNAAAAAAATATAAGTCAGAAATATTAAAATTGCAGTAATTGTGCTCCCAACTAACTTTGA
GTAACACAATTTTGCGTCCATCAGCAATTTTCAATAAACTCTCCTCTGTCAGCATTCATTTAATTTACCGTT
GTTGATTTGCCCTTGAAATTGTGTTGTTATTATTGTTGACAAGTATAGTCCATGAACAGCTCGTTTCTCAGG
TACAGAGACCGCTCTGCGAGGGGATCTGAGTCAGACGACTCCACCACCTTAGATAGGGAAGCCGTCCCGC
AAAAAATCGCTGACAACAGGTGTAAAATGGGAATGGAGCCTGAGCAGCTTAGGGCTTTGGTGCAGGCCG
CAGTGGCCTCCGCCTTAGAAGCACAGAGCGCAGTATTGAAGCAAAGAGATGCAGAGCTGAGGGCTTCCTT
AGACGCTCGGATACAAGAATTGGNAGGCAGAATATACTACCAGTAGTATAGAAACACCGCCGGTCCAGG
CATTTGAGCCGGTGGAAATCAGAAACGACGTCGAATGTAATGAACCGTTGGACGCCGTTAAATGCTTACC
AGAATTTACGGGGTGCCAGAGACGTACGCCTCTTGGAGACAAGCTGCGATAGCGGCATATAAAATTTACA
AGCCATACGACGGCAGCTCACGCCACTACCAGGCGGTCATTATTATTAAGAGCAAAATTAGAGGGGCAGC
TGACGGCGTTTTGGGTCAGTTTGGCACAGTAATGAATTTTGATGCCATAATAAACCGTCTCGATTTCACAT
ACAGGGATAAGAGAGGCATTCACGTCGTAGAGCAAGAGATGAGCACCCTAAGACAGGGCTCGTTAACAA
TTTTACAATACTATGACGAGGTTGAGAAAAAGCTTACTCTTCTCACCAATAAGGTCAACATGTCCTATGAA
CCAAGCATGGCACAAGGGCTCTGTACGAAGTTCAGAGATGATGCGTTGCGTGNTTTTGTCTCAGGCCTAA
AGCGTAGCCTGACGGATGTCCTGTTCGCGGCAAAGCCTAAGGACTTACCGTCGGCCCTAGCNCTAGCGCA
AGAGGTAGAGGCAAACCATGAGAGGTACGCCTTTGCCGCTAGCTTCGCTAAAGGGCAAGAAGATAGGGA
ACGAAAAGTGACCCCGAGGCCGCAGGTCCGTCAGCAAGATAGGGGTCAACAGCAGGGGGATCCGCAACC
CAGCGGTTCCAAAAATCCTCGCTTCACTAAACAACAGAAACAGCAACAAAGTCAATTTGAGCAACAGAGT
GGCAGGTGGAACAAAGGTGGCGGGTCACAGGAACCAATGGACGTCGATCCATCTGTGTCNAAAATTCTAC
AACCATCGCAAGCACCAGCCTACGTTAACTCTAGCGCTCCTCAAGCCTACAAAAGAACGGCCACGTCTGG
GCGTTTAAGCGGACAGAGGAGGCAGAGAGTCAATCACACAGCTCACGAGCCNGATCAAGATTCAGGTTC
GTATGCTAGCTCTGCAGCAGCAGCAGAACAGCAAATTGCCGACGACAATGGTAGCGGNTATGACTCAGAC
ACTCTCAATTTTTTAGGGGAAGCTCCCTGCTGCCCTACATCAGTAGGAGAGTAGCAGGGATCCAGATGAG
GTTTCTCATCGACACGGGCGCTTCCAAGAATTATATCAGGCCACATAAAAATCTAAAAGGTGTGTGCCCA
GTAAATTCTCCCTTCTCTACCCATTCTATACACGGCACCACGAAGATCACTCATAAGTGCCTTATATCTCTG
TTCGGCAAAACTGCCACATTTTTCTTACTCCCAAACCTGTCCACATTTGATGGAATTGTGGCCTCGACTT
GCTCACGCAAGCAGGAGTTTCGCTTTGTCTTTCTTCTGGCCAACTCAAATGTGGCGCAGAAACTGAACAAA
TCGAATTCCACAGGTGCGCGGATGTTAACTTTACCAATGTTGACTGCGCCGACGCGCCCGATGCTGTAAA
AGATGCATTCCTAAAGATGCTAAAAAGCAAACATAAGGCCTTCGCAGATCCGGAAGAAGCACTTCCCTAT
AACACATCAGTGGTGGCAACCATCAGGACCACTGATGAACAACCAGTTTATGTCCGACTGTACCCCTACC
CCATGGGAGCAGCGGAATTCGTTAATAAAGAGATTCAAAGCCTTCTGAAGAACGGTATCATCAAAAAATC
TGTCTCCCCTACAACAACCCCATTTGGGTGGTGGACAAGAAGGGGACGGACGAGTTTGGTAACAAGAATA
TGCGCCTNGTAATGGATTTTCGCAAACTTAACGAGAAAACCATCGCAGACAGATACCCTATGCCTAACAT
TCCAATGATTTTGGGGAATCTTGGCAAAGCTAAATACTTTTCGACATTGGACCTAAAATCCGGCTATCACC
AGATCACGCTAGCAGAGCGTGACCGTGAAAAACAGCCTTTGCAGTTAACGGGGGGAAGTACGAATTTTGT
CGACTTCCCTTTGGGTTGAAGAATGCGGCCAGCATCTTCCAAAGAACTGTTGACGACGTACTGCGTGAGC
AAATTGGCAAATCTTGCTATGTCTACGTTGACGATGTCATCGTCTTCTCAAAAGACGAAAATTCTCATGTC
CAACATGTAGAGTGGGTCTTAAAGAGCCTGTACGAGGCAAATATGAGAATCTCCAAGGAAAAATCGCGTT
TTTTTAAGAAAAGCGTAGGTTTTCTTGGTTTCGTGGTAACCAGTAATGGAGCTACAACGGACCCCGAAAA
AGTCAAAGCGATTCAGGAGTTCCCAGAACCAAAGAATATATTCGAGATCAGATCATTTTTAGGACTGGCC
AGTTACTATAGGTGTTTCATCAAAGACTTTGCCTCAATAGCAAAACCCATTTCTGATCTTCTAAAGGGCGA
GAATGGGTCAGTAAGTAAACATAGATCGCGAAACATCCCGGTAGAATTCTCTGAGNTTCAGCGTCACGCA
TTCCAAAAGCTACGCAACATTTTGTCATCAGAAAACGTCATGCTCAGATACCCTGACTTTAAGAAGCCGTT
TGATCTAACGACAGACGCTTCAGCTTACGGCATAGGGGCAGTGCTTTCTCAAGAGGGTCGCCCCATCTCA
ATGATTTCCAGAACATTGAAACCCCCTATATGGGGTACCTATATGGGGTAAAAGACATTAACATTTTCACG
GACCACCAACCGTTAACTTTTGCCGTTTCTGAGTCAAACCCGAACGCCAAAATTAAAAGATGGAAGGCGC
GCATTGAGGAAACGAATGCGCGAATATTTTATAAGCCAGGCAAGGAGAATTTAGTTGCTGATGCGCTATC
GCGACAACAGCTCAACGCTCTTGACGAGCTGAATACTGATTCCTGCGCAGCCACAGTCCACAGCGAGCTG
TCCTTGACCTATACAATTGAGTCGACCGAAAAACCACATTGTTTCCAAAATCAAATTGTCATTGAAGAGGC
ACGCGTCCCCACCAAACGCAACTTCATTCTTTTCGGGGACAAGAGGCGTCGTGAAGTGAGCTTCGCTGAT
CGCACAGCCTTTCTAGATGAACTCCTTGACTTGATTAATCCAAATGCGGTTAATGNCCTCTATTGCAACCT
GCACACACTANCACAGNTGAAGGATGGCTTAGTTCGCATGTTTCCAGGCACAAAGTTCTGGCACTGCAAA
AACCGAGTTGCAGATGTCTTCAACGTTGACGAGAGGAGAGAAATTCTCGTTGCCGAACATACTAGAGCGC
ACAGAGCAGCTCAAGAAAATGTTAAGCAAGTGTTATCAGAATACTATTTCCCTAAAATGGCNAAGTTGGC

TAAAGAAATTGCGGAAAGTTGTAGAATCTGCTNANCAGCCAAATACGATAGGCACCAGAAAAGACAGGA
GCTGGNAGAAACTTCGATCCNTGAGACTCCTGGGGAATTTCTACACATTGATATATTTCCCACGGACAAA
CAATTTTTTTGNCCTGTGTTGATAAGTTCTCTAAATTTGCAGTCGTACAACCAATTCAGTCGCGCACTATC
GGGGACTTAAAAGCTCCACTACTGCAGCNCACGAATTTCTTTCCACGAGCCGGAACAATTTATTGCGACA
ACGAGCCTTCGATGAATTCTCAAACGATAAAGACCCTGTTGTTAAACAACTATGGAATCCATATTGTTAAT
GCGCCGCCGCTGCATAGCACCTCCAATGGCCAAGTCGAGCGCTTCCATAGCACGCTAGCCGAGCTGGCAC
GTTGTCTCAAGCTGGAGAGAAACATCAGTGACTCAGTCGAAGTCATAATGCTAGCCACGATTGAGTATAA
CAAGACAATACATTCGGTCATCAACAAGAGACCTGTTGATGCCCTCCATGTGCCCNCAGGCGAACCCGAA
AGGGAAATATCAGCAAAGTTAAGGAGTGCGCAAGATGCGCTCCGGGCTAGGCTGAACGACCAGCGACAG
AATAGAGTCTTTGAAGTAGGCGAAAAAGTNCTAGTAAAAAGAAACCGCAGGCTTGGCAATAAACTCACG
CCCTTATGTGAAGAACGGATCGTAGAGGCAGACCTTGGTACGACCGTTCTCATTGGAGGGAGGGTGGTCC
ACAAGGACAACCTTAAGTAGGGCGAGAGCTGAAATAAAACATCGATTATATGCATCTCGAATTTAACTAT
AAGTTTCAACATTTGTGCCACTAGGCTTCACCTTCAAGTTTTAATATTTACGCTACTTGGCATTTCCTTTAG
GAAGACACGTTGTAATAGTTTGGTCCGTTTTGTCTGTCTTCCGCAGGTTCGGGTTTCTGCTATTTCTATTTT
TTGCGGCAGCGCGTGTGACTAATTACTCACACGCCTAAGTATATTCCCATTGTTGAGGGCCAAGTTCTGGT
CTGGGAGGAATTCAATTTCGTCAGGCACACAGCAAACATCTCTGAGTACGAGAAAATGGTAAACGAAAC
AATTGAGCTATGTGAAATGTTTCCGCAGTCACACATGAGAAAGCTTCTAAGCGTAGATGCGTCCCACCTC
AGAGATCTGTTAGAAGCTCTTGGCGTGCACCACAGGGTGGCAAGAAGTTTAGATTTCTTGGGCACAGCCC
TCAAAGTAGTTGCGGGAACACCTGACGCCAGTGACTTCGAGAAGATCAAATTCACCGAGTTACAGTTGGT
GAACTCTAACAACAGACAGGTAGCTATCAACACAAAAGTTCAGGAACAAATCAATCAACTCACTACAAGT
GTCAACACATTACTAAAAGCTGCAAAAAAGAATCAGATAGATTCCGGGCATTTATACGAAACTCTTTTAG
CTCGGAATAGAATGTTGCTGATGGAAATGCAGAATTTGATGCTCGCTGTTACCCTTGCAAAAATTAATATC
ATAAGCCCAAATATATTAGATCACGCAGATTTGAAATCTGTTTGGGTCGATGAACCCACAGGTACTTCAAT
AGCTGACCTTATGTCCGTTGCGTCCGTGAAAGTATTACAATCTGTTAATGCCCTTCACTTTATCATTAAGTT
CCCGAAAATCAAAATGGCTTGCAGAAAGGTGATGCTGTATCCAGTTGCACATGAGAATGCTGTGCTACAA
ATTGACGACAACATTATAGCTGAGTGCGGAGATCAAGTGCTTGCCCTTAAGAATTGCACTTCCACGCCGG
GAGGAACCTTTTGTCAACTTTCACCGACCAGCTCTTGTGCCAGAGAGCTGCATGCTGGTGG
>9_rnd-1_family-207__LTR___Pao
CTAATAATAGTATCCAGTTACTGACCAACGTAATTATTCGATGCCTTGAGATTGGACATTTGCATAAGCTT
ACTGGCTACTCTCTCTAAATTGAACGAAAAGAGTTTATTTACTCTATAGTGCTGCCAGTATTGAACGAGGC
TAGTTTGATCCCAGACCATCAGTTCGGCTTCAGGCGCATCCACGGAACACCGCAACAGTGTCATAGGATA
GTAGAGCGAATAATGCAGGCATTCGAAGAGAATAAGTACTGCTGTGCTGTCAAGCAAGCCTTTGATAGGG
TATGGCAGCCCGGTCTACTGCATAATGCATAAAGTCAGAGATTCTCTAGCTACAGTCGGCTGGTGAGGAC
GACGGCTTGGGTTTTAAAGTTCACGCGTTGGAGCCGTGGACAGAGGACTGAGCTTGAAAGATTCGGCTTC
ACCGCTACGGAGTGTGAGAATGCTGAGAACTTGCTGATACGGCGAGCGCAATGCGAAGCGTTGCCTGACG
AGGTTCGAGCCTCATACAATGAAGAATCAGTCGGTCATCGAAGAGACATAAGAGGGCTGGCGCCTTGCAG
TGTATACCAATACGCGTATACGCCCCACAGTGTAGGCCAATAATCCTGTCACACCGGCTCGCACTGACGA
AACTGATTGTGTATCACTACCATGTAAAAATGAAGCATCAATACATGGATGCAACGATAGGCGACATCAG
GACCAGATTTTGGATAACAAAGCTGCGTCGACTGTTGCGTACAGTAATATTCATAGTATATAGAGCGCAG
CCAGGGGCACCTATAATGGGTCATCTGCCAGAGGACCGACTGGAGGCCATTTGGACTATTTTGGACCACT
CCTTGTGACAATCGGACGTCGAAAAGAAAAGAGATGGGTCGCCTTGCTTACATGCCTGACAACAAGGGCC
ATACATTTGCACATAATCTGTCAACTAATTCCTGCATAATCGCGATCGGGAATTTTCTTTGCCGTCGCGGA
CCTGTACTGAGGTTGCGCAGTGACAACGGAAATAACTTTGTTGGAGCCGACAGAGAAGCCAAAAGGCTCA
TAGACGTATTCGAGNCGGAGAAGATTCAGGGCGAGCTATCTAAAGGCATTGATTGGATCTCCAATTGCCC
AGCGAACCCAGCTGAAGGGGGCGCTTGGGAAAGGATGGTGCAGTGTGTCAAGAGAGTGCTACGCCACAC
AGTAAGGGAGGTGGCACCAAACCAACACGTACTGGAGAGTTTCCTGATTGAGGCCGAGAGCGTTGTGGAT
TCTCGCCCGCTTACCCACTTACCGAACCAGGGAAACCCCTAACACCGAACGATCTACTCAAAGAAGTGGC
CAATCTGCCTGACACACCCACTTACAGCCGAACGAGAGACGGGCCACGAGGAAACAGTGGGACACGGCG
CGTCTGTTACGAGACCGTTTTTGGAAGCGGTGGGTCTTAGAGTACCTGCCTGCGCGCGTTGAACCGATACG
CCATGGTGATATGGTGTTTGTATGCGACCCGGCCGTGCCACGCAGAGAGTGGCGAAGGGGCATAGTGGAG
GAGCTCTACCCTGGACCACGCACCAGCGTGCGCGTGGCGGACAACAATCGAGTACGACTGATCACTCGTC
CTGTGGCTAAACTAGCCATACTGGATGTGTGTGAAGCAGGACCTTCACGGGGGCGGAGATGTTGCGGGAC
GAATTAAGTTATCGATCGTTTAGTATTAAGTATTACATTACTTAAAGGTTAAGCTCATTATTGTCGTGAGTT
CGAAGGCCGTTGGAGGCAAGACTGCGGTAAGATTCTAAGGGAATATTTATATCTANGTTTTAACCTATGT

AATCCGTTCAGAATCCGACTGCACTAGCACGTATTGCATTACGCTGTCTTTATTAACATCGGAGTAACATG
GCCGGGGGCCTTGCAGTGTTCAAATAGCAACTGCACCTTCGTTTGGCACGCGACACTTAGTAAAACTTTAT
CTCCCCTGAATAAAATTATGTCCTTAGTTTTAAGTCATATAGCTCTTTATTTTTTTGTTTGCGTAGTTCAAG
CATGTTTTTTGCTCTTTGGTATGCTACTTCTAATCAATACTTACTTTCTTTAGCGAAATCTTCTATGTTCTAT
TTAGGTTCTATATTACCTATGGTATCTAGTATTTCGGCAGATTACTAGTCTTACCAAAACTAACTCATATGG
ACAATAATTATGTGNCACATTTATGATTTATGATGATGATGCCATGAGTCACAATGCACCGCATCAAGCA
GGTGATACAGTGATGAGTACTGGACGGATGCCGCAGCCAGCGGATCCTCACACAAGGGCACCGCAGTCA
GTGGATGCTCAATTGACTCAATTGACAAAGGAACTTAAGTTGTGTCGATTCGGATAGAGAACGCCGACTT
TTCCGCATATTGCCAGTGACGTTGCACGGGGCCAATAAGCTGATCGATACGTACGCGCTCCTTGACGGTG
ACAAGCGCAAACGTCATGTGTTGCGAAATGTGTACGCCGTGTCCAACCTGAGCTTACCAATGCAAAGCCT
GCATCGACGTGACGTCCAAATGTGGGGCAACGGTGCACGNCTTCCAATGAAACCGTATCGCGGGGCGGTA
CCAAAACTTCTGATTGGACTTGACCATGCGCATCTTGGACTGCCAATGCAAACAAAAAGATTTGCACCAC
AGGGACCATATGCCGCAGCCACCGAACTTGGATGGGTGGTTTTTGGGCCGGTAAGAGGTCAACCGACGGC
AACGTCATCAAAGTCATGCCTCCTAGCAGTGTCGCAGGAGGATGCAATCCAAAAGATGGTAAACGACTAT
TTTGATATCGAAAACTTCGCCACCAGTCGCAGCCAGCGACGATGCACGAGCACAGAGGATACTCGAAGAC
ACCACGGTGAAAATAGGAGAACGTTACCAGACGGGTTTATTATGGAACCGTGATAATGTTGAGTTGCCAC
GAAGCTATGATATGGCATACAAAAGATTGGTCAACATTGAGAGAAAGATGAAGCGTAACGATCGGTTTA
AACAAGCCTATATGAAAATTATGGACGATTATGTTCATAAAGGATATGCTCGACGATTGAAGCAGGCACA
GGTCGCTTTAGCCAACAGCGACAAACTTTGGTATCTTCCGCACTTTGGAGTTGAAAATCCAAATAAGCCCG
GTAAGATAAGACTGGTGTTCGACGCGGCAAGCAAAGGTTGGTGATATTTCATTGAATTCGGCATTATCCA
AGGGGCCGCAGCACTATAAGTCGCTGCCGGCTGTTCTCTTCCATTTTCGGGAAGGTGCCGTTGGCGTTTGT
GGAGACATCCGAGAGATGTTCCATCAAGTGTTGATTCGACCGCAGGATAGATGCGCCCAGCGATTCCTGT
GGCGAAACGGCGATGATCGCCGGGAGCCTGATGTTTATGAGATGAGGGTAATGACGTTTGGAGCAGCATG
TTCGCCAAGCGCCGCGCACTATGTGAAGACCTTGAATGCCCTGCAGTTTCAGGATTCAGACCCTCGCGCG
GTTAAAGCTATTCTTGAATGCCACTATGTGGACGACTATGTGGATAGTTTTGATAGTGAAGGCGAAGCCAT
CACCATATCAACAAGAATAAGAGAAATACATGCAAATGCTGGATTTGAATTGTGCCAGTTTACTTCCAGT
TCGCCAACTGTAGCTGACGCGCTGGGCCAACATGGGGCTGCTAGGAGCATCGGATGGGGCGAAGCTGAA
GAAAAGATTCTAGGCATGTGTTGGCAACCAGCCACGGACGATTTCAAATTCAACATGAAGTACCACAAAG
TACCCCGATGTGTGCTAAATTTGGAGCGAATTCCTACGAAGAGGGAATTCTTGAGCTTGATCATGTCAACA
TTCGATCCTCTGGGATTTCTTAGCTGCCTCATGATAACTGGAAAGTTACTAATGCGTGAGATTTGGCGACG
AAATGTGCAATGGGATGAACCCTTACCAGATGAGATCGCCCGGGCCTTTAGCAAATGGCTTCAGGATATG
AACAACGTGGAAGGATTTCGGAGCTCGCGCCATTACTTCGGCCCAACACCTGTGCGGGCAATACAGCTGC
ACGTTTTTGTCGATGCTAGCCAGTCAGCATTCGCAGCCGTGACTTACTTGAGGGTCACCTACGACAACAAC
GACGTGCGAGTATGCTTCGTATGTGCCAAGACGAAGTGTGCCCCAATGAAGACGATGTCAATTCCACGAC
TGGAATTGCAAGCAGCCGTGTTAGGAACGAGGCTGATGGACACTGTCAAAAAGGAGCACAGTATAGCGA
TCAGCGACGCTATACTATGGACTGACTCCAAAACNGTGCTGCGTTGGATAGGCAGCACCCACCGTCGATA
TAAGCAGTTCGTCGGAAATCGAGTGGGAGAAATTTTGGAGTCAACAAAGGTTTCCCAATGGAGATGGGTA
CCTACTGCTGAAAATGCAGCAGACGACGCAACGAGGCCGCATTGCCACGTAGACCTCAGCCAACGGTCAC
GATGGTTAGACGGGCCAACATTTTTGCGGCAGCCGGAAAGCAGCTGGCCGCAGTCCGAACCGGGCACGG
AACACTCATCCTACGAAGATGAAGAAGAGATGCCGAGTGAATTTGCCTTAGTTGCAGCAAATAACTTTTT
CATTTCATTTCAGAGATTCTCTAGCTACAGTCGGCTGGTGAGGACGACGGCTTGGGTTTTAAGGTTCACGC
GCTGGAGCCGTGGACAGAGGACTGAGCTTGAGAGATTCGGCCTCACCGCGACGGAGTGTGAGAACGCTG
AGAACTTGCTGATACGGCGAGCGCAATGCGAAGCGTTCCCTGACGAGGTTCGAGCCTCATACAATGAAGA
ATCAGTCGGTCATCGAAGCGACATAAGAGGGCTGGCGCCNTACTTGGATGACAATGGAGTCTTGCGTGCG
TATGGCAGGATCGATGCCGCACTGTGTATACCGCATAGTGCGAGAAGGCCAATAATATTGTCACACCGGC
ACGCACTGACGGAACTGATTGTGTATCACTACCATGTGAAAATGAAGCATCAAAACGTGGATGCAACGAT
AGGCGACATCAGGACCAGATTTTTGGATAACAAAGCTGCGTCGACTGTTGCGTACAGTAATATCTGGATG
TAGTGTGTGCAAACTACATAGAGCGCAGCCAGCGCCACCTATAATGGGTCCTTTGCCAGAGGACAGACTG
GAGGCCAATGGATGGCCATTTAAGTTTACGGGTCTGGACTATTTTGGACCACTCCTTGTGACGATCGGACG
TCGAAAGGAAAAGAGATGGGTCGCCTTGTTTACGTGTCTGACGACAAGGGCCATACATTTGGAGCTGGCA
CATGACCTGTCAACTGATTCCTGCATAATCGTAATCAGGAACTTTCTTTGCCGTCGCGGACCTGTACTGAG
GTTGCGCAGTGACAACGGAAAGAACTTTGTTGGGGCCAACAGAGAAGCCAAAAGGCTAATAGATGTATT
CGAGCCGGAGAAGATTCAGGGTGAGCTATCTTCTAGAGGCATTGAATGGATCTTCAACTGCCCAGCGAAC
CCAGCTGAAGGGGGAGCCTGGGAAAGGATGGTGCAGTGTGTCAAGAGAGTGCTACGCCACACAGTAAAG

GAAGTGGCACCAAAGCAACACGTACTGGAGAGTTTCCTGATTGAGGCCGAGAACGTTGTGAATTCTCGCC
CGCTTACCCACTTGCCGGTGTCTGTGGACCAGGAAGCCCCCCTAACACCGAACGATCTACTCAAAGGAGT
GGCCAATCTGCCTGACACGCCCGCTATCCACGAGCAGCCGAACGAGAGATGCGTCACGAGGAAGCAGTG
GCGCATGGCGCGGCTGTTACGAGACCGTTTTTGGAAGCGGTGGGTCTTAGAGTACCTGCCTACACTTGTGC
GACGCGTGAAATGGTGTGCGCGCGTTGAGCCGATACGCCATGGTGATATGGTGTTTATATGCGACCCGGC
CGTGCCACGCAGAGAGTGGCGAAGGGGCATAGTGGAGGAGCTCTACCCTGGACCAGATGGAGTGCCTCG
ACGCGCCAGCGTCCGCGTGGCGGACAACAACCGAGTGCGACTGATGACTCGTCCCGTGGCTAAACTAGCC
ATACTGGATGTGAGTGAAGCAGGGCCTTCACGGGGGCGGGGATGTTGCGGGACGAATTAATTTATCGATT
GTTTAGTATTAAGTATATTCTGAAAGTATGTATATTTGCCCTCCGAATGTACGGGCACACTGTATTTGGCTT
GTTGCCGCCATTACTTAAATGTTAAGCTCATTATTGTCGTGAGTTCGAAGGCCGTAGGAGGAAAGACTGC
GGTAAGATTCCGAGGGAATATATATCTAAGTTCTAACCTATGTAATCCGTTTAGAATAAAACACTTGCACT
AGCACGTTTTGCATTACGCTGTCTCTAGTAACATCGGAGTAACATGGCCGGGGGCCAGGCGTAAACTTGC
AGTGTTCAAATTGCAACTCCACCTTCATTTGGCACGCGACA
>9_rnd-4_family-199__RC___Helitron
GCAGACAAGACNTAGTCTACCGGGCAAACGAACAAAGACAAAATACTGTAGACCACTCGACTCGACGAG
CTAATTCTCCAGTTAGAGAACAGGAGCAATCGGCTAATACTTCGCGTCGATCGACACGCAGACAAGACCC
AGTCTACCGGGCAAACGAACAAAGACAAAATACTGTAGACCACTCGACTCGACGAGCTAATTCTCCAGTT
AGAGAACAGGAGCAATTGGCTAATACTTCGCGTCGATCGACACGCAGACAAGACCCAGTCTACCGGGCA
AACGAACAAAGACAAAATACTGTAGACCACTCGACTCGACGAGCTAATTCTCCAGTTAGAGAACAGGAG
CAATTGGCTAATACTTCGCGTCGATCGACACGCAGACAAGACCCAGTCTACCGGGCAAACGAACAAAGAC
AAAATACTGTAGACCACTCGACTCGACGAGCTAATTCTCCAGTTAGACAACAGGAGCAATTGGCTAATAC
TTCGCGTCGATCGACACGCAGACAAGACCCAGTCTACCGGGCAAACGAACAAAGACAAAATACTGTAGA
CCACTCGACTCGACGAGCTAATTCTCCAGTTAGANAACAGGAGCAATTGGCTAATACTTCGCGTCGATCG
ACACGCAGACAAGACCCAGTCTACCGGGCAAACGAACAAAGACAAAATACTGTAGACCACTCGACTCGA
CGAGCTAATTCTCCAGTTAGACAACAGGAGCAATTGGCTAATACTTTGCGTCGATCGAGACGCAGAGAAA
ACCCAGTTTATAGGGCAGACGAACAAGAACAAGACAATATTCGTCGAGCTCGCCATCGACAAGACATAC
AACGACGACTTTTAGAAAGAGCTAGGGATAGGAATCAAAGATCACAGGTTAGAGAGAATCCGATTCGTA
GGATAATTGAGCAGGCACGTAACACAAGTGTGAGGCGAAGTGCTCGCATTCATGCACGACAGGAGGCTTT
TGACGCCAATAGAAGTTTGTCCCAGCGAGTTAGCCAGTATAGGGGTAATACAGGTAATAGGATAGCAGAG
AATAGGCGCAATGCTACGAGGAACAGGGGTGAACGTGCGATTAATTTTCAGAGGATAGAATCTCTCCAG
CTATTAGGAGTAGCTTAGATGGTCTGATAGCTGGATTCAATAGAACAGTTAAGCAGGGTCCCGATCAGGT
TTGCCTTTGTTGCAAAGGCTTATGGTTTCCACATCAAGTGGAAAAAATTTCTCGTGAGGTTATTGAAAGCA
AGTGTAGTGCAGAAGTAAGCTCCGATATTTTATATCTTTCCTCTATATTTCCCTCTGATAATAATAAATATG
ATTTTTGCAAAACGTGTAGTCGTAGCATTAAATTAGGCAAAAAACCTAAAACTTCTATTACAAATGGCCTA
GATTTTCCCGATCTGCCAGATTGTTTGAAAGGCTTAACGCCTTTAGAGGAAAGGCTGATTTCCCCTCGCCT
TCCTTTCATGATTATTAAGTCATTAGGATATGGTCGACAAAATGCAATTAAAGGAGCAGTGGTCAATGTAC
CCATTCCTGTGGCCAATGTTGTAACGTCCCTTCCGAGGGCGTTTAATGAGAGCGAGGTAGTGCAAATTCAT
CTCAAACGTAGGATGGAATATATCCATGATTTCATGGCTGAAACCATTAGGCCTTCTAAAGTTGCTGAAGC
AATAAAATTTTTAGTCAACACCGAGCTTTATAGAAAGCATCAAATTACTATTAATGAACAGTGGTTGTCGT
CTTTTTCGTCTGACGAGGTTCCGTTTGTTGCATCTGCTGATGACAGGGACTTAGTATCGCAGTTAGTGGCA
AGATCTGCTCAAGCCTCCATAGATGAGCAGAATAATATAGAAGAAGATATTAATCCAGGAGGCCAGGAA
ACATTATTAGAAAACGTCCCTGTCGAAAATATTGCCATGCAAAGAATAGTTATAGCCCCTGGAGAGGGTC
AGCGGCCTTTAGACATGATCGAAGATTGTGATTCAGAGGAGCTGGCCTTCCCAACTATTTTTGCTGGCATT
AAGAGAAAGTGTGCAGAAAGTTTTACAACCATAGTTAGGTCAGAACTTAGAAACTTTGATAGGAGAGGTT
GTAGAACAGATAAATTATTTCTCAATTTTAAAAAATTGGAAATGATTAATATAAGGAACAGTACTTCCATT
TGCTTACGTAAGCACAGCGCCACTCGAGGCATTACGGCAGCACAAGTCCTTAATGAGGAGTATTTAGAAA
ATTTAATACGTCACGATGATTGCTATAGGGTTTTGAAAAATATTCGCACTTCACCTGCTCATTGGGAAGGA
GAAAAGAAGAAGGTCATGGCGATGATAAGGCAGATTGGTCTTCCGACATTTTTTATAACCTTTTCGGCAG
CAGAGACTAGATGGGCAGAGCTGTTAGTTTTACTTTCTAAGAATGTAGATAAGCTAGAAATTTCAGAAGA
ATTGGCAGCTAATTTAAGTTTTGCCAATAAAGCCCGTCTAATTAGAGCCGACCCTGTTACTTGCGCTAGGT
ATTTTGATTTTCGTTACAGGCAGGTTTTAAAACTGATGAAAAAGGAAGGCGGTGTTTTTGGTAGTCACAGA
GTTATTAAGTATTATTGGCGAGTTGAGTTTCAGCAAAGAGGCTCCCCCACGTTCATGGCATGTTTTGGCTG
AAAGATGCTCCGATAGTTGACTTGGCTGATGAGTCAAGCATTCCGTCTGTAAAGTCCTTTATTGATCAGTT
TATCACAACTGAAACAAATAACCCGGATGTGGCTCCATTTTTGGAGTATCAAACTCACAATCACGGGCAC

TCATGTAGGCGAGAAGTTAGGGCACAAAATATATGTAGATTTGGTATTCCATACCCTCCCATGCCCCAAA
CAGAAATTCTATTCCCATTAGAAGAAGATGCTGAAAATAAAGAAGTGCATCGCAGAAATTTCGAAAAGAT
TCAGGGAGTTTTAGAATATCCGTTTGATCGAGAAAGTATAGATTACCTTAGCGATTTCAATAACTTTTTGA
CCCACGATCAACTTAACTTAAGTTATGAGTGCTATATAAATGCACTCAGGTCGAGTATAAAGAAGCCCAA
AATATATTTAAAGCGATCATTCGCAGCAATTAAGCTTAACGCTTATAATACTCACCTCCTTCTACTGCAGC
GCGCGAATATAGATATCCAGTTTATATTAGATCCCTATGCTTGCTGCAGTTACGTCGTTAATTATATTAAC
AAGTCCCAGAGAGGCATTTCAAAGCTAATGCGTGAAGCTGTAAGTGAGATAAGCAGGGGCAATGTAACTT
TAAAGCAAAGGTTACAGCATATAGGGCACAAGTTTATTTCGGGTACGGAAATATCCGCCCAGGAAGCCGT
TTACTGCTGTTTAGGTATGTCTTTGTCCGAAAGCAGTAACGCAGTGGTTTTTATTAATACCTCATTACCAGA
GAATCGGGTTGGGTTAAGGAAGTCTAAGCAACAGTTGCAAAACTTACCCGATGACTCAGTAGCAATATTG
GAAATTGGACTTATAGACCATTATGTTCAGCGCCCCGACGCTTTAGATAGTTTATGCCTAGCTGATTTTGC
TGCTTTTTACAGCTTTAGCAAGCGGCATAGTGGTACTCGCCAAAATATAGATGATGCTGATGACATCGAA
ACATCCAGGGCTTCCCAGCAATTAGTTTACGCACTTCGCGATGGTAGTGGGTTCGTAAAGAAGAGGAACA
CTCCTATTATAATTAGGTATAGGAGATTTAATATTAATACTGATAGGAGCAATTATTTTCGAGAATTAGTT
ATGTTGTATGTCCCATGGAGGGATGAAAATGTAGAACTAGTAGCAAGAGACTGCGAATCGTTTTGCGGCC
AGAATCATGAGTTAATTGAGAGCAATCGCAGGAAATACAATAGCTTAAATGACAGTGAGCTTCAGCATGC
TCTTGAAAATGCAGTGCAGGCAGAAAATGAAGAAAATGTTGAAAATCAAGAAGTAGTAGACGATAGATT
TCGCGTATTAGGATTACCAGAAATAAATCCTCATTTAAATGTCTTAAACATTAATGGAAATGACGAGAAT
CTAGATTCAGAAGATAACAATGTCCGATTAATAAAACTTCCAGCTATAGTTTCAGCTGAAAGCTTAGCAG
ATATAGTCAGGTCACTTAATTTATAAAGCAGAAAACTTATTTTACACATGTTTTTACACAATGCCAAAAAA
ACAAATCCGTCTTCTACGAATTTGTTGGAGGTGGTGCGGGTGTCGGAAAGAGCCGACTTATTTTAGCTCTT
TTTCAATCTTTGTCCGTTATGTACAATAGTAGGCCAGGAAGTGATCCAAATTCTCCTAAAAATAATATTGT
GTGCTCCAACAGGTAAAGCTGCTTTCGGAATCAGTGGCTCAACACTGCATTCCATGTTTTCCTTACCAGTA
AATCAATCTGGTTCCGATTTGAGGGCTTTAAGTAATGATTTAGTAAATACTTTGCATTCAAGGTTAATCGA
CCTTAAACTAATTATTATAGATGAAATATCTATGGTAGGCTCCAAAATGTTTTTCCCAGTTGGACTCCCCG
TTTAAAAACAAATTTTTAAAAATACTTCTCCATTCGGTGGTATTTCTGTCATAGGTTTTCGGGGATCTACGA
CAATTATCTCCAGTAGGTGACCGTTGGATTTTTCGAATCCAACTCTAATGATCCCTACAGTGCAATTTATG
CTTCATTGCTGTGGGACCTTTTCCAAGTATTTTTGAACTCACAGAAATAATGCGTCAACGGGAGGACCAAC
CGTTTGCTTCTGCTTTAAATAATATGGCATCGGGAAACATGACAGCCGATGATATTAGTCTTGTCCGATCT
CGCATTTGTCAGCAATCGGATGTGCCCGATGATGCCATTCATCTATTTAGTACAAATGCGGCGACAGATAG
TTATAACACGATAAAGCTAAACTCAATTCCGACTGATCAATTTATTTCTACCGCTGATGATTTTATAAAAG
CAACACATTTAACTGCGGCTTGTAGACAGAGAATTTTAGAAGCCGTTAGTAGCTTTAGGTCGTCCGAAAC
ACAGGGATTGTGCATGTCCCTTCTTCTAAAAACCACTGCGAAATATATGATTACGGTCAATGTAGATACTG
GGGATGGATTAGTTAATGGGGCAACAGGCATGTTAATGCAAATAGATTTCGATGCATCCAGCACTCCCAT
TATTTTATGGATGAAATTTTCAGATGATACCGTAGGAGTACTTGCTCGCTCAAAGTGTACACATCGTACAG
AACCTTTTCTGGACCCCAATTCAGAAAATAGTTAAAAGGTTTTCAATATAGAAGAAATGAGCAAGTTTCT
ATTGACCGCAGACAGTTTCCAGTTGTACCTGCAGAAGGTATAACTATTCACAAAAGTCAGGGAGCTACGT
ATAGCAAAGTGGTGGTTCACACTTGCTCTCGTATGCAGCGTGCTGCAATATACGTAGCTTGTAGTAGAGCA
ACAACTGCTTCCGGGTTGTTTATAATAGGAGATTTTGTTCCTCCTAAGCCTCGAAAAATGACGATAAGGTC
CAAAATGAGATAGAGAAATTGCGGACTGGAAAAACTACTTCGAACACATTATGATCACTTAGTTAACAGT
TCCGCCCTAGACATTTTCTTTCATAATACTGAAAGCATTCACTGTCATATTGAAGATATTCGATCAGATAA
GCTTATGCTTCAGTCGTCTGTTCTATGTTTCGTTGAGGCCTCAACATATTCAAATGAAAATATTGAAATTCC
AGGGTTTACTGTTGTTGTCCGTCTTGATTGCGAGGTAATACCGAATGCACCGCGACCTAAGAGGGGAATC
GTCATTTATATTAAAAATGCAATTTTAAACAATATATCTAATTGCTTTAGCGGTAGGCAAATTTCTGGGTC
ATTCGTTTTTGAGCATGCAATATTTCGTTATAAGTCTGTGTATGTTTTTAGTTATTTACAAAAGTCCGTCAT
ATCCGCTTGGTTTGTTTAAAACAGAATTTGAGGTTTTATTTCAGCAATATTTATTTCTAAGGGAGCGTTGCA
TTGTTTTAGGAGACTTTAATTTATGCTTGAGCAAATTTGATAGATGTAGGGCTCTTTACGATGAATTGCTC
AGAAAGGGATTAAGGTGCCTATTAGATTTAAAACGCCCACTACCAATTGTGACTCTCACATTGATTGGGCT
TTTTCAAATCTCTCCAGTGAGGAAGTTAGCGCTAGGACACTCGAGACAGTGTATAGTTACCACAGTGGTAT
CTCTCTAACGGTTCAGGATAGGGTAAATTAAGTTTCTATATATATGTCTATATAATTACAATATGTTTAATT
TAAACTTAAGAATTTTTTATGAATATAAATAAATATATTACCTATATTTATATTTAAAAGTTGTTTTGTGGT
CTTGTACTTGGGTTCAGGGTATTTTTCTAGTCGGGCACCCCCGACTAGAGCACTCTTACTTGTTTT
>160_rnd-1_family-354__LTR___Pao
TGTTACGGGGCGAAGTGAAAATAGCTGTTGACTAAGTCCTACTGGGTCTCGCATTTTTTCTGTAAGCAGCC

CTCGCTGCTTACACTGGAAGCGCACCGTTAGAATTAGTTTTACATGATAAGTCGATCGCACGCAGCTGCA
CAAGCTGCATTTTCGTCGAGGTTCATTTTCGTTTTGTGCTTGCAGCTGAGCAGAAGTGAAAGAGAACAGAA
GCGCGTGCGGAGCACGAATGTAAGTCTCATAATCATGTAAAATATAGGTTGTTAAATAATGAAAGAATAA
TAAAGATTTCAGCGTTCGAATTATATTATATCGTACCGCCTCGTACGAATCGAATCGAATCGAGTTGTTTC
TTTCGACGCTAGAAGTCCCTGCCGAATACGCGCCCCGGCGCGAGTCTCGGCGCCAACATTCTGAAATATTC
TCTGCAGCAGAGAGTGTCCGAGGGATTCTCTGCTACGGCGGAGATTTCTGCGAAGCAAGCGACTTGCAAA
GTCACAACCTTAGCTGCCTATAGAGAGACACGTGGTACAAGAGCTAGACTCACACTTAGCAGCCATAACC
TCAAAGCGGATTTCATAGTATTGGTGTGGCGAGATGGCTGACGGTGATGAACAAGATGCAAGTGCGAGAA
GGACGACAAGTAGTATTCAACCCGGTGGAACTGCAACGCTTGTAACGGCATGGTCAACGGGACAGGCTA
ACGGATCGCCTGTTCCTGCTGGAGTACAAGATAAAGTCGCCGGTGTAGAAAAAGGTAGCGGGGCAGCAA
TCATAGAAACTTATAGCGGCTGTCCGTCTACGTCGTCCGCTGCTACTGGTCCAAGGGAAAGTTCAACACCG
ACGCAGCAAAGAACTGAGAAAGTGGGAGCTCTTTTCGACCCTTTGGGATACCAGAACGTGATCTACAAGC
AGAAACAGGTTGCGGCTAATTGGAGTGTACACCCCAGTTTGCCGTATCAAGCAAATACGGCGGTGTTTAA
CCAGGCTACGGAGCCCAGTACTGCCGCGTACATGGGCCACACAGGTCTGGGAGCGCTCAATAGATCGATT
GAGCACGCGCCGCGGCAGCAATTCGACAATGTGCATTGTAATAATGCGACGCCGTGGAATGGATCGCTGA
CACCTGCGCAAATCAGCGCGCGACACGTCATCAGCAAAGATTTACCAATTTTTACAGGCAAACCAGAGGA
ATGGTTGATCTTTATCAGCAATTATGAGCAATCCACAGAGAGATGTGGATTCAGCAACGAGGAAAATCTC
ATCAGGCTTCAGAAGTGCCTCAGAGGGCAAGCCCTGGATGCGGTGCGCGGGGAAAACTTATGATCCCAGC
TACAGTGCCGTATGCTATAGGCACATTGCGAATGCTGTATGGCCGCTCTGAAATAGTACATGCTGCGCTGC
AGCAAAAGCTACGAGAGGAACCAGCAATAAAGTCGGACAATTTGAACACCGTTATCCGACTGGCCTTAGC
TGTCCAAAACTACTGCGCAACGATAGCGGCCATTGGTATGCACGAATATCTTTACGATCCCGCGCTGCTGA
ACGAGCTTGTTGCTAAGATGCCCAGCAATATGAAATTGGACTGGGGCCGGCATCGAATGTCGAATGANGG
TAGTAGTTTGGCCACCTTTGATAACTGGCTGTTTAATGTGGCTATGTGCGCCACTATGGTCACGCCCTACG
AGATACCACGGACTGATAACGAAAAGAAGAGCTCCACGAAAACCACTAGAGAGCGAATTTTTGTGCACA
ACACGGTAGATAACCCTGAGGGTTCGCAGCAACAGCGTTATCAACAGGAACAGGAGAGAGTTTCATGTGC
GAAGTGCGCCGGTAACCACCGTCTNTCAGATTGCGACGACTTTAGAGCCATGGGTACAAAACAGCGGTGG
GAACTTGTTAAGGAAAAGCGGCTCTGCTTTTGCTGCTTTATGCGACATCGTGCACAACAGTGTAAAACAA
AGAAGAAATGTCACGTGGATGGTTGCGAGTCAACGCACCATGTACTGCTACATACGCCTACCACGAGCGG
TAGTAAAGNCGAGCACACGGGCCAAGGTAGCAACAACCGGACTAAGTCAANGGAGTCTGCCGTTATGTTT
CACGGCAGCTCCGCAAGTAAGACACTGTTTCGATATGTTCCAATCACGCTCTATAGTAAATCAAAAAGCG
TGCGCGCATATGCATTAGTGGATGAAGGAGCTGAGTGTACGCTAATAGAAAGAGCGCTTGCAGACGAGCT
GGAACTAGATGGTCCNGCGACCCAGCTGTGTCTTAAATGGACAGGNGACATAACCAAGAACGAACCTGA
GTCGATGTCCGTGGCTGTGAGCATTTCAGCTCCAGAACATAACGCGATACAGTATGCCTTAAAAAACGTG
CGAACAATAGCGAACTTGGACTTACCAGAGCAAAGTATCGGGCCACAGTTGCTCAAGTCTCGGAAGCACT
TATCTACGCTTCCCATTCTGCCGTATAACAACGTCAAGGCTCAGCTTATCATCGGCTTAGACAACATCAAA
ATAACTGCACCCCTGGAGATACGAGAAGGTGAAAACGACGACGTCATAGCAGCGCGAAGCAGACTAGGA
TGGGCCGTATATGGCCGCCCCATNGTGGGAGACAGCACCACGCCTCGGCTGCTACATATTTGCCAGTGCA
GTGCGGCGCGTGGGATGGATGAAGCTCTGAAGAACTATTTTTCAATTGAGTCACTAGGTGTGAGCGTTTCC
TCACATCCGCTGAGATCCAAAGAGGACGAACGCTCGATGCAGATTATGGAGGCAACAACAACCTATCTGG
AAGACGAAAAGCGCTGGCAGACCGGCCTGTTATGGAGGTTCGATCGCATGCACCTGCCAGACTCTTATCA
GATGGCTGTTAAGCGCCTGAAATGTTTAGAGGCAAAGATGTCAAAAGACCCACCACTGAAAGAGTTCATG
ATGAACACGATGCACGATTACAAACAGAAGGGATATATCCGCCGGTTGAAGGATAGTGAGTTAGTAGCTA
ACACTACGTCGTGGTTTCTTCCTATCTTTACAGTCACCAATCCGAACAAGAAAAAGACGCGCCTGGTTTGG
GATGCGGCCGCCAAAGTTAGTGGCACGTCGCTGAACGATTGTTTGCTGAAAGGCCCAGATACACTAGCAT
CTTTGATGGGGGTGCTAATACGCTTCCGAGAGCGTCCTATCGCCGTATCCGGCGACATACGGGAAATGTTT
CATCAGGTCAAGGTGCGCCGAGAAGACCAACCGGCACAGAAGTTTCTCTGGCGTGATGGAGATTCGTCAC
GCTTCCCCGAAACGTACGTTATGCAAGTCATGACGTTCGGAGCATCCTGCTCGCCAGCCCTAGCAAATTAC
GTCAAGAATCGCAATGCCGAGCGGTTTGTAGCGGAGCATCCGGATGCCGTAAAGGCGATTTGCGAAAACA
CATTTGTCGACGACTGGCTGCAGTCGGTGGATACTGGAGCCGAAATGATGCAGTTAGCTGAGACTGTAAA
AAGGATTCATGCTAGTGGCGGCTTCGAGATGCGCCGCTGGACATCAAATTCGAAGCAAGTTATGCAGGCG
CTGGAAGACGACTGTGAGGAGTTGGACAAGCGTATCAGCGCTCAGGATGAAGCGCAAGAGAAGGTCCTA
GGCCTGTGGTGGCTACCTGGACAAGATCTGTTGACGTTTGTGGTGACGCCGAACTTGCTCGCGAAGGCAT
CTAAGGAGCGCCCAACTAAAAGACGCGTTCTGAGTGTGATCATGTCTATTTTCGACCCGCTCGGGCTGCTA
GGATTCTTTAATATACGCGCTAAGATCATCCTTCAGAACATATGGCGATCCAATATCGGATGGGATGACG

ACCTTACCAACGAAGACGAAGCCGATTGGCGACACTGGCTTGATCTAGTTTCAAATTTGAATGCCGTCCG
CATTCCACGGTGCATGAAGTGGGTGAGCCGAACCCAGGCTGTGCAGATGCATACATTCGTTGACGCCAGT
ATGAATGCTTACGCCGCAGTTGTATTTTTGCGGGCTGAACTTGATGGCCAAGTACATTGCAGTTTGGTCGC
CTCGAAAACGAGAGTAGCACCCCTAAAGCCCGTGTCCATACCTCGAATGGAACTGATGGCCGCGGTCTTA
GGTCTGAGACTGGCCAAATGTATCCAAAAGGAAATGTCGGTACGCATACATAGACGAACATTCTGGACAG
ATTCAAAGGATGTGCTCTACTGGATCCGTTCCGATGCTCGAAAGTTCCAGCAATTCGTAGCGCTTCGGATN
GGAGAGATTTTAGAAGAATCAGATGTGGACAGCTGGCGCTGGGTNCCATCGGCTCAAAACGTGGCAGAC
GATGGCACGAAATGGACCAAAACGCCCGAAATTCACGGCTCAACCAGGTGGTTCAATGGACCACCATTTT
TGTATCTAGAAGAATCGCAGTGGCCACAATCGGAAATAGGCCCTGCATTAAACATGTTGTACCATGCTGA
AGAACAACCCAATCACACGTCGTCCTGGAGATGTATTCTGCCGGATCTGCAGCGTTTCAGCAAGCTAGAA
AAATTGAGAGCCGTACAGCTATGCGTACTGGATTTCCTACGGAATATTACTAAGAAGGTCAGATTGGACG
GAGGACTGGATCTGCAGAAGACGCTGCTCCTTAAAAGAAGCAATGAAATGGATGTGATTTTTATCCGGAT
TTGTCAAGAAGAGGAGTTTTATAGTGAGATCACCTGCTTAAGGTCGGGGCGCCGCTTAACCGATCGCAAA
AGCTTGCTGTTTAAGTGCTCCCCTTATGTGGATGACTCGGGCATTCTACGTATTAAAGGACGGATAGACAA
TATAGAAGGAGTCGAAGTCTGCGTAAAACGCCCGATAATTCTGCCAAGACGACATCAATTGACGTATCTG
CTGGTTGAGTTTTATCACCGCAGATATCATCACCTACACAACGAAATCGTCGTAAATGAACTGCGGCAGC
GGTACTGGGTTTGTGGCTTACGAGCTTTGGTGAGAGAAGTTTCCAATACCTGCCCAGCGTGCCGCATACGA
CGCGCCCGCCCAAAGCCGCCAGGGATGGGCGACCTGCCAATCGAACGATTATCGCCCTATACTCCACCGT
TCACATATACTGGAGTGGATTACTTCGGCCCCTACGACATTGTCGTTGGACGGCGTCGTGAGAAGCGCTG
GGGCGTGCTATTTACATGCCTCACGGTGCGCGCCGTTCACCTAGATATAGCTACGTCGCTCTCAACTGACT
CATACTTATGCGTTCTGAAGTCGTTCGTGGCCAGACGCGGCTGCCCCCGCCGCATGCTGTCGGACAATGGC
ACAAATTTCAGAGGCGCTAGCAGAGTATTAAAAGATGAAATTGAACGTATATCGCCGGCTCTGATCGAGC
GACAGTACCCGGAACTCGAGTTTACCTTTATCCCGCCAGGATCACCCCACATGGGAGGATCGTGGAACG
AATGGTGCGCTCTACAAAGTCAATACTGTCGGAGATTCTGCCGCCCTCTGGGTTACGAGAGGAGGTGCTA
CGAGCAGCATTGGCTGACGTAGAAAGCGTGCTCAACTCAAGGCCGCTCACTTATGTTCCCTTGGAGACAT
CTGAGTCCGAAGCCCTGACTCCGAATCATTTTCTTATAGGTCATTCAAGCGGATTGCGTGAGAGAGGCTTA
CGGGAGCGAGGCGNAGCTAGCTTGGCACGAGGCTTCCGAGTCGCCAGTCAATTGGCTGACCAATTTTGGA
AGAGATGGCTGCGCGAGTATCTGCCTACACTCACCAGACGTACTAAGTGGTTTCAACCGCCACTCGAACC
AATATCAGTCGACGACATCGTCATAATAGTGGACGACGGTGCCAAGCGAAACTGTTGGACAAGAGGAGT
GGTCGTAGACGTGCACCGTTCGAAGGATGGCCAGGTCCGAAGTGCCGTGGTGCGAACCGCCGATGGAATT
ATTACTCGCCCCGCCGTCAAGCTAGCTAAGCTTGACATTAAAGTTGGTAACACACTGCACGGACGTTCGTG
AATTACGGGGTGGGGAATGTTACGGGGCGAAGTGAAAATAGCTGTTGACTAAGTCCTACTGGGTCTCGCA
TTTTTTCTGTAAGCAGCCCTCGCTGCTTACACTGGAAGCGCACCGTTAGAATTAGTTTTACATGATAAGT
CGATCGCACGCAGCTGCACAAGCTGCATTTTCGTCGAGGTTCATTTTCGTTTTGTGCTTGCAGCTGAGCAG
AAGTGAAAGAGAACAGAAGCGCGTGCGGAGCACGAATGTAAGTCTCATAATCATGTAAAATATAGGTCG
TTAAATAATGAAAGAATAATAAAGATTTCAGCGTTCGAATTATATTATATCGTACCGCCTCGTACGAATCG
AATTGAATCGAGTTGTTTCTTTCGACGCTAGAAGTCCCTGCCGAATACGCGCCCCGGCACGAGTCTCGGTG
CCAACA
>Ref_Telemac
CTGTGTAGTTGCAAGCTTTTTTGCAACCAATTAATTCTAACTGCTTGTGCAGCGAAATTCAAACACTTAGA
TTAGTTTTCAAATGGATTTATTTGCCGCGCAGGGCACTTATTAGAATTTATAAGTTTTAAATTGTTGCTTGG
CAGCCAACTCGAATTAGTTGTGCCTAGGTTGCGATCAAAACGAAAGACTGACTGGTTATAACAATATGTA
AAGCGAATCGAAACGAAACTCCAATAAGACGCTCGCCGGTTAGAGCGAAGGGCAGCAATGCAAGCAAAG
CAATTCAGAAGGAGAGTGGACAGCAGCTTACAAAGAGCGCGCAAAAAAAACTATTTGCTCTTAGGCTGTA
ATTATGATTGTTATTTTCTGCTGTTGGATGATGTCCGCAGCCGTCGGTGTTATCTCTGCTGTTACTTGACCA
AACATTCTCCCCCCGTTGGAAGGCAAGGGCTTTCAACATCATCCTGTTTGGGCAGCAAACACAGCTTTCGT
ACAGCTCTACGTATGACTCCAGTCGCAGTTTGAAGCACAGCAACTCTGGATACCCCATCAGATCCAGAGA
TCAGCTCTTGCACTCTGGCGAGTGGCCACTTCAGCGGCGGTAGATTCTCATCCTTTACAAGGACAACATCG
TTAATGGAGAGTCCAGGCTGAGGAGTGCGCCATTTGGAGCGCTGTTGAAGAAGCGTCAAATACTCTTCGC
GCCAACGGGCCCAGAAATACTTGTTGGGAGATATGTGACGGCGCTGAAAATAATTAAGCCCGATTGAAGT
TAAGCTGCCTTAGATCGGGCTCAATGTATGATGAAGATGGCGCTGTACCCAGGAAGTGTGCGGGTGTCAA
GACGTCAAGATCACCTGGATGCTCTGAAAGTGGAAGTAATGGTCGAGAATTAATAATTGCCGTGATGTGG
CAGACGAGCGTACGCAGCGAATCGAAATCCAAAATGGAAGAGCAAACAGATCGATAAAAGTGGTGCTTA
GCAGTCTTCACAGCGGCTTCCCATAGTCCACCAAAGTGCGGAGAGCGAGGAGGGATGAACAACCATTCAA

file:///E/10月/10.9/PDF/4. biology-1907542/biology-1907542-supplementary/SupplementalDocument2.refTElib_01_25_2022.fasta.txt[2022/10/9 15:38:02]

TTGATTCGGTTAGGCAAAACTCGTTAACGGCCTTGACATGCTCGTCTCTCAGGAACAGGCGGTTCAGATCT
GCTAGTTCGTTCTTGGCACCTACGAAGTTTGTCGCATTGTCAGACCAGATCCTTGAAGGTCGAGAGCGAGT
CAGGATGAAACGTTTTAGGGCGTTTAGAAACGATGTTGTGGACAAGTCCTTTACAAGCTCTAAGTGCACC
GCCTTTGTAGCGAAGCATATGAACAGACTGATATAGCATTTCACTGGTGGCCTGTTGCGCACTTCGTTCTT
GTAGTAAAATGGTCCACAGTAATCAACGCCAGTGACCCATAAAGGGGATACGATGTTATTAAGACGATCA
GCTTGGTAGGTTCTGCCATTATATGCTTCGGCCAATCGTGGCTTGGCACGGAAAACAGATTATGCATTTGG
CAACAATACTGGAGACTGTTTTTCGACCGCCAATGGGCCAGTACTGGAGCCGAATCGAAGACAATAAAGC
GCGAGGTCCAGCGTGTAAGTTTCGTTTATGAAAGTAAACTATGATTGCTCGCGTCACAGGATGCTGACGG
GGCAATATAATCGGATGTCGCGCTGAGAAGTCCAACGACGAGTTTTTCAGCCGTCCACCGATGCGAAGTA
GGCCGAAGTCGTCAAGGAATGGTGCAAGAGAGGCCATTGAGCTAGACGGTTTGACATGCCTTCCTTCCTT
CAATGCCTTGTAGTCATCGCTGAGAGTAGCCATTTGCACTGACCGCAGCAACCAATGTGTGCCATGATGC
AAGTGGTCAACGGTTAGTTCAGCTCCTCGGATTCGATTTACAAATTTGTAAACATAAGCAAAGACCCGCT
GCAGCTTGGAAAACGAATTGATGAACTTGCAACCAATCGAGAGATCCGGTTGCGCTGCAGTTCCGAGGAG
TACCTTGTGTCGAAGTTCTGGTAAGGATTCGACAGGTACACAGGAGGCAGGCCACTCTTCCTTTCCCTTAC
TGAGAAAACTCGGACCATGGGCCCATAATGGAGACCGGAAGAGCTCACTAGGCAGCGCTCCTCGTGAAA
TTATATCCGCCGGGTTTAATTCGGTGGGAATATGATGCCATTCCATTCCAGTGGTGAGCTCTTGGATGGCT
GCGACTCGGTTGGCAACGAAAACATTGAATTTCGAAGCATCATTGCGAATCCAAGCCAAAGTCACGGCAG
AGTCTGACCAACAGTAATACCGACAGTCGAACACTTTCATTTGGCATATTTCGCTGAGAAGTTGAGCCAGT
AATGCAGCTCCGCAGAGTTCCAGCTTTGGTACCGTGATGGTTTTCACAGGCGCGACACGCGATTTGGAGC
ATAAGAGACGCACGCTGGTGTTGCCATTGCACTTTGAGACTGTATAGACGCATGCTCCATAAGCGCTTAG
GCTGGCATCACAAAATCCGTGAATCTCCACTGTACTGTCTGATGAGAGTGCTCGACGGGGATACTGGAAT
TGCTGAGTATACTCAAAATCAGCGCAGAAGTTGACCCAGGCTGTGCTTAAGTGTACGGGCAGGCTCTCAT
CCCAGTCCAGCCTTTCTCTCCAGAGATCCTGCATAAAAATTTTCGATTTTGTTATTACGGGACCAACAAGA
CCAAGGGGGTCGTAAAAGCGAGCAATTGCGGAGAGTATTGACCGTTTTGTCAGTCTGGAGGGCAACCTCA
GTGGAGAGAAGGAGAAAAGGAAACAGTCTGAAGCGGGATCCCAAACGAGGCCTAACGTTTTCGTGATGT
CACTGCCATCGTCAAACTTAAGCAGCGTTTCTCTATCCTCTTCCGGATATGTTTTGGAGTACAGAATGTGT
CGCTAGAACACCATTTGCGCCAGCCTAAAGTTCCCCTTGGCGAGTAGTCCAGATGTTTGTTTTCAATATCT
CAATTGCTTCTTGAACACAGCTACCACCAGAAATGAGGTCATCCACGTAGAAATCTCTTTTAACAATGTCA
GAGCCAATAGGGAAAGTTTTCTGCTCATCCATGGCCAGTTGGTGCATAGCTCGCACTGAGAGAAACGATG
CTGGTTTTGTGCCGTAGGTGACGGTGTCTAATTTGTAAATCTGTATCTTCTGATGCTGAGACTCACGCCAC
AAGATACATTGAAAATAACTGTCTGCGGGTTCGACTCGTACGCATCGATACATTTTGCATATATCGCCTGT
CAGGGCGACTGCAAATGTACGAAACCGCATCAGTATCGAAAACAGCTTCGGTTGGATGGTAGGACCTGCC
ATCAGTGCATCATTCAGCGAGTGTCCAGAGGTAGTAACTGCTGAGCCATCAAACACGACCCTTAGCTTGG
TTGTAGTGCTATCCTCCTTCAGCACGCAGTGATGTGGCAGGTAGTACTTGCATTGGCCCAGTGCAGCTGAG
GTAACAAGTGACATGTGACCCAAGTCAAGATACTCCTTGATAAATGCTGCATACTGGGGTTTCAATAGTG
GATTACGGTCTAGTTTTCTTTCTAAGTTCAGGAATCGACGAACCGCCTGTTGATAGGAGTCACCCAGCGGA
TACATGCCTAGACGTAGCGGTAAACGAACTGAATAGTCGCCAGTCGACAGACGTTTAAAATTGGCCTGAA
AGTGTGCCTCACAGTCGCGTTCCTCCTTGTTTGGTAAGGACTCAGGGTCGGTGCAGCATTCCAATTCCCAA
AAACGACGGATTAATTCATCAATCTGTGTATTAGGCTGCAGATGCGAGTCAACCAGCAGATCTGGATTCT
CCATTGATCTCATGGCCATGAATGATGATTTTCCAGCATGTGAGGCACCTCCCGTAGCCACCCAACCAAGG
CGAGTCTTTTGCAATATCGGCAGTCCAGCAGCTAGTTTAATCTGGCCGACGCATAGCAGATCGAAGAACA
GACTGGCTCCAATCAACATGTCGATTTGCTGAGATTTAAAGAATTCAGGATCAGCTAGTTGTATATTTGAT
GGAATGTTCCATGATGCAGGGTCAAGAGTGAAGCCAGGCTGATTATCGGTGATGGATGGTGCAATCAAGG
CCGGGATGCATGTGGAGTACTCCGACACTCGAGATTTGACATTGATGTTGACCGAAAACCCATCGGACGC
AAATGCTGCATCACCAATGCCAGAGACGATTACTGTTGACTTGAATTTGCGCAGCTGGAGCTGATGAGCA
AGACGAGAGGTGATGATGTGCAACTGCGACCCAGAGTCGAGCAACGCACGGCAAGGCACCAAGGAGCCA
GCGCGATTTTTCACGTTGATGACGGCAGTGGCAAGCAGGACCACATCGTGCTTGAGATGCTGGGCGGTAG
TGAGAGGCGAAGATAAAAGTGCCGCCAAGGTGTTTTGGGATGACGAATAAGTTTCGGTATCTTGAGCATT
GTTTGGGCTAGAAGCAGCGATGTGACTGCTTGTGTTGCCAAGATGCAACAAGCTATGATGTTTGCTTCCAC
AAACTCTGCAAGCGCTGGAGCCGCAGACTCTCAGCTGATGTCCCCTTTGCAGGCAGTTTAGGCACAGCGC
AAGCCGCTTGGCTTCGTGAAGGCGCAGCAAGGGCGATAAATTTGCGAATTGCAAACATTTATAAATAGAG
TGGCCTGCGACTTCACAAAACACACAAGCACTCGTTCCATTCCTAGTCACTACAAACGTTCTTCTACTACT
GTTGTTCTGGCCCACCTGGGAGCTAGGCGTGTACATTGCCGTAGCGTGATCCGCATTTTCCAGCCTTTGGC
AACGCCTCTCTATGAAGGTTGTAAACCGCTCGCATGATGGTATGACGTCCAACGGCGCATCATCCTCCCAG

CTAGCCTGCGTAACGCTATCTAGTTTTTGCAGCAACGTATGTACTATGACGCAACCGGCGATCTGTTCCAC
ACTGCCCATCGATTTTAAAGCACGTAGATGAGAGTTGAGCTTATCTGAAAATTCGCGCAGCTGTGCAGTC
GCGCCCTTGTCCACCTTTCTCAAACCCAAAATTTCAGAGATGTGTGCCTGAAAAATAAGACGCTTGTTATT
AAAACGCTTATCAAGCAGTTCTAAAAGCGGCAGCATAATTTGCGTTTGAAATTTCCAATGAGCGAATTGT
ATCCAGCGCTGTTCCTTTAAGGCATGACCGTTAGATGCTGGAGTTTTTCAATGTTGGTCAAATACGGATCC
TTGTCAATGACCGTGGTGAACAGGTTCGAGAAATCGGCCCATTCTGTGTAGCCTCCACTAAACTCCGGCA
ATTTAAGTTGAGGCAATCGAGAGTGGTTTGGAACAACAAAGGTAGAACGCGAGTCATCGGCTGCGGCTGA
ATTGAAGGTGGAACAATGCGGAATCTGCTGAACCTTGCTTCGATTTCCGCGCTCGCGTTGAAGGGACAGC
CGTAGCTGCCTAATAGTTGACTCAAAACTTGTCTGAAGATCCTCATCAACGCTGGCATGATCCACCTCCAA
CAAACCATCACAGACTATCTCAAATTTCGCCTGCAAGTCAGATGCCATGCTTATGTAATCTTGCAGCTCGT
AGTCATCAACATCGCGAAGTCGAGCGGGATCAAGTTCCGAGGACAATCTATTGAGTCGATCAGTTAAATT
CTTGATCTTTATTTTAAAAACAGCAGCCTGGGTTCCAACCGCCACTTGACTAGCTGATGTCGCCTCATCAT
TTCCATTTGCACTTGCACCTTCATCAAACATTATGAATGTAGCGCACAACAAGGTTCACACAGCCGAACTA
CAATACAAGTCTGATCGCGCAAGTGTTCGTTCGAGCGAGATAGCGTATAGCGGGTTATAGCGCGCAACGA
TTAGCGGGGATTTGCTACGGCGTTTATGTATAACAACCAACTGTATGCAGATGCGCAAGCAAACGGCTCA
CCACACAGCTTTGCCCGAACTGATAACTGTCCGTTGACACGACAAGAACGAAGTACAAGAAAAAAATAGA
AAAAATGAAAGAAAGAAAAATCACGTCGGGGTCACCAAAATGTGTAGTTGCAAGCTTTTTTGCAACCAAT
TAATTCTAACTGCTTGTGCAGCGAAATTCAAACACTTAGATTAGTTTTCAAATGGATTTATTTGCCGCGCA
GGGCACTTATTAGAATTTATAAGTTTTAAATTGTTGCTTGGCAGCCAACTCGAATTAGTTGTGCCTAGGTT
GCGATCAAAACGAAAGACTGACTGGTTATAACAATATGTAAAGCGAATCGAAACGAAACTCCAATAAGA
CGCTCGCCGGTTAGAGCGAAGGGCAGCAATGCAAGCAAAGCAATTCAGAAGGAGAGTGGACAGCAGCTT
ACAAAGAGCGCGCAAAAAAAACTATTTGCTCTTAGGCTGTAATTATGATTGTTATTTTCTGCTGTTGGATG
ATGTCCGCAGCCGTCGGTGTTATCTCTGCTGTTACTTGACCAAACAG
>160_rnd-5_family-9342__LTR___Gypsy
GGAGCCGGAGGAAGGCCTCCCAAGGCATGGAAAGGGAGCACCTGCTCGGCTACAGAGGGAGTCGGAGGA
AGGCCTCCCAAGGCATGAAAAGGGAGCACCTGCTCGGCTACAGAGGGAGCCGGAGGAAGGCCTCCCAAG
GCATGGAAAGGGAGCACCTGCTCGGCTACAGAGGGAGCCGGAGGAAGGCCTCCCAAGGCATGGAAAGGG
AGCACCTGCTCGGCTACAGAGGGAGCCGGAGGAAGGCCTCCCAAGTCATGANGGGGGCACCTGCTCGGC
TACAGAGGGAGCCAAAGGCAGGCCTCCCAAGTCATGAGAAGGGAGTACCTGCTCGGCCTTCGACGGAAA
AGGTCAGGAAAAGGGGAGTAAAGTCCGGCCCGAATCGACAGCCTGGAGAACCCAAGAGGTGGCGCATAG
AGCTGCAGAGGCAGACTTCTAATGGCTACGGAAGCGGCACGGCGGGAACGTGCAAGAGAAAGGACGGAG
TAGGAGCGATGACCAGAATCCACACGGACATAGAAGGCCAGGAGCGATCGCCAGAATCGAGCGGCCACG
GAGTTCTGTCTGGAGTCGAAGTGGTCCCAGGATGGGGCGATAACGGCATCGAAGAGGCGGATTGGCCAG
CGGGTCTGGACCCAGAACTGAGGGAGTTCCTGGAGTCAGAGCTGGCTCTCTTCGAGGGGCTGCAGGGAAT
CTCGCACATCGCGGAGCACCGAATCCGGCTGAAGGATGACAAGCCGTTGAAACAAAGGTACTACCCCAA
GAATCCGGCCATGCAGAGGGTGATCGACGAGCAAGTGGATGAGTTGATCCGGGCAAATGCCATCGAGCC
GTCGCGTAGTCCGCACAGCGCCCCGATCGTCTTGGTCAAGAAGAAGACTGGCGAGTGGCGTATGTGCATT
GACTACCGACAACTCAACGCGCATTCCATTCCGGACGCTTATCCGGTGCCTCGTATTAACCACATCCTGGA
GCGGTTGCGCCACGCCCGGTTCATCTCCACTCTGGATTTGAAGCACGGGTACTGGCAGATACCGATGGCA
GCCGATAGCCGGGAGTGTACGGCGTTCACAGTCCCTGGAAGAGGTCTTTTCCAATGGCGGGTGATGCCAT
TCGGCTTACACTCGGCGTGTGCGACATTCCAGCGCGCTTTGGATACGGTAATAGGGCCGCAGATGGAGCC
TCATGCGTTCGCTTACCTAGACGACATCGTGGTAATAGGAGCGACGAAAACACAGCATGTAGAGAATTTG
AGGGAGGTATTCCGCCGGCTAAGGGCGGCCAATCTCCGGTTAAATAGAAAAAAATGCAGCTTCTTCCGGG
AGAGGTTAGTCTACCTAGGACATGTAATTTGCGGAGAAGGGATACGGACCGATCCGGAGAAAGTGGAGG
CGATACGAAACCTGAGGGCCCCAACAAATTGCAAAGAACTGCGACAGTGTCTCGGGATGGCGTCATGGTA
CAGGCGTTTCGTACCCAATTTCGCGACCCAGGTACAGCCACTCACAGCGCTCTTAAAGAAGGGGCAGAAA
TGGGCATGGGAGCAAGAACAGGAGGAGGCATTGCGTCAATTGAAGGAGAGCTTGACGACTGCCCCTGTG
TTGGCTTGCCCCGACTTTTCCGCCAAGTTCGTCTTGCAGACTGACGCGAGTGAGTATGGCCTCGGTGCAGT
GCTGACGCAGACGATCGATGGACAAGAGCGGGTGATAGCATACGCGAGCCGAAAGTTGCTGAAGGCCGA
GGTAAACTATTCCGCCACTGAGAAAGAGTGCCTGGCAATCGTGTGGTCGATCCGGAAGTTGCGTTGCTAC
CTAGAAGGGTACCGATTCGACGTAATAACGGACCATCTAGCGCTCAAGTGGCTGAATTCGATCGAGAGCC
CGTCAGGAAGGATTGCGCGATGGGCATTGGAACTGCAGCAATTCCAATTTGACGTTCATTATCGTCGAGG
GAAGCTCAACGTCGTGGCCGATGCCCTGTCGAGGCATCCTCTAGAGACCTGTCAACAAGCAGTGGTGGCC
GAGGCCCCCTGCAAATGGGTCGCGAGGATGCGTGTTAGAATCGCCGAAGAGCCAGCCAAGTTCCCGGATT

ACACCGAGGAAAACGGACAGCTATATAGGCATTTGGGTCACCGGATAGACGATGAGGACTACATCCCTTG
GAAACTATGCGTAGGCAGTGGTCAACGCCAGAGGGTACTGCGGGAATGTCATGATGCTCCCACGGCCGGA
CATCAAGGTGTCAGAAAAACGGTGGCGAGGTTAGCCCAGCGGTACTACTGGCCGGGTATGTTCCGCGATG
CCGCCAGATACGTGAAATGTTGCGAAGACTGCCAAAAATTCAAGAGTGAGCAACTCAAACCGGCTGGTCG
CATGCTGACGAGGCAGGTGGCAGAGCCGATGGCGGTACTTTGCGCGGACTTCGTCGGACCCCTTCCACGG
TCGAAACATGGAAACACGATGCTATTGGTGTTCCATGACGCTTTCTCGAAATGGGTGGAGTTGGTCCCTCT
CAGAAGAGCCACGGCAGCACACCTGCAAACTGCGTTTCGAGAGAGGATTCTAAGTAGGTTCGGGGTACCT
AGAAAGTTTGTGTGCGACAACGGGGTACAATTTACGAGTCGGAGCTTCAAGTCATTTTTGGGGTCCCTGG
GTGTAGAGATCCAGTATACCGCCCCGTACTGTCCGCAAGAGAATCCCACTGAGAGGACTAACCGGACTGT
AAAGACCATGATAGCGCAATTTATTGAGGGCCACCAGAGTTCATGGGATGAACTCTTGCCCGAGATCTCG
CTGGCGGTCAACACGAGCGTGGCAGATTCAACGGGCTTTAGTCCCGCTTTCCTGATGCAGGGCAGGGAAC
CTCGGTTACCCGCAGCTCTGTACGATCAGGTAACTCCGGGGTCCGCAACTAGTGCGCTCGACCCTAGGAC
AAAGGCGGATAGGCTAAAAGAAATTTTTGATATCGTCCACAGCAACCTCCAGCGCGCATCAAAGGACCAG
GGCAGGCATTACAACTTGCGGAGACGAGAGTGGCGGCCCTCGTTGGACTCGATGGTCCTGTTGAGGCAGC
ACCAGTTGTCCAACGCTGCCGAAGGTTTCGCCGCAAAGTTGGCACCCAAATTTGACGGCCCCTACAAAGT
CGTCAAATTCATATCCCCCAATATTGTGCGGCTGGTGAAATCAGACGACCGCAAGAAGAGGGTGGCCAAC
ATCTCACAATTGAAACCATTCTACAGAAAGACGACGAGCGACGAAGAAGACGAAGATCCAGGGCACGCA
AATGCCCACGAGTAAGCGAAGGATGAGAGCAACACCCGTCGCCCAACGAAACTATTATGATTAAGCTTAG
ATAAAACTAACATTAACAACATAATTCAGAATAGGCATAGTAAAGTGTGAGCCGCACTGAGAGAATAGG
GCATTAACAAATAAGTAAAGAGTAAGTTAACCAATAAGCTGAACTTTCAGATGGAACCCGAAGTTATCCG
ACATATCGTCAGACGAAGACAACAGCAGCGGGGACACCGAGCCTTTCCCCGGGCCGGGAGCCGCAGGCC
GTCCGCGCGACCCACGCCAGGGGCGTGAGTACTATCGAAGAGAACGGCAAGAAGGACCGCCTCGCCAGT
TCCGCCATACACTATTCTCCCGGCGGACCAGCATAATGGAGAGGCTAGACGGGATGGTCTTGCTGATGGC
AGTCGAGGTCACGACAATGTTCGTGGAGGCACCGTTCGCCCGGAACTCGCCACCGTACGCGGAAGAGCCT
GCATCTCCGCCCCTCGCATCCCACTCGGCACAATCATCAGAGCCGGCAACCCCAACCGCGGCCGGCGAGA
TGGACGAGGAGGAAGAGTCCGCACCGTTCGGAACGGAGGAGCACACACCACCAGCGCAGGACGTGGAGG
CGGTAGGAGAAGAGATATCCCCGTCGGCCGGAGACGCGGAGTCGCCACCGACGGAGGTGCCGAACGGCA
TCCGCGCAAACGACGGGGAAGCAGACCCGCTGAGCCTCGACCCAGAACCCTTAGTGGGAGGGGACCCGT
CAACGGAGGACGAGCCGGAATTCCGTCGCCGCTTTGCCACCCACCGTCTGGAGGTAGACCCGGGCAGCCA
GTTTCCCAGGATAACGCTGCGGTACCTACCAAAAGTCCCCGCGGTAGCCAACAAACCCCGTCTGAACCCC
GCAAAGCGCGAATGGGGGGGTGCCGAAGAAGGCCCCGGCGGAAAGCGCCGACGCAGCCGCCATCGTCGA
CGGAGAGCCGCCAGGGGCGAGCGTCGCCGGAGTGGAGGCCATCGCCGACCCAGTCCCGAGGGAGCATCC
TCGTCGGCCACGAAGAGGCTTCGCACGGAGACCGAGAAGCCCGAAGGATCGCAGCGGTGCGTGCCGCGT
TCGCCCCGCGAGCGCCAGGAGGGTCCTCCCCAGCTCGGGAAGGATAACCGCCGCTGCNAGATCGCTTCTC
GAGGAGGGCATCCCGAACCCTGGCCGATACCGATTGCGGTCTGCCAGATGGTCGTGGAGGAGCACCGTCT
CAGCACCAAGGACGCGGCGAACTCGGCCGTTTCCACAGCCCGCGCCGAGGCGCCAGACGAAACCTTTCCT
AAGGAATCGGAGGAGGAGTGGCTCGACGACGAGGACGAGATGCCCGCGCTCGTGCCTATTGCCCGCGCC
GAGACACCCGACACCATCGCCCGGCGGGAGTTAGAAGACGCCTGGTTCGGCCAAGAAGGCAAGGCGCCA
TCCCGAAAAGGTTCCTTCACGGTTCGCCAGGAAGGCCGGGATGAAGTCTGGNCCGAGGAAGAGGACGCG
GTGGTGACGGCTTCGTCGCCCAAGCTTTCGAGTCCAACCCAGGATTCCTCCCCCATGGCTCTAAAGACTCC
CACGGAGAGCGAGTTGTGGGACGACATAGTGGACCTTTTCGACGGCATCGACGGGCGGGACACTAGCCTG
GACTCAGTGCTGGGAGGAGGAACCCGACACCGAGGTCGACACGGTCGCCCGCAGTGCCGACGTGAGGGT
CACACCGGAGAGCCACCCCAAGAAACCCGACACCGAGGTCGAGGCGATCGCCCACAGTGCTGACGTTGG
GGTAAAACCGGAGGGCCCCGTCGAGGAACCCGTGCCCGATTCCACCCCCTCAGGCTGGAAGGTGTTTCCT
CTTGGAACACCGTTGTCGTCGGGCGTGGTGGACCTAATCGCCAGAGAGGCGCGAATCCGCCGCTTCCGGT
GCCGACGTACTCGGTTCCACGTGTCCGACGGGGAGAGCGAGTACAGCGTTACGCTGAACGCGAAAGGCG
ACGTGACCGTCACCTGTCCGAAGTGAGGGGGGGGTGTGAGGCGGCTAATGAGACCAGCCACCCCACAGTT
TTCACGGCAAGGAGCCGTCATCCCTTTGGCCAAATTACGGCAAGGAGCCGCTGGCGGGACAACGCATTAA
TTTTCTTTAGTTATAAGTATTAATTATATTAGGTTAAGATCTTAGTGAAATAAGAGGCAAGGAGCCATCAG
TAATATAAGCAGTTCGATTAGTAGCAAAGAGGCAAGGAGCCGCGAATATTATAATTGATTTAAATAGTGA
GTTTGAATTTAACCGCCGGCCGCCGCTGCAACCGTGCGTTGTCGATCGGTTGCTCAACACCGTCTTTGAGA
GCGCAACTTCAATTCGGCGGCGAAGAGCGAAAGCTCCGAGGCGAAGTTGCGAAAGCTCGCGGCGAAGTT
GAGCAGCCGAAGCGAGCGCATGGCTTGCCGGCGAGCGTGCCGGCACTTGTGATCGAACGTTAAGGGTGA
GTAGAAGTGCTGCGCGAGATAAACGAAATCATAGATGGTATAGTGGAGCGCTAGGGCTCCAGAAAACAG

TGCGGCAGGGTTTAGCTTAGGATTTACGATAAGGTCGGAATGAATAATAGTGAATAAAGTGCCAACGTTG
TAATTATCTAGAAAGTATTGGATTATTCCCTGTTTCCTGGTAACCCGGCTTACCTTAACATCGGAACTAAC
CGGTGTTCGACCTAACCTCGAACGAGGGGCATGCGGTACGAGATTGTGGCTCCGGGGTGGGCATTGCCGG
TTGGTGTTTAGGTAGTGGAGTTGGTGCGAGGCCACGGCCCGCTAGTGTAGTTGGTGCGAGGCCACGGCCC
GCTAGCGTAGTTGGTGCGAGGCCACGGCCCGCTAGCGTAGTGGTGCGAGGCCACGGCCCGCTAGTGTAGT
TGGTGCGAGGCCACGGCCCGCTAGCGTAGTGGTACGAGGCCACGGCCCGGCAGAGAGATAGGGTGTTTA
GGTAGTGGAGTTGGTGCGAGGCCACGGCCCGCTAGTGTGGTTGGAGCGAGGCCACGGCCCGCTAGCATAG
ATGGTACGAGGCCACGGCCCGATGGTGTAGGTGTGTAGAGGTAGATCTGGGTTTGGTTTGGTCAGGATAC
TCAC
>Ref_Het-A
TTGAGTCTCTTTTTGGTGCCTGAAGTGAACGGACGTGCTCGCGGAGAAATAAAAAGTAAAAATCATATAA
ATTCGGCAGCGCGCGCCAAAACGAACCGAACATAGTATATAGGGACACGATTGCAAAGTGCAAATTGCA
ATACATATCCCTCGGTTTTTTCATTCGGTTTTTCACTCGCTCAATACAGTGGCTTTTTTTTCCTTGTACATAT
ATATAGTGCACGTGGCCTGCTGTGACTTTTGCACAAAGTGTCGCCACGTGGACAATCTAGAACTTTTGCTT
TAGTTGTTCTGCTGCGCATCAGACGCTGGCGCACGGCGTGCAAAAACACAAAAGTGGGTTATGTATAACA
AATACATAACTGCCTAGTGCCTTATATGTACTAAAATTAACCACTATAACTCATACTCTCGTCAATAAATC
AATAATAATTGATATAACCTAAAATAAAATTACATATAAATCAATAAATTGCTATAGCTATATATATTTTC
AATTACTTATTAAATATACATATATACCTAGCATATATATTGCTACAAACTGTCCGTCTACGGACAATCAT
ACCCTCTTAGCTAAAAAGAGAGCAATTTAATCAAAAATTTATTACCTATAAATAAATTAATACATAACCA
AAAATAACAATTAACCCTTCAAATCTCTAAAAACAAGGTATTACATCAACTTTTGCCATAAAACTTCAAGC
CCAAAGGTGACCAATCAGATTAACCTAAAAATTTCTAGCAATTTCTCAGCAAAAGTTTATGCCAATATATG
ACGTGTGTGTGTGTGGGCAATTGTTCTGATTTTTCTTTGCACACCTATACAATCGCTAAAATAGCTGCTGTC
GCGCAACTTGACACACACACGCATACACACACTTATTACAGTTCATAAATTACACATATATACATCTATCA
AAACATATACATATATACTTTCACATACATATCTACAACTCCTGTCAAAACACATATATAAACAAACATAT
ATATCTACAGCAAACACATACAAACTCACTTATATACATTACTTCCCCTCTTTTTTGACGTCCATATAAGTG
ACGCGCGCATAGAAACCTGACGAAGCTGAGAGCAGGCGGAACTGTAGCTAGTCGACATTCTGGTTTGTGA
ATTGACTAAATCGGTCAACTTGGTGCGAAACCAAAAGCACACACTCACAAACAAATCACAACAAAGCGC
CATTTAGTCGTTTCATCCACCCATCTCCATTTTGCACTTTTCCTATTTCATATAAACTCAACCTTTGCAAAA
ACCAACAAAGAGCTCAAGCTTTTACACGTCCCCTTTGTAGGCTGCTCTTAATTTTTCGTAAAGGTTGACAC
AAACAAAATCAAAACTGGTGCTTCATCACCAATAAACTAAATAAATTAAAACTAAATACTTACCTTAATC
AAACCTATTAAAAAATTGAAGCTTAATCAGGTGTTTCGCCACCAACAATATTAAGCTACTATCACACATAC
ACTTACTTATACTCACCTTATCACACTTCTCATATACTTACCTATACTTACCTTATCACACCTTCATATTAC
ATATATTCATACTTACCTATACTTACCTTATCATACTCATATATAATTATAATTTCATACCGGTGTGCCATC
ACCAATCACAAACTATATATAATATATATACTTACCTCAACCATATACTCACCGTATACCAATACCTACCT
TTCACCCATATTTACCAATTACCACTATCAATATGTCAGATCCAAACACTACGCCCACTACTCACCTGTCC
GCCGAGCCTACAATATCGCCAGGACTCTTGTCAAACTTATCAGGCCTACTGCTCTCGCCGATACTGACAAT
GCCGGCATCAATGAACGGTCTACTATCAATTTCAGTAGACTCATTGGCGCCAGCAACAGCCCTGTCCCCCC
AGGCTCTCATATCGACAGCCAGTGAAACACCTGCGCAAAAAGGACAAGAACAGAAACGCGCTGCGGGCA
AAGAAACAGAAAACACTGTTTCGGAAACGACAACAACATCATCAATAACGGCAAAAACAACAGCTACAA
ACACGAATATCGAGAGTATTCATATACCAGCTGACGCTCAGGCACAACTAGCACATTTTAAACGCCTAAT
TGCCCAGCTTGAGTTGGATACTACTCCTCCAAACAGTCAAGCTGCCGACAACTTTACCAGCTACTACGATG
AACACTCCTCTAGTGATTCAATCGTAAATATTATGGATAGCGATGATGCCCCTTGCACACCAAAGCCAGCT
CAACCGCTGCCGATGAGCACCTTTCGCCACCATACTCCCACTTCGGTTGTTGCTACAAAAACCTCCACACA
AGTCATTTCGCCAACATATGCAGCCATTTTGGCTGGCAATGCTGCAAATCAAAAACCCAAAAATTCAACG
TGTATAAGCAGCAGCAGTGACAGCAGTTCCAACAATGCACCCGGTATTGGTGGTCCCGGCGGCAAAATCA
CCACCACAGCCACTACCGGTGCATCTCACTATAAGTTACTGCATCCCACAGGCAAAACAGCCAATCAAAT
TCGCAAAAATAATGCACGAAAGTCAAATCGCCAAAACTCGGCAATTTCTCGCCTATCCCAGCAAGATGCT
CAAAGACGCAACCAATTTGCAAATCGGTTCACATTATTATCTGAATCCGACTTTGAAGCGCCAGATACAC
ATCAGTCCCAACAGCTGCCGGCGAAAACTAACAATTTGGCCGTGCCGCTACAAGCTGCCCTGTCACCTGT
CAAACAGTCAACAACTACGACAACAACTCCAACTAACACAAGAGCAGCATTGACAAGTATCCATTTCGCC
ACTGCCGCCACGGCCGTCAATGCTACTCCTTCTAACTCTCCCGACTGTCAACAACAACAGCAAGCTGGTGC
GATCAATGTTTCTGCCCCTGCGGCGAAACACTACAAACCGCCACAGATATGCATTCAGCATTACAACCAG
CCAGATCAAAACGGCATCGATTCTGTAATTGCCAAATTAAACGGACAAAACCCGCCCATAATTGATTATG
GATTAAAATTGGGTGGTCCTGGCATATTAAGAATCCTGCCAAAAACTTTAGAGACTTACTCTAAAATCATA

AACGTCCTCAATACGGACAATTCAATCAATTATAACACGTACCAGAGGCGTGAGGAGCGCTCATTCAGGG
TTGTGGTGCGGGGCATTCACGCCTCCACAAGCACCGACGCAATCCGGAACGAGCTGACCTGCATGGGCTA
TACGATTCGCAACGTATATTGCCCAAAATATAAGAATCGCAGCGGCCCTGGTACATATCAACCGAATATA
TTCTTTGTGGAATTGGCTCCCGATTCCACAAAGAATCGATCTATATTCGAAATCAGAAATCTTTGCAAATA
CGTCGTCCGCTTCGAGTGGCCTAAAATAGATGGCAAGAATTTGCCACAGTGCCACCGATGCCAGCGCTTT
AACCACACGGCCCGTTATTGCCGCCACCCTGCTCGCTGTGTGAAGTGCGGTAATGAGCACCTCACACAGA
CTTGCGTCAAACCGGCAAATGTGCCAGCCACTTGTGCAAACTGCGGCTCGGACCATACAGCCAATTACAA
GGGCTGCCCCTTGTATCTGGATCTGCTACAAGCAAAACTGTTGTCCCTGCCAAACAGCAAAAATATCAGTC
CGAATGTTCGGCAGCCTCAGCCGAAATTACAGCGGCGGCAAAAGCAGCCTCAGCGCCAGCAATCTGCTCA
ACTGCAACTGCAACAGCAGCAGCAACAGCAAAAACAACAACAAGTTCAGCTGCAGCGCCAGCTGCCAAA
GCCTAAGCTAATCAACAACAAAAATAACAAGAAATCAAAACAAAATGGTGGACAGATAGGAACGCAACC
AATACAAAATCAAAAATTACAAACTAGCACTCCAGCTGGAGTTCCCCGAAAATCTACCACACCAATCACA
AACAACAATATCAATACGGCAAACAATGCCAATAACTCCAATCCCCCCACCGTCTCCTCATCCAACTCCTT
GGATCCACGCTCTAGATGGGCGCGGATCCAAAGTTTACAACAACAACAACAGCAGCAACGCCAACTTGA
GAGACCTGACTCTGTTCAAACCAGAGAACAGATCATCTCAAGGCAGCGCCAGATGCTAGAAAATTGGTCC
ATCCGGCAACAAGCCACCTCCGCCAATCAACCCGTCACACAACAACAATCCACGCCAATGCAAGTTGACG
TTGAGCAGCGACAGCTACTACCATCACCACAACAACAGCAACTTTACCAAACACCCTCCTCCAACGATCT
GCGAAACGATATACTGCTTCAAATGGCCAACAAGCAGCAAGAGCAGTTAGACGGTCTGATGGGCTCGGTG
TTGATATTGCAACAACAATTGCAAGGAGTGCTCCGCCTAGGCGCAGACTCTACACCGCTCGCTGTACCAT
GGTCAAACTCAAGCCAATAATTTGAATGAATTCAGAGCCCGTTTTCATTGGCTCATCTACTGCTCAATCAA
AATTAAAATCCTGGAAAAAATAGCAACTTTTACTTACCTCCAACTTACCTTTTATTAGTTAATTTCCCAAA
CCAAATCTTTATTTTAGTACTTACCCGTTACTTACCTCCTTATTATTTTTACCTACCTTATTTACTTACCTTTT
TAATTTATCCTCAAGCAGCTATTATTTTCCTAAAAATATACCAACCTCATCACAAATTGCATTTCTCAATGC
AACACAACACAATCATCAATGCAATTGTCAACAAGTTGCAAAATACAAAAATGCCACAACAAATTGCCAG
TCATGTTCCGCTATGTGCGCCTCTGTGTCTGCTCGCTCACAACACTCCAGCTTTTCCAATGCTCGAATACAG
TGAGCTTATGCACACACACGCGCACAATGAGCGCGCTTCCTATTCGTCAATGCGCTGGCGCACACTAAAA
CATAAGCAGACGAAACAAGATGTCTGTTCCACACTTGCCCACCCCATGACCAATGGCAACAGAAGCTGCT
ATTTGCATCCCTAACCGGCCTACGAGGGAAGACGGGCAACTTCCTTTTCAAGAAAAGCTCCAGAAATCCA
CATTTGGCAGCATCAAACATCATCACTCCTTTACACACACACACACACACAATCACATCGAGAAAAAGCA
ATGAACACAAACACAGCACACAATATGCTTATCATTGCCTCTTCACACCAATGAATCTGCCAATTCAAAAT
TCTACTTACTAAAAACTTCTCAATCTCAACCATCTTTTTTATCCCGCATCGCAAATATTTAATCATAGCCAA
CACGCATTTGATAACAGCCAGTGCAAATATATTTAAATTATTGAAATTTAAATGAACTGAGAGAAGGTAC
TCGGCTAAAATAACATCAATTTTCTTATCAACTGCGGTGTTATTTATCTGCTTTTGATGTGGGAAACAGAG
GATAGGGAGATTTTTTAAATTGGGAAAACAAAAAACCAACAATCAACACAGGACGGACATGACAAATCA
CAGTTGTGAATTGTGGAGTGGCCCTGTGTGTTCTCTGTTCATTTGCCAACCCTATGTCTCGGGCAATGCAG
ACACACACACCATACAATTGGGAACACACACACACTGTATATTCATGCGCCGAGTCCAATTCTCTATCAAT
AAGAAACAAGAAAACATTTCTCCTTATTTCGTACATCAAACACAGACAAATCACAACGAAAAATTATTAT
TTTCATAAACTCCATTAAATAAATTCTCTCATCATTTTGCTGTACTCTTATCAATAATGCTGGCTTATGGCG
AACCAGATTTGCTGACGCCGCACACCACTCCAATTGTATACTTCTTCTATACACTTGAATTTTTATTTACTA
AAAATATAGAGACATATCATTTTAGCTCTTATCATAAAGTTAATTTTTTGCACAAATGGTATGAATATTAA
GTTGGCAAATTCATATAATTTTGTAAAACATACACGACACACACAAATTCCTAAACATGTATATACACATA
AACATATCACAAAACACAAAAAATTTAAAATGTATAGAATTAAGGAAGTATATATCAATAAACTCTGGAGG
GGGGATTGGTTCGGTGTGTGACGGTGGGAGGGGGGATACTTTGTACCGCTGCAGGATAGTGGCCTGTGTG
TGGGTGTGCATACCCGTGATGGTTTGGAGCGGTTGACGAGCATGAGCTAGTAGGCACCTTTAATGCTGCCT
CGCCATGTCTCGAGCCCTCCAGGCCACCACGACCATGCATACTCAAACACTCGCTCGCTCTCTGTGCGATG
CGAATGCTCGATGCCCGACGCTCGGTACTCGGTACTCGGTACACGATGCTCGGTGTCCTCGATCCCGTTTC
CCATTCGTCTCATCCGACCCGGTCTCCTGCTCAACCTAAAACAGAAA
>9_rnd-4_family-2098__Unknown
TTATGTCGTTTTGAAGCGACATAGCTCCGCCGATCTAAAAATCATGGAGATATGACGTTTCAAAGCGACAT
AATTTCGCCGTCGATCATGGCGAGATTATGTCGTTTTGAAGCGACATAGCTCCGCCAGCTCTAAAAAAATC
TTGGAGATATGACGTTTCAAAGCGACATAACTTCGCCATTGATCAAGGCGAGATTATGTCGTTTTTGAAGC
GACTATGGCTCCGCCGATCTAAAAACAATCATGGAGATATGACGTTTCAATACGACATAACTTCGCCGTT
ATTCAGGGCGAGATTATGTCGTTTTGACGCGACATAGCTCCGCCGCTCTAAAAAAATCTTGGAGATATGA
CGTTTCAAAGCGACACAACTTCGCCGTTGATCATGGCGAGATTATGTCGTTTTGAAGCGACATAACTCCGC

CGCTCTAAAATCTTGGAGATATGACGTTTCAAAGCGACACAACTTCGCCGTTGATCATGGCGAGATTATGT
CGTTTTGAAGCGACATAGCTCCGCCGCTCTAAAAAATCTTGGAGATATGACGTTTCAAAGCGACACAACT
TCGCCGTTGATCATGGCGAGATTATGTCGTTTTGAAGCGACATAGCTCCGCCGCTCTAAAAATCTTGGAGA
TATGACGTTTCAAAGCGACACAACTTCGCCGTTGATCAAGGCGAGATTATGTCGTTTTGAAGCGACATAA
CTCCGCCGATCTAAAAATCATGGAGATATGACGTTTCAAAGCGACATAACTTCGCCGTCGATCATGGCGA
GATTATGTCGTTTTGAAACTGACATAACTCCGCCGCTCTAAAATCTTGGAATGATATGACGTTTCCAAAGC
GACATAACTTCGCCGTCGATCATGGCGAGATTATGTCGTTTTGAAGCGCGACATATCCGCCGCTCTAAAAT
CTTGGAGATATGACGTTTCAAAGCGACACAACTTCGCCGTTGATCAAGGCGAGATTATGTCGTTTTGAAGC
GACATAGCTCCGCCGCTCNAAAAAAAATCTCGGAGATATGACGTTTCAAAGCGACANAACTTCGCCGTCG
ATCATGGCGAGATTATGTCGTTTTGAAGCGACATAGCTCCGCCGCTCTAACAAAAATCTCGGAGATATGA
CGTTTCAAAGCGACATAACTTCGCCGTCGATCATGGCGAGATTATGTCGTTTTGAAGCGACATAGCTCCGC
CGCTCAAAAAAATNTCGGAGATATGACGTTTCAAAGCGACATAACTTCGCCGTCGATCATGGCGAGATTA
TGTCGTTTTGAAGCGACATAACTCCGCCGCTCTAAAAAAATCTTGGAGATATGACGTTTCAAAGCGACAT
AACTTCGCCGTCGATCACGGCGAGATTATGTCGTTTTGAAGCGACATAGCTCCGCCGCTCTAAAAAATCTC
GGAGATATGACGTTTCAAAGCGACATAACTTCGCCGTCGATCATGGCGAGATTATGTCGTTTTGAAGCGA
CATAGCTCCGCCGCTCTAAAAAATCTCGGAGATATGACGTTTCAAAGCGACATAACTTCGCCGTCGATCNT
GGCGAGATTATGTCGTTTTGAAGCGACATAGCTCCGCCGCCAAAAAATCTTGGAGATATGACGTTTCAAA
GCGACATAACTTCGCCGTCGATCATGGCGAGATTATGTCGTTTTGAAGCGACATAGCTCCGCCGCTAAAA
AATCTTGGAGATATGACGTTTCAAAGCGACATAACTTCGCCGTCGATCATGGCGAGATTATGTCGTTTTGA
AGCGACATAGCTCCGCCGCTCTAAAAAATCTTGGAGATATGACGTTTCAAAGCGACATAACTTCGCCGTC
GATCATGGCGAGATTATGTCGTTTTGAAGCGACATAGCTCCGCCGCTCTAAAAAATCTTGGAGATATGAC
GTTTCAAAGCGACATAACTTCGCCGTCGATCATGGCGAGATTATGTCGTTTTGAAGCGACATAGCTCCGCC
GCTCNAAAAATCTTGGAGATATGACGTTTCAAAGCGACATAACTTCGCCGTCGATCTTGGCGGATTCTACG
GACCTCCCCGAGTAGCCCCTCCCTTTTTGGCCCCNATCGGGCTTTATTTGGCCGAGATATGACCATTTAAA
GTTTTCCAAAAACGTGCGTGTGGTTGTGGACATATTAGTAAAAGAAAGCATTGCATACTTTCGAGCTGTCC
GCATAATATGCTTGAGCCTATTAGAATAACAGTTTTACTGCATACTCTTGGGGCGCGCATAAAATTAGCCC
TTTACAATAAGCACCGTGGCAGCGACATNGCTCCGCCGCTAAAAATCGGAGATATGACGTTTCAAAGCGA
CATAGCTTCGCCGTCGATCATGGCGAGATTATGTCGTTTTGAAGCGACATAGCTCCGCCGCTCTAAAAAAT
CTTGGAGATATGACGTTTCAAAGCGACATAACTTCGCCGTCGATCATGGCGAGATTATGTCGTTTTGAAGC
GACATAGCTCCGCCGCTCTAAAAATCTTGGAGATATGACGTTTCAAAGCGACATAACTTCGCCGTCGATC
ATGGCGAGATTATGTCGTTTTGAAGCGACATAGCTCCGCCGCTCTAAAAAATCTTGGAGATATGACGTTTC
AAAGCGACATAACTTCGCCGTCGATCATGGCGAGATTATGTCGTTTTGAAGCGACATAGCTCCGCCGCTCT
AAAAATCTTGGAGATATGACGTTTCAAAGCGACATAACTTCGCCGTCGATCATGGCGAGATTATGTCGTTT
TGAAGCGACATAGCTCCGCCGCTCTAAAAAATCTCGGAGATATGACGTTTCAAAGCGACATAACTTCGCC
GTCGATCATGGCGAGATTATGTCGTTTTGAAGCGACATAGCTCCGCCGCTCTAAAAAATCTTGGAGATATG
ACGTTTCAAAGCGACATAACTTCGCCGTCGATCATGGCGAGATTATGTCGTTTTGAAGCGACATAGCTCCG
CCGCTCTAAAAATCTTGGAGATATGACGTTTCAAAGCGACATAGCTTCGCCGTCGATCATGGCGAGATTAT
GTCGTTTTGAAGCGACATAGCTCCGCCGCTCTAAAAATCTTGGAGATATGACGTTTCAAAGCGACATAACT
TCGCCGTCGATCATGGCGAGATTATGTCGTTTTGAAGCGACATAGCTCCGCCGCTCTAAAAAATCTTGGAG
ATATGACGTTTCAAAGCGACATAACTTCGCCGTCGATCATGGCGAGATTATGTCGTTTTGAAGCGACATAG
CTCCGCCGCTCTAAAAATCTTGGAGATATGACGTTTCAAAGCGACATAACTTCGCCGTCGATCATGGCGA
GATTATGTCGTTTTGAAGCGACATAGCTCCGCCGCTCTAAAAAATCTTGGAGATATGACGTTTCAAAGCGA
CACAACTTCGCCGTCGATCATGGCGAGATTATGCCGTTTTGAAGCGACATAACCCCGCCGCTCTCGAAAAT
CTTGGAGATATGACGTTTCAAAGCGACACAACTTCGCCGTCGATCATGGCGAGATTATGTCGTTTTGAAGC
GACATANCTCCGCCGNTCTNAAAATCNTGGAGATATGACGTTTCAANGCGACACAACTTCGCCGTCGATC
ANGGCGAGATTATGTCGTTTTGAAGCGACATAGCTCCGCCGATCTAAAACAATCATGGAGATATGACGTT
TCAANGCGACATAACTTCGCCGTCGNTCATGGCGAGATTATGTCGTTTTGAAGCGACATATGCTCCGCCGC
TCTAAAAATCTTGGAGATATGACGTTTCAAAGCGACATAACTTCGCCGTCGATCATGGCGAGATTATGTCG
TTTTGAAGCGACATATAGCTCCGCCGCTCTAAAANCTTGGAGATATGACGTTTCGAAGCGACATAACTTCG
CCGTTGATCANGGCGAGATTATGTCGTTTTGAAGCGACATAGCTCCGCCGCTCTAAAAAATCNTGGAGAT
ATGACGTTTCAAAGCGACATAACTTCGCCGTCGATCATGGCGAGATTATGTCGTTTTGAAGCGACATAGCT
CCGCCGCTCTAAAAATCTTGGAGATATGACGTTTCAAAGCGACATAACTTCGCCGTCGATCATGGCGAGA
TTATGTCGTTTTGAAGCGACATAGCTCCGCCGCTCTAAAAATCTTGGAGATATGACGTTTCAAAGCGACAT
AACTTCGCCGTTGATCATGGCGAGATTATGTCGTTTTGAAGCGACATAGCTCCGCCGCTCTAAAAATCTTG

GAGATATGACGTTTCAAAGCGACATAACTTCGCCGTCGATCNTGGCGGGNTCTACGNACNTCCCGAAGCG
ACGTAGCCCCNCCCTCTNNGACCTATCGGGCTTTATTTGGAGATATGACNNTTCAAAGCGACATAACTTCG
CGTGCGATTNNGGCAGATTATGTCGTTTTGAAGCGACATAGCTCCGCCGCTCTAAAAAATCATGGAGATA
TGACGTTTCAAAGCGACATAACTTCGCCGTCGATCATGGCGAGATTATGNCGTTTCGAAGCGACGTAGCT
CCGCCGCTCTAAAAAATCTTGGAGATATGACGTTTTAAAGCGACATAACTTCGCCGTCGATCATGGCGAGA
TCTATGTCGTTTCGAAGCGACATAGCTCCGCCGCTCTAAAAAAATCTTGGAGATATGACGTTTCAAAGCGA
CATAACTTCGCCGTCGATCACGGCGAGATTATGTCGTTTTGAAGCGACATAGCTCCGCCGCTCTAAAAATC
TTGGAGATATGACGTTTCGAAGCGACATAACTTCGCCGTCGATCATGGCGAGATTATGTCGTTTTGAAGCG
ACATAGCTCCGCCGATCTAAAAAATCNTGGAGATATGACGTTTCAAAGCGACATAACTTCGCCGTCGATC
NTGGCGAGATTATGTCGTTTCGAAGCGACATAGCTCCGCCGATCTAAAAAACCTGGAGATATGACGTTTC
GAAGCGACATAACTTCGCCGTCGATCTTGGCGGATTCTACGGACCTCCCCGAGTAGCCCCTCCCTTTTTGG
CCCCTATCGGGCTTTATTTGGCCGAGATATGACCATTTAAAGTTTTCCAAAAACGTGCGTGTGGTTGTGGA
CATATTAGTAAAAGAAAGCATTGCATACTTTCGAGCTGTCCGCATAATATGCTTGAGCCTATTAGAATAAC
AGTTTTACTGCATACTCTTGGGGCGCGCATAAAATTAGCCCTTTACAATAAGCACGTGGCAGCAACTTTTC
GAATCCGCCAAGATCGACGGCGAAGTTATGTCGTTTTGAAGCGACATAGCTCCGCCGATCTAAAAATCAC
GGAGATATGACGTTTCAAAGCGACNTAACTTCGCCGTCGATCATGGCGAGATTATGTCGTTTTGAAGCGA
CATAGCTCCGCCGCTCTAAAAATCTTGGAGATATGACGTTTCAAAGCGACNTAACTTCGCCGTCGATCATG
GCGAGATTATGTCGTTTTGAAGCGACATAGCTCCGCCGATCTAAAAAATCNTGGAGATATGACGTTTCAA
AGCGACATAACTTCGCCGTCGATCATGGCGAGATTATGTCGTTTTGAAGCGACATAGCTCCGCCGCTCTAA
AAAATCNTGGAGATATGACGTTTCAAAGCGACATAACTTCGCCGTCGATCATGGCGAGATTATGTCGTTTT
GAAGCGACATAGCTCCGCCGNTCTAAAAAATCNTGGAGATATGACGTTTCAAAGCGACATAACTTCGCCG
TTGATCATGGCGAGATTATGTCGTTTTGAAGCGACATAGCTCCGCCGNTCTAAAAAAATCNTGGAGATAT
GACGTTTCAAAGCGACATAACTTCGCCGTCGATCTTGGCGAGATTATGTCGTTTTGAAGCGACATAGCTCC
GCCGCTCATAAAATATCCTGGAGATATGACGTTTCAAAGCGACGCAACTTCGCCGTCGNTCTTGGCGGGA
TTATGTCGTTTTGAAGCGACATAGCTCCGCCGCTCTAAAAAATCTTGGAGATATGACGTTTCAAAGCGACA
CAACTTCGCCGTTGATCATGGCGAGATTATGTCGTTTTGAAGCGACATAGCTCCGCCGCTCTAAAAATCTT
GGAGATATGACGTTTCAAAGCGACACAACTTCGCCGTTGATCAAGGCGAGATTATGTCGTTTTGAAGCGA
CATAACTCCGCCGATCTAAAAATCATGGAGATATGACGTTTCAAAGCGACATAACTTCGCCGTCGATCAT
GGCGAGATTATGTCGTTTTGAAACTGACATAACTCCGCCGCTCTAAAATCTTGGAATGATATGACGTTTCC
AAAGCGACATAACTTCGCCGTCGATCATGGCGAGATTATGTCGTTTTGAAGCGCGACATATCCGCCGCTCT
AAAATCTTGGAGATATGACGTTTCAAAGCGACACAACTTCGCCG
>9_rnd-1_family-168__LINE___I-Jockey
CACTTACGTTTGTGTTTCTCTGGAGATCGGACGTGAAAATTGCAGTCTCGTGTGTGTGTGCGCGTTAGTGC
ACCGCTGGCAGCAGGCATAAACCAAGAAAGTGACATTGACACTGTATAAAGCCCGCCCCAGGGCAATCC
ATACAAATCCCGCTTATAGTGGGTAAACTATACGCAACTGCTGCCTCTGGCAGCCGGAGTGTTATACAAA
TTAACAATATTTTAACAAAAAAATTACCGCGTGCAGTGCGTGAGAACAAGTTCTTGCAAAACTTAAGCTG
CGCTCAGCTGATCGCAACTCAAGTGCGTGTGTGTGTGCGCGAGTGCAGCAAAGCTTTCGCGAGAGAGCTT
TCAGCTTGCAGCTGCACTGATAGTGCTGGCAAAACAGTCACTGCAGTGACAGTGCTGACACTAAAGTCAC
AATTTGTGATCAATCACTTGTTACAAAAATACGGGTTATTTTTGTAATTAAGTGTAAAAACTTGTTTTGTTT
TTTTTTTTTTTTTTTTTTTCTCCCTGTGGGGAAAAAAATTATAACAAAGTAAAGAACAATGGCTACAAAAC
AACCGACCCCAAAACAACGAAGAAATTCATTCGCCAATCTACCCGCTGCCGAACAGCTCAATAATATCCC
CTACAAATATAGCCGCTAATGTCGATCGCCTCGACGGAAGCAGGCCACTGGAAGATTTTTACCAATATTA
CAACAACGTGCTCGCCAGTAGTGCAAACCGGAACGGCGCCTCCACCTCGCTGTCAGCCGGTACCCAGCAA
TGCTCGACTTGGCAACCGCAACAGCACGGCACTGTCAGCACAACGACAGCAACAACCACCACAACCACA
GCGTCATCCAGCAGCGGCTGCGGGACATCTGCCTCAACAGGCACAAGCCGCGCCGCTTTAGAGCCCATCG
GGACCCGCCCCAAAGCCGCCAATCCGACCTTGCAGTCCACCCCTTATGGCACCCAGGCAGCTGGTTCTTGT
GTCACAAACCCCCTCTGCCTCCCATAAAGAACGCAGGTGCTACGCTAGCCTTCGAAGAGCCACTGGTAGA
TTGGCAAACGGTTTCCGCAAAAACGGCGTGGGACTACATTATTATCCGAAGGCGTAAAAAAGGGAAGCA
ACTAAATAACCGCAACACCAGCCGCGCTGGAGCTAAGCCCGCAACTCCCAGTACCGCTTCGGCAATTAGC
AACAGATATGCCTCCCTTGAGCCTCAGGAAGACGCCTCTGTAATCGATGACGGCGAGATGGACACTGCCG
ACGAAGCGCCTCCAGTCACAGACCAACAACAATCAGCGAAAAGATCTCCAAACCCAGGCCGATAACAGT
CCCTGGAGTCAGCGATATTGTCGCAATGGAGCGAACCATAGATCAGGCAGTGGGCGCAGACGCCTATGAA
TTCAAGGTCTCTCCCTCTGGCTATCTAAGAATTTACGCCAAAGATGCCGACACCCACAGAGCAATTGTCCG
CAAGCTCACTGAAGTGAGAGTGCAATTTACCCACTTCTGCCTAAAGGAAGACAGACCATACCGCGTTGTG

GTTAAAAATCTGGCCGCTTCCACTCCCAGAAGCCAGATTGAAGCTGCCTTTACATCGCACGGCCATATTGT
GACGAACCTTTACAACCCGGGCGCCAGACAGCCAACAAGTGATGCAGACTCCACCAACGACTTTCCAAGT
CGCAACTTCTGGTTTGTCGATCTCAAGCAGTCTCTTAACAACAGGGAAGCGCTGCGGCTCAACAAGGTTG
GCCGCCAACGGGTCACTATTGAGCTTCCGAGGAAGAACAATAGTATAAGGCAGTGTTTCCGCTGCTTCGA
GTTCGACCACACCAAAACTACTGTCTCAAACAGCCCAAGTGTGGTAGGTGCGGTCAGTCCCATTGGACAA
ACTCCGAGCAGTGCCAGGCCAAAACAGTGGCAGACCTGCGATGCTCCAACTGCGAGGGAAACCATGCTG
CTTCCTATAAAGGATGCAAAAGCTATCAGGTGAGACTAAATGCCAGGCAGCCGGCCATCAATGTCTCCCC
TCCTACACGATATAGCCATCCTCAAAATACACGAAGAACCACTCAGCAGTTCGTACCTTCCCGAATTGTGC
AACAGGGCCTGTCGTATGCTGATGCGCTACGGCCAGGCCATCATGTACAAGTCCGCCAACCCCCAGCGCG
CACTCTTCAGCCCACCGTCAATCCAATGCAGCTCCAACAACCACTGCAAGGACCTCAGGAGCAAGACGCT
TCCCTTGGAGGCATCCTTCAAAACCTCCAGCAGTCCATTGCAGCACTGAATGCCGCAGTTGCAACCATTCT
GGTTAGCATTAAAGAGCTAAAGGATGCGCAAGGTTCCCCGCAGCTTAGATTCCCAAAAGCCGCAAATAAT
GGCTACCCAGTTACGTCTCGGATCGTGGAACGCTCGTGGCATCCTCCAAAATGCTAACGAGCTCAAGCTTT
TCTGAAAAAGCATGATGTAGACATCATGCTCATCTCAGAAACACATCTGCACACAAACTTGTACCTAACA
ATGGAGGGGTACGTCTGCTACCGTGCGGATCACCCTACAGGACGAGCCAGAGGTGGTGCTGTAGTGCTGG
CAAAGCAGGATTTAGCACACTACCATCTACACACAGTGACCTCCAGCGACACACAGCTGGCTGCTATCAT
GGTGGAGACTCCCTTGGGCGAAATCCTCGTTGCGGCAGCATACCTACCACCAAACATCCCCTGGAACCGA
GCGGAATTTGATTCCCTGTTTGGCCAGCTTGGCCCCAAATTCATCGTTGGAGGCGACTTCAACGCTAAGCA
CAGACTGTGGGGAAACTACAGAGCTGACAGCAGAGGCACTGCGCTCCATGAGGCGCTCACTCCCTGCTCA
GCCCAGGTACTGGCCACCGGTAGACCTACCCACTTTCCGTACAACCGGCAAAGTACGCCTTCCTGTATCGA
CTTTTTTATATTCAAGGGCATCCCCAACAGCGTACTATCGGTTCGCGAAGAGTATGATCTATCTTCCGACC
ATCTGCCACTCCTCACAACGATCAACACGGCTGCACAAAGGATCCCAAAAAGCGCAGGCTTGTCCTCCCT
GGGTCGAACATCCAACGATTTAAGGCCGAACTAAATAGCTTGATCAACCTAAACACGCAGATTCTGTCAG
TTGAGGACGTCGACGCTGCAGTACAAACCCTGTTGGACCAAGTACATGCCGCAGCCGCCAACGCTGCGCC
CGCACATATCTACTCAGCCCCTACAAGCCAGCGGCCAAGGACGTTTTCCCCATATTGGAAGGTCTTCTCGC
CCTAAAAAGCGACTGAGGAGGGAGTATGTCCGTACCCAGGACCCCACGATTGACAGGATCTATCGTCGTA
TTGCTAACAGGGTCAGGAAGGAGCTGATTATCTCGAAAAGAAATGCCACAGACACCATGCTGGAGGAAG
CAACGGCAGACGAGAGCTCCAAATTCGCGCTGTGGAAACTTAGCAGTCGATACAAGCGGCAAGCTGCGC
CAAAGTTCCCCGTTCGACTTCCCGATGACACCTGGGCCAGATCACCAATGGACAGAGCCGAAGCCTTTGC
CTGCAACTTGGAGGAGCGCTTCAAGCCGTTTGAGACCGCATCGCATGGAAGGATACAGCGAGTCGCGCAA
ATCCTGGAGGCGCCACTCCAAATGTCACTCCCTGTATCGCCAATCACCTTCCAAGAAGTAGAGCAGGAGA
TGAAGAGATTAAAAGCAGCAAAGCCCCTGGGGAAGATCGGTTGGACAACCAGACAATCAAGCTACTGCC
ATACAAGGCGGTGCTTTTTCTCGTCGCCATTTTTAATGCAGCTCTTCGGCTAGGTTACTTTCCAGTGGCTTG
GAAGAAGGCACATATAATCATGTTGCACAAGAGCGGAAAGCCCCCCTAATCAACTGGGATCCTATCGCCC
CATCAGCCTCCTGCCTGCCTTTGGTAAGATTCTGGAAAGGTGCATCCTCAGAAGGGTCCTGGATACCCCGG
AAATAAAACGCTCGATCCCGAAGTTCCAGTTTGGGTTTCGACTCAAACACGGTACCCCTGAGCAACTACA
TCGGGTCACAAATTATATTTTGGACGGCTTTCACAGGAAAGAGTATGTGGTGGCAGCATACCTCGACATC
CAAGAAGCCTTCGACCGGGTGTGGCACGCAGGACTCCTCTCCAAGATAAAGGACAAATTGAATCCGCAGC
TATTTAGCACCATTGCGAGCTTCCTAACTGAGAGAACCTTTCAAGTGGTGCAGGATGGAGTAGCCTCTCAA
ACCAAACGCATAAAAGCGGGCGTTCCACAAGGCAGCGTCTTGGGTCCCACGCTATATAGCCTGTTTACAC
ATGACATGCCCACAGCAAATGCAAGCAGACGGAATGATCGGCAGGACCTTCTGGTGGCCACGTTTGCTGA
CGACACGGCAATCCTTGCAAAAACAAATGTGTGTACGTGGCAACTGACCTCTTGCAGGAATACCTCAAGC
GATTTGAAAACTGGGCTTGTAGATGGAACATCGCAGTGAACCCTGGAAAGTGCGCCACGGTTACCCACAC
TCTACGCAAGGACTCATGCCCCGGACTTTTTCTTCATGGTGAGCTTCTAGAGCAATATCGTCAACACAAGT
ACCTAGGNGTCACTCTCGACAGACGACTTACATTTAGTAAGCACGTCTCTGCAGTAACGAGCAACATCAA
ATCTAAGATAAGGCGAATGAGCTGGCTCCTCAACTCCAAAACAAACTCTCGCTCCGAAACAAGGTCCTAA
TCTACAAGGTAATCCTTGCCCCAGTGTGGCGTTACGGGCTTCAAGTTTACGGTATAGCTGCAAAACACACC
TGAACAAAATTCGCATCATTCAGGCCAAAACCCTGCGCAAAATAACTGGAGCCGAATGGTATATTCGCAC
ACGCGACATCGCCAAGGACCTCAAGGTTCCAATGGTGGGCGACGTAGTCAACAGGCAAGCAGCGAGCTA
CTCAACAAAGCTCCACAACCATCCAAACATCCTGGCGAGGAGCCTGCTAAGGAGGCGAAGAAGAAGGCG
ACTGAAGAGGACCTACCCAACCGACCTAATCGATCGTTATGTATAGGTGACAAGCTTCGTTAATTACACC
AAAATATGTTTCTGTAATCTGTTAACCTAATTGTATAACTAATTGTAAAACCACAATCATCGCTAAGCTAA
GTTATTATGTCACTCTGATAAAAGTTGCTAACATAGAGTAACAGGATACCAGATTTCTTAATAAACAAAAT
ATAAAAAAAAAAAAAAAAAAAAAAAAATGATTACATATGTCAATCAATTATTTGCTATAAAGGGCCCTATT

CAAAAATTATTGAAATGTACATAATAAAGGTTGGAATACATACTCGTCAAAATATGAAATAAAATTCAAT
AAATTGTGACGTCATAATTTCTTATGTTAACGCGCCGTGAGCGTGACCCTTTGATTTGAATGCCATGGCCT
GCAGGCATTAAAATTTTCTACCTACTTGCGCCGAGACTCGGCTCTGCAACAACAACATTGGTATCCAAATT
GTGCTGTTGTTGTTTCTACGCGCTGCCTTGTAAGAACGACAGAGAAACCCGCAAAAAACACGCATTATCT
GTCCATTTTCCTTTGCGCTGAGTTTCATAAGTCCTCATCATGGCTCACAAACCTCTTATCGATCAAAACAA
AGGATGAAGAACAAATTGAGTTTCGTGCGCACTGTTGTTGTTTTCGCAAGTGATTTTTTNTTGCCGCACCC
GCCAATGCTGCGACATACGTAACCAGCCCCTACAAGCCAGCGCTCAAGGACATTTTTCCCTCATTTTGGAA
GGTCTACTTGCCCTAAAAAGCGATTGAGGAAGGAGTATGTCCGTACCCAGGACCCCACGATTGACAGGAT
CTATCGTCGTATTGCCAACAGGGTAGGGAAGGAGCTGATTATCCAGAAAAAAATGCCACAGACACCAAG
CTGGAGGAAGCAACAGTCGACGTGAGCTCGAATTTCGCGCTCTGGAACCTCACAAGGCAGTATGAACGGC
AAGCCGCACCCAAGTTTCCCGTGCGTCATGCCAACGGCACCTGGGCCAGATCGCCAAAGGACAGAGCAG
AAGCCTTCGTCAGCAATCTGGAGGAGCGATTCACGCCGTTCGAGACCGCATCACAAGACAGGCTACAGAG
TCGCGCAAACCCTGGAGCACCCCTTCCAAATGTCCCTCCCTGTGACACCAATCACAGTACACGAAGTGAA
GCAGGAGATCAAGAGTCTTAAAAACCGCAAAGCCCCCGGCGAAGACTGCTTTTTCATTTTGATTTTTCTTT
TGTCTCCATTTTTCAATGCAGCTCTTCGACTAGGCCACTTCCCAGCGGCCTGGAAGAAGGCGCAAATCGT
>9_rnd-1_family-423__LTR___Pao
TGTTCGGTCATATACCTCATATATACATTAGTCAGGGTATCAATAGCAATGTCAAAGCAGATTATGTTAAG
ATTATGCTAATTCTCTTCTATTTGAATGTCATAAGCACTCATACACATGTACGAAGCCTCGCTCTCTTTCGA
ACTCTGTAATTGCTCGGTCCCTTTAATCTTAATTTATGTTAAGAAATCAGTTCTGTAAGCATCATCATAGAA
GTCAGTCGTGTTGCTCATCAATTTGTAAAACCTTTATTAAAACTTCATACCATCCACACACGCCCACTTGA
GATAAGATTCTTAAAAGGTCAAAAAAACGCTTTTGATCCAACATTTTGGCGCCCAACGTTGGGGCTAGTG
TGTGGAAATTGACGGCATATCGTTGCCGCACAACATTTCGTTGGAAAAAGGTTTTATCGAACTCCTCGCAA
AATACAGCATCGACAGCAACATTTGGCCAGCTCTTCTTTCTTGTTGCCGTTGCTTCGTCGCTGGAAACTCG
CTACCTTCGCTTCGCTACTGCATTCTTGTAGCCGTTACTTCGCGCTGGATACGCTCTGAGTTTGATTGCTCA
TTGCCTTTGCCTCACATCTCCTTTCTCGTTGCCGTTGCTTCGTCGCTGGAAACTCGCTACCTTCGCTTCGCTG
CTGCATTCTCGTTGCCGTTGCTTCGTCGCTGGAAGCTCGCTGCCTCTGCTTGTTCACTGCATTTTGCTTCAT
CACTGCAAGCGACCCTCACATCGGTGAGCGCAACATTTACAAGTCTTAGCTGTTCTGTTAACAGTGGGAG
CATTTGAACTGTCGCTCGCTCACATTGGTGAGAGTGACATTATCAGGTTAAAGTTAGGCTGTGAAAATTTG
GCAGTATCAAAACTGTCGCTCGGTCACATCGGTGATCACAACATTCTCGATACATAATGTCAACTCTTGAA
GCAAACTTACGTAAGCGAGCGAACATAAAACGCAATATCGCACACTTAGAGGATGTTGTAAGAGTGTCTA
CTGAGGTCGATGGCAGTGAAAAAAGATTATCACACGGTGAGCTACAATTACGCTTGGACATGTTGGAGAT
ATACATTAAGGAGGTTTTGGAAGTACAATCTGAAATTGAATTGGAAGATCCTCAGGACAATGGACGTGCA
GAGGTGGAAGACACATATGTGAGTATCAAATTGTCCATCCAATCCCAACTGGATGATGTACTGCACAGCA
CCTCATTTTCTAACCCTGCTCCAATTCGCTCTTCGCCGCCATCGAGTCTTCCTCGATTGGCTCCGCCTGTAT
TTGATGGGAAACGAGCAGAGTTTAAAAACTTCATTACATCATTCAATCAGATTATCGGTCGTGAGTACTCT
CTTTCGAACATTGAAAAGTTTAATCATCTGCTGAATTGCTTGAAGGGCCCTGCCCTACAGGCAGTAAAGGC
ATTCCAAGTGACTCAGGAAAATTATCCCAAGGCTCTTGCTCGTTTAAGTGAACGTTATGACAATCCTACAC
TTACATTTTTAGACACAATAGAATCACTATTCAATCTACAGCCAGTCGACAAACCGGACGGAAATCAGCT
GCGCAGTCTGCTGGACAATGCGTCAGCCCTGTATTCATCATTATCGTCATTGGGTACCGGATCAGATATTG
CCCAAGCCATGTTGATATTCATCGTCATCGGAAAATGTGACCAGCAAACTCGTAACAAGTGGAATGAGTC
TCTTGACTTACGTACATTGCCAAATTGGGCTCACTGCACAACTATTTTGGAACGTCATTGTCAGTTTCTGCA
ATCAAGCGGCATTGCATCGTATCGAATGGCTGATTCTTCGAAACAAAAACACGTGTACAAGCCTAGCAAG
GGATTATCATTTGCTATTACTAATCCAACATGCATTGTTTGTTCCAGCTCCGACCATAAAATTGTACAATGT
TCTCAGTTTCAAGACATGAGTCCTAAACTCAAGTACGGAGTCGTAAAAAATGCTGGACTTTGCATTAATTG
CCTTCAATTAGGACATCGGATGGCTCAGTGTCCGTCTAACAATCGTTGTCGAAGCTGCAAGCATCGCCATC
ACACGCTTCTGCATTTTGGCCAACCGCCTGCATCATCATCGTCTACATCGCCATCATCCATAGCGCAAATG
TCCTCAACAACGACTCCTGCTGCAACGCACTCGCATATGAATCAATCGCCAGGCGGACAAATAATTCTTG
CTACTGCAAAGGTACTAGTGCGAGATTCATCAGGGGCGTATAAACAAGGCAGAGCATTGCTTGACTCATG
TTCCCAAGTCAATTTCATAACTGATCAATTCGCCCAACGGCTGCATCTACGCCGTGATAAGCATCATTTCG
AAATCCGCAGCATAGGGGAGACCTCGACTAAAATTAACAACTGTACAACTACTGGAATTAAATCTCGTTT
GTCACCATTTGAAGTAACACTACAGTTCTGCATAACGTCTCACATTGCCTACCAGCCGGATTCGGAAATCG
ACACCTCAGATTGGAGGTTGCCCGCGAATACACCACTTGCGGACGAAACTTTCCACAGGTCAGGTCGGAT
TGATCTCCTTCTCGGTACCGAGGCATTTTTCGAATGCCTGGCTGTTGGACAGATTAAGCTCGGAGACAATT
TGCCAGTCCTTCAGAAAACGTTGCTTGGATGGGTTGTGTCTGGCAAGTATCGTCATCAGTCAGCTAAGCAG

TCATCAACGTGCTTGCTTTCTGTGGAGGAGTCAATTGACAAAAATCTTCAATTACTGTGGAGCATTGATGC
TCTAGAAAATTCTCCAAAGTCAATCAATCCAGAACATCGGCTCTGTGAAGATATGTATTCAAGGACAACA
CAGATGAATAACGGCAGGATTGTTGTGCGTCTACCTTTCAAGGGAGATCCAATGTGCCTTGGCAATTCAA
GCGATGTTGCACGTCGTCGTTTTTATCGCTCGAGAGGCGCCTCAGTCAGCAGCCTACTGTTCAATCGCAAT
ATGTAACCTTCATGAAAGAATATGAGGATCTTGGCCATATGACTTTAGTGACGAATCCGAGATTAAGTGA
GCCACACTTTTATATACCTCACCATTGTGTTTTTAAACCCAGCAGTGCATCAACGAAGTTACGAGTCGTGT
TTGACGCCTCGTGCACTTCATCGTCCCAGCATTCATTGAATGATTTGCTTCTCGTTGGCCCAACACTCCAAG
AAGATCTGTATATACTTCTCCTTCGTTTTCGATTGGTTCGTTACGCCATCACAGCGGACATTACGAAAATG
TATAGACAAGTTTTAGTCAGCCCAGTGGATCGGAAATTTCAATATATATTTTGGAGAGCATCCCCTGATGC
TGATTTGCAGACCTTTGAGCTAAATACAGTGACGTATGGAACGGCCTCGGCCCCATACTTAGCAGTTCGCA
GTTTGCAATATCTGGCAGATCTGCATGTGAACGAGTTTCCCATTGGCGCATCCATTGTAAAATCATCGTTT
TATGTTGATGATCTGCTATGCGGAGCAAACGAGCTCATCGAGTTGCAGAACATCAAACACGAGGTCACCG
AGATACTTCGCCGAGGACACTTCCCATTGGTTAAGTGGCACTCCAATCATCCCGCCGTCAGGAACGACGA
ATCGGTAAAGGATTTAAACTTGGATGAAAACGCTGTCACTAGCGCACTTGGCGTAGCTTGGGATCAACGT
CGTGATGTGCTACTATTTGAATTTAAACCCAAAAAATTGTATTCAACAGTCACAAAGAGGACTATTCTCTC
GACTGCGTCTTCTCTGTTCGATCCATTAGGATTGCTGGCACCAATCATTATCGTCTCGAAAATCATCATAC
AGGAGCTATGGCTGCTAAAACTCGCCTGGGATGAATCTGTTCCGCAATCACTTCTACAAGCATGGAGAAA
CTGCGTAACGTCTCTCAAGGAATTGTCGTCGATTTCAGTACCCCGTTTTTGCTTGCAAGCTGAGCTCACAG
ACATACAACTGCACGCTTTCTGTGACGCATCAATACGTGCTTACGGCTGTTGCGTATATGTGCGTTCCACG
GACTCGTATGGCAATGTCCAGGTTCAGCTTCTGACTGCTAAATCTCGGGTGGCTCCTGTCAAGAAGCTGAC
GCTTCCTCGATTAGAGTTAAGCGGAGCTCAACTTCTGAGTAAGTTATATACAACTGTCAAAAACATATTCA
TCGGCAAGCCGCACAGCGCGCATTTCTGGTCTGATTCGCAGATTGTCTTGCACTGGCTGCAACAACACTCT
ATCACCTTGTCGACATTTGTTGGAAATAGAGTATCCATTATACAAGATCAAACAGCAGATGGAAAGTGGA
GACATGTACCGTCAAAATTGAACCCTGCTGACATCGTGTCACGTGGATGCCCAGCTAATGAGCTCCAGCA
ATCAATTTGGTTTACAGGACCTGCGTTTCTGCGTGATTCCGAGACTCAATGGCCCTGCCAACAATTCGACA
ATGATTTGGACATGGACATCATCGATGCTGAAAAACGGAAGTCTACATTTGCTGCAATTATCGAAAGTAA
TTCTATTCTGGACTGGCTGAACGGAACTAGTTCATACATTCGGGCGCTTCGAGTTATGGCATATATGTTCC
GGTTTTACCATATTGCCAAAAAGGAACACCCTGTCGAAAGTTCGCCGCCATGCCCAGAAGAACTTCGCCA
CGCGTTGCACTGCATCATCAGGATCATCCAACAGCACTATTTCAAGGAAGATTTGCAATTGCTAAGGAAA
CAAAGAGATTTGAAAAGCAATCTGAAATTTCTCAGCCCCTTCATAGATGACTCTTCTGGATACGAGCTCAT
TAAGGTTGGCGGCAGACTCGAGCATGCTGATATCCCCTCTCGTCAAAGGCATCCTTTACTTGTTCCCAAGG
AATCGCAATTTGTCCACAATTATGTTCGGCATCTTCACTTGCGCAATTACCATGCAGGACCAAAGGCATTA
GTTGGACTGCTGCGGCTTCAATTCTGGGTCATAAACGCAAGAGCTTTAGCACGCAGCATTGTTCGTTCTTG
TGTACACTGTGTCAGATATCGTCCAGTTCTACTTCGTCAAGTCATGGGTTCCCTGCCACCTGAGCGCTTATC
CACAGACAAGCCATTTCGCCACGTTGGAGTCGACTTCTGCGGTGTCCAGTGAACACCTATCTTCGAATACG
TGGCAAGGGTCCCACAAAATCCTATATCGCCATATATGTCTGCATGGCCGTAAAGGCCGTTCACATTGAA
ATCGTGTCAGATCTTTCAACGAATAAGTTTTTGGAATCGTTGAAACGCATGGTTGCCCGTCGTGGACCAGT
ATCGCACATCTATTGTGACAACGCCACAAATTTTGTCGGAGCATCCAACAAGCTACTCGAGTTAAAGAAG
TTCATGTTCAACACTAGCACTCAAGATGCTATCCTATCATATTGTTCCTCTGAAGAGATAACCTTTCATTTC
ATTCCACCTAGGGCTCCACACTTTGGCGGTCTTTGGGAGGCCGCTGTCAAGAGCGCAAAGAGTCTGCTGG
TGCGTACCCTCGCTAACACCCGGTTCACCTTTGAGGAACTGAACACCGCAGTCGTTGAGATAGAAGCAGT
ACTCAACTCTCGCCCGCTGTCACCGCTATCATCTGATCCCAACGACTTCAGCGCTCTCACTGCAGGACACT
TTCTAAATGGAGATGCACCGCGCGCTTTGCCCGAGCGTGAGATTATCACCAACAACATCTCCAACTTGGAT
CGTTACGAGCAGATCACAGCTGTTAAACAGCAATTCTGGCGTCGTTGGTCTGCGGAGTATGTGAACGAGC
TTCGAGCCAGGACAAAATGGACTTCGGCTTCCCCAAATCTCACAGTTGACTCTATGGTCATCATACATGAG
GACAACACACCGCCATTACACTGGAAATTAGGGCGTGTTGAGTCTGTTGTGAGTGGCAAAGATGACCGGG
TTCGTGTCGCATATATTCGCACTGCCTCTGGACTCTGTTGCCGGCCAGTACATAAGCTGGCTGTTCTACCTG
TCTCTTGAAAGATAATTCTTTCAAGGCGGCCGGGATGTTCGGTCATATACCTCATATATACATTAGTCAGG
GTATCAATAGCAATGTCAAAGCAGATTATGTTAAGATTATGCTAATTCTCTTCTATTTGAATGTCATAAGC
ACTCATACACATGTACGAAGCCTCGCTCTCTTTCGAACTCTGTAATTGCTCGGTCCCTTTAATCTTAATTTA
TGTTAAGAAATCAGTTCTGTAAGCATCATCATAGAAGTCAGTCGTGTTGCTCATCAATTTGTAAAACCTTT
ATTAAAACTTCATACCATCCACACACGCCCACTTGAGATAAGATTCTTAAAAGGTCAAAAAAACGCTTTT
GATCCAACA
>9_rnd-1_family-78__LTR___Gypsy

CTCGTTTCTCAGGTACAGAGACCGCTTTGCGAGGGGATCTGAGTCAGACGACTCCACCACCTTAGATAGG
GAAGCCGTCCNGCAAAAAATCGCTGACAACAGGTGTAAAATAGGAATGGAGCCTGAGCAGCTTAGGGCC
TTGCNCGGTGGCCGCCGCCTTAGAAGCACAGAGCGCAATATTGAGGCAAAGAGATGCAGAGCTGAGGGC
TTCCTTAGACGCTCAGATACAAGAATTGGTAGGCAGAATATCTACCAGTAGTATAGAAACACCGCCGGTC
CAGGCATTTGAGCCGGTGGAAATCAGAAACGACGTCCAATGTAATGAACCATTGGACGCCGTTAAATGCT
TACCAGAATTTACGGGGGTGCCAGAGACGTACGCCTCTTGGAGACAAGCTGCGATAGCGGCATATAAAAT
TTACAAGCCATACGACGGCAGCTCACGCCACTACCAGGCGGTCATTATTATAAAGAGCAAAATTAGAGGG
GCAGCTGACGGCGTTTTGGGTCAGTTTGGCACAGTAATGAATTTTGATGCCATAATAAACCGTCTCGATTT
CACATACAGGGATAAGAGAGGCATTCACGTCATAGAGCAAGAGATGAGCACCCTAAGACAGGGCTCGTT
AACAATTTTACAATACTATGACGAGGTTGAGAAAAGCTAACTCTTCTCACCAATAAGGTCAACATGTCTTA
TGAACCAAGCATGGCACAAGGGCTCTGTACAAAGTTTAGAGATGATGCGTTGCGTGTTTTTGTCTCAGGCC
TAAAGCGTAGCCTGACGGATGTCCTGTTCGCGGCAAAGCCTAAGGACTTACCGTCGGCCCTAGCGCTAGC
GCAAGAGGTAGAGGCAAACCATGAGAGGTACGCCTTTGCCGCTAGCTTCGCTAAAGGACAAGAAGATAG
GGAACGAAAAGTGACCCCGAGGCCGCAGGTCCGTCAGCAAGATAGGGGTCAACAGCAGGGGATCCGCAA
CCCAGCGGTTCCAAAAATCCTCACTTCACTAAACAACAGAAACAGCAACAAAGTCAATTTGAGCAACAGA
GTGGCAGGTGGAACAAAGGTGGCGGGTCACAGGAACCAATGGACGTCGATCCATCTGTGTCTAAAATTCT
ACAACCATCGCAAGCACCAGCCTACGTTAACTCTAGCGCTCCTCAAGCCTACAAAAGAACGGCCACGTCT
GGGCGTTTAAGCGGACAGAGGAGGCAGAGAGTCAATCACACAGCTCACGAGCCGGATCAAGATTCAGGT
TCGTATGCTAGCTCTGCAGCAGCAGCAGAACAGCAAATTGCCGACGACAATGGTAGCGGTTATGACTCAG
ACACTCTCANTTTTTAGGGGAAGCTCCCTGCTGCCCTACATCAGTAGAAGAGTAGCAGGGATCCAGATGA
GGTTTCTCATCGACACGGGCGCTTCCAAGAATTATATCAGACCACATAAAAATCTAAAAGGTGTGTGCCC
AGTAAATTCTCCCTTCTCTACCCATTCTATACACGGCACCACGAAGATCACTCATAAGTGCCTTATATCTCT
GTTCGGCAAAACTGCCACATTTTTCTTACTCCCAAACCTGTCCACATTTGATGGAATTGTGGGCCTCGACT
TGCTCACACAAGCAGGAGTTTCGCTTTGTCTTTCTTCTGGCCAACTCAAATGTGGCGCAGAAACTGAACAA
ATCGAATTCCACAGGTGCGCGGATGTTAACTTTACCAATGTTGACTGCGCCGACGCGCCCGATGCTGTAA
AAGATGCATTCCTAAAGATGCAGAAAGCAAACATAAGGCCTTCGCAGATCCGGAAGAAGCACTTCCCTAT
AACACATCAGTGGTGGCAACCATCAGGACCACTGATGAACAACCAGTTTATGTCCGACTGTACCCCTACC
CCATGGGAGCAGCGGAATTCGTTAATAAAGAGATTCAAAGCCTTCTGAAGAACGGTATCATCAAAAAATC
TGTCTCCCCCTACAACAACCCCATTTGGGTGGTGGACAAGAAGGGGACGGACGAGTTTGGTAACAAGAAT
ATGCGCCTAGTAATGGATTTTCGCAAACTTAACGAGAAAACCATCGCAGACAGATACCCTATGCCTAACA
TTCCAATGATTTTGGGGAATCTTGGCAAAGCTAAATACACTTTTCGACATTGGACCTAAAGTCGGGGTACC
ATCAAATTTACGCTTGCGGAGCGTGACCGCGAAAAGAGCTTCATTTTCAGTAAGCGGCGGCAAGTACGAG
TTCTGTCGCCTACCATTCGGCCTGCGCAATGCGAGCAGCATATTCCAAAGGGCAATAGACGATATCCTGA
GGGAACATATAGGAAAGATTTGCTTTGTCTATGTAGACGACGTCATAATTTTTCTAAAGACGAAACGGAG
CACCTTCAACACGTTAACATTGTGTTAAAATGTCTAATCGACGCAAACATGCGTGTAGGCCCCGGAAAAG
ACTCGTTTCTTCAAGGAAAGTATCGAATTCCTGGGTTTCGTTGTAACCAAAGACGGAGCAACATCCGACCC
CGAAAAAGTTAAAGCCATTCAGGAATTCCCTGAACCAAAGAATGTCTACGGCGTTAGGTCCTTCTTGGGG
TTAGCCAACTACTATAGGGTCTTTATTAAGGACTTTGCCGCTATAGCGCGTCCGATATCGGACATACTTAA
AGGGGAAAATGGTTCAGTTAGTAGGCACGTATCAAAGAAAATCCCTGTTGAATTTAATGAAGTTCAACGC
GATGCATTTCACCGTCTGCGGAGTATTTTGGCATCAGAAGACGTCATGTTAATGTATCCAGACTTCCAGAA
ACCGTTCGATTTAACCACTGACGCTTCGGCCAGCGGAATTGGCGCGGTTCTTTCCCAAGGCAATAGGCCA
ATAACAATGATTTCTCGCGCCTTAAAACAGGCCGAACGAAACTATGCCACTAACGAAAGAGAATTGCTGG
CCATTGTGTGGGCTTTAGGCAGATTGCAGAACTTTCTGTACGGTTCTAGGGAAATTAACATTTTCACGGAC
CACCAGCCGCTCACGTTCGCCGTGTCGGACAAGAACACGAATTCCAAGATTAAAAGATGGAAATCTTACA
TTGACCAGCACAATGCCAAAATGTTTATAAACCGGGCAAAGAGAATCTGGTGGCGGATGCCCTTTCAAG
GCAAAACATTAATGCTCTGCAAGATGGGCCCCGTTCGGACGCTGCAACCGTTCACAGCGAACTTTCCCTG
ACCTACACGGTCGAGACAACAGACAAACCTTTGAATTGCTTTAGGAACCAGATTGTCCTAGAGGAATCGC
GTTTTCCGCTAAAGCGAAACTTGTTGCTGTTTCGCAGCAAAACACGCCACTTGATCAGTTACACTGATCGC
ACAGCCCTTCTAGATGAACTCCTTGACTTGATTAATCCAAATGCGGTCAATGCCCTCTATTGCAACCTGCA
CACACTAGCACAGGTGCAGGATGGCTTAGTTCGCATGTTTCCAGGCACAAAGTTCTGGCACTGCAAAAAC
CGAGTTGCAGATGTCTTCAACGTTGACGAGAGGAGAGAAATTCTCGTTGCCGAACATAATAGAGCGCACA
AGAGCAGCTCAAGAAAATGTTAAGCAAGTGTTATCAGAATACTATTTCCCTAAAATGGCGAAGTTGGCTA
AAGAAATTGCGGAAAGTTGTAGAATCTGCTCAGCAGCCAAATACGATAGGCACCCGAAAAGACAGGAGC
TGGGAGAAACTCCGATCCCTGAGACTCCTGGGGAATTTCTACACATTGATATTTTTTCCACGGACAAACAA

TTTTTTTTTGACCTGTGTTGATAAGTTCTCTAAATTTGCAGTCGTACAACCAATTCAGTCGCGCACTATCGG
GGACTTAAAAGCTCCACTACTGCAGCTCACGAATTTCTTTCCACGAGCCAGAACAATTTATTGCGACAACG
AGNCTTCGATGAATTCTCAAACGATAAAGACCCTGTTGTTAAACAACTACGGAGTCCATATTGTTAATGCG
CCGCCGCTGCATAGCACCTCCAATGGCCAAGTCGAGCGCTTCCATAGCACTCTAGCCGAGCTGGCACGTT
GCCTCAAGCTGGAGAGAAACATCAGTGACTCAGTCGAAGTCATAATGCTAGCCACGATTGAGTATAACAA
GACAATACATTCGGTCATCAACAAGAGACCTGTTGATGCCCTCCATGTGCCCTCAGGCGAACCCGAAGGG
GAAATATCAGCAAAGTTAAGGAGTGCACAAGATGCGCTCCGGGCTAGGCTGAACGACCAGCGACAGAAT
AGAGTCTTTGAAGTAGGCGAAAAAGTCCTAGTAAAAAGAAACCGCAGGCTTGGCAATAAACTCACGCCCT
TATGTGAAGAACGGATCGTAGAGGCAGACCTTGGCACGACCGTTCTCATTGGAGGGAGGGTGGTCCACAA
GGACAACCTTAAGTAGGGCGAGAGCTGAAATAAAACATCGATTNTATGCATCTTGAATTTAACTATAAGT
TTCAACATTTGNGCCACTAGGCTTCACCTTCAAGTTTTAATATTTACGCCACTTGGCATTNCCTTTAGGAAG
ACACGTAGTAATAGTTTGGTCCGTTTTGTCTGTCTTCCGCAGGTTCGGGTTTCTGCTATTTCTATTTTTTGCA
AGTGCGGCAGCGCGTGTGACTAATTACTCACACGCCAAGTATATTCCCATTGTTGAGGGCCAAGTTCTGGT
CTGGGAGGAATTCAATTTCGTCAGGCACACAGCAAACATCTCTGAGTACGAGAAAATGGTAAACGAAAC
AATTGAGCTATGTGAAATGTTTCCGCAGTCACACATGAGAAAGCTTCTAAGCGTAGATGCGTCCCACCTC
AGAGATCTGTTAGAAGCTCTTGGCGTGCACCACAGGGTGGCAAGAAGTTTAGATTTCTTGGGCACAGCCC
TCAAAGTAGTTGCGGGAACACCTGACGCCAGTGACTTCGAGAAGATCAAATTCACCGAGTTACAGTTGGT
GAACTCTAACAACAGACAGGTAGCTATCAACACAAAAGTTCAGGAACAAATCAATCAACTCACTACAAGT
GTCAACACATTACTAAAAGCTGCAAAAAAGAATCAGATAGATTCCGGGCATTTATACGAAACTCTTTTAG
CTCGGAATAGAATGTTGCTGATGGAAATGCAGAATTTGATGCTCGCTGTTACCCTTGCAAAAATTAATATC
ATAAGCCCAAATATATTAGATCACGCAGATTTGAAATCTGTTTGGGTCGATGAACCCACAGGTACTTCAAT
AGCTGACCTTATGTCCGTTGCGTCCGTGAAAGTATTACAATCTGTTAATGCCCTTCACTTTATCATTAAGTT
CCCGAAAATCAAAATGGCTTGCAGAAAGGTGATGCTGTATCCAGTTGCACATGAGAATGCTGTGCTACAA
ATTGACGACAACATTATAGCTGAGTGCGGAGATCAAGTGCTTGCCCTTAAGAATTGCACTTCCACGCCGG
GAGGAACCTTTTGTCAACTTTCACCGACCAGCTCTTGTGCCAGAGAGCTGCATGCTGGTGGCTTGGCAAAC
TGCCAAATGCAGCCGAGTCACTTAGACCCGATCACATGGGTCGATGACGGAATTCTGATCATCAATAACC
GGCCTGCTAAAGTTTGCGTTGACAACGGTACTGAGACTTGGGTTCACGGAACGAATCTAATCACGTTCAA
TAATTGGGCTCTGATCAACGAAACTAAGTACTTGAATCGCAACAACGTCCAGAGTAAGTTCCCCGGCATC
GCGGCATCTCCCCTTCTGAACGTCACCGGCCACCAAGACGTGCTGAGTCTCCCCTACCTTCATCGCCTGAA
TGAACGTAACCTGGAGATCATCAAGGAAGTCAAGGACGAAATTGGAAGCGTCGACAGCGTGGTAATAGC
CTTCTTGTTGGGTGCTGTGTGTCGCACCATAATTTGCGTCGGAGTCACCTACCGTCGGTTCACCAAAAACA
TGGCATCGGCCAAGGAGATTAAGAAGGTGATCGCCGAGATAGGAGCGGCCGAGGGCGGCCTTAACTTAA
AAGGGGAGCAGTTAACAGAGGCCAACGACACCAGCTCCAGATGCTGATATAGCACTGGGCCGGATGTTCT
TACGAGTTAACAGGGGTCAGCAAAATGCAACATAACACCAGCCGCAGATGCTGACATAGCTCTGGGCCG
GATGTTCTTGCACGACGGCAGCGCACGAGGCGCAAGCTAAGCATTCTAGCGGCCGTTGTGAAAGCACACA
ATAGCTTAGGTATTTTTGATCAATCTTTTGTAAATTTAGTTTTTAATTCGACTTTCACTGCGGCACATCGGC
CCAGCGACTTCGCGTATAATTTAACTCACTCGCTACGGCGTGTCGGCCTAGTGTTT
>160_rnd-1_family-233__LTR___Gypsy
ATTGCGCAGTTGCATATAACTTTGGTTTTTTTTTTTACCGATCTCTATGAAATCTACAGTATATCTTATTGT
ATCATAGAATATTTGTGTATACTGAAAAAGTCATTTGCAAATTAATTTTGGCTTAAATTGGTTGGCAATAA
AAGTTGGGTTATTAACTTGATTTATAGCTCTAGGGTGGTAGCGGAGAGGTGGCGATAGGTATAAAATGAA
AGATACTATATNNTATTTTAGAAGCNACATGTCANGTTTNGTGACTCTAGCTCTTATTATTTACCAAAATT
GCCCAAAAAAACAGGATGTCGATATCGAGGTTCATCGATTGCTTGGAAACGGAGTAAGCTATCGATTATC
GGAAACAAACTCGATCTGCGCGGGCACTAGGAGCACCTNCATCTAATTTCAAGTCTATAGCACTTATATG
TTCTGAGATCCTTGCGTTCATACATGCGGATGGACGGACGGACAGACAGACGGACATGGCTAGATCGACT
CGGCTATTGATGCTGATCAAGAATATATATACTTTATGGAGTCGGAGATGCTTCCTTCTGCCTGTTACATA
CATTTGGATTTTGCACAAATACAATATACCCTTATACCCATTTTTAATGGGTTCAGGGTATAAAAATACAT
ATACATTGGCCTGATCAACCAACTGAAAAAAAAAATCAATCCTTGAAAAAAACAAATTACTATAATGAAT
TTTACATTTTTATATTTTATATCTTTATATTTTGAAATGTTTACATTAGTATATTCTCATAAATCTTAAATAA
TTTTTTTAAATTCATATTATTTTTATCATTTTTTTACATTTATTATTTTATAANAAAAAAAATTATATACAA
AAAATTTTTGTTCAAAATAATTAAGTTCATTAATAAATTAACTACACATCCATAATGTTACGTTATTTCTTT
GAAAAGGGAGGTGTAGTGTGTATATGCCTTAAAAGAACACCCTATATACAAATATAAAGTATGCGGCACT
TTGTCGCATACAATAACAATTATACATTAAACAAGCCCAGAGCCACTTTGTATATTGGCTCTGGCTCATAT
TACGCAGCGCAGTTCACTAGCCTCTCTAGCTGATGCAACGATGAACAACAGTAGCAGTTATATCCTTAATT

ACTTTTAATTATAAAAAAAAAATTTTGTAAAATTGAGCAGCATAGCCCGTAAANCTATGCGAAATGCTAG
GTAGTATTTTGTGTTATTAGCAATATTGAAACCTTGAAACTAAAAACATTGGCATTTAAAGCTAAAAACAC
TATATGTTACATAAAAACATTATATGAATTTAAAAACATTACATCAACTTNTGTAAGAAATTTTAATTAAA
ATGGGTTCGAACGAATCCAAGGTTGAAAAATCAGCCGCAAATGTTATAAATAACGTAAAAGTAATTGACC
ATAAAGAATAATTACTACAATATCTCTTGTATCACTAGCATTGAAAGTGTATGCAATGCATACAAAGAAA
ATTAAGAAACGCTATAGCAGTAGAGCAGACGGACTAGATAAGGTATGAATATATTTGAATATGTCAGACA
TAGAGGCAATCATTAGGTTTACGTATAGAGATGAGCCAGACTTTCGTACTCGCGTATTAAACGTACTGCG
ATTACATAGAGATTTTATAAAAGTGTGTGATTTCGAATACGAATACACGGACATGTTCAATACTCATATTA
ATGGTGCGACTGCATCGATAGATATCTATTATAATAATATGGAATGGAGTATATTAGTGAAAAATATCAA
TGACTTTAAACACCAATTTGACAAGTCATATAAATGCATCAATAAAGGCATACCTATAAAGGTAGAAACA
CTAATATACCATGTACAAATATTGATAAAACAATATAACAATATAGTACATGCAGTAGATTCCATTAAAG
AAAAACTAACACAAGAACATGCCAAACAATGCCTTAAAGTCATACAATCTTTAAATACAAGATTGAAAAA
CATTGGCATTAGACAGAATATCATAATAAATGTTCCAGAAGATCTATTTTTACCAGCAAAGTTTGAACCAA
ACCAGCTGCAAGATCTGAATGAGTCGAAGCACTAGCCAAAGAACATATAGACTGGACATGAGTACAGGA
AAGTAGGCACGAAAAGTATCGGTGGCATCTGGTCCAAAATCGCGAGTAAGGGAGTAAGATATACAAATG
ATGTATTTTTGAACCGCTTACTCTACAGGAAAGTGCTTGGCGCACTCTCTTCCATATGATTTGTGCGATAG
AACGACAACGCTGTTAACTTTTATCTCGCTCGCACTTACTCTCAGCTCTCAATGCTCTCTCTTTATGTTTAG
GGATCGTTTAAATGTGTGTGTGTATGTGTCCGACAAGCAAAACTGGGCAAAAATTCAATTTTTCCCAAATG
CTGAGGCATTTCTACCCTTTTGAAGGCACGAGTAATATGGAATAGAGAAAGGGTTTGCCACGTCGATGCG
AAATTCCTGCATTTTAGCTCGCTATCGTTGGTAAGGGTACCCAAGTGATGCCATGTGTGCATTTAAAATAT
TTTGGACTCGTTATTCACGCATACTTGCATATTTTGTATATGAATAGAAGCATTTATGCGCTTGTGTGCCTG
TTGCTTGCCTGTCCCCGATGCAAATTTAACAATATTTTGGGATTTCTTATTTTCGACCTCTACACATAGGCA
TAGACATACAAGTGTCTGTGAATGGGCAGATCATGGGCGCAAAAGAAAATATCATTATTGGCACGCGTCT
GACGCGCGCCCTTGAAATGAACAACAATAGCAATGGCAGAGGACATTATAGACGTAATGCCCGTGGTAAT
AACCGAGGAAGGAATAATTCCAACCGAGGTAACAACCAAAATAATAACAATAGAGGTAATGTCCGAGTA
ACTGAAGGTAATTCGGAAAACCATTGAGACCCCTCAGATGTTTAGAGCCAATAAATGGCACAATAAAAAC
CATCAATGATCCTCAACATCAGCATATTTGTAAAAATTAACAATGAAAGAACAGGTAAATCCTTTGCACT
GTTGCCTGATACTGGTGCAGATATATCTATATTAAAAGAAAATGTAGATATATTTGAAAACGTAAACGAA
AGAGTTACTACCCAAAAATCGGGAATTGGCGAAGGAACAATTTTTTCAAAATGCTTAGCCTTTATAGAAC
TCACTACAGGTAAATACATAATTCCGCATAACTTTCATTTAGTCGATCAAAAATTCCCAATACCTAGTGAT
GGCATACTAGGCATAGATTTCATAAAACAATATAATTGCCAATTAGATTTCACAAAGAGAGGCGACTCAC
TAATTCATCGCCCAAACAACTTAAATTGTCCGATTAAATTACAAATACTTCATACTTGTGACAATAATGCC
ACAGTACTCCCAGCTCGAAGCTGAAGTTCCGAAGTTGTCCGTGAAATACAAATACCCTCAATAAAAAATC
AAATTTTCGTATTAAATCAAGAAATCGATGAAGGAATTTATATCGCAAATACAAANATATCCAACCACAA
AAATACTTGTGTCCGAATTTTATACAACAAATACCAATAAAATCGTCAATTTAAATAAAATAAAATATGA
TAAAAGAAACTAACGAAGAAAGCGACAACCTCATTATGAATAAATTAATTAGAAACTTTCCGCCTTTATT
CAAAAGTCAATTAAAAGATTTATGTACCAGTACCAATTAAAAGATTATGTACGAAGAAGTCTCTTCCGGA
CTCTGAAAAGAAAAAATGGCGATTAGAAATTGACTATCGTCAAATTAATAAAACGCTATTGTCCGACAAA
TTTCCACTACCTAGAATTGATGATATTCTAGATCAACTAGGTAGAGCAAAATATTTTTCATGCCTAGACTT
AATGTGTGGCTTCCACCAAATTGAACTTGAAAAAAGTTCTAGAAATANAACGTAATTGTCAACAAGCAAT
GGCTCATATCGCTTCACGCGATTACCATTTGAATTAAAAATAGCATTTTCTGGTCTACAACCATCACAAGC
ATTTCTTAANATGGATGATCTAATAGTAATCGCTTNTTCCAAGAAACATATGGTCAAAAACTTAACTGATG
TTTTCGACCAATGTAAGAAACTCAACCTGAAACTACATCCGGAAAAATGTTCATTTTTTATGCATGAAGTC
ACATTTTTGGGACACAAATGCACTGATAAGGGAATCTTGCCAGACGACAAAAAATACGACGTGATTAAAA
ATTACCCACTCCCACACGATGTGGACAGCGCAAGACGATTCGTTGCATTTTGTAATTACTATACGCGCTTN
ATAAAGAACTACTCGCTAACTATTCACGTCACATAACAAGATTATGTAAAAAAGACGTAAGATTCGAATG
GACGCCTGAATGCCAGAACATCTCAAAACAGCACTAATAAACCTAAACTTGCTACAATATCCAGACTTCA
CCAAAGAATTCTGCATAATAACAGATGCAAGTGAGCAAGCGTGTGGAGCAGTTTTAACTTAAAACTTGAC
TGGACTCCAACNTCCAATTGCATACGCATCAAGATCCTTTACTGAAGGAGAAAGTAACAAGAGTACNACT
GAACAAGAATTAACAGCTNTTCNCTGGGTAATAACACACTTTCGACCATATATTTATGGTAACCATTTCAC
GGTAAAAACGGATCACAGACCACTAACATCTATTTTCAATGGTCAATCCAAGTTCACAATTAACACGTAT
GAGGCTTGATTTGCAGTAATATACCTTTACGGTAGAATATCCGAAGTGTAAAGACAGTTACGTAGCAGAT
GCGTTATCGAGAATAACCATCAAGGAGCTACAAAATATAACAGGAAACATAATGAAAGTCACTACAAGA
CAAGCACGTAGACAAGAATCCTGCGCAGGAGAAAAAGAAATAGAATTGCCTAGGCAATCTATAGAAAAA

GCTTCAAAGGCCAAAGTGTACCAAGTCATTAACAATGACGAGGTACGAAAAGTAGTGACCTTGCATGTAA
ACAATATGACATGTTTTTAAACATGGAAGACAAATTACAGCAAGATACGATGTTACCGATTTATATACTA
ATTAAACCATTGATTTGGATCAAATCTTTCAAAGGCTTGAAACGCAAGCCGGTATATATAAAATCAGTCA
ACTCAATGTGGCTTCGTGGGAAAATATCTCTGAACACGTTTCAATGGATAATTTATAGAAAATGGGCATTC
AAATATTAAAATCTTTAAGAGTTGCGCTACTCCACCCGGTGACCCAAATAAATTCTGAAAAATAAAAATA
AGCTATCTTGTCTAAACTACTTGATGATCCCATACTAGGAGGACAGGCATTATAAAAACCTTGGCCAAAG
TCAAACGACATTATTTTTGGAAAGGTATGTCTAAACAAATAAAAGAATACATCCGCAAATTTCCAAAATG
CCAGAAAGCAAAAACTACAAAGAACACAAAAACTCCTCTAACAATAACAGAAACACCAATATCCGCTTT
CGACAGAGTAATCGTTGACACAATTATTACCATTACCTAGAACAGAGAATGGAACGAGTATGCAGTTAC
ACTCATTTGTTATTTTACGAAATACCTCGTCGCAATTCCGATCCCAAATACAAGTGCAAACACTGTCGCTA
AATCTATATTCGAATCTTTTATTCTGAAGTACGGTCCAATGAAGACGTTCATTACGGACATGGGTACGGAA
TATAAGAATTCAATTATAAACAATTTGTGCAAGTATATGAAAATAAACAACTTAACATCAACTGCACATC
ATCACCAGACAGTTGGAACAGTAGAAAGAAGTCACAGGACTTTTAACGAATACATACGATCATATATATC
AGTTGATTGGGGCGTATGGATCAAATACATCGTATACTGTTTTAACACGACCCCCTCCATCGCACACAATT
ATTATGAGCAAATTTTTGGTAAACACCCAATTTACCAATATAAATAGAATCGACCCACCATATAATGTAG
AAGATTATGCTAAAGAAGTTAAGTTTAGATTAGAAATAGCATATAAAAGGTCTAGGATAATACTTGGAAA
ACATAAGCAAAAAGTAAGGAACTTTTTGATAGAAAAGTTATAGATTTTCTAGTAGAAATAGAAGTCAAAG
TCTTATTAAGAAACGAAACTGGCCATAAATTGGATCCAGAATATATAGGCCCATATACAATAGTTAACAT
AGAAAATAGGGATAACATTCTAATTTAAGATAACAAAAATAGGACCCAAAAAGTTTATAAGAATAGACT
AAAAACTTTTAACGAATAAAACATATAAATGTTCTATATGAA
>160_rnd-5_family-5029__LINE___R1
AAAACAAAAGATAAGGTGCAGGTGTTCGCTTTGGCGTAAACAAAGCCATAAAACTTAACGGGACAACTT
GCGGTAAATAGCAATAAAACTGCTTGTGCCCAAGACAGCGCGAAAGCAGTGCAAAGCAGTGCAAACGCG
TTCACTGCTTTATAAGCAGTGCACATAGTGCGAAGCAGTGCGAAATTTTAAAAAGCAGTGCAAGCAGCGC
AAAAAGAGCAGTGCGGTTCGTCAACCAGNTAAGCGGTTTGTGTCCGGGTTATCGCGTGGTGTTGCCGACG
GAGGGCGCCACGACAATGGACGCCAGAAGCGTGGCCGGCACGGCGTCTAGTGCCGAGTCGGTTGCCAGC
GTCGCCGCCGATGGAAGGAGGAGGGTGTGGAGAGGGAGGGGCCGTAGCACGNCGTCCCCCCCCTGCGNA
CCCGTTGAGCCGAAGAGGCAGGCGTTGGAAAGCGCGGATGAGTTTGCCAAGCCCGCCGCTAGGGCTGCTG
CAAAAAGAGCCGCTGATGCTGCCGCTGCAGAAGAAGACGTCAGAGCCGCTGCCGCTGCCGCCCCCATCAT
CGCGCTTGCCCCAGCATGGAGGATAATGGCCGAGGGGACAGGGGCGATTATAGCGGAGCTTTCGGCAGCT
GCCGAAATGACCCGCCTTGCCCTGGTGGGTGTCGCCCCCGAAGCCGCGCGCGAAGTGTCGGCAGCCGTTA
AGCGATTGGAGGAGCTGTCCACGTCGCTGGCAGTGCGGAACGCTCTGCTCGAGAGCCTGGCAGGCAGATC
GTCTGCAGCACAGCTCGCCGCCCCCACCCCAACTGCTGCTCACCAAGTCGTCCACCAAGTTGCCCACCATG
CTGCCCGCACCGCTGTCCCCGTCCCAGGAGTGCCGATCGCTGCCCCACGGATCAACAAGCCAGTTGAGAC
GTGGTCCGCGGTGGTCACGAGTCGGGACCCGAGCGAGTCGGGCGAAGTGGTGGCCAAACGGATCATGGA
GGAGGTCGCACCCACCCTTGGAGTGCGGGTGCACGAGGTGCGAGAGCTCAAGCGAGGCGGCGCGGTCAT
CCGCACACCATCGAAGGGCGAGCTCAACAGAGTGGTGGCAAACACCAAGTTCGCGGAGGTTGGACTCAA
CATCCAGCCGAACAAAGGATTGAGGCCGAGGATAACGGTGTTCGATGTGGACACATCGATCCTGCCCGAG
GTGTTCATGGAGGAGTTGTATACGAACAACTTCAAGGAGGAGATGACAGCAGCGGTGTTCAACAAGGCA
GTACACCTTGAGTCGAAGCCGTGGTCCGTCACTGATGGCGCCAGCGTTAATCTGACGCTAGAAGTTGACG
AGAGGGCCCTGGCGACTTTTGAAAAGACCGGACGGGTCTATATAAAGTGGTTTTCATACCGATGCCGGTC
CCTCGTCCGCACCTATGCTTGCCATAGGTGTGTCGGCTTCGACCACAAGGTGTCCGAGTGCCGCCTTAAGC
AGGACGTATGCCGACAGTGTGGACAGATGGGGCATATCGCAGCCAAGTGCGCAAATCCGGTGGACTGCC
GGAATTGCCGCTTCAAGGGGCTACCCTCGGGGCATTATATGCTTTCGGGGTCTTGCCCGATTTATGGCGCA
GTGTTGGCGAGGGTGCAAGCTAAACATTAATGTTTAGTTTCATCCAAGCCAACTGTGGCCGGGGCCGCGC
TGCGTGCATCGATATCGGTGTACGTATGCGTGACCACGGCCACTCCTTTGCGCTGCTGCAGGAGCCATACT
CGGACGTCGTTGGAAGGATTACGGGCTTCCCAGCCGGGATGCGCGTTTTCGGAAGCCACGCTGTCGAGGA
CCGGAAAAGGAGCGCTGCTGTCATCATCGACGACCCAGATGCCATCTGCCTACCAGTGGAGTCGCTGACC
ACCGAGGCTGGAGTATGTGTGAGGGTCACAGGTAAACATGGCACTGTTTACCTGACCTCCATATACTGCC
ATGCTCATGCTGACTTGACAAGGATATTGAGCTATATGGATACCATACTGCTACTGGCGAGCAGTACACC
GGTAATTCTCAGCCTGGACGCAAACGCTGTGTCTCCCTTGTGGTTCAGCAAACGGCACGAATTCTTTCGTG
GCCAGCTGAACTACAGAAGAGGCGAACAGCTAGATGAGTGGATACTCGGAAGTCGTGCCAACGTGCTAA
ATCAAGTCAGCGAGGTGTACACGTTCGATAATGGAAGAGGACAGAGCGATATCGACGTAACAATCGTGA
ACGAAGCAGCGTCTACGTGGGCCGCGTATGACTGGAGTGTGGACGAGTGGGATTTGAGCGACCACAACAT

GATTACTGTTGTGACTTCGCCATCCCCAGCAAATGCAGTTGAGAGCTATGCTCCTGTGCCGTCCTGGAAGC
TCGCGAATGCTAATTGGCGATTGTTCGACTCGGAGCTGGTAGAGGAAATCGAGGAGCACCTTTCCCAGGA
GGATATTAGGATGTTGCCGTTGGATACCGCGGTGTCTGCCCTACGCCTTGCCGTACAGTCTGTGTGCGACA
GAGTGTTAGGCCGCAGACCGTCGAGAGCCCAGGGAAAAGTTAAATGGTGGACTGCCGAACTGAGTACCA
AACGCCGGGAGGTCAGGAGACTGAGGCGGAGGCTCCAGGATGCACGGCGCAGAGGTCATGAATCTGCAC
CGTATCTTGCAGCTCATCTGCGGACAGCTGTCCGCGAGTTCAAGACGCTCATCCTGAAGACGAAGGAGGA
AAACTGGCGACGCTTCGTTGGAGACCACAGAGACGATCCGTGGGGGCACGTCTACCGGATATGCCGGGG
ACGAAAGCGGTCTACCGATTTGAGGTGCCTTCGTTCGGATGACGGGCTGGTCGTAACATGGCACGACTGC
GCGAGTCTCCTCCTCCACAGCTTTTTCCAGTTGCGGAGTCGAGTGAAGACATCGTCATTCCGGACGAAGC
TGCTCCAGCGCTCGAAGCTTTCGAGGTTGGAGTATGCGTCGCTCGGTTGAAGAGCAGGCGCTCTCCCGGG
CTTGACGGCATTACAGGTGGGATCTGCAAGGCGGTATGGCGTGCCATCCCCGAGCACCTGACAGCGTTGT
ATTCCCGCTGCCTGGCAGAAGGATACTTTCCCCTTGAGTGGAAGTGCCCGCGTGTGGTTGCGCTCCTCAAG
GGCCCCGACAAGGATAGGAGCGATCCAGCTTCCTATCGGGGCATCTGCCTGTTGCCAGTGTTTGGCAAAG
TGCTTGAGGGGATCATGGTGGATCGAATGAAGGAGGTGCTTCCGGATGGAAATAGATGGCAATTTGGCTT
TCGACCTGGACGCTGTGTCGAGGACGCCTGGAAGCACGTGATGACCAGCGTTGCCGCCAGCCAGGCGGTA
TACGTACTTGGTCTCTTCGTGGACTTTAAAGGCGCCTTTGACAACATTGAGTGGAGCGCCGCGATGCGCCG
CTTGATCGATGCGGGATGCAAGGAAGCCAGCTTGTGGAGAAGCTTCTTCTGTGGCAGGAAAGCCAGGATG
ACCAGCAGATGTGGCGAAGTCGAGGTGGAGGTCACGCGAGGGTGTCCCCAGGGATCCATCAGTGGGCCG
TTCATCTGGAACACCATGATGGATGTCCTGCTCCAACGCCTGGAGCCCTTGTGCACATTTAGTGCATATGC
CGATGACCTGCTGGTTCTTGTCGAGGGAAGGTCGAGAGCTGAACTGGAGCGTCGAGCAGCTGAGCTGATG
TCCGTCGTGGGAACTTGGGGAGCCGAGGTCGGAGTGTCGATCTCAACCAGCAAGACGGCAATGATGATGT
TGAAGGGGCGTTTTGCCGTTTCCAGGCCGCCCGTGGTACGGTTTGCAGGAGCAAGCCTACCATATGTCGA
CAAGNNCCGGTACCTTGGCATCACAGTCGGTGAACGGTTGAATTTTCAGCCGCATATCGCAGGATTGCGG
GATAAACTGACTGGAGTCGTCGGAGCATTAGGCCGCGTGCTACGGGTTGACTGGGGCCTCAGTCCCCGAG
CCAGACGCACTGTATATGCCGGACTCATGGTGCCTTGCGCACTGTTTGGTGCCTCGGTATGGTATTCCGCG
GTCGGGGGAGAGTCGGGGGCCAGGAGGCGCCTCATCTCCGGNATGCCGTACAGTGTCCACCGTGGCACTG
CAGGTGCTTGGCTGGAGCTCCGCCGTTGGATTTGGTTGCCAAGCAATTGGCAATCAGATTCAAGGTCAAG
CGTGCCCACCCGCTGGAGGATAGTGACTGGCTGTTCGGTCTAGATGTGGCAAATCTAGACCAGAGGCGGA
TGAAGGCGTTGCTGGACGAAGGCATGTGCTCCGAGTGGCAACGGAGATGGGATAGTGATGAGCACGGTC
GACCGACTCATCAGTTTATCCCGGACGCATGCTTCGTCTACAGCCGGCGAGACTTTGGATTCGGTCTTCAC
GCTGGATTCTTGCTGACGGGCCACGGGTCGCTAAATGCATTTCTGCATCAGAGAAGTCTGAGTGCAACGC
CCGCATGTCAATGCGGTGCGGAGCATGAAGACTGGCAGCACGTGCTAGTTGCTTGCCCACTCTATGCAGA
TCTGCGAGACCTCGACGGACTTGGTGTCAGGAGAGAAGGTACCCGTTGGGACTTTTCCCGGGTCCTGGAG
ACACCAGAGCGGCTGCTACTCCTGGAGATGTTTGCGCGAGCGGTGTTTGCTAGACGGCGGCGAGGGATGG
AGTGAAGTGCAACCACACAGGGCCGACCAGTGCCGGGCTGACGTTAGCCAAAACTGGGAGTGGCATGGA
CCGTGTCACGGAGCCGAACAGAGCTTAATCTGGTAGACAAACCCCAATTTGGGGTAAGTCGAACAATGGA
TGACCCATTCCGTGTTTGTCGTCAACAAAACTGGGGAGTGGCAATTGCTACGAACAGCCGACCAAAGCTG
TAATTTGGTACCACGGGTAACCAGGAGCCCACGGAACTCGTTCCGTCCTGGTTAGTTGGTGCGGCCCTTCG
GGGAGTATCGTGGTGGCTGTGGTTCGATACCCAATTGTGGGGAGAGTTTTCACTCGGCGTGGAGTTGCGT
GTCAACCGGGTGCTGTACCCATAGACCAGCGGAGGTATAGATAGACCTCACCTCCCACCAAGGGGGAATG
TCATGTTCGACAAGCATGCATTCAATTTGGTACCGAAGGGGCACTGCACATGCAGTGTCTTGGAGGGGCG
CTAGTCCGACGCTTTGTTTCGGCTCGCTGTATTTCGAATACCGTGGTAGTAAACCCCTACGGGGTACACGC
CACGTTAAACAAATATGTTTTGTTTTGTCCCTATCTACCAGTTTGTTCCAGACGNTCGTCGGGAGCAGACG
TGTCGCGGAGTTATTTTTGTGTCGAAAACAAAAGATAAGGTGCAGGTGTTCGCTTTGGCGTAAACAAAGC
CATAAAACTTAACGGGACAACTTGCGGTAAATAGCAATAAAACTGCTTGTGCCCAAGACAGCGCGAAAG
CAGTGCGAAGCAGTGCAAACGCGTTCACTGCTTTATAAGCAGTGCACATAGTGCGAAGCAGTGCGGAATT
TTAAAAAGCAGTGCAAACCTGAGTAAAACAGTGCAAGCAGCGCAAATAGAGCAGTGCGGTTCGTCAACC
AGATACCGACGGAGGGCGCCACGACAATGGACGCCAGAAGCGTGGCCGGCACGGCGTCTAGTGCCGAGT
CGGTTGCCAGCGTCGCCGCCGATGGAAGGAGGAGGGTGAGGAGAGGGAGGGGCCGTAGCACGATTTCCC
CCCCCTGCGTACCCGTTGACCCGAAGAGGCAGGCGTTGGAAAGCGCGGATGAGTTTGCCAAGCCCGCCAC
TAGGGCTGCTGCAAAAAGAGCCGCTGATGCTGCCGCTGCAGAAGAAGACGTCAGAGCCGCTGCCGCTGCC
GCCCCCATCATCGCGCTTGCCCCAGCATGGAGGATAATGGCCGAGGGGACAGGGGCGATTATAGCGGAG
CTTTCGGCAGCTGCCGAAATGACCCGCCTTGCCCTGGTGGGTGTCGCCCCCGAAGCCGCGCGCGAAGTGT
CGGCAGCCGTTAAGCGATTGGAGGAGCTGTCCACGTCGCTGGCAGTGCGGAACGCTCTGCTCGAGAGCCT

GGCAGGCAGATCGTCTGCAGCACAGCTCGCCGCCCCCACCCCAACTGCTGCTCACCAAGTCGTCCACCAA
GTTGCCCACCATGCTGCCCGCACCGCTGTCCCCGTCCCAGGAGTGCCGATCGCTGCCCCACGGATCAACA
AGCCAGTTGAGACGTGGTCCGCGGTGGTACGAGTCGGGACCCGAGCGAGTCGGGAAAAATGGTGGCCAA
ACGGATCATGG
>160_rnd-1_family-268__LTR___Gypsy
GAAATCCAAAAACAAGTAGGGAAATTAATAAACGACAAAATCGTAGAACCGTCTGTATCCGAGTATAAC
AGCCCACTCTTGCTAGTTCCGAAAATATCATTACCAAATTCGGAAGAAAAGAAATAGCGATTAGTAATTG
ACTATCGCCAAATCAACAAAAAATTACTTTCTGACAAATTAATTCTCCCCAGAATTGATGATATTCTAGAC
CAACTGGGCAGAGCTAAATACTTTTCATGCCTTGACTTAATGTCAGGCTTCCACCAAATTGAACTTGAAGA
AAATTCAAGAAATATAACATATTTTTCAACGAGCAATGGCTCATATCGCTTCACGCGATTACCATTTGGTC
TTAAAATAGCACCGAATGCATTTCAGAGAATGATGACTATAGCATTCTCGGGTTTAGAACCCTCTCAGGC
ATTCCTTTATATGGATGACTTAATGGTGATAGGATGTTCCGAAAAACACATGATTAAAAACTTAACTGATG
TTTTAAATGTATGTAGGAAATATAACCTAAAGTTGCATCCAGAAANATGTTAATTTTTCACGCATGAAGTG
ACATTCTTAGGTCACAAATGCACTGACAAAGGAGTATTGCCAGATGGCAAGAAATTCGACGTCATCAAAA
ATTATCCTATCCATGATGCGGACAGCGCGAGACGATTTGTAGCATTCTGCAATAATTATCGTCGATTTATA
AAAAACTTCGCCGATTATTCACGGCACATAACTAGATTATGTAAAAAGAATGTTCCTTTTGAATGGTCAAG
TGCCAAAACGCATTCCAATACCTCANAGAAAAGCTTATGCATCCCACATTATTACAATATACTGATCTTCG
CAAAGAATTTTGCATTATAACAGATGCTAGTAAACAAGCTTGCGGAACGGTTCTAACTCAGAACCGTGAC
GTAATTCAGCTCCCAATATTATGCATCTTATGCATCTCGTTCATTTACAAAAGTGGGAAAGCAATAAGAGC
ACAACAGAACAAGAGTTAGCGGCAATTCATTGGGCAATTACCCATTTTAGATCATATATTTATGACAAAC
ACTTTACTATTAAAACGGATCACAGACCATTGACATATCTTTTTCTATGACTAATCCCAGCTCGAAATTA
ACTCGCATGCGGCTAGAGCTTGAAGAAAACCACTTCACAGTAGAATACCTAAAGGGAAAAGATAATTTCG
TAGCAGACGCACTTTCGCGTATTAATATAAAAGAACTCAAGGATATGCAACATAAAGTCCTGAAAGTCAC
TACCAGGCGACAAATTAGACAAAAAAAACTGTACAGTAGAAAATTATGAACTTCTGCCAAGGCAAAGTC
ATAAAAATGTATCTAAGCCCAACGTACATGAAGTCATTACAAACGATGAAGTACGAAAAAAAGTGACCTT
GCGCATAACAGAATCTAAATGTTTATTTAAACATGGAAATAAAATTATCGCAAGAATTGAAGTTAGCGAT
TTAAATACTAATGGAATCCTCGACTTAGTTCAGTTCTTCCAAAGGCTTGAAACAGAAGCCGGTATATTAAA
AATCAGCCAACTCAAAGTGGCACCGAGTGAAAAGGTCTTTGAATCAGTTTCAATAGATTATTTTAAAAAT
TTGAGCAATATAATATTAAAATCTTTAAGAGTAGCGCCACTCCAGCCGGTGACCCAAATAAACGATGAAA
AAGAGATACAACAAATACTGTCTACATACCATGACGACCCAATTCAAGGAGGCCACAGTGGCATAACAA
GAACGCTAGCAAAAATAAAAAGACACTACTACTGGAAAAATATGACGGGTCATATAAAAGACGTACGTA
CGTGCAGATGCCAAATGTCTAAAATAAAGACACACACAACAAGTAGGAGCTTGTAGTCGGGAGCTCCCG
ACTAGGGGACACCCTGAACCCTCTACTTCCAACATCAAATGCATATATATATTCTATTTTAAAAGCTATAT
GTCAAGTTTGGTGACTCTAGCTTTTATTATTTTCCAAAATTGGCCAAAAAAATAGGATATCGATATCGTTT
CTTATCGATTGCTTGGAAACGGAGTAAGTTATCGATTATCGGAAACAAACTCGATTTGCGCAGGCACTAG
GAGCAACTACATCTAAAATTTCAAGTCTCTAGCTCTCATAGGTTCTGAGAACCTTGCGTTCATACATACGG
ACGGACGGACGGACGGACGGACGGATGGACAGACAGACGGACATGGCTAGATCGACTCGGCTATTGATG
CTGATCAAGAATATAAATACTTTATGGGGTCGAAGATGCTTCCTTTTGCCTGTTACATACATTTGGATTTA
AATAAATAATAATGTCAACAAAAAGTTAATAAAGAATATATATATTTAATGGAATGGAATGAAATTGTTT
CTATATTAAGCAAACGTTCTGAGCTTGATAGATCATTTAAGTGCCTCAATAAAAGTGCACTAATATCAAAC
CAAACAAAAAATAAAACATGTTCAAGTTCTGGTAGAATCATACAACAGAGTACGTGACATTGTAAACAACA
ATGGACGTAGGTTAAGCGATTTATATTTAACACAATCAATTGACGTGTTAAAAAGGCTACGTGAAAATAT
TAGACATATTTCGGAAAGACATAATTTTCAAATATTGTTCCGCATCGCGCTAAATACACCAGCAACATTCG
AAAATCACTGATTTAATGATATCAGTAGAAGAAGAACTTCGAACAAAAGCAAGAACAAATCGAAATAGT
CGACATGGCTCAAACACCAGTAGATTTTCTTAAGTTAGCCTCATCGCTCTTACCAGAGNTTGATGGCAAGG
CAGAAAATTTAAAAAGCTTCATAGATGCTCTGAATCTGGTAGATTCTCTAAAAGAAACACAACGAAGCAC
TTGCTGTAAGTCTTATTAAGACAAAACTAAAGAGCCATGCCAGGAACCTGATAAGTGATGAGAACACTAT
TGCAGCTCTCATATCGAAACTATCGGACAAAGTAAAATGAGAGTCAGTAGAAGTTATTTCTGCTAAACTT
CTAAACTNACAACAAAAGAGCAAAACGGCCAATCAATATACCCAAAAGGTAGAAAAATTGAAGGAGCCT
ATATCAGTGAAGGTCTAAGCCAATCACTTGCAAAACAAATACAGCACTACAACAGCTGTAAAGGCTATGA
CCCAAAATTGTTCGATCGATAAGGTAAAACTTATCATACAGGCAGGCACATTCACAAATATGAATGATGC
CGTCTCCAAATTTGTCAATAGTTGCACGGAGGTAACAGGTCAAAGTCAGCACCGCTCCGTACCNCCGANG
AGGTAAGACGTGTTACAGCAAAATAGTACACGAGGCCGAAGTTAATCGTGGCAGAAATTACGACCACAA
CAATTACAGCGGCGGCGTAATAANAACAACANCAATAACGACAACAATAACCGTGNAGGAAGACGAGGC

CAAAATCGGGGAAGAGGCCGCGGGAACTCCAACCAAGGTAACAATTATAACAATGTGAGAGTGGCGCAA
AATGCGTCGGAAAACTCTCAGAACCCTTTAGGAAACAATCAGTAAATGCAAAGGTTCATTGCATCAATTA
CAGTCTTAATATATTTGTCACTCTTTATAATCATTCAACTGAAAATAAACACACATTCCTAATAGATACTG
GTGCAGATATCTCACTTTTAAAAATAAATTCAGATAATTTCAAAATTCAAGAAGACAAAATTATAAACAT
CCAAGGCATCGGCCAGGGTGTAATAAAATCGCAAGGAACAACTTCAACAGAACTTCAACCAACAAAATA
TATTANTCCACACGAATTTCATTTAGTAGACTCCGATTTTTCAATACCGTGTGATGGAATAATAGGCATTG
ATTTTATTAAAAAATTCAATNGCCAATTAGACTTCAAACCAAGTGAAGACTGGTTTATAATTAGACCTCAA
AACTTAAATTATCCTATTTATGTTCCAATGCAACAATGCATTTCTTCTGCCAGCCAGATCCCAACTAATTCG
AAAAATAGACATTAATTCAGTAAACGATTATATATTTGTTCCAAATCAAGAAATACATAATGGAATTTAT
GTTGCAAATACAATAGCAGCATCCAAACATGTATACATTCGCCTTCTAAGCACAACTAATTTCGACCAATT
GGTCAAAGTTAACAAAATCGAATATGAAAATTTAACAGATTACGATATTCATAACACTAATCCAGAAAAT
AGAAGCTAACAAGTACTTTCAAAGTTAAAGAAAAATTTTCCAGAACAATTCAGAAATCAATTAACAGAAT
TATGCACCAAGTATAATGATGTGTTCGGACTGGGAACCGAACCAATAGCGACAGATCATTTTTATAAACA
AACATTAAAACTTAAAGATGATGAACCCATTTATATCAAAAACTACAGAAGCCCGCATAGTCAAGTTGAG
GAAATCCAAAAACAAGTAGGGAAATTAATAAACGACAAAATCGTAGAACCATCTGTATCCGAGTATAAC
AGCCCACTCTTGCTAGTTCCGAAAAAATTATTACCACATTCGGAGGAAAAGAAATGGCGATTAGTAATTG
ACTATCGCCAAATCAACAAAAAATTACTTTCTGACAAATTCCCACTCCCCAGAATTGATGATATTCTAGAC
CAACGGGGCAGAGCTAAATACTTTTCATGCCTTGACTTAATGTCAGGTTTTCACCAAATTGAACTTGAAGA
AAATTCAAGAAATATAACATCTTTTTCAACGAGCAATGCCTCATATCGCTTCACGCGATTACCATTTGGTC
TTAAAATAGCACCAAATTCATTTCAGAGAATGATGACAATAGCATTCTCGGGTTTAGAACCCTTTCAGGCA
TTCCTTTATGTGGATGATTTAATGGTGATCATGATTAAAAACTTAACTGATGTTTTTAATGTATGTAGATAA
TATAACCTAATGTTGCATCCAGAAAAATGTTCATTTTTCATGCATGAAGTACATTTTTAGGTCACAAATGC
ACTGACAAAGGAGTATTGCCAGATGACAAGAAATTTGATGTCATCAAAAATTATCCTGTCCCTCATGATG
CGGATAGCGCGAGACGATTTGTAGCATTCTGCAATTATTATCGTCGATTTATAAGAAACTTCGCCGATTAT
TCACGGCACATAACTAGATTATGTAAAAAGAATGTTCCTTTTGAATGGTCAAGTGAATGCCAAAACGCAT
TCCAATACCTTAAAGAAAAGCTTATGCATCCCACATTATTACAATATCCTGATATTCGCAAAGAATTTTGC
ATTATAACAGGTGCTAGTAAACAAGCCTGCGGAGCGGTTCTAACTCAGAACCGTGACGGAATTCAGCTCC
CAATAGCTTATGCATCTCGTTCGTTTACAAAAGGGGAAAGCAATAAGAGCACAACAGAACAAGAGTTAGC
GGCAATTCATTGGGCAATTACCCATTTTAGACCATATATTTATGGCAAGCAGTTTACTATTAAAACAGAAC
ACAGACCATTGACATATCTTTTTTCTATGACTAATCCCAGCTCGAAATTAATTCGCATGCGGCTAGAGCTT
GAAGAATACGACTTCACAGTAGAATACCTAAAGGGGAAAGATTATTTTGTAGCAGATGCACTTTCGCGTA
TTAATATAAAAGAACTCAAAGATATGCAACATAAAGTCCTGAATGTCACTACCAGGCGACAAAGTAAACA
AAATAAAAACTGTATAGTAGAAAATTATGAACTTCTGCCAAGGCAAAGTCATAAAAATGTATCCAAGCCC
AACGTACATGAAGTCATCACAAATGATGAAGTACGAAAAGTAGTGACCTTGCGCATAACAGAATCTAAAT
GTTTATTTAAACATGGAAATAAAATTATCGCAAGAATTAAAGTTAGCGATTTATATACCAATGGAATTCTC
GACCTAGGCTAGTTCTTCCAAAGGCTCGAAAAAGAGACCGGTATATTAAAAATCAGCCAACTCAAAGTGC
CGCCGAGCGAAAAGATCTTTGAATCAGTTTCAATAGATTGTTTTAAAAATATGGGCAATGAAATATTCAA
ATCTTTAAGAGTAGCGCTACTCCAGCTGGTGACCCAAATAACAGATGATAAAGAGATACAACAAATATTG
TCTACATACCATGACGACCCCCTATTACGACCTCAAGGAGGTCATAGCGGAATAAGAAGAACGCTAGCGA
AAATAAAAAGACACTACTACTGGTAAAATATGACACGTCATATAAAAGAGTACGTACGTAGATGTCAAA
AATGACAAA
>9_rnd-1_family-410__LTR___Pao
TGTTGGATTCTGCAGCAGCCTCATCAAATTAGCATAAGCAAACATTCATGTAAACATAAGAAGAGGTTGT
AATTTTGGTCTAGTGGAACAGCTGTTTATTATAAACAAGGTTGGCAAGCCTTTCTTGCACTTGTCGCAGCT
GCTATAAATTGCATATTCCTTTTTTGTCTGCTCTCTCAGTTCGTTGTTGATCGAGGCGAAGCGAAGAACCA
ATTCTGAGCCACAGCCAAATAACCTTATAATCTACATAAATTTGCCTAATAAACTGAATTATAACGTGTTG
TTGTGAAAGGCCAAAATTTACTTATTTCTTGGTTTAGGTTCTAAACAGTTGCTAAAAAACATTAGTAACTC
AACATTTGGAGGCCCCGGCGAGATTTAACATTGCAGTGCACACAAGCAAAATAAATCTATTGCAAATTAA
AATTGTGATTATTCAAGTAGAACATAATCATCGTAAATTGTTATAATTAGGCAAGTGCAAACCGCCTGTGT
GAGCTGACCATTTACAGCTCCCAAATATTCGCATATACATAAACGCCGCTAGTTGACTTTCGCGCTCTTCC
CCTTGCGCGGTCATTCGCCGGCTCAGCTTATATACATACGCTTGTACATAAGCAGCTGCACAATTCGTCGC
ATGTTGTGTGCTCAGTTTGGTGTTCTGATTTTGTGCTTGTGAAAAAATGATGCAAGGCGAGAACACAGATG
ATATGCAGTCAAACAGGGTGTCTTTTCTTAAGCGCAAAGCGGTTGCATTGTTCGAGCGAGCCAAGTGTATT
GAAAATGAATTATCTGAGGATAAAATTAACAACTCTTGTGCCGTCGACTTAGAAGTGCGCCTTTTGCTAAT

TGAACAAACTAAGTCTGCCTTTAATACCGCGCATAACTCGCTAGAGGAGCTTAATTTCGATGAAATTGGC
AGCAAACTACGCGATGATTTTGACGAGTTGATCGCCATGGTTGAAGCTCGCGTTCGCGTAAATATTCAGC
GTCGTCAATCTAACGGCCCGCCCCACTCAACCTTTCTTCGTCATGAGGATTTGCCAAACCAAACCTTGATC
TTAGCCAACAGAACGCGCCTGCCCGAGCTCAAATTACCAACATTTTCCGGTGGTTATACCGAATATTCGGA
CTTTATCGCGATGTTTGAGTCAGTTGTCGACAAGGATACCGAGCTGTCTAACATTGAAAGGTTGCAGCATC
TTCGTTCATGTTTGTCTGGCCCTGCGTTGGATTCGGTTCGATCTTTGGAGTTTTCCGGTGCCAATTATCCTG
TGGCTCTGGATATCCTAAATAGAAGATTTAGTAACAAACGTCTTGTTTTCCAGGCACACATCAATGAGATT
CTTGGGCTCAAGAGGGTAGATTCGGAGTCGGCTTCAAAACTGCGTGAGTTCTCGGACAAGGTCAATTCGC
ATATGCGTGCCTTACAGAATTTGGGCAGTTTGGAGCAAATTTCGGGATGCATAATAGTCCACACGCTGCTT
CAAAGGTTGGACACCCAGACTCAGACCAAATGGGAGGAGGCCGCAGGCATTGACAAAATCCCGACAGCC
GATGAGTTCTTCACATTTCTGGAAAGGCGTAGTCAAAGGCTGGAGAGCGTGCAACATGCCATGTCATCCC
ATATAACCAATTATCAGGTGGGCAAATCCAACCATAACACTAACCACAGCAAAAGAGTGCTTGTAGCTGA
GACTAACCAAAATGGGTGCAGTTTTTGTGATGCGTCTGGTCACGGGATCTACACATGTAGTAAATTCGGA
AATTTGTCTCCATCCCTTCGGCATAAGGAAGTCAAGAAACTGGCCCTTTGTTATAACTGCCTAAGGAAGGG
ACACCAAACTCGGTCTTGCAGTAGTAGTCATTGTCGTACTTGTGGAGGAAAGCATCATACACTGTTGCATT
TTGCCACTTCAAGAGAAGCTGACGCGCCGACACCATTACATACATCGGCTGATTTGGGCGCCGCCACTTC
GACGCACTCTCACTCCACTACCTCTCTTGCTGTCCAAGGTCTTCAGTCTGGTGTTGTGCTCCTGGCCACTGC
AGTGGTACTGGTAAAGAATAGTTCTGGTTCCTTGTTTCCCTGTAGGGCCTTATTAGACTCAGGCTCGCAGT
TGCACATGGTCACATCCCGTTTCGCACAGAGACTTCAGTTAAGGAAAACCAGGTCGTCGACAGTTGTCAT
GGGTCTCGGAGACACAAGCGTCTCTACTGAAGGATCAGCTGTTAGGATTAACATGAAGTCACAAGACTCA
GATTATTCAGCCACCTTTGATGCTCTTGTGACTCCAACAATTACCGATAGTCAGCCCAGCTTCGTCATCGA
TACATCAGACTGGAAAATTCCCTCGAACATAAGGTTGGCTGATCCTGCGTTCAACAAACCACAGCGAGTG
GATCTCTTAATTGGAGCGAGCCTTTTCTATGAGCTTTTGTCTGTTGGGCAAATTGTGCTTTTGCCAGGACTA
CCTCTTCTGCAAAAAACTCGTCTTGGCTGGGTCGTGACCGGTGGATTTGAGAGACCGCGCAGTACCTCGTT
GTTGGCTGCCGGCCGCACTCCTAATAGCGAAGCTAGTCAGTTGGACGTCCGTCTGGATGAGTTGGTTCGGC
GGTTTTGGGAAATTGAAAGCTTGGAAGAGCCTACAGTAAAGGCCACCAAAAAGAAGAGGCGGAATGCGA
GCAGCATTTTGCCAATAACTTCTCCCGTTTACCATCCGGCGAGTATTCAGTGCGGTTGCCTCTGAAGCGGA
GTGCAGAGCTTCTAGGAGAGTCTTATGGCCAGGCTCATCTTCGATTTCTCAGTCTTGGAGCGAAAATTGGG
TCGGAATTCGCATCTGAAGGAGCAGTATTCGGCATTCATCAAAGAGTTTCTGGATCTAAATCACATGTCGC
GAGTTAGTCCAGAGTTGAGTGGCTGTTGTAAATACTTCCTCCCACATCATTGCGTCTTGAAAGAAGACAGC
ACCACAACCAAGTTGCGTGTTGTGTTTGATGGCTCAGCAACTACTGATTCTGGACATTCTCTTAATGATGT
ACTGATGGCGGGACCAATCATTCAACCTAAGTTGTTTCACATTCTGATTCGCTTCCGCACATATCCTGTTG
CTCTCACAGCTGATATTTGCAAAATGTATCGTTGTGTCCGAGTGACTTCGCCTGATAGTTACTTCCAGTGC
ATCCTGTGGAGGGACTCGCCTCAGAAGGATATCGAAATCTTTAAATTAGATACAGTAACATATGGACCA
AATCAGCATCATTTCAGTCCGTGCGCGCAATGCACCAGTTGGCCTTTGATGAGCGTGATGCATTTCCAATT
GGATCGGATGCCATCAAGCAGGAGTTTTACGTTGACGATTTGATATCCGGAGCCAACTCAGTTGCTGAAG
CCAGAGAGAAAATTCGTCAGACGGCTGGGATATTGTCTCGTGGAAACTTCAAGCTTCGAAAGTGGTGTTC
CAGTCACCAAGAAGTACTCAATGGAATAGCTTATGAAGATAGGGAACAGTACTTAAAGTTCGATGATGGA
AGCGATGTCACCAAGGCCCTTGGTCTTGTATGGGATCCAGCGATCGACCAGTTGCTATTTTCATTTTCTGTT
CTAGATTGTACTGTTAAACCCTGCAGGCGATCAGTTCTGTCTATTGTTGCCCGGTTTTATGATCCGCTTGGA
CTTTTGGGACCAGTCATAACTAAGGCAAAGATATTTCTTCAACAGCTTTGTAAGGACAAATTAACTTGGGA
TGAAAGCTTGCCGATTGCACGTGATACGACCTGGAGGGAGCTATGCAGCAGTTTTCGCAACATTCGCCAT
GTATCGTTTCCCAGAGTAGTACTCTTCGAAGGAGCTGATGTTGAAATACATGGATTCTGCGATGCCAGTGT
GGAGGCCTATGGAGCCTGCATCTACGTAGTGGCCAGGAGTCCACTAGCCACAAGTCGTATCCTTTGCTCC
AAGTCTCGAGTTGCGCCATTAAAAACATTGACTGTCCCAAAACTGGAATTGTGTGGAGCGAAGCTGTTGG
CTAGTCTGATGCATGAAGTCAAGCGCACAGGCATCTTTGTCAGTCAGTTTTATTGCTGGTGCGATTCGTCC
GTTGTCCTATCCTGGATAAGAGATGAGCCCTTCAAATTTAACGTTTTCGTGGCAAATCGTGTGGCTGCCAT
ACAAGAACTCACTGCCAGTATGGAGTGGCGATATGTTCCGACGGACCGAAATCCAGCTGACATTCTTTCT
AGAGGCACAGTTCCCGACGTCCTAGCCCAGTCGGAGCTCTGGTTTCATGGCCCATCGTATCTATTAAATGG
TCGCCTAAATTGGCCGGCGACCTGTCTGCCTGAAGCTCCAACCATTGAATTGAGGAAAAGAGTACTAATT
CTTAAGTCTCCACATGCAGATTTAACTCTGGGATGCAAGCACGCCAACTCTTTTGGTTCCATGCAGCGCAC
ATTCGGCTATGTGTACAAATTTATGAAGCGTATAAGGCATCATGGACTCACTGTTGCAGACATTCATCACG
GCACTCGCATTCTTCTTCGGGTTGTACAGAGAGCGAACTTGTGGGATGACATCAAGGAGATTCATTCTCAT
GGAAATGTGAAAAAATCCAGCTCCGTTGCATCTCTTGCACCATTTCTGGATGAACGTGACCTCCTCCGCGT

CGGTGGCCGGCTTAACAATTCGTCCCTGGACTATGATGCGCAACACCCCATTATACTGCCTCGGCGTCATG
CAATCACCTTAGCTGTCATCGTTCAGTTTCATGAGAGGAATCTACACCCTGGTCCCCGTGCACTTCTANCC
AAAATTCGACTTAAATATTGGCCGATTGGTGGATTAAGAACAGTCTCTAAAGCAGTCAGTAGATGTGTGA
GGTGCTTCAGAGCGAGGCCCATTTTTGTCGAGCCTATGATGGCAAGTCTTCCAAGGGAACGTCTGGAAGT
CGTTCGAGCCTTCACCGTAACTGGAGTCGATTATTGCGGACCCTTCTTCTATAAGTCGGAAGTTCGTACGA
GGCCATCCATCAAGTGCTATGTCAGCGTCTTCATTTGTTTCACCAGCAAGGCAATGCACCTAGAGCTAGTC
AAGGACCTATCGACTGCTACATTTTTGAGTGCCCTGAAGCGATTCATCGCCACTCGTGGCAAACCCAGTCA
AATTTGGTCCGACAACGCAACAAACTTCGTCGGATCGAAGAATGAGCTGGTGGATTTGAAACGTCTCTTG
CTTAGCGATCCCCACATGGAAGCAGTTCGGCAGTTCTGCCTCGCCGATGACATCGAGTGGAAATTCATCCC
CCCCGTTCTCCGCACTTTGGTGGACTATGGGAGGCGGCAGTGAAGTTGGCCAAGCACCACTTCTATCGCTC
TGTCGGCCGCTCTTTACTTACTTTTGAAGAGCTTCGTACTCTTGTTTGCCAAATCACAGCTATTGTTAACTC
ACGCCCATTACTCTCGCTTTCAGAGAACCCAGCAGATCTAGATGTCCTGACTCCTGCTCATCTACTGCTTG
GTGGTCCTTCATCAGCCATCATCGAGCCAGACTTGACCAAATTAAACTACGACCGTTTGGATGGCTGGCA
GCGTGTGACGCAGCTACAACAGGTGTTCTGGGCTCGATGGCGTGAAGAGTACCTCACCTTGCTACAACAG
CGAGCTAAATGGCGCACTCAAACACAAGCGTTGTGCATCAACGATATGGTGCTCGTTAAAGACGAAAATC
TGCCTCCCATGCGGTGGCCGCTTGGTAGGATAGTTAACTTGATTCCTGGGAAGGATGGAGTCAATCGTGTA
GCCGATATGAAGACCACGGCTGGAATCATCCGAAGAGCCGTAAACAAGCTGTGTCTGCTGCCCCTTAAGG
ATTCTGTTGAAAGTCAGGCTTCCAACGGGGGGAGTATGTTGGATTCTGCAGCAGCCTCATCAAATNAGCA
TAAGCAAACATTCATGTAAACATAAGAAGAGGTTGTAATTTTGGTCTAGTGGAACAGCTGTTTATTATAA
ACAAGGTTGGCAAGCCTTTCTTGCACTTGTCGCAGCTGCTATAAATTGCATATTCCTTTTTTGTCTGCTCTC
TCAGTTCGTTGTTGATCGAGGCGAAGCGAAGAACCAATTCTGAGCCACAGCCAAATAACCTTATAATCTA
CATAAATTTGCCTAATAAACTGAATTATAACGTGTTGTTGTGAAAGGCCAAAATTTACTTATTTCTTGGTTT
AGGTTCTAAACAGTTGCTAAAAAACATTAGTAACTCAACA
>160_rnd-5_family-5240__LTR___Gypsy
ACGTAAATGGAACTCTTTTCATAAACCATAATAACATTTTGGATAGAATCCCAGGAACGGCGAACTCTCC
ACTGCTGAACATTACGGCTAGCCGAGATACTAAGTCTTCCATATCTCCATCTATTGAACGAACGTAATGTG
GAGGCAATTCAACAGTTCAGGGAGCAAATCAATGAGGACCGGGCCTACCGAGTTTCGTGCATAGCAGGA
GCTATCTGTTGCGCAACCGTTTTTATCGGGTTGACACTTTGGCGAATCACCAAGAAGAGGAAGACTGCAA
GGCGGCTGAGAGAGGTGATTGCCGGCATAAGGTCGACCGAGGATGGTCATAAACTCGAGGGGGGAATAG
TTAACACTTAAGAATTGCACCAAAATAACGTTGTATAAGAAACTTGCACAATACTTATATTGACCCATTTA
CAACCAAGCACATTCCGCTGACACATGAGTTCAGCGGCCTGACCGCAACGGTCAGCAGCGGTAACCTGAC
ACTTGACTATGCTGCAAGTATCCGCGTTGTCGCTTATGCTGAGTCGGCTTGCCGACTGTAGCTTTGTAGCTT
ATAGATCCAAGTTTTAGGTATAATTCCAATTCAATTTTATACCACAATTTAGTTTTGTATTCGACGTTTAAG
AATAAACTTGCACTACTCCGGCACACAGCCGTAAATATCATTTGTTATATATTACAAAAACATTGCTGAGC
CACAAGGCCAGTAATTAGCAAAGAGAGTGAAGTCAAACCCTCCACCATAATCGTCCTAAATATTACTGGA
GTAGGCATCGCTGAACCAGCAGATTGATATAATTGGCGCCCAACTCGTGGTAGTAACGAAAGAAAATTAC
CCACGCGAGCGTTCGTTAATAAATGAAACACGCTAACAAATACATTACGTGATTGCTGACCTACGTCAAT
AAAAGCTAGAAGTTCTTTCTTGAAAATGCTAAAGACCAGACAAAAGGCCTTTGCAGACCCAGACGAGGCG
TTGCCGTATAACACATCAGTCGTGGCTACCATTAGGACCACNGATGAGCAACCGATCTATGCCAAGCTTT
ACCCATATCCAATGGGAGCAGCTGATTTCGTTAATAAAGAAATCAAGGATCTGCTAAAAAATGATATAAT
TCAAAAGTCCGTCTCTCCCTACAATAACCCAATATGGGTAGTAGACAAGAAGGGCACTGATGAAGCAGGC
AATAGGAAAATGAGGTTGGTAATGGACTTTCGTAAACTTAACGAAAGAACCGTGCCTGACAAATACCCTA
TGCCAAATATATCAATGATATTGGGGAATTTGGGGAGAGCCAAATTCTTTTCAACATTAGACCTCAAGTCT
GGTTATCACCAGATCATACTCGCTGAACGCGACCGTGAAAAAACCTCTTTCTCTGTGAACGGGGGGAAAT
ACGAGTTCAAAAGACTCCCCATACTGCGAGAGCAGATTGGCAAATCTTGCTATGTTTATGTCGATGACGT
AATCATCTTTTCAGAAGATGAGGAATCCCACATTAAACACGTGGATTGGGTCCTAAAAAGCTTACACGAA
GCAAACATGAGGGTCTCCGTCGAAAAGTCACGTTTTTTAAGAAAAGCGTGAACTTTCTTGGTTTCATAGT
CACCAGCACTGGCGCCACGACGGATCCTGAAAAGGTTAGGGCAATACAGGAGTATCCAGAACCTAAAAC
ACTGTTCGAGGTCAGATCATTTCTAGGCCTCGCAAGTTATTACAGATGTTTCATTAGGGACTTCGCCGCAA
TAGCAAGACCCATCTCGAATATCTTGAAGGGTGAAAATGGAACAGTCAGTAAGCATAGGTCCAGAAATAT
AAAGGTTCAATTCTCTGAAGTGCAGCAAAGGGCGTTTCAGAAGTTGCGCGATATTTTGTCATCCGAGAAT
GTAATGCTCAGGTACCCCGATTTTAAGAAGCCATTCGATCTAACGACCGATGCTTCGGCTCACGGCATAG
GTGCAGTTTTGTCTCAAGAGGGTCGTCCTATAACAATGATTTCAAGAACATTGAAGGACAGCGAGGTTAA
CTATGCAACAAATGAGAGAGAGTTATTGGCCATCGTATGGTCACTTGCCAAGTTAAGGCACTATTTGTATG

GGGTGAAAGACATTAATATCTTCACTGACCATCAACCCTTGACCTTCGCAGTTTCGGAATCCAATCCAAAT
GCGAAGATTAAAAGGTGGAAAGCGCGCATTGACGAGTCTAATGCACGTATCTTCTATAAACCTGGCAAGG
ACAATCTGGTTGCGGATGCCTTGTCAAGACAGCAGCTCAACGCTGTAAATGAACAAGAAGTTGAGTCCTG
CGCAGCAACGATCCACAGTGAGGTATCTCTCACCAACACCATTGAGACAACAGACAAACCCTTGAATTGT
TTTCAAAACCAAATAGTGCTAGAAGAGGCGCGTTTCCCCTTAAAACGAAGTTTTATTCTTTTTGGAAATAA
GAAACGCTACTTGATCAGCTATACTTGCATCGAATCGCTGCTAGACGAGCTCGAAGATGTAGTTTCACCCA
AATGCGTAAATGCTCTCCACTGTAATTTACACACGCTTGCAATGATACAAGATGCATTGGTTCGAAGATAC
CCGGCCACAAAATTCTGGCACTGCAAAAATCGCGTAGTGGACGTATTTGCGGTTGGAGAAAGAAGGGAA
ATCCTCACAGCCGAGCATAATAGGGCCCATAGGTCTGCACAGGAAAATATTAAGCAGGTACTCTCGGAGT
ACTACTTTCCAAAGATGGCAAAAATAGCCAATGAAATAGTACAAAATTGCAAAACATGTGCTAGGGCTAA
ATATGATAGACACCCCAAAAAACAAGAGCTTGGGGAGACACCGATCCCCTCACATGTAGGAGAGATGTT
ACACATAGACATTTTCTCCACTGACAGGAAATACTTTCTCACTTGCATCGACAAGTTCTCTAAGTTTGCTA
TTGTCCAACCGGTCCCTTCAAGAACCATTGTGGATCTGAAGCCAGCTTTAATGCAACTAATGAATTTTTTT
CCAAAAGCCAGATCCATTTACTGTGACAATGAACCGTCATTGAATTCAGAGACCATCGTGACTATGGGGT
CAGCATTTCAAATGCACCACCATTGCATAGCATCTCGAATGGACAGGTTGAGCGCTTTCACAGCACTCTGT
TAGAACTAGCCAGATGCCTAAAGATCGATAAAGGCATAAGTGACACCGTAGAAGTCATTTTACTGGCTAC
AAGTGAATACAATAAGTCGATCCATTCGGTCATCGACNAGAGACCAGTCGACGTGGTTCAGGCGCACCCA
GACGAGCCACAAGAGAACATACAAGANAGGATCAGAGACGCCCAGACGGCGCTCAGAGCTAGGGAGAA
CGCNAANCGGCAGAATAGGGTCTTCGACGTTGGTGACAAAGTTTTGGTCAAGTCCAACCGAAGGCTCGGG
AACAAACTCACACCTTTATGTGATGAGAAGGCCGTTGAAGCAGACCTGGGGACCGAGGTCCTCATTAGAG
GGAGGGTGGTCCACAAGGACAACTTGCTAAGGTGATCACCACGAGAATAAGGCCCTTGAAGTTTTTTCTT
ATAATACCNATCTGTTTAGCCGCTTGGCATAGCTTCTTCNAATTAATTATAAGACTTAGATATTAGCGTGT
GCCCTCAGTTTAAGTTCTGGTGGTGGTGGGTTAAACAATATTAAAACACAAAAATAGCTAAAATTCCCATT
CCACAGGTTTGAACTAATTCTCATCTTGTCCCTGTCCCTGGCTTCGGCGCGCGTCACCGATTATTCAAACG
CNAGGTACATCCCGATAGTGGATGGACGAATCCTGGTGTGGGAGGATTTCGCCTACATTAAACACTCGGC
GAATCTTTCAGAATATGCGCGTGTGGTGGAGGAAACGGTCAGTTTGCTTGACATGTTCCCACAATCTCACA
TGAAGAAGCTCCTAAAGGTGGACACAGCTCACCTGCAGGACCTGCTGGAAGCGTTGGGCGTACACCATAG
GGTGGCTAGAAGTTTGGATTTCCTAGGGTCCATGCTGAAAGTAGTAGCAGGCACTCCGGATGCCAACGAT
TTAGAAAGAATAAGATTTACAGAACGGCAACTAATACATGAAAACAATAGACAAATAGAGATTAATTCT
AAAGTACAAGACCAAGTTAATAAGCTNACAATTACTGTCAATCAAATTCTCAAGGCCGCGAAAAACACAC
AAGTNAACACTGAACACTTGTATGAAACGCTATTGGCTAGAAATAGGATGCTGATGATGGAATTGCAAAA
TTTAATGCTTGCAATATCATTAGCGAAAGCAAACATTGTAAGCCCGAACATTTTAGACCATATNGACTTGA
AATCAGTTTGGCTTGAGGAGCCCACCGACACCCCCATAGCAGANCTTTTGTCCGTTTCGTCCGTTAAGATA
CTACAATCGAACAACGCTTTATACTTCATTATTAGGTTCCCAAAAGTTAAGCTAGCTTGTAAGAAAATCAC
GATTTATCCCGTCGTTCAGAAAGGCGTAATACTGCGAATAACAGACAACGCCGTGGCAGAGTGCGATGGA
NACGTTCACGCTCTCGAAAACTGCGCCGCATCCCCTGGAGCGACGTTCTGCAGNCTCTCANNAGAGAGCT
CATGCGCCAGAGAGCTACACGCCGGGGGGGCGGCTAACTGCGAAACCCAGCCNAGCAACCTGGAGCCAA
TTACTCAGGTGGACGAAGGGATGCTAATTATCAACGACGAGCTAGCAGAAGTGCAAGTTGATAACGGCCC
TAAGACCCGGCTCCAAGGCACGCACCTCATTACGTTCGCTGATCGGGCCGACGTAAACGGAACTCTGTTT
CATAAACCATAATAACGTNTTGGATAGAGTCCCGGGAACGGCGAACTCTCCACTGCTGAACATTACGGCC
AGCCGAGATATACTAAGTCTTCCATATCTCCATCTATTGAACGAACGTAATGTGGAGGCGATTCAACAGTT
CAGGGAGCACGTCGATGAGCTGACCGGGCCTACCGAGTTTCGTGCATAGCAGGAGCTATCTGTTGCGCAA
CCGTTTTTATCGGGTTGACACTTTGGCGAATCACCAAGAAGAGGAAGACTGCGAGGCGGCTGAGAGAGGT
GATCGCCGGCATAGGGTCGACCGAGGACGGTCATAAACTCGAGGGGGGAATAGTTAACACTTAAGAATT
GCACCAAAATAACGTTGTATAAGAAACTTGCACAATACTTATATTGACCCATTTACAACCAAGCACATTCC
GCTGACACATGAGTTCAGCGGCCTGACCGCAACGGTCAGCGGCGGTAACCTGACACTTGACTATGCTGCA
AGTATCCGCGTTGTCGCTTATGCTGAGTCGGCTTGCCGACCGTAGCTTTGTAGTTTATAGATCCGAGTTTTA
GGTATAATTCCAATTCAATTTTATACCACAATTCAGTTTTGTATTCGACGTTTAAGAATAAACTTGCACTAC
TCCGGCACGCTGCCGTAAATATCATTTGTTATATATTACAAAAACATTGCTGAGCCACAAGGCCAGTAATT
AGCAAAGAGAGTGAAGTCAAACTCTCCACCATAATCGTCCTAAATATCACTGGACTAGGCATCGCTGGAC
CAGCAGATCGATATAATTGGCGCCCAGCTCGTGGTAATAACGAAAGAAAATTACCCACGCGAGCGTTCGT
TAATAACTGAAACACGCTAACAAATACATTACGTGATTGCTGACCTGCGTCAATAGAGGCAGCAACAGTG
CAACAACTTTGCCCTCGTAAAGACACATTGGTAGAAGTGCACCGAGGCCACTGAAGCTATCTGGACAACG
AAGACTACGGCGTTGGAGCTTAATTCCCCTTTAGTAGTTGTTTTAGAATCATGTAAGTGGGCTTTTCGTTA

ACTCTTTTCAAGTCAATTCAAAAGAAATTTAAAAAAAAAGCACATTATATTTTGCATTACCTATTACTCGA
TTATAAATCTATAAAGAAAAATAATAATAATAAGTTATACGGCATTGAGAGCTTTGATTGATAGATAAGC
TGCATATTTGTCACTTGTATTTAAAGCAAATTGCAAGAACGCATATTTTGTCGGCTTGCTTCCCACTCTATT
TGTTAGTTGTCTTTATGTAACTTTGTAGTTAAACGCTCTGGCGTAACATGACACGCCAGCGTACAGGAACT
CACGCTTAATCATCAAAATATTGAATTGTTTATCTGTAGC
>9_rnd-5_family-2490__LINE___R1
ACACACACACACACACACACACACACACACACACACACACACACACACACACACACACACACACACACACA
CACACACACACACACACACACACACACACACACACACACACACACACACACACACACACACACACACACAC
ACACACACACACACACACACACACACACACACACACACGCACGCACACACACACACACACACACACACACA
CACACACACACACACATGAGTGAAAGTGAAAGTGAGTTGACGCGTCTCGACTGTCGCTCCGGCTGTTCTC
TGTCGTGGGCCGCAGCTCCGGCAGGGAAAGCAAAGCGATGATGTGCAATCCATTGGCGAAGGGGCTGAG
CACGTGGAGACCCACGCTCACATACGCGCTTCGCAAGCGGCGGATGCCAGGGTGCGCGTCAAACCAAAG
ATGACTACATACGCACACGGAGAGACGCAGGTGGCACGACTGGCACGACTGGTGGTACACCTGCAAGAC
TGTGGNGGTGAACACGAGAATGGAGCAAACCCGCGCACTCACCTGGATGACGGGGTGATGGCGAGGACT
ACCTGATGGTATATATCCAACCCTAAGCCTGATCCAAGTAAGCAAGTTGAGCACTTTCTTNGCGAANCTAT
CATGGCGACATTGTTTATGCTCAAGCACATGAGCAAGACAAAAGCTCTGGGACAGCTGGTTGGTTGTACA
GTGCTGCTCAACCGAGAAGTACATGCGTCAACGTAAAAAGCAAACACGCGCGAAAGTATGCCACGCTGT
GTGGGTATACATGAAGACAAAGGAAGATATTGCATGCTTTTAGGCTTTCACGTTCTACTCGCATTGCAGCT
GCAGACTCGAGGCAAGAAATTGTACAGAAGGCCACGGATCCGACCAAGTCAACTCCTGCCGCATAGCGG
TGTACTAAAAGGGGCGCTGGCGACCGAGCTGGAGCGGTTAAACTCCCATCTGTCAAACGCCAGTGAGAAA
CCATTGGCCACCGTTGGAGCCACAAATCAGCAGCGATGAGCGCCACAACTGGCAGCACGCCCGTCAATGC
AAGCAAGCAAACTCCACCCCCACACTGAGCCCAGTAAAGTGTGGAAGCGACCCACTGGACCCCCCTTACG
TAACTGTAGANATGGGCTGACGAGTCGGGCATTCTAGGACCTTAATAGGCAAGCTGACACCTTGTCGAAA
CGGCTCCTAGGCTAATGCTCGCCTCGCCCCCGTCCCGCTAAAACCTTGGCAGGCCTCGTAGAACGCTTATC
GTATGTCGCCATTCAGTTGGCCGTGCAGGCTCAGTTGATATTCCCTGACCAGCACTGATCTGGCTCTGAAC
TCCGTCTCCCATAAGGACCGGAGTCAACCCTCGCGTGGACCTCACTCCATGGAAAGGCAAACCACGGGAT
CGCGAAGGTGACTGCACAATACACACGACAACAACGGCTTCGAGTTGGGACCCAATTAATCACAATGATG
AACCGAAACAACAAAAGCACCACCCCACTAGGTGGACAAGAGGAAGAAGGAGTACGCCAGGCCGACCC
GTTCGCTAGGCGATCATGCCTGGCGAGCACGCCCCCGGCTACACCAGTAAGGGCCCCTCAGCCCACTGCA
CCATGCAGGGAAATTGAAGAGAAAGAGACCCCGAAAAGGCTTAGGGACCCGGCCAGCCCTACGAACAAG
CCACGGAGCACCCCGCCCAAGAAGAGCAAGGCAAATACCCAGGAGATCGAAGAGATTGGCTCGATACTG
GACGAGCTACTGTTCATGTATCATGGCAAGCAGCCAGCGATGAGGCACATAAACTTGGCCACTAAGAGCA
TGCTAGCGAGACTAAAAGAGCTGCAAGAAGTAGTCAAAGCGCAGCTCACGGAAAACCTGCGCATGCGAT
GCAGCGAAGAGTCCCATAAGCAGATGAGCCATGCTGAAGAAAGCGCCGGAAGCAATTCTAAATGCGACA
CGGCAACGCAGACGGAAGAAAGCGGTGCAGGCAGTAAACCACCGACAGCGGCGCAAAAACCGACGAAC
GAGGTTACAGGAAAAATCAGATCTTGGCCCCAAGCAACCCAAGCCGGCACCAACTCGCAAGCAGCAACG
TTCACAGCCGGCAGAGGGCAAGGTACCGACCCAAGCGCGAGAGGCAAAAACACCGGTCAAAAATACCCG
AGAGGTAAAAAAAAATGGACTGAGGTGAAGGGCAAGCTCACCAGGCGGAAACAGCCCAACCGACCGGA
TGCGATCCTGATCACGTGTACAGAGAGCATGCCATACGCAGATGTGCTCAAAATGGTGAAAACAGCTCCC
GCACTGCAGGCGCTCAAAAATAATGTACAGGGCATCCGCAAATCAGCAGCAGGAGAACTGATCCTCAGG
CTCCAGAAGCCATCGGACCCAGCTACCGACCAGTTGCGTGTGGCAATCAGCAGCGCACTGGCTGGAAAAG
CTGCCGTTAGAACCTTGCAGGAAACGGTGCAAATGGAGGTTCTAGATATCGATGAGTCCACGACAGCAGA
TGAGGTCCTCACAGCTCTCTCTGCCTCGGTGGAAGGAGACATACCAGTAGAAGCGACGCCCACTATGAGG
GCGGCCTACGGAGGAACACAGATCGCATCGCTGGCCCTGCAACCGAGCCTAGCCACGAAACTGCTCGAA
ACCGGCAAGGTCCGCATAGGGTGGGTTATATGCCGAATACGCCGAAAGATCGAGCCAAAGAGATGCTTC
AGATGCATGGGATTCGGCCATACATCCACCAGGTGCAAGAGCCAGCATCCGGTTGGAAAGGAGACATGCT
TTAAATGCGCAGGTACTGGGCACAAGTCTGGACAGTGCACGCATGAGGCAAAGTGCATCCTCTGCACAAG
AATGGGCACGGGCACTAACCAAGCCGCGCACTCAACTCTGAGCAGGAACTGCCCGGAGTATAAGAAGGC
CTACAAAGCGCTTAGAAATGGTTAAGTTCCTGCAACTTAACCTAAACCACTGCAGAGCAGCCCAGGATCT
GCTCGCACAAACGACCCGGGAGACGAGAATTGATGTAGCCATCATTAGCGAACCGTATACACCCAAACAT
ATCGGAGTGTGGCACGAAAGCCTAGACGCTGGAGCAGCACTATGGAGCTGCGGACAGGCACCATACCAG
CTCACTCAACGGATGGCTCGCAAAGGGTTTGCTAGGGCAAAATGTGGTCGCATATACGTTTATAGCTGCT
ACATACCACCAAGCATACCCCTGGCAGAGTACACAGCCATTATTGAAGACATCGCACAGGACGCGCACGG
AAAATCTCCAGTGATAATCGCCGGAGACTTCAACGCGTGGGCCACAGAATGGGGCAGCAGCAGAACGAC

ACCAAGGGGAACCATCCTGCTGGACATATTTGCTTCGCTAAATGTCTGTCTGCTGAACATCGGCACAAGA
AGCACTTACAGCAAAGCGGGTCGCGAGTCGATCATAGACCTGACGTTTGCCAGCCCAGACGTTGCCCGAG
ACGCCAAATGGCATGTATCGGATGTATACACCCACAGCGACCACTTTGCGGTGATAACGGACACCTATCA
ACTTGGAAGCAGAGGCGCCAGTAGAAGGCTCCAACACACGGGATACAAGATGAACACTATGGACCTGGA
AAGACTCCTCCCTATGGTAGAAGGACTTGGCATAAGAGGCGACGCCAACAGCTCTGCTGACGCGATAGCA
GAGACTATCACCAACGCATGTGACGCTATACTGACAAAAGTTCGCAAGGGTGGNAACGCACATAGACCG
GTAGCCTGGTGGAACGAGGACATAGCCAAAGCGCGCAAAGAGTGCCTCGCAGCTAGGCGGAAGAGCCAA
CGGTCAAGACCGTCCCCAATTTTGAGCACCATCTGCAGAACTTCAGAGCCAAGCGGAAAGTACTCAAGGA
AGCTATCAGACGCAGCAAATCCCGATACTTTCAGAAGCTCTGCGACGCTGCGGACGCGGAGCCCTTTGGA
CTGGCTTACAAGCTAGTCATGGGAAGGCTATGCAGGCAACCAATGCCAACAAACCCGGAACAGCTCAAA
CAAATAGTCCTAACGCTCTTTCCTCGACAGACACCTGGAATTGCACCGCAACTACCAGCAAACGGAATAA
ACGACTGTGCACCCACCGACGACAATGAGGTACTGAGCATTGCTGCGAAGCTAAAGGCGGACAAATCTCC
TGGACCAGACGGAATACCGAACGGGGTAATAAAAGCTGTCATTACTGCACAGCCCAGAAGCTTCGTGGA
GTTGTACAATCAATGTATAGCGGAATGCACAGTACCACGAAGATGGAAACTCCAGCGACTTGTGCTCATC
CCGAAGCCCGGCAAGGAACTAGATGATCCCTCCTCATACCGCCCCCTGTGTATGCTGGACACGATGGGAA
AAATATTTGAGCGGATAATATGCAATCGCCTAGAATGCAAGCTTGCTGAAGTGCGCGGTCTGTCTGAACG
ACAATTCGGCTTCAGGAAACACAGGAGCACCACGGATGCCATCAGGGCCGTGGCTGAACTAGCAGAAGA
CGCTATCCAAGGCACAAGATGGACAGGTGGCACCAAACAGTACTGCCTAGTCTGTACGTTGGATGTCAGG
AACGCGTTCAACTCAGCCAATTGGACAAAAATACTAGAAGCGCTAACGGCCAGACACATCCCGAGCCAA
CTAATAAAGTTAGTGACCAGCTACTTCCAGGACAGAGTGCTAAATTACGACACAGAAGACGGCCAACGAC
AGTACAGGGTAACTGGAGGCGTTCCTCAGGGCTCTGTTCTCGGGCCGCTACTGTGGAACGTCATGTACGA
CGGAGTACTGCGCCTTTGCCTACCAGAGGGCAACACAATCATTGGTTTCGCCGATGACATCGCTGTTGTTA
CTGTGGCGAAGAAACTCGATGAGGTCACTCTTAGGAGCAGTGCGGCCATCGACAGGATAATTGACTGGCT
ATCCCAGAACGGACTCACGCTAGCGGAGCAGAAGTCGGAGGCAGTCTTAATAAGCAGCAGAAAGATAGT
TGAGACGACATCGTTGCGCGTTGGCAGTGCTATCATTAAGTCCAAGCCCGCCATCAAATACCTGGGCGTC
ATGATAGACCACAGACTGTCGTATAAACAGCACCTGGAGTATATTAGCCACAAAGCGGCCAGTGTTACAA
CAGCCATTAGCCGAATGTTGGCGAACACCAAAGGACCAAGGCAGAGGAGCAGAAGGCTAATCGCTGGAG
TGGTGACGTCCACAATCCTCTACGGATCCAACATATGGGCCCCCGCAATGGAGGTTGCATCTTACAGCCG
AGGATGCAATGCTGCATACCGACGATGTGCATTGCGTGTAGCCACCTGCTTCCGCACTGTATCGGAAGAC
GCGGCCTTAGTACTGGCAGGCATGATCCCACTCGGCCTGATGGCTGCCGAAAGGCAAAGTGGGGCAACCA
CCAACAGGCAACTGAGGGATAACACCATCGATCAATGGCAAAGCAAATGGGATGCGAGCTCAAGTGGCA
GATGGACCCACAGACTAATCCCAAACCTGAAGCTTTGGCTACAGCGGCGACACGGCGATGTAGACCACTA
CCTGGCACAAATATTTACTGGGCATGGCTGCTTTAAAGCGTACCTGCACAGGTTTAATCATGAGGACGAC
CCCTTTTGCGAGCACTGTGGGTACGGTGTGATTGAGGACGCGGAGCACGCATTGTTCTACTGCCCACTCTA
TAGTCGCGAGCGAGACAAAGCGACGGCGGGCAGAAATGATTTGTCTCCGGAGAATCTCGTGGCGCGAAT
GGTGGCCACTGAAAATGGATGGACAGCCGTTGCTAACATGGCATCGGCTATAATGAAGGAGCTGCGACG
ACAGGAGAGGATCCGACGCGGCATCGACTGATGAGCGGCTGATATACAGCCGTACCCCGCCCTGCGAAGT
AGTACTTTGCGGTTGTCCCGCAGGAGCGGGCTACAACTTTCTACTCCCCACAAAATACCCTTTCCTTCTTTG
TTTGTCTTAGTTCCCCTTGTAAAATGTTAAGCCGTTATTTTTCTTTTCTTCCAAAATAAAAANAAAAACACA
CACACACACACACACACACACACACACACACACACACACACACACACACACACACACACACACACACAC
ACACACACACACACACACACACACACACACACACACACACACACACACACACACACACACACACACACA
CACACACA
>9_rnd-1_family-276__LTR___Pao
TGTTGGATTCTGCAGCAGCCTCATCAAATTAGCATAAGCAAACATTCATGTAAACATAAGAAGAGGTTGT
AATTTTGGTCTAGTGGAACAGCTGTTTATTATAAACAAGGTTGGCAAGCCTTTCTTGCACTTGTCGCAGCT
GCTATAAATTGCATATTCCTTTTTGTCTGCTCTCTCAGTTCGTTGTTGATCGAGGCGAAGCGAAGAACCAA
TTCTGAGCCACAGCCAAATAACCTTATAATCTACATAAATTTGCCTAATAAACTGAATTATAACGTGTTGT
TGTGAAAGGCCAAAAAATTTACTTATTTCTTGGTTTAGGTTCTAAACAGTTGCTAAAAAACATTAGTAACT
CAACATTTGGAGGCCCCGGCGAGATTTAACATTGCAGTGCACACAAGCAAAATAAATCTATTGCAAATTA
AAATTGTGATTATTCAAGTAGAACATAATCATCGTAAATTGTTATAATTAGGCAAGTGCAAACCGCCTGTG
TGAGCTGACCATTTACAGCTCCCAAATATTCGCATATACATAAACGCCGCTAGTTGACTTTCGCGCTCTTC
CCCTTGCGCGGTCATTCGCCGGCTCAGCTTATATACATACGCTTGTACATAAGCAGCTGCACAATTCGTCG
CATGTTGTGTGCTCAGTTTGGTGTTCTGATTTTGTGCTTGTGAAAAAATGATGCAAGGCGAGAACACAGAT
GATATGCAGTCAAACAGGGTGTCTTTTCTTAAGCGCAAAGCGGTTGCATTGTTCGAGCGAGCCAAGTGTA

TTGAAAATGAATTATCTGAGGATAAAATTAACAACTCTTGGTGCCGTCGACTTAGAAGTGCGCCTTTTGCT
AATTGAACAAACTAAGTCTGCCTTTAATACCGCGCATAACTCGCTAGAGGAGCTTAATTTCGATGAAATTG
GCAGCAAACTACGCGATGATTTTGACGAGTTGATCGCCATGGTTGAAGCTCGCGTTCGCGTAAATATTCA
GCGTCGTCAATCTAACGGCCCGCCCCACTCAACCTTTCTTCGTCATGAGGATTTGCCAAACCAAACCTTGA
TCTTAGCCAACAGAACGCGCCTGCCCGAGCTCAAATTACCAACATTTTCCGGTGGTTATACCGAATATTCG
GACTTTATCGCGATGTTTGAGTCAGTTGTCGACAAGGATACCGAGCTGTCTAACATTGAAAGGTTGCAGC
ATCTTCGTTCATGTTTGTCTGGCCCTGCGTTGGATTCGGTTCGATCTTTGGAGTTTTCCGGTGCCAATTATC
CTGTGGCTCTGGATATCCTAAATAGAAGATTTAGTAACAAACGTCTTGTTTTCCAGGCACACATCAATGAG
ATTCTTGGGCTCAAGAGGGTAGATTCGGAGTCGGCTTCAAAACTGCGTGAGTTCTCGGACAAGGTCAATT
CGCATATGCGTGCCTTACAGAATTTGGGCAGTTTGGAGCAAATTTCGGGATGCATAATAGTCCACACGCT
GCTTCAAAGGTTGGACACCCAGACTCAGACCAAATGGGAGGAGGCCGCAGGCATTGACAAAATCCCGAC
AGCCGATGAGTTCTTCACATTTCTGGAAAGGCGTAGTCAAAGGCTGGAGAGCGTGCAACATGCCATGTCA
TCCCATATAACCAATTATCAGGTGGGCAAATCCAACCATAACACTAACCACAGCAAAAGAGTGCTTGTAG
CTGAGACTAACCAAAATGGGTGCAGTTTTTGTGATGCGTCTGGTCACGGGATCTACACATGTAGTAAATTC
GGAAATTTGTCTCCATCCCTTCGGCATAAGGAAGTCAAGAAACTGGCCCTTTGTTATAACTGCCTAAGGAA
GGGACACCAAACTCGGTCTTGCAGTAGTAGTCATTGTCGTACTTGTGGAGGAAAGCATCATACACTGTTG
CATTTTGCCACTTCAAGAGAAGCTGACGCGCCGACACCATTACATACATCGGCTGATTTGGGCGCCGCCA
CTTCGACGCACTCTCACTCCACTACCTCTCTTGCTGTCCAAGGTCTTCAGTCTGGTGTTGTGCTCCTGGCCA
CTGCAGTGGTACTGGTAAAGAATAGTTCTGGTTCCTTGTTTCCCTGTAGGGCCTTATTAGACTCAGGCTCG
CAGTTGCACATGGTCACATCCCGTTTCGCACAGAGACTTCAGTTAAGGAAAACCAGGTCGTCGACAGTTG
TCATGGGTCTCGGAGACACAAGCGTCTCTACTGAAGGATCAGCTGTTAGGATTAACATGAAGTCACAAGA
CTCAGATTATTCAGCCACCTTTGATGCTCTTGTGACTCCAACAATTACCGATAGTCAGCCCAGCTTCGTCA
TCGATACATCAGACTGGAAAATTCCCTCGAACATAAGGTTGGCTGATCCTGCGTTCAACAAACCACAGCG
AGTGGATCTCTTAATTGGAGCGAGCCTTTTCTATGAGCTTTTGTCTGTTGGGCAAATTGTGCTTTTGCCAGG
ACTACCTCTTCTGCAAAAAACTCGTCTTGGCTGGGTCGTGACCGGTGGATTTGAGAGACCGCGCAGTACCT
CGTTGTTGGCTGCCGGCCGCACTCCTAATAGCGAAGCTAGTCAGTTGGACGTCCGTCTGGATGAGTTGGTT
CGGCGGTTTTGGGAAATTGAAAGCTTGGAAGAGCCTACAGTAAAGGCCACCAAAGAAGAGGCGGAATGC
GAGCAGCATTTTGCCAATAACTTCTCCCGTTTACCATCCGGCGAGTATTCAGTGCGGTTGCCTCTGAAGCG
GAGTGCAGAGCTTCTAGGAGAGTCTTATGGCCAGGCTCATCTTCGATTTCTCAGTCTTGAGCGAAAATTGG
GTCGGAATTCGCATCTGAAGGAGCAGTATTCGGCATTCATCAAAGAGTTTCTGGATCTAAATCACATGTCG
CGAGTTAGTCCAGAGTTGAGTGGCTGTTGTAAATACTTCCTCCCACATCATTGCGTCTTGAAAGAAGACAG
CACCACAACCAAGTTGCGTGTTGTGTTTGATGGCTCAGCAACTACTGATTCTGGACATTCTCTTAATGATG
TACTGATGGCGGGACCAATCATTCAACCTAAGTTGTTTCACATTCTGATTCGCTTCCGCACATATCCTGTTG
CTCTCACAGCTGATATTTGCAAAATGTATCGTTGTGTCCGAGTGACTTCGCCTGATAGTTACTTCCAGTGC
ATCCTGTGGAGGGACTCGCCTCAGAAGGATATCGAAATCTTTAAATTAGATACAGTAACATATGGACCA
AATCAGCATCATTTCAGTCCGTGCGCGCAATGCACCAGTTGGCCTTTGATGAGCGTGATGCATTTCCAATT
GGATCGGATGCCATCAAGCAGGAGTTTTACGTTGACGATTTGATATCCGGAGCCAACTCAGTTGCTGAAG
CCAGAGAGAAAATTCGTCAGACGGCTGGGATATTGTCTCGTGGAAACTTCAAGCTTCGAAAGTGGTGTTC
CAGTCACCAAGAAGTACTCAATGGAATAGCTTATGAAGATAGGGAACAGTACTTAAAGTTCGATGATGGA
AGCGATGTCACCAAGGCCCTTGGTCTTGTATGGGATCCAGCGATCGACCAGTTGCTATTTTCATTTTCTGTT
CTAGATTGTACTGTTAAACCCTGCAGGCGATCAGTTCTGTCTATTGTTGCCCGGTTTTATGATCCGCTTGGA
CTTTTGGGACCAGTCATAACTAAGGCAAAGATATTTCTTCAACAGCTTTGTAAGGACAAATTAACTTGGGA
TGAAAGCTTGCCGATTGCACGTGATACGACCTGGAGGGAGCTATGCAGCAGTTTTCGCAACATTCGCCAT
GTATCGTTTCCCAGAGTAGTACTCTTCGAAGGAGCTGATGTTGAAATACATGGATTCTGCGATGCCAGTGT
GGAGGCCTATGGAGCCTGCATCTACGTAGTGGCCAGGAGTCCACTAGCCACAAGTCGTATCCTTTGCTCC
AAGTCTCGAGTTGCGCCATTAAAAACATTGACTGTCCCAAAACTGGAATTGTGTGGAGCGAAGCTGTTGG
CTAGTCTGATGCATGAAGTCAAGCGCACAGGCATCTTTGTCAGTCAGTTTTATTGCTGGTGCGATTCGTCC
GTTGTCCTATCCTGGATAAGAGATGAGCCCTTCAAATTTAACGTTTTCGTGGCAAATCGTGTGGCTGCCAT
ACAAGAACTCACTGCCAGTATGGAGTGGCGATATGTTCCGACGGACCGAAATCCAGCTGACATTCTTTCT
AGAGGCACAGTTCCCGACGTCCTAGCCCAGTCGGAGCTCTGGTTTCATGGCCCATCGTATCTATTAAATGG
TCGCCTAAATTGGCCGGCGACCTGTCTGCCTGAAGCTCCAACCATTGAATTGAGGAAAAGAGTACTAATT
CTTAAGTCTCCACATGCAGATTTAACTCTGGGATGCAAGCACGCCAACTCTTTTGGTTCCATGCAGCGCAC
ATTCGGCTATGTGTACAAATTTATGAAGCGTATAAGGCATCATGGACTCACTGTTGCAGACATTCATCACG
GCACTCGCGTTCTTCNTCGGGGGAGAGGTGAAGATTCATTCTCATGGAAATGTGAAAAAATCCAGCTCCG

TTGCATCTCTTGCACCATTTCTGGATGAACGTGACCTCCTCCGCGTCGGTGGCCGGCTTAACAATTCGTCC
CTGGACTATGATGCGCAACACCCCATTATACTGCCTCGGCGTCATGCAATCACCTTAGCTGTCATCGTTCA
GTTTCATGAGAGGAATCTACACCCTGGTCCCCGTGCACTTCTAGCCAAAATTCGACTTAAATATTGGCCGA
TTGGTGGATTAAGAACAGTCTCTAAAGCAGTCAGTAGATGTGTGAGGTGCTTCAGAGCGAGGCCCATTTT
CGTCGAGCCTATGATGGCAAGTCTTCCAAGGGAACGTCTGGAAGTCGTTCGAGCCTTCACCGTAACTGGA
GTCGATTATTGCGGACCCTTCTTCTATAAGTCGGAAGTTCGTACGAGGCCATCCATCAAGTGCTATGTCAG
CGTCTTCATTTGTTTCACCAGCAAGGCAATGCACCTAGAGCTAGTCAAGGACCTATCGACTGCTACATTTT
TGAGTGCCCTGAAGCGATTCATCGCCACTCGTGGCAAACCCAGTCAAATTTGGTCCGACAACGCAACAAA
CTTCGTCGGATCGAAGAATGAGCTGGTGGATTTGAAACGTCTCTTGCTTAGCGATCCCCACATGGAAGCA
GTTCGGCAGTTCTGCCTCGCCGATGACATCGAGTGGAAATTCATCCCCCCCGTTCTCCGCACTTTGGTGGA
CTATGGGAGGCGGCAGTGAAGTTGGCCAAGCGCCACTTCTATCGCTCTGTCGGCCGCTCTTTACTTACTTT
TGAAGAGCTTCGTACTCTTGTTTGCCAAATCACAGCTATTGTTAACTCACGCCCATTACTCTCGCTTTCAGA
GAACCCAGCAGATCTAGATGTCCTGACTCCTGCTCATCTACTGCTTGGTGGTCCTTCATCAGCCATCATCG
AGCCAGACTTGACCAAATTAAACTACGACCGTTTGGATGGCTGGCAGCGTGTGACGCAGCTACAACAGGT
GTTCTGGGCTCGATGGCGTGAAGAGTACCTCACCTTGCTACAACAGCGAGCTAAATGGCGCACTCAAACA
CAAGCGTTGTGCATCAACGATATGGTGCTCGTTAAAGACGAAAATCTGCCTCCCATGCGGTGGCCGCTTG
GTAGGANAGGTTACTTGATTCCTGGGAAGGATGGAGTCAATCGTGTAGCCGATATGAAGACCACGGCTGG
AATCATCCGAAGAGCCGTAAACAAGCTGTGTCTGCTGCCCCTTAAGGATTCTGTTGAAAGTCAGGCTTCCA
ACGGGGGGAGTATGTTGGATTCTGCAGCAGCCTCATCAAATNAGCATAAGCAAACATTCATGTAAACATA
AGAAGGGGTTGTAATTTTGGTCTAGTGGAACAGCTGTTTATTATAAACAAGGTTGGCAAGCCTTTCTTGCA
CTTGTCGCAGCTGCTATAAATTGCATATTCCTTTTTGTCTGCTCTCTCAGTTCGTTGTTGATCGAGGCGAAG
CGAAGAACCAATTCTGAGCCACAGCCAAATAACCTTATAATCTACATAAATTTGCCTAATAAACTGAATT
ATAACGTGTTGTTGTGAAAGGCCAANATTTACTTATTTC
>160_rnd-6_family-453__Unknown
CCGCGAGCTTTTGAGCTTTAAGAGAATAAGCTTTATCATAACCATAGCTGACTTGCAGCATAAGTTCACAA
TTGTACTTTAGTTCTAATCTTGGATTCAAATAAATCGGAAGCCTTCAGCCTCCCACTTAAAAANATATTTTT
TTCCAATTAGTAGCCACTAATCGGTTAATTGGCGCCCAACGTGGGGCTCGCGATTATAAACACAAAAAAA
GGATAATTAAAAGAAACAAAATCCTTAGTGATTCGCGAGTGAGCAAAAGTGCCGATTTACCAGTCCAAGA
TTGTCTCAAAAGAGCATTAAAAATTTTCTAAGAACGAAAATATCGAGTGCCTCTAAAAGAAACATTAAAA
ATTTCCGACGAACGGAAATACCCTGTTTCTGAAAACTTGAAGAGTAGTGAAAATACCCTGAGCTGGAAAT
ATTGATTGTTCACAAGACTTAAAAGTGAAATCAAATTTTCCGATTCAAGTTAAATGAAAACATTCCAAAA
AATTAATCGATCAAAGAAGCGAAACGAATATTTTCGCCAACAAAAATTTNTTCACCAATCAAAAACTTTT
AACCAAATAAAAAATATCACGTCAACCTGAGACCCCCATAAAAGAAGACCCTAAAACCCAGAAGGAAGG
AAGGAAGGAAGACTTGAACGACATATTGTATAATCACCCATAAAGAAAGACCTTTTACGTGAGCCATCAT
CATTTACTTTAAAAAGAAATTTTTTTTTCTTAAGTAATGAGAGTTTAAATTTGTGTAAGCATTTGTTAGAAT
AATAAGTGTTATAAACTGGATAACCATATAGAAAGTTTAATTGAAGATTTTCAAAATTTAAAAATGGCTA
ACAATAATAACTTGACAGCTGATTTGGTAGCTCAGTTGATTGCAACTCAGACCAGAGCGCTTCAAGAGCA
AATTGCAGAATTGAAGTTACAAATCACTCTCTCATCTAATAATGAACAAGAATATAAGATTACTGAAATA
AATGACCTTATTCGTTGCGATGAATCTTTTGATGTTATCAAGTCCATACCAGAATTCTCAGGCAGCTATAA
TGCATATGTTAGTTGGCGCGAAGCCGCTTGCTGTGCCATGCGAATGTATAAAGAAGGAAGTAGAAAGTAC
GTAGGTGCTTTAACAATATTAAGAAATAAAATAACAGGTAAAGCTAATGACACCTTGACTAATCACGGTA
CCGTTCTTAATTTTGAAGCTATACTTGCAAGGTTGGATTTCGCCTACAGTGNTANACGACCAATCTATATA
ATAGAACAGGAGTTAAGTATTCTACGACAGGGTAACTTATCCGTTCTCGAATACTACAATTTAGTTAATAA
AAAATTAACCCTTTTGATAAACAAAACTATTATGACGTATGGTGACAAAGCCCNCATCATTACGGAAATG
AATAAGAAAACAAGTAAGAGGTTCTAGTCGGGAGCTCCCGACTAGGGGAACCCTGAACCCTCTTATTCCA
ACACCAAATAAATTAATAAAAAAAAGTTCCCTTTGCAGGGCTCGAATTTGCAACTGACCGCATTACTTACT
GTCGACTCTACCTAACAGCCACACTAGAAAAAAAAAAAAATAATAATACTTTCCTCGGTAGGGCTCGAACT
CGCGACAGACCGCACCACTTGCTATGCCTCTACTCAGCAGCCGCACCTACAATTAAGTACTTATTATAAAA
AAGGAACTAAAATAACATAANAAAAACGAGAAAGAAGGGCTATCTTCGGCACGCCAAAGATCTAATACC
CTTGCAGACCCAACCTTTTCATAATGCTCATAGTGCTTTGCCCCTATCTAAGAAGTACCGGGAAAATAGTA
AATGCCGAGTTTGGTCAAGATATCTTGATAAACAAAATGTTTTTTCATACAACTTAATTTTCGACCGATCG
TTCCTATGGCAGCTATATGATATAGTGGTCCGATCTTAATAGGATTTTGCATATATATGAGGAGCAAAGTA
AAACTAATAAATGCCGAGTTTGGTCAAGATATCTTGAAAAAACAAAATGTTTTTTCATACAAAAACTTAA
TTTTCGACCGATCGTTCCTATGGCAGCTATATGATATAGTGGTCCGATCTTAATAGGATTTTGCACATATAT

GAGGAGTAAAGTAAAACTAATAAATGCCGAGTTTGGTCAAGATATCTTGAAAAACAAAATGTTTTTTCAT
ACAAAAACTTAATTTTCGACCGATCGTTCCTATGGCAGCTATATGATATAGTGGTCCGATCTTAATAGGAT
TTTGCATATATATGAGGAGTAAAGTAAAACTAATAAATGCCGAGTTTGGTCAAGATATCTTGANAAACAA
AATGTTTTTTCATACAAAAACTTAATTTTCGACCGATCGTTCCTATGGCAGCTATATGATATAGTGGTCCG
ATCCAGCTGGTTTTCACATATGTGCTGCGTGCAACAGAAAGAGGGAGTTCTGCAAAGTTTCATTAAGATA
GCTTTAAAACTGAGGGACTAGTTCGCATAGAAACAGACAGACGGACAGACGGACATGGCTATATCGACTC
GGCTGTTGATGCTGATTAAGAATATATATACTTTATGGGGTCGGAGATGTCTCCTTCTATGCGTTACACAT
TTCACGACAAAATTATAATACCCTCTGCAAGGGTATAAAAAGATGCAATAACGNTGCTATTAGAAATGCG
AGCAGACGCGCATGAATGTGTGCGTGCCACATGTATACAGAAATTCTTAAAGCTGCTGCCCCATTCAATT
GCGCAGTTGCATATAACTTTGGTTTTTCTTAACCGATCTTTATGAAATTTTCTACAGTACTTCTTATCGTAC
CATTGGATATTTGTGTATTCTAAAAGTCAATTGCATTTAAATTGTGGTTTAAATTGGTTGGCAATAAAAGT
TGGGTTATTAACATGATTTATAGCTCTGTTGTGGTAGCGGGGAGGTGGCGATAGGTATGAAATGAAAGAT
ACTTGAAATATCTATAATATTCTAGAAGCAACATATGATGTTTGGTGGCTCTAGCTCGTATTATATATCCA
AATTTGCTGAAAAAAAAACAGGATGTCGATATCGATTTTTATCGATTGCTTGGAAACGGAGTAAGTTGTCG
ATTATCGGAAACAAACTCGATCTGCGCGGGAACTAGGAGCATCTACATCTAAAATTTCAGTCTCTAGCTCT
TATAGGTTCTGAGATCCTCGAGCTCATACGTACGGACGGACGGACGGACGGGCGGACGGACGGACGGAC
AGACGGACGGACATGGCTAGATCGACTCGGCTATTGATGCCGATCAAGAATATACATACTTTATGGGGTC
GGAGATGCTTCCTTCTGCCCGTTACATACGTTTGGATTTTGCACAAATACGATATACCCTTATACCTATTTT
TAATGGGTTCAGGGTATAAAAACAGAGACAACGCTTTAAGAATTTTCATCACAGGTCTTAACGGTTCTTTA
TCAGAAGTTTTGTTCTCCCTTAAACCTGAAAATTTGCCTAATGCTTTGGCAAAAGCTCAGGAACTGGAAGC
GAATTACCAAAGGGCTAACTTCGCTTACCACCATTTCGCAAATACCCATCAACGTAACAATCTAAGCCCA
GGGCATAACAATCAGCAAAAGAAAGCTGAATCCCCAAAACAACCTAATAAAAGATATGACAATCACTTA
AGGTTCAATCAAGTTCCGCAAAATCAAATTTTTGCGCAAAGCAACAAGACTCCTCAACCTCCACCGGAGC
CCATGGATGTAGACAAATCCATACAACTACAGAATACACCTTATCCGTATCCCCAAAATCGAAATTCGGC
TCAACCAAAGAAACGGGAATACGCTCCCCCTTCGGTTCAGCAACCTAACAAACTCATGCGTATCAATAAC
CTCGAGGAAGACTATTTTTTAGAAGAAGAGGGGGAGAACTCCCCTACATTTTCAGAATAGACAAAAAAAT
AAATAAAATTTTAAAAATATTAATTGACACCGGTGCCACCGCTTGTTATATAAAAGAGGGTATATACGAT
AACGCAGAAGAATTAGTTATAAAAAAAAAGAATTAAAACTGTAAATGGCTACAGTATCATAACTCATTTTC
ATAACATAAACATATTTGGCAAACGCCACAGATTTTATGAAATTAATAATCTAGACGCAGATCTATTAGTC
GGTTATAATCTACTGAAGGAGATTAAGGCAGTAATCGACTTGTCAAAGGGAACTTTGTTTTATAATAACA
AAAAGGAAAATATTAATTTTACAAAACATGTCCAAGAAACGGTTGGCCATTTAAAAAGTTCAGACGAAGC
CTATAACGTTGCTTCGTATTTGAACTACCAAAGTGGGAAATGTAAATCATTTTCTTTATTTAATGTATCCGA
TACAGAATCATCGTCTTTAAGTGACGCTGATAACCCACGTATCGAAAAAACTAAAGTTGACGATATTCTTA
ATGAAGGAAAGCTAAAGAAATTAAAACTCAAAAAGAGCAGACGTCGAAAGATGTCGGTATCCTCTACAA
CAAGTTCTTCTTTATGCAAAATAAATCATTCCATTAATGAAATGGATAATTTGAATATCCTTGAGCAACAA
GTCGTTCAACAAATAGAAACAAAGCATTCTCAGATTCAAACATGTCTCCCTTTTAGAACAGATATTAGAG
GAGAGATAAGAACATTAAACGAAAATGCAATTTACAGTAAACAATATCCTTATCCTCATTCAGCTAACAA
TTTTGTTAGTCAGGAAATAAACAGGATGTTAAATGAAAATATAATAAGGCCCAGCAGAAGTCCTTATAAT
GCCCCGGTATGGGTAGTAGGGAAAAAAGGTTTTAATGAAGATGGAACGCCTAAGAATCGATTGGTAATA
GATTTTAAGAAACTGAATAACGATACAATCCCCGATAGGTATCCAATGCCTGATCCTTCAGTCATTCTATC
CAACCTAGGCAAAGCCAAAATGTTTTCGACAATTGACTTAGAATCGGGATTTCATCAGATTCTAATGAAA
GAAGAAGACATCGAAAAAACAGCTTTTTCCATTAACAATGGAAAATATGAATTTCTTCGTTTGCCATTTGG
GCTTGCGAACGCGCCAAGCATCTTTCAGAGAGCAATGGACAATATATTACGTGAAAAAATCGGAAAAACT
TGTCACATTTATATGGACGACATAATAGTTTTTTCAAATACCCCGGAACAACATTTTGATGATTTACAAGA
AATCATTGAAATTCTATTCAAAGCAAACATGAAAATATCCTTGGAAAAATCAAAATTCTTTAGAAAAGAA
ATAGAATTTTTAGGATACATAGTATCATACAATATCATTAAAACTGATCCGAAAAAAATAGAATCAATCA
TGAAATACCCTCCTCCTAATAATTTAAGGGAATTACGAGGTTTTCTTGGCTTAACAGGCTACTACAGGAAG
TTTGTAAAAAACTACGCGACTATAGCAAAACCACTCACTAAATTGTTACAGGGACATAACGGTATGGTTT
CAAAACATATGTCCAAAAAAACAGAAATATCTCTAGACGAAGAAGCAATCTCTGCGTTTGAAAACTTAAA
ATCACACTTGATTGCAAACATCGAACTAACTCAGCCAGATTATACAAAAAAGTTCGTCCTCACCACAGAC
GCTTCCGACATCGCCATAGGCGCCGTCCTTTCGCAGGAAAACAAACCAATCACCTTTGTTTCAAAAACCTT
AAATAAAACAGAAAGAAATTACGCCACAAATGAAAGGGAATTATACGCCATTGTATGGGCATTAAAAAA
CCTTAGGAACTACTTGTATGGTGTTACAAATTTAGAAATCCAAACAGACCATCAACCACTTTCTTTTGCGG
TTTCTGCACGAAACCCTAACTCAAAAATGAAAAGATGGCATGCTTTCGTAGAAGAATTTTCCCCTAAAAT

ATTTTACAAACCAGGATCTACCAATGTCGTCGCTGACGCGTTATCAAGGGTAATGATCAATCATATTTCCA
ATGAAAGTAATCTCGTGTCCGAAGAAGAACCGTCGTCCGATAATGAAACTATCCATTCAGCCGACAGTAG
TTATAAAAGCGTAATTGAAGAAACACACAAACCATTGAACCAATTTAAACAACAATTACTTGTTTCTAAA
AACAACATAACAATTCATGAAAAATATGAAGTTTTCAACAAAACAAGACATAT
>160_rnd-1_family-276__LTR___Gypsy
GAGTAAATGGATGCGTTTTAACTTTAAATTGTTATAACTCTGTGATCTATAATTACAGAACAATGTAAATC
CCAGTATAATTGTTTGGTCGTGCGTAGTAGACTTTGGATATTTGATATACACGGAGAAATGTGCAGCGACA
ATCCCGTTTACTGCTACACCGATTCAATTATACCCTTCTGTTGCGCTATGGAGTTATCACTACTTTCATAAT
AAGTCGTTACCTAGGCAACGGCTATTTCTTGGATCCTAATCTTTTTTTTATGAGGGAGCTTCTGGAGCCTT
ACTGACGTGTGTTATACCAAGTGGATCAAACTGTTGTCTTTTGCCTTTTTGATCTCGTACTGGTCACATCAT
AAGATTAAGCCGGATCTCAACTCACTTGCCGCTTAATTATTAAATTTTTGTTTTCTCATCCCTATACCCTTC
ATTGTATTAGTTGATCAGTTTTGAAGCTGATGCAGATTATAACCTTTCATGAAAACGACTGCATGGCATCA
ACACATTTTTATGAAATATTGTTCTATATTTTCGATCTGTTAATCGATTTTGCACCTTTTTTTTGTAATTTTA
ATTCTTGTTAGTATTTTATTTTAAGCGTAGGTCAGAAATGTTAATACATCGCAGTTGCGAAAACTTAGCAG
CTGCTTCGATTAATAAGGAACTGCGCTATATTTGACTTAAAACGATTGAGTTTTCTACACAATTAGTAGCT
ACCAGTCGCGAGCACACATTCCATCGTGCATGCTATAATATTAGAGTAGTAAAGAAAAATATTTGCCCTG
TTTGCAAAACAACAATTTCACCGTCTAACCCATGTGTGGTAGATGACGCCCTCGCAAAGCAACCGACGAT
AGTTACTATGCCAATTGGAACCCGAAGTCAAGGCCCCCCAGAAGAGATTTTGTCCCAAGGCACCATGTCG
AAGAATCCTCGTCCGCCAAGTGTTCCGCCTTTGGCAGCCGAGCCAGATGCATGCTCTCGTGTATCCCCCAC
GAGAAGCGAAATACAGCAGTCAATTTCTGATTCAATGGCAGCAGCCACAGAAAGACAAACAGCCTCCCT
GACCCAAACTTTACAGTCAGGATTCCAAAATCGTTTGTCTGCTTTTACAGCTCAACTAGGCAGCTCCACAG
CTCAGCAACCAACTTCGAACATGAGTATTGTCGATCATCCTCAGCCCTGACCGGCTAATTCTATACGAAAC
GAGTCTAGAACTAGGAGCTCTCCAATGTTAGAAAACTTGCGTCCAGACCGACTTAGCCAAATAATCAGTA
ACTGGAAACTTAGATTTTCTGGTCGATCGGGCATGGGTATAGATGAGTTTATCTATCGCGTCGAGGCGTTA
ACAGCCGAGACATTNGATGGAAATAATGAGTTGTTGGCTCGTCATATAAGCCATCTATTTGAAGGCAGCG
CTAACGATTTCTTCTGGCGCTACCATAAGAGTGTCCCTATTATCTTATGGTCGGAATTATGTGTTGCTTTAC
GTGCTCAGAAGAAGTTCAAAGAAGAGCGACTTGATGTTGACATACGTGCGGCAATTGATCGTCGTAAACA
AAAAGAGGGTGAGTCTTTCGATGACTTTTACGATGCAATTATTATATTGACCGACAAACTGTCTCTACCCT
TATCGAATTCCTCCTTGCTAGAATCGTTGCGCTCTAATTTGCTGCTAGAAATTCAGCACGAGCTTCTTTATG
AGCCCATACATTCTATCGCGCAACTTAGACATTTAGTGCAAAGGCGGGAAGGTTTTATTCAGTCAATTTCT
AAAGCTCGAGTGTTTGCACCCCGCCCTGCGCCTCGTCCCTCATTGCACGCTATAGAAGTACAAACTGAAA
GCGAAGCAGAGTCCGATGCGATAGACCTTCTCGATAATGATGTATCAGCCATCAGTCTAGTCTGCTGGAA
CCATCGGTATCAAGATTGTGTCGCCGAACGCAAAGTGTTTTTCTATGGCTGTGGTCAGACAAACACTTACA
AGCCAACTTGTACCAAGTGTAACAATGTGCTAAAAAACTACCTGTCTCGTGTACCATTGACCGGTACACG
CAAACCAGTTGCATCCAAGGCAACAAATACCGACTAGAGGGAGAATTAGAATCCTCTAATCAATCTTTAC
CTGACTTTTCATTTAACCCAGAATTTACCTTATATACTCGTTTACAATCTCAATCTGTTAATAAAAATACTT
CTGTAAATCGCAAGATATTACGAGCGCGATTACGTCGAATAAATTTCTTTAAGCGATGTAAGATCGAGAA
ACAAGCTCTCGAGTCAGCGATCGTGTGTACCGATTCAAGCCCTTACGCTGAAGTAACCGTATTGGGAAAA
ACTGTTACAGGATTATTGGATACCGGTGCGTCGAAAAGTTGTTTTGGTGGTAGTTTCGCTGTGGAATTGTT
GAATACAAAAGTACTGTATAAACCGATTACTTCACAGGTGAAAACCGCCCATGGAAAAAACTCAATAAAT
ATTGAAACGGTTACGCACAGAAATTTATTATGGTGGAAAGGTTAAACGAATTACTTTTTATATAGTCCCTT
CTCTTATTCAACCGCTTTATTTGGGCATAGACCTTTGGTCAACGTTTGAGCTGTTGCCTCCTTGCTTGTTAA
GGCCATTGGAGGATTCGCTGCGAGCTTTAACTGCACACCAACAGCAACGTCTTGCAGAAAGTGTGAAGCT
ATTTCCTTCATTCGCGGAAAAAGAGTTAGGAAAAACCATCTAACTGTCTCATGTCATAGATGTAGGTAAC
GCAATTACTATCAAACAACGACACTATTCCGTATCTCCTGCAAGGGAGAAATTAATGTTCAAAAGAAATT
GATCGTATGCTTTCCCTTGGCGTTATTGAACAATCGGGAGAGTTCTTGGTCTTCCCCAGTAGTGCTCGTTTG
TAAACCAGGAAAGGTTCGTCTCTGTATTGATTGCAGAAAAGTTAACGAGGTTACTCGAAAGGATGCTTAT
CCGATGCCCCTAATTGATGGAATTATTAGTCGTCTTCCTAAGGCTGAATATATTACCAGTCTCGACTTAAA
AGACGCTTATTGGCAGATACCCTTAGAACCCAGTTCTCGTGACAAAACAGCGTTTGTAGTACCAGGTAGA
CCCCTTTACCAATACACGGTAATGCCATTTGGTTTGTCTAACGCCCCTGCTACGATGTCCAAATTGATGGA
CAGGATAATTCCAGCTTACTTAAGAAATGAAGTGTTTATATATTTAGATGATCTTCTTATAGTGTCTGATA
CATTCGACCGTCATCTTGCCGTGTTAAGTGAACTAGCCGGGAAAAATAAAGGATGCCGGGTTGACAATTA
ACCTTGAAAAAAAGCAAGTTTCGTCTAAAGGAAGTTAATTATCATGGTCATGTTGTTGGTCAGGGTATTAT
TCGGATGAATGCTGATAAGATAACTGCCATTACCGAATATCCAGCGCCTCGCTCGCTTAAACAATTGCGTC

GCTTTCTCGGTATGACTGGGTGGTACCATAAGTTCATTCGAAATTACACTTCTATAGTTTCGCCATTGACTG
ACGCCCTGACAAATCGAAAAAAGTTTGAATGGTCAGAAGCGGCAGAGCAAGCTTTCCAGGCGCTTAAAA
CTGAAATGTGCAAGGCCCCTGTGTGCATACACCTAACTTTGAAGCTTCCTTCTACATACATTGTGATGCTA
GTCACACTGGTGTGGGTGGTGTTCTCGTCCAAATTAATGATACAGGTGACGAGGTCCCCATAGCATTTATG
TCGCGAAAGTTGAACAAGTGCCAACGTAATTATTCAGTTACGGAGAAGGAATGTCTAGCAGCAATTTTAA
GCATCGCAAGTTTCGAGCTTACGTCGAAGGCCATCCGTTTACTGGTATTACCGACCACGCATCCCTTAAAT
GGCTCATGAATCAAGCAGATCTCAGTAGTCGTCTAGCTCGATGGGCTCTCAAGCTTCAAGGATATACATTT
TCTATACAACATCGGAAGGGCAGTAAGAATATTGTACCAGATGCTTTATCCCGTACTCACACGGAGGATT
TATCGGCTTTAGATGTCGATGACTTTATTGATATGCAGTCTCCATATTTTAAATCCCAGGGTTATGAGTTGT
TGAAGTCTAAAGTTTTGCAGAATCAGACTCAAATGCCGGACGTCAAAGTTATCGATGGCTTTATTTATCGA
AGGTCCGAGCATGCCTCAGGAGACAGAGTTGCGGATAACATGGTTTGAAAATTATGGCTACCCAAAGAGT
TGGTTCCCCATGCTTTGAAATTAGCTCATGATGATCCCTTAGCGTCGCATTGTGGAATGAATAAGACACTA
GAGAGATTACGACGATATTATTATTGGCCAAATTTAGTTTCAGACGTGAAAGAGTATATTAATAAATGTG
AAATTTGCAAAGCGACTAAACATCCGAATCGTGTCTTGAGACTCATCGATTCTTTCAAAAATTGTATGTTG
ATTGTCTTGGACCGCATCCCCGTTCCAAGGCTGGTCATATTGGCATATTCATTGTGCTCGACCATTTCACA
AAGTTTCCTTTTTTGAAACCTGTTCGCAAATTGACCGCAGATGCTATTCTACCGTTTTTGGAACAAGAGTT
GTTTCACTGCTGTTCAATGGTGTTCAATTTAAATCACAAATGTTTAACGATTTACTTCGAAAGTATTCGATA
CTCCACTCGGTTGATCCCGCTTGCTACTTCTAGAACGTACGAATAAGCAATAGAGTCTTAACCAGCGCTCA
CAATGGACGTCGAACCGATTTCGGACAAGTTATAGCCCGTCGAAAATCGATTGATGTGTGAAATATATGA
CGAACTGCAGATTTGCGTTTCCGATCCCCAGACTTTTTTCTATTCAAATATTAGTTTATTTTCAAATCTTAT
TTGAGAAAACTAACCTCTGACCCAGAATCTAACAGAGTCCTACCCAACTGGTAGCAAGCGGACAGATCCA
TGACACGAGTCCCCAGTGCTGCCAATATGACTGGACAATGTGGGCCATTCATTTCCTCTTGTTTCTTCGGG
ATCGACCAGAAAACGACAGCTCAGCTAAAAGGTGCCTAAACGCCTGCTGAGAGCAGTCTCGAAGTCGAA
CTATCAAAGAACGAAAAAAAGCCAATTGCCAAACTGCCTTTGGCTCCAAATTCCTCTATATTAATAAGGT
ATTGACCAAATTTTCAAAAAATCTGATAAAAGTTTTTATGTTGGTCATCAATTTCATATAACTTTGCCAAT
ATCAGTTTAAAATATCTTCTTGATACCTTGGTCCAGCCATGGTCATTAATTGTGTCGTTGTGTTGATGCAAT
GACTCTTTAAATCTGCTCTAGACGCATACGAAGGGTAAATTACGTCCGAAGGTACCAGCAATATAACGAA
TCAAAATTCTCAGCCTATAGGGGATAAATATTGCATGTACAAACTTTTATAATACCCAGAATATCAAACAC
ATTTTTATGTGGGAAGTAGACAATTCGCTTTGTTAGTAATGACACCTTTTGCATAGTAGAGCTTTCATCTTC
TTGCAGGTATATTTAAATTAGTCCAATTGTGCAATTGTGACAGATAATTAAAAACTTAAGAATGAAGCAA
TACACTGAAACAAATAGTTATGTATATTTTAGATTACCGTTGTTACATTCTCAGAAATAAAACCATAGATC
TAAGCACATTGCAGTGTCACAAATAAACTATGAATTAGAAGAAATTCTGCACGACTACAGCGTCCTGAAA
TCTCATGTAGTTTTTCACATGGTATTTGGTACAGTGGGGATTTCTAGTTCAATTTGCTGAACAAGTTTACTC
GTTCATTTTCTGGCCGAGAACTAAATCTGTCATTAAGTTCACTCGTTCAGTTATTCAGTCGGTTATGAACG
ATGAATTCTGCTCAGTAATTCAGTCGGTCATGAACTATTAGCTTAGTTCAGTTATTCAGTCAATCATGAAC
GATTATATTTCTGTTCAGTTATTTTTCAAGTTTTCAGTTGCAAAATATATGTTATACTTTTTACTTATTATAG
TGTACCATTGATAATAGATACAAATCAAAAGTGAAATTAAAATCCCGCTATCTTTTATTTATTTATTTTATT
TAGTACTGAACATCTCATAAGAATGTATCGTTCAGGAACGGCTGAACTGCTGAGTTGCAT
>160_rnd-1_family-253__LTR___Gypsy
CCGAGAAGGAATGCCTAGCCGCCAAACTGGCCATACATCGATTCCGACCGTACGTGGAGATGATGCCGTT
CACGGTAATCACCGATCATGCGAGCCTGCAGTGGCTTATGAGTCTCAAAGACTTGAGCGGGAGATTAGCC
AGTTGGTCCCTAGAACTGCAAGCGTTCCCTTTCTCCATGCAGTACCGCAAGGGAGCCGACAACGTCGTCG
CAGACACGTTGTCCCGAAGCGTGGAAGAGGTCGAACTGACCCCGGAGGATCTGTTGGGATTTCAGACCCC
GGAGGATCTGTTGGGATTTCAGACCCCGGAGTTCGAGAGCACCGAATATCGAGAGCTGATAAAAGAGGT
GATGAGCCAACAAGGGAAGTTGCCGGATCTTAGAGTGCAGGACGGACTTCTGTTCAAGAGGACCGTGAA
CGAGAGCTTGGAGGATGAGGTCGAAGGCACAAGTTGGAAGCTCTGGGTGCCGGAGTCGCTGACTGCAGG
ACTCATCCAACAAGCACACGCGGACGAGACGTCAGCTCATGGACGCTGCACGCGCTGCGCAGGCGATATT
ATTGGCCCGACATGGCCATACAAGTCAGGGACTATGTACGGAAATGCGATACATGCAAGGAGACCAAGG
CACAAAACTACCGGATGCAGGTCGGAATTGGGGAAGAAGTGCGCACCGACCGCCCCTTCCAGAAGCTAT
ATATCGACTTCTGGGAAAGTATCCACGGTCGAAACGAGGGCACGCGTGGATATTCGTCGTCGTAGACCAC
TTCTCGAAATTTACCTTCCTGAAGGCCATGAGGGNAGCCACCGCGGCGGATGTCGTGAACTTCCTAGTGC
ACGAGGTGTTCTTCAANTTTGGCGTGCCGGAAGTGATCCATTCGGATAATGGACGACAATTCGTGTCAAA
GTCGTTCGATGCGATGGTCCAGGCGTTTGGCATTACTCACCTGCGCACACCAGTGTATTCGCCTCAGAGCA
ACGCCGCCGAACGCGTGAGCCGCACAGTGCTGTCTGCAATCAGAACCTACCTNGGTCGGGATCATCGGGA

TTGGGACGCGTACCTACCGGAGGTGGAAGTGGCGATCCGGAATGCGGTCCACAGCGCTACGGAGTGACT
CCGTTCTTCGCGGTCTTCGGACAGCAGATGTACCTGAACGGTTCCAGTTACAAACTGGCCAGGAAGCTGA
GATCNTTGGCCGATCACGGTATTTCTGACCTTGACGCGAAGGACAGACTGGCCGTAATCCGAAGCCGAGT
CAAGGACCANCTGCACACCGCATACGAGCGAAGTCGCCGACGATACGACCACCGCGCGCGCCAGCTCCA
TCCGGAACCGGGACAGGAAGTGTGGAGGCGCAACTTCGCGCTAAGCAGCTTCGGCAAAGCCTTCAACGCC
AAGTTCGCCCGGAAATTCCTGAAGAGCCGAGTCGTCCGAGCCGTGGGCACCAACGCCTNCGAGTTAGAAG
ATCTCCAGGGACGCGCGCTAGGCGTCTTCCACGCAAAAGATATACGAACGTAATCGTAATTTGCTCACCT
CCCTACCAGGCAATGGCGGCGGCAACGCCCGTCCTCCCTTTTTTCTTTTGGGTCAGTGCGAAAATATAGAT
TACTTGTCGTGGGTGTGGCCTACGTTGGGCGTCGTTATTAGCCGGCTGGGTCAATCCATGAAATGCGCACG
CAGATTCCTTGTAACGGTCGTTCGCGTCGTTACAATAATCTGCGTGGTGATGTTGCATTTGACGTTATCGG
CGGCACCGAGGGTTTACTTAGTTGGTTGCGTGCGATATTATCGGCCACGCCGATATCTTTTTGTCATATGG
TCAGAGTATGCGGGGTGAAGCTGGGCCCACGGCGGAGCCCAAATTTTTGAAGGGTGGCAACGCCAGACTT
GCGATCCACGGCGCCAAAAGCGACGATTGCTATTGGCCGCCCGGTTCGCTCCCAAAGAGAATANGAGGA
GAGTGGATCGCATGCCCTTGGATATGCCCTTGGCCGTCATTTGGGCCGTTGACCAAAAAAAATGCAGCTG
TGCAGATGGGCAGCAACGGTGCCCGCCTCGCCAATGTATGCGTGTGCCGAAATTCGGCACCGATGATGTT
GCTGCCCAAATGTACGGGGAGTTCGACTTTGTCAGCGGTCCACAGCGGACGTACCGGCTCGCAGTTAAAA
ATTTTTGAAGTTGGATATTTTTTTTTTGCCGGCCACGGAGCATGTGCTTAACCAAAAAAAAAAGAAAAAAT
CAAAGGGCATACATTTGTTCATGACGGGCTTTCTTTATTATTTAATTGCCAAGATAATTCCTTATGTGCTAA
CGTATTGCAAAAGAAAATAGTGCACAAGAGCGATAATACACGAAACAATAATACACCAGAAACACACTG
TCGTCATTTAAAACCACTATAAAAGCGATACTCTATTAGAGCAGCCGCAGCCATTCGGTGGCACACNACG
CTTGAATCCGCGCCGGAGGACGCTGGCCAGATCAACCCAGATATTCACGGCCGGATTTTCAAAGCGCGG
CCAAGTGTCGGCGTAACCACGCGACGGAGAGACGAGCCATCACGGACGGGACTCACACGACGAAGAGGC
GGCCACGGGCGAGACCACGCCCATATCATCCGAGTGAGTCTGTTCCATTTCGTGCCTCCTCGCCTGAGTAA
ACGGCAGAGTCAGACGCAGACTTCCCATTGTTGCTCCCTGAAAGAGAACCGATTTTTTTTTGGTATTAAAT
ACATAAAAATACTCACGACTAGTTTAAATTAAGTAAATTTCCGAAAGTGCGAACCGGGAAGGAAAGGGC
CCAAAGATTATTGCGTCTGCCAAGTAAAGAATGAAGTTAAAAAGTTTAGATTTTGGTCAACTTATGCACG
AGTGATACCCACTTATACGTATATATATATATGTTACGCACCTCAGGTGCAGGTGCCCCCCACCCAGAGAG
CTCTTTAGGCAGGCAAGGTAATATAAGCGCTTTGACAAACAACGCCTTTCGTTGGAAACACATATGTATA
CACGCATTTATTTTAGCGGGTACTTGCTCCCCATTGGTGCAATAATTACCTGTGTTTATATTTCTCCCCTTG
TTTTCCTTTCCTTTGTTGACTGGCAATAACTTCTGGCCGTTTTTTTTTGGTCTCCGGTATTGTATGAAGGTCG
CCAACAAAAAAAAAGATATTCTGTACAAAAAAAAAGAAATTTAATATATTTAAGATTAATAGAAATTGTTA
TAAATATGAAATGATGCAACGCGTTATAGATAACTGTTGAAGCGAAGTTGAAAGATGGAAAAAAATTGA
AATTTAAAACTTATATCCATTATTCTAAATATAAGAATATACCAACTTATCGTATGCACCTGGTTTAAAAT
GCCGGAAATGTTCGCGAGTACGAAATTATAAATAACGGCTTGAAGATAACCTGCAGGGGAATAAGAAAT
AAGATTAAATGTTAATAACGATAAGAAATGCAACCAATTATATGCCGTATCTGAAGAGAGAAATGTCCGG
CTAAATTTTCTTTGATGGGGAGAGTTGGCATAACAGCCGGCACAGGCTGATGGCCATCTAATGGCGAAAN
GNGGGAGACGAGTGTAGGTGATCGTCTCATGGTNGGGCGGGTACGCTGGGTCCGGAACCGGGGNCCGAT
CTGGAAGAGTAAGGTGGATAAATAGTGTTTTTTGTTAATTTATATTGAGTGTCTCACCTTTGTTGTTTACAT
GTTCATAGTTGTTACAGCTGTTTATTTTGTTCATGAGTTAGAAGTTCCGATTGTTTGACGTTATCGATTTGT
GGTTCGCTTTGCATAGTGTTGTGTAAGTCTGTTTTGCTACAGACGTGTAGCTTCGTTGTTATCGATTGTGCG
GACTGCGGGCCAGGGATGGGACTACACCCAGAGAAACCGATGAGCCAGCCGGCGCGGCCCACCGAGAAA
CAAGCAGTTCCGTAACAATATGGCGCCCAATTTGAAGAAGCACTCGGCTTCTTTGAAGTCGGTGCGTAAT
CAAAGAAAAATAAATACGATATAAATGAAGGTACAGAGAAAAGGATTCCTTTGGCTTCAGGACCCACAC
CGCTGAAGTCTTATGTGGCACGAGTAGGTAGCTCATAATCGCGCAGCGTGTAGTGGAATCCGAGAAGCAC
GACGGAAGTAGAGAATATCCATAAACATGAGAAGTTTTCTTGTAGTGGTTGATGACGGTAAGTCACGTTC
GCATGTTGGCGAAACGTTCGCGACCGTTTATCGTAGCCTGGTGGTAGCCGCCACGACAAAATAACTTGTG
ATAGAATGTGATATTAGGGTAGGTCTTACTAGAGTTTGTTTAATGTTTTCTATTTGTTTTGTCCCACTCTGT
GCTAACGTTTGGGGTGTGCTGGCCGTAGTTAAATGAGCTTCGGAGACAGGTGACCCAGGACGATATAAAG
CCGATCCTGCGCCGCACCTGTCACACAGCATACCCTAAGGGTGCCCAGAAGTGCGACAGAAACCAAATAG
CCTTCAAGGTAGCGTTCCTTGAATGCCGTTCGTCAGTCAAACTGACGAACTGAGCATTTGACGAATGCTTA
AGTGTTGTCCACTGTCCAGATTATGCGTAGATTAGTTAGCCTGTGTTAGGATTTGTCTTCTCTCCCCCAAGT
ATTTGAAATTAATTAGAAAGAAAATTAGTAAGATTTGTTAGTGGGTGTTTATTTATTTATTAATTTATTAG
GAATATTGGTAAAGTTTTTTTTTATATANGGGTAGTAAGCCTTGTAGAAGAGAGGGGTACAGTTCTCAGA
AGGGATAAGTATGGCTGAGGAACAGGTGTCTGATTGGGCTTCTGACGCTGACGGGGCGTCGGCTCTGCTA

CCGCCGCGCCCGGCTGGGGCGCTGGTGCGAACGCCAATGAATTTCCGGGGATTTCCAGAGAGACAGATCC
CGGAAGAGGGTCACCTGGTAGACTTGGAGAAAGAATTGGCGCAACAAGATAAACCTCCACGGAACCTGG
AGTTCCCAAAAGCCCCGAAAGACTCAGTGCTCAAATCGGCGGCAAACGCGGCGATCGCTCAGGCCCACCA
AACGTATCGGCATGCTCTGCCATCTGGCATCAGCGAGTCCATCCGCGAGGAGATGCGAGCTGGCTTCATG
GAAATGATGAAGGGCATCATTGATATGATCAGGCCGCCAGGGAGCCAAGCCGCCGAGACTAACACTGCG
CCGAAGCCGCCGAGACATGCCGAGCCGGGACCACGAACAGGCGCCATCCCGAAAGAACGGAAGTGTTC
CAGCGCGAGGCATTTCCACCACCGGTGCCACCGAAGCCACATAGACAAGGCGCACGAGGGGCGGAGTAT
TCGTCCAGCAGCCCAGTTTACGTTGCAGATGCTCAGTCGAGGAACTGGCGTAACCCGAATAACCGCCATT
TCCGAGTAGAGGACCGGAGAGCGGATGATTTTCAACCGGAGGAGGCGTACGCCAACGTACGAGAAACGG
CGTACATGAAGCTCGAGCGGTGGAACGTCAAGTTCGGCGAGGACGCGATGAATTCGGTCGAGGACTTCGT
GTTCCGCCTGGAGTTCCTGCAGAGGCAGTATCAATGCCCGTGGAAGGAGGTCCTGCGCGGTTTCCATCTGC
TCCTGACCGGCCGCGCCCGCGAGTGGTATTGGATGCACGTGCGGCATTCTAGGGTCGACAGCTGGATGCA
ACTGCGGCATGCCCTCTTGGACAGGTTCCGGGGCTACCAGACGGAACACGATGTGATGCAGGAGCTATTA
CAACGAGAGCAACAGGCCAGCGAAGGGGTGGATGATTATATCCACCACGTGCGTCAACTCGCCGCGCGA
TTCCAGAAGCCGCTGAGAGACCGCGAGCTGGTGAAGATTATAAAGCGCGGTTTGAAAGAAAGTCTGGCG
AAATATATCTATGCCATGGACGTACTCACCGTGGATGAGCTGCGCCAAGAGTGCCTAGAAGTGGAGAGGC
ACATGGGTCGCAGAAGCCGGACGGGGTACCTGCCGCCGTCGCGTTGTCCACAAGGAACGAGGCCCGTAGT
CCACGAGGTTGAAGTACCACCACATCGTACAGAAACGCCGCCGGGAGAACTGGAGGAAGCATTCGTGCG
AGGCAGGAACTCATCCCGACCTGTATGCTGGAACTGCAGACAGTTCGATCA
>160_rnd-1_family-41__RC___Helitron
TTCATATAGAACATTTATATGTTTATTCGTTAAAAGTTTTAGTCTATTCTTATAAACTTTTTGGGTCCTAT
TTTTGTTATCTTAAATTAGAATGTTATCCCTATTTTCTATGTTAACTATTGTATATGGGCCTATATATTCTGG
ATCCAATTTATGGCCAGTTTCGTTTCTTAATAAGACTTTGACTTCTATTTCTACTAGAAAATCTATAACTTT
TCTATCAAAAAGTTCCTTACTTCTTGCTTATGTTTTCCAAGTATTATCCTAGACCTTTTATATGCTGNTTCTA
ATCTAAACTTAACTTCTTTAGCATAATCTTCTACATTATATGGTGGGTCGATTCTATTTATATTGGTAAATT
GGGTGTTTACCAAAAATTTGCTCATAATAATTGTGTGCGATGGAGGGGGTCGTGTTAAAACAGTATACGA
TGTATTTGATCCATACGCCCCAATCAGTTTTATCAACTGATATATATGATCGTATGTATTCGTTAAAAGTCC
TGTGACTTCTTTCTACTGTTCCAACTGTCTGGTGATGATGTGCAGTTGATGTTAAGTTGTTTATTTTCATAT
ACTTGCACAAATTGTTTATAATTGAATTCTTATATTCCGTACCCATGTCCGTAATGAACGTCTTCATTGGAC
CGTACTTCAGAATAAAAGATTCGAATATAGATTTAGCGACAGTGTTTGCACTTGTATTTGGGATCGGAATT
GCGACGAGGTATTTCGTAAAATAACAAATGAGTGTAACTGCATACTCGTTCCCATTCTCTGTTCTAGGTAA
TGGTAATAATTGTGTCAACGATTACTCTGTCGAAAGCGGATATTGGTGTTTCTGTTATTGTTAGAGGAGTT
TTTGTGTTCTTTGTAGTTTTTGCTTTCTGGCATTTTGGAAATTTGCGGATGTATTCTTTTATTTGTTTAGACA
TACCTTTCCAAAAATAATGTCGTTTGACTTTGGCCAAGGTTTTTATAATGCCTGTCCTCCTAGTATGGGATC
ATCAAGTAGTTTAGACAAGATAGCTTATTTTTATTTTTCAGAATTTATTTGGGTCACCGGGTGGAGTAGCG
CAACTCTTAAAGATTTTAATATTTGAATGCCCATTTTCTATAAATTATCCATTGAAACGTGTTCAGAGATAT
TTTCCCACGGNGCCACNTTGAGTTGGCTGATTTTATATATACCGGCTTGCGTTTCAAGCCTTTGAAAGATTT
GATCCAAATCAATGGTTTAATTAGTATATAAATCGGTAACATCGTATCTTGCTGTAATTTGTCTTCCATGTT
TAAAAAACATGTCATATTGTTTACATGCAAGGTCACTACTTTTCGTACCTAGTCATTGTTAATGACTTGGT
ACACTTTGGCCTTTGAAGCTTTTTCTATAGATTGCCTAGGCAATTCTATTTCTTTTTCTCCTGCGCAGGATT
CTTGTCTACGTGCTTGTCTTGTAGTGACTTTCATTATGTTTCCTGTTATATTTTGTAGCTCCTTGATGGTTAT
TCTCGATAACGCATCTGCTACGTAACTGTCTTTACACTTCGGATATTCTACCGTAAAGGTATATTACTGCA
AATCAAGCCTCATACGTGTTAATTGTGAACTTGGATTGACCATTGAAAATAGATGTTAGTGGTCTGTGATC
CGTTTTTACCGTGAAATGGTTACCATAAATATATGGTCGAAAGTGTGTTATTACCCAGTGAATAGCTGTTA
ATTCTTGTTCAGTTGTACTCTTGTTACTTTCTCCTTCAGTAAAGGATCTTGATGCGTATGCAATTGGAAGTT
GGAGTCCAGTAAAGTTTTAAGTTAAAACTGCTCCACACGCTTGCTCACTTGCATCTGTTATTATGCAGAAT
TCTTTGGTGAAGTCTGGATATTGTAACAAGGTTAGGTTTATTAGTGCTGTTTTGAGATGTTCTGGCATTCAG
GCGTCCATTCGAATTTTACGTCTTTTTTACATAATCTTGTTATGTGACGTGAATAGTTAGCGAAGTTCTTTA
TAAAGCGCGTATAGTAATTACAAAATGCAACGAATCGTCTTGCGCTGTCCACATCGTGTGGGAGTGGGTA
ATTTTTAATCACGTCGTATTTTTTGTCGTCTGGCAAGATTCCCTTATCAGTGCATTTGTGTCCCAAAAATGT
GACTTCATGCATAAAAAATGAACATTTTTCCGGATGTAGTTTCAGGTTGAGTTTCTTACATTGGTCGAAAA
CATCAGTTAAGTTTTTGACCATATGTTTCTTGGAACAAGCGATTACTATTAGATCATCCATNTTAAGAAAT
GCTTGTGATGGTTGTAGACCAGAAAATGCTATTTTTAATCCAAATGGTAATCGCGTGAAGCGATATGAGC
CATNGCTTGTTGACAATTACGTTATATTTCTAGAACTTTTTTCAAGNTCAATTTGGTGGAAGCCACACATT

AAGTCTAGGCATGAAAAATATTTTGCTCTACCTAGTTGATCTAGAATATCATCAATTCTAGGTAGTGGAAA
TTTGTCGGACAATAGCGTTTTATTAATTTGACGATAGTCAATTTCTAANCGCCATTTTTTTCAGAGTCCGGA
AGAGACTTCTTCGTACATAATCTTTTAATTGGTACTGGTACATAAATCTTTTAATTGACTTTTGAATAAAGG
CGGAAAGTTTCTAATTAATTTATTCATAATGAGGTTGTCGCTTTCTTCGTTAGTTTCTTTTATCATATTTTAT
TTTATTTAAATTGACGATTTTATTGGTATTTGTTGTTATNAGATTCGGACACAAGTATTTTTGTGGTTCAGN
ATGTTTTTGTACCTGCGACATAAATTCCTTCATCAGTCTCTTGATTTAATACGAAAATTTGATTTTTTATTG
AGGGTATTTGTATTTCACTGACAACTTCGGAACTTCAGCTTCGAGCTGGGAGTACTGTGGCATTATTGTCA
CAAGTATGAAGTATTTGTAATTTAATCGGACAATTTAAGTTGTTTGGGCGATGAATTAGTGAGTCGCCTCT
CTTTGTGAAATCTAATTGGCAATTAAAATGTTTTATGAAATCTATGCCTAGTATGCCATCACTAGGTATTG
GGAATATTTGATCGACTAAATGGAAGTTATGCGGAATTATGTATTTACCTGTAGTGAGTTCTATAAAGGCC
AAGCATTTTGAANAAATTGTTCCTTCGCCAATTCCCGATTTTTGGGTAGTAACCCTTTCGTTTACGTTTTCA
AATATATCTACATTTTCTCTTAATATAGATATATCTGCACCAGTATCAGGCAACAGTGCAAAGGATTTACC
TGTTCTTTCATTGTTAATTTTTACAAATATGCTGATGTTGATGATTATGGTTTTTATTGTGCCATTTATTGGC
TCTAAACATCTGAGGGGTCTCAATGGTTTTCCGAATTACCTTCAGTTACTCGGACATTACCTCTATTGTTAT
TATTTTGGTTGTTACCTCGGTTGGAATTATTCCTTCCTCGGTTATTACCACGGGCATTACGTCTATAATGTC
CTCTGCCATTGCTATTGTTGTTCATTTCAAGGGCGCGCGTCAGACGCGTGCCAATAATGATATTTTCTTTTG
CGCCCATGATCTGCCCATTCACAGACACTTGTATGTCTATGCCTATGTGTAGAGGTCGAAAATAAGAAATC
CCAAAATATTGTTAAATTTGCATCGGGGACAGGCAAGCAACAGGCACACAAGCGCATAAATGCTTCTATT
CATATACAAAATATGCAAGTATGCGTGAATAACGAGTCCAAAATATTTTAAATGCACACATGGCATCACT
TGGGTACCCTTACCAACGATAGCGAGCTAAAATGCAGGAATTTCGCATCGACGTGGCAAACCCTTTCTCT
ATTCCATATTACTCGTGCCTTCAAAAGGGTAGAAATGCCTCAGCATTTGGGAAAAATTGAATTTTTGCCCA
GTTTTGCTTGTCGGACACACACACACACACATTTAAACGATCCCTAAACATAAAGAGAGAGCATTGAGAG
CTGAGAGTAAGTGCGAGCGAGATAAAAGTTAACAGCGTTGTCGTTCTATCGCACAAATCATATGGAAGAG
AGTGCGCCAAGCACTTTCCTGTAGAGTAAGCGGTTCAAAAATACATCATTTGTATATCTTACTCCCTTACT
CGCGATTTTGGACCAGATGCCACCGATACTTTTCGTGCCTACTTTCCTGTACTCATGTCCAGTCTATATGTT
CTTTGGCTAGTGCTTCGACTCATTCAGATCTTGCAGCTGGTTTGGTTCAAACTTTGCTGGTAAAAATAGAT
CTTCTGGAACATTTATTATGATATTCTGTCTAATGCCAATGTTTTTCAATCTTGTATTTAAAGATTGTATGA
CTTTAAGGCATTGTTTGGCATGTTCTTGTGTTAGTTTTTCTTTAATGGAATCTACTGCATGTACTATATTGTT
ATATTGTTTTATCAATATTTGTACATGGTATATTAGTGTTTCTACCTTTATAGGTATGCCTTTATTGATGCAT
TTATATGACTTGTCAAATTGGTGTTTAAAGTCATTGATATTTTTCACTAATATACTCCATTCCATATTATTA
TAATAGATATCTATCGATGCAGTCGCACCATTAATATGAGTATTGAACATGTCCGTGTATTCGTATTCGAA
ATCACACACTTTTATAAAATCTCTATGTAATCGCAGTACGTTTAATACGCGAGTACGAAAGTCTGGCTCAT
CTCTATACGTAAACCTAATGATTGCCTCTATGTCTGACATATTCAAATATATTCATACCTTATCTAGTCCGT
CTGCTCTACTGCTATAGCGTTTCTTAATTTTCTTTGTATGCATTGCATACACTTTCAATACTAGTGATACAA
GAGATATTGTAGTAATTATTCTTTATGGTCAATTACTTTTACGTTATTTATAACATTTGCGGCTGATTTTTC
AANNTTGGATTCGTTCGAACCCATTTTAATTAAAATTTNTTACATAAGTTGATGTAATGTTTTTAAATTCAT
ATAATGTTTTTATGTAACATATAGTGTTTTTAGCTTTAAATGCCAATGTTTTTAGTTTCAAGGTTTCAATAT
TGCTAATAACACAAAATACTACCTAGCATTTCGCATAGNTTTACGGGCTATGCTGCTCAATTTTACAAAAA
TTTTTTTTTTATAATTAAAAGTAATTAAGGATATAACTGCTACTGTTGTTCATCGTTGCATCAGCTGAGAGG
CTAGTGAACTGCGCTGCGTAATATGAGCCAGAGCCAATATACAAAGTGGCTCTGGGCTTGTTTATACTTTA
TATTTGTATATAGGGTGTTCTTTTAAGGCATATACACACTACAGCTCCCTTTTCAAAGAAATAACGTAACA
TTATGGATGTGTAGTTATTTAATGAACTTAATTATTTTGAACAAAATCTTTTGTATATAATTTTTTTTTGCTA
TAAAATAATAAAATGTAAAAAAAATGATAAAAATAATATGAATTTAAAAAAATTATTTAAGATTTATGGGA
ATATACTAATGTAAACATTTCAAAGTATAAAGATATAAAATATAAAAATGTAAAATTCATTATAGTAATTT
GTTTTTTTCAAGGATTGATTTTTTTTTTCAGTTGGTTGATCAGGCCAATGTATATGTATTTTTATACCCTGAA
CCCATTAAAAATGGGTATAAGGGTATATTGTATTTGTGCAAAATCCAAATGTATGTAACAGGCAGAAGGA
AGCATCTCCGACCCCATAAAGTATATATATTCTTGATCAGCATCAATAGCCGAGTCGATCTAGCCATGTCC
GTCTGTCTGTCCGTCCGTCCGTCCGTCCGTCCGTATGTATGAACGCAAGGATCTCAGAACCTATAAGAGCT
AGAGACTTGAAATTTTAGATGTAGGTGCTCCTAGTTCCCGCGCAGATCGAGTTTGTTTCCGATAATCGATA
ACTTACTCCGTTTCCAAGCAATCGATAAAAATCGATATCGATATCCTGTTTTTTGGGCAATTTTGGTAAAT
AATAAGAGCTAGAGTCACCAAACNTGACATGTAGCTTCTAAAATANAATATATATATGCATTTGATGTTG
GAAGAAGAGGGTTCAGGGTATCCCCTAGTCGGGAGCTCCCGACTAGAACCTCTTACTTGTT
>160_rnd-1_family-120__LINE___R1
CACACACACACACACACACACACACACACACACACACACACACACACACACACACGCACACACACACACAC

ACACACACACACACACACACACACACACACACACACACACACACACACAGGAGAACATGAGTGAAAGTGAA
AGTGAGTTGACGCGTCTCGACTGTCGCTCCGGCTGTTCTCTGTCGTGGGCCGCAGCTCCGGCAGGGAAAG
CAAAGCGATGATGTGCAATCCATTGGCGAAGGGGCTGAGCACGTGGAGACCCACGCTCACATACGCGATT
CGCAAGCGGCGGATGCCAGGGTGCGTCAAACCAAAGATGACTACATACGCACACGGAGAGACGCAGGTG
GCACGACTGGCACGACTGGTGGTACACCTGCAAGACTGTGGAGGTGAACACGAGAATGGAGCAAACCCG
CGCACTCACCTGGATGACGGGATGATGGCGAGGACTACCTGATGGTATATATCCAACCCTAAACCTGATC
CAAGTAAGCAAGTTGAGCACTTTCTCAGCGAAACTATCATGGCGACATTGTTTATGCTCAAGCACATGAG
CAAGACAAAAGCTCTGGACAGCTGGTTGGTTGTACAGTGCTGCCAACCGAAGTACATGCGTCAACGAAAA
AAGCAATTACGCGCGAAAGTATGCCACGCTGTGTGGGTATACATGAGGACAAAGGAAGATATTGCATGCT
TTTAGGCTTTTACGTTTTACTCGCATTGCAGCTGAAGACTCGAGGCAAGAAATTGTACAGAAGGCCACGG
ATCCGACCAAGTCAACTCCTGCCGCATAGCGGTGTACTAAAAAGGGGCGCTGGCGACCGAGCTGGAGCG
GTTAAACTCCCATCTGTCAAACGCCAGTGAGAAACCATTGGCCACCGTTGGAGCCACAAATCAGCAGCGA
TGAGCGCCACAACTGGCAGCACGCCCGTCAATGCAAGCAAGCAAACTCCACCCCCACACTGAGCCCAGTA
AAGTGTGGAAGCGACCCACTGGACCCCCCTTACGTAACTGTAGAAATGGGCTGACGAGTCGGGCATTCTA
GGACCTTAATAGGCAAGCTGACACCTTGTCGAAACGGCTCCTAGGCTAATGCTAGCCTCGCCCCCGTCCC
GCTAAAACCTTGGCAGGCCTCGTAGAACGCTTATCGTATGTCGCCATTCAGTTGGCCGTGCAGGCTCAGTT
GATATTCCCTGACTAGCACTGATCTGGCTCTGAACTCCGTCTCCCATAAGGACCGGAGTCAACCCTCGCGT
GGACCTCACTCCATGGAAAGGCAACCACGGGATCGCGAAGGTGACTGCACAATACACACGACAACAACG
GCTTCGAGTTGGGACCCAATTAATCACAATGATGAACCGAAACAACAAAAGCACCACCCCACTAGGTGGA
CAAGAGGAAGAAGGAGTATGCNAGGCCGACCCGTTCGCTAGGCGATCATGCCTGGCGAGCACGCCCCCG
GCTACACCAGTAAGGGCCCCTCAGCCCACTGCACCATGCAGGGAAATTGAAGAGAAAGAGACCCCGAAA
AGGCTTAGGGACCCGGCCAGCCCTACGAACAAGCCACGGAGCACCCCGCCCAAGAAGAGCAAGGCAAAT
ACCCAGGAGATCGAAGAGATTGGCTCGATACTGGACGAGCTACTGTTCATGTATCATGGCAAGCAGCCAG
CGATGAGGCACATAAACTTGGCCACTAAGAGCATGCTAGCGAGACTAAAAGAGCTGCAAGAAGTAGTCA
AAGCGCAGCTCACGGAAAACCTACGCATGCGATGCAGCGAAGAGTCCCATAAGCAGATGAGCCATGCTG
AAGAAAGCGCCGGAAGCAATTCTAAATGCGACACGGCAACGCAGACGGAAGAAAGCGGTGCATGCAGTA
AACCACCGACAGCGGCGCAAAAACCGACGAACGAGGTTACAGGAAAAAATCAGATCTTGGCCCCAAGCA
ACTCCAAGCCGGCACCAACTCGCAAGCAGCAACGTTCACAGCCGGCAGAGGGCAAGGTACCGACCCAAG
CGCGAGAGGCAAAAACACCGGTCAAAAATACCCGAGAGGTAAAAAATAAATGGATTGAGGTGAAGGGC
AAGCTCACCAGGCGGAAACAGCCCAACCGACCGGATGCGATCCTGATCACGTGTACAGAGAGCATGCCA
TACGCAGATGTGCTCAAAATGGTGAAAACAGCTCCTGCACTGCAGGCGCTCAAAAATAATGTACAGGGCA
TCCGCAAATCAGCAGCAGGAGAACTGATCCTCAGGCTCCAGAAGCCATCGGACCCAGCTACCGACCAGTT
GCGTGTGGCAATCAGCAGCGCACTGGCTGGAAAAGCTGCCGTTAGAACCTTGCAGGAAACGGTGCAAAT
GGAGGTTCTAGATATCGATGAGTCCACGACAGCAGATGAGGTCCTCACAGCTCTCTCTGCCTCGGTGGAA
GGAGACATACCAGTAGAAGCGACGCCCACTATGAGGGCGGCCTACGGAGGAACACAGATCGCATCGCTG
GCCCTGCAACCGAGCCTAGCCACGAAACTGCTCGAAACCGGCAAGGTCCGCATAGGGTGGGTTATATGCC
GNATACGCGCAANAGATCGAGCCAAAGAGATGCTTCAGATGCCNGGGNTTCGGNCATACNNCCGCAAAC
TGCACCAGCGGAGACGATCTGAAAGGCGTTTGCTTCTGATGCGGGGATATAGGCCACGTAGCAAAGAGCT
GCTCCAAGTCACCAAAATGTAGCCTTTGCACACGAAAAGGCGAAAAATCTACGGACCACGCTACAGGCA
GCTTTAAATGTCCGGCGTACCGCGGGGCAGTACGTAAAATGAAATGGTAAGATTACTTCAATTAAATCTT
AACCACTGCATAGCTGCTCAAGCCCTTTTAGAGCAGACTGTTAGAGAAATGCGCATCGATATTGCCATTAT
CAGCGAACCCCACCATATAGACACCAACTGCCCGAGANTATGGACCGCGGATCAGGGCGGAAAAACTGC
TCTCTGGATATGTGGCGAGCCTTCGACGTCATTGGAGAACACAAAGCCCTTCACTGGGTTTGTACGCGCAC
GATATGGCAGCCTATGGATACACAGCTGCTACCTAGCCCGAGCATGCAGCTTAGCGAGTTTCAAGCTGCA
TTAGACGCATTTATAGACGACGCAAGGGGGCGGAAGGTCATAATTGCTGGTGACTTCAACGCCTGAGCAG
TGAAATGGGGCAGCCAAGCAACCAACGCAAGAGGTCGTGCCCTGTTGGAAAGCTTGGCAGTTCTGAATAT
TGGCTTGTTAAACTCCGGGACGGAAAACACCTTTAGTCGGGGCGGTATCGGATCAATAATCGATCTCACC
TTCGCAAGCTCGTCACTTTTCGGCGCAACGAAATGGAAATAAAGTGACGTATATACCGCAAGCGACCACT
CCGCAATAATANNNNNNNNNNNNNNNNNNNNNNNNNNNNNNNNNNNNNNNNNNNNNNNNNNNNNNNNNNN
NNNNNNNNNNNNNNNNNNNNNNNNNNNNNNNNNNNNNNNNNNNNNNNNNNNNNNNNNNNNNNNNNNNNNN
NNNNNNNNNNNNNNNNNNNNNNNNNNNNNNNNNNNNNNNNNNNNNNNNNNNNNNNNNNNNNNNNNNNNNN
NNNNNNNNNNNNNNNNNNNNNNNNNNNNNNNNNNNNNNNNNNNNNCTGGTGGAACGAAAATATTGCCGAAGCAC
GGCGGAACTGCATCCAAGCCAGGAGGAGATACCAGCGAGCCCGAGGTAGACCCAACTACGCCGAACAGC
AGCTAAGGTATCAGAGTGCCAGAAAAATGCTAAAAAAGTCCATAAGAGATAGCAAACGAGAATGTTTCC

TGGAGCTATGCGACTCAGCCGAATACGATCCTTGGGGCAAGGCGTACAAAACTGTCGTTAAAAGACTCTA
CGCCGGCAAACAAGCGCGGCCTTCCGACCCAAGTGAGCTGAAGTCGATTGTGGAAACGCTATTCCCAATC
AGACCCGACGTATTACACACGCGAGCCATACCATCGAGCCACGTAGCAGCCATGCCGTCCCTCATCGACG
AAGTCTCAGTCGAAGAGGTGCTGACGATCGCCAGAAGCCTAAAGCCCAGTAAGGCACCGGGTCCGGACG
GAATCCCCAACCGAGCACTGAAACTAGCCATGTCCTTGCGGCCCGCGCAGTTCGTGCAGCTTTTCAACAC
GTGCCTAATGGAGAGAACTTTCCCCACAATATGGAAACTCCAAACGCTAGTTTTATTACCGAAGCCTGGT
AAGCCGCCCGACGACCCATCGTCATACAGGCCGATTTGCCTCCTGGACACGTTAGGCAAAGTGCTGGAGA
AGCTAATCTGCAACAGGCTTCAGGTAGCTGTCGACAACAATGGTGGCCTGTCCCAAAGCCAATTTGGTTTC
ACCAAGGCAAAGTCTACAATAGACGCTCTGGAGAAAGCGGTCGGCATAGCAACAAACGCCATCCAAGGA
TCGAGATGGAAAAGCGGCACAAAAAAGTACTGCCTCATCGTCACGCTGGATGTTAAAAACGCCTTTAACA
CAGCCAGGTGGGACAAAATATTGGATGCGTTGTCAACGTTCCGCGTCCCATATTATCTTCAGGAGACGAT
ACGCAGCTACTTCACTGGAAGACTGCTGCAATACGACACCGCAGACGGCCCCACTACACATCAAGTGTCC
GCTGGAGTGCCCCAAGGCTCAGTTCTAGGACCCCTCCTCTGGAACATCATGTACGATGGCATCCTCCGACT
GGAACTCCCGATCGATACAAGTATCGTCGGCTTCGCGGACGACGTGGCCCTTGTAGTCGTCGGCAAGGAA
TTGAAGGACGTAGAGGAATCGTGCAACCACAGTATTGACCGTGTGCACCAATGGCTGGCAGCAGCTGGAC
TCGAGCTGGCTGCTCACAAAACAGAAGCGGTGTTAGTCAGCAGTCGAAAAAGAGTGGAAACAGCGCACA
TAAGAGTCGGCAGTGCGACAATAGAATCAGCAAGGGCGCTAAAGTACCTGGGTGTCATGCTAGACACAA
GAATGTCGTTCAAAGAGCACTTGGAGTACGTGCACAAGAAAGCAGCAGAGTCCTGTCGAGCGCTATCCAG
GATTATGCTGAACACACGGGGACCGAAACAGCAGAGACGCCGTCTCCTTATGACCGTAAACCGAGCGGTA
ATAATGTACGCGTCCCCCATCTGGAGCAGAGCTATGGCTGTTCCCAGCTACAGCCGGGGCGTACACTCTAT
ATATCGCCTAAGCGCTCTACGGGTCAGCTGCGCATTCCGGACGGTATCGGACGAAGCGGCGCTGGTTATC
GCAGGAATGGTCCCGCTAAGCGAACTGGCAACAGAAGCTGTAGTTTGCTATAGAGCCCACGATGGAAGC
AACACAGCAAACTCGATTCGCACTGAGGCAAGGATGCAGAGCGTAGCACGATGGCAAGCACGCTGGGAT
CGCGCATCTAAAGGACGCTGGACGCATAGGCTTATCCCAAACTTGGAAGCCTGGACAAATCGCAAGTATG
GCAATACTAATTTTTATTTAACGCAGCTCCTATCAGGCCACGGATGCTTCAGGAGCTACTTAAAGCGATTC
GGCCACGACAACACAGACGAGTGTTCTTGGTGCGGCAGAGGAATCATAGAGGACGCCAATCACGTCATAT
TCGAGTGTGGACGTTTCGCAGCGAACAGACAGGAACTGGAGGACATCATGGGCAGACCTATCACGGTGG
ACAACCTGGTCACGAGCATGATAGAGACCGCACAAAAGTGGGACGCTGCCAGCACCTTCGCAGCAGAAG
TAATGCGAGAACTCAGGAGAGTTGAGCGCAGCAGACGGAGAACCGCGGATATTTAGGTAGCAAGAACAT
GCCTGTGAAGCAATGCTTCGCGGCCGTTCCGCAGGCAATCCACGCTACCTAAACAATACCCCACCTATAA
CCTAAGATAAATAACTAAGGATAAATAAATAAACTGAATACAAAAAAAAAAAAAAAAAAAACACACACAC
ACACACACACACACACACACACACACACACACACACACACACACACACACACACACACACACACACACA
CACACACACACACA
>160_rnd-1_family-53__LTR___Gypsy
ACAATGATAAGCGACAAAATAAAATAAATAAATAAAATGAAATTTCAAGAAATATATATATATTCTTATT
ACCAGAAATGCCTATTAATAAGTTTGCGATAGAAGTATATTTAGAAGCAATATATAATAGCGACGCAGAC
TTTAGAATTCGTGCATTGAATATTCTACAACAATAAGATAAAAGGCATAATTTTAAATACACTGCCATATG
TTGGCATTAAAGAAATAGTGGTCCAGTGGACGGCGAGTGCCCAATTATAATGGTAAAACTTCATAAATAG
AGAACTATGGAATGGAGTGAATTAGCGAAAAAAATAAATAATTTCAAAGATAAATTCGAAAAATCTTATA
AGTGTGTTAATAAGAACGCACCTATAAGGGTTGAAACGCTAATTCACCACATAAACATATTAGTGCTAAA
CTATAATAACATAGTTCAACTTTATTTAAGTAAAGAAAATAGGCTTACAGAGCAGCATGTAAATCAATGT
AAGAAAATAATCAAATCACTTGGGACTAGAATATCGAATATCAGCACTAGGCACAACATAGTAATAGATA
TCCCAACAGATTTAAAATTAATGGCAACTTTTGAACCAGATCAACTAGAAGAACTGAATGAGTCTAAGCC
AACACTAAATAGTGACCCTGATATTGAAGCTGATAGCGACATTGANCCACTTGAATTAACAGAACCAAAC
ATGGCAGTAAATCAAGTGGCCTTAGACCGCGAATATGTAAGACAGGTATCGTCTACAGTCCCGGAATTTG
ACGGCGAAAAGCTGTCACTGGCCAGATTTCTAACGGCTTTAAGACTTGTCGAACGTACTAAAGGTACACG
AGAAGACTTAGCCGTAGAAGTCATAAAATCNAAAATAATTGGCCCAATCCTGTACAAAGTACAAAATGA
AACCNCNATTANCGGNATTATNAATAAATTAAANACCGATGTCAAAGGCGAGTCAACCGACGTCATCAA
GGCNAAGTTGCTTAACATAAAGCAGAAAGGCAAGNCAGCCNCGCAATNTACAGCTGAAATTGACAGTTT
GCGTAAGCAACTNGAAGCAGCCTATATTGACGATGGACTAGATTCTGATAACGCAGAGAAATTTNCTACT
AAAGAAACAATATCTGCNATGACAAAGAATCGCGAATACGAAAAGCTCCGCATAATATTAGAAGCTGGC
AATTTCAGCANTTTCGATGATGCCATGAGTAAATATATCCAGTGCAGCACTGAAATGACTGGAAGCGCGA
GTACAGTATTGTACTACAAAGGCAAAAACTATCGCGGNAATNACAGCCGTAACAACCAAAGAGGTAGAG
GCAATGGCCGCGGCAATAGTAACTTCCAANATAANGGNAATAACAACGACAGGGGTAATTNCAGAGGNA

ATGACCGTGGTGGCAACCGAGGAAGAAACAATTCCAACCGAGGTAACAACCAAAATAATAACAATAGAG
GTAATGTCCGAGTAACTCAAGGTAATTCGGAAAACCAACGAGACCCCTCAGATGTTCAGAGCCAATAAAT
GGCGCAATAAAAACCATCAACCTTAATCTCAGCATATTTGTAAAAATTAACAATGAAAGAACAGGTAAAT
CANTTGCACTGTTAGTAGATACTGGTGCAGATATATCCATATTAAAAGAAAATNCAGATATATTCGAAAA
CGTAAACGAAAGAATTACTACCCAAATATCGGGNATTGGCGAAGGNATAATTCGTTCAAAAGGCTTGGCC
TTTATAGAACTCACTACAGGTAAATACATAATTCCGCATAACTTTCATTTAGTCGATCAAAANTTCCCAAT
ACCTAGTGATGGCATACTAGGCATAGATTTCATAAAACAATATAATTGCCAATTAGATTTCACTCAAAGTG
GCGACTCACTNATTCTTCGCCCAAACAACTTAAATTATCCGATTNAATTACAAATACTTCATACTTGTGAC
AATAATGCCACAGTACTCCCAGCTCGTTCCGAAGTTGTCNGTGAAATACAAATACCCTCAATAAAAAATC
AAATTCTNATATTAAATCAAGAAATTGATGAAGGAATTTATATCGCAAACACNATATCCAACCACAAAAA
TACATATGTCCGATTCTAAATACAACAAATACCGATAAGATAGTGAANTTAAATAAAATAAAATATGAAA
CTTCGAATTACGACATAGTAAAAGAAACCAACGAAGAAAGAGACAACTTCGTTATGGATAAATTAATCAA
AAACTTTCCGCCTTTATTCAAAAGTCAATTAAAAGATTTATGCACCAGGTATACCGACATATTCGGACTAG
AAACCGAATCGATAACAACNAATAATTTTTACAAGCAAAAGCTTAGATTGAAGGATGATGAGCCCGTATA
CATAAAAAAATTATAGAAGTCCTCACAGCCAAGTACAAGAAATTCAAGCCCAAGTACAAAAATTAATAGA
CGATAAAATAGTCGAACCGTCAGTCTCANAATATAATAGCCCACTTTTATTAGTTCCGAAGAAGTCTCTTC
CGGACTCTGACAAGAAAAAATGGCGATTAGTAATTGACTATCGTCAAATTAATAAAAAGCTATTGTCCGA
TAAATTTCCGCTACCTAGAATTGATGATATTCTAGATCAACTAGGTAGAGCAAAATATTTTTCATGCCTAG
ACTTAATGTCTGGCTTCCACCAAATTGAACTCGAAAAAAGTTCTAGAGATATAACGTCATTNTCAACAAG
CAATGGCTCGTATCGCTTCACGCGATTACCATTTGGNTTAAAAATAGCCCCAAACTCNTTTCAAAGAATGA
TGACAATAGCATTTTCTGGTCTAGAACCATCNCAAGCNTTCCTTTATATGGATGATCTAATAGTAATCGGT
TGTTCCGAGAAACATATGGTCAAAAACTTAACTGATGTTTTCGACAAATGTAGGAAACTCAACCTGAAAC
TACATCCGGAAAAAATGTTCATTTTTCATGCATGAAGTCACATTTTTGGGACACAAATGCACTGATAAGGGA
ATCTTGCCAGACGACAAAAAATACGACGTCATNAAAAATTACCCAGTCCCACACGATGCGGACAGCGCN
AGACGATTCGTTGCATTTTGCAATTACTATAGGCGCTTTATNAAAAACTTTGCCGACTATTCACGTCACAT
AACAAGATTATGTAAAAAAGACGTAAAATTCGAATGGACGTCNGAATGCTTCGAACATCTCAAAACGGC
ACTAATAAACCCAACCTTGTTACAATATCCAGACTTCACCAAAGAATTCTGCATAATAACAGATGCAAGT
AAGCAAGCGTGTGGAGCAGTTTTAACTCAAAACTATAATGGACTCCAACTTCCAATTGCATACGCATCAA
GATCCTTTACTAAAGGAGAAAGTAACAAGAGTACAACTGAACAAGAATTAGCAGCTATTCACTGGGCAAT
AACACACTTTCGACCATATATTTATGGTAACCATTTCACGGTNAAAACGGATCACAGACCACTAACATCTA
TTTTCAATGGTCAATCCAAGTTCAAAATTAACACGTATGAGGCTTGAATTGGAGGAATATAACTTTACGGT
AGAATATCTGAAGGGTAAAGACAATTACGTAGCNGATGCGTTATCGAGAATAACCATCAAGGAGCTACA
AAANATAACAGGAAACATAATGAAAGTCACTACAAGACANGNAAGTAGACAAGAATCCTGCGCAGGAG
AAAAAGAAATAGAATTGCCTAGGCAATCTATAGAAAAAGCTTCAAAGCCCAANGTGTACCAAGTCATTA
ACAATGACGAGGTACGNAAAGTAGTGACCTTGCATGTAAACGATATGACATGTTTTTTTAAACATGGCAG
GAAAATTACAGCAAGATACGATGTTAGCGATTTATATACTAATGGAACCATTGACTTAGGTCAANTCTTCC
AAAGGCTTGAAACGCAAGCCGGTATATATAAAATCAGCCAACTCAAAGTGGCACCGTGGGAAAATATCTT
TGAACACGTTTCAATGGATAATTTCAAAAANATGGGCAATAAAATATTAAAATCATTAAGAGTAGCGCTA
CTCAACCCGGTGACCCAAATAAATAAAAAAGAAAAAGAAGCTATATTGTCTANACTACATGATGATCCAA
TACAAGGAGGTCATACAGGCATTACAAAAACCTTGGCCAAGGTCAAACGACATTATTTTTGGAAAGGTAT
GACTCGAGATATTACNGAGTACATCCGNAAATGTCCAAAATGCCAGAAAGCAAAAACTACAAAGAACAC
AAAAACTCCTCTAACAATAACNGAAACACCAATATCCGCTTTCGACAGAGTAATCGTNGACACAATTGGT
CCGCTACCTAGATCAGAGAATGGGAACGAGTACGCAGTCACACTCATTTGTGATTTNACGAAATACCTNG
TCGCGATTCCGATCCCAAATAAAAGCGCAAACACTGTCGCTAAAGCTATATTCGAATCTTTTATTCTGAAG
TACGGTCCAATGAAGACGTTCATTACGGACATGGGNACGGAATATAAGAATTCAATTATAAACGATTTGT
GCAAGTATATGAAAATAAACAACATAACATCAACTGCACATCATCACCAGACAGTTGGAACAGTAGAAA
GAAGTCACAGGACTTTTAACGAATACATACGATCATATATATCAGTTGATAAAACTGATTGGGACGTATG
GATCCAATACTTCGTNTACTGTTTTAACACGACCCCCTCTATGGCACACAATTATTGTCCATATGAGCTAG
TTTTTGGTAAACACCCAATTTACCAATATAAATAGNATAGANCCACTATATAACGTAGANGATTATGCTA
AAGAAGNTAAGTNTAGATTAGAANTAGCATATAAAAGGNCTAGGATAATGCTNGAAAAACATAAAGCAA
AAAGTAAGGAACTNTNTGATAAAAAAGTTATAGATTTTCAAGTANAAATAGGAGATAAAGTCTTATTAAG
AAACGAAACNGGCCATAAATTGGATCCAGAATATATAGGCCCATATACAGTAGTTAACATAGAAGATAG
GGATAACATTCTAATTAAAGATAACAAAAATAGGACCCAAAAAGTTCATAAGGATAGACTAAAAATTTTT
AACGAATAAAACATATAAATGTTCTATATGAAGTCTTTATGTTCCTTTATGAATTCACAACAAATAACAAG

TAAGAGGTTCTAGTCGGGAGCTCCCGACTAGGGGATACCCTGAAGAGGTTCTAGTCGGGAGCTCCCGACT
AGGGGATACCCTGAACCCTCTTCTTCCAACATCAAATGCATATATATATTCTATTTTAGAAGCTATATGTC
AAGTTTGGTGACTCTAGTCGGGAGCTCCCGACTATGGGATACCCTGAACCCTCTTCTTCCAACATCAAATG
CATATATATTCTATTTTAGAAGCTATATGTCAAGTTTGGTGACTCTAGCTCTTATTATTTACCAAAATTGCC
CAAAAAAGGATATCGATATTGATTTTTATCGATTGCTTGGAAACGGAGTAAGCTATCGATTATCGGAAAT
AAACTCGATCTGCGCGGGAACTAGGAGCACCTACATCTAAAATTTCAAGTCTCTAGCTCTTATATGTTCTG
AGATCCTTGCGTTCATACATACGGATGGACGGACGGACGGACAGACAGACGGACATGGCTAGATCGACTC
GGCTATTGATGCTGATCAAGAATATATATACTTTATGGGGTCGGAGATGCTTCCTTCTGCCTGTTACATAC
GTTTGGATTTTGCACAAATACAATATACCCTTATACCCATTTTTAATGGGTTCAGAGTATAAAAATACATA
TACATTGGCCTGATCAACCAACTATAAAAAAAAAAATCAATCCTTGAAAAATACAAGTTACTATAATGAAT
TTTACGTTTTTATATTTGATATCTTTATACTTTGAAATGTTTACATGAGTATATTCCCATAAATCTTAAATA
ATTTTTTTAAATTCATATTATTTTTATCATATTTTTACATTTTATTATTTTATAGCAAAAAAAA
>9_rnd-5_family-83__LTR___Gypsy
TTGTAGTGGCGCTTGAAGCAAGAACAATTTGTTTGAGTTTAAATAAACAGCCCAAAAATAGACAATGTAA
ATCCAGCCAACGTCTTGTACCTTGCTTACCCTAGTAGACCTCGCCATCTATGGCTCAACCCGGGCGTAATA
TCGCTTGTAACGAGTGAAAGAGAAAATAGTAGAGGAATGTTAATTTGATAATTGGAAATGGCGTCTGTCC
TGGTGTCGCTGTAAATAGCTTTCCTTAAGCCGATCGGAGCTCAGATCTTTCTTTTTACCTTTGGTCCACCGT
GGAGAAAGGTTGACTCAAAAAAGTAAGAAAGTGCGCCTATTTACAATCGCGCTTAACCTCTGAGAAACAG
TATTTTCTGAAGCATCATTTTTAATAGAAGTAATTTCAAAAAAATATCTAAGTGCCCGCATCGATGAACCA
CTTCTCAGCCGCCAGTTGGTCGCAAAATTAATTGAGAAACTAAAAAAAAATAAAAAGTGCCAGACCCAATC
AGGGGTAAGTCAGATTTGTTTTTTGTTGAACCAAAAAGACTAAAAAGCACTTATATTTCAACCTGTCTATT
GCAGGTATGGCGCTTCGTAATATTCTCATCAACAAAATATAATGAGATATTATAGTCTTTCGACTTTCTGC
AATTAGCAGCGGCAAACATTTCTTTTTGCCTGCCGAAGCCCAAAATATGCAGAGCGCCTGTAATTGTCATC
GTTAATTCGCCTTGTCGCAAATAGCTTAATCAGCTGCGCCCAGCTTTATCGCATCGTCAAATCTCGTTGTTT
CGTTTCGTGCTCTCTAGCCTGCTCGAAAATGGGAGTTCGAAAGACGAATTAGTAAGAGACGATCGCCGTC
GCCGAAACTCCAAAATTAGTTGGAAATCGTTCCCTGGTGGCTGACGTCGTCAAAGCAAGCTACATTTATCT
TGGTCGCGCAATCATTTTTTCATATAAGCGAAACGAAATCTTATTTGTGTTATTATTTTTGCTGGAAAAATG
AATAATTAGCTTAGCTAACAAAAAGTGAAAAACTGAATAAGTCCTATTGTGAACGCCATGGGCGTAGTCA
AAGTCGCCTTAAGCGCATAATTTAAAATTTTACATTAGTTATGTGCGAGTTTCTTTTCTTTTTCGGCAAAAA
GAGTGCAACCGCATGGTCAAATTCTTATTCCGCATTGTTTTTCCTTTAACTCCGTTTTGCCCTACATATTCT
TTTCCATTGGCCTATTTTTATCTCTCCTTCTCTCCTTCCATGCTCCGAAATTCGGATCGCCATATTGCTGGTG
AGAACCAGATNTTAAACAAATTCGAAAAGAAACGTGCNAAGAGAAAGANAGAGAATACGAGTCACATG
AAGTTCGAAAACGTTGTTATCTAGTGCCTTCTTTTTTTTTTCTAAATTTTTTTGTGTGCAATTCGAAGTGATT
TTTTTCAGCAGATCATCGCACGTTACATCGATGGAAGCGCTTTCGGAGATGGAGTGAATGCATGTAAGTG
AGCAAAAACTCTTAAATTTTATTTTTCTTTGCACTTAATTTCCGAGAAAATCAAAAGAGAATGGCCTAATA
ATAATTTAATGGATAATTTAATGCCGCGCTTAAAAGCAAAAAAATCGGTAAAAAGAAAAACGTTAAATTA
ATCTATTTCAAGTGAGCGTTACAGCTTTCGCTAAATGAATTAAAAATTTAAATGAATTTTTGTCGAACAAA
AAAAANAAAGGAATAATTTTTACTTTTTACCAATTTGGGTCNTAGACCAAATTAGAACCTCACCTAGAAA
AAAATTCTTTATCTNTGTGTAATATAAGTCTTTTATTAATTTTCTTTTCTTTTCTATTATAAAAAATGTATAA
TGGCTAAGCAAAATTTCGATTTAACTCCCCAATTCACCACAAAAATAAATATGTGTAACGAACTTATAATT
ATGTGCTAATTTGCCTTATTTATGTTGGAAGCCATGCGTCGTCGGAATGTTAGGGTAATGTAATCTGTGTT
GTTTTAGGGATCACCGACCATTTGTCTGGTCAAGCGGCCCCGGTGATCATGGTAGGGTGGGCAACAGCTG
TCGCATTGATACCAGCGCAGCATAACAACAACATATACAGCTACGGTCCCTAATCAATCTAATACAATTTC
GGTAACTCTGTAGATTTTTTCCTAACCCCCGTAATTTTGNNTGTGCAATCGAACTAGACATCTTAATAGCA
CGCGAAATTTTTATATATAATTATAAGAGCTTGCGAGCAGGTAGCTTAGCCATTTATGAGTCAACCCACCC
CAGCCGACTTCACTGTGCCGGAACCAGCGCGTGCTTCGCTAGACGCTACACGGACATCAGCTCGCTTACA
TACGTTGTATGTTACAGTATATATCATATATTTATATATTTGGCGCCCAACGTGGGGTATGAAGCAGCCAC
TCTGGCTGGTCCCGACTAATAACTCTTTACATTTGTCAACAATACCTGACAATACACAGCTAGATCGATTT
GTTTCGTCACTCCCCATTGTCCTGTTATTTGCAATACGTTTTTGGATCAGCTACAGCTTTGAATTAGTGTAA
CTGCCAAGACGTATTATAGCGAATAACACATGTTTTGTTTAGAGTAACTGGATGCGTTTTAACTTTAAATT
GTTATAACTCCGTGATCTATAATTACAGAACAATGTAAATCCCAGTATGATTGTTTGGTCGTGCGTAGTAG
ACTTTGGATATTTGTTATACGCGGAGAAATGTGCAGCGGCAATCCCGTTTACTGCTACACCGATTCAATTA
TACCCTTCTGTTGCNCTATGGAGTTAACACTACTTTCATANTAAGTCGTTACCTAGGCAACGGCTATCCCTT
GCATCCTAATCTTTTTTCTTATGAGGGAGCTTCTGGAGCCTTACTGACGTGTGTTATACCAAGTGGATCAA

ACTGTTGTCTTTCGCCTTTTTGATCTCGTACTGGTCACATCATAAGAGTAAGCCGGATCTCAANCTNACCA
TTGCCNCTTGNTGAGNAAGCNAATGTTTTCAGATATTAGTTACTGCTGGAGTCCAGTGTACTTGGTGTAGA
ACTGGCACTTGCCGCTTAATTATTAAATTTTTGTTTTCTCATCCCTATACCCTTCCTTGTATTAGTTGATCAG
TTTTGAAGCTGATGCAGATTATAACCTTTCATGAAAACGACTGCATGGCATCAACACATTTTTATGAAATA
TTGTTCTATATTTTCGATCTGTTAATCGATTTTGCACCTTTTTTTTTTAATTTTAATTCTTGTTAGTATTTTAT
TTTAAGCGTAGGTCGGAAATGTCAATACATCGCAGTTGCGAAAACTTAGCAGCTGCTTCGATTAATAAGG
AACNGCGCTATATTTGACTTAAAACGATTGAGTTTTCTACACAATTAGTAGCTACCAGTCGCGAGCACACA
TTCCATCGTGCATGCTATAATACTAGAGTAGTAAAGAAAAATATTTGCCCTGTTTGCAAAACAACAATTTC
ACCGTCTAACCCAAGTGTGGTAGACGACGCCCTCGCAAAGCAACCGACGATAGTTACTATGCCAATTGGA
ACCCGAAGTCAAGGCCCCCAGAAGAGATTTTTTCCCAAGGCACCATGTCGAAGAATCCTCGTCCGCCAAG
TGTTCCGCCTTTGGCAGCCGAGCCAGATGCATGCTCTCGTGTATCCCCACGAGAAGCGAAATACAGCAGT
CAATTTCTGATTCAATGGCAGCAGCCACAGAAAGACAAACAGCCTCCCTGACCCAAACTTTACAGTCAGG
ATTCCAAAATCGTTTGTCTGCTTTTACAGCTCAACTAGGCAGCTCCACAGCTCAGCAACCAACTTCGAACA
TGAGTATTGTCGATCATCCTCAGCCCNGACCGGCTAATTCTATACGAAACGAGTCTAGAACTAGGAGCTCT
CCAATGTTAGAAAACTTGCGTCCAGACCGACTTAGCCAAATAATCAGTAACTGGAAACTTAGATTTTCTG
GTCGATCGGGCATGGGTATAGATGAGTTTATCTATCGCGTCGAGGCGTTAACAGCCGAGACATTNGATGG
AAATAATGAGTTGTTGGCTCGTCATATAAGCCATCTATTTGAAGGCAGCGCTAACGATTTCTTCTGGCGCT
ACCATAAGAGTGTCCCTATTATCTTATGGTCGGAATTATGTGTCGCTTTACGTGCTCAGAAGAAGTTCAAA
GAAGAGCGACTTGATGTTGACATACGTGCGGCAATTGATCGTCGTAAACAAAAAGAGGGTGAGTNTTTCG
ATGACTTTNACGATGCAATTATTATATTGACCGACAAACTGTCTCTACCCTTATCGAATTCCTCCTTGCTGG
AATCGTTGCGCTCTAATTTGCTGCCAGAAATTCAGCACGAGCTTCTTTATGAGCCCATACATTCTATCGCG
CAACTTAGACATTTAGTGCGAAGGCGGGAAGGTTTTATTCAGTCAATTTCTAAAGCTCGAGTGTTTGCACC
CCGCCCTGCGCCTCGTCNCTCATTGCACGCTATAGAAGTACAAACTGAAAGCGAAGCAGAGTCCGATGCG
ATAGACNTTCTCGATAATGATGTATCAGCCATCAGTCTAGTCTGCTGGAACTGCAGGAAATCTGGCCATCG
GTATCGAGATTGTGTCGCCGAACGCAAAGTGTTTTNCTATGGCTGTGGTCAGACAAACACTTACAAGCCA
ACTTGTGCCAAGTGTAACAATGTGCCAAAAAACTACCTGTCTCGTGTACCATTGACCGGTACACGCAAAC
CAGTTGCATCCAAGGCAACAAATACCGACTAGAGGGAGAANTAGAATCCTCTAATCAATCTTTACCTGAC
TTGTCATTTAACCCAGAATTTACCTTACATACTCGTTTGCAATCTCAATCTGTTAATAAAAATACTTCTGTA
AGTCGCAAGATATTACGAGCGCGATTACGTCGAATAAATTTCTTCAAGCGATGTAAGATCGAGAAACAAG
CTCTCGTGNCAGCGATCGTGTGTACCGATTCAAGGCCTTACGCTGAAGTAACCGTATTGGGAAAAACTGTT
ACAGGATTATTGGATACCGGTGCGTCGATAAGTTGTTTTGGTGGTAGTTTCGCTGTGGAATTGTTGAATAC
AAANGTACTGTATAAACCGATTACTTCACAGGTGAAAACCGCCGATGGAAAAACCCAACAAATATTGGG
ACGGTTACGCACAGAAATTTATTATGGTGGAAAGGTTAAACGAATTACTTTTTATATAGTCCCTTCTCTTG
TTCAACCGCTTTGTTTGGGCATAGACTTTTGGTCAACGTTTGAGCTGTTGCCTCCTTGCTTGTTAAGGCCAT
TGAGGATTCGCTGCGAGCTTTAACTGCACACCAACAGCAACGTCTTGCAGAAAGTGTGAAGCTATTTCCTT
CATTCGCGGAAAAAGAGTTAGGAAAAACCATCTAACTGTCTCATGTCATAGATGTAGGTAACGCAATTAC
TATCAAACAACGACACTATTCCGTATCTCCTGCAAGGGAGAAATTAATGTTCAAAGAAATTGATCGTATG
CTTTCCCTTGGCGTTATTGAACAATCGGAGAGTTCTTGATCTTCCCCAGTAGTGCTCGTTTGTAAACCAGG
AAAGGTTCGTCTCTGTATTGATTGCAGAAAAGTTAACGAGGTTACTCGAAAGGATGCTTATCCGATGCCCC
TAATTGATGGAATTATTAGTCGTCTTCCTAAGGCTGAATATATTACCAGTCTCGACTTAAAAGACGCTTAT
TGGCAGATACCCTTAGAACCCAGTTCTCGTGACAAAACAGCGTTTGTAGTACCAGGTAAACCCCTTTACCA
ATACACGGTAATGCCATTTGGTTTGTCTAACGCCCCTGCTACGATGTCCAAATTGATGGACAGGATAATTC
CAGCTTACTTAAGAAATGAAGTGTTTATATATTTAGATGATCTTCTTATAGTGTCTGATACATTCGACCGTC
ATCTTGCCGTGTTAAGTGAACTA
>9_rnd-1_family-300__LTR___Pao
TGTTGCGGGACGAATTAATTTATCGATTGTTTAGTATTAAGTATATTCTGAAAGTATGTATNTTTGCCCTCC
GAATGTACGGGCACACTGTATTTGGCTTGTTGCCGCCATTACTTAAATGTTAAGCTCATTATTGTCGTGAG
TTCGAAGGCCGTAGGAGGAAAGACTGCGGTAAGATTCCGAGGGAATATATATCTAAGTTCTAACCTATGT
AATCCGTTTAGAATAAAACACTTGCACTAGCACGTTTTGCATTACGCTGTCTCTAGTAACATCGGAGTAAC
ATGGCCGGGGGCCAGGCGTAAACTTGCAGTGTTCAAATTGCAACTCCACCTTCGTTTGGCACGCGACATG
ATCTTTTATTCGCAGTTCTTATTGTAAGAAACTGCAAGGGCGACTTGCGCTGAAGACAAGACGTGGTGTAA
CNTTGGTGTTACAGTGTGTGTACATAGACACAGCACAGGCAGGGCCTCCACCCAGTTATAGAGTCACCAT
TAGTACGCAGCCAGCGTAAAATATAGCAGAAACCATCTCCGAGCTGAGTAGGATGACACAACGGTCACCC
AGGAAAAGCCCACGCTTAAACGATGGCCAGGAGATAGCTACCACGGCGAGTGGGACGCAGACAAAGCGG

ACAAGTAGAATGGTGAGCACTGTATCACAAATGCAAGTTCCCGCTGTTAGCGCACCACAAAGCCCCAGCA
CCGTGGATGTGGGCACAAGTGTACAACCTGCCGGCACTGTGGCCGCGGGAGTGAGTGCACGAGCCAGCA
CATTGACAGGAGCGCCAAGCCCAAGCACGCAACTCTCAAGTACGCAAGTCATGGATTTGATAAAGAGAGT
CGCTGCGCTAGAGCACGAGTTGCAGAGGTCAAAAACTGGTCAAGCACATGTGAGTACAGTTACTGATCCC
GTTAAGTTGAGTAGTGCTGGCGTGAGTGGTACAGCTAACCTGCCGCCATCTTTGATCGGAGCNTTGAGCG
GAGAGCCATCGTCGAGTGGAAGTTTGAGTGAAACTGCAACTATGAGTGTAGTCCCAAATTCAAGAGTAAG
TTTGAGTGAAACTGCAATTTTGAGTGGAACTACCATTTTGAGCAGATCGCCATCTTTGAGTGAGGCACTGC
CTACGTCATTGAGTGGAAATTTGGGAACACAGTTGCAAACGGTAGAACGGGCTACAGTTATGGATAACTG
CTCTACAACGTCAAGCCGCTCGACGCCATATTGCTTTGCAGGCATTGCACAGCCAACGCTCAGCTACACTC
CGGCACAGGAGAACCAACAAGCGATAATTTTGGAGCCAACGCAGGCAACTGGGAACTTCGCATGGACGA
CTCCCAGGAACGATGTATGCCTGCCACGCAAATTACCAGATCTACCCGAGTTTGGAGGCCAACCTGAGGA
TTGGCCTATATTCTTCTGCGCGTTTACGGAAACAACTTTGGCGTACAACTGTACGAATCTGGAGAATAACC
AGCGATTGTTGAAGGCACTCAAAGGCGAAGCACGTGACACTGTGAAGTCGTTGCTCATACATCCCAGGAA
TGTGAACAACGTAATGGAACAATTACGTTTTAGATACGGCCGCCCAGAACAGCTAATACGCAGCCAGCTA
ATTAGTATTCGAGAGGTGCAGCCAATTCAGGAGCACAGCATAGCTAAAATCGTGCCATATGCTACACGCG
TGAGTAACCTTGCCGCCTTTCTACAATCGGCTAACGGGGAACAGCATTTAGCGAATCCTACACTGATGGA
GGATTTGGTCGCTAAACTACCACCAAGCAAGAGGGTGGAATGGGCCAGACATGCAGCTACGATTAGGCCT
TTCCCGACGGTCGTACATTTTAGCGCGTGGTTAACGGACTACGCCAATGTGGTATGTACGATTGTGGACGT
CGATAGTAAGGAGCAAAGGCGTAGAGTGCTGCATGCCAGCATTGACCAGAATAATGAAGGGCGTCACCG
AGGTCGCACCAAACAGTGTCCAATCTGTAATGGACAACACGCAGTCAGGGACTGCAACGAGTTTAACTTA
GCCTCTCCGCTAAAGAGGTGGACAGTCGTAAAAAGGCACCGGCTTTGTTTTTCGTGTTTACGAGTTGGCCA
CATGGCAAGAGTCTGCAATAGGGCCAACGAATGCCAAGTTAATGGATGCCGGAAGAGACATCATCGACT
CTTACATGATCATGATGCCATGAGTCACAATGCACCGCANCCAGCAGGTGATAGAGTGATGAATACTGGA
CGGATGCCGCAGCCAGCGGATCCTCACACAAGGGCACCGCAGCCAGCGGATGCTCAATCGACGCAACAA
AGGAACTTAAGTTGTGTCGATTCGGATGGAGAACGCCTACTATTCCGCATATTGCCAGTGACGTTGCACG
GGGCCAATAAGCGGATCGATACGTACGCGCTCCTCGACGAGGGCTCGTCTGTAACGATGATCGACGACGA
GCTACTGCGCGATCTAGATATCAAAGGGGAACGCAGGCAACTAAATATACAGTGGTTTGGTGGAAGAGC
AACCAGAGAGCCGACCACAGTGGTAAGCATGGAAGTTAGTGGAGCGGACAAGCGCAAACGTCATGTGTT
GCGAAATGTGTACGCCGTGTCCAACCTGAGCTTACCAATGCAAAGCCTGCATCGACGTGACGTCCAAATG
TGGGGCAACGGTGCACGTCTTCCAATGAAACCGTATCGCGGGGCGGTACCAAAACTTCTGATTGGACTTG
ACCATGCGCATCTTGGACTGCCAATGCAAACAAAAAGATTTGCACCACAGGGACCATATGCCGCAGCCAC
CGAACTTGGATGGGTGGTTTTTGGGCCGGTAAGAGGTCAACCGACGGCAACGTCATCAAAGTCATGCCTC
CTAGCAGTGTCGCAGGAGGATGCAATCCAAAAGATGGTAAACGACTATTTTGATATCGAAAACTTCGGTG
TGAAGCTTACGCCACCAGTCGCAGCCAGCGACGATGCACGAGCACAGAGGATACTCGAAGACACCACGG
TGAAAATAGGAGAACGTTACCAGACGGGTTTATTATGGAACCGTGATAATGTTGAGTTGCCACGAAGCTA
TGATATGGCATACAAAAGATTGGTCAACATTGAGAGAAAGATGAAGCGTAACGATCGGTTTAAACAAGC
CTATATGAAAATTATGGACGATTATGTTCATAAAGGATATGCTCGACGATTGAAGCAGCATGAGGTCGCT
TTAGCCAACAGCGACAAACTTTGGTATCTTCCGCACTTTGGAGTTGAAAATCCAAATAAGCCCGGTAAGA
TAAGACTCAGCATTGATCATGTCAACATTCGATCCTCTGGGATTTCTTAGCTGCCTCATGATAACTGGAAA
GTTACTAATGCGTGAGATTTGGCGACGAAATGTGCAATGGGATGAACCCTTACCAGATGAGATCGCCCGG
GCCTTTAGCAAATGGCTTCAGGATATGAACAACGTGGAAGGATTTCGGAGCTCGCGCCATTACTTCGGCC
CAACACCTGTGCGGGCAATACAGCTGCACGTTTTTGTCGATGCTAGCCAGTCAGCATTCGCAGCCGTGACT
TACTTGAGGGTCACCTACGACAACAACGACGTGCGAGTATGCTTCGTATGTGCCAGGACGAAGTGTGCCC
CAATGAAGACGATGTCAATTCCACGACTGGAATTGCAAGCAGCCGTGTTAGGAACGAGGCTGATGGACAC
TGTCAAAAGGGAGCACAGTATAGCGATCAGCGACGCTATACTATGGACTGACTCCAAAACTGTGCTGCGT
TGGATAGGCAGCACCCACCGTCGATATAAGCAGTTCGTCGGAAATCGAGTGGGAGAAATTTTGGAGTCAA
CAAAGGTTTCCCAATGGAGATGGGTACCTACTGCTGAAAATGCAGCAGACGACGCAACGAGGCCGCATTG
CCACGTAGACCTCAGCCAACGGTCACGATGGTTAGACGGGCCAACATTTTTGCGGCAGCCGGAAAGCAGC
TGGCCGCAGTCCGAACCGGGCACGGAACACTCATCCTACGAAGATGAAGAAGAGATGCCGAGTGAATTT
GCCTTAGTTGCAGCAAATAACTTTTTCATTTCATTTCAGAGATTCTCTAGCTACAGTCGGCTGGTGAGGAC
GACGGCTTGGGTTTTAAGGTTCACGCGCTGGAGCCGTGGACAGAGGACTGAGCTTGAGAGATTCGGCCTC
ACCGCGACGGAGTGTGAGAACGCTGAGAACTTGCTGATACGGCGAGCGCAATGCGAAGCGTTCCCTGAC
GAGGTTCGAGCCTCATACAATGAAGAATCAGTCGGTCATCGAAGCGACATAAGAGGGCTGGCGCCGTACT
TGGATGACAATGGAGTCTTGCGTGCGTATGGCAGGATCGATGCCGCACTGTGTATACCGCATAGTGCGAG

AAGGCCAATAATATTGTCACACCGGCACGCACTGACGGAACTGATTGTGTATCACTACCATGTGAAAATG
AAGCATCAAAACGTGGATGCAACGATAGGCGACATCAGGACCAGATTTTGGATAACAAAGCTGCGTCGA
CTGTTGCGTACAGTAATATCTGGATGTAGTGTGTGCAAACTACATAGAGCGCAGCCAGCGCCACCTATAA
TGGGTCCTTTGCCAGAGGACAGACTGGAGGCCAATGGATGGCCATTTAAGTTTACGGGTCTGGACTATTTT
GGACCACTCCTTGTGACGATCGGACGTCGAAAGGAAAAGAGATGGGTCGCCTTGTTTACGTGTCTGACGA
CAAGGGCCATACATTTGGAGCTGGCACATGACCTGTCAACTGATTCCTGCATAATCGTAATCAGGAACTTT
CTTTGCCGTCGCGGACCCGTACTGAGGTTGCGCAGTGACAACGGAAAGAACTTTGTTGGGGCCAACAGAG
AAGCCAAAAGGCTAATAGATGTATTCGAGCCGGAGAAGATTCAGGGTGAGCTATCTTCTAGAGGCATTGA
ATGGATCTTCAACTGCCCAGCGAACCCAGCTGAAGGGGGAGCCTGGGAAAGGATGGTGCAGTGTGTCAA
GAGAGTGCTACGCCACACAGTAAAGGAAGTGGCACCAAAGCAACACGTACTGGAGAGTTTCCTGATTGA
GGCCGAGAACGTTGTGAATTCTCGCCCGCTTACCCACTTGCCGGTGTCTGTGGACCAAGAAGCCCCCCTAA
CACCGAACGATCTACTCAAAGGAGTGGCCAATCTGCCTGACACGCCCGCTATCCACGAGCAGCCGAACGA
GAGATGCGTCACGAGGAAGCAGTGGCGCATGGCGCGGCTGTTACGAGACCGTTTTTGGAAGCGGTGGGTC
TTAGAGTACCTGCCTACACTTGTGCGACGCGTGAAATGGTGTGCGCGCGTTGAGCCGATACGCCATGGTG
ATATGGTGTTTATATGCGACCCGGCCGTGCCACGCAGAGAGTGGCGAAGGGGCATAGTGGAGGAGCTCTA
CCCTGGACCAGATGGAGTGCCTCGACGCGCCAGCGTCCGCGTGGCGGACAACAACCGAGTGCGACTGATG
ACTCGTCCCGTGGCTAAACTAGCCATACTGGATGTGAGTGAAGCAGGGCCTTCACGGGGGCGGGGATGTT
GCGGGACGAATTAATTTATCGATTGTTTAGTATTAAGTATATTCTGAAAGTATGTATATTTGCCCTCCGAA
TGTACGGGCACACTGTATTTGGCTTGTTGCCGCCATTACTTAAATGTTAAGCTCATTATTGTCGTGAGTTCG
AAGGCCGTAGGAGGAAAGACTGCGGTAAGATTCCGAGGGAATATATATCTAAGTTCTAACCTATGTAATC
CGTTTAGAATAAAACACTTGCACTAGCACGTTTTGCATTACGCTGTCTCTAGTAACATCGGAGTAACATGG
CCGGGGGCCAGGCGTAAACTTGCAGTGTTCAAATTGCAACTCCACCTTCATTTGGCACGCGACA
>9_rnd-1_family-192__LTR___Gypsy
CACTTGCCGCTTAATTATTAAATTTTTGTTTTCTCATCCCTATACCCTTCNTTGTATTAGTTGATCAGTTTTG
AAGCTGATGCAGATTATAACCTTTCATGAAAACGACTGCATGGCATCAACACATTTTTATGAAATATTGTT
CTATATTTTCGATCTGTTNATCGATTTTGCACCTTTTTTTTGTAATTTTAATTCTTGTTAGTATTTTATTTTAA
GCGTAGGTCGGAAATGTCAATACATCGCAGTTGCGAAAACTTAGCAGCTGCTTCGATTAATAAGGAACNG
CGCTATATTTGACTTAAAACGATTGAGTTTTCTACACAATTAGTAGCTACCAGTCGCGAGCACACATTCCA
TCGTGCATGCTATAATACTAGAGTAGGAAAGAAAAATATTTGTCCTGTTTGCAAAACAACAATTTCACCGT
CTAACCCAAGTGTGGTAGACGACGCCCTCGCAAAGCAACCGACGATAGTTACTATGCCAATTGGAACCCG
AAGTCAAGGCCCCCAGAAGAGATTTTGTCCCAAGGCACCATGTCGAAGAATCCTCGTCCGCCAAGTGTTC
CGCCTTTGGCAGCCGAGCCAGATGCATGCTCTCGTGTATCCCCCACGGTAAGCGAAATACAGCAGTCAAT
TTCTGATTCAATGGCAGCAGCCACAGAAAGACAAACAGCCTCCCTGACCCAAACTTTACAGTCAGGATTC
CAAAATCTTTTGTCTGCTTTTACAGCTCAACTAGGCAGCTCCACAGCTCAGCAACCAACTTCGAACATGAG
TATTGTCGATCATCCTCAGCCCAGACCGGCTAATTCTATACGAAACGAGTCTAGAACTAGGAGCTCTCCAA
TGTTAGAAAACTTGCGTCCAGACCGACTTAGCCAAATAATCAGTAACTGGAAACTTAGATTTTCTGGTCGA
TCGGGCATGGGTATAGATGAGTTTATCTATCGCGTCGAGGCGTTAACAGCCGAGACATTAGATGGAAATA
ATGAGTTGTTGGCTCGTCATATAAGCCATCTATTTGAAGGCAGCGCTAACGATTTCTTCTGGCGCTACCAT
AAGAGTGTCCCTATTATCNTATGGTCGGAATTATGTGTCGCTTTACGTGCTCAGGAGAAGTTCAAAGAAG
AGCGACCTGATGTTGACATACGTGCGGCAATTGATCGTCGTAAACAAAAAGAGGGTGAGTCTTTCGATGA
CTTTTACGATGCAATTATTATATTGACCGACAAACTGTCTCTACCCTTATCGAATTCCTCCTTGCTGGAATC
GTTGCGCTCTAATTTGCTGCCAGAAATTCAGCACGAGCTTCTTTATGAGCCCATACATTCTATCGCGCAAC
TTAGACATTTAGTGCGAAGGCGGGAAGGTTTTATTCAGTCAATTTCTAAAGCTCGAGTGTTTGCACCCCGC
CCTGCGCCTCGTCCCTCATTGCACGCTATAGAAGTACAAACTGAAAGCGAAGCAGAGTCCGATGCGATAG
ACCTTCTCGATAATGATGTATCAGCCATCAGTCTAGTCTGCTGGAACTGCAGGAAATCTGGCCATCGGTAT
CAAGATTGTGTCGCCGAACGCAAAGTGTTTTNCTATGGCTGTGGTCAGACAAACACTTACAAGAGCCAAC
TTGTACCAAGTGTAACAATGTGCTAAAAAACTACCTGTCTCGTGTACCATTGACCGGTACACGCAAACCA
GTTGCATCCAAGGCAACAAATACCGACTAGAGGGAGAATTAGAATCCTCTAATCAATCTTTACCTGACTT
NTCATTTAACCCAGAATTTACCTTATATACTCGTTTGCAATCTCAATCTGTTAATAAAAATACTTCTGTAAA
TCGCAAGATATTACGAGCGCGATTACGTCGAATAAATTTCTTTAAGCGATGTAAGATCGAGAAACAAGCT
CTCGAGTCAGCGATCGTGTGTACCGATTCAAGGCCTTACGCTGAAGTAACCGTATTGGGAAAAACTGTTA
CAGGATTATTGGATACCGGTGCGTCGANAAGTTGTTTTGGTGGTAGTTTCGCTGTGGAATTGTTGAATACA
AAAGTACTGTATAAACCGATTACTTCACAGGTGAAAACCGCCGATGGAAAAAACCCAACAAATATTGAA
ACGGTTACGCACAGAAATTTATTATGGTGGAAAGGTTAAACGAATTACTTTTTATATAGTCCCTTCTCTTA

TTCAACCGCTTTGTTTGGGCATAGACTTTTGGTCAACGTTTGAGCTGTTGCCTCCTTGCTTGTTAAGGCCAT
TGAGGATCCGCTGCGAGCTTTAACTGCACACCAACAGCAACGTCTTGCAGAAAGTGTGAAGCTATTTCCTT
CATTCGCGGAAAAAGAGTTAGGAAAAACCATCTAACTGTCTCATGTCATAGATGTAGGTAACGCAATTAC
TATCAAACAACGACACTATTCCGTATCTCCTGCAAGGGAGAAATTAATGTTCAAAGAAATTGATCGTATG
CTTTCCCTTGGCGTTATTGAACAATCGGAGAGTTCTTGGTCTTCCCCAGTAGTGCTCGTTTGTAAACCAGG
AAAGGTTCGTCTCTGTATTGATTGCAGAAAAGTTAACGAGGTTACTCGAAAGGATGCTTATCCGATGCCCC
TAATTGATGGAATTATTAGTCGTCTTCCTAAGGCTGAATATATTACCAGTCTCGACTTAAAAGACGCTTAT
TGGCAGATACCCTTAGAACCCAGTTCTCGTGACAAAACAGCGTTTGTAGTACCAGGTAGACCCCTTTACCA
ATACACGGTAATGCCATTTGGTTTGTCTAACGCCCCTGCTACGATGTCCAAATTGATGGACAGGATAATTC
CAGCTTACTTAAGAAATGAAGTGTTTATATATTTAGATGATCTTCTTATAGTGTCTGATACATTCGACCGTC
ATCTTGCCGTGTTAAGTGAACTAGCGGGAAAAATAAAGGATGCCGGGTTGACAATTAACCTTGAAAAAAA
GCAAGTTTTGTCTAAAGGAAGTTAATTATCATGGTCATGTTGTTGGTCAGGGTATTATTCGGATGAATGCT
GATAAGATAACTGCCATTACCGAATATCCAGCGCCTCGCTCGCTTAAACAATTGCGTCGCTTTCTCGGTAT
GACTGGGTGGTACCATAAGTTCATTCGAAATTACACTTCTATAGTTNCGCCATTGACTGACGCCCTGACAA
ATCGAAAAAAGTTTGAATGGTCAGAAGCGGCAGAGCAAGCTTTCCAGGCGCTTAAAACTGAAATGTGCA
AGGCCCCTGTGTGCATACACCTAACTTTGAAGCTTCTTCTACATACATTGTGATGCTAGTCACACTGGTGT
GGGTGGTGTTCTCGTCCAAATTAATGATACAGGTGACGAGGTCCCCATAGCATTTATGTCGCGGAAGTTG
AACAAGTGCCAACGTAATTATTCAGTTACGGAGAAGGAATGTCTAGCAGCAATTTTAAGCATCCGCAAGT
TTCGAGCTTACGTCGAAGGCCATCCGTTTACTGTTATTACCGACCACGCATCCCTTAAATGGCTCATGAAT
CAAGCAGATCTCAGTAGTCGTCTAGCTCGATGGGCTCTCAAGCTTCAAGGATATACATTTTCTATACAACA
TCGGAAGGGCAGTAAGAATATTGTACCAGATGCTTTATCCCGTACTCACACGGAGGATTTATCGGCTTTAG
ATGTCGATGACTTTATTGATATGCAGTCTCCATATTTTAAATCCCAGGGTTATGAGTTGTTGAAGTCTAAA
GTTTTGCAGAATCAGACTCAAATGCCGGACGTCAAAGTTATCGATGGCTTTATTTATCGAAGGTCCGAGCA
TGCCTCAGGAGACAGAGTTGCGGATAACATGGTTTGAAAATTATGGCTACCCAAAGAGTTGGTTCCCCAT
GCTTTGAAATTAGCTCATGATGATCCCTTAGCGTCGCATTGTGGAATGAATAAGACACTAGAGAGATTAC
GACGATATTTATTATTGGCCAAATTTAGTTTCAGACGTGAAAGAGTATATTAATAAATGTGAAATTTGCAG
AGCGACTAAACATCCGAATCGTGTTTTGAGACTTCCTATGGGAAAAAGTGGAGAAACTCATCGATTCTTTC
AAAAATTGTATGTTGATTATCTTGGACCGTATCCCCGTTCCAAGGCTGGTCATATTGGCATATTCATTGTG
CTCGACCATTTCACAAAGTTTCCTTTTTTGAAACCTGTTCGCAAATTGACCGCAGATGCTATTCTACCGTTT
TTGGAACAAGAGTTGTTTCACTGCTGTTCAATGGTGTTCAATTTAAATCACAAATGTTTAACGATTTACTTC
GAAAGTATTCGATACNCCACTCGGTTGATCCCGCTTGCTACTTCTAGAACGTACGAATAAGCAATAGAGT
CTTAACCAGCGCTCACAATGGACGTCGAACCGATTTCGGACAAGTTATAGCCCGTCGAAAATCGATTGAT
GTGTGAAATATATGACGAACTGCAGATTTGCGTTTCCGATCCCCAGACTTTTTTCTATTCAAATATTAGTTT
ATTTTCAAATCTTATTTGAGAAAACTAACCTCTGACCCAGAATCTAACAGAGTCCTACCCAACTGGTAGCA
AGCGGACAGATCCATGACACGAGTCCCCAGTGCTGCCAATATGACTGGACAATGTGGGCCATTCATTTCC
TCTTGTTTCTTCGGGATCGACCAGAAAACGACAGCTCAGCTAAAAGGTGCCTAAACGCCTGCTGAGAGCA
GTCTCGAAGTCGAACTATCAAAGAACGAAAAAAAGCCAATTGCCAAACTGCCTTTGGCTCCAAATTCCTC
TATATTAATAAGGTATTGACCAAATTTTCAAAAAAATCTGATAAAGTTTTTATGTTGGTCATCAATTTCATAT
AACTTTGCCAATATCAGTTTAAAATATCTTCTTGATACCTTGGTCCAGCCATGGTCATTAATTGTGTCGTTG
TGTTGATGCAATGACTCATTAAATCTGCTCTAGACGCATACGAAGGGTAAATTACGTCCGAAGGTACCAG
CAATATAACGAATCAAAATTCTCAGCCTATAGGGGATAAATATTGCATGTACAAACTTTTATAATACCCA
GAATATCAAACACATTTTTATGTGGGAAGTAGACAATTCGCTTTGTTAGTAATGACACCTTTTGCATAGTA
GAGCTTTCATCTTCTTGCAGGTATATTTAAATTAGTCCAATTGTGCAATTGTGACAGATAATTAAAAACTT
AAGAATGAAGCAATACACTGAAACAAATAGTTATGTATATTTTAGATTACCGTTGTTACATTNTCAGAAAT
AAAACCATAGATCTAAGCACATTGCAGTGTCACAAATAAACTATGAATTAGAAGAAATTCTGCACGACTA
CAGCGTCCTGAAATCTCATGTAGTTTTTCACATGGTATTTGGTACAGTGGGGATTTCTAGTTCAATTTGCTG
AATAAGTTTACTCGTTCATTTTCTGGCCGAGAACTAAATCTGTCATTAAGTTCACTCGTTCAGTTATTCAGT
CGGTTATGAACGATGAATTCTGCTCAGTAATTCAGTCGGTCATGAACTATTAGCTTAGTTCAGTTATTCAG
TCAATCATGAACGATTATATTTCTGTTCAGTTATTTTTCAAGTTTTCAGTTGCAAAATATATGTTATACTTT
TTACTTATTATAGTGTACCATTGATAATAGATACAAATCAAAAGTGAAATTAAAATCCCGCTATCTTTTAT
TTATTTATTTTATTTAGTACTGAACATCTCATAAGAATGTATCGTTCAGGAACGGCTGAACTGCTGAGTTG
CAT
>Ref_dvir_728
ACTGAAAACAACGACCGCCCAAATAGCTAAGTCAGCGTTCTCACGCTTGACCGTCGTTGTGTATACATAT

GTGCGCGCATGACTCTCTCTGACTCTCTTGCTGTATATAATAAGCCTAAAAACCATGTATGTAAAACGGCT
GCTCACTGCTTCACGGTGGACAGCTCGAACTCCAAAAAAAAACAGATAATAAAGAACCATCAAGAAAGA
CGGACGTCTTTTAATCTTGGAACCATTACATGGCGACCGTGACAGGACGTCACAAAAACATGAAAACGTT
AAGATACATAACTTCAACTCTAATTTTAAACCAGTCATAAAAGTAATTAAAGGAAGTAAATTAAAAGGAA
ATATCACTCAAAAACAACTGAAACAACGAAGCATCTGGAACGGCACACACCCTGGAGAATCAAAATTAA
CTGAGTGAGTACAGGGGGCATGAGCTCTAAAAATACTTTAAGCTAAAAAGGCGCAGTGGCATTAAAGAG
CGGATCAGGCAAAGAAAGTCTGAGATCGTAAGACTCTTATATAATCCCAAAACCCAATACCTTGGGAAAC
TGCAGTCAAGCTACATGACGAACTCAAACATCTTGAGTAGTAAAGCCCCCTTCCCTCAGAGGAAAATTAT
TAATACATCGACCACAGCCACAACCGATCCCGAGAAGGCAACGTGAATGCCCAGCAGACTGCGAGGGAC
CGCTTTACAGCCAACATTGCCTGGGCACATGTCAGCGGCCACCCACGAGTAAAAACTAGTACGAGAGGCA
AACCGCCCCTTGTACCAACGCTGGAACAAGAAGAAGTACGAGAGGCAGACCGCCCCTTGTACCAACGCTG
GAAAACACCAAGGGTGAACTGACCGACAGCGCCACAGAGGGCGTACAGCAAAAAAAAAAATTTATAATTA
TAATTATTATTAAAATATTTGTAAAACTATTGTAATGCCCGACGACAATTGAGTCGACCGGCAAGACGTTT
GGCCCGTAAAGCCATTCGAAATACTAGGCAGGATAAAATGTACATTCAGTAAAAACTGAATTAATATTAA
ATTTCTCCCCGCCCACGGGCGTAAATACAAAAAATTTTTAAATATAAACTCAAACTACAAACCTTATAAA
GTTAATGAATAGCGTTAACAAAACTATAAGTTCATATATAAATCAAGGACCCAAGTATATATTAAGAATA
ACAACCCCAAATATGGGTTTGGTAGACGTTAAGACTGTTGACTCGACGGCTAACGTAATAAATAAAGTTG
AGGTCAGCAACACAGATGTAAAATTGATAACAATTTTTGTCGTTATGATATTTGTCATATTAGTAGGACAT
CTATTGTACAATTATATACGTACCAGAGTCACGCAAATGACCTGGATAAAATCTAAATTTTTCTTGAACAA
AATAAATAAATTATTCTTATTACCAGAAATGCCTATTAATAAGTTTGCGATAGAAGTATATTTAGAAGCAA
TATATAATAGCGACGCAGACTTTAGAATTCGTGCATTGAATATTCTACAACAATAAGATAAAAGGCATAA
TTTTAAATACACTGCCATAAGTTGGCATTAAAGAAATAGTGGTCCAGTGGACGGCGAGTGCCCAATTATA
ATGGTAAAACTTCATAAATAGAGAACTATGGAATGGAGTGAATTAGCGAAAAAAATAAATAATTTCAAA
GATAAATTCGAAAAATCTTATAAGTGTGTTAATAAGAACGCACCTATAAGGGTTGAAACGCTAATTCACC
ACATAAACATATTAGTGCTAAACTATAATAACATAGTTCAACTTTATTTAAGTAAAGAAAATAGGCTTAC
AGAGCAGCATGTAAATCAATGTAAGAAAATAATCAAATCACTTGGGACTAGAATATCGAATATCAGCACT
AGGCACAACATAGTAATAGATATCCCAACAGATTTAAAATTAATGGCAACTTTTGAACCAGATCAACTAG
AAGAACTGAATGAGTCTAAGCCAACACTAAATAGTGACCCTGATATTGAAGCTGATAGCGACATTGAGCC
ACTTGAATTAACAGAACCAAACATGGCAGTAAATCAAGTGGCCTTAGACCGCGAATATGTAAGACAGGTA
TCGTCTACAATTCCGGAATTTGATGGCAAAAAGCTATCACTGGCCAGATTTCTAACGGCTTTAAGACTAGT
CGATCGTACTAAAGGTACACAAGAAGACAGCCGTAGAAGTCATAAAGTCTAAAATAATTGGCCCAATTTT
GTACAAAGTACAAAATGAAACATCCATTATGGTATTATAAATAAATTAAATACCAATATCAAAGGCGAGT
CAACTGACGTTATCAAAGCTAAGTTGCTTAACATAAAGCAGAAAGGCAAGTCAGCCTCGCAATATACAGC
TGAAATTGACAGTTTGCGTAAGCAACTGGAAGCAGCCTATATTGACGATGGACTAGATTTTGATAACGCA
GAGAAATTTTCTACTAAAGAAACAATATCTGCCATGACAAAGAATTGCGAATACGAAAAGCTTCGCATAA
TATTAGAAGCGGGCAATTTCAGCAATTTCAATGATGCTATGGGTAAATATATCCAATGCAGCACTGAAAT
GACTGGAAGCCCAAGTACAATATTGTACTACAAAGGCAGAAATGATCGTGGAAATTACAACCGTAATAAC
CAAAGAGGTAGAGGCAATGGCCGCGGTAATAGTAATTTCCACTATAATAACAATAGCAACAATAGGGGT
AATTTCAGAGGAAATTACCGAGGTAGCAACCGAGGAAGAAATAATTCAAACCGAGGTGGCTACCAAAAC
CAAAATAATAACAATAGAGGTAATGTCCGAGTAACTCAAGGTAATTCGGAAAACCAACAAGACCCCTCA
GATGTTCAGAGCCAATAAATGGCACAATAAAAACCATCAACCTTAATCTCAGCATATTTGTAAAAATTAA
CAATGAAAGAACAGGTAAATCCTTTACACTGTTGCTTGATACTGGTGCAGATATATCTATATTAAAAGAA
AATGTAGATATATTTGAAAACGTAAACGAAAGAGTTACTACCCAAAAATCGGGAATTGGCGAAGGAACA
ATTTTTTCAAAAGGCTTGGCCTTTATAGAACTCACTACAGGTAAATACATAATTCCGCATAACTTCCATTT
AGTCGATCAAAAATTCCCAATACCTAGTGATGGCATACTAGGCATAGATTTCATAAAACAATATAATTGC
CAATTAGATTTCACTCAGAGTGGCGACTCACTAATTCTTCGCCCAAACAACTTAAATTATCCGATTAAATT
ACAAATACTTCATACTTGTGACAATAATGCCACAGTACTCCCAGCTCGTTCCGAAGTTGTCCGTGAAATAC
AAATACCCTCAATAAAAAATCAAATTTTGATATTAAATCAAGAAATTGATGAAGGAATTTATATCGCAAA
TACCATATCCAACCACAAAAATACTTATGTCCGATTTAAATACAACAAATCCAATAAGATGTAATTTAAAT
AAAATAAAATATGAAACTTAATTAAAAAAACAACGAAGAAAGAGACAACTTCGTTATGAAAAAATTAAT
TAAAAACTTTCCACCTTTATTCAAATCAATTAAAAGATTTATGCACCAGATATACTGACATATTCGGACTA
GAAACCGAATCAATAACAACATAATAATTTTTACAAACAAAAACTTAGATTGAAGGATGATGAGCCCGTA
TACATAAAAAAATTATAGAAGTCCTCATAGTCAAGTAGAAGAAATTCAAAAACAAGTAGAAAAAATTAATA
GACGATAAAATAGTCGAACCGTCTGTATCAGAATATAACAGCCCACTTTTACTAGTTCCGAAGAAATCAC

TTCCAGATTCTGAAAAAAAGAAATGGCGATTAGTAATTGACTATCGTCAAATTAATAAAAAACTATTGTC
TGATAAATTTCCACTCCCCAGAATTGAATGATATTCTAGATCAACTAGGCAGAGCAAAATATTTTTCATGC
CTTGACTTAATGTCAGGTTTTCACCAAATTGAACTTGAAGAAAGTTCAAGAAATATAACGTCTTTTTCAAC
GAGCAATGGCTCATATCGCTTCACGCGATTACCCATTTGGTTTAAAAATAGCACCAAATTCATTTCAGAGA
ATGATGACTATAGCATTTTCCGGGTTTAGAACCCTCTCAAGCATTCCTTTATATGGATGACTTAATAGTGA
TAGGTTGTTCCGGAAAAACACATGATTAAAAACTTAACTGATGTTTTTGATATATGTAGGAAAAACAAAC
CTTAAATGCTTACATCCATGAAAAATGTTCATTTTTTCATGCATGAAGTTGACATTTTTTAGGTCACAAATG
CACTGATAAAGGAATATTGCCAGATGACAAAAAATATGACGTCATCAAAAATTATCCAGTCCCACATGAT
GCGGACAGCGCGTAGACGATTCGTTGCATTCTGCAATTATTATAGACGTTTTATAAAAAACTTTGCCGATT
ATTCACGGCACATAACAAGATTATGTAAAAAAAGAATGTTCATTTTGAATGGACAGATGAATGCGCCCAA
AACGCATTCCAATATCTCAAAAAAAAACTTATGAACCCCACATTTATTACAATATCCAGATTTTAGCAAAG
AATTTTGCATAATAACAGATGCAAGTAAACAAGCTTGTGGAACGTTGCAGTTTTAACTCAAAACCATAAT
GGACTCCCCAACTTCCCAATTGCATACGCATCACGTTCATTTACAAAAGGAGAAAGCAATAAGAGCACAA
CAGAACAAGAATTAGCAGCAATTCATTGGGCAATAACCCATTTTAGACCATATATTTATGGCAAACACTTT
ACTATTAAAACAGATCACAGACCATTGACATATCTTTTTTCTATGACTAATCCCAGTTCAAAATTAACTCG
CATGCGGCTAGAGCTTGAAGAATACGACTTCACAGTAGAATACCTAAAGGGAAAAGATAATTTCGTAGCA
GATGCACTTTCGCGTATTAATATAAAAGAACTCAAAGATATGCAACATAAAGTCCTGAAAGTCACTACCA
GGCGACAAAGTAGACAAAATAAAAACTGTACAGTAGAAAATTATGAACATCTGCCAAGGCAAAGTCATA
AAAAAGTTTCTAAGCCCAACGTACATGAAGTCATTACAAATGATGAAGTACGAAAAGTAGTGACCTTGCA
CATAACAGATACTATATGTTTATTTAAACATGGAAAAAAAATTATCGCAAGAATTGAAGTTAGCGATTTA
TATACTAATGGAACCCTTGACTTAGGTCAATTCTTCCAAAGGCTTGAAACAGAAGCCGGTATATATAAAA
TCAGCCAACTTCAAAGTGGCACCGTGGGAAAATATCTTTTGAACACGTTTCAATAGATAATTTCAAAAAT
ATGGGCAATAAAATATTAAAATCTTTAAGAAGAGTAGCGCTACTCCACCCGGTGACCCACATAAAATATA
AAAAATAATAAAAAAAAAAAAAAAAAAAAGAAGCTATACTATCTATATTGTCTACACGACATGATAATAA
CAGTACACAAGGAGGTCACAAAATACCTTGGCCAAAGTCAAAAGACATTACTATTGGAAAAATATGACA
CGTCATATAACTGAGTACATACGTAAATGTCAAAAATGCCAAAAATCTAAAATAACAAAGCACACAAAG
ACTCCTTTAATTATAACTGATACACCTATACATGCTTTCGACAGGGTGATAGTGGACACTCCGCTACCCAA
ATCTGAAAATGGTAATGAATACGCAGTCACATTAATTTGTGAATAAACTA
>160_rnd-1_family-150__DNA
TTTATATGTNGGTCAGTCGAGATTAAATGTNAAATGTATCTCTTGTCATGTCCCTCTCCACCTTGCAGACTG
GTNAACGTTGCATAGTTTTAAGTGCTCCCAAAACTGTTCTAAAATCTCTCTGTGGCGGTCGAGGNGAGCGG
GCGTGTTTGCGGACAGCGCCCGGTCGGCCAATCAGACGAACAACTAAAAATGCCACCTTGTGAAAGTACA
TAAGTAAAAGTACACATGACGATGAAACATGTTATTTACATATATGTATGTTTATAAGTTAAACGTGTTAT
TTATATAAAATNAATACTTTAAATAGTTGTTAAAACATTCTACAATTTGTATTATTATCTGTGTATATATAA
ATCTATATACTTCGTTTTGAACACTCCCGTTCTCATTGCCTGCGTTGGCTGTACTGACGAATTCAACTTTTC
ACCGCACGCGAGTTGAATTCGTCCCACTGTACAGCCTCTGTCGCGAGCAATGAGAACGGGAGTGTTCAAA
ACGCACGCGAGTTGAATTCGTCCCACTGTACAGCCTCTGTCGCGACGCCTTAACGGCCCTTCGACGCCGAC
GTCGGCTGCGGCGCGCATCTGTGCACACGTATGCAAGCGTGGTCTCACTGTCGCCCGTCTGGCGCGCGCG
CGCTGTACTTTTTTGATCGCCGCTATATTCCCGATGTTCACTCTGTATGTGCGTCAACTTAGCGGCGGTGAC
TTAGCGTCGATTTTTGGTACTTGGTATCNGAAAAGGAATCGGTCACTTTTAAATGCATTTATTTATGTTTTT
AAGCACAGAACCTAATAAATTTAAATGCAGAATTGCAAAAAATTAAATTATTTACTGCCTACATTCATTTG
TTTTTGAATTTTAATTTATAATATGGGTAAACCAAGGGGTTNAAATATTAGGTTTCCACCATTCCATAAAT
ATCCAAATTTAGGGATGATGGCCCCGAAAACCGCATATACACGATTAAAGCCTATAGTTTCACCTAGNCA
ACTGTGAAAATTTGATTAAAATCGTCGGTACCATTTTTAAGATATTTGGAAAAGAGTGCAACAACCCCCAT
GTCAGCCCCATATAAAATGACCGCAAAAAATCAACCTTCTTATTTCTTAACTCAGAGACAATGGCCCTCAA
TGCACGCTATACCCTCTTGTAGATACATATTTTAAGAAGCTTTAGGCACTAAATTTAAGTAAATGGACGGT
ACAGATTTTCAGAAAATTAATTAATTGTAAACCAACCTCATATAGTTCATTCATAAAGTCTATCGAACTGC
TATTTCCTACGTGAACTGAATCACTGCCTGGCATCAGTGTTGAACAATTTTGTACAGAATTGGGCGGGAAC
CAATCCAATTTGAATAATATGTTATCATGAAAATTACTTATTGTTAACAATAAGTTTATAGAGCGAAATTA
GACAGATTTTCAACTGGCAGATAATGTTAAGAATCGAAAAATTATTACGAAAAGTTTTAAGTTCGTAAGA
AATATTAGAGCTTTAATATTTTGTATAACCCTTATTGGTGAGTTGTTTTACCCGTTTCAGGCTGCCTACTCA
AAAGAACGCCAGGACGTATCTAGTGGTAGTAATCATGTTAGCAGTAAACTAGAAATTAGCAATAATATTA
GTGATATGTCTAGCATAGAAAATAATAATTCTAATTATAGTCTTAATGAGCCTACAAATTGTCCGTCTGAG
TTTAATAATGACGCATGCTTTGTCGAAGCGACATTAAAATGCTTTCGGAAAAAAATACCACAATTTTCNTGA

TAGCTTAAACAGAGAAGTTCAGTTAGTTAGTAATTTGAAAGGATGCCGCGCAGCACTTTCAGGTTACAAC
AATTTGGATACTGAAATGAGTATCCAAGAATTTATGGCCACGTTAAATCAATTGTGTGCGATTTAAAAAA
AAAAAAAACATTGAACACATTATTCGAGAAGCATACACTCTTCATAGCAATGCATGTGTTTTGCACTTTAA
GTCAATTAGACTTAACACAAACTCTTTTGCTGCATATGGTTACTGCAAAGATCCATCATGCAACTACTTCT
TGTTCCATGGCAGCCAACATATCTCCCATACAATTGGCCTTAAAAAGTCAACGCAATGCAGAACAATAAA
TAGATTTATAGAAAAGGCTTCCATTAGAAACTCTTCTGCGTTCGAGTATAGATCAAAAAAAAAAAATAAC
AAAATCAATATGTCTCGAGCCAGAGTTGGTAATGATCAGATTACTAATGAGAAACTACCCTTTTATAGAA
ATTTAATTTCGGAGGAAAATTGACTTTGACAACTCACGGATAAATCGTCCTTGCTTTAAGCCGTTTTTATT
ATTTGTCTTTATAAAACTATATTTAAGCGTTTNAAAGAATGATTCTATAGGCCCATTACTGGGTCTTTTATT
AGATTTATCAATAAAAGTAATGATAATATTGGTTCAAAAAGGTGCGTAAACAATATATTTATTTAAAATTA
GATCCAGCATTTCTGCACTTAAATAAATATTTGACGTACAGAAGTTGCTTTTATATTGTGATACAAGTTCT
GTAACTTCTGTACGTATTTCTTGAAGATTAAGTTTAAATGGACTATTTTCGGCAATGGACAGCAACGTTGG
ATCATTGACGACTTCCTTACTTACTCCCAATTTTATACTGTCTATCAAATAAAATGTGGATGGCTCTTCACA
ATATTTAGTAAAATAATTAGCGGCATCATCCACAATATTATTAACATATGGAGAAAGAAGTATTATTGCA
ATATTTCGGAACCATTTTTCTAATTTTCCAATATTATTTATGGAATATGCACAACCCAAGGCATTTCTTAAA
AATGATTTAAGATCAATATTGTCGAAANCAACTACTTCATCTATGTTGCGACACTTAATTTTGCTGTAGTG
GCTCATACAAAACTGAACCCACACTTTTGGAAATGTGCGATTTAAATTATACAAAAAACTAAAACAAATT
TCATTGTACGTTGCAAGATCAATGTTGTTATATTCTTTTAAAATGGCTCCAATTATTGCAAAACTGACATC
AGTTATAATTCTTTTGCAAATAGGCCATTTGAATTTATTTTTTATGCAAAACTCGCGCAGCTCACATAGAA
ATCTGCCTATATCTGACGATTTATGATTGGAGAGAATCGCATGTGCGACAGGAAAAACGATTCGAAGCTC
TGGAATATAAGCAACCAATGAATATAAAAATGTACATTTTTTGATCAAGTTCATTGGCTGTACTACATTCC
CAGTCGCATCAATATGCAGTGTCTGTCCCATAAAAATATTTCTAGTTTCATAGAAAGTAACTGAATAAAGT
TGGCATACACATTAGTAATCATATCGAAGGTATCTTTAAATGGATCTTTGTCAAAATCAAATTTTCCATTTT
CCTTCGAAATTAAATTTCTATAAAAGGGTAGTTTCTCATTAGTAATCTGATCATTACCAACTCTGGCTCGA
GACATATTGATTTTTTTATTTTTCTTTTTTGATCTATATTCAAACGCAGAAGAGTTTCTAATGGAAGCCTTT
TCTATAAATCTATTTTTCGTTCTGCACCACGTTGACTTTTTAAGGCCAATTGTATGGGAGATATGTTGGCTG
CTTGTGTATACACTTACAACACCGTCAAATGTCCCATGGAACAAAACGTAATTGCATGACGGATCTTTGCA
GTAACCATATGCAGCAAAAGAATTTTTGTTAAATCTAATTGACTTAAAATACAAAATACATGCGTTGTTAT
GAGAAGTGTATGCTTCTCGAATANTGTGATCAATGCTTTTTTTTTTTAAATCACACACAATTGATTTAACGT
GGCCATAAATTCTTGGATACTCATTTCAGTATCCAAATTGTTGTAACCTGAAAGTGCTGCGCGGCATCCTT
TCAAATTACTAACTAACTGAACTTCTCTGTTTAAGCTATCATGAAAATTGTGGTATTTTTTCCGAAAGCATT
TTAATGTCGCTTCGACAAAGCATGCGTCATTATTAAACTCAGACGGACAATTTGTAGGCTCAATAAGACTA
TAATTAGAATTATTATTTTCTATGCTAGACATATGACTAATATTATTGCTAATTTCTAGTTTACTGCTTACA
TGATTACTACCACTAGATACGTCCTGGCGTTCTTTTGAGTAGGCAGCCTGAAAGGGGTAAAACAACTCAC
CAATTAGGGTTATAAAAAATATTAAAGCTTTATGATTACTTACGCATTTAAAACTTTTCGTAATAATTNTT
CGATTCTTAACATTATCTGCCAGTTGAAAATCTGTCTAATTTCGCTCTATAAACTTATTGTTAACAATAAGT
AATATAGATTGAAAATCAGTTGGACTTAAGTTTCCAACTGGAGATATTATGCTTCTAGCAATGGCATCACG
AAATCCAAGAGAGATTGTCCTTAAAAAATTGTTTATATATTAATGGTAAAATTAAATCGAATAAGTTAAG
CAACAATTTTATTATCTACCCTTGCAGAGGGTATTATAATTTTGTCGTGAAATGTGTAACGCATAGAAGGA
GACATCTCCGACCCCATAAAGTATATATATTCTTAATCAGCATCAACAGCCGAGTCGATATAGCCATGTCC
GTCTGTCCGTTTGTCTATTTCTATGCGAACTAGTCCCTCAGTTTTAAAGCTCTCTTAATGAAACTTTGCAGA
ACTCCCTCTTTCTGTTGCACGCAGCATATATGTGAAAACCAGCTGGATCGGACCACTATATCATATAGCTG
CCATAGGAACGATCGGTCGAAAATTAAGTTTTTGTATGAAAAAACATTTTGTTTATCAAGATATCTTGACC
AAACTCGGCATTTATTAGTGTTACTTTACTCCTCATATATATTCANAATCCTATTAAGATCGGACCACTATA
TCATATAGCTGCCATGGGAACGATCGGTCGAAAATTAAGTTTTTGTATGAAAAAACATTTTGTTTTTCAAG
ATATCTTGACCAAACTCGGCATTTATTAGTTTTACTTTACTCCTCATATATGTGCAAAATCCTATAAAGATC
GGACCACTATATCATATAGCTGCCATAGGAACGATCGGTCGAAAATTAAGTTTTTGTATGAAAAAACATT
TTGTTTATCAAGATATCTTGACCAAACTCGGCATTTATTAGTTTTACTTTACTCCTCATATATATGCAAAAT
CCTATTAAGATCGGACCACTATATCATATNGCTGCCATAGGANCGATCGGTCGAAAATTAAGTTTTTGTAT
GAAAAAACATTTTGTTTTTCAAGATATCTTGACCAAACTNGGCATTTATTAGTTTTACTTTACTCCTCATAT
ATGTGCAAAATCCTATAAAGATCGGACCACTATATCATATAGCTGCCATAGGAACGATCGGTCGAAAATT
AAGTTTTTGTATGAAAAAACATTTTGTTTTTCAAGATATCTTGACCAAACTCGGCATTTATTAGTTTTACTT
TACTCCTCATATATGTGCAAAATCCTATAAAGATCGGACCACTATATCATATAGCTGCCATAGGAACGATC
GGTCGAAAATTAAGTTTTTGTATGAAAAAACATTTTGTTTATCAAGATATCTTGACCAAACTCGGCATTTA

TTAGTTTCACTTTACTCCTCATATATGTGCAAAGCCCTATGAAGATCGGACCACTATATCATATAGCTGTC
ATAGGAACGATCGGTCGAAAATTAAGTTTTTGTATGAAAAAACATTTTGTTTATCAAGATATCTTGACCAA
ACTCGGCATTTACTA
>160_rnd-1_family-147__LINE___R1
ACACACACACACACACACACACACACACACACACACACACACACACGGACGACGCGAGATTGTTTTCATAG
AGTGCGGACGCGATTTGTAACTGCTCCGTGAAAATAAACAAATAAACCTGTGAAATTAAAGTGAAGTTAG
TGCACGTGTGCAGGGAACCGAGACAGGCCTAAGACACCAAAAGGTGCGTGGTGGTACTCACTGCGAAGC
CCGTGCAGACCATTCCTGGGGTGGCGTCTACAGTTCCACGATCCAGGCGAGGGGAGTGGAGGCGTCTACT
TGGCGTGTTTGTGCGCTTTTGTGAGTTTTACCACTCAATNGTATTAGCCGTCTCGCAAAGCTATTTGTGCAG
TTGGTTGCATTCACTCTAACAGCTGTTAGTAGTGTTGGCTAGCGCAGTTCACAACCAGCTGATCTGCACTA
TAGAGCCAGCTGCCAAACTGCGTAGCGGAGATACCAACCAGGCGATAAGCTGTAACTGGCAGAAAGTAC
AGCATACTTTCGGGCTTACGCTGTTCTTGCTAAGCATTTAAGCTTTGGAGAAAGAACAGGCAAAGAAGCC
GATATCGGTGCTCACTACAGGCTCCAGGGCAAAAAAGGAGCAAAAGGTCGCGGAACAGACCAATTTCCA
ACCCCCCCGCCGCGGCGGTGCCCATAAAAACGGGGCTCTGGTGACCGAGTCGGAGCGGTATAACTCCCAG
CGGTTAATAGCCAGAGGGAAACCGTTGGCCACCGAGGGACTTTCCTGTAGAGCCCTGATAGTTAGAGCAG
GTGCCCAGAGTAAAAAAATGGAGGATGAGCTGCATGCCTTTGCGCGCCGCTCTGCACTACAGCGATCACCG
TCACACCAGGCGGTCACGCCAGTGCAGCAAAGAGTGGCTGCGTCGTCGCCGGCAAAAGCGAGTGAGGAG
CNGCCAAGAACGCTAAGAGGCACGATTGAGAAGATGGGCTTAGTCCTAAACGGCTTAACGGCGCTGTTCA
ACGGACAACGCCACATTAACGGGCTGATGCGAGAGGAACTCGCGAGAGTAGAAAGCCTGCAGAAGGTGG
CGCTCAAGCTCTGTGCGCCGGAAACGAAGTGCGACGAGGCGCAAACGACGCCGTCATTAGCCAGACGTG
CGGAGCCGAACGTGCTAAAGACGCGCCCAACGAAGCTCGGGGGCCAAAAAGAGGCAAGCACGCCCAAAC
GGCCGAGGGATGGCGTGCCAGAGCAGCGCAGAACCCCCTCCAAGAAGGCTAAACGCCAGGGTCAAAACC
CAGCGAAGCAGGCGCTGCTCACGCATCAAAAGACAGCCCCTGCAAACTCCAACTTAGCTGGCCATAATGG
CAACCCAGAGAGCTGGACAGCAGTGAACGGACGTAAGCGAACGCAGAAGCGTCCCAGAGCCGACGCCAT
TACAGTCCACGGAGTGGGTGACTGCACATATGCCGATATGCTCAGGCTCGTGAAGAGCGACCCGGCCCTC
AAGCACCTAAGTGGCGATGTGCATGGCATTCGCAAGACCGCGAAGGGCGATCTTCTGCTGCGCCTCAATA
GGAAGCCGGAGCACTCCGCAGATGAGCTTCAAGAAGCTGTGGGAAAGGCATTGGGTGACCGGGCGGTAG
TAAGAACGCTGACCGAAACGTCCCAGGTGGAGATCAAGGACCTGGATGAGCTGGCGACTAAGGAGGAAG
TGCTCGAAGCGATTAAAAGTGCCTTACAAGACGACTCCTTGCCGATTGAAACGATCAAATCGCTGCGACC
AGCATTTGGCGGCCAGCAAAGGGCCACAGTTACTCTCCCAGCGCCAAAAGCCAGGACGCTTGTCGAGAAG
GCCAAGGTGAGGATCGGATGGGTTGTCTGCCGTATTCGTGCAATAGAGCAACCAAAGCGATGCTTTAAAT
GCCAGGGTTTTGGGCACATTGCAGCGAACTGCACCAGCGGAGACGATATGAAAGGCGCATGCTTCAGATG
CGGGGACACAGGTCACGAAGCAAAGAGCTGCTCCAAGCCACCAAAATGTAGCCTTTGCATACGGAAAGG
AGAAAAATCAACGGACCACGCTACAGGAAGCTTTAAATGTCCGGCGTATCGCGAGGCAGTACGCAAAAT
GAAATGTTAAGGTTACTCCAAATAAACCTTAACCACTGCATAGCTGCTCAAGCCATCTTGGAGCAGACTG
TTAGGGAGATGCGCATTGATATTGCCATTATCAGCGAACCCCACCACCGAGAAACCAACTGCCCGAGAAT
ATGGACCGAGGATCAGGATGGAAAAGCTGCGCTCTGGGTATGTGGCGACCCATCGACATCGCTGACAAA
AACAAAGTCCTTCACTGGGTTTGTACGCGCACAATACGGCAGCCTGTGGATATACAGCTGCTACCTGGCC
CCAAGCATGCAGCTCTGCGAATTTACANCTGCATTAGATGCATTAGTAGATGACGCAAGGGGGCGGAAGG
TCATTATTGCTGGTGACTTCAACGCCTGGGCAGTGGAATGGGGCAGCCAAGCAACTAACGCAAGAGGTCG
TGCCCTGTTGGAAAGCTTCGCAGCCCTAGATATAGCCCTGCTAAACTTCGGCTCGGAAAATACCTTCACTC
GAGGTGGTATCGGATCCATAATCGATCTTACCTTCGCAAGCTCGTCACTTTTTAGCGCAACAAAATGGCAA
CTAAGCGACTTATACACCGCAAGCGACCACTCCGCAATAATATGCACGATTGGTGAAGGAACTGCAGCCA
GCACTAGAAGCCTAAGTCGGAAAGCTTACCGCCTGGATACTTTCGACCCCGTGGCCTTTTCGAATGCAGTC
CACTCGCTTGTCACTGCCGGCAACGCAGAGAGAAGAGCCTCCCAAATGGCATGCCAGCTATCGCGTGCCT
GTGACGAGAGCATGCAAACCCGGAAAACGTTTCGGCGCCACCATGTACCCACGTACTGGTGGAACGAAA
ATATTGCCGAAGCACGGCGGAACTGCATCCAAGCCAGGAGGAGATACCAGCGAGCCCGAGGTAGACCCA
ACTATGCCGAACAGCAGCTAAGGTATCAGAGTGCCAGAAAAATGCTAAAAAAGTCCATAAGAGATAGCA
AACGAGAATGTTTCCTGGAGCTATGCGACTCAGCCGAATATGATCCTTGGGGCAAGGCGTACAAAACTGT
CGTTAAAAGACTCTACGCCGGCAAACAAGCGCAGCCTTCTGACCCAAGTGAGCTGAAGTTGATTGTGGAA
ACGCTATTCCCAATCAGACCCGACGTATTACACACGCAAGCCATACCATCGAGCCACGTAGCAGCCATGC
CGTCTCTCATCGACGAAGTCTCAGTCGAAGAGGTGCTGACGATCGCCAGAAGCCTAAAGCCCAGTAAGGC
ACCGGGTCCGGACGGAATCCCCAACCGAGCACTGAAACTAGCCATGTCCTTGCGGCCCGCGCAGTTCGTG
CAGCTTTTTAACATGTGCCTAATGGAGAGAACTTTCCCCACAATATGGAAACTCCAAACGCTAGTTTTATT

ACCGAAGCCTGGTAAGCCGCCCGACGACCCATCGTCATACAGGCCGATTTGCCTCCTGGACACGTTAGGC
AAAGTGCTGGAGAAGCTAATCTGCAACAGGCTTCAGGTAGCTGTCGACAACAATGGTGGCCTGTCCCAAA
GCCAATTTGGTTTCACCAAGGCAAAGTCTACAATAGACGCTCTGGAGAAAGTGGTCGGCATAGCAACAAA
CGCCATCCAAGGATCGAGATGGAAAAGCGGCACAAAAAAGTACTGCCTCATCGTCACGCTGGATGTTAAA
AACGCCTTTAACACAGCCAGGTGGGACAAAATATTGGATGCATTGTCAACGTTCCGCGTCCCATATTATCT
TCAGGAGACGATACGCAGCTACTTCACTGGAAGACTGCTGCAATACGACACCGCAGACGGCCCCACTACA
CATCAAGTGTCCGCTGGAGTGCCCCAAGGCTCAGTTCTAGGACCCCTCCTCTGGAACATCATGTACGATGG
CATCCTCCGACTGGAACTCCCGATCGATACAAGTATCATCGGCTTCGCGGACGACGTGGCCCTTGTAGTCG
TCGGCAAGGAATTGAAGGACGTAGAGGAATCGTGCAACCACAGTATTGACCGTGTGCACCAATGGCTGGC
AGCAGCTGGACTCGAGCTGGCTGCTCACAAAACAGAAGCGGTGTTAGTCAGCAGTCGAAAAAGAGTGGA
AACAGCGCACATAAGAGTCGGCAGTGCGACAATAGAATCAGCAAGGGCGCTAAAGTACCTGGGTGTCAT
GCTAGACACAAGAATGTCGTTCAAAAGAGCACTTGGAGTACGTGCACAAGAAAGCAGCAGAGTCCTGTC
GAGCGCTATCCAGGATTATGCTGAACACACGGGGACCGAAACAGCAGAGACGCCGTCTCCTTATGACCGT
AAACCGAGCGGTAATAATGTACGCGTCCCCCATCTGGAGCAGAGCTATGGCTGTTCCCAGCTACAGCCGG
GGCGTACACTCTATATATCGCCTAAGCGCTCTACGGGTCAGCTGCGCATTCCGGACGGTATCGGACGAAG
CGGCGCTGGTTATCGCAGGGATGGTCCCGCTAAGCGAACTGGCAACAGAAGCTGTAGTTTGCTATAGAGC
CCACGATGGAAGCAACACAGCAAACTCGATCCGCACAGCGGCAAGGATGCAAAGCGTGGAACGATGGCA
AGCACGTTGGGATCGCTCATCTAAAGGACGCTGGACGCATAGGCTTATCCCAAACTTGGAAGCCTGGATA
AACCGCAAGTATGGNAATACTAATTTTTACTTAACGCAGCTCCTATCTGGCCACGGATGCTTCAGGAGCTA
CTTGAAGCGATTCGGCCACGATAACACAGACGAGTGTTCTTGGTGCGGCAGTGGAATCGCAGAAAGGCTC
CTCACTGTAGGAGGGAAAAACCAGCAGTGCGGCATATCAACCTAGATGTGAAAGATGATATTGCCAGGGT
GAAGGTGATACAGATGGCAGCAACTGCTAAACTACCAAGTGAGCCAACTCCACTAGGAAGCCGACGCCA
GCAGATCCGAGAAAACCACACCGGCTAAATGTAGATAAACTCAGGATCACAGAAAAAAATCAGCTCACA
AAAAACAACAACAGAGGGCCTGGAAATCACAAATGTTTAAAAGGCGGTCCCAAGAGGGGTTGAAGCCTT
ACCCGAGCTAAGGAAAGCATATCGCAGAACACAGACGGCTACCATGCTTCTACCCAAGATCGCAGCAAA
CGAACTTCTGAAGAAAGCGAAGAAACGCATTGGCTGGGTTGTGTACCGTATATGCCAAAAATTGGAACCA
AGAAGATGTTTCCAGTGCCTGGACCTTGGGCACACGGCTGCGCGATGCATTAAAGGTTTGCATATGCAAG
CCAATGCATAATCTGCACAAGGCACGAGACTGCGAACACGGCAAACAACACCCTCAGCCATGCCTGCTCA
TGGTTAATCAAGGCGTCTCAGGCAACAATCAAAAATTAAGTTAATACAAATTAACCTGAACTATTGCAGA
GCGGTCCAGGACCTACTCGCTCAAACTACACGGGAAGAGAACGCTTACTTCGCCATCGTCAGTAAACCAC
ACCAGTCAAACATGATGGCATCTGGATTCAGAGCAACGGCGGCCAAGTATTGCGAAATATGTATATATAT
ATATATATATATAGTTGCTATATTGCACCCAGCATAGGAATTACCGCGTTCGCCGATATACTG
>9_rnd-4_family-255__LTR___Pao
CTTACATCTCCTTTCCCGTTGCCGTTGCTTCGTCGCTGGAAACTCGCTACCTTCGCTTCGCTGCTGCATTCT
CGTTGCCGTTGCTTCGTCGCTGGAAGCTCACGCTCTGNNTCTGNTTGTTCACTGCNTTTTGCTTCATCACTG
CAAGCGACCCTCACATCGGTGAGCGCAACATTTACAAGTCTTAGCTGTTCTGTTAACAGTGGGAGCATTTG
AACTGTCGCTCGCTCACATTGGTGAGAGTGACATTATCAGGTTAAAGTTAGGCTGTGAAAATTTGGCAGT
ATCAAANCTGTCNCTCGGTCACATCGGTGATCACAACATTCTCGATACGTAAGCGAGCGATGTCTACTGA
GGTCGATGGCAGTGAAAAAAGATTATCACACGGTGAGCTACAATTACGCTTGGACATGTTGGAGATATAC
ATTAAGGAGGTTTTGGAAGTACAATCTGAAATTGAATTGGAAGATCCTCAGGACAATGGACGTGCAGAGG
TGGAAGACACATATGTGAGTATCAAATTGTCCATCCAATCCCAACTGGATGATGTACTGCACAGCACCTC
ATTTTCTAACCCTGCTCCAATTCGCTCTTCGCCGCCATCGAGTCTTCCTCGATTGGCTCCGCCTGTATTTGA
TGGGAAACGAGCAGAGTTTAAAAACTTCATTACATCATTCAATCAGATTATCGGTCGTGAGTACTCTCTTT
CGAACATTGAAAAGTTTAATCATCTGCTGAATTGCTTGAAGGGCCCTGCCCTACAGGCAGTAAAGGCATT
CCAAGTGACTCAGGAAAATTATCCCAAGGCTCTTGCTCGTTTAAGTGAACGTTATGACAATCCTACACTTA
CATTTTTAGACACAATAGAATCACTATTCAATCTACAGCCAGTCGACAAACCGGACGGAAATCAGCTGCG
CAGTCTGCTGGACAATGCGTCAGCCCTGTATTCATCATTATCGTCATTGGGTACCGGATCAGATATTGCCC
AAGCCATGTTGATATTCATCGTCATCGGAAAATGTGACCAGCAAACTCGTAACAAGTGGAATGAGTCTCT
TGACTTACGTACATTGCCAAATTGGGCTCACTGCACAACTATTTTGGAACGTCATTGTCAGTTTCTGCAAT
CAAGCGGCATTGCATCGTATCGAATGGCTGATTCTTCGAAACAAAAACACGTGTACAAGCCTAGCAAGGG
ATTATTCATTTGCTATTACTAATCCAACATGCATTGTTTGTTCCAGCTCCGACCATAAAATTGTACAATGTT
CTCAGTTTCAAGACATGAGTCCTAAACTCAAGTACGGAGTCGTAAAAAATGCTGGACTTTGCATTAATTGC
CTTCAATTAGGACATCGGATGGCTCAGTGTCCGTCTAACAATCGTTGTCGAAGCTGCAAGCATCGCCATCA
CACGCTTCTGCATTTTGGCCAACCGCCTGCATCATCATCGTCTACATCGCCATCATCCATAGCGCAAATGT

CCTCAACAACGACTCCTGCTGCAACGCACTCGCATATGAATCAATCGCCAGGCGGACAAATAATTCTTGC
TACTGCAAAGGTACTAGTGCGAGATTCATCAGGGGCGTATAAACAAGGCAGAGCATTGCTTGACTCATGT
TCCCAAGTCAATTTCATAACTGATCAATTCGCCCAACGGCTGCATCTACGCCGTGATAAGCATCATTTCGA
AATCCGCAGCATAGGGGAGACCTCGACTAAAATTAACAACTGTACAACTACTGGAATTAAATCTCGTTTG
TCACCATTTGAAGTAACACTACAGTTCTGCATAACGTCTCACATTGCCTACCAGCCGGATTCGGAAATCGA
CACCTCAGATTGGAGGTTGCCCGCGAATACACCCTTGGTCAGGTCGGATTGATCTCCTTCTCGGTACCGAG
GCATTTTTCGAATGCCTGGCTGTTGGACAGATTAAGCTCGGAGACAATTTGCCAGTCCTTCAGAAAACGTT
GCTTGGATGGGTTGTGTCTGGCAAGTATCGTCATCAGTCAGCTAAGCAGTCATCAACGTGCTTGCTTTCTG
TGGAGGAGTCAATTGACAAAAATCTTCAATTACTGTGGAGCATTGATGCTCTAGAAAATTCTCCAAAGTC
AATCAATCCAGAACATCGGCTCTGTGAAGATATGTATTCAAGGACAACACAGATGAATAACGGCAGGATT
GTTGTGCGTCTACCTTTCAAGGGAGATCCAATGTGCCTTGGCAATTCAAGCGATGTTGCACGTCGTCGTTT
TTATCGCTCGAGAGGCGCCTCAGTCAGCAGCCTACTGTTCAATCGCAATATGTAACCTTCATGAAAGAATA
TGAGGATCTTGGCCATATGACTTTAGTGACGAATCCGAGATTAAGTGAGCCACACTTTTATATACCTCACC
ATTGTGTGTTTTTAAACCCAGCAGTGCATCAACGAAGTTACGAGTCGTGTTTGACGCCTCGTGCACTTCAT
CGTCCCAGCATTCATTGAATGATTTGCTTCTCGTTGGCCCAACACTCCAAGAAGATCTGTATATACTTCTCC
TTCGTTTTCGATTGGTTCGTTACGCCATCACAGCGGACATTACGAAAATGTATAGACAAGTTTTAGTCAGC
CCAGTGGATCGGAAATTTCAATATATATTTTGGAGAGCATCCCCTGATGCTGATTTGCAGACCTTTGAGCT
AAATACAGTGACGTATGGAACGGCCTCGGCCCCATACTTAGCAGTTCGCAGTTTGCAATATCTGGCAGAT
CTGCATGTGAACGAGTTTCCCATTGGCGCATCCATTGTAAAATCATCGTTTTATGTTGATGATCTGCTATGC
GGAGCAAACGAGCTCATCGAGTTGCAGAACATCAAACACGAGGTCACCGAGATACTTCGCCGAGGACAC
TTCCCATTGGTTAAGTGGCACTCCAATCATCCCGCCGTCAGGAACGACGAAATCGGTAAAGGATTTAAAC
TTGGATGAAAACGCTGTCACTAGCGCACTTGGCGTAGCTTGGGATCAACGTCGTGATGTGCTACTATTTGA
ATTTAAACCCAAAAAATTGTATTCAACAGTCACAAAGAGGACTATTCTCTCGACTGCGTCTTCTCTGTTCG
ATCCATTAGGATTGCTGGCACCAATCATTATCGTCTCGAAAATCATCATACAGGAGCTATGGCTGCTAAAA
CTCGCCTGGGATGAATCTGTTCCGCAATCACTTCTACAAGCATGGAGAAACTGCGTAACGTCTCTCAAGG
AATTGTCGTCGATTTCAGTACCCCGTTTTTGCTTGCAAGCTGAGCTCACAGACATACAACTGCACGCTTTC
TGTGACGCATCAATACGTGCTTACGGCTGTTGCGTATATGTGCGTTCCACGGACTCGTATGGCAATGTCCA
GGTTCAGCTTCTGACTGCTAAATCTCGGGTGGCTCCTGTCAAGAAGCTGACGCTTCCTCGATTAGAGTTAA
GCGGAGCTCAACTTCTGAGTAAGTTATATACAACCGTCAAAAACATATTCATCGGCAAGCCGCACAGCGC
GCATTTCTGGTCTGATTCGCAGATTGTCTTGCACTGGCTGCAACAACACTCTATCACCTTGTCGACATTTGT
TGGAAATAGAGTATCCATTATACAAGATCAAACAGCAGATGGAAAGTGGAGACATGTACCGTCAAAATT
GAACCCTGCTGACATCGTGTCACGTGGATGCCCAGCTAATGAGCTCCAGCAATCAATTTGGTTTACAGGA
CCTGCGTTTCTGCGTGATTCCGAGACTCAATGGCCCTGCCAACAATTCGACAATGATTTGGACATGGACAT
CATCGATGCTGAAAAACGGAAGTCTACATTTGCTGCAATTATCGAAAGTAATTCTATTCTGGACTGGCTGA
ACGGAACTAGTTCATACATTCGGGCGCTTCGAGTTATGGCATATATGTTCCGGTTTTACCATATTGCCAAA
AAGGAACACCCTGTCGAAAGTTCGCCGCCATGCCCAGAAGAACTTCGCCACGCGTTGCACTGCATCATCA
GGATCATCCAACAGCACTATTTCAAGGAAGATTTGCAATTGCTAAGGAAACAAAGAGATTTGAAAAGCAA
TCTGAAATTTCTCAGCCCCTTCATAGATGACTCTTCTGGATACGAGCTCATTAAGGTTGGCGGCAGACTCG
AGCATGCTGATATCCCCTCTCGTCAAAGGCATCCTTTACTTGTTCCCAAGGAATCGCAATTTGTCCACAAT
TATGTTCGGCATCTTCACTTGCGCAATTACCATGCAGGACCAAAGGCATTAGTTGGACTGCTGCGGCTTCA
ATTCTGGGTCATAAACGCAAGAGCTTTAGCACGCAGCATTGTTCGTTCTTGTGTACACTGTGTCAGATATC
GTCCAGTTCTACTTCGTCAAGTCATGGGTTCCCTGCCACCTGAGCGCTTATCCACAGACAAGCCATTTCGC
CACGTTGGAGTCGACTTCTGCGGTCCAGTGAACACCTATCTTCGAATACGTGGCAAGGGTCCCACAAAAT
CCTATATCGCCATATATGTCTGCATGGCCGTAAAGGCCGTTCACATTGAAATCGTGTCAGATCTTTCAACG
AATAAGTTTTTGGAATCGTTGAAACGCATGGTTGCCCGTCGTGGACCAGTATCGCACATCTATTGTGACAA
CGCCACAAATTTTGTCGGAGCATCCAACAAGCTACTCGAGTTAAAGAAGTTCATGTTCAACACTAGCACT
CAAGATGCTATCCTATCATATTGTTCCTCTGAAGAGATAACCTTTCATTTCATTCCACCTAGGGCTCCACAC
TTTGGCGGTCTTTGGGAGGCCGCTGTCAAGAGCGCAAAGAGTCTGCTGGTGCGTACCCTCGCTAACACCC
GGTTCACCTTTGAGGAACTGAACACCGCAGTCGTTGAGATAGAAGCAGTACTCAACTCTCGCCCGCTGTC
ACCGCTATCATCTGATCCCAACGACTTCAGCGCTCTCACTGCAGGACACTTTCTNAATGGAGATGCACCGC
GCGCTTTGCCCGAGCGTGAGATTATCACCAACAACATCTCCAACTTGGATCGTTACGAGCAGATCACAGC
TGTTAAACAGCAATTCTGGCGTCGTTGGTCTGCGGAGTATGTGAACGAGCTTCGAGCCAGGACAAAATGG
ACTTCGGCTTCCCCAAATCTCACAGTTGACTCTATGGTCATCATACATGAGGACAACACACCGCCATTACA
CTGGAAATTAGGGCGTGTTGAGTCTGTTGTGAGTGGCAAAGATGACCGGGTTCGTGTCGCATATATTCGC

ACTGCCTCTGGACTCTGTTGCCGGCCAGTACATAAGCTGGCCGTTCTACCTGTCTCTTGAAAGATAATTCT
TTCAAGGCGGCCGGGATGTTCGGTCATATACCTCATATATACATTAGTCAGGGTATCAATAGCAATGTCAA
AGCAGATTATGTTAAGATTATGCTAATTCTCTTCTATTTGAATGTCATAAGCACTCATACACATGTACGAA
GCCTCGCTCTCTTTCGAACTCTGTAATTGCTCGGTCCCTTTAATCTTAATTTATGTTAAGAAATCAGTTCTG
TAAGCATCATCATAGAAGTCAGTCGTGTTGCTCATCAATTTGTAAAACCTTTATTAAAACTTCATANCATC
CACACACGCCCA
>Ref_dvir_884
AAAGTTAAAGTGAGTTGACGCGTTTGGACTGTTGCTTCGGGCTGTTTTTGTTGTGGGCGGCAGTACGAGA
ATGGAGCAAACCCGCGCACTCACCTGGATGACGGGATGATGGCGAGGACTACCTGATGGTATATATCCAA
CCCTAAACCTGATCCAAGTAAGCAAGTTGAGCACTTTCTCAGCGAAACTATCATGGCGACATTGTTTATGC
TCAAGCACATGAGCAAGACAAAAGCTCTGGACAGCTGGTTGGTTGTACAGTGCTGCCAACCTCAACCGAA
GTACATGCGTCAACGAAAAAAGCAATTACGCGCGAAAGTATGCCACGCTGTGTGGGTATACATGAGGAC
AAAGAAAGATATTGCATGCTTTTAGGCTTTCACGTTTTACTCGCATTGCAGCTGAAGACTCGAGGCAAGA
AATTGTACAGAAGGCCACGGATCCGACCAAGTCAACTCCTGCCGCATAGCGGTGTACTAAAAAGGGGCGC
TGGCGACCGAGCTGGAGCGGTTAAACTCCCATCTGTCAAACGCCAGTGAGAAACCATTGGCCACCGTTGG
AGCCACAAATCAGCAGCGATGAGCGCCACAACTGGCAGCACGCCCGTCAATGCAAGCAAGCAAACTCCA
CCCCCACACTGAGCCCAGTAAAGTGTGGAAGCGACCCACTGGACCCCCCTTACGTAACTGTAGAAATGGG
CTGACGAGTCGGGCATTCTAGGACCTTAATAGGCAAGCTGACACCTTGTCGAAACGGCTCCTAGGCTAAT
GCCTCGCCCCCGTCCCGCTAAAACCTTGGCAGGCCTCGTAGAACGTTATCGTATGTCGCCATTCAGTTGGC
CGTGCAGGCTCAGTTGATATTCCCTGACTAGCACTGATCTGGCTCTGAACTCCGTTCTCCCATAAGGACCG
GAATCAAACCCTCGCGGGAACCTCACTCCATGGAAAGGCAACCCCGGGATCGCGAAGGTGACTGCACAA
TACACACGACAACAACGGCTTCGAGTTGGGACCCAATTAATCACAATGATGAACCGAAACAACAAAAGC
ACCACCCCACTAGGTGGACAAGAGGAAGAAGGAGTATGCAAGGCCGACCCGTTCGCTAGGCGATCATGC
CTGGCGAGCACGCCCCCGGCTACACCAGTAAGGGCCCCTCAGCCCACTGCACCATGCAGGGAAATTGAAG
AGAAAGAGACCCCGAAAAGGCTTAGGGACCCGGCCAGCCCTACGAACAAGCCACGGAGCACCCCGCCCA
AGAAGAGCAAGGCAAATACCCAGGAGATCGAAGAGATTGGCTCGATACTGGACGAGCTACTGTTCATGT
ATCATGGCAAGCAGCCAGCGATGAGGCACATAAACTTGGCCACTAAGAGCATGCTAGCGAGACTAAAAG
AGCTGCAAGAAGTAGTCAAAGCGCAGCTCACGGAAAACCTACGCATGCGATGCAGCGAAGAGTCCCATA
AGCAGATGAGCCATGCTGAAGAAAGCGCCGGAAGCAATTCTAAATGCGACACGGCAACGCAGACGGAAG
AAAGCGGTGCATGCAGTAAACCACCGACAGCGGCGCAAAAACCGACGAACGAGGTTACAGGAAAAAATC
AGATCTTGGCCCCAAGCAACTCCAAGCCGGCACCAACTCGCAAGCAGCAACGTTCACAGCCGGCAGAGG
GCAAGGTACCGACCCAAGCGCGAGAGGCAAAAACACCGGTCAAAAATACCCGAGAGGTAAAAAATAAAT
GGATTGAGGTGAAGGGCAAGCTCACCAGGCGGAAACAGCCCAACCGACCGGATGCGATCCTGATCACGT
GTACAGAGAGCATGCCATACGCAGATGTGCTCAAAATGGTGAAAACAGCTCCTGCACTGCAGGCGCTCAA
AAATAATGTACAGGGCATCCGCAAATCAGCAGCAGGAGAACTGATCCTCAGGCTCCAGAAGCCATCGGA
CCCAGCTACCGACCAGTTGCGTGTGGCAATCAGCAGCGCACTGGCTGGAAAAGCTGCCGTTAGAACCTTG
CAGGAAACGGTGCAAATGGAGGTTCTAGATATCGATGAGTCCACGACAGCAGATGAGGTCCTCCAGCTCT
CTCTGCCTCGGTGGAAGGAGACATACCAGTAGAAGCGACGCCCACTATGAGGGCGGCCAGATCGCATCGC
TGGCCCTGCAACCGAGCCTAGCCACGAAACTGCTCGAAACCGGCAAGGTCCGCATAGGGGTTTATATGCC
GAATACGCCAAAAGATCGAGCCAAAGAGATGCTTCAGATGCATGGGATTCGGCCATACATCCACCAGGTG
CAAGAGCCAGCATCCGGTTGGAAAGGAGACATGCTTTAAATGCGCAGGTACTGGGCACAAGTCTGGACA
GTGCACGCATGAGGCAAAAGTGCATCCTCTGCCACAAGAATGGGGCACGGGCACTAACCAAGCCGCGCA
CTCAACTCTGAGCAGGAACTGCCCCGGAGTATAAGAAGGCCTACAAAGCGCTTAGAAATGGTTAAGTTCC
TGCAACTTAACCTAAACCACTGCAGAGCAGCCCAGGATCTGCTCGCACAAACGACCCGGGAGACGAGAA
TTGATGTAGCCATCATTAGTGAACCGTATACACCCAAACATATCGGAGTGTGGCACGAAAGCCTAGACGC
TGGAGCAGCACTATGGAGCTGCGGACAGGCACCATACCAGCTCACTCAACGGATGGCTCGCAAAGGGTTT
GCTAGGGCAAAATGTGGTCGCATATACGTTTATAGCTGCTACATACCACCAAGCATACCCCTGGCAGAGT
ACACAGCCATTATTGAAGACATCGCACAGGACGCGCACGGAAAATCTCCAGTGATAATCGCCGGAGACTT
CAACGCGTGGGCCACAGAATGGGGCAGCAGCAGAACGACACCAAGGGGAACCATCCTGCTGGACATATT
TGCTTCGCTAAATGTCTGTCTGCTGAACATCGGCACAAGAAGCACTTACAGCAAAGCGGGTCGCGAGTCG
ATCATAGACCTGACGTTTGCCAGCCCAGACGTTGCCCGAGACGCCAAATGGCATGTATCGGATGTATACA
CCCACAGCGACCACTTTGCCGGTGATAACGGACACCTATCAACTTGGAAGCAGAGGCGCCAGTAGAAGGC
TCCAACACACGGGATACAAGATGAACACTATGGACCTGGAAAGACTCCTCCCTATGGTAGAAGGACTTGG
CATAAGAGGCGACGCCAACAGCTCTGCTGACGCGATAGCAGAGACTATCACCAACGCATGTGACGCTATA

CTGACAAAAGTTCGCAAGGGTGGAAACGCACATAGACCGGTAGCCTGGTGGAACGAGGACATAGCCAAA
GCGCGCAAAGAGTGCCTCGCAGCTAGGCGGAAGAGCCAACGGTCAAGACCGTCCCCCAATTTTGAGCACC
ATCTGCAGAACTTCAGAGCTAAGCGGAAATTACTCAAGGAAGCTATCAGACGCAGCAAATCCCGATACTT
TCAGAAGCTCTGCGACGCTGCGGACGCGGAGCCCTTTGGACTGGCTTACAAGCTAGTCATGGGAAGGCTA
TGCAGGCAACCAATGCCAACAAACCCGGAACAGCTCAAACAAATAGTCCTAACGCTCTTTCCTCGACAGA
CACCTGGAATTGCACCGCAACTACCAGCAAACGGAATAAACGACTGTGCACCCACCGACGACAATGAGG
TACTGAGCATTGCTGCGAAGCTAAAGGCCGACAAATCTCCTGGACCAGACGGAATACCGAACGGGGTAAT
AAAAGCTGTCATTACTGCACAGCCCAGAAGCTTCGTGGAGTTGTACAATCAATGTATAGCGGAATGCACA
GTACCACGTAGATGGAAACTCCAGCGACTTGTGCTCATCCCGAAGCCCGGCAAGGAACTAGATGATCCCT
CCTCATACCGCCCCCTGTGTATGCTGGACACGATGGGAAAAATATTTGAGCGGATAATATGCAATCGCCT
AGAATGCAAGCTTGCTGAAGTGCGCGGTCTGTCTGAACGACAATTCGGCTTCAGGAAACACAGGAGCACC
ACGGATGCCATCAGGGCCGTGGCTGAACTAGCAGAAGACGCTATCCAAGGCACAAGATGGACAGGTGGC
ACCAAACAGTACTGCCTAGTCTGTACGTTGGATGTCAGGAACGCGTTCAACTCAGCCAATTGGACAAAAA
TACTAGAAGCGCTAACGGCCAGACACATCCCGAGCCAACTAATAAAGTTAGTGACCAGCTACTCCAGGAC
AGAGTGCTAAATTACGACACAGAAGACGGCCAACGACAGTACAGGGTAACTGGAGGCGTTCCTCAGGGC
TCTGTTCTCGGGCCGCTACTGTGGAACGTCATGGTACGACGGGAGTACTGCGCCTTTGCCTACCAGAGGGC
AAAACAATCAATGGTTTCGCCGATGACATCGCTGTTGTTACTGTGGCGAAGAAACTCGATGAGGTCACTC
TTAGGAGCAGTGCGGCCATCGACAGGATAATTGACTGGCTATCCCAGAACGGACTCACGCTAGCGGAGAA
GTCGCTTAATAAGCAGCAGAAAGATAGTTGAGACGACATCGTTGCGCGTTGGCAGTGCTATCATTAAGTC
CAAGCCCGCCATCAAATACCTGGGCGTCATGATAGACCACAGACTGTCGTATAAACAGCACCTGGAGTAT
ATTAGCCACAAAGCGGCCAGTGTTACAACAGCCATTAGCCGAATGTTGGCGAACACCAAAGGACCAAGG
CAGAGGAGCAGAAGGCTAATCGCTGGAGTGGTGACGTCCACAATCCTCTACGGATCCAACATATGGGCCC
CCGCAATGGAGGTTGCATCTTACAGCCGAGGATGCAATGCTGCATACCGACGATGTGCATTGCGTGTAGC
CACCTGCTTCCGCACTGTATCGGAAGACGCGGCCTTAGTACTGGCAGGCATGATCCCACTCGGCCTGATG
GCGGCCGAAAGGCAAAGTGGGGCAACCACCAACAGGCAACTGAGGGATAACACCATCGAACAATGGCAA
AGCAAATGGGATGCGAGCTCAAGTGGCAGATGGACCCACAGACTAATCCCAAACCTGAAGCTTTGGCTAC
AGCGGCGACACGGCGATGTAGACCACTACCTGGCACAAATATTTACTGGGCATGGCTGCTTTAAAGCGTA
CCTGCACAGGTTTAATCATGAGGACGACCCCTTTTGCGAGCACTGTGGGTACGGTGTGATTGAGGACGCG
GAGCACGCATTGTTCTACTGCCCACTCTATAGTCGCGAGCGAGACAAAGCGACGGCGGGCAGAAATGATT
TGTCTCCGGAGAATCTCGTGGCGCGAATGGTGGCCACTGAAAATGGATGGACAGCCGTTGCTAACATGGC
ATCGGCTATAATGAAGGAGCTGCGACGACAGGAGAGGATCCGACGCGGCATCGACTGATGAGCGGCTGA
TATACAGCCGTACCCCGCCCTGCGAAGTAGTACTTTGCGGTTGTCCCGCAGGAGCGGGCTACAACTTTCTA
CTCCCCACAAAATACCCTTTCCTTCTTTGTTTGTCTTAGTTCCCCCTTGTAAAATGTTAAGCCGTTATTTTCT
TTTCTTCCAAAATAAAAAAAAAAAAAAAAAAAAAAAACGAAAAAACACACACACACACACACACAC
>160_rnd-1_family-75__LTR___Gypsy
AGATAGAGGTCAACAGCAGGGGGATCCGCAACCCAGCGGTTCCAAAAATCCTCACTTCACTAAACAACA
GAAACAGCAACAAAGTCAATTTGAGCAACAGAGTGGCAGGTGGAACAAAGGTGGCGGGTCAGAGGAACC
AATGGACGTCGATCCATCTGTGTCAAAAATTCNACAACCATCGCNGCACCAGCCTACGTTAACTCTAGCG
CTCCTCAAGCCTACAAAAGAACGGCCACGTCTGGGCGCTTAAGCGGACAGAGGAGGCAGAGAGTCAATC
ACACAGCTCACGAGCCGGATCAAGATTCAGGTTCGTATGCTAGCTCTGCAGCAGCAGCAGAACAGCAAAT
TGCCGACGACAATGGTAGCGGTTATGACTCAGACACTCTCAATTTTTTAGGGGAAGCTCCCTGCTGCCCTA
CATCAGTAGGAGAGTAGCAGGGATCCAGATGAGGTTTCTCATCGACACGGGCGCTTCCAANAATTATATC
AGGCCACATAAAAATCTAAAAGGTGTGTGCCCAGTAAATTCTTCCTTCTCTACCCATTCTATACACGGCAC
CACGAAGATCACTCATAAGTGCCTTATATCTCTGTTCGGCAAAACTGCCACATTTTTCTTACTCCCAGACC
TGTCCACATTTGATGGAATTGTGGGCCTCGACTTGCTCACGCAAGCAGGAGTTTCGCTTTGTCTTTCTTCTG
GCCAACTCAAATGTGGCGCAGAAACTGAACAAATCGAATTCCACAGGTGCGCGGATGTTAACTTTACCAA
TGTTGACTGCGCCGACGCGCCCGAGGCCGTAGCGGATCCGGAAGAAGCACTTCCCTATAACACATCAGTG
GTGGCAACCATCAGGACCACTGATGAACAGCCAGTTTATTTCCGACTGTACCCCTACCCCATGGGAGCAG
CGGAATTCGTTAATAAAGAGATTCAAAGCCTTCTGAAGAACGGTATCATCAAAAAATCTGTCTCCCCCTA
CAACAACCCCATTTGGGTGGCGGACAAGAAGGGGACGGACGAGTTTGGTAACAAGAATATGCGCCTAGT
AATGGATTTTCGCAAACTTAACGAGAAAACCATCGCAGACAGATACCCTATGCCTAACATTCCAATGATT
TTGGGGAATCTTGGCAAAGCTAAATACTTTTCGACATTTGACCTAAAATCCGGCTATCACCAGATCACGCT
AGCAGAGCGTGACCGTGAAAAAACAGCCTTTGCAGTTAACGGGGGGAAGTACGAATTTTGTCGACTTCCC
TTTGGGTTGAAGAATGCGGCCGGCATCTTCCAAAGAACTGTTGACGACGTACTGCGTGAGCAAATTGGCA

AATCTTGCTATGTCTACGTTGACGATGTCATCGTCTTCTCAAAAGACGAAAGTTCTCATGTCCAACACGTA
GAGTGGGTCTTAAAGAGCCTGTACGAGGCAAATATGAGAATCTCCAAGGAAAAATCGCGTTTTTTAAGA
AAAGCGTAGGTTTTCTTGGTTTCGTGGTAACCAGTAATGGAGCTACAACGGACCCCGAAAAAGTCAAAGC
GATTCAGGAGTTCCCAGAACCAANGAATATATTCGAGATCAGATCATTTTTAGGACTGGCTAGTTACTATA
GGTGTTTCATCAAAGACTTTGCCTCAATAGCAAAACCCATTTCTGATCTTCTAAAGGGCGAGAATGGGTCA
GTAAGTAAACACAGATCGCGAAACATCCCGGTAGAATTCTCTGAGATTCAGCGTCACGCATNCCAAAAGC
TACGCAACATTTTGTCATCAGAAAACGTCATGCTCAGATACCCTGACTTTAAGAAGCCGTTTGATCTAACG
ACAGACGCTTCAGCTTACGGCATAGGGGCAGTGCTTTCTCAAGAGGGTCGCCCCATCTCAATGATTTCCAG
AACATTGAAACCCCCGGAGGTGAACTACGCCACCAACGAGCGGGAACTTTTGGCTATAGTTTGGGCCCTT
AGCAAGCTACGTCACTACCTATATGGGGTAAAAGACATTAACATTTTCACGGACCACCAACCNTTAACTTT
TGCCGTTTCTGAGTCAAACCCGAACGCCAAAATTAAAAGATGGAAGGCGCGCATTGAGGAAACGAATGC
GCGAATATTTTATAAGCCAGGCAAGGAGAATTTAGTTGCTGATGCGCTATCGCGACAACAGCTCAACACT
CTTGACGAGCTGAATACTGATTCCTGCGCAGCCACAGTCCACAGCGAGCTGTCCTTGACCTATACAATTGA
GTCGACCGAAAAGCCACTTAATTGTTTCCAAAATCAAATTGTCATTGAAGAGGCACGCGTCCCCACCAAA
CGCAACTTCATTCTTTTCGGGGACAAGAGGCGTCATGAAGTGGGCTTCACTGATCACACAGCCCCTTTGGA
TGAACTGCTTGACTTGATTAATCCAAATGCGGTCAATGCCCTCTATTGCAACCTGCACACACTAGCACAGG
TGCAGGATGGCTTAGTTCGCATGTTTCCAGGCACAAAGTTCTGGCACTGCAAAAACCGAGTTGCAGATGT
CTTCAACGTTGACGAGAGGAGAGAAATTCTCGTTGCCGAACATAATAGAGCGCACAGAGCAGCTCAAGA
AAATGTTAAGCAAGTGTTATCAGAATACTATTTCCCTAAAATGGCGAAGTTGGCTAAGGAAATTGCGGAA
AGTTGTAGAATCTGCTCAGCAGCCAAATACGATAGGCACCCGAAAAGACAGGAGCTGGGAGAAACTCCG
ATCCCTGAGACTCCTGGGGAATTTCTACACATTGATATTTTTTCCACGGACAAAAAATTTTTTTGACCTGT
GTTGATAAGTTCTCTAAATTTGCAGTCGTACAACCAATTCAGTCGCGCACTATCGGGGATTTAAAAGCTCC
ACTACTGCAGCTCACGAATTTCTTTCCACGAGCCAGAACAATTTATTGCGACAACGAGCCTTCGATGAATT
CTCAAACGATAAAGACCCTGTTGTTAAACAACTATGGAATCCATATTGTTAATGCGCCGCCGCTGCATAGC
ACCTCCAATGGCCAAGTCGAGCGCTTCCATAGCACTCTAGCCGAGCTGGCACGTTGCCTCAAGCTGGAGA
GAAACATCAGTGACTCAGTCGAAGTCATAATGCTAGCCACGATTGAGTATAACAAGACAATACATTCGGT
CATCAACAAGAGACCTGTTGATGCCCTCCATGTGCCCTCAGGCGAACCCGAAAGGGAAATATCAGCAAAG
TTAAGAAGTGCACAAGATGCGCAACGGGCTAGGTTGAACGACCAGCGACAGAATAGAGTCTTTGAAGTA
GGCGAAAAAGTNCTAGTAAAAAGAAACCGCAGGCTTGGCAATAAACTCACGCCCTTATGTGAAGAACGG
ATCGTAGAGGCAGANCTTGGTACGACCGTTCTCATTGGAGGGAGGGTGGTCCACAAGGACAACCTTAAGT
AGGGCGAGAGCTNAAATAAAACATCGATTNTATGCATCTTGAATTTAACTATAAGTTTCAACATTTGNGC
CACTAGGCTTCACCTTCAAGTTTTAATATTTACGCCACTTGGCATTNCCTTTAGGAAGACACGTAATAGTT
TGGTCCGTTTTGTCTGTCTTCCGCAGGTTCGGGTTTCTGCTATTTCTATTTTTTGCAAGTGCGGCAGCGCGT
GTGACTAATTACTCACACGCCAAGTATATTCCCATTGTTGAGGGCCAAGTTCTGGTCTGGGAGGAATTCAA
TTTCGTCAGGCACACAGCAAACATCTCTGGGTACGAGAAAATGGTAAGCGAAACAATGGAGCTATGTGAA
ATGTTTCCGCAGTCACACATGAGAAAGCTTCTAAGCGTAGATGCGTCCCACCTCAGAGATCTGTTAGAAG
CTCTTGGCGTGCACCACAGGGTGGCAAGAAGTTTAGATTTCTTGGGCACAGCCCTCAAAGTAGTTGCGGG
AACACCTGACGCCAGTGACTTCGAGAAGATCGAATTCACTGAGTTACAGTTGGTGAACTCTAACAACCGA
CAGGTAGCTATCAACACAAAAGTTCAGGAACAAATCAATCAACTCACTACAAGTGTCAACACATTACTAA
AAGCTGCAAAAAAGAATCAGATAGATTCCGGGCATTTATACGAAACTCTTTTAGCTCGGAATAGAATGTT
GCTGATGGAAATGCAGAATTTGGTGCTCGCTGTTACCCTTGCAAAAATTAATATCATAAGCCCANATATAT
TAGATCACGCAGATTTGAAATCTGTTTGGGTCGATGAACCCACAGGTACTTCAATAGCTGACCTTATGTCC
GTTGCGTCCGTGAAAGTATTACAATCTGTTAATGCCCTTCACTTTATCATTAAGTTCCCGAAAATCAAAAT
GGCTTGCAGAAAGGTGATGCTGTATCCAGTTGCACATGAGAATGCTGTGCTACAAATTGACGACAACATT
ATAGCTGAGTGCGGAGATCAAGTGCTTGCCCTTAAGAATTGCGCTTCCACGCCGGGAGGAACCTTTTGTC
AACTTTCACCGACCAGCTCTTGTGCCAGAGAGCTGCATGCTGGTGGCTTGGCAAACTGCCAAATGCAGCC
GAGTCACTTAGACCCGATCACATGGGTCGATGACGGAATTCTGATCATCAATAACCGGCCTGCTAAAGTT
TGCGCTGACAACGGTACTGAGACTTGGGTTCACGGAACGAATCTAATCACGTTCAATAATTGGGCTCTGA
TCAACGAAACTAAGTACTTGAATCGCAACAACGTCCAGAGTAAGTTCCCCGGCATCGCGGCATCTCCCCT
TCTGAACGTCACCGGCCACCAAGACGTGCTGAGTCTCCCTTACCTTCATCGCTTGAATGAACGTAACCTGG
AGATCATCAAGGAAGTCAAGGACGAAATTGGAAGCGTCGACAGCGTGGTAATAGCCTTCTTGTTGGGTGC
TGTGTGTTGCACCATAATTTGCGTCGGAGTCACCTACCGTCGGTTCACCAAAAACATGGCATCGGCCAAG
GAGATTAAGAAGGTGATCGCCGAGATAGGAGCGGCCGAGGGCGGCCTTAACTTAAAAGGGGAGCAGTTA
ACAGAGGCCAACGACACCAGCTCCAGATGCTGATATAGCACTGGGCCGGATGTTCTTACGAGTCTAACAG

GGGTCAGCAAAATGCAACATAACACCAGCCGCAGATGCTGACATAGCTCTGGGCCGGATGTTCTTGCACG
ACGGCAGCGCACGAGGCGCAAGCTAAGCATTCTAGCGGCCGTTGTGAAAAGCACACAATAGCTTAGGTA
NTTTTGATCAATCTTTTGTAAATTTAGTTTTTAATTCGGCTTTCACTGAGGCACATCGGCCCAGCGACTTCG
CGTATAAATTAACTCACTCACTACGGCGTATCGGCCTAGTGTTTTATAAACCATAATTAATTAATTAATTTT
GTGTTTCTAAATAATCAAATAATCAAAATAAAGTCCTATAACGAAAATCCGCGAGGACTTNTTATTTAAAT
ATTAATTTGTACACACACATTCATGCGCGTCTGCTTCGCATTCTAACAGCATGGTAATGGCGACTTTTTTCT
GCAATGCTATTTNAGTNCCTTTTTTATCATAAGTACCTAATTATTGGTGTGGCTGCTGAGTAGAGGTATAG
CAAGTGGTGCGGTCTGTCGCG
>Ref_dvir_99
GTATTACCTCATTTGAAACTTAAGGGAATTTTTTATTTTATTGAATGCTTCGATCGCTTCTTCGTTGAGATT
AATGAGCTTTTTAAAGAGAGGTTAGGGGCTTTGCCAGCTTGTTGTCTGAGATGACAAATCCCAGGAATTCT
ACTCTGCTTTTAAAGAACTCGCATTTGTCCAACTGACATTTCATGTTGGCTTCTTGTAGAGTTCTGAATATG
GCGGCAAGGTGCTGCGCATGCGATTCATCATCTTTGCTAAAGATAATGATATCGTCAATATAAATGAAGC
ATATTTTTCCAATATGCTCACGAAGGATGTCGTCAAGAGCTCTCTGGAAAATTGAGGGCGCATTCTTGAGA
CCGAATGGGAGTCGTGTAAACTCGTATTTTCCGTTGTTAACGGAGAAGGCAGTTTTTTCTATGTCTGACTC
CTTAAGGGGTATTTGATGAAAGCCACTTTTCAAATCTAAGACAGAGAAAACTTTGTTATTGCCTAGCTGTG
CTAGTACCTCGTTGATTTCGGGTATAGGGTATTTGTCTGCGGTAGTTTCTTTATTTAGTTTCCTGTAGTCTA
TAACCATGCGATATTTCTTTTGCCCGGAGGCATCTAATTTTTTCGGAACAATCCACACAGGTGAATTGTAT
GGGGATCGGGAGGGTCGGATGATTCCGTCCTCTAAAAGTTCTTTGATTTGGCTGTGTACTTCGTCTTTTAA
AGACATTGGAAATTGGTAAAATTTTGAATATATGGGGGAGTCGGTTTTTGTTCGAATCTCTGCTCTGACGT
TGGTTGTGTATGTCAGTTTTTCGTCCGGATTGGCGAATAGGTGTGGGTGCATTTTAACTAGTTGATTAAGG
CTATTTTTTTGTTCGTTTGTCATATGTTCTGTCCGTGGTATTACCGTGTTGACCGCCTCTATTTTTTTCTGCTT
AATGGGTATGCAAATATTGTTCTTGATTATTAGTAGATTCTTGCTGCTTTGTATCACAGCATCTAGTTCCTT
TAGACTATCGTTACCTAATATGGCGTCGAATGATTTTAGGGTGGGTAAGAGAAAGAATTTTATTTTTATAT
CGTTTCTATTGAAAAGATTAGCGAACTTGTGGTGTGTAATTTTAATGCTTCCGCCGACTGTGACGGCTTCG
AAGGGTTTTTCGTTTGGAATAGGTTCGGACACGAATTTTGGTTGGACGTAATTTCGGTTTGATCCTGTATCT
ATTAGTATTTTGAGTAATTTTCCGCTCCCCATCTTACATTTCTATGTAAGGAAGAGAGGAGCTCTTTATCCT
AAAAAATTAATGTCTGAGTGTTCGTATTGGGTATTTTGTCCGTCATCTGTTTCTTCAATTTGTTGGGCGTAT
TCGTTTAATGGCTGCTCGTATTCAGTCTGCTGTTCGTTAATTTAGCATTGATTGTACGTATTCCTCCATAGT
CACATAGTTGTCGGTGTTTTCTGTCTCCGTCTGTTCTTCTCCTGTTTGTATGTTGAAATTTCTTTGTTTTTTGT
CCGTGGCGTTTGTTGGTGGTGGAGGTCTTTTCCCTACTTCAAAATTGGGTCTGTTCATGTAGTTTATCATCC
TCGTGCGGATGCTTTGGTCCACGTCCATTGGCTCAGGACGTGGTTGGGGTTTTGGTGCAGAAGGTCTATTG
GGTATGTATACCTGTTGTGGTTGTGTCTGGTAGGTTCGTTGGTTGGGATATTGTGGAATCGGTCCATTATTG
TTGTAAGTCGGTGGTCCATGGTTTGTATTGGTTGGCCAAGGTGTATGGATCGGGAGTTGGGGCCTGAAGCT
AATGGGTGGTGGCCTAAAAGTTTGAGCGAAAGAAGGCTGAGCTGTTCGAGGTGGCAGTGTAGGTGGTGGT
ATATTTCTATTTGGGTTTCTGGCATGTGTCTGTTGATTTCTATTCAAAGCCATGTTAGTGCGATAGCTTTGA
TTCTCTAGTTTTAAACAATAGTGAAGGGCTGAAGGTAGATCTGCTGGCTCCTTTATTCCAAGAATCCGTGG
TAAATCTCCCTTCAAACCTCTAATGAAGGTATCTAATGCTTTATCTCGGTATATTTTTGTCATAAGGGTTTC
GGATTCCCTATTTAATTCCATGCAGCCTACTTTATTTAATATCAGCGACAAGTGGTTGTAAACGACCTGGT
AGAATTCTTCTACTGTTTGGTTGTTACCTTGCACTAACATTGTCATCTGATATTCTAATGTTCCCAGATCTC
TTTTGTCTGCGTAGTGCAGCGTGAGACATTTGGAGATAGCGCTCCAATTTAGGGGAACAATTATACGACTC
GAGGGCCGTGTCGGCATTGGCCAGTGATTTTGTTTCTCACTGTATGCAATATGCCAAAGTATTAACCTGCC
TCAGCTGCATAGGCTGAGAGTATACGATCTACACTCTTCTTCCATGAGCTAAATTCCGCTGGATTACCGGA
GAATTCTCTGAGGCTTTTAACTATGTCCGGAACTCTGTCTAAGTCATTCAAATTGTGGTTGGCAGTTATTGT
TTGGTCGTCTACGTCACTAAACTCCCTAGAGGGGTTGACTAACTGCTTTACCAATGTCGGCAGTTGTTGTG
CCAAAATCTGGCTAACCGTGGCTGCTATTATCTGTTCTGCGTTATTCGCCATGACTCTATTTTGTAAATGTT
CGTTTTGTCTGTCGATATGTCGTTGTTGCTGTCTGTTCGAGTTATGTCCGCCTACCTGTGGTTGATCTGATA
CTATTGGGGGCCTAACTAAATTGCTTTCATTAGCCATAAACGAGATATATTACTTATATTGTATATTTTAA
GATATTTTCGAAGTATATTTAAATCTAGTGTTAGTTTTTTTACTCTATATTTTACTTGAATTTTTGATCGTTT
TGTTTTCTTTTTAAAACCAGTGGCTGGTTTCTTCCTTCTACTTTGGAGGGTCCCTTGGGAGTCGCAAAGTTG
GAAATAATTTATACTACGTTCGTAGTCTTTATATTTGTTTATCGTTTTACACTTTTTTATCTTGCTTATCGTT
CTACAATATTTAGTATTTATTATTCTTATAATTCTATTTCACTTTTGTGTTAGTTGTTTTTTTTTTTTTTTTTT
TTTGTTTCACTTAATTTCTAACTTACAGTACTATTCCTGATGTGGCAACCTCCCCTGCCGGTCCGGATTTCA
TCCTTTTTGAAGCCTTTGGGTATCTACTGGGGATCCTGCGACCAGTCTTTGTTGAGAGTATCCTGCGGGCG

GGCCTTACAAGATGCGTTGTCCACCGATGGGCAAAATTGAACAATTAAGTTCCAATTGCCTTTCGCGAATT
TAACCAATCGTTGAATTTAATCGACGAGAGTTGCACTGGTTTTATTTTCGCGTATAACCGACTAACGCGTA
CGTAGAAGCGTTTTGTTCTGTTGTAACACGAGCCGGTCCCTGCTCGGGCGCCAGTTAATTCCAAAGTTTAT
TAAACGATGAAAAAAATATAATTTTTAGGGAAGCAAGACTTCCTTTATTTGATGTTAATTTGCAACTGAAT
AAACTTAAATCAATTCTAGCCCTAGTAACCTGTGGCTTAGCGGCAGCGGCGTTGGCAGCGGCGTCGCTGC
TGATGTTGTTGGCCCGTTGGGCAATTCTTCTTGGAATTTTCGGCGGTTGCCAGTCGGCGCGTGGAGGAAAT
GGCACAGACACCAAGTCAGCATTTTGCTGCACTTCTTATGAGGTGTTGCAATGCTTCTTATTGGGACTGTA
TGAATTTATTTGTTGTTAACTCTTCCCCCTTTGATTTAAGCTTGTCCTCAAGCGATCATCAGAAGAAAGGTA
TGTTGTTGAACCTGAGGTTGTTGGCATCTGTATTTTGTATTTGGATATGTGCTGGTTCTTTTTTGGGTCTCTC
TTGTTCTTTGTTCAGTTGCAGTATTATATTTTTCTTATTTTTCTTCATTACGTGTAATACACAGGTAAGAAG
GATGGCAACTGTGAACAGAGTTATGAAACCGACTCTATTTGCTATAGAGTGCGTCTGAAGTTTTTGCATGT
TTTTTATGTTGTTGATGTGCAATTCTTCTAATGCTTCCAGAGAAAGAATCTTTATTCTTTCGTTTTCTATTGG
GGTTATTTGTATTGTGGGATTTCCTGGGGTCATGACGGTGACCTCTAGGTTGCTGAACTCTTGTTTGTTGAT
GAATAAGGTTTCGTTCCAGAAGTTAATCATGTAGGTGCCTCTCACGCGTTTCTTCTCTTCTTGCCATCTAAT
AGTGCCGTTGAAGTCGTTCACTAGTATAAGTCCCTCGTCGATTTCCTCGATCTCCGGGATATGATCTGCAT
TGCTAAAGCCACAGAGCGCTTGCTCTCCGCTAACTAACTTAGGTATACAATTATTAATTGTTCAAGTTTAC
AATTTTTGATTTGTTACAAATTTTAATATTATGGTATCTTTTACAATTGTTGTTTATGCCATAGATTGTTTTA
TTACAATGAAACAGTTCGTTAAATTCTAAATTTATTATATTGTTGTTTTTCACTACTGGCTTTATTATAGTA
TTTTTGCATTCTATTTCTTCTAAATTTGGAATTTTTACAATATAAAACAGAGTGGTTCCATTATGCAAAATG
GATACATCTGCGAATTCTAGCATTTCTTCAATATTTAAAGGTGGCATGTTGCTTTTATTAAAGTTATCGTTT
ACTATCTCTATTTCCTTTTTCGCCAAGTAGGAATTGTATTTATTAAATTCAATTTCGCCCAATTGTATGGCG
TACTTAATATTAATTATTTTCTTCTTTTATGAGCCTAATTTGATTTTGTAAGCTAATAGCTATCTCATCACTT
ATGAGAGATTCCTTTCTTATGCTATTGGTTAAATCGTTTGAGATTTTCGTGAGTTTATTAATTCGATCTTCT
AATTGCCTGTTTATGATAAGCTGTTTATTGTCGTTTTCTATAAGTTTGTTTATGTTGTCAGTAAGCATTATT
AAGTCGTCATTGTCCGGGCTGCCGGCTACATACTTCCAAGCTGTGCCTATAATGTTGATCGCTCTAGTCTTT
CGTTTATGGAAAAGTGATATTGTGTCGAAAGTGACAATAGCTTGCGTTATTTCGTGACGTAAAGTGTGGTA
TAAAGTATTGGTTGTTAAGTCTTTTTCGGTGTAAAAAAGGAGATTATCTAATGCGTTTTTAATCTGTTCTAG
CTCTATGACTTGTACGATTCGTGTTGTCGATGTCTGTAGTTTCCCTATTCCTTTTAGAATTGTAATTGTTTGT
GCGTCTGTGTAGTTCAAAATTTCCAAGTGTCCTTCTGTTGCTTTTATTGTCATCATTAATAGGAATCTGTAA
TTTTTATGTATTTTTTATATTGCTTTTATGTATTATTTTGTCAGTTGTTGTTAGTACGGTTGTGTTTCTATTTT
CTTTAACTATTTCCTTTTTGTATCTGGGTGACAGTTTGGTACCTAATCTGGTATTGACTTTGACGAATATTT
CTTGACCAGGTATGTAAGTTCGTATGTCGTTACGTTTTTTGTTATGATTCTGCATGTCAGTCGATTGTTTCT
GTTTGAGTCTGTCAATGTTCTCCTTCCTAGCTTTTTCGTATTGGTTCTGGGTCCGTAGTCACCATTTCTGCCC
AAAAATATATCTAATGGTCTCCTGGTAG
>9_rnd-1_family-350__LINE___R1
ACACACACACACACACACACACACACACACACACACACACACACACACACACAGNGACGACGAGATTGT
TTTCATAGAGTGCGGACGCGATTTGTAACTGCTCCGTGAAAATAAACAAATAAACCTGTGAAATTAAAGT
GAAGTTAGTGCACGTGTGCAGGGAACCGAGACAGGCCTAAGACACCAAAAGGTGCGTGGTGGTACTCAC
TGCGAAGCCCGTGCAGACCATTCCTGGGGTGGCGTCTACAGTTCCACGATCCAGGCGAGGGGAGTGGAGG
CGTCTACTTGGCGTGTTTGTGCGCTTTTTGTGAGTTTTACCACTCAATNGTATTAGCCGTCTCGCAAAGCTA
TTTGTGCAGTTGGTTGCATTCACTCTAACAGCTGTTAGTAGTGTTGGCTAGCGCAGTTCACAACCAGCTGA
TCTGCACTATAGAGCCAGCTGCCAAACTGCGTAGCGGAGATACCAACCAGGCGATAAGCTGTAACTGGCA
GAAAGTACAGCATACTTTCGGGCTTACGCTGTTCTTGCTAAGCATTTAAGCTTTGGAGAAAGAACAGGCA
AAGAAGCCGATATCGGTGCTCACTACAGGCTCCAGGGCNAAAAAGGAGCAAAAGGTCGCGGAACAGACC
AATTTCCAACCCCGCCGCGGCGGTGCCCATAAAAACGGGGCTCTGGTGACCGAGTCGGAGCGGTATAACT
CCCAGCGGTTAATAGCCAGAGGGAAACCGTTGGCCACCGAGGGACTTTCCTGTAGAGCCCTGATAGTTAG
AGCAGGTGCCCAGAGTAAAAAATGGAGGATGAGCTGCATGCCTTTGCGCGCCGCTCTGCACTACAGCGAT
CACCGTCACACCAGGCGGTCACGCCAGTGCAGCAAAGAGTGGCTGCGTCGTCGCCGGCAAAAGCGAGTG
AGGAGCTGCCAAGAACGCTAAGAGGCACGATCGAGAAGATGGGCTTAGTCCTAAACGGCTTAACGGCGC
TGTTCAACGGACAACGCCACATTAACGGGCTGATGCGAGAGGAACTCGCGAGAGTAGAAAGCCTGCAGA
AGGTGGCGCTCAAGCTCTGTGCGCCGGAAACGAAGTGCGACGAGGCGNAAACGACGCCGTCATTAGCCA
GACGTGCGGAGCCGAACGTGCTAAAGACGCGCCCAACGAAGCTCGGGGGCCAAAAAGAGGCAAGCACGC
CCAAACGGCCGAGGGATGGCGTGCCAGAGCAGCGCAGAACCCCCTCCAAGAAGGCTAAACGCCAAGGTC
AAAACCCAGCGAAGCAGGCGCTGCTCACGCATCAAAAGACAGCCCCTGCAAACTCCAACTTAGCTGGCCA

TAATGGCAACCCAGAGAGCTGGACAGCAGTGAACGGACGTAAGCGGACGCAGAAGCGTCCCAGAGCCGA
CGCCATTACAGTCCACGGAGTGGGTGACTGCACATATGCCGATATGCTCAGGCTCGTGAAGAGCGACCCG
GCCCTCAAGCACCTAAGTGGCGATGTGCATGGCATTCGCAAGACCGCGAAGGGCGATCTTCTGCTGCGCC
TCAATAGGAAGCCGGAGCACTCCGCAGATGAGCTTCAAGAAGCTGTGGGAAAGGCATTGGGTGACCGGG
CGGTAGTAAGAACGCTGACCGAAACGTCCCAGGTGGAGATCAAGGACCTGGATGAGCTGGCGACTAAGG
AGGAAGTGCTCGAAGCGATTAAAAGTGCCTTACAAGACGATTCCTTGCCGATTGAAACGATCAAATCGCT
GCGACCAGCATTTGGCGGCCAGCAAAGGGCCACAGTTACTCTCCCAGCGCCAAAAGCCAGGACGCTTGTC
GAGAAGGCCAAGGTGAGGATCGGATGGGTTGTCTGCCGTATTCGTGCAATAGACCAACCAAAGCGATGCT
TTAAATGCCAGGGTTTCGGGCACATTGCGGCGAACTGCACCAGCGGAGACGATATGAAAGGCGCATGCTT
CAGATGCGGGGACACAGGTCACGAAGCAAAGAGCTGCTCCAAGCCACCAAAATGTAGCCTTTGCATACG
GAAAGGAGAAAAATCAACGGACCACGCTACAGGAAGCTTTAAATGTCCGGCGTATCGCGAGGCAGTACG
CAAAATGAAATGTTAAGGTTACTCCAAATAAACCTTAACCACTGCATAGCTGCTCAAGCCATCTTGGAGC
AGACTGTTAGGGAGATGCGCATTGATATTGCCATTATCAGCGAACCCCACCACCGAGAAACCAACTGCCC
GAGAATATGGACCGAGGATCAGGATGGAAAAGCTGCGCTCTGGGTATGTGGCGACCCATCGACATCGCTG
ACAAAAACAAAGTCCTTCACTGGGTTTGTACGCGCACAATACGGCAGCCTGTGGATATACAGCTGCTACC
TGGCCCCAAGCATGCAGCTCTGCGAATTTACAGCTGCATTAGATGCATTAGTAGATGACGCAAGGGGGCG
GAAGGTCATTATTGCTGGTGACTTCAACGCCTGGGCAGTGGAATGGGGCAGCCAAGCAACTAACGCAAGA
GGTCGTGCCCTGTTGGAAAGCTTCGCAGCCCTAGATATAGCCCTGCTAAACTTCGGCTCGGAAAATACCTT
CACTCGAGGTGGTATCGGATCCATAATCGATCTTACCTTCGCAAGCTCGTCACTTTTTAGCGCAACAAAAT
GGCAACTAAGCGACTTATACACCGCAAGCGACCACTCCGCAATAATATGCACGATTGGTGAAGGAACTGC
AGCCAGCACTAGAAGCCTAAGTCGGAAAGCTTACCGCCTGGATACTTTCGACCCCGTGGCCTTTTCGAAT
GCAGTCCACTCGCTTGTCACTGCCGGCAACGCAGAGAGAAGAGCCTCCCAAATGGCATGCCAGCTATCGC
GTGCCTGTGACGAGAGCATGCAAACCCGGAAAACGTTTCGGCGCCACCATGTACCCACGTACTGGTGGAA
CGAAAATATTGCCGAAGCACGGCGGAACTGCATCCAAGCCAGGAGGAGATACCAGCGAGCCCGAGGTAG
ACCCAACTATGCCGAACAGCAGCTAAGGTATCAGAGTGCCAGAAAAATGCTAAAAAGTCCATAAGAGAT
AGCAAACGAGAATGTTTCCTGGAGCTATGCGACTCAGCCGAATACGATCCTTGGGGCAAGGCGTACAAAA
CTGTCGTTAAAAGACTCTACGCCGGCAAACAAGCGCAGCCTTCTGACCCAAGTGAGCTGAAGTTGATTGT
GGAAACGCTATTCCCAATCAGACCCGACGTATTACANACGCAAGCCATACCATCGAGCCACGTAGCAGCC
ATGCCGTCTCTCATCGACGAAGTCTCAGTCGAAGAGGTGCTGACGATCGCCAGAAGCCTAAAGCCCAGTA
AGGCACCGGGTCCGGACGGAATCCCCAACCGAGCACTGAAACTAGCCATGTCCTTGCAGCCCGCGCAGTT
CGTGCAGCTTTTTAACATGTGCCTAATGGAGAGAACTTTCCCCACAATATGGAAACTCCAAACGCTAGTTT
TATTACCGAAGCCTGGTAAGCCGCCCGACGACCCATCGTCATACAGGCCGATTTGCCTCCTGGACACGTTA
GGCAAAGTGCTGGAGAAGCTAATCTGCAACAGGCTTCAGGTAGCTGTCGACAACAATGGTGGCCTGTCCC
AAAGCCAATTTGGTTTCACCAAGGCAAAGTCTACAATAGACGCTCTGGAGAAAGTGGTCGGCATAGCAAC
AAACGCCATCCAAGGATCGAGATGGAAAAGCGGCACAAAAAAGTACTGCCTCATCGTCACGCTGGATGTT
AAAAACGCCTTTAACACAGCCAGGTGGGACAAAATATTGGATGCATTGTCAACGTTCCGCGTCCCATATT
ATCTTCAGGAGACGATACGCAGCTACTTCACTGGAAGACTGCTGCAATACGACACCGCAGACGGCCCCAC
TACACATCAAGTGTCCGCTGGAGTGCCCCAAGGCTCAGTTCTAGGACCCCTCCTCTGGAACATCATGTACG
ATGGCATCCTCCGACTGGAACTCCCGATCGATACAAGTATCATCGGCTTCGCGGACGACGTGGCCCTTGTA
GTCGTCGGCAAGGAATTGAAGGACGTAGAGGAATCGTGCAACCACAGTATTGACCGTGTGCACCAATGGC
TGGCAGCAGCTGGACTCGAGCTGGCTGCTCACAAAACAGAAGCGGTGTTAGTCAGCAGTCGAAAAAGAG
TGGAAACAGCGCACATAAGAGTCGGCAGTGCGACAATAGAATCAGCAAGGGCGCTAAAGTACCTGGGTG
TCATGCTAGACACAAGAATGTCGTTCAAAGAGCACTTGGAGTACGTGCACAAAAAAGCAGCAGAGTCCTG
TCGAGCGCTATCCAGGATTATGCTGAACACACGGGGACCGAAACAGCAGAGACGCCGTCTCCTTATGACC
GTAAACCGAGCGGTAATAATGTACGCGTCCCCCATCTGGAGCAGAGCTATGGCTGTTCCCAGCTACAGCC
GGGGCGTACACTCTATATATCGCCTAAGCGCTCTACGGGTCAGCTGCGCATTCCGGACGGTATCGGACGA
AGCGGCGCTGGTTATCGCAGGAATGGTCCCGCTAAGCGAACTGGCAACAGAAGCTGTAGTTTGCTATAGA
GCCCACGATGGAAGCAACACAGCAAACTCGATTCGCACTGAGGCAAGGATGCAGAGCGTAGCACGATGG
CAAGCACGCTGGGATCGCGCATCTAAAGGACGCTGGACGCATAGGCTTATCCCAAACTTGGAAGCCTGGA
CAAATCGCAAGTATGGCAATACTAATTTTTATTTAACGCAGCTCCTATCAGGCCACGGATGCTTCAGGAGC
TACTTAAAGCGATTCGGCCACGACAACACAGACGAGTGTTCTTGGTGCGGCAGAGGAATCATAGAGGACG
CCAATCACGTCATATTCGAGTGTGGACGTTTCGCAGCGAACAGACAGGAACTGGAGGACATCATGGGCAG
ACCTATCACGGTGGACAACCTGGTCACGAGCATGATAGAGACCGCACAAAAGTGGGACGCTGCCAGCAC
CTTCGCAGCAGAAGTAATGCGAGAACTCAGGAGAGTTGAGCGCAGCAGACGGAGAACCGCGGATATTTA

GGTAGCAAGAACATGCCTGTGAAGCAATGCTTCGCGGCCGTTCCGCAGGCAATCCACGCTACCTAAACAA
TACCCCAACTATAACCTAAGCTAAATAACTAAGCATAAATAAACTGAATACAAAAAAAACACACACAC
ACACACACACACACACACACACACACACACACACACACACACACACACACACACACACACACACACACA
CACACACACACACACACATACACATACACATACACATACACATACACATACACATACACATACACACA
CACATACACATACACATACACATACACATACACACACATACACATACACATACACATACACACACACA
CACACATACACATACACATACACATACACATACACATACACATACACATACACATACACATACACATACA
CATACACACACACATACACATACACATACACATACACATACACACACACACACACATACACATACACATA
CACATACACATACACATACACATACACATACACATAC
>160_rnd-1_family-98__LTR___Gypsy
ATATATATGTAGGAGTTGGAAACAGAACTAGGAGTGGTCGAACTTACAATCAGTTAAATTAAACAGGCGA
GTATAATGTTGGCATTATACTCATTTGGTAATATAAAATCACTTATCATATGCAACCCTGCTATGGATATTT
AATCAACGATAGATTTATCGATAAAAATACAAGAAGGAGAAGCTCAGTTGTAAGCTAGAAGAGTCAATG
AAAAGATGTGTTCGCTGTTGCTCGTTGTCTTTGAAATACATGTCGGAAAATGTACAATAATCATAAGTTTA
CGCTTGTTCACCAATTTCTCTAAATAAAATAATATGAACATAAAGAACTCAAGTGCTTGTGAAGGCCATCG
AATGGTCATCCCTACAAATTTGGTGTCAGAAGTGGGATAGACAGAAATTGTGAAATTCGTAAATTGATTA
ACGAGTACAAACGTATGCAAGACTATTAATCGGAGCATAAAACGAAGCGGGTGCATGAAAATTCATTAA
AATTCGTATGCCCTGGAAAAAATGGAAAAAGTCAAGAATCGTCTAGGATTAATGGCGACAGCGGCGAAG
TATGCCTAGAGAGCTCTAGTAAGAGCGAGACAAACAGTCGTGTATTGCGTGACAGTGGTCGATAAGCGCA
AGCGAGAAGGCGATGTTTTAAATTCTGCGTAGCAAAGGCTGGATAGCGCAAAATTTTCACTTCGTGATGA
AATAAGAAGTTGCAGATCAAAGAGGCGAAGAGCGAAGAGCGCGGAATCTTTCAATAAAAGAGCGTGTGA
CTGGAACGTGCTTCAACACGACAACGATGATCACAGGGTGTATCAAGTTACAGATCAACAACAGAGTATG
TTATTTCGTCATTATATGAAAATAATTAACTAGTTGTTGAGTAAAGAGTGTTAAATCAGTAAAAAATTTTC
AAAGAATCAAATTATAACAAAGAAGAAATTAAGTGTAAGCAAACACAAAGTGAAAGTATAAGTTAAGAC
AGACGAATATAATAATTTAATAATTTCTAAAGTTGCTGAAAAAAAAAAACTAAAATTTGATTCATAAGAG
TTTAATTACTTATACCATTAAGTTAAGTACATTGAATTGTTTATTTGTAAGATTCAATTTTAAGCATTGTGA
AAAGCGAGCAACGACGATAACAAAATGAACAGAGACGAAATTTTGGCGTTAAGAGTTAACGAATTAAAA
GAAAAGTTATCGGCACTTAGTTTGGTAACAACAGGTAGAAAGCAAACTTTGCAAGACCGACTTTTAGAAT
ATTATGGGTTGCAGGTTGACGATAATGAATCTGAGTATGACGATGCTGGGTCGGCTCATACCCGTCAAAG
CTCACCAATGCAGATAGTCCGAACAACGTTTACATTACGCGACATTGAAGATTCACTCTCGCAATTCAATG
GGTCAAATCAACCAGCTGTTGAAAAATGGATTCATGAATTTGAAGATAACGCTTTGGCAGTGCAGTGGAA
CGACCTTCAGAAATTTATTTACTCAAAGCAATTACTAAAAGGTGCAGCAAAATTATTCGTGCGCAGTCAG
CATGGAATTTCAGATTGGATATCTTTAAAGCAAGCGCTATTGGAAGAATTTGGGACGGTAGTTTCATCAAT
AGACGTCCACCGCATGTTGCGGAATAGGCGTAAGCGTCAAGGTGAAGATTATATAGAATATCTGTATAGC
CTTATGGAAATTGGCGGACCTCTTAACCTGGATGACTCAAGCCTGATAGAATATTTTATTGAGGGAATACC
TGACTCGAGGAGTAACAAAAGTAATCTGTATCAGGCCAAGACTCTGCAGCAACTAAAAGAACAAATCAA
GGTATATGAGAAAATCCGTAGTAGTCGCCCTCAAGCGTCTAATACGGGTAGGATGCATAACGATACCGAA
GAAAAGAAACCTGACTTGAATAAAGAACGAATTAAGAAATGTTTCAAATGCGGCGATCCATCACATCTAG
CCAGAGAATGTAGACAGCCAATAAAATGTTACAAGTGTGCTCAACCAGGGCATAAGGCAGCTAACTGTTC
AGGATCGCGTGCAGTGGAGAAGAAAGAAGGCCAAAATGCGAACACGATGATAAACAAGGCCAACAGTA
GTGGAAAATGCATTTTTAAGGAGTTGTCTTGTAAGAACATATGCTTCTCTGCATTAATCGATACAGGCTGT
GACCTCTGTCTGATACGCGATGATATCCTTAAAGAGCTAGGAAAAGTTGAACTGAATAATGAAAAACAGT
GTCTTGTTGGTATCTGTAATAGTGAGCTAAATACACTGGGAAGCTTTGTCACTCAAGTCGAAGTTGATGGT
ATCTTACTGGATATAACCTTTCATGTTACGAGCAGAGACGACATCCAATACTCAGCTGTAATAGGAAACA
GCGTATTGAAGCAAGCGGACTTAATAGTATCGGAAAATTTCGTAGAATTTCGACCCAAAGTGAAGGACAA
TGCAAACGAGAGAGTAATTGACAAGACATCAACAATAACAGAAGATACTGTCGAAAATAAGAAGCCAGA
CATAGAGCTTTTACAAGAATTCAAAGATGTATGTTTTTTTGCAGAAAATCAGGAGGCTGAGATCGATCTAC
ATCACTTAGAAAAACCTGTTGCAGACGCAGTTCAAGCAATAATTAAAAACTATTCACCTGAAATGAAGAA
GAAATGTCCAATTGAAATGAAAATTTTGTTAAATGATGAGCGTCCAGTTTTCGAACGCCCAAGGCGCATA
TCACATGCGGACAAGTCTATTGTCGATTCGCAAATCAAAACTTGGCTAGAAGAAAAGATCATACAGCCAA
GTACATCGGAGTATGCATCTCCTGTTGTCCTCGTTCCGAAAAAAGATGGTACCAAAAGATTGTGCTGTGAC
TACAGGCGTTTAAACGAAAAGATCGCGAGGGACAATTTTCCAATGCCCTTAATAGACGATGTGATTGAGA
GATTGCAGGGCCAAAGTGTGTTTACTACACTTGATCTCGCAAACGGATTTTTTCATGTTCCAATTGAGCCA
AATTCGAGAAAATTTACATCATTTGTAACAAGTGCAGGGCAATATGAATTCCTGTTCGTCCCTTTTGGAAT
ATCGAACTCCCCAGCTGTATTTTCCAGATTCATTCATGCAATTCTTGTAGATCTGGTACAGGATGGTACAG

TCGTAACTTATATGGATGATCTTATAATTCCCTCCAAGGATATAGATGAAGGNNNNNNNNNNNNNNNNNN
NNNNNNNNNNNNNNNNNNNNNNNNNNNNNNNNNNNNNNNNNNNNNNNNNNNNNNNNNNNNNNNNNNNNNNNN
NNNNNNNNNNNNNNNNNNNNNNNNNNNNNNNNNNNNNNNNNNNNNNNNNNNNNNNNNNNNNNNNNNNNNNNN
NNNNNNNNNNNNNNNNNNNNNNNNNNNNNNNNNNNNNNNNNNNNNNNNNNNNNNNNNNNNNNNNNNNNNNNN
NNNNNNNNNNNNNNNNNNNNNNNNNNNNNNNNNNNNNNNNNNNNNNNNNNNNNNNNNNNNNNNNNNNNNNNN
NNNNNNNNNNNNNNNNNNNNNNNNNNNNNNNNNNNNNNNNNNNNNNNNNNNNNNNNNNNNNNNNNNNNNNNN
NNNNNNNNNNNNNNNNNNNNNNNNNNNNNNNNNNNNNNNNNNNNNNNNNNNNNNNNNNNNNNNNNNNNNNNN
NNNNNNNNNNNNNNNNNNNNNNNNNNNNNNNNNNNNNNNNNNNNNNNNNNNNNNNNNNNNNNNNNNNNNNNN
NNNNNNNNNNNNNNNNNNNNNNNNNNNNNNNNNNNNNNNNNNNNNNNNNNNNNNNNNNNNNNNNNNNNNNNN
NNNNNNNNNNNNNNNNNNNNNNNNNNNNNNNNNNNNNNNNNNNNNNNNNNNNNNNNNNNNNNNNNNNNNNNN
NNNNNNNNNNNNNNNNNNNNNNNNNNNNNNNNNNNNNNNNNNNNNNNNNNNNNNNNNNNNNNNNNNNNNNNN
NNNNNNNNNNNNNNNNNNGATTTTGACTACAGTATTGAGCATCGAAATGGCAGCAAAATGAGGCATGTTG
ATGCTTTAAGCCGCGTATCCTGTCTTATGCTTGAAGACTCACTGCAGCACAGATTGCAGCAAGCACAAGC
GAGAGATGATTGGGTCCGAGCGGTGCTAACTGTTTTGGATTGTAGCACATACGAAGATTTTTATGTTAGGC
ATGGTATTTTATACAAGGATCCCATCAAGGAACTTATAGTAGTACCATCGCTGATGGAAGACGAAATCAT
AAGAATTGCACACAAGCAAGGGCATTTCTCAGCGAAACGAACTCAAGAAGCAATCGAGAAGACATTCTA
TATTCCGCAGCTATCGTCAAAAGTGCCAAAAATTGTAAAAAGTTGCGTAGAATGCATAATATCTGAGAGC
AAATCAGGCAAGAAAGAAGGGTTTTTGGCACCAATTGACAAAGAAGACAAGCCCTTAGGCACGTACCAT
GTGGATCATGTCGGTCCAATAGAACAGACAAGTAAAAACTATAACTATATTCTAGTGATAGTCGATGCGT
TTTCAAAATTTGTTTGGCTCTATCCAACAAAAAATACCACCGCAGAAGGAGTTGTAGATCGACTAAGACG
GCAGGCAGCAGTGTTTGGAAATCCTAAGCGCATAATATCAGATAGGGGTGCAGCTTTCGTTTCAAACCTG
TTCAAGGAGTACTGTGAAACAGAAGGTATACAGCATCTTACGATAGCTACAGGAGTTCCAAGAGGAAAC
GGCCAGGTAGAACGTATACACAAAATCGTAATACCAATGTTGTCTAAACTATGTCGTGAAAATCCTTGTA
GCTGGTATAAGCATATTGATATCGTTCAACAAACAATCAACAGTACGCCGCCAAGAAGCACTAAAGTTTC
ACCGTTTAGATTGTTGACTGGTGTGGAGATGAAACTTAAAAGAATATCGGACTTGCAAGAACTATTAGAA
GAAGCTGCTATAGAGGAGCTAAGTAACGAAAGAGAAGAAGTTCGTAAAGAAGCGCGGCAAAATATAGCA
AAAATACAGCAGGAAAACCGAAAATCTTTTAATAAACATCGCAAAAAAGAGTCCAATTATAAAATAAAC
GAACTTGTAGCCATTAAGAGAACACAGTATGGTACTGGTTTAAAATTGAAACCTAAATTCTTTGGTCCATA
TAAAATAGTAAAGATTCAAAACCATGGTAGATATGACGTAGAAAAGGTAGGCGACCACGAAGGTCCAGG
GAAAACTACAACTGTAGCAGAATACATGAAAAAATGGAGCCAAATGCTAATTTAACAGAAGAGTCGTC
ATTCGGGTCGAATGAAGAGTCAGGATGGCCGAGTGTAGGAGTTGGAAACAGAACTAGGAGTGGTCGAAC
TTACAATCAGTTAAATTAAACAGGCGAGTATAATGTTGGCATTATACTCATTTGGTAATATAAAATCACTT
ATCATATGCAACCCTGCTATGGATATTTAATCAACGATAGATTTATCGATAAAAATACAAGAAGGAGAAG
CTCAGTTGTAAGCTAGAAGAGTCAATGAAAAGATGTGTTCGCTGTTGCTCGTTGTCTTTGAAATACATGTC
GGAAAATGTACAATAATCATAAGTTTACGCTTGTTCACCAATTTCTCTAAATAAAATAATATGAACATAAA
GAACTCAAGTGCTTGTGAAGGCCATCGAATGGTCATCCCTACATATATNTATATATATAT
>9_rnd-1_family-401__LINE___R1
ACACACACACACACACACACACACACACACACACACACACACGGAGACGCGAGATTGTTTTCATAGAGTGC
GGACGCGATTTGTAACTGCTCCGTGAAAATAAACAAATAAACCTGTGAAATTAAAGTGAAGTTAGTGCAC
GTGTGCAGGGAACCGAGACAGGCCTAAGACACCAAAAGGTGCGTGGTGGTACTCACTGCGAAGCCCGTG
CAGACCATTCCTGGGGTGGCGTCTACAGTTCCACGATCCAGGCGAGGGGAGTGGAGGCGTCTACTTGGCG
TGTTTGTGCGCTTTTGTGAGTTTTACCACTCAATTGTATTAGCCGTCTCGCAAAGCTATTTGTGCAGTTGGT
TGCATTCACTCTAACAGCTGTTAGTAGTGTTGGCTAGCGCAGTTCACAACCAGCTGATCTGCACTATAGAG
CCAGCTGCCAAACTGCGTAGCGGAGATACCAACCAGGCGATAAGCTGTAACTGGCAGAAAGTACAGCAT
ACTTTCGGGCTTACGCTGTTCTTGCTAAGCATTTAAGCTTTGGAGAAAGAACAGGCAAAGAAGCCGATAT
CGGTGCTCACTACAGGCTCCAGGGCAAAAAAGGAGCAAAAGGTCGCGGAACAGACCAATTTCCAACCCC
CGCCGCGGCGGTGCCCATAAAAACGGGGCTCTGGTGACCGAGTCGGAGCGGTATAACTCCCAGCGGTTAA
TAGCCAGAGGGAAACCGTTGGCCACCGAGGGACTTTCCTGTAGAGCCCTGATAGTTAGAGCAGGTGCCCA
GCGNNAAAAATGGAGGATGAGCTGCATGCCTTTGCGCGCCGCTCTGCACTACAGCGATCACCGTCACACC
AGGCGGTCACGCCAGTGCAGCAAAGAGTGGCTGCGTCGTCGCCGGCAAAAGCGAGTGAGGAGCTGCCAA
GAACGCTAAGAGGCACGATCGAGAAGATGGGCTTAGTCCTAAACGGCTTAACGGCGCTGTTCAACGGAC
AACGCCACATTAACGGGCTGATGCGAGAGGAACTCGCGAGAGTAGAAAGCCTGCAGAAGGTGGCGCTCA
AGCTCTGTGCGCCGGAAACGAAGTGCGACGAGGCGCAAACGACGCCGTCATTAGCCAGACGTGCGGAGC
CGAACGTGCTAAAGACGCGCCCAACGAAGCTCGGGGCCAAAAAGAGGCAAGCACGCCCAAACGGCCGAG

GGATGGCGTGCCAGAGCAGCGCAGAACTCCCTCCAAGAAGGCTAAACGCCAGGGTCAAAACCCAGCGAA
GCAGGCGCTGCTCACGCATCAAAAGACAGCCCCTGCAAACTCCAACTTAGCTGGCCATAATGGCAACCCA
GAGAGCTGGACAGCAGTGAACGGACGTAAGCGGACGCAGAAGCGTCCCAGAGCCGACGCCATTACAGTC
CACGGAGTGGGTGACTGCACATATGCCGATATGCTCAGGCTCGTGAAGAGCGACCCGGCCCTCAAGCACC
TAAGTGGCGATGTGCATGGCATTCGCAAGACCGCGAAGGGCGATCTTCTGCTGCGCCTCAATAGGAAGCC
GGAGCACTCCGCAGATGAGCTTCAAGAAGCTGTGGGAAAGGCATTGGGTGACCGGGCGGTAGTAAGAAC
GCTAACCGAAACGTCCCAGGTGGAGATCAAGGACCTGGATGAGCTGGCGACTAAGGAGGAAGTGCTCGA
AGCGATTAAAAGTGCCTTACAAGACGATTCCTTGCCGATTGAAACGATCAAATCGCTGCGACCAGCATTT
GGCGGCCAGCAAAGGGCCACAGTTACTCTCCCAGCGCCAAAAGCCAGGACGCTTGTCGAGAAGGCCAAA
GTGAGGATCGGATGGGTTGTCTGCCGTATTCGTGCAATAGACCAACCAAAGCGATGCTTTAAATGCCAGG
GTTTTGGGCACATTGCGGCGAACTGCACCAGCGGAGACGATATGAAAGGCGCATGCTTCAGATGCGGGGA
CACAGGTCACGAAGCAAAGAGCTGCTCCAAGCCACCAAAATGTAGCCTTTGCATACGGAAAGGAGAAAA
ATCAACGGACCACGCTACAGGAAGCTTTAAATGTCCGGCGTATCGCGAGGCAGTACGCAAAATGAAATGT
TAAGGTTACTCCAAATAAACCTTAACCACTGCATAGCTGCTCAAGCCATCTTGGAGCAGACTGTTAGGGA
GATGCGCATTGATATTGCCATTATCAGCGAACCCCACCACCGAGAAACCAACTGCCCGAGAATATGGACC
GAGGATCAGGATGGAAAAGCTGCGCTCTGGGTATGTGGCGACCCATCGACATCGCTGACAAAAACAAAG
TCCTTTACTGGGTTTGTACGCGCACAATACGGCAGCCTGTGGATATACAGCTGCTACCTGGCCCCAAGCAT
GCAGCTCTGCGAATTTACAGCTGCATTAGATGCATTAGTAGATGACGCAAGGGGGCGGAAGGTCATTATT
GCTGGTGACTTCAACGCCTGGGCAGTGGAATGGGGCAGCCAAGCAACTAACGCAAGAGGTCGTGCCCTGT
TGGAAAGCTTCGCAGCCCTAGATATAGCCCTGCTAAACTTCGGCTCGGAAAATACCTTCACTCGAGGTGG
TATCGGATCCATAATCGATCTTACCTTCGCAAGCTCGTCACTTTTTAGCGCAACAAAATGGCAACTAAGCG
ACTTATACACCGCAAGCGACCACTCCGCAATAATATGCACGATTGGTGAAGGAACTGCAGCCAGCACTAG
AAGCCTAAGTCGGAAAGCTTACCGCCTGGATACTTTCGACCCCGTGGCCTTTTCGAATGCAGTCCACTCGC
TTGCCACTGCCGGCAACGCAGAGAGAAGAGCCTCCCAAATGGCATGCCAGCTATCGCGTGCCTGTGACGA
GAGCATGCAAACCCGGAAAACGTTTCGGCGCCACCATGTACCCACGTACTGGTGGAACGAAAATATTGCC
GAAGCACGGCGGAACTGCATCCAAGCCAGGAGGAGATACCAGCGAGCCCGAGGTAGACCCAACTATGCC
GAACAGCAGCTAAGGTATCAGAGTGCCAGAAAAATGCTAAAAAAGTCCATAAGAGATAGCAAACGAGAA
TGTTTCCTGGAGCTATGCGACTCAGCCGAATATGATCCTTGGGGCAAGGCGTACAAAACTGTCGTTAAAA
GACTCTACGCCGGCAAACAAGCGCAGCCTTCTGACCCAAGTGAGCTGAAGTTGATTGTGGAAACGCTATT
CCCAATCAGACCCGACGTATTACACACGCAAGCCATACCATCGAGCCACGTAGCAGCCATGCCGTCTCTC
ATCGACGAAGTCTCAGTCGAAGAGGTGCTGACGATCGCCAGAAGCCTAAAGCCCAGTAAGGCACCGGGT
CCGGACGGAATCCCCAACCGAGCACTGAAACTAGCCATGTCCTTGCAGCCCGCGCAGTTCGTGCAGCTTT
TTAACATGTGCCTAATGGAGAGAACTTTCCCCACAATATGGAAACTCCAAACGCTAGTTTTATTACCGAAG
CCTGGTAAGCCGCGCCCGACGACCCATCGTCATACAGGCCGATTTGCCTCCTGGACACGTTAGGCAAAGT
GCTGGAGAAGCTAATCTGCAACAGGCTTCAGGTAGCTGTCGACAACAATGGTGGCCTGTCCCAAAGCCAA
TTTGGTTTCACCAAGGCAAAGTCTACAATAGACGCTCTGGAGAAAGTGGTCGGCATAGCAACAAACGCCA
TCCAAGGATCGAGATGGAAAAGCGGCACAAAAAAGTACTGCCTCATCGTCACGCTGGATGTTAAAAACG
CCTTTAACACAGCCAGGTGGGACAAAATATTGGATGCATTGTCAACGTTCCGCGTCCCATATTATCTTCAG
GAGACGATACGCAGCTACTTCACTGGAAGACTGCTGCAATACGACACCGCAGACGGCCCCACTACACATC
AAGTGTCCGCTGGAGTGCCCCAAGGCTCAGTTCTAGGACCCCTCCTCTGGAACATCATGTACGATGGCATC
CTCCGACTGGAACTCCCGATCGATACAAGTATCATCGGCTTCGCGGACGACGTGGCCCTTGTAGTCGTCGG
CAAGGAATTGAAGGACGTAGAGGAATCGTGCAACCACAGTATTGACCGTGTGCACCAATGGCTGGCAGC
AGCTGGACTCGAGCTGGCTGCTCACAAAACAGAAGCGGTGTTAGTCAGCAGTCGAAAAAGAGTGGAAAC
AGCGCACATAAGAGTCGGCAGTGCGACAATAGAATCAGCAAGGGCGCTAAAGTACCTGGGTGTCATGCT
AGACACAAGAATGTCGTTCAAAGAGCACTTGGAGTACGTGCACAAGAAAGCAGCAGAGTCCTGTCGAGC
GCTATCCAGGATTATGCTGAACACACGGGGACCGAAACAGCAGAGACGCCGTCTCCTTATGACCGTAAAC
CGAGCGGTAATAATGTACGCGTCCCCCATCTGGAGCAGAGCTATGGCTGTTCCCAGCTACAGCCGGGGCG
TACACTCTATATATCGCCTAAGCGCTCTACGGGTCAGCTGCGCATTCCGGACGGTATCGGACGAAGCGGC
GCTGGTTATCGCAGGAATGGTCCCGCTAAGCGAACTGGCAACAGAAGCTGTAGTTTGCTATAGAGCCCAC
GATGGAAGCAACACAGCAAACTCGATTCGCACTGAGGCAAGGATGCAGAGCGTAGCACGATGGCAAGCA
CGCTGGGATCGCGCATCTAAAGGACGCTGGACGCATAGGCTTATCCCAAACTTGGAAGCCTGGACAAATC
GCAAGTATGGCAATACTAATTTTTATTTAACGCAGCTCCTATCAGGCCACGGATGCTTCAGGAGCTACTTA
AAGCGATTCGGCCACGACAACACAGACGAGTGTTCTTGGTGCGGCAGAGGAATCATAGAGGACGCCAAT
CACGTCATATTCGAGTGTGGACGTTTCGCAGCGAACAGACAGGAACTGGAGGACATCATGGGCAGACCTA

TCACGGTGGACAACCTGGTCACGAGCATGATAGAGACCGCACAAAAGTGGGACGCTGCCAGCACCTTCGC
AGCAGAAGTAATGCGAGAACTCAGGAGAGTTGAGCGCAGCAGACGGAGAACCGCGGATATTTAGGTAGC
AAGAACATGCCTGTGAAGCAATGCTTCGCGGCCGTTCCGCAGGCAATCCACGCTACCTAAACAATACCCC
AACTATAACCTAAGCTAAATAACTAAGCATAAATAAACTGAATACAAAAAAAAAACACACACACACACA
CACACACACACACACACACACACACACACACACACACACACACACACACACACACACACACACACACAC
ACACACACACACACACACACATACACATACACATACACATACACATACACATACACATACACATACACAT
ACACATACACATACACATACACATACACATACACACATACACATACACATACACATACACATACACATAC
ACACACACATACACATACACATACACATACACATACACATACACACACACATACACATACACATACACAT
ACACATACACATACACATACACATACACATACACATACACATACACACACACACACATACACATACAC
ATACACATACACATACACATACACATACACA
>160_rnd-1_family-159__LINE___I-Jockey
CACTTACGTTTGTGTTTCTCTGGAGATCGGACGTGAAAATTGCAGTCTCGTGTGTGTGTGCGCGTTAGTGC
ACCGCTGGCAGCAGGCATAAACCAAGAAAGTGACAGTGACACTGTATAAAGCCCGCCCCAGGGCAATCC
ATACAAATCCCGCTTATAGTGGGTAAACTATACGCAACTGCTGCCTCTGGCAGCCGGAGTGTTATACAAA
TTAACAATATTTTAACAAAAAAAATTACCGCGTGCAGTGCGTGAGAACAAGTTCTTGCAAAACTTAAGCT
GCGCTCAGCTGATCGCAACTCAAGTGCGTGTGTGTGTGCGCGAGTGCAGCAAAGCTTTCGCGAGAGAGCT
TTCAGCTTGCAGCTGCACTGATAGTGCTGGCAAAACAGTCACTGCAGTGACAGTGCTGACACTAAAGTCA
CAATTTGTGATCAATCACTTGTTACAAAAATACGGGTTATTTTTGTAATTAAGTGTAAAAAACTTGTTTTGT
TTTTATTTTTTATTCTTTTTTCTCCCTGTGGGGAAAAAATTATAACAAAGTAAAGAACAATGGCTACAAAC
AACCGACCCCAAAACAACGAAGAAATTCATTCGCCAATCTACCCGCTGCCGAACAGCTCAATAATATCCC
CTACAAATATAGCCGCTAATGTCGATCGCCTCGACGGAAGCAGGCCACTGGAAGATTTTACCAATATTA
CAACAACGTGCTCGCCAGTAGTGCAAACCGGAACGGCGCCTCCACCTCGCTGTCAGCCGGTACCCAGCAA
TGCTCGACTTGGCAACCGCAACAGCACGGCACTGTCAGCACAACGACAGCAACAACCACCACAACCACA
GCGTCATCCAGCAGCGGCTGCAGGACATCTGCCTCAACAGGCACAAGCCGCGCCGCTTTAGAGCCCATCG
GGACCCGCCCCAAAGCCGCCAATCCGACCTTGCAGTCCACCCCTTATGGCACCCAGGCAGCTGGTTCTTGT
GTCACAAACCCCCCTCTGCCTCCCATAAAGAACGCAGGTGCTACGCCAGCCTTCGAAGAGCCACTGGTAG
ATTGGCAAACGGTTTCCGCAAAAAAACGGCGTGGGACTACATTATTATCCGAAGGCGTAAAAAAAGGGA
AGCAACTAAATAACCGCAACACCAGCCGCGCTGGAGCTAAGCCCGCAACTCCCAGTACCGCTTCGGCAAT
TAGCAACAGATATGCCTCCCTTGAGCCTCAGGAAGACGCCTCTGTAATCGATGACGGCGAGATGGACACT
GCCGACGAAGCGCCTCCAGTCACAGACCAACAACAATCAGCGAAAAAGATCTCCAAACCCAGGCCGATA
ACAGTCCCTGGAGTCAGCGATATTGTCGCAATGGAGCGAACCATAGATCAGGCAGTGGGCGCAGACGCCT
ATGAATTCAAGGTCTCTCCCTCTGGCTATCTAAGAATTTACGCCAAAGATGCCGACACCCACAGAGCAATT
GTCCGCAAGCTCACTGAAGTGAGAGTGCAATTTACCCACTTCTGCCTAAAGGAAGACAGACCATACCGCG
TTGTGGTTAAAAATCTGGCCGCTTCCACTCCCAGAAGCCAGATTGAAGCTGCCTTTACATCGCACGGCCAT
ATTGTGACGAACCTTTACAACCCGGGCGCCAGACAGCCAACAAGTGATGCAGACTCCACCAACGACTTTC
CAAGTCGCAACTTCTGGTTTGTCGATCTCAAGCAGTCTCTTAACAACAGGGAAGCGCTGCGGCTCAACAA
GGTTGGCCGCCAACGGGTCACTATTGAGCTTCCGAGGAAGAACAATAGTATAAGGCAGTGTTTCCGCTGC
TTCGAGTTCGACCACACCAAAAACTACTGTCTCAAACAGCCCAAGTGTGGTAGGTGCGGTCAGTCCCATT
GGACAAACTCCGAGCAGTGCCAGGCCAAAACAGTGGCAGACCTGCGATGCTCCAACTGCGAGGGAAACC
ATGCTGCTTCCTATAAAGGATGCAAAAGCTATCAGGTGAGACTAAATGCCAGGCAGCCGGCCATCAATGT
CTCCCCTCCTACACGATATAGCCATCCTCAAAATACACGAAGAACCACTCAGCAGTTCGTACCTTCCCGAA
TTGTGCAACAGGGCCTGTCGTATGCTGATGCGCTACGGCCAGGCCATCATGTACAAGTCCGCCAACCCCC
AGCGCGCACTCTTCAGCCCACCGTCAATCCAATGCAGCTCCAACAACCACTGCAAGGGCCTCAGGAGCAA
GACGCTTCCCTTGGAGGCATCCTTCAAAACCTCCAGCAGTCCATTGCAGCACTGAATGCCGCAGTTGCAAC
CATTCTGGTTAGCATTAAAGAGCTAAAGGATGCGCAAGGTTCCCCGCAGCTTAGATTCCCAAAAAGCCGC
AAATAATGGCTACCCAGTTACGTCTCGGATCGTGGAACGCTCGTGGCATCCTCCAAAATGCTAACGAGCT
CAAGCTTTTTCTGAAAAAGCATGATGTAGACATCATGCTCATCTCAGAAACACATCTGCACACAAACTTGT
ACCTAACAATGGAGGGGTACGTCTGCTACCGTGCGGATCACCCTACAGGACGAGCCAGAGGTGGTGCTGT
AGTGCTGGCAAAGCAGGATTTAGCACACTACCATCTACACACAGTGACCTCCAGCGACACACAGCTGGCT
GCTATCATGGTGGAGACTCCCTTGGGCGAAATCCTCGTTGCGGCAGCATACCTACCACCAAACATCCCCTG
GAACCGAGCGGAATTTGATTCCCTGTTTGGCCAGCTTGGCCCCAAATTCATCGTTGGAGGCGACTTCAACG
CTAAGCACAGACTGTGGGGAAACTACAGAGCTGACAGCAGAGGCACTGCGCTCCATGAGGCGCTCACTCC
CTGCTCAGCCCAGGTACTGGCCACCGGTAGACCTACCCACTTTCCGTACAACCGGCAAAGTACGCCTTCCT
GTATCGACTTTTTTATATTCAAGGGCATCCCCAACAGCGTACTATCGGTTCGCGAAGAGTATGATCTATCT

TCCGACCATCTGCCACTCCTCACAACGATCAACACGGCTGCACAAAGGATCCCAAAAAAGCGCAGGCTTG
TCCTCCCTGGGTCGAACATCCAACGATTTAAGGCCGAACTAAATAGCTTGATCAACCTAAACACGCAGAT
TCTGTCAGTTGAGGACGTCGACGCTGCAGTACAAACCCTGTTGGACCAAGTACATGCCGCAGCCGCCAAC
GCTGCGCCCGCACATATCTACTCAGCCCCTACAAGCCAGCGGCCAAGGACGTTTTCCCCCATATTGGAAG
GTCTTCTCGCCCTAAAAAAGCGACTGAGGAGGGAGTATGTCCGTACCCAGGACCCCACGATTGACAGGAT
CTATCGTCGTATTGCTAACAGGGTCAGGAAGGAGCTGATTATCTCGAAAAGAAATGCCACAGACACCATG
CTGGAGGAAGCAACGGCAGACGAGAGCTCCAAATTCGCGCTGTGGAAACTTAGCAGTCGATACAAGCGG
CAAGCTGCGCCAAAGTTCCCCGTTCGACTTCCCGATGACACCTGGGCCAGATCACCAATGGACAGAGCCG
AAGCCTTTGCCTGCAACTTGGAGGAGCGCTTCAAGCCGTTTGAGACCGCATCGCATGGAAGGATACAGCG
AGTCGCGCAAATCCTGGAGGCGCCACTCCAAATGTCACTCCCTGTATCGCCAATCACCTTCCAAGAAGTA
GAGCAGGAGATGAAGAGATTAAAAAGCAGCAAAGCCCCTGGGGAAGATCGGTTGGACAACCAGACAATC
AAGCTACTGCCATACAAGGCGGTGCTTTTTCTCGTCGCCATTTTTAATGCAGCTCTTCGGCTAGGTTACTTT
CCAGTGGCTTGGAAGAAGGCACATATAATCATGTTGCACAAGAGCGGAAAGCCCCCTAATCAACTGGGAT
CCNNNNNNNNNNNNNNNNNNNNNNNNNNNNCATTTTTAATGCAGCTCTTCGGCTAGGTTACTTTCCAGTGG
CTTGGAAGAAGGCACATATAATCATGTTGCACAAGAGCGGAAAGCCCCCTAATCAACTGGGATCCTATCG
CCCCATCAGCCTCCTGCCTGCCTTTGGTAAGATTCTGGAAAGGTGCATCCTCAGAAGGGTCCTGGATACCC
CGGAAATAAAACGCTTGATCCCGAAGTTCCAGTTTGGGTTTCGACTCAAACACGGTACCCCTGAGCAACT
ACATCGGGTCACAAATTATATTTTGGACGGCTTTCACAGGAAAGAGTATGTGGTGGCAGCATACCTCGAC
ATCCAAGAAGCCTTCGACCGGGTGTGGCACGCAGGACTCCTCTCCAAGATAAAGGACAAATTGAATCCGC
AGCTATTTAGCACCATTGCGAGCTTCCTAACTGAGAGAACCTTTCAAGTGGTGCAGGATGGAGTAGCCTCT
CAAACCAAACGCATAAAAGCGGGCGTTCCACAAGGCAGCGTCTTGGGTCCCACGCTATATAGCCTGTTTA
CACATGACATGCCCACAGCAAATGCAAGCAGACGGAATGATCGGCAGGACCTTCTGGTGGCCACGTTTGC
TGACGACACGGCAATCCTTGCAAAAAACAAATGTGTGTACGTGGCAACTGACCTCTTGCAGGAATACCTC
AAGCGATTTGAAAACTGGGCTTGTAGATGGAACATCGCAGCGAACGCAACCNGGACGGAATGNNCCACG
GNCACGCACACCCTNCGCNGGCCACGTTTGCTGACGACACGGCAATCCTTGCAAAAAACAAATGTGTGTA
CGTGGCAACTGACCTCTTGCAGGAATACCTCAAGCGATTTGAAAACTGGGCTTGTAGATGGAACATCGCA
GTGAACCCTGGAAAGTGCGCCACGGTTACCCACACTCTACGCAAGGACTCATGCCCCGGACTTTTTCTTCA
TGGTGAGCTTCTAGAGCAATATCGTCAACACAAGTACCTAGGTGTCACTCTCGACAGACGACTTACATTTA
GTAAGCACGTCTCTGCAGTAACGAGCAACATCAAATCTAAGATAAGGCGAATGAGCTGGCTCCTCAACTC
CAAAAACAAACTCTCGCTCCGAAACAAGGTCCTAATCTACAAGGTAATCCTTGCCCCAGTGTGGCGTTAC
GGGCTTCAAGTTTACGGTATAGCTGCAAAAACACACCTGAACAAAATTCGCATCATTCAGGCCAAAACCC
TGCGCAAAATAACTGGAGCCGAATGGTATATTCGCACACGCGACATCGCCAAGGACCTCAAGGTTCCAAT
GGTGGGCGACGTAGTCAACAGGCAAGCAGCGAGCTACTCAACAAAGCTCCACAACCATCCAAACATCCT
GGCGAGGAGCCTGCTAAGGAGGCGAAGAAGAAGGCGACTGAAGAGGACCTACCCAACCGACCTAATCGA
TCGTTATGTATAGGTGACAAGCTTCGTTAATTACACCAAAATATGTTTCTGTAATCTGTTAACCTAATTGTA
TAACTAATTGTAAAACCACAATCATCGCTAAGCTAAGTAATTATGTCACTCTGATAAAAGTTGCTAACATA
GAGTAACAGGATACCAGATTTCTTAATAAACAAAATATAAAAAAAAAAAAAAAAAAAAAAAAAA
>Ref_NewTE4
CATGTAGGAGTTGGAAACAGAACTAGGAGTGGTCGAACTTACAATCAGTTAAATTAAACAGGCGAGTATA
ATGTTGGCATTATACTCATTTGGTAATATAAAATCACTTATCATATGCAACCCTGCTATGGATATTTAATC
AACGATAGATTTATCGATAAAAATACAAGAAGGAGAAGTTCAGTTGTAAGCTAGAAGAGTCAATGAAAA
GATGTGTTCGCTGTTGCTCGTTGTCTTTGAAATACATGTCGGAAAATGTACAATAATCATAAGTTTACGCT
TGTTCACCAATTTCTCTAAATAAATAATATGAACATAAAGAACTCAAGTGCTTGTGAAGGCCATCGAATG
GTCATCCCTACAAATTTGGTGTCAGAAGTGGGATAGACAGAAATTGTGAAATTCGTAAATTGATTAACGA
GTACAAACGTATGCAAGACTATTAATCGGAGCATAAAACGAAGCGGGTGCATGAAAATTCATTAAAATTC
GTATGCCCTGGAAAAAATGGAAAAAGTCAAGAATCGTCTAGGATTAATGGCGACAGCGGCGAAGTATGC
CTAGAGAGCTCTAGTAAGAGCGAGACAAACAGTCGTGTATTGCGTGACAGTGGTCGATAAGCGCAAGCG
AGAAGGCGATGTTTTAAATTCTGCGTAGCAAAGGCTGGATAGCGCAAAATTTTCACTTCGTGATGAAATA
AGAAGTTGCAGATCAAAGAGGCGAAGAGCGAAGAGCGCGGAATCTTTCAATAAAAGAGCGTGTGACTGG
AACGTGCTTCAACACGACAACGATGATCACAGGGTGTATCAAGTTACAGATCAACAACAGAGTATGTTAT
TTCGTCATTATATGAAAATAATTAACTAGTTGTTGAGTAAAGAGTGTTAAATCAGTAAAAAATTTTCAAAG
AATCAAATTATAACAAAGAAGAAATTAAGTGTAAGCAAACACAAAGTGAAAGTATAAGTTAAGACAGAC
GAATATAATAATTTAATAATTTCTAAAGTTGCTGAAAAAAAAAAACTAAAATTTGATTCATAAGAGTTTAAT
TACTTATACCATTAAGTTAAGTACATTGAATTGTTTATTTGTAAGATTCAATTTTAAGCATTGTGAAAAGC

GAGCAACGACGATAACAAAATGAACAGAGACGAAATTTTGGCGTTAAGAGTTAACGAATTAAAAGAAAA
GTTATCGGCACTTAGTTTGGTAACAACAGGTAGAAAGCAAACTTTGCAAGACCGACTTTTAGAATATTAT
GGGTTGCAGGTTGACGATAATGAATCTGAGTATGACGATGCTGGGTCGGCTCATACCCGTCAAAGCTCAC
CAATGCAGATAGTCCGAACAACGTTTACATTACGCGACATTGAAGATTCACTCTCGCAATTCAATGGGTC
AAATCAACCAGCTGTTGAAAAATGGATTCATGAATTTGAAGATAACGCTTTGGCAGTGCAGTGGAACGAC
CTTCAGAAATTTATTTACTCAAAGCAATTACTAAAAGGTGCAGCAAAATTATTCGTGCGCAGTCAGCATG
GAATTTCAGATTGGATATCTTTAAAGCAAGCGCTATTGGAAGAATTTGGGACGGTAGTTTCATCAATAGA
CGTCCACCGCATGTTGCGGAATAGGCGTAAGCGTCAAGGTGAAGATTATATAGAATATCTGTATAGCCTT
ATGGAAATTGGCGGACCTCTTAACCTGGATGACTCAAGCCTGATAGAATATTTTATTGAGGGAATACCTG
ACTCGAGGAGTAACAAAAGTAATCTGTATCAGGCCAAGACTCTGCAGCAACTAAAAGAACAAATCAAGG
TATATGAGAAAATCCGTAGTAGTCGCCCTCAAGCGTCTAATACGGGTAGGATGCATAACGATACCGAAGA
AAAGAAACCTGACTTGAATAAAGAACGAATTAAGAAATGTTTCAAATGCGGCGATCCATCACATCTAGCC
AGAGAATGTAGACAGCCAATAAAATGTTACAAGTGTGCTCAACCAGGGCATAAGGCAGCTAACTGTTCAG
GATCGCGTGCAGTGGAGAAGAAAGAAGGCCAAAATGCGAACACGATGATAAACAAGGCCAACAGTAGTG
GAAAATGCATTTTTAAGGAGTTGTCTTGTAAGAACATATGCTTCTCTGCATTAATCGATACAGGCTGTGAC
CTCTGTCTGATACGCGATGATATCCTTAAAGAGCTAGGAAAAGTTGAACTGAATAATGAAAAACAGTGTC
TTGTTGGTATCTGTAATAGTGAGCTAAATACACTGGGAAGCTTTGTCACTCAAGTCGAAGTTGATGGTATC
TTACTGGATATAACCTTTCATGTTACGAGCAGAGACGACATCCAATACTCAGCTGTAATAGGAAACAGCG
TATTGAAGCAAGCGGACTTAATAGTATCGGAAAATTTCGTAGAATTTCGACCCAAAGTGAAGGACAATGC
AAACGAGAGAGTAATTGACAAGACATCAACAATAACAGAAGATACTGTCGAAAATAAGAAGCCAGACAT
AGAGCTTTTACAAGAATTCAAAGATGTATGTTTTTTTGCAGAAAATCAGGAGGCTGAGATCGATCTACATC
ACTTAGAAAAACCTGTTGCAGACGCAGTTCAAGCAATAATTAAAAACTATTCACCTGAAATGAAGAAGAA
ATGTCCAATTGAAATGAAAATTTTGTTAAATGATGAGCGTCCAGTTTTCGAACGCCCAAGGCGCATATCAC
ATGCGGACAAGTCTATTGTCGATTCGCAAATCAAAACTTGGCTAGAAGAAAAGATCATACAGCCAAGTAC
ATCGGAGTATGCATCTCCTGTTGTCCTCGTTCCGAAAAAAGATGGTACCAAAAGATTGTGCTGTGACTACA
GGCGTTTAAACGAAAAGATCGCGAGGGACAATTTTCCAATGCCCTTAATAGACGATGTGATTGAGAGATT
GCAGGGCCAAAGTGTGTTTACTACACTTGATCTCGCAAACGGATTTTTTCATGTTCCAATTGAGCCAAATT
CGAGAAAATTTACATCATTTGTAACAAGTGCAGGGCAATATGAATTCCTGTTCGTCCCTTTTGGAATATCG
AACTCCCCAGCTGTATTTTCCAGATTCATTCATGCAATTCTTGTAGATCTGGTACAGGATGGTACAGTCGT
AACTTATATGGATGATCTTATAATTCCCTCCAAGGATATAGATGAAGGTATCCAAAAGTTAAAGCGCGTA
CTGGACAGAGCAGCAGAGTTTAACTTGCGTATTAAGTGGAGCAAGTGCCAGTTTCTCATGAAGGAAGTTG
ACTTTTTAGGGTACGTTATACAGGGAGGCACAATAAGACCATCTGCAAGAAAAACCCAGGCAGTTCAAAA
TTTTCCTATACCGCATGACCGAAAGAGTTTGCAGCGTTTTCTCGGATTAACATCGTACTTTAGGCGCTTTGT
AGAAGACTATGCGCTGATAGCCAAACCGCTCTCCGATTTGTTACGGAAAGACGTGCAATTTAAAGTTGAT
GAAGAACAACTAGTCGCATTCAGGCAATTAAAATCAGCATTAGTTAGTACTCCAGTATTGCGATTATATA
ATCCGAAAGCCAAGACTGAAGTGCATACGGACGCCTCAATGAATGGTTATGGTGGTGTACTCTTGCAAAA
AGACTCGGACGATCAATTGTTGCATCCAATTCAATATTTAAGTCGGAAAACAAAACCCTCAGAAGAGAAG
TATCATTCTTATGAGCTTGAGGTGCTGGCTATAGTCGAGGCCTTGAAAAAGTGGCGCGTTTACTTACTTGG
CATAAAATTTAAGATTGTGACCGACTGTAACGCTTTCGCTTTGACCATGAAAAAACGTGGAGATATTCCAT
TGAGAGTCTCGCGTTGGGCACTCTTCCTTCAAGATTTTGACTACAGTATTGAGCATCGAAATGGCAGCAAA
ATGAGGCATGTTGATGCTTTAAGCCGCGTATCCTGTCTTATGCTTGAAGACTCACTGCAGCACAGATTGCA
GCAAGCACAAGCGAGAGATGATTGGGTCCGAGCGGTGCTAACTGTTTTGGATTGTAGCACATACGAAGAT
TTTTATGTTAGGCATGGTATTTTATACAAGGATCCCATCAAGGAACTTATAGTAGTACCATCGCTGATGGA
AGACGAAATCATAAGAATTGCACACAAGCAAGGGCATTTCTCAGCGAAACGAACTCAAGAAGCAATCGA
GAAGACATTCTATATTCCGCAGCTATCGTCAAAAGTGCCAAAAATTGTAAAAAGTTGCGTAGAATGCATA
ATATCTGAGAGCAAATCAGGCAAGAAAGAAGGGTTTTTGGCACCAATTGACAAAGAAGACAAGCCCTTA
GGCACGTACCATGTGGATCATGTCGGTCCAATAGAACAGACAAGTAAAAACTATAACTATATTCTAGTGA
TAGTCGATGCGTTTTCAAAATTTGTTTGGCTCTATCCAACAAAAAATACCACCGCAGAAGGAGTTGTAGAT
CGACTAAGACGGCAGGCAGCAGTGTTTGGAAATCCTAAGCGCATAATATCAGATAGGGGTGCAGCTTTCG
TTTCAAACCTGTTCAAGGAGTACTGTGAAACAGAAGGTATACAGCATCTTACGATAGCTACAGGAGTTCC
AAGAGGAAACGGCCAGGTAGAACGTATACACAAAATCGTAATACCAATGTTGTCTAAACTATGTCGTGAA
AATCCTTGTAGCTGGTATAAGCATATTGATATCGTTCAACAAACAATCAACAGTACGCCGCCAAGAAGCA
CTAAAGTTTCACCGTTTAGATTGTTGACTGGTGTGGAGATGAAACTTAAAAGAATATCGGACTTGCAAGA
ACTATTAGAAGAAGCTGCTATAGAGGAGCTAAGTAACGAAAGAGAAGAAGTTCGTAAAGAAGCGCGGCA

AAATATAGCAAAAATACAGCAGGAAAACCGAAAATCTTTTAATAAACATCGCAAAAAAGAGTCCAATTA
TAAAATAAACGAACTTGTAGCCATTAAGAGAACACAGTATGGTACTGGTTTAAAATTGAAACCTAAATTC
TTTGGTCCATATAAAATAGTAAAGATTCAAAACCATGGTAGATATGACGTAGAAAAGGTAGGCGACCACG
AAGGTCCAGGGAAAACTACAACTGTAGCAGAATACATGAAAAAATGGGAGCCAAATGCTAATTCAACAG
AAGAGTCGTCATTCGGGTCGAATGAAGAGTCAGGATGGCCGAGTGTAGGAGTTGGAAACAGAACTAGGA
GTGGTCGAACTTACAATCAGTTAAATTAAACAGGCGAGTATAATGTTGGCATTATACTCATTTGGTAATAT
AAAATCACTTATCATATGCAACCCTGCTATGGATATTTAATCAACGATAGATTTATCGATAAAAATACAAG
AAGGAGAAGTTCAGTTGTAAGCTAGAAGAGTCAATGAAAAGATGTGTTCGCTGTTGCTCGTTGTCTTTGA
AATACATGTCGGAAAATGTACAATAATCATAAGTTTACGCTTGTTCACCAATTTCTCTAAATAAATAATAT
GAACATAAAGAACTCAAGTGCTTGTGAAGGCCATCGAATGGTCATCCCTACATTCATG
>9_rnd-1_family-224__LTR___Gypsy
ATATATGTAGGAGTTGGAAACAGAACTAGGAGTGGTCGAACTTACAATCAGTTAAATTAAACAGGCGAGT
ATAATGTTGGCATTATACTCATTTGGTAATATAAAATCACTTATCATATGCAACCCTGCTATGGATATTTA
ATCAACGATAGATTTATCGATAAAAATACAAGAAGGAGAAGCTGAGTTGTAAGCTAGAAGAGTCAATGA
AAAGATGTGTTCGCTGTTGCTCGTTGTCTTTGAAATACATGTCGGAAAATGTACAATAATCATAAGTTTAC
GCTTGTTCACCAATTTCTCTAAATAAAATAATATGAACATAAAGAACTCAAGTGCTTGTGAAGGCCATCG
AATGGTCATCCCTACAAATTTGGTGTCAGAAGTGGGATAGACAGAAATTGTGAAATTCGTAAATTGATTA
ACGAGTACAAACGTATGCAAGACTATTAATCGGAGCATAAAACGAAGCGGGTGCATGAAAATTCATTAA
AATTCGTATGCCCTGGAAAAAATGGAAAAAGTCAAGAATCGTCTAGGATTAATGGCGACAGCGGCGAAG
TATGCCTAGAGAGCTCTAGTAAGAGCGAGACAAACAGTCGTGTATTGCGTGACAGTGGTCGATAAGCGCA
AGCGAGAAGGCGATGTTTTAAATTCTGCGTAGCAAAGGCTGGATAGCGCAAAATTTTCACTTCGTGATGA
AATAAGAAGTTGCAGATCAAAGAGGCGAAGAGCGAAGAGCGCGGAATCTTTCAATAAAAGAGCGTGTGA
CTGGAACGTGCTTCAACACGACAACGATGATCACAGGGTGTATCAAGTTACAGATCAACAACAGAGTATG
TTATTTCGTCATTATATGAAAATAATTAACTAGTTGTTGAGTAAAGAGTGTTAAATCAGTAAAAAATTTTC
AAAGAATCAAATTATAACAAAGAAGAAATTAAGTGTAAGCAAACACAAAGTGAAAGTATAAGTTAAGAC
AGACGAATATAATAATTTAATAATTTCTAAAGTTGCTGAAAAAAAAACTAAAATTTGATTCATAAGAGTT
TTTAATTACTTATACCATTAAGTTAAGTACATTGAATTGTTTATTTGTAAGATTCAATTTTAAGCATTGTGA
AAAGCGAGCAACGACGATAACAAAATGAACAGAGACGAAATTTTGGCGTTAAGAGTTAACGAATTAAAA
GAAAAGTTATCGGCACTTAGTTTGGTAACAACAGGTAGAAAGCAAACTTTGCAAGACCGACTTTTAGAAT
ATTATGGGTTGCAGGTTGACGATAATGAATCTGAGTATGACGATGCTGGGTCGGCTCATACCCGTCAAAG
CTCACCAATGCAGATAGTCCGAACAACGTTTACATTACGCGACATTGAAGATTCACTCTCGCAATTCAATG
GGTCAAATCAACCAGCTGTTGAAAAATGGATTCATGAATTTGAAGATAACGCTTTGGCAGTGCAGTGGAA
CGACCTTCAGAAATTTATTTACTCAAAGCAATTACTAAAAGGTGCAGCAAAATTATTCGTGCGCAGTCAG
CATGGAATTTCAGATTGGATATCTTTAAAGCAAGCGCTATTGGAAGAATTTGGGACGGTAGTTTCATCAAT
AGACGTCCACCGCATGTTGCGGAATAGGCGTAAGCGTCAAGGTGAAGATTATATAGAATATCTGTATAGC
CTTATGGAAATTGGCGGACCTCTTAACCTGGATGACTCAAGCCTGATAGAATATTTTATTGAGGGAATACC
TGACTCGAGGAGTAACAAAAAGTAATCTGTATCAGGCCAAGACTCTGCAGCAACTAAAAGAACAAATCA
AGGTATATGAGAAAATCCGTAGTAGTCGCCCTCAAGCGTCTAATACGGGTAGGATGCATAACGATACCGA
AGAAAAGAAACCTGACTTGAATAAAGAACGAATTAAGAAATGTTTCAAATGCGGCGATCCATCACATCTA
GCCAGAGAATGTAGACAGCCAATAAAATGTTACAAGTGTGCTCAACCAGGGCATAAGGCAGCTAACTGTT
CAGGATCGCGTGCAGTGGAGAAGAAAGAAGGCCAAAATGCGAACACGATGATAAACAAGGCCAACAGT
AGTGGAAAATGCATTTTTAAGGAGTTGTCTTGTAAGAACATATGCTTCTCTGCATTAATCGATACAGGCTG
TGACCTCTGTCTGATACGCGATGATATCCTTAAAGAGCTAGGAAAAGTTGAACTGAATAATGAAAAACAG
TGTCTTGTTGGTATCTGTAATAGTGAGCTAAATACACTGGGAAGCTTTGTCACTCAAGTCGAAGTTGATGG
TATCTTACTGGATATAACCTTTCATGTTACGAGCAGAGACGACATCCAATACTCAGCTGTAATAGGAAAC
AGCGTATTGAAGCAAGCGGACTTAATAGTATCGGAAAATTTCGTAGAATTTCGACCCAAAGTGAAGGACA
ATGCAAACGAGAGAGTAATTGACAAGACATCAACAATAACAGAAGATACTGTCGAAAATAAGAAGCCAG
ACATAGAGCTTTTACAAGAATTCAAAGATGTATGTTTTTTGCAGAAAATCAGGAAGCTGAGATCGATCTA
CATCACTTAGAAAAACCTGTTGCAGACGCAGTTCAAGCAATAATTAAAAACTATTCACCTGAAATGAAGA
AGAAATGTCCAATTGAAATGAAAATTTTGTTAAATGATGAGCGTCCAGTTTTCGAACGCCCAAGGCGCAT
ATCACATGCGGACAAGTCTATTGTCGATTCGCAAATCAAAGACTATTTAAAAGATGGAATCATTAGGCCA
AGTTGTTCGGAGTATGCATCACCTATAGTACTAGTGAAGAAAAAAATGGAGACATGAGGTTATGTATTGA
TTTTAGAAGACTGAACGAAATTATATGTAGAGACAACTACCCGCTACCATTGATCGACGATTTATTAGAC
AGACTATGCGGAAAAACAGTATTTTCAAAGTTGGATCTAAAAACGGGTATTTTCATGTTTTTGTTAATGAG

CAATCTATTAAATACACTTCATTTGTTACCCCTTTAGGACAATATGAATTTCTTAGGATGCCCATGGGTTTA
AAAACTGCGCCACATGTATTTCAAAGATTTGTTAATAACATATTTTGTGATATGATAAAAGATAATAAGGT
AATTGTTTACATGGATGATATTATGATAGCAAGTACAAACATAAAGGAACACATGGAGACCTTAAAGGAA
GTGTTTATAAGACTGGTGCAAAACAAATTAGAGTTGAAATTAGATAAGTGTGAATTTTTNAAAAGCAGCG
TTAAATATTTAGGATTTGTGGTGGATAGTAAAGGCGTTAGGGCTGATGAAAAAGGGTTAGAAGCGGTTAA
ATCCTTTCCAATACCAAACAAAATTCAGGGAGTACAAAGTTTCCTAGGGTTATGCTCCTACTTCAGGAGGT
TCATTTTAAACTTTTCTATATTAGCTAAACCCTTATATGATTTATTGAAAAAAGATAAGAAATTCAAGTTT
GGAGAAGAGGAGTTTAAGTGTTTTAATATGTTAAAAGATAAATTGTTAGAGGCGCCAGTACTATCAATAT
ATAATTACAAAGAAGAAATAGAACTACATTGTGATGCCAGCGCAGTGGGATTTGGGGCTGTTTTATTTCA
AAAAAAGAAGANAGGAAATTACACCCGATTTTTTATTTTTCTAAAAGAACAACCGAAGTAGAGTCTAAAT
ATCATAGTTTTGAGTTAGAAACGCTAGCCATCATTTACGCATTACGCAGATTTCGTATATATCTGCAGGGA
AAACGATTTAAAATAATGACAGATTGCAACTCTTTAACTTTAACGCTTAACAAAATAGATTTAAATCCGA
GAATTGCTAGATGGGCGTTAGAACTACAAAATTACGATTTTGAGTTAGTGCATAGATCGGGCAAGCAAAT
GCAACATGTGGATGCGTTAAGCAGATGTTATGAGGACATTTTAGTTATTGAATCCAATAGTTTTGAGGACA
ATTTAGTTATTTGCCAAAGCAAAGATGACAAACTGGTAGATATTAGAAAGATGTTAGAAAAAGAAGAGC
ACAAGCTATTCGAAATGAGAAATGGGGTCATTTACAGAAAAACAAATGATGGCAGATTAGTTTTTATGTA
CCAAAGGATATGGAAGAACATGTACTTTACAAATATCATGATGAGTTGGGACATGCTGGGAGAGATAAG
ATGATCGATGCCATTGGAAAGAGTTACTGGTTCCCATATATTAAGGAAAAAGCAATAAAGCATATAGGGA
ATTGTTTAAAATGCATTGCCTTCGCCCCCAAGACGGGTAAGAGTGAAGGACTATTACATAGCATACCGAA
AGGAAACAAACCGTGGGAGATGATCCATATAGACCATTATGGACCAATTAATGCAGGAAGGGCTAATAA
ATATATATTGGCGGTGGTTGACGGATTTTCAAAATTTGTTAGACTATATACAGCAAAGACAACAAGTACG
AAAGAAGCGATTAAGGCATTAAAAGATTATTTCAGAGCGTATAGTAGACCTAAGTTCATTGTATCGGACA
GAGGGAGTTGTTTTACTGCAAAAGAGTTTGATGAATTTTTAAAAATGAGGGAGTAACGCATATTAAAATA
GCAACGGGTTGTCCGCAAGCAAATGGTCAGGTGGAAAGGTTGAATAGAGACTTGGGTCCGATGATGGCA
AAAATGGCGGAGCCAGAAAATGGTTTACATTGGGATGTTATTATAGAAAATGTTGAATATGCAATAAACA
ACACACAACACAAAAGCATAAAGAAATTTCCAAGCGAAATGTTATTCGGATTACAACAGAAAGGAAAAG
TTGTTGATGAATTAAAAGAAAAATTAGAAGAGTTTACACAGGTGAACACGTGTGATAAAAGAAATTTAGA
AAATATAAGAAAGAGTGGAGAAATATATCAAAAAGAAGCTCAAATCAGAAATGAAGAGCGGATTAATAA
AAAGAAGAAAGTATCAGTAGAATATAAAATAGGTGACTATGTCATGGTAAAAAATTTTGATAGCACACCA
GGAGTTTCAAAAAGTTAATACCAAGATATAAAGGGCCTTATAGTATAGAAAAGATTTTAAAAAAATGATA
GATACTTGTTAAAGGATGTAGAAGGCTTTCAATTATCTCGCAACCCGTACGTTGGTATCTGGAATAGCCAA
AACTTTAAGCATTGGATGCGAAAATAGGCAATAAGCATTAGTTATAAAGATAGATACACATATAAAAGAC
AGAGATCGGGAGCTCTCTTTGTCAGGATGGCCGAGTTGTAATAGGATAAGAGCATCATTGGTAACCCTAG
CCAGGGTATTATAGTGTCACTATCTATGTTAATTATAACAGTACGATAAGTAAATATCGATAAAGAAAAG
CGGCTGCAGAGAAGAAGTGGAAGGCAGTCTCTCTACAGTACTAGCAGTAAGAAGACGTGTTGTTAAAAAT
AAAAAGGCAACCCGAACATAAGTTAAATCAACGAAGAAAATCATACTATTACAATA
>160_rnd-1_family-38__RC___Helitron
TAAGTTTGTTAAATACTTGTAAGGTGCTCAATATGTTCTTGGAGCGATGTTGAAAATATAATGATTTCGTC
TACAATAGACAAAATATTCCTATTAGGTCTTGTAAGATATTTACAATAATTCTTTGAAATGTCTCTGGTAC
ATTTGTCACTCCGAAAGGCACACTCACTGTCAGTATGGAGTGGCGATATGTTCCGACGGACCGAAATCCA
GCTGACATTCTTTCTAGAGGCACTGTTCCTAGCCCAGTCGGAGCTCAGGTTTCATGGCCCATCGTATCTAT
TAAATGGTCGCCTAAATTGGCCGGCGACCTGTCTGCCTAAAGCTCCAACCATTGAATTGAGGAAAAGTGT
ACTAATTCTTAAGTCTCCACATGCAGATTTAGCTCTTGGATGCAAGCACGCCAACTCTTTTGGTTCCATGC
AGCGCACATTCGGATATGTGTACAAATTTATGAAGCATCATGGACTCACTGTTGCAGACATTCATCACGGC
ACTCGCATTCTGCTTCGGGGTGTACAGAGAGCGAACTTGTGGGGTGAAATTCCAGTGTCTTGAAATGCCA
GTGTCTTTTTATTTTAGCTAGCGTTCTTGAAATGCCAGTATGACCTCCGTGTATTGGATCTTCATGATATGT
AAACAGTATCGCTTCTAGATCTTTTTCATTTTTTATTGTGGTCAACGGCTTGAGTAGAGCTACTTGAAATGA
TTTTAATGTTTTGTTGCCCAGACTTTTAAAAGATTCAATTGAAATTAATTTAAATATCTTTTCGCTCGGTGC
CAGTTTGAGTTAGCTGATCTCAACTATACCGGCTTCTTTCTCAAGCTAAGTGACCTAAGTCAAATGTACCA
TTGGTGTACAGATCGCTAACATCAATTCTTGTTATAATTTCTCTTCCAGGCTTGAAAGAACATATATTGTTC
AATACAAGCAATAACACTACTTTTCGTACTTCGTCATTAGAAATGAATTCATATACTTTTGGCTAAGAAGC
GTTCTCAAGAGTTTGCATAGGCAAATCTACTTAATCTTTTGCTGCGCAGGAGTTTTTCTGTTTACTTTGGTG
CATGGTAGTGACTTTCAGGACCTTAACTTGTAAATCGTTAAGTTCAGATATTGTTATCCGGGACAATACAT
CGGCTACGAAATTATCTCTGCCTCTTAGGTACTGTACAGTGAACTGATACTCTTCTAAATCTAGTCTCATA

CTCGTTAATTTTGAAGTTGGGTTTACCATTGAAAACAAATATGTTAATGGTCAATGGTCTGTTTTAATTGTG
AAATGTTGACACTATATGTATGGTCGCAAATGCGTTATTGCCCAGTGGATTGCTGCCAACATTTGTTCAGT
TGTACTTTTATTGCTTTATCCTTTTGTAAATTAACGAGAAGCATATGCTACGGGCAATTGTTTATCTCCATA
CTTTTCAGTCAAAACTGCTGCGCAGGCTAGTTTACTTCGTTTACTTTTTTGCTTCTTTGCTAAAATTTGGGT
ATTTTAGTAAAGTAGGCTTCATAAGAGCTTTTTTTAAATATGCGAAAGCTTTTTGGCATTCATCGGATCAC
TCAAAAACTCTTCAAACTTTTTACATAATCTTGTTATGTGACGTAAATAATCTGCAACGTTCTTTATAAAAC
GTCTGTAATTAATGACCACTTATTTTTTGTCATCTGGCAAAATACCTTGATCCGTACAGTTATGACCTAGA
AAAGTCACTTTGTGAAGGTTCTAATCCAAAAAAGCGATGGTCATCATTCTTCGAAAAGAATTAGGCGCTA
TTTTTAGACGCTAAATTTCTAGAATTTTCATCTAATTCTGTTTGGTGAAAACCGGACATTAAATCTAAGCAT
GAAAAATATTTTGATCTGCCTAATTGATCGGGAATATTATCTATTCTTGGGAGTGGAAATTTGTCAGATAA
GAGTTCTTATAAATTTGACGATAGTCTATAACTAAGCGCCATCGCTTGGTATCGGAACAAGGTAATCCCCT
TTATGGGACAAGAAGTATAGGGCTGTTATATTCCGATACAGACGTTTCTACAATTTTATCCGAAATAAGTT
TTTCGACTTGATTATTAACTTCCACTTTATGAGTAAGAGGAGTTTTGTAGGTCTTAATGTAAACTGGCTCA
GTATATTTTAGTCTTAGTTTCTGTTTATAATACTTATTGGTTGTTGTGGTTTCGGTTTTGAGTCCGAATATGC
CTGTATACTTGGTGCAGAGATCTTTAAATTGTGTTTTGAATAATGGGGAAAAGTTCTTTTTTAGTTTTTTCA
TCTCCGTTTCTTGGCGATCTTTGCAAATTTCATAAACTCTATCATAGTGACATAGCTTTTAAATTTCAAGGT
CGTTAAGATTTACTATCTTTATTGAATTTGTTGTATTTAAAATTCGGACAAATGTTTGTTGATTATTGGAAA
TAATATTTCCACTGAATATTGCTTTATCAATTTCTTGATTTGAAATGAAAATATTGTTTTCTCGCGAAACAA
TATTGATTTGACGGACTACTTCAGAACGAGCTGGAAGTATTGTGGCATAATTATCACAAGTGTGAGTTATT
GGTAGTTTTATTGGAAAATTTAAATTATTTGGCCGAAGTATTAGAGAATCACATGTTTGTGTGAAATCTAA
TTGGCAAGTATACTTTTTTGGAATGACATATATTCCGGTAGATAATTCCACGAACGCAGGCAGAAGGAAG
CATCTACGACCCTATGAAGTATATATAGAAATATATATCAAAATTTTAAACGTAGGTGCTCCTAGTGCCTG
CGCAGATCGAGTTTGTTTCCNATAATCGATAACTTACTCCGTTTCAAGCAATCGATAAAAATCGATATCGA
TATCCTGCTTTTAGGGCAAATTTGGTAAATAATAAGAGCTAGAGTCACCAAACATGACATATAGCTTCTTA
AATAGAATATATATATATGCATTTGATGTTAGGAGAAGAGGGTTCAGGGTATCGTCTAATCGGAAGCTCCCG
ACAAGAACATCTTACTTGTTTAATTTATTTGATCTTGAAATAAGTGGGTTCAGGGTATTTCCTAGTCGGGA
GCTCCCGACTAGAACCTCTTACTTGTTGGGTTTTTCGCCGTTAAGACTTTTTGACTTGCTAAATCTGGGTGT
CATTTTATTACGTCGATTTTCTTATCTTCTATTTTATATTCTTTACGCTGTTATACCCTTGCAGAGGGTATTA
TAATTTTGTCGTGAAATGTGTAACGCATAGAAGGAGACATCTCCGACCCCATAAAGTATATATATTCTTAA
TCAGCATCAACAGCCGAGTCGATATAGCCATGTCCGTCTGTCCGTCTGTCTGTTTCTATGCGAACTAGTCC
CTCAGTTTTAAAGCTATCTTAATGAAACTTTGCAGAACTCCCTCTTTCTGTTGCACGCAGCACATATGTGA
AAACCAGCTGGATCGGACCACTATATCATATAGCTGCCATAGGAACGATCGGTCGAAAATTAAGTTTTTG
TATGAAAAAACATTTTGTTTATCAAGATATCTTGACCAAACTCGGCATTTATTAGTTTTACTTTACTCCTCA
TATATATGCAAAATCCTATTAAGATCGGACCACTATATCATATAGCTGCCATAGGAACGATCGGTCGAAA
ATTAAGTTTTTGTATGAAAAAACATTTTGTTTNTCAAGATATCTTGACCAAACTCGGCATTTATTAGTTTTA
CTNTACTCCTCATATATGTGCAAAATCCTATTAAGATCGGACCACTATATCATATAGCTGCCATAGGAACG
ATCGGTCGAAAATTAAGTTTTTGTATGAAAAAACATTTTGTTTNTCAAGATATCTTGACCAAACTCGGCAT
TTATTAGTTTTACTNTACTCCTCATATATATGCAAAATCCTATTAAGATCGGACCACTATATCATATAGCTG
CCATAGGAACGATCGGTCGAAAATTAAGTTTTTGTATGAAAAAACATTTTGTTTTTCAAGATATCTTGACC
AAACTCGGCATTTATTAGTTTCACTGTACTTCTCATATATATGCAAAATCCTATNAGGATCGGACCACTAT
ATCATATAGCTGCCATAGGAACGATCGGTCGAAAATTAAGTTTTTGTATGAAAAAACATTTTGTTTTTCAA
GATATCTTGACCAAACTCGGCATTTATTAGTTTTACTNTACTCCTCATATATGTGCAAAATCCTATTAAGAT
CGGACCACTATATCATATAGCTGCCATAGGAACGATCGGTCGAAAATTAAGTTTTTGTATGAAAAAACAT
TTTGTTTTTCAAGATATCTTGACCAAACTCGGCATTTATTAGTTTTACTNTACTCCTCATATATATGCAAAA
TCCTATTAAGATCGGACCACTATATCATATAGCTGCCATAGGAACGATCGGTCGAAAATTAAGTTTTTGTA
TGAAAAAACATTTTGTTTTTCAAGATATCTTGACCAAACTCGGCATTTATTAGTTTTACTTTACTCCTCATA
TATATGCAAAATCCTATTAAGATCGGACCACTATATCATATAGCTGCCATAGGAACGATCGGTCGAAAAT
TAAGTTTTTGTATGAAAAAACATTTTGTTTTTCAAGATATCTTGACCAAACTCGGCATTTATTAGTTTTACT
TTACTCCTCATATATATGCAAAATCCTATTAAGATCGGACCACTATATCATATAGCTGCCATAGGAACGAT
CGGTCGAAAATTAAGTTTTTGTATGAAAAAACATTTTGTTTTTCAAGATATCTTGACCAAACTCGGCGTTT
ATTAGTTTTACTTTACTCCTCATATATGTGCGAAATCCTATTAAGATCGGACCACTATATCATATAGCTGCC
ATAGGAACGATCGGTCGAAAATTAAGTTTTTGTATGAAAAAACATTTTGTTTTTCAAGATATCTTGACCAA
ACTCGGCATTTATTAGTTTTACTTTACTCCTCATATATATGCAAAATCCTATTAAGATCGGACCACTATATC
ATATAGCTGCCATAGGAACGATCGGTCCAAATTAAGTTTTTGTATGAAAAAACATTTTGTTTTTCAAGATA

TCTTGACCAAACTCGGCACTTATTAGTTTTACTTTACTCCTCATATATATGCAAAATCCTATTAAGATCGGA
CCACTATATCATATAGCTGNCATAGGAACGATCGGTCGAAAATTAAGTTTTTGTATGAAAAAACATTTTGT
TTTTCAAGATATCTTGACCAAACTCGGCATTTATTAGTTTTACTNTACTCCTCATATATGTGCAAAATCCTA
TTAANATCGGACCGCTATATCATANAGCTGCCATAGGAACGATCGGTCGAAAATTAAGTTTTTGTATGAA
AAAACATTTTGTTTTCAAGATATCTTGACCAAGCTCGGCATTTATTAGTTTTACTATGCTCCTCATATATAT
GCAAAATCCTATTAAGATCGGACCACTATATCATATAGCTGCCATAGGAACGATCGGTCGAAAATTAAGT
TGTATGAAAAAACATTTTGTTTATCA
>160_rnd-1_family-190__LTR___Gypsy
CCCAAATAAACGATGAAAAAGAGATACAACAAATACTGTCTACATACCATGACGACCCAATTCAAGGAG
GCCACAGTGGCATAACAAGAACGCTAGCAAAAATAAAAAGACACTACTACTGGAAAAATATGACGGGTC
ATATAAAAGAGTACGTACGTAGATGCCAAATGTATAAAATAAAGACACACACAACAAGTAGGAGCTTGT
AGTCGGGAGCTCCCGACTAGGGGACACCCTGAACCCTCTACTTCCAACATCAAATGCATATATATATTCTA
TTTTAAAAGCTATATGTCAAGTTTGGTGACTCTAGCTTTTATTATTTTCCAAAATTGGCCAAAAAAAATAGG
ATATCGATATCGTTTCTTATCGATTGCTTGGAAACGGAGTAAGTTATCGATTATCGGAAACAAACTCGATT
TGCGCAGGCACTAGGAGCAACTACATCTAAAATTTCAAGTCTCTAGCTCTCATAGGTTCTGAGAACCTTGC
GTTCATACATACGGACGGACGGACGGACGGAGGGACGGACGAATGGACAGACAGACGGACATGGCTAGA
TCGACTCGGCTATTGATGCTGATCAAGAATATAAATACTTTATGGGGTCGAAGATGCTTCCTTTTGCCTGT
TACATACATTTGGATTTAAATAAATAATAATGTCAACAAAAAGTTAATAAAGAATATATATATTTAATGG
AATGGAATGAAATTGTTTCTATATTAAGCAAACGTTCTGAGCTTGATAGATCATTTAAGTGCCTCAATAAA
AGTGCACTAATATCAAACCAAACAAAAATAAAACATGTTCGAGTTCTGGTAGAATCATACAACAGAGTAC
GTGACATTGTAAACAACAATGGACGTAGGTTAAGCGATTTATATTTAACACAATCAATTGACGTGTTAAA
AAGGCTACGTGAAAATATTAGACATATTTCGGAAAGACATAATTTTCAAATTTTGTTCCGCATGCGCTAAA
TACACCGGCAACATTCGAAATCACTGATTTAATGATATCAGTAGAAGAAGNAATTCGANCAAAAGCAAG
AACAAATCGAAATAGTCGACATGGCNCAAACAGCNGTAGANTTTNTCAAGCTAGCCNCATCTCTGATACC
AGAGTTTGACGGCAAGCCGGAAAATCTACAAAGCTTCCTAGATNCTCTGAATCTGNTAGANNCCCTAAAA
GGNACACACGAAGCTACCGGCCGTAAGCCTTATNAAGACAAAACTAAAAGGGCCATGCCAGGAACCTGA
TAAGTGATGAGNANACNATTGCNGCNNTCATNNCGNAACTATCGGACGNAGTAAAAGGAGAGTCGGTAG
AAGTTGTNTCTGCTAAGCTTCTAAACTTGCAACAGAAGAGCAAAACGGCCAACCAATACACCCAAGAGGT
GGAAAAACGGCAAAGGCCCTTGAAGGNGCCTATATCAGTGAAGGTCTAAGCCAATCACTTGCAAAACAA
ATACAGCTGTAAAAGCTATGACCCAAAATTGTTCGATTGATAAGGTAAAACTTATCATGCAGGCAGGCAC
ATTCACAAATATGAATGATGCCGTCTCCAAATTTGTCAATAGTTGCACGGAGGTAACAGGTCAAAGTAAC
ACTGTCCTGTATTATCGACGAGGTGCAAATNACAATAATAGAGGAGGCCGAGGTTATAATCGTGGCAGAA
ATTACAACCACAACAATTACAACCGAGGCGGTAACAACAACAACAACAATAATTACAATAACCGTGGAG
GAAGACGAGGCCAAAATCGAGGAAGAGGCCAACCAAGGTNACAATAATAACAATGTGAGAGTGGCGCA
AAATACGCCGGAAAACTCTCAGAACCCTTTAGGAAACAACCAGTAAATGCAAAGGTTCATTCCATCAATT
ACAGTCTTAATATATTTGTCACTTTTTATAATCATTCAACTGAAAATAAACTCACATTCCTAATAGATACTG
GTGCAGATATCTCACTTTTAAAAATAAATTCAGATAATTTCAAAATTCAAGAAGANAAAATTATAAACAT
CCAAGGCATNGGCCAGGGTGTAATAAAATCNCAAGGAACAACTTCAATAGAACTTCAATCAACAAAATA
TATTATTCCACACGAATTTCATTTAGTAGACTCNGATTTTTCAATACCGTGTGATGGAATAATAGGCATTG
ATTTTATTAAAAAATTCAATTGTCAATTAGATTTCAAACCAAGTGAAGACTGGTTTATAATTAGACCTCAA
AACTTAAATTATCCTATTTATGTTCCGATAACATATAGTGCTGGCAACAATGCATTTCTTCTGCCAGCCAG
ATCCCAAGTAATTCGAAAAATAGACATTAATTCAGTAAACGATTATATATTTGTTCCAAATCAAGAAATA
CATAATGGAATTTATGTTGCAAATACAATAGCAGCATCCAAACATGTATACATTCGCCTTCTAAACACAAC
TAATTTCGACCAATTGGTCAAAGTAAACAAAATCGAATATGAAAATTTAACAGATTACGATATTCATAAC
ACTAATCCAGAAAATAGAAGCGAACAAGTACTTTCAAAGTTAAAGAAAAATTTTCCAGAACAATTCAGAA
ATCAATTAACAGAATTATGCACCAAGTATAGTGATGTGTTCGGACTGGAAACCGAACCAATAGCGACAAA
TAATTTTTATAAACAAACATTAAGACTTAAAGATGATGAACCCATTTATATCAAAAACTACAGAAGCCCG
CATAGCCAAGTTGAGGAAATCCAAAAACAAGTAGGGAAATTAATAAACGACAAAATCGTAGAACCGTCT
GTATCCGAGTATAACAGCCCACTCTTGCTAGTTCCGAAAANATCATTACCAAATTCGGAAGAAAAGAAAT
GGCGATTAGTAATTGACTATCGCCAAATCAACAAAAAATTACTTTCTGACAAATTCCCGCTCCCCAGAATT
GATGATATTCTAGACCAACTGGGCAGAGCTAAATACTTTTCATGCCTTGACTTAATGTCAGGTTTCCACCA
AATTGAACTTGAAGAAAATTCAAGAAATATAACATCTTTTTCAACGAGCAATGGCTCATATCGCTTCACGC
GATTACCATTTGGTCTTAAAATAGCACCAAATTCATTTCAGAGAATGATGACTATAGCATTCTCGGGTTTA
GAACCCTCTCAGGCATTCCTTTATATGGATGACTTAATGGTGATAGGATGTTCCGAAAAACACATGATTAA

AAACTTAACTGATGTTTTNAATGTATGTAGGAAATATAACCTAANGTTGCATCCAGAAAAATGTTAATTTT
TCACGCATGAAGTGACATTCTTAGGTCACAAATGCACTGACAAAGGAGTATTGCCAGATGACAAGAAATT
CGACGTCATCAAAAATTATCCTGTCCATGATGCGGACAGCGCGAGACGATTTGTAGCATTCTGCAATNATT
ATCGTCGATTTATAAGAAACTTCGCCGATTATTCACGGCACATAACTAGATTATGTAAAAAGAATGTTCCT
TTTGAATGGTCAAGTGAATGCCAAAACGCATTCCAATACCTTAAAGAAAAGCTTATGCATCCCACATTATT
ACAATATNCTGATCTTCGCAAAGAATTTTGCATTATAACAGATGCTAGTAAACAAGCTTGCGGAGCGGTT
CTAACTCAGAACCGTGACGGAATTCAGCTCCCAATAGCTTATGCATCTCGTTCATTTACAAAAGGGGAAA
GCAATAAGAGCACAACAGAACAAGAGTTAGCGGCAATTCATTGGGCAATTACCCATTTTAGACCATATAT
TTATGGCAAACACTTTACTATTAAAACGGATCACAGACCATTGACATATCTTTTTTCTATGACTAATCCCA
GCTCGAAATTAACTCGCATGCGGCTAGAGCTTGAAGAATACGACTTCACAGTAGAATACCTAAAGGGGAA
AGATAATTTCGTAGCAGACGCACTTTCGCGTATTAATATAAAAGAACTCAAAGATATGCAACATAAAGTC
CTGAAAGTCACTACCAGGCGACAAAGTAGACAAAATAAAAACTGTACAGTAGAAAATTATGAACTTCTGC
CAAGGCAAAGTCATAAAAATGTATCTAAGCCCAACGTACATGAAGTCATTACAAATGATGAAGTACGAA
AAGNAGTGACCTTGCGCATAACAGAATCTAAATGTTTATTTAAACATGGAAATAAAATTATCGCAAGAAT
TGAAGTTAGCGATTTATATACTAATGGAATCCTCGACCTAGGTCAGTTCTTCCAAAGGCTCGAAANAGAG
GCCGGTATATTAAAAATCAGCCAACTCAAAGTGCCGCCGAGCGAAAAGGTCTTTGAATCAGTTTCAATAG
ATTATTTTAAAAATNTGGGCAATAAAATATTAAAATCTTTAAGAGTAGCGCCACTCCAGCCGGTGACCCA
AATAAACGATGAAAAAGAGATACAACAAATACTGTCTACATACCATGACGACCCAATTCAAGGAGGCCA
CAGCGGCATAACAAGAACGCTAGCGAAAATAAAAAGACACTACTACTGGAAAAATATGACGCGTCATAT
AAAAGAGTACGTACGTAGATGTCAAAAATGCCAAATGTCTAAAACAAAGACGCACACAAAAACTCCTTT
GACTCTCACAGAAACACCTGTGAATGCTTTCGATATAGTGATAGTGGATACAGTTGGTCCGCTACCTAAAT
CGGAAAATGGCAATGAATACATAGTCACATTAATATGCGACCTTACAAAATATTTAGTAGCCATTCCAAT
AAAAAGCAAAAGCGCAAATAATGTCGCAAAAGCTATATTCGAAAATTTCATACTCAAGTTTGGTCCAATG
AAGACGTTCATCTCGGACATGGGAACAGAGTATAAAAACCAGATTTTAACTGATTTATGCAAGTATATGA
AGATCAAGAATCTCACCTCTACTGCATATCATCATCAAACATTAGGGACAATAGAACGAAGTCATCGAAC
ATTTAATGAATACATTCGTTCATACATCTCTGCAGACAAAACTGACTGGGATGTCTGGATTCAATATTTCA
CATATTGCTTCAACACTTCGACACCATCAGCAACGCATGAGTATTGTCCATACGAACTAGTATTTGGCAGA
CTACCAAAACAGTTCATAGATTTAAATAAAACTAATTACATAGATCCTATTTATAATCTAGATGACTACTC
TAAGGAAATAAAGTTTCGATTAGAAATAGCATATAAAAGAGCTAGAATTATGTTAGAAAAAGCCAAGGC
TTATAGGAAATTAGCATACGATGAAAAAACAAAAAATTTTGAATTAAAAATAGGAGATAAAGTACTTAN
AAGAAACGAAACAGGCCACAAATTAGACCCAGTATATTTAGGACCATATATAGTAGAGAAAATAGAAGA
TAATGATAACATTTACATTAAAGATAGTAAGAATAAGAAACAAAAAGTACATAAGGATAGGTTAAAAAT
ATATAATC
>160_rnd-1_family-33__Unknown
CAATGCGTTAGCAATTGTCAAACATAAAGGCGTTTTGTTCACACATGGGGAGATATGTCGTTTCAAAAAC
GACATATCTCCATGATCTCCGGGATTCAAGAAGATATGTCGTTTCGAAACGACATATCTCCATGATCTCCG
GGATTCAAGAAGATATGTCGTTTCAAAACGACATATCTCCGCGATCTCCGGGATTCAAGGAGATATGTCG
TTTCAAAACGACATATCTCCGCGATCTCCGGTCACGGAGATATGACGTTCCAAAACGACATANCTCCGCG
NGGAGATATGNCGTTCTAAAACGACATATCTCCCCGNGGAGATATGTCGTTTCAAAACGACATATCTCCG
TGATCTCCGGGATTCANGGAGATATGNCGTTTCAAAACGACATANCTCCGCGATCTCCGGGANCGCGGAG
ATATGACGTTTTAAAACGACATAACTCCGCGCGGAGATATGACGTTTTAAAACGACATAATCTCCGCGAG
ATATGACCTCCGCGCGGAGATATGTCGTTTTAAAACCGNCATATCTCCGCGCGGAGGTCATATCTGCGTAG
GCTTCGTAACCGNTTACAAAAAGGTATAAAATTNTAAAATCCGACGATATTTACCCGAGATATTCAAAAA
AAATCATCGAAAAAGTTTTGATTTTCGAACCGACCCCGATTTTNAGAAAAAACGTGATGTAACCCCTTGCT
TTTTTTTGGTCGATTTNGGTCAAAATTTAGATTTTCAATTTTTGAACGCCAATCGATAGTATTGATCCCCAC
GAGTCGAAAATGGTATANCATTNCAAAATCGGACATTATTTGGCCGAGATATTCAANAAAATCATCAAAA
AAGGTNGACTTTACCAACGAATCGATTTTTGGTCAACAACTTTTTTTAGGCCATCGATTTTTGATTCGTTTG
AATGCAAATCGATAGTAGNGATCCGTACGAATTNAAAAAGGTATANCATTCCAAAATCCGACGATATTTA
CCCGAGATATTCAAAAAAAACATCAAAAAAGNTTTGACTTTCGAACCGACCTCCGATCTCCGCGAGAAGA
TATTGNCGTTCCAAAACGACATANCTCCGCGCGGAGATATGACGTTCCGAAACGACATAACTCCGCGCGG
AGATATGACGTTCCAAAACGACATAACTCCGCGCGGAGATATGACGTTCTAAAACGACATANCTCCGCGN
GGAGATATGAGAGATATGTCGTTTCGAAACGACATATCTTCTTGAATCCCGGGGATCGCGGAGATATGNC
GTTTTAAAACGACATATCTCCTCGAATCCCGGAGATCGCGGAGATATGTCGTTTCGAAACGACATATCTCC
TTGAATCCCGGAGATCGCGGAGATATGNCGTTTTGAAACGACATANCTCCTCGAATCCCGGAGATCGCGG

AGATATGTCGTTTTTGAAACGACATATCTCCNCGCGTATGACCTTCCAAATCATCACGATTTTTTTCAAAA
TCGAGGTCAGTGCGAAAATCGAAACTTTTTCGATGATTTTTTTAATATCTCGAGTAAATAACGCCTGATT
TTGGAATGTTATACTTTACCAACAAATCGATTTTTGGTCAACAACTTTTTTAAGGCCATCGATTTTTGATTT
ATTTGAATGCAAATTGAAAGTAGGGATCCGTACGAATTGAAAAAGGTATAAAATTATAAAATCCGACGAT
ATTTANCCGAGATATTCAAAAAAAACCATCGAAAAAGTTTTGATTTTCGAACCGACCCCGATTTTAAGAA
AAAACGTGATGTAACCCCTTGCTTTTTTGGTCGATTTTGGTCAAAATTTAGATTTTCAATTTTTGAACGCCA
ATCGATAGTATTGATCCTCACGAGTCAAAAATGGTACATCATTCCAAAATCGGACATTATTTGGCCGAGAT
ATTCAATAAAATCATCAAAAAAGGTGGACTTTACCAACAAATCGATTTTTGGTCAACAACTTTTTTTAGGC
CATCGATTTTTGATTTGTTTGAATGCAAATTGAAAGTAGGGATCCGTACGAATTGAAAAAGGTATAAAATT
ATAAAATCCGACGATATTTACCCGAGATATTCAAAAAAAACNATCGAAAAAGTTTTGATTTTCGAACCGA
CCCCGATTTTAAGAAAAAACGTGATGTAACCCCTTGCTTTTTTGGTCGATTTTGGTCAAAATTTAGATTTTC
AATTTTTGAACGCCAATCGATAGTATTGATCCTCACGAGTCAAAAATGGTACATCATTCCAAAATCGGAC
ATTATTTGGCCGAGATATTCAATAAAATCATCAAAAAAGGTGGACTTTACCAACAAATCGATTTTTGGTCA
ACAACTTTTTTTAGGCCATCGATTTTTGATTTGTTTGAATGCAAATTGAAAGTAGGGATCCGTACGAATTG
AAAAAGGTATANAATTATAAAATCCGACGATATTTACCCGAGATATTAAAAAAAAACTATCGAAAAAGTT
TTGATTTTCGAACCGAACTCCGAACTCCGCGCGGAGATATTGACGTTCCAAAACGACATAACTCCGCGCG
GAGATATGACGTTCCAAAACGACATAACTCCGCGCGGAGATATGACGTTCCAAAACGACATAACTCCGCG
CGGAGATATGACGTTCTAAAACGACATAACTCCGCGGGGAGACATGAGAGATATGTCGTTTTGAAACGGC
ATATCTTCTTGAATCCCGGAGATCATGGAGATATGTCGTTTTGAAACGACATATCTTCTTGAATCCCGGAG
ATCATGGAGATATGTCGTTTTGAAACGACATATCTCCCCGAGTCCCGGAGATCATGGAGATATGTCGTTTT
GAAACGACATATCTTCTTGAATCCCGGAGATCATGGAGATATGTCGTTTTCGAAGCGACATATCTCCCCAT
GTGTGAACAAAACGCCTTTATGTTTGACAATTGCTAACGCATTGCTTCTCTTATGAATAAAGGGTATCTTG
TGCAATTATGAAAGGATTTGCGATATCAGATAACGACGCGCGGAGGTAATATGTCGTTTCGAAACGACAT
ATCTTCTTGANTCCCGGAGATCATGCAGATATGTCGTTTCGAAACGACATATCTTCTTGAATCCCGGAGAC
GTTCCAAAACGACATAACTCCGCGCGGAGATATGACGTTCCAAAACGACATAACTCCGCGCGGAGATATG
NCGTTCTAAAACGACATANCTCCGCGGGGAGACATGAGAGATATGTCGTTTTAAAACGACATATCTCCNT
GAATCCCGGAGATCATGGAGATATGTCGTTTCGAAACGNCATATCTTCTTGATCCCGGAGATCATGGAGA
TATGTCGTTTCGAAACGACATATCTCCTTGAATCCCGGAGATCATGGAGATATGTCGTTTCGAAACGACAT
ATCTCCCCGNGTGTGAACAAAACGCCTTTATGTTTGACAATTGCTAACGCATTGCTTCTCTTATGAATAAA
GGGTATCTTGTGCAATTATAAAAGGATTTGCGATATCAGATAACGACGTGCGGAGGTACATATGTCGTTTC
GAAACGACATATCTTCTTGAATCGCGGAGATCGTAAGAGACCCCGATTTGAAGAAAAAACGTGATGTAAC
CCTTTGCTTTTTTGGTCGATTTTGGTCAAAATTTAGATTTTCTATTTTTGAACGCCAATCGATGGTATTGAT
CCTCACGAGTCGAAAATGGTACATCATTCCAAAATCGGACATTATTTGGCCGAGATATTCAANAAAATCA
TCAAAAAAGGTGGACTTTACCAACAAATCGATTTTTGGTCAACAACTTTTTTTAGGCCATCGATNTTTGAT
TTGTTTGAATGCAAATTGANAGTAGGGATCCGTACGAATTGAAAAAGGTATAAAATTATAAAATCCGACG
ATNTTTANCCGAGATATTAAAAAAAAACCGTCGAAAAAGTTTCGATTTTCGAANCGACATCTCCTGAACT
CCGGGATCGCGGAGATATGNCGTTTCGAAACGACATANCTCCGCGCGGAGATATGNCGTTCCGAAACGAC
ATAACTCCGCGCGGAGATATGNCGTTCCAAAACGACATANCTCCGCGCGGAGATATGNCGTTCTGAAACG
ACATATCTCCGCGNGGAGACATGAGAGATATGTCGTTTCGAAACGACATATCTTCTTGAATCCCGGAGAT
CGCGGAGATATGTCGTTTCGAAACGACATATCTCCTTGAATCCCGGAGATCGCGGAGATATGTCGTTTCGA
AACGACATATCTCCTTGAATCCCGGAGATCACGGAGATATGTCGTTTCGAAACGACATATCTCCTTGAATC
CCGGAGATCGCGGAGATATGTCGTTTCGAAACGACATATCTCCNCGCGGTGAATTACGCGNCTTCAAGAC
GACATATCGCTACGCATNGCTTCTCTTATGAATNNAGGANATCTCGTGCCGTTNTAAAANGATTTGCGATA
TCGNATAACGNCGCGCGGAGTAATATGTCGTTTCGAAACGACATATCTTCTTGAATCCCGGAGATCATGG
AGATATGTCGTTTCGAAACGACATATCTTCTCGAATCCCGGAGACGTTCCAAAACGACATAACTCCGCGC
GGAGATATGNCGTTCCAAAACGACATANCTCCGCGCGGAGATATGNCGTTCCGAAACGACATANCTCCGC
GNGGAGACATGAGAGATATGTCGTTTCGAAACGACATATCTTCTTGAATCCCGGAGATCACGGAGATATG
TCGTTTCGAAACGACATATCTTCTTGAATCCCGGAGATCATGGAGATATGTCGTTTTGAAACGACATATCT
TCTTGAATCCCGGAGATCATGGAGATATGTCGTTTTCGAAACGACATATCTCCCCGTGTGTGAACAAAACG
CCNTTATGTTTGACAATTGCTAACGACATTNCTTCTCTTATGAATAAAGGGTATCTTGTGCAATTATAAAA
GGATTTGCGATATCGGATAACGACGTGCGGAGGTAATATGTCGTTTCGAAACGACATATCTTCTTGAATCC
CGGAGATCATGGAGATATGTCGTTTCGAAACGACATATCTTCTTGAATCCCGGAGATCATGGAGATATGT
CGTTTCGAAACGACATANCTCCGCGCGGAGATATGACGTTCCGAAACGACATACTCCGCGCGGAGATATG
NCGTTCCGAAACGACATANCTCCGCGGGGAGACATGAGAGATATGTCGTTTTGAAACGACATATCTTCTT

GAATCCCGGAGATCATGGAGATATGTCGTTTTGAAACGACATATCTTCTTGAATCCCGGAGATCATGGAG
ATATGTCGTTTTGAAACGACATATCTTCTTGAATCCCGGAGATCATGGAGATATGTCGTTTTGAAACGACA
TATCTTCTTGAATCCCGGAGATCATGGAGATATGTCGTTTTGAAACGACATATCTTCTCGAATCCCGGAGA
TCATGGAGATATGTCGTTTCGAAACGACATATCTCC
>9_rnd-6_family-1139__LINE___R1
AAAACAAAAGATAAGGTGCAGGTGTTCGCTTTGGCGTAAACAAAGCCATAAAACTTAACGGGACAACTT
GCGGTAAATAGCAATAAAACTGCTTGTGCCCAAGACAGCGCGAAAGCAGTGCAAAGCAGTGCAAACGCG
TTCACTGCTTTATAAGCAGTGCACATAGTGCGAAGCAGTGCGGAATTTTAAAAGCAGTGCAAAGCTGAGT
AAAGCAGTGCAAGCAGCGCAAATAGAGCAGTGCGGTTCGTCAACCAGGTAAGCGGTTTGTGTCCGGGTTA
TCGCGTGGTGTTGCCGACGGAGGGCGCCACGACAATGGACGCCAGAAGCGTGGCCGGCACGGCGTCCAG
TGCCGAGTCGGTTGCCAGCGTCGCCGCCGATGGAAGGAGGAGGGTGGGGAGGGAGGGGCCGTAGTACGA
TTTCCNCCCCTGCGTACCCGTTGAGCCGAAGAGGCAGGCGTTGGAAAGCGCGGATGAGTTTGCCAAGCCC
GCCACTAGGGCTGCTGCAAAAGAGCCGCTGATGCTGCCGCCGCAGAAGAAGACGTCGGAGCCGCTGCCG
CTGCCGCCCCCATCATCGCGCTTGCCCCAGCATGGAGGATAATGGCCGAGGGGACAGGGGCGATTATAGC
GGAGCTTTCGGCAGCTGCCGAAATGACCCGCCTTGCCCTGGTGGGTGTCGCCCCCGAAGCCGCGCGCGAA
GTGTCGGCAGCCGTTAAGCGATTGGAGGAGCTGTCCACGTCGCTGGCAGTGCGGAACGCTCTGCTCGAGA
GCCTGGCAGGCAGATCGTCTGCAGCACAGCTCGCCGCCCCCACCCCAACTGCTGCTCACCGTCGTCCACC
AGTTGCCCACCATGCTGCCCGCACCGCTGTCCCCGTCCCAGGAGTGCCGATCGCTGCCCCACGGATCAAC
AAGCCAGTTGAGACGTGGTCCGCGGTGGTCACGAGTCGGGACCCGAGCGAGTCGGGCAAAACGGTAGCG
AAGAAGATCATGGAGGAGGTCGCACCATCACTTGGAGTGCGGGTGCACGANGTGCGAGAGCTCAAGCGA
GGCGGCGCGGTCATCCGCACGCCATCGANGGGCGAGCTCAACAGAGTGGTGGCGAACACCAAGTTCGCG
GAGGTTGGACTCAACATCCAGCCGAACAAAGGATTGAGGCCGAGGGTAACGGTGTTCGACGTGGACACG
TCGATCCTGCCCGAGGTGTTCATGGAGGAGTTGTATACGAACAACNTCAAGGAGGAGATGACAGCAGCG
GCGTTCAACAAGGCAGTACACCTTGAGTCGAAGCCGTGGTCCGTCACTGATGGCGCCAGCGTTAATCTGA
CGCTAGAAGTTGACGAGAGGGCCCTGGCGACTTTTGAAAAGACCGGACGGGTCTATATAAAGTGGTTTTC
ATACCGATGCCGGTCCCTCGTCCGCACCTATGCTTGCCATAGGTGTGTCGGCTTCGACCACAAGGTGTCCG
AGTGCCGCCTTAAGCAGGACGTATGCCGACAGTGTGGGCAGATGGGGCATATCGCAGCCAAGTGCGCAA
ATCCGGTGGACTGCCGGAATTGCCGCTTCAAGGGGNTACCCTCGGGGCATTATATGCTTTCGGGGTCTTGC
CCGATTTATGGCGCAGTGTTGGCGAGGGTGCAAGCTAAACATTAATGTTTAGTTTCATCCAAGCCAACTGT
GGCCGGGGCCGCGCTGCGTGCATCGATATCGGTGTACGTATGCGTGACCACGGCCACTCCTTTGCGCTGCT
GNAGGAGCCATACTCGGACGTCGNTGGAAGAGATTACGGGCTTCCCAGCCGCGCGTTTTCGGAAGCCACG
CTGTCGGGGACCGGAAAAGGAGCGCTGCTGTCATCATCGACGACCCGGATGCCATCNGCCTACCAGTGGA
GTCGCTGACCACCGAGGCTGGAGTATGCGTGAGGGTCACAGGTAAACATGGCACNGTTTACCTGACCTCC
ATATACTGCCATGCTCATGCTGACTTGACGAGGATATTGAGCTATATGATGCCNTACTGCTACTGGCGAGC
AGTACGCCGGTAATTCTCAGCCTGGGCGCAAACGCTGTGTCTCCCNTGTGGTTCAGCAAACGGCAATTCTT
TCGTGGCCAGCTGAGCTACAGAAGAGGCGAACAGCTGGTGAGTGGATACTCGGAAGTCGTGCCNACGTG
CCAAACCAAGTCAGCGAGGTGTACACGTTCGATAATGGAAGAGGACAGAGCGATATCGACGTAACAATC
GTGAACGAAGCAGCGTCTACGTGGGCCGCGTATGACTGGAGTGTGGACGAGTGGGATTTGAGCGACCAC
AACATGATCACTGTTGTGACGTCGCCATCCCCAGCAAATGCAGTTGAGAGCTATGCTCCTGTGCCGTCCTG
GAAGCTCGCGAATGCTAATTGGCGATTGTTCGACCCGGAGCTGGTAGAGGAAATCGAGGAGCACCTTTCC
CAGGAGGACATTAGGATGTTGCCGTTGGATNCCGCGGTGTCTGCCCTACGCCTTGCCGTACAGTCTGTGTG
CGACAGAGTGTTAGGCCGCAGNCCGTCGAGAGCCCAGGGAAAAGTTAAATGGTGGACTGCCGAACTGAG
TACCAAACGCCGGGAGGTCAGGAGACTGAGGCGGAGNCTCCAGGATGCACGGCGCAGAGGCCACGAATC
TGCGCCGTATCTTGCAGCTCATCTGCGGACAGCCGTCCGCGAGTTCAGGACGCTCATCCTGAAGACGAAG
GAGGAAAACTGGCGACGCTTCGTTGGAGACCACAGAGACGATCCGATGGGGGCACGTCTACCGGATATG
CCGGGGACGAAAGCGGTCTACCGATTTGAGGTGCCTTCGTTCGGATGGCGGGCTGGTCGTAACATGGCAC
GACTGCGCGAGTCTCCTCCTCCACAGCTTTTTTCCAGTTGCGGAGTCGAGTGAAGACATCGTCATTCCGGA
CGAAGCTGCTCCAGCGCTCGATGCAGCTTTCGAGGTCGGAGCATGCGTCGCTCGGTTGAAGAGCAGGCGC
TCTCCCGGGCTTGACGGCATTACAGGTGGATCTGCAAGGCGGTATGGCGTGCCATCCCCGAGCACCTGA
CAGCGTTGTATTCCCGCTGCCTGGCAGAAGGATACTTTCCCCTTGAGTGGAAGTGCCCGCGTGTGGTTGCG
CTCCTCAAGGGCCCCGACAAGGATAGGAGCGATCCAGCTTCCTATCGGGGCATCTGCCTGTTGCCAGTGTT
TGGCAAAGTGCTTGAGGGGATCATGGTGGATCGAATGAAGGAGGTGCTTCCGGATGGAAATAGATGGCA
ATTTGGCTTTCGACCTGGACGCTGTGTCGAGGACGCCTGGAAGCACGTGATGACCAGCGTTGCCGCCAGC
CAGGCGGTATACGTACTTGGTCTCTTCGTGGACTTTAAAGGCGCCTTTGACAACATTGAGTGGAGCGCCGC

GATGCGCCGCTTGATCGATGCGGGATGCAAGGAAGCTAGCTTGTGGAGAAGCTTCTTCTGTGGCAGGAAA
GCCAGGATGACCAGCAGATGTGGCGAAGTCGAGGTGGAGGTCACGCGAGGGTGTCCCCAGGGATCCATC
AGTGGGCCGTTCATCTGGAACACCATGATGGATGTCCTGCTCCAACGCCTGGAGCCCTTGTGCACATTTAG
TGCATATGCCGATGACCTGCTGGTTCTTGTCGAGGGAAGGTCGAGAGCTGAACTGGAGCGTCGAGCAGCT
GAGCTGATGTCCGTCGTGGGAACTTGGGGAGCCGAGGTCGGAGTGTCGATCTCAACCAGCAAGACGGCA
ATGATGATGTTGAAGGGGCGTTTTGCCGTTTCCAGGCCGCCCGTGGTACGGTTTGCAGGAGCAAGCCTAC
CATATGTCGACAAGTACCGGTACCTTGGCATCACAGTCGGTGAACGGTTGAATTTTCAGCCGCATATCGCA
GGATTGCGGGATAAACTGACTGGAGTCGTCGGAGCATTAGGCCGCGTGCTACGGGTTGACTGGGGCCTCA
GTCCCCGAGCCAGACGCACTGTATATGCCGGACTCATGGTGCCTTGCGCACTGTTTGGTGCCTCGGTATGG
TATTCCGCGGTCAGGGGAGAGTCGGGGGCCAGGAGGCGCCTCATCTCCTGCCAAAGAGTCATGCTCTTAG
GATGCCTACCGGTATGCCGTACAGTGTCCACCGTGGCACTGCAGGTGCTTGCTGGAGCTCCGCCGTTGGAT
TTGGTTGCCAAGCAATTGGCAATCAGATTCAAGGTCAAGCGTGCCCACCCGCTGGAGGATAGTGACTGGC
TGTTCGGTCTAGATGTGGCAAATCTAGACCAGAGGCGGATGAAGGCGTTGCTGGACGAAGGCATGTGCTC
CGAGTGGCAACGGAGATGGGATAGTGATGAGCACGGTCGACCGACTCATCAGTTTATCCCGGACGCATGC
TTCGTCTACAGCCGGCGAGACTTTGGATTCGGTCTTCACGCTGGATTCTTGCTGACGGGCCACGGGTCGCT
AAATGCATTTCTGCATCAGAGAAGTCTGAGTGCAACGCCCGCATGTCAATGCGGTGCGGAGCATGAAGAC
TGGCAGCACGTGCTAGTTGCTTGCCCACTCTATGCAGATCTGCGAGACCTCGACGGACTTGGTGTCAGGA
GAGAAGGTACCCGTTGGGACTTTTCCCGGGTCCTGGAGACACCAGAGCGGCTGCTACTCCTGGAGATGTT
TGCGCGAGCGGTGTTTGCTAGACGGCGGCGAGGGATGGAGTGAAGTGCAACCACACAGGGCCGACCAGT
GCCGGGCTGACGTTAGCCAAAACTGGGAGTGGCATGGACCGTGTCACGGAGCCGAACAGAGCTTAATCTG
GTAGACAAACCCCAATTTGGGGTAAGTCGAACAATGGATGACCCATTCCGTGTTTGTCGTCAACAAAACT
GGGGAGTGGCAATTGCTACGAACAGCCGACCAAAGCTGTAATTTGGTACCACGGGTAACCAGGAGCCCA
CGGAACTCGTTCCGTCCTGGTTGGTTGGTGCGGCCCTTCGGGGAGTATCGTGGTGGCTGTGGTTCGATACC
CAATTGTGGGGAGAGTTTTCACTCGGCGTGGAGTTGCGTGTCAACCGGGTGCTGTACCCATAGACCAGCG
GAGGTATAGATAGACCTCACCTCCCACCAAGGGGAATGTCATGTTCGACAAGCATGCATTCAATTTGGTA
CCGAAGGGGCACTGCACATGCAGTGTCTTGGAGGGGCGCTAGTCCGACGCTTTGTTTCGGCTCGCTGTATT
TCGAATACCGTGGTAGTAAACCCCTACGGGGTACACGCCACGTTAAACAAATATGTTTTGTTTTGTCCCTA
TCTACTCAGTTCGTTCCAGACGNTCGTCGCGAGCGGACGTGTCGCGGAGTTATTTCGTGTCGAAAACAAA
AGATAAGGTGCAGGTGTTCGCTTTGGCGTAAACAAAGCCATAAAACTTAACGGGACAACTTGCGGT
>Ref_dvir_71
CTCAATTTTTTAGGAGCAAATCCCTGCTACCCGTCATCCGACGAAGAGTAGCAGGGACTGATATGAAATT
CCTCATCGATACGGGCGCGTCGAAAAATTTCATTCGACCACATGAAGGATTAAAAGGCGTTTGCCCGGTT
GACAAAAATTTCACAATTCATTCAATCCATGGCACGACAAACATCACACAAAAATGCTTTGTGTCAATGTT
CAATACAAAAGCTACATTTTTTATTTTACCCAGATTTGTCAACATTTGATGGCATCATAGGCCTTGACCTGT
TAACACAGGTAGGAGCCTCACTTTGCTTAGCTTCAGGACAACTCAAGTGGGGTACGGAAGTCGAAAAAAT
CACCTTCCACACGTGCAAAAATGTAAATTTCACAAAAGTAGATTGTGCAGAGGCACCCCCATTAGTTAAA
AGTTCTTTCTTAAAAATGCTAAAGACCAGACAAAAGGCCTTTGCAGACCCAGATGAAGCGTTGCCGTATA
ACACATCAGTCGTGGCTACCATTAGGACCACTGATGAGCAACCCAGTCTATGCCAAGCTTTACCCATATCC
AATGGGAGCAGCTGATTTCGTTAATAAAGAAATCAAAGATCTTCTAAAAAATGATATCATTAAAAAATCT
GTCTCCCCCTACAATAACCCCATTTGGGTGGTGGACAAGAAGGGGACGGATGAGTTTGGGCAACAAGAAT
ATGCGGCTGGTAATGGATTTTCGCAAACTTAACGAAAAAACCATCCCAGACAGATACCCTATGCCTAACA
TTCCAATGATTTTGGGGAATCTTGGCAAAGCTAAATACTTTTCGACATTGGACCTAAAATCTGGCTATCAC
CAGATCACGCTAGCAGAGCGTGACCGTGAAAAAACCAGCCTTTGCAGTTAACGGGGGGGAAGTACGAAT
TTTGTCGGACTTCCCTTTGGGTTGAAGAATGCGGCCAGCATCTTCCAAAGAACTGTTGACGACGTACTGCG
TGAGCAAATTGGCAAATCTTGCTATGTCTATGTTGACGATGTCATCGTCTTCTCAAAAGACGAAAATTCTC
ATGTCCAACATGTAGAGTGGGTCTTAAAGAGCCTGTACGAGGCAAATATGAGAATCTCCAAGGAAAAATC
GCGTTTTTTTAAGAAAAGCGTAGGTTTTCTTGGTTTCGTGGTAACCAGTAATGGAGCTACAACGGACCCTG
AAAAAGTCAAAGCGATTCAGGAGTTCCCAGAACCAAAGAATATATTCGAGATCAGATCATTTTTAGGACT
GGCCAGTTACTATAGGTGTTTCATCAAAGACTTTGCCTCAATAGCAAAACCCATTTCTGATCTTCTAAAGG
GCGAGAATGGGTCAGTAAGTAAACATAGATCGCGAAACATCCCGGTAGAATTCTCTGAGCTTCAGCGTCA
CGCATTCCAAAAGCTACGCAACATTTTGTCATCAGAAAATGTCATGCTCAGATACCCTGACTTTAAGAAGC
CGTTTGATCTAACGACAGACGCTTCAGCTTACGGCATAGGGGCAGTTTTTTCTCAAGAGGGTCGTTCCATC
TCAATGATTTCCAGAACATTGAAGACAGCGAGGTTAACTATGCAACAAATGAGAGAGAGTTATTGGCCAT
CGTATGGTCACTTGCCAAGTTAAGGCACTATTTGTATGGGGTGAAAGACATTAATATCTTCACTGACCATC

AACCCTTGACCTTCGCAGTTTCGGAATCCAATCCAAATGCGAAGATTAAAAGGTGGAAAGCGCGCATTGA
CGAGTCTAATGCACGTATCTTCTATAAACCTGGCAAGGACAATCTGGTTGCGGATGCCTTGTCAAGACAG
CAGCTCAACGCTGTAAATGAACAAGAAGTTGAGTCCTGCGCAGCAACGATCCACAGTGAGGTATCTCTCA
CCAACACCATTGAGACAACAGACAAACCCTTGAATTGTTTTCAAAACCAAATAGTGCTAGAAGAGGCGCG
TTTCCCCTTAAAACGAAGTTTTATTCTTTTTGGAAATAAGAAACGCTACTTGATCAGCTATACTTGCATCG
AATCGCTGCTAGACGAGCTCGAAGATGTAGTTTCACCCAAATGCGTAAATGCTCTCCACTGTAATTTACAC
ACGCTTGCAATGATACAAGATGCATTGGTTCGAAGATACCCGGCCACAAAATTCTGGCACTGCAAAAATC
GCGTAGTGGACGTATTTGCGGTTGGAGAAAGAAGGGAAATCCTCACAGCCGAGCATAATAGGGCCCATA
GGTCTGCACAGGAAAATATTAAGCAGGTACTCTCGGAGTACTACTTTCCAAAGATGGCAAAAATAGCCAA
TGAAATAGTACAAAATTGCAAAACATGTGCTAGGGCTAAATATGATAGACACCCCAAAAAACAAGAGCT
TGGGGAGACACCGATCCCCTCACATGTAGGAGAGATGTTACACATAGACATTTTCTCCACTGACAGGAAA
TACTTTCTCACTTGCATCGACAAGTTCTCTAAGTTTGCTATTGTCCAACCGGTCCCTTCAAGAACCATTGTG
GATCTGAAGCCAGCTTTAATGCAACTAATGAATTTTTTTCCAAAAGCCAGATCCATTTACTGTGACAATGA
ACCGTCATTGAATTCAGAGACCATCGTGACTTTGCTCGCAAACAACTATGGGGTCAGCATTTCAAATGCAC
CACCATTGCATAGCATCTCGAATGGACAGGTTGAGCGCTTTCACAGCACTCTGTTAGAACTAGCCAGATG
CCTAAAGATCGATAAAGGCATAAGTGACACCGTAGAAGTCATTTTACTGGCTACAACTGAATACAATAAG
TCGATCCATTCGGTCATCGACCAGAGACCAGTCGATGTGGTTCAGGCGCACCCAGATGAGCCACAAGAAA
AGATACAAAACAGGATCAGAGACGCTCAGACGGCGCTCAGAGCTAGGGAGAACGCCAAACGGCAGAATA
GGGTCTTCGACGTTGGTGACAAAGTTTTGGTCAAGTCCAACCGAAGGCTCGGGAACAAACTCACACCTTT
ATGTGATGAGAAGGCCGTTGAAGCAGACCTGGGGACCGAGGTCCTCATTAAAGGGAGGGTGGTCCACAA
GGACAACTTGAGGTAATCACCACGAGAATAAGGCCCTTGAAGTTTTTTCTTATAATACCAATCTGTTTAGC
CGCTTGGCTCGCTTCTTCCAATTAATTATAAGACTTAGATATTAGCGTGTGCCCTCAGTTTAAGGTTCTGGT
GGTGGTGGGTTAAGCAATATTAAAACACAAAAATAGCTAAAATTCCCATTCCACAGGTTTGAACTAATTC
TCATCTTGTCCCTGTCCCTGGCTTCGGCGCGCGTCACCGATTATTCAAACGCAAGGTACATCCCGATAGTG
GATGGACGAATCCTGGTGTGGGAGGATTTCGCCTACATTAAACATCGGCGAATCTTTCAGAATATGCACG
TGTGGTGGAGGAAACGGTCAGTTTGCTTGACATGTTCCCACAATCTCACATGAAGAAGTTCCTAAAGGTG
GACACAGCTCACCTGCAGGACCTGCTGGAAGCGTTGGGCGTACACCATAGGGTGGCTAGAAGTTTGGATC
TCCTAGGGTCCATGCTGAAAGTAGTAGCAGGCACTACGGATGCCAACGATTTAGAAAGAATAAGATTTAC
AGAACAACAACTAATACATGAAAACAATAGACAAATAGAGATTAATTCAAAAGTACAAGACCAAGTTAA
TAAGCTTACAATTACTGTCAATCAAATTCTCAAGGCCGCGAAAAACACACAAGTAAACACTGAACACTTG
TATGAAACGCTATTGGCTAGAAATAGGATGCTGATGATAAAATTGCAAAATTTAATGCTTGCAATATCATT
AGCAAAAGCAAACATTGTAAGCCCGAACATTTTAGACCATATCGACTTGAAATCAGTTTGGCTTGAGGAG
CCCACCGACACCCCCATAGCAGAACTTTTGTCCGTTTCGTCCGTTAAGATACTACAATCAAACAACACCTT
ATACTTCATTATTAGGTTCCCAAAAGTTAAGCTAGCTTGTAAGAAAATCACAATTTATCCCGTCATTCAGA
AAGGCGTAATACTGCGAATAACAGACAACACTGTGGCAGAGTGTGATGGAAACGTTCACGCTCTCGAAA
ACTGCACCGCATCCCCTGGAGCGACGTTCTGCAGACTCTCAAAGGAAAGCTCATGCGCCAGAGAGCTACA
CGCCGGGGGGGTGGCTAACTGCGAAACCCAGCCTAGCAACCTGGAGCCAATTACTCAGGTGGACGAAGG
GATGCTAATTATCAACGACAAGCTAGCAGAAGTGCAAGTTGATAACGGCCCTAAGACCTGGCTCCAAGGC
ACACACCTCATTACGTTCGCTGATCGGGCTAACGTAAATGGAACTCTTTTCATAAACCATAATAACATCTT
GGATAGAATCCCAGGAACGGCGAACTCTCCACTGCTGAACATTACGGCTAGCCGAGATATACTAAGTCTT
CCATATCTCCATCTATTGAACGAACGTAATGTGGAGGCAATTCAACAGTTCAGGGAGCAAATCAATGAGG
ACCGGGCCTACCGAGTTTCGTGCATAGCAGGAGCTATCTGTTGCGCAACCGTTTTTATCGGGTTGACACTT
TGGCGAATCACCAAGAAGAGGAAGACTGCAAGGCGGCTGAGAGAGGTGATTGCCGGCATAGGGTCGACC
GAGGACGGTCATAAACTCGAGGGGGGAATAGTTAACACTTAAGATTGCACCAAATAACGTTGTAGAAACT
TGCACAATACTTATATTGACCCATTTACAACCAAGCACATTCCGCTGACACATGAGTTCAGCGGCCTGACC
GCAACGGTCAGCAGCGGTAACCTGACACTTGACTATGCTGCAAGTATCCGCGTTGTCGCTTATGCTGAGTC
GGCTTGCCGACTGTAGCTTTGTAGTTTATAGATCCAAGTTTTAGGTATAATTCCAATTCAATTTTATACCAC
AATTCAGTTTTGTATTTGACGTTTAAGAATAAACTTGCACTACTCCGGCACACTGCCGTAAATATCATTTG
TTATATATTACAAAAACATTGCTGAGCCACAAGGCCAGTAATTAGCAAAGAGAGTGAAGTCAAACTCTCC
ACCATAATCGTCCTAAATATCACTGGAGTAGGCATCGCTGGACGAGCAACGGATCAACTATTGCATCGCT
GGACCAGCATATGTTAATATTCTTTAGTAGTTGTTTTAGAATCATGTAAGTGGGCTTTTCGTTAACTCTTTT
CAAGTCCATTCAAAAGAAATTTAAAAAAAAAAAAAAGCACCTTATATTTTGCATTACCTATTACTCGATTAT
AAATCTATAAAAGAAAAATAATAATAATAATTTTTACGGGCTTGAGAAGCTTTGATTGATAGATAAGCTG
CATTTTTGGTCACTTTTATTTAAAGCAAA

>Ref_Nausicaa
TTAACCTGTGTAATAGGATAAGAGCATCATTGGTAACCCTAGCCAGGGTATTATAGTGTCACTATCTATGT
TAATTATAACAGTACGATAAGTAAATATCGATAAAGAAAAGCGGCTGCAGAGAAGAAGTGGAAGGCAGT
CTCGCTACAGTACTAGCAGTAAGAAGACGTGTTGTTAAAAATAAAAAGGCAACCCGAACATAAGTTAAAT
CAACGAAGAAAATCATACTATTACAATATCAGAAAGTGGGATTGACTCAGAAAAAAAAAAAAAAAACACA
ACAAAATGGAAAATATAAATATAAATGACGTGTCAATAGCGACATTGAAAAGTTGGTTGGCATTACTAAA
TTTGCCAACAGAGGGTACCAAAACTGAGCTGATGGCGAGATTAAATAAGGTACCAGTGGATATCCGAGAC
GATGCTGCAAAGGAACTTGAAGTTCAACGTAACAAGGAACAGACAATTGAGGCTCAAAATGAGTTGCAA
AACATAATGCAACAACAGCGTGACGAAATAGCAAATGGCGCCGAGATGCTGAAATTGATGCGTCTCGAA
ATTGAAGCGTCTCGAAAATTCCTAGAAGAATTTCAAGTAACGGTGAACCGCAACTCGCACATCGGCGGGA
GCGACGGGGGAGAGCAAGAAAGTGAAGTCGGCGATTTATTGCAGCTAGAGGATGGTCACACTGAAGAAA
GACCACGGTCGACGGATGGCAACGCTGGTGCAGCTGATTACACTGGAACGGATGGCAACGCTGGTGCAG
CTGATCACGCTGATGCAGCGGGCAACGCTGGGGCAGCTGGAAGGATCGACGAAAGTGGCAACGCTGTCG
GAGGCAACTTAAATAATTGCATCCAAACTGGAGCGATGATGTTAGCCAAGGAAATTTTATTAGAATTTAC
TGGAGAAAGTGAGGTGCGTAAATGGGTAATGCAATTCTTCAATGTGGCCAAGATCTACAGACTAAATGAT
ATGCAACAGCACTTGCTTTGTATAAGCAAATTGAAAGGCGGTGCTTTGAAGTGGTTGCATGCAGACCCTA
TGCGCATCATTGCTCCGATTGACGAGATGCTAAATCAATTGGTTTTGGCCTTCGGGGGAGGATTTTCGAAG
TCGGAACTACGACAGAAGTTCGAGGATCGGGTTTGGAAACCAGATGAGGTGTTCGCCACATATTTTAGCG
AAAAAAGCATATTGGCACAGGACATCAACATTGATGTAGAGGAGTTAATGGAGGGTATTATTCGAGGCAT
ACCTTGCGAAAACTTGCGCACTCAAGCTAGTATGCATTGCTTTACCAATCCGGCTCAAATTTTACGTGCGT
TTGCAGCTATAAAGTTGCCAATTAAACGGGTACGAAACCATGTAGTGAAACAAACTGCACAGGAAGCAC
AGGCGGACAAACAACAACGTTGCTACAATTGCAATGTTAAGGGCCATTGGGCCAAAGATTGTTTAAAGCC
CAAACGGGAGGCAGGATCTTGCTATGCGTGCGGCTCAAAGGATCATTTAATAGCAGGATGTCCAAATAAA
AAGTATGATATGGAAAACAAATATGTAAGATACTTAAGAATTCATTTTAGTAACTCATTTAACAAATCAA
TAATTGCAGAATGCCTTATAGACTCTGGCAGCCCTATTTCATTTTTAAAGGAAAAGTTTGTGCCATTAAAA
GTAAAGCGTATGCCAGATGCAAATTCGTATGTAGGTTTAAATGAAAGTAAATTGAAAAATTTTGGAAAAA
TTTTATGTTTTATGAAGAAAAAGTTAATAAATGTTTATTTTAATGTTATAATTGTGGCAAATGAGTCTATG
AGGTATGACGCTGTGCTTGGTAGAGATTTTATGGATTCTTTTGGTTTAAATATTGATTTAAGAACATTAAA
AATTGTGAACGGGCATAAAGTTGATCACCAGAATAATGGTAAAAATGATATCATAGCTACAAGCTACAAA
GAAAAACAAAAAAAAGTGGACGATGAAGATATCAAACACGAGCAAAATGAAACTATATTGTTAAATGAT
AAAATCGTTAATAAGGAAATAGTCAGTAATGAAACTGTTAGTGATGAATTAGTTAGTGAGAAAATTGTTA
GTCCGGAAACGCTTGGTGATAAAGTTGGTAGTGATGAGATAGTTAGAAATAAAATAATTAGTGGTGAAAT
AGTTAGAAATGAAGTGATTGGGATCGAAACTGTTAGTGATGAGATAGTTAGTTATGGAGAGCTTAAGCTC
GAACCTGATAGTGAAGAAGTTGTTAGTGATGAAATTATCTATAATCAAACCGGGATTGAAAAAGTGATTA
TAGAAGATATAGGAAACAGTGCAGTTAAAATTAATAATCATGTTAATGAAGCGCCAGTGAATAGCGAAAT
CATGGGAGTGTTAGAAGAGAATTTTGAGAAGGATATCTTAACAATAAGTTGGGAAGATTCTAACAAAAGA
GAAAATATATATATTTTAAGTGAAGATATTGATTACAGCACAAAGTGTGAATTTGAAAATTTATTTGAAA
ATACTTATGTAAAAGCGAAAAGGCCAAATCAGCCAAAATTAAGACATGAGTTAAGCATAAGATTAGAAA
ACGACAAAGTATTTAATTGTACACCTAGGCGGTTATCTTATATGGAAAAAGAAAAGTTGCAAGAATTATT
AGACGACTATTTAAAAGATGGAATCATTAGGCCAAGTTGTTCAGAGTATGCATCACCTATAGTACTAGTG
AAGAAAAAAACTGGAGACATGAGGTTATGTATTGATTTTAGAAGACTGAACAAAATTATATGTAGAGACA
ACTACCCGCTACCATTGATCGACGATTTATTAGACAGACTATGCGGAAAAACAGTATTTTCAAAGTTGGAT
CTAAAAAACGGGTATTTTCATGTTTTTGTTAATGAGCAATCTATTAAATACACTTCATTTGTTACCCCTCTA
GGACAATATGAATTTCTTAGGATGCCCATGGGTTTAAAAACTGCGCCACATGTATTTCAAAGATTTGTTAA
TAACATATTTTGTGATATGATAAAAGATAATAAGGTAATTGTTTACATGGATGATATTATGATAGCAAGTA
CAAACATAAAGGAACACATGGAGACCTTAAAGGAAGTGTTTATAAGACTGGTGCAAAACAAATTAGAGT
TGAAATTAGATAAGTGTGAATTTTTTAAAAGCAGCGTTAAATATTTAGGATTTGTGGTGGATAGTAAAGG
CGTTAGGGCTGATGAAAAAGGGTTAGAAGCGGTTAAATCCTTTCCAATACCAAACAAAATTCAGGGAGTA
CAAAGTTTCCTAGGGTTATGCTCCTACTTCAGGAGGTTCATTTTAAACTTTTCTATATTAGCTAAACCCTTA
TATGATTTATTGAAAAAAGATAAGAAATTCAAGTTTGGAGAAGAGGAGTTTAAGTGTTTTAATATGTTAA
AAGATAAATTGTTAGAGGCGCCAGTACTATCAATATATAATTACAAAGAAGAAATAGAACTACATTGTGA
TGCCAGCGCAGTGGGATTTGGGGCTGTTTTATTTCAAAAAAAAGAAGATAGGAAATTACACCCGATTTTTT
ATTTTTCTAAAAGAACAACCGAAGTAGAGTCTAAATATCATAGTTTTGAGTTAGAAACGCTAGCCATCATT
TACGCATTACGCAGATTTCGTATATATCTGCAGGGAAAACGATTTAAAATAATGACAGATTGCAACTCTTT

AACTTTAACGCTTAACAAAATAGATTTAAATCCGAGAATTGCTAGATGGGCGTTAGAACTACAAAATTAC
GATTTTGAGTTAGTGCATAGATCGGGCAAGCAAATGCAACATGTGGATGCGTTAAGCAGATGTTATGAGG
ACATTTTAGTTATTGAATCCAATAGTTTTGAGGACAATTTAGTTATTTGCCAAAGCAAAGATGACAAACTG
GTAGATATTAGAAAGATGTTAGAAAAAGAAGAGCACAAGCTATTCGAAATGAGAAATGGGGTCATTTAC
AGAAAAACAAATGATGGCAGATTAGTTTTTATGTACCAAAGGATATGGAAGAACATGTACTTTACAAAT
ATCATGATGAGTTGGGACATGCTGGGAGAGATAAGATGATCGATGCCATTGGAAAGAGTTACTGGTTCCC
ATATATTAAGGAAAAAGCAATAAAGCATATAGGGAATTGTTTAAAATGCATTGCCTTCGCCCCCAAGACG
GGTAAGAGTGAAGGACTATTACATAGCATACCGAAAGGAAACAAACCGTGGGAGATGATCCATATAGAC
CATTATGGACCAATTAATGCAGGAAGGGCTAATAAATATATATTGGGCGGTGGTTGACGGATTTTCAAAA
TTTGTTAGACTATATACAGCAAAGACAACAAGTACGAAAGAAGCGATTAAGGCATTAAAAGATTATTTCA
GAGCGTATAGTAGACCTAAGTTCATTGTATCGGACAGAGGGAGTTGTTTTACTGCAAAAGAGTTTGATGA
ATTTTTAAAAAATGAGGGAGTAACGCATATTAAAATAGCAACGGGTTGTCCGCAAGCAAATGGTCAGGTG
GAAAGGTTGAATAGAGACTTGGGTCCGATGATGGCAAAAATGGCGGAGCCTGAAAATGGTTTACATTGG
GATGTTATTATAGAAAATGTTGAATATGCAATAAACAACACACAACACAAAAGCATAAAGAAATTTCCAA
GCGAAATGTTATTCGGATTACAACAGAAAGGAAAAGTTGTTGATGAATTAAAAGAAAAATTAGAAGAGT
TTACACAGGTGAACACGTGTGATAAAAGAAATTTAGAAAATATAAGAAAGAGTGGAGAAATATATCAAA
AAGAAGCTCAAATCAGAAATGAAGAGCGGATTAATAAAAAGAAGAAAGTATCAGTAGAATATAAAATAG
GTGACTATGTCATGGTAAAAAATTTTGATAGCACACCAGGAGTTTCAAAAAAGTTAATACCAAGATATAA
AGGGCCTTATAGTATAGAAAAGATTTTAAAAAATGATAGATACTTGTTAAAGGATGTAGAAGGCTTTCAA
TTATCTCGCAACCCGTACGTTGGTATCTGGAATAGCCAAAACTTTAAGCATTGGATGCGAAAATAGGCAA
TAAGCATTAGTTATAAAGATAGATACACATATAAAAGACAGAGATCGGGAGCTCTCTTTGTCAGGATGGC
CGAGTTGTAATAGGATAAGAGCATCATTGGTAACCCTAGCCAGGGTATTATAGTGTCACTATCTATGTTAA
TTATAACAGTACGATAAGTAAATATCGATAAAGAAAAGCGGCTGCAGAGAAGAAGTGGAAGGCAGTCTC
GCTACAGTACTAGCAGTAAGAAGACGTGTTGTTAAAAATAAAAAGGCAACCCGAACATAAGTTAAATCA
ACGAAGAAAATCATACTATTACAC
>160_rnd-6_family-566__LINE___I-Jockey
CACTTACGTTTGTGTTTCTCTGGAGATCGGACGTGAAAATTGCAGTCTCGTGTGTGTGTGCGCGTTAGTGC
ACCGCTGGCAGCAGGCATAAACCAAGAAAGTGACAGTGACACTGTATAAAGCCCGCCCCAGGGCAATCC
ATACAAATCCCGCTTATAGTGGGTAAACTATACGCAACTGCTGCCTCTGGCAGCCGGAGTGTTATACAAA
TTAACAATATTTTAACAAAAAAAATTACCGCGTGCAGTGCGTGAGAACAAGTTCTTGCAAAACTTAAGCT
GCGCTCAGCTGATCGCAACTCAAGTGCGTGTGTGTGTGCGCGAGTGCAGCAAAGCTTTCGCGAGAGAGCT
TTCAGCTTGCAGCTGCACTGATAGTGCTGGCAAAACAGTCACTGCAGTGACAGTGCTGACACTAAAGTCA
CAATTTGTGATCAATCACTTGTTACAAAAATACGGGTTATTTTTGTAATTAAGTGTAAAAAACTTGTTTTGT
TTTTATTTTTTATTCTTTTTTCTCCCTGTGGGGAAAAAATTATAACAAAGTAAAGAACAATGGCTACAAAC
AACCGACCCCAAAACAACGAAGAAATTCATTCGCCAATCTACCCGCTGCCGAACAGCTCAATAATATCCC
CTACAAATATAGCCGCTAATGTCGATCGCCTCGACGGAAGCAGGCCACTGGAAGATTTTTACCAATATTA
CAACAACGTGCTCGCCAGTAGTGCAAACCGGAACGGCGCCTCCACCTCGCTGTCAGCCGGTACCCAGCAA
TGCTCGACTTGGCAACCGCAACAGCACGGCACTGTCAGCACAACGACAGCAACAACCACCACAACCACA
GCGTCATCCAGCAGCGGCTGCAGGACATCTGCCTCAACAGGCACAAGCCGCAGCACGGCACTGTCAGCAC
AACGACAGCAACAACCACCACAACCACAGCGTCATCCAGCAGCGGCTGCAGGACATCTGCCTCAACAGG
CACAAGCCGCGCCGCTTTAGAGCCCATCGGGACCCGCCCCAAAGCCGCCAATCCGACCTTGCAGTCCACC
CCTTATGGCACCCAGGCAGCTGGTTCTTGTGTCACAAACCCCCCTCTGCCTCCCATAAAGAACGCAGGTGC
TACGCCAGCCTTCGAAGAGCCACTGGTAGATTGGCAAACGGTTTCCGCAAAAAAACGGCGTGGGACTACA
TTATTATCCGAAGGCGTAAAAAAAGGGAAGCAACTAAATAACCGCAACACCAGCCGCGCTGGAGCTAAG
CCCGCAACTCCCAGTACCGCTTCGGCAATTAGCAACAGATATGCCTCCCTTGAGCCTCAGGAAGACGCCT
CTGTAATCGATGACGGCGAGATGGACACTGCCGACGAAGCGCCTCCAGTCACAGACCAACAACAATCAG
CGAAAAAGATCTCCAAACCCAGGCCGATAACAGTCCCTGGAGTCAGCGATATTGTCGCAATGGAGCGAAC
CATAGATCAGGCAGTGGGCGCAGACGCCTATGAATTCAAGGTCTCTCCCTCTGGCTATCTAAGAATTTACG
CCAAAGATGCCGACACCCACAGAGCAATTGTCCGCAAGCTCACTGAAGTGAGAGTGCAATTTACCCACTT
CTGCCTAAAGGAAGACAGACCATACCGCGTTGTGGTTAAAAATCTGGCCGCTTCCACTCCCAGAAGCCAG
ATTGAAGCTGCCTTTACATCGCACGGCCATATTGTGACGAACCTTTACAACCCGGGCGCCAGACAGCCAA
CAAGTGATGCAGACTCCACCAACGACTTTCCAAGTCGCAACTTCTGGTTTGTCGATCTCAAGCAGTCTCTT
AACAACAGGGAAGCGCTGCGGCTCAACAAGGTTGGCCGCCAACGGGTCACTATTGAGCTTCCGAGGAAG
AACAATAGTATAAGGCAGTGTTTCCGCTGCTTCGAGTTCGACCACACCAAAAACTACTGTCTCAAACAGC

CCAAGTGTGGTAGGTGCGGTCAGTCCCATTGGACAAACTCCGAGCAGTGCCAGGCCAAAACAGTGGCAG
ACCTGCGATGCTCCAACTGCGAGGGAAACCATGCTGCTTCCTATAAAGGATGCAAAAGCTATCAGGTGAG
ACTAAATGCCAGGCAGCCGGCCATCAATGTCTCCCCTCCTACACGATATAGCCATCCTCAAAATACACGA
AGAACCACTCAGCAGTTCGTACCTTCCCGAATTGTGCAACAGGGCCTGTCGTATGCTGATGCGCTACGGCC
AGGCCATCATGTACAAGTCCGCCAACCCCCAGCGCGCACTCTTCAGCCCACCGTCAATCCAATGCAGCTC
CAACAACCACTGCAAGGGCCTCAGGAGCAAGACGCTTCCCTTGGAGGCATCCTTCAAAACCTCCAGCAGT
CCATTGCAGCACTGAATGCCGCAGTTGCAACCATTCTGGTTAGCATTAAAGAGCTAAAGGATGCGCAAGG
TTCCCCGCAGCTTAGATTCCCAAAAAGCCGCAAATAATGGCTACCCAGTTACGTCTCGGATCGTGGAACG
CTCGTGGCATCCTCCAAAATGCTAACGAGCTCAAGCTTTTTCTGAAAAAGCATGATGTAGACATCATGCTC
ATCTCAGAAACACATCTGCACACAAACTTGTACCTAACAATGGAGGGGTACGTCTGCTACCGTGCGGATC
ACCCTACAGGACGAGCCAGAGGTGGTGCTGTAGTGCTGGCAAAGCAGGATTTAGCACACTACCATCTACA
CACAGTGACCTCCAGCGACACACAGCTGGCTGCTATCATGGTGGAGACTCCCTTGGGCGAAATCCTCGTT
GCGGCAGCATACCTACCACCAAACATCCCCTGGAACCGAGCGGAATTTGATTCCCTGTTTGGCCAGCTTG
GCCCCAAATTCATCGTTGGAGGCGACTTCAACGCTAAGCACAGACTGTGGGGAAACTACAGAGCTGACAG
CAGAGGCACTGCGCTCCATGAGGCGCTCACTCCCTGCTCAGCCCAGGTACTGGCCACCGGTAGACCTACC
CACTTTCCGTACAACCGGCAAAGTACGCCTTCCTGTATCGACTTTTTTATATTCAAGGGCATCCCCAACAG
CGTACTATCGGTTCGCGAAGAGTATGATCTATCTTCCGACCATCTGCCACTCCTCACAACGATCAACACGG
CTGCACAAAGGATCCCAAAAAAGCGCAGGCTTGTCCTCCCTGGGTCGAACATCCAACGATTTAAGGCCGA
ACTAAATAGCTTGATCAACCTAAACACGCAGATTCTGTCAGTTGAGGACGTCGACGCTGCAGTACAAACC
CTGTTGGACCAAGTACATGCCGCAGCCGCCAACGCTGCGCCCGCACATATCTACTCAGCCCCTACAAGCC
AGCGGCCAAGGACGTTTTCCCCCATATTGGAAGGTCTTCTCGCCCTAAAAAAGCGACTGAGGAGGGAGTA
TGTCCGTACCCAGGACCCCACGATTGACAGGATCTATCGTCGTATTGCTAACAGGGTCAGGAAGGAGCTG
ATTATCTCGAAAAGAAATGCCACAGACACCATGCTGGAGGAAGCAACGGCAGACGAGAGCTCCAAATTC
GCGCTGTGGAAACTTAGCAGTCGATACAAGCGGCAAGCTGCGCCAAAGTTCCCCGTTCGACTTCCCGATG
ACACCTGGGCCAGATCACCAATGGACAGAGCCGAAGCCTTTGCCTGCAACTTGGAGGAGCGCTTCAAGCC
GTTTGAGACCGCATCGCATGGAAGGATACAGCGAGTCGCGCAAATCCTGGAGGCGCCACTCCAAATGTCA
CTCCCTGTATCGCCAATCACCTTCCAAGAAGTAGAGCAGGAGATGAAGAGATTAAAAAGCAGCAAAGCC
CCTGGGGAAGATCGGTTGGACAACCAGACAATCAAGCTACTGCCATACAAGGCGGTGCTTTTTCTCGTCG
CCATTTTTAATGCAGCTCTTCGGCTAGGTTACTTTCCAGTGGCTTGGAAGAAGGCACATATAATCATGTTG
CACAAGAGCGGAAAGCCCCCTAATCAACTGGGATCCTATCGCCCCATCAGCCTCCTGCCTGCCTTTGGTAA
GATTCTGGAAAGGTGCATCCTCAGAAGGGTCCTGGATACCCCGGAAATAAAACGCTTGATCCCGAAGTTC
CAGTTTGGGTTTCGACTCAAACACGGTACCCCTGAGCAACTACATCGGGTCACAAATTATATTTTGGACGG
CTTTCACAGGAAAGAGTATGTGGTGGCAGCATACCTCGACATCCAAGAAGCCTTCGACCGGGTGTGGCAC
GCAGGACTCCTCTCCAAGATAAAGGACAAATTGAATCCGCAGCTATTTAGCACCATTGCGAGCTTCCTAA
CTGAGAGAACCTTTCAAGTGGTGCAGGATGGAGTAGCCTCTCAAACCAAACGCATAAAAGCGGGCGTTCC
ACAAGGCAGCGTCTTGGGTCCCACGCTATATAGCCTGTTTACACATGACATGCCCACAGCAAATGCAAGC
AGACGGAATGATCGGCAGGACCTTCTGGTGGCCACGTTTGCTGACGACACGGCAATCCTTGCAAAAAACA
AATGTGTGTACGTGGCAACTGACCTCTTGCAGGAATACCTCAAGCGATTTGAAAACTGGGCTTGTAGATG
GAACATCGCAGTGAACCCTGGAAAGTGCGCCACGGTTACCCACACTCTACGCAAGGACTCATGCCCCGGA
CTTTTTCTTCATGGTGAGCTTCTAGAGCAATATCGTCAACACAAGTACCTAGGTGTCACTCTCGACAGACG
ACTTACATTTAGTAAGCACGTCTCTGCAGTAACGAGCAACATCAAATCTAAGATAAGGCGAATGAGCTGG
CTCCTCAACTCCAAAAACAAACTCTCGCTCCGAAACAAGGTCCTAATCTACAAGGTAATCCTTGCCCCAGT
GTGGCGTTACGGGCTTCAAGTTTACGGTATAGCTGCAAAAACACACCTGAACAAAATTCGCATCATTCAG
GCCAAAACCCTGCGCAAAATAACTGGAGCCGAATGGTATATTCGCACACGCGACATCGCCAAGGACCTCA
AGGTTCCAATGGTGGGCGACGTAGTCAACAGGCAAGCAGCGAGCTACTCAACAAAGCTCCACAACCATCC
AAACATCCTGGCGAGGAGCCTGCTAAGGAGGCGAAGAAGAAGGCGACTGAAGAGGACCTACCCAACCGA
CCTAATCGATCGTTATGTATAGGTGACAAGCTTCGTTAATTACACCAAAATATGTTTCTGTAATCTGTTAA
CCTAATTGTATAACTAATTGTAAAACCACAATCATCGCTAAGCTAAGTAATTATGTCACTCTGATAAAAGT
TGCTAACATAGAGTAACAGGATACCAGATTTCTTAATAAACAAAATATAAAAAAAAAAAAAAAAAAAAAA
AAAAAAAAAAAAAAAAA
>160_rnd-1_family-345__LTR___Gypsy
TAAGNGAATAATAATAAAATTTTCTTTTATAAATAAAATTAANCGAACAAAAAAAAAAAAAAAAATAAAT
AAAAATAAAAAGTGAAAAGACACCAACACCAAGAAAAGGAGAGAGGAATAGTAAAGGGACCGACGGAA
CCCCTAGAACGGGAAGAGAGGGAAACAGCGACGTCGACTAGCAAGGAAGTATACAATAAAGGATATGGA

GTCAGGTGAGTGCCAAAACCCGGGGGTAGGCGTGCGTTGGATCCACCAGCGGCGCAAGGAGGAGTTGGT
GGCCATCGCCCAGGAGTTCGGGCTAGANAAGGAAGGGCGCGTGGAGGAGCTGCGTAGGCGGCTGTCCAC
CTACGTGCAGGAGGGCGGCCATTCGNCTGAGATCTGGGCTCGGCTTGCCGAGCTGGAGTCTCAGTTCGCG
AAAACGTTNGAGCCGCTATCCTGGGGCGTCCCATCCGACGTGGCTGGACGGCCGCGGCCAAAAGTCAGAG
CCTGATCNTAGGAGCTCACAATTGTACGGGCGTCCGCGGCCGCCTATCCCTGGTGTGGCCGTCACGGGCG
TGGAAGAGGCTCCCGATCCCGGGGAAAATGGAGACCGCCGNGCAACCGCGACCAACGTAGGNGTGGGGC
GGCCCCAACCGCCATATTCTCGGTCGGAGGTTAGGCCTTTCNCAAACGCTCCGGAACAAGTTCGGAGGTG
GGGCATGAGATACGATGGTCGCTCCGACCCCCTCGGGTTCTTGGAGGACCTAGAGGAACGCGCCATCACT
ATGGTATGCCCTCGATAGATTGCCCCGGCCGATGAGCGAGGTGTTCTGCGACAGGGCCGGTCGGTGGGCT
NAGAGATGTGCTATGGTCGGTGTTCAGGAGGGGTTTTTGGTTCTTCCTGCCCCCCCTGCCCCACGCTACTT
CGAAGGGCTTGAGGACGAAATCCGGTCCCGCAGGCAACGCGNGGGGGAGGAATTCANGGAGTACCTGNT
AGAGCTGAGAGCTCTGATGCATCACGCCAGATACGAACCGGCACAGGAGTTGCACCGGATCTACGAAAA
CGCCGCTCCCGAATACAAACTCTATATTGAATACAAAGTGCCAACTCAATTAATTTGATGTAACAACCACT
GTATTTGTATATTGACTTGTCCGTTATTTTTCGTTTTCGGCATCTCTTTATTCTCGGTTGTTTCCTAAATTTT
AAATGCACCATGAGAAATTCTTAACCGATTACACACAGCCAATGAAAAATGTATTAAATGTTTATGTGAC
AATTAAGAGAGTTGTATTCATTATTATATAATAAAGAAGTTATTCTGTGTTGTACCCAAAAAAAAAAAAA
CATCATAATGAAATATGTACATCGATACTTTATTTGTCAGTAGTAATCAAATTTTCATTGGCACGTGCCAA
AAGTGTGCTGCCTTGCACTGATCTTCGTTATTGCTTGATCTCATTTGGTTTCCTTTGCTCAAACTGTGCGGG
GGTTAACGAAACAAAAAATGTGAATTACTCAAATATTGAATTTGAATTAATAATAGGGAAGTCGTGCAGG
CTGTAGTTGTTCACTCTCAATGAGCAACAAATTTATATTAGAAGTAGGGTATTAAAGCTAGGTGACACGA
CTTACTTATGCCGTGTCGGATAAAATCAGTGGCCTTAGCGNCACGCCGAAGATCTGATAGCCTTGCCGACC
CAACCTTGTCATAATGCTTATAGTGCTTTGCCCGTATCTTAGAAGCAGAGGAAAATAGATAATGCCGAGTT
TGGTCAAGATATCTTGATAAACAAGAAAGGTTTTTAATCAAAAAACTTGTTTTCAACCCATCGTTCCTATG
CCAGATATATGATATAGTAGTCCGATCTAAATCGGATTTTGCATTTATATGAGGAGTACAATAAAACTAAT
AAATGTCGAGATTGGTTAAGATATCTTGATAAACAAAATGTTTTTTCATACAACAACTTAATTTTCGACCG
ATCGTTTGTAAGGCAGGTATATGATATAGTGGTGCGATCTTAATAGGATTTTGCATATATATGAGGAGTAT
AGTAAAACTAATAAATGCCGAGATTGGTCAAGGTATCTTAAAAACAAAATGTTTTTTCATACAACAATTT
AATTTTATATATAGTGGTGGGATATAGTGGTACGATCCAGCTTTTTTTGACATATGTTCTGCGTGCAACAG
AAAGAGGGACTTCTGCAAAGTTTCATTTAGATAGCTTTAAAATTGAGAGACTAGTTCGCATAGAAACAGA
CAGATGGACAGACAGACATGGCTATATCGACTCGGCTGTTGATGCTGATAAAGAATATGTATACTTTATG
GGGTCGGAGAAGTCTCCTTCTATGCGTTACACATTTTTCGACAAAGGATGCGTACATAATTGTGAGACATG
TACATACCATATGTGTGTATACATACATTCACGATAATACTATACCAAAGAAAAACTAAACACTAAGATG
AGTAACGCCAATTGCATGCAACGCAAAATTTATAGCCTGACTTTCTAAACTGGCTTTTACGATTTCGCACG
ACATGGATGCCAAAGTCGCGGCTCTCTGATTCTGACACGAGCAGAGAAATTAGTTGCCTACCTGCCTTCA
GCAAAGCTGGTCGATGGTGATTTGCATGGCCTCGTTTTCGAAGTACATTGAGCGAAAAAGTTTTGAACTTA
CAATCGACGCAAAGCACTTTTGATTTATCCATGAAACGCATGCTTTATTAAAATGATATAATACCCTAGAT
ACTAACGATAACTTTTACAAGAAAATTTGTGAGGACGCCACTAGGATAGCTACCCCACAGTTCATAATTG
TAATTATAGGGTATAAGCATTGTATAGTATATACAGTATGTCAAGAATGAGTTTACACATTGAATAAAAA
TATATTTTTTCATCTATTGGACTAAAAATATGAACACCCGCCTGACATTTTAAACTTGCTTTTGGGAATTTA
TAGGAAGTGATGATATTTTTAATGAAACAGTAGCATCAAAATGAAATGTTAACAATAAGAAACCAAAAA
AAAAAAAAACACAAATACAAAAATGACTCAGTTTGACCAAAGACAATATAAACTCTAAGATGACTAACG
CCATACAACAAAATGCTACTATGCAGCTTGATTTTTTGAAAATTGAGAACGAAAACGAAATCTGTAGCAT
TGCGCTCTCACAGCCGAGAGAACGAAAAAGCTAAAAATAGAATTTGCTCTCAGCTTCGGCTCTATGAGGG
CCGTGCAAGCAATTATGTTTGTATGCGTGTGAATATACATACATAACAGCATATTTGAATGTGTTGTTATC
CACNGCTCTGGCAAAGTGTCTGGCTGGCAAAGAAACAATTTTCTTNGTTTTTTAGCGCAGATCACGACCTA
CCCAAAAGTTCTGTGGATATACTTATGGGTGCATTTTGTGTACTGAGGTTGGCTCCGTACAAAAAAATTTG
TATATTTAAGTCTATGCAAATATTTATGTAATTTTTTACATTTGAAATTATTAATTAAATTATTATTATTTTT
AAGTAATACAAAATAAATTAAGGAAATAAAAGTGCAAATTTGTTGTTTTCAAAGATACATTTAATTAATTT
TTATTTNTTTTTATTTTATTATTTTTTTGTTCGAATTATAAAGAAGTACGCAGGGAAAACCGCGAGGAGGCC
ATTATAATTATAATAGGGTGATAACTAAATATGACCTTCGAGTCGACTTGAAATGTAATTGTTTTTTTAGC
ACTCTTTTATTTCTTAATTGGAGAATAAAAATAATATTAATTCAATTTAAAAAAATTATGTAAATATTTGC
ATCGACTTAGAAATACATATTTTCTGCGGAGCCAACCTCAGTACACAAAATGCATTCATATATCAACACA
AATTTGGGATAGGTCATGATCTGCGCTACAAAACAAACAAAATTAATTTCTTGCCAGCTGAAGCCAGAGC
AGTGAATAACAACACATACCTTAAAGTTCTTATGCATGTATAATTCACGCATACAACGAAAGGTGCACGC

ACGCTATTGGCTGAGACAAGAACTTTGCTCGAGCATACACTGTCCAAAAAGAAAAGTGAAATCAATCTAT
GTGTGCTATTTTCTTAACATAGTTATTGCGCAAGAAGCGCCTTGTGTAAAAATTTTATAAATTCTTAAATTT
TCCTGTAAAATTAATCGCTAATATCTAGGGTAATATATCATTTAAATAAAGCATGCATTTCATGGATAAAA
CACAAGCACTTTGCGTCGATCGTAAGTTCTAAACTTTTTCGCTCAATGTACTTCGAAAACGAAGCGACGCA
AAGCAGAATCCACCAGCTTTGCTGAAGGCAGGCTGGCGAATAACTTCGTAGCTCGTGTCAGCATCAGCAA
GCCACGACTTCGGCATTCGTTCGTCTGGTCTTTGGACTACGAACAACCAGTCGGCAGGCAAGACGTACGA
AGCCTCAAAGGCCAGATGAAAGGCCAGGCTATGTATGGGCGTTACTCATCTTAGTGTTTATATTGTCTTTG
GTTTGACTACTGTCAAGAAACTCTTTACACATTCTTTTCCTTCTCCCTTTTTTCAATATTCCAAGAACAACG
GTACATTTACCGATATCTATTCTTTGCTTTGCATTGTTTTCAATGCATTTCATTTCATTTAATTCGGCGATGC
TTTTCGATGATATCACGTGCTTTGAGAAGCTATTTGATTTTTAAAAGATGCCAGGCAAAAGATTATAATTT
GAAGTTGTCCAGCTTATATTCTATAACCACCAGATGGGAAAAAGTGTGCTTGAGCTATAAGCAATGTTTTC
TGTTACACGAAAGACTATATACAACGTCTTAAAACGAATTGAAAATGAAGGTACGTTGGACCCAAAAGAG
GAGGCGGACCTAAGCCTAAAATAGCGGAGCTTACAGAGCGTTTCATGATGAGGAAGGTGGCTCTAAATCC
TCAGATCTTAACCAGGGCTCTTCCCAGGGAGCTGAATGATAAATTTGAATTTGCGGTTTCTTATGAAACAG
TAAGGCAAGCAATAATTGGCAATGATTACAAGTAAAGAGCTGCGCGCAAGAAACCGCTCCTTTCTAAAAT
AAACATTGAAAAACGATTGTGCTTCGCCTATATGCACATTTCTTATCCAATTGATTATTGGGACAATCATA
TTTTATGATGAAACAAAAATGATGCTTTACTACAATGATGGGCCTAGTAGAGTATGGCGGATCAGCCACA
GACATGATCAGCCACAGACATTTGTATGTGTGTGTCTGTGTGTAGAGGTCGAAAATCAGAAATCCTAAAA
TCTTGTTAAATTTGCATCGGGGACAGGCAGGCAGGCAAGCAACAGGCACACAAGCGCATAAATGCTTCTA
TTCATATACAAAACATACAAGTATGCGTGAATAGCGAGTCC
>Ref_dvir.3.94.centroid
GTATATAAATTAAATATATATATGTCGTGTATCCAATAAATTATTGTGAGTATTATTTTGAATGCTAACGA
TTAAGTGTTAGTAAGAATCAGAGAATTTGACAGAAGGTACTGCTATTTAACATAACTGTCCTACTTGTATT
AGGGTTGCTACTGAAAGCATGGTGTGAGACAAAAAAAGATAGTCTCTGCCGAGCATGGCCCGAGTGTGTT
GTATAACGTCCAGTTTGCTATAGTGTTGAATGTTCACTAACAGTACTAAAAGTCAGAGAAGAATATAGAA
TGACAAGAGCATGAGTAGTATGCTCGAGGACGATGAAAATTATTCATTCTAAAAGTGGAAGTCATAGAGA
ACAGACGTGCTCGCTCCTAAAAATATAATTATTTTGTGACTATAACGACACTCGGGACTTGCTGTACTGCC
AGTATGCCTAATTGCAAAGAAAGTGATTTCTCTGTTCCGACATCAGAAGTGGGATTAGGCGATGCCCCTCC
ACGAGGCAATTCTGACGATTCGTGGCGTCAGCTTTTGGAATCTCAAAATCGAAATATTCTTGAGATTATTA
AAGCAATGCAGATGTCAAATTCAGCATCCTCAACAAAGAAGAGCATCTCGTTGCCAAAGTTCAACCCGGA
CATTGCAGGCGCAGATGCAAATGCCTGGTGCAAAACTGTGGACATAATACTTGACGATAATCCTATTGAG
GGCAGTACACTGGTGATGACCCTCAGCGGCTCCCTAAAAGGAAGCGCCTCGCCCTGGCTTTCTCAAATAT
GCTATACTGGAATGAACTGGCAAAATTTTAAGGAATTGTTTTTGCAACGTTTCGTGGGAATGGAAACATC
GGCGGCAACTTTAATGAACATTTTTAATGGCCGACCGAATGATGGCGAATGCCTCTCCGTATATGCGAGC
CGTCTTGTGACCTCACTTTTATCCAAATGGAAGACAGCGAACATGGAGGAAATTGCAGTCTCAGTTGTTTT
GGCACATACATCGAAAATCGACAAAAGTCTGCAACGTATGTTATATACGACTAATATTAAAAGTCAAGAC
GAGATGCAACAGCATCTGAAGGCCTATGCTTATGGAAGGTCAAATGGAGGTCTACAGCCAAATATCCCAA
TGGAACCAGATCGCAAGAGATCGAAAACATATGGCATTAAATGTCATCATTGTGGAAACCTCGGACACAA
AATGGTCGAATGTCGCAAGAGGAAGTATGAAGAAGGCAACGTGTCAAAAAACTCAGCATTACCTAAAGT
TCGATCTGCCGTTACCTGCTTCAAATGTGGCGATGCTGGCCACATTGCGTCTTCATGTACGAAAGGAGTTT
CTACGAGCGGTGTTGGATCACATGACAAGCGTATAAACATCTGTTGTGTGTCCGAGCCAAAAGGAATCCT
GCGTCAATCTGGTGAGCTCGTTCCATTTTGTTTTGACTCTGGAGCGGAATGTACGCTCATCAGAGAGAGTT
GTGCCGAGAAATTTTCCGGAAAAAGAATTAATAATTTGATAGTTTTGAGGGGTATTGGAGAAAATCCTGT
GTATAGCACTATGCAAATCTTGGCTACTGTTAAAATTGATCAGTACTCCTTAGAAATATTATTTCATGTAA
TAATGGATAAGTTTTTGAAATACGATGTATTGATAGGCCGTGATATACTCGGATTAGGCTTTGGAGTCTCC
ATCGAGGCCGACAAATTTGAAATGTACAAAACCAAAACCTGTTGATGTACTAATAAAAGTGCCAGATATA
AGTTACGTAGAGCATCTGAATAACGATAGAGCATTAAGCAACACTGATAAGAATCGTCTGCTAGAATTAT
TGCAAAACTATGCTGATAAATTTATTGAGGGTATTCCTCAAACGCGAGTGACTACGGGTGAACTTGAAAT
CCGCCTGCTTGACCCAAATAAAACTGTACAAAGGCGTCCGTATAGGTTAAGCATAGAAGAAAAAGAAGTC
GTGCGAAATAAAGTAAAAGAATTGTTAGACGCAAACGTTATACGTCCTAGTTGCTCTCCATTTGCAAGCC
CAATGCTGATGGTAAGAAAGAAAAATGGAACCGATCGACTATGTGTCGATTATCGGGAACTGAATGCTAA
TACGGTCGCTGACAAATATCCGCTGCCTTTGATTTCTGATCAAATTAATAGACTGAGTGAAGGCGAATATT
TCTCCAGTTTAGACATGGCAAGTGGTTTTTACCAAGTTCCAATACACCCAGACTCTATCGAGCGCACTGCT
TTTGTCACGCCAGAAGGACAGTATGAATTTTTGGCCATGCCTTTTGGATTAAAAAATGCTTCCTCGATTTT

file:///E/10月/10.9/PDF/4. biology-1907542/biology-1907542-supplementary/SupplementalDocument2.refTElib_01_25_2022.fasta.txt[2022/10/9 15:38:02]

CCAGCGCGCAATCGTGAGAGCATTGGGTGATTTGGCTCAAACGTATGCCATCGTCTATATAGATGACGTG
TTAATTCTAGCGAAGACAAAGGAAGAGGCACTTGAAAGGCTACAAACGGTCTTGGAAAACTTGTCTAGAG
CTGGATTCTCATTTAATATTACAAAATGCTCGTTCTTGAAAACAAGCATAGAATATTTAGGATACTTGGTT
GAAGCGGGTCAAATTCGACCAAATCCACGCAAGATACAAGCGTTGGCCGAATTGCCGTCACCGCAGTCAG
TAACGCAGGTGCGACAATTCATAGGCCTAGCCTCGTATTTTAGGCAATTCGTACCAAAATTTTCTGAGCAG
ATGAAAGCTTTATATAATCTCACCTCCAAGAATAATAAATTTGTCTGGAAGCCGGAATATGAGGAAATTC
GTCAAAAAATCGTATCAATTTTGACTAATGACTCCGTTTTGACAATTTTCGATCCACTAAAGCCAATTGAA
TTACATACAGACGCAAGTTCTATTGGATATGGTGCTATTTTAATGCACAAGATAGAAAATAGACCCAGAG
TCATTGAATATTACAGCAAATGTACGACAATGGCTGAAGCAAAATATCATTCATACGAGCTTGAGACCCT
CGCAGTTTACAACGCAGTAAGACACTTCCGCCATTATTTACATGGTCAAAAATTTATTGTGCATACAGATT
GCAACTCCCTGAAAGCAAGTATCAAAAAAATTAACCTTACCCCAAGGGTACATAGATGGTGGGCATATCT
GCAATCATTTGATTTCAGCATTGAATACAGGAAGGGAGAACGCATGTTACATGCTGATTTTTTCTCGCGCA
ATCCGTTGCCTACTGATTATAAATCCTCAGCGAAAAACGGTTGAAATGCACATAAAACCTAGCTGAGATA
ACCGACGATTGGGCTTCTAGCCGAACAGCAACGAGATTCTGACATTGCTTCCATAACCTTAAAATTGCGA
AACCATAAGATGGGAGAAGCTATCTCAGCGTTCGTACGAATTGAGAGCAGGAGTATTATATCGAAGGATT
CAAAGAAATGGCAGCACAAAATGTTTGCCTGTAATACCTAGAGCCTTTAGATGGTCAGTCATTAACAATG
TTCACGAAGCCATAATGCATTTAGGGTGGAAGAAAACTCTAGACAAAATGTATGCCAACTACTGGTTCGA
GAAGATGTCTAAGTATGTGAAAAAGTTTGTAGAAAATTGCATTACCTGTAGAGTGTCAAAACCACCATCT
GGTAAGCCACAAATAGAAATGCACCCGATTCCTAAAGATGACATACCCTGGCACACTGTGCATATCGATA
TAACCGGAAAATTAAGTGGCAAAAACGACTTAAAAGAGTACATCATAGTTCAAGTTGATGCATTCACAAA
ATTTGTTCACCTTCAACATACGCTGAACCTTAATACGGAAAACTGCATTAAGGCAGTCAAAGAAACTGTT
GCACTATTCGGAGTGCCGGCACGCATCATAGCTGATCAGGGTAAATGTTTTGCTAGCACAAGGTTTAGGG
AATTTTGTTCTTCGCAGAAGATAGGTTTACACCTGATTGCGACGGGCGCCAGTCGAGCAAACGGCCAAGT
AGAGCGTGTCATGAGCACCTTGAAGAGCATGCTCACAGCGGTTGAGAGTAGTCAGCGATCCTGGCAAGAC
GCTTTAAAAGACATACAGCTGGCAATAAATTGCACGACAAATCGTGTAACGAAATATAGTCCGTTAGAAT
TGCTAATCGGTAAAGAGGCAAGGCCGTTAGGAATGATAACAATATCAGATGAAAAAACAATTGATAAAG
ATTTAATAAGAGCACAAGCGAAAGATAACATGGTGAATAACGCTAAATATGATAAGGCTAGGTTTGATAA
AAATAAAGCAAAAATTGTAAAATATAAGCTTGGTGATCATGTCCTACTCAAGAGGGAAGAACGAAATCA
AACGAAGTTAGATCCCAAGTTTAAGGGACCTTTTGAGATAATCGAAATACTTGATGGAGACAGATATTTA
TTAAAGTCATTGACGAGTAAGCGCACGTTTAAGTATGCACATGAGTATTTGCGAGCTTTTCCAAGCAACA
ATGTATCGGAGGAGTTGATAGATTTGAGAGATGATGAGTGTGACAATGTTGAGGACATTGCTCAAGACAC
TGTGGCACTAAGAGAGAGTGCGGTTGGGCTCATACACGATGGCTAAGACCGCACCATAATACTCACGCCG
TGCACAAGTGCTCGATCATGGCGTGCTGAAGCGGGTGATATTATGGCGGGGGTCAGAGGTTGAAAGACCT
GTATGGATCCGCTCCATATTATCGTTGCTGGGCAGCCAAGAGCCTATAGACGTGTGATGACAGAACAACA
ACAAAAAAAAAAAAAAAAAAAAAAAAAAAAATAAAAAAAAAAATAAAACCTACAATGACCGATGATACTGC
TGTTTGTAAAGCTATTCTTAAAGTAAAGTAATGACTAATAAATTTTACCCAACAATTTTGACTATGGCATG
ATCTAAGCGAAATGTACACACGAGGACGTGTGAAATGTCAGGAAGGCCGTGTCGTGTATCCAATAAATTA
TTGTGAGTATTATTTTGAATGCTAACGATTAAGTGTTAGTAAGAATCAGAGAATTTGACAGAAGGTACTGC
TATTTAACATAACTGTCCTACTTGTATTAGGGTTGCTACTGAAAGCATGGTGTGAGACAAAAAAAGATAG
TCTCTGCCGAGCATGGCCCGAGTGTGTTGTATAACGTCCAGTTTGCTATAGTGTTGAATGTTCACTAACAG
TACTAAAAGTCAGAGAAGAATATAGAATGACAAGAGCATGAGTAGTATGCTCGAGGACGATGAAAATTA
TTCATTCTAAAAGTGGAAGTCATAGAGAACAGACGTGCTCGCTCCTAAAAATATAATTATTTGTGACTAT
AACGACACTCGGGACTTGCTGTACTGCCAGTATGCCTAATTGCAAAGAAAGTGATTTCTCTGTTCCGACAT
ATATATATTATGTATATTCACATATA
>160_rnd-1_family-111__LTR___Gypsy
ATATATATATATATATATAATATATATATATATATATGTCGGAACAGAGAAATCACTTTCTTTGCAATTAGGC
ATACTGGCAGCACAGCAAGTCCCGAGTGTCGTTATAGTCACAAAATAATTATATTTTTAGGAGCGAGCAC
GTCTGTTCTCTATGACTTCCACTTTTAGAATGAATAATTTTCATCGTCCTCGAGCATACTACTCATGCTCTT
GTCATTCTATATTCTTCTCTGACTTTTAGTACTGTTAGTGAACATTCAACACTATAGCAAACTGGACGTTAT
ACAACACACTCGGGCCATGCTCGGCAGAGACTATCTTTTTTGTCTCACACCATGCTTTCAGTAGCAACCCT
AATACAAGTAGGACAGTTATGTTAAATAGCAGTACCTTCTGTCAAATTCTCTGATTCTTACTAACACTTAA
TCGTTAGCATTCAAAATAATACTCACAATAATTTATTGGATACACGACACGGCCTTCCTGACATTTCACAC
GTCCTCGTGTGTACATTTCGCTTAGATCATGCCATAGTCAAAATTGTTGGGTAAAATTTATTAGTCATTACT
TTACTTTAAGAATAGCTTTACAAACAGCAGTATCATCGGTCATTGTAGGTTTTATTTTTTTTTTTTTTTTTT

TTTTTTTTTTTTTGTTGTTGTTCTGTCATCACACGTCTATAGGCTCTTGCTGCCCAGCAACGATAACATGGA
GCGGATCCATACAGGTCTTTCAACCTCTGACCCCCGCCATAATATCACCCGCTTCAGCACGCCATGATCGA
GCACTTGTGCACGGCGTGAGTATTATGGTGCGGTCTTAGCCATCGTGTATGAGCCCAACCGCACTCTCTCT
TAGTGCCACAGTGTCTTGAGCAATGTCCTCAACATTGTCACACTCATCATCTCTCAAATCTATCAACTCCTC
CGATACATTGTTGCTTGGAAAAGCTCGCAAATACTCATGTGCATACTTAAACGTGCGCTTACTCGTCAATG
ACTTTAATAAATATCTGTCTCCATCAAGTATTTCGATTATCTCAAAAGGTCCCTTAAACTTGGGATCTAACT
TCGTTTGATTTCGTTCTTCCCTCTTGAGTAGGACATGATCACCAAGCTTATATTTTACAATTTTTGCTTTATT
TTTATCAAACCTAGCCTTATCATATTTAGCGTTATTCACCATGTTATCTTTCGCTTGTGCTCTTATTAAATCT
TTATCAATTGTTTTTTCATCTGATATTGTTATCATTCCTAACGGCCTTGCCTCTTTACCGATTAGCAATTCTA
ACGGACTATATTTCGTTACACGATTTGTCGTGCAATTTATTGCCAGCTGTATGTCTTTTAAAGCGTCTTGCC
AGGATCGCTGACTACTCTCAACCGCTGTGAGCATGCTCTTCAAGGTGCTCATGACACGCTCTACTTGGCCG
TTTGCTCGACTGGCGCCCGTCGCAATCAGGTGTAAACCTATCTTCTGCGAAGAACAAAATTCCCTAAACCT
TGTGCTAGCAAAACATCTACCCTGATCAGCTATGATGCGTGCCGGCACTCCGAATAGTGCAACAGTTTCTT
TGACTGCCTTAATGCAGTTTTCCGTATTAAGGTTCAGCGTATGTTGAAGGTGAACAAATTTTGTGAATGCA
TCAACTTGAACTATGATGTACTCTTTTAAGTCGTTTTTGCCACTTAATTTTCCGGTTATATCAATATGCACA
GTGTGCCAGGGTATGTCATCTTTAGGAATCGGGTGCATTTCTATTTGTGGCTTACCAGATGGTGGTTTTGA
CACTCTACAGGTAATGCAATTTTCTACAAACTTTTTCACATACTTAGACATCTTCTCGAACCAGTAGTTGG
CATACATTTTGTCTAGAGTTTTCTTCCACCCTAAATGCATTATGGCTTCGTGAACATTGTTAATGACTGACC
ATCTAAAGGCTCTAGGTATTACAGGCAAACATTTTGTGCTGCCATTTCTTTGAATCCTTCGATATAATACTC
CTGCTCTCAATTCGTACGACGCTGAGATAGCTTCTCCCATCTTATGGTTTCGCAATTTTAAGGTTATGGAA
GCAATGTCAGAATCTCGTTGCTGTTCGGCTAGAAGCCAATCGTCGGTTATCTCAGCTAGGTTTATGTGCAT
TTCAACCGTTTTCGCTGAGGATTTATAATCAGTAGGCAACGGATTGCGCGAGAAAAAATCAGCATGTAAC
ATGCGTTCTCCCTTCCTGTATTCAATGCTGAAATCAAATGATTGCAGATATGCCCACCATCTATGTACCCTT
GGGGTAAGGTTAATTTTTTTGATACTTGCTTTCAGGGAGTTGCAATCTGTATGCACAATAAATTTTTGACC
ATGTAAATAATGGCGGAAGTGTCTTACTGCGTTGTAAACTGCGAGGGTCTCAAGCTCGTATGAATGATATT
TTGCTTCAGCCATTGTCGTACATTTGCTGTAATATTCAATGACTCTGGGTCTATTTTCTATCTTGTGCATTA
AAATAGCACCATATCCAATAGAACTTGCGTCTGTATGTAATTCAATTGGCTTTAGTGGATCGAAAATTGTC
AAAACGGAGTCATTAGTCAAAATTGATACGATTTTTTGACGAATTTCCTCATATTCCGGCTTCCAGACAAA
TTTATTATTCTTGGAGGTGAGATTATATAAAGCTTTCATCTGCTCAGAAAATTTTGGTACGAATTGCCTAA
AATACGAGGCTAGGCCTATGAATTGTCGCACCTGCGTTACTGACTGCGGTGACGGCAATTCGGCCAACGC
TTGTATCTTGCGTGGATTTGGTCGAATTTGACCCGCTTCAACCAAGTATCCTAAATATTCTATGCTTGTTTT
CAAGAACGAGCATTTTGTAATGTTAAATGAGAATCCAGCTCTAGACAAGTTTTCCAAGACCGTTTGTAGCC
TTTCAAGTGCCTCTTCCTTTGTCTTCGCTAGAATTAACACGTCATCTATATAGACGATGGCATACGTTTGAG
CCAAATCACCCAATGCTCTCACGATTGCGCGCTGGAAAATCGAGGAAGCATTTTTTAATCCAAAAGGCAT
GGCCAAAAATTCATACTGTCCTTCTGGCGTGACAAAAGCAGTGCGCTCGATAGAGTCTGGGTGTATTGGA
ACTTGGTAAAAACCACTTGCCATGTCTAAACTGGAGAAATATTTGCCTTCACTCAGTCTATTAATTTGATC
AGAAATCAAAGGCAGCGGATATTTGTCAGCGACCGTATTAGCATTCAGTTCCCGATAATCGACACATAGT
CGATCGGTTCCATTTTTCTTTCTTACCATCAGCATTGGGCTTGCAAATGGAGAGCAACTAGGACGTATAAC
GTTTGCGTCTAACAATTCTTTTACTTTATTTCGCACGACTTCTTTTTCTTCTATGCTTAACCTATACGGACGC
CTTTGTACAGTTTTATTTGGGTCAAGCAGGCGGATTTCAAGTTCACCCGTAGTCACTCGCGTTTGAGGAAT
ACCCTCAATAAATTTATCAGCATAGTTTTGCAATAATTCTAGCAGACGATTCTTATCAGTGTTGCTTAATG
CTCTATCGTTATTCAGATGCTCTACGTAACTTATATCTGGCACTTTTATTAGTACATCAACAGTTTTGGTTT
TGTACATTTCAAATTTGTCGGCCTCGATGGAGACTCCAAAGCCTAATCCGAGTATATCACGGCCTATCAAT
ACATCGTATTTCAAAAACTTATCCATTATTACATGAAATAATATTTCTAAGGAGTACTGATCAATTTTAAC
AGTAGCCAAGATTTGCATAGTGCTATACACAGGATTTTCTCCAATACCCCTCAAAACTATCAAATTATTAA
TTCTTTTTCCGGAAAATTTCTCGGCACAACTCTCTCTGATGAGCGTACATTCCGCTCCAGAGTCAAAACAA
AATGGAACGAGCTCACCAGATTGACGCAGGATTCCTTTTGGCTCGGACACACAACAGATGTTTATACGCT
TGTCATGTGATCCAACACCGCTCGTAGAAACTCCTTTCGTACATGAAGACGCAATGTGGCCAGCATCGCC
ACATTTGAAGCAGGTAACGGCAGATCGAACTTTAGGTAATGCTGAGTTTTTTGACACGTTGCCTTCTTCAT
ACTTCCTCTTGCGACATTCGACCATTTTGTGTCCGAGGTTTCCACAATGATGACATTTAATGCCATATGTTT
TCGATCTCTTGCGATCTGGTTCCATTGGGATATTTGGCTGTAGACCTCCATTTGACCTTCCATAAGCATAGG
CCTTCAGATGCTGTTGCATCTCGTCTTGACTTTTAATATTAGTCGTATATAACATACGTTGCAGACTTTTGT
CGATTTTCGATGTATGTGCCAAAACAACTGAGACTGCAATTTCCTCCATGTTCGCTGTCTTCCATTTGGATA
AAAGTGANGTCACAAGACGGCTCGCATATACGGAGAGGCATTCGCCATCATTCGGTCGGCCATTAAAAAT

GTTCATTAAAGTTGCCGCCGATGTTTCCATTCCCACGAAACGTTGCAAAAACAATTCCTTAAAATTTTGCC
AGTTCATTCCAGTATAGCATATTTGAGAAAGCCAGGGCGAGGCGCTTCCTTCTAGGGAGCCGCTGAGGGT
CATCACCAGTGTACTGCCCTCAATAGGATTATCGTCAAGTATTATGTCCACAGTTTTGCACCAGGCATTTG
CATCTGCGCCTGCAATGTCCGGGTTGAACTTTGGCAACGAGATGCTCTTCTTTGTTGAGGATGCTGAATTT
GACATCTGCATTGCTTTAATAATCTCAAGAATATTTCGATTTTGAGATTCCAAAAGCTGACGCCACGAATC
GTCAGAATTGCCTCGTGGAGGGGCATCGCCTAATCCCACTTCTGATGTCGGAACAGAGAAATCACTTTCTT
TGCAATTAGGCATACTGGCAGCACAGCAAGTCCCGAGTGTCGTTATAGTCACAAAATAATTATATTTTTAG
GAGCGAGCACGTCTGTTCTCTATGACTTCCACTTTTAGAATGAATAATTTTCATCGTCCTCGAGCATACTA
CTCATGCTCTTGTCATTCTATATTCTTCTCTGACTTTTAGTACTGTTAGTGAACATTCAACACTATAGCAAA
CTGGACGTTATACAACACACTCGGGCCATGCTCGGCAGAGACTATCTTTTTTTGTCTCACACCATGCTTTC
AGTAGCAACCCTAATACAAGTAGGACAGTTATGTTAAATAGCAGTACCTTCTGTCAAATTCTCTGATTCTT
ACTAACACTTAATCGTTAGCATTCAAAATAATACTCACAATAATTTATTGGATACACGACATATATATATA
TATATATATATAT
>Ref_snarf
CCTCACTTACGTTTGTGTTTCTCTGGAGATCGGACGTGAAAATTGCAGTCTCGTGTGTGTGTGCGCGTTAG
TGCACCGCTGGCAGCAGGCATAAACCAAGAAAGTGACAGTGACACTGTATAAAGCCCGCCCCAGGGCAC
ATCCATACAAAATCCCGCTTATAAGTGGGTAAACTATACGCAAACTGCTGCCTCTGGCAGCCCGGAGTGT
TATACAAATTAACAAATATTTTAACAAAAAAATTACCGCGTGCAGTGCGTGAGAACAAGTTCTTGCAAAA
CTTAAGCTGCGCTCAGCTGATCGCAACTCAAGTGCGTGTGTGTGTGCGCGAGTGCAGCAAAGCTTTCGCG
AGAGAGCTTTCAGCTTGCAGCTGCACTGATAGTGCTGGCAAAACAGTCACTGCAGTGACAGTGCTGACAC
TAAAGTCACAATTTGTGATCAATCACTTGTTACAAAAATACGGGTTATTTTTGTAATTAAGTGTAAAAAAC
TTGTTTTGTTTTTATTTTTTATTCTTTTTTCTCCCTGTGGGGAAAAAATTATAACAAAGTAAAGAACAATGG
CTACAAACAACCGACCCCAAAACAACGAAGAAATTCATTCGCCAATCTACCCGCTGCCGAACAGCTCAAT
TATATCCCCGTACAAATATAGCCGCTAATGTCGATCGCCTCGACGGAAGCAGGCCACTGGAAGATTTTTA
CCAATATTACAACAACGTGCTCGCCAGTAGTGCAAACCGGAACGGCGCCTCCACCTCGCTGTCAGCCGGT
ACCCAGCAATGCTCGACTTGGCAACCGCAACAGCACGGCACTGTCAGCACAACGACAGCAACAACCACC
ACAACCACAGCGTCATCCAGCAGCGGCTGCAGGACATCTGCCTCAACAGGCACAAGCCGCGCCGCTTTAG
AGCCCATCGGGACCCGCCCCAAAGCCGCCAATCCGACCTTGCAGTCCACCCCTTATGGCACCCAGGCAGC
TGGTTCTTGTGTCACAAACCCCCCTCTGCCTCCCATAAAGAACGCAGGTGCTACGCCAGCCTTCGAAGAGC
CACTGGTAGATTGGCAAACGGTTTCCGCAAAAAAACGGCGTGGGACTACATTATTATCCGAAGGCGTAAA
AAAAGGGAAGCAACTAAATAACCGCAACACCAGCCGCGCTGGAGCTAAGCCCGCAACTCCCAGTACCGC
TTCGGCAATTAGCAACAGATATGCCTCCCTTGAGCCTCAGGAAGACGCCTCTGTAATCGATGACGGCGAG
ATGGACACTGCCGACGAAGCGCCTCCAGTCACAGACCAACAACAATCAGCGAAAAAGATCTCCAAACCC
AGGCCGATAACAGTCCCTGGAGTCAGCGATATTGTCGCAATGGAGCGAACCATAGATCAGGCAGTGGGC
GCAGACGCCTATGAATTCAAGGTCTCTCCCTCTGGCTATCTAAGAATTTACGCCAAAGATGCCGACACCCA
CAGAGCAATTGTCCGCAAGCTCACTGAAGTGAGAGTGCAATTTACCCACTTCTGCCTAAAGGAAGACAGA
CCATACCGCGTTATGGTTAAAAATCTGGCCGCTTCCACTCCCAGAAGCCAGATTGAAGCTGCCTTTACATC
GCACGGCCATATTGTGACGAACCTTTACAACCCGGGCGCCAGACAGCCAACAAGTGATGCAGACTCCACC
AACGACTTTCCAAGTCGCAACTTCTGGTTTGTCGATCTCAAGCAGTCTCTTAACAACAGGGAAGCGCTGCG
GCTCAACAAGGTTGGCCGCCAACGGGTCACTATTGAGCTTCCGAGGAAGAACAATAGTATAAGGCAGTGT
TTCCGCTGCTTCGAGTTCGACCACACCAAAAACTACTGTCTCAAACAGCCCAAGTGTGGTAGGTGCGGTC
AGTCCCATTGGACAAACTCCGAGCAGTGCCAGGCCAAAACAGTGGCAGACCTGCGATGCTCCAACTGCGA
GGGAAACCATGCTGCTTCCTATAAAGGATGCAAAAGCTATCAGGTGAGACTAAATGCCAGGCAGCCGGCC
ATCAATGTCTCCCCTCCTACACGATATAGCCATCCTCAAAATACACGAAGAACCACTCAGCAGTTCGTACC
TTCCCGAATTGTGCAACAGGGCCTGTCGTATGCTGATGCGCTACGGCCAGGCCATCATGTACAAGTCCGCC
AACCCCCAGCGCGCACTCTTCAGCCCACCGTCAATCCAATGCAGCTCCAACAACCACTGCAAGGGGCCTC
AGGAGCAAGACGCTTCCCTTGGAGGCATCCTTCAAAACCTCCAGCAGTCCATTGCAGCACTGAATGCCGC
AGTTGCAACCATTCTGGTTAGCATTAAAGAGCTAAAGGATGCGCAAGGTTCCCCGCAGCTTAGATTCCCA
AAAAGCCGCAAATAATGGCTACCCAGTTACGTCTCGGATCGTGGAACGCTCGTGGCATCCTCCAAAATGC
TAACGAGCTCAAGCTTTTTCTGAAAAAGCATGATGTAGACATCATGCTCATCTCAGAAACACATCTGCAC
ACAAACTTGTACCTAACAATGGAGGGGTACGTCTGCTACCGTGCGGATCACCCTACAGGACGAGCCAGAG
GTGGTGCTGTAGTGCTGGCAAAGCAGGATTTAGCACACTACCATCTACACACAGTGACCTCCAGCGACAC
ACAGCTGGCTGCTATCATGGTGGAGACTCCCTTGGGCGAAATCCTCGTTGCGGCAGCATACCTACCACCA
AACATCCCCTGGAACCGAGCGGAATTTGATTCCCTGTTTGGCCAGCTTGGCCCCAAATTCATCGTTGGAGG

CGACTTCAACGCTAAGCACAGACTGTGGGAAACTACAGAGCTGACAGCAGAGGCACTGCGCTCCATGA
GGCGCTCACTCCCTGCTCAGCCCAGGTACTGGCCACCGGTAGACCTACCCACTTTCCGTACAACCGGCAA
AGTACGCCTTCCTGTATCGACTTTTTATATTCAAGGGCATCCCCAACAGCGTACTATCGGTTCGCGAAGA
GTATGATCTATCTTCCGACCATCTGCCACTCCTCACAACGATCAACACGGCTGCACAAAGGATCCCAAAA
AAGCGCAGGCTTGTCCTCCCTGGGTCGAACATCCAACGATTTAAGGCCGAACTAAATAGCTTGATCAACC
TAAACACGCAGATTCTGTCAGTTGAGGACGTCGACGCTGCAGTACAAACCCTGTTGGACCAAGTACATGC
CGCAGCCGCCAACGCTGCGCCCGCACATATCTACTCAGCCCCTACAAGCCAGCGGCCAAGGACGTTTTCC
CCCATATTGGAAGGTCTTCTCGCCCTAAAAAAGCGACTGAGGAGGGAGTATGTCCGTACCCAGGACCCCA
CGATTGACAGGATCTATCGTCGTATTGCTAACAGGGTCAGGAAGGAGCTGATTATCTCGAAAAGAAATGC
CACAGACACCATGCTGGAGGAAGCAACGGCAGACGAGAGCTCCAAATTCGCGCTGTGGAAACTTAGCAG
TCGATACAAGCGGCAAGCTGCGCCAAAGTTCCCCGTTCGACTTCCCGATGACACCTGGGCCAGATCACCA
ATGGACAGAGCCGAAGCCTTTGCCTGCAACTTGGAGGAGCGCTTCAAGCCGTTTGAGACCGCATCGCATG
CAAGGATACAGCGAGTCGCGCAAATCCTGGAGGCGCCACTCCAAATGTCACTCCCTGTATCGCCAATCAC
CTTCCAAGAAGTAGAGCAGGAGATGAAGAGATTAAAAAGCAGCAAAGCCCCTGGGGAAGATCGGTTGGA
CAACCAGACAATCAAGCTACTGCCATACAAGGCGGTGCTTTTTCTCGTCGCCATTTTTAATGCAGCTCTTC
GGCTAGGTTACTTTCCAGTGGCTTGGAAGAAGGCACATATAATCATGTTGCACAAGAGCGGAAAGCCCCC
TAATCAACTGGGATCCTATCGCCCCATCAGCCTCCTGCCTGCCTTTGGTAAGATTCTGGAAAGGTGCATCC
TCAGAAGGGTCCTGGATACCCCGGAAATAAAACGCTTGATCCCGAAGTTCCAGTTTGGGTTTCGACTCAA
ACACGGTACCCCTGAGCAACTACATCGGGTCACAAATTATATTTTGGACGGCTTTCACAGGAAAGAGTAT
GTGGTGGCAGCATACCTTGACATCCAAGAAGCCTTCGACCGGGTGTGGCACGCAGGACTCCTCTCTAAGA
TAAAGGACAAATTGAATCCGCAGCTATTTAGCACCATTGCGAGCTTCCTAACTGAGAGAACCTTTCAAGT
GGTGCAGGGATGGAGTAGCCTCTCAAACCAAACGCATAAAAGCGGGCGTTCCACAAGGCAGCGTCTTGG
GTCCCACGCTATATAGCCTGTTTACACATGACATGCCCACAGCAAATGCAAGCAGACGGAATGATCGGCA
GGACCTTCTGGTGGCCACGTTTGCTGACGACACGGCAATCCTTGCAAAAAACAAATGTGTGTACGTGGCA
ACTGACCTCTTGCAGGAATACCTCAAGCGATTTGAAAACTGGGCTTGTAGATGGAACATCGCAGTGAACC
CTGGAAAGTGCGCCACGGTTACCCACACTCTACGCAAGGACTCATGCCCCGGACTTTTTCTTCATGGTGAG
CTTCTAGAGCAATATCGTCAACACAAGTACCTAGGTGTCACTCTCGACAGACGACTTACATTTAGTAAGCA
CGTCTCTGCAGTAACGAGCAACATCAAATCTAAGATAAGGCGAATGAGCTGGCTCCTCAACTCCAAAAAC
AAACTCTCGCTCCGAAACAAGGTCCTAATCTACAAGGTAATCCTTGCCCCAGTGTGGCGTTACGGGCTTCA
AGTTTACGGTATAGCTGCAAAAACACACCTGAACAAAATTCGCATCATTCAGGCCAAAACCCTGCGCAAA
ATAACTGGAGCCGAATGGTATATTCGCACACGCGACATCGCCAAGGACCTCAAGGTTCCAATGGTGGGCG
ACATAGTCAACAGGCAAGCAGCGAGCTACTCAACAAAGCTCCACAACCATCCAAACATCCTGGCGAGGA
GCCTGCTAAGGAGGCGAAGAAGAAGGCGACTGAAGAGGACCTACCCAACCGACCTAATCGATCGTTATG
TATAGGTGACAAGCTTCGTTAATTACACCAAAATATGTTTCTGTAATCTGTTAACCTAATTGTATAACTAA
TTGTAAAACCACAATCATCGCTAAGCTAAGTAATTATGTCACTCTGATAAAAGTTGCTAACATAGAGTAA
CAGGATACCAGATTTCTTAATAAACAAAATATAAAAAAAAAAAAAAAAAAAAAAAAAAAAAAAAAAAGGA
G
>160_rnd-1_family-292__Unknown
GGAAGTTGCCGGATCTTAGAGTGCAGGACGGACTTCTGTTCAAGAGGACCGTGAACGAGAGCTTGGAGG
ATGAGGTCGAAGGCACAAGTTGGAAGCTCTGGGTGCCGGAGTCGCTGACTGCAGGACTCATCCAACAAGC
ACACGCGGACGAGACGTCAGCTCATGGACGCTGCACGCGCTGCGCAGGCGGTNTTACTGGCCCGGCATGG
CCGTACAGGTCAGGGACTGTGTGCGGNAATGCGATACATGCAAGGAGGCCAAGGCACNAAACTNCCGCA
TGCAAGTGGGTCTAGGAGGGGAGGTAGTGCACCGACCGNCCNTTCCAGAAGCTATATATCGACTTCTGGG
AAAGTACCCACGGTCGAAGCGAGGGCACGCGTGGATATTCGTNGTCGTNGACCATTTCTCGAAATTTACC
TTCCTGAAGGCNATGNGGGCGGCCACCGCGGCGGACGTCGTGGACTTCCTAGTGCGCGAGGTGTTCTNCA
ANTTTGGCGTGCCGGAGGTNATCCATTCGGATAATGGACGACAATTCGTGTCAAAGTCGTTCGATGCGAT
GGTCCAGGCGTTTGGCATTACTCACCCGCGCGCACCAGTGTATTCGCCTCAGAGCAACGNCGCCGAACGN
GTGAGCCGCACAGCGCTGTCTGCAATCAGAACNTACCTNGGNCGGGATCATCGCGATTGGGACGCGTACC
TNCCGGAGGTGGAGGTGGCGATCCGGAATGCCGTCCACAGCGCNACCGGGGTGACCCCGTTCTTCGCGGT
CTTCGGACAGCANATGTACCTGAACGGTTCCAGTTACAAACTGGCCAGGAAGCTGAGATCNTTGGCCGAT
CACGGTATTTCCGACCTTGACGCGAAGGACAGACTGNCCGTAATCCGAAGCCGAGTCAAGGACCANCTGC
ACACCGCATACGAGCGAAGTCGCCGACGATACGACCACCGCGCGCGNCAGCTCCATCCGGAACCGGGAC
AGGAAGTGTGGAGGCGCAACTTCGCGCTAAGAGACTTCGGCAAAGCCTTCAACGCCAAGTTCGCCCGGAA
GTTCCTNAAGAGCCAGAGTCGTCCGAGCCGTGGGCANCAACGCNTNCNNGCTGGAAGATCTNNAGGGAC

file:///E/10月/10.9/PDF/4. biology-1907542/biology-1907542-supplementary/SupplementalDocument2.refTElib_01_25_2022.fasta.txt[2022/10/9 15:38:02]

GCGCGCTAGGNGTNTTCCACGCAAAGGACATACGAACGTAATCGTAATTTGCTCACCTCCCTACCAGGCA
ATGGCGGCGGCAACGCCCGTCCTCCCTTTTTTCTTTTGGGTCAGTGCGAAAATATAGATTACTTGTCGTGG
GTGTGGCCTACGTTGGGCGTCGTTATTAGCCGGCTGGGTCAATCCATGAAATGCGCACGCAGATTCCTTGT
AACGGTCGTTCGCGTCGTTACAATAATCTGCGTGGTGATGTTGCATTTGACGTTATCGGCGGCACCGAGGG
TTTACTTAGTTGGTTGCGTGCGATATTATCGGCCACGCCGATATCTTTTTGTCATATGGTCAGAGTATGCGG
GGTGAAGCTGGGCCCACGGCGGAGCCCAAATTTTCGAAGGGTGGCAACGCCGGACTTGCGATCCACGGC
GCCAAAAGCGACGATTGCTATTGGCCGCCCGGTTCGCTCCCAAAGAGATAAAAGGGAGAGTGGATCGCAT
GCCCTTGGATATGCCCTTGGCCGTCATTCGGGCCGTTGACCCAAAAAAAATGCAGCTGTGCAGATGGGCA
GCAACGGTGCCCGCCTCGTCAATGTACGCGTGTGCCGAANTTCGGCACCNNTGATGTTGCTGCCCAATGT
ACGGGGAGTTCGACTTTGTCAGCGGTCCACAGCGGACGTACCGGCTCGCAGTTAAAAATTTTTGAAGTTG
GATATTTTTTTTGCCGGCCACGGAGCATGTGCTTAACCAAAAAGAAAAAATCAAAGGGCATACATTTGTT
CATGACGGGCTTTCTTTATTATTTAATTGCCAAGATAATTCCTTATGTGCTAACGTATTGCAAAAGAAATA
GTGCACAAGAGCGATAATACACGAAACAATAATACACCAGAAACACACTGTCGTCATTTAAAACCACTAT
AAAAGCGATACTCTATTAGAGCAGCCGCAGCCATTCGGTGGCACACCACGCTTGAATCCCGCGCCGGAGG
ACGCTGGCCAGATCAACCCAGGTATTCACGGGCCGGATTTTCAAAGCGCGGCCAAGTGTCGGCGTAACCA
CACGACGGAGAGACGAGCCATCACGGACGGGACTCACACGACGAAGAGGCGGCCACGGGCGAGACCAC
GCCCATATCATCCGAGTGAGTCTGTTCCATTTCGTGCCTCCTCGCCTGAGTAAACGGCAGAGTCAGACGCA
GACTTCCCATTGTGCTCCCTGAAAGAGAACCGATTTTTTTTTGGAATTGTTAACTCCAGTTTTTTTTTTTGT
AATTAAATACATAAAATACTCACGACTAGTTTAAATTAAGTAAATTTCCGAAAGTGCGAACCGGGAAGGA
AAGGGCCCAAAGATTATTGCGTCTGCCAAGTAAAGAATGAAATTAAAAGTTTAGATTTTGGTCAACTTAT
GCACGAGTGATACCCACTTATACGTATATATATATATATGTTACGCACCTCAGGTGCAGGTGCCCCCCACC
CAGAGAGCTCTTTAGGCAGGCAAGGTAATATAAGCGCTTTGACAAACAACGCCTTTCGTTGGAAACACAT
ATGTATACACGCATTTATTTAGCGGGTACTTGCTCCCCATTGGTGCAATAATTACCTGTGTTTATATTTCT
CCCCTTGTTTTCCTTTCCTTTGTTGACTGGCAATAACTTCTGGCCGTTTTTTTTTGGTCTCCGGTATCGTATG
AAGGTCGCCAACAAAAAAAAAGATATTCTGTACAAAAAAAAAAGAAATTTAATATATTTAAGATTAATAGA
AATTGTTATAAATATGAAATGATGCAACGCGTTATAGATAACTGTTGAAGCGAAGTTGAAAGATGGAAAA
AAATTGAAATTTAAAACTTATATCCATTATTCTAAATATAAGAATATACCAACTTATCGTATGCACCTGGT
TTAAAATGCCGGAAATGTTCGCGAGTACGAAATTATAAATAACAGCTTGAAGATAACCTGCAGGGGAATA
AGAAATAAGATTAAATGTTAATAACGATAAGAAATGCAACCAATTATATGCCGTATCTGAAGAGAGAAAT
GTCCGGCTAAATTTTCTTTGATGGGGAGAGTTGGCATAACAGCCGGCACAGGCTGATGGCCATCTAATGG
CGAAATGNGGGAGACGAGTAGGTGATCGTCTCATGGTAGGGCGGGTACGCTGGGTCCGGAACCGGGGCC
CGATCTGGAAGAGTAAGGTGGATAAATAGTGTTTTTTNTTAATTTATATTGAGCGTCTCACCTTTGTTGTTT
ACATGTTCATAGTTGTTACAGCTGTTTATTTTGTTCATGAGTTAGAAGTTCCGATTGTTTGACGTTATCGAT
TTGTGGTTCGCTTTGCATAGTGTTGTGTAAGTCTGTTTTGCTACAGACGTGTAGCTTCGTTGTTATCGATTG
TGCGGACTGCGGGCCAGGGATGGGACTACACCCAGAGAAACCGATGAGCCAGCCGGCGCGGCCCACCGA
GAAACAAGCAGTTCCGTAACAATATGGCGCCCAATGAAGAAGCACTCGGCTTCTTTGAAGTCGGTGCGTA
ATCAAAGAAAAATAAATACGATATAAATGAAGGTACAGAGAAAAGGATTCCTTTGGCTTCAGGACCCAC
ACCGCTGAAGTCTTATGTGGCACGAGTAGGTAGCTCATAATCGCGCAGCGTGTAGTGGAATCCGAGAAGC
ACGACGGAAGTAGAGAATATCCATAAACATGAGAAGTTTTCTTGTAGTGGTTGATGACGGTAAGTCACGT
TCGCATGTTGGCGAAACGTTCGCGACCGTTTATCGTAGCCTGGTGGTAGCCGCCACGACAAAATAACTTGT
GATAGAATGTGATATTAGGGTAGGTCTTACTAGAGTTTGTTTAATGTTTTCTATTTGTTTTGTCCCACTCTG
TGCTAACGTTTGGGGTGTGCTGGCCGTAGTTAAATGAGCTTCGGAGACAGGTGACCCAGGACGATATAAA
GCCGATCCTGCGCCGCACCTGTCACACAGCATACCCTAAGGGTGCCCAGAAGTGCGACAGAAACCAAATA
GCCTTCAAGGTAGCGTTCCTTGAATGCCGTTCGTCAGTCAAACNGACGAACTGAGCATTTGACGAATGCTT
AAGTGTTGTCCACTGTCCAGATTATGCGTAGATTAGTTAGCCTGTGTTAGGATTTGTCTTCTCTCCCCCAAG
TATTTGAAATTAATTAGAAAGAAAATTAGTAAGATTTGTTAGTGGGTGTTTATTTATTTATTAATTTATTAG
GAATATTGGTAAAGTTTTTTTTTTATATAAGGGTAGTAAGCCTTGTAGAAGAGAGGGGTACAGTTCTCAGA
AGGGATAAGTATGGCTGAGGAACAGGTGTCTGATTGGGCTTCTGACGCTGACGGGGCGTCGGCTCTGCTA
CCGCCGCGCCCGGCTGGGGCGCTGGTGCGAACGCCAATGAATTTCCGGGGATTTCCAGAGAGACAGATCC
CGGAAGAGGGTCACCTGGTAGACTTGGAGAAAGAATTGGCGCAACAAGATAAACCTCCACGGAACCTGG
AGTTCCCAAAAGCCCCGAAAGACTCAGTGCTCAAATCGGCGGCAAACGCGGCGATCGCTCAGGCCCACCA
AACGTATCGGCATGCTCTGCCATCTGGCATCAGCGAGTCCATCCGCGAGGAGATGCGAGCTGGCTTCATG
GAAATGATGAAGGGCATCATTGATATGATCAGGCCGCCAGGGAGCCAAGCCGCCGAGACTAACACTGCG
CCGAAGCCGCCGAGACATGCCGAGCAGGGACCACGAACAGGCGCCATCCCGAAAGAACGGAAGTGTTC

CAGCGCGAGGCATTTCCACCACCGGTGCCACCGAAGCCACATAGACGAGGCGCACGAGGGGCGGAGTAT
TCGTCCACCAGCCCAGATTACGTCGCAGATGCTCAGTCGAGGAACTGGCGTAACCCGAATAACCGCCATT
TCCGAGTAGAGGACCGGAGAGCGGATGATTTCAACCGGAGGAGGCGTACGCCAACGTACGAGAAATGG
CGTACATGAAGCTCGAGCGGTGGAACGTCAAGTTCGGCGAGGACGCGATGAATTCGGTCGAGGACTTCGT
GTTCCGCCTGGAGTTCCTGCAGAGGCAGTATCAATGCCCGTGGAAGGAGGCCCTGCGCGGTTTCCACCTG
CTCCTGACCGGCCGCGCCCGCGAGTGGTATTGGATGCACGTGCGGCA
>Ref_NewTE6
TATATATATGTGGGGTTTTATATTAAAAGTTTATTATAAACAAGTGTATTGTTGATTTAACAAATTGTATTG
CTTAGGATGATTATCATTCGAATCAAAGTAACACGTCGTTTCGTCGTTTTGTTCTCAGCCTTCATACCACAC
AGGTCCAGCGTTGAACTTTTCGTCTTAACGAGCAAACATCTTCTGCTCTACGTCCAGTACGTTGCCCTCGT
GGCTTTGCACTGTCCTCGTTGATCGTCGTTTTTGGATAACTCGCCGTCTGCGAATTCGCCGTCTTCTTTGCT
CGTTGTTACGCGTCGTGTATTCGTCGTTCGTCTTTTAGCGTGTTCGTTGTCTAGGGATTCTTGTGCTGTTGG
TTGTGCACAGTGGGATTTGGGTATACAGCATTTACCCAACCCACATTCGGCCATTCTGACTAAGCGTTCGG
CCCGAATGCTTCCTCGTCATCTTCATCATCGCTGCCAATGGACCATGGCTTCATATATTCGGCACACGTTG
CAGAATTCTTCGGCCCATCGCAATTACCTACTTTCTGGACATCATACGCGTTGTTATGCTTAATCTTAACGA
CTTGATAGGGCCCCAAAAACTTAGGCTTTAATTTTAGGCCTCTACCAAACTGTGTGCGTTTTATGGCAATA
AGGTCGCCAACATTATAACGTCTTGCATGCTTTCGTCTCAAATTGTATGTATTTCTATTTTCATTTTGAATT
TTTAAAATTTGTGTTTTTGCCTTTGCTCTTAATTCGGTTCGATCGTCATTAAATAAAGCTATTGTTTCTTTAT
TGATTATATCTCGAATCTTCCGATCTTCTTTTGTTTTCATTTTTACACCAACTAAAAGCTCAAAAGGTGTCG
CATCAATACTTCTGGAAAATGTCGAATTAATTGCCTGCTGTACCTGGTTGATGTGTCTGTACCATTTTGTTG
GGTCATCAATGCTCAATTTTGAGAGTACTGAAATAATTATAGCATTCAGCCTCTCGACTTGACCGTTCACT
CTTGGTAGGCCTGTAGTTGTCTTAACTAGTTTTATGCCTTCGTCGTCACAGTATTTAACAAAATCTTGCGAA
GTAAAGGCAGAGCCCCTATCCGTAATGATAAATGCTGGGTTACCAAATACAATACTTTGATTAGTTAACTT
TGTAATGACCTCGTTTGCATTAGTAGACTTCGTTGGATACAGCCAACAAAACTTGGTAAATGAGTCAATAA
TCGCCAAAATGTGTTTGTAGTCTTTATGCGTTGACTCAAGTGGACCCAAGAAATCGATGTGGTAAGTGTGT
AGGGGAACGTCTTCCTTCTGTAGTGGGTGTAGCAGCCCTTCTTGTTTGCCTCTTTTGCGATTTGTTAAAATG
CAGGGAACGCAACAATCTATATACTTTTTTATCTTCTCTTCGAGGTTAGGAATAAAGTATTCTCGGCAAAT
TAACTCTTTGGTCTTTTTTGCTGCAAAATGGCCTTTGTCATGAGCATAGCCAATAATTCTTTTTTGCATGTC
ACTGGGTACTACTATTAACTCATCATCTTTTATAAGTTTGTATAGAATACCTGATTTTATGAAGTAGTCGTC
GTATGCTTTATTTTTATCGGTTAAAACTTCCTTAATTGCTTTTATCTTTTCGTCTCGGGCTTGTGCATCTTTC
ATAGCAATACTGATACTATCTTCGTTGATTATCATTACTGGGTATCTGCTTAAAGCGTCGACGTGCTTCATT
CTTGTGCCAGCTCTATGTTCTACTGTATAATCGTATTCTTGTAGGAACATAATCCAGCGTGCCACCCTGGT
GCATAAACTTTGTTTCTCTAGAGTTTTTGCAAATGCATTACAGTCTGTTACAAGTTTAAAATGAATACCTA
GAAGATACACTCTAAACTTCGTCAATGCTTCTATGACTGCCAAAATTTCTAACTCATAGCTGCTGTATTTA
CGTTGGGCATCTGTAGTCTTTTTTGACATAAAGTAAACTGGATGCAGTTGACAATCTTCGGGGCTTTTCTG
CAATAAAACTGCTCCAAAGCCATCAATCGATGCGTCTGTGTGTACCTCTGTCTCACACGTTTGATTGTAAA
TACTTAACACAGGGTTTTCAGTAAGAAGCTTTTTCAATAAGTTAACCGAATTCTCCTCTGCTATTTCAATTC
TAAATTTCGCTTTATTCTTGGTCATCTCCGTTAAAGGTCTAGCTATTGTAGCATAATTTGGTATAAATTTTC
TAAAATAGCCTGTCAAGCCTAAGAAACTTTCTACCTGCTTTATTGTTTGTGGCATCTTAAACTTAGATACA
GCTTCAATTTTTTCTGGTGATGGGAATATCTTTTTATTACAGATCACATGACCAAGAAATTGAATACGTTTT
CTTTAAAAAAATTGCATTTTTTAATGTTAAGCTCCAACCCATACTCCTTGCATGTGCCAATAACTTCCTTTAA
ATGTTGCACCGCTTCTAGCTCGTTTTTGGCTGGTATGATAATATCATCTACATATGGCAAAGCAATTCCTCT
GCGTGACAGGTGACGAAATATCGCGTTAACATGGCGTTGAAAAACACCTGGTGAGTTTGAAAATCCGAAC
GGCACTCTCAGAAATTGATATTGTCCTCGATGTGTTACAAACGAAGTATATTTTCTACTTGATTCTGCCACT
GGTACGTGAAAAAATCCATTTTTTAGATCAATTGTACTAAAAATATTCGCTTCTTGTAATCGATCCAGCTG
ATCCTCGATTAGTGGTAAAGGAAAATGGTCTTTAACAATTACCTTGTTGATTCTTCTATAATCTATGCATA
GCCGGGTTGAACCATTGCGCTTTTTAACAAGTACTATCGGGCTAGTAAACTCGGATGTAGATTCTTCAATT
ATGCCATTTTCAAGCCACTCATCAATTTGCTCATCGACAATACTCATTTCCGTAAACGCCAATCTGCGCGG
CCGTGAAAATATTGGTGCCTCATCTTTTACTACAATGCGCATCTCTACATTTGTTGTCTTCGTCTTATTTGG
TTCATAGTTCGAAATAAGTTCTCGAACTTCTTGTTTAGCATACTCACTTGCTGTGTCTTCTATGTCGTATAC
GCAGTCGAAGTCTTCAGGGCAAATTCTTAGTATATTTATCTCTTCACTGTTTGACATTTTGCGAACTTGTAA
ACCGTTACGACTAAATACAATCTCAGCCTGTAAACATAGTTCTTCGCCAAGAATAACTTTGACATCAATAC
ATTTAATTGGAACCACGTAAAAATCAAGTATAAACTCTTCATCGTCAATAAGAATTAGATGTTTAAAATGT
CCAGTTGGTTTTATTTTATTCTCACTGCGCTCACTGCCGAATCCAATAAGAAAAACATTGCAGATTTCTAG

CTTCGGCTTGCCAATTTCACAATAAAAGTCTTCACGGATGATATTGAATTTGCTGCCAGTATCAAATAACG
CTACACATTTCTTATTTGAGAAACATACTTCTTTACAGGATAAATCGTTTTTGGCTTGCAAACTGCGTGTAT
TTGATATTACGGCCTCACTTTTCTCAATTTGTTCACAATCTTTCGAGATGTGACCGTATTGATTGCATCTGA
AACATTTTTTTCCTTTATTTTTATTTTGACAATAATTAGACGTATGCCCTTTTTCACCACAGTTAAAGCATTT
CACATCTTTTTTTGCATCTTTGCTTACGTTGAAAGCTTTTTTGTCTCTTTCTTTAACACTGCAATCGTTACGC
TTTGTTTTTTCCTTTTCGACTCGACACTCTTTTTCAATCATTGTTTTATAATATTTCAACTTTTCTTTGAATTG
ATTTAAATTTTTCGCACCGTATAATATGGATTTGTTTCCACCTCTCTCATCTATTCCGTCAATTAAATACTG
TATGAAAGCGTCATCTGCAATTTTACCTCGAGAAGCAATGTCTTTCATTCTATAAAAGAATTCGAATACAC
ACTCGTTGTTGCTGCGTTTCGTCTGCGCGAGCTGTTCGTGAATTTCTTTGCTGCTTACACTTGAACTGAATT
CTGCCAAGAGTGCTCGTTTGAGAGAGTTCCAAGTGCTTAAACCTCTCTCACTCAATACGAAGAGTTTCGCT
ATTCCTTTAAGTGCCTTTTTTGCGAACAGAAACTTCTGGAAATCGTTCCAATACGTGAGTGTTGCCTGCTCT
TCATATTCTTTCATATCCACACTTCTACTGGGAATAGTGTTTGTGCCGTCAAAACAACGAATGCTCTCTTCC
ACGTCGCGAAAGCTGATCGCAAAGCGAGGAGCAATTTCGTAATTTTCAGCAATTCTCCTAGTTTTCGAGTT
TTCCCTATTTCGAAGGTCAGCGGTCTGGAATGAAGCGTTGTCTGCTTCCTCTTCTATTTCATTTTCGTCGTC
AGTTGCTTCTTCGTCTACTTCGTTGAATTCCATCAAATTGTTTTTCATCAGATTACGAAAAATGTGTCCATG
CAAATCTGCGTGAACATATTCTATACCTAGAATGTTGCATATTGATACAAGGTCAGCCTGCGTTAAATTGT
TCAATGCGTATTCCTTCTTCGCACTATACTCTGCGTCATTTTCTGTATACTCGAAGCCAGAAAATTCGCGTA
GACGTTTGCGATTATTTCTGTCTCCCTCATTTTCAAAGATGAACTTGTGCAACGCAGTTATGCTCCCTTTTT
TGCTTGAGCGTAAGCTAGCACTTATTAAATCATTAAGGCACGACATTTTTTTTTTTGAATAGTTTTTTTTTT
TTTTTTGTTTTGCACAATTGTTGTTGTAACGTAAGATCGTTTTCAACTTTATTCACTCCTGTTCAATCTTGCA
CAATCTCGCTCACTTTTGCTCACACTTGCTCAATCTCGCTCAATCTTGCTCACACTTGCTCAATCTCGCTCA
ATCTTGCTCAATCCCGGACGAGCGCCCAAAATGTGGGGTTTTATATTAAAAGTTTATTATAAACAAGTGTA
TTGTTGATTTAACAAATTGTATTGCTTAGGATGATTATCATTCGAATCAAAGTAACACGTCGTTTCGTCGTT
TTGTTCTCAGCCTTCATACCACACAGGTCCAGCGTTGAACTTTTCGTCTTAACGAGCAAACATCTTCTGCTC
TACGTCCAGTACGTTGCCCTCGTGGCTTTGCACTGTCCTCGTTGATCGTCGTTTTTGGATAACTCGCCGTCT
GCGAATTCGCCGTCTTCTTTGCTCGTTGTTACGCGTCGTGTATTCGTCGTTCGTCTTTTAGCGTGTTCGTTGT
CTAGGGATTCTTGTGCTGTTGGTTGTGCACAGTGGGATTTGGGTATACAGCATTTACCCAACCCACATATA
TGTA
>9_rnd-1_family-355__LTR___Pao
GCCCGTTGGCGCGAAGAGTATTTAACGCTTCTTCAACAGCGCTCCAAATGGCGCACTCCTCAGCCAGGATT
CTCCATCAACGATGTCGTCCTTGTAAAGGATGAGAACCTACCGCCGCTGAAGTGGCCATTAGCTAGAGTT
CTAGAGCTGATCTCGGGATCTGATGGGGTATCTAGAGTTGCAGTGCTTCAGACTGCGACTGGAGTCATAC
GCAGAGCCGTACGAAAGCTGTGTTTGCTGCCAAAACAGGATGATGTTGAAAGTCCCTTGCCTTCCAACGG
GGGAGGATGTTTGGTCAAGTGACCGGTAGAGATAACAGCGACGGCTGCGTGCAGTAACAGCAGAAGACA
ATAANCATAATTACAGCTTAAGAGCAAATGATGTTTGCTCTTTGTAAGTTGCTGCCTACTCTCTTCATCGG
CCCTTTGCCTCCTGCATTGACGCCCGCTGCCCATCGTTCTGAGCGGAGCTCAATTTCGTTCGTTTTGCATAT
AGTTTAGTTTTTCGTTTGATCGCAAGCAAGGCACAGCTATTTCGAGTTGACTGCCGAGCAATATTATAACA
TTTCGTAAACTATAATAAAAGTGCCCTGTGCGGTAAATAAATACATTCGAAACAAAATATAAAACNTCGA
ATTTCGTTGCACACAAAGCAGTGCTAATTAATTAGTTGCAAAAAGGCTTGTAACTACACATTTTGGTGACC
CCGACGTGATTTTTTCTTTATTACTTTTTCGTTTCCCTTCTGTTCGCATTTTGTTTATAATTTCATTTTATAAT
TTCATTGCGCATATTTACAAAAGGTTGTGCGTTAAGTGCTGCGAGCCTCAACATAAAGTGATCAATTTAAC
CAAGTCTCTCTAATAACTGTACTTAACCGCTTGCTTGTGCATATGCATACAGTTTGTCGTTGTACATAGAC
GCCGTAGAAAAATCGCCGTTAATCGTCGCGCGCTTTAACCAGCTATACGCTATCTCGCTCGAACAAACACT
TGTGCGCGCAAATTTTGTATTGTAGTTCGGCTGTGTGAACTTTGTTGTGCGCTACATTAACAATGTTTGATG
AAGGTGCAAGTGCAAATGAAAATGAGGCGACATCTGCTAATCAAGTGGCGGTTGGAACACAGGCTGCAG
TTCTTAAAGTTAAGATTAAGAATTTAAGTGATCGAGTCAATAGATTGTCCTCTGAACTTGATCCCGCGCGA
CTTCGCGATGTTGATGACTACGAGCTGCAAGATTACATATGCATGGCATCTGACTTGCAGGCGAAATTTGA
AACANTCTATGATAGTTTGTTGGATGTGAGTCAGGCCAGCGTTGAAGAGGATCTTCAGTCAAGCTTTGAGT
CAACCATTAGGCGGCTACGAGTGTCCCTTCAACGCGAGCGCGGAAATCGAAGCAAGATTCAGCAGACTCC
ACATTGCTCCACTTTCAATTCCGCCGCAGCCGATGATCCGCGCTCTACTTTTGTTGTTCCAAACCACTCTCG
ATTGCCTCAACTTAAATTGCCGGAGTTTAGTGGAGGATACACAGAATGGTCTGACTTCTCGAATATGTTCA
CCACGGTCATTGATAAGGATCCGTATTTGACCAACATTGAAAAACTTCAGCATCTACGGTCATGCCTCAAA
GGAACAGCGCTGGATACAATTCGCTCGTTGGAAATGTCAAACGCTAATTATGCTGCCGCCCTAGAACTGC
TTGATAAGCGTTTTAATAACAAGCGTCTTGTTTTTCAGGCACATATTGCTGAAATTTTGGGTTTAAGAAAG

GTGGATAAGGGTGCGACGACACAACTGCGCGAATTCTCAGATAAGGTCAACTCTCATTTACGTGCGTTAA
AATCGATGGGCAGTGTGGAACAGATCGCCGGCTGCATCATAGTACACACACTGCTGCAAAAACTAGATAG
CGCCACGCAAGCTAGCTGGGAGGATGATGCGCCGTTGGACGTCATACCATCATGTGAGCGGCTTACAGCT
TTCATTGAGAAGCGTTGTCAAAGGCTGGAAAATGCGGATCACGCGACGGCAATGTACACGCCTGGCTCCC
AGGTGGGCCAGAACAACAGTAGTAGAAGAACGTTTGTGGTGACCAAGAATGGAACATATGCATGTGTATT
TTGTGAAGCCGCAGGCCACTCCATTTATAAATGTCTGCAATTCGCAAAATTATCGCCCTCGCTGCGCCTTC
ATGAAGCCAAACGGCTTGCGCTGTGCCTTAATTGCCTGCAAAGGGGACATCAGCTGAGAGTCTGCGGTTC
CAGCACTTGCAGAGTCTGTGGAAGCAAACATCATAGCCTGTTGCATCTTGGCAACACAAGCAGTCACAGC
GTTGCTTCTAGCTCACACAATGTTCAAGAAGCCGAAACTCATACGTCATCTCAAAACATCCTGGCGGCAA
CTTCATCGTCGACACTCTCCATCGACCAGCATCTTAAGCACGATGTCGTCCTACTTGCCACTGCCGTCATC
AATGTAAAAAATCGCGCTGGCTCCTTGGTGCCCTGCCGTGCGTTGCTCGACTCTGGGTCGCAATTGCACAT
CATCACCTCTCGTCTTGCTCATCAGCTCCAGCTGCGCAAATTCAAGTCCACAGCAGTCGTCTCTGGCATTG
GTGATGCAGCGTTTTCGTCAGATGGTTTTTCGGTAAACATCAATGTCCAATCTCGAGTGTCTGAATACTCC
ACATGCATCCCGGCATTGATTGCACCATCCATCACCGATAATCAGCCTGGCTTCACTCTTGACCCTGCATC
ATGGAACATTCCGTCAAACATACAACTAGCTGATCCTGAATTCTTCAAATCTCAGCAAATCGACATGTTGA
TCGGAGCCAGTCTGTTTTTCGATCTGCTCTGCGTTGGCCAGATCAAACTAGCTGCTGGACTGCCGATACTG
CAAAAGACTCGCTTTGGTTGGGTCGCGACTGGAGGTGCTCCACATGCAGGAAAATCATCGCTCGTGGCTA
GGCGATTAGTGATGAATCCAGATCTGCTGGTAGACTCGCATCTGCAGACGAATGCACAGATTGACGAATT
AATCCGTCGGTTTTGGGAGCTGGAATCTTGTACCGAGCCTGAGTCATCACCAAACAGGGAGGAGCGTGAC
TGCGAGGCACACTTTCAGGCCAATTTTAAGCGTCTGCCGACTGGCGAGTATTCAGTTCGCTTACCGCTTCG
ACTAAGCCTGGATCAGCTGGGTGACTCCTATCAACAGGCGCTTCGCCGATTCCTTAATTTAGAAAGGAAA
CTAGACCGCAATCCAACTTTGAAAACCCAGTATGCAGCATTTATCAAGGAATATCTTGACTTAAATCATAT
GTCACTTGTTAGCTTAGCTGCACTGGGCCAATGCAAATACTATCTGCCACATCACTGCGTGCTGAAGGAGG
ATAGTACTACAACCAAGCTAAGGGTCGTGTTTGATGGATCAGCAGCTACTACCTCTGGACACTCGCTGAA
TGATGCACTGATGGCAGGTCCTACCATCCAACCGAAACTGTTTTCGATACTGATGCGTTTTCGTACATTCG
CAGTCGCCCTCACAGGCGACATATGCAAAATGTATCGATGCGTACGGAGTCGAACCAGCAGACAGCTATC
TTCAATGTATCCTGTGGCGTGATTCTCAGCATCAGAAGATACAGACTTACAAATTAGACACCGTCACCTAC
GGCACAAAACCAGCATCGTTTCTCTCAGTGCGGGCTATGCACCAACTGGCCATGGATGAGCAGCAGGCAT
TTCCTATTGGCGCTGACATTATTAAAAGGGATTTCTACGTGGATGATCTCATCTCTGGTGGTAGCTGTGTTC
AGGAAGCAGTCGAGATATTGAAGCAAACGTCTGGACTACTCGCCAAGGGCAACTTTAAGCTGCGAAAAT
GGTGTTCTAGCGACGCTACTGTACTCCAAAGCATACCGGAAGAGGGATAGAGAATCGCTGCTTAAGTTCA
ACGATGGCAGCGATATCACGAAAACGTTAGGCCTCGTTTGGGATCCCGCCTCAGACTGTTTTCTTTTTCCT
TTCTCCACTGAGGCTACCCTCGAGACTGACAAAACGATCAATCCTCTCCTCAATTGCTCGTTTTTACGACC
CCTTGGTCTTGTTGGTCCTGTAATAACAAAATCTAAAATTTTTATGCAGGATCTCTGGAGAGAAAAACTGG
ACTGGGATGAGAGCTTGCCTGTACACTTAAGCACAGCCTGGGTCAACTTCTGCGCTGATTTTGAGCAGACT
CAGCAATTCCAGTATCCCCGTCGTGCACTCTCATCAGACAGCACAGTTGAGATTCACGGATTTTGTGATGC
CAGCCTGAGCGCTTATGGGGCATGCGTCTATACAGTTTCAAAGTGCCATGGCAACACAAGCGTACGTCTC
TTGTGTTCCAAGTCGCGTGTCGCGCCTGTGAAAACCATCACTGTACCAAAGCTGGAACTCTGCGGAGCTGC
ATTATTGGCTGAACTTCTCAGCGAAATCTGCCAACTGAAAGTGTTTAACTGTCGTTATTACTGTTGGTCAG
ACTCTGCTGTAGTTTTGGCTTGGATTCGCAATGATGCTTCCAAATTCAATATTTTCGTTGCCAATCGGGTCG
CAGCCATCCAAGAGCTCACCACTGGCATGGAATGGCATCATATTCCCACTGAATTAAACCCGGCGGATAT
AATTTCACGAGGAGCGCTGCCCAGTGAGCTTTCCAGGTCTCCGTTATGGGCCCATGGCCCGAGCTTTCTCA
GAAAGGAAAAGGAAGAGTGGCCTGCCTCCTGTGTACCTGTCGAATCCTTGCCAGAACTCCGACACAAGGT
ATTACTCGGAACTGCAGCGCAACTTGATCTCTCAATTGGCTGCAAGTTCATCAACTCGTTCTCCAAGCTGC
AACGCGTCTTTGCTTACATTTACAAGTTTGTAAATCGAATCCGAGGATCTGAGCTAACTGTAAGCCACGTG
CGTCATGGCACACATCTGTTGCTGCGGTCAGTGCAAATGGCTACTCTTGGCGATGACTCACAAGGGACTG
ATGGAAGGAAGGCCCGTCAAACGGTCTAGCTCAATGGCCTCTCTCGCGCCATTCTTTGACGACTTCGGCTT
ACTCCGCATCGGTGGACGGCTGAAAAAAATTCGTCGTTGGACTTCTCAGCGCGACATCCGATTATATTGCC
TCGCCAGCATCCTGTGACCCGAGCAATAATAATCCACTTTCATAAACGGAATTTGCATGCTGGACCTCGCG
CCGTATTGGCTTCGATTCGGCTCCAATACTGG
>9_rnd-4_family-24__LTR___Pao
GTTACACAGAATGGGCCGATTTTTATTCTATGTTCACAGCCGCCATAGAAAATCATCCTGATTTAGCAGAT
ATCGAAAAGCTCCAGCATCTTCTATCGATGCCTGAGAAGGATTCAGCGCTGGANGCGATTCGGATCATTG
GAAATAACAAACGATANTTATGCCATAGCACTTAAANTATTGGAGAAAAGGTTTAATAATAGACGGTTGG

TTTTCAGGCACACATACTCGAGATATTGGGAGGGCTCAAGAGGGTGGCAGGCGGATCGGCCGTGTCGCTT
AAAGAGAGTTATCCGACTTCAATTCAACGCACATATTCGCGCGTTAAAGATCATGGGCAACTCCGAACAG
ATCGCTGGATGCACCATTGTGCAGATTATTTACAAAAGCTGAACGTAACATCACACGCCAAGTGGGAAA
AAGGCTAAAGGCTTCGCCNNTCGTCAACTTAATACCGTCGTGGGAGCATATGGCTACGCTTCTGGAGCAG
AGCCATCAGGTGGGAAAAACTAGAGCAATTAACACNAGTAGATCGTCATTTATCGCCACTAACCACGANG
CCCCTGTCTGTTTCTTTTGTGACAGCCCTGGTCGTAGTATNTATAATTGTNCCCGATTTGGAAATTCGACCC
CCTACGGCTAGGGAGGCAAAACGAGTTGCATTGTGCTTGAATTGCCTAAAGANAGGTCACCAATTGCGAT
CATGCAGCTCTGGATGCTGTCGGACCTGCGGGGCGAAACATCATTCNCTNCTGCATTTGAATAATAATAAT
TCCGTATTTCCGTCTGGTCAGCTGACTGAGCCTGCNAATTCATCGNCGATANCCCATGCCTCTTTCGCTAA
GTCGTCGCCATCTANCTCCACATCTCTTGTTGCCCAGTATTCCAGTAGTGATGTCGTCNTCTTGGCTACNGC
TATGGTCTTCNTTAGAAATCGNTCCGGCACTTTGATACCTTGTCGTGCCCCTTTTGGATTCCGGCTCTCAGC
TTCACCTTATAACNTCGCGCCTTNCTCAACAGCTTCAGNTAAAGAAGCACAANTCCTCANCCATCGTCTCT
GGTCTAGGACAAGATTCTCGTTTTTGAAACAGAAGGAAACACGGTCACTATCACAATACAGTCTCAAGTC
TCTGAATACTCAACATGCCTCACAGCTCNGGTAGCGCCTAACAATAACTGATAGTCAACCGAGTTTTACTA
TCAACACGACGAATTGGAACATTCCCGATAACATAAAACTTNCAGATCCGTCTTTCTTTAAANCTCAACGC
GTGGACTTNCTANTTGGAGCTAGCCTGTTTTACGACCTTCTCAGNGTCGGCCAGATAAGGCTTCTCCCAGG
ACTACCGTGTCTTCAAAAAACACGGCTTGGTTGGGGTAGTTTCTGGAGGCGGACCNCGCTTGAGTGGTTCT
ACGTTAATGGCAAAGCANACTGCGGGCAAGCTTGAGTCAAGTTCTGAAGAACGTGCTGATGAATTGGTNC
GAAGATTTTGGGAGATAGAATCCTGCTTGGAAAGTACACCTACCACTTCAAAGGAAGAAAACATCTGTGA
NGAACATTTCGTAAAGACTCATATCAGATTATGCCTAGTGGCCAATATTCAGTTCGTTTACCCTTCAAGCG
AAGTTTGGAATGTCTGGGGACGTCATACCAACAAGCCTTTCGTCGNTTCCTGACCCTGGAGCGCAAACTA
CAAAATCGTCCAGATCTGAAGATTAAATTCAANNTTCCTAAAGGAATACTTGGATCTTNGTCATATGTCGC
NTATTGCTCCAGANTCTATNGGACTTTGTCAATACTTTCTTCCTCATCACTGTGTCTTTAAAGAAGACGGTT
CAACGACAAAATTGCGAGTCGTTTTCGACGGCTCAGCAGCTACCTCGTCAGGTTATTCATTAAATGAANTA
TTGATGGCTGGACCTGTTATTCAAAGCTCGCTGTTTCATATCCTTCTTCGCTTTCGTAATTTCCCGATCGCC
CTAACAGGAGATATACGGAAAAATGTATCGNTGCGTACGCGTTTCAGNACCGGGCGATTTTCTCCAATGC
ATTCTGTGGCGAGACTCGCCGAACAGGATATAAAGATTTTTAAGCTGGATACAGTTACATACGGAACAAA
GCCAGCTTCATTCCTCTCTATCCGTGCTATGCATCAGCTGTCTATGGATGAAGAANNTAATNTTCCAATTG
GATCTCGGACTCTGCGGCGNGACTTCTACGTTGATGACNTCATTTCAGGNGGTGATTCAATTCGAGAAGC
ANAGGAANTTCTTAATCAGACGGCTGGAATNTTGGCAAGTGGGGGCTTCAAGTTGCGAAAGTGGTGCTCA
AACAATNCTGCTGTACTGAAAGGGATACCCGTAGAGGAACAGAGAATCTTCCCTAAAGTTTGATGATGGT
AGCGACATCACCAAAACTCTTGGACTCGCTTGGGACTCAGCGTCGGATCGNTTGTTGTTTTCGTTTTCGTC
CATTCAGCAGAGATCTAAACCGTCCAAAAGGTCAATACTATCGACCATAGCTCGCTTGTATGACCCGCTTG
GACTCATAGGGCCGGTAATCGCCAAAGGCTAAAATCTTTATGCAGCAGATCTGGAAGGAGAAGCTTGAGT
GGGACGAGAGCTTGCCATCATCACTGGCAACAGCTTGGCATGATCTTTGCTGCAGTTTTGAGAAGGCACA
GTTTCTTAAATTTCCTCGAAGCATATGGGGCCTGTGTCTACGTCCTTTCTCGACGAACATCAGCCAGTTGTC
AGCTGTTGTGTTCCAAATCGCGTGTAGCTCCTCTGAAAACAATCACAGTTCCAAAGCTAGAGCTCTGTGGT
GCCGTCTTATTATCTAATCTCATGCGTGAGATATCTAAAATGAATCTGTTTGATGGAAAATTCTACTGTTG
GTCGGATTCTTCTGTATGCCTTTCCTGGATAAGGACCGAGCCCTGCAAGCTCAATGTGTTTGTGGCTAATC
GCGTCACAGCAGTTCAAGAGCTAACTAATGGCATGGAATGGCGTTACGTGCCTACTTCACTCAACCCGGC
AGATATACTCTCGAGAGGATCTACGCCCAGTGAATTAGATCAATCGTCACTGTGGTTCAACGGCCCAGAA
TTTCTAGTTAAAAACAAATTGGACTGGCCTGCTTCTTGCTTGTCAGAAAGGACAGAACTCGAACTTAAAA
AAAGGGTCCTTATTGCGTCATCTCCCTTCGTTGATATAACTGTCACTTGTAAATACGTCAACTCATTTGATA
CAATGCAGCGTATATTCGGATATATTTATAAATTCATACACAGAAGCAGGCATCCTGGACTAACAGTGTCT
GATATACAACATGGACCCAACTCTTACTAAGGCTCGTCCAGAGAGCACACCTGTGGGAAGAGATCAAAT
CATTGCAATCGAGAAGAGTTGTTAAACCGTCCAGTTGTATCGCATCACTTAACCCTTTTTTGATGATTTCG
GAATTCTTAGAGTTGATGGGCGCTTGAAAAACTCTTCGCTGGATTTCGACGCTCGCCATCCAATAATTCTC
CCGTGTCACCACCCCATCACAAACTCGGTCATCACACGGTTTCACAATCGGGACTTACACGCTGGTCCACG
GGCACTTCTTGCTCATATTCGGTTGCAGTATTGGCCGATTGGTGGAAGGAAGACCGTATCCAAGGTGGTTG
GAAAATGTGTGAGATGCTTTCGTGCCAAGCCTCGATTAATAGAGCATATAATGGGTGATCTGCCCAAAGA
ACGTTTGACTGCTTGTCGAGCCTTTGAAGTCTCTGGTGTTGACTATTGTGGACCATTTTCTATAAGCCAGAC
GTACGAAATAAAGCTCCAGTCAAATGTTACATGGCCGTCTTCGTCTGCTTCGCGACTAAAGCGGTTCACCT
GGAACTGGTCAAGGATCTGTCGACAGCTTCTTTCTTGAATGCGCTTAAGAGGTTTATATCGACTCGAGGCA
GGCCATCTCAGATATGGTCGGATAACGCTACCAACTTTGTCGGCGCCAAAAACGAGCTTCTGGATCTCAA

GCGTCTATTTACAAGTCGTAATCATATTGAGTCAGTGCATAATTTTTGCATGGCTGATTCCATTGATTGGA
AATTTATACCTCCTCGTTCACCGCATTTTGGCGGCCTTTGGGAAGCAGCAGTGAAGACAGCGAAACATCAT
TTTTATCGTGCTGTTGGATCTTGTGTTCTCAACTTCGAAGAGTTTCGCACCCTAGTCTGCCACATAGCATCG
ATAATTAATTCCAGGCCATTGTTTCCTCTTTCAGAAGATCCAGCAGATCTAGATGTGCTCACTCCAGCTCA
TTTCTTGATTGGTGGACCCAGCTCATCGTTCTTTGAACCTGATGTGACGCACCTTCAATTCAATCGTCTGGA
TGGTTGGCAACGCGTAACGTACCTCCAACAACTGTTTTGGTCTCGCTGGCGTGAAGAATACTTAACACTGC
TTCAGCAGCGCTCCAAATGGCGTACTCCGAAACCAGGCCTATGCGTCAACGACATAGTCCTCGTAAAGGA
TGAAAATCTGCCGCCTCTAAGGTGGCCACTGGCGAGAATCACAGAGTTAATCGTGGGAAAGGATGGAGTC
GCTCGAGTAGCCATTTTGCGCACCGCAACAGGAGAAACGAGAAGAGCTGCAAACAAGCTATGCCTATTGC
CCATCAGGGAATCTGTTGAAAGCCCATGCCTTCCAACGGGGGAGAATGTCTGGTTACGCAGCCAAAACAA
CTGGAGCGACCTCGCAACTGATAAGAGCAGCATCTTAATTTTATCTTATAATACTCGCTCTGCAAAGCAAG
TGCCTAAATTTCGCTCTTAAGAATTCATTTACATAGAGCTGCCGCTCTTCGCTGTAACGATCGTTTTATTGC
CCTAGCTTTACTTATGCGAAGGCCTCGATGCTGCGCATCGAGTTAGTTAAGTTCGTTCGCTTCATGCATTTG
GTACGCATCGGCCACTTGCATTACTCAAAATTATAACTAATAATTGTCTCTTCGCTAAAATTGGCACGTAC
TAATTCGGGTTCGCTTGCCTATATACAAACTATTGTTCTTAAATAATAAGCACAGACCAATTCGGGTCTGC
CTGCTTCTCGTAACTTTAAGAAATATAAAACAATTTTGAACACAAAAAATAAATAGTTAATTATTTCTATT
TGTGAAAGCTATTGAAAAAGCTCAATAGCGCAACA
>160_rnd-5_family-9199__LINE___R1
TACCTGGTTGACGAACCGCACTGCTCTATTTGCGCTGCTTGCACTGTTTTACTCAGGTTTGCACTGCTTTTT
AAAATTCCGCACTGCTTCGCACTATGTGCACTGCTTATAAAGCAGTGAACGCGTTTGCACTGCTTCGCACT
GCTTTCGCGCTGTCTTGGGCACAAGCAGTTTTATTGCTATTTACCGCAAGTTGTCCCGTTAAGTTTTATGGC
TTTGTTTACGCCAAAGCGAACACCTGCACCTTATCTTTTGTTTTCGACACAAAAATAACTCCGCGACACGT
CTGCTCGCGACGANCGTCTGGAACAAACTGGTAGATAGGGACAAAACAAAACATATTTGTTTAACGTGGC
GTGTACCCCGTAGGGGTTTACTACCACGGTATTCGAAATACAGCGAGCCGAAACAAAGCGTCGGACTAGC
GCCCCTCCAAGACACTGCATGTGCAGTGCCCCTTCGGTACCAAATTGAATGCATGCTTGTCGAACATGACA
TTCCCCCTTGGTGGGAGGTGAGGTCTATCTATACCTCCGCTGGTCTATGGGTACAGCACCCGGTTGACACG
CAACTCCACGCCGAGTGAAAACTCTCCCCACAATTGGGTATCGAACCACAGCCACCACGATACTCCCCGA
AGGGCCGCACCAACTAACCAGGACGGAACGAGTTCCGTGGGCTCCTGGTTACCCGTGGTACCAAATTACA
GCTTTGGTCGGCNGTTCGTAGCAATTGCCACTCCCCAGTTTTGTGTTGACGACAAACACGGAATGGGTCAT
CCATTGTTCGACTTACCCCAAATTGGGGTTTGTCTACCAGATTAAGCTCTGTTCGGCTTCGTGACACGGTC
CATGCCACTCCCAGTTTTGGCTAACGTCAGCCCGGCACTGGTCGGCCCTGTGTGGTTGCACTTCACTCCAT
CCCTCGCCGCCGTCTAGCAAACACCGCTCGCGCAAACATCTCCAGGAGTAGCAGCCGCTCTGGTGTCTCC
AGGACCCGGGAAAAGTCCCAACGGGTACCTTCTCTCCTGACACCAAGTCCGTCGAGGTCTCGCAGATCTG
CATAGAGTGGGCAAGCAACTAGCACGTGCTGCCAGTCTTCATGCTCCGCACCGCATTGACATGCGGGCGT
TGCACTCAGACTTCTCTGATGCAGAAATGCATTTAGCGACCCGTGGCCCGTCAGCAAGAATCCAGCGTGA
AGACCGAATCCAAAGTCTCGCCGGCTGTAGACGAAGCATGCGTCCGGGATAAACTGATGAGTCGGTCGAC
CGTGCTCATCACTATCCCATCTCCGTTGCCACTCGGAGCACATGCCTTCGTCCAGCAACGCCTTCATCCGC
CTCTGGTCTAGATTTGCCACATCTAGACCGAACAGCCAGTCACTATCCTCCAGCGGGTGGGCACGCTTGAC
CTTGAATCTGATTGCCAATTGCTTGGCAACCAAATCCAACGGCGGAGCTCCAGCAAGCACCTGCAGTGCC
ACGGTGGACACTGTACGGCATACCGGTAGGCATCCTAAGAGCATGACTCTTTGGCAGGAGATGAGGCGCC
TCCTGGCCCCCGACTCTCCCCTGACCGCGGAATACCATACCGAGGCACCAAACAGTGCGCAAGGCACCAT
GAGTCCGGCATATACAGTGCGTCTGGCTCGGGGACTGAGGCCCCAGTCAACCCGTAGCACGCGGCCTAAT
GCTCCGACGACTCCAGTCAGTTTATCCCGCAATCCTGCGATATGCGGCTGAAAATTCAACCGTTCACCGAC
TGTGATGCCAAGGTACCGGTACTTGTCGACATATGGTAGGCTTGCTCCTGCAAACCGTACCACGGGCGGC
CTGGAAACGGCAAAACGCCCCTTCAACATCATCATTGCCGTCTTGCTGGTTGAGATCGACACTCCGACCTC
GGCTCCCCAAGTTCCCACGACGGACATCAGCTCAGCTGCTCGACGCTCCAGTTCAGCTCTCGACCTTCCCT
CGACAAGAACCAGCAGGTCATCGGCATATGCACTAAATGTGCACAAGGGCTCCAGGCGTTGGAGCAGGA
CATCCATCATGGTGTTCCAGATGAACGGCCCACTGATGGATCCCTGGGGACACCCTCGCGTGACCTCCACC
TCGACTTCGCCACATCTGCTGGTCATCCTGGCTTTCCTGCCACAGAAGAAGCTTCTCCACAAGCTAGCTTC
CTTGCATCCCGCATCGATCAAGCGGCGCATCGCGGCGCTCCACTCAATGTTGTCAAAGGCGCCTTTAAAGT
CCACGAAGAGACCAAGTACGTATACCGCCTGGCTGGCGGCAACGCTGGTCATCACGTGCTTCCAGGCGTC
CTCGACACAGCGTCCAGGTCGAAAGCCAAATTGCCATCTATTTCCATCCGGAAGCACCTCCTTCATTCGAT
CCACCATGATCCCCTCAAGCACTTTGCCAAACACTGGCAACAGGCAGATGCCCCGATAGGAAGCTGGATC
GCTCCTATCCTTGTCGGGGCCCTTGAGGAGCGCAACCACACGCGGGCACTTCCACTCAAGGGGAAAGTAT

file:///E/10月/10.9/PDF/4. biology-1907542/biology-1907542-supplementary/SupplementalDocument2.refTElib_01_25_2022.fasta.txt[2022/10/9 15:38:02]

CCTTCTGCCAGGCAGCGGAATACAACGCTGTCAGGTGCTCGGGGATGGCACGCCATACCGCCTTGCAGA
TCCCACCTGTAATGCCGTCAAGCCCGGGAGAGCGCCTGCTCTTCAACCGAGCGACGCATACTCCAACCTC
GAAAGCTTCGAGCGCTGGAGCAGCTTCGTCCGGAATGACGATGTCTTCACTCGACTCCGCAACTGGAAAA
AAGCTGTGGAGGAGGAGACTCGCGCAGTCGTGCCATGTTACGACCAGCCCGTCATCCGAACGAAGGCACC
TCAAATCGGTAGACCGCTTTCGTCCCCGGCATATCCGGTAGACGTGCCCCCACGGATCGTCTCTGTGGTCT
CCAACGAAGCGTCGCCAGTTTTCCTCCTTCGTCTTCAGGATGAGCGTCTTGAACTCGCGGACAGCTGTCCG
CAGATGAGCTGCAAGATACGGTGCAGATTCATGACCTCTGCGCCGTGCATCCTGGAGCCTCCGCCTCAGT
CTCCTGACCTCCCGGCGTTTGGTACTCAGTTCGGCAGTCCACCATTTAACTTTTCCCTGGGCTCTCGACGGT
CTGCGGCCTAACACTCTGTCGCACACAGACTGTACGGCAAGGCGTAGGGCAGACACCGCGGTATCCAACG
GCAACATCCTAATATCCTCCTGGGAAAGGTGCTCCTCGATTTCCTCTACCAGCTCCGAGTCGAACAATCGC
CAATTAGCATTCGCGAGCTTCCAGGACGGCACAGGAGCATAGCTCTCAACTGCATTTGCTGGGGATGGCG
AAGTCACAACAGTAATCATGTTGTGGTCGCTCAAATCCCACTCGTCCACACTCCAGTCATACGCGGCCCAC
GTAGACGCTGCTTCGTTCACGATTGTTACGTCGATATCGCTCTGTCCTCTTCCATTATCGAACGTGTACACC
TCGCTGACTTGATTTAGCACGTTGGCACGACTTCCGAGTATCCACTCATCTAGCTGTTCGCCTCTTCTGTAG
TTCAGCTGGCCACGAAAGAATTCGTGCCGTTTGCTGAACCACAAGGGAGACACAGCGTTTGCGTCCAGGC
TGAGAATTACCGGTGTACTGCTCGCCAGTAGCAGTATGGTATCCATATAGCTCAATATCCTTGTCAAGTCA
GCATGAGCATGGCAGTATATGGAGGTCAGGTAAACAGTGCCATGTTTACCTGTGACCCTCACACATACTC
CAGCCTCGGTGGTCAGCGACTCCACTGGTAGGCAGATGGCATCTGGGTCGTCGATGATGACAGCAGCGCT
CCTTTTCCGGTCCTCGACAGCGTGGCTTCCGAAAACGCGCATCCCGGCTGGGAAGCCCGTAATCCTTCCAA
CGACGTCCGAGTATGGCTCCTGCAGCAGCGCAAAGGAGTGGCCGTGGTCACGCATACGTACACCGATATC
GATGCACGCAGCGCGGCCCCGGCCACAGTTGGCTTGGATGAAACTAAACATTAATGTTTAGCTTGCACCC
TCGCCAACACTGCGCCATAAATCGGGCAAGACCCCGAAAGCATATAATGCCCCGAGGGTAGCCCCTTGAA
GCGGCAATTCCGGCAGTCCACCGGATTTGCGCACTTGGCTGCGATATGCCCCATCTGTCCACACTGTCGGC
ATACGTCCTGCTTAAGGCGGCACTCGGACACCTTGTGGTCGAAGCCGACACACCTATGGCAAGCATAGGT
GCGGACGAGGGACCGGCATCGGTATGAAAACCACTTTATATAGACCCGTCCGGTCTTTTCAAAAGTCGCC
AGGGCCCTCTCGTCAACTTCTAGCGTCAGATTAACGCTGGCGCCATCAGTGACGGACCACGGCTTCGACTC
AAGGTGTACTGCCTTGTTGAACACCGCTGCTGTCATCTCCTCCTTGAAGTTGTTCGTATACAACTCCTCCAT
GAACACCTCGGGCAGGATCGATGTGTCCACATCGAACACCGTTATCCTCGGCCTCAATCCTTTGTTCGGCT
GGATGTTGAGTCCAACCTCCGCGAACTTGGTGTTTGCCACCACTCTGTTGAGCTCGCCCTTCGATGGTGTG
CGGATGACCGCGCCGCCTCGCTTGAGCTCTCGCACCTCGTGCACCCGCACTCCAAGGGTGGGTGCGACCT
CCTCCATGATCCGTTTGTCCACCATTTTGCCCGACTCGCTCGGGTCCCGACTCGTGACCACCGCGGACCAC
GTCTCAACTGGCTTGTTGATCCGTGGGGCAGCGATCGGCACTCCTGGGACGGGGACAGCGGTGCGGGCAG
CATGGTGGGCAACTTGGTGGACGACTTGGTGAGCAGCAGTTGGGGTGGGGGCGGCGAGCTGTGCTGCAG
ACGATCTGCCTGCCAGGCTCTCGAGCAGACCGTTCCGCACTGCCAGCGACGTGGACAGCTCCTCCAATCG
CTTAACGGCTGCCGACACTTCGCGCGCGGCTTCGGGGGCGACACCCACCAGGGCAAGGCGGGTCATTTCG
GCAGCTGCCGAAAGCTCCGCTATAATCGCCCCTGTCCCCTCGGCCATTATCCTCCATGCTGGGGCAAGCGC
GATGATGGGGGCGGCAGCGGCAGCGGCTCTGACGTCTTCTTCTGCAGCGGCAGCATCAGCGGCTCTTTTT
GCAGCAGCCCTA
>160_rnd-1_family-138__LTR___Gypsy
ACAATGATAAGCGACAAAATAAAATAAATAAATAAAATGAAATTTCAAGAAATATATATATATTCTTATT
ACCAGAAATGCCTATTAATAAGTTTGCGATAGAAGTATATTTAGAAGCAATATATAATAGCGACGCAGAC
TTTAGAATTCGTGCATTGAATATTCTACAACAATAAGATAAAAGGCATAATTTTAAATACACTGCCATATG
TTGGCATTAAAGAAATAGTGGTCCAGTGGACGGCGAGTGCCCAATTATAATGGTAAAACTTCATAAATAG
AGAACTATGGAATGGAGTGAATTAGCGAAAAAAATAAATAATTTCAAAGATAAATTCGAAAAATCTTATA
AGTGTGTTAATAAGAACGCACCTATAAGGGTTGAAACGCTAATTCACCACATAAACATATTAGTGCTAAA
CTATAATAACATAGTTCAACTTTATTTAAGTAAAGAAAATAGGCTTACAGAGCAGCATGTAAATCAATGT
AAGAAAATAATCAAATCACTTGGGACTAGAATATCGAATATCAGCACTAGGCACAACATAGTAATAGATA
TCCCAACAGATTTAAAATTAATGGCAACTTTTGAACCAGATCAACTAGAAGAACTGAATGAGTCTAAGCC
AACACTAAATAGTGACCCTGATATTGAAGCTGATAGCGACATTGACCCACTTGAATTAACAGAACCAAAC
ATGGCAGTAAATCAAGTGGCCTTAGACCGCGAATATGTAAGACAGGTATCGTCTACAGTCCCGGAATTTG
ACGGNAAGAAAAGCCGTCACTGGCCAGATTTCTAACGGCNTTAAGACTNGTCGACCGNACNAAGGGNAC
TTACGAGAAGACTTAGCCGTAGAAGTCATAAAGTCNAAAATAATTGGCCCAATCCTGTACAAAGTACAAA
ATGAAACNNCNATTANCGGNATTATNAATAAATTAAANACCGTAGTTGTCAAAGGCGAGTCAACCGACGT
CATCAAGGCNAAGNTGCTTAACACTTTAAAGCAGAAAGGCAAGNCAGCCNCGCAATNTACAGCTGAAAT

NGACAGTTTGCGTAAGCAACTNGAAGCAGCCTATATTGACGATGGCATACTAGATTCTGATAACGCAGAG
AAATTTTCCACTAAGGANACAATANNNGCCGTGACAAAGAATTGCGAGTACGAAAAGCCTCGCATAATGT
TAGAAGCGGGCAANTTCGGCANTTTCAATGATGCCATGGGTAAATNTGTCAATGCAGCACTGAAACGACC
GTGCCAGTACAATACTGTACTACGAATACAGAAACNACCGTGGAAATTGCGGNCGTAACAACCANAGAG
GTAGAGGCAATAGCCGCGGCAACAGTCGTTNCAANTACAATGGTAATAACAACAACAGAGGTAATTACA
GAGGNAATNACCGCGGTAGNGGCCGAGGAAGAAACAATTCNAACCGAGGCCAAAACCAAAATAATAAC
AATAGAGGTAATGTCCGAGTAACCCAAGGTAATTCGGAAAACCAACAAAACCCCTCAGATGTTCAGAGCC
AATAAATGGCGCAGTNAANACCATCAACCTCAGTCTNAGCATATTTGTAAAANTTAACAATGNGAGAACA
GGTAAANAACTTGCACTNTTAGTAGATACTGGTGCAGACATATCCATATTAAAAGAAAATNCNGATATAT
TCGANGACGTAAACGAGANAATTACTACCCAAATATCGGGNATNGGCGAAGGCATAATTCANTCCAAAG
GNTTGGCCTCTATAGAGNTACANACAGGTAAATACATAATTCCACACGATTTTCATTTAGTCGATCAACAN
TTCGCAATACCTNGTGATGGCATACTAGGCATAGATTTCATAAAACAATACAATTGCCAATTAGATTTCAA
NCAAAGTGAAGACTGGCTTATAATNAGACCAAACAACTTAAATTNTCCAATTTATGTTCCAATAATCATA
CAGTTCTGGCAATAATGCCACAGTACTCCCAGCNAGNTCCGAAGTTGTCAGNGAAATNCAAATACCNTCA
ANAGAAGATAGTGTNCTGATTCCAAATCAGGAAATTAANGAAGGNATTTACGTCGCAAACACNATTGCA
ACAACCAAAAATGCATTTGTCCGATTACTNAATACAACAAATACCGATCAAGTAGTNAATATAAANAATT
TANAATATGAACCACTTTCGAACTACGACATAGTCAAAANAATCCAGAAAATAGAGAGAAAACTGCGTT
ATCTNAATTAACNAAAAACTTTCCACCNCNATTCAAAAATCAATTAAAAGACTTATGCACCAAGTATACC
GACATATTCGGACTNGAAACCGAATCGATAACNACNAATAATTTTTACAAACAAAAACTNAGATTGAAGG
ATGATGAGCCCGTATACATAAAAAATTATAGAAGCCCTCACAGCCAAGTANANGAAATNCAAGNNCAAG
TACAAAAATTAATCGACGACAAAATAGTCGAACCGTCNGTCTCNGAATATAATAGCCCACTTTTATTAGTT
CCGAAGAAGTCNCTTCCGGGCTCTGAAAAGAAGAAATGGCGATTAGTAATTGACTATCGTCAAATTAATA
AAAAACTATTGTCCGATAAATTTCCGCTNCCTAGAATTGATGATATTCTAGATCAACTAGGNAGAGCAAA
ATATTTTTCATGCCTTGACTTAATGTCAGGNTTTCACCAAATTGAACTCGAAAAAAGTTCNAGAGATATAA
CGTCATTTTCAACGAGCAATGGCTCGTATCGCTTCACGCGATTACCATTCGGNTTAAAAATAGCGCCAAAT
TCATTTCAGAGAATGATGACNATAGCATTTTCTGGNCTAGAACCNTCNCAAGCATTCCTTTATATGGATGA
TCTAATAGTNATCGGTTGTTCCGAAAAACATATGGTCAAAAACTTAACTGATGTTTTCGACAAATGTAGGA
AATNCAACCTGAAACTACATCCGGAAAAATGTTCATTTTTCATGCATGAAGTCACATTTTTGGGACACAAA
TGCACNGATAAAGGAATCTTGCCAGACGACAAAAAATACGACGTCATTAAGAATTACCCAGTCCCACACG
ATGCGGACAGCGCTAGACGATTCGTTGCATTTTGCAATTACTATAGGCGCTTTATNAAAAATTTCGCCGAC
TATTCACGTCACATAACAAGATTATGTAAAAAAGACGTTAANTTCGAATGGACAGCTGAATGCCAGAACG
CNTTCGAACATCTCAAAACGGCACTAATAAACCCNACNTTGTTACAATATCCNGATTTCCGCAAAGAATTT
TGCATAATAACAGATGCAAGTAAGCAAGCGTGCGGAGCGGTTTTAACTCAGAACCGTAACGGACTCCAAC
TTCCAATTGCATACGCATCAAGATCNTTTACTAAAGGAGAAAGTAATAAGAGTACAACNGAACAAGAGTT
AGCGGCAATTCATTGGGCAATAACNCATTTTNGACCATATATTTATGGCAAACACTTCACNGTTAAAACG
GATCACAGACCATTAACATATCTNTTTTCAATGGTCAATCCAAGTTCNAAATTAACNCGTATGAGGCTNGA
GCTNGAGGAATACGACTTCACGGTAGAATATCTAAAGGGNAAAGACAATTNCGTAGCAGACGCGTTATC
GAGAATAACCATNAAAGAGCTACAAGATATAACGGAACAATAAAGAAAGTCACTACNAGGCGNCAAAGT
AGACAAAAATCCTGCGCAGGAGAAAANNAAATAGAATTGCCAAGGCAATCTATAGAAAAAGCNTCNAAG
CCCAACGTATACGAAGTCATTAACAATGACGAAGTACGAAAAGTAGTGACCTTGCGCGTAAANAATATGC
TATGTTTATTTAAACATGGNAANAAAATTACCGCAAGANNCGANGTTAGCGATTTATATACCAATGGAAT
CCTCGACTTAGGTCAATTCTTCCAAAGGCTCGAAACGGAAGCCGGTATATNNAAAATCAGCCAACTCAAA
GTGGCACCGTGGGAAAATATCTTTGAACACGTTTCAATAGATAATTTCAAAAATATGGGCAATAAAATAT
TAAAATCNTTAAGAGTAGCGCTACTCCACCCGGTGACCCAAATANAAACGATAAAAGAGAAANAAGCTA
TATTGTCTACATNCCATGACGACCCAATACAAGGAGGTCATACNGGCATNACAAGAACCTTGGCCAAAGT
CAAAAGACACTACTACTGGAAAAGGTATGACNCGNCATATAAAAGAGTACGTACGTAGATGTCAAAAAT
GCCAAANGTCTAAAACAAAGACGCACACAAAAACTCCTTTAACTATAACNGANACACCTGTANATGCTTT
CGACAGAGTGATAGTGGACACAATTGGTCCGCTACCCAAATCGGAAAATGGNAATGAATACGCAGTCAC
ATTAATNTGCGACCTNACNAAATATCTAGTCGCNATTCCAATACCAAACAAAAGCGCAAATACNGTCGCA
AAAGCTATATTCGAAAATTTTATACTCAAGTACGGTCCAATGAAGACGTTCATTACGGACATGGGAACAG
AATACAAGAACTCGATTATAAATGATTTGTGCAAGTATNTGAAAATNAAGAATATAACATCNACTGCACA
TCATCACCAGACAGTTGGAACAGTAGAAAGAAGTCACAGAACTTTTAACGAATACATACGNTCATATATA
TCGGTNGATAAAACTGATTGGGACGTATGGNTCCAATATTTCGTTTATTGCTTCAACACGACCCCATCNAC
GGCGCATGANTATTGTCCATACGAACTAGTATTTGGTAGACTACCGAAACAGTTCATAGATTTAAACAAA

ACTAATACATAGANCCTATATATAATNTAGATGACTATGCTAAGGAAAGTAAGTNTAGATTAGAAATAGC
ATACAAAAGAGCTAGAATTATGTTAGAANAAGATAAGGCATATAGGAAGTTAGCATATGACGAAAAAGC
AAAAAACTTTGAATTANAAATAGGAGATAAAGTCTTACTAAGAAACGAAACAGGNCATAAATTAGATCC
AGNATATTTAGGACCNTATANAGTAGAAAAAATAGAAGATAANGATAACATNTTAATTANGGATAGTAA
AAATAAAAAACAAANAGTNCATAAGGATAGATTAAAAATTTTTAATTCATAAT
>9_rnd-1_family-356__LINE___I-Jockey
CACTTACGTTTGTGTTTCTCTGGAGATCGGACGTGAAAATTGCAGTCTCGTGTGTGTGTGCGCGTTAGTGC
ACCGCTGGCAGCAGGCATAAACCAAGAAAGTGACATTGACACTGTATAAAGCCCGCCCCAGGGCAATCC
ATACAAATCCCGCTTATAGTGGGTAAACTATACGCAACTGCTGCCTCTGGCAGCCGGAGTGTTATACAAA
TTAACAATATTTTAACAAAAAAATTACCGCGTGCAGTGCGTGAGAACAAGTTCTTGCAAAACTTAAGCTG
CGCTCAGCTGATCGCAACTCAAGTGCGTGTGTGTGTGCGCGAGTGCAGCAAAGCTTTCGCGAGAGAGCTT
TCAGCTTGCAGCTGCACTGATAGTGCTGGCAAAACAGTCACTGCAGTGACAGTGCTGACACTAAAGTCAC
AATTTGTGATCAATCACTTGTTACAAAAATACGGGTTATTTTTGTAATTAAGTGTAAAAACTTGTTTTGTTT
TTTTTTTTTTTTTTTTTCTCCCTGTGGGGAAAAAAATTATAACAAAGTAAAGAACAATGGCTACAAAACA
ACCGACCCCAAAACAACGAAGAAATTCATTCGCCAATCTACCCGCTGCCGAACAGCTCAATAATATCCCC
TACAAATATAGCCGCTAATGTCGATCGCCTCGACGGAAGCAGGCCACTGGAAGATTTTTACCAATATTAC
AACAACGTGCTCGCCAGTAGTGCAAACCGGAACGGCGCCTCCACCTCGCTGTCAGCCGGTACCCAGCAAT
GCTCGACTTGGCAACCGCAACAGCACGGCACTGTCAGCACAACGACAGCAACAACCACCACAACCACAG
CGTCATCCAGCAGCGGCTGCGGGACATCTGCCTCAACAGGCACAAGCCGCGCCGCTTTAGAGCCCATCGG
GACCCGCCCCAAAGCCGCCAATCCGACCTTGCAGTCCACCCCTTATGGCACCCAGGCAGCTGGTTCTTGTG
TCACAAACCCCCTCTGCCTCCCATAAAGAACGCAGGTGCTACGCTAGCCTTCGAAGAGCCACTGGTAGAT
TGGCAAACGGTTTCCGCAAAAACGGCGTGGGACTACATTATTATCCGAAGGCGTAAAAAAGGGAAGCAA
CTAAATAACCGCAACACCAGCCGCGCTGGAGCTAAGCCCGCAACTCCCAGTACCGCTTCGGCAATTAGCA
ACAGATATGCCTCCCTTGAGCCTCAGGAAGACGCCTCTGTAATCGATGACGGCGAGATGGACACTGCCGA
CGAAGCGCCTCCAGTCACAGACCAACAACAATCAGCGAAAAGATCTCCAAACCCAGGCCGATAACAGTC
CCTGGAGTCAGCGATATTGTCGCAATGGAGCGAACCATAGATCAGGCAGTGGGCGCAGACGCCTATGAAT
TCAAGGTCTCTCCCTCTGGCTATCTAAGAATTTACGCCAAAGATGCCGACACCCACAGAGCAATTGTCCGC
AAGCTCACTGAAGTGAGAGTGCAATTTACCCACTTCTGCCTAAAGGAAGACAGACCATACCGCGTTGTGG
TTAAAAATCTGGCCGCTTCCACTCCCAGAAGCCAGATTGAAGCTGCCTTTACATCGCACGGCCATATTGTG
ACGAACCTTTACAACCCGGGCGCCAGACAGCCAACAAGTGATGCAGACTCCACCAACGACTTTCCAAGTC
GCAACTTCTGGTTTGTCGATCTCAAGCAGTCTCTTAACAACAGGGAAGCGCTGCGGCTCAACAAGGTTGG
CCGCCAACGGGTCACTATTGAGCTTCCGAGGAAGAACAATAGTATAAGGCAGTGTTTCCGCTGCTTCGAG
TTCGACCACACCAAAACTACTGTCTCAAACAGCCCAAGTGTGGTAGGTGCGGTCAGTCCCATTGGACAAA
CTCCGAGCAGTGCCAGGCCAAAACAGTGGCAGACCTGCGATGCTCCAACTGCGAGGGAAACCATGCTGCT
TCCTATAAAGGATGCAAAAGCTATCAGGTGAGACTAAATGCCAGGCAGCCGGCCATCAATGTCTCCCCTC
CTACACGATATAGCCATCCTCAAAATACACGAAGAACCACTCAGCAGTTCGTACCTTCCCGAATTGTGCA
ACAGGGCCTGTCGTATGCTGATGCGCTACGGCCAGGCCATCATGTACAAGTCCGCCAACCCCCAGCGCGC
ACTCTTCAGCCCACCGTCAATCCAATGCAGCTCCAACAACCACTGCAAGGACCTCAGGAGCAAGACGCTT
CCCTTGGAGGCATCCTTCAAAACCTCCAGCAGTCCATTGCAGCACTGAATGCCGCAGTTGCAACCATTCTG
GTTAGCATTAAAGAGCTAAAGGATGCGCAAGGTTCCCCGCAGCTTAGATTCCCAAAAGCCGCAAATAATG
GCTACCCAGTTACGTCTCGGATCGTGGAACGCTCGTGGCATCCTCCAAAATGCTAACGAGCTCAAGCTTTT
CTGAAAAAGCATGATGTAGACATCATGCTCATCTCAGAAACACATCTGCACACAAACTTGTACCTAACAA
TGGAGGGGTACGTCTGCTACCGTGCGGATCACCCTACAGGACGAGCCAGAGGTGGTGCTGTAGTGCTGGC
AAAGCAGGATTTAGCACACTACCATCTACACACAGTGACCTCCAGCGACACACAGCTGGCTGCTATCATG
GTGGAGACTCCCTTGGGCGAAATCCTCGTTGCGGCAGCATACCTACCACCAAACATCCCCTGGAACCGAG
CGGAATTTGATTCCCTGTTTGGCCAGCTTGGCCCCAAATTCATCGTTGGAGGCGACTTCAACGCTAAGCAC
AGACTGTGGGGAAACTACAGAGCTGACAGCAGAGGCACTGCGCTCCATGAGGCGCTCACTCCCTGCTCAG
CCCAGGTACTGGCCACCGGTAGACCTACCCACTTTCCGTACAACCGGCAAAGTACGCCTTCCTGTATCGAC
TTTTTATATTCAAGGGCATCCCCAACAGCGTACTATCGGTTCGCGAAGAGTATGATCTATCTTCCGACCAT
CTGCCACTCCTCACAACGATCAACACGGCTGCACAAAGGATCCCAAAAAGCGCAGGCTTGTCCTCCCTGG
GTCGAACATCCAACGATTTAAGGCCGAACTAAATAGCTTGATCAACCTAAACACGCAGATTCTGTCAGTT
GAGGACGTCGACGCTGCAGTACAAACCCTGTTGGACCAAGTACATGCCGCAGCCGCCAACGCTGCGCCCG
CACATATCTACTCAGCCCCTACAAGCCAGCGGCCAAGGACGTTTTCCCCATATTGGAAGGTCTTCTCGCCC
TAAAAAGCGACTGAGGAGGGAGTATGTCCGTACCCAGGACCCCACGATTGACAGGATCTATCGTCGTATT

GCTAACAGGGTCAGGAAGGAGCTGATTATCTCGAAAAGAAATGCCACAGACACCATGCTGGAGGAAGCA
ACGGCAGACGAGAGCTCCAAATTCGCGCTGTGGAAACTTAGCAGTCGATACAAGCGGCAAGCTGCGCCA
AAGTTCCCCGTTCGACTTCCCGATGACACCTGGCCAGATCACCAATGGACAGAGCCGAAGCCTTTGCCT
GCAACTTGGAGGAGCGCTTCAAGCCGTTTGAGACCGCATCGCATGGAAGGATACAGCGAGTCGCGCAAAT
CCTGGAGGCGCCACTCCAAATGTCACTCCCTGTATCGCCAATCACCTTCCAAGAAGTAGAGCAGGAGATG
AAGAGATTAAAAGCAGCAAAGCCCCTGGGGAAGATCGGTTGGACAACCAGACAATCAAGCTACTGCCAT
ACAAGGCGGTGCTTTTTCTCGTCGCCATTTTTAATGCAGCTCTTCGGCTAGGTTACTTTCCAGTGGCTTGGA
AGAAGGCACATATAATCATGTTGCACAAGAGCGGAAAGCCCCCTAATCAACTGGGATCCTATCGCCCCAT
CAGCCTCCTGCCTGCCTTTGGTAAGATTCTGGAAAGGTGCATCCTCAGAAGGGTCCTGGATACCCCGGAA
ATAAAACGCTTGATCCCGAAGTTCCAGTTTGGGTTTCGACTCAAACACGGTACCCCTGAGCAACTACATCG
GGTCACAAATTATATTTGGACGGCTTTCACAGGAAAGAGTATGTGGTGGCAGCATACCTCGACATCCAA
GAAGCCTTCGACCGGGTGTGGCACGCAGGACTCCTCTCCAAGATAAAGGACAAATTGAATCCGCAGCTAT
TTAGCACCATTGCGAGCTTCCTAACTGAGAGAACCTTTCAAGTGGTGCAGGATGGAGTAGCCTCTCAAAC
CAAACGCATAAAAGCGGGCGTTCCACAAGGCAGCGTCTTGGGTCCCACGCTATATAGCCTGTTTACACAT
GACATGCCCACAGCAAATGCAAGCAGACGGAATGATCGGCAGGACCTTCTGGTGGCCACGTTTGCTGACG
ACACGGCAATCCTTGCAAAAACAAATGTGTGTACGTGGCAACTGACCTCTTGCAGGAATACCTCAAGCGA
TTTGAAAACTGGGCCAAGCGTTGGAATATTGGCATTAATTTAAATAAATGCGCCAATGTGACCTATGCGCT
ACGGAGGGATATATGTCCGGGAGTATTCTTGCGCGGTCAATTGGTGACGCACCACAACAGCCACAAATAC
TTAGGGGTGCACTTAGACAGGCGCTTGAACTTTAACAAACACATCACAAGTGTCTCNGTAACCCTGAAGA
ACAGANTGGGAAAACTAAGATGGCTTCTGAGCAGTAGAAATGCGTTGTCTTTGAAAAATAAAGTAACCAT
TTATAAANCCTGCATAGCTCCCGCCTGGCGTTATGGTATTCAGGTATATGGCATTGCTGCAAAGTCCCGTC
TTACAAAAATCAGGGTGTTCCAAGCAAAAACGCTCCGATATCTGTCTGGCGCCCCCTGGTATATGAGAGC
TCCCGACTTGGAAAAAGACCTGGGAGTGCCCAAAGTCGGGGACGTTATAAATATACAGGCCGCAAATTAT
ATAGCGCGACTGAGAAACCATCCTAATCGTCTGGCTAGGCGTCTTCTGAATAGGCGTAGAATTGGAGGAC
TCCGAACGCGGAGGAGACTGCGAAGGTCTCACCCAATCGATCTCCCAGATCGATTTATATAGNCGCCACT
GTAAATTTGTATATTAGACTTACCAGTAGAATA
>160_rnd-1_family-214__LTR___Gypsy
ACAATGATAAGCGACAAAATAAAATAAATAAATAAAATGAAATTTCGAGAAATATATATATATTCTTATT
ACCAGAAATGCCTATTAATAAGTTTGCGATAGAAGTATATTTAGAAGCAATATATAATAGCGACGCAGAC
TTTAGAATTCGTGCATTGAATATTCTACAACAATAAGATAAAAGGCATAATTTTAAATACACTGCCATATG
TTGGCATTAAAGAAATAGTGGTCCAGTGGACGGCGAGTGCCCAATTATAATGGTAAAACTTCATAAATAG
AGAACTATGGAATGGAGTGAATTAGCGAAAAAAATAAATAATTTCAAAGATAAATTCGAAAAATCTTATA
AGTGTGTTAATAAGAACGCACCTATAAGGGTTGAAACGCTAATTCACCACATAAACATATTAGTGCTAAA
CTATAATAACATAGTTCAACTTTATTTAAGTAAAGAAAATAGGCTTACAGAGCAGCATGTAAATCAATGT
AAGAAAATAATCAAATCACTTGGGACTAGAATATCGAATATCAGCACTAGGCACAACATAGTAATAGATA
TCCCAACAGATTTAAAATTAATGGCAACTTTTGAACCAGATCAACTAGAAGAACTGAATGAGTCTAAGCC
AACACTAAATAGTGACCCTGATATTGAAGCTGATAGCGACATTGANCCACTTGAATTAACAGAACCAAAC
ATGGCAGTAAATCAAGTGGCCTTAGACCGCGAATATGTAAGACAGGTATCGTCTACAATTCCGGAATTTG
ATGGCAAAAAGCTATCACTGGCCAGATTTCTAACGGCTTTAAGACTAGTCGATCGTACTAAAGGTACACA
AGAAGACTTAGCCGTAGAAGTCATAAAGTCTAAAATAATTGGCCCAATTTTGTACAAAGTACAAAATGAA
ACATCCATTATTGGTATTATAAATAAATTAAATACCAATATCAAAGGCGAGTCAACTGACGTTATCAAAG
CTAAGTTGCTTAACATAAAGCAGAAAGGCAAGTCAGCCTCGCAATATACAGCTGAAATTGACAGTTTGCG
TAAGCAACTGGAAGCAGCCTATATTGACGATGGACTAGATTTTGATAACGCAGAGAAATTTTCTACTAAA
GAAACAATATCTGCCATGACAAAGAATTGCGAATACGAAAAGCTTCGCATAATATTAGAAGCGGGCAATT
TCAGCAATTTCAATGATGCTATGGGTAAATATATCCAATGCAGCACTGAAATGACTGGATGCCCAAGTAC
AATATTGTACTACAAAGGCAGAAACGATCGTGGAAATTACAGCCGTAACAACCAAAGAGGTAGAGGCAA
TAGCCGCGGCAATAGTAATTTCCACTATAACGGTAATAACAACGATAGGGGTAATTTCAGAGGAAATTAC
CGAGGTGGCAACCGAGGTAGAAACAATTCAAACCGAGGTGGCTACCAAAACCAGAACAGTAACAATAGA
GGTAATGTCCGAGTAACTCAAGGTAACTCGGAAAACCAACGAGACCCCCTCAGATGTTCAGAGCCAATAA
ATGGCTCAATAAAAACCATCAACCTTAATCTTAGCATGTTTGTAAAAATTAAGAATGAAAGAACAGGTAA
ATCAATTACACTGTTAGTAGATACTGGTGCAGATATATCCATATTAAAAGAAAATACAGATATATTCGAA
AACGTAAACGAAACAATTACTACCCAAATATCGGGTATTGGCGAAGGCATAATTCCTTCAAAAGGCTTAG
CCTTTATAGAACTCACTACAGGCAAATATATAATTCCGCATAACTTTCATTTAGTCGAACAAAAGTTCCCA
ATACCGAGTGATGGCATACTAGGCATAGATTTTATAAAACAATATAATTGCCAACTAGATTTCACTCAAA

GTGGCGACTCACTTATTCTTCGCCCAAACAACTTAAATTATCCGATTCGATTAAAAATACTTCATACTTGT
GACAATAATGCCACAGTACTCCCAGCTCGTTCCGAAGTTGTCCGTGAAATACAAATNCCCTCAATACAAA
ATCAAATTCTTATATTAAATCAAGAAATTGATGAAGGAATTTACATCGCAAACACGATATCCAATCACAA
AAATACATATGTCCGTATTATAAATACAACAAATGCCAATAAGATAGTAAACTTAAATAAAATAAAATAT
GAAACTTTGAACAATTATGATATAGTAAAAGAAACCAACGAAGGAAGAGACAACTTCGTTATGGAAAAA
TTAATCAAAAACTTTCCGCCTTTATTCAAAAGTCAATTAAAAGATTTATGCACCAGGTATACCGACATATT
CGGACTAGAAACCGAATCGATAACAACNAATAATTTTTACAAGCAAAAGCTTAGACTGAAGGATGATCA
GCCCGTATANATAAAAAATTATAGGAGTCCTCACAGCCAAGTACAAGAAATTCAAGCCCGAGTGCAAAA
ATTAATAGACGACAAAATAGTCGAACCGTCAGCCTCANAGTATAATAGCCCACTCTTACTAGTCCCGAAG
AAGTCCCTTCCGGACTCTGAAAAGAAGAAATGGCGATTAGTAATTGACTATCGTCAAATTAATAAAAAGC
TATTGTCCGACAAATTTCCGCTACCNAGAATTGATGATATTCTAGATCAGCTAGGTAGAGCAAAATATTTT
TCATGCCTAGACTTAATGTCTGGCTTCCACCAAATCGAACTCGAAAAAAGTTCTAGAAATATAACGTNATT
GTCAACGAGCNATGGCTCGTATCGCTTTACGCGATTACCATTTGGCTTGAAAATAGCCCCAAACTCATTTC
AAAGAATGATGACAATAGCATTTTCTGGTCTCGAACCATCNCAGGCNTTCCTTTATATGGATGATCTGATA
GTAATCGCTTGTTCCGAGAAACATATGGTCAAAAACTTAACTGANGTTTTCGACAAATGTAGGAAACTCA
ACCTGAAACTACATCCGGAAAAATGTTCATTTTTCATGCATGAAGTCACATTTTTGGGACACAAATGCACT
GATAAGGGAATCTTGCCAGACGACAAAAAATACGACGTGATAAAGAACTACCCAGTCCCACACGATGCG
GACAGCGCTAGGCGATTCGTTGCATTTTGCAATTACTATAGGCGCTTTATNAAGAACTTTGCCGACTATTC
ACGTCACATAACAAGATTATGTAAAAAAGACGTNAAGTTCGAATGGACGTCTGAATGCCAGAACATCTCA
AAACGGCACTAATAAACCTAACGTTGTTACAATATCCAGACTTCGCCAAAGAATTCTGCATAATAACTGA
TGCAAGTGAGCAAGCGTGTGGAGCGGTTTTAACTCAAAACCTTAATGGACTCCAACTTCCAATTGCATAC
GCATCAAGATCCTTTACTAAAGGAGAAAGTAACAAGAGTACAACTGAACAAGAATTAACGGCTATTCACT
GGGCAATAACGCACTTCCGACCATATATTTATGGTAACCATTTCACGGTNAAAACGGATCACAGACCGCT
AACATCTATTTTCAATGGTCAATCCAAGTTCACAATTAACACGTATGAGGCTTGANTTGGAGGAATATACC
TTTACGGTAGAATATCTGAAGGGTAAAGACAATTNCGTAGCCGATGCGTTATCGAGAATAACCATCAAAG
GAGCTACAAGATATAACAAGAAACATANTGAAAGTCACTACAAGACANGCACGTAGACAAGAATCCTGC
GCAGGAGAAAANGAAATAGAATTGCCNAGGCAATCTACAGAAAAAGCTTCANAGCCCAACGTATACNAA
GTCATTAACAATGACGAGGTACGAAAAGTAGTGACCTTGCATGTAAACGATACGACATGTTTCTTTTAAA
CATGGCAGACAAATTACAGCAAGATACGACGTTACCGATTTATATACTAATGAAACCATTGATTTAGATC
AANTCTTTCAAAGGCTCGAAACGCAAGCCGGTATATATAAAATCAGCCAACTCAAAGTGGCACCGTGGGA
AAATATCTTTGATACACGTTTCAATGGATAATTTCAAAAATATGGGCAATAAAATATTGACTAAATTAAG
GGTAGCGCTACTCAACCCGGTGACCACAATAAAAAATAATAAAGAAAAAGAAGCTATATTGTCTANACTA
CATGACGACCCAATACAAGGAGGTCATACTGGCATTACAAAAACCTTGGCCAAGGTCAAACGACATTACT
TTTGGAAAGGTATGACTCGAGATATTACTGAGTACATACGTAAATGTCACAAATGCCAGAAAGCTAAAAT
AACTAAGCACAGTAAGACCCCNCTAACNATAACNGANACACCNGTACCCGCNTTCGACAGGGTNATNGT
NGACACAATTGGTACCGCTACCCAGANCNGANAATGGNAACGAGTACGCAGTCACACTNATTTGTGACTT
NACNAAATACCTNGTCGCGATNCCGATNCCAAACANAAGCGCNAGTACNGTNGCCAAANNTATATTCNA
ATCTTTTATTCTGAAGTACGGTCCAATGAAGACGTTCATTACGGACATGGGNACGGAATACAAGAATTCA
ATTATAAACGATTTGTGCAAGTNTATGAAAATAGACAACNTAACATCAACTGCACATCATCACCAGACAG
TTGGAACAGTAGAAAGAAGTCACAGGACTTTTAACGAATACATACGATCNTATATATCAGTTGATAAAAC
TGATTGGGGCGTATGGATCAAATACNTCGTCTACTGTTTTAACACGACCCCCTCTACGGCACACAATTATT
GTGAGCATGAATTNGTATTTGGTAAGACACCCAATCTACCAANATACTTCAATAGAATCGACCCACTATA
TAACGTAGANGATTATGCTAAGGAGGGTAAGTNTAGATTAGAAGCAGCATACAAAAGGGCTAGGAATAT
GCTNGAATTAAACAAGATGAAACAAAAGAAGCGATATGACTTAAATAGTAAGGAATTAAAATTATCNAT
AGGAGATAAGGTCTTATTAAGAAACGAAGCNGGNCATAAACTGGATCCNGAATACACAGGCCCATATAG
AGTAGNNAANATAGAAGATAGGGATAACATNCTNATTNNNGGNAACAAGAATAGGACCCAAACAGTCCA
TAAGGACAGACTAAAAATTTTTAACGNATAATCAAGCATGCAAA
>Ref_dvir.9.78.centroid
ATTCTATAATTTTTTTTTTTTTTTTTTTTTTTTTTTTTTTTTTTTTTTATTTTGCGCTGGAATCGATCAGTGCCTTCTGA
TCTCTACCAAGCGGAGTTAAGGGCATAGTGTATAGTTATTATTTAATAACTTAAATGATAATTACAGACTT
AAAAATATATAACAATAATGGGAAAGGGATGTTTAAGTGATGTGCCTCGTTGGTAGGTCGTCGGGGTGTA
GCCTTCGGAGGCGCCTCTCACTGATGGGCTCAGCCAGGGCCCTTGCGGCCGCGTTCGGATGCTGCACCAA
TTTGTCGGAGTATCGGCTGCTGAAGAAGTTGAGCTGATCTCCGACCTTTGTTAGGCGGAGATCGCGTTCGA
TGGCGTCTGTTGTTACGTACCATGGCAGGCCAGCAACCTTGCGTGCTGCCTTGGCTTGGACCACTCTTATG

CGACGGAGCTGGTGTGCTGATGCCGTGCCCCAAACTTGGATCGCATATGTCCAAATAGGCGTTACCATCG
TTTTGATAAGCAGTGTCTTCGTGGATATGTGCAGCTTGCTTTGGGGCTTTAGTAGCCAATCGAGCTTTTGA
AGTCTTCTCAGGCATTTTGCTCTCGTTGCTGCGATGTGAGGCCCCCATATGAGCCTTCGATCCAGGTGGAC
ACCCAAGTAGCAGAAAGAGTTGGTCTGCTCAATGGTTCGTCCATCGAACCGTAGCCCCTGGCAGCTGTAT
GGGCGCAGCGTAAAAGTGACGCAGGCGGACTTGGCACAGTTTATAGCGATGTTCCACATAGCTGTCCAGG
TTTCGATCTGCAGCAGCCAGTCCTGCACTGCACTTGTAGCAGCTTGCAGATTTTTGTGGGAGGCTAGGAAG
GCAGTGTCGTCCGCATATGTTGCCACCAGCAGTCTCGCAGGGCTGAGGTCTTCGTTGCTTTGTCCTTGTGT
GGGCAGGTCCGCCGTATACAGCGTGTACAGCATCGGACCGAGGACGCTGCCTTGTGGGACACCTGCTCTG
ATGCCCTTAATTGCCGACCTTTGGTCTTTAACGGCCACATTAAAGGTGCGTCCTGCCAGAAAGGAGCTAAG
GATGCTGAAGTATGGCGCTGGGAGTATCAGTTTGATCTTGAACAGAAGACCATCGTGCCAGACCTTGTCA
AACGCTTGTTTGACATCGAGGAAAACGCCGACGCAGTATTGCTTCTGCTCAAAAGCTTCTAGTACGTGTTT
TACTGCCCGGTGGACCTGCTCGGGGCAGCCGTGAGACTTTCTGAAGCCAAACTGATGATCCGGGACCAGG
CGCGTGACCTCGTCGAGTTCTAAGAGACGGCTTAGCAGAAGTCGCTCAAAGACTTTTGAGAGCGTCGGCA
GAAGGCTTATAGGGCGGTAGGACTCAAGTTGAGTTAGCGGCTTACCGGGTTTGGCGATCATCACAATTTT
CGCATGTTTCCATTGCTTCGGAAAGTGGCCAAGGCGGAGCATGGAGTTGAAAATTGTGGCGAGCGCCATG
ACTCCTTTGCGTGGGAGAGCCTTAGCTGCTCTGTTGTCGATTTCGTCGCATCCTGGGGTCTTCTTTAGCTCA
AGCATTTTCAGCTGTGTCCCGATTTCCGCGGGGGAGATGCTTCTTATCGGGAGAGCCATCTGCAGTGGAGC
GTCCAGTGACTCGGCTACTTTACGGCGCTGTGCAACATCTGCGAAGTCGAATGGCTTGAAGGTTGTCTCCA
AGTAGTCAGCAAACGCGTTGGCCTTTTCCTCGTCCGATCGGAGCCAGAGGCCAGAGTGGTCCGTGATCGG
GGAGGTAAAAACTGGTTGCCTCTTAATGCTTCTGGTGAGCTTCCACACTGAAAAGGTGCTGTTGCCGTCAT
GCCCAGTTGTTTCGAGGAGGCTGTCTAGATTTTCCGTTCGAGTCCTAGCCAGCAGTCGTCTGAGCCGGTTG
GACGCGCTTACCATTTCTGCCTTGGTAGAGGGGTCTCCGTTGCGGATGTACAGCCTCCTTAGACGTCCTTT
TGTCGGCAAGGAGACTCCTGGCTTCCAGGGCTTAGTAAGGGGAACCTTCTGAAGGTACGAGAGTGAGGCA
GGGTTTGCGGGGCTGCACGTCTAGCAGCTTCCGTAATTTTCGCTGTGAGGTGGTCAACGTAGCTCTCCAGT
TCACTTGCCGTATTTATGGTGACGTTGAGCTCTAAATGTGTCCTCGAGGCAGGCCCGGTATAACTCCCAAG
TTAGAGCGCGGCGGGAGCATTTTGCGGATTCCAACTTCCCACTTGGGAGTCTCCTCGAGCTCAATCAGTAA
TGGGAGGTGATCCGACGTCAGGTCAAGGAGGCTTCGTATTCGCAGCTGTTCCTGTCTGAATCCCTTATAGA
GCCCGAAGTCAAGTGCACTTGGTGTTGCAGTAGCCCTATATGGGTAGCACGTTGCCTCTCCAGTGGCTATG
ACTTCGATGCCTTCATTCTCAATGGCAGAGAAGAGCGTTCTCCCTCGGGGGCACATGCGGTAGTTTCCCCA
CCAGCGATGTTTGGCATTCCAGTCTCCTCCTGCCAGGAATTTGGGGCCAAGCTGGTCGAAAAGGGCTCGA
AAGTGTGCCTCAGTCACTCTCAAGGCCGGAGGGCAGTATACAGAGGCGAGATTGAAGTCGCCTCTGCTGG
ATCCGAGTCGGATAACAGCAGCTTGCATATGCCAGTGGCAGGATGACTCAAGTGAGTGGTGCGACAAGG
ACGACCTGATCAATATTGCTGCTCCTCCATGTTGGCTGTTGTCTGGGTGGTTGGCGGTGTGGAGAGTGTAG
CCTGGCACTCTGAGGGTGATTCGAGAGCAGAAGTGAGTCTCCGTGAGCAGAAACACATCGATGTGGTGGA
GTTCGATGAAGGCTGTGATCTCATTCGCTTTCGAAAAGACTCCGTTGGCGTTCCATAGTGCTATCCTGAGG
GAGCTCATTTTAGGCACTGTTATTTTTGGCGCTAGTGTGCATTAGCGCTTGTAAAAGAGTCTGTATGAGAG
ACATCAGTTGGTCGAATCTGGCGAACATTTGCTTCATCATGAGCTCAAGGGCTGAGTTGGGGCAGTTGTCG
GCAACAGGTTGCGATTGGCTGGGAGTACTGCCAGTGTTCCTTCTGCGAGACGATGAGCGGGCACTGCGCT
CTGCGGGCTGGGTACCGCGGAGGACGCTGGCAAAGGATTGTTGGCCGGGCACGAAGCTGCAGGCAGGAG
CGGCTGTTTTGGGGCGGGGATCTCTGGAGCGGCTTGCATCTCTGCCATTTGCCTTGTGCTGGTTTTTCTGCT
GGAAAGCATATTGCAGGTACTTCTTGCAGCCTTTGAAGTTTGCTGCGTGTGCTTCTCCACAGTGGTGACAA
CACCGATTCTCGTCCTTGCCAAGTGTGCACTCGTTGCTTGGATGGTTGTCCCCACACTCCTTGCACACATA
GTGTCTCGAGCAGTAAGATTTTGTGTGCCCGTACTCTTGGCATCTGTTGCATTGTACAGGGCCCGACGTCT
TGCGAGGCTTCTCCCACGAAACTTTGTGCCTGCATAGTACTGTTATTGATTTGACAAGGTTATTGTTCTCCG
CTTTCCTCAGGTCAACAATTCTCATATTTAGAGGTTCGTTATTGACTCTGCTACGCGGGGTATATATGCGG
ACAACGTGGTGCCCCTTTTCTGCCAATTGCGCTGAAATATCTTCGGATGCCGACCTCGTGGTGGATATTTC
GTAGAACTATGCGGTACGGCCGGTCTTCTGCAAGCTGGTGGTGGTGAAACAGGCAGCCGTTCTCGCGAAG
GTGCCTTGCCAGCGTTCGGTATTCGTCTGAGTTGCGGCATTGGACGATGCAGTCGCCAGAATTAAGCGATC
TGACGGTGTAAGTGTCCGCGCCTAGGACGCTGTCTAGTTCCTTGCAGAACTTGGTGAAACTGAGGACGTTT
GGCACAGTTATTCTCGGCACCTTAGAGGCAGGCCGCTCATTGGGAGGCGCCGTCTGGGTGGGTTGCGGGT
GTGCGGCAAGCTTTTTATTTGCCGTTTGTTGCCTGCTGGCCCTTGCGCGAGCTGCGTGCGAGGTGGATGGC
CTATCATCTACATCCTCGTCGGCGCAAGCTTCCTTGTTGGTGGCTAGCTCTACTTCCGTGTCCATGTCGCTT
TCCTCTTCGTCACAGAGGGCCGCAAACCGGTTTTGGCTGGCTTTCTTGGTTTCGGTGGTGCTCGCCTTCTTA
CGGACGATATGGGGGGAGAGATTGCCACCTTCTCTTTTAGTGGCCTTAAACCATTGACCTTGCCAAGTACG

TGAAGTCTTGGGCGGGTAATTATTCAAAAACGCGTGGAGATCCGGGCTTGTAGCAGGATTCGCGCCAGGA
ATGGACGGGATTGCGGGGCCTTGGGAGCTGATGCCATGTGCGAGGGACGACGTTACCGTAGTCGTTCCTA
GGGAGCAGGCAGTTGTGGTGATGCACGACGTGGTGAGTAGCGTAGTACCCCCGGCACAGCGTGATGTATG
GCGGTAGCACATTATCCGACGGGTTGTATATAGGTGACGCAGGATAGTCGGGGATGAGTGGATTCGGCTT
GTTGGACGCCATTAGGTGAGATGTAGACCAAAATTTACTAGCTGCACTTATTTACAAAAATCTCCAGTATT
TTTGTGAAAAGTGACTTTGGTGAACGGGGAGCGAAACGGTGACTGGAAAGTGTCAGCACTGTCACGGTCA
GTGACTGACTTGCCAGCACTGATTGTTCTCCTATTGGCTAGTGCTTTACTTGTGTTAGTGAGTCTAGGCAA
AAGCTCTCTCGCGAAAGCTCTGCTCACTCACACGCACACACACACACTACAGCTGAGTTAAGGCGATCAG
CTGAGCGCAGTTTGAGCTTAGCAAAAGCTTGCTCTCTTACGCTCGCACGCGGTTGCACTTTTGTGTAAAAA
AAAAAAAATTTAAATGTTTATATATGTTTATATATATATATAGTTTAGCTGCCAGAGGCAGCACAATTGTT
TTTTTTCGCGCTCTGCAGGCGCGATTTGTTTGTTAAGCCCTGGGGCGGGCTTAGTTAGTGTCACTGTCACTT
TTTGTTTGTTTTTTGCCTGGGATGCACTGAACATACACACGCACGGGAATGCACAGAAAACACGACCGAT
CTCCGCGGAAGTTCAAGGCGA
>9_rnd-1_family-359__LINE___R1
AAACACACACACACACAGCCGTGTGGCAAGCGGCCCACAGAATACTACCACAGCTTGTCATTGCTTGTCG
TACGAGGCGACTGAAATGATACGGGGATGTAATTGCTCGGAGAGGTCCCTCTAGTAGGCGAAAAGATTCT
GACTGCCATTAACCTGGCAATGGCCAGATCGATTCCGTTCCTGGGCAATTAAAGCAACTCGTGACCTCCG
GCAGTGGCCAGGTATCCCCTGGGTAGGCGCTGCACACCCGTGACGCGCCTAGCTAATACGAGAAGGCGAC
TAAGACTGAGACCAGTAACGTCTTAGGTTGCAAACTCTGGACAACCCTACACGACAAGACGACCCCTGGG
CTGACGGCTGACAGTATTCTAGCATCTCAGTAAGCGGAGTGAAATCTGGCCGAAATGACTGCTAGGTTAA
TGCTGTTACCGCCCCCGTCCCGTTAAAACATGGCAAATCTCCTAGCACGCTCCTCGAATCGTCGCCATTTA
GTTGGCAGTATAGGTTCAGATGTGATATTCCTGATTAACGCGGATCTGGCTCTGAACACTCGACCCCATGA
AGGCGGGTGTCAACCCTCGCATGGCCTCACTGCTTGCAAAGACAACCATGAGATCTAAACGCGACTGTAC
AACGACCAAGGACAACGGCTTAGGAGTTGGGACCCAATAAAATGAGTACCAATGAAACCCCACAAATGA
AGGAGAGCTGCGATGCTTTTGCAAAGAGCAGCAGAATGGCGAGATCACCACTAGGTGTTCCAACAGCCGC
CCCGACAGAGACGAACTCAGGACGGGAGCCAACGACGGCAAACCAGAGCAGCTCAGCACTACGAGAACT
CGGGCAGGAGATCTCAAAGCTGGTAGTCATGCTCGAAGATGGCAAGCGACGCTCCATCCACCAGCCAATG
CGAGACTCGATAGAGAGCATCAGAGTGCTCTACGAGCTGGCGGCAATCCAGCTGCACGACGAAGGAGCC
AAGGAGATCATCAGGACGCACCAGTCGTCCCAAACATCGCCATTGTTCAGGACGACGATAGACGTCAAGC
GGAAGCAAGCCACAGCACGGACACCACAAACAAAACGAATCCGAGGACCAGCCGGTAGCCAGGCTACCG
TATCAACGGCGCAGGAGACACCGAGCCCCAAAGCGGATGGCGACAAGTGGAAGGAGGTCACNTACAAGA
AAAGGAAGGAGGTCACCAGGAAAAACAGCAAGGGATTGCTCAAGGAAAAGGCGCGACCGGATGCTATA
GTCATCTCGGCAAAAGGCGAAGCGAGCTACTCAGAGATACTGAGGAAAGTTAAAGCGGACGAAAAACTG
GGCGAGCTCGGAAAAGCGGTGACAAGGATAAGGAGGACACAAAAAGGAGACCTACTNCTCCAGCTTAAC
CACTCGGGAGAGAATACCATAGCATTTGGAAACCTGGTAGGCGAAACGTTAGGCGAAAATGCAGAGGTC
CGGACGCTGTCCCACCGAGTGACCGTTGAGTGCAAGGACCTGGACGAGATCACGACCAGGGAGGATATCT
GTGAGGCGCTGAGGGGTCAAATCCAAATACTGGACATAGCCCCTGAGAATATCAGCCTCCGAAAGGCGTA
CGGCCAAACACAGACGGCGACGATAAGACTGCCAGCGGGAAGCGCGAAAAAGGCCCTTTCAGTAGGTTT
CCTGAAAGTAGGCTGGGTTAACTCGAGAATCCGCGAACGGATNAACATCACCAGATGTTTCAGATGCCTC
GAGTTTGGACATCTAGCAAGACACTGCAAAAGTGATCTAGACAGATCGAATCTGTGCCGACGATGCGGAG
GAAAGGACCATCTGGCTAAGGACTGCAAACAAGAGCCACAGTGCATGCTCTGTAAGGAAAGGAATGCCG
ACTGCAAACACATTGCCGGCAGCGGCAGATGCCCCGTGTTTAGGAGCGCCCTGGCCAACAGAAAATGAA
GTGGACACAGCTGAACCTCAACCACTGCGAGGCTGCTCAGGCCCTGCTGACACAATCAATACGGGAGAGA
AATACGGACGTCGCAATAATCTGCGAGCCATACAAGCCTATCGCTGGAAGTGGCTGGGTGGCAAGTAAAT
GTGGAAAAGCGGCCATTTGGTCTCTCAAATCACCGGTCCAGGAGGTCAGAGCAGCAAACGACGGATTCGT
GAGAGCAAAAATTAATGGAATCACCGTTTACAGTTGCTACGCACCCCCAAGCTGGGAACTGCGGCAATTT
CAGACCATGCTGGAAGAAATATCAGAAGACGCTCGCAATAGAGCTCCTCTCGTCATAGCCGGAGACTTTA
ACGCCTGGGCCGTGGAATGGGGAAGCAAGGAAACTAACGCGAGAGGGACCACCCTGCTAGAAGAGTTCG
CGGCGCTCGACTTGGGCCTTGCAAACGACGGGCAGGAGCTCACGTACTGCAAGGCCGGTAGAGGCTCTAT
TATAGACCTTACCCTGCTCAGCAGCTCACTTTATAGGCTAACTACATGGAAAGTCAGTAATGAATTCACAT
TCAGCGACCACCGAGCAGTGCATTTCGAGATCCACGGGAGAGGACGGAGAAAACAGCAGCCCAAAACAA
CAGGCCCCAAATGGAAGGACAACGGCCTAGACGAGGAAACCTACACGGACTCCTTACGTCATGCTCAATG
GACTTGGGCCACGGAGTTAGCAGACGACATGGCGCGGCAGCTCATGGGAAACATCTCGGACGCGTGCGA
CTTATCCATGCCCAGGAGGATGCCAAACAGAAGAGGAGCACCTTGCTACTGGTGGAATGAGCAAATCAGC

GACCTAAGGAAGGCCTGCCTGCGTGCTAGAAGGCGCTCTCAGCGTGCCCGAGGCCACCCAGACTTTGCAT
CTAGGCTGGAACTCCTGCGGGCAGCCAAGCGAGACCTCAAGAGAAGCATTAAACGAAGCAAATCCAGCT
GCTTCCAGAAAATATGCGATGAGGCCGATGTTAACCCGTGGGCACGGCATACAAGGTCGTCACGAAGA
AGATCCGCAGCCAGGGAACAACCCAGGTGACCTGCCCTACCGCACTTGCCACTATAGTTGAGACACTGTT
CCCCCAACACCCACGACTGGAAGTAGCCAACCGCTCACTGAGCACGGAGCTAGTGCAGCAGGTGTCGGCA
GACGACGTGTTAGCGGCAAGCAAAAAACTAGGCGACAAAAAGGCTCCAGGACCAGATGGGGTGCCAAAC
AAGGCGCTAAAGTTAGCAATGCTAACGAAACCCGACGCCTTCGCCGAGACATATACTAGATGTCTACAGG
ACGGTGTATTCCCTAAGAGATGGAAGCGACAGAAACTAATCCTTCTACCGAAAGGGGAACAAGCCAATA
GAGGAACCCTCGGGATACAGGCCAATCTGCCTACTGGACACGGCGGGAAAGGTACTGGAAAGGATTGTG
TATGACCGACTACTTAGTGTCGTCGAAGAGAAAGAGGCCCTGTCCAAACAGCAATACGGATTTCGCAAGG
CAAGGTCTACGGTCGATGCAATTAAGGCGGTGGTAGATATTGCCGCTAAAGCAATAGAGGGAACCAGAT
GGAGGTGGGGAGACAAGGAGTATTGCGCGGTGATTACGTTGGACATCCGCAATGCCTTTAACTCAGCAAG
CTGGTCTCGTATACTGGAAGCCTTAGACCGAATAGGGATACCGTCCTCCCTTTTCCGTATAATATCCAACT
ACCTGTCCGATCGGGTATTGAGTTATGATACGGATGACGGTGCTAGGAGTTACACGGTCTCAGCAGGGGT
CCCACAGGGGTCGGTCCTGGGACCACTCCTGTGGAACATAATGTACGACGAGGTGCTCAGAGTGCAATTG
CCCCAGGGATGCAGAATCATAGGATTCGCGGATGACCTAGCTCTAGTAGTGGTGGGCAAAGAAATAGAA
GACGTGGAAGCGGCTGCGAATGCAGCCATAAGGATCGTGGCCAGCTGGATGCGTGGCGCTAGCCTGGAG
CTGGCAGGCCATAAAACAGAGGCGGTGCTCATCACAAGCCGCAAAATAGTCGAGTTTGCTAGATTTCAGG
TAGACAGCGTTAGTATCGAGTCAAAGGCTTCCATTAAGTATCTGGGGGTCATGTTAGACAACAGGCTTAG
CTTTCGACCGCACGTAGATTACGTTAGCCAGAAAGCAGCCAAAATGCAGGCGGCCCTTTCGAGGATGCTA
CCCAACATAGGAGGACCTAAAGCAGGTAGACGCCTGCTCCTCGCGAGAGTTGTAGCCTCCGTGCTGCTCT
ATGCTGCACCGATATGGGCTTCAGCGGTTGCAAGCCAGAAAGGCCTCCGAAGGAAGATGTCAATCCCCTA
TAGGCTCAGTGCTCTGCGCACCATTTCTGGATTCCGGACGGTGTCGGACGAGGCAGCCACAGTATTGGCA
GGAATGCTGCCAGTAGATATACTGGCCAAGGAAATGGAGAAAATTTACATGGCCCGAATAGCCACGGGA
GACGCCGTCTCAATGGACGTCATCCGGTCGGAGAGAGCAACATCAATGGCAACTTGGCAGGAGAGATGG
AACACGGCGAACAAGGGGAGATGGACACATAAACTGATCCCCAATCTGGAGCAATGGATTAGGAGACGG
AATGGCGAAATGAACTTCCACCTAACACAGTTTTTCACTGGACACGGCGGGTATAGGAAGTATCTCCATA
GGTTCAGGCATGAGGACTCTCCAGAGTGCCCGAACTGCCCAAATCTGGACGAAGACCCGGAACACGTGCT
GTACCAATGCTCGAGGTACAGAGAGGTAGTGGGCTCTGTACCTCCACCGGAGGGACTGGTAGCGTTCATG
CTGGAAGGAGAGGACAAGTGGAGAAATATAAGCCAGCTCATAATAGGCATCCAGAGGGATCTGAGACGA
TGCGAAAAGGAGCGACGACTAATGGATACTGCCTAAAATAGGCAACCCATCCCCGCGAAGTAATACTTTG
CGGTGATCCCGCGGGGAATGTGGTGTGTGAGTGGGGGGTGAGTTTAGTACGTAGGTGCTGACGGAGTCAG
CAAATCGTGCATACGGTTGGCCCGGGCCATCAGTATCTTACGAAGATTTTCACCTCCGTGCCGTAAGCAAA
AAAANACACACACACACACG
>9_rnd-5_family-539__LINE___R1
ACACACACACACACAGCCGTGTGGCAAGCGGCCCACAGAATACTACCACAGCTTGTCATTGCTTGTCGTA
CGAGGCGACTGAAATGATACGGGGATGTAATTGCTCGGAGAGGTCCCTCTAGTAGGCGAAAAGATTCTGA
CTGCCATTAACCTGGCAATGGCCAGATCGATTCCGTTCCTGGGCAATTAAAGCAACTCGTGACCTCCGGCA
GTGGCCAGGTATCCCCTGGGTAGGCGCTGCACACCCGTGACGCGCCTAGCTAATACGAGAAGGCGACTAA
GACTGAGACCAGTAACGTCTTAGGTTGCAAACTCTGGACAACCCTACACGACAAGACGACCCCTGGGCTG
ACGGCTGACAGTATTCTAGCATCTCAGTAAGCGGAGTGAAATCTGGCCGAAATGACTGCTAGGTTAATGC
TGTTACCGCCCCCGTCCCGTTAAAACATGGCAAATCTCCTAGCACGCTCCTCGAATCGTCGCCATTTAGTT
GGCAGTATAGGTTCAGATGTGATATTCCTGATTAACGCGGATCTGGCTCTGAACACCCGACCCCATGAAG
GCGGGTGTCAACCCTCGCATGGCCTCACTGCTTGCAAAGACAACCATGAGATCTAAACGCGACTGTACAA
CGACCAAGGACAACGGCTTAGGAGTTGGGACCCAATAAAATGAGTACCAATGAAACCCCACAAATGAAG
GAGAGCTGTGATGCTTTTGCAAAGAGCAGCAGAATGGCGAGATCACCACTAGGTGTTCCAACAGCCGCCC
CGACAGAGACGAACTCAGGACGGGAGCCAACGACGGCAAACCAGAGCAGCTCAGCACTACGAGAACTCG
GGCAGGAGATCTCAAAGCTGGTAGTCATGCTCGAAGATGGCAAGCGACGCTCCATCCACCAGCCAATGCG
AGACTCGATAGAGAGCATCAGAGTGCTCTACGAGCTGGCGGCAATCCAGCTGCACGACGAAGGAGCCAA
GGAGATCATCAGGACGCACCAGTCGTCCCAAACATCGCCATTGTTCAGGACGACGATAGACGTCAAGCGG
AAGCAAGCCACAGCACGGACACCACAAACAAAACGAATCCGAGGACCAGCCGGTAGCCAGGCTACCGTA
TCAACGGCGCAGGAGACACCGAGCCCCAAAGCGGATGGCGACAAGTGGAAGGAGGTCACATACAAGAA
AAGGAAGGAGGTCACCAGGAAAAACAGCAAGGGATTGCTCAAGGAAAAGGCGCGACCGGATGCTATAGT
CATCTCGGCAAAAGGCGAAGCGAGCTACTCAGAGATACTGAGGAAAGTTAAAGCGGACGAAAAACTGGG

CGAGCTCGGAAAAGCGGTGACAAGGATAAGGAGGACACAAAAAGGAGACCTACTACTCCAGCTTAACCA
CTCGGGAGAGAATACCATAGCATTTGGAAACCTGGTAGGCGAAACGTTAGGCGAAAATGCAGAGGTCCG
GACGCTGTCCCACCGAGTGACCGTTGAGTGCAAGGACCTGGACGAGATCACGACCAGAGAGGATATCTGT
GAGGCGCTGAGGGGTCAAATCCAAATACTGGACATAGCCCCTGAGAATATCAGCCTCCGAAAGGCGTAC
GGCCAAACACAGACGGCGACGATAAGACTGCCAGCGGGAAGCGCGAAAAAGGCCCTTTCAGTAGGTTTC
CTGAAAGTAGGCTGGGTCAACTCGAGAATCCGCGAACGGATAAACATCACCAGATGTTTCAGATGCCTCG
AGTTTGGACATCTAGCAAGACACTGCAAAAGTGATCTAGACAGATCGAATCTGTGCCGACGATGCGGAGG
AAAGGACCATCTGGCTAAGGACTGCAAACAAGAGCCACAGTGCATGCTCTGTAAGGAAAGGAATGCCGA
CTGCAAACACATTGCCGGCAGCGGCAGATGCCCCGTGTTTAGGAGCGCCCTGGCCAACAGAAAATGAAGT
GGACACAGCTGAACCTCAACCACTGCGAGGCTGCTCAGGCCCTGCTGACACAATCAATACGGGAGAGAA
ATACGGACGTCGCAATAATCTGCGAGCCATACAAGCCTATCGCTGGAAGTGGCTGGGTGGCAAGTAAATG
TGGAAAAGCGGCCATTTGGTCTCTCAAATCACCGGTCCAGGAGGTCAGAGCAGCAAACGACGGATTCGTG
AGAGCAAAAATTAATGGAATCACCGTTTACAGTTGCTACGCACCCCAAGCTGGGAACTGCGGCAATTTCA
GACCATGCTGGAAGAAATATCAGAAGACGCTCGCAATAGAGCTCCTCTCGTCATAGCCGGAGACTTTAAC
GCCTGGGCCGTGGAATGGGGAAGCAAGGAAACTAACGCGAGAGGGACCACCCTGCTAGAAGAGTTCGCG
GCGCTCGACTTGGGCCTTGCAAACGACGGGCAGGAGCTCACGTACTGCAAGGCCGGTAGAGGCTCTATTA
TAGACCTTACCCTGCTCAGCAGCTCACTTTATAGGCTAACTACATGGAAAGTCAGTAATGAATTCACATTC
AGCGACCACCGAGCAGTGCATTTCGAGATCCACGGGAGAGGACGGAGAAAACAGCAGCCCAAAACAACA
GGGCCCCAAATGGAAGGACAACGGCCTAGACGAGGAAACCTACACGGACTCCTTACGTCATGCTCAATG
GACTTGGGCCACGGAGTTAGCAGACGACATGGCGCGGCAGCTCATGGGAAACATCTCGGACGCGTGCGA
CTTATCCATGCCCAGGAGGATGCCAAACAGAAGAGGAGCACCTTGCTACTGGTGGAATGAGCAAATCAGC
GACCTAAGGAAGGCCTGCCTGCGTGCTAGAAGGCGCTCTCAGCGTGCCCGAGGCCACCCAGACTTTGCAT
CTAGGCTGGAACNCCTGCGGGCAGCCAAGCGAGACCTCAAGAGAAGCATTAAACGAAGCAAATCCAGCT
GCTTCCAGAAAATATGCGATGAGGCCGATGTTAACCCGTGGGGCACGGCATACAAGGTCGTCACGAAGA
AGATCCGCAGCCAGGGAACAACCCAGGTGACCTGCCCTACCGCACTTGCCACTATAGTTGAGACACTGTT
CCCCCAACACCCACGACTGGAAGTAGCCAACCGCTCACTGAGCACGGAGCTAGTGCAGCAGGTGTCGGCA
GACGACGTGTTAGCGGCAAGCAAAAACTAGGCGACAAAAGGCTCCAGGACCAGATGGGGTGCCAAACAA
GGCGCTAAAGTTAGCAATGCTAACGAAACCCGACGCCTTCGCCGAGACATATACTAGATGTCTACAGGAC
GGTGTATTCCCTAAGAGATGGAAGCGACAGAAACTAATCCTTCTACCGAAAGGGGACAAGCCAATAGAG
GAACCCTCGGGATACAGGCCAATCTGCCTACTGGACACGGCGGGAAAGGTACTGGAAAGGATTGTGTATG
ACCGACTACTTAGTGTCGTCGAAGAGAAAGAGGCCCTGTCCAAACAGCAATACGGATTTCGCAAGGCAAG
GTCTACGGTCGATGCAATTAAGGCGGTGGTAGATATTGCCGCTAAAGCAATAGAGGGAACCAGATGGAG
GTGGGGAGACAAGGAGTATTGCGCGGTGATTACGTTGGACATCCGCAATGCCTTTAACTCAGCAAGCTGG
TCTCGTATACTGGAAGCCTTAGACCGAATAGGGATACCGTCCTCCCTTTTCCGTATAATATCCAACTACCT
GTCCGATCGGGTATTGAGTTATGATACGGATGACGGTGCTAGGAGTTACACGGTCTCAGCAGGGGTCCCA
CAGGGGTCGGTCCTGGGACCACTCCTGTGGAACATAATGTACGACGAGGTGCTCAGAGTGCAATTGCCCC
AGGGATGCAGAATCATAGGATTCGCGGATGACCTAGCTCTAGTAGTGGTGGGCAAAGAAATAGAAGACG
TGGAAGCGGCTGCGAATGCAGCCATAAGGATCGTGGCCAGCTGGATGCGTGGCGCTAGCCTGGAGCTGGC
AGGCCATAAAACAGAGGCGGTGCTCATCACAAGCCGCAAAATAGTCGAGTTTGCTAGATTTCAGGTAGAC
AGCGTTAGTATCGAGTCAAAGGCTTCCATTAAGTATCTGGGGGTCATGTTAGACAACAGGCTTAGCTTTCG
ACCGCACGTAGATTACGTTAGCCAGAAAGCAGCCAAAATGCAGGCGGCCCTTTCGAGGATGCTACCCAAC
ATAGGAGGACCTAAAGCAGGTAGACGCCTGCTCCTCGCGAGAGTTGTAGCCTCCGTGCTGCTCTATGCTG
CACCGATATGGGCTTCAGCGGTTGCAAGCCAGAAAGGCCTCCGAAGGAAGATGTCAATCCCCTATAGGCT
CAGTGCTCTGCGCACCATTTCTGGATTCCGGACGGTGTCGGACGAGGCAGCCACAGTATTGGCAGGAATG
CTGCCAGTAGATATACTGGCCAAGGAAATGGAGAAAATTTACATGGCCCGAATAGCCACGGGAGACGCC
GTCTCAATGGACGTCATCCGGTCGGAGAGAGCAACATCAATGGCAACTTGGCAGGAGAGATGGAACACG
GCGAACAAGGGGAGATGGACACATAAACTGATCCCCAATCTGGAGCAATGGATTAGGAGACGGAATGGC
GAAATGAACTTCCACCTAACACAGTTTTTCACTGGACACGGCGGGTATAGGAAGTATCTCCATAGGTTCA
GGCATGAGGACTCTCCAGAGTGCCCGAACTGCCCAAATCTGGACGAAGACCCGGAACACGTGCTGTACCA
ATGCTCGAGGTACAGAGAGGTAGTGGGCTCTGTACCTCCACCGGAGGGACTGGTAGCGTTCATGCTGGAA
GGAGAGGACAAGTGGAGAAATATAAGCCAGCTCATAATAGGCATCCAGAGGGATCTGAGACGATGCGAA
AAGGAGCGACGACTAATGGATACTGCCTAAAATAGGCAACCCATCCCCGCGAAGTAATACTTTGCGGTGA
TCCCGCGGGGAATGTGGTGTGTGAGTGGGGGGTGAGTTTAGTACGTAGGTGCTGACGGAGTCAGCAAATC
GNGCATACGGTTGGCCCGGGCCATCAGTATCTTACGAAGATTTTCACCTCCGTGCCGTAAGCAAAAAAAA

AAAACACACACACACACAC
>160_rnd-1_family-363__LINE___I-Jockey
GAGTCGCTAATCTGCTCTCATCGAGATCGGTTGTGCCGCGCATAGACAAAGTACGAGTTGATCAATTGTGC
AATCTACGTGTGCTAAAAATAGCACAAATCGAACATTTCGGACGCTGTCTTGTGCAGTGTCGGCAGTTTGA
CAACAACAAAACGCAAGTGTAGTGCGATAAGACTGCATTCGTTGCCCGTTTGGGCAAAACATTACCCCTC
TCTCCGCGCGCAGCCGCACAAGAGGGGCAATAAACGCGGTGCTTATCAGCTAAGTTATCTTTTTATAAGCT
TAACTTTAACTTTAAAATTAACAAATATAAACTAATTTAATATGGACAAGAACGTTTTTTCTAACTCTCAG
CTTAGTAATAAGCCGATTGATTATGGAAAAATGCGCCAAGTAGCTGGCAAATCACCATTGGAAATCAGGA
CAGATTTTATTAATAGCCTGTTACGTACTGATACACAGAGCATCAGCACAATAACAACAACTTTAACATTC
ACCAGTGCCACTAGTAATTCTATTTACACCACGGCAACTGCTGCAACCAGCAGTGTGAGCCATTGCGGAA
GCATGGATAACCATGCAGCTAGCTTTACCCAATGCTCCTCTGACTCTACCTCTCCAACAATCAGTGGAGCT
TCTAAACTAGACTCCTTAGTTCTTAGTGAAGCTGCTACAAAATTAGTGGCTGCCCAAAAGAATTTGAAAA
ATAAGAGGAAGAAACCATTATCTCCCAATACTAGCTCTAAGAGGCCGAATAAAGATTTGTCGCTAGTCTC
GCCCTCAGCATCAACAAGTATGGCAATCTCGGGCAACAGGTTTGCCTTGCTAGACATGGACATGGACATT
GATGTAACAAACGAAAGCCATGTTGAAAGCCACAGAACTCCAAATGAAAGCCCTACTAGCACTTCTGAGG
CTGATCAGCAGAACAATACTAACGTCAGTATTGATGTAAGTGTCACAGCCAACAACCCACAACATAATGC
AACCAATATCAACGACCAGCTTCACGGTACCCGAAAGCCACCGCAAATAGTTGTGAGTGTTACTGACCTT
AATGATCTTTTTGAACTCATTATGGAGGTCACTAGTTTAGACAACGTGGCAGTGAAAGTCAACCAAGGAG
AAACGGCCAGAATATTTCCCAAAGACAGTGACACTTACAGAGCAATTGTCAATCATTTTGATGGTATTGG
TATTGAGTTCCATACCTATCAATTGAAGGAAGAGAAACCATACAGGATAGTAGTTAAGGGGCTTCACCAT
AGCACATTAAACAACGAAATTATTGAGAATTTTAAACAAAATGGCTTTGATGCTCTGCAGGTTCATAACC
CAAGACCAAGGAGTAACAATGATGGAAAACTCAATATATTTTTCATAAATATAAGGCCATGCGCAAAAAT
TAATGATATATATGATATCAAAACTCTTTGCCGACAAAAAGTGAGGATAGAGAGGATGCGCAAAGCATCT
GAAATCGCCCAATGCACACGATGTCAGGAATACGGACACACAGCCAAATACTGCCGTCGTCACCCCAATT
GTGCACGATGTGGTGAAGATCATCTCACGAAGCTATGCACACGTCCCCATGATGAACAGCCTTCCTGTTTG
CACTGTGGAGGGAATCATATGGCAAGCTACAAAGGTTGCCAATGGTACCAGGACTATCTACGACGATCAA
TGGGCTCTACAATGAAGCAACAGGCTCACACGCAACCGTCTAGACAGATCAGGAGGAATCAAGATCAAC
CAAAGCAGAAACAACCACAAAGGAAACAGCAGCAGCAGCAACCAACTCGACAGCAACAAATTCCTAACA
TCCCTAGAGTCAATACTGGAGGCAAGTCCTATGCCTCAATAGCAAGGAACGCCAACGAACCTGCCCAGCG
CCGTCTACATGGTTTGCAAGCGCAACATCAGCTACCAAAGCCAATAGAAAATATCAGGCAGCAGCAGCAT
CCAGCTGACGTTCAATCACTATTGGAACAACAGCAACAGCAATTTCTGAAATGGCAACAACAGCTGCAAC
TACAGCAGCAGCAGCAATTCCTCTTGTGGCTGCAGGAGCAGCAACGGGAACAACAACAACAAAACAAGC
AAAATAGTCAACGACTAGAACGGCTTGAAAAAATGGTCCACGAAATGGCCAGCATGTTAAAGCAATGGA
CAGGGGATGGATCGACTCCCCAGCTTCATAACAACGCCACAGCCTCCCAATGAATCCACTCAAGATCCTT
ATCTGGAATGTTAATGGCATCGCAGGAAAAGCCAGTGAAGTTGAGCTCTTCGCGCACAACAACAATGTCG
ACATTCTCCTTCTAAGTGAGATCAGACTAAACCGAGGACAAACAGTCAAAATACATGGGTACACCTTCTA
TCCAGCTTACAAGCCATCACGTCATAATCACGGTGTCGGAGGAGCGGCGGTATTCGTGAGGAGTTCTCTC
CGCCATTTTCCACAAAGAGTAATTGAAACGAGCACTATACAAATGTCTTCAATAAAAGTTGCCACTGGTTT
GGGAGACATGGAATTTAGCGCCATATACTGCCCACCAAGAAATAGAATTGAAGAAAGGCATTTCAGTGAC
ATACTCGCTTCTTGCGGCCAAAGGTATCTGGTTGGTGGAGACTGGAATGCGCGACATTGGCTGTGGGGAG
ATACCTACAATTCACCCAGGGGCCGAGAACTAGCCGAAGCCATTACAGCCAGAGGAGCTAATATTCTCGC
AACAGGATCACCAACTAGGTACCCATATGTGCCTAGTCACACACCTAGTTGCATTGATTTCGCATTATACC
ATGGGATACCGGATTCTCAAACAAATATCGGCCAAAGCTGGGATCTAGACTCTGACCATATAGCCCTTGT
TGTAAATTTGCAAACTGATGGTTCTTATGTCAGGCCAAACCCTCGATTAATTACCAACCGTACTGACATCA
TGGCTTTCAGACAACATCTTGAATGCTCGATTCAATTAAATCCCACCTTGGACTCTGGAGTAGACATTGAG
AATGCAGTAGATTTTCTAACTGAAAATATACATAGCGCTGCTACTGCCACAACTCCACCCAACACCGAGT
CACGCCCCATTCGTTATGGCATTGTTCTCACGAGGGAGGCTAAAGAGCTGATGAGAACAAAGAGGTGCCT
TCGAAGAAGAGCAATTCGATCACAAGATCCTTGGGACCGAATTTTGTGGAACCGAGCAGCAAAACAGCTA
CGTATCCTCCTAAGGGAACTCAGAAGTGATTTCTTCGAGCAAAAACTGGCTTCCATGGACTACACCGTTGA
TGCAAACTACTCTCTATGGAAATGTACAAAATCACTCAAACGACAACCCTTTAGACAGGTTCCTGTCCGAT
GTCCTGGTGGCGAACTTGCTAAAAACGAAAGGGAACAGGCTAATGCTTTCGGATATCATCTTGAGGAGCG
TTTCACTCCTTATGAATTTGCTACGATAGAGCAGACCAGGGAGACCCATCAGACTCTTCAGACCCCCCTGC
AGATGTCCCTGCCTATTGAGCCTATAAGGATTGAAGAAATAACTGACGCGATCCAGCTATTGCCGAAAAA
TAAAGCTCCGGGTATCGATAGAATCTGTAATGCTACGCTGAAAGCCCTGCCTACAAGCGCGCTTCTGTATA

TAATGCTAATTTTTAACGCTATTATCCGGCTCCAAGTGTTCCCAAAGCAATGGAAACTAGCAGCAATTATG
ATGATTCACAAGCCAGGCAAACCAGAAGCTGATCCTGAGTCGTATCGGCCAATAAGTCTCTTACCCTCCCT
GTCTAAACTATGGAGAGGACTATTGCCAAGCGGATAACTGCCATTACAAGCCAATGCAATATCTTGCCG
GATCACCAATTCGGATTTCGAGAGGGACACGGCACTGTGGAACAGGTCCACAGATTGGTGAAGCACATCA
TGCAGGCTTTTGACGACCTAGAGTTCTCAAACGCTGTCTTCATTGACATGCAGCAAGCGTTCGATAAGGTG
TGGCACGATGGATTACTGTACAAAATAAAAAACCTTTTACCCGCACCGTACTATGGCCTTTTAAGGTCATA
TCTGGAAGGACGCGAGTTCAAGGTCAAGGTAAGGGACACATACTCCTCCACCTATACTATGAGAGCAGGA
GTGCCACAGGGCAGTGTTCTTGGTCCGCTGCTCTACTCGTTGTATACCTCAGATATACCCAGCCCTACCTC
TCAACATATGGAAGATCCCTCTAAGGCTATCATTGCGACATATGCAGATGACATCGCAGTCATTTATAACT
GCGAAAGTTGCGTAGAAGCTAGCAATGGACTTCAGGGGTATCTGGATACTCTCGCAGCTTGGTGCAAACG
GTGGAACTTGAAGGTTAACCCGTTAAAAACTATTAACCCATGCTTCACCCTCAAAATGCGGATGCATATCC
CTCCAATTCAGCTTGAAGGAGTTACCCTGGAGCAGCCGCCACAAGCTAAGTATCTCGGCATCACCTTAGA
CAAGCGCCTTACCTTTGGGCCACACCTCAAAGCTACGGTAAAAAAATGTCGGCGCAGACTGCAACAACTG
CGGTGGCTCAACAACAAAAGGAGCACCCTGCCGCTGAGATGCAAAAGAGCTGTATATGTGCACTGTATTT
TGCCGATATGGCTCTATGGAGTGCAGATTTGGGGGATTGCAGCCAAATCAAATTATAAACGCATACAGGT
ACTGCAGAATCGGGTATTACGACAGATAACCAACTGTCCCTGGTACGTACGCGGTTCTACACTCCATAAA
GACCTCAAAGTGCACACAGTCGAAGAACAGATTGGAAGGCACACAAGCAGATACAGCGACAGACTGCTG
AGACACCGCAGCCTGCTCGCCAGAGGACTACTCCCTGCCCGACCTCTAAGGCGACTCAAACGGCAAGGTT
TTGCCAAGACGATTGGGCGCCAGTAAGACCACCCGCATTAAAATCCTCATACTACGTTATGGGGTTCGGC
TCTAAATAACATTTCTATCCATGTGTTGTTAAGGTACTAAATTATGATTGTACAGGTTCTACACTTAATAA
AAAAATAAAAAAAAAAAAAAAAAAAAAAAAAAAAAAAAAAAAAAAAAAAT
>Ref_dvir_442
GACACGAAAACAAGGCGACCCATCTCTTTTCTTTTCAACGTCCGATCGTCACAAGGAGTGGTTTAAGGTA
GTCCAGATCCGTAAACTTAAATGGCCATCCCATTGGCCTCCAGTATGTTCTTCGGCAAAGGACCCATTATA
GGTGGCGCTGGCTGCGCTCTGTGTAGCTTGCACACGCTACATCCAGAAATTACTGTACGCAACAGTCGAC
GCAGCTTTGTTAAATCTTGGTGATGTCGCCTAATCCACGTTTTGATGCTTCATTTTCACATGGTAGTGATAC
ACCATCAGTTCCGTCAGTGCGTGCCGGTGTGACAATATTATTGGCCTTCTCGCACTATGCGGTATACACAG
TGCGGCATCGATCCTGCCATACGCACGCAAGACTCCATTGTCATCCAAGTATGGCGCCAGCCCTCTGTAGC
TATGTCGCTTCGATGACCGACTGAATTCTTCATTGTATGAGGCTCGGGCAACCTCGTCAGGGAACGCTTCG
CATTGCGCTCGCCGTATCAGCAAGTTCTCAGCGTTCTCACACTCCGTCGCGGTGAGGCCGAATCTCTCAAG
CTCAGTCCTCTGTCCACGGCTCCAGCGCGTGAACCTTAAAACCCAAGCCGTCGTCCTCACCAGCCGACTGT
AGCTAGAGAATCTCTGAAATGAAATGTAAAAGCTATTTGCTGCAACTAAGGCAAATTCACTCGGCATCTC
TTCTTCATCTTCGTAGGATGAGTGTTCCGTGCCCGGTTCGGACTGCCAGCTGCTTTCTGGCTGCCGCAAAA
ATGTTGGCCCGTCTAACCATCGTGACCGTTGGCTGAGGTCTACGTGGCAATGCGGCCTCGTTGCGTCGTCT
GCTTACAGTTTTGGAGTCAGTCCATAGTATAGCGTCGCTGATCGCTATACTGTGCTCCTTTTTGACAGTGTC
CATCAGCCTCGTTCCTAACACGGCTGCTTGCAATTCCAGTCGTGGAATTGACATCGTCTTCATTGGGGCAC
ACTTCGTCTTGGCACATACGAAGCATACTCGCACGTCGTTGTTGTCGTAGGTGACCCTCAAGTAAGTCACG
GCTGCGAATGCTGACTGGCTAGCATCGACAAAAACGTGCAGCTGTATTGCCCGCACAGGTGTTGGGCCGA
AGTAATGGCGCGAGCTTCCGAAATCCTTCCACGTTGTTCATATCCTGAAACCATTTGCTAAAGGCCCGGGC
GAATCTCATCTGGTAAGGGTTCATCCCTTGCACATTTCGTCGCCAAATCTCACGCATTAGTAACTTTCCAG
TTATCATGAGGCAGCTAAGAAATCCCAGAGGATCGAATGTTGACATGATCAAGCTCAAGAATTCCCTCTT
CGTAGGAATTCGCTCCAAATTTAGCACACATCGGGGTACTTTGTGGTACTTCATGTTGAATTTGAAATCGT
CCGTGGCTGGTTGCCAACACATGCCTAGAATCTTTTCTTCAGCTTCGCCCCATCCGATGCTCCTCGCCCCAT
GTTGGCCCAGCGCGTCAGCTACAGTTGGCGAACTGGAAGTAAACTGGCACAATTCAAATCCAGCATTTGC
ATGTATTTCTCTTATTCTTGTTGATATGGTGATGGCTTCGCCTTCACTATCAAAACTATCCACATAGTCGTC
CACATAGTGGCATTCAAGAATAGCTTTAACCGCGCGAGGATCTGAATCCTGAAACTGCAGGGCATTCAAG
GTCTTCACATAGTGTGCGGCGCTTGGCGAACATGCTGCTCCAAACGTCATTACCCTCATCTCATAAACATC
AGGCTCCCGGCGATCATCGCCGTTTCGCCACAGGAATCGCTGGGCGCATCTATCCTGCGGTCGAATCAAC
ACTTGATGGAACATCTCTCGGATGTCTCCACAAACGCCAACGGCACCTTCCCGAAAATGGAAGAGAACAG
CCGGCAGCGACTTATAGTGCTGCGGCCCCTTGGATAATGCCGAATTCAATGAAATATCACCAACCTTTGCT
GCCGCGTCGAACACCAGTCTTATCTTACCGGGCTTATTTGGATTTTCAACTCCAAAGTGCGGAAGATACCA
AAGTTTGTCGCTGTTGGCTAAAGCGACCTCATGCTGCTTCAATCGTCGAGCATATCCTTTATGAACATAAT
CGTCCATAATTTTCATATAGGCTTGTTTAAACTGATCGTTACGCTTCATCTTTCTCTCAATGTTGACCAATC
TTTTGTATGCCATATCATAGCTTCGTGGCAACTCAACATTATCACGGTTCCATAATAAACCCGTCTGGTAA

CGTTCTCCTTTTTCACCGTGGTGTCTTCGATCCTCTGTGCTCGTGCATCGTCGCTGGCTGCGACTGGTGGCG
TAAGCTTCACACCGAAGTTTTCGATATCAAAATAGTCGTTTACCATCTTTTGGATTGCATCCTCCTGCGAC
ACTGCTAGGAGGCATGACTTTGATGACGTTGCCGTCGGTTGACCTCTTACCGGCCCAAAAACCACCCATCC
AAGTTCGGTGGCTGCGGCATATGGTCCCTGTGGTGCAAATCTTTTTGTTTGCATTGGCAGTCCAAGATGCG
CATGGTCAAGTCCAATCAGAAGTTTTGGTACCGCCCCGCGATACGGTTTCATTGGAAGACGTGCACCGTTG
CCCCACATTTGGACGTCACGTTGATGCAGGCTTTGCATTGGTAAGCTCAGGTTGGACACGGTGTACACATT
TCGCAACACATGACGTTTGCGCTTGTCCGCTCCACTAACTTCCATGCTTACCACTGTGGTCGGCTCTCTGGT
TGCTCTTCCACCAAACCACTGTATATTTAGTTGCCTGCGTTCCCCTTTGATATCTAGATCGCGCAGTAGCTC
GTCGTCGATCATCGTTACAGACGAGCCCTCGTCGAGGAGCGCGTACGTATCGATCCGCTTATTGGCCCCGT
GCAATGTCACTGGCAATATGCGGAATAGTAGATCTCCATCCGAATCGACACAACTTAAGTTCCTTTGTTGC
GTCAATTGAGCATCCACTGGCTGCGGTGCCCTTGTGTGAGGATCCGCTGGCTGCGGCATCCGTCCAGTATT
CATCACTCTATCACCTGCTGGCTGCGGTGCATTGTGACTCATGGCATCATGATCATGTAAGAGTCGATGAT
GTCTCTTCCGGCATCCATTAACTTGGCATTCGTTGGCCCTATTGCAGACTCTTGCCATGTGGCCAACTCGTA
AACACGAGAAACAAAGCCGGTACCTTTTTACGACTGTCCACCTCTTTAGCGGAGAGGCGAAATTAAACTC
GTTGCAGTCCCTGACTGCATGCTATCCATTACAGATTGTACACTGTTTGGTGCGACCTCAGTGACGCCCTT
TCATTATTCTGGTCAATGCTGGCATGTAGCACTCTACGCTTCTGCTGCCTACTATCCACGTCCACAATCGTA
CATACCACATTGGCGTAGTCCGTTAACCACGCGCTAAAATGTACGACAGTCGGGAAAGGCCTAATCGTAG
CTGCATGTCTGGCCCATTCCACCCTCTTGCTTGGTGGTAGTTTAGCGACCAAATCCTCCATCAGTGTAGGA
TTCGCTAAATGCTGTTCCCCGTTAGCCGATTGTAGGAAAGGCGGCAAGGTTACTCACGCGTGTAGCATATG
GCACGATTTTAGCTATGCTGTGCTCCTGAATTGGCTGCACCTCTCGAATACTAATTAGCTGGCTGCGTTATT
AGCTGTTCTGGGCGGCCGTATCTAAAACGGTAATTGTTCCATTACGTTGTTCACATTCCTGGGATGTATGA
GCAACGACTTCACAGTGTCACGTGCTTCGCCTTTGAGTGCCTTCAACAATCGCTGGTTATTCTCCAGATTT
GTACAGTTGTACGCCAAAGTTGTTTCCGTAAACGCGCAGAAGAATATAGGCCAATCCTCAGGTTGGCCTC
CAAACTCGGGTAGATCTGGTAATTTGCGTGGCAGGCATACATCGTTCCTGGGAGTCGTCCATGCGAAGTTC
CCAGTTGCCTGCGTTGGCTCCAAAATTATCGCTTGTTGGTTCTCCTGTGCCGGAGTGTAGCTGAGCGTTGG
CTGTGCAATGCCTGCAGAGCAATATGGCGTCGAGCGGCTTGACGTTGTAGAGCAGTTATCCATAACTGTA
GCCCGTTCTACCGTTTGCAACTGTGTTCCCAAATTTCCACTCAATGACGTAGGCAGTGCCTCACTCAAAGA
TGGCGATCTGCTCAAAATGGTAGTTCCACTCAAAATTGCAGTTTCACTCAAACTTACTCTTGAATTTGGGA
CTACACTCATAGTTGCAGTTTCACTCAAACTTCCACTCGACGATGGCTCTCCGCTCAAAGCTCCGATCAAA
GATGGCGGCAGGTTAGCTGTACCACTCACGCCAGCATTACTCAACTTAACGGGATCAGTAACTGTACTCA
CATGTGCTTGACCAGTTTTTGACCTCTGCAACTCGTGCTCTAGCGCAGCGACTCTCTTTATCAAATCCATGA
CTTGCGTACTTGAGAGTTGCGTGCTTGGGCTTGGCGCTCCTGTCAATGTGCTGGCTCGTGCACTCACTCCC
GCGGCCACAGTGCCGGCAGGTTGTACACTTGTGTCCACATCCACGGTGCTGGGGCTTTGTGGTGCGCTAAC
AGCGGGAACTTGCATTTGTGATACAGTGCTCACCATTCTACTTGTCCGCTTTGGCTGCGTCCCACTCGCCG
TGGTAGCCTCCTGGCCATCGTTTAAGCGTGGGGTGACCGTTGTGTCATCCTACTTAGCTCGGAGATGGTTT
CTACTATATTTTACGCTGGCTGCGTACCAATGGTGACTCTGCCTGTGCTGTGTCTATGTACACACACTGTA
ACACCAAAGTTACACCACGTCTTGTCTTCAGCGCAAGTCGCCCTTGCAGTTTCTTACAATAAGAACTGCGA
ATAAAAGATCATGTCGCGTGCCAAACGAAGGTGGAGTTGCAATTTGAACACTGCAAGGTTTACGCCTGGC
CCCCGGCCATGTTACTCCGATGTTAATAGAGACAGCGTAATGCAAAACGTGCTAGTGCAAGTGTTTTATTC
TGAACGGATTACATAGGTTAGAACTTAGATATATATCCCATCTTA
>160_rnd-6_family-2344__LTR___Gypsy
ATATAGGAATATGGGGGGAAAACAATCAAAGGCAGATGATAACACTGCCAATGTAGTTAACGTAGTAGA
AGTCGTAGACACCACTCCCCATGTGCAGTTAACAAACATACTGCTAATAATACTCATTGTAATATCTTCAG
TAAACATCCTTCTGAAGATTTATCTTCTTCATAAAAAATCACTGAGGAAGCGTTACATGAGTCGAGCCAAC
GACTTAGACAAGGTCTAAGCCACTGCTTAAATAAAATATTTAAGTAGCGGAAGCTTACATGGACTGGTCC
CAAATAGTAGAAAAGATAAAGCGGTATAGAGAAAATTTCGATAAATCATACAAGTGCCTCAATAAAAGC
GCACCAGTTTTAAGACCCACCANAATNAAACATGCTGAGATCCTTGTGTATGAGTATAATGAAATCAGGA
GTCCTTGACGTTGTAAACGACAATGTCAACGTAGGCTGAGCGGTTTACATNTAGCCCAAACTTCAATTGAC
GTGTTAAAGAGTCTAAGGGATAATCTGGTAGACATAAATCGGAAAGGCATGATCTCCAGCTTTATCTNCC
ATCGGCGCTAAATACACCGGTAACATTCGANAAGTCACTNACTGACATCAGTAGAAGAAAACTTGANCAA
ATAGAAGANACAAACGATTAGTAAACATGGCACAAACAGCTGTAGAATTTATCAAGCTAGCCNCATCTCT
GATACCAGAGTTTGACGGTAAGCCGGAAAATCTACAAAGCTTCNTAGATGCTCTGAATCTGNTAGANNCC
CTAAAAGGNACACACGAAGCAACGGCCGTAAGCCTTATAAAGACAAAGCTAAAAGGCCATGCCAGGAAC
CTGATAAGTGATGAGCAGACAATTGCNGCAATCATTACGCAACTATCGGACGCAGTAAAGGAGAGTCG

GTAGAAGTTGTATCTGCTAAGCTTCTNAACTTGCAACAGAAGAACAAAACGGCTAACCAATACACCCAAG
AGGTGGAAAAACTGGCAAAGGCCCTTGAAGGNGCCTATATCAGTGAAGGTCTAAGCCAATCACTTGCAA
ACAAATACAGCACTACAACAGCCGTAAAAGCTATGACCCAAAATTGTTCGATTGATAAGGTAAAACTTAT
CATGCAGGCAGGCACATTCACAAATATGAATGATGCCGTCTCCAAATTTGTCAATAGTTGCACGGAGGTA
ACAGGTCAAAGTAACACTGTCCTGTATTATCGACGAGGTGCAAATCACAATAATAGAGGAGGCCGAGGTT
ATAATCGTGGCAGAAATTACAACCACAACAATTACAACCGAGGCGGTAACAACAACAACAATAATTACA
ATAACCGTGGAGGAAGACGAGGCCAAAATCGAGGAAGAGGCCGAGGTAACTCCAACCAAGGTNACAATA
ATAACAATGTGAGAGTGGCGCAAAATGCGTCGGAAAACTCTCAGAACCCTTTAGGAAACAACCAGTAAA
TGCAAAGGTTCATTCCATCAATTACAGTCTTAATATATTTGTCACTTTTTATAATCATTCAACTGAAAATAA
ACTCACATTCCTAATAGATACTGGTGCAGATATCTCACTTTTAAAAATAAATTCAGATAATTTCAAAATTC
AAGAAGACAAAATTATAAACATCCAAGGCATCGGCCAGGGTGTAATAAAATCGCAAGGAACAACTTCAA
TAGAACTTCAATCAACAAAATATATTATTCCACACGAATTTCATTTAGTAGACTCCGATTTTTCAATACCG
TGTGATGGAATAATAGGCATTGATTTTATTAAAAAATTCAATTGTCAATTAGATTTCAAACCAAGTGAAGA
CTGGTTTATAATTAGACCTCAAAACTTAAATTATCCTATTCATGTTCCGATAACATATAGTGCTGGCAACA
ATACAGTTCTCCTGCCAGCCAGATCCCAAGTAATTCGAAAAATAGACATTAATNCAGTAAACGATTATAT
ATTTGTTCCTAATCAAGAAATACATAATGGAATTTATGTTGCAAATACAATAGCAGCATCCAAACATGTAT
ACATTCGCCTTCTAAACACAACTAATTTCGACCAATTGGTCAAAGTAAACAAAATCCAATATGAAAATTT
AACAGATTACGACATTCATAACACTAATCCAGAAAATAGAAGCGAACAAGTACTTTCAAAATTAAAGAA
AAATTTTCCAGAACAATTCAAAAATCAATTAACAGAATTATGCACCAAGTATAGTGACGTGTTCGGACTG
GAAACCGAACCAATAGCGACAAATAATTTTTATAAACAAACATTAAGACTTAAAGATGATGAACCCGTTT
ATATNAAAAACTACAGAAGCCCGCATAGCCAAGTTGANGAAATCCAAAAACAAGTAGGGAAATTAATAA
ACGACAAAATCGTNGAACCGTCTGTATCCGAGTATAACAGCCCACTCTTGCTAGTTCCGAAAAAATCATT
ACCAAATTCGGAAGAAAAGAAATGGCGATTAGTAATTGACTATCGCCAAATCAACAAAAAACTACTTTCT
GATAAATTCCCGCTCCCCAGAATTGATGATATTCTAGACCAACTGGGCAGAGCTAAATACTTTTCATGCCT
TGACTTAATGTCAGGTTTCCACCAAATTGAACTTGAAGAAAATTCAAGAAATATAACGTCTTTTTCAACGA
GCAATGGCTCATATCGCTTCACGCGATTACCATTTGGTCTTAAAATAGCACCAAATTCATTTCAGAGAATG
ATGACTATAGCATTCTCGGGTTTAGAACCCTCTCAGGCATTCCTTTATATGGATGACTTAATGGTGATAGG
ATGTTCCGAAAAACACATGATTAAAAACTTAACTGATGTTTTTAATGTATGTAGGAAATATAACCTAAAGT
TGCATCCAGAAAAATGTTCATTTTTCATGCATGAAGTGACATTTTTAGGTCACAAATGCACTGACAAAGGA
GTATTGCCAGATGACAAGAAATTCGACGTCATCAAAAATTATCCTGTCCCNCATGATGCGGACAGCGCGA
GACGATTTGTAGCATTCTGCAATTATTATCGTCGATTTATAAAAAACTTCGCCGATTATTCACGGCACATA
ACTAGATTATGTAAAAAGAATGTTCCTTTCGAATGGNCAAGTGAATGCCAAAACGCATTCCAATACCTTA
AAGAAAAGCTTATGCACCCCACATTATTACAATATCCTGATTTTCGCAAAGAATTTTGCATTATAACAGAT
GCTAGTAAACAAGCTTGCGGAGCGGTTCTAACTCAGAACCGTGACGGAATTCAGCTCCCAATAGCTTATG
CATCTCGTTCATTTACAAAAGGGGAAAGCAATAAGAGCACAACAGAACAAGAGTTAGCGGCAATTCATTG
GGCAATTACCCATTTTAGACCATATATTTATGGCAAACACTTTACTATTAAAACGGATCACAGACCATTGA
CATATCTTTTTTCTATGACTAATCCCAGCTCGAAATTAACTCGCATGCGGCTAGAGCTTGAAGAATACGAC
TTCACAGTAGAATACCTAAAGGGGAAAGATAATTTCGTAGCAGACGCACTTTCGCGTATTAATATAAAAG
AACTCAAAGATATGCAACATAAAGTCCTGAAAGTCACTACCAGGCGACAAAGTAGACAAAATAAAAACT
GTACAGTAGAAAATTATGAACTTCTGCCAAGGCAAAGTCATAAAAATGTATCTAAGCCCAACGTACATGA
AGTCATTACNAATGACGAAGTACGAAAAGTAGTGACCTTGCGCATAACAGAATCTAAATGTTTATTTAAA
CATGGAAATAAAATTATCGCAAGAATTGAAGTTAGCGATTTATATACCAATGGAATTCTCGACTTAGGTC
AGTTCTTCCAAAGGCTCGAAAAAGAAGCCGGTATATTAAAAATCAGCCAACTCAAAGTGGCACCGAGCG
AAAAGATCTTTGAATCAGTTTCAATAGATTATTTTAAAAATATGGGCAATGAAATATTAAAATCTTTAAGA
GTAGCGCTACTCCAGCCGGTGACCCAAATAAAAGATGAAAAAGAGATACAACAAATATTGTCTACATACC
ATGACGACCCAAATTCAAGGAGGTCATAGCGGCATAACAAGAACGCTAGCGAAAATAAAAAGACACTAC
TACTGGAAAAATATGACACGTCATATAAAAGAGTACGTACGTAGATGTCAAAAATGCCAAATGTCTAAAA
CAAAGACGCACACAAAAACTCCTTTGACTCTCACAGAAACACCTGTGAATGCTTTCGATATAGTGATAGT
GGACACAGTTGGTCCGCTACCCAAATCGGAAAATGGTAATGAATACGCAGTCACATTAATATGCGACCTT
ACAAAATATTTAGTAGCCATTCCAATAAAAAGCAAAAGCGCAAATANTGTCGCAAAAGCTATATTCGAAA
ATTTCATACTCAAGTTCGGTCCAATGAAGACGTTCATCTCGGACATGGGAACAGAATACAAAAACCCGAT
TNTAANTGATTTGTGCAAGTATNTGAAGATCAAGAATCTCACCTCTACTGCACATCATCACCAGACATTNG
GGACAATNGAACGAAGTCACCGAACTTTTAATGAATACATNCGTTCATACATNTCNGCNGACAAAACTGA
TTGGGACGTNTGGATCCAATATTTCGCTTATTGCTTCAACACGACCCCATCNACGGCGCATGANTATTGTC

CATACGAACTAGTATTTGGCAAACATACTAATCTACTAAAANATTTNAATAGNACNGATAACATAGANCC
TATNTATAATNTAGATGACTACGCTAAGGAAAGNAAGTNTAGATTAGAAATAGCATACAAAAGAGCTAG
AATTATGTTAGAANAAGACAAGGCGNAATAGGAAATTAGCATATGATNAAAAAGCNAAAAANTTTGAAT
TANAAGTAGGAGATAAAGTACTTATTAAGAAACGAAACAGGACACAAATTAGACCCAGTATATTTAGGA
CCATATATAGTAGAAAAAAATAGAAGATAACGATAACATATTATATTAAAGATAGTAAAAATAAAAAACA
AANAGTACATAAGGATAGATTAAAAATATTTTATAAT
>160_rnd-5_family-168__LINE___CR1
CTCTTCAATTTTCACCGCCAAATACACCACTTATAGATGTGACCGAGCTTTCCGTATCGGAGAAGGAGTGT
AGTATGTGCATATATTGAGGGTACACTGTACCAATAAAGTAACATCGGTTAAGCATAAATATATCAGCAA
CACATTGTTGCAGTACATAAACAAAACCATTTCAAGTGAGCGTGTAATATGATCACCCGCTTATGAGCTA
AGCTAAGTTAGCAGTCCCACGCTGANCACNTGTAGTATATATATATATATATATATATATATATTCATATATG
TACGTAAGACTCTCTCTCTCCCGATACTGCTGATAAGACAAAATATGTATGAAGCCGCTCCGCTGCTGGCG
TAACCGGCAGCGCAGCTATGCGGCCCTAGGCATAAGAATATATCGAATAAAACATTCTAAAAACTAAATT
CAAACGGCACAGACGTGCCCGTGTATTAAACTATTTGAATCTATCTCATGGCGACCGTGACAGTGGTTTTG
GTAAGAGGTTCTAGTCGGGAGCTCCCGACTAGGGGATACCTTGAACCCTCTTCTTCCAACATCAAATGCAT
ATATATATTTTATTTTAGAAGCTATATGTCAAGTTTGGTGACTTTAGCTCTTATTATTTACCAAAATCGCCC
AAAAAACAGGATATCGATATCGATANATTTTTATCGATTGCTTGGAAACGGAGTAAGCTAGCGATTATCG
AAAATAAACTCGATCTGCGCGGGAACTAGCAGCACTTACATTTCTGTTCAAATCGTTCAAATTCTGAATCT
GAATTGTCTTTTGGTCATCTACATCATCTAATTCTCTGGATGCGTTAACTAACTCCTTTACTAATGTTGGCA
ATTGTTACGCCAAAATTTGGCTTACTGTGGCTGTTATTATCTGGTCAGTCTCTATTTTGTAAGCCAAGATGT
CATTATTGTAGTCTGTTTATGCTATGTCCGCTTTACTGGTATTTGTCTACTTGGAGATCTAATCAAATTACG
TTCATTAGCCATTAAATTTGTGTAAGTTTGTTTTATTCTATATTTTCTCTTGATTGTTTTTCATTTGAAAACC
AGTGGCTTCCTTTTTTTTACTTTGGAGGGTCTCTTACGAATCGTAAAGCTTAAATTTCGTTATGCTACGTTT
GTCATTTTTATATTTCATTATCGTTTTAATCTTACATATTTTCCTTTTTTTCTTTTTGTTTTAAAATATTTGCA
TTGTTGATAATTTTTCTTTAAACGCTGAACCCATTAAAAATGGGCATAAGGGTATATTGTATTTGTGCAAA
ACCCAAATGTATGTAACAGGCAGAAGAAAGCATCTTCGACCCCACAAAGAACACATATTCTTGATCAGCA
TCAAGAGCCGTGTCGTTCATGCCATGTCCGTCTGTCCGTCCGTCCGTCCATCCGTCCGTTTGTATGAACGC
AAGGATCTCAGAACCTATAAGAGCTAGAGCTTGAAATTTTCGATGTAGGTGCTCCTAGTGCCCGCGCAGA
TCGAGTTTTTTTCTGCTAATCGGGAGCTCCCGATGTCAAGTTTGGTGACGCTAGCTCTTATTATTTACCAAA
ATTGCCCAATAAACAGGATATCGATATCGATTTTTGTCGATTGTTTGGAAACGGAGTAAGTAACGGAGTA
ACGGAGTAAGAAGACTGATTGGTTGGTATGACACGGGGTCATCCTCAGGTTTGCCAGGCTTATGGATCAT
TAATATTCTCGTCAGCCGCCATCTGATTGGAAAGTACTGTATCCTTAGTATAGTATTAAAAATCAATACAA
TTAAGAGAAACGCTCTTTTCGGCAAAGCCCTCTATCTCTATCTCTATTTCATGTTCATATATACCCCCTCTA
TATAACTTCTTAATGCTATTAAGTCTATCTCTTCCTTATTTTTTGATAGGGATCCTTTAGTTATCCTTGGGCA
CTTTGGTTATCCAAATATCGATTGGTCATCAGCATCGGACTGAAACTTTCTGTTGCCGCCTTGATCTTTCCT
GCTCAGAGATTGAGTTGGTTAGTTAGGTGGTGCATCGTTTATAATAAGTGTTCCGCCAGAAATATTTACTA
ACATTTATTGATAAGAGTCTTGGCCGGAACAATTAATAGTCAAAGAATATAAGGACAAACAGATTAATCG
GATTCCGATTTGATTGCCACCTGGTTCTAGTGGCGCATCAACATTAATTGCTTTTAATTTTTAAATAATTGT
TTTTACTTAGCCCCGGCTAAATCCGGACATCTCAGACTCTTCGTGATCGAGCTTCCCGTATTGGAGAAGGA
GTACTCATAGCTGTCGAGTCTGCTTTTTCGTCGGAACGCATTGATCTTCCCATCTCAGAGATTGAGTGGGT
GGCTGTAAGAGTTTCATTTGGAAGTTTTTCTATCTCTATTTCCTGTTCATGTTTTAGGTACCACTGCTAAAA
GTGTTCCCACTGTCCTACCTGTGGCGCCTAAGCCTCAAGTGGGTGTTCACACCCGAAGAGACATATTTTTG
TTTCTNGGCTTGCCCCTCACTTCTCATCAGATGATGNNAAAGCCTTTGTACAGGCAAAAATTCAAGCCNCA
GGTGNAACGGTGTAGAAATTNAAGTTCGCCTATGGAAGGTATATATTGCGTCCTTTATAATAAGTGTTCCG
CCAGAAATATTTACTAACATTTGTTTATAAGAGTCTTGGCCGGAACACTTAATAGTCAAAGAATATAAGG
CCAAAAAGATTAATCGGACTCCGATTTTATTGCCACCTGGTTCTAGTGGCGCATCAACTTTAGTTGCGTCA
AAAAACTAAATTCTTCTTTCGAGCTTGCCTACCAGAATGNTAGAGGTCTTAAGTCTAAGATCGTTAACATG
TATTTAGATTCTCTTGCNTTTAATTTTCAAATTATCGCTTTNACTGAGCCCCGGCTAAAGCCNGACATCTCA
GACTCTTCAATTTTCACCGCCAAATACACCACTTACAGATGTGACCGAGCTTTCCGTATCGGAGAAGGAGT
GTAGTATGTGCATATATTGAGGGTACACTGTACCAATAAAGTAACATCGGTTAAGCATAAATATATCAGC
AACACATTGTTGCAGTACATAAACAAAACCATTTCAAGTGAGCGTGTAATATGATCACCCGCTTATGAGC
TAAGCTAAGTTAGCAGTCCCACGCTGACCACTTGTAGCATATATATATATATATATATATATATATATNCATA
TATGTACGTAAGACTCTCTCTCTCCCGATACTGCTGATAAGACAAAATATGTATGAAGCCGCTCCGCTGCT
GGCGTAACCGGCAGCGCAGCTATGCGGCCCTAGGCATAAGAATATATTGAATAAAACATTCTAAAAACTA

AATTCAAACGGCACAGACGTGCCCGTGTATTAAACTATTTGAATCTATCTCATGGCGACCGTGACAGTGGT
TTTGGTAAGAGGTTCTAGTCGGGAGCTCCCGACTAGGGGATACCTTGAACCCTCTTCTTCCAACATCAAAT
GCATATATATATTTTATTTTAGAAGCTATATGTCAAGTTTGGTGACTTTAGCTCTTATTATTTACCAAAATT
GCCCAAAAAACAGGATATCGATATCGATAAATTTTTATCGATTGCTTGGAAATGGAGTAAGTTAGCGATT
ATCGAAAATAAACTCGATCTGCGCGGGAACTAGCGGCACTTACATTTCTGTTCAAATCGTTCAAATTCTGA
ATCTGAATTGTCTTTTGGTCATCTACATCATCTAATTCTCTGGATGCGTTAACTAACTCCTTTACTAATGTT
GGCAATTGTTACGCCAAAATTTGGCTTACTGTGGCTGTTATTATCTGGTCAGTCTCTATTTTGTAAGCCAAG
ATGTCATTATTGTAGTCTGTTTATGCTATGTCCGCTTTACTGGTATTTGTCTACTTGGAGATCTAATCAAAT
TACGTTCATTAGCCATTAAATTTGTGTAAGTTTGTTTTATTCTATATTTTCTCTTGATTGTTTTTCATTTGAA
AACCAGTGGCTTCCTTTTTTTTACTTTGGAGGGTCTCTTACGAATCGTAAAGCTTAAATTTCGTTATGCTAC
GTTTGTCATTTTTATATTTCATTATCGTTTTAATCTTACATATTTTCCTTTTTTTCTTTTTGTCTTAAAATATT
TGCATTGTTGATAATTTTTCTTTAAACGCTGAACCCATTAAAAATGGGCATAAGGGTATATTGTATTTGTG
CAAAACCCAAATGTATGTAACAGGCAGAAGAAAGCATCTTCGACCCCACAAAGAACATATATTCTTGATC
AGCATCAAGAGCCGTGTCGTTCATGCCATGTCCGTCTGTCCGTCCGTCCGTCCATCCGTCCGTTTGTACGA
ACGCAAGGATCTCAGAACCTATAAGAGCNAGAGCTTGAAATTTTCGATGTAGGTGCTCCTAGTGCCCGCG
CAGATCGAGTTTTTTTCTGCTAATCGGGAGCTCCCGATGTCAAGTTTGGTGACGCTAGCTCTTATTATTTAC
CAAAATTGCCCAATAAACAGGATATCGATATCGATTTTTGTCGATTGTTTGGAAACGGAGTAAGTAACGG
AGTAACGGAGTAAGAAGACTGATTGGTTGGTATGACACGGGGTCATCCTCAGGTTTGCCAGGCTTATGGA
TCATTAATATTCTCGTCAGCCGCCATCTGATTGGAAAGTACTGTATCCTTAGTATAGTATTAAAAATCAAT
ACAATTAAGAGAAACGCTCTTTTCGGCAAAGCCCTCTATCTCTATCTCTATTTCATGTTCATATATACCCCC
TCTATNTAACTTCTTAATGCTATTAAGTCTATCTCTTCCTTATTTTTTGATAGGGATCCTTTAGTTATCCNTG
GGCACTTTGGTTNTCCAAATATCGATTGGTCATCACCATCGGACTGAAACTTTCTGTTGCCGCCTTGATCTT
CCCTGCTCAGAGATTGAGTTGGTTAGTTAGGTGGTGCATCGTTTATAATAAGTGTTCCGCCA
>9_rnd-1_family-420__RC___Helitron
GATCGGACCACTATATCATATAGCTGCCATAGGAACGATCGGTCGAAAATTAAGTTTTTTGTATGAAAAA
AACATTTTTGTTTTTCAAGATATCTTGACCAAACTCGGCACTTATTAGTTTTACTNTACTCCTCATATATGT
GNAAAATCCTATTAAGATCGGACCACTATATCATATAGCTGCCATAGGAACGATCGGTCGAAAATTAAGT
TTTTGTATGAAAAAAACATTTTGTTTTTCAAGATATCTTGACCAAACTCGGCATTTATTAGTTTTACTTTAC
TCCTCATATATGTGCAAAATCCTATTAAGATCGGACCACTATATCATATAGCTGCCATAGGAACGATCGGT
CGAAAATTAAGTTTTTGTATGAAAAAAACATTTTNGTTTTTCAAGATATCTTGACCAAACTCGGCATTTAT
TAGTTTTACTTTACTCCTCATATATATGCAAAATCCTATTAAGATCGGACCACTATATCATATAGCTGCCAT
AGGAACGATCGGTCGAAAATTAAGTTTTTGTATGAAAAAAACATTTTGTTTTTTCAAGATATCTTGACCA
AACTCGGCACTTATTAGTTTTACTTTACTCCTCATATATGTGCAAAATCCTATTAAGATCGGACCACTATAT
CATATAGCTGCCATAGGAACGATCGGTCGAAAATTAAGTTTTTGTATGAAAAAACATTTTGTTTTTCAAGA
TATCTTGACCAAACTCGGCATTTATTAGTTTTACTNTGCTCCTCATATATATGCAAAATCCTATTAAGATCG
GACCACTATATCATATAGCTGCCATAGGAACGATCGGTCGAAAATTAAGTCGTATGAAAAAAACATTTTT
GNTTATCAAGATATCTTGACCAAACTCGGCATNTACTATTTTCCCCGTACTTCTTAGATAGGGGCAAAGCA
CTATGAGCATTATGAAAAGGTTGGGTCTGCAAGGGTATTAGATCTTTGGCGTGCCGAAGATAGCCCTTCTT
NCTTAAATTTTTTTATATTCCNTTTTTATACCCTTGCAGAGGGTATTATAATTTTGTCGTGAAATGTGTAAC
GCATAGAAGGAGACATCTCCGACCCCATAAAGTATATATATTCTTAATCAGCATCAACAGCCGAGTCGAT
ATAGCCATGTCCGTCTGTCCGTCTGTCTGTTTCTATGCGAACTAGTCCCTCAGTTTTAAAGCTATCTTAATG
AAACTTTGCAGAACTCCCTCTTTCTGTTGCACGCAGCACATATGTGAAAAACCAGCTGGATCGGACCACTA
TATCATATAGCTGCCATAGGAACGATCGGTCGAAAATTAAGTTTTTGTATGAAAAAAACNTTTTGTTTATC
AAGATATCTTGACCAAACTCGGCATTTATTAGTTTTACTTTACTCCTCATATATATGCAAAATCCTATTAAG
ATCGGACCACTATATCATATAGCTGCCATAGGAACGATCGGTCGAAAATTAAGTTTTTGTATGAAAAAAA
CATTTTGTTTTTCAAGATATCTTGACCAAACTCGGCATTTATTAGTTTTACTTTACTCCTCATATATGTGCA
AAAATCCTATTAAGATCGGACCACTATATCATATAGCTGCCATAGGAACGATCGGTCGAAAATTAAGTTT
TTTGTATGAAAAAAACATTTTGTTTTTTTCAAGATATCTTGACCAAACTCGGCATTTATTAGTTTTTACTTT
ACTCCTCATATATATGCAAAATCCTATTAAGATCGGACCACTATATCATATAGCTGCCATAGGANCGATCG
GTCGAAAATTAAGTTTTTGTATGAAAAAAACATTTTTTTTTTCAAGATATCTTGACCAAACTCGGCATTTAT
TAGTTTTNCTTTACTCCTCATATATGTGCAAAATCCTATTAAGATCGGACCACTATATCATATAGCTGCCAT
AGGAACGATCGGTCGAAAATTAAGTTTTTGTATGAAAAAAACATTTTGTTTTTCAAGATATCTTGACCAAA
CTCGGCATTTATTAATTTTTACTNTACTCCTCATATATGTGCAAAATCCTATTAAGATCGGACCACTATATC
ATATAGCTGCCATAGGAACGATCGGTCGAAAATTAAGTTTTTGTATGAAAAAAACATTTTGTTTTTCAAGA

TATCTTGACCAAACTCGGCATTTATTAGTTTTACTNTACTCCTCATATATATGCAAAATCCTATTAAGATCG
GACCACTATATCATATAGCTGCCATAGGAACGATCGGTCGAAAAATTAAGTTTTGTATGAAAAAAACATT
TTTGTTTNTCAAGATATCTTGACCAAACTCGGCATTTATTANTTTNCTNTACTCCTCATATATATGCAAAA
CCCTATTAAGATCGGACCACTATATCATATAGCTGCCATAGGAACGATCGGTCGAAAATTAAGTTTTTGTA
TGAAAAAAACATTTTGTTTTTCAAGATATCTTGACCAAACTCGGCATTTATTAGTTTTACTTTACTCCTCAT
ATATATGCAAAATCCTATTAAGATCGGACCGCTATATCATATAGCTGCCATAGGAACGATCGGTCGAAAA
TTAAGTTTTTGTATGAAAAAAACATTTTGTTTTTCAAGATATCTTGACCAAACTCGGCATTTATTAGTTTTA
CTTTACTCCTCATATATATGCAAAATCCTATTAAGATCGGACCACTATATCATATAGCTGCCATAGGAACG
ATCGGTCGAAAATTAAGTTTTTGTATGAAAAAAACATTTTGTTTNTCAAGATATCTTGACCAAACTCGGCA
TTTATTAGTTTTACTNTACTCCTCATATATATGCAAAAATCCTATTAAGATCGGACCACTATATCATATAGC
TGCCATAGGAACGATCGGTCGAAAAATTAAGTTTTTGTATGAAAAAAACATTTTGTTTTTTTCAAGATATC
TTGACCAAACTCGGCATTTATTAGTTTTACTTTACTCCTCATATATGTGCAAAATCCTATNAAGATCGGAC
CACTATATCATATAGCTGCCATAGGAACGATCGGTCGAAAATTAAGTTTTTGTATGAAAAAAACATTTTTT
TTTTTTCAAGATATCTTGACCAAACTCGGCATTTATTAGTTTTACTTTACTCCTCATATATGTGCAAAATCC
TATTAAGATCGGACCGCTATATCATATAGCTGCCATAGGAACGATCGGTCGGAAAATTAAGTTTTTGTATG
AAAAAAACATTTTTTTTTATCAAGATATCTTGACCAAACTCGGCATTTATTAGTTTTACTTTACTCNTCATA
TATATGCAAAATCCTATTAAGATCGGACCACTATATCATATAGCTGCCATAGGAACGATCGGTCGAAAAT
TAAGTTTTTGTATGAAAAAAACATTTTTGTTTTTCAAGATATCTTGACCAAACTCGGCACTTATTAGTTTTA
CTTTACTCCTCATATATATGCAAAATCCTATTAAGATCGGACCACTATATCATATAGCTGCCATAGGAACG
ATCGGTCGAAAATTAAGTTTTTGTATGAAAAAAACATTTTTTTTTTCAAGATATCTTGACCAAACTCGGCA
TTTATTAGTTTTACTTTACTCCTCATATATATGCAAAATCCTATTAAGATCGGACCACTATATCATATAGCT
GCCATAGGAACGATCGGTCGAAAATTAAGTTTTTGTATGAAAAAAACATTTTGTTTTTCAAGATATCTTGAC
CAAACTCGGCATTTATTAGTTTTACTTTACTCCTCATATATATGCAAAATCCTATTAAGATCGGACCACTAT
ATCATATAGCTGCCATAGGAACGATCGGTCGAAAATTAAGTTTTTTTGTATGAAAAAAACATTTTGTTTTT
TTCAAGATATCTTGACCAAACTCGGCATTTATTAGTTTTTACTTTACTCCTCATATATATGCAAAATCCTAT
TAAGATCGGACCACTATATCATATAGCTGCCATAGGAACGATCGGTCGAAAATTAAGTTTTTGTATGAAA
AAAACATTTTTNTTTTTCAAGATATCTTGACCAAACTCGGCACTTATTAGTTTTACTNTACTCCTCATATAT
ATGCAAAATCCTATTAAGATCGGACCACTATATCATATAGCTGCCATAGGAACGATCGGTCGAAAAATTA
AGTCGTATGAAAAAAACATTTTGNTTATCAAGATATCTTGACCAAACTCGGCATATACTATTTTCCCGGTA
CTTCTTAGATAGGGGCAAAGCACTATGAGCATTATGAAAAGGTTGGGTCTGCAAGGGTATTAGATCTTTG
GCGTGCCGAAGATAGCCCTTCTTTCTTTCTTTTTATACCCTTGCAGAGGGTATTATAATTTTGTCGTGAAAT
GTGTAACGCATAGAAAAAGGTGACATTTTCGACCCNATAAAATGTATATATTCCTTATCCGCATGGACAT
AGGAGTCCGTATTGTATTAATATTTTAAGCGTACGCAGTACTATGTTCAAGTTTATATTATATCTATTCTAA
AACTCTACAAGATTAAAATATATTCATAGATATCGATATCGATAAAAATATTTTTTTTTATACCCTGAACC
CATTAAAAATGGGTATAAGGGTATATTGTATTTGTGCAAAATCCAAATGTATGTAACGGGCAGAAGGAAG
CATCTCCGACCCCATAAAGTATATATATTCTTGATCAGCATCAATAGCCGAGTCGATCTAGCCATGTCCGT
CTGTCTGTCCGTCCGTCCGTCCGTATGTATGAACGCAAGGATCTCAGAACCTATAAGATCTAGAGACGAA
ATTAGATGTAGGTGCTCCTAGTTCCCGCGCAGATCGAGTTTGTTTCCGATAATCGATAACTTACTCCGTTTC
CAAGCAATCGATAAAAATCGATATCGGCATCCTGTTTTTTTAGCAAATTGGGTAAATAATAAGAGCTAGA
GCCACCAAA
>Ref_dvir_190
TCACCCCACAAGTTCGCTCTCTGTACACCCCGAAGCAGAATGCGAGTGCCGTGATGAATGTCTGCAACAG
TGAGTCCATGATACGCTTCATAAATTTGTACACATAGCCGAATGTGCGCTGCATGGAACCAAAAGAGTTG
GCGTGCTTGCATCCCAGAGCTAAATCTGCATGTGGAGACTTAAGAATTAGTACACTTTTCCTCAATTCAAT
GGTTGGAGCTTTAGGCAGACAGGTCGCCGGCCAATTTAGGCGACCATTTAATAGATACGATGGGCCATGA
AACCAGAGCTCCGACTGGGCTAGGATGTCGGGAACTGTGCCTCTAGAAAGAATGTCAGCTGGATTTCGGT
CCGTCGGAACATATCGCCACTCCATACTGGCAGTGAGTTCTTGTATGGCAGCCACACGATTTGCCACGAA
AACGTTAAATTTGAAGGGCTCATCTCTTATCCAGGATAGGACAACGGACGAATCGCACCAGCAATAAAAC
TGACTGACAAAGATGCCTGTGCGCTTGACTTCATGCATCAGACTAGCCAACAGCTTCGCTCCACACAATTC
CAGTTTTGGGACAGTCAATGTTTTTAATGGCGCAACTCGAGACTTGGAGCAAAGGATACGACTTGTGGCT
AGTGGACTCCTGGCCACTACGTAGATGCAGGCTCCATAGGCCTCCACACTGGCATCGCAGAATCCATGTA
TTTCAACATCAGCTCCTTCGAAGAGTACTACTCTGGGAAACGATACATGGCGAATGTTGCGAAAACTGCT
GCATAGCTCCCTCCAGGTCGTATCACGTGCAATCGGCAAGCTTTCATCCCAAGTTAATTTGTCCTTACAAA
GCTGTTGAAGAAATATCTTTGCCTTAGTTATGACTGGTCCCAAAAGTCCAAGCGGATCATAAAACCGGGC

AACAATAGACAGAACTGATCGCCTGCAGGGCTTAACAGTACAATCTAGAACAGAAAATGAAAATAGCAA
CTGGTCGATCGCTGGATCCCATACAAGACCAAGGGCCTTGGTGACATCGCTTCCATCATCGAACTTTAAGT
ACTGTTCCCTATCTTCATAAGCTATTCCATTGAGTACTTCTTGGTGACTGGAACACCACTTTCGAAGCTTGA
AGTTTCCACGAGACAATATCCCAGCCGTCTGACGAATTTTCTCTCTGGCTTCAGCAACTGAGTTGGCTCCG
GATATCAAATCGTCAACGTAAAACTCCTGCTTGATGGCATCCGATCCAATTGGAAATGCATCACGCTCATC
AAAGGCCAACTGGTGGCATTGCGCGCACGGACTGAAATGATGCTGATTTGGTCCCATATGTTACTGTATCT
AATTTAAAGATTTCGATATCCTTCTGAGGCGAGTCCCTCCACAGGATTGCACTGGAAGTAACTATCAGGCG
AAGTCACTCGGACACAACGATACATTTTGCAAATATCAGCTGTGAGAGCAACAGGATATGTGCGGAAGCG
AATCAGAATGTGAAACAACTTAGGTTGAATGATTGGTCCCGCCATCAGTACATCATTAAGAGAATGTCCA
GAATCAGTAGTTGCTGAGCCATCAAACACAACACGCAACTTGGTTGTGGTGCTGTCTTCTTTCAAGACACA
ATGATGTGGGAGGAAGTATTTACAACAGCCACTCAACTCTGGACTAACTCGCGACATGTGATTTAGATCC
AGCTCTTTGATGAATGCCGAATACTGCTCCTTCAGATGCGAATTCCGACCCAATTTTCGCTCAAGACTGAG
AAATCGAAGATGAGCCTGGCCATAAGACTCTCCTAGAAGCTCTGCACTCCGCTTCAGAGGCAACCGCACT
GAATACTCGCCGGATGGTAAACGGGAGAAGTTATTGGCAAAATGCTGCTCGCATTCCGCCTCTTCTTTGGT
GGCCTTTACTGTAGGCTCTTCCAAGCTTTCAATTTCCCAAAACCGCCGAACCAACTCATCCAGACGGACGT
CCAACTGACTAGCTTCGCTATTAGGAGTGCGGCCGGCAGCCAACAACGAGGTACTGCGCGGTCTCTCAAA
TCCACCGGTCACGACCCAGCCAAGACGAGTTTTTTGCAGAAGAGGTAGTCCTGGCAAAAGCACAATTTGC
CCAACAGACAAAAGCTCATAGAAAAGGCTCGCTCCAATTAAGAGATCCACTCGCTGTGGTTTGTTGAACG
CAGGATCAGCCAACCTTATGTTCGAGGGAATTTTCCAGTCTGATGTATCGATGACGAAGCTGGGCTGACT
ATCGGTAATTGTTGGAGTCACAAGAGCATCAAAGGTGGCTGAATAATCTGAGTCTTGTGACTTCATGTTAA
TCCTAACAGCTGATCCTTCAGTAGAGACGCTTGTGTCTCCGAGACCCATGACAACTGTCGACGACCTGGTT
TTCTAACTGAAGTCTCAAACGGGATGTGACCATGTGCAACTGCGAGCCTGAGTCTAATAAGGCCCTACAG
GGAAACAAGGAACCAGAACTATTCTTTACCAGTACCACTGCAGTGGCCAGGAGCACAACACCAGACTGA
AGACCTTGGACAGCAAGAGAGGTAGTGGAGTGAGAGTGCGTCGAAGTGGCGGCGCCCAAATCAGCCGAT
GTATGTAATGGTGTCGGCGCTCAGCTTCTCTTTTATGCTTTCTCCACAAGTACGACAATGACTACTACTGC
AAGACCGAGTTTGGTGTCCCTTCCTTAGGCAGTTATAACAAAGGGCCAGTTTCTTGGACTTCCTTATGCCG
AAGGGATGGAGACAAATTTCCGAATTTACTACATGTGTAGATCCCGTGACCAGACGCATCACAAAAACTG
CACCCATTTTGGTTAGTCTCAGCTACAAGCACTCTTTTGCTGTGGTTAGTGTTATGGTTGGATTTGCCCACC
TGATAATTGGTTATATGGGATGACATGGCATGTTGCACGCTCTCCAGCCTTTGACTACGCCTTTCCAGAAA
TGTGAAGAACTCATCGGCTGTCGGGATTTTGTCAATGCCTGCGGCCTCCTCCCATTTGGTCTGAGTCTGGG
TGTCCAACCTTTGAAGCAGCGTGTGGACTATTATGCATCCCGAAATTTGCTCCAAACTGCCCAAATTCTGT
AAGGCACGCATATGCGAATTGACCTTGTCCGAGAACTCACGCAGTTTTGAAGCCGACTCCGAATCTACCC
TCTTGAGCCCAAGAATCTCATTGATGTGTGCCTGGAAAACAAGACGTTTGTTACTAAATCTTCTATTTAGG
ATATCCAGAGCCACAGGATAATTGGCACCGGAAAACTCCAAAGATCGAACCGAATCCAACGCAGGGCCA
GACAAACATGAACGAAGATGCTGCAACCTTTCAATGTTAGACAGCTCGGTATCCTTGTCGACAACTGACT
CAAACATCGCGATAAAGTCCGAATATTCGGAAAACCACCGGAAAATGGTAATTTGAGCGCGTTCTGTTGG
CTAAGATCAAGGTTTGGTTTGGCAAGACGAAGAACGGTTGAGTGGGGCGGGCCGTTAGATTGACGACGCT
GAATATTTACGCGAACGCGAGCTTCAACCATGGCGATCAACTCGTCAAAATCATCGCGTAGTTTGCTGCC
ATTTCATCGAAATTAAGCTCCTCTAGCGAGTTATGCGCGGTATTAAAGGCAGACTTAGTTTGTTCAATTAG
CAAAAGGCGCACTTCTAAGTCGACGGCACAAGAGTTGTTAATTTTATCCTCAGATAATTCATTTTCAATAC
ACTTGGCTCGCTCAAACAATGCAACCGCTTTGCGCTTAAGAAAAGACACCCTGTTTGACTGCATATCATCT
GTGTTCTCGCCTTGCATCATTTTTTCACAAGCACAAAATCAGAACACCAAACTGAGCACACAACGTGCGA
CGAATTGTGCTGCTGCTTATGTACAAGCGTATGTATATAAGCTGAGCCGGCGAATGACCGCGCAAGGGGA
AGAGCGCGAAAGTCAACTAGCGGCGTTTATGTATATGCGAATATTTGGGAGCTGTAAATGGTCAGCTCAC
ACAGGCGGTTTGCACTTGCCTAATTATAACAATTTACGATGATTATGTTCTACTTGAATAATCACAATTTT
AATTTGCAATAGATTTATTTTGCTTGTGTGCACTGCAATGTTAAATCTCGCCGGGGCCTCCAAATGTTGAG
TTACTAATGTTTTTTAGCAACTGTTTAGAACCTAAACCAAGAAATAAGTAAATTTTGGCCTTTCACAACAA
CACGTTATAATTCAGTTTATTAGGCAAATTTATGTAGATTATAAGGTTATTTGGCTGTGGCTCAGAATTGG
TTCTTCGCTTCGCCTCGATCAACAACGAACTGAGAGAGCAGACAAAAAAGGAATATGCAATTTATAGCAG
CTGCGACAAGTGCAAGAAAGGCTTGCCAACCTTGTTTATAATAAACAGCTGTTCCACTAGACCAAAATTA
CAACCTCTTCTTATGTTTACATGAATGTTTGCTTATGCTAATTTGATGAGGCTGCTGCAGAATCCAACATAC
TCCCCCCGTTGGAAGCCTGACTTTCAACAGAATCCTTAAGGGGCAGCAGACACAGCTTGTTTACGGCTCTT
CGGATGATTCCAGCCGTGGTCTTCATATCAGCTACACGATTGAGTTGATGCACAACGCTTGTGTTTGAGTG
CTTTAGCTCGCTGAGCTGCGTCACACGCTGCAGCCATCCAAACGGTCGAGTTATTTGGTTCTGAAAGCGAG

AGTAATGGGCGTGAGTTAACAATAGCTGTGATTTGGCAAACAAGAGTACGAAGCTCTTCAAAAGTAACTA
AAGAGCGGCCGACAGAGTGATAGAAATGGTGCTTGGCCAACTTCACTGCCGCCTCCCATAGTCCACCAAA
GTGCGGGAGAACAGGGG
>9_rnd-1_family-277__LTR___Gypsy
TGTGAGGCGGCTAATGAGACCAGCCACCCCACAGTTTTCACGGCAAGGAGCCGTCATCCCTTTGGCCAAA
TTACGGCAAGGAGCCGCTGGCGGGACAACGTATTAATTTTCTTTAGTTATAAGTATTAATTATATTAGGTT
AAGATCTTAGTGAAATAAGAGGCAAGGAGCCATCAGTAATATAAGCAGTTCGATTAGTAGCAAAGAGGC
AAGGAGCCGCGAATATTATAATTGATTTAAATAGTGAGTTTGAATTTAACCGCCGGCCGCCGCTGCAACC
GTGCGTTGTCGATCGGTTGCTCAACACCGTCTTTGAGAGCGCAACTTCAATTCGGCGGCGAAGAGCGAAA
GCTCCGAGGCGAAGTTGCGAAAGCTCGCGGCGAAGTTGAGCAGCCGAAGCGAGCGCATGGCTTGCCGGC
GAGCGTGCCGGCACTTGTGATCGAACGTTAAGGGTGAGTAGAAGTGCTGCGCGAGATAAACGAAATCAT
AGATGGTATAGTGGAGCGCTAGGGCTCCAGAAAACAGTGCGGCAGGGTTTAGCTTAGGATTTACGATAAG
GTCGGAATGAATAATAGTGAATAAAGTGCCAACGTTGTAATTATCTAGAAAGTATTGGATTATTCCCTGTT
TCCTGGTAACCCGGCTTACCTTAACATCGGAACTAACCGGTGTTCGACCTAACCTCGAACGAGGGGCATG
CGATACGAGATTGTGGCTCCGGGGTGGGCATTGCCGGTTGGTGTTTAGGTAGTGGAGTTGGTGCGAGGCC
ACGGCCCGCTAGTGTAGTTGGTGCGAGGCCACGGCCCGCTAGCATAGTGGTACGAGGCCACGGCCCGGCA
GAAAGATAGAGCGTTTAGGTAGTGGAGTTGGTGCGAGGCCACGGCCCGCTAGTGTAGTTGGNGCGAGGC
CACGGCCCGCTAGCATAGATGGTACGAGGCCACGGCCCGATGGTGTAGGTGTGTAGAGGTAGATCTGGGT
TTGGTTTGGTCAGGATACCCCCCCCCTTTAAGCCTCTCTCCCAGCGAAATTATAAATAACTTGTAACTCGC
TAGACCTGGTAAGACTGAGAATTATACCCCGGCCAGAAGCTTCCTTACAAGTGGCGCCCGAGCAGGGACC
AAGAGGCGTTTAGGGGTGTGGCATGTCGGCGGATTAAAGCCCGCAAGCATGCCCGAATAGGTATTTTCCA
AAAAAAATANATATATAGGGTATTTCTAGTCGGGTATACCCTAGAGGGAAGGTAACCTAGCAGAGAGCA
AGAAAATGAGAAGGTCATGTATGTGCGAAATATGAAAATAGGGTATTAAGTAGTCCGGTATACCCCAGA
GAACATTCCTATACCTTGTGTTAGGCATAGCCAGAAAATGAACACGTGCAGCTGGTGAGTGACAAAAATG
CTAGCAGCAAGCCGGCAAGTAAGATAAAGAGACGAAAACATAACGGTCGGTCGTGAGAGTACAGCGCGC
GCGCCAGCCGGGCGACGTCGAGACCACGCTCGCACACGGGTGCCCACACGCGCGGCGCAGTCGACGTCG
GCGTCGAAGGACCGTTAAGGCGTCGCGGCAGCGGTTGTACAGTGGGGCGAATCCAACTCACGCGCGGTG
AAAAGTTGAATTCGTGAGTACAGCTGTCGGGGCAGTGAAGTGGTCACCCTTCAAGTGAATGCGAACAAAG
GGAACAATAAGAGCGGAACAAGTGCCAAATAAAAAAATAAGTGGATAATAATAAAATTTTTCTTTTATAA
ATAAAAATTAATTAAACAAAAAAATAAATAAAAAAATAAGTGGATAATAATAAAAATTTTCTTTTTATAA
ATAAAATTAATTAAACAAAAAAATAAATAAAAAAATAAGTGAATAATAATAAAATTTTTTTTTATAAATA
AAATTAATTAAACAAAAAAATAAATAAAAATGAGTGGATAATAATAAAATAAAAATAAACCGAGGTTCA
CAATAAAGATAAAGGCAGCGACCACATAAGGAACGGCCTAAAAAGCGGAAAAGTGAGAAAAGAAAAAT
TTTGAAATACCTTTTGTTGAGTTTTTTTACAAAAAAAGGGGGTAAATATTTTTGTGGAATATTTTTTTGTNT
TTTTGTTTGGTGGAAGANAGAAAGAAAAAAAAAAATAAAGAAAAAAAACTAAAAGGAACCAAAGTGGCTGA
GCAAGATCGGCGTAAAAGTGAAAAGACACCAACACCAAGAAAAGGGGAGAGGAATAGTAAAGGACCG
ACGGAACCCCTAGAACGGGAAGAGAGGGAAACAGCGACGTCGACTAGCAAGGAAGTATACAATAAAGG
ATGGAGGCAGGTGAGTGCCAAAACCCGGGGGTAGGCGTCCGCTGGATTCACCAGCGACGCAAAGAGGAG
TTAGTGGCGATCGCCCAGGAGTTTGGGCTGGAAAAGGAGGGGCGCGTGGAGGAGCTACGTAAGCGGCTG
TCCACCTACGTGCAGGAGGGCGGTCATTCGACTGGGATTTGGGCTCGGCTCGCCGAGCTGGAATCCCAGT
ATGCTAAAACTCTTGAGCCGCCATCTTGGGGCGTCCCACCCGACTTTGGTGGACGGCCACGGCAAAAGTC
GGAACCTGACCTCAGAAGCTCCCAGCTGTACGGGCTTTTGCAGGCGCCCACCCCGAGTTTGACCGTCTCG
GGCGTGGAGGCAGTTCCTGCTCTCGGTGGAAATGAAGACCGCCGAGCAACTGCGGTCAACGCAGAGGTG
GGGAGGTCCCCTGTTGAACCGGGTGGTGGTCCCTGCTACACTAGGCCGCACCATCCATCGGAGGCGAGGC
CACTTCCCAATGTCCCGGAGCAAGTTCGGAAGTGGGGTATGAGGTACGATGGACGCTCCGACCCCCTTGG
GTTCTTAGAGGACCTCGAGGAGCGCGCTATCACCTATGGTATAGCCCTCGACCGTATGCCCAGGGCAATG
AGCGAGGTGTTCTGCGATAGGGCTGCTCGTTGGTTTCTGAGCAGTGGGCTAAGGGATGTGCCGTGGGCCG
TGTTTAGGAGGGATTTCTTGGAGTTTTTCTTGCCCCCTCGTTACTTCGAGAGGCTTGAGGACGAGATCCGA
TCCCGGAGGCAGCGCGTAGGCGAAGCGTTCAAGGAATATTTGATAGAGCTGAGGGCTCTTATGCACCACG
CTAGATACGAGCCGGCACAAGAGCTGCATCGGATCTACGAGAACGCCGCTCCGGAATACAGGCTCTATGT
GCGACGGCAAGACTTTTCTACCCTATCCCAGCTAACTCAACTGGCCGTAGAATACGAGACCGTCGTGAGG
CAGAGGGCGGGGAGTACTCAGGCAGCCAACCCNTGGGCCNGGGTGTCTCNAGTTCGAATAGTGCAGAGT
GGGCGAGGACGGCGCAGTCCCCGGGGGGACATCTCAATCCGTTCCGGTCTGCTCCCAGCGGATCCGGGAC
GACCGTTGTGGACAACAGGGTCTCCGCCCCACCCGCTGCAACTCACGCAGAGGTGGCCCATCGGATGATG

AGCCAAGAGGCCGGGAACGGATATGATCCGCGACGGGCGTGTCGTAGGTGCGCACAGGAGGGGCACTTC
GCCAGGGAATGTCCCAACGCCCCCGTCTTATTTTGTTGGGTATGTGGAAGCCGGGGCCGAAGTAGTCGGG
AGTGTTGCCGCCGCGCGGAGACGGAAACGGCAACGGCGTCGTCCGGACCAGGATCGGGTCGGCGCCG
ATACACACGGAACGCAGCAATAGGACACCCGCTGAAGGTGGACGGTGGCCGGATCGTCGCTACGGTAGA
GATCGGCGGACGAACGATGCCCGCCACGATCGATACCGGAGCCACTCGTAGCTTCATGAGTGAGGACTGC
GTACGCAAATGGACGATCCGGGGAAGAGCGGAGGAAATACAGGCACGCATCCGCCTGGCGGATGGGTCC
ACCCTAGAGGTGGAGAAGGCTTTGAGAGTAGACGCCAGCTTAGGGGAAAAAAGATCAGCATGACGATGC
TCATCATGCCCTCAATGCTGGACCACGTAATCCTGGGTATGGATTTTTTGGGGGCGATGGATACCACAATA
CGCTGCGGCGAGGCGGAACTGGTCCTAGAAGCAGCGACGACACCGGGCGTGAGGCCAGGAACGAGCGAG
GAGTCGGAGGAAGGCCTCCCAGGGCATGAAAGGGGAGCACCTGCTCGGCTACAGAGGGAGCCGGAGGAA
GGCCTCCCGAGGCATGGAAAGGGAGCACCTGCTCGGCTACAGAGGGAGCCGGAGGAAGGCCTCCCAGGT
CATGGAAAGGGAGCACCTGCTCGGCTACAGAGGGAGCCGGAGGAAGGCCTCCCAAGTCATGGAAGGGGA
GCACCTGCTCGGCTACAGAGGGAGCCGGAGGAAGGCCTCCCAAGGCATGGAAAGGGAGCACCTGCTCGG
CTACAGAGGGAGCCGGAGGAAGGCCTCCCAAGNCATGANGGGGAGCACCTGCTCGGCTACAGAGGGAGC
CGGAGGAAGGCCTCCCAAGGCATGGAAAGGGGNACCTGCTCGGCTACAGAGGGAGCCGGAGGAAGGCCT
CCCAAGGCATGGAAAGGGGAGCACCTGCTCGGCTACAGAGGGAGCCGGAGGAAGGCCTCCCAAGGCATG
AAAAGGGAGCACCTGCTCGGCTACAGAGGGAGCCGGGAGGAAGGCCTCCCGAGGCATGGAAAGGGAGC
ACCTGCTCGGCTACAGAGGGAGCCGGGGGAAGGCCTCCCGAGGCATGGAAAGGGGAGCACCTGCTCGGC
TACAGAGGGAGCCGGAGGAAGGCCTCCCGAGTCATGANGGGGGAGCGCCTGCTCGGCTACAGAGGGAGC
CGGAGGAAGGCCTCCCGCGGCATGGAAAGGGGAGCACCTGCTCGGCTACAGAGGGAGCCGGAGGAAGGC
CTC
>9_rnd-1_family-188__LTR___Gypsy
ACACCAGCTCCAGATGCTGATATAGCACTGAGCCGGATGTTCTTACGAGCTAACAGGGGTCAGCAAAATG
CAACATAACACCAGCCGCAGATGCTGACATAGCTCTGGGCCGGATGTTCTTGCACGACGGCAGCGCACGA
GNCGCAAGCTAAGCATTCTAGCGGCCGTTGTGAAAAGCACACAATAGCTTAGGTANTGTTGATCAATCTT
TTGTAAATTTAGTTTTAATCCGGCTTTCACTGCGGCACATCGGCCCAGCGACTTCGCGTATAATTTAACTC
ACTNAATNCGGCGTGTCGGCCTAGTGTTTTACAAATAATAAAATAATCAAAATAAAGTCCTATAACGAAA
ATCCGCGAGGACTTNTTATTTAAATCCTAATTGGCGCCCAACGTTTTTCGGACCATTTTGTTCCATAATTAT
ATTGTGTGCGGTGCAGTGTAAAGAACTAATCTACCNGTGCAACACACTTCGCGTTGTGTAATAGCAATAC
CCAACCTACCAGTGCAAGCAATAAAAGCTGTGCCTGACCTACATCAACTTACGTCAGCGGCACAAGCCCA
ACAAAAGCCATTTGATACACCGAAGCCGCTGGAGCTCTTCGGCCAGCGACTTGTCACCAAACCAGGCGCA
AGCGGGTTTGGGCAGCGACGTAAGCCAACACCCACAGACTTCAGCGAGGGATCATAAAAAAGCATTCGC
GNCTGCAGCGTGGGAANCAGTAGTTTTANACGTAGAGCATACAACCCTTCTAGGATCAAATAAAATGTAA
GTGGGCCATCATTTGGCACAAACTTTTTTCAATTTGCGTGATTTATTTAAAATAAAATCCAAATAAAATNT
AAAGTATGAAATATTAAAATTGCAGTAATTGTGCTCCCAACTAACTTTGAGTAACACAATTTTGCGTCCAT
CAGCAATTTTCAATAAACTCTCCTCNGTCAGCATTCATTTAATTTACCGTTGTTGATTTGCCCTTGAAATTG
TGTTGTTATTATTGTTGACAGTATAGTNCATGAACAGCTCGTTTCTCAGGTACAGAGACAGCTCTGCGAGG
GGATCTGAGTCAGACGACTCCACCACCTTAGATAGGGAAGCCGTCCCGCAAAAAATCGCTGACAACAGGT
GTAAAATGGGAATGGAGCCTGAGCAGCTTAGGGCTTTGGTGCAGGCCGCAGTGGCCTCCGCCTTNGAAGC
ACAGAGCGCAGTATTGAAGCAAAGAGATGCAGAACTGAGGGCTTCCTTAAACGCTCGGATACAAGAATT
GGNAGGCAGAATATCTGCCAGTAGTATAGAAACNACCGCCGGTCCAGGCATTTGAGCCGGTGGAAATCA
GAAACGACGTCCAATGTAATGAACCGTTGGACGCCGTTAAATGCTTACCAGAATTTACGGGGGTGCCAGA
GACGTACGCCTCTTGGAGACAAGCTGCGATAGCGGCATATAAAATTTACAAGCCATACGACGGCAGCTCA
CGCCACTACCAGGCGGTCATTATTATNAAGAGCAAAATTAGAGGGGCANCTGACGGCGTTTTGGGTCAGT
TTGGCACAGTAATGAATTTTGATGCCATAATAAACCGTCTCGGATTTCACATACAGGGATAAGAGAGGCA
TTCACGTCGTAGAGCAAGAGATGAGCACCCTAAGACAGGGCTCGTTAACAATTTTACAATACTATGACGA
GGTTGAGAAAAAGCTNACTCTTCTCACCAATAAGGTCAACATGTCTTATGAACCAAGCATGGCACAAGGG
CTCTGTACGAAGTTTAGAGATGATGCGTTGCGTGTTTTTGTCTCAGGCCTAAAGCGTAGCCTGACGGATGT
CCTGTTCGCGGCAAAGCCTAAGGACTTACCGTCGGCCCTAGCGCTAGCGCAAGAGGTAGAGGCAAACCAT
GAGAGGTACGCCTTTGCCGCTAGCTTCGCTAAAGGGCAAGAAGATAGGGAACGAAAAGTGACCCCGAGG
CCGCAGGTCCGTCAGCAAGATAGGGGTCAACAGCAGGGGGATCCGCAACCCAGCGGTTCCAAAAATCCT
CACTTCACTAAACAACAGAAACAGCAACAAAGTCAATTTGAGCAACAGAGTGGCAGGTGGAACAAAGGT
GGCGGGTCACAGGAACCAATGGACGTCGATCCATCTGTGTCNAAAATTCTACAACCATCGCAAGCACCAG
CCTACGTTAACTCTAGCGCTCCTCAAGCCTACAAAAGAACGGCCACGTCTGGGCGCTTAAGCGGACAGAG

GAGGCAGAGAGTCAATCACACAGCTCACGAGCCTGATCAAGATTCAGGTTCGTATGCTAGCTCTGCAGCA
GCAGCAGAACAGCAAATTGCCGACGACAATGGTAGCGGNTATGACTCAGACACTCTCAATTTTTTAGGGG
AAGCTCCCTGCTGCCCTACATCAGTAGAAGAGTAGCAGGGATCCAGATGAGGTTTCTCATCGACACGGGC
GCTTCCAAGAATTATATCAGACCACATAAAAATCTAAAAGGTGTGTGCCCAGTAAATTCTCCCTTCTCTAC
CCATTCTATACACGGCACCACGAAGATCACTCATAAGTGCCTTATATCTCTGTTCGGCAAAACTGCCACAT
TTTTCTTACTCCCAAACCTGTCCACATTTGATGGAATTGTGGGCCTCGACTTGCTCACGCAAGCAGGAGTT
TCGCTTTGTCTTTCTTCTGGCCAACTCAAATGTGGCGCAGAAACTGAACAAATCGAATTCCACAGGTGCGC
GGATGTTAACTTTACCAATGTTGACTGCGCCGACGCGCCCGATGCTGTAAAAGATGCATTCCTAAAGATG
CTAAAAAGCAAACATAAGGCCTTCGCGGATCCGGAAGAAGCACTTCCCTATAACACATCAGTGGTGGCAA
CCATCAGGACCACTGATGAACAACCAGTTTATGTCCGACTGTACCCCTACCCCATGGGAGCAGCGGAATT
CGTTAATAAAGAGATTCAAAGCCTTCTGAAGAACGGTATCATCAAAAATCTGTCTCCCCTACAACAACCC
CATTTGGGTGGTGGACAAGAAGGGGACGGACGAGTTTGGTAACAAGAATATGCGCCTCGTAATGGATTTT
CGCAAACTTAACGAGAAAACCATCGCAGACAGATACCCTATGCCTAACATTCCAATGATTTTGGGGAATC
TTGGCAAAGCTAAATACTTTTCGACATTGGACCTAAAATCCGGCTATCACCAGATCACGCTAGCAGAGCG
TGACCGTGAAAAACAGCCTTTGCAGTTAACGGGGAAGTACGAATTTCGTCGACTTCCCTTTGGGTTGAAG
AATGCGGCCAGCATCTTCCAAAGAACTGTTGACGACGTACTGCGTGAGCAAATTGGCAAATCTTGCTATG
TCTACGTTGACGATGTCATCGTCTTCTCAAAAGACGAAAGTTCTCATGTCCAACATGTAGAGTGGGTCTTA
AAGAGCCTGTACGAGGCAAATATGAGAATCTCCAAGGAAAAATCGCGTTTTTCTAAGAAAAGCGTAGGTT
TTCTTGGTTTCGTGGTAACCAGTAATGGAGCTACAACGGACCCCGAAAAAGTCAAAGCGATTCAGGAGTT
CCCAGAACCAAAGAATATATTCGAGATCAGATCATTTTTAGGACTGGCTAGTTACTATAGGTGTTTCATCA
AAGGCTTTGCCTCAATAGCAAAACCCATTTCTGATCTTCTAAAGGGCGAGAATGGGTCAGTAAGTAAACA
TAGATCGCGAAACATCCCGGTAGAATTCTCTGAGCTTCAGCGTCACGCATTCCAAAAGCTACGCAACATTT
TGTCGTCAGAAAACGTCATGCTCAGATACCCTGACTTTAAGAAGCCGTTTGATCTAACGACAGACGCTTCA
GCTTACGGCATAGGGGCAGTGCTTTCTCAAGAGGGTCGCCCCATCTCAATGATTTCCAGAACATTGAAAC
CCCCGGAGGTGAACTACGCCACCAACGAGCGGGAACTTTTGGCTATAGTTTGGGCCCTTAGCAAGCTACG
TCACTACCTATATGGAGTAAAAGACATTAACATTTTCACGGACCACCAACCGTTAACTTTTGCCGTTTCTG
AGTCAAACCCGAACGCCAAAATTAAAAGATGGAAGGCGCGCATTGAGGAAACGAATGCGCGAATATTTT
ATAAGCCAGGCAAGGAGAATTTAGTCGCTGATGCGCTATCGCGACAACAGCTCAACGCTCTTGACGAGCT
GAATACTGATTCCTGCGCAGCCACAGTCCACAGCGAGCTGTCCTCGACCTATACAATTGAGTCGACCGAA
AAGCCACTTAATTGTTTCCAAAATCAAATTGTCATTGAAGAGGCACGCGTCCCCACCAAACGCAACTTCAT
TCTTTTCGGGGACAAGAGGCGTCGTGAAGTGGGCTTCACTGATCGCACAGCCCTTCTAGATGAACTCCTTG
ACTTGATTAATCCAAATGCGGTTAATGCCCTCTATTGCAACCTGCACACACTAGCACAGGTGCAGGATGG
CTTAGTTCGCATGTTTCCAGGCACAAAGTTCTGGCACTGCAAAAACCGAGTTGCAGATGTCTTCAACGTTG
ATGAGAGGAGAGAAATTCTCGTTGCCGAACATAATAGAGCGCACAGAGCAGCTCAAGAAAATGTTAAGC
AAGTGTTATCAGAATACTATTTCCCTAAAATGGCGAAGTTGGCTAAAGAAATTGCGGAAAGTTGTAGAAT
CTGCTCANCAGCCAAATACGATAGGCACCCGAAAAGACAGGAGCTGGNAGAAACTCCGATCCNTGAGAC
TCCTGGGGAATTNCTACACATTGATATATTTCCCACGGACAAACAATTTTTTTTGNCCTGTGTTGATAA
>160_rnd-4_family-321__LINE___CR1
TAACTGCTATAAAACCGCGCTTTATAACGAGCTGTGTTAACTGTTCGCGTACAGAGAGAGAGTTCTCAGTT
GAGAGTGATTGACGTGTTTTACTTGTGCTCCAAGATTTTATATATTTTTGCGCTTTTATCCTGCTTTATATT
TCAAAATTTTGCATTTTTCGGTGCTTTATTTAAAAAGCTTTTATAAACTGTGCAAAGTGTCATCTGTTGTGC
AGCGTGTCTGTTAATTATTACATTTTATAAAACGCGGCGTCAGTTCCGTGTTTGTTGTTATTGGCTGTTTTA
AGTCTTTTGCCTTTGTGCTCCCCTGTGCATACACTTGTTTGTGCGTGTGTTCGCTGCATACTTTCTGCTTGTG
CATCGCGCTCTCGCAAATTTCTTTCTATTGCTTTTCGCACCAAACAATTTGTTTGTGTACGAATAATTGTTT
TTTGAGTGTTTACTTGTGCACCGTTTTCGTTTCGTTTTGCTCTGCGCTCTCGCAAGTTGCTTGCGCATTTAAT
AATTATGTCGTGCTGTAAGAAACCGTGCACGTTCAAGCAGGCTGACGATTCTGCACTGCCGCAATTTGTTA
ATTGCTGGCTGTGTGAGAATATGATGCATGCTAAATGTGCAGGTCTCACTGGGCGTGATTGCGACAAGAT
CGCTGCTATTGCCAAGAAGGGCTGCGGTGGCTTGCGTTGGTCGTGCCCAGAATGCATCGACAAGCAAATT
GATTTCTCCAGAATGTATAATTCTGTGAGAAGTCAATTCTTAAAATTAAATAATGTGGCTAAGCAGCTCTC
GAGTAATTTTGACTCGAGTTTCGANTTGCTAGGCAAATATAAATTTGAGTCCCCAACCAATCGCCGGTTGG
AGCCTCAACTTTTGCAATTGCCCTCTACGTCTGTTCAGACNCCTACGACTTTCTTGTCTCTTCCTAGCAACT
TGGAGGTATCTCCAATGTTGTCTTTATCGCCTTCTCCAAGCGACTGTCCAATTACCCCGGCTTCAAGCCCTG
TACTAGACCCAGTAGAGCCCGTACCTGATTCCGTCCCAATTAATCAAACTGCCTCAGCCCCGTCAGCTCCT
GGCCGCAAAGGTAGACCTGCGCGCAATCTTGCGCAGATTGTTAAAAGCAATCATTGTGCCTCGTTAGCTC

CTCCAACTCCTGTATTACCTACAGCCCCATCAGCGCCTACTTTAACGGTAGTTGCCCCTCGTAGGCAAGTT
TTTGTGTCTCGACTTTCGTCGGACACCACTTCTGAGTCATTGGCAGCCTATATTGCTAGCAAATCTTCTGCT
AGCGTGTCAATAACTAAATTTAATTTCTCCACCCCTCGTGAAATATCATCATTCAAAATAAATGTTTCACA
TGATATGTTCCCAATCATTTGTGATCCCAAATTTTGGCCACCACACATGATTGTTCATGAGTTCAAGCCTA
AAAAGCGTAACCAGCCTGTCACCCTTCCAGTCCCTTCAATACAGGGTACCATTTCTGCCCCAAAAAACTAG
CTTCATCTTCTGACCAAATCTTTATTCATTATCAGAATGTCAGAGGTCTCACTTCAAAGCTAAAAAATCTTT
TTATTGCCAGCACTTCCTTCGATTCGGACATAATAGCATTTTCAGAAACATGGTTAAACTCAACCATATCT
GATTTTGAGATATTACCGAATAACTTTATTTTGTTTAGAAATGATCGGCCGACTCGAGGTGGTGGGGTGTT
AATTGCCGTTAAAGCCACTCTCCAGTCTGAACTATTCTGTTTTAGTACGCCTACTGACGCTGAGATTGTCTC
AGTTAAGGTGTCTTTTCCTACACGGAATATCTATGTAACATGCTCTTATGTCCCACCTTCTTCTGATATTCA
AGTTTATATGAATCATATTACTTGTATTAAAGAAGTTTCTTCACACGTGCGGGATAATGACCTATTATTGG
TGTTGGGTGATTTTAATTTGCCGAACATCTCTTGGTCATTATTCACTGCTGAAAATTATCTAGTGCCCTCAG
CACAGCACGATTTTTTAGACTGCATGCTTGATCTTTCGTTGTTTCAAATCAATTCAATTTCTAATCATATGA
ATAGAACACTTGACCTTGTATTTGTTAACGACTACCTTATTTCTAATGTGTCTAGGTCACCTCCTTTATCTC
TTCCTGAGGATGTCTATCATCCAACTTTAGAGATTGCAATGCTAAATTGTAATCCTTCCATTTCGTGCTCAT
CAGCCAATAAGCCCCGCTATTGCTTTCGCAAAGGAAATTATTCCTTGCTGAATCAGCTTATTTATTCAACC
GATTGGTCTTTCTTATATGACTCAGTTGATATGAATACAGCCATTAATTCTTTTTATATCACCCTAAACTCC
CTCTTAGATGTGTGTATTCCTAAGTCAATTCCTTCGACTACTTTTTCAAAACCACCCTGGTTTACTCCCAGA
TTATCACATCTAAAAAATGTAAAATCCAAATATTTTAAAAAGTTTAAAAAGTCTGGTTCGTGTACAGACTT
GGCTAAATATTCCATAGCCAAGTCGAACTTCTTTCTTCTTAATTCTCAATGCTATGAGAATTATCTAATTCG
TTGTAAAAGGCAATTCGCCCGAAATCCGAGACAGTTTTATAATTTTGTAAACTTGAAGCGTAAGTCTTCAA
GTTTGCCATCTTCTTTTTTTCTTGGGATGGATAAAGCTAGCAATGACACTGATTCTGCTAACCTCTTTGCTA
ATTTTTTCCAATCAACTTACTCTTCAGTCTGTCCTAATACTAATTCTTACCCTTATACTATTTCCTCCGCTAG
TTCTATACCTCCCCCCATTATTTCACACTCCTCTCTCCTATTAGCTATAAACTCTCTAAAATCTTCTTACTCT
CCTGGGCCGGACAATATTCCTAGTTGTCTACTCAAGGAGTGCAGTTCTTCCCTTTTCTATCCATTGCTAAAG
CTTTTTAATCTATCCCTTGAAACTTCTGTCTTTCCATCTCTTTGGAAAGAATCTTATATCATTCCTCTTCACA
AGAAAGGTGGGAAGTCTGATATACAAAATTACAGGGGCATTGCAAAACTGTCTGCTATTCCTAAATTATT
TGAAAAAATAATCACTTCGAATTTGCAGCATTTCTGCAAATCAGTTGTTTCCCCTGTTCAACATGGATTCG
TAAAGAATCGGTCAACTACGACCAATCTGCTTGAGTTTACTTCCTTGGTAAATGATGCTTTCCTTTCGAAA
ATGCAAACTGATGTCATTTACACTGACTTCAGTAAGGCGTTTGACTCTGTGCACCATGACATTTTACTTTTT
AAACTTGACCGAATAGGTTTCCCTCTGTCCCTTTTACTTTGGATTAATTCTTATTTAAAAGGTAGGACCCAA
AGAGTAATACTGAGGTTGACTACCTCTAAAAGGGTTCACGTTACTTCTGGTGTTCCTCAAGGTAGTCATCT
TGGACCTTTACTCTTTACACTTTTTATAAATGATCTTCCCTCTGTTATATCACATGCCCGTGTACTCATGTAT
GCGGACGATGTCAAACTTTTTCTATCATACACTAACCCCCTACATCATTCTCGTCTACAAGACGATTTGAA
CGCTATGCAACTTTGGTGTTCGGCCAATCAATTGCATCTAAATTGTTCAAAGTGTATGTTTATGACTTTTAA
TCGTACTACACCTTATCTTGTCAGTTATACAATCGACAATATCCCTTTGCAACGTATACTAACAGTTAAGG
ATCTAGGCGTTATTTTTGATTCTAAACTCTGTTTCAATCTTCATATAGATACCATTGTTAATGAGGCAAAAG
GTGTACTTGGATTCATTAAACGATGGTCTAAAGAGTTTAACGATCCTTTTATAACAAAACTTCTGTACATC
TCTCTTGTTCGCCCTATCCTTGAATACTGCTCTTGCATCTGGTCTCCACAGTATATCAACAGCCAGGATCGT
ATTGAATCTGTTCAGAAGCAATTTCTGCTTTTTGCCTTACGAGGTTTAAATTGGGACCCTAATCTTCGGTTG
CCGTCCTACTCCAGCAGACTGCTTCTTCTCAACCTTCCGTCGTTAATCAACCGTAGGACAATTCTCGGTGTT
GTCCTTCTACATAAGTTGATTATTGGCGACATTGATTCTCCTTTTCTGCTAGGGCGTCTTCAATTCTCTGTT
CCGGCTAGACCAACCAGAAATTATCGCCCCTTATTACTATCTACGTGCACAACTGATTACGCTATGCACAA
TTCTTTTCGCGTGCTATGTAAAAATTATAATAGTTTGCATCACGTTTTCTCTACCGAACTGTCTCTACCCCT
AATAAAATCGTTGCTTTCAGGATACCTTCGGGAAGTCGCTGACCATTCCCAGCTATGAGCAGGGGCATAG
CTAGCCGGACAGGCTGAAAAATTTTCCCCCACCGTGTCGGTGATTTCCCATTACTTTTCTCTTTGTCCTATC
CACTTAACACTCTTTATCTAACTTACATTGCTTTACTTTATTAACAATTTTCTTACTTTCTTTTTTCCTATTTA
TTGTATTTTCTTTATTAATNTAGCAAATTAAGATTATTTTTAATTTCACTTGAGATCACTTTTTTCCTACTTA
CAACCTTCTGCTTAGATGTAGGCTCTAATAGCGTCACTGACAAAATTAGCTGTGAAAAGGGGCACAGCTG
GCCGGACTGGCAAATAAAACACCTCCATCGGTCGGTGATGCTATTTAGAAAATTGTATCCTTTAACTAGGC
TTTTAGCATATANTTATATTGTACAACCTTTTGAATAATGAGGAAGACACTCGTTTTGTCGACCATTAATA
AATAAATAAATAAAAAAATAAATAAAAAAA
>9_rnd-1_family-490__Unknown
CACGCTGGTTCCTCTTTACTTTTAGTGCAGTCTGCTGAGCTGTGTTTATCCCAAACCCTTGACAGCGGAAG

CATTGAGTCAAATCATTTCTCTTTTCCTTACACCAAGTTCGGTTCCAGTACTGGAGTCATCAGATTCAGATG
TTTTTGCCATCGCCANTATTATCAGGCGAAGTTTGCCAGTCCATCGTGATGGAAAAAAAGGGGGGTCCTG
GGGCGTGAACGTTACCCAGCTACCCAGTATCAATCAAGACTCGGTTATTTTCTTGTAATTTATTTGTTTGTC
TACGTTATTCTTAATTGNGCACGTACGCGCGGGCGAATGAGTTGGTGAGNACGCGTGCTTCTAGCCAATA
GGGACGGTTGTTAAACGATTATTTTAATAATTTTAGGAGAATTAAATTTTGAGCTCAACGTTAACACTCAA
AATTATTATTATTATTATCTCTGAAATGAAATGAAAGAGTTATTTGCGCCCGGGCGATCTCATATGCTAAG
GGTTCATCCCGTTGCACATTTCGTCTTCAAATCTCAGGCATTAATAACTTTCCAGTTATCATGAGGCGGCT
AAGAAATCCCAGAGGATCGAATGTTGACATGATCAATGCTGAGTCGAACACCAGTCTTATCTTACCGGGC
TTATTTGGACTTTTAACTCAAAAGTGCGGAAGATACCAAAGTTTGTCGCTGTTGGCTAAAGCGACCTTATG
CTGCTTCAATCGTCGAGCATATCCTTTATGAACATAAACGTCCATAATTTTCATTTAGGCTTGTTTAAACCG
ATCGTTACGCTTTCTCTTTCTCTCAATGTTGACGAACCTTTTGTATTCCATATCATAGCTTCGTGGCAACTT
AACATTATCACGGTTCCATAATAAACCTGTCTGGTAACGTTCTCCTATTTTCACCATGGTGTCTTCGAGTAC
CTTCTGTGCTCGTGCATCGTCGCTGGCTTCGACTGGTGGCGAAGTTTTCGATATCAAAATAGTCGTTTACC
ATCTTTTGGATTGCATCCTCCTGCGACACTGCTAGGAGGCATGTTTTTGATGACGTTGCCGTCGGTTGACTT
CTTACCAGTCCAAAACCACCCATCCAAGTTCGGAGGCTGCGGCATATGGTCCCTGTGGTGCNAATATTTTT
GTTTGCATTGGCAGTTCAAGATGTGCGTGGTCAAGTCCAATCAGAAGTTTTGGTTCCGCACCGCGATACGG
TTTCATTGGAAGTCGTGCGCCGTTGCCCGACATTTGGACNTGACGTCGATGCAGGCTTTGCATTGGTAAGC
TCAGGTTGGACACAGCGTACACATTTCGCAATACATGACGCTTGCGTTTGTCACCGTCAAGGAGCGCGTA
CGTATCGGTCAGCTTATTGGCCCCGTGCAACGTCACTGGCAATATGCGGAAAAGTCGGCGTTCTCTATCCG
AATCGACACAACTTAAGTTCCTTTGTCAATTGAGTCAATTGAGCATCCACTGACTGCGGTGCCNTTGTGTG
AGGATCCGCTGGCTGCGGCATCCGTCCAGTACTCATCACTGTATCACCTGCTTGATGCGGTGCATTGTGAC
TCATGGCATCATCATCATAAATCATAAATGTGGCACATAATTATTGTCCATATGAGTTAGTTTTGGTAAGA
CTAGTAATCTGCCGAAATACTTAGATACCATAGGTAATATAGAACCTAAATAGAACATAGAAGATTTCGC
NAAAGAAGGTAAGTGTTGATTAGAAGTAGCATACCAAAGAGCAAAAAACATGCTTGAACTACGCAAACA
AAAAAATAAAGAGCTATATGACTTAAAACTAAGGACATAATTTTATTCAGGGGAGATAAAGTTTTACTAA
GTGTCGCGTGCCAAACGAAGGTGCAGTTGCTATTTGAACACTGCAAGGCCCCCGGCCATGTTACTCCGAT
GTTAATAAAGANAGCGTAATGCAATACGTGCTAGTGCAGTCGGATTCTGAACGGATTACATAGGTTAAAA
CATAGATATAAATATTCCCTTAGAATCTTACCGCAGTCTTGCCTCCAAAGGCCTTCGAACTCACGACAATA
ATGAGCTTAACCTTGTAATGTANTTAATACTAAACGATCGATAACTTAATTCGTCCCGCAACATCTCCGCC
CCCGTGAAGGTCCTGCTTCAAACACATTCAGTATGGCTAGTTAGCCACAGGACGAGTGATCAGTCGTACT
CGATTGTTGTCCGCCACGCGCACGCTGGTGCGTGGTCCAGGGTAGAGCTCCTCCACTATGCCCCTTCGCCA
CTCTCTGCGCGGCACGGCCGGGTCGCATACAAACACCATATCACCATGGCGTATCGGTTCAACGCGCGCA
GGCAGGTACTCTAAGACCCACCGCTTCCAAAAACGGTCTCGTAACAGACGCGCCGTGTCCCACTGTTTCCT
CGTGGCCCGTCTCTCGTTCGGCTGTAAGTGGGTGTGTCAGGCAGATTGGCCACTTCTTTGAGTAGATCGTT
CGGTGTTAGGGGTTTTCCTGGTTCGGTAAGTGGGTAAGCGGGCGAGAATCCACAACGCTCTCGGCCTCAA
TCAGGAAACTCTCCAGTACGTGTTGGTTTGGTGCCACCTCCCTTACTGTGTGGCGTAGCACTCTCTTGACA
CACTGCACCATCNTTTCCCAAGCGCCCCCTTCAGCTGGGTTCGCTGGGCAATTGGAGATCCAATCAATGCC
TTTAGATAGCTCGCCCTGAATCTTCTCCGCCTCGAATACGTCTATGAGCCTTTTGGCTTCTCTGTCGGCTCC
AACAAAGTNATTTCCGTTGTCACTGCGCAACCTCAGTACAGGTCCGCGACGGCAAAGAAAATTCCCGATC
GCGATTATGCAGGAATTAGTTGACAGATTATGTGCAAATGTATGGCCCTTGTTGTCAGGCATGTAAGCAA
GGCGACCCATCTCTTTTCTTTTCGACGTCCGATTGTCACAAGGAGTGGTCCAAAATAGTCCAAATGGCCTC
CAGTCGGTCCTCTGGCAGATGACCCATTATAGGTGCCCCTGGCTGCGCTCTATATACTATGAATATTACTG
TACGCAACAGTCGACGCAGCTTTGTTATCCAAAATCTGGTCCTGATGTCGCCTATCGTTGCATCCATGTAT
TGATGCTTCATTTTTACATGGTAGTGATACACAATCGGTTTTGTCAGTGCGAGCCGGTGTGACAGGATTAT
TGGCCTACACTGTAGGGCGTATACGCGCATTGGTATACACTGCAAGGCGCCAGCCCTCTTATGTCTCTTCG
ATGACCGACTGATTCTTCATTGTATGAGGCTCGAACCTCGTCAGGCAACGCTTCGCATTGCGCTCNCCGTA
CCAGCAAGTTCTCAGCATTCTCACACTCCGTAGCGGTGAAGCCGAATCTTTCAAGCTCAGTCATCTGTCCA
CGGCTCCAACGCGTGAACTTTAAAACCCAAGCCGTCGTCCTTACCAGCCGACTGAAGCTAGAGAATCTCT
GATTTTAAGCATTATGCAGTAGACCGGGCTGCCATACCCTATCAAAGGCTTGCTTGACAGCACAGCAGTA
CTTATTCTCTTCGAATGCCTGCATTATTCGCTCTACTATCCTATGACACTGTTGCGGTGTTCCGTGGATGCG
CCTGAAGCCGAACTGATGGTCTGGGATCAAACTAGCCTCGTTCAATACTGGCAGCACTATAGAGTAAATA
AACTCTTTCATTCAATTTAGAGAGAGTAGCCAGTAAGCTTATGCAAATGTCCAATCTCAAGGCATCGAATA
ATTACGTTGGTCAGTAACTGGATACTATTATTAGCTCCACACTTGGTAGCTCCAATAAAGGATAGNGTNNG
CTTTGTCCGTCAATGAGCAGCGATCAAACGGCATGAATGAGTCTTAAAGGGGCCTTTGCAAAGGCATCTG

CTCTTTCTTGGTCTGATCGGTACCAGTCCCCATTTGCTTTTCGAACAACAGCTGCCCTTCTAATAGGCCATT
TAATATGGCGTGTGACCTGCCATAAGCGATTTGGCGGGTCTCTGCCATTTAATATGGCGTGTGACCTGCCA
AAAGCGATTTGATGGGTNTCCTNGTTTGAAATCCTCGAGGAACCTCGACGCTTTTTTCTCGACCGGGTATT
GACATGTAATGTTACAGCGGTGTGGTCGGAGAATAAGTTCTCCTTCTATTTTATTGAAAGCTATACCTTTG
GAGATAGCAATGTATGTAGCAGTATGTCAGGGACCTTGCGCAGATTCGTGGGCCAGTATGTGGGTTCACC
AGGTGAGTGGCAGCAAAGTCCTTTCTTCCGTCCCTTCTTCTGTATGCAACGGAGCTTGGCCCTGGGGATGT
TGGTACGCGAGCCCTACCAGTAGTGTTTGGCATTGAAATCGCCACCAATTATAAACCAGGAGACAAGGCT
ACCTGGGTGGGAGATACGCAGCCGCGATTGTAAAAGGTCGCCGAGTGGGCTGGGCAGCCTGACCCGGCC
GCACTCCACTCAATCTTGGTATGCTGGCAGTTCAATATATTTTTATTCCTTGTTTGATGAGCACTGCAGCTC
CACCACAGGCTCTACCGTTTGGGTGGTTTCTGTAAATAATATCAAAACCTTTGATGGAGAACTACGATCTT
TGGGAGAAGAGAGTTTCTGATATAAGGAGTATATCTGTGTTGGTGGTCTTCACAAAGTTTTTAACCTCTGG
CCTGCTATTGCGCAGGCATTCCAGATGGTGAGGTTAAGCCTTACTTGCATAGGCTACCAACAAAAATGGC
TAGCACTTGCGTCATCTGGCTAAATAATTTCTTCATCATTTTTCCATCATCTACTCCAATCGAGTAACCA
>9_rnd-4_family-428__LINE___CR1
AGAGAGGAGAGTTCTCAGTTGAGAGTGATTGACGTGTTTTACTTGTGCTCCAAGATTTTTATATATTTTTG
CGCTTTTATCCTGCTTTNTATTTCAAAAATTTTGCATTTTTCGGTGCTTTATTTAAAAAGCTTTTATAAACTG
TGCAAAGTGTCATCTGTTGTGCAGCGTGTCTGTTAATTATTACATTTTATAAAACGCGGCGTCAGTTCCGT
GTTTGTTGTTATTGGCTGTTTTAAGTCTTTTGCCTTTGTGCTCCCCTGTGCATACACTTGTTTGTGCGTGTGT
TCGCTGCATACTTTCTGCTTGTGCATCGCGCTCTCACAAATTTCTTTCTATTGCTTTTCGCACCAAACAATT
TGTTTGTGTACGAATAATTGTTTTTGAGTGTTTACTTGTGCACCGTTTTCGTTTCGTTTTGCTCTGCGCTCTC
GCAAGTTGCTTGTGCATTTAATAATTATGTCGTGCTGTAAGAAACCGTGCACGTTCAAGCAGGCTGACGAT
TCTGCACTGCCGCAATTTGTTAATTGCTGGCTGTGTGAGAATATGATGCATGCTAAATGTGCAGGTCTCAC
TGGGCGTGATTGCGACAAGATCGCTGCTATTGCCAAGAAGGGCTGCGGTGGCTTGCGTTGGTCGTGCCCA
GAATGCATCGACAAGCAAATTGATTTCTCCAGAATGTATAATTCTGTGAGAAGTCAATTCTTAAAATTAAA
TAATGTGGCTAAGCAGCTCTCGAGTAATTTTGACTCGAGTTTCGAGTTGCTAGGCAAATATAAATTTGAGT
CCCCAACCAATCGCCGGTTGGAGCCTCAACTTTTGCAATTGCCCTCTACGTCTGTTCAGACACCTACGACT
TTCTTGTCTCTTCCTAGCAACTTGGAGGTATCTCCAATGTTGTCTTTATCGCCTTCTCCAAGCGACTGTCCA
ATTACCCCGGCTTCAAGCCCTGTACTAGACCCAGTAGAGCCCGTACCTGATTCCGTCCCAATTAATCAAAC
TGCCTCAGCCCCGTCAGCTCCTGGCCGCAAAGGTAGACCTGCGCGCAATCTTGCGCAGATTGTTAAAAGC
AATCATTGTGCCTCGTTAGCTCCTCCAACTCCTGTATTACCTACAGCCCCATCAGCGCCTACTTTAACGGTA
GTTGCCCCTCGTAGGCAAGTTTTTGTGTCTCGACTTTCGTCGGACACCACTTCTGAGTCATTGGCAGCCTAT
ATTGCTAGCAAATCTTCTGCTAGCGTGTCAATAACTAAATTTAATTTCTCCACCCCTCGTGAAATATCATC
ATTCAAAATAAATGTTTCACATGATATGTTCCCAATCATTTGTGATCCCAAATTTTGGCCACCACACATGA
TTGTTCATGAGTTCAAGCCTAAAAAGCGTAACCAGCCTGTCACCCTTCCAGTCCCTTCAATACAGGGTACC
ATTTCTGCCCCAAAAACTAGCTTCATCTTCTGACCAAATCTTTATTCATTATCAGAATGTCAGAGGTCTCAC
TTCAAAGCTAAAAATCTTTTTATTGCCAGCACTTCCTTCGATTCGGACATAATAGCATTTTCAGAAACATG
GTTAAACTCAACCATATCTGATTTTGAGATATTACCGAATAACTTTATTTTGTTTAGAAATGATCGGCCGA
CTCGAGGTGGTGGGGTGTTAATTGCCGTTAAAGCCACTCTCCAGTCTGAACTATTCTGTTTTAGTACGCCT
ACTGACGCTGAGATTGTCTCAGTTAAGGTGTCTTTTTCCTACACGGAATATCTATGTAACATGCTCTTATGT
CCCACCTTCTTCTGATATTCAAGTTTATATGAATCATATTACTTGTATTAAAGAAGTTTCTTCACACGTGCG
GGATAATGACCTATTATTGGTGTTGGGTGATTTTAATTTGCCGAACATCTCTTGGTCATTATTCACTGCTGA
AAATTATCTAGTGCCCTCAGCACAGCACGATTTTTAGACTGCATGCTTGATCTTTCGTTGTTTCAAATCAAT
TCAATTTCTAATCATATGAATAGAACACTTGACCTTGTATTTGTTAACGACTACCTTATTTCTAATGTGTCT
AGGTCACCTCCTTTATCTCTTCCTGAGGATGTCTATCATCCAACTTTTAGAGATTGCAATGCTAAATTGTAA
TCCTTCCATTTCGTGCTCATCAGCCAATAAGCCCCGCTATTGCTTTCGCAAAGGAAATTATTCCTTGCTGAA
TCAGCTTATTTATTCAACCGATTGGTCTTTCTTATATGACTCAGTTGATATGAATACAGCCATTAATTTTTT
TATATCACCCTAAACTCCCTCTTAGATGTGTGTATTCCTAAGTCAATTCCTTCGACTACTTTTTCAAAACCA
CCCTGGTTTACTCCCAGATTATCACATCTAAAAAAATGTAAAATCCAAATATTTTAAAAAGTTTAAAAAGT
CTGGTTCGTGTACAGACTTGGCTAAATATTCCATAGCCAAGTCGAACTTCTTTTCTTCTTAATTCTCAATGC
TATGAGAATTATCTAATTCGTTGTAAAAGGCAATTCGCCCGAAATCCGAGACAGTTTTATAATTTTGTAAA
CTTGAAGCGTAAGTCTTCAAGTTTGCCATCTTCTTTTTTTCTTGGGATGGATAAAGCTAGCAATGACACTG
ATTCTGCTAACCTCTTTGCTAATTTTTTCCAATCAACTTACTCTTCAGTCTGTCCTAATACTAATTCTTACCC
TTATACTATTTCCTCCGCTAGTTCTATACCTCCCCCCATTATTTCACACTCCTCTCTCCTATTAGCTATAAAC
TCTCTAAAATCTTCTTACTCTCCTGGGCCGGACAATATTCCTAGTTGTCTACTCAAGGAGTGTAGTTCTTCC

CTTTTCTATCCATTGCTAAAGCTTTTTAATCTATCCCTTGAAACTTCTGTCTTTTCCATCTCTTTTGGAAAGA
ATCTTATATCATTCCTCCTTCACAAGAAAGGTGGGAAGTCTGATATACAAAAATTACAGGGGCATTGCAA
AACTGTCTGCTATTCCTAAATTATTTGGAAAAATAATCACTTCGAATTTGCAGCATTTCTGCAAATCAGTT
GTTTCCCCTGTTCAACATGGATTCGTAAAGAATCGGTCAACTACGACCAATCTGCTTGAGTTTACTTCCTT
GGTAAATGATGCTTTCCTTTCGAAAATGCAAACTGATGTCATTTACACTGACTTCAGTAAGGCGTTTGACT
CTGTGCACCATGACATTTTACTTTTTAAACTTGACCGAATAGGTTTCCCTCTGTCCCTTTTTTACTTTGGATT
AATTCTTATTTAAAAGGTAGGGACCCAAAGAGTAATACTGAGGTTGACTACCTCTAAAAGGGTTCACGTT
ACTTCTGGTGTTCCTCAAGGTAGTCATCTTGGACCTTTACTCTTTTACACTTTTTATAAATGATCTTCCCTCT
GTTATATCACATGCCCGTGTACTCATGTATGCGGACGATGTCAAACTTTTTCTATCATATACACTAACCCC
CTACATCATTCTCGTCTACAAGACGATTTGAACGCTATGCAACTTTGGTGTTCGGCCAATCAATTGCATCT
AAATTGTTCAAAGTGTATGTTTATGACTTTTAATCGTACTACACCTTATCTTGTCAGTTATACAATCGACAA
TATCCCTTTGCAACGTATACTAACAGTTAAGGATCTAGGCGTTATTTTTGATTCTAAACTCTGTTTCAATCT
TCATATAGATACCATTGTTAATGAGGCAAAAGGTGTACTTGGATTCATTAAACGATGGTCTAAAGAGTTTA
ACGATCCTTTTATAACAAAACTTCTGTACATCTCTCTTGTTCGCCCTATCCTTGAATACTGCTCTTGCATCT
GGTCTCCACAGTATATCAACAGCCAGGATCGTATTGAATCTGTTCAGAAGCAATTTCTGCTTTTTGCCTTA
CGAGGTTTAAATTGGGACCCTAATCTTCGGTTGCCGTCCTACTCCAGCAGACTGCTTCTTCTCAACCTTCCG
TCGTTAATCAACCGTAGGACAATTCTCGGTGTTGTCCTTCTACATAAGTTGATTATTGGCGACATTGATTCT
CCTTTTCTGCTAGGGCGTCTTCAATTCTCTGTTCCGGCTAGACCAACCAGAAATTATCGCCCCTTATTACTA
TCTACGTGCACAACTGATTACGCTATGCACAATTCTTTTCGCGTGCTATGTAAAAATTATAATAGTTTGCA
TCACGTTTTCTCTACCGAACTGTCTCTACCCCTAATAAAATCGTTGCTTTCAGGATACCTTCGGGAAGTCGC
TGACCATTCCCAGCTATGAGCAGGGGCATAGCTAGCCGGACAGGCTGAAAATTTTCCCCACCGTGTCGGT
GATTTCCCATTACTTTTCTCTTTGTCCTATCCACTTAACACTCTTTATCTAACTTACATTGCTTTACTTTATT
AACAATTTTCTTACTTTCTTTTTTCCTATTTATTGTATTTTCTTTATTAATATAGCAAATTAAGATTATTTTT
AATTTCACTTGAGATCACTTTTTTCCTACTTACAACCTTCTGCTTAGATGTAGGCTCTAATAGCGTCACTGA
CAAAATTAGCTGTGAAAAGGGGCACAGCTGGCCGGACTGGCAAATAAAACACCTCCATCGGTCGGTGAT
GCTATTTAGAAAATTGTATCCTTTAACTAGGCTTTTAGCATATAATTATATTGTACAATCTTTTGAATAATG
AGGAAGACACTCGTTTTGTCGACCATTAATAAATAAATAAATAAATAAAAATAAATAAATAAAT
>Ref_dvir_495
GTTCATTGAGCTTCCCTTAAATTCGATTACTAATCCATTTTGTTTTTGTTGCCGTTGGGGTCGAAACCTTTTT
TGTCGATGACCCAAGAAGGGCTGTTGTATGGCGATTTGGATGGTCCAATTATACCATCTTTTAGCAATTGT
TTGATTTTGAAGTTTACGAAATCTGAAACTCCCATAGGGTGCGGGTATAGTTTTGAATACACCGGCTCGTT
GTCTGTGGTTCGAATAGTGGCAATGACAGAAGTGTTGAAAGGAAGTGCCTCGTTAGAGGTCGAAAATGCC
AAAGTCAAGTTTCAGCCATCCTCGGTCACCTTATAGCTACTGATGATCCTTTTCACCTTTCTCGAAGTCTGC
CATTACAACTAGCCAGATGATGATGAGCTGCTGATGGTAATGCTGAGAGCGGCGTCGACTGTGGCCAGGT
GCTTGGATGTTTCTGTGGTGCCAACTCATTTTTTAAGTTGTTGATGACATGGAGATTATGGTTATATGTTGA
GTGGAGGTGACCCCATGATGCCTGGTGCCTTCCCTATGATCTCGCGCTTATTCGCATATTTTGTGCCATTGA
TGGTAGCGGAGAAATGGAAGGTGACGAGGTGGGTTCCGTTTGTTACGACCCCTGGGCCATCGTCAGTGCT
CACTTTAGCTGTGCTTCCGTTGATCATGATGACATTACCGTCTATTAATGCGACTGGTTCGAGGTGGCTCG
GTTGTGTTCAGCAAGAGGACGTGCTGCCCGTATAATCCTCGTGCACAGAAGTCGTGTGAATTATACCCTAG
GATAGGCTATTAATATGTGGACTATATTTTCGGACTGGAGGACCTTAATTTAGACGCTTCTGTAATGCTAA
CTATGGGGACTTCTACTGGGTGTTCATTCAAAATAGTTTTAAAATCATTATGTTCAAAAATAGTTGGATAT
ATTATATTGGCTTTAGCTAATGTAATTATGAGAATTAGATTCGATATTTGATTATTAAATATTCTATTTCTC
GCTAATATTGTTTCATATAAATGCGGTGTGTCCACTAAACTATTCTTTTTTGCCTTTAGTATTTTATTTACTG
GTCAGTTAGTTTGTTGACTTGCCTTTGCATCTCTGAGTTGATAATCAATTGCCTATTGCATTGATTAATTGG
GATTCGGCCATCTAATCTTTTTGAAGTCGATCTAATAATTTCCGCATGTGAGATTGTGGGAATAGCTCTAT
TAGTTTTTCGGCTTAATCTGCAATGGCATTAAATTCCGTCAGGTTACTTGAATGCCTCAAGTAGTCCCTCTG
TTCCCATACCAATACCATTCCGTCTATTATCGGAATATAATTGGAATGCGAATAGTCGATAATCCGATTCT
GTACTAAACCTAGCATTAACAAGATTGCAGATTAAATCCTGTAAAAAAAAAACAGATAAGGTTTGTGCGG
TTAGTAATATGAACTTAGCTGCTAGCTCTTATTCTAACTGATTTTTCTGACTGCTACCTTAGATTGTCTTAT
GGACCACCCTCCCTTTAATGAGGGCGAAAATTTGTGCATGAGAATCTAGTGAAGTGTGTAAGGACCAGAG
GCTATAAAAACCTTTCGGTTAAAGAGGCAGGTGTATTAGCTTGTTATATTGTTGGCTATAATGTTTAAGTT
TTCCGTTCAGGGAAAATCGCTTTTTCCACTACCGATAACGTATGTGGACCTGAGAAAAAAAATTGAATTTG
ATTGTTAAAGAGGCGGCTGTCCTTATGAACAATTCTTACCCTAATGAGGACCGTTGTCCCCAGATCTGCCT
CGACTTTATTTTCAATATAGAGGGGCGATAGCTTGTTTCCCAATCTGCGGTTCGTCTTTACCAGAACCTATT

CGCCCACTTCAAAAGCCCTATACTGTCTGGCTGCATTACATCTTTCAAGTACAGCTTGCTGCACCTTTTCAG
TTGTGGCATGTATGACATCCGGCGGTTTTTGGTTCGTGACCGAGTGGTTTGACCTGTTATACTCCATAATA
CTCCTCATAATTAAGCTTAACTGTATCGCTGGCTTTCTTATCCATTTTTAGGCTTCGAGCTATGTCCGTCAA
GGGTGCTATGATAATCTTTCTACCTGACCATTGGAAACCGCTATGTAACGGTGGCGAGTTAAACGATGTGT
ATGTCGTAGTTATTTTTCAGAAGTGACACTATTGTTTCAGAATTGAATGCTGCTTCGTTATCGCAGAATATC
GTTTTCGATTTAGGGATTAATTCAGCTGATTTAAAGTCAATTTTTTGTGTGACGGGATTGGTTTGGACGAC
AGCGAATTTAGAGAATTTGTCTACACAAGTAAGGAAAAACTTCTTATCGGAGGAAAATGGAAAATGTCCA
TATGCAACATTTCCGCTAGCATATGAAGGTATTGGTGAGACTCCAAAAGTGTTTGTTTTTGTGGATACTTA
TCATATTTTGTTTTTGTGCATATTTTTTGCAATTTCTGACAACTACATTTGCCAAATTATTCATTTTGGGAAA
AATAGTAGTCACGAATTTGCTTAACATTTTCCTGTGCGGCTCTGTGCGCTCGGTTATGTTCTGTCGTAACGA
TTTCCATTTTGATCGTTTTTGTCATTAATATCTGACACATAGTTCTTCCAATGCCACAACTAAGGTGGCTGG
GAAGTTTAACTCGAGTTCGTGTTGAAAGCTAGCGAGCGTTGGAAGATCGTAGTGAATTACGTTGAACAGT
GAAGCTTTGTCCACGAAGCTAATGATGTGGCGAATTTTTTTGCCGAAGGTTATCATTTTCCTTTGCAGTGG
AAAACGTGCCTTTTCTAAGACAATTTGGGTTTAAAGCATTTTAATGGCTTGTCAGTTGCCTCTATCGTGTA
GGTTAACGAGGTCTCCCTGTGAATGGTTGCACCGTCGATAAAGGCTCATCTTGCACTGCGTTCACATTTTG
TCTGGATAGGGCATCGGCCACAAAATTTTCCTTGGCGTTGTGTTCGTCAATGAATACTTTCCATCTTTTGAT
TTTTGCATTTGTATTTCTGTCAGAGACAGCGAAAGTAAGGTGCAGATGTTCGGTGAATATATTTGTCCCAT
ATAGATAATTTTGTAACCTAACTATGGCCAGCAACTCTCGTTCATTTGTAGCGTAATGAGATTCGCCATCT
TTGAGGGTTCTGGAAATCATAGTTATTGGCCTACCATCTTGAGACAAAACGGCACCGATGCCGTCCATAG
AAGAGTCAGTGGTCAAATCAAAGCATATTTAAAGTCCGGGTACATGAGGATTACATCTTCAGATGCCAAA
ATACTCAAGGTTTCGAAAGCATTGCGTTGCGTTTCATCGAACTCAACTGAGATTTTTTTGGACATGTGTCT
ACTCACGGAGCCGTTTTCTCCTTTGAGAATGTTGGTCAGTGGCCTAGCTATTGCTGCAAAATCTTTAATAA
AGCCTCTATAAATAGTTGGCTAGACCAAGAAATGATCACAGGCAAGTTTTTTGGCTCAGGGCATTCCTGC
ATGGCTTTCACCTTTTCAGGGTCGGTTCTGGCCCCGTTTTATGTTACTACGAATCCCAGAAACTCGACGCTT
TCTTTGAAAAACTTTGTCTTTTCTTGAGCAACACACATGTTAGCATTGCACAGACATTCCAACACCGGGCT
TATGTTCCGGACATGATCAAAACTTCCCTGTGTGTTCTTCCCTATGTGTTCTCTGAGGACATCTTCGATGGC
CCTTTGAAAAATGCTTCCCGCATTTTTCAGTCCGAAAGGCAATCGGCAGAACTCGTATTTTCCCCCATTGA
CCGAGAACGAGATTTTTTCGCGAATTTGTTCTGCTAAATAAATTTGGTGGTAACCCGATTTTAAGTCGAGG
GTCGTGAAAAACTTTGCCTGTCCCAGATCATGGGAATACTCGGCATTGGGTATCGATCGGCTATAGTTCGT
TCATTGAGCTTCCTTAATTCGATTACTAATCATTTGTTTTTGTTGCCGTTGGGGTCGAAACCTTTTTTGTCG
ATGACCCAAGAAGGGCTGTTGTATGGCGATTTGGATGGTCCAATTATACCATCTTTTAGCAATTGTTTGAT
TTTGAAGTTTACGAAATCTGAAACTCCCATAGGGTGCGGGTATAGTTTTGAATACACCGGCTCGTTGTCTG
TGGTTTGAATAGTGGCAATGACAGAAGTGTTGAAAGGAAGTGCCTCGTTAGAGGTCGAAAATGCCAAATG
TCAAGTTTCAGCCATCCTCGGTCACCTTATAGCTACTGATGATCCTTTTCACCTTTCTCGAAGTCTGCCATT
ACAACTAGCCAGATGATGATGAGCTGCTGATGGTAATGCTGAGAGCGGCGCCGACTGTGGCCAGGTGCTT
GGATGTTTCTGTGGATGCCAACTCATTTTTTAAGTTGTTGATGACATGGAGATTATGGTTATTGATCCTGTG
GAGTTATGATTTATGATGTTGAGTAGAGGTGACCCATGATGCCTGGTGCCTTCCTATGATCTCGCGCTTAT
TCGTATTTTGTGCCATTGCTATTAATGCGACTGGTTCGAGGTGGCTCGGTTGTGTTCAGCACGAGGTCGTG
CTGCCATCGTATAATCGTCGTGCACAGTAGTCGTGTGCTGCGAGCTTACAATCAGAGGCGTAGGTTGTTTA
CACGCACTCGTTGACTGCTAGTTGCAATCGGCTACGGTGTTGTCTTGTATTTGTAGTGCGACATGTTGGTG
GGTCACGGGATAGATAGTAATCTTCTTGCAGATGAATTATACCCTAGGATAGGC
>160_rnd-1_family-112__LTR___Gypsy
GTGCCTCAATAAAAGTGCACTATTATCAAACAAACAAAAATAAAACATGTTCGAGTTCTAGTGGAATCAT
ATAACAAAGTCCTTGACATTGTAAACAACAATGAACGTAGGTTAAGCGATTTACATTTAACACAATCAAT
TGACGTGTTAAAAAGCCTGCGTGAAAATATTAGACATATTTCGGAAAGACATAATTTTCAAATATTTGTTC
CAAATGCGCTAAATACACCAGCAACATTCGAAATCACTGATTTCATGGTATCAGTAGAAGAGTAACTCTG
ACAAAAGACCAAANAACAGATACGAAATTAGTCGACATGGCNCAAACACCNGTAGAATTTCTCAAGCTA
GCCTCATCTCTGATACCAGAGTTTGACGGCAAGGCGGAAAATTTAAAAAGCTTCATAGATGCTCTGAATC
TGGTAGANTCTCTAAAAGGAACACACGAAGCACTTGCCGTAAGTCTTATTAAGACAAAACTAAAAGGCCA
TGCCAGGAACCTGATAAGTGATGAGAACACTATTGCAGCNCTCATATCGAAACTATCGGACGAAGTAAAA
GGAGAGTCGGTAGAAGTTGTNTCTGCTAAGCTTCTAAACTTACAACAAAAGAGCAAAACGGCCAACCAAT
ACACCCAAGAGGTGGAAAAACGCAAAGGCCCTTGAAGGNGCCTATATCAGTGAAGGTCTAAGCCAAACA
NTTACAGCACTACAACAGCCGTAAAAGCTATGACCCAAAATTGTTCGATTGATAAGGTAAAACTTATCAT
GCAGGCAGGCACATTCACAAATATGAATGATGCCGTCTCCAAATTTGTCAATAGTTGCACGGAGGTAACA

GGTCAAAGTAACACTGTCCTGTATTATCGACGAGGTGNAAGCCGNAATAACAGACGAGGCCGAAGTTATA
ATCGTGGCAAAAATCGCGGCAACAGCAATTGCAACCGCGGCGGTAATAACAACAATAACAATGGCAATA
ACCGTGGAGGCAAGAGGAGGCAACAATTCGAACCGAGGCGGNAACCACAACCAAAACAGTAACAATAG
AAACAATGTNAGAGTGGCNCAAGATACGTCGGAAAACTCTCAGAACCCCTCAGATATTCAACAGCCAGTA
AATGCAAAGGTTCATNCCATCAATCTCAGTCTTAATATATTTGTCACTTTTTATAATCANNCAACTGAAAA
TAAACTCGCATTCCTAATAGATACTGGTGCAGATATNTCACTNTTAAAAGAAAATTCAGATATATTTCAAA
ATNCAAGAAGANAAAATTATAAACATCCAAGGCATNGGCCAAGGCGTAATANANTCNAAAGGANTAACT
TCAATAGAACTTCAACCNACAAGCAAATATATAATTCCACACGANTTTCATTTAGTAGATTCGGATTTCNC
AATACCGTGTGATGGAATAATAGGCATTGATTTTATTAAAAAATTCAATTGCCAATTAGATTTCAAACCAA
GTGAAGACTGGCTTATAATTAGACCCCAAAACTTAAATTATCCAATTTATGTTCCAATAACATATAGTTCT
GGCAACAATGCCACAGTTCTGCCAGCCAGATCCCAAGTAGTCCGAAAAATACACATTAATTCAGTAAANG
ATNATATATTTGTTCCAAATCAAGAAATNCATGATGGAATTTACGTTGCAAACACAATAGCAGCATCCAA
ACATGCATACGTTCGCCTTCTAAACACAACTAATNTCGACCAATTGGTCAANGTAAACAAAATCNAATAT
GAAAATTTAACAGATTACGACATTCATAAAACTAATCCAGAAAATAGAAGNGAACAAGTACTTTCAAAAT
TAAAGAAAAATTTTCCACNACAATTCAAAAATCAATTAACAGANTTATGCACCAAGTATAGTGACGTGTT
CGGACTGGAAACCGAACCGATAGCGACAAATAATTTTTACAAACAAANACTAAGACTNAAGGATGATGA
ACCCATTTATATNAAAAACTACAGAAGCCCGCATAGCCAAGTNNAAGAAATTCAAAAACAAGTAGGGAA
ATTAATAAACGACAAAATCGTCGAACCGTCTGTATCCGAGTATAACAGCCCACTCTTGCTAGTTCCGAAA
AAATCATTNCCAGATTCGGAAGAAAAGAAATGGCGATTAGTAATTGACTATCGCCAAATCAACAAAAAA
CTACTNTCCGATAAATTCCCGCTCCCCAGAATTGATGATATTCTAGACCAACTGGGCAGAGCNAAATACTT
TTCATGCCTTGACTTAATGTCAGGCTTCCACCAAATTGAACTCGAAGAAAGTTCAAGAAATATAACGTCTT
TTTCAACGAGCAATGGCTCGTATCGCTTCACGCGATTACCATTTGGTCTNAAAATAGCGCCAAATTCATTT
CAGAGAATGATGACTATAGCATTCTCGGGTTTAGAACCCTCTCAGGCATTCCTTTATATGGATGACTTAAT
GGTGATAGGNTGTTCCGAAAAACACATGATTAAAAACTTAACTGATGTTTTCGACGNATGTAGGAAATAC
AACCTAAAGTTGCATCCAGAAAAAATGTTCATTTTTCATGCATGAAGTGACATTTTTGGGTCACAAATGCAC
TGACAAAGGAGTATTGCCAGACGACAAGAAATNCGACGTCATCAAAAATTACCCNGTCCCNCACGATGC
GGACAGCGCGAGACGATTCGTAGCATTCTGCAATTATTATCGNCGNTTTATAAAAAACTTCGCCGACTATT
CACGGCACATAACNAGATTATGTAAAAAGAATGTTCCTTTCGAATGGNCANNTGAATGCCAAAACGCATT
CCAATACCTCAAAGAAAAACTTATGNACCCNACNTTATTACAATATCCTGATTTCCGCAAAGAATTTTGCA
TNATAACAGATGCNAGTAAACAAGCNTGCGGAGCGGTTCTAACTCAGAACCGTGACGGANTCCAACTCCC
AATTGCNTACGCATCNCGNTCNTTTACNAAAGGGGAAAGCAATAAGAGCACAACAGAACAAGAGTTAGC
GGCAATTCATTGGGCAATNACCCATTTTAGACCATATATTTATGGCAAACACTTTACTGTTAAAACGGATC
ACAGACCATTGACATATCTNTTTTCNATGACNAATCCNAGCTCGAAATTAACNCGCATGNGGCTAGAGCT
NGAAGAATACGACTTCACGGTAGAATACCTAAAGGGNAAAGATAATTTCGTAGCAGACGCGTTNTCGNG
NATAACCATNAAAGAGCTNAAAGATATGCAACATAAAGTCCTGAAAGTCACTACCAGGCGACAAAGTAG
ACAAAAAANNCTGCGCAGNAGAAAATTATGAANTTCTGCCAAGGCAAAGTCTATAGAAAANGCNTCNAA
GCCCAACGTACATGAAGTCATTAACAATGACGAAGTACGAAAAGTAGTGACCTTGCGCGTAACAGAATAC
TAAATGTTTATTTAAACATGGNAANAAAATTACCGCAAGAATCGANGTTAGCGATTTATATACCAATGGA
ATCCTCGACTTAGGTCAATTCTTCCAAAGGCTCGAAACGGAAGCCGGTATATNNAAAATCAGCCAACTCA
AAGTGGCACCGTGGGAAAATATCTTTGAACAGTTTCAATAGATTAATTTCAAAAATATGGGCAATAAAAT
ATTAAANTCNTTAAGAGTAGCGCTACTCCACCCGGTGACCCAAATAAAAGATGAAAAAGAGATACAAGA
AGCTATTGTCTACATNCCATGACGACCCAATACAAGGAGGTCATAGCGGCATNACAAGAACGTTGGCNAA
GGTNAAAAGACACTACTACTGGAAAGGTATGACNCGNCATATAAAAGAGTACGTACGTAGATGTCAAAA
ATGCCAAANGTCTAAAACAAAGACGCACACAAAAACTCCTTTAACNATAACNGAAACACCTGTANACGC
TTTCGACAGAGTGATAGTGGACACAATTGGTCCGCTACCCAAATCGGAAAATGGNAATGAATACGCAGTC
ACATTAATNTGTGACCTTACNAAATATCTAGTCGCGATTCCAATNCCAAACAAAAGCGCAAACACNGTCG
CNAAAGCTATATTCGAAAATTTTATNCTCAAGTACGGTCCAATGAAGACGTTCATTACGGACATGGGAAC
AGAATACAAGAACTCGATTATAAACGATTTGTGCAAGTATNTGAAAATAAAGAACNTAACATCNACTGCA
CATCATCACCAGACAGTTGGAACAGTAGAAAGAAGTCACAGAACTTTTAACGAATACATACGNTCATATA
TATCGGTTGATAAAACTGATTGGGACGTATGGATCCAATATTTCGTTTACTGTTTCAACACGACCCCATCT
ACGGCGCATGANTATTGTCCGTACGAACTAGTATTTGGTAAACTACCAAAACACTTCAATAGAATAGAAC
AAACCTAGAACCTATATATAATATAGATGATTATGCTAAGGAAANNAAGTNTAGATTAGAAATAGCATAC
AAAAGAGCTAGAATTATGTTAGAAAAAAAATAAGGCGAATAGNAAGGAAGCATATGATAAAAAAGTAAN
AAACTTTGAANTAAAAATAGGAGATAAAGTCTTATTAAGAAACGAAACAGGNCATAAATTAGATCCAGN

ATATNTAGGACCATATANAGTAGAAAAAATAGAAGATAGNGATAACATTTTAATTAAGGATAATAAAAA
TAAAAAACAAAAAGTNCATAAGGATAGATTAAAAATTTTTAATNAATAA
>160_rnd-1_family-312__LTR___Gypsy
AAAATATATCCCACAATTGGCCGGCACACAAGAACACTATATCCTCTTGGCGACAGACAAGAATTGACGC
CTACGATATTTTCAAGCCATACTATGGAANTTCTTAACATGANCAGGCAGCTTCAATTATAAACAACAAA
GCACGTCTTAAATGAAAAATAAAGAAGTGAAGCTCTGCAAACATTCGTCTCTGGACTCAAGAAATCCCTT
AGGATTAAGATCTTCCCAGCCCAACCAAAAANTTTTCGAACAGGCCATGGCCCTTGCGCAGAAGGATAAG
TGAGCTACGATCGCTATGCGCTCACGGCAAATTATGCCAAATACAGTGAGGACAAAACTCATAAGCTCAN
AAAACAACTACTCCAGGGATGGCGTCAGAATGACTTCCGACAATCAACAGATAATCCTAATTACCAGGNC
AAAATGGTAATAATTCAGCCAAGTTGAAAACAATGCCCGTTTAGTCAGAANATTAACACGGCGACACGTC
GTCGCGCTGCAGACTGGCAACACAATTTTTTAGAACATCAGGTTCAGGGATTCCATGCAGTTTTTTTTTGT
AGTTTTTTTTCTGTTACTTTGCTTAGCAACTTTTATCAACGTGAGTAATTTCCGTGCAGAGTATTAACCATA
CGACCCAGGGTCAGGAAGAGAACAAGCGAGAGTTACGAGTCTGTAGAGCAGGCCGAAGCCGTTCGACCA
CAAGACGACTTGACCGAAATCGAAACGTGCAATTTTTGGAGGTGCATCTCAGCTACCAAAGTACAGTAGC
AGGAGAAACCATAAGGCCTACCTGCGTCCTATCGGCCGATCAGTGTCCTGCCGTCGATCGCGATGGTTTG
GGAGCGACTGGTCGCATCTAAGATGATTCTTGTCGTGGAATCCCGTGGACTCATTTCTGAATAACAACAGC
AACATTCCTCAATAACAGTACTACCGAACGAGCACGCCGGCTTTCGAACTACANTCTTCAGGTGTTTGACG
CAAAAGAGTATTGCAATGCAGTCTTCATTGACATAGAGCATAATTTGGACAGGTGAAGCTTCTGGGTCTC
CTGGTCAAGGTAAAATGTCGCCTACCCTCGATCGAACGATCCTTCAATGTCAAAGCGCGAGCGGGATTAT
CCGTCATAAATCCCCTTCTGGCAGAAGTACCTCAAGGCAGTGTACTCACTCCTATTCCGTTCTCGCTTCTCT
CTGCTTTACAGCATACATACCGTTCCCTGACGTGAACAAGTGGTGCATTGATCCGGGCAAACTGATGGTTA
CCTACGCTAATGATGTCGGAATCTTTTTCAGCTTCCGTTGCGAGTACGAAGCTGCAGATGGTATCCAGGAG
TACCTTCACTTTCTAGATAACTGGGCTAAGATGTGGAATACTAACGTGTGCTTTAATTTAAGGATAAATGA
GGGATAGCTCACTGGCAATACCGTTCCTCATGCCAACAAATTGAAATAATTAGGCTTGCCCTTTGAATAGA
GGCTAACTTTCATATCTCTCAAGTCAGTGGTTAATAGTTGCCGTACCAAAATATCAATTCTGAAATGGCTA
AATTGCATTGTTGCTCCCGTATGGATGCACGGCATTCCGATTTTGGAAACTGCTGCCCAGTCCCAATGAAA
GAGAGTGCAAATCATGCAAAAAAAACATTGCGCTTAATCAGCGACTATCCATGGTATGTCAGCAACTCAA
CTCTGCCTAAAGGCCTGAATCTTGCATTTTCACCATTTCCTTCGAGGGAATTCACGGCGCACTAGGCGTCA
ACCAGGCCTTTTTAGGCCGTAACACACCAGAGGTTATTACCTTAATAGATACGGCGGTCAACGCCGCGCC
TACACACCAATCAACATTCTTTAAGTCTGTAGCGGCGGAGATCCGCGCATTGAGTTACAAAATTAATTAG
AAAACATGAAATCTAGTGTAAGTATGAAGAGCGCAATAAAGCTTCATCACTCGCTTTGGGAACGAATTCG
CCTCGCTTCTCCGTCTTAGCGTTATGTAGGTCAGTTGTCGAATTAAATCGAAGCGATATACACCGAAACGA
ATAACTTGTCTCTTAAAGCCACCAAGTTTCGGTTTTGTNAATTATATAACATAATTNAATTCTTCAGGCTG
AATTAGGAACTATCACACAGAACAAAACATCAACTGTTAAAGAAGAAAACCAAGAACACTATATCCTCTT
GGCGACAGACAGGAATTGACGCCTACGATATTTTCAAGCCATACTATGGAATTTCTTAACATGAATAGGT
AGCTTCAATTATGAACAACAAAGCATGTCTTAAATGAAAAATAAAGAAGTGAAGCTCTGCAAACATTCGT
CTCTGGACTCAAGAAATCCATTAGGATTAAGATCTTCCCAGCCCAATCACAAATTTTTTCGAACCGGCTAT
GGCCCTTGCGCAGAAGAATCAGTGAGCTACGATCGCTATGCGCTCACGGCAAATTATGCCAAATACAGTG
AGGAACAAAACTCATAAGCTCAGAAAACAATTACTCCAGGGATGGCGTCAGAATGACTTCCGACAATCA
ACAGATAATCTTAATTACCAGGGCAAAATGGTAATACTTCAGCCAAGTAAACAGCCCGATTAGTCNGAAA
ATTACCAGGGCGACANGTCGTCGCGCTGCAGACTAGCAACACAATTTTTTNGAAACATCAGGTTCAGGGA
TTCCATGCAGTTCTTTTTTGTAGTTTTTTTTCTGTTNCTTTGCTTAGCAACTTTTATCAACGTGAGTAATTTC
CAGGCAGAGTATTAACCATACCACCCAGGGTCAGGAAGAGACAAGTGAGAGTTACGAGTCTGTAGAGCA
GGCCGAAGCCGTTCGACCACAAGACGACTTGACCGAAATCGAAACGTGCAATTTTTGGAGGTGCATCTCA
GCTACCAAAGTACAGTAGCAGGGCAAACCATAAGGCCTACCTGCGTCCTATCGGCCGATCAGTGTCCTGC
CGTCGATCGCGATGGTTTGGGAGCGACTGGTCGCATCTAAGATGATTCTTGTCGTGGAATCCCGTGGACTC
ATTTCTGAATAACAACAGCAACATTCCTGAATAACAGCACTACCGAACGAGCACGCCGGCTTTCAAACTA
CATTCTTGAGGTGTTTGACGCAAAAGAGTATTGCAATGCAGTCTTCATTGACATAGAGCATAATTTGGACA
GGTGAAGCTCCTGGGTCTCCTGGCCAAGGTAAAATGTCGCCTACCCTCGATCGAACGATCCTTCAATGTCA
AAGCGCGAGCGGGATTATCCGTCATANATCCCCTTCTGGCAGAAGTACCTCAAGGCAGTGTACTCACTCC
TATTCCGTTCTCGCTTCTCTCTGCTTTACAGCATACATACCGGTCCCTGACGTGAACAAGTGGTGCATTGAT
CCGGGCAAACTGATGGTCACCTACGCTGATGATGTCGGAATCTTTTTCAGCTTCCGTTGCGAGTACGAAGC
TGCAGATGGTATCCAGGAGTACCTTCACCTTCTAGACAACTGGGCTAAGATGTGGAATACTGAAATAAAT
CCTCCAAATAACTAACGTGTGCTTTAATCTGAGGATAAATGAGGGACAGCTCACTGGCAATACCGTTCCA

CATGCCAACAAAGTGAAATATTTAGGCTTGCCCTTTGANTAGAGGCTAACTTTCATATCTCACAAGTCAGT
GGTTAATAGTTGCCGTACCAAAATATCNANTCTGAAATGGCTAAATTGCATTGTTGCTCCCGTATGGATGC
ACGGCATTCCGATCTTGGAAACTGCTGCCCAGTCCCAACGAAAGAGAGTGCAAATCATGCAAAAAAAAA
ATTGCGCTTAATCAGCGACTATCCATGGTATGTCAGCAACTCAACTCTGCCTAAAGGCCTGAATCTTGCGG
TTNCACCATTTCCTTCGAGGGAATTCACGGCGCACTAGGCGTCAACCAGGCCTCTTAAGGCCGTAACACG
CCAGACGTCATCACCTTAATAGATACGGCGGTCAACGCCGCGCCTACACACCAATCAACATTCTTTAAGTC
TGTAGCGGCGGAGATCCGCGCATTGAGTTACAAAATTAATTAGAAAACATGAAATCTAGTGTAAGTNNGG
AGAGCGCAATAAAGCTTCATCACTCGCTTTGGGAGCGAATTCGCCTCGCTTCTCCGCCTTAGCGTTATGTA
GGTCAGTTGTCGAATTAAATCGAAGCGATACGCACCGAAACGCATAACTTGTCTCTNAAATCCACCAAAT
TTCGGTTTTGTTAATTGTATAACATAATAACAATTTTCAGGCTGAATTAGCAGCTATCACACAAAACACAA
CATCAACTGTTAAAGAAGAAAACCAAGAACACTATATCCTCTTGGCGACAGACAGGAATTGACGCCTACG
ATATTTTCAAGCCATACTATGGAATTTCTTAACATGAATAGGTAGCTTCAATTATGAACAACAAAGCATGT
CTTAAATGAAAAATAAAGAAGTGAAGCTCTGCAAACATTCGTCTCTGGACTCAAGAAATCCATTAGGATT
AAGATCTTCCCAGCCCAATCACAAATTTTTTCGAACTGGCTATGGCCCTTGCGCAGAAGAATCAGTGAGCT
ACGATCGCTATGCGCTCACGGCAAATTATGCCAAATACAGTGAGGACAAAACTCATAAGCTCAGAAAACA
ATTACTCCAGGGATGGCGTCAGAATGACTTCCGACAATCAACAGATAATCTTAATTACCAGGGCAAAATG
GTAATACT
>160_rnd-5_family-3466__LTR___Gypsy
GCAGCGTTGCTTTTAATGTTTTAATTTAAGCTTTGACCACCGTGTCCCCCATTTGCATATTTTTGTGTTCTTA
ATTCATCTTTATTTAATTCAGGTAATTTCACAAGTAGTGTGTTCAGCTATTTCATCATAAGAGAGTGATGCT
AGAGAGCGCAGTTCAATAGGGCGATAACTTACATGCTAAGAATATATATATCGATAGTAGCTTAAATTAA
AGTCATACATAACATTTCTTCCCCTTCTAAGGGACTGGTGAGAATATCCTTTTTGGTTTAATTACTCGAGTC
GAGCGACGTACACTTTGCTCTGTTCGCATTGGTGTTTCTATTGGTGTTGACGATCCCCGTAACATTGGCGA
TCCCCTTGGTGCTGACATAAGTTCGTCTTGATTTAGTGAACTATTATTTTCAGTTGTGCGCAAAGCTTTTGG
TGTTCTGAGGCTATCATTTTCATCGGCATGACCACCCTGCCTGTTGTCGGTCGTTACATCTGATCCAATGCT
GTACTCCCTTGGTCTCTGCTCTGTTGTGTTATGAGCAGGTGGTGAATCTCTAGTTTTGCTGATGTTCACTGC
TGTGTAGTAAGGTGTCTTTTCTGGGTTCCTTCCCTCTATGTTTTGTTGTTGTTGTCCTCCGTCCTTCCACCCT
GTGCCTCGTATCTGATTGATATGTCTTTTATGGTATTTATTGTTGTGTCGAATTTCATAATGCAGGTCACCC
AAACGTCTTACGATCTCTCCTCGTATCCACTTATCCATTCTCGGGTTTCCAGAAATGAAGTAAACTACATC
CTTAACCTCAAATTGGCGCCACGATTGCTTGCATTTTAAAAATTGTTTTGCTGACACCTTTTCCTTGGTGGT
TTCTGGTCTGATCAAATCCAGTCGTGTACGTAATTTTCGACCCAAGAAAAGTTTCGCTGGGGCTTCGCCAG
TTGTGCTATGCGGAGCGTTTCTGTATTGCTGTAAGAATTCGTTTACGTTTCTTTTAAGTGTCTTGGGTGTCG
TTCCCATTGCCTTTAGCGCACTTTTTACCGTTTGGACATTTCTTTCTGCTTGACCGTTCGTTGCGGGGTGAT
AGGGTGCTGATAACTTGTGGTATTTGACGCCAACAGATGTGAGGTATTGCTTGAATTCGGCCGAAGTGAA
ATTTGTCCCGTTATCGGACACTACCATTAGGGGGACGCCATACNCAGCAAATAAATCATCTAGTAATTCGC
TTGTTGTTGTTGCCGTAATTGACTTTGTGATCTTAACGTCCAACCACTTTGAGTATGCATCCGTAACAATCA
AAAGCATCGCACCTTCAACTGGTCCCGCGAAGTCGATGTGAATGCGCTCCCATGGCGCCTTAGGGTATTCC
CAATAGTGATCTGAGCATTTTGGAGGTCGATGCGCATGTTTTGCACAGAACTCGCATGTTCTTGCCGCATT
CTCGATGTCCTTATCGATTCCTGGCCAGTAGACGAAAGAGCGTGCAATACCCTTCATTTTTACTATACCCA
AGTGGGCCCTGTGTAGTTCGTCCAGGATCAGTTTTTGAAGTTTAGTCGGGATAACCACACGATGTTCGAAA
GTAATGCAGCCAGATGATATCTCATACTTGCACTCTGGTGATTTGTATCCATTTCTTGTGAGACATTGGCC
GCTCTCCAGTATTTGCAGGATTTTGCCCAAATGCTGGTCTTTTCTAGTTTCTCGAGCAACCAGCGAAGCTG
TAACTGGAAGCTGATTTATTTGTCGTATTATAAAACTATCGAATTCGTCTCGATTTGCTGTCTTAGAATGG
ATACCATTACTCGTGTTGAGTGGTAATCGGGAAAAGTAGTCAGCATTTACATTTTCCTTTGATGTTTTGGT
AGTTACTGTGAAATTGAAGTTGCTTAAAAAATCTGCATAGTTTGCCATCCTAGAGATGCATAGCGTCGGTA
GTGATTTAGTAGGGTGTAAAATTTGTGCGAGTGGTTTGTTATCGGTAACCAGTGTCCAACTTCTGGCGTAT
AAGCATTTGAAGAATCTTTGTACCGCCCATACAATTGCTAGAGCTTCCTTGTCGATTTGTGTATATCGCTGT
TCCGTGGGACTCATTGTACGGCTTGCGTATGCAATTGGTCGTTCGCCACCATTTTCCAAGCGATGCGATAG
TACAGCGCCTATACCAATTTTGCTGGCGTCCGTTGCTAGCACTAATGGAAGTGTTGGGTCATAAGGCATCA
GCACCTGCGGTGAAACTAAAATTTGTTTGATTCTGTTGTAAGCTAATTCCGTCTCTTCNGACCAGTAGAAT
TGTCGTCCTTTGAGTATATCACGCAGCGGTCTTTCCAAGGTTGACAGATTTGGTATGAACTTTGAGTAGTA
AGCTGCTTTCCCAAGAAACAGCTGCACGTGTTCGATTGTAGATGGTTTCGGTGCTTTTAAGATGGCCTCAA
TATGTTTAGTTGATTTATGTATGCCTTCTGCGTCAATCTTGTGTCCCAGAAACTCAACCGAAGATGCGGCG
AATACACATTTGGATTTGTTCAATTTAAATCCCAGGCTCTGTAATTTTTCTAATGTTGCTTCTAAGGTTCTT

AGCAACTCATCAACATCTTTTGCAAATATTAGAATATCGTCAAAGAAGTTACGTACGTTGGGAATATTTTT
CAGTGCGGCCTCCATTGTTCTCTGCCAAATTGCCGGTATGTTCGCTGCTCCGTACACTGCACGAGTCGGTC
GAATGAGCCCGTGTGTTGCCGTATTCAATGTCAGTACGTGTGCGAATTCTTCATCAATGCGCAAATGTGAA
TATGCATCAGTTACATCCAAGTGTGCGAACACTGTGCATCCTTTCATTTGGTTAAATATATCCTCTGTCCTC
GGAATCGGATGCTCATCGATGATCATCTGAGCATTTACGGTCGGCTTGTAGTTGCCAGTGATTCGTACGCC
TCCCGTTTTCTTCGTTACCACATGCGTTGTGGATGCCCATTCTGAGTAATCAACTCGTTTGTAGAAGCCTGC
AGCAATTTTTGCGTCGATCTCTTTCGCATAAGNATCACGCAGCGCGAATGGTACATCTCTTGCCCGAGCAA
AAATTGGTTTCGCGTCCGGTTTTAGGTGCACCTTAATAGCTGGTCCTTTGAAGGTTCCTGCTGCTGAGTCG
AATATCGCATCAAATTTTGTTAGTATTTGCTCTAGTTGTGTCCTCTTATTCGTAGTCAGTCCCGCCGCTTGT
ACTGCTATTGTTTTCACCGCGCTTCCTTTCGCCGAGAGCATTTCCAGATCTATGCCGTTGGCGTTGACAGA
ATTGTTTGTAAAGATCCCAGTAAAATCAATTTCTTTTGCGAATTGGGAGATCCATTCTCGCCCACACAGTG
TCTCCAGGTTTCCTTCTACTACATACAGATTTAGTTTTCGTGTAGTCTTTCCATAAGTCACGTTAACAACTG
CCCGTCCACAGCAGTTGATCCTATGCCCTGTATAACTCGAAAATTTGCGGTCAGTCTTCTTTAATCGTATTA
AAGGTTTAATTTTCCGAAGGGTCTTTTCATTCATTATGCTGCACGGAGCACCTGTGTCCAGTTCCATTGGTA
TTTTTATTCCATCAATCTTCATTGTTATGATTTTCCTACCTACTGACTTGATGGCGTTGGTATTCGGTTGTTG
AATAAATTGTATTTCTTCCGGGGCGTCAGAATCTGTTGACTCGGAAATCTGTTTCGTATTGGCTTTACATAC
CTTTCTTATATGCCCTACTTTACCACACACGTAGCATTTCTCGTTTTTGAATTTGCAATCTTTGCGCAAGTG
TTGCCCCCCACATCCGTAGCATTTGCTTCCTTGCTTCACTCCGCTTGACGGCTTCCTCCTGATTGGCGAGAA
GCCCAGTTTGCATGTAGGCTCTGTATCTGAAGCTTGGTTGTCATTAACCGTGTTTGCTGATCGTAGTAACA
ATTCCCTGGAATATGCGGCCTCGTATGCGTCTGCAAATGTTGTTGGCTGTTTTGCGATTACCTCATCGCAA
GTGCTGCGATTTTGCATGCCGAAAAGCATTTGTTCTGTTAGCATTCTGTCTAAAAACGAGTCGTACTTGCA
AAATGATGCTGCCTGTTTTAAGCGTAGAACAAACGCTGCTAGTGACTCCTCTGGTTCCTGGCGTAGTTGGC
GGAATTTGAAGCTCTCTGCGTAAATGTTTCGTTTGCCATCGAAGTGATTAATTAGCACTTCCTTAATCTCGC
TGAATTGTAGTGTCTCTGGTTGCCTCGGACTTATCAGAATACGTAGCGCTGAATTCAGTTCAGCTCCCATA
TGCACCCTTGCATAATGGTGGTGGTCAGCATCAGGAATTTTACTCAGTTGCAGTGCCCATTCGAATCGTTG
GAAGTAGTCTTGCACTGTACAGTCTTCTCCGCCCTTGAAGTTTGGCATTGCAAACGGCGGTACTTTCGTAG
AAATNGCTGCCCCACTTGCATTTTGACCTGCTTGTTGCACTGCTGACTGAATGGCGCTAGTCAGTGAACTG
ATGACCTCTCGTAGTTGTTCGTTAATAATGCTCATTTTGACCACTTCTGACACCACTTTTGTGTTCTTAATT
CATCTTTATTTAATTCAGGTAATTTCACAAGTAGTGTGTTCAGCTATTTCATCATAAGAGAGTGATGCTAG
AGAGCGCAGTTCAATAGGGCGATAACTTACATGCTAAGAATATATATATCGATAGTAGCTTAAATTAAAG
TCATACATAACAAT
>9_rnd-1_family-461__DNA___Crypton-V
GGAGCCCCCTGGCTCCAAACCTAGAACCTCTTAGTTTTTTATATCAAGTCAGTTTCCTATATAGGCTACAT
GGTTTAGATATATTTAATTTTTCGAATACCTCGGTACAAATTTTCAACAGTTGTTATAAAAGTAAGGATAT
GCATTACATAAGGTGAAGTGGGCAGACACTTCCACAATTTTGTCATAAAATATATTCATGAAAAACTCTTA
AGGCTTTTAATATAAAATATTCTTTACTTTACTTCTTTGCAAATATTATTTACACAACATCTGAATAACCAT
TTTTAATTACTTATTTTTATCGCACAGTAGACTTTACTTTTTAAATACTAAAATCTTATAATTTATGGAGTC
AACTGTTTCAGACATCACATACTATCCAGATAAATCATCGTCATTTATAAATTGTACATATTTTTCTCGCAA
ACCAATGGCACCGTTAGATGATGTTAGAAAAATTACTGTGCCAATTACTAAAAGTCAGAAACAATGTAGA
CGGCACTTTTACATATTTTGTAAAACATTGCAAACAAAATTTGCACGCCACATAGAGCTTAAATATAGAAT
GGAACCAGTTAGAGAGATCAGCAGTTTATCAAAAGACAAACAGCAAAGAATGAAATTGCTTGAGGGATT
ACGGAAAGACAAGGATGCTACTTACAATGAAGGAACCTTTTATAGTTTCACGTCNATTAAGGGAAAAACT
CAAAAAGAGGGCTTTGGATTTTGTGTGCTGCAATGGATGTAAAGCCTACTTACCGAAGACAACAACTCGG
AATCANATTAAAAATTAATGTAGTAATATCCATAAAATATGCAACCGAGATATATTAACCTCTGCACGAA
GTGCTGCAAATAATATTGCGAAATAAAATAATGCCTGTCCTTAATGACGTCGCCATTACTAGGACAATATT
GTATGATCACCTAATTATATTGTTTAAAAATTTAGAAGCAATGAAGCACACGGAAGTACAGCATGATATG
ATAACAGCAATTATGCGTTATCTAAGTCGCCTTTTGATAGTAATAAAATACGTTATTTCCCCTAAATATTA
TGATTCTGTTAAGTCTGGAATTCGTTATGTTGCTGAGTTTGATTATGATAATATTCGTTTTATGAAGCCTAC
TGCAGCGACAACTCTAACATGCTTGGTAAAAATAGTACATGTGCGAGAATATTAAAAGCACAGATCATAA
AAATATTGCGACAATAGCATAAAAATATTTAGAAAAAGAAAAGATTTTGTTCAGGATCTACCTGCTAAAG
CTTTGAATGAGCTAAACTAGAAATTCAACATATCACCATTAACCTCGTTATATCAGAGCAGAGTTAGCGA
ACTTGAGAGTCTTACTCTTAAGAGCTACAGAACTGCTCATGCAATTGGGGCTATCGACGATAGAGCTAATT
CTGACCTGTATGAAGGCACCTCAGCCAAAATGAAAAACATCACCAGGGTGATTATTCGTGCCAAAAAAAA
CCGAACATTTCCAGTTCTTTTTACACCATGCGATCGGTATTGCATAGCCAATATACTCGAATATGGCAAAC

GCACAATAAATAAACCAGCTACTACACTAAAGATATATTTTAGTGTGCCAAGCCCGTGGCCACTAAGAAA
AGAAAATATTCGTGAGTGTCCTATCCTAGAGAAATTCGCAAATGAATGTGGTACACGTTGCCCTGAGTCC
CTTTCAGGCACAGACCTGCGAAAGCAATTGACCACACATACCGCTTTGTTCAACGTTAAGGACATTCAAA
TTGATTATTTGGCGAGCTTAATGGGTGATCATAAAGACATTCATAAAAATTACTATCGTGTTGCTGCCTCT
GTAGCGGGGGTGACCCAAGTTTCTCAATTACTTATGTCAACAATGAACATGGAAGAGGAAAATGCTGAAA
TTGCAGATGCTAATCTCGAGAGTGAGTTAATTTATTTAGCCCGTCTGTTGCGAAGAAAATTTTAATAATCG
CTGCAAGTTTTGGACCTGGAACTGTCATGTTCGGAATACTTCAATAGAAGTTTTTCAAGTATCGGAACTTC
GATACAGTAGAAGATACATTCAGTATTTAAGAAATTAGTAATGGTTTTGGCGACCTATCCATATTTAAAAT
ATTTTGCTGGCGGGACCTCGGCCCGAGTTTTTTTTAATTAATAGATTAAAACAATCTAATTTAATACTTAT
ATTGCTTATTTTATTTAAAGCAAAATATTAAATAAAATATTTTTCAACTGTTTATTTAAACGAGCCCGAAC
CTCGACCACGCCAACATAAATATTTAAAAACATGCGCATTCCGGACGGTATCAGACGAAGCAGCGCTGGT
TATCGCAGGGATGATCCGCACAGCAGCAAGGATGCAAAGCATGGAACGATGGCAAGCACGTTGGGATCG
CTCATCTAAAGGACGCTGGACACATAGGCTTATCCCAAACTTGGAAGCCTGGATAAGCCGCAAGTATGGT
AATACTAATTTTTACTTAACGCAGCCTATTTGGCCACGGACGCTTCAGAAGCTACTTGAAGCGATTCGGCC
ACGATAACACAGACGAGTGTTCTTGGTGCGGCAGTGAAATCGCAGAGGACGCCAACCACATCATCTTTGA
ATGTGGAAGGTTCTCAGTGAACAGGCAGGGCCTGGAGGACAGCTTGAGGAGACCTATCACGGTGGACAA
CTTGTTCAGGAACAAGTTGAAGACCGCACAAAAATGGGAAGCTACCTACACTTTTGCAGCAGAAGTGATG
CTGGAACTCAGGAGAGTTGAGCGCAATAGATGGAGAGCGGAGGAAGTTTAGGTGGCAACAACATGCCTG
TGAAGCAATGCTTTGCGGCCGTTCCGCAGGCAATCCACGCCACCTAATCATCACGTACCCTCCTCCTCTAT
GTAATCTTGCTTCCCAAACAAAAATTGAAACATGCCGCTTACCTTCGTTGATATATCAGCTGTTTATGTTTA
CGGTACTTTACCATTTTTCAAACATCGATAGAAATCTCGATAAATCATCGTAAAAAACTCAATATGTTCCG
CGCGAATTTACACATTNCTCAAAAATGTGGCCTTTTTGAATGATGTGTGAAATGTGTTTTCTTATCTGGAA
ACGCACAGGGCGCATTTGTAGGGACCAACTGGCACAAAATTTATCATTTCATCCCATTACACCAGCACCC
GTTCACAGTCAAAAAGTCACCGAAATATTATGCATGCATCCCATCGTAGTCGCTATTTGCTTTCGCACGAC
GCTGCATGATTTCGCTATCTCAGCGTCGGTGCGTTTCTTCACTCGCACTTTCGTGAGAATGAAGGAATGCT
CGATAGATCAGGCCCCATCACCGTTATAATTTAGATGTGGTGAATAATAATTGTGTTTGTATTTGTGTTTTT
GCTGAAATTGAAGAAAAGTAGAAGGTCCGATTGCCACGCTTCGTTCTTTTTAATTCGACAGTGGAACAAG
CAAGTCACATATTTTGAGCGCGCATAAATCATAGTAATTAACGGTGCAGTGGCTGTACACCGCTTCGGATT
TTTAAGGGCATATCGAGCCCAACTCTTTTTAAAAGTCCCCAGTGCTCATACAGTTCGCGTTCAGCNGATGT
TATTCGCTTTTAATTAATCAAGCGCGTAATTCTGAATTAACCCCTTTTTTAGTGGATAAAATCTTACTTAGC
TACCGGCCAGCCATTGCGCAACGACGCGTTCGTGTATTCATATATATATACACATATAAATTCAGCGTGGC
TGGTCGGTCGTAGCCAATTTGTGAGTTGCAACGGCTCTAAGGGAGTGGAAAATATCAAAAGGGCCACGGA
TTTCTTAAGGATCACTACAGATTAACACTGCGGCGCCACCGGCACATAGCGGCTGAGTCTATACCCCGCG
ACCGCGTGTTTAGCCATTGATAACCAAGCTTTGCTTATCTACTTCTACGTTGCCGCCCCTTTTTGATCAACG
GAGTGTCTCCAACAAGGTATAGGACCGCTTAAGTGTGCGCAGATCGAGATCGAATATACGTGCTGGGCGA
TTGGTTGGAATTTTTGCGTACACGCTTCGATCTAAATGATCATGCTATTTAGCCATTGATAACCAAGCTTTG
CTGATCTACTTCTACTTTGCCGTCCCTTTTTGATCAACGGAGTGTCTTCAACAAGGAATCGGACCGCTTAA
GTGTGCGCAGATCGAGATCGAATATATGTGCTGGGCGATTGGTTGGAATTTTTGCGTACACGCTTCGATCT
AAATGCTCATGCCTTAATATTTAAAAACCTCTTTCGTTTTGCTCAATTTATGAAATAAAGAGAAAAGCTGC
TGCTTTAGAAACCTAATTTCTTTAGCCAAATCAATATAGTCAAGATCTATCGTATTTTATAATTCTATTTTT
TTGAAATCGACCATTTAAAGTAACTTGATAATGAGATGTTATACTTAAACTAAACGGAAAACTAAGTGGC
ATGACAATTAGGCGATGAGTGTACGGTATATCTCAAATTATTTTTTAACAGATATACTAATTCGAATTGCT
CCACAGATATTACGTACGCATGCGAAGGATTTTATTTGTTCAATTTTGTGAGTATGCCATGGCCACATAAG
CCAGCAAAAGAGTTGTAAATATTGAAAAATAATTGTAAATCGGCGAGCCTTCAGCACTACCCGACATAGG
AAAAAGCATATGATTTGAAACTACCGTCTCCCAGGGCCCATTATCTATTTTCAA
>9_rnd-6_family-1736__Unknown
TTTGGAACGTCATATCTCCGCGCGGAGTTATGTCGTTTTGGAACGTCATATCTCCGCGCGGAGTTATGTCG
TTTTGGAACGTCATATCTCCGCGCGGAGTTATGTCGTTTTGGAACGTCATATCTCCGCGCGGAGTTATGTC
GTTTTGGAACGTCATATCTCCGCGCGGAGCTATATTACCCGAGATATTAAAAAATCATCGAAAAAGTTTCG
ATTTTCGCACCGACCTCGATTTTGAAAAAAACGTGATGCAACCCTTGCCATTTTGTCGATTTGGGTCAAAA
TTTTGGATTTCCTTTTTGATGCCAATCGATAGAACTGATCCTTACGAGTCAAAAATGGTATAAAAATTCCA
AAATCGGACATTATTTGACGGAAATATTCCAAAAAAATCATCAAAAAGGAATTTACGAACGAATCGGATT
TTTCGACATCGACGATTATTTGTTTGAATGCCAATCGATGGTAGGGATCCGTACGCATTCAAAAAGAGGTA
TAACATTTTAAAAATCAGACATTATTTACCGAGATATTAAAAAAATCATCGAAAAAAAGTTTCGATTTTTC

GCACCGACCTCGATTTTGAAAAAATCGTGATGATTTTTGGAACGTCATATCTCCGCGCGGAGTTATGTCGT
TTTGGAACGTCATATCTCCGCGCGGAGTTATGTCGTTTTGGAACGTCATATCTCCGCGCGGAGTTATGTCG
TTTTGGAACGTCATATCTCCGCGCGGAGTTATGTCGTTTTGGAACGTCATATCTCCGCGCGGAGTTATGTC
GTTTTGGAACGTCATATCTCCGCGCGGAGCTATTACCCGAGATATTAAAAAAATCATCGAAAAAGTTTCG
ATTTTCGCACCGACCTCGATTTTGTCGNTTTGGGTGATNTTTTGGATTGCCGATTTTTGGATGNCAATCGAT
AGTCGNCATCCNCGCGAGTCAAAAAGGTATGNCGTTCCGAAATCGGACATNATTTGNCCGAGATATTCCA
AAAAATCATCGAAAAGGTTGATTTTCGCGANCGAATCGGTTTNCGAAAACGAAATTTTCCGCGATGATTT
TTCGTTTNAACGTCATTGATCTCCGCGCGGAGTTATGTCGTTTTGGAACGTCATATCTCCGCGCGGAGATA
TTAAAAAATCATCGAAAAAGTTTCGATTTTCGCACCGACCTCGATTTCGAAAAATCGTGATGATTTTTGGA
ACGTCATATCTCCGCGCGGAGTTATGTCGTTTTGGAACGTCATATCTCCGCGCGGAGTTATGTCGTTTTGG
AACGTCATATCTCCGCGCGGAGCTATTACCCGAGATATTAAAAAATCATCGAAAAAGTTTCGATTTTCGCA
CCGACCTCGATTTTNGAAAAACGTGATGTAACCCCTTGCCATTTTGTCGATTTGGGTCAAAATTTTTGGAT
TTCCTTTTTTTGATGCCAATCGATAGAACTGATCCTTACGAGTCAAAAATGGTATGAAATTNCAAAATCGG
ACATTATTTGACGGAAATATTCCAAAAAAATCATCAAAAAGGTTGANTTTACGAACGAATCGGTTTTTCGT
CAACAACATTTTTCAACCCATCGATGATTAATTTGTTTTGAATGCCAATCGATGGTAGGGATCCGTACGCA
TTCAAAAAGGTATAACATTTTAAAAATCGGACATTATTTACCCGAGATATTAAAAAAAATCATCGAAAAA
AAGTTTCGATTTTCGCACCGACCTCGATTTTTGAAAAAAATCGTGATGATTTGGAAGGTCATACCNCGCGG
AGTTATGTCGTTTTGGAACGTCATATCTCCGCGCGGAGTTATGTCGTTTTGGAACGTCATATCTCCGCGCG
GAGTTATGTCGTTTTGGAACGTCATATCTCCGCGCGGAGTTATGTCGTTTTGGAACGTCATATCTCCGCGC
GGAGTTATGTCGTTTTGGAACGTCATATCTCCGCGCGGAGTTATGTCGTTTTGGAACGTCATATCTCCGCG
CGGAGTTATGTCGTTTTGGAACGTCATATCTCCGCGCGGAGTTATGTCGTTTTGGAACGTCATATCTCCGC
GCGGAGCTATTACCCGAGATATTAAAAAAATCATCGAAAAAAAGTTTCGATTTTTCGCACCGACCTCGAT
TTTGAAAAAAAAACGTGATGTAACCCCTTGCCATTTTGTCGATTTGGGTCAAAATTTTGGATTTCCTTTTTTT
GATGCCAATCGATAGAACTGATCCTTACGAGTCAAAAATGGTATGAAATTCCAAAAATCGGACATTATTT
GACGGAAATATTCCAAAAAAATCATCAAAAAGGTTGAATTTACGAACGAATCGGTTTTTGTCAACAACAT
TTTTCAACCCATCGATGATTAATTTGTTTGAATGCCAATTGATGGTAGGGATCCGTACGCATTCAAAAAGG
TATAACATTTTAAAATCAGACATTATTTACCCGAGATATTAAAAAAAATCATCGAAAAAGTTTCGATTTTC
GCACCGACCTCGATTTTGAAAAAAATCGTGATGATTTGGAAGGTCATATCTCCGCGCGGAGTTATGTCGTT
TTGGAACGTCATATCTCCGCGCGGAGTTATGTCGTTTTGGAACGTCATATCTCCGCGCGGAGTTATGTCGT
TTTGGAACGTCATATCTCCGCGCGGAGTTATGTCGTTTTGGAACGTCATATCTCCGCGCGGAGTTATGTCG
TTTTGGAACGTCATATCTCCGCGCGGAGTTATGTCGTTTTGGAACGTCATATCTCCGCGCGGAGTTATGTC
GTTTTGGAACGTCATATCTCCGCGCGGAGTTATGTCGTTTTGGAACGTCATATCTCCGCGCGGAGTTATGT
CGTTTTGGAACGTCATATCTCCGCGCGGAGTTATGTCGTTTTGGAACGTCATATCTCCGCGCGGAGTTATG
TCGTTTTGGAACGTCATATCTCCGCGCGGAGCTATNTCGCTTTGGAACGTCATATCTCCGCGCGAAAAATT
TCGATTTTCGCACCGACCTCGATTTTGAAAAAAACGTGATGTAACCCCTTGCCATTTTGTCGATTTGGGTC
AAAATTTTGGATTTCCTTTTTTTGATGCCAATCGATAGAACTGATCCTTACGAGTCAAAAATGGTATAGAA
ATTCCAAAATCGGACATTATTTGACGGAAATATTCCAAAAAAATCATCAAAAAGGTTGANTTTACGAACG
AATCGGTTTTTTGTCAACAACATTTTTCAACCCATCGATGATTAATTTGTTTTGAATGCCAATTGATGGTAG
GGATCCGTACGCATTCAAAAAGGGGTATAACATTTTAAAAATCAGACATTATTTACCCGAGATATTAAAA
AAATCATCGAAAAAGTTTCGATTTTTCGCACCGACCTCGATTTTGAAAAAAATCGTGATGATTTGGAAGGC
CATGCGGGAGTTNTGTCGTTTTGGAACGTCATATCTCCGCGCGGAGTTATGTCGTTTTGAAACGTCATATC
TCCGCGCGGAGTTATGTCGTTTTGGAACGTCATATCTCCGCGCGGAGTTATGTCGTTTTGGAACGTCATAT
CTCCGCGCGGAGCTATGTCGTTTTGAAAGTCATATCTCCGCGCGGAGATATGTCGTTTGGAACGTCATATC
TCCGCGCGGAGTTATGTCGTTTTGGAACGTCATATCTCCGCGCGGAGTTATGTCGTTTTGGAACGTCATAT
CTCCGCGCGGAGCTATTACCCGAGATATTAAAAAAATCATCGAAAAAAGTTTCGATTTTCGCACCGACCT
CGATTTTGAAAAAAACGTGATGTAACCCCTTGCCATTTTGTCGATTTGGGTCAAAATTTTGGATTTCCTTTT
TTGATGCCAATCGATAGAACTGATCCTTACGAGTCAAAAATGGTATGAAAATTCCAAAATCGGACATTAT
TTGACGGAAATATTCCAAAAANATCATCAAAAAGGTNGAATTTACGAACGAATCGGTTTTTTGTCAACAA
CATTTTTCAACCCATCGATGATTNATTTGTTTGAATGCCAATCGATGGTGGGGATCCGTACGCATTCAAAA
AGGTATAGCATTTTAAAATCGGACATTATTTACCCGAGATATTAAAAAAANAATCGAAAAAGTTTCGATT
TTCGCACCGACCTCGATTTTGAAAAAAATCGTGATGATTTGGAAGGTCATATCTCCGCGCGGAGTTATGTC
GTTTTGGAACGTCATATCTCCGCGCGGAGTTATGTCGTTTTGGAACGTCATATCTCCGCGCGGAGCTATGT
CGTTTTGGAACGTCATATCTCCGCGCGGAGTTATGTCGTTTTGAAACGTCATATCTCCGCGCGGAGNTATG
TCGTTTTNGAACGTCATATCTCCGCGCGGAGTTATGTCGTTTTGGAACGTCATATCTCCGCGCGGAGTTAT

GTCGTTTTGGAACGTCATATCTCCGCGCGGAGTTATGTCGTTTTGGAACGTCATATCTCCGCGCGGAGCTA
TTACCCGAGATATTAAAAAAATCGAAAAAGTTTCGATTTTTCGCACCGACCTCGATTTTGAAAAAAACGT
GATGATTTGGAAGGTCATATCTCCGCGCGGAGTTATGTCGTTTTGGAACGTCATATCTCCGCGCGGAGTTA
TGTCGTTTTGGAACGTCATATCTCCGCGCGGAGCTATTACCCGAGATATTAAAAAATCATCGAAAAAGTTT
CGATTTTTCGCACCGACCTCGATTTTGAAAAA
>160_rnd-5_family-20753__Unknown
GTGTAGAGGTCCAAAATCAGAAATCCTAAAATCTTGTTAAATTTGCATCGGGGACGGGCAGGCAGGCAAG
CAACAGGCACACAAGCGCATAAATGCTTCTATTCATACACAAAATATGCAAGTATGCGTGTATAGCGAGT
CCAAAATATTCTAAATGCACACATGGCAACGCTTGGGTACCCTTACAAACGATAGTGAGCTAAAATGCAA
GAATTTCGCATCGACGTGGCAAACCCTTTCTCTATTTCATATTAGCCGTGCCTTCAAAAGGGTAGAAATGC
CTCAGCATTTGGGAAAAATTGAATTTGTGCGCAGTTTTGCTCGTCGGACAAATACACACACACACACATTG
AAACGATCCCTAAACATAGAGAGAGCATTGAAAGCTGCGAGTAAGTGCGAGCGAGATAAAAGTTAACAG
CGTTGCCGTTCTATCGCACAAATCATAAGGATGAGAGTGGGCCAAGCACTTTCCTGTAGAGTAAGCGGTT
CAAAATACATCATATGTATATCTTACTCCCTTACTCGCGATTTTGGACCAGATGCCTCTGATAGTTTTTGTG
ACAGCGCTGTTTATATGGGATGACCAGTCTATATGTTGTTTGGTTAGTGCTTCAGCAAAGCTGGTCGATGC
TTCTTTGCGTCGTTTCGTTATCGAAGTACATTGAGCGAAAAATTTTAGAACTTACGATCGACGCAAAGTGC
TTGTGCTTTATTGTGAAATGCATGCTTTATTTAAATGATTTATTACCATAGATACTAACGATTTTTTTACA
GGAAGATATAAGAATTAAATTATATTTACACAAGGCACATCTTGTGCAATAACTACGATAAGAATTTAGC
ATACATAGATTGATTTCACTTTCGTTTTTCGACAGAGAAAAGTTCTTGTCTCAGACAAAGGCGTGCAAGCA
GCTTTGGTTGAATGCTTTGAGTAATAAAGAGAAAGAGACGGCATTGGGATCAAAAGCTCTTTTTAGAAAT
ACTTCCTGAGCGAAAACGAGGTTGCAAACAAAAATTGCAAAGACAACACCCAGCTGTCTGCAGCTTCTTC
TGCCGATGCCCACAATTCAATGGCAACTTCAAAACATTTTCCATTAGAACCAACAACAACAACCATAATC
AAAGCAAATGACTCGACAGTGAAAAAAGCATACCACAAATTCTGTCCTTGCTATCCTTTTTTGTTTGAGGC
TACAGTCTATGATCTTAGCCTCTGCACCCATCCAACTCATTGTTGTGGTTTCTATGCACTGCTATGCCTATA
ATTCGCAAAGTCATGTTTTCTTCTTCTTCACTCGCTTTCAACTTGCAAGCAGATCGGCAGATTTTTATAATT
TTTCATTTTTCGACCAGCAAGGCACCTTTTCTCAAAATAGGGTATTTTAACTATTCATAAGCAATGTAACC
AAATAGCACAAACCATCTTATATATACATTTTTATTGAATGTCTAAAAAAATATAATTTTGTATTAAGTGT
GCAGCTATTAGTACAGTCAATAAAAATTGTTTTCTTTATAAATTATATACCATTCTTATTAAAATGAGTATC
AATGCCGCATCCTGCCAAATAGTTGCTTATGTGTGTCTCATCTCACACATATTGGTTCAAATCTGCATATTT
GTATGTGTCTGCCAAGCCAAGGGAACTCTATGAATTTAAGAAGATTGCAACGTTCTGTTGCGCATGTGAC
GTCACAGTCCAAGGCTTTTTATGTGCGATCATTTCGTACATAAAATTATTACATAAGTCAACAAATTATTA
TCTATAAAAGACCCCATTAATAAGGTATTTAAATGTACCTAATAAAGGTAGAAATATATGCTAAACATCA
CAAGAAATAAAATTCTATTAACTGTGACGTCATAATATATGGGCGCCGTGCGCGTAACCCTNTTATCTGAG
TGCCGTGGCTTGCTGATATTCAAATTTTCTACCTACTTGCGCCGAGACTCGGCTCTACAACAACAACATTA
GTACCCGAATTGTGCTGTTGTTGTTTATACGCGCTGCCTTGTAGGAACGACAGAGAAACCCGCAAAGAAC
ACTGTGGGATGAAGAACAAATTGGGTTTCGTGAGCACTGTTGTTGTTTTGCAAGTGATTTGTTGTTGCTTC
CTATGGTAAATGAGCTTTTTTATTGGGTGTGATTTTGCGTTTGCGGACCAACTCGATCTACGACTCGTGGTT
TTTAAAACGGTAAAAAAAGCTCAGAGCGTCAGTTCAGATCTTTCTCTTTATTACTCAATGACTAAAACTAT
ATACATAAGATAAGCAACATATATACATACATATACAGCATATAATTACATATCTACAACATAATACCGT
TTATGTCTAACTTAGGAAGGGTTAGGAGAATAATTCCTACAAGAGGTGCCAAACACACTAGTAGCATCCC
AGATATTAGAGAAGAAGAAGCATCCGAAACCATGTACATGGCTCAGGTAATACCCCCGGCACAACCTGCC
AACTTGCACATGTAAAAAATAATAGGTAGCTTTAAAATTGTCGATTTATTTAAAAACCTTCCCAACATACG
ACGGGAACATGGAAGGACTAAGGGAATATATTACAAAAGTTGAGTAGATATTAATGCTAATAAAAGGGG
CAGCCCAAACTCCAACCGGACTAATGATACTAAGGGCAATCAGAAACAAGATTCAGGGAAAAGCGCATG
AAGTACTATACCAATCGGGAACTGAGTTGAACTGGGACGAAATAAAGAATAAACTTATTACCCACTTCGC
GGACATGAGAACCGAACATAGCCTTATGGCAGACCTAGGCATCCTTTCACTTCAATGTTACCCAGTAGGA
ATTCTGTACGAAAAGATAACAGATATTCAAAGAGCTCTACTCAATATCGTAAAAGAAAAATAAGTGAATC
AAACGGTCATTGAAACCAAAAGAGAACTTTTCAACAAACAATGTTTTACAACATTTCTAAATGGACAAAT
CTGACTTTCTACAAAAAGAAATTGAATACCTCGGACACATTGTAACACTAGATGGCGTTAAACCAAACCC
GTCAAAAATGGAAGCTATAAAAAAATGTCCTTTACCGAAATCTTAAAAAGAAATTAAATGCTTCTTGGGT
TTAATGGGTTATTACAGGAAGTTTATGAAAGACTTCCCAAATATAACCCCTGACTAAACAATTAAAAGGG
AAAACGATTGTTGTTTTAAACGATGAATTTATAGAAACATCTAACTACTGTAAAACCTTGCTTTGTAACGA
CCAATTACTAATTTACCCCGACTTTACGAAACCATTCATACTAACAACAGATGCTAGCAATGTAGCGTTAT
GAGCTGTGTTATCACAAGGACCTTTAGGGTCTGATAAGCCAATTTTGTTCGCACGCCGAACACTAACCGAT

ACAGAAGAAAATTATTCCTATTCGGTCAAAAGTTTAAGATATTTACAGACCACAAGCCATTAACCTGGTT
AATGAGTTTAAAGGAACCCAACTCCAAATTAGTCAGATGGAAGCTTAGACTTGTGGCGCTATCTGGCAGC
ACAGAAATTGATACATTTTAAGTTTTTTGTCTTTGTTATTTCACCATATTACGAAAAGTATGTACACTTTCT
TCTAGCTCGAGCTCTCTAACCGTCAACATCTACGCATCTTAATATTCACTTTTTTGGTGTCTAATAAATAAA
GCTGCTATTNCTAAAATCGAAATATTATTATTACTTGTGTTAATGGATGTAGAATTTGACTTTTAAGTTCCA
TTCATATAACCAAAGACTGTTGGAATACGACTATGAAGTAATATACAAAAAGGGTTCGCAGAACGTTATA
GCAAACGCACTTTGTAGAACAACACTAGACGTAAACATGAATGACCCCGAATCAAACAAAGGAACTTGT
GGAGAGACAGTACATGTAATGTCTGTGAATGGGCTGATCATGGGCGCAAAAGAAAATACATTATTGGCAC
GCGTCTGACGAGCGCCCTTAGATTTTGATGAATGAATGTTGACCCCTTATTACCTGCACCAGTTCGTTGGG
TCAAGTGGGTCAACTTTTGGCAAGCACCTGCATATGGAGGTCAAGTGACTGATCACCAAAATTATTAAAA
CTATAAATATATAATCGCTCCTTTTTTTTTAATTTGGTATATTTTAATATTTTAAAGATTTTTCTTTTACTCA
AATTTCATTCAAAATCATTAAAAAGAAGGGCGCGCGTCAGACGCGTGCCAATAATGTATTTTCTTTTGCGC
CCATGATCAGCCCATTCACAGACATTTGTATGTGTGTGTCTGTGTGTAGAGGTCCAAAATCAGAAATCCTA
AAATCTTGTTAAATTTGCATCGGGGACAGGCAGGCAGGCAAGCAACAGGCACACAAGCGCATAAATGCTT
CTATTCATACACAAAATATGCAAGTATGCGTGTATAGCGAGTCCAAAATATTCTAAATGCACACATGCCA
ACGCTTGGGTACCCTTACCAACGATAGTGAGCTAAAATGCAAGAATTTCGCATCGACGTGGCAAACCCTT
TCTCTATTTCATATTAGCCGTGCCTTCAAAAGGGTAGAAATGCCTCAGCATTTGGGAAAAATTGAATTTGT
GCGCAGTTTTGCTCGTCGGACAAATACACACACACACACATTGAAACGATCCCTAAACATAGAGAGAGCA
TTGAAAGCTGCGAGTAAGTGCGAGCGAGATAA
>160_rnd-1_family-326__LTR___Gypsy
TGATGTCCAGTCTATATGTTGTTTGGTTAGTGGTCGAAGCCAATTACCGATATAAATATCGATATCGACAT
TGAAACACTGGACGATACAGAATCAAACATGCCAGACCAGGTAGCCTTAGATAGGGAATATGTAAGACA
AATTTCGTCTNCAGTCCCTGAATTTGACGGTAAAAAACTGTCACTAAATCGTTCTTTAACAGCCTTAAGGC
TTGTTGAACGAATAAAGGGCAACCAATAGGATTTGGCTGTAGAAGTCATAAAATCAAAACTAATTGGCCC
AATACTATACAAATGCCAAGACGAGACCACGATTAATGTCATTATTAATAAATTAAAAAGTAGCGTCAAA
GGTGAATCCATTGACTTGCTTAAGGCAAAGCTGTTAAACGCCCAACAAAAAGGCAAAACAGCAGCTCAAT
TTACGGCTGAAATCGATAACTTGCGTAAGCAACTCGAGGCAGCTTATATTGACGATGGCCTAGACTCGGA
TAATGCAGAGAAACTCTACCAAAGAGTCAATATCAACAATGACCAAAAATTGTGAATATGAAAAAAGCT
CCGCATAATACTAGAAGCTGGAAACTTCAGCACATTTAATGATGCAATGAGTAAATACATTCATTGCAGC
ACTGAAATGACTGGAAGTGTCAGCACAGTATTATATCAGCGAGGAAGACATAATAGAGGCATTGATTATC
GCAATTACCAAAGAGGTAGAGGCAATGGCCGCGGTAATCATAACGCAAACTATAACAACAATAGCAATG
GCAGAGGACATTATAGACGTAATGCCCGTGGTAATAACCGAGGAAGGAATAATTCCAACCGAGGTAACA
ACCAAAATAATAACAATAGAGGTAATGTCCGAGTAACTGAAGGTAATTCGGAAAACCANTGAGACCCCT
CAGATGTTTAGAGCCAATAAATGGCACAATAAAAACCATCAACCTCAACCTCAGCATATTTGTAAAAATT
AACAATGAAAGAACAGGTAAATCCTTTGCACTGTTGCTTGATACTGGTGCAGATATATCTATATTAAAAG
AAAATGTAGATATATTTGAAAACGTAAACGAAAGAGTTACTACCCAAAAATCGGGAATTGGCGAAGGAA
CAATTTTTTCAAAATGCTTGGCCTTTATAGAACTCACTACAGGTAAATACATAATTCCGCATAACTTCCAT
TTAGTCGATCAAAAATTCCCAATACCTAGTGATGGCATACTAGGCATAGATTTCATAAAACAATATAATTG
CCAATTAGATTTCACTCAGAGTGGCGACTCACTAATTCTTCGCCCAAACAACTTAAATTATCCGATTAAAT
TACAAATACTTCATACTTGTGACAATAATGCCACAGTACTCCCAGCTCGTTCCGAAGTTGTCNGTGAAATA
CAAATACCCTCAATAAAAAATCAAATTTTGATATTAAATCAAGAAATTGATGAAGGAATTTATATCGCAA
ATACCATATCCAACCACAAAAATACTTGTGTCCGAATTTTAAATACAACAAATACCAATAAGATCGTCAA
TTTAAATAAAATAAAATATGAAACCTTAAATGATAAAAGAAACTAACGAAGAAAGAGACAACTTCGTTAT
GAATAAATTAATTAAAAACTTTCCGCCTTTATTCAAAAGTCAATTAAAAGATTTATGTACCAGGTATACAG
ACATATTCGGACTAGAAACCGAATCAATAACAACAAATAATTTTTACAAGCAAAAGCTTAGACTGAAGGA
TGATCAGCCCGTATAAATAAAAAATTATAGGAGTCCTCATAGCCAAGTACAAGAAATTCAAGCCCGAGTA
CAAAAATTAATAGACAATAAAATAGTCGAACCGTCAGTCTCATAATATAATAGCCCACTCTTACTAGTCC
CGAAGAAGTCTCTTCCGGACTCTGAAAAGAAAAAATGGCGATTAGAAATTGACTATCGTCAAATTAATAA
AAAGCTATTGTCCGACAAATTTCCACCACCTAGAATTGATGATATTCTAGATCAACTATTTAGAGCAAAAT
ATTTTTCATGCCTAGACTTAATGTCTGGCTTCCACCAAATTGAACTTGAAAACAGTTCTAGAAATATAACG
TAATTGTCAACAAGCTATGGCTCATATCGCTTTACGCGATTACCATTTGGATTAAAAATAGCCCCAAACTC
ATTTCAAAGAATGATGACAATAGCATTTTCTGGTCTACAACCATCANAAGCATTTCTTTATATGGATGATC
TAATAGTAATCGCTTGTTCCAAGAAACATATGGTCAAAAACTTAACTGATGTTTTCGACAAATGTAAAAA
ACTCAACCTGAAACTACATCCGGTAAAATGTTCATTTTTTATGCATGAAGTCACATTTTTGGGACACAAAT

GCACTGATAAGGGAATCTTGCCAGACGACAAAAAATACGACGTGATTAAAAATTACCAACTCCCACACGA
TGTGGACAGCGCAAGACGATTCGTTGCATTTTGTAATTACTATACGCGCTTTATAAAGAACTTCGCTAACT
ATTCACGTCACATAACAAGATTATGTAAAAAAGACGTAAAATTCGAATGGACGTCTGAATGCCAGAACAT
CTCAAAACAGCACTAATAAACCTAACGTTGTTACAATATCCAGACTTCACCAAAGAATTCTGCATAATAA
CAGATGCAAGTGAGCAAGCGTGTGGAGCAGTTTTAACTTAAAACTTTACTGGACTCCAACTTCCAATTGC
ATACGCATCAAGATCCTTTACTAAAGGAGAAAGTAACAAGAGTACAACTGAACAAGAATTAACAGCTATT
CACTGGGTAATAACACACTTTCGACCATATATTTATGGTAACCATTTCACGGTAAAAACGGATCACAGAC
CACTAACATCTATTTTCAATGGTCAATCCAAGTTCACAATTAACACGTATGAGGCTTGATTTGCAGGAATA
TACCTTTACGGTAGAATATCCGAAGGGTAAAGACAGTTACGTAGCAGATGCGTTATCGAGAATAACCATC
AAGGAGCTACAAAATATAACAAGAAACATAATGAAAGTCACTACAAGACAAGCACGTAGACAAGAATCC
TGCGCAGGAGAAAAAGAAATAGAATTGCCTAGGCAATCTATAGAAAAAGCTTCAAAGGCCAAAGTGTAC
CAAGTCATTAACAATGACGAGGTACGAAAAGTAGTGACCTTGCATGTAAACAATATGACATGTTTTTTAA
ACATGGCAGACAAATTACAGCAAGATACGATGTTACCGATTTATATACTAATTAAACCATTGATTTAGATC
AAATCTTTCAAAGGCTTGAAACGCAAGCCGGTATATATAAAATCAGCCAACTCAAAGTGGCACCGTGGGA
AAATATCTTTGAACACGTTTCAATGGATAATTTATAGAAAATGGGCANTCAAATATTAAAATCTTTAAGA
GTTGCGCTACTCCACCCGGTGACCCAAATAAATTCTGAAAAAGAAAAATAAGCTATCTTGTCTAAACTAC
TTGATGATCCCATACTAGGAGGACATGCATTATAAAAACCTTGGCCAAAGTCAAACGACATTATTTTTGG
AAAGGTATGTCTAAACAAATAAAAGACTACATCCGCAAATTTCCAAAATGCCAGAAAGCAAAAACTACA
AAGAACACAAAAACTCCTCTAACAATAACAGAAACACCAATATCCGCTTTCGACAGAGTAATCGTTGACA
CAATTATTACCATTACCTAGAACAGAGAATGGGAACGAGTATGCAGTTACACTCATTTGTGATTTTACGAA
ATACCTCGTCGCAATTCCGATCCCAAATACAAGTGCAAACACTGTCGCTAAATCTATATTCGAATCTTTTA
TTCTGAAGTACGGTCCAATGAAGACGTTCATTACGGACATGGGTACGGAATATAAGAATTCAATTATAAA
CAATTTGTGCAAGTATATGAAAATAAACAACTTAACATCCACTGCACATCATCACCAGACAGTTGGAACA
GTAGAAAGAAGTCACAGGACTTTTAACGAATACATACGATCATATATATCAGTTGATAAAACTGATTGGG
GCGTATGGATCAAATACATCGTATACTGTTTTAACACGACCCCCTCTATCGCACACAATTATTATGAGCAA
ATTCTTGGTAAGCACCCAATTTACCAATATAAATAGAATCGACCCACTATATAATGTAGAAGATTATGCTA
AAGAAGNTAAGTTTAGATTAGAATCAGCATATAAAAGGTCTAGGATATTGCTTGAAAAACATAAAGCAA
GAAGTAAGGAACTTTTTGATAAAAAAGTTATAGATTTTCAAGTAAAAATAGGAGATAAAGTCTTATTAAG
AAACGAAACTGGCCATAAATTGGATCCAGAATATATAGGCCCATATACAATAGTTAACATAGAAAATAGG
GATAACATTCTAATTAAAGATAACAAAAATAGGACCCAAAAAGTTTATAAGAATAGACTAAAAATTTTTA
ACGAATAAAACATATAAATGTTCTATATGAAGTCTTTATGTTCCTTTATGAATTCACAACAAATAACAAGT
AAGAGGTTCTAGTCGGGAGCTCCCGACTAGGGGATACCCTGAACCCTCTTCTTCCAACATCAAATGCATAT
ATAATCTATTTTAGAAGCTATATGTCAAGTTTGGTTCTAG
>Ref_dvir_258
GTCTTTATTGGGGGACAGCACTTAGTGTAGTGGTTTGGAAACATCTGTTGGCGTCTCTCACTCGCGCCTTC
AATCGGCTCAATAGTTATGGATGGCCTGGAGGCAGGCCGGTAGCGATTCAGATTGCTAGATGGAGCCCAC
AAGGAGCTGCTGAGCTGTTGCATATTTTGTTTGAGTGGGCGATGCCGAAGCCGTCGCTCACTCCAAATTGA
GGTATTTAACCAGCAGGCCGTCGTGGAGCATATCATCTTCTTGGTAAGTATGTATTTTAAACAATGTTTAT
TTTGTTTTTGTTTATGTGCCTTTGCTTGCAGGACCAGCGCATCATCGTCGTGGTTTGGAAACATCCAGTGGC
GTCTCTCACCCTCCACAACTTGTCCAGGAATTGCATGTCAGCTGACTTGGCGGCCGCAGCAACGTATTGGG
TGGCCCGCAAACAGGCCGGTTGCAGTTCAGGGTGCTAGATGGAGCCCACAAGGAGCTGCTGAGCTGTTGC
ATATTTTATTTGAGTGGGCGATGCCGAGGCCGTCACTCACTCCAAATTGAGTTTTTAGCCAGCAGGCCGTC
GTGGAGCGCATCATCTTCTTGGTAAGTATGTATTTTAAACAATGTTTATTTATGTACCTTTGCTTGCAGGAC
AGCGCATCATCGTCGTGGTTTGGAAACATCTAGTGGCGTCTCTCACCCTCCACAACTTGTCCAGGAATTGT
TTTTTTTTTTTTTTTTTTTTTAACCACTTGCCCGCCAGAAAGATACTGACGGTGTCTTTATTTTTAATTTAA
CTCATTACTTTAACATAGCAACTTATTTTAAATTTAAATGGCAACAGAAGTTTTTATAATATGTGGGCAAA
ACATTTTGCAGCCCTGTTACAGCGTGTCGGTATTTTCTTATATACTAACTTATCGGCTAAACGCATTTTTGG
TATTTTAATTTGCAACGATAGTTTTCGTCCTATATTACTCATCTTTAGGAGCGGCATCAATGGCAGCGTTTC
CTGAGTTTGGCCTAGTAAATCGCCGTGGGAACCTGCGTCTTCTTGAAGCAGTGGCATCAATCTCTGTGGCG
GCTATCGCGCGTCGTGCTTCTCACTCAGCTTGCAGGTACGCTTATGATGGGTGGCCACTCGGGCAATTCAG
GAAGTAAGGGGAGCCCACATGGGGCTGCTGAGCTATACCGTCGCTCTTGCCGAATAGTGGTTTGGCTCAG
TGGGTCGTCGTGGAGCAGATCATCTTTTCTGATAGTTGCATGTCAGGGGACTTGGCGGTCACAGCAGCGC
AATTAAACCGGCTCAGTAGTAATGGATGGCCTTGGAAAACAGGCCGGTGGCGTTTCAGGGTGCTGGATAG
GACCCCCAAGAAGCTGTTGTGCTATTTATATTTTGTTTGGATGGACGATGCCTAGGCAGTCGCTCGCACCG

AATTGAGTATTTGGCCCCGTTGACCGTCGTAGGCGCATCGTCTTTATTTGCAGGACAGCAGCTTCAGCGTC
GTGGTTTGGAAACATCTAGTGGCGTCTCTCACCCTCCACAACTTGTCCAGGAATTGCATGTCAGCTGACTT
GGCGGCCGCAGCAACGTATTGGGTGGCCCGCAAAACAGGCCGGTAGCGATACAGATTGCTAGAATGGAG
CCCAACAGGGAGCTGCTGAGCTGTTGCATATTTTGTTTGAGAGGAGGATGCCGGAGCCGTCTCTTATTCCA
GATTGGGTATTTAACCAGCAGGCCTTCGTGGAGCATGTCATCTTCTTGGTAAGTATGTATTTTAAACAGTT
TTTTTATTTTTGTTTATGTGCCTTTGCTTGCAGGACAGCGCATCATCGTCGTGGTTTGGAAATATCTAGTGG
CGTCTCTCACCCTCCACAACTTGTCCAGGAATTGTTTTTTTTTTTTTTTTTTTTTTAACCACTTGCCCGCCAG
AAAGATACTGACGGTGTCTTTATTTTTAATTTAACTCATTACTTTAACATAGCAACTTATTTTAAATTTAAA
TGGCAACAGAAGTTTTTATAATATGTGGGCAAAACATTTTGCAGCCCTGTTACAGCGTGTCGGTATTTTCT
TATATACTAACTTATCGGCTAAACGCATTTTTGGTATTTTAATTTGCAACGATAGTTTTCGTCCTATATTAC
TCATCTTTAGGAGCGGCATCAATGGCAGCGTTTCCTGAGTTTGGCCTAGTAAATCGCCGTGGGAACCTGCG
TCTTCTTGAAGCAGTGGCATCAATCTCTGTGGCGGCTATCGCGCGTCGTGCTTCTCACTCAGCTTGCAGGT
ACGCTTATGATGGGTGGCCACTCGGGCAATTCAGGAAGTAAGGGGAGCCCACATGGGGCTGCTGAGCTAT
ACCGTCGCTCTTGCCGAATAGTGGTTTGGCTCAGTGGGTCGTCGTGGAGCAGATCATCTTGTCTGATAGTT
GCATGTCAGTGGACTTGGCGGTCACAGCAGCGCATTAAACCGGCTCAGTAGTAATGGATGGCCTGGAAAA
CAGGCCGGTGGCGTTTCAGGGTGCTGGATAGGACCCCCAAGAAGCTGTTGTGCTATTTATATTTTGTTTGG
ATGGACGATGCCTAGGCAGTCGCTCGCACCGAATTGAGTATTTGGCCCCGTTGACCGTCGTAGGCGCATC
GTCTTTATTTGCAGGACAGCACTTCAGCGTCATGGTTTGGAAGCATCTAGTGACGTCTCTCACACTCCACA
ACTTGTCCAGGAATTGCATGTCAGCTGACTTGGCGGCCGCAGCAACGTATTTGGGTGGCCCGCAAACAGG
CCGGTTGCAGTTCAGGATGCTAGATGGAGCCCACAAGGAGCTGCTGAGCTGTTGCATAGTTTGTTTGAGT
GGGCGATGCCGAGGCCGTCGCTCACTCCAAATTGAGATTTTAGCCAGCAGGCCGTCGTGGAGCATATCAT
CTTCTTGGTAAGTATGTATTTTAAACAATGTTTATTTATGTACCTTTGGCTTGCAGGACAGCGCATCATCGT
CGTGGTTTGGAAACATCTAGTGGCGTCTCTCACCCTCCACAACTTGTCCAGGAATTGCATGTCAGCTGACT
TGGCGGCCGCAGCAGCGTATTGGGTGGCCCGCAAACAGGCCGGTTGCAGTTCAGGATGCTAGATGGAGCC
CACAAGGAGCTGCCGAGCTGTTGCATACTTTGTTTGAGTGGGCGATGCCGAAGCCGTCGCTCACTCCAAA
TTGAGTATTTAACCAGCAGGTCGTCGTGGAGCATATCATCTTCTTGGTAAACAGTGTTTATTGTGTGCCTCT
GCTTGCAGGACAGCGCATCATCGTCGTGGTCTGGAAACATCCAGTAGCGTCTCTCACACGCCATGCAGTTT
GACCAGCAATTGTTTGGATGGACGATACCGAAGCAATCGCTCACCTCAAATTGAGTACTTGGCCCAGTCG
ACCGTCGTAGGCGCATCGTCTTTATTTGGGGGACAGCACTTAGTGTAGTGGTTTGGAAACATCTGTTGGCG
TCTCTCACTCGCCACGAAGCTTGCCTAGGAATTGCATGTCAGTTGGCTTGGCGGTCACAGCAACGCCTTCA
ATCGGCTCAATAGTTATGGATGGCCTGGAGGCAGGCCGGTAGCGATTCAGATTGCTAGATGGAGCCCAAC
AGTCGCTGCTGAGCTGTTGCATATTTTGTTTGAGAGGGCGATGCCGGAGCCGTCTCTTACTCCAGATTGGG
TATTTAACCAGCAGGCCTTCGTGGAGCATGTCATCTTCTTGGTAAGTATGTATTTTAAACAGTTTTTTTATT
TTTATTTATGTGCCTTTGCTTGCAGGACAGCGCATCATCGTCGTGGTTTGGAAACATCTAGTGGCGTCTCTC
ACCCTCCACACAACTTGTCCAGGAATTGTTTTTTTTTTTTTTTTTTTTTTAACCACTTGCCCGCCAGAAAGAT
ACTGACGGTGTCTTTATTTTTAATTTAACTCATTACTTTAACATAGCAACTTATTTTAAATTTAAATGGCAA
CAGAAGTTTTTATAAATATGTGGGCAAAACATTTTGCAGCCCTGTTACAGCGTGTCGGTATTTTCTTATAT
ACTAACTTATCGGCTAAACGCATTTTTGGTATTTTAATTTGCAACGATAGTTTTCGTCCTATATTACTCATC
TTTAGGAGCGGCATCAATGGCAGCGTTTCCTGAGTTTGGCCTAGTAAATCGCCGTGGGAACCTGCGTCTTC
TTGAAGCAGTGGCATCAATCTCTGTGGCGGCTATCGCGCGTCGTGCTTCTCACTCAGCTTGCAGGTACGCT
TATGATGGGTGGCCACTCGGGCAATTCAGGAAGTAAGGGGAGCCCACATGGGGCTGCTGAGCTATACCGT
CGCTCTTGCCGAATAGTGGTTTGGCTCAGTGGGTCGTCGTGGAGCAGATCATCTTGTCTGATAGTTGCATG
TCAGTGGACTTGGCGGTCACAGCAGCGCATTAAACCGGCTCAGTAGTAATGGATGGCCTGGAAAACAGGC
CGGTGGCGTTTCAGGGTGCTGGATAGGACCCCCAAGAAGCTGTTGTGCTATTTATATTTTGTTTGGATGGA
CGATGCCTAGGCAGTCGCTCGCACCGAATTGAGTATTTGGCCCCGTTGACCGTCGTAGGCGCATCGTCTTT
AATTTGCAGGACAGCACTTCAGCGTCCTGGTTGGAAACAA
>Ref_Uvir
CACCTCATCACACCTTCATATCTTTTATATTTACGCCCTATAAATATAAATTTATCTCTTTCTGGTGTACCA
TCACCAAAAACCATACTTTACTTACCTTCACACAAACTGCTCGCATGATTGGCCTCCGTATTGGCATTTGG
CATGCCAATGGTTTGTCAAACAAAACTGACGAGCTAGAGCAATTTATAATACGTCATAATGTCGATCTAA
TGCTTATAAGCGAGACTCGCTTCAACTTCAACTCTCGTGTCCAAATCCCTGGATACTCCATCATAACGGCC
AACTCACCCCAAACTCACTTATGCATTGGTGGTGCAGCCATCCTAATCTCAAACCACATCGAATATCAAGC
TCTTCCAGGCATATCACTGCCACAATTACAATGTGCTATTGTGCGCCTCCGTACAGATCTGGGTGGTGCCA
ACATTGCGTCCTGCTACTGGCCCCCCAATCACGCAGTCTTAGCAGACGACTATGTCTGCTTATTTAACCAA

TTGGGTGAAAACTTTCTGATTGGAGGGGATTGGAATGCCAAGCATCGCCTCTGGGGCAACATGCGACGTT
GTCCTCGTGGCTCGATCCTTGCAAACATTCTAATGGACTCCAACAAATATAACGTACTCACAACGGCTGAG
CCAACACACTTCCCATCTAATAGCAATCGTCCAAGTGTACTCGACTTTGGCATCTATGCAGGTATCTCGAG
TCATAGGCTTGCCATAAGTCGAGTCACAGAGCTCCATAGCGACCATCTGCCCTTGCTAGTCCAACTCAAAA
TCACCATTAACGGTCAAAGTCTATGGAATAATCCTAACCTAATTCATCGGTCACGCCCCCGCCGGCGAAA
CCGTCACCGTCTACTCACCGAACGCTCAAACCTGGAGATTTTTCATCAAAAATTGGAATCATCCATCAATC
TCAATATTGAGATTGATTGTATAAATGACATCGATGATATGCTCGAAAATTTTATGTCCAAGCTGCACATT
GCAGCCGAAGCGTCAAATTCTCGATCAGCCATGGCATCCATTATACCCCCGCATCAAAACAGCTCCAATA
ACACACTCAATAACGAAAACAATAACAATGTCAATCTCGACTTATCGTCACCTCAACCATTACCACTGTCC
GCTTTTATCAGAACAGACGAACATCGCGAATTGATGCGACTAAAACGCCGTCTCCGTAAACGCTGGGCAC
GCACGCGTCAACTTGATCACTGGCTTGAATGGCAGCGGGTTGGCCGACAACTGGCCAACCAGCTTGAACA
ACAACGCCGTGATTACGTTGACTACGTGCTGAGCAATGCAGACCCTCAAAAGAAGGGCGCCTTCAACCTG
TGGCATGCCACAAAATATTTAAAACGACAACCCCATGCTCAACCCAGTGTTCGCAACCTCAACGGTCACT
GGTGCCAGTCTGCCGAAGAGCAGGCTGAAGCATTTGCCGATGAGCTACAAGCACGTTTCACACCATTCAA
CCTTGCCCCATCAGGACAATGCGAACGTGTGAAGCGCACACTCAATCAACAAGTCAACTTATATGACAAC
ACTCTAAACGCAATCAATCCTCAATATCCCATTCTATCATCACACATACGCCATGTAACACTATGCGAGCT
AAATACTTACATTGGGCATCTACAAATGCGTAAAGCACCGGGGCTCGATCAAATTGATAACTATTTGATC
AGGTCCTTGCCTCAAAAAGCCAGACTTTTCTTGTTGCTACTATTCAATGGCATGCTTCGACTACGGTATTTT
CCATCAGCATGGAAATGTGCCGCCGTCCGGATGATATTGAAAAGTGGCAATCGGTCAGCTGCAAACCTCA
ACTCTTACCGGCCAATAAGCCTATTATCTACGATCTCGAAACTATTTGAGAAAGTGATATATGAGCGGCTA
AATGACGAATTACAATCTCCACTAGAGAGCGAATCTGAATTAGAGTGCTCCAGAGTGATCCCGAACCATC
AATTCGGTTTCCGCTCTGGTCACGGCACCATCCAACAAGTGCATCGGATTGTCGAGCACATAAACAACTC
ATTTGAAAGGGGCCACTGCACCTCGGGAGCTTTTCTCGATCTGCAGCAAGCTTTTGATCGTGTCTGGCATG
ACGGTTTACTATTCAAACTGCGCACACACACGAGTGAACCCCTCTATCAATTACTCAAATCATTCCTATCA
AATCGTAGTTTCTGTGTATTAAGCACAAATCCGACAACAAATGGCAACAGAGGTGCCGACGCCTGCCACT
CGACACTACGACCAATGGTGGCCGGTGTGCCGCAGGGCAGCGTGCTGGCTCCACTATTATTCAATATATTC
GTCTCAGACATGCCATGCATTGCTACGCACATACAACAAGTGTTCCAACCGCCGCCCGGCCAAACAAACA
CCAGCATAATTGGTCTAACAGCCACCTACGCCGATGACACAGCATTCTTATGCAGTGCCATGAACGCAGT
GGTGGCCACCACCATTCTGCAGGGTCATATGCATCGATTTGTTAAATGGGCCAATAACTGGAACATTCAA
ATCAATGACAACAAATCGGTGCATATTATCTTTACCCTAAGGCGCCAGATTGCCAATGCTGGCAGCCTAA
CAACACAACTAACAATAAACAACAATATCATACCGGCCAAGAGCTATGTCAAATATCTGGGTCTGAATTT
GGACAAAAAATTAAATTGGGCACGTCATGCCCGTTTACAGCACAAATTGTGCGCAAAAAACTGTTCAAAC
ACAAATGGCTACTATCTGCCAGCCAATCCAGATTATCATTAAAAAACAAATTGCTTATTTATCGACAGATC
ATAGCCCCCACCTGGCACTACGGCATTTGAACTCTATGGCTGTGCAGCTGACACGCATCTGCGCCGTCTAC
AAACTGCACAGTCGCGAGCTCTTCGCCTCATTAGCGGTGCACCATATTATATTACAAATCGCACACTGCAC
CGAGTAATGAAAGTGCCATCAATATGGGACCAGCTCAATCTAAACGCCAGTCGGCACAACGACCGACTGG
AAAAACACACTAACAAAATGGCTAGCACGCTATTGCTTTCAGGTGGACACCGAGCCCAACGCCGTCTCCG
ACGTATCTGGCCAGGCATTGAATTAATTCAACGCCAAGCGCCCGCCAACCTTTTTTAAATAACTACACTAA
TAATCAAAAATGTGACACCCCTCATGGGACACACATTCACCCACGAAAAATACTTTTTCCAAACATCATA
AATAAACACTTTAGTTTAGGTAACTCACTCACCTTATCCCATGGCGAGCCCATAAGAAACACTTGTCACAT
GGATTCAAAATAACGGCAAATATATTATGCCGCAATTATTTTTGATGACAGCCGCTAAGCCGCAGCTAAT
GACAACAAGAAACAGCAGCAGAAGAAGAAGCAGCGGCTAAAACAGCAGCAGAAGCATTGGTAGCAGAC
GAAGACAAAACGGCAGCAGAATGCAACAGCTACATCAACATCAACTATAAGCACGCCCAAATAACCATA
CCAATACAATCAAATCAGAAATTACACATGTAATATACACAATCACATTCATACCAAATATAATACAAAA
ATAATACATTCTCAATCAGCTGTAATACACACACTCACAATACTCACATAAGCTATAATACACCTATTAGA
TAAAATACACATATTTACTCTAACACTGGCATACACATATGCTCACACTCGAATCAAACAAAAAACCAAA
TTTTACTCACTCATCAAAAAAGACAATGAATATCAGGTAAAGTTATATCACCCGATTCCCCTCCCCCACCC
ACAAATCATAATGCCGATTGCTGGCGGCAACCAAATTACTGCCGCGAGAGAAGGCATTTCAAAAACGGCC
TGCTGCCTAGCTACAAATATATATATATATATGCGTACTCATACACATATATGTACATATTCTAAAAATAA
TCCCCTTTATCCCACCAAAACAATTGATAACGCAGTGGCAATGTCAATGTGTCAAAATTGCATAGTCTACG
AATGATGACAGCAGTATCAATTTTTGCTAAAAACTAATTAATATGCTGAATACATCTGGAGACAATTTGCA
CGCACACACACAAATCCGCTCGCATCAATATACTCCCACATATCACAACAAATTTACATCTCACAACATTC
TAGCAAATATAAACAATTAATTTCTCTCAAACATACATTCTTACAAAAAAGATACTCTATCAAAACTGCTG
GCTTATGGCGAACTAGTTTTGCTGATGCCGCACTTTCACCCTAAATTATATATTACACTAAACAATAATCA

TAAATAATCTTAACAAAAACTCTACCACATCAAGCCCATACAAAAAATATGGAGCATTCACTTGAAGACG
TGTGCAATCGCGACAAATGAATTATTCACTAAGTCAGTTCGGCCACTTAATACCCATATGTATACTCATTT
GTCTATACTGCGCACACACACACTCTCTTCACAACTTATGCCAAATTACACACAACCACCACAAAATATAA
CTTTATAACACTTTCAAATCAAAACCTCATGCACACATATACACATAAAAATTGTATAGCTTAAGAAATAC
TTTTTAAGAAATCAAAATTTATAACGTTATGCACAAATAGTAATATATAATTTTAAAG
>160_rnd-1_family-129__LTR___Gypsy
GACAAAAGCAAGAAGANACCGAANTAGTCGACATGGCNCAAACAGCTGTAGAATTTCTCAAGCTAGCCT
CATCGCTCTTACCAGAGTTTGACGGCAAGGCGGAAAATTTAAAAAGCTTCATAGATGCTCTGAATCTGGT
AGANTCCCTAAAAGAAACACACGAAGCACTNGCCGTAAGTCTTATTAAGACAAAACTAAAAGGCCATGC
CAGGAACCTGATAAGTGATGAGAACACTATTGCAGCNCTCATATCGAAACTATCGGACGAAGTAAAAGG
AGAGTCGGTAGAAGTTGTNTCTGCTAAGCTTCTAAACTTGCAACAAAAGAGCAAAACGGCCAACCAATAT
ACCCAAGAGGTGGAAAAAAAAAGGCCCTTGAAGGAGCCTATATCAGTGAAGGTCTAAGCCAATCACTTAC
AGCACTACAACAGCCGTAAAAGCTATGACCCAAAATTGTTCGATTGATAAGGTAAAACTTATCATGCAGG
CAGGCACATTCACAAATATGAATGATGCCGTCTCCAAATTTGTCAATAGTTGCACGGAGGTAACAGGTCA
AAGTAACACTGTCCTGTATTATCGACGAGGTGCAAANCAAAATAATAGACGAGGCGGAAGTTACAATCGT
GGCAGAAACTACAGCCGCAACAATTGTAACCGCGGCGGTGGTAACAACAACAACAATAATTACAATAAC
CGTGGAGGAAGACGAGGCCGAAATNGAGGAAGCAATTCAAGCCGAGGCAAANCCAACAACGGTAACAA
TAGNAACAATGTNAGAGTGGCNCAAAATACGTCGGAAAACTCTCAGAACCCTTTAGGAAACAACCAGTA
AATGCAAAGGTTCATACCATCAATCTCAGTCTTAATATATTTGTCACATTTTTATAATCATNCGACTGAAA
ATAAACTCGCATTCTTAATAGATACTGGTGCAGATATCTCACTNTTAAAAGAAAATTCAGATATATTTCAA
AATTCAAGAAGANAAAATTATAAACATCCAAGGCATNGGCCAAGGCGTAATAAAATCNAAAGGANCAAC
TTCAATAGAACTNCAGNCAACNAAATATATAATTCCACACGANTTTCATTTAGTAGATTCGGATTTCNCAA
TACCGTGTGATGGAATAATAGGCATTGATTTTATTAAAAAATTCAATTGCCAATTAGATTTCAAACCAAGT
GAAGACTGGCTTATAATTAGACCTCAAAACTTAAATTATCCAATTTATGTTCCAATAACATATAGTTCTGG
CAACAATGCAATTCTTCTGCCAGCCAGATCCCAAGTAATCCGAAAAATANACATTAATTCAGTAAAAGAT
NATATATTTGTTCCAAATCAAGAAATANATAATGGAATTTACGTTGCAAATACAATAGCAGCATCCAAAC
ATGCATACGTTCGCCTTCTAAACACAACNAATNTCGACCAATTGGTCAANGTAAACAAAATCGAATATGA
AAATTTAACGAATTACGATATTCATAAAACTAATCCAGAAAATAGAAGAGAACAAGTACTTTCAAAATTA
AAGAAAAATTTTCCACCACAATTCAAAAATCAATTAACAGANTTATGCACCAAGTATAGTGACGTATTCG
GACTGGAAACCGAACCAATAGCGACAAATAATTTTTATAAACAAAAACTAAGACTNAAAGATGATGAAC
CCGTNTATATAAAAAACTACAGAAGCCCGCATAGCCAAGTNGAAGAAATNCAAAAACAAGTAGGGAAAT
TAATAGACGACAAAATCGTCGAACCGTCTGTATCCGAGTATAACAGCCCACTCTTGCTAGTTCCGAAAAA
ATCATTTCCAGATTCGGAAGAAAAGAAATGGCGATTAGTAATTGACTATCGTCAAATTAATAAAAAACTA
CTNTCCGACAAATTCCCGCTCCCCAGAATTGATGATATTCTAGATCAACTGGGCAGAGCAAAATACTTTTC
ATGCCTTGACTTAATGTCAGGCTTTCACCAAATTGAACTCGAAAAAAGTTCAAGAAATATAACGTCTTTTT
CAACGAGCAATGGCTCGTATCGCTTCACGCGATTACCATTTGGTCTNAAAATAGCGCCAAATTCATTTCAG
AGAATGATGACAATAGCATTCTCNGGTTTAGAACCNTCTCAGGCATTCCTTTATATGGATGACTTAATAGT
GATNGGTTGTTCCGAAAAACACATGNTTAAAAACTTAACTGATGTTTTCGACGAATGTAGGAAATACAAC
CTAAAGTTACATCCAGAAAAATGTTCATTTTTCATGCATGAAGTGACATTTTTGGGTCACAAATGCACTGA
TAAAGGAGTATTGCCAGACGACAAGAAATNCGACGTCATCAAAAATTACCCAGTCCCACACGATGCGGA
CAGCGCGAGACGATTCGTAGCATTCTGCAATTATTATNGNCGNTTTATAAAAAACTTCGCCGACTATTCAC
GGCACATAACNAGATTATGTAAAAAGAATGTTCNTTTCGAATGGNCANNTGAATGCCAAAACGCATTCCA
ATACCTCAAAGNAGCGCTNATGNACCCNACNTTGTTACAATATCCNGATTTCCGCAAAGAATTTTGCATN
ATAACAGATGCNAGTAAGCAAGCGTGCGGAGCGGTTTTAACTCAGAACCGTAACGGACTCCAACTTCCAA
TTGCATACGCATCAAGNTCNTTTACTAAAGGGGAAAGCAATAAGAGTACAACAGAACAAGAGTTAGCGG
CAATTCATTGGGCAATAACNCATTTTNGACCATATATTTATGGCAAACACTTCACNGTTAAAACGGATCAC
AGACCATTAACATATCTNTTTTCAATGGTCAATCCNAGTTCGAAATTAACNCGTATGAGGCTNGAGCTNG
AAGAATACGACTTCACGGTAGAATACCTAAAGGGNAAAGACAATTTCGTAGCAGACGCGCTATCGAGAA
TAACCATNAAAGAGCTANAAGATATAACGGNAACATAAGTCCTGAAAGTCACTACCAGGCGNCAAAGTA
GACAAAAATCCTGCGCAGGAGAAAANNAAATAGAATTGCCAAGGCAATCTATAGAAAANGCNTCNAAGC
CCAACGTATACGAAGTCATTAACAATGACGAAGTACGAAAAGTAGTGACCTTGCGCGTAACAGAATATGA
TATGTTTATTTAAACATGGNAANAAAATTACCGCAAGANNCGANGTTAGCGATTTATATACCAATGGAAT
CCTCGACTTAGGTCAATTCTTCCAAAGGCTCGAAACGGAAGCCGGTATATNNAAAATCAGCCAACTCAAA
GTGGCACCGTGGGAAAATATCTTTGAACAGTTTCAATAGATAATTTCAAAAATATGGGCAATAAAATATT

AAANTCNTTAAGAGTAGCGCTACTCCACCCGGTGACCCAAATANAAACGATAAAAGAGANACAAGCNAT
ATTGTCTACATNCCATGACGACCCAATNCAAGGAGGTCATACCGGCATNACAAGAACCTTGGCNAAAGTN
AAAAGACACTACTACTGGAAAGGTATGACNCGNCATATNAAAGAGTACGTACGTAGATGTCAAAAATGC
CAAANGTCTAAAACAAAGACGCACACAAAAACTCCTTTAACTATAACNGANACACCTGTGAATGCTTTCG
ACAGAGTGATAGTGGACACAATTGGTCCGCTACCCAAATCGGAAAATGGTAATGAATACGCAGTCACATT
AATNTGCGACCTNACNAAATATCTAGTCGCGATTCCAATACCAAACAAAAGCGCNAATACNGTCGCAAAA
GCTATATTCGAAAATTTTATACTCAAGTACGGTCCAATGAAGACGTTCATTACGGACATGGGAACAGAAT
ACAAGAACTCGATTATAAATGATTTGTGCAAGTATNTGAAAATNAAGAATATAACATCNACTGCACATCA
TCACCAGACAGTTGGAACAGTNGAAAGAAGTCACAGAACTTTTAACGAATACATACGTTCATATATATCG
GTNGATAAAACTGATTGGGACGTATGGNTCCAATATTTCGTTTATTGCTTCAACACGACCCCATCNACGGC
GCATGANTATTGTCCGTACGAACTAGTATTTGGTAGACTACAAACAATTCATCAAAATATTNAATAGNAC
NGATAACATAGANCCTATATATAATNTAGATGACTATGCTAAGGAAAGTAAGTNTAGATTAGAAATAGCA
TACAAAAGAGCTAGAATTATGTTAGAANAAGATAAGGCNTATAGGAAATTAGCATATGACGAAAAAACA
AAAAACTTNGAATTANAAGTAGGAGATAAAGTNCTANTAAGAAACGAAACAGGNCATAAATTAGATCCA
GTATATTTAGGACCATATANAGTAGAAAAAATAGAAGATAANGATAACATNTTAATTANGGATAGTAAA
AATAAAAAACAAANAGTNCATAAGGATAGATTAAAAATTTTTAATTCATAATCGAANATTCTAATATGAA
TACAAAGAAAAATATAAAATAAAANAAAAAAATATAACGGAATAATAAAATGAAAAAAAAAAAAATNTA
AATAAAAATAAAAATTATNAATTNTTATAGAAAATAACTCTATTGTATTACGCTCCAACNTTTNAAA
>Ref_NewTE2
TTGTTAAAATAGTGACAATAAGCTGTTAAGCTTCTTTATAATCTGGCAACATTGTATCAGCACAGTACTCA
TTCTGTAATTGAGTACTTGTTCAGATTTTTGTACTCATGCTTTGAAGTTTCCATTAAACATTTAACCGAACC
ACTACGTGTGTTTTCTTTATGTGCATCTTAGGCACACTAGAAATTTCCCAACATTGGTTATGGGCCCAGGT
CTCGTTGTTTTCGGTTAAATAAGTACACAAAGTGTCTATGGTTTTGAAAGCTTCCTTTTGTTAAATTTTTAA
AAAGTTTATTTCGTGCGAAGAATAACGTGGTGAAATTGTCAAAATGAACATGTCGCATCAAATAGAAAAA
CTTGGTGAGGACAATTATGACGTATGGCATATGGCCATGAAAAGTGTCTTGGTGGTGGCGGACCTATGGA
ATGTTGTGTGTGGAAAATATGTTAAGCCAGAAGGAAATGGTGAGGATATCGAAAAATGGGTTAGTGTCGA
CCAAAAAGCGATGGCTTACATAATTTTAAATCTGAAGCCAACACAGTTGATGCATATCAAGTCATGCCCA
ACATCTGCAGCTGTATGGAAAAAACTAAAGGAAATTCATGTCTCAGTTGGACCGATTAGGAAGGTCCAAT
TATATCAAAAATTAATACGTTTCAATATGCAGCAGGGTGACGATGTAGTCGCATATGTCAATTCATTTGTT
GAGACAATTGAAAAGTTGGCAGAATTAGACATCAAACTCAATGACGAGTTGCAGGTCATCATGTTTTTGA
GCAGTCTACCAGACACATGGGAAAATTTTGTAATAGCGATTGAGACACGCGATGAGCTGCCCAAATTCGA
AGTCGTCAAGGTTAAAATGTTAGAAGAGGCCACGCGAAAGCAAGAAAGAGCTAGCAGAGACGGCATAGG
GACACCAGAAGCAGTGTATACACACAAACATAATGGACCGCGGGACAACAACAGTGTCGAAAGCAAAGG
CAGAAAGAATAGCAGTAAGCAGCCATTCCGAGGCAAATGTTTTAATTGCGAAAAAGAAGGGCATCGAGC
GAGCGATTGCCGAAATAAAAAGAAGGATGATAAAAAGAGCCAAAAGGACGAAAAGTCTCTATGCTTAAT
GCACACGTGTGTCAAGGAGAAGCAAAAGAGTTTTTGGTGCGTTGACAGTGGTGCAACATCGCACATGTGC
TGCGACCGCAGCATGTTCCTTACGTATGCTGATAAAAAGACGTCGATAATGTTGGCCGCAAATAAGTTTAT
CGATTCACCCGGCAGTGGCAGTGTTGAGTTGCACTGCAATAGAATAAAATTAAAGCTGCAAAATGTTTTA
TATGTCCCTGACTTAAGCATGAATTTTTTATCAGTAAGTGCTGCAGCAAAGTTTGAGAATTCTACCATATT
TGAAGGGAAAATTGCGCTGATAAAAGACAAAGAAGGCAACGAGATAATAAGGGCAAGACACGAAAATG
GTTTGTATGTTTACAAACATAGTCTTGATCAGGTTCATATGCTAAATAGTTCTGATTCATCTTCCATAAAAT
GGCACAATAGATTTGGCCACCTGAATTTTAAGGATTTGAAATTGCTTAATGATAAGCAACTGGTGCGTGGT
ATGAAAGTTGAAAATGTACAATTAGAAATAAATTGCGATACATGTAATAAAGCAAAAATTTGTGCATTGC
CATTCCCACAGAAAGCGAATCGTGTCTCAAAGCAAGTACTGGACTTAGTACATACAGACGTATGTGGTCC
GATGAATGTTAAGTCGCTTGCAGGCAATCGATATTTCGTAACTTTTATTGATGACTACTCACGCAAGATAT
TTGTTTACCTTATGCACTCAAAAAGCCAAGTTTTTGAAAAGTTTAAAATGTTTAAAACTTTTGTAGAATGC
CAAACAGGAAATAAAATAAAGGCTGTGCGCAGCGACAATGGAACAGAGTATGTGAATAAGCAATTCACT
GAATTTTTGATCGAGTGCGGTATAAAGAGGCAATTAACAGTTCCTTACACTCCGCAGCAAAATGGTGTGG
CCGAACGTGCTAATCGGACCATCGTTGAAATGGCAAAAAGTCTTCTAATTCATTCAAAGCTTGAAGAATTT
TTGTGGGCAGAGGCTGTTGCAACAGCAGTTTATTTGCGCAACAGATGTCCCACAAAAGCATTAGATGGAA
GCACACCATTTGAGGTATGGAAGGATCGCAAGCCATCAGTACAGCACTTGAGAGTATTTGGCTCGCGGGC
ATTTGCACTCGACAAAGTCAGAAAAAGCAAGTTCCAAGCAAAGGGCAAGGAGTACATTTTTGTTGGTTAC
TCCTTTACAGCAAAGGCATATCGCCTATATGATCGCGAGAAGCGAACAGTTATTGAAAGAAGGGATGTCA
AATTCATTGAAGGTGAGTTTGACATAGCAACAACTGATGCATGCAGTATTTCAGACAGAAACACCTTCAC

GACGTACATTATCCCGTTTGAGTCAAATCATGAGGTTGCAAAAAACCACCCAGAGCAACCTGCCGAGGTG
CAAGAGTACAACAGTGACTCAAATGAAGAAGAAGAGGAGGATTTTGTGAGCGCAAGCGATCCAGGAGAA
GATGGCAACGATGACGACAATGACCAAGTAGCGCTGCCACAAGTTGAAGCTGTAGTAAAACATGGGCGT
GGCAGGCCAAAAATTATACGTACAGGTCAGCCAGGCAGGCCCAGAAAGCAGTACCAATTGATCAATAGT
CTTAATCAGATTGAAGACATTGAAACTCCGCAGTCAGTTGCAGAAGCATTACATGGCCAAAATGCGCAGG
ATTGGGAAAAGTCTATGCAAATAGAGTACGACGCTTTACAAGCAAATGAGACATGGTCAGTGTGTGAGCT
ACCTCCTGGACAAAAGGCCATTGGTTCAAAATGGGTTTACCGAATCAAGAGAGATAAAGATGGTAATATT
GAAAAATTTAAGTCTCGACTAGTTGCACAAGGTTGTGGTCAACAATTTGGTGTCAATTACTGGGAAACGTT
TTCTCCCGTAATTCGATATGAAACCATTCGCATGTTATTCGCAATAGCTGCCGAAAAAGAGCTGTATATGC
ATCAAGTCGACATTTCAAACGCATACTTAAATAGTAAGCTTAATGAAACTGTGTACATGAAACAGCCGCC
GAATTTTATTGACAAGCAGCATCCAAATAAAGTCCTCAAACTAGAAAAGGCACTTTACGGGCTTAAACAA
TCCGGACGAGTTTGGAACAATACGCTCGATGAGGTATTGAGAGGCATGGATTTTAAAAGATGCAAAAATG
AAGCATGCTTATACGTCAAGCAAAAGCAACAACAATTCAGCTACATCGCTGTCTACGTCGACGATTTAAT
CATAGTGTGTCCCAGCGAAGAAGACATAGCCGTGATTAAGAAGAAGATTGCATCAAATTTTAAAATGCAC
GATGGAGGCCCCATTAGCTATTTCCTAGGCATGGAAATCCAACGCGACGGCGATCGAGGTTCCGTTTCGC
TGTGCCAGAAGACGCACATTCAAGGCCTATTGGACAAGTATGGCATGAGCAATTGCCGTCCAGTAAGCAC
ACCTCTTGACCCAGGTTTTCAAGTGGACTGCAACGACGATACTTGTGTAAAGGTAAATATAACGCAATAT
CAGTCATTAATTGGTTCGCTTATATACTTAGCAGTTTTGAGCCGACCAGACATTTTGCATGCTGTAAACAA
ATTATCACAAAGAAATGCTAATCCTCATGGAGAACACCAGTCTGCAGCAAAGCACATTCTAAGATATCTT
GCGGGTACAAAGAATATGAAATTAGTATACCGTAAAAATGGCAAACCACTAAAAGGCTATGCAGATGCA
GATTGGGGCAACGATCGGCTGGACAGGAGATCCTATAGTGGCTACGCATTTTTCCTGGCAGGCAGTGCGT
TCTCTTGGACTTCATCAAAGCAAAGCGTTGTCGCATTGAGCAGCACTGAAGCAGAATATATTTCTTTGTCC
ATGGCTGCCAAAGAAGCAGTTTATCTACGCAGGCTACTTAGTGAGATGGGATGGTCAAAGGATACAGAGC
CGTTGACTATATGTGGCGACAACCTAAGCGCGCAACATATTGCGCAAAATCCAGTCCATCATAAGCGCAC
TAAGCACATAGACATAAGGTATCATTTTATACGTGAAAAGGTACAATGTAATGATATTGCATTAGAATAC
GTACCTACAAATAATAATGTTGCAGATATACTAACAAAATGTTTAAGTAAGCAAAAGCACGTAGGCTTTG
TAAAAACACTTGGATTGAATTAAATTTGTTATTTTGCAACTGATTATAATTAGCTTAAGAAGAAGTGTTAA
AATAGTGACAATAAGCTGTTAAGCTTCTTTATAATCTGGCAACATTGTATCAGCACAGTACTCATTCTGTA
ATTGAGTACTTGTTCAGATTTTTGTACTCATGCTTTGAAGTTTCCATTAAACATTTAACCGAACCACTACGT
GTGTTTTCTTTATGTGCATCTTAGGCACACTAGAAATTTCCCAACAAATTTGACAT
>160_rnd-1_family-346__LTR___Gypsy
TGTGAGGCGGCTAATGAGACCAGCCACCCCACAGTTTTCACGGCAAGGAGCCGTCATCCCTTTGGCCAAA
TTACGGCAAGGAGCCGCTGGCGGGACAACGTATTAATTTTCTTTAGTTATAAGTATTAATTATATTAGGTT
AAGATCTTAGTGAAATAAGAGGCAAGGAGCCATCAGTAATATAAGCAGTTCGATTAGTAGCAAAGAGGC
AAGGAGCCGCGAATATTATAATTGATTTAAATAGTGAGTTTGAATTTAACCGCCGGCCGCCGCTGCAACC
GTGCGTTGTCGATCGGTTGCTCAACACCGTCTTTGAGAGCGCAACTTCAATTCGGCGGCGAAGAGCGAAA
GCTCCGAGGCGAAGTTGCGAAAGCTCGCGGCGAAGTTGAGCAGCCGAAGCGAGCGCATGGCTTGCCGGC
GAGCGTGCCGGCACTTGTGATCGAACGTTAAGGGTGAGTAGAAGTGCTGCGCGAGATAAACGAAATCAT
AGATGGTATAGTGGAGCGCTAGGGCTCCAGAAAACAGTGCGGCAGGGTTTAGCTTAGGATTTACGATAAG
GTCGGAATGAATAATAGTGAATAAAGTGCCAACGTTGTAATTATCTAGAAAGTATTGGATTATTCCCTGTT
TCCTGGTAACCCGGCTTACCTTAACATCGGAACTAACCGGTGTTCGACCTAACCTCGAACGAGGGGCATG
CGGTACGAGATTGTGGCTCCGGGGTGGGCATTGCCGGTCGGCGTCTAGGTAGTGGAGTTGGTGCGAGGCC
ACGGCCCGCTAGTGTAGTTGGTGCGAGGCCACGGCCCGCTAGCATAGTGGTACGAGGCCACGGCCCGGCA
GAAAGATAGAGCGTTAGGTAGTGGAGTTGGTGCGAGGCCACGGCCCGCTAGTGTGGTTGGNGCGAGGCC
ACGGCCCGCTAGCATAGATGGTACGAGGCCACGGCCCGATGGTGTAGGTGTGTAGAGGTAGATCTGGGTT
TGGTTTGGTCAGGATACTCCCCCCCCCTTTAAGCCCTCTCTCCCAGCGAAATTATAAATAACTTGTAACTC
GCTAGACCTGGTAAGACTGAGAATTATACCCCGGCCAGAAGCTTCCTTACAAGTGGCGCCCGAGCAGGGA
CCAAGAGGCGTTTAGGGGTGTGGCATGTCGGCGGATTAAAGCCCGCAAGCATGCCCGAATAGGTATTTTC
CAAAAAAAAAAAAAAATATATATAGGGTATTTCTAGTCGGGTATACCCTAGAGGGAAGGTAACCTAGCA
GAGAGCAAGAAAATGAGAAGGTCATGTATGTGCGAAATATGAAAATAGGGTATTAAGTAGTCCGGTATA
CCCCAGAGAACATTCCTATACCTTGTGTTAGGCATAGCCAGAAAATGAACACGTGCAGCTGGTGAGTGAC
AAAAATGCTAGCAGCAAGCCGGCAAGTAAGATAAAGAGACGAAAACATAACGGTCGGTCGTGAGAGTAC
AGCGCGCGCGCGCCAGCCGGGCGACGTCGAGACCACGCTCGCACACGGGTGCCCACACGCGCGGCGCAG
TCGACGTCGGCGTCGAAGGACCGTTAAGGCGTCGCGGCAGCGGTTGTACAGTGGGGCGAATCCAACTCAC

GCGCGGTGAAAAGTTGAATTCGCGAGTACAGCTGTCGGGGCAGTGAAGTGGTCACCCTTCAAGTGAATGT
GAACAAAGGGAACAATAAGAGCGGAACAAGTGCCAAATAAAAATAAGTGGATAATAATAAAATTTTCTT
TTATAAATAAAATTAATTAAACAAAAATAAATAAAAATAAGTGGATAATAATAAAATTTTCTTTTATAAA
TAAAATTAATTAAACAAAAATAAATAAAAATAAGTGAATAATAATAAAATTTTCTTTTATAAATAAAATT
AATTAAACAAAAATAAATAAAAATGAGTGGATAATAATAAAATTAAAATAAACCGAGGTTCACAATAAA
GATAAAGGCAGCGACCACATAAGGAACAGCCTAAAAGCGGAAAAGTGAGAAAAGAAAATTTTGAAATAC
CTTTTGTTGAGTTTTTTTTTTTTAAAAAAGGGGGGTAAATACTTTTGTGGAATATTGTTTTGTATTTTTGTTT
GGTGAAAGATATAAAGACAAGAAAATAATAATAAAATTTTNTTTTATAAANAAAANNAACTAAAAAGAA
AACTAAAAGGAACCAAAGTGGCTGAGNAAGATCGGCGTAAAAAAAGTGAAAAGACACCAACACCAAGA
AAAGGAGAGAGGAATAGTAAAGGGACCGACGGAACCCCTAGAACGGGAAGAGAGGGAAACAGCGACGT
CGACTAGCAAGGAAGTATACAATAAAGGATGGAGGCAGGTGAGTGCCAAAACCCGGGGGTAGGCGTCCG
CTGGATCCACCAGCGACGCAAGGAGGAGTTGGTGGCCATCGCCCAGGAGTTCGGGCTGGAAAAGGAAGG
GCGCGTGGAGGAGCTGCGTAGGCGGCTGTCCACCTACGTGCAGGAGGGCGGCCATTCGNCTGGGATCTGG
GCTCGGCTCGCCGAGCTGGAGTCTCAGTTCGCGAAAACGTTNGAGCCGCCATCCTGGGGCGTCCCACCCG
ACGTGGGTGGACGGCCGCGGGCCAAAAGTCGGAGCCTGACCTCAGGAGCTCNCAGCTGTACGGGCGTCC
GCGGCCGCCCACCCCNGGTNTGGCCGTCNCGGGCGTGGAGGCGGCTCCCGCTCCCGGGGGAAATGGAGA
CCGCCGAGCAACCGCGGCCAACGCAGGGGTGGGGCGGTCCCCTGTTGAACCGGGTGGTGGTCCCTGCTAC
ACTAGGCCGCACCATCCGTCGGAGGCGAGGCCACTTCCCAACGTCCCGGAGCAAGTTCGGAAGTGGGGCA
TGAGGTACGATGGNCGCTCCGACCCCCTCGGGTTCTTGGAGGACCTAGAGGAGCGCGCCATCACCTATGG
TATAGCCCTCGACCGTATGCCCAGGGCGATGAGCGAGGTGTTCTGCGACAGGGCCGCTCGTTGGTTTCTG
AGCAGTGGGCTAAGGGATGTGCCGTGGGCCGTGTTCAGGAGGGGTTTCTTGGAGTTCTTCCTGCCCCCNC
GCTACTTCGAGAGGCTTGAGGACGAGATCCGGTCCCGGAGGCAGCGCGTGGGNGAGGAGTTCAAGGAGT
ACCTGATAGAGCTGAGGGCTCTGATGCATCACGCCAGATACGAGCCGGCACAGGAGCTGCACCGGATCTA
CGAGAACGCCGCTCCGGAATACAGGCTCTATGTGCGACGGCAAGACTTTTCTACCCTATCCCAGCTAACTC
AACTGGCCGTAGAATACGAGACCGTCGTGAGGCAGAGGGCGGAGTACTCAGGCAGCCAACCCTTGGGTC
AGGGTGTCTCTAGTTCAAATAGTGCAGAGTGGGCGAGGACGGCGCAGTCCTCGGGACATCTCAATCCGTT
CCGGTCTGCTCCCAGCGGATCCGGGACGACCGTTGTGGACAACAGGGTCTCCACCCCACCCGCTGCAACT
CACGCGGAGGTGGCCCATCAGATGATGAGCCAAGAGGCCGGGAACGGATATGATCCGCGACGGGCGTGT
CGTAGGTGCGCACAGGAGGGGCATTTTGCCAGGGAATGTCCCAACGCCCCTGTCTTATTTTGTTGGGTATG
TGGAAGCCGGGGCCGAAGTAGTCGGGAGTGTTGCCGCCGCGCGGAGACGGGAAACGGGCAACGGCGTCG
TCCGGACCAGGATCGGGTCGGCGCCAATACACGCGGAACGCAGCAATAGGACACCCGCTGAAGGTGGAC
GGTGGCCGGATCGTCGCTACGGTAGAGATTGGCGGACGAACGATGCCCGCCACGATCGATACCGGAGCC
ACTCGTAGCTTCATGAGTGAGGACTGCGTACGCAAATGGACGATCCGGGGAAGAGCGGAGGAAATACAG
GCACGTATCCGCCTGGCAGATGGGTCCACCCTAGAGGTAGAAAAGGCTTTGAGAGTAGACGCCAGCTTAG
GGGGAAAAACGATCAGCATGACGATGCTCATCATGCCCTCAATGCTGGACCACGTAATCCTGGGTATGGA
TTTTTTGGGGGCGATGAATACCACAATACGCTGCGGCGAGGCGGAACTGGTCCTAGAAGCAGCGACGACA
CCGGGCGTGAGGCCAGGAACGAGCGAGGAGTCGGAGGAAGGCCGCCCAAGGCATGAAAAGGGAGCACC
TGCTCGGCTACAGAGGGAGCCGGAGGAAGGCCTCCCAAGGCATGGAAAGGGAGCACCTGCTCGGCTACA
GAGGGAGCCGGAGGAAGGCCTCCCAAGGCATGGAAGGGGAGCACCTGCTCGGCTACAGAGGGAGCCGG
AGGAAGGCCTCCCAAGTCATGGNAAGGGAGCACCTGCTCGGCTACAGAGGGAGCCGGAGGAAGGCCTCC
CAAGTCATGGAAAGGGAGCACCTGCTCGGCTACAGAGGGAGCCGGAGGAAGGCCTCCCAAGTCATGANG
GGGGAGCACCTGCTCGGCTACAGAGGGAGCCGGAGGAAGGCCTCCCAAGTCATGGGAAGGGAGCACCTG
CTCGGCTACAGAGGGAGCCGGAGGAAGGCCTCCCAAGGCATGGAAAGGGAGCACCTGCTCGGC
>9_rnd-1_family-97__LTR___Gypsy
AATTTTTTAGGGGAAGCTCCCTGCTGCCCTACATCAGTAGGAGAGTAGCAAGGATCCAGATGAGATTTCT
CATCGACACGGGCGCTTCCAAGAATTATATCAGGCCACATAAAAATCTAAAAGGTGTGTGCCCAGTAAAT
TCTTCCTTCTCTACCCATTCTATACACGGCACCACGAAGATCACTCATAAGTGCCTTATATCTCTGTTCGGC
AAAACTGCCACATTTTTCTTACTCCCAGACCTGTCCACGTTTGATGGAATTGTGGGGCTCGACTTGCTCAC
GCAAGCAGGAGTGTCGCTTTGTCTTTCTTCTGGCCAACTCAAATGTGGCGCAGAAACTGAACAAATCGAA
TTCCACAGGTGCGCGGATGTTAACTTTACCAATGTTGACTGCGCCGACGCGCCCGATGCTGCAAAAGATG
CATTCCTAAAGATGCTAAAAAGCAAACATAAGGCCTTCGCGGATCCGGAAGAAGCACTTCCNTATAACAC
ATCAGTGGTGGCCACTATCAGGACCACTGATGAACAACCAGTTTATGTCCGACTGTACCCCTATCCCATGG
GAGCAGCGGAATTCGTTAATAAAGAGATTCAAAGCCTTCTGAAGAACGGTATCCTAAAAAATCTGTTTGC
CCCTACAANAACCCCATTTGGGTGGTGGACAAGAAGGGGACGGACGAGTTTGGTAACAAGAATATGCGC

CTCGTAATGGATTTTCGCAAGCTTAACGAGAAAACCATCGCAGACAGATACCCTATGCCTAACATTCCAA
TGATTTTGGGGAATCTTGGCAAAGCTAAATACTTTTCGACATTGGACCTAAAATCCGGCTATCACCAGATC
ACGCTAGCAGAGCGTGACCGTGAAAAAACAGCCTTTGCAGTTAACGGGGGAAGTACGAATTTCGTCGACT
TCCCTTTGGGTTGAAGAACGCGGCCAGCATCTTCCAAAGAACTGTTGACGACGTACTGCGTGAGCAATTT
GGCAAATCTTGCTATGTCTATGTTGACGATGTCATCGTCTTCTCAAAAGACGAAAATTCTCATGTCCAACA
TGTAGAGTGGGTCTTAAAGAGCCTGTACGAGGCAAATATGAGAATCTCCAAGGAAAAATCGCGTTTTTTA
AGAAAAGCGTAGGTTTTCTTGGTTTCGTGGTAACCAGTAATGGAGCTACAACGGACCCTGAAAAAGTCAA
AGCGATTCAGGAGTTCCCAGAACCAAAGAATATATTCGAGATCAGATCATTTTTAGGACTGGCCAGTTAC
TATAGGTGTTTCATCAAAGACTTTGCCTCAATAGCAAAACCCATTTCTGATCTTCTAAAGGGCGAGAATGG
GTCAGTAAGTAAACATAGATCGCGAAACATCCCGGTAGAATTCTCTGAGCTTCAGCGTCACGCATTCCAA
AAGCTACGCAACATTTTGTCATCAGAAAACGTCATGCTCAGATACCCTGACTTTAAGAAGCCGTTTGATCT
AACGACAGACGCTTCAGCTTACGGCATAGGGGCAGTGCTTTCTCAAGAGGGTCGCCCCATCTCAATGATT
TCCAGAACATTGAAACCCCGGAGGTGAACTACGCCACCAACGAGCGGGAACTTTTGGCTATAGTTTGGGC
CCTTAGCAAGCTACGTCACTACCTATATGGGGTAAAAGACATTAACATTTTCACGGACCACCAACCGTTA
ACTTTTGCCGTTTCTGAGTCAAACCCGAACGCCAAAATTAAAAGATGGAAGGCGCGCATTGAGGAAACGA
ATGCGCGAATATTTTATAAGCCAGGCAAGGAGAATTTAGTTGCTGATGCGCTATCGCGACAACAGCTCAA
CGCTCTTGACGAGCTGAATACTGATTCCTGCGCAGCCACAGTCCACAGCGAGCTGTCCTCGACCTATACAA
TTGAGTCAACCGAAAAGCCACTTAATTGTTTCCAAAATCAAATTGTCATTGAAGAGGCACGCGTCCCCAC
CAAACGCAACTTCATTCTTTTCGGGGACAAGAGGCGTCGTGAAGTGAGCTTCGCTGATCGCACAGCCCTTC
TAGATGAACTCCTTGACTTGATTAATCCAAATGCGGTTAATGCCCTCTATTGCAACCTGCACACACTAGCA
CAGGTGCAGGATGGCTTAGTTCGCATGTTTCCAGGCACAAAGTTCTGGCACTGCAAAAAACCGAGTTGCA
GATGTCTTCAACGTTGACGAGCGGTTAGAAATCATCGCTGCCGAGCACAATCGAGCGCACAGAGCAGCTC
AGGAAAACGTTAAACAAGTACTTCGGGACTATTATTTTCCCAATATGGCCAGTCTGGCCAAAGAAGTGGT
TGCAAACTGCAGGGTCTGCACTAAAGCCAAATACGATAGGCACCCGAAGAAACAAGAACTCGGGGAAAC
GCCTATACCAAGTTATACTGGCGAAATGTTGCATATTGACATTTTCTCGACAGACAAGAAACAGTTCTTGA
CCTGCGTAGACAAATTCTCAAAATTTGCAGTCGTCCAACCGATATTATCCAGAACGATAATCGATGTCACA
AGCCCTCTACTGCAAATCGTTAACCTTTTTCCCGCAACCAAGACAAGAGATATGGACTACCGCAAACTTCG
AGAACTTGTCAACGCAAGTAAGGAAGAACTTTTTGTCCGTGGAGAAAATGTCAATGTGCAACATTTCTCC
AGTCTGTGAGGGAAGTGGCGTCTGGCCGAGTTCCTGCTTCTTCGGGTGTCTGTCGTATTTTGCTTTCGTGCA
AACTCTACAGTTAAGCACCACTTCGTTAGCTAATTTGGTCATTTTAGGGAAATAGTACTCCGTCACGACCT
GTTTTACAAGTCCCTTAGTAACTNGTATTGGAAACTAGCCAAAGCTGGAAGACAACATTTAAATGCATTA
ACCACGTTTGGGTTAATGACGATGTTTGGTCAAGTAACCATCAGAGACAACACCGACTGCGTGCAGTAAC
AGCAGAAGACAACAATCATACTTGCAAACTAGAGCAAATATTGTTTGGGAGCTGTTTGTAAGCTGCTGCC
CATTCTATTTCGTAGGCCCTTTGCCTTCTGCACGAATGCCCGCTGCCCACCGTTTTGAGCGCAGCTCCATTT
CGTTACAAGTCATTAGTTTTTCATTTGATCGCAACCGAGGCACAACTATTTCGAGTTGGCAAACAATATTA
ACAATAATTGCTAATAAGTGTAAGTCGAAACTAAGAATATTGTTCATTTGATTGCAAAAAGGCTTGCAAC
TACAAGTTAACAGAGCCCAAGTACACCAGCTCCAGATGCTGATATAGCACTGTGCCGAATGTTCTAACGA
GTTAACAGGGGTCAGCAAAATGCAATTTAACACCAGTCACAGATGCTGACATAGCTCTGCGCCGGATGTT
CTTGCACGAGGGAAGCGCATGAGCCGCAAGCGGCCGTTGTGAAAAGCACACAATAGCTTAGGTAATGTTG
ATCAATCTTTTGTAAATTTAGTTTTAATCCGGCTTTTACTGAGGCACATCTCAGCGACTTTGCGCATAAATT
TAGCGTATCGGCCTAGTGTTTTACAAATAATAAAATAATCAAAATAAAGTCTTGTAACGAAAATCCGCGA
GGACTTCTTATTTAAATTTTAATTTGCGCCAAACGTTTTTCGCTTTTTGTGCTATAATTATATTGTGTGCGGT
GCAGTGTAAAGAACTAATCTACCTGTACAACACACTTCGCGTTGTGTAATAGCAATACCCAACCTACCAG
TGCAAGTAAAAAAGCTGTGCCTGACCTATTCTATTTACAGCACAAGCCCAACAAAAAGGCATTTGATAGC
GACTTGTCACCAAGCAAGCGGGTTTGGGCAGCGAAGTAAGCCAACACCCATAGACTTCAGCGAGGGATC
ATAAGAAAGCATTCACGTCTGCAGCGTGGGAAGCAGTAGTTTTTAGACGTAGAGCATACTGCCCTTGTAG
GATCAAAAAAATGTAAGTGGCCCATCATTTGGCACAAACTTTTTTCAATATGATTGATTTATTTCAAATAA
AATCCAAAGTCTGAAATATAAAAAATTGCAGTAATTGTGCTACCAACTAATTTTGAGTAACACAATTTTGC
ATCCATCAGCAATTTTCAATAAACTCTCCTAATTCAGCATTCATTTAATTTATTGTTGTTGATTTGCCCTTG
AAATTGTGTTGTTATTATTGTTGACAGTATAGTGCATGAACAGCTCGTTTCTCAGGTACAGAGACAGCTCT
GCGAGGGGATTTGAGTAAAACGACTTCAACGCCTTAAATAGGGAAGCCGTCCCGCAAGAAATCGGTGAC
AACAGGTGTAAAATGGAAATGGAGCCTTAGCAGCCTAGGGCTTTGGTGCAGGCCGNAGTGGCCTCCGCCT
TTGAAGCCGAAAGCGCAGTATTGAAGCAAAGAGATGTAGAACTGAGGGNTTCCTTAAACGCTTGGATACA
AGAATTGGGAGGCAGAATATCTGCCAGTAGTATAGAAACACAGTAGTATAGAAACCACCGTCCAGGCATT

TGAGCCGGTGGAAATCAGAAAGGACGTCGAATGTAATGAACCGTTGGACGCCGTTAAATGCTTACCA
>9_rnd-5_family-2111__Unknown
GTCCAGGCGCATGGTGGCTTATGAAGCCAAGCTAGCACAATTGTGGAATCTGTCCAATAGAACCAGTCGC
CGCCAGCGGAAGGTAGCTGGGGACTGAGTGCAGCGGATAACTCGGACAGAAGTGCTGCTCCGCACAGCT
CTAGTCGTGGCAAGGATACCGTCTTGACCGGAGCGACCCTCGTTTTCGCTGTTAGGAGATGTACTGAGATG
CGATCTTGTATCTCAGTGCGCACGTAAATGGCGGCCCCATATGCACGCTGCGAAGCGTCACAAAACCCGT
GGTATTGAATAGTCCCCTGTGGGCGGAATTCGACCCATCTAGGGATGCGAATCGCACTGAGGCCCGGATA
CTCTTGCAGAAATAAATTCCACTTACTGAGAAGACTCTCAGGTAGCGGATCGTCCCAGCCCAGTTCTTGGA
GCCAGATCTCCTGCATGAATATTTTTGCCTGAATAATGACTGGACCCAGCCAACCTGCTGGATCGAACAGT
TTGGCAATCTGTGAAAGGACTTCACGTTTGGAGACGGACTGTTTGGTTGTTAGCTCGGATGGAGCGAAAA
AGAACTCATCGGTGTCAGCTTTCCATCTGATCCCGAGAGTCTTTGCCGAACAGGCTTCTTCGATCTCTAAG
AAGTCGGCGCGAAGCAAGTGGTCCTTGGGAATCGCTTTTAGCAAGTCCTTTTCATTGGATGTCCATTTTCG
CAAGGGGAACCCGGCAGATTCAAGGGCTTTGCGGAGTTCTGCAATTGCTTGATCTGCCGTTGATTTGGAAT
GGGCTCCCGCTAGTACATCGTCGACATACATATTGTTTGCGATAATGTGACTTGCTAAGGGATAGAGCGA
ACGAACGTCATCAGATAACTGATGTAGTACCCGGATTGCCAAAAAGGGGCACAGTTAACTCCAAACGTGA
CTGTGCTGAGTTCGAAGTCACGAAGCTCGTTGTCCTTTGTTCGGAACAGAATTCTCTGATAGGGCGTGTGT
TTGGGATGCACAAGTATCTGCCGATACATTTTCGTGATATCGGCATTGAAGACATATCGGAAGAAGCGCC
ATCTTAGAATTTGAATGGTTAAGTCGGATTGCAGAACGGGCCCTGCATGTAGCACATCGTTCAGACCTTTA
CCATTCGATGACGGGTGGGAGGCGTTAAATACTACGCGGAGCTTGGTCGTGGTGCTGTCAGGCTTGTGAA
CAGCATGGTGTGGTAGATAAAAGTTATCCGAACCATTGCTTGGAGGGACTTGTCGCATGTGACCTAAGTC
CAGATATTCCTGGATCACCGCATCGTACTGCTCCTTAAGGGGGGCATTTTTTGCCAGACGATTCTCATTTC
GTAGAAATTGAGCTAGTGCGATGGACCTTGAATGGCCTAACTGGATGTGATCGGAATTCTTGAATGGTAG
TGTGACCAAGTATCTGCCGTTTTTATCCCTTTTAGTGTTTTTTTGGAAATTGCGCTCACAAAACAGGTCGGA
TTCCTTTTCCAGCTTTACTGGAATATCCTCCACCTCCCAAAATTTTGTGAGCAGTTTATCCAAGCTCGCATT
TGGATTCAGCGAGACTTGAGTCGAAAAGGATGCGACCGATTTGGCACTGGTCTTAGAAACGGGGCCTGTA
AGGATCCAGCCGAAAATCGTTTCTTGACCAAGGAGAGTGCCACATATGTTGGGATAGGAGCCACCTAACA
ATATGGATGGGAGGATATCGGCGCCGAGAAGCACGTCTATCTGTGAGCTCCTATAGAAAGAGGGATCGGC
CAAGTGTAATTTTGGAAGTTCTTCCAAAGCGGTTTGGGGATGGTGTAGGAGGGCAAATTGCCTGCTAGCTT
TGGAAGAACATAAGCCGAGGTTTCAATATGAACCCTGGGCTTTATCGGAGAGCCAATATTAAAATGGCAA
AGTTTTTTTGAGGTTGAGCCGAAATAGCTTGATTTAAACCGGAGACCTGTGCGTCGACGGGTTGATAAGG
AAGTTTAACCCTTTGAACAATCGCTCTGAAATAAAGGTCGCTTCAGACCCAGAGTCGATTAATGCCCGAG
CTGTATACTTTGTGCCCAAATGACACACTTGGATGACAGCCGTGCCGAGGAGAACACCATGTGAGTTTGC
GGAAAAGTAGTTTTGAACGGTAGCTTGGGTTGAAGTAGAAGTGGACTGTATAGACGAAGGAGGGTTCAA
AACTATTGGTGTTGTGGAAGCTGAGGGCACCTGGGGTCTGTCGCGGTGGAGCAGCGTGTTGTGACGTCCC
TTGCACGTGAAACAACTATGTGCGCTGGTGCAGTCTTTCAACTGGTGCTGTCTGGCGAAGCAGTTTAAGCA
CAACTTTTGTTGCTTAATGACGGAAACTCTCTCGGGTACGGACATTTGTAGAAAACGTGGGCATAGACGC
ACGGGATGGTTTTCCTTCTGACACAATTTGCAGTGTTGTGACTTTGTAGTCACTGTTGCTTCGAAGGTTCTC
AGGTGGTTGGACTCTTTTCTTGTGGGTTTGGACTGGGGTGGCGTATGTGCGGTAGGTCGTACATCCTCTAT
CGCCTCCAAGGTGCGATATCGGTCGATCAGAAATGAATTCATCTCGGACCATTGAGGTATCTCTGCCTTAT
CGTGGAGCGATTGTTCCCATAACGACAGCGTGAGCTTAGGCAATTTAGAGGAGCACAAGAAAAATAAGC
AGACAATCCCAATTTTCGGTGCTCACCCTGGACAAGTCGAGAGCCGTTAAACATCCCTGGATTGTGCCCTG
AAGCTCTTTAAGGGCGGCTCCAGACTCTTGTGTTGCTGAGGGGAGATTGAAAAGAATTTTCAATTGACTAT
TGACTAATAGTCGCTTATTCTCAAATCTCTCCGTCAGGCTCGCCCACGCTGACTGAAAGCCGTCATTCGTG
AGCGGGGACTTGGAGACAATTGCCCTAGCTTCGCCGCTGGTTTTGGAATTTAGATGGAACAATTTTTCCAC
CGGCGTCAATCTTGGATTGTTAATATAAACCGCTGTAAACAAGTCTCGGAAAGTAGGCCAGCGTAGGTAA
TCCCCACTGAAAACTTCAGTGTCGCATGGCGGGAGACTGCAACCCCTGGACATGGAGAAGTTGGAGGGGG
TTTGCTCAGCTTGTCTATTTAAAGCCGTGGCTCTTTCTATTTTTTCACTTAGTTGAGCCGCACATCGCTCGT
AAACGGAGTACGAGTAATCATATTTAGCTTGCATAGCCACCAGGTCACTGCTGCTGCAAGCGCCTTGGTT
GAGAGCTTTGGAACATAACTCGTACTCGCGTTCTACCTTTTCCCATAAAGTCTTAATCTGATCTCGTCGGA
CTTGGAGAGTGAACAGTGACACGTCACTGTCCGCTGCGTCATTGATCTTCACTTCAAACTGGCCTAGTCGG
TCAGTTATAGAAACGAAGTCGTTCAGTGCCATTTCGCTTGACTTCTGCATTTTTTGCGTAGTCGAACGGGT
AATTTTTGGGGTAATATCTCTTGTTGGAATCGGACTCACAGTCTTGCGGACGGTTGTGCGCTGGTACCCTC
GACCTTGAGTTTCCCGAAAACCGCTCTAGGTGAGGTTAAAGGTCGCTTTGACTCCTTANCGGACGGTGCTG
TGAGTCGTTTNTGGCTGTTTTGATCACCACCGATTGGCATACTAGGCTCTTCACAGAGCCAAAAATTGTAA

TAACCGGGTGAGATGTGATCAAATACAAATAANTTGAATCAGCCGAGCGAAAACGGAATGAGAAATGGC
ACAGGAACGATGGAATCGAGATATCAGAATAAAATGGAATGACCGAAATGGTGTTCACTATTGAGTCCA
ACGAAATCGGAAAATTTTGTTAAATTAATTATTTTACTAATAATTTACTAAATTGATGCTTATTTAGCTTCC
TTTACTTGAGAAACAATCTAATTATTTCTTTATTGAAACACCAGAGAAAAGATTAATTCTCTTTTGACAAG
GCGGCCGTGTAGTGGAAACGGATCGCGCAAAAAAAATTGATGCTTATTGCTTATTACTTGTTGAGCTCAA
GAAGATTCTAAACGCAGCTCAATGGAAGGTTTTTTGTCATAGAATTATTTGTATAAATTGTTATTTTTAAG
AAAAATCGCACGGATATTCGATTTATTACGCAACTTGGAAACGTCTTAACTTATAATTATGATTTATAAAT
TTTTATAAGAATAAAGAAAAGTAATGCATATGTTTGTATTTTATTAAGTAAACGCAGCTCAATGGCAAAGT
GGAAACACACAAAAATATAAAGTTTCTCAGAATCAGTTTATCGAAAACCGATCTTGATACTTTACAACCC
TGATGTTTGTTTACCTTTTGCACTCGCCAATTAATTGGTAAAAATAAATATATAAATTGATCTTGAAGCTG
CTGTGGAGGAAAAATAGCAAACGATACACATATATATATGATGTGCGTGTGTGTGTATACATGCAGGTGA
ATTTGCACAGTGTGGTTTTAATTAACTAAATACCAATCAGAATTGTGTGTCTTTTTTTTATATTTAGGTATG
TGATTATTTGGTGAAGGTTAGAATGGAGAAGCGCATACGCTT
>9_rnd-4_family-2491__LTR___Gypsy
ATGAGGATGCACGTGAGATGGAAACAGCCAGCTTAAAGATATCCAAAACAATCATGGATGAACCGGATT
GGTTATTTGCTATACAACTTCAAGATGAAAAGATCCAAAAACTTGTAACCGATATGAAATCCGCCAAAGT
AACCGAAAATGACGAGTATGTCATTGAACTAGATCGGCTCTATCGCAAATACAACAACAAATTCTTGTGG
GTTGTGCCTAAGCAGCTACGGTATCAGATCCTTTATGAATGTCATGATAAATCAGGTCATATGAGCGTTGA
GAAAACAATGAGCCGTATNACAAATTTATTTTGGTTTCCACGCATGCGAAATTATGTCAAAGGCTATATA
AAGTCCTGTGTGGGCTGCGCATTTTTTAAGCAACGAGGTGGTCGCAAAGAGGGACAATATCATTATGACG
ACATCAAGCCAATACCATTTTCAACGGTTCATATCGACCACCTGGGACCATTTCCAAAGAGTTGAAACGC
AACGAGCNCATACTTGTTTGTGTCGATGCATTTACCAAATTTACGATTATGCGCGCCGTTAAAAGCACAGC
AACCAAGCACGTCATCGATATCCTCAAAGAAGTAACCAGCTATCTGGGGATGCCACAAAGGATTGTCTCC
GATCGAGGTACAGCTTTCACCTCAAAAGATTTTGGAAAGTTCTGTACAGACAATAATATTAAACACGTCCT
TAATGCTGTTCGAACACCCAGAGCCAACGGACATGCAGAACGCACTAACAGAATCATCTTGTCAATGTTG
CTGCCTATGTCCGAAAATGAGAAGCGTTGGGATGAGAAGATGCACTCCATCCAGTGGAGTATTAACACGA
TGGTTAACAAATCTACTAACTGTTCTCCGTTCCAGCTATTATACGGTTACGAGCCTCGGGACATACTACAA
AACACTTTAGTGAAATTGCTCCAAAACCAGAATCCTACTATGCTGACAGATACCGAATTACAACAACTAA
GAAGCGACGCTGCCCATCGGGTCAATGATCATCGAGCTGAAGCCAAAAGACGATATGACGCAAGACACG
CCAAGCCGACAGTGTATAAACCTGGAGATATTGTTTTGGTCGAAAATGAACCATGCAGCACGGGCACGTC
ACGCAAATTAGAGCCACATTTCAAAGGACCCTTTATAATCAGCAAAGTTTTGCCAAAGGACCGATATCTT
GTTGAGGATATCCCGAATGCTGAGCGAACACAAAACATTATAAATCGGTGTATTCTTCAGACAAAATCAA
ACCATGNCAGTTACCGCCAGAGGAGCCCGACGAGGATGAAGACTCCGTTGATGACCAAGTAAACTCATCT
CGCGAGGACGCTTCGAATGTCAGGAAAGCCGAATGTAAGCGTCTGGCAATCTCAACCCATTGCCATTCAC
AGCGAATCGATAATTAGGCGAAGAAGTATCGAGTTAAGCATCACNAGAGAAAAAGGAGGACGGACGCTT
ATTAAGAATCTAAGACATAAGCAAATCTTTGTGAAGTTAACGCATTAAACTATTCTATCTTTACTTATGTT
TGTATAAGCGTATCTCACTCAGTCGACCTGGACCGATTCTTACAGTTCAGAAGTGGATAGATAGATCTATG
TATGTACATAGATATGCCGTGTTCGCCTACACGCATATACAAACACATGCAGAGAAGAAACGCATAGAAG
TCTGGATGAGAAGTTTGCAGTGGGATATGTTCCCAAAGGTCTAAGTGACAAAGATTACTGCAAGCTGGGA
TAAGTTCCCTCGATCTTAGCGTCAGACTGTTTCATAAGTTGGAATACGTTTCCNGCAGCGTCGGTGTGTAT
AAGTACAGATAGAAGCCCTGTATGTACGCATGTGTTGGTGAATTTCACAAGGAATTTCACCATCGAATTTC
ACAACTTGAGCTAACATTGCGATTGCTGTTGCGGAGAAGCAATTCACAGAAGAGTTGGCATCATTTTGTA
CGAGCACTTCGTAGACAAAAATTGAAAAGAGATAATAACAAATTTGGAAATATGCAAAGGATAGGAGAT
TTAAAAGTTGATCAGCTAAAATTCCTGTTGCGAGAGCGAGAATTGTCGACAAGTGGCACACGGGATGAAT
TAATTCGTCGATTACAGGAGCATGAAGGCTCAGATGAAGTTGAAATGCCACATGCCCCTCGGCAAAGCCA
TGGGAGAAGAGGGAGGATCACTTTATCAGCTACAGAATGAAATAAAAAGGTTAAGCGAAATTATGAGTC
NGTTTATAAGCATGCAATGCGCTAGTGCTAGTGGACAAATGTCGAATTCGCAACCGGCTATAAACAACTT
AGTGCAAGATACAGAAAGCGAGGNGGATGATGAGTATCAACAAAGCACAGCCACACGTCAAGCTTCGGT
CAAGGAAATTGCTAACACGTTGCCTGAGTTTGACCCGACAGATGAGAATGCCATTTCAGTCAATCAGTTC
ATTGATCGTGTCAACAAAGTGGTTGAGGCATATCAATGGGACGAAAAGTTTTTAATGCTCGCAATCTACA
CTCGACTCAAAGGTCCAGCCAGAATGTGGTTTGACTCTTCGCCAAGACTTCATACGACATGGCACGACTTT
GCAGACGCACTTCGCGACGAATTTGGTGTTACACCAGATGAAGCAGAAATCCACTACGCCATGGCAAAAT
CGATAAGACGTCCAAAAGAGACAATAAAGGAATATTGTTTCCGTATGTCCGCCCTGGGAGTTCGATTTAA
TCTTAGCGAGGCAGCTGTAATACGATACACACGATCAGGTCTTCAGCACCGTGAACTGCAGCAGAGTATC

GCAGCAATGCACTTCTCAACAATGAAGCAGATGCGAGAAACGGCGGAATCCTATTTTGCCAATCGAGGTA
GACAACATGAAGTCAGAGACAGCACAGCTCTAAAGTATAGCAACGTTGAACAAAGGCCTGAGAAAGATT
CAAAGACAATGAAACCAAAGGCAACACTCAAATGTTATAATTGCAACGAGTTTGGTCACTTTGCAAATCA
GTGTCCAAAAGAACCGAAATGTCAGCGCTGTACCAATTGCAACAAATTCCACCCGAGTAACGTCAAGTGC
AAGTCAGCTGAAGTAAATGTAAATCGTCTTGGTGCCATAAATAGAGACAAATGGTTCTCTAAACAGATAA
TCGTCCAAAAACAACCGTACGTCGCTTTTATAGATACTGGTAGCCAAGTGAGTATGGTTCGCAAGTCGGTT
GCTCAGTATATTAATGGGGATCGTCAAAAATGCTCAATTCGTATAAGAGGCATTTGCGGTGGTACACGAT
TTCTAACTGAATCTAAGATTTTGGATATATGGATCGACGGAAGGATCGTTAAGGCCCAAGTTTACATCGCC
GATGATGAGATCTTACAGGAAGACTTCTTGCTGGGACAGGATGTTATAATCGATGCGCACCTCACGTTTAC
CTTCACAAATGGAAAAACGGCTTTTGAGAGCAAAAGAGCAGTTCAGCATACAGAAACAAGTGATTCCAA
GTTTGCTAAGCTTCTTTCTAATTTTAAGCTCGAAACTGATAAAGTCCGAATGCACACATTATTGGACAAGT
ATACTGATGTGTTTTCAAGTGGTCTAGAAGGAATTGGCCGCACAAACGTCATACAANCAAGCCTTGAGGG
AGGACATATTGTTTCTCAAGCACCATACCGCATACCTGAACCGAAGCGAGATATTGTCACCGCCATGATT
GACGAACTCTTGCAAAAAGATATAATCGCTATGTCAACATCCGAGTATGTCAGTCCTATTGTACTAATCAA
GAAGCAGAATGGAGGTGATAGACTATGTGTTGACTACCGTCGTCTCAATAAAGCTTCATTAAGAAGGAAA
ACTTTTCAGTTCCCAACAACATCGAAGAGCGTTTACAAGAAGCCAAACGATTTAAATATTTCTCATCGTTG
GATTTTAAATAGCGGATATTATCAGATTGAAATAGCCCCTGATAGTCGGACGTATACAGCATTCATTACAA
CAGATGGACTTTTTGAATTCAAACGCACGCCATTTGGATTGAAGAACGCGCCAACTGTGTTTAACCGACTT
ATGGCGAAAATTCGTAAAGCGCGTTCGTAAAGGTGATATGCTGCACTATATNGATGATATTCTTATTGGA
AGCAAACTCATTGGACGGAGTGCATGAAAAGCTTGCGCGTGTCCTACAGGTTTTGAAAGAATGCGGCTTG
ACATTGAATATCGACAAATGTGAGTTATATAAGCAGACAACAACATTTTTAGGACATCAGATACATCCAA
AAGGTATCAGTCCAGGTGAAATAAAGACCAATGCCATCTCTTTGTTTGCTGAGCCAAGCTAACGTGAATG
AAGTAAGATAGTTTTTGNGTCTCAGTGGATATTTTCGAAAATTTGTAGCAGGTTATGCTATAATATCTGAG
CCATTACGTATGCTACTACGTAAAAATCAGCAGCTTATATGGAATCAACAACAGAAGGATGCATTCAACG
AATTGAAGCAATGCCTCGTATCTAAGCCAGTGCTTACAAGTT
>160_rnd-1_family-132__LTR___Gypsy
GATTCAAAGCCTTCTGAAGAACGGTATCATCAAAAAATCTGTCTCCCCCTACAACAACCCCATTTGGGTGG
TGGACAAGAAGGGGACGGACGAGTTTGGTAACAAGAATATGCGCCTAGTAATGGATTTTCGCAAACTTAA
CGAGAAAACCATCGCAGACAGATACCCTATGCCTAACATTCCAATGATTTTGGGGAATCTTGGCAAAGCT
AAATACTTTTCGACATTGGACCTAAAATCCGGCTATCACCAGATCACGCTAGCAGAGCGTGACCGTGAAA
AAAACAGCCTTTGCAGTTAACGGGGGGAAGTACGAATTTTGTCGACTTCCCTTTGGGTTGAAGAATGCGG
CCAGCATCTTCCAAAGAACTGTTGACGACGTACTGCGTGAGCAAATTGGCAAATCTTGCTATGTCTACGTT
GACGATGTCATCGTCTTCTCAAAAGACGAAAGTTCTCATGTCCAACATGTAGAGTGGGTCTTAAAGAGCC
TGTACGAGGCAAATATGAGAATCTCCAAGGAAAAATCGCGTTTTTTTAAGAAAAGCGTAGGTTTTCTTGG
TTTCGTGGTAACCAGTAATGGAGCTACAACGGACCCCGAAAAAGTCAAAGCGATTCAGGAGTTCCCAGAA
CCAAAGAATATATTCGAGATCAGATCATTTTTAGGACTGGCTAGTTACTATAGGTGTTTCATCAAAGACTT
TGCCTCAATAGCAAAACCCATTTCTGATCTTCTAAAGGGCGAGAATGGGTCAGTAAGTAAACATAGATCG
CGAAACATCCCGGTAGAATTCTCTGAGATTCAGCGTCACGCATTCCAAAAGCTACGCAACATTTTGTCGTC
AGAAAACGTCATGCTCAGATACCCTGACTTTAAGAAGCCGTTTGATCTAACGACAGACGCTTCAGCTTAC
GGCATAGGGGCAGTGCTCTCCCAAGAGGGTCGCCCCATCTCAATGATTTCCAGAACATTGAAACCCCCGG
AGGTGAACTACGCCACCAACGAGCGGGAACTTTTGGCTATAGTTTGGGCCCTTAGCAAGCTACGTCACTA
CCTATATGGGGTAAAAGACATTAACATTTTCACGGACCACCAACCGTTAACTTTTGCCGTTTCTGAGTCAA
ACCCGAACGCCAAAATTAAAAGATGGAAGGCGCGCATTGAGGAAACGAATGCGCGAATATTTTATAAGC
CAGGCAAGGAGAATTTAGTTGCTGATGCGCTATCGCGACAACAGCTCAACACTCTTGACGAGCTGAATAC
TGATTCCTGCGCAGCCACAGTCCACAGCGAGCTGTCCTTGACCTATACAATTGAGTCGACCGAAAAGCCA
CTTAATTGTTTCCAAAATCAAATTGTCATTGAAGAGGCACGCGTCCCCACCAAACGCAACTTCATTCTTTT
CGGGGACAAGAGGCGTCATGAAGTGGGCTTCACTGATCGCACAGCCCTTTTAGATGAACTGCTTGACTTG
ATTAATCCAAATGCGGTCAATGCCCTCTATTGCAACCTGCACACACTAGCACAGGTGAAGGATGGCTTAG
TTCGCATGTTTCCAGGCACAAAGTTCTGGCACTGCAAAAACCGAGTTGCAGATGTCTTCAACGTTGATGAG
AGGAGAGAAATTCTCGTTGCCGAACATAATAGAGCGCACAGAGCAGCTCAAGAAAATGTTAAGCAAGTG
TTATCAGAATACTATTTCCCTAAAATGGCGAAGTTAGCTAAAGAAATTGCGGAAAGTTGTAGAATCTGCT
CAGCAGCCAAATACGATAGGCACCCGAAAAGACAGGAGCTGGGAGAAACTCCGATCCCTGAGACTCCTG
GGGAATTGCTACACATTGATATTTTTTCCACGGACAACAAATTTTTTTTGACCTGTGTTGATAAGTTCTCTA
AATTTGCAGTCGTACAACCAATTCAGTCGCGCACTATCGGGGATTTAAAAGCTCCACTACTGCAGCTCACG

AATTTCTTTCCACGAGCCAGAACAATTTATTGCGACAACGAGCCTTCGATGAATTCTCAAACGATAAAGA
CCCTGTTGTTAAACAACTATGGAATCCATATTGTTAATGCGCCGCCGCTGCATAGCACCTCCAATGGCCAA
GTCGAGCGCTTCCATAGCACTCTAGCCGAGCTGGCACGTTGTCTCAAGCTGGAGAGAAACATCAGTGACT
CAGTCGAAGTCATAATGCTAGCCACGATTGAGTATAACAAGACAATACATTCGGTCATCAACAAGAGACC
TGTTGATGCCCTCCATGTGCCCTCAGGCGAACCCGAAAGGGAAATATCAGCAAAGTTAAGAAGTGCGCAA
GATGCGCTCCGGGCTAGGCTGAACGACCAGCGACAGAATAGAGTCTTTGAAGTAGGCGAAAAAGTNCTA
GTAAAAAGAAACCGCAGGCTTGGCAATAAACTCACGCGCTTATGTGAAGAACGGATCGTAGAGGCAGAC
CTTGGTACGACCGTTCTCATTGGAGGGAGGGTGGTCCACAAGGACAACCTTAAGTAGGGCGAGAGCTGAA
ATAAAACATCGATTATATGCATCTTGAATTTAACTATAAGTTTCAACATTTGTGCCACTAGGCTTCACCTTC
AAGTTTTAATATTTACGCCACTTGGCATTTCCTTTAGGAAGACACGTTGTAATAGTTTGGTCCGTTTTGTCT
GTCTTCCGCAGGTTCGGGTTTCTGCTATTTCTATTTTTTGCAAATGCGGCAGCGCGTGTGACTAATTACTCA
CACGCCAAGTATATTCCCATTGTTGAGGGCCAAGTTCTGGTCTGGGAGGAATTCAATTTCGTCAGGCACAC
AGCAAACATCTCTGAGTACGAGAAAATGGTAAACGAAACAATGGAGCTATGTGAAATGTTTCCGCAGTCA
CACATGAGAAAGCTTCTAAGCGTAGATGCGTCCCACCTCAGAGATCTGTTAGAAGCTCTTGGCGTGCACC
ACAGGGTGGCAAGAAGTTTAGATTTCTTGGGCACAGCCCTCAAAGTAGTTGCGGGAACACCTGACGCCAG
TGACTTCGAGAAGATCAAATTCACTGAGTTACAGTTGGTGAACTCTAACAACCGACAGGTAGCTATCAAC
ACAAAAGTTCAGGAACAAATCAATCAACTCACTACAAGTGTCAACACATTACTAAAAGCTGCAAAAAAG
AATCAGATAGATTCCGGGCATTTATACGAAACTCTTTTAGCTCGGAATAGAATGTTGCTGATGGAAATGC
AGAATTTGGTGCTCGCTGTTACCCTTGCAAAAATTAATATCATAAGCCCAAATATATTAGATCACGCAGAT
TTGANATCTGTTTGGGTCGATGAACCCACAGGTACTTCAATAGCTGACCTTATGTCCGTTGCGTCCGTGAA
AGTATTACAATCTGTTAATGCCCTTCACTTTATCATTAAGTTCCCGAAAATCAAAATGGCTTGCAGAAAGG
TGATGCTGTATCCAGTTGCACATGAGAATGCTGTGCTACAAATTGACGACAACATTATAGCTGAGTGCGG
AGATCAAGTGCTTGCCCTTAAGAATTGCACTTCCACGCCGGGAGGAACCTTTTGTCAACTTTCACCGACCA
GCTCTTGTGCCAGAGAGCTGCATGCTGGTGGCTTGGCAAACTGCCAAATGCAGCCGAGTCACTTAGACCC
GATCACATGGGTCGATGACGGAATTCTGATCATCAATAACCGGCCTGCTAAAGTTTGCGTTGACAACGGT
ACTGAGACTTGGGTTCACGGAACGAATCTAATCACGTTCAATAATTGGGCTCTGATCAACGAAACTAAGT
ACTTGAATCGCAACAACGTCCAGAGTAAGTTCCCCGGCATCGCGGCATCTCCCCTTCTGAACGTCACCGGC
CACCAAGACGTGCTGAGTCTCCCCTACCTTCATCGCCTGAATGAACGTAACCTGGAGATCATCAAGGAAG
TCAAGGACGAAATTGGAAGCGTCGACAGCGTGGTAATAGCCTTCTTGTTGGGTGCTGTGTGTTGCACCAT
AATTTGCGTCGGAGTCACCTACCGTCGGTTCACCAAAAACATGGCATCGGCCAAGGAGATTAAGAAGGTG
ATCGCCGAGATAGGAGCGGCCGAGGGCGGCCTTAACTTAAAAGGGGAGCAGTTAACAGAGGCCAACGAC
ACCAGCTCCAGATGCTGATATAGCACTGAGCCGGATGTTCTTACGAGCTAACAGGGGTCAGCAAAATGCA
ACATAACACCAGCCGCAGATGCTGACATAGCTCTGGGCCGGATGTTCTTGCACGACGGCAGCGCACGAGG
CGCAAGCTAAGCATTCTAGCGGCCGTTGTGAAAGCACACAATAGCTTAGGTATTTTTGATCAATCTTTTGT
AAATTTAGTTTTTAATTCGACTTTCACTGCGGCACATCGGCCCAGCGACTTCGCGTATAATTTAACTCACTC
ACTACGGCGTGTCGGCCTAGTGTTTTATAAATCATTATTAAAATAAAGTTCTATCAAAATAATCCGCGAGG
ACTTATTATTTAAATCCTAATTTTGCACA
>9_rnd-1_family-409__LTR___Copia
TAAAATAGTGACAATAAGCTGTTAAGCTTCTTTATAATCTGGCAACATTGTATCAGCATAGTACTCATTCT
GTAATTGAGTACTTGTTCAGATTTTGTACTCATGCTTGAAGTTTCCATTAAACATTTAACCGAACCACTAC
GTGTGTTTTCTTTATGTGCATCTTAGGCACACTAGAAATTTCCCAACATTGGTTATGGGCCCAGGTCTCGTT
GTTTTCGGTTAAATAAGTACACAAAGTGTCTATGGTTTTGAAAGCTTCCTTTTGTTAAATTTTTAAAAAAGT
TTATTTCGTGCGAAGAATAACGTGGTGAAATTGTCAAAATGAACATGTCGCATCAAATAGAAAAACTTGG
TGAGGACAATTATGACGTATGGCATATGGCCATGAAAAGTGTCTTGGTGGTGGCGGACCTATGGAATGTT
GTGTGTGGAAAATATGTTAAGCCAGAAGGAAATGGTGAGGATATCGAAAAATGGGTTAGTGTCGACCAA
AAAGCGATGGCTTACATAATTTTAAATCTGAAGCCAACACAGTTGATGCATATCAAGTCATGCCCAACAT
CTGCAGCTGTATGGAAAAACTAAAGGAAATTCATGTCTCAGTTGGACCGATTAGGAAGGTCCAATTATAT
CAAAAATTAATACGTTTCAATATGCAGCAGGGTGACGATGTAGTCGCATATGTCAATTCATTTGTTGAGAC
AATTGAAAAGTTGGCAGAATTAGACATCAAACTCAATGACGAGTTGCAGGTCATCATGTTTTTGAGCAGT
CTACCAGACACATGGGAAAATTTTGTAATAGCGATTGAGACACGCGATGAGCTGCCCAAATTCGAAGTCG
TCAAGGTTAAAATGTTAGAAGAGGCCACGCGAAAGCAAGAAAGAGCTAGCAGAGACGGCATAGGGACAC
CAGAAGCAGTGTATACACACAAACATAATGGACCGCGGGACAACAACAGTGTCGAAAGCAAAGGCAGAA
AGAATAGCAGTAAGCAGCCATTCCGAGGCAAATGTTTTAATTGCGAAAAGAAGGGCATCGAGCGAGCGA
TTGCCGAAATAAAAAGAAGGATGATAAAAAGAGCCAAAAGGACGAAAAGTCTCTATGCTTAATGCACAC

GTGTGTCAAGGAGAAGCAAAAGAGTTTTGGGGTGCGTTGACAGTGGTGCAACATCGCACATGTGCTGCGA
CCGCAGCATGTTCCTTACGTATGCTGATAAAAAGACGTCGATAATGTTGGCCGCAAATAAGTTTATCGATT
CACCCGGCAGTGGCAGTGTTGAGTTGCACTGCAATAGAATAAAATTAAAGCTGCAAAATGTTTTATATGT
CCCTGACTTAAGCATGAATTTTTATCAGTAAGTGCTGCAGCAAAGTTTGAGAATTCTACCATATTTGAAGG
GAAAATTGCGCTGATAAAAGACAAAGAAGGCAACGAGATAATAAGGGCAAGACACGAAAATGGTTTGTA
TGTTTACAAACATAGTCTTGATCAGGTTCATATGCTAAATAGTTCTGATTCATCTTCCATAAAATGGCACA
ATAGATTTGGCCACCTGAATTTTAAGGATTTGAAATTGCTTAATGATAAGCAACTGGTGCGTGGTATGAAA
GTTGAAAATGTACAATTAGAAATAAATTGCGATACATGTAATAAAGCAAAAATTTGTGCATTGCCATTCC
CACAGAAAGCGAATCGTGTCTCAAAGCAAGTACTGGACTTAGTACATACAGACGTATGTGGTCCGATGAA
TGTTAAGTCGCTTGCAGGCAATCGATATTTCGTAACTTTTATTGATGACTACTCACGCAAGATATTTGTTTA
CCTTATGCACTCAAAAGCCAAGTTTTTGAAAAGTTTAAAATGTTTAAAACTTTTGTAGAATGCCAAACAGG
AAATAAAATAAAGGCTGTGCGCAGCGACAATGGAACAGAGTATGTGAATAAGCAATTCACTGAATTTTTG
ATCGAGTGCGGTATAAAGAGGCAATTAACAGTTCCTTACACTCCGCAGCAAAATGGTGTGGCCGAACGTG
CTAATCGGACCATCGTTGAAATGGCAAAAGTCTTCTAATTCATTCAAAGCTTGAAGAATTTTTGTGGGCAG
AGGCTGTTGCAACAGCAGTTTATTTGCGCAACAGATGTCCCACAAAAGCATTAGATGGAAGCACACCATT
TGAGGTATGGAAGGATCGCAAGCCATCAGTACAGCACTTGAGAGTATTTGGCTCGCGGGCATTTGCACTC
GACAAAGTCAGAAAAAGCAAGTTCCAAGCAAAGGGCAAGGAGTACATTTTTGTTGGTTACTCCTTTACAG
CAAAGGCATATCGCCTATATGATCGCGAGAAGCGAACAGTTATTGAAAGAAGGGATGTCAAATTCATTGA
AGGTGAGTTTGACATAGCAACAACTGATGCATGCAGTATTTCAGACAGAAACACCTTCACGACGTACATT
ATCCCGTTTGAGTCAAATCATGAGGTTGCAAAAACCACCCAGAGCAACCTGCCGAGGTGCAAGAGTACAA
CAGTGACTCAAATGAAGAAGAAGAGGAGGATTTTGTGAGCGCAAGCGATCCAGGAGAAGATGGCAACGA
TGACGACAATGACCAAGTAGCGCTGCCACAAGTTGAAGCTGTAGTAAAACATGGGCGTGGCAGGCCAAA
AATTATACGTACAGGTCAGCCAGGCAGGCCCAGAAAGCAGTACCAATTGATCAATAGTCTTAATCAGATT
GAAGACATTGAAACTCCGCAGTCAGTTGCAGAAGCATTACATGGCCAAAATGCGCAGGATTGGGAAAAG
TCTATGCAAATAGAGTACGACGCTTTACAAGCAAATGAGACATGGTCAGTGTGTGAGCTACCTCCTGGAC
AAAAGGCCATTGGTTCAAAATGGGTTTACCGAATCAAGAGAGATAAAGATGGTAATATTGAAAAATTTAA
GTCTCGACTAGTTGCACAAGGTTGTGGTCAACAATTTGGTGTCAATTACTGGAAACGTTTTCTCCCGTAA
TTCGATATGAAACCATTCGCATGTTATTCGCAATAGCTGCCGAAAAAGAGCTGTATATGCATCAAGTCGA
CATTTCAAACGCATACTTAAATAGTAAGCTTAATGAAACTGTGTACATGAAACAGCCGCCGAATTTTATTG
ACAAGCAGCATCCAAATAAAGTCCTCAAACTAGAAAAGGCACTTTACGGGCTTAAACAATCCGGACGAGT
TTGGAACAATACGCTCGATGAGGTATTGAGAGGCATGGATTTTAAAAGATGCAAAATGAAGCATGCTTAT
ACGTCAAGCAAAAGCAACAACAATTCAGCTACATCGCTGTCTACGTCGACGATTTAATCATAGTATGTCC
CAGCGAAGAAGACATAGCCGTGATTAAGAAGAAGATTGCATCAAATTTTAAAATGCACGATGGAGGCCC
CATTAGCTATTTCCTAGGCATGGAAATCCAACGCGACGGCGATCGAGGTTCCGTTTCGCTGTGCCAGAAG
ACGCACATTCAAGGCCTATTGGACAAGTATGGCATGAGCAATTGCCGTCCAGTAAGCACACCTCTTGACC
CAGGTTTTCAAGTGGACTGCAACGACGATACTTGTGTAAAGGTAAATATAACGCAATATCAGTCATTAAT
TGGTTCGCTTATATACTTAGCAGTTTTGAGCCGACCAGACATTTTGCATGCTGTAAACAAATTATCACAAA
GAAATGCTAATCCTCATGGAGAACACCAGTCTGCAGCAAAGCACATTCTAAGATATCTTGCGGGTACAAA
GAATATGAAATTAGTATACCGTAAAAATGGCAAACCACTAAAAGGCTATGCAGATGCAGATTGGGGCAA
CGATCGGCTGGACAGGAGATCCTATAGTGGCTACGCATTTTTCCTGGCAGACAGTGCGTTCTCTTGGACTT
CATCAAAGCAAAGCGTTGTCGCATTGAGCAGCACTGAAGCAGAATATATTTCTTTGTCCATGGCTGCCAA
AGAAGCAGTTTATCTACGCAGGCTACTTAGTGAGATGGGATGGTCAAAGGATACAGAGCCGTTGACTATA
TGTGGCGACAACCTAAGCGCGCAACATATTGCGCAAAATCCAGTCCATCATAAGCGCACTAAGCACATAG
ACATAAGGTATCATTTTATACGTGAAAAGGTACAATGTAATGATATTGCATTAGAATACGTACCTACAAA
TAATAATGTTGCAGATATACTAACAAAATGTTTAAGTAAGCAAAAGCACGTAGGCTTTGTAAAAACACTT
GGATTGAATTAAATTTGTTATTTTGCAACTGATTATAATTAGCTTAAGAAGAAGTGTTAAAATAGTGACAA
TAAGCTGTTAAGCTTCTTTATAATCTGGCAACATTGTATCAGCATAGTACTCATTCTGTAATTGAGTACTTG
TTCAGATTTTTGTACTCATGCTTGAAGTTTCCATTAAACATTTAACCGAACCACTACGTGTGTTTTCTTTAT
GTGCATCTTAGGCACACTAGAAATTTCCCAACA
>160_rnd-1_family-331__LTR___Gypsy
TGATGTCCAGTCTATATGTTGTTTGGTTAGTGGTCGAAGCCAATTACCGATATAAATATCGATATCGACAT
TGAAACACTGGACGATACAGAATCAAACATGCCAGACTAGGTAGCCTTAGATAGGGAATATGTAAGACA
AATTTCGTCTTCGGTCCCTGAATTTGACGGTAAAAAACTGTCACTAAATCGTTCTTTAACAGCCTTAAGGC
TTGTTGAACGAATAAAGGGCAACCAATAGGATTTGGCTGTAGAAGTCATAAAATCAAAACTAATTGGCCC

AATACTATACAAATGCCAAGACGAGACCACGATTAATGTCATTATTAATAAATTAAAAAGTAGCGTCAAA
GGTGAATCCATTGACTTGCTTAAGGCAAAGCTGTTAAACGCCCAACAAAAAGGCAAAACAGCAGCTCAAT
TTACGGCTGAAATCGATAAACTTGCGTAAGCAACTCGAGGCAGCTTATATTGACGATGGCCTAGACTCGG
ATAATGCAGAGAAATTCTACCAAAGAGTCAATATCAACAATGACCAGAAATTGTGAATATGAAAAGCTCC
GCATAATACTAGAAGCTGGAAACTTCAGCACATTTAATGATGCAATGAGTAAATACATTCATTGCAGCAC
TGAAGTGACTGGAAGTGTCAGCACAGTATTATATCAGCGAGGAAGACATAAAGNGGCATTGATTATCGCA
ATTACCAAAGAGGTAGAGGCAATGGNCGCGGTAATCATAACGCAAACTATAACAACAATAGCAATGGCA
GAGGACATTATAGACGTAATGCCCGTGGTAATAACCGAGGAAGGAATAATTCCAACCGAGGTAACAACC
AAAATAATAACAATAGAGGTAATGTCCGAGTAACTGAAGGTAATTCGGAAAACCAATGAGACCCCTCAG
ATGTTTAGAGCCAATAAATGGCACAATAAAAACCATCAACCTCAACCTCAGCATATTTGTAAAACTTGAC
AATGAAAGAACAGGTAAATCCTTTGCACTGTTGCTTGATACTGGTGCAGATATATCTATATTAAAAGAAA
ATGTAGATATATTTGAAAACGTAAACGAAAGAGTTACTACCCAAAAATCGGGAATTGGCGAAGGAACAA
TTTGTTCAAAAGGCTTGGCCTTTATAGAACTCACTACAGGTAAATACATAATTCCGCATAACTTCCATTTA
GTCGATCAAAAATTCCCAATACCTAGTGATGGCATACTAGGCATAGATTTCATAAAACAATATAATTGTC
AATTAGATTTCACTCAGAGTGGCGACTCACTAATTCTTCGCCCAAACAACTTAAATTATCCGATTAAATTA
CAAATACTTCATACTTGTGACAATAATGCCACAGTACTCCCAGCTCGTTCCGAAGTTGTCAGTGAAATACA
AATACCCTCAATAAAAAATCAAATTTTGATATTAAATCAAGAAATCGATNAAGAAATTTATATCGCAAAT
ACCATATCCGACCACAAAAATACTTATGTACGAATTTTAAATACAACAAATACCAATAAGATCGTCAATT
TAAATAAAAAAAATATGAAACCTTAAATGATAAAAGAAACCAACGAAGAAACAAACAACTTCGTTATGA
ATAAATTAATTAAAAATTTTCCGCCTTTATTCAAAAGTCAATTAAAAGATTTATGTACCAGGTATACAGAC
ATATTCGGACTAGAAACCGAATCAATAACAACAAATAATTTTTACAAGCAAAAGCTTAGACTGAAGGATG
AATCAGCCCGTATAAATAAAAAATTATAGGAGTCCTCATAGCCAAGTACAAGAAATTCAAGCCCGAGTAC
AAAAATTAATAGACAATAAAATAAGTCGAACCGTCAGTCTCATAATATAATAGCCCACTCTTACTAGTCC
CGAAGAAGTCTCTTCCGGACTCTGAAAAGAAAAAATGGCAATTAGAAATTGACTATCGTCAAATTAATAA
AAAGCTATTGTCCGACACCTAGAATTGATGATATTCTAGATCAACTATTTAGAGCAAAATATTTTTCATGC
CTAGACTTAATGTCTGGCTTCCACCAAATTGAACTTGAAAACAGTTCTAGAAATATAACGTAATTGTCAAC
AAGCTATGGCTCATATCGCTTTACGCGATTACCATTTGGATTAAAAATAGCCCCAAACTCATTTCAAAGAA
TGATGACAATAGCATTTTCTGGTCTACAACCATCAGAAGCATTTCTTTATATGGATGATCTAATAGTAATC
GCTTGTTCCAAGAAACATATGGTCAAAAACTTAACTGATGTTTTCGACAAATGTAAAAAACTCAACCTGA
AACTACATCCGGTAAAATGTTCATTTTTTATGCATGAAGTCACATTTTTGGGACACAAATGCACTGATAAG
GGAATCTTGCCAGACGACAAAAAATACGACGTGATTAAAAATTACCAACTCCCACACGATGTGGACAGCG
CAAGACGATTCGTTGCATTTTGTAATTACTATACGCGCTTTATAAAGAACTTTGCTAACTATTCACGTCAC
ATAACAAGATTATGTAAAAAAGACGTNAAATTCGAATGGACGTCTGAATGCCAGAACATCTCAAAACAG
CACTAATAAACCTAACGTTGTTACAATATCCAGACTTCACCAAAGAATTCTGCATAATAACAGATGCAAG
TGAGCAAGCGTGTGGAGCAGTTTTAACTTAAAACTTTACTGGACTCCAACTTCCAATTGCATACGCATCAA
GATCCTTTACTAAAGGAGAAAGTAACAAGAGTACAACTGAACAAGAATTAACAGCTATTCACTGGGTAAT
AACACACTTTCGACCATATATTTATGGTAACCATTTCACGGTAAAAACGGATCACAGACCACTAACATCTA
TTTTCAATGGTCAATCCAAGTTCACAATTAACACGTGTGAGGCTTGATTTGCAGGAATATACCTTTACGGT
AGAATATCTGAAGGGTAAAGACAATTACGTAGCAGATGCGCTATCGAGAATAACCATCAAGGAGCTACA
AAATATAACAAGAAACATAATGAAAGTCACTACAAGACAAGCACGTAGACAAGAATCCTGCGCAGGAGA
AAAAGAAATNGAATTGCCTAGGCAATCTATAGAAAAAGCTTCAAAGCCCAGAGTGTACCAAGTCATTAAC
AATGACGAGGTACGAAAAGTAGTGACCTTGCATGTAAACAATATGACATGTTTTTTAAACATGGCAGAC
AAATTACAGCAAGATACGATGTTACCGATTTATATACTAATNAAACCATTGATTTAGATCAAATCTTTCAA
AGGCTTGAAACGCAAGCCGGTATATATAAAATCAGCCAACTCAAAGTGGCACCGTGGGAAAATATCTTTG
AACACGTTTCAATGGATAATTTATAGAAAATGGGCAATCAAATATTAAAATCTTTAAGAGTTGCGCTACTC
CACCCGGTGACCCAAATAAATTCTGAAAAAGAAAAATAAGCTATCTTGTCTAAACTACATGATGATCCCA
TACTAGGAGGACCTACAGGCATTATAAAAACCTTGGCCAAAGTCAAACGACATCATTTTTGGAAAGGTAC
GTCTAAACAAATAAAAGACTACATCCGCAAATGTCCAAAATGCCAGAAAGCAAAAACTACAAAGAACAC
AAAAACTCCTCTAACAATAACAGAAACACCAATATCCGCTTTCGACAGAGTAATCGTTGACACAATTATT
ACCATTACCTAGAACAGAGAATGGGAACGAGTATGCAGTTACACTCATTTGTGATTTTACGAAATACCTC
GTCGCAATTCCGATCCCAAATACAAGTGCAAACACTGTCGTTAAATCTATATTCGAATCTTTTATTCTGAA
GTACGGTCCAATGAAGACGTTCATTACGGGTACACGGGCACGGAATATAAGAATTCAATTATAAACAATT
TGTGCAAGTATATGAAAATAAACAACTTAACATCCACTGCACATCATCACCAGACAGTTGGAACAGTAGA
AAGAAGTCACAGGACTTTTAACGAATACATACGATCATATATATCAGTTGATAAAACTGATTGGGGCGTA

TGGATCAAATACATCGTATACTGTTTTAACACGACCCCCTCTATCGCACACAATTATTATGAGCAAATTCT
TGGTAAGCACCCAATTTACCAATATAAATAGAATCGACCCACTATATAATGTAGAAGATTATGCTAAAGA
AGTTAAGTTTAGATTAGAATCAGCATATAAAAGGTCTAGGATATTGCTTGAAAAACATAAAGCAAGAAGT
AAGGAACTTTTTGATAAAAAAGTTATAGATTTTCAAGTAAAAATAGGAGATAAAGTCTTATTAAGAAACG
AAACTGGCCATAAATTGGATCCAGAATATATAGGCCCATATACAATAGTTAACATAGAAAATAGGGATAA
CATTCTAATTAAAGATAACAAAAATAGGACCCAAAAAGTTTATAAGAATATACTAAAAATTTTTAACGAA
TAAAACATATAAATGTTCTATATGAAGTCTTTAT
>160_rnd-5_family-4735__LTR___Gypsy
GTTTGGATTTGTCCTTCCCTTCTTCCTATCCTTGACTTCGGCACGCATCACGGACTATTCTCAGTCCAAATA
CATCCCCATTGTCGATGGCCGTGTCCTAGTGTGGGAAGAATACGCCTACGTTAAACATTCAGCGAATCTCT
CAGAATACATGCGTGTGGTAGAAGAAACGGTCAAATTGAATGACATGTTCCCACAGTCGCATATGAAAAA
GCTTCTGAGCGTGGATGCAGATCACCTCCGAGATTTGCTAGAGGCGTTGGGCGCGCACCACAGAGTGGCC
AGGAGCTNAGACTTCTTAGGTTCCATGTTGAAGGTTGTGGCAGGCACACCGGATGCCGGCGATTTAGAAA
AAACTCGGTTCATTGAGGCACAATTAATTAATTCAAACAATAAGCAAATAGAAATTAACACAAGAATCCA
GGTACAAATTAATCAACTTACTTCTACAGTCAATCAAATTCTCGAAACAACTAAAGACACTCAAGCCGAT
ACTGGCCATTTATATGAGACATTGCTAGCCAGGAATCGAATGTTAATGATGGAGTTGCAAAATCTAATGC
TGGCAATAACTCTTGCAAAGGTTAACATTGTTAGCCCAAACATTCTCGATCATTCAGATTTGGCATCAGTT
TGGCTGGAGGAACCCACTAACACTCCCATAGGGGATCTTATGTCCGTTGCGTCCGTGAAAGTTTTACAGTC
CGTTAACATCCTTTACTTTATTATTAAATTTCCGAAAATTAAATTTGCTTGCAAAAAGGTCGCGATTTTCCC
GGTCACACATGAGAATTTCATACTACGCATGATGAATAACGTCGTGGCAGATTGCGATGGGGAAACGCAT
GCTCTCGAAAATTGCACCGCCTCTCCGGGGGCCACATTTTGTCATCTAGCATCCGGGAGTTCATGCGCCAG
AGAGCTCCACGCAGGCGGGATAGCGAATTGCGAAATCCAACCAAGCAATTTAGACGCTATCACTTTCGTA
GATGACGGAATCCTCATCGTAAATGACAGGCCAGCGAAAGTGCAAGTGGACAACGGCACCGAAATCTGG
GTACATGGCACGCACCTCATCACCTTCATCGACCGCGCTGCGGTGAACGGAACTTTATTCATTAACCATAA
CAACGTGCAGAATAAGGTCCCGGGTACTGCTAACACCCCCTTACTAAATATCACGGCCAGCCGGGATATC
CTCAGTCTTCCGTACCTTCACCGACTGAGTGAAAGTAACCTGGAGGAGATCCAGAAATTCAAAGAGGCCC
CGGTAGCAAAGCCGTATACCAGATAATATGCATCGTGGGAGTGGTATGCGTTTTGCTCATCGGCATCGGT
CTTGCGTTTTGGCGAATCACCAGCGCCAGAAAGTCTGCGCTACAGTTAAGAGAAGTGATCGCAGAAATAG
GGTCGGCCGAGGGCGGCCTTGGTCTTAAAGGGGGATAAGTTAAGCGGAGTCAACGAACGGTTGGCCAGA
ATATGACTCCGACCACAATTGCTGAATAAGCATTGGGCCGGATGTCGTTGCATAGCCGGCAGACACAGCA
TATTTGCGTGGCCGCTATGAACGAAGAAATAATTTAAGAATCACATATTCTGTTAGCTTTAAGCCCTAAGA
ATTGTATCGCATGTTTTTAGTTCTGAAATAAACGTCGTTAGCTCAACACGTCAGTACTCCGGCACCAAGTC
GTCAGCGTAAAACATAATTGCCAAACGGGCAACAAATAAATGTCACTTAATCAATACACTCAAAAGACTC
ATAATTGAGTCGTCAGCGTAAAACATAATTGCCAAACGGGCAACAAATAAATATCACTTAATCAATACAC
TCAAAAGACTCATGATTGGCGCCCAACTAGTGGTAGTTACGATTACCCACGCGATTGCAAAGAAAAAAAT
TCTTACAAAGTGAACATTAAGTCGCGGTCACACCAACAGTGCCGCGCAGTGAACACTACAANAATAAGAA
ACAATAAGCTGCAGTCACCAGTGAATTAGTGATAATAGTGACAGTGAGTTAACAAAGTGCGGTCGCGCAG
TGAACACTACAACAAGAAGCAATAAGCTGCAGTTACAGTGAATTAGCGATCATAATCTCGTGATTCACAA
AGGTGCGCTCAAGCGCAGTGCGATTTACATATATTTTTACACCGAAGCCGCTGGAGCTCTTCGGGCAGCG
GAGACTGCGGCGAAGGGAGCGACTACGAATAACAAGTGCGTGACTGCGGCGTTGGACCCGCAAACGTGG
CTGGTGTAGTGACTAAAATAAATCATAAAAATCATGTAAGTGGGCACTTTAGCCAAATCTGTTATTAAATC
TGATCGAATTAAAAAAAAAAAAAAAAAAAAAAACAAAAGAAACAAAGAGAGGAGAAATCTTGCATGCGT
TATATTTTGCAACAAATCATCTGCTAGAGTAAGAACAAAAGTTTTTGAGAATTCGATTTGACAGCAGTAA
ACGAATTTTGTAAAAAAAAAAATTACTTTGTGTTACTTTTTATGCCACTCTAAAAATTGTGCTGCAACGGC
ACATACTTTCTTGCTTATGCGCTTCTCGTGGTTCAGAGCTGAGGCGTAATTCGACCCATCTTTCGACTTTCC
GTTTTGTTTTTGCGTCCGCTAGACCGCGCGTGCATTTATTTGTTTTTGCTTTGAATGCCCATGAAATCGTCT
TTCGGTCAGTATAGGACCCCCCGCAAGAACTCTAGGCGACTCGGATTCTGACTGTGACAACCGCATCAGA
ATCCCGGCTCCACGACTTGCCCCTACAGTCCCCCCAACTTCGAACAAAATGGATCCAGAGCAACTTAGAG
AGATAATTCAAACGGCCATAAGCAATGCCCTAACAGAACAAGCCAATGCAAACCAGCAAAGAGAGCAGC
TATTAGTGCAGAGAATTAATGAATTATCCGAACAAATGGCAGCAGCAAACGTTGCTGCCCCACAAGTAGT
AGCGTATGCTGCTATTGAGATTAGAAATGACATTCGCTGCGACGAGCCACTAGACGCCGTCAAATGCTTA
CCTGAGTTTGCAGGAATACAGGAATCTCGGCATTATCGGGCCGTNANAATTATAGGAAGCAAAATAAAGG
GCCCCGCGGATGCGGTGCTGGCCTCATTTGGCACTGTGATNTCACATACAGTGACAAAAGGCCGATCCAT
GTAATTGAACAAGAGTTAGGCACCTTGAGGCAGGGCGGCTTGCCCCTACTCCAATATTTTGACGAAGTGG

AGAAAAAACTAACCCTACTGACCAACAAGGTCAATATGTCTTACGAGCCCGTTATGGCAAAAGGTCTTTG
TGAGAAGTTCCGTGACGACGCGTTGCGCGTTTTCGTATCGGNGCTTAGGCGCAATCTCACGGATGTGCTCT
TCGCAGCCAAGCCTAGAGATCTGCCTTCGGCACTGGCGCTTGCTCAGGAGGTGGAGTCAAACCACGAAAG
ATATGCTTTCGCGGCTAATTTTGCCAAAAGCCAAGAAGAAAGAGAGCGTAGGCCGAATACATAGACACA
GGGCCGACGGCAAGATAGACATTCTGAGCCTCAGACAAATGGCGCAAAAAACCCTCATTTCAACAGGCA
GCAGAAACAACAGGGTCAGTCCGATCAACGAAGTGGTCGGCAGCAAAACAAAATGGTGGACAGGAACCC
ATGGAGGTAGATCCTTCATTTTCAAGGATCCTCCAACCCACACAGGCACCGGGGTATCTGAATAGAAAAC
AGGCAACATCTGATCACACGGGCTTTCAAAAAAGGCAACAAAGGGTGAATCATGTAACGCAGGACGCGG
AACAAGGATCGCAAATGTATGCTAATGCCGCCTCTAGCGCAGAGGCGCAAATTGAGGATGACGTGAGCG
CTAGCTATGATTCAGATGCTGTTAATTTTTTAGGGGTAGATCCCTGTTACCCGTCATCGGGCGAAGAGTAG
CAGGGGCCGATATGAAATTCCTCATAGACACGGGCGCGTCTAAAAACTTTATCAGACCACATGAAGGGTT
AAAAGGCGTCCGACCAGTAGATTGTCCATTCACAATCCACTCGATTCATGGTACAACTAGAATCACGCAA
AAATGTTTTGTCTCAATGTTCAACATAAAGGCAACATTTTTTCTTTTATCGGACCTGTCAACTTTCGATGGA
ATAATAGGCCTCGACCTGTTAACACAGGCAGGGGCATCGCTTTGCTTAGCGTCAGGTCAGCTTAAATGGG
GTTCAGAAATTGAACAAATTTCTTTTCACGCATGCACTAATGTCAATTTCACTAACGTAGATTGCTCAGAT
GCACCTCCTTTAGTAAAGAACAAATTCCTGAAACTGCTAAAAGACAGGAAAAGG
>9_rnd-1_family-308__DNA
TTAAAATATGGCTGCCATTTAACAGTATGATATATAATATTGCCAGGTTGTTTTTCTGCATTAACATAGGA
TGTTATGAGAGAAATAGAAAAATGAGAATTTCTCAAAATCTGTACCGATGATTTTAATGAAATAAATAGC
AAAATGATTGATAAATCGTGCTCTATATGCACAACTACCGTGCATTTGGGNCCGCGTTCGCAGAAATTAA
ATAATAAAGCTTAAAATATGGCTGCCATTTAACAGTATGATATATAATATTGCCAGGTTGTTTTTGCATTA
ACATAGGATGTTATGAGAGAAATAGAAAAATGAGAATTTCTCAAAATCTGTACCGATGATTTTAATGAAA
TAAATAGCAAAATGATTGATAAATCGTGCTCTATATGCACAACTACCGTGCATTTGGGGCCGCGTTCGCA
GAAATTAAATAATAAAGTTTAAAATATGGCTGCCATTTAACAGCGCGATATATAATATTGCCATGTTTTTT
TTGCATTAACATAGGATGTTATGAGAGAAATAGAAAAATGAGAATTTCTCAAAATCTGTACCGATGATTT
TAATGAAATAAATAGCAAAATGATTGATAAATCGTGCTCTATATGCACAACTACCGTGCATTTGGGGCCG
TTTTCGGAGAAATNAAATAATAAAGNTTAAAATATGGCTGCCATTTAACAGTATGATATATAATATTGCC
AGGTTGTTTTTTTGCATTAACATGGGATGTTATGAGAGAAATAGAAAAATGAGAATTTCTCAAAATCTGTA
CCGACGATTTTAATGAAATAAATAGCAAAATGATTGATAAATCGTGCTCTATATGCACAACTACCGTGCA
TTTGGGGCCGTTTTCGGAGAAATAAAATAATAAAGNTTAAAATATGGCCGCCATTTAACAGTATGATATC
TACATTAACATTTTTGCATTAACATAGGATGTAAAGAAAAAATTGGAAAAATGCAATTTTTCCATAAACTG
CGCTATTAAGTATGACTTAATACTTCTCATATAATTTGCTTTGACTTATTAATTTAATTNACTGTGAGTCCT
TTCCGTTTTTCATGATAACATATTATTCAAATTGGATTGGTTCCCGCCCAATTCTGTACAAAATTGTTCAA
CACTGATGCCAGGCAGTGATTCAGTTCACGTAGGAANTAGCAGTTCGATAGACTTTATGAATGAACTATA
TGAGGTTGGTTTACAATTAATTAATTTTCTGAAAATCTGTACCGTCCATTTACTTAAANTTAGTGCCTAAA
GCTTCTTAAAATATGTATCTACAAGAGGGTATACCGTGCATTGAGGGCCATTGTCTCTGAGTTAAGAAATA
AGGGCTCGAGACAAGGTTGATTTTTTTTGCGGTCATTTTATATGGGGCTGACATGGGGGTTGTTGCACTCT
TTTCCAAATATCTTAAAAATGGTACCGACGATTTTAATCAAATTTTTCACAGTTGGCTAGGTGAAACTATA
GGCTTTAATCGTGTATATGCGGTTTCGGGGCCATCATCCCTAAATTTAGATATTTATGGAATGGTGGAAA
CCTAATATTTNAACCCCTTGGTTTACCCATATTATAAATTAAAATTCAAAAACAAATGAATGTAGGCAGTA
AATAATTTAATTTTTTGCAATTCTGCATTTAAATTTATTNGGTTCCGTGCTTAAAAACATAAATAAATGCAT
TTAAAAGTGACCGATTCCTTTTCCGATACCAAGTACCAAAATTCGACGCTAAGTCACCGCCGCTAATTTGA
CGCACATACAGAGTGAACATCGGGAATATAGCGGCGATCAAAAAAGTACAGCGCGCGCGCGCCAGACGG
GCGACAGTGAGACCACGCTTGCATACGTGTGCACAGATGCGCGCCGCAGCCGACGTCGGCGTCGAAGGG
CCGTTAAGGCGTCGCGACAGAGGCTGTACAGTGGGACGAATTCAACTCGCGTGCGGTGAAAAGTTGAATT
CGTCAGTACAGCCAACGCGGGCAATGAGAACGGGAGTGTTCAAAACGAAGTATATAGATTTATATATACA
CAGATAATAATACAAATTGTAGAATGTTTTAACAACTATTTAAAGTATTTATTTTTATATAAATAACATGT
TTAACTTATAAACATATATGTAAATAACATGTTTCATCGTCATGTGTACTTTTACTTATGTACTTTCACAAT
TTTTAGTTGTTCGTCTGATTGGCCGACCGGGCGCTGTCCGCAAACACGCCCGCTCGCCTCGACCGCCACAG
AGAGATTTTTATTGTTGCTCATTTCTACATATGATTTCAATTGCAAACAGAAATTTTAGAACAGTTTTGGG
AGCACTTAAAACTATGCAACGTTCACCAGTTTGCAAGGTGGAGAGGGACATGACAAGAGATACATTTAAC
ATTTAATCTCGACTGACCTACATATAAACATAACATGACATAACAGACTCTGTACATTTAACATATTGTCT
TAGGTCAGTTAGCTGGGGAAATTTCTGCACGATCAGCTGGCAATGCTGTCGTGTGAATGGCAAGAATACA
TAAAAATCAAATGGGCACGCTAAAGACATGTAAGTAGAAAATTATTTTAAATTTAACAATTTTTTGGCTA

AATATACTAACAATGTCAATGTACTTCCACAAGGTGGCATCTTTATGTTCCCATAGGTTAGCATTCTACAT
AAGTTGGCAGCCCCTAACATAGCGTCACTTTTGGATTCCATGCTATTTAATTCAAATGAAATTTATATGCA
TATTTATACATATGAACATATATATTTATAACATTTGTTATTTATTTGTTTATATTTTAATCTAACAAGTTTG
AAATGGCTCCAGGAAACGCAACACGGCAAGATTTTTATTTATTGACTGATTGTGTTCGCCTTTTTAAAAGC
CTGAACCCATTAAAAATGGGTAAAAGGGGTATAATGTATTTGTGCAGCCGTATGTAACAGGCAGAAGAAA
GGATCTTCAACCACTTAAAGTATGTATTCTTGATCAGCATCAATAGCCTAGTCGATCTAGCCATGTGCCTC
TGCCTGTCCATGCAAATCAGGTATCTTTTATTCTATAGCTATAGCGGCGTTTGTTGTTGGTAGGAATGGTAT
CCCCTAGCCGTGCACTCCCAACTANAGCATTCTTACTTGTTTTTNTACCTAAAAGCTATTGAAAATGAATG
AACATTTTTTCAAACGAACTGGTAAATAAAGGAATTTTAAATTTTGACATAACTTAGTCGTTAGTACATTG
TTAAAATTTTTTTGAAATTGTACTGAGTTTTTAAAACTAAAAACTAACGAGAAAGAAGGGCTATCTTCGGC
ACGCCAAAGATCTAATACCCTTGCAGACCCAACCTTTTCATAATGCTCATAGTGCTTTGCCTCTATCTAAG
AAGTACCGGGAAAAATAGTAAATGCCGAGTTTGGTCAAGATATCTTGATAAACAAAATGTTTTTTTCATA
CAACTTAATTTTCGACCGATCGTTCCTATGACAGCTATATGATATAGTGGTCCGATCTTTATAGGATTTTGC
ACATATATGAGGAGTAAAGTAAAAACTAATAAATGCCGAGTTTGGTCAAGATATCTTGAAAAACATAATG
TTTTTTTCATACAAAAACTTAATTTTCGACCGATCGATCCTATGGCAGCTATATGATATAGTGGTCCGATCT
TAATAGGATTTTGCATATATATGAGGAGCTTAGTAAAACTAATAAATGCCGAGTTTGGTCAAGATATCTTG
AAAAAAACAAAAATGTTTTTTTCATACAAAAAACTTAATTTTCGACCGATCGTTCCTATGGCAGCNATATG
ATATAGTGGTCCGATCTTTATAGGATTTTGCACATATATGAGGAGTAAAGTAAAACTAATAAATGCCTAGT
TTGGTCAAGATATCTTGATAAACAAAATGTTTTTTTCATACAAAAAAACTTAATTTTCGACCGATCGNTCC
TATGGCAGCTATATGATATAGTGGTCCGATCTTAATAGGATTTTGCATATATATGAGNAGTAAAGTAAAA
CTAATAAATGCCGAGTTTGGTCAAGATATCTTGAAAAAAACAAAAATGTTTTTTTCATACAAAAACTTAAT
TTTTCGACCGATCGTTCCCATGGCAGCTATATGATATAGTGGTCCGATCTTAATAGGACTTATGAACATAT
ATGAGGAGTAAAGTAACACTAATAAATGCCGAGTTTGGTCAAGATATCTTGAAATAAACAAAAATGTTTT
TTTCATACAAAAAAACTTAATTTTCGACCGATCGTTCCTATGGCAGCTATATGATATAGTGGTCCGATC
>9_rnd-5_family-2324__LTR___Pao
GAGCTTAATTTCGATCAACATTTTCCGGTGGTTATACCGTATATTCGNACTTTATCGCGATGTTTGAGTCAG
TTGTCGACAAGGATGCCGAGCTGTCTAACATTGAAAGGTTGCAGCATCTTCGTTCATGTTTGNCTGGCCCT
GCGTTGGATTCGGTTCGANCCTTGGAGTTTTCCGGTGCCAATTATCCTGTGGCTCTGGATATCCTNAATAG
AAGATTTAGTNACAAACGNNTTGTTTTCCAGGCACACATCAATGAGATTCTTGGGCTCAAGAGGGTTGAT
TCGGAGTCGGCTTCAAAACTGCGTGAGTTCTCGGACAAGGTCAATTCGCATATGCGTGCCTTACNGAATTT
GGGCAGTTTGGAGCAAATTTCGGGATGCATAATAGTCCACGCGCTGCTTCAAAGGTTGGACACCCAGACT
CAGACCAAATGGGAGGAGGCCGCAGGCATTGACAAAATCCCGACAGCCGATGAGTTCTTCACATTTCTGG
AAAGGCGTAGTAAGGCTGGAGAGCGTGCAACATGCCATGTCATCCCATATAACCAATTATCGGGTGGGCA
AATCCAACCATAACACTAACCACAGCAAAAGAGTGCTTGTAGCTGAGACTAACCAAAATGGGCGCAGTTT
TTGTGATGCGTCTGGTCACGGGATCTACACATGTAGTAAANTCGAGAAACTTGTCTCCATCCCTTTGGCTG
CCNNAGGAAGGGACACCAAACTCGGTCTTGCAGTAGTAGTCATTGTCGTACTTGTGGAGGAAAGCATCAT
ACACTGTTGCATTTTGCCACTTCAAGAGAAGCTGACGCGCCGACACCATTACATACATCGGCTGATTTGGG
CGCCGCCACTTCGACGCACTCTCACTCCACTACCTCTCTTGCTGTCCAAGGTCTTCAGTCTGGTGTTGTGCT
CCTGGCCACTGCAGTGGTACTGGTAAAGAATAGTTCTGGTTCCTTGTTTCCCTGTAGGGCCTTATTAGACT
CAGGCTCGCAGTTGCACATGGTCACATCCCGTTTCGCACAGAGACTTCAGTTAAGGAAAACCAGGTCGTC
GACAGTTGTCATGGGTCTCGGAGACACAAGCGTCTCTACTGAAGGATCAGCCGTTAGGATTAACATGAAG
TCACAAGACTCAGATTATTCAGCCACCTTTGATGCTCTTGTGACTCCAACAATTACCGATAGTCAGCCCAG
CTTCGTCATCGATACATCAGACTGGAAAATTCCCTCGAACATAAGGTTGGCTGATCCTGCGTTCAACAAAC
CACAGCGAGTGGATCTCTTAATTGGAGCGAGCCTTTTCTATGAGCTTTTGTCTGTTGGGCAAATTGTGCTTT
TGCCAGGACTACCTCTTCTGCAAAAAACTCGTCTTGGCTGGGTCGTGACCGGTGGATTTGAGAGACCGCG
CAGTACCTCGTTGTTGGCTGCCGGCCGCACTCCTAATAGCGAAGCTAGTCAGTTGGACGTCCGTCTGGATG
AGTTGGTTCGGCGGNTTTGGGAAATTGAAAGCTTGGAAGAGCCTACAGTAAAGGCCACCAAAGAAGAGG
CGGAATGCGAGCAGCATTTTGCCAATAACTTCTCCCGTTTACCATCCGGCGAGTATTCAGTGCGGTTGCCT
CTGAAGCGGAGTGCAGAGCTTCTAGGAGAGTCTTATGGCCAGGCTCATCTTCGATTTCTCAGTCTTGAGCG
AAAATTGGGTCGGAATTCGCATCTGAAGGAGCAGTATTCGGCATTCATCAAAGAGTTTCTGGATCTAAAT
CACATGTCGCGAGTTAGTCCAGAGTTGAGTGGCTGTTGTAAATACTTCCTCCCACATCATTGCGTCTTGAA
AGAAGACAGCACCACAACCAAGTTGCGTGTTGTGTTTGATGGCTCAGCAACTACTGATTCTGGACATTCTC
TTAATGATGTACTGATGGCGGGACCAATCATTCAACCTAAGTTGTTTCACATTCTGATTCGCTTCCGCACA
TATCCTGTTGCTCTCACAGCTGATATTTGCAAAATGTATCGTTGTGTCCGAGTGACTTCGCCTGATAGTTAC

TTTCCAGTGCATCCTGTGGAGGGACTCGCCTCAGAAGGATATCGAAATCTTTAAATTAGATACAGTAACAT
ATGGGACCAAATCAGCATCATTTCAGTCCGTGCGCGCAATGCACCAGTTGGCCTTTGATGAGCGTGATGC
ATTTCCAATTGGATCGGATGCCATCAAGCAGGAGTTTTACGTTGACGATTTGATATCCGGAGCCAACTCAG
TTGCTGAAGCCAGAGAGAAAATTCGTCAGACGGCTGGGATATTGTCTCGTGGAAACTTCAAGCTTCGAAA
GTGGTGTTCCAGTCACCAAGAAGTACTCAATGGAATAGCTTATGAAGATAGGGAACAGTACTTAAAGTTC
GATGATGGAAGCGATGTCACCAAGGCCCTTGGTCTTGTATGGGATCCAGCGATCGACCAGTTGCTATTTTC
ATTTTCTGTTCTAGAATTGTACTGTTAAACCCTGCAGGCGATCAGTTCTGTCTATTGTTGCCCGGTTTTATG
ATCCGCTTGGACTTTTGGGACCAGTCATAACTAAGGCAAAGATATTTCTTCAACAGCTTTGTAAGGACAAA
TTAACTTGGGATGAAAGCTTGCCGATTGCACGTGATACGACCTGGAGGGAGCTATGCAGCAGTTTTCGCA
ACATTCGCCATGTATCGTTTCCCAGAGTAGTACTCTTCGAAGGAGCTGATGTTGAAATACATGGATTCTGC
GATGCCAGTGTGGAGGCCTATGGAGCCTGCATCTACGTAGTGGCCAGGAGTCCACTAGCCACAAGTCGTA
TCCTTTGCTCCAAGTCTCGAGTTGCGCCATTAAAAACATTGACTGTCCCAAAACTGGAATTGTGTGGAGCG
AAGCTGTTGGCTAGTCTGATGCATGAAGTCAAGCGCACAGGCATCTTTGTCAGTCAGTTTTATTGCTGGTG
CGATTCGTCCGTTGTCCTATCCTGGATAAGAGATGAGCCCTTCAAATTTAACGTTTTCGTGGCAAATCGTG
TGGCTGCCATCAAGAACTCACTGCCAGTATGGAGTGGCGATATGTTCCGACGGACCGAAATCCAGCTGAC
ATTCTTTCTAGAGGCACAGTTCCCGACGTCCTAGCCCAGTCGGAGCTCTGGTTTCATGGCCCATCGTATCT
ATTAAATGGTCGCCTAAATTGGCCGGCGACCTGTCTGCCTGAAGCTCCAACCATTGAATTGAGGAAAAGA
GTACTAATTCTTAAGTCTCCACATGCAGATTTAACNCTGGGGATGCAAGCACGCCAACTCTTTTGGTTCCA
TGCAGCGCACATTCGGCTATGTGTACAAATTTATGAAGCGTATAAGGCATCATGGACTCACTGTTGCAGA
CATTCATCACGGCACTCGCATTCTTCTTCGGGTACAGAGAGCGAACTTGTGATGACATCAAGGAGATTCAT
TCTCATGGAAATGTGAAAAAATCCAGCTCCGTTGCATCTCTTGCACCATTTCTGGATGAACGTGACCTCCT
CCGCGTCGGTGGCCGGCTTAACAATTCGTCCCTGGACTATGATGCGCAACACCCCATTATACTGCCTCGGC
GTCATGCAATCACCTTAGCTGTCATCGTTCAGTTTCATGAGAGGAATCTACACCCTGGTCCCCGTGCACTT
CTANCCAAAATTCGACTTAAATATTGGCCGATTGGTTGGATTAAGAACAGTCTCTAAAGCAGTCAGTAGA
TGTGTGAGGTGCTTCAGAGCGAGGCCCATTTTTGTCGAGCCTATGATGGCAAGTCTTCCAAGGGAACGTCT
GGAAGTCGTTCGAGCCTTCACCGTAACTGGAGTCGATTATTGCGGACCCTTCTTCTATAAGTCGGAAGTTC
GTACGAGGCCATCCATCAAGTGCTATGTCAGCGTCTTCATTTGTTTCACCAGCAAGGCAATGCACCTAGAG
CTAGTCAAGGACCTATCGACTGCTACATTTTTGAGTGCCCTGAAGCGATTCATCGCCACTCGTGGCAAACC
CAGTCAAATTTGGTCCGACAACGCAACAAACTTCGTCGGATCGAAGAATGAGCTGGTGGATTTGAAACGT
CTCTTGCTTAGCGATCCCCACATGGAAGCAGTTCGGCAGTTCTGCCTCGCCGATGACATCGAGTGGAAATT
CATCCCCCCCGTTCTCCGCACTTTGGTGGACTATGGGAGGCGGCAGTGAAGTTGGCCAAGCACCACTTCTA
TCGCTCTGTCGGCCGCTCTTTACTTACTTTTGAAGAGCTTCGTACTCTTGTTTGCCAAATCGCAGCT
>160_rnd-5_family-743__RC___Helitron
ATATTTTAATATTTTAAAGATTTTTATTTTTACAGAAATTTCATTGCTTGTCGGACACACACACACACACAC
TTAAACGATCCCTAAACATAAAGAGAGAGCATTGAGAGCTGAGAGTAAGTGCGAGCGAGATAAAAGTTA
ACAGCGTTGTCGTTCTATCGCACAAATCATATGGAAGAGAGTGCGCCAAGCACTTTCCTGTAGAGTAAGC
GGTTCAAAAATACATCATTTGTATATCTTACTCCCTTACTCGCGATTTTGGACCAGATGCCACCGATACTTT
TCGTGCCTACTTTCCTGTACTCATGTCCAGTCTATATGTTCTTTGGCTAGTGCTTCGACTCATTCAGATCTT
GCAGCTGGTTTGGTTCAAACTTTGCTGGTAAAAATAGATCTTCTGGAACATTTATTATGATATTCTGTCTA
ATGCCAATGTTTTTCAATCTTGTATTTAAAGATTGTATGACCTTAAGGCATTGTTTGGCATGTTCTTGTGTT
AGTTTTTCTCTAATGGAATCTACTGCATGTACTATATTGTTATATTGTTTTATCAATATTTGTGCATGGTAT
ATTAGTGTTTCTACCTTTAGAGGTATGCCTTTATTGATGCATTTATATGACTTGTCAAATTGGTGTTTAAAG
TCATTGATATTTTTCACTAATATACTCCATTCCATATTATTGTAATAGATATCTATCGATGCAGTCGCACCA
TTAATATGAGTATTGAACATGTCCGTGTATTCGTATTCGAAATCACACACTTTTATAAAATCTCTATGTAAT
CGCAGTACGTTTAATACGCGAGTACGAAAGTCTGGCTCATCTCTATACGTAAACCTAATGATTGCCTCTAT
GTCTGACATATTCAAATATATTCATACCTTATCTAGTCCGTCTGCTCTACTGCTATAGCGTTTCTTAATTTT
CTTTGTATGCATTGCATACACTTTCAATACTAGTGATACAAGACATATTGTAGTAATTATTCTTTATGGTCA
ATTACTTTTACGTTATTTATAACATTTGCGGCTGATTTTTCAANCTTGGATTCGTTCGAACCCATTTTAATT
AAAATTTATNACATAAGTTGATGTAATGTTTTTAAATTCATATAATGTTTTTATGTAACATATAGTGTTTTT
AGCTTTAAATGCCAATGTTTTTAGTTTCAAGNTTTCAATATTGCTAATAACACAAAATACTACCTAGCATT
TCGCATAGCTTTACGGGCTATGCTGCTCAATTTTACAAAAATTTTTTTTTTATAATTAAAAGTAATTAAGGA
TATAACTGCAACTGTTGTTCATCGTTGCATGCAGCTGAGAGGCTAGTGAACTGCGCTGCGTAATATGAGCC
AGAGCCAATATACAAAGTGGCTCTGGGCTTGTTTATACTTTATATTTGTATATAGGGTGTTCTTTTAAGGC
ATATACACACTACAGCTCCCTTTTCAAAGAAATAACGTANCATTATGGATGTGTAGTTATTTAATGAACTT

AATTATTTTGAACAACAAAATCTTTTGTATATAATTTTTTTTTTGCTATAAAATAATAAAATGTAAAAAAA
TGATAAAAATAATATGAATTTAAAAAAATTATTTAAGATTTATGGAATATACTAATGTAAACATTTCAA
GTATAAAGATATAAATATAAAAACGTAAAATTCATTATAGTAATTTGTNTTTTCAAGGATTGATTTTTTT
TTATAGTTGGTTGATCAGGCCAATGTATATGTATTTTTATACCCTGAACCCATTAAAAATGGGTATAAGGG
TATATTGTATTTGTGCGAAATCCAAATGTATGTAACAGGCAGAAGGAAGCATCTCCGACCCCATAAAGTA
TATATATTCTTGATCAGCATCAATAGCCGAGTCGATNTAGCCGTCTGTCCGTCCGTCCGTCCGTCCGTCCG
TCCGTATGTATGAACGCAAGGATCTCAGAACCTATAAGAGCTAGAGACTTGAAATTTTAGATGTAGGTGC
TCCTAGTTCCCGCGCAGATCGAGTTTGTTTCCGATAATCGATAACTTACTCCGTTTCCAAGCAATCGATAA
AAATCGATATCGATATCCTGTTTTTTGGGCAATTTTGGTAAATAATAAGAGCTAGAGTCACCAAACTTGAC
ATATAGCTTCTAAAATAGAATATATATATATGCATTTGATGTTGGAAGAAGAGGGTTCAGGGTATCCCCTAGT
CGGGAGCTCCCGACTAGAACCTCTTACTTAGTTTCTATAATAGATTATATATGCATTTGATGTTGGAAGA
AGAGGGTTCAGGGTATCCCCTAGTCGGGAGCTCCCGACTAGAACCTCTTACTTGTTATTTGTTGTGAATTC
ATAAAGGAACATAAAGACTTCATATAGAACATTTATATGTTTTATTCGTTAAAAATTTTTAGTCTATTCTTA
TAAACTTTTTGGGTCCTATTTTTGTTATCTTTAATTAGAATGTTATCCCTATTTTCTATGTTAACTATTGTAT
ATGGGCCTATATATTCTGGATCCAATTTATGGCCAGTTTCGTTTCTTAATAAGACTTTATCTCCTATTTTTA
CTTGAAAATCTATAACTTTTTTATCAAAAAGTTCCTTACTTCTTGTTTTATGTTTTTCAAGCAATATCCTAG
ACCTTTTATATGCTGATTCTAATCTAAACTTAACTTCTTTAGCATAATCTTCTACATTATATAGTGGGTCGA
TTCTATTTATATTGGTAAATTGGGTGCTTACCAAGAATTTGCTCATAATAATTGTGTGCGATAGAGGGGGT
CGTGTTAAAACAGTATACGATGTATTTGATCCATACGCCCCAATCAGTTTTATCAACTGATATATATGATC
GTATGTATTCCTTAAAAGTCCTGTGACTTCTTTCTACTGTTCCAACTGTCTGGTGATGATGTGCAGTGGATG
TTAAGTTGTTTATTTTCATATACTTGCACAAATTGTTTATAATTGAATTCTTATATTCCGTANATATGTCCG
TGTACCCATGAACGTCTTCATTGGACCGTACTTCAGAATAAAAGATTCGAATATAGATTTAACGACAGTCT
TTGCACTTGTATTTGGGATCGGAATTGCGACGAGGTATTTCGTAAAATCACAAATGAGTGTAACTGCATAT
TCGTTCCCATTCTCTGTTCTAGGTAATGGTAATAATTGTGTCAACGATTACTCTGTCGAAAGCGGATATTG
GTGTTTCTGTTATTGTTAGAGGAGTTTTTGTGTTCTTTGTAGTTTTTGCTTTTTGGCATTTTGGACATTTGCG
GATGTAGTCTTTTATTTGTTTAGACATACCTTTCCAAAAATAATGTCGTTTGACTTTGGCCAAGGTTTTTAT
AATGCCTGTAGGTCCTCCTAGTATGGGATCATCATGCAGTTTAGACAATATAGCTATATATATAAATTATA
TATATAAATATATATATATAAATTATCCNTTGAAACGTGTTCAAAGATATTTTCCCACGGTGCCACTTTGA
GTTGGCTGATTTTATATATACCGGCTTGCGTTTCAAGCCTTTGAAAGATTTGATCTAAATCAATGGTTTAAT
TAGTATATAAATCGGTAACATCGTATCTTGCTGTAATTTGTCTGCCATGTTTAAAAAAACATGTCATATTG
TTTACATGCAAGGTCACTACTTTTCGTACCTCGTCATTGTTAATGACTTGGTACACTTTGGGCTTTGAANCT
TTTTCTATAGATTGCCTAGGCAATTCAATTTCTTTTTCTCCTGCGCAGGATTCTTGTCTACGTGCTTGTCTTG
TAGTGACTTTCATTATGTTTCTTGTTATATTTTGTAGCTCCTTGATGGTTATTCTCGATACCGTCGTTATCCT
TCAGATATTCTACCGTAAAGGTATATTCCTGCAAATCAAGCCTCATACGTGTTAATTGTGAACTTGGATTG
ACCATTGAAAATAGATGTTAGTGGTCTGTGATCCGTTTTTACCGTGAAATGGTTACCATAAATATATGGTC
GAAAGTGTGTTATTACCCAGTGAATAGCTGTTAAATCTTGTTCAGTTGTACTCTTGTTACTTTATCCTTTAG
TAAAGGATCTTGATGCGTATGCAATTGGAAGTTGGAGTCCAGTAAAGTTCTGAGTTAAAACTGCTCCACA
CGCTTGCTCACTTGCATCTGTTATTATGCAGATTTGGTGAAGTCTGGATATTGTAACAACGTTAGGTTTATT
AGTGCTGTTTTGAGATGTTCTGGCATTCAGACGTCCATTCGAATTTTACGTCTTTTTACATAATCTTTTTAT
GTGACGTGAA
>160_rnd-1_family-187__LTR___Gypsy
GAGCTAACGGCCATAAGCCTTATAAAAACAAAGCTAAAAGGCCATGCGAGAAATTTAATAAGTAATGAG
CAGACAATTGCTGCAATCATTACGCAATTATCAAATGCAGTAAATGGAGAATCGGTAGAAGTTGTATCTG
CTAAGCTTCTTAATTTGCAACAGAAAAATAAAACGGCTAACCAATACACCCAAGAGGTGGAAAAACTGGC
AAAGGCCCTTGAAGGCGCCTATATCAGTGAAGGTCTAAGCCAATCACTAGCAAACAAATACAGCACTACA
ACAGCTGTAAAAGCTATGACCCAAAATTGTTCAATTGATAAGGTAAAACTTATCATGCAGGCAGGCACAT
TCATAAATATGAATGATGCAGTCTCCAAATTTGTTAATAGCTGCACGGAGATAACAGGTCAAAGTAACAC
TGTGCTGTATTATCGACGAGGTGCAAACAATAATAATAGAGGAGGCCGAGGTTATAATCGTGGCAGAAAT
TACAACGACAACAATTATAGCCGAGGCGGTAACAATAATAACAATAATTACAATAACCGTGGAGGAAGA
CGAGGCCAAAATAGAGGAAGAGGCCGAGGTAACTCCAACCAAGGTTATAATAGTAAAAATGTGAGAATG
GCGCAAAATCCGGAAAACCAAAAGAACCCTTTAGGAAACAACCAGTAAATGTAAAGGTTCATTCCATCA
ATTACAGTCTTAATATATTTGTCACTTTTTATAATCATTCAACTGAAAATAAACTCACATTCCTAATAGATA
CTGGTGCAGATATCTCACTTTTAAAAATAAATTCAGACAATTTCAAAATTCAAGAAGAAAAAATTATAAA
CATCCAAGGCATAGGCCAAGGTGTAATAAAATCTCAAGGAACAACTTCAATAGAACTTCAATCCACAAAA

TATATTATTCCACACGAATTTCATTTAGTAGATTCCGATTTTTCAATACCGTATGATGATTGATTTTATTAA
AAAATTCAATTGTCAATTAGATTTCAAACCAAGTGAAGACTGGTTTATAATTAGACCTCAAAATTTAAATT
ACCCAATTCATGTTCCGATAACATATAGTGCTGGCAACAATACAGTTCTCTTGCCAGCCAGATCCCAAGTA
ATTCGAAAAATAGACATTAATACAGTAAATGATTATATATTTGTTCCTAATCAAGAAATACATAATGGAA
TTTATGTTGCAAATACAATAGCAGCATAAAAACATGTATACATTCGCCTTCTAAACACAACTAATTTCGAC
CAATTGGTCAAAGTAAACAAAATCCAATATGAAAATTTAACAGATTACGACATTCATAGCACTAATCCAG
AAAATAGAAGCGAACAAGTACTTTCAAAATTAAAGAAAAATTTTCCAGAACAATTCAAAAGTCAACTAAC
AGAATTATGCACCAAGTATAGTGATGTGTTCGGACTCGAAACCGAACCAATAGCGACAAATAATTTCTAT
AAACAAACATTAAGACTTAAAGATGATGAACCCATTTATATTAAAAACTACAGAAGCCCGCATAGCCACG
TCGAAGAAATTCAAAAACAAGTAGGGAAATTAATAAACGACAAAATTCGTAGAACCGTCTGTATCCGAGT
ATAACAGCCCACTCTTGTTAGTTCCGAAAATATCATTACCAAATTCGGAAGAAAAGAAATGGCGATTAGT
AATTGACTATCGCCAAATCAACAAAAAACTACTTTCTGATAAATTCCCGCTCCCCAGAATTGATGATATTC
TAGACCAACTGGGCAGAGCTAAATACTTTTCATGCCTTGACTTAATGTCAGGTTTCCACCAAATTGAACTT
GAAGAAAATTCAAGAAATATAACATCTTTTTCAACGAGCAATGGCTCATATCGCTTCACGCGATTACCATT
TGGTCTTAAAATAGCACCAAATTCATTTCAGAGAATGATGACTATAGCATTCTCGGGTTTAGAACCCTCCC
AGGCATTCCTTTATATGGATGACTTAATGGTGATAGGATGTTCCGAAAAACACATGATTAAAAACTTAACT
GATGTTTTTAATGTANGTAGGAAATATAACCTAAAGTTGCATCCAGAAAAATGTTCATTTTTCATGCATGA
AGTGACATTTTTAGGTCACAAATGCACTGATAAAGGAGTATTGCCAGATGACAAGAAGTTTGACGTCATC
AAAAATTATCCGTCCCCACGATGCGGACAGCGCGAGACGATTTGTAGCATTCTGCAATTATTATCGTCGAT
TTATAAAAAACTTCGCCGATTATTCACGGCACATAACTAGATTATGTAAAAAGAATGTTCCTTTTGAATGG
TCAAGTGAATGCCAAAACGCATTCCAATACCTTAAAGAAAAGCTTATACACCCCACATTATTACAATATC
CTGATTTTCGCAAAGAATTTTGCATTATAACAGATGCCAGTAAACAAGCTTGCGGAGCGGTTCTAACTCAG
AACCGTGACGGAATTCAGCTCCCAATAGCTTATGCATCTCGTTCATTTACAAAAGGGGAAAGCAATAAGA
GCACAACAGAACAAGAGTTAGCGGCAATTCATTGGGCAATTACCCATTTTAGACCATATATTTATGGCAA
ACATTTTACTATTAAAACGGATCACAGACCATTGACATATCTTTTTTCTATGACTAATCCCAGTTCNAAATT
AACTCGCATGCGGCTAGAGCTTGAAGAATACGACTTCACAGTAGAATACCTAAAGGGGAAAGATAATTTC
GTAGCAGACGCACTTTCGCGTATTAATATAAAAGAACTCAAAGATATGCAACATAAAGTCCTGAAAGTCA
CTACCAGGCGACAAAGTAGACAAAATAAAAACTGTACAGTAGAAAATTATGAACGTCTGCCAAGGCGAA
GTCAAAAAAATGTATCTAAGCCCAACGTACATGAAGTCATTACAAATGATGAAGTACGAAAAGTAGTGAC
CTTGCGCATAACAGAATCTAAATGTTTATTTAAACATGGAAATAAAATTATCGCAAGAATTGAAGTTAGC
GATTTATATACTAATGGAATTCTCGACCTAGGTGAGTTCTTCCAAAGGCTCGAAAAAGAGGCCGGTATATT
AAAAATCAGCCAACTCAAAGTGGCACCGAGCGAAAAGATCTTTGAATCAGTTTCAATAGATTCTTTTNAA
AATATGGGCAATGAAATATTAAAATCTTTAAGAGTAGCGCTACTCCAGCCGGTGACCCAAATAAAAGATG
AAAAAGAGATACAACAAATATTGTCTACATACCATGACGACCCAATTCAAGGAGGTCATAGCGGCATAAC
AAGAACGTTAGCGAAAATAAAAAGACACTACTACTGGAAAAATATGACACGTCATATAAAAGAGTACGT
ACGTAGATGTCAAAAATGCCAAATGTCNAAAACAAAGACGCACACAAAAACTCCTCTGACTCTCACAGA
AACACCTGTGAATGCTTTCGATATAGTGATAGTGGATACAGTTGGTCCGCTACCCAAATCGGAAAATGGT
AATGAATACATAGTCACATTAATATGCGACCTTACAAAATATTTAGTAGCCATTCCAATAAAAAGCAAAA
GCGCAAATAATGTCGCAAAAGCTATATTCGAAAATTTCATACTCAAGTTTGGTCCAATGAAGACGTTCATC
TCGGACATGGGAACAGAGTATAAAAACCAGATTTTAACTGATTTATGCAAGTATTTGAAGATCAAGAATC
TCACCTCTACTGCATATCATCATCAAACATTAGGGACAATAGAACGAAGTCNTCGAACTTTTAATGAATAC
ATTCGTTCATACATCTCTGCAGACAAATCTGATTGGGATGTCTGGGTTCAATATTTCACTTATTGCTTCAAC
ACGACACCATCAGCAACGCATGAGTATTGTCCATACGAACTAGTATTTGGCAGACTACCGAAACAGTTCA
TAGACTTAAACAAAACTAATTACATAGATCCTATTTATAATTTAGATGACTATTCTAAGGAAATAAAGCTT
AGATTAGAAATAGCATACAAAAGAGCTAGAATTATGTTAGAAAAAGCCAAGGCTTATAGGAAATTAGCA
TATGACGAAAAAACAAAAAACTTTGAATTAAAAGTAGGAGATAAAGTACTTATAAGAAACGAAACAGGG
CACAAATTAGACCCAGTATATTTAGGACCATATATAGTAGAGAAAATAGAAGATAATGACAACATTTTTA
TTAAAGATAGTAAAGATAAAAAACAAAAAGTTCATAAGAATAGATTAAAAATTTTTAATTCTTAATAA
>9_rnd-5_family-816__LINE___I-Jockey
GTCGCTAATCTGCTCTCATCGAGATCGGTTGTGCCGCGCATAGACAAAGTACGAGTTGATCAATTGTGCAA
TCTACGTGTGCTAAAAATAGCACAAATCGAAAATTTCGGACGCTGTCTTGTGCAGTGTCGGCAGTTTGACA
ACAACAAAACGCAAGTGTAGTGCGATAAGACTGCATTCGTTGCCCGTTTGGGCAAAACATTACCCCTCTC
TCCGCGCGCAGCCGNACAAGAGGGGCAATAAACGCCGCGCTTATCAGCTAAGTTTTCTGTTTATAAGCTT
AACTTTAACTTTAAAGTTAACAAATATAAACTAAATTAATATGGATAAGAACCTTTTTTCTAACTCTCAGC

TTAGTAATAAGCCGACTGATTATGGAAAAAATGCGCCAAGTAGCTGGAAAATCACCATTGGAGATCAGGA
CAGATTTTTATTAGTACTGATACTCAGAGCATCAGCACAATAACAACAACTTTAACATTTACTAGTGCCAC
TAGTAATTCTATTTACACCACGGCAACTGCTGCAACCAGCAGTGTGAGCCATAGCGGAAGCATGGATAAC
CATGCAGCTAGCTTTACCCAATGCNCNTCAGACATTACCTCTCCAACAATCAGTGGAGCTTCTAAAATAGA
CTCCTTAGTTCTTAGTGAAGCTGCTACAAAAATTGGTGGCAGCCCAAAAAGAATTTGAAAAATAAGAGGA
AGAAACCATTATCTCCCAATACTAGCTCTAAGAGGCCGAATAAAGATTTGAAGCTAGTCTCGCCCCTCAG
CATCAACAAGTATGGCAATCTCTGGCAACAGGTTTGCCTTGCTAGACATGGACATGGACATGGAGGTAAC
AAACGAAAGCCATGTTGAAAGTTACAGAGCTCCAAATGAAAGCCCTACTAGCGCTTCTGAGGCTGATCAG
CAGAACAATACTAACGTCAGTATTGATGTAAGTGACACAGCCAACAACCCACACAACATAACGCAACCA
ATACCAACGACCAGCTTCACGGTACCCCAAAGCCACCGCAAATAGTTGTGTGTTACTGACCTTAATGATCT
CTTTGAACTCATTATGGAGGTCACTAGTTTAGATAACGTGGCAGTGAAAGTCAACCAAGGAGAAACGGCC
AGAATATTTCCCAAAGACAGTGACACTTACAGAGCAATTGTCAANCATTTTGATGGTATTGGTATTGAGTT
CCATACCTATCAATTGAAGGAAGAGAAACCATACAGGATAGTAGTTAAGGGGCTTCACCATAGCACTTTA
AACAACGAAATTATTGACAATTTAAAAAAAAATGGCTTTGATGCTCTGCAGGTACATAACCCAAGACCAA
GGAGTAAACAAAGATGGAAAAACTCAATATATTTTTATTAATATAAGGCCATGCGCAAAAATTAATGATA
TATATGATATCAAAACTCTTTGCCGACAAAAAGTGAGGATAGAAAGGATGCGCAAAACATCTGAAATCGC
CCAATGCACACGATGTCAGGAATACGGACACACAGCCAAATACTGCCGTCGTCATCCCAATTGTGCACGA
TGTGGTGAAGATCATCTCACCAAGCTATGCGCACGTCCCCATGATGAACAGCCTTCCTGTTTGCACTGTGG
AGGGAATCATATGGCAAGCTACAAAGGTTGCCAATGGTACCAGGACTATCTACGACGATCAATGGGCTCT
TCAATGAAGCAACAGGCTCACACGCAACCGTCTAGACAGATCAGGAGGAATCAAGATCGACCAAAGCAG
AAACAACCACAAAGGAAACAGCAGCAGCAACCAACTCGACAGCAACAAATACCTAACATTCCTAGAGTC
AATACCGGAGGCAAGTCCTATGCCTCAATAGCAAGGAACGGCAACGAACCTGCCCAGCGCCGTCTACATG
GTTTGCAAGCGCAACAACAGCTACTNAAGCCAATAGAAAATATCAGGCAGCAGCAGCATCCAGCTGACG
TTCAATCATTATTGGAACAACAGCAACAGCAATTTAAACAACAGCTGCAACTACAGCAGCAGCAGCAATT
CCTCTTGTGGCTTCAGGAGCAGCAACGGGAACAACAACAACAAAACAAGCAAAATAGTNAACGACTAGA
ACGGCTGGAAAAAATGGTCCATGAAATGGCCAGCATGATAAAGCAATGGACAGGGGATAGATCGACTCC
CCAGCTTCATAGCAACGCCTCAGCCTCCCAATGAACCCACTCAAGATCCTTATCTGGAATGTTAATGGCAT
CGCAGGAAAAGCCAGTGAAGTTGAGCTCTTCGCGCACAACAACAATGTCGACATTCTCCTTCTAAGTGAG
ATCAGACTAAACCGAGGAGAAACAGTCAAAATACACGGGTACACCTTCTATCCAGCTTACAAGCCATCAC
GTCACAGTCACGGTGTCGGAGGAGCGGCGGTATTCGTGAGGAGTACTCTCCGCCATTTTCCACAAAGAGT
AATTGAAACGAGCACCATACAAATGTCTTCAATAAAAGTTGCCACAGGTTTGGGAGACATGGAATATAGC
GCCATATACTGCCCACCAAGAAATAGAATTGAAGAAAGGCATTTCAGTGACATACTCGCTTCTTGCGGCC
AAAGGTATCTGGTTGGTGGAGACTGGAATGCGCGACATTGGCTGTGGGGAGATACCTACAATTCACCCAG
GGGCCGAGAACTAGCCGAAGCCATTACAGCCAGAGGAGCTAATATTCTCGCAACAGGATCACCAACTAG
ATACCCATATGTGTCTAGTCACACACCTAGTTGCATTGATTTCGCATTATACCATGGGATACCGGATTCTC
AAACAAATATCGGCCAAAGCTGGGATCTAGACTCTGACCATATAGCCCTTGTTGTAAATTTGCAAACGGA
TGGTTCTTATGTCAGGCCAAACCCTCGATTAATTACCAACCGTACTGACATCATGGCTTTCAGACAACATC
TTGAATGCTCGNTTCAATTAAATCCGACCTTGGACTCTGGAGTAGACATTGAGAATGCAGTAGATTTTCTA
ACTGAAAATATACATAGCGCTGCTGCTGCCACAACTCCACCCATCACCGAGTCACGTCCCATTCGTTATGG
CATTGTTCTCACGAGGGAGGCTAAGGAGCTGATCAGAACAAAGAGGTGCCTTCGAAGAAGAGCAACGCG
ATCACAAGATCCTTGGGACCGAATTTTGTGGAACCGAGCAGCAAAACAGCTACGTAACCTCCTAAGGGAA
CTCAGAAGTGATTTCTTCGAGCAAAAACTGGCTTCCATGGACTACACCGTTGATGCGAACTACTCTCTACG
GAAATGTACAAAATCACTCAAACGACAACCCTTTAGACAGGTACCTGTCCGATGTCCTGGTGGCGAACTT
GCNAAAAACGAAGGGGAACAGGCTAATGCTTTCGGATATCATCTTGAGGCTCGTTTCACTCCTTATGAATT
TGCTACGATAGAGCAGACCAGGGAGACCCATCAGACTCTGCAGACCCCCTGCAGATGTCCCTGCCTATTG
AGCCTATAAGGATTGAAGAAATAACTGACGCGATCCAGCTATTGCCAAAAATAAAGCTCCGGGTATCGAT
AGGATCTGTAATGCTACGCTGGAAAGCCCTGCCTACAAGAGCGCTTCTGTATATAGTGCTAATTTNTAATG
CTATTATCCGGCTCCAAGTGTTCCCAAAGCAATGGAAACTAGCAGCGATTATGATGATTCACAAGCCAGG
CAAACCAGAAGCTGATCCTGAGTCGTATCGGCCAATAAGTCTCTTACCCTCCCTGTCTAAACTATGGAGA
GGACTATTGCCAATCGGATTACTGCCATTACAAGCCAATGCAATATCTTGCCGGATCACCAATTCGGATTT
CGAGAGGGACACGGCACTGTGGAACAGGTCCACAGATTNGTGAAGCACATCATGCAGGCTTTTGACGACC
TAGAGTTCTCAAACGCTGTCTTCATTGACATGCAGCAAGCGTTCGATAAGGTGTGGCACGATGGATTACTG
TGCAAAATAAAAACCTTTTACCTGCACCGTACTATGGCCTTTTAAGGTCATATCTGGAAGTACGAGAGTTC
AAGGTCAAGGTAAGGGACACATACTCGTCCACCTATACTATGAGAGCAGGAGTGCCACAGGGCGGTGTTC

TTGGTCCGCTGCTNTACTCGTTGTATACCTCAGATATACCCAGCCCTACCTCTCAACATATGGANGATCCC
TCTAAGGCTATTATTGCG
>9_rnd-5_family-2112__Unknown
GGAGGTTGCCGGATCTTAGAGTGCAGGACGGACTTCTGTTCAAGAGGACCGTGAACGAGAGCTTGGAGG
ATGAGGTCGAAGGCACAAGTTAGAAGCTCTGGGTGCCGGAGTCGCTGACTGCAGGACTCATCCAACAAGC
ACACGCGGACGAGACGTCAGCTCAGGCGCTGCACGCGCTGCGCAGGCGATATTATTGGCCCAACATGGCC
ATACAAGTCAGGGACTATGTACGGAAATGCGATACATGCAAGGAGACCAANGCACAAAACTACCGGACG
CAGGTCGGAATTGGGGAAGAAGTGCGCACCGACCGCCCCTTCCAGAAGCTATATATCGACTTCTTGGGGA
AGTATCCACGGTCGAAACGAGGGCATGCGTGGATATTCGTCGTCGTAGACCACTTCTCGAAATTTACCTTC
CTGAAGGCCATGAGGGAAGCCACCGCGGCGGATGTCGTGAACTTCCTAGCGCATGAGGTGTTCTTCAAAT
TTGGCGTGCCGGAAGTGATCCATTCGGATAATGGACGACAATTCGTGTCAAAGTCGTTCGATGCGATGGT
CCGGCGTTTGGCATTACTCACCTGCGCACACCAGTGTATTCGCCTCAGAGCAACGCCGCCGAACGCGTGA
ACCGCACAGTGCTGTCTNCAATCAGAACCTACCTGGGTCGGGATCATCGGGATTGGGACGCGTACCTACC
AGAGGTGGAAGTGGCGATCCGGAATGCGGTCCACAGCGCTACNGGAGTGACTCCGTTCTTCGCGGTCTTT
GGACAGCAGATGTACCTGAATGGTTCCAGTTACAAACTGGCCAGGAAGCTGAGATCNTTGGCCGATCACG
GTATTTCTGACCTTGACGCGAAGGACAGACTGGCTGTAATCCGAAGCCAAGTCAAGGACCACCTGCACAC
CGCATACGAGCGAAGTCGCCAACGATACGACCACCGCGCGCGCCAGCTCCATCTGGAACCGGGACAGGA
AGTGTGGAGGCGCAACTTCGCACTAAGCAGCTTCGGCAAAGCCTTCAATGCCAAGTTCGCCCGGAAATTC
TTGAAGAGCCGAGTCGTCCGAGCCGTGNGCACCAACGCCTACGAGTTAGAAGATCTCCAGGGACGCGCGC
TAGGCGTCTTCCACGCAAAAGATATACGAACATAATCGTAATTTNCTCACCTCCCTGCCAGGCAATGGCG
GCGGCAGCGCCCGTCCTCCCTTTTTTCTTTTGGGTCAGTGCGAAAATATAGATTACTTGTCGTGGGTGTGG
CCTACGTTGGGCATCGTTATTAGCCGGCTTGGTCAATCCATGAAATGCGCACGGAGATTCCTTGTAACGGT
CATTCATGTCGTTACAATAATCTGCGTGGTGATGTTGCATTTGACGTTATCGGCAGCACCGAGGGTTTACT
TAGTTGGTTGCGTGCGATATTATCGGCCACGCCGATATCTTTTGTCATATGGTCAGAGTATGCGGGGTGAA
GCTGGGCCCACGGCGGAGCCCAAATTTTTGAAGGGTGGCAACGCCAGACTTGCGATCCACGGCGCCAAAA
GCGACGATTGCTATCGGCCGCCCAGTTCGCTCTTAAAGAGAATATGAGGAGAGTGGATCGCATGCCCTTG
GATATGCCCTTGGCCGTCATTTGGGCCATTGACCCAAAAAATGCAGCTGTGCAGATGGGCAGCAACGGTG
CCCGCCTCGCCAATGTATGCGTGTGCCGAAATTCGGCACCGATGATGTTGCTGCCCAAATGTACGGGGAG
TTCGACTTTGTCAGCGGTCCACAGCGGACGTACCGGCTCACAGTTAAGAATTTTTGGAAGTTGGATATTTT
TTTTGCCGGCCACGGAGCATGTGCTTAACCAAAAAGAAAAAAAAAATCAAAGGGCATACATTTGTTCATG
ACGGGCTTTCTTTATTATTTAATTGCCAAGATAATTCCTTATGTGCTAACGTATTGCAAAAGAAATAGTGC
ACAAGAGCGATAATACGCGAAACAATAATACACCAGAAACACACTGTCGTCATTTAAAACCACTATAAA
AGCGATACTCTATTAGAGCAGCCGCAGCCATTCTGTGGCACACCACGCTTGAATCCCGCGCCGGAGGACG
CTGGCCAGATCAACCCAGATATTCACGGGCCGGATTTTCAAAGCGCGGCCAAGTGTCGGCGTAATCCACG
CGACGGAGAGACGAGCCATCACGGACGGGACTCACACGACGAAGAGGCGGCCACGGGCGAGACCACGC
CCATATCATCCGAGTGAGTCTGTTCCATTTCGTGCCTCCTCGCCTGAGTAAACGGCAGAGTCAGACGCAGA
CTTCCCATTGTGCTCCCTGAAAGAGAACCGATTTTTTGNAATTTTTAACTCCAGTTTTTTTTGCTATTAAAT
ACANAAAAATACTCACGACTAGTTTAAATTAAGTAAATTTCCGAAAGTGCGAACCGGGAAGGAAAGGGC
CCAAAGATTATTGCGTCTGCCAAGTAAAGAATGAAATTAAAAAGTTTAGATTTTGGTCAACTTATGCACG
AGTGATACCCACTTATGCGTATATATATATATATATATACGTACCTCAGGTGCAGGTGCCCCCCACCCAGA
GAGCTCTTTAGGCAGGCAAGGTAATATAGCGCTTTGACAAACAACGCCTTTCGTTGGAAACACATATGTA
TACACGCATTTATTTAGCGGGTACTTGCTCCCCATTGGTGCAATAATTACCTGTGTTTATATTTCTCCCCT
TGTTTTCCTTTCCTTTGTTGACTGGCAATAACTTCTGGCCGTTTTTTTTGGTCTCCGGTATCGTACGAAGGTC
GCCAACAGAAAAAGATATTCTGTACAAAAAAAAGGAAATTTAATATATTTAAGATTAATAGAAATTGTTA
CAAATATGAAATGATGCAACGCGTTATAGATAACCGTTGAAGCGAAGTTGAAAGANGGAAAAAAATTGA
AATTTAAAACTTATATCCATTATTTTAAATATAAGAATATACCAACTTATCGTATGCACCTGGTTTAAAAT
GCCGGAAATGTTCGCGAGTACGAAATTATAAATAACGGCTTGAAGATAACCTGCAGGGGAATAAGAAAT
AAGATTAAATGTTAATAACGATAAGAAATGCAACCAATTATATACCGTATCTGAAGAGAGAAATGTCCGG
CTAAATTTTCTTTGATGGGGAGAGTTGGCATAACAGCCGGCACAGGCTGATGGCCATCTAATGGCAGAAN
GTGGGAGACGAGTAGGTGATCGTCTCATGGTTGGGCGGGTACGCTGGGTCCGGAACCGGGGCCCGATCTG
GAAGAGTAAGGTGGATAAATAGTGTTTTTGTTAATTTATATCGAGCGTCTCACCTTTGTTGTTTACATGTTC
ATAGCTGTCACAGCTGTTTATTTTGTTCATGAGTTAGAAGTTCCGATTGTTTGACGTTATCGATTTGTGGTT
CGCTTTGCATAGTGTTGTGTAAGTCTGTTTTGCTACAGACGTGTAGCTTCGTTGTTATCGATTGTGCGGACT
GCGGGCCAGGGATGGACTACACCCAGAGAAACCGATGAGCCAGCCGGCGCGGCCCACCGAGAAACAAG

CAGTTCCGTAACAATATGGCGCCCAATTTTTGAAAAGGCACTCGGCTTCTTTGAAGTCGGTGCGTACATCA
AAGATAAAAATAATANGATATNAATGAAGGTACAGAGAAAAGGACTCCTTTGGCTTCAGGACCCACACC
GCTGAAGTCTTATGTGGCACGAGTNGGTAGCCCATAATCGCGCGGCGTGTAGTGGAATCCGAGAAGCACG
ACGGAAGTAGAATTATCAGAATATCCATAAACATGAGAAGTTTTCTTGTAGTGGTTGATGGCGGTAAGTC
ACGTTCGCATGTTGGCGAAACGTTCGCGACCGTTCATCGTTAGCCTGGTGGTAGCCGCCACGACAAAGGC
ANCTCGTGATAAGAATGTGATATTAGGGTAGGTCGTNCTAGAGTTTGTTTAATGTNTTCATATTTGTTTTGT
TGGTCCCACTCTGTGCTAACGCTTGGGGTGTGCTGGCCGTNGNTAAANGAGCTTCGGAGACAGGTGACCC
AGGACGATGTAAAGCCGATCCTGCGCCGCACCTGTCGCACAGCATACCCCAAGGGTGCCCAGAAGTGCGA
CAGAAACCAAATAGCCTTCAAGGTAGCGTTCCTTGAATGCCGTTCGTCAGCCAANCNGACGAACTGAGCA
TTTGACGAATG
>Ref_dvir_1031
TAGTTATATTGGGCATTTAAAAAGGTAACGGTTGAAATCGCCAATTTGGTATCTGAATCGTCAGATCGAAT
TGGAGTTACGGGTCCCGTGTGTAAGGATATCATTTTAGACTTTTGCCATTGGAAGAAGGGCTGGAGGCGG
CTCTTTCAAAGGAGGGTTTCTTTTTAAACGATTCTCGTTCTTTAGAACTGAGCTAGTGCGATAGATCTTGA
ATGTCCTAAGTCTATGTGATTAGGTTCTTGGAAAGGTAGGGTGACTACATATCTGCCATTCTCGTTCCTTGT
TGTTGTTCGGATGAAATTACTCTCACAAAAAGAGTCGGAGCTCTTTACCAGTTTGACTGGAATGTCCTCCA
CCTCCCAAAATTTTGTGAGAATGACGTCTAATTGCTCTTCTTGAGAGACAGATATTCGAGTCGAGAATGAC
GAGATTGCGGTGGATGCGGTCTTTGAAACTGGGCCTGTTAAAATCCACCCGAAAATAGTTTCCTGGCCCA
GCAACGTGCCACATATATTAGGATGGGAGCCGCCCGTTAGTATGGACGGTAGGATATCAGCGCCAATCAG
CACATCTATCTGCGAACTCTTGTGGAAATTAGGGTCCGCTAACTGGAGTTTTGGCAGGGTTGTCAATAAAC
TTTGGGGGATCGAACATGATGGTAGATTCCCTGCCAAATGTGGTAGGACGTAGGCTGATGCCTCGATTTG
GATACGAGGCTTGGAAGGAGAGCCAATATTAAAATGGCAGAGCTTGCGAGGCTGGGCTGCAACAGCTTG
ATTTAATCCCTTGTGCTGTACGGATTTGTACGGCAGCTTTATCAGGTGGAAGAGCGCTCGGAAATAAATGT
TGCCTCTGATCCCGAGTCGATCAGTGCACGAGCTGTATAATTACACCCAAATGGCGAACATATATAACAG
CCGTGCTTAGGAGAACACCGTGGGTGTTTAATTGCAACATAATTGTGAACATTCGTTTTGTGGGAAGGAA
GTGGACTGTATGGATTGATTGGAGGGAGCGGTTGCGTATGGCATGGAGACTACTGTAGGGCTCAATTGTG
GAGTGTCACTACGATGCAAGAGCGTATTGTGACGACCCTTGCACGTAAAACAATTGTGAGCACTTGTGCA
ATCCCTTAACTGATGACCCCTAGCGAAACAGTTCAAACATAGTTTTTGCTGTTTGATGTAAGTTGCCCGTT
CGCTGACGGGCATTTGGAGAAATCGTGGGCACAGACGGATAGGGTGATTCTCCCTGGAGCACAATTTGCA
GGCTTGCGGCTTGTTAGCGACCTTATTCTCAAATGAGTTAAGTTTCTGGAGTGCGCTGGAGTATTTGCGCT
TGGCTTAAGCTCTTCTATCGCTTCTAGAGTGCGATAGCGTTCTAAGAGAAAGGCGTTCATCTCAAGCCAAG
ATGAGATGTCGTTTTTTCTGGAGATGGACTGTTCCCATAAGGAGAGTGTTAATTTGGGTAATTTGGAGGAA
CATAGGAACACGAGGATACAGTCCCAGTTTTCTATGTTGATTTCGGATAACTCCAGAGCTGTTAAGCAACC
TTGAATGGTGCTCTGAAGTTCCTTAATTGCCGTTCCAGACTCTTGGGAGATTGGCTGCAGGTTGAAAAGAA
TCTTGAGCTGGCTGTTGACTAACAATTTTCGGTTCTCAAAACGCTCAGTCAGATTGGCCCAGGCTGATCGG
AAGCCATCGTTAGTCAAAGGTGACTTCGATACAATCGCCTGAGCCTCACCGCTGGTTTTCGCATTGAGATG
GAATAATTTCTCGACCTCTGATAACCTAGGATTATTTATGTAAATTGCGGTAAAGAGGTCTCGAAATGTGG
GCCAACGTATGTAATCTCCGCTGAAGATTTCTGTGTCGCAAGGAGGTAAGCGACACCCTGTGGGAACATA
TGTCGGAATGGTGGACTGAGCGGATGGTCTATTTTGGGCGTTAGCGGTATCGATTTGCTCGCTAAGTTTAG
CGCGCATCTCATATACCATGTAACAATAGTCGTACTTGGACTGCATAACTGAAATGGTTTCCATTGAACCG
GCTTCCGAGATTATTGTTGAGCCGACCTCGTACTCTTTTTCAACCTTGTCCACAAGGGTCGCACTTGGTCCC
GACGGACCTGGCACGTATAGAGAGACGGGTCAGAGCCTTCAGGTAATGTTTTTTTTTTTCCCTCAAACTGA
CTTAGCCTATCAGTGACAGCAATAAACTTGATTAAGGCCGCGTCTGTCATATTTTGGGCCGTTTTGGTATT
AGCTCGCGTGGAAATCGGACTAGATCTTGTGCTAATGGTTGCCTTAGTGCGTCCGACTGCAGTGGTCAGG
GATGCTTCGCTTTTAGCTTTGGACGGCGGGCGACTGATTTTTATCAGTGGACTTGGCCTCGAACTAAGAGG
TGCCTTAACAGTTCCCCTTGGCGTGGACGCGGATGATTCGCTTTTAGTCCGGGACGATGTTTCCCTTTCGA
CTCTAAGGGCTGCTGTTGGAGACGGAGAAGTTCTGGAGGTGCTTGCGGACGGAACTGCTAATTGATGCTT
TGTTTCTTCCCCTTTGCCAGTTGGCATACTAGGCGGAAGCGCTTGGGTAGGAAAACAAAGGTGCTCGTTTC
TAGTAGTAGAGGTTGCGATGCTTAAAACAAAGAAACACTGGAATGCCGGTACAGGTAAATAATCGAGTTT
ATAATTCGATTGAGATTGCGGAAGATTTAAATAATTTTGCTTAAAATCAAAATTTATTTGATTTAATTAAT
TTAATTAATAAATTTATTTGACTTGGTAATTTAAAAAAAATACGATTATTTATTAAACACGGGAAATAATA
ATAAAGAATTCGATGCTCGTTTTCGATAATTTTATTATTATATTAGTCTTTGAATACGGTAACGGTTGGTTT
ATTTTTACTTTTTTGTGGTACTCATAAAATACCCACTATTGTGGCTGAGTACACACCGCTGACAAATGTAG
AACTTCGAAGTTAATGTGTAGTGTTTTTGTTTATTTACAATAAATTAGTAGTATTATTAATCTATTAATTAT

GATTGTTGTGTTTTTATTTGGTGCGTCTCGGAACAAGAGTAATTGTATGGGAATGTGTGCAATTGCATAAA
AACGTGTAATTGGAGTTCGGATCGGCAGCTGTGGTTTTTACATATATGTGTGTGGTGTGTACATACGCACA
TACGCCGGTTGAAATTGCAATTTATTACTATAATATTGCTGTGTTATAATATTGCTGTGGATTGTGCACTAA
AAAATATTATATGTGGACACACATAGTACATATGATTTGTGGATTTATTTATCGGATGTATGTACTATGTG
GTTGCACAAATATATTCGTTTGCACTATGCACAATTATAAGTTAAGAAACTGAATTTGGGTTTGTGTTTAT
TTAATAAAGTAAAACACAGTGCAATAAATTATAAGTTAAGAGAATGAATTGGAGTTTGTGTTTATGTTTAT
TAAATAAAAAGGTTAAATATACCTGGTGTATGTTTGTGTGTCGGACCAAGAACATGAATTAACATATTAA
TTAGGAAACAATATTGTGAAGCAACTTGTCGGATTAAGTGCGGACACGTAATTTTTTTATAGTTAGGAAA
GTGCACGAGTGCGTACGTATGTACATACAAAAAATGTTGAAGTGGATTTCTTTTGTAGTTGGAAGTAACTA
ACACAAAATTTAACTAAACTGTTTTTAGTTTTGTGGTCGGATAATTTATATATATTTTATATATATATAATA
TAGAAAATTGCCAATCAGTTTTCTTAAAGAAAAAAATAGGCGGAAAAATATGGCGGATATTGCCGAATAT
ATATAGAAAGGAAATTTTGTATTTACTTATCTTTGCCAAAAAACTTGTGTAGGGGCGATCGGTTTATATAT
AATCCGGCTCGAAGGACCAATGTTTGTGGGGCGTCAGATAAATAATCCTTGATTGTTATTGGTGAAACACT
TTATTAGAATTTTCTGCTGCATGTAACAAACTCTGCTGGTTAAGTGATTTTACATATTTTTGGTTGGAGTGA
TGGAGGTGGAAAGGAGACCACAAGGAGAGTGCTGAGCGCATCTGGGATCGAATTAGTGAGTGAGTCTCT
CTCAGTGAGTGAGTCTGTCTCACTGAGTGACTCTCTCACACTGAGTGAGGCTCAGTCAATGCTGTGTTCAC
ACTAGCACTGAACTGGCATGAACAATGAACA
>Ref_dvir_316
CCCCCACAGCACAGGCCAACAGCATTATGCCGAAGGAAATCGCCAATGTGTTTCCGATTGCGATACTGTC
TCTAACCTCCTTGATGTTGGTTAAGGTTGTTTTCCTCAGGCGGTGAATGTAGGGAAGGCTGAGTATCGTGT
CATGGCCGCTGATGTTAAGCAACGGTGATGCCGCTACCACCGGGAACCGATTTTGCACGCTCTTGCGATTG
AAGTAGTTTGTCCCATCTATCTTCACGCAGTGCAATCGTCGTAGGTGAGTATTGCGCTACGATGTTAATGA
CGATGATGCCGTCGTCCACTATGGTGACAGGGGCAAGATGACTCGGCTGCGTTTGGCAATGGGCTGTACC
CCCTGCATGGAGGCCTTGTGCGCAACTGATGCTCTTGGAACGCTTGCAGAATGTGGCAGTCGCAGTTTGTG
AGCAATTGGCTACGGCCAACATCTGTCCGTCGCAATCGGCCACAAGATTGTCGTGGAGTTGGAGCACAGA
ACCGTTGTGGGCGACTGGGAAAATTGTTATCTTCCTGCATTGCTTACTTATTATAGGGAATTCGATTATCA
AACTTATTACATTATCTCTACCTTAATATTAGATACTTTCAATATGTCAGTGATAGGGGTCCCTGTGATTTG
GTCAGTGAGAATATTCATAAGATCAGAATGATCTATTATTGTTGGGTTTATTACGTATAATTTTTTCTAGG
GTTAAAGTAAGCATAAGGTTCCCTATTTCTGTTATTATCATTCTATTGCGTGTGCTTAAGGTTTCAAAAAG
GTGGCCAGTGTCGGCCTGCTTATCTTTTGAGGAATTAAGAATTTTGTTTACAGTGTCGGTCAGTTGGTTAA
TCTTTTCTTGTACTTTGGAATTTATAGAAATCTGCCTATTGCTTGCAGTGACCAGCTGCGTCTGCGTTAGTT
CTATTTTCCTGAAGTCGTTTGCGTCCGGAGTGCCCGCTACAACCTTAAGGGCTGTTCCCAAGAAGTCAAGG
CTTCTTACCTTGCGATGGTGCACATCGAGAGCGGACAACATGTCCAGTGTCAATTTCCAGCAATTTCCTCA
TATGGGATTGTGGAAACATTCCCGTCAGCTTCATGGTCTCCTCAGTCATTGTCCTATAGTCCGTGAGATTG
GTAGTGTGACGTAAGTATCCGTAACTGTCCCAAAGTAGAATGTCCCCGTCCATAATCGGTATGTAGTCCGC
GTGCGAGTAGTCCGTCACCCGAGCCGTTGTCATGGACATGATTGAAAGTATGGTGAACGTAATCCTAAAA
TGTTGAAACTACTGTTATCGGAATGGTAATGAAAAAAACTGTGCTTTCAATTCAACGGAGGTTGTCCTTGT
GGACCACCCTCCCCCCAATGAGGACCGTTGTCCCCAAGTCCGCTTCAACCTTTTTTTCAATGCACAAAGGC
GATAGCTTGTTGCCTAGTCTGCGGTTCGTCTTTACCAGGACCTTTTCTCCTACCTCGAAGACTCTGTTCCGA
CGCATTTTATTAACCCTAGCGTTCTGCTGGATTTGGGCCCTTTCTAACCTCGTGGGTGGATTCCGGAAGCG
AGTCAAGCAGGATGTCCTTAGGCTTCTTCTCAACCACTGAGTGAAAGCGCTCTACCTGACCATTGGACGTG
CTATGAAGGGAAGGCGCATTTACCACGCTTACGCCGTAGTTATTCAATAGCAGAGACCTGATAGTACTTG
AATGCGAGCTCATTGTCGCAATACACTTGCTTAGCCGAAGGGAAAATATTCATTACCAGCAGAGGTGGTC
TCGTGTCTTCTATCGTTCGAGATGCGATAGGCTGGACTACCGCAAACTTCGAGAACTTGTCAACACAAGTA
AGGAAGAACTTTTGTCCGTGGAGAAAATGTCAATGTGCAACATTTCTCCAGTCTGTGAGGGAAGGGCGTC
GGCCGAGTTCCTGCTTCTTTGGGTGTCTGTCGTATTTTGCTTTCGTGCAAATTCTACAGTTAAGCACCACTT
CATTAGCTAATTTGGTCATTTTAGGGAAATAGTACTCCGTCATGACCTGTTTTACATTCTCCTGAGCCGCCC
TATGGGCTCGATTGTGTTCCCTAGTGATGATTTCTCTTCTATCACTGACGTCGAACACGTCGGCCACGAAG
TTTTTGCAATACCAAAACTTCGTAGTCGGAAAGGCTCTTACCAACTCGTGCTGGAAACTGGCCAAGATTGG
GAGAGTACAGTGAAACGCATTAACCACGTTTGGGTTAATGATGGCCTTTATCGTTTCGATGAGGGCGACC
GTATCGGTAAAGTTTATCAAATGGCGGGTTTTTCGCCGAAAAGTATGAAATTTCTTTTTAAAGGAAAGCGA
GATTCCTCTAATACAACCTGGTTTCGGAAGCAGTTTAAGGGCTTCTCCGTCGAATCGATTGTGTAAGACAG
GGACAATTCACTGTGAATCGTGGCCGCGTCAGATTCTGGTTCATCGCGGCTACCCTGCAGGGCGTTCACGT
TCTGGCGCGACAGCGCGTCCGCTACAAGCTTGGCCCCTGTTTCATCAATGCGCGCTTTCCAGCGCTTTATT

TTTGCATTGGGATTGCTCTCGATACCGAGAACGTAAGAGGCTGGTGGTCAGTGAAAATGTTTATGTCCCTA
ACCCCGTAAAGGTGCCGTAACTTACCCAATCCCCAAACGATGGCAAGAAACTCCCGCTCGTTAGTAGGCG
AAGTTTGCTTCCCTTATCTTTTAAGGACCGGAATATCATAATAATGGGGCGACTCATTTTGCGACAAGACA
GCTACCTATGCCATAGGGCTGATAGCGTCTGTGAGCTAAGTCAAAAGGCTTTTTATAGTCCGGGTACAGTA
TGACGTCTTCCGACGCTAATATGTTCCTAAGCTTCTGGAACGCGTTGCGTTCATCGTTGAATTGTATAGGG
ACCTTTCTAGACCTGTACTTGCTAATGGATCCGTTTTCCCCTTTCAGGATGTCGGAGATTGGTTTCGCTATT
GCTGCAAAATCTTTGATAAAGCAGCGGTAATAGCTTGCCAACCCCAAAAAGGATCTTACTTCAAACACCG
TGGTCGGCTCAGCATATTCCCGAATGGCCTTTACCTTTTCTGGATCTGACTTTGCACCGTCTTTAGTCACTA
TGAAGCCGAGGTACTCGACGCTTTCTTTAAAAAACTGTGACTTCTCGTGCGCCACGCGCATGTTCGCATTT
AAAAGGCTCCCTAATACCCAGTCGACATGTCTGACATGGTCCGACTCGTTTTCCGAAAAGACGATGACGT
CATCTACATAAACGTAGCAAGATTTTCCAATCTGCTCCCGCAGGACGTCATCTATGGTTCTCTGAAAAATG
CTTGCTGCATTCTTCAAACCGAAGGGAAGTCGACAGAATTCGTACTTCCCGCCATTGATCGAAAAGGCCG
TCTTCTCACGATCCTGCTCCGCCAGCGTAATCTGGTGATAACCTGACTTTAGGTCGAGCGTCGTGAAGTAC
TTGGCTTTACCCAGATTAGCCAGTATCATTGTGATTACTTGGCATTGGATATTTGTCGGCAACCGTCTTGA
GGTTGAGCTTCCTAAAGGTCTATTACGAGACGTTTATTTCTATTGCCTACCTCGTCTGTGCCTTTCTTATCG
ACCACCCACAGTGGATTGTTGTAGGGCGATTTCGACTTTCTAATTATACCGTTTTTTAGTAGTTCGTTGACT
TCTGAAACGACAAAATCCTCTACACCCCTAGGGTAGGGGTATAGCTTTGAGTAGACCACATTTTCGTCGGT
CGTACGGATCGTGGCCACAACAGCCGTGTTATAAGGCAGCGCCTCGTTGGAGTCCGCAAAAGCCTTGTTG
TTTCGATGAAGTACTCGCAGGAAATCTGCCTTCACCGCAGGTGGTGCCTCTACTTCCTTATTTTTCGTGTGA
TTAACGCTCACACATTTGTGGTACTGCAACGCTTCCGAAGTCGCACCAAATATTAACATACCTTTTTTTAG
GCATAAAACCGCTCCCACCTGCTTTAGCAGTCCAGCCCTAATTCCATCAAAAGTGGATAAAAATCTAGTA
GGAAAAATATGTGTCGCACCCAAGCATGGATAACTTGCATTTTTCGGCGATGTTGTTGGACCCATGAATG
GAGTGGAACACGAAAAGGGATCGACCCGAACGAGCACGAAGCCGTTTGGCGGCCGATGGATTTTTT
>160_rnd-5_family-7852__Unknown
TGGGCAGTGCGTTGATCACCAATCAGTCAGTCAGTCAGTCAGTCAGTGAGGACAAACAGTTTTATATATA
GAGATAAATAAATATAAATTTTGACTCAATATTTAGTTAGATATTTTATTAATTCAATTTAGATTATTGCGC
TTGCAACTCTATTCCAAAAAAGGCGCCAAGCTTTAAGAATATTGAATTCGTGTCAGCAACTTAACGAAAA
ATTAGTGTGCATTTAAAACCTAGTGTTATGTGCCAAAATTGCAAAAAATAAAATTCCAAAGCATAAATAC
TTTTGGCGCAAATTTCGGGTGCTAGTGTGTCGCGTTCTTTCAAGAGAATTCTGCATGCCACTTTTAGCCGTT
GCTGCTGTTTGAAATAATTGCCTCTTTTAAGGCTTTACTGAATTATTATATACCATTAATATTGTCAATTAT
TTAATTAAATTTATTACATATGTATATTGTATCTGTATATCAGTATATATTATAAATTGCAATATATATTGT
ATATACTTATTAATATATATATAATTTAGAGTATCTGGAGTATTTTAAGATTGGTGTAAATAAATGTTTGTCG
ATGTAAACTTTTAGTTTTGCTACCATTTCACATCCGAAAGTTTATAAAGCTGCATTGTATACATACATAAT
GTATATGTGCAAATGTATATAACAGGTAAAAGGAAGCAACTCCGACCCCATAAAGTATATTATTCTTGAT
CAGCATGAATAATCGAGTTGATGTAGCGAAGTCCGTCTGTTCGTTCGTTCGTCCGTCAGTCCGTATATACA
TACGAACGCAAGGATATTAGATCTATACAAGCTAGAAATTTGAAATTTGCTACGTAGGCCCTTCTAGTGCC
CGAGCAGCACGTGTTTGTTTCCGATAATCGATAAATTGTCCCGTTTCCAAGTAATCGATAAAAATCAATAT
CACAATCGTGTTTTTTTTTACCAAATTTAGTAAAAATTATGATCCAAGGCCACAAAATTTACATGTAGCTT
CTAGAATAGAATATATACGAACAATGCCTTCTCAAAGCTTCTAAAATATTCTTTGGTAACGAAAATAACTT
GGTGGCAGAAGAGCAACAAAAAAAGTTTAAATGCAGTTTTCTTTATTCATTCATTATTTTTACTTCACAAA
AAAGATATTCAATATGTTTAATAGTATAAAAGTTATAAAGACAAAAAATTACACGTGTACTATTCTAGGA
CTTAAGCTGCATACGATATGCTAAATAAGGTAACGGTAGTTTGTGAATGTTTATATGTATACATACATGTA
CATACATATGCATTTTTGGCACATCTTGGAAGCAAGTTTCTGAGATTTTTTTTTAAATTTGCTTAATACGTG
GTAAGATAGTGTTTCAAAATGCCTAAAATTTATTTAAGCATTGGGGAAGGGCAGCTAATTTTAAATAGAA
TATTCGTACAAGCTTAGATAACAGAGTTTCATAATAAATGTTTGTGCGACGCGCTGATCACTGGTAGCACT
AAAGATAAGTCAAAATAAGTTAGTAGGTGTTGTAGATTACAAATATAACGCTGGTTTTGGGTTTTGCAATT
TGAATTGTGTAACAGTTTCAGCGATTGTGTATCATTAGCAATGTCAGCTGAGGATGTCTTAATTTAATTTA
AAAATGCCTTGTGCCAATCAACACACTTTCTTCGCTTATTAATTTACGACAGGTTCTCTTTACTTTGGTTTT
GGGTTGGCTTGTGGATAATAGTGTAATAATGCGTTAACAGCTTGTACTTTCGTTAAAAATGCTCATAAAAG
TGGTTGTATAATGATATGTCGACATACATATTTTCAGCTTGTACCTAATCATTTTAGTGGTATACGCCAGTA
TATAACCATAGAGCTATGTAGGCCCCATTTTTATATACTCTAATGCTGTGTATGAATTTACTTCTCCTACTC
CTTAAAGTACAAATATTATTTAGCTAGATGGCAAACTAGGTCAATTTATCTGACTGCTATTCTAGCAAACA
AGTTGACTATAATCAAATTTTAGAACCTTAGAGCTGTCTTAAAACGAACGAAATATGAATTTTACAATAG
GTAAATAATCTATAATGATTTTGTGAAAATGTATGTTGCATACAATAAGGTGTCTCCAGCCCCATAAAGTA

TATGAACGTTAATTTAGAAACCTGTAAGGTTTACAAACATGCATTTTCGAATATTGTTGTTTCTAGTGCCC
GCACAGTGTTAGTTTGCTTAATAAACGATAACTTATTCTCTATCAATATTGGGGGGTTTTTTTTTGAGCA
TTATAAAGCTAGAGACATTATTCAGACTGTCGTTTAGTGTACGCAGATCCTCTTTCGTGAATACCAACCCC
CATCACCCTAAATTTTAACTGAATATCATTCTTAGAGAAGCAGACTGGGTCTTTGTTATTTTCACACATTTG
GATGCCGTTCTTGTGCCCTTATAATATGATAAAGATTCAACCATAAACACCGAAGTCAATCATGAAAGAA
TTTACCATAAGTGGGGCTAAGTGCTTTAATGTGCCAGTATGCTAATAAGGTCTTAATGTGGTTTATTAAAG
TTTTTGAGGGTAAATAATGCTCACAATTTTGAAGAATACGAATCACTCTTCATTATTATTATCGTTATTCAG
TGGATTGATCGTTCAGTCAACATTATAAATTGATTTAATTTTAAGTTTTACCTGAGGTTTTGACTTTACCAC
GTTATTTCCTCGATTACACGAAAAGTGTTCAATAAAATCAGAATATGCGTTTAAGTTTTTAAACTTTAATC
ACTTTACCAAACAGTAATGACTAATTTGAATCTGACTTTCCATCTTACTTGCAACTTCACACTTAATGTTTC
CAGCTGTGTCTGCTAAATGCGATTAAACAAGATTGTCGAAACCCATGCCCATTTTAATGGGCAACATGAA
ACAAGCGGCAATCTGAAAGCTGATATTGTTATGTGGATTGTGCGAGTGGCCATAAAGCAATATAAACAAG
ATCGTTTAAATCAGCTGAAACCGTTTGCGTAATACTCTGCTTATAATAGTCTATCCAACTATTTAGTGCTTC
ATGCAGCGACTCTCAAAAATGAACATATGCAATTTATAAGGAGTGTGCGTGATTTCAATAGTTTTCTGTCG
ATCTGTTTTCTGCAGGACATTGAACCAAAGCCAATTTACTCCTTTTCGCTACTTGAACGAAGTTAAAACAT
AAGAAACACGTTTTCGTGAAAAATAATGAAGATAGCCATTATCAACGAGATCAGAGAGCATTTGGTAAGC
GGTGTTTATCATCTTTGCGACACGTCTTATGGACAACTGACGCTTTATATGAGCGTATATGGGTTTATTAA
GTCTAAGTTATCCCGTACAATTTGAGGGAAACCAAATGTTTTTATACTTAGACGTGGACATTGCGGAATAG
TTAGAGGTTCGTTGACCATAAAGATTAAAGTTTTAATAAATTTACAAACAATAAGACTCATCCGAACAAA
CGGATGGGCGGAAAGCAATAGGGACGTTACACAAAACTAATAAACTAATTGAAAATGTTGTTAAAAAAT
TAAGTAAGCATAGGCATAGCCTTAAGCTTTACCATACGCAAAAATGCTTATATGGAGGCTCTTCCGCAATT
GCCACACTTAACTTCTTAAATTTTAAGAGCGTAAATATGGATCTAAATTGATCGATGCTGCTTACGAATAT
ATATTTACTTAATGGGATCGAAGATAGTTCTTCTGCATACATACATTTGTACAAATAAATTCTATCCGTTTT
AAATAAGTTCAGGGTATGGAAAACAGATGTGGAAATCGAGGACTATTAAGATTCAAAAACTAGTTGCGA
ACACTACGCCGAACAAAGCAGGCTTGTAAGACAAGCATTGTAAGTATTTTAAATTGTGAATATACCTGTG
TTTATATACCTCAAACGCTATTGACTGTGACAAGGGCATCTCACTACCAATAAGTCCCGATTTCAGTATTT
GTTTGTCAAGCTGTTAAAGAGTGCAGAACCAAAGACAATATAAACACTAAGATGAGTAACGCCATACATT
TGAATGCGACGCAAAATTTGTAGCCTGGCCTTTCATCTGGCCTTCGAAGCTTCGCACGAGATGACTGCCGA
CGGCTATTCGCTCTC
>9_rnd-1_family-287__LTR___Gypsy
CCGCCGGCCGCCGCTGCAACCGTGCGTTGTTGATCGGTTGCTCAACACCGCATTCAAGAGCGCAACTCCA
ATTCGGCGGCGAAGAGCGAAAGCTTCGAGGCGAAGTTACGAAAGCTCGCGGCGAAGTTGAACGGCCGAG
GCAAGCGCATGGCTTGCTGGCGATCGCGCCGGGACTTTGGAATGAACGCTAAGGGTGAGTAGACGTGCAT
TGTGAGACGAGGAAGGTCATAGAGTGTACATCGGAGCGCTAGGGCTCCAGAAAACAGTGCGGGTTCAGG
GTTCAGGGTTCAGCTCGTGAGGTACTATAAAGTAGCAATAAGCGAGGAGTGAATCAAGTACATTTGTTAT
AATTATCGAGAGGAAACCGGATAATTCTTTGTTTTTTGGTAACCTGGCTTACCTTAACATCGGAATTAACC
GGCGTTCGAGCTAATCTCGATCAGATCAGATTGTGGCCCCGCGGTGGGCATTACCGGTTGGTGTTTCGATA
GAGTAGTGGTACGAGGCCACGGCCCGATAGAGCGTTTGGGTAGTGGAGTTGGCGCGAGGCTCTGGCCCGA
TAGAGTAGTGGTACGACGCCACGGCCCTCAAGTGTAGTTGGTACGAGGCTACGNCCCGCTAGTGTAGTTG
GTACGAGAACACGGCCCGGTAGTGTAGGCGTGCAAAAATAGCTCTAGGCTTGGTCTGGTCAGGAAACTCA
CCCCCCTTCCCCCTTAAGCCCTCCTTCCCAGCGAAATTGAGGGGCAGAGGGCGGAGTACTCAGGCAGCCA
GCCCGTAGCCCCGGGTGTCTCGAGTTCGAATAGTGCAGAATGGGCGAGGACGGCACAGTCCCCGGGGGG
ACATCTCAATCCGCTCCGGTCTGCTCCCAGCGGATCCGGAACGACCGTTGTGGACAACAGGGTCTCCGCC
CCACCCGCTGCAACACACGCAGAGGTGGCCCATCGGATGATGAGCCAAGAGGCCGGGAACGGATATGAT
CCGCGACGCGCGTGTCGTAGGTGCGCACAGGAGGGGCACTTCGCCAGAGAATGTCCCAACGCCCCCGTCT
TNTTTTTGTTGGGTATGTGGAAGCCGGGGCCGAAGTAGTCAGGACCAGGATCGGGTCGGCGCCGATACAC
ACGGAACGCAGTAGTAGGACACCCGCTGAANGTGGACGGTGGCCGGATCGTCGCTACGGTAGAGATCGG
CGGACGAACGATGCCTGCCACGATCGATACTGGAGCCACTCGTAGCTTCATGAGTGAAGACTGCGTACGC
AAGTGGACGATTCGGGAAGAGTGGAGGAAATACAGGCACGCATCCGCCTGGCGAATGGGTCCACCATAG
AGGTGGAGAAAGCCTTGAGAGTAGACGTCAGCTTAGGGGAAAAAAGATCAGCATGACGATTCTCATCAT
GCCCTCAATGTTGGACCACGTAATCCTGGGTATGGATTTCTTGGGTGCAATGGGCACTACAATACGCTGCG
GCGAGGCAGAACTGGTCCTAGAAGCGGTGACGACACCGGGCGTGGGGCCAGGACCGAGCGAGGAGTCGG
AGAAAGGCCTCCCGGGTCGTGAAAGGGGAGCACCTGCTCGGCTACAGAGGGAGCCGGAGGAAGGCCTCC
CGGGTCATGAAAGGGGAGCACCTGCTCGGCTACAGAGGGAGCCGGAGGAAGGCCTCCCAGGTCATGGAA

GGGGAGCACCTGCTCGGCACCCGATGGAAAAGGTCAGGAAAAGCGAAGTAAAGTCCGGCTCAAATCGAG
AGCCCGAAGAACCCAGGAGGGCGGCGTAGAGCTGCAAAGGCAGACTTATGTTGGCTACGGAAGCGGC
ACGGCGGGAACGTGCCAGGGAAAGGAGGGAGTAGGAGCAATGGCCAGAATCCACACGGACACAGGGGG
CCAGGAGCGACTGCCAGAATCGAGCGGTCATGGAGTTCTGTCTGGAGTCGAAGTGGTGCCAGGAGGGGG
CGAGAACGGCATCGAAGAGGCGGAGTGGCCAGCGGATCTGGATCCAGAACTGAGGGAGTTCTGGAGTCA
GAGCTGGCTCTCTTCGAGGGGCTGCGGGGAGTCTCACACATCGCGGAGCACAGAATCCGGCTGAGGGATG
ACAAGCCGTTGAAACAGAGATACTATCCCAAGAATCCGGCCATGCAGAGGGTGATCGACGAGCAAGTGG
ATGAGCTGATCCGGGCAAATGCCATCGAGCCGTCGCGTAGTCCGCACAGCGCCCCGATCGTCTTAGTCAA
GAAGAAGACTGGCGAGTGGCGTNTGTGTATTGACTACCGACAACGCAACGCGCATTCCATTCCGGACGCT
TATCCGGTGCCTCGTATTAACCACATCCTGGAGCGGTTGCGTCACGCCCGGTTCATCTCCACTCTGGATTT
GAAGCACGGGTACTGGCAGATACCGATGGCAGCCGATAGTCGGGAGTGTACGGCGTTCACAGTCCCTGAA
AGAGGTCTTTTCCAATGGCGGGTGATGCCATTTGGTCTACACTCGGCGTGTGCGACCTTTCAGCGAGCATT
GGATACAGTAATAGGACCGGAAATGGAGCCCCATGCGTTCGCCTACCTAGACGACATCGTGGTAATAGGA
GCAACGAAAGAACAGCATGTAGAGAATTTGAGGGAGGTATTCCGCCGGCTANGGGCGGCCAATCTCAGG
TTAAATAGGAAAAAATGCAGCTTCTTCCGGGAGAGGTTGGTCTACCTAGGACATGTGATTTGCGGGGAAG
GAATAGGTACTGATCCGGAGAGAGTGGAGGCGATACGTAGACTGAGGGCCCCGACAAATTGCAAAGAAC
TGCGGCAGTGTCTCGGGATGGCGTCATGGTACAGGCGCTNCGTCCCTAACTTCGCGACGCAAGTACAGCC
AATCACAGCGCTCTTAAAGAAGGGGCAGAAATGGGCATGGNGGCAAGAACAGGAGGAGGCATTGCGTCA
ATTGAAGGATAGCTTGACGACTGCCCCTGTGTTGGCTTGTCCCGACTTTTCCGCCAAGTTCGTCTTGCAGA
CTGACGCGAGTGAGTATGGCCTCGGCGCAGTGCTGACGCAGACGATTGATGGCCAAGAGCGAGTGATAG
CGTATGCGAGCCGGAAGTCGCTGAAGGCCGATGTGAACTATTCCGCCACTGAGAAAGAGTGCCTGGCAAT
AGTGTGGTCGATCCGGAAGTTGCGCTGCTACTTAGAAGGGTACCGGTTCGACGTGATAACTGACCATCTA
GCACTGAAGTGGCTCAACTCGATCGAGAGTTCGTCCGGAAGAATCGCGCGATGGGCGTTAGAACTGCAAC
AGTTCCAATTTGACGTCCGTTATCGACGGGGGAAGCTCAACGTCGTGGCCGATGCGCTATCGAGGCATCC
TCTAGAGACCTGTCAGCAAGCAGTGGTGGCCGAGGCCCCTTGCAAATGGGTCGCGAGTGTGCGTGCCAGA
ATCGCCGAAGAGCCATGCAAGTTCCCGGATAACATCGAGGAAAACGAACAGCTATATAGGCATTTGGGTC
ACCGGATAGACGATGAGGACTACATCCCCTGGAAACTATGCGTAGACAGTGGTCAACGCCAGAGGGTACT
GAGGGAATGTCATGATGCTCCCACGGCCGGACATCAAGGTGTCAGGAAAACGGTGTCGAGGTAAGCCCA
GCGGTACTACTGGTCGGGTATGTTCCGCGATGCCACCAGATACGTGAGATGTTGCGAAGACTGTCAAAAA
TTCAAGAGTGAGCAACTCAAACTGGCTGGTCGCATGCTGACGAGGCAGGTGGCAGAGCCGATGGCGGTA
CTTTGCGCGGACTTCGTCGGACCCCTTCCACGGTCGAGACATGGAAACACGATACTACTGGTGTTCCATGA
CACTTTCTCAAAGTGGGTGGAATTGGTCCCTCTCAGGAGAGCCACAGCAGAACACCTGCAGACTGCGTTT
CGAGAGAGGATTCTAAGTAGGTCCGGGTTAGAAAGTTTGTGTGCGACAACGGGATTCAGTTTACGAGGCG
GAGCTTCAAGTCGTTTTAGGGGTCCCTGGGTGTAGAGATCCAGTAAACCGCCCCGTACTGTCCGCAAGAG
AATCCCACTGAGAGGACCAACAGGACTGTAAAGACCATGATAGCGCAATTTATTGAGGGCCACCAGAGTT
CATGGGACG
>9_rnd-5_family-2109__LTR___Pao
CCGGCAACGGAATCGTTCCGATGCCGAGTATGCNGAGTCCANGCGCTGCGNAAGTGCCAGCGGTTCATGC
GGCTGTCTCCAGAGAAGAGACTCCGAGTAGTTCTGGTCAACAAATACTGCGCAAACTGCCTGCCTCACCA
ACATTCCGGGGAGTCCTGTCGCCGCAAGGACAAGTGCCGCAAGTGCCAGGGCGACCATCACACCTTGCTT
CATTTCCAAGAAGCGCGTCGCATGCCATCGCGATCTCCAGAGCGATCCCAAACAATGCGCAGCACCAAGG
CCCAAGCGCGACGCTCCGCGCCGCCTTCATCCGCTGGTTCCATCTCCTTGTCGACTTTAATGGAACACAAA
GGACAACCGTGCTCCCGAGAGCACTCATCCGACTTGGAAACGGCAAGGCGACCCGCGACACTCGGGCACT
TTTAGACCCGTGCTGTCCGGTAAGTCTTATCGACGCATCCTTGGCAAAGGCCTCCGGGCTATCCGTCACCC
GCGTCGGTAAAGACAACGCNTGCTCGGCCATTATAAGTTCCNGGACCTCTAGCTTCCAGCGAGAGGTCAT
CCTGCAGGTGATGCCAAATCTCCGGTATCGAACTCCAACGAAGCTCGNCAGCGAGAAACTGAACGAGCCT
TTCAACAACGTTCGATTGGCAGACGATCGCTGGTTCCAGCCGGAAATCTCCAATCTGGTTTTGGGCTCGGA
TATCTACCCAGACTTAATTCTCCAAGGCGTCCTGCCGAGCCATGGCGGCGCACCACTGGCCCAAAACACG
GTCTTCGGGTGGATACTGTCCGGCTCCTGCGCTCACCAGTAGGAACAGAGCCACTTCCCTCTCCCTTTTTCT
TTTTGCAATACTGCAAGGCGGGCGGCATGTTCACACAAGAAGTGTATCTCCGGCAGTCGCCATCCAACCG
CCATGTAACTCCGGAACCAGTACATGGAACATGAAATTCACCTCACTTACACTAATACATTGTAATGTACT
CTGAAAATATAGAACAGAAGAAAAATAACCTGCAAGCGAACAAACGTGTTTTTTTCAAACCTTCTTAAGT
AAATTTGTGCAGAGCAGAAACGCTCCCCCAACGATACAGAAATTCGTAAAAGTTCATCTGGAAGCCCTGC
GGACGCCCTACTACATCATTTGGTCCTTCGAGCCGGATNTCTCATCAACATTTGGCATATCCAGTTCGCCC

CAGCAAGCATTGGTGGATCTGCAATAAAAGGTTCCAGGAACAGCTACAAATTCAAACAAGATAAGTAGC
AGTGCACCTACCTCAACTCGTCCATTGCCAAATTTCCGGCAACTCCAGTCCGATTAATTCATATTTGTGTGT
CTGAACATATTTTTTCGCTTCACATCGTAAGGGCATCTAAAAAGTGACAAATATTAAAACAAATAAAACA
GAAAGTGACTCCGATAAGGTCGCCATATTGGTGTCTGTACATATGTATGTATATATATATGTACAAGTGCA
TACCCAAAAATTTAACCGATCGCCATATTGATGTACGCAAGTACATCAGACATATCTGCACACATAGGCTT
TCGCTCCGTTGAACAACAAGTGTATGCGCTTCTCCATTCTAACCTTCACCAAATAATCACATACCTAAAAT
ATAAAAAAAAGACACACAATTCTGATTGGTATTTAGTTAATTAAAACCACACTGTGCAAATTCACCTGCA
TGTATACACACACACGCACATCATATTATATGTNTTCTATTTTATCGTTTGCTATTTTTCCTCCACAGCAGC
TTCAAGATCAATTTATATATTTATTTTTACCAATTAATTGGCAAGTGCAAAAGGTAAACAAACATCAGGGT
TGTAAAGTATCAAGATCGGTTTTCGATAAACTGATTCTGAGAAACTTTTATATTTTTGTGTGTTTCCACTTT
GCCATTGAGCTGCGTTTACTTAATAAAATACAAATCGAATATCCGTGCGNTTTTTCTTAAAATATAACAAT
TTATACAATATAATTATAAGNNAAAACCTTCCATTGAGCTGCGTTTAGAATAAACGTACTTCTCCTGAGCT
CAACAAGTAATAAGCAATAAGCATCAATTTTTTTCGCGATCCGTTTCCACTACACGGCCGCCTTGTCAAAA
GAGAATTAATTCTTTTCTCTGGTGTTTCAATAAAGAAATAATTAGATTGTTTCTCAAGTAAAGGAAGCTAA
ATAAGCATCAATTTAGNAAATTATTAGTAAAATAATTAATTTAACAAAATTTTCCGATTTCGTTGGACCCA
ATAGTGAACACCATTTCGGTCATTCCATTTTATTCTGATATCTCGATTCCATCGTTCCTGTGCCATTTCTTAT
TCCGTTTTCGCTCGGCTGATTCAAATTATTTGTATTTGATCACATTTCACCTGGTTATTACAATTTTTGGCTC
TGTGAAGAGCCTAGTATGCCAATCGGTGGTGATCAAAGCAGCCACAAGCGACTCACAGCACCGTCCGTTA
AGGAGTCAAAGCGACCTTTAACCTCACCTAGAGCAGTTTTCGGGAAACTCAAGGTCGAGAGTACCAGCAC
AACCGTCCGCAAGACTGTGAGTCCGATTCCAACAAGAGATATTACCCCAAAAATTACCCGTTCGACTACG
CAAAAAATGCAGAAGTCAAGCGAAATGGCACTGAACGACTTCGTTTCTATAACTGACCGACTAGGCCAGT
TTGAAGTGAAGATCAATGACGCAGCGGACAGTGACGTGTCACTGTTCACTCTCCAAGTCCGACGAGATCA
GATTAAGACTTTATGGGAAAAGGTAGAACGCGAGTACGAGTTACGTTCCAAAGCTCTCAACNAAGGCGCT
TGCAGCAGCAGTGACCTGGTGGCTATGCAAGCTAAATATGATTACTCGTACTCCGTTTACGAGCGATGTGC
GGCTCAACTAAGTGAAAAAATAGAAAGAGCCACGGCTTCAAATAGACAAGCTGAGCAAACCCCTCCAAC
TTCTCCATGTCCAGGGGTTGCAGTCTCCCGCCATGCGACACTGAAGTTTTCAGTGGGGATTACCTACGCTG
GCCTACTTTCCGAGACTTGTTTACAGCGGTTTATATTAACAATCCAAGATTGACGCCGGTGGAAAAATTGT
TCCATCTAAATTCCAAAACCAGCGGCGAAGCTAGGGCAATTGTCTCCAAGTCCCCGCTCACGAATGACGG
CTTTCAGTCAGCGTGGGCGAGCCTGACGGAGAGATTTGAGAATAATCGACTATTAGTCAATAGTCAATTG
AAAATTCTTTTCAATCTCCCCTCAGCAACACAAGAGTCTGGAGCCGCCCTTAAAGAGCTTCAGGGCACAA
TCCAGGGATGTTTAACGGCTCTCGACTTGTCCAGGGTGAGCACCGAAAATTGGGATTGTCTGCTTATTTTC
TTGTGCTCCTCTANATTGCCTAAGCTCACGCTGTCGTTATGGGAACAATCGCTCCACGATAAAGCAGAGAT
ACCTCAATGGTCCGAGATGAATTCATTTCTGATCGACCGGATATCGCACCTTGGAGGCGATAGAGGATGT
ACGACCTACCGCACATACGCCACCCCAGTCCAAACCCACAAGAAAAAGAGTCCAACCACCTGAGAACCTT
CGAAGCAACAGTGACTACAAAGTCACAACACTGCAAATTGTGTCAGAAGGAAAACCATCCCGTGCGTCTA
TGCCCACGTTTTCTACAAATGTCCGTACCCGAGAGAGTTTCCGTCATTAAGCAACAAAAGTTGTGCTTAAA
CTGCTTCGCCAGACAGCACCAGTTGAAAGACTGCACCAGCGCACATAGTTGTTTCACGTGCAAGGAACGT
CACAACACGCTGTCTCCACCGTGACAGACCCCAGGTGCCCTCAGCTTCCACAACACCAATAGTTTTGAACC
CTCCTTCGTCTATACAGTCCACTTCTNCTTCACCCAAGCTCCGTTCAAAACTACTTTTCCGCAAACTCACAT
GGTGTTCTCTCTCGGCACGGCTGTCATCCAAGTGTGTCATTTGGGCACAAAGTATACAGCTCGGGCATTAA
TCGGACTTCTGGGTCTGAAGCGACCTTTGGCTTCAGAGCGATTGTTCAAAGNGGTTAAACTTCTTTATCAA
CCCGTCGACGCACAGGTCTCCGG
>9_rnd-1_family-293__DNA
ATATATAATATTGCCAGGTTGTTTTTGCATTAACATAGGATGTTATGAGAGAAATAGAAAAATGAGAATTT
CTCAAAATCTGTACCGATGATTTTAATGAAATAAATAGCAAAATGATTGATAAATCGTGCTCTATATGCAC
AACTACCGTGCATTTGGGGCNNGTTCGCAGAAATTAAATAATAAAGTTTAAAATATGGCTGCCATTTAAC
AGCGCGATATATAATATTGCCATGTTGTTTTTTGCATTAACATAGGATGTTATGAGAGAAATAGAAAANTG
AGAATTTCTCAAAATCTGTACCGATGATTTTAATGAAATAAATAGCAAAATGATTGATAAATCGTGCTCTA
TATGCACAACTACCGTGCATTTGGGGCCGCGTTCGNAGAAATTAAATAATAAAGNTTAAAATATGGCTGC
CATTTAACAGTATGATATATAATATTGCCAGCNTGTTTTTTTGCATTAACATAGGATGTTATGAGAGAAAT
AGAAAAATGAGAATTTCTCAAAATCTGTACCGATGATTTTAATGAAATAAATAGCAAAATGATTGATAAA
TCGTGCTCTATATGCACAACTACCGTGCATTTGGGGCCGTTTTCGGAGAAATNAAATAATAAAGTTTAAAA
TATGGCTGCCATTTAACAGTNTGATATATAATNTTGCCAGCTANTTTTTCGCATTAACATAGGATGTAAAG
AAAAAATTGGAAAAATGCAATTTTTCCATAAACTGCGCTATTAAGTATGACTTAATACTTCTCATATAATT

TGCTTTGACTTATTTTAATTTAATTNACTGTGAGTCCTTTCCGTTTTTCATGATNACATATNATTCAAATTG
AATTGGTTCCCGCCCAATTCTGTACAAAATTGTTCAACACTGATGCCAGGCAGTGATTCAGTTCACGTAGG
AACTAGCAGTTCGATAGACTTTNTGAATGAACTATATGAGGTTGGTTTACAATTAATTAATTTTCTNAAAA
TCTGTACCGTCCATTTACTTAAANTTAGTGCCTAAAGCTTCTTAAAATATGTATCTACAAGAGGGTATANC
GTGCATTGAGGGCCATTGTCTCTGAGTTAAGAAATAAGGGCTCGAAAACAAGGTTGATTTTTTGCGGTCAT
TTTATATGGGGCTGACTTGGGGTTGTTGCACTCTTTTCCAAATATCTTAAAAATGGTACCGACGATTTTAAT
CAAATTTTCACAGTTGGCTAGGTGAAACTATAGGCTTTAATCGTGTATATGCGGTTTTCGGGGCCATCATC
CCTAAATTTAGATATTTATGGAATGGTGGAAACCTAATATTTTAACCCCTTGGTTTACCCATATTATAAATT
AAAATTCAAAAAAACAAATGAATGTAGGCAGTAAATAATTTAATTTTTTGCAATTCTGCATTTAAATTTAT
TNGGTTCCGTGCTTAAAAACATAAATAAATGCATTTAAAAGTGACCGATTCCTTTTCCGATACCAAGTACC
AAAATTCGACGCTAAGTCACCGCCGCTAATTTGACGCACATACAGAGTGAACATCGGGAATATAGCGGCG
ATCAAAAAAGTACAGCGCGCGCGCGCCAGACGGGCGACAGTGAGACCACGCTTGCATACGTGTGCACAG
ATGCGCGCCGCAGCCGACGTCGGCGTCGAAGGGCCGTTAAGGCGTCGCGACAGAGGCTGTACAGTGGGA
CGAATTCAACTCGCGTGCGGTGAAAAGTTGAATTCGTCAGTACAGCCAACGCGGGCAATGAGAACGGGA
GTGTTCAAAACGAAGTATATAGATTTATATATACACAGATAATAATACAAATTGTAGAATGTTTTAACAA
CTATTTAAAGTATTTATTTTTATATAAATAACATGTTTAACTTATAAACATATATGTAAATAACATGTTTCA
TCGTCATGTGTACTTTTACTTATGTACTTTCACAATTTTTAGTTGTTCGTCTGATTGGCCGACCGGGCGCTG
TCCGCAAACACGCCCGCTCGCCTCGACCGCCACAGAGAGATTTTTATTGTTGCTCATTTCTACATATGATT
TCAATTGCAAACAGAAATTTTAGAACAGTTTTGGGAGCACTTAAAACTATGCAACGTTCACCAGTTTGCA
AGGTGGAGAGGGACATGACAAGAGATACATTTAACATTTAATCTCGACTGACCTACATATAAACATAACA
TGACATAACAGACTCTGTACATTTAACATATTGTCTTAGGTCAGTTAGCTGGGGAAATTTCTGCACGATCA
GCTGGCAATGCTGTCGTGTGAATGGCAAGAATACATAAAAATCAAATGGGCACGCTAAAGACATGTAAGT
AGAAAATTATTTTAAATTTAACAATTTTTTGGCTAAATATACTAACAATGTCAATGTACTTCCACAAGGTG
GCATCTTTATGTTCCCATAGGTTAGCATTCTACATAAGTTGGCAGCCCCTAACATAGCGTCACTTTTGGATT
CCATGCTATTTAATTCAAATGAAATTTATATGCATATTTATACATATGAACATATATATTTATAACATTTGT
TATTTATTTGTTTATATTTTAATCTAACAAGTTTGAAATGGCTCCAGGAAACGCAACACGGCAAGATTTTT
ATTTATTGACTGATTGTGTTCGCCTTTTTAAAAGCCTGAACCCATTAAAAATGGGTAAAAGGGGTATAATG
TATTTGTGCAGCCGTATGTAACAGGCAGAAGAAAGGATCTTCAACCACTTAAAGTATGTATTCTTGATCAG
CATCAATAGCCTAGTCGATCTAGCCATGTGCCTCTGCCTGTCCATGCAAATCAGGTATCTTTTATTCTATAG
CTATAGCGGCGTTTGTTGTTGGTAGGAATGGTATCCCCTAGCCGTGCACTCCCAACTANAGCATTCTTACT
TGTTTTTNTACCTAAAAGCTATTGAAAATGAATGAACATTTTTTCAAACGAACTGGTAAATAAAGGAATTT
TAAATTTTGACATAACTTAGTCGTTAGTACATTGTTAAAATTTTTTTGAAATTGTACTGAGTTTTTAAAACT
AAAAACTAACGAGAAAGAAGGGCTATCTTCGGCACGCCAAAGATCTAATACCCTTGCAGACCCAACCTTT
TCATAATGCTCATAGTGCTTTGCCTCTATCTAAGAAGTACCGGGAAAAATAGTAAATGCCGAGTTTGGTCA
AGATATCTTGATAAACAAAATGTTTTTTTCATACAACTTAATTTTCGACCGATCGTTCCTATGACAGCTATA
TGATATAGTGGTCCGATCTTTATAGGATTTTGCACATATATGAGGAGTAAAGTAAAAACTAATAAATGCC
GAGTTTGGTCAAGATATCTTGANAAACANAATGTTTTTTTCATACAAAAACTTAATTTTCGACCGATCGNT
CCTATGGCAGCTATATGATATAGTGGTCCGATCTTAATAGGATTTTGCATATATATGAGGAGCNNAGTAA
AACTAATAAATGCCGAGTTTGGTCAAGATATCTTGAAAAAAACAAAAATGTTTTTTTCATACAAAAAACT
TAATTTTCGACCGATCGTTCCTATGGCAGCTATATGATATAGTGGTCCGATCTTNATAGGATTTTGCACAT
ATATGAGGAGTAAAGTAAAACTAATAAATGCCTAGTTTGGTCAAGATATCTTGATAAACAAAATGTTTTTT
TCATACAAAAAAACTTAATTTTCGACCGATCGATCCTATGGCAGCTATATGATATAGTGGTCCGATCTTAA
TAGGATTTTGCATATATATGAAGAGTAAAGTAAAACTAATAAATGCCGAGTTTGGTCAAGATATCTTGAA
AAACAAAAATGTTTTTTTCATACAAAAACTTAATTTTTCGACCGATCGTTCCTATGGCAGCTATATGATAT
AGTGGTCCGATCTTAATAGGATTTTGCATATATATGAGGAGTAAAGTAANACTAATAAATGCCGAGTTTG
GTCAAGATATCTTGANAAANACAAAATGTTTTTTTCATACAAAAAAACTTAATTTTCGACCGATCGTTCCT
ATGGCAGCTATATGATATAGTGGTCCGATC
>9_rnd-5_family-10772__LINE___I
ATGCGCGAAGCCAAACTACTGTTCAANGGAACAACAACTAAGCAATCCAACCCTTCTTCTACGCTGCAGT
NGCGAATGCGGGACACTATAGTAACTCAAAAACATCCGAACNAAACAAAACAAAAACACTAGTCCAAAT
CACAACAAACCAACCAAAGATAACACAATGCCTTATGCTAATAAAGAAACAACAACAACAACACCACTG
AACACCTCCTCCACGCCGCTGACTTATCAGCAAAATCAACAGAAAAAACAACACAAACGGAACATTCGAT
GATGACTACGAGAGTCTTGGCCTCTCGCCTAAAACTACTCAATTCCTAAAACAACTTAAATCTAACAAAA
ATAAAAATAATTCTAATTCAAACATCCTAACTACTTCTACCAATTTAAAACAAAATGAATATATCAACATA

TCAGATAATCCTATGGATGATTCTGCTATATAAACTGAACCTAACTTTTTCCATTGCATATAATATCCACTT
TATCTCTTCCAATGACCCTTAAGATTCTACAATGGAACATAAACATAAATGTATACTTCAACATTACATTG
AACTTGCATTATTAATTAAACAAACAAACAAACAATCCGNATATTAATTGCCTNCAAGAGACTAATCTTA
AATTTAATAATTCTCAAGTTGCAACCCCCAAACCATTTATTGGTTATTTTTTCAACAATTCTTCCATTCTCC
ATGCGAAGTATGGCGTTGGTATACTTATACGGCAAAACATCCNACACAAAACTATCAATTTGATCTCACC
CACTTGCTCTATGGGCATTGANCTTTTATCCCACCCGAATATAATAATATTTAATTCCTTATATACCCCCAA
CCAAAGACTATTAGCTACAGATTTAATAAATATTATAGAGTCAGCCCAAAAACCTATAGTTTTTGTAGGG
GACTTTAACGCTTGGAGTCCGCTTTGGGGGTCCTGCTCCCGGAACCCCAAAGGTTATNAAGTTGAAGATCT
TATAGTCAAAACAAACTTAGCCATTCTAAATGACGGCTCTGCCACCCACCATTCTACCCACAACTCCTTTA
CTAACATCGACCTAAGTATGGCGTCCGCAACCCTGGTACCATTCTGTAAATGGTCCATTGGCCAAAACTTA
CGCGGGAGCGATCATTTCCCTATTACTATTAACCTTCGCACACATATTAAACCTGAAAAATTTCCGCTTCC
AATTAGGTTCAAAACCGACCATGCCAACTGGAGTACCCTTCCAATCTGTCTGTAAAAATCACTTTCAGTTT
CCCTCGACCAACGATCTGAACCTCTTCGCCGCCAGAATTACGAAAACCATAAAAACAGCAGCCAACGTAG
CCATTCCACAAACTAATCCCAAATAACAGATAAGGTACCCTGCTTGGTTCAGCCCAAGTCTCCAAGATCTG
AGAAAACAAAAGCAAGTTNTCTGGCACATCTATAAATCCTCTCAAAACTGTANCAATCTAGTAGCTTACA
AAAAAGCGAGAGCCCTGTTTAAAAGAGAAGCTATTGGGGCAAAACGAGAATCTTTTAGCAGTTTCACCTC
TAAAATAAACCCACTTTCCACAACCAAAAAAATGTGGGCCGATATCAACAAATTAGNAGTGATTTTCCCA
GGGCCTTTTAAATCCATCAGAGACAACAATGTAGAGCTCCAAAATTCAGTAGACATTGGCAATGCATTTG
GAGCTATGTGGTCCAATTATTCCAGGGATGAAAACTTCTCAAATGCGTATAAAGTTAATAAGGAAAACGT
TCTCTCTCAGGCATATCTGCCCAACCACATATCCTCAAATTCTAAGTACCTTGAATCTCAGTTCACACTGAT
GGAACTAGAATTAGCCCTTCAACACGCTAAAGGGAAAACTCCTGGGTATGACCGCATCTCGTACAATATG
CTTCGAAACTTACCAATAGAAGGAAAGAAAAGAATACTAGAAATGTACAACATAATGTTAGATGGGGGT
AACTATCCGGAAAACGGCTACAATTATCCCAATCCTTAAGCCAAACAAACCTTCCCACGAATTATCATCCT
TTCGTCCCATATCCCTACTCTCCTGCTTGAGCAAAACACTGGAAAAAATTCTTGCCAAAAGGATCATGTGG
TTCATATCCAAAAATAAACTTATCAGCCCCAACCAAACTGCATTCAAACAACAGCACAGTACCATTGACT
CCCTATTGCATATCCATCATTACGCGTCAGATGCCCTTTCAACCAGAAATCATGTAACCATCCTGGCTACT
GATTTCGAAAGGGCATTTGAGCGCGTTGGAGTGCACACAGTACTGGAGCAACTTTCCCGGTGGGGGTTGG
TCCTAAGACCTTCAACCTCGTGAAGGCTTTCATGACCAAGCGATCCATCCGTGTTAGAGTTAATAACACCT
TATCTAAGTTCTATAAATTACACATCGGCATACCACAAGGGTCTCCACTCTCGGTGGTTTTGTTCATGATT
GCCTTCAACTCCCTCAATAACATAATACTAAAACATAAGAAAATACAACTCTCCGCGTACGCGGACGATC
ATTATCTTTAGTAACTGCAAAAATACAAATTTACTCTTCCTTACGTTTAAACAAATACTTAATGATATCGT
AGAATGGGGAAAACTCTCAGGAGCCACTCTTGCTTTAAACAAATTTGTTCCCCCCGCATACCATGCAGCA
GTACGGCGTAGCATTGACGCCTTTCCCACTACTCCGACGAAATGCCTTTTAGCTGAAGCGGGTCTTCCTGA
AATACAGACGAGAACTGCGCAACTGACTTTCAAACTCATCCCTAAAATTCTTTGCTCGACCAACCCAATTC
TGCTTAAAGACTTTAACAGAACGGTTAAGCAGCGCCGAAGATTTAAAGTCTTGTCTACTATCCGTAGATGT
TGCAGTTTTTTCAAAAAACATCAATTGGATATGCCATATCGTTGGAACAAAATCAGCCCATCCCCCCCTTG
GCAACTACAGGACCACACCGTGAACACCTCTCTCCACTTATTAAAAAAATCAGAAACACCTAAAAGTATA
TTTCAGCAAATGCTAGCGCCGAATCGGAAAAATTCCCCAACTGGAGTTGGCTCTACAGTGACGGATCGAA
AACAATTGACTCCACTAGCTTCGCAGTTGTAAGCCAATCAGGAACTGTGGTCGCATCAGGAGTGCTCCCT
GCATACTTTTCTATATATTCCGCCGAAGCTGTAGCTGTTCTAAAAGCAATTAAACATGCTCTCGAGCTTAA
GGGAAAATTTATAATATGCAGCGACAGTCTTTCTACTCTCAATGCCATTAACAACGCATACAACCAAAAC
AGCCTGATATGCGAAATTCGACATCTTTGCATCAAACACGATACCAAAATAAAACTCCTTTGGGCGCCAG
GTCACATGGGCATAAAAGGTAACGAACTGGCAGATGCAGCGGCTAAACTGGCNGGACAGTCTCCTTGCCT
AACAGTCATCCCTGGTGTCAGAGAAGACTTATGGCGCAACATTGAGGATATCCTAAAAAGGGACGAAATG
ACACAATGGGACACTTACGTCCATAGATTTAAGCAATTTAATCCGAACAAGGAACCCGTCCGATATCCAG
CTTCATGGCACAGAAGACATTGCAGTGTTATCACCCGGCTCAGAACTGGGCACACCGCCTTAACCCACGA
CCACCTGCTTAAAGGAAAAGCCAATCCCAACTGCCCAAAATGCGGGGGAGAATTATCAATCCAACACATA
TTGAATAACTGCCCACAACTTAATAACATTAGGACTTGTCTATTTGGCCAAATCCAACCTACCTCTCTTCTT
ACCACACCTACTCACAATAATGTAAAATTGCTAATCAAATTCTTAGACACCAATAACATACTAAATAGCA
TATAATAATATATATATAATATAATATACATATAATACTATAAATAATTTAACTGTCGAGTCGATGGCCTT
AGTAGCTAGCACTCCCTCCCGTTAGTTATTATATTATTTTTTTGTATAATAATTAATTACGTAAANTAAATA
AATA
>160_rnd-5_family-151__LINE___I
CAACAAACCAACCAAAGATAACACAATGCCTTATGCTAATAAAGAAACAACAACAACAACACCACTGAA

CACCTCCTCCACGCCGCTGACTTATCAGCAAAATCAACAGAAAAACAACACAAACGGAACATTCGATGAT
GACTACGAGAGTCTTGGCCTCTCGCCTAAAACTACTCAATTCCTAAAACAACTTAAATCCAACAAAAATA
AAAATAATTCTAATTCAAACATCCTAACTACTTCTACCAATTTAAAACAAAATGAATATATCAACATATCA
GATAATCCTATGGATGATTCTGATATATAAACTGAACCTAACTTTTTCCATTGCATATAATATCCACTTTAT
CTCTTCCAATGACCCTTAAGATTCTACAATGGAACATAAACGGATACTTCAACAATTACATTGAACTTTCA
TTATTAATTAAACAAGCCAATCCGTATATTANNTGCCTNCAAGAGACTAATCTTAAATTTAATAATTCTCA
AGTTGCAACCCCCAAACCATTTATTGGTTATTTTTTCAACAATTCTTCCATTCTCCATGCGAAGTATGGCGT
TGGTATACTTATACGGCAAAACATCCCACACAAAACTATCAATTTGATCTCACCCACTTGCTCTATGGGCA
TTGAGCTTTTATCCCACCCGAATATAATAATATTTAATTCTCTTATATACCCCCCAACCAAAGACTATTAGC
TACAGATTTGATAAATATTATAGAGTCAGCCCAAAAACCTATAGTTTTTGTAGGGGACTTTAACGCTTGGA
GTCCGCTTTGGGGGTCCTGCTCCCGGAACCCCAAAGGTTATNAAGTTGAAGATCTTATAGTCAAAACAAA
CTTAGCCATTCTAAATGACGGCTCTGCCACCCACCATTCTACCCACAACTCCTTTACTAACATCGACCTAA
GTATGGCGCCCGCAACCCTGGTACCATTCTGTAAATGGTCCATTGGCCAAAACTTACGCGGGAGCGATCA
TTTCCCTATTACTATTAACCTTCGCACACATATTAAACCTGAAAAATTTCCGCTTCCAATTAGGTTCAAAAC
CGACCATGCCAACTGGAGTACCTTCCAATCTGTCTGTAAAAATCACTTTCAGTTTCCCTCGACCAACGATC
TGAACCTCTTCGCCGCCAGAATTACGAAAACCATAAAAACAGCAGCCAACGTAGCCATTCCACAAACTAA
TCCCAAATAACAGATAAGGTACCCTGCTTGGTTCAGCCCAAGTCTCCAAGATCTGAGAAAACAAAAGCAA
GTTNTCTGGCACATCTATAAATCCTCTCAAAACTGTACCAATCTAGTAGCTTACAAAAAAGCGAGAGCCCT
GTTTAAAAGAGAAGCTATTGGGGCAAAACGAGAATCTTTTAGCAGTTTCACCTCTAAAATAAACCCACTT
TCCACAACCAAAAAAAATGTGGGCCGATATCAACAAATTAGNAGTGATTTTCCCAGGGCCTTTTAAATCC
ATCAGAGACAACAATGTAGAGCTCCAAAATTCGGTAGACATTGGCAATGCATTTGGAGCTATGTGGTCCA
ATTATTCCAGGGATGAAAACTTCTCAAATGCGTATAAAGTTAATAAGGAAAACGTTCTCTCTCAGGCATAT
CTGCCCAACCACATATCCTCAAATTCTAAGTACCTTGAATCTCAGTTCACACTGATGGAACTAGAATTAGC
CCTTCAACACGCTAAAGGGAAAACTCCTGGGTATGACCGCATCTCGTACAATATGCTTCGAAACTTACCA
ATAGAGGGAAAGAAAAGAATACTAGAAATGTACAACATAATGTTAGATGGGGGTAACTATCCGCACGCT
GGAGAACGGCTACAATTATCCCAATCCTTAAGCCAAACAAACCTTCCCACGAATTATCATCCTTTTCGCCC
CATATCCCTACTCTCCTGCTTGAGCAAAACACTGGAAAAAATTCTTGCCAAAAGGATCATGTGGTTCATAT
CCAAAAATAAACTTATCAGCCCCAACCAAACTGCATTCAAACAACAGCACAGTACCATTGACTCCCTATT
GCATATCCATCATTACGCGTCAGATGCCCTTTCAACCAGAAATCATGTAACCATCCTGGCTACTGATTTCG
GAAAGGGCATTTGAGCGCGTTGGAGTGCACACAGTACTGGAGCAACTTTCCCGGTGGGGGGTTGGTCCTA
AGACCTTCAACCTCGTGAAGGCTTTCATGACCAAGCGATCCATCCGTGTTAGAGTTAATAACACCTTATCT
AAGTTCTATAAATTACACATCGGCATACCACAAGGGTCTCCACTCTCGGTGGTNTTGTTCATGATTGCCTT
CAACTCCCTCAATAACATAATACTAAAACATAAGAAAATACAACTCTCCGCGTACGCGGACGATCATTAT
CTTTAGTAACTGCAAAAATACAAATTTACTCTTCCTTACGTTTAAACAAATACTTAATGATATCGTAGAAT
GGGGAAAACTCTCAGGAGCCACTCTTGCTTTAAACAAATTGTTCTCCCCCGCATACCATGCAGCAGTACG
GCGTAGCATTGACGCCTTTCCCACTACTCCGACGGAATGCCTTTTAGCTGAAGCGGGTCTTCCTGAAATAC
AGACGAGAACTGCGCAACTGACTTTCAAACTCATCCCTAAAATTCTTTGCTCGACCAACCCAATTCTGCTT
AAAGACTTTAACAGAACGGTTAAGCAGCGCCGAAGATTTAAAGTCTTGTCTACTATCCGTAGATGTTGCA
GTTTTTTCAAAAAACATCAATTGGATATGCCATATCGTTGGAACAAAATCAGCCCATCCCCTCCTTGGCAA
CTACAGGACCACACCGTGAACACCTCTCTCCACTTATTAAAAAAATCAGAAACACCTAAAAGTATATTTC
AGCAAATGCTAGCGCCGAATCGGAAAAATTCCCCAACTGGAGTTGGCTCTACAGTGACGGATCGAAAACA
ATTGACTCCACTAGCTTCGCAGTTGTAAGCCAATCAGGAACTGTGGTCGCATCAGGAGTGCTCCCTGCATA
CTTTTCTATATATTCCGCCGAAGCTGTAGCTGTTNTAAAAGCAATTAAACATGCTCTCGAGCTTAAGGGAA
AATTTATAATATGCAGCGACAGTCTTTCTACTCTCAATGCCATTAACAACACATACAACCAAAACAGCCTG
ATATGCGAAATTCGACATCTTTGCATCAAACACGATACCAAAATAAAACTCCTTTGGGCGCCAGGTCACA
TGGGCATAAAAGGTAACGAACTGGCAGATGCAGCGGCTAAACTGGCNGGACAGTCTCCTTGCCTAACAGT
CATCCCTGGTGTCAGAGAAGACTTATGGCGCAACATTGAGGATATCCTAAAAAGGGACGAAATGACACA
ATGGGACACTTACGTCCATAGATTTAAGCAATTTAATCCGAACAAGGAACCCGTCCGATATCCAGCTTCAT
GGCACAGAAGACATTGCAGTGTTATCACCCGGCTCAGAACTGGGCACACCGCTTTAACCCACGACCACCT
GCTTAAAGGAAAAGCCAATCCCAGCTGCCCAAAATGCGGGGGAGAATTATCAATCCAACACATATTGAAT
AACTGCCCACAACTTAATAACATTAGGACTTGTCTATTTGGCCAAATCCAACCTACCTCTCTTCTTACCAC
ACCTACTCACAATAATGTAAAATTGCTAATCAAATTCTTAGACACCAATAACATACTAAATAGCATATAAT
AATATATATATAATTTAANATAATATACATATAATACTATAAATAATTTAACTGTCGAGTCGATGGCCTTA
GTAGCTAGCACTCCCTCCCGTTAGTTATTATATTATTTTTTTGTATAATAATTAATTACGTAAACTAAATAA

ATANTTTATTGCCTATTAATNTTTGTACTTGGGCTTGAATTTCTTGTACTTGGCTATGAGGACTCATATAAT
TTTTGATTTATCCGGGCTGATCATCCTT
>9_rnd-6_family-1040__LTR___Gypsy
GAATCGGTAGAAGTTGTATCTGCTAAGCTGTAAAAGCTATGACCCAAAATTGTTCGATTGATAAGGTAAA
ACTTATCATGCAGGCAGGCACATTCACAAATATGAATGATGCCGTATCCAAATTTGTCAATAGCTGCACG
GAGGCAACAGGTCAAAGTAACACTGTCCTGTATTATCGACGAGGCGCAAACAATAATAATAGAGGAGGC
CGAGGTTATAATCGTGGCAGAAATTACGACCGCAACAATTAAAACCGAGGCGGTAACAACCAATAACAA
TAATTACAATGAGAATGGCGCAAAATACGCCGGAAAACTCCCAGAACCCTTTAGGAAACAACCAGTAAA
TGTAAAGGTTCATTCCATCAATTACAGTCTTAATATATTTGTCANTTTTTATAATCATTCAACTGGAAAATA
AACTCACATTCCTAATAGATACTGGTGCAGATATCTCACTTTTTAAAAAAATAAATTCAGATAATTTCAAA
ATTCAAGAAGANAAAAATTATAAACATCCAAGGCATAGGCCAAGGTGTAATAAAATCTCAAGGAACAAC
TTCAATAGAACTTCAATCNACAAAATATATTATTCCACACGAATTTCATTTTAGTAGATTCGGATTTTTCA
ATACCGTGTGATGGAATAATAGGCATTGATTTTATTAAAAAATTCAATTGTCAATTAGATTTCAAACCAAG
TGAAGACTGGTTTATAATTAGACCTCAAAATTTAAATTATCCAATTCATGTTCCGATAACATATAGTGCTG
GCAACAACACAGTTCTCTTGCCAGCCAGATCCCAAGTAATTCGAAAAATAGACATTAATACGGTAAATGA
TTATATATATTTGTTCCNAATCCAAGAAATACATAACGGAATTTATGTTGCAAAATACAATAGCAGCATCC
AAACATGCATACGTTCGCCTTCTAAACACAACTAATTTCGACCAATTGGTTAAAGTAAACAAAATCNAAT
ATGAAAATTTAACAGATTACGACATTCATAGCACTAATCCAGAAAATAGAAGCGAACAAGTACTTTCAAA
ATTAAAGAAAAAATTTTCCANAACAATTCAAAAATCAATTAACAGAATTATGCACCAAGTATAGCGATGT
GTTCGGACTGGAAACCGAACCAATAGCGACAAATAATTTTTATAAACAAACGTTNAGATTGAAAGATGAT
GAACCCGTTTATATNAAAAACTACAGAAGCCCGCATAGCCAAGAAGAAACTCAAGCAAGTAGGGAAATT
AATAAACGACAAAATCGTNGAACCGTCTGTNTCCGAGTATAACAGCCCACTCTTGTTAGTTCGAAGAAAT
CATTACCAAATTCGGAAGAAAAAAATGGCGATTAGTAATTGACTATCGTCAAATCAACAAAAAAGCNCTT
TCTGATAAATTCCCGCTTCCCAGAATTGATGATATTCTAGACCAACTGGGCAGAGCNAAATACTTTTTCAT
GCCTTGACTTAATGTCAGGTTTCCACCAAATTGAACTTGAAGAAAATTCAAGAAATATAACATCTTTTTTC
AACGAGCAATGGCTCATATCGCTTCACGCGATTACCATTTGGTCTNAAAATAGCGCCAAATTCATTTCAGA
GAATGATGACTATNGCATTCTCNGGTCTAGAACCCTCCCAGGCATTCCTTTATATGGATGACCTAATAGTG
ATNGGNTGTTCCGAAAAACACATGATTAAAAACTTAACNGATGTTTTTAACGTATGTAGGAAATACAACC
TGAAGTTGCATCCAGAAAAATGTTCATTTTTCATGCATGAAGTGACATTTTTAGGNCACAAATGCACCGAC
AAAGGAATNTTGCCAGACGACAAGAAATNCGACGTCATCAAAAATTATCCNGTCCCACATGACGCGGAC
AGCGCTAGACGNTTCGTTGCATTCTGCGATTACTATAGACGNTTTATAAAAAACTTCGCCGATTATTCACG
GCACATAACNAGATTATGTAAAAAGAATGTTCCNTTCGAATGGACAGNTGAATGCCAAAACGCATTCCAA
TACCTTAAAGAAAGCCTNATGAACCCACATTGTTACAATATCCNGATTTTCGCAAAGGAATTTTGCATTAT
AACAGATGCNAGTAAGCAAGCTTGCGGAGCGGTTCTAACTCAAAACCGTAACGGANTCCAACTCCCAATN
GCTTACGCATCNNGNTCATTTACCAAAGGGGAAAGCAATAAGAGCACAACGGAACAAGAGTTAGCGGCA
ATTCATTGGGCAATTACNCATTTTNGACCATATATTTATGGCGAACATTTTACNGTTAAAACGGATCACAG
GCCATTAACATATTTGTTTTCAATGGCNAATCCCGGTTCTAAAGTAACNCGNATGNGGCTNGAGTTAGAG
GAATACGATTTCACGGTAGAATACCTAAAGGGTAAAGATAATTNCGTAGCCGATGCGTTATCAAGAATAA
CCATCAAAGAACTAAAAGANATACAAGATAAAGTCCTGAAAAAGTCACTACCAGGCANCAAAGTAGACA
GAAAAANCCCTGCGCAGGAGAAAATCATGAANTTCTGCCNAGGCAAATCCGTGAAAANGCTTCTAAGCC
CCAACGTATACGAAGTCATTACGAATGACGAAGTACGAAAAGTAGTGACCTTGCGTATAAAANAATNTAA
ATGTTTCTTTAAACACGGAANAAAATTATNGCAAGAATTGATGTTAGCGATCTGTATACCAATGGAATTCT
CGACCTAGGTCAGTTCTTCCAAAGGCTCGAAAAANAAGCCGGTATATTATAAAATCAGCCAACTCAAAAG
TGGCACCGAGCGGAAAAAGATCTTTGAATCAATTTCAATNGATTCTTTTAAAAATATGGGCAACGAAATA
TTAAAATCNTTAAGAGTAGCGCTACTCCAGCCGGTGACCCAAATAAAAGATAAAAAGAGATACAGCAAA
TATTGTCTACATACCATGACGACCCAATTCAAGGAGGCGATAGCGGCATNACAAGAACGTTGGCGAAAAT
AAAAAGACACTACTTACTGGAAAGATATGACNCGTCATATAACNGAGTACGTACGTAAATGTCAAAAATG
CCAAAAGTCTAAAACAACGACGCACACNAAGACCCCNTTGACCANTAACNGAAACGCCTATAAATGCTTT
CGATAGAGTGATAGTGGACACTATTGGTCCGCTACCAAAATCGGAAAATGGCAATGAATATGCAGTCACG
CTAATATGCGACCTTACNAAATATTTAGTAGCCATTCCAGTNCCAAATAAAAAGCGCGAACACTGTCGCN
AAAGCNATATTCGAANATTTTATNCTGAAGTNCGGTCCAATGAAGACGTTCATTNCGGACGTGGGAACAG
AATATAAGAACTCGATTATAAATGATTTGTGCAAATATNTAAAGATNGANAATNTAACGTCTACNGCACA
TCACCACCAGACAGTTGGGAACAATAGAACGAAGTCATNGAACNTTCAATGAATACATACGNTCNTATAT
ATCNGTTGATAAAACTGATTGGGACGTATGGCTTCAATATTTACTGTTTTTGCTTCAACACGACACCCTCN

ATGGCACATAATTATTGTCCATACGAGCTAGTATTCGGCAGACTACCAAAACACTTCAAAGATTTTAACA
AAGNTAATATAGANCCCATATATAATATAGATGACTACGCTAAGGAAATAAAGTNTAGATTAGAAATAGC
ATACAAAAGAGCTAGAATNATGTTAGAAAAAGCTAAGGCTATAAGAAATTAGCATATGACGAAAAAAAC
AAAACAACTTCGAATNAAAAGTAGGAGATAAAGTTTTATAAGAAACGAAACAGGNCATAAATTAGAACN
AGTATATTCAGGNCCGTATANAGTAAAAAAAAATAGAAGATAATGACATNATAATNAAAGATAATAAAGA
TAAAAAACAAAAAGTTCATAAGGATAGATTAAAAATTTTTATTTCATAATCATAATAAA
>9_rnd-5_family-1151__DNA___Maverick
ATAATGCCTGTAACATAAATTACAAAGTTGCCTCATTTATCCCCGTACTTTTTCATAATTTTTCACACTATG
ATGCTCATTTGTTTGTAAGAGAGTTGTCAAAAGTTGAAGGTGAAATTAAGGTTCTACCTCTCAATAAAGAG
ATTTACATATCCCTTTCAAAATACACATATATTACAGAGCATAATACAAATTTGGANATACGTTTTTAGAC
TCATTTCGATTTATGGCNGCAAGTTTAGATAGTCTTAGCTCTAATTTAGAACATAATAATTTCACAACTGTT
AAACGGTTTTCCCGGACGAAACACACTTTAATCTAGTACATAGGAAGGGAGTTTTCCCATATGAATACTTT
ACTAAATTTGAATGTTTGCAAGAAACTGCTCTTCCACCAAGGGAAAAATTTTTCAACAGTCTTGCAAATAG
AGAATGTTTAGATGATGATTATTTACACGCTCAGAACGTGTGGAGAGTTTTTAATTGTAAAACCCTCAAAG
ATTATATGGAGCTGTATTTAAAGACAGATGTACTTTTGCTTGCAGATGTTTTCGAAAATTTTCGTTTAATTT
GCAAGAATATTTATTCATTGGACCCAGCCCATTATTACACAGCTCCGGGGTTATCTTGGGATGCTATGCTA
AAAACAACTGGAATAGAATTAGAATTATTTACAGACATAGATATGCAAAGATTTATACAAAATGGTATTC
GTGGGGGATTAGTACAATGTTGTCAGAGATATTCAATTGCTAATCATAAGTATATGAATGATTATGATAGC
ACAAAACCCTCTCAATATTTAATGTATTTGGATTCAAATAATCTATATGGGTGGGCGATGTCACAATATCT
TCCATATGGTGATTTCCAGTGGTTAAGTGAGGATGAAATACGATCATTTCATTTATTTTCAATTTCTGAAA
CGTATGAATATGGATATATTCTTGAAGTAGATTTAGAATACCCAGCATCCCTTCATGATATGCATAACGAC
TTGCCGTTTTGTCCAAGCAATCAAAACCGACTGCAAAGTGTACAGAGTTAAAAAGCTTATCGCTGACCTA
AGACCGAAATATAAATATCCCATACATTATAGAAATCTTCAACAATGTATTCATAATGGATTGCGGTTAAC
TAAGATTTACCGTATATTACGGTTTAAGCAGTCTCCTTGGTTAAGAAAATATATTGATATTAATACATATC
ATAGAACGCAGGCGAAAAAAATAATTTCGAAAAAGATTTTTTTAAGCTATTAAATAATGCAGTATATGGG
AAAACCATGGAAAACGTTGAAAAACGTAAAAAACATTAAATTGGTAACTGCATGGGAAACAACGTCTTG
CAATAGATTGGGAGCAGGGGCATTAATCGCAAAATCAAACTTTCATAGCTCAGTGCAATTCTCGAATAAT
ATGTATGCAATCCAAATGAAAAGATTGCATAACTATTATAATAAGCCCATATATTTAGGTTTTACAGTATN
AGATCTTTCGAAATTGTTGATGTATGATTTTCATTATGATTATATGGTAGCAAAGTTTAAATCTGATCTAGC
ATTAAATTATATGGATACAGACTCCTTAATATATACAATTAAAACTGAGGATTTTATGCAGATATTAGAGA
TGATCTCGAAATGAAATTCGACACATCTGAATATTCTGATGATCATGCTCATAAATATGCCCTAAAGAGA
GTTAATAAAAAAAGTCTTGGGCATGATGAAAGATGAGAATAATGGTAGGATAATGAAAGAATTCATAGG
ACTTCGACCCAAAATGTACTCATTGAAACTTGAGGACGGAGGTTGTGTTAAAAAGGCAAAGGGTATTAAA
AATCAATTTTGGAAAACATCAATTAAATGCTTTAAGATTGTCTTTTCAATAATAAATCATATACAACGAAT
AATATGTTNATTTTNAGATCTAACTTACATAAATTGTAGACTCAANAAATAAAAAGTAGCTTTAAGTTCTC
AAGATGATAAGAGAATAAGAGATGATTGTATGCATACTTATGCATGGGGTCACTATAAGATTGTAAATTA
ATTGGTTAAATAAAACATTAAAAAAACGGATTCGACTCCTTTTNATTTTATTCTGAAATTGTATATAAATT
TGAGAGAGAGAAAGAGTTAAACGGAGTTACCTTTAAAAAATTAANTTAAATTTAAAGATTNAGATTTATA
AAACATCGTGAGATTCAAGAAAATATAATATCATCTACAACTCGTGNGCTTTTTGATGAGGATGATGATA
CCGTTGAAGTCCCTGAAACAGNCATTGAAGATGAGGAGGACGATATATTTAACAATANACANCATTATTT
GAATCGAGAAGCTCAACCAATANNATTATCAGATAGTGAGGNAGAACTNTTTGAATACCAAAATTTATTT
GATTAATAAAATCATATATATTTTACAAAACTATATCCTTCTTGTNTATTATATATATATATATATATTTTA
CAANACTATATCCTTCTTGTTTATTCTATTTTCATTAAAGAAGTTTTTGCGAATAATTTTTTAAATTAATCA
ATCGCGTTTTCAGATCTTAAGNATTCCTCAAATATTNAAACTCATTCAACCTCTCCTCTTATATCTTATCTT
TCAGCTTCTTTAACAGGTAAATTTCAGATGATCTGCATGCTACTTTTATAGGTACACCTTTTGTTGAGACAT
ATTCATCGTATTGAATCATAAAAATGTCTATAAAACGATGTATTACTCTAAAAATTGCAGAAAGAGGCAA
ACATCTTTCTGAACTAGCTCTTGCCTACACATCTTTTGCGCTTTGAATTCTCATCAAGTTGAGATGGGAATT
AGTAGCGTATTATAACGTTGAGCTACTAAATTAACATATAATATTTATATAGATGAGTGCACTTTATATTT
TCAGAGCAAACGATAAAATATTCATTTATATTAAATCTATAAATCAAACCTAGTCATAACTCTAATTTCAA
AAAAAAATTTTCACATAAAACTTTATTGCTTCCGAGAAGAACGTCGATCTGTGAGCTGCTATAGAAAGAG
AGATCGGCCCAGTGTAATTTTGGAAGTTCTTTCAAAACATTTTGGGGGATGGTGTGGGGCAAATTGCCAG
CTAGCTTTGGAAGAACATAAGCCGAGGTTTCAATATTAGCCGGGGCGTTATCGGAGAGCCGATATTAAAA
NGACAGAGTATTTGGGGCCGAGCCGAAATAGCTTGATTTAAACCGGAGACCTGTGCGTCGACGGGTTGAT
ATGGAAGTTTAACCCTTTTGAATAATCGCTCTGATAAAAAGCTCGCTTCTGACCCTGTGTTGATTAGTGCC

TGAGCTGTATATTTTGTGCCCAAATGACACACTTTGATGACAGCCGCTCCGAGGAGAACACCATGTTAGTT
TGCGGAAAAGTAGTTTTGAACGGTAGCTTTGGTGGAACTAGAAGTAGACTGTATAGACGAAGGGGAGCTC
AAAACCACTGGTATTGCAGAAACGGAGGGCACCTGGGGTCTCTCACGGTGGAGCAGCATGTTGTGACGTC
CCTTGCACGTGAAACAACAATGCCCACTCGTGCAATCCCTTATCTGGAGCTGTCTGACGAAGCAGTTTAAG
CACAACTTTTGTTGTTTGATGACAGAAAGTCTCTCGGGTACGGACATTTGTAAAAAGTGGGCATAGACGC
AACGGATGGTTTTCCTTCTGACACAATTCGCAGTGTTGTGTCTTTGTAGTCACTGTTTCCTCGAAGGTTTTG
CAGGTATTTATTACTATTTATTTGTGGGCTTGGACTGAAATGGCGTATGTGCGGTAGGTCGTACATCCGCT
ATCGCCTCCAGGTGCGATATCGGTCGATCAGAATTAAATTCATCTCGGACCATTGAGGTATCTCTGCCTTA
TTGTGGAGCGATTGTTCCCATAGTGNCAGCAATTTATAGGAGCACAAGAAAATGAGTAGACAATCCC
>9_rnd-1_family-332__LTR___Gypsy
CAATTGAAGGATAGCTTGACGACTGCCCCTGTGTTGGCTTGTCCCGACTTTTCCGCCAAGTTCGTCTTGCA
GACTGACGCGAGTGAGTATGGCCTCGGCGCAGTGCTGACGCAGACGATTGATGGCCAAGAGCGAGTGAT
AGCGTATGCGAGCCGGAAGTTGCTGAAGGCCGATGTGAACTATTCCGCCACTGAGAAAGAGTGCCTGGCA
ATAGTGTGGTCGATCCGGAAGTTGCGCTGCTACTTAGAAGGGTACCGGTTCGACGTGATAACTGACCATC
TAGCACTGAAGTGGCTCAACTCGATCGAGAGTTCGTCCGGAAGAATCGCGCGATGGGCGTTAGAACTGCA
ACAGTTCCAATTTGACGTCCATTATCGACGGGGGAAGCTCAACGTCGTGGCCGATGCGCTATCGAGGCAT
CCTCTAGAGACCTGTCAGCAAGCAGTGGTGGCCGAGGCCCCTTGCAAATGGGTCGCGAGTGTGCGTGCCA
GAATCGCCGAAGAGCCATGCAAGTTCCCGGATAACATCGAGGAAAACAAACAGCTATATAGGCATTTGG
GTCACCGGATAGACGATGAGGACTACATCCCCTGGAAACTATGCGTAGACAGTGGTCAACGCCAGAGGGT
ACTGAGGGAATGTCATGATGCTCCCACGGCCGGACATCAAGGTGTCAGGAAAACGGCGTCGAGGTAAGC
CCAGCGGTACTACTGGTCGGGTATGTTCCGCGATGCCACCAGATACGTGAGATGTTGCGAAGACTGTCAA
AAATTCAAGAGTGAGCAACTCAAACTGGCTGGTCGCATGCTGACGAGGCAGGTGGCAGAGCCGATGGCG
GTACTTTGCGCGGACTTCGTCGGACCCCTTCCACGGTCGAGACATGGAAACACGATACTACTGGTGTTCCA
TGACACTTTCTCAAAGTGGGTGGAATTGGTCCCTCTCAGGAGAGCCACAGCAGAACACCTGCAGACTGCG
TTTCGAGAGAGGATTCTAAGTAGGTCCGGGTTAGAAAGTTTGTGTGCGACAACGGGGTTCAGTTTACGAG
NCGGAGCTTCAAGTCGTTTTAGGGGTCCCTGGGTGTAGAGATCCAGTAAACCGCCCCGTACTGTCCGCAA
GAGAATCCCACTGAGAGGACCAACAGGACTGTAAAGACCATGATAGCGCAATTTATTGAGGGCCACCAG
AGTTCATGGGACGAACTCTTGCCCGAGATCTCGCTGGCGGTCGACACGAGCGTGGCAGATTCAACGGGTT
CTAGTCCCACTTTCCTGATGCAGGGCAGGGAACCTCGGTTACCCGCAGCTCTGTACGATCAGATGACTCCG
GGGTCCGCCACTAGTGCGCTCGACCGTAAGACTAAGGCGGATAGGCTAAAAGAAATTTTTGATATCGGGC
CGGTATTACAACAGGGCCGGTATTACAACTTGCGAAGACGATAGTGGCGGCCTTCGCTGGACTCGATGGT
CCTGTTGAGGCAGCACCAGTTGTCCAACGCAGCTGAAGGTTTCGCCGCAAAGTTGGCACCCAAAGATGAC
GGCCTCTACAGGGTCGTCAAAGTCATGTTCCCCAATATTGTAGGGCTGGTGAAATCAGGCGAGCATAAGA
AGAGGGTGGCCAACATTTCACAGTTGAAACCATTCTACCAAAAGNCGACGGGCGACGAAGAAGACGAAG
ATCCAGGGCACGTAAATGNCCACGACTAGGCGAAGGATGAGAGCGACTCCCGTCGCCCAACGAAACCAT
TATAATAAAGTTTAGATAAGACTAACACTAATAACATAATTGAGAATAGGCCTAGTAAAATGTGAGCCGC
ACAGAGAAGATAGGGCATTAACAAATAAGTAACGAGTAAGTTAACCAATAAGCTGAATTTACTTTACCGA
ATTTACATCTTGCCGACGCAGAGAGGCGACGTGACCGTCACCTGCTCGAAGTGAGGGGGGGGTGTGAGGC
GGCTAGTGAGGTCCGCCGTCCCACAGTTTTCTTGGCGAGGAGCCATCGTTCCCATGGCCGAATCACGGCG
AGGAGCCGCTGGCGGGACAACCCAACAATGTGTTTATAAGTCTTGATGGTATTAGTTANATAAGAGGCAA
GAAGCCGCCAATGNTACAAGTAGCAATTGTATTAGTTATATAAGAGGCAAGGAGCCGACAATATTATAAG
CCGCAGTGTATTAATTATATTATAATCACCAATTATATTAGTGAGTAAGGAGGCGAGGAGCCACGAAGGT
TATGGGAGCGAATTTGAATTTAACCGCCGGCCGCCGCTGCAACCGTGCGTTGTTGATCGGTTGCTCAACAC
CGCATTCAAGAGCGCAACTCCAATTCGGCGGCGAAGAGCGAAAGCTTCGAGGCGAAGTTACGAAAGCTC
GCGGCGAAGTTGAACGGCCGAGGCAAGCGCATGGCTTGCTGGCGATCGCGCCGGGACTTTGGAATGAAC
GCTAAGGGTGAGTAGACGTGCATTGTGAGACGAGGAAGGTCATAGAGTGTACATCGGAGCGCTAGGGCT
CCAGAAAACAGTGCGGGTTCAGGGTTCAGGGCTCAGCTCGTGAGGTACTATAAAGTAGCAATAAGCGAG
GAGTGAATCAAGTACATTTGTTATAATTATCGAGAGGAAACCGGATAATTCTTTGTTTTTTGGTAACCTGG
CTTACCTTAACATCGGAATTAACCGGCGTTCGAGCTAATCTCGATCAGGAGCATCAGATTGTGGCCCCGCG
GTGGGCATTACCGGTTGGTGTTTCGATAGAGTAGTGGTACGAGGCCACGGCCCGATAGAGCGTTTGGGTA
GTGGAGTTGGCGCGAGGCTCTGGCCCGATAGAGTAGTGGTACGACGCCACGGCCCTCAAGCGTAGTTGGT
ACGAGGCCACGGCCCGCTAGTGTAGTTGGTACGAGGACACGGCCCGGTAGTGTAGGCGTGCAAAAATAG
CTCTAGGCTTGGTCTGGTCAGGANACTCACCCCCCTTCCCCCTTAAGCCCTCCTTCCCAGCGAAATTGAGG
GGCAGAGGGCGGAGTACTCAGGCAGCCAACCCGTAGCCCCGGGTGTCTCGAGTTCGAATAGTGCAGAAT

GGGCGAGGACGGCACAGTCCCCGGGGGACATCTCAATCCGCTCCGGTCTGCTCCCAGCGGATCCGGAAC
GACCGTTGTGGACAACAGGGTCTCCGCCCCACCCGCTGCAACACACGCAGAGGTGGCCCATCGGATGATG
AGCCAAGAGGCCGGGAACGGATATGATCCGCGACGCGCGTGTCGTAGGTGCGCACAGGAGGGGCACTTC
GCCAGAGAATGTCCCAACGCCCCCGTCTTATTTTGTTGGGTATGTGGAAGCCGGGGCCGAAGTAGTCAGG
ACCAGGATCGGGTCGGCGCCGATACACACGGAACGCAGTAGTAGGACACCCGCTGAACGTGGACGGTGG
CCGGATCGTCGCTACGGTAGAGATCGGCGGACGAACGATGCCTGCCACGATCGATACTGGAGCCACTCGT
AGCTTCATGAGTGAAGACTGCGTACGCAAGCGGACGATTCGGGAAGAGTGGAGGAAATACAGGCACGCA
TCCGCCTGGCGAATAGGTCCACCATAGAGGTGGAGAAAGCCTTGAGAGTAGATGTCAGCTTAGGGGAAA
AAAGATCAGCATGACGATTCTCATCATGCCCTCAATGTTGGACCACGTAATCCTGGGTATGGATTTCTTGG
GTGCAATGGGCACTACAATACGCTGCGGCGAGGCAGAACTGGTCCTAGAAGCAGTGACAATACCGGGCG
TGGGGCCAGGATCGAGCGAGGGGTCGGAGAAAGACCTCCCAGGTCATGAAAGGGAGGCACCTGCTCGGC
TGCAGAGAGAGTCGGAGAAAGGCTTCCCGAGGGGAGCACCTGCTCGGCTACAGAGGGAGCCGGAGGAAA
GCCTCCCAGGTCATGAAAAGGGAGCACCTGCTCAGCACCCGATGGAA
>160_rnd-1_family-328__RC___Helitron
AATATATATATATTCAAACTAACAATTAAATTATCAAATAAGTTAATATTATAATTATTTTCTTTTTCAGTA
TCCATCTTAAGTTTCGATTCATACTTAATTATTCTTTGTTATCTTAACATAACTTTAATTTTCGAAATATTTG
GGAATAATTTCTTCAGTTTTTAAAAATGCCGCGCGCGTTTAAAAAATCCGCTAATCAAAGGCGCCAAGGT
GCCATTAATCGTAGTCGTGTCTACTACATAGAGAATACCGAAAGCGTTTGCCTTCGAAGTTCTCAAGGTTA
AGCTGACACTCGTGAGTGCAACGGTGATATTCGTGAAGTAGACCGTAATACTGACGCCCTGGCCCGATCT
AAAAGAAGAAGAAGTGCTCGATTTCGACAGGATGAAAGGGATCTCGATGCAGCAGCTCGCGCAACGCAA
AGACAGGATGAGGATATGCGAGCTCTGGAGCAGACACAAAATACAGCGGACCACGCTGTTCGGCGACAA
AATCTGGAGTACCGTACCCTAGAGCGCGAGCGCGATGCAGAAGCCCACGCTACAAGGAGACAGGACTTA
GAAATNAGACTGCAAGAGCAGGAATTGAACACAGTGGAACACACTGTGCGTCGCCAAAACGTGGAGTAC
CGCGAATCAGAACGCCAGCGAGATGCAGCAGCCCACGTTACAAGGCGACAGGATTTAGAAATAAGACTG
CAAGAGCAGGAATCGAACACAGTGGAACACACTGTGCGACGCCAAAACGTGGAGTACCGCGAATCAGAA
CGCCAGCGAGATGCAGGCGACAGAACTTAGAANTGAGAGTGCAAGAGCAGGAAATTAACACAGTGGAAC
ACACTGTGCGTCGACAAAATGAAGAATATCGTGCATCTGAGCGGTATACGTGATGCTTCTGCTCACGTAG
CGCGAAGACTGAATGTTAATGTTCGAGAGCTAGAGCAGGACGCTAATACAGTTCAACATAACGTTCGTCG
GCAAAATGAAGCATATCGCACTAGAGAGCGTGAAAGAGACGTCTTAAATCGGCGAAATGCTCGTCAAAA
TATTATCGCAAGACGCAGAGAACAAGTCGTCAATTCCTCTCAAAGACGAGTTACTAGAAACCAGGATCGT
ATAAGAGAGAACGAATTACGAAATATGGCTGAAGCTCGAGTTATAAACTTTAGAAATTAGTTTTCAAAAT
CGTGCCATACAGTCAGCTCAGCAATCTCAGAGAAATAGAGATGCTAGAGAAGAAATGTCAACTGAACAG
CGCGAGCAACAGCGTGACTTGCAAAGACAGCGTAGAGTTTTTTCTGCCAGAGATAAATACCTGGAAAATA
TTAAGAAAGGCCCTACAGAAATATGCATATCATGCGGAGGAACTTGGTTTCCTTTCCAAGTAAAGCTTTTG
AATAAAATAAATCTATCGGCAAAGTTTCCAAACATGGATATTTCAAAAGCATTTTATCTGTCCAATAAGTT
CCCAAGTCTATCTGAAGACTATTTGTTTTGCTCCACTTGTCGCCGACACATATCGTCCGGCAAGATTCCGA
GTCTCTGTCTGTCCGAGGGGTTCAACTTTCCTGAGATTCCAGACTGTCTTAAAGACTTAACGTGTCTTGAA
GAGCGTTTAATTTCGCCAAGAATCCCATTTATGCGGATAATTTCTCTTGGCTATGAGCGGCAATGTGCAGT
TAGAGGAGCTGTGGTCAACGTACCAATTTCTGTTTCGGAAACTGTGACAGCTCTTCCGCGCACCTTTGAAA
ACAGTCATGTAGTACAAATTCATTTGAAGCGTAAGCTGGAATATGCGCATAATTTCATGACTGAGACAAT
TAGGCCCGCTCGTGTGCTCGAAGCGTTGCGGTATTTAATAAACACAGAATTATATCTTAAGCACAATATTT
CAGTTGCTTCCAACTGGCTAGGTGTAAATGCTCACAATGAAGAAGTCCCTTTTGTTGCTGATCCAGCTGAT
ATAACAGCTGTGGATAGCCTTCTTCGCTGCCGAATGGAAACAGATTCAGAAGGACACGANACTTCTGATC
TTAATCCAGGTGGACAGGAAACTTTGTTGGATAACGAGTCAATAGAAAACAATGCTATAATTGGTCGTAT
TGCAATGGCTCCAGGTGAGGGTCGACGACCAATTGATATGACGACAGATGAGGATGCTGAAGAATTATCG
CTCCCAACCATTTCATGTGGCGAAATTTGTAAAAGCAAAAGTACTTATTGTCGAAAGGCCAAATCACAGA
TTCGGTATTACGATNGTCGTTGCGCGAAAGTACCAAAAATTCTGTATATGTATAAGTGTTATGAGCTCACA
CGCATAAAAAGTTCTATTTCTATCTGCCTTCGGAAAAAATCAAGAAACGCCAATATCACCGTTGGTAACTT
GAGAGATGAAGGTTTTGTCAGCAATCTTGTGCAGCATGATGACGGATATCATGTTCTTAAGGGCCTGCGA
TCTGCTCCAGCACATTGGGAGGCTGAGAAGAAAAAAGTGCTGGCAATGATTCGTCAGTTTGGACTACCAA
CTTTTTTCATAACGCTTTCTGCTGCTGAAACCAAATGGAGTGAGCTGCTGGTCATTCTATCTCTCATTTTGG
ATAATAGAGTCCTTAGTGAGGAAGAAGCAGCGAATCTTTCAAGTTCCGAAAAAGCTCGACTAATTCGGTC
TGATCCTGTGACTTGCTCGAGATATTTTGACTATAGGTTTAGGCAGCTGTTGAAATTATTTAAAACAGATA
TTTTTGGAGAATATCAACTCTCTCACTTTTATTGGAGAATTGAATTCCAACATAGGGGATCGCCACATTCA

CATGGCATGTATTGGCTAAGCAGCGCTCCTAAACTTAATGAGAATAGTTCCCAAAGTAGCCGTCAGGTTA
TAGAATTTATTGACAGATTCGTTACAACAAACGGAGATAATCCAGATTTGGAAGAGGTAATTAAGTACCA
ACAGCACAGACATAGCTTTTCATGCCAAAGAGAAAAAAATAGGCATAAAATTTGTCGTTTTAACATGCCA
TACCCTCACATGCCTCAGACTGAGATACTATACCCCCTAGAAGAGGGAGATTCAAATCTGAGAAGGCATC
AAGACATGTGGAAGAAAATCAGAGAGCTACTGGATAGTTCAGTTTCGGGCGCTGAAGCCTTAAGCTTAAA
TAATTTTGATAGTTTTATTGTACTGGATATAAATNCGCACTGAGATCGAGTCTTAAAAAGCCGCAAATATT
TTTGCGTAGAAAATACTGTGACAAAATGTTGAATGCATATAATAAAGATATATTGAAGCTATATAGAGCC
AACATGGATATTCAATACATACTAGATGCGTTTGCTTGTTGAAGTTATATAATAAACTACATAAACAAATC
GAATAGAGGGGTTTCTCAATTATTGCGAGAAGCCATGAGCGAGATAAGTAGTAATAACATTTCAGTTAAG
CAAAAACTACAACATATCGGCAATAAGTTCATCAATGGAACAGAAATATCTGCGCTGGAGGCTTCATACA
ACATCCTTGGACTCCGTATGTCAGGATGTAGCCATGGCGAGATTTTTGTAAACACTTCGCATCCTGATCAA
AGAGTTGGTATGGTGAAAGCCCAGAGAGATTTAGAAACAGTACCTTCAGATTCAAAAGATTATTTCTTAC
CAAACTTGCTAGATCATTATGTTCAAAGACCGGACGAATTAAATAATTTGTGCCTAGCTGACTTTGCTGCC
AAATTTAAATATTCTAAGTCGCTAAATGCCCATTCAACAGGAGACTACGAGGAGGAAAATTATGATGAAG
AGCGGGAGGACAATGCCAATGACGGGAGTCCCATGGTACTCAAAAATAGATCAGGAATTTTGAGAGAAC
GTAGGCACTCTCTTATTCTTCGGTGGAGAAGGTATAGCATAAATATATCCCGATCTGATTATTTCGACAA
CTTGTAA
>Ref_dvir_463
ACACGATTCGGATGTTTAGTCGCTTTGCAAATTTCACATTTATTAATATGCTCTTTCACGTCTGAAACTAAA
TTTGGCCAATAATAATATAGTCGTAATCTCTCTAGTGTCTTATTCATTCCACAATGCGACGCTAAGGGATC
ATCATGAGCTAATTTTAAAGCATGGGGTACCAACTTCCAAACATGTTATCGGCAACTCTGTCTCCTGAGGC
ATGCTCGGACCTTCGATATATAAAGCCATCGATAACTTTGACGTCCGGCATTTGAGTCAGATTCTGCAAAA
CTTTAGACTTCAACAAGTCATAACCCTGGGATTTAAAATATGGAGACTGCATATCAATAAAGTCATCGAC
ATCTAAAGCCGATAAATCCTCCGTGTGAGTACGGGATAAAGCATCTGGTACAATATTCTTACTGCCCTTCC
GATGTTGTATAGAAAATGTATATCCTTGAAGCTTGAGAGCCCATCGAGCTAGACGACTACTGAGATCTGC
TTGATTCATGAGCCATTTAAGGGATGCGTGGTCGGTAATACCAGTAAACGGATGGCCTTCGACGTAAGCT
CGAAACTTGGATGCTTAAAATTGCTGCTAAAGTAGGTGTATGCACACAGGGGCTTGCACATTTCAGTTTTA
AGCGCCTGGAAAGCTTGCTCTGCCGCTTCTGACCATTCAAACTTTTTTGAGTTGTCAGGGCGTCAGTCAAT
GGCGAAACTATAGAAGTGTAATTTCGAATGAACTTATGGTACCACCCAGTCATACCGAGAAAGCGACGCA
ATTGTTTAAGCGAGCGAGGCGCTGGATATTCGGTAATGGCAGTTATCTTATCAGCATTCATCCGAATAATA
CCCTGACCAACAACATGACCATGATAATTAACTTCCTTTAGACAAAACTTGCTTTTTTTCAAGGTTAATTG
TCAACCCGGCATCCTTTATTTTTCCCGCTAGTTCACTTAACACGGCAAGATGACGGTCGAATGTATCAGAC
ACTATAAGAAGATCATCTAAATATATAAACACTTCATTTCTTAAGTAAGCTGGAATTATCCTGTCCATCAA
TTTGGACATCGTAGCAGGGGCGTTAGACAAACCAAATGGCATTACCGTGTATTGGTAAAGGGGTCTACCT
GGTACTACAAACGCTGTTTTGTCACGAGAACTGGGTTCTAAGGGTATCTGCCAATAAGCGTCTTTTAAGTC
GAGACTGGTAATATATTCAGCCTTAGGAAGACGACTAATAATTCCATCAATTAGGGGCATCGGATAAGCA
TCCTTTCGAGTAACCTCGTTAACTTTTCTGCAATCAATACAGAGACGAACCTTTCCTGGTTTACAAACGAG
CACTACTGGGGAAGACCAAGAACTCTCCGATTGTTCAATAACGCCAAGGGAAAGCATACGATCAATTTCT
TTGAACATTAATTTCTCCCTTGCAGGAGATACGGAATGTAATTGCGTTACGTACATCTATGACATGAGACA
GTTAGATGGTTTTTCCTAACTCTTTTTCCGCAAATGAAGGAAATAGCTTCACACTTTCTGGCAAGACGTTG
CTGTTTGTGTGCAGTTAAAGCTCGCAGCGAATCCTCAATGGCCTTAACAAACAAGGAGGAAACAGCTCAA
ACGTTGACCAAAGGTCTAGGCCAAATAAAGCGGTGGAATAAGAGAAGGGACTATATAAAAGTAATTCG
TTTAACCTTTCCACCATAATAAATTTCTGTGCGTAACCGTTTCAATATTTATTGGGTTTTTTCCATCGGCGG
TTTTCACCTGTGAAGTAATCGGTTTATACAGTACTTTTGTATTCAACAATTCCACAGCGAAACTACCACCA
AAACAACTTTTCGACGCACCGGTATCCAATAATCCTGTAACAGTTTTTCCCAATACGGTTACTTCAGCGTA
AGGCCTTGAATCGGTACACACGATCGCTGACACGAGAGCTTGTTTCTCGATCTTACATCGCTTAAAAAATT
TATTCGACGTAATCGCGCTCGTAATATCTTGCGATTTACAGAAGTATTTTTATTAACAGATTGAGATTGCA
AACGAGTATATAAGGTAAATTCTGGGTTAAATGACAAGTCAGGTAAAGATTGATTAGAGGATTCTAATTA
TCCCTCTAGTCGGTATTTGTTGCCTTGGATGCAACTGGTTTGCGTGTACCGGTCAATGGTACACGAGACAG
GTAGTTTTTTGGCACATTGTTACACTTGGTACAAGTTGGCTTGTAAGTGTTTGTCTGACCACAGCCATAGA
AAAACACTTTGCGTTCGGCGACACAATCTTGATACCGATGGTTCCAGCAGATTAGACTGATGGCTGATAC
ATCATTATCGAGAAGGTCTATCGCATCGGACTCTGCTTCGCTTTCAGTTTGTACTTCTATAGCGTGCAATG
AGGGACGAGGCGCAGGGCGGGGTGCAAACACTCGAGCTTTAGAAATTGACTGAATAAAACCTTCCCGCCT
TTGCACTAAATGTCTAAGTTGCGCGATAGAATGTATGGGCTCATAAAGAAGCTCGTGCTGAATTTCTAGCA

GCAAATTAGAGCGCAACGATTCTAGCAAGGAGGAATTCGATAAGGGTAGAGACAGTTTGTCGTTCAATAT
AATAATTGCATCGTAAAAGTCATCGAAAGACTCACCCTCTTTTTGTTTACGACGATCAATTGCCGCACGTA
TGTCAACATCAAGTCGCTCTTCTTTGAACTTCTTCTGAGCACGTAAAGCACACATAATTCCGACCATAAGA
TAATAGGGACACTCTTATGGTAGCGCCAGAAGAAATCGTTAGCGATGCCTTCAAATAGATGGCTTATATG
ACGAGCCAACAACTCATTATTTCCATCGAATGTCTCGGCAGTTAACGCCTCGACGCGATAGATAAACTCAT
CTATACCCATGCCCAATCGACCAGAAAATCTAAGTTTCCAGTTACTGATTATTTGGCTAAGTCGGTCTGGA
CGCAAGTTTTCTAACATTGGAGAGCTCCTAGTTCTAGACTCGTTTCGTATAGAATTAGCCGGTCAGGGCTG
AGGATGATCGACAATACTCATGTTCGAAGTTGGTTGCTGAGCTGTGGAGCTGCCTAGCTGTGCTGTAAAA
GCAGACAAAAGATTTTGGAATCCTGACTGTTAAGTTTGGGTCAGGGAGGCTGTTTGTCTTTCAGTGGCTGC
TGCCATTGAATCAGAAATACACGAGAGCATGCATCTGGCTCGGCTGCCAAAGGCGGAACACTTGGCGGAC
GAGGATTCTTCGACATGGTGCCTTGGGACAAAATCTCTTCTGGGGGGCCTTGACTTCGGGTTCCAATTGGC
ATAGTAACTATCGTCGGTTGCTTTGCGAGGGCGTCATCTACCACACATGGGTTAGACGGTGAAATTGTTGT
TTTGCAAACAGGGCAAATATTTTTCTTTACTACTCTAATATTATAGCATGCACGATGGAATGTGTGCTCGC
GACTGGTAGCTACTAATTAGAAAACTCAATCGTTTTAAGTCAAATATAGCGCAGTTCCTTATTAATCGAAG
CAGCTGCTAAGTTTTCGCAACTGCGATGTATTAACATTTCTGACCTACGCTTAAAATAAAATACTAACAAG
AATTAAAATACAAAAAAAAGGTGCAAAATCGATTAACAGATCGAAAATATAGAACAATATTTCATAAAA
ATGTGTTGATGCCATGCAGTCGTTTTCATGAAAGGTTATAATCTGCATCAGCTTCAAAACTGATCAACTAA
TACAATGAAGGGTATAGGGATGAGAAAACAAAAATTTAATAATTAAGCGGCAAGTGCCAGTTTCTATATC
AAAGAACACTGGGACTCCCAGCAGTAA
>9_rnd-1_family-312__LTR___Gypsy
TATATAAGTTAAGCGGAGTCAACGAACGGTTGGCCAGAATATGACTCCGACCACAATTGCTGAATAAGCA
TTGGGCCGGATGTCGTTGCATAGCCGGCAGACACAGCATATTTGCGTGGCCGCTATGAACGAAGAAATAA
NTTAAGAATCATATTCTGTTAGCTTTAAGCCCTAAGAATTGTATCGCATGTTTTTAGTTCTGAAATAAACG
TCGTTAGCTCAACACGTCCGTACTCGGGCACCAAGTCGTCAGCGTAAAACATAATTGCCAAACGGGCAAC
AAATAAATATCACTTAATCAATACACTCAAAAGACTCATAATTGGCGCCCAACTAGTGGTAGTTACGATT
ACCCACGCGATTGCAAAGAAAAAAATTCTTACAAAGTGAACATTAAGTCGCGGTCACACCAACAGTGCCG
CGCAGTGAACATTACAAAAATAAAATAATAAAAATAATAAGCTAATCACTAATAATAATAATAGTGACA
GTGAGTTAACAAATGCAGTTGCGCAGTGAACACTACAACAAGAAGCAATAAGCTGCAGTTACAGTGAATT
AGCGATCATAATCTCGTGATTCACAAAGGTGCGCTCAAGCGCAGTGCGATTTACATATATTTTTACACCGA
AGCCGCTGGAGCTCTTCGGGCAGCGGAGACTGCGGCGAAGGGAGCGACTACGAATAACAAGTGCGTGAC
TGCGGCGTTGGACCCGCAAACGTGGCTGGTGTAGTGACTAAAATAAATCATAAAAATCATGTAAGTGGGC
ACTTTAGCCAAATCTGTTATTAAATCTGATCGAATAAAAAAAAAAAAAAAAAACAAAAGAAACAAAGAGA
GGAGAAATCTTGCATGCGTTATATTTTGCAACAAATCATCTGCTAGAGTAAGAACAAAAGTTTTTGAGAA
TTCGATTTGACAGCAGTAAACAAATTTTGTAAAAATAAAAAAAAATTACTTTGTGTTACTTTTTATGCCAC
TCTAAAAAAATTGTGCTGCAACGGCACATACTTTCTTGCTTATGCGCTTCTCGTGGTTCAGAGCTGAGGCG
TAATTCGACCCATCTTTCGACTTTCTGTTTTGTTTTTGCGTCCGCTAGACCGCGCGTGCATTTATTTGTTTTT
GCTTTNAATGCCCATGAAATCGTCTTTCGGTCAGTATAGGACCCCGCAAGAACTCTAGCGACTCGGATTCT
GACTGTGACAACCGCATCAGAATCCCGGCTCCACGACTTGCCCCTACAGTCCCCCCAACTTCGAACAAAA
TGGATCCAGAGCAACTTAGAGAGATAATTCAAACGGCCATAAGCAATGCCCTAACAGAACAAGCCAATG
CAAACCAGCAAAGAGAGCAGCTATTAGTGCAGAGAATTAATGAATTATCCGAACAAATGGCAGCAGCAA
ACGTTGCTGCCCCACAAGTAGTAGCGTATGCTGCTATTGAGATTAGAAATGACATTCGCTGCGACGAGCC
ACTAGACGCCGTCAAATGCTTACCTGAGTTTGCAGGAATACAGGAATCTTTATGTTTCTTGGAGGCAAGCG
GCATTAGCCGCCTACCACATTTTCAAGCCATATAATGGCAGTTCTCGGCACTATCAGGCCGTTATAATTAT
AGGAAGCAAAATAAGGGGCCCCGCGGATGCGGTGCTGGCCTCATTTGGCACTGTGATAAATTTCGCATGC
GATAACAAATCGCCTTGATTTCACATACAGTGACAAAAGGCCGATCCATGTAATTGAACAAGAGTTAGGC
ACCTTGAGGCAGGGCGGCTTGCCCCTACTCCAATATTTTGACGAAGTGGAGAAAAAACTAACCCTACTGA
CCAACAAGGTCAATATGTCTTACGAGCCCGTTATGGCAAAAGGTCTTTGTGAGAAGTTCCGTGACGACGC
GTTGCGCGTTTTCGTATCGGNGCTTAGGCGCAATCTCACGGATGTGCTCTTCGCAGCCAAGCCTAGAGATC
TGCCTTCGGCACTGGCGCTTGCTCAGGAGGTGGAGTCAAACCACGAAAGATATGCTTTCGCGGCTAATTTT
GCCAAAAGCCAAGAAGAAAAAGAGCGTAGGCCGAATACANAGACACAGGGCCGACGGCAAGATAGACA
TTCTGAGCCNCAGACAAATGGCGCAAAAAACCCTCATTTCAACAAACAGCAGAAACAACAGGGTCAGTC
CGATCAACGAAGTGGTCGGCAGCAAAACAAATGGTGGACAGGAACCCATGGAGGTAGATCCTTCATTTTC
AAGGATCCTCCAACCCACACAGGCACCGGGGTATCTGAATAGAAAACAGGCAACATCTGATCACACGGG
CTTTCAAAAAGGCAACAAAGGGCGAATCATGTAACGCAGGACGCGGAACAAGGATCGCGAACGTATGCT

AATGCCGCCTCTAGCGCAGAGGCGCAAATTGAGGATGACGTGAGCGCTAGCTNTGATTCAGATGCTGTTA
ATTTTTTAGGGGTAGATCCCTGTTACCCGTCATCGGGCGAAGAGTAGCAGGGGCCGATATGAAATTCCTC
ATAGACACGGGCGCGTCTAAAAACTTTATCAGACCACATGAAGGGTTAAAAGGCGTCCGACCAGTAGATT
GTCCATTCACAATCCACTCGATTCATGGTACAACTAGAATCACGCAAAAATGTTTTGTCTCAATGTTCAAC
ATAAAGGCAACATTTTTTCTTTTATCGGACCTGTCAACTTTCGATGGAATAATAGGCCTCGACCTGTTAAC
ACAGGCAGGGGCATCGCTTTGCTTAGCGTCAGGTCAGCTTAAATGGGGTTCAGAAATTGAACAAATTTCT
TTTCACGCATGCACTAATGTCAATTTCACTAACGTAGATTGCTCAGATGCACCTCCTTTAGTAAAGAACAA
ATTCCTGAAACTGCTAAAGGACAGGAAAAGGGCCTTTGCAGNNCCCAATGAGGCCTTACCATACANTACG
TCGGTGGTGGCCACTATAANAACAATAAATGAGGAACCCAAAAGCTTTACCCATATCCTATGGGGGCGGC
TGACTTCGTCAATAAAGAGATTCAAANCCTGCTGAAAAATGGCGTCATACAGAAATCAGTATCTCCCTAC
AATAATCCTATTTGGGTTGTGGATAAAAAAGGTACAGATGAGGCGGGTAATCGAAACATGCGATTAGTAA
TGGACTTTCCCAAACTTAACGAGGTAACGATACCCGATAAATATCCCATGCCAAATATTTCAATGATATTG
GGAAACTGGGGAAAAGCGAAATTTTTCTCNACGTTGGACCTCAAGTCTGGCTACCATCAAATCATGCTCG
CAGAANGCGATCGTGAGAAAACTTCCTTCTCAGTAAATGGGGGAGTATGAGTTTCGAAGACTCCCGTTCG
GTCTGAAAAATGCAGCCAGCATCTTTCAGNGGACCATTGACGATATCTTGCGGGAGCAGATCGGCAAATT
TTGCTATGTCTACGTGGATGACGTGATNATCTTCTCTGAAGATGAAGAGTCACATGTCAAACACGTAGACT
GGGTCCTGAAGAGNTTATATGAGGCAAATATGAGGGGTGTCAGTAGAAAAATCACGCTTCTTTAAGAAAA
GTGTGAACTTCCTTGNGCTTATAGTCACTTGCAACGGCGCGACAACAGACCCGGAAAAGGTGAAAGCCAT
ACAGGACTTCCCAGAGCCGAAAACGNTTTTCGAAGCCAGGTCATTTCTGGGTCTCGCAAGTTATTATAGAT
GTTTCATAAAAGACTTCGCAGCAATAGCAAGGCCTATTTCGGAAATTCTAAAAGGGGAAAATGGAACAAT
CAGTAGGCATAGATCGCGGAACATTCAGGTGCAGT
>9_rnd-6_family-1385__LTR___Pao
ATAAATCGAATATCCGTGCGATTTTTCTTAAAAATAACAATTTATACAAATAATTCTATGACAAAAAACCT
TCCATTGAGCTGCGTTTAGAATAAACGTACTTCTTCTCGAGCTCAACAANTAATAAGCAATAAGCATNTTT
TTTTTGCGCGATCCGTTTCCACTACGCGGCCGCCTTGTAAAAAGAGAATTAATCCTTTTCTCTGGTGTTTCA
ATAAAGAAATAATTAGATTTTTTCTCAAGTAAAGGAAGCTAAATAAGCATCAATTTAGTAAATTATTAGT
AAAATAATTAATTTAACAAAATTTTCCGATTTCGTTGGACTCAATAGTGAACACCATTTCGGTCATTCCAT
TTATTCTGATATCTCGATTCCATCGTTCCTGTGCCATTTCTCATTCCGTTTTCGCTCGGCTGATTCAANTTAT
TTGTATTTGATCACATCTCACCCGGTTACTACAATTTTTGGCTCTGTGAAGAGCCTAGTATGCCAATCGGT
GGTGATCAAAACAGCCANAAACGACTCACAGCACCGTCCGNTAAGGAGTCAAAGCGACCTTTAACCTCAC
CTAGAGCAGTTTTCGGGAAACTCAAGGTCGAGGGTACCAGCACAAACCGTCCGCAAGNCTGTGAGTCCGA
TTCCAACAAGATACGACTACGCAAAAGATGCAGAAGTCAAGCGAAATGGCACTGAACGACTTCGTTTCGA
TAACTGACCGACTAGGCCAGTTTGAAGTGAAGATCAATGACGCAGCGGACAGTGACGTGTCACTGTTCAC
TCTCCAAGTCCGACGAGATCAGATTAAGACTTTAATGGGAAAAGGTAGAACGCGAGTACGAGTTATGTTC
CAAAGCTCTCAACCAAGGCGCTTGCAGCAGCAGTGACCTGGTGGCTATGCAAGCTAAATATGATTACTCG
TACTCCGTTTACGAGCGATGTGCGGCTCAACTAAGTGAAAAAATAGAAAGAGCCANGGCTTTAAATAGAC
AAGCTGAGCAANCCCCCTCCAACTTCTCCATGTCCAGGGGTTGCAGTCTCCCGCCATGCGACACTGAAGTT
TTCAGTGGGGATTACCTACGCTGGCCTACTTTCCGAGACTTGTTTACAGCGGTTTATATTAACAATCCAAG
ATTGACGCCGGTGGAAAAATTGTTCCACCTAAATTCCAAAACCAGCGGCGAAGCTAGGGCAATTGTCTCC
AAGTCCCCGCTCACGAATGACGGCTTTCAGTCAGCGTGGGCGAGCCTGACGGAGAGATTTGAGAATAAGC
GACTATTAGTCAATAGTCAATTGAAAATTCTTTTCAATCTCCCCTCAGCAACACAAGAGTCTGGAGCCGCC
CTTAAAGAGCTTCAGGGCACAATCCAGGGATGTTTAACGGCTCTCGACTTGTCCAGGGTGAGCACCGAAA
ATTGGGATTGTCTGCTTATTTTCTTGTGCTCCTCTAAATTGCCTAAGCTCACGCTGTCGTTATGGGAACAAT
CGCTCCACGATAAGGCAGAGATACCTCAATGGTCCGAGATGAATTCATTTCTGATCGACCGATATCGCAC
CTTGGAGGCGATAGAGGATGTACGACCTACCGCACATACGCCACCCCAGTCCAAACCCACAAGAAAAGA
GTCCAACCACCTGAGAACCTTCGAAGCAACAGTGACTACAAAGTCACAACACTGCAAATTGTGTCAGAAG
GAAAACCATCCCGTGCGTCTATGCCCACGTTTTCTACAAATGTCCGTACCCGAGAGAGTTTCCGTCATCAA
GCAACAAAAGTTGTGCTTAAACTGCTTCGCCAGACAGCACCAGTTGAAGGACTGCACCAGCGCGCATAGT
TGTTTCACGTGCAAGGGACGTCACAACACGCTGCTCCACCGTGACAGACCCCAGGTGCCCTCAGCTTCCA
CAACACCAGTAGTTTTGAACCCTCCTTCGTCTATACAGTCCACTTCTACTTCAACCNAAGCTACCGTTCAA
AACTACTTTTCCGCAAACTCACATGGTGTTCTCCTCGGCGCGGCTGTCATCCAAGTGTGTCATTTGGGCAC
AAAGTATACAGCTCGGGCACTAATCGACTCTGGGTCNGAAGCGACCTTTATTTCAGAGCGATTGTTCAAA
AGGGTTAAACTTCCNTATCAACCCGTCGACGCACAGGTCTCCGGTTTGAATCAAGCTATTTCGGCTCAGCC
CCAAAAACTCTGCCATTTTAATATCGGCTCTCCGATAAAGCCCNGGGTTCATATTGAAACCTCGGCTTATG

TTCTTCCAAAGCTAGCAGGCAATTTGCCCTCCTACACCATCCCCCAAAACGCTTTGGAAGAACTTCCAAAA
TTACACTTGGCCGATCCCTCTTTCTATAGGAGCTCACAGATAGACGTGCTTCTCGGCGCCGATATCCTCCC
ATCCATATTGTTAGGTGGCTCCTATCCCAACATATGTGGCACTCTCCTTGGTCAAGAAACGATTTTCGGCT
GGATCCTTACAGGCCCCGTTTCTAAGACCAGTGCCAAATCGGTCGCATCCTTTTCGACTCAAGTCTCGCTG
AATCCAAATGCGAGCTTGGATAAACTGCTCACAAAATTTTGGGAGGTGGAGGATATTCCAGTAAAGCTGG
AAAAGGAATCCGACCTGTTTTGTGAGCGCAACTTCCAAAAACCACTAAAAGGGATAAAAACGGCAGATA
CTTGGTCACACTACCATTCAAGAATTCCGATCACATCCAGTTAGGCCATTCAAGGTCCATCGCACTAGCTC
AATTTCTACGAAATGAGAATCGTCTGGCAAAAAATGCCCCCTTAAGGAGCAGTACGATGCGGTGATCCAG
GAATATCTGGACTTAGGTCACATGCGACAAGTCCCTCCAAGCAATGGTTCGGATAACTTTTATCTACCACA
CCATGCTGTTCACAAGCCTGACAGCACCACGACCAAGCTCCGCGTAGTATTTAACGCCTCCCACCCGTCAT
CGAATGGTAAAGGTCTGAACGATGTGCTACATGCAGGGCCCGTTCTGCAATCCGACTTAACCATTCAAAT
TCTAAGATGGCGCTTCTTCCGATATGTCTTCAATGCCGATATCACGAAAATGTATCGGCAGATACTTGTGC
ATCCCAAACACACGCCCTATCAGAGAATTCTGTTCCGAACAAAGGACAACGAGCTTCGTGACTTCGAACT
CAGCACAGTCACGTTTGGAGTTAACTGTGCCCCTTTTTGGCAATCCGGGTACTACATCAGTTATCTGATGA
CGTTCGTTCGCTCTATCCCTTAGCAAGTCACATTATCGCAAACAATATGTATGTCGACGATGTACTAGCGG
GAGCCCATTCCAAATCAACGGCAGATCAAGCAATTGCAGAACTCCGCAAAGCCCTTTGAATCTGCCGGGT
TCCCCTTGCGAAAATGGACATCCAATGAAAAGGACTTGCTAAAAGCGATTCCCAAGGACCACTTGCTTCG
CGCCGACTTCTTAGAGATCGAAGAAGCCTGTTCGGCAAAGACTCTCGGGATCAGATGGAAAGCTGACACC
GATGAGTTCTTTTTCGCTCCATCCGAGCTAACAACCAAACAGTCCGTCTCCAAGCGTGAAGTCCTTTCACA
GATTGCCAAACTGTTCGATCCAGCAGGTTGGCTGGGTCCAGTCATTATTCAGGCAAAAATATTCATGCAG
GAGATCTGGCTCCAAGAACTGGGCTGGGACGATCCGCTACCTGAGAGTCTTCTCAGTAAGTGGAATTTAT
TTCTGCAAGAGTATCCGGGCCTCAGTGCGA
>160_rnd-5_family-9448__LTR___Pao
GTACGCAATAGAGTCCCGGGTATTGCCAATTCTCCCTTGCTGAACATCACTGCCAGCCAAGATGTCCTCAG
CCTCCCTTACCTTCATCGTTTGAGTGAACGTAATTTGGAGGTGATACAGCAATTCAAGAAAGACGCCGGA
ACTGATCAAGCTTATCGTGTGGCATTCATCATTGGAGCCATATGCTGTGCTTTAATCTGCATTGGCCTCACT
CTTTGGTGGATCATCGACGCAAAGAAGTCTGCGAAGCAGTTACGAGAAGTCGTCGCCGGGATTGGGTCGG
CCGAGGACGGCCTTATAATTGAAGGGGGAGTAGTTACCACATAACACTTAATATAGGATATTTCATAAGT
TATGATCAACCCTCGCTATCGCGTGCTTCATAAGTTAAGGAACAGCCCCCGCAATTGCGACACTGCATACT
TTGTATCAGCGCGCAATAGCTGCAATGGTCAGCGGCGGTAACCGGACACTTTTGTTTGCTGCAAGAGTTTC
CGTTATTGCCTAAGATTTTGCTGAGTCGGCTTGCCGACCAAGACATTGTGGCGTGATGCGAGTTTTGGCTT
AGCTTAAGTAAAATTTTCTGTGCATTAAGTTTTTCAGTTTTGTATTTGACGTTTAAGAATAAACTTGCACTA
CTCCGGCACGCTGCCGTAAATATTATTTGTTATATATTACAAAAACATTGCTGAGCCACAAGGCCAGTAAT
TAGCAAAGAGAGTGAAGTCAAACTCTCCACCATAATCGTCCTAAATATCACTGGACTAGACATCGCTGGA
CGAGAGACGGATCAACCATCGCCTCATTGGACCAGCAGATCGACTTAATTTATACATGATTCTATTAGCTA
ACAGGTATGTTGAACCAAAGAGATTTTCTCAAAAAGCAGAATTGTGATATAGATTTTTATTGATTTATAGA
CTCGTACTGTGCCAGCATTAGAAGAACCTATGTTCACACTTCAACTCTTAAAGTTCATATAGACGGGCAGG
CGGATGGACAGGCAAGCGGACATGTCTACATCGATTCGGCTAAAAAATAGGTTATTATACTGTGTTTGTA
GCATTACTTATTTGTGTGCCACATTGGTAAACTTGTTTAAATAACTGAGTAAATGACCGATTCTCGAACGC
GAACAAAACGAAGTCGACTATGGACTAGTCGATAAAAATATGCCTGGTCCCATTCTTTTACTCTAGTTGTA
CAGTCAATATGTTCTATGGTTAGCGTTTGTATCAGTTGGTTGCCAGTTATTGAGATTGGTGTATTTGTGCAA
CTTGAGAATATTTTTGTTCAAATCTTGTCAAATTTGCATTGGACAGGCAGGCAACAAACAAAAAAAAAAC
TGAATTTGTGCGCAATGTTGCTTGTGCGGACACATACACATGCATAGCCAAAGCAGCTTCCCGTAGTGGG
AGCGAACGAAAAACACATAAATCAAATGAATTTCTTACTCTCTTGCTATTTGAACAAATGACCAAGTCTC
GGATCCGAACAAAAACCAAGTCGACTTTGGACCAGAAATCTAAAAAATGAATGGCCACAAGCATTTGTTC
TATCTGTCCCATCTATATGTTGTACGGCAGTGGTAATACCCAACCTAAATATTTAAGTTTCATTTAGCTAAT
TGTTGAAAATATAATATAAGCATGTAATATTCACGTATTTATATGAATGCGATGGGGCGCAACATTTTATG
TTATTTGTATAGTATCTGCTTTTGGTACTAAAAATCCTTTATAAGTTTTGTTTTTATTGCCTATATTATTCGG
CAATCGATTGAGTGTATCATTCAAAAGTGTTGTTATCTTCATCAATGTCTGATCCGTTTTGTGTGATTTCCT
GAATAATTGATTTGTATATTTTCTTTTTCTTCTGATGTGCCCTTCTATAATTATATTGTGGTCCGGATTTCTA
TTTCATATCGTTTAATGTCTATTTGTCGTATATTTGTAATTATATGCCCTGAACCCATTGAAGATGGTTAAA
ATGGATAGAATTTATTTATGCAACTGTATGTATCCGGCAGAAGGTATTTTCTCCTCTCGACCTCTTAACGT
ATATATTTTTTTTAATCAGCATCAGAAGCCGAGTCCATCTACCCAAGTCAGTATGTCCGTCCATTCGTCCGT
ATATCCGTCCGTAATTATAAACGTAATAATTTCAGAAGCCTTTTGATTTTTAAACTAATCCTTAGGTATTAA

GCTAGCTTCTTCTGTAGCTTCTTGTATAGCATATACAGATTAAATAGTGTTCGGCTCAGCAGCAAAAAAAT
AAACTTGTATTATGTTAGAATAACATTTCAGTTACATATTGCCCTGCGAGGCAAGCAATGTCACTATTTTG
TTCGCTTTGCGCTCATCGCAGTTTCTGTTATCGCGCTTTNTTACCTTTTGCACTTTGTCTTAAGCGGCTAGC
GCATCAATGCTAGCGACGCCGCAATGCACTCTTGTCTCGTATGTTCGCTATATCGGATCTCTTATCGATCG
CTTCGCTAAAAATTGTAAATTTTCGCATATTGTCAATACGCTCGCTATAAGCATATAAATAAANCAACCCG
TTTGCATGAACTTATAAGAAAAAGCGGAATTTTTATAACATCCTTAACTTCTCGCTATTAAGTGCTAATAA
AAAAGCTTGTTAGCCCAACATTTTGGTGACCCCGACGTGATTTCTTTTATTTAACATTATANTGTTGCATAA
AATTATTTAAATTAAAATTGAGCTAGTTGTTACTAGTTTCGGCAGCAATTAAAACCGCTCTTATATACATA
CATATGTACGAGCAAGTGTCATGCGGTAAATTGCAGTAAAGATGACCCTCGCTATTTTTACTTGTGTTTTG
ATTCGACTAACTGTTGCGCGCGCTTAAGAGTACAGTGCTGACAATAAGTCAACGGTCAACGAACTTTCGT
GTGCAGTTTTANTTCGGTTTTCTTTTACCTTTTTTCGTTTNCTTCGGTTGCCATGGAANGTTCTAGTGCTGAA
TCGCTGGCCCAGCCTGCAAACAGGGTGTCATTTTTAAAGCGCAAGGTGGCGTCCTCGTATGAGCGGTTGC
AGGCGCTTCAATCTAGCCTCAACCAAACGCCCCTTGCCAATAATGATGAAATTGCATTGTCGGTGCTGCTC
AAGCGGATTGAAAAAATGGAATCTGCATTCGAAGACGCTCAAAACAAATTTGAAGAACTAGACTTTGACG
AAATCGGCAGTGAGTTACGCGAGGCTTTTGATGAGCTTTCAGTAACTTTGGAGACTTCGGTGCGGTTTGAA
ATCGCTAAGCGCAGTAGAGCATTGCCGTCCAGCTCCACATTTGTAAATGAGCCTATTGCTGCTGCGTCAAT
GACTAGATCCAGAGCGTTACCGACCTNACCGGCCAATCAAATTGCCAACGTTTTCTGGAGGTTATACGGA
GTGGTCAGATTTCTTTTCGATGTTCACAACTTNAATCGAAAATCATCCCGAACTCACAAACATTGAAAAAA
TGCAGCATTTGCGGCTCTGCTTGAAGGACTCTGCGCTGGATACAATTCGGTCATTGGAAATATCAGAGGCT
AATTATTCCATAGCATTAAATTTATTACAAAAACGATTTGATAACCGTCGATTAGTATTTCAGGCACACAT
CGTTGAACTGCTGGGGCTCAAGAAGGTAGAGAAAGGCTCCGCTGGCATGC
>160_rnd-5_family-9379__LINE___I-Jockey
GCTCGTTGCATTCGGACGTGCGCGCTTGACAAGTTATTTTCGCAAGTGATCAACACAATATCTTTACCTCC
TTATCTCTTTGTGCTCTTCTTGCGCTATTTTTAGCCACTGCTTGCGGTGGCGGCAGCTTATTGCGGTACTGT
ACCTAAGATAAGCCAGACTTAAACGGTGCTAAGTTTGCCCCTCGCCCTGCGCNCTGCGCAAGCGAGAGGT
TGGTTCCGTTAATTCTTATCTTTTGATCTGGAAAAGTGGACGACAATTGCATTATTTGACTCTAAATAATA
ACAATTGACGNTATGGACAATAACTCATNGAATCAGTCGCTACCTAGTAATAGGCAAGCTGTACTGGAGG
AGATGAGAAAAGCAGCAAGGGTAAGCCCTGAAACTCTACGCTCTCAAATCCGCGGTAGCGTAGCATCCAT
TTCTGCGGAAAACAACAGCTTATTGCCAAATCGGGTGATAACTGGCCCGACGGTAACAACAAGCACTGNT
ACCAGCGTTACCTGCACTATCTCTATGTCGCAAGCAAGTTTGACATCTACATCTACGTATACTGTAGCGGC
AACTGTGGGGCCTAACATTTCCTCGACTACGTCACTTGACAACACATTGGGTGAAATCTTGTCAGCAGCAA
CTCGTAAGACGAAGTGCTCGTTTCAAGACCGCGGCCTGCCTGCTAGTAAAAAACATAAAGGAGTTATAAA
CACNTCTAAATCTATACAGAAAGCTAAGCCGAAGCCCGCTGAGCTGCACAAGGAAGTAGCCAAAGACAC
GGCATCCACCTCGACTGCAGCAGCACTGAGTAAAAACCGTTTTGCAGCTCTGGACGAAGAAGCGGACAAC
ATGATGGAGCAAGGATTGTCAGAAAGCGATTCTGACGACGAAGATGAAGCTCGTGCTGTTGGCAACGGA
AACAAACAATCCTCAAAAACGCTCTCAAAGCCTCCAGCAATATGTGTACCACAAGTCAACAATCCCTCAG
GCATGGAACAAGCACTAGACTCGTGCGTGGGTGCCAACAACTATAGCATTCGCACCTCTAAGTTCGGCGT
ATCACGGATATACACCGCAAACTCTGACGCTTTCCGAGCAGCTGTAAGACATTTGACAAAACTTAACTGT
CAATTCTGGCACCATCAACTTAGGGAGGAAAAGCCTTACAGGGTTGTCGTCAAGGGCGTTAATGCCAACG
TCCCCAAACCACAAATTGCGCAAGCTTTTACTGACCAGGGTTTCGAAACTCTCAATATATATTGCCCAAGA
AAGGCAGATTGGCGTAACGTTCAGGTCAACGAGACAGACGATGAAGCGACGATCAACTACAAGACTAGG
CAAAATCAATTTTTTGTCAATCTAAAACAAGGACCCGATGTGTCTAATGCTTTGAAAATTACACAACTCGG
CAGATACAGAGTCTCAGTGGAGCGTGCTCCTCGCAGAAAAGAAGTTCTACAATGTCTGAGATGCCACACC
TTCGGTCATTCCAAAAATTATTGCCTCCGGGACCCAATCTGTGGCAAATGCGCTGGCTCTCATATGACTGG
TTCTCTGCTTTGCACGAGTGAAAATTTCCTGTGCGTCAACTGCGGTGGTGATCACGCGTCGACCGACAAAG
ACTGCCCCGTCAGGATTGAGAAAAGCAAAAAAATGAAACCGATCCCAAGGCTCCCTGCTACTGGCAACAC
TGCCAGGCCAAAATATAACCAAGGCCCNTCACGTGGCTTCATCCCGGCTGAGACTGTCAGATGCAGTATG
TCTTACCGCGGCGGAATCTGGTCCAAATGCTGACAAGCATCTTCAGCGAGAANCAAACTCACTGCCTGAC
AAGAAATTGGATGCGCTGGAGAGCTCCATACGGGATCTCAACTCCAGAATGGACCAATTATTCAAGTTGG
TTCAAGAGACTATGGAGGCTAACAAGGCCTTCCGAGAATTGGTCCAGGTTCTCATTTCAAGTATCCAAAA
ATGATGCAACCGATCATTAAAATCGGATTGTGGAACGCACGGGGACTAGTTAGGGGCTCTGGAGAGCTCC
GGCTGTTTCTCAGTGCAAATAATATAGATGTAATGTTTGTCACAGAGACACACATGAAAGAGGGACAGCG
TGTCTTCCTGCCAGGCTACTCCACCTACCATGCCTTCCACCCCAGCGGTAACAGCAGAGGTGGCGCCACAG
TCATTGTGCGCTCCAGCATGTGCCACATTCCTCAGCCTCCCATCTCAACAAACAACATACAGCTAGCAAGA

GTGAATCTGCAGACATCGGAAGGGCCCGTCGCTCTAGCATCTTTGTATCTTCCTCCCAAAGAGCCTTGTAC
TAAAGCGGAATTTGCAGGTGGTGACTACAACGCCAAGCACTCATGGTGGGGCAACTCAACAAATTGCTCC
AGAGGCAAAATATTGCAGGATGTAGTGGCGAATGATCGGTACCAAATTCTTGCAACGGGTGAGCCTACAT
TCTACTCATCCAACTCCCTAGTGTCAACATCAGCGCTAGATTTTTTTGTCATTAATGGATACGCAATGAGC
AGGCTTAACGTGAAAACGCTTCATGAGCTCTCTTCGGATCACACACCACTTATAGCTGATTTACACGCCAC
TCCGATAAGCAAGTCACAGCGCTCTTGTTTACTAGCACGCGGTGCTGATACAGAAATTTTCAGGGCCCATC
TGGAGCAGCTGGTTGATCTAAATATGGAAATTCAAGACGCAGATGATATTGATAACGCAATCGATATTTT
TATGAGCAAGCTGAAAGATGCTGCAACAAGAGCTGCTCCTAGGAATCAACAACATCTGCATGGAGATAG
ATCGCCTCAGCTTCCTCCTATAATCGTTGAGCTCCTGCGTCTCAAGCGTAAAGTAAGAAAGGAGTATGCAA
GAACAGGAGATGCCCGAATCCAACAGATTCACAACAGGCTGGCTAATCGTCTTCACAAAGTTCTGGCTCG
CAGGAAAAGAATTCAAATTGACAACATGTTGGAAAACTTGGGTACCGATGTTTCTACTAATTTCTCTCTGT
GGAAAATTACTAAGAGATATAAGACGTTGGCAACACCGAAATCGGCTATTAGGGACCCTTCAGGGAGCTG
GTGCCGCACTAGTCTTGACAAAACGGAAGTATTTGCAAACAATCTCGAGCAACGATTCAGGCCGCACTAC
TTTGCACCCGAGAGTCACTGCAGGATAGTTGCGGAGGCATTGGAAACACCGTTTCAAATGGCTTTGCCTTC
AGATACTGTGACACTGGACGAAGTGAAGCAGCTGGTGTCAAAGCTTAAACCCAATAAGGCCCCTGGCGA
AGACTTACTCGATAACAGAACCATAAGACTCCTACCAGATCAAGCACTGTTGTTTTTGGTGCTTATTTTCA
ACAGCGTCCTACGTGTGGGATACTTTCCGAGAGCCTGGAAGTCGGCTAACATTATTATGATCCTCAAGCCT
GGAAAGCAACCAACTGATGTGGACTCTTATAGGCCGATCAGCCTTCTTCCTTCACTGAGCAAAATAATGG
AGAGGTTGATTCTAAACAGAATGCTGGATCTCGAGACTGTTACCCTTGCGATCCCTAAATTTCAATTTGGA
TTTCGCAAGCAGCATGGCACTCCAGAACAGCTGCATAGAGTTGTCAACTTTGCGCTAGAGGCCATAGAGA
AGAAAGAGTATG
>9_rnd-5_family-1804__RC___Helitron
AAAAATCAAGCTGCATAGTAGCATTTTGTTGTATGGCGTTACTCATCTTAGTGTTTATATTGTCTTTGATGT
ATATATGTTCTTGATCAGAGCGACGAGCTGAATCGATTGAGACATATTCACCTCACCGTTTCTCTGTCTGG
TGGTATGACATATACGTACTATATATATACATATGTGCAAATATTGATACATTTGCGTATATACGTACTAA
TACTATTTAAGCCAAACTCTNCGACCAGGAAGAAATGTATAAATGATGTTTAATAATTATTAATATTATTA
TTGAGAACATTATTAACTTATTTACTTGAAGTTTTTAAAATAAAATCATCATTTATTTTGGTGGTCTTGATT
TCAAAACATAAATAGTGACGCGCCTTACGTCCAAAAAAGAGCACGGACATTTTGAAGCGGCGAANTGAC
AAATTGAGAGGGCATGCTTTGCAGCAAATTTTTAAGTCATCGATTTTCGATTGTCGCTGTAAAAAGTTTCA
CTGAGAGCCCTAAAAANAATTGTCCCATGGAATTAAATTTTAAAATGGTAATCAAGGAAGGAGTCGGTTT
TGACCCCATAAAGTATATATATTCTTGATTAGGACGGCAAGCTGAATCGACTAAGTAATATCCGCCTACCT
GTTTTTCTGTCCGGCGGTATGTCATATGTATNATATAAGTTATGTTCACATACATATATAAATTATATATGT
TCAAAAATTGGTCAAAAATTACCTATACATGGAAGTCCTATCGTAAATCNTTTTTTAATCTATATAAACAA
ATTAAAATATGTTGTTTTTATGTTTAATAATATTATTTAATTTAATAATATATATCTACACTTACGACCGAA
GCGCGNATGCNTTNAGTTGTACANCCGTAATATGAAACTATTATATTATTGATTCGATAAATGATCTTTTG
CATTCAAATGTCGCTGGAAGTGCGCGCAATGAAAATGTTCAGTGAACTTTCAAAGGGAGCTTTTGAAAAA
AGCGAATTCGCCTATTGTCAGGCGAGACAAAACGAAATTGCCATTCACTCTTCAACCAACCATCGATTTGT
GCGGTCGTCGATTCTCGATTGCGATTACGATTCTAAGAATTTGCGTTTTCTTAGATTTAAAACAAATAAAA
CTTAATATTCGTTTTAATAAGATCCAGTGCAACTGAAATTAAACAATTCTTCGGTTATTTCAGTGCTTTGAA
GAATATTTAATGTAACAATCTATAATCGGTGGTTTGCCGCGNAAAATCGNCAATTATTCCCCGCCAGTGCT
AGTGAACATTTGTGTGTGTATGTGTGCGTTGACTCGTCGTTAGACCTCACCTCCCCTTAGACTAACAACCT
GTGTGTGTGCGTGTGTAAACATTTTGTGCAAGAGTGTGAGGCGCACTTGCTAGCGGTGGACCAATAATAC
GTGAGTGTTGGTGTTCGTGTAAGCGTTGTGACTCTGGCATTGCTTTGCAATATATTTTCTGGATGTTTGCAC
TACACCTGTTGTACTGGATTTTTATCTAACTGGAAAAATCGCAAGTTTATTCGAAAAGCTTAGATAGTCTT
TTTAAAAACTTATTGATTTATAATATTTTCCTTTTTCAATACATATTTATATATATTTAAATATAATAATAT
ATATANAATATATATATATTCAAACTAACAATTAAATTATCAAATAAGTTAATATTATAATTATTTTTCTTT
TTCAGTATCTATCTTAAGTTTCGATTCATATTTAATTATTCTTTGTTATCTTAACATAACTTTAATTTTCGAA
ATATTTGGGAATAATTTCTTCAGTTTTTTAAAATGCCGCGCGCGTCTAAAAAATCCGCTAATCAAAGGCGT
CAAGATGCCATTAATCGTAGTCGTGTCTACTACATAGAGAATACTGAAAGCGTTTGCCTTCGAAATTCTCA
AGGTTTAGCTGACACTCGTGAGTGCAACGGTGATATTCGTGAAGTAGAGCGTAATACTGACGCCCCGGCC
CGATCTAAAAGAAGAAGAAGTGCTCGATTTCGACAGGATGAAAGGGATCTTGATGCAGCAGCTCGCGCA
ACGCAAAGACAGGATGAGGATATGCGAGCTCTGGAGCAGACACAAAATACAGCAGACCATGCTGTTCGG
CGACAAAATCTGGAGTACCGTACCCTAGAGCGTGAGCGCGATGCAGAAGCCCACGCTACAAGGAGACAG
GACTTAGAAATTAGACTGCAAGAGCAGGAATTGAACACAGTGGAACACACTGTGCGTCGCCAAAACGTG

GAGTACCGCGAATCAGAACGCCAGCGAGATGCAGCAGCCCACGTTACAAGGCGACGGGATTTAGAAATN
AGACTGCAAGAGCAGGAATTGAACACAGTGGAACACACTGTGCGTCGCCAAAACGTGGAGTACCGCGAA
TCAGAACGCCAGCGAGATGCAGCAGCCCACGTTACAAGGCGACAGGACTTAGAANTNAGAGTGCAAGAG
CGGGGAATTAAACACAGTGGAACACACTGTGCGTCGACAAAATGAAGAATATCGTGCATCTGAGCGTATA
CGTGATGCTTCTGCTCATGTAGCGCGAAGACTGAATGTTAATAGTCGNGAGCTAGAGCAGGAAGCTAATA
CAGTTCAACATAACATTCGTCGNCAAAATGAAGCATATCGCACTGGAGAGCGTGAAAGAGACGTCTTAAA
TCGGCGAAATGCTCGTCAAAATATTATCGCAAGACGCAGAGAACAAGTCGTCAATTTCTCTCAAAGACGA
GTTACTAGAAACCAGGCTCGTATAAGAGAGAACGAATTNCGAAATATGGCTGAAGCTCGAGTTATAAACT
TTAGAAATAGTTTTCAAAATCGTGCCATACAGTCAGCTCAGCAATCTCAGAGAAATAGAGATGCTAGAGA
AGAAATGTCACCTGAACAGCGCGAGCAACAGCGTGACTTGCAAAGACAGCGTAGAGTTTTTCTGCCAGA
GATAAATACCTGGAAAATATTAAGAAAGGCCCTACAGAAATATGCATATCATGCGGAGGAACTTGGTTTC
CTTTCCAAGTAAAGCTTTTGAATAAAATAAATGTATCGGCAAAGTTTNCAAACATGGATATTTCAAAAGC
ATTTTATCTGTCCAATAAGTTCCCAAGTCTATCTGAAGACTATTTGTTTTGCTCCACTTGTCGCCGACACAT
ATCGTCCGGCAAGATTCCGAGTCTCTGTCTGTCCGAGGGGTTCAACTTTCCTGAGATTCCAGACTGTCTTA
AAGACTTAACGTGTCTTGAAGAGCGTTTAATTTCGCCAAGAATCCCATTTATGCGGATAATTTCTCTTGGC
TATGAGCGGCAATGTGCAGTTAGAGGAGCTGTGGTCAACGTACCAATTTCTGTTTCGGAAACTGTGACAG
CTCTTCCGCGCACCTTTGAAAACAGTCATGTAGTACAAATTCATTTGAAGCGTAAGCTGGAATATGCGCAT
AATTTCATGACTGANACAATNAGGCCGGCTCGTGTGCTCGAAGCGTTGCGGTATTTAATAAACACAGAAT
TATATCTTAAGCACAATATTTCAGTTGCTTCCAACTGGCTAGGTGTAAATGCTCACAATGAAGAAGTCCCT
TTTGTTGCTGATCCAGCTGATATAACAGCTGTGGATAGCCTTCTTCGCT
>9_rnd-4_family-1038__LINE___R1
CACACACACACACACACACACACACACACACACACACACACACACACACACACACACACACACACACACAC
ACACACACACACACACGCACACACACACACACACAGGGCACACGAGTGAAAGTGAAAGTGAGTTGACGC
GTCTCGACTGTCGCTCCGGCTGTTCTCTGTCGTGGGCCGCAGCTCCGGCAGGGAAAGCAAAGCGATGATG
TGCAATCCATTGGCGAAGGGGCTGAGCACGTGGAGACCCACGCTCACATACGCGATTCGCAAGCGGCGG
ATGCCAGGGTGCGTCAAACCAAAGATGACTACATACGCACACGGAGAGACGCAGGTGGCACGACTGGCA
CGACTGGTGGTACACCTGCAAGACTGTGGAGGTGAACACGAGAATGGAGCAAACCCGCGCACTCACCTG
GATGACGGGGTGATGGCGAGGACTACCTGATGGTATATATCCAACCCTAAACCTGATCCAAGTAAGCAAG
TTGAGCACTTTCTCAGCGAAACTATCATGGCGACATTGTTTATGCTCAAGCACATGAGCAAGACAAAAGC
TCTGGACAGCTGGTTGGTTGTACAGTGCTGCCAACCGAAGTACATGCGTCAACGAAAAAAGCAATTACGC
GCGAAAGTATGCCACGCTGTGTGGGTATACATGAGGACAAAGGAAGATATTGCATGCTTTTAGGCTTTTA
CGTTTTACTCGCATTGCAGCTGAAGACTCGAGGCAAGAAATTGTACAGAAGGCCACGGATCCGACCAAGT
CAACTCCTGCCGCATAGCGGTGTACTAAAAGGGGCGCTGGCGACCGAGCTGGAGCGGTTAAACTCCCATC
TGTCAAACGCCAGTGAGAAACCATTGGCCACCGTTGGAGCCACAAATCAGCAGCGATGAGCGCCACAACT
GGCAGCACGCCCGTCAATGCAAGCAAGCAAACTCCACCCCCACACTGAGCCCAGTAAAGTGTGGAAGCG
ACCCACTGGACCCCCCTTACGTAACTGTAGAAATGGGCTGACGAGTCGGGCATTCTAGGACCTTAATAGG
CAAGCTGACACCTTGTCGAAACGGCTCCTAGGCTAATGCTAGCCTCGCCCCCGTCCCGCTAAAACCTTGGC
AGGCCTCGTAGAACGCTTATCGTATGTCGCCATTCAGTTGGCCGTGCAGGCTCAGTTGATATTCCCTGACT
AGCACTGATCTGGCTCTGAACTCCGTCTCCCATAAGGACCGGAGTCAACCCTCGCGTGGACCTCACTCCAT
GGAAAGGCAACCACGGGATCGCGAAGGTGACTGCACAATACACACGACAACAACGGCTTCGAGTTGGGA
CCCAATTAATCACAATGATGAACCGAAACAACAAAAGCACCACCCCACTAGGTGGACAAGAGGAAGAAG
GAGTACGCNAGGCCGACCCGTTCGCTAGGCGATCATGCCTGGCGAGCACGCCCCCGGCTACACCAGTAAG
GGCCCCTCAGCCCACTGCACCATGCAGGGAAATTGAAGAGAAAGAGACCCCGAAAAGGCTTAGGGACCC
GGCCAGCCCTACGAACAAGCCACGGAGCACCCCGCCCAAGAAGAGCAAGGCAAATACCCAGGAGATCGA
AGAGATTGGCTCGATACTGGACGAGCTACTGTTCATGTATCATGGCAAGCAGCCAGCGATGAGGCACATA
AACTTGGCCACTAAGAGCATGCTAGCGAGACTAAAAGAGCTGCAAGAAGTAGTCAAAGCGCAGCTCACG
GAAAACCTACGCATGCGATGCAGCGAAGAGTCCCATAAGCAGATGAGCCATGCTGAAGAAAGCGCCGGA
AGCAATTCTAAATGCGACACGGCAACGCAGACGGAAGAAAGCGGTGCAGGCAGTAAACCACCGACAGCG
GCGCAAAACCGACGAACGAGGTTACAGGAAAAATCAGATCTTGGCCCCAAGCAACCCAAGCCGGCACCA
ACTCGCAAGCAGCAACGTTCACAGCCGGCAGAGGGCAAGGTACCGACCCAAGCGCGAGAGGCAAAAACA
CCGGTCAAAAATACCCGAGAGGTAAAAAATAAATGGACTGAGGTGAAGGGCAAGCTCACCAGGCGGAAA
CAGCCCAACCGACCGGATGCGATCCTGATCANGTGTACAGAGAGCATGCCATACGCAGATGTGCTCAAAA
TGGTGAAAACAGCTCCCGCACTGCAGGCGCTCAAAAATAATGTACAGGGCATCCGCAAATCAGCAGCAG
GAGAACTGATCCTCAGGCTCCAGAAGCCATCGGACCCAGCTACCGACCAGTTGCGTGTGGCAATCAGCAG

CGCACTGGCTGGAAAAGCTGCCGTTAGAACCTTGCAGGAAACGGTGCAAATGGAGGTTCTAGATATCGAT
GAGTCCACGACAGCAGATGAGGTCCTCACAGCTCTCTTTGCCTCGGTGGAAGGAGACATACCAGTAGAAG
CGACGCCCACTATGAGGGCGGCCTACGGAGGAACACAGATCGCATCGCTGGCCCTGCAACCGAGCCTAGC
CACGAAACTGCTCGAAACCGGCAAGGTCCGCAAGAGCCAGCATCCGGTTGGAAAGGAGACGTGCTTTAA
ATGCGCAGGTACTGGGCACAAGTCTGGACAGTGCACGCATGAGGCAAAGTGCATCCTCTGCACAAGAATG
GGCACGGGCACTAACCAAGCCGCGCACTCAACTCTGAGCAGGAACTGCCCGGAGTATAAGAAGGCCTAC
AAAGCGCTTTAGAAATGGTTAAGTTCCTGCAACTTAACCTTAAACCACTGCAGAGCAGCCCAGGATCTGC
TCGCACAAACGACCCGGGAGACGAGAATTGATGTAGCCATCATTAGCGAACCGTATACACCCAAACATAT
CGGAGTGTGGCACGAAAGCCTAGACGCTGGAGCAGCACTATGGAGCTGCGGACAGGCACCATACCAGCT
CACTCAACGGATGGCTCGCAAAGGGTTTGCTAGGGCAAAATGTGGTCGCATATACGTTTATAGCTGCTAC
ATACCACCAAGCATACCCCTGGCAGAGTACACCAGCCATTATTGAAGACATCGCACAGGACGCGCACGGG
AAAATCTCCAGTGATAATCGCCGGGGACTTCAACGCGTGGGCCACAGAATGGGGCAGCAGCAGAACGAC
ACCAAGGGGAACCATCCTGCTGGACATACTTGCCTCGCTAAATGTCTGTCTGCTGAACATCGGCACAAGA
AGCACTTACAGCAAAGCGGGTCGCGAGTCGATCATAGACCTGACGTTTGCCAGCCCAGACGTTGCCCGAG
ACGCCAAATGGCATGTATCGGATGTATACACCCACAGCGACCACTTTGCGGTGATAACGGACACCTACCA
ACTTGGAAGCAGAGGCGCCAGTAGAAGGCTCCAACACACNGGATACAAGATGGACACTATGGACCTGGA
AAGACTCCTCCCTATGGTAGAAGGACTTGGCATAAGAGGCGACGCCAACAGCTCTGCTGACGCGATAGCA
GAGACTATCACCAACGCATGTGACGCTATACTGACAAAAGTTCGCAAGGGTGGAAACTCCCCAATNCNGA
CCGGCANCNCCGGCGGAACNTGGNCGNAGCCAAACCGGAAGTGCGCTCAAGGA
>9_rnd-1_family-346__LTR___Pao
TGCAAGCAGCCGTGTTAGGAACGAGGCTGATGGACACTGTCAAAAAGGAGCACGGTATAGCGATCAGCG
ACGCTATACTATGGACTGACTCCAAAACTGTGCTGCGTTGGATAGGCAGCACCCACCGTCGATATAAGCA
GTTCGTCGGAAATCGAGTGGGAGAAATTTTCGGAGTCAACAAAGGTTTCCCAATGGAGATGGGTACCTAC
TGCTGAAAATGCAGCAGACGACGCAACGAGGCCGCATTGCCACGTAGACCTCAGCCAACGGTCACGATG
GTTAGACGGGCCAACATTTTTGCGGCAGCCAGAAAGCAGCTGGCCGCAGTCCGAACCGGGCACGGAACA
CTCATCCTACGAAGATGAAGAAGAGATGCCGAGTGAATTTGCCTTAGTTGCAGCAAATAGCTTTTTCATTT
CATTTCAGAGATTCTCTAGCTACAGTCGGCTGGTGAGGACGACGGCTTGGGTTTTAAGGTTCACGCGCTGG
AGCCGTGGACAGAGGACTGAGCTTGAGAGATTCGGCCTCACCGCGACGGAGTGTGAGAACGCTGAGAAC
TTGCTGATACGGCGAGCGCAATGCGAAGCGTTCCCTGACGAGGTTCGAGCCTCATACAATGAAGAATCAG
TCGGTCATCGAAGCGACATAAGAGGGCTGGCGCCATACTTGGATGACAATGGAGTCTCGCGTGCGTATGG
CAGGATCGATGCCGCACTGTGTATACCGCATAGCGCGAGAAGGCCAATAATNTTGTCACACCGGCACGCA
CTGACGGAACTGATTGTGTATCACTACCATGTGAAAATGAAGCATCAAAACGTGGATGCAACGATAGGCG
ACATCAGGACCAGATTTTGGATAACAAAGCTGCGTCGANTGTCTGCGTACAGTAGTATNCAAACTACATA
GAGCGCAGCCAGCGNCACCTATAATGGGTCNTCTGCCAGAGGACNGACTGGAGGCCAATGGATGGCCAT
TNAAGTTTACGGGTCTGGACTATTTTGGACCACTCCTTGTGACGATCGGACGTCGAAAGGAAAAGAGATG
GGTCGCCTTGCTTACGTGTCTGACGACAAGGGCCATACATTTGGAGCTGGCACATGACCTGTCAACTGATT
CCTGCATAATCGCGATCGGGAACTTTCTTTGCCGTCGCGGACCTGTACTGAGGTTGCGCAGTGACAACGG
AAAGAACTTTGTTGGGGCCGACAGAGAAGCCAAAAGGCTCATAGACGTATTCGAGCCGGAGAAGATTCA
GGGTGAGCTATCTTCTAGAGGCATTGANTGGATCTCCAACTGCCCAGCGAACCCAGCTGAAGGGGGAGCC
TGGGAAAGGATGGTGCAGTGTGTCAAGAGAGTGCTACGCCACACAGTAAGGGAGGTGGCACCAAAGCAA
CACGTACTGGAGAGTTTCCTGATTGAGGCCGAGAGCGTTGTGGATTCTCGCCCGCTTACCCACTTGCCGGT
GTCTGTGGACCAGGAAGCCCCCCTAACACCGAACGATCTACTCAAAGGAGTGGCCAATCTGCCTGACACG
CCCGCTACCCACNNGCAGCCGAACGAGAGACGNGCCACGAGGAAGCAGTGGCGCACGGCGCGNCTGTTA
CGAGACCGTTTTTGGAAGCGGTGGGTCTTAGAGTACCTGCCTACACTTGTGCGACGCGTGAAATGGTGTG
CGCGCGTTGAGCCGATACGCCATGGTGATATGGTGTTTATATGCGACCCGGCCGTGCCACGCAGAGAGTG
GCGAAGGGGCATAGTGGAGGAGCTCTACCCTGGACCAGATGGAGTGCCTCGACGCGCCAGCGTCCGCGT
GGCGGACAACAACCGAGTGCGACTGATGACTCGTCCCGTGGCTAAACTAGCCATACTGGATGTGAGTGAA
GCAGGGCCTTCACGGGGGCGGAGATGTTGCGGGACGAATTAAGTTATCGATCGTTTAGTATTAAGTATAT
TTCTGAAAGTATGTATATTTGCCCTCCGAATGTACGGGCACACTGTATTTGGCTTGTTGCCGCCATTACTTA
AATGTTAAGCTCATTATTGTCGTGAGTTCGAAGGCCGTNGGAGGNAAGACTGCGGTAAGATTCCGAGGGA
ATATATATCTAAGTTCTAACCTATGTAATCCGTTCAGAATAAAACACTTGCACTAGCACGTTTTGCATTAC
GCTGTCTCTANTAACATCGGAGTAACATGGCCGGGGGCCAGGCGTAAACTTGCAGTGTTCAAATNGCAAC
TNCACCTTCGTTTGGCACGCGACACTTAGTAAAACTTTATCTCCTCTGAATAAAATTATGTCCTTAGTTTTA
AGTCATATAGCTCTTTATTTNTTTGTTTGCGTAGTTCAAGCATATTTTTGCTCTTTGGCATGCTACTTCTAAT

CAATACTTACTTTCTTTAGCGAAATCTTCTATGTTCTATTTAGGTTCTATATTACCTATGGTATCTAAGTATT
TCGGCAGATTACTAGTCTTACCAAAACTAACTCATATGGACAATAATTATGTGACACATTTATGATTTATG
ATGATGATGCCATGAGTCACAATGCACCGCATCAAGCAGGTGATATAGTGATGAGTACTGGACGGATGCC
GCAGCCAGCGGATCCTCACACAAGGGCACCGCAGTCAGTGGATGCTCAATTGACTCAATTGACAAAGGAA
CTTAAGTTGTGTCGATTCGGATAGAGAACTCCGACTTTTCCGCATATTGCTAGTGACGTTGCACGGGGCCA
ATAAGCTGATCGATACGTACGCGCTCCTTGACGGTGACAAACGCAAGCGTCATGTATTGCGAAATGTGTA
CGCTGTCCAACCTGAGCTTACCAATGAAAAGCCTGCATCGACGTCAAGTCCAAATGTCGGGCAACGGCGC
ACGACTTCCAATGAAACCGTATCGCGGTGCGGAACCAAAACTTCTGATTGGACTTGACCATGCACAGCTT
GAACTGCCAATGCAAACAAAAATATTTGCACCACAGGGACCATATGCCGCAGCCTCCGAACTTGGATGGG
TGGTTTTTGGACTGGTAAGAAGTCAACCGACGGCAACGTCATCAAAAACATGCCTCCTAGCAGTGTCGCA
GGAGGATGCAATCCAAAAGATGGTAAACGACTATTTTGATATCGAAAACTTCGCCACCAGTCGAAGCCAG
CGACGATGCACGAGCACAGAAGGTACTCGAAGACACCATGGTGAAAATAGGAGATCGTTACCAGACAGG
TTTATTATGGAACCGTGATAATGTTAAGTTGCCACGAAGCTATGATATGGAATACAAAAGGTTCGTCAAC
ATTGAGAGAAAGAGAAAGCGTAACGATCGGTTTAAACAAGCCTAAATGAAAATTATGGACGTTTATGTTC
ATAAAGGATATGCTCGACGATTGAAGCAGCATAAGGTCGCTTTGCAACAGCGACAAACTTTGGTATCTTC
CGCACTTTTGAGTTAAAAGTCCGAATAAGCCCGGTAAGANAAGACTGGTGTTCGACTCAGCATTGATCAT
GTCAACATTCGATCCTCTGGGATTTCTTAGCCGCCTCATGATAACTGGAAAGTTATTAATGCCTGAGATTT
GAAGACGAAATGTGC
>Ref_dvir_652
TAACCTGGCCGTTCCTGGGCAATTAAAGCAACTCGTGACCTCCGGCAGTGGCCAGGTATCCCCTGGGTAG
GCGCTGCACACCCGTGACGCGCCTAGCTAATACGAGAAGGCGACTAAGACTGAGACCAGTAACGTCTTAG
GTTGCAAACTCTGGACAACCCTACACGACAAGACGACCCCTGGGCTGACGGCTGACAGTATTCTAGCATC
TCAGTAAGCGGAGTGAAATCTGGCCGAAATGACTGCTAGGTTAATGCTGTTACCGCCCCCGTCCCGTTAA
AACATGGCAAATCTCCTAGCACGCTCCTCGAATCGTCGCCATTTAGTTGGCAGTATAGGTTCAGATGTGAT
ATTCCTGATTAACGCGGATCTGGCTCTGAACACCCGACCCCATGAAGGCGGGTGTCAACCCTCGCATGGC
CTCACTGCTTGCAAAGACAACCATGAGATCTAAACGCGACTGTACAACGACCAAGGACAACGGCTTAGGA
GTTGGGACCCAATAAAATGAGTACCAATGAAACCCCACAAATGAAGGAGAGCTGTGATGCTTTTGCAAAG
AGCAGCAGAATGGCGAGATCACCACTAGGTGTTCCAACAGCCGCCCCGACAGAGACGAACTCAGAACGG
GAGCCAACGACGGCAAACCAGAGCAGCTCAGCACTACGAGAACTCGGGCAGGAGATCTCAAAGCTGGTA
GTCATGCTCAAAGATGGAAAGCTCCATCCACCAGCCAATGCGACCGGTAGCCAGGCTACCGTATCAACGG
CGCAGGAGACACCGAGCCAAAGCGGATGGCGACAAGTGGAAGGAGGTCACATACAAGAAAAGGAAGGA
GGTCACCAGGAAAAACAGCAAGGGATTGCTCAAGGAAAAGGCGCGACCGGATGCTATAGTCATCTCGGC
AAAAGGCGAAGCGAGCTACTCAGAGATACTGAGGAAAGTAAAGCACGAAAAACTGGGCGAGCTCGGAA
AAGCGGTGACAAGAATGAGGACACAAAAAGGAGACCTCAGCTTAACAACTCGGGAGAGAATACCATAGC
ATTTGGAAACCTGGTAGGCGAAACGTTAGGCGAAAAGGCAGAGGTCCGGACGCTGTCCCACCGAGTGAC
CGTTGAGTGCAAGGACCTGGACGAGATCACGACCGAGAGGATATCGAGGCGCTGAGGGGTCAAATCCAA
ATACTGGACATAGCCCCTGAGAATATCAGCTCCGAAAGGCGTACGGCCAAACACAGACGGCGACGATAA
GACTGCCAGCGGGAAGCGCGAAAAAGGCCCTTTCAGTAGGTTTCCTGAAAGTAGGCTGGGTTAACTCAAG
AATCCGCGAACGGATAAACATCACCAGATGTTTCAGATGCCTCGAGTTTGGACATCTAGCAAGACACTGC
AAAAGTGATCTAGACAGATCGAATCTGTGCCGACGATGCGGAGGAAAGGACCATCTGGCTAAGGACTGC
AAACAAGAGCCACAGTGCATGCTCTGTAAGGACAGGAATGCCGACTCCAAACACATTGCCGGCAGCGGC
AGATGCCCCGTGTTTAGGAGCGCCCTGGCCAACAGAAAATGAAGTGGACACAGCTGAACCTCAACCACTG
CGAGGCTGCTCAGGCCCTGCTGACACAATCAATACGGGAGAGAAATACGGACGTCGCAATAATCTGCGA
GCCATACAAGCCTATCGCTGGAAGTGGGGCGGACATTTGGTACGCACCCACAAGCTGGAAACTGCGGAAT
TTCAGACCATGGAAGAAATATCAGAGACGCAAGAGAGCTCCTCTCGTCATAGCCGGAGACTTTAACGCCT
GGGCCGTGGAATGGGGAAGCAAGGAAACTAACGCGAGAGGGACCACCCTGCTAGAAGAGTTCGCGGGGC
CTTGCAAACGACGGGCAGGAGCTCACGTACTGCAAGGCCGGTAGAGGCTCTATTATAGACCTTACCCTGC
TCAGCAGCTCACTTTATAGGCTAACTACATGGAAAGTCAGTAATGAATTCACATTCAGCGACCACCGAGC
AGTGCATTTCGAGATCCACGGGAGAGGACGGAGAAAACAGCAGCCCAAAACAACAGGCCCCAAATGGAA
GGACAACAGCCTAGACGAGGAAACCTACACGGACTCCTTACGTCATGCTCAATGGACTTGGGCCACGGAG
TTAGCAGACGACATGGCGCGGCAGCTCATGGGAAACATCTCGAACGCGTTGCGACTTATCCATGCCCAGG
AGGATGCCAAACAGAAGAGGAGCACCTTGCTACTGGTGGAATGAGCAAATCAGCGACCTAAGGAAGGCC
TGCCTGCGTGCTAGAAGGCGCTCTCAGCGTGCCCGAGGCCACCCAGACTTTGCATCTAGGCTGGAACTCCT
GCGGGCAGCCAAGCGAGACCTCAAGAGAAGCATTAAACAAAGCAAATCTAGCTGCTTCCAGAAAATATG

CGATGAGGCCGATGTTAACCCGTGGGGCACGGCATACAAGATCGTCACGAAGAAGATCCGCAGCTAGGG
AACAACCCAGGTGACCTGCCCTACCGCACTTGCCACTATAGTGGAGACACTGTTCCCCCAACACCCACGA
CTGGAAGTAGCCAACCCCTCACTGAGCACGGAGCTAGTGCAGCAGGTGTCGGCAGACGACGTGTTAGCGG
CAAGCAAAAAACTAGGCGACAAAAAGGCTCCATGACCAGATGGGGTGCCAAACAAGGCGCTAAAGTTAG
CTATGTTAACAAAACCAGACGCCTTCGCCGAGACATATACTAGATGTCTACAGGATTGTGTATTCCCTAAG
AGATGGAAGCGACAGAAACTAATCCTTCTACCGAAAGGGGACAAGCCAATAGAGGAACCCTCGGGATAC
AGGCCAATCTGCCTACTGGACACGGCGGGAAAGGTACTGGAAAGGATTGTGTATGACCGACTACTTAGTG
TCATCGAAGAGAAAGAGGCCCTGTCCAAACAGCAATATGGATTTCGCAAGGCAATGTCTGCGGTCGATGC
AATTAAGGCGGTGGTAGATGTTGCCGCTAAAGCAATAGAGGGAACCAGATGGAGGTGGGGAGACAAGGA
GTATTGCGCGGTGATTACGTTGGACATCCGCAATGCCTTTAACTCAGCAAGCTGGTCTCGTATACTGGAAG
CCTTGGACCGAATAGAGATACCGTCCTCCCTTTTCCGTATAATATCCAACTACCTCTCCAATCGGGTATTG
AGTTATGATACGGATGACGGTGCTAGGAGTTACACGGTCTCAGCAGGGGAACATAATGTACGACGAGTTG
CTCAGAGTGTAATTGCCCCAGTGATGCACATAGGATTCACGGATGACCTAGCTCTAGTATTGGTGGGCAA
AGAAATAGAAGACGTGGAAGCGGCAGCGAATGCAGCCATAAGGATCGTGGCCAGCTGGATGCGTGGCGC
TAGCCTGGAGCTTGCAGGCCATAAAACAGAGGCGGTCCTCATCACAAGCCGCAAAATGTCGAGTTTGCTA
GATTACAGGTAGACAGCGTTAGTAAAGAGTCAAAAAACAACAGGATAACAGGCTTAGCTTTCGACCGCA
CGTGGAGTACGTAAGCCGGAAAACAGCCATAATGCAGGCG
>Ref_dvir_934
TTTGTGTTAAACCCGAAAGGTACTGGTTCATTTTTTTTTAGGGGGCCGTTTGTCCCAGACTGTATTGAAAG
CTTTTTTGAAGTTCAAGGGCACAGGCATTGTAGCACGCTGTCTAGCGATGTTGCTAGATATAAAGGAATTT
GCGACAGCTAATGGGTTGCTAATAGATAGGTTGTCTCTAAAACCAAACTGATAGGGCTTAAGTATTTGAT
GGGAGTCTAAGTGTTTTTTGATTTGTGCCATTAACATTTTTTCAAATAAGAGGCTAATAGCGGAGAGGATT
GAGATTGGCCTGTACCCTTGTAGGCTTTGTGGTTTTGCTTTAGGTTTTAGTATGGGAGTTATTCTGCTGCAT
TTCCAGGCATTTGGGAAGTAGCCTGAATTAAAGCAATGATTGAAAATAACTGCTAGGTGTTTGAGGACTA
ACATATTGCAATGTTTTAGGAGGTAATTACTACTGCCATCTGGTCCCATGGTTTTTTTGCTATTTTTCAATT
TGACCCACTCGAGAAGTTGCTTGGCGTTTGTGAAGCCAAGCTCTTCTGAGTTTTCCCAGTTGCCGTCTGCT
CTTCGGCTGGGATCAAAGTTGACTATGTAGTTTGGTATGTGCAAGGGATTTTGTGCTATAGTGCCGACTCG
CCTATTTATGCGGGCAGTGTGTTGCCTAAGCTCGGGGCTGCCATTAGACATGTCGTCATTCTGCTTATGAA
TTTGCTCGAATGACCACGCTAGGGCATTGACTTTTTCAAATTTCGACTTAATAACGGTGGAAACGTTAATG
TCGATTTCATTATGCTCAGTGGCTGTTAGGTGGATGTCGTGTTTAAGCGGCTCGTCTGTTTGGTGGTTTGGG
TTACTATGTCTTAGGGATTTTAAGCTTACCTTACTTGGCCTTATGTATTTCAGGCGGCTTTCTACATGTCGT
AGGTTGCTAGTGTGGATATGACTATTGATTTGGATTGTGAGGTCGGAGATAAGGGATTTAAGCTGTGACTT
TTCGTCAAATGACAGTGAGTGTTGGCGCCTAACGAGGTATCTTCTAATGATGCGCTTCTGCTTGATAAGAC
CAAGGCATTGTGCGCTTAATTTAACTGTATTATAGGGCTTGGATGGGTGTATAGTTCTTTTGGGGATGGCA
TCTGGCGAGTCCAGTGCAATATTAAGTGCGGTATTAAGTTCTTCAATGTGTGCGTCTATTTCTTGAGGGCT
TAGGTTGGAAGATATCGTAATTGGTCTGAGATTTCTTAAGAGAACAGAGTTCCATATGTCTGTGTTGATAC
TGTCGAATTTATAGACTTTTTTTGGATCGGTGCTTATAAGTGTATATTGGCCTAGTTGTATTTTTAGGGTTA
CCCCCGAGATGATCTGATGGGAATTCATCTATTGTGTTGCAGAGGGAACTATGTTCTGTCTGGAGGTCTAT
AGAGCATATAAAGAAATCTATTGAGGATGGGTTGTTAGAGTTATTGGGACGACTAGGTTCTTTGGGGGGG
CAAAGAACGAAGTTGGGTTGTCGTAGAAGCCAGGTTTGAAGGTTTTTACCGGCGTTATTTATGTGGGCTG
GGCTATAGTGGGGCACATGCCAACTAGGGTGTTTGGCGTTTAGGTCGCAACCTATGATTATTTGGCAACCA
TTTCCAATATGGTGTAAAGAGTTGAATAGCGTATTCATGTCATTAGTAAGAAGCGGGTCTTTAGGGCGTTT
GTATAATGAGACTGCTATAAAGCTTTGGGCGTTCCTGAGCGAGTCTTTGTATGAGAGTTTAATTGCTACGG
CTTCGATATAGCATAGTGGCGGGATATCTACGCGTTCAAACTTTATGCCGTTTTTAACGCATATAGCGACA
GACGTACCATCATTTTCAGGGTGGCGTTGCGCATGTCTTGTGTGGGTGGCTTTCACATTTTGCCTTATAAAT
TGGTATTGGGATAGCTTTGCGGTATTTCTGTTGCCTAGACGATGCTCTGCTATGAGCATTATGTCTGGCCTA
TGTCTATTTAGGAATTGGTCGAACTCATGTCTCTTCTGTAGTGATATGAGTGAATTGACGTTTTGGAAGAT
ACATTTAAGGGAGGTCATTTTTATTGTGCGATTTGGATGAGAAAGGGCTTGTTTTATTTGGCGAGCTTCTG
ACTCATCTTGGGCTTGGCTGGTGGGGTATTTTTTGGTAAGTTTCTGCATGATGTCTATGCAGGTGAAAATA
TCTTTGCCAAAGGCTTGGACACTATCGCTGTTGATGGTCTCAAGCGAGAGATTTGTTGTTTGTGGGCCATT
TGGGCCAAATTGTTAGCAAGATGCTTCTTTAGTACCGGGTTGGTGACAACAATGTTTTTTTGGAGCTGTGG
CTGACGGGAGTCGGGCCTAACCTCATCTGTTTTTTTTTTAAGAGTGAGGCGAATGGGACATTATTTTAAAT
CTTGCTTACAGTTAATGCTGTGGTGGCTTTGGTAATTTTTTTTTACATTTCTCTGTTCGGTGCTATTTTGCTT
ATTTTCGATTTTGGCCGTGATTGCTACTCTGACCGGGCAGATTTTAGCATTTGCTGGGATGTTTTGCCCCTT

GCATTTGAAACATTGTGTTTTATTGTTTTCTGATGGGTTGTCAGTCTTTTTGATACTGCATTGCCCGGGGGC
GTGGTCAGCAGTGCATTTGATGCATCTATACTGCATTTTGCAATTGCTTGCGACATGGCAATACCGTTGGC
AGTTGTGGCATTGTTTTATTTTCCTTTCGGCTTTGAAGGCCTCAATTTTTATTTTGCAGTTAAGTAGACTTTT
GATGGAGTATATTGTTTTGGCCCTTTCTGTTTTTTCCATTGGACCAGCCAAAATGACGCTAATGATTTTCAG
GTCAGGGCAAGTTTCTTGTACTGCTATCTTAACTTCATCTTCTGTGAAGATTTGCTGAGGTAATTTTTTTAT
AACTATGGAGTAAGGCTTAAGTTCTTTGGGGGTGTAAGTATAGTAGCTTATTTGATTAAGGTCTAATAGTT
TCTTAGTCTCTTGGAAATCTTCGAGGGTAAAGACATTGATATGGGTTCTATTTTTGTTGATTATATTAAGTC
TAAAACGCTCATTTTTTATTATTTGTCGCAACATACCTGTCAGTTCCTTTGGATTCACATTATATGTCGCTA
TAATGGGTAGTTTTGACGTCTTGGGTTTCCTCTCTTTTGTTTTGGGTGGTCCAGGTAATTCCTTCCTGGGTT
CAGTTGGGGCTATCGCGGTGGTTTCCTCTGGGTCATCATCGATGTCCATATCGTCAGCTACTTTTGGAGTG
GCTGGTTTCTGGCCCTTTGATTTCTTGTTTTTCTTGGGCTTGGACGGGCCCAGTTCTGGGAACTCGTCGTTC
CTGCGTGGCTTCCTTCTCGTCTAGTTGGGAGAAAGCCGCAAATTTGTTGCCATCCAATGCCTTTGCCGTTG
CTTGGATTTTTAGGTGTTTCAGCCGGGCTTGAATTCCTTGATGTTTCTTCCAAATTGCTCCTGACACGCTAG
CAGAACCGGCTGGCTTGTGCTCCGAAAACCATTAACTCCTTCCAAAATTAAATGGTATTCTGGTGGATTTC
CTCCACACCCCCTCC
>9_rnd-1_family-387__Unknown
GGCCACCTCAAGGACCACTCTGATGACCTTTGGACAACATGCAGTCCTTGCTCTGCCAATTTAGAATATGT
NATTTTTGTCGCAGAGATAAAGGGTGTAGATCGGCCGTATCCTTGCCGATCATGATGTCCNTTGGCCAAC
CGTAAGTCCTCATCCTCCCAATTCAGAATATNTAATGAAAAGGACGAAAGTCCTTCAGACAGCCNAGATA
TGAGTACAATTCTTTCGTAAATTANCGAAGCCATAACTCGGCCGTATCCTTGCAGATCCTGACAAGTCCTG
TGTCAAACAAATCGTNTGAACAAGGACCTAAACTGTCCAAAGGACATCAAAATCGTCCTTGCCGTTTCAG
AGATATGGCCAATTTTCTAAAAAGGACATTTTTTAATTAGCCATAACTCGGCCAAATCCTTAAGGATATCG
GAAGGATATACCGTTTTGAACTCGTGGNGAGCAGGACTATCGATTGGCATTCAAGGATTTCCAGGATTCG
ACAAAAAATTTCGTCGAAATTTATCCCAGGGGGTTAGTCGCAAAAGTGGCNAAAATCGAAAAAATTCGGGA
CTAACTTGGCCATTTGAAGGACTATCGGGATGTCCTTTGGACAGTAGATAGTGCTCGTCCTCCCATTTAAG
AAAATATAACAAAAAGGACGAAAGTCCTTCAAACGGCGAAGTTTTAAGGACAATTTTTGCAAGAGAAAA
TAGGCCATAGCTCGGCCGTATCCTGGCGTATCCTGACAAGTCCTGTGGCAAATAAATTGTATTAACCAGG
ACTACCAACTGGCCAAAATTCATTAAGATCGTCCTGNTAGTTTTGAAGATATTCAAGGATTTGTGTTTTCG
GGCCATTTCCGTGCGCTCCGGGATGAAAATTTCACAAGTAAAAATACAAGTCCTGGGGTTCTTAGATGAC
CCCATATTTTTTGATGCAGATCGATGCAGGGGGTCNATACGATCCTAACGCACCATGTCCTTTGAAAATCT
GCAAGGATATGACCGAGTTATGTCCTTTTTTTTCCTTACATAAAATGCTTTTATATATTTTTCGCCTTGTTTA
ATTTAAATGTGTTTTTATCCTTTCAGACTGACAAATTAAGGACTACAATGAGCTCTAAGGACCTCCACGAC
TGACAAAGCAAATTTTTCCAGGATCGGACTTTAATTAAGCCAGATATAGTTATTTTAAGTTTCAATAAGCT
ANAATTATAATTTATGCTCGATTTGTATAATTTTAAGAGAAATAATTAAGAAAACTAGCATAAAAACAAC
AATTTTATATATTTGAACATAAATAAATCAATTATTTTAAAATAAAAGAAGAAAATCCAGTTTTTTTAATT
AAAAAATTCAAAAATCAATATTCCAACGCTTGGCCCACATTTAAAATTTGTGAAGATATGAAAGATGTTA
TGTCGTTCTAAAACGACATAACTCCGCGCGGAGATATGACGTTCTAAAACGACATAACTCCGCGCGGAGA
TATGACGTTCTAAAACGACATAACTCCGCGCGGAGATATGACGTTCTAAAACGACATAACTCCGCGCGGA
GATATGACGTTCTAAAACGACATANCTCCGCGCGGAGATATGACGTTCTAAAACGACATAACTCCGCGCG
GAGATATGACGTTCTAAAACGACATANCTCCGCGCGGAGATATGACGTTCTAAAACGACATAACTCCGCG
CGGAGATATGACGTTCTAAAACGACATAACTCCGCGCGGAGATATGACGTTCTAAAACGACATAACTCCG
CGCGGAGATATGNCGTTCTAAAACGACATANCTCCGCGCGGAGATATGNCGTTCTAAAACGACATAACTC
CGCGCGGAGATATGACGTTCTAGAACGNCATATCTCCGCGCGGAGNTATGACGTTCTAAAACGACATANC
TCCGCGCGGAGNTATGNCGTTCTAGAACGNCATATCTCCGCGCGGAGNTATGNCGTTTTAGAACGNCATA
NCTCCGCGCGGAGNTATGNCGTTTTAGAACGNCATANCTCCGCGCGGAGATATGNCGTTCTAAAACGNCA
TANCTCCGCGCGGAGNTATGNCGTTCTAGAACGACATANCTCCGCGCGGAGNTATGNCGTTCTAGAACGN
CATANCTCCGCGCGGAGNTATGNCGTTCTAGAACGNCATANCTCCGCGCGGAGNTATGNCGTTCTAAAAC
GACATAACTCCGCGCGGAGATATGACGTTCCGGAACGNCATANCTCCGCGCGGAGATATGTCGTTTTAGA
ACGNCATANCTCCGCGCGGAGNTATGTCGTTTTAGAACGTCATATCTCCGCGCGGAGNTATGNCGTTCTA
GAACGTCATATCTCCGCGCGGAGTTATGTCGTTTTAGAACGTCATATCTCCGCGCGGAGTTATGTCGTTTT
AGAACGTCATATCTCCGCGCGGAGTTATGTCGTTTTAGAACGTCATATCTCCGCGCGGAGTTATGTCGTTT
TAGAACGTCATATCTCCGCGCGGAGTTATGTCGTTTTAGAACGTCATATCTCCGCGCGGAGTTATGTCGTT
TTAGAACGTCATATCTCCGCGCGGAGTTATGTCGTTTTAGAACGTCATATCTCCGCGCGGAGTTATGTCGT
TTTAGAACGTCATATCTCCGCGCGGAGTTATGTCGTTTTAGAACGTCATATCTCCGCGCGGAGTTATGTCG

TTTTAGAACGTCATATCTCCGCGCGGAGTTATGTCGTTTTAGAACGTCATATCTCCGCGCGGAGTTATGTC
GTTTTAGAACGTCATATCTCCGCGCGGAGTTATGTCGTTTTAGAACGTCATATCTCCGCGCGGAGTTATGT
CGTTTTAGAACGTCATATCTCCGCGCGGAGTTATGTCGTTTTAGAACGTCATATCTCCGCGCGGAGTTATG
TCGTTTTGGAACGTCATATCTCCGCGCGGAGTTATGTCGTTTTAGAACGTCATATCTCCGCGCGGAGTTAT
GTCGTTTTAGAACGTCATATCTCCGCGCGGAGTTATGTCGTTTTAGAACGTCATATCTCCGCGCGGAGTTA
TGTCGTTTTAGAACGTCATATCTCCGCGCGGAGTTATGTCGTTTTAGAACGTCATATCTCCGCGCGGAGTT
ATGTCGTTTTAGAACGTCATATCTCCGCGCGGAGTTATGTCGTTTTAGAACGTCATATCTCCGCGCGGAGT
TATGTCGTTTTAGAACGTCATATCTCCGCGCGGAGTTATGTCGTTTTAGAACGTCATATCTCCGCGCGGAG
TTATGTCGTTTTAGAACGTCATATCTCCGCGCGGAGTTATGTCGTTTTAGAACGTCATATCTCCGCGCGGA
GTTATGTCGTTTTAGAACGTCATATCTCCGCGCGGAGTTATGTCGTTTTAGAACGTCATATCTCCGCGCGG
AGTTATGTCGTTTTAGAACGTCATATCTCCGCGCGGAGTTATGTCGTTTTAGAACGTCATATCTCCGCGCG
GAGTTATGTCGTTCTAGAACGTCATATCTCCGCGCGGAGTTATGTCGTTTTAGAACGTCATATCTCCGCGC
GGAGTTATGTCGTTTTAGAACGTCAT
>9_rnd-5_family-2959__LINE___R1
GGACATGCAACGAGCCTGCGATGCAGCGATGGTGAGGAAAAAGAAAGGAGGTAACAAACACAAGCCAG
TCCGATGGTGGAATGGCGAAATAGAGGATGCGCGTAATAGGTGCCACACAGCAAGACGCATATACCAGC
GCGACGGGAAACAGCAAACCTGGCCCTGGAGCACGAAGCCGAAGAAGCTCGCGCAGCCTACCACTCCTG
GCGAGCTATGCTCACCAAGGCTATCAAGCGAAGCAAGCGGCAATGCTGGACCAACCCGATACCCAGCCTT
CGGACAAGCCTACAAAACGGTAATGGGCAAACTCGCAAGAGGACCCGTACCGACCTGCCCAGAGCATCT
AAAGCAGATAGTGGAGACACTGCTCCCGCGCCAGATAGAGCTAGCCCACAACCCTCCAGCCGTAGGCAC
GTCGGTGGTGCTTCCCATCGACACAGAAGACATAAGTGCCGCGCTAAAGCGTATTAAATCAAACAAAGCC
AGGGTCCGACGGCATCCCCAATGCAGTGCTGAAGACCATNGCTAGCGCTCACGCAGACACAGTCGTAAAT
GTGTTCAACCTATGCATTGCCGAAAGAACCTTTCCAACGTGCTGGAAGTTGCAGAGACTGGTCCTTATCCC
TAAAGCCGGAAAGGACCCGCCTACAGGCCACTCTGCATGCTGGACTCCACGGGCAAGCTGTTCGAGCGCA
TCATCTGCGATCGTCTGAAGAAGGAGATCGCTGATCGCTACGGACTGTCGGACCAGCAGTTCGGCTTCCG
TAAACACAGAGCACTGTCGNCGCTATCAAATTGGTAGCGGACACGGCAGGGAAGGCCATATGAGGCACG
CGATGTCGCGGCGGATCCAAAAAGTATTGCCTTGTGAATATACTCCTGGCACTCTCTTGGCTGCAAATCTC
CGAGTACCTCATCAGTCTTGTTGCCGGATACTTCAAGGACCGGGTACTCATATACGACACGACTGCAGGA
CGACGCCAGTTCGCTATTACTGGGGCGTTCCTCGCAGACGACATCGAAATCGTCACGGTGGCGAAAGAGA
AAGCGCAGGTGGAGAGAAAAACTAATGAAGCAGCCCATAAGGTGACTAGGTGGCTTGTAGAAAATGGCC
TCAACTTAGCTGCGCAAAAAACAGAGGCAGTTCCTATCAGCAGCCGGAAGCAAATGGAAGCCGCCAACA
TCACAGTGGACGGAACTAAGGTAGTTTCCAAACCTTCTATAAAATATCTAGGCGTGTTGCTGGAACACAG
GCTCAGCTTCCAGGAAAGGCGAGTAGAGCTATAAATGCACTATCTGGCATAATGGCGAACACTGGAGGCC
CTAGACAGCGCCGCAGACGACTGATCTGCAACGTGGTCCAGTAGATGCTCCTCTACGCTTCATCGATCTGG
GGACCAGCAATGAGGATCCCCTCCTACAGCCACAAATGGGCATCGTAAAGAAGATGCGCACTCCGAGTCC
TCTGCTCTTTTCGGACCGTCTCAGACGACGCAGCTCTAATGCTGGCCGGAATGCCCCCGCTTGGATCTACT
TTCAACAGAAGANCGCACCGTAGCACGCAGCCGGGAGACAACACTAGNAGAATGGCAAGCACGATGGGC
GTCTTCCTCCAATGGCAGCTGGACTAAACGCATAGTCCCGGACCTACGGCCGTGGGTNAGCAGGAAGCAC
GGACAAACCGACTTCTACTTGACCCAGCTACTGACAGGACATGGATGCTTTAAATCCTATCTCTTTAGGTT
TAGGCATGAGACGGATCCGTTTTGCACACACTGTGCGCGGACCATAGAGGACGCAGAACACGTCTTTTTT
GTCTGTCCCAGATTTTCTGCTGAACGGGAAGAGCTAAGCCAAAGTATTGGGAGAAATTTTACAGTCACTG
ACTTAATAGATATCCTACTTAGCTCGCAAAACAATTGGGTTGCAGTAACCCACATGGCTGCAGAAATNAT
CAAATCCTTGCGTCGCGCTGAAAGACAGCGCAATTCAAGTTCTNACTCTAGATAAGATCCTAGAAGAGGC
TTTCATGCCATCTCCCCCTCGTGNNGTAATACTTAGCGGTCGTTCCGCNANAGGGAAACGGAGAGTAGGT
GGTGGTTTCAGTGGGTGAANGTCCCACTTTTCGGCNTTAAATGCNTTCCTCCTATTAAAATAAAAAAAAA
GCACGCACACACACACAAACACACATTCAGCGACCACCGAGCAGTGCATTTCGAGATCCACGGGAGAGG
ACGGAGAAAACAGCAGCCCAAAACAACAGGCCCCAAATGGAAGGACAACGGCCTAGACGAGGAAGCCT
ACACGGGCGAAAGCCTTACGTCATGCTCAATGGACTTGGGCCACGGAGTTAGCAGACGACATGGCGCGGC
AGCTCATGGGAAACATCTCGGACGCGTGCGACTTATCCATGCCCAGGAGGATGCCAAACAAAAGAGGAG
CACCTTGCTACTGGTGGAATGAGCAAATCAGCGACCTAAGGAAGGCCTGCCNGCGTGCTAGAAGGCGCTC
TCAGCGTGCCCGAGGCCACCCAGACTTTGCATCTAGGCTGGAACTCCTGCGGGCAGCCAAGCGAGACCTC
AAGAGAAGCATTAAACGAAGCAAACCCAGCTGCTTCCAGAAAATATGCGATGAGGCCGATGTTAACCCG
TGGGGCACGGCATACAAGGTCGTCACGAAGAAGATCCGCCGCCAGGGAACAACCCAGGTGACCTGCCCT
ACCGCACTTGCCACTATAGTTGAGACACTGTTCCCCCAACACCCGCGGCTGGNGTAGCCAACCGCTCACT

GAGCACGGAGCTAGTGCAGCAGGTGTCGGCAGACGACTGTTAGCGGCAAGCAAAAAACTAGGCGACAAA
AAGGCTCCAGGACCAGATGGGGTGCCAAACAAGGCGCTAAAGTTAGCAATGCTAACGAAACCGACGCCT
TCGCCGAGACACTATACTAGATGTCTACAGGACGGTGTATTCCCTAAGAGATGGAAGCGACAGAAACTAA
TCCTTCTACCGAAAGGGGACAAGCCAATAGAGGAACCCTCGGGATACAGGCCAATCTGCCTACTGGACAC
GGCGGGAAAGGTACTGGAAAGGATTGTGTATGACCGACTACTTAGTGTCGTCGAAGAGAAAGAGGCCCT
GTCCAAACAGCAATACGGATTTCGCAAGGCAAGGTCTACGGTCGATGCAATTAAGGCGGTGGNAGATATT
GCCGCTAAAGCAATAGAGGGAACCAGATGGAGGTGGGGAGACAAGGAGTATTGCGCGGTGATTACGTTG
GACATCCGCAATGCCTTTNACTCAGCAAGCTGGTCTCGTATACTGGAAGCCTTAGACCGAATAGGGATAC
CGTCCTCCCTTTTCCGTATAATATCCAACTACCTGTCCGATCGGGTATTGAGTTATGATACGGATTACGGT
GCTAGGAGTTACACGGTCTCAGCAGGGGTCCCACAGGGGTCGGTCCTGGGATCACTCCTGTGGAACATAN
TGTACGACGAGGTGCTCAGAGTGCAATTGCCCCAGGGATGCAGAATCATAG
>Ref_dvir_514
AAACAGCAGAGACACGGTCTTCTCATGACAGTAAACAAAGCGGTAATAATGTACCCCATCTGAAGCAGAC
CAAGAGCATAAATTCTATATATCGCCTAAGCGCTCTACGGGTCAGCTGCGCATTCCGGACGGTATCAGAC
GAAGCAGCGCTGGTTATCGCAGGGATGGTCCGCACAGCAGCAAGGATGCAAAGCATGGAACGATGGCAA
GCACGTTGGGATCGCTCATCTAAAGGACGCTGGACACATAGGCTTATCCCAAACTTGGAAGCCTGGATAA
ACCGCAAGTATGGAAATACTAATTTTTACTTAACGCAGCTCCTATCTGGCCACGGATGCTTCAGGAGCTAC
TTGAAGCGATTCGGCCACGATAACACAGACGAGTGTTCTTGGTGCGGCAGTGAAATCGCAGAGGACGCCA
ACCACATCATCTTTGAATGTGGAAGGTTCTCAGCGAACAGGCAGGGCCTGGAGGACAGCTTGAGGAGACC
TATCACGGTGGACAACTTGGTCAGGAACATGTTGAAGACCGCACAAAAATGGGAAGCTACCTACACTTTT
GCAGCAGAAGTGATGCTGGAACTCAGGAGAGTTGAGCGCAATAGATGGAGAGCGGAGGAAGTTTAGGTG
GCAACAACATGCCTGTGAAGCAATGCTTTGCGGCCGTTCCGCAGGCAATCCACGCCACCTAATCATCACG
TACCCCCCTACTATCTACATCATAAGCATAAATTATTTATTATTTATATTATATTGTGCAAATAAACTACAA
AAAAAAAATATATTCATAGATATCGATATCGATAACAAAATTTTTTTTTATATCAAGTCAGTTTCCTATA
TAGGCTACATGGTTTAGATATATTTAATTTTTTGAATACCTCGGTACAAATTTTCAACAGTTGTTATAAAA
GTAAGGATATGCATTACATAAGGTGAAGTGGGCAGACACTTCCACAATTTTGTCATAAAATATATTCATG
AAAAACTCTTAAGGCTTTTAATATAAAATATTCTTTACTTTACTTCTTTGCAAATATTATTTACACAACATC
TGAATAACCATTTTTAATTACTTATTTTTATCGCACAGTAGACTTTACTTTTTTAAATACTAAAATCTTATA
ATTTATGGAGTCAACTGTTTCAGACATCACATACTATCCCAGATAAATCATCGTCATTTATAAATTGTACA
TATTTTTCTCGCAAACCAATGGCACCGTTAGATGATGTTAGAGAAAAATTACTGTGCCAATTACTAAAAGT
CTCAGAAACAATGTAGACGGCACTTTTACATATTTTGTAAAACATTGCAAACAAAATTTGCACGCCACAT
AGAGTTTAAATATAGAATGGAACCAGTTAGAGAGATCAGCAGTTTATCAAAAGACAAACAGCAAAGAAT
GAAATTGCTTGAGGAGGGATGGATGCTACTTACAATGAAGGAACTTTTATAGTTTCACGTCAATTAAGGG
AAAACTCAAAAAGAGGGCTTTGGATTTTGTGTGCTGCAATGGATTGTAAAGCCTACTTACCGAAGACAAC
AACTTCGGAATCAAATTAAAAATTAATGTAGTAATATCCATAAAATATGCAACCGAGATATATTAACCTC
TGCACGAAGTGCTGCGCAAATAATATTGCGAAATAAAATAATGCCTGTCCTTAATGACGTCGCCATTACT
AGGACAATATTGTATGATCACCTAATTATATTGTTTAAAAATTTAGAAGCAATGAAGCACACGGAAGTAC
AGCATGATATGATAACAGCAATTATGCGTTATCTAAGTCGCCTTTTGATAGTAATAAAATATGTTATTTCC
CCTAAATATTATGATTCTGTTAAGTCTGGAATTCGTTATGTTGCTGAGTTTGATTATGATAATATTTCGTTT
TATGAAGCCTACTGCAGCGACAACTCTAACATGCTTGGTAAAAATAGTACATGTGCGAGAATATTAAAAG
CACAGATCATAAAATATTGCGACAATAGCATAAAATATTTAAAAAAAGAAAAGATTTTGTTCAGGATCTA
CCTGCTAAAGCTTTGAATGAGCTAAACTAGAAATTCAACATATCATCATTAACCTCGTTATATCAGAGCAG
AGTTAGCGAACTTGAGAGTCTTACTCTTAAGAGCTACAGAACTGCTCATGCAATTGGGGCTATCGACGAT
AGAGCTAATTCTGACCTGTATGAAGGCACCTCAGCCAAAATGAAAAACATCACCAGGGTGATTATTCGTG
CCAAAAAAACCGAACATTTCCAGTTCTTTTTACACCATGCGATCGGTATTGCATAGCCAATATACTCGAAT
ATGGCAAACGCACAATAAATAAACCAGCTACTACACTAAAGATATATTTTAGTGTGCCAAGCCCGTGGCC
ACTAAGAAAAGAAAATATTCGTGAGTGTCCTATCCTAGAGAAATTCCCAAATGAATGTGGTACACGTTGC
CCTGAGTCCCTTTCAGGCACAGACCTGCGAAAGCAATTGACCACACATACCGCTTTGTTCAACGTTAAGG
ACATTCAAATTGATTATTTGGGGCAGAAGCTTAATGGGTGATCATAAAGACATTTATAAAAATTACTATCG
TGTTGCTGCCTCTGTAGCGGGGGTGACCCAAGTTTCTCAATTACTTATGTCAACAATGAACATGGAAGAGG
AAAATGCTGAAATTGCAGATGCTAATCTCGAGAGTGAGTTAATTTATTTAGCCTGTCTGTTGCGAAGAAA
ATTTTAATAATCGCCTGCAAGTTTTGGACCTGGAACTGTCATGTTCGGAATACTTCAATAGAAGTTTTTCA
AGTATTGGAACTTCGATACAGTAGAAGATACATTCAGTATTTAAAAAATTAGTAATGGTTTTGGCGACCTA
TCCATATTTAAAATATTTTGATGGCGGGACCTCGGTCCGAGTTTTTTTTTTTAATTAATAGATTAAAACAAT

ATAATTTAATACTTATATTGCTTATTTTATTTAAAGCAAAATATTAAATAAAATATTTTTCAACTGTTTATT
TAAACGAGCCCGAACCTCGACCAAGCCAACATAAATATTTAAAAACATAAAGCGCTCTACGGGTCAGCTG
CGCATTCCGGACGGTATCAGACGAAGCAGCGCTGGTTTCGCAGGGATGGTCACAGCAGCAAGGATGCAA
AGCATGGAACGATGGCAAGCACGTTGGGATCGCTCATCTAAAGGACGCTGGACACATAGGCTTATCCCAA
ACTTGGAAGCCTGGATAAACCGCAAGTATGGAAACACTAATTTTTACTTAACGCAGCTCCTATCTGGCCAC
GGATGCTTCAGGAGCTACTTGAAACGATTCGGCCACGATAACACAGACGAGTGTTCTTGGTGCGGCAGTG
AAATCGCAGAGGACGCCAACCACATCATCTTTGAATGTGGAAGGTTCTCAGCGAACAGGC
>Ref_dvir_956
CAGCTGTGTCAGCGTGGAGAAATCTTGCCGCCGCAACGTAGAGTTTGTATTCGGAAGCAGCGTTTTGGTA
GATCCGGTGCAACTCCTGTACCGGCTCGTACCTGGCGTGGTGCATCAGAGCTCTCAGCTCTATCAGGTACT
CCTTGAACTCCTCCCCCACGCGTTGCCTGCGGGACCGGATCTCGTCCTCAAGCCTTTCGAAGTAACGTGGG
GGCAGGAAGAACTCCAAGAAACCCCTCCTGAACACCGACCATGGCACATCCCTTAGCCCACTGCTCAGAA
ACCACCGAGCAGCCCTATCGCAGAATACCTCGCTCATTGCCCTGGGCATACGGTCGAGGGCTATACCATA
GGTGATAGCGCGCTCCTCGAGGTCCTCTAAGAACCCAAGGGGGTCGGAGCGTCCATCGTACCTCATACCC
CACTTCCGAACTTGCTCCGGGACATTAGGAAGTGGCCTCGCCTCCGATGGATGGTGCGGCCTAGTGTAGC
AGGGACCACCACCCGGTTCAACAGGGGACCTCCCCACCTCTGCGTTGACCGCAGTTGCTCGGCGGTCTTC
ATTTCCACCGAGAGCAGGAACTGCCTCCACGCCCGAGACGGTCAAACTCGGGGTGGGCGCCTGCAAAAGC
CCGTACAGCTGGGAGCTTCTGAGGTCAGGTTCCGACTTTTGCCGTGGCCGTCCACCAAAGTCGGGTGGGA
CGCCCCAAGATGGCGGCTCAAGAGTTTTAGCATACTGGGATTCCAGCTCGGCGAGCCGAGCCCAAATCCC
AGTCGAATGACCGCCCTCCTGCACGTAGGTGGACAGCCGCTTACGTAGCTCCTCCACGCGCCCCTCCTTTT
CCAGCCCAAACTCCTGGGCGATCGCCACTAACTCCTCTTTGCGTCGCTGGTGAATCCAGCGGACGCCTACC
CCCGGGTTTTGGCACTCACCTGCCTCCATCCTTTATTGTATACTTCCTTGCTAGTCGACGTCGCTGTTTCCC
TCTCTTCCCGTTCTAAGGGGTTCCGTCGGTCCCTTTACTATTCCTCTCTCCTTTTCTTGGTGTTGGTGTCTTT
TCACTTTTACGGATCTTGCTCAGCCACTTTGTTCCTTTTAGTTTTCCCCTTTGTTCACCACTTGAAGGGTGAC
CACTTCACTGCCCCCGACAGCTGTACTCACGAATTCAACTTTTCACCGCGCGTGAGTTGGATTCGCCCCAC
TGTACAGCCGCTGCCGCGACGCCTTAACGGCCCTTCGACGCCGACGTCGACTGCGCCGCGCGTGTGGGCA
CCCGTGTGCGAGCGTGGTCTCGACGTCGCCCGGCTGGCGCGCGCGCTGTACTCTCACGACCGACCGTT
ATGTTTTCGTCTCTTTATCTTACTTGCCGGCTTGCTGCTAGCATTTTTGTCACTCACCAGCTGCACGTGTTCA
TTTTCTGGCTATGCCTAACACAAGGTATAGAAATGTTCTCTGGGGTATACCGGACTACTTAATACCCTATT
TTCATATTTCGCACATACATGACCTTCTCATTTTCTTGCTCTCTGCTAGGTTACCTTGGAAAAACCTAGTAA
GGAAGCTTCTGGCCGGGGTATAATTCTCAGTCTTACCAGGTCTAGCGAGTTACAAGTTATTTATAATTTCG
CTGGGAGAGAGGGCTTAAAGGGGGGGTGAGTATCCTGACCAAACCAAACCCAGATCTACCTCTACACACC
TACACCATCGGGCCGTGGCCTCGTACCATCTATGCTAGCGGGCCGTGGCCTCGCTCCAACCACACTAGCG
GGCCGTGGCCTCGCACCAACTCCACTACCTAAATACTCTATCTTTCTGCCGGGCCGTGGCCTCGTACCACT
ATGCTAGCGGGCCGTGGCCTCGCACCAAAACTACACTAGCGGGCCGTGGCCTCGCACCAACTCCACTACC
TAAACACCAACCGGCAATGCCCACCCCGGAGCCACAATCTCGTACCGCATGCCCCTCGTTCGAGGTTAGG
TCGAACACCGGTTAGTTCCGATGTTAAGGTAAGCCGGGTTACCAGGAAACAGGGAATAATCCAATACTTT
CTAGATAATTACAACGTTGGCACTTTATTCACTATTATTCATTCCGACCTTATCGTAAATCCTAAGCTAAAC
CCTGCCGCACTGTTTTCTGGAGCCCTAGCGCTCCACTATACCATCTATGATTTCGTTTATCTCGCGCAGCAC
TTCTACTCACCCTTAACGTTCGATCACAAGTGCCGGCACGCTCGCCGGCAAGCCATGCGCTCGCTTCGGCT
GCTCAACTTCGCCGCGAGCTTTCGCAACTTCGCCTCGGAGCTTTCGCTCTTCGCCGCCGAATTGAAGTTGC
GCTCTCAAAGACGGTGTTGAGCAACCGATCGACAACGCACGGTTGCAGCGGCGGCCGGCGGTTAAATTCA
AACTCACTATTTAAATCAATTATAATATTCGCGGCTCCTTGCCTCTTTGCTACTAATCGAACTGCTTATATT
ACTGATGGCTCCTTGCCTCTTATTTCACTAAGATCTTAACCTAATATAATTAATACTTATAACTAAAGAAA
ATTAATACGTTGTCCCGCCAGCGGCTCCTTGCCGTAATTTGGCCAAAGGGATGACGGCTCCTTGCCGTGAA
AACTGTGGGGTGGCTGGTCTCATTAGCCGCCTCACATTCACTTCGGACAGGTGACGGTCACGTCGCCTTTC
GCGTTCAGCGTAACGCTGTACTCGCTCTCCCCGTCGGACACGTGGAACCGAGTACGTCGCACCGGAAGCG
GCGGATTCGCGCCTCTCTGGCGATTAGGTCCACCACGCCCGACGACAACGGTGTTCCAAGAGGAAACACC
TTCCAGCCTGAGGGGGTGGAATCGGGCACGGGTTCCTCGACGGGGCCCTCCGGTTTTACCCCAACGTCAG
CACTGTGGGCGATCGCCTCGACCTCGGTGTCGGGTTTCTTGGGGTGGCTCTCCGGTGTGACCCTCACGTCG
GCACTGCGGGCGACCGTGTCGACCTCGGTGTCGGGTTCCTCCTCCAGCACTGAGTCCAGGCTAGTGTCCCA
CCCGTCGATGCCGTCGAAAAGGTCCACTATGTCGTCCCACAACTCGCTCTCCGTGGGAGTCTTTAGAGCCA
TGGGGGAGGAATCCTGGGTTGGACTCGAAAGCTTGGGCGACGAAGCCGTCACCACCGCGTCCTCTTCCTC
GGACCAGACTTCATCCCGGCCTTCCTGGCGAACCGTGAAGGAACCTTTTCGGGATGGCGCCTTGCCTTCTT

GGCCGAACCAGGCGTCTTCTAACTCCCGCCGGGCGATGGTGTCGGGTGTCTCGGCGCGGGCAATAGGCAC
GAGCGCGGGCATCTCGTCCTCGTCGTCGAGCCACTCCTCCTCCGATTCCTTAGGTTCGTCCTGGCGCCTCG
GCGCGGGGTGGGAAAA
>Ref_Penelope
GATCGAAAAAAGCAACGTCGTTTACCAAATACCATGTGGCGGGGATAACAACAACAAGTGCAATAGTGT
CTACATAGGTACAACAAAATCGAAGCTAAAAACAAGGATTAGTCAACATAAATCGGACTTCAAACTAAG
ACATCAAAATAATATACAGAAAACAGCACTTATGACCCATTGTATAAGAAGCAACCCACACACCAAATTT
TGATGAAACAACAATCTTACAACAAGAACAACACTATAACAAGCGACACACATTGGAAATGCTACACAT
AATTAACACACCAACCTACAAACGACTAAACTACAAGACAGACACAGAAAATTGCGCTCACTTGTACAGA
CACCTCTTAAACAGTCAAACAACCTCAGTAACAATCTCCACGTCAAAAAGCGCAGACGTGTAAAATAATG
TATGTAAAATGTTCGAAATAATGTTTAATTTATTGTATTATAATTGTTAATTGTTTTTTGTATCTTGGTGTTA
GTGCCCTGAAGACGGTTTGCCGATGTGCAACCGAAATATATCGGAAGAGAATTGAATAAAATTGTTTTTC
ATTGTTTGTTTTAACAAACTCGGACCTCGAGCCAGCCAACAAATAAATATTGAAATATGGAAAGGTCGCC
AGAGCCATCAATAAATATCAACGGAAGGCACGCCGTATGCACAGCAACCAACATGAGCTACGCAAAAAT
AAAAACTAAATACAAGGATTCGAAAAGAACAATTAATAAATTCCAACTAACACTGGTAAAATTAACTAA
ACTTAAATCTAGTTTAAAATTTTTGTTAAAATGTAGAAAATCAAATTTAATACCTAACTTCATCAAAAACT
TGACACAGCATTTGACCATACTGACCACTGACAATAAAACCCACCCTGACATAACAAGAACATTGACTAG
ACACACACATTTTTACCATACCAAAATATTAAACTTACTTATAAAACACAAACACAACCTATTACAAGAA
CAAACAAAACATATGGAAAAAGCAAAAACAAACATAGAACAACTGATGACCACAGATGACGCAAAAGC
GTTTTTTGAGAGCGAGAGAAATATAGAAAACAAAATAACAACAACACTCAAGAAAAGACAAGAAACGAA
ACACGATAAGTTACGAGATCAACGGAACCTAGCCTTAGCGGATAACAACACGCAAAGAGAGTGGTTTGT
AAACAAAACAAAAATAGAATTCCCGCCAAACGTCGTAGCGTTACTCGCAAAAGGGCCGAAGTTCGCTCTC
CCAATCAGCAAGAGAGAGATTTTCCTCTCTTGAAATACATCGCAGACGGTGAGGAGCTAGTGCAAACAATAA
AAGAAAAGGAAACACAAGAGTCGGCGCGCACAAAATTCTCTTTGTTAGTCAAAGAGCATAAAACCAAGA
ACAACCAAAACAGTAGGGATCGAGCAATACTGGACACAGTGGAACAGACACGAAAATTACTGAAAGAAA
ATATAAATATTAAAATTCTATCGTCGGATAAGGGCAACAAAACCGTAGCAATGGATGAGGATGAATATAA
AAATAAAATGACAAATATTTTAGACGACTTATGCGCGTATAGAACATTGAGACTGGATCCGACATCAAGA
CTACAGACAAAGAATAACACCTTCGTAGCACAATTATTCAAGATGGGTCTTATTTCAAAGGACGAAAGAA
ATAAGATGACTACAACAACAGCGGTACCTCCGAGGATATATGGACTACCAAAAATACACAAGGAAGGAA
CTCCACTGAGACCAATATGTTCTTCCATAGGATCTCCATCTTACGGGCTGTGCAAATATATAATACAAATA
TTAAAAAATCTGACAATGGACTCTAGGTACAACATCAAGAACGCGGTAGATTTTAAAGACAGAGTCAACA
ACTCCCAGATTAGAGAAGAGGAAACATTAGTATCTTTTGACGTAGTATCCTTATTTCCCAGCATACCAATA
GAATTAGCACTTGACACAATAAGACAAAAATGGACCAAATTAGAAGAGCACACGAATATACCGAAACAA
CTATTTATGGACATAGTTAGATTTTGCATAGAGGAAAACAGATATTTCAAATACGAAGACAAAATATACA
CACAACTTAAGGGAATGCCAATGGGATCACCGGCTTCCCCAGTAATCGCAGATATATTAATGGAGGAACT
GTTGGACAAGATTACAGATAAATTAAAAATTAAACCAAGACTCTTGACCAAATATGTAGATGACCTTTTT
GCCATAACGAACAAAATAGACGTGGAAAATATTCTAAAAGAATTGAATTCCTTCCACAAACAGATAAAAT
TTACAATGGAATTAGAAAAGGACGGAAATTACCATTTTTAGACTCTATTGTAAGCAGAATGGACAACAC
ACTCAAAATAAAGTGGTATAGGAAACCCATAGCCTCCGGACGAATACTCAACTTCAATTCAAACCACCCA
AAGAGTATGATAATCAATACAGCACTAGGCTGTATGAATAGAATGATGAAAATATCGGACACAATATACC
ACAAAGAAATTGAACATGAAATCAAAGAACTTTTGACCAAAAATGACTTCCCCCCAAATATAATCAAAAC
ATTATTAAAAAGACGACAAATCGAAAGAAAAAAGCCAACAGAACCTGCTAAAATATACAAATCACTAAT
ATATGTACCACGACTATCAGAACGCCTCACAAACTCAGACTGTTATAACAAACAAGATATAAAAGTAGCA
CACAAACCGACGAATACATTACAAAAATTCTTCAACAAGATAAAGTCGAAAATCCCGATGATCGAAAAA
AGCAACGTCGTTTACCAAATACCATGTGGCGGGGATAACAACAACAAGTGCAATAGTGTCTACATAGGTA
CAACAAAATCGAAGCTAAAAACAAGGATTAGTCAACATAAATCGGACTTCAAACTAAGACATCAAAATA
ATATACAGAAAACAGCACTTATGACCCATTGTATAAGAAGCAACCACACACCAAATTTTGATGAAACAAC
AATCTTACAACAAGAACAACACTATAACAAGCGACACACATTGGAAATGCTACACATAATTAACACACCA
ACCTACAAACGACTAAACTACAAGACAGACACAGAAAATTGCGCTCACTTGTACAGACACCTCTTAAACA
GTCAAACAACCTCAGTAACAATCTCCACGTCAAAAAGCGCAGACGTGTAAAATAATGTATGTAAAATGTT
CGAAATAATGTTTAATTTATTGTATTATAATTGTTAATTGTTTTTTGTATCTTGGTTGTTAGTGCCCTGAAG
ACGGTTTGCCGATGTGCAACCGAAATATATCGGAAGAGAATTGAATAAAATTGTTTTTCATTGTTTGTTTT
AACAAACTCGGACCTCGAGCCAGCCAACAAATAAATATTGA
>160_rnd-4_family-2259__LTR___Gypsy

TTATCTCGCTCGCACTTACTCGCAGCTTTCAATGCTCTCTCTATGTTTAGGGATCGTTTCAATGTGTGTGTG
TGTGTATTTGTCCGACGAGCAAAACTGCGCACAAATTCAATTTTTCCCAAATGCTGAGGCATTTCTACCCT
TTTGAAGGCACGGCTAATATGAAATAGAGAAAGGGTTTGCCACGTCGATGCGAAATTCTTGCATTTTAGC
TCACTATCGTTGGTAAGGGTACCCAAGCGTTGGCATGTGTGCATTTAGAATATTTTGGACTCGCTATACAC
GCATACTTGCATATTTTGTGTATGAATAGAAGCATTTATGCGCTTGTGTGCCTGTTGCTTGCCTGCCTGCCT
GTCCCCGATGCAAATTTAACAAGATTTTAGGATTTCTGATTTTGGACCTCTACACACAGACACACACATAC
AAATGTCTGTGAATGGGCTGATCATGGGCGCAAAAGAAAATACATTATTGGCACGCGTCTGACGCGCGCC
CTTCTTTTTAATGATTTTGAATGAAATTTGAGTAAAAGAAAAATCTTTAAAATATTAAAATATACCAAATT
AAAAAAAAAGGAGCGATTATATATTTATAGTTTTAATAATTTTGGTGATCAGTCACTTGACCTCCATATGC
AGGTGCTTGCCAAAAGTTGACCCACTTGACCCAACGAACTGGTGCAGGTAATAAGGGGTCAACATTCATT
CATCAAAATCTAAGGGCGCTCGTCAGACGCGTGCCAATAATGTATTTTCTTTTGCGCCCATGATCAGCCCA
TTCACAGACATTACATGTACTGTCTCTCCACAAGTTCCTTTGTTTGATTCGGGGTCATTCATGTTTACGTCT
AGTGTTGTTCTACAAAGTGCGTTTGCTATAACGTTCTGCGAACCCTTTTTGTATATTACTTCATAGTCGTAT
TCCAACAGTCTTTGTTTATATGAATGGAACTTAAAAGTCAAATTTTACATCCATTAACACAAGTAATAACA
ATATTTCAATTTTAGAAATAGCAGCTTTATTTATTAGACACCAAAAAAGACACCAAACTTATCAAAGACA
AAAAACTTAAAATGTATCAATTTCTGTGCTGCCAGATAGCCACAAGTCTAAGCTTCCATCTGACTAATTTG
GAGTTGGGTTCCTTTAAACTCATTAACCAGGTTAATGGCTTGTGGTCTGTAAATATCTTAAACTTTTGACC
GAATAGGAATAATTTTCTTCTGTATCGGTTAGTGTTCGGCGTGCGAACAAAATTGGCTTATCAGACCCTAA
AGGTCCTTGTGATAACACAGCTCATAACGCTACGTTGCTAGCATCTGTTGTTAGTATGAATGGTTTCGTAA
AGTCGGGGTAAATTAGTAATTGGTCGTTACAAAGCAAGGTTTTACAGTAGTTAGATGTTTCTATAAATTCA
TCGTTTAAAACAACAATCGTTTTCCCTTTTAATTGTTTAGTCAGGGGTTATATTTGGGAAGTCTTTCATAAA
CTTCCTGTAATAACCCATTAAACCCAAGAAGCATTTAATTTCTTTTTAAGATTTCGGTAAAGGACATTTTTT
TATAGCTTCCATTTTTGACGGGTTTGGTTTAACGCCATCTAGTGTTACAATGTGTCCGAGGTATTCAATTTC
TTTTTGTAGAAAGTCAGATTTGTCCATTTAGAAATGTTGTAAAACATTGTTTGTTGAAAAGTTCTCTTTTGG
TTTCAATGACCGTTTGATTCACTTATTTTTCTTTTACGATATTGAGTAGAGCTCTTTGAATATCTGTTATCTT
TTCGTACAGAATTCCTACTGGGTAACATTGAAGTGAAAGGATGCCTAGGTCTGCCATAAGGCTATGTTCG
GTTCTCATGTCCGCGAAGTGGGTAATAAGTTTATTCTTTATTTCGTCCCAGTTCAACTCAGTTCCCGATTGG
TATAGTACTTCATGCGCTTTTCCCTGAATCTTGTTTCTGATTGCCCTTAGTATCATTAGTCCGGTTGGAGTT
TGGGCTGCCCCTTTTATTAGCATTAATATCTACTCAACTTTTGTAATATATTCCCTTAGTCCTTCCATGTTCC
CGTCGTATGTTGGGAAGGTTTTTAAATAAATCGACAATTTTAAAGCTACCTATTATTTTTTACATGTGCAA
GTTGGCAGGTTGTGCCGGGGGTATTACCTGAGCCATGTACATGGTTTCGGATGCTTCTTCTTCTCTAATATC
TGGGATGCTACTAGTGTGTTTGGCACCTCTTGTAGGAATTATTCTCCTAACCCTTCCTAAGTTAGACATAA
ACGGTATTATGTTGTAGATATGTAATTATATGCTGTATATGTATGTATATATGTTGCTTATCTTATGTATAT
AGTTTTAGTCATTGAGTAATAAAGAGAAAGAGCTGAACTGACGCTCTGAGCTTTTTTTACCGTTTTAAAAA
CCACGAGNCGTAGATCGGGTTGGTCCGCAAACGCAAAATCAAACCAAAAAAAAGCTCATTTACCATAGG
AAGCAACAACAAATCACTTGCAAAACAACAACAGTGCGCACGAAACCCAATTTGTTCTTCATCCCCCGTT
TTGATCGACAAGAGGTTTGTGAGCCATGATGAGGACATAGGAAACTCAGTGAAAGGGGACAAATAATGC
CACNGTGTTCTTTGCGGGTTTCTCTGTCGTTCCTACAAGGCAGCGCGTTAGAAACAACAACAGCACAATTC
GGGTACTAATGTTGTTGTTGCAGAGCCGAGTCTCGGCGCAAGTAGGTAGAGAAAATTTGAATGCCAGCAG
GCCATGGCATTCAGATCAAAGGGTTACGCGCACGGCGCNCCATCATATATTATGACGTCACAGTTNATAG
AATTTTATTTCTTGTGATGTTTAGATATATTTCTACCTTTATTAGGTACATTTAAATAACTTATGAATGGGG
TCTTTTATAGNAAATAATTCGTTGACATATGTAATNATTTTATATACGAAATGATCGCACATAAAAAGCCT
TGGACTGTGACGTCACATGCGCAACAGAACGTTGCAATCTTCTTAAATTCATAGACTTCCCTTGGCTTGGC
AGACACATCATATATGCAGATTTGCACCAATATGTGTGAGATGAGACACACATACAACAACTATTTGGCG
GGTCGCGGCATTGATACTCTTTTTATAAGAATGGTATATAATTTATAAAGAAAACAATTATTATTGACTGT
ACTAATAGTTGCACACTAAATACGAAATTATATTTTTCATAGACATTGAATAAAAATGTATATATAAGAN
GGTTTGTGCTATTTGGTTACATCGCTTATGAATAGTTAAAATACCCTATTTTGAGAAAAGGTGCCTTGCTG
GTCGAAAAATGATAAAAATCTGCCGAT
>9_rnd-5_family-2925__DNA___Maverick
ATATATATAGTTACATACTCTATTATATGTCTTTAATATTCAGTCATCTCTTTAACTGCTAACAGGGCAGTC
AAGCTGTATATCAGCCATTCNTAACGGGCTGTTTAACTGCTATAAAACCGTTCATAACAGGCTGTTTAACT
GCTATAAAACCGAATAACAGATAAGTTACTGCTCTAAAACTGTATAGCAGATCAGAAAATGGATGAGAAT
TTACTAAAGGAATTATCAAAGACTAGAAATGTTTTGAAAAAAATAAAATTTCAAAGTATTAAATTAGGTA
AAGCTGAATCCGCTTTACAACTGGAAGAAACATTAAGGCCCATAACAGCACCATTGCAGAAACTCGTTAC

TAAGAGTAATAAATTAAATAATGCATTTTTGAAACCGAAGAAAGAAGAGCAGTCGTACACGCCACTTATT
TCAAATACTGGAATTAAGCCAGAGTTAGATAGTTCGCATTTTAGACGACGGAAACTAGATGATGATGATG
ATGATAATACGAGAATCAATCAAACTGATGTCTATGCTGATGCGGATGATGATGATGATGATGATGATAA
TACAGTTAATCAACTCTACTCTCAATCTAATGTTGGAGTTGAAAATATGTTTAATCTAAGTGAATTAGCTA
GAAAAAAGATACTTGATAAAGCGTATGGACCATATAAGATTTAAATGGTGAATTAAGGTTAGGAAATTCT
ATTCATATATCAGAAGAAAAATTTATTCTAGACAGTGGTGAAACATGGGATCTCACTACAGGCTTATATTC
TTTAATTTATGAAAATACTCTCAGAAATTATACGATCTAGACCACAAATTATAAAGATATTCTAATAAAAA
CTTCTGCTCACAGAAAACATAACAAACCTGATGGACAACTTAAAGGAAATCGTTCGAAAAAATATTTAAC
TATAATTAAACATNTTTTTCCAAAATTATCTCAANAAGTTCTTTTTCATAAAACAGTACCNATTGCCATATC
AACTCCAAATCAATCAAAATCATCTATATCCGGTAGTGGTATAATGAAGCTCAATACAAATAAACCAAAC
TATATTTACTGGGATGATCCTAATGAAATTGTTGATCGATTAAAGCTTCTAATCGCCTCACAAACAGCTGG
TCATAGTAATCATAACAATGAGATAATTTCAATAGTTGAAGAACTTCGGGAAGCAAAAAATTATCGAATA
AATTTATAAAAGGGAAAAATATTTGTATTTAACTGACATTATTAAAATGAGTATCGACAAGTTTGGACATT
ACTCCTCAGCTAAAAAGAGGGATTCTGTTGACAATAAANTTTATGTTNAAAATATATTATACAAATCAAA
AGAGGATTTGGAGCTTCTCATACATAATAAAGTTGATGAAGTAGATAAATCTATAAACGAACGTTTAAAT
AAAGTTAATATTGAAATTCCAAAACTGAGAAGTGATATTAATTTATATAATAATACGATTTTTGGAAAACT
TGAAGAGAATAANATAAGTTTTGAGAAAATCTTACAAAAATCAAAAAACTATTTGTATACGTATATTAAT
AGTGAAAATAATGAATTGAGAGAATATCTTATAAGTGAAAATGTTAAATTAAAGAGTATAAATACAAAAT
AGCTTGAATGAACTTAAGACGTTTTTGATNACTTATATCAATAGTGAAAATATTGCGTTGAGAGAGTATAT
TAATAAGGAAAAATGCTAAAATNAGGGAGTCAATTACATTGTTGAAAAGTTGTGTAGCATATCTACCCTA
CCTTATCATAAATCAAAGGGTATAACAATCCGTTAATACATTGTGACACCTTGTCATAATAAATGCAAATA
TACAAATGCCCAAAGTACATTAAAAGACCGCTTAGAATTACATAAACGTTCCGGCGCACAAGCTNTACGA
TCCNTCGCTTATACATAAGCCGATCGTGGCGCGTAGGAATGCTGAGCATGCGCTTTGCAGCTCAAGCGGT
CAAACCATACANAACATATNCATGCCTTCTGCATACATATGTATATACCAACANACTTATACATACATATA
AACCCAACATGAACATATAAGAACGATTCTCAATAAAGTAACAGAACGGAGCAGACGCTCCAATCTAAA
ACGGTCTGTGCTCGAACTTATTATTTCGAAGTTATTGCCTGGCGATCCTGCCAGGAGTTATTACATGGCGA
ACGTGACAGGACTCACAGGCTGCAGTCTAGTTGAACAAGTGCGAAGTGCATAATGGCATAACGCCAAGTA
AAAAGCATTAAAACATTGCAAAAATCAAAGTGGAAATTTTGAAAGTCCCAGGCACAACTAAAAAATTAC
ATTGAATTCGTTGGCGGTGGAAAGTATCCTAAGGCAGCTGAAGACTTGAAAANCCAAATAATTGATTGCA
GAGGAGCCCATGTTCGAGAAAGCTGTTCCGATTGAACAAAGGAATTTGCGGACGTTGCAAAAGTCCTTGC
AGGCAAATCCAGCCGGTGGACCACTCCCACTCACAATAGCTAAGTACCGGGCTCGACAAGAAAAGAAAG
CGATACCAAAGCACAAAAGGAGTGAATGGTGAGTGAAACTCTTGCAGCAACGGCGACTGGTCAAGGAAA
TGATTCTGTTAGCCAAAGCTACACAGAGCGTCTTCAACACCTAGATCAGCAGCTTCGCGTTGGTGCGAAA
CAACGCAAACGGGCTGCATAATTACGAACACCCCAATTTGCTTTAACATTAAGTTCTAAAAGCCTATGTG
AGAAACCGCTGCTTGAAAAANACTAAACACTGATAGGCCTTACACTAACAAATACGGTTAGAGAAAGAG
AATATTTCTCTTTAATTGAATTGCTTTTATATATATTCGATAAGAAAACAAAATTTTTTTAACGTTCATGA
AATACATTTTTTAACTGCAAGAGGTTCTTGTCGGGAGCTTCCTGAACCCTCTTATTCGAACATGAAATGCA
TATACATATTATATTTATGAAGCTATATGTCAAGTTTGGTTACTCTAGCTCTTGTTATTTACCAAAATTGCA
CAAAAAACAGGATATCGATATCGATTGCTTGGAAACGGAGTAAACTATCGATTATCGGAAACAAACTCGA
TCTGCGCAGGCACTAGGAGCATCTACACCTAAAATTTCAATTTCAATTTCTAGCTCTTATAAGTTCTGAGA
TCCTTGCGNTCGTACATACGGACTGACGGACAGACGGACTGACGGACTGACGGACAGACGGACAGACGG
ACAGACGGACAGACGGACATGGCTAGTTCGAGTGGCTATTGGTGCTGATCAAGAATATATATACTTTATG
GGGTCGGAGATGCTTCCTTCTGCCTGTTACATACATT
>9_rnd-1_family-450__DNA___Maverick
AAACGGCATCAACATCAGCAAACAATAACCAAAATGCACAACGCTTTTGTGAGGTTTGTGGAGTAACGGT
GGCTCATAATCGCTGGGCACAACATTTGCGGACAAATGAGCACAAAAATAAGGCCTTCACACCGTTGGAT
GGCTATGTTCAACTTGCGGCATCCGGATTCCAAAACCGGATTAACACCTATTCATATAGCAATGTTTCGGA
TAACGTTATGTCCCTGAAGAGTTCCTATTGGAAGCAGGAAGTTCATTACTTCCAATAATGGAAAACGTTTT
AGAGGTGCACACCTCATATAAAAATCAACTTTGAACTTTTCGCTGAATATTTGAAAATAAATAATAATAAT
ACTGATGGTGAAGATTTTGAAATAATGATTAAATCCCATCAAACTGAAATGTGTGTGGCTACGAAGTCTTC
TGACTTGGAAGAAATATTCGTAAGCCACTCATACATCATAAGTTCAAAGATGGATGAATTCCAAGAGAGA
GATAGTGGTTGGACTCTCATAAAAATTATACGTTTGGAAATGAACGTCAATAAATATAAGCCGCTAAGAG
GCTCTAGTTTTATTCCATTACCACGGAAATTGAGCTTGAAGAAAGCTATTATTAATATAAAAAACGACGAT
GAGTTTTGCTTTAAGTGGGCCTTAATAGCAGCCCTAAAGAACTTGCAACATAATGCTGATAGGTGTGCCCC

ATATAAAATCGATGATATAAGGAGTCCAATTATTGAAATTGATGAGATATTCTCACTCAAATTTCATAATA
TGGAATTTCCATTAAAGCTCAAAGACATTAAATTGTTTGAGCAACAAAATACTTACTTAAGTATAAATGTC
TTTGGCTATGATGAAGACATTAATGAAATTATTGGACCACTCTATCTAACCAAAGAGCACCGGGGAAACA
TATTAATCTATTGCTTTTGGAAAAGCAAGATATATCCCATTATACCTGGATTAAGAATCTATCAAGGTAAG
TAAAGAANACAAAAAAATATACATTTCTTTTTTAATATCAATCATATTTTCTCCTCCAGGCTTACACACAA
TCAGTTGACTTCGCATACGAGGAAATTACATTTCTGTAATGTATGCTTAAACTATTCGCATAACGACAGGA
TGCATGGCGAGCAGTGTGGAAAAGTGGTGGCGAAGCTTCCAACAGCGGATAAGGCGAGTGTAAAATTTA
AGAATTATGCAAATAGTTTAGATATACCTTTTATAATTTATGCAGATTTCGAGTGCGTCTTAAAACCTATC
AACAGGAATGCGAGCGAGAATGTAATAAGCATTCAAGAACACATTCCTGTTGCATACGCATATTTCGTAA
AATCTGCATATGATTCTAGTTTAGATAAATTCGTTATATATAGTGGAGAAAGTGTAGGATTACATTTTGTA
ACATCTCTCATTGCTGATGCAAGAGAGATTTATAGCAAATATTTATCTACTGTAAAGCCAATCGAAATGAG
CTTAGAAGATGAGCATAAATTTAAAAATGCTATAAATTGCCACATTTGCACTAAATTTTAAACTTCGATAG
AGTACGGGATCATTGCCACTTAACTGGTAAATTTAGAGGGGCAGCTCATAATGCCTGTAACATAAATTAC
AAAGTTGCCTCATTTATCCCCGTACTTTTTCATAATTTTTCACACTATGATGCTCATTTGTTTGTAAGAGAG
TTGTCAAAAGTTGAAGGTGAAATTAAGGTTCTACCTCTCAATAAAGAGATTTACATNTCCCTTTCAAAATA
CACATATATTACAGAGCATAATACAAATTTGGAAATACGTTTTTTAGACTCATTTCGATTTATGGCTGCAA
GTTTAGATAGTCTTAGCTCTAATTTAGAACATAATAATTTCACAACTGTTAAACGGTTTTCCCGGACGAAA
CACACTTTAATCTAGTACATAGGAAGGGAGTTTTCCCATATGANTACTTTACTAAATTTGAATGTTTGCAA
GAAACTGCTCTTCCACCAAGGGAAAAATTTTTCAACAGTCTTGCAAATAGAGAATGTTTAGATGATGATT
ATTTACACGCTCAGAACGTGTGGAGAGTTTTTAATTGTAAAACCCTCAAAGATTATATGGAGCTGTATTTA
AAGACAGATGTACTTTTGCTTGCAGATGTTTTCGAAAATTTTCGTTTAATTTGCAAGAATATTTATTCATTG
GACCCAGCCCATTATTACACAGCTCCGGGGTTATCTTGGGATGCTATGCTAAAAACAACTGGAATAGAAT
TAGAATTATTTACAGACATAGATATGCAAAGATTTATACAAAATGGTATTCGTGGGGGATTAGTACAATG
TTGTCAGAGAATATTCAATTGCTAATCATAAGTATATGAATGATTATGATAGCACAAAACCCTCTCAATAT
TTAATGTATTTGGATTCAAATAATCTATATGGGTGGGCGATGTCACAATATCTTCCATATGGTGATTTCCA
GTGGTTAAGTGAGGATGAAATACGATCATTTCATTTATTTTCAATTTCTGAAACGTATGAATATGGATATA
TTCTTGAAGTAGATTTAGAAATACCCAGCATCCCTTCATGATATGCATAACGACTTGCCGTTTTGTCCAAG
CAATCAAAAACCGACTGCAAAGTGTACAGTTAAAAAGCTTATCGCTGACCTAAGACCGAAATATAAATAT
CCCATACATTATAGAAATCTTCAACAATGTATTCATAATGGATTGCGGTTAACTAAGATTTACCGTATATT
ACGGTTTAAGCAGTCTCCTTGGTTAAGAAAATATATTGATATTAATACATATCATAGAACGCAGGCGAAA
AATAATTTCGAAAAGATTTTTTAAGCTATTAAATAATGCAGTATATGGAAAACCATGGAAAACGTTGAA
AAACGTAAAAACATTAAATTGGTAACTGCATGGGAAACAACGTCTTGCAATAGATTGGGAGCAGGGGCA
TTAATCGCAAAATCAAACTTTCATAGCTCAGTGCAATTCTCGAATAATANGTATGCAATCCAAATGAAAA
GATTGCATAACTATTATAATAAGCCCATATATTTAGGTTTTACAGTATTAGATCTTTCGAAATTGTTGATGT
ATGATTTTCATTATGATTATATGGTAGCAAAGTTTAAATCTGATCTAGCATTAAATTATATGGATACAGAC
TCCTTAATATATATACAATTAAAACTGAGGACTTTTATGCAGATATTAGAGATGATCTTGAAACGAAATTCGA
CACATCTGAATATTCTGATGATCATGCTCATAAATATGCCCTAAAGAGAGTTAATAAAAAGTCTTGGGCAT
GATGAAAGA
>160_rnd-1_family-322__LTR___Gypsy
GACCTTGCATATAAAAGATATGCTATGTTTCTTTAAACATGGTAAGAAAATTATAGCAAGAATTGATATTG
GNGATTTATATACTAATAATGGAAATATTGACTTAGGACAACTATTTCAAAGGCTCGCAAAAGAAGCCGG
TATATTAAAAATCAGCCAATTAAAAATGGNACCGTNGGAAAATATCTTTGAAAAAGTTTCAATAGTTAAT
TTCAAAATTATGGGCAATAAAATATTGAACACATTGAGAGTAGCGCTACTCAACCCGGTGACCACATTAA
CAAATGTTAAAGAGAAAGAAGCCATATTGTCTACACTACATGATGATCCAGTACAAGGAGGTCACACAGG
CATTGCAAAAACCTTGGCCAAAGTCAAAAGACATTATTACTAGAAAGTCATACCTTTCTCGTGATATAAC
AGAGTACAACCAGAAATGTCAAAAAGCAAAAATAACAACGAATATTAAAACCTCTTTAACAATTACTGA
AACCCCGTAAAGGCTTTCGACAGAGTGATAGTGGATACAATTGGTCCACTACCAAAATCAGAACAAGGCA
ATGAATATGCAGTCACGCTCATATGTGACTTGACTTAATATTTAGTTACTATACCTGTCGCAAATAAAAGC
GCAGTAGCTGTTGCAACAGCATTATTCGAATTATTTATTCTCAAGAACGGTCCAATGAAGACGTTCATTTC
GGACATGGGAATGGAATCATAAAATTCAATTATAAATGATTTGTGCAAATATTTAAAAATATAACATCAA
CTGCACATCATCATCAAACTGCTGGAACAGTAGAAAGAAGCCACAGAACATTTAACGAATATATACGATC
CTTTATATCAGTAAATAAAACTGATTCGGATGTATGGCCTCAATATTTTGTTTATTGCTTCAATACTACCCC
CTCTATGGCACATAATTTTTGTCCATATGAGTTAGTATTTGGTAAGACTAATAATCTACCAAAGTATNTTA
ACAGCATAAGTAATATAGAACCTATATAACGTAGAAGACTACGCTAAAGAAAGTAGGTATAGATAAGAA

GTAGCATACAAAAGAGCAAGAAATATGCTCGAATTAAATAAATAGAAAAATAAGGAATTATATGATTTA
AAAACTAGGAAATAAATTTGTACTAAGAAATGAAGTAGGCCATAAGCTAGATTTTAAAAACACTGGGCCC
TATAGAGTTGAAAAAATAGAAGATAATGATAAATAAGAAAATAAGAAATAAGAGTAAAAAACAAACAGT
TCATAAAGAAAGGTTAAAAATCTTTAATTCATAAAAGACACACAAGAAAAAAATGTAATTATCACAAGGT
ACGCAAAGAAAAAATTAAAAAAACTAAATACATTATAAATACATTACAAATATAATAAATCTTCAAATAT
CAAAATNATTTTAATATAAAATAAATTTAAGTAAAATAAATCCACTAGTTACGTTATTTTTCGNATAGGAG
GGAGATGTAGTATGTGCATATATTGAGGGTACACTGTACCAATAAAGTAACATCGTTTAAGCATAAATAN
ATCAGCAACACATTGTTGCAGTACACAAACAAAACCNTTTCGAGTGAGCGTGTAATATGATCGCCCGCTT
ATGAGCTACGCTAAGTCAGCAGTCCCACGCTGACCATTCGTAGCATATATATATATACATACATGCAAAT
ATGTACATAAGACTCTCTCTCTCCCGATTATGCTGATAAGACAAAATATGTATGAAGCCGCTCCGCTGCTG
GCGTAACCGGCAGCGCAGCTATGCGGTCTTAGCCATAAGAATATATTGAATAAAACATTCTAAAAACTAA
AGTCAAACGGCACAGACGTGCCAGATTCATTAGCTCGTACCTATTACGTACTACCTCGAGTTTACAAGAG
AGCACTTGGAGTACGTGCACAAAAAAGCAGCAGAATCCTGTCGAGCGCTATCCAGGATTATGCTCAACAC
TCGAGGTCCAAAACAGCAGAGACGCAGTCTTCTCATGACAGTAAACAAAGCGGTAATAATATACGCGTCC
CCCATCTGGAGCAGAGTAATGGCTGTTCCTAGCTACAGCCGGAGTATAAATTCTATATATCGCCTATGCGC
TCTACCGGTCAGCTGCGCATTCTGGACGGTATCAGGATAAGCAACGCTGGTTTTCGCAGGGATGGTCCCGT
TCAATGAACTGGCAACAGAAGCGGTAGTTTTCTACAGAGCTCTGAATGGAAACAGCACAGCAAACTCGGT
TCGCACAGCAGCAAGGATGCAAAGCATGGAACGATGGCATCCACGTTGGGATCGCTCATCTAAAGGACG
CTGGACACATAGGCTTATCCCTAACTTGGAAGCCTGGATAAACCGCAAGTATGGAAACACTAATTTCCAC
TTAACGCAGCTCCTATCTGGCCACGGATGCTTCTAGAGCTACTTGAACCGATTCGGCCTCGATAACACAGA
CGAGTGTTCTTGGTGCGGCAGTGAAATCGCAGAAAGGCTCCTCACTGTAGGAGGGAAAAACCAGCAGTGC
GGCATATCAACCTAGATGTGAAAGATGATATTGCCAGGGTGAAGGTGATACAGATGGCAGCAACTGCTAA
ACTACCAAGTGAGCCAACTCCACTAGGAAGCCGACGCCAGCAGATCCGAGAAAACCACACCGGCTAAAT
GTAGATAAACTCAGGATCACAGAAAAAAATCAGCTCACAAAAAACAACAACAGAGGGCCTGGAAATCAC
AAATGTTTAAAAGGCGGTCCCAAGAGGGGTTGAAGCCTTACCCGAGCTAAGGAAAGCATATCGCAGAAC
ACAGACGGCTACCATGCTTCTACCCAAGATCGCAGCAAACGAACTTCTGAAGAAAGCGAAGAAACGCATT
GGCTGGGTTGTGTACCGTATATGCCAAAAATTGGAACCAAGAAGATGTTTCCAGTGCCTGGACCTTGGGC
ACACGGCTGCGCGATGCATTAAAGGTTTGCATATGCAAGCCAATGCATAATCTGCACAAGGCACGAGACT
GCGAACACGGCAAACAACACCCTCAGCCATGCCTGCTCATGGTTAATCAAGGCGTCTCAGGCAACAATCA
AAAATTAAGTTAATACAAATTAACCTGAACTATTGCAGAGCGGTCCAGGACCTACTCGCTCAAACTACAC
GGGAAGAGAACGCTTACTTCGCCATCGTCAGTAAACCACACCAGTCAAACATGATGGCATCTGGATTCAG
AGCAACGGCGGCCAAGTATTGCGAAATATGTATATATATATATATATATATAGTTGCTATATTGCACCCAG
CATAGGAATTACCGCGTTCGCCGATATACTG
>9_rnd-1_family-357__DNA___Maverick
TCGCTGGGCACAACATTTGCGGACAAATGAGCACAAAAATAAGGCCTTCACACCGTTGGATGGCTATGTT
CAACTTGCGGCATCCGGATTCCAAAACCGGATTAACACCTATTCATATAGCAATGTTTCGGATAACGTTAT
GTTCCTGAAGAGTTCCTATTGGAAGCAGGAAGTTCATTACTTCCAATAATGGAAAACGTTTTAGAGGTGC
ACACCTCATATAAAAATCAACTTTGAACTTTTCGCTGAATATNTGAAAATAAATAATAATAATACTGATGG
TGAAGATTTTGAAATAATGATTAAATCCCATCAAACTGAAATGTGTGTGGCTACGAAGTCTTCTGACTTGG
AAGAAATATTCGTAAGCCACTCATACATCATAAGTTCAAAGATGGATGANTTCCAAGAGAGAGATAGTGG
TTGGACTCTCATAAAAATTATACGTTTGGAAATGAACGTCAATAAATATAAGCCGCTAAGAGGCTCTAGT
TTTATTCCATTACCACGGAAATTGAGCTTGAAGAAAGCTATTATTAATATAAAAAACGACGATGAGTTTTG
CTTTAAGTGGGCTTTAATAGCAGCCCTAAAGAACTTGCAACATAATGCTGATAGGTGTGCCCCATATAAN
ATCGATGATATAAGGAGTCCAATTATTGAAATTGATGAGATATTCTCACTCAAATTTCATAATATGGAATT
TCCATTAAAGCTCAAAGACATTAAATTGTTTGAGCAACAAAATACNTACTTGAGTATAAATGTCTTTGGCT
ATGATGAAGACATTAATGAAATTATTGGACCACTCTATCTAACCAAAGAGCACCGGGGAAACATATTAAT
CTATTGCTTTTGGAAAAGCAAGATATATCCCATTATACCTGGATTAAGAATCTATCAAGGTAAGTAAAGA
AAATAAAAAAAAGATATACATTTCTTTTTTAATATCAATCATATTTTCTCCTCCAGGCTTACACACAATCA
GTTGACTTCGCATACGAGGAAATTACATTTCTGTAATGTATGCTTAAACTATTCGCATAACGACAGGATGC
ATGGCGAGCAGTGTGGAAAGGTGGTGGCGAAGCTTCCAACAGCGGATAAGGCGAGTGTAAAATTTAAGA
ATTATGCAAATAGTTTAGATATACCTTTTATAATCNATGCAGATTTCGAGTGCGTCTTAAACCTATCAACA
GGAATGTGAGCGAGAATGTAATGAGCATTCAAGAACACATTCCTGTTGCATACGCATATTTCATAAAATC
TGCATATGATTCTAGTTTAGATAAATTCGTTATATATAGTGGAGAAAATGTANGATTACATTTTGTAACAT
CTCTCATTGCTGATGCAAGAGAGATTTATAGCAAATATTTATCTACTGTAAAGCCAATCGAAATGAGNTTA

GAAGATGAGCATAAATTTAAAAATGCTATAAATTGCCACATTTGCACTAAGATTTTAAACTTAGATAGAG
TACGGGATCATTGCCACTTAACTGGTAAATTTAGGGGCAGCTCACAATGCCTGTAACATAAATTACAAAG
TTCTCCCCGTACCTTTCCATAAATTTTCACACTATGATGCTCATTTGTTTGTAAGAGAGTTGTCAAAAGTTG
AAGGTGAAATTAAGGTTCTACCTCTCAATAAAGAGATTTACATTTCCCTTTCAAAATACACATATATTACA
GAGCATAATACAAATTTGGAAATACGTTTTTTAGACTCATTTCGATTTATGGCTGCAAGTTTAGATAGTNT
TAGCTCTAATTTAGAACATAATAATTTCACAACTGTTAAACGGNTTTTCCCGGACGAAACACACTTTAATC
TAGTACATAGGAAGGGAGTTTTCCCATATGAATACTTTACTAAATTTGAATGTTTGCAAGAAACTGCTCTT
CCACCAAGGGAAAAATTTTTCAACAGTCTTGCAAATAGAGAATGTTTAGATGATGATTATTTACACGCTCA
GAACGTGTGGAGAGTTTTTAATTGTAAAACCCTCAAAGATTATATGGAGCTGTATTTAAAGACAGATGTA
CTTTTGCTTGCAGATGTTTTCCGAAAATTTTCGTTTAATTTGCAAGAATATTTATTCATTGGACCCAGCCCA
TTATTACACAGCTCCGGGGTTATCTTGGGATGCTATGCTAAAAACAACTGGAATAGAATTAGAATTATTTA
CAGACATAGATATGCAAAGATTTATACAAAATGGTATTCGTGGGGATTAGTACAATGTTGTCAGAGATAT
TCAATTGCTAATCATAAGTATATGAATGATTATGATAGCACAAAACCCTCTCAATATTTAATGTATTTGGA
TTCAAATAATCTATATGGGTGGGCGATGTCACAATATCTTCCATATGGTGATTTCCAGTGGTTAAGTGAGG
ATGAAATACGATCATTTCATTTATTTTTCAATTTCTGAAACGTATGAATATGGATATATTCTTGAAGTAGAT
TTAGAATACCCAGCATCCCTTCATGATATGCATAACGACTTGCCGTTTTGTCCAAGCAATCAAAACCGACT
GCAAAGTGTACAGTTAAAAAGCTTATCGCTGACCTAAGACCGAAATATAAATATCCCATACATTATAGAA
ATCTTCAACAATGTATTCATAATGGATTGCGGTTAACTAAGATTTACCGTATATTACGGCTTAAGCAGTCT
CCTTGGTTAAGAAAATATATTGATATTAATACATATCATAGAACGCAGGCGAAAAAAAAATTTCGAAAAAG
ATTTTTTAAGCTATTAAATAATGCAGTATATGGGAAAACCATGGAAAACGTTGAAAAACGTAAAAACATT
AAATTGGTAACTGCATGGGAAACAACGTCTTGCAATAGATTGGGAGCAGGGGCATTAATCGCAAAATCAA
ACTTTCATAGCTCAGTGCAATTCTCGAATAATATGTATGCAATCCAAATGAAAAGATTGCATAACTATTAT
AATAAACCCATATATTTAGGTTTTACAGTATTAGATCTTTCGAAATTGTTGATGTATGATTTTCATTATGAT
TATATGGTAGCAAAGTTTAAATCTGATCTAGCATTAAATTATATGGATACAGACTCCTTAATATATACAAT
TAAAACTGAGGACTTTTATGCAGATATTAGAGATGATCTCGAAATGAAATTCGACACATCTGAATATTCTG
ATGATCATGCTCATAAATATGCCCTAAAGAGAGTTAATAAAAAAGTCTTGGGCATGATGAAAGATGAGAA
TAATGGTAGGATAATGAAAGAATTCATAGGACTTCGATCCAAAATG
>9_rnd-1_family-407__LTR___Gypsy
AAGTTAACACATAACACTTAATATAGGATATTTCATAAGTTTATAATCAACCCTCGCTATCGCGTGCTTCA
TAAGTTAAGGAACAGCCCTCGCAATTGCGACACTGCATACTTTGTATCAGCGCGCTATAGCTGCAATGGTC
AGCGGCGGTAACCGGACACTTTTGTTTTGCTGCAAGAGTTTCCGTTATTGCCTAAGATTTTGCTGAGTCGG
CTTGCCGACCATGACATTGTGGCGTGATGCGAGTTTTGGCTTAGCTTAAGTGAAATATTCTGTGTATTAAG
AAATTAGTTTTTAATTCGGCTTTCACTGAGGCACATCGGCCCAGGGACTTCGCGCTTAAATTAACTCACTC
ACTACGGCGTATCGGCCTAGTGTTTTATAAATAACCAAAATAAAGTCCTATAACGAAAATCCGCGAAGAC
CTTTTTATTTNAATATTAATTGGCGCCCAACTAGTGGTAGTTACGATTACCCACGCGATTGCAAAGAAAAA
AATTCTTACAAAGTGAACATTAAGTCGCGGTCACACCAACAGTGCCGCGCAGTGAACATTACAAAAATAA
AATAATAAAAAATAATAAGCTAATCACTAATAATAATAATAGTGACAGTGAGTTAACAAATGCAGTTGCG
CAGTGAACACTACAACAAGAAGCAATAAGCTGCAGTTACAGTGAATTAGCGATCATAATCTCGTGATTCA
CAAAGGTGCGCTCAAGCGCAGTGCGATTTACATATATTTTTACACCGAAGCCGCTGGAGCTCTTCGGGCA
GCGGAGACTGCGGCGAAGGGAGCGACTACGAATAACAAGTGCGTGACTGCGGCGTTGGACCCGCAAACG
TGGCTGGTGTAGTGACTAAAATAAATCATAAAAATCATGTAAGTGGGCACTTTAGCCAAATCTGTTATTA
AATCTGATCGAATTAAAAAAAAAAAAAAAAAAAAAAAAAGAAACAAAGAGAGGAGAAATCTTGCATGCGTT
ATATTTTGCAACAAATCATCTGCTAGAGTAAGAACAAAAGTTTTTGAGAATTCGATTTGACAGCAGTAAA
CAAATTTTGTAAAAAAAAAAAAAAATACTTTGTGTTACTTTTTATGCCACTCTAAAAATTGTGCTGCAACGG
CACATACTTTCTTGCTTATGCGCTTCTCGTGGTTCAGAGCTGAGGCGTAATTCGACCCATCTTTCGACTTTC
TGTTTTGTTTTTGCGTCCGCTAGACCGCGCGTGCATTTATTTGTTTTTGCTTTNAATGCCCATGAAATCGTC
TTTCGGTCAGTATAGGACCCCCCGCAAGAACTCTAGCGACTCGGATTCTGACTGTGACAACCGCATCAGA
ATCCCGGCTCCACGACTTGCCCCTACAGTCCCCCAACTTCGAACAAAATGGATCCAGAGCAACTTAGAGA
GATAATTCAAACGGCCATAAGCAATGCCCTAACAGAACAAGCCAATGCAAACCAGCAAAGAGAGCAGCT
ATTAGTGCAGAGAATTAATGAATTATCCGAACAAATGGCAGCAGCAAACGTTGCTGCCCCACAAGTAGTA
GCGTATGCTGCTATTGAGATTAGAAATGACATTCGCTGCGACGAGCCACTAGACGCCGTCAAATGCTTAC
CTGAGTTTGCAGGAATACAGGAATCTTATGTTTCTTGGAGGCAAGCGGCATTAGCCGCCTACCACATTTTC
AAGCCATATAATGGCAGTTCTCGGCATTATCAGGCCGTTATAATTATAGGAAGCAAAATAAGGGGCCCCG
CGGATGCGGTGCTGGCCTCATTTGGCACTGTGATAAATTTCGATGCGATAATAAATCGCCTTGATTTCACA

TACAGTGACAAAAGGCCGATCCATGTAATTGAACAAGAGTTAGGCACCTTGAGGCAGGGCGGCTTGCCCC
TACTCCAATATTTTGACGAAGTGGAGAAAAAACTAACCCTACTGACCAACAAGGTCAATATGTCTTACGA
GCCCGTTATGGCAAAAGGTCTTTGTGAGAAGTTCCGTGACGACGCGTTGCGCGTTTTCGTATCGGGGCTTA
AGCGCAATCTCACGGATGTGCTCTTCGCAGCCAAGCCTAGAGATCTGCCTTCGGCACTGGCGCTTGCTCAG
GAGGTGGAGTCAAACCACGAAAGATATGCTTTCGCGGCTAATTTTGCCAAAAGCCAAGAAGAAAAAGAG
CGTAGGCCGAATACAAAGACACAGGGCCGACAGCAAGATAGACATTCTGAGCCGCAGACAAATGGCGCA
AAAAACCCTCATTTCAACAAACAGCAGAAACAACAGGGTCAGTCCGATCAACGAAGTGGTCGGCAGCAA
AACAATAATGGTGGACAGGAACCCATGGAGGTAGATCCTTCATTTTCAAGGATCCTCCAACCCACACAGG
CACCGGGGTATCTGAATAGAAAACAGGCAACATCTGATCACACGGGCTTTCAAAAAAGCAACAAAGGGT
GAATCATGTAACGCAGGACGCGGAACAAGGATCGCAAACGTATGCTAATGCCGCCTCTAGCGCAGAGGC
GCAAATTGAGGATGACGTGAGCGCTAGCTATGATTCAGATGCTGTTAATTTTTTAGGGGTAGATCCCTGTT
ACCCGTCATCGGGCGAAGAGTAGCAGGGGCCGATATGAAATTCCTCATAGACACGGGCGCGTCTAAAAA
CTTTATCAGACCACATGAAGGGTTAAAAGGCGTCCGACCAGTAGATTGTCCATTCACAATCCACTCGATTC
ATGGTACAACTAGAATCACGCAAAAATGTTTTGTCTCAATGTTCAACATAAAGGCAACATTTTTTCTTTTA
TCGGACCTGTCAACTTTCGATGGAATAATAGGCCTCGACCTGTTAACACAGGCAGGGGCATCGCTTTGCTT
AGCGTCAGGTCAGCTTAAATGGGGTTCAGAAATTGAACAAATTTCTTTTCACGCATGCACTAATGTCAATT
TCACTAACGTAGATTGCTCAGATGCACCTCCTTTAGTAAAGAACAAATTCCTGAAACTGCTAAAGGACAG
GAAAAGGGCCTTTGCAGACCCCAATGAGGCCTTACCATACAATACGTCGGTGGTGGCCACTATAAGAACA
ATAAATGAGGAACCCATTTATGCAAAGCTTTACCCATATCCTATGGGGGCGGCTGACTTCGTCAATAAAG
AGATTCAAAACCTGCTGAAAAATGGNATCATACAGAAATCAGTATCTCCCTACAATAATCCTATTTGGGTT
GTGGATAAAAAAGGTACAGATGAGGCGGGTAATCGAAACATGCGATTAGTAATGGACTTTC
>160_rnd-5_family-4267__LTR___Gypsy
AAACGGAGTGAACTATAAAANAACGAAGTGTAAGACAAGTTAAAGGAGCGAGAAGTTAAAGTTGATGAA
ACAGAAAGTGACTGATTTTGTTTTACTTTTTTTGGTGGCGTGACCGGAAAAAAAAAAATAATAAAACAGA
ACTCTNANAATAATAACAATTGTCAATAATTGGCGGAGTTTTTTTAAACAATATTAACGAATCGAAAATA
ATNATTGACAATAATCAGTGAACTATTTTGTTTATTTTTTTTTGGTGGAGTGACCAACAAAAAAAAAAAAA
AAAAAACNCAGAGTGAGCGGAGAGCCTAAAATGGAATCAGGTGAGTGCCAAAACCCGGGGGTAGGCGTG
CGTTGGATCCACCAGCGACGCAAGGAGGAGCTGGTGGCCATCGCCCAGGAGTTCGGGCTAGAAAAGGAA
GGGCGCGTGGAAGAGCTGCGTAGGCGGCTGTCCACCTACGTGCAGGAGGGCGGCCATTCGCCATTCGGAT
CTGGGCTCGTCTTGCCGAGCTGGAGTCTCAGTTCGCGAAAACGTTGGAACCGCTACCCTGGGGTGTTCCAT
CTGACGTGGACGACCGCGCCAAAAGTCAGAGCCTGACCTTAGGAGCTCGCAATTATACGGGCGTCCGCAG
CCGCCTATCCCTGGCGTGGCCGTCACGGGGGTGGAAGAGGCTCNCGATCCCGGGGAAAACGGGGATCGC
CGAGCNACCACGACCAACGNGGGTATGGGGCGGAACCCGACCGACCCTAATGACGTCCCCTACGCTCCCC
AACCATATTCTCGGCCGGAGGTTAGGCCTTTCNCAAACGTTCCGGAACAAGTTCGGAAGTGGGGCATGAG
ATACGATGGCCGCTCCGACCCCCTCGGGTTCTTGGAGGACCTAGAGGAACGAGCCATTACGTATGGTATC
GCCCTCGATAGATTGCCCCGGGTAATGAGCGAGGTGTTCTGCGACAGGGCCGCTCGGTGGTTCTTGAGCA
GCGGGCTTAGAGATGTGCCATGGTCGGTGTTCAGGAGGGGCTTCTTGGAGTTCTTCCTGCCCCCACGCTAC
TTCGAAAGGCTTGAGGCGTGGGGGAGGAGTTCAAGGAGTACCTGATAGAGCTGAGAGCTCTGATGCATCA
CGCCAGATACGAACCGGCACAGGAGTTGCACCGGATCTACGAAAACGCCGCTCCCGAATACAAACTCTAC
GTGCGTCGGCAAGATTTCTCCACCCTGTCNCAGCTGACCCAGCTGGCNGTGGAGTACGAAACGGTGATGA
GACAGCGAGCGGAATACTCCGGAAGCAGGGCAGGGAGTCAGGTGGTCGCGAGCTCAAGTAGCGCGGAGT
GGGAAAGGACGGTACAGCCCCCGGGGGGACACCTTAACCCGTTCCGGTCTACTCCCATCGGATCCGGAGC
GATCGTTGTGGATAGACAAGGTCTCCGCCCCGCCGGCAACGGCCCCTGCGGAAGTGGCCCACCGAATGAT
GAACCAAGAGGCCGGAACCGGGTATGATCCGCGACGAGCATGTCGCAGGTGCGCACAGGAGGGGCACTT
TGCCAGGGAATGTCCCAATGCGCCCGTTTTGTTTTGTTGGGTATGTGGGCACCGGGGCAGAAGTAGCCAG
GATTGTTGTCGCCGCACGGAGGCGGGAAACGGGCAACGGCGTCGCCCAGGTCAGGGTCGGGTCGACGCC
GACACACACGGGACTCGGTAGTAGTACACCCGCTTAAGGTGGAGGGTGGCCGGATCGTCGCCACGGTGG
AGATTGGCGGGCGGGCGATGCCTGCTACTATCGACACAGGGGCCACTCGCAGCTTCATGAGTGAAGACTG
CGTACGCAAGTGGGCGATTCGGGGGAGAGTGGAGAACGTACAGGCGCGCATTCGTCTGGCAGATGGGTC
CACGCTCGAAGTAGGGAAGGCATTGAGGATAGACGTCGGCCTAGCGGGGAAGGCGGTCAGCATGTTAAT
GCTTATCATGCCCGCAATGCTGGACCACGTCATCCTGGGGATGGACTTCTTGGGTGCGATGGGCACTACG
ATACGCTGCGGCGAGGCGGAGACGTCTCCGGCCGCGGGGCCTGGAACCANGAAAGAGCCGAAAGAAGGC
CTCTCGCGCCAAGGAGCGGGAGCGCCTGCTCGGCAACCGACGGCAGAAGAGACNAAGAAGGAACCGAAG
GAAGGCCTCCCAGGTCAGGGAAGGGGAGCACCTGCTCGGCAACAGAGAGAGGACACCAAGAGGGAACC

GGAGGAAGGCCTCCCAGGTCAGGAAGAGGGAGCACCTGCCCGGCAACCGATGGAAAGGGGAACGAGAA
AGAGACCGTCGAAGGGGTCTCACGCNGGATTGGACCAAGGCGCTTAAAGAGTCCAAAAGGCGGCGGGTA
GACTTTCAGCGACAGGTCTACGCCGGTCATGGAGGCGGCACGGAAAGGTGCCAGATAACGGAGGACTA
GGAGCAATGGCTAGAATCCATACGGACACAGGGGATCAGGAGCGACTGCCAGAGGGTAGCGAGAGAGG
GTTGTGGTGCCCGGAGGAGGCGAGAAGCGAGCCCAGCATCGAAGAGGCAGAGTGGCCCGCGGATCTGGA
TCCGGAACTTAGGGAGTTCCTGGAGTCGGAACTGGCACTCTTCGAGGGGCTGCAGGGAGTCTCGCATATC
GCGGAGCACAGGATCCGGCTAAAGGACGACAANCCATTAAAACAGCGATATTATCCGAAGAATCCGGCC
ACGCAGAGGGTGATTGACGAACAGGTAGATGAGCTGATCCGGGCAGGTGCCATCGAGCCGTCGCGTAGT
CCGCACAGTGCCCCAATCGTCTTAGTCAAAAAGAAGACTNGCGAATGGCGCATGTGCATCGACTACCGGC
AACTCAACGCGCATTCCATCCCAGATGCTTATCCTGTTCCCAGGATTAATCACATCCTTGAGCGGTTGCGT
CACGCCCGGTTCATCTCCACTCTGGATTTGAAGCACGGGTACTGGCAGATACCGATGGCAGCCGATAGTC
GGGAATGTACGGCGTTCACGGTCCCCGGAAGAGGTCTTTTTCCATGGCGGGTGATGCCATTCGGTTTACAC
TCGGCGTGTGCGACCTTCCAGCGAGCCTTAGACACGGTTATAGGGCCGGAAATGGAGCCTCATGCGTTCG
CCTACCTAGACGACATCGTGGTAATAGGGGCAACGAGAGAGCAACATGTAGAGAACTTGAGGGAGGTGT
TCCGCCGCTTAAGGGCGGCTAATCTCAGGCTGAATAGGAAGAAGTGCAGTTTCTTCAGGGAGAGGTTGGT
TTACCT
>9_rnd-5_family-2230__LTR___Gypsy
TATAAAATTGTGCGGAACCTTCTCCGCTCAATTAGAAGTATAGCCCGTAAAGCTATGCGAAACAATAGGT
AGGAAAACTGTAAACATAAATGCAAAATAATTCAATTAGCAAATTTGATGAAAATTTACCTAATAATTAT
AATTATAATTTAATTATATATAAACCCAAAATGGGACTAGTAGACTTAAAGACAGTGGATTCCACTGCTA
ATGTCATTAATAAAGTAGAGTCAAGTAACATGGATTTACAATTAGTAAATATACTATTGATAATAATTGTT
GTTATAATGTGCGCCAATTGTTTTTATAAATTATACAAGTTGCATAACAAATGCTTAAAAAGAGGTATATG
AGCCAGGCAAATGATCTGGACAAGATTTAAATTAAAAAAAAAAGAGCGCAAACAAAAAAAAAAAAAAA
ATTTAACAATAATTCCGTAATTTACTTTGTAACCATTGAGAAAAATGAAATAAAATTAATAAAATCAAAT
ACAAATAAAAAATATATATAAATATGGCTAAACTAAGGACAATTAGAGAAACTTATAATGGAATGGAAT
GAATTATATCAAGAACTAAATAAAAATAAAAAAATTGACTTTGGTAAATCATATAAATCACTTAACCAAA
AATAGGCCAATTCAGAAGAATACTGTTAAGAAACATGCAGAAATATTAGTAAAGTGCTTTAATGAAGCAC
GTATATTAATACATGGCCAAAGAGGAAAATTAAATAAAGATCATTGGTCCAGAGTATCCAAATTTTTGAT
CAGACTTCGAGCGAATTTAATATCAATTAAGCAAAAATTCGATATTAGATATATCAGTCCCGACCATACTT
AATACTTCTCTAACAGTTGCAACTGATGATGAACCGGAATCAACAGAACCGGCTGACATTGAACCAATAT
ATTTAGAAGAGTTAGAGTTAGAGTCAGAAGAGTTAGAGACAAACCCCAAAATAGAAGAGCAAGATTTAA
ATAACCTTACTATTCCAGCAGTTTTAATGTCAGATCTGGATGATAATACAGATTCTGATACTAGTTCAAGC
ATAATAGAGAATAAATTCAGTAATAATATCACAAATGGCACAATCCAATATTGATTTCATTAATACTGCAT
CAAAGCTTATACCAGTTTTCGATGGTAAAGCTGAAAACTTAACAAGTTTTATTGATGCATTGCACATAATA
GAATCAATAAAAGGCGAACACGAACAATTAGCAGTTTCAATAATAAAAACCAAGCTAAAAGGTGTTGCC
AGAAATATAATCGGAAATGAAACAACAGTAACTGAAGTCATTACCAGATTGAAAAGCAATGTCAAAGGC
GAAACTGTAGAAGTATTGTCAGCAAAACTGATGAACCTACAGCAACGCAACAAAACTGCAAACCAATAT
ACATTAGAGATTGAGCAGATGACAAAAGCTTTAGAAGGTGCGTATATAACAGACGGTTTAACCCTAGAGT
TAGCCAGAAGTTATTCCACTCAGCATGCAGTCAAAGCAATGATTAAGAATTGCACGGTTGATAAGGTAAA
ACTTATCATGCAAGCTGGTACCTTCAATACAATGAATGATGCCGTATCCAAATTTGTAAATAGTTGCACAG
AAGCAACTGGACAGCCGAACACTGTCCTATATTACAAAAATTATTCACAGCGCGGCGGAAACCGCGGACG
AGGAAATNATAGAGGTAACTTTCGTGGTCGTAACAACTACAATAGTACCTATTATAACAATGGACGAGGC
CAATATAGAGGAAACTCAAGAGGCCGTGGCAATTCGAATCGAGGCCACAACAGCAATACCTCAAATAGT
GTCCGAGTAGTCAACAATACATCGGAAAACTCACAAAACCCTTTAAATGCTCAACAGTAGAAGATGCAAG
GGTTCACACCATTAATCTCAGTCAGAACATATTCGTATCATTCAANCATGTGGCTACAGGAAAAGAACTT
GTCTTTTTAGTAGACACAGGTGCAGACATTTCAATAGTTAAAGAAAATTCTGATGTCTTTCAAGACATACA
AGATAATTACATTATAGACATAAAAGGAATAAGTGAAGAGGTTATCAAGTCCAAAGGATTAACCTCTATT
GAGATACAGACCACTAAGTATATAATCCCACACGATTTTCATATCGTGGATTTACAATTCGCTATTCCTTG
TGATGGCATAATAGGAATCGATTTTATTAAAAAATACAACTGTCAATTAGACTTCAAGCAATCTGAAGAT
TGGCTTATAATTAGACCTAATAACTTAAAATTTCCAATTTATGTTCCGATAACATACAGTTCTGGCAACAA
CTCAATACTTCTGCCAGCAAGATCCCAAGTTATTAGAAAAATTCAACTAAATTCAAAAGAGGACAGTGTA
TTGATTCCAAATCAAGAAATTAAACATGGTATTTACATAGCAAGTACCATAGCAACAACTGAAANNCGTT
TTATAAGACTACTCAATACAACAAATAAAGATCAAGTAGTCAGTGCAAACAACTTAACGTATGAATCACT
TTCGAACTACGAAGTAGTCAAAACTAATACAGAAGATAGGGAAAAGTCTGTATTATATCAACTTTCAAAA

AACTTTCCACCACAATTCAAGTCACAGCTTTCAAGCTTATGCACCNAATATAATGATATATTCGGATTGGA
AACAGAACCAATAAGCACAAATAATTTTATAAACAAAAACTGAGATTAAAAGATGATGAACCAGTGTAT
ATAAAAAAACGACAGAAGCCCACACAGTCAGGTTAACGAAATACAGAAGCAAGTCGGAAAATTGATTCA
CCGACGGGATTGTTGAACCATCCGTATCTGAATATAATAGCTCCATTATTACTTGTCCCCAAAAAGCCATC
TCCAGGTTCAGAAGAAAAGAAATGGCGATTAGGTGATTGACTATCGTCAAATTAATAAAAAATTACTGTC
CGATAAATTTCCACTCCCNAGAATTGATNATATCCTAGAACAACNNGGACGAGCAAAATATTTTTCATGC
CTTGACTTAATGTCAGGATTTCATCAAATTGAACTCGAAAAAGTTCAAGAGATATAACGTCNTCNTCAAC
GAGCAATGGCTCGCATCGCGCTTCACGCGATTGCCTCTTCGGACTAAAAAATGACAGCGCCTAATTCATTT
CAAAGAATGATGACTNTTGTCATTCCTCTGGACTAGAACCTTCACAAGCATTCCTTTTATATGGGTGACTT
AATAGTTATAGGTTGTTCCGAAAAACATA
>9_rnd-6_family-395__LTR___Gypsy
GAAGAGGACCTGTTTCATTGTTTCGGCGCTCCCGAAACTGTAGTCTCTGATAATGGAGTACAGTTCAAGTC
GCATGCCTTTAATAATTCAATGCAAAAGTATAGAATAAACCATGCCTATACAGCGGTCNACGCACACNGG
CTAATGCCTCCGANCNAGTTANTAGNTCTGTGATTTCTGCCATNANGGCTATATTTATCNCAATCAATCGA
ACTGGGACGAAAATTTGAGTNAAATTTGCTGCTCCTTACGGCTCAAAGCTTACATACAGCAGTAAATAAT
ACCCCGTATAGACTTGTCTTTGGTCAACATATGATAACAAATGGTTCTACCTTATCGGCTTTTACGTGACCT
AAACTTACTCGAGGATCGTGCCGTGTCATTCAACAGAGACGATTCTTTCGATTTAATGAGGAACAAAGCG
GTCAACGTGATGCACAATCAACATTCACGAAACGAAAAGCTTTACAATTTGAGAAGCCGANAGGTCTCCT
ATGTCGTTGGACAGGAAGTCTTTCGCAGAAACTTTCAACAAAGTAATTTTGCCAAGGGGTTTAATGCGAA
GCTCGCCCCGACATTCGTAAAAGCAAGAATTCGTCTAAGCTAGGAAACAGTTATTATGAGATTGAAGATA
TGCAGGGTAATCTAATTGGAAAATATCATGCAAAAGATATTGAACAATAACTTCTCTTTCAGTCCTTGTCA
CAAGCGCTTATCCCCCTTGTGGTACCCCAAGTGCGATTTTTGGTCGGGGAGGCTTGTAGTGGCGCTTGTTA
NCAAAAATTANTTGACTATTTTTTTGTGTGTGCCCAAAAGTAGGCAATGGAATTTTCGTTAATGCAGTTNT
GACTAACAATTGGTTTGACACTTAGTAGTAGATTTCTTTTTAGTTTTTTAGTAGTATTTTTGTTAATTATTGT
AGTTTGCCTACCTACGCAGAAAATGACAACAAAAATAAAATATAAATATAAAAATAATATAATATATATA
ATATAATATATANATATATTTATATTTATTTAAAAATGTTAGCTGAAATATAAAACTACTCGATTACACTT
GGAAATTGCACACTTGTGTTCGATCTACAAGAAAAAAATGCGTGTCTGCGAATTTTCGACACGGTGAGGT
TAGATGACACTTCCGCAAGCCAGACTCGACCTTTCTACCAGTGATTGTCAACCAGAGCAGTCGCATTTAAT
AATTTTGCNTAAAGCTTAGCCTAACCTGCAAGCACGTTTTTGTATTATTTTTTTGTGCTAAATTTAAAAATA
CAAGGCTAACTCCCATTGGCTTGAGTCCATGCAGGCAGCTATCCAATTGGTACATAGCTGACCGTAGTTTG
AAAAAAGCAAAGCCCACAGTCGGACGGAACAACCTTTCCATGTTTTNATTTTTGGTGCCAATCCCAAAGG
GTAAATATTTCTTTCCTCTTGCGGACCAGCCGCTCGTTGCTTTTGCGCTGTGTACTTTTTCTAATTATCATTT
TTCTGGTGTACACAGATTGGCACTTATACAGAAACTCCTAAGCTAAGTTTCTCACTCTCTGAAATAATCGA
GCAGAANCGGAATTACGGGCACAGGGAGCATGGCAGATCATCGCCACCTGACACAGTAGTGTTCGGCAT
GTGTTAAACAAGCACGGATCGACGCACACAAAAAAGAAAAAAAATGTTATAAGTTAGCAAACGAAAGTG
CCGCCGTTTGTAATAGTCCGAGCCATAACCTGCGGTGAAAAATATTTTTGATTTAACAAGTTTGCTATGCT
CGAATAATTTAATTGAATAAGTTATAATAGTTAATTACCGAGCGGCCAACCTGGCGACTAAGAAGTTGCA
TATTTTTTTGTTTTGGTTTCGATAATATTCCCCTTTTATTTTTTTTGTTTTTTTGCGTTGGGTATAATGTGCTA
AAGCGAAAAGTTGCCGATTTAACAATAAGTGAAAAGCAAAGTGATTTTTTGTCGGCAAATGAAANTTTTA
AAACTTTAATTCATCTAAAACAATGCGTCAGAAAAATAAACCCTGCCTTTAAACATATTTATAAATACCAC
TCGTATGGACACCGCCGAAAGTACTTTCGTCGCTGGATCTGCTAAATGGTAAGCTGGAGCACCCCATTATA
ATTTACAATTGTTCTGTTTGCCGAGGGTATTGCTTGGCAGGTTCACGAGCCAAAATAAGTTTTTATTTCAA
CAGCCAAATGAGCATGCGAACGACAATTTTAAACAGTTGTTGTTGTTTTATCGTCTTGTTGTGCCAAAGCG
TGAACCGAGCGTATGTGAAACCTGAGTAAACAACGGTAAAAAAAAAATTGATAACAAATTGGCGAGCGTT
TGATCAGTCGCATGCAATGCATATCAGCTGTGCACCGTACGGTAGTGGCACTAAATATTAAGCACACCTC
CGGTAAATTTATTAAACAGCTAAAATGGCCTTTAGCCGCATAATATAATCTGCATGGCTTAATCCTACTTT
TATTATNACGATACATATTTGTGTCTTTTGACATATTTGNATATATAAAATATACATATTAACCCTTACTGT
TTTCAGCAACTTTTTCGCTTCGGTATAGTAATAAAATAAATCAACTAGTTTTTGGCCTAGAGTAAGCAAAA
GATATGTAAAATTTGGTTAGTGGTAGGGAAGCAACTATAATATAATAAATGATCATTGCTTCTAATTTAAA
GTAAAATTAACTCATTTATATGTTGTAATAGTTTTTGGAATGTTAAGGAATAACCACAACTTAAATGAAAG
ATCTAATTGACATAAATAAAGAAGTTACCATGAATTCTAACTAAGACCCTTTCCCATTCCGGGATTTATGA
AAAGCAGACTTGAGTCAATGTAAACAATATCGAGGGTGTACAAGGGATCGTCTCAAACATGTCTTGGCCC
TTGGACGATCATGGTAGGGTGGGCATACGCTGCTTCCAATTTCCATCTCTGGGGTGGCTATCGTACACTAT
ACATTCATAAATAAGTGCAGACCTACTTTTTTTTTCTTCTCTTTTCTTTGTCTATGATGTCGTTCCAACCGAT

TGATAGGCGACCTGTTCCCCAAAACAACACGCGAAATTTTAATAATTAATAATTTTCGAACGAGCATTTGA
CATCTCGGAATTTTTCTGAACCCCCATCGCCGACGAGATAGAGCCGGAAAACTAACGCGTGCTCCGTAAG
GTGTCTATACTTCTATCAGCTCGACTCTGCAACTATCCTACGTTGTATGTTATA
>9_rnd-1_family-338__RC___Helitron
TTTTTATACCCTGAACCCATTAAAAATGGGTACAAGGGTATATTGTATTTGTGCAAAATCCAAATGTATGT
AACAGGCAGAAGGAAGCTTCACCGACCCCATAAAGTATATATATTCTTGATCAGCATCAATAGCCGAGTC
GATCTAGCCATGTCCGTCCGTCCGTCCGTCCGTCCGTCCGTCCGTCCGTCCGTCCGTATGTATGAACGCAC
GGATCTCAGAACCTATAAGAGCTAGAGACTTGAAATTTTAGATGTAGATGCTCCTAGCTCCCGCGCAGAT
CGAGTTTGTTTCCGATAATCGATAACTTGCTCCGTTTCCAAGCAATCGATAAAAATCGATATCGATATCCT
GTTTTTGGGCAATTTTGGTAAATAATAAGAGCTGGAGTCACCAAACATGATATGTTGCTTCTAGAATATTA
TATATATGTCAAGTATCTTTCATTTTATACCTATCGCCACCCCCGCTACCACCGCAGAGCTATAAATCAAG
TTAATAACCCAACTTGTATCGCCAACCAATATAAGCTAGAATTTAAATGCAATTGACTTTTTCAGAATACA
CAAATATTCTATGGTACAATAAGATATACTGTATAAAATTTCATCAAGATCGGTTAAGATACATAGAAGTT
ATTAAAGAAACCATGTGGTTATATGTATATGTACATATATATTGGCAGGCCACGAGTCAGCGGCAGCTGG
CACAAATTTCTGTATACATGTGCACTAATACACACAAAAAGAATTACAATTGCGATTGCATCGTATGCTCT
ATGTACATATGTGGTCCTATTTTAGTTCAGTTTTTTTCAATTAGTAGCAACTTTCGGTATGGCTGCAAGGTA
AAAAGCCACGGGAAGCAGTTCAATTGTTATATGTCTGGAATAAGCGGCGCAAGTTCAATTCCAATGGGGA
AAAAGAATTTTTTGACTATTTATTATTTGTCTAATAATGTTAGTTTTATTCCTTCACCCATTAAAAACGGAA
ATAAGTCAAGAGAATGNCTTTTTTGCTTAAAGCCCTAGTAAGCTTGGCAAGTGCGTAGTGCGAGCTATTTC
AGGTAAGGTATGCATAAATATCCATGCATCTTAATCACTAAAATGTCAATTTACGCAAAATTTAATTTCCT
CATTCAGAAATCATATTCTTTATTAAATCTTACAAAAGAATGTAATCATATGTATTTATATCTATTTTCATA
CATACATACGTTTTAGGCTTAGGCCTAACCAGTGCGTATACACATACGTATATACATTATAATAATTTACA
TATGTACATATGTGTGTTTGTACACATTATTTTTTCTTCCCGCAAGTAGTCGAAGACCACGAAGTGGAACT
TTTCAACACTGTTACTATAAATCTGCGCGCGTAATTTGAGAATTTTATTTCGTAAATATTTATTATAGTTAC
ATGATTTTAATAATTCATAAGACAGTCTTAAGGAGGTTGACTGATGCATTAATAAATATTATCATTAGATT
AATGTTATTATATATGTGATTATAATAATAAATAAAGTAGCGCGCGCATTTCGATATGTATACTAAATCGT
GGCGTTAGGAGAGTGAATATTTCCCAACACTCTCTCTTCAAATGTGCTCAGAAGAAGCAGTAATCGATTTA
GTCTGTAGCCTGCGATTGCCATCAAACTACCACTAACCAAACAGCATATAGATGGAACGTGCCGTAGAAG
CAAAATGGGCACAAAAAGTGCCTGTTTTGGTTTTGTGATTTGTGATTTTTGCGTTTCTTACTGATCTAACAA
CGATTTTTATTCGAATAAACTATATTCTATCGGTTCCGAGCGGTTGAAAATAAATAAATTGCAGTGTTTTT
GTGACCATTTTAATAAAAACAGTGAATTATCGATGAATTACCGATAAAAATAGTTTTCGGCCGTTACAACT
TGGCGTGTATGTGCAAGTGTGTGATTGACACTTATTAGGAATCGTATTTGTGCGAATGCAATTCACACAGC
TGGTGTTGTAAGGGAAGTTTTGGAGAACCATTTTAGCTGAAATCGCAAAAATCGCAAGTTTTAAATATAA
TTATAATTTTAAAGTCTTTTTAAAGACTATACAAATATTACCTACTTTTTATTTATTCCAGACATATATATA
TATCTATATATACATATAATATATATAAATTATATAAATTAATTTTAATAAATTTTATTGCAAATAATTTAT
TGGAATTAATTAATTTAAATTACGAATTTTACTATATTACATTTTTTTTTTTTTAAAGTTTTAAAGTTAAGT
AAAATGCCTAGGACAAAAAGTTGTCAGTTTTGAATAGGGCTCGTAGACAACAAGCTAGAAATGAGCTAA
ATAGGTCTAATTCTGAATATGCTGAGCATGAACGTATCAACAATATAGAGTCCCAACGAGGGCGTAGGGA
AAATGAAGGTTTTAGCCAAACTGAGAGAAATAGGAATAGGCAAAGAAACCAAGTTCGCAGACAATACAT
AGTTTACAGGGCAAACGAACAAAGACAAAATACTGTAGACCACTCGAGTCGACGAGCTAATTCTCCAGTT
AGAGAACAGGAGCAATTGGCTAATACTTCGCGTCGATCGACACGCAGACAAGACCCAGTCTACCGGGCA
AACGAACAAAGACAAAATACTGTAGACCACTCGACTCGACGAGCTAATTCTCCAGTTAGACAACAGGAG
CAATTGGCTAATACTTCGCGTCGATCGACACGCAGACAAGACCCAGTCTACCGGGCAAACGAACAAAGAC
AAAATACTGTAGACCACTCGACTCGACGAGCTAATTCTCCAGTTAGACAACAGGAGCAATTNGCTAATAC
TTCGCGTCGATCGACACGCAGACAAGACCCAGTCTACCGGGCAAACGAACAAAGACAAAATACTGTAGA
CCACTCGACTCGACGAGCTAATTCTCCAGTTAGAGAACAGGAGCAATTGGCTAATACTTCGCGTCGATCG
ACACGCAGACAAGACCCAGTCTACCGGGCAAACGAACAAAGACAAAATACTGTAGACCACTCGACTCGA
CGAGCTAATTCTCCAGTTAGACAACAGGAGCAATTGGCTAATACTTTGCGTCGATCGACACGCAGACAAG
ACCCAGTCTACCGGGCAGACGAACAAGGACAAAATACTGTAGAACACTCGACTCGACGAGCTAATTCTCC
AGTTAGACAACAGGAGCAATTGGCTAATACTTCGCGTCGATCGACACG
>Ref_dvir_953
CTGGAAACAACAGGAAACCAACGGGGTCTACCATTTCTTTGAATTTTACGATACAGCAAGCCTAGTCTCA
ACTCATACATACTGGCAACATTCTCGTTTAATTCATTGTTTTGTTTAGTTTAGAGATTAAAGATGTCATTTC
GAGATCTCGCTGCTGTTCAGCTATAAGCCAGGTGTCACTTAATTCAGTTAAATCTACGCGCATTTCAGGAG

file:///E/10月/10.9/PDF/4. biology-1907542/biology-1907542-supplementary/SupplementalDocument2.refTElib_01_25_2022.fasta.txt[2022/10/9 15:38:02]

CTTTGACAGCAAACTGTAACTTTTTTGTTATTGGGTTTCTTGAGAAGAAGTCTACATGCGCCATGCGTTTTC
CCTTTCTATACACAATATCGAATTCAAAAGATTGCAAGTACAACCACCATCGATATACCTTGTCATCTAAC
TCAATCTTTTGTTGACTTGCCTTTAAAGAGTTACAGTCAGTATATACTGTAAATTTGATACCTTTTAAATAA
TGTTGATAATATTTTACTGCGTTAAATACTGCCAAGGTTTCCAAAAGATATGAGTTATACCTGGACTCTGC
TGGGGTTGTACATTTACTGTAATACTCGATGACATGGTTTCTATCATTTATTTTGTGCATGAGTATTGCACC
ATAACCCACAGAGCTAGCATCTGTATGCAGCTCAACCGGATATTGTGGATCGAAAATGGTTAATACCGGG
CTACGGGTCAGTGCAAAGATTATCTCCTTTCTCACTATTTTCATGACTTTCTTTCCAATCAAAACTATTATT
GCCTTTAGACGTAAGACAAAATAGAGGCTTTAAAGTTTCGGAGAATTTCGGCACGAATTGTCTAAAGTAT
GATGCTAATCCAATGAACTGTCTTAGCTGCGTAACCGGTTTGCGGCGGTGGCCAATTCAACTAATGCCTGC
AATTTTCGAGGATTAGGTCTAAGCTCACCTGCCTCTACTTCGTAACCCAAATACTCTATTTTTGATTTTAAA
AATGTACATTTTTCGAAATTAAATGAGAATCCAGCTTGAATAAGAATTTCTATGACTTTCTGCAATCTTGA
AATTGCCTCATCTTTAGTTTCGGCCACGACCAATACATCATCTATGTAAACCACAACATACGAATACGAAA
GTGGCCCCAACGCTTTTATTATTGCCCGCTGAAACACCGACCGGAGCATTTTTTAAACCAAACGGCATTGC
CAAAACTCAAATTGCCCTTCTGGCGTTACAAATGCTGTACGCTCTACTGAATCTGGGTGAATAGGTATTTG
GTAAAATCCGCTGGCCATGTCCAAACAAGAAAAATACCTACCCCCACGTAACCTTGCAATTTGGTCGGAG
ATAAGAGGCAATGGAAATTTATCCGAAACTGTGTTAGCGTTAAGCTCCCTATAGTCGACGCATAAGCGAT
CAGTTCCGTTTTTCTTTCTTACCAATAAAATCGGACTCGCAAATGGGGAACAGCTCGGTCTTATAATATTT
GCCGCCATCAACTCATTAATTTTTTCGCGAACAGTTTGCTTCTCATCTACACTAAGCCTATATGGTCGCCTT
TGTACCGTTTTATTTGGATCTATTAACCGAATTTCCAACGGCCTGTGTTAACCTGTTTCTTTGGACCCAGAG
ATAAAATGTTCCATCTATGGTTTCAAAACGTTTATTAAACGGGTTAATACATTCTTCATATGTTTTGGGTTC
AGTAACAACATTAATCAGCTTTACTTTATAAATGCAGAACCTGTCTGACTCAATGGTCACTCCGAAATCTT
GGCTTAAAATTTCCCTGCCTATCATAACATCGTGTTTCATATATTTGTCCATAAACGACATGAAATAACAT
TTCCAAAATAAAATCATTAATGGTTACATTGGTCAAAATTTGCGTGGAGCTACATACAGAATCGTTTCCAA
CACCCTTGAGCATTACTACGTTAAAAAGTCTTTTTCCAGAGAATTTTCCAGCTGCGCTTTCCTTAATGAGTG
AACACTCGGCTCCAGAGTCAAAACAAAAATCAACGGACTCACCAGAATGAACGAGCACACCTTTTTGTTC
CATTACCGAACACATGTTCACTCTCTTTTCAGAGCTTTGTCCAGTCGAAGGGCACCCAGTGGAAATGTGTC
CAGCCTGTCCACACTTGTAGCATATTACGTTGGATTTTTGCTCTTTTGGCCTCATACCAATGCGTCCAGCAT
GCCCGCTTCCCAATGGTTGTGATTCGCGATTTTTCTTCTGGCGGCAGTCTGCGATTTTGTGCCCTGTCTTTC
CACAATAATGGCAAACGATGTTTGAAAACTTCTGCTTCTTCCTTTCAGGTCCCACAGTAGTTTCTGCTTTCG
AAGTCTCTTGACGCTGCCCGATTTCATATACCTGCAATTGTTGCTGTAATTCGTTTCGCGATTTGATGTTTG
GTGGTGCACACAAGCTTTTTTAGTCTGCTGTCCAGTTGCGCAGTATGCGCAAGCACAACTGACACCAACA
ATTTCCTCCAGCTCCATATTTTTCATTTTCGTCAATTAACGAAGTGACGAGTCGGGCTTGCATACGACGAC
ATGCACTCTCCAGTCCCTGGGCGTCCGTCTAGCAGCTTAAGAAATGTTGCTGTGGTTGTCTCTACGCCGAC
AAATCTTTGCGTAAAAAGTTGCTTGAACTCAACCCAAGTCATGCCAGGAAAACAGATTTGTGAAAGCCAT
TGCGATGCACTGCCCTCCAAAGCTTTACTGAGGGCAATCACGAGCGCACAACCCTCAAGTGGATTTTCAA
TAAAAATTATATCGGCCGTAATACTCCAAGCACATGCATCAGCACCGGTAGCTTCGGGGTTGTATTTGGGT
AAAAACACCGTCTTTGACTCACGAGGCGCTGCGCTTCTATTTTGTTGCTCCAAAATTTGACACAACTGAGA
GTTAGGAGGATCCAATTCCACTTATGTCGAAGCGGCCGTAGGGTCAGAATTTTTTACACTTTGAGCAGCGA
GTCCCGGCATCTTTGTTGGTGGCATTTGCTTCTCTTTTCTAAACTTCACAGTGCACAATCACGTCCGTTCGC
TCAGACCATTTACTTCTGTCTGTCCACATCAATCTAACGAAAGGTAACCGCTTTTGCTTCTTGCAACGCGCT
TCTTGCAACGCAACGAAAACCAGCCGTAACCGCTTTTACTTCTTGCAACGCTTTCTTCGTGTCCTTACACG
AATCAGCCGACAAAGAACTACACGCACACCAACAACTCTATGTACGGGCCACACTCAGCGAGAAAAAGA
AAAAAATCGCTCTCTCTCTCTCTC
>9_rnd-5_family-11410__LTR___Gypsy
AAATGCCGAGTTTGGTCAAGATATCTTGANAANCAAAAATGNTTTTTCATACGAAAACTTAATTTTTCGAC
CGATCGTTCCTATGGCAGCTATATGATATAGTGGTCCGATCTTAATAGGATTTTGCATATATATGAGGAGT
AAAGTAAAAACTAATAAATGCCGAGTTTGGTCAAGATATCTTGAAAAACAAAAATGTTTTTCATACAAAA
ACTTAATTTTCGACCGATCGNTCCTATGGCAGCTATATGATATAGTGGTCCGATCTTAATAGGATTTTGCA
TATATATGAGGAGTAAAGTAAAACTANTAAATGCCGAGTTTGGTCAAGATATCTTGATAAAACAAAAATG
TTTTTTTCATACAAAAAACTTAATTTTCGACCGATCGTTCCTATGGCAGCTATATGATATAGTGGTCCGATC
TTAATAGGATNTTGCATATATATGAGGAGCATAGTAAAAACTAATAAATGCCGAGTTTGGTCAAGATATC
TTGATAAACAAAATGTTTTTCATACAAAAACTTAATTTTCGACGACTCGCATATGATGTCCGATCTTAATA
GGATTTCGCATATATATGAGGATATAATGGTCCGATCTTAATAGGAGATTGCATATATATGAGGAGCATA
GTAAAAACTAATAAATGCCGAGTTTGGTCAAGATATCTTGATAAACAAAATGTTTTTCATACAAAAACTT

AATTTTCGACCGATCGTTCCTATGGCAGCTATGTATATGATATAGTGGTCCGATCTTAATAGGATTTTGCA
TATATTGTAGTATGTGCATATATTGAGGGTACACTGCACCAATAAAGTAACATCGTTTAAGCATAAATAA
ATCAGCAACCCATTGTTGCAGTACACAAACAAAACCNTTTCGAGTGAGCGTGTAATATGATCGCCCACTT
ATGAGCCACGCTGACCATTCGTAGCACATATATACATACATGCAAATATGTACATAAGACCCTCTCTCTCC
CGATTATGCTGATAAGACAAAATATGTATGAAGCCGCTCCGCTGCTGGCGTAACCGGCAGCGCAGCTATG
CGGTCTTAGCCATAAGAATATATTGAATAAAACATTGAAAAAGCTAAAGTCAAACGGCACAGACGTGCCC
GAGTGTTTAACTATTTGGAATCGACTTTAATAACTATTACATGGCGACCGTGACTAAAAGGTCAAAACAG
ACCTGGACATGGAAGGACAGCAGCAACGGCAGTAACAAAAACACACAACAACAACTATACAAAAACAAC
AACAACTATGAAAAGTGTTACAAGTGATGACCAGCAGTCAACAAGTGTGAGAGGCAATCCGTCCCTTACG
CCAGCGCGGGAGACTTNGAGATTGAAAGAAAAAACAAAGTCTGAGTGAGTAGAGTGCGAGTAATGGGCA
AACGCGGAGGCCAGAAAAACAAAAATAAATAAAAAGAATAGCACACATTAAGTGGCTATTATACACGAA
TACGCCGCCCACATGGGCAACGGCACAAAAATTAACTGCCCGAACTTGATTTTCTGAAAGAAAGCCTCCA
GCAAAGAAAGCCAAATACCGCAACTCACTCAGCAAATATCAAAGTTATAAATGAAAAACTAATGAAAAA
ACCACCATCTCCAAAGCCGTTGCCGAAAAACCTGACGTCTGCCCGAACGACTGCCCTGGACCACTATACG
CTCCAGCATGCAAATTCGCATGCTTGGGACCAAAGACCAGTGCGAAGACCGGCACCGGCCCCGCCTGAGC
CAATAGGCCACGGACATCAGNAGCAGTGAAGGCAACAGCGCGAGGGGCACTCCGCGACTTTCGCCTACG
AAGGAGCGTCAGCGTAGCCAAACCGGCGACGACCAGCTGACGCGGAGGACAACTGGACATCTGGAGCAA
CAGTTANCATTATTATAATAAAATTCGTAAAATTGTGCGGAACCATCTCCGCTCAATTAGAAGTATAGCCC
GTAAAGCTATACAAACCAATAGGTAGAAAAACTTGTTAACGTAAATGCAAAGCAATGCAATAGTAAAATT
TACTGAAGATACAACAAATAGTTATAAATACACTCTAAAAATATATACGCCCAAAATGGCAATAGTAGAC
GTTAAGTCAGTGGACTCCACTGCTAATGTCATTAATAAAGTAGAGTCAAATAATAATGACTTAGAACTAA
TACACACACTGTTAATAATAATTGTTGTTATTATGTGCGCAAATNGTTTTTATAAACTATATAAATTACAT
AACAAATGTTTAAAAAAGAAATATATGAGCCAGGCAAATGACCTAGACAAANTTTAATTTAAACGAGAA
AGAAGGGCTATCTTCGGCACGCCACAGATCTAATACCCTTGCAGACCCAACCTTTCCATAATGCTCATAGT
GCTTTGCCCCTATCTAAGAAGTACCGGGAAAATAGTAAATGCCGAGTTTGGTCAAGATATCTTGATAATC
AAAAATATTTTTTCATACGACTTAATTTTTCGACCGATCGTTCCTATGGCAGCTATATGATAGAGTGGTCC
GGTCTTAATAGGATTTTGCATATATATGAGGAGCATAGTAAAACTAATAAATGCCGAGTTTGGTCAAGAT
ATCTTGAAAAACAAAATGTTTTTCATACAAAAACTTAATTTTCGACCGATCGNTCCTATGGCAGCTATATG
ATATAGTGGTCCGATCTTAATAGGATTTTGCATATATATGAGGAGCANAGTAAAACTAATAAATGCCGAG
TTTGGTCAAGATATCTTGANAAACAAAATGTTTTTTCATACAAAAACTTAATTTTCGACCGATCGTTCCT
ATGGCAGCTATATGATATAGTGGTCCGATCTTAATAGGATTTTGCATATATATGAGGAGTANAGTAAAAA
CTAATAAGTGCCGAGTTTGGTCAAGATATCTTGANAAACAAAAATGTTTTTCATACAAAAAACTTAATTTT
CGACCGATCGTTCCTATGGCAGCTATATGATATAGTGGTCCGATCTTAATAGGATTTTGCATATATATGAG
GAGTAAAGTAAAAACTAATAAATGCCGAGTTTGGTCAAGATATCTTGAAAAACAAAAATGTTTTTCATAC
AAAAACTTAATTTTCGACCGATCGTTCCTATGGCAGCTATATGATATAGTGGTCCGATCTTAATAGGATTT
TGCATATATATG
>9_rnd-5_family-1769__LINE___I-Jockey
CGGTGGTAACCGCCCCGCCAATTATAAAGGCTGCGAGTGGTTCACCGAATATACAAAGCGTGCGGGAGCC
CCAAAGCCCGCCAAAATGAAAAATCTCCCACCGACTCAAGCGAACCGAAGCCGCAGCAATAAAGTTCAT
GGGCCTACACCTGTTGACTTCCCCCGTACAGCACCCACTGAACAAGTAGAAGGGCTTTCATACGCCGCTG
CCACCAGAAGAGGACTAGCTGCATTCAGCAAAGAGAACAACGTACCCAGCGCTAGCCAGCGAGAAGGAC
CAGCGCAAGCTAGGCTACATAGATTGCTGAGGGAACAGCAACAGGAGAGGCTGACTGCGCAAGAACAGC
AACAGCAGACATCTGGAAACGGGGAAGGACTTTCCTCTGCCATCAAGGACATGAGCCGAGCTATCGAGA
GGATGGCAGTAATGCAACAGCAGGCTTTGGGTATGCAACAGCAAATCCTTAACCTTTTGACCGCGTTGCT
ATCCAAATGAACACACCACTGAAGGTGTTAATCTGGAATGCCAATGGCATTTCAGGTAAAGTACTAGAGC
TGGAGGCTCTAGTACAAAAACACGCTATCGGAATAATTCTGCTGTCAGAAACTAGAACCAACGGTCCTAC
TTTCCCCAGAATCTTTGGCTTCGAGGCGTACCCTGTACACTGGACTCAAAATCGCTCAGCTAGTGGCGGCT
GCGCTGTCCTCGTCCGGAGTAACTTGGTTCACTTCCCGCAATTGAGCATTGCAAATCGTGTTTACCAATAT
TCGACTGTGAAAGTCTCCACTGATGCTGGTAGCGTGGACTTTGGAGCAGTCTATTGCAGGCCAAATGAGC
AGACGTCGACTGATGACTTCAACGTCATACTCAACTCGGCTGGAAGCAGATATCTNGTGGGAGGCGACTG
GAATGCACATCACTGGCTGTGGGGCGCAAATAGCAGCAATGCGCGCGGCCTTCAACTAGCTAACAGCCTA
GCGCATCGTCAAGCGAAGGTACTGGCCACAGGTTCTCCCACAAGATACCCGTACGGCAGCAGAGGCACTC
CGTCTTGCATAGACTTCGCTATCTACCAGGGAATCCCTGACCAGCTCCTTTCAATCGCTGACGATTGGGAC
CTCCCGAGCGACCACCTGGCACTGATAGTTACCCTGGAGAGCAGGGCCAGAGTGGTCAGCGCAATACCGA

GGCTGATCAACTCACGCACTGATCTCGGTGTCTTCCAGAGACAGATGAGTGATAGTATACACCTCAATAC
CGAAATCGGCACTGGACTGGATGTAGAGGACGCCATTGAGCTGCTTAACGCAAAGATTCGCGGCAGCAGC
AGCCGAAGCCACCCCTCCCCCCACCAGTTGCCAGGCGGCTGGATAGTGCTGGCAACGGACTGCCCGCAGA
TATAGCCAACCTGCTCAGGATCAAGAGGCGGTGCAGACGCAGGTTCCAGACGACTCAGCATCCGTGGGAC
AAGGCGGAGCGGCGAAAACTCAAGGAGGCCCTTCGCAGACACAAAGACGCATCCTTCTCCAGAAGGTTG
GAGAGCATGGACTATACCCATGACTCAGGATACTCGCTCTGGCGAGCAACTAAAAACCTGAAGCGCCAAC
CCTGGAGAACCGTTCCCCTTCTTCGCCACAATGGAACATACTGCCTTACAGACGAAGAGCAAGCTGATAC
CTTCGCCAGCCACCTGGAGGAACGGTTCACACCTTCGATGAGACTACCCGAGCGTTGGAGACTCCGCTTC
AGATGTCACCACCTATCGGCCCCATCCGCGTGGAGGAGGTGATCAACGTCGTCAAAGCCCTTCCCAGGGC
TAAAGCACCGGGCCATGATGGAATATGCCACACCACTCTTGAAGGCCCTGCCAATGCGCGCGCTCCTTTTT
ATAACGTTGATCTTCAACGCCATATTAAGGACGCAACACTTCCCTGCCCAGTGGAAGTTAGCGAGGATCC
ATATGATCCATAAGTCGGGCAAGCCGGAATATGACCCTGAGTCATATCGGCCGATCAGTCTCCTGCCTTCC
TTGTCGAAGGTTTGGGAAAGACTGATTGTCGCCCGACTTGCTAGGCTTATGGATCAAATGGCCATTCTTCC
TGATCACCAATTTGGGTTCAGGGGNGGGCATGGCGCTGTTGAACAGGTTCACAGAGTGATTAACCATATT
CTTAAGGCTTACGACGACAAAGACTATTGCTATGGAGTCTTTGTAGACGTTAAGCAGGCCTTCGACCGAG
TCTGGATNGATGGATTGCTCCAGAAAATCAAGATGAGTCTCCCCGCCCCCTTCTTTCTACTTCTGCGCTCTT
ATCTAATGGAGCGTAAGTTCGAAGTCAAAGTCAGGACAGCAATCTCTCCGGTTCGCTATATGAAGGCGGG
TGTCCCTCAAGGCAGCGTACTGGGTCCCTTCCTCTACAATTTGTACACGGCAGACATCCCCACTCCGGATG
ACCTACATGAGTTCGCCACCCCAGGCAAACACTTGATGGCCACATTTGCCGATGACGTGGCAATATTTTAC
TCCGCCCACTGTGAGTACGAAGCCACTGATGGGGCCCAGGAATACATCCACTTGCTTGAGAGCTGGGCTC
GCAGGTGGAACATCAAGCTCAATGCCTCGAAGTGCCAGGCGGTCAACTACACACTGAGGCGTGGCAAGG
CTCCAGCAGTCTGGATAGATGGCAGCCCCCTTCACCNTGGCANAAGCGCCAAGTATCTTGGAATTGTACT
GGATAAGAGGCTTACCTTTCGGGCGCACATCGACGGCCTTCTCAAGCAGTGCCGCTTTAAGATGGCCAAG
GCCAACTGGCTACTAAGCCCCAACAGCCCATTGCCACTTCGCTGCAAACGGGCAGTTTACAACGTCGTGG
TGGCGCCAACTTGGAGATACGGTATCCAGGTTTGGGGAATTGCATCTCGGACAAATCGAAGACGGATTCA
GACACTCCAAAACTTTGCCTTGCGCCGAATGACCGGTGCTGACTGGTACGTCAGAAATTCTACCTTGCACA
GAGACCTCAAAATGGAGACTATAGACAGCCAAGTCGAAGCATTCGCCAACCGATACAACGAACGCTTGC
AGCGACACTGTAACCGGGTAGCAAGAGACCTCGCTGGGAGGTGTGTAGTCAGGAGGCTCAAGAGAAGAT
CCTTTAAGGACCT
>160_rnd-1_family-135__LTR___Pao
TGTTGGATTCTGCAGCAGCCTCATCAAATTAGCATAAGCAAACATTCATGTAAACATAAGAAGAGGTTGT
AATTTTGGTCTAGTGGAACAGCTGTTTATTATAAACAAGGTTGGCAAGCCTTTCTTGCACTTGTCGCAGCT
GCTATAAATTGCATATTCCTTTTTTGTCTGCTCTCTCAGTTCGTTGTTGATCGAGGCGAAGCGAAGAACCA
ATTCTGAGCCACAGCCAAATAACCTTATAATCTACATAAATTTGCCTAATAAACTGAATTATAACGTGTTG
TTGTGAAAGGCCAAAATTTACTTATTTCTTGGTTTAGGTTCTAAACAGTTGCTAAAAAACATTAGTAACTC
AACATTTGGAGGCCCCGGCGAGATTTAACATTGCAGTGCACACAAGCAAAATAAATCTATTGCAAATTAA
AATTGTGATTATTCAAGTAGAACATAATCATCGTAAATTGTTATAATTAGGCAAGTGCAAACCGCCTGTGT
GAGCTGACCATTTACAGCTCCCAAATATTCGCATATACATAAACGCCGCTAGTTGACTTTCGCGCTCTTCC
CCTTGCGCGGTCATTCGCCGGCTCAGCTTATATACATACGCTTGTACATAAGCAGCTGCACAATTCGTCGC
ATGTTGTGTGCTCAGTTTGGTGTTCTGATTTTGTGCTTGTGAAAAAATGATGCAAGGCGAGAACACAGATG
ATATGCAGTCAAACAGGGTGTCTTTTCTTAAGCGCAAAGCGGTTGCATTGTTCGAGCGAGCCAAGTGTATT
GAAAATGAATTATCTGAGGATAAAATTAACAACTCTTGTGCCGTCGACTTAGAAGTGCGCCTTTTGCTAAT
TGAACAAACTAAGTCTGCCTTTAATACCGCGCATAGCTCGCTAGAGGAGCTTAATTTCGATGAAATTGGC
AGCAAACTACGCGATGATTTTGACGAGTTGATCGCCATGGTTGAAGCTCGCGTTCGCGTNAATATTCAGC
GTCGTCAATCTAACGGCCCGCCCCACTCAACCTTTCTTCGTCATGAGGATTTGCCAAACCAAACCTTGATC
TTAGCCAACAGAACGCGCCTGCCCGAGCTCAAATTACCAACATTTTCCGGTGGTTATACCGAATATTCGGA
CTTTATCGCGATGTTTGAGTCAGTTGTCGACAAGGATACCGAGCTGTCTAACATTGAAAGGTTGCAGCATC
TTCGCTCATGTTTGTCTGGCCCTGCGTTGGATTCGGTTCGATCTTTGGAGTTTTCCGGTGCCAATTATCCTG
TGGCTCTGGATATCCTAAATAGAAGATTTAGTAACAAACGTCTTGTTTTCCAGGCACACATCAATGAGATT
CTTGGGCTCAAGAGGGTAGATTCGGAGTCGGCTTCAAAACTGCGTGAGTTCTCGGACAAGGTCAATTCGC
ATATGCGTGCCTTACAGAATTTGGGCAGTTTGGAGCAAATTTCGGGATGCATAATAGTCCACACGCTGCTT
CAAAGGTTGGACACCCAGACTCAGACCAAATGGGAGGAGGCCGCAGGCATTGACAAAATCCCGACAGCC
GATGAGTTCTTCACATTTCTGGAAAGGCGTAGTCAAAGGCTGGAGAGCGTGCAACACATGCCATGTCATC
CCATATAACCAATTATCAGGTGGGCAAATCCAACCATAACACTAACCACAGCAAAAGAGTGCTTGTAGCT

GAGACTAACCAAAATGGGTGCAGTTTTTGTGATGCGTCTGGTCACGGGATCTACACATGTAGTAAATTCG
GAAATTTGTCTCCATCCCTTCGGCATAAGGAAGTCAAGAAACTGGCCCTTTGTTATAACTGCCTAAGGAAG
GGACACCAAACTCGGTCTTGCAGTAGTAGTCATTGTCGTACTTGTGGAGGAAAGCATCATACACTGTTGC
ATTTTGCCACTTCAAGAGAAGCTGACGCGCCGACACCATTACATACATCGGCTGATTTGGGCGCCGCCACT
TCGACGCACTCTCACTCCACTACCTCTCTTGCTGTCCAAGGTCTTCAGTCCGGTGTCGTGCTCCTGGCCACT
GCAGTGGTACTGGTAAAGAATAGCTCTGGTTCCTTGTTTCCCTGTAGGGCCTTATTAGACTCAGGCTCGCA
GTTGCACATGGTCACATCCCGTTTCGCACAGAGACTTCAGTTAAGGAAAACCAAGTCGTCACGGTGTCTC
GGAGACGCAAGCGTCTCTACTGAAGGATCAGCCGTTAGGATTAACGTGAAGTCACGAGACTCAGATTATT
CAGCCACCTTTGATGCTCTTGTGACTCCAACAATTACCGATAGTCAGCCCAGCTTCGTCATCGATACATCA
GACTGGAAAATTCCCTCGAACATAAGGTTGGCTGATCCTGCGTTCAACAAACCACAGCGAGTGGATCTCT
TAATCGGAGCGAGCCTTTTCTATGAGCTTTTGTCTGTTGGGCAAATTGTGCTTTTGCCAGGACTACCTCTTC
TGCAAAAAACTCGTCTTGGCTGGGTCGTGACCGGTGGATTTGAGAGACCGCGCAGTACCTCGTTGTTGGCT
GCCGGCCGCACTCCTAATAGCGAAGCTAGTCAGTTGGACGTCCGTCTGGATGAGTTGGTTCGGCGGTTTTG
GGAAATTGAAAGCTTGGAAGAGCCTACAGTAAAGGCCACCAAAGAAGAGGCGGAATGCGAGCAGCATTT
TGCCAATAACTTCTCCCGTTTACCATCCGGCGAGTATTCAGTGCGGTTGCCTCTGAAGCGGAGTGCAGAGC
TTCTAGGAGAGTCTTATGGCCAGGCTCATCTTCGATTTCTCAGTCTTGAGCGAAAATTGGGTCGGAATTCG
CATCTGAAGGAGCAGTATTCGGCAGCACCACAACCAAGTTGCGTGTTGTGTTTGATGGCTCAGCAACTAC
TGATTCTGGACATTCTCTTAATGATGTACTGATGGCGGGACCAATCATTCAACCTAAGTTGTTTCACATTCT
GATTAGAGGAACCCTCGGGATACAGGCCAATCTACCTACTGGACACGGCGGGAAAGGTCCTGGAAAGGA
TTGTGTATGACCGTTTACTTAGTGTCGTCGAAGAGAAAGAAGCTCTGTCCAAACAGCAATACGGATTTCGC
AAGGCAAGGCTTACGGTCGATGCAATTAGGGCGGCGGTAGATAT
>9_rnd-1_family-292__RC___Helitron
TTTTTATACCCTGAACCCATTAAAAATGGGTACAAGGGTATATTGTATTTGTGCAAAATCCAAATGTATGT
AACAGGCAGAAGGAAGCTTCACCGACCCCATAAAGTATATATATTCTTGATCAGCATCAATAGCCGAGTC
GATCTAGCCATGTCGTCCGTCTGTCCGTCCGTCCGTCCGTCCGTCCGTCCGTCCGTATGTATGAACGCACG
GATCTCAGAACCTATAAGAGCTAGAGACTTGAAATTTTAGATGTAGATGCTCCTAGCTCCCGCGCAGATC
GAGTTTGTTTCCGATAATCGATAACTTGCTCCGTTTCCAAGCAATCGATAAAAATCGATATCGATATCCTG
TTTTTGGGCAATTTTGGTAAATAATAAGAGCTGGAGTCACCAAACATGATATGTTGCTTCTAGAATATTAT
ATATATGTCAAGTATCTTTCATTTTATACCTATCGCCACCCCCGCTACCACCNCAGAGCTATAAATCAAGT
TAATAACCCAACTTGTATCGCCAACCAATATAAGCTAGAATTTAAATGCAATTGACTTTTCAGAATACACA
AATATTCTATGGTACAATAAGATATACTGTATAAAATTTCATCAAGATCGGTTAAGATACATAGAAGTTAT
TAAAGAAACCATGTGGTTATATGTATATGTACATATATATTGGCAGGCCACGAGTCAGCGGCAGCTGGCA
CAAATTTCTGTATACATGTGCACTAATACACACAAAAAGAATTACAATTGCGATTGCATCGTATGCTCTAT
GTACATATGTGGTCCTATTTTAGTTCAGTTTTTTTCAATTAGTAGCAACTTTCGGTATGGCTGCAAGGTAAA
AAGCCACGGGAAGCAGTTCAATTGTTATATGTCTGGAATAAGCGGCGCAAGTTCAATTCCAATGGGGAAA
AAGAATTTTTTGACTATTTATTATTTGTCTAATAATGTTAGTTTTATTCCTTCACCCATTAAAAACGGAAAT
AAGTCAAGAGAATGTTTTTTTTGCTTAAAGCCCTAGTAAGCTTGGCAAGTGCGTAGTGCGAGCTATTTCAG
GTAAGGTATGCATAAATATCCATGCATCTTAATCACTAAAATGTCAATTTACGCAAAATTTAATTTCCTCA
TTCAGAAATCATATTCTTTATTAAATCTTACAAAAAAGAATGTAATCATATGTATTTATATCTATTTTCATA
CATACATACGTTTTAGGCTTAGGCCTAACCAGTGCGTATACACATACGTATATACATTATAATAATTTACA
TATGTACATATGTGTGTTTGTACACATTATTTCTTCCCGCAAGTAGTCGAAGACCACGAAGTGGAACTTT
TTCAACACTGTTACTATAAATCTGCGCGCGTAATTTGAGAATTTTTATTTCGTAAAATATTTATTATAGTTA
CATGATTTTTAATAATTCATAAGACAGTCTTAAGGAGGTTGACTGATGCATTAATAAATATTATCATTAGA
TTAATGTTATTATATATGTGATTATAATAATAAATAAAGTAGCGCGCGCATTTCGATATGTATACTAAATC
GTGGCGTTAGGAGAGTGAATATTTCCCAACACTCTCTCTTCAAATGTGCTCAGAAGAAGCAGTAATCGATT
TAGTCTGTAGCCTGCGATTGCCATCAAACTACCACTAACCAAACAGCATATAGACGGAACGTGCCGTAGA
AGCAAAATGGGCACAAAAAGTGCCTGTTTTTGGTTTTGTGATTTGTGATTTTTGCGTTTCTTACTGATCTAA
CAACGATTTTTATTCGAATAAACTATATTCTATCGGTTCCGAGCGGTTGAAAATAAATAAATTGCAGTGTT
TTGTGACCATTTTAATAAAAACAGTGAATTATCGATGAATTACCGATAAAAATAGTTTTCGGCCGTTACAA
CTTGGCGTGTATGTGCAAGTGTGTGATTGACACTTATTAGGAATCGTATTTGTGCGAATGCAATTCACACA
GCTGGTGTTGTAAGGGAAGTTTTGGAGAACCATTTTAGCTGAAATCGCAAAAATCGCAAGTTTTAAATAT
AATTATAATTTTAAAGTCTTTTTAAAGACTATACAAATATTACCTACTTTTTATTTATTCCAGACATATATA
TATATCTATATATACATATAATATATATAAATTATATAAATTAATTTTAATAAATTTTATTGCAAATAATTT
ATTGGAATTAATTAATTTAAATTACGAATTTTACTATATTACATTTTTTTTTTTATTAAAGTTTTAAAGTTA

AGTAAAATGCCTAGGACAAAAAGTTGTCAGTTTTGAATAGGGCTCGTAGACAACAAGCTAGAAATGAGCT
AAATAGGTCTAATTCTGAATATGCTGAGCATGAACGTATCAACAATATAGAGTCCCAACGAGAGCGTAGG
GAAAATGAAGGTTTTAGCCAAACTGAGAGAAATAGGAATAGGCAAAGAAACCAAGTTCGCAGACAATAC
ATAGTTTACAGGGCAAACGAACAAAGACAAAATACTGTAGACCACTCGAGTCGACGAGCTAATTCTCCAG
TTAGAGAACAGGAGCAATTGGCTAATACTTCGCGTCGATCGACACGCAGACAAGACCCAGTCTACCGGGC
AAACGAACAAAGACAAAATACTGTAGACCACTCGACTCGACGAGCTAATTCTCCNGTTAGACAACAGGA
ACAATTGGCTAATACTTCGCGTCGATCGACACGCAGACAAGACCCAGTCTACCGGGCAAACGAACAAAG
ACAAAATACTGTAGACCACTCGACTCGACGAGCTAATTCTCCAGTTAGACAACAGGAGCAATTCGCTAAT
ACTTCGCGTCGATCGACACGCAGACAAGACCCAGTCTACCGGGCAAACGAACAAAGACAAAATACTGTA
GACCACTCGACTCGACGAGCTAATTCTCCAGTTAGAGAACAGGAGCAATTGGCTAATACTTCGCGTCGAT
CGACACGCAGACAAGACCCAGTCTACCGGGCAAACGAACAAAGACAAAATACTGTAGACCACTCGACTC
GACGAGCTAATTCTCCAGTTAGACAACAGGAGCAATTGGCTAATACTTTGCGTCGATCGANACGCAGACA
AGACCCAGTCTACCGGGCAGACGAACAAGGACAA
>9_rnd-6_family-1543__RC___Helitron
TTTTTATACCCTGAACCCATTAAAAATGGGTACAAGGGTATATTGTATTTGTGCAAAATCCAAATGTATGT
AACAGGCAGAAGGAAGCTTCACCGACCCCATAAAGTATATATATTCTTGATCAGCATCAATAGCCGAGTC
GATCTAGCCATGTCCGTCCGTCCGTCCGTCCGTCCGTCCGTCCGTCCGTCCGTCCGTATGTATGAACGCAC
GGATCTCAGAACCTATAAGAGCTAGAGACTTGAAATTTTAGATGTAGATGCTCCTAGCTCCCGCGCAGAT
CGAGTTTGTTTCCGATAATCGATAACTTGCTCCGTTTCCAAGCAATCGATAAAAATCGATATCGATATCCT
GTTTTTGGGCAATTTTGGTAAATAATAAGAGCTGGAGTCACCAAACATGATATGTTGCTTCTAGAATATTA
TATATATGTCAAGTATCTTTCATTTTATACCTATCGCCACCCCCGCTACCACCNCAGAGCTATAAATCAAG
TTAATAACCCAACTTGTATCGCCAACCAATATAAGCTAGAATTTAAATGCAATTGACTTTTTCAGAATACA
CAAATATTCTATGGTACAATAAGATATACTGTATAAAATTTCATCAAGATCGGTTAAGATACATAGAAGTT
ATTAAAGAAACCATGTGGTTATATGTATATGTACATATATATTGGCAGGCCACGAGTCAGCGGCAGCTGG
CACAAATTTCTGTATACATGTGCACTAATACACACAAAAGAATTACAATTGCGATTGCATCGTATGCTCTA
TGTACATATGTGGTCCTATTTTAGTTCAGTTTTTTTCAATTAGTAGCAACTTTCGGTATGGCTGCAAGGTAA
AAAGCCACGGGAGAAGCAGTTCAATTGTTATATGTCTGGAATAAGCGGCGCAAGTTCAATTCCAATGGGG
AAAAAGAATTTTTTGACTATTTATTATTTGTCTAATAATGTTAGTTTTATTCCTTCACCCATTAAAAACGGA
AATAAGTCAAGAGAATGCTTTTTTTGCTTAAAGCCCTAGTAAGCTTGGCAAGTGCGTAGTGCGAGCTATTT
CAGGTAAGGTATGCATAAATATCCATGCATCTTAATCACTAAAATGTCAATTTACGCAAAATTTAATTTCC
TCATTCAGAAATCATATTCTTTATTAAATCTTACAAAAGAATGTAATCATATGTATTTATATCTATTTTCAT
ACATACATACGTTTTAGGCTTAGGCCTAACCAGTGCGTATACACATACGTATATACATTATAATAATTTAC
ATATGTACATATGTGTGTTTGTACACATTATTTTCTTCCCGCAAGTAGTCGAAGACCACGAAGTGGAACTT
TTCAACACTGTTACTATAAATCTGCGCGCGTAATTTGAGAATTTTATTTCGTAAATATTTATTATAGTTACA
TGATTTTAATAATTCATAAGACAGTCTTAAGGAGGTTGACTGATGCATTAATAAATATTATCATTAGATTA
ATGTTATTATATATGTGATTATAATAATAAATAAAGTAGCGCGCGCATTTCGATATGTATACTAAATCGTG
GCGTTAGGAGAGTGAATATTTCCCAACACTCTCTCTTCAAATGTGCTCAGAAGAAGCAGTAATCGATTTAG
TCTGTAGCCTGCGATTGCCATCAAACTACCACTAACCAAACAGCATATAGATGGAACGTGCCGTAGAAGC
AAAATGGGCACAAAAGTGCCTGTTTTGGTTTTGTGATTTGTGATTTTTGCGTTTCTTACTGATCTAACAACG
ATTTTTATTCGAATAAACTATATTCTATCGGTTCCGAGCGGTTGAAAATAAATAAATTGCAGTGTTTTGTG
ACCATTTTAATAAAAAAGTGAATTATCGATGAATTACCGATAAAAATAGTTTTCGGCCGTTACAACTTGGC
GTGTATGTGCAAGTGTGTGATTGACACTTATTAGGAATCGTATTTGTGCGAATGCAATTCACACAGCTGGT
GTTGTAAGGGAAGTTTTGGAGAACCATTTTAGCTGAAATCGCAAAAATCGCAAGTTTTAAATATAATTAT
AATTTTAAAGTCTTTTTAAAGACTATACAAATATTACCTACTTTTTATTTATTCCAGACATATATATATATC
TATATATACATATAATATATATAAATTATATAAATTAATTTTAATAAATTTTATTGCAAATAATTTATTGGA
ATTAATTAATTTAAATTACGAATTTTACTATATTACATTTTTATTTTTATTAAAGTTTTAAAGTTAAGTAAA
ATGCCTAGGACAAAAAGTTGTCAGTTTTGAATAGGGCTCGTAGACAACAAGCTAGAAATGAGCTAAATAG
GTCTAATTCTGAATATGCTGAGCATGAACGTATCAACAATATAGAGTCCCAACGAGAGCGTAGGGAAAAT
GAAGGTTTTAGCCAAACTGAGAGAAATAGGAATAGGCAAAGAAACCAAGTTCGCAGACAATACATAGTT
TACAGGGCAAACGAACAAAGACAAAATACTGTAGACCACTCGAGTCGACGAGCTAATTCTCCAGTTAGA
GAACAGGAGCAATTGGCTAATACTTCGCGTCGATCGACACGCAGACAAGACCCAGTCTACCGGGCAAAC
GAACAAAGACAAAATACTGTAGACCACTCGACTCGACGAGCTAATTCTCCNGTTAGACAACAGGAGCAAT
TGGCTAATACTTCGCGTCGATCGACACGCAGACAAGACCCAGTCTACCGGGCAAACGAACAAAGACAAA
ATACTGTGGACCACTCGACTCGACGAGCTAATTCTCCAGTTAGACAACAGGAGCAATTGGCTAATACTTC

GCGTCGATCGACACGCAGACAAGACCCAGTCTACCGGGCAAACGAACAAAGACAAAATACTGTAGACCA
CTCGACTCGACGAGCTAATTCTCCAGTTAGAGAACAGGAGCAATTGGCTAATACTTCGCGTCGATCGACA
CGCAGACAAGACCCAGTCTACCGGGCAAACGAACAAAGACAAAATACTGTAGACCACTCGACTCGACGA
GCTAATTCTCCAGTTAGANAACAGGAGCAATTGGCTAATACTTCGCGTCGATCGANACGCAGACAAGACC
CAGTCTACCGGGCAAACGAACAAGGACAA
>160_rnd-5_family-10722__RC___Helitron
TCAAACTGTAGATACTGGGGATGGATTAGCTATATGGGGCAACAGGCATGCCAACGCAAATAGATTTCGA
TGCATCCAGCACTCCCATTACTTTATGGATGAAATTTTCAGATGATTCTGGACCCCAATTCAGAAAATAGT
TAAAAGTTTTCAGGCATAGCGGAAATNAGCAAGTGACAGTTTCNAGTCGTACCTGCAGAAGGTATAACTA
TTCACAAAAGTTAGGGAGCTACGTATAGCAAAGTGGTGGTTCACACTTGCTCTCGTATGCAGCGTGCTGC
AATAAACGTAGCTTGTAGTAGAGCAACGACTGCTTCCGNGTTGTTTATNATAGNAGATTTTNTTCCTCCTA
AGCNTCCNAAAAATGACGATAAGGTCCAAAANGAGATAGAGAAATTGCGGACTGGNAAACTACTTCGAA
CACATTATGATCACTTAGATAACAGTTCCGCCCTAGACATTTCCTTTATCATAATACTGAANGCATTTACN
GTCATATTAAAGATATTCGATCAGACAGGCTNATGCTTCAGTCGTCTGTTCTATGTTTCGTTGAGGCCTCA
ACATACTCAAATGAAAATATTGAGATTCCAGGGTTTACCGTTGTTGTCCGTCTTGATTGCGAGGTAATACC
TAATACACCGCGACCTAAGAGGGGAATCGTCATTTATATTAAGAATGCAATTTTAAACAATATATCTAATT
GCTTAAGCGGTAGGAAAATATCTGGGTCAGTCGTTTTTGAGTATGCCATNTTTCTAAANGTCTGTAGGTNT
TTTAGTTANTAATAATAATTAGTCCCTCATATCCGCTTGGTTTGTTTAAAACAGAATTTGAGGATTTATTTC
AGCAANATTTATTTCAAAGGGACAGTTGCATTATTTTAGGAGACTTTAATTTATGCTTGAGCAAATTTGAT
AGATGTAGGGCCCTTTACGATGAATTGCTCAGGAAGGGATTAGGTGCCTATTAGATTTAAAAACGCCCAC
TACCAATTGTGACTCTCACATTGATTGGGCTTTTTCAAATAGTTCCAGTAACGAAGTTAGCGCTAGCACCG
AGACAGTGTACAGTTACCACAGTGGCATCTCTTTAAATGTTCAGGATAAGGTAAATTAAGTTTCTATNTAT
AGGTTTATATAATAAAAATACGTTTAATTTAAACTAAGGAATTATTTGATGAATATAAATAAATAAATATA
TTACCTACATTTATATTTAAAAGAAGTTTAGTGATCTTGTACTTGGGTTCAGGGTATTTTCAAGTCGGGCA
CCCCGGGCGACCATTTTAGTGTGCCCAGATAGAGGTTTATGCTTGTGCAGAGTATGCCTTGCTCTCGCTTA
TTGCTCATTGCGCACTTGTGTGCATCTTTTTCTCTCTTCTTCCCACAAAACCCCGAGATCTACAAACATTCA
TATGTTTATCACGGCGTTGGTCTTCAAACATTCATATGTTGGTGTTGTGTGGATGTGCGCACAAGTAGATC
ACGGCGATTGTCGCGAGACAAAGGTTTTTCTTCAATTGTGGGCCTTTTGTGATTGTGGTAGCAAAAGGCAT
TCAAATTCAAATGCTTTATCATGTATTTTTTCGCGCAGTCAGGCATAATGATAAGAATCTGATATATTTTTA
TTCAATAAAAACTTACATGACCATAATGAAATAGTTGTAGTTGTATGAAATTATTTAAGGGCTTGACCTGT
TCATGTGCAAAATGCTATTTTTTACCTACTTTTGCGTGTTGTGCAAGTTGGTTGAATACCTGCCCGATTGTG
CACCAGCAAGTGCACAGCTTTTTTGCTGCACCAGCAAGTCGCAAAAATTGAACATTGTCAATTTTTGGTCT
TTTCGCAAAACACTTCGACAAGCCGACGATGAATTGCCCTTGCAGAAATGCGACTTAGTTTTTTTTTATTT
AAAGCAAAAAGTAAGCATACCAAGTAAGAGGTACTAGTAGAGAGCTCCCGACTAGGGAACCCTCTTATTC
CAATAATAACATAAAAATAAAGATAAAATAAGAAAAAAAAAGTTCTCCTGACAGGGTGTGAGCTCGCAGC
TAACCGCATTACTTGCTGACGTATCTACTCAACAGTCACACCAGCCAAAACAAAAAAACAAGTTAGAGGT
TCCAATCAGGAGCTCCCGACTAGGGAATACCCTGAACCCTCTTATCCCAACACCAAATTCAGCTCGTGCTA
TGTGCTCTTTTGCTTAAGTTGTAACAAAGATAAATAAAACAAAATAAAATTCCCTTGCCAGGGTTCGAAAT
CCCAACTAACTGCTCTACTTTCCGCCGTCACTACTCAACAGCCACACCAGCCACAAAAATAAAATTTCTTC
GGTAGGTCTCGAACTCGCGACGGATAACACCACTTGCCACAACACAATCGCAATTATAATGCTGTTGATG
AGTACGAAGCAGATGTGCATGAATGTGTGTATGCGAACAGTTATTCTTCGAATTTGCGCTGATGCCGCAA
ACACGTGTTTCTTAACCGATCTTCATGAAAATTTTACAGTATATCTTATTGTACCATAAAATATTTGTGTAT
TCCCAAAAAGTAAATTGCATAAGAATTGTGGCTTAAGTTGTTGGACAATATAAGTGGGCTTATTAACTTGA
TAACTATTCGAGAGTATGCTATCATTTTTGGTGCCCCTAGCTCTTGTTATTAACTGGATTTAGATATCGTTT
TCTATTGGTCGCTTGGAATCGGAGTAAGTTATCGATAACCGGAAACAAACTCGATTTGCGCAGGCAGCTA
CAGCTAAAATTTCATGTCTCTAGCTCCTAGGACGGACGGACAGACGGACATGGCTAGGTCGACTCGGCTA
TTGATGCTGATCAAGAATATATATACTTGTTACATACTATACCTGTTACATTCATTTACATTTTTAACAGAT
ACAATATACCCTTTTTATCCATTTTGAATGAGTTCAGGGTATATAAACAGTTTCCCCGGTAGGGCTTGAAT
CGCGACAGACCGCAGCACTTGCTATATCTCTACTCAACAGCCACACCAATATAGACAGTTATATATCAGA
CTTTCTTTCTTGTGAAGAGAAATGATATAAGATGCTTTCCGAAAAGATGGAAAGACAGAAGTTTCAAAGG
AATAGATTAA
>9_rnd-5_family-540__LINE___R1
AAACACACACACACACACAGCCGTGTGGCAAGCGGCCCACAGAATACTACCACAGCTTGTCATTGCTTGTCG
TACGAGGCGACTGAAATGATACGGGGATGTAATTGCTCGGAGAGGTCCCTCTAGTAGGCGAAAAGATTCT

GACTGCCATTAACCTGGCAATGGCCAGATCGATTCCGTTCCTGGGCAATTAAAGCAACTCGTGACCTCCG
GCAGTGGCCAGGTATCCCCTGGGTAGGCGCTGCACACCCGTGACGCGCCTAGCTAATACGAGAAGGCGAC
TAAGACTGAGACCAGTAACGTCTTAGGTTGCAAACTCTGGACAACCCTACACGACAAGACGACCCCTGGG
CTGACGGCTGACAGTATTCTAGCATCTCAGTAAGCGGAGTGAAATCTGGCCGAAATGACTGCTAGGTTAA
TGCTGTTACCGCCCCCGTCCCGTTAAAACATGGCAAATCTCCTAGCACGCTCCTCGAATCGTCGCCATTTA
GTTGGCAGTATAGGTTCAGATGTGATATTCCTGATTAACGCGGATCTGGCTCTGAACACCCGACCCCATGA
AGGCGGGTGTCAACCCTCGCATGGCCTCACTGCTTGCAAAGACAACCATGAGATCTAAACGCGACTGTAC
AACGACCAAGGACAACGGCTTAGGAGTTGGGACCCAATAAAATGAGTACCAATGAAACCCCACAAACGA
AGGAGAGCTGTGATGCTTTTGCAAAGAGCAGCAGAATGGCGAGATCACCACTAGGTGTTCCAACAGCCGC
CCCGACAGAGACGAACTCAGGACGGGAGCCAACGACGGCAAACCAGAGCAGCTCAGCACTACGAGAACT
CGGGCAGGAGATCTCAAAGCTGGTAGTCATGCTCGAAGATGGCAAGCGACGCTCCATCCACCAGCCAATG
CGAGACTCGATAGAGAGCATCAGAGTGGCTCTACGAGCTGGCGGCAATCCAGCTGCACGACGAAGGAGC
CAAGGAGATCATCAGGACGCACCAGTCGTCCCAAACATCGCCATTGTTCAGGACGACGATAGACGTCAAG
CGGAAGCAAGCCACAGCACGGACACCACAAACAAAACGAATCCGAGGACCAGCCGGTAGCCAGGCTACC
GTATCAACGGCGCAGGAGACACCGAGCCCCAAAGCGGATGGCGACAAGTGGAAGGAGGTCACATACAAG
AAAAGGAAGGAGGTCACCAGGAAAACAGCAAGGGATTGCTCAAGGAAAAGGCGCGACCGGATGCTATA
GTCATCTCGGCAAAAGGCGAAGCGAGCTACTCAGAGATACTGAGGAAAGTTAAAGCGGACGAAAAAACT
GGGCGAGCTCGGAAAAGCGGTGACAAGGATAAGGAGGACACAAAAAGGAGACCTAACTTCTCCAGCTTA
ACCACTCGGGAGAGAATACCATAAGCATTTGGAAACCTGGTAGGCGAAACGTAGGAAAATGCAGAGGTC
CGGGACGCTGTCCCACCGAGTGACCGTTGAGTGCAAGGACCTGGACGAGATCACGACCAGAGAGGATAT
CTGTGAGGCGCTGAGGGGTCAAATCCAAATACTGGACATAGCCCCTGAGAAATATCAGCCTCCGAAAGGC
GTACGGCCAAACACAGACGGCGACGATAAGACTGCCAGCGGGAAGCGCGAAAAAGGCCCTTTCAGTAGG
TTTCCTGAAAGTAGGCTGGGTTAACTCGAGAATCCGCGAACGGATNAACATCACCAGATGTTTCAGATGC
CTCGAGTTTGGACATCTAGCAAGACACTGTAAAAGTGATCTAGACAGATCGAATCTGTGCCGACGATGCG
GAGGAAAGGACCATCTGGCTAAGGACTGCAAACAAGAGCCACAGTGCATGCTCTGTAAGGAAAGGAATG
CCGACTGCAAACACATTGCCGGCAGCGGCAAGATGCCCCGTGTTTAGGAGCGCCCTGGCCAACAGAAAAT
GAAGTGGACACAGCTGAACCTCAACCACTGCGAGGCTGCTCAGGCCCTGCTGACACAATCAATACGGGAG
AGAAATACGGACGTCGCAATAATCTGCGAGCCATACAAGCCTATCGCTGGAAGTGGCTGGGTGGCAAGTA
AATGTGGAAAAGCGGCCATTTGGTCTCTCAAATCACCGGTCCAGGAGGTCAGAGCAGCAAACGACGGATT
CGTGAGAGCAAAAATTAACGGAATCACCGTTTACAGTTGCTACGCACCCCCAAGCTGGGAACTGCGGCAA
TTTCAGACCATGCTGGAAGAAATATCAGAAGACGCTCGCAATAGAGCTCCTCTCGTCATAGCCGGAGACT
TTAACGCCTGGGCCGTGGAATGGGGAAGCAAGGAAACTAACGCGAGAGGGACCACCCTGCTAGAAGAGT
TCGCGGCGCTCGACTTGGGCCTTGCAAACGACGGGCAGGAGCTCACGTACTGCAAGGCCGGTAGAGGCTC
TATTATAGACCTTACCCTGCTCAGCAGCTCACTTTATAGGCTAACTACATGGAAAGTCAGTAATGAATTCA
CATTCAGCGACCACCGAGCAGTGCATTTCGAGATCCACGGGAGAGGACGGAGAAAACAGCAGCCCAAAA
CAACAGGCCCCAAATGGAAGGACAACAGCCTAGACGAGGAAACCTACACGGACTCCTTACGTCATGCTC
AATGGACTTGGGCCACGGAGTTAGCAGACGACATGGCGCGGCAGCTCATGGGAAACATCTCGGACGCGT
GCGACTTATCCATGCCCAGGAGGATGCCAAACAGAAGAGGAGCACCTTGCTACTGGTGGAATGAGCAAA
TCAGCGACCTAAGGAAGGCCTGCCTGCGTGCTAGAAGGCGCTCTCAGCGTGCCCGAGGCCACCCAGACTT
TGCATCTAGGCTGGAACNCCTGCGGGCAGCCAAGCGAGACCTCAAGAGAAGCATTAAACGAAGCAAATC
CAGCTGCTTCCAGAAAATATGCGATGAGGCCGATGTTAACCCGTGGGGCACGGCATACGAGGTCGTCACG
AAGAAGATCCGCAGGNAACAACCCAGGTGACCTGCCTACCGCACTTGCCACTATAGTTGAGACACTGTTC
CCCCAACACCCACGACTGGAAGTAGCCAACCGCTCACTGAGCACGGAGCTA
>9_rnd-5_family-303__Unknown
TAGGGACGAGCGNTATTTACTCAACCTGTTACAGTACAGTATTTAAAAGTAACTAGTTACTGCTGTTACAC
CTTTCAAAAGTAACAGCGACAAAAATCATCCAATGACATGCACGGATAGTAAAGCAAGGGTCTACATTTT
GGTCGTTGTTTACTCAAAAGATTGACACCGAAAATTTCACAAGAGATTACTACGACGATATAACAAATGA
CTGGATCAATGCTGATGGCTTAATTTGTATTCACTTTACACGAAGATATACTTTTTAATGTTTATTTTTATT
TTTACTTTGTCACAGGCCTGTACTCAGGCTGTTCCTTTCGTACTGGCTACAAAGCTAAGAACTATGAAAAT
GATGTGTACCTGGCTGTTTATTTTGNACTTTGTCACAGGCCTGTACTCAGGCTGTTCCTTTCGTACTGGCTA
CAAAGCTAAGAACTATGAAAATGATGTGTACCTGGCTGTTATTTTGAACTTGCAACTGCACAGCCTCACTT
ACAAAAATCGTTTAAATTTGTGCGAACGTTTTTACCTTATCTTAAAATGGATAAAAAAACGAATGAAATTT
GGTCCTTATTTAGGGCTGACGACGACAAATTTGCAAAATGCATTATTTTCAAAGAAAAGTAATCCTATAGT
TTCCTACAAATTTAATAAAATAAAAAAAAATTGTTATACAGCCTAACAATATTAAATTTTAATTGGTTTCC

TTATATATATTAAAAATTGCTGCGCGCCCAAGGTCAAGTAAAAGTCGCTGTTATATGTAACTGTCACAGTT
GTCACTGTGACACAAATATAACATTATCGATCATCTCTAGATTGANACAGCTGATTAGTGATGAGCTTATT
TGATGTAGTGTTTTGTAATTGTCCGCAAGGTGTACACGCGAAGCCGACAGGTAAGAAAAATCTATGAGTA
GGATACATTTTTCATTGATAAGTTTATTGCATAGTTTGTAAACCCTAAATAAAACAGCTTCAAAGGCACGT
TTTTTTAGCTAAATTTAGTAACACTGTCTAAAAGCCTTTGTCCACTGTCCAACTTTAGCAAAATGTGTGAA
ACGCGCCCTGTGTGTTTCCCCATAAGATAACACATTTCACTCATCTTTCAAAACAGGCAAATAATGTGTAA
ATTCACCCCGAACATTTCGTGAATTTAGCGATGACTAATCGAGATTTCTATCGATGCTGTGAAAAATGGTA
AAGTATCGTTAACATAAACAGCTGATATATCAAACTTTAAAATGTGCGTTAAATTAGCGGTGGTGACTTTA
GGCTTTAGGCACTAATTTTAACTAAATGGACGGTACAGATTTTGAGAAAATTAACTAATTGTAAACCAAC
CTCATATAGTTCATTCATAAAATCTATCGAACTGCTAGTTCGTACGAGAACTGAATCACTGCCTGGAATTA
GTGCTGAACAATTTTGTACAGAATTGGGCGGGAACCAATTCAATTTGAATTATATATAATCATGAACAAC
GGAAAGGACTCATAGTTAATTAAATTAATAAGTCAAAGCAAAAAATGAGAAACATTAAGTCATACTTAAT
AGCGCAGTTTATGGAAAAATTGCATTTTTCCATTTTTTTCATTACATCCTATGTTATTGCAAAAAACATGCT
GGCAATATTAGATATCATACTGTTAAATGGCAGCCATATTTTAAACTTTATTGTTTCATTTCTCCGAAAAC
GGCCCCAAATGCACGGTAGTTGTGCATATATAGAGCACGATTTATCAATTATTTTGCTATTTATTTCATTA
AAATCATCGGTACAGATATTGAGAAATTCTCAATTTTCTATTTCTCTCATAACATCCCATGTTAATGCAAA
AAAACATACAGGCAATATTAGATATCATACTGTTAAATGGCAGCCATATTTTAAACTTTGTTATTTAATTT
CTCCGAAAACGGCCCCAAATGTACGATGGTTGTGCATATATAGAGCACGATTTATCAATCATTTTGCTATT
TATTTCATTAAAACCATCGGTACAGATTTTGAGAAATTCTCATATTTCTATTTCTCTCATAACATCCTATGT
TAATGCAAAAATACATATGTATATATTGCGCTGTTTTCTTGAATACATAAAAGTACCGTTATAATAGACCC
TGCCAACCCATTATAAGCCTAGCATATAAAAGTGTCAATCTCTGAAAATAAGTGAATAATTAAAATATCG
GTCACTCTTTCTATACATGGAGATGAATAGTTTGTAATTGTGCTCACAATAAAAATGTAAATATTGTATCT
ATATTTATATATACACAGATAATAATACAAATTGAAGAATGTTTTAACGACTATACTAACTATTTATTTTA
TATAAATAACATTTTTAGATAATATTCATCGTCATGTGTACTTTTACTTATTTTCACAAGGTGGCATCTTTA
TGTTCCCATAGGTTAGCTTTCTTCATAAGTTGGCATCCCCTAACATAGCGGCACTTTTGCATTCCATGCTTA
TATCAAATAAGTTTTTTAATTTGTAGTTCTGTACATCACACAACTGCTCAATTGGGCCATATATGTACATCA
TGCACAGCAACCGCCTGTCGCCACTGGTCTACGATCGGGAAACGAATACAACGGTTGTCGATGATGTGAC
CACGGCTGCCGCCGATGATAAACAGCACTCGTAGAAGAGTGCCGATCCGCTTAGTGCGCTGCTAATAGCC
TGTGGGCTTAGTTTATTGATTAGCTTAATACACTCGCAGATTGTTGCAACAAATACGTCGCCGAGCGACAG
CAACAAGAACAAATTGCGTCTCATTTCCATGTGCGCTTTTGGCGAGAAACCGAATAGGCCTAGAGAGCAT
CGATTGCGGCGACGCAAAAGTTTGTACAGGGCGTTACCAAACCATAATTGCACCCTAACTAATTAGTATTT
TATTTCACAGCCCAGCAGCAATCATTTATTGAGCGCACGACGTAGCAAACCGACTCAGATTTAATGCCCTA
CAAGGCTACGAGTGCATCGATTCGCTGGGCATGACCAAGGATTACATTTACATAGGCCTCGTCTGTACAT
ATATATATATATATATATATAT
>9_rnd-4_family-1027__LINE___Penelope
ACTTCGCAACTGATAAGAGCAGCATCTTAATTTTATCTTATAATACTCGCTCTGCAAAGCAAGGGCCTAAA
TTTCGCTCTTAAGAATTCATTTACATAGAGCTGCCGCTCTTCGCTGTAACGATCGTTTTATTGCCCTAGCTT
TACTTATGCGAAGGCCTCGATGCTGCGCATCGNGTTAGATTAAGTTCGTTCGCTTCATGCATTTGGTACGC
ATCGGCCACTTGCATTACTCAAAATTATAACTAATAATTGTCTCTTCGCTAAAATTGGCACGTTCTTATTCG
GGTTCGCTTGCCTATATACAAACTATTGTTCTTAAATAATAAGCACAGACCAATTCGGGTCTGCCTGCTTC
TCGTAACTTTAAGAAATATAAAACAATTTTGAACACAAAAAATAAATAGTTCAATCTTTCTATTTGTGAAA
GCTATTGAAAAAGCTCAATAGCGCAACAACTACAATACATAGAAGTAATGTAATTTACAAAATACATTGT
TGCGGGGTCACCAACAATAATTTCAATAGAGTGTAAACTAAAAACAATGACAATGCGACACAAATCGGA
CTTCAGATTAAGAATTCTCGAATTCGCGACAGACCGCACCACCTCTACTCAACAGTCACACCAATAATTGT
GTCTTATTGAATTAAAATATCAGCACAAGAAACAATCGCAATCGCAATGCTGTTGACAGATTTCGCAGCA
GATGCGCATGAATGTGTGTGTGTGTACATGTATACAGAAATTCTTGGAATTTGACCCCATCCAACTGCGCA
GTAAGACACATAACTTATGGGATTTTCTTAACCAATCTAAATAAAATGTTCTACAGTACATCTTATTGAAC
CATCAGAACATCTTATTGAACTCAAAAAGAAAATTGCATTTAAAATGTGGCTCAAATTCATGGGCAATAT
AAGTGTGGTTATTAACTTGATTTATAGCTCTGCGGTGGTAGCGGAAAGGTGGCAATAGGTATTAACTGAG
AGGACCTTGATATATATATATATATATATGTATATATATATATATATATATATACTAGTCTAAAAGCAATATA
TCAATTATTTTACTCTAGCTCTTATTATTTACCAAAATTGGTAAAAAAAAAAGGATTTTGATGTCGATTCTT
ATCGATTACTTGGAAACGGACCAAGTTATCGAATATCGGAAAATCTGTCTCTAACTGCTATAGTTTCTGAT
ATCCTCGCGTTCATACGTTCGGATAGACGGATGGACGGTTAGACAGACAGACGGACGGACATGGCTGGAT
TGATTCGGCTGTTGATGCTGATCAAGAATATGTATGTACTTTATGGGATCGGTGCTTCTNCCTTCTGCCTGT

TTCATATATTTACATAAATACATTATACTTGTTTTACGCATTTTAATGGGTTCAGGGTATAAAANTAGCAC
CAATGACCGACTGTATGACAAGGAAACAACAATAATCCTGCACAACACTTCAACATACAGCATACANTAA
AATAGCTACACAACAAACTAAAGAGTAAACTTAAACAAATACACATAAAATTGCGCTCGCTTGTGTTGAG
ACTTATTAAATAGCCAAAGAATTTCAATCTCCAAGTCAAGAAGCGCAGACGTATGTTAAATGTTTAATTTA
TTGCTTAAATTAATTTATTTTAATGTTATTTGTTTATTGTTTCTTGGTGTTAGTGCCCTGAAGACGGTTGCCG
ATAAGCAATCGAATAAAGCCAATTTTTTTTAACTTTTGTTTTCAATAAACTAAACCCTGGCCCTCGAGCTA
GCAAATCAAAATACAAAAAAAATTAAAATTAAATTGTTTATTAAAAAAATTATTTAATGACTGATAATTTT
CCCTAGCACTATATTTTACACGATTTGGGTCGAGCAAGAGAAATGTTAGCACGCGAAAAGTAAACGAACA
GTTCGAAAGTAGCACCACGCTTAGACATAGCAGCGAGTAAACGGTTTATTGCTGCAGAAATGCACACACC
ATTTGTAGATATGGAAGGCGTCATGGTTACCGAAGCTCAATATATGCGTAGCCTGACGGAGGCAAGCGGC
TCCAATCTCCAATAATCTTGGAACATGAAAATCGTTAAATAAAAAATATGTTTAAGTAACACAAAGGTTA
ACCCCGTAAAATAACAGTGTTGACTCTTAAATGGTCTTACAAACAATATACAAATATATTGCTGTATATAC
TATATATTACGCAGGAATATGTTGCTAGATTAATATAATTGCGGATAGGTTGCAATTGGATTTAGAATAAA
GAACGCGGAATGAAATGTTTTCTGTTTGAATTGTTTGCATTATGTCGGGCATGGACTTTACCACCGCATAC
AAAGCANCGAACCTGCTTTCGAAATTTTAAAATAATAAGTAAATTAATCTATACCTACACTTATTATATTG
ATAAAAGGGAGTCAATACGGTCTATTGTACCTAGTGCTCGCTAATGAACAAGAGTTGTTCGACTTTCCCTA
TGAAGAGGAGCCGTGCCTTTCTAAAAAGTTTTTATTTCACCTTTCCCAACTTTCACTGAGAAAATACATAA
GTGTTTTTGTGAAGAAGAACGTACAATAAGATATTTAAGAGGTGTATTCTGTGAAGGTGATATTTCGCTGT
GTGGTGCCCTACCAGGTAGGCGAACAACAGTTTAAGCCATCTCTTCTCTTCGCAATTGCTGTAGTTGTTGC
CATTTTCCGAGGTCCTGCAGACGAATTTACGAGATCCNTTTTATATATTAGCAAATATGGATGCAGAACAG
GCTTAATGTGGCAATCTTTTTCACGTGTAAACACAATAGAGTGAGCCCAATTTTATGGCAGTGGCTCGTAA
CCAACCAGGAGCGGCCTGAGTTGCCCCTGGTTAGGCAAGTCCAGACAACATGTGTAGATTTAGTTAACGT
ATTGTCTCGATAGTGGGTCTGTTATCCAGAACTACTATAAAGATTTGACCAATATCATCTAGGTACTATTC
AGAAAAGAGCACAATATTT
>9_rnd-1_family-103__Unknown
CGATCGGTCGAATACTTAGTTGTTGTATGAAAAAACATTTTGTTTTTCAAGATATCATGACCAAAGTCGGA
ATTCATTACTTGTACTACTTCTCATATATATGCAAGATCCTATTCAGATTAGACCACTATATCATATAGATG
CCATAGGAACGATCGGTCGTAAATTATATTTTGTTGTATGAAAAACATTTGGTTTTCAAGATACCTTGACC
AAACTCGGCATTTATTAGTTTTACTATACTCCTCATATATAGGCAAAATCATATTTAGATCGGAACACTAT
ATCCTAAAGCCGCCAAAGGAACGATCTGTCGAAAACTAAGTTGTATGAAAAAACATTTTGTTTTTCAAGA
TATCTTAACACTTTACATTTATTAGTTGTACTTTACTCTTATATATAGGCAAAATCTTATTAAGATCGGTCC
ACTATATCATAAAGCTGCCATAGGAACCATCGGTCGAAAACAAAGTTGTTGTATGCGAAAACATTTTGTTT
TTCAAGATATCTTGACCAATTCGACATTTATTAGTTTTACTATACTCCTCATATATAGGCAAAATCATATNA
AGATCGGANCACTATATCATAAAGCTGCCATAGGAACGATCGGTCGAAAACTAAGTTGTTGTATGAAANA
ACATTTTTTTTTTCAAGATATCTTGACCAAACTCGGCATTTATTAGTTTTACTAAACTCCTCATATATAGGC
AAAATCGTATTAATATGGGACCACTATATCACATAGCTGCCATAGGAACGATCGGTCGAAAACTAAGTTG
TTGTANGAAAAAAACTTTTGTTTTTCAAGATATCNCGACCAAACTCGGCATTTATTAGTTTTACTATACTCC
TCATATATATGCAAAATCATATTAGCATCGGACCACTATATCATATAGCTGCCATAGGAACGATCGGTCGT
AAATTAAGTTCTTATAGGAAAAAACATTTTGTTTTTCAAGATATCTTGACCAAACTCGGCATTTATTAGTTT
TACTAAACTCCTCATATATAGGTCAAATCATATTAATATGGAACCATTAAATCATATAGCAGTTATAGGAA
CGATCGGTCGAAAACTCAGTTGTTGTAGNGAAAAACATTTTGTTTTTCAAGATATCTTGACCAATGTCGAC
ATTTATTACTTTTACTATACTTCTCATATATATGCAAGATCCTATTAAGATCGGACCACTATATCATATAGC
TGCCATAGGAACGATCGGTCGAAAATTAAGTTTTGTTGTATGAAAAACCATTTGGTTTTTCAAGATATCTT
NACCAAACTCGGCATTTATTAGTTTTACTAGGCTCCCTTCATTTTTCAAATTCTTATTTACATCGGACTACT
ATATCATATATCTGCCATAGGAACGATGCGTCGAAAACTAGGCTTTTGATTCAAAAACTTGTTTGTTTATC
AAGATATATTAACCAAACTCGGCATTAACTATTTACCCTGTGCTTGTTAGATACGGGCAAAGCACTATAAG
CATTATGAAAAGGTTGGTTCTGCAATGGTATTAGATCTTCGGCGTGCCGAAGATAGCCTTTCTTTCTCGTTT
TGTAACACTTGTATTTCTGTTTATCGCAACTTTTTATACTCTGTTCTCACAAGAAGTATATCTGGCAGTCGC
TACCCAACCGCCANCGTTATCTCGTAATCACAACAAGAAAAAGTGATAGTCACTACACCTACTCTAAAAA
CATTTTAACATATATTCGGAAAAAGAAGACAGACGAAAATATAACCGAATGGTGAACAAACGAGTNTTTC
TTCGGCTTCGGAAAAAATCAATTTTTCAGAGCAGAAACGCTCCCCCAATTGCCNGCGATTCAACCAGCATT
CGCTCCGCTTGTATAATATACTTAAGAAAATAATTACTATTCGATTTGTATTTATGGAATTCAAAAAGAAT
CACTGTGCATATTCACCAGCATGTACACACACACACACACACACACACACATCACATTATATGTGTATCGT
GTTTTATTTTTCCCACAACAGTTTTAAGAACACTTTTTATTAGTTCTTTGCGCAAATTAATTGGCAAGTGCA

AAAGTAAACAAACACCAGGTTGTAAAATATGAAGCTAAGTCTGANCCTAACTAAGTCTGAGCCTNACATA
TTTATATTTTTTTCTATGTTTCCATCTTGCTATTGAGCTTACTACTTAACAAAATGCAAGTANATTTTCATA
AATAATAATTGCCTTTTAAGGAAGTGTCCACTGAGCGGCTTTTTGCTTAATAAATCAAAACATACTTAAGT
TTTTCTTATTTATAACAAATNATTTACAATTATATGTGAAAAACTACCAAGTGAGTTNCGTTTAGAACAAA
TGTGTTTCTTTCTGAGTTCGACAGTTAATGAACCATAAGCATGCATTTTTATACCCTTGCAGAGGGTATTAT
AATTTTGTCGTGAAATGTGTAACGCATAGAAGGAGACATCTCCGACCCCATAAAGTATATATATTCTTGAT
CAGCATCAACAGCCGAGTCGATAGAGCCATGTCCGTCTGTCCGTCTGTCTGTTTCTATGCGAACTAGTCTC
TCAGTTTTAAAGCTATCTTAATGAAATTTCGCAGAAGTCCCTCTTTCTGTTGCACGCAGCACATATGTGAA
AACCAGCTGGATCGGACCACTATATCATATAGCTGCCATAGGAACGATCGGTCGAAAATTAAGTTTTTGT
ATGAAAAACACTTTTGTTTTTCAAGATATCTTGACCAAACTCGGCATTTATTAGTTTTACTTTACTCCTCAT
ATATATGCAAAATCCTATTAAGATCGGACCACTATATCATATAGCTGCCATAGGATCGATCGGTCGAAAA
TTATGTTTTTGTNTGAAAAAAACATTTTGTTTTTCAAGATATCCTGACCAAACNAGGCATTTCCCTTCCTAG
CAACTTGGATGTATCTCCAATGTTGTCTTTATCGCCTTCTCTAAGCGACTGTCCAATTACCC
>9_rnd-1_family-471__LINE___I-Jockey
GTCAAGGGTNTGGCGATAAACACAGCTCAGCAGACTGCACTAAAAGTAAAGAGGAACCAGCGTGCTGTG
TACACTGTGGAGGCGACCATGCTGCTTCACATAAAGGCTGCGCCATTTATAAAACGGCCAAGGCGCAGGC
GTTCCCNAATGCTTTTCGTCACCCAAATGTCACGGCCAGGCGTACCTTAGGATCACCCATCTGCTAAAATC
GACCCACAAGTCAGCTTCTCGGAAGCGCTTAGAGAAGGAAATAATAATTCACAAAGCCAAGCCCGCTAAT
CTGAGCAGTCAGCAGCAGAAGCTACGGAAACCATGGTTACTCGATTGGAGTAGATGATGGAAAAATGAT
GAAGAAATTATTTAGCCAGATGACGCAAGTGCTAGCCATTTTGTTGGTAAGCCTATGCAAGTAAGGCTTA
ACCTCACCATCTGGAATGCCTGTGCAATAGCAGGCCAGAGGTCGAAAACTTTGTGAAGACCACCAACACA
GATATACTCCTAATATCAGAAACTCACTTCTCCCAAAGATCGTAGTTCTCCATCAAAGGCTTTGATATTAT
TTACAGCAACCACCCAAACGGTAGAGCCCGTGGTGGAGCTGCAGTGCTCATCAAACAAGGAATAAAATAT
ATTGAACTGCCAGCATACCACAAGATTGAGTGGAGTGCGGCCAGGTCAGGCTGCCCAGCCCGCTCGGCGA
CCTTTACAATCGCGGCTGCGTATCTCCCACCCAGGTAGCCTTGTCCCCTGGTTTATAATTGGTGGCGATTTC
AATGCCAAACACTCCTGGTAGGGCTCGCGTACCAACATCCCCAAGGCCAAGCTCTTTACCGTTGCATACA
GAAAAGAAAGGACTTTGCTGCCACTCACCTGGTGAACCCACATACTGGCCCCACGGATCTGCGCAAGGTC
CCTGACATACTGCTACATACATTGCTATCTCCAAAGGTATAGCTTCCAATAAAATAGAACGAGAACTTATT
CTCCGACCACACCGCTGTAACATTACATGTCAATACCCGGTCGAGAAAAAAGCGTCGAGGTTCCTCGAGG
ATTTCAAACCAGGAGACCCATCAAATCGCTTTTGGCAGGTCACACGCCATATTAAACGGCAGAGACCCAT
CAAATCGCTTATGGCAGGTCACACGCCATATTAAATGGCCTATTAGAAGGGCAGCTGTGGTTCGAAAAGC
AAATGGGGACTGGTGCCGATCAGACCAAGANAGAGCAGATGCCTTTGCAGAGCCCCTTTAAGACTCATTC
ATGCCGTTTGATCGCTGCTCATTGACGGACAAAGCGGACACTATCCTTTATTGGAGCTACCAAGTGTGGAG
CTAATAATNGTATCCAGTTACTGACCAACGTAATTATTCGATGCCTTGAGATTGGACATTTGCATAAGCTT
ACTGGCTACTCTCTCTAAATTGAACGAAAGAGTTTATTTACTCTATAGTGCCGCCAGTATTGAACGAGGCT
AGTTTGATCCCAGACCATCAGTTCGGCTTCAGGCGCATCCACGGAACACCGCAACAGTGCCATAGGATAG
TAGAGCGAATAATGCAGGCATTCGAAGAGAATAAGTACTGCTGTGCTGTCAAGCAAGCCTTTGATAGGGT
ATGGCAGCCCGGTCTACTNCATAATGCNTAAAGTCAGAGATTCTCTAGCTNCAGTCGGCTGGTGAGGACG
ACGGCTTGGGTTTTAAGGTTCACGCGTTGGAGCCGTGGACAGAGGACTGAGCTTGAAAGATTCGGCTTCA
CCGCTACGGAGTGTGAGAATGCTGAGAACTTGCTGGTACGGCGAGCGCAATGCGAAGCGTTGCCTGACGA
GGTTCGAGCCTCATACAATGAAGAATCAGTCGGTCATCGAAGAGACATAAGAGGGCTGGCGCCTTGCAGT
GTATACCAATGCGCGTATGCGCCCNACAGTGTAGGCCAATAATCCTGTCACACCGGCTCGCACTGACGAA
ACCGATTGNGTATCACTACCATATAAAAATGAAGCATCAATACATGGATGCAACGATAGGCGACATCAGG
ACCAGATTTTGGATAACAAAGCTGCGTCGACTGTTGCGTACAGTAATATTCATAGTATATAGAGCGGAGC
CAGGGGCACCTATAATGGGTCATCTGCCAGAGGACCGACTGGAGGCCATTTGGACTATTTTGGACCACTC
CTTGTGACAATCGGACGTCGAAAAGAAAGAGATGGGTCGCCTTGCTTACATGCCTGACAACAAGGGCCAT
ACATTTGCACATAATCTGTCAACTAATTCCTGCATAATCGCGATCGGGAATTTTCTTTGCCGTCGCGGACC
TGTACTGAGGTTGCGCAGTGACAACGGAAATNACTTTGTTGGAGCCGACAGAGAAGCCAAAAGGCTCATA
GACGTATTCGAGNCGGAGAAGATTCANGGTGAGCTATCTAAAGGCATTGATTGGATCTCCAATTGCCCAG
CGAACCCAGCTGAAGGGGGCGCTTGGGAAANGATGGTGCANTGTGTCAAGAGAGTGCTACGCCACACAN
TAAGGGAGGTGGCACGAAAGCAACACGTACTGGAGAGTTTCCTGATTAAGGCCGAGAGCGTTGTGGATTC
TCGCCCGCTTACCCACTTACCGAACCAGGAAAACCCCCTAACACCGAACGATCTACTCAAAGAAGTGGCC
AATCTGCCTGACACACCCACTTACAGCCGAACGAGAGACGGGCCACGAGGAAACAGTGGGACACGGCGC
GTCTGTTACGAGACCGTTTTTGGAAGCGGTGGGTCTTAGAGTACCTGCCTGCGCGCGTTGAACCGATACGC

CATGGTGATATGGTGTTTATATGCGACCCGGCCGTGCCGCGCAGAGAGTGGCGAAGGGGCATAGTGGAGG
AGCTCTACCCTGGACCACGCACCAGCGTGCGCGTGGCGGACAACAATCGAGTACGACTGATCACTCGTCC
TGTGGCTAAACTAGCCA
>9_rnd-1_family-294__LINE___R1
CTAGAACCTCTTANTTGTTTTTATATCAAGTCAGTTTCCTATATAGGCTACATGGTTTAGATATATTTAATT
TTTCGAATACCTCGGTACAAATTTTCAACAGTTGTTATAAAAGTAAGGATATGCATTACATAAGGTGAAGT
GGGCAGACACTTCCACAATTTTGTCATAAAATATATTCATGAAAAACTCTTAAGGCTTTTAATATAAAATA
TTCTTTACTTTACTTCTTTGCAAATATTATTTACACAACATCTGAATAACCATTTTTAATTACTTATTTTTAT
CGCACAGTAGACTTTACTTTTTAAATACTAAAATCTTATAATTTATGGAGTCAACTGTTTCAGACATCACA
TACTATCCAGATAAATCATCGTCATTTATAAATTGTACATATTTTTCTCGCAAACCAATGGCACCGTTAGA
TGATGTTAGAAAAATTACTGTGCCAATTACTAAAAGTCAGAAACAATGTAGACGGCACTTTTACATATTTT
GTAAAACATTGCAAACAAAATTTGCACGCCACATAGAGCTTAAATATAGAATGGAACCAGTTAGAGAGAT
CAGCAGTTTATCAAAAGACAAACAGCAAAGAATGAAATTGCTTGAGGGATTACGGAAAGACAAGGATGC
TGCTTACAATGAAGGAACTTTTATAGTTTCANGTCNATTAAGGGAAAACTCAAAAAGAGGGCTTTGGATT
TTGTGTGCTGCAATGGATGTAAAGCCTATTTACCGAAGACAACAACTCGGAATCATATTAANAATTAATG
TAGTAATATCCATAAAATAGGCAACCGAGATATATTAACCTCTGCACGAAGTGCTGCAAATAATATTGCG
AAATAAAATAATGCCTGTCCTTAATGACGTCGCCATTACTAGGACAATATTGTATGATCACCTAATTATAT
TGTTTAAAAATTTAGAAGCAATGAAGCACACGGAAGTACAGCATGATATGATAACAGCAATTATGCGTTA
TCTAAGTCGCCTTTTGATAGTAATAAAATATGTTATTTCCCCTAAATATTATGATTCTGTTAAGTCTGGAAT
TCGTTATGTTGCTGAGTTTGATTATGATAATATTCGTTTTATGAAGCCTACTGCAGCGACAACTCTAACAT
GCTTGGTAAAAATAGTGCATGTGCGAGAATATTAAAAGCACAGATCATAAAATATTGCGACAATAGCATA
AAATATTTAGAAAAAGAAAAGATTTTGTTCAGGATCTACCTGCTAAAGCTTTGAATGAGCTAAACTAGAA
ATTCAACATATCACCANTAACCTCGTTATATCAGAGCAGAGTTAGCGAACTTGAGAGTCTTACTCTTAAGA
GCTACAGAACTGCTCATGCAATTGGGGCTATCGACGATAGAGCTAATTCTGACCTGTATGAAGGCACCTC
AGCCAAAATGAAAAACATCACCAGGGTGATTATTCGTGCCAAAAAAACCGAACATTTCCAGTTCTTTTTA
CACCATGCGATCGGTATTGCATAGCCAATATACTCGAATATGGCAAACGCACAATAAATAAACCAGCTAC
TACACTAAAGATATATTTTAGTGTGCCAAGCCCGTGGCCACTAAGAAAAGAAAATATTCGTGAGTGTCCT
ATCCTAGAGAAATTCGCAAATGAATGTGGTACACGTTGCCCTGAGTCCCTTTCAGGCACAGACCTGCGAA
AGCAATTGACCACACATACCGCTTTGTTCAACGTTAAGGACATTCAAATTGATTATTTGGCGAGCTTAATG
GGTGATCATAAAGACATTCATAAAAATTACTATCGTGTTGCTGCCTCTGTAGCGGGGGTGACCCAAGTTTC
TCAATTACTTATGTCAACAATGAACATGGAAGAGGAAAATGCTGAAATTGCAGATGCTAATCTCGAGAGT
GAGTTAATTTATTTAGCCCGTCTGTTGCGAAGAAAATTTTAATAATCGCTGCAAGTTTTGGACCTGGAACT
GTCATGTTCGGAATACTTCAATAGAAGTTTTTCAAGTATCGGAACTTCGATACAGTAGAAGATACATTCAG
TATTTAAGAAATTAGTAATGGTTTTGGCGACCTATCCATATTTAAAATATTTTGATGGCGGGACCTCGGTC
CGAGTTTTTTTTAATTAATAGATTAAAACAATCTAATTTAATACTTATATTGCTTATTTTATTTAAAGCAA
AATATTAAATAAAATATTTTTCAACTGTTTATTTAAACGAGCCCGAACCTCGACCAAGCCAACATAAATAT
TTAAAAACATGCGCATTCCGGACGGTATCAGACGAAGCAGCGCTGGTTATCGCAGGGATGGTCCGCACAG
CAGCAAGGATGCAAAGCATGGAACGATGGCAAGCACGTTGGGATCGCTCATCTAAAGGACGCTGGACGC
ATAGGCTTATCCCAAACTTGGAAGCCTGGATAAACCGCAAGTATGGNAATACTAATTTTTACTTTAACGCA
GCTCCTATCTGGCCACGGATGCTTCAGGAGCTACTTGAAGCGATTCGGCCACGATAACACAGACGAGTGT
TCTTGGTGCGGCAGTGGAATCGCAGAGGACGCCAACCACATCATCTTCGAATGTGGAAGGTTCTCAGCGA
ACAGGCAGGGACTGGAGGACAGCTTGGGGAGACCTATCACGGTGGACAACTTGGTCAGGAACATGTTGG
AGACCGCACAAAAATGGGAAGCTACCTGCACTTTCGCAGCAGAAGTGATGCTGGAACTCAGGAGAGTTG
AGCGCAATAGACGGAGAGCGGAGGAAGTTTAGGTAGCAACAACATGCCTGTGAAGCAATGCTTTGCGGC
CGTTCCGCAGGCAATCCACGCCACCTAATCATCACGTACCCCCCTACTATATACACCTAAGCTAAATAACT
AATCATNAATANACTTTATATTTGTGCAAATAAACAGAATACAAAAAAAAAACACACACACACACA
>160_rnd-1_family-358__Unknown
ATGGAACTACAAGTAGTACCTACAACATGAAGGTGTTTCTAACAATTCTCGCTTTGGCTGTTGCCTCCGCC
TCGGCATTCGAGTCGGTCGTCCACCCTAAGGACATTCCCAAGGTCAACAAGATCGAGGGCCGTATCACCA
ATGGTTACCCAGCACCTGAGGGCAAGGCCCCCTATACCGTTGGCCTGGGCTTCAGCGGTGGCTGGTGGTG
CGGTGGTTCTATCATTGGAAACACCTGGGTCCTGACTGCCGAGCACTGCACGGGATCTGACGTCACCGTCT
ACTTCGGAGCCACCTGGCGCACCAACGCCCAGTTCACCCACTGGGTCAGCGGCAATGACATCATCAACCA
CCACAACGCTGACATCGCTCTGATCCGCATTCCTCATGTGGACTTCTGGCACATGGTTAACAAGGTGGAGC
TGCCCAGCTACAACGATCGCTACAACGACTACAACAATAACTGGGCCGTTGCCTGCGGCTGGGGCGGTAC

CTATGATGGCAGCCCACTGCCCGACTATCTTCAGTGCGTCGACCTGCAGATCATGCACAACTCCGAGTGCC
AGCAGACTTACGGCTCTGCAACTGTTGGTGACAACATCCTCTGTGTGAGAACTCCCGACGGCAAGTCCAC
CTGCGGTGGTGACTCCGGTGGTCCATTGGTTACACACGACGGCAACAAACTGGTCGGTGTTACCAACTGG
GTTTCTTCTGCCGGTTGCCAATCTGGACACCCATCTGGCTTCCAGCGTGTCACCTACCACTTGGACTGGATT
CGTGACCACACTGGCATTTCTTACTAATAAGCTACTGCTTCCGATATGACCTAAATAAAGCATACATATAA
AAAATGAATACATTATACTCTTTTGTCTTGTTTGACGCCTATTTTACGTCAGAGAGCATAACGCAACGTAA
ATAGATCCAGCTTCCTGCTAAGGCTCGTTGACAACCACTCAATGAGTCAGTTACTCAGTTAGCCAGTGAGT
CAATCAGAAGATGTAGACACAACATACTAGGAAACGAGAAAAAAAGGTTGCAGACCCAACCTTTACATA
ATGCTCATAGTGCTTTGACCGTATCTATGAAGCACAGGGAAAATAGTTAATGCCGAGTTTGGTCAAGATA
TCTTGAAAAACAAAAAAGGTCTTTAATCAAACACCAAGTTTGCGACCGATCGTTCCTATGACAGATATGT
GATATGTAGTCCGATCTAAAAAAGATTTTTCAAAATTGAAGGGAGTATAGTGATGACTTTAAGTGACGTG
CTTAATCAAACTATTTTTTTAATGACAAAAACGTTTTACATAAAACAACTTAATTTTCCACCGATCATTCCT
ATGGCAGCTATATGTTATAGTGGTCCGATCTTAATAGGGTTTTGAATATATATAAGAAGCATCGAAAAACT
AATAAATTCCAAGTTTGGTCAAAATATCTAAATAAACAAAAATTTTTATCACACAACAACTTAATTTCGAC
CAATCGTTCCTATGCCAGCTATGTGATATAGTGTTCCGATCCAGTTGGTTTTGACATATGTGCTGCGTGCA
ACAGAAAGAAGGACTTCTGCAAAGTTTCATAAAAATAGCTTTAAAACTGAGAGATTAGTTCGCATAGAAA
CAGACAGACGGACTGACGGACATGACTATATCGACTCGGCTGTTTATACTGATTAAGAATATAAATACTT
TATGGGGCCGAGATGTCTTCTTCTAGGCGTTACACATTTTACGACAAACTTATAATCCCCTTTCCAAGGGT
ATAAAGGAGATTTTTGAAATAAAAATATATTTCATAGAATAAAAATGAAGGATTAAAAACACTAACCCAA
GAACCATAAGCCTTTGTGCTCATGCATTTTAAAATCTGTTCTGGCTTCTTAAAACTTGACTTTGGAACTCAA
TACACAAAATTTACATAAGACACGATTATCAAATGCATTCAAATCAATTTTATTTGATGTTTAATTTTAAA
TCTATTAGTAAGCGATGCCAGTGTGGTCGCGAATCCAGTCCAAGTGGTAGGTGACACGCTGGAAGCCAGC
TGGAGCGCCAGATGTGCACGAGGAAGTGCCAAAGTTGGTAACGCCCACCAATTTGTTGCCGTCGTGTGTA
ACCAATGGACCACCGGAGTCACCACCGCAGGTGGACTTGCCGTCGGGAGTTCTTACACAGAGGATGTTGT
CTCCTACAACGGAAGAGCCATAGGTCCTCTGACATTCGGAGTTGTGCATGATCTGCAGGTCGACGCACTG
CAGCCAGTCGGGCAGTGGGCTGCCATCGTAGGTACCGCCCCAGCCGCAAGCAACAGCCCAGCGTTCATTG
AAGTCGTTGTAGCGATCGTTGTAGCTGGGCAGCTCCACCTTGTTAACCATGTGCCAGAAGTCCACATGGG
GAATACGGATCAGAGCGATATCAGCGTTGTGGTGGTTGATGATGTCATTACGGCTGACCCAGTGAGTAAA
CTGGGCGTTGGTGCGCCAGGTGGCACCGAAGTAGACGGTGACGTCAGAGCCCGTGCAGTGCTCGGCAGTC
AGGACCCAGGTGTTTCCAATGATGGAACCACCGCACCACCAGCCACCGCTGAAGCCCAAGCCAACGGTAT
AGGGGGCCTTGCCTTCATAGGCTGGGTAGCCATTGGTGATACGGCTGGACATGTCCTTCATATGGACAGC
CTTCTGCGTGGCGGCAGGCACCGCAGCTCCAGTGGCGGAGGCAACAGCCAAAGCCAAAATTGTAATGAA
CACCTTCATATTGCAGGTACAACTTGGGCCTCGGTGGACCCTACTGTTGCTTTTATACGAAAATCAGGCAC
AAACTCTATTCGCCATTATCTTAATTTGAGTGGTTCGTAAATTAAATATATAATCCGATCATAAAAAATGA
ATTTTACAAGCCGATAACGAGTCACGATTTATGCAAAGTCTTTCGAATTTATGCGCATTT
>9_rnd-1_family-252__LTR___Gypsy
GAATGATGACAATAGCATTTTCTGGTCTACAACCATCANAAGCATTTCTTTANATGGATGATCTAATAGTA
ATCGCTTGTTCCAAGAAAACATATGGTCAAAAAACTTAACTGATGTTTTCGACAAATGTAAAAAACTCAA
CCTGAAACTACATCCGGTAAAATGTTCATTTTTTATGCATGAAGTCACATTTTTGGGACACAAATGCACTG
ATAAGGGAATCTTGCCAGACGACAAAAAAATACGACGTGATTAAAAAAATTACCAACTCCCACACGATGT
GGACAGCGCAAGACGATTCGTTGCATTTTGTAATTACTATATGCGCTTTATAAAGAACTTTGCTAACTATT
CACGTCACATAACAAGATTATGTAAAAAAGACGTNAAATTCGAATGGACGTCTGAATGCCAGAACATCTC
AAAACAGCACTAATAAACCTAACGTTGTTACAATATCCAGACTTCACCAAAGAATTCTGCATAATAACAG
ATGCAAGTGAGCAAGCGTGTGGAGCAGTTTTAACTCAGAACTTTACTGGACTCCAACTTCCAATTGCATAC
GCATCAAGATCCTTTACTAAAGGAGAAAGTAACAAGAGTACAACTGAACAAGAATTAACAGCTATTCACT
GGGTAATAACACACTTTCGACCATATATTTATGGTAACCATTTCACGGTAAAAACGGATCACAGACCACT
AACATCTATTTTCAATGGTCAATCCAAGTTCACAATTAACACGTATGAGGCTTGATTTGCAGGAATATACC
TTTACGGTAGAATATCTGAAGGATAACGACAGTTACGTAGCAGATGCGCTATCGAGAATAACCATCAAGG
AGCTACAAAATATAACAAGAAACATAATGAAAGTCACTACAAGACAAGCACGTAGACAAGAATCCTGCG
CAGGAGAAAAAGAAATTGAATTGCCTAGGCAATCTATAGGAAAAAGCTTCAAAGCCCAAAGTGTACCAA
GTCATTAACAATGACGAGGTACGAAAAGTAGTGACCTTGCATGTAAACAATATGACATGTTTTTAAACAT
GGCAGACAAATTACAGCAAGATACGATGTTACCGATTTATATACTAATTAAACCATTGATTTAGATCAATT
CTTTCAAAGGCTTGAAACGCAAGCCGGTATATATAAAATCAGCCAACTCAAAGTGGCACCGTGGGAAAAT
ATCTTTGAACACGTTTCAANGGATAATTTATATATANGCAATTAAATATATAAAATATATATATAGCTATA

TTGTCTAAACTGCATGATGATCCCATACTAGGAGGACCTACAGGCATTATAAAAACCTTGGCCAAAGTCA
AACGACATTATTTTTGGAAAGGTATGTCTAAACAAATAAAAGACTACATCCGCAAATGTCCAAAATGCCA
GAAAGCAAAAACTACAAAGAACACAAAAACTCCTCTAACAATAACAGAAACACCAATATCCGCTTTCGA
CAGAGTAATCGTTGACACAATTATTACCATTACCTAGAACAGAGAATGGGAACGAGTATGCAGTTACACT
CATTTGTGATTTTACGAAATACCTCGTCGCAATTCCGATCCCAAATACAAGTGCAAACACTGTCGCTAAAT
CTATATTCGAATCTTTTATTCTGAAGTACGGTCCAATGAAGACGTTCATGGNCACACGGACATGTGTACGG
AATATAAGAATTCAATTATAAACAATTTGTGCAAGTATATGAAAATAAACAACTTAACATCCACTGCACA
TCATCACCAGACAGTTGGAACAGTAGAAAGAAGTCACAGGACTTTTAACGAATACATACGATCATATATA
TCAGTTGATAAAACTGATTGGGGCGTATGGATCAAATACATCGTATACTGTTTTAACACGACCCCCTCTAT
CGCACACAATTATTATGAGCAAATTCTTGGTAAGCACCCAATTTACCAATATAAATAGAATCGACCCACT
ATATAATGTAGAAGATTATGCTAAAGAAGTTAAGTTTAGATTAGAATCAGCATATAAAAGGTCTAGGATA
NTGCTTGAAAAAACATAAAGCAAGAAGTAAGGAACTTTTTGATAAAAAAAAGTTATAGATTTTCAAGTAA
AAATAGGAGATAAAGTCTTATTAAGAAACGAAACTGGCCATAAATTGGATCCAGAATATATAGGCCCATA
TACAATAGTTAACATAGAAAATAGGGATAACATTCTAATTAAAGATAACAAAAATAGGACCCAAAAAGT
TCATAAGGATAGACTAAAAATTTTTAACGAATAAACATATAAATGTTCTATATGAAGTCTTTATGTTCCTT
TATGAATTCACAACAAATAACAAGTAAGAGGTTCTAGTCGGGAGCTCCCCGACTAGGGGATACCCTGAAC
CCTCTTCTTCCAACATCAAATGCATATATAATCTATTTTAGAAGCTATATGTCAAGTTTGGTTCTAGTCGGG
AGCTCCCGACTATGGGATACCCTGAACCCTCTTCTTCCAACATCAAATGCATATATATTCTATTTTAGAAG
CTATATGTCAAGTTTGGTGACTCTAGCTCTTATTATTTACCAAAATTGCCCAAAAAAAGATATCGATATTG
ATTTTTATCGATTGCTTGGAAACGGAGTAAGCTATCGATTATCGGAAATAAACTCGATCTGCGCGGGAACT
AGGAGCACCTACATCTAAAATTTCAAGTCTCTAGCTCTTATATGTTCTGAGATCCTTGCGTTCATACATAC
GGATGGACGGACGGACGGACAGACGGACATAGCTAGATCGACTCGGCTATTGGTGCTGATCAGGAATAT
ATATACTTTATGGGGTCGGAGATGCTTCCTTCTGCCTGTTACATACGTTTGGATTTTGCACAAATACAATAT
ACCCTTATACCCATTTTTAATGGGTTCAGGGTATAAAAA
>9_rnd-5_family-311__DNA___P
TGGCCCCATTAGTCAACAGGGCATGNTGTTGTGCTGCGCGAGTTANTCTATTGAGCCCGTTATTGTTTCGT
GAATGCGATGNATAAGTAGATAAGANCCTACACGCAAATACACATACGCACGTGCATATTCGGCGATTCC
CTCTGCATANNTACGTATGTTTGGGTGNGTGTTCTGGCTTGCGGTTGNCAATTGCCCATGCTTGCTGTGCG
TATAATTATGCGTTATCTACCTTTGAACGTCGCTCACTCGAGTTGCTGCCGCCGACTTTTGTCCACTTTGCG
TCCGCGCAGCAGATTCTGTTTTAGTTAAACGGCAGCAGTCGAGTAAAGCTGTTTTANAAAAATTATAAGT
GCAAATTCTCANGTGAATGCCATCGTATCTCTAATGCGAAAGTATAAAAGTCGACGAAAAAGTGAAATCA
AAGCTTGAAGATGTCCATCGAATTTATGGACTTTCACGCATTTTAAAGTGTTTATTNTNTTGTAATTTTCTC
TCACCTNGCCAAANAAGTCATGGGAGCGCGATGAACAAGAAGATAAGAAAATAATACAAGCATACACAT
ACGCATGCANATANTTGGCGATTCTNNCTGCATACACATGTTTGNGTGNGTGTTCGCGCAATTGATATCTA
TTTATGTGTGCTTGTGCGTTATCTACCTTTGAGCAGACTCCCGCTTACTCCAGTTGCTGTGCGCCGACTTTT
ATCTACTCTGCGTCCGCGCAAGATGATTCTGTTTTAGTTAAACGGCAGCAGTCGAGTGAATGTAAGTAAA
AATTCTCATGGTGTACGTTACGGATCGGAAGTATTAATAGGCATTAAAAAAATTATAATATCAAAGTTTG
AAGAAGTGCATCGATTTTATAGACTTTCATAAATGTTAATTTTTTGCTCACTTCTGAGAGCACTATGAAAA
AGAAGATGAGNAGATAACTTGCAAATACACACATACATGTGCGTACATATTTGGCGATTCTCTCTTCATAC
ATACGTATGTGCGTGTGTGCTTNCGAGTTATCTATTATGCTTGAATGTGTCGNTATCNACTCTTGATCCGCT
TGCTCGATTGCTGTGCGCCGACTTCTGTCCCGCATCGATTCTGCTTTAGTTAAACGCCAGCAGTCGAGTCG
GGCAAANCTGTTTTAAANGAGTTGTACGTGNANATATCNCAANTAAANGCACAGCCGTNTGTNTAGTGGT
CTGGCTAAGTTGTCGGANTCAAGAATGNATCAGGTGATTATTTAATTGTNATTTCGCAATATCAGAAGGA
AATGCACAAGCAATCATATACACATACATACATATGCAAGTACATATTTGGACTTGTAAGAGTGGAAAGT
CTAAGTAGCCATAAAAAAAGTCGTATTCTAAAGTTTAACAAAAGTGAAATCAAAATTAGAAGAAGGCCAT
CGATTTTATAGACTTTTCAATTTTAAAGTGCTTATTTTATTGTAATTAGTCTCCTTTGCAAAAATTTCGTGA
GAAACCCGCGAGCAGANAATNCACNAGCATGCATACATGTATAAACACATACATCTGTACGTATGCATTT
GGCNATCCCCTCTCTTCTTCTTCTTCTTCTGCATATGTGCGAGTGTGTGTTCTCGCAATTCATATCTACTTAT
GCATGCTCGGTGCTTCCTATGCGTTATCTGCTTTGAACGCCGCTCGTCGGGTTTCTGTGCGCCGACTTTTGT
TCACTTTGCGTCCGCGCAGACAGGCTTCTGTGTAAAGCTGTTTTAATAAAATTACAAGTGCAAATTATCAA
GTAAATTCCAATCGGGTCGGAAGTATTAATAGCCATAGAAACTCGTAAAATCAAAACTAAAGAAGTCTAG
TGTGTTTCTTTAATAATTTACAATCAATTATATACACATGTGTATGTGTGTACACCTTTTTACTTGTACGTC
TCGGAAGTATTAAAAGCCATAAAAAAATTGTATTCTAAGGTTTGACGAAAATGNAATCGTAAAATTTAAAG
AAGTCACANCGNTTTNATAGACTTTCACAAATGTTTAAGTGCTTANTTTACCGTAATTTCCATCTCGCTTCG

CAAATAAGTTCGTGGGNGCGCGATAAATAAGAAGATAANGCACCGCCNCNCATATANACATGCGTACAT
ACATACGTATGTATGTCCGTATGCATACATTTGNCGATTCTCTCTNCTTTCTACATATGTTCGGTGCTGTGT
TTTCGCAATTCATATCTACTTATGCTTGCTTGTGCGTTATCTNACTCGAGTTCGTGGATTGCTCGCTCAGTT
TTCTGTGCGCCGGCAGTCGAGTNAAGCTNTTTGAAATGTTAAAGGCAAAATTCTCGGGCTAATATCGTCCG
AATCGGGAATATCAATTGACATGTTANAAAATCGCCTTCTGAGGTTTCGACGAGGTCAAAGTGGTTGCTA
AATTNTTTCGGTTCCGAAATCAAAATAAAACTCTAACTCTAAAAAAACGTACTCAATTTTCTTCCAAATTG
GAACTTTGCTTTCTATCTGCGTCGAACAAACCAANATTTNNTAGNNCTCCGTCGATTTCGAAGNCTCTCNA
ATCCTAAAAGGTACGTCTATTTCGAAAAGTTTACAAAATACTAATTCATTTTTTATTTGNATTTTGCAGCC
AATCTGNCATGGACAANAAAACATTCANTANCGTTGGTACCTAGATGACCTTGGTATTTCATGCCATGGA
TATACCTTAAACAAAATCGNATTTAAATTTTGACCAAAATGGCAATGGTTATAACAAATTGTTGCTAATTT
ATGTGGAATTTAAAGACTCGCGAATCCTTAAAGGTATGTATTTCCGAAATCGGACGTATTTGTTTACAAA
AACTAATATATTTTTCATTTTATATNTTTCAGCCGAATCTGCCAACGTTCATGGACAA
>Ref_dvir_1002
GATTCGACCGAGTATGTCAAAGCCGTCTATATTATATACTTGGCCTTTTCTACTCCAAGTCTCTACGAAAC
ATAAAATATCTGCTGCTTTTATTATAAAATCATTTGAAATGTCTAAACTATCATTATGCAAGCCCTCAATA
TTATGGAAATATGTCAAAATATTTCCTTCTACTCTCTCAAAATATATCTGATGGTGGTGGTCAATTTTTTCT
CGCCCCGGAGTTTTTCCAACTCAATATAGACGGGGTCGGTAGCTTGTATTTCTCTAGGCGGATTAAACTTT
CCTATAAGGAAAAGCCCAGATGCATTAGTCGCTGTGCTACAAGCTACGTATAATGCTGCACGGCTCATGC
CATCTTGCAAATGCACTGCAACTTTTAGGTAGGTTGCTCCCTGACTCTTGTGGATGGTTATGGCTTCAGCA
GGAACCACTGGAAATTGCAAGCGTTCAATTGTTACTTGGTCATTTTTCCTATATTGAAATGTTCTTAAAAC
TCTTTCAATCGGTGTCCAATCAGAGTGCAAAGGATTTGTTTTTTTTTGGCTCTTGCTTGCGCTCCCACAGTT
GCTTCTGTAAATTTAAGCCAAAGCGTCGAAACTAACACATCAGATCCGCAGGCTGTATAGTCAATATGCA
TAAGCTGTCCACTTGCTCCATTGACCAAACCAACATTGGTATTTATATTGACGGTAATCATATAAATATTG
AGCTGGGAAGGCCTTGGGTTTCGGAAGTCTTGAATGCCTTAATATTTTTTAAAATATTTAGCTTGTTCCTTT
CAGATATTGATTTGGCCTTCAGCATATCTTTAGATTTTGAAGTATATTCTTCAGTAATAATTTGGCTAAGTC
GGATATTATTGTAGTCGCTCGCTTTCTCATTTGAGTAGAACAATCTTATAGCATCGTTGGGAATATTATTTA
TTTCGGAAGGCAAAACAATTCTCTGTTGAATAAGCAGCTTGTCCGTGTCAGTCATTTTCCTTCTGACATATT
ATTTAATGCGATGGCAAAGGCGCAATCCTCACGTTGCCGCATTATTTCGGTCAATTCAAAATATTGAAATA
AATCCCAAAGTGGTGCTCCCAAAATTATGCTATATGGGTCTCTCGAGTTTGGAGCAAAAATCCATCTATCG
CCAACTGGTGGAAGCTGCTTTAGATCACCAAATGCCAATATTGAAATTCCGCCAAAGTATGTATTATTTCT
AAAAATTTGCTTTAGACGAAGATCCAAGTACGACAACATTTTTGCACCCACCATTGAAATTTCATCAATAA
TAATAAGCTTTAAGTCTATAAGCTTTGAGTGCATAGTGTTGATTGCGTCGTTGCTAAGGGGTCTTAATTCC
ATTGAATATTGGTTAACAGGCAAAGAGAACACGGAATGGAGAGTGAGTCCACCAATTCCAAAGGCTGCTT
TGCCAGTTGGGGCACACAGCAATATCTTTGCAGAATCAGAGTCAAAACCAACTCTGGTGTTAAATCTTAA
AGTTAGTGACTGATATATAGTTGATATCAGCCTACTTTTTCCAACACCCGCCCCACCTCCAACGTATTCAT
AAAAATATTATGCATAAAGTGAGCGTAATATTGTCGTTGTTTACTATTAAATGATCGCACCATTAAATTAA
GCTCATTTTCTGGAACTTGTGCAGGTAATTTAATCATTCGGATTGCATTTTCATCACAGTTGCCTTCATGCA
CATTATCTTCAAATACGTTAATGTTTCAAGCAACTTCTGATACTGCAAAACCCCTGAATTCTTCACCAAGC
AACTGAGATGCTGAAATTTCTTGTCCATCATTATTGTCATTGTTGACTTCTTGGATCTCAGCTAAGACTTCT
TGAAAATGAGCCTCATCCAAATATTCATATCGTTTTCTATTTGATTCGATGATTTGAAATTGTGCATTGAAC
GTTTGCTCATAATCGGTGCTAAGAATATCCACTTGCTCATTTCGCCAAGGGAAAAAGAGCATTACAAGTTC
TCGAAAATAATAGATCGGGATATATTTATGCTATACCTCTCCACCGAAAAATAGAGAGTGCCTACGTTCTC
TCAAAATTCCTGATCTATTTTTGAGTACCATGGGAATCCCGTCATTGGCATTGTCCTCCCGCTCTTCATCAT
AATTTCCCTCCTCGTAGTCTCCTGTTAAATGGGCATTTAGCGACTTAGAATATTTAAATTTGGCAGCAAAG
TCAGCTAGGCACAAATTATTTAATTCGTCCGGTCTTTGAACATAATGATCTAGAAAGTTTGGTAAGAAATA
ATCTTTTGAATCTGAAGATACTGTTTCTAAATCTCTCTGGGCTTTCACCATACCAACTCTTTGATCAGGATG
CGAAGTGTTTACAAAAATCTCGCCATGGCTACATCCTGACATACGGAGTCCAAGGATGTTGTATGAAGCC
TCCAGCGCAGATATTTCTGTTCCATTGATGAACTTATTGCCGATATGTTGTAGTTTTTGCTTAACTGAATTG
TTATTACTACTTATCTCGCCCATGGCTTCTCGCAATAATTGAGAAACCCCTCTATTCGATTTGTTTATGTAG
TTTATTATATAACTTCAACAAGCAAACGCATCTAGTATGTATTGAATATCCATGTTGGCTCTATATAGCTTC
AAATATCTTTATTATATGCATTCACCATTTTGTCACATATTTTCTACGCAAAATATTTGCGGCTTTTAAACT
CGATCTCGTGCAAATTATATCCAATATAATAAAACTATCAAAATTATTTAAGCTTAAGGCTTCAGCGCCCG
AAACTGAACTATCCAGTAGCTCTTTGATTTTCTTCCACATGTCTTGATGCCTTCTCAGATTTGAATCTTCCT
CTTCTAGG

>160_rnd-5_family-7347__LTR___Pao
GGGATGACGTAGTGCCGTTAGAAGAGAATGGGAGAAGTTTTTAACGGAGTTGCCCAATATTTCAGACATA
CGCATACCACGATATATAACATGGGGATTATATAACGCAGCAGCAGAACTACATATCTTTTGTGATGCAT
CAACTCAAGCCTANGGAGCGGTTGCGTATTTACGTGTTCCAACCGGGGATCACGCATTTCATACGGAGTT
GCTGCTGTCAAAATGTAAGGTCTCGCCTGTTAAGCCGCAATTAACCATACCCAGAGCCGAGTTGTGTGCTG
CTCTTGAAGCTGCAAAAATGTATAAGTATTTAAAACAGAATTTGCGAATTAATATAAACTTCTGCAGTACT
ATTTTTTGGTCCGACTTAATGATAGCCATCGCGGAGGAGGTGCAAACTGGCAGGTATTTGTTTCAAATCGC
ATAAACAAAATTCTGNAGATCATCTGAGTCGGGGAATTAGCGTATCTAAGTTGACGGCATGTGAAATGTG
GTGGCATGGACCGCAGTGGTTACAAGGNCCGGAAGAAGGATGGCCTTGCAATAACATTGATCAACAGTCC
TCTCACATGTGTGTATTGAGACAAGAAGCTTGACTTGCCACAAAATATCATTGGAAGAGATGCCTAGCTC
GATGTTCATAAGTCAATATTCATCGTATACCCGACTTCTTNGAGTATGTTAAACGTTTTATTCGAAACTGTC
GCATAATAGAGTCTGAGCGCAAGCGAAGTGAGTTATCAACAGATGAACTAGAGGAAAGCCTCAGAGAAG
TAATAAGATTGGTGCAAAAGGACTGTTTTGAAAANGAATGGAAGGCACTCTCTTCTAGCAAGCCGGTCCC
AAGAGATAGCAGTTTATTACCTCTCAACCCATATTTTGATTTGGGGGAGCTCGTCTTACGGCTCGGAGGAA
GATTGCGCGACGCCTTGTTGCCAGTGAATGAGAAACATCCAAATATATTGCCCTATAAACATATTTCACGA
GTCTTGTCGTAAATTATAGTCATGCTTGCACCTTGCATGGAGGCTCATCGCTCACACTCAATTATATAAGA
AAGAAATTTTGGATCATCAATGGGCGTAACGCAGTTCGTTTTGGTGTACATAAATGTGTCACGTGTTTTAA
AATGAAGCCTNAGTTTGCAAGTCAACAAATGGGAAATTTGCCAGCGGTGCGTGTGCGGCCCTCAAGAGCT
TTCTCATCTGCCGGAGTTGACTATGCTGGACCTATTAATATTTCGCCATCCAAGGGAAGAGGACGTGTAGC
ACAAAAGGGATACGTTGCCGTGTTTATATGTTTGGTGACCAAGGCTTTACATTTAGAGCCAGTTGGAGACT
TAACGTCAGATGCGTTTTTAGGAGCCTTACATCGATTCATAGGCAGACGTGGTCTATGTACAGATATTTAC
AGTGACTGTGGTACAAATTTTGTATGAGCCAACGAAAAACTACGAGCTGATAGGGAGGCATACTACTCAT
ACATTTCAAGATTAATCGCGCCAAAATTGCTGAATAAGGGAATCACTCGGCATTTCAACCCTCCATCAGCT
CCACACTTTGGTGGATTATGGGAAGCCGGAGTTAAGTCCATGAAGTATCATCTCAAGAGAACCATTGGAG
AGCGAGTGTTAACCTTTGAGGAAATTGCGACAGTGTTAGCACAAATTGAGTCTTGTCTTAATTCAAGGCCT
CTATGTCCTATTTCGAATGATCCTGAGGATTTGATGGTGTTAACCCCCGGTCACTTTCTCACTGGTGATGCG
CTCTTGTCGCCTCCTACANCTGAAGTACCTCAACTGAGTTTAGTGCATCGGTGGAGAAACTGTCAATACTA
TTCACAACAATTTTGGAAAAGGTGGAACTCTGAATACTTGACACGGTTGCAGAGAAGACCAAAATGGCTG
CAGATGAAGAGAAACCTAGATGTTGGATGCATGGTGTTAATNAAGGATGAACGATTCCCTTCGCATCGGT
GGCCTTTGGGTCGGATTACAAGCACTCATGCTGGTGCAGATGGATTAGTTAGAGTTGTGACTCTAAAAAC
AAGCAGTGGTGTTATCAAACGGCCAGTAACAAAGCTCTGTCCATTACCCATACAAGAGAATTGGCTTGAT
AATGACACCTAACCTTTACGTAAATGTTAAATTGTACTCTTCTATTTTCAGTTTAAGTTAAGGTACCAAGC
CCGAGACTCTGAAAGNTGGCGGACGTAAATTTGATGATGAATGCTGCAATCATCAAAGGGGGAGAATGTT
TGGGCTACAGCCNACCAGCAAGAGCAATCGCGCCGGCCTAAGAGCGGATATTATAAGAGAGCGCATGAA
AAGAGCGATTAACGAAAATGCACCTGCGCTTAGATCAGCCGNATTTAAGTAATGTCTTATCACTCAATGG
ATCACGCGCTATCACGGTTGAGTTGAGTTGAATTTAGTGGCTCTCTCAAGCGCTCGAGCNTTCAGTCCTGT
TACTTTAAGTTATCTTACTTTCTTGTATTTCGTAATTCTATTGAGCTTTGAATTTGCCCGCTTAGTAACAGC
GGAATTTGCGACACATGTCGAAGTCTGTGAGCGTCAGTCCAAGTCAACGGACGTACGGCTCTGTGTAGGT
CAAATTAATAATTATAGTATTTTTAGGTTAAGTTCAATAAAAGTTGAAGCCTTAAAATGAACAGCCCCCTC
GTGAACTGGGAGTCTTTTGCTAATCTCAACATTCCCANCAATCGTGCAAAACCCTCAAAGCCGCCTCGGTT
GGTAACCACGCCGCAGGCCGCATCATATCTACGGGNAGTTTGGGGCTCCGCACCTGGCGCAGCCGATATC
CAACA
>Ref_dvir_847
GCTCGTGCGTATATCTGACAACAAATACATACCAGCAAGTTTGTGCGCTTGCAAGTACATATATATGTATG
TGTGAATATATAATTGCGACGAGTGTGCAGCGATAGGTAAATTGCCACCGTGTGATAGGATTACATGGCG
ACTTGTTATGATATGGACACTATAATTGGCAGCAGCGATAATATTACATCATCGAGGCGTGCCATTTGATC
GTCAAGTGAGATTCTATATAAGATTTGTTTTAAATTTAGGACTGGATCCCTCGAAAATATTTCAATGGAGA
AAGTTACTATATAAACATGTGTGCAATTGCAATAGTAAGATTTGAGGCCGGTGCCATTCTCTCACACTCGA
AAAGGCAGTTTCCTTGGTAGAAAACATTGGCATTAGCTGAGCAATCCGGGAACCTAAGTGGCTCTTGTAT
TACAATCTGCCACATATAGAAGTATTAAGGAGAGTATTTAAATTATATGTAATTAATGCACATATATATAC
ATACATGTATGTACATATATGCGCATACATGCATGTATATACATACATGCACGCACATACATACATATATA
TAAGTAGTGAATGAATCTTATTGATTATAGATGAAGCGGTTTTTGATGTTGTTGCTGGGGCTGCTATTCGG
CGGCGGCTGCTGGTGTTGCTAAACGGCTGCTAATGAAGATAGTTGCCAGCTTCGTGCAGCAGCACTTTGTT
GTTGATGTTGATTGTTGTTTTTGCAGCCGCTGGTTGACATGTTTTGCTGGTGACGGTAGCGAGGATGATGA

TGGTGAAGTTCAATCAGCTGGGCTATACTTGGGCTGAGTGCGTTGTCTTTGTGGTTGCGTGTGCGTGTAAG
TGTATGATAGTAGCGGCGTTATGCGTCTGTGTGCGCACTGGAGGGGATGCAAATAGGCGGCAATGGATGG
GCCGGCGGCAGGCGGGCTGGCGCGTCTGTGTGCGAGTATATAATTCTGTGATGGTGAGGCAACGTTTGCT
GGGCATTGGTTATGTGAGTGTGTGCCAGCATACGGAAGTGTGTGCTGGATATTTATTTGGCACGCGTTTGA
GTGTGAGCGAGTGCGTGCATCTGATGTTGGGCGCAATTGCGAAGGATGACAATAAGCGAGCAGGCAAAC
TAGAAAGGTAAGTAAGTAAAGGTAGGTTTGAAAGATGTTTATGAAAGAGGTAAGTTGGAGTTTCTTTTCT
TTTCAGTTAGTTGATAGGTTATTTGTTCAGAGCCTTAGATGGAGCTGGATGAGGGTGAAGCAGCTATTTGA
ATGGAATCGTCTCCATGTCGGAAAATTCCTTGCATTTTCTGCTGCACATCACTCATGTAGCGAGTCAACTT
GTCCAGTTGTTCTTGTTGCCTTTGGGCAAGCTTAACCAATAGTTGGGTGGTTGAGTTATGCTCAGTGGAAG
GAAGAATAGGGGTTTGATGATGTTGTTGTTGTGGTGGCTGTTGCTGAAACTCAGCATCGATTTGCATTGGC
GTAGGCTCGTGCTGGTTCAAGTGGTGAGCAGAGGCAGCATTATGTCGTGTGGACGTAAGTGTATCTAGGT
GTTGGCGCTGCATAAGAACAGTTTGATCACGATTGATCATCGAACCAAACTGTTCAATTTGACGCTGCTGT
TGCTGTTGTTGGATGTTTTGAGTTTTGGGCCATCTTAGGCGCGGGTCCAAATTTGCGGAAGTTCTTGATAT
ATTTCTCGTTGCAGCTGCTGTTGATGTTGTTGTTACTGCTGCTATTGAGGATGAAAGATTGGCGCTTGGTGC
CGGTTTGATAGAGTTATGAACAGGAATGGTGTCAGAATTAACATTGTTAATTGGGATATTGTTGTAATTAT
TAATGATGGGAATATTTGCGGCTTTGATAACATTTGATCGTTGCCTTTGCGCAAGCGCACGTTCCATTTGT
GCTCGCTTGTGTTGTTTTGATTTTTGCTTGCGCGCTTTGGCTTTTTGTTTTTTATTTGTTGGCTTGGGCAAAG
ACAGCAATGTCGGCAGCTGTTGTTGCAATGGCAATTGCAATTGCAGTGGCATATGAGCCTGTTGACGTTGC
TGCGAGCGTATTTGTCGCCTTTGCTGCCGCTGAATATTCGGATTGCTTTGTCTCGGTAACAGTTGCTGCCTT
GACTGAACGTTCCGAATGTTTGTATTTGATATTAGTTTTGATTTCAGCAAGTCAAGATATAAGGGGCAGCC
CTTATAATTGGCCGTGTGTGATTGGGAGCAGTTGGCACAGTTTGCCGGTAAATTGATAGGCTTAGTACATT
GTTGCGATAAATGCTCCTGGCCGCATTTCACGCAGCGCGGATTGTGACGACAGTAGCGCGCCGTGTGATT
GTATCGTTGGCAGCGGTGGCATTGAGGCAGTTTCCTGTCGTCGGTTTTTGGCCACTCAAAACGGACTAAGA
ATTTGCATAATTCTTGGACGTTGAAAATTGTTGTTGGATTCTTTATTGAATTATGAGCTAATTCAATAAAG
AATAGATTCGGGCAGTGATGACCGGGATTACTGCGATTCCGATAAACAGGGTTATATACATTACGTATTA
TGTAGCCCATATCGGATAGCTCATTTCAATTTTGGTCGTGCTAATGGGAGGCTGAATGCCTCGTACGACAA
CCCGAATTGATCACGTTTCTGGTAAGTATTGAAGTTAATGGACCTGTCCAGTTCCACGATATGTATGATAT
CTTCCTTGTGTTTGAGTCCGATTTGGGAAAGATTCGTAGGATGCCAGGGCCCCCAATTTTACGCCGTATCC
ACTATGGGGGATTGGCCCTTTTATTTGGCCACTACAATCAAGGCATTCCAAT
>Ref_dvir_718
CGCCAAAAGAGAAGTAAGAGACACAAGAAGAACGCCAAGTGCCAACGAAATCGGCACAGGCATACTAC
AATAGTGTGCAGAACAAATACACACACAGGGCAAAGGAACTGCTTTACCGACAAGTGCCAACAATTTCG
GCACACACATAACGCAGCAAGAACTAAACGCTGCTTTGCAGTCCTCCTTTACCAATACACTCATAAATTCT
AACATAATTGGCACTCAGCAACAACTATTACAGCAACAGCCAATACAGCATCAACAACAAGTTAAACCTT
GGAAGCTATTTCTCCAACAACTCTATCCCTGTGGCAGCAGCAACAGCTACAACAACCAGAAGAAATTTGA
TGCCATCATTAACAAAATCGCCACCAACACAGACGCTAACGACAACAATAGAAACTACCAGCAATGACA
AAGCAACACTGCTACAAAACTGGCTATCAGGTGTGCCACCACTGCTGCCCAGCGAACTACCAAAGCCTCA
ACTAGCAACGATAAATACCTCCGCCACAACGTGGCGATCACCTCTGAAGAGAAGATGTGTGGATATGGAC
GAGTCTGACTATGATCCCGCTCTCCAAATACTACCGTTAACGACGCCCATCTTTAAGAGTGGTTTCGCTAT
CAAGCTTCACCACGAGGCACGCCGCCAACAAATGCTCTAAAGTGAGGCAGAGCAACACGGACATCAGAC
AGCACAAAGAGGGCAGCAGCCACAGAAATGCCAGCAACTGCAACCGCTACAACAACAACAATCGCAACA
ACATCAGGACGAGAGTCTGAAGCAGGAATCGAAAGAGAAACTTCCCCAGCCAATCCAGCAACAGGCAAC
ACAAGGCAAGAAAGTGCCTGTGCTCTTCTTGCCAATGGTGCCCGCAATTATCTGGATAAACTGAAAAGCA
ATCCAGGCGTGCGAAAATTCTGGACAAAGGCAACCTCTGGCACTGGACTAAGTGTTCAATGTGAAGATCT
AGCCACGCATAAAGCAATTCTATCCGCCCTGGCTGAAGAAGGCACTCATCTGCATACGCACCAACTGCGC
AGCGAAAAGGAATATCGCATAATCTTACGGCATATCCACCACTCTACAACCTGTGAATGGTTAGCGGCCA
ATCTAGAACAAATGGGCTTTGTCACTCGTTTCATCCGAGTCAAAAAACACCGGTTCACAGGCAGGCCGCT
AAATTTGTTTGAAACCGAACTGCAGACCAAAGCGGATGGGAGCCATGAAGGCATCCTCAACCTGAAAGA
GCTGGGCAGCCAATCCATCACGGTCGAGCGGCAAGCAAAACCTCGAGATCCAGTGCAGTGCCATCGGTGC
CTTTGGTCTTTGGTCACACGAAAAAATATTGCAGGCGGCCCTTCGTCTGCATGAAATGCACAGGCGAGCA
CATGTCAACTGCATGTACCAAACCCAGAAAGGTGGAGCCTAAGGCGCAACGTACGTAACCCGGCCTAAA
GTCATTTGGCTCCTTCCAGCGGCAACTACGTTTGCACAGCATCAAAAACAACATCCAAATCTTTCCCCGGG
GCCAGCCGAAAGATCCAGCAATATGTCCTATAGCCGGGTTGTACGAGATGGCGGCAATGCCACTGTCCGT
GAACGAAACCCAGCCTAAAAGCATTTGAGAGCTCTCCAGCAGCCACTACACAGACGGCAGAAGCAACAA

CAACAACATCATCTGTACAATTTCAGGAAGCAAGCAACATCAACAAATAGAAGAGAGCTACTCCAGCCA
GTGGTGCCTAGTTGACGCACAGCCGTGTCGACCCTGCCTCCTTGCGTAATCCTTTCGGAAATCAAATGGTA
ACTACCCAACGGGGAGTTCCCCAACGGCATAAATGGACGAATAACAGCAGCTGGTAGGGCAACAATCA
CCAATGTCCAAAAGCAATATAATCCAGAACTTGGAACAAAACATGGCAATGTTCACTCGCATGAAACGAG
GAATATATACTTTATTAAATGTCCTTTTGCAATTGCCACTCATTATAAAAACAATAACACAAATACCAATA
ATCATGTGTTGTAAAACACAGCTCAACAACAATCAGACAGCAAAACAGTCCAAATGGACTGCTCTATGGA
AGCTGCCATCGCCAATGAATCCATCAATTATGTATAGTCATCCACACGGTAGTACCGCCGCTCAATCGACT
TTAGCCACTAATACTTCCGCTGAAAATGTCCGCAGTCCGGCGCCAATCAGTACAGTAAATTCACATCTGAC
CATCCACAATGCAGCCAAAATAGCAACCCATTTAAAATTGCTTTCTGGAATGCATGTGGCATAGGCAACA
AAATGACGGAACTCGAGCTGTACATGCGACGCGAGTCCATACAAATCATGATGGTCATCGAAACTCTGGT
CACGACACTAACATACAAAGCGTATCAGGCTACCACACGTACAGTGTAACCCATCCACGCATGCATTGTA
GAGGGGGGGTCGCTATCTTTGTGCGTAACGATATCAGGCACGCTGCCCTCCAGCCAATTAACGCGAACTT
TCTCCAGAGAATGCCTATTGCCCTGTTGCTTGAAAATGAGAATAAAAGGGAAATAATTATAATTGCCCCA
ATCTATTGCCCACCAGTGTTCAAATGGTCCGTCGAACACTTCACAAAACTATTCAAACATTTTGACAACTT
CATCGATCCGGCTCCATCTAACTTTATTTCTGCGGCGATTGGAACGCCCAGCACTCATGGGGGGCACTGT
CCTTTGTCGTAAAAATGTCCTTGCCACC
>Ref_Helena_2_2014
ATGAATCCACTCAAGATCCTTATCTGGAATGTTAATGGCATCGCAGGAAAAGCCAGTGAAGTTGAGCTCT
TCGCGCACAACAACAATGTCGACATTCTCCTTCTAAGTGAGATCAGACTAAACCGAGGACAAACAGTCAA
AATACATGGGTACACCTTCTATCCAGCTTACAAGCCATCACGTCATAATCACGGTGTCGGAGGAGCGGCG
GTATTCGTGAGGAGTTCTCTCCGCCATTTTCCACAAAGAGTAATTGAAACGAGCACTATACAAATGTCTTC
AATAAAAGTTGCCACTGGTTTGGGAGACATGGAATTTAGCGCCATATACTGCCCACCAAGAAATAGAATT
GAAGAAAGGCATTTCAGTGACATACTCGCTTCTTGCGGCCAAAGGTATCTGGTTGGTGGAGACTGGAATG
CGCGACATTGGCTGTGGGGAGATACCTACAATTCACCCAGGGGCCGAGAACTAGCCGAAGCCATTACAGC
CAGAGGAGCTAATATTCTCGCAACAGGATCACCAACTAGGTACCCATATGTGCCTAGTCACACACCTAGT
TGCATTGATTTCGCATTATACCATGGGATACCGGATTCTCAAACAAATATCGGCCAAAGCTGGGATCTAG
ACTCTGACCATATAGCCCTTGTTGTAAATTTGCAAACTGATGGTTCTTATGTCAGGCCAAACCCTCGATTA
ATTACCAACCGTACTGACATCATGGCTTTCAGACAACATCTTGAATGCTCGATTCAATTAAATCCCACCTT
GGACTCTGGAGTAGACATTGAGAATGCAGTAGATTTTCTAACTGAAAATATACATAGCGCTGCTACTGCC
ACAACTCCACCCAACACCGAGTCACGCCCCATTCGTTATGGCATTGTTCTCACGAGGGAGGCTAAAGAGC
TGATGAGAACAAAGAGGTGCCTTCGAAGAAGAGCAATTCGATCACAAGATCCTTGGGACCGAATTTTGTG
GAACCGAGCAGCAAAACAGCTACGTATCCTCCTAAGGGAACTCAGAAGTGATTTCTTCGAGCAAAAACTG
GCTTCCATGGACTACACCGTTGATGCAAACTACTCTCTATGGAAATGTACAAAATCACTCAAACGACAAC
CCTTTAGACAGGTTCCTGTCCGATGTCCTGGTGGCGAACTTGCTAAAAACGAAAGGGAACAGGCTAATGC
TTTCGGATATCATCTTGAGGAGCGTTTCACTCCTTATGAATTTGCTACGATAGAGCAGACCAGGGAGACCC
ATCAGACTCTTCAGACCCCCCTGCAGATGTCCCTGCCTATTGAGCCTATAAGGATTGAAGAAATAACTGAC
GCGATCCAGCTATTGCCGAAAAATAAAGCTCCGGGTATCGATAGAATCTGTAATGCTACGCTGAAAGCCC
TGCCTACAAGCGCGCTTCTGTATATAATGCTAATTTTTAACGCTATTATCCGGCTCCAAGTGTTCCCAAAG
CAATGGAAACTAGCAGCAATTATGATGATTCACAAGCCAGGCAAACCAGAAGCTGATCCTGAGTCGTATC
GGCCAATAAGTCTCTTACCCTCCCTGTCTAAACTATGGGAGAGGACTATTGCCAAGCGGATAACTGCCATT
ACAAGCCAATGCAATATCTTGCCGGATCACCAATTCGGATTTCGAGAGGGACACGGCACTGTGGAACAGG
TCCACAGATTGGTGAAGCACATCATGCAGGCTTTTGATGACCTAGAGTTCTCAAACGCTGTCTTCATTGAC
ATGCAGCAAGCGTTCGATAAGGTGTGGCACGATGGATTACTGTACAAAATAAAAAACCTTTTACCCGCAC
CGTACTATGGCCTTTTAAGGTCATATCTGGAAGGACGCGAGTTCAAGGTCAAGGTAAGGGACACATACTC
CTCCACCTATACTATGAGAGCAGGAGTGCCACAGGGCAGTGTTCTTGGTCCGCTGCTCTACTCGTTGTATA
CCTCAGATATACCCAGCCCTACCTCTCAACATATGGAAGATCCCTCTAAGGCTATCATTGCGACATATGCA
GATGACATCGCAGTCATTTATAACTGCGAAAGTTGCGTAGAAGCTAGCAATGGACTTCAGGGGTATCTGG
ATACTCTCGCAGCTTGGTGCAAACGGTGGAACTTGAAGGTTAACCCGTTAAAAACTATTAACCCATGCTTC
ACCCTCAAAATGCGGATGCATATCCCTCCAATTCAGCTTGAAGGAGTTACCCTGGAGCAGCCGCCACAAG
CTAAGTATCTCGGCATCACCTTAGACAAGCGCCTTACCTTTGGGCCACACCTCAAAGCTACGGTAAAAAA
ATGTCGGCGCAGACTGCAACAACTGCGGTGGCTCAACAACAAAAGGAGCACCCTGCCGCTGAGATGCAA
AAGAGCTGTATATGTGCACTGTATTTTGCCGATATGGCTCTATGGAGTGCAGATTTGGGGGATTGCAGCCA
AATCAAATTATAAACGCATACAGGTACTGCAGAATCGGGTATTACGACAGATAACCAACTGTCCCTGGTA
CGTACGCGGTTCTACACTCCATAAAGACCTCAAAGTGCACACAGTCGAAGAACAGATTGGAAGGCACACA

AGCAGATACAGCGACAGACTGCTGAGACACCGCAGCCTGCTCGCCAGAGGACTACTCCCTGCCCAACCTC
TAAGGCGACTCAAACGGCAAGGTTTTGCCAAGACGATTGGGCGC
>9_rnd-5_family-1262__LTR___Pao
ATTTCGTGGGTGCTCGAAATGAGCTGAAGGAACTGCGCGACCTGTTTATAAGCGATCCCCAGCGCAATGA
GATTGTTCGCAACTGCCTAGCTCTGGGAATCAACTGGAAATTCATTCCGCCCCGTTCGCCACATTTTGGAG
GATTGTGGGAGGCCGCTGTCAAGTCAGCAAAATATCATTTTTACCGCGTCGTTGGAAATTCTCTTCTCTCA
TTCCACGAATTCCGAACTCTCATCTGTCAAATATCCGCAATGCTTAACTCAAGACCGCTTTGCTATATTAC
AGAAAACCCGAATGACTTGGAGATACTCACGCCTGGGCACTTCATCTGGGGCAAAGCCAATGCCACTATC
GACGAGCCAGACATAACTCACCTAAGCATAGGTCGCCTCAGCCGTTGGCAGCGAATATGTCACATGCAGC
AATCATTTTGGAAGAAATGGAGTACATCGTATTTGTCGTTGCTCCAAGAGCGCGGGAAATGGCGATCGTC
CAAGCAAAATCTTCAGGCCGGCAGCATCGTGCTGCTCAAGGAGGACAATGCACCACCACTCAGGTGGCCG
CTTGGTCGAGTCGACAGCGTCATCACGGGTGACGACGGCATTGTGAGAGTAGCAGTCATTCGTACACAAA
ATGGTCTGGTCAAGAGAGCCATTGCCAAAATCGCCGTTCTGCCAATCGAGACCACATCGGTTGAAAGCTT
GTATCTTCCAACGGGGGAGCATGTTCGCAGCAGAACGCCCTCTGAATAATATTATATCTGAATTACAATAT
ATCTTTACTTCTCAAATCACGATATTGAAGTCACATACTATTATTTTTAAACCACCATAAAATACCATTGTA
AATTGCTCATCACTAATCTATCGATAGTTATTGTTGAAAGTTCGAGAAGTAAATCACGCATTACAAGCAGT
CTAAAGGCTCCTTTGATTTTTATTAAAATTTATAAAACATAAAAGGGGCCGAAGTGGGGTGCAAACTAGC
ATCTCCTCAAAACATTGGTCGCCTCGCCGCGAATCTTNCTTTCGTTGTTCATCGCTCTTTTGCTGCTGCAGC
ATAGGCACTAAACTCAAGTACAGACATATGTACATATGTATGTACGTATTTCGCACACCGGCTCGAGTGC
GCGCTTCTCTCTAACGATCTTATCGCGTTATTTAGCAGATCGGCTATTATAACCAAGCTGTTTGGTGCATAT
CGGTGCCAAGCACAACCGTTTGCTTAGAAAGGAGCCGTCGAGCAGATACCTACATACTTACATACATACA
TATGAATGTGAATGCAAGTGCCGCTACAATTGATCTCGATACATACACATACACAAGCCGCTGCACACAT
AAAACGGTAATCGGTCAAGGGCAAAGCTCTTCTCCATAAGTGAATTTTGGTGACAGGCCGGAGTACAGTG
AGCACTCAACAAAACGTACCAACCGAACATTACCTCGCCAACAACAGAATGGAAAATTCGACAAGTGAA
AAAGACTTTTGCAAAGCCAAATGCGCTTCTGTTCAACGCCAACTATTAGCAATGAACGAATTCTTCAGCGC
TGGTGAGATGATCAGCTTTGACGAGGCCGATCTGATGGTGCGTATAGAATCTGCGGAGAGCATGAAGGCC
GAGTTTGATATCAGCCAGTCAAGGCTAGAAAAATTAGATTTGCTCGAGCTTTCAACGGACGCACGCTCAG
ACTTCGAGAAGGTTTACTGGAAGATCAAATCCAATCTCGCTCGTCAAATGGATGTAGTCACTAAGGGACG
TATGACTGACGCCAATTCAACAACTATCCCACGCTTTGCTGAAAGTACGCACACATTTGCTCACTTGTTCA
ACCGTAAGTCTCGCCTACCGGAATTGCAGGCTGCCCAGGTTCAGCGGCTCCTACGAGGATTGGCCGGATT
TTTACGCCATGTACAAGACTGTCATTGGCAGCAATGAGGACCTAAGTAAGATTGAGAAATTACAACATTT
ACGTGCGTGCTTGGATGGTGCTGCTCTCAACACAATCAAATCGTTAGAGCCTGTCGACGTGAATCACGAC
AAGGCGTTGGAGTTACTCATAAAGCGTTTTGATAATAAGTTGCTACATTTTCAGGGCCACGTGCGGGCTAT
AGTCGGGCTGCCAGGTGTTGAAAAGGGATCAGCAGAGGCGTTGCGTGAGTTGAGTGACAAAATTAACTCG
CATCTTCGGGCGCTTAGCACCATGTCAACAACTGAGGAGCTGGCTGACGGATTACTCATACACACAATAT
CGCACAAGCTCGATCACCACACCCAGGAAAAGTGGCAGGAGGCGCTTGCAACGGATAAGCTACCCAGGT
GGACGGATATGTCGGCGTTCTTAGAGAAGAGATGCCGCATGATGGAAAACTTGAAAGGCGCTACGGCAC
CTACGCCTAGCCACCAGGTTACCAGAAATGTACAAAAATTGAATCGTAACACATTCATTGCATCCTCATCT
AACAATTACCAGCAAAATTGTAATTTTTGCGACTCAAAAGAGCATTTTCTTGTTAAATGCCCGTTGTTTTT
GAATCTGTCCCCAAGTTTGCGCCACAAGGAGGCCAAGCGATTGCATCTTTGCCTAAACTGCTTGCGCAAA
GGCCATGGCTTGCAGCAATGTCGCTCAGGTCACTGCAGGCATTGTCAACAAAAACATCATTCGTTGCTGC
ACATGAGCCCCATCACAACTCAATCATC
>Ref_dvir_326
GTTACCACGGCCCCTAGAGTTTCTTCAATATTTGCCTCTATTTCTATTGCTGCCATCATTATTATAATTATT
ATTATTATTATATCTCTTACTATTATTTTGGTAATTTCCACGGTATTTTCCGTGGTTATACCATCGGTTAGGT
CTATATCGTTGTTGCTGCGCAGCATATAGGACAGTATTTGGGTGTCCTACTGCGTCTGTGCAACTATCGAC
GAACACAGTTGTAATCTCACTCATTGTATTGAAATTTCGTGATTTCATTACAGACCTAACTTCGTCAATTGT
GCTGTTTGCTATGATTGATTTTAGGCTCAGTTCTTCGGCATATTCTTGCGATACACCGTTAATCATATGTGC
TGATTGCAAAACCCTTGTCAACTGTTCAACTTCTTGAATATATTGACTTGCTGTCTTTTGCTTTTGCTGCAG
ATTGAACAATTTAGCGGATAATGCTTTAGCTGATTCGCCTTTTATGCCGCTTTTTAGTTTGTCTATTATGGA
CACTATTGTTTGTTCAGTATTGATTAAGTTTCGAACATGGCCTTTGAGTCTAGTTTTTATTAGGCTGACTGC
TGTAGTTTCATGTGTTCCTTTTAGTGAATCCACTAAACTAAGAGCATCAATGAAACTTTGTAGATTCTCAG
ACTTTCCACTAAACTCAGGTATGAGTGAAGAGGCTAGCTTAAGAAATTCTACTTGTGCCATGGCCATATTT
ACAGTTTTTACTTCAGTAATAGACTCTGGTTCAGACTCAGATAGTTCGGCTAGTGTGATTACCGCTGGTAT

CGTAAGATCGCTTAGATCTTCTTCTTTTATATGTTTATCTGAACCGCTTGCGATTTCAGATTCGCTTTCTGTC
TCTAAGTCTGTGTTTGCTTCTTCCTCCTCTAATGCTGGAGTATTCAAAATCGAAGGTATAGTTATGTCTATC
GAATATTTTAATTTAATGCTATTTAGGTTTGTGTTTAATCTATGCAAAAATCTTGATACATACGACCAATGC
TGAGTATTTAACTTTTCTTTATTGTCGTGTATCAGGGAACGAGCTTGGTTAAAACATGCAACTCGTATAGC
TGCGTGTCTACTTATAGTCTCGTTTGAAATATTCCTATTTTGTGCTAGACACTTGTATGATTTATCAAAATT
TTTTTTATTTTAATTAATGCGGCATTAATGTTTTGCCATTCCATTTCATTTTAAAATAAATTATTAAAATGT
GCAAAGACATATACAAATTTTCAATCAAATAGTTATATATTTTTAATTAATTTATTTTCAATAACTTTAAGC
AATCTGTTGTTGATTATTAGTAATTTTATTTTAAATTTTATGCAGATCGTTTGCCTGGCTTTGATATCTCTTT
TTAATGCATTTATTATGGTACTTATACAATTTATATAATAGATGTACTGGTATTATAATACTTGTTATATAA
AAATTGTAACAATAAGTATGCAAGGGAAAAAATGCGCTCTTGGAAGCGCTGTCGGTTCATTTATTTTTGGC
CTGACTTGGTGCGTCAGACGGGATCGGTGCTGGCTGCACATGTTGTTAATTCTTTGATGGGGCGCCAGTGC
TGGTTGTTACTGCCGTTGCTGCTATCCTTCCATGTCCAGGTCTGTTTTGACCTTAGTCACGGTCGCCATGTA
ATGGTAAATGTAATCGATTCAAATAGTTCAACACACGGGCACGTCTGTGCCGTTTGACTTTAGTTTTTTGA
ATGTTTTATTCAATATATTCTTATGGCTAAGACCGCATAGCTGCGCTGCCGGTTACGCCAGCAGCGGAGCG
GCTTCATACATATTTTGTCTTATCAGCATAATCGGGAGAGAGAGAGTCTTATGTACATATTTGCATGTATG
TATATATGTGCTACGAATGGTCAGCGTGGGACTGCTGACTTAGCGTAGCTCATAAGTGGGCGATCATATTA
CACGCTCACTTGAAAAGGTTTTGTTTGTGTACTGCAACAATGTGTTGCTGATATATTTATGCTTAAACGAT
GTTACTTTTTATTGGTACAGTGTACCCTCCAATATATGCACATACTACACACCTTCCTAATTATTAAAGGA
AGTTATTTAACTCCAATTTGTTTAATATTAAATTAATTTTGATATTCGAGGATTTATAATATTGGTAAGGTA
TTTATAAGGTATTTAGTTTTTTTTATTTTTTCTAGGCGTACCTGGTATAATTCATTTTTTTCTTGTAGGTCTTT
TACGAATTAAAGATTTTTAACCTATCTTTATGAACTGTTGGTTTTTTACTCTTATTTCTTATTTTCTTATTTA
TCATTATCTTCTATTTTTTCAACTCTATAGGGCCCAGTGTTTTTAAAATAGAGCTTATGGCTTACTTCATTTC
TTAGTACAAATTTATTTCCTAGTTTTTAAATCATATAATTCCTTATTTTTCTATTTATATAATTCGAGCATAT
TTCTTGCTCTTTTGTATGCTACTTCTTATCTATACTTACTTTCTTTAGCCGTAGTCTTATATGTTATATAGGT
TATCTATTACTTATGCTGTTAACATACTTTGGTCGATTATTAGTCTTACCAAATACTAACATCGACACCGAC
AAAAATTATGTGCCATAGAGGGGGTAGTATGGAAGCAATAAACAAAATGTAGAGGCCATACGATCGGAA
TCAGTTTTATGTTACTGGATATCAAGCGCATCGTATATATT
>9_rnd-3_family-319__Unknown
CAAAAATCATCGAAAAAAGGTTTCGATTTTTCGCGCCGACCTCGATTTTGAAAAANAANCGTGATGTNAC
CCCTTGCCATTTNTGGCCGATTTNGGTCAAAANTTTTGGATTTTTTTATTTTTTTGAATGCCAGTNGATAGN
ACTGATCCTTACGAGTCNAAAATGGTATGAAAATTTGNAAAACCGGACATTATTTGGNNGAGATATTCNA
AAATAATCATNAGGAAAGGTTGACTTNACGAACNAATCGTTTTTCGGNTCCACAACNTTTTGTAACCCATC
GATNTTTTTAATTTGTTTGAATGCCAATTGATGGTAGGGATNCGTACAAATTTAAAGAGGTATAACATTTG
CAAAATCAGACGTTATTTACCCGAGATATTAAAAAAATCATCGAAAAAAAGTTTCGATNTTTCGCACCGA
CCTCGATTTTGAAAAAAAACGTGATGAATGTCGTTTTGGAGCGTCATATCTCCGCGCGGAGCTATTACCCG
AGATCTTAAAAAAATCATCGAAAAAGTTTCGATATTCGCACCGACCTCGATTTTTGAAAAAACGTGATGTT
TTGGAACGTCATATCTCCGCGCGGAGTTATGTCGTTTTGGAACGTCATATCTCCGCGCGGAGTTATGTCGT
TTTGGAACGTCATATCTCCGCGCGGAGCTATGTCGTTTTGGAACGTCATATCTCCGCGCGGAGCTATTACC
GACGTTTCGAAACGACATAACTCCGAAAAGGTTTCGATATTCGCGACGTTCCAAANCATCATTTTTTTTTT
CAAAATNAAGGTCGGCGCAAAAATCGAATCATTTTCGGGTCCACATCCTCTGTAACCCGTCGTCCTAATGT
TGAACGCCAATTGCATAAGGGNATGCGTACAAATTTAAAAAGGTATAACGTCGAANATCGGAGCTATTGC
CCGAGATATTAAAAAAATCATCGAAAAAAGGTTTCGATTTTCGNACCGACCCCGATTTTGAAAAAAAAAA
CGTGATGNAACCCTTGCCATTTTGGCCGATTTGGGTCAAAATTTGGATTTTCTATTTTTGATGCCAGTNGAT
AGNACTGATCCTTACGAGTCCAAAATGGTATAAAATTTGAAAATCGGACATTATTTGGCCGAGATATTCG
AAAAATCATAAGGAAAGGTTGACTTAACAAACGAATCATTTTCGGGTCCACGTCTTTTGTAACCCATCGAT
TATTAATTTGTTTGAATGCCAATTGATAATAGGGATGCGTACGAATTTAAAAAGGTATAATATTTGAAAAT
CAGACTTTATTTACCCGAGATATTAAAAAAATCATCGAAAAGGTTTCGATTTTCGCACCGACCTCGATTTT
GAAAAAAACGTGATGTAACCCCTTGCCATTTNGTCGATTTGGGTCCAAATTTTGGACATTATTCACGGAAA
TTTCAAAAATCATCAAAAAGGTTGATTTTACGAACGAATCGGTTTTGTCGACAACNTTTTNNAANATAANC
GATGTTTAATTTNTTTGAATGCCAATTTATGGTAGGGATCCGTACGAATTCAAAAAGGTATAACATTTCAA
AATCAGACGNTATTTACCCGAGATATTAAAAAAAATCATCGAAAAAGTTTCGATTTTCGCACCGACCTCG
ATTTTGAAAAAAACGTGATGATTTTTGGAAGGTCATATCCGCGCGGAGTTATGTCGTTTTGGAACGTCATA
TCTCCGCGCGGAGCTATGATATTATAAAAATCATCGAAAAATTTGATTTTCGCGCCGACCTATTGTTGGCA
GATAAAAATCATCAGAAAGGTTGACTTNACGAACGAATNGTCGTTTTTGGTCAACATCTTTTTTCAACCCA

TCGATTTTTAATTTGTTCGAATGCCAATTGATAATAGGGATCCGTACGANTTTAAAAGGTATAACATTTNA
AAATCAGACGTTATTTGCCCGGACTAAAAAAATCATCGAAAAGGTTTCGATTTTCGAACCGACCCCGATTT
TGAAAAAAACGTGATGTGACCCCTTGGTCCACTTTGGCCGATTTGGGTCAAATTTAGATTTTCATTGTTTTT
GATGNCAGTGGATAGTACTGATCCTTATGAGTCCAAAATGGTATAAAATTCTNGCACCNANCTAGATTGA
CTGGNCGGAAGATATTTCAAAAACCGTCAAGGAAAGGTTGACTTAACGAACGAANNNCATTTTCGGGTCN
ACANCNTTTNCAACCCATCGATNNTTAATTTTGCCTGAATGCCAATTGATGATAGGGATNCGTTACGCAAA
TTNAAAAAGGTATAACATTTNAAAATCAGACNTTATTTGCCCNAGGGATTTNAAAAAATCATCGGGAAGG
TTTCGATTTTTAACGAANCGGCCNCGGTCTTGNCTAAATGTGTTGCGTGATTGAACCCCTTGTCATTTAGTC
GATTTGGGTCCAAATTTTGGACATTATCAACGGAAATATTCAAAAATCATCAAAAAGGTTGACTTTACAG
ACGAATCGGTTTTGTCTACAACGTTACAAAAAAAAAAATAAACGATGTTTAATTTGCTTGAATGCCAATTG
ATGGTAAGGGATCCATTACGCATTCAAAAGGGTATAACATTTTTAAAATGAGACATTATTTACCCGAGAT
ATTANATAAANCATCGNAAAGTTTCGATTNTCGCANNGACCTCGATTTNGAAAAACAACGTGATGATTTA
TGGAA
>Ref_dvir_508
TTCAACAAATGATTGAGGCCAAGCGGCTGATTAAACGCCACATACAGGCATACCACAACCCGGCTGCTAG
GCAAGCACACAACAGATTAGCCAACAGCATTAGCAGCGAAATCAAGCAGGCCAAGGCAAGAAGACTTGA
TGAATTGCTGGAATCTATCGATGCAAGAGATAGATACAGTATGCAAAAGTTATGGCGGCTAACGGGGAAC
ATCAAAAGGCAGCCTGCACCTAATTTACCAGTGAGGAAGCACACTGAGGGAACTGGGGCTTCCCAGTGGT
GCAAGACCACCGAAGAAAAAGCCGAAGCCTTTGCGCAGCACCTTGAACAAAGGTTAAGGCCAATGAACA
CCACACTGCCCTCTGACAAGAGCCATATCGAGATGCAATTATCGCTACCGCTAGCCCAAGGTGAACTCAG
CTTCCCTACAGTCACGCTAGGGGAAGTACAAACTATTATCAAAACATTGCAAACAAAAAAGTCTCCGGGG
ATTGACCGAATCGATAATCGCACGATCAAGGAACTGCCACAGTCAGCTGTAATGTACCTAATGCATTTTTA
ACCTTTGGACACTTTCCTTACAAGTGGAAATGTGCCAAAATAACAATGATTCAAAAGCCAGGCAAGCCTT
CTGATCAGCTTGGATCATACAGACCAATAAGCTTGCTTGCTGGCTTTTCAAAAATCTTTGAACGACTGCTG
CTGCGCCGGATGTTCCAATTGGAGAACATCATCAATGCAATTCCGAGCCACCAATTCGGTTTCAGACGTG
AGCATGCTGCTGAACAACAACTGGGTCGAGTTACGCAGTTTATACTAGAAGCGTATGAAGACAATAAATA
CTGCTCTGCGGTCTATATGGACATACAGGAGGCGTTTGATCGTGTCTGGCATGACGGACTGCGATTCAAGC
TCAAACAACTATTGCCGGAAGTCCTCTACCGAGTTATGAACAGCTATCTAAGCGAACGCACATTCATTGTG
GACTGCTGTGGAGGTACACGTTCCAGCCTAAAAGAAATCTGCGCCGGCGTTCCGCAGGGTAGTGTTCTAG
GGCCTGTATTGTACACCCTATACACATCCGACATGCCCCTACCTGCACCGCTACGTCATAAACTGATGCTG
GCAACTTATGCAGATGACACGGCCATCTTGTCATCGGCAAACTCGCAAGAAGATGCCATTGCCAATTTGG
ATTTGTTCCTTACCACCATAACAACATGGACACAAAAGTGGAGCATTAGCATCAACATTACCAAAACCAA
GCACATAATGCACACTTGGAGAAACTTGCAGATGCCAATTCTTTCCCCAAGAATTTTGGATCAACGGCTTA
CCACAGAAGAAAGGATCTCATACCTGGGAGTACAAGTCGATAAAAAGCTTCTTTTGAGCAGCCACGTCAC
CACCATTGTCGCCAGGGTTAGAGACAGGGCCAAAAAGCTGCACTGGTTAATATCAGAAAGGAGCAAGCT
GCCATTAAAATGCAAGGTAACACTTTATAAGCAGCTCATAGCCCCAATATGGCAGTTCGCACTTCCGGTCT
GGGGATCACTTCTATGCGAAGGCCAATTTGAACGTATTCAAGTTGCGCAAAATAAGATACTGCGAACCAT
TGCTGGAGCACCTTGGTACGTGCGGAACCAAAGCATCCACACTGATCTGAAGGTCGTGCCGGTGGAAAAA
GTGTACCAGGATGCTTGCTCCAGGTACTGGGCTCGGCTCAGCAACCACCAGCAACGAAGAAGCCAAAAAT
ATTGCCCTAAAGCAAGACTCCCACACGTCACCTAGGTGTCAGAAGAAGATACGCTGAAATGCTTCGGAAA
CACCTACCTCTGGGTCGCCTAGCACAGCAGCAGCGATCAGCAGGTCCAGCAGCAGGAACTACCTTCTCAA
GGGCAGTCAGGTCCGCTCCAACGCCCAACCCAGCCTCAGATACTACCTTGTATTTGACATACAAGAACTG
CTTGAGTCAAACAATGGTGCAGCCCAACAACCTCCAGGATGAACGAAGCAGCCCGTCTCATATGCAGCTG
TGCTCAACTCTCGCTGCCCCTACAAGGCCAGGAGCCCTCATTCCTCCCAGTCCAGAGACACCAAGCTCGCA
TCCAAATCCACAGACGCAGCGGGACGTGGCCACCGGTGCGAATCATGAAGCAGTGTGCAACACCATGTGC
ACACTTGCTACACTATTCATTATGGCAGCCAACCTCTATGTACTAGTACCACAGCGAGGGCCTAAGACTAG
GACTATTTGCAGGACACGGCAACTCATCCAATTGCGAATGGCTGAAAGGCAGATAAAACAAAGATCGCGT
TACGCTAACGAACCACTCAGATGGCTCCATACAACTCGTTGTCAAAGGGTTGCCTACTTACAGAAATTTAG
ACTGCGACATCCCACACAATGGAAAAAGGCCTTGATGCTTCGTTTCTCAAGCACCCATACTAATGGCATCT
GGCGACAGGATCAACAGTGGTCACGCTGGAGACTCCAGCTTATTCAGATGCAACGGCTATCAGACTGGGT
AGCTTCCAGACAATTGGGAATTTTCCCAGTCCTCAAATAATAATTTGACAGCAATCCCCATGCCAAGGCTG
GGCAACTTCACTTGATTTTGTATATAAACACATGCCACTAAATTCAATGTAAAAACTCCGGGGTAATAAAT
GAAAAAAAAAAAACACTC
>Ref_dvir_786

TATATGAAATATATATATATTCAAACTAACAATTAAATTATCAAATAAGTTAATATTATAATTATTTTACTT
TTCAGTATCCATTTTAAGTTTCGATTCATACTTAATTATTCTTTGTTATCTTAACATAACTTTAATTTTCGAA
ATATTTGGGAATAATTTCTTCAGTTTTTTAAAAATGCCTCGCGCGTTTAACAAATCCGCTTATCAAAGGCG
CCAAGGTGCCATTAATCGTAGTCGTGTCTACTACATTGAGAATACCGAAAGCGTTTGCCTTCGAAGTTCTC
AAAGTTAAGCTGACTGTCGTGAGTGCAACGGTGATATTCGTGAAGTAGACCGTAATACTGACGCTCTGGC
CCGATCTAAAAGATGAAGAAGTGGTCGATTTCGACAGGATGAAAGGGATCTCGATGCAGCAGCTCGCGC
AACGCAAAGACAGGATGAGGAAATGCGAGCTCTGGAGCAGACACAAAATACAGCGGACCATACTGTTCG
GCGACAAAATCTGGAGTACCGTACCCTAGAACGTGAGCGCGATGCAGAAGCCCACGCTACAAGGAGACA
GGACTTAGAAATAAGACTGCAAGAGCAGGAATTAAACACAGTGGAACACACTGTGCGTCGCCAAAACGT
GGAGTACCGCGAATCAGAACGCCAGCGAGATGCAGCAGCCCACGTTACAAGGCGACAGGATTTAGAAAT
AAGACTGCAAGAGCAGGAATTGAACACAGTGGAACACACTGTGCGTCGCCAAAACGTGGAGTACCGCGA
ATCAGAACGCCAGCGAGATGCAGCAGCCCACGTTACGACAGGATTTAGAAATAAGACTGCAAGAGCAGG
AAATGCGTCGACAAATCGTCAATTCCTCTCAAAGACGAGTTACTAGAAACCAGGATCGTACAAGAGAGAA
CGAATGAAAAAATATGACTGAAGCTTATAAACTTTAGAAATAGTTTTCAAAATCGTGCAATACAGTCAGC
TCAGCAATCTCAGAGAAATAGAGATGCCAGAGAAGAAATGTCAACTAAACAGCGCGAGCGTAGAGTTTT
TTCTGCCAGAGATAATACCTGGAAAAAATTAAGAAAGGCCCTACAGAAATATGCATATCATGCGGAGGA
ACTTGGTTTCCTTTCCAAGTAAAGCTTTTGAATAAAATAAATCTATCGGCAAAGTTTCCAAACATGGATAT
TTCAAAAGCATTTTATCTGTCCAATAAGTTCCCAAGTCTATCTGAAGACTATTTGTTTTGCTCCACTTGTCG
CCGACACATATCGTCCGGCAAGATTCCGAGTCTCTGTCTGTCCGAGGGGTTCAACTTTCCTGAGATTCCAG
ACTGTCTTAAAGACTTAACGTGTCTTGAAGAGCGTTTAATTTCGCCAAGAATCCCATTTATGCGGATAATT
TCTCTTGGCTATGAGCGGCAATGTGCAGTTAGAGGAGCTGTGGTCAACGTACCAATTTCTGTTTCGGAAAC
TGTGACAGCTCTTCCGCGCACCTTTGAAAACAGTCATGTAGTACAAATTCATTTGAAGCGTAAGCTGGAAT
ATGCGCATAATTTCATGACTGAGACAATTAGGCCCGCTCGTGTGCTCGAAGCGTTGCGGTATTTAATAAAC
ACAGAATTATATCTTAAGCACAATATTTCAGTTGCTTCCAACTGGCTAGGTGTAAATGCTCACAATGAAGA
AGTCCCTTTTGTTGCTGATCCAGCTGATATAACAGCTGTGGATAGCCTTCTTCGCTGCCGAATGGAAACAG
ATTCAGAAGGACACGACACTTCTTATATTAATCCAGGTGGACAGGAAACTTTGTTGGATAACGAGTCAAT
AGAAAACAATGCTATAATTGGTCGTATTGCAATGGCTCCAGGTGAGGGTCGACGACCAATTGATATGACG
ACAGATGAGGATGCTGAAGAATTATCGCTCCCAACATTTGTAAAAGCAAAAGTACTTATTGTCGAAAGGC
CAAATCACAGATTCGGTATTACGATAGTCGTTGCGCGAAAGTACCAAAAATTCTGTATATGTATAAGTGTT
ATGAGCTCACACGCATAAAAAGTTCTATTTCTACCTGCCTTCGGAAAAAATCAAGAAACGCCAATATCAC
CGTTGGTAACTTGAGAGATGAAGGTTTTGTCAGCAATCTTGTGCAGCATGATGACGGATATCATGTTCTTA
AGGGCCTGCGATCTGCTCCAGCACATTGGGAGGCTGAGAAGAAAAAAGTGCTGGCAATGATTCGTCAGTT
TGGACTACCAACTTTTTTCATAACGCTTTCTGCTGCTGAAACCAAATGGAGTGAGCTGCTGGTCATTCTAT
CTCTCATTTTGGATAATAGAGTCCTTAGTGAGGAAGAAGCAGCGAATCTTTCAAGTTCCGAAAAAGCTCG
ACTAATTCGGTCTGATCCTGTGACTTGCTCAAGATATTTTGACTATAGGTTTGGGCAGCTGTTGAAATTATT
TAAAACAGATATTTTTGGAGAATATCAACTCTCTCACTTTTATTGGAGAATTGAATTCCAACATAGGGGAT
CGCCACATTCAACATGGCATGTATTGGCCAAGCAGCGCTCCTAAACTTAATGAGAATAGTTCCCAAAGTA
GCCGTCAAGTTATAGAAATTATTGACAGATTCATTACAACAAACGGAGATAATCCAAATTTG
>9_rnd-1_family-233__LTR___Pao
AAATTTACTTATTTCTTGGTTTAGGTTCAAAACAGTTGCTAAAAAAACATTAGTAACTCAACATTTGGAGG
CCCCGGCGAGATTTAACATTGCAGTGCACACAAGCAAAATAAATCTATTGCAAATTAAAATTGTGATTAT
TCAAGTAGAACATAATCATCGTAAATTGTTATAATTAGGCAAGTGCAAACCGCCTGTGTGAGCTGACCAT
TTACAGCTCCCAAATATTCGCATATACATAAACGCCGCTAGTTGACTTTCGCGCTCTTCCCCTTGCGCGGT
CATTCGCCGGCTCAGCTTATATACATACGCTTGTACATAAGCAGCTGCACAATTCGTCGCATGTTGTGTGC
TCAGTTTGGTGTTCTGATTTTGTGCTTGTGAAAAAATGATGCAAGGCGAGAACACAGATGATATGCAGTC
AAACAGGGTGTCTTTTCTTAAGCGCAAAGCGGTTGCATTGTTCGAGCGAGCCAAGTGTATTGAAAATGAA
TTATCTGAGGATAAAATTAACAACTCTTGTGCCGTCGACTTAGAAGTGCGCCTTTTGCTAATTGAACAAAC
TAAGTCTGCCTTTAATACCGCGCATAACTCGCTAGAGGAGCTTAATTTCGATGAAATTGGCAGCAAACTAC
GCGATGATTTTGACGAGTTGATCGCCATGGTTGAAGCTCGCGTTCGCNAATATTCAGCCGTCGTCAATCTA
ACGGCCCGCCCCACCAAACCTTGATCTTAGCCAACAGAACGCGCCTGCCCGAGCTCAAATTACCAACATT
TTCCGGTGGTTATACCGAATATTCGGACTTTATCGCGATGTTTGAGTCAGTTGTCGACAAGGATACCGAGC
TGTCTAACATTGAAAGGTTGCAGCATCTTCGTTCATGTTCGTCTGGCCCTGCGTTGGATTCGGTTCGATCTT
TGGAGTTTTCCGGTGCCAATTATCCTGTGGCTCTGGATATCCTAAATAGAAGATTTAGTAACAAACGTCTT
GTTTTCCAGGCACACATCAATGAGATTCTTGGGCTCAAGAGGGTAGATTCGGAGTCGGCTTCAAAACTGC

GTGAGTTCTCGGACAAGGTCAATTCGCATATGCGTGCCTTACAGAATTTGGGCAGTTTGGANCNAATTTCG
GGATGCATAATAGTCCACGCGCTGCTTCAAAGGTTGGACACCCAGACTCAGACCAAATGGGAGGAGGCC
GCAGGCATTGACAAAATCCCGACAGCCGATGAGTTCTTCACATTTCTGGAAAGGCGTAGTCAAAGGCTGG
AGAGCGTGCAACATGCCATGTCATCCCATATAACCAATTATCAGGTGGGCAAATCCAACCATAACACTAA
CCACAGCAAAAGAGTGCTTGTAGCTGAGACTAACCAAAATGGGTGCAGTTTTTGTGATGCGTCTGGTCAC
GGGATCTACACATGTAGTAAATTCGGAAATTTGTCTCCATCCCTTCGGCATAAGGAAGTCAAGAAACTGG
CCCTTTGTTATAACTGCCTAAGGAAGGGACACCAAACTCGGTCTTGCAGTAGTAGTCATTGTCGTACTTGT
GGAGGAAAGCATCATACACTGTTGCATTTTGCCACTTCAAGAGAAGCTGACGCGCCGACACCATTACATA
CATCGGCTGATTTGGGCGCCGCCACTTCGACGCACTCTCACTCCACTACGTCTCTTGCTGTCCAAGGTCTTC
AGTCTGGTGTTGTGCTCCTGGCCACTGCAGTGGTACTGGTAAAGAATAGTTCTGGTTCCTTGTTTCCCTGTA
GGGCCTTATTAGACTCAGGCTCGCAGTTGCACATGGTCACATCCCGTTTCGCACAGAGACTTCAGTTAAGG
AAAACCAGGTCGTCGACAGTTGTCATGGGTCTCGGAGACACAAGCGTCTCTACTGAAGGATCAGCCGTTA
GGATTAACATGAAGTCACAAGACTCAGATTATTCAGCCACCTTTGATGCTCTTGTGACTCCAACAATTACC
GATAGTCAGCCCAGCTTCGTCATCGATACATCAGACTGGAAAATTCCCTCGAACATAAGGTTGGCTGATC
CTGCGTTCAACAAACCACAGCGAGTGGATCTCTTAATCGGAGCGAGCCTTTTCTATGAGCTTTTGTCTGTT
GGGCAAATTGTGCTTTTGCCAGGACTACCTCTTCTGCAAAAAACTCGTCTTGGCTGGGTCGTGACCGGTGG
ATTTGAGAGACCGCGCAGTACCTCGTTGTTGGCTGCCGGCCGCACTCCTAATAGCGAAGCTAGTCAGTTG
GACGTCCGTCTGGATGAGTTGGTTCGGCGGTTTTGGGAAATTGAAAGCTTGGAAGAGCCTACAGTAAAGG
CCACCAAAGAAGAGGCGGAATGCGAGCAGCATTTTGCCAATAACTTCTCCCGTTTACCATCCGGCGAGTA
TTCAGTGCGGTTGCCTCTGAAGCGGAGTGCAGAGCTTCTAGGAGAGTCTTATGGCCAGGCTCATCTTCGAT
TTCTCAGTCTTGAGCGAAAATTGGGTCGGAATTCGCATCTGAAGGAGCAGTATTCGGCAGCACCACAACC
AAGTTGCGTGTTGTGTTTGATGGCTCAGCAACTACTGATTCTGGACATTCTCTTAATGATGTACTGATGGC
GGGACCAATCATTCAACCTAAGTTGTTTCACATTCTGAT
>Ref_dvir_745
AAAAAAAAATAGAAATAGATTTCACACCTCTATAAGCACCTCTTGAATTAACAAAAATAAATATCACAAC
ATCATTGTTCATATAATTTTCCAAAATAACTAATTAAATTAATAAATTAAATAGTGGCATATTCGCACGTA
TGCACAGTTATAGTGACAAATATAAGAAGGCAGCATAATTGCAGCTGCGAATCTGCAAGCGCTTATCGCT
TGCAAAACCATAAATACAGTTAAGCAAAGCAATAAAACATTATTCAGATAATTGCAGCTGCATCCATACA
GATAAGCAAAGCAATTAACAATTAACTTAGATAATTGCAGCTGCACTTGTACAGTTACACAAAGCAATAG
ACACCTCACTCAGATGATTGCAGCTGCGCGTCTTTACGTAGAAATAAGCTTCTCAACATAAAATAAGTTCT
AATGAATTTTACTCTTAAGTTGTTCTTCAATAAATGAACTCGTCGTGAAATATCGCCTTGTCTTTCATTCAA
TTTAACCGTTAGAGCTGTGCGCGGTTTTTATAAGATTCAAACTCACAGTAAATAAAAAAAACAAATGCGC
AGGATCCTGATGCAATAGCTAAGTTATTAATTATTGTTTCCCGTTTACAGTTATCCGCTAAGACAACCAAC
GCGAAGGCAACCCAAGCGATGGTCAATATCGCGTCAAGTTTTTATCAACCTCGACAGTGACGTGTACACC
AAAATTCGCTTGTCAAAGCCGCGGAATTCAAGAAGACGCGATAGAATTCGAAGAGAAAATAATTCCAGA
AACTTCTGGTTATCTCAAAAGATTCTAGTGCTATCTGTGCACAGCAGTGAAGTGATGTTAGCCCTTGGCGT
AAGCAACTTGATGACCTGGACGGATATTCGGAAGCAACAAATATTGAACTACAAGACGCAAACCAAGAA
CCACATCCTACTGGAGGAATTCAGGAGCACCCCGTTTAAAGGCAACGTGCGAGCTTTTCTGGAAGAAGCA
GAACACCGTAGGCAGACACTAATGAGTAAGCTATAAATTGCGAAATTCCCGATTTAAGATGTCTAATTAA
TATTTTGCAAGAAAAAAATCTTTATAACGAACCCTGTGTTAATCCAACCCCAAATAAAGTAAAACAAGAA
AAATGTTTAAAGCCATTGTATCAACCACACCCAAATATTCAAAGATTCCCCACTTTCCTTCAACCAAATCA
ACCCTATGTCCCATATCGACCCCATCAACCTTACATTTAACAACCATATCATCAACCATCACCCAATTATA
CTAACAACAACACTTTTAAACCTAATAATTGTTCACCTCAACCGATGCAACAAATACTTAGTCCAATCAGA
CCAACCTAATTTACACCCAGACCAAACTTCCGACAAACCCAATTCGACAACAATCGTTTCGGACAAGTAA
CCCCACAACAAAACTCTTTCAATAACTCTGGCGTAGCCAACCACCGACTAAACGAGTAGACCTGCAAGTG
GACAGAGTAAGATGAGTATTGACGATGAAGCTAGAAATCAGGAAGAGCTAAACTTTCATGATTACATGCA
ACAAGAATACTATAATCAATGCGCTGCTTATCCTGAATATTATTCACAATATTATCAAGCAGAAAGTTACC
CACAAAGTCCAGACTTTACATACACACAATCAGCCGAACACGAGACAGAACAACCAAATGAAATAGAAA
TCGAGCAAGCCGAAAATTTTCAGCTTCCAGCCTTGGAAACCAACTGTACATAGTAATCACTTAAAAAGCC
AAAAATCTGAAATGTTTAATAGATACAGGATCTACTGTAAATATGATGACATCAAATGTTTTAATTAACCG
ACTGTTTCATTCAAACCAGCAACGGACCTTTAACTATCACAAAGTAAGTTAACGTCCTTTCTAGTAGAGTA
ATAATGAATTTCTTCTACACCAATTCTCCTCAGACTATGATCTATTAATTGGTAGAAAAATGCTTTCAGAA
GCACAAGCCATTATAGATTATAAAACCCAAACTATTACTTTATACAATAAAAGGTATAAACTAACCAACG
ATATTCCAATCACAAATCAGAGCCACTTCTTAGCCCAACCAAATCCTGAAACTAATCAACAGTTTAAAAA

TCCAATAGAGAAATTCCGATAACCAAGAATCTGCAAACTCAAGCCTTACCGATTAGATCATTTAAATGAA
GAAGAAAGAATCAATTTAATCAGTTTGTTGAAACAATATCAACACATACAATATAAAGAAGGCGACCAAC
TTAGTTTCAGTAATCAAGAAAAACACATTATAAATACTACCCACAATTCAGCCTTACATACAAAACCATA
CAGTTATCCACAAGCATATGAACAGGAAGTTGAACATCAAATTCAAGACATGCTTAATCAAGGTATCATA
AGGCCTAGCAATTCTTCTTATTGAAGTCCTATATGGATCGTACAGAAAAAGCAAGATGCTTCAGATAAAC
AAAAATTCAAAATCGTAATTGACTACAGGAAATTAAATGAAGTAAACCATAAGCGATAGGCACCCAATA
CCAAATATGGACGAGATTTTAGGAAAACTAGGTAGATGCAATTATTTCACTACAATTGACCTTGCAAAAG
GATTCCACCAACTTGAAATGCATCCAGATTCCTTTCAAAAACAGC
>9_rnd-5_family-3379__LTR___Gypsy
TGATTGGTTTTGGTAATGATCGCAACGCGAATAAAACAAAAGCCGATTGGGCAGGTTAATTATACGGTTC
ACATTGATAAAAAAATGAGTGTTTTGNATTTTTACGTGGTAACGNAAAGTTGTATGGACATTGAACTAAT
AATTGGAGAAGAACTTTTGTATGCGGAAATATCATTTAATCGCAACGGTGTAAGCGTACGAAAAAATTGA
AGCCGTTTCAAAATTTANGACTGGCCCTAAAGAAGAAAGCGCTATCGAAACCCTGAAAAAATGTTTGACC
TCTAACCCGGTATTAGCTATATACAATCAAAAATACGAGACCGAAGTACATACTGATGCATCAATAGACG
GTTTTGGAGCTGTGCTCATGCAAAAGTCACCAGACGATCGACAGTTGCATCCAGTATACTTCATGAGTAA
GAAAACGACGGACGCACAGCGAAAATATTCAAGTTATGAGCTTGAGATATTAGCTGTCATCGAAGCGTTA
AACAAGTTTCGAGTTTACCTTTTAGGCATCCCATTTAAGTTGATCACAGACTGCAATGCATTTACAAAGAC
GTTAGAAAAGCAGAATCTGTGCACAAGGGTGGCCGTGGGTGTTGTTTTTACAAGAGTTTAACTATGAAGT
CGAGCACCGATCCGGTTCACGCATGAAACATGTAGATGCGCTCTCGAGATATCCAATAATGNCAATAAAT
GATGAAAGCATATCGATTCGGATAGGGAATGCACAAAAACAAGATGACCAATTGAAATCAATCATAAAA
ANTATCGATGACGAAAAGAAGAATTATGAAGATTATTTCATAAAGGCAGGCATTCATTCTTTACTCATGG
TAGTTAAAAAAAAGTTTGTAACACCGATGGCCCAAGTAACTCTTCGACTTGTGCAAAGTATCTAAAGCCA
TGGTCGATTCCGGAAAAATTCGCCAGTCGGCGAATCCGCTAAAACAGAAGACGAAAACGTGCACGATGC
AAAAGAAGAGCAGCGTGCCGAAAAACGACGAAGACGGGAGTTGGAGTTCGGCCTTAGACGAGAAAGGTT
GTGAGAGCTGCTTCCCAAACGAGGAAAGACGACGATTCGCTGATCTATNACTTAATCCAACTATTTGTAA
CTTTAAACATTACAATAATATTTGTATAACCTTATCCAAAATAAAACCCCACACTGCTGATATATATATAG
AGAAGAGAAATAAATTGAATATGAATACATGCACAGGTATGACCTAGCTTTTTTCCACTGTAAGAAATTG
TAGAGTATTCTTCGAAAAATACATAGACGAATTGTTAGGACAAATTATTTTACTAAAAATTATGAAAAAA
AATAAAAAAAAAAATTATAAATCTTTAGTATGGAATGGGAGCAACTAACTCTCCATATCCGTGAAATTAA
AGAAACTTCTGATAAGTTTTACAAATGTGTTGGCCAGAATAGGGTAAGACACGATAAATAAACATGCAAA
GATTGTTGTCGATTGCTTTAACGAGGCAGCACCGATGGGTATCAGTTCACGAGGGGGAGATGGCATGAAA
GCCTCAGCTAGATTCTCATGATTGACGGATTGCCATCTTCTTTTTCTTGGGATGGATAAAGCTAGCAATGA
CACTAATTCTGCTCTTTGCTAATTTTTCCAATCAACTTACTCTTCAGTCTGTCGTAATACTAATTCTTACCCT
TATACTATTTCTTCCGCTAGTTCTATACCTCCTCCCATTATTTCACACNCCTCTCTCCTATTAGCTATAAACT
CTTTAAAATCTTCTTACTCTCCTTGGNCGGACAATATTCCTAGTTGTCTACTCAAGGAGTGTAGTTCTTCCC
TTTTTATCCATTGCTAAAGCTTTTTTTAAAACTTCTGTCTTTCCATCTCTTTGGAAAGAATCTTATATCATTC
CTCATCACAAGAATGGTGGGAAGTCTGATATACAGAATTACAGGGGCATTCCAAAACTGTCCGCTATTCC
TAAATTTTTTAAAAAATAATCACTTCGAATTTGCAGCATTTGTGCAAATCAGTTGTTTCCCCTGTTTAACAT
GGATTCGTAAAGAATCGGTCAACTACGACCAATCTGCTTGAGTTTACTTCTTTGGTAAATGATGCTTTCCT
TTCGAAAATGCAAACTGATGTCATTTACACTGACTTCAGTAAGGCGTTTGACTCTGTGCACCATGACATTT
TACTTTTTTAAACTTGACCGAATAGGTTTCCCTCTGTCCCTTTTACTTTGGATTAATTCTTATTTAAAAGGT
AGGACCCAAAGAGTAAACTGAGGTTGACTACCTCTAAAAGGGTTCACGTTACTTCTGGTGTTCCTCAAGG
TAGTCATCTTGGACCTTTACTCTTTATTTTTNTATAAATGATCTTCCCTCTGTTATATCACATGCCCGTGTAC
TCATGTATGCGGACGATGTCAAACTTTTTCTATCATACACTAACCCCCCACATCATTCTCGTCTACAAGAC
GATTTGAACGCTATTCCACTTTGGTGTTCGGCCAATCGATTGCATCTAAATTGTTCAAGGTGTATGTTTATG
ACATTTAATCGTACTACATCTTATCTTGTCAGTTATACAATCGACAATATCC
>160_rnd-5_family-2389__LTR___Gypsy
AGTTTGCCTACCTACGCAGAAAATGACAACAAAAATAAAATATAAATATAAAATAATATAATATATATAA
TAATATATATATATATTTATATTTATTTAAAATGTTAGCTGAAATATAAAATTACTCGATTACACTTGGAA
ATTGCACACTTGTGTTCGATCTACAAGAAAAATGCGTGTCTGCGAATTTTCGACACGGTGAGGTTAGATGA
CACTTCCGCAAGCCAGACTCGACCTTTCTACCAGTGATTGTCAACCAGAGCAGTCGCATTTAATAATTTTG
CGTAAAGCTTAGCCTAACCTGCAAGCACGTTTTTGTATANTATTTTTTTTGCTAAATTTAAAAATACAAGG
CTGTAAGTTTAAATACTACAGAAAAGAAATTCATGCTTAATGGCATTGGTCCACAGAACTCCCATTGGCTT
GAGTCCATGCAGGCGGCTATCAATTGGTGCATAGCTGACCGTAGTTTGAAAAAGCAAAGCCTACAGTCGG

ACGGAACAACATTTCCACGTTTTAATTTTGGTGCCAATCCCGAAAGGGTAAATATTTCTCTCCTCTTGCGG
ACCAGCCGCTCGTTGCTTTTGCGCTGTGTACTTTTTTTCTAATTATCATTTTCTGGTGTACACAGATTGGCA
CTTATACAGAAACCCCTAAGCTAAGTTTCTCACTCTCTGAAATAATCGAGCAGAATCGGAATTACGGGCA
CAGGGAGCATGGCAGATCATCGCCACCTGACACAGTAGTGTTCGGCATGTGTTAAACAAGCACGGATCGA
CGCACACAAAAAAAAAAAAAAAAATGTTATAAGTTAGCAAACGAAAGTGCCGCCGTTTGTAATAGTCCG
AGCCATAACCTGCAGTGAAAAAATATTTTTGATTTAACAAGTTTGCTATGCTCGAATAATTTAATTGAATA
AGTTATAATAGTTAATTACCGAGCGGCCAACCTGGCGACTAAGAAGTTGCATATTTTTTTGTTTTGGTTTA
TCCGCATAATATTCCCTTTTATTTTTTTTATTGTTTTTTTTTGCGTTGGGTATAANGTGCTAAAGCGAAAAG
TTGCCGATTTAACAATAAGTGAAAAGCAAAGTGATTTTTGTCGGCAAATGAAATATTTTAAAACTTTAATT
CATCTAAAACATGCGTCAGAAAAATAAACCCTGCCTTTAAACATATTTATAAATACCACTCGTATGGACA
CCGCCGAAAGTACTTTCGTCGCTGGATCTGCTAAATGGTAAGCTGGAGCACCCCATTATAATTTACAATTG
TTCTGTTTACCCAGGGTATTACTTGGCAGGTTCACGAGCCAAAATAAGTTTTTTTTTTTTTCAACAGCCAAA
TGAGCATGCGTACGACAATTTTAAACAGTTGTTGTTGTTTTATCGTCTTGTTGTGCCAAAGCGTGAACCGA
GCTTATGTGAAACCTGAGTAAACAACGGTAAAAAAAAAAATTGATAACAAATTGGCGAGCGTTTGATCAGT
CGCATGCAATGCATATCAGCTGTGCACCGTACAGTAGTGGCGCTAAATATTANGCACACCTCCGGTAANT
TTATTAAACAGCTAAAATGGCCTTTAGCCGCATAATATAATCTGCATGGCTTAATCCTACTTTTATTATAA
CGATACATATTTGCGTCTTTGACATATTTGCATATATAAAATATACATATTAACCCTTANTGTTTTCAGCAA
CTTTTCGCTTCGGTATAGTAATAAAATAAATCAACTAGTTTTTGGCCTAGAGTAAGCAAAAGATATGTAAA
ATTTGGTTAGTGGTAGGGAAGCAACTATAATATAATAAAATATCATTTTTGGAATGTTAAGGAATAACCA
CAACTTAAATGAAAGATCTAATTGACATAAATAAAGAAGTTACCATGAATTCTAACTAAGACCCTTTCCC
ATTCCGGGATTTATGAAAAGCAGACTTGAGTCAATGTAAACAATATCGAGGGTGTACAAGGGATCGTCTC
AAACATGTCTTGGCCCTTGGACGATCATGGTAGGGTGGGCATACGCTGCTTCCAATTTCCATCTCTGGGGT
AGCTATCGTACACTATACATTCATAAATAAGTGCAGACCTACTTTTCTTTTTCTTTTTCTTCTCTTTTCTTTG
TCTATGATGTCGTTCCAACCGATTGATAGGCGACCTGTTCCCCCAAAACAACACGCGAAATTTTAATAATT
AATAATTTGCAAACGAGCATTTGACATCTCGGAATTTTTTTCTGAACCCCCCCATCGCCGACGAGATAGAG
CCGGAAAACTAGCGCGTGCTCCGTAAGGTGTCTATACTTCTATCAGCTCGACTCTGCAACTATCCTACGTT
GTATGTTATAGTTATTATTAATTATTTTTGGCCCGCCAATGTGGAGCCCGGTCTGGCAAGAAACCCTGATC
TTAAATACAATAGAGCAATTTTTTTTCTCAGTCGATAACATCTGNCTNTACTCAATCAGTTGGAACATGGT
TCCATTTCCCTTGTCCTNGTTTATTGTTGAAAGGCTATGGATTACCCAGNATTTCTAAATAGCTCAGGTCCG
AGCCTACAAAACAACAAATAAGCCCATGAATTGTTTAAA
>Ref_dvir_693
GTTGTTAAGAAAAAAACTTGTTTAAAGTGGAATTATTTAAAATGGTGGCCCGGTAAGTTATTTCGGGAGC
ATAACGGGAATGGAATCTTTGCGTGAATTTTGGACAATTAGTATTAAGCGATTGAAATGAATCGTTTATCG
TGGAATGATTTTGATTTTTTGCCGATGAATCTATCCTATCTTAAAGATCCACTATTAGCCGAGGAATCTATT
TTAATTATTAATGGACGTAATTCAATATTTTAAGTAAATATTTTGTATTCAATATAGTAGCACTAGGAGCA
GTGCCATATACATATTTAAGAACATTTATTTTGCTTAACTTTCAGACCTTTAAGGATATGGCATAGTGTAT
ACCTCCACTTCAGTTGCAGTTAATTGGCATTTGCTATGATCATTCGGATGTGTCTCTATTGCATCTTATATT
TTAATACAATCATTTTTATATAACGTTAAGCAGCATGAATGTTGTTAAAAAAATGTATCTCTGTACTGATCA
TGATAAGGATTAAATTTATATATATAAAAAAAAATGTATTTATACTTTTCATTACCTTTGTTAGAAATATA
TAAATATAAGACGAGCACAAGACAGTTGAAACGAACACAAACTAGCTGGAATAAACACGAATCAGTTAA
CAAAATAAAATTTGAGTAACATCAAGTATATAAAAATAGCCTTAGTAAATAAATAAAAAAATTAACAAA
AACGGTTTAAATACGTCAGGAGGCATATAGCTTGCAAGAGAACGTCACTTGCACTTTTATGCATAGAGCC
GGTTTTAGAGCGACCTTGCTTAAGCAAGTCAGGAGTTCCTTTCTTATGATCGCCGCCTATGCAATTATTTAT
ATGACTGCGTATTGCACTTTTGGGGAAAAAATCCAGAACATCCCTTGCAACATATGACAACATATTCGTTT
TTAGAGTTAATTAGCTTTAAAATTTCGTTATTTAACGATGCATCGATATTGTCGATTTAATCGCCATTACTC
CAAATCTCATTAATAACTACTTTTCGAGTTTTGTCATTTTTCGAGGCATTAACACTTTTTTACTTGTGGCTT
ATTATTTGGAAGTGACGAAGAATCAAATCCCTTATATCACTTAGTGGAGTTACATTGTGTAAGTTAAACTT
GGAAGTATATAAGTGCTGGACTGATTAGTTATATCCGGAGTATAGTTGGAATCGACTCCTTGAATAGAAT
GTGGAAAATTACCAAACGATTTAAATGTATAAAATGCTTATGCTGGGGAATCTAGCCATTTAGAGGCCAG
TCTTTGTCTATTATGACGGGCAAAAGACACCTGAAACGAACACAAAATAGCTGGTACTAGCACAAATTAG
GTAACAATCCAAAAGGCGAGTATTATCAGTATACAATGTTAGCTTTAGTTAAAATACAAATTAGCAAAAA
CTGTTTGAATACTAAAAAAGGTTTGTACGTCCGGAGGCATATAGCGGACAAGAGAATACACTATTCCTAG
AAAAGTAATGACCGATTGTGTCTTTTGTGTAGCTTTTTATAATAAATTGATCTGATTTTGCATATATGATTA
ACTCATAATACAAATGATAATTAAATTGTTTTCAACAACTAATAAGGCTATAAATCTTTTGATATTTTCCG

ACTCTGGCCTTAAAACTGTGGTACTCACAATTATGCATATTTAAAACTTAACATATAATAATAAATACATT
ACAAAACATAACTCTGTCAGAAGTGTAATGCAAATGATTTTTTATTTAGTCCTGGCTTAGTTTTAATAATT
GCACACATACCGAAAGCTTCAAATTGTCCCAGGTTGCTTTTATTTGTTGTGTCAGTATTTGACTTATAAGCT
GCTAATGGCGCCTCTTTTAAAAGACAATGCTCATTAATTTATTTCAAAGGCACATTAGTATTGTACTTACG
GATTTATCATCGTTCGTTGTTGCTTCACTTTCATTTAACTTAGTATCTGCTTCTAGTAAGTCTATAAATATTT
AATGTATTATTAATTATAATTTAAATTAAAAAACGATTATTGCATTGCCTATGTACATATGTATAGTTATTG
ATTTATGTACATACCCTTATTAAGTTATTGACATTGCTGGTCCAGTATTTCACGATCTTCTAATGAAATAAA
AGTACATATACAGACAAATATAATTATTGAAATGTCAAGCTCAACTTACCTCGCGAATCAATAATCTCCGC
TTCGAAACTGCCTTTGCTAAATTTTGCAATGCACATTTTACTTCCTTCCACTATTTTAATTTTTTTTATGCA
ATACATAATATTGCTCGCACTTAATTAAGAAATACATCCTCTCGCAGTTTCCAGAAATTTAATGGCGCCAA
GTTTTCGAGATACGCGACGAGCAAGTCAAGTCATTTCTTCAAATGTACTTTTAAGGCAGCTCAGCACTATC
AAATTGAATACTCGAAACGCGTTCATTTTGCTTGCGTTATGGTAAAAACTAGTCGATATTTATGCAATTAA
AATGAAGATGTGAGAGAGATATTAAATTGATGATGGATTGTAAAAATGAAAGCGGTCAAATAGAATAAC
TGTTAAGAAGAAATAACTGTTTTTAATTGGA
>9_rnd-5_family-2852__LINE___I-Jockey
CAGTCGCGATCGAACACTCGTTGAGTAGAGACGTGCCTGCGGACCGCTGCGAAGCATTCCAAATACACAA
GCAACATAAACAACAGTTANTTGCGGCGAAATTNTCAGGCCAGCTGCGCTCTGATTGGTCGCTGCAACCG
TTACTGCGCTCTTTCAGCTTGCGTGCCGTGCGCAAATAACGGCGCACTAATACTTACACACGCACACGCTT
ACACGCTTGTGCGCGTTATTTCGACACACACACGACAACAAGAATACAACAACAAACAACAACACAGACT
AGCACTCGCTGCTTACGCTCGCCAGACGGGCAGCTTGTTTGCTCNCGCCCCCCGTACCCGTCACCAACAAG
ATGGACAAGATGGACTGGCAAGCCGAGCCACGTGCTATACAGCTCATTAAAACTAGCAGCACCAAGAGG
AAGAAGACCAACATCAGAGCTAGCAACCCTGACTCTGAGAATTCGAGTTGCACTAGCGAACCTGGAGAA
ATAAGGAGGAAGCCCGTGCGCAAACAGCGCAAACAGCCTTCCCGCGTACACCTACCAGACGGACCAAGC
ACCAGCAAAGCTGCTAGAGAAAATCTAACAAGTAACAGATTTGCTTCACTCTCTACAGATGAAGACGACG
AAGAATCTTCGGCCAGCGATGACTCCACTGAAGTCATAGGACTAGCTAAGGGCCAACCAAATAAGGAGC
CTGCTAAAGAACCTAAGACGATCAGGCCACCACCGATTTTTATTCCAGACGTGACCAATATCAAAGCGCT
TACCAATGCCATAACTGGCGCCCTTGGCAAAGCAGTAGAAGTTACATATAAAGCCACTAAAAACAACAAC
ATTAGAGTAATGACGCCAGATAAAGCTAGCTTCACTGCACTTAAGGCATTTCTCAATGAAAATAACAAAC
GGTATCAGACCTTTCAGCCCAGAGACGAAAGAGCCTACCGGATAGTGGTCAAAGGCCTGCACCACTCTAC
TGAGCTGGTCGAGATCAAGGAAGGTTTAAGCAACTACGGGCACGAAGTAAGAGACCTGTATAACCTCAC
ACAAAAAAGCACAAAAAAACCCCTAAACATATTCTTCGTAAATCTCGAGCCGGCGAAAAAAATAACAAA
GAGGCATACAAAATCAAAAGGCTTTGCTGCTCTGTGGTTACAGTCGAGCCCCCACTGAAGTTTAACGATG
TCCCCCAATGCTATCGCTGCCAAGGATACGGACACACTCAAAGATACTGTCACCTGGAACATAGATGTGT
GAAATGTGGAGGCGGACACTCCACGTCGGAATGCTCAAAGAAGAGCAGTGACCCGGCCTTCTGCCTGCAC
TGCCAGGGTCCCCATGTGGCCTCATACAAAGGATGCCCAGCCTATAAGAAGGCAAAGAGCCTCTTAACGG
CAAACAGACAGACAGCTCCAAGAGATGGACTGCCCCTAAACTCCAGCAAAGTGAATGCAAACATTAGTTT
TGCTAAGGCTGCAAAGAGCACCTACGAGGCAAATCAAGCTCCCTGCCCGGATTCACAGCCGGACAATCAA
CTTAATGCGATAGTGACAAGGCTAGAGGTCATGTTTGAAAAAATGATGGATAAAATACTAGGTCAAATGA
CACAGATGCTGGAAACTGTTTGCTCCACATTATGCAAGAAGGTTTAGAGATCACGATCTGGAATGCTAAC
GGCCTGTGCAAAAACAGGCCTGAGGTTGAGCTTTACTGCAAGACCAACAATACCGAAATTCTCCTCATAT
CTGAAACCCATTTCTCTGAAAGATCTTACTTCTGCCTGCGAGGCTACGACCCGATCTCAGCCAATCACCCC
AACGGGAGAGCCCGCGGCGGAGCTGCCATTCTAATTCGTAGCAACATAAGTTACGAGATCCTCCGGAGGA
GTGGATCCAATGCGCAAAAATAAGAGTGAGTACCACGCTGGGAGACGTCACAATAGCCGCTGCCTACTGC
CCCCTGCGCTAAGCACAATGCTAGGGACGAAGAATTCGGGACGCTAATGAACAGCCTAGGCACAAGGTTT
CTAATAAGCGGAGACTTCAACGCCAAGCACACCTGGTGGGGATCCAGAACAAACAACCCTAAAGGCTAC
GCGCTGTTCAGGCAAACCCGGTTAAGAAACCTCGACTGCCACAGTACAGGCGCACCTACATACTGGCCTA
CAGACCCATATATAGCGGTGCCAGCTACGTCCTGGACATCGCGATAACTCGTGGTTTGAATACTGGCAGA
ATCAAAGTCAGAGAAGTAGAGGACCTCCTATCTGATCACAGCGCCATAGCCATCTCTATATTTGCTGCAG
CCATGCGGAAGTCTGCCCGCAAGAAGCTTATATATAAAGCCACTGATATTGGCAAATATCAAACCTGGCT
GGACAACTGCACCAATCCCAGCCCCCCACTGAATAGCCCGGAGGACATCGAAGCGGCAGTCACAGAGCT
AACAACTCATATCCACAATGCGGCGTCACATGCGGCTCCAAGACTCTNAAGCCGGCCCAGAGTTGAAGTA
ACGGAGACGTTCACCTCTGGTCACAACAAGTGGCCGAGCTGC
>9_rnd-1_family-486__DNA___Maverick
TAATATACGGTAAATCTTGGTTAACCGCAATCCATTATGAATACATTGTTGAAGATTTCTATAATGTATGG

GATATTTATATTTCGGTCTTAGGTCAGCGATAAGCTTTTTAACTGTACACTTTGCAGTCGGTTTTGATTGCT
TGGACGAAACGGCAAGTCGTTATGCATATCGTGAAGGGATGCTGGGTATTCTAAATCTACTTCAAGAATA
TATCCATATTCATACGTTTCAGAAATTGAAAAAAATGAAATNATCGTATTTCATCCTCACTTAACCACTGG
AAATCACCATATGGAAGATATTGTGACATCGCCCACCCATATAGATTATTTGAATCCAAATACATTAAATA
TTGAGAGGGTTTTGTGCTATCATAATCATTCGTATACTTATGATTAGCAATTGAATATCTCTGACAACATT
GTACTAATCCCCACGAATACCATTTTGTATAAATCTTTGCATATCTATGTCTGTAAATAATTCTAATTCTAT
TCCAGTTGTTTTTAGCATANCATCCCAAGATAACCCCGGNGCTGTGTAATAATGGGCTGGGTCCAATGAAT
AAATATTAAACGAAAATTTTCGAAAACATCTGCAAGCAAANGTACATCTGTCTTTAAATACAGCTCCATA
TAAATCTTTGAGGGTTTTACAATTAAAAACTCTCCACACGTTCTGAGCGTGTAAATAATCATCATCTAAAC
ATTCTCTATTTGCAAGACTGTTGAAAAATTTTTCCCTTGGTGGAAGAGCAGTTTCTTGCAAACATTCAAAT
TTAGTAAAGTANTCATATGGGAAAACTCCCTTCCTATGTACTAGATTAAAGTGTGTTTCGTCCGGGAAAAC
CCGTTTAACAGTTNTGAAATTATTATGTTCTAAATTAGAGCTAAGACTATCTAAACTTGCAGCCATAAATC
GAAATGAGTCTAAAAAACGTATTTCCAAATTTGTATTATGCTCTGTAATATATGTGTATTTTGAAAGGGAA
ATGTAAATCTCTTTATTGAGAGGTAGAACCTTAATTTCACCTTCAACTTTTGACAACTCTTACAAACAAAT
GAGCATCATAGCGTGAAAAATTATTAAAAAAGTACGGGGATAAATGAGGCAACTTTGTAATTTATGTTAC
AGGCATTATGAGCTGCCCCTCTAAATTTACCAGTTAAGTGGCAATGATCCCGTACTCTATCTAAGTTTAAA
ATCTTAGTGCAAATGTGGCAATTTATAGCATTTTTAAATTTATGCTCATCTTCTAAGCTCATTTCGATTGGC
TTTACAGTAGATAAATATTTGCTATAAATCTCTCTTGCATCNGCAATGAGAGATGTTACAAGATGTAATCN
TACATTTTCTCCACTATATATAACGAATTTATCTAAACTAGAATCATATGCAGATTTTATGAAATATGCGT
ATGCAACAGGAATGTGTTCTTGAATGCTCATTACATTCTCGCTCACATTCCTGTTGATAGGTTTTAAAACG
CACTCGAAATCTGCATAGATTATAAAAGGTATATCTAAACTATTTGCATAANTCTTAAATTTTACACTCGC
CTTATCCGCTGTTGGAAGCTTCGCCGCCACTTTTCCACACTGCTCGCCATGCATCCTGTCGTTATGCGAATA
GCTTAAGCATACATTACAGAAATGTAATTTCCTCGTATGCGAAGTCAACTGATTGTGTGTAAGCCTGGAGG
AGAAAATATGATTGATATTAAAAAAGAAATGTATATTTTTTTGTATTCTTTACTTACCCTTGATAGGTTCTT
AATCCAGGTATAATGGGATATATCTTGCTCTTCCAAAAGCAATAGATTAATATGTTTCCCCGGTGCTCTTT
GGTTAGATAGAGTGGTCCAATAATTTCATTAATGTCTTCATCATAGCCAAAGACATTTTATACTCAAGTAA
GTATTTTGTTGCTCAAACAATTTAATGTCTTTGAGCTTTAATGGAAATTCCATATTAGATCTTTCTCTTGGA
ATTCATCTATCTTTGAACTTATGATGTATGAGTGGCTTACAANTATTTCTTCTAAGTCAGAAGACTTAGTA
GCCACACACATTTCAGTTTGATGGGATTTAATCATTATTTCAAAATCTTCGCCNTCAGTATTCTTATTATTT
ATTTTCAAAATTCAGCGAAAAGTTCAAAGCTGATTTTATACGAGGTGTGCACCTCTAAGACGTTTTCGATT
GTTGGAAGTAATAAGCTTCCTGCTTCCGATAGAAACTTTTCAGGGAACATAATGTTATCCGAAACATTGCT
AAATAANCAGGGTGTTAGTCCTGTTTTGGAATCCGAATGCCGCAAGTTGAACAGAGCCATCCAACGGTGT
GAAGGCCTTATTTTGTGCTTATTTGTCCGCAAATGTTGTGCCCAGTGATTATGAGCCACCGTTACTCCACA
AACCTTTTCACTTTAAAACTTTCAGATAACTAAATAAATTTTATTTAAAAANAATACTCACTTCACTTCAGT
TTCCCCCAGCCACAGCTCGCTGATAACATAGCACATAGCACACACATACACACACACACAC
>160_rnd-1_family-298__Unknown
CATTGAGTAATAAAGAGAAAGAGCAGGCGTTTGGATCAAAAGCTCTTTTTAGAAATACTTCCTGAGCGAA
AACGAGGTTGCAAACAAAAATTGCAAACACAACACCCGGCTGTCTGCAGCTACTTCTGCCGATGCCCACA
ATTCAATGGCAACTTCAAAACATTTTCCATAAGAACCAACAACAACCATAATCAAAGCAAACGACTCGAC
AGTGAAAAAAGCATACCACAAATTCTGTCCTTGCTATCCCTTTTTGTTTGAGGCTACAGTCTATGATCTTA
GCTTCTGCACCCTTCCAACTCATTGTTGTGGTTTCTAAGCACTGCTATGCCTATAATTCGCAAAGTCATGTC
TTCTTCTTCTTCACTCGCTTTCAACTTGCAAGCAGATCGGCAGATTTTTATAATTTTTCATTTTTCGACCAGC
AAGGCACCTTTTCTCAAAATAGGGTATTTTAACTATTCATAAGCAATGTAACCAAATAGCACAAACCATCT
TATATATACATTTTTATTTAATGTCTATAAAAAATATAATTTTGTATTTAGTGTGCAACTATTAGTACAGTC
AATAATAATTGTTTTCTTTATAAATTATATACCATTCTTATAAAAAAGAGTATCAATGCCGCGNCCCGCCA
AATAGTTGTTTATGTGTGTCTCATCTCACACATATTGGTGCAAATCTGCATATATGTATGTGTCTGCCAAGC
CAAGGGAAGTCTATGAATTTAAGAAGATTGCAACGTTCTGTTGCGCATGTGACGTCACAGTCCAAGGCTT
TTTATGTGCGATCATTTCGTATATAAAATTATTACATATGTCAACAAATTATTTNCTATAAAAGACCCCATT
AATAAGNTATTTAAATGTACCTAATAAAGGTAGAAATATATGCTAAACATCACAAGAAATAAAATTCTAT
TAACTGTGACGTCATAATATATGATGTTGGGCGCCGTGCGCGTAACCCTTTGATCTGAGTGCCGTGGCTTG
CTGATATTCAAATTTTCTACCTACTTGCGCCGAGACTCGGCTCTACAACAACAACATTAGTACCCGAATTG
TGCTGTTGTTGTTTCTACGCGCTGCCTTGTAGGAACGACAGAGAAACCCGCAAAGAACACTGTGGCATTNT
TTGCCCTTTTCTTTGCACTGAGTTTCCTATGTCCTCATTATGGCTCACAAACCTCTTGTCGATCAAAACGGG
GGATGAAGAACAAATTGGGTTTCGTGCGCACTGTTGTTGTTTTGCAAGTGATTTGTTGTTGCTTCCTATGGT

AAATGAGCTTTTTTATTGGGTGTGATTTTGCGTTTGCGGACCAACTCGATCTACGACTCGTGGTTTTTAAAA
CGGTAAAAAAAGCTCAGAGCGTCAGTTCAGATCTTTCTCTTTATTACTCAATGACTAAAACTATATACATA
AGATAAGCAACATATATACATACATATACAGCATATAATTACATATCTACAACATAATACCGTTTATGTCT
AACTTAGGAAGGGTTAGGAGAATAATTCCTACAAGAGGTGCCAAACACACTAGTAGCATCCCAGATATTA
GAGAAGAAGAAGCATCCGAAACCATGTACATGGCTCAGGTAATACCCCCGGCACAACCTGCCAACTTGCA
CATGTAAAAAATAATAGGTAGCTTTAAAATTGTCGATTTATTTAAAAACCTTCCCAACATACGACGGGAA
CATGGAAGGACTAAGGGAATATATTACAAAAGTTGAGTAGATATTAATGCTAATAAAAGGGGCAGCCCA
AACTCCAACCGGACTAATGATACTAAGGGCAATCAGAAACAAGATTCAGGGAAAAGCGCATGAAGTACT
ATACCAATCGGGAACTGAGTTGAACTGGGACGAAATAAAGAATAAACTTATTACCCACTTCGCGGACATG
AGAACCGAACATAGCCTTATGGCAGACCTAGGCATCCTTTCACTTCAATGTTACCCAGTAGGAATTCTGTA
CGAAAAGATAACAGATATTCAAAGAGCTCTACTCAATATCGTAAAAGAAAAATAAGTGAATCAAACGGT
CATTGAAACCAAAAGAGAACTTTTCAACAAACAATGTTTTACAACATTTCTAAATGGACAAATCTGACTTT
CTACAAAAAGAAATTGAATACCTCGGACACATTGTAACACTAGATGGCGTTAAACCAAACCCGTCAAAGA
TGGAAGCTATAAAAAAATGTCCTTTACCGAAATCTTAAAAAGAAATTAAATCCTTCTTGGGTTTAATGGGT
TATTACAGGAAGTTTATGAAAGACTTCCCAAATATAACCCCTGACTAAACAATTAAAAGGGAAAACGATT
GTTGTTTTAAACGATGAATTTATAGAAACATCTAACTACTGTAAAACCTTGCTTTGTAACGACCAATTACT
AATTTACCCCGACTTTACGAAACCATTCATACTAACAACAGATGCTAGCAACGTAGCGTTATGAGCTGTGT
TATCACAAGGACCTTTAGGGTCTGATAAGCCAATTTTGTTCGCACGCCGAACACTA
>160_rnd-5_family-148__LINE___I
ATATCAACAAATTAGTAGGGATTTTCCCAGGGCCTTTTAAATCCATCAGAGACAACAATGTAGAGCTCCA
AAATTCAGTAGACATTGGCAATGCATTTGGAGCTATGTGGTCCAATTATTCCAGGGATGAAAACTTCTCAA
ATGCGTATAAAGTTAATAAGGAAAACGTTCTCTCTCAGGCATATCTGCCCAACCACATATCCTCAAATTCT
AAGTACCTTGAATCTCAGTTCACACTGATGGAACTAGAATTAGCCCTTCAACACGCTAAAGGGAAAACTC
CTGGGTATGACCGCATCTCGTACAATATGCTTCGAAACTTACCAATAGAGGGAAAGAAAAGAATACTAGA
AATGTACAACATAATGTTAGATGGGGGTAACTATCCGCACGCTTGGAAAACGGCTACAATTATCCCAATC
CTTAAGCCAAACAAACCTTCCCACGAATTATCATCCTTTCGCCCCATATCCCTACTCTCCTGCTTGAGCAA
AACACTGGAAAAAATTCTTGCCAAAAGGATCATGTGGTTCATATCCAAAAATAAACTTATCAGCCCCAAC
CAAACTGCATTCAAACAACAGCACAGTACCATTGACTCCCTATTGCATATCCATCATTACGCGTCAGATGC
CCTTTCAACCAGAAATCATGTAACCATCCTGGCTACTGATTTCGAAAGGGCATTTGAGCGCGTTGGAGTGC
ACACAGTACTGGAGCAACTTTCCCGGTGGGGGGTTGGTCCTAAGACCTTCAACCTCGTGAAGGCTTTCATG
ACCAAGCGATCCATCCGTGTTAGAGTTAATAACACCTTATCTAAGTTCTATAAATTACACATCGGCATACC
ACAAGGGTCTCCACTCTCGGTGGTTTTGTTCATGATTGCCTTCAACTCCCTCAATAACATAATACTAAAAC
ATAAGAAAATACAACTCTCCGCGTACGCGGACGATCATTATCTTTAGTAACTGCAAAAATACAAATTTAC
TCTTCCTTACGTTTAAACAAATACTTAATGATATCGTAGAATGGGGAAAACTCTCAGGAGCCACTCTTGCT
TTAAACAAAAATTGTTCTCCCCCGCATACCATGCAGCAGTACGGCGTAGCATTGACGCCTTTCTCACTACT
CCGACGGAATGCCTTTTAGCTGAAGCGGGTCTTCCTGAAATACAGACGAGAACTGCGCAACTGACTTTCA
AACTCATCCCTAAAATTCTTTGCTCGACCAACCCAATTCTGCTTAAAGACTTTAACAGAACGGTTAAGCAG
CGCCGAAGATTTAAAGTCTTGTCTACTATCCGTAGATGTTGCAGTTTTTTCAAAAAACATCAATTGGATAT
GCCATATCGTTGGAACAAAATCAGCCCATCCCCTCCTTGGCAACTACAGGACCACACCGTGAACACCTCT
CTCCACTTATAAAAAAAAATCAGAAACACCTAAAAGTATATTTCAGCAAATGCTAGCGCCGAATCGGAAA
AATTCCCCAACTGGAGTTGGCTCTACAGTGACGGATCGAAAACAATTGACTCCACTAGCTTCGCAGTTGTA
AGCCAATCAGGAACTGTGGTCGCATCAGGAGTGCTCCCTGCATACTTTTCTATATATTCCGCCGAAGCTGT
AGCTGTTCTAAAAGCAATTAAACATGCTCTCGAGCTTAAGGGAAAATTTATAATATGCAGCGACAGTCTTT
CTACTCTCAACGCCATTAACAACACATACAACCAAAACAGCCTGATATGCGAAATTCGACATCTTTGCATC
AAACACGATACCAAAATAAAACTCCTTTGGGCGCCAGGTCACATGGGCATAAAAGGTAACGAACTGGCA
GATGCAGCGGCTAAACTGGCTGGACAGTCTCCTTGCCTAACAGTCATCCCTGGTGTCAGAGAAGACTTAA
TGGCGCAACATTGAGGATATCCTAAAAAGGGACGAAATGACACAATGGGACACTTACGTCCATAGATTTA
AGCAATTTAATCCGAACAAGGAACCCGTCCGATATCCAGCTTCATGGCACAGAAGACATTGCAGTGTTAT
CACCCGGCTCAGAACTGGGCACACCGCCTTAACCCACGACCACCTGCTTAAAGGAAAAGCCAANCCCAAC
TGCCCAAAATGCGGGGGAGAATTATCAATCCAACACATATTGAATAACTGCCCACAACTTAATAACATTA
GGACTTGTCTATTTGGCCAAATCCAACCTACCTCTCTTCTTACCACACCTACTCACAATAATGTAAAATTG
CTAATCAAATTCTTAGACACCAATAACATACTAAATAGCATATAATAATATATATATAATTCAANATAATA
TACATATAATACTATAAATAATTTAACTGTCGAGTCGATGGCCTTAGTAGCTAGCACTCCCTTCCGTTAGT
TATTATATTATTTTTTTGTATAATAATTAATTACGTAAACCAAATAAATATTTTATTGCCTATTAATTTTCGT

ACTTGGGCTTGAATGTCTTGTACTTGGCTATGAGGACTCATATAATTTTT
>160_rnd-4_family-254__RC___Helitron
TTATTGTTGTTGCTTTCGACAGCGTCGGTTTTACTAGCGCCGTTTGTATCGGTGTATCTGCATGCTCTATTG
TTGTTTTTATACCCTGAACCCATTAAAATGGGTAAAAAGGGTATATTGTATTTGTGCAAAATGTATGTAAN
AGGCAGAAGGAAGCATCTCCGACCCCATAAAGTATATATATTCNTGATCAGCATCAATAGCCGAGNCGAT
CTAGCCATGTCCGTCTGTCCGTCCGTCCGTCTGTCTCAGAACCTATAAGAGCTAGAGACCTGAAATTTTGA
GATGTAGGTCCTCCTAGTGCCTGCGNAGATCNAGTTTGTTTCCGATAATCGATAACTTACTCNGTTNCCAA
NCAATCGATAAAAATCGGTATCGACATCCTNNTTTTTNGNNAANTTNGGTAAATAATAAGANCTGGAGT
CACCAAACNTNATNTGTTGNTTCTAGAATATTATAAATATGTATCGAGTATATTTCATTTTATACCTATCGC
CACCTCCCCGCTANCACNGCANAGCTATAAATNAAGTTAAAAACCCGACTTNTATCGCCAACCAATCTAA
GCCACAATTTTAATGCTATTGACTTNTTATAAGCACACAAATATTCTATGGCACAATAAGATATACTGTAG
AAAATTTCATTAAGATCGGTTAANAAAAGGCCAAAGTTATATGCAACTGCGCAATTGAATAGAGCAGCAT
CTTTAAGAATTTCTGTATACATGTACANACANACATTCATGCGCGTCTGCTTNGCATTCTAGCAGCATNGT
NATTGCGACTNTTTTCTGTAATGCTATTTTAGTCCCTTTTTNTACCCTGAATAATACTTAATTATTGGNGTG
GCTGTTGAGTAGGGGCTGTGGCAAGNGNNGCGGCCTNTCGCGNGTTCGAGCNCCACCTGGNNAACTTTTC
TTTTTTTTATACCCTGAACCCATTNAAAATGGGTACAAGAGCATATTGCAGNTGCGCGAAATATTATGCAA
CAGGCAGAANGAAGCATCTCCGANCCCATAGNGTATATATATTCTCGATCGGCATTAANAACCGAGTCGA
TCTAGNCANGTCTNTCCGTCCGTCCGTATGAATGAACGCAAGGATCTCAGAACCTATAAGAGCTTGAAAG
TTTCGATGAAGGTGCTCCTAGTTCCCGCGCAGANCTAGANTANTTCCGATNATCGATAACCTACCCCGTTN
CCGAGCAGTCGANAGGAATCGATANCGGCANCCTGTTTNTTGGGCATATATTTAAGCCTTTAAGCCAGAG
NTACCAAANTNGGCACGATANCTCTCNNAAAAGGAATATATATGCGTTTGACGTTGGAGGAAGGCGGTTC
AGGGTATCCGCTAGTCGGGCGCCNCCGACTAGAACTTCTTACTTGTTTTGATATGTTCGCCTGTNNAGTAC
AGCCGTCAGCAAGTAATACAGGTCACTTTACAACGTNGAGCCTTGCCAAGGGAAGTTCCTTTTTATACCCT
GAACCCATTAAAAATGGGTATAAGGGTATATTGTATTTGTGCAAAATCCAAATGTATGTAACAGGCAGAA
GGAAGCATCTCCGACCCCATAAAGTATATATATTCTTGATCAGCACCAATAGCCGAGTCGATCTAGCCAT
GTCCGTCTGTCTGTCCGTCCGTCCGTCCGTATGTATGAACGCAAGGATCTCAGAACCTATAAGAGCTAGAG
ACTTGAAATTTTAGATGTAGGTGCTCCTAGTGCCCGCGCAGATCGAGTTTGTTTCCGATAATCGATAACTT
ACTCCGTTTCCAAGCAATCGATAAAAATCGATATCGATATCCTGTTTTTTGGGCAATTTTGGTAAATAATA
AGAGCTAGAGTCACCAAACTTGACATATAGCTTCTAAAATAGAATATATATATATATGCATTTGATGTTGG
AAGAAGAGGGTTCAGGGTATCCCCTAGTCGGGAGCTCCCGACTAGAACCTCTTACTTGTTTAATATNGTTT
CTAAAATAGATTATATTTGCATTTGATGTTGGAAGAAGAGGGTTCAGGGTATCCCCTAGTCGGGAGCTCCC
GACTAGAACCTCCGTCACTTNTTANTNGATTNTGCAATNCATAANGGAACTATATGCTAAAAGCCTAGTT
AAAGAACATAATTTTTTAAATAGCATCACGGACCGATGGAGGTGTTTTATTTGCCAGCTGTCCCCCTCTCA
CAGTTAATATTGTCAGTGACGCTATTAGAGCCAACATCTAAGCAGAAGAAAGTATTCTTAAGTAAAATTA
AAAATAATCTTAATTGGCTATATTAATAGACAAATACAAAAGAAATGTAAGCTAGATGTATAGAGTAGAT
AGGACAAAGAGAAAAGTAATGCGAAATCACCGAAATCAATTTACAGCCTGTGCGGCAAGCAATACCCCT
GCTCATAGGTAGGAATGGTCAGTGACTTTCCGAAGGTATCCTGAAAGCATCGATTTTATTAGGGGTAGGT
AAACAAACAATTGAAGACGCCCTAGCAGAAAATGAGATTCTATA
>9_rnd-5_family-2824__LTR___Pao
TAATATAAACTTCTGCAGTACTATTTTTTGGTCCGACTCAATGATAGCCATCAGTTGGATCCGTGGAGGAG
GTGCAAACTGGAAGGTATTTGTTTCAAATCGCATAAACAAAATTCTGGAGATCTCGAATGGAAGACAATG
GAGACATGTTCCCTCGATTGATAATCCTGCCGACCATCTGAGTCGGGGAATTAGCGTATCTAAGTTGACGG
CATGTGAAATGTGGTGGCATGGACCGCAGTGGTTACAAGGTCCGGAAGAAGGATGGCCTTGCAATAACAT
TGATCAACAGTCACTCTCACAAGAAGCTTGACTTGCCACAAAATATCATTGGAAGAGATGCCTAGCTCGA
TGTTCATAAGTCAATATTCATCGTATACCCGACTTCTTCGAGCTATGGNATATGTTAAACGTTTTATTCGAA
ACTGTCGCATAATAGAGTCTGAGCGCAAGCGAAGTGAGTTATCAACAGATGAACTAGAGGAAAGCCTCA
AAGAAGTAATAAGATTGGTGCAAAAGGACTGTTTTGAAAACGAATGGAAGGCACTCTCTTCTAGCAAGCC
GGTCCCAAGAGATAGCAGTTTATTACCTCTCAACCCATATTTTGATTTGGGGGAGCGTCGTCTTACGGCTC
GGAGGAAGATTACGCGATGTCTTGTTGCCAGTGAATGAGAAACATCCAAATATATTGCCCTATAAACATN
ATTTCACGAGTCTTGTCGTAAATTATAGTCATGCTTGCACCTTGCATGGAGGCTCATCGCTCACACTCAAT
TATATAAGAAAGAAATTTTGGATCATCAATGGGCGTAACGCAGTTCGTTTTGGTATACATAAATGTATCAA
ATGTTTTAAAATGAAGCCTAAGTTTGCAAGTCAACAAATGGGAAATTTGCCAGCGGTGCGTGTGCGGCCC
TCAAGAGCTTTCTCATCTACCGGAGTTGACTATGCTGGACCTATTAATATTTCGCCATCCAAGGGAAGAGG
ACGTGTAGCACAAAAGGGATACGTTGCCGTGTTTATATGTTTGGTGACCAAGGCTATACATTTAGAGCCA

GTTGGAGATTTAACGTCAGATGCGTTTTTAGGAGCCTTACATCGATTCATAGGCAGACGTGGTCTATGTAC
NGATATTTACAGTGACTGTGGTACAAATTTTGTTGGCAAAAAACTACAAGCTGATAGGGAGGCATACTAC
TCATACATTTCAAGATTAATCGCGCCAAAATTGCTGAATCCACTTTGGTGGATTATGGGAAGCCGGAGTTA
AGTCCATGAAGTATCATCTCAAGAGAACCATTGGAGAGCGAGTGTTAACCTTTGAGGAAATGGCGACAGT
ATTAGCACAAATTGAGTCTTGTCTTAATTCAAGGCCTCTATGTCCTATTTCGAATGATCCTGAGGATTTGAT
GGTGTTAACCCCCGGTCACTTTCTCACTGGTGATGCGCTCTTGTCGCCTCCTACAACTGAAGTACCTCAAC
TGAGTTTAGTGCATCGGTGGAGAAACTGTCAATACTATTCACAACAATTTTGGAAAAGGTGGAACTCTGA
ATACTTGACACGGTTGCAGAGAAGACCAAAATGGCTGCAGATGAAGAGAAACCTAGATGTTGGATGCAT
GGTGTTAATTAAGGATGAACGATTCCCTTCGCATCGGTGGCCTTTGGGTCGGATTACAAGCACTCATGCTG
GTGCAGATGGATTAGTTAGAGTTGTGACTCTAAAAACAAGCAGTGGTGTTATCAAACGGCCAGTAACAAA
GCTCTGTCCATTACCCATACAAGAGAATTGGCTTGATAATGACACCTAACCTTTACGTAAATGTTAAATTG
TACTCTTCTATTTTCAGTTTAAGTTAAGGTACCAAGCCCGAGAATCTGACAGTTGGCGGACGTAAATTTGA
TGATGAATGCTGCAATCATCAAAGGGGGGAGAATGTTTGGGCTACAGCCTATCAGCAAGAGCAATCGCGC
CAGCCTAAGAGCGGATATTATAAGAGAGCGCATGAAAAGAGCGATTAACGAAAATGCACCTGCGCTTAG
ATCAGCCGCATTTGAGTAATGTCTTATCACTCAATAGATCACGCGCTATCACGGTTGAGTTGAGTTGAATT
TAGTGGCTCTCTCAAGCGCTCGAGCATTCAGTCCTGTTACTTTAAGTTATCTTATTTTCTTGTATTTCGTAA
TTCTATTGAGCTTTGAATTTGCACGCTTAGTAACAGCGGAATTTGCGACATATGTTGAAGTCTGTGAGCGT
CAGTCCAAGTCAACGGACGTATGCTCTGTGTAGGTCAAATTAATAATTATAGTTTTTAGGTTAAGTTCAAT
AAAGGTTGAAGTTTAAAATGAACAACCCCGCTCGTGAACTGGGATTCTTTTGCTAACCTCATCATTCCCAC
CAATCGTGCAAAACCCTCAAAGCCGCCTCGGTTGGTAACCACGCCGCAGGCCGCATCATATCTACGGGCA
GTTTGGGGCTCCGCACCTGGCGCAGCCGATATCACCAACA
>160_rnd-5_family-1549__Unknown
ACCACTAACCATACAATGTATAGACATACCAAATGGCACTTTTAATCGAAACAAGAAAAATCGAAACGAG
GCGACCCCAATGGTACCTGCGACCATTTTAGTGTGCCCAGACACTGCTGAGACTGCTGAGAGTGGCAGCC
GAACGCCGGCCTCAAAAGCCGAACGTTCAGTATGAAGCCGCATCTCGCTCAGCGCACAACGTCGCGGCTG
ATGCGTTTTCTTAATATATGTATGTATGCCATTTTTAAATCCTTACAGCCCAAAAACATATATGTGTATGCA
TGTGCCTCGCTCTCACTCATTGCTTCGCGCACTTGCGTCCATCTCTTTCTCCCTCCATAGCTTCCCACAAAA
CCCCGAGATCTACAAACATTCATACGTTTGTGTTGTGTGGATGTGCGCACAAGTAGATCGCGGCGTTTGTC
GCGAGCCAGAGGTTATTTCTTCAATTGTGTGCCTTTTGTGCCTGTGGTAGCAATAGGCACTCACATTTAAA
TGCTCTATCATGTATTTCTTCGTCCAGTCAGGCATGATGAGAAGAATTTGATCAAATTTTATTTAATACAC
AATTAATTGACCTAAACAAAATATTTATATTTATTTTCTGTTTACTGTAGGGACCTATTTTGCTTAAAATAC
AAAAATATCTAAGTCGTATTTCTGCAAGGGTAATTCATCGTCGGCTTGTCGAAGAATTTAAGAAAAGACC
AAAAATTGACGCACACTCTTTCAAGTAAGCAGTTGTGTGAAATTATTAAAGGGTTTGGCCTGTTCATGTGC
AAAATTGCTATTTTCCATGCTTGTTGTAGGGGTAAGGGTACCTGATCGCTGGCATGTGTGCATGATAACTT
TTTGGGTCGGCCAACCACGCACACTAACACACTTTTCTATATGGATGGAAGCATTAATGCGTTTGTGTGCG
TGTTGGTTGCCTGCCTGCCTGCCCGATGCAAATTTGACAAAATTTGAAGTTTTTGGCATTTGTGAATTTCGA
GCTCTACACACACACATATACACGCNCACGAATGTGTGCGAATGAGCTGATCAAGGGTTTGTCGTGAGGC
AGAGATATTTTTATCAATGGGTGCGTAAATTTGGGCGACTGGCACGGTCTTTGTTTTTTTGGCATGCCTGC
GGTGGGCTTGTGAATGAAACAGGTGGATCGCACTTGTCGCTCGTTTGCACTTTGATATGGAAAAAATGCA
AGTAATCTTTTATTGGCATTATTATAATCTTAGGTTAAAGGGTTTTATGACAGGCAATTAACGATCAGAAT
GTATAGCATGCGTCTGGATCCATACCATTTTCATTGCCTTTTTATACATTTGTGCCTTTTGCGGCTGCAAGC
CCACCGCAGGCATGCCAAAAAAACAAAGACCGTGCCAGTCGCCCAAATTTACGCACCCATTGATAAAAAT
ATCTCTGCCTCACGACAAACCCTTGATCAGCTCATTCGCACACATTCGTGTGTGTGTATATGTGTGTGTGT
AGAGCTCGAAATTCACAAATGCCAAAAACTTCAAATTTTGTCAAATTTGCATCGGGCAGGCAGGCAGGCA
ACCAACACGCACACAAGCGCATTAATGCTTCCATCCATATAGAAAAGTGTGTTAGTGTGCGTGGTTGGCC
GACCCAAAAAGTTATCATGCACACATGCCAGCGATCAGGTACCCTTACCCCTACAACAAGCATGGAAAAT
AGCAATTTTGCACATGAACAGGCCAAACCCTTTAATAATTTCACACAACTGCTTACTTGAAAGAGTGTGCG
TCAATTTTTGGTCTTTTCTTAAATTCTTCGACAAGCCGACGATGAATTACCCTTGCAGAAATACGACTTAG
ATATTTTTGTATTTTAAGCAAAATAGGTCCCTACAGTAAACAGAAAATAAATATAAATATTTTATTTAGGT
CAATTAATTGTGTATTAAATAAAATTTGATCAAATTCTTCTCATCATGCCTGACTGGACGAAGAAATACAT
GATAGAGCATTTAAATGTGAGTGCCTATTGCTACCACAGGCACAAAAGGCACACAATTGAAGAAATAACC
TCTGGCTCGCGACAAACGCCGCGATCTACTTGTGCGCACATCCACACAACACAAACGTATGAATGTTTGT
AGATCTCGGGGTTTTGTGGGAAGCTATGCAGGGAGAAAGAGATGGACGCAAGTGCGCGAAGCAATGAGT
GAGAGCGAGGCACATGCATACACACATATGTTTTTGGGCTGTAAGGATTTAAAAATGGCATACATACATA

TATTAAGAAAACGCATCAGCCGCGACGTTGTGCGCTGAGCGAGATGCGGCTTCATACTGAACGTTCGGCT
TTTGAGGCCGGCGTTCGGCTGCCACTCTCAGCAGTCTTACTCTGTCTGGGCACACTAAAATGGTCGCAGGT
ACCATTGGGGTCGCCTCGTTTCGATTTTTCTTGTTTCGATTAAAAGTGCCATTTGGTATGTCTATACATTGT
ATGGTTAGTGCT
>9_rnd-1_family-209__Unknown
CATCCGTTTACTGTTATTACCGACCACGCATCCCTTAAATGGCTCATGAATCAAGCAGATCTCAGTAGTCG
TCTAGCTCGATGGGCTCTCAAGCTTCAAGGATATACATTTTCTATACAACATCGGAAGGGCAGTAAGAAT
ATTGTACCAGATGCTTTATCCCGTACTCACACGGAGGATTTATCGGCTTTAGATGTCGATGACTTTATTGA
TATGCAGTCTCCATATTTTAAATCCCAGGGTTATGAGTTGTTGAAGTCTAAAGTTTTGCAGAATCAGACTC
AAATGCCGGACGTCAAAGTTATCGATGGCTTTATTTATCGAAGGTCCGAGCATGCCTCAGGAGACAGAGT
TGCGGATAACATGGTTTGAAAATTATGGCTACCCAAAGAGTTGGTTCCCCATGCTTTGAAATTAGCTCATG
ATGATCCCTTAGCGTCGCATTGTGGAATGAATNAGACACTAGAGAGATTACGACGATATTATTATTGGCC
AAATTTAGTTTCAGACNTGAAAGAGTATATTAATAAATGTGAAATTTGCAGAGCGACTAAACATCCGGAT
CGTGTTTTGAGACTTCCTATGGGAAAAAGTGGAGAAACTCATCGATGTTGATTATCTTGGACCGTATCCCC
GTTCCAAGGCTGGTCATATTGGCATATTCATTGTGCTCGACCATTTCACAAAGTTTCCTTTTTTGAAACCTG
TTCGCAAATTGACCGCAGATGCTATTCTACCGTTTTTGGAACAAGAGTTGTTTCACTGCTGTTCAATGGTG
TTCAATTTAAATCACAAATGTTTAACGATTTACTTCGAAAGTATTCGATACTCCACTCGGTTGATCCCGCTT
GCTACTTCTAGAACGTACGAATAAGCAATAGAGTCTTAACCAGCGCTCACAATGGACGTCGAACCGATTT
CGGACAAGTTATAGCCCGTCGAAAATCGATTGATGTGTGAAATATATGACGAACTGCAGATTTGCGTTTC
CGATCCCCAGACTTTTTTCTATTCAAATATTAGTTTATTTTCAAATCTTATTTGAGAAAACTAACCTCTGAC
CCAGAATCTAACAGAGTCCTACCCAACTGGTAGCAAGCGGACAGATCCATGACACGAGTCCCCAGTGCTG
CCAATATGACTGGACAATGTGGGCCATTCATTTCCTCTTGTTTCTTCGGGATCGACCAGAAAACGACAGCT
CAGCTAAAAGGTGCCTAAACGCCTGCTGAGAGCAGTCTCGAAGTCGAACTATCAAAGAACGAAAAAAAG
CCAATTGCCAAACTGCCTTTGGCTCCAAATTCCTCTATATTAATAAGGTATTGACCAAATTTTCAAAAAAT
CTGATAAAAGTTTTTATGTTGGTCATCAATTTCATATAACTTTGCCAATATCAGTTTAAAATATCTTCTTGA
TACAGCTTGGTCCAGCCATGGTCATTAATTGTGTCGTTGTGTTGATGCAATGACTCTTTAATATCTGCTCTA
GACGCATACGAAGGGTAAATTACGTCCGAAGGTACCAGCAATATAACGAATCAAAATTCTCAGCCTATAG
GGGATAAATATTGCATGTACAAACTTTTATAATACCCAGAATATCAAACACATTTTTATGTGGGAAGTAG
ACAATTCGCTTTGTTAGTAATGACACCTTTTGCATAGTAGAGCTTTCATCTTCTTGCAGGTATATTTAAATT
AGTCCAATTGTGCAATTGTGACAGATAATTAAAAACTTAAGAATGAAGCAATACACTGAAACAAATAGTT
ATGTATATTTTAGATTACCGTTGTTACATTGTCAGAAATAAAACCATAGATCTAAGCACATTGCAGTGTCA
CAAATAAACTATGAATTAGAAGAAATTCTGCACGACTACAGCGTCCTGAAATCTCATGTAGTTTTTCACAT
GGTATTTGGTAGAGTGGGGATTTCTAGTTCAATTTGCTGAATAAGTTCACTCGTTCATTTTCTGGCCGTGA
ACTAAATCGGTCATTNAGTTCACTCGTTCAGTTATTCAGTCGGTCATGAACGATGAATTCTGCTCAGCTAT
TCAGTCGGTCATGAACTATTCGCTTAGTTCAGTTATNCAGTCAATCATGAACGATTATATTTCTGTTCAGTT
ATTTTTCANGTTTTCAGTTGCAAAANATATGTTATACTTTTTACTTATTATAGTGTACCATTGATAATAGAT
ACAAATCAAAAGTGAAATTAAAATCCCGCTATCTTTTATTGTTTATTTTAAGAACTGAACATCTCAAAAGA
ATGTATCGTTCAGGAACGGCTGAACTGCTGAGTTGCATACANCGTTCATGACCGACTGAACGAGTATCAA
AAGTACAGAATACATCGTTCATGACCGACTGAATAGCTGAACGAGTGAACTAAATGACCGATTTAGTTCA
CTAATCACTGAATNAGTTCAGTAGTTCATTTTAATGACCCGTTCAATCGAACTTGGTCTGAACTAAACGAC
ACAACTCT
>160_rnd-1_family-49__RC___Helitron
TTTTTATACCCTTGCAGAGGGTATTATAATTTTGTCGTGAAATGTGTAACGCATAGAAGGAGACATCTCCG
ACCCCATAAAGTATATATATTCTTAATCAGCATCAACAGCCGAGTCGATATAGCCATGTCCGTCTGTCCGT
CTGTCTGTTTCTATGCGAACTAGTCCCTCAGTTTTAAAGCTATCTTAATGAAACTTTGCAGAACTCCCTCTT
TCTGTTGCACGCAGCACATATGTGAAAACCAGCTGGATCGGACCACTATATCATATAGCTGCCATAGGAA
CGATCGGTCGAAAATTAAGTTTTTGTATGAAAAAACATTTTGTTTATCAAGATATCTTGACCAAACTCGGC
ATTTATTAGTTTTACTTTACTCCTCATATATGTGCAAAATCCTATTAAGATCGGACCACTATATCATATAGC
TGCCATAGGAACGATCGGTCGAAAATTAAGTTTTTGTATGAAAAAACATTTTGTTTTTCAAGATATCTTGA
CCAAACTCGGCATTTATTAGTTTTACTTTACTCCTCATATATGTGCAAAATCCTATTAAGATCGGACCACTA
TATCATATAGCTGCCATAGGAACGATCGGTCGAAAATTAAGTTTTTGTATGAAAAAACATTTTGTTTNTCA
AGATATCTTGACCAAACTCGGCATTTATTAGTTTCACTTCTCATATATGTGCAAAATCCTATTAAGATCGG
ACCACTATATCATATAGCTGCCATAGGANCGATCGGTCGAAAATTAAGTTTTTGTATGAAAAAACATTTTG
TTTNTCAAGATATCTTGACCAAACTCGGCATTTATTAGTTTTACTNTACTCCTCATATATATGCAAAATCCT

ATNAAGATCGGACCACTATATCATATAGCTGCCATAGGAACGATCGGTCGAAAATTAAGTTTTTGTATGA
AAAAACATTTTGTTTTTCAAGATATCTTGACCAAACTCGGCATTTATTAGTTTTACTTTACTCCTCATATAT
GTGCAAAATCCTATTAAGATCGGACCACTATATCATATAGCTGCCATAGGAACGATCGGTCGAAAATTAA
GTTTTTGTATGAAAAAACATTTTGTTTATCAAGATATCTTGACCAAACTCGGCATTTATTAGTTTTACTTTA
CTCCTCATATATGTGCAAAATCCTATTAAGATCGGACCACTATATCATATAGCTGCCATAGGAACGATCGG
TCGAAAATTAAGTTTTTGTATGAAAAAACATTTTGTTTTTCAAGATATCTTGACCAAACTCGGCATTTATTA
GTTTTACTTTACTCCTCATATATATGCAAAATCCTATTAAGATCGGACCACTATATCATATAGCTGCCATA
GGAACGATCGGTCGAAAATTAAGTTTTTGTATGAAAAAACATTTTGTTTTTCAAGATATCTTGACCAAACT
CGGCATTTATTAGTTTTACTTTACTCCTCATATATATGCAAAATCCTATTAAGATCGGACCACTATATCATA
TAGCTGCCATAGGAACGATCGGTCGAAAATTAAGTTTTTGTATGAAAAAACATTTTGTTTTTCAAGATATC
TTGACCAAACTCGGCGTTTATTAGTTTTACTTTACTCCTCATATATGTGCGAAATCCTATTAAGATCGGACC
ACTATATCATATAGCTGCCATAGGAACGATCGGTCGAAAATTAAGTTTTTGTATGAAAAAACATTTTGTTT
TTCAAGATATCTTGACCAAACTCGGCATTTATTAGTTTTACTTTACTCCTCATATATATGCAAAATCCTATT
AAGATCGGACCACTATATCATATAGCTGCCATAGGAACGATCGGTCCAAAATTAAGTTTTTGTATGAAAA
AACATTTTGTTTNTCAAGATATCTTGACCAAACTCGGCACTTATTAGTTTTACTTTACTCCTCATATATGTG
CAAAATCCTATTAAGATCGGACCACTATATCATATAGCTGCCATAGGAACGATCGGTCGAAAATTAAGTT
TTTGTATGAAAAAACATTTTGTTTNTCAAGATATCTTGACCAAACTCGGCATTTATTAGTTTTACTNTGCTC
CTCATATATATGCAAAATCCTATTAAGATCGGACCACTATATCATATAGCTGCCATAGGAACGATCGGTCG
AAAATTAAGTTTTTGTATGAAAAAACATTTTGTTTATCAAGATATCTTGACCAAACTCGGCATTTATTAGT
TTTACTNTACTCCTCATATATATGCAAAATCCTATTAAGATCGGACCACTATATCATATAGCTGCCATAGG
AACGATCGGTCGAAAATTAAGTTGTATGAAAAAACATTTTGTTTATCAAGATATCTTGACCAAACTCGGC
ATTTACTATTTTCCCGGTACTTCTTAGATAGGGGCAAAGCACTATGAGCATTATGCAAAGGTTGGGTCTGC
AAGGGTATTAGATCTTTGGCGGGCCGAAGATAGCCCTTCTTTCTCGTTA
>Ref_dvir_413
TATTATAATTATAAGAAATATTAAAGCTTTAATTTTTTTTAAAACCCTAATTGGTGAGTTGTTTTACCCGTT
TCAGGCTGCCTACTCAAAAGAACGCCAGGACGTATCTAGTGGTAGTAATCATGTAAGCAGTAAACTAGAA
ATTAGCAATAATATTAGTGATATGTCTAGCATAGAAAATAATAATTCTAATTATAGTCTTAATGAGCCTAC
AAATTGTCCGTCTGAGTTTAATAATGACGCATGCTTTGTCGAAGCGACATTAAAATGCTTTCGGAAAAAAT
ACCACAATTTTCATGATAGCTTAAACAGAGAAGTTCAGTTAGTTAGTAATTTGAAAGGATGCCGCGCAGC
ACTTTCAGGTTACAACAATTTGGATACTGAAATGAGTATCCAAGAATTTATGGCCACGTTAAATCAATTGT
GTGCGATTTAAAAAAAAAAAGCATTGAACACATTATTCGAGAAGCATACACTTTTCATAACAATGCATGT
GTTTTGTACTTTAAGTCAATTAGATTTAACAAAAACTCTTTTGCTGCATATGGTTACTGCAAAGATCCGTC
ATGCAATTACTTTTTGTTCCATGGGACATTTGACGGTGTTGTAAGTGTATACACAAGCAGCCAACATATCT
CCCATACAATTGGCCTTAAAAAGTCAACGCGATGCAGAACAAAAAATAGATTTATAGAAAAGGCTTCCAT
TAGAAACTCTTCTGCGTTTGAATATAGATCAAAAAAAAAAAAAAAATCAATATGTCTCGAGCCAGAGTTG
GTAATGATCAGATTACTAATGAGAAACTACCCTTTTATAGAAATTTAATTTCGGAGGAAAATTGACTTTGA
CAACTCACGGATAAATCGTCCTTGCTTTAAGCGTTTTTATTATTTGTCTTTATAAAACTATATTTAAGCGTT
TTAAAGAATGATTCTATAGGCCCATTACTGGGTCTTTTATTAGATTTATCAATAAAAGTAATGATAATATT
GGTTCAAAAAGGTGCGTAAACAATATATTTATTTAAAATTAGATCCAGCATTTCTGCACTTAAATAAATAT
TTGACGTACAGAAGTTGCTTTTATATTGTGATACAAGTTCTGTAACTTCTGTACGTATTTCTTGAAGATTAA
GTTTAAATGGACTATTTTCGGCAATGGACAGCAACGTTGGATCATTGACGACTTCCTTACTTACTCCCAAT
TTTATACTGTCTATCAAATAAAATGTGGATGGCTCTTCACAATATTTAGTAAAATAATTAGCGGCATCATC
CACAATATTATTAACATATGGAGAAAGAAGTATTATTGCAATATTTCGGAACCATTTTTCTAATTTTCCAA
TATTATTTATGGAATATGCACAACCCAAGGCATTTCTTAAAAATGATTTAAGATCAATATTGTCCAAACCA
ACTACTTCATCTATGTTGCGACACTTAATTTTGCTGTAGTGGCTCATACAAAATTAAACCCCACACTTTTGG
AAATGTGCGATTTAAATTATACAAAAAACTAAAACAAATTTCATTGTACGTTGCAAGATCAATGTTGTTAT
ATTCTTTTAAAATGGCTCCAATTATTGCAAAACTGACATCAGTTATAATTCTTTTGCAAATAGGCCATTTG
AATTTATTTTTTATGCAAAACTCGCGCAGCTCACATAGAAATCTGCCTATATCTGACGATTTATGATTGGA
GAGAATCGCATGTGCGACAGGAAAAACGATTCGAAGCTCTGGAATATAAGCAACCAATGAATATAAAAA
TGTACATTTTTTGATCAAGTTCATTGGCTGTACTACATTCCCAGTCGCATCAATATGCAGTGTCTGTCCCAT
AAAAATATTTCTAGTTTCATAGAAAGTAACTGAATAAAGTTGGCATACACATTAGTAATCATATCGAAGG
TATCTTTAAATGGATCTTTGTCAAAATCAAATTTTCCATTTTCCTTCGAAATTAAATTTCTATAAAAGGGTA
GTTTCTCATTAGTAATCTGATCATTACCAACTCTGGCTCGAGACATATTGATTTTTTTATTTTTCTTTTTTGA
TCTATATTCAAACGCAGAAGAGTTTCTAATGGAAGCCTTTTCTATAAATCTATTTTTCGTTCTGCACCACGT

TGACTTTTTAAGGCCAATTGTATGGGAGATATGTTGGCTGCTTGTGTATACACTTACAACACCGTCAAATG
TCCCATGGAACAAACGTAATTGCATGACGGATCTTTGCAGTAACCATATGCAGCAAAAGAATTTTTGTTA
AATCTAATTGACTTAAAAATGCTTCTCGAATAATGTGATCAATGCTTTTTTTAAAATCACACAACAATTG
ATTTAACGTGGCCATAAATTCTTGGATACCCATTTCAGTATCCAAATTGTTGTAACCCGAAAATGCCGCCC
GGCATCCTTTCAAAATTACTAACTAACTGAACTTCTCTGTTAAGCTATC
>9_rnd-4_family-489__LINE___R1
GACTCACTGCGAAGCCCGTGCAGACCATTCCTGGGGTGTCGTCTACAGTTCCACGGTGGTGTCAAAACCC
AGCGAAGCAGGCGGTGCTCACGCATCAAAAAGACAGCCCCTGCAAACTCCAACTTAGCTGGCCATAATGG
CAACCCAGAGAGCTGGACAGCAGTGAACGGACGTAAGCGAACGCAGAAGCGTCCCAGAGCCGACGCCAT
TACAGTCCACGGAGTGGGTGACTGCACATATGCCGATATGCTCAGGCTCGTGAAGAGCGACCCGGCCCTC
AAGCACCTAAGTGGCGATGTGCATGGCATTCGCAAGACCGCGAAGGGCGATCTTCTGCTGCGCCTCAATA
GGAAGCCGGAGCACTCCGCAGATGAGCTTCAAGAAGCTGTGGGAAAGGCATTGGGTGACCGGGCGGTAG
TAAGAACGCTAACCGAAACGTCCCAGGTGGAGATCAAGGACCTGGATGAGCTGGCGACTAAGGAGGAAG
TGCTCGAAGCGATTAAAAGTGCCTTACAAGACGACTCCTTGCCGATTGAAACGATCAAATCGCTGCGACC
AGCATTTGGCGGCCAGCAAAGGGCCACAGTTACTCTCCCAGCGCCAAAAGCCAGGACGCTTGTCGAGAAG
GCCAAAGTGAGGATCGGATGGGTTGTCTGCCGTATTCGTGCAATAGACCAACCAAAGCGATGCTTTAAAT
GCCAGGGTTTTGGGCACATTGCGGCGAACTGCACCAGCGGAGACGATATGAAAGGCGCATGCTTCAGATG
CGGGGACACAGGTCACGAAGCAAAGAGCTGCTCCAAGCCACCAAAATAGCCTTTGCATACGGAAAGGAG
AAAAATCAACGGACCACGCTACAGGAAGCTTTAAATGTCCGGCGTATCGCGAGGCAGTACGCAAAATGA
AATGTTAAGGTTACTCCAAATAAACCTTAACCACTGCATAGCTGCTCAAGCCATCTTGGAGCAGACTGTA
GGGAGATGCGCATTGATATTGCCATTATCAGCGAACCCCACCACCGAGAAACCAACTGCCCGAGAATATG
GACCGAGGATCAGGATGGAAAAGCTGCGCTCTGGGTATGTGGCGACCCATCGACATCGCTGACAAAAAC
AAAAGTCCTTTACTGGGTTTGTACGCGCACAATACGGCAGCCTGTGGATATACAGCTGCTACCTGTGGCCC
CAAGCATGCAGCTCTGCGAATTTACACAGCTGCATTAGATGCATTAGTAGACGACGCAGGGGGCGGAAGG
TCATTATTGCTGGTGACTTCAACGCCTGGGCAGTGGAATGGGGCAGCCAAGCAACTAACGCAAGAGGTCG
TGCCCTGTTGGAAAGCTTCGCAGCCCTAGATATAGCCCTGCTAAACTTCGGCTCGGAAAATACCTTCACTC
GAGGGTGGTATCGGATCCATAATCGATCTTACCTTCGCAAGCTCGTCACTTTTAGCGCAACAAAATGGCA
ACTAAGCGACTTATACACCGCAAGCGACCACTCCCGCAATAATATGCACGATTGGTGAAGGAACTGCAGC
CAGCACTAGAAGCCTAAGTCGGAAAGCTTACCGCCTGGATACTTTCGACCCCGTGGCCTTTCGAATGCAG
TCCACTCGCTTGTCACTGCCGGCAACGCAGAGAGAAGAGCCTCCCAAATGGCATGCCAGCTATCGCGTGC
CTGTGACGAGAGCATGCAAACCCGGAAAACGTTTCGGCGCCACCATGTACCCACGTACTGGTGGAACGAA
AATATTGCCGAAGCACGGCGGAACTGCATCCAAGCCAGGAGGAGATACCAGCGAGCCCGAGGTAGACCC
AACTACGCCGAACAAGGCAGCCTTCTGACCCAAGGTGAGCTGAAGTTGATTGTGGAAACGCTATTCCCAA
TCAGACCCGACGTATTACACACGCAAGCCATACCATCGAGCCACGTAGCAGCCATGCCGTCTCTCATCGA
CGAAGTCTCAGTCGAAGAGGTGCTGACGATCGCCAGAAGCCTAAAGCCCAGTAAGGCACCGGGTCCGGA
CGGAATCCCCAACCGAGCACTGAAACTAGCCATGTCCTTGCAGCCCGCGCAGTTCGTGCAGCTTTTTAACA
TGTGCCTAATGGAGAGAACTTTCCCCACAATATGGAAACTCCAAACGCTAGTTTTATTACCGAAGCCTGGT
AAGCCGCCCGACGACCCATCGTCATACAGGCCGATTTGCCTCCTGGACACGTTAGGCAAAGTGCTGGAGA
AGCTAATCTGCAACAGGCTTCAGGTAGCTGTCGACAACAATGGTGGCCTGTCCCAAAACCAATTTGGTTTC
ACCAAGGCAAAGTCTACAATAGACGCTCTGGAGAAAGTGGTCGGCATAGCAACAAACGCCATCCAAGGA
TCGAGATGGAAAAGCGGCACAAAAAAGTACTGCCTCATCGTCACGCTGGATGTTAAAAACGCCTTTAACA
CAGCCAG
>9_rnd-5_family-1950__LTR___Pao
CTGGTGCTGCATGCGTAGAGAACCTTCAGACAATTAAGTCTGCAGCGTCGCAAATACTCGAAAGCGCAGG
TTTCGAACTAACCAAATGGTTTTCGAATTCTCCTGAGTTCTCCGCAACAAACGCAGCTAAGCCGATAACGA
TCTCAGAGTCTGACACAACCAAAACACTTNGCGTGTTGTGGTTGCCGAATGAAGATGCCTTTCAATTCAAG
ATCGACGATTCATTCATGGGTCTTAGAGCGACAAAACGGAACGTTCTCTCGGTGACACGCGGCTNTTTGA
CCCGCNTGGCCNGTTGAGCCTGATAATGAAGGCAAACATTTTACTCCAAGAGCTCTGGCTTCAGAAGTTA
GACTGCCGCCGACGCCTCAATAAGAGCGTTTGGCGCATGCGTATATATACGCAGCCATACTGCAGAAGGT
TGTCGTATTTCGTTGTTGACATCCAAATCTAAAGTAGCCCCCCTCAAGACGAAGACTCTTCCCCGACTTGA
GTTGTGCGCGGCTCACCTTCTCGCAGATCTTTGTCATCGAGTCAGAAATCTCATAAGATTACCGATTGAAC
GGACGGTTCTTTGGTCTGATTCTGAAGTCACGCTTCATTGGATTCGGTCACATCCTTCGTCTTTGGCAACAT
TTGTGTCAAATCGAGTGGCTGAAATCCAAGAATGGACAGAGAAAGCTGAATGGCGCCATGTACCTACAAA
GCAAAACCCAGCAGATATTGTTTCAAGAGGCTGCGATGTTGATGAATTACTTGCGTCGATGTGGTTCGATG

GGCCTTCATTTTTGAAGGAACCAAGAGAATCCTGGCCGGTTAACCAGCATCTGATGACAGATGAGATGCG
AACTCTAGAAGTACGCAAAACAGCTGTTGGACTAGCAGCTATGGTAGACCGACCAGATATCTTGGAAACT
ATCGATAGTTTCTCATCGCATGCCAGGCTGCTGAGAGTGTTTGCTTATGTGTTCCGCTTTATTNAAAAATGT
AAAAATCGAGAAGTACAATTTGAGAGTATCCCTACGGCGCTCGAAAGGAAAGAAGCTTTCGAGAAAATA
GTTGAAGTCATCCAACGAGTTGAATTTAAAGATGACAAGGTCATAGCCTGCAGCGTCTCAATCCATTTATA
CGTGAGTAATGATCTTGGTGCTCCTTTTCGTTGTTGCGAGTAGGGGGACGACTTGTAAATTTCATATGACG
CCAAGTTTCCGCTACTTAAAAAAAGCTCGCAATTCGCTAGAAGCTTATTAGATACCTGCATTTGACGAATT
GTCATGCTGGCCCAAGAGCACTCGTGAGTCTTCTTCGTGAGCGACTTTGGCTAATCAACGCTCAAGAGGTC
TGTCGTCAGACAGTGAGATCATGTATCCGTTGCTTTAAATGCAAACCTCGTCTGCTAACACAAATTATGGG
AAATTTACCAGCAGATCGACTCCGAGCGCTCCGCCCATTTNCTATCTGTGGCGTCGATTTTTGTGGCCCAA
TTAGCACGACCTTTCGAATGCGTGGAAAGCCTACGTATAAGTCGTACATAGCACTGTTTGTTTGCTTCGCC
CCCCAAAGCGGTTCACTTAGAACTAGTATCTGACTTATCCACTAATACATTTATAATGGCTTTTCAAAGGT
TCGTCGGCCGTCGAGGAGTCCCACAGACCGTCCATTGCGACAACGCGACGAACTTCNTAGGAGCAAGTCG
CCAGTTCCTGGAGTGCGAGCTCAATCGGGGAAGAGGCGGACGCAATTCGCGAATTCGCATCAAAAACGG
ATGCGAACTTGCGTTCATACCGCCAAGAGCTNCGCACTTCGGGGGTCTGTGGGAGGCAGGTGTGGAGTCT
GCAAAGCACCTACTCCTACGGACGGTAGGCAGCCTGCTGCTAACGGCCGAGGAGCCGCAGACGACTCTTG
TCGCAGTGGAAGCCGTCCTCAATTCCAGGCCCCTGGGAGCAATAAGCGAGGACCCAAACGACGGCGAGG
CTCTAACGCCAGGCCACCTACTGGTGGCGGGGCTCTGCCAGCAGAGGGGACCCCGGACCAGCAGGGTCTC
ACCTGNTTGAAGGATCAAAGGAGTATATGCTCGGACTCCAAGTGCGGAGCAAGTGGCACCGTCGCCTGCC
AAACGTNGTCGTGGGAGAGCTGGTGGTCGTCGCCGAGGANAATCTCGGCCGCCCCAGCAGTGGCTGATCG
GCAGAGTGACGGCAGTGCACGCAGGAGAGGACGGCGTCGTTAGAGTGGCAGANATTAGGACGGCTAGTG
GCGGAAATTTTAAAAGGCCGATCCACAAGTTGGCTCGGCTACCCGTGGAATGAAGCCCCCAAGCGCTTCA
TGGTGGCCGGTGTTGGCGCAGATCCGCGCATTGAATTAAAAAATGAAAANGAAACATGAAGTCTAGTGTA
AATTGGNTTTAGGGCGGAGCGAGACCGCAATAAAGCTTTA
>9_rnd-1_family-321__LTR___Gypsy
TGGAGGACCTAGAGGAGCGAGCCATTACGTACGGTATCGCCCTCGATAGATTGCCCCGGGTAATGAGCGA
GGTGTTCTGCGACAGGGCCGCTCGGTGGTTCTTGAGCAGTGGGCTTAGAGATGTGCCATGGTCGGTGTTCA
GGAGGGGCTTCTTGGAGTTCTTCCTGCCCCCACGCTACTTCGAAAGGCTTGAGGACGAAATCCGGTCCCGC
CGGCAGCGCGTGGGGGAGGAGTTCAAGGAGTACCTGATAGAGCTGAGAGCTCTGATGCATCACGCCAGA
TACGAGCCGGCGCAGGAGTTGCACCGGATCTACGAGAACGCCGCTCCCGAATACAGGCTTTACGTGCGAC
GGCAAGATTTTTCCACCTTGTCCCAGCTGACTCAGCTNGCNGTGGAGTACGAAACGGTGATGAGACAGCG
AGCGGAATACTCCGGAAGCAGGGCAGGGAGTCAGGTGGTCGCGAGCTCAAGTAGCGCGGAGTGGGAAAG
GACGGTACAGCCCCCGGGGACACCTTAACCCATTCCGGTCTACTCCCATCGGATCCGGAGCGATCGTTGT
GGATAGACAAGTCTCCGCCCCGCCGGCAACGGCCCCTGCGGAAGTGGCCCACCGAATGATGAACCAAGA
GGCCGGAACCGGGTATGATCCGCGACGAGCATGTCGCAGGTGCGCACAGGAGGGGCACTTTGCCAGGGA
ATGTCCCAATGCGCCCGTTTTGTTTTGTTGGGTATGTGGGGCACCGGGGCAGAAGTAGCCAGGATTGTTGT
CGCCGCACGGAGGCGGGAAACGGGCAACGGCGTCGCCCAGGTCAGGGTCGGGTCGACGCCGACACACAC
GGGACTCGGTAGTAGTACACCCGCTTAAGGTGGAGGGGTGGCCGGATCGTCGCCACGGTGGAGATTGGCG
GGCGGGCGATGCCTGCTACTATCGATACAGGGGCCACTCGCAGCTTCATGAGTGAAGACTGCGTACGCAA
GTGGGCGATTCGGGGGAGAGTGGAGGACGTACAGGCGCGCATTCGTCTGGCAGATGGGTCCACGCTCGA
AGTAGGGAAGGCCTTGAGGGTAGACGTCGGCCTAGCGGGGAAGGCGGTCAGCATGTTAATGCTTATCATG
CCCGCAATGCTGGACCACGTCATCCTGGGGATGGACTTCTTGGGTGCGATGGGCACTACGATACGCTGCG
GCGAGGCGGAGCTGGTCCTAGAGGCGGGGACGGCACCGGCCGCGGGGCCGGGAACCGGGAAAGAGCCG
AAAGAAGGCCTCCCGCGCCANGGAGCGGGAGCGCCTGCTCGGCAACCGATGGCAGAGGAGACGAAGAA
GGAACCGAAGGAAGGCCTCCCAGGTCATGGAAGGGGAGCACCTGCTCGGCAACAGAGAGAGGACACCAA
GAGGGAGGAGGAAGGCCTCCCAGGTCANGAAGGGGGAGCACCTGCCCGGCAACCGATGGAAAGGGGAA
CGAGAAAGGGANCGTCGAAGGGANCTCACGTAGGATTGGACCAAGGCGGCGCGTAGACTTTCAGCGGCA
GGTCTACGCCGGTCACGGAGGCGGCACGGAAAGGTGCCAGGTAACGGAGGGANTAGGAGCAATGGCTAG
AATCCATACGGACACGGGGGATCAGGAGCGACTGCCAGAGGGGAGCGAGAGGGGGTTGTGGTGCCCGGA
GGAGGCGAGACGCGGGGCGAGCNCAGCATCGAAGAGGCAGAGTGGCCAGCGGATCTGGATCCGGAACTT
AGGGAGTTCCTGGAGTCGGAACTGGCACTCTTCGAGGGGCTGCAGGGAGTCTCGCATATCGCGGAGCACA
GGATCCGGCTGAAGGACGACAAGCCATTAAAACAGCGATATTACCCGAAGAATCCGGCCATGCAGAGGG
TGATTGACGAACAGGTAGATGAGCTGATCCGGGCAGGGCCATCGAGCCGTCGCGTAGTCCGCACAGCGCC
CCAATCGTCTTAGTCAAAAAGAAGACTGGCGAATGGCGCATGTGCATCGACTACCGGCAACTCAACGCGC

ATTCCGTCCCAGATGCTTATCCTGTTCCCAGGATTAATCACATCCTTGAGCGGTTGCGTCACGCCCGGTTC
ATCTCCACTCTGGATTTGAAGCACGGGTACCGGCAGATACCGATGGCAGCCGATAGTCGGGAATGTACGG
CGTTCACGGTCCCCGGAAGAGGTCTTTTCCAATGGCGGGTGATGCCATTCGGTTTACACTCGGCGCGTGCG
ACCTTCCAGCGAGCCTTAGACACGGTCATAGGGCCGGAAATGGAGCCTCATGCGTTCGCCTACCTAGACG
ACATCGTGGTAATAGGGGCAACGAAAGAGCAACATGTAGAGAACTTGAGGGAGGTGTTCCGCCGGTTAA
GGGCGGCCAATCTCAGGCTGAATAGNAAGAAGTGCAGTTTCTTCCGGGAGAGGTTGGCTTACC
>9_rnd-5_family-1958__LTR___Pao
ACTGTGCTAAAAATTGAAAGCCCAACTCTATTGGTTTGGTTGCGCTAATGAAGCNAACGCTTTCAATTCNC
GGCACATATGTGAAGTAGTTGAGCATAATTAAAATTTGCGCGCACTTACTAACCGCCTTGGANATTCCTAC
ATATAATTTAGTATTCGTGCGAATTAGTGCTTCTGAGCGAATGATAATTAGTGTCGAAAATTAATTGTCAC
TNCAATTTNATTTCGCTTAGCTTTGTCGTTCNCTCCTTCTTTCCATCACTCGCAACCACTGTNAATTATACA
GTTGCCCGAGACCAAGCGCTTGTATATAAGTGCAGTACACGCGTACATAGCAAGATCGGTCAAGAGACGC
TCGCTTCCCTACGTTTNTTGATCAGGCATATCAGTTTTTGAATTCGTTTGGCAAATTCGATTTTAATTTAAA
TTAATAATTATTAAATCGAATTTAGAGTCAAATATGGCTACTAAGGAAAGTTTTGACGATTCAAAAAAGA
AACGCAATATGTCCGTTAAAAGCATAATGCGTTTAAATGATCAAATAATAAAGGGTAAATTAAACCTAGA
GTCTACAGAGTTAGAATGTAGATTAGAAATGCTGGCCTCACATATAGATACAGCGTTAAAATATCAGACT
CGAGTAGAAACTTTTGACCCAGATGATGAGTATCGTGCCGAGCTCGAAGAACTTTGTATAACGACAAAGG
CTCGTCTAAGGTCCGTTATCAAGACATTAAGTCCTGCAACGCCTACAGGCATGCCCGTTGCGGCTCCACGC
ACACGNCTTCCGCATCTGGCGCTTCCCAAGTTTAGTGGAAAATACTCCGAGCTTAAGAATTTTATTAGCCT
GTTCGAAACTCTTGTTCATAACGATNATGCGATCCCGAGCATAGAGAAATTCAATCATCTGATCTCATGTC
TCTCTGGTGAAGCTCTAGGCACTGTTAGAGCGTTTCAAGTCACCGAGGACAATTATTTAAAAGCATTGGAC
AGCCTAAAGAGAGTATACGACAATGAATGTCTAATATTTAATGAAAATATTTCGGCTCTCTTTAGCCTGCC
AAAAATAACGCAGCCTTCTGCGGCATCATTGAGATCAATGATTGACACTGTCTCCGCCATCTATGGCTCGC
TCCTGTCAATAGGTGATGACAAAAAGATTTCTAACGCGATTCTCATTCACCTCGTTATGAACAAAGTTGAC
CCAATGCCAGACGAAAATGGGAGGATCAACTCGATTATGCAATCATTCCACTATGGAGTGATTGTGAAAC
AGCCTTGAATCGTAGATATCAACACCTTTCTGCTGAAGAATCAGCCAAAGCTTCGTCCGATGGCCCGAAG
CCAGAGTCAAAGGCTGTTTCCAAAAACAATAATCGAGGTTCGTTTTCGTGTTCTGTGGCTAGTGGAAGAA
ACGATCAAGTGACCCCAAAATGTTTATATTGCGGTTCCGAAGGCCATTCGATATTGAATTGCTCAGCATAT
GCCGCGTTATCAGTACAGCAGCGGTTNGAGTTCGCTAAGGCGGCTCCTTTATGCATCAATTGCCTTCGGAA
GGGCACACTGTTGCCAAATGTAAGGCAAACAAGTGCAGAGTATGCAGTCGTTCTCATAACACACTGTTGC
ATAGATACTCTGCCGTTCTAACACTCGAGCGTTACCACCACCCCTTGACTCCGCACAGGATCAACCATCGA
CTTCACATGCTATGCATGCGAATGTGTCTGATAGTGTAATCCTAGCTACCGCAGTCGTTAGCGTTAAAACG
AGAAGGGCGAGTTCGTTTTGGCTAGAGCTTTACTCGATTCTGGATCACAAATTAATTTTGTAACAGACGAG
CTAGCACAACGATTGCAGCTGCGTAGAGAAGAGGATAGTATCAGCTTACTTGGGATTGCTCAATGCAATA
CTNAAGTAAGAAAGAAGATTCATACAGTGGTGAAGTCGCGAGTCAATTGCAGTGAGTTCTCGTTAGATTT
GTGGATTTTNAAGACCGTTTCGGGTTACCAGCCTGACCATACGGTCAATGTCAGTGGTTGGAAAATCCCTG
AGAACCTGCAATTGGCTGATCCATATTTTCATAAGCCACAGAAGATCGACATGCTCATCGGAGCTGAGAC
GTTCTTTGAGCTTCTGTCTGTCGGTCAGATTAAACAGCTGACAGCTTGGAAACTCCTTCGAGTTTGCAAAA
CGTCGTTATTTGTTACTTGAAAGACGCTTGTCTCGAGATCCCGAGGTTACGCGCATGTACTTGAAATTCAT
GGAGGAGTATTTAACTTAAGGTCACATGTCTGAAATAGATGATCAGATTCCAAGTACTCCACACTACTAG
CACGTCGACGCCGTCTCAAATTGCGTTGAATGACCTAATGGTTGGCGCGACTATTCAAGAGTAGCTGAAC
TCAATTCTGGTTTTTGAATGGTTT
>9_rnd-1_family-54__LTR___Gypsy
TATATATATATATATATATATATATATATATCAAGTTCCTCTCAGTTAATACCTATTGCCACCTTTCCGCTA
CCACCGCAGAGCTATAAATCAAGTTAATAACCCAACTTATATTGCCCATGAATTTGAGCCACATTTTAAAT
GCAATTTTCTTTTTGAGTTCAATAAGATGTTCTGATGGTTCAATAAGATGTACTGTAGAACATTTTATTTAG
ATTGGTTAAGAAAATCCCATAAGTTATGTGTCTTACTGCGCAATTGGATGGGGTCAAATTCCAAGAATTTC
TGTATACATGTACACACACACATTCATGCGCATCTGCTGCGAAATCTGTCAACAGCATTGCGATTGCGATT
GTTTTTTGTGCTGATATTTTAATTCAATAAGACACAATTATTGGTGTGACTGTTGAGTAGAGGTGGTGCGG
TCTGTCGCGAATTCGAGAATTCTTAATTTGAAGTCCGATTTGTGTCGCATTATCATTGTTTTTAGTTTACAC
TCTATTGAAATTATTGTTGGTGACCCCGCAACAATGTATTTTGTAAATTACATTACTTCTATGTATTGTAGT
ATTGTTGAAAATTTTGTTGTAATGTTTTCGTAGGGTTGTGTGCTACTTTTATTTCTTGCTTGTTGTAGTATTC
TGAGTTCGTGAGACGTACTGATAATTGTGGTACNTGTATTNAAGACATATATATTTTGANAAGTTCTGTAG
GCTATTGTGTTCTACATTGTTGTCTATTTAAAAATGTATTAATTATATTCTGTGAGAAGTCGTTTTTGGTCA

AATGTTGTTTAATATCGTGTCCAATTTCTTTGTGGTCTCTTGGTTAGTGCTCTGAAGATGGTTCCCGAGATT
TAGCACTCAAGGTTATATCAGGCTAGACGCCTCTGAATTCGAAAATGTAAGGTGGTCCGAGTCAAATTTG
GTGGAGGCTAACTCCAAACAGATACTAATAAACTCCGAAACTCAAAAACAAATAAACAGACTAACGGAC
ACTATCAATAAAATTATCAGTTCCCGCAAAGGCGATTTGGTTGACACTCCTCATTTATTCGAGACCCTGTT
GGCAAGAAATAGAATACTGAACACAGAAATTCAAAATTTAGTACTCACCATAACGTTGGCAAAAGCAAG
CATAGTAAATCCTACAATCCTTGATCATACCGATCTAAAATCACTTATCGAACAAGATACCCCAATAGTTA
GCTTAATAGAAGCTTCTAAGATCAAGGTCCTTCAGTCCGAGAATATTATCCACATATTAATAGCCTACCCT
AAAGTTGAGTTTAGGTGCCAAAAGGTCTCCGTCTACCCCGTTTCACACCAACAAACCATTCTGCGACTCGA
CGAAGACACGTTGGCGGAATGCGAAAAGGATACCTTTGCAGTTACTGGATGCACCGTAACTACGCATAAC
ACCTTCTGCGAACGAGCACGGCGCGAAACCTGTGCTAGCTCCCTTCACGCGGGAAACATCGCCAACTGCC
ACACTCAACCTAGCCACCTGAAAGCGATAATGCCTGTAGATGACGGCGTCGTGGTTATCAACGAAGCGAC
AGCGCGCGTCCGAACAGATGATGACGCAGAGGTAACCGTCTCAGGAACCTTCCTGATAACTTTCGAACGG
TCTGCAACTATCAATGGCACAGAATTTGTAAACCTGCGCAAATCACTAAGCAAGCAGCCTGGCATCGTGA
GGTCACCGCTCCTAAACATCATCGGACATGACCCGGCACTAAGCATACCACTCCTGCATCGTATGAACAT
CAACAACTTGCTATCAATTCGAGATTTCAAGGAGGAGGTAGTTGCGGCTGGCTCGCCTAAATTCTGGTTCG
CGATTGGCGCGGTCCTGAATGTCGGACTAATTGGCTCATTCTTCCTTTTCCTGGTGCTAAGGAAAGACGAG
CCTCCATGCAGATGCAGAAGGCGATTGACAAATACGACATGCCCGAGGACGGCCATCATCCTGAGGGGG
GAGTTAACAACTAAGCATAAATATATTGCCCTAGCAACTAAGTAGCTTCGTATTAACAATGCTGACGCGC
CAGAACTGAGTTCAGCGCTCTGCGCTAAGAACGGCCAGCAGCGGTAAGCTGACACTGCATGGAATGCTGA
GTCGGCTTGCCGACCATGCTTTGATGGCGTGATGCATTGAGGCTTAGGGTATTTGTATAATTTGCTTGTCC
CCTAGTTTTAGTTTTATATGAGGCGCGCAACACATAAGTCGCCGAAATAAATATACTGCTCCGGCATTGC
CGATAAAACATATCTTTCGTNATNTTACTGGACCAAGGCCAGAACATATTTTGATCAAGGGCAGCGCGGG
ATCAACGCAACTTACCCAAGATAACATTGTGTTACATCGCAGAACATACATCAATGGATAACTTATAT
>9_rnd-4_family-181__LINE___CR1
TTTAATTTTCAAATTATCGCCTTTACTGAGACCTGGCTGAAGCCGGACATCTCAGACTCTTCAATTTTTACC
TTCAAATACACACTTACAGGTGTGATCGAGCTTCCCGNATCGGTGGAGGAGTACTTATAGCGGTCGAGTC
TGCTCTTTCGTCGGAACGCATTGATCTTTCCATCTCAGAGATTGAGCTGGTGGCTGTAAGAGTTGTATTTG
GAAGTTTTTCTATCTTTATTTCATGTTCTTATATNCCTTNTATCTAACTTGTCCACCTATCAAACCCATNTTA
ATGCTATTAAATCTATCTCCTCCCTACTTTCTGATAGAGACCTTTTAGTAGTTCTTGGGGACTTTAATCTTC
CAGATGTCGAGTGGTCATCGCCATCAGACTCAAACTTTCTGTTGCCGTCTTCGTCNAAACGAATTTATTGA
TGGTCTACATGAAATGTCCTTACGGCAGGTTAATCGTGTGCGTAATTCAAGAAACAGATCTCTTGATCTTA
TTTTCGTGTTGGATTCGTTCAACAGTGTTGTGCACAGGATCAATCCGCTTACGAATTTTACCAGCCCGAGT
CCCTATCAAGGAACCGGAATTAGCTGAACGTACGCGTTGTTTCCGAAAGGCTGATTTTCATAAGCTAAAT
GATCTTATTCTGCAAGTCGACTGGTCCCAACTGTACAATTGTGCAGACATTGATGTTGCCATTAAACTTTTT
TATGATACCTTGTACTCGATTTTTGATGTCTGCGTTCCTCTTGATCACCCAGCGAGGCCTGATAGGCCTCCC
TGGTTCTCAAAGAACTTATGAGGTTAAAAAACGTGAAGTCGAGGTGTTATAAAAAGTTTAAGAAATCGGG
AAATCATTCTGATTTCTCCCGTTATGCAGTTGCCCGATCGAATTTTTCCACTCAAAATTCCATCTGTTATAA
GGATTACCTTCGGCGTGTCGCCTTCAATTTTCACATAATCAGATGAGAGCGCGAACACTGATCCAGGCATA
GCCGATCTGTTCGCAAACTTTTTTCAAACTACCTACTCCTCCTCTAGGTATAATAAAAATCAGCCTTATCCT
TATCAGTTACAGAGGTCAAACTGCATCTTAAATCCCATTATTACCGAAAGTTCGCTTCTTAGCGACCTTAA
CACTATCAAGCCGGTTTACTCACCGGGCCCTGATGGTGTTCCTGCTTGCGTGCTTAGATACTGCGCCTGCA
GCATTATGTAAACCTGTTTTAAAACTTTTTGAATTGTCCGTAAACACTTCATCTTTTCCGACCATTTGGAAG
GAATCATACATTATCCCTCTCCATAAGAAAGGGAGTAAGTCCGATGTCGAAAACTATAGAGGTATTTCCA
AATTATCCGCTATACCTAAAGCTTTTGAGAGAATTATAACTTCTCAACTGCAACATTTATGTAGTTCTATTA
TTTCACCTTCCCAGCATGGGTTCGTGAAGCGTAGATCTACAACCACTAACCTTCTTGAGTTTACTTCGTTTG
TAATTGATGGGTTTAAGAAAAGAATGCAGACTGACGTCATCTACACTGATTTCAGTAAGGCTTTCGACTCT
GTCAACCACTCCTTGCTGCTTCATAAATTATGCCTTCTCGGCTTCCCAGTCAATCTTTTGAACTGGATTTCA
GAGTATTTGCGTGATAGAACCCAAAAGGTTNTTTTCAGAAATTCGTTTTCTAAACGTATTCTGGTGACTTC
CGGTGTTCCTCAAGGTAGTCATCTTGGCCCGCTACTCTTTACTCTTTTCATCAATGACCTTCCCTCGATACT
GTCTCACTNTCGAGTATTAATGTATGCTGATGATGTAAAATTATGCCTTGCATATAAAAATATGGCATCAT
GTTATCTCTTACAAGCTGACATTGATGCGTTTCAGGCTTGGTGTCTAGCCAACCTTTTAAACTTAAACTGCT
CTAAATGTAAGGTAATGACCTTCTATAGAAGCCCTCCTCAATTGGTTTCTTACATCCTTGATAGCGTACCG
CTCGAGCGTGTTAACAGTATTTCTAATGAAGTTAATGATCTAGGTGTCATCCTTGATCATAAACTTAAATT
CGACTCTCATGTTAAGACCGTGGTCAATAAGGCCAGAATAGTTATGGGTTTCATCAAGCGANGGTCGAAG

GAGTTCGACGATCCGTACACGACAAAAACACTTTATATATCTTTGGTACGACCTATTCTTGAGTATGCCTC
ATGTATCTGGTCACCACAATATGCCATCCACCAGGACAGAATTAAATCCGTTCAGAAACAATTCTTGTTGT
TTGCCCTTCGTGGTTTAAATTGGGATCCTCGCGTGCGGTTACCGTCATACTCCAGCAGACTTCTGATGATT
AACCTTCCTTCTTTGATTAATCGTAGAACCATGTTGGGT
>160_rnd-1_family-137__Unknown
TTTCATTTTGTATTTTTCAGCCAANTCTGCCACGCCCATGGACAACATTTAATCAGCAAATGATCGCTATTC
ATGGTACCTAGTTGACCTATATATCCTTGGTTATTCCATATCATGGATATAGTCCTAGCGAACCTAAAGGA
CNAAGTTCTCGCAAAATGCGTAGGAACAGGGTCCAAAAATATGTATGCTTTTCTTTTACAGCCGCCCCCAG
CAACAGCCCCGCCCCCTGGATGGTTTCTAGTTGACTAGCATATCCTTGGTTATACCATACCATCGATATAG
TCCTTGGGAACCTAAAGGATCAGATCCCTGCAAAATGTGCATTGAAATGGTCCAAAAACATGTATGCTTTT
TCTTTCAGGAGCCCTTTTACATTAGCTGCAGCTGCAGCTGTTGTTTAACAGCCGGCTTACAGCGCTCATTTT
ACATAAACTGCTANTTAACAGCACTCATTTTAATTGAGTTACATTTTTGCCCCTTAATGCTATGCAATGAA
AATTTTTATTCAAACCACTCGTCGACCTTCTGGGCATTTCTGTTCGCCTGGTATTTGAGTGGAATTTTAGGG
CAAATTTGGGGCCCAGCCGCCGACCAGGGCGCCCCTTGNGGGTATGCAATGAAATTTTTCGGCCGCTAAA
AATATTNTTCGTCGACCTTCTGGGCATTTCTGTTCGCCTGGTATTTTGGCGGATTTTAGGAAAAATTTGGGC
AGCCAGCGGCCGCCGACGGGCGCCCCTTTCGGGTATGCAATGAAATTTTTTNGGCACGCTAAAAATATTTT
TTGTCGACCTTCTGGGCATTTCTGTTCGCCTGGTATTTTGGCAGATTTTAAGAAAAATCTCGGCAGCCAGC
GGTTGCCAACAGACGCCCCTTNCGGGTATGCAATGGATTTCTAAAGACCGTTGATTTTTGAATTTTTAAAT
AAAAAAACAGGAATTTCTCTTTTATTTNAANTAATTGATTTATTTATGTTTNAATTTATAAAATTGTTGTTT
TTATGCTAATTTTCTTAATTATTTCTCTTAAAATTATACAAATCGAGCATAAATTATAATTTTAGCTTATTT
AAACTTAAAATAACTATATCTGGCTTAATTAAAGTCCGATCCTGGAAAAATCTGCTTTGTCAGTCGTGGAG
GTCCTTAGAGCTCATTGTAGTCCTTAATTTGTCAGTCTGAAAAGATAAAAACACATTTAAATTAAACATAT
AGAAAATTATATAAANGCGGAAAATAGGCCATAACTCGGGCATGTCCTTGCAGATTTTCAAAGGACATGG
TGCGTTAGGATCGTATAGACCCCCTGCATCGATCTGCATCANAAAAATATGGGGTCATCTAAGAATCATA
TGACTCGAAATTGGACTTGTAAAATTTCNGTCCCGGAGCGCACGGAAATGGCCCGAAAACACAAATCCTT
GAATATCTCCAAAACTACAAGGACGATCTTAATGAAATTTGGCCAGTTGGTAGTCCTGGTCAACACAATT
ATTTTGACATATGACTCGCCAGGATACGCAAGGATATGGTCGAGTTATGGCTTATTTTCTTCAAACAAAAT
CGTCCTTAAAACTTTTTTGTCTGAAGGACTTTCGTCCTTTGGGTTGTAAATTCTTNAATGGGAGGGCGAGG
ACTATCTACTGGCCAAAGGACATCCCGATAGTCCTTCAAATGGCAAAGTTAGCCCGGAATTTTTCGATTTT
GGCCAATTTTGCGACTAACCCCCTAGGANAAATTTCGATGAAATTTTTTGTCGAATCCTGGAAATCCTTGA
ATGCCAATCGATAGTCCTGCTCCCCACGAGTTCAAAANGGTATATCCTTCCGATATCCTTAAGGATTTGGC
CGAGTTATGGCTAATTAAAAAATGTCCTTTTTAGAAAATNGGCCATATCTCGGAAACGGCAAGGACGATT
TTGTTGTCCTTTGGACAGTTTATGGTCCTTGTTCATACGATTTGTTTGACACAGGACTTGTCAGGATCCGCA
AGGATACGGCCGAGTTATGGCTTTGTTAATTTACGAAAGAATTGTACTCATATCTAGGCTGTCTAAAGGAC
TTTCGTCCTTTTCATTATATATTCTGAATTGGGAGGATGAGGACTAACGGTTGGCAAATGGACATCATGAT
CGGCAAGGATACGGCCGATCTACAGCCTTTTATCTCTACAGACAAAAATCTCTTTTCCTCAGCTCCTTGAA
GGATTTTCGTCCTTTTTATTACATATTCTAAGTTGGCAGGACAAGGACTACATGTTGTCCAAAGGACATCA
GAATGGTCCTTAAGGTGGCCAATTTAATGTCCTTTAAAAAGACAATNCCCATATCTCGGACGCCATAAGG
ACGATTTTGATGTCCTT
>Ref_dvir_620
CATTTAGAAAGTATCCTGCAGTGAGAGCGCTAAAGTCATTGGGATCAGATGATAGCGGTGACAGCGGGCG
AGAGTTGAGTACTGCTTCTATCTCAACGACTGCAGTGTTCAGCTTCTTTGCGCTCTTGACAGCGGCCTCCC
AAAGTGTGGAGCCCTAGGTGGAATGAAATGAAAGGTTATCTCTTCAGAGGAACAATATGATAGGATAGC
ATCTTGAGTGCTAGTGTTGAACATGAACTTCTTTAACTCGAGTAGCTTGTTGGATGCGTTGTCACAATAGA
TGTGCGATACTGGTCCGGGCAACCATGCGTTTCAACGATTCCAAAAACTTATTCGTTGAAAGATCTGACCG
ATTTCAATGTGAACGGCCTTTACGGCCATGCAGACATATATGGCGATATAGGATTTTGTGGGACCCTTGCC
ACGTATTCGAAGATAGGTGTTCACTGGACCGCAGAAGTCGACTCCAACGTGGCGAAATGGCTTGTCTGTG
GATAAGCGCTCAGGTGGCAGGGAACCCATGACTTGACGAAGTAGAACTGGACGATATCTGACACAGTGT
ACACAAGAACGAACAATGCTGCGTGCTAAAGCTCTTGCGTTTATGACCCAGAATTGAAGCCGCAGCAGTC
CAACTAATGCCTTTGGTCCTGCATGGTAATTGCGCAAGTGAAGATGCCGAACATAATTGTGGACAAATTG
CGATTCCTTGGGAACAAGTAAAGGATGCCTTTGACGAGAGGGGATATCAGCATGCTCGAGTCTGCCGCCA
ACTTAATGAGCTCGTATCCAGAAGAGTCATCTATGAAGGGGCTGAGAAATTTCAGATTGCTTTTCAAATCT
CTTTGTTTCCTTAGCAATTGCAAATCTTCCTTGAAATAGTGCTGTTGGATGATCCTGATGATGGTGGCGAA
GTTCTTCTGGGCATGGCGGCGAACTTTCGACAGGGTGTTCCTTTTTGGCAATATGGTAAAACCGGAACATA

TATGCCATAACTAGCGCCCGAATGTATGAACTAGTTCCGTTCAGCCAGTCCAGAATAGAATTCTGAATTGA
TGGCAGGGCCATTGAGTCTCGGAATCACGCAGAAAAGCAGGTCCTGTAAACCAAATTGATTGCTGGAGCT
CATTAGCTGGGCATCCACGTGACACGATGTCAGAAGGGTTCAATTTTGACGGTACATGTCTCCACTTTCCA
TCTGCTGTTTGATCTTGTAAAATGGATACTCTATTTCCAACAAAGTCGACAAGGTGATAGAGTGTTGTTGC
AGCCAGTGCAAGACAATCTGCGAATCAGACCAGAAATGCGCGCTGTGCGGCTTGCCGATGAATATGTTTT
TGACAGTTGTATATAACTTACTCAGAAGTTGAGCTCCGCTTAACTCTAATCGAGGAAGCGTCAGCTTCTTG
ACAGGAGCCACCCGAGATTTAGCAGTCAGAAGCTGAACCTGGACATTGCCATACGAGTCCGTGGAACGCA
CATATACGCAACAGCCGTAAGCACGTATTGATGCGTCACAGAAAGCGTGCAGTTGTATGTCTGTGAGCTC
AGCTTGCAAGCATACAATTTTTTGGGTTTAAATTCAAATAGTAGCACATCACGACGTTGATCCCAAGCTAC
GCCAAGTGCGCTAGTGACAGCGTTTTCATCCAAGTTTAAATCCTTTACCGATTCGTCGTTCCTGACGGCGG
GATGATTGGAGTGCCACTTAACCAATGGGAAGTGTCCTCGGCGAAGTATCTCGGTGACCTCGTGTTTGATG
TTCTGCAACTCGATGAGCTCGTTTGCTCCGCATAGCAGATCATCAACATAAAACGATGATTTTACAATGGA
TGCGCCAATGGGAAACTCGTTAACATGCAGATCTGCCAGATATTGCAAACTGCGAACTGCTAAGTATGGG
GCCGAGGCCGTTCCATACGTCACTGTATTTAGCTCAAAGGTCTGCAAATCAGCATCAGGGGATGCTCTCCA
AAATATATATTGAAATTTCCGATCCACTGGGCTGACTAAAACTTGTCTATACATTTTCGTAATGTCCGCTG
TGATGGCGTAACGAACCAATCGAAAACGAAGGAGAAGTATATACAGATCTTCTTGGAGTGTTGGGCCAAC
GAGAAGCAAATCATTCAATGAATGCTGGGACGATGAAGTGCACGAGGCGTCAAACACGACTCGTAACTTC
GTGATGCACTGCTGGGTTTAAAAACACAATGGTGAGGTATATAAAAGTGTGGCTCACTTAGTTATATGGC
CAAGATCCTCATATTCTTTCATGAAGGTTACATATTGCGATTGAACAGTAGTCTGCTGACTGGAGGCGACT
CTCGAGCGATTAAAAACAACGACATGCAACATCG
>160_rnd-5_family-1391__LTR___Gypsy
TTTATATATTAGCAAATAGGGATGCAGNACAGGCCTANTGTGGCAACCTAGTGCTCGCTNGTGAACACAA
TAGAGTGAGCCCGACTTTATGGCAGTGGCTCGTAACCAACCAGGAGCGGCGTGAGTTGCCCCTGATTAGG
CAAGTCCAGAAAACATGTGTAGATTTAGTTAACGTATTGTCTCGATAGTGGGTCTGTTATCCAGAACTACT
ATAAAGATTTGACCAATATCATCTAGGTACTATTTAGAAAAGATCACAATATTTTTCATTTAATGTTGAGG
CCTTGGTAACAAGCACCTGCAGTTTCCGCATTCTCTTCTGGAGCTTGTTTACCTCTAATATGTTCGGCGCCT
AGCACACCGACTTTGGTCACAGGTCATTGAAGGATCTCGATAGCTATCCCGACGACTGTTCGTATTGTCTC
CTTGTCTTTTTCCAGGATGGTTTGCTTGATAATGATCACGTTTGCTTACAGGACGATGGAGGTGTTCGTTTG
ACAAATCACTTCATGTACCGCGCAAGATCACGCACGTATTCATACACCCAATATGGCCAGAATTGACTGA
TGAACAGTTGTCTGTCTTCCGTCGTTGGTACCTAATAGCAGCTGCGAAATTCGGTGGTGCGTGCGCAGCAA
CTTTTATAAATTAGTATCCAGTAGCATATAAGCTTTTTACCTGAATAATATAGCTCACACCTGTAAGTTAT
GTGATTTTTTCAATTGTAGATGTTGATGTATATTCAGACCCTCGTCACAAGTGCTGAGATTGGTAAACTTG
TAGATTATGTTCGGAAAGCAACTAGCCTTCCAAATCACTGGCCTTGTGGCTCAGCAATGAAACAACTAGT
AACGGACTGATAACGGCAAGTGCCGGAGCAGTATATTTGAAGAAGTTTGTTCCTACAAAGTTGGNTACAA
AACCAAACTAGAATTAAAACATTGAATTGGATCTGAACCCTAAGGCTAGGCGCATAAGTCTTAGCTGCTA
TATCCGGCAAGCCGANTGGGCATACNCCAACACTGTCGCTTGCGGCATAGGCAAGTGTCAGGTTACCGCT
GCTGGCCGCAGTGGCCGGGCACTGAACTCGTAGNTCAGCAGAATGTCGCAGTGTAAGCAAAGTGCACTGC
AACATTCTTGCGTTCTTATAAACAAAAGTGTGTTCTTATGACATAAATATGAAGTGCAATTCTTATGTGTT
GACCTACTCCTCTCTCAAGCTTTTGACCGTTCCCGGTTATTTTATAGCTGTTAATGGTTTTTATTCCACCTTC
TGTACTTCCTTCGATGCTTGCCTTCTCTTTACAATTTGCCAAACGATGTGAAAGCCAATGCCAACGACACT
GAGGATGATGCCTGCTGCAATCCAAATCTTATGAGACCCAGAGGCCTTCAATTCGTCCTCGAAACTTCTGA
TGACGCGCAAATTGCGGTTGTTCATCCTTTGTAGTTGGGGCATGCTTAGCACTGGATCGTGGCTGATGATG
CTGAGTAGAGGCGACCCTGCCACTTCTGGCGATCGGGCTAACATCTCGCGCTGGTTTACATATTTTGTGCC
GTTGATTATGGCCGAAGAGAGGAATGTGGGGAGGTGTGTCCAGTTTGTAGTAATCTCTGGACTATCGTCT
GTGCTGACCTTGGCAGCACTCTCGTTGATAATGATAATACCGTCATCGACTTGTGTGATGGGCTTTAGGTG
GTGCGTGCGGCAAAGGGCTGTGCTGCCGGCGTCTAGTGCTTGTGCGCAGGTGTCTTGCGCTGCCAATTTAC
AGACGGCGGCGTAGGTCGTCGTTACTCATTCATTGACCGCTAGAATGTCATCGTCGCATTCGGCTATGGTA
TCATCTTGTAGCTGTAGTGTGACGTGTTGGTGTGCCACCGGAAAGATGGTGATTTTCTTGCAAAAGAGCTT
GACCCTGGGATACGCTATTAATATATGGACAATGTTCTCGGACTGGAGGACCTTGATTTTAGACGCTTCGG
TTGGGTTAACTATGGGAATTTCGACAGGATGTTTATTTATAATTGATTTCAAATCATCGTGGTCAAATATT
GTGGGGTTTATTATACCCGCTTTGGCTAGTGTAATTGTAAGTATCAAGTTCGATATTTGATTTATNAGTATT
ATATTTCTTACTAATAGTGTTTCATATAAATGCGGTGTGTTTACCAATTAATTTCTTTTACATTTAATTATTT
CGTTCACCGTGTCGGTGAGTTTGTTAATTTGTGTCTGTGTTTGTGTATTAATGACTACCTGCCTATTGTTCG
CATCAATCAAATTTGACTCGGACCACCTTACATTTTCGAATTCAGAGGCGTCTAGCCTGATATAACCTTGA

GTGCTAAA
>Ref_dvir_967
GTCAAGCCAAGAAAACGTTTGAACACCTTTTTTATCGACGGGTATGGGAAAAGGTGACACCGCCTGCGTT
TTCCAATCACTTGGTTTAATAGTACCGTTTTCCAAAATGTACGCTAAAAAATGCATCTTCTGCAAAACCTA
ACACTGGCTCCAATTAATACGCAAGCCATTTCTCGCTGCCCATCAAAACTCTCTGCAACTTTTGTAAGCCT
TCCTCAACAATAATGCTTGAAATGACAATATCATCCATGTATACGACTGCATTACCATTATCCTAAGTTCA
CACTCCAGATAAGTTTGAGATACCAAATGGTACAAAAAACGCATACTGCACTTTCTGCATTACAAAAGAT
GTATATTTCTGTGACTGTGCCTCGATTGGTAGTTGAAAGTATCCATTCGTCAAATCCAATGTAGTAAAAAT
CAGTGCTCCCTGCAACTTGTCAATAACACTGTCCATTTGCCTAATCGAAAAGCTATCGCGCACTATCTTTT
CATTCAACCTGCGAGAATGACAACATTGCCGCTTTTAACGTTTTTTTTTTGCTACCAGAAAAATGGGTTAA
GCATATTCGGATGTTCTGGGTTTTATAAACTTATCCGCAAGCCACTATTGATCTTAATCGTATGCATCTTTC
TGCTTACTGAGCGCTAAGCGCCTCGGATGCTGGTGAACTGGTATCTCATTCCGTTAAAACTATCTTCATTT
CTACGGGACAAGTGACACTTTTCTTCAGACTATACCCAATTATCATCCTTCTCACTCTATTTGACTGTGTCT
GTGCTAAACCTGACAGGAAAACCTCCTCACCTGCCTCACTTTGCTCTGAGCTCAGCATACATTGGATTTTG
AATGCATTATTTAAATCGGCGCTTGAGCCCTGAGCTTTCTGATTACCATAGCTTTAATTCCTGTATTCACTT
TTGCTGACCAATCTGCGTACAAACTCTGTTCCATGCTTGGTCACTTTCATATCGATAGTTTCCAAAATCTTT
CTGCCCAAAAGATCGTCAAATGCCATGCCTTCATCGGAAATGTCATGAAACTCGAAATACATATCGATTTT
ATCTATCTGCACTGGGATTTACAAATATCCAAATGGTACTACATGACTTTCGCCAATGCCGGTCAAAAATT
CTAAACACGATAACTTGCCACCCTTTGCTAAAAAATTTCGGCGCAGCAAAATCTGCTCCGTATCCACAAGT
CCTTTAAAAAAATAAGCTGCTCTATGCACTGGTTGGTAAGGGGTAAAAAAACTTGCTGGCTTTATTTTTCT
CTCATCCTGAACAAGATTTGTTCCTTTATCTTGCTTTAGATGCCCGTCCATTTTGCACTGAGCGGCTCTATG
CACTGGTTGAGTGCAACGAAAACACTTGAATCCAGTTTGCGTAAATTGATAGATCACCACACTTGAAAGA
TTTCCTTACTGGTTCCGATGTTTTTCCTGCGACAGTCTCATTTTTGCCGACCTGCGAGTTATTTTTATCATCT
AGCTTTAACGACCCTCGCATCTCTTGGTACCCTTCAATCTTCAACTGCCTAGTTGCTCTGGTATCCGGTATA
CCATCTATGAAATATTCAATTAAGCTTTCATCTTCCAAATCTATCGGCTTCGCTATCTCCATCTGCGGATGA
AAATATTCATGCAGTGTTTCGCCCTTCTTCTGCTGACGCCTGCTAAGCCTACTCATCGTACAATGCAACTTT
CAACGTACACCAATCGAGAATATCTCGTTGGCTGGGTATAAATAATTTCGCCACACCAACGAGTAACTGC
TCTGCATAAATGAACAACTGCAACTGGCTCCAGTCTACCTCAAGTGCACGTTCCTCAAGTTCTTTAATCCA
CTGATTTATGTCTTGGTTGTTCGACCCAGAGAATTGTGTCACACTACCCTCGACATCCTTCAACGTGAACC
ATGACCTAGCCGGTTCTGACCTACATCACAAGGATTTCTACCCTGCGCGATATATATTGCTTACCGTTATA
AATGAACCCTCATCTCCATCAGCACAGTTTTCTTGATCATAATTAACTGGAAATCCATATTGAGGAAGCAG
CCTACATTGCAATTCCACTTTTCTACCAAAATCGCGTCTCTACTCATTTTACAACTATATTATTTAATGTAA
ATGAATTGAAAATAAGTATATGAGAATTAAATTAAATGCGTTTAAGCTTTTGTTCAAAACTTATTGCTGGT
AACTTTAAAAATATCCTCAACATAAAATACAAATGTTACTTACAGTAAGTCAATAGCAAATACAAAATTA
ATTTTCGGTTGTTCTCCTTAAATTTGTCTCCCCGATATCGCGAAACTCA
>160_rnd-1_family-338__LTR___Gypsy
GGAAACGCTAGAGACCTTACGTAGGCGGTTTTACTGGCCCGGCATGGTTNGTCAGGTAAGGGATTGGGTG
AGACGATGTGTGACATGCAAGGAAGTCAAGGCACCGAACTTTCGCATGCAAGTGGGTCTAGGAGAAGAG
GTAGTGACCGAGCGACCATTCCAAAAACTATATATTGACTTTCTGGGAAAGTACCCACGTTCGAAGCAAG
GGCACGCGTGGATNTTCGTGGTAGTCGACCATTTTTCAAAATTTACTTTCTTGAAAGCGATGCGGGAGGCT
ACGGCTCTTAACGTGGTGGAGTTTTTGGTCAGCGAGGTTTTTTATAAGTTTGGAGTACCCGAGGTCATCCA
CTCTGATAACGGACGACAGTTCATATCCAGTACGTTCCAAAACATGATCTCAGCTTTCAACATCTCGCACA
TACGCACCGCAGTCTACGCACCACAGAGCAATGCGAGCGAGCGAGTTAATCGTACCGTGTTGTCGGCCAT
CCGGGCGTATCTAGAGCAAGATCACCGCGAGTGGGACCTCTACCTTCCGGAAATAGAAGGTGTCCATACG
AAATGCCATACACACCGCGACCGGGGTGACCCCCTTCTTTGCCGTTTTCGGACATCATATGTTCCTTAACG
GGGCATGCTACAGATTGGCTCGTAGGTTGGCGTCTCTGAGTGATCATGAGATCTCCCATTTGCACCCNGCG
GACAAAATACACCTGATTAGACGGCGAGTCCAAGGCCAACTACATCAGGCTTACGAAAGAAGTCAGCGG
CAGTACAATGCTCGAGCCCGGGTGTTCCAGGCGGAACCCGGCCAAGAAGTTTACCGACGCAATTTTGTGC
TGAGCGACTTTGGGAAAGCTTTNAACGCCAAGTTTGCAAGAAAGTTCCTTAAGGCCAGAGTAGTGCGTCG
AGTAGGCAACAACTCGTACCTGCTGGAAGATTTGAAAGGGAAGCCCTTAGGAGTGTTCCATGCGAAGGAC
ATACGGGTGTAGATCAGGGTCGTGGGTGTGGCCTACGTTGGGCGTTCTTATTTGGCCGGCTGGGCGAACC
GGATAGGANATGCGCACGCAGATTTATAGTAACGGTCGGGTTCGCGCTTACAATAATCTGCGTGGCGATG
TCGCATTTGACATATCGACGACATCGATATTGCCCGTAATTGGTTGCGTTCTTTATTATCGGCCGCGCCGA
TATCTTTGGTCATAGGGGCAGAGTATCGGGGTGAAGCCGGACACACAACGGCGCTTAAAATATCGAGGAG

TGGCAACACCGGACTTGCGATCCACGACGCCAAAAGAGGCGATTGCTATTGGCTGCCCGATTCGCNCCCA
AAGAGATAAAAGAGAGAGTNGATCGCATGAAGATGCCCTTGGCCGTGNTTCGAGCCGTTGACCCAAAAA
AAGAAAAAAAGGAAGAAAAAGAACAGCTGTGCCGATGAGCAGCAACGGTAACCATATTTAAATGTACGC
GGGTNCCGAATTTCGGCACCTTTGATGTGCTGCCCAATGTACAGCGAGGGAGTTCGACTTTGTCAGCGGTC
CACAGCGGACGTACCGGCTCGCAGTTAGCATTTTTGAAGTTGGATATATGTTTTTTGCCGGCCACGGAGCA
TGCGCTTAAACAAATCAAAGCGCATACATTTGTTCATGACGGGCTTTCTTTATTAGTTAATTGACAAGATA
ATTCCTTATGTGCTAACGTACTGCAAAAGAAATAGTGCACAAGAGCGATAATACACGAAGCAATAATACA
CTAGAAACACACTGTCGTCATTTAAAACCACTATAAAAGCGATACTCTATTAGAGCAGCCGCAGCGATTC
TGTGTCACACAACGCCTGAAACCCGCGCCGGAGGACGCTGGCCAGATCAACCCAGATATTCACGGGCCGA
ATTTTCAAGGCACGGCCAAGTATCGGCGTAATCGCGCGACGGAGAGACGAGCCATCACGGACGGGACTC
NCACGACGAAGAGGCGGCCACGGGCGAGACCACGCCCATATCATCCGAGTGAGTCTGTTCCATTTCGTGC
CTCCTCGCCTGAGTAAACGGCAGAGTCAGAAGCAGACCCCTCATTGTGCTCCCTGAAAGAGAACNGATTT
TTTTTCGGAATTTTTAACTCTAGTTTTTTTTTGTTATTAAATACATAAAAATACTCACGACTAGTCTAAATT
AAGTAAATTTCCGAAAGTGCGAACNGGGAAGGAAAGGGCCCAAAGATTATTGCGTCTGCCAAGTAAGGA
ATGAAATTAAAAATTTAGATTTTGGTCAACTTATGCACGAGTGATACCCACNTATACGTATATATATATAT
>160_rnd-1_family-59__Unknown
AAACTAATATATTTTTCATTTTGTATNTTTCAGCCAAATCTGCCACGCCCATGGACAACATTTAATCAGCA
AATGATCGCTATTCATGGTACCTAGTTGACCTATATATCCTTGGTTATTCCATATCATGGATATAGTCCTAG
CGAACCTAAAGGACCGAGTTCTCGCAAAATGCGCAAGAACATGGTCCAAAAATATGTATGCTTTTCTTTT
ACAGCCGCCCCCAGCAACAGCCCCGCCCCCTGGATGGTTTCTAGTTGACTAGCATATCCTTGGTTATACCA
TACCATCGATATAGTCCTTGGGAACCTAAAGGATCAGATCCCTGCAAAATGTGCATTGAAATGGTCCAAA
AACATGTATGCTTTTTCTTTCAGGAGCCCTTTTACATTAGCTGCAGCTGCAGCTGTTGCTTAACAGCCGGCT
TACAGCGCTCATTTNACATGAACTGCTANTTAACAGCACTCATTTTAATTGAGTTACATTTTTGCCCCTTNA
TGCTATGCAATGAAAATTTTTATTCGCAAAAACACTCGTCGACCTTCTGGGCATTTCTGTTCGCCTGGTATT
TNAGCGGAATTTTAGNGCAAATTTGGGACCCAGCCGGCGACGAGGGCGCCCCTTNNGGGTATGCAATGAA
TTTTTTCGGCAGCTAAAAATATTTTTCGTCGACCTTCTGGGCATTTCTGTTCGCCTGGTATTTTGGCAGATT
TTAAGAAAAATCTCGGCAGCCAGCGGTTGCCAACGGACGCCCCTTGCGGGTATGCAATGGATTTCTAAAG
ACCGTTGATTTTTGAATTTTTAAATAAAAAAACAGGAATTTCTTCTTTTATTTTAAATAATTGATTTATTTA
TGTTTAAATTTACAAAATTGTTGTTTTTTATGCTAATTTTCTTAATTATTTCTCTTAAAATTATACAAATCGA
GCATAAATTATAATTTTAGCTTATTTAAACTTAAAATAACTATATCTGGCTTAATTAAAGTCCGATCCTGG
AAAAATTTGCTTTGTCAGTCGTGGAGGTCCTTAGAGCTCATTGTAGTCCTTAATTTGTCAGTCTGAAAAGA
TAAAAACACATTTAAATTAAANAAGGCAAAAAATATATAAAGGCATTTTATGTGAAGAGAAAAANGGCC
ATAACTCGGCCATATCCTTGCAGATTTTCAAAGGACATGGTGCGTTAGGATCGTATAGACCCCCTGCATCG
ATCTGCATCAAAAAANTATGGGGTCATCTAAGAATCCCGGGACTTGTATTTTACTTGTAAAATTTTCAGTC
GCGGAGCGCACGGAAATGGCCCGAAAACACAAATCCTTGAATATCTCCGAAACTACAAGGACGATCTTA
ATGAAATTTGGCCAGTTGGTAGTCCTGGTCAATACAATTTTTTTGACACATGACTCGCCAGGATACGCAAG
GATACGGCCGAGTTATGGCCTATTTTCTNTTGCAAAAATCGTCCTTAAAACTTTTCCGTCTGAAGGACTTT
CGTCCTTTGGGTTGTANATTCTTNAATGGGAGGGCGAGGACTATCTGCTGGCCAAAGGACATCCCGATAG
TCCTTCAAATGGCNAAGTTAGNCCCGATTTTTTCGATTTTGGCCAATTTTGCGACTAACCCCCTAGGAAAA
ATTTCGGTGAAATTTTTTGTCGAATCCTGGAAATCCTTGAATGCCAATCGATAGTCCTGCTCCCCACGAGT
TCAAAAAGGTATATCCTTCCGATATCCTTAAGGATTTGGCCGAGTTATGGCTAATTAAAAAATGTCCTTTT
TAGAAAATTGGCCATATCTCGGAAACGGCAAGGACGATTTTGATGTCCTTTGGNCAGTTTATGGTCCTTGT
CCATACGATTTGTTTGACACAGGACTTGTCAGGATCCGCAAGGATACGGCCGAGTTATGGCTTTGTTAATT
TACGAAAGAATTGTACTCATATCTNGGCTGTCTGAAGGACTTTCGTCCTTTTCATTATATATTCTGAATTGG
GAGGACGAGGACTANCGGTTGGCCAAAGGACATCATGATCGGCAAGGATACGGCCGATCTACAGCCTTTT
ATCTCTACAGACAAAAATCTCTTTTCCTCAGCTCCTTGAAGGATTTTCGTCCTTTTTATTACATATTCTAAG
TTGGCAGGACAAGGACTGCATGTTGTCCAAAGGACATCAGAATGGTCCTTAAGGTGGTCAATTTAATGTC
CTTTAAAAAGAAAATACCCATATCTCGGAAACCATAAGGACGATTTTNATGTCCTTTGCGCAGATTATAGT
CCTTGTC
>9_rnd-1_family-226__Unknown
TTTCAGCCNAATCTGCCACGCCCATGGACAACATTTAATCAGCAAATGATCGCTATTCATGGTACCTAGTT
GACCTATATATCCTTGGTTATTCCATATCATGGATATAGTCCTAGCGAACCTAAAGGACCGAGTTCTCGCA
AAATGCGCAGGAACANGGTCCAAAAATATGTATGCTTTTCTTTTACAGCCGCCCCCAGCAACAGCCCCGC
CCCCTGGATGGTTTCTAGTTGACTAGCATATCCTTGGTTATACCATACCATCGATATAGTCCTTGGGAACC

TAAAGGATCAGATCCCTGCAAAATGTGCATTGAAATGGTCCAAAAACATGTATGCTTTTTCTTTCAGGAGC
CCTTTTACATTAGCTGCAGCTGCAGAGCTGTTGTTTAACAGCCGGCTTACAGCGCTCATTTTACATAAACT
GCTAATTAACAGCACTCATTTTAATTGAGTTACATTTTTGCCCCTTAATGCTATGCAATGAAAATTTTTATT
CAAACCACTCGTCGACCTTCTGGGCATTTCTGTTCGCCTGGTATTTGAGCGGAATTTTAGGGCAAATTTGG
GCCGCCGNCGACCAGGGCGCCCCGTGAGGGTATGCAATGAATTTTTCGGCCGCNAAAATTTTCGTCGACC
TTCTGGGCATTTCTGTTCGCCTGGTATTTTGGNAAATTTTAGNNAAAATTTGGGACCCAGCCGGCGACGAG
GGCGCCCCTTTGGGTATGCAATGAAATTTTTTCGGCAGCTAAAAATATTTTTGTCGACCTTCTGGGCATTTC
TGTTCGCCTGGTATTTTGGCAGATTTTAAGAAAAATCTCGGCAGCCAGCGGTTGCCAACAGACGCCCCTTN
CGGGTATGCAATGGATTTTAAANNCCGTTGATTTTTGAATTTTAAAAAAAAAACAGGAATTTCTCTTTTTT
TATTTGAAATAATTGATTTATTTATGTTTAAATTTATAAAATTGTTTATTTTTATGCTAATTTTCTTAATTAT
TTCTCTTAAAATTATACAAANCGAGCATAAATTATAATTTTAGCTTATTTAAACTTAAAATAACTATATCT
GGCTTAATTAAAGTCCGATCCTGGAAAAATCTGCTTTGTCAGTCGTGGAGGTCCTTAGAGCTCNTTGTAGT
CCTTAATTTGTCAGTCTGAAANGATAAAAACACATTTAAATTAAACATANACAAATAATATATAAAAGCG
GAAAATAGGCCATAACTCGGGCATGTCCTTGCAGATTTTCAAAGGACATGGTGCGTTAGGATCGTATAGA
CCCCCTGCATCGATCTGCATCATAAAAATATGGGGTCATCTAAGAATCATATGACTCGAAATTGGACTTGT
AAAATTTCCGTCCCGGAGCGCACGGAAATGGCCCGAAAACACAAATCCTTAAATATCTCCAAAACTACAA
GGACGATCTTAATGAAATTTGGCCAGNTGGTAGTCCTGGTCAACACAATTATTTTGACATATGACTCGCCA
GGATACGCAAGGATATGGTCGAGTTATGGCTTATTTCTTCAAACAAAATCGTCCTTAAAACTTTTTTGTC
TGAAGGACTTTCGTCCTTTGGGTTGTAAATTCTTTAATGGGAGGGCGAGGACTATCTACTGGCCAAAGGAC
ATCCCGATAGTCCTTCAAATGGCAAAGTTAGCCCGGAATTTTTCGATTTTGGCCAATTTTGCGACTAACCC
CCTAGGANAAATTTCGATGAAATTTTTGTCGAATCCTGGAAATCCTTGAATGCCAATCGATAGTCCTGCTC
CCCACGAGTTCAAAACGGTATATCCTTCCGATATCCTTAAGGATTTGGCCGAGTTATGGCTAATTAAAAAA
TGTCCTTTTTAGAAAATNGGCCATATCTCGGAAACGGCAAGGACGATTTTGTTGTCCTTTGGACAGTTTAT
GGTCCTTGTTCATACGATTTGTTTGACACAGGACTTGTCAGGATCCGCAAGGATACGGCCGAGTTATGGCT
TTGTTAATTTACGAAAGAATTGTACTCATATCTAGGCCGTCTGAAGGACTTTCGTCCTTTTCATTATATATT
CTGAATTGGGAGGATGAGGACTAACGGTTGGCNAATGGACATCATGATCGGCAAGGATACGGCCGATCT
ACAGCCTTTTATCTCTACAGACAAAAATCTCTTTTCCTCAGCTCCTTGAAGGATTTTCGTCCTTTTTATTAC
ATATTCTAAGTTGGCAGGACAAGGACTACATGTTGTCCAAAGGACATCAGAATCGTCCTTAAA
>9_rnd-1_family-453__LINE___CR1
TTGCTTTTAATTTTTAAATAATTGTTTTTACTTAGCCCCGGCTAAATCCGGACATCTCAGACTCTTCGTGAT
CGAGCTTCCCGTATTGGAGAAGGAGTACTCATAGCTGTCGAGTCTGCTTTTTCGTCGGAACGCATTGATCT
TCCCATCTCAGAGATTGAGTGGGTGGCTGTAAGAGTTTCATTTGGAAGTTTTTCTATCTCTATTTCCTGTTC
ATGTTTTAGGTACCACTGCTAAAAGTGTTCCCACTGTCCTACCTGTGGCGCCTAAGCCTCAAGTGGGTGTT
CAACCGAAGAGACATATTTTTGTTTCTTGGCTTGCCCCCCACTTTCTCATCAGATGATGGTGAAGCCTTTGT
ACAGGCAAAAATTCAACCACAGGTAAAACGGTGNAGAAATTNAAGTTCGCCTATGGAAGATATATATTGC
ATCCTTTATAATAAGTGTTCCGCCAGAAATATTTACTAACATTTGTTTATAAGAGTCTTGGCCGGAACACT
TAATAGTCAAAGAATATAAGGCCAAAAAGATTAATCGGACTCCGATTTTATTGCCACCTGGTTCTAGTGG
CGCATCAACTTTAGTTGCGTCAAAAAACTAAATTCTTCTTTCGAGCTTGCCTACCAGAATGNTAGAGGTCT
TAAGTCTAAGNTCGCTAACATGTATTTAGATTCTCTTGCNTTTAATTTTCAAATTATCGCTTTAAGTAACCT
GAGACCCGGCTGAAGCCGGACATCTCAGACTCTTCAATTTTCACCGCCAAATACACCACTTATAGATGTG
ACCGAGCTTTCCGTATCGGAGAAGGAGTGTAGTATGTGCATATATTGAGGGTACACTGTACCAATAAAGT
AACATCGGTTAAGCATAAATATATCAGCAACACATTGTTGCAGTACATAAACAAAACCATTTCAAGTGAG
CGTGTAATATGATCACCCGCTTATGAGCTAAGCTAAGTTAGCAGTCCCACGCTGACCACTTGTAGCATATA
TATATATATATATATATATATATATATATATATATATATATATATATATTCATATATGTACGTAAGACTCTCTC
TCCCGATACTGCTGATAAGACAAAATATGTATGAAGCCGCTCCGCTGCTGGCGTAACCGGCAGCGCAGCT
ATGCGGCCCTAGGCATAAGAATATATTGAATAAAACATTCTAAAAACTAAATTCAAACGGCACAGACGTG
CCCGTGTATTAAACTATTTGAATCTATCTCATGGCGACCGTGACAGTGGTTTTGGTAAGAGGTTCTAGTCG
GGGGCTCCCGACTAGGGGATACCTTGAACCCTCTTCTTCCAACATCAAATGCATATATATATTTTATTTTTA
GAAGCTATATGTCAAGTTTGGTGACTTTAGCTCTTATTATTTACCAAAATTGCCCAAAAACAGGATATCGA
TATCGATAAATTTTTATCGATTGCTTGGAAATGGAGTAAGTTAGCGATTATCGAAAATAAACTCGATCTGC
GCGGGAACTAGCGGCACTTACATTTCTGTTCAAATCGTTCAAATTCTGAATCTGAATTGTCTTTTGGTCATC
TACATCATCTAATTCTCTGGATGCGTTAACTAACTCCTTTACTAATGTTGGCAATTGTTACGCCAAAATTTG
GCTTACTGTGGCTGTTATTATCTGGTCAGTCTCTATTTTGTAAGCCAAGATGTCATTATTGTAGTCTGTTTA
TGCTATGTCCGCTTTACTGGTATTTGTCTACTTGGAGATCTAATCAAATTACGTTCATTAGCCATTAAATTT

GTGTAAGTTTGTTTTATTCTATATTTCTCTTGATTGTTTTCATTTGAAAACCAGTGGCTTCCTTTTTTTCTT
TGGAGGGTCTCTTACGAATCGTAAAGCTTAAATTTCGTTATGCTACGTTTGTCATTTTTATATTTCATTATC
GTTTTAATCTTACATATTTTCCTTTTTTTCTTTTTGTTTTAAAATATTTGCATTGTTGATAATTTTTCTTTAAA
CGCTGAACCCATTAAAANCGGGCATAAGGGTATATTGTATTTGTGCAAAACCCAAATGTATGTAACAGGC
AGAAGAAAGCATCTTCGACCCCACAAAGAACACATATTCTTGATCAGCATCAAGAGCCGTGTCGTTCATG
CCATGTCCGTCTGTCCGTCCGTCCGTCCATCCGTCCGTTTGTATGAACGCAAGGATCTCAGAACCTATAAG
AGCNAGAGCTTGAAATTTTCGATGTAGGTGCTCCTAGTGCCCGCGCAGATCGA
>Ref_dvir_424
CCTTTCAACGTTGCATGAACAATGTTTTACGACCGTTACTAAATAAACACTGTCTGGTATATCTTGATGAT
ATAATTGTATTGTCAACATCCTTAGAAGAACATTTGCAAACTTGCAAAAATCAAATTAGCTTTCGAGAAGC
TTTCTGAAGCCAATCTCAAATTACAACTAATAAATGCGAGTTTCTAAAACAAGAAAATTCATTCCTAACTA
AGGATGGAATAAAACCGAATCCTGATAAAATCAAAGCAATTTAGAAATATCCAATTCCCACAAAAACTAA
AGAAATAAAAGGTTTTCTTGGATTACTACAGAAAGTTCATTCCAAATTTTGCAGACATAGCCAAATCAAT
GACAGCAATGCTTAGAAAAGACTCAAAAATCGGCATTAAAAATACTGAATACATTTAAGCTTTTGAAAAA
TTTAAACAACTAATTTCTAATGACCCAATTCTAAAAATTCCAGACTTTAAAAGTAAATTCGTTTAAACCAC
TGACGCAAGTAATGTAGCTCTTGGAGCAGTACTCTCTCATGACGGACATCCCATTTGTTTTATTAGCAGAA
CTCTCAATGACCACGAAACTTACTACAGTGCCATAGAAAAAGAGCTTTAAGCCATTGTATGGGCAACAAA
ACCTTTAAGACATTACCTACTAGGACGACATTTCGAAATCTATAGTGACCATCAACCATTATGTTGGCTGT
ACAAAATGAAAGAACCAAATTCAAAATTAGCCCGATGGAGAGTTAAAATATCTGAATATGACTTTGACAT
AAAACATATAAAAGGAAAAAAAAACCACATAGCTGACGCTCTATCACGAGTACAAATAGAACAGACTTA
TTTCGGAGAAGAAACACAACATAGCGCTGACGAAGACAATAGTAACTTAATATTCACTTCAGAGAAACCA
ATAAACACATTTAAAAGACAAATTATATTTTCTAAAGGCATTCCAAACATAAAAAAATAAAACTACTTCA
AAAAATTTTAATTCACATTACATACGATAACATGACTAAAGATAATAATAGACCATTTTTGCACAAAAAC
CAGTGCAATATTGATAGACAATGATGCAGATTTTGAAATAATTCAAAACGCACACAAGGAAATTATTAAT
TAGCCAAAATTCTTCGAAGTCTTATTCAATTAAAAAACATTAACTGCTATGCCGAATTCAAAGAACTTATT
TTGAAAACCCATGAAAAATTCCTACACCCCGAAATCAAAAAACAAATAAACGTGAAACCTATTATTTCCC
ATATATTAACACAAAACATAATAAACAAATGTCATATTTGCAACTTAGCAAAATCAGAACATAGAAACAC
AAAAATGCCAATGAAAATTACACCTGAGCCCTAACACTGTCGTGACAAATTTGTTATTGACATTTATTCTT
CTGAATGCAAACATTATCTTAGCTGCATGGACATTTATTCAAAATTCGCTACTCTTGAAAATATTAAAACA
AAAGACTGGATAGAATGCAAAAATGCATTAATGCGTATATTTAATCAGTTAGGAAAACCAATATTATTAA
AAGCTGACCGAGACGGCGCACTTTCCAGCTTAGCACTAAAAGAAATGGCTTGAAAGTGAAGGAGTAGAA
ATACAATTAAATACAACAAAAACAGGAATAGCAGACATCGAAAGACTACATATAACAATAAATTAAAAA
TCAGAATAATTAACACTTTAAATGACGAAGAAAATAAACTCAGCAAAATAGAAACAATTCTTTACATTTA
CAATCACAAAACAAAACATGACACAACTGGACAAACTCCTGCACACATTTTCTTATATGCTGGACAACTA
AATTTAGATACCCAAGAAAAAGAGGAAAAAATAATAAATAAAATTAACAGTGACCGACAAGAATACGAA
GTTGATACTAGATATCGAAAAGGACCATTACAAAAAGGAAAATTAGAAAACCCATTTAAAGCTAATAAA
AACGTTGAACAAACAGACCCAGACCATTATCAAATTACTAACAGAAATTTCTATTTCTAAAACACAATTT
AAAAAACGAAAGAAATTTAATCAAATCTTAATGCCACAAGTCTATTAAATACAATGGTGATGTTAGTTCT
ATTAATCACCACATGCCCCAAAAAAAATAATTAATTTGACAATTAACAGTACAACTAACTGCAATAGAAT
ACCGGAATAATCCTACACCAAGAAAATAGACAGAAACAAAGACTGTTTAACATA
>Ref_dvir_633
TCCTGGGGCCCAAGGTGTAACGTCCCTCCCGAGGGGCGTTAATGAGAGCGAGGTAGTGCAAATTCATCTC
AAACGTAGGATGGAATATAGCCATGATTTCATGGCTGAAACCATTAGGCCTTCTAAAGTTGCTGAAGCAA
TAAAATTTTTAGTCAACACCGAGCTTTACAGAAAGCATCAAATTACTATTAATGAACAGTGGTTGTCGTCT
TTTTCGTCTGACGAGGTTCCGTTTGTTGCATCTGCTGATGACAGGGACTTAGTAGTTAGTGGCAAGATCTG
CTCAAGCCTCCATAGATGAGCAGAATAATATAGAAGAAGATATTAATCCAGGAGGCCAGGAAACATTATT
AGAAAACGTCCCTGTCGAAAATATTGCCATGCAAAGAATAGTTATAGCCCCTGGAGAGGGTCAGCGGCCT
TTAGACATGATCGAAGATTGTGATTCAGAGGAGCTGGCCTTCCCAACTATTTTTGCTGGCATTAAGAGAAA
GTGTGCAGAAAGTTTTACAACCATTTGTAGAACAGATAAATTATTTCTCAATTTTAAAAATTGGAAATGAT
TAATATAAGGAACAGTACTTCCATTTGCTTACGTAAGCACAGCGCCACTCGAGGCATTACGGCAGCACAA
GTCCTTAATGAGGAGTATTTAGAAAATTTAATACGTCACGATGATTGCTATAGGGTTTTGAAAAATATGCA
CTTCACCTGCTCATTGGGAAGGAGAAAAGAAGAAGGTCATGGCGATGATAAGGCAGATTGGTCTTCCGAC
ATTTTTTATAACCTTTTCGGCAGCAGAGACTAGATGGGCAGAGCTGTTAGTTTTACTTTCAAAGAATGTAG
ATAAGCTAGAAATTTCAGAAGAATTGGCAGCTAATTTAAGTTTTGCCAATAAAGCCCGTCTAATTAGAGC

CGACCCTGTTACTTGCGCTAGGTATTTTGATTTTCGTTACAGGCAGGTTTTAAAACTGATGAAAAAGGAAG
GCGGTGTTTTTGGTAGTCACAGAGTTATTAAGTATTATTGGCGAGTTGAGTTTCAGCAAAGAGGCTCCCCC
CACGTTCATGGCATGTTTTGGCTGAAAGATGCTCCGATAGTTGACTTGGCTGATGAGTCAAGCAAAGTCCT
TTATGATCAGCTCAGGTCGAGTATAAAGAAGCCCAAAATATTCGCAGCAATTAAGCTTAACGCTTATAAT
ACTCACCTCCTTCTACTGCCGCGCGCCAATATAGATATCCAGTTTATATTAGATCCCTATGCTTGCTGCAGT
TACGTCGTTAATTATATTAACAAGTCCCAGAGAGGCATTTCAAAGCTAATGGGCAATGTAACTTTAAAGC
AAAGGTTACATATAGCCCAGGAAGCCGTTTACTGCTGTTTAGGTATGTCTTTGTCCGAAAGCAGTAACGCA
GTGGTTTTTATTAATACCTCATTACCAGAGCATCGGGTTGGGTTAAGGAAGTCTAAGCAACAGTTGCAAA
ACTTACCCGATGACTCAGTAGCAATATTGGAAATAGGACTTATAGACCATTATGTTCAGCGCCCGACGCTT
TAGATAGTTTATGCCTAGCTGATTTTGCTGCTTTTTACAGTTTTAGCAAACGGCATAGTGGTACTCGCCAA
AATATAGATGATGCACCCAGGGCTTCCCAGCAATTAGTTTTATTTTCGAGAATTATTTATATTGTATGTTCC
ATTTAGGGATGTTAATGTGGAACTAATAGTAATGGATTGCGAATAGTTTTGCAGCCAGAATCAGGAGCTA
TTCGAGAGCACTCGCAGTAAATGTAATAGCTTAAATGCCAGTGAGCTTCAGCATACTCTTGAAAATGCAG
TAGAGGCACAAATTTTAGAAAATGTTAAAAATTAAGAAATAGTAGACGATAGATATTAATCCTCGTTTAA
ATGTCTTAAACACAAATAGAAATGACGAGAATCTAGATCCAGAACAATGCCCGATAAATAAAACTTCCAG
CTATAGTTTCAGCTGAAAGTTTAGATGTATTCAGGTAACTTCATTTAAAGCAGAAAACATATGTTGAAACA
TATGCACAATACGAAAAACTAATCCGTCTTCTACGAATTTGTTGGAGGTGGTGCGGGTGTCGGAAAGAGC
CGACTTATCTCAGCGCTTTTTTTCAATCTTTGTGCGTACTGTACAATAGAAAGCCAAGGAAGTGATCCAAA
TTCTCCTAAAATAATATTGTGTGCTCC
>Ref_dvir_1012
TCAGTTCTGTAAGCATCACCATAGAAGTCAGTCGTGTTGCTCATAAATTTGTAAAACCTTTATTAAAACTT
CATACCACCCACACACGCCCACTTGAGATAAGATTCTTAAAAGGTCAAAAAAACGCTTTCGATCCAACAT
TTTGGCGCCCAACATTGGGGCTAGTGTGTGGAAATTGACGGCATATCGTTGCCGCACAACATCTCGTTGGA
AAAAGGTTTTATCGAACTCCTCGCAAAATACAGCATCGACAGCAATATTTGGCCAGCTCTTCATTCTTGTA
GCCGTTACTTCGTGCTGGGTTCGCTCTGAGTTTGATTGCTCATTGCCTTTGCCTCACATCTCCTTGCTCGTT
GCCGTTGCTTCGTCGCTGGAAACTCGCTACCTTCGCTTTGCTGCTGCATTCTCGTTGCCGTTGCTTCGTCGA
TGGAAGCTGGCTGCCTCTGCTTGTTCACTGCATTTTGCTTCATCACTTCAAGCGACCCTCACATCGGTGAG
CGCAACATTTACAAGTCTTAGCTGTTCTGTTAACAGTGGGAGCATTTGAACTGTCGCTCGCTCACATTGGT
GAGAGTGACATTATCAGGTTAAAGTTAGGCTGTGAAAATTTGGCAGTATCAAAACTGTCGCTCAAGAGTG
TCTACTGAGGTCGATGGCAGTGAAAAAAGATTATCACACGGTGAGCTACAATTACGCTTGGACATGTTGG
AGATATACATTAAGGAGGTTTCGGAAGTACAATCTGAAATTGAATTGGAAGATCCTCAGGACAATGGACG
TGCAGAGGTGGAAGACACATATGTGAGTATCAAATTGTCCATCCAATCCCAACTGGATGATGTACTGCAC
AGCACCTCATTTTCTAACCCTGCTCCAATTCGCTCTTCGCCGCCATCGAGTCTTCCTCGATTGGCTCCGCCT
GTATTTGATGGGAAACGAGCAGAGTTTAAAAACTTCATTACATCATTCAATCAGATTATCGGTCGTGAGTA
CTCTCTTTCGAACATTGAAAAGTTTAATCATCTGCTGAATTGCTTGAAGGGCCCTGCCCTACAGGCAGTAA
AGGCATTCCAAGTGACTCAGGAAAATTATCCCAAAGGCTCTTGCTCGTTTAAGTGAACGTTATGACAATCC
TACACTTACATTTTTAGGACTCAATAGAATCACTATTCAATCTACAGCCCAGTCGACAAACCGGACGGAA
ATCAGCTGTCGCAGTCTGCTGGACAATGCGTCACGCCCTGTAATCATCATTATCCGCCATTGGGTACCGGA
TCAGATATTGCCCAAGCCATGTTGATATTCATCGTCATCGGAAAATGTGACCAGCAAACTCGTAACAAGT
GGAATGAGTCTTCTTGACTTACGTACATTGCCAAATTGGGCTCACTGCACAACTATTTTGGAACGTCATTG
TCAGTTTCTGCAATCAAGCGGCATTGCATCGTATCGAATGGCTGATTCTTCGAAACAAAAACACGTGTACA
AGCCTAGCAAGGGATTATCATTTGCTATTACTAATCCAACATGCATTGTTTGTTCCAGCTCCGACCATAAA
ATTGTACAATGTTCTCAGTTTCAAGACATGAGTCCTAAACTCAAGTACGGAGTCGTAAAAAATGCTGGAC
TTTGCATTAATTGCCTTCAATTAGGACATCGGATGGCTCAGTGTCCGTCTAACAATCGTTGTCGAAGCTGC
AAGCATCGCCATCACACGCTTCTGCATTTTGGCCAACCGCCTGCATCATCATCGTCTACATCGCCATCATC
CATAGCGCAAATGTCCTCAACAACGACTCCAGCTACAAAGCGCTCGCATATGAATCAATCGCCAGGCGGG
CAAATAATTCTTCTACTGCAAAGGTACTAGTGCGGCTGCATCTACGCCGTGATAAGCATAATTTCGAAATC
CGCAGCATAGGGGAGACCTCGACTAAAATTAACAACTGTACAACTAATGGAATTAAATCTCGTTTGTCAC
CATTTGAAGTAACACTACAGTTCTGCATAACGTCTCACATTGCCTACCAGCCGGATTCGGAAATCGACACC
TCAGATTGGAGGTTGCTCGCGAACACCACTTGCGGACGAAACTTTCCACAGGTAAGGTCGGATTGATCTC
CTTATCGGTACCGAGGTATTTTTCGAATGCCTGGCTGTTGAACAGATTAAACTCGAAGACAATTTTTCCAG
TCCTTCAGA
>Ref_dvir_840
TTCTTCTACGCTGCAGTTGCGAATGCGGGACACTATAGTAACTCAAAAACATCCGAACAAACAAAAACAA

AAACACTAGTCCAAATCACAACAAACCAACCAAAGATAACACAATGCCTTATGCTAATAAAGAAACAAC
AACAACAACACCACTGAACACCTCCTCCACGCCGCTGACTTATCAGCAAAATCAACAGAAAAACAACACA
AACAGAACATTCGATGATGACTACGAGAGTCTTGGCCTCTCGCCTAAAACTACTCAATTCCTAAAACAAC
TTAAATCTAACAAAAATAAAAATAATTCTAATTCAAATATCCTAACTAATATCACATATCAGATGATCCTA
TGGATGATTCTGATATATAAACTGAACTTAACTTTTTCCATTGCATATAATATCCACTTTATCTCTTCCAAT
GACCCTTAAGATTCTACAATGGAACATAAACGGATACTTCAACAATAACATTGAACTTTCATTATTAATTA
AACAAGCCAATCCAGATATTATATGCCTACAAGAGACTAATCTTAAATTTAATAATTCTCAAGTTGCAACC
CCCAAACCATTTATTGGTTATTTTTTCAACAATTCTTCCATTCTCCATGCGAAGCATGGCGTTGGTATACTT
ATACGGCAAAACATCCCACACAAAACTATCAATTTGATCTCACCCACTTGCTCCATGGGCAGCTTTTATCC
CACCCGAATATAATAATATTTAATTCTTATATACCCCCCAACCAAAGACTATTAGCTACAGATTTAATAAA
TATTATAGAGTCAGCCCAAAACCTATAGTTTTTGTGGGGACTTTAACGCTTGGAGTCCGCTTTGGGGGTCC
TGCTCCCGGAACCCCAAAGGTTATCAAGTTGAAGATCTTATAGTCAAAACAAACTTAGCCATTCTAAATG
ACGGCTCTGCCACCCACCATTCTACCCACAACTCCTTTACTAACATCGACCTAAGTATGGCGTCCGCAACC
CTCGCTTCCAATTAGTTCAAAACCGACCATGCCACTGGAGTACCTTCCAATCTGTCTGCAAAAATCACTTT
CAGTTTCCCTCGGCCAACGATCTGAACCTCTTCGCCCATTCACAAACTAATACCAAAAACAGATAATGTAC
CCTGCTTGGTTTAGCTCAAGTCTCCAAGATCTGAGAAAACAAAAGCAAGTTCTTTGACACATCTATAAATC
TTCTCAAACTGTACCAATCTAGTAGCTTACAAAGAAGCAAGAGCCCTGTTTAAAAGAGAAGCTATTAGGG
CAAAACGAGAATCTTTTAGCAGTTTCATCTCTAAAATAAACCCACTTTCCACAACCAAAAAAAAATGGGC
AGATATCAACAAATTAGTAGGGATTTTCCCAGGGCCTTTTAAATCCATAGATAATAATGTAGAGCTCCAA
AATTCAGTAGACATTGGCAATGCATTTGGCTATGTGGTCCAATTATTCCAGGGATGAAAACTTCTCAAATG
CGTATAAAGTTAATAAGGAAAATCTCAGGCATATCTGCCCAACACATATCCTCAAATGCTAAGTACCTTG
AATCTCAGTTCATAGAATTAGCCCTAAAGGGAAAACTCCTGGGTATGACCGCATCTCGTACAATATGGTTC
GAAACTTACCAATAGAAGGAAAGAAAAGAATACTAGAAATGTACAACATAATGTAAGATGGGGGTAACT
ATCCGCACGCGTGGAAAACGGCTACAACTATCCCAATCCTTAAACCAAACAAACCTTCCCACGAATTATC
ATCCTTTTCGTCCCATATCCCTACTCTCCTGCTTGAGCAAAACACTGGAAAAAATTCTTGCCAAAAGGATC
ATGTGGTTTATATCCAAAAATAAACTTATCAGCCCCAACCAAACTGCATTCAAACAACAGCACAGTACCA
TTCACTCCCTATTGCATATCCATCATTACGCGTCAAATGCCCTTTCAACCAGAAATCATGTAACCATCCTG
GCTAATAATTTCGAAAGGGCATTTGAGCGCGTTGGAGTGCACACAGTACTGGAGCAACTTTACCGGTGGG
GGGGTGGGTCCTAAAACCTTCAACCTCGTAAAGGCTTTCATGGACAAGCGATCCATTTCGTGTTAAAGTTT
AAAACACCCTTACCTAAGTTCCTATAAATTACACA
>9_rnd-5_family-2214__LTR___Gypsy
GTGGGTGTGTATTACTCAAGGCAGGCCATTGCCCACNTTCGACGTACTCATAACAACAACGAGATCCCTA
GACAACAACCATTCAACACACAACGAAGGCGAATTCGCCAGTCGGCGAATCCGCTAAAACAGAAGACGA
AAACGTGCACGATGCAAAGAAGAGCAGCGTGCCGAAAACGACGAAGACGGGAGTTGGAGTTCGGCCTTA
GACGAGAAAGGTTGTGAGAGCTGCTTCCCAAACGAGGAAAGACGACGATTCGCTGATCCTATAACTTAAT
CCCAACTATTTGTAACTTTAAACATTACAATAATATTTGTATAACCTTATCCAAAATAAAACCCCACATAT
TTGGGAGCTCATCCGGGATAAAAAGAAGTAAAGTGGTTAAAAAGGCTCATCAAAGACGATTAACGAAGT
AAAGTGANTTGTGATAGTGAACATTTTTTGTAGACAACGTAAGAAGTGGAAGGCTGCAAAATTGTTTCAG
AGGTGTAAAAGAGGAAGGACTGAAGTGTTGAAATCGTGTGAGAAATAAAAGATAGTACTACTTTTTTTCT
CCACATCTCATTTGTAAATGGCAAATTTAAACGAGTTAATTCCAAGAAGTTTGAAGCCGAAGGTGACAGG
AATAACAGACGCCGTCTGCGTGAATTTAGTTTTTAAATATAATGAAAACGACGCAACGTTTTTGGCGAAA
AGCGTATACATACAAGAAAAATTGTCTGAGCATGACCTTGTATCGATTTGCACAGTGTTGGGAATAAACC
ACAGCGTAAAAACATTCCGTTACATATTTTTCGAAATTTGCTCGCCGGACAACCGCTTCGGCACAACAATA
ACGCAGACGAGGGAAGCAGCGATGAAGAAAATGAAGACCTTGAGAGCGGCGCGCACGGATTTGACGATT
TCGCACACGACGAAGAGGAAGTAGCAGAAGAAGCAACGGTTGAAAACGTACGAGCAGGCGCAGCTCGA
AACACAAAGCGAAACGACGATTGCCGACAACGCGTCAACGAATTGCAAGCACAACGCTTTGCTATAAGTT
TCCGCGACGTGGAAGAGTCCGTTCGAGTATTTGATGGTAGTGAAGAAATNTCTATGAAGATGCGATCGAA
GAACAAGCTTTGCTTTATGTGTTGGAGTGATTTGCAAACGCTCATTTTCGCAAAGAAATCATTGCGAGGCA
TTGCGAAATTATTTGTTTTGAGCGAAAAGCATCTCAATTCATGGGTTGCTCTGAAGAGTGCATTGTTGAGC
GAATTCAAATCTGGAGTGAACAGCAAGCGAATACACTCACAGTTGACTGAGAGCAAGCGTCGAAATGGT
GAGAGACATTGCATCTCGTGGAAATGTGGAGGACGAAGCACTCATGCAATACGTGATCGATGGTATAGAT
GATTTTTCCAATAACAAATCGATTTTATATGGTGCAAAAAAACGAGTTTAAAAACAAATTAAAGGACTAT
GAAAAATTGAGTGGAAAAAAAAATAAGAAAAGTGTAAAATTGACAAAGAGAAAAAAGGTGCAAAATGAT
GCACAGTGGTACGCGCAGGAAAAAAGAAATCAATTGTTTTAGTTGTGGAGAAAAGGGGCATTTAGCCAA

CAATTGCATGAATAAACAAAAGGGAAGAAAGTTTTAATTGTAACCAATTCGACACATATCGAAATATTGT
AAGGAAAAATGTGAAAATCAAAATGAAGTCAAATCAAATATTCGTGTTGTAAAGAACAAAACCGCGCTA
ACGTCAAAAGAAGTTTATTTCAAAAACAAGAAGTATTTAGCATTGTTTGATACCGGTAGCAAGTTTAATGT
TTTACGTGAAGATCTTTATAAGGAACTTTCCCTTCCAAAATTAGAAATCTGCAATGTTTTTCTTATTGGATT
CGGCAGTGAGCGCAGTGAGAATAAAAATGAAGAGTTTATACTTGATTTTTACGTGGTTCCAATTAAATGT
ATTGATGTCAAAGTTATTCTTGGCGAAGAACTATGTTTACAGGCTGAGATTGTATTTAGTCGTAACGGTAA
GCGTAATAAAAATTGATGCGGTAATCGATAATGATCAAGTCGATATAAACTCTCAGATTGGTGATGTTAC
AAAAAAACAGAGTAC
>Ref_dvir_679
TGCTCGTCATCGCTCACACCAACCCCACGATTCGTCACCACATGCTCGTCATCGCCCAACACTGGGCTTCA
CGAATTCATAAACCACATTGGTCGTTCATGGCCCAACAATTGGCTTCACGAACTCATCAACCACATTGCTC
GTTCATCGCCCAACACATGGGCTTCACGAATTCATCAACCACATTGCTCGTTCATCGCTCAACACTTGGCT
TCACGAATTCATCAACCACATTGCTCGTTCATCGCCCAACACTTGGCTTCACGAATTCATCAACCACATTG
CTCGTTCATCGCCCAACACTTGGCTTCACGAATTCATCAACCACATTGCTCGTTCATCACCCAACACTTGG
CTTCACGAATTCATCAACCACATTGCTCGTTCATCGCCCAACACTTGGCTTCACGAATTCATCAACCACAT
TGCTCGTTCATCGCCCAACACTTGGCTTCACGAATTCATCAACCACATTGCTCGTTCATCGCCCAACACGG
GCTTCTGCACGAATTCATCAACCACATTGCTCGTTCATCGCCCAACACTTGGCTTCACGAATTCATCAACC
ACATTGCTCGTTCATCGCCCAACACTTGGCTTCACGAATTCATCAACCACATTGCTCGTTCATCGCCCAAC
ACTTGGCTTCACGAATACATCAAGCTCTTCTGGAATTTGCACATCCGTAACTTCCGCAAACTTCGTGTGGA
TATTTGTAACGTCTATTGTTCTGTGTAGACTTTAAAACGTGTCTCCGTCCAATACCTCGACCACAAAAAAG
GGCCTTTGAACTTTGGATCTAATTTTGTTTGCTGTCTCTCACTGTTTTTAAGCAAAACAAAGTCACCCACAC
AGAACCTAATTAGCTTAGCCATATTTTTATCAAAATTTCAGTTTCATAACGAGATAACGCTAACTTATTAG
AAACGCTTTCTTTATATAATTAGCACTCAGCTACCGAATCAATGTAATCGGAAATCTCTCACCATTGACCA
GCATACTCGATTGAAGCTCCACTATAGCGCAAAGCTCCACTCTTTTTTCCGATATTACTCCAAATCCGGAT
TCTGCACTTTTTTTTTGGAATTAGTGGACATTTGAGTTCCATACTTTAGCACGCTTGGTCTCTGGTTTCCAGT
TTGATGTAATAACACGCTCTTTCAACATTGAGAATACTTGCAGCTCCGCTTGCAGTTCACGTCGACTGTAT
ACATGCGTTGTGTATATCATTCGTTGCAAACGCCGGTCGAAATTAGCCATGTGCCCAAGAACCAGCGCGA
TAGAAATTTGTTCAGAATTCAAATTACGCAATTTTGTGCACAGTGGCAAACTTTCACCGTTGCTTGGACGA
CTATTAAGAAAATCAAAATAACAGCGGCTGGCGTCTCCGATGTCTCGTATCGCTGCTCAAACAATTGCCTG
AACTGTTGCCAAGTTATTCCTGGGTAGCATATTTGTGATAACCACTGGGCCGCTGCGCCTTCCAAAGCTTT
ACTCAACGCTAATATAAGAGCGCTGTCACTTAACGGTTTTCTTGTAAAATGATGTCGACCGTAGCGCACCA
CTGCGTGGCATCCACCCCAGCAATGTCCGGATTATATTTCGGCAAGCAAATCTCGCTAGTCTGTTGTAACT
CGCTCGCCGGCATCTGTAGCCTCTTAACCAGTTCAAGAAAATTCCGGTTTTGCTGCTCTAACAACGCGAAG
ACATTCCTTGAAGAAGTTTGGTTAGGGCCGAATCCCACTTCTGATGTCGGAGAATTAATGTCATCTCCCTG
CGGGTCGTCATTATTGGTCGCTTGGTCGCACGGATTCTGGTGTTCCATACTCAGTCCTGGCGAATGCAGCG
AGTCTTCGTTGCCTTTGTTGCTCATTATTCTCTTAAATACACACACGTGACCAACTCACGTCACGCCGGAA
ACACAGGACAGCCACAAGAGAGAGACCAGATCTAGCAACGCATACGAAGCCACACACAGCCTCAGTTCG
CAAACTGAGTCCCCATTCTACCAGAACTAGTATCTACCAGCACATCCCATGGTCACACCTTAACGTCTCAT
GCCTATCGAGATGCTACAGTACTTTCCTCTCCGAC
>9_rnd-5_family-2333__Unknown
ATAGATGTAGGAACCTCTAGAAGGCCCGAGCGACAGAGAATAACACGCACCGGTCGTTGTGTCTGATCGC
CTCTCTTCGCTTATTTATAATCTCTCCGGTTCTCGCGTCTGATAAAGCAGGCTGCGCAACGCGAAAAGAAA
AGGAGCGACACTTTGCATTTAGTAGTTGAATGAAAAACTAGTCGTCCGTCGAATAGTTGTGTAAAGTTTTA
ATAATAACTTAAATCATCCCGTAATAACTTAAAAATAATAATAATCAATCATCTTATAATATTAAACATAT
TTGTAAGAGTTAACCACTTTGAAACAAATTATAATAAGAAACTTCTATAGTAATAAAGAACCTACAAATT
GGGGGCTCAACCGGGATTATCCAAGTTACAAAAACAATGAGACAAAGTGAGTGAATTTTAAAGAAAAAA
ACATCATCGTAAAGGTCGATATAAAAGACGTGTGCAGTTGGAAAAACAGAGTGCAAAGTAAAGTGTGAG
CGAGATAGCAANAGGCGCTCAGTAAAAAACGTTATTCCAAAAAATAAATTTCTGGTGAAATCCGGACTTT
GTGTGTATGCATCGCCAAAAGCGAAGCAGACAGAAAGAACGAGAAGAGAGAAGTGCAAAGTAAAGTGT
GAGCGAAATGGCAACAGACGCTCAGTGAAACGTTATTCCAAAAAATAAATTTCTGGTGAAATCCGGNCTT
TGTGTGTATGCATCGCCAAAAGCGAAGCAGACAGAAAGAACGAGAAGAGAGAAGAGAAGAGAAAAGTA
ATAGAAGGCTGGAGTAGAGTTGGCGTTTTCTGTGGGCCGGTGCGTAAGGGAAACCAAAACGATCAAAGC
AGCTGGTCATCTATGAGGAACGCAGGCAAGTTGGCCAAGGCGTGAAATAGACAGCAGCAATTTCTTGGTA
CGCTGGCAAGGTGGTCGAGCGTAATAGAAGGCTGGAGCAGAGTCGGTGTTAGCAGTTACGCAGGCGGAT

GAGCCAGTGCGACAGAGTCCTTCGTGGGAGTGTTGGGAAACGTTCAAATCGTGTGGTGAGCTGCAGCAGC
GCTGCAAAATAAAATTTTACTATTTGTGCTGACCTTAGCAAGTGGTGTATCTTGCGCAGACAGATCGTACA
TTATTAACTAAGAAATTTAGTCACGGACGGTGACACGAAACACGGAAGAAANTTAAANTNNTNTTTGCAT
AATTGTTTTATGTTCATCGACATTATTCATTAACATAGTTTTAACATTTTGATAACNGCTGAGAACAAATTC
AAAAACAAACGGCCCTTAGCGCTGTTAAACTCAAGATACAAAAGAGTGTCCGTCGAGGCTGTTAAACTTA
AGCTACAAAAGAGAGCAGCCGCTGTTATTACTAATTAACATAGGACAAACTGAAGCTGTTGTGCTGAAAA
TTTACATCTAAGAATATCAAATCTGAAAATAGTAAATAAGAATAATTTGAAAACCTAAAATCTAAGTAAC
CGTGTGNCAATTTTTGTAAGATGGAGAGAAACGAGATTCTAAGTCTTCGTGTCGATCAATTACGCGAAAA
ATNAACAGATTTGCAATTGAGTACTGCAGGACGCAAAGCTGAACTGCAAGCAAGACTATTTGAACANTAT
GGCATGGAAATAGAAGAAGAGGGTTCAGGTGATGATACGTCGGTTAATGCAGCACCTTTGATGCAGAGTC
CTGAAAGAGCTAGGTCGTGGTTTACGCTGAAAGATGTTGAGGGTAGCGTGTATAAATTTGATGGGTCGAG
TTCGAGAGATATTGAACAGTGGGTGAGTGAGTTAGAAGAATGCGCTATAACAGTACAGTGGAGCGACTTA
CAGTTATTTATCTATGCCAAGCAGTTGCTTGAGGGTGCCGCTAAATCATTTATTCGTAGTCAGCGGGGTAT
TAATAACTGGGAAGCGCTAAAAAACGCATTGAAAAATGAATTTGAGGTAAGATTGTCATCAGCAGAAGTT
CATAGACGNTTGGGAAAGCGACAGCAGAAAAAGGGCGAGACGTTGTTAGAATTTTTATATGCGTTGATGG
AAATAGCAAAATCTATCGATCTCGAGGATGAGAGTTTGATTGACTATTTTATAGAAGGCAT
>Ref_dvir_995
GGGCCCTGCATATAGCACATCGTCCAGACCTTTACCGTTCGATGACGGGTGGAAGCCGTTAAATACTACA
CGGAGCTTGGTCGTGGTGCTGTCAGGCTTGTGAACAGCATGGTGTGGTAGATAAAAGTTATCCAAACAAT
TGCTTGGAGGGACTTGTCGCATGTGACCTAAGTCCAGATATTCCTGGATCACCGCATCGTACTGCTCCTTA
AGGGGGGCATTTTTTGCCAGCTAGTGCGATGGACCTTGAAAGCCTAACTGGATGTGATCGGAATTCTTGA
ATGGGACCAAGTATCTGCCGTTTTTATCTCTTTTAGTGTTCTTTTGGAAATTGCGCTAGGTCGGATTCCTTT
TCCAGCTTTACTGGAATATCCTCCACCTCCCAAAATTTTGTGAGCAGTTTATCCAAGCTCGCATTTGGATTC
AGCGAGACTTGAGTCGAAAAGGATGCGATCGATTTGGCACTGGTCTTAGAAACGGGGCCTGTAAGGTCCA
TCGTTTCTTGACCAAGGAGAGTGCCACATATGTTGGGATAGGAGCCACCTAACCGAGAAGCACGTCTATC
TGTGAGCTCCTATAGAAAGAGGGATCGGCCAAGTGTTATTTTGGAAGTTCTTCCAAAGCGGTTTGGGGGA
TGGTGTAGGAGGGCAAATTGCCTGCTAGCTTTGAAAGAACATAAGCCGACGTTTCAATATAAGCCCTGGG
CGGAGAGCCAATATTAAAATGGCAAAGTTTTTGAGGTTGAGCCGAAATAGCTTGATTCAAACCGGAGACC
TGTGCGTCGACGGGTTGATAAGGAAGTTTAACCCTTTTGAACAATCGCTCTGAAATAAAGGTCGCTTCAG
ACCCAGAGTCGATTAATGCCCGAGCTGTATACTTTGTGCCCAAATGACACACTTGGATGACAGCCGTGCC
GAGGAGAACACCATGTGAGTTTGCGGAAAAGTAGTTTTGAACGGTAGCTTGGGTCGAAGTAGAAGTGGA
CTGTATAGACGAAGGAGGGTTCAAAACTATTGGTGTTGTGGAAGCTGAGGGCACCTGGGGTCTGTCACGG
TGGAGCAGCGTGTTGTGACGTCCCTTGCACGTGAAACAACTATGTGCGCTGGTGCAGTCTTTCAACTGGTG
CTGTCTGGCGAAGCAGTTTAAGCACAACTTTTGTTGCTTAATGACGGAAACTCTCTCGGGTACGGACATTT
GTAGAAAACGTGGGCATAGACGCACGGGATGGTTTTCCTTCTGACACAATTTGCAGTGTTGTGACTTTGTA
GTCACTGTTGCTTCGAAGGTTCTCAGGTGGTTGGACTCTTTTCTTGTGGGTTTAGACTGGGGTGGCGTATGT
GCGGTAGGTCGTACATCCTCTATCGCCTCCAAGGTGCGATATCGGTCGATCAGAAATGAATTCATCTCGGA
CCATTGAGGTATCTCTGCCTTATCGTGGAGCGATTGTTCCCATAACGACAGCGTGAGCTTAGGCAATTTAG
AGGAGCACAAGAAAATAAGCAGACAATCCCAATTTTCGGTGCTCACCCTGGACAAGTCGAGAGCCGTTAA
ACATCCCTGGATTGTGCCCTGAAGCTCTTGGCCTGACTGAAAGCCGTCATTCGTGAGCGGGGACTTGGAG
ACAATTGCCCTAGCTTCGCCGCTGGTTTTGGAATTTAGATGGAACAATTTTTCCACCGGCGTCAGTCTTGG
ATTGTTAATATAAACCGCTGTAAACAAGTCTCGGAAAGTAGGCCAGCGTAGGTAATCCCCACTGAAAACT
TCAGTGTCGCATGGCGGGAGACTGCAACCCCTGGACATGGAGAAGTTGGAGGGGGGTTGCTCAGCTTGTC
TATTTAAAGCAGTGGCTCTTTCTATTTTTTCACTTAGTTGAGCCGCACATCGCCCGTAAACCAAGTACGAG
TAATCATATTTAGGTTGCATAGCCACAAGGTCACTGCTGCTGCAAGCGCCTAGGTTGAGAGCTTTACAACA
TAAATCGTACTCGCGTTCCACCTCTTCCCATAAAGTCTTAATCTGATCTCACCGGACTTGGAGAGTGAACA
GTGACACGTCACTACCCGCTGCTTCATTGATCTTT
>160_rnd-4_family-335__RC___Helitron
ACCACTAACCAAACAACATATAGACTGGACATGAGTACAGAGAAGTAGNCACAAAANGTATCGGTGGCA
TCTGGTCCAAANTCGCGAGTAAGGGAGTAAGATATACATATGATGTATTTTTGAACCGCTTACTCTACAGG
AAAGTGCTTGGCGCACTCTCTTCCATATGATTTGTGCGATAGAACGGCAACGCTGTTAACTTTTATCTCGC
TCGCACTTACTCTCAGCTCTCAATGCTCTCTCTATGTTTAGGGATCGTTTCAATGTGTGTGTGTGTGTATTT
GTCCGACNAGCAAAACTGCGCACAAATTCAATTTTTCCCAAATGCTGAGGCATTTCTACCCTTTTGAAGGC
ACGGCTAATATGAAATAGAGAAAGGGTTTGCCACGTCGATGCGAAATTCTTGCATTTTAGCTCGCTATCGT

TGGTAAGGGTACCCAAGCGTTGCCATGTGTGCATTTAGAATATTTTGGACTCGCTATNCACGCATACTTGC
ATATTTTGTGTATGAATAGAAGCATTTATGCGCTTGTGTGCCTGTTGCTTGCCTGCCTGCCTGTCCCCGATG
CAAATTTAACAAGATTTTAGGATTTCTGATTTTGGACCTCTACACACAGACACACACATACAAATGTCTGT
GAATGGGCTGATCATGGGCGCAAAAGAAAATACATTATTGGCACGCGTCTGACGCGCGCCCTTCTTTTTTG
AGATTTTGATGAATGAATGTTGAACCCCTTATTACCTGCAGCAGCTCGTTGGGTCGAATGGGTCAACTTTT
GGCAAGCACCTGCATATGGAGGTCAAGTGACTGATCACCAAAATTATTAAAACCATAAATATATAATCGC
TCCTTTTTTTTAATTTGGTATATTTTAATATTTTAAAGATTTTTATTTTTACTCAAATTTCATCAAAATCATN
AAAAAGAAGGGCGCGCGTCAGACGCGTGCCAATAATGATATTTTCTTTTGCGCCCATGATCAGCCCATTC
ACAGACATTTGTATGTGTGTGTCTGTGTGTAGAGGTCCAAAATCAGAAATCCTAAAATCTTGTTAAATTTG
CATCGGGGACAGGCAGGCAGGCAAGCAACAGGCACACAAGCGCATAAATGCTTCTATTCATATACAAAA
TATGCAAGTATGCGTGAATAGCGAGTCCAAAATATTTTAAATGCACACATGGCAACGCTTGGGTACCCTT
ACCAACGATAGCGAGCTAAAATGCAAGAATTTCGCATCGACGTGGCAAACCCTTTCTCTATTTCATATTAG
CCGTGCCTTCAAAAGGGTAGAAATGCCTCAGCATTTGGGAAAAATTGAATTTGTGCGCAGTTTTGCTCGTC
GGACAAATACACACACACACACATTGAAACGATCCCTAAACATAAGAGAGAGCATTGAGAGCTGAGAGT
AAGTGCGAGCGAGATAAAAGTTAACAGCGTTGCCGTTCTATCGCACAAATCATATGGAAGAGAGTGCGCC
AAGCACTTTCCTGTAGAGTAAGCGGTTCAAAAATACATCATATGTATATCTTACTCCCTTACTCGCGAGTT
TGGACCAGATGCCTCCGATACNTTTTGTGNCTACTTTCCGGTACTCATGTCCAGTCTATATGTTGTTTGGTT
AGTGGTATAAGGGTATATTGTATTTGTGCACAAATGTATGTAACAGGCAGAAGGAAGCATCTCCGACCCC
ANAAAGTATATATATTCTTGATCAGCATCAATAGCCGAGTCGATCTGGCCATGTCCGTCTGTCTGTCCGTC
CGTCCGTCCGTATGTATGAACGCAAGGATCTCAGAACCTATAAGGGCTAGAGACTTGAAATTTTATCCTGT
TTTTTGGGCAATTTTGGTAAACAATAAGAGCTAGAGTCACCAAACTTGACATATANCTTCTGATATAGAAT
ATATATGTGCATTTGATGTTGGAAGANGAGGGTTCAGGGTATCCCCTAGTCGGGAGCTCCCGACTAGAAC
NTCTTACCTGTTTTAATTCAGACATGTCGGGTTGTAGTTTTAGTTTTGCTTCCGTATGTTTGTAAAATACTT
GTTTTATTCAATGTGTTCT
>160_rnd-5_family-4918__LINE___I-Jockey
CAATATTTTACTCCGCCCACTGTGAGTACGAAGCCACTGACGGGGCCCAGGAATGAGAGCAACAATGCCT
CGAAGTGCCAGGCGGTCAACTACACACTGAGGCGTGGCAAGGCTCCAGCAGTCTGGATAGATGGCAGCC
CCTTCACCCTGGCATAAGCGCCAAGTATCTTGGAATTGTACTGGATAAGAGGCTTACCTTCGGGCGCACAT
CGACGGCCTTCTCAAGCAGTGCCGCTTTAAGATGGCCAAGGCCAACTGGCTACTAAGCCCCAACAGCCCA
TTGCCACTTCGCTGCAAACGGCAGTTTACAACGTCGTGGTGGCGCCAACTTGGAGATACGGTATCCAGGTT
TGGGGAATTGCATCTCGTACAAATCGAAGACGGATTCAGACACTCCAAAACTTTGCCTTGCGCCGAATGA
CCGGTGCTGACTGGTACGTCAGAAATTCTACCTTGCACAGAGACCTCAAAATGGAGACTATAGACAGCCA
AGTCGAAGCATTCGCCAACCGATACAACGAACGCTTGCAGCGACACTGTAACCGGGTAGCAAGAGACCTC
GCTGGGAGGTGTGTAGTCAGGAGGCTCAAGAGAAGATCCTTTAAGGACCTCCTCCAACCACGACACAGGA
GACGCCCCAGAGCCCCCGACTACCTACGTGACGATGCAAAGAAGACGCCTTTGACGTTTGTCCACGATAA
AGCCTGAGGACCCAACCAGGCTTGGCGAAATACCACCTAAGTTAACTGTGTTGTAATAAAAATGTATGCT
ATTTTTGATTCAATACAATAAACATATACAAAAAAAATAATAATAAAAGTGACAAATGACTATGTCGAAC
AGTCTTCTGACAAAATTATTTAAGATAGGACCTATTTCAAGGAATAAAAGAAATAAGATGATTACTTCTA
CATCGGGACCTCCAAGAAGATATAGACGACCAAAAATTCACAAGAAAGGAATATCTCTGAGACCACAAT
GCTCCTCTATCGGTTCCCCATCAAATGAACTATACACATATATTACAAAATTATTAGGAAACTTGACAACT
AATATAACATTAAGAATTCGTTAGAGTTCAAAGACAGAGTCAATAGGGAAGACGATATATTGGTATCTTT
TGAAGTCGTCCCTGTTTCCCAGCATACCAATAGAACTTGTACTGTACACAAATTGAAAGGGAACAGGTAT
TTAAAATATGAACACAGAAAATACATTCTGTAAATGGGAATGGCAATGGGATCACCGGCCTCTGCATGTT
ACTGATAAATTGACTACAAAGCTAAAACTTCTGGCAAAATAAGTAGACATCCACTTTGTAACTGTAACGG
ACAAAACTGAAGTATAAAACATTCTAGTAGAGTTAAATTCTTTTCACAAGCACATCAAATTTACAGTGGA
ACTAGAGCAATTTCAAATCTTGTCAAATTTGCAAGGAACGGCCTGGCAGGCCTTCAATAGGCACACAAGT
GCATAAATTGTTCTATTCAAAATATGTAAGTATGTGTGAGTAGCTAGTCGAAAATATTTTAAATACACACA
TGGTTACGCTTAGGTACCCTTACCAACGATAACGAGCTAAATTGCAAGAATTTCGCAACGACGTGGCAAA
CGCTTTCATTATTTCATATTACTCGTCCCTTCAAAAGGGAAGAAAGCCACCGACTTTTGAGAAAAATTGAA
TTTGTGGGCATTTTTGCTTATGCGGACAAATACACATACAGACAGTACATACAAAATTTGAACGAGCACT
GATACTAAGCCAAAGCAGTTTCCTATAGTGCGAGGAAATGAAAAAAAAAACAACAAAGCCTTTCTATCAC
GCAAGTCATAGGTATTTCTTACTCTCTTGCTGTCTGAGCGCGTGAGCGTGTCTCGAACCCGAAGAAAAACG
ATCTGTCGTGGCACCTCTGCTGGTGACTATGAATCCTAGAAAGCTAACGCTTTTCTAAAAAATACTCGATA
TCTCGAATGAGACCCTCATGTTTGCCTCGTGCAATCTTTTAAGGACCCAGTCCACATATTAAATGTGGGAC

TCTTCATCTTCTGAGAAGATAATTACGTCATCGACA
>Ref_dvir_690
CCTAATAATGATACGCTGAAAATCAAGCAAATAGGGCGACAAAGAGTCTTTGGTTGAAATACCAAAGAA
AAATTTGAATGCCATCCTGTGCCACAGATGCCAAGAATTTGGACATACAAAAAACTACTGTATGAGGCCT
TATATCTGTGGAAAGTGTGCTCGAAATCATCCAACAGCTCAATGCAAAGAAACTTTGGTTGAGAATCTTTG
CTGTATCAACTGCGGTGGAAACCATGAGGCTTGGCATAAGGGATGCAATGTGTTCAAAGCTAGAACGCAA
GAAAGAAGACCAAAATTAAGTATTGGATTGAAAAGTCATGTCAGTAGCAAAAAAGACCATCAGCAACAC
ATTCCAGCATCCTTAGTGCGAGGAAATTTTTCGTACGCCGATACTCTGCGTACTGGGCTGGAAAAGTTCAA
TAAAGAGACGTCAATTAAGAACCCTACCCCCAAGCACCAAGAAACCCCACCCCACATTAGTTCAAGGCTG
GAGGCTGCAATAGAAGCACTCACTGTGAAAGTCGACACTCTACAAGCTATGTTTAAGGAACTTCAGGAGA
CGAATCAGTTCCTTAAACAAATGTGTCTGAGTCTATTGAACGCCAAAAAATGATGCACAGTTCACTAAGC
CTCGGATTGTGGAATGCCCGTGGTATCTCTGGGCATGTGGAGGAGCTAAAGCACTTCCTAACTGAACATA
ATATTGATGTGATGCTAATTACATGGCTACGGCGGTGTATCCATCTTTATTCAGGCTACAAAATTCACAAA
GCGGATCATCCTAGCGTAGAGCAAGAGCAGGAGCGGCGGTGTTAAGCAGGAGATCGAATATTTCCAAAA
TGAAGGAGAAACAGATTGCAACTAGCCGGGGTGAATTAACCATTGCTTCGACATATCTCCCGCCGCAACT
GTCGTGGTCAGCAATGGAGTTCCAGCAACTTTTCAGCGGACTAGGAAGAATGTTCCTTATGGGAGGCGAT
TTTAACGCCAAAAACAGATGCTGGGGTTGCTTTAGAGCTAACGCAAGAGGCACCTGCTTACTGGACGCGG
TGCAAAACTCATCATCTGAAATACTAGCGACTGGACAAGCCACTCATTTCCCGTTTGCCCAAAGTTACACG
CCGAGTGCCATTGACTTCTTTATTTATAACGGACTTCACCCAGATGGCTTAAGTATAAGGCCTGAGTTTCA
GCTATCCTCTGATCATATCCCACTAGTAGTTGGTTACGACTGTAAAAGGGCAAAACAATTAAAGGTCGTCT
GCTGCCTCCTGGAGCCAACATTGCTGCTTTTCGACGAGAGAGGAAACTCTAGACCTAAACTTGGAAATAC
TTGCACCAGAAGACATCGAAGATGTATATGCGCTATTCCTGAACAAGGTTCACTCGGCACCCCTCATTTCT
GCGCCAGTAAACGACCACAAGTGAGACTTCCGCAGCAGGAGATCCAATTGTGGACAGGATCTACCGCAG
AGTGTCGAACCGTGTCAAAAAGCTGCTGCAGCTGAATAAAAGGGAAACCGACCGTTTTTTGGAAAATGTT
TCTCCAGATGAATCTTCAAGTCACTCCCTATGGAGACTGACGAGAAGGTACAAACAGCAAGTGATCCCCA
AATTTCCGATCAGAAAGCCTGATGGATCTTGGGGCGATCCCCAAAGAACGGGCTGATGCATTCGGATCGC
TTCAAGCCTTTTAATACAAGCTCGGCAAGCATGAACAGAAGGGTTCAACTCACACTGGAGAGCCCAGGCC
AGCTAAGCCTCCCAGTTGCTCCTATCACTGCCAACGAAGTATCAGACCACATAGCAGCACTTAAGACAAG
AAAAGCTCCTGGCGAAGATGGGATCGACAACATAACACTTAAAGCATTGCCGGTCAGAGCAGTCCTATTT
TTAGTGTTAATCTTTAATGCGGTATTGAGACTGGGGCATGTTCCAAGCCAATGGAAAAAGGCGAGGATAT
GCATGATCCATAAGGCCGGAAAGAGCCCCTCGTCCCTAGACCTCATTCAGGCCTATTAGTTTTGTTGCCCA
CGAATGGAAAACTCTTCGAACGTAGTCTGCT
>Ref_dvir_672
TAATACGTCTTGGCAGTTACACTAATTCAAAGCTGTAGCTGATCCAAAAAAACGTATTGCAAATAACAGG
ACAATGGGGAGTGACGAAACAAATCGATCTAGCTGTGTATTGTCAGGCATTGTTGACAAATGTAAAGGGT
TATTAGTCGGGACCAGCCAGAGTGGCTGCTTTGTTGGGCGCCAAATATATAAATATATGATACATACTGTA
ACATACAACGTATGTAAGCGAGCTGATGTCCGTGTAGCGTCTAGCGAAGCACGCGCTGGTTCCGGCACAG
TGAAGTCGGCTGGGGTGGGTTGACTCATAAATGGCTAAGCTACCTGCTCGCAAGCTCTTATAATTATATAT
AAAAATTTCGCGTGCTATTAAGATGTCTAGTTCGATTGCACATTCAAACTTACGGGGGTTAGGAAAAAAT
CTACAGAGTTACCGAAATTATATTAGATTGATTAGGGACCGTAGCTGTATATGTTGTTGTTATGCTGCGCT
GGTATCAATGCGACAGCTGTTGCCCACCCAACCATGATCACCGGGGCCGCTTGACCAGACAAATGGTCGG
TGATCCCTAAAACAACACAGATTACATTACCCTAACATTCCGACGACGCATGGCTTCAACATAAATAAGG
CAAATTAGCACATAATTATAAGTTCGTTACACATATTTATTTTTGTGGTGAATTAGGGAGTTAAATCGAAA
TTTTGCTTAGCCATTATACATTTTTTTTATAATAGAAAAGAAAAGAAAATTAATAAAAAATTTATATTACA
CAAAGATAAAGCATTTTTTTTCTAGGTGAGGTTCTAATTTAGTCTAGGACCCAAAATTGGTAAAAGTAAAA
ATTATTCCTTTATTTTTTTTTTTGTTCGACAAAAATTCATTTAAATTTTTAATTCATTTAGCGAAAGCTGTAAC
GCTCACTTGAAATAGATTATTTTTTTAACGTTTTCTTTTTACCGATTTTTTTGCTTTTAAGCGCAGCATTAAA
TTATCCATTAAATTATTATTAGGCCATTCTCTTTTGATTTTCTCGGAAATTAAGTTTAAAGAAAAATAAAAT
TTAAGAGTTTTTTTTGCTGTACTTACATACATTCACTCCATCTCCGAAAGCGCTTCCATCGATGTAACGGGC
GATGATCTGCTGAAAAAAAAAAAAAAAAATTTAGAAATATTTTAAAATCTGGCTCTCACCAGCAATATGGC
GATCCGAATTTCGGAGCATGGAAGGAGAAAAGGAGAGTAAAAAATATAGGCCAATGGAAAGAATATGTA
GGGCAAGAAAAAAAAACGGAGTTAAAGAAAAAAATGCGGAATAAGAATTTGACCATGCGGTTGCACTCT
TTTGCCGAAAAAAAAAAAAAAAAAAAAGAAACTCGCACATAACTAATATAAAATTTTAAATTATGCGCTTA
AGGCGACTTGACTACGCCCATGGCGTTCACAATAGGACTTATTCAGTTTTTTCACTTTTTTTGTTAGCTAAG

CTAATTATTCATTTTTCCAGCAAAAAAATAACACAAAAAAAAAATAAGATTTCGTTTCGCTTAAAAAAAA
AGATAAATGTAGCTTGCTTGACGACGTCAGCCACAAGGAACGATTCCATCAGCGACGGCGATCGTCTCTT
ACTATTCGTCTTTCGAACTCCCATTTTCGAGCAGGCTAGAGAGCACGAAACGAAACAACGAGATTTGACT
GCGATAAAGGCGATTGTAAATAGGCGCACTTCCTTATTTTTTTGAGTCAACCTTTCTCCACGGTGGACCAA
AGGTAAAAGAAAGATCTGAGCTCCGATCGCCTTAGGGAAAGCTATTTACAGCGACACCAGGACAGACGC
CATTTCCAATTATCAAATTAACATTCCTCTACTATTTTTCTCTTTCACTCGTTACAAGCGATATCACGCCCG
GGTTGAGCCATAGATGGCGAGATCTACAAGGGTAAGCAAGGTACAAGACCTTGACTGGATTTACATTGTC
TATTTTTGGGCTGTTTATTTAAACTCAAACAAATTGTTCTTGCTTCAAGCGCCACTACAAT
>160_rnd-1_family-61__Unknown
ATTTTTCATTTTGTATTTTTCAGCCAAATCTGCCACGCCCATGGACAACATTTNATCAGCAAATGATCGCTA
TTCATGGTACCTAGTTGACCTATATATCCTTGGTTATTCCATATCATGGATATAGTCCTAGCGAACCTAAA
GGACNAAGTTCTCGCAAAATGCGCAGGAACATGGTCCCAAAATATGTATGCTTTTCTTTTACAGCCGCCCC
CAGCAACAGCCCCGCCCCCTGGATGGTTTCTAGTTGACTAGCATATCCTTGGTTATACCATACCATCGATA
TAGTCCTTGGGAACCTAAAGGATCAGATCCCTGCAAAATGTGCATTGAAATGGTCCAAAAACATGTATGC
TTTTTCTTTCAGGAGCCCTTTTACATTAGCTGCAGCTGCAGCTGTTGCTTAACAGCCGGCTTACAGCGCTCA
TTTNACATGAACTGCTANTTAACAGCACTCATTTTAATTGAGTTACATTTTTGCCCCTTNATGCTATGCAAT
GAAAATTTTTACGAAAACCACTCGTCGACCTTCTGGGCATTTCTGTTCGCCTGGTATTTNAGCGGAATTTT
AGGGCAAATTTGGGGCCCAGCCGGCGACCAGGGCGCCCCGTNNGGGTATGCAATGAATTTTTTCGGCAGC
TAAAAATATTTTTCGTCGACCTTCTGGGCATTTCTGTTCGCCTGGTATTTTGGCAGATTTTAAGAAAAATCT
CGGCAGCCAGCGGTTGCCAACAGACGCCCCTTTGGGTNTGCAANGACCGTTGATTTTTGAATTTTTAAATA
AAAAAACAGGAATTTCGCTTTTATTTAAATAATTGATTTATTTATGTTTAAATTTACAAAATTGTTGTTTT
TATGCTAATTTTCTTAATTATTTCTCTTAAAATTATACAAATCGAGCATAAATTATAATTTTAGCTTATTTA
AACTTAAAATAACTATATCTGGCTTAATTAAAGTCCGATCCTGGAAAAATTTGCTTTGTCAGTCGTGGAGG
TCCTTAGAGCTCATTGTAGTCCTTAATTTGTCAGTCTGAAANGATAAAAACACATTTAAATTAAANAAGGC
GAAAAATATATAAAAGCATTTTATGTAGAAAANGGCCATAACTCGGCCATATCCTTGCAGATTTTCAAA
GGACATGGTGCGTTAGGATCGTATAGACCCCCTGCATCGATCTGCATCAAAAAAATATGGGGTCATCTAA
GAATCCAGGACTTGTATTTTGACTTGTAAAATTTCAGTCGCGGAGCGCACGGAAAAATGGCCCGAAAACA
CAAAATCCTTGATATCTCCGAAACTACAAGGACGATCTTGATGNANTTTGGCCAGTTGGTAGTCCTGGTCA
ATACAATTTTTTTGACACAGGACTCGCCAGGATACGCAAGGATACGGCCGAGTTATGGCCTATTTTCTGTT
CGCAAAATTGTCCTTAAAACTTCGCCGTTTGAAGGACTTTCGTCCTTTGGGTTGTANATTCTTAAATGGGA
GGNCGAGGACTATCTGCTGGCCAAAGGACATCCCGATAGTCCTTCAAATGGCGAAGTTAGCCCGGATTTT
TTCGATTTTGGCCAATTTTGCGACTAACCCCCTAGGAAAAATTTCGGTGAAATTTTTTGTCGAATCCTGGA
AATCCTTGAATGCCAATCGATAGTCCTGCTCCCCACGAGTTCAAAAAGGTATATCCTTCCGATATCCTTAA
GGATNTGGCCGAGTTATGGCTANNTAAAAAATGTCCTTTTTAGAAAATTGGCCATATCTCGGAAACNACA
AGGACGATTTTGATGTCCTTTGGCCAGTTTATAGTCCTNGTCCATACGATTCGTTTGACACAGGACTCGTC
AGGATCCGCAAGGATACGGCCGAGCTACGGCCTTTTTCTNTACACGAAAGAATGTCCTTATATCTCGGCTN
CCTGAAGGACTTTCGTCCTTTTCATTGCATATTCTGAATNGGNAGGACGAGGACTATCNGTTGGCCAAAGG
ACATCANGATCGTCCTTGAAATGGCCGATTTACGGCCGATTTTGTGT
>9_rnd-5_family-2924__RC___Helitron
TTGTTTTTGTTTTTACTTTCTGTTTGGTTGTGGTTTGTGTTATTTTTTGTTTGTTGCTCCTCTTGGTTGCAAGA
CATTTTTTGTTTTGTGAATTGACTTTAATCACTAAGTGGCCTTTTATTTGAGATTCTCCTTGATCGGGAGTT
GTTTTCCTTCTCTATATGAACTTTCTTTTTGTTTATTTGTAATCGCCACTCGTGGCATGATTCTTTAATATTN
TGGCTGTGTACATTTATTATACCCTGAACCCATTGAAAACGGGTATAAGGGTATATTGTATTTGTAGCAAT
TCAAATGTATNTAACAGGCGGAAGGAAGCATCTCCGACCCCTAATGTGTATATATTCTTGATCGGCACCA
ANAGCCGAGTCGATTTAGCCATGTCCGTCATACGAGTCCAGATCTCGGAAACTATAAGAGCTAGAGACTT
GAAATTTAAGATGAGTTTGTTTCCGGTAATCTAAAAACACTCCGTNTCCAAGCAATCGGTAAGAGTCGAT
ATCGANATCNTNTTTTCCGAGTTTAATCCACAATTGTGTAAACNACTTTTTTGCAGAATACACAAACANTN
TATGGCACANTAANATNTACTATAGACANTTCCNTTAAGATCGGTTAAGAAAAAAAAAAAAAAAGTACCT
AATTATTGGTGTGGCTGCTGAGTAGAGGCATAGCAAGTGGTGCGGTCTGTCGCGGGTTTAAGTCCTACCG
GTGTAGTATTTTTTTTTGTTTTATTGCTGGTATAGCTGTTGAGTAGAGTTCACAGCAAGTGATGCTGTCAGG
GTCAGTTGCGAGTTCGAACCCTGCCAAGGCAACCTTTTTATACCCTTGCAGAGGGTATTATAATTTTGTCG
CGAAATGTGTAACGCATAGAAGGAGACATCTCCGACCCCATAAANTATATATATTCTTGATCNGTATCAA
CAGCCGAGTCGATATAGCCATGTCCGTCTGTCCGTCTGTCTGTTTCTATGCGAACTAGTCTCTCAGTCTTAA
AGCTATCTTGACCAAACTTTGCAGAACTCCCTCTTTCTGTTGCAGGCAGCATATATGTGAAAATCGGTCAA

GATCGGACCACTATATCATATAGCTGCCATAGGAACGATCGGTCGAAAATTAAGTTNTTGTATGAAAAAC
ATTTTGTTTATCAAGATATCTTGACCAAACTCGGCATTTATTAGTTTTACTATGCTTCTCATATATATGCGA
AATCCCGTTAAAAATTAAGTTTTTTGTATGAAAAAAACATTTTGTTTTTTCAAGATATCTTGACCAAACTC
GGCATTTATTAGTTTTTACTATGCTCCTCATATATATGCGAAAATCCTATTAAGATCGGACCACTATATCAT
ATAGCTGCCATAGGAACGATCGGTCGAAAAATTAAGTTTTTGTATGAAAAAAACATTTTTGTTTTTTCAAG
ATATCTTGACCAAACTCGGCATTTATTAGTTTTACTATGCTCCTCATATATATGCGAAAATCCTATTAAGAT
CGGACCACTATATCATATAGCTGCCATAGGAACGATCGGTCGAAAAATTAAGTTTTTGTATGAAAAAAAC
ATTTTGTTTNTCAAGATATCTTGACCAAACTCGGCATTTATTAGTTTTACTATGCTCCTCATATATATGCAA
AATCCTATTAAGATCGGACCACTATATCATATAGCTGCCATAGGAACGATCGGTCGAAAAATTAAGTTTTC
GTATGACCAAACTCGGCATNTACTAGTTTNACTATGCTNCTTATATATGGGCAGAATCCTATCAGCATTAT
GAAAAGGTTGGGTCTGGGTATTAGATCTTTGGCGTGCCGAAGATAGCCCTTCTTTCTCGTTTTAAATTTATT
TGGTGTTGGAATAAGANGGTTCCTTAGTCGGGAGCTCCCGACTAGAACCTCTTACTCTGTTTTTATTTGTG
GTCAGTCTTGAATAAATATTTTATTATTCACTGTGTGGTTAGTTGCGTTTTTCTTCACTATGTGAATTTTTTT
ATATTATGGTACATAGTGTGATTAGGACTAGGAC
>9_rnd-4_family-690__RC___Helitron
TCATGATAACAATCCAACTTTAAAAGATTTAAATCATTTATACCGCGGATTTTATACCCTGAACCCATTGA
AAATGGGTANGAGGGTGCATTGTCCATTTGTGCAAATGTATGTAACAGGCAGAAGGAAGCATCTCCGACC
CCATAAAGTATATATATTCTTGATCAGCATCAATAGCCGAGTCGATCTAGCAATTGTAATAGCAAAAGAT
CGTCATGGGTAGCCCTAGCTATGTTATGATAGTGTCGTTATCAATGTTAAGTAAAACAGCATCATAAGTGT
TTATCGATATTACGTCTGTCCGTCCGTCCGTACGTATGAACGCAAGGATATCAGAACCTATAAGAGCTAGA
GACTTGAAATTTTAGATGNAGGACCTCCTATTGCCTGCGCAGATCGAGTTTGTTTTCGATATTAGATAACT
TGCCCCGTTTCCAAGTAATCGATAAGAATCGATATCCGTTTATTTTGAACAATTTTGGTAAATANTAAGAG
CTAGAGTCACCAAACTTGACATATNGCTTCTCGANTAGNATGTATCAAGTATCCTTCATTATCTCTCCGCT
ACTACCTCATAGCTATAAATCAAGGTAATAAAACCAATATACAACACAAATAAATGCAATTGACTTTTTG
AGAATACAAAAATATTCTATGGCAGAATGAAATAAATATACCGTAGAGAATTTAAATCTAAAATNAAGAC
CGGTTAAGAAAAAACCAAAGCTNCTGTTTTGCTGCGCACATTCGATGGGGCAGCTAGCGCAAATCGCAAA
AATTTCTTTATACACACACACACACATTCATGCGCGTCCGCTTCGAATTCTATCAACAGCATTGCAATCGC
GATTGTTTTCGGTGCTGCTATTTTAATTAGTTTTTCTCAATAATACACAAATATTGGTATGGCTGTTGAGTA
GAGGCATAGCAGGTGGTGCGGTCTGTCGCGAGTTCGANCCCTACCGGGGGAAANTATTATTTTTTTNTTC
GCTGGTGTGGGAGCTTTTTTTCTTAAGATATCCTTTTTACTTAAAACAAGCGCGTTGCGAGAACTTCATTTT
GTGTTGGAATAAGACGGTTCAGGGTATCCCCTAGTCGGGAGCTCCCGACTAGAACCTCTTGCTTAATTTGT
TTCTTACCTCCAAAATTGAATAGGTTATTCTTTTAGTTAACATAGCTGTTACACATAATTATTTCTACTATT
TTATTACTTTTAAACTTGGATTGCATATCCTCTTTTTATACCCTTGCAGAGGGTATTATAATTNTGTCGTGA
NNTGTGTAACGCATAGAAGGAGACATCTCCAACCTCAGTACACAAAATGCATTCATATATCAACACAAAT
TTGGGATAGGTCATGATCTGCGCTAGAAAACTAACAAAATTAATTTCTTGCCAGCTAAAGCCAGAGCAGT
GAATAACAACACATACATACATACTTTAAAGTTCTTATGCATGTATAATTCACGCATACAACGAAAGGTG
CACGCACGCTATTGGCTGAGACAAGAACTTTTCTCGAGCATACACTGTCCAAAAAGAAAAGTGAAATCAA
TCTATGTGTGCTATTTTCTTAACATAGTTATTGCGCAAGAAGCGCCTTGTGTAAAAATTATATAAGTTCTTA
AATTTTCCTGTAAAATTAATCGTTAATATCTAGGGTAATATATCATTTAAATAAAGCATGCATTTCATGGA
TAAAGCACAAGCACTTTGCGTCGATCGTAAGTTCTAAACTTTTTCGCTCAATGTACTTCGAAAACGAAGCG
ACGCAAAGCAGCATCGACCAGCTTTGCTGAAGGCAGGCTGGCGACTAACTTCGTAGCTCGTGTCAGCATC
AGCAAGCCGCGACTTCGGCATTCGTTCGTCTGGTCTTTGGAGAACGAACAACCAGTCGGCAGGCATGCCG
TACGAAGCCTCAAAGGCCAGATGAAAGGCCAGGCTAGAAATTTTGCGTCGCATTCAAATGTATGGGCGTT
ACTCATCTTAGTGTTTATATTGTCTTTGGT
>9_rnd-5_family-2101__LINE___I-Jockey
AGTAACAACAAAAGTAGCAACGGAAATACCTNCAACAACAACGNGCTGCAACCGCATGGAAGNAANA
AGCNANAANACAAAAANAAAATNATTACAACAAAATACGTCGAGGGAAATTAATCTGGCGGAGGAAAC
ACAAAGGTATACTGAGTGAGTAAAGGTGTTTTAAAAAGTGTTCGCAAATGTCCATACGCATCACATTAGA
TGAGTATAGGGCAAGANGACTAACAGCCAGTGCCCCGGCCGTCAATNAAGAAAGCGTTGGAAAAAAAAC
CAAGAAATCGTGGCGGCATACGGCTGCGTATTAAAAAGCGCAAAGCCGAAATATGCNAGNTATTATATA
ATAGCACAAAGCCCCCGACTTGGGAAAACGCGAACAGACTAGCAAGAGAGCTAGATTTCCTCACAGAGG
AGGAAGAGCGCAAATATAAAACACAAAACACCTTCTCCTCAACACATTAAAAAATTTATTAAAAAACTTC
ACCGCCAGAGANAAAGAAGCTGCCCAACGAACAAAGAAGTGCCCTGACGACTGCGCTGGACCACTGTAC
ACCCCGGCCTGTGCGACGACCTGCACCGGCAACGCTGACCGCTCCAAGGCAGCCAAATAAAAAAGGGAA

CAACACGACATCTTCACGCAGCAGCAGCAGAAGAAGCAGCATTATTTAAAGATAATATTAANACTTTATA
TAATTTGTAATGACGCCCACTGTGTTGTGTGCGTCAAGGCAGAGTACAACCTAAAAAGTTGTACGACACA
TTAGAGAGGAGATCACTGCGTTGATCGAAGTAGGATTATTATATATTAATATACANAAACGGAAAAGGCC
TTCTGCCAAAAATATATATTTAAATGAAAAAAATTTTCCAAATCGATCAAATTAAGGAAAATTTGAAAAA
GTAATCTAAAAAGAAATTAGATGAAGTTATATATTTGTGTTTCGTATTTGTATGGGNACTTCAAAAATNGA
AAAANAAAATTTTTTTAAAATAAAAAATAAAACTCTCGATANAAATACATGCCCCGTTNGCTTCATTAGG
TGTTTGAACAATAATGTACATTCAACGTAACACTTCGCAACACTTCGTAAATACGTCATGGTTGAAGACAA
GATCAATCAGGTNTACAGAGACAATCCAAACTTTCGTATGCGAGCCATACANATTTTTGAAAGGCATAGG
GAATTCATACGAGTATATGAATTCGAAGATGAATATGTTGAAACATACGGAACAACAACAAGCCCACTGT
TCTCATGTTCTGAAATAGAGAGGGGAATAGTCTTGCCTAATCTACCATACGAAATCATAAAAACCAGTGT
TGACGAGCATNCCATCCTAGGGCNAACACCNATTCTTCTAATACGTCTGCACGGGTAGAAAAATAAGAGA
GGCATATATGTATAATTAAAAAAAAAACATAAAATATGGGTGGAAAACAATTAAAGGCAGATGATAACA
CTGCCAATGTAGTTAACGTAGTTGAAGTTTTAGACACCACCCCCCATGTGCAGTTAACAAACATACTGTTA
ACTATACTCTTTGTTGTAGCCTCAGTTAACATCCTTCTGAAGATTTATCTTCTGCATAAAAAATTACTGAAA
AAGCGGTACATGAGTCCAGCCAACGACTTAGACAAGGTCTAAGCCGCCGCTTAAATACAAATTTGAATAT
GCCAGATATAGACGCATTGCGCTTACGTATAGAGATGAGCGAGATCTTCGTACTCGCGCACTAAAAGTGC
AGCGACTACGTAAATTGACAAAACAATGGCCCAAACAGCTATAAGTTTCTTAAACTATCCTCATCGCTCT
TGCCAGACTTTGATGGAAAGGCAGAAAACTTACAAAGCTTCAATGATGCTTTCAGTCTTATAGATTACTAA
AAGAAGATCATGAAGCAACAGCTGTCANTCTTATGAAGACAAAATTAAAAGGCCATGAACGAAACTTCG
TAAGCGATGAAAAACAATCGCCGAAATAACATCAAAA
>9_rnd-1_family-60__RC___Helitron
AAGATATCTTGACCAAACTCGGCATTTATTAGTTTTACTATGCTCCTCATATATATGCGAAATCCTATTAA
GATCGGACCACTATATCATATAGCTGCCATAGGAACGATCGGTCGAAAATTAAGTTTTTGTATGAAAAAA
CNTTTTGTTTTCAAGATATCTTGACCAAACTCGGCATTTATTAGTTTTACTATGCTCCTCATATATATGCAA
AAATCCTATTAAGATCGGACCACTATATCATATAGCTGCCATAGGAACGATCGGTCGAAAATTAANTTTT
GTATGAAAAACATTTTGTTTTTCAAGATATCTTGACCAAACTCGGCATTTATTAGTTTTACTATGCTCCTCA
TATATNTGCGAAATCCTATTAAGATCGGACCACTATATCATATAGCTGCCATAGGAACGATCGGTCGAAA
ATTAAGTTTTTGTATGAAAAAAACATTTTGTTTTTCAAGATATCTTGACCAAACTCGGCATTTATTANTTTT
ACTTTGCTCCTCATATATATGCGAAATCCTATTAAGATCGGACCACTATATCATATAGCTGCCATAGGAAC
GATCGGTCGAAAATTAAGTTTTTGTATGAAAAAAACATTTTGTTTTTCAAGATATCTTGACCAAACTCGGC
ATTTATTAGTTTTACTNTACTCCTCATATATATGCAAAATCCTATTAAGATCGGACCACTATATCATATAGC
TGCCATAGGANCGATCGGTCGAAAATTAAGTTTTTGTATGAAAAACATTTTGTTTTTCAAGATATCTTGAC
CAAACTCGGCATTTATTAGTTTTACTTTGCTCCTCATATATATGCGAAATCCTATTAAGATCGGACCACTAT
ATCATATAGCTGCCATAGGAACGATCGGTCGAAAATTAAGTTTTTGTATGAAAAAAACATTTTGTTTNTCA
AGATATCTTGACCAAACTCGGCATTTATTAGTTTTACTNTGCTCCTCATATATATGCAAAATCCTATTAAG
ATCGGACCACTATATCATATAGCTGCCATAGGAACGATCGGTCGAAAATTAAGTTTTTGTGAGAAAAACA
TTTTGTTTTTCAAGATATCTTGACCAAACTCGGCATTTATTAGTTTTACTNTGCTCCTCATATATATGCAAA
ATCCTATTAAGATCGGACCACTATATCATATAGCTGCCATAGGAACGATCGGTCGAAAATTAAGTTTTTGT
ATGAAAAAACATTTTGTTTTTCAAGATATCTTGACCAAACTCGGCATTTATTAGTTTTACTATGCTCCTCAT
ATATATGCGAAATCCTATTAAGATCGGACCACTATATCATATAGCTGCCATAGGAACGATCGGTCGAAAA
TTAAGTTTTTGTATGAAAAAACATTTTGTTTTTCAAGATATCTTGACCAAACTCGGCATTTATTAGTTTTAC
TNTACTCCTCATATATATGCAAAATCCTATTAAGATCGGACCACTATATCATATAGCTGCCATAGGAACGA
TCGGTCGAAAATTAAGTTTTTGTATGAAAAAACATTTTGTTTTTCAAGATATCTTGACCAAACTCGGCATT
TATTAGTTTTACTATGCTCCTCATATATATGCGAAATCCTATTAAGATCGGACCACTATATCATATAGCTGC
CATAGGAACGATCGGTCGAAAATTAAGTTTTTGTATGAAAAACTTTTTGTTTTTCAAGATATCTTGACCAA
ACTCGGCATTTATTAGTTTTACTATGCTCCTCATATATATGCAAAATCCTATTNAGATCGGACCACTATATC
ATATAGCTGCCATAGGAACGATCGGTCGAAAATTAGGTTTTTGATTAAAAACNTTTTTGTTTATCAAGATA
TCTTGNCCAAACTCGGCATTAACTATTTTCCCTGTGCTTCTTAGATACGGGCAAAGCACTATAAGCATTAT
GAAAAGGTTGGGTCTGCAAGGGTATTAGATCTTCGGCGTGCCGAAGATAGCCTTTCTTTCTCGTTTT
>9_rnd-1_family-46__RC___Helitron
ATATCCAATAGCTCTTTTTGCTATTCTAGCTGAAAATGTCTCAATAGGCAAACGCGTACAGTTTTTTCGAG
CATGATACTGCAAGTGAAATTCGGTGAGATAGAAAATATGTAATTTGTTTTTAGATTATTCCTATCACAGT
GTTGTCTTTTTTGGTATGATGGACAGATTTTTGTACATTAAATTCAAGATGTATATAGGAAAATATCAATC
TCTTTGTTTAAAATATTTTGTGGTCCTGAAAAGGACCGATTTTTTTTATATTTATGTATTAGAGACGAGGTA

GACTTAAGCGCGCTCCCCACGAATACGTCTGGCCAGTTGAATGTCCTTGGGCATGATGGTGACACGCTTA
GCGTGGATAGCACACAAATTTGTATCTTCGAATAGGCCAACTAGATAGGCTTCGCTAGCTTCCTGCAAGG
CCATCACAGCCGAGCTCTGGAAACGTAAGTCGGTCTTGAAGTCTTGAGCAATTTCGCGTACTAAACGCTG
GAACGGCAACTTGCGAATAAGCAGTTCCGTGCTCTTCTGGTAGCGACGAATTTCACGCAGGGCAACAGTG
CCTGGCCTATAGCGATGAGGTTTCTTCACACCACCAGTTGCTGGAGCACTTTTACGTGCTGCCTTAGTAGC
TAACTGTTTCCGAGGGGCCTTTCCTCCAGTTGATTTACGAGCAGTTTGCTTAGTACGAGCCATCTTTCTTTC
ACAATTCCAACTTCACACTGTCACTGTTAAAAACACAATAATCGTGCGTCTCGTTTGTGTATCGCTTATAT
AGAAAATGAGTGATAAGTTAGAAAGAGATCGATATATTCGCTGTTCGTCGCTTTGCCGAATATGTTACGA
GGGTAGTGTGTGAGCGAGATGGAAATATTCTTGTACTCTTTCGAGTTTTGGATATTTTTGGTATAAATATA
AGCGCTGCCCGAAGAGCTAGCATTAGTACCTCAGTGACATTCGTGGTGTTAAGTTAAATAAAAAATATAA
AGTGAAGAATGACTGGTCGTGGTAAAGGTGGCAAAGGCTTGGGTAAAGGCGGCGCNAAACGTCATCGCA
AAGTGTTGCGTGATAATATCCAGGGCATCACGAAACCAGCTATCCGGCGTTTAGCTCGTCGTGGTGGCGT
AAAACGTATTTCTGGTCTTATTTATGAGGAAACTCGCGGTGTTCTAAAGGTTTTCTTGGAGAATGTCATCC
GTGATGCGGTCACATACACCGAACACGCTAAGAGGAAGACAGTCACAGCCATGGATGTCGTNTATGCGCT
GAAAAGACAAGGCCGTACCCTGTACGGTTTTGGCGGTTAAAAAGAAAATGTAGAATTTATGTTGACACCA
ATCGGTCCTTTTCAGGACCACCAATCCGTTTTATTAAAAGAGATNAAAAACATTAATNATTCAACACTAAC
GAAACGTGATGCTGTTAAGCGTCTCCAACTCAGATTCTGAGATCGACCGTGATCACAGATGTTCGTTTTTT
TTATACCCTGAACCCATTAAAAATGGGTATAAGGGTATATTGTATTTGTGCGAAATCCAAATGTATGTAAC
AGGCAGAAGGAAGCATCTCCGACCCCATAAAGTATATATATTCTTGATCAGCATCAATAGCCGAGTCGAT
CTAGCCATGTCCGTCTGTCTGTCCGTCCGTCCGTCCGTATGTATGAACGCAAGGATCTCAGAACCTATAAG
AGCTAGAGACTTGAAATTTTAGATGTAGGTGCTCCTAGTGCCCGCGCAGATCGAGTTTGTTTCCGATAATC
GATAACTTACTCCGTTTCCAAGCAATCGATAAAAATCGATATCGATATCCTGTTTTTTGGGCAATTTTGGT
AAATAATAAGAGCTAGAGTCACCAAACTTGACATATAGCTTCTAAAATAGAATATATATATGCATTTGAT
GTTGGAAGAAGAGGGTTCAGGGTATCCCCTAGTCGGGAGCTCCCGACTAGAACCTCTTACTTGTTTTT
>Ref_dvir_39
TCGCAGCCAGATCCAATTGTGCCCAAACACACCAAATATGCCCATTTAACAATAACAACACAGCCGATTT
ATATTGTGCACTACCAATAATAACACTTTTTCTCTATCCCACAAATACCCAGCATACCAAAAGTGCGCATT
CCGGAGCCAAATCGGAACATTCTCTCTGAGATAAGTATCTAGCCGAGGACTGCCAGGTGAGTTGAAGCTG
GGACTTTGTGCCCAAGCAGTAAATTTACATGTATGGTGCCTGGCTAATACCTTTCTATATTCAGATCGTCC
TAAAGAGCCCAGCATTGCCAACCTCATGATCACCAGCCAATTTAAATGTAAACAATGCATTTGCCTGCTGC
CGATCCAAAATATGCTTGTGTGTAAAATGTGCGCACACGCATATGAGTTGCAATCGCCCCCGCTCCAGCG
GATACCACCAGATGCCGGCCTAAAATGAGACTGCGCTGAAGGTGATACCGCTCCAAAGGACTCAAATCCA
TCTCCATATCCCCACTCGTCTCAAAATTAGGCCATTTAAATTGTTTATCTATACCCTGTCTGCTGCTGGCCA
CTGCCATGCAAATCAATTTGTTAGCCAATATTCCATAGGCCCAATTAATTGTACATACACTTGCATACCCA
TCTCTGGGCAAATGTCAGTGTCTGTCTACATACAAATTTCAAACTTATTCCAAAATTTAATGCATTCCCAT
ATATGTATAGTTCGAATAGGCCCGTCTCAAAGTACCAATCTGTTTAAAATTCTCAATTCTGATTGCCATGT
CATACCAGATACCACAACTCAGCCAAAACGAGCCAAATATCAGCCACCTGACCATCAAACAACTAGCTTC
AGCTAAAACAAACGCCAAACCACTAACAATATGCAAAACAAGCTATTTACAAACATGTATAATACAAAA
AGAAGCCATCTACAAACATGTATAATGAGAATGAAAGCCAAGGGTGGGTGCATGTGAGGAAACATAGTG
GTGCAGTGCCTCGTGTACGGCATCGCTATCACTGACAGTACCTACCCGGAAGCTCTCGACCCCGCGACTGC
CCCAAACCAGCCGCAAACAGCACAGCCGATTTATATTGAGCCTAAAATCCATATATGCCCATTTAAACCA
ACATACAACTGCGCAGCCAGATCCAATTGTGCCCAAAACACACCAAATATGCCCATTTAACAATAACAAC
ACAGCCGATTTATATTGTGCACCGCCAATAATAACACTTTTTCTCTATCCCACAAAACACCCAGCATACCA
AAAGTGCGCATTCCGGAGCCAAATCGGAACATTCTCTCTGAGATAAGTATCTAGCCGAGGACTGCCAGGT
GAGTTGAAGCTGGGACTTTGTGCCCAAGCAGTAAATTTACATGTATGGTGCCTGGCTAATACCTTTCTATA
TTCAGATCGTCCTAAAGAGCCCAGCATTGCCAACCTCATGATCACCAGCCAATTTAAATGTAAACAATGC
ATTTGCCTGCTGCCGATCCAAAATATGCTTGTGTGTAAAATGTGCGCACACGCATATGAGTTGCAATCGCC
CCCGCTCCAGCGGATACCACCAGATGCCGGCCTAAAATGAGACTGCGCTGAAGGTGATACCGCTCCAAAG
GACTCAAATCCATCTCCATATCCCCACTCGTCTCAAAATTAGGCCATTTAAATTGTTAATCTATACCCTGTC
TGCTGCTGGCCACTGCCATGCAAATCAATTTGTTAGCCAATATTCCATAGGCCCAATTAATTGTACATACA
CTTGCATACCCATCTCTGGGCAAATGTCAGTGTCTGTCTACATACAAATTTCAAACTTATTCCAAATTTAAT
GCATTCCCATATGTATAGTTCGAATAGGCCCGTCTCAAAGTACCAATCTGTTTAAAATTCTCAATTCTGAT
GCCATGTCATACCAGATACCACAACTCAGCCAAAACGAGCCAAATATCAGCCACCT
>9_rnd-5_family-1253__Unknown

TGCGTCCTTTAAAATAAGTGTTCCGCCAGAAATATTTACTGACATTTGTTGTAAAGAGTTTTGGCCGGAGC
ANTTAATAGTAAAAGAATATAAGGCCAAAAAGAAAAATCGGACTCCGATTTTATTACCACCTATTTCTAG
TGGCGCANCAACTTTAGTTGCTGCGTCAAAAAACTAATTTCTTCTTTCGAGCTTGCCTACCAGAATGTTAG
AGGTCTTAAATCTAAGCTCGCTAACCTGTATTNAGACTCTTTAGCTTTTAATTTTCAAATNATCGCCTTCAC
TGAGACCTGGCTGAAGCCGGACATCTCAGACTCTTCAATTTTTACCTCCAAATACACCACTTACAGGTGTG
ATCGAGCTTCCCGNATCGGAGGAGGAGTACTCATTATAGCTGTCGAGTCTGCTCTTTCGTCGGAACGCATT
GATCTTCCCATCTCAGAGATTGAGTTGGTGGCTGTAAGAGTTGTATTTGGAAGTTTTTCTATCTTTATTTCA
TGTTCTTATATACCCCCTCTATCTAACTTGTCCACCTATCAAACCCATCTTAATGCTATTAAATCTATCTCC
TCCCTACTTTCTGATAGAGACCTTTTAGTAGTTCTTGGGGACTTTAATCTTCCAGATGTCGAGTGGTCATCG
CCATCAGACTCAAACTTTCTGTTGCCGTCTTCGTCAAACGAATTTATTGATGGTCTACATGAAATGTCCTTA
CGGCAGGTTAATCGTGTGCGCAACTCAAGAAACAGATCTCTTGATCTNATTTCGTGTTGGATTCGTTCAA
CAGTGTTGTGCACAGGATCAATCCGCTTACACTTCCTGAAGACCCTTTTCATCCAACTCTGGAAATGGTCA
TTGATTTACCAGCCCGAGTCCCTATCAAGGANCCGGAATTAGCTGAACGTACGCGTTGTTTCCGAAAGGCT
GATTTTCATAAGCTAAATGATCTTATTCTGCAAGTCGACTGGTCCCAACTGTACAATTGTGCAGACATTGA
TGTTGCCATTAAACTTTTTTATGATACCTTGTACTCGATTTTTGATGTCTGCGCTCCTCTTGATCACCCAGC
GAGGCCTGATAGGCCTCCCTGGTTCTCAAATGAACTTATGAGGTTAAAAAACGTGAAGTCGAAGTGTTAT
AAAAAGTTTAAGAAATCGGGAAATCACTCTGATTTCTCCCGTTATGCAGTTGCCCGATCGAATTTTTCCAC
TCAAAATTCCATCTGTTATAAGGATTACCTTCGGCGTTGTCGCCTTCAATTTTCACATAATCCTAAGCAATT
TTTAACTTTGTCAATTCTAAGCGAAAGACATATATTTTGCCTTCTTCGGCTAAGTTTGCAGATGAGAGCGC
GAACACTGATCCAGGCATAGCCGATCTGTTCGCAAACTTTTTTCAAACTACCTACTCCTCCTCTAGGTATA
ATAAAAATCAGCCTTATCCTTATCAGTTGCAGAGGTCAAACTGCATCTTAAATCCCATTATTACCGAAAGT
TCGCTTCTTAGCGACCTTAACACTATCAAGCCGGCTTACTCACCGGGCCCTGATGGTGTTCCTGCTTGCGT
GCTTAGATACTGCGCTGCAGCATTATGTAAACCTGTTTTAAAACTTTTTGAATTGTCCGTAAACACTTCATC
TTTTCCGACCATTTGGAAGGAATCATACATTATCCCTCTCCATAAGAAAGGGAGTAAGTCCGATGTCGAA
AACTATAGAGGTATTTTCAAATTATCCGCTATACCTAAAGCTTTTGAGAGAATTATAACTTCTCAACTGCA
ACATTTATGTAGTTCTATTATTTCACCTTCCCAGCATGGGTTCGTGAAGCGCAGATCTACAACCACTAACC
TTCTTGAGTTTACTTCGTTTGTAATTGATGGGCTTAAGAAAAGAATGCAGACTGACGTCATCTACACTGAT
TTCAGTAAGGCTTTCGACTCTGTCAACCACTCCTTGCTG
>160_rnd-5_family-170__LTR___Pao
CTAAGGCGGCTCCTTTATGCATCAATTGCCTTCGGAAGGGGCACACTGTTGCCAAATGTAAGGCAAACAA
GTGCAGAGTATGCAGTCGTTCTCATAACACACTGTTGCATAGATACTCTGCCGTTTCTAACACTCGAGCGT
TACCACCACCCCTTGACTCCGCACAGGATCAACCATCGACTTCACATGCTATGCATGCGAATGTGTCTGAT
AGTGTAATCCTAGCTACCGCAGTCGTTAGCGTTAAAACGAGAAGTGGCGAGTTCGTTTTGGCTAGAGCTTT
ACTCGATTCTGGATCACAAATTAATTTTGTAACAGACGAGCTAGCACAACGATTGCAGCTGCGTAGAGAA
GAGGCTAGTATCAGCTTACTTGGGATTGCTCAATGCAATTTCAAGCTACGGAAGATCGACATGCTCATCGC
CGAGACAAAAGACACTTATTGGCTGGATTGTATCAGGGCGTTACACGCCGTCAAGTCGTGAGAAGACTCA
TGCTGTCAATCTGAGTTGCCAAGAAGAGTCTTATACCTCGATAGACAAAACATTGCAGAAATTTTGGTCAT
TGGAAGAGTTGCCTTCAGCCAAAAAGGTCCGAACAGTTGAACAGCAGTTATGTGAGAGCCACTTTCTAGA
TAATTTGGAAATACTGCCGTCAGGTCGATTTAAAGTAAGACTGCCATTTAAATCAGATCCCAATCAGCTTG
GTAACTCCTTCGAGGTTGCTAAGCGTCGTTTTTTGTCGCTTGAAAGACGCTTGTCTCGAGATCCCGAGCTT
AAGCGCATGTACTTGGAATTCATGGAGGAATATTTAACGTCGGCACATCGTCTNAAATTGCGTTGAATGA
CCTTCTAATGGTTGGACCGACCATTCAAGAGGAGCCNNACTCGACTCTGCTTCGATTTNGACTCCACAAAT
ATGCGTTGACAGCTGACATCAAAAAGATGTATAGGCAGGTCATGGTGCATGAAGCAGATCGAGATTCCAG
CTCGTAGTGCGGAGAAAGAACCCATCTGACTCTCTGAGGTCCTTCGGGTTAAACACCGTAACTTACGGCA
CTGCGCCTGCCCCGGTTTTGGCGATGTTNAACAAAGCTTGCTNACGATATGGGGTGCAACTTTCACGTCGA
TGATTTACTAAGCCTGGTGGCGTAGAGAACCTTCAGACGATTGAGTCTGAAGCGTCGCAAACACTCCAAA
GGTTTCGAACTAACCAAATGGTTTTCGAATTCTCCTGAGTTCTCCGCAACAAACGCAGCTAAGCCGATCNC
AGNGTCTGACACAAAACACTTGGCGTGTTGTGGTTGCCGAATGNAGATGCCTTTCAATTCAAGNTNGACG
ATTCATTTCATGGGCCTTAGAGCGACAAAACGGAACATTCTCTCGGTGACATCGAGGCCGTTTGACCCGCT
TGGCCTGTTAAGCCCTCTGATAATCAAGGCAAAAATCCTACTCCAAGAGCTCTGGCTTCAGAAGTTAGACT
GGGATGAATCTNTCCCTCAGAGTTCGGATAGTTCGTGGCGTGAACCGTAAAGATTGGGTTCAGTCACATC
ATTCGTCTTTGGCAACATTTGTGTCGAATCGAGTGGCTGAAATCCAAGAGTGGACAGAGAACGCAGAATG
GCGCCATGAACCTGCAGAGCAAAACCCAGCAGATGTCTTTTCGAGAGGCCGTCGATATTGGCGAGTTACT
TGCGTCGATGTGGTTCGATGGACCTTCATTTTAGATGGAACAAAGAAAATCCTGGTCGGTTAACCAGCATC

TGATGACAGATAGCTGTTGCACTTGCAGCTATGATAGACCAACCAGATATCTTGGAAACTATCGGTAGTTT
TTCATCACATGCCAAGCTGCTGAGAGTGTTTTCTTATCTGTTCCGCGCTCGACAAAAAAGAGGCTTTCGAA
AAAAAAGTTGAAGTCAAGAGCTCCGCACTTCGGGAGTTTCTGGAAGGCAAGT
>9_rnd-1_family-430__Unknown
ACCACTAACCATACAATGTATAGACATACCAAATGGCACTTTTAATCGAAACAAGAAAAATCGAAACGAG
GCGACCCCAATGGTACCTGCGACCATTTTAGTGTGCCCAGACAGAGTAAGACTGCTGAGAGTGGCAGCCG
AACGCCGGCCTCAAAAGCCGAACGTTCAGTATGAAGCCGCATCTCGCTCAGCGCACAACGTCGCGGCTGA
TGCGTTTTCTTAATATATGTATGTATGCCATTTTTAAATCCTTACAGCCCAAAAACATATATGTGTATGCAT
GTGCCTCGCTCTCACTCATTGCTTCGCGCACTTGCGTCCATCTCTTTCTCCCTGCATAGCTTCCCACAAAAC
CCCGAGATCTACAAACATTCATACGTTTGTGTTGTGTGGATGTGCGCACAAGTAGATCGCGGCGTTTGTCG
CGAGCCAGAGGTTATTTCTTCAATTGTGTGCCTTTTGTGCCTGTGGTAGCAATAGGCACTCACATTTAAAT
GCTCTATCATGTATTTCTTCGTCCAGTCAGGCATGATGAGAAGAATTTGATCAAATTTTATTTAATACACA
ATTAATTGACCTAAATAAAATATTTATATTTATTTTCTGTTTACTGTAGGGACCTATTTTGCTTAAAATACA
AAAATATCTAAGTCGTATTTCTGCAAGGGTAATTCATCGTCGGCTTGTCGAAGAATTTAAGAAAAGACCA
AAAATTGACGCACACTCTTTCAAGTAAGCAGTTGTGTGAAATTATTAAAGGGTTTGGCCTGTTCATGTGCA
AAATTGCTATTTTCCATGCTTGTTGTAGGGGTAAGGGTACCTGATCGCTGGCATGTGTGCATGATAACTTT
TTGGGTCGGCCAACCACGCACACTAACACACTTTTCTATATGGATGGAAGCATTAATGCGTTTGTGTGCGT
GTTGGTTGCCTGCCTGCCTGCCCGATGCAAATTTGACAAAATTTGAAGTTTTTGGCATTTGTGAATTTCGA
GCTCTAACACACACACATATACACACNCACAAATGTGTGCGAATGAGCTGATCAAGGGTTTGTCGTGAGG
CAGAGATATTTTTATCAATGGGTGCGTAAATTTGGGCGACTGGCACGGTCTTTGTTTTTTTGGCATGCCTG
CGGTGGGCTTGAATGAAACAGGTGGATCGCACTTGTCGCTCGTTTGCACTTTGATATGGAACAAATGCAA
GTAATCTTTTATTGGCATTATTATAATCTTAGGTTAAAGGGTTTTATGACAGGCAATTAACGATCAGAATG
TATAGCATGCGTCTGGATCCATACCATTTTCATTGCCTTTTTATACATTTGTGCCTTTTGCGGCTGCAAGCC
CACGGCAGGCATGCCAAAAAAATAAGTTGCCTCGGCAGGGTTTGAGCTCGGAACTAACCGCACCACTTGC
CATCGCCTCCACTCAACAACCACAATAAAAATCGAGTATAAGTGAGAAAAANAAACAACGGAACGGCTT
CCTTCTACCTGTTACAGACATTAACATTTTGCATAAATACAATATACCCTTTTTTCCATTTTCAATGGGTTC
AGGGTATACAAAATACGGACAACAATACAATCGCAATTATAATGCTGTTGATAAATACGAAGCAGACGC
GCATAAATGCGTGTGTACATGCATACAGATATTCTTCGAATTTGCGCTGGTGCCGCAAACACGTGTTTCTT
AATGGATCTTCATGAAGTGTTCTATAGTATTCCTTAATGTACCATAGAATATCTGTGTATTCTCTGAAAGT
AAATTGCATTAAAATTGTAGCTTTAATTGGTGAGCAATGGAAGTTGGGCTATTAACTTGATTTATAGCTAT
CAGGTAGTAGCGGGAAGGTCGCGATGGGTATATGAATGATAGTTGATATAAATATACTATTATATTGTAG
ACATGTCAATTTTGGTGGATCTAGCTNTTACTGTTTACCAA
>160_rnd-1_family-176__DNA___P
TACCANCCACTAACCAAACAACATATAGACTGGACAATCAGTGNACGGCGATGGGGCCNCTCGTTTTCTG
TGCGGCTGGTCCAAAGTCGCCTTCGTTTTTGTTCGGGTTCGAGACACGCTCGCGCGCTCAGACAGCAAGAG
AGTAAGAAATACATATGACTTTGTGCGATAGAACGGCATCGCTGCTTTTTTTTATTCCCTCGCACTACAGG
AAACTGCTTTGGCTTACTCTCAGTGCTCTTTCAATTTGTGTATTTACCGTTGTACTGTCTGTGTGTGTATTTG
TCCGCACAAGCAAAAATGCCCACAAATTCAATTTTTCTCAAAAGTCGGTGGCTTTCTTCCCTTTTGAAGGG
ACGAGTAATATGAAATAATGAAAGCGTTTGCCACGTCGTTGCGAAATTCTTGCANTTTAGCTCGTTATCGT
TGGTAAGGGTACCTAAGCGTGACCATGTGTGTATTTAAAATATTTTCGACTCGCTACTCACGCATACTTGC
ATATTTTGAATAGAACAATTTATGCGCTTGTGTGCCTGTTGGAGGCCTGCCTGGCCGTTCCTTGCAAATTT
GACAAGATTTGAAATTGCTCTAGTTCCACTGTAAATTTGATGTGCTTGTGAAAAGAATTTAACTCTACTAG
AATGTTTTATACTTCAGTTTTGTCCGTTACAGTTACAAAGTGGATGTCTACTTATTTTGCCAGAAGTTTTAG
CTTTGTAGTCAATTTATCAGTAACATGCAGAGGCCGGTGATCCCATTGCCATTCCCATTTACAGAATGTAT
TTTCTGTGTTCATATTTTAAATACCTGTTCCCTTTCAATTTGTGTACAGTACAAGTTCTATTGGTATGCTGG
GAAACAGGGACGACTTCAAAAGATACCAATATATCGTCTTCCCTATTGACTCTGTCTTTGAACTCTAACGA
ATTCTTAATGTTATATTAGTTGTCAAGTTTCCTAATAATTTTGTAATATATGTGTATAGTTCATTTGATGGG
GAACCGATAGAGGAGCATTGTGGTCTCAGAGATATTCCTTTCTTGTGAATTTTTGGTCGTCTATATCTTCTT
GGAGGTCCCGATGTAGAAGTAATCATCTTATTTCTTTTATTCCTTGAAATAGGTCCTATCTTAAATAATTTT
GTCAGAAGACTGTTCGACATAGTCATTTGTCACTTTATTTTTGTTTATTTTTGTATATGTTTTTTGTATTGA
ATCAAATATGGCATTCATTCTAATTACAACACAGTTAACTTAGGTGGTATTTCGCCAAGCCTGGTTGGGTC
ATCAGACTTTATCGTGGGCAAACGTCAACGGCGTCTTCTTTGAGTACGGTCGCATCGTCACGTAGGTAGTC
GGGGCCGTCTCCTGTATTGTAGTTGGAGGAGGTCCTTAAAGAATCTTCTCTTGAGCCTCCTGACCACATAC
CTCCCAGCGAGATCTCTTGCTTCCCGGTTACAGTGTCACTGCAGTGTCTGGATCCTTTGAAGTGTCTGAAT

CCGTCTTCGATTTGTACGCGAGGCAATTCCCCACATCTGGATACCGTATCTCCATGTTGGCGCCACCACGA
CATTGTAAACTGCCCGTTTGCAGCGAAGTGGCAATGGGCTTCTGGAGCTCAGTAGCCAGTTGGCCTCAGC
CATTTTATGGCGGCACTGCTTCAGTAGGCCGTCGATGGTCGACCGAAGTTAAGCCTTTCATCATGTACGAT
TCCAAGATACTTTGCGCTTATGCCAAGGTTAAGGGGGCTGCCATCTATCCAGACTGCTGAAGCCTTGCCAC
GCACTTTGAGGCATTGAACTTGATGTCCCATTTGCGAGCCCAGCTCTCAAGCAAGTGGATGTATTCCCTTG
CCCCATCGGTGACTTCGTACTT
>Ref_dvir_390
TTTAAAATTTGTTCTTGTGTATTTTTGTTTGTGCGCTCGCGCTATTAGCTGTGCGGGTTAGCTTCTTGTTGTT
GTATGTTTAACTGCACATTGCCCCTCTGGTGAGCAAGTTTTCGTTAGCAAGCAAGCAATCTATGAATAGAC
TACTCTCGCGCTCTCTACCGTTTTTCAACTTCCGCTTTGAAATTGTTCTACTTTCTGCCGCTTAGTGCTCCAT
ACGCTATTAATCTAACTCTCTTTTTTTATTTGGCTGCGTTATTACAGTAGAGACCCACAATAATGCACCATA
AAATTGTATGTTAAAAGAAAACTTGCCAAATGCAAATTACGGCAAGTCAATCCAGCATACCTTGTTGGCT
GTATGATAATGTCGTGCATTCTAAATGTACGGGTTACACTGGCCTAGTGGGGGATGCTTTTGCCAAACGAA
ATGGTTTGCACTATAGTTGCGACTCGTGTAGGGAGATTAATGGTAAAATGCGGGCGATTATACGTCAGAC
CAGAAGTGGCTTTAGGGGTCTGATTGTTGGTTTTCGTAAACTAAACGATCAGCTCACAGCCTTGGATACCC
AATTTAATAGCCCAAAACTGTTGGATGAGTCTCCTGGGCCTAAAAAGCCCATTCCTTGTGATTTGACATTG
CCTAATACCCCTCAGCCTCCAAATGCAAATCAGCAGCCTACATCTCGTTTATTACTCCGGTAGCTGGGAGG
TCTGCTGTGGACACTGATTCTGACCTAAGCTGTGTGAAATCGGCACGAGAAAACCAGTCAATTTATCCGTG
TCGTCCGCTGTCATAGAAGTTACGAAAGGGCGATGGCAACAATTAAATTTACAACAGCCCCTAGAAGGCT
CGTCGTCCGGACTACCGGCTGCTTTAGGTACCACAGCTAATAGTGTTCCCACTGCCCTACCTGTGGCGCCT
ACGCCTGTAGTGGGTTTTGTTTCTAGGCTTTCCCCTTACTTCTCATGAGATGGTGTAAAAGCCTTTATACAG
GTAAAAATGCAAGCCCCAGGTATAAAGGTGGGGAATTTTAAACGCGCCTATGGACCTGGCTGAAGCAGG
ACATCTCAGACTCTTCAATTTTTACCTTCAAATACACAACTTACAGATGTGATCGAGCTTCCCGTATCGGT
GGAGGAGTACTCAAGCTGTCGATTTTGCTCTTTCGTGGGAACGCACTGATCTTTCCATCTCAGAGATTGAG
TGGTGGCTGTAAGAGTTGTATTTGGAAGTTTTTATTTCATTGTCCCTTTCTGATAGAGACCTTTTAGTAGTT
CTTGGGGACTTTCATCTTCCATATGTGGAGTGGTCATCGCCATCAGACTCAAACTTTCTGTTGCCGTCTTCG
TCTAACAAATTTATTGATGGTTTACATGAAATGTCCTTACGGCAGGTTAATCGTGTGCATAATTCAAGAAA
CAGATCTCTTGATCTTATTTTCGTGTTGAATTCGTTCAACAGTGTTGTGCACTTCCTGAAGACCCTTTTCAT
CCAACTCTGGAAATGGCATTGATTTACCAGGCCGAGTCCCTATCAAGGATCCGGAATTAGTTGAACGTTTG
CGTTGTTTCCAAAAGGCTTATTTTCATAAGCCAAATGAACTTATTCTACAAGTTGTACTTAAACTTTTTTAT
CTCTATTTTTGATGTCGTACCTCTTGATCACATAGCGAGGCCTGAAGGCCTTCGTGGTTTTCAAAGGAACT
TATAAGGTAAAAAGGAACGTCGAAGTGTTATAAAAAGTTTAAGAAATCGGGAAATCACTCTGATTCATCA
CGTTATGCAGTTGCCCGATAAAATTTTTCCACTCAAAATTCCATCTGTTTTAAGGATTACCTTCGGCGTTGT
CGGCTTCAATTTTCACACAATCCT
>160_rnd-1_family-287__LTR___Gypsy
AGTGAGCGGAGAACCTAAAATGGAATCAGGTGAGTGCCAAAACCCGGGGGTAGGCGTGCGTTGGATCCA
CCAGCGGCGCAAGGAGGAGTTGGTGGCCATCGCCCAGGAGTTCGGGCTAGANAAGGAAGGGCGCGTGGA
GGAGCTGCGTAGGCGGCTATCCACCTNCGTGCAGGAGGGCGGCCATTCGTCTGAGATCTGGGCTCGGCTT
GCCGAGCTGGAGTCTCAGTTCGCGAAAACGTTAGAGCCGCTATCCTGGGGCGTCCCATCTGACGTGGCTG
GACGGCCGCGCCAAAAGTCAGAGCCTGATCNTAGGAGCTCGCAATTGTACGGGCGTCCGCGGCCGCCTAT
CCCTGGTGTGGCCGTCACGGGTGTGGAAGAGGCTCCCGATCCCGGGGAAAACGGAGACCGCCGNGCAAC
CACGACCAACGTAGGCGTGTGGGCCCCAACCCACCATATTCTCGGTCGGAGGTTAGGCCTTTCNCAAACG
CTCCGGAACAAGTTCGGAGGTGGGGCATGAGATACGATGGCCGCTCCGACCCCCTCGGGTTCTTGGAGAC
CTAGAGGAACGCGCCATCACTATGGTATCGCCCTCGATAGATTGCCCCGGGCGATGAGCGAGGTGTTCTG
CGACAGGGCCGCTCGGTGGGCTTAGAGATGTGCCATGGTCGGTGTTCAGGAGGGGTTTCTTGANTTGTTCT
TCCTGCCCCACGCTACTTCGAAAGGCTTGAGGACGAAATCCGGTCCCGCAGGCAACGCGTGGGGGAGGAG
TTCAAGGAGTACCTGNTAGAGCTGAGAGCTCTGATGCATCACGCCAGATACGAACCGGCACAGGAGTTGC
ACCGGATCTACGAAAACGCCGCTCCCGAATACCAAACTCTATGTGCGACGGCAAGATTCCACCCCANTCA
ANCTGACGCAACTGNCACTGTAGTACGAGACGGTCATGAGACAGAGGGCGGAGTACTCGGGAAGCAGGC
CNGGGGGTCAGGTGGTCNCGAGCTCGAATAGCGCGGAGTGGGCGAGGACGGCACAGCCCCCGGGGGGAC
ACCTCAACCCGTTCCGGTCTGCTCCCATCGGATCCGGAACGACCGTTGTGGACAGNAGAGTCTCCGCCCC
GCCGGCAGCGGCTCCCGCGGAGGTGGCCCATCGGATGATGAGCCAAGAGGCCGGGANCGGTATGATCC
GCGACGAGCGTGTCGCAGGTGCGCACAGGAGGGGCACTTCGCCAGGGAATGTCCCAACGCNCCCGTTTTG
TTTTGTTGGGTATGTGGGCGCCGGGGCAGAAGTAGCCAGGATTGTTGTCGCCGCACGGAGGCGGGAAACG

GGCAACGGCGTCGCCCAGGTCAGGGTCGGGTCGGCGCCGACACACACGGGACTCGGTAGTAGTACACCC
GCTTAAGGTGGAGGGTGGCCGGATCGTCGCCACGGTGGAGATCGGCGGGCGGGCGATGCCTGCCACTATC
GACACAGGGGCCACTCGCAGCTTCATGAGTGAAGACTGCGTACGCAAGTGGGCGATTCGGGGGAGAGTG
GAGAACGTACAGGCGCGCATTCGTCTGGCAGATGGGTCCACCNTNGAGGTAGGGAAGGCCTTGAGGATA
GACGTCGGCCTAGCGGGGAAGACGGTCAGCATGTTGATGCTTATCATGCCCGCAATGCTGGACCACGTCA
TCCTGGGGATGGACTTCTTGGGTGCGATGGGCACTACGATACGCTGCGGCGAGGCGGAGCTGGTCCTAGA
GGCGGTGACGTCTCCGGCCGCGGGGCCAGGAACCAGGGAAGAGCCGGAGGAAGGCCTCCCGGGTCANGA
AGNGGGAGCGCCTGCTCGGCAACAGAGGCGGAAGAGACGAAGAAGGAACCGAAGGAA
>160_rnd-5_family-5434__LINE___I-Jockey
CTCTTGTTAATTACCTCTAATAGTAAGGGAAGTATACAGTTTTCATGACTCACTCTGTCAAATGAGCCTAT
CAATTCGTCACATTAGCTTCGAGGAACTTGCAACAGAGATTTAACACACAAACAATAAAAAAGCAGCAGG
ATATAAAAAAAATCGATGGCTTAACCATACAAATCTTCCTCCAAAATGCATTCGTTTCCTAACTTATATATT
CAACACAATACTCAGACTAGATCACTTCCCGAGACAATGGATAAGCGTAGAGATAATCAATATTCTAAAG
ACAAACAAACTTGAAAATAAGCTCTCGTCGTACTGTCCTATTAGCCTAATGACAACATTTTCTAAAGTATT
TGAAACAATACTTTTAAAGAGAATGTTGCCAACTTTGGACGAACCTGCCATCATTCCTGAACATCACTTTG
GAAGAAGAGGCCACGGAACTCCTGAGCAGTGCCACAGAGTAATAAATAAAATCATAGCTTTTGAAAACA
AAACAATCCATTACTGCTGCAAAAATGTGAATCGTTGCCACTGTTTCTCATAAATGCCTGGTTCCTCCATT
AAAAAATGAAAGTAAGCTCCCCAAAATCTGTTCAAATAACAAGTATCTTGGCTTCCATCTAGGATCGGAG
ACTTACATAGAAAAATCATTAATAAATAAAGACAACGAGTGTTCTGGATAAGAGACCAAAAGTCTTAAAC
TAGCTTAGCAAACAATGTTCGCCTCTATAAATCCATACTAAAGCCTGTGTGGATATATGATTTACAACTGT
GGGGTTCTGCTAGCAACTCTAATATTGAAATTATATTATATTATAAATAATTTTTAGCGCTCAAGAAATTA
TTCAGCAGTCAAAAAAAAAAATGCGATCCATAGTAAATGCTCCTTTTTAACGTTCTAATGCAAACATACG
CAAAGACCTAAAAATTGGTTATGTAAAAGAAGAATTAAAGAAAAATAGTACAAAATACATTGATAAACT
TCACAACCACCAAAATAACATAGCCGTAAATCTACTAGATAAGGATAGAAGACTTAAAAGAGTGCATATT
TCTCCCGGATAAATAAATTAAATAAGTACGACTTCAGCCTAACATACGAAATAAGTCAAATATAATAGTA
GATTTCACTGGAAACTTGCTATTCCTGTTGAATTTAATTATAGTTAACAATATTAACTTATTGTCTTAAAGC
AGAATGTAAATAAATAAAATGTAAAAATGAAAATCTTACAAATAAATAGGAGTGGTATGCATAACCGTA
GTAAGATCGGTATTCTACATACCAGACGTAAAATTGGCGAATTCTAGCAAGCAGTTTGCCCAAGAAACAC
CAATCATGTCAATCTGTATTGAGAAAACACTACATGCAAGTGCAATATGTCGGAATCATCAGATAAGCAA
AACTATCTGGATTAAACAGATGATCAGTTGTGACATTATTTAAAAGTATGAAAGCTCCCGCAAGCATGAC
AGCAGACTTCATTATCATTGAGTTTTTTTGACAGCCAAATGTAGTCTTAGGTTCCTAACAGGAAAGAGGAG
TCAAATGTTGAAGATTATATAAACGTAGTTATAAGTCAGCCAGATACTAATTAATACAATTTTTCAATAAA
TGTACTTGTCACGAAGCCAGCACAATTATACATTCCCGCTGCGACATGCCGCCTAAGAAAGATAGCTTAA
TGCTGCATTGCGGCTGCTACCCGTCTATTACATTGCCACCACGCCAGTATTGTGATCTGCCATTAACAATA
GTTAACAATATGGCTATCACAGTTTTCATTATAATAAAATAAAACTTTATGATTTATATTGTCTGCATTTGA
GTTGTATCGACAGTCTATTAAACTTCAGAAAA
>Ref_dvir_741
AAAAATTCAACTGCACCAAATTCATCATATCTACAAGCCCTTAAATGAGAACCCATCCCATCCGAATGCTC
AATCGATCAATCCTAATCCATGGTCACAATCGGTTGGTTCAATATTAAATCCAGACAAATGGAAGAAATA
CGTCTTTTTGAAAGACGCATCCTACGCCGTGCCTTAGGGAAATGCTCCCTCATACCCCACTCACACAATTA
CATATCAAACTCAAAGCTATACAAAATCTCAAAGACCAAGCGAGTAGACACCCTCATATTTAAGATAATT
GAAAAGTTCCTAGAACGAAACCGCAACAGTACAAACCAACAAATATTAGATAGCATAGAGCAGATCCGG
ATACCGGTCGGATTCTCAACAAACCACCTAACCATAAACCATATAAACAGTCTCAAAGAGAAGGGCATAA
TACGCAACTCTCAGAACAATCTAATATTCTACCATAGAAAAAGGCAGTCTATCGACCCTAACGATGGACT
TGCTTACAACATTAGACAATAAAAAACGAAATGCTCATCTTATTATTTGTCGCAACATATCTCCCCCCTTT
CTCCCTATCTTCTATTGTATAAATATTTCCTACCTCCTATCTCTTCCTCAATCCAGTTAACTAGTTCCTAAGC
CCCTTGTTATTACTAATTAAGCTAGTTATAAGACAAAAACCAACAAAACCAAATTAACACAATGAAATCC
AGGTTTTTACTCAATACGAGTCTTTCCTAGGAATATATATATACACAATAAAATTACACAACTACACCAAA
ACATAAACCTTAAGAACTATAAAATAAATAAAATTAAGTAATAGAATTAACGCCTGGATAAGCTAGAAGC
GCAAGGCAACATGAAAATGTAAAACCTAAAATTAAGTTAACTTGACTTTGTCAATCAAGACTGTACTAAT
AAATAAGGTACCAAAATGTATTACCATGTAACCTACTGTGACCAATAAAAAAAGAAGAAAGAAGAAGTT
TTCACACCAAAATAGTGTTTTCAGTTCACAAAAGTAAAGTAAAGCGCACGAAATGTCTGATTCTGCAGTT
GCAACGTCCGCATCTCCTGTACAGCAGTCCGCCACGCACCACCAACTCAGCAAATGGTAGATGCATCGAT
TAAAAATTTGAAGGAACGTGGTGGCTCATCCCTTCTGGCAATTAAAAAATATATCGGTGCTACATACAAG

TGCGATGCCCAGAAGCTAGCCCCGTTCATTAAAAAGTACCTTAAGAATGCAGTTGCGAATGGAAAGCTGA
TCCAAACAAAGGGCAAGGGTGCCTCCGGTTCGTTTAAACTATCTGCATCCGCTAACAAGGATGCCAAGCC
AAAGGCCTCCGCTGTCGAGAAGAAAACCAAGAAGGTAAATGCTTCGGCGGCAGCAGCAACTAAAAGGAA
GAGTAGTACGTCTACCACGAAGAAAGCAGCGGGTGCCGCTGATAAAAAGCTATCAAAATCCGCAGCAGC
CAAAAAAAAATGTTGAGAAAAAGAAGGCTGACAAAGAAAAGGCTAAGGATGCGAAGAAAACGGGAACCA
TAAAGGCCAAGCCTACAACAGCAAAGGCTAAGTCAAGCGCAACAAAGCCAAAGACCCCTAAACCTAAGA
CCACAAGTGCTAAACCGAAAAAAGTCGTGTCGGCTACGACCCCCAAGAAAACTGCTGTCAAGAAGCCAA
AAGCGAAGACTGCATCTGCAACGAAGAAATAATTTGAAATTGCTAACTGCTTGTCAGCATATGCAAATTT
CTAATAATGAAATTTGTTTTCCTACAAAGCCCTTTTAAGGGCTACAAATTACGATTTTCTAAGAGAAAGAA
TTTTTCCTTTTGTGTACAACAGCAAATA
>9_rnd-5_family-2817__LTR___Gypsy
TGTAGTGTGTATATGCCTTAAAAAGAACACCCTATATACAAATATAAAGTATGCGGCACTTTGTCGCATAC
AATAACAATTATACATTAAACAAGCCCAGAGCCACTTTATATATTGGCTCTGGCTCATATTACGCAGCGCA
GTTCACTAGCCTCTCTAGCTGACCGTTTATGCTTACGCTGCATTTCCCACGCTCCGTCTGAGCGCATACCAT
GAGCAACATATATGCTCACTAACACAGGCATATGCAAGCTACTCACTCTCCCTCTCTTTGTAGCTGTTAAC
CAATAACAGCATAAGTATATACATGTATCCATAGGTTAAGCGCAATATTTTACTTTGAATAAAATCATTCT
AAAAACTAAAGTCAAACGGCACAGACGTGCCAGTGTATTAAATTAAATTGNATATCAGTAACGACATTAC
ATGGCGACCGTGACAATTGGTCTCACCAACCTGCAGTGAATAAAAAACATCCCAAAAACTAGTGGAAAG
GCAGCCAAGAAGGCTGGCAACAAACAACAACAAACTTCAACAAAAACAACAGCAACAGCAGATGCCATT
TACTACAATTCTGGCAACAAATACAAAGACAGCAGCGATGGAGGGATAGCTGTGGACAATAAATAAATA
TCTGAGTGAGTAGAGTGCGGATGGATAAGAAACCAAAGAAACAGAAAAAGCGCGGCGGTGTCAAACAAA
GACTGAGGTCGGAAATTGACCGTCTCAGCAAAGACTTGAACGATCAAGATAACCCGCCAGCATGGAACG
ATTCTGTTTCCATGCATGAAAAGCGCTCGTTTCGTGAAGCACAACTAGTGCAATTAATAAACAAAAAACA
AAATCCATCCGTCTCCTACCGCCAGATACAAACTATAAACAAGAAAGTAACTCCTCCTATTCAAACAGCT
ATCCCAGCATATAAGAGACAATGTCCGGCGGACTGTGAGGGTCCATTCTACAACATTGGCTGCGCACTGA
CTTGCGAGATTGCCAAACCGACAGCGCTACAAAGAGCGCAATAGAGGGCAACATACAATCATCCCGGCCT
GTGCGAGGACCAGCACCGGCAACATGAACGAGTTTCGACAGTGGACCGACAGCGCTACAAAGAGCGCAC
TACGAATTTTTTGTTTTATATATATAATGATTTTANAAAATTGAGCGAGAACTCGCCCGATCAGAGTACAT
GTTGGAAGGCTGTACAAAATATAGGCAGGAAAAATTGTAAATTCACCTGCGAGTGAATAATAAAAAGAA
AAGACATATAGCCAAAGGGCAATATAAGAGACAAATAAAATAAAATTAAGTTAAAACGAGAAAGAAGG
GCTATCTTCGGCACGCCAAAGATCTAATACCCTTGCAGACCCAGCTTTTTCATAATGCTCATAGTGGCTTG
CNCCATATCTAAGAAGTACCGGGAAAATANTAAATGCCGAGTTTGGTCAAGATATCTTGATAATCAAAAT
GTATTTTCATACGACTTAATTTTCGACCGATCGTTCCTATGGCAGCTATATGATAGAGTGGTCCGATCTTA
ATAGGATTTTGCATATATATGAGGAGTAAAGTAAAACTAATAAGGTGCCGAGTTTGGTCAAGATATCTTG
GAAAAACAAAATGTTTTTTCATACAAAAACTTAATTTTCGACCGATCGTTCCTATGGCAGCTATATGATA
TAGTGGTCCGATCTTAATAGGATTTTGCATATATATGAAGAGTAAAGTAAAAACTAATAAATGCCGAGTT
TGGTCAAGATATCTTGAAAAACAAAANTGTTTTTCATNCAAAAACTTAATTTTCGACCGATCATTCCTATG
GCAGCTATATGAT
>160_rnd-1_family-70__DNA___P
TTTAAATGCACACATGGCAACGCTTGGGCACCCTTACCAACGATAGCGAGCTAAAATGCAAGAATTTCGC
ATCGACGTGGCAAACCCTTTCTCTATTTCATATTGCTCGTACCTTTAAAAGGGTAGAAATGCCTCAGCATT
TGAGAAAAATTGAATTTGTGCGCAGTTTTGCTTGTCGGACACATACACACACACACACACATTTAAACGA
TCCCTAAACATAAAGAGAGCATTGAGAGCTGAGAGTAAGTGCGAGCGAGATAAATGTTAACAGCGTTGC
CGTTCTATCGCCCAAATCATATGGAAGAGAGTGCGCCAAGCACTTTCCTGTAGAGTAAGCGGTTCAAAAA
TACATCATATGTATATCTTACTCCCTTACTCGCAAGTTTGGACCAGATGCCTCCGATACATGTTGTGCCTTC
TTTCCGGTACTCATTTCTAGTCTATATGTTGTTTGGTTAGTGCTGGCAAAGACAATATAAACACTAAGATG
AGTAACGCCCATACATTTGAATGGGACGCAAAATTTCTAGCCGGGCCTTTCATCTGGCCTTTGAGGCTTCG
TACGGCATGCCTGCCGACTGGTTGTTCGTTTTCCAAAGACCAGACGAACGAATGCCGAAGTCGCGGCTTG
CTGATGCTGACACGAGCTACGAAGTTAGTCGCCAGCCTGCCTTCAGGAAAGCTGGTCGATGCTGCTTTGC
GTCGGTTCGTTTTCGAAGTACATTGAGCGAAAAAGTTTAGAACTTACGATCGACGCAAAGTGCTCGTGCTT
TATCCATAATATGCATGCTTTATATAAATGATATATTACCTTAAATATTAACAAATAATTTTACAGGAAAA
TTTAAGAACTTATATAATTTTTACACAAGGCGCTTCTTGCGCAATAACTATGTTAAGAAAATAGCACACAT
AGATTGATTTCACTTTTCTTTTTGGACAGTGTATGCTCGAGAAAAGTTCTTGTCTCAGCCAATAGCGTGCGT
GCACCTTTCGTTGTATGCGTGAATTATACATGCATAAGAACTATAATGTATGTATGCATGTGTTGTTATTC

ACTGCTCTGGCTTTAGCTGGCAAGAAATTAATTTTGTTAGTTTTCTAACGCAGATCATGACCTATCCCAAA
TTTGTGTTGACATATGAATGCATTTTGTGTACTGAGGTTGGCTCCGCAGAAAATGTGTATTTCTAAGTCGA
TGCAAATATTTACATAATTTCTTTAAATTAAATTAATATTATTTTTATTCTCCAATTAANAAAATAAAAAN
AAATTAAAATTAATTAAATGTATCTTTGAAAACAACAAATTTGCACTTTTATTTCCTTAATTTATTTTGTAT
TACTTAAAAATAATAATAATTTAATTAATAATTTCAAATATAAAAAATTACATAAATATTTGCATCGACTT
AAAAATACAAATTTTTTTGTACGGAGCCAACCTCAGTACACAAAATGCATTCATATATCAACAGAAATTTT
GGGTAGGTCGTGATCTGCGCTAAAAAACTAAGAAAATTGTTTCTTTGCCAGCCAGAGACTTTGCCAGAGC
AGTGGATAACAACACATTCAAATATGCTGTTATGTATGTATATTCACACGCATACAAACATAATTGCTTGC
ACGGCCCTCATAGAGCCGAAGCTGAGAGCAAATTCTATTTTTAGCTTTTTCGTTCTCTCGGCTGTGAGAGC
GCAATGCTACAGATTTCGTTTTCGTTCTCAATTTTCAAAAAATCAAGCTGCATAGTAGCATTTTGTTGTATG
GCGTTACTCATCTTAGTGTTTATATTGTCTTTGGTGCNGGGGTGTAGCGAGCAAATGCAAA
>9_rnd-1_family-87__Unknown
ATGATTTTGCCTATATATGAGGCGAGTATAGTAAAACTAATAAATGCCGAGTTTNGTCAAGATATCTTGAG
AANCAAAATGTTTTTTTATAAACAACTTAATTTTCGACCGATCGTTCCTATGGCAGCTATATGATATAGTG
GTCCGATCTTAATAGNATTTAGCATATATATGAGGAGCATAGNAAAACTAATAAATGCCGAGTTTGGTCA
AGATATCTTGAAAAACNAAACGTTTTTTCATAAAACAACTTAATNTTCGACCGATCGTTCCTATGGCAGCT
ATATGATATAGTGGTCCGATNTTAATAGGATTTTGCATATATATGAGGAGNATAGTAAAACCAAAGACAA
TATAAACACTAAGATGAGTAACGCCATACAACAAAATGCTACTATGCAGCTTGATTTTTTGAAAATTGAG
AACGAAAACGAAATCTGTAGCATTGCGCTCTCACAGCCGAGAGAACGAAAAAGCTAAAAATAGAATTTG
CTCTCAGCTTCGGCTCTATGAGGGCCGTGCAAGCAATTATGTTTGTATGCGTGTGAATATACATACATAAC
AGCATATTTGAATGTGTTGTTATCCACTGCTCTGGCAAAGTCTCTGGCTGGCAAAGAAACAATTTTCTTAG
TTTTTTAGCGCAGATCACGACCTACCCAAAATTTCTGTTGATATATGAATGCATTTTGTGTACTGAGGTTG
GCTCCGTAAAAAAATTTGTATTTTTAAGTCGATGCAAATATTTATGTAATTTTTTATATTTGAAATTATTAA
TTAAATTATTATTATTTTTAAGTAATACAAAATAAATTAAGGAAATAAAAGTGCAAATTTGTTGTTTTCAA
AGATACATTTAATTAATTTTTTTTTTTTTTATTTTATTNTTTTTGTTCGAATTATAAAGAACAAAGTACGCA
GGGAACACCGCGAGGAGGCCATTATAATTATAATGGGGTCATAACTAAATATGACCTTCGAGTCGACTTG
AAATATGTAATTGTATTTTTTAGCACTCTTTTTATTTCTTAATTGGAGAATAAAAATAATATTAATTTAATT
TAAAGAAATTATGTAAATATTTGCATCGACTTAGAAATACATATTTTTCTGCGGAGCCAACCTCAGTACAC
AAAATGCATTCATATATCAACACAAATTTGGGATAGGTCATGATCTGCGCTAGAAAACTAACAAAATTAA
TTTCTTGCCAGCTAAAGCCAGAGCAGTGAATAACAACACATACATACATACTTTAAAGTTCTTATGCATGT
ATAATTCACGCATACAACGAAAGGTGCACGCACGCTATTGGCTGAGACAAGAACTTTTCTCGAGCATACA
CTGTCCAAAAAGAAAAGTGAAATCAATCTATGTGTGCTATTTTCTTAACATAGTTATTGCGCAAGAAGCGC
CTTGTGTAAAAATTATATAAGTTCTTAAATTTTCCTGTAAAATTAATCGTTAATATCTAGGGTAATATATCA
TTTAAATAAAGCATGCATTTCATGGATAAAGCACAAGCACTTTGCGTCGATCGTAAGTTCTAAACTTTTTC
GCTCAATGTACTTCGAAAACGAAGCGACGCAAAGCAGCATCGACCAGCTTTGCTGAAGGCAGGCTGGCG
ACTAACTTCGTAGCTCGTGTCAGCATCAGCAAGCCGCGACTTCGGCATTCGTTCGTCTGGTCTTTGGAGAA
CGAACAACCAGTCGGCAGGCATGCCGTACGAAGCCTCAAAGGCCAGATGAAAGGCCAGGCTAGAAATTT
TGCGTCGCATTCAAATGTATGGGCGTTACTCATCTTAGTGTTTATATTGTCTTTGGT
>9_rnd-1_family-488__LTR___Gypsy
TGTAGGCAGTTCGATCATGCCTTTAGATACTGCCTATCTAAGCGGAAGATATTCTGCTACAGATGCGGAAA
GCCGAACACATTCTGCTCGAAATGCGAAACTATCCGGGAAACCCCAGAGGGAGCGCGATAGTAGCGGGA
CAGGCGCGTTCAGGGACGGAGAACGCGGAGAAGCAGGAGAGGAGCCCCTACTAAAGCCAATAGTCGTTG
GAAAGGAGTCGGATATTTGAGATAAGGACCAGGATAAGGGTATTTATAAGATATAAACGAAGGAGGTAC
ATTAAGGGGCTTACCATACGAAGAACGTGTCCGGGAATACATGTCGGTCCGAAATAGATTTTTTGGTATG
CGACAGTTTGATGGGATGACGCTGGCCACCAAAAGAGTGGTGAAAGCTCGGGCGCGCTTCCGTAGACGCA
GGATGACGAGGCGCCAGGTCATCGAAACTGTAGCCGGGAGGATCCAAAANGAAAGGGTTACTAGACACC
GGAGCCTCGGCCAGCCTGCTGGGAAAAGGTGGTCGAGAATTAGTTATTTAGTTAAGNATTAGGAGTTACG
GTGCAACCGTACGTTTCGGTAGTCACGACCGCTGGAGGGGAGGATAAATCAATCATAGGTAGGGTAGAGC
TCCTAATCTCGTACAGGGAGCAGCATAAAAACATAACGTTTTTATCTGCCCCTACCTGAAACAGCGAGCCT
ATTTTGGAATTGATTTCTGGCGAGCTTTTAACCTAGCCCCTGATATTTTGGGCCCGAGAAGGTAACCTCCA
CGGAACACCAAGGGGAAGAGCTCTTGGCAGTGTCGATGACACACTATGCGGAAGAGGACGAAGAANGCG
AAAACGAGCCAGATTCATGGAGTTGTCTCGGGAGGAAAGGGTAGAGTTGGAGGCGGTAAAGCAGGGGT
TTTGTCTTTGAGCATAGCGGATTAGGAACGACAGACTTAGAGAAGCACACTATCGACTTAGTGGAAGGTG
CCCAACCGTTCAAAGATAGACCCTACCCATTATCGCCAGCGATGCAGAAAGTAGTGGATGACGAAGTAGA

CAAAATGTTGAGTTTGAAGGTTATAGAAGTGCAACAGCCCATGGAGTAACCGCACAACCGTAGTGCGAAG
ACCGGGGAAACCCCGTTTTTGCCTCGATGCACGCAAAGCTCAACAATTTGACGGTGAAAGACGCGTATCC
ACTACCCTGTATAGAAGGCATCCTATCTAGAATAGACCAGACCCACTACATCTCCAGTGCGGATCTGAAG
TTCGCGTATTGGCAGGTCGAACTAGACGACAAAAGCAAGCCCTATACCGCATTTACCGTACCAGGTAGGC
CGCTATACCAGTTTCGCGTCATGCCGTTTGGTTTATGCAATGCAGCCCAACGTCTAGTTCGTCTGATGGAC
AAGGTAATTCCCAACGAGTTGCGGTCCAACGTCTTTGTGTACTTGGGACTTATTAGTCATTTCCCCTGATTT
CCCAACGCACTTGAAATATTTGCGACAAGTGGGCAAATGCTTAAGAGAAGCCAAACTCTCTATCGGACTG
CAAAAATCCAAATTTTGCTTCCGGGCATTAAAATACCTAGGATTCATTATTGGTGGTGGCACGCTTCGCAT
GGACCCCGAACGGGTTATAGCTNTTAAGCGAATCCCCGTGCCTCGGTCGCGCAAAGAGGTTCGCTCTTTTT
GGGTACCGCAGGTTGGTACCGGCGCTTTATGCGAGATTTCGCGGCAATGGCCGCCCCACTGACTGAAATG
CTAAAAAAGGATAGCGGAAAGTTTGTATTGAGTGACGAGGCAACACGAGCCGTTNANCAGTTGAAGNCC
GC
>Ref_dvir_635
CCACCACTATCTCTAGTCTTTCTGACGCTTCATCTTTCGTTTTAGCTACTTACCATAACATTCGTCAAATGT
AAAACAATTGCGAAAGTGTGGGCCAACGGACCCAGGGCTTTTATTATTGCACGCTGAAACACAGAAGGTG
CGTTCTTCAGACCAAAGGGCATGGTTAAGAATTCGTACTGTCCATCAGGTGTAACGAAAGCCGTTCGCTC
AATTGAATCTGGGTGTACTGGGATTTGGTAAAATCCACTTGCCATATCTATACACGAAAAGTACCTTGCAC
CACGAAGACGAGCTATTTGATCCGAAATAAGAGGCAAAGGATATTTATCGGCAATTGTATTGTCATTTAA
AACGCGGTAATCTACACAAAGACGATCTGTGCCATTTTTCTTCTTAACCAGCATCATGGGACTAGCGAATG
GTGAACAGCTGGGACGAATAATTTTTGATGTAAGCAATTCATCAATTTTGCACCGAACGTGCTTCCTCTCA
TCTTCGCTAAGCCTATAAGGTCGGCGTTGCACAGTCTTTTGTGGATCTATTAGCCTAATTTCTAATTGGCCT
GTCGTCACACGAGTCATTGGAATTCCGTCGATAAACCTATCAGAGTGACATCTCAACATATTGTTCAATAT
CGTCTTATCAGAATCATTTAAATTGGTATCAGAATTTATGATTTCTTCATACTTCACTAGACTCAGAACATT
TACTCTTTTTGTCTTATAAAAGCAAAATTTATCTGCATCAATTGATACACCAAAGCCCTGGCTTAAAACCT
CACGGCCAATCAACACTTCATGCTTCAAATATTTGTCCATACACACATGTAATAAAACTTGTAAACAATAT
TGTTCAATATTAATATTACTCAGAACTTGCAATGTGCTACAAATTGAATCGTTATGCCATTTAAAGTTACA
ATATTGTGAAATCGTTTTCCGGTGAATTTTTCGACAACACTCTCCTTGACCAGTGAACATTCGGCTCCAGA
ATCGAAACAATATGGAATAGACTCACCGGATAAGGTATTGGTCCACTTGGTGGAGCTACTGTACAAACGT
CAACTCGCTTCTAACGCTACTGATACAGCAATCTCCTCCAAATTCTTTGCCTTCCACTTAGAGAGCAATAG
GGTGACTATGCGACTGCCGTACTCAGCAAAACTCTCTCCAGTTTTGGGACGTCCGTTCAAAATGTTCATCA
GTATGGCAGAAGTTGTCTCAATGCCCACGAAACGTTGGATGAAGAGTTCCTTATACTGCGGCCATGTGAT
GCCAGCGAAGCATATTTGGAATAGCCATTGCGATGCACTGCCTTCTAATGCTTTGCTTAACGCAATCACAA
GCGCACTGCCTTCGAGAGCATTATCCGCGAAGGTTAGGTCCACTGTAGTACACCAAGCTGATGCATCTGCT
CCAGCGGTGTCAGGGCAAAACTTGGGTAGAGTGACGTGGAGTGCTTTCCCGTCTATTGCCGGTGCTCTTGG
TGCCTGCATGGATTTTATTATTTCCATTAGATTACGATTTTGCTCTTCTAAGGCTGCCCTTAATAAGTTAGA
GCTGTCCTCTTTTGGCGGAGCTTATCCCACTTCTGATGTCGGCGGCAGGGATATGTCTGTCGGCGGCAAAG
GTATGTCATCCATGTTGATACTTCTGATGATTGACGCGGCTGCACAGCGAGTTCCGATCGGTTCAAATACC
CAAGCTCAACTGACCATGTCAGCCTACGCACTCATGTCGTAAACTCACTCATGTCGTAAACTCACTCATGT
CGAGTGTATGTAGCCATCTCTGTCTTACTCAAGACTTCTTGGAATCTTA
>9_rnd-1_family-389__Unknown
ACGATTTGCGCCTTCTTCCAGGCCGCTGGGAAGTGGCCTAGTCGAAGAGCTGCATTGAAAAATGGAGACA
AAAGAAAAATCAAAATGAAAAAGCAGTCTTCGCCGGGGGCTTTGCGGTTTTTAAGACTCTTGATCTCCTG
CTTCACTTCGTGTACTGTGATTGGTGTCACAGGGAGGGACATTTGGAAGGGGTGCTCCAGGGTTTGCGCG
ACTCTGTAGCCTGTCTTGTGATGCGGTCTCGAACGGCGTGAATCGCTCCTCCAGATTGCTGACGAAGGCTT
CTGCTCTGTCCTTTGGCGATCTGGCCCAGGTGCCGTTGGCATGACGCACGGGAAACTTGGGTGCGGCTTGC
CGTTCATACTGCCTTGTGAGGTTCCAGAGCGCGAAATTCGAGCTCACGTCGACTGTTGCTTCCTCCAGCTT
GGTGTCTGTGGCTTTTTTTCTGGATAATCAGCTCCTTCCCTACCCTGTTGGCAATACGACGATAGATCCTGT
CAATCGTGGGGTCCTGGGTACGGACATACTCCTTCCTCAATCGCTTTTTAGGGCAAGTAGACCTTCCAAAA
TGAGGGGAAAATGTCCTTGAGCGCTGGCTTGTAGGGGCTGGTTACGTATGTCGCAGCATTGGCGGTGCG
GCAACAANAAATCACTTGCAAAACAACAACAGTGCGCACGAAACTCAATTTGTTCTTCGTCCTCCGTTTTG
ATCGATAAGAGGTTTGTGAGCCATGATGAGGACNTANGAAACTCAGCGCAAAGAAAATGGACAGATAAT
GCCACTGTGTTTTTTGCGGGTTTCTCTGTCGTTCCTACAAGGCAGCGCGTAGAAACAACAACAGCACAATT
TGGGTACCAATGTTGTTGTTGCAGAGCCGAGTCTCGGCGCAAGTAGGTAGAAAAATTTGAATGCCAGCAG
GCCATGGCATTCAAATCAAAGGGTCACGCGCACGGCGCGCTAACATNATANATTATGACGTCACAATTTA

TNGAATTTTATTTCTTGTTNTGACGAGTATATATTTCNACCTTTATTACGTACATTTAAATAATTTATGAAT
AGGGTCCTTTATAGCAAATAATTGATTGACATATGTTAATCATTTTANATACGAAATGATCGCACACAAAA
AGCCTTGGGCTGTGACGTCACATGCGCAACAGAATATTGCAATCTTCTTAAATTCATAGACTTCCCTTGGC
TTGGCAGACACATACATATATGCAGATTTGCACCAATATGTGTGAGATGAGACACACATACAACAACTAT
TTGGCGGGTCGCGGCATTGATACCCTTATTTATAAGAATGGTATATAATTTATAAAGAAAACAATTATTAT
TGACTGTACTAATAGTTGCACACTAAATACGAAATTATATTTTTCATAGACATTGAATAAAAATGTATATA
TAAGANGGTTTGTGCTATTTGGTTACATCGCTTATGAATAGTTAAAATACCCTATTTTGAGAAAAGGTGCC
TTGCTGGTCGAAAAATGAAAAATGATAAAAATCTGCCGATCTGCTTGCAAGTTGAAAGCGAGTGGAGAAG
AAGAAGACATGACTTTGCGAATTATAGGCATAGCAGTGCTTAGAAACCACAACAATGAGTTAGAAGGGT
GCAGAAGCTAAGATCATAGGCTGTAGCCTCAAACAAAAAGGGATAGCAAGGACAGAATTTGTGGTGTGC
TTTTTTCATTGTCGAGTCGTTTGTTTTGATTATGGTTGTTGTTGGTTCTTATGGAAAATGTTTTGAAGTTGTC
ATTGAATTNTGGGCATCGGCAGAAGTAGCTGCAGACAGCCGGGTGTTGTGTTTGCAA
>160_rnd-5_family-3646__LTR___Gypsy
GGTATCCACTTCCACACATACAAGACTGTACACACATTTTTCATGGCAAGAAAATATTCTCAAAAATTGAT
CTTAACCGTGCGTATCACCAGATCCCCATCGAACCAGGCGACGTCCCAAAGACTGCAATAACCACACCAT
TTGGATTATTTGAGTTTACACATGACATTTGGATTGTGCAACGCTGCACAAACCTTCCAAAGGTACATGCA
TTCCGCACTGAGGGATTTAGACTTTGTTTTCGTCTATATTGACGATATCGCCGTTATCAACACCAAATCCA
CCTTCGGCAAGTGTTTGAACGACTTCGACGTTTTCGTCTCAAAATCAACAATCGAGTTTCTTGGTCATTTG
ATCGATCATAGTGGCATCAAACCATTACCAGACAAAGTCAAAACCATCATCGAGTTTCCCTTGCCCAAAG
TCGCAAAAGAACTGAAACGTTTTTTGGCAACGATTAACTTTTATCGAAGATTCCTGCCAAAAGCAATCACT
CATCGAGCACCACTGGTANAATTAATACCGGGTAACAAAAAAAACGACTCAACTCCAATAACTTGGACAG
GTGACACGCTAAAACATTTTGAGGAATGCAAGCGTTCCCTAGCTGCAGCAACACTTTTGGTACATCCTGCT
CCAGATGCCCAGTTATCACTCAGCACGGACGCAAGCGATACAGCCGTTGGCGCTGTACTGCACCAGATCG
TCAACAACGAACTTCAGCCAATGGGATTTTTTAGCGTTAAACTTACTGACGCACAACGCAAGTACAGTAC
TTACGATCGAGAGCTTCTTGCAATTTACCTTAGTATCAAGCATTTCCGTTACATGCTCGAAGGTAAAATTT
TTTATGTCCACACTGACCATAAACCATTGGTATACGCTTTTACTCAAAAATCAGAAAAAGCTTCTCCACGT
CAGCTTCGACAGCTAGATTTTATCGGACAATTCACGACATCTATTCTGCACATAGCAGGTGTCGATAACAT
TACTGCTGATACACTCTCTCGCATCGAAACTATTGAAGCCAACATCATCGACTACAATAAGATTGCTGCTT
CACAAGCAAGTTGTCCAGAGCTGAAAAAGCTACTTAATGACAACACAACTTCACTTCAAATCAAATCTAT
CAACTTACCGCAATCAAGTACTCCAATCTTATGCGATTTTTCTACTTCCACAGTGCGGCCATTCATACCGC
AGCCACATCGAAAAAATATTATCAGCAAGATACATGGCATTGCCCATGGAGGAGTTGGATCAACAATTAA
AATTTGGACCTCATTGGAGCATCGCTACACAAGATGGCCAGATGCAATCGCCATCGAGGATATCCAAGCT
GAAACAGTAGCCACAGCACTCATCAACACATGGATTTCTCGCTTTGGAGTTCCAGCACGCATTACCACGG
ACCAAGGCAGACAATTTGAATCCAGGTTATTTAGCGAGCTATCGCGCCTCCTCGGCATTGACCATTTACGA
ACAACCGCCTACCATCCACAGGCAAACGGAATCATCGAACGTTGGCACCGCGCGATTGAGGCAGCTATAA
TGTGTCACANCAGGATCGGCTGGCCATCCAAATTGCCAATGATTCTCCTCGGCCTACGCTCAGCATATAAG
CCCGACATCCGAACGACAGCAGCACAGCTTGTTTACGGCACAACTCTTCGTCTTCCTGGAGAATTTTTCAG
CAACTCCGGTCAACTTCAACCACANACTGATTTCGCCAAGGATCTAGCAGAGATGATGCGCGACATCCGA
CCAACTCAAACTTCCAACCACGACTCATCAAAGCCTTTCATCTTCAAGCAATTAAG
>9_rnd-5_family-8689__LTR___Gypsy
AGAAGACCGACAGCGCCACATAGAGCGCATCAAAGAAAATAAAAGCTGAATTAAGACTTCGCAACCGAG
CGAAGCCATGCGAGGCCGCAAGTATGCGTAAGCCTTAACTTGCCATAGATTTAATTTCTACATGCGATTTG
ACGCACGTCAGTGCGAGTCGCAAAATACTAAACCTCTTAGACTTTCATTACTAACATATCTGGTTGGACAA
TTTGTTTTTATATATATAAAATGTAATTTGAATTGTGAGCCAACCACATAAACTAAATAACTAACTTTTTCC
ACGTCTCTGGCGCTGAGTATTTATACTTAGCTGCGTTTAGAATCTTAGTGTAATTGATAAATCTTGCAATA
AGTTCATTGCAATGAAAATAACACTCAAGATTTTGGAAAATAAAAATTCCATTTTCTTTCATGAACGTTAG
ATCGATTNTTTTCATTGAATATATAAAGACAATTAAGATTACATAATGGGATGGTTTAGTGATTCTAGTGA
GGCAAAAGACAATACTGCCAACGTTGTAAACAACGTCAAGATAATTGATCACACGGACGATATAATTGTG
GATCCTGTTATTAATTATCACGATAGTTAACACTTTCATAATAAAATTATTAAGAAAAGATACATGAGTAG
GGCAAATAACTTAGATAAGATCTAAAAATGAATAAGGAGAAAATAGATCACGCTTACGAAAGCTTTCCA
AATTTAAGCATTAATGCGATTAGAGTTTTACATTCCCATAGCGAGTACGTATCAGTATATCAGTTCGAGAC
TGATTATATAGAAGCTTTTCGGCGGAATCTGTAGCCCACTTATNCACTGCGATAAAGCAGAAAAGGCAAT
GGTACTTTCAACACTACNATATGTCAGGGTAAAATTAACAAAAGAAGACTACATGGTTAGTACGCTTACA
NAAGTAAGAAAAGTCCAGTAAAAAGAATGGAATGGAACGAAATCGCGAAACATATCGACGCATTTCGTT

ACAAATTCGATAAATCTTACAAATGCATCAATAAAGATGCAGTTATAAAAGCGGAGACACTAAAAAACC
ATAGAAATTATTAGTAAGGGAATATAATAATATAATAGTTACCTTTAGTAAATAGNTACGAANAATAAAT
TCACATCTGAGCACAANAGTAAATGTTTAAGGGTAATAAAATCCCTAAACACAAGATTGATTACTGTCAC
CAATAGGCGACATATTCAGATAAACGTGCCAGAAAGTCTAAATCAATTGGCGGAGTTTGATCCCGACCAA
TTAAAAGAATNAAACGAATCAATTCAACCAAGCGACGCTGAATCTGATAGTGACATTGAAACACTGGAA
GAAAGCGATCCAATCGAAACCAAACAGTAATAANAATCTTTGAAATGGCACAAACAGTTGTAGAATTTAT
CAAGATAGCCACATCTCTGATACCAGAGTTTGACGGTAAGNCGGAAAATCTACAAAGTTTTCTAGATTCTT
TGAATCTATTAGAAACCTTAAAGGGCACATATGAGCTAACAGCCTCGTAAAAACANAGCTAAAAGGCCA
NGCGAGAAATTTAATAGGCAATGAGCAGACAATTGCTGCAATCATCACGCAACTANCAAATGCAGTAAA
AGGAGAATCGGNAGAAGTTGTATCTGCTAAGCTTCTAAATCTGCAACAGAAAAATAAAACGGCTAACCA
ATACGCCCAAGAGGTGGAAAAACTGGCAAAGGCCCTTGAAGGCGCCTATATCAGTGAAGGTCTAAGCCA
ATCACTTGCAAACAAATACAGCACTACAACAGCTGTAAAAGCTATGACCCAAAATTGTTCAATTG
>9_rnd-5_family-2674__DNA___Maverick
GGCATCAACATCAGCAAACAATAATCCAAAATGCACAACGCTTTTGTGAGGTTTGTGGAGTAACGGTGGC
TCATAATCGCTGGGCACAACATTTGCGGACAAATGAGCACAAAAATAAGGCCTTCACACCGTTGGATGGC
TATGTTCAACTTGCGGCATCCGGATTCCAAAACCGGATTAACACCTATTCATATAGNAATGTTTCGGATAA
CGTTATGTTCCCTGAAGAGTTCCTATTGGAAGCAGGAAGTTCATTACTTCCAATAATGGAAAACGTTTTAG
AGGTGCACACCTCATATAAAAATCAACTTTGAACTTTTCGCTGAATATTTGAAAATAAATAATAATAATAC
TGATGGTGAAGATTTTGAAATAATGATTAAATCCCATCAAACTGAAATGTGTGTGGCTACGAAGTCTTCTG
ACTTAGAAGAAATATTCGTGAGCCACTCATACATCATAAGTTCAAAGATGGATGAATTCCAAGAGAGAGA
TAGTGGTTGGACTCTCATAAAAATTATACGTTTGGAAATGAACGTCAATAAATATAAGCCGCTAAGAGGC
TCTAGTTTTATTCCATTACCACGGAAATTGAGCTTGAAGAAAGCTATTATTAATATAAAAAACGACGATGA
GTTTTGCTTTAAGTGGGCTTTAATAGCAGCCCTAAAGAACTTGCAACATAATGCTGATAGGTGTGCCCCAT
ATAAAATCGATGATATAAGGAGTCCAATTATTGAAATTGATGAGATATTCTCACTCAAATTTCATAATATG
GAATTTCCATTAAAGCTCAAAGACATTAAATTGTTTGAGCAACAAAATACTTACTTAAGTATAAATGTCTT
TGGCTATGATGAAGACATTAATGAAATTATTGGACCACTCTATCTAACCAAAGAGCACCGGGGAAACATA
TTAATCTATTGCTTTTGGAAAAGCAAGATATATCCCATTATACCTGGATTAAGAATCTATCAAGGTAAGTA
AAAAAAAAAAAAAAAATATACATTTCTTTTTTTAATATCAATCATATTTTCTCCTCCAGGCTTACACACAA
TCAGTTGACTTCGCATACGAGGAAATTACATTTCTGTAATGTATGCTTAAACTATTCGCATAACGACAGGA
TGCATGGCGAGCAGTGTGAAAAGTGGTGGCGAAGCTTCCAACAGCGGATAAGGCGAGTGTAAAATTTAA
GAATTATGCAAATAGTTTAGATATACCTTTTATAATTTATTCAGTGCGTTTTAAAACCTATCAACAGGAAT
GTGAGCGAGAATGTAATGAGCATTCAAGAACACATTCCTGTTGCATACGCATATTTCATAAAATCTGCAT
ATGATTCTAGTTTAGATAAATTCGTTATATATAGTGGAGAAAATGTAGGATTACATTTTGTAACATCTCTC
ATTGCNGATGCAAGAGAGATTTATAGCAAATATTTATCTACTGTAAAGCCAATCGAAATGAGCTTAGAAA
AATTGCCACATTTGCACTAAGATNTTAAACTTANATAGAGTACGGGATCATTGNCACTTAACTGGTAAATT
TAGAGGGGCAGCTCATAGTGCCTGTAATATANATTACAAAATTGCCNCATTCATCCCCGTACTTTTTATAA
TTTTTCACGCTATAATGCTCATTTGTTTGTAAGAGAGTTGTCAAAAGTTGAAGGTGAAATTAAGGTTCTAC
CCTCTCAATAAAGAGATTTACATTTCCCTTTCAAAATACACATATATTACAGAGCATAATACAAATTTGGA
AATACGTTTTTAGACTCATTTCGATTTATGGCT
>Ref_dvir_532
ATTAATTACAAAGTATTTTAAAGTGTCAATGTGAGTGCAGTGCAACATATTATATGTTTGATACTAAATCA
ATTGTCGTTCCGCTGCGAACAGGCTCCTCCCGTCCGAAGTCGAAGACTTTCAACCGATGTGTTCTCGATAG
GCAGAACGGCGATCTTTGTAATAGCTCTCTTGTAAAGTCCGCTTGCAGTTTGATCATCTCCATCATCTCCAT
TTATTGATCTGTCAACTCGGCCGAGAGGTCATCTTAGAGGGGACCGTTATCCTCCTTGAGCAGCAAGTTGT
GGCAAGGCTGCAAATTTTTCTTAGGAGCTCGCCACTTTCCACGCTAAGCGATGCTCCACTTTTGTCAAAAG
GTCTGCTGCATGTGGCAAACACGCTGCCAGCGGCTAGGACGATTGATGTTCAGATGCGTCAAGTCGGGTT
CGTCAGTAGAAGTGTTGGACTTCCCCCAGAGAGCGTGGCCAGACGAAAGTACCTACAAGTCACCTGGATT
TTCTGTAATAAAAACAGAATATAACATGGCGCATATATGGCAAATAAAGGTTCAGCATTGCCAACGACGC
GGTAAAAATGTTGTTTGGCTGACTTCACTGCGGCTTGCCACCAAAATGTGGCGATTGTGGCGGAGTAAAG
TGCATTCGTTGACAGGCTACGTTCAGGCTCACTCATGAAAAGGTTAGACTGATGAATCTTTTTAACGCAGC
AATAAAAGACGGAGTAGACAGATCTTCTATTAGCTCTAAGTTGAGAAGCAAACAAAAATGGAGATATAG
CATTTTGTTGACGACCGATTTCTGACCCTACTGGATGCTGTTTATGAGTTCTTTGAGATGTGTTGCTTGTTT
AATCTCGTCCAGAGTTAAGAAATCTTTGGACCGAAGTGAGTTCGTTTGAATGTAATAAATCGAAAAATAT
ATGCGAAAATACGCTGGAGCTTTCCAAATGAATTTTTAAATTTACACGATGAGGTCAAATCCCTTTGAATT

GATAGGACTAGTGTATTGTGACGACGCTCTGGAAGCTCCAGTTGATAAACACTGTCTTCAGGCCGTTCTCA
GCCGCTGGAGCGCAGGAATGTCGGACCGTTGTGCCTAGGATGCGATTATATGAGATCCAACGGCGTGCAT
CCACGTAATAAGATGTCAGCAGGGCTCTGCTGGAACGGCACGTAAGTGCGTTCCTGGATTTGTGATTCCC
GGTTAGGAACGAAAACATTGAAATTAGACGGCCTTTCTCGTATCCATATACCATATATTTCATAATTTGCT
AGCGGTGTCATAGCAAACCGTGGAAATTTCAGACTACGAACATATGAGAGTTACGAAGAGAGCTGAAAC
CATACTGAATGCAATGACTGCGGAAGGCTTTCGTCCCACCTGACCTTGTCCTTCTAAGGTGCTTGAAGAAA
AATCTAAAAAAAATAATCCGAGCGGATCGTAAAATCTGGCTATTGTTGCCTTTTGCTAACAGCTGCTTTAT
TTGATGTCGAATTTGCCGGGTTCCTCGATGGAATCAACTACAGAGACATTGAAGTCACGGCGGATGCTCTT
GGCAGCCATTGGGAAAATGCGCTCCTCGTCGTAAGTAAGCAGTTGCATCGTGCGGATGGCAATAAACGCA
GCTGGTTTAGTTCCGTATGTAACTGTATCCAAGGAGTATGTTTTAATCCGGGCTTGATTTTCGCGCCATAA
GATGCATTGCAGGAACTGGTCTGGAGGTGTTATGCGTACCGCAATGATACATCCTACAGATGTCACCGCA
AGATGCAAACTTA
>9_rnd-1_family-428__LTR___Gypsy
ATTCAAGCTAAAGAAATCGTAAAAGATCACACAAAAATTTACAATACCTTTCAAATTGTAATAGTTACAA
CACTTTGCATAATTATAAGTGCCATAATATTGTATATTATCTATAGGTTCAAAGCATTACCCACAAGAATA
ACTGTCAAATATAAGAAACAAAAAGAAGATCAATCGTTCCTATAGCAGATATGTGCTGTAGTATTCCGTA
TGTAAATACGATTTTGCAAAAATGAAGGGAGCATAGTGAAATCTTTAAATGCCGAGTTTAATCAAAACAA
GAAGAATAAAATTCTGAAATCGGAGCCGTACGATTGGCAATGAAATACACTGAAATATTAAGGAAAATC
AAAGAGCGAATTAATTTCCCTATTGGGCGCCCACAGTGTCTAGTTTGGAGAAAGCGTCTCAATTCATCAAT
TGCGCTCAGCTCAGCCCGCCAGCCAAAGAGGCCAGATGGGGCGTGCTTCAAGTGCTGGAGTATGGATCAT
CAGTATGCGCGGTGTCCTAAGCGCAGTACTACAACAACAATTGCAGCAGTGCAAGGCAACGAGGGCACG
GGTGAGATGAATAGTAACGAGTTAGCAGCTGTTCAATTAATACAATTATGAGTTCCGTTTGGTGAAGAAA
GAGGCACAAGTGTTTTTNGTTTATTCGATACGGGGAGTCCCGTAAGCTTTATTCATAAGGCATTAAGGCAT
TAATACCAAAAAATATAAAGCTAAAATCTGAAGTCAGTCAATACCATGGATTAGGAGGACGGCCTATCCC
AACTCGGGGAAAGTTGAAATGCCGAATTAATTTTCTTATGAGAACCTATATAATTTCACTTAATATTGTAG
ACGATAAGATATTACCTCTACCAGTATTATTAGGAAGAGATACTTTAGAAATCATTGAAGNAAAATTAGT
TCATAAGAAAATTATTAATGCTGAATATCATCTTTTGAGTAGACACAGGTGCAGACATTTCAATAGTTCAT
ATCGTGGATTTACAATTCGCTATTCCTTGTGATGNCATAATAGGAATCGATTTTATTAAAAAAATATAACT
GTCAATTAGACTTCAAGCAATCTGAAGATTGGCTTATAATTATACCTAATAACTTAAAATTTTCAATCTAT
AACATACAGTTCTGGCAACAACTCAATACTTCTGCCAGCAAGATCCCAAGTTATTAGAAAAATTCAACTA
AATTCAAAAGAGGACAGTGTATTGATTCCAAATCAACAAATTAAACGTGGTATTTACATTGCAAGTACCA
TAGCAATAACTGAAAACGCTTTCATAAGAATACTTAATACAACAAATAAAGATCAAGTAGTCAGTGCAAA
AAACTTAAAGTATGAATCACTTTCGAACTACGAAGTAGTCAAAACTAATACAGAAGATAGGGAAAAGTCT
GTATTATCTCAACTTTCAAAAAACTTTCCACCACAATTCAAGTCACAGCTTTCAAACTTATGAACCAAATA
TAAAGATATATTCGGATTGGAAACAGAACCAATAAGCACAAATANTTTTTATAAACAAAAACTGAGATTA
AAAGACCAGTGTATACAAAAACTACAGAAGCCCACACAGTCAGGTGAACGAAGCACACAGAAGCAAGTT
GGAAAATTAATCACCGACGGGATTGTTGAACCATCCGTATCTGAATATAATAACCCATTATTACTTGTCCC
TAAAAACCATCTCCAGGTTCAGAAGAAAAGAAATGGCGATTAGTGACTGACTATCGTCAAATTAATAAAA
AATTATTGTCCGATAAGTT
>Ref_dvir_1071
CTTCCGATAGACACACCTACGCATAAGACACAGGTGCGCTCCGGTATCCACTAAACCTTTGCAAATCACA
TCTTTTAATTCTAAGCCAGAAGATATGAATGTGCTTAATGACTTTAACATCCACTTTTCGTATCGGACAAC
ATTCGTCGACTTACCACATTTAAAACAAGAAAAAGTATATTTGGGACAGTCCTTTATTGCATGTGAGTCGG
CACCACATTTAAAACATTTACGGCTTTTGTTTGCTTGAAAGTATTGACCCACGGTAGGCTCTCTTTTTGCAA
TTGTCGATCCAGGTTTACTGGGCGGTGCGCAAGAACCACGAGCTTTCTGATAGGTTTCTATCTGAGCTTTC
AACTGTTTGATTGTTTGAGCTTGATACAATAGTGATTTGTTAGTTTTAGAATCCGGTATGCCTTCTATAAAA
TAGTCAATCAAACTCTCATCCTCGAGATCGATAGATTTTGCTATTTCCATCAACGCATATAAAAATTCTAA
CAATGTCTCGCCCTTTCTCTGCTGTCGCTTTCCCAAACGTCTATGAACTTCTGCTGATGACAATCTTACCTC
AAATTCATTTTTCAATGCGTTCTTTAGCGCTTCCCAGTTATTAATACCCCGCTGACTACGAATAAATGATTT
AGCGGCACCCTCAAGCAACTGTTTGGCATAGATAAACAACTGTAAGTCGCTCCACTGTACTGTTATAGCG
CATTCTTCTAACTCACTCACCCACTGTTCAATATCTCTCGAACTCGACCACGCTCAACATCTTTCTGTTCAA
ATAGTCTTGCTTGCGTTTTACTTTGCGTCCTGCAGTACTCAATTGCAAATCTGTTAATTTTTCGCGTAATTG
ATCGACACGAATACTTAGAATCTCGTTTCTCTCCATCTTACAAAATTGTCACACGGTTACTTAGATTTTAG
GTTTTCAAATTATTCTTATTTACCATTTTCAGATTTGATATTCTTACATGTAAATTTTCAGCACAACAGCTT

CAGTTTGTCCTATGTTAATTAGTAATAACAGCGGCTGCTCTCTTTTGTAGCTTAAGTTTAACAGCCTCGACG
GACACTCTTTTGTATCTTGAGTTTAACAGCGCTAAGGGACGTTTGTTTTTGAATTTGTTCTCAGCAGTTATC
AAAATGTTAAAACTATGTTAAGGAATAATGTCGATGAACATAAAACAATTATGCTAACAAATAATTTAAA
TTTTCTTCCGTGTTTCATGTTTCGTGTCACCGTCCGTGGCTAAATTTCTTAGTTAATAATGTAGGATCTGTCT
GCGCAAGATACAACACTTGCTAAGGTCAGCACAAATAGTAATTTGCAGCGCTGCTGCAGCTCACCACACG
ATTTTAACGTTTCCCAACACTCCCACGATGGACTCTGTCGCTAACTCTAACTCTGACTCTGACTCCGACTCT
GCTCCAGCCTTCTATTACGCTCGACCACCTTGCCAGCGTACCAAGAAATTGCTACTGTCTATTTCACGCCTT
GGCCAACTTGCCTGCGTTCCTCATAGATGACCAGCTGCTTTGATCGTTTTGGTTTCCCTTACGCACCAGCA
AAACGCCAACTTTACTACAGCCTTCTATTACTTTTCTCTTCTCTTCTCTCTTCTCGTTCTTTCTGTATGCTTT
ACATTTGGCGATGCATACACACAAAGGCCGGATTTCACCAGAAATTTATTTTTTGGAATTACGTTTCAGTG
AGCGTCTGTTGCTATTTCGCTCACTACTTTACTTTGCACTTCTCTCTTCTCGTT
>9_rnd-1_family-440__LINE___CR1
TCCTCTAGGTATAATAAAAATCAGCCTTATCCTTATCAGCTACAGAGGTCAAACTGCATCTTAAATCCNAT
TATTACCGAAAGTTCGCTTCNTAGCGACCTTAACACTATCAAGCCTGGTGGTGTTCCTGCTTGCGTGCTTA
GGTACTGCGCTGCAGCATTATGTAAACCTGTTTTAAAACTTTTTGAATTGTCCGTAAACACTTCATCTCNTC
CGACCATTTGGAAGGAATCATACATTATCCCTCTCCANAAGAAAGNGACTAAGTCCGATGTTGAAAACTA
TAGAGGTATTTTCAAATTATCCGCTATACCTAAAGCTTTTGAGAGAATTGNAGCTATTCAACTGCAACATT
TATGTAGTTCTATTATTTCACCTTCCCAGCATGGGTTCGTGAAGCGCAGATCTACAACCACTAACCTTCTTG
AGTTTACTTCGTTTGTAATTGATGGGTCTAAGAAAAGAATGCAGACTGACGCCATCTACACTGATNTCAGT
AAGGCTTTCGACTCTGTCAACCANTCCTTGCTGCTTCATAAATTATGCCTTCTCGGCTTCCCAGTCAATCCT
TTGAACTGGATTTCAGAGTATTTGCGTGATAGAACCCAAAAGGTTCTTTTCAGAAATTCGTTTTCTAAACG
TATTCTGGTGACTTCCGGTGTTCCTCAAGGTAGTGATCTTGGCCCGTTACTCTTTTCATTAATGACCTTCCC
TCGATACTGTCTCACTCTCGAGTATTAATGTATGCTGATGATGTAAAATTATGCCTTGCGTATAAAGATAT
GGCATCATGTTATCATCTTACAAGCTGACATTGATGCGTTTCAGGCTTGGTGTCTAGCCAACCTTTTAAAC
TCANACTGCTCTAAATGTAAGGTAATGACCTTCTATAGAAGCCCTCCTCAATTGGTTTCTTACATCCTTGAT
AGCTTACCGCTCGAGCGTGTTAACAGTACTTCTAATGAAGTTAATGATCTAGGTGTCATCCTTGATCATAA
ACTTAAATTCGACTCTCATGTTAAGACCGTGGTCAATAAGGCCAGAATAGTTATGGGTTTCATCAAGCGAT
GGTCGAAGGAGTTCGACGATCCGTACACGACAAAAACACTTTATATATCTCTGGTACGACCTATTCTTGAG
TATGCCTCATGTATCTGGTCACCACAATATGCTATCCATCAGGACAGAATTGAATCCGTTCAGAAACAATT
CTTGTTGTTTGCCCTTCGTGGTTTAAATTGGGATCCTCGCGTGCGGTTACCGTCATACTCCAGCAGACTTCT
GATGATTAACCTTCCTTCTTTGATTAATCGTAGAACCATGTTGGGTGTAATATTTATTCATAAATTAATTAC
TGGTGAAATCGANTCTTCTGTGTTGACTAGCCAATTAAATTTCTCTATCCCTACTAGACTAACACGTAATTT
TGTACCCCTATCGCTTCAATTTTGCTCAACTAATTATGCTTTGCATGAACCGTTCAGGGTTCTCTGTGATGA
CTATAACAAGCATTACAGAGTATTCTGCTCGGAGTCTTCTCTTCCTCGTATAAAGTCTTCTATTCTAAACTA
TTTAGCAAGTAGATAGTCTTATTTTTATTTGTAATTGTATTTGTACACATACTCGTAGTTTGTATCGTTTTTT
TTTTTTTTTTTATTTTTACTTTGCATTTCGTGTCGTACGTTACACGACTGCGCCCCCCGGTCGGTTGGGCGG
GAGGTGGGATGCNGTACGTTGCATCGGGATTCGCGTAANAAAAAAAA
>Ref_dvir_656
AGCAACAAACACGGCGACCCAAGATAGCCGGCTAAATGAAAAACTTAAAAAATTGAAGGTGTAAGTGCA
GCAACTTCCATCGCCGGTTGAACGGCAACTAGTAGTAAATGCTCGGCAATACTCGCAGATCAAAGCTCCG
AAGTTCAACAATTGCAATTCTCAACTTTGGTTTGCAAATTGGAGCGCTTGTTTTTTTTGCACAATGTTATAA
CTGACAATCGCAAGTTCGACTTGGTGTCGGTACAGTTCGAGAAGGATGTCGCTCTCACCATCGAGGATCTC
ATCTGAAATCCACCTCTGAATAACAGATTCGGCGAATTGAAGCAACGACTCCCCGAAACATTTGCGGAAA
CAGAGGACTCAAAATTAAAGAGACTACTGCAGGGTGGTGGTATGACCGGGAAGCCGTCGGTAATCCCTAC
ACACATGCTTCGTTTAGCACCCACTACTGGCTGCGAAGCCGTTATGTGCACTCTATTTATCGCCAAGCTGT
CAATGTCGGTTCGTCCGCTAATTTTTATCTGGGAGGAAGACGACCTAAATAAGTTGGCAAAAATCGCGGA
CAAGATGCTGGAAAATAGTGGAATGGAACCGACTTACGTAATGCCGACACAAACTGTAACTGCTGAGCA
AAAACAGCAGAATATTGTCAGTAACAATAGTCTCAGTTTAACTGATTTAGCGGCCGCCTTGCACGCGCATT
CGCAGAAAGTCGATACGGACGAATGTGGTAGCTCTACACGTACAGGATCTGTATACTAATCATATCTTCCT
CGTTGATTCTGGATCTGTGGTATCAGTTGTGCCTTGAACAATGACAAACAGAAAACTTCAAAAAGCTAAA
CTGCAATTATATGCCGTAAGCCGAACCATAATACAAACATATGGCAACCTATACCTAGAGCTCAACTGGA
GCCTGGGTAGTGCTTTTAAGTGGTCCTTTATCATTGTTGACTCGCAAGCGGCAATAATTAGCGCTGACTTT
CTGTTACCGTTCCACTTGCTCATAGACATCAAGCAAAAACGTCTTATCTCCACACAGGAGCACCTAACTTT
CGGCAGTTCAATCAACCCGTAGCATTTCGAAGCAGCAAATAATGCACGAAAACCACATTGACGTAGGCCT

CACGCAAAACATACAGAAGCGTTCAGCAAGGCCAACAACTCACACAAAAAGCGCGTATCACATGCATCA
CAACTGGAGCACCAGTAGCAGATTGTCCACGGCGACTGATGGGTGAGAAACTGTCATCAGCGAGAGCAC
AAATCCATCAACTTATCGACGAAGGAATCTGTCGCGCAAGCCTGCTGCATATGATAGCAAAGACCAGTGA
TGGTTGGCGGGCTTGTGGCGATTACAGGAAGCTTAATGCGATAACAACTCCTGATCGTTATCCGATACCAC
ACATACACGACTTCGTAGACCGGTTACAGGGTAAACTCATTTTCACAAATTAAGACTTAAGAACGCGCAT
ACATACACAGCCATCGGTGCAGTACTGGAAAAATTCAACAGAGGTACACGAGAACCCCTAAGGTTTCTCT
TCAAAATAACTAAGCGCAACGAAAGGCACATATGACAGGGAGCTACTAGCAATCTTTGAAGCGATGATGT
ATTTCAAGCATATGCTAGAAGCAAGGGTTTTCGGGCAATAAAATCTGATCACAAGCTGCTGATATACGCA
TTCCTTAAAAAAACCTGGAAAAGCATCGGAGCGCCAACTCCGCCAATTAGGATTTATCTCCCAATACTCC
ACAAA
>160_rnd-5_family-4071__LTR___Gypsy
GTAGTGGCGCTTGAAGCAAGAACAATTTGTTTGAGTTTAAAATAAACAGCCCAAAAATAGACAATGTAAA
TCCAGCCAANGTCTTGTACCTTGCTTACCCTAGTAGATCTCGCCATCTATGGCTCANCCCGGGCGTAATAT
CGCTTGTAACGAGTGAAAGAGAAAAAAAATAGTAGAGGAATGTTAATTTGATAATTGGAAATGGCGTCTG
TCCTGGTGTCGCTGTAAATAGCTTTCCCTAAGCCGATCGGAGCTCAGATCTTTCTTTTACCTTTGGTCCACC
GTGGAGAAAGGTTGACTCAAAAAAAAAGTAAGAAAGTGCGCCTATTTACAATCGCGCTTAACCTCTGAGA
AACAGTATTTTCTTTTGGAAGCATCATTTTTAATAGAAGTAATTTCAAAAAAATATCTAAGTGCCCGCATC
GATGAACCACTTCTCAGCCGCCAGTTGGTCGCAAAATTAATTGAGAAACTAAAAATAAAATAAAAAAAGT
GCCAGACCCAATCAGGGGTAAGTTAGATTTTTGCTAGACCGTTTTTTTTTTTTTAAAAAGCACTTATATTTC
AACCTGTCTATTGCAGGTATGGCGCTTCGTAATATTCTCATCAACAANATATAATGAGATATTATAGTCTT
TCGACTTTCTGCAATTAGCAGCGGCAAACATTTTTTTTTTTTTTGCCTGCCGAAGCCCAAAATATGCAGAG
CGCCTGTAATTGTCATCGTTAATTCGCCTTGTCGCAAATAGCTTAATCAGCTGCGTCCAGCTTTATCGCATC
TCGTCAAATCTCGTTGTTTCGTTTCGTGCTCTCTAGCCTGCTCGAAAATGGGAGTTCGAAAGACGAATTAG
TAAGAGACGATCGCCGTCGCCGAAACTCCGAAAATTAGTTGGAGATCGTTCCTTGGTGGCTGACGTCGTC
AAAAGCAGCTACATTTATCTTGGTCGCGCAATCATTTTTTNCATATAAGCGAAACGAAATCTTATTTTTTTT
TTTGTAGTGTNATATTTTTTTGCTGAAAAATGAATAATTAGCTTAGCTAACAAAAAAGTGAAAAAACTGA
ATAAGTCCTATTGTGAACGCCATGGGCGTAGTCAAGTCGCCTTAAGCGCATAATTTAAAATTTTACAATTA
GTTATGTGCGAGTTTCTCTTTTTCTTTTTTTCGGCAAAAGAGTGCAACCGCATGGTCAAATTCTTATTCCGT
CGTTTTTTCTTTAACTCCGTTTTTTTTCTCGCCTTCCATGCTCCGAAATTCGGATCGCCATATTGCTGGTGAG
AGCCAGATTTCAAACAAATTCGAAAAGCAACGTATAAAGAGAAAGAGAGAGAATACAAGTCACATGAAG
TTCGAAAAAATTTTATCTAGTGCCTTCTTTTTTTTTCTTCTAAATTTTTTTTTGTGCAATTCGAAGTGATTTT
TTTTNCAGCAGATCATCGCACATTACATCGATGGAAGCGCTTTCGGAGATGGAGTGAATGCATGTAAGTG
AAGCAAAAAAAACTCTTAAATTGTATTTTTCTTTGCACTTAATTTCCGAGAGATAAAGAGAATGGCCNAAT
AATAAGTTAATGGATAATTTAATGCCGCGTTNAAGAGCAAACGAATCGGCGAAGAGAAAACGTTAAGAG
AATAATCTACTTCAAGTGAGCGTTAAAGCTTTCGCTAAATGAATCAAAAATTAAATGAAAGGAATAATTT
NTACTTTTACCGATTCGGGTCACAGACCAAATTAGAACCTCCCCGTC
>9_rnd-5_family-3263__DNA___TcMar-Tc1
ACATACCAGTTTGTCAAGTAATTGTTTGCAAAANGAAAAACAGCACACAAATTCATTTTTAAACTAATATT
TTACCGAATTAAACTATTTCTTCTTAATTTTTTTTTTGCATATTATTATTATTCTAAGTATTCTTCATAGATA
TAAGGCACAAACATAACAAAATTCAAAACGCAAATGTAAAAACAGACAAAACTAAAATGAGGCAATTTT
TGCGCTGTCAATTAATTCTTTGCAAAATGCAAACACAATTCGAAATAGAGCCCAAGCTACCTAACGGTAC
TCCAAATTCGAAGAAATTTGAATGCGTGAGTCATTGATATGCAGTGTGCGAACCTTCTAAGCGATTTTGCG
TTTTTGACAGTTTACATCTTGCCTAATTTAGAGCTTTTTGCAATGCCTGGTCAACGAGTTAATGAAAATCTA
ATTCAATTAGTTTATTTCCACTACCACAAGGGGAAATCTGCTAAAGAAATTGGCGGAAATGTTCGCAATTA
AGCTAAGAACTATTTATAATATTATTAACCGCGCCGAAAAAGAAAACAGATTGGAATTAAAGCACTCCGG
AGGCAGACCAGCGAAGCTCACTAGACGCGATCATGGAAAAATTTTAAAACAGATCAACGATCAACCCTC
AAACTAGCCTCCGACAATTGGCATCGGACTTAAAAAATGACTGCAATAAGACAGTGAGCCACGAGACAG
TTCGTAAAGTCTTAAAAGTGCATAAATACTCCTCGCAAATAGCTAGGAAGAAGCCACTACTGTCCGCAGT
AAATATTCAGAGGCGGCTTANTTTTTCTATTGCCAATGTCAACAAGCCCGCGGAGTACTGGGATGACGTTA
TTTTTTGCGACGAGACAAAAATAATGTTATATTATCATGACGGACCCAGCAAAGTTTGGAGAAAGCCCAA
TGCAGCTCTGGAATCGAAGAATATAATACCAACAGTAAAGTTTGGCAAGCTTTCGGTTATGGTGTGGGGC
TGCATATCGTCGAAAGGTGTTGGTGAGCTGCGCATTTTCAATGATGTGATGACGAAAGAGTTTTACTTGGA
CATTCTAAAGAATGAATTGTCAAGGAGCGCAATAAAGTTTGGCTTCGAAGACCCCCAAAACCCCAATAAA
CAGAGGTACAAGCTTTATCAGGACAACGACCCCAAACACAAATCGTTTTTGTGCAGGACTTGGCTACTGT

ATAATTGCAGCAAAGTCATTGACACCCCTGCCCAGAGTCCTGGACTTAAACCCAATTGAAAATTTGTGGG
CTTTCCTAAAGAAGCGCGTAGGGAAACGGAGCCCAACTAANAAAAACGCTTCTCATCAAGGCCATTCAAG
AAGAATGGATAAAAATACCCGAAATCTATGACCTGAATAACCTTATTCACTCAATGTCACGTCGTCTGCG
GGCTGTAGTGGATGCTAATGGCCAATATACCAAATATTAGTACCACCATNGCTATAGTTTCCTAGATTTAG
TTATTATGTATATAGTAATATTTTGCATTTTGCAAAGAATTAATTGACAGCGCAAAAAATTGCCTCATTTT
AGTTTTGTCTGTTTTTTACATTTGCGTTTTGAATTTTGTTATGTTTGTGCCTTATATCTATGAAGAATACTT
AGAATAATAATAATATGCAAAAAAAAATTAAGAAGAAATAGTTTAATTCGGTAAAATATTAGTTTAAAAA
TGAATTTGTGTGCTGTTTTTCATTTTGCAAACAATTACTTGACAAACTGTATGT
>160_rnd-1_family-210__Unknown
GTCCTGTGTCTAACAAATCGAATGATCCAGGGGTTATCCNGGCAGCTTACGAGATATCAAGCCGATTTCG
CATCTCACCCAAATGCGGTCGCTTGGTTGCAATTAATATTTCAAATTTAACGGCCGCCAAATTCCATTCGG
CATGTCGGCTGTGTCACAAAATTNNAACCAAATTCAGCTGAATTACCAGGCGAACAGAAATGTCAGCAGG
TAGCCAAAAGTCCGCGACACCAGAACAAAATTAGATTGTGCGCCGGCGCGCCATTGGTCAAGCGCAAATC
CATGATGGCTGCTCGGCGAAATATGTAACTGTAACTATCCTTTTGCTTTTGATTCCTATGCATATTCGTCAA
TATATAGTGATGCGTAAGCACGCAGGTGATGCGCCGGATTTGGCTGAATAATGCGATATCCGCTAAATCT
AGCCGTTTATTTTGACGGATGTCCGCAAATTTAGTAAATTGAGTCCTATCTTGGGCAGATGATATACTCCG
ACTTGAGAAAATAGCTATATACATATGCTATGTCATACGCTTGCAAACTCCGTCCTGTGCCTAACAGATCG
GATTATCCAGGCAGCGTACGAGATATAAAACCGCTATCGCATCTTACTTAAATGCGGGGTTGCTTGGTTGC
AATTAATAATTTAAATTTAACGGCCGCCAAATTCCATTCGGCATGTCGGCTGTGTCACAAAATTTGAACCA
AATTCAGCTGAANTACCAGGCGAACAGAAATGCCCAGCAGGTAGNCAAAAGTCCGCGACACCAGAACAA
AATTCGATTGTGCGCCGGCGCGCCATTGGTCAAGCGCAAATCCATGATGGCTGCTCGGCGAAATATGTAA
CTGTAACTATCCTTTTGCTTTGATTCCTATGCATATTCGTCAATATATAGTGATGCGCAAGCACGCAAGTG
ATGCGGCGGATTTGGCTGAATAATGCGATATCCGCTAAAACTAGCCATTTATTTTGGCGGATGTCTGGATA
TATATTAAATTGAGTTCCTATCNTGGGCAGATGATTATTTTTGGACTCGCGAGAGTATTTTAATTTTATTAA
ATACTGTCAAATTTTAAATTGGAATTTTTNTTGCCAATCGGACATTTTATCCGCATGTGTCAACTAAACTAT
ATATTTTCTCGATCGGCGTATATTTATTCTATATTTAGCTTTATTTGGCATGCATATACACATATTTCTTATA
ATAAACATATAGAGTGAAGGAAGATATGTCCTGCTTAAGAAAATAGCTATATTCATATGGTTATGTCAGA
CGCTAGCAAACTCCGTCTTGTGCCTAACAGATCGATTGATACAGGGGTTATCCAGACAGCATACGAGATA
TAAAACCGCTATCATATTTTACTTAAATGCGGTTGCTTGGTTGCAATTAATAAATATTTAAATTTAACGGC
CGCCAAATTCCATTCGGCATGTCGGCTGTGTCACAAAATTGGAACCAAATTCAGCTGAATTACCAGGCGA
ACAGAAATGTCAGCAGGTAGCCAAAAGTCCGCGACACCAGAACAAAATTCGATTGTGCGCCGGCGCGCC
ATTGGTCAAGCGCAAATCCATGATGGCTGCTCGGCGAAATATGTAACTGTAACTATCCTTTTGCTTTGATT
CCTATGCATATTCGTCAATATATAGTGATGCGCAAGCACGCAAGTGATGCGGCGGATTTGGCTGAATAAT
GCGGTATCCGCTAAAACTAGCCATTTATTTTGGCGGATGTC
>Ref_Paris
CAGTTTGTCAAGTAATTGTTTGCAAAGTGAAAAACAGCACACAAATTCATTTTTAGGCTTATATTTTACCG
AATTAAACTATTTCTTCTTCATTTTTTTTGCATATAATTATTATTCTAAGTATTCTTAATAGATATAAGGCA
CAAACATAACAAAATTCAAAGCGCAAATGTAAAAAAAACAGTCAAAACTAAAATTAGGCAAATATTGCG
CTGTCAATTAATTCTTTGCAAAATGCAAACACAATTCGAAATAGAGCCCAAGCTACCTAACGGTACTTCA
AATTCGAAGAAATTTGAATGCATGAGTCATTGGTATGCAGTGTGCGAATGCCTTCTAAACGATTTTGCGAT
TTTAACAGTTCACATCTTGCCTAATTGAGAGCTTCTTACAATGCCTGGCCAACGAATTAATGAAAACGTTA
TTCAATTAGTTTATTTCCACTACCACAAAGGAAAATGTGCTAAGGAATTGGCGGAAATGTTCTCAATTAAG
CTAAGAACTATTTATAATATTATTAACCGCGCCGAAAAAGAAAACAGATTGGAATTAAAGCACTCCGGAG
GCAGGCCAGCAAAGCTCAGTAGACGCGATCATTCAAAAATTTTAAAACAGATTAATGAAAATCCTCAAAC
TAGCCTCAGACAGTTAGCATTGGACTTGAAAAATGACTGCAATAAGACAGTGAGCCACGAGACAGTTCGT
AAAGTTTTAAAAATGCATAAATACTCCTCGCAAATAGCTAGAAAGAAGCCACTACTATCTGCAGTAAATA
TTCAGAGGCGGCTTAATTTCTCTATTACGAATGTCAACAAGCCCGCGGAGTACTGGGATGACGTTATTTTT
TGCGACGAGACTAAAATAATGTTATACTATCATGACGGACCCAGCAAAGTTTGGAGAAAGCCCAACACAG
CTCTGGAACAAAAGAATATAATACCAACAGTAAAGTTTGGCAAGCTGTCTGTAATGGTGTGGGGCTGCAT
ATCGTCGAAAGGTGTTGGTGAGCTGCGCATTTTCAACGATGTGATGACGAAGGAATTTTACTTGGACATTC
TGAAGAATGAATTGTCAAGGAGCGCAATAAAGTTTGGCTTCGTAGACCCCCAAAACCCCAGTAAACAGAG
GTACAAGCTTTATCAGGACAACGACCCCAAGCACAAATCGTTTTTGTGCAGGACTTGGCTGCTGTATAATT
GCAGCAAAGTCATTGACACCCCTGCCCAGAGTCCTGACTTAAACCCGATTGAAAATTTGTGGGCTTTCCTA
AAGAAGCGCGTCGGGAAACGGAGCCCAACTAACAAAAACGCTCTCATCAAGGCCATTCAAGAAGAATGG

ATAAAAATACCCGAAATATATGACCTGCATAACCTTATTCAGTCAATGTCTCGTCGTCTACGGGCTGTAAT
GGATGCTAATGGCCAATATACCAAATATTAGTACCACAATAGCTATAATTTCCTAGATTTAGTTATGTATA
CAGTACTAGTTTGCATTTTGCAAAGAATTAATTGACAGCGCAATATTTGCCTAATTTTAGTTTTGACTGTTT
TTTTTACATTTGCGCTTTGAATTTTGTTATGTTTGTGCCTTATATCTATTAAGAATACTTAGAATAATAATT
ATATGCAAAAAAAATGAAGAAGAAATAGTTTAATTCGGTAAAATATAAGCCTAAAAATGAATTTGTGTGC
TGTTTTTCACTTTGCAAACAATTACTTGACAAACTG
>9_rnd-6_family-1025__RC___Helitron
TCCAATGGCAGCTATGATATAGTGGTCCGATCCAGCTGGTTTTTCACATATGAGCTGCGTGCAACGAAAG
AGGGACTTCTGCTAGATTTCCTTTAAAGATATAGCTTTAAAACCCCAGAGACTAGTTCGCATAGAAACAC
ACAGACGGGCAGACGGACATGGCTCTATCGAATCGCGGGCTGCACTGATGCATTTATGTATCAAGAATAT
ATATATCATGAGNNGGATTTGTGTATCTTGGATNTTNTTCAGNATGCGTTACATATNCCACGAACAAAATT
ATANTACCCNCTGCAAGNGTAAAAGGCGGTCGAAAGCCTCTCGCTTGCAAGCATTGTCCAAGCGCGATCT
GCACCTACACACCAATTCTAGCTGGAAAGCCTATGTATGGATATTATATGGATGTTTGCAGATGCGATCAC
GCAACGAGAAAGTCAGAACGCCGGACACGCCCAATAAATGTAATAGAGGAAAAAGAAGTGTGGTGACTT
ACATGCATCGGCGGCGAAAGGCCTTCCAACGCTGGCGACGGTGTTGTTTGGTTCATGAGTTGTGGGTTGGT
ATGGTATAGATCTTCTTGNTCTCGTTATTTTTTTATACCCTTGCAGAGGGTATTATAATTTTGTCGTGAAAT
GTGTAACGCATAGAAGGAGACATCTCCGACCCCATAAAGTATATATATTCTTGATCAGCATCAACAGCCG
AGTCGATATAGCCATGTCCGTTGTCCGTCTGTCTGTTTCTATGCGAACTCGTCTCTCAGTTTTAAAGCTATC
TTAATGAAACTTTGCAGAAGTCCCTCTTTCTGTTGCACGCAGCGCATATGTGAAAACCAGCCGGATCGGAC
TNCTATATCATATAGCTGCCATAGGAACGATCGGTCGAAAATTAAGTTTTTATATGAAAAAAACTTTTGTT
TATCAAGATATCTTGACCAAACTCGGCATTTATTAGTTTCACTNTTNCTCCTCGTATATNTGCGAAAATNCT
ATTAAGATATCTTGACCAAACTCGGCGTTTATTAGTTTTACTATGTCTCTTCATATATATGCAAAAATCCTA
TTAAGATCGGANCACTATATCATATGGCTNCCATAGGAACGATCGGTCGAAAGTTAAGTTNTTGTATGAA
AAAAACATTTTGTTTTTTTCAAGNTATCTTGACCAAACTCGGCATTTATTAGTTTTACTTTACTTCTCATAT
ATGTGCAAAATCCTATTAATATCGGACCACTATATCATATAGCTGCCATAGGAACGATCGGTCGAAAAAT
TAAGTTTGTTGNATGAAAAAAACATTTTTTTCAAGATATCTTGACCAAACTCGGCATTTATTAGTTTTACTA
TGCTCCTCATATATATGCAAAATCCTATTAAGATCGGACCACTATATCATATAGCTGCCATAGGAACGATC
GGTCGAAAAATTAAGTTGTTATGAAAAACATTTTGATTATCAAGATATCTTGACCAAACTCGGCATTTACT
ATTTTCCCCTTCTTAGATAGGGGCGAAGCACTATGAGCATTATGAAAAGGTTGGGTCTGCAAAGGGTATT
ATTAGATCTTTGGCGCGCCGAAGATAGCCNTTCTTTCTCGTTATTTCTTTGTTTNCCCTTTTGCGGGTGCAC
TCCACAAAAGTAAAAACAAAAATNGTTNACTACTTATGCATGTNNATANTTATTTTATTTTTCGATCCTTA
TTGTTATTATTTTTTTGTTATGTTTGTA
>9_rnd-5_family-1695__LINE___I-Jockey
ATTCTTATCAGGAATAAACTAAATTACATTTAAGAGGATTGGATCCAATGTGCACTTATTAAAGTCAATAC
TGTGCTGGGCGATCTGAAAATTGCTGCCGTTTACTGTCCACCGAGACATAATGCTAGAGATGACAAGTTTA
ATAGGCTGATACATACCCTTGGCCGCAGGTTTGTCATAGGCGGTGACTTTAACGCCAAACTTTGCTGGTGG
GGCTCTAGAAGAAANAACCCAAAGGGACAAGCGCTNATTATGTAGATTCAGACCGAAAACNTCAACCAG
GGCACTTAGTCATAGCCCGAGGATTCCAGACAGGGAAATCCATCATTGGAGCCTCCCATGTTGCTGAGCT
CCTCCACGAGAGACGGAGACTCCAGGNGCGTCTGGTCCCGNCAAAAAAACTCCAGGGGCTAAAGACCGC
GCTTANTGGAGCCACCAAGGCTTNAAAGGGCTTAAAAAGATTGTAAGAGAAGCCAACAAGGTGGAGCAG
CTCTTCCAATGGATGCCTCGATCTCAGGCATTTTTCCTAATCCTGGCAAACCNGAAGCCAATCTCGCCTCA
TACCGTCTGGATTAGATTACTAACAATACTCTCCAAAATTCTGGAAAGAGNCGTCCTGCGTAGAGTGCTCT
CAGTCTTGGACGAGGCTGGCCAAATTCTCGATTACCAATTTGGTTTCAGGTTTGCAACGCATCTTGGAGGC
GTTTTTAAAATAGAAAATACTGCTGCGCCGTAAGTCTGGCATCCTGGGCTTTTACTCAAAAATCAGGAGCA
ACCTGTGCCTGCCGCAGGCAGTTCCATGCAAATGTGGAGAAGCTATCTCTCGAATTAAACTCATTAGAGC
GGGAGTTCCCCCAAGGCAGCGTGCTGGANCCCCTACTATATANCATCTATAGTGCGGACCTTCCTGTTATT
GGGAACCCTAACNTTCTAGTCGCCACATATGCTGATGACACGGCATTCCTCGCCACTGCTGACACACTTAA
CGAATGGCAGAGGAGATGGAACATCTTGGTGAACAACGAAAAGTCCACAGCCACCACTTTCTCTCTCAGG
CCTGGTAACTGCCCACCTGTCTACCTAGGCGGAGCTGTTATACCAGAAGAAGACGACGCCGAAATACCTT
GGCATGACTCTCNACNGGAGGTTAACTTGGAGAGCTCACCTTTTAAAGAAAAGGAAAAGAGCTGAAGTC
AAGCTAAAGTCNTATTACTGGCTTATTGGCCGTAAGTCCAAGCTTAGTACTCGGTCAAAGCTCCTTGTCTA
CAAGGCAATTATTAAACCGATTTCGACTTACGGCATTCAGTTATGGGGTACTGCCAGCCGAAGCAATCTA
AGNTTATTTCAGGTTTTTCAAAACANGGCACTGCGCATCGTATCATGACAACGCTTCCATCCACAGTGCTC
TCAAGATCCCATGGGTCAGTGACGAGATAGCCAAAAGCTGCAGTAGATACCTGCATAGGCTCCACCTGCA

CAACAATAACCNAGCAGTTGGCCTGCTGGACAACAGCGACACCATCAGAAGACTCAAGAGAGTACACCC
TCTGGNTCTTCCAAGGGAACCAAAATAATTCAATCATTTACTCCCAACACTACCGAACCACCAACCATTAA
TTAAAAATCGACATATATTACAATAGGAAATATCTACAAATGAAACACACCTAGNTAAGTACCCTTCAAA
ACATTTAATAATTGTCGACGCGTGACAGATTTAAA
>160_rnd-1_family-232__DNA___TcMar-Tc1
ACACATACAGTCACGGACAAAAGTATTGGCAATTTAATATTATCTTGTTTTATTGTCGGTTTTGTCCAATAT
ACCGTCTAATATTTAATAATTTTATATTTTTCATACGATTATTAGCTATTTGTGCATGCAATCTAATTATTTT
TATAAACTTTGGCGGCATGGTTAATTTTTAATAATTATTTAATTCCAAGTACCCGTAATTTTATAGGGACA
AAAGTATTGGCAATTTTCAATATAAAGTTGTTATTTTAATATTATTTTTATATGGTCACACTTTTCTTTGCA
AAAATGNAACTCTGTAAGCACCGTTATTTGAATCTTAAAAAAAAAATACTTAGAAAAAATTGTTTAAGCAT
CAATTTATATATTATTTTGCAAAAATGGCCGGTAAGAAGTGTGAAACCACCGTAGAGCAGCGAAAAATTA
TTTTAAATTTACGCAAACAAGGCAAATCCTTTAAAGAAATTGGCGAGCTATTGGACAGAAGTCAATTTAC
CATTCGGAGCATAGTCCATAGATTTTCAGACGACCCTACGGTAGAAAACGCTGAACGCACCGGCCGCCCA
CGAAAGTTAACCGATCGTGATTGCACCCAAATTCTGAGAAAAATCGAGAAAAATCCACAAATTTCGCTTA
GGGATATTGCTCAGGAGTTCAAGGAAGAAAGTGGTGTTGATGTGTCACACGAAACTGTCCGAAAATTACT
GAATTCCAATGACTACACATCACGAGTTGCCAGGAAAAAACCTCTACTATCGGCGGCGAATATTTTGAAG
CGGCTATCGTTCGCTCAAGTCCATGTAAATAGTTCGAACGATTTTTGGAGTAACGTCATATTCTGTGACGA
AAGCAAGATGATGCTATTCTACAACGATGGGCCATCCAGAGTCTGGAGAAAGCCACTAACAGCGTTGGAA
AACCGCAATATTATTCCAACCGTTAAATTCGGAAAACTATCAGTGATGGTGTGGGGGTGTATTTCGAGCA
AGGGTGTAGGAGATTTGACCTTCATTGAGAATACCATGGATGCTAGACAATATCTGAGCATTTTACAGAC
ACATCTCGTCAGTAGTGCACAGAAATTTGGATTCTATGAGGACAATAAGCCAATTTTTAAATTTTACCAGG
ACAATGACCCGAAGCATAAAGCTCATATGGTAAGAGTTTGGCTGTTGTACAACTGTGGAAAGGTGTTAGA
CACTCCACCACAAAGTCCAGATATGAATCCAATAGAAAATGTGTGGTCATATTTGAAAAAAAAAGTCGCA
AAGCGGAGTCCAAAATCAAAAACTGATTTAAAAGCTGCTGTACTGGAAGAATGGCAGAAGATTCCAGAA
ACATACATACAAAACCTTATTGTTTCAATGAAACGGCGATTGCAGGCTGTATGTGACGCTAATGGCAGCC
ACACAAAGTATTAAAAAAAAATATTCCTGTTTTTTTAACATTGTTATTTAGTAATAAAGTTTCGTGTAAAA
ACACTTTATTGACAGCATAAAATTTGAATGTTTTTAGTTTTTTAATTTTATAAAATGTTTGGGTAATGTTAT
TTAACAATTCTTTTTGCCATTAAGTTCTAGGAAAAATATAGAATCGAAATAAATTAGAATTGAAAAAAAA
TTCTCACTTTTAGGTACATTTATTTTCAACTAAACCCAAAAAATTAGTTTTTTTCGTTAGTCAAAACACTT
TATTGACATACTGTATAT
>Ref_Polyphemus
ATACAGTATGTCAATAAAGTGTTTTTACTAACAAAAAAAAACTAATTTTTTGGGTTTAGTTGAAAATAAAT
GTACCTAAAAGTGAGAATTTTTTTTCAATTATAATTTATTTCGATTCTATATTTCTCCTAGAACTTAATGGC
AAAAAGAATTGTTAAATAACATTACCCAAACATTTTATAAAATTAAAAAACTAAAAACATTCACATTTTA
TGCTGTCAATAAAGTGTTTTTACAGCTACGGTAAATTGTTTTGCCGTTCTCTTTTGGGTGATCCCGTTAGAT
TTGGGCCCATGCGTTTTGGCTTCTCTTGTGTCTTCGACTTTTCTTGTCATTTTCTATTGCAATTTATAACGTT
TTGCTTTAATTTTATGAAAAATAAAATGCCAGGAAAAAGAACAACTTTTGAAGTCGCTCAGCTGGTGTATT
ATAACCATCAGATGGGACGTCAAGTCTCGGAACTGGCTGATATGTTTAATTTATCAAAAGCAACGATATA
CAACATATTAAACAGAGCCAATAAGGAAGATAGGCTAGAGGCAAAGCCAGTATGTGGTCGACCCTGCAA
AATTTCTGACAGAGATAAGCGAAAAATTCTGAGAAAAATTGAGAAAAATCCACAAATTTCGCTTAGGGAT
ATTGCTCAGGAGTTCAAGGAAGAAAGTGGTGTTGATGTGTCACATGAAACTGTCCGAAAATTACTGAATT
CCAATGACTACACATCACGAGTTGCCAGGAAAAAACCTCTACTATCGGCGGCGAATATTTTGAAGCGGCT
ATCGTTCGCTCAAGTCCATGTAAATAGTTCGAACGATTTTTGGAGTAACGTCATATTCTGTGACGAAAGCA
AGATGATGCTATTCTACAACGATGGGCCATCCAGAGTCTGGAGAAAGCCACTAACAGCGTTGGAAAACCG
CAATATTATTCCAACCGTTAAATTCGGAAAACTATCAGTGATGGTGTGGGGGTGTATTTCGAGCAAGGGT
GTAGGAGATTTGACCTTCATTGAGAATACCATGGATGCTAGACAATATCTGAGCATTTTACAGACACATCT
CGTCAGTAGTGCACAGAAATTTGGATTCTATGAGGACAATAAGCCAATTTTTAAATTTTACCAGGACAAT
GACCCGAAGCATAAAGCTCATATGGTAAGAGTTTGGCTGTTGTACAACTGTGGAAAGGTGTTAGACACTC
CACCACAAAGTCCAGATATGAATCCAATAGAAAATGTGTGGTCATATTTGAAAAAAAAAGTCGCAAAGC
GGAGTCCAAAATCAAAAACTGATTTAAAAGCTGCTGTACTGGAAGAATGGCAGAAGATTCCAGAAACAT
ACATACAAAACCTTATTGTTTCAATGAAACGGCGATTGCAGGCTGTATGTGACGCTAATGGCAGCCACAC
AAAGTATTAAAAAAAAATATTCCTGTTTTTTTAACATTGTTATTTAGTAATAAAGTTTCGTGTAAAAACAC
TTTATTGACAGCATAAAATTTGAATGTTTTTAGTTTTTTAATTTTATAAAATGTTTGGGTAATGTTATTTAA
CAATTCTTTTTGCCATTAAGTTCTAGGAAAAATATAGAATCGAAATAAATTAGAATTGAAAAAAAATTCTC

ACTTTTAGGTACATTTATTTTCAACTAAACCCAAAAAATTAGTTTTTTTCGTTAGTCAAAACACTTTATTG
ACATACTGTACATA
>Ref_NewTE7
TGTATATAAGGTTCGTAACGAAATTATACGAAACTATCAAAATATTACTTCAAACACTCGACAAATCGAA
AATGTTGTTAAATCAACTCGCACGATGGAATAAAATGAGAATTCAAAGCATAATGTGCAATAGAGAACCG
TTACATTCTATATTTAGCCTATGCTGGAAATGTCCCGTTAGATTTGCTTCTAAAAGCCGTTGTTTTGCTGAA
ATTTTATTGTAACGAAATTATACGAAACAATCGAAAAACTGATCTTTAGGCGTGCGTATCAACTTTGTTTG
ATTTGTAGTTTTGAATTTTATCAATTTTATTTAAAAAATCATTGAAATGCCACGAGGAACGCGTTTGACTG
AAGAGGAAAAAGGGAAAATTCTCGCTTTTAAAGCCTCTGGCTTATTAATTAACCAAATTGGGAAATTAAA
TTGGTCGAACATGCTGCGTAATCAATAATTTTTTACAAAATGAGGAAGGATACGGCAGAAATAATAATGC
TGGAAGAAAATCCAAACTGTCTGGCCGTAACAAGCGTCAAATTTTGAAAAAAGCAAGTAATTCGACCTTG
TCGTGTCGTCAACTTGCTGCATACAGCAGCATTCCAGTGTCACCAATGTGCGTTTGGCGTACTTTAAAAAC
ATCCAAACATATAGTGCGAGGAGTAATGAAGAAGCAGCCAACTCTCAAAAATCACCACAAGGCAGCACG
ACTTGAATTCGCTTTTAAAAACCTTGATACGTGCTGGGACTTGGTAAGGAATCGTTGTCTTGTAATGTTGC
ATGCAACATGCAGATGCAACACATGCAACATAAGATTTTTAAATATTTTTTTTTAAAAAGGTTATTTTTTC
GGATGAGAAAAAGTTTAATTTAGATGGTCCAGATGGATTTCGCGGCTATTGGCACGATTTGCGTAAGGAA
CCCCTCGTATTTTCAAAACGGAATTTTGGAGGTGGTTCTGTTATGGTATGGGGTGCATTTACCAAGTGTAG
CACCCTTGAACTCCGCTTTGTTACTTCAAGAATGACAAGCGTAGATTATGTGGAAGTGTTGGACAACACTC
TCCTACCCTTCCTACAGGATAACAGGGAAAGTCCTTACATTTTCATGCAAGATAACGCAAGAATTCACACC
AGTCGTCACACGATGGCCTACCTATTGTCATCCTCAGTCAATACTTTGGACTGGCCTGCGTGTTCACCTGA
TCTGAACCCTATTGAAAACCTCTGGGGAATTCTTGTACGTCGTGTTTACGCCGAAAATAAGAAATTTGATA
GTGTTACTGAACTAAGAAGGAGGATTTTAAGAGAATGGACAAAATTGGATCAAACCCTTTTAGCAAATTT
AATAAACTCAATGAAAAGAAGGTTATTCAATGTAGGAAAAGAACAAGGAGGCCCTATTTAATGTTCAAAT
TTTTTTTTAAGCCATTATCTCAACGACTTATGGTTTTATAAGCTAAAAAAACAAGTTTTCGTATAATATCGT
TACACTCGAAAAGTGTAATTTTTCTTATTTTTTCAAATATTTTAAAACCTATGAACATTTAAATGCCTATGT
TATTATATAATGTTGCTTTCATATACATTTTCGAAAATAAAGCATTACTATACTTCTAAGATTACTCAAACA
AAAAAAAGTTAACAAATAAAAAGTTTCGTATAATTTCGTTACGCACCTTATATATATATATA
>Ref_dvir_964
CATACATATTAGGTTCATAAGCCTGGTCGGCAACCTGCCAAGAATTAAAAAAACACAATTGCAAATAATC
GAATTTAACCAATTGACAAGCCTCTACAGACAATATGTATCAACATTCAAATTGCTAGCGAATTATATATT
GTACACATATGAACACACTGGTCTGCTGGGTGGTTGCCCACCCAAATTTGTTAGTGTAAAAATTGGTAGA
AATCGTTCAGCTGGCACTTATACATATACATATCCAAATTTTCCCAACAATTGTGATTGACACTGCTAAAC
TGAGCATAATTTCCTGACTCTTCACCAATTTAAATATAATTCATAATCCATATTTTGCTTGATAACATTTTG
CTAGGTGATCGTCGATATATCGATACAGCGACAGGCGTGCTGATCTCAAACGATACTTTTCTCTCTGCCGC
CGCTAGGCGATAGTCTCGAAATTGTCATAAGCTGCGAGTTGTCTCTATAAAGTGGTCGTGGGTTTAAAATT
GGCGGTAATTGAAAAAGACATCGAGCAAGTTTAAACTTATTAAAACACTAACAGTGGAAGCATTTATTGC
TGATATAAACCTAAAGCGTGTTCAATAAGCAAATTAAGGTATTTCAAAAAATTCCTCAAATTAGTGTGTG
AAAATTATTTTGCAATTAACAAATAACCGGTGTCCGTCTGTGAATTGCCAAGCAACAAATTGAAGGCCTA
ATTACCACCAAAAGTGAAAGACATATAATAAAGACCAAAGTCCCAATTCAAATCTGAGAATAACTGGGGT
AAGGCTCAAACCAAAAAATTTTTACATATATAACCCCAAATTTGCCCAGTTGAGTCTCGAAATGGAGCGC
GCATAATAATTTCATCACAAGCGTCACCTATACATGCACAAACTTGCAGCTGCAGACAATTTTGGCGACG
GCCACAGCGGCGCCCTGCCAGACTGACGCACACACACACACACAAATTTTCTAAGAAATTTTCTACCCAA
CATTGCCAGGCCTAAAAACCACATACGGACAGAACTATATTCTACGCTAGCATCTATTATTTACACTTTCT
TATTTTTTGCTAACTTTAAACATTTTTGCAAATTTAAAACATACAATTAAACATTACTGCCAAGCGATCATC
GATACATCGATACATATACGCGCGCACTGATCTCAATACTATACCTTCCACTTTGCCGTCACTAAGTGATA
TTCTTGAAATTGTCATGAGCTGCGAGTTGTGTACTACGATATGGTTGCGAATGTGAAACTGTTCAAACTTA
TAAATACCCTAATAGTGAAAAGCATTTAATGCTTATAAGGAACTAAAGTACGAATTAAACAAATTTAGTG
AATTTCAATCAAGTGTAAGTCTTTGAAACTAACAGTGCCTATAAAAGCCCCAAATAGCCGGTGTCTGTCTG
TGTGTAAATAGGCACATATGCAATTAATTGTAAGTGAAACATAATTCAGGGCTTCTGACAAGTGAAATTT
AGTCCTGCATGTCCAAAAAGAGTCCCAAAATAGTGTGTGCCAAGTGTGTCCACCTGCATGAATAAATGCA
TATATCCAAACACAAATTTAATCTAGTGAATAATCCACCAAATTATGCCATACAACTCCAATTTTGTCCAT
AAAAGTCTGAAAATAGTGCGCGATCAATTATTCCACAATTTTAGAGGAACCAATTTAAAGGCCTAAC
>160_rnd-1_family-350__Unknown
ATATACATACAGTCACGGACAAAAGTATTGGCAATTTAATATTATCTTGTTTTATTGTCGGTTTTGTCCAAT

ATACCGTCTAATATTTAATAATTTTATATTTTTCATACGATTATTAGCTATTTGTGCATGCAATCTAATTAT
TTTTATTAAACTTTGGCGGCATGGTTAATTTTTAATAATTATTTAATTCCAAGTACCCGTAATTTTATAGGG
ACAAAAGTATTGGCAATTTTCAATATAAAGTTGTTATTTTAATATTATTTTTATATGGTCACACTTTTCTTT
GCAAAAATGNAACTCTGTAAGCACCGTTATTTGAATCTTAAAAAAAAAACTTAGAAAAAATTGTTTAAGC
ATCAATTTATATATTATTTTGCAAAAATGGCCGGTAAGAAGTGTGAAACCACCGTAGAGCAGCGAAAAAT
TATTTTAAATTTACGCAAACAAGGCAAATCCTTTAAAGAAATTGGCGAGCTATTGGACAGAAGTCAATTT
ACCATTCGGAGCATAGTCCATAGATTTTCAGACGACCCTACGGTAGAAAACGCTGAACGCACCGGCCGCC
CACGAAAGTTAACCGATCGTGATTGCACCCAAATAATAAGAAAGATCAAGAAAGACCCAAAAATCACAT
CATCCAAAATAGTATCAGAGGTTAAAGAAGAGACTGGGAAAGAAATTCACTCCAAAACGGTACGGCGGG
TACTACACAAGGCTGGGTACAATGCGAGAATTGCAAGACGCAAACCACTCATTTCGAGTGTTAACCAATC
GAAGAGATTGGCATTCGCCAAAGAATTTAAATCGAAGCCATTAACTTACTGGAACGATGTCCTATTCTCC
GACGAAAGTAAGTTTAATATTTTTCGCTCAGATGGCCGCATTAAAGTGTGGAGAAAACCCAATACGGCAT
ACAATAGTTGCAACTTAATGCCCACAGTAAAGCACGGTGGTGGAGGTGTTATGGTGTGGGGTTGTATGTC
CGCAGCTGGTGTGGGTGAACTAGTTTTCATCGAAGGAACAATGGACAAGCGTGTTTACTTGGAAATATTG
AAGGCAAATTTAAAAAAAAGCGCTGAAAAGTTGATGTTAAGCAGTCAGTTCACTTTTCAACAGGACAATG
ATCCAAAGCACACGGCCCATGATGTGCGAATGTGGCTTGCATACAATACAGCTCATGTTCTTCCACATCCT
CCTCAATCACCAGACCTCAATCCAATCGAGCACCTATGGGAGGAACTAGAAAAAAGGCTTAGAAAGCAT
ACATTTTCAACAAAGACTGAGCTTAAAGAAGCCCTAATGGAAGAGTGGCACAAAATTGAGCCAAATGTTG
CCCGAAAATTGGTGCATTCGATGCCAAATCGCCTGGAAAGCGTTATAAAACAAAAAGGCATGCCGACTAA
GTATTAATTTTTATCTCATGTTTTATTTTTTTAAATTTTAACACAATTATTTAAGTTTTTGCCAATACTTTTG
TCCCTATGAAAATTACCATATTTTTTGTTTTTTATTTGTAAGGCTTGAAGCAACAAAAAATGTTCCTTTTTT
AATACTTTTAAGTGAGTTAGATATTTAAAAGATCATAAAAAAAATATATTTCAATAAAATAATAAGTTGG
TTATTCTCAAATTTCGAATTTTTAGTTGACAACACCACAATTGCCAATACTTTTGTCCGTGACTGTA
>Ref_dvir_887
AATAATGGGTCCCATTTGGAGTTGGGATTCCCGTTGGGAGCTCTCCAGCGCAGCCAATTTCTTGTCAGGCA
AGTTTGATTCTCGCTGAAGCTGCTTGTCACAATTTGACCAGATTCCGCCCTTTTGGGCCGAGCAACGTCGG
CGTAAGACATACTGCATCTGACAGTCTCAGCCGGGATGAATCCACGTGAAGGGCCTTGGTTATATTTTGGC
CTGGCAGTGTTGCCAGTAGCAGAGAGCCTTGGGATCGGTTTCATTTTTTTGCTTTTCTTAATCCTGACGGG
GCAGTCTTTGTCGGTCGACGCGTGATCACCACCGCAATTGACGCACAGGAAATTTTCACTCGTGCAAAGC
AGAGAACCAGTCATATGAGAGCCAGCGCATTTGCCACGGATTGGGTCCCGAAGGCAATAATTTTTGGAAT
GACCGAAGAAGAACTTCTTTTCTGCGAGGAGCACGCTCCACTGAGACTCTGTATCTGCCGAGTTGTGTAAT
TTTCAAAGCATTAGACACATTGGGTCCTTGTTTTAGATTGACAAAAAATTGATTTTGCCTAGTCTTGTAGTT
GATCGTCGCTTCATCGTCTGTCTCGTTGACCTGAACGTTACGCCAATCTGCCTTTCTTGGGCAATATATATT
GAGAGTTTCGAAACCCTGGTCAGTAAAAGCTTGCGCAATTTGTGGTTTGGGGACGTTGGCATTAACGCCCT
TGACGACAACCCTGTAAGGCTTTTCCTCCTAAGTTGATGGTGCCAGAATTGACAGTTAAGTTTTGTCAAAT
GTCTTACAGCTGCTCGGAAAGCGTCAGAGTTTGCGGTGTATATCCATGATACACCGAACTTAGAGGTGCG
AATGCTATAGTTGTTGGCACCCACGCACGAGTCTAGTGCTTGTTCCATGCCTAAGGGATTGTTGACTTGTG
GTACAAATATTGCTGGAGGCTTTGAGAGCGTTTTTGAGGATTGTGTGTTTCCGTTGCCAACAGCACGAGCT
TCATCTTCGTCGTCAGAATCGCTTTCTGACAATCCTTGCTCCATCATGTTGTCCGCTTCTTCGTCCAGAGCT
GCAAAACGGTTTTTACTCAGTGCTGCTGCAGTCGAGGTGGATGCCGTGTCTTTGGCTACTTCCTTGTGTAG
CTCAGCGGGCTTCGACTTAGCTTTCTGTATAGATTTAGATGTGTTTATAACTCCCTTATGTTTTTTACTAGC
AGGCAGGCCACGGTCTTGAAACGAGCACTTCGTCTTACGAGATGCTGCTGACAAGAATTCACCCAATGTG
TTGTCAAGTGACGTAGTCGAGGAAATGTTAGGCCCCACAGTTGCCGCTACAGTATACGTAGATGTAGATG
TCAAACTTGCTTGCGACATAGAGATAGTGCAGGTAACGCTGGTATCAGTGCTTGGTGTTACTGTCGGCCCA
GTTATCACCCGATTTGACAATAAGCTGTTGTTTTCTGCAAAAATGGATGCTACGCTACCGCGGATTTGAGA
GCGTAGAGTTTCAAGGCTTATCCTTGCTGCTTTTCTCATCTCCTCCAGTACAGCTTGCCCAATACTAAGTAG
CGACTGATTCCATGAGTTAATGTCCAAAACGTCAAATTGTTATTAATTAGGAGTCCAATAATGGCATTGTC
GTCCACCTTTTCCGAATCAAAAGAATAAGAAATTACGGAAACCAACCTCTC
>Ref_dvir_942
GTGAAAAAAACAAGCAGTAATAAGTTTATGTTGATGATATTTTACTTGCCAGGTCAAATAAAGACGAATT
GTTGAAAATCAAAGCATCCATTGCCAGGGAATTCGACATTGTTGATGGCGGCCAGCTCAACCATTTCTTGG
GCATAGAAATTGAGCGCGAAGGGGAGACTGGAAGCATAACAATCGGCCACAAGCAGTATGTGGAACAAA
TGCTACATACTTGTGGCATGTCAACGTGTAGGCCTGTAGCTACACCACTGGATGCAAGTCACCAAGTCAA
ATGTGATGACGTCAACTGCGAGATGGTTAATGAAAAGAGCTACCAATCATTGATAGGTTCTTTGATGTATT

TAGCCATAACAACCAGGCCGGACATACTACATTCGGTAGCGAAACTGGCACAACGAAATGCCAGCCCTCA
CTAGGAGCATGAAGTTGCAGCGAAGCGTGTGCTAAGGTATTTAAAAGGCACGGCTAATATGAGACTTCAT
TATCAGGATACTGGCAAGCAGATCTATGGATACGTCGACGCAGACTGGGCGAACGATGCTGCAGATCGAA
AATCATATAGTGGATATGTCTTTATTGGTGCGGCAGCGCGATTTCGTGGGAATCGAAGAAACAAAGTCTG
GTGGCGTTAAGCAGCACTGAAGCCGAATATATTGCCTTATCTACCGCAGCAAGGGAAGCAACATATATTA
AAAAATTGTTGCATGATATGGGATTTATAGAAACCAAGCCTATTTTATTGTATTGCGATAATCAAAGCGCA
CAGTGCTTAGCTGAAAATCCAATTTTACATAAACGTAGTAAGCACATCGATATAAAGTTTCACCATGTACG
CGAGCTATATTCTAAGAACGAAATAGATGTTAAATATATAGGTACTAATGATATGATTTCCGATGTACTAA
CTAAAAATTTGCAAAAGAACAAGCATGTAAAATGTATTAATGAAATGAATTGTTATTAATAAGTTTATGTT
GATGATATTTTACTTGCCAGGTCAAATAAAGACGAATTGTTGAAAATCAAAGCATCCATTGCCAGGGAAT
TCGACATTGTTGATGGCGGCCAGCTCAACCATTTCTTGGGCATAGAAATTGAGCGCGAAGGGGAGACTGG
AAGCATAACAATCGGCCACAAGCAGTATGTGGAACAAATGCTACATACTTGTGGCATGTCAACGTGTAGG
CCTGTAGCTACACCACTGGATGCAAGTCACCAAGTCAAATGTGATGACGTCAACTGCGAGATGGTTAATG
AAAAGAGCTACCAATCATTGATAGGTTCTTTGATGTATTTAGCCATAACAACCAGGCCGGACATACTACA
TTCGGTAGCGAAACTGGCACAACGAAATGCCAGCCCTCACTAGGAGCATGAAGTTGCAGCGAAACGTGTG
CTAAGGTATTTAAAACGCACGGCTAATATGAGACTCCATTATCAAGGATACTGGCAAGCAGATCTATGGA
TACGTCGACGCAGACTGGGCGAACGAATGCTGCAAATCGAAAATCATAAAGTGGAAATGTACTTTTTTGG
TGCAAGGCACCCCGATTTCCGTGGGAATCCAAAACACAAAGTCTGGTGGCGGTAAACCACCATGAAAGCC
GATTAAATTGCCTTATCTAACCCAGCAGGGGAAGCAACTAAATTAAAAAA
>160_rnd-1_family-222__LINE___R1
ACACACACACACACACACACACACACACACACACACACACACACACACACACACACACACACACACACACA
CACACACACACACACACACACACACACACACACACACACACACGCACACACACATACACACACACACAC
ACATACACACACACACACACACACACACACACACACACACACGGACGACAGATTGTTTTGATAGAGTGCGG
ACGCGTTTGTAACTGCTCCGCTCGAAAAATAANCAAATCGGTGGTTTTTCTGAGAAAATTATAGTGAAGTT
AGTGCACGTGTGATCCGTGTCATAGGCCTTATACACCAAAAGGTGCGTGGCGGTACTCACTGCGAAGCCC
GTGCAGAGCGTTCCTGGTGGTGGCGTCTACTGCTCCACGATCCAGGCGAGGGGAATGGAGGCGTCTCCTT
GGCGTGTTTTTTGCGCTTATTGTGAGTTTTATCACTCAAGTTTAGTAGCCGGCGCGCGAAGCTATTTGTGA
AGTTGGTTGCATTCACTCTAACAGCTGTNAGTAGTGTTGGCTAGCGCAGCTCACAACCAGCTAATCTGCAC
TAGTGAACCAGCGGCCAAACTGCGTAGCGGAGATACCAACCAGGCGATAAGCTGTAACTGGCAGAAAGT
ACGGCATACTTTCGGGCTTACGCTGTTCTTGCTAAGCATTTAAGCTTTGGAGAAAGAACAGGCAAAGAAG
CCGATATCGGTGCTCACTACAGGCTCCAGGGCAAAAAAGGAGCAAAAGGTCGCGGAACAGACCAATTTC
CAACCCCCCGCCGCGGCGGTGCCCATAAAAACGGGGCTCTGGTGACCGAGTCGGAGCGGTATAACTCCCA
GCGGTTAATAGCCAGAGGGAAACCGTTGGCCACCGAGGGACTTTCCTGTAGAGCCCTGATAGTTAGAGCA
GGTGCCCAGAGTGAAAAATGGAGGATGAGCTGCATGCCTTTGCGCGCCGCTCTGCACTACAGCGATCACC
GTCACACCAGGCGGTCACGCCAGTGCAGCAAAGAGTGGCTGCGTCGTCGCCGGCAAAAGCGAGTGAAGA
GCTGCCAAGAACGCTAAGAGGCACGATTGAGAAGATGGGCTTAGTCCTAAACGGCTTAACGGCGCTGTTC
AACGGACAACGCCACATTAACGGGCTGATGCGAGAGGAACTCGCGAGAGTAGAAAGCCTGCAGAAGGTG
GCGCTCAAGCTCTGTGCGCCGGAAACGAAGTGCGACGAGGCGCAAACGACGCCGTCATTAGCCAGACGT
GCGGAGCCGAACGTGCTAAAGACGCGCCCAACGAAGCTCGGGGGCCAANAAGAGGCAAGCACGCCCAA
ACGGCCGAGGGATGGCGTGCCAGAGCAGCGCAGAACTCCCTCCAAGAAGGCTAAACGCCAGGGTCAAAA
CCCAGCGAAGCAATGAGCATGGCATTCGCAAGACCGCGAAGGGCATTGGGTGACCGGACGGAAGTAAGA
ACGCTAACCGAAACGTCCCAGGTGGAGATCAAGGACCTGGATGAGCTGGCGACTAAGGAGGAAGTGCTC
GAAGCAATTAAAAGCGCCTTAAATGACGACTTCCTGTTGATCAAAGCGATCAATCGCGGCGAACTGCATT
TGGCGGCCAGCAAAGGGCCACNGTTACTCTCTCAGCGCCAANAGCCAGGAAGCTTGT
>9_rnd-1_family-315__LINE___CR1
TCGCTTCTTAGCGACCTTAACACTATCAAGCCTGGTGGTGTTCCTGCTTGCGTGCTTAGGTACTGCGCTGC
AGCATTATGTAAGCCTGTTTTACAACTTTTTGAATTGTCCGTAAACACTTCATCATCCGACCATTTGGAAG
GAATCATACACTATCTCTCTCCAAAAGAAAGTGACTAAGTCCTATGTCGAAAACTANAGAGGTTTTTTCAA
ATTATCCGCTATACCTAAAGCTATTTAGCCATTTATGTAGTTCTATTATTTCACCTTCTCAGCACGGGTTCG
TGAAGCGCAGATCTACAACCACTAACCTTCTTAAGTTTACTTCGTTTGTAATTGATGGGTCTAAGAAAAGA
ATGCAGACTGACGCCATCTACACTGATATCAGTAAGGCTTTCGACTCTGNCAACCAATCCTTGCTGCTTCA
TAAATTATGCCTTCTCGGCTTCCCAGTCAATCCTTTGAACTGGATTTCAGAGTATTTGCGTGATAGAACCC
AAAAGGTTCTTTTCAGAAATTCGTTTTCTAAACGTATTCTGGTGACTTCCGGTGTTCCTCAAGGTAGTGATC
TTGGCCCGCTACTCTTTTCATTAATGACCTTCCCTCGATACTGTCTCACTCTCGAGTATTAATGTATGCTGA

TGATGTAAAATTATGCCTTGCGTATAAAGATATGGCATCATGTTATCTCTTACAAGCTGACATTGATGCGT
TTCAGGCTTGGTGTCTAGCCAACCTTTTAAACTTANACTGCTCTAAATGTAAGGTAATGACCTTCTATAGA
AGCCCTCCTCAATTGGTTTCTTACATCCTTGATAGCTTACCGNTCGAGCGTGTTAACAGTACTTCTAATGA
AGTTAATGATCTAGGTGTCATCCTTGATCATAAACTTAAATTCGACTCTCATGTTAAGACCGTGGTCAATA
AGGCCAGAATAGTTATGGGTTTCATCAAGCGATGGTCGAAGGAGTTTGACGATCCGTACACGACAAAAAC
ACTTTATATATCTTTGGTACGACCTATTCTTGAGTATGCCTCATGTATCTGGTCACCACAATATGCTATCCA
TCAGGACAGAATTGAATCCGTTCAGAAACAATTCTTGTTGTTTGCCCTTCGTGGTTTAAATTGGGATCCTC
GCGTGCGGTTACCGTCATACTCCAGCAGACTTCTGATGATTAACCTTCCTTCTTTGATTAATCGTAGAACC
ATGTTGGGTGTAATATTTATTCATAAACTAATTACTGGTGAAATCGANTCTTCTGTGTTGACTAGCCAATT
AAATTTCTCTATCCCTACTAGACTAACACGTAATTTTGTACCCCTATCGCTTCAAATTTTGCTCAACTAATT
ATGCTTTGCATGAACCGTTCAGGGTTCTCTGTGATGACTATAACAAGCATTACAGAGTATTCTGCTCGGAG
TCTTCTCTTCCTCGTATAAAGTCTTCTATTCTAAACTATTTAGCAAGTAGATAGTCTTATTTTTATTTGTAAT
TGTATTTGTACACATACTTGTAGTTTGTATCGTTTTTTTTTTTTTTTTTATTTTCATTTCGTGTCGTACGTTAT
ACGACTGCGCCCCTCGGTCGGTTGGGCGGGAGGTGGGATGCAGTACGTTGCATCGGGATTCGCGTAAAAA
AAAAAAA
>160_rnd-4_family-287__RC___Helitron
CTCAAGTATTTCAAATATTTATCAAACCTATATTGTTATACCCTTGCAGAAGGTTGTTGTTGNANGAAAAN
CNTTTTGTCTNNCGAGATATCTTGACCGANCCCGGCANTTACTAGTCTTACNATACGCCTCATATTAAGTG
CAAAAAAACGGATCTATATTAAGATCGGANCACNATATCATAAAGCTGCCATAGGAACGATCGGNNGAA
AATTAAGTTCTCTGTTAATGGGNNAATATNCTGTTTTAANCGAGATNTCTTGACCAANCNCGGCGCTTACG
AGCTNTACTATNCTCCTTATGTATAGGAAATATTCTATTAAGATCGGACTACTATGTNATATAGCTGCCAT
AGGAACGATCGGTCGAAATTAAGTTGTTGTATCGAAACTCCCGTTTTCAATATATCGACCAAACTCGGCAT
TTNTCAGTTTCGTGTATNATGCTNCTCATACGGACGATCGATNAAAAAATAGATCCATATTTAGATCGGAT
CACTATATCATATAGCTGCCATAGGAACGATCGGTCGAAAATGAAGTTGTTGTGTGCAAAACCTTTTTGCT
CATTCAATATACACTGCATTTAAAGGCTCCTTTCATTTTCGCAAAATCTTAATTACATCGGACTACTATATA
ATACATCTGCCATCGGAACGATCGGTCGAAAACTAGGTTTTTGATAAACCTTTTTTTTACCCTCGCAGAGG
GTATTACAATTTTGTCGCGGAATGTGTAACGCATGGAGGAGACATCTCGANCCCATAGAGTATGTATATN
CTTGATCAGCATCAACAGCCGAGTGGATGGAGCACATGTNCGCCTGTCCGATNTGCATCTGTTGTCTANGC
GAACTAGTCTCTCAGTTTTAAAGCATCTTATGAAATTTGCAGGTCCGTCTTTCTGTTACAGGCAGCACATA
TGTCGNAACCGGCCGGATCGGGCCACTATATCATANAGCTGCCATTGGAACGATCGGTCGAAAATTAAGT
TTGGTTTTTCGTTTATCAAGATGTCTTGACCAAACTCGGCATTTATNAGTTTTACTTTATTCCTCATATATAT
GCGAGNTCCNACCAGAGATCAGACCACTATATCGTATTAAGCTGCCATAGGAACGATCGGTCGAAAGNG
ATGTTTNNGTATGAAANAACNTTTTGGACTCTTTACAANGCATCTTGNCCAGGCTCGGCACTTATTAGGTT
TACTTTACTCCTCATATATATGCGAAATCCTACTAAGATCGGACCGTATATCATTAGCTGCCATAGGAACG
ATCGNTCGAAAATTAAGTTTTTGTACGAAAAACTCTTTATCTTTATCGAGATATCTTGACCGAACNTANCA
TTTACTTCTTTACTTTATNCTTCATNTANATGCGAAAAAACGGNCCTATTAAGATCGCACTACTATATTATA
AAGCTCCATAGNAAGNATCGGNCGAAAATTAAGTTNTTGTATGGNAAACTTTAACATTACTGTTTTCCAC
GTANTTCTTAGATCGAGCANAGCACTATAAGNNTTACAAAGGTTGGTCTACGAGGGTATTAAATNTTCGG
CGTGCCGAAGATATCCTTTNTTTCTCGTTAAATATGATTTANNAACTTATACACNTGGACAACATTATGAT
AGGAAATAAGAACATAT
>160_rnd-5_family-5230__LTR___Gypsy
TGACTATTATTATTTAAAAAAAATTTTTTTTTTTNATGATCTGTTTGCAATATAACTAATACTCNCTAGTA
CCAAAGACAATATAAACGCTAAGATGAATAACGCGANNCAGCAGAATGCTACTATGCAGNNTTGCGCTCT
CACAGCCGNGAGAGCGAANAATGCTAAAAATAAAATATTGNTTTCAGCTTCGGCTCTATGAGGGCCGTGC
GAGCANNTATGTTTGTATGCGTGTGAATTATACATGCATAAGACAAGCATATNTGNATGTGTTGTTATCCA
CTGCTCTGGCAAAGTCTCTGGCTNGCAAGGAANCAATNTTNTTAGTTTTCTAGNGCAGATCACGACCTACC
CNAAATTTCTGTTGATATATGAATGCATTTTGTGTACTGAGGTTGGCTCCGTACAAAAAATTTGTATTTTTA
AGTCGATGCAAATATTTACGTAATTTTCTATGTTTGAAATTATTAATTAAATTATTATTATTTTTAAGTNAT
ACAAAATAAATTAAGGAAATAAAAGTGCAAATTNGTTGTTTTCAAAGATACATTTAATTAATTGTTTTTTT
TATTTTATTATTTTTTTGTTCGAATTATAAAGAACAAAGCGCTCTTTTATTTCTTAATTGGAGAATAAAAATA
ATATTAATTTAATTTAAAGAAATTATGTAAATATTTGCATCGACTTAGAAATACATATTTTCTGCGGAGCC
AACCTCAGTACACAAAATGCATTCATATATCAACACAAATTTGGGATAGGTCATGATCTGCGCTAGAAAA
CTAACAAAATTAATTTCTTGCCAGCTAAAGCCAGAGCAGTGAATAACAACACATACGTACATACTTTAAA
GTNTTATGCATGCATAATTCACGCATACAANCANAGGTGCNCGCACGCCATTGGCTGAGACAAGAACTTC

TCTCGAGCATACACTGTNCAAAAANAAAAGTGAAATCAATCTATGTGTGCTATTTTCTTAACATAGTTATT
GCGCAAGAAGCGCCTTGTGTAAAAATTATATAAGTTCTTAAANTTTCCTGTAAAATTAATCGTTAATATCT
AGGGTAATATATCATTTAAATAAAGCATGCATTTCATGGATAAAGCACAAGCACTTTGCGTCGATCGTAA
GTNCTAAACTTTTTCGCTCAATGTACTTCGAAAACGAAGCGACGCAGAGCACCATCGACCAGCTTTGCTG
AAGGCAAGCCGCGACTTCGGCATTCGTTCGTCTGGTCTTTGGAGAACGAACAACCAGTCGGCAGGCATGC
CGTACGAAGCCTCAAAGGCCAGATGAAAGGCCAGGCTAGAAATTTTGCGTCGCATTCAAATGTATGGGCG
TTACTCATCTTAGTGTTTATATTGTCTTTGGTAGCACTAACTAATTTTTTATAAAATAAACTTAAAAATTTT
TGTGTTTGAAGAAGAGTTTTGGCTAGAATATCCACTTACATGATTCAATTAACACAAAAAAATGTTCTTTA
TAAAATTGGTCACTCGCAGCTCCCGCGCTGCTGTCTTAATTGTTGTTGGTTGTTGCAGCTCCCGCGCTGCTG
TCTTAATTGTTGTTGGTTGTTGCAGCTCCTGTGCTGCTGTTTTACTTGTTGTTGGTTGTGGAGTCCGACCAC
TAGTTGGGATTAATA
>9_rnd-6_family-1345__LINE___I-Jockey
CTGAAGCTTCCATTGGTGTTCGAAGTGAACGGACGTACTCGCGGAATAAAATCAAATATCGAAATTCGAG
TGCACGCCTAAACGGACAATCGCGGCTTTTGCAAGCCGGCACAAAGATTGCAACGCATCAAGTATTGTGA
ACCAACAACAAACAGAAAAAAAAACAATGACGTCAAAAATTAAAACAAAACTACTTGCTGACAAAGCAA
TTTTTTTTAGTGAGCAAGTAGTCTTCGCTGTCTTGCTGACATGCACCCAGCCAGTGGCAATGCCAATAGAG
CCACCATACCAGAGAGTGCTGAGATCAGCAACGCGCCCACAATGAGCAACAAGGGGTCGTCTCGAACTA
ACGAGGTAGGCCAACTATATGCTGACGCCGCTACGCCGCCAAGCACCTCAATGCTTAACAGCCGCTTAGC
AACAGCTGCCAATAAATTCTCCGCGCTGATGTCTGCAAGAAGTACTCCAGTTTCACCTTCCTACCCTTTAA
TGGTCTCCGACGACCCGGCAGCAGTCCGCATGCTGGTGCAAGAGATATCGTCAAACCCGATTCACAGGCG
AAAAAACGATAAGCGCAAAGTCTTAGACAGCAGGTCCCCACCTGCCGCGCGGCGAAAACAGAACAAGAC
TGACACCAGTCCAGTTCAAATACATACATCCAATAGATATGCTCTCTTGGCGCCACCCGAAAACGACGAA
GACATGGAGACTGATGAATCTGAGGCCGAGCAGATAAGCTCAGCCCAAGAAGAGAACTTGCCTGCCNCA
GCGACTCCTCAGGTTGAGGGCGGCAGCCAGCCCAACGCCGTCGCCCATGCCAGCACCAGCTCCTCCGTGC
CCGAAGAAAGGGCCAACAACAATGCCCCCACTGGAGGAAAGAAGCACCCCCATCGTCCTAAATGACATG
ATGACATCAACGACGTGTGGCTCATACTATCTGAAGTAGTGGACCTGGAACCTGACCATCAAGTCCAACC
TAGGTCGGACTATGCGACTCCTAGCCAATGATGTGCCCACATACAGGGCCATCATAAAGAAACTGGAAGA
ACAAAAAATCCAATTCTATTCATACCAGCTGCAGGAGGAGAAGCCCTACCGCATAGTCATCAAGGCCTTC
ACCACTCCACACTCAACCGAGAGCTCATCGACGACCTGCGGAGCCAGGGCCATACCGCTCTCCATGTGCA
CAATCCGAACAGCAAAGCCACCAAGGAGCCCATGAACATCCACTTCATTAACTTGAAGCCGGCGGCCAAC
AACAAGGAGGCTCTTACCATCAGGAAGCTGTGCAAGCAGGTGGTCAAGGTGGAGACTGCACGACTCAGTT
CCGGNAACGTGCGCCCACTGCGGTGGTAACCACCCTGCCAACTATAAAGGCTGCGAGTGGTTCACCGAAT
ATACAAAGCGTGCGGGAGCCCCAAAGCCCGCCAAAACGAAAGATCTAGCTGCATTCAGCAAAGAGAACA
ACGCACCCAGCGCTAGCCAGCGAGAAGGACCAGCGCAAGCTAGGCTACATAGATTGCTGAGGGAACAGC
AACAGGAGAGGCTGACTGCGCAAGAACAGCAACAGCAGACATCTGGAAACGGGGAAGGACNTTCCTCTG
CCATCAAGGACATGAGCCGAGCTATCGAGAGAATGGC
>Ref_dvir_299
ATTAAAAAAAAATTAATTCATCGACCTGCTGGGCATTTCTGTTCGCCTGGTATTTCAGGGAAATTTTAGCT
AAAATTTGGGCAACCCAGCCGGCGACGAGGGCGCCCTTTTGGGTATGCAATGAAATTTTTTTGGCACGTA
AAAATATTTTTTGTCGACCTTCTGGGCATTTCTGTTCGCCTGGTATTTTGGCAGATTTTTAGAAAAATCTCG
GCAGCCAGCGGCTGCCAAAGACGCACCGTGCAGGTATGAAATGAAGTTCTAAAGTTTAAATAAAAAAAC
AGACTTCTTTTTAAATAATTGTTATTTATGTTAAAATTTAAAAAATGGTTGTTTTTATGCTAATTTTCTTAAT
TATTTCTCTTAAAATTATACAAATCGAGCATAAATTATAATTTTAGCTTATTTAAACTTAAAATAACTATAT
CTGGCTTAATTAAAGTCCGATCCTGGAAAAATTTGCTTTGTCAGTCGTGGAGGTCCTTAGAGCTCATTGTA
GTCCTTAATTTGTCAGTCTGAAAAGATAAAAACACATTTAAATTAAAAAAAGAAAAAAAAATATAAAGG
AAGAAAAAAGGCCATAACTCGGCCATATCCTTGCAGATTTTCAAAGGACATGGTGCGTTAGGATCGTATA
GACCCCCTGCATCGATCTGCATCAAAAAAATATGGGGTCATCTAAGAATCCTAGGACTTGTATTTTTACTT
GTGAAATTTTTGTCGCGGAGCGCACGGAAATGGCCCGAAAACACAAATCCTTGAATATCTCCAAAACTAC
AAGGACGATCTTAATGAACTTTGGGCCAGTTGGGTAGTCCTGGTCAATTACAATTTTTTTGACACAGGACT
CGTCAGGATACGCAAGGAATATGGCCGAGTTATGGCCTATTTTCTGTTTACAAAATTGTCCTTAAAACTTT
GCTGTTTGAAGGACTTTCGTCCTTTGGGTTGTATATTCTTAAATGGGAGGACGAGGACTATCTACTGGCCA
AAGGACATCCCGATAGTCCTTCAAATGGCCAAGTTAGTCCCGAATTTTTCGATTTTTGCCAATTTTGCGAC
TAACCCCCTAGGAAAAATTTTGGTGAAATTTTTTGTCGAATCCTGGAAATCCTTGAATGCCAATCGATAGT
CCTGCTCCCCACGAGTTCAAAAAGGTATATCCTTCCGATATCCTTAAGGATTTGGCCGAGTTATGGCTATT

AAAAAAATGTCCTTTTTAGAAAATTAGCCATATCTCGGAAAACGACAAGGACGATTTTGATGTCCTTTGGC
CAGTTTATAGTCCTTGTCCATACGATTTGTTTGACACAGGACTTGTCAGGATCTGCAAGGATACGGCCGAG
TTATGGCTTTGTTAATTTACAAAAGAATTGTACTCATATCTTGGCTGTCTGAAGGACTTTCGTCCTTTTCAT
TATATATTCTGAATTGGGAGGATAAGGACTATCGGTTGGCCAAAGGACATCATGATTCCGCAAGGATACG
GCCGATCTACAGCCTTTTATCTTTACAGACAAAAATCTCTTTTACTCAGCTCCTTGAAGGATTTTCGTCCTT
TTTATAACATATTCTATATTTGCAAGACTAGGACTTAATGTTTTCCAAAGTATTTCAGGATTGTGCTTGAAA
TGGCCAA
>9_rnd-1_family-186__Unknown
ACCACTAACCAAACAACATATAGACTGGACATGAGTACCAGAAGTAGNCACAAAATGTATCGGAGGCAT
CTGGTCCAAACTCGCGAGTAAGGGAGTAAGATATACATATGATGTATTTTTGAACCGCTTACTCTACAGG
AAAGTGCTTGGCGCACTCTCTTCCATATGATTTGTGCGATAGAACGGCAACGCTGTTAACTTTTATCTCGC
TCGCACTTACTCTCAGCTCTCAATGCTCTCTCTATGTTTAGGGATCGTTTCAATGTGTGTGTGTGTGTATTT
GTCCGACNAGCAAAACTGCGCACAAATTCAATTTTTCCCAAATGCTGAGGCATTTCTACCCTTTTGAAGGC
ACGGGTAATATGAAATAGAGAAAGGGTTTGCCACGTCGATGCGAAATTCTTGCATTTTAGCTCGCTATCGT
TGGTAAGGGTACCCAAGCGTTGCCATGTGTGCATTTAGAATATTTTGGACTCGCTATTCACGCATACTTGC
ATATTTTGTGTATGAATAGAAGCATTTATGCGCTTGTGTGCCTGTTGCTTGCCTGCCTGCCTGTCCCCGATG
CAAATTTAACAAGATTTTAGGATTTCTGATTTTGGACCTCTACACACAGACACACACATACAAATGTCTGT
GAATGGGCTGATCATGGGCGCAAAAGAAAATACATTATTGGCACGCGTCTGACGCGCGCCCTTCTTTTAA
TGATTNTGAATGAAATTTGAGTAAAAATAAAAATCTTTAAAATATTAAAATATACACCAAATTAAAAAAA
AGGAGCGATTATATATTTATAGTTTTAATAATTTTGGTGATCAGTCACTTGACCTCCATATGCAGGTGCGT
TGCCAAAAGTTGACCCATTCGACCCAACGAGCTGCTGCAGGTAATAAGGGGTCAACATTCATTCATCAAA
ATCNTAAAAAAGAAGGGCGCGCGTCAGACGCGTGCCAATAATGATATTTTCTTTTGCGCCCATGATCAGC
CCATTCACAGACATTTGTATGTGTGTGTCTGTGTGTAGAGGTCCAAAATCAGAAATCCTAAAATCTTGTTA
AATTTGCATCGGGGACAGGCAGGCAGGCAAGCAACAGGCACACAAGCGCATAAATGCTTCTATTCATACA
CAAAATATGCAAGTATGCGTGAATAGCGAGTCCAAAATATTTTAAATGCACACATGGCAACGCTTGGGTA
CCCTTACCAACGATAGCGAGCTAAAATGCAAGAATTTCGCATCGACGTGGCAAACCCTTTCTCTATTTCAT
ATTAGCCGTGCCTTCAAAAGGGTAGAAATGCCTCAGCATTTGGGAAAAATTGAATTTGTGCGCAGTTTTGC
TCGTCGGACAAATACACACACACACACNTAAACGATCCCTAAACATAGAGAGAGCATTGAGAGCTGAGA
GTAAGTGCGAGCGAGATAAAAGTTAACAGCGTTGCCGTTCTATCGCACAAATCATATGGAAGAGAGTGCG
CCAAGCACTTTCCTGTAGAGTAAGCGGTTCAAAAATACATCATATGTATATCTTACTCCCTTACTCGCGAG
TTTGGACCAGATGCCTCCGATACTTTTTGTGNCAACGCTTCTTGTACGTGATGTCCAGTCTATATGTTGTTT
GGTTAGTGGT
>9_rnd-1_family-102__LTR___Gypsy
AAATACATTATAAATACATTACAAATATAATAAATCTNAAAATCAAAAAATATTTTAATATAAAAATAAA
TTTAAGTAAAATAACTCCACTTAGTTACGTTATTTTTCAAAAGGAGGGAGATGTGTAGTATGTGCATATAT
TGAGGGTACACTGTACCAATAAAGTAACATCGGTTAAGCATAAATANATCAGCAACACATTGTTGCAGTA
CACAAACAAAACCNTTTCGAGTGAGCGTGTAATATGATCGCCCGCTTATGAGCTACGCTAAGTCAGCAGT
CCCACGCTGACCACTCGTAGCACATATATATATATATATAATATATATATATATATATATATATACATACA
TGCAAATATGTACATAAGACTCTCTCTCCCGATTATGCTGATAAGACAAAATATGTATGAAGCCGCTCCGC
TGCTGGCGTAACCGGCAGCGCAGCTATGCGGTCTTAGCCATAAGAATATATTGAATAAAACATTCTAAAA
CTAAAGTCAAACGGCACAGACGTGCCCGTGTATTGAACTATTTGAATCTATTACATGGCGACCGTGACCT
AAGGTCAAAACAGACCTGGACATGGAAGGACAGCAGCAACGGCAGTAACAAAAACACACAACAACAAC
CANACAAAAAAAAAAAAACAACTATGAAAAGTCTTACAAGTGATGACCAGCAGTCAACAAGTGTGAGAG
GCAATCCGTCCCTTACGCCAGCGCGGGAGACTTTGAGATTGAAAGAAAAAACAAAGTCTGAGTGAGTAG
AGTGCGAGTAATGGGCAAACGCGGAGGCCTGAAAAACAAAAATAAATAAAAGAATAGCACACATTAAGT
GGCTATTATACACGAATACGCCGCCCACATGGGCAACGGCACAAAAATTAACTACCGAACTTGATTTTCT
GAAAGCCTCCAGCAAAGAAAGCCAAATACCGCAACTCACTCAGCAAATATCAAAGTTATAAATGAAAAA
CTAATGAAAAAACCACCATCTCCAAAGCCGTTGACGAAAAAACCCGACGTCTGCCCGAACGACTGCCCTG
GACCACTATACGCTCCAGCATGCAAATTCGCATGCTTGGGACCAAAGACCAGTGCGAAGACCGACACCGG
CCTCGCCTGAGCCAATAGGCCACGGACATCAGAAGCAGTGAAGGCAACAGCGCAAGGGGCACTCCGCGA
CTTTCGCCTACGAAGGAGCGTCAGCGTAGCCAAACCGGCGATTATAAATTATGCGGAACCATCTCCGCTC
AATTAGAAGTATAGCCCGTAAAGCTATACAAACCAATAGGTAGAAAAATTTGTTAACGTAAATGCAAAGC
AATGCAATAGTAAAATTTACTGAAGATACAACAAATAGTTATAAATACACTCTAAAAATATATACGCCCA
AAATGGCAATAGTAGACGTTAAGTCAGTGGACTCCACTGCTAATGTCATTAATAAAGTAGAGTCAAATAA

TAATGACTTAGAACTAATACACACACTGTTAATAATAATTGTTGTTATTATGTGCGCAAATNGTTCTTATA
AACTATATAAATTACATAACAAATGTTTAAAAAAGAAATATATGAGCCAGGNAAATGACCTAGACAAAN
TTTAATTTAAA
>9_rnd-1_family-443__LTR___Pao
ACAACAAATGNCTNATATTTTTGGATACGATTTCTCAACTATCTGAAATTCCTCACATGACAAAACCTTCG
AACTTAAGTCNTAGNCACCATATCGGCATTATATTCGTCTCTGCAGTCTCTAGGCGATGACAAGGATATTG
TAAGTGCAATGTTAATGCACNTGGCAATGTCAANAGTAGACCAGGCAACAAAATCTCGATTCGAGGAACA
GCTTGACTATGATAGTTTGCCATCTTGGTCAGATTGCGCTGCTAAATTGAATCGGCGCTATCAAGATTTAG
CTGCCGAAGAGTCAACTAGGGGCGAAACGAAACCAAGGCACGAGGCTAAAAAGGGACATANCAGGTCAT
CGTTTTCGTGTTCTAAGGGTGATCAGAAAACAGGTACCAAATGCAGCTATTGTTCAGTCGCTGATAGTCTC
GCAAACTGTTTAGCGCTCTACCTGTTTATCAACGCTTTGGGTTTGTGAAGCAACACAACTTGTGCATCAAC
TGTTTAAAACAGGAGCATGCAGTAAAAAAATNTGGAGGATCGAAATGCCGNGCGTGTTCAAAGCCACAC
CACACTGTACTTCACAGGTATGAGAGCGAGAATTCGCAAACGCAAGCATCNAGTTCCGACAGCTTCTCTC
CACAGCAACAGGCTTATGTAAGTCATGTCCTTAGTGCCGAACAAGTAATCCTAGCCACAGCTGTAGCAAA
GTTAAAAGATAGGTTAGGGCATTTCTAGCCCGCGAGAGCGCTTTTAGATTCCGGATCGCAAATAAACTTT
ATATCAGAGGACTTAGCAAAACGCTTACGTCTACGTCGTGATGATGCGTCATTGAAAATTGTCNCAGTGG
GAGCCACTGCTTCGAGCATCAAAAACAAAGTCTACACATCGGCTCAACCGCGATTGACTTTTCATACTTTT
GGGGCTGATTTTTGGATTTTGAGTTCTACTTCTATATACCAACCTGATCAGAAGATTCACTTTTCACAGTGT
ATACAGTTGTCACAGAATAAATATCCAAATATTGAACTGGCCGACCCCCGATTCCCCAAACCTGCCAGAG
TAGACTTGCTTTTAGGTGCTGGAAAGCTTTTCGACCTCCTTTGTATTGGCCAAATAAACCTGTCTCCTGAAC
ATCCGATTCAGAAAACATTTTTAGGATGGGCCGTGTCGCAAGGCACATCATCGCAAGCACAAATTAGCAC
TNTCGCAATTGATTTTGGGACTAAGAACAGACTCCACAGAAGTCGAAAGCTGCATATACAAGTTATTTGA
TNTCTCAATTGCAGATGTTGATGTTGTATATTCAGTTTCTCGTCACAAGTGCTGAGATTGGTAAGGTTATAT
TCCTTGTAGATTATATTCGGAAGCAACTAGCTTTCCAAATCACTGGCCTTGTGGCTCGGCAATGAAACAAT
TAGTATAACGAATTGATGACGGAAAGTGCCGGAGCAGTATATTTAATGAAGTTCTTTTTAAAAGTTGGAT
ATAAAACTAAACTAGAATTGAAACATAGAATTGGATCTGAGCATACGCCAACACTGTCGTTTGCGACATA
GGCAAGTGTCAGGTTACCGCTGCTGACCGCAGTGGCGGGCACTGAACTCATGC
>160_rnd-4_family-304__RC___Helitron
TCACACATATTGGTGCAAATCTGCATATATGTATGTGTCTGCCAAGCCAAGGGAAGTCTATGAACTTAAG
AAGATTGCAACGTTCTGTTGCGCGTGTGACGTCACAGCCCAAGGCTTTTTACGCGCATCATTTCGTATTTA
AAATGATTACATATGCCANTCAATTATTTACTATAAAGGACCCTATTCATAAATTATTTAAATGTACCTAA
TAAAGGTAGAAATATATACTAAACATCACAAGAAATAAAATTCTATTAACCGTGACGTCATAATATATGA
TGTTGGCGCGCCGTGCGCGTAACCCTTTGATCTGAATGCCATGGCCTGCTGGCATTCAAATTTTCTACCTA
CTTGCGCCGAGACTCGGCTCTGCAACAACAACTTAGTACCCAAATTGTGTGTTGTTGTTTCTACGCGCTGC
CTTGTAGGAACGACAGAGAAACCAGCAACGAACACAGTGGCATTATCTGTCCCTTTTCTTTGCACCGAGTT
TCCTATGTCCTCGATCTCGCAAACTCTTGNCGATCAAAACGGGCGACGAAGAAAAAATTGGGTTTCGTGC
GCACTGTTGTTGTTTTGCAGGTGATTTGTTGTTGCTTCCTATGGTAAACGAGCTTTTTATACCCTGAACCCA
TTAAAAATGGGTATAAGGGTATATTGTATTTGTGCAAAATCCAAATGTATGTAACAGGCAGAAGGAAGCA
TCTCCGACCCCATAAAGTATATATATTCTTGATCAGCACCAATAGCCGAGTCGATCTAGCCATGTCCGTCC
GTCCGTNCGTATGNACGCAAGGATCTCAGAACCTATAAGAGCTAGACTCGAAATTTTAGATGTAGGTGCT
CCTAGTTCCCGCGCAGATCGAGTTTGTTTCCGATAATCGATAACTTACTCCGTTTCCAAGCAATCGATAAA
AATCGATATCGACATCCTGTTTTTTTGGCNAATTGGGTAAATAATAAGAGCTAGAGCCACCAAACATGAT
ATGTTGCTTCTAGAATATTATATATATGTCAAGTATCTTTCATTTTATACCTATCGCCACCTCCCCGCTACC
ACCGCAGAGCTATAAATCAAGTTAATAACCCAACTTTTATTGCCAACCAATTTAAACCACAATTTAAATGC
AATTGACTTTTTCAGAATACACAAATATTCTATGGTACAATAAGATATACTGTAGAAAATTTCATAAAGAT
CGGTTAAGAAAAACCAAAGTTATATGCAACTGCGCAATTGAATGGGGCAGCAGCTTTAAGAATTTCTGTA
TACATGTACACACACACATTCATGCGCGTCTGCTTCGCATTCTAACAGCATGGTAATTGCGATCTTTTTTTT
GTAATGTTATTTTAGTTCCTTTTTTATCATAAGTACTTAATTGTTGGTGTGGCTGCTGAGTAGAGGCGATAG
CAAGTGGTGCGGTCNGTCGCGAGTTCGAGCCCTACCGGGGAAAGTATTTTTTTTATACCCTGAACCCATTA
AAAATGGGTATAAGGGTATATTGTATTTGTGCAAAATCCAAATGTATGTAACAGGCAGAAGGAAGCATCT
CCGACCGTCCGTCCGTCCGTCCGTACGTATGAACGC
>Ref_dvir_833
ATTAAAAAAAGTGTAATTTTTTAAAAACTAGAATCCAGTTTTTGGGTCATATTATAGAATTTAAAAAGTTG
TACCCGATACCGCAAAAAATTGAAGCCGGTTCAAAATTTGACACAAAACAGACACTAAAGCAGCTTGAA

AGTTTTTTAGGCCTTACTGGTTATTTCCGAAAATGTATCCAGAATTATGCCATTATTTCAAGGCCCTTATCA
GATTTGACAAAATGCAACACAAAATTTATGATTGGTCCTTAAGAAGAAAGCGCTATCGAAACCCTGACCC
GGTATTAGCTATATACAATCAAAAATACGAGACAGAAGTACATACTGATGCATCAATAGACGGTTTTGGA
GCTGTGCTCATGCAAAAGTCACCAGACGATCGACAGTTGCATCCAGTATACTTCATGATTAAGAAAACGA
CGGGCGCACAGCGAAAATATTCAAGTTATGAGCTTGAGATAATAGATGTCATCGAAGCGTTAGACAAGTT
TCGAGTTTACATTTAAGCATCCCATTTGAGTTGATCAATGACTGCAATGCATTTACAAAGACGATAGAAAA
GCAGAATCTGTGCACAAGGGTGGCCCGTTATTTTACAAGAGTTTAACTATGAAGTCGAGCACCGATCCGG
TTCACGCATGAAACATGTAGATGCGCTCTCGAGATATCCAATAATGCCAATAAATGATGAAAGCATATCG
ATTTCGGATAAGGAATGCACAAAAACAAGATGACCAATTGAAATCAATCATAAAAATTATCGATGACGA
AAAGAAGAATTATGAAGATTATTTCATAAAGGCAGGCATTCTTTACAAGCTAGTCGACGATGTTGAACTC
ATAGTTGTTCCAAGGGATATGCAAAGACAAAATATCAGTAAAGCCCAAAAAGACAAAAGAGTTGATATC
CAAAGAGTATTTTATACCGAACGTTGAAGAAAAAATTAAGAAATTAAATGACTGCTGTATACCCTGCATT
TTTACAAATCGTAAGCGAGGGAAACAAGAAGGTTTGTTACATCCATTGCAAATAAGATGTCCCATTGCAA
ACATATCACATTGATTTTTTGGGTCGGCTATAATCGACACACAAGGATTATAAACACATATTAGCTGTAAT
CGATGCTTTTACGGAGTTTTGTTGGCTTTATCCCACAAAGTAGACTACAGCAAATGAAGTCATTTCGAAAT
TGCGCTGCCAGAGCGTAGTATTTGGAAATCCAGCCTGTATAATTTCTGACAGCGGCTCAGCATTTACAGGT
CCGAAATACTGTGTCGAAGAAAATATCAAACTCATCAAGTCGACTACTGGAACTACCCCGAGTGAATGGT
CAAGTGGAACGACTTAATGCAGTTATTATATCTGTACTGTCAAAACTAAGTATCGAAGACCCAACGAAAT
GGTATAAACCTGTAGAAAAAGTGCAAAGCGCTATAAACTAGACGTATACCAGGAGCATTGACACCACTCC
TTTTGATGTTTTGATGGGAGTCAAGATGCGAACTAAAGATGATCATCAACTGCATGAAATGTTAAGAAAA
GAGTCCGCAGTTGCATTCAACGAAGTCCGCAACCAACTA
>9_rnd-5_family-12145__LINE___R1
AACGAGATAGGCAGAGTCTGCTATCCAGTGCGAAGATGGTCTCGACTTCGCGAAATCCAGTAGAGTTAAG
CTAGAGCTCCACTTATCAACTGCTAAAATCACTGCAACAACTGCAGGAGCCAATCAAACCAAAACAGCCA
GAGAGATACGAGCCGACGTGGCACAAATCCGAAGAGAGTTGGGTCCACAAGGTTACACCGACTGGGGAA
GTGCCGGGACGAACCCGCAAGGTTACACGCTGCTAGACGACTTCGAAGCCCTTAATGTAACACTGCTGAA
CACCGGGACCGAGCACACCTTCACCAAGGCAGGACGCGGCTCAATTATTGACCCTGACGTTTTGCCAGCC
CCTCAATAGCAAGACGGGCCACATGGAGGTGTCCCAGCTGTANACGCATAGCGACCACCAGGCCATTGT
CAGCAATACTGATGGTGGACAACTAAGCCCGACGGGCAGAACACACCACATCGCTTGGGACCCTGAGGG
GCTCTTAGCGNTTTTTGAACCCACCGCAATCGCTGGAAACGCAGAACAGCTCGCTAACGNCCTAACACGG
GANATGCAACGAGCCTGCGATGCAGCGATGGCGAGGAAAAAGAAAGGAGGTAACAAACACAAGCCAGT
CCGATGGTGGAATGGCGAAATAGAGGATGCGCGTAATAGGTGCCACACAGCAAGACGCATATACCAGCG
CGCAGAAGGCGAGCGCGACGGGAAACAGCAAACCTGGCCCTGGAGCACGAAGCCGAAGAAGCTCGCGC
AGCCTACCACTCCTGGCGAGCTATGCTCACCAAGGCTATCAAGCGAAGCAAGCGGCAATGCTGGACGCAA
CTCTGCGACGAAGCCGACACCCAGCCCTTGGACAAGCCTACAAAACGGTAATGGGCAAACTCGCAAGAG
GACCCGTACCGACCTGCCCAGAGCATCTAAAGCAGATAGTGGAGACACTGTTCCCGCGCCAGATAGAGCT
ACGCCACAACCCTCCAGCCGTAGGCACGTCGGTGGTGCCTCCCATCGACACAGAAGACATAAGTGCCGCG
CTAAAGCGCATTAAATCAAACAAAGCCAGGGCCCGACGGCATCCCCAATGCAGTGCTGAAGACCATCGCT
AGCGCCCACGCAGACACAGTCGTAAATGTGTTCAACCGATGCATTGCNGAGAGAACCTTTCCAACGTGCT
GGAAGTTGCAGAGACTGGTCCTTATCCCTAAAGCCGGAAAGGCAGGACCCCTCTGCCTACAGGCCACTCT
GCATGCTGGACTCCACGGGCAAGCTNTTCGAGCGCATCATCTGCGATCGNCTGGAGAAGGAGATCGCTGA
TCGCTACGGACTCTCGGACCAGCAGTTCGGCTTCCGTAAACACAGGAGCACTGTCGACGCTATCAAGTTG
GTAGCGGACACGGCACGGAAGGCCATANGAGGCACGCGATGNCGCGGCGGATCCAAAAAGTATTGCCTT
GTGACNACGCTNCTGGCACANACAAGGCTNCNAACTCGGCAAGCTGGCCAAACNTACTCCTGGCACTCTC
CAGGCTGCGGGTCTCCGTGTACNTCATCAGTCTTGTTGCCGGACGACTCCA
>Ref_dvir_1036
TGGATTCTGGTTTGTAAAAAATGGCACTTTTTAAATTCAGCTCAAGCCCGTACTCTTTACAGGTATTCATA
ACTTCCATCAGATTTTCCAATGCCTCCTTTTCGTCTTTAGTCGGTATGATTACGTCGTCTACGTAGGGTAGT
GCAATATTTCTACGAGGTAGCGGTCTAAAATTGCATTTATGTGCCGTTGGAATATTCCAGGTGAATTTGAT
AAACCAAAAGGCACATTGAGGAACTGATATTGTCCGCTGTGCGTAACAAATGAAGTGTACTTCTTACTGT
CCTCGGCTACTGGCACATGGAAGAAGCCATTCTTCAAGTCGATTGTGCTAAATATCGAAGCCTCTTGCAGG
CGATCGATCTGATCATCTATTAGTGGCAATGGGAAATGATCTTTTATGATTACCTTATTTATTCTTCTGTAA
TCGATACAGAGTCGTGATGTACCATCAGTCTTTTTTACGAGAACCACTCGACTCGTAAACTCCGAATCTGA
CTCTTCGATAATACCATTTTTTAGCCATTCATCAACCTGCGTTTCAATGATGCGTGCCTCTGTAAATGCAAA

TCTTCTTTGCCACGAAAAAATTTGAGTTTCATCTTTTAAAATTATTTTCATCTCAACATTTGTTGTTTTAAA
ATTTTCCGGCTTATAGTTCTGAACCATCTCAAGTACTTTGTTTTTTGTAACATCACCAACCTGAGTGTTTAT
ATCGACTTGATCATTATCAATTACCGCATCAATTTTCATTATATTACTTTCGTCACTATCGTTTTCTTCATAT
GTCTTTCGTACGCTTACACGCGATTAAATGATATTTCCGCATACAAACAAAGTTCTTCTCCAATTATTAGTT
TAACGTCCATACAACTTTGCGTTACCACGTAAAAATTCAAAACATATTCATTTTTATCAATCTGAACCGTA
TAATTAAACTGCCCAATCGGCTTTATTTTATTCGCGTTACGACCAAAACCAATCAAATACACGTTACAGTC
TTTTAATTTTGGAAGGGAAAGTTCCTTATAAAGATCTTCACGTAAAACATTAAACTTGCTACCGGTATCAA
ACAATGCTAAGCACTTCTTGTTTTTGAAATAAACTTCTTTTGACGTTAGCGCAGTTTTGTTCTTTACAACAC
GAATATTTGATTTGACTTCATTTTGATTTTCACATTTTTCCTTACAATATTTCGATATGTGTCCGAATTGGTT
ACAATTAAAACATTTTCTTCTTTTTGTTTATTCATGCAATTGTTGGCTAAATGCCCCTTTTCTCCACAACTA
AAACAATTGATTTCTTTTTTTTCCCTGCGCGTACCACTGTGCATCATTTGCACCTTTTTCTCTTTGTCAATTT
TATCACTTTTCTTATTTTTTCCACTCAATTTTTCATAGTCCTTTAATTTGTTTTTAAACTCGTTCATGTTTATT
GCACCATATAAAATCGATTTGTTATTGGAAAAATCATCTATACCATCGATCACGTATTGCATGAGTGCTTC
GTCCTCCACATTTCCACGAAATGCAATGTCTTTCATACGAAAGAAATATCGTAGGCGCTCCCCCCAAT
>160_rnd-5_family-1543__LTR___Gypsy
ATAGTGACATATTCACAACAACAGCATTTTGAACCACTGTTATTCATAAGCTACATTGCATGGCATAACCC
ATTGTGTGTTTACAAACTGAATAATCATAACAATAGCATGCCTTCTGCATCCGAATTCAAATAACGCTAAC
AGCAGCAAAAATCCTAAGAGCAGAATGCCTTCTGCAGCCAAGCTCACATATTGTCAACAATCATTTATAA
ACAAAAACTGATCAACAGAAAGGCGCTAGCATAAACAAGCTGCAGCCGCTGTCCCTTTGCAAAAAGTCAC
GTACTCCAATTATTATTTAGCTTTAAATATAGCGTAAGCCAATTTTCGTNCTCAGTCTTANTCCAGATATTC
AACATAATAAACCGATACAGATTCACGGTCGGGAAATATAGTCTTTTTATTTCCAAACTTATAACTACGTT
ATTGGCGCAGCCGGTAGGATAGCGTACATTTTGTGAAACGAAATAAATTAAAGTTCGTGAGTGATACGAT
CAATTAAAGAGAGGCTTATCCATACAACTGGTGTCTTCTCCGATCAACAAGTGTAATTGTGCACAGTATTA
CAACTAAATCGAATATATTTCAACTCCGAAATTCGCTATCCGCAAAAGAACTTAACGTGTNNAAACAATT
CAAGCAACGTATAAATCCAATTCGGTCGTTGCAACAAAGTGAATTTCGTGACACAAACAAAAAACCTAAA
ATNATTAAAAAATGGAGCAACCAGAAGGCTCTCATGTTGCTGCACTTCCAGCAATGAATAAATTAAACGA
CCAAAATTTAAACCAATTGCTTCAACAAATACGGAACGTGCCAGCCTTTGAGGGAGACCCTATAACCCTG
GGTCCATTTATCAGAAGGATGGATTACCTGCTTCAACTGTACTCTACTCACAATGAACGACAACAATGCAT
CATATTCGGGGCGATTGAGCTCCAGATATCCGGAGATGCCCAGAAGGTTCCTAGAATGCTGCAAATCAAC
AACTGGTCCCAACTCAGGGAAGCCTTAATCTCCGAATACAAGACGCAAACACCCGTGGAAGAACTACTGC
GAAGACTATATAATACCAATTATCAAGGTAATTTACGTAAATTTTGTAGTGAGGTAGAGGCTAAGATAAT
AATTATAACTGACAAGTTAGAATTAGAAGGGAATTTAGATAATTCCAGATTGTACACAAACGCTATGCAT
AACACTGTAAAAAACTTGATAAATAAAAAATTACCAGATAGACTCTTTATGTCACTCGCAAGACACGATA
TATCCACTATTTCCAAATTAAAACAAGTAGCTCAAATGGAGGGCTACTATGAGGAAAATTATTATAACAA
TAATAAAAAACAAAATACTAACAATGCTATAAATTATACAGGCAATACTAAACCTCAATTTACCCCAAGT
TACATAACTAACAATAATCAAAGCCACAATTTTTCACATCACCCTCCGAACAACCAAACTTATTTTAACCA
AAATAAGCAACTAGCTCAAGAATTCAAACAGAATTTACAACAAGCACAAATAAATAACCCCATAAATCA
AAATATTTCTCAAGGTAGATATATA
>160_rnd-1_family-30__RC___Helitron
TTTTTATACCCTTGCAGAGGGTATTATAATTTTGTCGTGAAATGTGTAACGCATAGAAGGAGACATCTCCG
ACCCCATAAAGTATATATATTCTTAATCAGCATCAACAGCCGAGTCGATATAGCCATGTCCGTCTGTCCGT
CTGTCTGTTTCTATGCGAACTAGTCCCTCAGTTTTAAAGCTATCTTAATGAAACTTTGCAGAACTCCCTCTT
TCTGTTGCACGCAGCACATATGTGAAAACCAGCTGGATCGGACCACTATATCATATAGCTGCCATAGGAA
CGATCGGTCGAAAATTAAGTTTTTGTATGAAAAAACATTTTGTTTNTCAAGATATCTTGACCAAACTCGGC
ATTTATTAGTTTTACTTTACTCCTCATATATATGCAAAATCCTATTAAGATCGGACCACTATATCATATAGC
TGCCATAGGAACGATCGGTCGAAAATTAAGTTTTTGTATGAAAAAACATTTTGTTTTTCAAGATATCTTGA
CCAAACTCGGCATTTATTAGTTTTACTTTACTCCTCATATATATGCAAAATCCTATTAAGATCGGACCACTA
TATCCATATAGCTGCCATAGGAACGATCGGTCGAAAATTAAGTTTTTGTATGAAAAAACATTTTGTTTTTC
AAGATATCTTGACCAAACTCGGCATTTATTAGTTTTACTNTACTCCTCATATATATGCAAAATCCTATTAA
GATCGGACCACTATATCATATAGCTGCCATAGGAACGATCGGTCGAAAATTAAGTTTTTGTATGAAAAAA
CATTTTGTTTNTCAAGATATCTTGACCAAACTCGGCATTTATTAGTTTTACTNTACTCCTCATATATATGCA
AAATCCTATTAAGATCGGACCACTATATCATATAGCTGCCATAGGAACGATCGGTCGAAAATTAAGTTTTT
GTATGAAAAAACATTTTGTTTTTCAAGATATCTTGACCAAACTCGGCATTTATTAGTTTTACTNTGCTCCTC
ATATATATGCAAAATCCTATTAAGATCGGACCACTATATCATATAGCTGCCATAGGAACGATCGGTCGAA

AATTAAGTTTTTGTATGAAAAAACATTTTGTTTATCAAGATATCTTGACCAAACTCGGCATTTACTATTTTC
CCGGTACTTCTTAGATANGGGCAAAGCACTATGAGCATTATGAAAAGGTTGTCGGTGCTGCAAGGGTATT
AGATCTTCGGCGTGCCGAAGATAGCCCTTCTTTCTCGTTTGAAAAAACATTTTGTTTTTCAAGATATCTTGA
CCAAACTCGGCATTTATTAGTTTTACTNTACTCCTCATATATATGCAAAATCCTATTAAGATCGGACCACT
ATATCATATAGCTGCCATAGGAACGATCGGTCGAAAATTAAGTTTTTGTATGAAAAAACATTTTGTTTATC
AAGATATCTTGACCAAACTCGGCATTTATTAGTTTCACTGTACTCCTCATATATGTGCAAAATNCTATGAG
CATCATGAAAAGGTTGGGTCTGCAAGGGTATTAGATCTTTGGCGTGCCGAAGATAGCCCTTCTTTCTCGTT
TCTTT
>160_rnd-6_family-866__Unknown
TGGGCAAAGCCCACTGATTCCATTGATTTCTTGACTTATAAAAGTGNATAATGGAGTTTGTTAAAATATCG
TATATTTCGTGGTATTGTATATATTTTCAAAAACTTTTACAATTTTCNACAGCTCTTGGGNCGTATAAGCAT
TTTGACTGTTTTCAAAGAGNCGATGCTATCCCAAAATATGGCATCNNTTCTTTNCACCTCATGATCATAGT
ATTTGCCGTTTAAATTGCTAAAAAGAAAGCAATTAAATTTCAATAATTCTTTTTTTTTTCTGCGGAATTCAT
ACCTNCCTGCACAGNCACTGTACCACCAGCCACCACAAATGNNTGCTTGCGCAGTTGCCATNACCCCGNN
CATCATTATCACGATCGTATGTGCTGAATTTCATGTTTNTGTGGTAACTCAANAAATCGCCTGTTGCATTG
CCCTCAGCCTTACCCAAGGCGATCAGTCTGTATTGATCGTCCTCGCCAGCGATGGCAAATTGTTCATATCT
GTAGTATTCGATGATACCATCAAATCCTTCCATATGTATGTAGAGCTCATGGGGCTGGGCGCTCGTCAAGC
GATGAATCTTCTCNAGTCCTAAGAAAAAGTCACCATCAAAGTCGCCAAAGCCGTCGCGGTATGCGGCCCA
ATTCCTATNGAAATCCTCCTTACCATCGATTCGCTGTTGGATAACCGTCCAGCCAGGACCTGCCGTCGCAC
TGTCGCANAGTACATCGAACGGATCTTGATCCGGTTTAATCTTATGAATACCCGGANCATCTCCAAAGTGA
GTGCAATCACTGNAAACGGGCTTCTCTTCGCAGCTTTTTAATTTCTTGGCACAAATTGTTGATTTCGTTCTT
GATTTCGGATATTTGTTGGTCCTTTTGATCGATTGNCTTCTTTTGTTTCTCCAGCTGCTCTTCATAGTCCTGG
ATTTTCAAGGNATTGGAANTGAGTTTACCCATTTCCTCTTTTATTGATAAAACGTTCTTCGTTAGCTCTTTG
ANCGCCTCGATGANGAATTCATTACGTTGGTTCAGTTTTTCCTGCAGTTGCTTGATTTCTCTGTCTTTGCGT
TCAACTNTATTGTTATTGTTTTCAATTTCCTCCTTCATCTGATGTATATACTTTAATAATGGTCTCACAGCCT
TATAGGTGTGCTCTCCGCATTGCTCCTCTGCTATTCTGGTTGCCGNACAAGACTAATAAGAGAGTACGATT
TATTTTCTGCAGTGCACACAGACATTGATAAATAATTTCTTTTACCTCATTTTCGTTAGNCTCTGCTTCAGC
TATGATCAGTATNAGGCTTATATATAGAATATATAGAAACANAACACATTTCATTTTGCACTNTGATTNTG
GAATGAAATCGCGAATCTCAACTGNACTCAGCACGCNCGAAAATAGACTGAAATATGGAAATNAATTGG
AAGCTCGGGTNCCCAAAATTTCCGAAACTNATAAGCGATNAGCAGTAGGTCAGGAACTCAATATAAATAA
ACANAAGGCATGGATCAGGCTTTAAGTGAGCTTCATTGAACAGCTGANAGATTGCACATA
>Ref_dvir_1003
CAATATAATGGCATTTTTTTACTTCTGGATGGTTAGTACGAATTTCTTTTGATGGCCCCTTGGATTAAAGAA
CGGCACCCTCTGTGTTTCAAAGAGCTGTATGAAAAGCACTTGACAGTCTTGCTCATTCGTTTGTAATCGTT
TATATGGACGATATATTAATTGTTTCAGAAACGAAGGAAGAAGCGTATGAGCGGTTGCAACTTGTATGAG
ACGTTCTAAGAAAGGTTTCTCATTTAAAATTTCGAAGTGCTTTTTCTTGAGATCTACATTTCGCTACCGTGG
CTATGAGATAAAAGCGGGGGAGAGGCGATACGTCCAAATTTAAGCAAAATAAAATCGCTAGTGGCGTTG
CCTCCACCGAAGTCAGTTGCTACTCTAAGACAGTTCATTGGGTTAGTGTCATATTTTCGTCAATTCATTCAT
TTTCTCACATCTGAAAAGAATAGTTTTGATTGGCAGCCACAGCATGAAGAAATTCGCAGTAAAATCAGCA
GTGTATTAACAAACAAGCCCGTTTTAATAATTTTTGAACCCCAGTTTCCGATAGAACTGCATACCGACGCC
AGCTCTATCGGGTATGGTGCTATCCTTTTGCACCGCATAAACACTAAGCCTCATGTGGTTAAATACTATAG
TAGAGCAACTAGCTCCTGTGAGTCCAGGTACACATCCTATGAATTGGAAACGCTGGCTGTCGTATCTGCAT
GGGAGAAAATTTGTGATTTTTACTGACTGTAATTCAATCAAGTCTTCTCGAAATAAAGTCAAATGGCACCT
AAGGTTTATCGTTGGTGGGAGTTTTTACAAACATTTGAATTCGAAATTGAATACAGAGAAGGTAAACGCA
TAGCACACGTGGACTTTTTATCTAGGAATCCGGTGTGTCAAAAGAATAAAACAGAAGAGAAGCGAATTGA
TCTAACAGAAATATCGGAAAACTGGATACTGGTGGAACAACAAAAGGATCCAGAAGTACCAGAAATCTT
AACTAAGTTGAATAATGACGATTTACCATTGGCATTAATTGCCACTTAGGAAATTAGATCAAATGTTTTGT
ATAAAAAGATTCAGAGGAACTCAAGAACTCGTTGTTTACCGGTAGTTCCTCGTGCATTCCGCTGGTCAGTT
ATAGATCAGGTACATGAGTCTATCATGCATCTACGTTGGGAGAAAACGCTAGACAAGGTGTACGATCATT
ACAGGTTTTCTAGTATGACAAAATATGTTCGAGATTTTTGGAGAACTGCATTACATGCAGAGTTTCTAAGG
GTACGTCAGGAAAACCTCAAATGGATGTAAATCCAATTCCAAAAAATAGCATTCCTTGGCATACCGTTCA
CATAGATATATCGGGAAAACTTAGCGGAAAAACTGATCGTAAAGAATACATCATTGTACAAATCAACGCT
TTTACTAAATACGTTTACCTGTTTCACAACTTTTTTTTGGGTGCCAATACTTGTATAAACGCAATGAAATCT
GTATTTTTGTGTACTGAGATTGGCTCCGCAAAATATAACCCGAGGTCATATTTAAGTATGACCCCC

>9_rnd-5_family-3470__LINE___Penelope
GCATAGCAGCCAACATGAGCTACGCAAAAATCAATACTAAATACAAGGATTCCAAAAGAACAACAACAA
CAACAAAAGAACAATTAAGAAATTCCAGCTAACATTGGTAAAAATTAATCCAAACTTAAATCCAGCTAAN
AATTTTTGTTAAAATGTAGAAAATCTAATTNAATACCTAACTTCATCAAAAACTTGACACAGCATTTTGCC
TATACTGACCACCAACAAAAACCAACCCCGACATAAAAAGAACATNGACTAGACACACCCATTTATACCA
TACCAAAATATTAAACTTANTTNTTAAAACACAAAACACAACCTATTACAAGAAGAAACAAAAACATATG
GAAAAAGANAAAAAAGATAGAACAACTGATGACCACAGATGACGCCAAATCGTTTTTCGAGAGCGGCAG
GAACATAGAAAAGTGTTGTTATTACAACATTTAAAGAATAGACAAGAAACGAAACNCGATAGGATTACG
AGATCAATGAAACCTTACCATAGCGGACAGCAGGAGATCAAAAAAACTGGTCCATAAACATAAGCAAAA
CAAAAAACAGAATTCCTGCCAAGCGTAGAAGCGTTATCACTCGCTAAACGGCCGCAACTTCGTGGCACNA
CTATTCAAGATGGGTCTTATTTCGAAGGACGAAAGAAATAAGATGACTACAACAACAGCGGTACCTCCGA
GGATATACGGACTACCGAAAATGCATAAGGAAGGAACACCACTGAGACCGATATGTTCCTCCATCGGATC
TCCATCTTACGGGCTGTGCAAATACATGATAAAAATACTAAGAAATCTGACAATGGACTCTAGATACAAC
ATTAAAAACGCGGTAGATTTCANAGACAAAGTTAACAATTCCANTATCAGGGAAGACGAAATATTAGTAT
CATTTGACGTAGTATCCTTGTTTCCCAGCATACCAATGGAACTAGCACTTGACACAATAAGACGAAAATG
GACCAAATTANAGGAACACACCAGTATACCAAAACAACTATTTATGGACATAGTAAATTCTGCATTCAGG
AAAACAGATATTTNAAATACGAGGACAAAATATACACACAACTAAAAGGAATGCCAATGGGATCACCGG
CCTCCCCAGTAATNGCNGACATAATAATGGAGGAACTNTTGGACAATATTACNGATAAATTGACAATCAA
ACCAAGACTTTTGACNAAATATGTAGATGACCTTTTTGCCGTAACGGAAAAAACAGAAGTACACAAAATT
CTAAAAGAATTGAATTCGTTTCACAAACATATAAATTCACCGTGGAATTAGAANAAGACGGAAAACTAC
CATTTTTAGACTATATAGTTAACAGATTGGACAACTCACTCAAAATTAAGTGGTATAAGAAACCCATAGC
CTCCGGACGANTACTCAACTTCAATTCAAACCACCCAAAGAGTATGATAATCAATACAGCACTAGGCTGT
ATAAAAAGAATGATTAACATATCGGACNCAACATACCACAAAGAAATTGAANACGAAATTAANAAGCTC
TTGACGAAAAA
>9_rnd-5_family-17__Unknown
CACCCATAACTGTGGGTAATTAGCGGAAAATGTTTTTATGAAACTTATGTGCTTACCGTTCGATCGGCCGC
AAACTCATATTTTCAACGAAATGNGAAAATCTGTNCTCNTTGGTGGNAATGGGGNCCAAAAATTTGGTGN
GTGACGTTTCNCNCTNAAAATTAATTTTCAGAGATCTATAATGGNAAATNATCTAGGANCGNGTTTCACAT
TTTTCGNANAATCACANACTCCTTCGGTAGAAGTGCGATTCCAAAGACTTTTATGGGCTAAGTTTTAAGTG
GTGTCCGATTTGCTTCAAACTTATTTTCAAAGCTCTACCTGCAAAAGAACTTAATGNATACGCGTTTCAGC
GATTTGGAAAATTCCGCACGCAANTGTGGTTAATTAGAGAAAACGTTTGTATGAAACTTTTTTGTTTATCA
TCCGATCGGNNTCGAACTTATTTTTCAAAGATNTTTCTNAAAATTCATTTGAGGCGTNTCGCACCTTTCGG
AAAATCCNTCCTCCTTGGTGGAAATGGGAGTCAAAGATTTNATGGNTGGCCTGTTTCGCGCTNAAATTNAT
TTTAAAATCTCTTAGAAAAATAATTTAGGTTGTGCTTCACATTTTTTCGGAGAAAAAAGTGTGATTCTAAG
AAATTTTTGTGAAGTTTTAAATATAGTACGATTTTGTTCAAGCTTATTTTCAGAGCTTCACCTGCATAAAAA
CTTAATAAATAGGTATATCCAGCGGGTGGAGANATTCCACNCACATAATATGTNNGNAANTGGGGAGAA
AATGTTNCAACGATTGTGGCAANCTNNTTTGCTTATCATCCGNTNGGCCTNAAACTCATTGCCATTTTCAG
GAAAATCCGTTGTTCGCTGATTTTTTGCGNAGCATTTCANGCTTTTGANCAAANATTTNATGGGTGNCGTT
TCGCGNTNAAATNAACTTTCAGGGATCTCTNNGGAAAATTAATTTNAGACGTATTTNACACTTTTCGNANN
ATTATANCGTCCTCGCTGGAAAATGCGATTGTAAGANTTTTCGTAAAATTTTAAGTTTGTCCGATCGGCTT
CAAATTTATTTTTAAGCTTCACCNTGTGCGCAAGAANTTAATGAATANGTATATCAGCGATTTNGGAAATT
CCACCCGCAANTGTGGANAATTAGCGGAAAACGTTTGTTATGAAACTNTTTTGNTTACCATCCGATCGGCC
TCCAAGCTCATTTTCAACGANCTNTCTGTTCAAATTCATTTTAGAGGTTTTTCACATTTTTCGGANAATCCA
TCCCTCTTTGGTGGAAACGGGNGTCAAAAATTTGGTGGATGACGTTTCGCGCTAAAATNAATTTTCAAGAT
CTATAAGAAAATCATTAAACGTGTTTCACGTTTTTCGCAAATTNAAACNCCTTCGGTAGAAGTGCGATTCA
AAGATTTTTTGNGCTAAGTTTTAAGTNGTGTCCGATTTGCCTCAAACTTANCCTTAAGGCTCTACATTGAA
AGAACTCAATATATAAATTAGTGCATTAATGTCTACAACTTTGG
>9_rnd-1_family-361__Unknown
GAAGTGTCACTTTTTAGTGAGTGAGTGTGTAAGGGTAAGCGTATATGTGTATGAGTGCGAATGTATGTGG
AGCTCGCTATAGAGCTAGTTGCGCGACGGCAACTGTATTATAGCTAATCTGTACAGCATATGTATGTATAC
ACATGCATATGTTGTTTTATTGGGTTATTGTGTTGGGTGAGCTGTTAGGTTATGGCAATTGAGGGGAAAAG
AAATGTATATATGTATGGAAAAAGTAATTGAGTACCTTGTGTGTCCTTTTTGTTTTTGTTTAGTTGTATATT
TGTGTTTTTGTGTCAATGAAGTTATGTGTGTGTGATTTTTGACAGTTGTTAAAAAGGAAATTGCTCTTTTGT
TGTGTGTGTATGTATATATAGAGGTATATATATGTATATATACATCTACATATGGATATACATATATGCAT

GTGATGACGGAGTAATTTGATTATAGTTGAAAAAATATGAGCACACAGCAATTTATTGTTTGNTGTTGTTA
GGTTATGTTATATTTAGTCGTTGTTTAGTAAGTTAGGATGAAGGTTAGGTACGAGTTAATGTCAAATAACA
GTGACGTTTATGGCGAGGGTTAGCGGCAGAAAGTGGCGAAAAACAGAGAACTCAGCCGCGTATATGTGC
GGCATACGTACGTATGTGTATACAAGTTTGTAAAATTAGCAAGCGTATAGACATATGCATGCATACATGC
GGCATGAAAGAGCAAAGGAAGAGAAAGAAGAAAGGAATATACTAGAGACACGCGTACGCATACGCGTA
TGAAATACGCATGCGCTAAGTGTTGCACATACACAAATGCACAGAAAAGTACACGTGTACAATTCCTTTC
CCTCTATCTTTTCTCGACCACTATCCTCTCAGTTCCNCTGTCCACACTATTTCACTGTTCCGTTGATTGCTAT
TTCACAATTCCACTATTTCACGTTACTCGGAGTCTCAAAATATAATTTAGTTTAACTTTTACTTTAACTTTA
ACGATTTTCAGTTTTTTTCAATTTTCCGTCGACCGGCTAAGAGGAACAAGACTGTTCTGGGGTAAGTGGCT
TTATGCGAATGGGAATCGGGGATCGAGCAGCAGGTTGCCAGGAGCTGGCAGGCAAGCGAGCGAGCGAAC
GAGCGAGCAGGAGAGGCAGCGAGACAGCAAGGCAGCAAGGCGGCGAGACAGCAAGGCAGCGAGACAGC
TAGGCAGCAGGACAGCAGGACATCAAGACAGCAAGGCANCGAGACATCGAGACACTCCACTCCAGTTCC
AACTCCGGTTCCGACTCCAGATTCAGACTCCGACTCCAACTCAATGGCCGCTCACTCACTCGCCCAGTCGG
CCGCTTCCAATTGGGAACGTGAATGCGCTCAATCTCCAACNCCCAGACCAGAAATTTATTTATTGATATAT
ATTACTTTAGAGATGGATAAACGTTACAAATGTTAGTACAGGGAGACAGATAGACAGACAAACAGACGG
ATAGACAGACAGACAGATAGACAGACAGACAGACAGACAGACAGACAGACAGACAGA
>9_rnd-5_family-1255__LTR___Gypsy
CCAGATTGGAGACGTGAATAAAAAATGGATTCGCAGAGANTAGGGNAAANNAGAAGAAANATTTTAATA
TAAATNATAGNAGCCGANTATAAACTCGGCACTAGAATTGGAGTGGTTGGTGTTACCCCAGATGGTGTGN
ACTCAATCTTACAAACTGAGCAGATGCNAGATNAAGACGTAGAAAAAGTTAGAGAAGTAATAGAGGAAG
GTCGCGAGACTTTAGAATTNGANATNATAGATGNAATAATATACAAAGGAGATAGTGATGNCAAGTTAG
GCNTATATGTTCCGAAGTGNATGGAAGAGCAGTTAATCAGAGTAGCACACGAGAAGTTAGGACANCTAG
CGGCGGACAAGTGTGTTAATGACCTAAAAANAACATANTGGTTCCCGTCAATGCGTAGTAAAGTATNACA
GTTTATTAGAAATTGTTTGCCGTGCATATTACACTCTATGCCCAAAACCATACATAATAGAACATTACATA
GCATACCGAAGTCAGAAGTACCCTTTCATACGTTACACATAGACCATCTAGGCCCTTTACCGAGTATCACA
TCAAGAAGAAAACATATTTTGGTAATCGTAGGTGCCTTCACAAAATTTGTGAAATTATTTCCGGTAAATAG
TACCAGTACACGCGAAGCCAAAGACGCCTTGAGTAAATACTTCGAATTTTACAGTNGAGTAAGAATAATC
TCGGACCGAGGCACTGTTAAATTGCGAATTTCTNGCAAGAACGCGGTATAAGAGCATATCAAAAGTTGCC
ACTGGGGCACCACAGGCAAATGGCCATGTTGANAGAGTTAATCGTGTTCTCACCCCCATGTTAGGTAAAC
TCTCAGAGCCCATTCAGCAGGCAGATTGGTATAGTTGATGAACAAAGTCGAGCATGCCATAAATAACGCT
ATTCATAGTAGCACGCAGAAAACACCCAGTATCTTGTTATTTGATGATCCAAAAGGACAATAGTTGACGT
AATTAACTGAATATTTAGAGGATAAATTAAACATAGAGGAGAGAAATCTTAGTGCGGTGCGAGAGAAAG
CTAGTGAAANTATTTTGTGCTCACAGCTAAGAAATGAGAAAATATATGGCAAGAAACATAAAGCCCCGTT
AACGTATCAAATAGGTGATTACGTGGCCATTTCGTAATGTTGACACTAGCAATAGGAGCTAATAAGAAAT
TCGCACCAAAATATCGCGGCCCGTATAAGATAAGCAGAGCCNTTGACAACGACCGTTATGAAGTGGTTGA
TATTGAAGATTGCCAGTTGACTCAGATGCCATACTGTAGTATATTAGAGCCAGCTAGGCTCAAGCCATGG
GTTCAGTGTGTATCCGACACGATAGATGTATGTCGTTAACCTACATATATAGATAACCTAAGATCATATAA
ATAAGCGAGACGTATTGGTAACCCCAAAATTGTAAGATCGTGGGCGATCTGTCCTCGGGTTAGCCGAATG
TAAGATCGACAACCAATCGAATGTAAATTTAAATGTAATCAATGTAACCATATTATAGGATA
>9_rnd-5_family-1956__LTR___Pao
ATAAGTGCAGTACAAGCAAAGATACAGCGTACATAGCAAGCCAGCTGCAGCCCGTTTATTTTCGTTCCGC
TTTGTGCTAAGCGGTGGCTGTGTACGCATTTTTCGTTGGCGNGTTTCGGTAAAAGACGCTCGCTCGCNTAC
GCTTTATTGATCGGGCATATCAGTTTTTCGAATCGATTGGGATATTCGACTTTAATTTAAATTAATANTTAT
TATATCGAATTNAGAGTCAAATATGGCTACTAAGGAAAGTTTCGACGATTCAAAAAAGAAAACNAAATTG
AATATGTCCGTGAAAAGCATAATGCGTTTAAATGATNAAATAATAAANGGTAAATTAAACCTAGAATCTA
CAGAGTTAGAATGTAGATTAGAAATGCTGGCCTCACATATAGATACAGCTTTAAATATCAGACGAGTAGA
AACTTTTGACCCAGATGATGAGTATCGTGCCGAGCTCGAAGAACTTTGTATAACGACAAAAGCTCGTCTA
AGGTTCGGCATGCCCGTTGNGGCTCCACGCACACGTCTTCCGCATCTGGCACTTCCCAAGTTTAGTGGAAA
ATACTCCGAGTTTAAATTTGATTAGCCTNTTCGAAACTCTTGTTCACAACGATCATNAGATCCGAGCATAG
AGAATTCGATCATCTGACTCATGTCCTGTGGAGAATCTAGGCACCGTTAGAGCGTTTCAAGTCACCGAGG
ACAATTATTTAAACATTGGCAGCCTAAAGAGTATTCGACAATGAATTTCTAATATNCATAAAAATATTTCG
NCTCTCTNNAGCCTGCCAAANGTANTTTCGCAGCCTTCTGCGGCACCATTGAGATCAANGATTGANNCCG
TCTCCGCNANCTATGGNTCGCTCCTGTCAATAGGTGATGACAGNNAGGTTTNTAACGCGATTCTCGTTCAC
CTCGTTACGAACAAAGTTGACCCAATGACCCGACGAAAATGGGAGGATCGAATCGTAGGTATCAGCACCT

TTCTGCTGAAGAAGCCGGAGTCAAAGGCTGTTTCCAAAAATAATAATCGAGGTTCGTTTTCGTGTTCCGTG
GCTAGTGGAAGAAACGATCAANTGACCCCAAAATNCTTATATTGCGGTTCCAAANGCCATTCGATATTAA
ATTGNTCAGCNTATGCCGCGTTACAGTACTAAGNCGGCTCCTTTATGTATCAATTGCCTTCGGAAGGGGCA
CACTGTTGCCAAATGTAAGGCAAACAAGTGCAGAATATGAAGTCGTTCTCATAACACACTGTCGCATAGA
TACTCTGCCGTTTCTATCACCACCCCTTGACTCCGCACAGGATCAACCATCGACTTCACTGCTATGCATGC
GAATGTGTCTGATAGTGAAACGAAGTTGGCGAGTTCGTTTTGGCTAGATCTTTACTCGATTCTGGATCACA
AATTAATTTTNTAACAGACGAGNTAGCACAACGATTGCANCTGCGTAGAGAAGAGGCTCGTATCAGCTTA
CTTGGGATTNCTCAATGCAATACAGT
>Ref_dvir.36.87.centroid
TTCGACGTATCAAAGATATCCGCACTTTGTAGTTTTAAGTTTATACAGTCGTAATCGAAAGTACCTTTTTCT
ATAAAAGGCAGAGCACGAAAAAAGCATTTAAGTATTCGAGCTCTTGCCGCGTTCGGAAAGCATCGGCATC
ATCTTTAGTGGATTATTAAAAGTTTATCCTTCGCGATATTCTAAGCCTAAAATGGGTTCAAATATTACTGA
ATTTTATAAAAATAAGACTATTTTCTTGACCCGGTGGCAGCGGATTCCTTGGAAAATGTAGGTAAACCTTG
ATATATCAAGGACTATGCCGGACACAGTTTATCGAGTACAACTCGATTGCGTTTAGTGTGTGTGATACTTA
ATTAAGTTTAAAATGTGATTTTTTCTTATTGATGTGTGTCCCTCAGTGATAATTGAGAAACTGTTGCGATCA
ACGGAGGTGGAACGTATCTATATGCTAATACGGCCCAAACGTGGCGAGCAAATCCAGAGTCGGATGGCTG
TTCTTCGAAGCAATTTTGTAAGTCGCCCATGATCCGAAGAAATAATTGAACTCTTACTCAATGCTCCTGGA
AATAGATGTTCAGCGAACTGCTTAAATTGAAGGCCAACTCCCTGGAGAAGGTAATACCCATAGCTGGGGA
CTGTGCACTACCGGACCTGGGATTGAGCGAGGCAGACAGGCAGGTGCTGACTGAGGAGGTTGCAAGTGG
TGGTGCACAGTGCAGCCACGGTGAACTTTGTGGAGCCGCTTAGCAGCGCGTTGAGCATCAACACGCGAGC
AACTCGTCTACTTGTGCAGCTAGCCAAGCAGATGGGCCGCCTGGAGGCATTTGTCCATGTGTCCACGGCGT
TCTCTAACTGCCCGGTCGAGCACATCCGCGAGTGCTTCTATCCCGAGCTATTGAGTTGCTCGGCTGACAAG
GTTTTGGCGCTGCAGGATCAGCTGAGCTGCGAGCTGATTGACCAAATGACACCAGCCCTGCTTGGCAAGT
TTCCCAATACCTACACCTACACAAAGGCGCTGGCGGAGCAGGTGCTGCAAAGGGAGGCTGCCGATCTACC
CATATGCATATTTCGGCCAGGCATGAGTATGTGAGGATCGATAGCCAAGCTTCCTTTTGATAATATTTTTG
TCTTCCTTTGGCTTAAGTTCTTCCCAGCTATAAGGAGCCATTTAAAGGATGCATTGACAACCTTTATGGAC
CCATTGCCATTTCATATGGTTGTGCCGCTGGCGTTGTACGCGTCGTGTCCATAAGAGAAGAAGCCCGAACC
AATATTGTGCCAGTTGACTATTGTGTCAATTTAGTCCTGTCCTGCGCCTGGCAAGCAGCTGTGGCAACTGC
TCAGCGCCGGGTTCCTGCACAAGATCCAACTATTTATAATTTTGCTCCGTCCGCTCAAAACCCAATAACAT
GGAGGAAATACACGGATTTGTATGCAAACAATAAGCATATTTATCCAGTTAATCAAATGATATGGTTCCC
GTT
>9_rnd-6_family-1293__LINE___CR1
TAGGTATAATAAAAATCAGCCTTATCCTTATCAGTTACAGAGGTGAAACTGCATCTTAAATCCCATTATTA
CCGAAAGTTCGCTTCTTAGCGACCTTAACACTATCAAGCCTGGTGGTGTTCCTGCTTGCGTGCTTAGGTAC
TGCGCTGCAGCATTATGTAAGCCTGTTTTACAACTTTTTGAATTGTCCGTAAACACTTCATCATCCGACCAT
TTGGAAGGAATCATACACTATCCCTCTCCAAAAGAAAGTGACTAAGTCCTATGTTGAAAACTATAGAGGT
NTTTTCAAATTATCCGCTATACCTAAAGCNATTCAGCTGAACATTTATGTAGTTCTATTATTTCACCTTCCC
AGCATGGGTTCGTGAAGCGCAGATCTACAACCACTAACCTTCTTGAGTTTACTTCGTTTGTAATTGATGGG
TCTAAGAAAAGAATGCAGACTGACGTCATCTATACTGATNTCAGTAAGGCTTTCGACTCTGTCAACCANTC
CTTGCTGCTTCATAAATTATGCCTTCTCGGCTTCCCAGTCAATCTTTTGAACTGGATTTCAGAGTATTTGCG
TGATAGAACCCAAAAGGTTCTAAACGTATCTGGTGACTTCCGGTGTTCCTCGATACTGTCTCGCTCTCGAG
TATTAATGTATGCTGATGATGTAAAATTATGCCTTGCATATAAAGATATGGCATCATGTTATCTCTTACAA
GCTGACATTGATGCGTTTCAGGCTTGGTGTCTAGCCAACCTTTTAAACTTAAACTGCTCTAAATGTAAGGT
AATGACCTTCTATAGAAGCCCTCCTTGATAGCTTACCGCTCGAGCGTGTTAACAGTACTTCTAATGAAGTT
AATGATCTAGGTGTCATCCTTGATCATAAACTTAAATTCGACTCTCATGTTAAGACCGTGGTCAATAAGGC
CAGAATAGTTATGGGTTTCATCAAGCGATGGTCGAAGGAGTTTGAACGATCCGTACACGACAAAAACACT
TTATATATCTTTGGTACGANCTATTCTTGAGTATGCCTCATGTANCTGGTCACCACAATATTCCGTCCACCA
GGACAGAATTGAATCCGTTCAGAAACAATTCTTGTTGTTTGCCCTTCGTGGTTTAAATTGGGATCCTCGCG
TGCGGTTACCGTCATACTCAGGATACAGCAGACTTCTGATGATTAACCTTCCTTCTTTGATTAATCGTAGA
ACCATGCTGGGTGTAATATTTATTCATAAACAAATTACTGCTGAAATCAAATCTTCTGTGTTGACTAGCCA
ATTAAATTTCTCTATACCTAATAGACTAACACGTAATTTTGTACCCCTATCGCTTCAATTTGGCTCAACTAA
TTATGCTTTGCATGAACCGTTCAGGGTTCTCTGTGATGACTATAACAAGCATTACAGAGTANTTTGNTCGG
TATCTTCACTTCCTCGTATAAAGTCTTCTATTCTAAACTATTTAGCAAGTAGATAGTCTT
>160_rnd-5_family-152__LINE___I

CGCAACATTGAGGATATCCTAAAAAGGGACGAAATACACAATGGGACACTTACGTCCATAGATTTAAGCA
ATGTAATCCGAACAAGGAACCCGAACTAGGCACACCGCTTTAACCCACGACCACCTGCTTAAAGGAAAAG
CCAATCCCAGCTGCCCAAATTGCGGGGCAGAATTATCAATCCAACACATATTGAATAACTGCCCACAACT
TAATAACATTAGGACTTGTCTATTTGGCCGAATCCAACCTACCTCTCTTCTTACCACACCTACTCACTATAA
TGTAAAATTGCTAATCAAATTATTAGACACCGATTACATACTAAAGAGCATATAATAATATATATATAATT
TAAAATAATATACAAATAATACTATAAATAATTTAACTGTCGAGTCGATGGCCTTAGTAGCTAGCACTCCC
TTCCGTTAGTTATTATATTAATTTTTTGTACGNTGGACCCANTGAAAATGGGTTAAAAACGGNTNCANNGT
ATTTGTGCAAAATCTAAATNTATGTNACTANGCAGTAAAAGGAAGCATCCCCGGCCCCATAAATAGTATA
TATTCCTGATCAGCATCTGTAGCCGAGCTAGATCTAGCCATGTCTATCCGTATGTATGAACGCAAGCATCA
TAGNACCTGTAAGAGCTAGAGACTTCGAAAAGTCATTTTTAACTTGGGATGTAGGTCCTCNTANTGCCTCA
GNCCGAGTTCGTTTCCGAGCAATCGNTTAGCANCTGTACTCNGCNTCTGTACGAGCNAGAGACTTCGATA
AAAATCGACTAGNGNCNTCNTGTTNCTTGCCCCGTTTCCAAGCAAATCGCTTAGGTTTTAAAAAATGGGA
GCTNGAGTCACCAAGCNTGATANGTNGCTTCTAGAATATATATGTACCTACATATNTGCATATCAAGTATN
TTTCATTTTATANCTATCGACCACCNCCCCGCTACCANCNCANAGNTATACAGTNATGTNAATAANCCGA
NTNNNACCGCCNANCAATTTAAGNCACNATTTNNATGCAATTGACTTTTGTCAGAATACACAAATATTCT
AAGGTACACTAAGATATACTGTAAAAAATTGCATAAAGATCGGTTAAGGAAAAAACAAAGTTATATGGA
ACTTTAAGAATTTCTGTATACATGTACACACACACATTTATGCGCGTCTGCTTCGCATTCTAACAGCATGA
TGGTAATTGCGACTTATTTCTGTAGTGCTATTTTAGTTCCTTTTTANACCCCGAACCCATTGAGANTGAGTA
GNAACGGTTTATTGTATTTGTNCGAAAACCAAATGTATGTAACAGGCAGAATAGAATATAAGTATATATA
TATTTGATGTTGGAAGAAGANGGTACAGGGTATCCNCTAGTCGNGCGCTCCCGNCTGGAGCACTCTCGCT
TGTTTCTTTTTAATATAATAATTAATCACGTAAATAAATAATGAGAAAAAGACATGAAGTTCAGATAGAA
T
>9_rnd-1_family-41__RC___Helitron
ACTAGCGGGTATAGCCCGGCCTTGCCCGTGGCACTTATGTTATAAAATCAATGTTCTCGAATAGCTTTGAA
AATGATTTTGAAACTGAGCGGACAGTAAACAAACTACTCTCATACAAACGTTTTCCGTCATTTTACCACTG
TTTCGAGTGGAATTTCCCAAATCGCTGAAACACGTATTCATTATATTCTTATATAGAGCTTTGAAAATAAG
TTTGATGCAAATCGGACACAGCTTAAAATTCTCAAAAAATTACTTTAAAATGCACTTTCCACCGAAGGAG
CTTAGATTTTGCAAAAAATGTGAAACACGTCTCAAATGATTTTTTATGTAGATCCATGCAAATGAATTTGA
AGGCAAAAGGCCACCCACCAAATCTTTGACTCCCATTTCCACCAAGGAGGAATGGATTTTTCGAAAAGTG
TGAAACACCCCTCTAATGAATTTTAGCAATGATCTTTGAAAATAAGTTTGAGGCCGATCCGATGGTTAACA
AAAAAAGTTTCATACCAACGTTTTTCGCTAATTAACCACTGTTGCGGGTGGAATTTTCCAAATCGCTGAAA
CACGTATTCATTAAGTTCTTATGTAGAGCTTTAAAAATAAGTTTGAAGCAAATCGGACACNACTTAAAACT
TCGCCCAAAAAATCTTTGAATCGCATTTCTACCGAAGGAGCTTAGATTTTGCGAAAAATGTGAAACACGT
CTCAAATGATTTTTCATATAGAGCTTTGAAAATGAATTTGAGCGCGAAACGTCACCCACCAAATCTTTGAC
CCCCATTTCCACCAAGGAGGAATANATTTTCGAAAAGTGTGAAACACCCCTCAAATGAATTTGAACAAAG
ANCTTTGAAAATGAGTTTGAGGCCGATCGGATGGTAAACAAAAAAGTTTCATACAAACGTTTTTCGCTAN
TTNACCACTGTTGCGGGTGGAATTTCCCAAATCGCTGAAACACGTATTCATTAAGTTCTTATGTAGAGCTT
TAAAAATAAGTTTGAAGCAAATCGGACACCACTTAAAACTTCGCCCAAAAAATCTTTGAATCGCATTTCT
ACCGAAGGAGTTTAGATTTTGCGAAAAATGTGAAACACGTCTCAAATGATTTTTCATATAGAGCTTTGAA
AATGAATTTGAGCGCGAAACGTCACCCACCAAATCTTTGACCCCCATTTCCACCAAGGAGGAATACATTTT
TGGAAAAGTGTGAAACACCCCTCAAATGAATTTGAACAAAGAACTTTGAAATTGAGTTTGAGGCCAATCG
GATGGTAAACAAAAAAGTTTCATACAAACGTTTTTCGCGATTTTTCCATACTTGCGGGTGGAATTTCCCGA
AACCCCGTCTTAACGTGCACCTTCATCACATAAGGTAACTACAGTGAAAATTTCAGCTTCCTAGCTCTTAC
GGTTTGGGCTGTGCGTTGATCACCAATCAGTCAGTCAGTCAGTCAGNCAGTTTTATATATAGAGAT
>9_rnd-1_family-39__LTR___Gypsy
TCGATCGCTTGGAAACGGAGTAAGTTATCGATTATCGGAAATAAACTCGATCTGCGCGGGAACTAGGAGC
ATCTACATCTAAAATTTCAAGTCTCTATCTTTTATAGGTTCTGAAATCCTTGCGTTCATACATACGGACGGA
CGGAAGGACGGACGGACGGACAGACAGACGGACATGACTAGATCGACTCGGCTATTGATGCTGATTAAG
AATATATATACTTTATAGGGTCTGAGGTGCTTCCTTCTGCATGTTACATACATTTGGATTTTGCACAAATAC
AATATACCCTTATACCCCATTTTTCATGGGTTCAGGGTATAAAAAGGGACGAGGAGAGTCTGTCCAAAAA
ACGATAACGGTAGACTCAAAATGTCACCTGGATATAGCATGTCGATCCTTTAGAAGGATTATCAAACACT
GTTTAAAAAAAAATAACCGTAAATAAGTGTGTTAACATGTTTCTTAAATATCAAAAAACAAAAACCACTA
AGCATACTAAAACGCCAATAACCAGAACACAAACACCAACAACAGCTTTTGATCGATATCATTAGTCCAA
ATTTAGTAAGTTCTACCAAACAAAAGTGCAACAACCTTTGCTAAGACGTTAATAACGGACATGTGTACAG

AATACAAAATTTCTTTAATATCGGATTTAATAAGTCTATGAAAATAAATAACATAAAATCAACCGTTGGTC
AAAACCACGCACTTGGTACCATAGAACGATATACAAATTTTAACGAATATGTTCGTATGAAAGACTCGAT
TGACAAAAGTGATTAAGACGTATTTAAAAGCAAAGCCTTTTATTTTTTACTAGCGGGTATAGCCCCGCCTT
GGCCATGTATGCATGTATGTTATGCAAATTGGACACAACTTAAAATTCAGCAAAAAATCTTAAGAAAGCA
TTTCCCATCGAAGGAGTTTAAATCTTACGAAAAATGTAATATACGTCTTAAATGATTTTTATACCCTGAAC
CCATTAAAAATGGGTATAAGGGTATATTGTATTTGTGCAAAATCCAAATGTATGTAACAGGCAGAAGGAA
GCATCTCCGACCCCATAAAGTATATATATTCTTGATCAGCATCAATAGCCGAGTCGATCTAGCCATGTCCG
TCTGTCTGTCCGTCCGTCCGTCCGTATGTATGAACGCAAGGATCTCAGAACCTATAAGAGCTAGAGACTTG
AAATTTTAGATGTAGGTGCTCCTAGTGCCCGCGCAGATCGAGTTTGTTTCCGATAATCGATAACTTACTCC
GTTTCCAAGCAATCGATAAAAATCGATATCGATATCCTGTTTTTTGGGCAATTTTGGTAAATAATAAGAGC
TAGAGTCACCAAACNTGACATGTAGCTTCTAAAATAGAATATATATATGCATTTGATGTTGGAAGAAGAG
GGTTCAGGGTATCCCCTAGTCGGGAGCTCCCGACTAGAACCTCTTACTTGTT
>9_rnd-5_family-353__RC___Helitron
ATATACAAAGTATGCAAGTATGCGTGAATAGCGAGTCCAAAATATTTTAAATGCACACATGGCAACGCTT
GGGTACCCTTACCAACGATAGCGAGCTAAAATGCAAGAATTTCGCATCGACGTGGCAAACCCTTTCTCTA
TTTCATATTAGCCGTGCCTTCAAAAGGGTAGAAATGCCTCAGCATTTGGGAAAAATTGAATTTNTGCGCAG
TTTTGCTCGTCGGACAAATACACACACACACACATTNAAACGATCCCTAAACATAGAGAGAGCATTGAGA
GCTGAGAGTAAGTGCGAGCGAGATAAAAGTTAACAGCGTTGCCGTTCTATCGCACAAATCATATGGAAGA
GAGTGCGCCAAGCACTTTCCTGTAGAGTAAGCGGTTCAAAAATACATCATATGTATATCTTACTCCCTTAC
TCGCGAGTTTGGACCAGATGCCTCCGATACTTTTTGTGACAACGCTGTTTGTATGTGATGTCCAGTCTATAT
GTTGTTTGGTTAGTGGTATAAGGGTATATTGTATTTGTGCAAAATCCAAATGTATGTAACAGGCAGAAGG
AAGCATCTCCGACCCCATAAAGTATATATATTCTTGATCAGCATCAATAGCCGAGTCGATCTAGCCATGTC
CGTCTGTCTGTCCGTCCGTCCGTCCGTCCGTCCGTCCGTCCGTCGCTCGTCCGTATGTATGAGCNCAAGGA
TCTCAGAACCTATAAGAGCTAGAGACTTGAAATTTTAGATGTAGATGCTCCTAGTTCCCGCGCAGATCGA
GTTTGTTTCCGATAATCGATAACTTACTCCGTTTCCAAGCAATCGATAAAAATCGATATCGACATCCTGTT
TTTTTAGCAAATTGGGTAAATAATAAGAGCTAGAGCCACCAAACATGATATGTTGCTTCTAGAATATTATA
GATATTTCAAGTATCTTTCATTTCATACCTATCGCCACCTCCCCGCTACCACCCCAGAGCTATAAATCAAG
TTAATAACCCAACTTTTATTGCCAACCAATTTAAACCACAATTTAAATGCAATTGACTTTTTCAGAATACA
CAAATATTCTATGGTACAATAAGATATACTGTAGAAAATTTCATAAAGATCGGTTAAGAAAAAACCAAAG
TTATATGCAACTGCGCAATTGAATGGGGCAGCAGCTTTAAGAATTTCTGTATACATGTACACACACACATT
CATGCGCGTCTGCTTCGCATTCTAACAGCATGGTAATTGCATCTTTTTTTGTAATGTTATTTTAGTTCCTTTT
TTATCATAAGTACTTAATTGTTGGTGTGGCTGCTGAGTAGAGGCGACAGCAAGTGGTGCGGTCTGTCGCG
AGTTCGAGCCCTGCCGGGGGAAGTTTATTTTATTAATTTNTTTGGTGTTGGAATAAGAGGGTTCAGGGTAT
CCCCTAGTCGGGAGCTCCCGACTAGAACCTCTTACTTGTTATTTTT
>9_rnd-5_family-7__LTR___Gypsy
ACAATCAGTATATACAGGCCCATTTGAAGTAATTAGTATAGAGGGATAATAATAACATAATAATAAGAAA
TGCTCGAGAAAGAANGGCTATCANCGGCACGCCGAAGATCTAATACCCTTTGCAGAGCGAACCATTNCAT
TATGCTCATAGTGCTTTAATATGNCTCTGTCTAAGNAGCACCGGGAGAAATAGTGAANNCCGTAACAGCT
NGGTCAAGATATCTTGAAAAAATAAACAGAANGATGTNTCATACAAAAACTTANTNTTCGACCGNTCGTT
CCNATAAAGCTATATNATATAGTAGTCCGATTTATAATAGGATTTTGCNCATATNTGAGGAGCATGGTGG
ANNTCGTAAATGCCGGNATGGTCNGGNTATCTTAGAAAACAAAATGNTTTTTTANACAAGGGTGAGNTTT
TCGANNGATTGTTCCTATGGCAGCTGGCANGATATAGTAGTCCGATCTATAGATTTGCACATATATNAAG
AGCAAAGTGANACTATTGAATGCTGAGCTTGGTAAGATATCTTGAAAAAACAAAATGTTTTTTTCATACA
AAAACTTAATTTTCGACCGATCGTTCCTATGGCAGCNATATGATATAGTGGTCCGATCTTAATAGGATTTT
GCATATATATGAGGAGCATAGTAAAAACTAATAAAGTGCCGAGTTTGGTCAAGATATCTTGATAAAACAA
AAACGTTTTTTTCATACAAAAAAACTTAATTTTCGACGATCGTTCCTATGGCAGCTNTATGATATAGCGGT
CCGATCCAGCTGGTTTTNACATACGTGCTGCNTGCAACANAAAGAGGGAGTTCTGCAAAGTTTCATTAAG
ANAGCTTTGAAACTGAGAGATTAGTTCGCATAGAAACAGACAGACGGACAGGCGGACAGACGGACACGG
CTATATCGACTCGGCTGTTGATCCTGATTAAGAATATATATACTTTATGGGGTCGCAGATATCTTCTTCTAT
GCGTTACACATTTCGTGACNAAATTATAATACCCTCTGCAAGGGTATAAAAAGTTCTAGATATATATAACGTC
CTTTTCAACGAGCANTGGCTCATATCGCTTCACACGATTACCATTTGGTCTCAAAATAGCACCAAACTCAT
TTCAGAGAATGGTGACTATNGCGTNCTGCGGGTCTANAACCCTCNCAGNCATTCCTTTATATGGACGACTT
TAACGNTGNTAGGATGTTCCGAAAAACACATGATTAAAAACTTAANAGACGTTTTTCGAAAAATATAATC
CAAAGTTACATCCAGAAAAATGTTCATTTTTCATGCACGAAGTAACATTTCTAGGTCACAAGTGCACTGAC

AAAGGAATNTTGCCAGATGACAAGAAATACGACGTCATCAAAAATTATCCAGTCCCACATGATGCGGACA
GCGCGAGACGATTCGTTGCATTCTGCAATTACCGTAGACGATTTATAAAA
>9_rnd-5_family-5878__LTR___Gypsy
GTTACGGATCATAGGCCATTGACTTGGCTTATGAACTTCAAGGAACCAAATTCAAAATTGGTTCGATGGA
AACTACAGCTACTCGAGTACGACTACGAGATAATATACAAGAAAGGACCCCAAAACGTAGTAGCCGACG
CCCTTAGCCGCGTCACTGGCGACGTAAATATTAACGACGTTGTTCTCCCCATAAACAAGAATAACATTAAT
ACGATTCCTATTTCCAACAAACCTTTAAACGAATTCAATGTACAGACTGTATTCAAAGTTGGAACACAAGC
TGAAATCTTGACTTCTTCCGTACCTTTCAAAAACAAAAATCAGAAGAACGATATCAGAACACAGTACGAT
AAAAAAACAATACGACTAATATTTTGAAAAAGATCCTTAAACNTAACAAAACATGCGCTATTTTTGCACC
TGACGANATTTTTTTAAAACTATTGAGGAAACTTTTAAAAGAATTCTTCCTACCAACTCAGTTATATAAGG
TAATTAGATGTGTCACGCTACTAAGAGACATAACCGACCCAAACGAACAAGAACTAGAAATACGCCAAT
ACCACGTGGCAAACAACCACAGGGGAATAGACGAGACCAGTAGCTCACCTAAGAAGATCCATATATTTCC
CGTTATTAAAGACCAAANACTCAGGAACAACAGGCAACCACAGAAACTAGAATATTCTGAAACAGAAAC
CCCGTCATGCCCTNTGCAGATAATACACACAGACATTTATGCNATTAACGGAAAAACCATCTTGACTATC
ATTGACAAATTCTCAAAATTTGCTTCAGGCTATACAATTCCGACCAGAGATAGTTTAAAACATAGTTAAAT
CCATGAGGAATTTCCTCCCTACATTCGGAATCCCGGAAAAANCTTATTTGCGATCAAGGTTCCGAATTTAC
GNAAAAATTTTTAAAGATTTTTGCGCACAATATAACATATTAGTGCATTTTACTTTATTTCAACAATCTTCG
AGTAATTCCCCAGTGGAAAGACTCCATTCGTCTCTAACAGAGATATACAGAATAATTTTAAGCAAAAGAA
ATACAGAAAAANTCAACTGCGACCATGAAGACATTTTCNTCGAGACGCTGACAACATACAATAACTCTAT
CCATTCAACAACAAAATATACACCCTACGAGATCTTCCACGGAAGACCGCACATACTAACTCAAAACGCT
TCCCAATAACGACACGACATTATCTCCAAAAACTTAACGAATTCCGAAATATCATCTACCCCGAGCGATA
GAAAANAAAACGAGAAAATAAAATGACAGAACTGAACGAAAACAGAGATTCCCCAGAAACCCTCAATG
AAGGCGACACAGTTTTTAGAAAAGAAAATAGGAGAAATAAAAATAGTCTCCAATTTTTGCGCGGTGAATA
ACTTTGAGACGACGTTATATTATTTAAATGTTTATTTATAATATTTGGATAA
>160_rnd-1_family-90__Unknown
TCGAACCAAAGACAATATAAACACTAAGATGAGTAACGCCCATACATTTGAATGCGACGCAAAATTTCTA
GCCTGGCCTTTCATCTGGCCTTTGAGGCTTCGTACGGCATGCCTGCCGACTGGTTGTTCGTTCTCCAAAGA
CCAGACGAACGAATGCCGAAGTCGCGGCTTGCTGATGCTGACACGAGCTACGAAGTTAGTCGCCAGCCTG
CCTTCAGCAAAGCTGGTCGATGCTGCTTTGCGTCGCTTCGTTTTCGAAGTACATTGAGCGAAAAAGTTTAG
AACTTACGATCGACGCAAAGTGCTTGTGCTTTATCCATGAAATGCATGCTTTATTTAAATGATATATTACC
CTAGATATTAACGATTAATTTTACAGGAAAATTTAAGAACTTTATATAATTTTTACACAAGGCGCTTCTTG
CGCAATAACTATGTTAAGAAAATAGCACACATAGATTGATTTCACTTTTCTTTTTGGACAGTGTATGCTCG
AGAAAAGTTCTTGTCTCAGCCAATAGCGTGCGTGCACCTTTCGTTGTATGCGTGAATTATACATGCATAAG
AACTTTAAAGTATGTATGTATGTGTTGTTATTCACTGCTCTGGCTTTAGCTGGCAAGAAATTAATTTTGTTA
GTTTTCTAGCGCAGATCATGACCTATCCCAAATTTGTGTTGATATATGAATGCATTTTGTGTACTGAGGTTG
GCTCCGCAGAAAATATGTATTTCTAAGTCGATGCAAATATTTACATAATTTCTTTAAATTAAATTAATATT
ATTTTTATTCTCCAATTAAGAAATAAAAGAGTGCTAAAAAAATACAATTACATTTCAAGTCGACCCGAAGG
TCATATTTAGTTATGACCCCATTATAATTATAATGGCCTCCTCGCGGTGTTCCCTGCGTACTTTGTTCTTTA
TAATTCGAACAACAAAAATAATAAAATAAAAAAAAAATAAAATTAATTAAATGTATCTTTGAAAACAACA
AATTTGCATTNNTANTTCCTAAATNTANATTANATTACATAAATATTTGCATCGACTTAAAAATACAAATT
TTTTTGTACGGAGCCAACCTCAGTACACAAAATGCATTCATATATCAACAGAAATTTTGGGTAGGTCGTGA
TCTGCGCTAAAAAACTAAGAAAATTGTTTCTTTGCCAGCCAGAGACTTTGCCAGAGCAGTGGATAACAAC
ACATTCAAATATGCTGTTATGTATGTATATTCACACGCATACAAACATAATTGCTTGCACGGCCCTCATAG
AGCCGAAGCTGAGAGCAAATTCTATTTTTAGCTTTTTCGTTCTCTCGGCTGTGAGAGCGCAATGCTACAGA
TTTCGTTTTCGTTCTCAATTTTCAAAAAATCAAGCTGCATAGTAGCATTTTGTTGTATGGCGTTACTCATCT
TAGTGTTTATATTGTCTTTGGT
>Ref_dvir_1052
CCTGGAAAGGTCATCTGTCGCGTGGGGGTGAACTAGTGGTGGTTGTTGTTTTTCAGACTTGGAATATGAAA
TCATGGTTGTTGAGCGTAATTAAATAGACAGCGTGAAATCAAAAGAGGTTAAACTGTTACGCTAAAACCG
GCGGCTCATATATCTGTTACATGACCTATCATATTGGGCGCCTCAGTCTATTCTCATGCAATATGAGACTC
TTCCCATTAAACTACTTCACTTTCAGTATATCAGTTGCTTGAGTCATCGGAGCCGAGGCAATACAAAGCGT
TCGGCAAATGGGAGTGGCGTGTCACTACTTTAAGCGGCTCCGATGCCGTGTCCTCGATTGGGATTCAGTGG
CCCGTCGATTTCTGCAAGCAGAGGAATAAACAGCTTGTCTTCGGACGTAATACAAATGCACACATTGCTC
ATTAATGTGGACGAATGGAATATCTTTTGTGAGGAGCATGTCTCTTAAATAGATAGGGTGTTGGTCCACCT

TCAAAATGCTTTCGGATGCAATAATAGATCATGATCATTTTTTTGATTTGTTAGAATGTCTATCTCGAGCG
GAATAGTAAATAGAATACAATGAAATGCTTGACACGATCTTTGTTTATCATTAAGCTGGCAACAGTGTTG
AAAGAAGAGTGAAAGTTTGTACATCTGTGTGAGCAGTAATGTCATTACTGACGCAAATCGTTAACATTTG
TTATTGAAATTCGGTGGAATTTAAAAATTATATTAATATATATTGGATGAGAAAAAACACCGGATCCCAG
CTTGTCTTCTTGGGATTATATGAAATATATTGTAGGTCTGCCTGGCCTGAAAAATGGGAGGTTGTGGTCTA
GGACCTATTTAGTGGCTGTCTTATAACCTCAAGCAACTCTTATTGCTCTTCAACTGCTTTAAGCAATGACTG
CATGACTACATAGACACCTATAAATTTTTATATTTTATTTTCTATCCTGCCTACCACACTGAAAGCCGTGTT
TTTTTGTATTGCCTTGCTGAAGGTACGCAACATAGAACAAGGCGAATTGAAAAACAGATGATAAGATAAG
GTTATATTTGCATTCGCTGAATCCATTAAAAATGAGTAAAAGGGGTATAGTGTATTTGTGCAAATGTAGGT
AACAGACGGAAGATGCTTCACAACCAATCATAACCAATATCGATGCAAAAAGATATTGAAATTCGCAAAT
ACCTGTTTCGCGTATGGGCCTGCACATTTGGTTATAGGTATATTTATCGAGCCCATAGTGAGTATCGCCGT
TAAAGTTTGCTGAACGACACGTTTATAAGCAAAAGCACTACCTGTGCGAATTTTAACCGAACAACCAAAA
AAGAATTTTAATTAAATCACAGGTAATTGTGAAAGCATTAAAAAATTAAATAACCTTCCTGCCAAATTTCA
GAATCTGTATAATAAAAACCTACCAA
>9_rnd-1_family-258__LTR___Gypsy
TGTTNAAAATTTTGTTGTCATGTTTTCGTAGGGTTGTGTGCTACTTTTATTTCTTGCTTGTTGTAGTATTCTG
AGTTCGTGAGACGTACTGATAATTGTGGTACNTGTATTNAAGACATATATATTTTGANAAGTTCTGTAGGC
TATTGTGTTCTACATTGTTGTCTATTTAAAAATGTATTAATTATATTCTGTGAGAAGTCGTTTTTGGTCAAA
TGTTGTTTAATATCGTGTCCAATTTCTTTGTGGTCTCTTGGTTAGTGCTCTGAAGATGGTTCCCGAGATTTA
GCACTCAAGGTTATATCAGGCTAGACGCCTCTGAATTCGAAAATGTAAGGTGGTCCGAGTCAAATTTGAT
TGATGCGAACAATAGGCAGGTAGTCATTAATACACAAACACAGACACAAATTAACAAACTCACCGACAC
GGTGAACGAAATAATTAAATGTAAAAGAAATTAATTGGTAAACACACCGCATTTATATGAAACACTATTA
GTAAGAAATATAATACTTATAAATCAAATATCGAACTTGATACTTACAATTACACTAGCCAAAGCGGGTA
TAATAAACCCCACAATATTTGACCACGATGATTTGAAATCAATTATAAATAAACATCCTGTCGAAATTCCC
ATAGTTAACCCAACCGAAGCGTCTAAAATCAAGGTTCTTCAGTCCGAGAACATTGTCCATATATTAATAGC
GTATCCCAGGGTCAAGCTCTTTTGCAAGAAAATCACCATCTTTCCGGTGGCACACCAACACGTCACACTAC
AGCTACAAGATGATACAATAGCCGAATGCGACAATGACATTCTAGCGGTCAATGAATGAGTAACGACGA
CCTACGCCTAAAAACCATTAACAGCTATAAAATAACCGAGGACGGTCAAAAGCTTGAGGGGGGAGTAGT
TAACACATAAGAATTNCACTTCATATTTATGTCATAAGAACACACTTTTGTTTATAAGAACGCAAGAATGT
TGCAGTGCACTTTGCTTACATTGCGACATTCTGCTGACGCATGAGTTCAGTGCCCGGCCACTGCGGCCAGC
AGCGGTAACCTGACACTTGCCTATGTCGCAAGCGACAGTGTTGGCGTATGCTGANTCGGCTTGCCGGATA
TAGCAGCTGAGACTTATGCGCCTAGCCTTTGGGTTCAGATCCAATTCAATGTTTCAATTCTAGTTTAGTTTT
GTATCCAACTTTGTAAGAACAACTTCTTCAAATATACTGCTCCGGCACTTGCCGTCATCAATCCGTTATAC
TAATTGTTTCATTGCTGAGCCACAAGGCCAGTGATTTGGAAGGCTAGTTGCTTTCCGAACATAATCTACAA
GGAATATAACCTTACCAATCTCAGCACCTGTGACGAGGATCTGAATATACAACATCAACATCTACAATTG
AGAAATCAAATAACTTGCA
>Ref_dvir_640
ATGTACGCATGGTGTTGGAGAATTTCACAAGGGAATTTCACCATCGAATTTCACAACTTGAGCTAACAGT
GCGATTGCTGTTGCGGAGAAGCAATTCACAGAAGAGTTGGCATCATTTTGTACGAGCACTTCGTAGACAA
AAATTGAAAAGAGATAATAACAAATTTGGAAATATGCAAAGGATAGGAGATTTAAAAGTTGATCAGCTA
AAATTCCTGTTGCGAGAGCGAGAATTGTCGACAAGTGGCACACGGGATGAATTAATTCGTCGATTACAGG
AGCATGAAGGCTCAGATGAAGTTGAAAGGCCACATGCCCTATGGAGAAGAGGGAGGATCACTTTATCAG
CTACAGAATGAAATAAAAAGGTTAAGCGAAATTTGTCAGTTTATAAGCATGCAATGCGCTAGTGCTAGTG
GACAAATGTCGAATTCGCAACCGGCTATAACAACTTAGTGCAAGATACAGAAAGCGAGGTGCATGATGA
ATATCAACAAAGCACAGCCACACGTCAAGCTTCGGTCAAAGAAATTGCTAACACGTTGCCTGAGTTTGAC
CCGACAGATTAGAATGCCATTTCAGTCAATCAGTTCATTGATCGTGTCAACAAAGTGGTTGAGGCATATCA
ATGGGACGAAAAGTTTTTAATGCTCGCAATCTACACTCGACTCAAAGGTCCAGCCAGAACCCGGTTTGAC
TCTTCGCCAAGACCTAATACGACATGTGCAGACGCACTTCGCGACGAATTTGGTGTTACACCAGATGAAG
CAGAAATCCACTACGCCATGGCAAAATCGACAAGACGTCCAAAAGAGACAATGGTCTTCAGCACCGTGA
ACTGCAGCAGAGTATCGCAGCAATGCACTTCTCAACAATGAAGCAGATGCGAGAAACGGCGGAATCCTAT
TTTGCCAATCGAGGTAGACAACATGAAGTCAGAGACAGCACAGCTCTAAAGTATAGCAACGTTGAACAA
AGGCCTGAGAAAGATTCAAAGACAATGAAACCAAAGGCAACACTCAAATGTTATAATTGCAACGAGTTT
GGTCACTTTGCAAATCAGTGTCCAAAAGAACCGAAATGTCAGCGCTGTACCAATTGCAACAAATTCCACC
CGAGTAACGTCAAGTGCAAGTCAGCTGAAGTAAATGTAAATCGTCTTGGTGCCATAAATAGAGACAAATG

GTTCTCTAAACAGATAATCGTCCAAAAACAACCGTACGTCGCTTTTATAGATACTGGTAGCCAAGTGAGT
ATGGTTCGCAAGTCGGTTGCTCATTATTGGACAAGTATACTGATGTGTTTTCAAGTGGTCTAGAAGGAATT
GGCCGCACAAACGTCATACAAGCCAGAATTACCCTTGACGGAGGACATGTTGTTTCTCAAGCACTATACT
GCATACCTGAACCGAAATGAGATATTGTCACCGTCAT
>9_rnd-1_family-67__Unknown
ACCAAAGACAATATAAACACTAAGATGAGTAACGCCCATACATTTGAATGCGACGCAAAATTTCTAGCCT
GGCCTTTCATCTGGCCTTTGAGGCTTCGTACGGCATGCCTGCCGACTGGTTGTTCGTTCTCCAAAGACCAG
ACGAACGAATGCCGAAGTCGCGGCTTGCTGATGCTGACACGAGCTACGAAGTTAGTCGCCAGCCTGCCTT
CAGCAAAGCTGGTCGATGCTGCTTTGCGTCGCTTCGTTTTCGAAGTACATTGAGCGAAAAAGTTTAGAACT
TACGATCGACGCAAAGTGCTTGTGCTTTATCCATGAAATGCATGCTTTATTTAAATGATATATTACCCTAG
ATATTAACGATTAATTTTACAGGAAAATTTAAGAACTTATATAATTTTTACACAAGGCGCTTCTTGCGCAA
TAACTATGTTAAGAAAATAGCACACATAGATTGATTTCACTTTTCTTTTTGGACAGTGTATGCTCGAGAAA
AGTTCTTGTCTCAGCCAATAGCGTGCGTGCACCTTTCGTTGTATGCGTGAATTATACATGCATAAGAACTT
TAAAGTATGTATGTATGTGTTGTTATTCACTGCTCTGGCTTTAGCTGGCAAGAAATTAATTTTGTTAGTTTT
CTAGCGCAGATCATGACCTATCCCAAATTTGTGTTGATATATGAATGCATTTTGTGTACTGAGGTTGGCTC
CGCAGAAAATATGTATTTCTAAGTCGATGCAAATATTTACATAATTTCTTTAAATTAAATTAATATTATTTT
TATTCTCCAATTAAGAAATAAAAGAGTGCTAAAAAATACAATTACATTTCAAGTCGACCCGAAGGTCATA
TTTAGTTATGACCCCATTATAATTATAATGGCCTCCTCGCGGTGTTCCCTGCGTACTTTGTTCTTTATAATT
CGAACAACAAAAATAATAAAATAAAAAAAAAAAAATTAATTAAATGTATCTTTGAAAAANAATAATTTAA
TTAATAATTTCAAATATAAAAAATTACATAAATATTTGCATCGACTTAAAAATACAAATTTTTTTGTACGG
AGCCAACCTCAGTACACAAAATGCATTCATATATCAACAGAAATTTTGGGTAGGTCGTGATCTGCGCTAA
AAAACTAAGAAAATTGTTTCTTTGCCAGCCAGAGACTTTGCCAGAGCAGTGGATAACAACACATTCAAAT
ATGCTGTTATGTATGTATATTCACACGCATACAAACATAATTGCTTGCACGGCCCTCATAGAGCCGAAGCT
GAGAGCAAATTCTATTTTTAGCTTTTTCGTTCTCTCGGCTGTGAGAGCGCAATGCTACAGATTTCGTTTTCG
TTCTCAATTTTCAAAAAATCAAGCTGCATAGTAGCATTTTGTTGTATGGCGTTACTCATCTTAGTGTTTATA
TTGTCTTTGGT
>9_rnd-6_family-70__RC___Helitron
GTGCAAATTGANTAANTGTACGATTNTTTTCATACCCTCGNAGAGGGTATTATAATTTTACATAGAGACCT
GTGTAACGCATGAAGGAAACATCTNCGACCCCACAAGTGTANATCTATACGTATTATATAAAATCATATT
TTAATTAATATTTTTGATAAGACCGCAAATTGACTAAATGCGAAAACTGTGTTTTTATACCCTTGCAGAGG
GTATTATAATTTTGTCGCGAAATGTGTAACGCATAGAAGGAGACATCTCCGACCCCATGAAGTATATATAT
TCTTGATCAGCATCAACAGCCGAGTCGATATAGCCATGTCCGTCTATCCGTCTGCTGTCCGTCTGTCTGTTT
CTATGCGAACTAGTCTCTCAGTTTNAAAGCTATCTTAATGAAACTTTGCAGAGNTTCGTCTTTCTGTTGCAC
GCAGNACGTATGTCAGAACCGGCCGGATCGGACCACTATATCATATAGCTGCCATAGGAACGATCGGTCG
AAAATTAAGTTTTGTATGAAAAAAAACATTTTGTTTTTAAGATATCTTGACCAAACTCGGCATTTATTAA
GANTACATTATACTCCTACGTGTATGCAAAATCCTATTAAGATCGGACCACTATATCATATGTATCGACAT
AAATTNTCGGTCGAAANTTAAGTTTTTGTATAAAGCATGTTTCGTTTAACAAGATATCTTGACCAAACTCG
ACATTTATTAGTTTNACTATACTCCTCATATATGGTGCAAAATCTATTAAGATCGGACCACTATATCATAT
AGCCGCCATAGGAACGATCGGTCGAAAATTAGTTGTTGTATGAAAAACATTTTGTTTTTCAAGATATCTTG
ACCAAACTCGGCGTTTATTAGTTTNACTATAAATCTNCTCATNTATGTGCTAAANTCNTATTAGGATCGGA
CCACTATATCATATAACCAAGCNANAGGAACGATNGGNCGCAAATNAAGTAATTGTGCGATATGTNTTNG
TACCCTTGCAGAAGGCATTATAATTTCGACGCGAGACTTGTGTGACGNNTANAAGNAGACANCTTCGACC
CCACNAAGTATATATTTATACGTATGTNTGAAATCATGTCTTGATCAGTATCTTGATAAGGCCACAAAACA
ATGCGAAACTGCACGTTATTATGCCCTCGCAGAGGGCATTANAATTTNGTCGCGAAATGTGTAACGCATA
GAAAGCGACGTCTTGACTCCATTAAGCATATATATTTTTGATCAGTATCAACTGCCGGCTCGATATAGCCA
TATNCATCTNTCCAAGTCTGACCACCCGTCCGTCTGNCTGTCTTGGCGTGAACTAGTCTCTCAGTTTGAAA
GTTATCTTAATGAAATTTGCTGAAGTCCTTCTTTCTTTTGCACGCAGCACATATGTCAAACCAGC
>9_rnd-1_family-331__Unknown
CATNGAGTAATAAANAGAAAGAGANGGCGTTTGGATCAAAAGCTCTTTTTAGAAATACTTCCTGCGAGAN
AACGAGGTTGCAAACAAAAATTGCAAACACAACACCCGGCTGTCTGCAGCTACTTCTGCCGATGCCCACA
ATTCAATGACAACTTCAAAACATTTTCCATAAGAACCAACAACAACCATAATCAAAACAAACGACTCGAC
AATGAAAAAAGCACACCACAAATTCTGTCCTTGCTATCCCTTTTTGTTTGAGGCTACAGCCTATGATCTTA
GCTTCTGCACCCTTCTAACTCATTGTTGTGGTTTCTAAGCACTGCTATGCCTATAATTCGCAAAGTCATGTC
TTCTTCTTCTCCACTCGCTTTCAACTTGCAAGCAGATCGGCAGATTTTTATAATTTTTCATTTTTCGACCAG

CAAGGCACCTTTTCTCAAAATAGGGTATTTTAACTATTCATAAGCGATGTAACCAAATAGCACAAACCNTC
TTATATATACATTTTTATCCAATGTCTAAAAAATATAATTTCGTATTTAGTGTGCAACTATTAGTACAGTCA
ATAATAATTGTTTTCTTNATAAATTATATACCATTCTTATAAAAAAGGGTATCAATGCCGCGACCCGCCAA
ATAGTTGTTGTATGTGTGTCTCATCTCACACATATTGGTGCAAATCTGCATATATGTATGTGTCTGCCAAGC
CAAGGGAAGTCTATGAATTTAAGAAGATTGCAACGTTCTGTTGCGCATGTGACGTCACAGTCCAAGGCTT
TTTNCGTGCGATCATTTCGTATATAAAATGATTACATATGTCAATCAATTATTTGCTATAAAGGACCCTATT
CATAAATTATTTAAATGTACGTAATAAAGGTAGAAATATATACTAAACATCACAAGAAATAAAATTCTAT
NAACTGTGACGTCATAATATATGATGTTGGCGCGCCGTGCGCGTAACCCTTTGATCTGAATGCCATGGCCT
GCTGGCATTCAAATTTTTTCTACCTACTTGCGCCGAGACTCGGCTCTGCAACAACAACATTAGTACCCAAA
TTGTGCTGTTGTTGTTTCTACGCGCTGCCTTGTAGGAACGACAGAGAAACCCGCAAAGAACACAGTGGCA
TTATCTGTCCCTTTTCTTTGCACTGAGTTTCTATGTCCTCATCATGGCTCACAAACCTCTTGTCGATCAAAA
CGGGGGATGAAGAACAAATTGGGTTTCGTGCGCACTGTTGTTGTTTTGCAAGTGATTTGTTGTTGCCTTCC
TATGGTAAATGAGCTTTTTTTTTGGGTTTGATTTTGCGTTTGCGGACCAACCCGATCTACGTCTCGTGGTTT
TTAAAACGGTAAAAAAAGCTCAGAGTGTCAGTTCAGCTCTTTCTCTTTATTACTCAATG
>160_rnd-1_family-32__RC___Helitron
TTTTTATACCCTTGCAGAGGGTATTATAATTTTGTCGTGAAATGTGTAACGCATAGAAGGAGACATCTCCG
ACCCCATAAAGTATATATATTCTTAATCAGCATCAACAGCCGAGTCGATATAGCCATGTCCGTCTGTCCGT
CTGTCTGTTTCTATGCGAACTAGTCCCTCAGTTTTAAAGCTATCTTAATGAAACTTTGCAGAACTCCCTCTT
TCTGTTGCACGCAGCACATATGTGAAAACCAGCTGGATCGGACCACTATATCATATAGCTGCCATAGGAA
CGATCGGTCGAAAATTAAGTTTTTGTATGAAAAAACATTTTGTTTATCAAGATATCTTGACCAAACTCGGC
ATTTATTAGTTTTACTTTACTCCTCATATATATGCAAAATCCTATTAAGATCGGACCACTATATCATATAGC
TGCCATAGGAACGATCGGTCGAAAATTAAGTTTTTGTATGAAAAAACATTTTGTTTTTCAAGATATCTTGA
CCAAACTCGGCATTTATTAGTTTTACTTTACTCCTCATATATATGCAAAATCCTATTAAGATCGGACCACTA
TATCATATAGCTGCCATAGGAACGATCGGTCGAAAATTAAGTTTTTGTATGAAAAAACATTTTGTTTTTCA
AGATATCTTGACCAAACTCGGCATTTATTAGTTTTACTNTACTCCTCATATATATGCAAAATCCTATTAAG
ATCGGACCACTATATCATATAGCTGCCATAGGAACGATCGGTCGAAAATTAAGTTTTTGTATGAAAAAAC
ATTTTGTTTTTCAAGATATCTTGACCAAACTCGGCATTTATTAGTTTTACTNTACTCCTCATATATATGCAA
AATCCTATTAAGATCGGACCACTATATCATATAGCTGCCATAGGAACGATCGGTCGAAAATTAAGTTTTTG
TATGAAAAAACATTTTGTTTTTCAAGATATCTTGACCAAACTCGGCATTTATTAGTTTTACTNTGCTCCTCA
TATATATGCAAAATCCTATTAAGATCGGACCACTATATCATATAGCTGCCATAGGAACGATCGGTCGAAA
ATTAAGTTTTTGTATGAAAAAACATTTTGTTTTTCAAGATATCTTGACCAAACTCGGCATTTATTAGTTTTA
CTNTGCTCCTCATATATATGCAAAATCCTATTAAGATCGGACCACTATATCATATAGCTGCCATAGGAACG
ATCGGTCGAAAATTAAGTTTTTGTATGAAAAAACATTTTGTTTATCAAGATATCTTGACCAAACTCGGCAT
TTACTATTTTCCCGGTACTTCTTAGATAGGGGCAAAGCACTATGAGCATTATGAAAAGGTTGCGGTGCTGC
AAGGGTATTAGATCTTTGGCGTGCCGAAGATAGCCCTTCTTTCTCGTT
>Ref_dvir_548
CTGTAGAATCCAACATACTCCCCCTGTTGGAAGCCTGACTTTCAACAGAATCCTTAAGGGCAGCAGACAC
AGCTTGTTTACGGCTCTTCGGATGATTCCAGCCGTGGTCTTCAGATCAGCTACACGATTGACTCCATCCTTC
CCAGGAATCAAGTTAATTACCCTACCAAGCGGCCACCGCATGGGAGGCAGATTTTCGTCTTTAACGAGCA
CCATATCGTTGATGCACAACGCTTGTGTTTGAGTGCGCCATTTAGCTCGCTGTTGTAGCAAGGTGAGGTAC
TCTTCACGCCATCGAGCCCAGAACACCTGTTGTAGCTGCGTCACACGCTGCCAGCCATCCAAACGGTCGTA
GTTTAATTTGGTCAAGTCTGGCTCGATGATGGCTGATGAAGGACCACCAAGCAGTAGATGAGCAGGAGTC
AGGACATCTAGATCTGCTGGGTTCTCTGAAAGCGAGAGTAATGGGCGTGAGTTAACAATAGCTGTGATTT
GGCAAACAAGAGTACGAAGCTCTTCAAAAGTAAGTAAAGAGCGGCCGACAGAGCGATAGAAATGGTGCT
TGGCCAACTTCACTGCCGCCTCCCATAGTCCACCAAAGTGCGGAGAACGGGGGGGATGAATTTCCACTCG
ATGTCATCGGCGAGGCAGAACTGCCGAACTGCTTCCATGTGGGGATCGCTAAGCAAGACCAGCTCATTCT
TCGATCCGACGAAGTTTGGGACCAAATTTGACTTTAGAAGGTCCGCAATAATCGACTCCAGTTACGGTGA
AGGCTCGAAGACTTCCAGACGTTTTGGAAGACTTGCCATCATAGGCTCGACAAAAATAGGCCTCGCCTGA
AGCACCTCACACATCTACTGACTGCTTTAGAGACTGTTCTTAATCCACCAATCGGCCAATATTTAAGTCGA
ATTTTGGCTAGAAGTGCACGGGGACCAGGGTGTAGATTCCTCTCATGAAACTGAACGATGACAGCTAAGG
TGATTGCATGACGCCGAGGCAGTATAATGGGGTGTTGCGCATCATAGTCCAGGGACGAATTGCTTAGCCG
GCCACCGACGCGGAGGAGGTCACGTTCATCCAGAAATGGTGCAAGAGATGCAACGGAGCTGGATTTTTTC
ACATTTCCATGAGAATGAATCTCCTTGATGTCATCCCACAGAAGAATGCGAGTGCCGTGATGAATGTCTGC
AACAGTGAGTCCATGATGCCTTATACGCTTCATAAATTTGTACACATAGCCGAATGTGCGCTGCATGGAAC

CAAAAGAGTTGGCGTGCTTCATCCAGATAAATCTGCATGAGCTCCGACTGGGCTAGGAACAGTGCCTCTA
GAAAGAATGTCAGCTGGATTTCGGTCCGTCGGAACATATCGCCACTCCATACTGACAGTGAGT
>160_rnd-1_family-97__Unknown
ACCAAAGACAATATAAACACTAAGATGAGTAACGCCCATACATTTGAATGCGACGCAAAATTTCTAGCCT
GGCCTTTCATCTGGCCTTTGAGGCTTCGTACGGCATGCCTGCCGACTGGTTGTTCGTTCTCCAAAGACCAG
ACGAACGAATGCCGAAGTCGCGGCTTGCTGATGCTGACACGAGCTACGAAGTTAGTCGCCAGCCTGCCTT
CAGCAAAGCTGGTCGATGCTGCTTTGCGTCGCTTCGTTTTCGAAGTACATTGAGCGAAAAAGTTTAGAACT
TACGATCGACGCAAAGTGCTTGTGCTTTATCCATGAAATGCATGCTTTATTTAAATGATATATTACCCTAG
ATATTAACGATTAATTTTACAGGAAAATTTAAGAACTTATATAATTTTTACACAAGGCGCTTCTTGCGCAA
TAACTATGTTAAGAAAATAGCACACATAGATTGATTTCACTTTTCTTTTTGGACAGTGTATGCTCGAGAAA
AGTTCTTGTCTCAGCCAATAGCGTGCGTGCACCTTTCGTTGTATGCGTGAATTATACATGCATAAGAACTT
TAAAGTATGTATGTATGTGTTGTTATTCACTGCTCTGGCTTTAGCTGGCAAGAAATTAATTTTGTTAGTTTT
CTAGCGCAGATCATGACCTATCCCAAATTTGTGTTGATATATGAATGCATTTTGTGTACTGAGGTTGGCTC
CGCAGAAAATCGACCCGAAGGTCATATTTAGTTATGACCCCATTATAATTATAATGGCCTCCTCGCGGTGT
TCCCTGCGTACTTTGTTCTTTATAATTCGAACAAAAAAAATAAAAAAAAAAAATAAAAATTAATTAAATG
TATCTTTGAAAACAACAAATTTGCACTTTTATTTCCTTAATTTATTTTGTATTACTTAAAAATAATAATAAT
TTAATTAATAATTTCAAATATAAAAAATTACGTAAATATTTGCATCGACTTAAAAATACAAATTTTTTTGT
ACGGAGCCAACCTCAGTACACAAAATGCATTCATATATCAACAGAAATTTTGGGTAGGTCGTGATCTGCG
CTAAAAAACTAAGAAAATTGTTTCTTTGCCAGCCAGAGACTTTGCCAGAGCAGTGGATAACAACACATTC
AAATATGCTGTTATGTATGTATATTCACACGCATACAAACATAATTGCTTGCACGGCCCTCATAGAGCCGA
AGCTGAGAGCAAATTCTATTTTTAGCTTTTTCGTTCTCTCGGCTGTGAGAGCGCAATGCTACAGATTTCGTT
TTCGTTCTCAATTTTCAAAAAATCAAGCTGCATAGTAGCATTTTGTTGTATGGCGTTACTCATCTTAGTGTT
TATATTGTCTTTGGTATTACCTTGTCGGGAGCTCCCGACTAGAGGTCTT
>9_rnd-1_family-14__RC___Helitron
TTTTTTATACCCTGAACCCATTAAAAATGGGTATAAGGGTATATTGTATTTGTGCAAAATCCAAATGTATG
TAACAGGCAGAAGGAAGCATCTCCGACCCCATAAAGTATATATATTCTTGATCAGCATCAATAGCCGAGT
CGATCTAGCCATGTCCGTCTGTCCGTCCGTCCGTCCGTCCGTCCGTCCGTATGTATGAGCTCAAGGATCTC
AGAACCTATAAGAGCTAGAGACTTGAAATTTTAGATGTAGATGCTCCTAGTTCCCGCGCAGATCGAGTTT
GTTTCCGATAATCGATAACTTACTCCGTTTCCAAGCAATCGATAAAAATCGATATCGACATCCTGTTTTTT
AGCAAATTGGGTAAATAATAAGAGCTAGAGCCACCAAACATGATATGTTGCTTCTAGAATATTATAGATA
TTTCAAGTATCTTTCATTTCATACCTATCGCCACCTCCCCGCTACCACCNCAGAGCTATAAATCAAGTTAAT
AACCCAACTTTTTATTGCCAACCAATTTAAACCACAATTTAAATGCAATTGACTTTTTCAGAATACACAAA
TATTCTATGGTACAATAAGATATACTGTAGAAAATTTCATAAAGATCGGTTAAGAAAAAACCAAAGTTAT
ATGCAACTGCGCAATTGAATGGGGCAGCAGCTTTAAGAATTTCTGTATACATGTACACACACACATTCAT
GCGCGTCTGCTTCGCATTCTAATAGCATGGTAATTGCATCTTTTTTTGTAATGTTATTTTAGTTCCTTTTTTT
ATCATAAGTACTTAATTGTTGGTGTGGCTGCTGAGTAGAGGCATAGCAAGTGGTGCGGTCTGTCGCGAGTT
CGAGCCCTACCGGGGAAAGTATTATTATTTTTTGCTGGTGTGGCTGTTGAGTAGAGTCGACAGTAAGTAAT
GCGGTCAGTTGCGAATTCGAGCCCTGCCAAGGGAACTTTTTTTATTAATTTATTTGGTGTTGGAATAAGAG
GGTTCAGGGTATCCCCTAGTCGGGAGCTCCCGACTAGAACCTCTTGCTTTATTAATTTATTTGGTGTTGGA
ATAAGAGGGTTCAGGGTATCCCCTAGTCGGGAGCTCCCGACTAGAACCTCTTACTTGTTTTATCATAAGTA
CTTTATTGTTGGTGTGGCTGCTGAGTAGAGGCATAGCAAGTGGTGCGGTCTGTCGCGAGTTCGAGCCCTAC
CGGGGAAAGTATTATTATTTTTGTTTGCTGGTGTGGCCTGTTGAGTAGAGTCGACAGTAAGTAATGCGGTC
AGTTGCGAATTCGAGCCCTGCCGAGGGAACTTTTTTTATTAATTTATTTGGTGTTGGAATANGAGGGTTCG
GGGTATCCCCTAGTCAGGGGCTCCCGACTAGAACCTCTTACTTGTT
>160_rnd-4_family-309__LINE___CR1
GTGGTCCTGCTTTTTTTTTAAATATTTTGAATTNTCCGTAAACACTTAATCTTTTCCGACCATTTGNAANGA
ATCGTATATTATTCCTTCCATNAGAAAGGTAGTAAGTCTAACGTCGAAAACTATAGAGGTATTTCCAAATT
ATCCGCTATACCTAAAGCTTTTAAGAAATCGTAACATCTCAACTGCAACATTTATGTAGCTCTATTATTTC
ACCNTCCCAGCAGGGTTCGTGAGGCAGATCTACAACCACTAACCTTCTTGACCTTACTTCGTTTGAAGNAA
NTGATGGGTTTAAGAAGAGAACGCNGACTAGCGTCGTCTNTACTGATTTCAGTAAAGCTTTTGGCTCTGTC
AACCACTCCTTGCTGCTTCANAAATTATGCCTTCTCGGCTTCCCAGTCAATCTTTTGAACTGGATTTCAGAG
CATTCGATGATAGANCCAAAAGGTTNTTTTCAGAAATTCATGTTCTAAACGTATTCTACTGATTTCCGGTG
TTCCTCAAGACAGTCATCTTGGATCGTTACTCGTTACTCTTTTCTTACGGCCTTCCCTCGATANTGTCTCAC
TCTCGAGTATTAATGTATGCTGAAGATGTANAGTGATGCCTTGCATATAAAAATATGGCATCATGTTATCT

NTTACAAGCCGACATTGATGCCTTTCAGACTTGGTGTCTAGCCAACGTTTCTTACATCCTTAATNGCTTACC
GCTCGAGCGTGTTAACAGTACTTCAAATGAAGTTACTGATCTAGGTGTCATCCTTGATCATAAACTTAAAN
NCGANTCTCATGTTAAGACTGTGGTCAATAAGGACGGAATNGTTTTGGGCTTCATCAAGCGATGNTCGAA
GGAATTCGACACCGTACACGACAAANNCACTTTATATATCTCTATCGGCCTATTCTTGAGTATGCCTCATN
TNTCTGGTCGCCACAATAGCTTTCCATCAGGACAGAATTGAATCTGTACAGAAACAGTATTGCTGTTTGCC
CTTCGTNGATTAAACTGGGATCCTGGTGTGCGGTTACCGTCATANTCCAGCAGACTTCNGATGATTAATCT
TCCCTCTTTAATTAATCGTAGAACTATGCTTGGTGTAATATTTATTCATAAGNTAATTACGGGCGAAATCG
ACTCTTCTGNGTTGACTAGCCAATTAAACTTCTCTGTCCCTACTAGACTAACACTGTACACCTTTATCGCTT
CNATTTTGTTNAACTAACTATGCCTTGCATGAACCGTTCAGGGTTCTCTGTGATGATTATAACAAGCATTA
CAGAGTATTCTGCTCGGAGTCTTCCCTTCCTCGTATAAAGTCNTCTATTCTNAACTATACATTCATGTNAAT
TTATTGTTCTTTGTCTCTTGGTGCTAGTG
>Ref_dvir_608
CTAGAAGCTAATTGACACGTACGACTGGCTCAGGCCTAACTAGACTATTACACCAGAGACTTTTCAACCTT
CCGAAAAGAAGTTGCCTTCATGGAACAAACCAACTGCCAAAACTTCCACTACCAACTGCACGAAGACACC
CCTTTTCGCATTGTCATCAAACAGATGGCGTCTTCTGCGGCCTAAGGGCCCAAGGCCATATAATTGCAAGA
TAAAGGACACAAGGTAGTAAATATCTATAACCCTAAAGGCAAGGCCACAAATGGCACGCAGCAAAGCAG
GCTACATATGTGACATCAAACCGGCCCCCAACAATAAGGAAGCGCTGAAAATAAACCTTATATGCCGGCA
AGAGTCTTAGTGCAGCGCCCCGCCGATCTGCTGACGTTATCCAATGCCACCGCTGCCAAGATTATGGACAT
TCAAAGAATTACTGTATGAAGAAATTCGTCTGTGGTAGATGTGCTGAAGAGCACCCAACAGGATCTCCTC
CTTGCAAAGCAGTCCAGGAAGCCGACTTCTTCTACGCCAACTGCGGCGGAAACCACTCTGCCAATTACAA
AGGCTGTAAATGGTACATAGACCGAATCAAGGTACGCAAGCCGGCCCCCAATATCTCGGCTCCCTCCACA
AGGCGCATATAACCACGCTCGGCAAAATAGGAATGCAATGCAATTTATCCCTCCTCCCATGGTAGCTGGC
AAATCCGTATCATTTGCGGACATGCTCAGATCGGGCAACCAACAAACAGCCAGCGAAGAGACACCACCC
AACGCATACCGGATGAAAAAGCGTACGAAAGGCAGCCCAACAACATTACCGGAAACCAACCGAGCGACG
ATACTAACTCGAGAATTGCGGCTCTTGAAGAGGCTATGCTAAGCCTTACCTAAGAATGGACCAGATCCTT
AATACACTTCATAAAAACTCCGAAACTCAGCACATCCTGATGTCGCTAATTTCTCGCCTTATCCCATCAAA
ATGAGCCAATTAAGGATCGGATTATGGAACGCGGATGGTCTTAATAGAAAAATCATTGACATAATGCTCC
TAACGGAAACGCACATGCACATGTGCTCAAAAATATATGTCCCCGGTTATACTACATATATAACTCAACA
TCCCTGCAACTACCGCAGAGGAGGTGCCGGCATCTTAATCCGCCATAGCCTGAGCCACCACCCCCTGGAA
CACATTTCCCAACCACATCTGCAGATGGCCGCAGTCAAGATAAACACTAACAGAGGAGACTTCACAATTG
CTCTCTATACCTCCCGCCCGGCTGTGCCATGGACCAAAGCAAATTTTGAGTTCCTAATAATGAACTTAGGA
CAAAAATTCATTGCCGGAGGGGACTGGAATGCGAA
>160_rnd-5_family-2104__RC___Helitron
AAGAAACTGAGATACAATCAAATATTTAGTATAATTTCTGGATAGAATATCTCTTGGCACATTTNCGCAAT
TTTATGTCGTTTGATTAAATATTTTTATACCCTGAACCCATTAAAAATGTATGTAACAGGCAGAAGGAAGC
ATCTCCGACCCCATAAAGTATATATATTCTTGATCAGCATCAATAGCCGAGTCGATCTAGCCATGTCCGTC
CGTCCGTCCGTCCGTCCGTAAGAGCTAGAGACTTGAAATTTTAGATGTAGGTGCTCCTAGTGCCCGCGCAG
ATCGAGTTTGTTTCCGATAATCGATAACTTACTCCGTTTCCAAGCAATCGATAAAAATCGATATCGACATC
CTGTTTTTTGGGCAANTTNGGTAAATAATAAGAGCTAGAGTCACCAAACATGATATGTTGCTTCTAGAATA
TTATATATNTGTCAAGTATCTTTCATTTTATACCTATCGCCACCTCCCCGCTACCACCCCAGAGCTATAAAT
CAAGTTAATAACCCAACTTTTATTGCCAACCAATTTAAGCCANAATTTAAATGCAATTGACTTTTTCAGNA
TACACAAATATTCTATGGTACAATAAGATATACTGTAGAAAATTTCATAAAGATCGGTTAAGAAAAAACC
AAAGTTATATGCAACTGCGCAATTGAATGGGGCAGCANCTTTAAGAATTTCTGTATACATGTACANACAC
ACATTCATGCGCGTCTGCTTCGCATTCTAACAGCATGGTNATTGCGACTTTTTTCTGTAATGCTATTTAAGT
TCCTTTTTTATCATAAGTAATCGACAAAATGGCAAGGGGTTACATCACGTTTTTTTTTCAAAATCGAGGTC
GGTGCGAAAATCGAAACTTTTTCGATGATTTTTTTTTAATATCTCGGGTAATAGCTCCGCGCGGAGATATG
ACGTTCCAAAACGACATAACTCCGCGCGGAGATATGACGTTCCAAAACGACATAACTCCGCGCGGAGATA
TGACGTTCCAAAACGACATAATATCCGGGTATAGCTCCGCGCGGAGATATGACGTTCCAAAACGACATAA
CTCCCCGCGGAGATATGACGTTCCAAAACGACATAATATCTCGGGTAATAGCTCCGCGCGGAGATATGAC
GTTCCAAAACGACATAACTCCGCGCGGAGATATGACGTTCCAAAACGACATAACTCCGCGCGGAGATATG
ACGTTCCAAAACGACATAACTCCGCGCGGAGATATGACGTTCCGAAACGACATAACTCCGCGCGGAGATA
TGACGTTCCGAAACGACATAACTCCGCGCGGAGATATGACGTTCCGAAACGACATAACTCCGCGCGGAGA
TATGACGTTCCAAAACGACATAA
>9_rnd-5_family-8__LTR___Gypsy

TATTTTCTTTNTACTTTTGATCTCTCCTAAATATACTCAATGGCTACGAACAAAGCGATAGCTGAAGTTTAT
TTACGAGACCCATATTATTAGGGAGAAAGAGTTCAGCAATTAGTAACTGCAACTCCAATAAGTATATACG
CAAAAGGAATAGTATTAAATAACGCTCCCGGTATACAAGTGAAATCGGTAATTAAAGAACACCCAATACT
AGGNAGAACGTTAGATTTTGTAGCATTAAAAAAACAACAACAATTAGAACACCCTATTTTAGGACCCATC
GACATTATTATTGTGCGTTTGCACAGATAAATTAAAAATGTCTATAAATGGAATGGACGCAAATCGTTACC
ATTCTTAGGGAACAACGTGAGGAGTTTAATAAATCTTTCAAGTGCCTTAACAAAAGCGCACCTGTCTCAA
ACGAGACAAAGTTAAAACACGTTAAAATTCTTGTAGATTCCTACAATATCAGCCGTCAACTTATATGTGAC
AATGCAACAAAACTAAAAGATTTGCATTTAACACAGACAGTTGACGTATTAAGCACGTTAAGAGAAAAAC
ATTAGACGAATTTCGGAAAGGCATAATATAACAATATTCCTTCCAAATTCTCTTAACACTTCAGCGTCATT
CGACGAGAAACAACTTTCGTTGCNAGCTGATATTAAACCATCAGATTCTGAAATAAATACCCATTCAATT
GACAACACAATGGCCCAAACAGCTATAAGTTTTCTCAAACTNGCCTCATCGCTCTTACCAGAGTTTGATGG
CAAGGCAGAAAACTTACAAAGCTTCAAAGATGCTTTGAGTCTTATAGACTCACTAAAAGAAGATCATGAA
GCAACAGCTGTCAGTCTTATAAAGACAAAACTAAAAGGCCATGCACGAAACTTGATAAGCGATGAAAAA
ACAATCGCCGAAATAATTGCAAAATTATCAAGAACGGTAAAAGGAGAATCAGTTGAAGTACTATCAGCTA
AACTCCTAAGCCTACAGCAAAAGAGCAAAACTGCCAACACTTATACACAAGAAGTTGAGCAACTGGCAA
AAGCTCTGGCAGGTGCTTATATTAATGATGGCTTAAATTCAGATTTAGCCACCAAATACAGCACAGCCCA
AGCTGTAAAGGCCATGACCAAGAACTGCGCTATAGATAAGGTAAAACTTATCATGCAGGCAGGCACATTC
CATAACATGAATGATGCCATATCAAAGTTTGTCGATAGTTGCACAGAAGCAACCGGACAAACTAATACTG
TCCTTTATTACCAAAATTACTCTCATCGTGGTGGAAACCGCGGTAGAGGTAATTATAGAGGCCGACCATAC
AACAGAGGCGGCTACAATAATAACAATAG
>Ref_dvir_805
AGTAAAATACTGACATTTACAGAGTTTGTCTAATACACTATATATTGGTGGTATAGTGTATTTGTCATAAG
TCCTTACGTCGTTAAGATTTCGATAGTCAATGACTATCCTAAATTTTTGTTTGCCGGATGCATCCCTCTTTT
TGGGTATGATCCAAATTGGCGAACAGTAAGGGGATTTCGACTTGCGAATTATCCCTTGTTTGATCATATCT
TTGATCTGGTTTTCGACTTCTTGGTCGAATGTTTGGGCATATTTGTATGGTTTTTTATAAACCGGATCTTCA
TGTATGGTTTGGATGACATGTTTAACCGTACTTGTGAAGGTCAAATTTTCACCTTCTCGGTATTGAATATCA
CCAAATTCATGTAAGATTTTTGTTAGTTTACCTTTCTCTTCATCGTTTAGATGATCAAGGCGGAATTCGTTG
AACTCTGAGAGTTCGTTGTTATGGCGAACACAAATTGTTCGTCGGGGGCGGAACGAGTACTCAATTTTTGA
CTCATTCGCTGGCAATTCGATTTCCGAATTGTTTTGCATGGTAAATGTATATTCATTTATCCGTATGGTTTT
ATCTTTGTAATCTATTAGGGCTTGAGTACTCTCTAAGAAGTCTCTACCTAATAACACGTCATAATTTTGTGA
AAAATTATGTAAATAAAATTTCTGATTGGTGGGGCAAAGTTTGCTGGGTTTCAGCGAAATACTTTTATTTA
GTACTATTGTACCATTAATTGTTTGAACTTCCAATTGGCAAGTTCTTATGGGGAAATTTAGGAATTTTTCTT
TCATTAAGTTAATAGACGACCCCGTGTCTATTACACATTTTAGGATTTTATTATTCATGGTTAGTTTGATAA
AGGGGTTGGTTCTTCTTCGGTTTCTGACTCCGAGGCAGTTTGAAGAAAATTTTCGGACGTGTCCATTTTCTT
TGACCACTACCGCTTTGCGTTTCCTTTTTGTTGGCGGATTGGGGGCGGATAAATGTTTTTGGTGATTTTGGT
AATTAAGAGGGTTTTGTGCCGCTTTGTTTAGATTTTGACTAAACTCTAAGTTTATCTGTGGACTACAGTCA
ACTTGTGATTTGGTTTTTTGTATAGTGTTACTATAGTGTTGTTGTTTTGTTGATTGATTATAATTTGGAGTTT
TCCCGTATTCAGTGTTACCTCGATTTTTGTTGGAAGTAGCTGGATTGGTTTCATTGCTTGGGTTTTTATTTCT
ATCGGATAATGCTGCATCATAGACGCCTTCTCTCTGAGCTACCTGTTTTAATTTTGAAACTGTCGTTATATC
ATGACGAGCTAATACAACAAAGAACCCACCGGACATGTCCCCCAATAATAACGCATT
>9_rnd-4_family-27__Unknown
CCATTGAGTAATAAAGAGAAAGAGCGGCGTTTGATCAAAAGCTCTTTTTCAGAAATACTTCCTGAGCGAA
AACGAGGTTGCAAACAACTTCAAAACATTTTCCATAAGAACCAACAACAACCATAATCAAAACAAACGA
CTCGACAATGAAAAAGCACACCACAAATTCTGTCCTTGCTATCCCTTTTTGTTTGAGGCTACAGCCTATGA
TCTTAGCTTCTGCACCCTTCTAACTCATTGTTGTGGTTTCTAAGCACTGCTATGCCTATAATTCGCAAAGTC
ATGTCTTCTTCTTCTCCACTCGCTTTCAACTTGCAAGCAGCTCGGCAGATTTTTATAATTTTTCATTTTTCGA
CCAGCAAGGCACCCTTTCTCAAAAAATAGGGTATTTTAACTATTCATAAGCGATGTAACCAAATAGCACAA
ACCNTCTTATATATACATTTTTATCCAATGTCTATGAAAAATATAATTTCGTATTTAGTGTGCAACTATTAG
TACAGTCAATAATAATTGTTTTTTTANAAATTATATACCATTCTTATAAAAAAGGGTATCAATGCCGCGAC
CCGCCAAATAGTTGTTGTATGTGTGTCTCATCTCACACATATTGGTGCAAATCTGCATATATGTATGTGTCT
GCCAAGCCAAGGGAAGTCTATGAATTTAAGAAGATTGCAACGTTCTGTTGCGCATGTGACGTCACAGTCC
AAGGCTTTTTATGTGCGATCATTTCGTATATAAAAATTATTACATATGTCAACAAATTATTTACTATAAAA
GACCCCATTCATAAGTTATTTAAATGTACNTAATAAAGGTAGAAATATATACTAAACATCACAAGAAATA
AAATTCTATTAACTGTGACGTCATAATATATGATGTTGGGCGCCGTGCGCGTAACCCTTTGATCTGAATGC

CATGGCTTGCTGGCATTCGAATTTTCTACCTACTTGCGCCGAGACTCGGCTCTGCAACAACAACATTAGTA
CCCGAATTGTGCTGTTGTTGTTTCTACGCGCTGCCTTGTAGGAACGACAGAGAAACCCGCAAAGAACACA
GTGGCATTATTTGTCCCTTTTCTTTGCACTGAGTTTCCTATGTCCTCATCATGGCTCACAAACCTCTTGTTG
ATCAAAACGGGGGATGAAGAACAAATTGGGTTTCGTGCGCACTGTTGTTGTTTTGCAAGTGATTTGTTGTT
GCTTCCTATGGTAAATGAGCTTTTTTATTGGGTNGATTTTGCGTTTGCGGACCAACTCGATCTACGACTCG
TGGTTTTTAAAACGGTAAAAAAAGCTCAGAGCGTCAGTTCAGCTCTTTCTCTTTATTACTCAATG
>Ref_dvir_5
GCCAAAAATGTTCAGATTTATGCAAATTCATTAGAGGGAAAACCATGAAAAAAAGCCAAACCTCTGAAA
ATCGGATGAGATTTTGACCGAGTTATGGGGGTGGGAAATTTTGACCATGTCTATATAGGAAATTTGGGGG
TAAGATTTTGACATGAATTTCGACCAAAAATTATGTTGTTCAGATTTAAATGCCAAATTAATCTAGAGGCA
AAACCATGAACAAAAAGCCAAACCTCGTGAAAATCGGATGAGATTTGACCGAGTTATGGGGGTGGGAAA
TTTTGACCCATGTCTATCTATAGGATATTTGTCAAGTAGGGTAAGATTTTGACATGAATTTCGACCAAAAA
TTATGTTGTCAGATTTAAATGCCAAATTGATCTAGAGGCCAAACCATGAACAAAAAGCCAAACCTCGTGA
AAATCGGATGAGATTTGACCGAGTTATGGGGGTGGGAAATTTTGACCCATGTCTAATATAGGAATATTTG
ACCAAGTCCGGGTAAGATTTTGACATGAATTTCGACCAAAAATTATGTTGTCAGATTTAAATGCCAAATTA
ATCTAGAGGCAAAACCATGAACAAAAAGCCAAACCTCGTGAAAATCGGATGAGATTTGACCGAGTTATG
GGGGTGGGAAATTTTGACCCATGTCTATATAGGATCTATTTGGGGGTAAGATTTTGACATGAATTTCGACC
AAAAATTATGTTGTCAGATTTAAATGCCAAATTAATCTAGAGGCCAAACCATGAACAAAAAGCCAAACCT
CGTGAAAATCGGATTGAGATTTGACCGAGTTATGGGGGTGGGAAATTTTGACCCATGTTCTATATATGAG
AATCTGAGGGTAAGATTTTGACATGAATTTTCGACCAAAAAAAAGGTTGTCAGATTTAAATGCCAAATTT
AATCTAGAGGGCAAACCATGAACAAAAAGCCAAACCTCGTGAAAATCGGTTGAGTTTTGACCGAGTTATG
GGGGTGGGAAATTTTGACCTATTCTAGTCTATATAATGATAGTCATTGGATATTTGAGGGGTACCATTTTG
ACATGAATTTTGACCAAAAATAATGTTGCCAGATTTAAATGCCAAATTGATCTAGAGGCCAAACCATGAA
CAAAAAGCCAAACCTCGTGAAAATCGGTTGAGTTTTGACCGAGTTATGGGGGTGGGAAATTTATGCCATT
TGACCTATGTCTATATAGGATTGAGGGTATTGGATTGGATTTACATGAATTTGACCAAAAAAGTTCAGATT
TAATGCCAATTATTAGAGGCCAAACCATGAAAAAAACCAAACCTCGTGAAAATCGGTTGAGTTTTGACCG
AGTTATGGG
>Ref_dvir_697
TTTTTATTAAAAAGTTTTGGGCATATTTTTACTAATCACCGTCACTGGAAGTGTATTGTTATTGCTTTCAAT
TGTGAGAATCAGAGTGATGAGTATTCGTTCTATCAAATTATCTATGATGAAATCAACAATGTCCTCAAGCT
CGTATGATCCTACAGGTATCTCAAACGTATGTTGCCCAAAATGAAATAGATGATTAGTCCGATCAACATTT
GGAATTGAATTGTACATATGAATACAACTAGACAGCATTCGTAATCTTTTGACAATTCAATGGAAGGAAA
GCAATAAGTATGCAGTTAAAATTGCCCCTTTCCCAGTTAAAGTTATTGTTGTTACCATTTTGAGAAACGAA
TGTTAAATGAATCAAAATTTATTTTTTTACAGTTTTATACTAGAACTTTATTATAATGAATTATTTAAATTA
TAATGAAATGTATCAAATCCTATTCTATATCTTCCATTGTCTATTGCATCATCCTTGCTAATCAAAAGAAAT
CCATATTTATTTTTCCAACATAATTGACAAATTTTCATAAATGTTTCGAATTCATATCGACACCCACATGAT
CTTGATAAATGTGCTTCATATTAAGTTGATCTTGATTGAATATTATTATTAAATTGGCATTATCGCGCACAA
GATGCTTTGGTATGCGTGTATACGTTTGGCATAAATAAAATGAGTCAATGTTTTTATGTCGACCCATGCAA
AAGTATGAACGTATATTATCTTGTTTTTCACATGCTACATCATCAAATAGCATTATCGAACTATCCTTAGCT
TTCTCAGGTGATAACACCTTCGTTCTCAGAAAACGTGAAGTACCCTATTCCTTTTATGGGAGTGACAAGAT
TTTTTAAGTATTCATATTTCGGTTGAAATAATGATTTTGAGTATAAGTAAATATTTTTAAATTTTAATCCAT
TTGGACTCTCTATTAAGCTGATCATGAGATTTGTCTTTCCGCAATTAGATGTACCTACAATTAATCCTCTAA
TTGAGTTTGATAATAGCACACTGTGTCTAATATTAGTTTTACTGGGCGAAAAATCAATATTTCTCACTTCTA
TATTCTTTTTTTGTTTCTTTAAATGCATATTTAATGATTTTATTCTCGCAAATGAATATCCTTCACTGCTATA
CCTTTAACGATTAAGTATCAATTAAACTGCCCATTTATTTTTATAAAAGATTCAGTCGTAAACCAAACGCC
TATTGTGGAGGTAGCTAGTTATAAAATAATAATAGCCTTCCGAGTGAATTCCCCAGCGGGGTGAAAAAGG
TATTAACCCCTTTAGACGCAGCTTGCAGGGAGCACGATATAGCATATTCAG
>Ref_dvir_915
CTGCGACTTTCCGACACTAGCTTGTGTTCAGCATGCCTTGAGGCAGGAGTTCCCTGCAACGAAATTTTGGC
ATTGTAAAAATTTTGTAACTGACATTACAAATACCAATGAACAGCTGGAAATTGTTAATGTCGAACACAA
CCGCGCCCACAGGGCTGCGCAGGAAAACGCCAAGCAAATTCTTCGCGACTACTATTTCCCCAACATGTCA
AAAGTGGCCAGCGAAGTCGTCGCGAATTGCAAAGTCTGCAATAAAGCCAAATACGATCGCCACCCCAAG
CGACAGGAGTTAGGAAGCACCCCAATTCCTTCATATGTAGGGGAGATGTTACATATTGACATTTTTTCTAC
CGACAAAAAGATCTTCCTAACTTGCATCGATAAGTTCTCAAAGTTCGCCATCGTACAGCCCTTATCTTCTA

GGGCAATAGTTGATGTACGGGCCCCTATACTCCAATTGGTAAATTTCTACCACAAAACTAAAACCATCTAT
TGTGACAATGAGGCTTCCTTCAATTCTGAAACGATTACGTCTTTGCTGAAAAACCAATACAGCATCGATGT
CATTAATGCGCCACCGCTTCACAGTAGCTCCAATGGCCAAGTGGAAAGATTCCACAGTACCTGGCAGAAA
TAGCCAGGTGCCTCAAAATCGATAAAAAAAAATTGACGACACTGTGGAATTAATTTTGCGGGCTACAGTA
GAATATAATAGATCGTTGCACTCAGTCACGGATTGTAGGCCAGCGGAAGTTATCCATGCCAGCAGCGACG
AGGTTAACTCGGCTATTAGAGCCAAGATTGACAAGGCGCAACAAACCAACCTCGATAGAATCAACACTTC
AAGACAAAACAGAGTCTTTGAAGTTGGGGAAAAGGTCCTCGTGCGCAATAATAAAAGACTCGGAAATAA
GCTGACGCCGCTCTACTCAGAAGAGCGCATAGAAGCAGACCTAAAAACGTCTGTTCTCATTAAAGGGAGG
GTGGTCCATAAGGACAATATAAAATAAACACCTCCGCCATCCATACTCGCCCTTATGTTATTCAGAACTCA
ACATTACTACAATCTCCACAGGCTTGCCGTTATACTCATCTTACTGCTGTCCATAGCGAATGCGAGAATTA
CCGACTTCTCTCACGCGAAGAATATTCCGGTACTGGACGGGAATGTCCTGGTGTGGGAACAATATGGCCT
AGTTAGGCACTCGACAAATTTGTCTGAGTTCTCCAGCATAATTGACTCTACGGTCAGAATGCTGGAGCTGT
TCCCACAGTCGCATATGAGGAAGTTATTGGAAGTGGACGTCGATCATGCCCCAGAACATTCTGG
>160_rnd-1_family-131__Unknown
TATCTATGCCATACGCTTGCAAGCTCCGACGANTCCTGTGTCCAACAAATCGAATGATCCAGGGGTTATCC
AGGCAGCNTACGAGATATAAAACCGCTNTCACATCTTACTCAAATGCGGTTGCTTGGTTGCAATTAATATT
TNAAATTTAACGGCCGCCAAATTCCATTCGGCATGTCGGCTGNGTCGCGAAATTTGAACNAAATTCAGCT
GAAGTACCAGGCGAACAGAAATGCCCAGCAGGTAGACAAAAGTCCGCGACACCAGAACAAAATTCGATT
GCGCGCCGGCGCGCCATTGGTCAAGCGCAAATCCATGATGGCTGCTCGGCGAAATATGTAACTGTAACTA
TCCTTTTGCTTTGATTCCTATGCATATTCGTCAATATATAGTGATGCGCAAGCACGCAAGTGATGCGGCGG
ATTTGGCTGAATAATGCGATATCCGCTAAAACTAGCCATTTATTTTGGCGGATGTCTGCANATNTATTAAA
TTGAGTTCCTATCNTGGGCAGATGATTATTTTTGGACTCGCGAGAGTATTTTAATTTTATTAAATACTGTCA
AATTTTAAATTGGAATTTTTNTTGCCAATCGGACATTTTATCCGCATGTGTCAACTAAACTATATATTTTCT
CGATCGGCGTATATTTATTCTATATTTAGCTTTATTTGGCATGCATATACACATATTTCTTATAATAAACAT
ATAGAGTGAAGGAAGATATGTCCNACTTGAGAAAATAGCTATATNCATATGGTTATGTCATACGCTTGCA
AACTCCGTCCCGTGCCTAACAGATCGAATGATCCAGGGGTTATCCAGACAGCGTACGAGATATNAAACCG
CTATCGCATCTNACCCAAATGCGGTTGCTTGGTTGCAATTAATATATTCAAATTTAACGGCCGCCAAATTC
CATTCGGCATGTCGGCTGTGTCACAAAATTNGAACCAAATTCAGCTGAATTACCAGGCGAACAGAAATGT
CCAGCAGGTAGCCAAAAGTCCGCGACACCAGAACAAAATTAGATTGTGCGCCGGCGCGCCATTGGTCAA
GCGCAAATCCATGATGGCTGCTCGGCGAAATATGTAACTGTAACTATCCTTTTGCTTTGATTCCTATGCAT
ATTCGTCAATATATAGTGATGCGCAAGCACGCAAGTGATGCGCCGGATTTGGCTGAATAATGCGATATCC
GCTAAAACTAGCCGTTTATTTTGGCGGATGCCNGCAAGTNTAGTAAATTGAGTCCGATCTTGGGCAGATG
ATATTTTTGGACTCGTGAGAGTATTTAAATTNTATNNAATACGTTTGCAAAATT
>160_rnd-5_family-1379__LTR___Gypsy
TAACCAAGAACAGGGTATAATTAATAATTTCATTAAAAATTATAAAAATAACATTTACAAAACATTAAAC
GTAGAACATAATGAAATTCACCTACTTCAGTATATAACAAGAATAAATTTTAATATAGATGTATTATACAA
CCACATAGATAATATAGCAGAAAGATATTCTGCTAACCAAATTGAATATAATACCCAAATTCATACTATC
CGAAAAAGAAATTAGATTTATAAACGAATTCATTTCAAGCCAAAATGTCTTAGTAGAATCCGATGAACAC
TTATATAGATTGTTAACATTAGAAACTTACATAAATAACTTTAATATAACGTTCCATGTAAAAATTCCGAT
ATTTAACAAACAAGNATATAAATTAGCACACATTGTCCCCCTACCGTTAGACCACACTAAATTCCTGATAG
TACCAAACTACATCTTGTACGACCAAAACGGTCAAATCTTGTACCTTGAAAACAAATGTCCCAAAGTCCA
TCAGACATTTATATGCGAAAACGCATCTACACAAACGAACTTAGTCAATATCCACCGCCTTAAGGGCATT
CTCTTTGACCAAACGGCAACATGCGACATCAAGGATGTCGGCCGTCAAGAGGTCATCAGAGAAGTGGAAC
GACGACACATCTTCGTCTTTAACACCATNAACACCAATGTCACGTCTTCATGTCACAGACCCTTATCATAC
GATGATACTGTAATGGAGTTCGAACAAAGGACAGAACTGTCCATAGAATCGTTCAAACAAACGAACATA
AACAACACGTTCGAAGATTTAAATCTGCAAAAACTCCATTTACAGACATTATCAAGCCGAAACGACCTAA
TTGTCGGAAGACGACAGCGACCACCTTTTCACACTTTATATACTGCTNACTATATTGACAATATCTGCAAT
ATGCGTCTTGACACTCGGCGGAGCCTCCAGCGACGTGCGTTCCGGCCATTCCATCGATGTGGCCATCACTT
CATTCCAGGGGGGGAGGAGTTACCGAATATACCATACAGGGAGAAACGGCGGCGGCAGCACAGCATTGT
GACACTATGTTGCAATGCTGATCCAGCACTTTAACAGGATGTTGGTTGCGACAGTTCTCGTGCAAGACACT
TGAACTGCTGCAACGGCAATTTGTCAGATACATGTTGCTAGCTTTTAAGAAAACCATTGTATACATTTAGC
TTTACTGAAGCTCGAACAATAAATAGATATTCAGTTCAGTTTGGAACATTCAAAGAAAACGTTGCAGTGTT
TATTGAATTCTTATTTCATTTACATCAAATCAACATATATATATA
>9_rnd-1_family-65__DNA___P

ACCAAAGACAATATAAACACTAAGATGAGTAACGCCCATACATTTGAATGCGACGCAAAATTTCTAGCCT
GGCCTTTCATCTGGCCTTTGAGGCTTCGTACGGCATGCCTGCCGACTGGTTGTTCGTTCTCCAAAGACCAG
ACGAACGAATGCCGAAGTCGCGGCTTGCTGATGCTGACACGAGCTACGAAGTTAGTCGCCAGCCTGCCTT
CAGCAAAGCTGGTCGATGCTGCTTTGCGTCGCTTCGTTTTCGAAGTACATTGAGCGAAAAAGTTTAGAACT
TACGATCGACGCAAAGTGCTTGTGCTTTATCCATGAAATGCATGCTTTATTTAAATGATATATTACCCTAG
ATATTAACGATTAATTTTACAGGAAAATTTAAGAACTTATATAATTTTTACACAAGGCGCTTCTTGCGCAA
TAACTATGTTAAGAAAATAGCACACATAGATTGATTTCACTTTTCTTTTTGGACAGTGTATGCTCGAGAAA
AGTTCTTGTCTCAGCCAATAGCGTGCGTGCACCTTTCGTTGTATGCGTGAATTATACATGCATAAGAACTT
TAAAGTATGTATGTATGTGTTGTTATTCACTGCTCTGGCTTTAGCTGGCAAGAAATTAATTTTGTTAGTTTT
CTAGCGCAGATCATGACCTATCCCAAATTTGTGTTGATATATGAATGCATTTTGTGTACTGAGGTTGGCTC
CGCAGAAAATACCCGAAGGTCATATTTAGTTATGACCCCATTATAATTATAATGGTCTCCTCGCGGTGTTC
CCTGCGTACTTTGTTCTTTATAATTCGAACAACAAAAATAATAAAATNAAAAAAATTAATTAAATGTATCC
TTNAAAAAATAATAATTTAATTAATAATTTCAAATATAAAAAATTACGTAAATATTTGCATCGACTTAAAA
ATACAAATTTTTTTGTACGGAGCCAACCTCAGTTACACAAAATGCATTCATATATCAACAGAAATTTTGGG
TAGGTCGTGATCTGCGCTAAAAAACTAAGAAAATTGTTTCTTTGCCAGCCAGAGACTTTGCCAGAGCAGT
GGATAACAACACATTCAAATATGCTGTTATGTATGTATATTCACACGCATACAAACATAATTGCTTGCACG
GCCCTCATAGAGCCGAAGCTGAGAGCAAATTCTATTTTTAGCTTTTTCGTTCTCTCGGCTGTGAGAGCGCA
ATGCTACAGATTTCGTTTTCGTTCTCAATTTTCAAAAAATCAAGCTGCATAGTAGCATTTTGTTGTATGGCG
TTACTCATCTTAGTGTTTATATTGTCTTTGG
>9_rnd-5_family-3463__DNA___TcMar-Tc1
CCAGTTAGTCTATTTTAATCACCAATTGGGTAAAAGTGCGCAGGAATTGTCAGAAATGTTTCCTGTAACCC
GCAAGACCATTTATAATATTATAAATCGCGCTGAAAACGAGGGAAGATTGGAGCNAAAGAAAGGAGCCG
GTCGGAAGCAAAAAATAAATAAACGAGCGGAGCGCGCATTGGTAAGAAAACTGGACCAAAATCCGATGN
TTTCGACGAGAACTTCTTGCAAGAGANCTNAAGGAAGAGTTGGATCTGGATGTATCCCACGAAACTGTAC
GTCAGGCCATACTACGACGNGAGTACAAATCAAGAGCTGCTCGCAAGAAAACCAATGCTCTCTGATGTTA
ATGTTGGAAAACGNTTGTCCTTTGCCACAATGTACCACAATCAACCTGCCGAATATTGGGATGACGTCATC
TTCTGCGATGAGACGAAAATGATGCTATATTATAATGATGGGCCAACAAGGGTGTGGCGTAAGCCCTTAA
CATCTTTAGAAAACCGAAACATCATTCCGACAGTCAAATTTGGCAAACTGTCCATAATGATTTGGGGGTG
CATCTCAAGCCAAGGAGTCGGAGATTTGGCTTTTATTGAAAACACGATGGACGCACAACAATATTTAAGC
ATTTTAAAGAACCAGTTGACAAGCAGCGCGAATAAATTTGGGTTTATAAACAATAATATGCCGAATTTTA
AATTTTATCAGGACAACGACCCAAAGCATAAGGCGCATATGATACGCTTGTGGTTACTTTATAACTGCGGT
AAAGTCTTCGATACACCTGCGCAAAGTCCTGATCTCAATCCCATCGAAAATGTNTGGGCTTTGTTAAAGAA
AAGAGTGGCTAAAAGGCATCCGAAGAACCGAACAGCACTNAAAACTGCTATACTAGAAGAGTGGCAGCA
GTTGCCGAGCCATTATGATTTTAAAAGCTTATTCATTCAATGAAAAGCGCCTGNACGAAGTCATTAATGCC
AATGGTGGCCATACAAAATATTAAATATAAGAAAAATATGCNATTTCAGTGCCTTAAATTAATTTAATTAT
TACTTATGCCAAATGTGTAAACAATTGTTTGACAACGAAAAAACCGACTATTTTTTGTTTTTGTATTATTTC
TCACATATCATTCTACTTTCTATTACTATTTTTATGAATTCTATTAGTAAATACTATAAGCAACTATAATAA
ATTNAAAAAAAAATATTTACAATATTTGAAAGTATTTATTTTTGCTAAGAAAGTCGAAAAATCATTTTTTT
TTCTAATGTGAAAACAATTTCTTGACATACTGTATGTTA
>Ref_dvir.14.34.centroid
CCAAAGACAATATAAACACTAAGATGAGTAACGTCCATACATTTGAATGCGACGCAAAATTTCTGGCCTG
GCCTTTCATCTGGCCTCTGAGGCTTCGTACGGCATGCCTGCCGACTGGTTGTTCGTTCTTCAAAGACCAGA
CGAACGAATGCCGAAGTCGCGGCTTGCTGATGCTGGACACGAGCTACGAAGTTAGTCGCCAGCCTGCCTT
CAGCAAAGCTGGTCGATGCTGCTTTGCGTCGCTTCGTTTTCGAAGTACATTGAGCGAAAAAGCTTAGCACT
AACGATCGACGCAAAGTGATTGTGCTTTATTAATGACATGCATGCTTTATTTAAATGATTTATTACCCTAG
ATATTAACGATTAATTTTACAGGAAAGTTTAAGAACTTATATAACTTTTACACAAGGCGCTTCTTGCGCAA
TAACTATTTTAAGAAAATAGCGCACATAGATTGATTTCACTTTTCTTTTTGGACAGTGTATGCCCGAGAAA
AGTTCTTGTCTCAGCCAATAGCATGCGTGCACCTTTCGTTGTATGCGTGAATTATACATGCATAAGATCTTT
AAAGTATGTATGTATGTGTTGTTATTCACTGCTCTGGCTTTAGCTGGCAAGAAATTAATTTTGTTAGTTTTC
TAGCGCAGATCATGACCTATCCCAAATTTGTGTTGATATATGAATGCATTTTGTGTACTGAGGGTTGGCTC
CGCAGAAAATACCCGAAGGTCATATTTAGTTATGACCCCATTATAATTATAATAGTCTCCTCGCGGTGTTC
CCTGCGTACTTTGTTATTTTTAATTCGAACAACAAAAATAATAATATAAAAAAATAAATAATTAAAAGTTT
TTTTGAAAAAATAATACTTTAATTAATAATTTCAAATATAACAAATTACATAAATATTTGCATCGACTTAA
AAATACAATTTTTTTTGTACGGAGTACACAAAATGCATTCATATATCAACAGAAATTTTGGGTAGGTTGTG

ATCTGCGCTAAAAAACTAAGAAAATTGTTTCTTTGCCAGCCAGCCAGAGACTTTGTCAGAGCAGTGGATA
ACAACACATTCAAATATGCTGTTATGTATGTATATTCACACGCATACAAACATAATTGCTTGCACAGCCGT
CACAGAGCCAAAGCTGAGACCAAATTCTATTTTTAGCTTTTTCGTTCTCTCGGCTGTGAGAGCGCAATGCT
ATTGATTTCGTTTTCTTTCTCAATTTTCAAAAAAATCAAGCTGCATAGTAGCATTTTGTTGTATGGCGTTAC
TCATCTTAGTGTTTATATTTTCTTTG
>Ref_dvir_572
AGGGTAGAATGCCTCAGCATATATTATTAGATTGCTGCATCTGCTAAGAAAACACCTCATATCAATCGGTC
ATGACCGCTTTTTGGGTTGTTTGGGAGTCAAAATACTTTATATGCAAATGATATATTGTTACTGAAGCCTA
GCACTTTTTATCAGAGTGAGGAGATCACTACAGCACCCTAACATATAAATCATATTAGTGTTCTGGATATA
GGCAAAAAAGAGAGCCAATCTCCCGCGATAAGAGGGAAAGTATAGAAAACAAATACAGAATCGATTTCG
AGCAGACCTCAAAGAAAGCAGGGAACCACCACAATGTAGGCTGACTTTAGCACAGATTAGAAGTAATCA
CCCGCAATAATAAACGCTACGCCGATTTTCTGGATTAGCGGTGAAAGATTTTAAGCAATTTGTTGATAATA
TTAAATCTTAAAATTGTACCTAAATCTCTGTAAAACAAAAAGAAGAATATTAATCCACCAAATGCGAAAC
TAAGGGAGAGCCAAGGTTTGAAGCTTTTTATTGATTATAGTAGATCGCTCAAAAAGACTCTCGAATAAAC
TTCAGGCAAGAGAGCCATGACAAAACCGTGCCGGAGGTAGCCGACAGTGCATCGTCGTCAGTGGAGTGT
AGCCGCACCACTAACTCAGGTTGTAATTTTCCCTCGTAGTGCTATTTCTCCTGACTGTCCTAGCGGACTGG
CTGAACGCACTGTTGCCGCATCTAACACCATGGAAAGTTAAAATTCTGGAGGCACTACCAGAGGAAATAA
ATACCCGGACTCACATTTTTGCCAGCTACTGCCCAGCGTCTGAAAAGGACAAGAAGACCTTTCGACTGTTA
ATTAACGAGTTCTCTACAGAGAGCAGAGGCTGTTACAATCGCCATGGGCCTGCTAAGAAACCAGAAATTA
ATATTTGAACTTTGCTTGGGTAAAAAAGACTGCAGCACAGTGTGGCGAGCTCCGTCGCCGAAAAGGCGCT
TATATTAAATAAACGAGTCGCTTATACAGCCCTTGTATATATATGAAATCAATTTTCCACTGGAATCACTT
TGCCCAAAATTCAAGCAACAAATTTGAGACCCATTAAAGGTGCACACAAAAGATCCGGACGGTTAATCGA
TATATCCCCTGGTGGAATAACACTACAACATTCTATAACCTCTATAACAAAGATCTAAGAAGAACCACTG
TGGAAGAATCAAAAGGCAATTGACAGATATTGCTCAAAAGGGATATGTACACTTAAGAAGCCAGAAGGT
AAACAAATGAAGGCAAACTGCTGGAAACTCATTTTCCGG
>Ref_dvir_1027
ATTATATATGTGCGAAAATTGTTCAAATTACCTAAACGTGGAAGTCCTATCGTAAATCAATTTTTAATCTA
TGTAAACAAATTAAAATATGTTGTTTTATGTTTTAATATTATTTTATTTAATATTATTTACATTTATGACAG
AAGCGCAAATACATTTAGTTGTATATTCGCAAAATAAATATCTACACTTACGCATAAGTATATATGCTTTT
AGTTATACAACTGTAATATGAAACTATTGTATTATTGATTCGATAAATGATCTTTTGCATTCAAATGTCGCT
GAAAGTGCGCGCAATGAAAATATTAATTGAACTTTGAAAGGGAGCTATTGTCAGGCGAGACAAACGAAA
TTGCCATTCACTTTTCAACCAACCATCTATTTGTGCGGTCGTCGATTCTCGATTGCGATTCAGCGGAACAA
CACGAGAGCCTTTAGAGAGAGTCTCGCAAAATGCTCACAAAGAGCTCGCATGTGAGCAGAAAACGAATG
GGATATTTTTCTTTTTCGCTAATCACTTGCACACGAGCGTTGCAGTCAGATCCTTGCGATAAAAATGCGTG
TACAGTAGAGAGAAGTTTTTGTTTCAAAAGTTAATTGTGTTCAGAGAGTAAAACATTGTCATGAGAACGA
GAGATAAAAACGACGTGGCATTCGGATGCTGTGTTGTTTTTGTTATTTTTTCCTACTCAGACCATTTTTTGA
TGGTTGAAAAATTCCCTTGTTCTCTTTGCTAGAGAGCTAAGTCTTTAACAAGTGTAAATTAAAAACTAAAC
TATTCCCTTTCAGAGAATAATTAAATCATTTTTGCGACCATTTGTCTGTGAATTATCGGAATTGATCACAG
AGGGGCATGATATTTCGAGATACGCAATTCAAAATATCTATTAGATCATTTATTTGCGATGCGCCGGCCCG
CGCCTTCATTTGGGTGTGAAATACCACTCTGAGTTTTCCTCATGCAACTATTGCGGTCAGAAGGGAGAAAC
TCTTAACAGGGCAAGTAAAATCATGTAATCCTCCAATCAACCAAAGCACCACAAAATACAAAGCAAATTC
ATTCCTGAAAATTTGTGATTGATTTTGTACATCAGTTTCCACTAGATTATATGCACGTAGTTTGTCTTGGCG
TGACAAAATCAGTTTTAGTGGGTTTAGTGAGAGGACATTCCTTTTCTTTGAAAAGACAGTAACTAACCTCG
CTTAATAATAAACCAATACGCATCTTACCAAGAGAATTCAATAGAGAAGTTAGATCTCTAGACAATCTGG
ACAGATGTAAGGCAACGGAGTTT
>Ref_dvir_356
CATTGAGTAATAAAGAGAAAGAGCAGGCGTTTGGATCAAAAGCTCTTTTTAGAAATACTTCCTGAGCGAA
AACGAGGTTGCAAACAAAAATTGCAAACACAACACCCAGCTGTCTGCAGCTACTTCTGCCGATGCCCACA
ATTCAATGACAACTTCAAAACATTTTCCATAAGAACCAACAACAACCATAATCAAAACAAACGACTCGAC
AGTGAAAAAAGCATACCACAAATTCTGTCCTTGCTATCCCTTTTTGTTTGAGGCTACAGTCTATGATCTTA
GCTTCTGCACCCTTCTAACTCATTGTTGTGGTTTCTAAGCACTGCTATGCCTATAATTCGCAAAGAAAGTC
ATGTCTTCTTCTTCTCCACTCGCTTTCAACTTTGCAAGCAGATCGGCAGATTTTTATATAAATTTTTCATTTT
TCATAAGGACCAGCAAGGCACCTTTTCTCAAAAAAGGGTATTTTAACTATTCATAAGCGGATGTAACCAA
ATAGCACAAACCTTCTTATATATACATTTTTATTTAATGTCTAAAAAAAAATAATTTTGTATTTAGCAATTA

TTAAATAATTTCTATAAATTATTTCTATAAAATACCTAATTAACCATTTTTATAAAAATGAGTATCAATGC
CGCAACGCCAAATAGTTGTTTATGTGTCTCATCTCACACATATTGGAATCTGCATATGTATGTGTCTGCCA
AGCCAAGGGAATCTATGAATTTAAGAAGATTGCAACATTCTGTTGCGCATGTGACGTCACAGTCCAAGGC
TTTTTATGTGCGATCATTTCGTATTTAAAATGATTACATATGTCAATCAATTATTTACTATAAAAGACCCTA
TTCATAAATTATTTAAATGTACATAATAAAGGTAGAAATATATACTAAACATCACAAGAAATAAAATTCT
ATAAACTGTGACGTCATAATATATTATGTTAACGCGCCGTGCGCGTAACCCTTTGATTTGAATGCCATGGC
CTGCTGGCATTCAAATTTTCTACCTACTTGCGCCGAGACTCGGCTCTACAACAACAACATTAGTACCCAAA
TTGTGCTGTTGTTGTTTCTACGCGCTGCCTTGTAGGAACGACAGAGAAACCCGCAAAGAACTCCTATGGTA
AATGAGCTTTTCTTTGGATTTTGATTTTGCGTTTGCGGACCAGCCCGATCTATGTCTCGTGGTTTTTAAAAC
GGTAAAAAAAGCTCAGAGTGTCTAGTTTAGCTCTTTCTCTTTATTTCTCAATG
>Ref_dvir_401
CTCCCCCGGGTAGCCCCCCCCCTTTTTTGGCCCCCTTATCGGGGCTTTATTTGTGCCCGGAAATATACCCAT
TTAAAAGTTTTCGGGTTATGTCTTTTGAAGCGACAACTCCGCCGATCTAAAAAAATCATGGAGATATGACG
CCCTATCGGGCTTTATTTGGCCGAGTTATGTCGTTTTGAAGCGACATTCCGCCGATCTAAAAAAATCATGG
AGATATGACGTTTCAAAGCGACATAACTTCGCCGTCGATCTTGGCGGATTCTACGGACCTCCCCGAGTAGC
CCCTCCCTTTTTGGCCCCTATCGGGCTTTATTTGGCCGAGATATAGCCATTTAAAGTTTTCGGGGGGGTAGT
TATGTCGTTTTGAAGCGACATAGCTCCGCCGATCTAAAAAAATCATGGAGATATGACGTTTCAAAGCGAC
ATAACTTCGCCGTCGATCTTGGCGGATTCTACGGACCTCCCCGAGTAGCCCCTCCCTTTTTGGCCCCTATCG
GGCTTTATTTGGCCGAGATATAGCCATTTAAAGTTTTCCGAAAAAAATCGTGTGGGTGGGGTAGTTATGTC
GTTTTGAAGCGACATAGCTCCGCCGATCTAAAAAAATCATGGAGATATGACGTTTCAAAGCGACATAACT
TCGCCGTCGATCTTGGCGGATTCTACGGACCTCCCCGAGTAGCCCCTCCCTTTTTGGCCCCTATCGGGCTTT
ATTTGGCCGAGATATAGCCATTTAAAGTTTTCGGGGGTAGTTATGTCGTTTTGAAGCGACATAGCTCCGCC
GATCTAAAAAAATCATGGAGATATGACGTTTCAAAGCGACATAACTTCGCCGTCGATCTTGGCGGATTCT
ACGGACCTCCCCGAGTAGCCCCTCCCTTTTTGGCCCCTATCGGGCTTTATTTGGCCGAGATATAACCATTT
AAAGTTTTTCCAAAAAATTCGTGTGGTTGTGTGACATAGAAGTAACGCAAAGCATTGCATACTTTCGAGCT
GTTCACATAAAAAAAAAAATGCAGAACGACATAATCTCGCCATGATCAACGGCGGAGTTATGTCGTTTTG
AAGCGACATAGCTCCGCCGTCTAAAAAAATCATGGAGATATGACGTTTCAAAGCGACATAACTTCGCCGT
CGATCTTGGCGGATTCTACGGACCTCCCCGAGTTTTTGGCCCCTATCGGGCTTTATTTGGCCGAGATATAG
CCATTTGTTTTCGGAGTTATGTCGTTTTGAAGCGACATAACTCCGCCGATC
>Ref_dvir_904
AAGCTGGTTTTGTGACATACGTAACTGTGTCCAACTTGAAGACACTTAATTCAAAATCAGTAGAACTTCGC
CACACGATGCATTGTAGGTTGTTATCTGGGGCCGTAACTCTGACACAGCGATACATCTTGCTAATGTCTCC
AGTCAGGGCAACATGGATCGTGCGAAATCGCATTAAAATGTTTAACGGTTTTTGACTGCCCAACCATCAA
TACTTCATTCAGCGAACGCTGGTGATAGCTGAACCATCAAACAAGACACAAAGTTTGGTGCTTTTGTTCCT
TCAGCACGCAATGATGAGGGAGAAAATATTTGCATAAACGTAACGACTTTGGAGGGACAAGAGTCATAT
GTCCGAGCTCCATATACTCTTGAACGCGCAATACTGGGATTTAAGGTTGGGATGACGATGCAACTTTCTCT
CAAGCGATATAAATCTGCGAAATGCCTGTTGGTAAGATTCACCTAATTGATCCAGAGTGAGTTTGAGAGG
GAGTCGCACGGAATATGCTCCTGTTGGCAACCTTTGGAAATTCTTAACAAAATGCTCCTCGCAGTCAAGCT
CTTCCTTTGTTGCTTTAGCAACAGGGTCTGGAAAACTTTCTACTTCCCAAAATCGTTGAATTAACTCATCGG
TTCGGCATCAGAATCAGTGCGGGCATCAGACGATACACGATTGGCCAAAGGAGAGGTACCGCCTTGCTGG
GAACCGCCACCTGCAACTATCCATCCAAGCAAGTCTTCTGCAGAAGAGATAAGTCAACTCGCTGCGGCTT
GTAAAAGCAAGGATCACGAGGCGAATATTTCACATTCCAATTAAGGGCGATGCTTGGCTGGCGATCTGCT
ATAGTGGAAGCTACGATGGCTGTGATGATTGCAGAGTACTCTGACGTTTGAGATTGTAGATTGACGGTCA
CAGTGCATCCATCAGTAGCGAAAGGGGCGTTTTCCCAGGCCAGCAACTAAGGATGAAGATGGACTTTTCT
TTAACTGAAGCTGCAGAGCGAATCGGGATGTAATTTAATGTAGCCGTGACCCACTATCCAATACAGCACG
ACACGGAACTAGAGTTCCAGACCGATTTTTAACTAGTACAATAGCAGTGGCCAGGAACACAACATCGCTG
CTGAGGTTGTGGGCAACAAGAGAGGTAAAGACGGGGGCTGAACAAGCTGTAGTAGGAGTGACAGAATGA
TGAACCGAATGCAAAACGTCGAAGTGCAGAAGTGTGTGGTGTTTGGAGC
>Ref_dvir_689
GTTCTCGTCCGATCACCGAAATTAAGCAGCGTCGGGCGCGGTTAGTACTTAGATGGGGGACCGCTTGGGA
ACACCGCGTGTTGTTGGCATCGTCCACAACTTTTTTTATAGCTCGAACAAATTGTATATTACTTCAGAATC
AGTATCTCATTTTTCTCTATCTAACATTATTTATCTGGTTATATATGGATTAAAGATTTTTTAAAATATAGT
TCAGGTAAATTCCGTATTAGTTAATATTTTAATTTTCCCCTTTCCCGACTTCTTGCCGTCTTGTCGTTCTGAC
TTATATATAAAATAGAAACTTTTCTTGCGTTCGTTTGCCGACTCTTGCAAATGCTGAGCCCCAAACGTCAC

ACTTGTAGCATAACAACAATTTTTAAATCCAGCAAAAATGCACTCCTCATGATTTTGAAATTCCAGCTACA
AGTTTGTGCTCTAAGCACTCGACATTCGCCAACGACCATACCACGCTGAATACATCGGTTCTCGTCCGATC
ACCGAAATTAAGCAGCGTCGGGCGCGGTTAGTACTTAGATGGGGGACCGCTTGGGAACACCGCGTGTTGT
TGGCATCGTCCACAACTTTTTTTATAGCTCGAACAAATTGTATATTACTTCAGGTTATCTATCGTTTAAATT
GCTCAAAAAGTACCTCATTTTTCTCTATCTAACATTATTTCTCTGGTTATATATGGATTAAAGATTTTTAAA
ATATAGTTCAGGTAAATTCCGTATTAGTTAATATTTTAATTTTCCCCTTTCCCGACTTCTTGCCGTCTTGTCG
TTCTGACTTATATATTAAAATAGAAACTTTTCTTGCGTTCGTTTGCCGACTCTTGCAAATGCTTAGCCCCAA
ACGTCACACTTGTAGCATAACAACAATTTTTAAATCCAGCAAAAATGCACTCCTCATGATTTTGAAATTCC
AGCTACAAGTTTGTGCTCTAAGCACTCGACATTCGCCAACGACCATACCACGCTGAATACATCGGTTCTCG
TCCGATCACCGAAATTAAGCAGCGTCGGGCGCGGTTAGTACTTAGATGGGGGACCGCTTGGGAACACCGC
GTGTTGTTGGCATCGTCCACAACTTTTTTATATTACTTCAGGTTATCTATCGTTTAAATTGCTCAATCAGTA
TCTCATTTTTCTCTATCTAACATTATTTCTCTGTTATATATGGATTAAAGATTTTAAAATATAGTCAGGTAA
ATTCCGGTTAATATATAAAAAAAA
>Ref_dvir_610
TGCAATTATTGTTATAATTGTTATTATAACCACGAGAGTTTTCTCTGCCTCGGTAATTACCACGATAGTTAA
CAATACAAAAGTTTTTAGCTTCAGCAAGTCCAAAACACGTCTACTTTACTCGCTTCTCTGCTAAGGTCTGA
CTCCAAGATCTTTTACAATTGTCTAACACTCTTTTCTTATTCTTAGTTATCGAACAATACTTAACAGCTACC
TATGGATAGGACGTGCAGCTTCACTGTTGTGTTGCTAGCAGCCTGCTACAGTTATTACAATACAAGAATAC
AATACCGAGCATGCATTGCCTGTCATAACTATACTGTATTGGATATATTTTGGCATCGTTTTGAAAGTGCC
TGCGTAAAGTATTGCTTTTAGTTTGTCATGCTCACAATTTGATCATGACTGAAATGGCTTCTTTGGTACAGA
ACGTATCAGCATGTTCTGCCTTTCGTCCTTCATCGATATAAGAAGATTCAAATTATCGATTTAGTTTGCGA
GTTGTGTGGCTAATTTACATTTTGGTAGAAATAAGCCGTTTTTGCCTCTACTACATCGGAAAATCGCCGAA
AAATCATAGTCTGCAATTTAGATAGCATCGCGTTTATTGTGATTTCATCTTGTATTTTATAAAGAGTTGAAC
CAACTATTTTGGATTTTATTACTTCAATTACAATTTCTTCGTATGGACCTTTTGTTAGGTTTATTAATTATAT
TGCAGTAATAAATTTGTAGATGGATTTTATTCCATCGATTTCTGGTGTGGCCTGTACAATTTCTTTTATGTA
AGCCCTTGAGGCTGCAGAAATGTCTTCCATCGTAATAGGTTTTAATTCTATGTTTGCTTCAGAATCAGAAC
ACTCTGACAATTCAGGTAATGCAATTATCGCTGAAGTTGTAAGATTATGTATGTCTTTAACTTCTAGTTCC
ATGTCAGTGACTGTATCAGATTCACTTTCTTTCTTTTCTTTATCTTCAGGCTGTTTAGCTGTTTCGATTGTTA
AAGCTGTGTTCAGTATAGTTGGAATCGATTTATCCAAATTATTCTTTACTCTTATCGCAACTTAATTTATTC
GAAATTTGGTTAAGACCTTTGAGGCTTGTGACAAATGTTGATTGGTAATCTTTCTCTATGGTCGAATATAA
TGTTCATGCTTCATTTAAAAACCTTACTAATATATTGCGTGGTAGTTATTGTACTTGCTGTGTTCTTTGATT
GCGT
>160_rnd-6_family-1820__RC___Helitron
GCTCTTAAAAACTGGTTTTAAATATGACCAAAATATATTTCCTTATAATATTTAATAATCAAGATATTGTA
TTCGTAGATTCGTACAGTGGCAACACAATTTGTTTCTTAAAGTACAACAAGAAGTCGTGCACTTTNATAGN
GCATTTAATNTTCGGCNTCGCCGAGAACATGCTTTTTTATACCCTTNCAGCGNGTANTTTANTTTTGTCGCG
AAATGTGTAACGCATAGAAGGAGACATCCCGTATCATGAAGCATATATATTCNTGATCGGCATCAACAGN
CAGAGTCGATATAGCCATGTCCGTCTGTCTTTCTAGCAACTANTCCCTCAGTTTAAAGCTATCCTAATGAA
ACTTTGCANANATCCTTTCTGTTGCGTGNAGNANATATGTGAAAACCGGCTGGATCGGANCACTATATCG
TATAGCTGTCGCGGGAACNATCGTTCGGAGAATTGAGTTTTTGATATGNNAAACGTNATGTTTATCAAGAT
ATNTTGAGCAAACTCGGNATTTACGAGCTTTNCCGNGCTCCTCATATATATGCAGGNTCCAATATTAACGA
TCGGACCACTATATCATATAGCTGCCATAGGAACGATCGGTCGAAAATTAAGTTNTTGTCTGAAAAACAT
TTCGTTTTTCAAGATATCTTGAGCAAACTCGGCATTTGCGAGTTTTAATATANTCCTTATATATAAGCNAA
ATCCTATTAAGATCGGACCACTATATCGTATAGCTGCTATAGGAAGAATCGGTCGAAAATTAAGTTTTTGT
ATGAGATTTGTTTATCAAGNTATCTTGANCAAACTCGGNANTTACNAGCTTCACTGCGCTCCTCGCGTATC
TGCAAAGTCGTANTAAGATCGGACCACCATATCATGTGGCTGNCATAGGAACGATCNNTCGNAAATNAAG
CTAGTGTNTAAAAACACTTTGTTTATCAAGATATCTTGACCAAACTCGGCATTTACTGTTTTCCCGGTACTT
CTTAGATAGGGGCAAAGCACTATGAGCATTATGAAAACCGTTTAGCAAATGTCTGCAAGGGTATTAAATC
TTCGGCGTGCCGAANATGCCCTTCGTTCTCGTTTTGTTAANTTTGNCTTCAAAACGCTTTGACTCCATGCCA
CTTATCCATTGNATATNTANGAAACNCCAGAGTNNTGCANTAATTAAANCTTTGCGCCTACTCCATAATGG
GTAATTATTTTGTAT
>9_rnd-1_family-266__LTR___Pao
AAAAGTTAACACATAAGAATTTCACTTCATATTTATGTCATAAGAACACACTTTTGTTTATAAGAACGCAA
GAATGTTGCAGTGCACTTTGCTTACACTGCGACATTCTGCTGACGCACGAGTTCAGTGCCCGGCCACTGCG

GCCAGCAGCGGTAACCTGACACTTGCCTATGTCGCAAGCGACAGTGTTGGCGTATGCCGATTCGGCTTGC
CGGATATAGCAGCTGAGACTTATGCGCCTAGCCTTTGGGTTCAGATCCAATTCAATGTTTCAATTCTAGTT
TAGTTTTGTATCCAACTTTGTAAGAACAACTTCTTCAAATATACTGCTCCGGCACTTGCCGTCATCAATCCG
TTATACTAATTGTTTCATTGCTGAGCCACAAGGCCAGTGATTTGGAAAGCTAGTTGCTTTCCGAACATAAT
CTACAAGGAATATAACCTTACCAATCTCAGCACCTGTGACGAGGATCTGAATATACAACATCAACATCTA
CAATTGAGAAATCAAATAACTTACANGTGCGAGCTATATTATTCAGGTAAAAAGCTTATATGCTACTGGA
TGCTAATTTATAAAAGTTGCTGCGCACGCACCACCGAATTTCGCAGCTGCTATTAGGTACCAACGACGGA
AGACAGACAACTGTTCGTCAGTCAATTCTGGCCATATTGGGTGTATGAATACGTGCGTGATCTTGCGCGGT
ACATGAAGTGATTTGCCAAACGANCACCTTCATCGTCCTGTAAGCAAACGTGATCATTATCAAGCAAACC
ATCCTGGAAAAAGACAAGGAGACAATACGAACAGTCGTCGGGATAGCTATCGAGATCCTTCAATGACCTG
TGACCAAAGTCGGTGTGCTAGGCGCTGAACATATTAGAGGTAAACAAGCTCCAGAAGAGAATGCGGAAA
CTGCAGGTGCTTGTTACCAAGGCCTCAACATTAAATGAAAAATATTGTGCTCTTTTCTAAATAGTACCTAG
ATGATATTGGTCAAATCTTTATAGTAGTTCTGGATAACAGACCCACTATCGAGACAATACGTTAACTAAAT
CTACACATGTTGTCTGGACTTGCCTAACCAGGGGCAACTCAGGCCGCTCCTGGTTGGTTACGAGCCACTGC
CATAAAATCGGGCTCACTCTATTGTGTTTACACGCGAGAAAGATTGCCACATTAAGCCTGTTCTGCATCCA
TATTTGCTAATATATAAA
>Ref_dvir_846
AAAAATTAGATTAATATTATGCGAAAGCAGCAAATGCCAATATTTTGTTTGTTGCCCGCTCCTCGCATATC
ATAAAATCGCTCAAATATTATTACCTATTAACTATCTAGCGCTCTTTTACCTTTTATACTTTTGTCTTAAGC
GGCTAGCGCATCAATGCTAGCGACGCAGCAATTTATTCTTGTCTCGTATTTCTTATCGATCGCTTCGTTGAA
CATCGTGCATTTCGCATAATTTCAATGCGCTCGCTATAAGCAAATAAATAAATCAAACTATTGCATGATTT
TATAAGAAAAAGACGTATATTTGTTAGCCCAACAGTTATAATCGCGTATGCATCAGCAATACTTTGTCCGC
ACCTTATGGGAGTTGTCGTGGTCTACTTCTACTTCTATATAACATGTTTATTATCATCGCTGGAAGATAGA
AGAGTTATAACAGTGCTGCATTTTTAAACACCTGTAAGAATTATTGACTGCTCGTCTCTCAAGCATGTTCT
AACTTTAATGTGCCGAGCAGGGACTCATAGAGTTGCTTGTGCCAGCAACGATCATTAAAGCTTTAAAAGA
ATCTCCCAACTAAAATGCTCTTTGATTCTTCTGCCAATAGCGCTCTGGTCAACATGAGCTTCAAGAATTCTT
TCTTCAAAACTAATAAATTTATATATTCTTTATGTTTCATGGTTTCTTTTTACCTATATTAATTATAATGTAA
TATTAGAGATAGCCTGAAAGCTACCAGAATTGGTTGATAAATAAATTAATATTTGTGTATTAATACAAAG
GAGTTCTTAACACTGAACCCTAGCTGTAAGTTGCGGTAGCGTGATTGTGCCTCGTCCAAGTCGTAAAGTAT
CATAAACTGTCGCTAACTACTGCAATATTGACTGTTGCTTATGTTTTGTCAGCTAATATACAACAAGTTCTT
GAAGGCTGCCGTTGTGATGCTGGTGAAACCGCCATAGCATCAGCAGTTAACTGCTTTCCCGTTGGAGGCA
TGTTGAGACAAATACATGCAAATTTTATGCTACCAATTTGCTGATTTTACGCTTCTCTTGCCTCCTTCTCAA
ATACCGTTTGAGGAGGGCAAGACCGCTGACGGTATCACCGCTTATTAGCGCTTGGAAAAATACCCCTTAG
GTACGGAGTTCGTTAACGCGTCGGCCTTAACCTCCTGTGTAAACTCAAATTATTGCAGGAATGGTATAGTC
AGATA
>9_rnd-1_family-398__LINE___I-Jockey
GGTTCCGAAAANCTCACGGCACACCGGAACAGCTAAATAGGGTGGTGAATTTTATCCTCGAGGGTTTCGA
AGCCAAAGAGTACGNAGTGGCAGCGTACTTAGATATAAAACAAGCCTTCGACAGAGTCTGGCATGATGG
ACTACTCTGCAAAATCAAGCCCTTGTTGCCACCTCAACTATATTTGATTGTGCAAAGTTATTTGACAAACC
GGTCATTTTCGGTAGTACTCGATGGAGTTGAGTCCAACATGAAGGCCATCGGGGCTGGCGTTCCTAAGGG
AGTGTGCTAGGGCCCACGCTCTACACCCTCTTTACCTACGACATGCCCACTGGCTCTACAATTGAAGTCGC
GGATCCACANGACCTTACCATCGCCACTTTTGCAGATGACGCAGCCATCTTATCAAGAAATGAATGTGTCT
ACGAAGCTACCGACGTCCTGCAGCAATATCTTCACAAATTTGAAATGTGGGCCAAGCGTTGGAATATTGG
CATTAATTTAAATAAATGCGCCAATGTGACCTATGCGCTACGGAGGGACATGTGTCCGGGAGTTTTCTTGC
GCGGTCAATTGGTGACGCACTACAACAGCCACAAATACTTAGGTGCACTTAGACAGGCGCTTGAACTTTA
ACAAACACATCACAAGTGTCTCTGTAACCCTGAAGAACAGAATAGGAAAACTAAGATGGCTTCTGAGCAG
TAGAAATGCGTTGTCTTTGAAAAATAAAGTAACCATTTATAAATCCTGCATAGCTCCCGCCTGGCGTTATG
GTATTCAGGTATATGGCATTGCTGCAAAGTCCCGTCTTACAAAAATCAGGGTGTTCCAAGCAAAAACGCT
CCGATATCTGTCTGGTGCCCCCTGGTATATGAGAACTTCCGACTTGGAAAAAGACCTAGGAGTGCCCAAA
GTCGGGGACGTNATAAATATACAGGCCGCAAATTATATAGCGCGACTGAGAAACCATCCTAATCGTCTGG
CTAGGCGTCTTCTGAATAGGCGTAGAATTGGAGGACTCCGAACGCGGAGGAGACTGCGAAGGTCTCACCC
AATCGATCTCCCAGATCGATTTATATAGTCGCCACTGTAAATTTGTATATTAGACTTACCAGTAGAATAAG
TAGTTTGTAAATATTCATATAAATTTAATTTAAGTTGTTCCTGATAATAACAGAAAATTAAAATAAAAAGC
AAATTTAAATAAAAAAA

>Ref_dvir_807
CTTAATAGCTTTCACTTTATCGGGTGGGGACTGAATCCCGCCTCTGCACACCGTAAATACCAAATATTTCA
CGCTTTTTGGAAAAACTTGGATTTTTCTTGAGACACTCGTATGCCAGCACCCCTAAGGTTTCTCAAGACC
CACTCAATGTCCCTGATATGGTCTTCTTTGGACTTGGAGAAGATTATTACGTCATCAACGTAAACATAACA
TATCTTACCAATTTGTTCGCGGAGGACATCATCAATGGCTCGTTGATAAATACTTGGGGCATTTTTCAGCT
CGAAAGGGAGTCTACAAAGCTCATACTTCCCGTTGTTAATAGAGAAAGCTGTCTTTTTCCTGTCTCTTTCA
GCCAATTTAATTTGGAGAAACCCTGACTTGAGGTGTACCGTAGTGAAATAATGAGCCTCGCCCAAGTTTG
ACAATATAGTCGAAATGGATGGGATAGGGTATTTGTCATCGGTTGTCTTCTGATTTAGTTTCCTAAAATCA
ATAACCAGACTTCTGTTTGTGCCCCGCTTCGTCGATCCTTTTTTTGTCGACAACCTATAACGGGTTAATTTA
AGGGGACTTTGACGGTCGTATTATGCCATTCGCTAGAAATTGTGTAACCTCTGCATTGACAAAGTCGGACA
CGCCCTTTGGATACGGATAAAGTTTTGAGTAAACCGGTTCGTCATCCGTTGGTATAGTGGCAACCGTATTT
ATGTTGTACGGCAGGGATTCGTTAACGTCCGCAAATGCTTTTACCATTTTGTTGATCATACTATCAAAGGT
GGATCTGACAGCAGGAGGGACCTCCGTGTCATTAATCCGAATGTGGTTCACCTGTTGACATTTAGTGAACT
CAATTTTTTTAATCCCATGGTCATGTTTAATGACGTTGTTTGTCAAATCTATTAAGGCATTAATCTGTTTTA
ACAGATCAAGGCCAATTATGCCGTCAAAGTCATTAAGTGACTCCAACATGAACAAGTGGGACTTTACGCT
GAAAAGGTAGATGAGGCATTTCTGCTCAAACTTAGTGTGGCCGTGAATTGATTTGACTTGAAAAGGGCTG
TCAACTGGCACCCAATGTGTCAGTTCGGGAAGGGGTTTTCTATAATTTTTCGAGGCCCCTGTGTCTATCAA
AAGTCTGATAACTCAACCTGCAACCATTTGTCTTATAAACGGAAGCAGGGAGCTTCCGCTAAAAAATTAC
AACTTTC
>Ref_dvir_702
AATTATTCCGTCACTGAAAAGGAGTGCTAAGCGTCGATACTGGCCATAAAAAATTTCAGACGCTTTATAA
GTAGAAGGGATGCCGTTCACTTGCATCACAGACCACACCAGCCTAAAATGGCTGATGTCTATGAAGGACC
TAGCACGTTGGACATTGCAGCTGCAAGGCTACAACTTTACCATCGAGCACCGTAAAGGCAGTGAGAACGT
AGTAGCCGACACCTTATCTCGCTGTATTGAAGCCGTAGGAAAGGTTACGGAAAACCTTCTCGAATTTGAG
ACCACCGAGCTTGACTCCGCGAAACGCTCGAGACCTTACGTAGGCGGTTTTACTGGCCCGGCATGGTTAG
TCAGGTAAGGGATTGGGTGAGACAATGTGTGACATGCAAGGAAGTCAAGGCACCAAACTTTCGCATGCA
AGTGGGTCTAGGAGAAGAGGTAGTGACCGAGCGACCATTCCAAAAACTATATATTGACTTTCTGGGAAAG
TACCCACGTTCGAAGCAAGGGCACGCGTGGATCTTCGTGGTAGTCGACCATTTTTCAAAATTTACTTTCTT
AAAAGCGATGCGGGAGGCTACGGCTCTTAACGTGGTGGAGTTTTTGGTCAGCGAGGTTTTTTATAAGTTG
ACATGATCTTAGCTTTCAACATCTCGCACATGCGCACCGCAGTCTACCACAGAACAAGGCGAGCGAGCGA
GTTAATCGTACCGCGTTGTCGGCCATCCGGGCGTATCTAGAGCAAGATCACCGCGAGTGGGACCTCTACC
TTCCGGAAATAGAGGTGTCCATACGAAATGCCATACACACCGCGACCGGGGTGACCCCTTTCTTTGCCGTT
TTCGGACATCATATGTTCCTTAACGGGGCATGCTACAGATTGGCTCGTAGGTTGGCGTCTCTGAGTGATCA
TGAGATCTCCCATTTGCACCCAGCAGACAAAATACACCTGATTAGACAGCGAGTCCAAGGCCAACTACAT
CAGGCTTACGAAAGAAGTCAGCGGCAGTACAATGCTCGAGCCCGGGTGTTCCAGGCGGAAACCCGGACA
AGAAGTTTACCGACGCAATTTTGTGCTGAGCGATTTTGGGAAAGCTTTCAACGCCAAGTTTGCAAGAAAA
GTTTCTTAAGGCCAGAGTAGTGCGTCGAGTAGGGCAACAACTCGTACCTGCTGGAAAAATTGAAAAGCAA
AA
>Ref_dvir_979
TTTTGGTAGCAAACCGGTGCTGTATCATTTGTATCACTTGGGCTTAGCCGTTTCTCAGGCTCTAAAATAGA
TGTACTTGTTGGGCTGAGTGGCTTCATATTTGCTTCCTCAAGCAAAATTTTCATTGGGCTTATAATAGATTC
TGAAACTAATTCATTATTTTCACCAATTGGGCTTTTTAGTGATGGTATGATGACTCCCGCTTGCGACTCTTC
TATAGATTCCAAGCTATCTATTTGAGGGCTAAGTTTTGTAATGGATTCTTTTATATTAATGTCGTCTTGAAC
TGTTTTAATATTGTCCTCGGCTCGCTCATTTACATTAACATTATATTCGGTAATATTATTGTTATCTATTTGA
GTTTGAAGCTCTGAAGTTTTGTCACCCGCATCTGTTTTTGTATCATTCTGCCGATTCTTTTGTCCATTTGTAT
TTTCATCTCCATGGTCTTCTAATAGTGGTTATTTAAGGTATCATGAACATTTAAGACTGAAAAAAAAAAAA
CCAAAAAAAATTTTAATAATCACCTGCCAGTCATGATTGCGGTTTAGGAAGGGGATTCCTATGCAATATCTA
AGCACAGGCTCGCTTACCTAAGTTATTAGCATTGACAATATTCTCATTGTTACATTTAGCTGAAGTGGTCG
CGGTCAGAATTGATGGATTCTTAAAAAGAGAAGTTTTAGATTACATTACGTTGTAGTCAATACTACAAAA
AAATTGAAAGTGGTTTACCTGATCGACTTTATCATAATCACCACTTTCCATAGAAGAATTGGTTACTTCTA
AAGGTGCATTTTGGTAGCAAACCGGTGCTGTATCATTTGTATCACTTGGGCTTAGCCGTTTCTCAGGCTCT
AAAATAGATGTACTTGTTGGGCTGAGTGGCTTCATATTTGCTTCCTCAAGCAAAATTTTCATTGGGCTTAT
AATAGATTCTGAAACTAATTCATTATTTTCACCAATTGGGCTTTTTAGTGATGGTATGATGACTCCCGCTTG
CGACTCTTTCATAGATTCCAAGCTATCTATTTGAAGGGGCAAGTTTTGTAATGGGATTCTTTTATATTAATG

TCGTCCTGAACTGTTTTAATATTGTCCTCGGCTTGCTCATTTACCTTAAAATTAATTCCGGAAAATTATTTT
ATCTTTTTTAGTTTTAAATTCTAAGTTTTTTCCCCC
>160_rnd-4_family-17__RC___Helitron
TATAAACCTTTTTGTCCGTATGTATGAACGCGTGGATCTCATAACCTATAAGAGCTAGAGACTGAAATTTT
AGATGTAGGTGCTCCTAGTGCCGGCGCAGATCGAGTTTGTTTCCGATAATCGATCACTTACTCCGTTTCCA
AGAAATCGATAAGAATCGATATCGATATCCTGTTTTGTTCGGAAAGGGTATTAGATCTTCGGCGTGCCGA
AGATAGCCTTTCTTTCCCGTTTTTATACCCTTGCAGAGGGTATTATAATTTTGTCGTGAAATGTGTAACGCA
TAGAAGGAGACATCTCCGACCCCATAAAGTATATATATTCTTAATCAGCATCAACAGCCGAGTCGATATA
GCCATGTCCGTCTGTCCGTCTGTCTGTTTCTATGCGAACTAGTCCCTCAGTTTTAAAGCTATCTTAATGAAA
CTTTGCAGAACTCCCTCTTTCTGTTGCACGCAGCACATATGTGAAAACCAGCTGGATCGGACCACTATATC
ATATAGCTGCCATAGGAACGATCGGTCGAAAATTAAGTTTTTGTATGAAAAAACATTTTGTTTNTCAAGAT
ATCTTGACCAAACTCGGCATTTATTAGTTTTACTTTACTCCTCATATATATGCAAAATCCTATTAAGATCGG
ACCACTATATCATATAGCTGCCATAGGAACGATCGGTCGAAAATTAAGTTTTTGTATGAAAAAACATTTTG
TTTTTCAAGATATCTTGACCAAACTCGGCATTTATTAGTTTTACTTTACTCCTCATATATATGCAAAATCCT
ATTAAGATCGGACCACTATATCATATAGCTGCCATAGGAACGATCGGTCGAAAATTAAGTTTTTGTATGA
AAAAACATTTTGTTTTTCAAGATATCTTGACCAAACTCGGCATTTATTAGTTTTACTNTGCTCCTCATATAT
ATGCAAAATCCTATTAAGATCGGACCACTATATCATATAGCTGCCATAGGAACGATCGGTCGAAAATTAA
GTTTTTGTATGAAAAAACATTTTGTTTATCAAGATATCTTGACCAAACTCGGCATTTACTAGTTTTCCCGTA
CTTCTTAGATANGGGCAAAGCACTATGAGCATTATGAAAAGGTTGGGTCTGCAAGGGTATTAGATCTTTG
GCGTGCCGAAGATAGCCCTTCTTTCTCGTTTTAATTTTTT
>9_rnd-4_family-368__RC___Helitron
TTAAGTGAAAATAATAAATTGAAATATAATTTTTTTTATAGTAACCTAACGTGTAAAACTTAATTATATAT
ATATATTTTCCTTTGCTTACATATTAATAATTCTGGTNTATTAAAGCCGATGCTATTTTTTTGTTTTTTATAC
CCTTTGCGGAGGGTATTATAATTTTGTCATGAAATGTGTAACGCATAGAAGGAGACATCTCCGANCCCAT
AAAGTATATATATTCTTAATCAGCATCAACAGCCGAGTCGATGTAGCCATGTCCGTCTGTCCGTCTGTCTG
TTTCTATGCGAACTAGTCCCTCAGTTTTAAAGCTATCTTAACGAAACTTTGCAGAACTGCCTCTCTTTCTGT
TGCAACGCAGCACATATGTGAAAACCAGCTGGATCGGACCACTATATCATACAGCTGCCATTAAGTTTTT
GTATGAAAAAACATTTTTGTTTATCAAGATATCTTGACCAAACTCGGCATTTATTAATTTTACTTTTACTCC
TCATATATATGCAAAATCCTATTAAGATCGGACCACTATATNATATGCATAGCTGCCATAGGANCGATCG
GTCGAAAATTAAGNTTTTTTGTATGAAAAAAACATTTTTGTTTTTCAAGATATCTTGACCAAACTCGGCAT
TTATTAGTTTTACTTTACTCCTCATATATATACGAAAATNCTATTAATATCGGACCGCTATANNATATACAT
AGCTGCCATAGGAACGATCNGTCGANAATTAAGTTTTTTTGNATGAAAAAAACATTTTTGTTTNTCAAGAT
ATCTTGACCAAACTCGGCATTTANNAGTTTTNNTNTGCTCCTTATATATATGCAAAATCCTATTAAGATCG
GACCACTATATCATATAGCTCCATNGGAATAATGAGGTCGAAANTTAAGTCTTTTAATAANAATATATTTG
NTTNNCGAGATAATATCTTGACCAAACTCGGCATTTACTTTTTCCCGGTACTTCTTAGATAGGGGCAAAGN
ACTATGAGCATTATGAAAAGGGTTCGGGTCTGCAAAGGGTATTAGATCTTTGGCGTGCCGAAGATAGCCC
TTCTTTCTCGTTTTTCAATTCAATTCACTGCATATGGCTAGTCTTAAACAATTATCAGCTTAATTTTTATTTT
TAATTGTCTTTTCAATATTTT
>Ref_dvir_868
TGAAAATTGAAATGTTTTATACAATTATAATCATAAGCTAGAAAAGAACTCCACTGGTAAAGAGACAAAA
ATAAACAATCTTAATAAAAACCGAAATGATTTCGAAATAAATACTAATTTGGAAAGCACCCATAATGCAC
GGTAAATTACAAAACCACTGGGAAAATAGAAAAAGTTGACGAAGATCATTTTAAAGAGACTAACAGAGG
TCGAAAAATTAAACATTATAAAACAAATTATCAAAAACAAAAGAAATTAAATAAGAGCAAATATTTACA
GGAGGACGCAATTCCAAAACAGGACCTATAACTTTCATTTTTCTGGCATTAATGATGTCAACTATATTACC
AGGAAGTAATAACCAGTTTATAGATATCAACCCTATTAAATCAAATAATGGATACTTACTCTTTAAAATAG
GCATGATAGACAACTACGAGCACCACTGCCTCACAATAAATTTACAAAGACAGAAGACACATATGACAA
ATTAATGTTACAAACAAACAAATATAAAGACAGAATACAAATTCAATATTTAACAGAAAAGTTGCAAAG
AGAAATAAACGGTATCCGAATAGTAAAAAGAAGAAGAGAGGATTACTTAATTTCGTAGGAACAACATAC
AAATACTTGTTTGGAACTTTAGATAACAACGATAAGGAGGAACTTGAAGAAAAAATGAATTCAATAGCTC
AAAATAGTATTCAAATAAACGAACTCAACCATGTTATTGAAGCAGTCAACCAAGGAATAGAAATAGTAA
ACAAAGGAGAAGAATTTAAATACTGGAACTACATTCAACTTACAACAATTCACAGAATATATTGAAGATT
TAGAAATGGGAATGCAACTAACTAGATTGGGAATTTTTAATCCAAAATTATTAAAACACGACTATCTCAC
AAACGTTAATTCCGAAAAGCTACTTAACATAAAAACATCTACTTGGCTTAGATCAGATACGAATGAAATT
TTGATTATTTCACACATTCCTAGAGAAAATAAATAAAACCCCATTATACGAAATTCTACCCTACCCGGACG

AATGATAAAAATATTTTACAAGACGAAACACAATACAAATACTACTCACATAATAAAAAATCAACACAAC
AAGGACAAAAAAAAACACAATAATAAACCAAAGATTAGGA
>Ref_dvir_1037
TCGACGAATGTTGTCCGAGACGAAAAGTGGATGTTAAAGTCATTAAGCACATTCACTTCTTCTGACTTAGA
ATTAAAAGATGTCGTTTGTGGCGCAGTGATTTGCAAAGGTTTAGTGGATACCGGAGCGGACCTGTGTCTTA
TGCGTAGGCGTGCCTATCGGAAGTTAGGAATCACACAATTAGCCGGATGTTTTAAGACTCTGACGGGTAT
AGCTGACAGCCAAATTCTGACGTTTGGAAGCTTTATAATATCTGTCAAGATTGATTACATTCAAATGGAAA
TCGAATTTCATGTCGTACCAGATGAGGATATAAGTTTCGAAGCAATTCTTGGCAGAACCATTTTGGATTAT
GTTGACTTGCAAGTGACTAAAAAGGGGATTTTACTAAAACCGAAAGTTGATGTAGAAAATAAAAAGATCA
GCGCTAACAATAGTTCTTGTTTTGAACTGTTTCATGAGTTTGAAAGTATGTGCATGTTAGCTATGGACCAA
GAGGCGGTTACGATCGAATTAGATCTTTCACATTTGAGAAAGTTGCAACAAAAAGAGTTTAAGCAACTAC
TACATTCGTATAAACCAATCAGAAATCCAAGCTTTCTAATTGAGATGAAGATTCTTTTAACAGACGAGCTG
CCAGTATTTCAGCATCCGAGACGTATAGCCGTGTGTGATCAGAAAATTATTAATGAGTAAGTGAAAGAAT
GGCTAGCAGAGAAAATTATCAAGCCCAGTATGTCAGAGTATGCCTCACCAGTTGTTTTAGTGGTAAAAAA
GAATGGCAAAAATCGTTTGTGATGCGACTACCGTAAGCCAAATGAAAAGGTTATTCATGATAATTTTCCT
ATGCCTTAAATGGATACTGTAAATTGAGAAGCTGCAAGGTGGGTCAATTTTCACTACATTAGAATTTGACA
AATGGATTTTTCCATGTACCCAGTTGAAACAGGCTCGCAAAAATATACTTCATTTGTGAACCAAAATGGGC
AGTATGAATTTTTATACGTGACTTTCGGCATTTCAAACTCTCCAGCAGTGTTCACTAGATTTATAATGGCA
ATTATGCGAGAATTAATCCAAAATAATGATGGGTCTGTTGAAGCTGAAAAGAGTGTTGCAAGTTGCAAGA
ATAAATTGGTCAAAATATCAC
>Ref_dvir_938
ACCAATTTGTCACGGGTTCATAAACGCAATTATATTAACCATTCGTAATTAAGCTTAAAAATTATACAAAA
ATAAAATTTTTTTTTGAAAATCTTCAAAGTACTTCATTAATATATCTGGGGCTTATGAGCCACTTTCCTGAG
TAATGATAACACTGTTCATAAATGTGCAACAAGTAAAGGCATTTATAATATACGATATCTACAAAATACG
ATAGTTTCGTTGGGATATGTTCATAAAGCAACGATCAGCCGCATAGCAAACGACAGTATGCCTGATCGCC
AGAATTATCAAGTATCAGATAGGTATGCTTCCGTATTTCAGTTAAAATCAGCGGTATACTGTAATAAAAG
GGTAATCAATCCAATTGCCATCCTAGTAAAGAAAAGCATCAGTAACACTAAGAAGACCAGGATCACTAGC
CACCAGTATATTATCCATAATGTATCGATGTACGATAGATGGAAATTGGGAGCAGCGTATGCCCACCCTA
CCATGATCGTCCAAGAGGCAAGCCATGTTTGAGACGATCCCTTGTACACCCTTGATCTTGTTTACATTGAC
TCAAGTCCGCTTTTCATAAATCCCGGATTGGGAAAGGGTCCTAGTTAGAATTAATGGTAACTTCTTTATTT
ATACCAATAAGATCTTTCATTTAAGTTGTTGTTATTCCGTAACATTTCAAAACTATTACATCTTATAGATGA
GTTAGTTTTACTATAAGTAAGAAGCGATAGTCCTTTCTTAAATTAGAGTTCTTCCCTACCACTAACTGAAG
TTTTATACCCTAGGCAAAAATTTTTTTTTTACCGTTGTTTACTCAGGGTTCTCATAAGCTCGGTTCACGCCT
TGAAACAACAAGACGATAATAAAACAACAACTGTTTAAAATTATCGTACGCATGCTAATTTGGCTGTTAA
AAGAAAAAAAAACTTATTTTGGCTCGTGAACCTGCCAAGTAATACCAAAAGTAAACAGAACAATTGTAAA
TTATAATGGGGGCTCCAGCTTACCATTTAGCAGATCCAGCGACGAAAGTACTTTCGGCGGTGTCCATACG
AGTGGTATTTATAAATATGCTTAAAGGCTTATTTTTCTAACGCATTGTTTTAGATGAATTAAAGTTGTAAAT
ATCACTTGCCAACCAAAA
>Ref_dvir_808
CAACATTAATAATATTAAGAAAAAGAAAAACGAAGGACAATCTTCAAGTGACCTTTTTATTGATATTGAG
GGTATAGATAGTGGACCATCTACATCAGCAGCTAATCTACTGATGATTTCTGCATCGCGAAAGATAGTCA
ACACACCAGCTCCACCTGCTACAGCCGTTGCTCCCCCTTTTTTAACAACAGCAACGGCAACAACAACAAC
AACAACAGAAACTCCATCGTTCCCATCATACGCAGCCGCACTGGCTCACAATAATGCTGCTCCCTTGGCAC
AAATCACAATTATTCGCAGCGGAACCAGCAGCAGCCCCACCAGCAGCAACAACGATGATTGCAACAATA
GCAATTTGCCCATATCTGGCGAAAAGCTGTTCCATAAGCAACAACTCATATCAACTAAAGCTGTTAGTCA
GACTCGGCGGAAGACTGCACGCATCCTAAACCGAAAAATCTCAGCCAAATCCCGTCTGACGCAACAATAT
ACATCTAGCAATGCACGACTAGCATCAAATCGATTCAGTCTTCTATCCGAATCCGATCTAGATGCGTCCGA
CCATCAGCTACAACCAAAATCGTAATCGCCGCCTAAACAGCGACAAGCGAACGCTATGATCAATAGCCTC
GCTTCTGCTGTACAATCAGCTCTGACAGCATGTCCACCATCCTCACCAATGTTAGCTCCCACAACAACAAC
CTCAACTCACAACGCACCGACGGCTCAAACAGCAGCAATAGCAGCTACCCCAAAAACAGCTTCAATTCCG
GCTGCTTCAACAACAATCCCCTTTTACAATGCTGTCACCAACGCTCCCCCCAATATTGTGACTAACTTCCTT
TCTGCCCCCATCCAACAACAACACGAGCAACATACAAACTTTAATCCTGTTCCAAAACAGTTCAAGCCCC
CACAAATATGCGTTCAACATAATGAATCCAATTGGAATGGCATTGATTCGTTAGTGGCCAAATTAAACGG
GCACAATCCACCCATAGTCGACTACGGCCTAAAATTGGGTGGCCCTGGCATCCTACGAATCATACCAAAA

ACGACACAAACCCAAGTCATCACTGCCCGAACATATCCTTCTTTGAATTAGCTCATATTTAAATAAAGAAT
CAAAACAACACTCTAAAAGCACAA
>9_rnd-3_family-201__RC___Helitron
ATCTCTATATATAAAACTGTTTGTCCTGACTGACTGACTGATTGGTGATCAACGCACAGCCCAAACCGTAA
GAGCTAGGAAGCTGAAATTTTCACTGTAGTTACCTTATGTGATGAAGGTGCACGTTAAGACGGGGTTTCG
GGAAATTCCACCCGCAAGTATGGAAAAAAAATCGCGAAAAACGTTTGTATGAACTTTTNGCCATCCGATT
GGCCTCAAACTCAATTTCAAAGTTCTTTGTTCAAATTCATTTGAGGGGTGTTTCACACTTTTCCAAAAATGT
ATTCCTCCTTGGTGGAAATGGGGGTCAAAGATTTGGTGGGTGACGTTTCGCGCTCAAATTCATTAGATGAA
AAATCATTTGAGACGTGTTTCACATTTTTCGCAAAATCTAAACTCCTTCGGTAGAAATGCGATTCAAAGAT
TTTTTGGGCGAAGTTTTAAGTGGTGTCCGATTTGCTTCAAACTTATTTTAAAGCTCTACATAAGAACTTAAT
GAATACGTGTTTCAGCGATTTGGAAAATTCCACCCGCAACAGTGGTTAATTAGCGAAAAACGTTGGTATG
AAANTTTTTGTTAACCATCGGATCGGCCTCAAACTTATTTCAAAGATCATTGCTAAAATTCATTAGAGGG
GTGTTTCACACTTTTCGAAAAATCCATTCCTCCTTGGTGGAAATGGGAGTCAATTTGGTGGGTGGCCTTTT
GCCTTCAAATTCATTTGCATGGATCTACATAAAAAATCATTTGAGACGTGTTTCACATTTTTTGCAAAATCT
AAGCTCCTTCGGTGGAAAGTGCATTTTAAAGTAATTTTTTGAGNAAGTCCGATTTGCGTCAAACTTATTTT
CAAAGCTTCCCAACTCTTACGGTTTGAGGCTGTGCTTTCATCGCCAATCAGTCAGTCAACAGNACCAAACA
GCTCTATATAAGAATATAATGAATACGTGTTTCAGCGATTTGGGAAATTCCACTCGAAACAGTGGTAAAA
TGACGGAAAACGTTTGTATGAGAGTAGTTTGTTTTACCGTCCGCTCAGTTTCAAAATCATTTTCAAAGCTA
TTCGAGAACATTGATTTTATAACATAAGTGCCACGGGCAAGGCCGGGCTATACCCGCTAGTGTNTATATAT
ATGTGTAGTTAC
>9_rnd-4_family-46__RC___Helitron
TTATATANTATAATTTTTGCATATATTTCGGCAGAAGGACTTTNTATTTGTTTTACTAATATTTGCCTACTT
CGATCAACGCAGTGATCTCCTCTCNATTACGTCGTACAATTTTTATACCCTAAACCCATTGAAAATGGGTA
AAAAGGGTATNATGCAAATGTATGTAACAGGCAGAAGGAGGCATCTCCGACCCCATAAGTATATATATTC
TTGATCAGCANCAANAGCCGAGTCGATCTAGCCATGTCCGTCTGTCCGTCCTCCGCGCCCGACCGTATGTA
TGAACGCGTCCATCTCAGAGACTATAAGAACTAGAGACTGAAGCTTAAATGTAGTTCCTGTTTGTTTCCGA
TANTCGATAACTTGNCCCGTTTCCACGNAATCGATAAGAATCGNTATCGAGATCCCGTTTTTTGAGCAAAT
CTCGTAAATTATAAGAGCTAGAGTCGCCAAACTTTACATGTAGCTTCTNGAATAGTATATACATNTGTNAA
AAGTATCTTTCACTTTACANCTATCGCCACCTCCCCGCTACCACCCCAGAGCTATAAATCANGTTAATAGC
CCAACTTGTATTGCCAACCAATTCAAGGCACAATTTNAATGCAATTGACATTTTTGAGTACACGCGAATAT
TGTATGGTACAATAATANNATATACTNTAGAAAATTTCATAAAGATCGGTTAAGAAACAACCAAAGTTAT
ATGCAACTGCGCAAATTGACAGCTAGCGAATCGCTAATCAAGCAATNTNTGTATGCACGTACANACACAC
ACGTTCATGCGCTTCGCTTCGCATTCTATCGCATTCTAACAGCATGGCTGTTGCGATTTTTCTGTAATGGTG
TGGCTGCTGAGTAGAGGCATAGCAAGTGGTGCGGTCTGTCGCGAGTTCGAGCCCTACCGGGGAAACTATT
TTATTTGCTGGTGTGGCTGTTGAGTGGAGTCGACAGCAAGTAATTCGGTTAGTTGCGAGCTCGAGCCCTGC
CAAGGGAACTTTTTCTAATATTTTGGTTTTGGAATAAAAGGGTCAGGGTATTTCGTAGTCCGGAGCTCCAG
ACTAGGACCTTTTACTTGTTTCTTACTTATTTGGTTCCCTCGTTGNTTGACACTTGTCACGGTCGCCATGTA
A
>9_rnd-1_family-69__DNA___P
CTTTCATCTGGCCTTTGAGGCTTCGTACTGCATGCCTGCCGACAGGTTGTTCGTTCTCCAAAGACCAGACG
AACGAATGCCGAAGTCGCGGCTCGCTGATGCTGACACGAGCTACGAAGTTAGTCGCCAGCCTGCCTTCCG
CAAAGCTTTGCGTCACTTCGTTTTCGAAGTACATTGAGCGAAAAAGTTTAGAACTTACGATCGACTGCTCT
GGCTTTAGCTGGCAAGAAATTAATTTTGTTAGTTTTCTAGCGCAGATCATGACCTATCCCAAATTTGTGTT
GATATATGAATGCATTTTGTGTACTGAGGTTGGCTCCGCAGAAAATATGTATTTCTAAGTCGATGCAAATA
TTTACATAATTTCTTTAAATTAAATTAATATTATTTTTATTCTCCAATTAAGAAATAAAAGAGTGCTAAAA
ATACAATTACATTTCAAGTCGACTCGAAGGTCATATTTAGTTATGACCCCATTATAATTATAATGGCCTCC
TCGCGGTGTTCCCTGCGTACTTTGTTCTTTATAATTCGAACAAAAATAATAAAATAAAAAAAATTAAAATT
AATTAGTGTTTATATTGTCTTTGGTCAAGTCCATTTATCTGTTTGCTTAATCGATGAAAGTAGTAAAATAGG
GGGAATAGAATCTGAGTCAGTCAATGATGCATCAGTCCAATTATTCGAAAAGCCTGATTCACGGTCTTAG
AAATTTACTCACATATTTTTCCCNTTGAACGCGTAATCTCCCTTCATAGATTAAAATACATCGCAGCCGAA
GCAATAATAATGTGGACTGAACAAAGTGTGTTAAATTTTGTTTTATTACGTTAGCGCCATGTATGCAATTT
TTAATAGGAGTTCTCTTTTCTTGTATTTTTATTCTACTTTTTCAACTTCTTAAAATTGAAAAGCGTTTTGGAC
TCGTTGGCTGTGCAGCTGGAAATCCTTGCGTGGCTGAGGTAGTTTTGCTAGGTTATTTGCTAGTGTGGCGG
CTGTATAAAGAACGTATAAACTTGCCATTACTTTGGCGGCGTCGTCGACGCTGGGCGGGCACATCGTAGC

CCGGATCGTAGTCATAGTCCTTTGAGTCCTCCTCCAAGTGGGCTGGAGGCGAAGGCTTACGTTTGGGCA
>9_rnd-2_family-62__RC___Helitron
CAAAAAGAATACAAAATTAAGTTTAATATAAAGAAATTCAATAATTTTTAAATAGAGTTTCTATGTATAA
AGCTGAAAAGTTTGATGTCTTCGCCCGTTTCTGTGTGTTTTTCTACACAGAAATTATAATGTCATATTCAAG
GACCACTTTAAAGCGGCATTCTCTGTTGTTTCAGTTTGTATGCCCTTGTAGAGGGTATTATAGTTTTGTCAT
GAAATGTGTAACACTTAAAAGGAGGCATCTCTGACTATTTAAATTGTATAAATGTTAGATCAGCATGAAT
AGAAGAGTTGATATAGTCATGTCCGTCTCTTGGTTTCTATGCGAAATAGTCTCTCAGCTATCTATGTTGAT
ATTTATGTCGGAACCAGCTGGATCGGACCACTATATCATATAGCTGGCATAGTTTTATGGAAAACTTGTTT
ATTTATCTAATTACATTACACAAGCTTGGCATTCATAGGTTTTACTGTGCCCCCTTCATATATCTTATATGC
CAAAATATTATATTTTCAAAGATATTTTTAACAACCTAAGCATTTTTAATCTGCACTATGCTCACATATCTG
TCAACGGGATACAAGGGTATTAAATCTTCGGCGTGCCGAAAATATCCTTTCTTTCTTGTTTCTTTTTTTATA
CCCTGAACCCATTAAAAATGGGTATAAGGGTATATTGTATTTGTGCGAAATCCAAATGTATGTAACAGGC
AGAAGGAAGCATCTCCGACCCCATAAAGTATATATATATATTCTTGATCAGCATCAATAGCCGAGTCGAT
CTAGCCATGTCCGTCTGTCCGTCCGTCCGTCCGTCCGTATGTATGAACGCAAGGATCTCAGAACCTATAAG
AGCTAGAGACTTGAAATTTTAGATGTAGGTGCTCCTAGTNCCCGCGCAGATCGAGTTTGTTTCCGATAATC
GATAACTTACTCCGTTTCCAAGCAATCGATATCGATATCCTGTTTTTTGGGCAATTTTGGTAAATAATAAG
AGCTAGAGTCACCAAACTTGACATATAGCTTCTAAAATAGAATATATATATGCATTTGATGTTGGAAGAA
GAGGGTTCAGGGTATCCCCTAGTCGGGAGCTCCCGACTAGAACCTCTTACTTGTTTTTTATT
>Ref_dvir_625
AAAATTAACCATTTTTAAGAATTCTAGATTTAAAATTGGAATATTGCCCAAATTTATCTAGGGGAACAAAA
ACCGTATAAAAACGTCATTCACATTGAACGGCGGCAAATATGAGTTTTGCCGTCTGCCTTTTGGCTTAAAA
AACGCAGGAAGCATTTTTCAAAGAGCCATCGATGACATCCTGAGGGAACACATAGGAAAAATATGTTACG
TTTATGTGGATGACGTAATAATCTTTTCTAAAAACGAAACTGACCATGTGCTACATATTGACACAATTTAA
AACAAGAGAAAACCAAGTTCTTTAAAGAGAGCGTGGAATTCTTAGGGTTTGTGGTGACCCAAAACGGGGC
AAAAACTGACCCTGAAAAGGTCAGGGCGATACAAGAGTATACTGAGCCGAAAAATCTGTTCAGCCTGCG
ATCATTCCTGGGGCTAGCTAGTTACTACAGGGGGTTTATTAAGGACTTCGCAGCAATAGCAAGGCCTCTA
ACCAATATTCTCAAAGGAGAAAATGTTTCGGTGAGCAAGCATATGTCACGATTGAATTTGACGAAACTCA
ACGTAATGCTTTCGAAAGACTGAGGAATATCCTGGCATCAGAAGATGTCATACTTATATACTTAGACTTCA
AGCACCCTCAGACCTAACTACGCCTCAATGGATGGCATCGGTGCAGTCCTATTTCCATCATTTTATGCACG
CTAAAGCAAAGCGAGACAGCCGCGAATAATCGAGAATTGTTAGCTATTGTCTGGGCTCTGGATAAACTGC
AAAATTACCTCTATGGGACAAAAGAAATAAATACTTAACTGATCATCAACCCTTTACTAATGCTGTATCAG
ATAGAAACACGAATGCAAAAATCAAACGATGGAAGGCCTTCATCTTCTGTCCCGTTACACCTTACAAAAC
GATATACAGTCAGACGCCAACGACTCACAGCGAAATGTCATTCACCTACACGATAAAAAAAACTGAAAA
GCCAACTGTTTCAAAAATCAAATTGTTATACTGGGGGAGACACAAATTCCATCAAACACGGAAAAAAAC
TCCACATTAAAATCTTTTCACCGAAAAAAAAAAAATTTCCTTACTTCAATGGACAAAAATATCCAAA
>9_rnd-1_family-55__RC___Helitron
TATATATAATACTAGCGGGTATAGCCCGGCCTTGCCCGTGGCACTTATGTTATAAAATCAATGTTCTCGAA
TAGCTTTGAAAATGATTTTGAAACTGAGCGGACAGTAAACAAACTACTCTCATACAAACGTTTTCCGTCAT
TTTACCACTGTTTCGAGTGGAATTTCCCAAATCGCTGAAACACGTATTCATTATATTCTTATATAGAGCTTT
GAAAATAAGTTTGATGCAAATCGGACACAGCTTAAAATTCTCAAAAAATTACTTTAAAATGCACTTTCCA
CCGAAGGAGCTTAGATTTTGCAAAAAATGTGAAACACGTCTCAAATGATTTTTTATGTAGATCCATGCAA
ATGAATTTGAAGGCAAAAGGCCACCCACCAAATCTTTGACTCCCATTTCCACCGGGTATGGNCCGGCCTTT
CGAAAAGCGCGNGACACCTNTCAAATGAATTTTAGCAANGATCTTTGAAAATGAGTTTGAGGCCGATCCG
ACGGTNAACAAAAAAACTTTCATACAAACGTTTTTCGCCAATTNACCACTGTTGCGGGTGGAATTTCCCAAA
TCGCTGAAACACGTATTCATTAAGTTCTTATGTAGAGCTTTGAAAATAAGTTTGAAGCAAATCGGACANC
ACTTAAAACTTCGCCCAAAAAATCTTTGAATCGCATTTCTACCGAAGGAGTTTAGATTTTGCGAAAAATGT
GAAACACGTCTCAAATGATTTTTCATATAGANCCTTGAAAATGAATTTGAGCGCGAAACGTCACCCACCA
AATCTTTGACCCCCATTTCCACCAAGGAGGAATACATTTTTGGAAAAGTGTGAAACACCCCTCAAAAATG
AATTTGAACAAAGAACTTTGAAATTGAGTTTGAGGCCGATCGGATGGTAAACAAAAAAGTTTCATACAAA
CGTTTTTCGCGATTTTTCCATACTTGCGGGTGGAATTTCCCGAAACCCCGTCTTAACGTGCACCTTCATCAC
ATAAGGTAACTACAGTGAAAATTTCAGCTTCCTAGCTCTTACGGTTTGGGCTGTGCGTTGATCACCAATCA
GTCAGTCAGTCAGTCAGTCAGGACAAACAGTTTTATATATAGAGATATTA
>9_rnd-6_family-1496__RC___Helitron
TTTTAAAAGAAATAACTTAACAAAATGGATGTGTAGTTATTAAATGAAATTGATTGTTTATGTTGATTACA

AAGATATTTTTATANCCNATGCAGNGGGTATTNTGATNCTGCCGTCAAATGTGTAACGCATAGAAGGAGA
CATCTCCGACCCCATAAATTATATATATTCTTAATCAGCATCAACAGCCGAGTCGATATAGCCATGTCCGT
CTGCCCGTCNNTCTGTTCAAAACTATGCGAGCCAGCCCCTCAGTTTCAAANCTATNTNANTCGAACTTTGA
AACAGANNTCCGTCTTTCTGCTGCACGNAGCNCATAAACTTCGGAANCCAGCTGGATCGGACCACTATAN
CATATAGCTGCCATAGGAACGATCGGTCGAAAATTAAGTTTTTTTGTATGAAAAACATTTTTGTTTNTCAA
GATATCTTGACCAAACTCGGCATTTATTAGTTTTACTTTGCTCCTCATATATNTGCAAAATCCTATTAAGAT
CGGACCACTATATCATATAGCTGCCATAGGAACGATCGGTCGAAAATTAAGTTGTTGTATGAAAAACATT
TTTGTTTTTTCAAGATATCTTGACCAAACTCGGCATTTATTGGTTTTACTTACTCCTCATATATATGCAAAA
TTCTATTAATATCGGACCACTATATCATATAGCTGCCATAGGAACGATCGGTCGAAAATTAAGTTGTTTTG
TATGAAAAACATTTTGTTTTTCAAGATATCTTGACCAAACTCGGCATTTATTAGTTTTACTATACTCCTCAT
ATATATGCAAAATCCTATTAAGATCGGACTACTATATCATATAGCTGCATAGGAACGATCGNTCGAAAAT
NAAATCGTATGAAAAACATTTTTGATTATCAAGNTATCTTGACCAAACTCGGCATACACNAGTTTTACCCG
GTACTCCTCGNANANNCGCAAGNCACTATNAGCNTTATGAAAAGGTGTGAGTCTGCAAGGGTATTAGATC
TTTGGCGTGCCGAAGATAGCCCTTCTTTCTTGTTGCATATATATGTTATAGAATAATAAAATGTAATAAAA
TGATAAAAAAATAATATAAATTTAAAAATTTTTAATTTTATTATTTT
>9_rnd-5_family-12813__LTR___Gypsy
TCACTCAAATATAAAAGTTCTAATAAACCAAACAACTAATAAAGTTACACCCATTGACGCAACCAGCCTA
CCAAAAGAAAAACGAATGTGCCCACAGGATTGCGAAGGCCCACTCGACAGCCCCTTCTGCGAATATACGT
GCAGACACTGCACAGCCAGCACCTCCAAACATTACATTACANCCAACAAATAAACACCTGTGCGAAGACC
AGCACCGGTACCGCCAGCGCAACAAGCTGTGCCATCGGAATAAGNGCGAGAGGCACACCGCCACTTGCG
CATACGAGGGAGCATCAGCGTGCCACATCCGGCGACGCCCAGCTGATGCAACGATGAACAACAGTTGCA
GCTATATCCTTAACTACTTTTAATTTTAAATATAAAAAAATTTTTGTAAAATTGAGCGGAAACACCTCCGC
CCGATTAGCAGCATAGCCCGTAAAGCTATGCGAAATGCTAGGGTAGTATTTTGTGTTATTAGCAATATTGA
AACCTTGAAACTAAAAACATTATTGGCATTTAAAGCTAAAAACACTATATGTTACATAAAAACATTATAT
GAATTTAAAAACATTACATCAACTTTTGTAAGAAATTTTAATTAAAATGGGTTCGAACGAATCCAAGGTTG
AAAAATCAGCCGCAAATGTTATAAATAACGTAAAAGTAATTGACCATAAAGAACANTTAGAAAATATTAC
TATTTTGCTAATTATAATTACTACAGTATCTCTTGTATCACTAGCATTGAAAGTGTATGCAATGCATACAA
AGAAAATTAAGAAACGCTATAGCAGTAGAGCAGACGGACTAGATAAGGTATGAATATATTTGAATATGTC
AGATATAGAGGCAATCATTAGGTTTACGTATAGAGATGAGCCAAACTTTCGTACTCGCGCACTAAACGTA
CTGCGATTACATAGAGATTTTATAAAAGTGTGTGATTTCGAATACGAATACACGGACATGTTCAATACTCA
TATGGGTCCATTGATACATTGTTCAAAAATCGAAAAGGGAATCATACTAAGTACGTTGCCATATGTCTCAG
TAAAACTAGTAACTGAAGAACACCCAATTTTGGGACCAGCAACGATACTAATGG
>160_rnd-5_family-2070__RC___Helitron
ACACTCGGGACTTGCTGTGCTGCCNGTATGCCTAATTNCAGGAGAAAGTGATTATATATATAAGGCTAAA
TTTTAGACCTTCCTGTTTTAATAAATTGAATGATATAATTTGATCATTTGTAATTAAAAATAAAATAATTGT
TTCACATAGCGCACCATGTAGTTATTAAACATATTTAGCTTCANANTTTGATTTTCCNCAAACCATACATG
AAGATGNATGTTAGAACNATGCNGAAGACTTTAAAGCNGCTCCGNCCCCANAAAGTGTATATACNCCTGN
TCAGTGTTAACACCTGAGTCCGTGTAAGCCTANCCGACCGNCCGGCCGTCCGTATGTATGACCGCAAGGA
TCTATTAACCTATAAAAGATATAGTAGTAGTACCCTATGGGGCTAGTTATCGGTCAAAATCGATATCGGG
ACCGTGATTNNTGNGCAAATCTCTTAGGTTTTTAGAGCTAGAGTCACCAAATTTGACAAGTAGCTTCTAGA
ATAGTCTATATAAATCAAGTATCTTTTATTTTNTAGCTATCCCCAACCCGCTACCACCATATAGCTATAAAT
CAAGTAAATAAACTAATTTGTATTGCACAACAAATATACGCCACAACCTTAATGCAATTGTNTTTTAGAGA
ATACANGTATATTCTATATCAATAAGATCTACTACTACTTGAAATTTACATCAAGATCGGTTAAGAAACAC
AGGAGTTATTAATGAAAATGTGTTTCGGCTGGCCACGTGGTTGGGGCAGCTAGCGCAAATTGCGAGATTT
TCTATATACATACACACACACACACACATTCATGCGCGTCTCATTGAATTCTATAAACAGCATTGCAATTG
CGATTGTATNGATTTCCGAGNGTGTNTATGTGTTGCTATTTTTATTCATTTTGTTTTCATTGGCATTTAATTG
TTGCTTTGGCGGAGAGTAGCGGTATATTACGAGTTCGAATCATATCGGATCTATTTTTTGTTGTTGAATTA
ATTTTCTGCATTTGGTGTTGGTNGAAGAGGGTTCAGGGTATCCCCTAGTCGGGAACTTCCGACTAGGACTC
TTACTTTGTTTTCGTTCTCTGNAGTGTATGCTAAAAAGAAA
>160_rnd-1_family-58__RC___Helitron
ATCTCTATATATAAAACTGTTTGTCCTGACACTGACTGACTGACTGATTGGTGATCAACGCACAGCCCAAA
CCGTAAGAGCTAGGAAGCTGAAATTTTCACTGTAGTTACCTTATGTGATGAAGGTGCACGTTAAGACGGG
GTTTCGGGAAATTCCACCCGCAAGTATGGAAAAATCGCGAAAAACGTTTGTATGAAACTTTTTTGTTTACC
ATCCGATTGGCCTCAAACTCAATTTCAAAGTTCTTTGTTCAAATTCATTTGAGGGGTGTTTCACACTTTTCC

AAAAATGTATTCCTCCTTGGTGGAAATGGGGGTCAAAGATTTGGTGGGTGACGTTTCGCGCTCAAATTCAT
TTTCAAAGCTCTATATGAAAAATCATTTGAGACGTGTTTCACATTTTTTCGCAAAATCTAAACTCCTTCGGT
AGAAATGCGATTCAAAGATTTTTTGGGCGAAGTTTTAAGTGGTGTCCGATTTGCTTCAAACTTATTTTTAA
AGCTCTACATAAGAACTTAATGAATACGTGTTTCAGCGATTTGGAAAATTCCACCCGCAACAGTGGTTAAT
TAGCGAAAAACGTTGGTATGAAACTTTTTTGTTAACCGTCGGATCGGCCTCAAACTCATTTTCAAAGATCA
TTGCTAAAATTCATTNGAGGGGTGTTTCACACTTTTCGAAAAATCCATTCCTCCTTGGTGGAAATGGGAGT
CAAAGATTTGGTGGGTGGCCTTTTGCCTTCAAATTCATTTGCATGGATCTACATAAAAAATCATTTGAGAC
GTGTTTCACATTTTTTGCAAAATCTAAGCTCCTTCGGTGGAAAGTGCATTTTAAAGTAATTTTTTGAGAATT
TTAAGCTGTGTCCGATTTGCATCAAACTTATTTTCAAAGCTCTATATAAGAATATAATGAATACGTGTTTC
AGCGATTTGGGAAATTCCACTCGAAACAGTGGTAAAATGACGGAAAACGTTTGTATGAGAGTAGTTTGTT
TACTGTCCGCTCAGTTTCAAAATCATTTTCAAAGCTATTCGAGAACATTGATTTTATAACATAAGTGCCAC
GGGCAAGGCCGGGCTATACCCGCTAGTATATATATATA
>Ref_dvir_912
AAATTTACATGTAGCTTCTAGAATAGAATATATACGAACAATACCTTCTCAAAGCTTCTAAAATATTCTTT
GGTAACGAAAATAACTTGGTGGCAGAAGAGCAACAAAAAAAGTTTAAATGCAGTTTTCTTTATTCATTCA
TTATTTTTACTTCACAAAAAAAATATTCAATATGTTTAATAGTATAAAAGTTATAAAGACAAAAAATTGCA
CGTGTACTATTCTAGGACTTAAGCTGCATACGATATGCAAAATAAGGTAACAGAGTTTCTGAGATTTTTTT
TTAAATTTGCTTAATACGTGGTAAGATAGTGTTTCAAAATGCCTAAAATTTATTTAAGCATTGGGGAAGGG
CAGCTAATTTTAAATAGAATATTCGTACAAGCTTAGATAACAGAGTTTCATAATAAATGTTTGTGCGACGC
GCTGATCACTGGTAGCACTAAAGATAAGTCAAAATAAGTTAGTAGGTGTTGTAGATTACAAATATAACGC
TGGTTTTGGGTTTTTTTGGTTTTGGGTTGGCTTGTGGATAATAGTGTAATAATGCGTTAACAGCTTGTACTT
TCGTTAAAAATGCTCATAAAAGTGGTTGTATAATGATATGTCGACATACATATTTTCAGCTTGTACCTAAT
CATTTTAGTGGTATACGCCAGTATATAACCATAGAGCTATGTAGGCCCCATTTTTATATACTCTAATGCTG
TATATGAATTTACTTCTCCTACCCCTTAAACTACAAATATTATTTAGCTAGATGGCAAACTAGGTCAATTT
ATCTGACTGCTATTCTAGCTGTCTTAAAACGAACGAAATATGAATTTTACAATAGGTAAATAATCTATAAT
GATTTTGTGAAAATGTATGTTGCAGACAATAAGGTGTCTCCAGCCCCATAAAGTATATGAACGTTAATTTA
GAAACCTGTAAGGTTTACAAACATGCATTTTCGAATATTGTTGTTTCTAGTGCCCGCACAGTGTTAGTTTG
CTTAATAAACGATAACTTATTCTCTATCAATATTGGGGGGGTTTTTTTTTTGAGCATTATAAAGCTAGAGA
CATTATTCAGACTGTCGTTTAGTGTACGCAGAT
>9_rnd-4_family-2091__LTR___Gypsy
ATATATATAGTGACATATGTACCACACCCACAACATATACTATAAGTACACATACACTCATGTATCCTAAT
ACAGCATATATAACATATGTCCACAGCACCTAATGCCAACCCCTCACACATAAGCCCTAGCAACTAAGCG
CACCACATATGTAACATGATACACCCCAATTTGTAAACAATAAACCCTAGAGCCCCAAAATCATGAATTC
TTGGTGAAAATAACAATTCTGGCGTGGGACCGATCATAAAATCGAAAACCAGTAGACATAAACATCCGCC
ACAATCGTTATTCCCACCAAANGCCCAAATTGCCAACGCAGCACAAATTCAGGCTCTTAGAATATTCTAG
AAACTCATCAAGAGAATGAGAGCATTGTTTAAAGAGAGCGAGCTCATGATCTTGAGCCCTCAGCCGAGGT
TTGCTAAGCGTTGACAATATTTTAGAAGTTGAGTTTTACTGAGGACGTTAAACCGTCAAGTTGCATCGTGC
ACAAATTGTAAATTCCAATAAGTCACGCCTGTTNAAATAAAGTTATATTTTTTTCTAACAACGCGTATTGG
CTTATGTAATTGGCGCAGCCGGTAGGATNCGCGAGTGATATAAGACCATTAAAGGACAATCGTTGTCCCA
GGCCTTTAANGCAAATNGGTCGGCGCTGATGATCGACGCGAACGCACAATCATTCGAAAATCGTATCCGC
AAAAGACACACGCGAAACTTGCCGCGAGTGCCCATTGACCNTTCACGGATTATAATCCCAGCAATTATCT
AGTACTCAACTAAGTATTGATCCTTTGGACAATCAGAGTTACTAGGTAGTGAATTTTATTCACTATCTAAC
AGTGAGGGTGCCCTTGGTCTTGCGCATCCTTCGATTCCTGCAAGTCGCAGTCATTGGTAACGCGCGAAATA
ACGCCATACCTAAACCAAAAGACTACACCAAGAGCAATAACAACAAANAAGTGAANGTGAACATCATGG
CACAATCAAATGAGCCAGTATTGCAACTTAGTGAGCAACACCTTTCACAAGCACNAGGCCAAATAAGGCA
AGTGCCAACGTTTCACGGAGCCACCGAGGAGCTAA
>Ref_dvir_889
TTTACCGGGCAAAGGAACAAAGACCAAAATACCGAACACTGGACTCGACGAAATTCTCCAGTTAGACAA
GGAGCAATTGGTAATACTTCGCGTCGATCGACACGCAGACAAGACCCAGTCTACCGGGCAAACAAACAA
AGACAAAATACTGTAGACCACTCGACTCGACGAGCTAATTCTCCAGTTAGACAACAGGAGCAATTGGCTA
ATACTTCGCGTCGATCGACACGCAGACAAGACCCAGTCTACCGGGCAAACGAACAAAGACAAAATACTG
TAGACCACTCGACTCGACGAGCTAATTCTCCAGTTAGACAACAGGAGCAATTGGCTAATACTTCGCGTCG
ATCGACACGCAGACAAGACCCAGTCTACCGGGCAAACGAACAAAGACAAAATACTGTAGACCACTCGAC
TCGACGAGCTAATTCTCCAGTTAGAGAACAGGAGCAATTGGCTAATACTTCGCGTCGATCGACACGCAGA

CAAGACCCAGTCTACCGGGCAAACGAACAAAGACAAAATACTGTAGACCACTCGACTCGACGAGCTAAT
TCTCCAGTTAGACAACAGGAGCAATTGGCTAATACTTCGCGTCGATCGACACGCAGACAAGACCCAGTCT
ACCGGGCAAACGAACAAAGACAAAATACTGTAGACCACTCGACTCGACGAGCTAATTCTCCAGTTAGACA
ACAGGAGCAATTGGCTAATACTTCGCGTCGATCGACACGCAGACAAGACCCAGTCTACCGGGCAAACGG
GAGCAATTGGCTAATACTTCGCGTCGATCGACACGCAGACAAGACCAACAAAGACAAAATACTGTAGAC
CACTCGACTCGACGAGCTAATTCCTCCAGTTAGACAACAGGAGCAATTGGCTAATACTTCGCGTCGATCG
ACACGCAGACAAGACCCAGTCTACCGGGCAAACGAACAAAGACAAAATACTGTAGACCACTCGACTCGA
CGAGCTAATTCTCCAGTTAGAGAACAGGAGCAATTGGCTAATACTTCGTCGATCGACACCGCAAACAAGA
CCCAGTCTACCGGGGCAAAAAAAAAAAAAAACAAAAT
>9_rnd-1_family-105__Unknown
ATTTTTATACCCTGAACCCATTGAAAATGGGTAAAAAGGGTATNATGTATTTGTGCAAATGTATGTAACAG
GCAGAAGGAAGCATCTCCGACCCCATAAAGTATATATATTCTTGATCAGCATCAATAGCCGAGTCGATCT
AGCCATGTCCGTCTGTCCGTCTGTCCGTCCGTCCGTATGTATGAACGCAAGGATCTCAGAACCTATAAGAG
CTAGAGACTGAAATTTTAGCTGTAGGTCCTCCTAGTGCCTGCGCAGATCGAGTTTGTTTCCGATAATCGAT
AACTTACTCCGTTTCCAAGCAATCGATAAAAATCGATATCGACATCCTGTTTTTTGAGCAAATTGGGTAAA
TAATAAGAGCTAGAGTCACCAAATTGATATGTTGCTTCTAGAATATTATATATATATCAAGTATCTTTCAT
TTTATACCTATCGCCACCTCCCCGCTACCACCNCATAGCTATAAATCAAGTTAATAACCCAACTTATATTG
CCCACCAATTTAAGCCACAATTTNAATGCAATTGACTTTTTGAGAATACACAAATATTCTATGGTACAATA
AGATATACTGTAGAAAATTTCATTAAGATCGGTTAAGAAAAATATGTTTACTGCGCACGTGTTTGCGGCA
GCTAGCGCAAATTCCAAGAATTTCTGTATACATGTACACACACACACATTCATGCGCGTCTGCTTCGAATT
CTATCAACAGCATTGCAATTGCGATTGTATTGTTTCTGTGCTGCTATTTTAATTCGTTTTTCTCANTAAN
ACTTAATTATTGGTGTGGCTGTTGAGTAGAGGCGGCGGCAAGTGGTGCGGTCTGTCGCGAGTTCGAGCCC
TACCGAGGAAACTATTTTTTTTTTTTGCTNGTGTGGCTGTTGAGTAGAGNCGNCAGCAAGTAATGCGGTCA
GTTGCGAGTTCGAACCCTGCCAAGGGAACTTTTTTTTATATTTATCTTTATTACTTCAAACGAGCGCGTAGC
GCGANCTGCATTTNGTGTTGGAATAAGAGGGTTCAGGGTATTCCCTAGTCGGGAGCTCCCGACTAGAACC
TCTTACTTGTT
>160_rnd-5_family-3283__RC___Helitron
CGTACATCTCTATATATAAAACTGTTTGTCCTGACCGACTGATCGCACAGCCCAAACCGTAAGAGCTAGA
AAGCTGAAATTTTCACTGTAGTTAGTTTATGTGATGACGGTTTCGGNANATNCCNNCCGCAACTATGGAA
AAATCGCGAAAAAAATTTGTATGAAACTTTTTTGATCACCATCTAATCGGCCTCAAACTCNTTTTCNAAGA
TCCCTGCTTCAAANTCATCTNAGGGGNGTTCCACACATTTTCGCAAAATNCACCCCTCCTTGGTNGATTGA
ATAGGTGGNCAACGNTTTGGTTCGTCGGGTGACGGTTCGTTTTAGAAGTGTTTCACACTTTTCGCAGAATA
AAACATCTTCGGTAAAATATGGATAGTACGATTTTTTTGATAAAGTTTTTAATATTGTCCGATTTGCATTAA
ATTTATTTTACTGATCTGTTCAAAGAANAAGAACTTANTGAATACGCGTTTCAGCGATTTNGGAATTCCAC
CCACAACAGGGACTAATTAGCGAAAGACAGTTTTTTTTGCTTATCCTCCGGGCATTGTGAGAATTTATTTG
TTACGTTCTTGGGTGGCAAATGAAGGTCAGATGTTTAATTAACGAAAAATGTTTGTATGAAACTTTTATGT
TTAGCATCTGACCGGTCTCAAAAAAATTGTTCGAATTCATTTCACGGGTGTCTCTCACTTTTTTAGTATCCA
TTCCTCCTTGGTGGAAATTTGGGTCAANGATTTGGTGNGTGANGTTTCGCGNTNAAATTNATTTCCAAAGA
TCTNTNTGGGAAATCATTTGGGACGTGTTTCNCNCTATCGCAGATCCAAGCTTCTTCGGTGGAAAATTTAA
GTTTAGCNAAGTTTTAAGTTTTGTCCGATTNGCTTTAAACTTATTTTCAAAGCCCTACATAGGAACTTAATG
ATTACNTGGTTCAGCGATTTGCAAAATCCNCCAAGACTGTNGTAAATTTGCGGNAAACGATTGTATGAGA
ACTATTTGATTATCGTCTCAATATTTTCAAAATCATTTTCAAGGCTTTCTTACAAAATTTATTTTGTATGTG
CCACGGG
>160_rnd-3_family-1__RC___Helitron
TTTTTATACCCTTGCAGAGGGTATTATAATTTTGTCGTGAAATGTGTAACGCATAGAAGGAGACATCTCCG
ACCCCATAAAGTATATATATTCTTGATCAGCATCAACAGCCGAGTCGATATAGCCATGTCCGTCTGTCTGT
TTCTATGCGAACTAGTCCCTCAGTTTTAAAGCTATCTTAATGAAACTTTGCAGAACTCCCTCTTTCTGTTTG
CACGCAGCACATATGTGAAAACCAGCTGGATCGGACCACTATATCATATAGCTGCCATAGGAACGATCGG
GTCGAAAATTAAGTTTTTGTATGAAAAAACATTTTGTTTTCCAAGATATCTTGACCAAACTCGGCATTTAT
TAGTTTTACTATCCTCATATATATGCAAAATCCTTATTAAGATCGGACCACTATATCATATAGCTGCCATA
GGAACGATCGGTCGAAAATTANGTTGTATGAAAAAACATTTTGTTTATCAAGATATCTTGACCAAACTCG
GCATTTACTATTTTCCCTGTACTTCTTAGATACGGGCAAAGCACTATGAGCATTATGAAAAGGTTGGGTCT
GCAAGGGTATTAGATCTTCGGCGTGCCGAAGATAGCCCTTCTTTCTCGTTTTTTTTCGCTGATGTGGCTGTT
GAGTAGAGTCGACAGCAAGTAATGCGGCCAGTTGCGAGTTCGAGCCCTGCCAAGGGAACTTTTTTTTATA

CCCTGAACCCATTGAAAATGGATACAAAGGGTAAATTGTATTTGTGCAAAATGCAAATGTATGTAACAGG
CAGAAGGAAGCATCTCCGACCCCATAAAGTATATATATTCTTGATCACCAACAATACGTCGTTATTGGATT
AAACGCCTACCTCCTGCAGCTCGCGCCGTCATTGCAGGCATTCTTGAGACTTCCCCAAATACAACATTAAC
GCAACTTGCAACAGCCGCTGACGCTGTAATGGATACACTCAGTTATACTGCATGTGATAACGACGCAGTG
CTTTCACGTAACAACACAGCAACTGAGCATCTAATTGACACCCACATCACTAAGATAGAAAAACGTCTGG
ATGCCCA
>9_rnd-5_family-1148__Unknown
CAGTGGGTCATTTGTTTTCTGTATATTATTTTGATCTTGTTCGGCTCAGCAGCTAAAAATTAAACTAGTATT
ATGTTAGCATAACATTTCAGTTACATATTGCCCTGCGAGGCAAGCAATGTCACTATTTTGTTTTGTTTGGCG
CTTCTCGCATGTCATAAAATAGCTCAATATTATTTGCTGTTAACTATCTAGCGCTTATCGCTTTGCGCTCAT
CGTAGTTTCTGTTATCGCGCTCTTTTACCTTTTGCACTTTGTCTTAAGCGGCTAGCGCATCAATGCTAGCGA
CGCCGCAATGCACTCTTGTCTCGTATGTTCGCTATATCGGATCTCTTATCGATCGCTTCGCTAAAAATTGTA
CATTTTCGCATATTGTCAATACGCTCGCTATAAGCATATAAATAAATCAACCCGTTGCATGAACTTATAAG
AAAAAGCGGAATTTTTATAACATCCTTAACTTCTCGCTATTAAGTGCTAATAAAAAAGCTTGTTAGCCCAA
CACATTTTGGTGACCCCGACGTGATTTTTTTATTTAACATTATAGTGTTGCATAAAATTATTTAAATTAAA
ATTGAGCTAGTTGTTACTAGTTTCGGCAGCAATTAAAAACCGCTCTTATATACATACATATGTACGAGCAA
GTGTCATGCGGTAAATTGCAGTAAAGAGGAGTCTGTGTACATAAGCATATATACNCCTACATACAGACGC
GTTGGCTCTCGCTATCTGCATTTTACTTGTGTTTTTATTCGACTAACTGTTGCGCGCGCTTAAGAGTACAGT
GCTGACAATAAGTCAACGGTCAACGAACTTTCGTGTGCAGTTTTAATTCGGTTTTCTTTGACCTTTTTCGTT
TGCTTCGGTTGCCATGGAAAGTTCTAGTGCNGAATCGCTGGCCCAGCCTACAAACAGGGTGTCATTTTTAA
AGCGCAAGGTGGCGTCCTCGTATGAGCGGTTGCAGGCGCTTCAATCTAGCCTCAACCAAACGCCCCTTGC
CAATAATGATGAAATTGCATTGTCGGTGCTGCTCAAGCGGATTGAAAAATGGAATCTGCATTCGAA
>9_rnd-4_family-938__RC___Helitron
TTTTATAATTCATAATACGCATTCAAATTTTAATATTAAGCCAATATNAGATGCTGACANATNTATAATGA
TACTAGCGGGTATAGCCCGGCCTTGCCCGTGGCACTTAATGTTATAAAATCAATGTTCTCGAATAGCTTTG
AAAATGATTTTGAAACTGAGCGGACGGTAAACAAACTACTCTCATACAAACGTTTTTCGTCANTTTACCAC
TGTTTCGAGTGGAATTTCCCAAATCGNTGAAACACGTTTTCGCAAAAATGTGAAACACGTCTCAAATGATT
TTTTATGTAGATCCATGCAAATGAATTTGAAGGCAAAAGGCCACCCACCAAATCTTTGACTCCCATTTCCA
CCAAGGAGGANTGGATTTTTCGAAAAGTGTGAAACACCCCTCTAATGAATTTTAGCAATGATCTTTGAAA
ATGAGTTTGAGGCCGATCCGACGGTNTCAAAAAAGTTTCATACNAACGTTTTTCGCTAATTAACCACTGTT
GCGGGTGGAATTTCCCAAATGCTGAAACACGTATTCATTAAGTTCTTATGTAGAGCTTTGAAAATAAGTTT
GAAGCAAATCGGACACNACTTAAAACTTCGCCAAAAAAATCTTTGAATCGCATTTCTACCCACGAAGGAGC
TTAGATTTTGCGAAAAATGTGAAACACGTCTCAAATGATTTTTCATATAGANCCTTGAAAATGAATTTGAG
CGCGAAACGTCACCCACCAAATCTTTGACCCCCATTTCCACCAAGGAGGAATACATTTTTGGAAAAGTGT
GAAACACCCCTCAAATGAATTTGAACAAAGAACTTTGAAATGAGTTTGAGGCCGATCGGATGGTAAACAA
AAAAGTTTCATACAAACGTTTTTCGCGATTTTTCCATACTTGCGGGTGGAATTTCCCGAAACCCCGTCTTA
ACGTGCACCTTCATCACATAAGGTAACTACAGTGAAAATTTCAGCTTCCTAGCTCTTACGGTTTGGGCTGT
GCGTTGATCACCAATCAGTCAGTCAGTCAGTCAGTCAGTCAGGACAAACAGTTTTATATATATAGAGA
>9_rnd-4_family-178__LINE___CR1
TGAAGCGAACAGGCGTATTTTTTGTTTGCCTCTCCGTTTTTTATTTATATTTCGCGTGTACATGTAAATCAT
TTAATATTGTTTTTTATGTTTATCGCGAGTGCATTCTTGTGGCGCGCTTGCGCTTAATAAATATTGTTTCTTT
TAAATTTGTGTTNTTGTGCATTTTTATTTGGTAGCGCTTGCGCCGTTAGCTCTGTGGGTAGGCTTCTTGTTG
TTGTTTGTTGTTCATTCTTAACTGCGCATCGCCTCNCTGGCGNGCAAGATTTCGTGAGCAAGCGAACAATC
TATAAATAAACTACTCTCGACTCTCGCGCTCTCTCGCCTTTTTTCAACTTGTGCTTTGATCTTTTTTTATTCC
GCCGCTTGTTGGTGTTCTATACGCTATTAACAATTCTATAACTCTCTTTTTACATTTGGCTGCGTTATGTAC
AGTAGAGACCCATAATAATGGACCATAAAATTGTCTGCTTCAAAAAACTTGCCAAANGCAAATTACGGCA
AGTCAATCCAGCATACCTTGTTGGCTGTGTGATAATGTCGTGCATTCTAAATGCGGGTTACGTGGGGGACG
CTATTGCCAAACGAAATGGCTTGCACTATAGTTGCGACTCGTGTAGGGAGATTGATGGCGAAATGCGNNC
GTTTATGCGTCAGACCAGGAANGGCTTTAGGGGTCTGATTGTTGGTTTTCGTAAACTNAACGATCAGCTCA
CAGCCCTGGATACCCAATTTAACAGCCTAAAACTGTTGGATGAGTCTCCTGGGCCTAAAAAGCACCCACT
CCTTGTGATTTGCCGTTGCCTAATAACCTTCAGCCTCCGACTGCAATTCAGCAGCCTACNACACCGATTAC
ACAGCAGCTATCTCGTTTGTTACTCCGGTAGCTGTGAGGTCGCTGTGGACNCTGATTCTGTCCCGAAATGT
GTGACATCGGCACGAGAAAAACCAGTCGAATTATCCGTGTCGTCCGCTGTCGTAGGTGTTCCGAAAGGGC
GACAGCAACAATTAAATTTACAACAGCCGCTAGAAGGCTCGTCGGTCGCA

>Ref_dvir_941
AGGTACCAGAGGGGTGGTGGAGGGAAGGAGCTTGTCTTGAGGCTTTTCCAAACAAATCTCTTCATCACTT
TCATCATCAGAATATTCCTTAAGAGTTTGCTTGGATGCATCAATGGGGGACTTGGGTTTCTCATCTAATTT
AGCTTGTGGCTTATCGTCCTTCAATTCAGTCAATGATCAGTTTTCTGCAAGGTCTTGTCTGCAAGTGGCAG
CAGAGGAGTGGTTGAAGGAAAAGGCTTATCTAGTGGCTTGACAATTCCAAAGTCGTCATCACTCTCATCG
TCTGAATACCCCTTCGAAACTGTCTTAGCTACATCAGTTGGGGATTTGGGTTTCTCAACCGATTTTAAAGG
ATCCTTGGGCAACTTAACTTCCTCTTTGATGTGAATATCAACAGACTCGCGTGTTTCACTCACAGAAGTAA
TTGTGCTTGTAGTCATTGCTACAGGTACCAGGGGTATGGCTGGGGAAAGGAGCTTCTCTTTAGGCTTATCA
AATCCAATATCTTCATCACTTTCATTATCGGAATATTCCTTAAGAGTTTGCTTGGATGCATCAATGGGGGA
CTTGGGTTTCTCATCTAATTTAGCTTGTGGCTTATCGTCCTTCAATTCAGTCAATGGTTTAACAATATCGTC
AGTTTTCTGCAAGGTCTTGTCTGCAAGTGGCAGCAGAGGAGTGGTTGAAGGAATAGGCTTATCTAGTGGC
TTAACAATTCCAAAGTCGTCATCACTCTCATCGTCTGAATACCCCTTTGAAACTGTCTTAACTACATCAGTT
GGGGATTTGGGTTTCTCAACCGATTTTAAAGGATCCTCGGGCAGCTTAACTTCCTCTTTGATGTGAATATC
AACAGACTCGCGTGTTTCACTCACAGAAGTAATTGTGCTTGTAGTCATTGCTACAGGTACCAGGGGTATGG
CTGGGGAAAGGGGCTTCTCTTTAGGTTTATCAAAATCCAATATCTTCATCACTTTTCATAATCGGAAAATT
TCCTTAAAAAGTTTCCTTGGATGCATCAATGGGGGAACTTGGGGTT
>160_rnd-5_family-171__RC___Helitron
CTGCTCACGAATAGTTGGCGCGCTTTTTATACNCTGAACCCATTGAAAATGGGTTAAAAAAAAGGGTATA
ATGTTTTTGTGCAAATGTATGTAACAGGCAGAAGGAAGCACCTCCGACCCCATAAAGTATATATATACTT
GATCAGCGTCTGTCCGTATGTATGAACGCGACGATCTCAGAACCTATAAGAGCTAGAGACTTGAAATTTG
ACGTAAATCCCCTTTTTACTTATCCGTTTTNAAGCAATCGATAAAAATCGATATCGANATCCTGTTTTGAG
CAATTGTGGTAAGTNTTNAGAGCTAGAGTCACCAAATTTGNAACGTAGCTTCTAAAATAGATTATATATAT
GTATATGTATATAAATCCAGTTAATAACCCAACTTATATTGGCCACCACTTTAATGCAATTGACTTTCTGA
GAATACACAAATATTCTATGGTACAATAAGATATATAAAAACCCAAAGTTATGTTTTACTGCGCAATTGG
ATGGGGCGCAAATTCCAAGAATTTCTGTATNCATGTACACACACANACATTCANCCATGCGANTCTGNTT
CAAATTCTATCAANAGNATTGCAATTGCGATTGTNTTATGTGCTGCTATTTTAATAGGGTTTTCTCTCTCAA
TAATACCCAATAATTGGTATGGCTGTCGNGTANAGGATAGCATGTNCTGCGNTCTGTTACGGGCTCAAGC
TCGACCGAAACTTTTTTTTTTANATTTATATTTATTGCTAAAGCAAACGAGCNCGTAGCGCGAACTGCATT
TGGTGTTGGTAGAAGAGGGTNCANGGTATCCCCTANTCGGGAGCTCCCGACTAGAGCCATTCTTTTCATTT
TACCGTTTCTTTTTTTCTANNANAACTATCGTAAATGAATATATTACATATATATGTTTAGTGTGTTTCACG
TTGTATTTTATGCTTTGCTTTAAAAAGCAACTCGTATAAACGTTTTGTGGTTTAACAATATTAGGAAACGT
GTAATGAATACAATGAGTTATATTTAATTGGTTTTTGTTTTCGAGTAT
>9_rnd-6_family-18__RC___Helitron
CGAAGGTATAGTTATGTCTAGCGAGTACTTTGACTTANCGCTATTTATACCCTGAACCCATTGAAAATGGG
TANAAGGGTATATTGTATTTGTGCGAAATCCAAATGTATGTAACAGGCAGAAGGAAGCATCTCCGACCCC
ATAAAGTATATTCTTGATCAGCATCAATAGCCGAGTCGATCTAGCCATGTCCGTCTGTCTGTCCGTCCGTC
CGTCCGTCCGTCCGTCCGTCCGTCCGTCCGTACGTATTATAGCTAGAGAGAAATTTTAGATGTAGGTGCTC
CTAGTTCCCGCGCAGATCGAGTTTATTTCCGATAATCGATAACTTACTCCGTTTCCAAGCAATCGATAAAA
ATCGATATCGATATCCTGTTTTGGGCAATTTTGGTAAATAATAAGAGCTAGAGTCACCAAACTTGACATAT
AGCTTCTAAAATAGAATATATATATGCATTTGATGTTGGAAGAAGAGGGTTCAGGGTATCCCCTAGTCGG
GAGCTCCCGACTAGAACCTCTTACTTGTTTTTANATAGTATGAATGAAAATAATGTACAATTGAGCGATAT
ACCCTGAACCCATTGAAAATGGGTATAAGGGTGTAACGCAGAAGGAAGCATCTCCGACCCCATNAAGTAT
ATATATTCTTGATCAGCATCAACAGCCGAGTCGATCTAGCCATGTCCGTCTGTCCGTCCGTCCGTCTGTCC
GTCCGTCCGTNTGTATGAACGCAAGGATCTCAGAGAACCTATAAGAGCTAGAGACTTGAAATTTTAGATG
TAGGTGCCTNCGCAGATCGAGTTTGTTTCCGATAATCGATAACTTGCCCGTTTCCAAGCAATCGATAATCG
ATATCCTGTTTTTGAGCAAATCTCTTTGGTTTAAGAGCTAGAGTCACCAAATTTGACATNTAGCTTCTAGA
ATANTATATATATATGCATTTGNTGTTGGNGGAAGTAGGGTNTAGGGTATTCCCTAGTCGGGAGCTCCCGACT
AGAACCTCTTACTTGTTTAAAATTATTTTTATTATTGCTATAGAAA
>Ref_dvir_592
ATAATTTAATGCTAGATGAGATTTAAACGTTGCTACCATATAATCATAATGAAAAACATACATCAACAATT
TCGAAAGTAGTAAACTAGTAAAACCTAAATATATAGGCTTACTATAATAGTTATGCAATCTTTTCATTTGG
ATTGCATACATTTTATTCGAGAATTACACTGAGCTCCCAATCTATTGCAAGACGTTGTTTCCATGCAGTTA
CCAATTTAATGTTTTTACGTTTTTCAACGTTTTCCATGGTTTTCCCATATACTGCAAATTAAAAAAATCTTT
TTCGAAATTATCGCCTGCGTTCAATGATATGTATTAATATCAATATATTTTCTTAACCAAGGAGACTGCTT

AAACCGTAATATACGGTAAATTTAGTTAACCGCAATCCATTATGAATACATTGTTGAAGATTTCTATAATG
TATGGGATATTTATATTTCGGTCTTAGGTCAGCGATAAGCTTTTTAACTGTACACTTTGCAGTCGGTTTTTG
ATTGCTTGGACAAAACGGCAAGTCGTTATACATATCATGAAGGGATTGGTTATTCTAAATCTACTTCAAGA
ATATATCCATATTCAACGTTTCAGAAATTGAAAATAAATGAAATGATCGTATTTCATCCTCACTTAACCAC
TGGAAATCACCATATGGAAGATATTGTGACATCGCCCACCCATATAGATTATTTAAATCCAAATACATTAA
ATATTGAGAGGGTTTTGTGCTATCATAATCATTCATATACTATGATTAGCAATTGAATATCTCACCATTTTG
TATAAATCTTTTGCATATCTACTTATATCTGTAAATAATTCTAATTCTATTCCTGTTAATTTTAGCATAGCA
TCCCAAGATTACCCCGGAGCTGTGTAATAATGGGCTCGGTCCAATGAATAAATATTCTCGCAAACTAAAT
GGAAATTTTCGAAAACATCTGCAAGCAAAAATATATCTGTCTTTAAATACAACTCTATATAATCTTTAAGG
GTTTTACAATTAAAAACTCTCCACACGTTCT
>Ref_dvir_931
CCGCTTGATAGCTGCACAAATCATGCTGATTTGAATGGGAACCACACTTTTTATAGCCGCTGATTTGATCA
GATGAAAGCACTGGAGCATTCATATAATCATTATAGTCTTCGACGCTTGTTTTTAGCATATATTGTAAGAT
GTCTTTTTCCTGACATCCGTACGTAATTATACGTTTGCCTTTAGTTTTTTCGCGAAGCCATCTTTTAGTTGA
GTTGTAATCGTCCTCACCGATCTCCCACGAGAGCACTGCATCTGCCCGTGAATAGGTTTGCTGAAGCTTGT
TTAAGTCCCTAAAGGGGTATGGAGGTTTTATTCGAAATACTCGATCGAGCATGGTGTCCGGTGTAAAGAA
GATTCTTTGACAATAATCTCACATTGTTTTCCGCCAGTAAATGTAAGATATATGACAGCGGTATTAATTTT
CTTGGGATCGCTACGAATACATCCGATCGGTATCAGACTATGCGTTGAACTAAAGAGGTTAACGGTGAAT
ACTCAACCAAGCGGTCATGGATAAGTAGACAGTAAGCATCACATGTTGAAGTATTTCCTGTTGCTAAGTCT
ATAGAAATTCGAACATCAATGGGTCCGGTTTTAACTGCCTCATTCTGATATGATAAGTCCACAACAACAAT
GGGGGCATTTTGTTCAAATTCTTTAGGTGTTAGCAGTGGTTGAGGTTCTTTCATATAATAGCTTCTTGAAAT
TAGCGTACATATCATACAAAACAGCAAATCGTCCGCGCTCAAAATTTAAATTTAAACATATGTTTCTGAGT
TCAAATGAACTTTTAAATTTAATAAATTGTTTGTAATCATTTTTCCCTCCGAAATAAATACGAATAATGCA
AATCGAGGATGTTCTCTATTAGCTGCTAGCTTTACATTCCAAGTGTGTTGAGCGCCAACTGTGAGTGATGG
ATTCATATAACAGTCCCAGCTGCAGAATGCCATTGGGAGAGAGCTTCCAATTTTTAACCACATTAAACATT
TGCAGTTTTGC
>Ref_dvir_662
AGGATGATGAGAGAAATAGAAAAATGAGAATTTCTCAAAATCTGTACCGATGATTTTAATGAAATAAATA
GCAAAATGATTGATAAATCGTGCTCTATATGCACAACTACCGTGCATTTGGGGCCGTTTTCGGAGAAATTA
AATAATAAAGTTTAAAATATGGCTGCCATTTAACAGTATGATATATAATATTGCCAGCTTGTTTTTTTTGC
ATTAACATAGGATGTTATGAGAGAAATAGAAAAATGAGAATTTCTCAAAATCTGTACCGATGATTTTAAT
GAAATAAATAGCAAAATGATTGATAAATCGTGCTCTATATGCACAACTACCGTGCATTTGGGGCCGTTTTC
GGAGAAATTAAATAATAAAGTTTAAAATATGGCTGCCATTTAACAGTATGATATATAATATTGCCAGCTT
GTTTTTTTTGCATTAACATAGGATGTTATGAGAGAAATAGAAAAATGAGAATTTCTCAAAATCTGTACCGAT
GATTTTAATGAAATAAATAGCAAAATGATTGATAAATCGTGCTCTATATGCACAACTACCGTGCATTTGGG
GCCGTTTTCGGAGAAATTAAATAATAAAGTTTAAAATATGGCTGCCATTTAACAGTATGATATATAATATT
GCCAGCTTGTTTTTTTTGCATTAACATAGGATGTTATGAGAGAAATAGAAAAATGAGAATTTCTCAAAATC
TGTACCGATGATTTTAATGAAATAAATAGCAAAATGATTGATAAATCGTGCTCTATATGCACAACTACCGT
GCATTTGGGGCCGTTTTCGGAGAAATTAAATAATAAAGTTTAAAATATGGCTGCCATTTAACAGTATGATA
TATAATATTGCCAGCTTGTTTTTTTTGCATTAACATAGGATGTTATGAGAGAAATAGAAAAATGAGAATTTC
TCAAAATCTGTACCGATGATTTTAATGAAATAAATAGCAAAATGATTGATAAATCGTGCTCTATATGCACA
ACTACCGTGCATT
>9_rnd-1_family-472__Unknown
TACAATACCTTTCAAATTGTAACAGTAACAACACTTTGCATAATTATAAGTGCCATAATATTGTATATTAT
TTATAAGTTCAAAGCATTACCCCAAAGAATAATTGTCAAATATAAGAAACAAAAAGAAGAACCACAAGA
ACCAATTAATATACAACCACATGAAAATATTATAAGCAGCACCAACTTAAATGAAACCGATAATATTGNA
ATNAGCAATAATCCAACATTATACCCGAAAATGTCCGCCTGAGGACAGGCTAATTTCTTAGGGATGGGGA
AGTGACATATGTACCACACCCACAACATATACTATAAGTACACATACACTCATGTATCCTAATACAGCAT
ATATAACATATGTCCACAGCACCTAATGCCAACCCCTTACACATAAGCCCTAGCAACTAAGCGGCACCAC
ATATGTAACATGAGACACCCCAATTTGTAACAATAAACCCTAGAGCCCCAAAATCATGAATTCTTGGTGA
AAATAACAATTCTGGCGTGGGACCGATCATAAAATCAAAAACCANTAGACATAAACATCCGCCACAATCG
TTATTCCCACCAAATGCCCAAATTGCCAACGCAGCNCAAATTCAGGCTCTTAGAATATTCTAGAAACTCAT
CAAGAGAATGAGAGCATTGTTTAAAGAGAGCGAGCTCATGATCTTGAGCCCTCAGCCGAGGTTTGCTAAG
CGTTGACAANATTTTAGAAGTTGAGTTTTACTGAGGACGTTAAACCGTCAAGTTGCATCGTGCACAAACTG

TAAATTCCAATAAGCCACGCCTGTCNAAATAAAGTTATATTTTTTTAACAACGCGTATTGGCTTATGTAA
TTGGCGCAGCCGGTGGGATGCGCCGGTGATATAAGACCATTAACGGACAATCGTTGCCCCAGGTCTTTAA
AGCAAATCGTGGTCGGCGCTGATGATCGACGCGAACGCACAATCATTNGAAAATCGTATCCGCAAAAGA
ACACACGCGAAACTTGCCG
>160_rnd-6_family-2217__RC___Helitron
ATTCTCAATTTTCAAAAAATCAAGCTGCATAGTAGCATTTTGTTGTATGGCGTTACTTATCTAACTGTTTAT
ATTGTCTTTGGTACAACGTATCCCCTAGTAGGGAGCTCNTGGCTNGAACATCTTACTTGTTATACCCTTGC
AGAGGGTATTATAATTTTGTCGCGAAATGCGTAATCCATAGAAGGAGACATCTCCGACCCCATAAAGTAT
ATATATTCTTGATCAGCGTCAACAGCCGAGCCGATATAGCCATGTCCGTCTGTCTGTTTCTATGCGAACTA
GTCCCTCGGTTTTAAAGCTATCNTAATGAAACTTTGCAGATCGCTTTCTTTTGCCAAAGCAGCATATATGT
AAAAACTGGCCGGATCGGACCACTATATCATATAGCTNCCATAGGAACAATCGGTCGAAAATAAGGTTTT
TGCATNAAAAAACATTTTGTTTATCAAGATATCTCGACTAAACTCAGCATTTATTAGTTTTACTTTACTCCT
CATACATATGCAAAATCCTACTAAGATCGGACCACTATATCATATAGCTGTATAGGAACGATCGGTCGAA
AATTAAGTTTTTGTNTGAANAAACTTTNTGTTTTTCNAATATCTTGACCAAACTCGGCATTTACGAGTTTTA
CTATGCTTCTCATATGTATGCCGAATCCTGTCAATATCNGACCACTNTATCATATAGCTGCCATGGAACG
ATCNGTCGANCGTTAAGTTCTTGTNTGAAAAAGCATTTTGCTTATCAAGATATCTTGNCCAAANTCGGCAT
CCACTATTTTGTATTCNACTACTGTAGCTGTCGAGTATGGTCGGCAGCAAGTAATGCGGCCANTTGCGGGT
TCGAACCATGCCAAGGGAACATTTTTTTGAATTTATTTAGTGTTGGAATAGGAGNGAAATNCAGGGTATN
CCCNAGTCGGGCGCTCCCGANTGGAACACTNTTANTTGTNATATCTATCCTAAATCATTCGTATTATTCAG
TTTTCTC
>Ref_dvir_920
TAAGAGCTAGAGACTTAAATTTTTAGATGTAGGTGGCTTCTTATAATAATAATGTGATGTTTAATATGTGA
TTATAATTATAAAGCTAAGTAGCGGCGCGCAGTTCGATTATGTATACTAAATCGTGGCGGTAGGAGAGGG
AATATTTCCGAACACTTTCTTTCAATGTGCTCCAAGAAGCAGTAATCGATTAGTCTGTAGCCTGCGATGCC
ATCACACTACCACTAACCCAAAGGGTACAAAAAAGTTGTGCCTTTTGGTGCCTGCCTACCTGTCCAATGCG
CATTTGACTTTATTTTGGGATTTCTGATTTACGACACACACACCCATAAATGTGTGAATGGGCAGATCATG
GGTGCATGACCTGTATCTGTATCAGCATTTTGGGCCGAAAGGGTCAACTTATGGAAAGCACCTGCATCTGC
ATCTGACCAGCTGCAAATTCATAAATTTGCCATAAAATCTGGGAAATCAAAAACAATGTTTGAAAAACTA
CTAAAACTACTACAAATTAGACTTTCTGTTTTATGCTGTTTGGCTAGTGACACTACAGAATTTTGCGTGTTG
TCGATTTTTGCGTTTCTTACTGATCTAACAACGATTTTTATTCAAATAAACTTTTCTATCGGGTTCAAGCGG
TTGAAAATAAATAAATTGAAGTGTTTTGTGACCATTTAAAAAAAAAGAAGTGAATTATCGACAAACATAA
TTTTCGACCGTTACAATTTGGCGTGTATGTGCAAGTATTTATGCACTTATGCGAAATAGAAAAAATCTCAA
GTTCTTAAACATAACTTTAGTTTTAGTCTTTTTTTTAAAAACTATCCAGACATATAACAAAATAATAAATAT
AATATAATCAATACATACATATAGTATAATTTTAAATAAATTATATGGAAAATAATTTATTGGAAATTAA
TTAATTTAAATTACAAATTTTACTATATGACATTTTTATGCCCCTGCAGAGATATTATAATTTTGGTCGTGA
AAT
>160_rnd-5_family-4736__LTR___Pao
GTTTCGAGATTCGCCACTGGACATTAACTTCGAAGCNAGTTAAACAGGCGCTAGAAGACGGCTGCGAGGA
TTTGGACAAGAGAAGGTCCTGGGCCTGTGTGGCTACCTGGNCAAGATCTGTTGACNTTTGTGGTGAAGCC
GAACTTGCTTGCGAAAGCATCTAAGGAGCGCCNAATTAAAAGAAGCGTTCTAAGCGTGGTCATGTCTATT
TTCGACCCGCTCGGGCTGCTAGGATTCTTTAATATACGCGCCAAGATCATCCTTCAGAACATATGGCGACC
CAACATCGGGATGGGATGACGACCTCACCAACGAAGACGAAGCCGATTGGCGACACTGGCTAGATCTAG
TTTCAAANTTGAATGCCGTCCGCATTCCACGGTGTATGAAGTGGGTGAGCCGAACCCAGACTGTACAGAT
GCATACATTCGTGGACGCCAGTATTAATGCTTACGCCGCAGTTGTATTTTTGCGGGCTGAACTCGATGGCC
AAGTACATTGCAGTTTGGTCGCCTCGANAACGCGNATGGATCTGATGCCCGCTGTCTTAGNTCTGAGACTG
CCCTAGATCTGAGAGGAAATGTATCCAAAAGGAAATGTCGGTACGCATACATAGACGAACATTTTGGACG
CATTCGAAGGTTCCAGCGATTCGTAGCGCTNCGCATCGGAAAGATTTTAGAAGAATCAGATGTGGACAGC
TGGCGCTGGGTCNCATCAGCTCAAAACGTGGCAGACGATGGCACGAANTGGACCCAAGCCACCATTTTTG
TATTTAGAAGAATCGCAGTGGCCACACACGGAAATAGGGCCCTGCAATAAACATGTTGTACCATGCTGAA
GAACAACCCAATCACACGTCGTCCTGGAGATGTATCCTGCCGGATCTGCAGCGCTTCAGCAAGCTGGANA
AATTGAGAGCCGTACAGCTATGCGTACTGGATTTCCTACGGAATATTACTAAGAAGATCAGATTGGACGG
GCGATT
>160_rnd-5_family-3380__DNA___hAT-Blackjack
TCAGTGTTGCCANTTCAGATTTTTTCCGTCAGATNTGGCTTTTTTGAAAGTCTGACTGCCGGAAAAAATTG

AAAACAGCGGGCAGCGGGAATTCTGGCTTTTTAATAAGAAGATTAACTTGTTTTTTCGCTTTTTTATAACA
AATTTTTTAATATTAACAAAAATGTGTCTAAACTTGAAATGAGCGAAAGTGTGTATCATTATTGAAAGGA
AAGCGTTGGTGTAACTATCACTATTGGTTTCANATTGCTTAGTGCGATTTACGCGCGNCGCACACCACTAA
GGCAATGAATTTCACAACTNGATAAANTTTTGACTTGTNAAATGNCGAAAGCTGTTTATAAGCGAAAAGT
GCGTNAAGTACCGAGCTTANAAGCTGGCTCGGCAAGGATGTTACTGACGATACANGGGCTTATTGCAGCT
ATTGCAAAAGCAATATAACGGCCAAGTTATCCGATTTAAAAGCACATGCAGCCATACAACATTTAAGTAT
GGTTGGTGGACATACNCAAAAAAAAAAACAAAATAGATTTTGCANNTGTAGCCCAAAACACTGCAGTGG
CTCCAGTGGATCACTTGAGCAGTTTGTATGCTATGCAATTCGATGACAGCAGCACGGCTAGCCGAATANA
AATGCANCGAANCAAATGCACAAATATATTTAAAAATGCTTTGGCTCCTCATTTGTGGTTCCTCATTGCTG
TTGGATGAAACAACAGACAGAAATTATTTGCATTTAGATGAGGATGAAAATCTGGATTATGTAATAAACA
TCTTATNATTATNTGCTTTAATTAATTTAATTATACGAATTTTTGAATTTGTTGTATACTCTATTACTTTTGA
AAATATCAAACAAAAAATACCTTTAATCAGCTTTTTGTTCAGCTTTTTTTTAAACAACATTTGACTGTTTTG
CGCTTTTTTAAAGCAGATCTAGCTTTTTTGGTTGCTCAATAAATGGCAACACTA
>9_rnd-1_family-467__Unknown
CCCAGCGGACCAACACGCGAGCCTTTAGAGAGAGTCTCGCAAAATGCTCACAAATGAGCTCGCATGTGAG
CAGAAAACGAGTGGGATATATTTTTATCGCAAAAACACTCGCACACGAGCGTTGCAGTGAGATCCTTGCG
ATAAAAATGCGTGCACAGTAGAGAGAATGGTGTGTTGTTTTTGTTTCAAATGTTAATCGTGCTCAGAGAGT
GCAACATTGTCATGAGAGCGAGAGAGTAAAAACGACGTGGCATTCGGATGCTGTGTTGTTTTTGTTGTTTT
TTTCCTACTCAGACCGTTTTTTGGTGGTTGAAAAACTCCTTTGTTCTCTTTTCTATTGTCCGCCGAAGCGAG
CAGACACGTGGTCAAAGTGATAAGTGATTTGTTGAAATTTGNATTTCTATAGAGAGGTAAGTAATTAACA
AGTGTAAATTAATAACTAAACTATTCCCTTTCAGAATATATTTAAATAAATATACAAAAAAATNAAAACA
CATTAAGTTTTGTTTTAAATAGTTGTAAATAAAATTATTTTATATTATGTATTCACTTTTTATAATTATAAG
ATTGCTTTAAAAATGCCTTTTAATTTTAAGTTAAATTAAATGTTATTTATTTTTAAAATACAATTTGAATTA
AAATGAATATTAAAAAAAAAAAAAACAGCAACAAACATTTTTATGGGAACAAAATGGGCATGGAACAAAA
AAAGTTTCGCAAATCATATTTCATGAGAGCCTTTTGCGATATCTTTTAAGAGAGTTTTTAAAATATCACAA
AACGCTCGCTAGTCGTATTTCTTGCGACAGTCGTAAATGAAACTTTGACGGCCACAAACTGAACGACATA
TGATTCATATTGCTATCGTTTCCCGATGTGTTTGCGAGAGTTTTGTCGGGCAAAGTCTCGCATTTTACCAGT
TTGCGATATCATTTGAGACAGTTTGGTCCGCTGG
>Ref_dvir_586
ATTTAAAGCAGAAAACTTATTTTAAACTATGTTTTACACAATGCCAAAAACAAATCCGTCTTCTACGAATT
TGTTGGAGGTGGTGCGGGTGTCGGAAAGAGCCGACTTATTTTAGCTCATTTTCAATCTTTGTCCGTTATGT
ACAATAGTAGGCCAGGAAGTGATCCAAATTCTCCTAAAATAATATTGTGTGCTCCACAGGTAAGCTGCTTT
CGGAATCAGTGGCTCAAACTGCATCCATGTTTTCCTTACCAGTAAATCAATCTGGTTCCGATTTGAGGGCT
TTAAGTAATGATTTAGTAAATACTTTGCATTCAAGGTTAATCGACCTTAAACTAATTATTATAGATGAAAT
ATCTATGGTAGGCTCCAAAATGTTTTCCCAGTTGGACTCCCGTTTAAAACAAATTTTCTTCTCCATTCGGTG
GTATTTCTGTCATAGTTTTCGGGGATCTACGACAATTATCTCCAGTAGGTCCGTTGCATTTTCGAATCCAAC
TTAATGATCCCTACAGTGCAATTTATGCTTCATTGCTGTGGGACCTTTTCAAGTATTTTGAACTCAAAGAA
ATAATGCGTCAACGGGAGGACCAACCGTTTGCTTCTGCTTTAAATAATATGGCATCGGGAAACATGACAG
CCGATGATATTAGTCTTGTCCGATCTCGCATTTGTCAGCAATCGGATGTGCCCGATGATGCCATTCATCTA
TTTAGTACAAATGCGGCGACAGATAGTTATAACACGATAAAGCTAAACTCAATTCCGACTGATCAATTTA
TTTCTACCGCTGATGATTTTATAACTGCGGCTTGTAGACAGAGAATATATGATTACGGTCAATGTAGATAC
GGGGGAGGGATTAGTTAATGGGGGCAACAGGCATGGCTAATGCAAATAGATTTCGATGCATCCAGCACTC
CCATTATTTTATGGATGAAATTTTCAGATGA
>Ref_dvir_533
CACAGAAAAATTCCTAAGCGGCATTTGCGAGATTTGTGGCACGACGAGGGTGCCCAAGTCATATGCATTC
CGACAGCGGCAAAACCTTCGTCGGAGCGGCCAAAGTCCTCTCGCGCGATTTTGTAGACTAAACTCCAGGC
CACTTACAGTCTCCTGTCCTAGCACTTCAACCCTCCTAGTGCACCGCATATGGGTGGCCTGTGGTAGGCAG
GTTAAAGAGTTTCAAAAATCGACCTCCAAATACACCTTCGAAGAGCTTTCCACGCTCCTATCCAAAGTTGA
AGCATGCCTGAATTCAAGACCGATATCTCCAATGTCGGAAGATCCTACTGATCTCCTACTCTGACTCCCGG
TCATTTTCTAGTCGGGGGTCCTCTACTTTCCGCCACAGAGCCAGAGATCAAGGAAAACGCCGCGTCCATCA
TCAACCGCTGGCAGCGCTTGAAGGCTTTGCAACAACAGTTCTGTCTCCGTTGGAAACATGAGTATCTTTTA
GAGTTACACAAAAGAAACAAATGGCAAACTCCAACGCGAGACTTACAACGGGACGATCTAGTCATTATTA
AAGAAGATAATCTTCCAGTTAATGACTGGCGTCTAGGTCGAATCCAAACGACCTATCCTGGCGACGACCA
AAGAGTCCGAGGGGTCGACGTGTTAACAGCCCGAGGTGTCTCCAAGAGACCTATCTCTAAGCTGATCCTG

CTCCCGATCTCAATTCTTCATTCTCTATTCTCCAGTAGAATCTCCAGTAAAGACTAGAATACTAGATAAAT
AGGTCTGCGGACCATCCGTATAAAGCCGCATCAGTAATGTCTATTATGTTTTTTTCCATCTTTTTCACGTA
ACAGCACGATGTCTCCACGCCAACAACATGGCCGATCTAAGAGCCGCCTGGCAACGGGATCGTTCTGATG
CCGAGTATGTCTAGAAGTCCACGCGCTACGGAA
>9_rnd-5_family-2110__LTR___Pao
CGGCAACGGAATCGTTCCGATGCCGAGTATGCCGAGTCCACGCGCTGCGNAAGTGCCAGCGGTTTATGCG
GCTGTCTCCAGAGAAGAGACTCCGAGTAGTTCTGGTCAACAAATACTGCGCAAACTGCCTGNCTCACCAA
CATTCCGGGGAGTCCTGTCGCCGCAAGGTCAAGTGCCGNAAGTGCCAGGGCGACCATCACACCTTGCTTC
ATTTCCAAGAAGCGCGTCGCATGCCATCGCGATCTCCAGAGCGATCCCAAACAAAAATGCGCACCACCAA
GGCCCAAGCGCGACGCTCCGCGCCGCCTTCATCCGCCGGCTCCATCTCTTGTCNACCTTGATGGAGCACAA
AAGGACAACCGTGCTCCCGAGAGCACTAATCCGACTTGGAAACGGCAAGGCGACCCGCGACACTCGGGC
ACTTTTAGACCCGTGCTGTCCGGTAAGTCTTATCGACGCATCTTTGGCAAAGGCCTTCGGGCTATCCGTCA
CCCGCGTCGGTACAGACAGCGCTTGTTCGGCCGTCATCCGTTCCAGGACCTCCAGCTTCCAGCGAGAGGTC
ATCCTGCAGGTGATGCCTAACCTCCGGTATCGGACTCCANCGAAGCTCGTCAGCGAGAAACTGAACGAGC
CTTTCAACAACGTCCGATTGGCAGACGATCGCTGGTTCCAGCCGGAAATCTCCAATCTGGTGTTGGGCTCG
GATATCTACCCNGACTTAATTCTCCAAGGCGTCCTGCCGAGCCATGGCGGCGCACCACTGGCCCAAAACA
CGGTCTTCGGGTGGATACTGTCCGGCTCCTGCGCTCACCGGTAGGAACAGAGCCACTTCCCTCTCCCTTTT
CTTTTTGCAATACTGCAAGGCGGGCGGCATGTTCACACAAGAAGTGTATCCGGCAGTCGCTATCCAACCG
CCATGTAACTCCGGAACCAGTACATGGAACATG
>9_rnd-1_family-118__LTR___Gypsy
AAAATAATTAATTTCATTAATTAAAAATAACTACATTATGTTACGTTATTTCTCTAAAAGGGAGGTGTAGT
GTGTATATGCCTTAAAAGAACACCCTATATACAAATATAAAGTATGCGGCACTTTGTCGCATACAATAAC
AATTATACATTAAACAAGCCCAGAGCCACTTTATATATTGGCTCTGGCTCATATTACGCAGCGCAGTTCAC
TAGCCTCTCATGCTTACGCTGCATTTCCCACGCTCCGTCTGAGCGCATTCCATGAGCAACATATATGCTCA
CTAACACAGGCATATGCAAGCTACTCACTCTCCCTCTCTTTGTAGCTGTTAACCAATAACAGCATAAGTAT
ATACATGTATCCATAGGCTAAGCGCAATATTTTACTTTGAATAAANTCATTCTGAAAACCNAAGTCAACCA
GCACTGACGTGCTTTNTTAAATCATTATTGAAAATAAACATTACATGGCGACCGTGACTTGGTCACGCATC
CCTGCCTGGAGGCAGTAGACCAAGTGAAAAGGAACTTCATGGCCAACAAAGAGGCCATGTATAATATAC
AATTTGAAAAGGAACTTCATGGCCAACAAAGAGGCCATCTATAATATCCAAGTGAAAAAGGAACTTCATG
ATCAACGAGAGGCATCCCGTATTCTGAGTGAGTAGAGAGTGAGTGTTATGGTTCATAAACGCGGTGGCTT
AAAAAATAAAATTAATAAAAGAATAGCGCACCTAAGGTGGCTGCTATACACAAAACAGCCAACACCCGC
ATGGTCGACAGCTCAGAAGTTACAAAATGAGCTCGACTATCTGCAAAATNAAATTAAGCAGAGAAAACC
AAACACCACTACTCACTCAAATATAAAAGTTCTAATAAGCCAAACAACTAATGAAGTTACACCCATTGCC
GCAACCAGCCTACCAAAAGAAAA
>Ref_dvir_710
CCGGGAATTCCCTTGAAGGAAAATGGTGAAAAATCCTTTTTTGAGACGCCGCATAGGTGTAGGCGATGTG
ATGCTCTGCAACCAGATTCAGGCCTTTAGGCAGAGTTGAGTTGCTGACATACCATGGATAGTCGCTGATTA
AGCGCAATGTTTTTTTTGCATGATTTGCACTCTCTTTCATTGGGACTGGGCAGCAGTTTCCAAAATCGGAA
TGCCGTGCATCCATACGGGAGCAACAATGCAATTTAGCCATTTCAGAATTGATATTTTGGTACGGCAACTA
TTAACCACTGACTTGAGAGATATGAAGTTAGCCTCTATTCAAAGGGTCCACATCTTAGCCCAGTTATCTAG
AAAGTGAAGGTACTCCTGGATACCATCTGCAGCTTCGTACTCGCAACGGAAAAAGATTCCGACATCATCA
GCGTAGGTAACCATCAGTTTGCCCGGATCAATGCACCACTTGTTCATGCTGGTGCGACCAGTCGCTCCCAA
ACCATCGCTATCGACGGCAGGACACTGATCGGCCGATAGGACGCAGGTAGGCTTTATGGTTTGTCCTGCTT
CTGTACTTTGGTAGCTGAGATGCACCTCCAAAAATTGCACGTTTCGATTTCGGTCAAGTTGTCTTGTGGTC
GAACGGCTTCGGTCTGCTCTACAGACTCGTAACTCTCACTTTTCTCTTCCTGACCCTGGGTCGTATGGATAA
TACTCTGCATGGAAATTACTCACGGTGATAAAACTTGCTAAGCAAAGTAACAGAAAAAAATCTACAAATA
AGAACTGCATGGAATCCCTGAACCGGATGTTCTAAAAAATTGTGTTGCTAGTCTGCAGAGCGACGACTTG
TTTTCTGACTAATCGGGCTATTTACTTGGCTGAAGTATTAACAATTTGCCCTGGTAATTTAAGATTATCTGT
TGATGTCGGAAGTCA
>9_rnd-5_family-219__RC___Helitron
TGTATTTGTGTTTTTTCCGTATATATATATTTGCTGTGAATATTTTATGGATTTTTTTAATAAATTTATTTGT
TTTCTTTTCGTATATTTATTTTTTAAATAATTTTATGTTTTTTTTTAAAGACTTTTATGATTTTGACATTTTTA
TACAGGATCTGCTCTCTTAAGTTTTTTAGTGCTTTTGTTGTTTTTCTTCATTTGTTTGCTAAATATAATTTAC
GACATGTTTTACAAAGCGTTTCTAGATGAAATATTGTATTTTTTTTATACCCTGAACCCATTGAAAATGGG

CAAAAAGGGTATAATGGTTTTGTGCAAATGTATGTAACAGGCAGAAGAAGGCATCTTCGGCCTCAAAAAG
TANANATATTATTAATCAGCATCANTAGCCGAATTGNTCNAGCCATGTCCGTCTGTCCGTCTATCCGTCTG
CTCGTCCGTCNGTCTGTCCGNACGTATGAACGCAAGGNTNTCANAACCNATAGGAGCTAGAGAAATTTTA
ACGTAGTAGGATCCTTCTAGTGCCTGCGCGGNNCGAGCTTGTTTCCGATANTCGATAAGCTACCCNGTTTC
CAAGCAACTGNTAAAAATCNATATCGAAATTTTTTTAGAACTTGGGTTTAAGAGCTGGAGTCATCAAACT
CGATATGTGGTATCGTTTATTTTACAGCTATCGCAACTTCCAAGAAGAAGCTATATAATGCGATCAGTTGT
TTTTGTGCCTTGTAGAGGGTATTCTAATTTTGTCGTGAAATGTCTAACGCATAGAGGAGGCATCTCCGACC
NCATAAAGTATATATATTATTGATCAGNANCAACCGTCGAGTCGATATAGCCGTGTCCGTCTTCCGTCTGT
GTTTCTACGCGAACTAGTCCCCAATTTTAAACCTGAAACTTTGNANAAGTCGNCAAAAAGCCNATAANTC
GTTTC
>Ref_dvir_593
GTCCTGTTGAAAAGTGAACTGACTGCTTAACATCAACTTTTCAGCGCTTTTTTTAAAATTTGCCTTCCATAT
TTCCAAATAAACACGCTTGTCCCTTGATGAAAATTAATTTACCCACACCAGCTGCGGACATACAACCCCTT
TGGCGAATGCCAATCTCTTCGATTGGTTAACACTCGAAATGAGTGGTTTGCGTCTTGCAATTCTCGCATTG
TACCCAGCCTTGTGTAGTACCCGCCGTACCGTTTTGGAGTGAATTTCTTTCCCAGTCTCTTCTTTAACCTCT
GATACTATTTGGGATGATGTGATTTTTGGGTCTTTCTTGATCTTTCTTATTATTTGGGTGCAATCACGATCG
GTTAACTTTCGTGGGCGGCCGGTGCGTTCAGCGTTTTCTACCGTAGGGTCGTCTGAAAATCTATGGACTAT
GCTCCGAATGGTAAATTGACTTCTGTCCAATAGCTCGCCAATTTCTTTAAAGGATTTGCCTTGTTTGCGTAA
ATTTAAAATAATTTTTCGCTGCTCTACGGTGGTTTCACACTTCTTACCGGCCATTTTTGCAAAATAATATAT
AAATTGATGCTTAAACAATTTTTTCTAAGTATTTTTTTTTAAGATTCAAATAACGGTGCTTACAGAGTTACA
TTTTTGCAAAGAAAAGTGTGACCATATAAAAATAATATTAAAATAACAACTTTATATTGAAAATTGCCAA
TACTTTTGTCCCTATAAAATTACGGGTACTTGGAATTAAATAATTATTAAAAATTAACCATGCCGCCAAAG
TTTAAAAAATAATTAATTGCATGCACAAATAGCTAATAATCGTATGAAAAATATAAAATTATTAAATATT
AGACGGATTGGACAAAACCGACAATAAAACAAGATAATATTAAATTGCCAATACTTTTGTCCGTGACTGT
A
>9_rnd-1_family-220__LTR___Gypsy
CATACGGACGGACGGACGGACGGACGGACAGACAGACGGACATGGCTAGATCGACTCGGCTATTGATGC
TGATCAAGAATATATATACTTTATGGGGTCGGAGATGCTTCCTTCTGCCTGTTACATACATTTGGATTTTGC
ACAAATACAATATACCCTTATACCCATTTTTAATGGGTTCAGAGTATAAAAATACATATACATTGGCCTGA
TCAACCAACAAAAAAAAATCAATCCTTGAAAAAAACAAATTACTATAATGAATTTTACATTTTTATATTTT
ATATCTTTATATTTTTGAAATGTTTACATTAGTATATTTTCATAAATCTTAAATAATTTTTTAAATTCATATT
ATTTTTTATCATTTTNTTACATTTTTATTATTTTATAACAAAAAAAATATTATAACAAAAATTTTGTTCAAA
ATAATTAAGTTCATTAAATAACTACACATCCATAATGTTACGTTATTTCTTTAAAAAGGAGGTGTAGTGTG
TATATGCCTTAAAAGAACACCCTATATACAAATATAAAGTATGCGGCACTTTGTCGCATACAATAACAATT
ATACATTAAACAAGCCCAGAGCCACTTTATATATTGGCTCTGGCTCATATTACGCAGCGCAGTTCACTAGC
CTCTCTAGCTGACCGTTTATGCTTACGCTGCATTTCCCACGCTCCGTCTGAGCGCATTCCATGAGCAACAT
ATATGCTCACTAACACAGGCATATGCAAGCTACTCACTCTCCCTCTCTTTGTAGCTGTTAACCAATAACAG
CATAAGTATATACATGTATCCATAGGTTAAGCGCAATATTTTACTTTGAATAAAATCATTCTNAAAACTNA
AGTCAACCAGCACTGACGTGCTTTCGTTAAAATCATTATTAAATATAAACATTANATGGCGACCGTGACA
>9_rnd-5_family-546__DNA___CMC-EnSpm
ATATATATTTGTAATTACGTACTCACCTGTAAATTCACACGCACGATTATTTTATCGTCATCTATCAGTAGG
CGGGAGACGAACTTTTATATAAACACATCACTGAAAGAACAGAAAAGCAGAGANAATGTTTATTAGCTTA
AAACGCAGCTTGCTTGTTAAGTGAGAATCGCGTAGTAGAGCAATATTTATAAATANGTTTTNTGTTCACTC
AATACACACGTCTCAACACACACACATGTACATACATACATGTATGCAAATACATGCAAACTAACATGCA
TACATACGATTGTAATGTTTTTTAAATACAATAAGACACAGTTTTGNTNATNATACATACCAGAGCCGCCG
NCNCCTGCGTTGCCCAAACTGCAGCCTCCNCCATAAAGATAGCNAGNNCGGTGNCGNCTCCTCCATANTC
GAAGCNGCCGCCTCTTGCTTTGCTCAATCTGCAGACTCCTCTATAGTTGAAGCCGCCACCTCCTGCTTGGC
CCAGGCTGCCGCCAACTCCTGTATAGTTGAAGTCGCCTTCTCCTTCTATGTCCAAGCCGCCGATTCCTGCTT
GGTCCACACTGCCGCCGCCTCCTGCTTGGTCCATGCTGCCGCCGCCTCCTGCTTGGTCCATGCTGCCGCCG
ACTCCAGCTTTGCTTCATCTGCCAACTCCTGTATAGTTGAATCGCCTTCTCCTCCTATGCCCAAGCCGCTGC
CTCCTGCTTTGCTCAATCTGCAAACTCCTCTATAGATGAAGCAGCCTCCTGCTTTGCCACAGCCGCCGCCT
CCTGCTTTGCTTAGTCCACCGACTCCTCTATAGTGGAAGCCGCCTCCTTCTGTGACCAAGCCGCCGACTCC
TGTGTTGCTTTAGCCGCCTCTGATAGTAATGCCGGCGCCGACTCCTCTATAGTTGAAAT
>Ref_dvir_810

ATTATTTCAAACATTGGAGTTGATATGGAATCGTAATATTTTTCAATTTCACTCAGTGAAATAGTTGACCA
GGCTTTTTTTATCGCTTCAACCAGGCCGAGTCCTGAAATTGTCTTCCCCTTCATACACTCTCCTGGATAAAA
GTCCCCAAATGTTCTCGATAATATTTATGTCTGGGGAGTATGGCGGCCACTCGAGCATGTTGACATTCTGG
CCCTGAATATTACTGTTTTGTAACCCGGGCTGTATGGATGGGGGCGTTGTCTTGTTTGTCCATTGAAAGGT
CCAAAAATTCGGAAAACTGGGGAAAAACCTTTTGGAGCACAGTCTTGTAGACATTTGCATTCATCTTTGA
GGAAACAAACTGCATCTCGCAAGTTCCATAAAACGATATGGCTCCCCAAACCATTACGCCTCCTGTACAG
GAGTGATGAGGACTCAAGAAGCATTCCTCATTGCGCATATCGTGAAATAAAAGTTGTAGCCATACGGTCC
GGCGAGATTAAACTTTTTGTCTTCGGAGAAGACCACAGCCTTCCACTCCTTGTTGCATGTCATGTGATCCC
GCGCAAATCGAAGTAGCGCTTCCTTCCGTGTATCATTGAGAGGAGGTTTTTTATTTTTAATCTCTTTAACTG
TTCGAATAACTCGTTTTACCGTTGCCAAACTAGCATTTACACCGCATTCATCCCTGAATTTTACTGGCAGA
GAGGTGGGAATTCGAAGCACTTCGAAGGATTAGTAGCCTCTCTGATGCCGATGTAGCAGAATTTTTTCCTG
CTTTCATTTTTAGGAACAAAGAGTAGAAGAATAAATATAAGAAATCAGTATCTTGCTGGAGGGCAGAGTT
GTTAAGACTAAAAAATAAAATAAATAATAAAAAAAAAAAAGTGGTTAAGATAAAAAAAA
>Ref_dvir_993
TCAGTCTCTACTTTACTTGGCCTTATTGAGTTGTCTTCAATTATGTATCCCAGAAACTGTAGTTTTATCTGC
AACAACTGGCACTTGGTCCAATTTATGCGCAATCCATTCTTTGCAGCCACCGTCAGAACTCAACTTAGTGT
CTGTACACCCCCGTTTACATCCTTACTTGAAATGATAACGTCATCCATGTATACAATTGCGTCATTACTCCT
AACTAAGTCGCGCAAAACAGCCATTATGAATCGTGTAAATACTGCTGGGGAGTTTGAAATTCCAAACGGT
ACATACAGAAACTCATATTGACCGTTCTGAGTAACAAAGGAAGTGTATTTTTGCGAGTCTTCATCTACTGG
GACGTGAAAGAAACCATTGGTCAAGTCAAGTGTGGTAAACACCTTTGCACCCCGAAGCTTTTCTAATACT
GTGTCCATGTGTGCCATTGGAAAATTATCTCGAACAATTTTTTCGTTAAGTTTCCTGTAGTCGCAACATAAT
CGTTCTGCCCATTTTTCTTTGCAACCAAAACCACTGGAGAGGCAAATTCTGATACGCTCGGTTTAATAATC
TTCTGCATTTGCCTCTGATCTTGCGGCTGCCTATCAATTTTAACGTTATTGTCGAAGATATGACGAATAAAC
TGCGTTCCGTTTTTAGTGACCTGCATGTCAATATATTCAAGAATAGTTCTTCCTAAAATTGCTTCAAACCTA
ACGTCCTCATCTTGCATAACGTGGAATTCTATCTGCATTTCAATCGTGTCAATGGTGACGGGTATGGTAAT
GCTTCCGAAGGTTAGAACCTGACTTTCCCCGATACCTGTTAGACATTTTTAAAGGCCACTTACTCTTAAAT
TTCCAAATTTTAAGAATATGCTTCTGCGAAGTAAACACAAATCGGC
>Ref_dvir_1062
TTAATAAGCTGTTGTATTCTCCTTACGTGCTCTTACCAAGACAATTGGATCTCTAACTTAATTTTGTTAACA
TGGGCACCACAATTTTATGCATTCGCTCGACCTGTCCGTTACCGCGTAGAACACCTGTGGTTACATCTCTT
CTTGGTTATTTTCATTCGCCCCACGTGATAAATATGATACGTTACTAATGGCTTATCACCCTTATCTATACT
GCCTAGGTATCCTTCATGTCTTCCAGCCTTTGTATTAACCAGAATGCACTGGACACAACTACCGACTACCC
GAGCCACTTTTTCCTTCAGTTTTGGAATAAAATATGACTTTTCTACCAAGCATTGCGTTTTCTTTGCTGAAA
AATGTCCCTGTCTGTGAGCAATTTGGATTATCTCTGTCTCCATTTGAGATGGAACAACAATCAACTCTCGA
TCTGGATCCTTGAACAAAATCTGATTCGCAATGTAAAAGTTCTCGTACCCTTTACTCTCCACCAAATCTCTT
ACTACTTTTGTCCAATCGTCAGCTAACTGCGCTTCCTTTAACCGATGCATCACACTGTCAGTCAGAACGAA
ACAAGCTATTTTGCTTAATGCATCTACATGACTCATTTTCGTACCTGAGCCGGTGCTCTATTTCGTAATCAA
AGTCTTGTAGAAACATTGACCATCGTGCTACTCTCAAAGGCACTTCCTTCTTTTTCATCGTCATGGCGAAC
GCATTGCAATCGGTTACGATCTTAAATTTCGTTCCCAAAAGATAAACTCGCCATTTTGTCAGAGCTCCTAT
GACTGCAAGCACTTTCGAGCTCGAATGAGTGATACTTTTCCTCACTAGGCCTTTGTTTTACGACTCATGTA
CATTTCAGGATGTAACAAACGATCTTCCGAGTTTCTCTGCAATA
>9_rnd-1_family-143__Unknown
ATGGTTCCTAGTTGACCCATATATCCTTGGTTATTCCATACCATGGATATAGTCCTGGCGAACCTAAAGGN
CCGNATCGCTGCGAAATNCGCACGNAAATAGTNCAGAATTATATCATGCATGCATTTTTCAGCCGCCCCN
NNNNNNNNNNNNNNNGTCCTTAAAAGTCTTTTTTTCTCAGCCGTCTGAAGGACTTTCGTCCTTTGGGTTGT
ATATTCTTAAATGGGAGGACGAGGACTATGCTCTGGCCAAAGGACATCCCGATAGTCCTTCAAATGGCNA
AGTTAGCCCGGANTTTTTCGATTTTNGCCAATTTTGCGACTAACCCCCTAGGANAAATTTCGGCGAAATTT
TTTGTCGAATCCTGGAAATCCTTGAATGCCAATCGATAGTCCTGCTCCCCACGAGTTCAAAAAGGTATATC
CTTCCGATATCCTTAAGGATTTGGCCGAGTTATGGCTAATTAAAAAATGTCCTTTTTAGAAAATTGGCCAT
ATCTCGGAAACGACAAGGACGATTTTGNTGTCCTTTGGACAGTTTATGGTCCTTGTCCATACGATTTGTTT
GACACAGGACTTGTCAGGATCCGCAAGGATACGGCCGAGTTATGGCTTTGTTAATTTACGAAAGAATTGT
ACTCATATCTNGGCTGTCTGAAGGACTTTCGTCCTTTTCATTATATATTCTGAATCGGGAGGATGAGGACT
NACGGTTGGCAAATGGACATCATGATCGGCAAGGATACGGCCGATCTACAGCCTTTTATCTCTACAGACA
AAAATCTCTTTTCCTCAGCTCCTTGAAGGATTTTCGTCCTTTTTATNACATATTCTAAGTTGGCAGGACAAG

GACTGCATGTTGTCCAAAGGNCATCAGAATGGTCCTTGAGGTGGCCAAT
>160_rnd-1_family-307__LINE___I-Jockey
AAAACCTCAAGCGCCAACCCTGGAGAACCGTTCTCCTTCTTCGCCACAATGGAAAATACTGCCTTAAAGA
CGAAGAGCAAGCTGATACCTTCGCCAGCCACCTGGAGGAACGGTTCACACCTTTCGATTTGGCCTCCCCG
GAAGAGATCGATGAGACTACCCGAGCGTTGGAGACTCCGCTTCAGATGTCACCACCTATCGGCCCCATCC
GCGTGGAGGAGGTGATCAACGTCGTCAAAGCCCTTCCCAGGGCTAAAGCACCGGGCCATGATGGAATATG
CCACACCACTCTGAAGGCCCTGCCAATGCGCGCGCTCCTTTTTATAACGTTGATCTTTAACGCCATATTAA
GGACGCAACACTTCCCTGCCCAGTGGAAGTTAGCGAGGATCCGTATGATCCATAAGTCGGGCAAGCCGGA
ATATGACCCTGAGTCATATCGGCCGATCAGTCTCCTGCCTTCCTTGTCGAAGGTCTGGGAAAGACTGATTG
GTCGCCCGACTTGCAAGGCTTATGGATCAAATGGCCATTCTTCCTGATCACCAATTTGGGTTCAGGGGTGG
GCATGGCGCTGTTGAACAGGTTCACAGAGTGATTAACCATATTCTTAAGGCTTACGACGACAAAGACTAT
TGCTATGGAGTCTTTGTAGACGTTAAGCAGGCCTTCGACCGAGTCTGGATNGACGGATTGCTCCAGAAAA
TCAAGATGAGTCTTCCCGCCCCCTTCTTTCTACTTCTGCGCTCTTATCTAATGGAGAGTAAGTTCGAAGTCA
AAGTCAGGACAGCAATCTCTCCTGTTCGTNATATGAAGGCGGGTGTCCCTCAAGGCAGCGTACTGGGTCC
CTTCCTCTACAATTTGTACACGGCAGACATCCCCACTCCGGATGACCTACA
>160_rnd-5_family-484__RC___Helitron
TTTTTATACCCTTGCAGAGGGTATTATAATTTTGTCGTGAAATGTGTAACGCATAGAAGGAGACATCTCCG
ACCCCATAAAGTATATATATTCTTAATCAGCATCAACAGCCGAGTCGATATAGCCATGTCCGTCTGTCCGT
CTGTCTGTTTCTATGCGAACTAGTCCCTCAGTTTTAAAGCTATCTTAATGAAACTTTGCAGAACTCCCTCTT
TCTGTTGCACGCAGCACATATGTGAAAACCAGCTGGATCGGACCACTATATCATATAGCTGCCATAGGAA
CGATCGGTCGAAAATTAAGTTTTTGCATATGAGGAACATTGATCGTTTGTCGAAGATTAAGTTTCTATGCG
AACTAGTCCCTCGGTTTTAAAGCTATCTTAATGAAACTTTGCAGAACTCCCTCTTTCTGTTGCACGCAGCA
CATATGTGAAAACCAGCTGGATCGGACCACTATATCATATAGCTGCCATAGGAACGATCGGTCGAAAATT
AAGTTTTTGTATGAAAAAACATTTTGTTTTTCAAGATATCTTGACCAAACTCGGCATTTATTAGTTTTACTN
TACTCCTCATATATATGCAAAATCCTATTAAGATCGGACCACTATATCATATAGCTGCCATAGGAACGATC
GGTCGAAAATTAAGTTTTTGTATGAAAAAACATTTTTGTTTNTCAAGATATCTTGACCAAACTCGGCATTT
ATTAGTTTTACTNTACTCCTCATATATATGCAAAATCCTATTAAGATCGGACCACTATATCATATAGCTGC
CATAGGAACGATCGGTCGAAAATTAAGTTTTTGTATGAAAAAACATTTTGTTTTTCAAGATATCTTAAACT
CGGCATTAATTANTTTTACTTTGCTCCTTTGTTGTTTCTTTA
>160_rnd-4_family-450__RC___Helitron
TTCGTATATTTATTTTTTAAATAATTTTATGTTTTTTTTTTAAAGACTTTTATGATTTTGACATTTTTATACA
GGATCTGCTCTCTTAATTTTTTTAGTGCTTTTGTTGTTTTTCTTCATTTGTTTGCTAAATATAATTTACGACA
TGTTTTACAAGGCGTCTCTAGATGAAATATTATATTTTTTTTATACACCGAACCCATTGAAAATGGGCAAA
AAGGGTATTGTGTATTCGTGCAAATGTATGTAACAGGCAGAAGAAGGCATCTCCGACCTTAAAAAGTATA
TATATTNTTGATCAGACATCAANAGCCGANTCAATCTAGCCATGTCCGTCTGTCCGTCTATCCGTCTGCTC
GTCCGTCCGTCTGTCCGTATGTATAAACGCAAGGTTATCATAACCAATAAGAGCTAGAGAAATTTTAGCA
GTAGGTCCTCCTAGTGCCTTTAAAAGCTGGAGATGTCGANCTTGNTTCCGATAACCGATAAGTTACTCAAT
TTCCAAGCAATCGATAANAATCGATATCGANNTNCTGTTTTTTAGAGCAAATTTCTTGGGTAAATATTAAG
AGCNGGAGCCGGGTTTCAAGCTTTTGGATTAGATGTTGCTANCTTTCANTTTACANCTATCGCAAATNCCA
AGACAGAAACTGTATAATGCGAGCAGTTATTTTTATACCCTTGCAGAGGGTATTATAATTTTGTCATGAAA
TGTGTAACGCATAGAAGGAGACATCTCCGACCCCATAAAGTGTATATATTCTTGACGATAGCAACANCCG
AGTCGATATAGCCATGTCCGTCTGTCCGTCTGTGTTTCTGTGCGAACAGTCTCTCAGTTTTAAAGCCTGAA
ACTTTGCANAGGTCACTAAAGAGTCCATAACTGT
>Ref_dvir_1029
CAGAAGGCGTGAGCACCAGTAGGGTAGGGGCACAACCACTTAAACCACAAAATCCACACAAACATTTTT
GCAAACAAATAGTTTATCCCATTTTTCTCGTTCGCCCTCCCATTGCCCACTGAATTATTTAGAGCAAAGAA
ACACACGAAATATTAACAGGAATTTATTAAAACATCAGTCGAAGCAGCACATGTGAGCTTCAATCAAAAG
AGGAACTCCTGCTGTTCCCTTTTCCCTTTCGCCTTTCTGTTGGACGATTCTCGAAGCTGTTAATCTTTTTTCA
ACTTTGTAATTACTTTTGCAGGCAGAAAACTTTCTTTGAGCTGCACATTCACAAATTATTTTCGAAACAAA
CAAACAACAAAGCCACTCAACCACAGCACCACACACACCTCTTACACACACTCCCCTCAAACACACTCTC
GCACCACACATCACAGAGTGTCACAGACTCTGCATCTTCAGTCCGAAAGTCAACTAAATCACTTTCTATTC
AACAGGACTGCGAGCAGACGGCACAAAAAGGCCACCAGCACAAGGAGGACAACCTGGACACGTGCTCGA
CACCAACAACTGGCACAAGCCACTGACCAGCGGGCGAGACGTTGCCAACGTGACGAGCTGAACCACCAG
CAGCAGGAGTGGAAGCAGCTAGGCAAGGGGGGAAGAAGTGGCCAATAAAAAAGCTAACGAAATGCTGA

TGCCGCTTGCAGTCGACAGGCGAACTCGGCAACAACAACAAAAACAACCGAAGCAACCGCATTAAAAAC
AAAACTGGAAAAGTTGAACCCAGTCCGTAGAAAGGACAGCCAGAGTAAAAGTTCCAAATTTGCCAGAC
AGAGTCCACGTAGAGCTGCTCGTCGCTTTGTGGCCGCCATC
>160_rnd-5_family-471__RC___Helitron
AAGCAGCGTAATGCACGCCTGCGTGCNTGNAACAGGCANAAGGNAGCATCTCCNACCCCNTAAGGNGCA
TANATTCTTGATCCGCACCAATANCCGAGTCAATCTANCCATNTCCGTCTGTGGACAGCTAGAGACTTTAA
ATCTAGTGNCCGCGCANNNCGAGTTCGTTTNCGGTAATCGATAACTTNCNCCGTTTCCAAGCAATCGATA
AGANTCGATATCGACGTCCTNTCTNTTGAGCATAAAACAATCTATATGTGTCAAGTANCTTTCATTTTATT
TTTTACCTAATTGCCGCCTCCCCGCTACCACCAAAGAGCTATAAATCAAGTNAATAANCCAACTTTTATTG
CCAACCAATTTAAGNNACAATTTAAATGCAATTNNCTCTTTTTTTTTAGANTNCACGAATATTNTACAGCT
AAAAATTTAATTGTTCTAATATTGACACGTGATTGCGTCAGCTAGCGCAAATTGCAAGAATTTCTGTGTAC
ATGTACACACACACNTTCANGCGCGTNTNGNTCGNATTCAATAACGGGAAATAATAATTGCGATCGTATT
GCATGCCGTGCTGTGTGTATGTGCTGCTATTTTGGTTTAGTTTTTNNTCGTTGAAACTGAATTTTGACATGG
CTGTGAGGTGGTAAGCAATTGNTGCGATTTGTNACGGGTTCGAGTCCTACTAAGGAATTTTTCTTAATTTA
TTTTTATTACTGCTNAAGCAAACGAGCGCGTAGCGCGAGCTGCATTTGGTGTTGGTAGANGANGTTTCAG
GGTATCCTCCTAGTCGGGAACTCCCGACTAAAGCCTTTTTTTNNTTTAGTTGTTCCATCTACATATGTACTG
GCTGGCATTTGGTCTTATTCGGCTTGCATT
>9_rnd-6_family-523__LINE___I-Jockey
TAAGCCCCTTCCCAGGGCTACAGCACGGGATGTCGCCCGTCTTGCTAGGCTATCAAATGGCCATTCTTCCT
GATCACCAATTTGGGTTCAGGGATGGGCATGGCGCTGTTGAACAGGTTCACAGAGTGATTAACCATATTC
TTAAGGCTTACGACGACAGGCCTTCGACCGAGTCTGGATAGATGGATTGCTCCAGAAAATCAAGATGAGT
CTTCCCGCCCCCTTCTTTCTACTTTTGCGCTCTTATCTAATGGAGCGTAAGTTCGAAGTCAAAGTCAGGACA
GCAATCTCTCCGGTTCGTNATATGAAGGCGGGTGTCCCTCAAGGCAGCGTACTGGGTCCCTTCCTCTACAA
TTTGTACACGGCAGACATCCCCACTCCGGATGACCTACATGAGTTCGCCACCCCAGGCGAGTACGAAGCC
ACCGATGGGGCCCAGGAATACATCCACTTGCTTGAGAGCTGGGCTCGCAGGTGGAACATCAAGCTCAATG
CCTCGAAGTGCCAGGCGGTCAACTACCCGAGCGTGGCAAGGCTCCAGCAGTCTGGATAGATGGCAGCCCC
CTTCACCCTGGCATAAGCGCCAAGTATCTTGGAATTGTACTGGATGAGAGGCTTACCTTTCGGGCGCACAT
CGACGGCCTTCTCAAGCAGTGCCGCTTTAAGATGGCCGAGGCCAACTGGCTACTAAGCCCCAACAGCCCA
TTGGCCACTTCGCTGCAAACGGGCAGTTTACAACGTCGTGGTGGCGCCAACTTGGAGATACGGTATCCAG
GTTTGGGGAATTGCNTCTCGTACAAATCGAAGACGGATTCAGACACTCCAAAACTTTGCCTTGCGCCGAA
TGACCGGTGCTGACTGNTACGTCAGAAA
>Ref_dvir_500
GTTTGGAAATCAACTGACGGATATCTCTCTGTCGGAATGGTAGTCAGTCCAGGCATAGTTCCATGCTGAGC
TATTGTTGATGAAGTGGTGGTTTCCCCGGATGCAGTCACTAAGGTGGTAGCCTCTGAAGGTATTCTACCTG
TTTGAGTAGTATTTGAAGTTTCAAGGCTAGTCGGTTCTGTTTGAGTTACTAGTACTGTGGATTCTTCTGTGA
CTGTTGGTACAGAAGTACTAGCGAAAGTTGTTTGGGTAGTTTGAGAAACAACTGACGGATATCTCTCAGT
CGGAATGGTAGTCAGTCCAGGCATAGTTCCATGCTGAGCTATTGTTGATGAAGTGGTGGTTTCCCCGGATG
CAGTCACTAAGGTGGTAGCCTCTGAAGGTATTCTATCTGTTTGAGTAGTTTTTGAAGTTTCAAGACTAGTC
GGTTCTGTTTGAGTTACTAGTACTGTGGATTCTTCTGTGACTGTTGGTACAGAAGTACTAGCGAAAGTTGT
TTGGGTAGTTTGAGAAACAACTGACGGATATCTCTCTGTCGGAATGGTAGTCAGTCCAGGCATAGTTCCAT
GCTGAGCTATTGTTGATGAAGTGGTGGTTTCCCCGGATGCAGTCACTAAGGTGGTAGCCTCTGAAGGTACT
CTACCTGTTTGAGTAGTTTTTGAAGTTTCAAGACTAGTCGGTTCTGTTTGAGTTACTAAGACTGTGGATTCT
TCTGTGACAGTTGGTACAGAAGTACTAGTGAAAGTTGTTTGGGTAGTTGTCGGTATGTCATTTGAAGAGGA
CGTTTGAGAATCAACTGACGGATATCTCTCTGTCGATAGTAGTCAGTCCAGGCATAGGTTCCATGCTGAGC
TATTGTTGATGAAGT
>9_rnd-4_family-44__RC___Helitron
CGAAATTATACGAAACTTTTTGTTNCTTAACTTTCTTTTGTTTNAGTAATCTTAGAAGTATAGTAATGCTTT
ATTTTCGAAAAATGTATATGAAAGCAACATTATATAATAACATAGGCATTTAAATGCTCATAGGTTTTTTA
AAATATTTGAAAAAATAAGAAAAAAATTACACTTTTCGAGTGTAACGATATTATACTAAAACTTGTTTTTA
TACCCTGAACCCATTAAAAAATGGGTAAAAGGGTATATTGCATTTGTGCAAATGCAAATGTATGTAACAG
GCAGAAGGAAGCATCTCCGACCCCATAAAGTATATATATTCTTGATCAGCATCAATAGCCGAGTCGATCT
AGCCATGTCCGTCTGTCTGTCCGTCCGTCCGTCCGTATGTATGAACGCAAGGATCTCAGAACCTATAAGAG
CTAGAGACCGAAATTTTAGATGTAGGTGCTCCTAGCGCCTGCGCAGATCGAGTTTGTTTCCGATAATCGAT
AACTTACTCCGTTTCCAAGCAATCGATAAAAATCGATATCGATATCCTGTTTTTTGGGCAATTTCGGTAAA

TAATAAGAGCTAGAGTCACCAAACTTGACATATAGCTTCTAAAATAGAAAATACATATATGCATTTGATG
TTGGAAGAAGAGGGTTCAGGGTATCCCCTAGTCGGGAGCTCCCGACTAGAACCTCTTACTTGTTTCTTTTG
CCACATTGCATATTTGCATCGAGTTTGTTAAATTTGCGAAAACGATGTGGTCNACTTTTCCCATTCTCTNAA
AANTCNTACCTTCTTNGTTCAGTAACANTATCNAATNTCTNTTTTTCNGCGTAAACACGACGTACAAGAAT
TTCCCCAGCTAGGC
>9_rnd-1_family-301__LTR___Pao
ATATCCAAATATTGAACTGGCCGACCCCCGATTCCNCAAACCTGCCAGAGTAGACTTGCTTTTAGGTGCTG
GAAAGCTTTTCGACCTCCTTTGTGTTGGCCAAATAAACCTGTCTCCTGAACATCCGATTCAGAAAACATTT
TTAGGATGGGCCGTGTCGCAAGGCACATCATCGCAAGCACAAATTAGCACTNTCGCAATTGATTTTGGGA
CTAAGAACAGACTCCANAGGAGTCGAAAGCTGCATATACAAGTTATTTGATTTCTCAATTGCAGATGTTG
ATGTTGTATATTCAGTTCCTCGTCACAGGTGCTGAGATTGGTAAGGTTATATTCCTTGTAGATTATGTTCGG
AAAGCAACTAGCTTTCCAAATCACTGGCCTTGTGGCTCAGCAATGAAACAATTAGTATAACGGATTGATG
ACGGNAAGTGCCGGAGCAGTATATTTAAAGAAGTTGTTCTTACAAAGTTGGATACAAAACTAAACTAGAA
TTGAAACATAGAATTGGATCTGAGCATACGCCAACACTGCCGTTTGCGACATAGGCAAGTGTCAGGTTAC
CGCTGCTGACCGCAGTGGCGGGCACTGAACTCATGCGTCAGCAGAATGTCGCAATGTAAGCAAAGTGCAC
TGCAACATTCTTGCGTTCTTATAAACAAAAGTGTGTTCTTATGACATAAANATGAAGTGAAATTCTTACGT
GTTAACCTGTCCCCCCTCAAGCNTTTGACCGNCCGCGGTTATTTTATAGCTGTAAATGGCCTNTGTACTTCC
TTCGAATTGCATTCTCTTTACAAATTGTCAAACGATGAGAAAGCCAATGCCAACGAAACTGAGGATGATG
CTTGCTGCAATTC
>Ref_dvir_869
GGCTTTGCCGAGGGGGCATGGGGCATTTCAACTTCATTTGAGCCTTCAAGGTCCTGTAATCGACGAAATTA
ATTTCATCCCCGTGTGCCACTGGTCGACAAATCTCGCTCGCGCAGTGCTCGTACAAAAGATGCCAACTCTT
CTGTGAATTGCTTCGCAACAGCAATCGCAATGTTAGCTCAAGTTGTGAAATTCGATGGTGAAATTGTGAA
ATTCACCAACACATGCGTACATACAGGGCTTCTATCTGTACTTATACACACCGACGCTGCAGGAAACGTAT
TCCAACTTATGAAAAGTCTGACGCTAGATCGAGGGAACTATCCCAGCTGCAGTAATCTTTGTCACTTAGAC
CTTGGGAACATATCCCACTGCAAACTTCTCACCAGACTTTTATGCGTTTCTTCTCTGCATGTGTTTGTATAT
GCGTGTAGGCGAACACGGCATATCTATGTACATACATAGATCTATCTACCCACTTCTGAACTGTAAGAATC
GGTCAGGTCGACTGAGTGAGATACGCTTATACAAACATAAGTAAAGATAGAATAGTTTAATGCGTTAACT
TCACAAAGATTTGCTTATCTCTTAGATTCTTAATAAGCGTCCGTCCTCCTTCTTTATTGGCTTGCTGCTTTTT
CTCTAGTGATGCTTAACTCGATACTTCTTGCCTAATTATCGATTCGCTGTGAATGGCAATGGGTTGAGATT
GCCAGACGCTTACATTCGGCTTTCCTAATATTCGAAGCGTCCTCGCGAGATGAGTTTACTTGGTCATCAAC
GGAGTCTTCATCCTCGTCGGACTCCTCTGTTGGTAACTGTCATTGTTTGATTTTATCTGAAGAATACACCGA
TTTA
>Ref_dvir.23.26.centroid
TAAACCTTGAGAGAAATCGCGCTGTTGGAAGTTCCTTCGAAAAACTTCTTGTCCTACAGTAAACAATACTG
GAGTACACCTTAGATTGTAGGAGTTTTCATTTCGTAGGTGCTTCTTTTGAGTAGCTTTGGCAGCCTGATTTC
TCATTAAATCAAAGGAATCATCTCTGCTAAATTGCACTGTTCGATCTTCTAATAAATTCAAGTCTCGTATT
AATTTATATGTTGACCCATTGGTAATCATATTCTGACCAAAAGCCAAGCGATACGGACTATGGGTAATAG
CTGAATGCCAACTGGATCACAGTGAACAACAGATACTGCTGAGTTTTTCATCCCTATTAGACTGATTAGGA
TTTATATAAGCTTTCATGGCTGCTATAACAGATCTATTGACACGCTCGGATGCGTTTGCTTGAGGTGCGTG
AACTGCAGTGTACGAGTGGTGTATCGAATACTTTTGAAGTAAATCGTTAAACATTTGTGATTTAAATTGAA
CACCATTGTCCGACACAATCACTTCTGGGACCCTGAAGCAGTGAAACAACTCTTTTTCCAAAAACGGTAG
AATAGCATCTGCGGTCAATTTGCGAACCGGTTTCAAAAAAGGAAACTTTGTGAAATGGTCGAGCACAATA
AATATGCCAATATGACCGGCCTTGGAACGGGGATACGGTCCAAGATAATCAACATACAATTTTTGAAAGA
ATCGATAAGTTTCTCCACTTTTTCCCATAGGAGGTCTCAAAACACGATTCGGATGTTTAGTCGATTTGCAA
ATTTCACATTTATTGATATACTCTTTCACGTCTGAAACTAAATTTGGCCAATAATAATATCGTCGTAATCTC
TCTAGTG
>Ref_dvir_632
TGAGCGAGAGAGCGTGAGCGAGAGAGCGTGAGCGAGAGAGCTGAGCGAGAGAGCGTGAGCGAGAGAGC
GTGAGCGAGAGAGCGTGAGCGAGAGAGCGTGAGCGAGAGACTACAGAGAGCTACAAGAGCTGACGGAG
AGAGCTGAGCGAGAGAGCGTGAGCGAGAGAGCATGAGCGAGAGAGCTGAGCTAGAGAGCGTGAGCTAG
AGAGCTGAGCGAGAGAGCGTGAGCGAGAGAGCGTGAGCGAGAGAGCGTGAGCGAGAGAGCGTGAGCGA
GAGAGCGTGAGCGAGAGAGCGTGAGCGAGAGAGCGTGAGCGAGAGAGCGTGAGCGAGAGAGCGTGAGC
GAGAGAGCGTGAGCGAGAGAGCGTGAGCGAGAGAGCGTGAGCGAGAGAGCGTGAGCGAGAGAGCGTGA

GCGAGAGAGCGTGAGCGAGAGAGCGTGAGCGAGAGAGCGTGAGCGAGAGAGCGTGAGCGAGAGAGCGT
GAGCGAGAGAGCGTGAGCGAGAGAGCGTGAGCGAGAGAGCGTGAGCGAGAGAGCGTGAGCGAGAGAGC
GTGAGCGAGAGAGCGTGAGCGAGAGAGCGTGAGCGAGAGAGCGTGAGCGAGAGAGCGTGAGCGAGAGA
GCGTGAGCGAGAGAGCGTGAGCGAGAGAGCGTGAGCGAGAGAGCGTGAGCGAGAGAGCGTGAGCGAGA
GAGCGTGAGCGAGAGAGCGTGAGCGAGAGAGCGTGAGCGAGAGAGCGTGAGCGAGAGAGCGTGAGCGA
GAGAGCGTGACCGAGAGAGCGTGAGCGAGAGAGCGTGAGCGAGAGAGCGTGAGCGAGAGAGCGTGAGC
GAGAGAGCGTGAGCGAGAAGCTGAGCGAGGA
>Ref_Dv
AATCATCGAAAAAGTTTCGATTTTCGCACCGATTTTGATTTTGAAAAAAAAAACGTGACCCCTTGCCATTT
TGTCGACTTGGGTCGGGGTTTAGGTTTCATTTTTTTTATGCCAACCGATAGAACTAACCCTTACGGTTCAA
AATGGTGTGAATACAAAACTGGCATATTGGGAATATTCAAAACAACAAAAGTTAATTTCGAACTATGGTT
TTGTAGTCTTTAACATTCGTTTTGGAACGTCATATCTCCGCGCGGAGTTATGTCGTTTTGGAACGTCATATC
TCCGCGCGGTGTAATGTCGTTTTGGAACGTCATATCTCCGCGCGGAGCTTTGTCGTTTTGGAACGTCATAT
CTCCGCGCGGAGTTATGTCGTTTTTGAACGTCATATCTCCGCGGGGAGCTATGTCGTTTTGGAACGTCATA
TCTCCGCGGGGAGTTATGTCGTTTTGGAACGTCATATCTCCGCGTGGAGTTATGTCGTTTGGGAACGTCAT
ATCTCCGCGGGGAGTTATGTCGTTTGGGAACGTCATATCTCCACGCGGAGTTATGTCGTTTTGGAACGTCA
TATCTCCGCGCGGAGTTATGTCGTTTTGGAACGTCATATCTCCGCGTGGAGTTATGTCGTTTGGGAACGTC
ATATCTCCGCGGGGAGTTATGTCGTCTTGGAACGTCATATCTCCGCGGGGAGTTATGTCGTTTTGGAACGT
CATATCTCCACGCGGAGTTATGTCGTTTTGGAACGTCATATCTTGGGCGGAGCTATTACCCGAGATATTGA
AAAAAAAAAATCATCGAAAAAGGTTCAATTTTCACACCGACCTCGATTTTGAAAAAAAAAACGT
>160_rnd-5_family-4254__RC___Helitron
TTTTACTCTTAGCTTTTTTCTGCATTAACANTTCNGCTNTTCAATTTCTGTGTTCAGTTGTTTGTATACCCTT
GCAGAGGNTATTATAATTTTGTCNNNAAATGTGTAACGCATAGAAGGAGACATCTCCGACCCCATAAGGT
ATATATATTTGTCCGTCTGTTTGTCTCTTTGCGAACAAGTCTCTCAGCTTTAAAGCTATCCTTACGAAACNC
TGCANNAGTCCGNCTTTCTGTACAATTGCNGGATCTGCACATATANNGGGACCTGCTGNATCGCACCGGA
TCGGGTCGAAGAGTAGGTTCTTGTATAGAAANCATTTATTANTTCTACCGAGCTCCCCTTACGTGCGCAAA
ATCNTATCAACATCGGACCTATAAANCATATAGCTTNCTGNGGAACGACCGGGCAGAAATAAAGTTGTTC
TAAGCAAACCTTTTTTTGTTTTTCAAAATTTCGTGGCCAAGCTTGGCATTGATAAGTTTCACTACGCTCCCT
ACATTTATGCAAAGTCTTAAATAGCTGTCATATGAACGATCATTGAAAAATAGATTCTTGTATGAAAAAAT
CGTTGTTTTTCAAGAAATCCTAGCCATATTCGGTATATCATATTTTTTCCTGTGCCTCTTAGATACGTGCAT
AGCATTATGAGCATTATAAAAAGAATGGACCTACAAGGGTATGGAGTCTTCGGCGTGCCGAAGAAAGGTC
TTCCTTCAGGATTTTTTCATGTTTATATTTGTCATATTTGCCTTGTCTTTGGGGATTCCTCTTATGAGCAGCA
TTGGTCAACAGTGGGCGTGTCTCAGCTGTTACCACCCACACTTGGAGTCATTG
>160_rnd-5_family-2728__LINE___I-Jockey
CAGCAGCAAAATGCACCGGCAGGCGTTGGTGTTGGCGTGATGATGCCCCAAAACCAAGTTGGGNCCACG
GCTGCAAGTCCATCGGCATCCGCAAATGCAGCAGCAGCAACAACAGCAACAACAACACATGTACNCAAA
TACGCCACAGCGTCATCAGGCACAATAAATTCNCCGCCGCAGACCTATTTGCCGGCGGGCGCTGTACAGC
AGCAGCAACAAGTTCTTTGCAGCAATCGGCCGCACCAGCAGCAGCGACAGCCGCAGCAACAACAACAGC
ATCGGCAACGAGCGCAGCAGCCGACACAATCACGGCAGCAGCAGCAGCACAATCACGACGGCAGCAGCA
ACAGCAAACGGCACAACATNTTACGGCGCCGACACACAGCCAGCAACAGCCGCGTCAGCGGCGTCACCA
CAGCAGCGCTTGCANNCGAACAGNCCCGCATCGGATGNATCAGCTGTCGCTCGCTGGTCGATGGCCATGG
GCGTGGGCGTCGGGCAGCCAAGCAACGGCAGCAGCTGCTGCCGGCAGNGCGGCGACGGCAATAAGCGCC
NCGCATAGNGCTGCAACGGCAGCGGCAGCAACAACAACAACAACAACAGCAGCAGCAGCAGCAGCTTGC
AACCGCAATCGCAGCTGCAGCAGCAGCAGCCACAACGANCTGTAGCAGCCCTTATGCAACCAGCAACAG
CAGCAAAATTTNCNAGTGCCNGCGGCGGGNGCTGCAGNCACNGCNGCAGGCGCCGCNGNTGGGCCCGGC
ACNGCNGACAGCGGTGCCAGNGCCACAGTCCANGCAGCATGCGCCGCCAACAGCCGCATATCCACAGAC
CCA
>Ref_dvir_271
TAGACAGTTTCGGTCCGGATTCCCGGGAATCCCCTTTTTTTTTTTTTTTTTTTTTTTTTATTTTAGAATATTAA
AAATAATTATATAAGAAAAAGTTTTAGTATTGTTTAAAGAAAAAATAATTTTAATAATAGTTTAATAGTAT
TGTAAAAGAAAATTGAAATATTTTGAAAAATTTTTATTTTAAAAGAAAATTTAATTTATTGTACCTTGTGT
ATCAGCGTTTATTAAATAAAAAATATTTAAATATATTTTTCTCGATTTTAAAAGAGTTAATATAATATTAG
AGTTAATGTAATAAAATTATTTTAAATATTATATTAGAAATGAAATGTTATTCGTTTTTAAAGGTATCTAG
TTTTTCAAGAAATAAATTTAATTTAGAAATTATAAATTTATTTAAAAAATTTTTTAATTAAATAATTTATAA

TTTTAATATTTTATGGGATAAGCTATAAAATAAATTTTTAAAAATAATAAATAAATTTAAAATATATATGC
TTAGACCATTAGCAATTATTAAAAATTGTGTTATAATTTATTTTATAAATTAAATTATTTATTATATTTTAA
ATTTTTATTGATAATATTAATTTTAATTTTGATGTTAAATTAAGAATAATGATAGAATTAGTATATTTTAT
TGTAAAATAAATTTAAATGATAAGTTTTAGTAAAGAATTCGGCAAAACCAATATTCGCCTGTTTAGTTAAA
ACCTGTAATTTTGAATAAAATTTAAGTCCGAAACGGCCACTAAAATTAAAATAATTTTATTTTTATTTAGA
ATAATATTAATTAATATAAAAAAAAAAAAAAAAAAAAAA
>160_rnd-5_family-1171__DNA___CMC-EnSpm
TTCTTCAATCTGAGAGAATCGTTGGCCATTCAGGCCAAACAGTTATATATNTATAAATTACGTACTCACCT
GTAAATTCACACGCACGATTATTTCATCATCTATCAGTAGGCGGGAGACGAACTTTTATATAAACACATCA
CTGAAAGAACAGAAAAGCAGAGATAATGTTTATTAGCTTAAAAACGCAGCTGCTTGCTAAACCTTACTTG
AATTAGTGAGAATCGCGTAGNAGAGCAATATATGTTCATATACATGCAACATGCATACATACGATTGTAT
GTAATAGTTGTTGCTATTATTTTAAATATAATAAGACATAGTTATGTTTATAATACGTACCATGTTGATCCG
TCCCATCCTTTGCTCAATCCGCCGCCTCCTCCANTGTTNAAGCCGCCGCCTCCTCCNTAGTTCAGACGCCG
CCTCCTCCAATGTTAAAGAGCCGCCGCCTCCTCCATAGTTGAAGCCGCCGTCTCCTCCTTTGCTNAAGCCG
CCGCCTCCTTCCATAGTTGAAGCCCGCCTCCTCCTTTGCTCAAGCCGCCGTCTCCTCCAATATTAAAGCCGC
CGCCTCCTCCNTTGCTNAAGCCGCCGCCTCCTCCATAGTTGAAGCCGCCTCCTCCATTGTTGAAGCCGCCG
CCGCCTCCTNCGTNGTTGAAGCCGCCGCCTCCTCCTATATTAAAGCCGCCGCCTCCTCCATNGTTGAAGCC
GCCGCCTCCTCCATTGCTNAAGCCGCCGCCTCCTCCATTGCTGAAGCCGCCGCCTCCTCCATNGTTGAAGC
CGCCTCCTCCTGCGTNGAAGCCGCCGCCGCCGCCGCCGCCGC
>Ref_dvir_1007
ATAAGGTGCGCAACGAGATTATACGAAACTATCAAAATCTTACTTTAAACCCCCGTCAAATCGAAAATGT
TGTTTTATCAACTCACACGATGGAATACAATGAGAATGCCAAGCGTAATGTGAAATATGCTGGAAAAGTC
TCGTTAGATTTGCTACTGAAAGTTGTTATATTGCAAAATTTTATTTTAACGAAATTATAAAAAATTAATTTG
AAACTTTAAAAGGAAGTTGAAACTGGAGATGTTGTCCGCTTTCGATGTGCATCAAAAGGTGAGAAGGTCT
TGACTTCTTAGGAACAACCCTTAAGGTTGTAGCGGGCACTCTGGACATAAATTTCAAATTAATTCCATAGG
GCAAGAAAAGATTAACCAACTGACCGACACAGTAAACAAAATTCTCAAATCCTCATAAAGCAGGCGGAT
ACAGGCCAAATTTTTTTAGACCCAGCACACGCTACAAAATGGTGAACAGAACAGAAATAGGACAATTATG
CTTACTTTTACCTTATAAAAATTTAAATGAAATAAATCCAACAAAAATTGATCACTTGAATATTCTCACTA
ACGAATTTACAGGACGCATAACACACATTTTGAAAGTATCTAATATTAAAGTAGAACATGAGAACAAAGT
AATAACTTTCGTAATTCAGTTCCCTATAGTAAGCAAGCAACACAGAAAGATAAACATTTTCTCAATCGCCT
TCAATGGTTTCGTACTTCACCTCCACGGCAAATTTAACCGGAATCAATTGGTCCCAAACAACAACTGCCAC
ATTCTGCCAACAACCCCTAAAGAACCATGCAACCAAAGGC
>Ref_dvir_1069
GAAGTAACTTTCTCAACTGCTGCCCGTGGAGGACTCCTCGGCAGCAGCAGTTACTTCTGCCGGAGTAACTA
TCTCAACTGCTGGCACTGGAACTTCCTGAGAAGCAGTTACTTCTGCCGGAGTAACTATCTCAACTGCTGGC
ACTGGAACTTCCTCGGAAGCAGTTACTTCTGCCGGAGTAACTATCTCAACTGCTGGCTCTGGAACTTCCTC
GGGAGCAGCAGTTACTTCTGCCGGAGTAACTATCTCAACTGCTGGCGCTGGAGATTCCTCGGGAGCAGCA
GTTACTTCTGCCGGAGTAACTATCTCAGCTGCTGGCACTGAAACTTCCTGAGATGCAGTTACTTCTGCCGG
AGTCACTATCTCAACTGCCGGCACTGGAACTTCCTCGGAAGGAGTCACTTCTGAAGGAGTAACTATCTCA
ACTGCTGGCGCTGGAGACTCCTCGGGAGCAGCAGTTACTTCTGCCGGCGTAACTATCTCAGCTGCTGGCAC
TGTAACTTCCTGAGAAGCAGTTACTTCTGCCGGAGTCACTATCTCAACTGCTGGCGCTGGAACTTCCTCAG
AAGCAGTTACTTCTGCCGGAGTAACTATCTCACTTGCGGGCACTGGAACTTCCTCGGGAGCAGCAGTCACT
TCTGCTGGAGTAACTTTCTCAACTGCTGCCGTTGGAGACTCCTCGGGAGCAGCAGTTACTTCTGCCGGCGT
AACTATCTCAGCTGCTGGCACTGTAACTTCCTGAGATGCCGTTACTTCTGCCGGGAGTCACTATCTCAACT
GCTGGCGACTGGAACGTCCTCAGAAG
>Ref_dvir_552
CAAGTCTTTATTGTATTGAATCTAAAATAGCATTCATTTTGATTAAAACACAGTTAACTTAGGTGGTATTTC
GTCTAGCCTGGTTGGGTCCTCAAACGTTGTAAACTGCCCGAATGGCTTTTTCGGGGACAATCGCCAAATGC
GTCTTCTTTGAGTAAGGCGGCATCGCTCACGTAGGTAGTCGGTGGGCTCTGGGGCGTCTCCTGTGTCTAGG
CTGGAGGAGGTCCTTAAAAGATCTTCTCTTGGGCCTCCTGACTACACACCTCCCAGCGAGGTCTCTTGCTT
CCCGGTTACAGTATCGCTGCAAGCGTTCATTGTATCGGTTGGCGAATGCTTCGACTTTGCTGCATATAGTC
TGTATTTTGAAGTCTTTGCCTGGGGGTGCCAACTCATGTAGGTAATCCGGAGTGGGGATGTCTGCCGTGCA
CAAATTGTAGAGGAAGGGACCCAGTACGCTGCCTTGAGGGACACCCGCCTTCATATTGCGAACCGGAGAG
ATTGCTGTCCTGACTTTGACTTCGAACTTACGCTCCATTAGATAAGAGCGCAAAAGTAGAAAGAAGGGGG

CGGGAAGACTCATCTTGATTTTCTGGAGCAATCCATCAATCCAGACTCGGTCGAAGGCCTGCTTAACGTCT
ACAAAGACTCCATAGCAATAGTCTTTGTCGTCGTAAGCCTTAAGAATATGGTTAATCACTCTGTGAACCTG
TTCAACAGCGCCGCCCACCCCTGAACCCAAATGGGTGATCAGGAAGAATGGCCATGTGATCCATAAGCCT
AGCAAGTCGGGCGACAATCAGTCTT
>Ref_dvir_466
AAAAACAAAAGAGGTCTTCGGAAAAAACATATCTCCGCCGTTTTAAAAAAGGAAAATAAGATATGTTTTG
AAACGACATATCTCCGCCGTTTTAATATCGAAAATATATGTCGTTCTGAAACGACATATCTCCGCCGTTTT
AATAACGAAAATAAGATATGTCGTTCTGAAACGACATATCTCCGCCGTTTTAATAACGAAAATAAGATAT
GTCGTTCTGAAACGACATATCTCCGCCGTTTTAATAACGAAAATAAGATATGTCGTTCTGAAACGACATAT
CTCCGCCGTTTTAATAACGAAAATAAGATATGTCGTTCTGAAACGACATATCTCCGCCGTTTAAATATCGA
AAATAAGATATGTCGTTCTGAAACGACATATCTCCGCCGTTTTAATAACGAAAATAAGATATGTCGTTCTG
AAACGACATATCTCCGCCGTTTTAATAACGAAAATAAGATATGTCGTTCTGAAACGACATATCTCCGCCGT
TTTAATATCGAAAATAAGATACGTTCTGAAACGACATATCTCCGCCGTTTTAATAACGAAAATAAGATAT
GTCGTTCTGAAACGACATATCTCCGCCGTTTAAATAACGAAAATAAGATATGTCGTTCTGAAACGACATAT
CTCCGCCGTTTTAATATCGAAAATAAGATATGTCGTTCTGAAACGACATATCTCCGCCGTTTAAATATCGA
AAATAATATGTCGTTCTGAAACGCCTTTTTAAATACAAAAAAAAAAATGGGCTTTTGAAAACAAATTTTCC
CCCCCTTTTAAAAAAAAAAAAAAAAA
>160_rnd-1_family-327__Unknown
GCTTATTAGTAAGAAATGCCAGTGTGGTCGCGAATCCAGTCCAAGTGGTAGGTGACACGCTGGAAGCCAG
ATGGGTGTCCAGATTGGCAACCGGCAGAAGAAACCCAGTTGGTAACGCCGACCAGTCTGTCGCCGTCGTG
TGTAACCAATGGACCACCGGAGTCACCGCCGCAGGTGGACTTGCCGTCGGGNGTGCTNACGCAGAGGATG
TTGTCACCAACGGCGCAGAGCCGTAGGTCTGCTGGCACTCGNNGTTGCGNATGATCTGCAGGTCGACGCA
CTGCAGCCAGTCGGGCAGTGGGCCGCCATCGTAGGTACCGCCCCAGCCGCAGGCAACGGCCCAGCGGTCG
TCGTAGTCGTTGTAGCGGTCGTTGTAGCTGGGCAGCTCCACCTTGTTGACCATGCGCCAGAAGTCCACATG
AGGAGTGCGGATCAGAGCGATGTCAGCGTTGTGGTGGTTGATGATGTCGTCGCCGCCGACCCGGTGGGTG
AACTGGGCGTTGGTGCGCCAGGTGGCACCGNAGTAGACGGTNACGTCAGAGCCCGTGCAGTGCTCGGCA
GTCAGGACCCAGGTGTTTCCAATGATGGAACCACCGCACCACCAGCCACCGCTGAAGCCCAGGCCAACGG
TGTAGGGCGCCTTGCCCTCGTAGGCTGGGTAGCCATTGGTGATACGNCCCTCGATCTTGTTGACCTTGGGC
ATGTCCTCGGGGTGGACGGCAGGCGCCGCGGCCGCAGNGGCGGCGGCAACAGCCAAAGCCAGAATTGTN
ACGAACACCTTCATGTTGCAGGTACNACTTGG
>160_rnd-5_family-1376__LINE___I-Jockey
CGCAGCGAGAGGATGGCAGTAATGCAACAGCAGGCTTTGGGTATGCAACAGCAAATCCTTAACCTTTTGA
CCGCGTTGCTATCCAAATGAACACACCACTGAAGGTGTTAATCTGGAATGCCAATGGCATTTCAGGCTCTA
GTACAAAAACACGCTATCGGAATAATTCTGCTGTCAGAAACTAGAACCAACGGTCCTACNTTCCCCAGAA
TCTTTGGCTTCGAGGCGTACCCTGTACACTGGACTCAAAATCGCTCAGCTAGTGGCGGCTGCGCTGTCCTC
GTCCGGAGTAACTTGGTTCACTTCCCGCAATTGAGCATTGCAAATCGTGTTTACCAATATTCGACTGTGAA
AGTCTCCACTGATGCTGGTAGCGTGGACTTTGGAGCAGTCTATNGCAGCAATGCGCGCGGCCTTCAACTA
GCTAACAGCCTAGCGCATCGTCAAGCGAAGGTACTGGCCACAGGTTCTCCCACAAGATACCCGTACGGCA
GCAGAGGCACTCCGTCTCGCATAGACTTCGCTATCTACCAGGGAATCCCTGACCAGCTCCTTTCAATCGCT
GACGATTGGGACCTCCCGAGCGACCACCTGGCACTGATAGTTACCCTGGAGAGCAGGGCCAGAGTGGTCA
GCGCAATACCGAGGCTGATCAACTCACGCACTGATCTCGGTGTCTTCCAGAGACAGATGAGTGANAGTAT
ACACCTCAATACTGAAATCGGCACTGGACTGGATGTAGAGGACGCCATTGAGCTGCTTAACGCAAAGATT
CGCGCAGCAGCAGCCGAAGCCA
>Ref_dvir_481
AGATAACGCACAAGCAAGCATAAGTAAATATGAATTGCGAAAACAGACACCCAAACATGTGTATGCATA
CAGAGAGAATCGTCGAATATGTACATACATACACATGTATGTGTCTATGATTGCTTGTGCATTGCCTTCTT
ATTTATCGCGCTGACACTAATATTTGGCAAAGTGTCGAGAAAATTACAATAAAACAAACACTTTAACATT
CTTGGAATCGATGGACTTCTTCAAATTTTGATTAAACTTTTGTACGATTTCTTTTATGGCTATTAATGCTTA
TTAGTTATACCATTAGAGATAAGGATGGCACTTACTTGAGAATTTGCACTTATAATTGCACTTTACTTATG
CATCACATCCAAAAACACACTAACATAGAAAGTTGAGCTGATCCATAAAAACGGCGCTCAGAAAAAAGA
GCACTCAATCGACCAGGCGAGAACATCACTGCCAGCAGATATTGAGTGGACCAATCAGCGATCAGCACCA
AATTGAATCGTGAGCGCGTGAGCGTAAGCAGAAAAATGACAGCGAACACACACACGAACAGGTGACTGC
TTATCTCTGGCCTTGGCTCATCTCTGCGCATACACGTGTTTGTGTGGGTGTGTGCATGCTAAGCAATGTGG
CGCATCGTTCTCACTCGTATTTCGATCGCTTGCTTATTTTCTGGGCGCCGGTTTTTTCGTCCGCTTGACTCAC
[truncated: 76,945 more chars]
